# CircRNAs in the tree shrew (*Tupaia belangeri*) brain during postnatal development and aging

**DOI:** 10.18632/aging.101437

**Published:** 2018-04-30

**Authors:** CaiXia Lu, XiaoMei Sun, Na Li, WenGuang Wang, DeXuan Kuang, PinFen Tong, YuanYuan Han, JieJie Dai

**Affiliations:** 1 Center of Tree Shrew Germplasm Resources, Institute of Medical Biology, Chinese Academy of Medical Science and Peking Union Medical College, Kunming, China; 2 Yunnan Key Laboratory of Vaccine Research and Development on Severe Infectious Diseases, Kunming, China; 3 Yunnan Innovation Team of Standardization and Application Research in Tree Shrew, Kunming, China

**Keywords:** circRNAs, RNA-sequencing, tree shrew, ubiquitin-mediated proteolysis

## Abstract

Circular RNAs (circRNAs) are a novel type of non-coding RNA expressed across different species and tissues. At present, little is known about the expression and function of circRNAs in the tree shrew brain. In this study, we used RNA-seq to identify 35,007 circRNAs in hippocampus and cerebellum samples from infant (aged 47–52 days), young (aged 15–18 months), and old (aged 78–86 months) tree shrews. We observed no significant changes in the total circRNA expression profiles in different brain regions over time. However, circRNA tended to be downregulated in the cerebellum over time. Real-time RT-PCR analysis verified the presence of circRNAs. KEGG analysis indicated the occurrence of ubiquitin-mediated proteolysis, the MAPK signaling pathway, phosphatidylinositol signaling system, long-term depression, the rap1 signaling pathway, and long-term potentiation in both brain regions. We also observed that 29,087 (83.1%) tree shrew circRNAs shared homology with human circRNAs. The competing endogenous RNA networks suggested novel_circRNA_007362 potential functions as a 24-miRNAs sponge to regulate UBE4B expression. Thus, we obtained comprehensive circRNA expression profiles in the tree shrew brain during postnatal development and aging, which might help to elucidate the functions of circRNAs during brain aging and in age-related diseases.

## Introduction

Circular RNAs (circRNAs) are a novel type of non-coding RNA with a covalently closed-loop structure generated by back splicing. CircRNAs were first identified approximately 37 years ago [1], but they have only recently attracted the attention of researchers. CircRNAs are considerably more stable and more resistant to RNase R than linear RNAs [2]. However, the functions of most circRNAs remain unclear. Previous studies have shown circRNAs can act as “sponges” for microRNAs via the competing endogenous RNA (ceRNA) network in order to regulate target gene expression [3,4]. CircRNAs may also serve as sponges for RNA-binding proteins to post-transcriptionally modulate mRNA expression [4].

Recent studies have suggested that circRNAs can have critical effects on the modulation of cellular processes, such as apoptosis, proliferation, development, cancer, and aging [5–7]. Studies have also shown that circRNAs accumulate in the brain during aging, and thus they constitute a novel biomarker for aging [8]. For example, aged mice exhibit increased expression of circRNAs in the cortex and hippocampus [9]. It has been reported that FOXO3, a member of the forkhead family of transcription factors, plays an important role in cellular senescence and aging, and it is associated with hippocampal neuronal injury [5,10,11]. Furthermore, the circ-Foxo3 (the circRNA generated from Foxo3) is present in aged patients and murine cardiovascular disease models [5].

Many circRNAs have been detected in different tissues in humans, rhesus macaques, mice, sheep, pigs, and zebrafish [2,12–15]. It has also been reported that circRNAs are more enriched in neuronal tissues compared with other tissues [2,8,16,17]. In mice, researchers have identified 1,212 circRNAs in the hippocampus and 2,407 circRNAs in the cerebellum [2]. In the human brain, 65,731 circRNAs have been identified [2]. Ninety percent of mouse circRNAs and 73% of human circRNAs remain unannotated [18].

The tree shrew (*Tupaia belangeri*) is a novel and valuable laboratory animal due to its inexpensive maintenance, small body size, short reproductive cycle, and high brain-to body mass ratio [19,20]. This model has been used to study human diseases, such as herpes simplex viruses [21], hepatitis C viruses [22], and Coxsackie virus A16 [23], especially in the nervous system, as well as Alzheimer's disease [24], brain development and aging [25,26], social stress and depression [27,28]. Whole genome sequencing of the tree shrew has been completed, but only a limited number of transcriptome profiles are available for of various tissues in the tree shrew. Furthermore, very little information is available regarding circRNAs in the tree shrew.

In this study, we investigated the effects of age on the circRNA expression profiles in the tree shrew hippocampus and cerebellum using high-throughput sequencing. We also constructed the ceRNA networks based on target analysis. Numerous previously unreported circRNAs were identified in the tree shrew brain with increasing age, which may improve our understanding of the roles of circRNAs during brain aging and in age-related brain diseases.

## RESULTS

### Overview of circRNA-Seq

**Figure 1:**
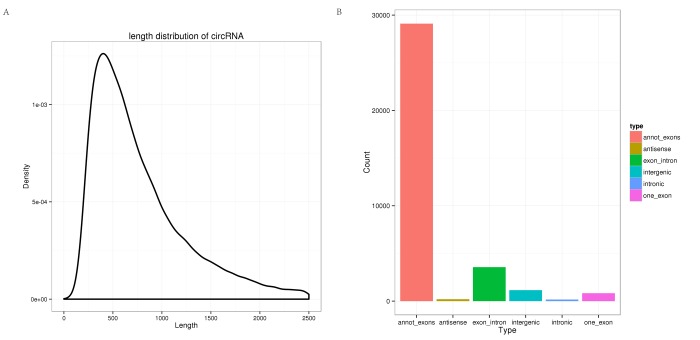
**Characterization of circRNAs in the tree shrew brain.** (**A**) Length distribution of circRNAs. (**B**) Type distribution of circRNAs. Red indicates annot_exon, brown represents antisense, green indicates exon_intron, light blue shows intergenic, blue represents intronic, and pink indicates one_exon.

In total, 465,791,754 clean reads (235,543,658 for hippocampus and 230,248,096 for cerebellum) were generated. After removing the low quality, poly-N-containing, and adapter-containing reads from the clean reads, 433,887,754 high quality clean reads (216,130,360 from the hippocampus and 217,757,394 from the cerebellum) were obtained from the two tissues (Table S1). The high quality clean reads from which the mapped rRNA reads had been removed were mapped to the tree shrew genome using TopHat2, and the unmapped reads were selected. Based on theoretical predictions, 35,007 circRNAs were detected using *find_circ*. All of the identified circRNAs were novel. As shown in Fig. 1A, most of the circRNAs were approximately 400 nt in length, as found in previous studies [29]. In addition, different types of circRNAs were identified (summarized in Fig. 1B), where the most common type was annot_exon followed by exon_intron.

### CircRNA expression profiles in different brain regions over time

**Figure 2:**
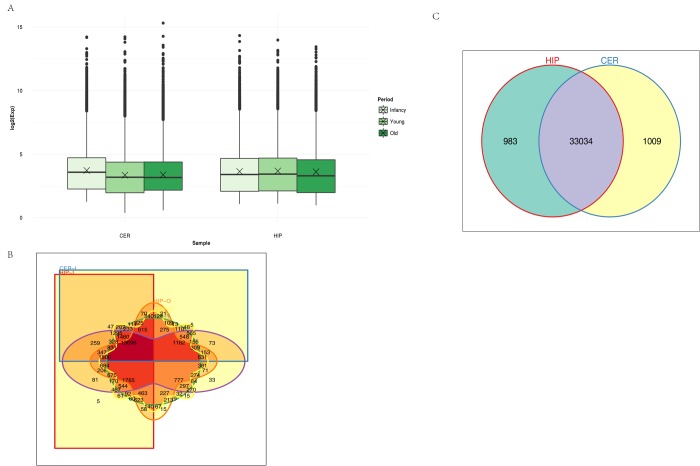
**Characteristics of circRNA expression.** (**A**) Box plots comparing circRNAs measured based on the log of the expression level (base 2) in the two brain regions over time. CER: cerebellum, HIP: hippocampus. (**B**) Venn diagram showing the overlapping circRNAs identified in the hippocampus and cerebellum. (**C**) Venn diagram showing the overlapping circRNAs detected in different brain regions over time. Different colors and shapes represent different brain regions and time points. HIP-I, HIP-Y, and HIP-O represent the three ages in the hippocampus. CER-I, CER-Y, and CER-O represent the three ages in the cerebellum.

Unsupervised hierarchical clustering and principal component analysis (PCA) were performed to understand the relationships between circRNA expression and spatiotemporal dynamics in the tree shrew brain. The circRNA expression patterns in the two brain regions comprised relatively independent clusters, and the first two principal coordinates corresponded to the brain region and age (Fig. S1). A box view was used to compare the expression distributions in the samples in order characterize the changes in circRNA expression over time. As shown in Fig. 2A, there were no significant changes in total circRNA expression in different brain regions over time. The majority of the circRNAs annotated in the hippocampus were also detected in the cerebellum, and vice versa (Fig. 2B). In total, 983 circRNAs were expressed exclusively in the hippocampus and 1009 were expressed exclusively in the cerebellum. A small number of circRNAs were expressed exclusively in each age group (Fig. 2C).

### CircRNAs expressed in the cerebellum tended to be downregulated with increasing age

**Figure 3:**
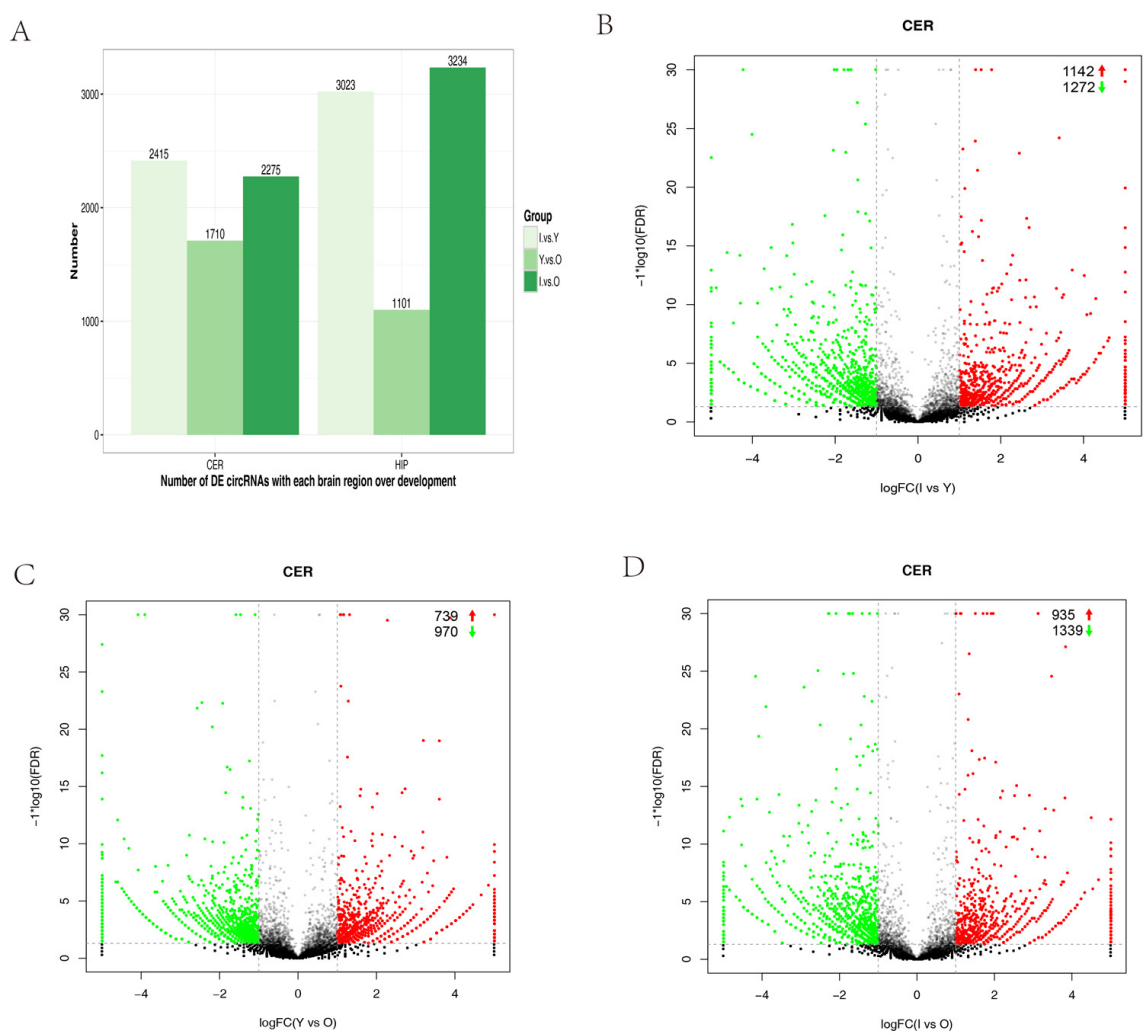
**Differential circRNA expression in the cerebellum with aging.** (**A**) Bar plot representation of differentially expressed circRNAs in different age groups. (**B**–**G**) Volcano plots showing the dysregulated circRNAs in the cerebellum in different age groups. The log of FC (base 2) is plotted on the x-axis and the negative log of FDR (base 10) is plotted on the y-axis (FC >2 and *p* < 0.05). The green and red points on the graph represent downregulated and upregulated circRNAs, respectively.

We performed expression profiling to determine whether circRNAs were differentially expressed in the hippocampus and cerebellum during development. Differences in circRNA expression between samples were calculated based on the back-spliced reads per million (RPM) mapped reads method. Only differentially expressed circRNAs with a fold change (FC) ≥ 2 and *p*-value < 0.05 were considered. We compared infants (I) versus young (Y) animals, Y versus old (O) animals, and I versus O for each brain region. The numbers of differentially expressed circRNAs were high in the group comparison, where they ranged from 1,101‒3,234 with increasing age in the different brain regions (Fig. 3A). Volcano plots indicated that circRNAs tended to be downregulated with age in cerebellum samples (Fig. 3B–D). However, in the hippocampus, the tendency to be downregulated with increasing age was only found when I was compared with Y (Fig. S2A–C). In the cerebellum, we found that 1,339 (4.1%) circRNAs were significantly downregulated and 935 (2.9%) were significantly upregulated in I compared with O animals (Fig. 3D). In I vs Y, 1,272 (3.9%) were significantly downregulated and 1,142 (3.5%) were significantly upregulated (Fig. 3B). In Y vs O, 970 (2.9%) were significantly downregulated, and 739 (2.2%) were significantly upregulated (Fig. 3C).

In the hippocampus, we observed that 1,636 (5.0%) circRNAs were significantly downregulated and 1,386 (4.3%) were significantly upregulated in I compared with Y animals (Fig. S2A). However, in Y vs O, 512 (1.7%) were significantly downregulated, and 588 (1.9%) were significantly upregulated (Fig. S2B). In I vs O, the numbers of significantly downregulated and upregulated circRNAs were almost unanimously (Fig. S2C). These results indicated that circRNA expression could be spatially and temporally specific.

### Dynamics of circRNA expression in the brain over time

**Figure 4:**
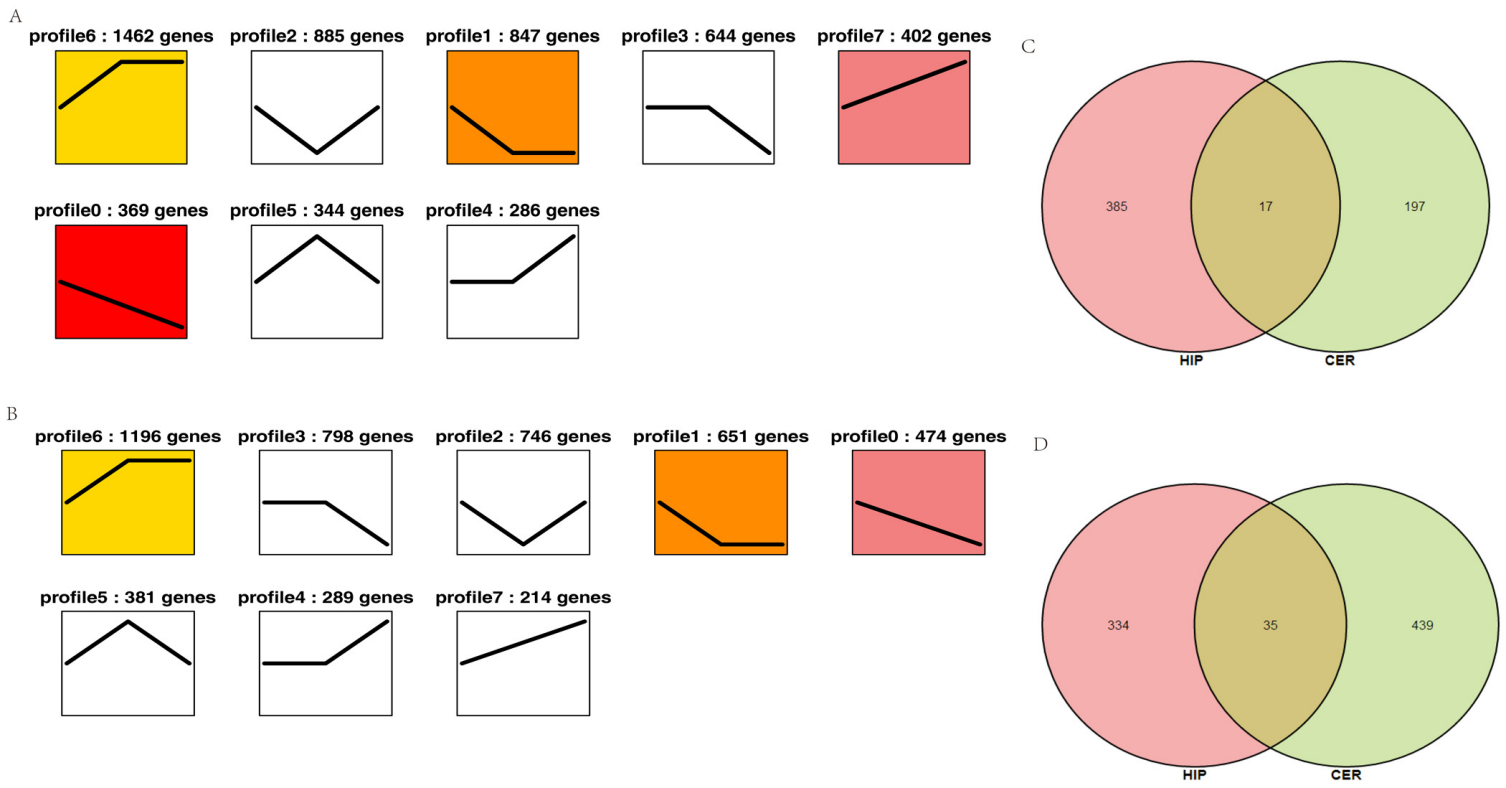
**Overall expression patterns of circRNAs in different regions of the brain.** (**A**) Expression patterns of circRNAs in the hippocampus. (**B**) Expression patterns of circRNAs in the cerebellum. The colored profile represents significant enrichment (*p* < 0.05). The uncolored profiles denote no significant enrichment. The profile ID and the number of genes in the profile are shown above the chart. (**C**–**D**) Venn diagram showing the up- and downregulated circRNAs in both the hippocampus and cerebellum. C: upregulated circRNAs. D: downregulated circRNAs.

To characterize the changes in circRNA expression, we performed trend analysis to identify the predominant differentially expressed circRNAs in each brain region over the course of development. We identified seven main circRNA profiles in the hippocampus and cerebellum, where each represented a characteristic expression pattern (Fig. 4A–B). Four patterns (profiles 0, 1, 6, and 7) were significant in the hippocampus and three patterns were significant in the cerebellum (profiles 0, 1, and 6). Among these patterns, profile 0 was significantly downregulated in both the hippocampus and cerebellum. We also analyzed the up- and downregulated circRNAs in both the hippocampus and cerebellum. We found that 385 circRNAs were increased in the hippocampus and 197 circRNAs in the cerebellum, whereas 334 circRNAs were decreased in the hippocampus and 439 circRNAs in the cerebellum (Fig. 4C). These findings suggested that the age-associated dysregulation of circRNAs in the tree shrew brain may play roles in brain development and aging.

### Enrichment of differentially expressed circRNAs

**Figure 5:**
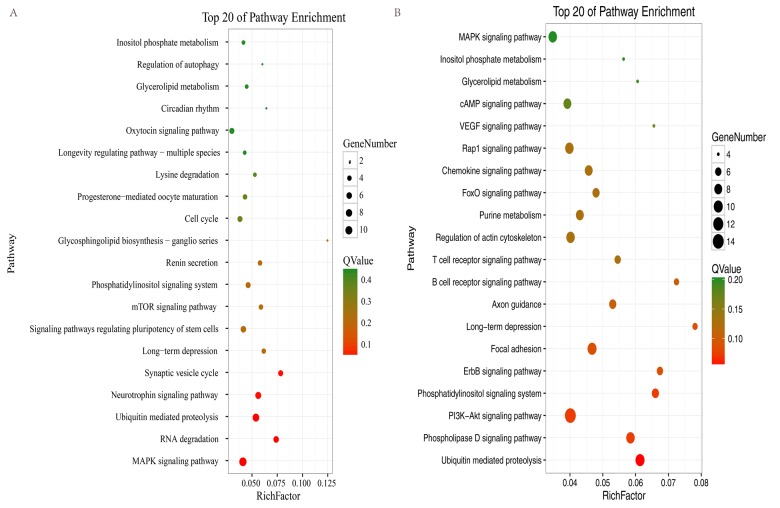
**KEGG analysis of profile 0 in the hippocampus and cerebellum.** The bubble chart shows enriched differentially expressed genes in signaling pathways. (**A**) Bubble chart of the top 20 pathways in the hippocampus. (**B**) Bubble chart of the top 20 pathways in the cerebellum. The Y-axis label represents the pathway and the X-axis label represents the rich factor (rich factor = amount of differentially expressed genes enriched in the pathway/amount of all genes in background gene set). The color and size of the bubble represent enrichment significance and the amount of differentially expressed genes enriched in the pathway, respectively.

To elucidate the possible roles of the up- and downregulated circRNAs in different brain regions, enriched Gene Ontology (GO) and Kyoto Encyclopedia of Genes and Genomes (KEGG) pathways were obtained for the circRNAs in profile 0 (downregulated circRNAs) and profile 7 (upregulated circRNAs). For profile 0, GO analyses showed that the circRNAs in the two brain regions were enriched in terms of similar functions (Fig. S3A). However, for profile 7, GO analyses showed that the circRNAs in the two brain regions were enriched in terms of quite divergent functions (Fig. S3B). In profile 0, 14 pathways in the hippocampus and 30 pathways in the cerebellum satisfied the requirement of *p* < 0.05 (Table S4, Table S5). We used bubble charts to represent the top 20 pathways in profile 0 in each brain region (Fig. 5A–B). In profile 7 (upregulated circRNAs), seven and 24 KEGG pathways (*p* < 0.05) were identified in the hippocampus and cerebellum, respectively (Table S6, Table S7). We used bar plots to represent the KEGG pathways in profile 7 for each brain region (Fig. S4). In profile 0, RNA degradation, the neurotrophin signaling pathway, and synaptic vesicle cycle were identified in the hippocampus. The phosphatidylinositol signaling system, phospholipase D signaling pathway, PI3K-Akt signaling pathway, and ErbB signaling pathway were identified in the cerebellum. The ubiquitin-mediated proteolysis (UMP) pathway, MAPK signaling pathway, phosphatidylinositol signaling system, and long-term depression were identified in both brain regions (Fig. 5A–B). In profile 7, the synaptic vesicle cycle, UMP, and the Fanconi anemia pathway were identified in the hippocampus. The cAMP signaling pathway, phosphatidylinositol signaling system, and insulin signaling pathway were identified in the cerebellum. The rap1 signaling pathway and long-term potentiation were identified in both brain regions (Fig. S3C). These results suggested that the circRNAs involved in these pathways may be related to aging in the hippocampus and cerebellum of the tree shrew. Furthermore, most of the pathways in the two brain regions were classified into environmental information processing, genetic information processing, and organismal systems (Tables S4–7).

### CircRNA-miRNA-mRNA co-expression

**Figure 6:**
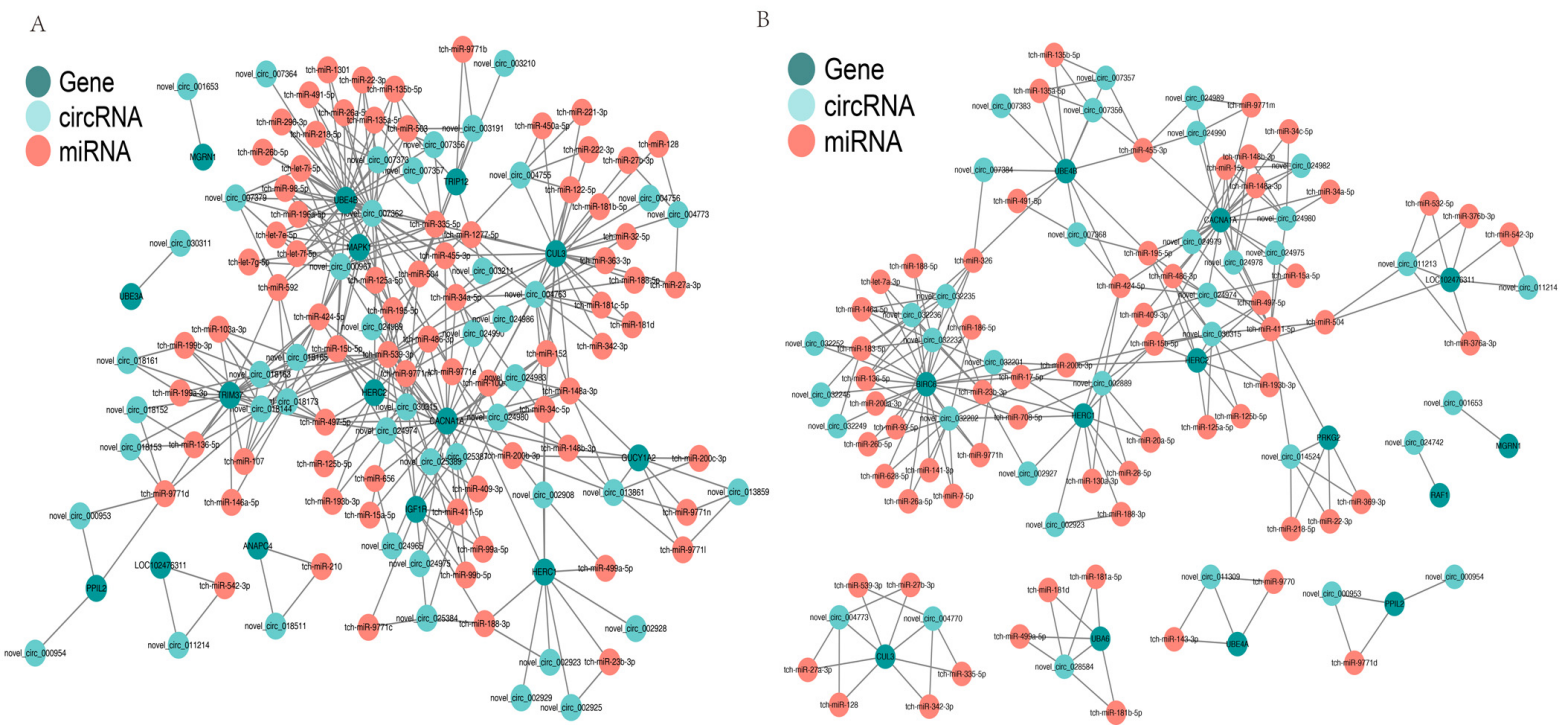
**Competing endogenous RNA network based on circRNA–miRNA–mRNA interactions.** (**A**) Network in the cerebellum. (**B**) Network in the hippocampus. Red circles represent miRNAs. Blackish green circles represent genes. Pink blue circles represent circRNAs.

CircRNAs can serve as “sponges” for microRNAs to regulate host gene expression, so we constructed a co-expression network based on the UMP pathway and long-term depression. In the cerebellum, novel_circRNA_007362 was predicted to combine with 24 miRNAs (tch-let-7e-5p, tch-let-7i-5p, tch-let-7f-5p, tch-miR-125a-5p, tch-miR-1301, tch-miR-135a-5p, tch-miR-135b-5p, tch-miR-15b-5p, tch-miR-195-5p, tch-miR-1-5p, tch-miR-218-5p, tch-miR-22-3p, tch-miR-26a-5p, tch-miR-26b-5p, tch-miR-296-3p, tch-miR-335-5p, tch-miR-34a-5p, tch-miR424-5p,tch-miR-491-5p, tch-miR-455-3p,tch-miR-503, tch-miR-592, tch-miR-9771e, and tch-miR-98-5p). UBE4B was predicted to be targeted by the same 24 miRNAs and six circRNAs (novel_circRNA_007356, novel_circRNA_007357, novel_circRNA_007362, novel_circRNA_007364, novel_circRNA_007373, and novel_circRNA_007379) (Fig. 6A). In the hippocampus, UBE4B was predicted to be targeted by seven miRNAs (six of these miRNAs were the same as those in the cerebellum) and five circRNAs (two circRNAs were the same as those in the cerebellum), as shown in Fig. 6B. This complicated ceRNA network suggested that novel_circRNA_007362 and UBE4B might play regulatory roles in the UMP pathway via the miRNAs mentioned above and their targets during brain development and aging.

### Validation of tree shrew circRNAs

**Figure 7:**
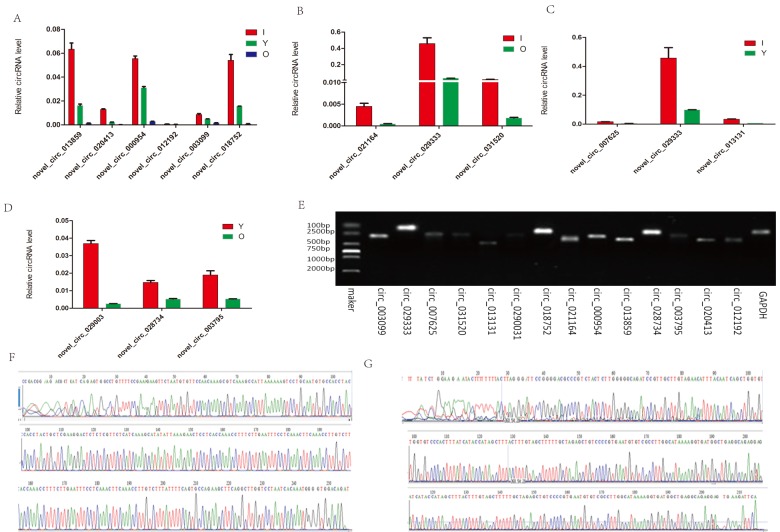
**RT-PCR and Sanger sequencing validation of the selected circRNAs.** (**A**) Six circRNAs from the cerebellum were quantified by RT-qPCR analysis. (**B**–**D**) Expression level changes for eight circRNAs from the hippocampus using RT-qPCR between I vs O, I vs Y, and Y vs O. (**E**) RT-qPCR products were visualized using electrophoresis. (**F**–**G**) Two representative examples of qPCR products confirmed by Sanger sequencing. F: novel_circ_013859. G: novel_circ_007625.

To validate the circRNAs generated by RNA-seq, we randomly selected eight circRNAs from the hippocampus and six circRNAs from the cerebellum. We then designed appropriate primers (Table S2) and amplified the circular junctions. Similar to our RNA-seq data, the expression levels of the six circRNAs (novel_circ_013859, novel_circ_020413, novel_circ_000954, novel_circ_012192, novel_circ_003099, and novel_circ_018752) decreased with age (Fig. 7A). The FCs detected in the hippocampus by RNA-seq were in agreement with the RT-PCR quantification results (Fig. 7B–D). The expression of novel_circ_029333 was higher in both I vs O and I vs Y. In addition, the PCR products were analyzed using agarose gels to validate that a single DNA was amplified (Fig. 7E). In particular, two of the selected circRNAs, i.e., novel_circ_013859 and novel_circ_007625, were further confirmed by Sanger sequencing (Fig. 7F–G), where the real-time RT-PCR results were in agreement with the RNA-seq data, thereby demonstrating that the identified circRNAs had different expression levels in vivo.

### Homology of tree shrew circRNAs

**Figure 8:**
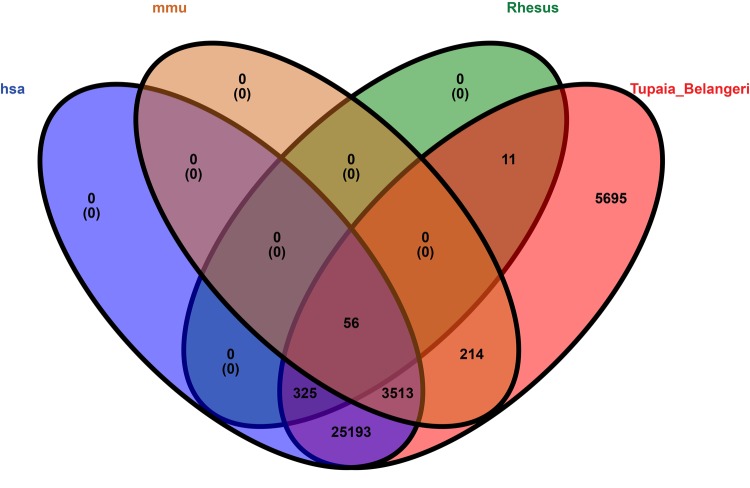
**Venn diagram representation showing the homology among four species, i.e., human, mouse, rhesus macaque, and tree shrew.** The alignment threshold was e-value < 1e-10 by BLASTN (Table S8). Rhesus: rhesus macaque, has: human, mmu: mouse, Tupaia_Belangeri: tree shrew.

We compared tree shrew circRNAs with human, mouse, and rhesus macaque circRNAs. The results indicated that 29,087 (83.1%) tree shrew circRNAs shared homology with human circRNAs (Table S8), 3,783 (10.8%) with mouse circRNAs, and 392 (1.1%) with rhesus macaque circRNAs. Only 56 tree shrew circRNAs (0.2%) shared homology with all three species and 5,695 (16.3%) of the tree shrew circRNAs did not share homology with any of the other three species (Fig. 8).

## DISCUSSION

In the present study, we identified 35,007 circRNAs in the tree shrew brain using high-throughput sequencing. We found that most of the circRNAs measured approximately 400 nt in length, as also reported in previous studies [29]. In the tree shrew, the major circRNAs were annot_exon, whereas in the sheep pituitary, the major circRNAs were intergenic [13]. This finding may suggest that the distribution of circRNAs can exhibit species and tissue specificity.

We observed that abundant circRNAs were expressed in the tree shrew hippocampus and cerebellum. A previous study that compared circRNA expression in the human frontal cortex, liver, thyroid gland, and muscle found that circRNAs were most abundant in the brain [2]. Our results were consistent with these findings. Agnieszka et al. [2] also reported enriched circRNA expression in the mouse cerebellum compared with other brain regions, where the ratio of circRNA expression to linear RNA expression was also higher in the cerebellum. However, in our study, we found no significant difference in total circRNA expression levels in the hippocampus and cerebellum (Fig. 2A). We removed the linear RNA when sequencing, so there were no data concerning the ratio of circRNA relative to linear RNA in the tree shrew.

We found that the downregulation of circRNAs tended to increase with age in the cerebellum. However, in the hippocampus, the downregulation of circRNAs only tended to occur in I versus Y. Clearly, some circRNAs were downregulated whereas others were upregulated with increasing age in the tree shrew brain. It is unclear why this phenomenon occurred. In aged mice, Gruner et al. [30] found a striking trend where circRNAs were upregulated in the cortex and hippocampus, but not in the heart. It has also been shown that in the rhesus macaque, the abundance of most skeletal muscle circRNAs did not change with increasing age, although 19 circRNAs were downregulated with age [12], possibly due to tissue or species specificity. Furthermore, we employed reverse transcription followed by real-time quantitative PCR (qPCR) to identify the expression levels in the brain over time, and the qPCR results were in agreement with the RNA-sequencing, thereby confirming that our RNA-sequencing results were high quality. However, it was unclear whether the corresponding targets of these circRNAs also changed.

To determine the circRNAs related to brain development and aging, we compared the circRNA expression levels in the cerebellum and hippocampus of I, Y, and O animals. We focused on the upregulated and downregulated circRNAs in the hippocampus and cerebellum based on GO and KEGG analyses. In profile 0 (downregulated circRNAs), GO analyses showed that circRNAs in the two brain region were enriched in terms of similar functions (Fig. S3A). Furthermore, the UMP pathway, MAPK signaling pathway, phosphatidylinositol signaling system, and long-term depression were identified in the hippocampus and cerebellum. Other pathways such as RNA degradation, the ErbB signaling pathway, and PI3K-Akt signaling pathway were also incorporated in the four pathways mentioned above (Fig. 4C–D). In profile 7 (upregulated circRNAs), GO analyses showed that circRNAs in the two brain regions were enriched in terms of quite divergent functions (Fig. S3B), and KEGG analyses identified the rap1 signaling pathway and long-term potentiation in both brain regions. Other pathways such as the Fanconi anemia pathway, cAMP signaling pathway, and insulin signaling pathway were also incorporated in the two pathways mentioned above (Fig. S3C). These findings demonstrated the involvement of pathways related to brain development and aging. Previous studies have shown that the UMP pathway was important for primary and secondary hair follicle development in cashmere goats [31], and that UBE2O played an important role in controlling development-associated exon expression in the primary and secondary hair follicles [31]. Ham et al. also found that UMP was involved with normal and pathological brain aging [32]. UMP can degrade damaged and aggregated proteins that increase with age [33]. Furthermore, we constructed ceRNA networks comprising circRNA-miRNA-mRNA that significantly participated in UMP and long-term depression. We found that a ceRNA, novel_circRNA_007362, competed to bind 24 miRNAs, thereby affecting UBE4B expression. In addition, novel_circRNA_032232 affected BIRC6 expression by competing to bind nine miRNAs (Fig. 6B), and it indirectly influenced the expression levels of UBE4B and HERC1. According to KEGG analysis, UBE4B, BIRC6, and HERC1 are important elements of UMP. UBE4B is an E3/E4 ligase that triggers substrate polyubiquitination and it regulates neuronal death and survival [34]. HERC1 (HECT domain and RCC1 domain) is an E3 ubiquitin ligase protein and previous studies have shown that the HERC1 E3 ubiquitin ligase-*tbl* mutation affects neurons in the cerebral neocortex, the CA3 region of the hippocampus, and the ventral horn of the spinal cord [35]. BIRC6 (Baculoviral IAP Repeat Containing 6), a member of the inhibitors of apoptosis proteins (IAPs) family, encodes an inhibitor of apoptosis and a chimeric E2/E3 ubiquitin ligase in mammals, which regulates p53 and the mitochondrial pathway of apoptosis and it is essential for mouse embryonic development [36,37]. In summary, the ceRNA network suggested that novel_circRNA_007362 and novel_circRNA_032232 might interact with 24 miRNAs (Fig. 6A) as well as tch-let-7a-3p, tch-miR-136-5p, tch-miR-146a-5p, tch-miR-17-5p, tch-miR-183-5p, tch-miR-188-5p, tch-miR-23b-3p, tch-miR-326, and tch-miR-93-5p (Fig. 6B) to regulate development and aging of the cerebellum and hippocampus. In general, the regulation of brain development and aging is complex and multifaceted, and many of the details remain unknown.

Analyzing the homology of circRNAs can help to understand common characteristics. Previous studies indicated that circRNAs were highly conserved in the mouse and human brains. For example, Agnieszka et al. [2] showed that 4,522 out of 15,849 mouse circRNAs were conserved in humans. Guo et al. found that 20% of mouse circRNAs were orthologous with human circRNAs [38]. However, in the tree shrew, 83.1% of the circRNAs shared homology with human circRNAs, probably because tree shrews are more closely related to primates than rodents. Furthermore, the conserved expression of circRNAs in humans and tree shrews suggests that their functions and mechanisms of biogenesis might also be similar.

In summary, we identified abundantly expressed circRNAs in the tree shrew hippocampus and cerebellum. Our results may provide a valuable platform for identifying circRNAs that influence brain development, normal aging, synapse regeneration and remodeling, Alzheimer's disease, and Parkinson’s disease. Further studies should focus on the functions of these circRNAs in brain development and age-related disease.

## MATERIALS AND METHODS

### Animals and tissue collection

All of the tree shrews were the first filial generation, and they were raised at the Institute of Medical Biology, Chinese Academy of Medical Science and Peking Union Medical College in Kunming, China. The tree shrews were healthy and consistent with the group standards for tree shrews (T/CALAS 08-2017 and T/CALAS 09-2017), without visible signs of tumorigenesis or disease. Nine male tree shrews in three different stages of development (I, Y, and O) were used. The I group comprised three tree shrews aged 47–52 days and weighing 100–110 g, the Y group comprised three animals aged 15–18 months and weighing 120–150 g, and the O group comprised three animals aged 78–86 months and weighing 120–150 g (Table S3). The life span of tree shrews in captivity is up to 10 years, and their lifespan in approximately one-eighth of the human lifespan [26,39]. Thus, the ages of our youngest and oldest animals corresponded to human ages of about 1 and 57 years, respectively. Tree shrews were sacrificed and the hippocampus and cerebellum were dissected immediately, and stored at -80°C until use. All of the animal procedures were approved by the ethical committee for Animal Research in the Institute of Medical Biology, Chinese Academy of Medical Science and Peking Union Medical College.

### RNA extraction and RNA-Seq

Total RNA was isolated from each sample using TRIzol reagent (Invitrogen) according to the manufacturer’s protocol. The quality of the RNA was assessed using an Agilent 2100 Bioanalyzer (Agilent Technologies).

For the circRNA-seq, after isolating the total RNA, we used RNase R to degrade the linear RNA, and purification was performed using the RNeasy MinElute Cleanup Kit (Qiagen). Next, we removed the ribosomal RNA. We then added fragmentation buffer to fragment the circRNA into short pieces, and random primers were used to synthesize the first-strand cDNA. Subsequently, the second-strand cDNA was synthesized using dNTPs, RNase H, DNA polymerase I, and buffer. The library fragments were purified with VAHTSTM DNA Clean Beads, before repairing the ends, adding poly-(A), and ligating to Illumina sequencing adapters. Next, we used uracil-N-glycosylase to digest the second-strand cDNA. The digested products were purified with VAHTSTM DNA Clean Beads and PCR amplified, thereby completing the preparation of the entire library. CircRNA sequencing was performed using the Illumina HiSeqTM2500 platform by Gene Denovo Biotechnology Co. (Guangzhou, China).

### CircRNA sequencing analysis

Using the raw FASTQ files, we removed adapter contamination, reads containing more than 10% unknown nucleotides, and low quality reads containing more than 50% low quality (Q-value ≤ 20) bases. Next, we used the short reads alignment tool Bowtie2 to map reads to the rRNA database and removed the rRNA mapped reads. The sequences were then aligned with the tree shrew genome using TopHat2 [40] (version 2.0.3.12). Mapped reads were discarded and unmapped reads were collected for circRNA identification. The two ends of unmapped reads were intercepted (default = 20 bp) and aligned with the tree shrew genome to find unique anchor positions within a splice site. Anchor reads that aligned in the reverse orientation (head-to-tail) indicated that circRNA splicing had occurred. The anchor reads were mapped to the tree shrew genome again and the results were submitted to find_circ [3] software to identify circRNAs.

The identified circRNAs were subjected to statistical analysis based on their type, chromosome distribution, and length distribution. To quantify circRNAs, back-spliced junction reads were scaled to RPM mapped reads. The RPM method can eliminate the effects of different amounts of sequencing data on the calculation of circRNA expression levels. Therefore, the calculated expression levels could be used directly to compare differential expression among samples. circRNAs were compared with circBase [18] using BLAST for annotation. The circRNAs that could not be annotated were defined as novel circRNAs. Box plots and Venn diagrams were generated using online tools (www.omicshare.com/tools/Home/Soft/box and www.omicshare.com/tools/Home/Soft/venn).

### Unsupervised hierarchical clustering and PCA

Unsupervised hierarchical clustering and PCA were performed to explore the relationships between samples. Unsupervised hierarchical clustering analyses were performed using gmodels in R (https://cran.r-project.org/web/packages/gmodels/index.html) and PCA was conducted using ggplot2 in R (https://cran.r-project.org/web/packages/ggplot2/index.html).

### Analysis of differentially expressed circRNAs

The edgeR package (http://www.r-project.org/) was used to identify differentially expressed circRNAs between samples. We identified circRNAs with FC ≥ 2 and *p* < 0.05 in comparisons of samples as significant differentially expressed circRNAs. Volcano plots were generated using gglpot2 in R.

### Trend analysis

In order to examine the patterns of differentially expressed circRNAs, the expression data for each sample were normalized to 0, log2 (v1/v0), and log2 (v2/v0), and then clustered using Short Time-series Expression Miner (STEM) software [40]. The clustered profiles with *p* ≤ 0.05 were considered to be significant profiles.

### GO and KEGG pathway analyses

GO annotations are useful for predicting the functions of gene products across numerous species [41]. The GO categories were derived from the GO database (http://www.geneontology.org), which is a community-based bioinformatics resource for classifying gene product functions using structured, controlled vocabularies, and it includes three domains: Molecular Function, Biological Processes, and Cellular Components [42,43]. The differentially expressed circRNAs (profile 0 and profile 7) were mapped to GO terms in the GO database, where FDR ≤ 0.05 was taken as the threshold. GO terms that satisfied this condition were defined as significantly enriched GO terms. KEGG is a major public pathway-related database [44]. KEGG analysis can help to understand the biological roles of differentially expressed genes. We selected the differentially expressed circRNAs in profile 0 and profile 7 obtained by trend analysis for GO and KEGG analyses (http://kobas.cbi.pku.edu.cn/home.do), respectively. Pathways with *p* < 0.05 were considered significant pathways. Bubble charts were generated using gglpot2 in R.

### Construction of co-expression network

We identified the interactions among circRNAs, miRNAs, and mRNAs by constructing co-expression networks. We selected genes involved with UMP and long-term depression (*p* < 0.05, and downregulated in profile 0 in both the hippocampus and cerebellum), and used Cytoscape (V3.2.0) to establish a circRNA–miRNA–mRNA pathway regulation network.

### Data access

RNA-seq data were submitted to the NCBI Gene Expression Omnibus (https://www.ncbi.nlm.nih.gov/geo/) under accession number SRP 132147.

### Homology analysis

The numbers of noncoding circRNAs in the human, mouse, and rhesus macaque genomes that shared homology with tree shrew brain circRNAs were identified based on a threshold (e-value < 1e-10) using BLASTN (Table S8). The human, mouse, and rhesus macaque data were downloaded from: http://www.ncbi.nlm.nih.gov.

### Quantitative real-time RT-PCR

To confirm the individual circRNAs, total RNA was extracted from samples using TRIzol reagent (Invitrogen) and reverse transcribed into cDNA with random primers by a RevertAid First Stand cDNA Synthesis Kit (GeneCopoeia, Cat AORT-0020), according to the manufacturer’s instructions. qPCR analysis was performed using the SYBR Green Kit (GeneCopoeia, Cat AORT-0020) with the PikoReal PCR system (Thermo, USA) under the following conditions: 95°C for 10 min, followed by 45 cycles at 95°C for 15 s and 60°C for 60 s. All of the primers used are listed in Table S2. Relative expression levels were calculated using the 2^–△△Ct^ method. GAPDH was used as a reference. PCR products were tested by electrophoresis on 2% agarose gels, then gel extracted and Sanger sequenced.

## Supplementary Material

Supplementary File

Table S4

Table S5

Table S6

Table S7

Table S8
