# Supplementary material for: CircRNAs in the tree shrew (Tupaia belangeri) brain during postnatal development and aging
Source: Aging (Albany NY). 2018 Apr 30;10(4):833–52. doi: 10.18632/aging.101437 (PMC5940110; doi:10.18632/aging.101437)
Supplement: Supplementary File [file aging-10-101437-s001.pdf]

SUPPLEMENTARY MATERIAL

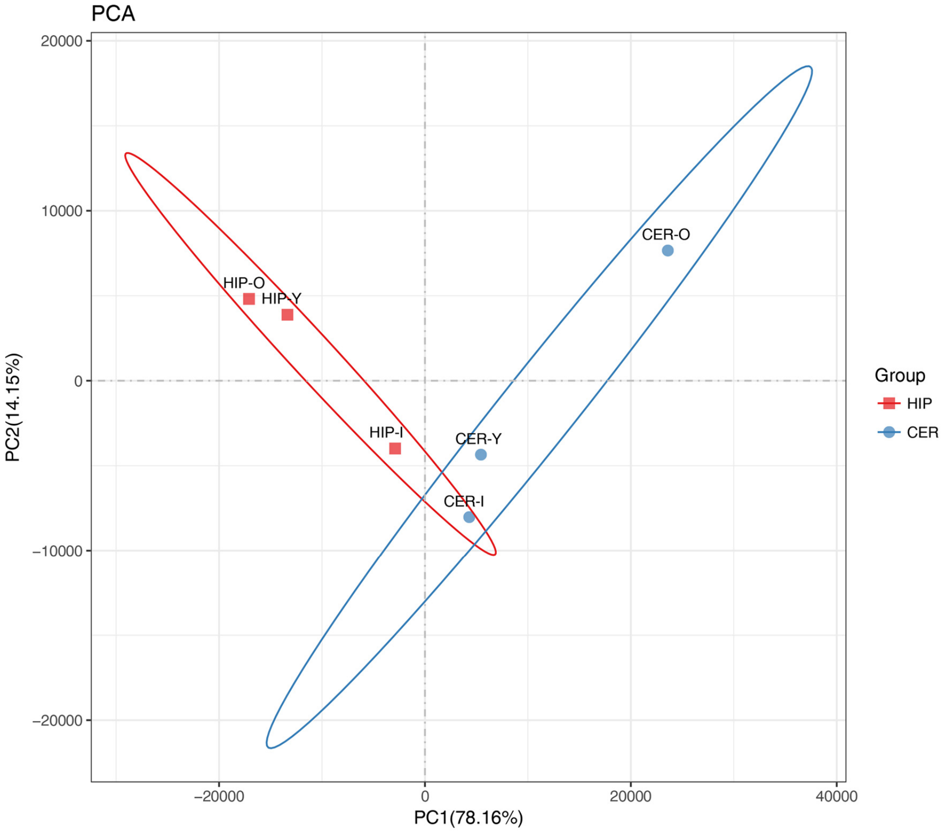

Figure S1. Principal Component analysis (PCA) of different samples among the three ages based on the normalized circRNA expression levels. Brain regions are illustrated by different shapes, and the ellipse for each group represent the confidence ellipse. The x- and y-axes represent the first and second PCs, respectively. HIP-I, HIP-Y, and HIP-O represent the three ages in the hippocampus. CER-I, CER-Y, and CER-O represent the three ages in the cerebellum.

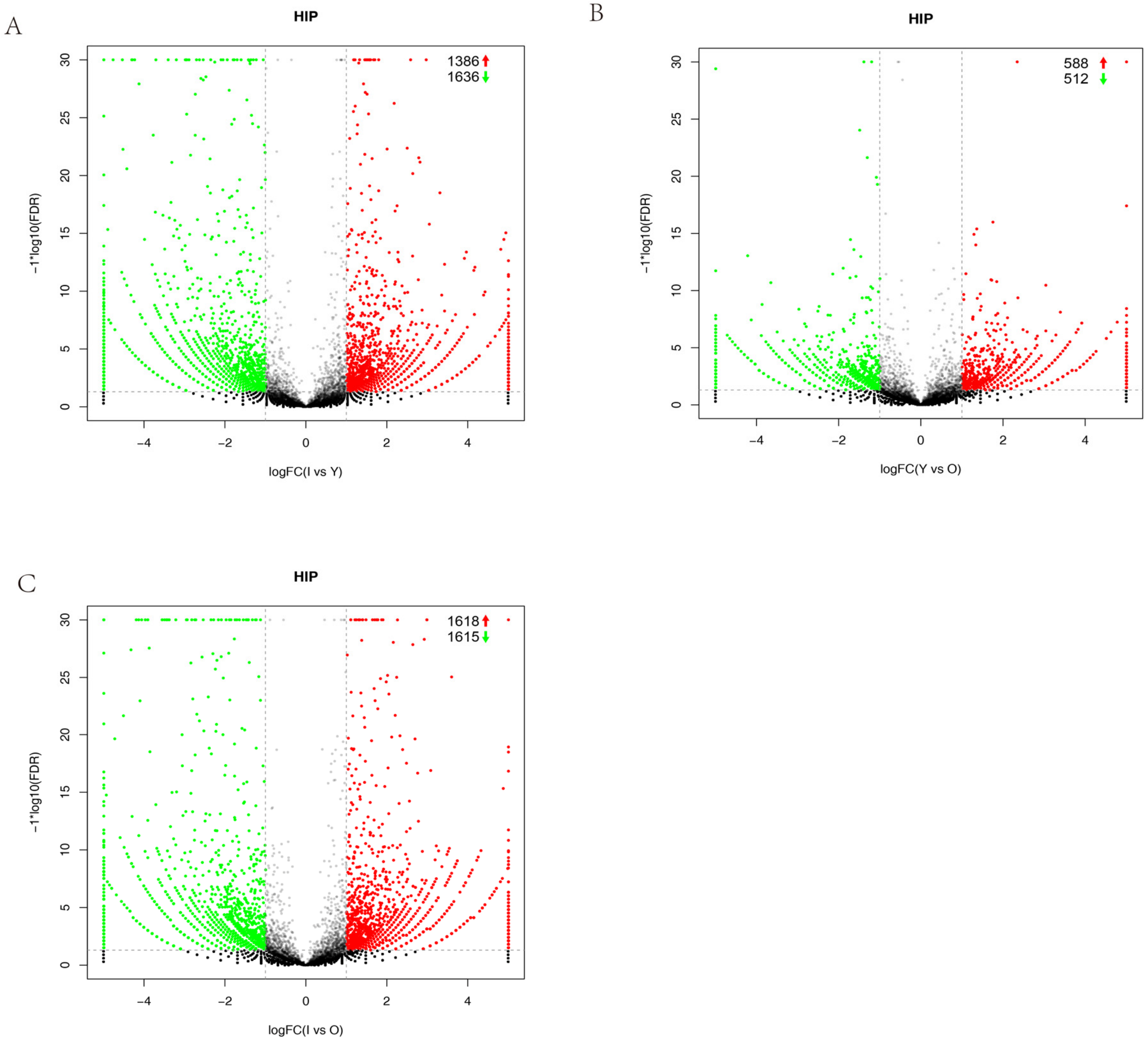

Figure S2. Differential circRNA expression in the hippocampus with aging. (**A**–**C**) Volcano plots illustrating the dysregulated circRNAs in the hippocampus in different age groups. The log of FC (base 2) is plotted on the x-axis and the negative log of FDR (base 10) is plotted on the y-axis (FC > 2 and $p < 0.05$). The green and red points on the graph represent downregulated and upregulated circRNAs, respectively.

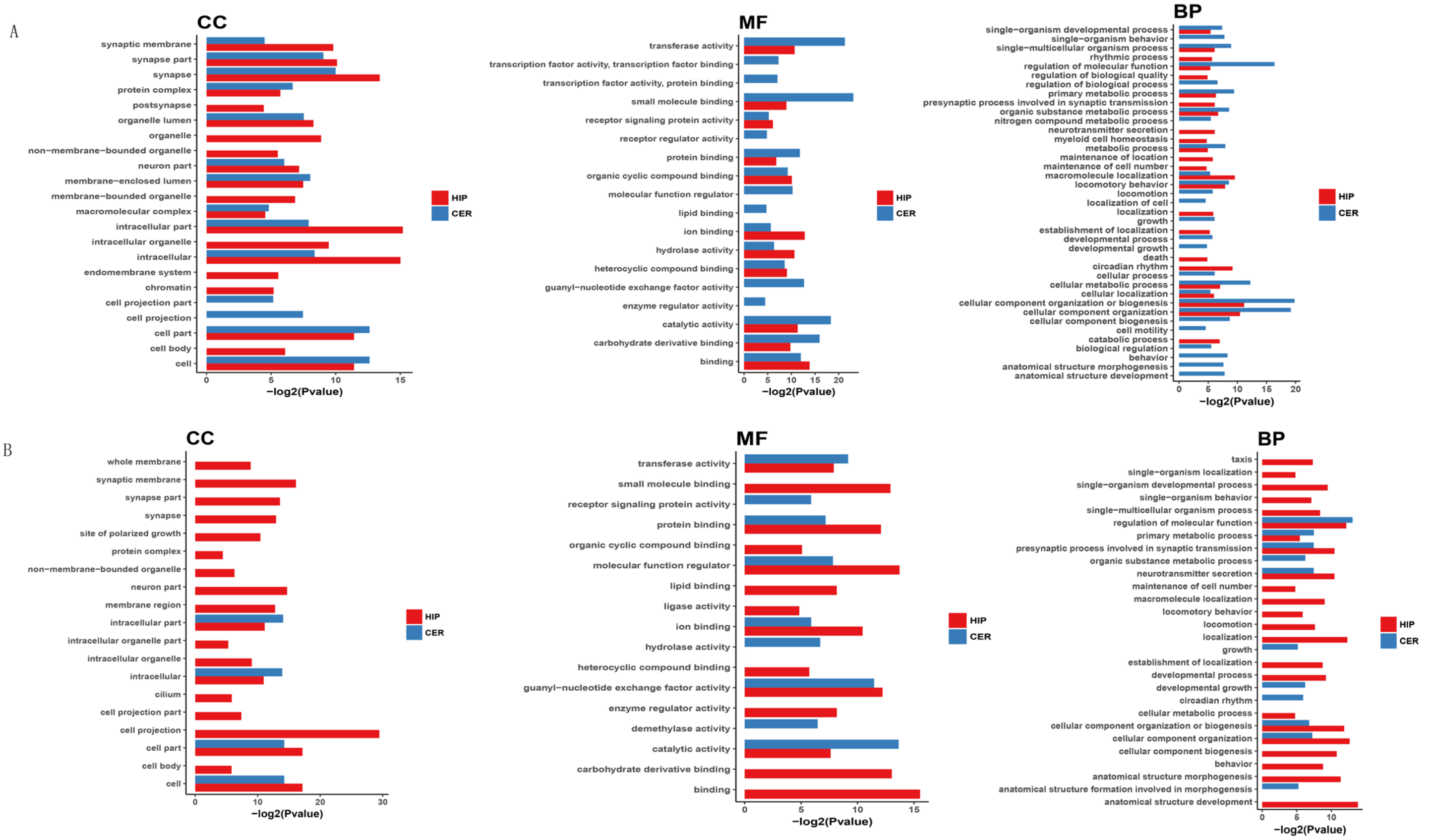

Figure S3. Bar plot representation of the GO terms and KEGG pathways associated with circRNAs in profile 0 and profile 7. (**A**) GO terms in profile 0 for the circRNAs in the two brain regions enriched in terms of similar functions. (**B**) GO terms in profile 7 for the circRNAs in the two brain regions enriched in terms of quite divergent functions. The length of the bar represents the statistical significance of the pathway. Red bar represents the hippocampus. Blue bar represents the cerebellum.

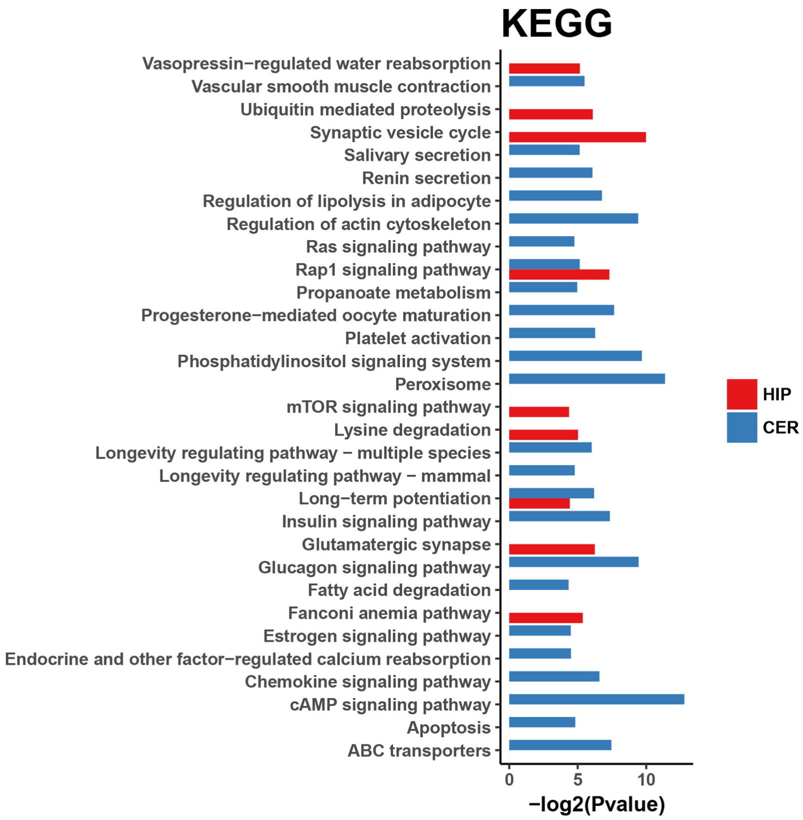

Figure S4. KEGG analysis of profile 7 in the hippocampus and cerebellum. The rap1 signaling pathway and long-term potentiation are observed in both brain regions. The length of the bar represents the statistical significance of the pathway. Red bar represents the hippocampus. Blue bar represents the cerebellum.

Table S1. Details of circRNA sequencing information and subsequent data analysis. Indicated from left to right are the numbers of clean reads high-quality(HQ) clean reads, HQ Clean reads mapped rRNA, and HQ Clean reads unmapped rRNA, the number of unmapped rRNA that could be unmapped(unmapped Reads) or maped (Unique Mapped Reads) to known *Tupaia belangeri* genome, respectively.

Sample	Clean reads	HQ Clean reads(%)	mapped rRNA(%)	unmapped rRNA(%)	Unmapped Reads(%)	Unique Mapped Reads(%)
HIP_I	83948726	78569560 (93.59%)	7143774 (9.09%)	71425786 (90.91%)	13391569 (18.75%)	57780955 (80.90%)
HIP_Y	73971998	65528234 (88.59%)	5922582 (9.04%)	59605652 (90.96%)	11446979 (19.20%)	47938237 (80.43%)
HIP_O	77622934	72032566 (92.8%)	5889928 (8.18%)	66142638 (91.82%)	12923717 (19.54%)	52958183 (80.07%)
CER_I	76720460	72713298 (94.78%)	4249316 (5.84%)	68463982 (94.16%)	10320524 (15.07%)	57944454 (84.63%)
CER_Y	84019284	79491078 (94.61%)	4699098 (5.91%)	74791980 (94.09%)	12482094 (16.69%)	62055254 (82.97%)
CER_O	69508352	65553018 (94.31%)	3073322 (4.69%)	62479696 (95.31%)	10544559 (16.88%)	51714495 (82.77%)

CER: cerebellum. HIP: hippocampus. I: Infants. Y: Young. O: Old.

Table S2. PCR primers used in this study.

	Forward	Reverse
circ_003099	5’TAACCCCCCTCGCTTTCCT 3’	5’ACTTGCCCCTTCGCCTGAC 3’
circ_029333	5’AAGCCTTCCTTTCCACCCT3’	5’ATCCGATTTCCTCTCCTGC3’
circ_021164	5’CCCGGTCATTCTGTGTCCTT3’	5’AGCTGTCCTGCATGCTTCAA3’
circ_031520	5’GAATCTTCACTACCTCTGCCTC3’	5’TTCTTTACTGTTTTGGTCCTCC3’
circ_007625	5’GAATCTTCACTACCTCTGCCTC3’	5’TTTACTGTTTTGGTCCTCCTTT3’
circ_013131	5’TCAATGAAAACAAACCCCAC3’	5’CAGCTGACATCAAGGTAGCG3’
circ_029003	5’TCTCTTCTCACTCTCCTCTACTG3’	5’CTGGTTCATCTAACGCCTTCTGT3’
circ_028734	5’GCAAAAGAAGCAGAAAATGAGAAG3’	5’AGCAAGGGTCAGGAAAGAATAAAG3’
circ_003795	5’AATGAGAGGCAAGGAGGAAAAGA3’	5’CCACCTGGCAAGCAGTAAATAGA3’
circ_013859	5’CTGTCCACCCCATTTGTGATTAG3’	5’GAGAGTACCTTCAGGGAGCTCTT3’
circ_020413	5’GAGGAAAGGGGAAGTGGGC3’	5’AATTGGTGCGTGAGCGAAA3’
circ_000954	5’ACCCCACCAATCTGGACAAGTT3’	5’TCGGTCAGCAGGTCTCGTAAGT3’
circ_012192	5’TCGGTCAGCAGGTCTCGTAAGT3’	5’TATGTCAAGAGGAAAACCAACG3’
circ_018752	5’CGTAAAGGCCAAAAAGCAA3’	5’CACACATTCGGGGGCAGAG3’
GAPDH	5’GCTGGTGCCGAGTATGTTGTG3’	5’AGTGATGGCGTGGACTGTGGT3’

Table S3. Ages distribution in different stage of tree shrew brain.

	Age(months)	Age(months)	Age(months)
Infants (I)	1.57	1.73	1.63
Young (Y)	18	18	15
Old (O)	86	85	78

Please browse the Full Text version to see Supplementary Data related to this manuscript.

Table S4. KEGG analysis of profile 0 in the cerebellum.

Table S5. KEGG analysis of profile 0 in the hippocampus.

Table S6. 6 KEGG analysis of profile 7 in the hippocampus.

Table S7. KEGG analysis of profile 7 in the cerebellum.

Table S8. The BLASTN alignment result between our predicted tree shrew circRNAs with human circRNAs from NONCODE.
