# Supplementary material for: CircRNAs in the tree shrew (Tupaia belangeri) brain during postnatal development and aging
Source: Aging (Albany NY). 2018 Apr 30;10(4):833–52. doi: 10.18632/aging.101437 (PMC5940110; doi:10.18632/aging.101437)
Supplement: Table S4 [file aging-10-101437-s002.docx]

Table S 4 KEGG analysis of profile 0 in the cerebellum

| KEGG_A_class | KEGG_B_class | Pathway | profile0 (134) | All (8016) | P value | Pathway ID |
| --- | --- | --- | --- | --- | --- | --- |
| Genetic Information Processing | Folding, sorting and degradation | Ubiquitin mediated proteolysis | 10 | 163 | 0.000383 | ko04120 |
| Environmental Information Processing | Signal transduction | Phospholipase D signaling pathway | 9 | 154 | 0.001066 | ko04072 |
| Environmental Information Processing | Signal transduction | Phosphatidylinositol signaling system | 7 | 106 | 0.001924 | ko04070 |
| Environmental Information Processing | Signal transduction | PI3K-Akt signaling pathway | 14 | 349 | 0.002016 | ko04151 |
| Cellular Processes | Cellular commiunity | Focal adhesion | 10 | 214 | 0.003043 | ko04510 |
| Environmental Information Processing | Signal transduction | ErbB signaling pathway | 6 | 89 | 0.003645 | ko04012 |
| Organismal Systems | Nervous system | Long-term depression | 5 | 64 | 0.004194 | ko04730 |
| Organismal Systems | Immune system | B cell receptor signaling pathway | 5 | 69 | 0.005786 | ko04662 |
| Organismal Systems | Development | Axon guidance | 7 | 132 | 0.006509 | ko04360 |
| Organismal Systems | Immune system | Chemokine signaling pathway | 8 | 175 | 0.008894 | ko04062 |
| Organismal Systems | Immune system | T cell receptor signaling pathway | 6 | 110 | 0.010119 | ko04660 |
| Environmental Information Processing | Signal transduction | FoxO signaling pathway | 7 | 146 | 0.011058 | ko04068 |
| Metabolism | Nucleotide metabolism | Purine metabolism | 8 | 186 | 0.012566 | ko00230 |
| Cellular Processes | Cell motility | Regulation of actin cytoskeleton | 9 | 224 | 0.0126 | ko04810 |
| Environmental Information Processing | Signal transduction | Rap1 signaling pathway | 9 | 226 | 0.013294 | ko04015 |
| Environmental Information Processing | Signal transduction | VEGF signaling pathway | 4 | 61 | 0.018697 | ko04370 |
| Environmental Information Processing | Signal transduction | cAMP signaling pathway | 8 | 204 | 0.020817 | ko04024 |
| Metabolism | Lipid metabolism | Glycerolipid metabolism | 4 | 66 | 0.024236 | ko00561 |
| Organismal Systems | Endocrine system | Renin secretion | 4 | 68 | 0.026701 | ko04924 |
| Environmental Information Processing | Signal transduction | cGMP - PKG signaling pathway | 7 | 177 | 0.028651 | ko04022 |
| Environmental Information Processing | Signal transduction | MAPK signaling pathway | 9 | 259 | 0.029288 | ko04010 |
| Metabolism | Carbohydrate metabolism | Inositol phosphate metabolism | 4 | 71 | 0.030672 | ko00562 |
| Organismal Systems | Endocrine system | Prolactin signaling pathway | 4 | 74 | 0.034974 | ko04917 |
| Organismal Systems | Endocrine system | Thyroid hormone signaling pathway | 5 | 114 | 0.041895 | ko04919 |
| Organismal Systems | Nervous system | Glutamatergic synapse | 5 | 115 | 0.043235 | ko04724 |
| Metabolism | Metabolism of other amino acids | Selenocompound metabolism | 2 | 20 | 0.043302 | ko00450 |
| Cellular Processes | Cell growth and death | Oocyte meiosis | 5 | 117 | 0.04599 | ko04114 |
| Environmental Information Processing | Signal transduction | Sphingolipid signaling pathway | 5 | 118 | 0.047407 | ko04071 |
| Metabolism | Glycan biosynthesis and metabolism | N-Glycan biosynthesis | 3 | 49 | 0.048197 | ko00510 |
| Genetic Information Processing | Transcription | Basal transcription factors | 3 | 49 | 0.048197 | ko03022 |
| Environmental Information Processing | Signal transduction | Ras signaling pathway | 8 | 243 | 0.050602 | ko04014 |
| Organismal Systems | Endocrine system | Oxytocin signaling pathway | 6 | 161 | 0.05264 | ko04921 |
| Organismal Systems | Nervous system | Neurotrophin signaling pathway | 5 | 122 | 0.05333 | ko04722 |
| Organismal Systems | Development | Osteoclast differentiation | 5 | 124 | 0.056447 | ko04380 |
| Organismal Systems | Endocrine system | Insulin secretion | 4 | 87 | 0.057472 | ko04911 |
| Environmental Information Processing | Signal transduction | AMPK signaling pathway | 5 | 125 | 0.058044 | ko04152 |
| Organismal Systems | Immune system | Natural killer cell mediated cytotoxicity | 5 | 126 | 0.059667 | ko04650 |
| Environmental Information Processing | Signaling molecules and interaction | ECM-receptor interaction | 4 | 89 | 0.061487 | ko04512 |
| Organismal Systems | Endocrine system | Progesterone-mediated oocyte maturation | 4 | 91 | 0.065647 | ko04914 |
| Organismal Systems | Development | Dorso-ventral axis formation | 2 | 27 | 0.074205 | ko04320 |
| Cellular Processes | Cell growth and death | Apoptosis | 4 | 95 | 0.074396 | ko04210 |
| Organismal Systems | Sensory system | Taste transduction | 4 | 97 | 0.078982 | ko04742 |
| Metabolism | Lipid metabolism | Glycerophospholipid metabolism | 4 | 98 | 0.081327 | ko00564 |
| Organismal Systems | Nervous system | Synaptic vesicle cycle | 3 | 63 | 0.088043 | ko04721 |
| Organismal Systems | Endocrine system | Estrogen signaling pathway | 4 | 104 | 0.096109 | ko04915 |
| Organismal Systems | Immune system | Fc epsilon RI signaling pathway | 3 | 66 | 0.097948 | ko04664 |
| Genetic Information Processing | Translation | RNA transport | 6 | 193 | 0.10417 | ko03013 |
| Organismal Systems | Aging | Longevity regulating pathway - multiple species | 3 | 69 | 0.108282 | ko04213 |
| Organismal Systems | Circulatory system | Adrenergic signaling in cardiomyocytes | 5 | 156 | 0.120033 | ko04261 |
| Environmental Information Processing | Signal transduction | HIF-1 signaling pathway | 4 | 114 | 0.12331 | ko04066 |
| Metabolism | Carbohydrate metabolism | Propanoate metabolism | 2 | 37 | 0.126637 | ko00640 |
| Cellular Processes | Cellular commiunity | Adherens junction | 3 | 76 | 0.133924 | ko04520 |
| Organismal Systems | Immune system | Leukocyte transendothelial migration | 4 | 118 | 0.135022 | ko04670 |
| Organismal Systems | Immune system | Platelet activation | 4 | 120 | 0.141044 | ko04611 |
| Organismal Systems | Excretory system | Aldosterone-regulated sodium reabsorption | 2 | 42 | 0.155355 | ko04960 |
| Organismal Systems | Nervous system | GABAergic synapse | 3 | 83 | 0.161436 | ko04727 |
| Environmental Information Processing | Signaling molecules and interaction | Cell adhesion molecules (CAMs) | 5 | 175 | 0.168877 | ko04514 |
| Metabolism | Lipid metabolism | Ether lipid metabolism | 2 | 45 | 0.173133 | ko00565 |
| Genetic Information Processing | Translation | Aminoacyl-tRNA biosynthesis | 2 | 46 | 0.179132 | ko00970 |
| Organismal Systems | Immune system | Fc gamma R-mediated phagocytosis | 3 | 89 | 0.186253 | ko04666 |
| Environmental Information Processing | Membrane transport | ABC transporters | 2 | 48 | 0.191222 | ko02010 |
| Metabolism | Lipid metabolism | Sphingolipid metabolism | 2 | 48 | 0.191222 | ko00600 |
| Cellular Processes | Cellular commiunity | Gap junction | 3 | 91 | 0.19474 | ko04540 |
| Metabolism | Amino acid metabolism | Cysteine and methionine metabolism | 2 | 50 | 0.203419 | ko00270 |
| Organismal Systems | Endocrine system | GnRH signaling pathway | 3 | 94 | 0.207646 | ko04912 |
| Metabolism | Lipid metabolism | Fatty acid biosynthesis | 1 | 14 | 0.21038 | ko00061 |
| Organismal Systems | Aging | Longevity regulating pathway - mammal | 3 | 96 | 0.216356 | ko04211 |
| Metabolism | Glycan biosynthesis and metabolism | Glycosaminoglycan biosynthesis - keratan sulfate | 1 | 16 | 0.236606 | ko00533 |
| Organismal Systems | Environmental adaptation | Circadian entrainment | 3 | 101 | 0.238451 | ko04713 |
| Metabolism | Amino acid metabolism | Lysine degradation | 2 | 56 | 0.240446 | ko00310 |
| Genetic Information Processing | Translation | mRNA surveillance pathway | 3 | 102 | 0.242918 | ko03015 |
| Organismal Systems | Nervous system | Retrograde endocannabinoid signaling | 3 | 102 | 0.242918 | ko04723 |
| Environmental Information Processing | Signal transduction | Calcium signaling pathway | 5 | 202 | 0.248984 | ko04020 |
| Organismal Systems | Immune system | Toll-like receptor signaling pathway | 3 | 105 | 0.256398 | ko04620 |
| Organismal Systems | Endocrine system | Insulin signaling pathway | 4 | 154 | 0.256695 | ko04910 |
| Metabolism | Lipid metabolism | Steroid biosynthesis | 1 | 20 | 0.286492 | ko00100 |
| Organismal Systems | Nervous system | Cholinergic synapse | 3 | 112 | 0.288216 | ko04725 |
| Organismal Systems | Immune system | RIG-I-like receptor signaling pathway | 2 | 66 | 0.302619 | ko04622 |
| Organismal Systems | Nervous system | Long-term potentiation | 2 | 66 | 0.302619 | ko04720 |
| Cellular Processes | Transport and catabolism | Endocytosis | 6 | 273 | 0.305946 | ko04144 |
| Organismal Systems | Immune system | NOD-like receptor signaling pathway | 2 | 67 | 0.308812 | ko04621 |
| Environmental Information Processing | Signal transduction | mTOR signaling pathway | 2 | 67 | 0.308812 | ko04150 |
| Metabolism | Energy metabolism | Sulfur metabolism | 1 | 22 | 0.310207 | ko00920 |
| Metabolism | Metabolism of cofactors and vitamins | One carbon pool by folate | 1 | 23 | 0.32177 | ko00670 |
| Environmental Information Processing | Signal transduction | TNF signaling pathway | 3 | 120 | 0.324925 | ko04668 |
| Metabolism | Metabolism of terpenoids and polyketides | Terpenoid backbone biosynthesis | 1 | 25 | 0.344321 | ko00900 |
| Metabolism | Glycan biosynthesis and metabolism | Glycosaminoglycan biosynthesis - heparan sulfate / heparin | 1 | 26 | 0.355316 | ko00534 |
| Organismal Systems | Endocrine system | Renin-angiotensin system | 1 | 26 | 0.355316 | ko04614 |
| Genetic Information Processing | Folding, sorting and degradation | Protein processing in endoplasmic reticulum | 4 | 181 | 0.358583 | ko04141 |
| Cellular Processes | Cell growth and death | Cell cycle | 3 | 128 | 0.36166 | ko04110 |
| Organismal Systems | Nervous system | Dopaminergic synapse | 3 | 130 | 0.37081 | ko04728 |
| Cellular Processes | Transport and catabolism | Lysosome | 3 | 131 | 0.375377 | ko04142 |
| Metabolism | Glycan biosynthesis and metabolism | Glycosylphosphatidylinositol(GPI)-anchor biosynthesis | 1 | 28 | 0.37676 | ko00563 |
| Genetic Information Processing | Translation | Ribosome biogenesis in eukaryotes | 2 | 82 | 0.399755 | ko03008 |
| Cellular Processes | Transport and catabolism | Peroxisome | 2 | 82 | 0.399755 | ko04146 |
| Cellular Processes | Cell growth and death | p53 signaling pathway | 2 | 83 | 0.405639 | ko04115 |
| Metabolism | Metabolism of cofactors and vitamins | Nicotinate and nicotinamide metabolism | 1 | 32 | 0.417547 | ko00760 |
| Organismal Systems | Circulatory system | Vascular smooth muscle contraction | 3 | 141 | 0.420616 | ko04270 |
| Cellular Processes | Cellular commiunity | Signaling pathways regulating pluripotency of stem cells | 3 | 141 | 0.420616 | ko04550 |
| Metabolism | Glycan biosynthesis and metabolism | Other types of O-glycan biosynthesis | 1 | 33 | 0.427322 | ko00514 |
| Genetic Information Processing | Folding, sorting and degradation | SNARE interactions in vesicular transport | 1 | 35 | 0.446388 | ko04130 |
| Genetic Information Processing | Replication and repair | Base excision repair | 1 | 35 | 0.446388 | ko03410 |
| Cellular Processes | Cellular commiunity | Tight junction | 3 | 147 | 0.447255 | ko04530 |
| Metabolism | Glycan biosynthesis and metabolism | Mucin type O-glycan biosynthesis | 1 | 36 | 0.455683 | ko00512 |
| Metabolism | Carbohydrate metabolism | Glyoxylate and dicarboxylate metabolism | 1 | 37 | 0.464823 | ko00630 |
| Organismal Systems | Digestive system | Fat digestion and absorption | 1 | 41 | 0.499885 | ko04975 |
| Environmental Information Processing | Signal transduction | Jak-STAT signaling pathway | 3 | 160 | 0.503198 | ko04630 |
| Organismal Systems | Digestive system | Carbohydrate digestion and absorption | 1 | 44 | 0.524677 | ko04973 |
| Metabolism | Metabolism of cofactors and vitamins | Porphyrin and chlorophyll metabolism | 1 | 44 | 0.524677 | ko00860 |
| Metabolism | Carbohydrate metabolism | Pyruvate metabolism | 1 | 45 | 0.532666 | ko00620 |
| Metabolism | Nucleotide metabolism | Pyrimidine metabolism | 2 | 108 | 0.542383 | ko00240 |
| Genetic Information Processing | Folding, sorting and degradation | Proteasome | 1 | 47 | 0.548248 | ko03050 |
| Organismal Systems | Endocrine system | Glucagon signaling pathway | 2 | 110 | 0.552358 | ko04922 |
| Environmental Information Processing | Signal transduction | Hedgehog signaling pathway | 1 | 49 | 0.563314 | ko04340 |
| Metabolism | Global and Overview | Fatty acid metabolism | 1 | 50 | 0.570658 | ko01212 |
| Metabolism | Amino acid metabolism | Valine, leucine and isoleucine degradation | 1 | 51 | 0.577881 | ko00280 |
| Genetic Information Processing | Replication and repair | Fanconi anemia pathway | 1 | 51 | 0.577881 | ko03460 |
| Organismal Systems | Digestive system | Mineral absorption | 1 | 52 | 0.584982 | ko04978 |
| Organismal Systems | Excretory system | Vasopressin-regulated water reabsorption | 1 | 54 | 0.598831 | ko04962 |
| Organismal Systems | Endocrine system | Ovarian Steroidogenesis | 1 | 54 | 0.598831 | ko04913 |
| Metabolism | Carbohydrate metabolism | Amino sugar and nucleotide sugar metabolism | 1 | 55 | 0.605583 | ko00520 |
| Organismal Systems | Nervous system | Serotonergic synapse | 2 | 124 | 0.617878 | ko04726 |
| Organismal Systems | Endocrine system | Regulation of lipolysis in adipocyte | 1 | 57 | 0.61875 | ko04923 |
| Environmental Information Processing | Signaling molecules and interaction | Neuroactive ligand-receptor interaction | 5 | 322 | 0.63034 | ko04080 |
| Metabolism | Metabolism of other amino acids | Glutathione metabolism | 1 | 61 | 0.643789 | ko00480 |
| Organismal Systems | Immune system | Cytosolic DNA-sensing pathway | 1 | 63 | 0.655689 | ko04623 |
| Environmental Information Processing | Signal transduction | Wnt signaling pathway | 2 | 135 | 0.664053 | ko04310 |
| Organismal Systems | Endocrine system | Adipocytokine signaling pathway | 1 | 73 | 0.709523 | ko04920 |
| Environmental Information Processing | Signal transduction | Hippo signaling pathway | 2 | 149 | 0.71626 | ko04390 |
| Genetic Information Processing | Folding, sorting and degradation | RNA degradation | 1 | 80 | 0.74215 | ko03018 |
| Environmental Information Processing | Signal transduction | TGF-beta signaling pathway | 1 | 83 | 0.754994 | ko04350 |
| Genetic Information Processing | Transcription | Spliceosome | 2 | 163 | 0.761538 | ko03040 |
| Organismal Systems | Digestive system | Salivary secretion | 1 | 87 | 0.771136 | ko04970 |
| Organismal Systems | Immune system | Hematopoietic cell lineage | 1 | 87 | 0.771136 | ko04640 |
| Organismal Systems | Digestive system | Pancreatic secretion | 1 | 90 | 0.782546 | ko04972 |
| Organismal Systems | Digestive system | Protein digestion and absorption | 1 | 91 | 0.786222 | ko04974 |
| Environmental Information Processing | Signal transduction | NF-kappa B signaling pathway | 1 | 93 | 0.793391 | ko04064 |
| Organismal Systems | Circulatory system | Cardiac muscle contraction | 1 | 96 | 0.803699 | ko04260 |
| Organismal Systems | Endocrine system | Melanogenesis | 1 | 104 | 0.828758 | ko04916 |
| Organismal Systems | Sensory system | Inflammatory mediator regulation of TRP channels | 1 | 111 | 0.848063 | ko04750 |
| Metabolism | Global and Overview | Carbon metabolism | 1 | 124 | 0.878364 | ko01200 |
| Environmental Information Processing | Signaling molecules and interaction | Cytokine-cytokine receptor interaction | 2 | 259 | 0.934416 | ko04060 |
| Cellular Processes | Transport and catabolism | Phagosome | 1 | 184 | 0.956636 | ko04145 |
| Metabolism | Energy metabolism | Oxidative phosphorylation | 1 | 185 | 0.957377 | ko00190 |
| Organismal Systems | Sensory system | Olfactory transduction | 1 | 1466 | 1 | ko04740 |
