# Supplementary material for: CircRNAs in the tree shrew (Tupaia belangeri) brain during postnatal development and aging
Source: Aging (Albany NY). 2018 Apr 30;10(4):833–52. doi: 10.18632/aging.101437 (PMC5940110; doi:10.18632/aging.101437)
Supplement: Table S5 [file aging-10-101437-s003.docx]

Table S5 KEGG analysis of profile 0 in the hippocampus.

| KEGG_A_class | KEGG_B_class | Pathway | profile0 (134) | All (8016) | P value | Pathway ID |
| --- | --- | --- | --- | --- | --- | --- |
| Genetic Information Processing | Folding, sorting and degradation | Ubiquitin mediated proteolysis | 9 | 163 | 0.000467 | ko04120 |
| Genetic Information Processing | Folding, sorting and degradation | RNA degradation | 6 | 80 | 0.000884 | ko03018 |
| Environmental Information Processing | Signal transduction | MAPK signaling pathway | 11 | 259 | 0.00102 | ko04010 |
| Organismal Systems | Nervous system | Neurotrophin signaling pathway | 7 | 122 | 0.001622 | ko04722 |
| Organismal Systems | Nervous system | Synaptic vesicle cycle | 5 | 63 | 0.001868 | ko04721 |
| Organismal Systems | Nervous system | Long-term depression | 4 | 64 | 0.012434 | ko04730 |
| Cellular Processes | Cellular commiunity | Signaling pathways regulating pluripotency of stem cells | 6 | 141 | 0.014465 | ko04550 |
| Environmental Information Processing | Signal transduction | mTOR signaling pathway | 4 | 67 | 0.014526 | ko04150 |
| Organismal Systems | Endocrine system | Renin secretion | 4 | 68 | 0.01527 | ko04924 |
| Environmental Information Processing | Signal transduction | Phosphatidylinositol signaling system | 5 | 106 | 0.016734 | ko04070 |
| Metabolism | Glycan biosynthesis and metabolism | Glycosphingolipid biosynthesis - ganglio series | 2 | 16 | 0.020778 | ko00604 |
| Cellular Processes | Cell growth and death | Cell cycle | 5 | 128 | 0.034332 | ko04110 |
| Organismal Systems | Endocrine system | Progesterone-mediated oocyte maturation | 4 | 91 | 0.039264 | ko04914 |
| Metabolism | Amino acid metabolism | Lysine degradation | 3 | 56 | 0.044095 | ko00310 |
| Organismal Systems | Nervous system | Long-term potentiation | 3 | 66 | 0.065891 | ko04720 |
| Metabolism | Lipid metabolism | Glycerolipid metabolism | 3 | 66 | 0.065891 | ko00561 |
| Organismal Systems | Environmental adaptation | Circadian rhythm | 2 | 31 | 0.070283 | ko04710 |
| Organismal Systems | Aging | Longevity regulating pathway - multiple species | 3 | 69 | 0.073233 | ko04213 |
| Organismal Systems | Endocrine system | Oxytocin signaling pathway | 5 | 161 | 0.076817 | ko04921 |
| Organismal Systems | Endocrine system | Thyroid hormone signaling pathway | 4 | 114 | 0.077019 | ko04919 |
| Metabolism | Carbohydrate metabolism | Inositol phosphate metabolism | 3 | 71 | 0.078322 | ko00562 |
| Cellular Processes | Transport and catabolism | Regulation of autophagy | 2 | 33 | 0.078386 | ko04140 |
| Organismal Systems | Nervous system | Serotonergic synapse | 4 | 124 | 0.097587 | ko04726 |
| Organismal Systems | Digestive system | Salivary secretion | 3 | 87 | 0.12412 | ko04970 |
| Organismal Systems | Digestive system | Pancreatic secretion | 3 | 90 | 0.133602 | ko04972 |
| Cellular Processes | Cellular commiunity | Tight junction | 4 | 147 | 0.153261 | ko04530 |
| Organismal Systems | Sensory system | Taste transduction | 3 | 97 | 0.156653 | ko04742 |
| Metabolism | Lipid metabolism | Glycerophospholipid metabolism | 3 | 98 | 0.160044 | ko00564 |
| Organismal Systems | Environmental adaptation | Circadian entrainment | 3 | 101 | 0.170347 | ko04713 |
| Organismal Systems | Nervous system | Retrograde endocannabinoid signaling | 3 | 102 | 0.173824 | ko04723 |
| Metabolism | Carbohydrate metabolism | Amino sugar and nucleotide sugar metabolism | 2 | 55 | 0.18149 | ko00520 |
| Cellular Processes | Transport and catabolism | Endocytosis | 6 | 273 | 0.187345 | ko04144 |
| Organismal Systems | Endocrine system | Regulation of lipolysis in adipocyte | 2 | 57 | 0.191688 | ko04923 |
| Organismal Systems | Endocrine system | Glucagon signaling pathway | 3 | 110 | 0.202321 | ko04922 |
| Metabolism | Glycan biosynthesis and metabolism | Glycosphingolipid biosynthesis - globo series | 1 | 16 | 0.203373 | ko00603 |
| Organismal Systems | Nervous system | Glutamatergic synapse | 3 | 115 | 0.220661 | ko04724 |
| Environmental Information Processing | Signal transduction | cGMP - PKG signaling pathway | 4 | 177 | 0.239352 | ko04022 |
| Metabolism | Glycan biosynthesis and metabolism | Other glycan degradation | 1 | 20 | 0.247445 | ko00511 |
| Metabolism | Lipid metabolism | Steroid biosynthesis | 1 | 20 | 0.247445 | ko00100 |
| Genetic Information Processing | Folding, sorting and degradation | Protein processing in endoplasmic reticulum | 4 | 181 | 0.251635 | ko04141 |
| Metabolism | Glycan biosynthesis and metabolism | Glycosaminoglycan degradation | 1 | 21 | 0.258081 | ko00531 |
| Environmental Information Processing | Signal transduction | AMPK signaling pathway | 3 | 125 | 0.258257 | ko04152 |
| Metabolism | Nucleotide metabolism | Purine metabolism | 4 | 186 | 0.267175 | ko00230 |
| Organismal Systems | Excretory system | Proximal tubule bicarbonate reclamation | 1 | 23 | 0.278906 | ko04964 |
| Organismal Systems | Development | Axon guidance | 3 | 132 | 0.285079 | ko04360 |
| Metabolism | Glycan biosynthesis and metabolism | Glycosaminoglycan biosynthesis - heparan sulfate / heparin | 1 | 26 | 0.309062 | ko00534 |
| Environmental Information Processing | Signal transduction | Calcium signaling pathway | 4 | 202 | 0.31792 | ko04020 |
| Organismal Systems | Nervous system | GABAergic synapse | 2 | 83 | 0.327246 | ko04727 |
| Environmental Information Processing | Signal transduction | FoxO signaling pathway | 3 | 146 | 0.339265 | ko04068 |
| Metabolism | Carbohydrate metabolism | Ascorbate and aldarate metabolism | 1 | 30 | 0.347335 | ko00053 |
| Organismal Systems | Immune system | Fc gamma R-mediated phagocytosis | 2 | 89 | 0.358143 | ko04666 |
| Cellular Processes | Cellular commiunity | Gap junction | 2 | 91 | 0.368335 | ko04540 |
| Organismal Systems | Endocrine system | Insulin signaling pathway | 3 | 154 | 0.370205 | ko04910 |
| Environmental Information Processing | Signal transduction | Phospholipase D signaling pathway | 3 | 154 | 0.370205 | ko04072 |
| Metabolism | Glycan biosynthesis and metabolism | Other types of O-glycan biosynthesis | 1 | 33 | 0.374653 | ko00514 |
| Genetic Information Processing | Replication and repair | Homologous recombination | 1 | 33 | 0.374653 | ko03440 |
| Organismal Systems | Endocrine system | GnRH signaling pathway | 2 | 94 | 0.383504 | ko04912 |
| Metabolism | Carbohydrate metabolism | Fructose and mannose metabolism | 1 | 34 | 0.383505 | ko00051 |
| Genetic Information Processing | Replication and repair | Base excision repair | 1 | 35 | 0.392233 | ko03410 |
| Organismal Systems | Aging | Longevity regulating pathway - mammal | 2 | 96 | 0.393532 | ko04211 |
| Metabolism | Glycan biosynthesis and metabolism | Mucin type O-glycan biosynthesis | 1 | 36 | 0.400838 | ko00512 |
| Metabolism | Carbohydrate metabolism | Propanoate metabolism | 1 | 37 | 0.409322 | ko00640 |
| Metabolism | Carbohydrate metabolism | Glyoxylate and dicarboxylate metabolism | 1 | 37 | 0.409322 | ko00630 |
| Metabolism | Carbohydrate metabolism | Pentose and glucuronate interconversions | 1 | 38 | 0.417688 | ko00040 |
| Genetic Information Processing | Translation | mRNA surveillance pathway | 2 | 102 | 0.423164 | ko03015 |
| Organismal Systems | Endocrine system | Estrogen signaling pathway | 2 | 104 | 0.432879 | ko04915 |
| Organismal Systems | Immune system | Chemokine signaling pathway | 3 | 175 | 0.449832 | ko04062 |
| Organismal Systems | Excretory system | Endocrine and other factor-regulated calcium reabsorption | 1 | 44 | 0.46547 | ko04961 |
| Organismal Systems | Nervous system | Cholinergic synapse | 2 | 112 | 0.470849 | ko04725 |
| Genetic Information Processing | Translation | Aminoacyl-tRNA biosynthesis | 1 | 46 | 0.480517 | ko00970 |
| Cellular Processes | Cell growth and death | Oocyte meiosis | 2 | 117 | 0.493807 | ko04114 |
| Environmental Information Processing | Membrane transport | ABC transporters | 1 | 48 | 0.495144 | ko02010 |
| Environmental Information Processing | Signal transduction | Sphingolipid signaling pathway | 2 | 118 | 0.498324 | ko04071 |
| Metabolism | Glycan biosynthesis and metabolism | N-Glycan biosynthesis | 1 | 49 | 0.502303 | ko00510 |
| Metabolism | Amino acid metabolism | Cysteine and methionine metabolism | 1 | 50 | 0.509363 | ko00270 |
| Environmental Information Processing | Signal transduction | Notch signaling pathway | 1 | 50 | 0.509363 | ko04330 |
| Metabolism | Amino acid metabolism | Valine, leucine and isoleucine degradation | 1 | 51 | 0.516322 | ko00280 |
| Organismal Systems | Digestive system | Mineral absorption | 1 | 52 | 0.523184 | ko04978 |
| Metabolism | Carbohydrate metabolism | Starch and sucrose metabolism | 1 | 53 | 0.52995 | ko00500 |
| Organismal Systems | Excretory system | Vasopressin-regulated water reabsorption | 1 | 54 | 0.53662 | ko04962 |
| Organismal Systems | Nervous system | Dopaminergic synapse | 2 | 130 | 0.550498 | ko04728 |
| Environmental Information Processing | Signal transduction | cAMP signaling pathway | 3 | 204 | 0.552464 | ko04024 |
| Environmental Information Processing | Signal transduction | VEGF signaling pathway | 1 | 61 | 0.580757 | ko04370 |
| Organismal Systems | Immune system | Fc epsilon RI signaling pathway | 1 | 66 | 0.609707 | ko04664 |
| Cellular Processes | Cell motility | Regulation of actin cytoskeleton | 3 | 224 | 0.616399 | ko04810 |
| Metabolism | Metabolism of cofactors and vitamins | Retinol metabolism | 1 | 68 | 0.620724 | ko00830 |
| Environmental Information Processing | Signal transduction | Rap1 signaling pathway | 3 | 226 | 0.622449 | ko04015 |
| Environmental Information Processing | Signal transduction | Hippo signaling pathway | 2 | 149 | 0.62514 | ko04390 |
| Organismal Systems | Immune system | B cell receptor signaling pathway | 1 | 69 | 0.626116 | ko04662 |
| Organismal Systems | Digestive system | Bile secretion | 1 | 71 | 0.636674 | ko04976 |
| Organismal Systems | Endocrine system | Adipocytokine signaling pathway | 1 | 73 | 0.646936 | ko04920 |
| Organismal Systems | Endocrine system | Prolactin signaling pathway | 1 | 74 | 0.651959 | ko04917 |
| Organismal Systems | Digestive system | Gastric acid secretion | 1 | 75 | 0.656911 | ko04971 |
| Environmental Information Processing | Signal transduction | Jak-STAT signaling pathway | 2 | 160 | 0.663833 | ko04630 |
| Environmental Information Processing | Signal transduction | Ras signaling pathway | 3 | 243 | 0.67126 | ko04014 |
| Genetic Information Processing | Translation | Ribosome biogenesis in eukaryotes | 1 | 82 | 0.689673 | ko03008 |
| Cellular Processes | Transport and catabolism | Peroxisome | 1 | 82 | 0.689673 | ko04146 |
| Cellular Processes | Cell growth and death | p53 signaling pathway | 1 | 83 | 0.694093 | ko04115 |
| Environmental Information Processing | Signal transduction | TGF-beta signaling pathway | 1 | 83 | 0.694093 | ko04350 |
| Organismal Systems | Immune system | Hematopoietic cell lineage | 1 | 87 | 0.711157 | ko04640 |
| Organismal Systems | Endocrine system | Insulin secretion | 1 | 87 | 0.711157 | ko04911 |
| Environmental Information Processing | Signaling molecules and interaction | Cytokine-cytokine receptor interaction | 3 | 259 | 0.712892 | ko04060 |
| Environmental Information Processing | Signal transduction | ErbB signaling pathway | 1 | 89 | 0.719331 | ko04012 |
| Environmental Information Processing | Signal transduction | NF-kappa B signaling pathway | 1 | 93 | 0.734999 | ko04064 |
| Cellular Processes | Transport and catabolism | Phagosome | 2 | 184 | 0.737173 | ko04145 |
| Organismal Systems | Circulatory system | Cardiac muscle contraction | 1 | 96 | 0.746178 | ko04260 |
| Genetic Information Processing | Translation | RNA transport | 2 | 193 | 0.760981 | ko03013 |
| Organismal Systems | Endocrine system | Melanogenesis | 1 | 104 | 0.773755 | ko04916 |
| Organismal Systems | Immune system | T cell receptor signaling pathway | 1 | 110 | 0.792469 | ko04660 |
| Organismal Systems | Sensory system | Inflammatory mediator regulation of TRP channels | 1 | 111 | 0.795435 | ko04750 |
| Environmental Information Processing | Signal transduction | HIF-1 signaling pathway | 1 | 114 | 0.804084 | ko04066 |
| Environmental Information Processing | Signal transduction | TNF signaling pathway | 1 | 120 | 0.820309 | ko04668 |
| Organismal Systems | Immune system | Platelet activation | 1 | 120 | 0.820309 | ko04611 |
| Metabolism | Global and Overview | Carbon metabolism | 1 | 124 | 0.830379 | ko01200 |
| Organismal Systems | Immune system | Natural killer cell mediated cytotoxicity | 1 | 126 | 0.835202 | ko04650 |
| Cellular Processes | Transport and catabolism | Lysosome | 1 | 131 | 0.846672 | ko04142 |
| Environmental Information Processing | Signal transduction | Wnt signaling pathway | 1 | 135 | 0.855276 | ko04310 |
| Organismal Systems | Circulatory system | Vascular smooth muscle contraction | 1 | 141 | 0.867293 | ko04270 |
| Environmental Information Processing | Signal transduction | PI3K-Akt signaling pathway | 3 | 349 | 0.875894 | ko04151 |
| Organismal Systems | Circulatory system | Adrenergic signaling in cardiomyocytes | 1 | 156 | 0.893179 | ko04261 |
| Environmental Information Processing | Signaling molecules and interaction | Cell adhesion molecules (CAMs) | 1 | 175 | 0.918899 | ko04514 |
| Environmental Information Processing | Signaling molecules and interaction | Neuroactive ligand-receptor interaction | 2 | 322 | 0.945444 | ko04080 |
| Cellular Processes | Cellular commiunity | Focal adhesion | 1 | 214 | 0.954021 | ko04510 |
| Organismal Systems | Sensory system | Olfactory transduction | 2 | 1466 | 1 | ko04740 |
