# Supplementary material for: CircRNAs in the tree shrew (Tupaia belangeri) brain during postnatal development and aging
Source: Aging (Albany NY). 2018 Apr 30;10(4):833–52. doi: 10.18632/aging.101437 (PMC5940110; doi:10.18632/aging.101437)
Supplement: Table S6 [file aging-10-101437-s004.docx]

Table S 6 KEGG analysis of profile 7 in the hippocampus

| KEGG_A_class | KEGG_B_class | Pathway | profile7 (98) | All (8016) | Pvalue | Pathway ID |
| --- | --- | --- | --- | --- | --- | --- |
| Organismal Systems | Nervous system | Synaptic vesicle cycle | 5 | 63 | 0.000989 | ko04721 |
| Environmental Information Processing | Signal transduction | Rap1 signaling pathway | 8 | 226 | 0.006284 | ko04015 |
| Organismal Systems | Nervous system | Glutamatergic synapse | 5 | 115 | 0.013138 | ko04724 |
| Genetic Information Processing | Folding, sorting and degradation | Ubiquitin mediated proteolysis | 6 | 163 | 0.01459 | ko04120 |
| Genetic Information Processing | Replication and repair | Fanconi anemia pathway | 3 | 51 | 0.024168 | ko03460 |
| Organismal Systems | Excretory system | Vasopressin-regulated water reabsorption | 3 | 54 | 0.028045 | ko04962 |
| Metabolism | Amino acid metabolism | Lysine degradation | 3 | 56 | 0.030802 | ko00310 |
| Organismal Systems | Nervous system | Long-term potentiation | 3 | 66 | 0.046633 | ko04720 |
| Environmental Information Processing | Signal transduction | mTOR signaling pathway | 3 | 67 | 0.0484 | ko04150 |
| Genetic Information Processing | Folding, sorting and degradation | RNA degradation | 3 | 80 | 0.074242 | ko03018 |
| Organismal Systems | Nervous system | Dopaminergic synapse | 4 | 130 | 0.074578 | ko04728 |
| Metabolism | Carbohydrate metabolism | Propanoate metabolism | 2 | 37 | 0.074762 | ko00640 |
| Organismal Systems | Development | Axon guidance | 4 | 132 | 0.077916 | ko04360 |
| Organismal Systems | Nervous system | GABAergic synapse | 3 | 83 | 0.080925 | ko04727 |
| Cellular Processes | Cellular commiunity | Signaling pathways regulating pluripotency of stem cells | 4 | 141 | 0.09384 | ko04550 |
| Genetic Information Processing | Translation | Aminoacyl-tRNA biosynthesis | 2 | 46 | 0.108386 | ko00970 |
| Environmental Information Processing | Membrane transport | ABC transporters | 2 | 48 | 0.116325 | ko02010 |
| Cellular Processes | Transport and catabolism | Endocytosis | 6 | 273 | 0.116853 | ko04144 |
| Organismal Systems | Environmental adaptation | Circadian entrainment | 3 | 101 | 0.125991 | ko04713 |
| Cellular Processes | Cell motility | Regulation of actin cytoskeleton | 5 | 224 | 0.138938 | ko04810 |
| Environmental Information Processing | Signal transduction | Phosphatidylinositol signaling system | 3 | 106 | 0.13984 | ko04070 |
| Organismal Systems | Immune system | Chemokine signaling pathway | 4 | 175 | 0.165985 | ko04062 |
| Genetic Information Processing | Replication and repair | Non-homologous end-joining | 1 | 15 | 0.168626 | ko03450 |
| Metabolism | Glycan biosynthesis and metabolism | Glycosphingolipid biosynthesis - ganglio series | 1 | 16 | 0.178809 | ko00604 |
| Genetic Information Processing | Folding, sorting and degradation | Protein processing in endoplasmic reticulum | 4 | 181 | 0.180386 | ko04141 |
| Organismal Systems | Nervous system | Long-term depression | 2 | 64 | 0.184088 | ko04730 |
| Organismal Systems | Nervous system | Neurotrophin signaling pathway | 3 | 122 | 0.187225 | ko04722 |
| Organismal Systems | Nervous system | Serotonergic synapse | 3 | 124 | 0.193425 | ko04726 |
| Environmental Information Processing | Signal transduction | AMPK signaling pathway | 3 | 125 | 0.196545 | ko04152 |
| Cellular Processes | Cell growth and death | Cell cycle | 3 | 128 | 0.205977 | ko04110 |
| Metabolism | Metabolism of cofactors and vitamins | Pantothenate and CoA biosynthesis | 1 | 19 | 0.208624 | ko00770 |
| Metabolism | Carbohydrate metabolism | Inositol phosphate metabolism | 2 | 71 | 0.215297 | ko00562 |
| Environmental Information Processing | Signal transduction | Wnt signaling pathway | 3 | 135 | 0.228372 | ko04310 |
| Environmental Information Processing | Signal transduction | cAMP signaling pathway | 4 | 204 | 0.23902 | ko04024 |
| Environmental Information Processing | Signal transduction | PI3K-Akt signaling pathway | 6 | 349 | 0.254256 | ko04151 |
| Metabolism | Metabolism of terpenoids and polyketides | Terpenoid backbone biosynthesis | 1 | 25 | 0.265075 | ko00900 |
| Genetic Information Processing | Translation | Ribosome biogenesis in eukaryotes | 2 | 82 | 0.265138 | ko03008 |
| Cellular Processes | Cellular commiunity | Focal adhesion | 4 | 214 | 0.265826 | ko04510 |
| Environmental Information Processing | Signal transduction | TGF-beta signaling pathway | 2 | 83 | 0.269687 | ko04350 |
| Cellular Processes | Cell growth and death | p53 signaling pathway | 2 | 83 | 0.269687 | ko04115 |
| Metabolism | Glycan biosynthesis and metabolism | Glycosaminoglycan biosynthesis - heparan sulfate / heparin | 1 | 26 | 0.274088 | ko00534 |
| Organismal Systems | Endocrine system | Aldosterone synthesis and secretion | 2 | 85 | 0.278782 | ko04925 |
| Genetic Information Processing | Folding, sorting and degradation | Protein export | 1 | 27 | 0.282991 | ko03060 |
| Metabolism | Lipid metabolism | Fatty acid elongation | 1 | 27 | 0.282991 | ko00062 |
| Environmental Information Processing | Signal transduction | ErbB signaling pathway | 2 | 89 | 0.29695 | ko04012 |
| Environmental Information Processing | Signaling molecules and interaction | ECM-receptor interaction | 2 | 89 | 0.29695 | ko04512 |
| Organismal Systems | Circulatory system | Adrenergic signaling in cardiomyocytes | 3 | 156 | 0.297771 | ko04261 |
| Organismal Systems | Environmental adaptation | Circadian rhythm | 1 | 31 | 0.317537 | ko04710 |
| Cellular Processes | Cell growth and death | Apoptosis | 2 | 95 | 0.32407 | ko04210 |
| Cellular Processes | Transport and catabolism | Regulation of autophagy | 1 | 33 | 0.334187 | ko04140 |
| Metabolism | Lipid metabolism | Glycerophospholipid metabolism | 2 | 98 | 0.337539 | ko00564 |
| Environmental Information Processing | Signal transduction | Ras signaling pathway | 4 | 243 | 0.346023 | ko04014 |
| Organismal Systems | Nervous system | Retrograde endocannabinoid signaling | 2 | 102 | 0.355374 | ko04723 |
| Genetic Information Processing | Translation | mRNA surveillance pathway | 2 | 102 | 0.355374 | ko03015 |
| Environmental Information Processing | Signaling molecules and interaction | Neuroactive ligand-receptor interaction | 5 | 322 | 0.35837 | ko04080 |
| Metabolism | Amino acid metabolism | Alanine, aspartate and glutamate metabolism | 1 | 36 | 0.358411 | ko00250 |
| Environmental Information Processing | Signaling molecules and interaction | Cell adhesion molecules (CAMs) | 3 | 175 | 0.361617 | ko04514 |
| Metabolism | Carbohydrate metabolism | Glyoxylate and dicarboxylate metabolism | 1 | 37 | 0.366291 | ko00630 |
| Metabolism | Carbohydrate metabolism | Citrate cycle (TCA cycle) | 1 | 38 | 0.374074 | ko00020 |
| Environmental Information Processing | Signal transduction | MAPK signaling pathway | 4 | 259 | 0.390777 | ko04010 |
| Organismal Systems | Nervous system | Cholinergic synapse | 2 | 112 | 0.399178 | ko04725 |
| Organismal Systems | Excretory system | Endocrine and other factor-regulated calcium reabsorption | 1 | 44 | 0.418826 | ko04961 |
| Metabolism | Lipid metabolism | Ether lipid metabolism | 1 | 45 | 0.425971 | ko00565 |
| Metabolism | Amino acid metabolism | Tryptophan metabolism | 1 | 45 | 0.425971 | ko00380 |
| Metabolism | Lipid metabolism | Sphingolipid metabolism | 1 | 48 | 0.446886 | ko00600 |
| Metabolism | Global and Overview | Carbon metabolism | 2 | 124 | 0.449877 | ko01200 |
| Environmental Information Processing | Signal transduction | Hedgehog signaling pathway | 1 | 49 | 0.453689 | ko04340 |
| Metabolism | Glycan biosynthesis and metabolism | N-Glycan biosynthesis | 1 | 49 | 0.453689 | ko00510 |
| Metabolism | Amino acid metabolism | Cysteine and methionine metabolism | 1 | 50 | 0.460409 | ko00270 |
| Environmental Information Processing | Signal transduction | Notch signaling pathway | 1 | 50 | 0.460409 | ko04330 |
| Metabolism | Global and Overview | Fatty acid metabolism | 1 | 50 | 0.460409 | ko01212 |
| Metabolism | Amino acid metabolism | Valine, leucine and isoleucine degradation | 1 | 51 | 0.467047 | ko00280 |
| Cellular Processes | Transport and catabolism | Lysosome | 2 | 131 | 0.478341 | ko04142 |
| Metabolism | Amino acid metabolism | Arginine and proline metabolism | 1 | 55 | 0.492801 | ko00330 |
| Metabolism | Metabolism of other amino acids | Glutathione metabolism | 1 | 61 | 0.52914 | ko00480 |
| Environmental Information Processing | Signal transduction | FoxO signaling pathway | 2 | 146 | 0.536252 | ko04068 |
| Cellular Processes | Cellular commiunity | Tight junction | 2 | 147 | 0.539956 | ko04530 |
| Environmental Information Processing | Signal transduction | Hippo signaling pathway | 2 | 149 | 0.547303 | ko04390 |
| Metabolism | Lipid metabolism | Glycerolipid metabolism | 1 | 66 | 0.557445 | ko00561 |
| Organismal Systems | Immune system | NOD-like receptor signaling pathway | 1 | 67 | 0.5629 | ko04621 |
| Environmental Information Processing | Signal transduction | Phospholipase D signaling pathway | 2 | 154 | 0.565315 | ko04072 |
| Organismal Systems | Endocrine system | Renin secretion | 1 | 68 | 0.568289 | ko04924 |
| Organismal Systems | Immune system | B cell receptor signaling pathway | 1 | 69 | 0.573612 | ko04662 |
| Organismal Systems | Endocrine system | Thyroid hormone synthesis | 1 | 72 | 0.589194 | ko04918 |
| Organismal Systems | Endocrine system | Adipocytokine signaling pathway | 1 | 73 | 0.594262 | ko04920 |
| Organismal Systems | Endocrine system | Prolactin signaling pathway | 1 | 74 | 0.599268 | ko04917 |
| Organismal Systems | Digestive system | Gastric acid secretion | 1 | 75 | 0.604213 | ko04971 |
| Cellular Processes | Cellular commiunity | Adherens junction | 1 | 76 | 0.609097 | ko04520 |
| Cellular Processes | Transport and catabolism | Peroxisome | 1 | 82 | 0.637176 | ko04146 |
| Organismal Systems | Immune system | Hematopoietic cell lineage | 1 | 87 | 0.659042 | ko04640 |
| Organismal Systems | Endocrine system | Insulin secretion | 1 | 87 | 0.659042 | ko04911 |
| Cellular Processes | Transport and catabolism | Phagosome | 2 | 184 | 0.66258 | ko04145 |
| Organismal Systems | Endocrine system | Progesterone-mediated oocyte maturation | 1 | 91 | 0.675592 | ko04914 |
| Environmental Information Processing | Signal transduction | NF-kappa B signaling pathway | 1 | 93 | 0.683566 | ko04064 |
| Organismal Systems | Circulatory system | Cardiac muscle contraction | 1 | 96 | 0.695165 | ko04260 |
| Organismal Systems | Aging | Longevity regulating pathway - mammal | 1 | 96 | 0.695165 | ko04211 |
| Organismal Systems | Immune system | Antigen processing and presentation | 1 | 97 | 0.698937 | ko04612 |
| Organismal Systems | Sensory system | Taste transduction | 1 | 97 | 0.698937 | ko04742 |
| Organismal Systems | Endocrine system | Estrogen signaling pathway | 1 | 104 | 0.724078 | ko04915 |
| Organismal Systems | Endocrine system | Melanogenesis | 1 | 104 | 0.724078 | ko04916 |
| Organismal Systems | Immune system | T cell receptor signaling pathway | 1 | 110 | 0.743965 | ko04660 |
| Organismal Systems | Endocrine system | Glucagon signaling pathway | 1 | 110 | 0.743965 | ko04922 |
| Environmental Information Processing | Signal transduction | Sphingolipid signaling pathway | 1 | 118 | 0.76829 | ko04071 |
| Environmental Information Processing | Signal transduction | TNF signaling pathway | 1 | 120 | 0.774005 | ko04668 |
| Organismal Systems | Development | Osteoclast differentiation | 1 | 124 | 0.78502 | ko04380 |
| Organismal Systems | Immune system | Natural killer cell mediated cytotoxicity | 1 | 126 | 0.790326 | ko04650 |
| Organismal Systems | Circulatory system | Vascular smooth muscle contraction | 1 | 141 | 0.8262 | ko04270 |
| Organismal Systems | Endocrine system | Insulin signaling pathway | 1 | 154 | 0.852329 | ko04910 |
| Environmental Information Processing | Signal transduction | Jak-STAT signaling pathway | 1 | 160 | 0.863038 | ko04630 |
| Organismal Systems | Endocrine system | Oxytocin signaling pathway | 1 | 161 | 0.864746 | ko04921 |
| Genetic Information Processing | Transcription | Spliceosome | 1 | 163 | 0.8681 | ko03040 |
| Environmental Information Processing | Signal transduction | cGMP - PKG signaling pathway | 1 | 177 | 0.889382 | ko04022 |
| Metabolism | Energy metabolism | Oxidative phosphorylation | 1 | 185 | 0.899977 | ko00190 |
| Metabolism | Nucleotide metabolism | Purine metabolism | 1 | 186 | 0.901229 | ko00230 |
| Genetic Information Processing | Translation | RNA transport | 1 | 193 | 0.909567 | ko03013 |
