# Supplementary material for: CircRNAs in the tree shrew (Tupaia belangeri) brain during postnatal development and aging
Source: Aging (Albany NY). 2018 Apr 30;10(4):833–52. doi: 10.18632/aging.101437 (PMC5940110; doi:10.18632/aging.101437)
Supplement: Table S7 [file aging-10-101437-s005.docx]

Table S 7 KEGG analysis of profile 7 in the cerebellum

| KEGG_A_class | KEGG_B_class | Pathway | profile7 (61) | All (8016) | Pvalue | Pathway ID |
| --- | --- | --- | --- | --- | --- | --- |
| Environmental Information Processing | Signal transduction | cAMP signaling pathway | 8 | 204 | 0.000142 | ko04024 |
| Cellular Processes | Transport and catabolism | Peroxisome | 5 | 82 | 0.000376 | ko04146 |
| Environmental Information Processing | Signal transduction | Phosphatidylinositol signaling system | 5 | 106 | 0.001216 | ko04070 |
| Organismal Systems | Endocrine system | Glucagon signaling pathway | 5 | 110 | 0.001434 | ko04922 |
| Cellular Processes | Cell motility | Regulation of actin cytoskeleton | 7 | 224 | 0.001465 | ko04810 |
| Organismal Systems | Endocrine system | Progesterone-mediated oocyte maturation | 4 | 91 | 0.004953 | ko04914 |
| Environmental Information Processing | Membrane transport | ABC transporters | 3 | 48 | 0.005686 | ko02010 |
| Organismal Systems | Endocrine system | Insulin signaling pathway | 5 | 154 | 0.006143 | ko04910 |
| Organismal Systems | Endocrine system | Regulation of lipolysis in adipocyte | 3 | 57 | 0.009166 | ko04923 |
| Organismal Systems | Immune system | Chemokine signaling pathway | 5 | 175 | 0.010395 | ko04062 |
| Organismal Systems | Immune system | Platelet activation | 4 | 120 | 0.012936 | ko04611 |
| Organismal Systems | Nervous system | Long-term potentiation | 3 | 66 | 0.01366 | ko04720 |
| Organismal Systems | Endocrine system | Renin secretion | 3 | 68 | 0.014801 | ko04924 |
| Organismal Systems | Aging | Longevity regulating pathway - multiple species | 3 | 69 | 0.015392 | ko04213 |
| Organismal Systems | Circulatory system | Vascular smooth muscle contraction | 4 | 141 | 0.022092 | ko04270 |
| Environmental Information Processing | Signal transduction | Rap1 signaling pathway | 5 | 226 | 0.028231 | ko04015 |
| Organismal Systems | Digestive system | Salivary secretion | 3 | 87 | 0.028291 | ko04970 |
| Metabolism | Carbohydrate metabolism | Propanoate metabolism | 2 | 37 | 0.031988 | ko00640 |
| Cellular Processes | Cell growth and death | Apoptosis | 3 | 95 | 0.035409 | ko04210 |
| Organismal Systems | Aging | Longevity regulating pathway - mammal | 3 | 96 | 0.036358 | ko04211 |
| Environmental Information Processing | Signal transduction | Ras signaling pathway | 5 | 243 | 0.03693 | ko04014 |
| Organismal Systems | Excretory system | Endocrine and other factor-regulated calcium reabsorption | 2 | 44 | 0.043929 | ko04961 |
| Organismal Systems | Endocrine system | Estrogen signaling pathway | 3 | 104 | 0.04442 | ko04915 |
| Metabolism | Lipid metabolism | Fatty acid degradation | 2 | 47 | 0.049479 | ko00071 |
| Organismal Systems | Sensory system | Inflammatory mediator regulation of TRP channels | 3 | 111 | 0.052145 | ko04750 |
| Organismal Systems | Nervous system | Cholinergic synapse | 3 | 112 | 0.053298 | ko04725 |
| Metabolism | Global and Overview | Fatty acid metabolism | 2 | 50 | 0.055269 | ko01212 |
| Organismal Systems | Immune system | Leukocyte transendothelial migration | 3 | 118 | 0.060477 | ko04670 |
| Organismal Systems | Excretory system | Vasopressin-regulated water reabsorption | 2 | 54 | 0.063339 | ko04962 |
| Metabolism | Carbohydrate metabolism | Amino sugar and nucleotide sugar metabolism | 2 | 55 | 0.065416 | ko00520 |
| Organismal Systems | Nervous system | Neurotrophin signaling pathway | 3 | 122 | 0.065504 | ko04722 |
| Metabolism | Amino acid metabolism | Lysine degradation | 2 | 56 | 0.067515 | ko00310 |
| Organismal Systems | Nervous system | Serotonergic synapse | 3 | 124 | 0.068088 | ko04726 |
| Environmental Information Processing | Signal transduction | AMPK signaling pathway | 3 | 125 | 0.069398 | ko04152 |
| Organismal Systems | Immune system | Natural killer cell mediated cytotoxicity | 3 | 126 | 0.070718 | ko04650 |
| Cellular Processes | Cellular commiunity | Focal adhesion | 4 | 214 | 0.079406 | ko04510 |
| Organismal Systems | Nervous system | Synaptic vesicle cycle | 2 | 63 | 0.082812 | ko04721 |
| Organismal Systems | Nervous system | Long-term depression | 2 | 64 | 0.085078 | ko04730 |
| Organismal Systems | Immune system | Fc epsilon RI signaling pathway | 2 | 66 | 0.089666 | ko04664 |
| Cellular Processes | Cellular commiunity | Signaling pathways regulating pluripotency of stem cells | 3 | 141 | 0.091862 | ko04550 |
| Environmental Information Processing | Signal transduction | mTOR signaling pathway | 2 | 67 | 0.091987 | ko04150 |
| Organismal Systems | Immune system | B cell receptor signaling pathway | 2 | 69 | 0.096682 | ko04662 |
| Organismal Systems | Endocrine system | PPAR signaling pathway | 2 | 70 | 0.099056 | ko03320 |
| Environmental Information Processing | Signal transduction | FoxO signaling pathway | 3 | 146 | 0.099437 | ko04068 |
| Cellular Processes | Cellular commiunity | Tight junction | 3 | 147 | 0.100982 | ko04530 |
| Metabolism | Carbohydrate metabolism | Inositol phosphate metabolism | 2 | 71 | 0.101445 | ko00562 |
| Metabolism | Lipid metabolism | Fatty acid biosynthesis | 1 | 14 | 0.101503 | ko00061 |
| Organismal Systems | Endocrine system | Thyroid hormone synthesis | 2 | 72 | 0.103851 | ko04918 |
| Organismal Systems | Digestive system | Gastric acid secretion | 2 | 75 | 0.111162 | ko04971 |
| Environmental Information Processing | Signal transduction | Phospholipase D signaling pathway | 3 | 154 | 0.112064 | ko04072 |
| Metabolism | Glycan biosynthesis and metabolism | Glycosphingolipid biosynthesis - ganglio series | 1 | 16 | 0.11515 | ko00604 |
| Organismal Systems | Endocrine system | Oxytocin signaling pathway | 3 | 161 | 0.123595 | ko04921 |
| Genetic Information Processing | Folding, sorting and degradation | Ubiquitin mediated proteolysis | 3 | 163 | 0.126968 | ko04120 |
| Organismal Systems | Endocrine system | Aldosterone synthesis and secretion | 2 | 85 | 0.136431 | ko04925 |
| Organismal Systems | Endocrine system | Insulin secretion | 2 | 87 | 0.141631 | ko04911 |
| Metabolism | Lipid metabolism | Steroid biosynthesis | 1 | 20 | 0.141836 | ko00100 |
| Organismal Systems | Immune system | Fc gamma R-mediated phagocytosis | 2 | 89 | 0.146874 | ko04666 |
| Environmental Information Processing | Signal transduction | ErbB signaling pathway | 2 | 89 | 0.146874 | ko04012 |
| Organismal Systems | Digestive system | Pancreatic secretion | 2 | 90 | 0.149511 | ko04972 |
| Environmental Information Processing | Signal transduction | cGMP - PKG signaling pathway | 3 | 177 | 0.151474 | ko04022 |
| Cellular Processes | Cellular commiunity | Gap junction | 2 | 91 | 0.152157 | ko04540 |
| Cellular Processes | Transport and catabolism | Endocytosis | 4 | 273 | 0.153395 | ko04144 |
| Genetic Information Processing | Folding, sorting and degradation | Protein processing in endoplasmic reticulum | 3 | 181 | 0.158742 | ko04141 |
| Organismal Systems | Endocrine system | GnRH signaling pathway | 2 | 94 | 0.160152 | ko04912 |
| Metabolism | Lipid metabolism | Biosynthesis of unsaturated fatty acids | 1 | 24 | 0.16773 | ko01040 |
| Metabolism | Nucleotide metabolism | Purine metabolism | 3 | 186 | 0.167978 | ko00230 |
| Metabolism | Lipid metabolism | alpha-Linolenic acid metabolism | 1 | 25 | 0.174082 | ko00592 |
| Metabolism | Glycan biosynthesis and metabolism | Glycosaminoglycan biosynthesis - heparan sulfate / heparin | 1 | 26 | 0.180387 | ko00534 |
| Organismal Systems | Nervous system | Retrograde endocannabinoid signaling | 2 | 102 | 0.181832 | ko04723 |
| Genetic Information Processing | Folding, sorting and degradation | Protein export | 1 | 27 | 0.186644 | ko03060 |
| Environmental Information Processing | Signal transduction | Calcium signaling pathway | 3 | 202 | 0.198535 | ko04020 |
| Organismal Systems | Immune system | T cell receptor signaling pathway | 2 | 110 | 0.203928 | ko04660 |
| Genetic Information Processing | Transcription | RNA polymerase | 1 | 31 | 0.211207 | ko03020 |
| Organismal Systems | Endocrine system | Thyroid hormone signaling pathway | 2 | 114 | 0.215093 | ko04919 |
| Organismal Systems | Nervous system | Glutamatergic synapse | 2 | 115 | 0.217894 | ko04724 |
| Cellular Processes | Cell growth and death | Oocyte meiosis | 2 | 117 | 0.223507 | ko04114 |
| Metabolism | Glycan biosynthesis and metabolism | Mucin type O-glycan biosynthesis | 1 | 36 | 0.240887 | ko00512 |
| Metabolism | Metabolism of other amino acids | beta-Alanine metabolism | 1 | 36 | 0.240887 | ko00410 |
| Metabolism | Amino acid metabolism | Alanine, aspartate and glutamate metabolism | 1 | 36 | 0.240887 | ko00250 |
| Organismal Systems | Nervous system | Dopaminergic synapse | 2 | 130 | 0.260214 | ko04728 |
| Organismal Systems | Development | Axon guidance | 2 | 132 | 0.26588 | ko04360 |
| Organismal Systems | Excretory system | Aldosterone-regulated sodium reabsorption | 1 | 42 | 0.275055 | ko04960 |
| Organismal Systems | Digestive system | Carbohydrate digestion and absorption | 1 | 44 | 0.286105 | ko04973 |
| Metabolism | Lipid metabolism | Ether lipid metabolism | 1 | 45 | 0.291567 | ko00565 |
| Metabolism | Xenobiotics biodegradation and metabolism | Drug metabolism - other enzymes | 1 | 47 | 0.302369 | ko00983 |
| Environmental Information Processing | Signal transduction | Hedgehog signaling pathway | 1 | 49 | 0.31301 | ko04340 |
| Metabolism | Glycan biosynthesis and metabolism | N-Glycan biosynthesis | 1 | 49 | 0.31301 | ko00510 |
| Environmental Information Processing | Signal transduction | Hippo signaling pathway | 2 | 149 | 0.313959 | ko04390 |
| Environmental Information Processing | Signal transduction | MAPK signaling pathway | 3 | 259 | 0.315236 | ko04010 |
| Metabolism | Amino acid metabolism | Valine, leucine and isoleucine degradation | 1 | 51 | 0.32349 | ko00280 |
| Organismal Systems | Circulatory system | Adrenergic signaling in cardiomyocytes | 2 | 156 | 0.333607 | ko04261 |
| Organismal Systems | Endocrine system | Ovarian Steroidogenesis | 1 | 54 | 0.338916 | ko04913 |
| Environmental Information Processing | Signal transduction | VEGF signaling pathway | 1 | 61 | 0.373578 | ko04370 |
| Organismal Systems | Immune system | Cytosolic DNA-sensing pathway | 1 | 63 | 0.383149 | ko04623 |
| Metabolism | Lipid metabolism | Glycerolipid metabolism | 1 | 66 | 0.397236 | ko00561 |
| Organismal Systems | Immune system | NOD-like receptor signaling pathway | 1 | 67 | 0.401861 | ko04621 |
| Cellular Processes | Transport and catabolism | Phagosome | 2 | 184 | 0.41034 | ko04145 |
| Organismal Systems | Digestive system | Bile secretion | 1 | 71 | 0.420014 | ko04976 |
| Organismal Systems | Endocrine system | Adipocytokine signaling pathway | 1 | 73 | 0.428887 | ko04920 |
| Organismal Systems | Endocrine system | Prolactin signaling pathway | 1 | 74 | 0.433273 | ko04917 |
| Cellular Processes | Cellular commiunity | Adherens junction | 1 | 76 | 0.441946 | ko04520 |
| Genetic Information Processing | Folding, sorting and degradation | RNA degradation | 1 | 80 | 0.458901 | ko03018 |
| Genetic Information Processing | Translation | Ribosome biogenesis in eukaryotes | 1 | 82 | 0.467188 | ko03008 |
| Organismal Systems | Nervous system | GABAergic synapse | 1 | 83 | 0.471285 | ko04727 |
| Environmental Information Processing | Signal transduction | TGF-beta signaling pathway | 1 | 83 | 0.471285 | ko04350 |
| Environmental Information Processing | Signaling molecules and interaction | ECM-receptor interaction | 1 | 89 | 0.495221 | ko04512 |
| Environmental Information Processing | Signal transduction | PI3K-Akt signaling pathway | 3 | 349 | 0.499699 | ko04151 |
| Environmental Information Processing | Signal transduction | NF-kappa B signaling pathway | 1 | 93 | 0.510583 | ko04064 |
| Organismal Systems | Sensory system | Taste transduction | 1 | 97 | 0.525485 | ko04742 |
| Organismal Systems | Immune system | Antigen processing and presentation | 1 | 97 | 0.525485 | ko04612 |
| Metabolism | Lipid metabolism | Glycerophospholipid metabolism | 1 | 98 | 0.52914 | ko00564 |
| Organismal Systems | Environmental adaptation | Circadian entrainment | 1 | 101 | 0.53994 | ko04713 |
| Organismal Systems | Endocrine system | Melanogenesis | 1 | 104 | 0.550497 | ko04916 |
| Organismal Systems | Immune system | Toll-like receptor signaling pathway | 1 | 105 | 0.553962 | ko04620 |
| Metabolism | Nucleotide metabolism | Pyrimidine metabolism | 1 | 108 | 0.564202 | ko00240 |
| Environmental Information Processing | Signal transduction | HIF-1 signaling pathway | 1 | 114 | 0.583993 | ko04066 |
| Environmental Information Processing | Signal transduction | Sphingolipid signaling pathway | 1 | 118 | 0.596693 | ko04071 |
| Environmental Information Processing | Signal transduction | TNF signaling pathway | 1 | 120 | 0.602899 | ko04668 |
| Organismal Systems | Development | Osteoclast differentiation | 1 | 124 | 0.615031 | ko04380 |
| Cellular Processes | Transport and catabolism | Lysosome | 1 | 131 | 0.635391 | ko04142 |
| Environmental Information Processing | Signal transduction | Wnt signaling pathway | 1 | 135 | 0.646545 | ko04310 |
| Environmental Information Processing | Signal transduction | Jak-STAT signaling pathway | 1 | 160 | 0.709035 | ko04630 |
| Genetic Information Processing | Transcription | Spliceosome | 1 | 163 | 0.715762 | ko03040 |
| Genetic Information Processing | Translation | RNA transport | 1 | 193 | 0.775149 | ko03013 |
| Organismal Systems | Sensory system | Olfactory transduction | 1 | 1466 | 0.999996 | ko04740 |
