# Supplementary material for: CircRNAs in the tree shrew (Tupaia belangeri) brain during postnatal development and aging
Source: Aging (Albany NY). 2018 Apr 30;10(4):833–52. doi: 10.18632/aging.101437 (PMC5940110; doi:10.18632/aging.101437)
Supplement: Table S8 [file aging-10-101437-s006.doc]

The BLASTN alignment result between our predicted tree shrew circRNAs with human circRNAs from NONCODE.

Query_id

novel_circ_000001 novel_circ_000005 novel_circ_000006 novel_circ_000007 novel_circ_000008 novel_circ_000009 novel_circ_000010 novel_circ_000011 novel_circ_000012 novel_circ_000013 novel_circ_000014 novel_circ_000015 novel_circ_000016 novel_circ_000017 novel_circ_000018 novel_circ_000019 novel_circ_000020 novel_circ_000042 novel_circ_000043 novel_circ_000044 novel_circ_000046 novel_circ_000051 novel_circ_000052 novel_circ_000053 novel_circ_000054 novel_circ_000056 novel_circ_000057 novel_circ_000058 novel_circ_000059 novel_circ_000060 novel_circ_000062 novel_circ_000063 novel_circ_000064 novel_circ_000065 novel_circ_000066 novel_circ_000067 novel_circ_000068 novel_circ_000069 novel_circ_000070 novel_circ_000071 novel_circ_000072 novel_circ_000073 novel_circ_000074 novel_circ_000075 novel_circ_000076 novel_circ_000077 novel_circ_000078 novel_circ_000079 novel_circ_000080 novel_circ_000081

| Subject_id | Identi | Q_start Q_end | | S_star S_end | | Gap | E_value |
| --- | --- | --- | --- | --- | --- | --- | --- |
|  | ty(%) |  |  |  |  |  |  |
| hsa_circ_0017968 | 93 | 4 | 300 | 541 | 245 | 0 | 7.00E-122 |
| hsa_circ_0017974 | 89 | 1 | 493 | 1 | 493 | 0 | 3.00E-172 |
| hsa_circ_0017977 | 92 | 1 | 274 | 1293 | 1020 | 0 | 1.00E-105 |
| hsa_circ_0017977 | 94 | 236 | 525 | 1457 | 1168 | 0 | 5.00E-121 |
| hsa_circ_0017982 | 91 | 1 | 205 | 580 | 379 | 0.01 | 4.00E-73 |
| hsa_circ_0017983 | 89 | 1 | 710 | 710 | 1 | 0 | 0 |
| hsa_circ_0017984 | 90 | 1 | 425 | 425 | 1 | 0 | 4.00E-156 |
| hsa_circ_0017985 | 88 | 1 | 260 | 299 | 40 | 0 | 1.00E-84 |
| hsa_circ_0017990 | 89 | 1 | 1897 | 1897 | 1 | 0 | 0 |
| hsa_circ_0017990 | 90 | 1 | 2154 | 2154 | 1 | 0 | 0 |
| hsa_circ_0017990 | 96 | 1 | 134 | 2061 | 1928 | 0 | 1.00E-56 |
| hsa_circ_0017990 | 96 | 1 | 227 | 2154 | 1928 | 0 | 9.00E-100 |
| hsa_circ_0017993 | 93 | 1 | 375 | 375 | 1 | 0 | 3.00E-153 |
| hsa_circ_0017993 | 93 | 1 | 375 | 375 | 1 | 0 | 2.00E-153 |
| hsa_circ_0017994 | 93 | 1 | 357 | 357 | 1 | 0 | 4.00E-145 |
| hsa_circ_0017995 | 93 | 1 | 291 | 291 | 1 | 0 | 9.00E-115 |
| hsa_circ_0017995 | 93 | 1 | 291 | 291 | 1 | 0 | 7.00E-117 |
| hsa_circ_0018002 | 92 | 100 | 459 | 23 | 382 | 0 | 1.00E-141 |
| hsa_circ_0018002 | 92 | 100 | 681 | 23 | 604 | 0 | 0 |
| hsa_circ_0018002 | 92 | 100 | 683 | 23 | 606 | 0 | 0 |
| hsa_circ_0018002 | 94 | 85 | 451 | 240 | 606 | 0 | 6.00E-159 |
| hsa_circ_0018043 | 95 | 677 | 782 | 574 | 469 | 0 | 3.00E-40 |
| hsa_circ_0018043 | 87 | 677 | 1248 | 574 | 6 | 0.01 | 0 |
| hsa_circ_0018043 | 87 | 677 | 1248 | 574 | 6 | 0.01 | 0 |
| hsa_circ_0018043 | 87 | 483 | 1054 | 574 | 6 | 0.01 | 0 |
| hsa_circ_0018043 | 95 | 335 | 440 | 574 | 469 | 0 | 2.00E-40 |
| hsa_circ_0018043 | 87 | 335 | 711 | 574 | 201 | 0.01 | 4.00E-118 |
| hsa_circ_0018043 | 87 | 335 | 906 | 574 | 6 | 0.01 | 0 |
| hsa_circ_0018043 | 87 | 335 | 906 | 574 | 6 | 0.01 | 0 |
| hsa_circ_0018043 | 87 | 335 | 906 | 574 | 6 | 0.01 | 0 |
| hsa_circ_0018034 | 88 | 6 | 431 | 95 | 520 | 0 | 5.00E-145 |
| hsa_circ_0018029 | 96 | 138 | 295 | 167 | 10 | 0 | 3.00E-66 |
| hsa_circ_0018027 | 94 | 138 | 394 | 257 | 1 | 0 | 4.00E-106 |
| hsa_circ_0018026 | 93 | 138 | 504 | 518 | 152 | 0 | 7.00E-154 |
| hsa_circ_0018024 | 93 | 138 | 1199 | 1062 | 1 | 0 | 0 |
| hsa_circ_0018027 | 94 | 6 | 262 | 257 | 1 | 0 | 2.00E-106 |
| hsa_circ_0018026 | 93 | 6 | 372 | 518 | 152 | 0 | 5.00E-154 |
| hsa_circ_0018024 | 94 | 6 | 607 | 1062 | 461 | 0 | 0 |
| hsa_circ_0018024 | 94 | 6 | 689 | 1062 | 379 | 0 | 0 |
| hsa_circ_0018024 | 93 | 6 | 781 | 1062 | 287 | 0 | 0 |
| hsa_circ_0018024 | 93 | 6 | 1067 | 1062 | 1 | 0 | 0 |
| hsa_circ_0018024 | 93 | 6 | 1067 | 1062 | 1 | 0 | 0 |
| hsa_circ_0018024 | 93 | 6 | 1067 | 1062 | 1 | 0 | 0 |
| hsa_circ_0018026 | 92 | 4 | 209 | 357 | 152 | 0 | 4.00E-78 |
| hsa_circ_0018024 | 93 | 4 | 444 | 901 | 461 | 0 | 0 |
| hsa_circ_0005125 | 93 | 1 | 345 | 428 | 84 | 0 | 2.00E-143 |
| hsa_circ_0005125 | 93 | 1 | 427 | 428 | 2 | 0 | 1.00E-180 |
| hsa_circ_0006534 | 94 | 378 | 860 | 484 | 2 | 0 | 0 |
| hsa_circ_0018024 | 94 | 1 | 133 | 133 | 1 | 0 | 6.00E-51 |
| hsa_circ_0018023 | 97 | 134 | 307 | 332 | 159 | 0 | 2.00E-78 |

novel_circ_000082 novel_circ_000083 novel_circ_000084 novel_circ_000085 novel_circ_000086 novel_circ_000087 novel_circ_000089 novel_circ_000090 novel_circ_000091 novel_circ_000093 novel_circ_000094 novel_circ_000095 novel_circ_000096 novel_circ_000097 novel_circ_000098 novel_circ_000099 novel_circ_000100 novel_circ_000101 novel_circ_000102 novel_circ_000104 novel_circ_000105 novel_circ_000106 novel_circ_000107 novel_circ_000108 novel_circ_000109 novel_circ_000110 novel_circ_000111 novel_circ_000112 novel_circ_000113 novel_circ_000114 novel_circ_000115 novel_circ_000116 novel_circ_000117 novel_circ_000118 novel_circ_000119 novel_circ_000120 novel_circ_000121 novel_circ_000122 novel_circ_000123 novel_circ_000124 novel_circ_000125 novel_circ_000126 novel_circ_000127 novel_circ_000128 novel_circ_000129 novel_circ_000130 novel_circ_000131 novel_circ_000132 novel_circ_000133 novel_circ_000134 novel_circ_000135 novel_circ_000136 novel_circ_000137 novel_circ_000138

| hsa_circ_0006534 | 94 | 134 | 616 | 484 | 2 | 0 | 0 |
| --- | --- | --- | --- | --- | --- | --- | --- |
| hsa_circ_0006534 | 94 | 134 | 616 | 484 | 2 | 0 | 0 |
| hsa_circ_0018023 | 97 | 1 | 174 | 332 | 159 | 0 | 9.00E-79 |
| hsa_circ_0006534 | 94 | 1 | 483 | 484 | 2 | 0 | 0 |
| hsa_circ_0006534 | 94 | 1 | 483 | 484 | 2 | 0 | 0 |
| hsa_circ_0006534 | 94 | 1 | 483 | 484 | 2 | 0 | 0 |
| hsa_circ_0018011 | 85 | 24 | 296 | 727 | 455 | 0.02 | 5.00E-71 |
| hsa_circ_0018011 | 85 | 24 | 296 | 727 | 455 | 0.02 | 5.00E-71 |
| hsa_circ_0018011 | 88 | 1 | 202 | 656 | 455 | 0.03 | 3.00E-60 |
| hsa_circ_0018009 | 76 | 1 | 444 | 1062 | 607 | 0.05 | 3.00E-53 |
| hsa_circ_0018009 | 76 | 1 | 714 | 1062 | 325 | 0.05 | 9.00E-95 |
| hsa_circ_0018009 | 78 | 1 | 1038 | 1062 | 1 | 0.04 | 0 |
| hsa_circ_0018007 | 84 | 1 | 447 | 447 | 1 | 0.02 | 2.00E-118 |
| hsa_circ_0000226 | 95 | 1 | 259 | 1 | 259 | 0 | 9.00E-114 |
| hsa_circ_0018046 | 90 | 149 | 1372 | 1224 | 1 | 0 | 0 |
| hsa_circ_0018046 | 90 | 149 | 1255 | 1224 | 118 | 0 | 0 |
| hsa_circ_0018047 | 91 | 1 | 806 | 808 | 3 | 0 | 0 |
| hsa_circ_0018047 | 91 | 1 | 637 | 808 | 172 | 0 | 0 |
| hsa_circ_0018047 | 90 | 1 | 300 | 808 | 509 | 0 | 3.00E-105 |
| hsa_circ_0018056 | 95 | 1 | 331 | 1 | 336 | 0.01 | 2.00E-148 |
| hsa_circ_0018057 | 96 | 1 | 533 | 1 | 538 | 0.01 | 0 |
| hsa_circ_0018057 | 96 | 1 | 779 | 1 | 784 | 0.01 | 0 |
| hsa_circ_0008473 | 98 | 1 | 309 | 1 | 309 | 0 | 2.00E-153 |
| hsa_circ_0018056 | 92 | 1 | 119 | 1164 | 1282 | 0 | 2.00E-39 |
| hsa_circ_0018077 | 80 | 1777 | 2184 | 400 | 2 | 0.03 | 2.00E-76 |
| hsa_circ_0018071 | 88 | 2186 | 2849 | 757 | 94 | 0 | 0 |
| hsa_circ_0018077 | 80 | 1 | 406 | 398 | 2 | 0.03 | 4.00E-76 |
| hsa_circ_0018076 | 83 | 1 | 632 | 623 | 1 | 0.02 | 7.00E-155 |
| hsa_circ_0018071 | 88 | 408 | 1071 | 757 | 94 | 0 | 0 |
| hsa_circ_0018071 | 89 | 408 | 1164 | 757 | 1 | 0 | 0 |
| hsa_circ_0018071 | 88 | 5 | 668 | 757 | 94 | 0 | 0 |
| hsa_circ_0018071 | 89 | 5 | 761 | 757 | 1 | 0 | 0 |
| hsa_circ_0018071 | 88 | 1 | 664 | 757 | 94 | 0 | 0 |
| hsa_circ_0018071 | 89 | 1 | 757 | 757 | 1 | 0 | 0 |
| hsa_circ_0018064 | 82 | 97 | 1071 | 1102 | 128 | 0.01 | 0 |
| hsa_circ_0018064 | 82 | 97 | 1178 | 1102 | 24 | 0.01 | 0 |
| hsa_circ_0018064 | 82 | 97 | 1178 | 1102 | 24 | 0.01 | 0 |
| hsa_circ_0018062 | 88 | 1 | 448 | 748 | 301 | 0 | 3.00E-152 |
| hsa_circ_0073514 | 90 | 3 | 407 | 123 | 523 | 0.01 | 3.00E-147 |
| hsa_circ_0073513 | 96 | 1 | 535 | 1026 | 1560 | 0 | 0 |
| hsa_circ_0073506 | 94 | 1 | 1258 | 542 | 1799 | 0 | 0 |
| hsa_circ_0073500 | 95 | 2 | 1885 | 9739 | 11622 | 0 | 0 |
| hsa_circ_0073506 | 94 | 1 | 892 | 542 | 1433 | 0 | 0 |
| hsa_circ_0073500 | 96 | 1 | 1373 | 9451 | 10823 | 0 | 0 |
| hsa_circ_0073506 | 95 | 1 | 375 | 542 | 916 | 0 | 2.00E-168 |
| hsa_circ_0073506 | 95 | 1 | 189 | 542 | 731 | 0.01 | 2.00E-80 |
| hsa_circ_0073506 | 95 | 1 | 541 | 1 | 541 | 0 | 0 |
| hsa_circ_0073501 | 96 | 1 | 914 | 1 | 914 | 0 | 0 |
| hsa_circ_0073500 | 92 | 1 | 1313 | 9053 | 10364 | 0.01 | 0 |
| hsa_circ_0073504 | 95 | 2 | 505 | 2 | 505 | 0 | 0 |
| hsa_circ_0006499 | 90 | 1 | 644 | 655 | 12 | 0 | 0 |
| hsa_circ_0006499 | 90 | 1 | 644 | 655 | 12 | 0 | 0 |
| hsa_circ_0073494 | 90 | 1 | 1038 | 50 | 1084 | 0 | 0 |
| hsa_circ_0073494 | 90 | 1 | 1087 | 1 | 1084 | 0 | 0 |

novel_circ_000139 novel_circ_000140 novel_circ_000141 novel_circ_000147 novel_circ_000148 novel_circ_000149 novel_circ_000150 novel_circ_000151 novel_circ_000152 novel_circ_000153 novel_circ_000154 novel_circ_000155 novel_circ_000156 novel_circ_000157 novel_circ_000158 novel_circ_000159 novel_circ_000160 novel_circ_000161 novel_circ_000162 novel_circ_000163 novel_circ_000164 novel_circ_000165 novel_circ_000166 novel_circ_000167 novel_circ_000168 novel_circ_000169 novel_circ_000172 novel_circ_000173 novel_circ_000174 novel_circ_000175 novel_circ_000176 novel_circ_000177 novel_circ_000178 novel_circ_000179 novel_circ_000180 novel_circ_000181 novel_circ_000182 novel_circ_000184 novel_circ_000185 novel_circ_000186 novel_circ_000187 novel_circ_000188 novel_circ_000190 novel_circ_000191 novel_circ_000192 novel_circ_000193 novel_circ_000194 novel_circ_000195 novel_circ_000196 novel_circ_000197 novel_circ_000199 novel_circ_000201 novel_circ_000205 novel_circ_000206

| hsa_circ_0073494 | 91 | 1 | 581 | 380 | 957 | 0.01 | 0 |
| --- | --- | --- | --- | --- | --- | --- | --- |
| hsa_circ_0009146 | 90 | 1 | 327 | 1 | 327 | 0 | 4.00E-115 |
| hsa_circ_0073494 | 90 | 1 | 376 | 1 | 376 | 0 | 1.00E-135 |
| hsa_circ_0084779 | 98 | 1 | 310 | 2718 | 2409 | 0 | 6.00E-154 |
| hsa_circ_0084779 | 96 | 4 | 641 | 3046 | 2409 | 0 | 0 |
| hsa_circ_0084779 | 83 | 1368 | 2817 | 1471 | 4 | 0.04 | 0 |
| hsa_circ_0084779 | 98 | 1 | 308 | 2718 | 2411 | 0 | 6.00E-153 |
| hsa_circ_0084779 | 97 | 7 | 501 | 2905 | 2411 | 0 | 0 |
| hsa_circ_0084779 | 96 | 4 | 639 | 3046 | 2411 | 0 | 0 |
| hsa_circ_0084779 | 95 | 1 | 1120 | 3530 | 2411 | 0 | 0 |
| hsa_circ_0084779 | 94 | 1 | 810 | 3530 | 2721 | 0 | 0 |
| hsa_circ_0084779 | 93 | 1 | 751 | 3530 | 2780 | 0 | 0 |
| hsa_circ_0084779 | 94 | 1 | 887 | 3666 | 2780 | 0 | 0 |
| hsa_circ_0084779 | 94 | 1 | 619 | 3530 | 2912 | 0 | 0 |
| hsa_circ_0084779 | 95 | 1 | 755 | 3666 | 2912 | 0 | 0 |
| hsa_circ_0084779 | 94 | 1 | 915 | 3826 | 2912 | 0 | 0 |
| hsa_circ_0084779 | 95 | 1 | 617 | 3666 | 3050 | 0 | 0 |
| hsa_circ_0084779 | 95 | 1 | 777 | 3826 | 3050 | 0 | 0 |
| hsa_circ_0084779 | 94 | 1 | 268 | 3530 | 3263 | 0 | 8.00E-111 |
| hsa_circ_0084779 | 96 | 1 | 404 | 3666 | 3263 | 0 | 0 |
| hsa_circ_0084779 | 95 | 1 | 564 | 3826 | 3263 | 0 | 0 |
| hsa_circ_0084487 | 86 | 7949 | 8962 | 1196 | 185 | 0.04 | 0 |
| hsa_circ_0084781 | 96 | 1 | 296 | 296 | 1 | 0 | 1.00E-134 |
| hsa_circ_0084781 | 96 | 1 | 427 | 427 | 1 | 0 | 0 |
| hsa_circ_0084781 | 96 | 1 | 493 | 493 | 1 | 0 | 0 |
| hsa_circ_0007554 | 95 | 1 | 232 | 232 | 1 | 0 | 7.00E-101 |
| hsa_circ_0030407 | 92 | 1 | 417 | 135 | 551 | 0 | 2.00E-163 |
| hsa_circ_0030406 | 95 | 2 | 610 | 872 | 1480 | 0 | 0 |
| hsa_circ_0030406 | 95 | 1 | 511 | 970 | 1480 | 0 | 0 |
| hsa_circ_0030408 | 96 | 1 | 345 | 1 | 345 | 0 | 8.00E-157 |
| hsa_circ_0008556 | 98 | 1 | 683 | 683 | 1 | 0 | 0 |
| hsa_circ_0008556 | 98 | 71 | 753 | 683 | 1 | 0 | 0 |
| hsa_circ_0030416 | 97 | 1 | 547 | 547 | 1 | 0 | 0 |
| hsa_circ_0030416 | 97 | 71 | 617 | 547 | 1 | 0 | 0 |
| hsa_circ_0030416 | 97 | 170 | 716 | 547 | 1 | 0 | 0 |
| hsa_circ_0091206 | 88 | 586 | 3071 | 2 | 2493 | 0.01 | 0 |
| hsa_circ_0091206 | 88 | 2 | 2487 | 2 | 2493 | 0.01 | 0 |
| hsa_circ_0087632 | 88 | 18 | 653 | 14 | 650 | 0.01 | 0 |
| hsa_circ_0087632 | 88 | 15 | 650 | 14 | 650 | 0.01 | 0 |
| hsa_circ_0087641 | 93 | 1 | 1183 | 1183 | 1 | 0 | 0 |
| hsa_circ_0005789 | 94 | 1 | 337 | 337 | 1 | 0 | 1.00E-145 |
| hsa_circ_0029596 | 100 | 170 | 197 | 154 | 181 | 0 | 9.00E-06 |
| hsa_circ_0052054 | 75 | 1016 | 1310 | 1181 | 887 | 0.05 | 6.00E-25 |
| hsa_circ_0087419 | 90 | 1 | 458 | 590 | 1047 | 0 | 2.00E-169 |
| hsa_circ_0087412 | 92 | 2 | 1540 | 2 | 1540 | 0 | 0 |
| hsa_circ_0030920 | 77 | 1 | 895 | 1799 | 902 | 0.05 | 2.00E-137 |
| hsa_circ_0087408 | 90 | 1 | 477 | 935 | 459 | 0 | 5.00E-175 |
| hsa_circ_0087408 | 90 | 1 | 566 | 935 | 370 | 0 | 0 |
| hsa_circ_0087408 | 91 | 1 | 618 | 935 | 318 | 0 | 0 |
| hsa_circ_0087408 | 93 | 1 | 458 | 458 | 1 | 0 | 0 |
| hsa_circ_0087405 | 93 | 502 | 591 | 90 | 1 | 0 | 8.00E-30 |
| hsa_circ_0087404 | 94 | 264 | 863 | 716 | 117 | 0 | 0 |
| hsa_circ_0087399 | 93 | 379 | 1359 | 1 | 981 | 0 | 0 |
| hsa_circ_0087399 | 93 | 899 | 1879 | 1 | 981 | 0 | 0 |

novel_circ_000207 novel_circ_000208 novel_circ_000209 novel_circ_000210 novel_circ_000211 novel_circ_000212 novel_circ_000213 novel_circ_000214 novel_circ_000215 novel_circ_000216 novel_circ_000217 novel_circ_000218 novel_circ_000219 novel_circ_000220 novel_circ_000221 novel_circ_000222 novel_circ_000223 novel_circ_000224 novel_circ_000225 novel_circ_000226 novel_circ_000227 novel_circ_000228 novel_circ_000229 novel_circ_000230 novel_circ_000231 novel_circ_000232 novel_circ_000233 novel_circ_000234 novel_circ_000235 novel_circ_000236 novel_circ_000237 novel_circ_000238 novel_circ_000239 novel_circ_000240 novel_circ_000241 novel_circ_000242 novel_circ_000243 novel_circ_000244 novel_circ_000245 novel_circ_000246 novel_circ_000247 novel_circ_000248 novel_circ_000249 novel_circ_000250 novel_circ_000251 novel_circ_000252 novel_circ_000253 novel_circ_000254 novel_circ_000255 novel_circ_000256 novel_circ_000257 novel_circ_000258 novel_circ_000259 novel_circ_000260

| hsa_circ_0087399 | 95 | 295 | 806 | 1 | 512 | 0 | 0 |
| --- | --- | --- | --- | --- | --- | --- | --- |
| hsa_circ_0087395 | 97 | 1 | 768 | 1 | 768 | 0 | 0 |
| hsa_circ_0087395 | 97 | 1 | 354 | 415 | 768 | 0 | 1.00E-166 |
| hsa_circ_0087399 | 98 | 1 | 164 | 1 | 164 | 0 | 1.00E-76 |
| hsa_circ_0087395 | 97 | 191 | 958 | 1 | 768 | 0 | 0 |
| hsa_circ_0087395 | 97 | 1 | 354 | 415 | 768 | 0 | 8.00E-167 |
| hsa_circ_0087395 | 97 | 1 | 708 | 1 | 708 | 0 | 0 |
| hsa_circ_0087395 | 97 | 191 | 898 | 1 | 708 | 0 | 0 |
| hsa_circ_0087395 | 97 | 1 | 529 | 1 | 529 | 0 | 0 |
| hsa_circ_0087395 | 97 | 191 | 719 | 1 | 529 | 0 | 0 |
| hsa_circ_0087395 | 97 | 1 | 212 | 68 | 279 | 0 | 4.00E-98 |
| hsa_circ_0087395 | 96 | 1 | 279 | 1 | 279 | 0 | 1.00E-128 |
| hsa_circ_0006687 | 91 | 196 | 598 | 403 | 1 | 0 | 2.00E-155 |
| hsa_circ_0087391 | 92 | 196 | 824 | 629 | 1 | 0 | 0 |
| hsa_circ_0087388 | 93 | 196 | 1071 | 2182 | 1307 | 0 | 0 |
| hsa_circ_0087388 | 93 | 196 | 1071 | 2182 | 1307 | 0 | 0 |
| hsa_circ_0087388 | 89 | 1192 | 2497 | 1306 | 1 | 0 | 0 |
| hsa_circ_0006687 | 91 | 2 | 404 | 403 | 1 | 0 | 1.00E-155 |
| hsa_circ_0087391 | 92 | 2 | 630 | 629 | 1 | 0 | 0 |
| hsa_circ_0087388 | 93 | 2 | 877 | 2182 | 1307 | 0 | 0 |
| hsa_circ_0087388 | 89 | 998 | 2303 | 1306 | 1 | 0 | 0 |
| hsa_circ_0006687 | 91 | 3 | 211 | 209 | 1 | 0 | 9.00E-75 |
| hsa_circ_0087388 | 87 | 330 | 1315 | 1306 | 321 | 0.01 | 0 |
| hsa_circ_0087388 | 89 | 330 | 1635 | 1306 | 1 | 0 | 0 |
| hsa_circ_0087384 | 91 | 267 | 2740 | 2474 | 1 | 0 | 0 |
| hsa_circ_0087388 | 88 | 1 | 928 | 1248 | 321 | 0.01 | 0 |
| hsa_circ_0087388 | 89 | 1 | 1248 | 1248 | 1 | 0 | 0 |
| hsa_circ_0087384 | 90 | 1 | 1481 | 2416 | 936 | 0 | 0 |
| hsa_circ_0087384 | 91 | 1 | 2416 | 2416 | 1 | 0 | 0 |
| hsa_circ_0087388 | 87 | 1 | 830 | 1150 | 321 | 0.01 | 0 |
| hsa_circ_0087388 | 88 | 1 | 1150 | 1150 | 1 | 0.01 | 0 |
| hsa_circ_0087384 | 88 | 1 | 1153 | 2318 | 1166 | 0.01 | 0 |
| hsa_circ_0087384 | 90 | 1 | 1383 | 2318 | 936 | 0 | 0 |
| hsa_circ_0087384 | 91 | 1 | 1980 | 2318 | 339 | 0 | 0 |
| hsa_circ_0087384 | 91 | 1 | 2318 | 2318 | 1 | 0 | 0 |
| hsa_circ_0087388 | 86 | 1 | 713 | 1033 | 321 | 0.01 | 0 |
| hsa_circ_0087388 | 88 | 1 | 1033 | 1033 | 1 | 0.01 | 0 |
| hsa_circ_0087387 | 89 | 1 | 1266 | 1731 | 466 | 0 | 0 |
| hsa_circ_0087384 | 91 | 1 | 2201 | 2201 | 1 | 0 | 0 |
| hsa_circ_0087387 | 96 | 1 | 233 | 698 | 466 | 0 | 4.00E-103 |
| hsa_circ_0087386 | 95 | 1 | 830 | 830 | 1 | 0 | 0 |
| hsa_circ_0087384 | 94 | 1 | 1007 | 1168 | 162 | 0 | 0 |
| hsa_circ_0087384 | 94 | 1 | 1008 | 1168 | 161 | 0 | 0 |
| hsa_circ_0087384 | 94 | 1 | 1168 | 1168 | 1 | 0 | 0 |
| hsa_circ_0087387 | 96 | 1 | 480 | 610 | 131 | 0 | 0 |
| hsa_circ_0087384 | 94 | 1 | 1080 | 1080 | 1 | 0 | 0 |
| hsa_circ_0087384 | 93 | 1 | 935 | 935 | 1 | 0 | 0 |
| hsa_circ_0087384 | 91 | 1 | 600 | 600 | 1 | 0 | 0 |
| hsa_circ_0087384 | 92 | 1 | 470 | 470 | 1 | 0 | 0 |
| hsa_circ_0074240 | 93 | 7240 | 7284 | 7589 | 7633 | 0 | 3.00E-08 |
| hsa_circ_0014798 | 85 | 1 | 154 | 242 | 395 | 0 | 1.00E-36 |
| hsa_circ_0087380 | 91 | 1 | 408 | 1 | 408 | 0 | 1.00E-156 |
| hsa_circ_0087379 | 93 | 1 | 539 | 1 | 539 | 0 | 0 |
| hsa_circ_0087379 | 93 | 190 | 728 | 1 | 539 | 0 | 0 |

novel_circ_000261 novel_circ_000262 novel_circ_000263 novel_circ_000264 novel_circ_000265 novel_circ_000266 novel_circ_000267 novel_circ_000268 novel_circ_000269 novel_circ_000281 novel_circ_000284 novel_circ_000285 novel_circ_000286 novel_circ_000287 novel_circ_000288 novel_circ_000289 novel_circ_000299 novel_circ_000300 novel_circ_000301 novel_circ_000302 novel_circ_000303 novel_circ_000304 novel_circ_000305 novel_circ_000306 novel_circ_000308 novel_circ_000309 novel_circ_000310 novel_circ_000311 novel_circ_000312 novel_circ_000313 novel_circ_000314 novel_circ_000315 novel_circ_000316 novel_circ_000317 novel_circ_000318 novel_circ_000331 novel_circ_000332 novel_circ_000333 novel_circ_000334 novel_circ_000335 novel_circ_000336 novel_circ_000337 novel_circ_000338 novel_circ_000339 novel_circ_000340 novel_circ_000341 novel_circ_000342 novel_circ_000343 novel_circ_000344 novel_circ_000345 novel_circ_000346 novel_circ_000347 novel_circ_000348 novel_circ_000349

| hsa_circ_0087379 | 95 | 1 | 304 | 1 | 304 | 0 | 2.00E-132 |
| --- | --- | --- | --- | --- | --- | --- | --- |
| hsa_circ_0004848 | 82 | 7383 | 7479 | 4765 | 4857 | 0.04 | 4.00E-13 |
| hsa_circ_0078140 | 97 | 1 | 359 | 359 | 1 | 0 | 6.00E-173 |
| hsa_circ_0078140 | 97 | 883 | 1244 | 362 | 1 | 0 | 5.00E-174 |
| hsa_circ_0078142 | 93 | 2 | 472 | 912 | 442 | 0 | 0 |
| hsa_circ_0078144 | 89 | 1 | 543 | 1595 | 1026 | 0.05 | 0 |
| hsa_circ_0078144 | 89 | 1 | 1534 | 2586 | 1026 | 0.02 | 0 |
| hsa_circ_0078144 | 85 | 1 | 662 | 4196 | 3532 | 0.03 | 0 |
| hsa_circ_0078144 | 85 | 144 | 805 | 4196 | 3532 | 0.03 | 0 |
| hsa_circ_0005144 | 79 | 26133 | 26282 | 11853 | 12001 | 0.03 | 2.00E-17 |
| hsa_circ_0066599 | 95 | 1 | 490 | 1 | 490 | 0 | 0 |
| hsa_circ_0066599 | 95 | 1 | 490 | 1 | 490 | 0 | 0 |
| hsa_circ_0066601 | 90 | 1 | 297 | 1 | 294 | 0.01 | 1.00E-105 |
| hsa_circ_0066601 | 91 | 1 | 755 | 1 | 752 | 0 | 0 |
| hsa_circ_0066601 | 92 | 1 | 1099 | 1 | 1096 | 0 | 0 |
| hsa_circ_0066601 | 91 | 1 | 458 | 295 | 752 | 0 | 4.00E-176 |
| hsa_circ_0030163 | 92 | 3 | 685 | 742 | 61 | 0 | 0 |
| hsa_circ_0066602 | 92 | 181 | 607 | 1 | 427 | 0 | 4.00E-167 |
| hsa_circ_0066602 | 92 | 1 | 321 | 1 | 321 | 0 | 2.00E-123 |
| hsa_circ_0066602 | 92 | 1 | 427 | 1 | 427 | 0 | 3.00E-167 |
| hsa_circ_0066602 | 91 | 1 | 317 | 5 | 321 | 0 | 3.00E-121 |
| hsa_circ_0066602 | 91 | 3 | 250 | 74 | 321 | 0 | 2.00E-91 |
| hsa_circ_0082866 | 84 | 632 | 988 | 167 | 523 | 0 | 6.00E-93 |
| hsa_circ_0082866 | 83 | 632 | 1041 | 167 | 574 | 0 | 2.00E-103 |
| hsa_circ_0082857 | 89 | 1 | 441 | 271 | 690 | 0.05 | 3.00E-147 |
| hsa_circ_0082851 | 89 | 1 | 345 | 710 | 366 | 0 | 4.00E-120 |
| hsa_circ_0082843 | 93 | 1 | 878 | 899 | 1 | 0.02 | 0 |
| hsa_circ_0082843 | 94 | 1 | 646 | 646 | 1 | 0 | 0 |
| hsa_circ_0082826 | 92 | 1 | 1244 | 1 | 1244 | 0 | 0 |
| hsa_circ_0082830 | 94 | 1 | 418 | 1 | 418 | 0 | 0 |
| hsa_circ_0082826 | 92 | 1 | 1113 | 1 | 1113 | 0 | 0 |
| hsa_circ_0082826 | 96 | 1 | 474 | 477 | 950 | 0 | 0 |
| hsa_circ_0082826 | 91 | 1 | 643 | 1 | 643 | 0 | 0 |
| hsa_circ_0082824 | 88 | 9851 | 10152 | 189 | 490 | 0 | 1.00E-94 |
| hsa_circ_0082826 | 88 | 1 | 283 | 1 | 283 | 0 | 9.00E-91 |
| hsa_circ_0082823 | 94 | 1 | 243 | 243 | 1 | 0 | 3.00E-100 |
| hsa_circ_0082821 | 92 | 1 | 355 | 2078 | 1724 | 0 | 5.00E-139 |
| hsa_circ_0047737 | 96 | 1 | 471 | 706 | 236 | 0 | 0 |
| hsa_circ_0047737 | 93 | 1 | 193 | 706 | 514 | 0 | 6.00E-76 |
| hsa_circ_0047737 | 96 | 94 | 271 | 884 | 707 | 0 | 2.00E-77 |
| hsa_circ_0047738 | 92 | 8 | 358 | 206 | 556 | 0 | 9.00E-137 |
| hsa_circ_0047738 | 92 | 8 | 533 | 206 | 731 | 0 | 0 |
| hsa_circ_0047738 | 93 | 1 | 361 | 371 | 731 | 0 | 2.00E-147 |
| hsa_circ_0047739 | 92 | 1 | 1233 | 80 | 1312 | 0 | 0 |
| hsa_circ_0047739 | 91 | 58 | 896 | 253 | 1091 | 0 | 0 |
| hsa_circ_0047739 | 92 | 1 | 446 | 255 | 700 | 0 | 8.00E-178 |
| hsa_circ_0047739 | 91 | 1 | 837 | 255 | 1091 | 0 | 0 |
| hsa_circ_0047739 | 92 | 1 | 1058 | 255 | 1312 | 0 | 0 |
| hsa_circ_0047741 | 91 | 1 | 640 | 121 | 760 | 0 | 0 |
| hsa_circ_0047741 | 93 | 1 | 861 | 121 | 981 | 0 | 0 |
| hsa_circ_0047741 | 91 | 1 | 494 | 267 | 760 | 0 | 0 |
| hsa_circ_0047741 | 93 | 1 | 715 | 267 | 981 | 0 | 0 |
| hsa_circ_0047741 | 90 | 1 | 391 | 370 | 760 | 0 | 3.00E-142 |
| hsa_circ_0047742 | 92 | 1 | 1270 | 1 | 1270 | 0 | 0 |

novel_circ_000350 novel_circ_000351 novel_circ_000352 novel_circ_000353 novel_circ_000354 novel_circ_000355 novel_circ_000356 novel_circ_000357 novel_circ_000358 novel_circ_000359 novel_circ_000360 novel_circ_000362 novel_circ_000363 novel_circ_000364 novel_circ_000365 novel_circ_000366 novel_circ_000367 novel_circ_000368 novel_circ_000369 novel_circ_000370 novel_circ_000371 novel_circ_000372 novel_circ_000373 novel_circ_000374 novel_circ_000375 novel_circ_000376 novel_circ_000377 novel_circ_000380 novel_circ_000381 novel_circ_000382 novel_circ_000383 novel_circ_000386 novel_circ_000387 novel_circ_000388 novel_circ_000389 novel_circ_000390 novel_circ_000391 novel_circ_000392 novel_circ_000393 novel_circ_000394 novel_circ_000395 novel_circ_000396 novel_circ_000397 novel_circ_000398 novel_circ_000399 novel_circ_000400 novel_circ_000401 novel_circ_000402 novel_circ_000403 novel_circ_000404 novel_circ_000405 novel_circ_000406 novel_circ_000407 novel_circ_000408

| hsa_circ_0047742 | 92 | 1 | 1612 | 1 | 1612 | 0 | 0 |
| --- | --- | --- | --- | --- | --- | --- | --- |
| hsa_circ_0047742 | 92 | 1 | 1612 | 1 | 1612 | 0 | 0 |
| hsa_circ_0047746 | 92 | 3 | 1074 | 3 | 1074 | 0 | 0 |
| hsa_circ_0047746 | 91 | 3 | 1416 | 3 | 1416 | 0 | 0 |
| hsa_circ_0047746 | 91 | 3 | 1525 | 3 | 1525 | 0 | 0 |
| hsa_circ_0009038 | 91 | 1 | 431 | 1 | 431 | 0 | 8.00E-168 |
| hsa_circ_0047746 | 91 | 1 | 540 | 986 | 1525 | 0 | 0 |
| hsa_circ_0009038 | 91 | 51 | 396 | 86 | 431 | 0 | 6.00E-129 |
| hsa_circ_0047746 | 90 | 51 | 505 | 1071 | 1525 | 0 | 2.00E-169 |
| hsa_circ_0009038 | 91 | 1 | 342 | 90 | 431 | 0 | 8.00E-127 |
| hsa_circ_0047746 | 90 | 1 | 451 | 1075 | 1525 | 0 | 3.00E-167 |
| hsa_circ_0047749 | 90 | 3 | 352 | 3 | 352 | 0 | 1.00E-124 |
| hsa_circ_0047749 | 90 | 3 | 352 | 3 | 352 | 0 | 2.00E-124 |
| hsa_circ_0047750 | 93 | 115 | 336 | 1 | 222 | 0 | 2.00E-88 |
| hsa_circ_0047750 | 93 | 1 | 222 | 1 | 222 | 0 | 1.00E-88 |
| hsa_circ_0006389 | 94 | 225 | 760 | 3 | 538 | 0 | 0 |
| hsa_circ_0006389 | 94 | 3 | 538 | 3 | 538 | 0 | 0 |
| hsa_circ_0047751 | 96 | 119 | 451 | 1 | 333 | 0 | 1.00E-151 |
| hsa_circ_0047753 | 89 | 1 | 479 | 479 | 1 | 0.01 | 9.00E-168 |
| hsa_circ_0047756 | 92 | 1 | 404 | 404 | 1 | 0 | 7.00E-158 |
| hsa_circ_0047775 | 93 | 1 | 982 | 982 | 1 | 0 | 0 |
| hsa_circ_0047775 | 91 | 1 | 2371 | 2371 | 1 | 0 | 0 |
| hsa_circ_0047777 | 94 | 1 | 513 | 513 | 1 | 0 | 0 |
| hsa_circ_0047780 | 88 | 2 | 402 | 409 | 9 | 0 | 8.00E-133 |
| hsa_circ_0047778 | 88 | 1 | 593 | 1678 | 1086 | 0 | 0 |
| hsa_circ_0047777 | 90 | 1 | 1644 | 2953 | 1310 | 0 | 0 |
| hsa_circ_0047777 | 90 | 1 | 576 | 2953 | 2378 | 0 | 0 |
| hsa_circ_0013828 | 73 | 44005 | 46403 | 24098 | 26496 | 0.04 | 0 |
| hsa_circ_0013828 | 73 | 44005 | 46403 | 24098 | 26496 | 0.04 | 0 |
| hsa_circ_0013828 | 73 | 44005 | 46403 | 24098 | 26496 | 0.04 | 0 |
| hsa_circ_0047788 | 97 | 1 | 74 | 1 | 74 | 0 | 1.00E-27 |
| hsa_circ_0003954 | 90 | 1 | 385 | 384 | 768 | 0 | 6.00E-139 |
| hsa_circ_0007510 | 96 | 1 | 581 | 1 | 581 | 0 | 0 |
| hsa_circ_0007510 | 95 | 1 | 642 | 1 | 642 | 0 | 0 |
| hsa_circ_0047791 | 95 | 1 | 1060 | 1022 | 2081 | 0 | 0 |
| hsa_circ_0047791 | 95 | 1 | 1131 | 1022 | 2152 | 0 | 0 |
| hsa_circ_0047791 | 95 | 1 | 1131 | 1022 | 2152 | 0 | 0 |
| hsa_circ_0047791 | 94 | 1 | 879 | 1274 | 2152 | 0 | 0 |
| hsa_circ_0047791 | 94 | 1 | 418 | 1664 | 2081 | 0 | 0 |
| hsa_circ_0047791 | 94 | 1 | 489 | 1664 | 2152 | 0 | 0 |
| hsa_circ_0000853 | 91 | 168 | 418 | 1 | 251 | 0 | 4.00E-91 |
| hsa_circ_0047800 | 87 | 290 | 725 | 1 | 427 | 0.02 | 7.00E-136 |
| hsa_circ_0047806 | 89 | 1 | 511 | 179 | 689 | 0 | 0 |
| hsa_circ_0047816 | 89 | 3 | 1766 | 3 | 1775 | 0.01 | 0 |
| hsa_circ_0047816 | 90 | 3 | 2354 | 3 | 2363 | 0 | 0 |
| hsa_circ_0047820 | 94 | 180 | 983 | 1 | 804 | 0 | 0 |
| hsa_circ_0047820 | 93 | 1 | 359 | 1 | 359 | 0 | 1.00E-149 |
| hsa_circ_0047820 | 94 | 1 | 522 | 1 | 522 | 0 | 0 |
| hsa_circ_0047820 | 94 | 1 | 804 | 1 | 804 | 0 | 0 |
| hsa_circ_0004371 | 94 | 1 | 811 | 1 | 811 | 0 | 0 |
| hsa_circ_0047820 | 94 | 1 | 917 | 1 | 917 | 0 | 0 |
| hsa_circ_0047820 | 93 | 1 | 1065 | 1 | 1065 | 0 | 0 |
| hsa_circ_0047820 | 93 | 1 | 1065 | 1 | 1065 | 0 | 0 |
| hsa_circ_0047823 | 89 | 183 | 3555 | 1 | 3389 | 0.03 | 0 |

novel_circ_000409 novel_circ_000410 novel_circ_000411 novel_circ_000412 novel_circ_000413 novel_circ_000414 novel_circ_000415 novel_circ_000416 novel_circ_000417 novel_circ_000418 novel_circ_000419 novel_circ_000420 novel_circ_000421 novel_circ_000422 novel_circ_000423 novel_circ_000424 novel_circ_000425 novel_circ_000427 novel_circ_000428 novel_circ_000429 novel_circ_000431 novel_circ_000432 novel_circ_000433 novel_circ_000434 novel_circ_000435 novel_circ_000436 novel_circ_000437 novel_circ_000438 novel_circ_000439 novel_circ_000440 novel_circ_000441 novel_circ_000442 novel_circ_000443 novel_circ_000444 novel_circ_000445 novel_circ_000446 novel_circ_000447 novel_circ_000448 novel_circ_000449 novel_circ_000450 novel_circ_000451 novel_circ_000452 novel_circ_000453 novel_circ_000454 novel_circ_000455 novel_circ_000456 novel_circ_000457 novel_circ_000458 novel_circ_000459 novel_circ_000460 novel_circ_000461 novel_circ_000462 novel_circ_000463 novel_circ_000464

| hsa_circ_0047820 | 94 | 1 | 801 | 4 | 804 | 0 | 0 |
| --- | --- | --- | --- | --- | --- | --- | --- |
| hsa_circ_0047823 | 94 | 1 | 622 | 1 | 622 | 0 | 0 |
| hsa_circ_0004371 | 94 | 1 | 629 | 183 | 811 | 0 | 0 |
| hsa_circ_0004371 | 94 | 1 | 629 | 183 | 811 | 0 | 0 |
| hsa_circ_0047823 | 93 | 1 | 735 | 1 | 735 | 0 | 0 |
| hsa_circ_0047823 | 92 | 1 | 883 | 1 | 883 | 0 | 0 |
| hsa_circ_0047823 | 92 | 1 | 886 | 1 | 886 | 0 | 0 |
| hsa_circ_0047823 | 95 | 84 | 528 | 178 | 622 | 0 | 0 |
| hsa_circ_0047823 | 95 | 1 | 445 | 178 | 622 | 0 | 0 |
| hsa_circ_0047824 | 95 | 1 | 282 | 1 | 282 | 0 | 7.00E-122 |
| hsa_circ_0047828 | 92 | 1 | 1349 | 4588 | 3242 | 0 | 0 |
| hsa_circ_0047828 | 92 | 1 | 1282 | 4588 | 3307 | 0 | 0 |
| hsa_circ_0047829 | 93 | 1 | 581 | 735 | 155 | 0 | 0 |
| hsa_circ_0047828 | 93 | 1 | 1006 | 4588 | 3583 | 0 | 0 |
| hsa_circ_0047828 | 93 | 27 | 1032 | 4588 | 3583 | 0 | 0 |
| hsa_circ_0047828 | 92 | 1 | 549 | 4588 | 4040 | 0 | 0 |
| hsa_circ_0047828 | 92 | 1 | 425 | 4588 | 4164 | 0 | 2.00E-169 |
| hsa_circ_0000498 | 93 | 1 | 477 | 566 | 90 | 0 | 0 |
| hsa_circ_0000498 | 93 | 1 | 566 | 566 | 1 | 0 | 0 |
| hsa_circ_0000498 | 94 | 4 | 338 | 335 | 1 | 0 | 6.00E-143 |
| hsa_circ_0030529 | 92 | 153 | 931 | 237 | 1015 | 0 | 0 |
| hsa_circ_0030529 | 92 | 443 | 1221 | 237 | 1015 | 0 | 0 |
| hsa_circ_0030529 | 92 | 458 | 1236 | 237 | 1015 | 0 | 0 |
| hsa_circ_0030529 | 92 | 3 | 498 | 237 | 732 | 0 | 0 |
| hsa_circ_0000497 | 92 | 1 | 648 | 141 | 788 | 0 | 0 |
| hsa_circ_0000497 | 92 | 145 | 794 | 139 | 788 | 0 | 0 |
| hsa_circ_0000497 | 92 | 228 | 877 | 139 | 788 | 0 | 0 |
| hsa_circ_0000497 | 92 | 289 | 938 | 139 | 788 | 0 | 0 |
| hsa_circ_0000497 | 92 | 304 | 953 | 139 | 788 | 0 | 0 |
| hsa_circ_0000497 | 92 | 145 | 638 | 139 | 632 | 0 | 0 |
| hsa_circ_0000497 | 92 | 1 | 492 | 141 | 632 | 0 | 0 |
| hsa_circ_0000497 | 92 | 289 | 782 | 139 | 632 | 0 | 0 |
| hsa_circ_0030529 | 93 | 147 | 300 | 1 | 154 | 0 | 2.00E-57 |
| hsa_circ_0000497 | 93 | 289 | 440 | 139 | 290 | 0 | 4.00E-56 |
| hsa_circ_0030516 | 95 | 1 | 370 | 2561 | 2192 | 0 | 1.00E-165 |
| hsa_circ_0030520 | 95 | 1 | 822 | 1989 | 1168 | 0 | 0 |
| hsa_circ_0030520 | 95 | 1 | 978 | 1989 | 1012 | 0 | 0 |
| hsa_circ_0030520 | 95 | 1 | 978 | 1989 | 1012 | 0 | 0 |
| hsa_circ_0030520 | 95 | 1 | 625 | 1792 | 1168 | 0 | 0 |
| hsa_circ_0030524 | 95 | 1 | 310 | 387 | 78 | 0 | 4.00E-134 |
| hsa_circ_0030520 | 96 | 467 | 1148 | 1038 | 357 | 0 | 0 |
| hsa_circ_0030504 | 95 | 467 | 3486 | 3242 | 223 | 0 | 0 |
| hsa_circ_0030520 | 95 | 370 | 1299 | 1038 | 109 | 0 | 0 |
| hsa_circ_0030520 | 96 | 157 | 838 | 1038 | 357 | 0 | 0 |
| hsa_circ_0030520 | 95 | 1 | 364 | 720 | 357 | 0 | 1.00E-160 |
| hsa_circ_0030516 | 95 | 1 | 763 | 763 | 1 | 0 | 0 |
| hsa_circ_0030512 | 95 | 1 | 881 | 881 | 1 | 0 | 0 |
| hsa_circ_0030522 | 94 | 1 | 248 | 248 | 1 | 0 | 5.00E-103 |
| hsa_circ_0030516 | 95 | 1 | 558 | 558 | 1 | 0 | 0 |
| hsa_circ_0030509 | 94 | 1 | 1062 | 1316 | 255 | 0 | 0 |
| hsa_circ_0030506 | 94 | 1 | 1408 | 1917 | 510 | 0 | 0 |
| hsa_circ_0030504 | 94 | 1 | 2192 | 2414 | 223 | 0 | 0 |
| hsa_circ_0030516 | 95 | 1 | 310 | 310 | 1 | 0 | 4.00E-139 |
| hsa_circ_0030512 | 95 | 1 | 320 | 320 | 1 | 0 | 1.00E-139 |

novel_circ_000465 novel_circ_000466 novel_circ_000467 novel_circ_000468 novel_circ_000469 novel_circ_000470 novel_circ_000471 novel_circ_000472 novel_circ_000473 novel_circ_000474 novel_circ_000475 novel_circ_000476 novel_circ_000477 novel_circ_000478 novel_circ_000479 novel_circ_000480 novel_circ_000481 novel_circ_000482 novel_circ_000483 novel_circ_000484 novel_circ_000485 novel_circ_000486 novel_circ_000487 novel_circ_000488 novel_circ_000489 novel_circ_000490 novel_circ_000491 novel_circ_000492 novel_circ_000493 novel_circ_000494 novel_circ_000495 novel_circ_000496 novel_circ_000497 novel_circ_000498 novel_circ_000499 novel_circ_000500 novel_circ_000501 novel_circ_000502 novel_circ_000503 novel_circ_000504 novel_circ_000505 novel_circ_000506 novel_circ_000507 novel_circ_000508 novel_circ_000509 novel_circ_000510 novel_circ_000511 novel_circ_000512 novel_circ_000513 novel_circ_000514 novel_circ_000515 novel_circ_000516 novel_circ_000520 novel_circ_000521

| hsa_circ_0030498 | 93 | 1 | 4178 | 4178 | 1 | 0 | 0 |
| --- | --- | --- | --- | --- | --- | --- | --- |
| hsa_circ_0030492 | 93 | 1 | 4922 | 4922 | 1 | 0 | 0 |
| hsa_circ_0030509 | 94 | 1 | 359 | 786 | 428 | 0 | 1.00E-154 |
| hsa_circ_0030506 | 92 | 1 | 519 | 1028 | 510 | 0 | 0 |
| hsa_circ_0030506 | 92 | 1 | 835 | 1028 | 194 | 0 | 0 |
| hsa_circ_0030504 | 94 | 1 | 1303 | 1525 | 223 | 0 | 0 |
| hsa_circ_0030509 | 93 | 1 | 254 | 254 | 1 | 0 | 5.00E-103 |
| hsa_circ_0030506 | 93 | 1 | 565 | 855 | 291 | 0 | 0 |
| hsa_circ_0030502 | 93 | 1 | 1544 | 1883 | 340 | 0 | 0 |
| hsa_circ_0030502 | 94 | 4 | 1290 | 1626 | 340 | 0 | 0 |
| hsa_circ_0030506 | 94 | 1 | 316 | 509 | 194 | 0 | 5.00E-134 |
| hsa_circ_0030500 | 94 | 1 | 1541 | 2179 | 639 | 0 | 0 |
| hsa_circ_0030500 | 93 | 1 | 2179 | 2179 | 1 | 0 | 0 |
| hsa_circ_0030504 | 96 | 1 | 468 | 690 | 223 | 0 | 0 |
| hsa_circ_0030499 | 93 | 1 | 1828 | 1878 | 51 | 0 | 0 |
| hsa_circ_0030498 | 93 | 1 | 1264 | 1264 | 1 | 0 | 0 |
| hsa_circ_0030497 | 94 | 1 | 215 | 216 | 2 | 0 | 4.00E-88 |
| hsa_circ_0030492 | 93 | 1 | 744 | 744 | 1 | 0 | 0 |
| hsa_circ_0030492 | 93 | 1 | 472 | 472 | 1 | 0 | 0 |
| hsa_circ_0030492 | 92 | 1 | 356 | 356 | 1 | 0 | 3.00E-141 |
| hsa_circ_0000495 | 98 | 214 | 441 | 228 | 1 | 0 | 5.00E-110 |
| hsa_circ_0000495 | 98 | 73 | 247 | 228 | 54 | 0 | 8.00E-81 |
| hsa_circ_0000495 | 98 | 73 | 300 | 228 | 1 | 0 | 3.00E-110 |
| hsa_circ_0000495 | 98 | 73 | 300 | 228 | 1 | 0 | 6.00E-110 |
| hsa_circ_0030481 | 97 | 76 | 556 | 481 | 1 | 0 | 0 |
| hsa_circ_0030480 | 97 | 76 | 560 | 2295 | 1811 | 0 | 0 |
| hsa_circ_0030481 | 97 | 1 | 481 | 481 | 1 | 0 | 0 |
| hsa_circ_0030480 | 97 | 1 | 485 | 2295 | 1811 | 0 | 0 |
| hsa_circ_0030480 | 95 | 1 | 260 | 2070 | 1811 | 0 | 2.00E-114 |
| hsa_circ_0030480 | 93 | 1 | 1814 | 1814 | 1 | 0 | 0 |
| hsa_circ_0030475 | 94 | 1 | 569 | 921 | 353 | 0 | 0 |
| hsa_circ_0030475 | 94 | 1 | 918 | 921 | 1 | 0 | 0 |
| hsa_circ_0030474 | 93 | 1 | 1079 | 1239 | 158 | 0 | 0 |
| hsa_circ_0030475 | 93 | 1 | 505 | 508 | 1 | 0.01 | 0 |
| hsa_circ_0030472 | 94 | 1 | 1048 | 1117 | 67 | 0 | 0 |
| hsa_circ_0030472 | 94 | 1 | 1114 | 1117 | 1 | 0 | 0 |
| hsa_circ_0030472 | 94 | 1 | 893 | 959 | 67 | 0 | 0 |
| hsa_circ_0004850 | 94 | 1 | 543 | 543 | 1 | 0 | 0 |
| hsa_circ_0004850 | 96 | 1 | 382 | 382 | 1 | 0 | 1.00E-175 |
| hsa_circ_0030468 | 91 | 1 | 681 | 971 | 285 | 0.02 | 0 |
| hsa_circ_0030465 | 93 | 1 | 1546 | 1845 | 294 | 0.01 | 0 |
| hsa_circ_0030468 | 91 | 1 | 351 | 632 | 285 | 0.01 | 5.00E-129 |
| hsa_circ_0030465 | 94 | 1 | 1216 | 1506 | 294 | 0 | 0 |
| hsa_circ_0030467 | 94 | 1 | 768 | 768 | 1 | 0 | 0 |
| hsa_circ_0030465 | 95 | 1 | 484 | 777 | 294 | 0 | 0 |
| hsa_circ_0030456 | 94 | 1 | 647 | 644 | 1 | 0 | 0 |
| hsa_circ_0030455 | 89 | 2 | 392 | 2 | 392 | 0 | 6.00E-134 |
| hsa_circ_0030451 | 85 | 1205 | 2510 | 773 | 2084 | 0.01 | 0 |
| hsa_circ_0030451 | 85 | 719 | 2024 | 773 | 2084 | 0.01 | 0 |
| hsa_circ_0030454 | 84 | 1 | 997 | 1 | 1003 | 0.01 | 0 |
| hsa_circ_0029815 | 92 | 37184 | 37719 | 10 | 543 | 0 | 0 |
| hsa_circ_0030433 | 93 | 1 | 420 | 1 | 420 | 0 | 1.00E-176 |
| hsa_circ_0030430 | 86 | 1 | 786 | 910 | 134 | 0.02 | 0 |
| hsa_circ_0030430 | 87 | 1 | 919 | 910 | 1 | 0.01 | 0 |

novel_circ_000522 novel_circ_000523 novel_circ_000524 novel_circ_000525 novel_circ_000526 novel_circ_000527 novel_circ_000528 novel_circ_000529 novel_circ_000533 novel_circ_000534 novel_circ_000535 novel_circ_000536 novel_circ_000537 novel_circ_000538 novel_circ_000539 novel_circ_000540 novel_circ_000541 novel_circ_000542 novel_circ_000543 novel_circ_000544 novel_circ_000545 novel_circ_000546 novel_circ_000547 novel_circ_000548 novel_circ_000549 novel_circ_000550 novel_circ_000551 novel_circ_000552 novel_circ_000553 novel_circ_000554 novel_circ_000555 novel_circ_000556 novel_circ_000557 novel_circ_000558 novel_circ_000559 novel_circ_000560 novel_circ_000561 novel_circ_000562 novel_circ_000563 novel_circ_000570 novel_circ_000571 novel_circ_000572 novel_circ_000573 novel_circ_000574 novel_circ_000575 novel_circ_000576 novel_circ_000577 novel_circ_000578 novel_circ_000579 novel_circ_000580 novel_circ_000581 novel_circ_000583 novel_circ_000585 novel_circ_000586

| hsa_circ_0030427 | 88 | 1 | 1121 | 1535 | 424 | 0.01 | 0 |
| --- | --- | --- | --- | --- | --- | --- | --- |
| hsa_circ_0030427 | 89 | 1 | 1242 | 1535 | 303 | 0.01 | 0 |
| hsa_circ_0030427 | 89 | 1 | 1243 | 1535 | 302 | 0.01 | 0 |
| hsa_circ_0030427 | 89 | 1 | 1544 | 1535 | 1 | 0.01 | 0 |
| hsa_circ_0030430 | 92 | 4 | 328 | 325 | 1 | 0 | 2.00E-127 |
| hsa_circ_0030427 | 93 | 4 | 651 | 950 | 303 | 0 | 0 |
| hsa_circ_0030420 | 90 | 1 | 928 | 1264 | 331 | 0.01 | 0 |
| hsa_circ_0030421 | 91 | 1 | 316 | 318 | 3 | 0.01 | 1.00E-115 |
| hsa_circ_0078382 | 83 | 29964 | 30672 | 77124 | 77838 | 0.02 | 3.00E-180 |
| hsa_circ_0078370 | 93 | 1 | 1693 | 2111 | 3800 | 0.01 | 0 |
| hsa_circ_0078370 | 93 | 1 | 2607 | 1197 | 3800 | 0 | 0 |
| hsa_circ_0078370 | 93 | 1 | 3227 | 577 | 3800 | 0 | 0 |
| hsa_circ_0078370 | 93 | 3709 | 7432 | 80 | 3800 | 0 | 0 |
| hsa_circ_0078370 | 93 | 4 | 3803 | 4 | 3800 | 0 | 0 |
| hsa_circ_0078380 | 94 | 1 | 720 | 440 | 1159 | 0 | 0 |
| hsa_circ_0078376 | 93 | 1 | 1357 | 625 | 1981 | 0 | 0 |
| hsa_circ_0078370 | 93 | 3709 | 5739 | 80 | 2110 | 0 | 0 |
| hsa_circ_0078380 | 94 | 1 | 845 | 246 | 1090 | 0 | 0 |
| hsa_circ_0078380 | 94 | 1 | 700 | 246 | 945 | 0 | 0 |
| hsa_circ_0078380 | 94 | 1 | 945 | 1 | 945 | 0 | 0 |
| hsa_circ_0078370 | 94 | 4 | 1762 | 4 | 1762 | 0 | 0 |
| hsa_circ_0078377 | 93 | 2 | 360 | 82 | 440 | 0 | 3.00E-146 |
| hsa_circ_0078377 | 93 | 1 | 440 | 1 | 440 | 0 | 0 |
| hsa_circ_0078376 | 92 | 1 | 748 | 317 | 1064 | 0 | 0 |
| hsa_circ_0078370 | 93 | 3709 | 4822 | 80 | 1193 | 0 | 0 |
| hsa_circ_0078370 | 93 | 4 | 1193 | 4 | 1193 | 0 | 0 |
| hsa_circ_0078377 | 92 | 1 | 198 | 1 | 198 | 0 | 2.00E-75 |
| hsa_circ_0078376 | 93 | 1 | 822 | 1 | 822 | 0 | 0 |
| hsa_circ_0078376 | 94 | 1 | 447 | 1 | 447 | 0 | 0 |
| hsa_circ_0078370 | 94 | 3709 | 4205 | 80 | 576 | 0 | 0 |
| hsa_circ_0078370 | 96 | 3709 | 3920 | 80 | 291 | 0 | 2.00E-93 |
| hsa_circ_0078370 | 97 | 4 | 291 | 4 | 291 | 0 | 3.00E-135 |
| hsa_circ_0078338 | 88 | 1 | 825 | 834 | 13 | 0.01 | 0 |
| hsa_circ_0078338 | 86 | 1 | 655 | 664 | 13 | 0.01 | 0 |
| hsa_circ_0078338 | 84 | 1 | 581 | 590 | 13 | 0.02 | 1.00E-156 |
| hsa_circ_0078338 | 82 | 1 | 509 | 518 | 13 | 0.02 | 3.00E-118 |
| hsa_circ_0078338 | 80 | 1 | 450 | 459 | 13 | 0.02 | 2.00E-90 |
| hsa_circ_0078338 | 79 | 3 | 361 | 371 | 13 | 0.01 | 2.00E-64 |
| hsa_circ_0065012 | 81 | 13508 | 13681 | 315 | 488 | 0.01 | 3.00E-28 |
| hsa_circ_0078330 | 87 | 1 | 645 | 10206 | 9562 | 0 | 0 |
| hsa_circ_0078330 | 86 | 6 | 307 | 10022 | 9721 | 0 | 6.00E-88 |
| hsa_circ_0078330 | 87 | 6 | 466 | 10022 | 9562 | 0 | 4.00E-146 |
| hsa_circ_0078330 | 90 | 6 | 769 | 10022 | 9259 | 0 | 0 |
| hsa_circ_0078330 | 91 | 6 | 1048 | 10022 | 8980 | 0 | 0 |
| hsa_circ_0078330 | 93 | 1 | 690 | 9669 | 8980 | 0 | 0 |
| hsa_circ_0078330 | 94 | 1 | 582 | 9561 | 8980 | 0 | 0 |
| hsa_circ_0078330 | 95 | 1 | 279 | 9258 | 8980 | 0 | 7.00E-122 |
| hsa_circ_0078330 | 91 | 1 | 1095 | 8976 | 7882 | 0 | 0 |
| hsa_circ_0078330 | 91 | 1 | 1763 | 8976 | 7214 | 0 | 0 |
| hsa_circ_0078330 | 91 | 1 | 839 | 8879 | 8041 | 0 | 0 |
| hsa_circ_0078330 | 91 | 1 | 998 | 8879 | 7882 | 0 | 0 |
| hsa_circ_0078330 | 87 | 1 | 777 | 7716 | 6940 | 0 | 0 |
| hsa_circ_0078330 | 86 | 7 | 442 | 7207 | 6772 | 0 | 3.00E-127 |
| hsa_circ_0078330 | 88 | 7 | 1066 | 7207 | 6148 | 0 | 0 |

novel_circ_000587 novel_circ_000588 novel_circ_000589 novel_circ_000590 novel_circ_000591 novel_circ_000592 novel_circ_000593 novel_circ_000594 novel_circ_000595 novel_circ_000596 novel_circ_000598 novel_circ_000599 novel_circ_000600 novel_circ_000601 novel_circ_000602 novel_circ_000603 novel_circ_000604 novel_circ_000605 novel_circ_000606 novel_circ_000607 novel_circ_000608 novel_circ_000609 novel_circ_000611 novel_circ_000612 novel_circ_000613 novel_circ_000614 novel_circ_000615 novel_circ_000616 novel_circ_000617 novel_circ_000618 novel_circ_000619 novel_circ_000620 novel_circ_000621 novel_circ_000622 novel_circ_000623 novel_circ_000624 novel_circ_000625 novel_circ_000626 novel_circ_000627 novel_circ_000628 novel_circ_000629 novel_circ_000630 novel_circ_000633 novel_circ_000634 novel_circ_000636 novel_circ_000637 novel_circ_000638 novel_circ_000640 novel_circ_000641 novel_circ_000642 novel_circ_000643 novel_circ_000644 novel_circ_000645 novel_circ_000646

| hsa_circ_0078330 | 89 | 7 | 1393 | 7207 | 5821 | 0 | 0 |
| --- | --- | --- | --- | --- | --- | --- | --- |
| hsa_circ_0078330 | 88 | 7 | 2835 | 7207 | 4379 | 0 | 0 |
| hsa_circ_0078330 | 88 | 1 | 480 | 6939 | 6460 | 0 | 2.00E-158 |
| hsa_circ_0078330 | 89 | 1 | 641 | 6939 | 6299 | 0 | 0 |
| hsa_circ_0078330 | 89 | 1 | 2081 | 6459 | 4379 | 0 | 0 |
| hsa_circ_0078330 | 92 | 1 | 478 | 6298 | 5821 | 0 | 0 |
| hsa_circ_0078330 | 88 | 1 | 4342 | 6298 | 1957 | 0 | 0 |
| hsa_circ_0078330 | 88 | 1 | 2262 | 6147 | 3886 | 0 | 0 |
| hsa_circ_0078330 | 90 | 2 | 310 | 4377 | 4069 | 0 | 8.00E-112 |
| hsa_circ_0078330 | 88 | 2 | 1007 | 4377 | 3372 | 0 | 0 |
| hsa_circ_0078330 | 90 | 3 | 475 | 3369 | 2897 | 0 | 9.00E-173 |
| hsa_circ_0078330 | 89 | 3 | 940 | 3369 | 2432 | 0 | 0 |
| hsa_circ_0078330 | 89 | 3 | 1415 | 3369 | 1957 | 0 | 0 |
| hsa_circ_0078330 | 89 | 1 | 621 | 3052 | 2432 | 0.01 | 0 |
| hsa_circ_0078330 | 90 | 1 | 1147 | 2431 | 1285 | 0 | 0 |
| hsa_circ_0078330 | 90 | 1 | 672 | 1956 | 1285 | 0 | 0 |
| hsa_circ_0078330 | 92 | 4 | 315 | 1596 | 1285 | 0 | 4.00E-120 |
| hsa_circ_0078330 | 86 | 1 | 492 | 801 | 310 | 0.01 | 6.00E-150 |
| hsa_circ_0078326 | 89 | 4 | 318 | 7401 | 7087 | 0 | 4.00E-105 |
| hsa_circ_0078326 | 90 | 4 | 972 | 7401 | 6433 | 0 | 0 |
| hsa_circ_0078326 | 88 | 4 | 6288 | 7401 | 1117 | 0 | 0 |
| hsa_circ_0078326 | 91 | 4 | 321 | 6429 | 6112 | 0 | 8.00E-117 |
| hsa_circ_0078326 | 90 | 1 | 448 | 6111 | 5664 | 0 | 3.00E-167 |
| hsa_circ_0078326 | 88 | 1 | 443 | 5661 | 5219 | 0 | 4.00E-151 |
| hsa_circ_0078326 | 88 | 1 | 2604 | 5661 | 3058 | 0 | 0 |
| hsa_circ_0078326 | 86 | 1 | 695 | 3057 | 2363 | 0 | 0 |
| hsa_circ_0078326 | 87 | 1 | 1941 | 3057 | 1117 | 0 | 0 |
| hsa_circ_0078326 | 88 | 1 | 579 | 2355 | 1777 | 0 | 0 |
| hsa_circ_0078326 | 88 | 1 | 1053 | 2355 | 1303 | 0 | 0 |
| hsa_circ_0078326 | 89 | 4 | 660 | 1773 | 1117 | 0 | 0 |
| hsa_circ_0078326 | 89 | 4 | 774 | 1437 | 667 | 0 | 0 |
| hsa_circ_0078326 | 89 | 1 | 636 | 1302 | 667 | 0 | 0 |
| hsa_circ_0078326 | 89 | 1 | 450 | 1116 | 667 | 0 | 1.00E-156 |
| hsa_circ_0078323 | 90 | 4 | 1179 | 1368 | 193 | 0 | 0 |
| hsa_circ_0078326 | 94 | 1 | 336 | 666 | 331 | 0 | 2.00E-143 |
| hsa_circ_0078326 | 94 | 1 | 337 | 666 | 330 | 0 | 3.00E-143 |
| hsa_circ_0078326 | 92 | 1 | 666 | 666 | 1 | 0 | 0 |
| hsa_circ_0078323 | 90 | 1 | 1227 | 1227 | 1 | 0 | 0 |
| hsa_circ_0078326 | 90 | 1 | 330 | 330 | 1 | 0 | 2.00E-118 |
| hsa_circ_0078323 | 88 | 1 | 699 | 891 | 193 | 0 | 0 |
| hsa_circ_0078323 | 88 | 1 | 891 | 891 | 1 | 0 | 0 |
| hsa_circ_0078323 | 89 | 1 | 723 | 723 | 1 | 0 | 0 |
| hsa_circ_0078320 | 88 | 4 | 960 | 1455 | 499 | 0 | 0 |
| hsa_circ_0078315 | 89 | 4 | 1680 | 4574 | 2898 | 0 | 0 |
| hsa_circ_0078320 | 88 | 1 | 596 | 1263 | 668 | 0 | 0 |
| hsa_circ_0078320 | 89 | 1 | 1053 | 1263 | 211 | 0 | 0 |
| hsa_circ_0078315 | 89 | 1 | 1485 | 4382 | 2898 | 0 | 0 |
| hsa_circ_0078320 | 90 | 1 | 288 | 498 | 211 | 0 | 2.00E-103 |
| hsa_circ_0078318 | 90 | 1 | 720 | 1740 | 1021 | 0 | 0 |
| hsa_circ_0078318 | 89 | 1 | 507 | 1020 | 514 | 0 | 0 |
| hsa_circ_0078317 | 90 | 1 | 1503 | 1964 | 462 | 0 | 0 |
| hsa_circ_0078317 | 89 | 1 | 663 | 1457 | 795 | 0 | 0 |
| hsa_circ_0078317 | 90 | 1 | 996 | 1457 | 462 | 0 | 0 |
| hsa_circ_0078317 | 89 | 1 | 466 | 1260 | 795 | 0 | 3.00E-167 |

novel_circ_000647 novel_circ_000648 novel_circ_000649 novel_circ_000650 novel_circ_000651 novel_circ_000652 novel_circ_000654 novel_circ_000655 novel_circ_000656 novel_circ_000657 novel_circ_000658 novel_circ_000659 novel_circ_000660 novel_circ_000661 novel_circ_000662 novel_circ_000663 novel_circ_000664 novel_circ_000665 novel_circ_000666 novel_circ_000667 novel_circ_000668 novel_circ_000669 novel_circ_000670 novel_circ_000671 novel_circ_000672 novel_circ_000673 novel_circ_000674 novel_circ_000675 novel_circ_000676 novel_circ_000677 novel_circ_000680 novel_circ_000682 novel_circ_000683 novel_circ_000684 novel_circ_000685 novel_circ_000688 novel_circ_000689 novel_circ_000690 novel_circ_000691 novel_circ_000694 novel_circ_000695 novel_circ_000696 novel_circ_000697 novel_circ_000698 novel_circ_000699 novel_circ_000700 novel_circ_000701 novel_circ_000702 novel_circ_000703 novel_circ_000704 novel_circ_000705 novel_circ_000706 novel_circ_000707 novel_circ_000708

| hsa_circ_0078317 | 92 | 1 | 333 | 794 | 462 | 0 | 2.00E-128 |
| --- | --- | --- | --- | --- | --- | --- | --- |
| hsa_circ_0078317 | 91 | 1 | 660 | 794 | 135 | 0 | 0 |
| hsa_circ_0078314 | 89 | 1 | 1947 | 3625 | 1676 | 0 | 0 |
| hsa_circ_0078314 | 90 | 1 | 2273 | 3625 | 1350 | 0 | 0 |
| hsa_circ_0078314 | 90 | 1 | 2274 | 3625 | 1349 | 0 | 0 |
| hsa_circ_0078314 | 89 | 3 | 1776 | 3452 | 1676 | 0 | 0 |
| hsa_circ_0078315 | 88 | 1 | 633 | 728 | 96 | 0 | 0 |
| hsa_circ_0078314 | 90 | 1 | 963 | 3115 | 2153 | 0 | 0 |
| hsa_circ_0078314 | 91 | 1 | 1767 | 3115 | 1349 | 0 | 0 |
| hsa_circ_0078314 | 91 | 1 | 975 | 2932 | 1958 | 0 | 0 |
| hsa_circ_0078314 | 91 | 1 | 1416 | 2932 | 1517 | 0 | 0 |
| hsa_circ_0078314 | 92 | 1 | 1584 | 2932 | 1349 | 0 | 0 |
| hsa_circ_0078314 | 92 | 1 | 525 | 2482 | 1958 | 0 | 0 |
| hsa_circ_0078314 | 92 | 1 | 651 | 2482 | 1832 | 0 | 0 |
| hsa_circ_0078314 | 91 | 1 | 556 | 2387 | 1832 | 0 | 0 |
| hsa_circ_0078314 | 91 | 1 | 871 | 2387 | 1517 | 0 | 0 |
| hsa_circ_0078314 | 92 | 1 | 1038 | 2387 | 1350 | 0 | 0 |
| hsa_circ_0078314 | 92 | 1 | 1039 | 2387 | 1349 | 0 | 0 |
| hsa_circ_0078314 | 90 | 1 | 636 | 2152 | 1517 | 0 | 0 |
| hsa_circ_0078314 | 91 | 1 | 803 | 2152 | 1350 | 0 | 0 |
| hsa_circ_0078314 | 91 | 1 | 804 | 2152 | 1349 | 0 | 0 |
| hsa_circ_0078314 | 92 | 995 | 1819 | 1351 | 527 | 0 | 0 |
| hsa_circ_0078314 | 92 | 1 | 608 | 1957 | 1350 | 0 | 0 |
| hsa_circ_0078314 | 92 | 800 | 1624 | 1351 | 527 | 0 | 0 |
| hsa_circ_0078314 | 92 | 359 | 862 | 1351 | 848 | 0 | 0 |
| hsa_circ_0078314 | 91 | 1 | 339 | 1186 | 848 | 0.01 | 4.00E-130 |
| hsa_circ_0078314 | 91 | 1 | 321 | 847 | 527 | 0 | 8.00E-122 |
| hsa_circ_0078313 | 90 | 4 | 1620 | 1617 | 1 | 0 | 0 |
| hsa_circ_0078313 | 90 | 1 | 882 | 882 | 1 | 0 | 0 |
| hsa_circ_0078313 | 93 | 3 | 342 | 340 | 1 | 0 | 2.00E-137 |
| hsa_circ_0078308 | 88 | 1 | 517 | 301 | 817 | 0 | 2.00E-175 |
| hsa_circ_0078305 | 85 | 4 | 1019 | 1013 | 1 | 0 | 0 |
| hsa_circ_0078305 | 91 | 7 | 498 | 492 | 1 | 0 | 0 |
| hsa_circ_0078303 | 92 | 1 | 471 | 471 | 1 | 0 | 0 |
| hsa_circ_0078299 | 77 | 43 | 2226 | 4136 | 1914 | 0.03 | 0 |
| hsa_circ_0078296 | 88 | 1 | 547 | 1 | 535 | 0.02 | 0 |
| hsa_circ_0078281 | 90 | 1 | 742 | 205 | 946 | 0 | 0 |
| hsa_circ_0078281 | 89 | 1 | 476 | 1 | 476 | 0 | 9.00E-168 |
| hsa_circ_0078266 | 89 | 1 | 553 | 372 | 924 | 0 | 0 |
| hsa_circ_0003249 | 85 | 1 | 639 | 639 | 1 | 0.01 | 0 |
| hsa_circ_0078249 | 92 | 1 | 651 | 1 | 651 | 0 | 0 |
| hsa_circ_0078249 | 93 | 1 | 448 | 1 | 448 | 0 | 0 |
| hsa_circ_0078249 | 94 | 1 | 363 | 1 | 363 | 0 | 2.00E-153 |
| hsa_circ_0078249 | 95 | 1 | 242 | 1 | 242 | 0 | 1.00E-104 |
| hsa_circ_0078249 | 95 | 1 | 137 | 1 | 137 | 0 | 5.00E-55 |
| hsa_circ_0002101 | 89 | 6 | 498 | 483 | 1 | 0.02 | 9.00E-168 |
| hsa_circ_0078222 | 90 | 6 | 646 | 4342 | 3712 | 0.02 | 0 |
| hsa_circ_0078222 | 93 | 6 | 3030 | 4342 | 1325 | 0 | 0 |
| hsa_circ_0078226 | 96 | 1 | 873 | 873 | 1 | 0 | 0 |
| hsa_circ_0078226 | 95 | 1 | 290 | 290 | 1 | 0 | 3.00E-126 |
| hsa_circ_0078218 | 92 | 1 | 333 | 333 | 1 | 0 | 8.00E-132 |
| hsa_circ_0078218 | 92 | 1 | 333 | 333 | 1 | 0 | 1.00E-131 |
| hsa_circ_0078216 | 93 | 515 | 901 | 387 | 1 | 0 | 3.00E-161 |
| hsa_circ_0078216 | 93 | 515 | 901 | 387 | 1 | 0 | 3.00E-161 |

novel_circ_000709 novel_circ_000710 novel_circ_000711 novel_circ_000712 novel_circ_000713 novel_circ_000714 novel_circ_000715 novel_circ_000716 novel_circ_000717 novel_circ_000718 novel_circ_000719 novel_circ_000720 novel_circ_000721 novel_circ_000722 novel_circ_000723 novel_circ_000724 novel_circ_000725 novel_circ_000726 novel_circ_000727 novel_circ_000728 novel_circ_000732 novel_circ_000733 novel_circ_000734 novel_circ_000735 novel_circ_000736 novel_circ_000739 novel_circ_000740 novel_circ_000742 novel_circ_000744 novel_circ_000745 novel_circ_000746 novel_circ_000747 novel_circ_000748 novel_circ_000749 novel_circ_000750 novel_circ_000751 novel_circ_000752 novel_circ_000753 novel_circ_000754 novel_circ_000755 novel_circ_000756 novel_circ_000757 novel_circ_000758 novel_circ_000759 novel_circ_000760 novel_circ_000761 novel_circ_000762 novel_circ_000763 novel_circ_000764 novel_circ_000765 novel_circ_000766 novel_circ_000767 novel_circ_000768 novel_circ_000769

| hsa_circ_0078216 | 93 | 515 | 901 | 387 | 1 | 0 | 3.00E-161 |
| --- | --- | --- | --- | --- | --- | --- | --- |
| hsa_circ_0078217 | 90 | 1 | 339 | 339 | 1 | 0 | 5.00E-120 |
| hsa_circ_0078216 | 93 | 182 | 568 | 387 | 1 | 0 | 2.00E-161 |
| hsa_circ_0078216 | 93 | 1 | 387 | 387 | 1 | 0 | 2.00E-161 |
| hsa_circ_0078216 | 93 | 1 | 387 | 387 | 1 | 0 | 2.00E-161 |
| hsa_circ_0078216 | 93 | 1 | 265 | 265 | 1 | 0 | 2.00E-107 |
| hsa_circ_0078216 | 93 | 1 | 265 | 265 | 1 | 0 | 4.00E-107 |
| hsa_circ_0078211 | 90 | 5 | 767 | 5 | 761 | 0.01 | 0 |
| hsa_circ_0001650 | 89 | 1 | 446 | 1 | 440 | 0.01 | 8.00E-153 |
| hsa_circ_0078211 | 91 | 5 | 466 | 5 | 466 | 0 | 5.00E-175 |
| hsa_circ_0078206 | 92 | 1 | 251 | 912 | 662 | 0 | 5.00E-98 |
| hsa_circ_0078206 | 92 | 1 | 254 | 912 | 659 | 0 | 5.00E-98 |
| hsa_circ_0078206 | 91 | 1 | 406 | 912 | 519 | 0.03 | 6.00E-149 |
| hsa_circ_0078206 | 92 | 1 | 620 | 912 | 305 | 0.02 | 0 |
| hsa_circ_0078206 | 93 | 1 | 745 | 912 | 180 | 0.02 | 0 |
| hsa_circ_0078204 | 92 | 69 | 1169 | 1089 | 1 | 0.01 | 0 |
| hsa_circ_0078207 | 95 | 1 | 482 | 482 | 1 | 0 | 0 |
| hsa_circ_0078204 | 91 | 1 | 549 | 549 | 1 | 0 | 0 |
| hsa_circ_0078204 | 90 | 1 | 424 | 424 | 1 | 0 | 1.00E-155 |
| hsa_circ_0078204 | 90 | 1 | 357 | 357 | 1 | 0 | 9.00E-127 |
| hsa_circ_0085375 | 95 | 1 | 473 | 633 | 161 | 0 | 0 |
| hsa_circ_0085375 | 94 | 1 | 680 | 840 | 161 | 0 | 0 |
| hsa_circ_0085375 | 94 | 1 | 1017 | 1177 | 161 | 0 | 0 |
| hsa_circ_0085375 | 94 | 1 | 1192 | 1353 | 161 | 0 | 0 |
| hsa_circ_0004116 | 95 | 1 | 264 | 1 | 264 | 0 | 6.00E-117 |
| hsa_circ_0085386 | 90 | 1 | 324 | 67 | 390 | 0 | 2.00E-118 |
| hsa_circ_0085386 | 90 | 1 | 324 | 67 | 390 | 0 | 2.00E-118 |
| hsa_circ_0085387 | 99 | 1760 | 1917 | 267 | 110 | 0 | 2.00E-75 |
| hsa_circ_0085397 | 96 | 1 | 514 | 514 | 1 | 0 | 0 |
| hsa_circ_0085397 | 96 | 1 | 1019 | 1019 | 1 | 0 | 0 |
| hsa_circ_0085396 | 96 | 1 | 1787 | 2017 | 231 | 0 | 0 |
| hsa_circ_0085396 | 95 | 1 | 2592 | 2809 | 231 | 0.01 | 0 |
| hsa_circ_0085401 | 95 | 1 | 1367 | 1577 | 211 | 0 | 0 |
| hsa_circ_0085401 | 95 | 1 | 2272 | 2469 | 211 | 0.01 | 0 |
| hsa_circ_0085401 | 95 | 1 | 1693 | 2208 | 516 | 0 | 0 |
| hsa_circ_0085404 | 94 | 1 | 253 | 394 | 142 | 0 | 2.00E-107 |
| hsa_circ_0085401 | 93 | 1 | 805 | 2469 | 1678 | 0.02 | 0 |
| hsa_circ_0085401 | 93 | 1 | 966 | 2630 | 1678 | 0.01 | 0 |
| hsa_circ_0085401 | 92 | 1 | 572 | 2350 | 1792 | 0.02 | 0 |
| hsa_circ_0085401 | 93 | 1 | 691 | 2469 | 1792 | 0.02 | 0 |
| hsa_circ_0085407 | 91 | 1 | 387 | 374 | 1 | 0.03 | 7.00E-143 |
| hsa_circ_0085407 | 92 | 1 | 506 | 493 | 1 | 0.03 | 0 |
| hsa_circ_0085413 | 94 | 243 | 473 | 1 | 231 | 0 | 3.00E-93 |
| hsa_circ_0085413 | 93 | 243 | 914 | 1 | 672 | 0 | 0 |
| hsa_circ_0057970 | 91 | 1 | 430 | 1170 | 741 | 0 | 6.00E-164 |
| hsa_circ_0057970 | 91 | 123 | 565 | 1183 | 741 | 0 | 1.00E-167 |
| hsa_circ_0057972 | 95 | 1 | 625 | 150 | 771 | 0 | 0 |
| hsa_circ_0057972 | 94 | 1 | 923 | 150 | 1069 | 0.01 | 0 |
| hsa_circ_0057972 | 94 | 1 | 1062 | 150 | 1208 | 0 | 0 |
| hsa_circ_0057972 | 94 | 1 | 1220 | 150 | 1366 | 0 | 0 |
| hsa_circ_0057972 | 94 | 1 | 1480 | 150 | 1626 | 0 | 0 |
| hsa_circ_0057972 | 95 | 1 | 1648 | 150 | 1794 | 0 | 0 |
| hsa_circ_0057972 | 95 | 1 | 1648 | 150 | 1794 | 0 | 0 |
| hsa_circ_0057972 | 95 | 1 | 1648 | 150 | 1794 | 0 | 0 |

novel_circ_000770 novel_circ_000771 novel_circ_000772 novel_circ_000773 novel_circ_000774 novel_circ_000775 novel_circ_000776 novel_circ_000777 novel_circ_000778 novel_circ_000779 novel_circ_000780 novel_circ_000781 novel_circ_000782 novel_circ_000783 novel_circ_000784 novel_circ_000785 novel_circ_000786 novel_circ_000787 novel_circ_000788 novel_circ_000789 novel_circ_000799 novel_circ_000801 novel_circ_000803 novel_circ_000804 novel_circ_000805 novel_circ_000806 novel_circ_000807 novel_circ_000808 novel_circ_000809 novel_circ_000810 novel_circ_000811 novel_circ_000812 novel_circ_000813 novel_circ_000814 novel_circ_000815 novel_circ_000816 novel_circ_000817 novel_circ_000818 novel_circ_000819 novel_circ_000820 novel_circ_000821 novel_circ_000822 novel_circ_000823 novel_circ_000824 novel_circ_000825 novel_circ_000826 novel_circ_000827 novel_circ_000828 novel_circ_000829 novel_circ_000830 novel_circ_000831 novel_circ_000832 novel_circ_000833 novel_circ_000834

| hsa_circ_0057972 | 95 | 1 | 1648 | 150 | 1794 | 0 | 0 |
| --- | --- | --- | --- | --- | --- | --- | --- |
| hsa_circ_0057972 | 95 | 1 | 1648 | 150 | 1794 | 0 | 0 |
| hsa_circ_0057972 | 95 | 1 | 1648 | 150 | 1794 | 0 | 0 |
| hsa_circ_0057979 | 95 | 628 | 2339 | 3 | 1714 | 0 | 0 |
| hsa_circ_0057976 | 95 | 1 | 881 | 1 | 878 | 0.01 | 0 |
| hsa_circ_0057976 | 98 | 1 | 291 | 151 | 441 | 0 | 1.00E-143 |
| hsa_circ_0057976 | 96 | 1 | 728 | 151 | 878 | 0 | 0 |
| hsa_circ_0057972 | 96 | 1 | 1314 | 481 | 1794 | 0 | 0 |
| hsa_circ_0057972 | 96 | 1 | 1314 | 481 | 1794 | 0 | 0 |
| hsa_circ_0057979 | 95 | 294 | 2005 | 3 | 1714 | 0 | 0 |
| hsa_circ_0057977 | 95 | 1 | 470 | 1 | 470 | 0 | 0 |
| hsa_circ_0057976 | 95 | 1 | 609 | 270 | 878 | 0 | 0 |
| hsa_circ_0057986 | 94 | 1 | 190 | 1 | 190 | 0 | 1.00E-77 |
| hsa_circ_0057986 | 95 | 1 | 554 | 1 | 554 | 0 | 0 |
| hsa_circ_0057984 | 92 | 1 | 2327 | 317 | 2643 | 0 | 0 |
| hsa_circ_0057984 | 92 | 1 | 2881 | 317 | 3197 | 0 | 0 |
| hsa_circ_0057984 | 91 | 1 | 3159 | 317 | 3490 | 0.01 | 0 |
| hsa_circ_0057984 | 92 | 1 | 3723 | 317 | 4054 | 0.01 | 0 |
| hsa_circ_0057988 | 95 | 1 | 554 | 346 | 899 | 0 | 0 |
| hsa_circ_0057989 | 90 | 1 | 653 | 1 | 668 | 0.03 | 0 |
| hsa_circ_0057996 | 92 | 1279 | 1652 | 3 | 376 | 0 | 2.00E-145 |
| hsa_circ_0057996 | 92 | 922 | 1295 | 3 | 376 | 0 | 1.00E-145 |
| hsa_circ_0057996 | 91 | 627 | 828 | 3 | 204 | 0 | 3.00E-70 |
| hsa_circ_0057996 | 92 | 627 | 1000 | 3 | 376 | 0 | 9.00E-146 |
| hsa_circ_0057996 | 91 | 627 | 1214 | 3 | 590 | 0 | 0 |
| hsa_circ_0057996 | 92 | 627 | 1469 | 3 | 845 | 0 | 0 |
| hsa_circ_0057996 | 92 | 627 | 1469 | 3 | 845 | 0 | 0 |
| hsa_circ_0057999 | 91 | 2054 | 3234 | 648 | 1831 | 0 | 0 |
| hsa_circ_0057996 | 91 | 478 | 679 | 3 | 204 | 0 | 2.00E-70 |
| hsa_circ_0057996 | 92 | 478 | 851 | 3 | 376 | 0 | 8.00E-146 |
| hsa_circ_0057996 | 91 | 478 | 1065 | 3 | 590 | 0 | 0 |
| hsa_circ_0057996 | 92 | 212 | 585 | 3 | 376 | 0 | 5.00E-146 |
| hsa_circ_0057999 | 92 | 1 | 468 | 1 | 468 | 0 | 0 |
| hsa_circ_0057999 | 92 | 1 | 468 | 1 | 468 | 0 | 0 |
| hsa_circ_0057999 | 92 | 1 | 468 | 1 | 468 | 0 | 0 |
| hsa_circ_0057999 | 92 | 335 | 501 | 483 | 649 | 0 | 8.00E-61 |
| hsa_circ_0057999 | 90 | 369 | 671 | 648 | 950 | 0 | 2.00E-106 |
| hsa_circ_0058000 | 91 | 1 | 630 | 449 | 1081 | 0 | 0 |
| hsa_circ_0058003 | 90 | 1 | 316 | 1 | 319 | 0.01 | 1.00E-114 |
| hsa_circ_0058003 | 93 | 1 | 648 | 1 | 651 | 0 | 0 |
| hsa_circ_0058003 | 93 | 1 | 1940 | 1 | 1943 | 0 | 0 |
| hsa_circ_0058003 | 95 | 1 | 464 | 188 | 651 | 0 | 0 |
| hsa_circ_0058003 | 95 | 1 | 743 | 188 | 930 | 0 | 0 |
| hsa_circ_0058003 | 95 | 1 | 461 | 191 | 651 | 0 | 0 |
| hsa_circ_0058003 | 95 | 1 | 740 | 191 | 930 | 0 | 0 |
| hsa_circ_0058003 | 95 | 1 | 611 | 320 | 930 | 0 | 0 |
| hsa_circ_0058003 | 95 | 1 | 467 | 464 | 930 | 0 | 0 |
| hsa_circ_0058003 | 95 | 1 | 366 | 565 | 930 | 0 | 2.00E-163 |
| hsa_circ_0058003 | 93 | 1 | 821 | 1123 | 1943 | 0 | 0 |
| hsa_circ_0058003 | 94 | 1 | 236 | 1708 | 1943 | 0 | 5.00E-98 |
| hsa_circ_0058003 | 95 | 1 | 264 | 1944 | 2207 | 0 | 3.00E-115 |
| hsa_circ_0058003 | 93 | 1 | 519 | 1944 | 2462 | 0 | 0 |
| hsa_circ_0058003 | 95 | 1 | 331 | 2914 | 3244 | 0 | 5.00E-144 |
| hsa_circ_0057999 | 95 | 5 | 624 | 4946 | 5565 | 0 | 0 |

novel_circ_000835 novel_circ_000836 novel_circ_000837 novel_circ_000838 novel_circ_000839 novel_circ_000841 novel_circ_000842 novel_circ_000843 novel_circ_000844 novel_circ_000845 novel_circ_000846 novel_circ_000847 novel_circ_000848 novel_circ_000849 novel_circ_000850 novel_circ_000851 novel_circ_000852 novel_circ_000853 novel_circ_000854 novel_circ_000857 novel_circ_000858 novel_circ_000859 novel_circ_000860 novel_circ_000868 novel_circ_000869 novel_circ_000870 novel_circ_000871 novel_circ_000872 novel_circ_000873 novel_circ_000874 novel_circ_000875 novel_circ_000876 novel_circ_000877 novel_circ_000879 novel_circ_000884 novel_circ_000885 novel_circ_000886 novel_circ_000887 novel_circ_000888 novel_circ_000889 novel_circ_000890 novel_circ_000891 novel_circ_000892 novel_circ_000893 novel_circ_000894 novel_circ_000895 novel_circ_000896 novel_circ_000897 novel_circ_000898 novel_circ_000899 novel_circ_000900 novel_circ_000901 novel_circ_000902 novel_circ_000903

| hsa_circ_0007225 | 90 | 216 | 715 | 190 | 697 | 0.03 | 0 |
| --- | --- | --- | --- | --- | --- | --- | --- |
| hsa_circ_0007225 | 83 | 216 | 1183 | 190 | 1152 | 0.06 | 0 |
| hsa_circ_0007225 | 83 | 216 | 1183 | 190 | 1152 | 0.06 | 0 |
| hsa_circ_0007225 | 90 | 136 | 635 | 190 | 697 | 0.03 | 0 |
| hsa_circ_0058008 | 91 | 1 | 370 | 367 | 1 | 0.02 | 4.00E-136 |
| hsa_circ_0058008 | 90 | 1 | 715 | 846 | 129 | 0.02 | 0 |
| hsa_circ_0058008 | 92 | 341 | 830 | 968 | 476 | 0.01 | 0 |
| hsa_circ_0058008 | 94 | 27566 | 27772 | 848 | 642 | 0 | 4.00E-83 |
| hsa_circ_0058008 | 94 | 1 | 327 | 968 | 642 | 0 | 8.00E-142 |
| hsa_circ_0058008 | 94 | 1262 | 1588 | 968 | 642 | 0 | 4.00E-141 |
| hsa_circ_0058010 | 89 | 1 | 462 | 462 | 1 | 0 | 7.00E-165 |
| hsa_circ_0058010 | 90 | 1 | 1576 | 1579 | 1 | 0 | 0 |
| hsa_circ_0002617 | 87 | 1 | 340 | 340 | 1 | 0.01 | 2.00E-107 |
| hsa_circ_0002617 | 89 | 1 | 1454 | 1457 | 1 | 0 | 0 |
| hsa_circ_0002822 | 90 | 1 | 1235 | 1238 | 1 | 0 | 0 |
| hsa_circ_0002822 | 90 | 1 | 1256 | 1259 | 1 | 0 | 0 |
| hsa_circ_0002822 | 90 | 1 | 1121 | 1259 | 137 | 0.01 | 0 |
| hsa_circ_0008459 | 90 | 1 | 1114 | 1117 | 1 | 0 | 0 |
| hsa_circ_0058013 | 92 | 8 | 250 | 243 | 1 | 0 | 1.00E-93 |
| hsa_circ_0058031 | 91 | 1 | 541 | 541 | 1 | 0 | 0 |
| hsa_circ_0058031 | 92 | 1 | 355 | 541 | 187 | 0 | 2.00E-137 |
| hsa_circ_0058031 | 92 | 76 | 430 | 541 | 187 | 0 | 3.00E-137 |
| hsa_circ_0058031 | 92 | 703 | 1057 | 541 | 187 | 0 | 7.00E-137 |
| hsa_circ_0058041 | 90 | 102 | 353 | 6 | 257 | 0 | 2.00E-88 |
| hsa_circ_0058041 | 90 | 102 | 649 | 6 | 553 | 0 | 0 |
| hsa_circ_0058041 | 89 | 102 | 748 | 6 | 652 | 0 | 0 |
| hsa_circ_0058041 | 90 | 6 | 553 | 6 | 553 | 0 | 0 |
| hsa_circ_0058041 | 89 | 6 | 652 | 6 | 652 | 0 | 0 |
| hsa_circ_0058041 | 89 | 1 | 434 | 120 | 553 | 0 | 8.00E-148 |
| hsa_circ_0058041 | 88 | 1 | 533 | 120 | 652 | 0 | 2.00E-179 |
| hsa_circ_0058041 | 89 | 1 | 395 | 258 | 652 | 0.01 | 2.00E-134 |
| hsa_circ_0071629 | 89 | 1 | 476 | 812 | 1287 | 0 | 9.00E-168 |
| hsa_circ_0071629 | 88 | 1 | 357 | 931 | 1287 | 0 | 2.00E-118 |
| hsa_circ_0071647 | 88 | 1 | 1021 | 1 | 1021 | 0 | 0 |
| hsa_circ_0077500 | 96 | 1 | 226 | 562 | 787 | 0 | 1.00E-98 |
| hsa_circ_0077500 | 96 | 1 | 226 | 562 | 787 | 0 | 7.00E-99 |
| hsa_circ_0077500 | 96 | 1 | 226 | 562 | 787 | 0 | 3.00E-99 |
| hsa_circ_0077500 | 98 | 1 | 561 | 1 | 561 | 0 | 0 |
| hsa_circ_0077498 | 97 | 1 | 768 | 1611 | 2378 | 0 | 0 |
| hsa_circ_0077498 | 95 | 1 | 321 | 1497 | 1817 | 0 | 4.00E-140 |
| hsa_circ_0077498 | 95 | 1 | 573 | 1245 | 1817 | 0 | 0 |
| hsa_circ_0004589 | 96 | 1 | 366 | 1 | 366 | 0 | 9.00E-167 |
| hsa_circ_0004589 | 94 | 1 | 252 | 1 | 252 | 0 | 6.00E-107 |
| hsa_circ_0077499 | 96 | 1 | 410 | 1 | 410 | 0 | 0 |
| hsa_circ_0077498 | 96 | 1 | 836 | 409 | 1244 | 0 | 0 |
| hsa_circ_0004139 | 95 | 1 | 426 | 1 | 426 | 0 | 0 |
| hsa_circ_0013210 | 95 | 1 | 487 | 797 | 1283 | 0 | 0 |
| hsa_circ_0013209 | 96 | 1 | 1246 | 266 | 1511 | 0 | 0 |
| hsa_circ_0013209 | 96 | 117 | 1627 | 1 | 1511 | 0 | 0 |
| hsa_circ_0013209 | 94 | 117 | 381 | 1 | 265 | 0 | 1.00E-110 |
| hsa_circ_0000093 | 94 | 1 | 378 | 1395 | 1772 | 0 | 9.00E-162 |
| hsa_circ_0013193 | 97 | 1 | 289 | 628 | 916 | 0 | 1.00E-134 |
| hsa_circ_0013193 | 98 | 1 | 916 | 1 | 916 | 0 | 0 |
| hsa_circ_0013193 | 98 | 1 | 627 | 1 | 627 | 0 | 0 |

novel_circ_000905 novel_circ_000906 novel_circ_000907 novel_circ_000908 novel_circ_000909 novel_circ_000910 novel_circ_000911 novel_circ_000912 novel_circ_000913 novel_circ_000914 novel_circ_000915 novel_circ_000916 novel_circ_000917 novel_circ_000918 novel_circ_000919 novel_circ_000922 novel_circ_000923 novel_circ_000924 novel_circ_000925 novel_circ_000926 novel_circ_000927 novel_circ_000929 novel_circ_000930 novel_circ_000931 novel_circ_000932 novel_circ_000933 novel_circ_000935 novel_circ_000936 novel_circ_000937 novel_circ_000938 novel_circ_000941 novel_circ_000942 novel_circ_000943 novel_circ_000944 novel_circ_000945 novel_circ_000946 novel_circ_000947 novel_circ_000948 novel_circ_000949 novel_circ_000950 novel_circ_000956 novel_circ_000957 novel_circ_000958 novel_circ_000959 novel_circ_000960 novel_circ_000961 novel_circ_000963 novel_circ_000965 novel_circ_000966 novel_circ_000967 novel_circ_000968 novel_circ_000969 novel_circ_000970 novel_circ_000971

| hsa_circ_0013173 | 95 | 1337 | 1828 | 492 | 1 | 0 | 0 |
| --- | --- | --- | --- | --- | --- | --- | --- |
| hsa_circ_0013173 | 95 | 279 | 770 | 492 | 1 | 0 | 0 |
| hsa_circ_0013173 | 95 | 1 | 415 | 490 | 76 | 0 | 0 |
| hsa_circ_0013173 | 95 | 1 | 490 | 490 | 1 | 0 | 0 |
| hsa_circ_0013172 | 95 | 1 | 948 | 948 | 1 | 0 | 0 |
| hsa_circ_0013168 | 95 | 1 | 1009 | 1741 | 733 | 0 | 0 |
| hsa_circ_0013168 | 96 | 1 | 519 | 1251 | 733 | 0 | 0 |
| hsa_circ_0013168 | 96 | 1 | 393 | 1125 | 733 | 0 | 0 |
| hsa_circ_0013168 | 95 | 1 | 249 | 981 | 733 | 0 | 1.00E-108 |
| hsa_circ_0013168 | 93 | 235 | 816 | 678 | 97 | 0 | 0 |
| hsa_circ_0013165 | 94 | 235 | 1008 | 774 | 1 | 0 | 0 |
| hsa_circ_0013168 | 93 | 1 | 678 | 678 | 1 | 0 | 0 |
| hsa_circ_0013165 | 94 | 1 | 774 | 774 | 1 | 0 | 0 |
| hsa_circ_0013165 | 93 | 1 | 732 | 732 | 1 | 0 | 0 |
| hsa_circ_0005123 | 92 | 8976 | 9028 | 2913 | 2965 | 0 | 2.00E-11 |
| hsa_circ_0013154 | 86 | 1 | 928 | 1 | 931 | 0.01 | 0 |
| hsa_circ_0013153 | 86 | 259 | 1218 | 5 | 967 | 0.01 | 0 |
| hsa_circ_0013153 | 86 | 374 | 1333 | 5 | 967 | 0.01 | 0 |
| hsa_circ_0013153 | 86 | 259 | 1218 | 5 | 967 | 0.01 | 0 |
| hsa_circ_0013153 | 86 | 5 | 964 | 5 | 967 | 0.01 | 0 |
| hsa_circ_0013150 | 91 | 1 | 211 | 47 | 257 | 0 | 1.00E-75 |
| hsa_circ_0013147 | 95 | 5 | 475 | 400 | 870 | 0 | 0 |
| hsa_circ_0013147 | 91 | 1 | 870 | 1 | 870 | 0 | 0 |
| hsa_circ_0013147 | 92 | 1 | 781 | 1 | 781 | 0 | 0 |
| hsa_circ_0013147 | 90 | 1 | 684 | 1 | 684 | 0 | 0 |
| hsa_circ_0013147 | 90 | 1 | 603 | 1 | 603 | 0 | 0 |
| hsa_circ_0006622 | 81 | 39 | 178 | 136 | 3 | 0.04 | 2.00E-21 |
| hsa_circ_0013132 | 87 | 1 | 1549 | 1552 | 1 | 0 | 0 |
| hsa_circ_0013127 | 93 | 1 | 845 | 399 | 1243 | 0 | 0 |
| hsa_circ_0013127 | 92 | 1 | 464 | 179 | 635 | 0.02 | 0 |
| hsa_circ_0006643 | 73 | 20851 | 21162 | 3284 | 2979 | 0.08 | 2.00E-16 |
| hsa_circ_0006643 | 73 | 20851 | 21162 | 3284 | 2979 | 0.08 | 3.00E-16 |
| hsa_circ_0013123 | 99 | 1 | 148 | 1 | 148 | 0 | 3.00E-70 |
| hsa_circ_0013120 | 90 | 1 | 2766 | 1 | 2747 | 0.02 | 0 |
| hsa_circ_0013119 | 92 | 65 | 2384 | 5 | 2318 | 0 | 0 |
| hsa_circ_0013119 | 92 | 1 | 2165 | 154 | 2318 | 0 | 0 |
| hsa_circ_0013119 | 92 | 65 | 2384 | 5 | 2318 | 0 | 0 |
| hsa_circ_0013116 | 92 | 1430 | 1534 | 2006 | 2110 | 0 | 3.00E-33 |
| hsa_circ_0062401 | 94 | 1 | 466 | 1 | 466 | 0 | 0 |
| hsa_circ_0062399 | 86 | 1 | 379 | 345 | 726 | 0.01 | 2.00E-114 |
| hsa_circ_0062428 | 88 | 1 | 419 | 1 | 419 | 0 | 2.00E-139 |
| hsa_circ_0062436 | 93 | 1 | 228 | 236 | 9 | 0 | 3.00E-90 |
| hsa_circ_0062436 | 91 | 1 | 475 | 483 | 9 | 0 | 0 |
| hsa_circ_0062436 | 92 | 1 | 592 | 600 | 9 | 0 | 0 |
| hsa_circ_0062436 | 92 | 1 | 688 | 696 | 9 | 0 | 0 |
| hsa_circ_0062436 | 93 | 1 | 965 | 973 | 9 | 0 | 0 |
| hsa_circ_0062438 | 92 | 1 | 570 | 570 | 1 | 0 | 0 |
| hsa_circ_0062439 | 91 | 1 | 364 | 364 | 1 | 0 | 1.00E-135 |
| hsa_circ_0062439 | 92 | 1 | 460 | 460 | 1 | 0 | 0 |
| hsa_circ_0062439 | 93 | 1 | 737 | 737 | 1 | 0 | 0 |
| hsa_circ_0004872 | 95 | 1 | 490 | 490 | 1 | 0 | 0 |
| hsa_circ_0008241 | 94 | 1 | 533 | 1 | 533 | 0 | 0 |
| hsa_circ_0008241 | 94 | 1 | 702 | 1 | 702 | 0 | 0 |
| hsa_circ_0008241 | 94 | 1 | 702 | 1 | 702 | 0 | 0 |

novel_circ_000972 novel_circ_000973 novel_circ_000974 novel_circ_000975 novel_circ_000976 novel_circ_000977 novel_circ_000978 novel_circ_000979 novel_circ_000982 novel_circ_000983 novel_circ_000984 novel_circ_000985 novel_circ_000986 novel_circ_000987 novel_circ_000988 novel_circ_000989 novel_circ_000990 novel_circ_000991 novel_circ_000992 novel_circ_000993 novel_circ_000994 novel_circ_000995 novel_circ_000996 novel_circ_000997 novel_circ_000998 novel_circ_000999 novel_circ_001000 novel_circ_001001 novel_circ_001002 novel_circ_001003 novel_circ_001004 novel_circ_001005 novel_circ_001006 novel_circ_001007 novel_circ_001008 novel_circ_001009 novel_circ_001010 novel_circ_001011 novel_circ_001012 novel_circ_001013 novel_circ_001014 novel_circ_001015 novel_circ_001016 novel_circ_001017 novel_circ_001018 novel_circ_001019 novel_circ_001020 novel_circ_001021 novel_circ_001022 novel_circ_001023 novel_circ_001024 novel_circ_001025 novel_circ_001026 novel_circ_001027

| hsa_circ_0004417 | 90 | 103 | 268 | 3 | 168 | 0 | 4.00E-54 |
| --- | --- | --- | --- | --- | --- | --- | --- |
| hsa_circ_0016482 | 91 | 1 | 372 | 372 | 1 | 0 | 2.00E-138 |
| hsa_circ_0016480 | 90 | 1 | 2014 | 2682 | 663 | 0 | 0 |
| hsa_circ_0016483 | 90 | 1 | 472 | 478 | 1 | 0.01 | 7.00E-174 |
| hsa_circ_0016480 | 91 | 1 | 930 | 3140 | 2205 | 0.01 | 0 |
| hsa_circ_0016484 | 93 | 1 | 573 | 576 | 1 | 0.01 | 0 |
| hsa_circ_0016485 | 94 | 6 | 321 | 316 | 1 | 0 | 5.00E-134 |
| hsa_circ_0016485 | 93 | 1 | 458 | 458 | 1 | 0 | 0 |
| hsa_circ_0016491 | 92 | 1 | 350 | 354 | 5 | 0 | 6.00E-136 |
| hsa_circ_0016494 | 91 | 284 | 1212 | 1 | 929 | 0 | 0 |
| hsa_circ_0016494 | 92 | 161 | 560 | 1 | 400 | 0 | 4.00E-157 |
| hsa_circ_0016494 | 91 | 161 | 676 | 1 | 516 | 0 | 0 |
| hsa_circ_0016494 | 91 | 161 | 937 | 1 | 777 | 0 | 0 |
| hsa_circ_0016494 | 91 | 161 | 1089 | 1 | 929 | 0 | 0 |
| hsa_circ_0016494 | 91 | 161 | 1250 | 1 | 1090 | 0 | 0 |
| hsa_circ_0016499 | 89 | 1 | 361 | 110 | 470 | 0 | 1.00E-125 |
| hsa_circ_0016505 | 92 | 1 | 281 | 281 | 1 | 0 | 2.00E-109 |
| hsa_circ_0016506 | 91 | 1 | 1172 | 1175 | 1 | 0 | 0 |
| hsa_circ_0016505 | 92 | 1 | 281 | 281 | 1 | 0 | 1.00E-109 |
| hsa_circ_0016506 | 89 | 1 | 387 | 390 | 1 | 0.01 | 5.00E-137 |
| hsa_circ_0016506 | 91 | 1 | 1172 | 1175 | 1 | 0 | 0 |
| hsa_circ_0016506 | 91 | 1 | 1533 | 1536 | 1 | 0 | 0 |
| hsa_circ_0016507 | 92 | 1 | 946 | 946 | 1 | 0 | 0 |
| hsa_circ_0016507 | 91 | 4 | 440 | 704 | 268 | 0 | 2.00E-169 |
| hsa_circ_0016507 | 91 | 1 | 531 | 798 | 268 | 0 | 0 |
| hsa_circ_0016507 | 92 | 1 | 679 | 946 | 268 | 0 | 0 |
| hsa_circ_0016507 | 92 | 1 | 1040 | 1307 | 268 | 0 | 0 |
| hsa_circ_0016509 | 91 | 4 | 342 | 343 | 5 | 0 | 8.00E-127 |
| hsa_circ_0016509 | 91 | 1 | 433 | 437 | 5 | 0 | 1.00E-165 |
| hsa_circ_0016509 | 92 | 1 | 581 | 585 | 5 | 0 | 0 |
| hsa_circ_0016509 | 91 | 1 | 942 | 946 | 5 | 0 | 0 |
| hsa_circ_0016507 | 93 | 83 | 1908 | 2191 | 366 | 0 | 0 |
| hsa_circ_0016509 | 92 | 1 | 868 | 1086 | 219 | 0 | 0 |
| hsa_circ_0016509 | 94 | 1 | 239 | 585 | 347 | 0 | 1.00E-99 |
| hsa_circ_0016509 | 92 | 1 | 600 | 946 | 347 | 0 | 0 |
| hsa_circ_0016510 | 92 | 1 | 649 | 649 | 1 | 0 | 0 |
| hsa_circ_0016510 | 91 | 1 | 501 | 649 | 149 | 0 | 0 |
| hsa_circ_0016507 | 93 | 1 | 1019 | 1965 | 947 | 0 | 0 |
| hsa_circ_0025780 | 93 | 262 | 865 | 1732 | 1129 | 0.01 | 0 |
| hsa_circ_0025780 | 93 | 262 | 966 | 1732 | 1028 | 0.01 | 0 |
| hsa_circ_0025780 | 93 | 57 | 660 | 1732 | 1129 | 0.01 | 0 |
| hsa_circ_0025780 | 93 | 3 | 351 | 1312 | 964 | 0.01 | 1.00E-145 |
| hsa_circ_0025776 | 95 | 1 | 239 | 1510 | 1272 | 0 | 5.00E-103 |
| hsa_circ_0025774 | 94 | 1 | 611 | 611 | 1 | 0 | 0 |
| hsa_circ_0025772 | 92 | 288 | 1345 | 1585 | 528 | 0 | 0 |
| hsa_circ_0025772 | 94 | 4 | 520 | 1585 | 1069 | 0 | 0 |
| hsa_circ_0025772 | 94 | 4 | 744 | 1585 | 845 | 0 | 0 |
| hsa_circ_0025772 | 92 | 4 | 1061 | 1585 | 528 | 0 | 0 |
| hsa_circ_0025772 | 90 | 1 | 608 | 1135 | 528 | 0 | 0 |
| hsa_circ_0025772 | 89 | 1 | 541 | 1068 | 528 | 0 | 0 |
| hsa_circ_0025772 | 87 | 7 | 317 | 838 | 528 | 0 | 1.00E-94 |
| hsa_circ_0025772 | 91 | 1 | 527 | 527 | 1 | 0 | 0 |
| hsa_circ_0025764 | 92 | 185 | 376 | 192 | 1 | 0 | 1.00E-71 |
| hsa_circ_0025760 | 93 | 185 | 666 | 482 | 1 | 0 | 0 |

novel_circ_001028 novel_circ_001029 novel_circ_001030 novel_circ_001031 novel_circ_001034 novel_circ_001035 novel_circ_001036 novel_circ_001038 novel_circ_001039 novel_circ_001040 novel_circ_001041 novel_circ_001042 novel_circ_001043 novel_circ_001044 novel_circ_001045 novel_circ_001046 novel_circ_001047 novel_circ_001048 novel_circ_001049 novel_circ_001050 novel_circ_001051 novel_circ_001052 novel_circ_001053 novel_circ_001054 novel_circ_001055 novel_circ_001056 novel_circ_001057 novel_circ_001058 novel_circ_001059 novel_circ_001060 novel_circ_001061 novel_circ_001062 novel_circ_001063 novel_circ_001064 novel_circ_001065 novel_circ_001066 novel_circ_001067 novel_circ_001068 novel_circ_001069 novel_circ_001071 novel_circ_001072 novel_circ_001073 novel_circ_001074 novel_circ_001075 novel_circ_001076 novel_circ_001077 novel_circ_001078 novel_circ_001079 novel_circ_001082 novel_circ_001083 novel_circ_001084 novel_circ_001085 novel_circ_001086 novel_circ_001087

| hsa_circ_0025760 | 93 | 1 | 480 | 480 | 1 | 0 | 0 |
| --- | --- | --- | --- | --- | --- | --- | --- |
| hsa_circ_0025759 | 94 | 1 | 282 | 426 | 145 | 0 | 1.00E-118 |
| hsa_circ_0025758 | 92 | 1 | 253 | 253 | 1 | 0 | 8.00E-96 |
| hsa_circ_0025756 | 90 | 1 | 631 | 631 | 1 | 0 | 0 |
| hsa_circ_0025747 | 92 | 1 | 656 | 1 | 653 | 0 | 0 |
| hsa_circ_0025747 | 91 | 1 | 470 | 1 | 467 | 0.01 | 1.00E-176 |
| hsa_circ_0006584 | 91 | 367 | 838 | 1022 | 1490 | 0.01 | 2.00E-177 |
| hsa_circ_0025737 | 91 | 1 | 320 | 1 | 320 | 0.01 | 1.00E-119 |
| hsa_circ_0025734 | 93 | 1 | 549 | 1 | 549 | 0 | 0 |
| hsa_circ_0025732 | 91 | 1 | 369 | 250 | 618 | 0 | 9.00E-137 |
| hsa_circ_0025731 | 93 | 73 | 636 | 73 | 636 | 0 | 0 |
| hsa_circ_0025731 | 93 | 164 | 727 | 73 | 636 | 0 | 0 |
| hsa_circ_0025725 | 92 | 115 | 246 | 1 | 132 | 0 | 3.00E-45 |
| hsa_circ_0025722 | 91 | 1 | 1254 | 296 | 1552 | 0.01 | 0 |
| hsa_circ_0025722 | 90 | 1 | 808 | 745 | 1552 | 0 | 0 |
| hsa_circ_0025722 | 91 | 3 | 664 | 891 | 1552 | 0 | 0 |
| hsa_circ_0025722 | 91 | 3 | 664 | 891 | 1552 | 0 | 0 |
| hsa_circ_0025722 | 91 | 3 | 664 | 891 | 1552 | 0 | 0 |
| hsa_circ_0025722 | 90 | 1 | 808 | 745 | 1552 | 0 | 0 |
| hsa_circ_0025722 | 91 | 6 | 1096 | 459 | 1552 | 0.01 | 0 |
| hsa_circ_0025722 | 91 | 1 | 1254 | 296 | 1552 | 0.01 | 0 |
| hsa_circ_0025722 | 91 | 3 | 497 | 891 | 1385 | 0 | 0 |
| hsa_circ_0025722 | 90 | 6 | 432 | 459 | 888 | 0.02 | 8.00E-158 |
| hsa_circ_0025722 | 91 | 3 | 585 | 303 | 888 | 0.02 | 0 |
| hsa_circ_0025722 | 91 | 1 | 588 | 298 | 888 | 0.02 | 0 |
| hsa_circ_0025722 | 91 | 1 | 590 | 296 | 888 | 0.02 | 0 |
| hsa_circ_0025722 | 91 | 1 | 632 | 254 | 888 | 0.01 | 0 |
| hsa_circ_0025722 | 89 | 1 | 888 | 1 | 888 | 0.02 | 0 |
| hsa_circ_0025722 | 93 | 6 | 288 | 459 | 744 | 0.02 | 5.00E-113 |
| hsa_circ_0025722 | 93 | 1 | 446 | 296 | 744 | 0.01 | 0 |
| hsa_circ_0025722 | 89 | 1 | 456 | 1 | 453 | 0.01 | 1.00E-155 |
| hsa_circ_0005234 | 98 | 115 | 242 | 195947 | 196074 | 0 | 4.00E-56 |
| hsa_circ_0005234 | 98 | 280 | 407 | 195947 | 196074 | 0 | 6.00E-56 |
| hsa_circ_0005234 | 94 | 1 | 156 | 192559 | 192714 | 0 | 1.00E-59 |
| hsa_circ_0005234 | 98 | 115 | 242 | 195947 | 196074 | 0 | 2.00E-56 |
| hsa_circ_0005234 | 98 | 280 | 407 | 195947 | 196074 | 0 | 4.00E-56 |
| hsa_circ_0005234 | 94 | 1 | 156 | 192559 | 192714 | 0 | 7.00E-60 |
| hsa_circ_0005234 | 96 | 1 | 126 | 186394 | 186519 | 0 | 5.00E-52 |
| hsa_circ_0005234 | 95 | 193 | 323 | 186389 | 186519 | 0 | 9.00E-53 |
| hsa_circ_0025715 | 91 | 219 | 370 | 427 | 276 | 0 | 4.00E-51 |
| hsa_circ_0025715 | 91 | 1398 | 1550 | 428 | 276 | 0 | 5.00E-51 |
| hsa_circ_0025715 | 85 | 219 | 524 | 427 | 122 | 0.01 | 6.00E-85 |
| hsa_circ_0025715 | 87 | 219 | 645 | 427 | 1 | 0.01 | 1.00E-133 |
| hsa_circ_0005824 | 86 | 219 | 810 | 777 | 186 | 0.01 | 0 |
| hsa_circ_0005824 | 86 | 219 | 993 | 777 | 3 | 0.01 | 0 |
| hsa_circ_0025709 | 87 | 219 | 1163 | 1098 | 154 | 0.01 | 0 |
| hsa_circ_0025709 | 87 | 219 | 1163 | 1098 | 154 | 0.01 | 0 |
| hsa_circ_0025709 | 87 | 219 | 1163 | 1098 | 154 | 0.01 | 0 |
| hsa_circ_0025700 | 89 | 168 | 500 | 3151 | 2819 | 0 | 3.00E-113 |
| hsa_circ_0025700 | 88 | 168 | 663 | 3151 | 2656 | 0 | 4.00E-167 |
| hsa_circ_0026148 | 91 | 7 | 410 | 2455 | 2860 | 0 | 2.00E-151 |
| hsa_circ_0026148 | 91 | 7 | 410 | 2455 | 2860 | 0 | 1.00E-151 |
| hsa_circ_0025693 | 95 | 1 | 589 | 589 | 1 | 0 | 0 |
| hsa_circ_0025691 | 93 | 13 | 304 | 561 | 270 | 0 | 1.00E-120 |

novel_circ_001088 novel_circ_001089 novel_circ_001090 novel_circ_001091 novel_circ_001092 novel_circ_001093 novel_circ_001094 novel_circ_001095 novel_circ_001096 novel_circ_001097 novel_circ_001098 novel_circ_001099 novel_circ_001100 novel_circ_001101 novel_circ_001102 novel_circ_001103 novel_circ_001104 novel_circ_001105 novel_circ_001106 novel_circ_001107 novel_circ_001108 novel_circ_001109 novel_circ_001110 novel_circ_001111 novel_circ_001112 novel_circ_001113 novel_circ_001114 novel_circ_001115 novel_circ_001116 novel_circ_001117 novel_circ_001118 novel_circ_001119 novel_circ_001120 novel_circ_001121 novel_circ_001122 novel_circ_001123 novel_circ_001124 novel_circ_001125 novel_circ_001126 novel_circ_001127 novel_circ_001128 novel_circ_001129 novel_circ_001130 novel_circ_001132 novel_circ_001134 novel_circ_001135 novel_circ_001136 novel_circ_001137 novel_circ_001138 novel_circ_001139 novel_circ_001143 novel_circ_001144 novel_circ_001145 novel_circ_001146

| hsa_circ_0025691 | 90 | 13 | 412 | 561 | 180 | 0.05 | 6.00E-139 |
| --- | --- | --- | --- | --- | --- | --- | --- |
| hsa_circ_0025691 | 92 | 13 | 591 | 561 | 1 | 0.03 | 0 |
| hsa_circ_0025684 | 94 | 1 | 545 | 545 | 1 | 0 | 0 |
| hsa_circ_0025683 | 92 | 4 | 426 | 468 | 46 | 0 | 1.00E-171 |
| hsa_circ_0025680 | 92 | 4 | 884 | 1023 | 143 | 0 | 0 |
| hsa_circ_0025675 | 93 | 4 | 2215 | 3024 | 813 | 0 | 0 |
| hsa_circ_0025681 | 92 | 10 | 701 | 692 | 1 | 0 | 0 |
| hsa_circ_0025681 | 91 | 1 | 458 | 458 | 1 | 0 | 4.00E-176 |
| hsa_circ_0025679 | 91 | 1 | 717 | 1448 | 732 | 0 | 0 |
| hsa_circ_0025675 | 91 | 1 | 719 | 812 | 91 | 0 | 0 |
| hsa_circ_0025671 | 92 | 1 | 1887 | 2081 | 192 | 0 | 0 |
| hsa_circ_0025671 | 93 | 1 | 918 | 1359 | 442 | 0 | 0 |
| hsa_circ_0025671 | 94 | 4 | 441 | 438 | 1 | 0 | 0 |
| hsa_circ_0025669 | 93 | 4 | 584 | 690 | 110 | 0 | 0 |
| hsa_circ_0025669 | 93 | 4 | 693 | 690 | 1 | 0 | 0 |
| hsa_circ_0025669 | 94 | 1 | 334 | 443 | 110 | 0 | 2.00E-142 |
| hsa_circ_0025669 | 94 | 1 | 443 | 443 | 1 | 0 | 0 |
| hsa_circ_0025669 | 93 | 1 | 252 | 252 | 1 | 0 | 3.00E-100 |
| hsa_circ_0025667 | 91 | 1 | 406 | 406 | 1 | 0 | 3.00E-157 |
| hsa_circ_0025660 | 92 | 1 | 1375 | 1378 | 1 | 0 | 0 |
| hsa_circ_0025660 | 91 | 1 | 1266 | 1269 | 1 | 0 | 0 |
| hsa_circ_0025660 | 92 | 1 | 785 | 788 | 1 | 0 | 0 |
| hsa_circ_0025647 | 93 | 1 | 1749 | 1845 | 94 | 0 | 0 |
| hsa_circ_0025647 | 92 | 1 | 1842 | 1845 | 1 | 0 | 0 |
| hsa_circ_0025644 | 92 | 1 | 2139 | 2142 | 1 | 0 | 0 |
| hsa_circ_0025644 | 92 | 1 | 1570 | 1573 | 1 | 0 | 0 |
| hsa_circ_0025644 | 92 | 1 | 1570 | 1573 | 1 | 0 | 0 |
| hsa_circ_0025644 | 91 | 1 | 751 | 751 | 1 | 0 | 0 |
| hsa_circ_0025643 | 91 | 1 | 912 | 912 | 1 | 0 | 0 |
| hsa_circ_0025643 | 91 | 1 | 912 | 912 | 1 | 0 | 0 |
| hsa_circ_0025644 | 90 | 1 | 586 | 586 | 1 | 0 | 0 |
| hsa_circ_0025643 | 91 | 1 | 747 | 747 | 1 | 0 | 0 |
| hsa_circ_0025642 | 91 | 1 | 751 | 5041 | 4291 | 0 | 0 |
| hsa_circ_0025644 | 92 | 46 | 390 | 345 | 1 | 0 | 2.00E-133 |
| hsa_circ_0025643 | 92 | 46 | 551 | 506 | 1 | 0 | 0 |
| hsa_circ_0025642 | 92 | 46 | 555 | 4800 | 4291 | 0 | 0 |
| hsa_circ_0025644 | 93 | 1 | 297 | 297 | 1 | 0 | 3.00E-120 |
| hsa_circ_0025643 | 93 | 1 | 458 | 458 | 1 | 0 | 0 |
| hsa_circ_0025642 | 93 | 1 | 462 | 4752 | 4291 | 0 | 0 |
| hsa_circ_0025638 | 92 | 1 | 1241 | 1 | 1246 | 0 | 0 |
| hsa_circ_0025638 | 93 | 1 | 1096 | 1 | 1101 | 0 | 0 |
| hsa_circ_0025638 | 93 | 533 | 1628 | 1 | 1101 | 0 | 0 |
| hsa_circ_0025636 | 88 | 529 | 738 | 92 | 305 | 0.03 | 3.00E-64 |
| hsa_circ_0082489 | 83 | 916 | 1819 | 1138 | 223 | 0.03 | 0 |
| hsa_circ_0007579 | 90 | 1 | 445 | 445 | 1 | 0 | 6.00E-164 |
| hsa_circ_0025624 | 98 | 3080 | 3522 | 979 | 537 | 0 | 0 |
| hsa_circ_0025624 | 98 | 258 | 700 | 979 | 537 | 0 | 0 |
| hsa_circ_0025624 | 98 | 1 | 443 | 979 | 537 | 0 | 0 |
| hsa_circ_0025624 | 96 | 1 | 211 | 747 | 537 | 0 | 1.00E-92 |
| hsa_circ_0025624 | 96 | 367 | 488 | 210 | 89 | 0 | 2.00E-49 |
| hsa_circ_0086460 | 93 | 725 | 1402 | 3 | 683 | 0 | 0 |
| hsa_circ_0005684 | 95 | 323 | 575 | 1 | 253 | 0.01 | 9.00E-109 |
| hsa_circ_0005684 | 95 | 323 | 575 | 1 | 253 | 0.01 | 1.00E-108 |
| hsa_circ_0005684 | 95 | 323 | 575 | 1 | 253 | 0.01 | 1.00E-108 |

novel_circ_001147 novel_circ_001148 novel_circ_001149 novel_circ_001150 novel_circ_001151 novel_circ_001152 novel_circ_001153 novel_circ_001154 novel_circ_001155 novel_circ_001156 novel_circ_001157 novel_circ_001158 novel_circ_001159 novel_circ_001160 novel_circ_001161 novel_circ_001162 novel_circ_001163 novel_circ_001164 novel_circ_001165 novel_circ_001166 novel_circ_001167 novel_circ_001168 novel_circ_001169 novel_circ_001170 novel_circ_001171 novel_circ_001172 novel_circ_001173 novel_circ_001174 novel_circ_001175 novel_circ_001176 novel_circ_001177 novel_circ_001178 novel_circ_001179 novel_circ_001180 novel_circ_001181 novel_circ_001182 novel_circ_001183 novel_circ_001184 novel_circ_001185 novel_circ_001186 novel_circ_001187 novel_circ_001188 novel_circ_001189 novel_circ_001190 novel_circ_001191 novel_circ_001192 novel_circ_001193 novel_circ_001194 novel_circ_001195 novel_circ_001196 novel_circ_001197 novel_circ_001198 novel_circ_001199 novel_circ_001200

| hsa_circ_0086460 | 92 | 648 | 1121 | 3 | 479 | 0.01 | 0 |
| --- | --- | --- | --- | --- | --- | --- | --- |
| hsa_circ_0086460 | 93 | 648 | 1325 | 3 | 683 | 0 | 0 |
| hsa_circ_0086460 | 94 | 648 | 1499 | 3 | 857 | 0 | 0 |
| hsa_circ_0086464 | 95 | 1055 | 2134 | 1 | 1080 | 0 | 0 |
| hsa_circ_0005684 | 95 | 1 | 253 | 1 | 253 | 0.01 | 8.00E-109 |
| hsa_circ_0086460 | 92 | 326 | 799 | 3 | 479 | 0.01 | 0 |
| hsa_circ_0086460 | 93 | 326 | 1003 | 3 | 683 | 0 | 0 |
| hsa_circ_0086460 | 94 | 326 | 1177 | 3 | 857 | 0 | 0 |
| hsa_circ_0086460 | 94 | 326 | 1177 | 3 | 857 | 0 | 0 |
| hsa_circ_0086464 | 94 | 1 | 271 | 1 | 271 | 0 | 9.00E-116 |
| hsa_circ_0086464 | 95 | 1 | 700 | 68 | 767 | 0 | 0 |
| hsa_circ_0086464 | 95 | 1 | 846 | 68 | 913 | 0 | 0 |
| hsa_circ_0086464 | 95 | 1 | 496 | 272 | 767 | 0 | 0 |
| hsa_circ_0086464 | 95 | 1 | 642 | 272 | 913 | 0 | 0 |
| hsa_circ_0086464 | 95 | 1 | 809 | 272 | 1080 | 0 | 0 |
| hsa_circ_0086464 | 95 | 1 | 942 | 272 | 1213 | 0 | 0 |
| hsa_circ_0086468 | 90 | 1568 | 4098 | 779 | 3306 | 0 | 0 |
| hsa_circ_0086466 | 93 | 1 | 320 | 1 | 320 | 0 | 6.00E-133 |
| hsa_circ_0086464 | 94 | 1 | 633 | 448 | 1080 | 0 | 0 |
| hsa_circ_0086468 | 89 | 1392 | 3676 | 779 | 3060 | 0 | 0 |
| hsa_circ_0086469 | 92 | 1 | 481 | 1 | 481 | 0 | 0 |
| hsa_circ_0086468 | 90 | 1 | 2528 | 782 | 3306 | 0 | 0 |
| hsa_circ_0086469 | 89 | 6 | 1316 | 610 | 1917 | 0.01 | 0 |
| hsa_circ_0086468 | 89 | 6 | 1604 | 910 | 2505 | 0.01 | 0 |
| hsa_circ_0086468 | 89 | 1 | 2012 | 1052 | 3060 | 0 | 0 |
| hsa_circ_0086470 | 92 | 1 | 919 | 289 | 1207 | 0 | 0 |
| hsa_circ_0086470 | 91 | 1 | 743 | 465 | 1207 | 0 | 0 |
| hsa_circ_0086470 | 91 | 1 | 743 | 465 | 1207 | 0 | 0 |
| hsa_circ_0007110 | 96 | 1 | 364 | 1 | 364 | 0 | 1.00E-165 |
| hsa_circ_0007110 | 96 | 1 | 364 | 1 | 364 | 0 | 2.00E-165 |
| hsa_circ_0086485 | 95 | 1 | 255 | 1 | 255 | 0 | 6.00E-112 |
| hsa_circ_0086485 | 92 | 1 | 395 | 1 | 395 | 0 | 2.00E-159 |
| hsa_circ_0086488 | 96 | 1 | 450 | 450 | 1 | 0 | 0 |
| hsa_circ_0086490 | 96 | 1 | 306 | 306 | 1 | 0 | 3.00E-140 |
| hsa_circ_0003212 | 96 | 196 | 816 | 618 | 1 | 0 | 0 |
| hsa_circ_0003212 | 96 | 279 | 899 | 618 | 1 | 0 | 0 |
| hsa_circ_0086499 | 95 | 1 | 319 | 1 | 319 | 0 | 2.00E-142 |
| hsa_circ_0086499 | 94 | 1 | 424 | 1 | 424 | 0 | 0 |
| hsa_circ_0086499 | 94 | 1 | 526 | 1 | 526 | 0 | 0 |
| hsa_circ_0086499 | 93 | 1 | 731 | 1 | 731 | 0 | 0 |
| hsa_circ_0086499 | 92 | 1 | 1224 | 1 | 1224 | 0 | 0 |
| hsa_circ_0086499 | 92 | 1 | 1224 | 1 | 1224 | 0 | 0 |
| hsa_circ_0086499 | 90 | 1 | 1487 | 1 | 1487 | 0 | 0 |
| hsa_circ_0086502 | 94 | 1 | 437 | 1 | 437 | 0 | 0 |
| hsa_circ_0086502 | 89 | 1 | 1398 | 1 | 1398 | 0 | 0 |
| hsa_circ_0086526 | 90 | 1 | 698 | 1 | 698 | 0 | 0 |
| hsa_circ_0086526 | 90 | 1 | 698 | 1 | 698 | 0 | 0 |
| hsa_circ_0086526 | 88 | 1 | 961 | 1 | 961 | 0 | 0 |
| hsa_circ_0086526 | 89 | 1 | 1426 | 1 | 1426 | 0 | 0 |
| hsa_circ_0086526 | 91 | 2 | 493 | 207 | 698 | 0 | 0 |
| hsa_circ_0086526 | 91 | 2 | 493 | 207 | 698 | 0 | 0 |
| hsa_circ_0086537 | 91 | 1 | 465 | 1 | 465 | 0 | 1.00E-176 |
| hsa_circ_0086537 | 91 | 1 | 600 | 1 | 600 | 0 | 0 |
| hsa_circ_0086537 | 91 | 1 | 1170 | 1 | 1170 | 0 | 0 |

novel_circ_001201 novel_circ_001202 novel_circ_001203 novel_circ_001204 novel_circ_001205 novel_circ_001207 novel_circ_001208 novel_circ_001209 novel_circ_001210 novel_circ_001211 novel_circ_001212 novel_circ_001213 novel_circ_001215 novel_circ_001216 novel_circ_001217 novel_circ_001218 novel_circ_001219 novel_circ_001220 novel_circ_001223 novel_circ_001224 novel_circ_001225 novel_circ_001226 novel_circ_001227 novel_circ_001228 novel_circ_001229 novel_circ_001230 novel_circ_001231 novel_circ_001232 novel_circ_001233 novel_circ_001248 novel_circ_001249 novel_circ_001250 novel_circ_001251 novel_circ_001252 novel_circ_001253 novel_circ_001254 novel_circ_001255 novel_circ_001256 novel_circ_001257 novel_circ_001258 novel_circ_001259 novel_circ_001260 novel_circ_001264 novel_circ_001265 novel_circ_001266 novel_circ_001267 novel_circ_001268 novel_circ_001269 novel_circ_001270 novel_circ_001271 novel_circ_001272 novel_circ_001273 novel_circ_001274 novel_circ_001275

| hsa_circ_0086559 | 92 | 1 | 705 | 1 | 705 | 0 | 0 |
| --- | --- | --- | --- | --- | --- | --- | --- |
| hsa_circ_0086559 | 92 | 1 | 570 | 136 | 705 | 0 | 0 |
| hsa_circ_0086561 | 93 | 1 | 435 | 85 | 519 | 0 | 0 |
| hsa_circ_0006136 | 93 | 1 | 490 | 1 | 490 | 0 | 0 |
| hsa_circ_0006136 | 93 | 1 | 304 | 187 | 490 | 0 | 2.00E-122 |
| hsa_circ_0058788 | 84 | 1 | 1324 | 1 | 1324 | 0 | 0 |
| hsa_circ_0058793 | 85 | 7597 | 7932 | 59 | 382 | 0.04 | 2.00E-85 |
| hsa_circ_0058795 | 84 | 1 | 335 | 1 | 323 | 0.04 | 6.00E-86 |
| hsa_circ_0005003 | 88 | 1 | 235 | 1 | 235 | 0 | 8.00E-74 |
| hsa_circ_0058797 | 87 | 231 | 881 | 4 | 654 | 0 | 0 |
| hsa_circ_0058807 | 89 | 1 | 377 | 1 | 377 | 0 | 7.00E-128 |
| hsa_circ_0058807 | 89 | 1 | 481 | 1 | 481 | 0 | 1.00E-165 |
| hsa_circ_0058803 | 92 | 1 | 595 | 513 | 1107 | 0 | 0 |
| hsa_circ_0058811 | 93 | 1 | 326 | 1 | 326 | 0 | 3.00E-131 |
| hsa_circ_0058811 | 92 | 1 | 490 | 1 | 490 | 0 | 0 |
| hsa_circ_0003272 | 95 | 1 | 321 | 1 | 321 | 0 | 8.00E-142 |
| hsa_circ_0058814 | 92 | 3 | 469 | 3 | 469 | 0 | 0 |
| hsa_circ_0007947 | 89 | 1 | 491 | 1 | 491 | 0 | 5.00E-176 |
| hsa_circ_0084504 | 91 | 4 | 235 | 234 | 1 | 0.02 | 2.00E-86 |
| hsa_circ_0084501 | 93 | 4 | 533 | 532 | 1 | 0.01 | 0 |
| hsa_circ_0084499 | 93 | 4 | 736 | 1336 | 602 | 0.01 | 0 |
| hsa_circ_0084499 | 93 | 4 | 1334 | 1336 | 1 | 0.01 | 0 |
| hsa_circ_0084497 | 93 | 4 | 3982 | 4603 | 620 | 0 | 0 |
| hsa_circ_0084499 | 93 | 1 | 1099 | 1102 | 1 | 0 | 0 |
| hsa_circ_0084499 | 92 | 1 | 801 | 804 | 1 | 0 | 0 |
| hsa_circ_0084498 | 93 | 2 | 1114 | 1220 | 105 | 0 | 0 |
| hsa_circ_0084498 | 95 | 1 | 791 | 791 | 1 | 0 | 0 |
| hsa_circ_0084497 | 91 | 1 | 571 | 619 | 49 | 0 | 0 |
| hsa_circ_0084495 | 91 | 1 | 813 | 815 | 3 | 0 | 0 |
| hsa_circ_0001801 | 95 | 1092 | 1463 | 372 | 1 | 0.01 | 8.00E-168 |
| hsa_circ_0001800 | 96 | 1092 | 1566 | 475 | 1 | 0 | 0 |
| hsa_circ_0001801 | 95 | 136 | 507 | 372 | 1 | 0.01 | 3.00E-168 |
| hsa_circ_0001800 | 96 | 136 | 610 | 475 | 1 | 0 | 0 |
| hsa_circ_0001800 | 89 | 4672 | 5370 | 669 | 1 | 0.05 | 0 |
| hsa_circ_0001802 | 95 | 60 | 415 | 356 | 1 | 0.01 | 2.00E-159 |
| hsa_circ_0001801 | 95 | 60 | 431 | 372 | 1 | 0.01 | 2.00E-168 |
| hsa_circ_0001800 | 96 | 60 | 534 | 475 | 1 | 0 | 0 |
| hsa_circ_0001800 | 96 | 60 | 534 | 475 | 1 | 0 | 0 |
| hsa_circ_0001800 | 96 | 60 | 534 | 475 | 1 | 0 | 0 |
| hsa_circ_0084491 | 89 | 722 | 1693 | 3438 | 2464 | 0.02 | 0 |
| hsa_circ_0084491 | 88 | 722 | 1940 | 3438 | 2217 | 0.02 | 0 |
| hsa_circ_0084492 | 91 | 1 | 296 | 296 | 1 | 0 | 1.00E-109 |
| hsa_circ_0085341 | 93 | 1 | 720 | 720 | 1 | 0 | 0 |
| hsa_circ_0085341 | 93 | 1 | 1623 | 1623 | 1 | 0 | 0 |
| hsa_circ_0085341 | 93 | 1 | 2343 | 2343 | 1 | 0 | 0 |
| hsa_circ_0085341 | 93 | 1 | 543 | 720 | 178 | 0 | 0 |
| hsa_circ_0085343 | 96 | 1 | 918 | 918 | 1 | 0 | 0 |
| hsa_circ_0085345 | 97 | 1 | 324 | 324 | 1 | 0 | 8.00E-152 |
| hsa_circ_0085345 | 95 | 1 | 723 | 723 | 1 | 0 | 0 |
| hsa_circ_0085345 | 95 | 1 | 1029 | 1029 | 1 | 0 | 0 |
| hsa_circ_0085345 | 95 | 4 | 1231 | 1228 | 1 | 0 | 0 |
| hsa_circ_0085345 | 95 | 1 | 1770 | 1770 | 1 | 0 | 0 |
| hsa_circ_0085348 | 92 | 1 | 306 | 516 | 211 | 0 | 7.00E-122 |
| hsa_circ_0085348 | 94 | 1 | 411 | 621 | 211 | 0 | 2.00E-178 |

novel_circ_001276 novel_circ_001277 novel_circ_001278 novel_circ_001279 novel_circ_001280 novel_circ_001281 novel_circ_001282 novel_circ_001283 novel_circ_001284 novel_circ_001285 novel_circ_001286 novel_circ_001287 novel_circ_001288 novel_circ_001289 novel_circ_001290 novel_circ_001291 novel_circ_001292 novel_circ_001293 novel_circ_001294 novel_circ_001295 novel_circ_001296 novel_circ_001297 novel_circ_001298 novel_circ_001299 novel_circ_001300 novel_circ_001301 novel_circ_001302 novel_circ_001305 novel_circ_001306 novel_circ_001307 novel_circ_001308 novel_circ_001309 novel_circ_001310 novel_circ_001311 novel_circ_001312 novel_circ_001313 novel_circ_001314 novel_circ_001319 novel_circ_001320 novel_circ_001321 novel_circ_001322 novel_circ_001323 novel_circ_001335 novel_circ_001336 novel_circ_001337 novel_circ_001338 novel_circ_001339 novel_circ_001340 novel_circ_001341 novel_circ_001342 novel_circ_001343 novel_circ_001344 novel_circ_001345 novel_circ_001346

| hsa_circ_0085350 | 97 | 4 | 202 | 199 | 1 | 0 | 1.00E-92 |
| --- | --- | --- | --- | --- | --- | --- | --- |
| hsa_circ_0085349 | 97 | 1 | 741 | 930 | 190 | 0 | 0 |
| hsa_circ_0085349 | 95 | 1 | 639 | 1569 | 931 | 0 | 0 |
| hsa_circ_0085349 | 95 | 3 | 831 | 1759 | 931 | 0 | 0 |
| hsa_circ_0085351 | 94 | 1 | 732 | 1773 | 1042 | 0 | 0 |
| hsa_circ_0085351 | 95 | 1 | 849 | 1890 | 1042 | 0 | 0 |
| hsa_circ_0085347 | 95 | 1 | 1226 | 4025 | 2800 | 0 | 0 |
| hsa_circ_0085357 | 92 | 1 | 405 | 405 | 1 | 0 | 1.00E-161 |
| hsa_circ_0085357 | 93 | 1 | 522 | 522 | 1 | 0 | 0 |
| hsa_circ_0085357 | 94 | 1 | 899 | 899 | 1 | 0 | 0 |
| hsa_circ_0085357 | 94 | 3 | 1038 | 1036 | 1 | 0 | 0 |
| hsa_circ_0085357 | 95 | 1 | 1743 | 1743 | 1 | 0 | 0 |
| hsa_circ_0085357 | 92 | 1 | 397 | 405 | 9 | 0 | 3.00E-157 |
| hsa_circ_0085357 | 93 | 1 | 514 | 522 | 9 | 0 | 0 |
| hsa_circ_0085357 | 94 | 1 | 891 | 899 | 9 | 0 | 0 |
| hsa_circ_0085357 | 94 | 3 | 1030 | 1036 | 9 | 0 | 0 |
| hsa_circ_0085357 | 95 | 1 | 1735 | 1743 | 9 | 0 | 0 |
| hsa_circ_0085357 | 95 | 1 | 377 | 899 | 523 | 0 | 1.00E-169 |
| hsa_circ_0085357 | 96 | 3 | 516 | 1036 | 523 | 0 | 0 |
| hsa_circ_0085357 | 95 | 1 | 1038 | 1560 | 523 | 0 | 0 |
| hsa_circ_0085357 | 95 | 1 | 1221 | 1743 | 523 | 0 | 0 |
| hsa_circ_0085357 | 97 | 3 | 327 | 1036 | 712 | 0 | 1.00E-155 |
| hsa_circ_0085357 | 95 | 1 | 844 | 1743 | 900 | 0 | 0 |
| hsa_circ_0085358 | 96 | 1 | 213 | 439 | 227 | 0 | 1.00E-93 |
| hsa_circ_0085357 | 97 | 1 | 312 | 2685 | 2374 | 0 | 1.00E-146 |
| hsa_circ_0085357 | 98 | 112 | 279 | 2375 | 2208 | 0 | 2.00E-78 |
| hsa_circ_0085357 | 95 | 1 | 336 | 3537 | 3202 | 0 | 4.00E-150 |
| hsa_circ_0085362 | 91 | 1 | 604 | 604 | 1 | 0 | 0 |
| hsa_circ_0085362 | 91 | 1 | 604 | 604 | 1 | 0 | 0 |
| hsa_circ_0085362 | 93 | 4 | 1734 | 1731 | 1 | 0 | 0 |
| hsa_circ_0085362 | 94 | 1 | 2663 | 2663 | 1 | 0 | 0 |
| hsa_circ_0085362 | 95 | 1 | 2821 | 2821 | 1 | 0 | 0 |
| hsa_circ_0085363 | 94 | 4 | 1130 | 1127 | 1 | 0 | 0 |
| hsa_circ_0085363 | 95 | 1 | 2059 | 2059 | 1 | 0 | 0 |
| hsa_circ_0085363 | 95 | 1 | 2217 | 2217 | 1 | 0 | 0 |
| hsa_circ_0006950 | 97 | 1 | 1087 | 1087 | 1 | 0 | 0 |
| hsa_circ_0085365 | 93 | 3 | 418 | 416 | 1 | 0 | 2.00E-169 |
| hsa_circ_0050976 | 89 | 1 | 492 | 1 | 471 | 0.04 | 7.00E-169 |
| hsa_circ_0051054 | 93 | 1 | 470 | 1 | 470 | 0 | 0 |
| hsa_circ_0003712 | 94 | 262 | 612 | 3892 | 3543 | 0 | 1.00E-147 |
| hsa_circ_0021558 | 88 | 1 | 151 | 1 | 151 | 0 | 7.00E-44 |
| hsa_circ_0021558 | 88 | 1 | 151 | 1 | 151 | 0 | 2.00E-43 |
| hsa_circ_0030221 | 93 | 1 | 573 | 1573 | 1001 | 0 | 0 |
| hsa_circ_0030221 | 94 | 1 | 836 | 1836 | 1001 | 0 | 0 |
| hsa_circ_0030229 | 92 | 1 | 1156 | 276 | 1431 | 0 | 0 |
| hsa_circ_0030229 | 92 | 1 | 520 | 344 | 863 | 0 | 0 |
| hsa_circ_0030229 | 93 | 1 | 607 | 344 | 950 | 0 | 0 |
| hsa_circ_0030229 | 92 | 1 | 1088 | 344 | 1431 | 0 | 0 |
| hsa_circ_0030232 | 92 | 1 | 756 | 1 | 756 | 0 | 0 |
| hsa_circ_0030232 | 92 | 1 | 568 | 189 | 756 | 0 | 0 |
| hsa_circ_0030232 | 91 | 1 | 280 | 277 | 556 | 0 | 4.00E-104 |
| hsa_circ_0030232 | 92 | 1 | 480 | 277 | 756 | 0 | 0 |
| hsa_circ_0030232 | 91 | 1 | 410 | 757 | 1166 | 0 | 2.00E-159 |
| hsa_circ_0030232 | 90 | 1 | 291 | 876 | 1166 | 0 | 3.00E-105 |

novel_circ_001347 novel_circ_001348 novel_circ_001349 novel_circ_001350 novel_circ_001355 novel_circ_001356 novel_circ_001357 novel_circ_001358 novel_circ_001359 novel_circ_001360 novel_circ_001361 novel_circ_001362 novel_circ_001363 novel_circ_001364 novel_circ_001365 novel_circ_001368 novel_circ_001369 novel_circ_001370 novel_circ_001371 novel_circ_001372 novel_circ_001373 novel_circ_001374 novel_circ_001375 novel_circ_001376 novel_circ_001377 novel_circ_001379 novel_circ_001380 novel_circ_001381 novel_circ_001382 novel_circ_001383 novel_circ_001384 novel_circ_001385 novel_circ_001386 novel_circ_001387 novel_circ_001388 novel_circ_001390 novel_circ_001391 novel_circ_001392 novel_circ_001393 novel_circ_001394 novel_circ_001395 novel_circ_001396 novel_circ_001397 novel_circ_001398 novel_circ_001399 novel_circ_001400 novel_circ_001401 novel_circ_001402 novel_circ_001403 novel_circ_001404 novel_circ_001405 novel_circ_001406 novel_circ_001407 novel_circ_001408

| hsa_circ_0030260 | 77 | 626 | 1583 | 1223 | 266 | 0.02 | 4.00E-143 |
| --- | --- | --- | --- | --- | --- | --- | --- |
| hsa_circ_0030260 | 77 | 626 | 1583 | 1223 | 266 | 0.02 | 4.00E-143 |
| hsa_circ_0030235 | 93 | 2 | 316 | 317 | 3 | 0 | 7.00E-130 |
| hsa_circ_0030260 | 77 | 626 | 1583 | 1223 | 266 | 0.02 | 3.00E-143 |
| hsa_circ_0030243 | 92 | 1 | 641 | 1 | 641 | 0 | 0 |
| hsa_circ_0030244 | 91 | 1 | 564 | 1 | 564 | 0 | 0 |
| hsa_circ_0030245 | 90 | 1 | 326 | 1 | 326 | 0 | 3.00E-116 |
| hsa_circ_0030245 | 92 | 1 | 801 | 330 | 1127 | 0 | 0 |
| hsa_circ_0030247 | 94 | 1 | 1189 | 61 | 1249 | 0 | 0 |
| hsa_circ_0030248 | 95 | 2 | 609 | 264 | 871 | 0 | 0 |
| hsa_circ_0030247 | 94 | 2 | 1371 | 642 | 2008 | 0 | 0 |
| hsa_circ_0030249 | 96 | 1 | 282 | 1 | 282 | 0 | 7.00E-127 |
| hsa_circ_0030249 | 94 | 1 | 1044 | 1 | 1041 | 0 | 0 |
| hsa_circ_0005263 | 94 | 1 | 762 | 1 | 759 | 0 | 0 |
| hsa_circ_0005263 | 93 | 2 | 648 | 116 | 759 | 0 | 0 |
| hsa_circ_0021572 | 82 | 342 | 4019 | 274 | 3960 | 0.01 | 0 |
| hsa_circ_0021578 | 87 | 142 | 789 | 145 | 792 | 0.01 | 0 |
| hsa_circ_0021578 | 87 | 142 | 789 | 145 | 792 | 0.01 | 0 |
| hsa_circ_0021580 | 94 | 1 | 251 | 1 | 251 | 0.01 | 1.00E-104 |
| hsa_circ_0021580 | 89 | 1 | 469 | 1 | 469 | 0 | 3.00E-162 |
| hsa_circ_0021580 | 89 | 1 | 469 | 1 | 469 | 0 | 5.00E-162 |
| hsa_circ_0021580 | 89 | 1 | 469 | 1 | 469 | 0 | 7.00E-162 |
| hsa_circ_0021581 | 92 | 1 | 347 | 357 | 703 | 0 | 7.00E-138 |
| hsa_circ_0021583 | 86 | 1 | 1264 | 1 | 1264 | 0 | 0 |
| hsa_circ_0021583 | 87 | 2 | 555 | 711 | 1264 | 0 | 0 |
| hsa_circ_0021584 | 80 | 138 | 330 | 1 | 193 | 0.04 | 1.00E-30 |
| hsa_circ_0021585 | 82 | 1 | 355 | 1 | 355 | 0 | 6.00E-79 |
| hsa_circ_0021590 | 94 | 2633 | 3102 | 470 | 1 | 0 | 0 |
| hsa_circ_0021590 | 95 | 1 | 595 | 595 | 1 | 0 | 0 |
| hsa_circ_0021591 | 89 | 101 | 442 | 334 | 1 | 0.04 | 3.00E-114 |
| hsa_circ_0008719 | 93 | 60 | 266 | 207 | 1 | 0 | 3.00E-80 |
| hsa_circ_0008719 | 93 | 17723 | 17929 | 207 | 1 | 0 | 2.00E-78 |
| hsa_circ_0051093 | 88 | 1 | 387 | 647 | 264 | 0.01 | 1.00E-125 |
| hsa_circ_0051094 | 92 | 1 | 705 | 1839 | 2543 | 0 | 0 |
| hsa_circ_0051094 | 93 | 1 | 502 | 2042 | 2543 | 0 | 0 |
| hsa_circ_0056674 | 90 | 76 | 322 | 248 | 1 | 0 | 5.00E-84 |
| hsa_circ_0056673 | 92 | 80 | 685 | 606 | 1 | 0 | 0 |
| hsa_circ_0056673 | 92 | 80 | 685 | 606 | 1 | 0 | 0 |
| hsa_circ_0056673 | 92 | 80 | 685 | 606 | 1 | 0 | 0 |
| hsa_circ_0056674 | 90 | 4 | 242 | 239 | 1 | 0 | 5.00E-83 |
| hsa_circ_0056673 | 92 | 4 | 605 | 602 | 1 | 0 | 0 |
| hsa_circ_0056673 | 92 | 4 | 605 | 602 | 1 | 0 | 0 |
| hsa_circ_0056673 | 92 | 4 | 605 | 602 | 1 | 0 | 0 |
| hsa_circ_0056673 | 93 | 1 | 531 | 531 | 1 | 0 | 0 |
| hsa_circ_0056673 | 93 | 1 | 531 | 531 | 1 | 0 | 0 |
| hsa_circ_0056673 | 92 | 1 | 253 | 454 | 202 | 0 | 2.00E-97 |
| hsa_circ_0056673 | 93 | 1 | 454 | 454 | 1 | 0 | 0 |
| hsa_circ_0056673 | 94 | 1 | 363 | 363 | 1 | 0 | 4.00E-155 |
| hsa_circ_0056673 | 94 | 1 | 363 | 363 | 1 | 0 | 6.00E-155 |
| hsa_circ_0056673 | 94 | 1 | 363 | 363 | 1 | 0 | 7.00E-155 |
| hsa_circ_0056673 | 93 | 3 | 287 | 285 | 1 | 0 | 1.00E-116 |
| hsa_circ_0056670 | 96 | 1 | 617 | 1 | 617 | 0 | 0 |
| hsa_circ_0056670 | 96 | 1 | 617 | 1 | 617 | 0 | 0 |
| hsa_circ_0056670 | 96 | 1 | 617 | 1 | 617 | 0 | 0 |

novel_circ_001409 novel_circ_001410 novel_circ_001415 novel_circ_001416 novel_circ_001417 novel_circ_001418 novel_circ_001419 novel_circ_001420 novel_circ_001421 novel_circ_001422 novel_circ_001423 novel_circ_001424 novel_circ_001425 novel_circ_001426 novel_circ_001427 novel_circ_001428 novel_circ_001431 novel_circ_001432 novel_circ_001433 novel_circ_001434 novel_circ_001436 novel_circ_001437 novel_circ_001438 novel_circ_001439 novel_circ_001440 novel_circ_001441 novel_circ_001442 novel_circ_001443 novel_circ_001444 novel_circ_001445 novel_circ_001449 novel_circ_001450 novel_circ_001451 novel_circ_001452 novel_circ_001453 novel_circ_001454 novel_circ_001455 novel_circ_001456 novel_circ_001457 novel_circ_001458 novel_circ_001459 novel_circ_001460 novel_circ_001461 novel_circ_001462 novel_circ_001463 novel_circ_001464 novel_circ_001465 novel_circ_001466 novel_circ_001468 novel_circ_001469 novel_circ_001470 novel_circ_001471 novel_circ_001472 novel_circ_001473

| hsa_circ_0056670 | 96 | 1 | 617 | 1 | 617 | 0 | 0 |
| --- | --- | --- | --- | --- | --- | --- | --- |
| hsa_circ_0056670 | 96 | 1 | 473 | 1 | 473 | 0 | 0 |
| hsa_circ_0030250 | 94 | 1 | 307 | 307 | 1 | 0 | 6.00E-132 |
| hsa_circ_0030250 | 94 | 1 | 307 | 307 | 1 | 0 | 4.00E-132 |
| hsa_circ_0002670 | 92 | 3 | 192 | 3 | 192 | 0 | 1.00E-70 |
| hsa_circ_0030256 | 84 | 1 | 473 | 1 | 497 | 0.05 | 4.00E-126 |
| hsa_circ_0030255 | 81 | 1 | 1012 | 565 | 1612 | 0.04 | 0 |
| hsa_circ_0030258 | 89 | 14963 | 15323 | 1 | 361 | 0 | 5.00E-124 |
| hsa_circ_0030255 | 82 | 47 | 814 | 922 | 1701 | 0.03 | 0 |
| hsa_circ_0030258 | 89 | 1 | 361 | 1 | 361 | 0 | 1.00E-125 |
| hsa_circ_0030260 | 93 | 1 | 410 | 410 | 1 | 0 | 7.00E-173 |
| hsa_circ_0030260 | 94 | 1 | 1496 | 1496 | 1 | 0 | 0 |
| hsa_circ_0030260 | 94 | 1 | 1499 | 1499 | 1 | 0 | 0 |
| hsa_circ_0030265 | 93 | 1 | 644 | 644 | 1 | 0 | 0 |
| hsa_circ_0030265 | 92 | 1 | 318 | 644 | 327 | 0 | 8.00E-122 |
| hsa_circ_0030260 | 92 | 1 | 321 | 1499 | 1179 | 0 | 2.00E-123 |
| hsa_circ_0030273 | 95 | 1 | 387 | 760 | 374 | 0 | 4.00E-170 |
| hsa_circ_0030273 | 94 | 1 | 284 | 760 | 477 | 0 | 5.00E-118 |
| hsa_circ_0000487 | 89 | 1039 | 1503 | 487 | 20 | 0.01 | 5.00E-161 |
| hsa_circ_0068512 | 72 | 7128 | 7727 | 28279 | 27684 | 0.03 | 3.00E-42 |
| hsa_circ_0080989 | 74 | 1632 | 2215 | 17694 | 18290 | 0.05 | 2.00E-49 |
| hsa_circ_0000489 | 88 | 256 | 937 | 1 | 677 | 0.03 | 0 |
| hsa_circ_0000489 | 88 | 1 | 271 | 1 | 257 | 0.05 | 7.00E-82 |
| hsa_circ_0000489 | 89 | 1 | 386 | 1 | 372 | 0.04 | 3.00E-127 |
| hsa_circ_0000489 | 88 | 1 | 557 | 1 | 552 | 0.04 | 0 |
| hsa_circ_0000489 | 88 | 1 | 639 | 1 | 634 | 0.04 | 0 |
| hsa_circ_0000489 | 88 | 1 | 682 | 1 | 677 | 0.03 | 0 |
| hsa_circ_0000489 | 88 | 1 | 682 | 1 | 677 | 0.03 | 0 |
| hsa_circ_0000489 | 88 | 1 | 682 | 1 | 677 | 0.03 | 0 |
| hsa_circ_0000489 | 89 | 1 | 368 | 258 | 634 | 0.02 | 4.00E-125 |
| hsa_circ_0030292 | 90 | 1 | 734 | 1 | 734 | 0 | 0 |
| hsa_circ_0030293 | 94 | 1 | 609 | 4747 | 4139 | 0 | 0 |
| hsa_circ_0030293 | 94 | 1 | 1957 | 6238 | 4282 | 0 | 0 |
| hsa_circ_0030293 | 94 | 1 | 502 | 5249 | 4748 | 0 | 0 |
| hsa_circ_0030293 | 94 | 1 | 718 | 5465 | 4748 | 0 | 0 |
| hsa_circ_0030293 | 94 | 4 | 829 | 5573 | 4748 | 0 | 0 |
| hsa_circ_0030293 | 95 | 1 | 1491 | 6238 | 4748 | 0 | 0 |
| hsa_circ_0030293 | 95 | 4 | 1675 | 6419 | 4748 | 0 | 0 |
| hsa_circ_0030293 | 93 | 4 | 327 | 5573 | 5250 | 0 | 4.00E-130 |
| hsa_circ_0030293 | 93 | 1 | 422 | 5671 | 5250 | 0 | 2.00E-174 |
| hsa_circ_0030293 | 95 | 1 | 989 | 6238 | 5250 | 0 | 0 |
| hsa_circ_0030293 | 95 | 4 | 1173 | 6419 | 5250 | 0 | 0 |
| hsa_circ_0030294 | 94 | 1 | 492 | 492 | 1 | 0 | 0 |
| hsa_circ_0030294 | 95 | 1 | 773 | 773 | 1 | 0 | 0 |
| hsa_circ_0030297 | 96 | 1 | 567 | 567 | 1 | 0 | 0 |
| hsa_circ_0030297 | 96 | 1 | 567 | 567 | 1 | 0 | 0 |
| hsa_circ_0030297 | 98 | 4 | 465 | 748 | 287 | 0 | 0 |
| hsa_circ_0030301 | 98 | 147 | 332 | 186 | 1 | 0 | 2.00E-88 |
| hsa_circ_0034348 | 94 | 803 | 1420 | 901 | 284 | 0 | 0 |
| hsa_circ_0034348 | 92 | 4 | 242 | 712 | 474 | 0 | 2.00E-91 |
| hsa_circ_0034348 | 92 | 4 | 243 | 712 | 473 | 0 | 5.00E-91 |
| hsa_circ_0034346 | 96 | 1 | 316 | 316 | 1 | 0 | 7.00E-142 |
| hsa_circ_0034346 | 96 | 1 | 316 | 316 | 1 | 0 | 8.00E-142 |
| hsa_circ_0034345 | 94 | 1 | 233 | 233 | 1 | 0 | 2.00E-95 |

novel_circ_001474 novel_circ_001475 novel_circ_001484 novel_circ_001485 novel_circ_001486 novel_circ_001487 novel_circ_001495 novel_circ_001496 novel_circ_001497 novel_circ_001498 novel_circ_001499 novel_circ_001500 novel_circ_001501 novel_circ_001502 novel_circ_001503 novel_circ_001504 novel_circ_001505 novel_circ_001510 novel_circ_001512 novel_circ_001513 novel_circ_001514 novel_circ_001515 novel_circ_001516 novel_circ_001526 novel_circ_001527 novel_circ_001529 novel_circ_001530 novel_circ_001533 novel_circ_001534 novel_circ_001535 novel_circ_001536 novel_circ_001538 novel_circ_001539 novel_circ_001540 novel_circ_001541 novel_circ_001542 novel_circ_001543 novel_circ_001544 novel_circ_001545 novel_circ_001546 novel_circ_001547 novel_circ_001548 novel_circ_001549 novel_circ_001550 novel_circ_001551 novel_circ_001552 novel_circ_001553 novel_circ_001554 novel_circ_001555 novel_circ_001556 novel_circ_001566 novel_circ_001567 novel_circ_001568 novel_circ_001569

| hsa_circ_0034345 | 93 | 1 | 175 | 175 | 1 | 0 | 2.00E-65 |
| --- | --- | --- | --- | --- | --- | --- | --- |
| hsa_circ_0034343 | 95 | 5 | 452 | 448 | 1 | 0 | 0 |
| hsa_circ_0087517 | 86 | 3 | 198 | 218 | 26 | 0.02 | 5.00E-53 |
| hsa_circ_0087517 | 86 | 3 | 198 | 218 | 26 | 0.02 | 6.00E-52 |
| hsa_circ_0050775 | 81 | 905 | 1165 | 4069 | 3809 | 0.02 | 7.00E-51 |
| hsa_circ_0082725 | 88 | 1141 | 1191 | 20193 | 20243 | 0 | 3.00E-07 |
| hsa_circ_0030200 | 95 | 3 | 269 | 3 | 269 | 0 | 7.00E-117 |
| hsa_circ_0030208 | 91 | 1 | 226 | 1 | 229 | 0.01 | 4.00E-83 |
| hsa_circ_0030208 | 92 | 1 | 640 | 1 | 640 | 0.01 | 0 |
| hsa_circ_0030207 | 93 | 1 | 993 | 1 | 993 | 0.01 | 0 |
| hsa_circ_0030207 | 93 | 1 | 993 | 1 | 993 | 0.01 | 0 |
| hsa_circ_0030207 | 94 | 1 | 556 | 300 | 855 | 0 | 0 |
| hsa_circ_0030207 | 94 | 1 | 694 | 300 | 993 | 0 | 0 |
| hsa_circ_0030215 | 94 | 1 | 326 | 1 | 326 | 0 | 6.00E-138 |
| hsa_circ_0030215 | 88 | 1 | 629 | 1 | 632 | 0.03 | 0 |
| hsa_circ_0030215 | 94 | 1 | 216 | 111 | 326 | 0 | 5.00E-87 |
| hsa_circ_0013829 | 85 | 12944 | 13038 | 113053 | 113146 | 0.01 | 5.00E-17 |
| hsa_circ_0005447 | 97 | 6951 | 7062 | 7469 | 7356 | 0.02 | 1.00E-45 |
| hsa_circ_0021547 | 97 | 1 | 409 | 638 | 230 | 0 | 0 |
| hsa_circ_0021551 | 95 | 1 | 226 | 226 | 1 | 0 | 2.00E-97 |
| hsa_circ_0021551 | 97 | 1 | 408 | 408 | 1 | 0 | 0 |
| hsa_circ_0021551 | 95 | 1 | 657 | 657 | 1 | 0 | 0 |
| hsa_circ_0021553 | 95 | 1 | 431 | 431 | 1 | 0 | 0 |
| hsa_circ_0071392 | 92 | 1 | 116 | 874 | 989 | 0 | 7.00E-39 |
| hsa_circ_0071392 | 95 | 1 | 241 | 749 | 989 | 0 | 1.00E-101 |
| hsa_circ_0071392 | 92 | 1 | 116 | 874 | 989 | 0 | 6.00E-39 |
| hsa_circ_0071392 | 95 | 1 | 241 | 749 | 989 | 0 | 1.00E-101 |
| hsa_circ_0071392 | 92 | 1 | 116 | 874 | 989 | 0 | 6.00E-39 |
| hsa_circ_0071392 | 95 | 1 | 241 | 749 | 989 | 0 | 1.00E-101 |
| hsa_circ_0071392 | 94 | 1 | 989 | 1 | 989 | 0 | 0 |
| hsa_circ_0071392 | 95 | 1 | 241 | 749 | 989 | 0 | 8.00E-102 |
| hsa_circ_0071392 | 92 | 1 | 116 | 874 | 989 | 0 | 2.00E-39 |
| hsa_circ_0071392 | 95 | 1 | 241 | 749 | 989 | 0 | 4.00E-102 |
| hsa_circ_0071392 | 94 | 1 | 526 | 464 | 989 | 0 | 0 |
| hsa_circ_0071392 | 94 | 3 | 797 | 195 | 989 | 0 | 0 |
| hsa_circ_0071392 | 94 | 1 | 878 | 112 | 989 | 0 | 0 |
| hsa_circ_0071392 | 94 | 1 | 989 | 1 | 989 | 0 | 0 |
| hsa_circ_0071392 | 92 | 1 | 116 | 874 | 989 | 0 | 1.00E-39 |
| hsa_circ_0071392 | 94 | 3 | 797 | 195 | 989 | 0 | 0 |
| hsa_circ_0071392 | 95 | 1 | 241 | 749 | 989 | 0 | 2.00E-102 |
| hsa_circ_0071392 | 94 | 1 | 526 | 464 | 989 | 0 | 0 |
| hsa_circ_0071392 | 94 | 1 | 989 | 1 | 989 | 0 | 0 |
| hsa_circ_0071392 | 95 | 1 | 410 | 464 | 873 | 0 | 0 |
| hsa_circ_0071392 | 94 | 1 | 762 | 112 | 873 | 0 | 0 |
| hsa_circ_0071392 | 94 | 1 | 873 | 1 | 873 | 0 | 0 |
| hsa_circ_0071392 | 94 | 1 | 286 | 464 | 749 | 0 | 2.00E-122 |
| hsa_circ_0071392 | 94 | 1 | 285 | 464 | 748 | 0 | 7.00E-122 |
| hsa_circ_0071392 | 94 | 44 | 329 | 463 | 748 | 0 | 2.00E-122 |
| hsa_circ_0071392 | 94 | 1 | 748 | 1 | 748 | 0 | 0 |
| hsa_circ_0071392 | 94 | 1 | 460 | 1 | 460 | 0 | 0 |
| hsa_circ_0071379 | 92 | 3 | 570 | 1016 | 1583 | 0 | 0 |
| hsa_circ_0071379 | 92 | 639 | 1371 | 851 | 1583 | 0 | 0 |
| hsa_circ_0071381 | 95 | 1 | 604 | 108 | 711 | 0 | 0 |
| hsa_circ_0071384 | 95 | 1 | 319 | 1 | 319 | 0 | 4.00E-142 |

novel_circ_001570 novel_circ_001571 novel_circ_001572 novel_circ_001573 novel_circ_001574 novel_circ_001576 novel_circ_001578 novel_circ_001579 novel_circ_001580 novel_circ_001581 novel_circ_001582 novel_circ_001583 novel_circ_001584 novel_circ_001585 novel_circ_001586 novel_circ_001587 novel_circ_001588 novel_circ_001589 novel_circ_001590 novel_circ_001591 novel_circ_001592 novel_circ_001593 novel_circ_001594 novel_circ_001595 novel_circ_001596 novel_circ_001597 novel_circ_001598 novel_circ_001599 novel_circ_001600 novel_circ_001601 novel_circ_001602 novel_circ_001603 novel_circ_001604 novel_circ_001605 novel_circ_001606 novel_circ_001607 novel_circ_001608 novel_circ_001609 novel_circ_001610 novel_circ_001611 novel_circ_001612 novel_circ_001613 novel_circ_001614 novel_circ_001615 novel_circ_001616 novel_circ_001617 novel_circ_001618 novel_circ_001619 novel_circ_001620 novel_circ_001621 novel_circ_001624 novel_circ_001625 novel_circ_001626 novel_circ_001627

| hsa_circ_0071381 | 95 | 1 | 604 | 108 | 711 | 0 | 0 |
| --- | --- | --- | --- | --- | --- | --- | --- |
| hsa_circ_0071381 | 95 | 1 | 512 | 108 | 619 | 0 | 0 |
| hsa_circ_0071379 | 96 | 1 | 627 | 1 | 627 | 0 | 0 |
| hsa_circ_0071379 | 96 | 1 | 355 | 1 | 355 | 0 | 2.00E-162 |
| hsa_circ_0071379 | 97 | 1 | 233 | 1 | 233 | 0 | 2.00E-106 |
| hsa_circ_0083679 | 92 | 1 | 659 | 1452 | 794 | 0 | 0 |
| hsa_circ_0056008 | 90 | 2 | 1131 | 1130 | 1 | 0 | 0 |
| hsa_circ_0001061 | 91 | 1 | 370 | 118 | 487 | 0 | 4.00E-137 |
| hsa_circ_0001061 | 90 | 1 | 487 | 1 | 487 | 0 | 4.00E-177 |
| hsa_circ_0056001 | 90 | 1 | 958 | 470 | 1427 | 0 | 0 |
| hsa_circ_0001061 | 91 | 1 | 370 | 118 | 487 | 0 | 3.00E-137 |
| hsa_circ_0001061 | 91 | 4225 | 4598 | 114 | 487 | 0 | 2.00E-138 |
| hsa_circ_0001061 | 90 | 1 | 487 | 1 | 487 | 0 | 3.00E-177 |
| hsa_circ_0056001 | 89 | 1 | 594 | 834 | 1427 | 0 | 0 |
| hsa_circ_0056001 | 89 | 8 | 652 | 783 | 1427 | 0 | 0 |
| hsa_circ_0056001 | 89 | 1 | 838 | 590 | 1427 | 0 | 0 |
| hsa_circ_0056001 | 90 | 1 | 958 | 470 | 1427 | 0 | 0 |
| hsa_circ_0056001 | 89 | 1 | 1299 | 129 | 1427 | 0 | 0 |
| hsa_circ_0056001 | 89 | 1 | 1424 | 1 | 1427 | 0 | 0 |
| hsa_circ_0056001 | 89 | 122 | 1545 | 1 | 1427 | 0 | 0 |
| hsa_circ_0056001 | 89 | 180 | 1603 | 1 | 1427 | 0 | 0 |
| hsa_circ_0056001 | 89 | 823 | 2246 | 1 | 1427 | 0 | 0 |
| hsa_circ_0056001 | 89 | 1 | 781 | 470 | 1250 | 0 | 0 |
| hsa_circ_0056001 | 89 | 1 | 1122 | 129 | 1250 | 0 | 0 |
| hsa_circ_0056001 | 89 | 122 | 1368 | 1 | 1250 | 0 | 0 |
| hsa_circ_0056001 | 89 | 180 | 1426 | 1 | 1250 | 0 | 0 |
| hsa_circ_0056001 | 89 | 1 | 929 | 129 | 1057 | 0 | 0 |
| hsa_circ_0056001 | 89 | 1 | 812 | 129 | 940 | 0 | 0 |
| hsa_circ_0056001 | 89 | 122 | 1058 | 1 | 940 | 0 | 0 |
| hsa_circ_0056001 | 89 | 180 | 1116 | 1 | 940 | 0 | 0 |
| hsa_circ_0056001 | 89 | 1 | 461 | 129 | 589 | 0 | 8.00E-163 |
| hsa_circ_0056001 | 89 | 122 | 707 | 1 | 589 | 0.01 | 0 |
| hsa_circ_0056001 | 89 | 180 | 765 | 1 | 589 | 0.01 | 0 |
| hsa_circ_0056001 | 88 | 122 | 587 | 1 | 469 | 0.01 | 1.00E-152 |
| hsa_circ_0056001 | 88 | 180 | 645 | 1 | 469 | 0.01 | 1.00E-152 |
| hsa_circ_0055999 | 83 | 2 | 428 | 2 | 425 | 0.02 | 4.00E-101 |
| hsa_circ_0055999 | 83 | 60 | 486 | 2 | 425 | 0.02 | 5.00E-101 |
| hsa_circ_0055999 | 84 | 2 | 246 | 2 | 243 | 0.04 | 1.00E-58 |
| hsa_circ_0055997 | 91 | 1 | 609 | 912 | 1520 | 0 | 0 |
| hsa_circ_0055997 | 87 | 1 | 896 | 1 | 911 | 0.02 | 0 |
| hsa_circ_0055997 | 87 | 1 | 750 | 1 | 750 | 0.01 | 0 |
| hsa_circ_0055997 | 89 | 1 | 443 | 1 | 443 | 0.01 | 8.00E-153 |
| hsa_circ_0002728 | 89 | 1 | 3241 | 1 | 3269 | 0.01 | 0 |
| hsa_circ_0008364 | 92 | 1 | 847 | 1 | 847 | 0 | 0 |
| hsa_circ_0008364 | 92 | 1 | 711 | 1 | 711 | 0 | 0 |
| hsa_circ_0009078 | 91 | 1 | 327 | 2 | 328 | 0 | 8.00E-122 |
| hsa_circ_0055987 | 90 | 1 | 452 | 531 | 982 | 0.01 | 2.00E-164 |
| hsa_circ_0055987 | 90 | 1 | 982 | 1 | 982 | 0.01 | 0 |
| hsa_circ_0055987 | 89 | 1 | 570 | 154 | 723 | 0.01 | 0 |
| hsa_circ_0055985 | 93 | 1 | 333 | 1 | 333 | 0 | 4.00E-135 |
| hsa_circ_0055965 | 90 | 2 | 590 | 2 | 590 | 0 | 0 |
| hsa_circ_0055965 | 90 | 2 | 200 | 2 | 200 | 0 | 1.00E-67 |
| hsa_circ_0007432 | 91 | 113 | 573 | 1 | 461 | 0 | 5.00E-176 |
| hsa_circ_0007432 | 92 | 113 | 281 | 1 | 169 | 0 | 2.00E-62 |

novel_circ_001628 novel_circ_001633 novel_circ_001634 novel_circ_001635 novel_circ_001636 novel_circ_001646 novel_circ_001649 novel_circ_001650 novel_circ_001651 novel_circ_001652 novel_circ_001653 novel_circ_001656 novel_circ_001657 novel_circ_001658 novel_circ_001659 novel_circ_001660 novel_circ_001661 novel_circ_001662 novel_circ_001663 novel_circ_001664 novel_circ_001665 novel_circ_001666 novel_circ_001667 novel_circ_001668 novel_circ_001669 novel_circ_001671 novel_circ_001672 novel_circ_001673 novel_circ_001674 novel_circ_001678 novel_circ_001679 novel_circ_001680 novel_circ_001681 novel_circ_001682 novel_circ_001683 novel_circ_001684 novel_circ_001685 novel_circ_001686 novel_circ_001687 novel_circ_001688 novel_circ_001689 novel_circ_001690 novel_circ_001691 novel_circ_001692 novel_circ_001693 novel_circ_001694 novel_circ_001695 novel_circ_001696 novel_circ_001697 novel_circ_001698 novel_circ_001699 novel_circ_001700 novel_circ_001701 novel_circ_001702

| hsa_circ_0007432 | 92 | 113 | 189 | 1 | 77 | 0 | 4.00E-23 |
| --- | --- | --- | --- | --- | --- | --- | --- |
| hsa_circ_0063057 | 85 | 1 | 208 | 648 | 441 | 0 | 9.00E-54 |
| hsa_circ_0063057 | 85 | 4 | 262 | 699 | 441 | 0 | 4.00E-69 |
| hsa_circ_0063057 | 84 | 76 | 406 | 772 | 441 | 0 | 3.00E-87 |
| hsa_circ_0063057 | 84 | 319 | 649 | 772 | 441 | 0 | 5.00E-87 |
| hsa_circ_0007133 | 89 | 15216 | 15271 | 393176 | 393121 | 0 | 4.00E-09 |
| hsa_circ_0037766 | 93 | 1 | 861 | 1 | 861 | 0 | 0 |
| hsa_circ_0037766 | 93 | 289 | 1149 | 1 | 861 | 0 | 0 |
| hsa_circ_0037764 | 96 | 1 | 382 | 382 | 1 | 0 | 5.00E-179 |
| hsa_circ_0002241 | 94 | 1 | 747 | 751 | 2 | 0 | 0 |
| hsa_circ_0037735 | 92 | 1 | 352 | 1 | 352 | 0 | 1.00E-140 |
| hsa_circ_0007637 | 91 | 1 | 710 | 713 | 1 | 0.01 | 0 |
| hsa_circ_0037673 | 90 | 3 | 777 | 826 | 46 | 0.01 | 0 |
| hsa_circ_0037675 | 89 | 1 | 534 | 540 | 1 | 0.01 | 0 |
| hsa_circ_0037675 | 90 | 2 | 290 | 289 | 1 | 0 | 9.00E-101 |
| hsa_circ_0037671 | 88 | 2 | 649 | 639 | 1 | 0.01 | 0 |
| hsa_circ_0037661 | 88 | 1 | 787 | 1316 | 530 | 0 | 0 |
| hsa_circ_0037661 | 90 | 1 | 448 | 529 | 82 | 0 | 3.00E-167 |
| hsa_circ_0037658 | 92 | 1 | 586 | 586 | 1 | 0 | 0 |
| hsa_circ_0037652 | 92 | 1 | 1144 | 1478 | 334 | 0 | 0 |
| hsa_circ_0037661 | 91 | 4 | 329 | 407 | 82 | 0 | 3.00E-121 |
| hsa_circ_0037658 | 93 | 4 | 467 | 464 | 1 | 0 | 0 |
| hsa_circ_0037657 | 95 | 1 | 146 | 146 | 1 | 0 | 6.00E-60 |
| hsa_circ_0037652 | 92 | 1 | 558 | 892 | 334 | 0 | 0 |
| hsa_circ_0037653 | 91 | 3 | 412 | 410 | 1 | 0 | 4.00E-156 |
| hsa_circ_0037605 | 88 | 3 | 905 | 3 | 905 | 0 | 0 |
| hsa_circ_0037605 | 89 | 3 | 557 | 3 | 557 | 0 | 0 |
| hsa_circ_0044686 | 89 | 121 | 813 | 1 | 693 | 0 | 0 |
| hsa_circ_0044686 | 89 | 1 | 498 | 1 | 498 | 0 | 1.00E-171 |
| hsa_circ_0044696 | 85 | 134 | 631 | 1 | 486 | 0.02 | 3.00E-138 |
| hsa_circ_0044696 | 85 | 134 | 631 | 1 | 486 | 0.02 | 4.00E-138 |
| hsa_circ_0044696 | 85 | 134 | 631 | 1 | 486 | 0.02 | 6.00E-137 |
| hsa_circ_0044696 | 85 | 134 | 631 | 1 | 486 | 0.02 | 4.00E-138 |
| hsa_circ_0044696 | 85 | 134 | 631 | 1 | 486 | 0.02 | 5.00E-138 |
| hsa_circ_0044698 | 92 | 632 | 1454 | 1 | 823 | 0 | 0 |
| hsa_circ_0044696 | 87 | 1 | 383 | 1 | 371 | 0.03 | 8.00E-118 |
| hsa_circ_0044696 | 85 | 1 | 498 | 1 | 486 | 0.02 | 3.00E-138 |
| hsa_circ_0044696 | 85 | 1 | 498 | 1 | 486 | 0.02 | 3.00E-138 |
| hsa_circ_0044696 | 85 | 1 | 498 | 1 | 486 | 0.02 | 6.00E-137 |
| hsa_circ_0044696 | 85 | 1 | 498 | 1 | 486 | 0.02 | 4.00E-138 |
| hsa_circ_0044696 | 85 | 1 | 498 | 1 | 486 | 0.02 | 4.00E-138 |
| hsa_circ_0044698 | 93 | 499 | 843 | 1 | 345 | 0 | 1.00E-139 |
| hsa_circ_0044698 | 93 | 499 | 1020 | 1 | 522 | 0 | 0 |
| hsa_circ_0044698 | 92 | 499 | 1321 | 1 | 823 | 0 | 0 |
| hsa_circ_0044698 | 91 | 1 | 634 | 190 | 823 | 0 | 0 |
| hsa_circ_0080878 | 96 | 1 | 507 | 565 | 59 | 0 | 0 |
| hsa_circ_0080877 | 97 | 309 | 492 | 184 | 1 | 0 | 4.00E-82 |
| hsa_circ_0080877 | 97 | 18216 | 18399 | 184 | 1 | 0 | 1.00E-80 |
| hsa_circ_0080877 | 97 | 251 | 434 | 184 | 1 | 0 | 3.00E-82 |
| hsa_circ_0080875 | 96 | 435 | 1073 | 1437 | 799 | 0 | 0 |
| hsa_circ_0080877 | 97 | 239 | 422 | 184 | 1 | 0 | 3.00E-82 |
| hsa_circ_0080875 | 96 | 1 | 639 | 1437 | 799 | 0 | 0 |
| hsa_circ_0080875 | 95 | 1 | 766 | 766 | 1 | 0 | 0 |
| hsa_circ_0080875 | 95 | 1 | 576 | 576 | 1 | 0 | 0 |

novel_circ_001703 novel_circ_001704 novel_circ_001705 novel_circ_001706 novel_circ_001707 novel_circ_001708 novel_circ_001709 novel_circ_001710 novel_circ_001711 novel_circ_001712 novel_circ_001713 novel_circ_001714 novel_circ_001715 novel_circ_001716 novel_circ_001717 novel_circ_001718 novel_circ_001719 novel_circ_001720 novel_circ_001721 novel_circ_001722 novel_circ_001723 novel_circ_001725 novel_circ_001726 novel_circ_001727 novel_circ_001728 novel_circ_001729 novel_circ_001730 novel_circ_001731 novel_circ_001732 novel_circ_001733 novel_circ_001734 novel_circ_001735 novel_circ_001736 novel_circ_001737 novel_circ_001738 novel_circ_001739 novel_circ_001741 novel_circ_001742 novel_circ_001743 novel_circ_001744 novel_circ_001745 novel_circ_001746 novel_circ_001747 novel_circ_001748 novel_circ_001749 novel_circ_001750 novel_circ_001751 novel_circ_001752 novel_circ_001753 novel_circ_001754 novel_circ_001755 novel_circ_001756 novel_circ_001757 novel_circ_001758

| hsa_circ_0080875 | 94 | 1 | 534 | 534 | 1 | 0 | 0 |
| --- | --- | --- | --- | --- | --- | --- | --- |
| hsa_circ_0080875 | 94 | 1 | 534 | 534 | 1 | 0 | 0 |
| hsa_circ_0080875 | 93 | 1 | 442 | 442 | 1 | 0 | 0 |
| hsa_circ_0003002 | 93 | 362 | 725 | 364 | 1 | 0 | 5.00E-152 |
| hsa_circ_0003002 | 93 | 1 | 364 | 364 | 1 | 0 | 2.00E-152 |
| hsa_circ_0080872 | 96 | 1 | 492 | 1 | 492 | 0 | 0 |
| hsa_circ_0080870 | 96 | 1 | 840 | 1221 | 2060 | 0 | 0 |
| hsa_circ_0080870 | 94 | 1 | 691 | 878 | 1568 | 0 | 0 |
| hsa_circ_0080870 | 94 | 1 | 1308 | 261 | 1568 | 0 | 0 |
| hsa_circ_0080870 | 94 | 4 | 1041 | 183 | 1220 | 0 | 0 |
| hsa_circ_0080870 | 94 | 1 | 617 | 261 | 877 | 0 | 0 |
| hsa_circ_0080852 | 90 | 1 | 1262 | 331 | 1586 | 0 | 0 |
| hsa_circ_0080852 | 91 | 1 | 1154 | 331 | 1478 | 0.01 | 0 |
| hsa_circ_0080858 | 91 | 1 | 1123 | 1 | 1117 | 0.01 | 0 |
| hsa_circ_0080858 | 91 | 1 | 1055 | 1 | 1049 | 0.01 | 0 |
| hsa_circ_0080855 | 91 | 1 | 1063 | 1 | 1057 | 0.01 | 0 |
| hsa_circ_0080839 | 95 | 78 | 817 | 1 | 740 | 0 | 0 |
| hsa_circ_0080844 | 96 | 1 | 275 | 1 | 275 | 0 | 1.00E-124 |
| hsa_circ_0080843 | 96 | 1 | 314 | 1 | 314 | 0 | 3.00E-141 |
| hsa_circ_0080836 | 94 | 1 | 487 | 1 | 487 | 0 | 0 |
| hsa_circ_0080835 | 94 | 1 | 596 | 1 | 596 | 0 | 0 |
| hsa_circ_0081724 | 91 | 6 | 342 | 6 | 342 | 0 | 2.00E-127 |
| hsa_circ_0081724 | 91 | 6 | 342 | 6 | 342 | 0 | 3.00E-127 |
| hsa_circ_0081725 | 87 | 112 | 642 | 2 | 505 | 0.05 | 2.00E-165 |
| hsa_circ_0081725 | 87 | 112 | 642 | 2 | 505 | 0.05 | 2.00E-165 |
| hsa_circ_0081725 | 93 | 2 | 232 | 2 | 232 | 0 | 6.00E-92 |
| hsa_circ_0081725 | 87 | 2 | 532 | 2 | 505 | 0.05 | 2.00E-165 |
| hsa_circ_0081725 | 87 | 2 | 532 | 2 | 505 | 0.05 | 2.00E-165 |
| hsa_circ_0081725 | 86 | 2 | 439 | 95 | 505 | 0.06 | 3.00E-122 |
| hsa_circ_0081725 | 86 | 2 | 439 | 95 | 505 | 0.06 | 4.00E-122 |
| hsa_circ_0008271 | 83 | 1 | 300 | 1 | 273 | 0.09 | 5.00E-64 |
| hsa_circ_0008271 | 81 | 1 | 258 | 43 | 273 | 0.1 | 2.00E-42 |
| hsa_circ_0081730 | 87 | 1 | 846 | 1010 | 165 | 0 | 0 |
| hsa_circ_0081730 | 88 | 1 | 627 | 1010 | 384 | 0 | 0 |
| hsa_circ_0081730 | 89 | 3 | 757 | 1138 | 384 | 0 | 0 |
| hsa_circ_0081730 | 89 | 1 | 911 | 1294 | 384 | 0 | 0 |
| hsa_circ_0058662 | 74 | 14603 | 14954 | 4894 | 4545 | 0.03 | 1.00E-31 |
| hsa_circ_0081738 | 94 | 1 | 644 | 647 | 4 | 0 | 0 |
| hsa_circ_0081738 | 93 | 2 | 954 | 956 | 4 | 0 | 0 |
| hsa_circ_0081741 | 93 | 1 | 358 | 1 | 358 | 0 | 2.00E-147 |
| hsa_circ_0081742 | 93 | 142 | 1055 | 1 | 914 | 0 | 0 |
| hsa_circ_0081745 | 91 | 1 | 418 | 81 | 498 | 0 | 3.00E-162 |
| hsa_circ_0081747 | 93 | 1 | 305 | 1 | 305 | 0 | 3.00E-126 |
| hsa_circ_0081762 | 92 | 8 | 837 | 830 | 1 | 0 | 0 |
| hsa_circ_0081763 | 92 | 1 | 378 | 378 | 1 | 0 | 9.00E-152 |
| hsa_circ_0081763 | 92 | 1 | 702 | 1316 | 615 | 0 | 0 |
| hsa_circ_0081763 | 92 | 1 | 2020 | 2634 | 615 | 0 | 0 |
| hsa_circ_0081768 | 92 | 1 | 925 | 925 | 1 | 0 | 0 |
| hsa_circ_0081768 | 92 | 1 | 1552 | 2477 | 926 | 0 | 0 |
| hsa_circ_0081768 | 92 | 1 | 2139 | 3064 | 926 | 0 | 0 |
| hsa_circ_0081768 | 92 | 2 | 3437 | 4361 | 926 | 0 | 0 |
| hsa_circ_0081768 | 92 | 1 | 1970 | 3064 | 1095 | 0 | 0 |
| hsa_circ_0081768 | 92 | 1 | 726 | 2477 | 1752 | 0 | 0 |
| hsa_circ_0081768 | 92 | 1 | 1313 | 3064 | 1752 | 0 | 0 |

novel_circ_001759 novel_circ_001760 novel_circ_001761 novel_circ_001762 novel_circ_001763 novel_circ_001764 novel_circ_001765 novel_circ_001766 novel_circ_001767 novel_circ_001768 novel_circ_001769 novel_circ_001770 novel_circ_001771 novel_circ_001772 novel_circ_001773 novel_circ_001774 novel_circ_001777 novel_circ_001784 novel_circ_001787 novel_circ_001788 novel_circ_001789 novel_circ_001790 novel_circ_001791 novel_circ_001792 novel_circ_001793 novel_circ_001794 novel_circ_001795 novel_circ_001796 novel_circ_001797 novel_circ_001798 novel_circ_001799 novel_circ_001800 novel_circ_001801 novel_circ_001802 novel_circ_001803 novel_circ_001804 novel_circ_001805 novel_circ_001806 novel_circ_001807 novel_circ_001808 novel_circ_001809 novel_circ_001811 novel_circ_001812 novel_circ_001813 novel_circ_001814 novel_circ_001817 novel_circ_001818 novel_circ_001819 novel_circ_001820 novel_circ_001821 novel_circ_001822 novel_circ_001823 novel_circ_001824 novel_circ_001825

| hsa_circ_0081768 | 92 | 1 | 951 | 2923 | 1973 | 0 | 0 |
| --- | --- | --- | --- | --- | --- | --- | --- |
| hsa_circ_0081768 | 92 | 1 | 1092 | 3064 | 1973 | 0 | 0 |
| hsa_circ_0081768 | 93 | 1 | 587 | 3064 | 2478 | 0 | 0 |
| hsa_circ_0081768 | 93 | 1 | 403 | 3064 | 2662 | 0 | 6.00E-164 |
| hsa_circ_0081772 | 95 | 7 | 348 | 616 | 275 | 0 | 8.00E-152 |
| hsa_circ_0081772 | 93 | 2 | 676 | 1297 | 623 | 0 | 0 |
| hsa_circ_0081772 | 95 | 1 | 373 | 1671 | 1299 | 0 | 2.00E-167 |
| hsa_circ_0081773 | 93 | 1 | 1261 | 1705 | 445 | 0 | 0 |
| hsa_circ_0081773 | 93 | 1 | 1413 | 1857 | 445 | 0 | 0 |
| hsa_circ_0081774 | 93 | 2 | 396 | 632 | 238 | 0 | 3.00E-161 |
| hsa_circ_0081773 | 92 | 1 | 780 | 1857 | 1078 | 0 | 0 |
| hsa_circ_0081779 | 95 | 3 | 338 | 336 | 1 | 0 | 2.00E-148 |
| hsa_circ_0081779 | 94 | 1 | 487 | 487 | 1 | 0 | 0 |
| hsa_circ_0081779 | 93 | 1 | 670 | 670 | 1 | 0 | 0 |
| hsa_circ_0081782 | 92 | 1 | 332 | 332 | 1 | 0 | 6.00E-128 |
| hsa_circ_0081796 | 93 | 3 | 385 | 383 | 1 | 0 | 5.00E-161 |
| hsa_circ_0005490 | 90 | 1 | 423 | 423 | 1 | 0 | 1.00E-151 |
| hsa_circ_0052806 | 91 | 139 | 669 | 531 | 1 | 0.01 | 0 |
| hsa_circ_0052800 | 93 | 558 | 1411 | 1 | 854 | 0 | 0 |
| hsa_circ_0052785 | 92 | 4 | 629 | 765 | 140 | 0 | 0 |
| hsa_circ_0052788 | 91 | 1 | 367 | 367 | 1 | 0 | 2.00E-137 |
| hsa_circ_0052783 | 90 | 1 | 575 | 575 | 1 | 0 | 0 |
| hsa_circ_0008061 | 90 | 1 | 704 | 704 | 1 | 0 | 0 |
| hsa_circ_0052771 | 91 | 1 | 1958 | 1960 | 3 | 0 | 0 |
| hsa_circ_0052783 | 90 | 2 | 208 | 207 | 1 | 0 | 5.00E-72 |
| hsa_circ_0008061 | 90 | 2 | 337 | 336 | 1 | 0 | 8.00E-122 |
| hsa_circ_0052771 | 91 | 2 | 1591 | 1592 | 3 | 0 | 0 |
| hsa_circ_0052774 | 92 | 1 | 1143 | 1143 | 1 | 0 | 0 |
| hsa_circ_0052771 | 92 | 1 | 612 | 614 | 3 | 0 | 0 |
| hsa_circ_0052763 | 91 | 1 | 690 | 698 | 9 | 0 | 0 |
| hsa_circ_0052762 | 90 | 1 | 852 | 852 | 1 | 0 | 0 |
| hsa_circ_0052757 | 90 | 1 | 1212 | 1346 | 135 | 0 | 0 |
| hsa_circ_0052757 | 90 | 1 | 1346 | 1346 | 1 | 0 | 0 |
| hsa_circ_0052754 | 89 | 1 | 1532 | 1532 | 1 | 0 | 0 |
| hsa_circ_0052762 | 88 | 1 | 480 | 480 | 1 | 0 | 5.00E-160 |
| hsa_circ_0052757 | 89 | 1 | 840 | 974 | 135 | 0 | 0 |
| hsa_circ_0052757 | 89 | 1 | 974 | 974 | 1 | 0 | 0 |
| hsa_circ_0052757 | 89 | 4 | 732 | 729 | 1 | 0 | 0 |
| hsa_circ_0052754 | 88 | 1 | 834 | 834 | 1 | 0 | 0 |
| hsa_circ_0052757 | 90 | 7 | 494 | 488 | 1 | 0 | 9.00E-178 |
| hsa_circ_0052754 | 89 | 7 | 680 | 674 | 1 | 0 | 0 |
| hsa_circ_0052736 | 89 | 4 | 1242 | 1239 | 1 | 0 | 0 |
| hsa_circ_0052741 | 89 | 1 | 560 | 560 | 1 | 0 | 0 |
| hsa_circ_0052736 | 90 | 1 | 674 | 922 | 249 | 0 | 0 |
| hsa_circ_0052736 | 90 | 1 | 922 | 922 | 1 | 0 | 0 |
| hsa_circ_0052716 | 89 | 4 | 1545 | 1542 | 1 | 0 | 0 |
| hsa_circ_0052724 | 89 | 1 | 445 | 445 | 1 | 0 | 6.00E-159 |
| hsa_circ_0052719 | 89 | 1 | 556 | 556 | 1 | 0 | 0 |
| hsa_circ_0052716 | 90 | 1 | 1142 | 1142 | 1 | 0 | 0 |
| hsa_circ_0052716 | 91 | 1 | 586 | 586 | 1 | 0 | 0 |
| hsa_circ_0052693 | 89 | 2 | 598 | 603 | 7 | 0 | 0 |
| hsa_circ_0052702 | 90 | 8 | 279 | 272 | 1 | 0.01 | 3.00E-96 |
| hsa_circ_0052693 | 88 | 8 | 402 | 401 | 7 | 0.01 | 4.00E-131 |
| hsa_circ_0052693 | 89 | 1 | 262 | 268 | 7 | 0.01 | 9.00E-86 |

novel_circ_001826 novel_circ_001827 novel_circ_001828 novel_circ_001829 novel_circ_001830 novel_circ_001834 novel_circ_001835 novel_circ_001836 novel_circ_001837 novel_circ_001838 novel_circ_001839 novel_circ_001840 novel_circ_001841 novel_circ_001842 novel_circ_001845 novel_circ_001846 novel_circ_001847 novel_circ_001848 novel_circ_001849 novel_circ_001850 novel_circ_001851 novel_circ_001852 novel_circ_001853 novel_circ_001854 novel_circ_001855 novel_circ_001856 novel_circ_001857 novel_circ_001858 novel_circ_001862 novel_circ_001863 novel_circ_001864 novel_circ_001865 novel_circ_001866 novel_circ_001867 novel_circ_001868 novel_circ_001869 novel_circ_001881 novel_circ_001882 novel_circ_001883 novel_circ_001884 novel_circ_001885 novel_circ_001886 novel_circ_001887 novel_circ_001888 novel_circ_001889 novel_circ_001890 novel_circ_001891 novel_circ_001892 novel_circ_001893 novel_circ_001894 novel_circ_001895 novel_circ_001896 novel_circ_001897 novel_circ_001898

| hsa_circ_0052678 | 89 | 1 | 236 | 128 | 363 | 0 | 1.00E-79 |
| --- | --- | --- | --- | --- | --- | --- | --- |
| hsa_circ_0052670 | 86 | 4 | 731 | 4 | 731 | 0 | 0 |
| hsa_circ_0052660 | 90 | 4 | 1400 | 2197 | 3593 | 0 | 0 |
| hsa_circ_0052661 | 89 | 4 | 844 | 1622 | 2462 | 0 | 0 |
| hsa_circ_0052648 | 98 | 1 | 55 | 188 | 134 | 0 | 1.00E-18 |
| hsa_circ_0007494 | 94 | 1 | 186 | 183 | 1 | 0.02 | 3.00E-74 |
| hsa_circ_0052645 | 95 | 1 | 324 | 3307 | 2987 | 0.01 | 1.00E-140 |
| hsa_circ_0052645 | 95 | 1 | 585 | 3307 | 2726 | 0.01 | 0 |
| hsa_circ_0052645 | 95 | 1 | 730 | 3307 | 2581 | 0 | 0 |
| hsa_circ_0052645 | 94 | 1 | 869 | 3307 | 2442 | 0 | 0 |
| hsa_circ_0052645 | 94 | 1 | 870 | 3307 | 2441 | 0 | 0 |
| hsa_circ_0052645 | 95 | 1 | 399 | 3124 | 2726 | 0 | 9.00E-177 |
| hsa_circ_0052646 | 92 | 1 | 325 | 1997 | 1673 | 0 | 3.00E-127 |
| hsa_circ_0052622 | 89 | 143 | 325 | 423 | 241 | 0 | 2.00E-58 |
| hsa_circ_0052576 | 89 | 1 | 1090 | 192 | 1281 | 0 | 0 |
| hsa_circ_0052577 | 90 | 1 | 269 | 469 | 737 | 0 | 5.00E-93 |
| hsa_circ_0052575 | 87 | 1 | 379 | 226 | 604 | 0 | 8.00E-119 |
| hsa_circ_0052575 | 86 | 149 | 752 | 1 | 604 | 0 | 0 |
| hsa_circ_0052573 | 85 | 8 | 584 | 107 | 689 | 0.02 | 5.00E-167 |
| hsa_circ_0003233 | 90 | 1349 | 1701 | 353 | 1 | 0 | 3.00E-127 |
| hsa_circ_0003233 | 90 | 1 | 353 | 353 | 1 | 0 | 7.00E-128 |
| hsa_circ_0003233 | 90 | 1 | 353 | 353 | 1 | 0 | 2.00E-127 |
| hsa_circ_0052569 | 90 | 1 | 860 | 1094 | 235 | 0 | 0 |
| hsa_circ_0052558 | 90 | 1 | 299 | 1587 | 1289 | 0 | 3.00E-106 |
| hsa_circ_0052556 | 89 | 1 | 282 | 1 | 282 | 0 | 3.00E-95 |
| hsa_circ_0052555 | 90 | 1 | 391 | 1 | 391 | 0 | 1.00E-140 |
| hsa_circ_0052550 | 90 | 1 | 841 | 242 | 1082 | 0 | 0 |
| hsa_circ_0052550 | 88 | 1 | 335 | 242 | 576 | 0 | 1.00E-109 |
| hsa_circ_0052533 | 86 | 1 | 285 | 135 | 419 | 0 | 7.00E-82 |
| hsa_circ_0052522 | 90 | 1 | 707 | 88 | 794 | 0 | 0 |
| hsa_circ_0052517 | 90 | 1 | 1211 | 220 | 1430 | 0 | 0 |
| hsa_circ_0052529 | 89 | 3 | 350 | 3 | 350 | 0 | 4.00E-120 |
| hsa_circ_0008768 | 90 | 1 | 438 | 1 | 438 | 0 | 1.00E-161 |
| hsa_circ_0052527 | 89 | 1 | 612 | 1 | 612 | 0 | 0 |
| hsa_circ_0052520 | 89 | 1 | 775 | 87 | 861 | 0 | 0 |
| hsa_circ_0052517 | 90 | 1 | 1116 | 220 | 1335 | 0 | 0 |
| hsa_circ_0052496 | 83 | 1 | 663 | 1338 | 676 | 0 | 3.00E-173 |
| hsa_circ_0052496 | 84 | 1 | 953 | 1338 | 386 | 0 | 0 |
| hsa_circ_0052496 | 88 | 1 | 318 | 993 | 676 | 0 | 8.00E-102 |
| hsa_circ_0052496 | 87 | 1 | 667 | 675 | 9 | 0 | 0 |
| hsa_circ_0006786 | 92 | 1 | 309 | 309 | 1 | 0 | 8.00E-122 |
| hsa_circ_0084572 | 86 | 492 | 583 | 500 | 409 | 0.04 | 3.00E-19 |
| hsa_circ_0084572 | 91 | 492 | 991 | 500 | 1 | 0.01 | 0 |
| hsa_circ_0084572 | 91 | 1 | 498 | 498 | 1 | 0.01 | 0 |
| hsa_circ_0084572 | 93 | 1 | 235 | 408 | 174 | 0 | 3.00E-94 |
| hsa_circ_0084572 | 92 | 1 | 307 | 408 | 102 | 0 | 2.00E-117 |
| hsa_circ_0084572 | 92 | 1 | 408 | 408 | 1 | 0 | 4.00E-160 |
| hsa_circ_0084567 | 93 | 376 | 635 | 344 | 85 | 0 | 1.00E-102 |
| hsa_circ_0003822 | 90 | 1 | 189 | 67 | 255 | 0 | 1.00E-63 |
| hsa_circ_0084563 | 93 | 2 | 587 | 2 | 587 | 0 | 0 |
| hsa_circ_0084563 | 94 | 1 | 483 | 105 | 587 | 0 | 0 |
| hsa_circ_0084563 | 94 | 1 | 332 | 256 | 587 | 0 | 3.00E-141 |
| hsa_circ_0084563 | 93 | 2 | 587 | 2 | 587 | 0 | 0 |
| hsa_circ_0084561 | 96 | 319 | 544 | 6260 | 6035 | 0 | 9.00E-99 |

novel_circ_001900 novel_circ_001901 novel_circ_001902 novel_circ_001903 novel_circ_001904 novel_circ_001905 novel_circ_001906 novel_circ_001907 novel_circ_001910 novel_circ_001913 novel_circ_001914 novel_circ_001915 novel_circ_001916 novel_circ_001917 novel_circ_001918 novel_circ_001920 novel_circ_001921 novel_circ_001922 novel_circ_001923 novel_circ_001924 novel_circ_001925 novel_circ_001926 novel_circ_001927 novel_circ_001928 novel_circ_001929 novel_circ_001930 novel_circ_001931 novel_circ_001932 novel_circ_001933 novel_circ_001934 novel_circ_001935 novel_circ_001936 novel_circ_001937 novel_circ_001940 novel_circ_001941 novel_circ_001942 novel_circ_001943 novel_circ_001944 novel_circ_001945 novel_circ_001946 novel_circ_001947 novel_circ_001948 novel_circ_001949 novel_circ_001950 novel_circ_001951 novel_circ_001952 novel_circ_001953 novel_circ_001954 novel_circ_001955 novel_circ_001956 novel_circ_001957 novel_circ_001958 novel_circ_001959 novel_circ_001960

| hsa_circ_0084552 | 92 | 1 | 260 | 1 | 260 | 0 | 5.00E-98 |
| --- | --- | --- | --- | --- | --- | --- | --- |
| hsa_circ_0007920 | 93 | 3 | 303 | 206 | 506 | 0 | 1.00E-120 |
| hsa_circ_0007920 | 92 | 1 | 506 | 1 | 506 | 0 | 0 |
| hsa_circ_0084547 | 92 | 1 | 795 | 1 | 795 | 0 | 0 |
| hsa_circ_0084543 | 83 | 2 | 459 | 2 | 462 | 0.01 | 1.00E-111 |
| hsa_circ_0084541 | 86 | 2 | 262 | 1116 | 1376 | 0 | 3.00E-75 |
| hsa_circ_0084539 | 82 | 1 | 1064 | 1 | 1061 | 0.03 | 0 |
| hsa_circ_0009077 | 88 | 136 | 451 | 24463 | 24778 | 0 | 3.00E-102 |
| hsa_circ_0088277 | 92 | 37323 | 37439 | 343 | 460 | 0.01 | 4.00E-36 |
| hsa_circ_0007810 | 93 | 1 | 257 | 257 | 1 | 0 | 5.00E-103 |
| hsa_circ_0084522 | 93 | 388 | 780 | 393 | 1 | 0 | 2.00E-161 |
| hsa_circ_0084522 | 93 | 325 | 717 | 393 | 1 | 0 | 2.00E-161 |
| hsa_circ_0084516 | 94 | 219 | 802 | 2022 | 1440 | 0 | 0 |
| hsa_circ_0084522 | 93 | 134 | 526 | 393 | 1 | 0 | 1.00E-161 |
| hsa_circ_0084522 | 93 | 131 | 523 | 393 | 1 | 0 | 1.00E-161 |
| hsa_circ_0084513 | 88 | 31 | 341 | 311 | 1 | 0 | 3.00E-101 |
| hsa_circ_0084511 | 90 | 141 | 455 | 574 | 258 | 0.01 | 3.00E-112 |
| hsa_circ_0084508 | 94 | 342 | 905 | 1085 | 522 | 0 | 0 |
| hsa_circ_0084508 | 92 | 342 | 1210 | 1085 | 217 | 0 | 0 |
| hsa_circ_0084508 | 94 | 1 | 345 | 866 | 522 | 0 | 4.00E-150 |
| hsa_circ_0084510 | 94 | 1 | 291 | 698 | 408 | 0 | 7.00E-122 |
| hsa_circ_0084510 | 91 | 1 | 596 | 698 | 103 | 0 | 0 |
| hsa_circ_0084510 | 91 | 1 | 698 | 698 | 1 | 0 | 0 |
| hsa_circ_0084508 | 91 | 1 | 812 | 812 | 1 | 0 | 0 |
| hsa_circ_0084510 | 89 | 1 | 407 | 407 | 1 | 0 | 2.00E-139 |
| hsa_circ_0084508 | 90 | 1 | 521 | 521 | 1 | 0 | 0 |
| hsa_circ_0084510 | 93 | 2 | 228 | 227 | 1 | 0 | 2.00E-91 |
| hsa_circ_0084508 | 94 | 2 | 342 | 341 | 1 | 0 | 6.00E-143 |
| hsa_circ_0020949 | 93 | 1 | 1042 | 543 | 1584 | 0 | 0 |
| hsa_circ_0007923 | 93 | 1 | 631 | 1 | 631 | 0 | 0 |
| hsa_circ_0020940 | 96 | 1 | 356 | 1 | 356 | 0 | 3.00E-166 |
| hsa_circ_0020938 | 96 | 3 | 584 | 3 | 584 | 0 | 0 |
| hsa_circ_0020938 | 95 | 3 | 228 | 3 | 228 | 0 | 2.00E-97 |
| hsa_circ_0020919 | 92 | 1 | 277 | 279 | 3 | 0 | 4.00E-109 |
| hsa_circ_0020918 | 92 | 1 | 418 | 419 | 2 | 0 | 3.00E-167 |
| hsa_circ_0004960 | 94 | 1 | 248 | 248 | 1 | 0 | 5.00E-103 |
| hsa_circ_0004637 | 94 | 1 | 429 | 429 | 1 | 0 | 0 |
| hsa_circ_0020917 | 94 | 1 | 593 | 593 | 1 | 0 | 0 |
| hsa_circ_0020906 | 96 | 1 | 234 | 234 | 1 | 0 | 1.00E-103 |
| hsa_circ_0020905 | 95 | 1 | 368 | 368 | 1 | 0 | 1.00E-164 |
| hsa_circ_0020903 | 94 | 1 | 556 | 556 | 1 | 0 | 0 |
| hsa_circ_0020893 | 93 | 1 | 972 | 972 | 1 | 0 | 0 |
| hsa_circ_0020888 | 93 | 1 | 1225 | 1225 | 1 | 0 | 0 |
| hsa_circ_0020885 | 93 | 1 | 1568 | 1568 | 1 | 0 | 0 |
| hsa_circ_0020883 | 93 | 1 | 1908 | 1908 | 1 | 0 | 0 |
| hsa_circ_0020883 | 93 | 1 | 1908 | 1908 | 1 | 0 | 0 |
| hsa_circ_0020867 | 92 | 1 | 2568 | 2568 | 1 | 0 | 0 |
| hsa_circ_0020865 | 91 | 1 | 2993 | 2993 | 1 | 0 | 0 |
| hsa_circ_0020858 | 91 | 1 | 3500 | 3502 | 3 | 0 | 0 |
| hsa_circ_0020851 | 91 | 1 | 3885 | 4011 | 127 | 0 | 0 |
| hsa_circ_0020903 | 95 | 1 | 463 | 463 | 1 | 0 | 0 |
| hsa_circ_0020885 | 93 | 1 | 1475 | 1475 | 1 | 0 | 0 |
| hsa_circ_0020903 | 93 | 3 | 322 | 320 | 1 | 0 | 6.00E-133 |
| hsa_circ_0020893 | 93 | 3 | 738 | 736 | 1 | 0 | 0 |

novel_circ_001961 novel_circ_001962 novel_circ_001963 novel_circ_001964 novel_circ_001965 novel_circ_001966 novel_circ_001967 novel_circ_001968 novel_circ_001969 novel_circ_001970 novel_circ_001971 novel_circ_001972 novel_circ_001973 novel_circ_001974 novel_circ_001975 novel_circ_001976 novel_circ_001977 novel_circ_001978 novel_circ_001979 novel_circ_001980 novel_circ_001981 novel_circ_001982 novel_circ_001983 novel_circ_001984 novel_circ_001985 novel_circ_001987 novel_circ_001988 novel_circ_001989 novel_circ_001993 novel_circ_001994 novel_circ_001995 novel_circ_001996 novel_circ_001997 novel_circ_001998 novel_circ_001999 novel_circ_002000 novel_circ_002001 novel_circ_002002 novel_circ_002003 novel_circ_002004 novel_circ_002005 novel_circ_002006 novel_circ_002007 novel_circ_002008 novel_circ_002009 novel_circ_002011 novel_circ_002012 novel_circ_002013 novel_circ_002014 novel_circ_002015 novel_circ_002016 novel_circ_002017 novel_circ_002018 novel_circ_002019

| hsa_circ_0020888 | 93 | 3 | 253 | 251 | 1 | 0 | 2.00E-101 |
| --- | --- | --- | --- | --- | --- | --- | --- |
| hsa_circ_0020865 | 87 | 1 | 425 | 425 | 1 | 0 | 2.00E-134 |
| hsa_circ_0020853 | 89 | 1 | 605 | 619 | 15 | 0 | 0 |
| hsa_circ_0020853 | 88 | 5 | 383 | 393 | 15 | 0.01 | 3.00E-127 |
| hsa_circ_0023409 | 96 | 1 | 384 | 1 | 384 | 0 | 4.00E-175 |
| hsa_circ_0023412 | 95 | 2 | 102 | 2 | 102 | 0 | 2.00E-38 |
| hsa_circ_0023474 | 94 | 1 | 664 | 664 | 1 | 0 | 0 |
| hsa_circ_0023474 | 93 | 1 | 402 | 664 | 263 | 0 | 2.00E-168 |
| hsa_circ_0023474 | 93 | 1 | 519 | 781 | 263 | 0 | 0 |
| hsa_circ_0023474 | 93 | 292 | 810 | 781 | 263 | 0 | 0 |
| hsa_circ_0023476 | 93 | 1 | 267 | 267 | 1 | 0 | 1.00E-108 |
| hsa_circ_0023476 | 93 | 1 | 384 | 384 | 1 | 0 | 9.00E-157 |
| hsa_circ_0023476 | 93 | 292 | 675 | 384 | 1 | 0 | 2.00E-156 |
| hsa_circ_0000338 | 97 | 1 | 441 | 441 | 1 | 0 | 0 |
| hsa_circ_0005918 | 96 | 1 | 318 | 318 | 1 | 0 | 8.00E-147 |
| hsa_circ_0000338 | 97 | 1 | 463 | 586 | 124 | 0 | 0 |
| hsa_circ_0000338 | 96 | 1 | 540 | 663 | 124 | 0 | 0 |
| hsa_circ_0000338 | 96 | 1 | 356 | 663 | 308 | 0 | 3.00E-166 |
| hsa_circ_0023493 | 93 | 1 | 318 | 318 | 1 | 0 | 1.00E-131 |
| hsa_circ_0023493 | 93 | 1 | 318 | 318 | 1 | 0 | 1.00E-131 |
| hsa_circ_0023502 | 88 | 114 | 2811 | 2704 | 1 | 0.04 | 0 |
| hsa_circ_0023506 | 97 | 1 | 425 | 425 | 1 | 0 | 0 |
| hsa_circ_0023508 | 97 | 1 | 331 | 331 | 1 | 0 | 2.00E-157 |
| hsa_circ_0023508 | 97 | 2541 | 2872 | 332 | 1 | 0 | 6.00E-157 |
| hsa_circ_0032072 | 90 | 3845 | 4492 | 2 | 649 | 0.02 | 0 |
| hsa_circ_0023522 | 92 | 4 | 282 | 4 | 282 | 0 | 7.00E-107 |
| hsa_circ_0005914 | 88 | 1 | 359 | 359 | 1 | 0 | 1.00E-119 |
| hsa_circ_0023548 | 88 | 6 | 1643 | 1992 | 358 | 0 | 0 |
| hsa_circ_0023565 | 94 | 1 | 187 | 201 | 387 | 0 | 6.00E-76 |
| hsa_circ_0023565 | 93 | 1 | 543 | 201 | 743 | 0 | 0 |
| hsa_circ_0023573 | 92 | 1 | 223 | 223 | 1 | 0 | 8.00E-84 |
| hsa_circ_0023573 | 92 | 1 | 465 | 465 | 1 | 0 | 0 |
| hsa_circ_0023573 | 92 | 2 | 629 | 628 | 1 | 0 | 0 |
| hsa_circ_0023573 | 92 | 2 | 629 | 628 | 1 | 0 | 0 |
| hsa_circ_0023574 | 91 | 4 | 225 | 222 | 1 | 0 | 6.00E-82 |
| hsa_circ_0023574 | 92 | 2 | 566 | 565 | 1 | 0 | 0 |
| hsa_circ_0023575 | 91 | 2 | 406 | 405 | 1 | 0 | 1.00E-156 |
| hsa_circ_0023577 | 92 | 2 | 341 | 340 | 1 | 0 | 5.00E-134 |
| hsa_circ_0023581 | 94 | 1 | 663 | 7650 | 6988 | 0 | 0 |
| hsa_circ_0023581 | 93 | 1 | 747 | 7734 | 6988 | 0 | 0 |
| hsa_circ_0023584 | 94 | 1 | 276 | 1 | 276 | 0 | 7.00E-117 |
| hsa_circ_0023585 | 91 | 1 | 471 | 41 | 514 | 0.01 | 3.00E-177 |
| hsa_circ_0023588 | 86 | 3 | 748 | 3 | 741 | 0.01 | 0 |
| hsa_circ_0023588 | 87 | 1 | 469 | 274 | 741 | 0.01 | 1.00E-146 |
| hsa_circ_0023590 | 96 | 3 | 340 | 3 | 340 | 0 | 1.00E-154 |
| hsa_circ_0023633 | 93 | 1 | 390 | 1 | 390 | 0 | 2.00E-158 |
| hsa_circ_0023633 | 92 | 1 | 476 | 1 | 476 | 0 | 0 |
| hsa_circ_0023637 | 93 | 3 | 237 | 4 | 237 | 0 | 1.00E-93 |
| hsa_circ_0023637 | 93 | 3 | 323 | 4 | 323 | 0 | 1.00E-129 |
| hsa_circ_0023640 | 93 | 107 | 467 | 1 | 361 | 0 | 2.00E-145 |
| hsa_circ_0023640 | 93 | 1 | 361 | 1 | 361 | 0 | 2.00E-145 |
| hsa_circ_0023640 | 93 | 1 | 361 | 1 | 361 | 0 | 2.00E-145 |
| hsa_circ_0023641 | 95 | 1 | 315 | 1 | 315 | 0 | 8.00E-137 |
| hsa_circ_0023641 | 94 | 1 | 227 | 89 | 315 | 0 | 4.00E-93 |

novel_circ_002020 novel_circ_002021 novel_circ_002022 novel_circ_002023 novel_circ_002024 novel_circ_002025 novel_circ_002026 novel_circ_002027 novel_circ_002028 novel_circ_002029 novel_circ_002030 novel_circ_002031 novel_circ_002032 novel_circ_002033 novel_circ_002034 novel_circ_002035 novel_circ_002036 novel_circ_002039 novel_circ_002040 novel_circ_002041 novel_circ_002042 novel_circ_002043 novel_circ_002044 novel_circ_002045 novel_circ_002046 novel_circ_002047 novel_circ_002049 novel_circ_002053 novel_circ_002054 novel_circ_002055 novel_circ_002056 novel_circ_002057 novel_circ_002058 novel_circ_002059 novel_circ_002061 novel_circ_002062 novel_circ_002063 novel_circ_002066 novel_circ_002067 novel_circ_002068 novel_circ_002069 novel_circ_002070 novel_circ_002071 novel_circ_002072 novel_circ_002073 novel_circ_002074 novel_circ_002076 novel_circ_002077 novel_circ_002078 novel_circ_002084 novel_circ_002086 novel_circ_002088 novel_circ_002089 novel_circ_002091

| hsa_circ_0023642 | 94 | 1 | 166 | 1 | 166 | 0 | 8.00E-66 |
| --- | --- | --- | --- | --- | --- | --- | --- |
| hsa_circ_0008467 | 99 | 1 | 179 | 1 | 179 | 0 | 4.00E-86 |
| hsa_circ_0023644 | 95 | 218 | 806 | 1 | 586 | 0.01 | 0 |
| hsa_circ_0023644 | 95 | 1 | 589 | 1 | 586 | 0.01 | 0 |
| hsa_circ_0008467 | 94 | 1 | 866 | 176 | 1038 | 0 | 0 |
| hsa_circ_0023649 | 94 | 330 | 1439 | 1 | 1113 | 0 | 0 |
| hsa_circ_0023649 | 95 | 330 | 1949 | 1 | 1623 | 0 | 0 |
| hsa_circ_0008467 | 94 | 2 | 690 | 350 | 1038 | 0 | 0 |
| hsa_circ_0023649 | 95 | 154 | 1773 | 1 | 1623 | 0 | 0 |
| hsa_circ_0023649 | 93 | 1 | 789 | 1 | 792 | 0 | 0 |
| hsa_circ_0023651 | 93 | 1 | 529 | 1 | 532 | 0.01 | 0 |
| hsa_circ_0023654 | 96 | 1 | 573 | 1 | 576 | 0.01 | 0 |
| hsa_circ_0023654 | 96 | 1 | 710 | 1 | 713 | 0 | 0 |
| hsa_circ_0023654 | 96 | 1 | 1083 | 1 | 1086 | 0 | 0 |
| hsa_circ_0023657 | 97 | 1 | 632 | 1 | 632 | 0 | 0 |
| hsa_circ_0023657 | 97 | 1 | 831 | 1 | 831 | 0 | 0 |
| hsa_circ_0023658 | 97 | 1 | 694 | 138 | 831 | 0 | 0 |
| hsa_circ_0027718 | 86 | 8230 | 8526 | 307 | 1 | 0.04 | 1.00E-85 |
| hsa_circ_0027718 | 86 | 8230 | 8526 | 307 | 1 | 0.04 | 1.00E-85 |
| hsa_circ_0027718 | 86 | 8234 | 8530 | 307 | 1 | 0.04 | 1.00E-85 |
| hsa_circ_0020821 | 89 | 1 | 286 | 286 | 1 | 0 | 1.00E-94 |
| hsa_circ_0020821 | 91 | 1 | 719 | 719 | 1 | 0 | 0 |
| hsa_circ_0020821 | 91 | 1 | 878 | 878 | 1 | 0 | 0 |
| hsa_circ_0020821 | 91 | 1 | 573 | 719 | 147 | 0 | 0 |
| hsa_circ_0020821 | 92 | 1 | 433 | 719 | 287 | 0 | 1.00E-170 |
| hsa_circ_0020821 | 92 | 1 | 533 | 819 | 287 | 0 | 0 |
| hsa_circ_0023347 | 92 | 1 | 284 | 284 | 1 | 0 | 3.00E-111 |
| hsa_circ_0075660 | 90 | 1 | 787 | 789 | 3 | 0 | 0 |
| hsa_circ_0075656 | 90 | 1 | 791 | 937 | 147 | 0 | 0 |
| hsa_circ_0075651 | 89 | 1 | 419 | 422 | 1 | 0.01 | 1.00E-146 |
| hsa_circ_0075642 | 87 | 1 | 802 | 39449 | 38648 | 0 | 0 |
| hsa_circ_0002268 | 92 | 1 | 405 | 1 | 405 | 0 | 1.00E-161 |
| hsa_circ_0002268 | 92 | 350 | 754 | 1 | 405 | 0 | 2.00E-161 |
| hsa_circ_0002268 | 91 | 1 | 248 | 1 | 248 | 0 | 1.00E-89 |
| hsa_circ_0061589 | 79 | 19861 | 19985 | 107473 | 107351 | 0.03 | 1.00E-13 |
| hsa_circ_0061589 | 79 | 11214 | 11338 | 107473 | 107351 | 0.03 | 9.00E-14 |
| hsa_circ_0061589 | 79 | 19861 | 19985 | 107473 | 107351 | 0.03 | 1.00E-13 |
| hsa_circ_0075602 | 86 | 1 | 235 | 494 | 728 | 0 | 7.00E-67 |
| hsa_circ_0075598 | 90 | 9 | 416 | 974 | 567 | 0.01 | 2.00E-148 |
| hsa_circ_0075598 | 92 | 9 | 777 | 974 | 206 | 0.01 | 0 |
| hsa_circ_0075598 | 93 | 9 | 982 | 974 | 1 | 0.01 | 0 |
| hsa_circ_0075598 | 94 | 1 | 483 | 688 | 206 | 0 | 0 |
| hsa_circ_0075598 | 94 | 1 | 688 | 688 | 1 | 0 | 0 |
| hsa_circ_0075598 | 94 | 1 | 688 | 688 | 1 | 0 | 0 |
| hsa_circ_0075598 | 93 | 1607 | 1812 | 206 | 1 | 0 | 8.00E-79 |
| hsa_circ_0029815 | 91 | 1825 | 2098 | 5 | 279 | 0.01 | 1.00E-97 |
| hsa_circ_0007133 | 82 | 1 | 305 | 1E+06 | 1228320 | 0.04 | 3.00E-69 |
| hsa_circ_0075597 | 93 | 1 | 272 | 272 | 1 | 0 | 2.00E-111 |
| hsa_circ_0075597 | 92 | 1 | 189 | 189 | 1 | 0 | 1.00E-68 |
| hsa_circ_0007133 | 77 | 5396 | 5638 | 650392 | 650639 | 0.06 | 5.00E-26 |
| hsa_circ_0075572 | 88 | 1 | 1020 | 246 | 1268 | 0.01 | 0 |
| hsa_circ_0075566 | 89 | 6 | 714 | 709 | 1 | 0 | 0 |
| hsa_circ_0075566 | 89 | 6 | 714 | 709 | 1 | 0 | 0 |
| hsa_circ_0075558 | 87 | 1 | 279 | 591 | 869 | 0.01 | 3.00E-85 |

novel_circ_002092 novel_circ_002093 novel_circ_002094 novel_circ_002095 novel_circ_002096 novel_circ_002099 novel_circ_002100 novel_circ_002106 novel_circ_002107 novel_circ_002108 novel_circ_002110 novel_circ_002111 novel_circ_002112 novel_circ_002113 novel_circ_002114 novel_circ_002115 novel_circ_002116 novel_circ_002117 novel_circ_002118 novel_circ_002119 novel_circ_002120 novel_circ_002121 novel_circ_002122 novel_circ_002124 novel_circ_002125 novel_circ_002126 novel_circ_002127 novel_circ_002129 novel_circ_002130 novel_circ_002131 novel_circ_002132 novel_circ_002133 novel_circ_002134 novel_circ_002135 novel_circ_002136 novel_circ_002138 novel_circ_002139 novel_circ_002141 novel_circ_002142 novel_circ_002143 novel_circ_002144 novel_circ_002145 novel_circ_002146 novel_circ_002147 novel_circ_002148 novel_circ_002149 novel_circ_002150 novel_circ_002151 novel_circ_002152 novel_circ_002153 novel_circ_002154 novel_circ_002155 novel_circ_002156 novel_circ_002157

| hsa_circ_0075558 | 88 | 1170 | 1615 | 424 | 869 | 0.01 | 1.00E-150 |
| --- | --- | --- | --- | --- | --- | --- | --- |
| hsa_circ_0075558 | 90 | 1 | 533 | 337 | 869 | 0.01 | 0 |
| hsa_circ_0075557 | 85 | 14 | 697 | 16 | 709 | 0.03 | 0 |
| hsa_circ_0003086 | 85 | 12013 | 12113 | 341822 | 341922 | 0.04 | 7.00E-19 |
| hsa_circ_0003086 | 85 | 12013 | 12113 | 341822 | 341922 | 0.04 | 7.00E-19 |
| hsa_circ_0055813 | 88 | 647 | 966 | 390 | 68 | 0.01 | 6.00E-102 |
| hsa_circ_0011612 | 76 | 50018 | 50747 | 5438 | 4708 | 0.06 | 3.00E-88 |
| hsa_circ_0075536 | 92 | 3 | 132 | 3 | 132 | 0 | 6.00E-43 |
| hsa_circ_0075536 | 92 | 9270 | 9399 | 3 | 132 | 0 | 2.00E-42 |
| hsa_circ_0075535 | 90 | 1 | 292 | 1 | 292 | 0 | 2.00E-102 |
| hsa_circ_0075526 | 94 | 1 | 124 | 1319 | 1196 | 0 | 3.00E-46 |
| hsa_circ_0075526 | 78 | 7 | 442 | 1067 | 628 | 0.03 | 7.00E-73 |
| hsa_circ_0075522 | 91 | 1 | 430 | 1 | 430 | 0 | 1.00E-165 |
| hsa_circ_0007577 | 87 | 1 | 667 | 1 | 667 | 0 | 0 |
| hsa_circ_0075518 | 87 | 8669 | 9339 | 221 | 891 | 0 | 0 |
| hsa_circ_0008694 | 91 | 1 | 368 | 368 | 1 | 0 | 1.00E-139 |
| hsa_circ_0060898 | 89 | 480 | 1182 | 703 | 1 | 0 | 0 |
| hsa_circ_0060898 | 89 | 112 | 814 | 703 | 1 | 0 | 0 |
| hsa_circ_0060903 | 89 | 4 | 395 | 392 | 1 | 0 | 7.00E-138 |
| hsa_circ_0060898 | 89 | 4 | 651 | 648 | 1 | 0 | 0 |
| hsa_circ_0060893 | 90 | 4 | 1026 | 1023 | 1 | 0 | 0 |
| hsa_circ_0060898 | 89 | 1 | 494 | 494 | 1 | 0 | 9.00E-173 |
| hsa_circ_0060849 | 92 | 1 | 1202 | 1202 | 1 | 0 | 0 |
| hsa_circ_0060844 | 92 | 238 | 721 | 649 | 163 | 0.01 | 0 |
| hsa_circ_0060844 | 92 | 138 | 621 | 649 | 163 | 0.01 | 0 |
| hsa_circ_0060844 | 95 | 104 | 383 | 649 | 370 | 0 | 1.00E-120 |
| hsa_circ_0060844 | 92 | 104 | 587 | 649 | 163 | 0.01 | 0 |
| hsa_circ_0060838 | 82 | 1 | 1031 | 224 | 1276 | 0.02 | 0 |
| hsa_circ_0005024 | 92 | 1 | 291 | 1 | 291 | 0 | 7.00E-112 |
| hsa_circ_0060821 | 93 | 23 | 481 | 1 | 459 | 0 | 0 |
| hsa_circ_0060821 | 93 | 23 | 373 | 1 | 351 | 0 | 2.00E-143 |
| hsa_circ_0060820 | 93 | 1 | 589 | 1 | 589 | 0 | 0 |
| hsa_circ_0006626 | 96 | 1 | 445 | 145 | 589 | 0 | 0 |
| hsa_circ_0006626 | 93 | 1 | 389 | 1 | 389 | 0 | 2.00E-159 |
| hsa_circ_0060806 | 96 | 1 | 249 | 4418 | 4170 | 0 | 6.00E-112 |
| hsa_circ_0004969 | 90 | 1 | 546 | 546 | 1 | 0 | 0 |
| hsa_circ_0060796 | 91 | 1 | 803 | 4706 | 3907 | 0 | 0 |
| hsa_circ_0060785 | 92 | 326 | 1191 | 1 | 866 | 0 | 0 |
| hsa_circ_0060785 | 93 | 152 | 805 | 1 | 654 | 0 | 0 |
| hsa_circ_0060776 | 92 | 155 | 462 | 308 | 1 | 0 | 9.00E-118 |
| hsa_circ_0060773 | 92 | 155 | 561 | 407 | 1 | 0 | 2.00E-164 |
| hsa_circ_0060776 | 95 | 1210 | 1353 | 310 | 167 | 0 | 8.00E-58 |
| hsa_circ_0060751 | 94 | 1 | 883 | 148 | 1030 | 0 | 0 |
| hsa_circ_0060751 | 95 | 1 | 699 | 148 | 846 | 0 | 0 |
| hsa_circ_0060741 | 93 | 1 | 590 | 1 | 590 | 0 | 0 |
| hsa_circ_0060731 | 94 | 1 | 391 | 1 | 391 | 0 | 3.00E-167 |
| hsa_circ_0060719 | 92 | 1 | 595 | 153 | 747 | 0 | 0 |
| hsa_circ_0060710 | 91 | 1 | 1156 | 1 | 1156 | 0 | 0 |
| hsa_circ_0060696 | 95 | 1 | 606 | 1 | 606 | 0 | 0 |
| hsa_circ_0060670 | 89 | 125 | 1032 | 1 | 908 | 0 | 0 |
| hsa_circ_0060670 | 91 | 1 | 530 | 1 | 530 | 0 | 0 |
| hsa_circ_0007141 | 91 | 1 | 685 | 1 | 685 | 0 | 0 |
| hsa_circ_0007133 | 93 | 22068 | 22134 | 1E+06 | 1082442 | 0.01 | 1.00E-16 |
| hsa_circ_0007133 | 79 | 1962 | 2089 | 201882 | 202015 | 0.09 | 2.00E-12 |

novel_circ_002158 novel_circ_002159 novel_circ_002160 novel_circ_002161 novel_circ_002162 novel_circ_002163 novel_circ_002165 novel_circ_002166 novel_circ_002167 novel_circ_002168 novel_circ_002169 novel_circ_002170 novel_circ_002171 novel_circ_002172 novel_circ_002173 novel_circ_002174 novel_circ_002175 novel_circ_002176 novel_circ_002177 novel_circ_002178 novel_circ_002179 novel_circ_002180 novel_circ_002181 novel_circ_002182 novel_circ_002183 novel_circ_002184 novel_circ_002185 novel_circ_002186 novel_circ_002188 novel_circ_002189 novel_circ_002190 novel_circ_002191 novel_circ_002192 novel_circ_002193 novel_circ_002194 novel_circ_002195 novel_circ_002196 novel_circ_002197 novel_circ_002198 novel_circ_002199 novel_circ_002200 novel_circ_002201 novel_circ_002202 novel_circ_002203 novel_circ_002204 novel_circ_002206 novel_circ_002207 novel_circ_002208 novel_circ_002209 novel_circ_002212 novel_circ_002213 novel_circ_002214 novel_circ_002215 novel_circ_002216

| hsa_circ_0054221 | 92 | 1 | 252 | 1 | 252 | 0 | 3.00E-95 |
| --- | --- | --- | --- | --- | --- | --- | --- |
| hsa_circ_0054221 | 92 | 1 | 252 | 1 | 252 | 0 | 3.00E-93 |
| hsa_circ_0054222 | 90 | 2928 | 3115 | 188 | 1 | 0 | 2.00E-63 |
| hsa_circ_0054222 | 90 | 88 | 275 | 188 | 1 | 0 | 2.00E-64 |
| hsa_circ_0054223 | 96 | 88 | 160 | 73 | 1 | 0 | 5.00E-26 |
| hsa_circ_0054223 | 83 | 12 | 404 | 811 | 413 | 0.04 | 5.00E-95 |
| hsa_circ_0054226 | 95 | 1 | 376 | 376 | 1 | 0 | 2.00E-167 |
| hsa_circ_0054226 | 95 | 3 | 507 | 505 | 1 | 0 | 0 |
| hsa_circ_0054226 | 93 | 1 | 734 | 713 | 1 | 0.03 | 0 |
| hsa_circ_0054226 | 93 | 108 | 841 | 713 | 1 | 0.03 | 0 |
| hsa_circ_0054226 | 93 | 108 | 565 | 713 | 277 | 0.05 | 0 |
| hsa_circ_0054226 | 92 | 108 | 465 | 713 | 377 | 0.06 | 9.00E-133 |
| hsa_circ_0000994 | 94 | 1 | 1829 | 1829 | 1 | 0 | 0 |
| hsa_circ_0000994 | 94 | 1 | 1832 | 1832 | 1 | 0 | 0 |
| hsa_circ_0000994 | 94 | 1 | 1829 | 1829 | 1 | 0 | 0 |
| hsa_circ_0000994 | 94 | 1 | 1832 | 1832 | 1 | 0 | 0 |
| hsa_circ_0000994 | 94 | 1 | 1827 | 1829 | 3 | 0 | 0 |
| hsa_circ_0000994 | 94 | 1 | 1830 | 1832 | 3 | 0 | 0 |
| hsa_circ_0054230 | 91 | 1 | 313 | 1 | 313 | 0 | 5.00E-119 |
| hsa_circ_0054232 | 92 | 1 | 332 | 131 | 459 | 0.01 | 3.00E-128 |
| hsa_circ_0054236 | 95 | 1 | 274 | 1 | 274 | 0 | 9.00E-121 |
| hsa_circ_0054234 | 93 | 1 | 455 | 27 | 481 | 0 | 0 |
| hsa_circ_0054234 | 94 | 1 | 551 | 27 | 577 | 0 | 0 |
| hsa_circ_0054234 | 94 | 1 | 822 | 27 | 848 | 0 | 0 |
| hsa_circ_0054240 | 93 | 1 | 548 | 1 | 548 | 0 | 0 |
| hsa_circ_0054245 | 94 | 1 | 367 | 1 | 367 | 0.01 | 2.00E-157 |
| hsa_circ_0054249 | 96 | 1 | 478 | 272 | 749 | 0 | 0 |
| hsa_circ_0054255 | 89 | 1 | 187 | 1 | 187 | 0 | 3.00E-59 |
| hsa_circ_0071435 | 95 | 1 | 176 | 1 | 176 | 0 | 3.00E-73 |
| hsa_circ_0071435 | 89 | 1 | 248 | 1 | 250 | 0.02 | 9.00E-81 |
| hsa_circ_0071436 | 94 | 653 | 963 | 1 | 311 | 0 | 4.00E-129 |
| hsa_circ_0071436 | 94 | 653 | 1170 | 1 | 518 | 0 | 0 |
| hsa_circ_0071436 | 94 | 653 | 2600 | 1 | 1948 | 0 | 0 |
| hsa_circ_0071436 | 94 | 259 | 569 | 1 | 311 | 0 | 2.00E-129 |
| hsa_circ_0071436 | 94 | 259 | 776 | 1 | 518 | 0 | 0 |
| hsa_circ_0071436 | 94 | 259 | 2206 | 1 | 1948 | 0 | 0 |
| hsa_circ_0071436 | 94 | 101 | 411 | 1 | 311 | 0 | 2.00E-129 |
| hsa_circ_0071436 | 94 | 101 | 699 | 1 | 599 | 0 | 0 |
| hsa_circ_0071436 | 94 | 1 | 1349 | 600 | 1948 | 0 | 0 |
| hsa_circ_0071438 | 94 | 1 | 586 | 1 | 586 | 0 | 0 |
| hsa_circ_0071436 | 94 | 1 | 803 | 1146 | 1948 | 0 | 0 |
| hsa_circ_0071436 | 94 | 1 | 616 | 1333 | 1948 | 0 | 0 |
| hsa_circ_0071441 | 93 | 1 | 261 | 503 | 243 | 0 | 1.00E-102 |
| hsa_circ_0071441 | 93 | 1 | 261 | 503 | 243 | 0 | 1.00E-103 |
| hsa_circ_0071441 | 93 | 1 | 600 | 1103 | 504 | 0 | 0 |
| hsa_circ_0047094 | 79 | 4795 | 5179 | 27940 | 28330 | 0.03 | 7.00E-69 |
| hsa_circ_0071443 | 89 | 11 | 456 | 508 | 63 | 0.01 | 4.00E-156 |
| hsa_circ_0071443 | 90 | 11 | 272 | 508 | 247 | 0.02 | 2.00E-92 |
| hsa_circ_0071443 | 90 | 219 | 480 | 508 | 247 | 0.02 | 4.00E-92 |
| hsa_circ_0005121 | 88 | 7 | 633 | 1 | 627 | 0 | 0 |
| hsa_circ_0005121 | 88 | 7 | 633 | 1 | 627 | 0 | 0 |
| hsa_circ_0017451 | 88 | 342 | 1463 | 3 | 1118 | 0.01 | 0 |
| hsa_circ_0005121 | 85 | 7 | 380 | 1 | 374 | 0 | 4.00E-106 |
| hsa_circ_0017462 | 86 | 1 | 502 | 1 | 496 | 0.01 | 7.00E-149 |

novel_circ_002217 novel_circ_002218 novel_circ_002219 novel_circ_002220 novel_circ_002221 novel_circ_002222 novel_circ_002223 novel_circ_002224 novel_circ_002226 novel_circ_002227 novel_circ_002228 novel_circ_002229 novel_circ_002230 novel_circ_002231 novel_circ_002232 novel_circ_002233 novel_circ_002234 novel_circ_002235 novel_circ_002236 novel_circ_002237 novel_circ_002238 novel_circ_002239 novel_circ_002240 novel_circ_002241 novel_circ_002242 novel_circ_002244 novel_circ_002245 novel_circ_002246 novel_circ_002247 novel_circ_002248 novel_circ_002249 novel_circ_002250 novel_circ_002251 novel_circ_002252 novel_circ_002253 novel_circ_002254 novel_circ_002256 novel_circ_002257 novel_circ_002258 novel_circ_002259 novel_circ_002260 novel_circ_002261 novel_circ_002262 novel_circ_002263 novel_circ_002264 novel_circ_002265 novel_circ_002266 novel_circ_002267 novel_circ_002268 novel_circ_002269 novel_circ_002270 novel_circ_002271 novel_circ_002272 novel_circ_002273

| hsa_circ_0017444 | 88 | 243 | 1210 | 1 | 962 | 0.01 | 0 |
| --- | --- | --- | --- | --- | --- | --- | --- |
| hsa_circ_0017444 | 90 | 243 | 830 | 1 | 588 | 0 | 0 |
| hsa_circ_0017444 | 89 | 243 | 712 | 1 | 469 | 0 | 2.00E-164 |
| hsa_circ_0017444 | 89 | 243 | 708 | 1 | 466 | 0 | 2.00E-165 |
| hsa_circ_0017444 | 90 | 243 | 631 | 1 | 389 | 0 | 6.00E-141 |
| hsa_circ_0017444 | 93 | 243 | 500 | 1 | 258 | 0 | 6.00E-105 |
| hsa_circ_0005772 | 84 | 83 | 484 | 404 | 3 | 0 | 6.00E-105 |
| hsa_circ_0017410 | 86 | 83 | 702 | 620 | 1 | 0.01 | 0 |
| hsa_circ_0017410 | 85 | 10 | 505 | 496 | 1 | 0.01 | 7.00E-139 |
| hsa_circ_0017409 | 87 | 10 | 609 | 711 | 112 | 0.01 | 0 |
| hsa_circ_0003023 | 94 | 2278 | 2531 | 254 | 1 | 0 | 5.00E-107 |
| hsa_circ_0003023 | 94 | 2278 | 2531 | 254 | 1 | 0 | 3.00E-106 |
| hsa_circ_0003023 | 94 | 2278 | 2531 | 254 | 1 | 0 | 4.00E-106 |
| hsa_circ_0067863 | 90 | 19443 | 20552 | 1133 | 1 | 0.03 | 0 |
| hsa_circ_0067863 | 90 | 5763 | 6872 | 1133 | 1 | 0.03 | 0 |
| hsa_circ_0067863 | 90 | 5763 | 6872 | 1133 | 1 | 0.03 | 0 |
| hsa_circ_0067863 | 90 | 5763 | 6872 | 1133 | 1 | 0.03 | 0 |
| hsa_circ_0017379 | 92 | 8393 | 8596 | 314 | 111 | 0 | 2.00E-73 |
| hsa_circ_0017376 | 95 | 4707 | 4839 | 133 | 1 | 0 | 8.00E-53 |
| hsa_circ_0017376 | 95 | 4707 | 4839 | 133 | 1 | 0 | 4.00E-52 |
| hsa_circ_0017360 | 93 | 395 | 1632 | 283 | 1519 | 0 | 0 |
| hsa_circ_0017360 | 99 | 1 | 296 | 1 | 296 | 0 | 1.00E-147 |
| hsa_circ_0017360 | 99 | 1 | 296 | 1 | 296 | 0 | 1.00E-146 |
| hsa_circ_0017360 | 99 | 1 | 296 | 1 | 296 | 0 | 1.00E-147 |
| hsa_circ_0017360 | 99 | 1 | 295 | 1 | 295 | 0 | 2.00E-147 |
| hsa_circ_0027821 | 93 | 22245 | 22351 | 145 | 251 | 0 | 6.00E-36 |
| hsa_circ_0027821 | 92 | 68812 | 69019 | 690 | 897 | 0 | 5.00E-75 |
| hsa_circ_0027821 | 93 | 888 | 994 | 145 | 251 | 0 | 6.00E-37 |
| hsa_circ_0027821 | 93 | 888 | 994 | 145 | 251 | 0 | 8.00E-37 |
| hsa_circ_0027821 | 92 | 47455 | 47662 | 690 | 897 | 0 | 4.00E-75 |
| hsa_circ_0027821 | 92 | 47455 | 47662 | 690 | 897 | 0 | 6.00E-75 |
| hsa_circ_0027822 | 92 | 79653 | 79876 | 7 | 231 | 0 | 8.00E-84 |
| hsa_circ_0027822 | 92 | 79653 | 79896 | 7 | 251 | 0 | 1.00E-91 |
| hsa_circ_0027821 | 92 | 1 | 205 | 693 | 897 | 0 | 7.00E-76 |
| hsa_circ_0027821 | 92 | 1 | 205 | 693 | 897 | 0 | 1.00E-73 |
| hsa_circ_0027826 | 93 | 1 | 180 | 123 | 302 | 0 | 4.00E-70 |
| hsa_circ_0027830 | 88 | 1 | 557 | 1 | 557 | 0.01 | 0 |
| hsa_circ_0027830 | 89 | 1 | 747 | 1 | 747 | 0.01 | 0 |
| hsa_circ_0027830 | 90 | 1 | 996 | 1 | 996 | 0 | 0 |
| hsa_circ_0027830 | 92 | 1 | 1834 | 1 | 1834 | 0 | 0 |
| hsa_circ_0027830 | 92 | 1 | 1961 | 1 | 1961 | 0 | 0 |
| hsa_circ_0027830 | 93 | 1 | 2087 | 1 | 2087 | 0 | 0 |
| hsa_circ_0027830 | 93 | 1 | 2338 | 1 | 2338 | 0 | 0 |
| hsa_circ_0027830 | 93 | 1 | 2507 | 1 | 2507 | 0 | 0 |
| hsa_circ_0027830 | 93 | 1 | 2636 | 1 | 2636 | 0 | 0 |
| hsa_circ_0027829 | 92 | 1 | 3125 | 537 | 3661 | 0 | 0 |
| hsa_circ_0027829 | 92 | 1 | 3371 | 537 | 3907 | 0 | 0 |
| hsa_circ_0027831 | 94 | 2 | 429 | 2 | 429 | 0 | 0 |
| hsa_circ_0027830 | 94 | 2 | 1940 | 569 | 2507 | 0 | 0 |
| hsa_circ_0027834 | 95 | 1 | 972 | 301 | 1272 | 0 | 0 |
| hsa_circ_0027836 | 96 | 1 | 504 | 1 | 504 | 0 | 0 |
| hsa_circ_0027836 | 95 | 1 | 673 | 1 | 673 | 0 | 0 |
| hsa_circ_0027834 | 92 | 1 | 1537 | 600 | 2136 | 0 | 0 |
| hsa_circ_0027836 | 94 | 1 | 546 | 128 | 673 | 0 | 0 |

novel_circ_002274 novel_circ_002275 novel_circ_002276 novel_circ_002277 novel_circ_002278 novel_circ_002279 novel_circ_002280 novel_circ_002281 novel_circ_002282 novel_circ_002283 novel_circ_002284 novel_circ_002285 novel_circ_002286 novel_circ_002287 novel_circ_002288 novel_circ_002289 novel_circ_002290 novel_circ_002291 novel_circ_002292 novel_circ_002293 novel_circ_002294 novel_circ_002295 novel_circ_002296 novel_circ_002297 novel_circ_002298 novel_circ_002299 novel_circ_002300 novel_circ_002301 novel_circ_002302 novel_circ_002303 novel_circ_002304 novel_circ_002305 novel_circ_002306 novel_circ_002307 novel_circ_002308 novel_circ_002309 novel_circ_002310 novel_circ_002311 novel_circ_002312 novel_circ_002313 novel_circ_002314 novel_circ_002315 novel_circ_002316 novel_circ_002317 novel_circ_002318 novel_circ_002319 novel_circ_002320 novel_circ_002321 novel_circ_002322 novel_circ_002323 novel_circ_002324 novel_circ_002325 novel_circ_002326 novel_circ_002327

| hsa_circ_0027838 | 94 | 364 | 969 | 1450 | 845 | 0 | 0 |
| --- | --- | --- | --- | --- | --- | --- | --- |
| hsa_circ_0027838 | 94 | 364 | 801 | 1450 | 1013 | 0 | 0 |
| hsa_circ_0027838 | 94 | 1 | 309 | 1321 | 1013 | 0 | 2.00E-133 |
| hsa_circ_0027838 | 94 | 1 | 438 | 1450 | 1013 | 0 | 0 |
| hsa_circ_0027838 | 94 | 73 | 510 | 1450 | 1013 | 0 | 0 |
| hsa_circ_0027838 | 94 | 76 | 513 | 1450 | 1013 | 0 | 0 |
| hsa_circ_0027838 | 94 | 256 | 693 | 1450 | 1013 | 0 | 0 |
| hsa_circ_0027838 | 94 | 364 | 801 | 1450 | 1013 | 0 | 0 |
| hsa_circ_0027839 | 97 | 1 | 291 | 501 | 210 | 0 | 4.00E-138 |
| hsa_circ_0027839 | 97 | 1 | 291 | 501 | 210 | 0 | 3.00E-138 |
| hsa_circ_0027839 | 96 | 1 | 252 | 753 | 502 | 0 | 3.00E-115 |
| hsa_circ_0027840 | 90 | 2 | 660 | 662 | 4 | 0 | 0 |
| hsa_circ_0027840 | 90 | 2 | 660 | 662 | 4 | 0 | 0 |
| hsa_circ_0027840 | 90 | 2 | 649 | 662 | 15 | 0 | 0 |
| hsa_circ_0027840 | 92 | 1 | 296 | 981 | 686 | 0.01 | 3.00E-116 |
| hsa_circ_0027840 | 93 | 1 | 462 | 1147 | 686 | 0 | 0 |
| hsa_circ_0027840 | 94 | 1 | 303 | 1147 | 845 | 0 | 7.00E-127 |
| hsa_circ_0069749 | 80 | 9911 | 10456 | 527963 | 528521 | 0.07 | 8.00E-103 |
| hsa_circ_0069749 | 80 | 9911 | 10456 | 527963 | 528521 | 0.07 | 3.00E-103 |
| hsa_circ_0069749 | 80 | 9911 | 10456 | 527963 | 528521 | 0.07 | 2.00E-103 |
| hsa_circ_0027840 | 93 | 3 | 425 | 1570 | 1148 | 0 | 1.00E-176 |
| hsa_circ_0027840 | 94 | 1 | 603 | 1750 | 1148 | 0 | 0 |
| hsa_circ_0007294 | 95 | 1 | 459 | 459 | 1 | 0 | 0 |
| hsa_circ_0027840 | 92 | 1 | 756 | 2047 | 1292 | 0 | 0 |
| hsa_circ_0027840 | 93 | 1 | 913 | 2204 | 1292 | 0 | 0 |
| hsa_circ_0027840 | 94 | 1 | 994 | 2285 | 1292 | 0 | 0 |
| hsa_circ_0027841 | 94 | 1 | 292 | 292 | 1 | 0 | 4.00E-124 |
| hsa_circ_0027841 | 91 | 1 | 589 | 589 | 1 | 0 | 0 |
| hsa_circ_0027841 | 92 | 1 | 746 | 746 | 1 | 0 | 0 |
| hsa_circ_0027841 | 93 | 1 | 827 | 827 | 1 | 0 | 0 |
| hsa_circ_0027841 | 93 | 1 | 632 | 746 | 115 | 0 | 0 |
| hsa_circ_0027841 | 94 | 1 | 713 | 827 | 115 | 0 | 0 |
| hsa_circ_0027842 | 91 | 1 | 454 | 454 | 1 | 0 | 1.00E-175 |
| hsa_circ_0027842 | 93 | 1 | 535 | 535 | 1 | 0 | 0 |
| hsa_circ_0027846 | 92 | 1 | 363 | 363 | 1 | 0 | 2.00E-143 |
| hsa_circ_0027846 | 91 | 1 | 657 | 657 | 1 | 0 | 0 |
| hsa_circ_0027846 | 90 | 1 | 2532 | 2532 | 1 | 0 | 0 |
| hsa_circ_0027846 | 91 | 390 | 3060 | 2671 | 1 | 0 | 0 |
| hsa_circ_0027846 | 91 | 571 | 3241 | 2671 | 1 | 0 | 0 |
| hsa_circ_0027848 | 91 | 1 | 2319 | 2319 | 1 | 0 | 0 |
| hsa_circ_0027848 | 91 | 1 | 2403 | 2403 | 1 | 0 | 0 |
| hsa_circ_0027848 | 91 | 1 | 2755 | 2755 | 1 | 0 | 0 |
| hsa_circ_0027846 | 90 | 4 | 2160 | 2671 | 515 | 0 | 0 |
| hsa_circ_0027848 | 91 | 1 | 2403 | 2403 | 1 | 0 | 0 |
| hsa_circ_0027848 | 91 | 1 | 2755 | 2755 | 1 | 0 | 0 |
| hsa_circ_0027848 | 90 | 3 | 1681 | 1679 | 1 | 0 | 0 |
| hsa_circ_0027848 | 90 | 1 | 1875 | 1875 | 1 | 0 | 0 |
| hsa_circ_0027848 | 91 | 4 | 2017 | 2014 | 1 | 0 | 0 |
| hsa_circ_0027848 | 91 | 1 | 2136 | 2136 | 1 | 0 | 0 |
| hsa_circ_0027848 | 91 | 1 | 2319 | 2319 | 1 | 0 | 0 |
| hsa_circ_0027848 | 91 | 1 | 2403 | 2403 | 1 | 0 | 0 |
| hsa_circ_0027848 | 91 | 1 | 2584 | 2584 | 1 | 0 | 0 |
| hsa_circ_0027848 | 92 | 1 | 3178 | 3178 | 1 | 0 | 0 |
| hsa_circ_0027848 | 92 | 4 | 3217 | 3214 | 1 | 0 | 0 |

novel_circ_002328 novel_circ_002329 novel_circ_002330 novel_circ_002331 novel_circ_002332 novel_circ_002333 novel_circ_002334 novel_circ_002335 novel_circ_002336 novel_circ_002337 novel_circ_002338 novel_circ_002339 novel_circ_002340 novel_circ_002341 novel_circ_002342 novel_circ_002343 novel_circ_002344 novel_circ_002345 novel_circ_002346 novel_circ_002347 novel_circ_002348 novel_circ_002349 novel_circ_002350 novel_circ_002351 novel_circ_002352 novel_circ_002353 novel_circ_002354 novel_circ_002355 novel_circ_002356 novel_circ_002357 novel_circ_002358 novel_circ_002359 novel_circ_002360 novel_circ_002361 novel_circ_002362 novel_circ_002363 novel_circ_002364 novel_circ_002365 novel_circ_002366 novel_circ_002367 novel_circ_002368 novel_circ_002369 novel_circ_002370 novel_circ_002371 novel_circ_002372 novel_circ_002373 novel_circ_002374 novel_circ_002375 novel_circ_002376 novel_circ_002377 novel_circ_002378 novel_circ_002379 novel_circ_002380 novel_circ_002381

| hsa_circ_0027848 | 91 | 1 | 2227 | 2403 | 177 | 0 | 0 |
| --- | --- | --- | --- | --- | --- | --- | --- |
| hsa_circ_0027848 | 91 | 1 | 2579 | 2755 | 177 | 0 | 0 |
| hsa_circ_0027848 | 94 | 1 | 638 | 2319 | 1682 | 0.01 | 0 |
| hsa_circ_0027848 | 94 | 1 | 722 | 2403 | 1682 | 0.01 | 0 |
| hsa_circ_0027848 | 93 | 1 | 1074 | 2755 | 1682 | 0 | 0 |
| hsa_circ_0027848 | 94 | 1 | 444 | 2319 | 1876 | 0.01 | 0 |
| hsa_circ_0027848 | 94 | 1 | 528 | 2403 | 1876 | 0.01 | 0 |
| hsa_circ_0027848 | 93 | 1 | 709 | 2584 | 1876 | 0.01 | 0 |
| hsa_circ_0027848 | 93 | 1 | 880 | 2755 | 1876 | 0 | 0 |
| hsa_circ_0027848 | 94 | 1 | 1505 | 3380 | 1876 | 0 | 0 |
| hsa_circ_0027848 | 95 | 1 | 302 | 2319 | 2018 | 0.01 | 6.00E-133 |
| hsa_circ_0027848 | 93 | 1 | 567 | 2584 | 2018 | 0.01 | 0 |
| hsa_circ_0027848 | 94 | 1 | 738 | 2755 | 2018 | 0.01 | 0 |
| hsa_circ_0027848 | 94 | 4 | 1200 | 3214 | 2018 | 0 | 0 |
| hsa_circ_0027848 | 93 | 1 | 577 | 2896 | 2320 | 0 | 0 |
| hsa_circ_0027848 | 93 | 1 | 859 | 3178 | 2320 | 0 | 0 |
| hsa_circ_0027848 | 94 | 1 | 1061 | 3380 | 2320 | 0 | 0 |
| hsa_circ_0027849 | 93 | 1 | 493 | 493 | 1 | 0 | 0 |
| hsa_circ_0027849 | 93 | 1 | 775 | 775 | 1 | 0 | 0 |
| hsa_circ_0027849 | 93 | 4 | 814 | 811 | 1 | 0 | 0 |
| hsa_circ_0027849 | 94 | 1 | 977 | 977 | 1 | 0 | 0 |
| hsa_circ_0027849 | 94 | 149 | 1125 | 977 | 1 | 0 | 0 |
| hsa_circ_0027850 | 94 | 4 | 633 | 630 | 1 | 0 | 0 |
| hsa_circ_0027849 | 94 | 1 | 796 | 977 | 182 | 0 | 0 |
| hsa_circ_0027849 | 94 | 149 | 944 | 977 | 182 | 0 | 0 |
| hsa_circ_0027852 | 95 | 1 | 330 | 331 | 2 | 0 | 2.00E-143 |
| hsa_circ_0027852 | 95 | 1 | 422 | 423 | 2 | 0 | 0 |
| hsa_circ_0027852 | 95 | 4 | 461 | 459 | 2 | 0 | 0 |
| hsa_circ_0027849 | 95 | 1 | 624 | 977 | 354 | 0 | 0 |
| hsa_circ_0027849 | 95 | 149 | 772 | 977 | 354 | 0 | 0 |
| hsa_circ_0027853 | 94 | 4 | 321 | 318 | 1 | 0 | 4.00E-135 |
| hsa_circ_0027849 | 94 | 1 | 484 | 977 | 494 | 0 | 0 |
| hsa_circ_0027849 | 95 | 1 | 294 | 977 | 684 | 0 | 7.00E-127 |
| hsa_circ_0027858 | 95 | 1 | 564 | 119 | 682 | 0 | 0 |
| hsa_circ_0027858 | 95 | 1 | 495 | 188 | 682 | 0 | 0 |
| hsa_circ_0027858 | 95 | 1 | 495 | 188 | 682 | 0 | 0 |
| hsa_circ_0027865 | 95 | 1 | 508 | 1 | 508 | 0 | 0 |
| hsa_circ_0027865 | 95 | 1 | 1143 | 659 | 1789 | 0.01 | 0 |
| hsa_circ_0027868 | 95 | 1 | 366 | 1 | 366 | 0 | 9.00E-162 |
| hsa_circ_0027865 | 93 | 1 | 168 | 1790 | 1957 | 0 | 2.00E-63 |
| hsa_circ_0027870 | 92 | 157 | 632 | 121 | 596 | 0 | 0 |
| hsa_circ_0027872 | 94 | 298 | 593 | 1 | 296 | 0 | 1.00E-122 |
| hsa_circ_0027872 | 94 | 298 | 593 | 1 | 296 | 0 | 1.00E-121 |
| hsa_circ_0027871 | 95 | 106 | 502 | 17090 | 17486 | 0 | 3.00E-177 |
| hsa_circ_0027871 | 96 | 106 | 679 | 17090 | 17663 | 0 | 0 |
| hsa_circ_0027871 | 95 | 1 | 397 | 17090 | 17486 | 0 | 3.00E-177 |
| hsa_circ_0027871 | 95 | 1 | 398 | 17090 | 17487 | 0 | 2.00E-177 |
| hsa_circ_0027871 | 95 | 1 | 442 | 17090 | 17531 | 0 | 0 |
| hsa_circ_0027871 | 96 | 1 | 574 | 17090 | 17663 | 0 | 0 |
| hsa_circ_0027873 | 99 | 1 | 278 | 1 | 278 | 0 | 2.00E-141 |
| hsa_circ_0027877 | 91 | 68 | 1304 | 1 | 1237 | 0 | 0 |
| hsa_circ_0027883 | 83 | 1 | 242 | 1 | 245 | 0.01 | 6.00E-57 |
| hsa_circ_0027883 | 87 | 1 | 348 | 1 | 351 | 0.01 | 1.00E-110 |
| hsa_circ_0027883 | 88 | 1 | 499 | 1 | 502 | 0.01 | 2.00E-169 |

novel_circ_002382 novel_circ_002383 novel_circ_002384 novel_circ_002385 novel_circ_002386 novel_circ_002387 novel_circ_002388 novel_circ_002389 novel_circ_002390 novel_circ_002394 novel_circ_002395 novel_circ_002396 novel_circ_002397 novel_circ_002398 novel_circ_002399 novel_circ_002400 novel_circ_002401 novel_circ_002402 novel_circ_002403 novel_circ_002406 novel_circ_002407 novel_circ_002408 novel_circ_002409 novel_circ_002410 novel_circ_002411 novel_circ_002412 novel_circ_002413 novel_circ_002414 novel_circ_002415 novel_circ_002416 novel_circ_002417 novel_circ_002418 novel_circ_002419 novel_circ_002420 novel_circ_002421 novel_circ_002422 novel_circ_002423 novel_circ_002424 novel_circ_002425 novel_circ_002426 novel_circ_002427 novel_circ_002428 novel_circ_002429 novel_circ_002430 novel_circ_002431 novel_circ_002432 novel_circ_002433 novel_circ_002434 novel_circ_002435 novel_circ_002436 novel_circ_002437 novel_circ_002440 novel_circ_002441 novel_circ_002442

| hsa_circ_0027895 | 90 | 1 | 1243 | 225 | 1467 | 0 | 0 |
| --- | --- | --- | --- | --- | --- | --- | --- |
| hsa_circ_0027895 | 90 | 1 | 581 | 452 | 1032 | 0 | 0 |
| hsa_circ_0027895 | 91 | 1 | 1016 | 452 | 1467 | 0 | 0 |
| hsa_circ_0027895 | 91 | 1 | 1016 | 452 | 1467 | 0 | 0 |
| hsa_circ_0027893 | 91 | 1 | 1239 | 1040 | 2278 | 0 | 0 |
| hsa_circ_0027902 | 95 | 1 | 332 | 511 | 180 | 0 | 1.00E-149 |
| hsa_circ_0027904 | 92 | 1 | 375 | 1 | 375 | 0 | 2.00E-148 |
| hsa_circ_0027904 | 92 | 1 | 507 | 1 | 507 | 0 | 0 |
| hsa_circ_0027904 | 92 | 1 | 507 | 1 | 507 | 0 | 0 |
| hsa_circ_0027905 | 90 | 1 | 2831 | 2831 | 1 | 0 | 0 |
| hsa_circ_0027905 | 90 | 1 | 2831 | 2831 | 1 | 0 | 0 |
| hsa_circ_0027905 | 90 | 201 | 3031 | 2831 | 1 | 0 | 0 |
| hsa_circ_0027905 | 89 | 1 | 2478 | 2831 | 354 | 0 | 0 |
| hsa_circ_0027908 | 91 | 1 | 713 | 713 | 1 | 0 | 0 |
| hsa_circ_0027908 | 91 | 1 | 1081 | 1081 | 1 | 0 | 0 |
| hsa_circ_0027908 | 92 | 1 | 513 | 513 | 1 | 0 | 0 |
| hsa_circ_0027910 | 91 | 1 | 568 | 568 | 1 | 0 | 0 |
| hsa_circ_0027910 | 91 | 87 | 654 | 568 | 1 | 0 | 0 |
| hsa_circ_0004925 | 92 | 1 | 368 | 368 | 1 | 0 | 1.00E-144 |
| hsa_circ_0027928 | 82 | 2 | 525 | 5778 | 5263 | 0.03 | 4.00E-122 |
| hsa_circ_0027928 | 90 | 2 | 310 | 6153 | 5845 | 0 | 8.00E-112 |
| hsa_circ_0027937 | 93 | 1 | 399 | 1 | 399 | 0 | 9.00E-167 |
| hsa_circ_0027937 | 94 | 1 | 915 | 1 | 915 | 0 | 0 |
| hsa_circ_0027940 | 95 | 1 | 516 | 1 | 516 | 0 | 0 |
| hsa_circ_0027944 | 91 | 1 | 455 | 1 | 455 | 0 | 2.00E-174 |
| hsa_circ_0008166 | 93 | 71 | 556 | 1 | 486 | 0 | 0 |
| hsa_circ_0008166 | 92 | 71 | 682 | 1 | 612 | 0 | 0 |
| hsa_circ_0027947 | 95 | 1 | 751 | 150 | 900 | 0 | 0 |
| hsa_circ_0027947 | 95 | 1 | 751 | 150 | 900 | 0 | 0 |
| hsa_circ_0027947 | 95 | 1 | 590 | 311 | 900 | 0 | 0 |
| hsa_circ_0027947 | 94 | 1 | 296 | 605 | 900 | 0 | 6.00E-126 |
| hsa_circ_0027951 | 92 | 897 | 1294 | 1 | 398 | 0 | 1.00E-160 |
| hsa_circ_0027961 | 88 | 1 | 234 | 1 | 234 | 0 | 6.00E-72 |
| hsa_circ_0027966 | 94 | 4 | 509 | 506 | 1 | 0 | 0 |
| hsa_circ_0027966 | 93 | 4 | 801 | 798 | 1 | 0 | 0 |
| hsa_circ_0027966 | 91 | 1 | 1703 | 1703 | 1 | 0 | 0 |
| hsa_circ_0027969 | 95 | 4 | 228 | 225 | 1 | 0 | 3.00E-95 |
| hsa_circ_0027969 | 93 | 1 | 592 | 592 | 1 | 0 | 0 |
| hsa_circ_0027969 | 91 | 1 | 1422 | 1422 | 1 | 0 | 0 |
| hsa_circ_0027971 | 90 | 1 | 1194 | 1194 | 1 | 0 | 0 |
| hsa_circ_0003012 | 88 | 1 | 830 | 830 | 1 | 0 | 0 |
| hsa_circ_0006260 | 87 | 1 | 722 | 722 | 1 | 0 | 0 |
| hsa_circ_0027972 | 94 | 1 | 199 | 199 | 1 | 0 | 2.00E-80 |
| hsa_circ_0027974 | 93 | 129 | 638 | 510 | 1 | 0 | 0 |
| hsa_circ_0027976 | 90 | 874 | 1359 | 614 | 129 | 0 | 3.00E-176 |
| hsa_circ_0027977 | 91 | 103 | 545 | 443 | 1 | 0 | 1.00E-172 |
| hsa_circ_0027977 | 91 | 649 | 1091 | 443 | 1 | 0 | 2.00E-172 |
| hsa_circ_0027977 | 91 | 103 | 453 | 443 | 93 | 0 | 1.00E-131 |
| hsa_circ_0027977 | 91 | 2831 | 3181 | 443 | 93 | 0 | 8.00E-131 |
| hsa_circ_0027977 | 91 | 504 | 854 | 443 | 93 | 0 | 2.00E-131 |
| hsa_circ_0027977 | 91 | 649 | 999 | 443 | 93 | 0 | 3.00E-131 |
| hsa_circ_0027978 | 94 | 1 | 1138 | 1 | 1138 | 0 | 0 |
| hsa_circ_0027980 | 95 | 1 | 1226 | 44 | 1269 | 0 | 0 |
| hsa_circ_0027981 | 94 | 1 | 534 | 105 | 638 | 0 | 0 |

novel_circ_002443 novel_circ_002444 novel_circ_002445 novel_circ_002446 novel_circ_002447 novel_circ_002448 novel_circ_002449 novel_circ_002450 novel_circ_002451 novel_circ_002452 novel_circ_002453 novel_circ_002454 novel_circ_002458 novel_circ_002459 novel_circ_002460 novel_circ_002461 novel_circ_002463 novel_circ_002464 novel_circ_002470 novel_circ_002471 novel_circ_002472 novel_circ_002474 novel_circ_002475 novel_circ_002476 novel_circ_002477 novel_circ_002478 novel_circ_002479 novel_circ_002482 novel_circ_002483 novel_circ_002484 novel_circ_002485 novel_circ_002486 novel_circ_002487 novel_circ_002488 novel_circ_002489 novel_circ_002490 novel_circ_002491 novel_circ_002492 novel_circ_002493 novel_circ_002494 novel_circ_002495 novel_circ_002497 novel_circ_002498 novel_circ_002499 novel_circ_002500 novel_circ_002501 novel_circ_002502 novel_circ_002503 novel_circ_002504 novel_circ_002505 novel_circ_002506 novel_circ_002507 novel_circ_002508 novel_circ_002509

| hsa_circ_0027981 | 94 | 1 | 787 | 105 | 891 | 0 | 0 |
| --- | --- | --- | --- | --- | --- | --- | --- |
| hsa_circ_0027980 | 95 | 1 | 1122 | 148 | 1269 | 0 | 0 |
| hsa_circ_0027983 | 95 | 1 | 414 | 129 | 542 | 0 | 0 |
| hsa_circ_0027983 | 96 | 1 | 588 | 290 | 877 | 0 | 0 |
| hsa_circ_0027983 | 96 | 1 | 694 | 290 | 983 | 0 | 0 |
| hsa_circ_0027983 | 94 | 1 | 748 | 1101 | 1848 | 0 | 0 |
| hsa_circ_0027983 | 95 | 1 | 899 | 1101 | 1999 | 0 | 0 |
| hsa_circ_0027983 | 94 | 1 | 579 | 1270 | 1848 | 0 | 0 |
| hsa_circ_0027983 | 95 | 1 | 730 | 1270 | 1999 | 0 | 0 |
| hsa_circ_0027986 | 94 | 1 | 596 | 1 | 596 | 0 | 0 |
| hsa_circ_0027987 | 93 | 1 | 442 | 88 | 529 | 0 | 0 |
| hsa_circ_0027987 | 93 | 1 | 294 | 236 | 529 | 0 | 2.00E-118 |
| hsa_circ_0069633 | 88 | 539 | 1047 | 18 | 526 | 0 | 3.00E-172 |
| hsa_circ_0069633 | 88 | 18 | 526 | 18 | 526 | 0 | 2.00E-172 |
| hsa_circ_0069633 | 86 | 1 | 321 | 206 | 526 | 0 | 9.00E-95 |
| hsa_circ_0069633 | 86 | 1 | 235 | 292 | 526 | 0 | 1.00E-63 |
| hsa_circ_0069654 | 82 | 1 | 1684 | 1681 | 1 | 0 | 0 |
| hsa_circ_0069654 | 82 | 1 | 1592 | 1681 | 93 | 0 | 0 |
| hsa_circ_0069672 | 83 | 7 | 414 | 612 | 205 | 0 | 2.00E-98 |
| hsa_circ_0069677 | 90 | 1 | 327 | 327 | 1 | 0.01 | 8.00E-117 |
| hsa_circ_0069680 | 85 | 1 | 655 | 1209 | 555 | 0 | 0 |
| hsa_circ_0069698 | 93 | 1 | 501 | 501 | 1 | 0 | 0 |
| hsa_circ_0069698 | 92 | 1 | 763 | 763 | 1 | 0 | 0 |
| hsa_circ_0069700 | 90 | 1 | 463 | 463 | 1 | 0 | 7.00E-169 |
| hsa_circ_0069701 | 89 | 1 | 357 | 361 | 5 | 0 | 9.00E-122 |
| hsa_circ_0069701 | 90 | 1 | 261 | 361 | 101 | 0 | 7.00E-92 |
| hsa_circ_0069699 | 84 | 53 | 548 | 1451 | 966 | 0.02 | 2.00E-129 |
| hsa_circ_0069699 | 83 | 53 | 494 | 1451 | 1020 | 0.02 | 5.00E-106 |
| hsa_circ_0069705 | 83 | 53 | 294 | 232 | 1 | 0.04 | 1.00E-54 |
| hsa_circ_0069705 | 83 | 53 | 294 | 232 | 1 | 0.04 | 4.00E-55 |
| hsa_circ_0001409 | 90 | 64 | 558 | 1 | 489 | 0.01 | 3.00E-178 |
| hsa_circ_0001409 | 94 | 1 | 238 | 82 | 319 | 0 | 4.00E-99 |
| hsa_circ_0069718 | 94 | 3 | 318 | 3 | 318 | 0 | 5.00E-134 |
| hsa_circ_0069718 | 94 | 3 | 388 | 3 | 388 | 0 | 7.00E-163 |
| hsa_circ_0069718 | 93 | 3 | 478 | 3 | 478 | 0 | 0 |
| hsa_circ_0069718 | 94 | 3 | 590 | 3 | 590 | 0 | 0 |
| hsa_circ_0069718 | 93 | 1 | 277 | 112 | 388 | 0 | 9.00E-111 |
| hsa_circ_0069718 | 93 | 1 | 367 | 112 | 478 | 0 | 1.00E-150 |
| hsa_circ_0069718 | 93 | 1 | 479 | 112 | 590 | 0 | 0 |
| hsa_circ_0069718 | 92 | 1 | 252 | 227 | 478 | 0 | 1.00E-98 |
| hsa_circ_0069718 | 94 | 3 | 272 | 321 | 590 | 0 | 7.00E-112 |
| hsa_circ_0090870 | 92 | 7 | 185 | 407 | 229 | 0 | 1.00E-63 |
| hsa_circ_0008423 | 92 | 7 | 180 | 174 | 1 | 0 | 7.00E-63 |
| hsa_circ_0008423 | 92 | 7 | 180 | 174 | 1 | 0 | 1.00E-63 |
| hsa_circ_0008423 | 92 | 1 | 372 | 372 | 1 | 0 | 2.00E-148 |
| hsa_circ_0008423 | 95 | 1 | 552 | 552 | 1 | 0 | 0 |
| hsa_circ_0008423 | 96 | 1 | 372 | 552 | 181 | 0 | 2.00E-173 |
| hsa_circ_0066708 | 83 | 2574 | 2692 | 56192 | 56309 | 0.01 | 5.00E-21 |
| hsa_circ_0057098 | 96 | 1 | 281 | 1 | 281 | 0 | 1.00E-129 |
| hsa_circ_0057082 | 92 | 3 | 850 | 909 | 1756 | 0 | 0 |
| hsa_circ_0057082 | 90 | 1 | 724 | 344 | 1067 | 0.01 | 0 |
| hsa_circ_0057082 | 91 | 1 | 562 | 344 | 905 | 0.01 | 0 |
| hsa_circ_0057082 | 92 | 1 | 402 | 344 | 745 | 0 | 9.00E-162 |
| hsa_circ_0057082 | 93 | 3 | 613 | 135 | 745 | 0 | 0 |

novel_circ_002510 novel_circ_002511 novel_circ_002512 novel_circ_002513 novel_circ_002514 novel_circ_002515 novel_circ_002516 novel_circ_002517 novel_circ_002519 novel_circ_002520 novel_circ_002521 novel_circ_002522 novel_circ_002523 novel_circ_002524 novel_circ_002525 novel_circ_002526 novel_circ_002528 novel_circ_002529 novel_circ_002530 novel_circ_002531 novel_circ_002532 novel_circ_002535 novel_circ_002536 novel_circ_002537 novel_circ_002538 novel_circ_002539 novel_circ_002540 novel_circ_002541 novel_circ_002542 novel_circ_002543 novel_circ_002544 novel_circ_002545 novel_circ_002546 novel_circ_002547 novel_circ_002548 novel_circ_002549 novel_circ_002550 novel_circ_002551 novel_circ_002552 novel_circ_002553 novel_circ_002554 novel_circ_002555 novel_circ_002556 novel_circ_002557 novel_circ_002558 novel_circ_002559 novel_circ_002560 novel_circ_002561 novel_circ_002562 novel_circ_002563 novel_circ_002564 novel_circ_002565 novel_circ_002566 novel_circ_002567

| hsa_circ_0057068 | 95 | 1 | 259 | 1380 | 1122 | 0 | 2.00E-112 |
| --- | --- | --- | --- | --- | --- | --- | --- |
| hsa_circ_0057068 | 95 | 363 | 883 | 1122 | 602 | 0 | 0 |
| hsa_circ_0057068 | 99 | 634 | 750 | 1238 | 1122 | 0 | 5.00E-53 |
| hsa_circ_0057068 | 95 | 220 | 825 | 1122 | 517 | 0 | 0 |
| hsa_circ_0057068 | 95 | 1 | 520 | 1121 | 602 | 0 | 0 |
| hsa_circ_0057070 | 92 | 1 | 460 | 460 | 1 | 0 | 0 |
| hsa_circ_0057062 | 95 | 1 | 632 | 1 | 632 | 0 | 0 |
| hsa_circ_0057058 | 82 | 218 | 675 | 9 | 469 | 0.04 | 4.00E-103 |
| hsa_circ_0057057 | 94 | 6 | 428 | 112 | 534 | 0 | 0 |
| hsa_circ_0057055 | 95 | 1 | 301 | 1 | 301 | 0 | 9.00E-131 |
| hsa_circ_0057052 | 92 | 1 | 216 | 1 | 216 | 0 | 5.00E-82 |
| hsa_circ_0057040 | 88 | 93 | 717 | 625 | 1 | 0 | 0 |
| hsa_circ_0057040 | 88 | 93 | 717 | 625 | 1 | 0 | 0 |
| hsa_circ_0057040 | 88 | 1 | 625 | 625 | 1 | 0 | 0 |
| hsa_circ_0004384 | 93 | 51 | 427 | 377 | 1 | 0 | 7.00E-156 |
| hsa_circ_0004384 | 93 | 1 | 377 | 377 | 1 | 0 | 6.00E-156 |
| hsa_circ_0057036 | 98 | 1 | 191 | 191 | 1 | 0 | 6.00E-89 |
| hsa_circ_0057033 | 96 | 1 | 256 | 256 | 1 | 0 | 2.00E-117 |
| hsa_circ_0057030 | 90 | 6712 | 7544 | 857 | 1 | 0.04 | 0 |
| hsa_circ_0057029 | 96 | 1 | 354 | 436 | 83 | 0 | 8.00E-162 |
| hsa_circ_0057014 | 92 | 1 | 593 | 373 | 953 | 0.02 | 0 |
| hsa_circ_0057004 | 93 | 1 | 350 | 1 | 350 | 0 | 3.00E-141 |
| hsa_circ_0056977 | 95 | 1 | 530 | 4032 | 4561 | 0 | 0 |
| hsa_circ_0056977 | 93 | 3 | 539 | 3675 | 4211 | 0 | 0 |
| hsa_circ_0056977 | 93 | 1 | 643 | 3569 | 4211 | 0 | 0 |
| hsa_circ_0056977 | 94 | 1 | 746 | 3466 | 4211 | 0 | 0 |
| hsa_circ_0056977 | 94 | 2 | 1654 | 2559 | 4211 | 0 | 0 |
| hsa_circ_0057000 | 91 | 1 | 463 | 104 | 566 | 0 | 6.00E-179 |
| hsa_circ_0057000 | 92 | 1 | 566 | 1 | 566 | 0 | 0 |
| hsa_circ_0056989 | 94 | 1 | 2356 | 98 | 2453 | 0 | 0 |
| hsa_circ_0057000 | 95 | 1 | 207 | 1 | 207 | 0 | 5.00E-87 |
| hsa_circ_0056989 | 95 | 1 | 1997 | 98 | 2094 | 0 | 0 |
| hsa_circ_0056989 | 95 | 1 | 2094 | 1 | 2094 | 0 | 0 |
| hsa_circ_0056997 | 95 | 1 | 772 | 151 | 922 | 0 | 0 |
| hsa_circ_0056997 | 95 | 3 | 922 | 3 | 922 | 0 | 0 |
| hsa_circ_0056992 | 95 | 2 | 1011 | 742 | 1751 | 0 | 0 |
| hsa_circ_0056989 | 95 | 1 | 1893 | 98 | 1990 | 0 | 0 |
| hsa_circ_0056997 | 99 | 441 | 528 | 732 | 819 | 0 | 4.00E-37 |
| hsa_circ_0056997 | 95 | 1 | 669 | 151 | 819 | 0 | 0 |
| hsa_circ_0056997 | 94 | 3 | 819 | 3 | 819 | 0 | 0 |
| hsa_circ_0056989 | 95 | 1 | 1790 | 98 | 1887 | 0 | 0 |
| hsa_circ_0056997 | 94 | 3 | 733 | 3 | 733 | 0 | 0 |
| hsa_circ_0056997 | 94 | 3 | 395 | 3 | 395 | 0 | 9.00E-167 |
| hsa_circ_0056989 | 95 | 1 | 1366 | 98 | 1463 | 0 | 0 |
| hsa_circ_0056977 | 95 | 1 | 2139 | 792 | 2930 | 0 | 0 |
| hsa_circ_0056990 | 95 | 1 | 971 | 1 | 971 | 0 | 0 |
| hsa_circ_0056977 | 95 | 1 | 2596 | 51 | 2646 | 0 | 0 |
| hsa_circ_0056973 | 95 | 141 | 2118 | 1 | 1978 | 0 | 0 |
| hsa_circ_0056981 | 96 | 1 | 544 | 918 | 1461 | 0 | 0 |
| hsa_circ_0056981 | 96 | 1 | 700 | 762 | 1461 | 0 | 0 |
| hsa_circ_0056981 | 96 | 1 | 787 | 675 | 1461 | 0 | 0 |
| hsa_circ_0056979 | 96 | 1 | 1528 | 1 | 1528 | 0 | 0 |
| hsa_circ_0056973 | 95 | 1 | 1722 | 160 | 1881 | 0 | 0 |
| hsa_circ_0056973 | 95 | 141 | 2021 | 1 | 1881 | 0 | 0 |

novel_circ_002568 novel_circ_002569 novel_circ_002570 novel_circ_002571 novel_circ_002572 novel_circ_002573 novel_circ_002574 novel_circ_002575 novel_circ_002576 novel_circ_002577 novel_circ_002578 novel_circ_002579 novel_circ_002580 novel_circ_002581 novel_circ_002582 novel_circ_002583 novel_circ_002584 novel_circ_002585 novel_circ_002586 novel_circ_002587 novel_circ_002588 novel_circ_002589 novel_circ_002590 novel_circ_002591 novel_circ_002592 novel_circ_002593 novel_circ_002594 novel_circ_002595 novel_circ_002596 novel_circ_002597 novel_circ_002598 novel_circ_002599 novel_circ_002600 novel_circ_002601 novel_circ_002602 novel_circ_002603 novel_circ_002604 novel_circ_002605 novel_circ_002606 novel_circ_002607 novel_circ_002608 novel_circ_002609 novel_circ_002610 novel_circ_002611 novel_circ_002612 novel_circ_002613 novel_circ_002614 novel_circ_002615 novel_circ_002616 novel_circ_002617 novel_circ_002618 novel_circ_002619 novel_circ_002620 novel_circ_002621

| hsa_circ_0056983 | 96 | 1 | 243 | 135 | 377 | 0 | 3.00E-110 |
| --- | --- | --- | --- | --- | --- | --- | --- |
| hsa_circ_0056979 | 96 | 1 | 984 | 1 | 984 | 0 | 0 |
| hsa_circ_0056973 | 95 | 141 | 1477 | 1 | 1337 | 0 | 0 |
| hsa_circ_0056973 | 95 | 141 | 1387 | 1 | 1247 | 0 | 0 |
| hsa_circ_0056981 | 97 | 1 | 401 | 361 | 761 | 0 | 0 |
| hsa_circ_0056981 | 96 | 1 | 761 | 1 | 761 | 0 | 0 |
| hsa_circ_0056979 | 96 | 1 | 828 | 1 | 828 | 0 | 0 |
| hsa_circ_0056973 | 95 | 1 | 1022 | 160 | 1181 | 0 | 0 |
| hsa_circ_0056973 | 95 | 141 | 1321 | 1 | 1181 | 0 | 0 |
| hsa_circ_0056981 | 96 | 1 | 674 | 1 | 674 | 0 | 0 |
| hsa_circ_0056979 | 96 | 1 | 741 | 1 | 741 | 0 | 0 |
| hsa_circ_0056977 | 96 | 8 | 791 | 8 | 791 | 0 | 0 |
| hsa_circ_0056973 | 95 | 1 | 935 | 160 | 1094 | 0 | 0 |
| hsa_circ_0056973 | 95 | 141 | 1234 | 1 | 1094 | 0 | 0 |
| hsa_circ_0056981 | 96 | 1 | 360 | 1 | 360 | 0 | 4.00E-165 |
| hsa_circ_0056977 | 95 | 8 | 477 | 8 | 477 | 0 | 0 |
| hsa_circ_0056975 | 94 | 1 | 621 | 1 | 621 | 0 | 0 |
| hsa_circ_0056973 | 94 | 1 | 780 | 1 | 780 | 0 | 0 |
| hsa_circ_0056973 | 94 | 141 | 920 | 1 | 780 | 0 | 0 |
| hsa_circ_0056973 | 93 | 141 | 691 | 1 | 551 | 0 | 0 |
| hsa_circ_0056975 | 90 | 1 | 261 | 1 | 261 | 0 | 1.00E-93 |
| hsa_circ_0002603 | 95 | 1 | 493 | 1 | 493 | 0 | 0 |
| hsa_circ_0002603 | 95 | 1 | 493 | 1 | 493 | 0 | 0 |
| hsa_circ_0002603 | 95 | 1 | 443 | 1 | 443 | 0 | 0 |
| hsa_circ_0002603 | 98 | 1 | 299 | 1 | 299 | 0 | 1.00E-144 |
| hsa_circ_0002603 | 98 | 607 | 905 | 1 | 299 | 0 | 4.00E-144 |
| hsa_circ_0030821 | 92 | 1 | 1155 | 1 | 1156 | 0 | 0 |
| hsa_circ_0006288 | 93 | 1 | 423 | 1 | 423 | 0 | 1.00E-176 |
| hsa_circ_0030821 | 92 | 1 | 726 | 1 | 727 | 0.01 | 0 |
| hsa_circ_0030815 | 91 | 3227 | 4007 | 778 | 1547 | 0.01 | 0 |
| hsa_circ_0030819 | 91 | 1 | 719 | 1 | 708 | 0.02 | 0 |
| hsa_circ_0030815 | 94 | 1 | 560 | 219 | 778 | 0 | 0 |
| hsa_circ_0030817 | 94 | 1 | 325 | 1 | 325 | 0 | 2.00E-140 |
| hsa_circ_0030815 | 94 | 1 | 560 | 219 | 778 | 0 | 0 |
| hsa_circ_0030808 | 92 | 1 | 782 | 250 | 1031 | 0 | 0 |
| hsa_circ_0030797 | 95 | 1 | 612 | 126 | 737 | 0 | 0 |
| hsa_circ_0030797 | 94 | 2 | 521 | 2 | 521 | 0 | 0 |
| hsa_circ_0030795 | 93 | 226 | 619 | 1 | 394 | 0 | 2.00E-165 |
| hsa_circ_0030791 | 90 | 1 | 699 | 1 | 699 | 0 | 0 |
| hsa_circ_0030791 | 91 | 1 | 552 | 1 | 552 | 0 | 0 |
| hsa_circ_0030789 | 93 | 4305 | 4451 | 330 | 184 | 0 | 6.00E-54 |
| hsa_circ_0030789 | 92 | 4305 | 4634 | 330 | 1 | 0 | 1.00E-130 |
| hsa_circ_0030788 | 94 | 4635 | 4987 | 508 | 156 | 0 | 5.00E-150 |
| hsa_circ_0030788 | 94 | 4635 | 5142 | 508 | 1 | 0 | 0 |
| hsa_circ_0030788 | 94 | 331 | 683 | 508 | 156 | 0 | 6.00E-151 |
| hsa_circ_0030788 | 94 | 331 | 838 | 508 | 1 | 0 | 0 |
| hsa_circ_0030788 | 94 | 331 | 838 | 508 | 1 | 0 | 0 |
| hsa_circ_0030788 | 94 | 331 | 838 | 508 | 1 | 0 | 0 |
| hsa_circ_0030788 | 94 | 303 | 810 | 508 | 1 | 0 | 0 |
| hsa_circ_0030788 | 94 | 184 | 536 | 508 | 156 | 0 | 5.00E-151 |
| hsa_circ_0030788 | 94 | 184 | 691 | 508 | 1 | 0 | 0 |
| hsa_circ_0030788 | 94 | 1 | 508 | 508 | 1 | 0 | 0 |
| hsa_circ_0030788 | 93 | 4 | 269 | 421 | 156 | 0 | 5.00E-108 |
| hsa_circ_0030786 | 93 | 1 | 622 | 622 | 1 | 0 | 0 |

novel_circ_002622 novel_circ_002623 novel_circ_002624 novel_circ_002625 novel_circ_002635 novel_circ_002637 novel_circ_002638 novel_circ_002639 novel_circ_002640 novel_circ_002641 novel_circ_002642 novel_circ_002643 novel_circ_002644 novel_circ_002645 novel_circ_002646 novel_circ_002647 novel_circ_002648 novel_circ_002649 novel_circ_002650 novel_circ_002651 novel_circ_002652 novel_circ_002653 novel_circ_002654 novel_circ_002655 novel_circ_002656 novel_circ_002657 novel_circ_002658 novel_circ_002659 novel_circ_002660 novel_circ_002661 novel_circ_002662 novel_circ_002663 novel_circ_002664 novel_circ_002665 novel_circ_002666 novel_circ_002667 novel_circ_002668 novel_circ_002669 novel_circ_002670 novel_circ_002671 novel_circ_002672 novel_circ_002673 novel_circ_002674 novel_circ_002676 novel_circ_002677 novel_circ_002678 novel_circ_002679 novel_circ_002680 novel_circ_002681 novel_circ_002683 novel_circ_002684 novel_circ_002685 novel_circ_002686 novel_circ_002687

| hsa_circ_0030787 | 94 | 9 | 335 | 327 | 1 | 0 | 2.00E-138 |
| --- | --- | --- | --- | --- | --- | --- | --- |
| hsa_circ_0030786 | 93 | 9 | 467 | 459 | 1 | 0 | 0 |
| hsa_circ_0030786 | 93 | 9 | 467 | 459 | 1 | 0 | 0 |
| hsa_circ_0030786 | 93 | 9 | 467 | 459 | 1 | 0 | 0 |
| hsa_circ_0030783 | 92 | 217 | 831 | 983 | 369 | 0 | 0 |
| hsa_circ_0030778 | 92 | 1 | 330 | 558 | 229 | 0 | 8.00E-127 |
| hsa_circ_0030774 | 90 | 1 | 316 | 1 | 316 | 0.01 | 2.00E-112 |
| hsa_circ_0030768 | 90 | 1 | 461 | 1 | 461 | 0 | 8.00E-173 |
| hsa_circ_0030764 | 92 | 1 | 680 | 1 | 680 | 0 | 0 |
| hsa_circ_0030761 | 92 | 1 | 831 | 1 | 831 | 0 | 0 |
| hsa_circ_0030774 | 94 | 1 | 214 | 1 | 214 | 0 | 1.00E-87 |
| hsa_circ_0030770 | 93 | 1 | 290 | 1 | 290 | 0 | 3.00E-116 |
| hsa_circ_0030764 | 94 | 1 | 578 | 1 | 578 | 0 | 0 |
| hsa_circ_0030761 | 93 | 1 | 729 | 1 | 729 | 0 | 0 |
| hsa_circ_0030764 | 94 | 1 | 470 | 1 | 470 | 0 | 0 |
| hsa_circ_0030761 | 93 | 1 | 621 | 1 | 621 | 0 | 0 |
| hsa_circ_0030757 | 93 | 1 | 819 | 1 | 819 | 0 | 0 |
| hsa_circ_0030764 | 94 | 1 | 364 | 1 | 364 | 0 | 1.00E-155 |
| hsa_circ_0030755 | 93 | 3 | 792 | 3 | 792 | 0 | 0 |
| hsa_circ_0030759 | 92 | 1 | 92 | 1 | 92 | 0 | 3.00E-28 |
| hsa_circ_0030759 | 90 | 1 | 246 | 1 | 246 | 0 | 6.00E-87 |
| hsa_circ_0030757 | 91 | 1 | 349 | 1 | 349 | 0 | 2.00E-132 |
| hsa_circ_0030755 | 92 | 3 | 428 | 3 | 428 | 0 | 2.00E-173 |
| hsa_circ_0030754 | 93 | 1 | 465 | 1 | 465 | 0 | 0 |
| hsa_circ_0030750 | 93 | 1 | 597 | 169 | 765 | 0 | 0 |
| hsa_circ_0030750 | 93 | 31402 | 31999 | 168 | 765 | 0 | 0 |
| hsa_circ_0030750 | 94 | 1 | 765 | 1 | 765 | 0 | 0 |
| hsa_circ_0030750 | 95 | 1 | 351 | 169 | 519 | 0 | 4.00E-155 |
| hsa_circ_0030750 | 95 | 1 | 519 | 1 | 519 | 0 | 0 |
| hsa_circ_0030750 | 96 | 1 | 248 | 169 | 416 | 0 | 1.00E-109 |
| hsa_circ_0030750 | 95 | 1 | 300 | 1 | 300 | 0 | 2.00E-133 |
| hsa_circ_0030743 | 95 | 1 | 120 | 388 | 507 | 0 | 1.00E-46 |
| hsa_circ_0030743 | 89 | 292 | 678 | 1 | 387 | 0 | 2.00E-131 |
| hsa_circ_0030738 | 94 | 1 | 436 | 339 | 774 | 0 | 0 |
| hsa_circ_0030738 | 94 | 1 | 596 | 179 | 774 | 0 | 0 |
| hsa_circ_0030738 | 93 | 1 | 774 | 1 | 774 | 0 | 0 |
| hsa_circ_0030738 | 95 | 1 | 424 | 179 | 602 | 0 | 0 |
| hsa_circ_0030738 | 95 | 1 | 482 | 121 | 602 | 0 | 0 |
| hsa_circ_0030738 | 94 | 1 | 602 | 1 | 602 | 0 | 0 |
| hsa_circ_0030736 | 94 | 1 | 735 | 190 | 924 | 0 | 0 |
| hsa_circ_0030737 | 93 | 1 | 471 | 1 | 471 | 0 | 0 |
| hsa_circ_0030733 | 91 | 1 | 815 | 95 | 909 | 0.01 | 0 |
| hsa_circ_0030733 | 93 | 1 | 256 | 95 | 350 | 0.01 | 8.00E-101 |
| hsa_circ_0030721 | 91 | 1 | 358 | 1 | 358 | 0 | 5.00E-134 |
| hsa_circ_0030717 | 91 | 1 | 243 | 243 | 1 | 0 | 1.00E-86 |
| hsa_circ_0030717 | 91 | 1 | 243 | 243 | 1 | 0 | 2.00E-86 |
| hsa_circ_0030716 | 92 | 634 | 1376 | 743 | 1 | 0 | 0 |
| hsa_circ_0030717 | 96 | 1 | 70 | 70 | 1 | 0 | 8.00E-24 |
| hsa_circ_0030717 | 96 | 1 | 70 | 70 | 1 | 0 | 1.00E-23 |
| hsa_circ_0030716 | 92 | 250 | 992 | 743 | 1 | 0 | 0 |
| hsa_circ_0030712 | 92 | 7 | 950 | 946 | 3 | 0 | 0 |
| hsa_circ_0030712 | 92 | 7 | 752 | 748 | 3 | 0 | 0 |
| hsa_circ_0030712 | 92 | 1 | 521 | 523 | 3 | 0 | 0 |
| hsa_circ_0030709 | 93 | 1 | 839 | 1956 | 1118 | 0 | 0 |

novel_circ_002688 novel_circ_002689 novel_circ_002690 novel_circ_002691 novel_circ_002692 novel_circ_002693 novel_circ_002694 novel_circ_002695 novel_circ_002696 novel_circ_002697 novel_circ_002698 novel_circ_002701 novel_circ_002702 novel_circ_002703 novel_circ_002704 novel_circ_002705 novel_circ_002706 novel_circ_002707 novel_circ_002708 novel_circ_002709 novel_circ_002710 novel_circ_002711 novel_circ_002712 novel_circ_002713 novel_circ_002714 novel_circ_002715 novel_circ_002716 novel_circ_002717 novel_circ_002722 novel_circ_002723 novel_circ_002724 novel_circ_002725 novel_circ_002726 novel_circ_002727 novel_circ_002728 novel_circ_002729 novel_circ_002730 novel_circ_002731 novel_circ_002732 novel_circ_002738 novel_circ_002739 novel_circ_002740 novel_circ_002741 novel_circ_002742 novel_circ_002743 novel_circ_002744 novel_circ_002745 novel_circ_002746 novel_circ_002747 novel_circ_002748 novel_circ_002749 novel_circ_002750 novel_circ_002751 novel_circ_002752

| hsa_circ_0030712 | 91 | 3 | 409 | 409 | 3 | 0 | 4.00E-156 |
| --- | --- | --- | --- | --- | --- | --- | --- |
| hsa_circ_0030711 | 95 | 1 | 249 | 364 | 116 | 0 | 1.00E-108 |
| hsa_circ_0030711 | 95 | 1 | 316 | 364 | 49 | 0 | 1.00E-140 |
| hsa_circ_0030709 | 90 | 1 | 709 | 885 | 177 | 0 | 0 |
| hsa_circ_0030708 | 91 | 57 | 802 | 2443 | 1702 | 0.01 | 0 |
| hsa_circ_0030708 | 91 | 57 | 995 | 2443 | 1509 | 0 | 0 |
| hsa_circ_0003170 | 94 | 1 | 231 | 14559 | 14329 | 0 | 1.00E-93 |
| hsa_circ_0003170 | 77 | 1 | 1118 | 14559 | 13416 | 0.06 | 2.00E-168 |
| hsa_circ_0005148 | 92 | 1 | 287 | 555 | 269 | 0 | 5.00E-113 |
| hsa_circ_0005148 | 92 | 1 | 555 | 555 | 1 | 0 | 0 |
| hsa_circ_0003170 | 93 | 1 | 96 | 139 | 48 | 0.04 | 1.00E-28 |
| hsa_circ_0030705 | 90 | 1 | 330 | 330 | 1 | 0 | 8.00E-117 |
| hsa_circ_0030697 | 89 | 1 | 412 | 1 | 412 | 0 | 1.00E-145 |
| hsa_circ_0030686 | 88 | 1 | 1655 | 1 | 1655 | 0 | 0 |
| hsa_circ_0030686 | 92 | 1 | 592 | 326 | 917 | 0 | 0 |
| hsa_circ_0030686 | 91 | 2 | 408 | 442 | 848 | 0 | 2.00E-154 |
| hsa_circ_0030686 | 91 | 1 | 523 | 326 | 848 | 0 | 0 |
| hsa_circ_0030686 | 93 | 1 | 227 | 1 | 227 | 0 | 2.00E-91 |
| hsa_circ_0030684 | 93 | 16 | 193 | 17 | 194 | 0 | 1.00E-67 |
| hsa_circ_0030684 | 93 | 13751 | 13928 | 17 | 194 | 0 | 1.00E-65 |
| hsa_circ_0030677 | 95 | 1 | 572 | 997 | 1568 | 0 | 0 |
| hsa_circ_0030678 | 94 | 1 | 392 | 134 | 525 | 0 | 7.00E-168 |
| hsa_circ_0030677 | 94 | 1 | 339 | 220 | 558 | 0 | 8.00E-142 |
| hsa_circ_0030663 | 93 | 1 | 742 | 1 | 742 | 0 | 0 |
| hsa_circ_0030663 | 93 | 1 | 498 | 1 | 498 | 0 | 0 |
| hsa_circ_0006833 | 81 | 557 | 708 | 139564 | 139715 | 0 | 1.00E-25 |
| hsa_circ_0030641 | 96 | 25690 | 25856 | 777 | 943 | 0 | 6.00E-71 |
| hsa_circ_0030641 | 88 | 1 | 780 | 1 | 778 | 0.01 | 0 |
| hsa_circ_0017921 | 89 | 1 | 359 | 1 | 359 | 0 | 1.00E-124 |
| hsa_circ_0017921 | 90 | 1 | 429 | 1 | 429 | 0 | 1.00E-156 |
| hsa_circ_0017925 | 94 | 1 | 242 | 1 | 242 | 0 | 1.00E-99 |
| hsa_circ_0017925 | 94 | 1 | 342 | 1 | 342 | 0 | 8.00E-147 |
| hsa_circ_0017925 | 93 | 1 | 520 | 1 | 520 | 0 | 0 |
| hsa_circ_0017926 | 93 | 1 | 278 | 1 | 278 | 0 | 5.00E-113 |
| hsa_circ_0017921 | 91 | 1 | 690 | 672 | 1361 | 0 | 0 |
| hsa_circ_0017932 | 92 | 109 | 330 | 333 | 112 | 0 | 4.00E-85 |
| hsa_circ_0017932 | 92 | 214 | 435 | 333 | 112 | 0 | 5.00E-85 |
| hsa_circ_0017932 | 92 | 637 | 858 | 333 | 112 | 0 | 1.00E-84 |
| hsa_circ_0017932 | 94 | 637 | 747 | 333 | 223 | 0 | 1.00E-39 |
| hsa_circ_0007197 | 81 | 12662 | 12791 | 465070 | 465199 | 0.02 | 3.00E-18 |
| hsa_circ_0007197 | 81 | 32346 | 32475 | 465070 | 465199 | 0.02 | 3.00E-18 |
| hsa_circ_0017931 | 94 | 1 | 111 | 280 | 170 | 0 | 3.00E-38 |
| hsa_circ_0017931 | 94 | 1 | 111 | 280 | 170 | 0 | 5.00E-39 |
| hsa_circ_0017938 | 95 | 1 | 165 | 1 | 165 | 0 | 9.00E-69 |
| hsa_circ_0017938 | 96 | 1 | 269 | 1 | 269 | 0 | 2.00E-121 |
| hsa_circ_0017936 | 95 | 1 | 363 | 81 | 443 | 0 | 2.00E-163 |
| hsa_circ_0017936 | 96 | 1 | 459 | 81 | 539 | 0 | 0 |
| hsa_circ_0017939 | 96 | 1 | 500 | 1 | 500 | 0 | 0 |
| hsa_circ_0017941 | 96 | 1 | 390 | 1 | 390 | 0 | 4.00E-180 |
| hsa_circ_0017941 | 95 | 1 | 1216 | 1 | 1216 | 0 | 0 |
| hsa_circ_0017941 | 96 | 1 | 1292 | 1 | 1292 | 0 | 0 |
| hsa_circ_0017941 | 95 | 2 | 1112 | 106 | 1216 | 0 | 0 |
| hsa_circ_0017945 | 95 | 1 | 922 | 1 | 922 | 0 | 0 |
| hsa_circ_0017946 | 95 | 1 | 826 | 1 | 826 | 0 | 0 |

novel_circ_002753 novel_circ_002754 novel_circ_002755 novel_circ_002756 novel_circ_002757 novel_circ_002758 novel_circ_002759 novel_circ_002760 novel_circ_002761 novel_circ_002762 novel_circ_002763 novel_circ_002765 novel_circ_002766 novel_circ_002767 novel_circ_002772 novel_circ_002773 novel_circ_002774 novel_circ_002775 novel_circ_002776 novel_circ_002777 novel_circ_002778 novel_circ_002779 novel_circ_002780 novel_circ_002781 novel_circ_002782 novel_circ_002783 novel_circ_002784 novel_circ_002785 novel_circ_002786 novel_circ_002787 novel_circ_002789 novel_circ_002790 novel_circ_002791 novel_circ_002792 novel_circ_002795 novel_circ_002796 novel_circ_002797 novel_circ_002798 novel_circ_002799 novel_circ_002800 novel_circ_002801 novel_circ_002802 novel_circ_002803 novel_circ_002804 novel_circ_002805 novel_circ_002806 novel_circ_002808 novel_circ_002809 novel_circ_002816 novel_circ_002817 novel_circ_002818 novel_circ_002819 novel_circ_002820 novel_circ_002821

| hsa_circ_0017951 | 97 | 1 | 76 | 1 | 76 | 0 | 1.00E-29 |
| --- | --- | --- | --- | --- | --- | --- | --- |
| hsa_circ_0017952 | 91 | 78 | 257 | 81 | 260 | 0 | 7.00E-62 |
| hsa_circ_0017956 | 93 | 450 | 1234 | 2 | 795 | 0.01 | 0 |
| hsa_circ_0007084 | 89 | 1 | 361 | 1 | 355 | 0.02 | 6.00E-124 |
| hsa_circ_0017956 | 93 | 372 | 1156 | 2 | 795 | 0.01 | 0 |
| hsa_circ_0017956 | 93 | 372 | 1253 | 2 | 892 | 0.01 | 0 |
| hsa_circ_0017959 | 94 | 1 | 882 | 1374 | 499 | 0.01 | 0 |
| hsa_circ_0017962 | 93 | 1 | 613 | 607 | 1 | 0.01 | 0 |
| hsa_circ_0017962 | 94 | 1 | 762 | 756 | 1 | 0.01 | 0 |
| hsa_circ_0017962 | 95 | 1 | 598 | 756 | 159 | 0 | 0 |
| hsa_circ_0083255 | 83 | 51 | 417 | 1516 | 1147 | 0.01 | 2.00E-87 |
| hsa_circ_0002333 | 94 | 1 | 247 | 247 | 1 | 0 | 8.00E-101 |
| hsa_circ_0084187 | 87 | 4027 | 4517 | 112 | 601 | 0 | 7.00E-153 |
| hsa_circ_0084187 | 88 | 162 | 762 | 1 | 601 | 0 | 0 |
| hsa_circ_0084184 | 81 | 1 | 187 | 71 | 257 | 0 | 6.00E-36 |
| hsa_circ_0084183 | 86 | 3 | 189 | 3 | 189 | 0 | 6.00E-51 |
| hsa_circ_0002201 | 93 | 1 | 381 | 116 | 496 | 0 | 4.00E-160 |
| hsa_circ_0002201 | 93 | 1 | 496 | 1 | 496 | 0 | 0 |
| hsa_circ_0084174 | 93 | 1 | 582 | 1 | 582 | 0 | 0 |
| hsa_circ_0084165 | 96 | 1 | 361 | 124 | 484 | 0 | 5.00E-164 |
| hsa_circ_0084161 | 91 | 1 | 722 | 1 | 722 | 0 | 0 |
| hsa_circ_0084161 | 93 | 1 | 508 | 1 | 508 | 0 | 0 |
| hsa_circ_0084153 | 93 | 1 | 765 | 1 | 765 | 0 | 0 |
| hsa_circ_0084153 | 95 | 1 | 257 | 1 | 257 | 0 | 2.00E-111 |
| hsa_circ_0084150 | 96 | 1 | 343 | 1 | 343 | 0 | 1.00E-155 |
| hsa_circ_0084139 | 91 | 260 | 590 | 405 | 75 | 0 | 2.00E-125 |
| hsa_circ_0084141 | 91 | 1 | 329 | 329 | 1 | 0 | 1.00E-124 |
| hsa_circ_0084139 | 92 | 1 | 403 | 403 | 1 | 0 | 3.00E-157 |
| hsa_circ_0084135 | 91 | 1 | 514 | 514 | 1 | 0 | 0 |
| hsa_circ_0084139 | 89 | 1 | 259 | 259 | 1 | 0 | 2.00E-87 |
| hsa_circ_0084129 | 87 | 1782 | 3571 | 1787 | 1 | 0 | 0 |
| hsa_circ_0084132 | 89 | 1 | 877 | 877 | 1 | 0 | 0 |
| hsa_circ_0084129 | 87 | 1 | 1790 | 1787 | 1 | 0 | 0 |
| hsa_circ_0084130 | 89 | 3 | 441 | 439 | 1 | 0 | 3.00E-152 |
| hsa_circ_0002754 | 95 | 477 | 594 | 1057 | 938 | 0.02 | 7.00E-46 |
| hsa_circ_0084090 | 94 | 1 | 307 | 307 | 1 | 0 | 2.00E-132 |
| hsa_circ_0084089 | 94 | 1 | 760 | 882 | 120 | 0 | 0 |
| hsa_circ_0084089 | 94 | 1 | 879 | 882 | 1 | 0 | 0 |
| hsa_circ_0084087 | 94 | 1 | 995 | 2580 | 1583 | 0 | 0 |
| hsa_circ_0084089 | 94 | 6 | 770 | 768 | 1 | 0 | 0 |
| hsa_circ_0084089 | 93 | 1 | 572 | 575 | 1 | 0.01 | 0 |
| hsa_circ_0084087 | 93 | 1 | 1387 | 2137 | 745 | 0 | 0 |
| hsa_circ_0084087 | 89 | 1 | 437 | 1184 | 745 | 0.01 | 5.00E-155 |
| hsa_circ_0084087 | 89 | 1 | 439 | 1184 | 743 | 0.01 | 9.00E-156 |
| hsa_circ_0084079 | 92 | 1 | 559 | 941 | 383 | 0 | 0 |
| hsa_circ_0084079 | 92 | 1 | 559 | 941 | 383 | 0 | 0 |
| hsa_circ_0007366 | 88 | 1 | 225 | 225 | 1 | 0 | 1.00E-71 |
| hsa_circ_0084070 | 81 | 1 | 1010 | 1 | 1013 | 0.03 | 0 |
| hsa_circ_0006828 | 94 | 89 | 697 | 609 | 1 | 0 | 0 |
| hsa_circ_0006828 | 94 | 89 | 697 | 609 | 1 | 0 | 0 |
| hsa_circ_0006828 | 94 | 1 | 609 | 609 | 1 | 0 | 0 |
| hsa_circ_0006828 | 94 | 1 | 609 | 609 | 1 | 0 | 0 |
| hsa_circ_0005054 | 95 | 801 | 1350 | 550 | 1 | 0 | 0 |
| hsa_circ_0006828 | 95 | 2 | 458 | 457 | 1 | 0 | 0 |

novel_circ_002823 novel_circ_002824 novel_circ_002825 novel_circ_002826 novel_circ_002827 novel_circ_002828 novel_circ_002829 novel_circ_002830 novel_circ_002831 novel_circ_002832 novel_circ_002833 novel_circ_002834 novel_circ_002835 novel_circ_002836 novel_circ_002837 novel_circ_002838 novel_circ_002839 novel_circ_002840 novel_circ_002842 novel_circ_002843 novel_circ_002844 novel_circ_002846 novel_circ_002847 novel_circ_002848 novel_circ_002849 novel_circ_002850 novel_circ_002851 novel_circ_002852 novel_circ_002853 novel_circ_002854 novel_circ_002855 novel_circ_002856 novel_circ_002857 novel_circ_002859 novel_circ_002860 novel_circ_002861 novel_circ_002862 novel_circ_002863 novel_circ_002864 novel_circ_002865 novel_circ_002866 novel_circ_002867 novel_circ_002868 novel_circ_002869 novel_circ_002870 novel_circ_002871 novel_circ_002872 novel_circ_002873 novel_circ_002874 novel_circ_002875 novel_circ_002877 novel_circ_002878 novel_circ_002879 novel_circ_002880

| hsa_circ_0034364 | 94 | 1 | 556 | 744 | 189 | 0 | 0 |
| --- | --- | --- | --- | --- | --- | --- | --- |
| hsa_circ_0034364 | 94 | 16 | 777 | 950 | 189 | 0 | 0 |
| hsa_circ_0034369 | 95 | 1 | 594 | 594 | 1 | 0 | 0 |
| hsa_circ_0034369 | 95 | 16 | 705 | 690 | 1 | 0 | 0 |
| hsa_circ_0034369 | 94 | 4 | 1164 | 1161 | 1 | 0 | 0 |
| hsa_circ_0034369 | 93 | 1 | 1823 | 1823 | 1 | 0 | 0 |
| hsa_circ_0034370 | 95 | 16 | 357 | 342 | 1 | 0 | 8.00E-152 |
| hsa_circ_0034370 | 93 | 1 | 1475 | 1475 | 1 | 0 | 0 |
| hsa_circ_0034371 | 93 | 1 | 850 | 850 | 1 | 0 | 0 |
| hsa_circ_0034370 | 93 | 1 | 1339 | 1475 | 137 | 0 | 0 |
| hsa_circ_0034371 | 92 | 1 | 328 | 438 | 111 | 0 | 2.00E-127 |
| hsa_circ_0034371 | 93 | 1 | 740 | 850 | 111 | 0 | 0 |
| hsa_circ_0034379 | 93 | 1 | 901 | 901 | 1 | 0 | 0 |
| hsa_circ_0034380 | 93 | 1 | 659 | 659 | 1 | 0 | 0 |
| hsa_circ_0034382 | 93 | 1 | 487 | 489 | 3 | 0 | 0 |
| hsa_circ_0034388 | 94 | 1 | 503 | 766 | 264 | 0 | 0 |
| hsa_circ_0034389 | 94 | 1 | 400 | 400 | 1 | 0 | 6.00E-169 |
| hsa_circ_0034388 | 94 | 1 | 909 | 1275 | 367 | 0 | 0 |
| hsa_circ_0008295 | 95 | 19103 | 19472 | 1 | 370 | 0 | 6.00E-164 |
| hsa_circ_0008295 | 94 | 1 | 198 | 1 | 198 | 0 | 2.00E-80 |
| hsa_circ_0008295 | 95 | 1 | 370 | 1 | 370 | 0 | 1.00E-165 |
| hsa_circ_0003140 | 95 | 1 | 393 | 1 | 390 | 0.01 | 1.00E-175 |
| hsa_circ_0003140 | 94 | 1 | 553 | 1 | 550 | 0.01 | 0 |
| hsa_circ_0003140 | 94 | 1 | 553 | 1 | 550 | 0.01 | 0 |
| hsa_circ_0000616 | 92 | 1 | 379 | 379 | 1 | 0.01 | 4.00E-147 |
| hsa_circ_0004374 | 95 | 3 | 274 | 277 | 5 | 0 | 2.00E-116 |
| hsa_circ_0035881 | 95 | 3 | 636 | 1623 | 990 | 0 | 0 |
| hsa_circ_0035881 | 96 | 3 | 1232 | 1623 | 394 | 0 | 0 |
| hsa_circ_0035881 | 96 | 1 | 798 | 1191 | 394 | 0 | 0 |
| hsa_circ_0035884 | 96 | 1 | 596 | 596 | 1 | 0 | 0 |
| hsa_circ_0035882 | 95 | 1 | 896 | 896 | 1 | 0 | 0 |
| hsa_circ_0035882 | 95 | 1 | 896 | 896 | 1 | 0 | 0 |
| hsa_circ_0035881 | 97 | 1 | 93 | 93 | 1 | 0 | 5.00E-37 |
| hsa_circ_0035841 | 91 | 1 | 2338 | 1 | 2341 | 0 | 0 |
| hsa_circ_0035840 | 91 | 1 | 2426 | 1 | 2429 | 0 | 0 |
| hsa_circ_0031595 | 86 | 7790 | 8332 | 1310 | 1853 | 0.04 | 9.00E-162 |
| hsa_circ_0031595 | 86 | 7889 | 8431 | 1310 | 1853 | 0.04 | 9.00E-162 |
| hsa_circ_0000615 | 98 | 1 | 874 | 1 | 874 | 0 | 0 |
| hsa_circ_0000615 | 98 | 1 | 874 | 1 | 874 | 0 | 0 |
| hsa_circ_0000615 | 98 | 27849 | 28722 | 1 | 874 | 0 | 0 |
| hsa_circ_0035834 | 95 | 1 | 214 | 741 | 954 | 0 | 1.00E-90 |
| hsa_circ_0035834 | 92 | 1 | 517 | 426 | 954 | 0.02 | 0 |
| hsa_circ_0035834 | 91 | 1 | 390 | 553 | 954 | 0.03 | 4.00E-150 |
| hsa_circ_0035831 | 92 | 1 | 729 | 1 | 726 | 0 | 0 |
| hsa_circ_0035831 | 92 | 1 | 729 | 1 | 726 | 0 | 0 |
| hsa_circ_0035831 | 92 | 1 | 599 | 1 | 596 | 0.01 | 0 |
| hsa_circ_0007949 | 86 | 1 | 400 | 405 | 1 | 0.02 | 1.00E-116 |
| hsa_circ_0004256 | 87 | 1 | 441 | 516 | 71 | 0.02 | 2.00E-139 |
| hsa_circ_0004256 | 88 | 1 | 511 | 516 | 1 | 0.02 | 6.00E-175 |
| hsa_circ_0004256 | 88 | 1 | 511 | 516 | 1 | 0.02 | 4.00E-174 |
| hsa_circ_0001955 | 97 | 71 | 457 | 815 | 429 | 0 | 0 |
| hsa_circ_0001955 | 97 | 1 | 387 | 815 | 429 | 0 | 0 |
| hsa_circ_0001955 | 97 | 1 | 473 | 815 | 343 | 0 | 0 |
| hsa_circ_0001955 | 96 | 1 | 815 | 815 | 1 | 0 | 0 |

novel_circ_002881 novel_circ_002882 novel_circ_002883 novel_circ_002884 novel_circ_002885 novel_circ_002886 novel_circ_002888 novel_circ_002889 novel_circ_002890 novel_circ_002891 novel_circ_002892 novel_circ_002893 novel_circ_002894 novel_circ_002895 novel_circ_002896 novel_circ_002897 novel_circ_002898 novel_circ_002899 novel_circ_002900 novel_circ_002901 novel_circ_002902 novel_circ_002903 novel_circ_002904 novel_circ_002905 novel_circ_002906 novel_circ_002907 novel_circ_002908 novel_circ_002909 novel_circ_002910 novel_circ_002911 novel_circ_002912 novel_circ_002913 novel_circ_002914 novel_circ_002915 novel_circ_002916 novel_circ_002917 novel_circ_002918 novel_circ_002919 novel_circ_002920 novel_circ_002921 novel_circ_002922 novel_circ_002923 novel_circ_002924 novel_circ_002925 novel_circ_002926 novel_circ_002927 novel_circ_002928 novel_circ_002929 novel_circ_002930 novel_circ_002931 novel_circ_002932 novel_circ_002933 novel_circ_002934 novel_circ_002935

| hsa_circ_0035828 | 94 | 1 | 234 | 234 | 1 | 0 | 1.00E-98 |
| --- | --- | --- | --- | --- | --- | --- | --- |
| hsa_circ_0035827 | 95 | 1 | 344 | 449 | 106 | 0 | 1.00E-152 |
| hsa_circ_0002779 | 92 | 10 | 572 | 10 | 572 | 0 | 0 |
| hsa_circ_0002779 | 91 | 10 | 493 | 10 | 493 | 0 | 0 |
| hsa_circ_0035818 | 90 | 1 | 195 | 1 | 195 | 0 | 5.00E-67 |
| hsa_circ_0002779 | 91 | 10 | 307 | 10 | 307 | 0 | 2.00E-112 |
| hsa_circ_0007112 | 94 | 95 | 1049 | 955 | 1 | 0 | 0 |
| hsa_circ_0007112 | 94 | 2 | 956 | 955 | 1 | 0 | 0 |
| hsa_circ_0035811 | 94 | 2 | 1052 | 1051 | 1 | 0 | 0 |
| hsa_circ_0035808 | 94 | 1 | 748 | 1328 | 581 | 0 | 0 |
| hsa_circ_0035808 | 94 | 1 | 876 | 1328 | 453 | 0 | 0 |
| hsa_circ_0035804 | 95 | 1 | 1299 | 2347 | 1049 | 0 | 0 |
| hsa_circ_0035804 | 95 | 1 | 987 | 2035 | 1049 | 0 | 0 |
| hsa_circ_0035809 | 95 | 3 | 445 | 443 | 1 | 0 | 0 |
| hsa_circ_0035805 | 95 | 1 | 618 | 1252 | 635 | 0 | 0 |
| hsa_circ_0035805 | 95 | 1 | 473 | 1107 | 635 | 0 | 0 |
| hsa_circ_0035805 | 94 | 1 | 820 | 1107 | 288 | 0 | 0 |
| hsa_circ_0035805 | 95 | 1 | 301 | 935 | 635 | 0 | 2.00E-132 |
| hsa_circ_0035805 | 93 | 1 | 347 | 634 | 288 | 0 | 1.00E-139 |
| hsa_circ_0035802 | 92 | 1 | 396 | 1290 | 895 | 0 | 2.00E-158 |
| hsa_circ_0035802 | 94 | 1 | 842 | 894 | 53 | 0 | 0 |
| hsa_circ_0035802 | 94 | 1 | 894 | 894 | 1 | 0 | 0 |
| hsa_circ_0035798 | 94 | 1 | 896 | 1379 | 484 | 0 | 0 |
| hsa_circ_0035798 | 94 | 1 | 1163 | 1379 | 217 | 0 | 0 |
| hsa_circ_0035802 | 95 | 1 | 486 | 486 | 1 | 0 | 0 |
| hsa_circ_0035798 | 95 | 1 | 488 | 971 | 484 | 0 | 0 |
| hsa_circ_0035798 | 94 | 1 | 755 | 971 | 217 | 0 | 0 |
| hsa_circ_0035798 | 93 | 1 | 269 | 485 | 217 | 0 | 5.00E-108 |
| hsa_circ_0035789 | 92 | 1 | 551 | 791 | 241 | 0 | 0 |
| hsa_circ_0035789 | 93 | 1 | 395 | 635 | 241 | 0 | 7.00E-163 |
| hsa_circ_0035788 | 91 | 2 | 612 | 1132 | 522 | 0 | 0 |
| hsa_circ_0035788 | 94 | 1 | 521 | 521 | 1 | 0 | 0 |
| hsa_circ_0035760 | 91 | 1 | 1496 | 1496 | 1 | 0 | 0 |
| hsa_circ_0035718 | 91 | 1 | 3476 | 3476 | 1 | 0 | 0 |
| hsa_circ_0035718 | 91 | 1 | 3452 | 3452 | 1 | 0 | 0 |
| hsa_circ_0035755 | 92 | 1 | 635 | 635 | 1 | 0 | 0 |
| hsa_circ_0035728 | 93 | 1 | 1554 | 1554 | 1 | 0 | 0 |
| hsa_circ_0035714 | 92 | 1 | 2833 | 3033 | 201 | 0 | 0 |
| hsa_circ_0035755 | 90 | 1 | 357 | 357 | 1 | 0 | 9.00E-132 |
| hsa_circ_0035728 | 93 | 1 | 1044 | 1044 | 1 | 0 | 0 |
| hsa_circ_0035716 | 94 | 1 | 457 | 457 | 1 | 0 | 0 |
| hsa_circ_0035716 | 94 | 1 | 329 | 329 | 1 | 0 | 3.00E-141 |
| hsa_circ_0035713 | 92 | 1 | 437 | 437 | 1 | 0 | 4.00E-171 |
| hsa_circ_0035700 | 92 | 1 | 1485 | 1485 | 1 | 0 | 0 |
| hsa_circ_0035706 | 91 | 1 | 729 | 729 | 1 | 0 | 0 |
| hsa_circ_0035702 | 90 | 1 | 486 | 486 | 1 | 0 | 0 |
| hsa_circ_0035691 | 92 | 1 | 396 | 396 | 1 | 0 | 4.00E-160 |
| hsa_circ_0035690 | 92 | 1 | 535 | 875 | 341 | 0 | 0 |
| hsa_circ_0035688 | 90 | 3 | 506 | 504 | 1 | 0 | 0 |
| hsa_circ_0004866 | 93 | 1 | 335 | 1 | 335 | 0 | 6.00E-138 |
| hsa_circ_0035658 | 94 | 1 | 449 | 1 | 449 | 0 | 0 |
| hsa_circ_0035654 | 93 | 1 | 563 | 250 | 812 | 0 | 0 |
| hsa_circ_0035654 | 94 | 1 | 728 | 85 | 812 | 0 | 0 |
| hsa_circ_0035654 | 94 | 1 | 812 | 1 | 812 | 0 | 0 |

novel_circ_002936 novel_circ_002937 novel_circ_002938 novel_circ_002939 novel_circ_002940 novel_circ_002941 novel_circ_002942 novel_circ_002943 novel_circ_002944 novel_circ_002945 novel_circ_002946 novel_circ_002947 novel_circ_002948 novel_circ_002949 novel_circ_002950 novel_circ_002951 novel_circ_002952 novel_circ_002953 novel_circ_002954 novel_circ_002955 novel_circ_002956 novel_circ_002957 novel_circ_002958 novel_circ_002959 novel_circ_002960 novel_circ_002963 novel_circ_002964 novel_circ_002965 novel_circ_002966 novel_circ_002967 novel_circ_002968 novel_circ_002969 novel_circ_002970 novel_circ_002971 novel_circ_002972 novel_circ_002973 novel_circ_002974 novel_circ_002975 novel_circ_002976 novel_circ_002977 novel_circ_002979 novel_circ_002980 novel_circ_002981 novel_circ_002982 novel_circ_002983 novel_circ_002984 novel_circ_002985 novel_circ_002986 novel_circ_002987 novel_circ_002988 novel_circ_002989 novel_circ_002990 novel_circ_002991 novel_circ_002992

| hsa_circ_0035651 | 94 | 1 | 944 | 62 | 1005 | 0 | 0 |
| --- | --- | --- | --- | --- | --- | --- | --- |
| hsa_circ_0035651 | 94 | 141 | 1086 | 60 | 1005 | 0 | 0 |
| hsa_circ_0035651 | 94 | 2902 | 3847 | 60 | 1005 | 0 | 0 |
| hsa_circ_0035651 | 94 | 2991 | 3936 | 60 | 1005 | 0 | 0 |
| hsa_circ_0035651 | 94 | 3012 | 3957 | 60 | 1005 | 0 | 0 |
| hsa_circ_0035654 | 95 | 1 | 624 | 1 | 624 | 0 | 0 |
| hsa_circ_0035651 | 95 | 1 | 756 | 62 | 817 | 0 | 0 |
| hsa_circ_0035651 | 95 | 141 | 898 | 60 | 817 | 0 | 0 |
| hsa_circ_0035651 | 95 | 3012 | 3769 | 60 | 817 | 0 | 0 |
| hsa_circ_0035654 | 94 | 1 | 393 | 85 | 477 | 0 | 9.00E-172 |
| hsa_circ_0035654 | 95 | 1 | 477 | 1 | 477 | 0 | 0 |
| hsa_circ_0035653 | 95 | 1 | 609 | 1 | 609 | 0 | 0 |
| hsa_circ_0035651 | 95 | 141 | 751 | 60 | 670 | 0 | 0 |
| hsa_circ_0035651 | 95 | 2902 | 3512 | 60 | 670 | 0 | 0 |
| hsa_circ_0035651 | 95 | 3012 | 3622 | 60 | 670 | 0 | 0 |
| hsa_circ_0035653 | 95 | 1 | 488 | 1 | 488 | 0 | 0 |
| hsa_circ_0035651 | 95 | 141 | 630 | 60 | 549 | 0 | 0 |
| hsa_circ_0035651 | 95 | 3012 | 3501 | 60 | 549 | 0 | 0 |
| hsa_circ_0035654 | 96 | 1 | 249 | 1 | 249 | 0 | 6.00E-112 |
| hsa_circ_0035651 | 94 | 141 | 274 | 60 | 193 | 0 | 3.00E-51 |
| hsa_circ_0035645 | 84 | 2581 | 2822 | 3466 | 3694 | 0.06 | 2.00E-56 |
| hsa_circ_0035645 | 84 | 2691 | 2932 | 3466 | 3694 | 0.06 | 3.00E-56 |
| hsa_circ_0035645 | 84 | 2691 | 2932 | 3466 | 3694 | 0.06 | 2.00E-56 |
| hsa_circ_0035634 | 93 | 1 | 194 | 1 | 194 | 0 | 3.00E-76 |
| hsa_circ_0035634 | 93 | 1 | 194 | 1 | 194 | 0 | 2.00E-76 |
| hsa_circ_0035600 | 92 | 1 | 595 | 2764 | 3358 | 0 | 0 |
| hsa_circ_0035602 | 93 | 1 | 325 | 1 | 325 | 0 | 5.00E-134 |
| hsa_circ_0035600 | 93 | 39 | 826 | 1 | 788 | 0 | 0 |
| hsa_circ_0035600 | 92 | 39 | 402 | 1 | 364 | 0 | 3.00E-142 |
| hsa_circ_0009078 | 87 | 48915 | 49292 | 475475 | 475101 | 0.01 | 2.00E-113 |
| hsa_circ_0000609 | 94 | 3 | 524 | 582 | 61 | 0 | 0 |
| hsa_circ_0035590 | 92 | 1 | 1146 | 6831 | 5686 | 0 | 0 |
| hsa_circ_0000609 | 92 | 1 | 261 | 497 | 237 | 0 | 6.00E-102 |
| hsa_circ_0000609 | 94 | 1 | 437 | 497 | 61 | 0 | 0 |
| hsa_circ_0035598 | 93 | 1 | 696 | 1979 | 1284 | 0 | 0 |
| hsa_circ_0035598 | 92 | 1 | 470 | 1283 | 814 | 0 | 0 |
| hsa_circ_0035598 | 92 | 1 | 695 | 1283 | 589 | 0 | 0 |
| hsa_circ_0035597 | 92 | 1 | 1407 | 1525 | 119 | 0 | 0 |
| hsa_circ_0035597 | 91 | 1 | 1525 | 1525 | 1 | 0 | 0 |
| hsa_circ_0035597 | 92 | 4 | 937 | 1052 | 119 | 0 | 0 |
| hsa_circ_0035597 | 91 | 1 | 712 | 830 | 119 | 0 | 0 |
| hsa_circ_0035597 | 91 | 1 | 830 | 830 | 1 | 0 | 0 |
| hsa_circ_0035593 | 89 | 1 | 480 | 820 | 341 | 0 | 1.00E-166 |
| hsa_circ_0035591 | 92 | 1 | 694 | 996 | 303 | 0 | 0 |
| hsa_circ_0035590 | 88 | 1 | 1106 | 2216 | 1108 | 0 | 0 |
| hsa_circ_0035590 | 88 | 1 | 1751 | 2216 | 460 | 0 | 0 |
| hsa_circ_0035591 | 90 | 1 | 452 | 607 | 156 | 0 | 2.00E-164 |
| hsa_circ_0035590 | 87 | 1 | 758 | 1220 | 460 | 0 | 0 |
| hsa_circ_0035587 | 89 | 1 | 1186 | 1191 | 6 | 0 | 0 |
| hsa_circ_0035585 | 88 | 4 | 576 | 696 | 123 | 0 | 0 |
| hsa_circ_0035578 | 91 | 1 | 435 | 801 | 367 | 0 | 5.00E-170 |
| hsa_circ_0035578 | 93 | 1 | 152 | 518 | 367 | 0 | 1.00E-56 |
| hsa_circ_0035578 | 93 | 1 | 518 | 518 | 1 | 0 | 0 |
| hsa_circ_0035576 | 94 | 1 | 190 | 524 | 335 | 0 | 6.00E-76 |

novel_circ_002998 novel_circ_003001 novel_circ_003002 novel_circ_003003 novel_circ_003006 novel_circ_003007 novel_circ_003008 novel_circ_003009 novel_circ_003010 novel_circ_003011 novel_circ_003012 novel_circ_003013 novel_circ_003014 novel_circ_003015 novel_circ_003016 novel_circ_003017 novel_circ_003018 novel_circ_003019 novel_circ_003020 novel_circ_003021 novel_circ_003022 novel_circ_003023 novel_circ_003024 novel_circ_003025 novel_circ_003026 novel_circ_003027 novel_circ_003028 novel_circ_003029 novel_circ_003030 novel_circ_003031 novel_circ_003032 novel_circ_003033 novel_circ_003034 novel_circ_003035 novel_circ_003036 novel_circ_003037 novel_circ_003038 novel_circ_003039 novel_circ_003040 novel_circ_003041 novel_circ_003042 novel_circ_003043 novel_circ_003044 novel_circ_003045 novel_circ_003046 novel_circ_003047 novel_circ_003048 novel_circ_003049 novel_circ_003050 novel_circ_003051 novel_circ_003052 novel_circ_003053 novel_circ_003054 novel_circ_003055

| hsa_circ_0035572 | 87 | 2 | 1767 | 1896 | 131 | 0.01 | 0 |
| --- | --- | --- | --- | --- | --- | --- | --- |
| hsa_circ_0035536 | 95 | 108 | 764 | 843 | 187 | 0 | 0 |
| hsa_circ_0035533 | 94 | 4 | 226 | 350 | 128 | 0 | 2.00E-92 |
| hsa_circ_0035533 | 94 | 4 | 353 | 350 | 1 | 0 | 1.00E-148 |
| hsa_circ_0035519 | 90 | 1 | 316 | 316 | 1 | 0 | 5.00E-114 |
| hsa_circ_0035480 | 91 | 1 | 444 | 476 | 895 | 0.05 | 5.00E-165 |
| hsa_circ_0035480 | 98 | 1 | 189 | 603 | 791 | 0 | 1.00E-88 |
| hsa_circ_0035480 | 96 | 1 | 316 | 476 | 791 | 0 | 5.00E-144 |
| hsa_circ_0035480 | 96 | 1 | 349 | 1 | 349 | 0 | 2.00E-160 |
| hsa_circ_0035478 | 93 | 1 | 605 | 810 | 1417 | 0 | 0 |
| hsa_circ_0035478 | 93 | 1 | 608 | 807 | 1417 | 0 | 0 |
| hsa_circ_0035480 | 96 | 1 | 349 | 1 | 349 | 0 | 2.00E-160 |
| hsa_circ_0035475 | 94 | 3 | 2310 | 2 | 2316 | 0 | 0 |
| hsa_circ_0035478 | 92 | 1 | 579 | 487 | 1068 | 0.01 | 0 |
| hsa_circ_0035478 | 94 | 1 | 1065 | 1 | 1068 | 0 | 0 |
| hsa_circ_0035475 | 94 | 3 | 1961 | 2 | 1967 | 0 | 0 |
| hsa_circ_0035475 | 94 | 3 | 1702 | 2 | 1705 | 0 | 0 |
| hsa_circ_0035475 | 94 | 3 | 1023 | 2 | 1026 | 0 | 0 |
| hsa_circ_0035475 | 94 | 3 | 896 | 2 | 899 | 0 | 0 |
| hsa_circ_0000605 | 95 | 6120 | 6430 | 311 | 1 | 0 | 1.00E-136 |
| hsa_circ_0000605 | 95 | 1 | 311 | 311 | 1 | 0 | 6.00E-138 |
| hsa_circ_0035469 | 98 | 66 | 977 | 1166 | 255 | 0 | 0 |
| hsa_circ_0000605 | 96 | 1 | 246 | 246 | 1 | 0 | 6.00E-112 |
| hsa_circ_0035467 | 92 | 1 | 671 | 1769 | 1099 | 0 | 0 |
| hsa_circ_0035467 | 95 | 1 | 727 | 1348 | 622 | 0 | 0 |
| hsa_circ_0035462 | 95 | 263 | 579 | 1 | 317 | 0 | 5.00E-141 |
| hsa_circ_0035462 | 97 | 104 | 246 | 1 | 143 | 0 | 2.00E-61 |
| hsa_circ_0035460 | 98 | 1 | 262 | 124 | 385 | 0 | 1.00E-125 |
| hsa_circ_0035460 | 98 | 3 | 327 | 61 | 385 | 0 | 7.00E-159 |
| hsa_circ_0035460 | 98 | 1 | 385 | 1 | 385 | 0 | 0 |
| hsa_circ_0035460 | 98 | 1 | 262 | 124 | 385 | 0 | 8.00E-126 |
| hsa_circ_0035460 | 98 | 9659 | 9987 | 57 | 385 | 0 | 4.00E-158 |
| hsa_circ_0035460 | 98 | 1 | 385 | 1 | 385 | 0 | 0 |
| hsa_circ_0035457 | 94 | 1 | 151 | 270 | 120 | 0 | 2.00E-59 |
| hsa_circ_0035456 | 99 | 152 | 429 | 529 | 252 | 0 | 2.00E-139 |
| hsa_circ_0035456 | 97 | 152 | 523 | 529 | 158 | 0 | 6.00E-180 |
| hsa_circ_0035455 | 96 | 152 | 773 | 806 | 185 | 0 | 0 |
| hsa_circ_0035455 | 97 | 152 | 957 | 806 | 1 | 0 | 0 |
| hsa_circ_0035452 | 96 | 430 | 1305 | 876 | 1 | 0 | 0 |
| hsa_circ_0035447 | 95 | 533 | 1970 | 1438 | 1 | 0 | 0 |
| hsa_circ_0035455 | 96 | 1 | 622 | 806 | 185 | 0 | 0 |
| hsa_circ_0035455 | 97 | 1 | 806 | 806 | 1 | 0 | 0 |
| hsa_circ_0035455 | 96 | 1 | 503 | 687 | 185 | 0 | 0 |
| hsa_circ_0035455 | 97 | 1 | 687 | 687 | 1 | 0 | 0 |
| hsa_circ_0035455 | 94 | 1 | 344 | 528 | 185 | 0 | 1.00E-149 |
| hsa_circ_0035455 | 96 | 1 | 528 | 528 | 1 | 0 | 0 |
| hsa_circ_0035452 | 96 | 1 | 876 | 876 | 1 | 0 | 0 |
| hsa_circ_0035447 | 95 | 104 | 1541 | 1438 | 1 | 0 | 0 |
| hsa_circ_0035455 | 97 | 3 | 243 | 425 | 185 | 0 | 8.00E-111 |
| hsa_circ_0035452 | 97 | 1 | 532 | 532 | 1 | 0 | 0 |
| hsa_circ_0035452 | 96 | 1 | 348 | 348 | 1 | 0 | 8.00E-162 |
| hsa_circ_0035447 | 94 | 1 | 1013 | 1013 | 1 | 0 | 0 |
| hsa_circ_0035447 | 93 | 1 | 665 | 665 | 1 | 0 | 0 |
| hsa_circ_0035447 | 94 | 1 | 514 | 514 | 1 | 0 | 0 |

novel_circ_003056 novel_circ_003059 novel_circ_003060 novel_circ_003061 novel_circ_003063 novel_circ_003064 novel_circ_003065 novel_circ_003066 novel_circ_003067 novel_circ_003068 novel_circ_003069 novel_circ_003070 novel_circ_003071 novel_circ_003072 novel_circ_003073 novel_circ_003075 novel_circ_003076 novel_circ_003077 novel_circ_003078 novel_circ_003082 novel_circ_003083 novel_circ_003088 novel_circ_003089 novel_circ_003090 novel_circ_003091 novel_circ_003092 novel_circ_003093 novel_circ_003094 novel_circ_003095 novel_circ_003096 novel_circ_003097 novel_circ_003098 novel_circ_003106 novel_circ_003107 novel_circ_003108 novel_circ_003109 novel_circ_003110 novel_circ_003111 novel_circ_003112 novel_circ_003113 novel_circ_003114 novel_circ_003115 novel_circ_003116 novel_circ_003117 novel_circ_003118 novel_circ_003119 novel_circ_003120 novel_circ_003121 novel_circ_003122 novel_circ_003123 novel_circ_003124 novel_circ_003125 novel_circ_003126 novel_circ_003127

| hsa_circ_0035447 | 93 | 1 | 330 | 330 | 1 | 0 | 2.00E-133 |
| --- | --- | --- | --- | --- | --- | --- | --- |
| hsa_circ_0035445 | 94 | 1 | 376 | 376 | 1 | 0 | 2.00E-162 |
| hsa_circ_0035443 | 95 | 1 | 681 | 681 | 1 | 0 | 0 |
| hsa_circ_0008799 | 97 | 14736 | 14770 | 269459 | 269494 | 0.03 | 5.00E-06 |
| hsa_circ_0035437 | 86 | 1 | 645 | 1213 | 1857 | 0.02 | 0 |
| hsa_circ_0035435 | 90 | 7 | 600 | 7 | 600 | 0.01 | 0 |
| hsa_circ_0035427 | 96 | 1 | 353 | 1 | 353 | 0 | 3.00E-161 |
| hsa_circ_0035423 | 96 | 1 | 432 | 457 | 888 | 0 | 0 |
| hsa_circ_0035423 | 96 | 1 | 888 | 1 | 888 | 0 | 0 |
| hsa_circ_0035423 | 96 | 332 | 1219 | 1 | 888 | 0 | 0 |
| hsa_circ_0035423 | 97 | 1 | 535 | 1 | 535 | 0 | 0 |
| hsa_circ_0035423 | 97 | 1 | 456 | 1 | 456 | 0 | 0 |
| hsa_circ_0035423 | 97 | 332 | 787 | 1 | 456 | 0 | 0 |
| hsa_circ_0035416 | 95 | 112 | 218 | 71 | 177 | 0 | 2.00E-41 |
| hsa_circ_0035416 | 95 | 1259 | 1365 | 71 | 177 | 0 | 1.00E-40 |
| hsa_circ_0071040 | 93 | 1 | 517 | 1 | 517 | 0 | 0 |
| hsa_circ_0071041 | 89 | 603 | 1594 | 1 | 992 | 0 | 0 |
| hsa_circ_0071041 | 89 | 308 | 1299 | 1 | 992 | 0 | 0 |
| hsa_circ_0071041 | 89 | 1 | 992 | 1 | 992 | 0 | 0 |
| hsa_circ_0071045 | 93 | 1 | 408 | 1 | 408 | 0 | 4.00E-170 |
| hsa_circ_0071046 | 93 | 1 | 286 | 1 | 286 | 0 | 4.00E-114 |
| hsa_circ_0047911 | 91 | 1 | 629 | 255 | 883 | 0 | 0 |
| hsa_circ_0047911 | 91 | 1 | 883 | 1 | 883 | 0 | 0 |
| hsa_circ_0047911 | 91 | 357 | 1239 | 1 | 883 | 0 | 0 |
| hsa_circ_0047911 | 91 | 772 | 1654 | 1 | 883 | 0 | 0 |
| hsa_circ_0047911 | 92 | 1 | 631 | 1 | 631 | 0 | 0 |
| hsa_circ_0047911 | 92 | 357 | 987 | 1 | 631 | 0 | 0 |
| hsa_circ_0047911 | 92 | 772 | 1402 | 1 | 631 | 0 | 0 |
| hsa_circ_0047911 | 92 | 357 | 610 | 1 | 254 | 0 | 6.00E-96 |
| hsa_circ_0047911 | 92 | 772 | 1025 | 1 | 254 | 0 | 1.00E-95 |
| hsa_circ_0074783 | 86 | 11783 | 12237 | 27352 | 26901 | 0.01 | 3.00E-131 |
| hsa_circ_0047911 | 92 | 1177 | 1430 | 1 | 254 | 0 | 1.00E-95 |
| hsa_circ_0003774 | 91 | 4 | 309 | 498 | 193 | 0 | 8.00E-112 |
| hsa_circ_0047893 | 91 | 1 | 294 | 1586 | 1879 | 0 | 3.00E-110 |
| hsa_circ_0047893 | 91 | 1 | 747 | 1133 | 1879 | 0 | 0 |
| hsa_circ_0047896 | 92 | 1 | 252 | 810 | 1061 | 0 | 1.00E-98 |
| hsa_circ_0047896 | 91 | 1 | 516 | 546 | 1061 | 0 | 0 |
| hsa_circ_0047896 | 92 | 1 | 894 | 168 | 1061 | 0 | 0 |
| hsa_circ_0047896 | 91 | 1 | 1061 | 1 | 1061 | 0 | 0 |
| hsa_circ_0047893 | 92 | 1 | 1384 | 1 | 1384 | 0 | 0 |
| hsa_circ_0047896 | 91 | 1 | 642 | 168 | 809 | 0 | 0 |
| hsa_circ_0047893 | 92 | 1 | 487 | 1 | 487 | 0 | 0 |
| hsa_circ_0047887 | 91 | 1 | 1494 | 848 | 2341 | 0 | 0 |
| hsa_circ_0047887 | 92 | 1 | 1623 | 719 | 2341 | 0 | 0 |
| hsa_circ_0047885 | 88 | 1 | 901 | 1 | 907 | 0.01 | 0 |
| hsa_circ_0047885 | 91 | 1 | 547 | 1 | 553 | 0.01 | 0 |
| hsa_circ_0047885 | 93 | 1 | 351 | 113 | 463 | 0 | 4.00E-145 |
| hsa_circ_0047885 | 91 | 1 | 463 | 1 | 463 | 0 | 1.00E-180 |
| hsa_circ_0047885 | 93 | 1 | 374 | 1 | 374 | 0 | 7.00E-153 |
| hsa_circ_0047885 | 93 | 1 | 261 | 1 | 261 | 0 | 3.00E-105 |
| hsa_circ_0047878 | 94 | 3 | 206 | 3 | 206 | 0 | 2.00E-83 |
| hsa_circ_0047878 | 94 | 857 | 1060 | 3 | 206 | 0 | 7.00E-83 |
| hsa_circ_0047878 | 94 | 3 | 206 | 3 | 206 | 0 | 1.00E-83 |
| hsa_circ_0047878 | 94 | 496 | 699 | 3 | 206 | 0 | 4.00E-83 |

novel_circ_003128 novel_circ_003131 novel_circ_003132 novel_circ_003133 novel_circ_003135 novel_circ_003136 novel_circ_003138 novel_circ_003139 novel_circ_003140 novel_circ_003141 novel_circ_003142 novel_circ_003143 novel_circ_003144 novel_circ_003145 novel_circ_003146 novel_circ_003147 novel_circ_003148 novel_circ_003149 novel_circ_003150 novel_circ_003151 novel_circ_003152 novel_circ_003153 novel_circ_003154 novel_circ_003155 novel_circ_003156 novel_circ_003159 novel_circ_003161 novel_circ_003162 novel_circ_003163 novel_circ_003164 novel_circ_003165 novel_circ_003167 novel_circ_003178 novel_circ_003180 novel_circ_003182 novel_circ_003183 novel_circ_003184 novel_circ_003185 novel_circ_003186 novel_circ_003187 novel_circ_003188 novel_circ_003189 novel_circ_003190 novel_circ_003191 novel_circ_003192 novel_circ_003193 novel_circ_003194 novel_circ_003195 novel_circ_003196 novel_circ_003197 novel_circ_003198 novel_circ_003199 novel_circ_003200 novel_circ_003201

| hsa_circ_0047875 | 94 | 1 | 582 | 1 | 582 | 0 | 0 |
| --- | --- | --- | --- | --- | --- | --- | --- |
| hsa_circ_0047877 | 91 | 1 | 129 | 282 | 410 | 0.02 | 2.00E-41 |
| hsa_circ_0047877 | 95 | 1 | 253 | 158 | 410 | 0.01 | 3.00E-110 |
| hsa_circ_0047875 | 94 | 1 | 582 | 1 | 582 | 0 | 0 |
| hsa_circ_0047877 | 91 | 1 | 129 | 282 | 410 | 0.02 | 2.00E-41 |
| hsa_circ_0047875 | 94 | 1 | 582 | 1 | 582 | 0 | 0 |
| hsa_circ_0047875 | 94 | 1 | 582 | 1 | 582 | 0 | 0 |
| hsa_circ_0047875 | 94 | 1 | 582 | 1 | 582 | 0 | 0 |
| hsa_circ_0047877 | 95 | 1 | 253 | 158 | 410 | 0.01 | 1.00E-110 |
| hsa_circ_0047875 | 94 | 1 | 582 | 1 | 582 | 0 | 0 |
| hsa_circ_0047871 | 92 | 1 | 1034 | 243 | 1279 | 0 | 0 |
| hsa_circ_0047875 | 95 | 1 | 165 | 418 | 582 | 0 | 3.00E-68 |
| hsa_circ_0047875 | 94 | 1 | 582 | 1 | 582 | 0 | 0 |
| hsa_circ_0047875 | 94 | 1 | 582 | 1 | 582 | 0 | 0 |
| hsa_circ_0047875 | 94 | 1 | 417 | 1 | 417 | 0 | 4.00E-180 |
| hsa_circ_0047875 | 94 | 1 | 275 | 1 | 275 | 0 | 1.00E-114 |
| hsa_circ_0047875 | 93 | 1 | 204 | 1 | 204 | 0 | 1.00E-78 |
| hsa_circ_0047871 | 92 | 1 | 1001 | 1 | 1004 | 0 | 0 |
| hsa_circ_0047869 | 93 | 1 | 1091 | 1 | 1094 | 0 | 0 |
| hsa_circ_0047872 | 90 | 1 | 587 | 1 | 590 | 0.01 | 0 |
| hsa_circ_0047871 | 92 | 1 | 829 | 1 | 832 | 0 | 0 |
| hsa_circ_0047872 | 91 | 1 | 296 | 1 | 296 | 0 | 6.00E-108 |
| hsa_circ_0047864 | 92 | 3 | 569 | 567 | 1 | 0 | 0 |
| hsa_circ_0047854 | 88 | 5 | 595 | 792 | 202 | 0 | 0 |
| hsa_circ_0047845 | 95 | 1 | 96 | 96 | 1 | 0 | 1.00E-35 |
| hsa_circ_0061017 | 84 | 640 | 1048 | 1 | 409 | 0 | 2.00E-108 |
| hsa_circ_0060985 | 90 | 11927 | 12292 | 150 | 515 | 0 | 7.00E-132 |
| hsa_circ_0060985 | 90 | 1 | 515 | 1 | 515 | 0 | 0 |
| hsa_circ_0060980 | 85 | 1 | 660 | 1 | 661 | 0 | 0 |
| hsa_circ_0060980 | 85 | 1 | 651 | 1 | 652 | 0 | 0 |
| hsa_circ_0060980 | 90 | 1 | 289 | 1 | 289 | 0 | 9.00E-101 |
| hsa_circ_0060964 | 91 | 1 | 340 | 342 | 3 | 0 | 1.00E-130 |
| hsa_circ_0060910 | 95 | 1 | 225 | 2198 | 1974 | 0 | 3.00E-95 |
| hsa_circ_0058615 | 76 | 4 | 695 | 723 | 1432 | 0.06 | 7.00E-86 |
| hsa_circ_0058588 | 99 | 1 | 147 | 937 | 791 | 0 | 9.00E-69 |
| hsa_circ_0003273 | 96 | 271 | 1076 | 806 | 1 | 0 | 0 |
| hsa_circ_0007381 | 96 | 271 | 1182 | 1049 | 138 | 0 | 0 |
| hsa_circ_0007381 | 96 | 271 | 1319 | 1049 | 1 | 0 | 0 |
| hsa_circ_0058583 | 94 | 271 | 1499 | 1360 | 150 | 0.01 | 0 |
| hsa_circ_0058583 | 94 | 271 | 1648 | 1360 | 1 | 0.01 | 0 |
| hsa_circ_0058577 | 94 | 271 | 2035 | 1747 | 1 | 0.01 | 0 |
| hsa_circ_0003273 | 96 | 124 | 929 | 806 | 1 | 0 | 0 |
| hsa_circ_0007381 | 96 | 124 | 1035 | 1049 | 138 | 0 | 0 |
| hsa_circ_0007381 | 96 | 124 | 1172 | 1049 | 1 | 0 | 0 |
| hsa_circ_0058583 | 94 | 124 | 1352 | 1360 | 150 | 0.01 | 0 |
| hsa_circ_0058583 | 94 | 124 | 1501 | 1360 | 1 | 0.01 | 0 |
| hsa_circ_0007381 | 96 | 1 | 1046 | 1046 | 1 | 0 | 0 |
| hsa_circ_0007381 | 95 | 1 | 243 | 243 | 1 | 0 | 3.00E-105 |
| hsa_circ_0058585 | 91 | 1 | 423 | 405 | 1 | 0.04 | 5.00E-155 |
| hsa_circ_0058583 | 91 | 1 | 572 | 554 | 1 | 0.03 | 0 |
| hsa_circ_0058585 | 89 | 1 | 317 | 299 | 1 | 0.06 | 3.00E-101 |
| hsa_circ_0058548 | 93 | 1 | 329 | 3344 | 3016 | 0 | 3.00E-136 |
| hsa_circ_0058548 | 93 | 1 | 461 | 3344 | 2884 | 0 | 0 |
| hsa_circ_0058548 | 94 | 1 | 716 | 3344 | 2629 | 0 | 0 |

novel_circ_003202 novel_circ_003203 novel_circ_003204 novel_circ_003205 novel_circ_003206 novel_circ_003207 novel_circ_003208 novel_circ_003209 novel_circ_003210 novel_circ_003211 novel_circ_003212 novel_circ_003213 novel_circ_003214 novel_circ_003215 novel_circ_003216 novel_circ_003217 novel_circ_003218 novel_circ_003219 novel_circ_003220 novel_circ_003221 novel_circ_003224 novel_circ_003225 novel_circ_003226 novel_circ_003227 novel_circ_003228 novel_circ_003229 novel_circ_003230 novel_circ_003231 novel_circ_003232 novel_circ_003233 novel_circ_003234 novel_circ_003235 novel_circ_003236 novel_circ_003237 novel_circ_003238 novel_circ_003239 novel_circ_003240 novel_circ_003241 novel_circ_003242 novel_circ_003243 novel_circ_003244 novel_circ_003245 novel_circ_003246 novel_circ_003247 novel_circ_003248 novel_circ_003249 novel_circ_003250 novel_circ_003251 novel_circ_003252 novel_circ_003253 novel_circ_003254 novel_circ_003255 novel_circ_003258 novel_circ_003259

| hsa_circ_0058583 | 93 | 3 | 311 | 309 | 1 | 0 | 8.00E-127 |
| --- | --- | --- | --- | --- | --- | --- | --- |
| hsa_circ_0058577 | 94 | 1 | 536 | 536 | 1 | 0 | 0 |
| hsa_circ_0058577 | 96 | 1 | 255 | 255 | 1 | 0 | 1.00E-113 |
| hsa_circ_0058549 | 95 | 1 | 1234 | 1236 | 3 | 0 | 0 |
| hsa_circ_0058549 | 95 | 1 | 725 | 727 | 3 | 0 | 0 |
| hsa_circ_0058546 | 95 | 1 | 1278 | 1278 | 1 | 0 | 0 |
| hsa_circ_0058549 | 95 | 1 | 573 | 575 | 3 | 0 | 0 |
| hsa_circ_0058546 | 95 | 1 | 870 | 1126 | 257 | 0 | 0 |
| hsa_circ_0058549 | 94 | 1 | 558 | 560 | 3 | 0 | 0 |
| hsa_circ_0058546 | 95 | 1 | 855 | 1111 | 257 | 0 | 0 |
| hsa_circ_0058546 | 95 | 1 | 1111 | 1111 | 1 | 0 | 0 |
| hsa_circ_0058545 | 95 | 1 | 1546 | 1546 | 1 | 0 | 0 |
| hsa_circ_0058543 | 94 | 1 | 1920 | 1920 | 1 | 0 | 0 |
| hsa_circ_0058546 | 95 | 1 | 879 | 879 | 1 | 0 | 0 |
| hsa_circ_0058545 | 96 | 1 | 526 | 691 | 166 | 0 | 0 |
| hsa_circ_0058544 | 95 | 1 | 600 | 714 | 115 | 0 | 0 |
| hsa_circ_0058543 | 93 | 4 | 209 | 206 | 1 | 0 | 2.00E-81 |
| hsa_circ_0002054 | 91 | 1 | 574 | 571 | 1 | 0.01 | 0 |
| hsa_circ_0058537 | 91 | 1 | 369 | 1639 | 1271 | 0 | 4.00E-140 |
| hsa_circ_0006005 | 80 | 22569 | 22815 | 12580 | 12344 | 0.04 | 4.00E-39 |
| hsa_circ_0058536 | 83 | 1 | 4029 | 4280 | 276 | 0.02 | 0 |
| hsa_circ_0058536 | 82 | 2 | 3773 | 4023 | 276 | 0.02 | 0 |
| hsa_circ_0058536 | 92 | 1 | 273 | 273 | 1 | 0 | 6.00E-103 |
| hsa_circ_0058519 | 96 | 1 | 1283 | 1 | 1283 | 0 | 0 |
| hsa_circ_0058519 | 96 | 1 | 1283 | 1 | 1283 | 0 | 0 |
| hsa_circ_0058519 | 96 | 1 | 1283 | 1 | 1283 | 0 | 0 |
| hsa_circ_0058519 | 96 | 1 | 1283 | 1 | 1283 | 0 | 0 |
| hsa_circ_0058530 | 96 | 1 | 564 | 1 | 564 | 0 | 0 |
| hsa_circ_0058529 | 97 | 1 | 756 | 1 | 756 | 0 | 0 |
| hsa_circ_0058525 | 96 | 1 | 910 | 1 | 910 | 0 | 0 |
| hsa_circ_0058522 | 96 | 1 | 1073 | 1 | 1073 | 0 | 0 |
| hsa_circ_0058521 | 96 | 1 | 1189 | 1 | 1189 | 0 | 0 |
| hsa_circ_0058519 | 96 | 1 | 1283 | 1 | 1283 | 0 | 0 |
| hsa_circ_0058519 | 96 | 1417 | 2699 | 1 | 1283 | 0 | 0 |
| hsa_circ_0058529 | 97 | 1 | 583 | 1 | 583 | 0 | 0 |
| hsa_circ_0058522 | 96 | 1 | 900 | 1 | 900 | 0 | 0 |
| hsa_circ_0058519 | 96 | 1 | 1110 | 1 | 1110 | 0 | 0 |
| hsa_circ_0058519 | 97 | 1 | 929 | 1 | 929 | 0 | 0 |
| hsa_circ_0058521 | 96 | 1 | 433 | 1 | 433 | 0 | 0 |
| hsa_circ_0058519 | 96 | 1 | 527 | 1 | 527 | 0 | 0 |
| hsa_circ_0058519 | 97 | 1 | 373 | 1 | 373 | 0.01 | 5.00E-179 |
| hsa_circ_0058519 | 98 | 1 | 210 | 1 | 210 | 0.01 | 2.00E-100 |
| hsa_circ_0058505 | 92 | 191 | 471 | 713 | 993 | 0 | 2.00E-109 |
| hsa_circ_0058505 | 94 | 1 | 493 | 222 | 714 | 0 | 0 |
| hsa_circ_0058505 | 94 | 1 | 714 | 1 | 714 | 0 | 0 |
| hsa_circ_0058505 | 94 | 289 | 1002 | 1 | 714 | 0 | 0 |
| hsa_circ_0058505 | 94 | 1 | 493 | 222 | 714 | 0 | 0 |
| hsa_circ_0058505 | 94 | 1 | 493 | 222 | 714 | 0 | 0 |
| hsa_circ_0058505 | 94 | 1 | 714 | 1 | 714 | 0 | 0 |
| hsa_circ_0058505 | 93 | 1 | 256 | 222 | 477 | 0 | 8.00E-101 |
| hsa_circ_0058505 | 94 | 1 | 477 | 1 | 477 | 0 | 0 |
| hsa_circ_0058502 | 93 | 1 | 679 | 1 | 681 | 0 | 0 |
| hsa_circ_0058495 | 88 | 48 | 937 | 54 | 946 | 0.01 | 0 |
| hsa_circ_0058495 | 89 | 21 | 623 | 54 | 656 | 0 | 0 |

novel_circ_003260 novel_circ_003261 novel_circ_003262 novel_circ_003263 novel_circ_003264 novel_circ_003266 novel_circ_003268 novel_circ_003269 novel_circ_003270 novel_circ_003271 novel_circ_003273 novel_circ_003276 novel_circ_003277 novel_circ_003278 novel_circ_003279 novel_circ_003281 novel_circ_003282 novel_circ_003284 novel_circ_003287 novel_circ_003288 novel_circ_003289 novel_circ_003290 novel_circ_003291 novel_circ_003292 novel_circ_003293 novel_circ_003294 novel_circ_003295 novel_circ_003296 novel_circ_003297 novel_circ_003298 novel_circ_003299 novel_circ_003300 novel_circ_003301 novel_circ_003302 novel_circ_003303 novel_circ_003305 novel_circ_003306 novel_circ_003307 novel_circ_003308 novel_circ_003309 novel_circ_003310 novel_circ_003311 novel_circ_003312 novel_circ_003313 novel_circ_003314 novel_circ_003316 novel_circ_003317 novel_circ_003318 novel_circ_003319 novel_circ_003320 novel_circ_003321 novel_circ_003322 novel_circ_003323 novel_circ_003324

| hsa_circ_0058495 | 89 | 48 | 650 | 54 | 656 | 0 | 0 |
| --- | --- | --- | --- | --- | --- | --- | --- |
| hsa_circ_0051259 | 94 | 1 | 644 | 1458 | 818 | 0 | 0 |
| hsa_circ_0051259 | 95 | 660 | 1229 | 1611 | 1042 | 0 | 0 |
| hsa_circ_0051277 | 94 | 273 | 383 | 111 | 1 | 0 | 5.00E-40 |
| hsa_circ_0051295 | 90 | 1 | 1329 | 1 | 1326 | 0 | 0 |
| hsa_circ_0051364 | 95 | 3 | 294 | 399 | 108 | 0 | 9.00E-126 |
| hsa_circ_0051374 | 85 | 67 | 261 | 94 | 287 | 0.02 | 7.00E-48 |
| hsa_circ_0051374 | 89 | 8 | 142 | 153 | 287 | 0 | 3.00E-40 |
| hsa_circ_0051372 | 86 | 44 | 314 | 359 | 629 | 0.01 | 1.00E-75 |
| hsa_circ_0066097 | 87 | 60 | 233 | 463 | 636 | 0 | 2.00E-49 |
| hsa_circ_0050289 | 79 | 1698 | 1819 | 6984 | 7105 | 0.06 | 1.00E-13 |
| hsa_circ_0016756 | 77 | 1196 | 1329 | 511 | 644 | 0.01 | 5.00E-12 |
| hsa_circ_0016756 | 77 | 1073 | 1206 | 511 | 644 | 0.01 | 5.00E-12 |
| hsa_circ_0051420 | 94 | 1 | 277 | 1 | 277 | 0 | 2.00E-117 |
| hsa_circ_0051420 | 94 | 1 | 277 | 1 | 277 | 0 | 1.00E-116 |
| hsa_circ_0051555 | 92 | 1 | 403 | 591 | 189 | 0 | 1.00E-160 |
| hsa_circ_0051558 | 93 | 1 | 196 | 614 | 419 | 0 | 1.00E-77 |
| hsa_circ_0051572 | 91 | 1419 | 1853 | 435 | 1 | 0 | 1.00E-162 |
| hsa_circ_0051588 | 92 | 1 | 270 | 1 | 270 | 0 | 3.00E-105 |
| hsa_circ_0051617 | 92 | 1 | 257 | 1333 | 1077 | 0 | 5.00E-98 |
| hsa_circ_0051632 | 90 | 5 | 330 | 326 | 1 | 0 | 3.00E-116 |
| hsa_circ_0000943 | 92 | 1 | 3876 | 1 | 3869 | 0 | 0 |
| hsa_circ_0000943 | 92 | 1 | 4021 | 1 | 4014 | 0 | 0 |
| hsa_circ_0051645 | 90 | 79 | 210 | 1 | 132 | 0 | 6.00E-42 |
| hsa_circ_0007022 | 93 | 1 | 286 | 1 | 286 | 0 | 4.00E-114 |
| hsa_circ_0007022 | 94 | 1 | 429 | 1 | 429 | 0 | 0 |
| hsa_circ_0007022 | 94 | 1 | 529 | 1 | 529 | 0 | 0 |
| hsa_circ_0007022 | 93 | 1 | 635 | 1 | 635 | 0 | 0 |
| hsa_circ_0007022 | 93 | 1 | 635 | 1 | 635 | 0 | 0 |
| hsa_circ_0051660 | 94 | 1 | 317 | 1 | 317 | 0 | 3.00E-136 |
| hsa_circ_0051660 | 94 | 1 | 417 | 1 | 417 | 0 | 2.00E-178 |
| hsa_circ_0007022 | 93 | 1 | 523 | 113 | 635 | 0 | 0 |
| hsa_circ_0051660 | 95 | 1 | 243 | 175 | 417 | 0 | 1.00E-103 |
| hsa_circ_0007022 | 93 | 1 | 349 | 287 | 635 | 0 | 5.00E-144 |
| hsa_circ_0051678 | 92 | 1 | 356 | 313 | 668 | 0 | 7.00E-143 |
| hsa_circ_0071254 | 95 | 225 | 304 | 109 | 188 | 0 | 5.00E-27 |
| hsa_circ_0009114 | 84 | 23680 | 24099 | 407 | 1 | 0.06 | 5.00E-101 |
| hsa_circ_0009114 | 97 | 1 | 123 | 123 | 1 | 0 | 6.00E-52 |
| hsa_circ_0020124 | 94 | 126 | 389 | 264 | 1 | 0 | 2.00E-108 |
| hsa_circ_0020120 | 92 | 1 | 683 | 3273 | 2588 | 0.01 | 0 |
| hsa_circ_0020120 | 92 | 1 | 937 | 3273 | 2334 | 0.01 | 0 |
| hsa_circ_0020120 | 92 | 1 | 1184 | 3273 | 2087 | 0.01 | 0 |
| hsa_circ_0020120 | 91 | 1 | 588 | 3178 | 2588 | 0.01 | 0 |
| hsa_circ_0020120 | 92 | 1 | 324 | 2911 | 2588 | 0 | 8.00E-127 |
| hsa_circ_0020120 | 92 | 1 | 743 | 2829 | 2087 | 0 | 0 |
| hsa_circ_0020116 | 92 | 1 | 623 | 623 | 1 | 0 | 0 |
| hsa_circ_0020116 | 92 | 1 | 623 | 623 | 1 | 0 | 0 |
| hsa_circ_0020100 | 93 | 1 | 764 | 764 | 1 | 0 | 0 |
| hsa_circ_0015018 | 94 | 12 | 271 | 12 | 271 | 0 | 5.00E-108 |
| hsa_circ_0015018 | 89 | 12 | 827 | 12 | 827 | 0 | 0 |
| hsa_circ_0015020 | 89 | 1 | 750 | 1 | 750 | 0 | 0 |
| hsa_circ_0015020 | 88 | 1 | 662 | 89 | 750 | 0 | 0 |
| hsa_circ_0015025 | 87 | 1 | 555 | 1 | 555 | 0 | 3.00E-178 |
| hsa_circ_0015025 | 88 | 1 | 833 | 1 | 833 | 0 | 0 |

novel_circ_003325 novel_circ_003326 novel_circ_003327 novel_circ_003328 novel_circ_003329 novel_circ_003331 novel_circ_003332 novel_circ_003333 novel_circ_003334 novel_circ_003335 novel_circ_003336 novel_circ_003337 novel_circ_003338 novel_circ_003339 novel_circ_003340 novel_circ_003341 novel_circ_003342 novel_circ_003343 novel_circ_003344 novel_circ_003345 novel_circ_003346 novel_circ_003347 novel_circ_003348 novel_circ_003349 novel_circ_003351 novel_circ_003352 novel_circ_003353 novel_circ_003354 novel_circ_003355 novel_circ_003356 novel_circ_003357 novel_circ_003358 novel_circ_003359 novel_circ_003360 novel_circ_003361 novel_circ_003362 novel_circ_003363 novel_circ_003364 novel_circ_003365 novel_circ_003366 novel_circ_003367 novel_circ_003368 novel_circ_003369 novel_circ_003370 novel_circ_003371 novel_circ_003372 novel_circ_003373 novel_circ_003374 novel_circ_003375 novel_circ_003376 novel_circ_003377 novel_circ_003378 novel_circ_003379 novel_circ_003380

| hsa_circ_0015025 | 88 | 1 | 425 | 131 | 555 | 0 | 2.00E-139 |
| --- | --- | --- | --- | --- | --- | --- | --- |
| hsa_circ_0015035 | 89 | 1 | 804 | 1 | 810 | 0.01 | 0 |
| hsa_circ_0008857 | 88 | 6 | 526 | 6 | 532 | 0.02 | 2.00E-179 |
| hsa_circ_0008857 | 91 | 5 | 394 | 137 | 532 | 0.02 | 4.00E-150 |
| hsa_circ_0008857 | 90 | 1 | 180 | 347 | 532 | 0.03 | 2.00E-60 |
| hsa_circ_0015044 | 92 | 1 | 359 | 1 | 359 | 0 | 7.00E-143 |
| hsa_circ_0015047 | 90 | 1 | 891 | 1 | 891 | 0 | 0 |
| hsa_circ_0015047 | 90 | 1 | 1226 | 1 | 1226 | 0 | 0 |
| hsa_circ_0015047 | 90 | 1 | 1415 | 1 | 1415 | 0 | 0 |
| hsa_circ_0007398 | 93 | 1 | 554 | 1 | 554 | 0 | 0 |
| hsa_circ_0007398 | 92 | 1 | 889 | 1 | 889 | 0 | 0 |
| hsa_circ_0007398 | 93 | 1 | 349 | 206 | 554 | 0 | 1.00E-145 |
| hsa_circ_0015067 | 95 | 246 | 572 | 1 | 327 | 0 | 7.00E-145 |
| hsa_circ_0015067 | 96 | 246 | 732 | 1 | 487 | 0 | 0 |
| hsa_circ_0015067 | 97 | 246 | 935 | 1 | 690 | 0 | 0 |
| hsa_circ_0015067 | 96 | 1 | 487 | 1 | 487 | 0 | 0 |
| hsa_circ_0015067 | 97 | 1 | 690 | 1 | 690 | 0 | 0 |
| hsa_circ_0015068 | 99 | 1 | 499 | 1 | 499 | 0 | 0 |
| hsa_circ_0015068 | 99 | 1 | 363 | 137 | 499 | 0 | 0 |
| hsa_circ_0015071 | 86 | 150 | 1026 | 2065 | 1189 | 0 | 0 |
| hsa_circ_0003014 | 87 | 1 | 749 | 749 | 1 | 0 | 0 |
| hsa_circ_0003014 | 87 | 1 | 411 | 749 | 339 | 0 | 1.00E-131 |
| hsa_circ_0015077 | 89 | 1 | 260 | 260 | 1 | 0 | 2.00E-86 |
| hsa_circ_0015077 | 89 | 61 | 320 | 260 | 1 | 0 | 3.00E-86 |
| hsa_circ_0004977 | 87 | 139 | 494 | 233 | 588 | 0 | 5.00E-111 |
| hsa_circ_0015395 | 93 | 1 | 937 | 218 | 1170 | 0.02 | 0 |
| hsa_circ_0015395 | 93 | 1 | 1488 | 1755 | 3242 | 0 | 0 |
| hsa_circ_0015395 | 93 | 1 | 1467 | 1776 | 3242 | 0 | 0 |
| hsa_circ_0015398 | 91 | 1 | 293 | 1 | 296 | 0.01 | 3.00E-110 |
| hsa_circ_0015398 | 91 | 1 | 294 | 1 | 297 | 0.01 | 6.00E-109 |
| hsa_circ_0015398 | 91 | 1 | 294 | 1 | 297 | 0.01 | 6.00E-109 |
| hsa_circ_0015398 | 92 | 1 | 377 | 1 | 380 | 0.01 | 2.00E-148 |
| hsa_circ_0015398 | 93 | 1 | 467 | 1 | 470 | 0.01 | 0 |
| hsa_circ_0015398 | 94 | 1 | 560 | 1 | 563 | 0.01 | 0 |
| hsa_circ_0015398 | 95 | 1 | 687 | 1 | 690 | 0 | 0 |
| hsa_circ_0015398 | 95 | 1 | 689 | 1 | 692 | 0 | 0 |
| hsa_circ_0015399 | 93 | 1 | 157 | 1 | 157 | 0 | 3.00E-57 |
| hsa_circ_0015399 | 93 | 1 | 240 | 1 | 240 | 0 | 6.00E-97 |
| hsa_circ_0015399 | 96 | 1 | 550 | 1 | 550 | 0 | 0 |
| hsa_circ_0015401 | 98 | 7939 | 8332 | 1 | 394 | 0 | 0 |
| hsa_circ_0015401 | 98 | 1 | 394 | 1 | 394 | 0 | 0 |
| hsa_circ_0015403 | 99 | 1 | 310 | 1 | 310 | 0 | 9.00E-156 |
| hsa_circ_0015403 | 98 | 1 | 220 | 91 | 310 | 0 | 7.00E-106 |
| hsa_circ_0015403 | 97 | 1 | 356 | 184 | 539 | 0 | 3.00E-171 |
| hsa_circ_0015403 | 95 | 1 | 712 | 184 | 895 | 0 | 0 |
| hsa_circ_0015406 | 96 | 1 | 229 | 1 | 229 | 0 | 2.00E-102 |
| hsa_circ_0015406 | 94 | 1 | 585 | 1 | 585 | 0 | 0 |
| hsa_circ_0015406 | 94 | 1 | 640 | 1 | 640 | 0 | 0 |
| hsa_circ_0015403 | 95 | 1 | 917 | 311 | 1227 | 0 | 0 |
| hsa_circ_0015403 | 94 | 1 | 1002 | 311 | 1312 | 0 | 0 |
| hsa_circ_0015408 | 93 | 2 | 356 | 2 | 356 | 0 | 1.00E-145 |
| hsa_circ_0015408 | 94 | 2 | 595 | 2 | 595 | 0 | 0 |
| hsa_circ_0015408 | 94 | 1 | 569 | 205 | 773 | 0 | 0 |
| hsa_circ_0015410 | 96 | 1 | 239 | 1 | 239 | 0 | 5.00E-108 |

novel_circ_003381 novel_circ_003382 novel_circ_003383 novel_circ_003384 novel_circ_003385 novel_circ_003386 novel_circ_003387 novel_circ_003388 novel_circ_003389 novel_circ_003390 novel_circ_003391 novel_circ_003392 novel_circ_003393 novel_circ_003394 novel_circ_003395 novel_circ_003396 novel_circ_003397 novel_circ_003398 novel_circ_003399 novel_circ_003400 novel_circ_003401 novel_circ_003402 novel_circ_003403 novel_circ_003404 novel_circ_003405 novel_circ_003406 novel_circ_003407 novel_circ_003408 novel_circ_003409 novel_circ_003410 novel_circ_003411 novel_circ_003412 novel_circ_003413 novel_circ_003414 novel_circ_003415 novel_circ_003416 novel_circ_003417 novel_circ_003418 novel_circ_003419 novel_circ_003420 novel_circ_003421 novel_circ_003422 novel_circ_003423 novel_circ_003424 novel_circ_003425 novel_circ_003426 novel_circ_003427 novel_circ_003428 novel_circ_003429 novel_circ_003430 novel_circ_003431 novel_circ_003432 novel_circ_003433 novel_circ_003434

| hsa_circ_0015410 | 96 | 1 | 332 | 1 | 332 | 0 | 6.00E-153 |
| --- | --- | --- | --- | --- | --- | --- | --- |
| hsa_circ_0015410 | 94 | 1 | 417 | 1 | 417 | 0 | 1.00E-177 |
| hsa_circ_0015411 | 92 | 1 | 284 | 1 | 284 | 0 | 2.00E-110 |
| hsa_circ_0015413 | 92 | 157 | 863 | 159 | 867 | 0 | 0 |
| hsa_circ_0015413 | 92 | 13742 | 14449 | 158 | 867 | 0 | 0 |
| hsa_circ_0015414 | 92 | 1 | 508 | 1 | 510 | 0 | 0 |
| hsa_circ_0015414 | 92 | 1 | 701 | 1 | 703 | 0 | 0 |
| hsa_circ_0015414 | 92 | 1 | 705 | 1 | 707 | 0 | 0 |
| hsa_circ_0015417 | 94 | 1 | 424 | 111 | 534 | 0 | 1.00E-180 |
| hsa_circ_0015426 | 92 | 3 | 243 | 241 | 1 | 0 | 8.00E-91 |
| hsa_circ_0015426 | 92 | 1 | 964 | 964 | 1 | 0 | 0 |
| hsa_circ_0015426 | 92 | 1 | 448 | 691 | 244 | 0 | 1.00E-175 |
| hsa_circ_0015462 | 95 | 13 | 133 | 1 | 121 | 0 | 2.00E-47 |
| hsa_circ_0015463 | 92 | 87 | 405 | 1 | 319 | 0 | 3.00E-122 |
| hsa_circ_0015463 | 88 | 87 | 1028 | 1 | 942 | 0 | 0 |
| hsa_circ_0015463 | 89 | 87 | 1145 | 1 | 1059 | 0 | 0 |
| hsa_circ_0015463 | 89 | 87 | 1259 | 1 | 1173 | 0 | 0 |
| hsa_circ_0015470 | 88 | 1 | 895 | 1 | 895 | 0 | 0 |
| hsa_circ_0015470 | 89 | 1 | 1012 | 1 | 1012 | 0 | 0 |
| hsa_circ_0015470 | 89 | 1 | 1126 | 1 | 1126 | 0 | 0 |
| hsa_circ_0015472 | 88 | 3 | 780 | 3 | 780 | 0 | 0 |
| hsa_circ_0015474 | 88 | 1 | 737 | 1 | 737 | 0 | 0 |
| hsa_circ_0015479 | 89 | 1 | 925 | 1 | 925 | 0 | 0 |
| hsa_circ_0015479 | 90 | 1 | 2310 | 1 | 2304 | 0.01 | 0 |
| hsa_circ_0015479 | 89 | 1 | 2497 | 1 | 2479 | 0.01 | 0 |
| hsa_circ_0007133 | 76 | 2051 | 2277 | 2E+06 | 1643188 | 0.1 | 5.00E-22 |
| hsa_circ_0015481 | 85 | 1 | 572 | 1499 | 2058 | 0.03 | 1.00E-157 |
| hsa_circ_0015486 | 92 | 1 | 252 | 1 | 252 | 0 | 1.00E-98 |
| hsa_circ_0015487 | 88 | 1 | 493 | 279 | 768 | 0.01 | 9.00E-163 |
| hsa_circ_0015487 | 89 | 1 | 678 | 279 | 953 | 0 | 0 |
| hsa_circ_0015487 | 90 | 3 | 385 | 574 | 953 | 0.01 | 4.00E-140 |
| hsa_circ_0015488 | 94 | 1 | 390 | 1 | 390 | 0 | 2.00E-168 |
| hsa_circ_0015489 | 94 | 1 | 302 | 1 | 302 | 0 | 1.00E-129 |
| hsa_circ_0015474 | 92 | 1 | 862 | 4868 | 5729 | 0 | 0 |
| hsa_circ_0015474 | 93 | 1 | 1023 | 4868 | 5890 | 0 | 0 |
| hsa_circ_0015474 | 93 | 1 | 1023 | 4868 | 5890 | 0 | 0 |
| hsa_circ_0007652 | 92 | 1213 | 1930 | 1 | 718 | 0 | 0 |
| hsa_circ_0007652 | 97 | 1 | 161 | 558 | 718 | 0 | 4.00E-71 |
| hsa_circ_0015515 | 95 | 1 | 316 | 1 | 316 | 0 | 5.00E-139 |
| hsa_circ_0015518 | 96 | 1 | 543 | 1 | 543 | 0 | 0 |
| hsa_circ_0015518 | 96 | 1 | 543 | 1 | 543 | 0 | 0 |
| hsa_circ_0015521 | 93 | 1 | 307 | 1 | 307 | 0 | 1.00E-125 |
| hsa_circ_0015525 | 92 | 1 | 391 | 391 | 1 | 0 | 6.00E-154 |
| hsa_circ_0015525 | 93 | 5 | 656 | 652 | 1 | 0 | 0 |
| hsa_circ_0015527 | 93 | 5 | 561 | 557 | 1 | 0 | 0 |
| hsa_circ_0015528 | 90 | 1 | 189 | 189 | 1 | 0 | 1.00E-63 |
| hsa_circ_0015533 | 97 | 1 | 267 | 195 | 461 | 0 | 3.00E-123 |
| hsa_circ_0015533 | 96 | 1 | 513 | 195 | 707 | 0 | 0 |
| hsa_circ_0015537 | 95 | 1 | 439 | 1 | 439 | 0 | 0 |
| hsa_circ_0015536 | 95 | 1 | 540 | 247 | 786 | 0 | 0 |
| hsa_circ_0015536 | 93 | 115 | 965 | 958 | 1808 | 0 | 0 |
| hsa_circ_0015542 | 94 | 1 | 479 | 1 | 479 | 0 | 0 |
| hsa_circ_0015542 | 94 | 1 | 662 | 112 | 773 | 0 | 0 |
| hsa_circ_0015542 | 95 | 1 | 258 | 388 | 645 | 0 | 3.00E-110 |

novel_circ_003435 novel_circ_003436 novel_circ_003440 novel_circ_003441 novel_circ_003442 novel_circ_003443 novel_circ_003444 novel_circ_003446 novel_circ_003447 novel_circ_003448 novel_circ_003449 novel_circ_003450 novel_circ_003451 novel_circ_003452 novel_circ_003453 novel_circ_003454 novel_circ_003455 novel_circ_003456 novel_circ_003457 novel_circ_003458 novel_circ_003459 novel_circ_003470 novel_circ_003471 novel_circ_003472 novel_circ_003473 novel_circ_003474 novel_circ_003475 novel_circ_003476 novel_circ_003477 novel_circ_003478 novel_circ_003479 novel_circ_003480 novel_circ_003481 novel_circ_003482 novel_circ_003483 novel_circ_003484 novel_circ_003485 novel_circ_003486 novel_circ_003487 novel_circ_003488 novel_circ_003489 novel_circ_003490 novel_circ_003491 novel_circ_003492 novel_circ_003493 novel_circ_003494 novel_circ_003495 novel_circ_003496 novel_circ_003498 novel_circ_003499 novel_circ_003500 novel_circ_003501 novel_circ_003502 novel_circ_003503

| hsa_circ_0015542 | 93 | 1 | 386 | 388 | 773 | 0 | 7.00E-158 |
| --- | --- | --- | --- | --- | --- | --- | --- |
| hsa_circ_0015544 | 97 | 113 | 241 | 1 | 129 | 0 | 5.00E-55 |
| hsa_circ_0015550 | 94 | 2 | 272 | 2 | 272 | 0 | 9.00E-116 |
| hsa_circ_0015550 | 90 | 2 | 389 | 2 | 386 | 0.01 | 7.00E-138 |
| hsa_circ_0015559 | 92 | 652 | 1447 | 197 | 992 | 0 | 0 |
| hsa_circ_0015567 | 92 | 1 | 225 | 1 | 225 | 0 | 3.00E-85 |
| hsa_circ_0015570 | 95 | 1 | 258 | 1 | 258 | 0 | 6.00E-112 |
| hsa_circ_0015577 | 92 | 1 | 542 | 564 | 1105 | 0 | 0 |
| hsa_circ_0015263 | 93 | 1 | 275 | 870 | 1144 | 0 | 1.00E-109 |
| hsa_circ_0015263 | 94 | 1 | 576 | 569 | 1144 | 0 | 0 |
| hsa_circ_0015263 | 94 | 13 | 588 | 569 | 1144 | 0 | 0 |
| hsa_circ_0015261 | 90 | 10198 | 11304 | 109 | 1201 | 0.01 | 0 |
| hsa_circ_0015261 | 90 | 1583 | 2583 | 109 | 1095 | 0.01 | 0 |
| hsa_circ_0015261 | 90 | 1583 | 2475 | 109 | 987 | 0.02 | 0 |
| hsa_circ_0015261 | 90 | 10198 | 11090 | 109 | 987 | 0.02 | 0 |
| hsa_circ_0015261 | 90 | 1583 | 2270 | 109 | 794 | 0 | 0 |
| hsa_circ_0015261 | 90 | 10198 | 10885 | 109 | 794 | 0 | 0 |
| hsa_circ_0015261 | 89 | 1 | 555 | 116 | 670 | 0 | 0 |
| hsa_circ_0015261 | 89 | 1583 | 2146 | 109 | 670 | 0 | 0 |
| hsa_circ_0015261 | 89 | 8607 | 9170 | 109 | 670 | 0 | 0 |
| hsa_circ_0015261 | 89 | 10198 | 10761 | 109 | 670 | 0 | 0 |
| hsa_circ_0015239 | 90 | 1 | 656 | 1 | 656 | 0 | 0 |
| hsa_circ_0015236 | 91 | 1 | 758 | 94 | 851 | 0 | 0 |
| hsa_circ_0015236 | 91 | 1 | 851 | 1 | 851 | 0 | 0 |
| hsa_circ_0015227 | 92 | 1 | 1289 | 991 | 2282 | 0 | 0 |
| hsa_circ_0015227 | 92 | 1 | 1375 | 905 | 2282 | 0 | 0 |
| hsa_circ_0015239 | 91 | 1 | 406 | 1 | 406 | 0 | 3.00E-152 |
| hsa_circ_0015227 | 92 | 1 | 1039 | 991 | 2032 | 0 | 0 |
| hsa_circ_0015227 | 92 | 1 | 1125 | 905 | 2032 | 0 | 0 |
| hsa_circ_0015227 | 93 | 1 | 719 | 905 | 1626 | 0 | 0 |
| hsa_circ_0015227 | 93 | 1 | 617 | 905 | 1524 | 0 | 0 |
| hsa_circ_0015227 | 93 | 1 | 524 | 905 | 1431 | 0.01 | 0 |
| hsa_circ_0015227 | 85 | 104 | 1335 | 153 | 1431 | 0.05 | 0 |
| hsa_circ_0015224 | 88 | 1 | 2831 | 1 | 2834 | 0.01 | 0 |
| hsa_circ_0015223 | 88 | 3 | 643 | 3 | 628 | 0.02 | 0 |
| hsa_circ_0015218 | 89 | 1 | 843 | 1 | 843 | 0 | 0 |
| hsa_circ_0015214 | 87 | 1 | 287 | 1 | 287 | 0 | 6.00E-88 |
| hsa_circ_0015208 | 89 | 1 | 504 | 1 | 504 | 0 | 0 |
| hsa_circ_0015195 | 94 | 4 | 1091 | 216 | 1306 | 0 | 0 |
| hsa_circ_0015208 | 88 | 1 | 450 | 1 | 450 | 0 | 2.00E-153 |
| hsa_circ_0015195 | 94 | 4 | 1091 | 216 | 1306 | 0 | 0 |
| hsa_circ_0015204 | 92 | 1 | 438 | 1 | 441 | 0.01 | 6.00E-174 |
| hsa_circ_0015199 | 93 | 1 | 564 | 1 | 567 | 0.01 | 0 |
| hsa_circ_0015197 | 94 | 1 | 674 | 179 | 855 | 0 | 0 |
| hsa_circ_0015195 | 95 | 4 | 1012 | 216 | 1227 | 0 | 0 |
| hsa_circ_0015196 | 95 | 4 | 795 | 7 | 801 | 0.01 | 0 |
| hsa_circ_0015196 | 97 | 4 | 574 | 7 | 577 | 0 | 0 |
| hsa_circ_0015196 | 96 | 4 | 338 | 7 | 341 | 0.01 | 6.00E-153 |
| hsa_circ_0017184 | 85 | 969 | 1725 | 1335 | 2079 | 0.02 | 0 |
| hsa_circ_0017184 | 85 | 821 | 1577 | 1335 | 2079 | 0.02 | 0 |
| hsa_circ_0017184 | 86 | 821 | 1665 | 1335 | 2167 | 0.02 | 0 |
| hsa_circ_0017184 | 85 | 765 | 1521 | 1335 | 2079 | 0.02 | 0 |
| hsa_circ_0017184 | 85 | 765 | 1523 | 1335 | 2081 | 0.02 | 0 |
| hsa_circ_0017184 | 86 | 765 | 1609 | 1335 | 2167 | 0.02 | 0 |

novel_circ_003504 novel_circ_003505 novel_circ_003506 novel_circ_003507 novel_circ_003508 novel_circ_003509 novel_circ_003512 novel_circ_003513 novel_circ_003515 novel_circ_003516 novel_circ_003517 novel_circ_003518 novel_circ_003519 novel_circ_003520 novel_circ_003522 novel_circ_003523 novel_circ_003526 novel_circ_003527 novel_circ_003528 novel_circ_003529 novel_circ_003531 novel_circ_003532 novel_circ_003533 novel_circ_003534 novel_circ_003535 novel_circ_003536 novel_circ_003537 novel_circ_003538 novel_circ_003539 novel_circ_003544 novel_circ_003545 novel_circ_003546 novel_circ_003547 novel_circ_003548 novel_circ_003549 novel_circ_003550 novel_circ_003551 novel_circ_003552 novel_circ_003553 novel_circ_003554 novel_circ_003555 novel_circ_003556 novel_circ_003557 novel_circ_003558 novel_circ_003560 novel_circ_003562 novel_circ_003564 novel_circ_003565 novel_circ_003566 novel_circ_003567 novel_circ_003568 novel_circ_003569 novel_circ_003570 novel_circ_003571

| hsa_circ_0017184 | 87 | 765 | 1671 | 1335 | 2229 | 0.02 | 0 |
| --- | --- | --- | --- | --- | --- | --- | --- |
| hsa_circ_0002027 | 94 | 1 | 700 | 1 | 700 | 0 | 0 |
| hsa_circ_0002027 | 94 | 1 | 793 | 1 | 793 | 0 | 0 |
| hsa_circ_0002636 | 94 | 1 | 550 | 1 | 550 | 0 | 0 |
| hsa_circ_0002636 | 94 | 1 | 643 | 1 | 643 | 0 | 0 |
| hsa_circ_0003095 | 94 | 1 | 695 | 63 | 757 | 0 | 0 |
| hsa_circ_0017186 | 95 | 1 | 174 | 174 | 1 | 0 | 4.00E-73 |
| hsa_circ_0017186 | 93 | 6 | 299 | 393 | 100 | 0 | 2.00E-118 |
| hsa_circ_0017190 | 95 | 4 | 423 | 973 | 554 | 0 | 0 |
| hsa_circ_0017191 | 90 | 18271 | 18842 | 572 | 1 | 0 | 0 |
| hsa_circ_0032147 | 94 | 1 | 427 | 427 | 1 | 0 | 0 |
| hsa_circ_0032148 | 96 | 1 | 869 | 869 | 1 | 0 | 0 |
| hsa_circ_0032153 | 99 | 4 | 300 | 297 | 1 | 0 | 3.00E-148 |
| hsa_circ_0032153 | 99 | 199 | 495 | 297 | 1 | 0 | 4.00E-148 |
| hsa_circ_0032153 | 99 | 4 | 300 | 297 | 1 | 0 | 2.00E-148 |
| hsa_circ_0032155 | 100 | 1 | 197 | 197 | 1 | 0 | 2.00E-99 |
| hsa_circ_0003051 | 89 | 1 | 1145 | 1216 | 72 | 0 | 0 |
| hsa_circ_0032159 | 88 | 382 | 579 | 3 | 200 | 0.01 | 2.00E-61 |
| hsa_circ_0032159 | 86 | 3 | 338 | 3 | 341 | 0.01 | 1.00E-95 |
| hsa_circ_0032160 | 88 | 617 | 1046 | 1 | 430 | 0 | 3.00E-140 |
| hsa_circ_0032160 | 87 | 279 | 873 | 1 | 595 | 0 | 0 |
| hsa_circ_0032160 | 88 | 114 | 543 | 1 | 430 | 0 | 2.00E-140 |
| hsa_circ_0032160 | 87 | 1 | 595 | 1 | 595 | 0 | 0 |
| hsa_circ_0032160 | 85 | 1 | 727 | 1 | 727 | 0.01 | 0 |
| hsa_circ_0032160 | 85 | 1 | 869 | 1 | 869 | 0.01 | 0 |
| hsa_circ_0032160 | 84 | 1 | 3006 | 1 | 2988 | 0.01 | 0 |
| hsa_circ_0032160 | 83 | 1 | 439 | 431 | 869 | 0.02 | 2.00E-110 |
| hsa_circ_0032160 | 83 | 1 | 957 | 431 | 1375 | 0.02 | 0 |
| hsa_circ_0032160 | 82 | 1 | 651 | 596 | 1234 | 0.03 | 3.00E-153 |
| hsa_circ_0032162 | 92 | 1 | 973 | 5970 | 6942 | 0 | 0 |
| hsa_circ_0032162 | 91 | 1 | 630 | 6619 | 7248 | 0 | 0 |
| hsa_circ_0032162 | 85 | 762 | 3604 | 7249 | 10115 | 0.01 | 0 |
| hsa_circ_0032162 | 92 | 1 | 492 | 6757 | 7248 | 0 | 0 |
| hsa_circ_0032162 | 90 | 1 | 306 | 6943 | 7248 | 0 | 2.00E-108 |
| hsa_circ_0032162 | 85 | 11 | 524 | 7775 | 8288 | 0 | 8.00E-144 |
| hsa_circ_0032167 | 83 | 1 | 453 | 232 | 693 | 0.02 | 2.00E-115 |
| hsa_circ_0032167 | 84 | 1 | 270 | 415 | 693 | 0.03 | 1.00E-65 |
| hsa_circ_0032168 | 88 | 1 | 611 | 1144 | 1754 | 0 | 0 |
| hsa_circ_0032162 | 86 | 1 | 511 | 11367 | 11877 | 0.01 | 2.00E-155 |
| hsa_circ_0032170 | 89 | 1 | 1133 | 146 | 1278 | 0 | 0 |
| hsa_circ_0032170 | 93 | 1 | 482 | 797 | 1278 | 0 | 0 |
| hsa_circ_0032170 | 94 | 1 | 326 | 953 | 1278 | 0 | 6.00E-138 |
| hsa_circ_0032186 | 91 | 1 | 1224 | 231 | 1454 | 0 | 0 |
| hsa_circ_0032203 | 93 | 5402 | 5545 | 254 | 111 | 0 | 3.00E-52 |
| hsa_circ_0032240 | 99 | 1 | 124 | 124 | 1 | 0 | 9.00E-57 |
| hsa_circ_0032247 | 92 | 3090 | 3519 | 2 | 430 | 0 | 9.00E-171 |
| hsa_circ_0032247 | 92 | 542 | 971 | 2 | 430 | 0 | 2.00E-171 |
| hsa_circ_0032247 | 92 | 442 | 871 | 2 | 430 | 0 | 2.00E-171 |
| hsa_circ_0032247 | 92 | 348 | 777 | 2 | 430 | 0 | 2.00E-171 |
| hsa_circ_0032247 | 92 | 2 | 431 | 2 | 430 | 0 | 1.00E-171 |
| hsa_circ_0032247 | 93 | 2 | 710 | 2 | 709 | 0 | 0 |
| hsa_circ_0032247 | 93 | 2 | 825 | 2 | 824 | 0 | 0 |
| hsa_circ_0032247 | 94 | 2 | 1063 | 2 | 1062 | 0 | 0 |
| hsa_circ_0032247 | 94 | 1 | 394 | 431 | 824 | 0 | 3.00E-172 |

novel_circ_003572 novel_circ_003573 novel_circ_003574 novel_circ_003575 novel_circ_003576 novel_circ_003577 novel_circ_003580 novel_circ_003583 novel_circ_003584 novel_circ_003585 novel_circ_003586 novel_circ_003587 novel_circ_003588 novel_circ_003590 novel_circ_003595 novel_circ_003596 novel_circ_003597 novel_circ_003598 novel_circ_003599 novel_circ_003600 novel_circ_003601 novel_circ_003602 novel_circ_003603 novel_circ_003604 novel_circ_003605 novel_circ_003606 novel_circ_003607 novel_circ_003608 novel_circ_003609 novel_circ_003610 novel_circ_003611 novel_circ_003612 novel_circ_003613 novel_circ_003614 novel_circ_003615 novel_circ_003616 novel_circ_003617 novel_circ_003618 novel_circ_003619 novel_circ_003620 novel_circ_003621 novel_circ_003622 novel_circ_003623 novel_circ_003626 novel_circ_003627 novel_circ_003628 novel_circ_003629 novel_circ_003630 novel_circ_003631 novel_circ_003632 novel_circ_003633 novel_circ_003634 novel_circ_003635 novel_circ_003636

| hsa_circ_0032247 | 95 | 1 | 632 | 431 | 1062 | 0 | 0 |
| --- | --- | --- | --- | --- | --- | --- | --- |
| hsa_circ_0032247 | 95 | 1 | 632 | 431 | 1062 | 0 | 0 |
| hsa_circ_0032247 | 95 | 1 | 632 | 431 | 1062 | 0 | 0 |
| hsa_circ_0032248 | 95 | 1 | 353 | 1 | 353 | 0 | 1.00E-159 |
| hsa_circ_0032249 | 94 | 1 | 575 | 1 | 575 | 0 | 0 |
| hsa_circ_0032249 | 94 | 1 | 575 | 1 | 575 | 0 | 0 |
| hsa_circ_0073022 | 81 | 3691 | 4676 | 1018 | 53 | 0.05 | 0 |
| hsa_circ_0001095 | 91 | 1 | 936 | 936 | 1 | 0 | 0 |
| hsa_circ_0057966 | 91 | 1 | 939 | 1082 | 144 | 0 | 0 |
| hsa_circ_0057966 | 94 | 30574 | 30722 | 149 | 1 | 0 | 7.00E-56 |
| hsa_circ_0057955 | 93 | 1 | 305 | 4329 | 4025 | 0 | 3.00E-126 |
| hsa_circ_0057950 | 98 | 1 | 412 | 1 | 412 | 0 | 0 |
| hsa_circ_0057950 | 97 | 1 | 269 | 1 | 269 | 0 | 5.00E-128 |
| hsa_circ_0057944 | 89 | 1034 | 1230 | 1 | 200 | 0.02 | 8.00E-63 |
| hsa_circ_0057943 | 89 | 6 | 498 | 838 | 346 | 0 | 3.00E-172 |
| hsa_circ_0057943 | 90 | 1 | 192 | 537 | 346 | 0 | 2.00E-65 |
| hsa_circ_0057939 | 93 | 2 | 289 | 566 | 853 | 0 | 1.00E-116 |
| hsa_circ_0057939 | 94 | 1 | 510 | 344 | 853 | 0 | 0 |
| hsa_circ_0057939 | 93 | 1 | 853 | 1 | 853 | 0 | 0 |
| hsa_circ_0057937 | 93 | 1 | 613 | 413 | 1025 | 0 | 0 |
| hsa_circ_0057934 | 94 | 1 | 302 | 75 | 376 | 0 | 6.00E-128 |
| hsa_circ_0057933 | 91 | 148 | 360 | 1 | 213 | 0 | 4.00E-75 |
| hsa_circ_0057916 | 94 | 1 | 811 | 1201 | 391 | 0 | 0 |
| hsa_circ_0057919 | 94 | 1 | 476 | 476 | 1 | 0 | 0 |
| hsa_circ_0057919 | 94 | 2 | 399 | 398 | 1 | 0 | 7.00E-173 |
| hsa_circ_0057916 | 94 | 2 | 534 | 923 | 391 | 0 | 0 |
| hsa_circ_0057916 | 94 | 2 | 924 | 923 | 1 | 0 | 0 |
| hsa_circ_0057912 | 94 | 2 | 1054 | 1214 | 162 | 0 | 0 |
| hsa_circ_0057906 | 94 | 2 | 1546 | 1753 | 209 | 0 | 0 |
| hsa_circ_0057916 | 94 | 1 | 711 | 711 | 1 | 0 | 0 |
| hsa_circ_0057912 | 94 | 1 | 841 | 1002 | 162 | 0 | 0 |
| hsa_circ_0057912 | 94 | 1 | 655 | 816 | 162 | 0 | 0 |
| hsa_circ_0057913 | 93 | 1 | 520 | 520 | 1 | 0 | 0 |
| hsa_circ_0057912 | 94 | 1 | 681 | 681 | 1 | 0 | 0 |
| hsa_circ_0057913 | 94 | 1 | 405 | 405 | 1 | 0 | 4.00E-175 |
| hsa_circ_0057910 | 93 | 1 | 622 | 622 | 1 | 0 | 0 |
| hsa_circ_0057902 | 96 | 92 | 1101 | 1482 | 473 | 0 | 0 |
| hsa_circ_0057902 | 95 | 92 | 1573 | 1482 | 1 | 0 | 0 |
| hsa_circ_0057902 | 97 | 1 | 1099 | 1571 | 473 | 0 | 0 |
| hsa_circ_0057902 | 95 | 1 | 1571 | 1571 | 1 | 0 | 0 |
| hsa_circ_0057905 | 96 | 1 | 746 | 746 | 1 | 0 | 0 |
| hsa_circ_0057904 | 96 | 1 | 852 | 855 | 4 | 0 | 0 |
| hsa_circ_0057902 | 94 | 1 | 1324 | 1324 | 1 | 0 | 0 |
| hsa_circ_0057887 | 90 | 1 | 751 | 177 | 927 | 0 | 0 |
| hsa_circ_0021468 | 92 | 67 | 485 | 693 | 275 | 0 | 2.00E-169 |
| hsa_circ_0021468 | 92 | 1 | 693 | 693 | 1 | 0 | 0 |
| hsa_circ_0021468 | 92 | 1 | 693 | 693 | 1 | 0 | 0 |
| hsa_circ_0021468 | 92 | 1 | 393 | 393 | 1 | 0 | 2.00E-153 |
| hsa_circ_0021465 | 91 | 123 | 564 | 509 | 65 | 0.01 | 1.00E-167 |
| hsa_circ_0021465 | 89 | 123 | 625 | 509 | 1 | 0.02 | 1.00E-177 |
| hsa_circ_0021465 | 89 | 123 | 625 | 509 | 1 | 0.02 | 1.00E-177 |
| hsa_circ_0021465 | 89 | 123 | 625 | 509 | 1 | 0.02 | 2.00E-177 |
| hsa_circ_0021468 | 92 | 4 | 274 | 271 | 1 | 0 | 4.00E-104 |
| hsa_circ_0021465 | 91 | 4 | 445 | 509 | 65 | 0.01 | 8.00E-168 |

novel_circ_003638 novel_circ_003641 novel_circ_003642 novel_circ_003643 novel_circ_003644 novel_circ_003645 novel_circ_003646 novel_circ_003647 novel_circ_003648 novel_circ_003649 novel_circ_003650 novel_circ_003651 novel_circ_003652 novel_circ_003654 novel_circ_003655 novel_circ_003657 novel_circ_003658 novel_circ_003659 novel_circ_003660 novel_circ_003661 novel_circ_003666 novel_circ_003669 novel_circ_003670 novel_circ_003672 novel_circ_003673 novel_circ_003674 novel_circ_003675 novel_circ_003676 novel_circ_003677 novel_circ_003678 novel_circ_003679 novel_circ_003680 novel_circ_003681 novel_circ_003682 novel_circ_003683 novel_circ_003684 novel_circ_003685 novel_circ_003686 novel_circ_003687 novel_circ_003688 novel_circ_003689 novel_circ_003690 novel_circ_003691 novel_circ_003692 novel_circ_003693 novel_circ_003694 novel_circ_003695 novel_circ_003696 novel_circ_003697 novel_circ_003698 novel_circ_003699 novel_circ_003700 novel_circ_003701 novel_circ_003702

| hsa_circ_0021458 | 92 | 1 | 295 | 295 | 1 | 0 | 9.00E-116 |
| --- | --- | --- | --- | --- | --- | --- | --- |
| hsa_circ_0021444 | 93 | 3 | 414 | 544 | 954 | 0 | 1.00E-171 |
| hsa_circ_0021444 | 91 | 1 | 630 | 325 | 954 | 0 | 0 |
| hsa_circ_0021442 | 93 | 1 | 1197 | 170 | 1366 | 0 | 0 |
| hsa_circ_0021445 | 89 | 1 | 423 | 231 | 653 | 0 | 5.00E-150 |
| hsa_circ_0021445 | 92 | 1 | 653 | 1 | 653 | 0 | 0 |
| hsa_circ_0021442 | 93 | 1 | 1106 | 170 | 1275 | 0 | 0 |
| hsa_circ_0021445 | 87 | 1 | 216 | 231 | 446 | 0 | 6.00E-62 |
| hsa_circ_0021442 | 94 | 1 | 899 | 170 | 1068 | 0 | 0 |
| hsa_circ_0021442 | 96 | 1 | 683 | 170 | 852 | 0 | 0 |
| hsa_circ_0021423 | 87 | 214 | 2331 | 2505 | 379 | 0.01 | 0 |
| hsa_circ_0021423 | 84 | 1 | 1308 | 1695 | 379 | 0.01 | 0 |
| hsa_circ_0021423 | 85 | 1 | 1686 | 1695 | 1 | 0.01 | 0 |
| hsa_circ_0021426 | 84 | 3 | 1080 | 1194 | 108 | 0.01 | 0 |
| hsa_circ_0021423 | 85 | 3 | 1458 | 1465 | 1 | 0.01 | 0 |
| hsa_circ_0021416 | 90 | 1 | 826 | 1068 | 243 | 0 | 0 |
| hsa_circ_0021419 | 91 | 1 | 625 | 625 | 1 | 0 | 0 |
| hsa_circ_0021412 | 92 | 14482 | 14596 | 335 | 221 | 0 | 5.00E-37 |
| hsa_circ_0021412 | 92 | 14482 | 14596 | 335 | 221 | 0 | 1.00E-36 |
| hsa_circ_0021412 | 92 | 14482 | 14596 | 335 | 221 | 0 | 1.00E-36 |
| hsa_circ_0013763 | 75 | 26509 | 26875 | 17708 | 17332 | 0.06 | 4.00E-33 |
| hsa_circ_0021409 | 92 | 328 | 619 | 292 | 1 | 0 | 2.00E-115 |
| hsa_circ_0021409 | 92 | 7 | 298 | 292 | 1 | 0 | 9.00E-116 |
| hsa_circ_0021401 | 94 | 253 | 379 | 1 | 127 | 0 | 3.00E-47 |
| hsa_circ_0003577 | 90 | 3 | 1131 | 1128 | 1 | 0.01 | 0 |
| hsa_circ_0021400 | 90 | 3 | 1235 | 1511 | 280 | 0.01 | 0 |
| hsa_circ_0021400 | 92 | 1 | 262 | 383 | 122 | 0 | 8.00E-101 |
| hsa_circ_0021398 | 93 | 1 | 495 | 639 | 145 | 0 | 0 |
| hsa_circ_0021392 | 92 | 1 | 1043 | 1875 | 833 | 0 | 0 |
| hsa_circ_0021398 | 93 | 1 | 391 | 535 | 145 | 0.01 | 3.00E-162 |
| hsa_circ_0021398 | 94 | 1 | 372 | 377 | 6 | 0.01 | 9.00E-157 |
| hsa_circ_0021392 | 91 | 1 | 781 | 1613 | 833 | 0.01 | 0 |
| hsa_circ_0021392 | 93 | 1 | 1072 | 1613 | 542 | 0 | 0 |
| hsa_circ_0021392 | 93 | 1 | 1330 | 1613 | 284 | 0 | 0 |
| hsa_circ_0021396 | 90 | 1 | 404 | 527 | 124 | 0 | 4.00E-146 |
| hsa_circ_0021395 | 92 | 1 | 695 | 695 | 1 | 0 | 0 |
| hsa_circ_0021393 | 92 | 1 | 953 | 953 | 1 | 0 | 0 |
| hsa_circ_0021392 | 92 | 1 | 1235 | 1236 | 2 | 0 | 0 |
| hsa_circ_0021382 | 92 | 1 | 1331 | 2304 | 974 | 0 | 0 |
| hsa_circ_0021382 | 92 | 1 | 1512 | 2304 | 793 | 0 | 0 |
| hsa_circ_0021382 | 92 | 1 | 1617 | 2304 | 688 | 0 | 0 |
| hsa_circ_0021382 | 92 | 1 | 1710 | 2304 | 595 | 0 | 0 |
| hsa_circ_0021382 | 92 | 1 | 1840 | 2304 | 465 | 0 | 0 |
| hsa_circ_0021382 | 92 | 1 | 1977 | 2304 | 328 | 0 | 0 |
| hsa_circ_0021380 | 93 | 1 | 2747 | 2747 | 1 | 0 | 0 |
| hsa_circ_0021393 | 92 | 1 | 809 | 809 | 1 | 0 | 0 |
| hsa_circ_0021392 | 91 | 1 | 540 | 541 | 2 | 0 | 0 |
| hsa_circ_0021382 | 92 | 1 | 1015 | 1609 | 595 | 0 | 0 |
| hsa_circ_0021382 | 92 | 1 | 1145 | 1609 | 465 | 0 | 0 |
| hsa_circ_0021382 | 93 | 1 | 1282 | 1609 | 328 | 0 | 0 |
| hsa_circ_0021392 | 89 | 1 | 282 | 283 | 2 | 0 | 7.00E-97 |
| hsa_circ_0021385 | 90 | 1 | 378 | 1194 | 817 | 0 | 9.00E-137 |
| hsa_circ_0021385 | 93 | 1 | 1194 | 1194 | 1 | 0 | 0 |
| hsa_circ_0021381 | 93 | 1 | 1461 | 1461 | 1 | 0 | 0 |

novel_circ_003703 novel_circ_003704 novel_circ_003705 novel_circ_003706 novel_circ_003707 novel_circ_003708 novel_circ_003709 novel_circ_003710 novel_circ_003712 novel_circ_003713 novel_circ_003714 novel_circ_003715 novel_circ_003716 novel_circ_003717 novel_circ_003718 novel_circ_003719 novel_circ_003720 novel_circ_003721 novel_circ_003722 novel_circ_003723 novel_circ_003724 novel_circ_003725 novel_circ_003726 novel_circ_003727 novel_circ_003728 novel_circ_003729 novel_circ_003730 novel_circ_003731 novel_circ_003732 novel_circ_003733 novel_circ_003734 novel_circ_003735 novel_circ_003736 novel_circ_003737 novel_circ_003738 novel_circ_003739 novel_circ_003740 novel_circ_003741 novel_circ_003742 novel_circ_003743 novel_circ_003744 novel_circ_003745 novel_circ_003746 novel_circ_003747 novel_circ_003748 novel_circ_003749 novel_circ_003750 novel_circ_003751 novel_circ_003752 novel_circ_003753 novel_circ_003754 novel_circ_003757 novel_circ_003758 novel_circ_003759

| hsa_circ_0021387 | 92 | 1 | 198 | 328 | 131 | 0 | 2.00E-75 |
| --- | --- | --- | --- | --- | --- | --- | --- |
| hsa_circ_0021387 | 94 | 1 | 328 | 328 | 1 | 0 | 1.00E-140 |
| hsa_circ_0021385 | 94 | 1 | 465 | 635 | 171 | 0 | 0 |
| hsa_circ_0021381 | 94 | 1 | 902 | 902 | 1 | 0 | 0 |
| hsa_circ_0021381 | 95 | 1 | 704 | 704 | 1 | 0 | 0 |
| hsa_circ_0021378 | 95 | 1 | 900 | 901 | 2 | 0 | 0 |
| hsa_circ_0021378 | 94 | 1 | 633 | 634 | 2 | 0 | 0 |
| hsa_circ_0021378 | 94 | 1 | 300 | 301 | 2 | 0 | 2.00E-128 |
| hsa_circ_0021370 | 92 | 1 | 476 | 651 | 176 | 0 | 0 |
| hsa_circ_0021352 | 97 | 1 | 241 | 241 | 1 | 0 | 8.00E-111 |
| hsa_circ_0021349 | 97 | 1 | 449 | 902 | 454 | 0 | 0 |
| hsa_circ_0021350 | 97 | 1 | 242 | 242 | 1 | 0 | 2.00E-111 |
| hsa_circ_0021345 | 95 | 4 | 564 | 7785 | 7225 | 0 | 0 |
| hsa_circ_0021345 | 95 | 4 | 673 | 7785 | 7116 | 0 | 0 |
| hsa_circ_0021343 | 92 | 115 | 680 | 1 | 566 | 0 | 0 |
| hsa_circ_0021343 | 92 | 365 | 930 | 1 | 566 | 0 | 0 |
| hsa_circ_0021343 | 92 | 2201 | 2766 | 1 | 566 | 0 | 0 |
| hsa_circ_0021338 | 85 | 1 | 1021 | 301 | 1342 | 0.03 | 0 |
| hsa_circ_0021343 | 92 | 115 | 680 | 1 | 566 | 0 | 0 |
| hsa_circ_0021343 | 92 | 365 | 930 | 1 | 566 | 0 | 0 |
| hsa_circ_0021343 | 92 | 2201 | 2766 | 1 | 566 | 0 | 0 |
| hsa_circ_0021343 | 92 | 514 | 1079 | 1 | 566 | 0 | 0 |
| hsa_circ_0021343 | 92 | 778 | 1343 | 1 | 566 | 0 | 0 |
| hsa_circ_0021338 | 85 | 1 | 1021 | 301 | 1342 | 0.03 | 0 |
| hsa_circ_0021338 | 86 | 1 | 1321 | 1 | 1342 | 0.03 | 0 |
| hsa_circ_0021343 | 93 | 365 | 768 | 1 | 404 | 0 | 3.00E-164 |
| hsa_circ_0021343 | 93 | 2201 | 2604 | 1 | 404 | 0 | 1.00E-163 |
| hsa_circ_0021341 | 89 | 1 | 513 | 393 | 905 | 0.01 | 0 |
| hsa_circ_0021341 | 85 | 1 | 777 | 108 | 905 | 0.03 | 0 |
| hsa_circ_0021338 | 85 | 1 | 1021 | 301 | 1342 | 0.03 | 0 |
| hsa_circ_0021341 | 93 | 1837 | 2200 | 542 | 905 | 0 | 2.00E-151 |
| hsa_circ_0021341 | 89 | 1 | 513 | 393 | 905 | 0.01 | 0 |
| hsa_circ_0021338 | 85 | 1 | 1021 | 301 | 1342 | 0.03 | 0 |
| hsa_circ_0021341 | 93 | 1 | 364 | 542 | 905 | 0 | 2.00E-152 |
| hsa_circ_0021341 | 89 | 1 | 513 | 393 | 905 | 0.01 | 0 |
| hsa_circ_0021338 | 85 | 1 | 1021 | 301 | 1342 | 0.03 | 0 |
| hsa_circ_0021338 | 86 | 1 | 1270 | 52 | 1342 | 0.03 | 0 |
| hsa_circ_0021341 | 94 | 1 | 250 | 542 | 791 | 0 | 4.00E-104 |
| hsa_circ_0021341 | 94 | 1837 | 2086 | 542 | 791 | 0 | 3.00E-103 |
| hsa_circ_0021341 | 88 | 1 | 399 | 393 | 791 | 0.01 | 2.00E-133 |
| hsa_circ_0021341 | 84 | 1 | 663 | 108 | 791 | 0.04 | 4.00E-177 |
| hsa_circ_0021341 | 84 | 1 | 767 | 1 | 791 | 0.04 | 0 |
| hsa_circ_0021338 | 84 | 1 | 907 | 301 | 1228 | 0.04 | 0 |
| hsa_circ_0021338 | 86 | 1 | 1156 | 52 | 1228 | 0.03 | 0 |
| hsa_circ_0021338 | 83 | 1 | 795 | 301 | 1116 | 0.04 | 0 |
| hsa_circ_0021338 | 85 | 1 | 1095 | 1 | 1116 | 0.03 | 0 |
| hsa_circ_0021342 | 77 | 1 | 262 | 1 | 283 | 0.07 | 1.00E-34 |
| hsa_circ_0021340 | 81 | 1 | 506 | 1 | 527 | 0.05 | 1.00E-111 |
| hsa_circ_0021338 | 84 | 1 | 755 | 52 | 827 | 0.03 | 0 |
| hsa_circ_0021340 | 86 | 1 | 243 | 1 | 243 | 0.02 | 1.00E-68 |
| hsa_circ_0021338 | 88 | 1 | 492 | 52 | 543 | 0.01 | 3.00E-168 |
| hsa_circ_0021328 | 98 | 1 | 285 | 516 | 232 | 0 | 7.00E-137 |
| hsa_circ_0021328 | 94 | 1 | 231 | 231 | 1 | 0 | 4.00E-93 |
| hsa_circ_0021326 | 96 | 607 | 1131 | 1078 | 554 | 0 | 0 |

novel_circ_003761 novel_circ_003762 novel_circ_003763 novel_circ_003764 novel_circ_003765 novel_circ_003770 novel_circ_003771 novel_circ_003772 novel_circ_003773 novel_circ_003774 novel_circ_003775 novel_circ_003776 novel_circ_003777 novel_circ_003778 novel_circ_003779 novel_circ_003780 novel_circ_003781 novel_circ_003782 novel_circ_003783 novel_circ_003784 novel_circ_003786 novel_circ_003787 novel_circ_003788 novel_circ_003789 novel_circ_003790 novel_circ_003791 novel_circ_003792 novel_circ_003793 novel_circ_003794 novel_circ_003799 novel_circ_003800 novel_circ_003801 novel_circ_003802 novel_circ_003803 novel_circ_003807 novel_circ_003808 novel_circ_003809 novel_circ_003810 novel_circ_003811 novel_circ_003812 novel_circ_003814 novel_circ_003815 novel_circ_003816 novel_circ_003817 novel_circ_003818 novel_circ_003819 novel_circ_003820 novel_circ_003821 novel_circ_003822 novel_circ_003823 novel_circ_003824 novel_circ_003825 novel_circ_003834 novel_circ_003835

| hsa_circ_0004429 | 96 | 36848 | 37038 | 300 | 110 | 0 | 8.00E-81 |
| --- | --- | --- | --- | --- | --- | --- | --- |
| hsa_circ_0004429 | 96 | 1 | 191 | 300 | 110 | 0 | 2.00E-82 |
| hsa_circ_0004429 | 97 | 1850 | 2061 | 212 | 1 | 0 | 2.00E-95 |
| hsa_circ_0004429 | 97 | 1850 | 2061 | 212 | 1 | 0 | 2.00E-95 |
| hsa_circ_0004429 | 97 | 1850 | 2061 | 212 | 1 | 0 | 5.00E-95 |
| hsa_circ_0021320 | 95 | 197 | 894 | 1 | 698 | 0 | 0 |
| hsa_circ_0021320 | 95 | 1 | 342 | 357 | 698 | 0 | 1.00E-149 |
| hsa_circ_0021320 | 95 | 1 | 342 | 357 | 698 | 0 | 4.00E-150 |
| hsa_circ_0021318 | 94 | 436 | 635 | 710 | 511 | 0 | 6.00E-81 |
| hsa_circ_0021318 | 95 | 436 | 921 | 710 | 225 | 0 | 0 |
| hsa_circ_0021318 | 95 | 436 | 1024 | 710 | 122 | 0 | 0 |
| hsa_circ_0021318 | 95 | 1 | 483 | 707 | 225 | 0 | 0 |
| hsa_circ_0021318 | 95 | 1 | 586 | 707 | 122 | 0 | 0 |
| hsa_circ_0021318 | 95 | 1 | 707 | 707 | 1 | 0 | 0 |
| hsa_circ_0021318 | 95 | 1 | 707 | 707 | 1 | 0 | 0 |
| hsa_circ_0021318 | 95 | 1 | 389 | 510 | 122 | 0 | 2.00E-174 |
| hsa_circ_0021318 | 96 | 1 | 224 | 224 | 1 | 0 | 2.00E-99 |
| hsa_circ_0021316 | 93 | 1 | 347 | 1144 | 1490 | 0 | 1.00E-144 |
| hsa_circ_0021316 | 96 | 1 | 291 | 459 | 749 | 0 | 3.00E-130 |
| hsa_circ_0021316 | 90 | 257 | 810 | 6 | 538 | 0.04 | 0 |
| hsa_circ_0021310 | 95 | 1 | 543 | 1 | 543 | 0 | 0 |
| hsa_circ_0036344 | 87 | 1 | 1102 | 1102 | 1 | 0 | 0 |
| hsa_circ_0036345 | 86 | 1 | 744 | 744 | 1 | 0 | 0 |
| hsa_circ_0036345 | 87 | 1 | 928 | 928 | 1 | 0 | 0 |
| hsa_circ_0036431 | 91 | 1 | 436 | 1 | 430 | 0.01 | 6.00E-164 |
| hsa_circ_0036427 | 89 | 1 | 799 | 242 | 1049 | 0.03 | 0 |
| hsa_circ_0036427 | 88 | 2 | 1040 | 2 | 1049 | 0.02 | 0 |
| hsa_circ_0036429 | 87 | 1 | 363 | 1 | 378 | 0.04 | 1.00E-110 |
| hsa_circ_0036428 | 86 | 12 | 511 | 12 | 526 | 0.03 | 6.00E-150 |
| hsa_circ_0036422 | 87 | 309 | 3749 | 3435 | 1 | 0 | 0 |
| hsa_circ_0036423 | 87 | 11 | 3257 | 3241 | 1 | 0 | 0 |
| hsa_circ_0036422 | 87 | 11 | 3451 | 3435 | 1 | 0 | 0 |
| hsa_circ_0000641 | 91 | 1 | 606 | 606 | 1 | 0 | 0 |
| hsa_circ_0036417 | 90 | 1 | 257 | 254 | 1 | 0.02 | 1.00E-86 |
| hsa_circ_0000640 | 94 | 1095 | 1250 | 299 | 144 | 0 | 2.00E-59 |
| hsa_circ_0000640 | 91 | 1095 | 1393 | 299 | 1 | 0 | 2.00E-110 |
| hsa_circ_0000640 | 91 | 1 | 299 | 299 | 1 | 0 | 6.00E-111 |
| hsa_circ_0000640 | 91 | 1 | 299 | 299 | 1 | 0 | 7.00E-111 |
| hsa_circ_0000640 | 91 | 1 | 299 | 299 | 1 | 0 | 9.00E-111 |
| hsa_circ_0000640 | 91 | 1 | 299 | 299 | 1 | 0 | 1.00E-110 |
| hsa_circ_0036416 | 95 | 331 | 570 | 629 | 390 | 0 | 7.00E-105 |
| hsa_circ_0036416 | 95 | 1 | 240 | 629 | 390 | 0 | 3.00E-105 |
| hsa_circ_0036416 | 93 | 1 | 629 | 629 | 1 | 0 | 0 |
| hsa_circ_0036414 | 88 | 1 | 394 | 544 | 151 | 0 | 3.00E-132 |
| hsa_circ_0036408 | 90 | 1 | 412 | 412 | 1 | 0 | 1.00E-150 |
| hsa_circ_0036405 | 90 | 1 | 625 | 625 | 1 | 0 | 0 |
| hsa_circ_0002226 | 90 | 1 | 694 | 694 | 1 | 0 | 0 |
| hsa_circ_0036400 | 90 | 1 | 776 | 777 | 2 | 0 | 0 |
| hsa_circ_0036389 | 90 | 1 | 973 | 1234 | 262 | 0 | 0 |
| hsa_circ_0036400 | 90 | 1 | 464 | 465 | 2 | 0 | 2.00E-169 |
| hsa_circ_0036389 | 81 | 1 | 530 | 540 | 7 | 0.03 | 3.00E-111 |
| hsa_circ_0036389 | 79 | 4 | 447 | 454 | 7 | 0.03 | 4.00E-80 |
| hsa_circ_0036375 | 92 | 1 | 508 | 307 | 814 | 0 | 0 |
| hsa_circ_0036378 | 93 | 1 | 356 | 287 | 642 | 0 | 1.00E-144 |

novel_circ_003836 novel_circ_003837 novel_circ_003838 novel_circ_003839 novel_circ_003840 novel_circ_003841 novel_circ_003844 novel_circ_003845 novel_circ_003846 novel_circ_003847 novel_circ_003848 novel_circ_003849 novel_circ_003850 novel_circ_003851 novel_circ_003852 novel_circ_003853 novel_circ_003854 novel_circ_003855 novel_circ_003856 novel_circ_003857 novel_circ_003858 novel_circ_003859 novel_circ_003861 novel_circ_003862 novel_circ_003863 novel_circ_003864 novel_circ_003865 novel_circ_003866 novel_circ_003867 novel_circ_003868 novel_circ_003869 novel_circ_003870 novel_circ_003871 novel_circ_003872 novel_circ_003873 novel_circ_003877 novel_circ_003878 novel_circ_003882 novel_circ_003884 novel_circ_003885 novel_circ_003886 novel_circ_003887 novel_circ_003888 novel_circ_003889 novel_circ_003890 novel_circ_003891 novel_circ_003892 novel_circ_003893 novel_circ_003894 novel_circ_003895 novel_circ_003896 novel_circ_003897 novel_circ_003898 novel_circ_003899

| hsa_circ_0036378 | 92 | 1 | 441 | 202 | 642 | 0 | 1.00E-176 |
| --- | --- | --- | --- | --- | --- | --- | --- |
| hsa_circ_0036378 | 92 | 1 | 582 | 61 | 642 | 0 | 0 |
| hsa_circ_0036375 | 91 | 1 | 744 | 1 | 747 | 0 | 0 |
| hsa_circ_0036476 | 87 | 173 | 1184 | 1429 | 2440 | 0 | 0 |
| hsa_circ_0003590 | 89 | 11 | 219 | 11 | 219 | 0 | 7.00E-68 |
| hsa_circ_0003590 | 89 | 295 | 503 | 11 | 219 | 0 | 1.00E-67 |
| hsa_circ_0036472 | 95 | 1 | 271 | 1 | 271 | 0 | 2.00E-117 |
| hsa_circ_0036464 | 93 | 1 | 1843 | 524 | 2366 | 0 | 0 |
| hsa_circ_0036464 | 92 | 1 | 1301 | 918 | 2218 | 0 | 0 |
| hsa_circ_0036464 | 92 | 1 | 1441 | 778 | 2218 | 0 | 0 |
| hsa_circ_0036464 | 93 | 1 | 502 | 1468 | 1969 | 0 | 0 |
| hsa_circ_0036464 | 93 | 1 | 779 | 1191 | 1969 | 0 | 0 |
| hsa_circ_0036468 | 94 | 1 | 413 | 274 | 686 | 0 | 2.00E-179 |
| hsa_circ_0036464 | 93 | 1 | 1080 | 524 | 1603 | 0 | 0 |
| hsa_circ_0036468 | 94 | 1 | 277 | 274 | 550 | 0 | 9.00E-116 |
| hsa_circ_0036468 | 93 | 1 | 550 | 1 | 550 | 0 | 0 |
| hsa_circ_0036464 | 93 | 1 | 690 | 778 | 1467 | 0 | 0 |
| hsa_circ_0036464 | 93 | 1 | 944 | 524 | 1467 | 0 | 0 |
| hsa_circ_0036464 | 93 | 1 | 1163 | 305 | 1467 | 0 | 0 |
| hsa_circ_0036467 | 93 | 1 | 413 | 185 | 597 | 0 | 3.00E-171 |
| hsa_circ_0036464 | 94 | 91 | 1007 | 1 | 917 | 0 | 0 |
| hsa_circ_0036449 | 91 | 85 | 521 | 1 | 437 | 0 | 1.00E-167 |
| hsa_circ_0036438 | 83 | 1 | 1101 | 1092 | 1 | 0.02 | 0 |
| hsa_circ_0036436 | 83 | 1 | 1596 | 1587 | 1 | 0.01 | 0 |
| hsa_circ_0036438 | 82 | 1 | 743 | 928 | 195 | 0.02 | 0 |
| hsa_circ_0036436 | 83 | 7 | 689 | 683 | 1 | 0 | 0 |
| hsa_circ_0000643 | 98 | 199 | 369 | 1 | 171 | 0 | 2.00E-78 |
| hsa_circ_0000643 | 95 | 199 | 577 | 1 | 379 | 0 | 2.00E-170 |
| hsa_circ_0000643 | 95 | 1 | 379 | 1 | 379 | 0 | 1.00E-170 |
| hsa_circ_0036510 | 93 | 1 | 208 | 1 | 208 | 0 | 1.00E-82 |
| hsa_circ_0036516 | 90 | 1 | 377 | 1 | 377 | 0 | 7.00E-138 |
| hsa_circ_0036516 | 92 | 1 | 591 | 1 | 591 | 0 | 0 |
| hsa_circ_0036518 | 91 | 116 | 838 | 1 | 723 | 0 | 0 |
| hsa_circ_0036519 | 92 | 1 | 428 | 1 | 428 | 0 | 4.00E-171 |
| hsa_circ_0036519 | 92 | 1 | 416 | 13 | 428 | 0 | 8.00E-168 |
| hsa_circ_0036537 | 92 | 1 | 397 | 193 | 589 | 0 | 3.00E-157 |
| hsa_circ_0036541 | 81 | 59558 | 59965 | 1 | 408 | 0.01 | 4.00E-86 |
| hsa_circ_0036543 | 88 | 110 | 360 | 1 | 254 | 0.01 | 2.00E-78 |
| hsa_circ_0036546 | 88 | 1 | 1165 | 1144 | 1 | 0.02 | 0 |
| hsa_circ_0036545 | 88 | 2030 | 4707 | 3002 | 343 | 0.01 | 0 |
| hsa_circ_0036545 | 88 | 1 | 2814 | 3138 | 343 | 0.01 | 0 |
| hsa_circ_0036550 | 88 | 1 | 980 | 959 | 1 | 0.03 | 0 |
| hsa_circ_0036550 | 89 | 3 | 1260 | 1237 | 1 | 0.02 | 0 |
| hsa_circ_0036550 | 89 | 9 | 1399 | 1370 | 1 | 0.02 | 0 |
| hsa_circ_0036545 | 88 | 2030 | 4522 | 3002 | 528 | 0.01 | 0 |
| hsa_circ_0036545 | 88 | 1 | 2629 | 3138 | 528 | 0.01 | 0 |
| hsa_circ_0036552 | 87 | 1 | 1194 | 1477 | 281 | 0 | 0 |
| hsa_circ_0036552 | 87 | 1 | 1231 | 1514 | 281 | 0 | 0 |
| hsa_circ_0036552 | 87 | 2030 | 3262 | 1516 | 281 | 0 | 0 |
| hsa_circ_0036552 | 88 | 1 | 1369 | 1652 | 281 | 0 | 0 |
| hsa_circ_0036552 | 87 | 2030 | 3116 | 1516 | 427 | 0 | 0 |
| hsa_circ_0036552 | 88 | 1 | 1223 | 1652 | 427 | 0 | 0 |
| hsa_circ_0036552 | 89 | 2030 | 2707 | 1516 | 839 | 0 | 0 |
| hsa_circ_0036552 | 90 | 1 | 814 | 1652 | 839 | 0 | 0 |

novel_circ_003918 novel_circ_003919 novel_circ_003920 novel_circ_003922 novel_circ_003923 novel_circ_003924 novel_circ_003925 novel_circ_003926 novel_circ_003927 novel_circ_003945 novel_circ_003946 novel_circ_003947 novel_circ_003948 novel_circ_003949 novel_circ_003950 novel_circ_003951 novel_circ_003952 novel_circ_003953 novel_circ_003954 novel_circ_003955 novel_circ_003956 novel_circ_003957 novel_circ_003958 novel_circ_003959 novel_circ_003960 novel_circ_003961 novel_circ_003962 novel_circ_003963 novel_circ_003964 novel_circ_003965 novel_circ_003966 novel_circ_003967 novel_circ_003968 novel_circ_003969 novel_circ_003970 novel_circ_003971 novel_circ_003972 novel_circ_003977 novel_circ_003978 novel_circ_003979 novel_circ_003980 novel_circ_003981 novel_circ_003982 novel_circ_003983 novel_circ_003984 novel_circ_003985 novel_circ_003986 novel_circ_003987 novel_circ_003988 novel_circ_003989 novel_circ_003990 novel_circ_003991 novel_circ_003992 novel_circ_003993

| hsa_circ_0036656 | 89 | 2 | 989 | 138 | 1125 | 0 | 0 |
| --- | --- | --- | --- | --- | --- | --- | --- |
| hsa_circ_0036648 | 91 | 10 | 735 | 761 | 1486 | 0 | 0 |
| hsa_circ_0036650 | 90 | 10 | 351 | 22 | 363 | 0.01 | 2.00E-123 |
| hsa_circ_0036648 | 88 | 1 | 476 | 438 | 925 | 0.03 | 4.00E-156 |
| hsa_circ_0036648 | 87 | 1 | 913 | 1 | 925 | 0.02 | 0 |
| hsa_circ_0036648 | 88 | 1 | 738 | 1 | 738 | 0 | 0 |
| hsa_circ_0036648 | 87 | 1 | 630 | 1 | 630 | 0 | 0 |
| hsa_circ_0036648 | 87 | 1 | 371 | 1 | 371 | 0 | 7.00E-113 |
| hsa_circ_0036640 | 88 | 1 | 629 | 1 | 629 | 0 | 0 |
| hsa_circ_0036949 | 94 | 1 | 466 | 1 | 466 | 0 | 0 |
| hsa_circ_0036967 | 94 | 1 | 381 | 1 | 381 | 0 | 9.00E-167 |
| hsa_circ_0036967 | 94 | 1 | 1938 | 1 | 1938 | 0 | 0 |
| hsa_circ_0036974 | 91 | 1 | 826 | 1 | 826 | 0 | 0 |
| hsa_circ_0036973 | 91 | 1 | 951 | 109 | 1059 | 0 | 0 |
| hsa_circ_0036973 | 94 | 1 | 1449 | 109 | 1557 | 0 | 0 |
| hsa_circ_0036973 | 94 | 1 | 1638 | 109 | 1746 | 0 | 0 |
| hsa_circ_0036973 | 93 | 1 | 1954 | 109 | 2062 | 0 | 0 |
| hsa_circ_0036973 | 94 | 1 | 2026 | 109 | 2134 | 0 | 0 |
| hsa_circ_0036973 | 94 | 1 | 2322 | 109 | 2430 | 0 | 0 |
| hsa_circ_0036973 | 94 | 1 | 2322 | 109 | 2430 | 0 | 0 |
| hsa_circ_0036973 | 93 | 1 | 2557 | 109 | 2665 | 0 | 0 |
| hsa_circ_0036978 | 91 | 1 | 676 | 1 | 676 | 0 | 0 |
| hsa_circ_0036978 | 94 | 1 | 1174 | 1 | 1174 | 0 | 0 |
| hsa_circ_0036978 | 94 | 1 | 1363 | 1 | 1363 | 0 | 0 |
| hsa_circ_0036985 | 97 | 1 | 687 | 1 | 687 | 0 | 0 |
| hsa_circ_0036985 | 96 | 1 | 850 | 1 | 850 | 0 | 0 |
| hsa_circ_0036985 | 95 | 1 | 1003 | 1 | 1003 | 0 | 0 |
| hsa_circ_0036986 | 97 | 1 | 470 | 1 | 470 | 0 | 0 |
| hsa_circ_0036986 | 95 | 1 | 633 | 1 | 633 | 0 | 0 |
| hsa_circ_0036986 | 95 | 1 | 786 | 1 | 786 | 0 | 0 |
| hsa_circ_0036985 | 94 | 1 | 873 | 499 | 1371 | 0 | 0 |
| hsa_circ_0036985 | 93 | 1 | 1108 | 499 | 1606 | 0 | 0 |
| hsa_circ_0036990 | 93 | 1 | 489 | 1 | 489 | 0 | 0 |
| hsa_circ_0036993 | 94 | 230 | 650 | 299 | 719 | 0 | 9.00E-179 |
| hsa_circ_0036994 | 92 | 1 | 218 | 1 | 218 | 0 | 4.00E-83 |
| hsa_circ_0036998 | 92 | 3 | 290 | 3 | 290 | 0 | 7.00E-112 |
| hsa_circ_0037006 | 93 | 1 | 314 | 180 | 493 | 0 | 1.00E-129 |
| hsa_circ_0087897 | 91 | 1 | 1062 | 1062 | 1 | 0 | 0 |
| hsa_circ_0087897 | 92 | 1 | 1250 | 1250 | 1 | 0 | 0 |
| hsa_circ_0087897 | 89 | 1 | 1976 | 2015 | 1 | 0.02 | 0 |
| hsa_circ_0087899 | 91 | 1 | 867 | 867 | 1 | 0 | 0 |
| hsa_circ_0087899 | 92 | 4 | 950 | 947 | 1 | 0 | 0 |
| hsa_circ_0087899 | 92 | 1 | 1055 | 1055 | 1 | 0 | 0 |
| hsa_circ_0087912 | 91 | 1 | 269 | 269 | 1 | 0 | 1.00E-99 |
| hsa_circ_0087912 | 92 | 4 | 500 | 497 | 1 | 0 | 0 |
| hsa_circ_0087912 | 92 | 1 | 591 | 591 | 1 | 0 | 0 |
| hsa_circ_0087912 | 93 | 4 | 674 | 671 | 1 | 0 | 0 |
| hsa_circ_0087912 | 93 | 1 | 779 | 779 | 1 | 0 | 0 |
| hsa_circ_0087912 | 89 | 1 | 1505 | 1544 | 1 | 0.03 | 0 |
| hsa_circ_0087927 | 93 | 4 | 231 | 228 | 1 | 0.01 | 3.00E-90 |
| hsa_circ_0087927 | 93 | 1 | 322 | 322 | 1 | 0.01 | 2.00E-132 |
| hsa_circ_0087927 | 94 | 4 | 405 | 402 | 1 | 0 | 9.00E-172 |
| hsa_circ_0087927 | 94 | 1 | 510 | 510 | 1 | 0 | 0 |
| hsa_circ_0087927 | 89 | 1 | 1236 | 1275 | 1 | 0.03 | 0 |

novel_circ_003994 novel_circ_003995 novel_circ_003996 novel_circ_003997 novel_circ_003998 novel_circ_003999 novel_circ_004002 novel_circ_004003 novel_circ_004004 novel_circ_004005 novel_circ_004006 novel_circ_004007 novel_circ_004008 novel_circ_004010 novel_circ_004011 novel_circ_004016 novel_circ_004017 novel_circ_004018 novel_circ_004021 novel_circ_004022 novel_circ_004023 novel_circ_004024 novel_circ_004025 novel_circ_004027 novel_circ_004028 novel_circ_004029 novel_circ_004030 novel_circ_004031 novel_circ_004032 novel_circ_004033 novel_circ_004034 novel_circ_004035 novel_circ_004036 novel_circ_004037 novel_circ_004041 novel_circ_004042 novel_circ_004043 novel_circ_004044 novel_circ_004045 novel_circ_004046 novel_circ_004047 novel_circ_004050 novel_circ_004051 novel_circ_004052 novel_circ_004053 novel_circ_004054 novel_circ_004055 novel_circ_004056 novel_circ_004057 novel_circ_004058 novel_circ_004059 novel_circ_004060 novel_circ_004061 novel_circ_004062

| hsa_circ_0087927 | 95 | 1 | 190 | 322 | 133 | 0 | 3.00E-79 |
| --- | --- | --- | --- | --- | --- | --- | --- |
| hsa_circ_0087927 | 95 | 1 | 378 | 510 | 133 | 0 | 4.00E-170 |
| hsa_circ_0087927 | 85 | 1 | 694 | 1055 | 323 | 0.05 | 0 |
| hsa_circ_0087932 | 88 | 1 | 726 | 741 | 1 | 0.02 | 0 |
| hsa_circ_0087936 | 89 | 1 | 554 | 571 | 3 | 0.03 | 0 |
| hsa_circ_0087939 | 95 | 1 | 220 | 220 | 1 | 0 | 7.00E-96 |
| hsa_circ_0009016 | 76 | 53 | 291 | 217281 | 217054 | 0.06 | 3.00E-23 |
| hsa_circ_0087941 | 90 | 1 | 256 | 256 | 1 | 0 | 9.00E-89 |
| hsa_circ_0087941 | 90 | 10 | 265 | 256 | 1 | 0 | 1.00E-88 |
| hsa_circ_0087941 | 90 | 1 | 256 | 256 | 1 | 0 | 7.00E-89 |
| hsa_circ_0087941 | 90 | 10 | 265 | 256 | 1 | 0 | 7.00E-89 |
| hsa_circ_0087941 | 88 | 1 | 642 | 892 | 257 | 0.01 | 0 |
| hsa_circ_0087941 | 88 | 1 | 468 | 892 | 431 | 0.01 | 1.00E-151 |
| hsa_circ_0087941 | 92 | 1 | 480 | 1372 | 893 | 0 | 0 |
| hsa_circ_0087941 | 94 | 417 | 1073 | 1549 | 893 | 0 | 0 |
| hsa_circ_0087946 | 92 | 1 | 396 | 396 | 1 | 0 | 9.00E-158 |
| hsa_circ_0087946 | 92 | 1 | 298 | 298 | 1 | 0 | 4.00E-115 |
| hsa_circ_0087946 | 92 | 1 | 298 | 298 | 1 | 0 | 3.00E-115 |
| hsa_circ_0087946 | 92 | 1 | 298 | 298 | 1 | 0 | 3.00E-115 |
| hsa_circ_0087946 | 92 | 1 | 351 | 351 | 1 | 0 | 2.00E-136 |
| hsa_circ_0087946 | 92 | 1 | 396 | 396 | 1 | 0 | 5.00E-158 |
| hsa_circ_0087946 | 92 | 1 | 298 | 298 | 1 | 0 | 2.00E-115 |
| hsa_circ_0002963 | 98 | 1 | 179 | 1 | 179 | 0 | 1.00E-84 |
| hsa_circ_0087951 | 87 | 2 | 968 | 2565 | 1599 | 0 | 0 |
| hsa_circ_0087951 | 85 | 3 | 351 | 2118 | 1770 | 0.01 | 5.00E-99 |
| hsa_circ_0087951 | 87 | 2 | 797 | 2565 | 1770 | 0 | 0 |
| hsa_circ_0087951 | 86 | 2 | 620 | 2565 | 1947 | 0 | 0 |
| hsa_circ_0087951 | 87 | 3 | 916 | 6945 | 6032 | 0 | 0 |
| hsa_circ_0087951 | 88 | 3 | 294 | 6945 | 6654 | 0 | 2.00E-92 |
| hsa_circ_0087951 | 88 | 3 | 2031 | 8682 | 6654 | 0 | 0 |
| hsa_circ_0087953 | 92 | 1 | 249 | 249 | 1 | 0 | 1.00E-93 |
| hsa_circ_0087953 | 90 | 1 | 425 | 425 | 1 | 0 | 8.00E-158 |
| hsa_circ_0087951 | 87 | 3 | 1315 | 8682 | 7370 | 0 | 0 |
| hsa_circ_0087951 | 86 | 3 | 1141 | 8682 | 7544 | 0 | 0 |
| hsa_circ_0087956 | 84 | 1 | 317 | 317 | 1 | 0.02 | 1.00E-82 |
| hsa_circ_0087956 | 84 | 1 | 317 | 317 | 1 | 0.02 | 1.00E-82 |
| hsa_circ_0087956 | 85 | 780 | 1269 | 490 | 1 | 0.01 | 4.00E-140 |
| hsa_circ_0087957 | 94 | 593 | 772 | 180 | 1 | 0 | 3.00E-71 |
| hsa_circ_0087957 | 94 | 160 | 339 | 180 | 1 | 0 | 9.00E-72 |
| hsa_circ_0087957 | 94 | 337 | 516 | 180 | 1 | 0 | 1.00E-71 |
| hsa_circ_0087957 | 94 | 593 | 772 | 180 | 1 | 0 | 2.00E-71 |
| hsa_circ_0004362 | 94 | 1 | 746 | 748 | 3 | 0 | 0 |
| hsa_circ_0004362 | 94 | 132 | 905 | 776 | 3 | 0 | 0 |
| hsa_circ_0087973 | 93 | 6 | 585 | 580 | 1 | 0 | 0 |
| hsa_circ_0087980 | 93 | 1 | 895 | 895 | 1 | 0 | 0 |
| hsa_circ_0087980 | 94 | 2 | 1367 | 1366 | 1 | 0 | 0 |
| hsa_circ_0087978 | 94 | 1 | 1551 | 1804 | 254 | 0 | 0 |
| hsa_circ_0087988 | 93 | 1 | 2349 | 2346 | 1 | 0 | 0 |
| hsa_circ_0087990 | 95 | 1 | 977 | 977 | 1 | 0 | 0 |
| hsa_circ_0087992 | 95 | 2 | 642 | 641 | 1 | 0 | 0 |
| hsa_circ_0087992 | 95 | 1 | 826 | 826 | 1 | 0 | 0 |
| hsa_circ_0087992 | 94 | 1 | 1027 | 1027 | 1 | 0 | 0 |
| hsa_circ_0087992 | 93 | 1 | 2042 | 2039 | 1 | 0 | 0 |
| hsa_circ_0087992 | 92 | 1 | 2351 | 2348 | 1 | 0 | 0 |

novel_circ_004063 novel_circ_004064 novel_circ_004065 novel_circ_004066 novel_circ_004067 novel_circ_004068 novel_circ_004069 novel_circ_004071 novel_circ_004072 novel_circ_004073 novel_circ_004074 novel_circ_004075 novel_circ_004076 novel_circ_004077 novel_circ_004078 novel_circ_004079 novel_circ_004080 novel_circ_004081 novel_circ_004082 novel_circ_004083 novel_circ_004086 novel_circ_004091 novel_circ_004092 novel_circ_004093 novel_circ_004094 novel_circ_004095 novel_circ_004096 novel_circ_004097 novel_circ_004098 novel_circ_004099 novel_circ_004100 novel_circ_004101 novel_circ_004102 novel_circ_004103 novel_circ_004104 novel_circ_004106 novel_circ_004107 novel_circ_004108 novel_circ_004109 novel_circ_004110 novel_circ_004112 novel_circ_004113 novel_circ_004114 novel_circ_004116 novel_circ_004117 novel_circ_004118 novel_circ_004119 novel_circ_004120 novel_circ_004121 novel_circ_004122 novel_circ_004123 novel_circ_004124 novel_circ_004125 novel_circ_004126

| hsa_circ_0087996 | 94 | 1 | 857 | 905 | 49 | 0 | 0 |
| --- | --- | --- | --- | --- | --- | --- | --- |
| hsa_circ_0088005 | 93 | 1 | 385 | 385 | 1 | 0.01 | 5.00E-159 |
| hsa_circ_0088005 | 92 | 1 | 1400 | 1397 | 1 | 0.01 | 0 |
| hsa_circ_0088005 | 93 | 1 | 201 | 385 | 185 | 0 | 5.00E-77 |
| hsa_circ_0088005 | 91 | 1 | 1216 | 1397 | 185 | 0.01 | 0 |
| hsa_circ_0088005 | 92 | 1 | 1807 | 2313 | 510 | 0 | 0 |
| hsa_circ_0088008 | 91 | 1 | 318 | 315 | 1 | 0.02 | 1.00E-115 |
| hsa_circ_0088005 | 93 | 1 | 915 | 2313 | 1399 | 0 | 0 |
| hsa_circ_0088009 | 94 | 1 | 711 | 835 | 125 | 0 | 0 |
| hsa_circ_0088011 | 94 | 4 | 525 | 522 | 1 | 0 | 0 |
| hsa_circ_0088011 | 94 | 1 | 607 | 607 | 1 | 0 | 0 |
| hsa_circ_0088017 | 94 | 1 | 1031 | 1193 | 166 | 0 | 0 |
| hsa_circ_0088017 | 94 | 103 | 1135 | 1195 | 166 | 0 | 0 |
| hsa_circ_0088024 | 93 | 1 | 462 | 459 | 1 | 0.01 | 0 |
| hsa_circ_0088024 | 94 | 1 | 581 | 578 | 1 | 0.01 | 0 |
| hsa_circ_0088024 | 94 | 1 | 698 | 695 | 1 | 0 | 0 |
| hsa_circ_0088024 | 95 | 1 | 829 | 826 | 1 | 0 | 0 |
| hsa_circ_0088017 | 95 | 103 | 933 | 1195 | 368 | 0 | 0 |
| hsa_circ_0088024 | 96 | 1 | 689 | 826 | 141 | 0 | 0 |
| hsa_circ_0088017 | 96 | 103 | 793 | 1195 | 508 | 0 | 0 |
| hsa_circ_0001962 | 92 | 612 | 806 | 1 | 195 | 0 | 5.00E-73 |
| hsa_circ_0088031 | 95 | 1 | 460 | 389 | 848 | 0 | 0 |
| hsa_circ_0004830 | 94 | 143 | 726 | 1 | 584 | 0 | 0 |
| hsa_circ_0004830 | 94 | 143 | 726 | 1 | 584 | 0 | 0 |
| hsa_circ_0004830 | 94 | 1 | 266 | 319 | 584 | 0 | 4.00E-111 |
| hsa_circ_0088064 | 95 | 1 | 484 | 484 | 1 | 0 | 0 |
| hsa_circ_0088064 | 95 | 1 | 267 | 484 | 218 | 0 | 3.00E-115 |
| hsa_circ_0003458 | 96 | 82 | 680 | 599 | 1 | 0 | 0 |
| hsa_circ_0088077 | 95 | 19928 | 20351 | 424 | 1 | 0 | 0 |
| hsa_circ_0003500 | 96 | 1 | 171 | 317 | 147 | 0 | 7.00E-74 |
| hsa_circ_0003500 | 96 | 82 | 229 | 148 | 1 | 0 | 2.00E-62 |
| hsa_circ_0003500 | 96 | 19928 | 20075 | 148 | 1 | 0 | 2.00E-60 |
| hsa_circ_0088084 | 96 | 16009 | 16179 | 171 | 1 | 0 | 2.00E-73 |
| hsa_circ_0088084 | 96 | 35855 | 36025 | 171 | 1 | 0 | 5.00E-73 |
| hsa_circ_0002925 | 92 | 5 | 1195 | 5 | 1195 | 0 | 0 |
| hsa_circ_0088104 | 93 | 193 | 1098 | 1 | 906 | 0 | 0 |
| hsa_circ_0088104 | 94 | 1 | 666 | 1 | 666 | 0 | 0 |
| hsa_circ_0088106 | 94 | 1 | 555 | 1 | 555 | 0 | 0 |
| hsa_circ_0088106 | 94 | 1 | 642 | 1 | 642 | 0 | 0 |
| hsa_circ_0088106 | 93 | 1 | 795 | 1 | 795 | 0 | 0 |
| hsa_circ_0088138 | 87 | 97 | 370 | 277 | 1 | 0.01 | 4.00E-81 |
| hsa_circ_0088152 | 89 | 1 | 670 | 1 | 670 | 0 | 0 |
| hsa_circ_0088159 | 96 | 369 | 444 | 3 | 78 | 0 | 4.00E-27 |
| hsa_circ_0088183 | 89 | 1 | 343 | 453 | 111 | 0 | 4.00E-117 |
| hsa_circ_0088186 | 89 | 196 | 939 | 3 | 743 | 0 | 0 |
| hsa_circ_0088186 | 89 | 3 | 746 | 3 | 743 | 0 | 0 |
| hsa_circ_0088186 | 89 | 3 | 746 | 3 | 743 | 0 | 0 |
| hsa_circ_0088262 | 92 | 1 | 519 | 1149 | 631 | 0 | 0 |
| hsa_circ_0088262 | 93 | 1 | 661 | 1291 | 631 | 0 | 0 |
| hsa_circ_0088262 | 93 | 1 | 285 | 1149 | 865 | 0 | 3.00E-115 |
| hsa_circ_0088265 | 95 | 1 | 283 | 283 | 1 | 0 | 4.00E-124 |
| hsa_circ_0088263 | 95 | 1 | 549 | 691 | 143 | 0 | 0 |
| hsa_circ_0088269 | 94 | 1 | 410 | 410 | 1 | 0 | 2.00E-174 |
| hsa_circ_0088272 | 96 | 1 | 189 | 189 | 1 | 0 | 5.00E-82 |

novel_circ_004127 novel_circ_004128 novel_circ_004129 novel_circ_004130 novel_circ_004131 novel_circ_004132 novel_circ_004133 novel_circ_004134 novel_circ_004135 novel_circ_004136 novel_circ_004137 novel_circ_004138 novel_circ_004139 novel_circ_004140 novel_circ_004141 novel_circ_004146 novel_circ_004147 novel_circ_004148 novel_circ_004149 novel_circ_004150 novel_circ_004151 novel_circ_004152 novel_circ_004153 novel_circ_004154 novel_circ_004155 novel_circ_004156 novel_circ_004157 novel_circ_004158 novel_circ_004159 novel_circ_004160 novel_circ_004161 novel_circ_004162 novel_circ_004163 novel_circ_004164 novel_circ_004165 novel_circ_004166 novel_circ_004167 novel_circ_004168 novel_circ_004169 novel_circ_004170 novel_circ_004171 novel_circ_004172 novel_circ_004173 novel_circ_004177 novel_circ_004178 novel_circ_004179 novel_circ_004180 novel_circ_004181 novel_circ_004182 novel_circ_004183 novel_circ_004184 novel_circ_004185 novel_circ_004186 novel_circ_004187

| hsa_circ_0088272 | 95 | 12686 | 12883 | 197 | 1 | 0.01 | 3.00E-81 |
| --- | --- | --- | --- | --- | --- | --- | --- |
| hsa_circ_0088272 | 95 | 1 | 356 | 356 | 1 | 0 | 6.00E-158 |
| hsa_circ_0088272 | 94 | 1 | 507 | 507 | 1 | 0 | 0 |
| hsa_circ_0088272 | 94 | 1146 | 1654 | 509 | 1 | 0 | 0 |
| hsa_circ_0088273 | 95 | 1 | 313 | 613 | 301 | 0 | 1.00E-135 |
| hsa_circ_0088275 | 90 | 1 | 385 | 385 | 1 | 0 | 6.00E-139 |
| hsa_circ_0088275 | 90 | 3129 | 3514 | 386 | 1 | 0 | 2.00E-138 |
| hsa_circ_0088275 | 96 | 1 | 189 | 573 | 385 | 0 | 8.00E-83 |
| hsa_circ_0088275 | 96 | 1 | 188 | 573 | 386 | 0 | 4.00E-83 |
| hsa_circ_0006833 | 73 | 24246 | 24626 | 139524 | 139899 | 0.05 | 3.00E-21 |
| hsa_circ_0007966 | 95 | 1 | 716 | 1 | 716 | 0 | 0 |
| hsa_circ_0007966 | 95 | 1 | 812 | 1 | 812 | 0 | 0 |
| hsa_circ_0007966 | 96 | 1 | 1069 | 1 | 1069 | 0 | 0 |
| hsa_circ_0069393 | 87 | 1 | 4332 | 218 | 4546 | 0.04 | 0 |
| hsa_circ_0069393 | 99 | 1 | 217 | 1 | 217 | 0 | 3.00E-105 |
| hsa_circ_0038225 | 91 | 2138 | 2411 | 40 | 314 | 0.01 | 2.00E-100 |
| hsa_circ_0027616 | 95 | 1 | 804 | 804 | 1 | 0 | 0 |
| hsa_circ_0027616 | 95 | 1 | 1267 | 1267 | 1 | 0 | 0 |
| hsa_circ_0027616 | 96 | 1 | 424 | 804 | 381 | 0 | 0 |
| hsa_circ_0027616 | 96 | 1 | 521 | 901 | 381 | 0 | 0 |
| hsa_circ_0027616 | 95 | 1 | 887 | 1267 | 381 | 0 | 0 |
| hsa_circ_0027617 | 94 | 1 | 873 | 1070 | 198 | 0 | 0 |
| hsa_circ_0027617 | 94 | 1 | 776 | 1070 | 295 | 0 | 0 |
| hsa_circ_0027617 | 93 | 1 | 423 | 898 | 476 | 0 | 1.00E-176 |
| hsa_circ_0027617 | 97 | 1 | 205 | 1359 | 1155 | 0 | 5.00E-93 |
| hsa_circ_0027618 | 96 | 147 | 321 | 175 | 1 | 0 | 1.00E-75 |
| hsa_circ_0079763 | 94 | 16677 | 16726 | 324362 | 324411 | 0 | 1.00E-10 |
| hsa_circ_0027621 | 96 | 1 | 227 | 1 | 227 | 0 | 2.00E-101 |
| hsa_circ_0027621 | 97 | 1 | 450 | 1 | 450 | 0 | 0 |
| hsa_circ_0027625 | 97 | 228 | 1667 | 1 | 1440 | 0 | 0 |
| hsa_circ_0027622 | 97 | 2 | 346 | 2 | 346 | 0 | 4.00E-165 |
| hsa_circ_0027621 | 96 | 2 | 700 | 106 | 804 | 0 | 0 |
| hsa_circ_0027625 | 98 | 1 | 223 | 1 | 223 | 0 | 1.00E-107 |
| hsa_circ_0027625 | 96 | 1 | 499 | 79 | 577 | 0 | 0 |
| hsa_circ_0027625 | 97 | 1 | 711 | 79 | 789 | 0 | 0 |
| hsa_circ_0027625 | 97 | 1 | 931 | 79 | 1009 | 0 | 0 |
| hsa_circ_0027625 | 97 | 1 | 1025 | 79 | 1103 | 0 | 0 |
| hsa_circ_0027625 | 97 | 1 | 1362 | 79 | 1440 | 0 | 0 |
| hsa_circ_0027625 | 96 | 1 | 228 | 224 | 451 | 0 | 6.00E-102 |
| hsa_circ_0027625 | 95 | 1 | 354 | 224 | 577 | 0 | 8.00E-157 |
| hsa_circ_0027625 | 96 | 1 | 880 | 224 | 1103 | 0 | 0 |
| hsa_circ_0027625 | 98 | 1 | 526 | 578 | 1103 | 0 | 0 |
| hsa_circ_0027625 | 97 | 1 | 239 | 865 | 1103 | 0 | 4.00E-113 |
| hsa_circ_0027639 | 97 | 1 | 637 | 1 | 637 | 0 | 0 |
| hsa_circ_0027639 | 93 | 1 | 1661 | 1 | 1661 | 0 | 0 |
| hsa_circ_0027639 | 92 | 1 | 1780 | 1 | 1780 | 0 | 0 |
| hsa_circ_0008877 | 96 | 1 | 257 | 1 | 257 | 0 | 1.00E-114 |
| hsa_circ_0008877 | 92 | 1 | 1609 | 1 | 1609 | 0 | 0 |
| hsa_circ_0008877 | 92 | 1 | 1536 | 74 | 1609 | 0 | 0 |
| hsa_circ_0027639 | 92 | 1 | 2149 | 245 | 2393 | 0 | 0 |
| hsa_circ_0027647 | 91 | 1 | 1233 | 1 | 1233 | 0 | 0 |
| hsa_circ_0027647 | 91 | 1 | 1352 | 1 | 1352 | 0 | 0 |
| hsa_circ_0027647 | 92 | 1 | 1965 | 1 | 1965 | 0 | 0 |
| hsa_circ_0027653 | 91 | 1 | 1896 | 1 | 1896 | 0 | 0 |

novel_circ_004188 novel_circ_004189 novel_circ_004190 novel_circ_004191 novel_circ_004192 novel_circ_004193 novel_circ_004194 novel_circ_004195 novel_circ_004196 novel_circ_004197 novel_circ_004198 novel_circ_004199 novel_circ_004200 novel_circ_004201 novel_circ_004202 novel_circ_004203 novel_circ_004204 novel_circ_004205 novel_circ_004206 novel_circ_004207 novel_circ_004208 novel_circ_004209 novel_circ_004210 novel_circ_004211 novel_circ_004212 novel_circ_004213 novel_circ_004214 novel_circ_004215 novel_circ_004216 novel_circ_004217 novel_circ_004218 novel_circ_004221 novel_circ_004222 novel_circ_004223 novel_circ_004224 novel_circ_004225 novel_circ_004226 novel_circ_004227 novel_circ_004228 novel_circ_004240 novel_circ_004241 novel_circ_004242 novel_circ_004243 novel_circ_004244 novel_circ_004245 novel_circ_004246 novel_circ_004247 novel_circ_004248 novel_circ_004249 novel_circ_004250 novel_circ_004251 novel_circ_004252 novel_circ_004253 novel_circ_004254

| hsa_circ_0027654 | 90 | 1 | 1024 | 1 | 1024 | 0 | 0 |
| --- | --- | --- | --- | --- | --- | --- | --- |
| hsa_circ_0027654 | 90 | 1 | 1143 | 1 | 1143 | 0 | 0 |
| hsa_circ_0008600 | 92 | 2 | 729 | 2 | 729 | 0 | 0 |
| hsa_circ_0027656 | 93 | 1 | 613 | 1 | 613 | 0 | 0 |
| hsa_circ_0027656 | 92 | 1 | 504 | 110 | 613 | 0 | 0 |
| hsa_circ_0027656 | 91 | 1 | 900 | 110 | 1009 | 0 | 0 |
| hsa_circ_0027656 | 90 | 1 | 1608 | 110 | 1726 | 0.01 | 0 |
| hsa_circ_0027656 | 89 | 1 | 1104 | 614 | 1726 | 0.01 | 0 |
| hsa_circ_0027657 | 92 | 3 | 1392 | 274 | 1672 | 0.01 | 0 |
| hsa_circ_0027659 | 93 | 1 | 428 | 204 | 631 | 0 | 0 |
| hsa_circ_0027659 | 93 | 1 | 351 | 393 | 743 | 0 | 4.00E-145 |
| hsa_circ_0027654 | 92 | 1 | 1223 | 4102 | 5345 | 0.02 | 0 |
| hsa_circ_0027654 | 94 | 3 | 391 | 5112 | 5500 | 0 | 2.00E-169 |
| hsa_circ_0027662 | 93 | 4 | 829 | 2 | 827 | 0 | 0 |
| hsa_circ_0027662 | 94 | 1 | 644 | 184 | 827 | 0 | 0 |
| hsa_circ_0027662 | 94 | 1 | 760 | 184 | 943 | 0 | 0 |
| hsa_circ_0027663 | 93 | 1 | 459 | 1 | 459 | 0 | 0 |
| hsa_circ_0027663 | 93 | 1 | 575 | 1 | 575 | 0 | 0 |
| hsa_circ_0027663 | 93 | 1 | 566 | 10 | 575 | 0 | 0 |
| hsa_circ_0027663 | 93 | 1 | 452 | 124 | 575 | 0 | 0 |
| hsa_circ_0027666 | 95 | 1 | 2016 | 2019 | 1 | 0 | 0 |
| hsa_circ_0027668 | 94 | 1 | 447 | 447 | 1 | 0 | 0 |
| hsa_circ_0027666 | 95 | 1 | 521 | 1948 | 1428 | 0 | 0 |
| hsa_circ_0027666 | 95 | 1 | 592 | 2019 | 1428 | 0 | 0 |
| hsa_circ_0027666 | 94 | 1237 | 1990 | 2181 | 1428 | 0 | 0 |
| hsa_circ_0027666 | 94 | 1 | 871 | 2298 | 1428 | 0 | 0 |
| hsa_circ_0027666 | 94 | 132 | 1002 | 2298 | 1428 | 0 | 0 |
| hsa_circ_0027669 | 95 | 1 | 424 | 424 | 1 | 0 | 0 |
| hsa_circ_0027669 | 96 | 1 | 555 | 555 | 1 | 0 | 0 |
| hsa_circ_0027669 | 94 | 1 | 279 | 424 | 146 | 0 | 3.00E-115 |
| hsa_circ_0027669 | 95 | 1 | 410 | 555 | 146 | 0 | 0 |
| hsa_circ_0002335 | 94 | 15300 | 15347 | 50502 | 50549 | 0 | 8.00E-10 |
| hsa_circ_0027678 | 91 | 1 | 610 | 1 | 610 | 0 | 0 |
| hsa_circ_0027678 | 91 | 1 | 465 | 146 | 610 | 0 | 0 |
| hsa_circ_0027678 | 91 | 1 | 465 | 146 | 610 | 0 | 0 |
| hsa_circ_0027678 | 91 | 1 | 465 | 146 | 610 | 0 | 0 |
| hsa_circ_0027678 | 91 | 1 | 465 | 146 | 610 | 0 | 0 |
| hsa_circ_0027679 | 92 | 1 | 177 | 141 | 318 | 0.01 | 5.00E-65 |
| hsa_circ_0027679 | 92 | 1 | 177 | 141 | 318 | 0.01 | 1.00E-64 |
| hsa_circ_0069910 | 81 | 1 | 947 | 1204 | 234 | 0.05 | 0 |
| hsa_circ_0069910 | 81 | 1 | 947 | 1204 | 234 | 0.05 | 0 |
| hsa_circ_0069910 | 86 | 1 | 586 | 1204 | 609 | 0.03 | 1.00E-176 |
| hsa_circ_0069912 | 93 | 1 | 496 | 395 | 890 | 0 | 0 |
| hsa_circ_0069912 | 93 | 1 | 667 | 395 | 1061 | 0 | 0 |
| hsa_circ_0069912 | 93 | 1 | 244 | 647 | 890 | 0 | 8.00E-96 |
| hsa_circ_0069912 | 92 | 1 | 415 | 647 | 1061 | 0 | 3.00E-167 |
| hsa_circ_0069912 | 93 | 1 | 513 | 647 | 1159 | 0 | 0 |
| hsa_circ_0069915 | 90 | 1 | 333 | 333 | 1 | 0.01 | 8.00E-122 |
| hsa_circ_0069916 | 92 | 145 | 723 | 579 | 1 | 0 | 0 |
| hsa_circ_0069917 | 97 | 1 | 144 | 144 | 1 | 0 | 7.00E-62 |
| hsa_circ_0069917 | 95 | 1 | 300 | 300 | 1 | 0 | 5.00E-130 |
| hsa_circ_0069920 | 95 | 1 | 261 | 1 | 261 | 0 | 3.00E-115 |
| hsa_circ_0069920 | 95 | 1 | 395 | 1 | 395 | 0 | 3.00E-176 |
| hsa_circ_0069920 | 95 | 1 | 559 | 1 | 559 | 0 | 0 |

novel_circ_004255 novel_circ_004256 novel_circ_004258 novel_circ_004259 novel_circ_004260 novel_circ_004261 novel_circ_004262 novel_circ_004263 novel_circ_004264 novel_circ_004265 novel_circ_004266 novel_circ_004267 novel_circ_004272 novel_circ_004275 novel_circ_004280 novel_circ_004283 novel_circ_004288 novel_circ_004289 novel_circ_004291 novel_circ_004292 novel_circ_004293 novel_circ_004294 novel_circ_004295 novel_circ_004296 novel_circ_004297 novel_circ_004298 novel_circ_004301 novel_circ_004302 novel_circ_004303 novel_circ_004304 novel_circ_004305 novel_circ_004306 novel_circ_004307 novel_circ_004308 novel_circ_004309 novel_circ_004310 novel_circ_004311 novel_circ_004312 novel_circ_004313 novel_circ_004314 novel_circ_004315 novel_circ_004316 novel_circ_004317 novel_circ_004318 novel_circ_004319 novel_circ_004320 novel_circ_004321 novel_circ_004322 novel_circ_004323 novel_circ_004324 novel_circ_004325 novel_circ_004326 novel_circ_004327 novel_circ_004328

| hsa_circ_0069920 | 95 | 1 | 392 | 168 | 559 | 0 | 3.00E-176 |
| --- | --- | --- | --- | --- | --- | --- | --- |
| hsa_circ_0069920 | 95 | 1 | 298 | 262 | 559 | 0 | 9.00E-131 |
| hsa_circ_0069923 | 93 | 1 | 254 | 1 | 254 | 0 | 2.00E-101 |
| hsa_circ_0069924 | 93 | 1 | 475 | 1 | 475 | 0 | 0 |
| hsa_circ_0069924 | 93 | 1 | 554 | 1 | 554 | 0 | 0 |
| hsa_circ_0069924 | 93 | 1 | 418 | 137 | 554 | 0 | 1.00E-170 |
| hsa_circ_0069924 | 95 | 1 | 77 | 478 | 554 | 0 | 2.00E-26 |
| hsa_circ_0069924 | 95 | 1 | 77 | 478 | 554 | 0 | 6.00E-26 |
| hsa_circ_0069924 | 95 | 1 | 77 | 478 | 554 | 0 | 8.00E-26 |
| hsa_circ_0069924 | 95 | 1 | 77 | 478 | 554 | 0 | 1.00E-25 |
| hsa_circ_0069924 | 95 | 1 | 77 | 478 | 554 | 0 | 1.00E-25 |
| hsa_circ_0069924 | 95 | 1 | 77 | 478 | 554 | 0 | 1.00E-25 |
| hsa_circ_0069925 | 91 | 1168 | 1359 | 1 | 192 | 0 | 4.00E-66 |
| hsa_circ_0069925 | 91 | 1010 | 1201 | 1 | 192 | 0 | 3.00E-66 |
| hsa_circ_0069925 | 91 | 922 | 1113 | 1 | 192 | 0 | 3.00E-66 |
| hsa_circ_0069925 | 91 | 767 | 958 | 1 | 192 | 0 | 3.00E-66 |
| hsa_circ_0069925 | 91 | 478 | 669 | 1 | 192 | 0 | 2.00E-66 |
| hsa_circ_0069925 | 92 | 478 | 945 | 1 | 468 | 0 | 0 |
| hsa_circ_0069925 | 91 | 344 | 535 | 1 | 192 | 0 | 1.00E-66 |
| hsa_circ_0069925 | 92 | 344 | 811 | 1 | 468 | 0 | 0 |
| hsa_circ_0069925 | 92 | 344 | 811 | 1 | 468 | 0 | 0 |
| hsa_circ_0069926 | 93 | 1 | 276 | 1 | 276 | 0 | 1.00E-113 |
| hsa_circ_0069926 | 93 | 1 | 276 | 1 | 276 | 0 | 2.00E-113 |
| hsa_circ_0069926 | 93 | 1 | 276 | 1 | 276 | 0 | 3.00E-113 |
| hsa_circ_0069926 | 93 | 1 | 276 | 1 | 276 | 0 | 3.00E-113 |
| hsa_circ_0069926 | 93 | 1 | 276 | 1 | 276 | 0 | 5.00E-113 |
| hsa_circ_0069929 | 93 | 4 | 299 | 3358 | 3063 | 0 | 3.00E-121 |
| hsa_circ_0069929 | 91 | 1 | 845 | 4040 | 3196 | 0 | 0 |
| hsa_circ_0069929 | 90 | 1 | 580 | 4186 | 3607 | 0 | 0 |
| hsa_circ_0069929 | 91 | 541 | 1234 | 4300 | 3607 | 0 | 0 |
| hsa_circ_0069929 | 96 | 541 | 654 | 4300 | 4187 | 0 | 9.00E-45 |
| hsa_circ_0069935 | 94 | 1 | 344 | 344 | 1 | 0 | 6.00E-148 |
| hsa_circ_0069942 | 96 | 1 | 2138 | 2138 | 1 | 0 | 0 |
| hsa_circ_0069943 | 93 | 1 | 451 | 754 | 304 | 0 | 0 |
| hsa_circ_0069943 | 94 | 1 | 754 | 754 | 1 | 0 | 0 |
| hsa_circ_0069943 | 93 | 1 | 451 | 754 | 304 | 0 | 0 |
| hsa_circ_0069943 | 94 | 1 | 604 | 754 | 151 | 0 | 0 |
| hsa_circ_0069947 | 96 | 1 | 552 | 552 | 1 | 0 | 0 |
| hsa_circ_0069947 | 96 | 1 | 1167 | 1167 | 1 | 0 | 0 |
| hsa_circ_0069947 | 96 | 1 | 1316 | 1316 | 1 | 0 | 0 |
| hsa_circ_0069943 | 97 | 1 | 3167 | 4093 | 927 | 0 | 0 |
| hsa_circ_0069943 | 97 | 1 | 3854 | 4780 | 927 | 0 | 0 |
| hsa_circ_0069943 | 97 | 1 | 4008 | 4934 | 927 | 0 | 0 |
| hsa_circ_0069949 | 96 | 1 | 1050 | 1050 | 1 | 0 | 0 |
| hsa_circ_0069951 | 96 | 1 | 338 | 339 | 2 | 0 | 6.00E-158 |
| hsa_circ_0069951 | 96 | 1 | 695 | 696 | 2 | 0 | 0 |
| hsa_circ_0069951 | 96 | 1 | 953 | 954 | 2 | 0 | 0 |
| hsa_circ_0069957 | 98 | 4 | 1365 | 1362 | 1 | 0 | 0 |
| hsa_circ_0069957 | 97 | 1 | 1667 | 1667 | 1 | 0 | 0 |
| hsa_circ_0069957 | 97 | 1 | 2000 | 2000 | 1 | 0 | 0 |
| hsa_circ_0069957 | 97 | 1 | 2530 | 2530 | 1 | 0 | 0 |
| hsa_circ_0069964 | 97 | 1 | 957 | 957 | 1 | 0 | 0 |
| hsa_circ_0069964 | 97 | 1 | 302 | 390 | 89 | 0 | 6.00E-143 |
| hsa_circ_0069964 | 97 | 1 | 635 | 723 | 89 | 0 | 0 |

novel_circ_004329 novel_circ_004330 novel_circ_004331 novel_circ_004332 novel_circ_004333 novel_circ_004334 novel_circ_004335 novel_circ_004338 novel_circ_004339 novel_circ_004340 novel_circ_004341 novel_circ_004342 novel_circ_004343 novel_circ_004344 novel_circ_004345 novel_circ_004347 novel_circ_004353 novel_circ_004354 novel_circ_004355 novel_circ_004362 novel_circ_004363 novel_circ_004364 novel_circ_004365 novel_circ_004366 novel_circ_004367 novel_circ_004368 novel_circ_004369 novel_circ_004370 novel_circ_004371 novel_circ_004372 novel_circ_004373 novel_circ_004374 novel_circ_004375 novel_circ_004376 novel_circ_004377 novel_circ_004378 novel_circ_004379 novel_circ_004380 novel_circ_004381 novel_circ_004382 novel_circ_004383 novel_circ_004384 novel_circ_004385 novel_circ_004386 novel_circ_004387 novel_circ_004388 novel_circ_004389 novel_circ_004390 novel_circ_004391 novel_circ_004392 novel_circ_004393 novel_circ_004394 novel_circ_004395 novel_circ_004396

| hsa_circ_0069960 | 97 | 1 | 1476 | 1939 | 464 | 0 | 0 |
| --- | --- | --- | --- | --- | --- | --- | --- |
| hsa_circ_0069965 | 96 | 1 | 333 | 333 | 1 | 0 | 4.00E-155 |
| hsa_circ_0069964 | 96 | 1 | 453 | 1410 | 958 | 0 | 0 |
| hsa_circ_0069960 | 98 | 17232 | 17391 | 1946 | 1786 | 0.01 | 1.00E-70 |
| hsa_circ_0069960 | 98 | 66229 | 66388 | 1946 | 1786 | 0.01 | 5.00E-70 |
| hsa_circ_0069983 | 89 | 1 | 662 | 1 | 662 | 0 | 0 |
| hsa_circ_0069987 | 89 | 3 | 354 | 3 | 354 | 0 | 2.00E-122 |
| hsa_circ_0069998 | 96 | 2 | 631 | 630 | 1 | 0 | 0 |
| hsa_circ_0069998 | 96 | 1 | 734 | 734 | 1 | 0 | 0 |
| hsa_circ_0070000 | 96 | 1 | 466 | 750 | 285 | 0 | 0 |
| hsa_circ_0070007 | 93 | 1 | 707 | 180 | 886 | 0 | 0 |
| hsa_circ_0070008 | 94 | 1 | 244 | 1 | 244 | 0 | 2.00E-102 |
| hsa_circ_0070007 | 94 | 1 | 566 | 321 | 886 | 0 | 0 |
| hsa_circ_0070010 | 92 | 1923 | 2334 | 1 | 412 | 0 | 7.00E-165 |
| hsa_circ_0070010 | 92 | 1 | 412 | 1 | 412 | 0 | 1.00E-165 |
| hsa_circ_0070011 | 88 | 1 | 812 | 424 | 1235 | 0 | 0 |
| hsa_circ_0070036 | 93 | 1 | 346 | 346 | 1 | 0 | 5.00E-144 |
| hsa_circ_0070039 | 94 | 1 | 455 | 643 | 189 | 0 | 0 |
| hsa_circ_0070041 | 94 | 1 | 228 | 228 | 1 | 0 | 3.00E-95 |
| hsa_circ_0008537 | 90 | 262 | 502 | 243 | 3 | 0 | 2.00E-85 |
| hsa_circ_0079950 | 89 | 3 | 552 | 553 | 1 | 0.01 | 0 |
| hsa_circ_0079949 | 91 | 3 | 1020 | 1022 | 2 | 0 | 0 |
| hsa_circ_0079949 | 94 | 1 | 670 | 671 | 2 | 0 | 0 |
| hsa_circ_0014355 | 86 | 13249 | 13534 | 1 | 301 | 0.05 | 8.00E-83 |
| hsa_circ_0087834 | 75 | 2213 | 2510 | 22490 | 22193 | 0.04 | 4.00E-27 |
| hsa_circ_0087834 | 75 | 3146 | 3443 | 22490 | 22193 | 0.04 | 4.00E-27 |
| hsa_circ_0079943 | 96 | 1 | 121 | 264 | 384 | 0 | 6.00E-49 |
| hsa_circ_0079943 | 95 | 1 | 384 | 1 | 384 | 0.01 | 1.00E-171 |
| hsa_circ_0079943 | 95 | 1 | 384 | 1 | 384 | 0.01 | 1.00E-171 |
| hsa_circ_0079943 | 95 | 1 | 384 | 1 | 384 | 0.01 | 9.00E-172 |
| hsa_circ_0079934 | 94 | 1 | 909 | 1 | 909 | 0 | 0 |
| hsa_circ_0079939 | 93 | 1 | 426 | 1 | 426 | 0 | 2.00E-178 |
| hsa_circ_0079935 | 94 | 1 | 673 | 1 | 673 | 0 | 0 |
| hsa_circ_0079934 | 94 | 1 | 909 | 1 | 909 | 0 | 0 |
| hsa_circ_0079933 | 93 | 1 | 1572 | 1 | 1569 | 0.01 | 0 |
| hsa_circ_0079935 | 94 | 1 | 427 | 1 | 427 | 0 | 0 |
| hsa_circ_0079934 | 94 | 1 | 909 | 1 | 909 | 0 | 0 |
| hsa_circ_0079933 | 95 | 3350 | 4261 | 658 | 1569 | 0 | 0 |
| hsa_circ_0079933 | 93 | 1 | 1572 | 1 | 1569 | 0.01 | 0 |
| hsa_circ_0079935 | 95 | 1 | 247 | 1 | 247 | 0 | 2.00E-107 |
| hsa_circ_0079934 | 95 | 1 | 729 | 1 | 729 | 0 | 0 |
| hsa_circ_0079933 | 95 | 3350 | 4081 | 658 | 1389 | 0 | 0 |
| hsa_circ_0079933 | 94 | 1 | 1392 | 1 | 1389 | 0.01 | 0 |
| hsa_circ_0079933 | 94 | 1484 | 2875 | 1 | 1389 | 0.01 | 0 |
| hsa_circ_0079934 | 95 | 1 | 484 | 1 | 484 | 0 | 0 |
| hsa_circ_0079933 | 93 | 1 | 1147 | 1 | 1144 | 0.01 | 0 |
| hsa_circ_0079933 | 93 | 1 | 1147 | 1 | 1144 | 0.01 | 0 |
| hsa_circ_0079934 | 95 | 1 | 482 | 1 | 482 | 0 | 0 |
| hsa_circ_0079933 | 95 | 3350 | 3834 | 658 | 1142 | 0 | 0 |
| hsa_circ_0079933 | 93 | 1 | 1145 | 1 | 1142 | 0.01 | 0 |
| hsa_circ_0079928 | 93 | 247 | 1394 | 1491 | 2635 | 0.01 | 0 |
| hsa_circ_0079928 | 93 | 1481 | 2628 | 1491 | 2635 | 0.01 | 0 |
| hsa_circ_0079928 | 93 | 1671 | 2818 | 1491 | 2635 | 0.01 | 0 |
| hsa_circ_0079934 | 95 | 1 | 311 | 1 | 311 | 0 | 1.00E-134 |

novel_circ_004397 novel_circ_004400 novel_circ_004401 novel_circ_004403 novel_circ_004404 novel_circ_004405 novel_circ_004406 novel_circ_004407 novel_circ_004408 novel_circ_004409 novel_circ_004410 novel_circ_004411 novel_circ_004412 novel_circ_004413 novel_circ_004414 novel_circ_004415 novel_circ_004416 novel_circ_004417 novel_circ_004418 novel_circ_004423 novel_circ_004424 novel_circ_004427 novel_circ_004428 novel_circ_004429 novel_circ_004430 novel_circ_004432 novel_circ_004436 novel_circ_004438 novel_circ_004439 novel_circ_004442 novel_circ_004449 novel_circ_004450 novel_circ_004451 novel_circ_004452 novel_circ_004453 novel_circ_004454 novel_circ_004455 novel_circ_004456 novel_circ_004457 novel_circ_004458 novel_circ_004459 novel_circ_004465 novel_circ_004466 novel_circ_004467 novel_circ_004468 novel_circ_004469 novel_circ_004470 novel_circ_004471 novel_circ_004472 novel_circ_004473 novel_circ_004474 novel_circ_004475 novel_circ_004476 novel_circ_004477

| hsa_circ_0079933 | 93 | 1 | 974 | 1 | 971 | 0.01 | 0 |
| --- | --- | --- | --- | --- | --- | --- | --- |
| hsa_circ_0003163 | 80 | 26133 | 26502 | 757484 | 757859 | 0.03 | 7.00E-67 |
| hsa_circ_0007133 | 79 | 58496 | 58587 | 2E+06 | 1693825 | 0 | 4.00E-07 |
| hsa_circ_0001698 | 92 | 1 | 538 | 538 | 1 | 0 | 0 |
| hsa_circ_0001698 | 92 | 1 | 396 | 463 | 68 | 0 | 9.00E-157 |
| hsa_circ_0001698 | 92 | 1 | 463 | 463 | 1 | 0 | 0 |
| hsa_circ_0001698 | 92 | 2 | 267 | 333 | 68 | 0 | 2.00E-101 |
| hsa_circ_0001698 | 91 | 2 | 334 | 333 | 1 | 0 | 8.00E-127 |
| hsa_circ_0079904 | 91 | 1 | 638 | 638 | 1 | 0 | 0 |
| hsa_circ_0079901 | 91 | 4 | 504 | 979 | 479 | 0 | 0 |
| hsa_circ_0079901 | 92 | 4 | 837 | 979 | 146 | 0 | 0 |
| hsa_circ_0079901 | 91 | 1 | 145 | 145 | 1 | 0 | 7.00E-49 |
| hsa_circ_0079901 | 91 | 1 | 145 | 145 | 1 | 0 | 5.00E-48 |
| hsa_circ_0008224 | 92 | 4 | 194 | 618 | 428 | 0 | 8.00E-70 |
| hsa_circ_0008224 | 91 | 4 | 492 | 618 | 130 | 0 | 0 |
| hsa_circ_0008224 | 91 | 4 | 621 | 618 | 1 | 0 | 0 |
| hsa_circ_0008224 | 91 | 4 | 621 | 618 | 1 | 0 | 0 |
| hsa_circ_0008224 | 91 | 4 | 351 | 348 | 1 | 0 | 2.00E-128 |
| hsa_circ_0008224 | 91 | 4 | 351 | 348 | 1 | 0 | 3.00E-128 |
| hsa_circ_0079898 | 83 | 2 | 652 | 80 | 736 | 0.03 | 7.00E-165 |
| hsa_circ_0004698 | 89 | 1 | 707 | 1 | 707 | 0 | 0 |
| hsa_circ_0079891 | 92 | 1 | 636 | 637 | 2 | 0 | 0 |
| hsa_circ_0079887 | 92 | 1 | 742 | 851 | 110 | 0 | 0 |
| hsa_circ_0079891 | 92 | 1 | 536 | 537 | 2 | 0 | 0 |
| hsa_circ_0079887 | 91 | 1 | 642 | 751 | 110 | 0 | 0 |
| hsa_circ_0079880 | 93 | 1 | 468 | 546 | 79 | 0 | 0 |
| hsa_circ_0079874 | 88 | 5380 | 5749 | 2009 | 2378 | 0 | 2.00E-119 |
| hsa_circ_0079860 | 92 | 1 | 357 | 1 | 357 | 0 | 4.00E-140 |
| hsa_circ_0079860 | 92 | 344 | 700 | 1 | 357 | 0 | 8.00E-140 |
| hsa_circ_0079858 | 81 | 1 | 359 | 347 | 1 | 0.03 | 2.00E-69 |
| hsa_circ_0064067 | 92 | 1 | 453 | 668 | 1120 | 0 | 0 |
| hsa_circ_0064071 | 92 | 1 | 250 | 1 | 250 | 0 | 2.00E-97 |
| hsa_circ_0064072 | 92 | 1 | 324 | 1 | 324 | 0 | 8.00E-127 |
| hsa_circ_0001262 | 91 | 1 | 435 | 1 | 435 | 0 | 5.00E-170 |
| hsa_circ_0064066 | 92 | 1 | 1264 | 2578 | 3841 | 0 | 0 |
| hsa_circ_0005046 | 94 | 1 | 249 | 1 | 249 | 0 | 3.00E-105 |
| hsa_circ_0005046 | 95 | 1 | 388 | 1 | 388 | 0 | 1.00E-175 |
| hsa_circ_0064089 | 90 | 1 | 292 | 1 | 292 | 0 | 9.00E-106 |
| hsa_circ_0064092 | 93 | 24292 | 24514 | 788 | 1010 | 0 | 4.00E-87 |
| hsa_circ_0064092 | 94 | 1 | 217 | 794 | 1010 | 0 | 1.00E-87 |
| hsa_circ_0064092 | 96 | 3 | 141 | 1310 | 1448 | 0 | 8.00E-59 |
| hsa_circ_0075448 | 93 | 34876 | 35301 | 426 | 1 | 0 | 4.00E-173 |
| hsa_circ_0075449 | 92 | 1 | 298 | 298 | 1 | 0 | 4.00E-114 |
| hsa_circ_0075448 | 93 | 1 | 426 | 426 | 1 | 0 | 5.00E-175 |
| hsa_circ_0075443 | 91 | 35 | 699 | 746 | 82 | 0 | 0 |
| hsa_circ_0075443 | 92 | 1 | 320 | 320 | 1 | 0 | 6.00E-123 |
| hsa_circ_0075435 | 91 | 1 | 896 | 896 | 1 | 0 | 0 |
| hsa_circ_0075432 | 91 | 1 | 1021 | 1087 | 67 | 0 | 0 |
| hsa_circ_0075421 | 91 | 2 | 1638 | 1816 | 180 | 0 | 0 |
| hsa_circ_0075431 | 91 | 1 | 224 | 224 | 1 | 0.01 | 9.00E-80 |
| hsa_circ_0075424 | 90 | 1 | 795 | 795 | 1 | 0 | 0 |
| hsa_circ_0075423 | 91 | 1 | 937 | 937 | 1 | 0 | 0 |
| hsa_circ_0075428 | 89 | 1 | 269 | 270 | 2 | 0 | 1.00E-89 |
| hsa_circ_0075424 | 90 | 1 | 387 | 387 | 1 | 0 | 9.00E-142 |

novel_circ_004478 novel_circ_004479 novel_circ_004480 novel_circ_004481 novel_circ_004482 novel_circ_004493 novel_circ_004494 novel_circ_004495 novel_circ_004496 novel_circ_004497 novel_circ_004499 novel_circ_004500 novel_circ_004501 novel_circ_004502 novel_circ_004503 novel_circ_004504 novel_circ_004505 novel_circ_004506 novel_circ_004507 novel_circ_004508 novel_circ_004509 novel_circ_004510 novel_circ_004515 novel_circ_004517 novel_circ_004518 novel_circ_004519 novel_circ_004520 novel_circ_004521 novel_circ_004522 novel_circ_004524 novel_circ_004525 novel_circ_004526 novel_circ_004527 novel_circ_004528 novel_circ_004529 novel_circ_004530 novel_circ_004531 novel_circ_004534 novel_circ_004535 novel_circ_004536 novel_circ_004537 novel_circ_004538 novel_circ_004539 novel_circ_004540 novel_circ_004541 novel_circ_004542 novel_circ_004543 novel_circ_004544 novel_circ_004545 novel_circ_004546 novel_circ_004547 novel_circ_004548 novel_circ_004549 novel_circ_004550

| hsa_circ_0075423 | 94 | 1 | 259 | 259 | 1 | 0 | 2.00E-107 |
| --- | --- | --- | --- | --- | --- | --- | --- |
| hsa_circ_0066214 | 95 | 1 | 148 | 148 | 1 | 0 | 3.00E-58 |
| hsa_circ_0066214 | 95 | 1 | 148 | 148 | 1 | 0 | 1.00E-56 |
| hsa_circ_0066213 | 96 | 1 | 135 | 135 | 1 | 0 | 5.00E-53 |
| hsa_circ_0066213 | 96 | 1 | 135 | 135 | 1 | 0 | 1.00E-52 |
| hsa_circ_0086593 | 97 | 1 | 348 | 502 | 155 | 0 | 4.00E-165 |
| hsa_circ_0086593 | 97 | 92 | 439 | 502 | 155 | 0 | 5.00E-165 |
| hsa_circ_0086593 | 97 | 155 | 502 | 502 | 155 | 0 | 6.00E-165 |
| hsa_circ_0086593 | 96 | 92 | 335 | 502 | 259 | 0 | 5.00E-109 |
| hsa_circ_0021776 | 83 | 9573 | 9708 | 17104 | 17237 | 0.04 | 2.00E-23 |
| hsa_circ_0086598 | 93 | 1 | 1406 | 1406 | 1 | 0 | 0 |
| hsa_circ_0086598 | 93 | 1 | 686 | 686 | 1 | 0 | 0 |
| hsa_circ_0086598 | 93 | 1 | 619 | 686 | 68 | 0 | 0 |
| hsa_circ_0086598 | 93 | 1 | 617 | 686 | 70 | 0 | 0 |
| hsa_circ_0086598 | 93 | 1 | 548 | 686 | 139 | 0 | 0 |
| hsa_circ_0086598 | 94 | 1 | 1268 | 1406 | 139 | 0 | 0 |
| hsa_circ_0086598 | 94 | 1 | 328 | 686 | 359 | 0 | 1.00E-140 |
| hsa_circ_0086600 | 93 | 1 | 890 | 890 | 1 | 0 | 0 |
| hsa_circ_0086600 | 94 | 1 | 720 | 890 | 171 | 0 | 0 |
| hsa_circ_0086600 | 94 | 1 | 584 | 890 | 307 | 0 | 0 |
| hsa_circ_0086602 | 93 | 1 | 416 | 416 | 1 | 0 | 7.00E-173 |
| hsa_circ_0086603 | 92 | 1 | 273 | 265 | 537 | 0 | 1.00E-106 |
| hsa_circ_0086607 | 92 | 85 | 243 | 159 | 1 | 0 | 1.00E-58 |
| hsa_circ_0086609 | 91 | 1 | 426 | 1 | 426 | 0 | 1.00E-161 |
| hsa_circ_0086609 | 90 | 3 | 307 | 122 | 426 | 0 | 1.00E-109 |
| hsa_circ_0086609 | 93 | 2 | 532 | 290 | 820 | 0 | 0 |
| hsa_circ_0086610 | 91 | 139 | 1218 | 1 | 1080 | 0 | 0 |
| hsa_circ_0086610 | 91 | 1 | 1080 | 1 | 1080 | 0 | 0 |
| hsa_circ_0086611 | 91 | 118 | 655 | 1 | 538 | 0 | 0 |
| hsa_circ_0086627 | 94 | 1 | 1264 | 6957 | 5694 | 0 | 0 |
| hsa_circ_0086627 | 95 | 1 | 885 | 6746 | 5862 | 0 | 0 |
| hsa_circ_0086627 | 94 | 1 | 1096 | 6957 | 5862 | 0 | 0 |
| hsa_circ_0086631 | 95 | 1 | 360 | 360 | 1 | 0 | 4.00E-160 |
| hsa_circ_0086627 | 95 | 1 | 724 | 6957 | 6234 | 0 | 0 |
| hsa_circ_0086627 | 95 | 1 | 604 | 6957 | 6354 | 0 | 0 |
| hsa_circ_0086627 | 95 | 1 | 475 | 6957 | 6483 | 0 | 0 |
| hsa_circ_0086627 | 94 | 1 | 364 | 6957 | 6594 | 0 | 2.00E-157 |
| hsa_circ_0086640 | 87 | 1 | 1005 | 1 | 1008 | 0.01 | 0 |
| hsa_circ_0086639 | 88 | 1 | 1164 | 179 | 1345 | 0.01 | 0 |
| hsa_circ_0086639 | 88 | 1 | 1242 | 179 | 1423 | 0.01 | 0 |
| hsa_circ_0086647 | 92 | 1 | 530 | 1 | 530 | 0 | 0 |
| hsa_circ_0086651 | 92 | 1 | 318 | 1 | 318 | 0 | 8.00E-122 |
| hsa_circ_0086651 | 94 | 1 | 527 | 1 | 527 | 0 | 0 |
| hsa_circ_0086651 | 94 | 1 | 636 | 1 | 636 | 0 | 0 |
| hsa_circ_0086655 | 96 | 1 | 318 | 1 | 318 | 0 | 8.00E-147 |
| hsa_circ_0086655 | 95 | 1 | 438 | 1 | 438 | 0 | 0 |
| hsa_circ_0004000 | 94 | 1 | 309 | 1 | 309 | 0 | 3.00E-130 |
| hsa_circ_0004000 | 94 | 1 | 389 | 1 | 389 | 0 | 2.00E-164 |
| hsa_circ_0086662 | 92 | 1 | 305 | 1 | 305 | 0 | 6.00E-118 |
| hsa_circ_0003558 | 98 | 1 | 185 | 1 | 185 | 0 | 2.00E-86 |
| hsa_circ_0003558 | 98 | 1 | 320 | 1 | 320 | 0 | 1.00E-154 |
| hsa_circ_0086691 | 85 | 1 | 336 | 336 | 1 | 0 | 4.00E-95 |
| hsa_circ_0086712 | 90 | 1 | 866 | 873 | 8 | 0 | 0 |
| hsa_circ_0086712 | 91 | 1 | 1056 | 1063 | 8 | 0 | 0 |

novel_circ_004551 novel_circ_004552 novel_circ_004553 novel_circ_004554 novel_circ_004555 novel_circ_004556 novel_circ_004557 novel_circ_004558 novel_circ_004559 novel_circ_004560 novel_circ_004561 novel_circ_004562 novel_circ_004563 novel_circ_004564 novel_circ_004565 novel_circ_004566 novel_circ_004567 novel_circ_004575 novel_circ_004576 novel_circ_004577 novel_circ_004578 novel_circ_004579 novel_circ_004580 novel_circ_004581 novel_circ_004582 novel_circ_004583 novel_circ_004584 novel_circ_004585 novel_circ_004586 novel_circ_004587 novel_circ_004588 novel_circ_004589 novel_circ_004599 novel_circ_004602 novel_circ_004603 novel_circ_004604 novel_circ_004605 novel_circ_004606 novel_circ_004607 novel_circ_004608 novel_circ_004609 novel_circ_004610 novel_circ_004611 novel_circ_004612 novel_circ_004613 novel_circ_004614 novel_circ_004615 novel_circ_004616 novel_circ_004617 novel_circ_004618 novel_circ_004619 novel_circ_004620 novel_circ_004621 novel_circ_004622

| hsa_circ_0086712 | 91 | 3 | 1480 | 1485 | 8 | 0 | 0 |
| --- | --- | --- | --- | --- | --- | --- | --- |
| hsa_circ_0086712 | 90 | 1 | 1634 | 1641 | 8 | 0 | 0 |
| hsa_circ_0086705 | 91 | 79 | 1904 | 1873 | 48 | 0 | 0 |
| hsa_circ_0086705 | 91 | 218 | 2043 | 1873 | 48 | 0 | 0 |
| hsa_circ_0086715 | 93 | 1 | 404 | 404 | 1 | 0 | 3.00E-166 |
| hsa_circ_0086715 | 92 | 4 | 472 | 469 | 1 | 0 | 0 |
| hsa_circ_0086715 | 92 | 1 | 525 | 525 | 1 | 0 | 0 |
| hsa_circ_0086715 | 91 | 3 | 828 | 826 | 1 | 0 | 0 |
| hsa_circ_0086715 | 91 | 1 | 982 | 982 | 1 | 0 | 0 |
| hsa_circ_0086705 | 91 | 1 | 1171 | 1870 | 700 | 0 | 0 |
| hsa_circ_0086705 | 91 | 79 | 1252 | 1873 | 700 | 0 | 0 |
| hsa_circ_0086705 | 91 | 218 | 1391 | 1873 | 700 | 0 | 0 |
| hsa_circ_0086722 | 90 | 1 | 768 | 768 | 1 | 0 | 0 |
| hsa_circ_0086722 | 91 | 218 | 1177 | 960 | 1 | 0 | 0 |
| hsa_circ_0086733 | 89 | 3 | 237 | 235 | 1 | 0 | 8.00E-76 |
| hsa_circ_0086733 | 88 | 1 | 391 | 391 | 1 | 0 | 3.00E-132 |
| hsa_circ_0086737 | 92 | 1 | 218 | 329 | 111 | 0 | 8.00E-83 |
| hsa_circ_0086743 | 94 | 1 | 1046 | 1 | 1046 | 0 | 0 |
| hsa_circ_0086744 | 93 | 1 | 921 | 1 | 921 | 0 | 0 |
| hsa_circ_0086744 | 94 | 1 | 1209 | 1 | 1209 | 0 | 0 |
| hsa_circ_0007356 | 91 | 1 | 310 | 304 | 1 | 0.02 | 5.00E-114 |
| hsa_circ_0086750 | 92 | 1 | 408 | 630 | 229 | 0.01 | 4.00E-160 |
| hsa_circ_0086750 | 93 | 1 | 598 | 820 | 229 | 0.01 | 0 |
| hsa_circ_0086750 | 93 | 643 | 1240 | 820 | 229 | 0.01 | 0 |
| hsa_circ_0086778 | 94 | 1 | 406 | 779 | 374 | 0 | 3.00E-172 |
| hsa_circ_0086790 | 93 | 1 | 144 | 144 | 1 | 0 | 4.00E-53 |
| hsa_circ_0086800 | 96 | 1 | 474 | 31 | 504 | 0 | 0 |
| hsa_circ_0086811 | 90 | 511 | 1151 | 1 | 641 | 0.01 | 0 |
| hsa_circ_0086802 | 96 | 1 | 374 | 1 | 374 | 0 | 1.00E-174 |
| hsa_circ_0086811 | 90 | 1 | 641 | 1 | 641 | 0.01 | 0 |
| hsa_circ_0086816 | 96 | 1 | 341 | 761 | 1101 | 0 | 1.00E-154 |
| hsa_circ_0086975 | 92 | 1 | 208 | 208 | 1 | 0 | 1.00E-77 |
| hsa_circ_0070431 | 86 | 1 | 367 | 367 | 1 | 0 | 1.00E-110 |
| hsa_circ_0070430 | 90 | 6 | 362 | 1063 | 707 | 0.01 | 2.00E-128 |
| hsa_circ_0070430 | 90 | 1 | 706 | 706 | 1 | 0 | 0 |
| hsa_circ_0070430 | 90 | 1 | 305 | 485 | 181 | 0 | 3.00E-106 |
| hsa_circ_0070430 | 90 | 1 | 485 | 485 | 1 | 0 | 9.00E-178 |
| hsa_circ_0070430 | 92 | 13 | 180 | 168 | 1 | 0 | 1.00E-61 |
| hsa_circ_0070426 | 89 | 1 | 147 | 2282 | 2428 | 0 | 6.00E-45 |
| hsa_circ_0070426 | 94 | 2 | 1176 | 1107 | 2281 | 0 | 0 |
| hsa_circ_0070429 | 94 | 1 | 468 | 113 | 580 | 0 | 0 |
| hsa_circ_0070429 | 94 | 2 | 580 | 2 | 580 | 0 | 0 |
| hsa_circ_0070426 | 95 | 1 | 765 | 921 | 1685 | 0 | 0 |
| hsa_circ_0070426 | 95 | 1 | 842 | 844 | 1685 | 0 | 0 |
| hsa_circ_0070426 | 94 | 1 | 564 | 844 | 1407 | 0 | 0 |
| hsa_circ_0070426 | 95 | 1 | 374 | 844 | 1217 | 0 | 3.00E-166 |
| hsa_circ_0070424 | 93 | 1 | 255 | 138 | 392 | 0 | 6.00E-102 |
| hsa_circ_0070412 | 84 | 1 | 571 | 1010 | 1580 | 0 | 2.00E-150 |
| hsa_circ_0070412 | 84 | 1 | 665 | 916 | 1580 | 0 | 0 |
| hsa_circ_0070408 | 94 | 1 | 267 | 2894 | 2628 | 0 | 7.00E-112 |
| hsa_circ_0070408 | 92 | 1 | 311 | 2793 | 2483 | 0 | 6.00E-123 |
| hsa_circ_0070408 | 93 | 1 | 446 | 2793 | 2348 | 0 | 0 |
| hsa_circ_0070407 | 87 | 329 | 1284 | 1116 | 158 | 0 | 0 |
| hsa_circ_0070402 | 91 | 115 | 2305 | 1 | 2191 | 0 | 0 |

novel_circ_004623 novel_circ_004624 novel_circ_004625 novel_circ_004626 novel_circ_004627 novel_circ_004628 novel_circ_004629 novel_circ_004630 novel_circ_004631 novel_circ_004632 novel_circ_004633 novel_circ_004634 novel_circ_004635 novel_circ_004636 novel_circ_004637 novel_circ_004638 novel_circ_004639 novel_circ_004640 novel_circ_004641 novel_circ_004644 novel_circ_004645 novel_circ_004646 novel_circ_004649 novel_circ_004650 novel_circ_004651 novel_circ_004652 novel_circ_004653 novel_circ_004654 novel_circ_004655 novel_circ_004656 novel_circ_004657 novel_circ_004658 novel_circ_004659 novel_circ_004660 novel_circ_004661 novel_circ_004662 novel_circ_004663 novel_circ_004664 novel_circ_004665 novel_circ_004666 novel_circ_004667 novel_circ_004668 novel_circ_004669 novel_circ_004670 novel_circ_004671 novel_circ_004672 novel_circ_004673 novel_circ_004674 novel_circ_004675 novel_circ_004676 novel_circ_004677 novel_circ_004678 novel_circ_004679 novel_circ_004680

| hsa_circ_0070402 | 91 | 115 | 2075 | 1 | 1961 | 0 | 0 |
| --- | --- | --- | --- | --- | --- | --- | --- |
| hsa_circ_0070402 | 92 | 1 | 1649 | 1 | 1649 | 0 | 0 |
| hsa_circ_0070399 | 92 | 1 | 1763 | 683 | 2445 | 0 | 0 |
| hsa_circ_0006645 | 95 | 1 | 220 | 351 | 570 | 0 | 2.00E-92 |
| hsa_circ_0006645 | 93 | 1 | 402 | 169 | 570 | 0 | 2.00E-163 |
| hsa_circ_0006645 | 92 | 1 | 570 | 1 | 570 | 0 | 0 |
| hsa_circ_0004050 | 92 | 1 | 799 | 477 | 1275 | 0 | 0 |
| hsa_circ_0004050 | 92 | 1 | 1024 | 252 | 1275 | 0 | 0 |
| hsa_circ_0004050 | 92 | 1 | 1275 | 1 | 1275 | 0 | 0 |
| hsa_circ_0070402 | 92 | 1 | 1409 | 1 | 1409 | 0 | 0 |
| hsa_circ_0070399 | 92 | 1 | 1523 | 683 | 2205 | 0 | 0 |
| hsa_circ_0006645 | 90 | 1 | 350 | 1 | 350 | 0 | 3.00E-126 |
| hsa_circ_0070402 | 91 | 1 | 1189 | 1 | 1189 | 0 | 0 |
| hsa_circ_0070399 | 91 | 1 | 1303 | 683 | 1985 | 0 | 0 |
| hsa_circ_0070399 | 92 | 1 | 1121 | 683 | 1803 | 0 | 0 |
| hsa_circ_0070402 | 92 | 1 | 833 | 1 | 833 | 0 | 0 |
| hsa_circ_0070399 | 92 | 1 | 947 | 683 | 1629 | 0 | 0 |
| hsa_circ_0070402 | 92 | 1 | 610 | 1 | 610 | 0 | 0 |
| hsa_circ_0007076 | 92 | 1 | 724 | 1 | 724 | 0 | 0 |
| hsa_circ_0070396 | 92 | 1 | 527 | 1 | 527 | 0 | 0 |
| hsa_circ_0070396 | 92 | 1 | 527 | 1 | 527 | 0 | 0 |
| hsa_circ_0070396 | 95 | 1 | 183 | 1 | 183 | 0 | 2.00E-75 |
| hsa_circ_0005384 | 91 | 18 | 1238 | 1230 | 1 | 0.01 | 0 |
| hsa_circ_0070389 | 92 | 18 | 1679 | 1671 | 1 | 0.01 | 0 |
| hsa_circ_0005384 | 94 | 1 | 880 | 880 | 1 | 0 | 0 |
| hsa_circ_0070390 | 94 | 1 | 992 | 1161 | 170 | 0 | 0 |
| hsa_circ_0005384 | 95 | 7 | 331 | 325 | 1 | 0 | 2.00E-142 |
| hsa_circ_0070389 | 94 | 7 | 772 | 766 | 1 | 0 | 0 |
| hsa_circ_0070384 | 88 | 262 | 1129 | 1564 | 2431 | 0 | 0 |
| hsa_circ_0070384 | 87 | 1 | 347 | 1861 | 2207 | 0.01 | 1.00E-109 |
| hsa_circ_0070384 | 88 | 262 | 905 | 1564 | 2207 | 0 | 0 |
| hsa_circ_0070378 | 85 | 2 | 2455 | 2 | 2428 | 0.02 | 0 |
| hsa_circ_0070378 | 86 | 2 | 1260 | 2 | 1239 | 0.03 | 0 |
| hsa_circ_0070370 | 88 | 1 | 1722 | 2651 | 4360 | 0.01 | 0 |
| hsa_circ_0070370 | 90 | 4 | 1265 | 1952 | 3210 | 0.01 | 0 |
| hsa_circ_0070370 | 90 | 3321 | 4582 | 1952 | 3210 | 0.01 | 0 |
| hsa_circ_0070372 | 89 | 2643 | 3590 | 910 | 1854 | 0.01 | 0 |
| hsa_circ_0070372 | 89 | 1 | 1191 | 664 | 1854 | 0.01 | 0 |
| hsa_circ_0070372 | 90 | 4 | 702 | 910 | 1608 | 0.01 | 0 |
| hsa_circ_0070372 | 90 | 2643 | 3341 | 910 | 1608 | 0.01 | 0 |
| hsa_circ_0070372 | 90 | 3321 | 4019 | 910 | 1608 | 0.01 | 0 |
| hsa_circ_0070372 | 90 | 1 | 942 | 664 | 1608 | 0.01 | 0 |
| hsa_circ_0070372 | 90 | 1 | 1032 | 574 | 1608 | 0.01 | 0 |
| hsa_circ_0070372 | 94 | 1 | 661 | 1 | 661 | 0 | 0 |
| hsa_circ_0070365 | 92 | 1 | 1285 | 1 | 1291 | 0.01 | 0 |
| hsa_circ_0070362 | 90 | 1 | 1224 | 1 | 1224 | 0.01 | 0 |
| hsa_circ_0070362 | 89 | 1 | 974 | 1 | 974 | 0.02 | 0 |
| hsa_circ_0070360 | 89 | 1 | 1062 | 1 | 1062 | 0.01 | 0 |
| hsa_circ_0070362 | 88 | 1 | 567 | 1 | 561 | 0.01 | 0 |
| hsa_circ_0070360 | 89 | 1 | 655 | 1 | 649 | 0.01 | 0 |
| hsa_circ_0070356 | 91 | 121 | 1206 | 1 | 1080 | 0.01 | 0 |
| hsa_circ_0070352 | 95 | 1 | 639 | 1 | 639 | 0 | 0 |
| hsa_circ_0070352 | 95 | 1 | 551 | 1 | 551 | 0 | 0 |
| hsa_circ_0070352 | 97 | 1 | 299 | 1 | 299 | 0 | 5.00E-143 |

novel_circ_004682 novel_circ_004683 novel_circ_004684 novel_circ_004685 novel_circ_004686 novel_circ_004687 novel_circ_004688 novel_circ_004689 novel_circ_004690 novel_circ_004691 novel_circ_004692 novel_circ_004693 novel_circ_004694 novel_circ_004695 novel_circ_004696 novel_circ_004697 novel_circ_004698 novel_circ_004699 novel_circ_004700 novel_circ_004701 novel_circ_004702 novel_circ_004703 novel_circ_004704 novel_circ_004705 novel_circ_004706 novel_circ_004707 novel_circ_004708 novel_circ_004709 novel_circ_004710 novel_circ_004711 novel_circ_004712 novel_circ_004713 novel_circ_004714 novel_circ_004715 novel_circ_004716 novel_circ_004717 novel_circ_004718 novel_circ_004719 novel_circ_004720 novel_circ_004721 novel_circ_004722 novel_circ_004723 novel_circ_004724 novel_circ_004725 novel_circ_004726 novel_circ_004727 novel_circ_004728 novel_circ_004729 novel_circ_004730 novel_circ_004731 novel_circ_004732 novel_circ_004733 novel_circ_004734 novel_circ_004735

| hsa_circ_0070350 | 94 | 243 | 570 | 874 | 547 | 0 | 2.00E-140 |
| --- | --- | --- | --- | --- | --- | --- | --- |
| hsa_circ_0070350 | 93 | 1 | 364 | 744 | 381 | 0 | 2.00E-152 |
| hsa_circ_0070350 | 90 | 1 | 421 | 546 | 126 | 0 | 1.00E-155 |
| hsa_circ_0070350 | 91 | 1 | 546 | 546 | 1 | 0 | 0 |
| hsa_circ_0070350 | 93 | 12593 | 12919 | 327 | 1 | 0 | 2.00E-133 |
| hsa_circ_0070350 | 93 | 12593 | 12919 | 327 | 1 | 0 | 2.00E-133 |
| hsa_circ_0002221 | 91 | 8 | 94 | 187 | 101 | 0 | 2.00E-25 |
| hsa_circ_0008165 | 93 | 164 | 359 | 196 | 1 | 0 | 3.00E-77 |
| hsa_circ_0070344 | 95 | 16 | 607 | 1709 | 1118 | 0 | 0 |
| hsa_circ_0070344 | 94 | 16 | 1154 | 1709 | 571 | 0 | 0 |
| hsa_circ_0070342 | 94 | 16 | 1918 | 3183 | 1281 | 0 | 0 |
| hsa_circ_0070344 | 94 | 1 | 819 | 1389 | 571 | 0 | 0 |
| hsa_circ_0070344 | 94 | 1 | 709 | 1279 | 571 | 0 | 0 |
| hsa_circ_0070344 | 93 | 1 | 547 | 1117 | 571 | 0 | 0 |
| hsa_circ_0070344 | 94 | 1 | 1117 | 1117 | 1 | 0 | 0 |
| hsa_circ_0070342 | 93 | 1 | 1311 | 2591 | 1281 | 0 | 0 |
| hsa_circ_0070344 | 95 | 3 | 924 | 922 | 1 | 0 | 0 |
| hsa_circ_0070342 | 94 | 3 | 1837 | 2396 | 562 | 0 | 0 |
| hsa_circ_0070342 | 94 | 3 | 2048 | 2396 | 351 | 0 | 0 |
| hsa_circ_0070342 | 94 | 3 | 2166 | 2396 | 233 | 0 | 0 |
| hsa_circ_0070333 | 93 | 3 | 3749 | 4078 | 332 | 0 | 0 |
| hsa_circ_0070344 | 95 | 2 | 635 | 736 | 103 | 0 | 0 |
| hsa_circ_0070344 | 95 | 2 | 737 | 736 | 1 | 0 | 0 |
| hsa_circ_0070342 | 94 | 2 | 931 | 2210 | 1281 | 0 | 0 |
| hsa_circ_0070342 | 94 | 2 | 1650 | 2210 | 562 | 0 | 0 |
| hsa_circ_0070344 | 95 | 1 | 570 | 570 | 1 | 0 | 0 |
| hsa_circ_0070342 | 94 | 1 | 1483 | 2044 | 562 | 0 | 0 |
| hsa_circ_0070342 | 92 | 1 | 296 | 1576 | 1281 | 0 | 6.00E-113 |
| hsa_circ_0070342 | 93 | 1 | 913 | 1474 | 562 | 0 | 0 |
| hsa_circ_0070342 | 94 | 1 | 719 | 1280 | 562 | 0 | 0 |
| hsa_circ_0070318 | 92 | 1 | 5334 | 5334 | 1 | 0 | 0 |
| hsa_circ_0070342 | 95 | 1 | 177 | 738 | 562 | 0 | 2.00E-75 |
| hsa_circ_0070342 | 94 | 1 | 388 | 738 | 351 | 0 | 1.00E-165 |
| hsa_circ_0070342 | 94 | 1 | 506 | 738 | 233 | 0 | 0 |
| hsa_circ_0070342 | 94 | 1 | 738 | 738 | 1 | 0 | 0 |
| hsa_circ_0070340 | 92 | 1 | 1056 | 1633 | 578 | 0 | 0 |
| hsa_circ_0070340 | 92 | 1 | 1240 | 1633 | 394 | 0 | 0 |
| hsa_circ_0070340 | 92 | 1 | 1633 | 1633 | 1 | 0 | 0 |
| hsa_circ_0070338 | 92 | 1 | 1743 | 1745 | 3 | 0 | 0 |
| hsa_circ_0070334 | 93 | 1 | 2089 | 2325 | 237 | 0 | 0 |
| hsa_circ_0070329 | 93 | 1 | 2759 | 2995 | 237 | 0 | 0 |
| hsa_circ_0070340 | 92 | 1 | 878 | 1455 | 578 | 0 | 0 |
| hsa_circ_0070334 | 92 | 1 | 1911 | 2147 | 237 | 0 | 0 |
| hsa_circ_0070340 | 92 | 1 | 663 | 1240 | 578 | 0 | 0 |
| hsa_circ_0070340 | 91 | 1 | 550 | 1127 | 578 | 0 | 0 |
| hsa_circ_0070340 | 88 | 8 | 318 | 888 | 578 | 0 | 6.00E-103 |
| hsa_circ_0070340 | 89 | 8 | 502 | 888 | 394 | 0 | 3.00E-178 |
| hsa_circ_0070340 | 90 | 8 | 734 | 888 | 162 | 0 | 0 |
| hsa_circ_0070340 | 90 | 8 | 895 | 888 | 1 | 0 | 0 |
| hsa_circ_0070338 | 90 | 8 | 1005 | 1000 | 3 | 0 | 0 |
| hsa_circ_0070334 | 92 | 8 | 1351 | 1580 | 237 | 0 | 0 |
| hsa_circ_0070340 | 91 | 1 | 401 | 794 | 394 | 0 | 7.00E-153 |
| hsa_circ_0070336 | 92 | 1 | 1097 | 1097 | 1 | 0 | 0 |
| hsa_circ_0070334 | 93 | 1 | 1031 | 1267 | 237 | 0 | 0 |

novel_circ_004736 novel_circ_004737 novel_circ_004738 novel_circ_004739 novel_circ_004740 novel_circ_004741 novel_circ_004742 novel_circ_004743 novel_circ_004744 novel_circ_004745 novel_circ_004746 novel_circ_004747 novel_circ_004748 novel_circ_004753 novel_circ_004754 novel_circ_004755 novel_circ_004756 novel_circ_004757 novel_circ_004758 novel_circ_004759 novel_circ_004760 novel_circ_004761 novel_circ_004762 novel_circ_004763 novel_circ_004764 novel_circ_004765 novel_circ_004766 novel_circ_004767 novel_circ_004768 novel_circ_004769 novel_circ_004770 novel_circ_004771 novel_circ_004772 novel_circ_004773 novel_circ_004777 novel_circ_004778 novel_circ_004779 novel_circ_004780 novel_circ_004781 novel_circ_004782 novel_circ_004783 novel_circ_004784 novel_circ_004785 novel_circ_004786 novel_circ_004787 novel_circ_004788 novel_circ_004789 novel_circ_004790 novel_circ_004791 novel_circ_004792 novel_circ_004793 novel_circ_004794 novel_circ_004796 novel_circ_004804

| hsa_circ_0070340 | 91 | 1 | 393 | 393 | 1 | 0 | 9.00E-152 |
| --- | --- | --- | --- | --- | --- | --- | --- |
| hsa_circ_0070331 | 93 | 1 | 670 | 670 | 1 | 0 | 0 |
| hsa_circ_0070328 | 93 | 3 | 1124 | 1122 | 1 | 0 | 0 |
| hsa_circ_0070326 | 92 | 1 | 1040 | 1150 | 111 | 0 | 0 |
| hsa_circ_0070327 | 94 | 1 | 675 | 675 | 1 | 0 | 0 |
| hsa_circ_0070326 | 93 | 1 | 725 | 835 | 111 | 0 | 0 |
| hsa_circ_0070320 | 92 | 1 | 1254 | 1254 | 1 | 0 | 0 |
| hsa_circ_0070318 | 92 | 1 | 1482 | 1482 | 1 | 0 | 0 |
| hsa_circ_0070310 | 94 | 1 | 437 | 492 | 56 | 0 | 0 |
| hsa_circ_0070299 | 94 | 10 | 379 | 370 | 1 | 0 | 1.00E-160 |
| hsa_circ_0070293 | 95 | 10 | 684 | 675 | 1 | 0 | 0 |
| hsa_circ_0070288 | 93 | 10 | 1326 | 1597 | 281 | 0 | 0 |
| hsa_circ_0070288 | 91 | 1 | 642 | 922 | 281 | 0 | 0 |
| hsa_circ_0058474 | 91 | 1 | 469 | 249 | 717 | 0 | 3.00E-177 |
| hsa_circ_0058474 | 90 | 1 | 645 | 249 | 893 | 0 | 0 |
| hsa_circ_0058478 | 98 | 546 | 1018 | 4371 | 3899 | 0 | 0 |
| hsa_circ_0058478 | 95 | 1 | 322 | 4693 | 4372 | 0 | 5.00E-144 |
| hsa_circ_0058478 | 96 | 1 | 544 | 4915 | 4372 | 0 | 0 |
| hsa_circ_0058478 | 96 | 1 | 823 | 5194 | 4372 | 0 | 0 |
| hsa_circ_0058478 | 96 | 1 | 1651 | 6022 | 4372 | 0 | 0 |
| hsa_circ_0058478 | 96 | 115 | 1765 | 6022 | 4372 | 0 | 0 |
| hsa_circ_0058480 | 97 | 1 | 357 | 357 | 1 | 0 | 2.00E-168 |
| hsa_circ_0058480 | 96 | 3 | 959 | 957 | 1 | 0 | 0 |
| hsa_circ_0058480 | 96 | 1 | 1464 | 1464 | 1 | 0 | 0 |
| hsa_circ_0058480 | 96 | 115 | 1578 | 1464 | 1 | 0 | 0 |
| hsa_circ_0058481 | 96 | 3 | 824 | 822 | 1 | 0 | 0 |
| hsa_circ_0058481 | 97 | 1 | 278 | 501 | 224 | 0 | 2.00E-131 |
| hsa_circ_0058481 | 96 | 3 | 601 | 822 | 224 | 0 | 0 |
| hsa_circ_0058481 | 96 | 1 | 830 | 1053 | 224 | 0 | 0 |
| hsa_circ_0058481 | 96 | 1 | 1106 | 1329 | 224 | 0 | 0 |
| hsa_circ_0058482 | 95 | 3 | 323 | 321 | 1 | 0 | 8.00E-142 |
| hsa_circ_0058482 | 95 | 1 | 552 | 552 | 1 | 0 | 0 |
| hsa_circ_0058482 | 95 | 1 | 505 | 828 | 324 | 0 | 0 |
| hsa_circ_0003591 | 99 | 1 | 199 | 312 | 114 | 0 | 7.00E-99 |
| hsa_circ_0058487 | 95 | 1 | 566 | 942 | 377 | 0 | 0 |
| hsa_circ_0058491 | 91 | 1 | 1679 | 1679 | 1 | 0 | 0 |
| hsa_circ_0058487 | 91 | 4 | 748 | 1980 | 1236 | 0 | 0 |
| hsa_circ_0058491 | 91 | 3 | 1379 | 1474 | 98 | 0 | 0 |
| hsa_circ_0058491 | 90 | 4 | 522 | 700 | 182 | 0 | 0 |
| hsa_circ_0058491 | 91 | 3 | 1295 | 1474 | 182 | 0 | 0 |
| hsa_circ_0058491 | 91 | 1 | 1498 | 1679 | 182 | 0 | 0 |
| hsa_circ_0058491 | 91 | 1 | 585 | 1288 | 704 | 0 | 0 |
| hsa_circ_0058491 | 92 | 3 | 773 | 1474 | 704 | 0 | 0 |
| hsa_circ_0058491 | 91 | 1 | 492 | 1288 | 797 | 0 | 0 |
| hsa_circ_0058491 | 91 | 3 | 680 | 1474 | 797 | 0 | 0 |
| hsa_circ_0058491 | 92 | 1 | 883 | 1679 | 797 | 0 | 0 |
| hsa_circ_0058491 | 92 | 1 | 391 | 1679 | 1289 | 0 | 1.00E-155 |
| hsa_circ_0058491 | 91 | 1 | 1049 | 2337 | 1289 | 0 | 0 |
| hsa_circ_0058491 | 91 | 1 | 1059 | 2347 | 1289 | 0 | 0 |
| hsa_circ_0058491 | 91 | 1 | 1145 | 2433 | 1289 | 0 | 0 |
| hsa_circ_0058491 | 91 | 1 | 791 | 2347 | 1557 | 0 | 0 |
| hsa_circ_0058491 | 90 | 1 | 877 | 2433 | 1557 | 0 | 0 |
| hsa_circ_0087123 | 84 | 3796 | 3884 | 4445 | 4357 | 0 | 5.00E-14 |
| hsa_circ_0071873 | 96 | 305 | 478 | 748 | 575 | 0 | 9.00E-75 |

novel_circ_004805 novel_circ_004806 novel_circ_004807 novel_circ_004808 novel_circ_004811 novel_circ_004812 novel_circ_004813 novel_circ_004814 novel_circ_004816 novel_circ_004817 novel_circ_004818 novel_circ_004819 novel_circ_004820 novel_circ_004821 novel_circ_004822 novel_circ_004823 novel_circ_004824 novel_circ_004825 novel_circ_004826 novel_circ_004827 novel_circ_004828 novel_circ_004830 novel_circ_004831 novel_circ_004832 novel_circ_004837 novel_circ_004838 novel_circ_004839 novel_circ_004844 novel_circ_004845 novel_circ_004846 novel_circ_004847 novel_circ_004848 novel_circ_004849 novel_circ_004851 novel_circ_004852 novel_circ_004853 novel_circ_004854 novel_circ_004855 novel_circ_004856 novel_circ_004857 novel_circ_004858 novel_circ_004859 novel_circ_004860 novel_circ_004861 novel_circ_004862 novel_circ_004863 novel_circ_004864 novel_circ_004865 novel_circ_004866 novel_circ_004867 novel_circ_004868 novel_circ_004869 novel_circ_004870 novel_circ_004871

| hsa_circ_0071873 | 96 | 187 | 360 | 748 | 575 | 0 | 7.00E-75 |
| --- | --- | --- | --- | --- | --- | --- | --- |
| hsa_circ_0071873 | 96 | 1 | 174 | 748 | 575 | 0 | 5.00E-75 |
| hsa_circ_0071873 | 95 | 74 | 224 | 576 | 426 | 0 | 2.00E-62 |
| hsa_circ_0071873 | 94 | 1 | 127 | 127 | 1 | 0 | 2.00E-47 |
| hsa_circ_0071868 | 93 | 1 | 359 | 318 | 676 | 0 | 1.00E-149 |
| hsa_circ_0071868 | 93 | 1 | 494 | 183 | 676 | 0 | 0 |
| hsa_circ_0071868 | 93 | 1 | 676 | 1 | 676 | 0 | 0 |
| hsa_circ_0071864 | 94 | 1 | 746 | 206 | 951 | 0 | 0 |
| hsa_circ_0071867 | 94 | 1 | 610 | 1 | 610 | 0 | 0 |
| hsa_circ_0071868 | 94 | 1 | 317 | 1 | 317 | 0 | 6.00E-133 |
| hsa_circ_0071867 | 94 | 1 | 387 | 1 | 387 | 0 | 9.00E-167 |
| hsa_circ_0071867 | 95 | 1 | 252 | 1 | 252 | 0 | 3.00E-110 |
| hsa_circ_0071862 | 93 | 1 | 595 | 102 | 696 | 0 | 0 |
| hsa_circ_0071857 | 93 | 1 | 1026 | 1 | 1026 | 0 | 0 |
| hsa_circ_0071841 | 92 | 1 | 1552 | 387 | 1958 | 0.01 | 0 |
| hsa_circ_0071857 | 92 | 1 | 774 | 1 | 774 | 0 | 0 |
| hsa_circ_0071857 | 93 | 1 | 431 | 1 | 431 | 0 | 8.00E-178 |
| hsa_circ_0071857 | 92 | 1 | 330 | 1 | 330 | 0 | 4.00E-130 |
| hsa_circ_0071847 | 94 | 43 | 546 | 1 | 504 | 0 | 0 |
| hsa_circ_0071847 | 95 | 43 | 408 | 1 | 366 | 0 | 4.00E-165 |
| hsa_circ_0071845 | 91 | 1 | 598 | 170 | 787 | 0.03 | 0 |
| hsa_circ_0071825 | 88 | 1145 | 1334 | 3483 | 3667 | 0.05 | 2.00E-53 |
| hsa_circ_0050564 | 77 | 5216 | 5431 | 58608 | 58819 | 0.05 | 3.00E-24 |
| hsa_circ_0007133 | 76 | 21632 | 21875 | 393368 | 393124 | 0.02 | 2.00E-23 |
| hsa_circ_0071820 | 88 | 1 | 301 | 301 | 1 | 0.01 | 5.00E-99 |
| hsa_circ_0071819 | 90 | 1 | 347 | 347 | 1 | 0.01 | 7.00E-123 |
| hsa_circ_0006634 | 88 | 1 | 376 | 376 | 1 | 0 | 3.00E-122 |
| hsa_circ_0071785 | 92 | 1 | 844 | 390 | 1233 | 0 | 0 |
| hsa_circ_0071774 | 89 | 7289 | 7487 | 277 | 475 | 0 | 3.00E-64 |
| hsa_circ_0008903 | 94 | 1 | 741 | 1 | 741 | 0 | 0 |
| hsa_circ_0008903 | 92 | 1 | 292 | 1 | 292 | 0 | 4.00E-114 |
| hsa_circ_0071766 | 92 | 1 | 211 | 283 | 73 | 0 | 3.00E-79 |
| hsa_circ_0071758 | 87 | 10 | 556 | 547 | 1 | 0 | 4.00E-172 |
| hsa_circ_0077110 | 89 | 223 | 485 | 263 | 1 | 0 | 3.00E-89 |
| hsa_circ_0077110 | 96 | 1 | 317 | 577 | 261 | 0 | 3.00E-144 |
| hsa_circ_0077110 | 95 | 1 | 714 | 974 | 261 | 0 | 0 |
| hsa_circ_0077110 | 95 | 1 | 1601 | 1861 | 261 | 0 | 0 |
| hsa_circ_0077110 | 89 | 223 | 485 | 263 | 1 | 0 | 2.00E-89 |
| hsa_circ_0077110 | 96 | 1 | 317 | 577 | 261 | 0 | 3.00E-144 |
| hsa_circ_0077110 | 95 | 1 | 714 | 974 | 261 | 0 | 0 |
| hsa_circ_0077110 | 95 | 1 | 991 | 1251 | 261 | 0 | 0 |
| hsa_circ_0077116 | 95 | 1 | 397 | 397 | 1 | 0 | 3.00E-177 |
| hsa_circ_0077116 | 95 | 1 | 674 | 674 | 1 | 0 | 0 |
| hsa_circ_0077116 | 94 | 1 | 848 | 848 | 1 | 0 | 0 |
| hsa_circ_0077116 | 94 | 1 | 1284 | 1284 | 1 | 0 | 0 |
| hsa_circ_0077118 | 95 | 1 | 196 | 196 | 1 | 0 | 3.00E-84 |
| hsa_circ_0077120 | 94 | 1 | 403 | 403 | 1 | 0 | 1.00E-175 |
| hsa_circ_0077121 | 94 | 1 | 277 | 277 | 1 | 0 | 9.00E-116 |
| hsa_circ_0077121 | 93 | 1 | 451 | 451 | 1 | 0 | 0 |
| hsa_circ_0077121 | 95 | 1 | 2659 | 2662 | 1 | 0 | 0 |
| hsa_circ_0077123 | 93 | 1 | 174 | 174 | 1 | 0 | 2.00E-65 |
| hsa_circ_0077125 | 95 | 1 | 208 | 208 | 1 | 0 | 3.00E-89 |
| hsa_circ_0077125 | 95 | 1 | 436 | 436 | 1 | 0 | 0 |
| hsa_circ_0077128 | 95 | 1 | 2327 | 2558 | 229 | 0 | 0 |

novel_circ_004872 novel_circ_004873 novel_circ_004874 novel_circ_004875 novel_circ_004876 novel_circ_004877 novel_circ_004878 novel_circ_004879 novel_circ_004880 novel_circ_004881 novel_circ_004882 novel_circ_004883 novel_circ_004884 novel_circ_004885 novel_circ_004886 novel_circ_004887 novel_circ_004888 novel_circ_004889 novel_circ_004890 novel_circ_004891 novel_circ_004892 novel_circ_004893 novel_circ_004894 novel_circ_004895 novel_circ_004896 novel_circ_004897 novel_circ_004898 novel_circ_004899 novel_circ_004901 novel_circ_004902 novel_circ_004904 novel_circ_004906 novel_circ_004907 novel_circ_004908 novel_circ_004909 novel_circ_004910 novel_circ_004911 novel_circ_004912 novel_circ_004916 novel_circ_004917 novel_circ_004920 novel_circ_004921 novel_circ_004922 novel_circ_004923 novel_circ_004924 novel_circ_004925 novel_circ_004926 novel_circ_004927 novel_circ_004928 novel_circ_004929 novel_circ_004930 novel_circ_004931 novel_circ_004933 novel_circ_004934

| hsa_circ_0077131 | 95 | 1 | 1860 | 1860 | 1 | 0 | 0 |
| --- | --- | --- | --- | --- | --- | --- | --- |
| hsa_circ_0077131 | 95 | 1 | 2021 | 2021 | 1 | 0 | 0 |
| hsa_circ_0077131 | 95 | 1 | 2271 | 2271 | 1 | 0 | 0 |
| hsa_circ_0077132 | 96 | 1 | 1207 | 1325 | 119 | 0 | 0 |
| hsa_circ_0077132 | 96 | 1 | 1601 | 1719 | 119 | 0 | 0 |
| hsa_circ_0077139 | 97 | 586 | 941 | 356 | 1 | 0 | 2.00E-172 |
| hsa_circ_0077139 | 96 | 1 | 743 | 743 | 1 | 0 | 0 |
| hsa_circ_0077139 | 96 | 1 | 885 | 885 | 1 | 0 | 0 |
| hsa_circ_0077139 | 96 | 1 | 1279 | 1279 | 1 | 0 | 0 |
| hsa_circ_0077139 | 96 | 1 | 1440 | 1440 | 1 | 0 | 0 |
| hsa_circ_0077139 | 96 | 1 | 1690 | 1690 | 1 | 0 | 0 |
| hsa_circ_0077139 | 96 | 1 | 517 | 743 | 227 | 0 | 0 |
| hsa_circ_0077139 | 96 | 1 | 1214 | 1440 | 227 | 0 | 0 |
| hsa_circ_0077144 | 95 | 1 | 289 | 289 | 1 | 0 | 2.00E-127 |
| hsa_circ_0077144 | 96 | 1 | 924 | 924 | 1 | 0 | 0 |
| hsa_circ_0077144 | 96 | 1 | 1085 | 1085 | 1 | 0 | 0 |
| hsa_circ_0077144 | 96 | 1 | 1335 | 1335 | 1 | 0 | 0 |
| hsa_circ_0077145 | 96 | 1 | 142 | 395 | 254 | 0 | 4.00E-61 |
| hsa_circ_0077146 | 96 | 1 | 555 | 656 | 102 | 0 | 0 |
| hsa_circ_0077146 | 96 | 1 | 805 | 906 | 102 | 0 | 0 |
| hsa_circ_0077148 | 96 | 1 | 484 | 484 | 1 | 0 | 0 |
| hsa_circ_0077150 | 96 | 1 | 383 | 383 | 1 | 0 | 1.00E-174 |
| hsa_circ_0077150 | 95 | 1 | 633 | 633 | 1 | 0 | 0 |
| hsa_circ_0077151 | 96 | 1 | 411 | 411 | 1 | 0 | 0 |
| hsa_circ_0003810 | 94 | 1 | 250 | 250 | 1 | 0 | 8.00E-106 |
| hsa_circ_0003163 | 93 | 16530 | 16588 | 374339 | 374281 | 0 | 3.00E-14 |
| hsa_circ_0077154 | 96 | 393 | 916 | 524 | 1 | 0 | 0 |
| hsa_circ_0077154 | 96 | 2692 | 3215 | 524 | 1 | 0 | 0 |
| hsa_circ_0077154 | 96 | 393 | 916 | 524 | 1 | 0 | 0 |
| hsa_circ_0077154 | 96 | 2692 | 3215 | 524 | 1 | 0 | 0 |
| hsa_circ_0077154 | 96 | 2692 | 3215 | 524 | 1 | 0 | 0 |
| hsa_circ_0077154 | 96 | 393 | 916 | 524 | 1 | 0 | 0 |
| hsa_circ_0077154 | 96 | 2692 | 3215 | 524 | 1 | 0 | 0 |
| hsa_circ_0077154 | 96 | 7 | 530 | 524 | 1 | 0 | 0 |
| hsa_circ_0077154 | 96 | 393 | 916 | 524 | 1 | 0 | 0 |
| hsa_circ_0077154 | 96 | 2692 | 3215 | 524 | 1 | 0 | 0 |
| hsa_circ_0077155 | 88 | 2 | 617 | 226 | 838 | 0.02 | 0 |
| hsa_circ_0077155 | 88 | 2 | 619 | 226 | 840 | 0.02 | 0 |
| hsa_circ_0077165 | 96 | 1 | 399 | 1 | 399 | 0 | 0 |
| hsa_circ_0077165 | 93 | 1 | 608 | 1 | 608 | 0 | 0 |
| hsa_circ_0077173 | 89 | 3 | 543 | 2072 | 1532 | 0 | 0 |
| hsa_circ_0077173 | 91 | 1 | 930 | 2458 | 1532 | 0 | 0 |
| hsa_circ_0077173 | 91 | 1 | 1109 | 2637 | 1532 | 0 | 0 |
| hsa_circ_0077173 | 92 | 2 | 1308 | 2835 | 1532 | 0 | 0 |
| hsa_circ_0077173 | 92 | 8 | 2593 | 4117 | 1532 | 0 | 0 |
| hsa_circ_0077174 | 90 | 3 | 401 | 399 | 1 | 0 | 4.00E-145 |
| hsa_circ_0077174 | 92 | 1 | 788 | 785 | 1 | 0 | 0 |
| hsa_circ_0077174 | 92 | 1 | 967 | 964 | 1 | 0 | 0 |
| hsa_circ_0077174 | 92 | 1 | 1069 | 1066 | 1 | 0 | 0 |
| hsa_circ_0077173 | 93 | 2 | 1166 | 2835 | 1674 | 0 | 0 |
| hsa_circ_0077173 | 93 | 1 | 1287 | 2957 | 1674 | 0 | 0 |
| hsa_circ_0077175 | 89 | 3 | 257 | 255 | 1 | 0 | 3.00E-85 |
| hsa_circ_0077175 | 92 | 1 | 730 | 820 | 94 | 0 | 0 |
| hsa_circ_0077175 | 94 | 1 | 387 | 641 | 258 | 0.01 | 5.00E-164 |

novel_circ_004935 novel_circ_004936 novel_circ_004937 novel_circ_004938 novel_circ_004939 novel_circ_004940 novel_circ_004941 novel_circ_004942 novel_circ_004943 novel_circ_004944 novel_circ_004945 novel_circ_004946 novel_circ_004947 novel_circ_004948 novel_circ_004949 novel_circ_004950 novel_circ_004951 novel_circ_004952 novel_circ_004953 novel_circ_004954 novel_circ_004955 novel_circ_004956 novel_circ_004957 novel_circ_004958 novel_circ_004959 novel_circ_004960 novel_circ_004961 novel_circ_004962 novel_circ_004963 novel_circ_004964 novel_circ_004965 novel_circ_004966 novel_circ_004967 novel_circ_004968 novel_circ_004969 novel_circ_004970 novel_circ_004971 novel_circ_004972 novel_circ_004973 novel_circ_004974 novel_circ_004975 novel_circ_004976 novel_circ_004977 novel_circ_004978 novel_circ_004979 novel_circ_004980 novel_circ_004981 novel_circ_004982 novel_circ_004983 novel_circ_005002 novel_circ_005003 novel_circ_005004 novel_circ_005005 novel_circ_005006

| hsa_circ_0077175 | 94 | 2 | 765 | 1018 | 258 | 0 | 0 |
| --- | --- | --- | --- | --- | --- | --- | --- |
| hsa_circ_0077177 | 92 | 1 | 1539 | 1542 | 1 | 0 | 0 |
| hsa_circ_0077177 | 95 | 2 | 317 | 377 | 62 | 0 | 5.00E-139 |
| hsa_circ_0077177 | 93 | 1 | 1478 | 1542 | 62 | 0 | 0 |
| hsa_circ_0077177 | 92 | 1 | 1161 | 1542 | 379 | 0 | 0 |
| hsa_circ_0077180 | 92 | 1 | 1870 | 1956 | 84 | 0 | 0 |
| hsa_circ_0077180 | 93 | 4 | 705 | 1828 | 1127 | 0 | 0 |
| hsa_circ_0077180 | 93 | 1 | 830 | 1956 | 1127 | 0 | 0 |
| hsa_circ_0077180 | 93 | 98 | 927 | 1956 | 1127 | 0 | 0 |
| hsa_circ_0077180 | 93 | 810 | 1639 | 1956 | 1127 | 0 | 0 |
| hsa_circ_0077181 | 93 | 4 | 581 | 578 | 1 | 0 | 0 |
| hsa_circ_0077181 | 93 | 1 | 803 | 803 | 1 | 0 | 0 |
| hsa_circ_0077181 | 93 | 713 | 1515 | 803 | 1 | 0 | 0 |
| hsa_circ_0077185 | 89 | 1 | 809 | 775 | 1 | 0.05 | 0 |
| hsa_circ_0077187 | 86 | 1 | 1072 | 1072 | 1 | 0 | 0 |
| hsa_circ_0077187 | 87 | 1 | 784 | 784 | 1 | 0 | 0 |
| hsa_circ_0077187 | 86 | 1 | 1072 | 1072 | 1 | 0 | 0 |
| hsa_circ_0004908 | 85 | 7 | 273 | 267 | 1 | 0 | 3.00E-70 |
| hsa_circ_0004908 | 86 | 1 | 343 | 343 | 1 | 0 | 5.00E-99 |
| hsa_circ_0004908 | 88 | 1 | 645 | 645 | 1 | 0 | 0 |
| hsa_circ_0077187 | 88 | 3050 | 3696 | 786 | 140 | 0 | 0 |
| hsa_circ_0077187 | 87 | 1 | 736 | 875 | 140 | 0 | 0 |
| hsa_circ_0077187 | 87 | 1 | 933 | 1072 | 140 | 0 | 0 |
| hsa_circ_0004908 | 88 | 1 | 481 | 645 | 165 | 0 | 1.00E-160 |
| hsa_circ_0077187 | 86 | 1 | 769 | 1072 | 304 | 0 | 0 |
| hsa_circ_0077187 | 87 | 1 | 518 | 1072 | 555 | 0 | 4.00E-161 |
| hsa_circ_0077190 | 93 | 1 | 734 | 94 | 827 | 0 | 0 |
| hsa_circ_0077190 | 93 | 1 | 734 | 94 | 827 | 0 | 0 |
| hsa_circ_0077190 | 93 | 1 | 734 | 94 | 827 | 0 | 0 |
| hsa_circ_0077197 | 93 | 933 | 1745 | 127 | 939 | 0 | 0 |
| hsa_circ_0077197 | 93 | 100 | 912 | 127 | 939 | 0 | 0 |
| hsa_circ_0077197 | 93 | 3 | 620 | 322 | 939 | 0 | 0 |
| hsa_circ_0077197 | 92 | 2 | 1121 | 442 | 1561 | 0 | 0 |
| hsa_circ_0077203 | 92 | 1736 | 3705 | 830 | 2799 | 0 | 0 |
| hsa_circ_0077202 | 94 | 1 | 786 | 857 | 1642 | 0 | 0 |
| hsa_circ_0077203 | 92 | 325 | 2294 | 830 | 2799 | 0 | 0 |
| hsa_circ_0077202 | 96 | 1 | 1326 | 3931 | 5256 | 0 | 0 |
| hsa_circ_0077202 | 96 | 1 | 1544 | 3931 | 5474 | 0 | 0 |
| hsa_circ_0077202 | 96 | 1 | 1677 | 3931 | 5607 | 0 | 0 |
| hsa_circ_0077202 | 96 | 1 | 830 | 4645 | 5474 | 0 | 0 |
| hsa_circ_0077202 | 96 | 1 | 720 | 4755 | 5474 | 0 | 0 |
| hsa_circ_0077202 | 96 | 1 | 853 | 4755 | 5607 | 0 | 0 |
| hsa_circ_0077205 | 96 | 1 | 290 | 1 | 290 | 0 | 3.00E-131 |
| hsa_circ_0077202 | 96 | 1 | 351 | 5257 | 5607 | 0 | 2.00E-163 |
| hsa_circ_0077212 | 94 | 1 | 664 | 1087 | 424 | 0 | 0 |
| hsa_circ_0077214 | 94 | 1 | 452 | 452 | 1 | 0 | 0 |
| hsa_circ_0077216 | 92 | 1 | 522 | 522 | 1 | 0 | 0 |
| hsa_circ_0077219 | 92 | 1 | 284 | 284 | 1 | 0 | 1.00E-109 |
| hsa_circ_0077221 | 94 | 1 | 285 | 285 | 1 | 0 | 3.00E-120 |
| hsa_circ_0077223 | 84 | 395 | 933 | 548 | 1 | 0.02 | 2.00E-146 |
| hsa_circ_0077224 | 83 | 7 | 283 | 286 | 1 | 0.04 | 6.00E-63 |
| hsa_circ_0077225 | 86 | 1 | 388 | 391 | 1 | 0.01 | 1.00E-112 |
| hsa_circ_0077232 | 89 | 1 | 810 | 190 | 999 | 0 | 0 |
| hsa_circ_0077233 | 94 | 1 | 534 | 534 | 1 | 0 | 0 |

novel_circ_005007 novel_circ_005008 novel_circ_005009 novel_circ_005010 novel_circ_005011 novel_circ_005012 novel_circ_005013 novel_circ_005014 novel_circ_005015 novel_circ_005016 novel_circ_005017 novel_circ_005018 novel_circ_005019 novel_circ_005020 novel_circ_005021 novel_circ_005022 novel_circ_005023 novel_circ_005024 novel_circ_005025 novel_circ_005026 novel_circ_005027 novel_circ_005028 novel_circ_005029 novel_circ_005030 novel_circ_005031 novel_circ_005032 novel_circ_005033 novel_circ_005034 novel_circ_005035 novel_circ_005036 novel_circ_005037 novel_circ_005038 novel_circ_005039 novel_circ_005040 novel_circ_005041 novel_circ_005042 novel_circ_005043 novel_circ_005044 novel_circ_005045 novel_circ_005046 novel_circ_005047 novel_circ_005048 novel_circ_005049 novel_circ_005050 novel_circ_005051 novel_circ_005052 novel_circ_005053 novel_circ_005054 novel_circ_005055 novel_circ_005056 novel_circ_005062 novel_circ_005063 novel_circ_005065 novel_circ_005066

| hsa_circ_0077233 | 95 | 1 | 1194 | 1194 | 1 | 0 | 0 |
| --- | --- | --- | --- | --- | --- | --- | --- |
| hsa_circ_0077235 | 95 | 1 | 2417 | 2417 | 1 | 0 | 0 |
| hsa_circ_0077233 | 94 | 1 | 477 | 534 | 58 | 0 | 0 |
| hsa_circ_0077233 | 94 | 3 | 638 | 693 | 58 | 0 | 0 |
| hsa_circ_0077233 | 95 | 1 | 1137 | 1194 | 58 | 0 | 0 |
| hsa_circ_0077233 | 95 | 4 | 1270 | 1324 | 58 | 0 | 0 |
| hsa_circ_0077235 | 95 | 1 | 1449 | 1449 | 1 | 0 | 0 |
| hsa_circ_0077234 | 96 | 1 | 385 | 385 | 1 | 0 | 1.00E-175 |
| hsa_circ_0077235 | 95 | 1 | 1449 | 1449 | 1 | 0 | 0 |
| hsa_circ_0077235 | 95 | 1 | 2417 | 2417 | 1 | 0 | 0 |
| hsa_circ_0077235 | 95 | 1 | 949 | 949 | 1 | 0 | 0 |
| hsa_circ_0077235 | 95 | 1 | 1690 | 1690 | 1 | 0 | 0 |
| hsa_circ_0077235 | 96 | 1 | 782 | 949 | 168 | 0 | 0 |
| hsa_circ_0077235 | 96 | 4 | 915 | 1079 | 168 | 0 | 0 |
| hsa_circ_0077235 | 95 | 1 | 1282 | 1449 | 168 | 0 | 0 |
| hsa_circ_0077237 | 96 | 1 | 660 | 660 | 1 | 0 | 0 |
| hsa_circ_0077237 | 96 | 4 | 793 | 790 | 1 | 0 | 0 |
| hsa_circ_0077235 | 95 | 1 | 2128 | 2417 | 290 | 0 | 0 |
| hsa_circ_0001616 | 95 | 6 | 213 | 208 | 1 | 0 | 3.00E-89 |
| hsa_circ_0001616 | 94 | 1 | 297 | 297 | 1 | 0 | 2.00E-123 |
| hsa_circ_0001616 | 96 | 1 | 499 | 499 | 1 | 0 | 0 |
| hsa_circ_0001616 | 96 | 4 | 632 | 629 | 1 | 0 | 0 |
| hsa_circ_0077238 | 95 | 1 | 999 | 1040 | 42 | 0 | 0 |
| hsa_circ_0077238 | 95 | 1 | 1240 | 1281 | 42 | 0 | 0 |
| hsa_circ_0077235 | 95 | 1 | 1473 | 1923 | 451 | 0 | 0 |
| hsa_circ_0077235 | 95 | 1 | 1558 | 2008 | 451 | 0 | 0 |
| hsa_circ_0077235 | 95 | 1 | 1967 | 2417 | 451 | 0 | 0 |
| hsa_circ_0077235 | 95 | 1 | 1855 | 2417 | 563 | 0 | 0 |
| hsa_circ_0077240 | 95 | 1 | 1059 | 1446 | 388 | 0 | 0 |
| hsa_circ_0077235 | 95 | 1 | 1468 | 2417 | 950 | 0 | 0 |
| hsa_circ_0077243 | 95 | 1 | 340 | 340 | 1 | 0 | 2.00E-152 |
| hsa_circ_0077243 | 95 | 1 | 840 | 899 | 60 | 0 | 0 |
| hsa_circ_0077243 | 95 | 1 | 1249 | 1308 | 60 | 0 | 0 |
| hsa_circ_0077246 | 95 | 1 | 559 | 559 | 1 | 0 | 0 |
| hsa_circ_0077243 | 95 | 1 | 968 | 1308 | 341 | 0 | 0 |
| hsa_circ_0077246 | 95 | 1 | 555 | 559 | 5 | 0 | 0 |
| hsa_circ_0077246 | 95 | 1 | 318 | 559 | 242 | 0 | 2.00E-138 |
| hsa_circ_0077243 | 94 | 1 | 727 | 1308 | 582 | 0 | 0 |
| hsa_circ_0001617 | 95 | 1 | 494 | 494 | 1 | 0 | 0 |
| hsa_circ_0007300 | 94 | 1 | 409 | 409 | 1 | 0 | 3.00E-177 |
| hsa_circ_0004990 | 94 | 1 | 277 | 277 | 1 | 0 | 9.00E-116 |
| hsa_circ_0005820 | 97 | 1 | 614 | 614 | 1 | 0 | 0 |
| hsa_circ_0006768 | 99 | 1 | 370 | 1 | 370 | 0 | 0 |
| hsa_circ_0006768 | 98 | 1 | 573 | 1 | 573 | 0 | 0 |
| hsa_circ_0006768 | 97 | 1 | 710 | 1 | 710 | 0 | 0 |
| hsa_circ_0006768 | 97 | 1 | 711 | 1 | 711 | 0 | 0 |
| hsa_circ_0006768 | 96 | 1 | 852 | 1 | 852 | 0 | 0 |
| hsa_circ_0006768 | 96 | 1 | 852 | 1 | 852 | 0 | 0 |
| hsa_circ_0077266 | 93 | 235 | 2977 | 1 | 2749 | 0 | 0 |
| hsa_circ_0006768 | 98 | 1 | 486 | 20 | 505 | 0 | 0 |
| hsa_circ_0077271 | 92 | 1 | 343 | 439 | 97 | 0 | 2.00E-137 |
| hsa_circ_0077276 | 93 | 1 | 359 | 359 | 1 | 0.01 | 1.00E-149 |
| hsa_circ_0077283 | 90 | 1 | 446 | 106 | 551 | 0 | 2.00E-164 |
| hsa_circ_0077283 | 91 | 1 | 1089 | 106 | 1194 | 0 | 0 |

novel_circ_005067 novel_circ_005068 novel_circ_005069 novel_circ_005070 novel_circ_005071 novel_circ_005072 novel_circ_005073 novel_circ_005074 novel_circ_005075 novel_circ_005079 novel_circ_005080 novel_circ_005081 novel_circ_005082 novel_circ_005083 novel_circ_005084 novel_circ_005085 novel_circ_005086 novel_circ_005087 novel_circ_005088 novel_circ_005089 novel_circ_005090 novel_circ_005091 novel_circ_005092 novel_circ_005093 novel_circ_005094 novel_circ_005095 novel_circ_005096 novel_circ_005097 novel_circ_005098 novel_circ_005099 novel_circ_005100 novel_circ_005101 novel_circ_005102 novel_circ_005103 novel_circ_005104 novel_circ_005105 novel_circ_005106 novel_circ_005107 novel_circ_005108 novel_circ_005109 novel_circ_005110 novel_circ_005113 novel_circ_005114 novel_circ_005115 novel_circ_005116 novel_circ_005117 novel_circ_005118 novel_circ_005119 novel_circ_005120 novel_circ_005121 novel_circ_005122 novel_circ_005123 novel_circ_005124 novel_circ_005125

| hsa_circ_0077283 | 91 | 1 | 1089 | 106 | 1194 | 0 | 0 |
| --- | --- | --- | --- | --- | --- | --- | --- |
| hsa_circ_0077283 | 91 | 1 | 294 | 258 | 551 | 0 | 7.00E-112 |
| hsa_circ_0077283 | 90 | 1 | 427 | 1285 | 1708 | 0.02 | 8.00E-158 |
| hsa_circ_0042696 | 86 | 1191 | 1665 | 415 | 890 | 0.01 | 2.00E-138 |
| hsa_circ_0042696 | 86 | 1191 | 1665 | 415 | 890 | 0.01 | 5.00E-139 |
| hsa_circ_0042696 | 86 | 1191 | 1665 | 415 | 890 | 0.01 | 4.00E-139 |
| hsa_circ_0005234 | 74 | 23357 | 23722 | 13497 | 13865 | 0.05 | 5.00E-32 |
| hsa_circ_0091001 | 81 | 4722 | 5855 | 1350 | 181 | 0.03 | 0 |
| hsa_circ_0071127 | 89 | 3 | 1756 | 1757 | 1 | 0.01 | 0 |
| hsa_circ_0071119 | 85 | 1 | 337 | 912 | 1252 | 0.02 | 2.00E-93 |
| hsa_circ_0071123 | 83 | 1 | 302 | 1 | 302 | 0.01 | 1.00E-74 |
| hsa_circ_0071119 | 88 | 1 | 715 | 188 | 902 | 0.01 | 0 |
| hsa_circ_0071121 | 89 | 1 | 564 | 1 | 564 | 0 | 0 |
| hsa_circ_0071119 | 91 | 1 | 639 | 113 | 751 | 0 | 0 |
| hsa_circ_0071122 | 91 | 1 | 266 | 60 | 325 | 0 | 1.00E-99 |
| hsa_circ_0071122 | 91 | 2 | 325 | 2 | 325 | 0 | 4.00E-120 |
| hsa_circ_0071121 | 91 | 1 | 413 | 1 | 413 | 0 | 4.00E-156 |
| hsa_circ_0007265 | 92 | 1 | 488 | 1 | 488 | 0 | 0 |
| hsa_circ_0071119 | 92 | 6 | 600 | 6 | 600 | 0 | 0 |
| hsa_circ_0071116 | 92 | 1 | 682 | 1 | 682 | 0 | 0 |
| hsa_circ_0071112 | 92 | 1 | 884 | 1 | 884 | 0 | 0 |
| hsa_circ_0071112 | 92 | 1 | 718 | 1 | 718 | 0 | 0 |
| hsa_circ_0071119 | 93 | 6 | 331 | 6 | 331 | 0 | 6.00E-133 |
| hsa_circ_0071112 | 92 | 1 | 615 | 1 | 615 | 0 | 0 |
| hsa_circ_0071110 | 92 | 1 | 850 | 112 | 961 | 0 | 0 |
| hsa_circ_0071112 | 92 | 1 | 559 | 1 | 559 | 0 | 0 |
| hsa_circ_0071112 | 92 | 1 | 471 | 1 | 471 | 0 | 0 |
| hsa_circ_0071116 | 91 | 1 | 194 | 1 | 194 | 0 | 4.00E-68 |
| hsa_circ_0071112 | 91 | 1 | 396 | 1 | 396 | 0 | 4.00E-150 |
| hsa_circ_0071110 | 91 | 1 | 526 | 217 | 742 | 0 | 0 |
| hsa_circ_0071110 | 92 | 1 | 631 | 112 | 742 | 0 | 0 |
| hsa_circ_0071112 | 91 | 1 | 199 | 1 | 199 | 0 | 1.00E-72 |
| hsa_circ_0071110 | 91 | 1 | 329 | 217 | 545 | 0 | 3.00E-126 |
| hsa_circ_0071110 | 92 | 1 | 434 | 112 | 545 | 0 | 2.00E-174 |
| hsa_circ_0071110 | 91 | 1 | 545 | 1 | 545 | 0 | 0 |
| hsa_circ_0071108 | 92 | 1 | 647 | 1 | 647 | 0 | 0 |
| hsa_circ_0071103 | 92 | 1 | 877 | 1 | 877 | 0 | 0 |
| hsa_circ_0071110 | 92 | 1 | 338 | 112 | 449 | 0 | 1.00E-134 |
| hsa_circ_0071110 | 93 | 1 | 235 | 112 | 346 | 0 | 2.00E-92 |
| hsa_circ_0071103 | 93 | 1 | 230 | 1 | 230 | 0 | 2.00E-91 |
| hsa_circ_0071103 | 91 | 1 | 158 | 1 | 158 | 0 | 7.00E-55 |
| hsa_circ_0047734 | 98 | 1 | 180 | 180 | 1 | 0 | 2.00E-85 |
| hsa_circ_0047734 | 98 | 1 | 177 | 177 | 1 | 0 | 9.00E-84 |
| hsa_circ_0047727 | 97 | 11712 | 12406 | 695 | 1 | 0 | 0 |
| hsa_circ_0047731 | 97 | 1 | 373 | 373 | 1 | 0 | 5.00E-179 |
| hsa_circ_0047727 | 97 | 107 | 801 | 695 | 1 | 0 | 0 |
| hsa_circ_0047727 | 97 | 1 | 695 | 695 | 1 | 0 | 0 |
| hsa_circ_0047727 | 97 | 4 | 561 | 558 | 1 | 0 | 0 |
| hsa_circ_0076415 | 87 | 19811 | 20541 | 727 | 1 | 0.01 | 0 |
| hsa_circ_0076415 | 87 | 19811 | 20541 | 727 | 1 | 0.01 | 0 |
| hsa_circ_0047722 | 90 | 1 | 996 | 292 | 1287 | 0 | 0 |
| hsa_circ_0047722 | 90 | 1 | 996 | 292 | 1287 | 0 | 0 |
| hsa_circ_0003428 | 91 | 1 | 792 | 1 | 792 | 0 | 0 |
| hsa_circ_0047722 | 91 | 2 | 1080 | 2 | 1083 | 0 | 0 |

novel_circ_005126 novel_circ_005127 novel_circ_005128 novel_circ_005129 novel_circ_005150 novel_circ_005151 novel_circ_005152 novel_circ_005153 novel_circ_005154 novel_circ_005155 novel_circ_005156 novel_circ_005157 novel_circ_005158 novel_circ_005159 novel_circ_005160 novel_circ_005161 novel_circ_005163 novel_circ_005165 novel_circ_005166 novel_circ_005167 novel_circ_005168 novel_circ_005169 novel_circ_005170 novel_circ_005171 novel_circ_005172 novel_circ_005173 novel_circ_005174 novel_circ_005175 novel_circ_005176 novel_circ_005177 novel_circ_005178 novel_circ_005179 novel_circ_005180 novel_circ_005181 novel_circ_005182 novel_circ_005183 novel_circ_005184 novel_circ_005185 novel_circ_005186 novel_circ_005187 novel_circ_005190 novel_circ_005191 novel_circ_005192 novel_circ_005193 novel_circ_005194 novel_circ_005195 novel_circ_005196 novel_circ_005197 novel_circ_005198 novel_circ_005199 novel_circ_005200 novel_circ_005201 novel_circ_005202 novel_circ_005203

| hsa_circ_0002825 | 84 | 159 | 1453 | 1300 | 1 | 0.07 | 0 |
| --- | --- | --- | --- | --- | --- | --- | --- |
| hsa_circ_0005584 | 96 | 1 | 706 | 706 | 1 | 0 | 0 |
| hsa_circ_0002825 | 84 | 1 | 1293 | 1298 | 1 | 0.07 | 0 |
| hsa_circ_0005584 | 96 | 1 | 408 | 408 | 1 | 0 | 0 |
| hsa_circ_0071095 | 94 | 1 | 213 | 213 | 1 | 0 | 5.00E-87 |
| hsa_circ_0071091 | 92 | 1 | 292 | 292 | 1 | 0 | 4.00E-114 |
| hsa_circ_0006204 | 92 | 1 | 1047 | 1050 | 1 | 0.01 | 0 |
| hsa_circ_0002293 | 94 | 1 | 1701 | 1704 | 1 | 0.01 | 0 |
| hsa_circ_0071089 | 94 | 1 | 1935 | 1938 | 1 | 0.01 | 0 |
| hsa_circ_0071083 | 94 | 1 | 2337 | 2817 | 478 | 0 | 0 |
| hsa_circ_0002293 | 98 | 1 | 654 | 654 | 1 | 0 | 0 |
| hsa_circ_0071089 | 96 | 1 | 888 | 888 | 1 | 0 | 0 |
| hsa_circ_0071083 | 95 | 1 | 1290 | 1767 | 478 | 0 | 0 |
| hsa_circ_0001448 | 92 | 1 | 532 | 636 | 105 | 0 | 0 |
| hsa_circ_0001448 | 92 | 1 | 636 | 636 | 1 | 0 | 0 |
| hsa_circ_0001448 | 92 | 1 | 402 | 402 | 1 | 0 | 9.00E-162 |
| hsa_circ_0071075 | 88 | 1 | 503 | 1 | 502 | 0.01 | 2.00E-170 |
| hsa_circ_0071075 | 86 | 1 | 1867 | 1 | 1874 | 0.02 | 0 |
| hsa_circ_0071075 | 86 | 1 | 1867 | 1 | 1874 | 0.02 | 0 |
| hsa_circ_0071075 | 86 | 1 | 1867 | 1 | 1874 | 0.02 | 0 |
| hsa_circ_0071075 | 83 | 1 | 1239 | 628 | 1874 | 0.03 | 0 |
| hsa_circ_0071075 | 86 | 1 | 1867 | 1 | 1874 | 0.02 | 0 |
| hsa_circ_0071075 | 89 | 1 | 1496 | 1 | 1498 | 0.01 | 0 |
| hsa_circ_0071075 | 91 | 1 | 794 | 1 | 793 | 0 | 0 |
| hsa_circ_0071075 | 90 | 1 | 628 | 1 | 627 | 0 | 0 |
| hsa_circ_0071075 | 88 | 1 | 503 | 1 | 502 | 0.01 | 3.00E-172 |
| hsa_circ_0071074 | 93 | 3 | 854 | 579 | 1430 | 0 | 0 |
| hsa_circ_0071074 | 92 | 32 | 1435 | 30 | 1430 | 0 | 0 |
| hsa_circ_0071074 | 89 | 32 | 581 | 30 | 576 | 0.01 | 0 |
| hsa_circ_0071069 | 94 | 1 | 386 | 630 | 245 | 0 | 3.00E-166 |
| hsa_circ_0071064 | 91 | 1 | 959 | 1830 | 866 | 0.01 | 0 |
| hsa_circ_0071068 | 89 | 1 | 386 | 386 | 1 | 0 | 3.00E-131 |
| hsa_circ_0073245 | 92 | 6 | 782 | 777 | 1 | 0 | 0 |
| hsa_circ_0073245 | 92 | 6 | 782 | 777 | 1 | 0 | 0 |
| hsa_circ_0073245 | 93 | 1 | 486 | 486 | 1 | 0 | 0 |
| hsa_circ_0073245 | 93 | 1 | 486 | 486 | 1 | 0 | 0 |
| hsa_circ_0073240 | 82 | 23 | 5681 | 35 | 5732 | 0.02 | 0 |
| hsa_circ_0073235 | 82 | 1269 | 8633 | 1578 | 8987 | 0.02 | 0 |
| hsa_circ_0073240 | 81 | 23 | 5211 | 35 | 5262 | 0.02 | 0 |
| hsa_circ_0073234 | 88 | 1 | 678 | 77 | 754 | 0 | 0 |
| hsa_circ_0073226 | 92 | 1 | 99 | 1 | 99 | 0 | 8.00E-31 |
| hsa_circ_0073225 | 93 | 1 | 238 | 109 | 346 | 0 | 5.00E-93 |
| hsa_circ_0074029 | 77 | 11070 | 11367 | 207744 | 207446 | 0.04 | 2.00E-35 |
| hsa_circ_0001509 | 80 | 3161 | 5047 | 4828 | 2913 | 0.07 | 0 |
| hsa_circ_0001509 | 80 | 1578 | 3464 | 4828 | 2913 | 0.07 | 0 |
| hsa_circ_0073222 | 95 | 1 | 219 | 220 | 2 | 0 | 3.00E-95 |
| hsa_circ_0073198 | 92 | 1 | 685 | 633 | 1317 | 0 | 0 |
| hsa_circ_0073198 | 92 | 1 | 601 | 633 | 1233 | 0 | 0 |
| hsa_circ_0073199 | 92 | 1 | 433 | 384 | 816 | 0 | 6.00E-174 |
| hsa_circ_0073199 | 92 | 1 | 323 | 384 | 706 | 0 | 3.00E-126 |
| hsa_circ_0073199 | 92 | 2 | 326 | 382 | 706 | 0 | 2.00E-127 |
| hsa_circ_0073193 | 92 | 1 | 1409 | 1 | 1403 | 0.01 | 0 |
| hsa_circ_0073199 | 92 | 1 | 217 | 384 | 600 | 0 | 8.00E-81 |
| hsa_circ_0073197 | 91 | 1 | 937 | 1 | 931 | 0.01 | 0 |

novel_circ_005204 novel_circ_005205 novel_circ_005206 novel_circ_005207 novel_circ_005208 novel_circ_005209 novel_circ_005210 novel_circ_005211 novel_circ_005212 novel_circ_005213 novel_circ_005214 novel_circ_005215 novel_circ_005216 novel_circ_005217 novel_circ_005218 novel_circ_005219 novel_circ_005220 novel_circ_005221 novel_circ_005222 novel_circ_005223 novel_circ_005224 novel_circ_005228 novel_circ_005229 novel_circ_005230 novel_circ_005231 novel_circ_005232 novel_circ_005233 novel_circ_005234 novel_circ_005235 novel_circ_005236 novel_circ_005237 novel_circ_005238 novel_circ_005239 novel_circ_005240 novel_circ_005241 novel_circ_005242 novel_circ_005244 novel_circ_005246 novel_circ_005247 novel_circ_005248 novel_circ_005249 novel_circ_005250 novel_circ_005251 novel_circ_005252 novel_circ_005253 novel_circ_005254 novel_circ_005255 novel_circ_005256 novel_circ_005257 novel_circ_005258 novel_circ_005259 novel_circ_005260 novel_circ_005261 novel_circ_005262

| hsa_circ_0073193 | 92 | 1 | 1302 | 1 | 1296 | 0.01 | 0 |
| --- | --- | --- | --- | --- | --- | --- | --- |
| hsa_circ_0073183 | 93 | 1 | 2297 | 108 | 2398 | 0 | 0 |
| hsa_circ_0073183 | 93 | 1 | 2078 | 108 | 2179 | 0 | 0 |
| hsa_circ_0073184 | 96 | 1 | 766 | 1 | 766 | 0 | 0 |
| hsa_circ_0073183 | 96 | 1 | 873 | 1 | 873 | 0 | 0 |
| hsa_circ_0001991 | 80 | 42782 | 43715 | 37896 | 38843 | 0.06 | 0 |
| hsa_circ_0073172 | 93 | 1 | 560 | 1081 | 1640 | 0 | 0 |
| hsa_circ_0073172 | 93 | 1586 | 2312 | 914 | 1640 | 0 | 0 |
| hsa_circ_0073172 | 93 | 1 | 917 | 724 | 1640 | 0 | 0 |
| hsa_circ_0073170 | 87 | 1 | 985 | 122 | 1103 | 0.01 | 0 |
| hsa_circ_0073170 | 87 | 4 | 1106 | 4 | 1103 | 0.01 | 0 |
| hsa_circ_0073168 | 91 | 1 | 218 | 293 | 510 | 0 | 7.00E-78 |
| hsa_circ_0073168 | 91 | 1 | 510 | 1 | 510 | 0 | 0 |
| hsa_circ_0073168 | 91 | 1 | 510 | 1 | 510 | 0 | 0 |
| hsa_circ_0073165 | 93 | 718 | 1231 | 1 | 514 | 0 | 0 |
| hsa_circ_0073165 | 96 | 562 | 642 | 1 | 81 | 0 | 9.00E-30 |
| hsa_circ_0073165 | 96 | 718 | 798 | 1 | 81 | 0 | 1.00E-29 |
| hsa_circ_0073165 | 96 | 254 | 334 | 1 | 81 | 0 | 4.00E-30 |
| hsa_circ_0073165 | 96 | 562 | 642 | 1 | 81 | 0 | 9.00E-30 |
| hsa_circ_0073165 | 96 | 718 | 798 | 1 | 81 | 0 | 1.00E-29 |
| hsa_circ_0073164 | 90 | 3 | 305 | 3 | 305 | 0 | 4.00E-106 |
| hsa_circ_0073164 | 92 | 6 | 143 | 168 | 305 | 0 | 2.00E-47 |
| hsa_circ_0073164 | 92 | 6 | 143 | 168 | 305 | 0 | 7.00E-48 |
| hsa_circ_0073164 | 90 | 3 | 305 | 3 | 305 | 0 | 3.00E-106 |
| hsa_circ_0073164 | 90 | 100 | 402 | 3 | 305 | 0 | 3.00E-106 |
| hsa_circ_0073163 | 84 | 3 | 2457 | 3 | 2458 | 0.02 | 0 |
| hsa_circ_0073164 | 90 | 100 | 402 | 3 | 305 | 0 | 2.00E-106 |
| hsa_circ_0073163 | 84 | 3 | 2457 | 3 | 2458 | 0.02 | 0 |
| hsa_circ_0073163 | 84 | 3 | 2360 | 3 | 2361 | 0.02 | 0 |
| hsa_circ_0073161 | 93 | 1 | 262 | 1043 | 782 | 0 | 4.00E-104 |
| hsa_circ_0073161 | 92 | 1 | 356 | 1043 | 688 | 0 | 7.00E-138 |
| hsa_circ_0073161 | 91 | 1 | 566 | 1043 | 478 | 0 | 0 |
| hsa_circ_0073161 | 89 | 1 | 791 | 1043 | 253 | 0 | 0 |
| hsa_circ_0073161 | 88 | 2 | 441 | 692 | 253 | 0 | 8.00E-143 |
| hsa_circ_0073161 | 88 | 1 | 435 | 687 | 253 | 0 | 1.00E-141 |
| hsa_circ_0073161 | 87 | 1 | 687 | 687 | 1 | 0 | 0 |
| hsa_circ_0073161 | 86 | 1 | 379 | 379 | 1 | 0 | 1.00E-110 |
| hsa_circ_0073141 | 95 | 1 | 1325 | 1 | 1337 | 0.01 | 0 |
| hsa_circ_0073156 | 94 | 1 | 181 | 1 | 181 | 0 | 1.00E-72 |
| hsa_circ_0002260 | 94 | 1 | 731 | 1 | 743 | 0.02 | 0 |
| hsa_circ_0073141 | 95 | 1 | 1233 | 1 | 1245 | 0.01 | 0 |
| hsa_circ_0073153 | 97 | 3 | 276 | 3 | 276 | 0 | 2.00E-127 |
| hsa_circ_0073150 | 97 | 1 | 343 | 1 | 343 | 0 | 5.00E-164 |
| hsa_circ_0002260 | 94 | 1 | 550 | 1 | 562 | 0.02 | 0 |
| hsa_circ_0073145 | 95 | 1 | 788 | 1 | 800 | 0.02 | 0 |
| hsa_circ_0073141 | 95 | 1 | 1052 | 1 | 1064 | 0.01 | 0 |
| hsa_circ_0002260 | 93 | 1 | 344 | 1 | 356 | 0.03 | 1.00E-144 |
| hsa_circ_0073145 | 95 | 1 | 582 | 1 | 594 | 0.02 | 0 |
| hsa_circ_0073141 | 95 | 1 | 846 | 1 | 858 | 0.01 | 0 |
| hsa_circ_0073141 | 95 | 3 | 772 | 7 | 788 | 0.02 | 0 |
| hsa_circ_0073141 | 95 | 1 | 776 | 1 | 788 | 0.02 | 0 |
| hsa_circ_0073141 | 96 | 1 | 709 | 1 | 709 | 0 | 0 |
| hsa_circ_0073141 | 95 | 1 | 264 | 1 | 264 | 0 | 3.00E-115 |
| hsa_circ_0073132 | 97 | 1 | 382 | 382 | 1 | 0 | 0 |

novel_circ_005263 novel_circ_005264 novel_circ_005265 novel_circ_005266 novel_circ_005267 novel_circ_005268 novel_circ_005269 novel_circ_005270 novel_circ_005271 novel_circ_005272 novel_circ_005273 novel_circ_005274 novel_circ_005276 novel_circ_005277 novel_circ_005278 novel_circ_005279 novel_circ_005280 novel_circ_005281 novel_circ_005282 novel_circ_005283 novel_circ_005284 novel_circ_005285 novel_circ_005286 novel_circ_005287 novel_circ_005288 novel_circ_005289 novel_circ_005290 novel_circ_005291 novel_circ_005292 novel_circ_005293 novel_circ_005295 novel_circ_005296 novel_circ_005300 novel_circ_005301 novel_circ_005302 novel_circ_005303 novel_circ_005306 novel_circ_005307 novel_circ_005308 novel_circ_005309 novel_circ_005310 novel_circ_005311 novel_circ_005312 novel_circ_005313 novel_circ_005314 novel_circ_005315 novel_circ_005316 novel_circ_005317 novel_circ_005318 novel_circ_005319 novel_circ_005320 novel_circ_005321 novel_circ_005322 novel_circ_005323

| hsa_circ_0006916 | 97 | 1 | 522 | 522 | 1 | 0 | 0 |
| --- | --- | --- | --- | --- | --- | --- | --- |
| hsa_circ_0006916 | 96 | 1 | 365 | 365 | 1 | 0 | 7.00E-168 |
| hsa_circ_0008147 | 92 | 3582 | 3655 | 950 | 1022 | 0.01 | 2.00E-19 |
| hsa_circ_0073124 | 89 | 1 | 897 | 538 | 1443 | 0.02 | 0 |
| hsa_circ_0073124 | 90 | 1 | 993 | 442 | 1443 | 0.01 | 0 |
| hsa_circ_0073124 | 90 | 1 | 1434 | 1 | 1443 | 0.01 | 0 |
| hsa_circ_0073124 | 87 | 1 | 682 | 442 | 1132 | 0.02 | 0 |
| hsa_circ_0073123 | 92 | 326 | 936 | 1 | 611 | 0 | 0 |
| hsa_circ_0073122 | 94 | 175 | 495 | 1 | 321 | 0 | 3.00E-138 |
| hsa_circ_0073119 | 90 | 1 | 586 | 586 | 1 | 0 | 0 |
| hsa_circ_0008469 | 90 | 6 | 315 | 310 | 1 | 0 | 1.00E-110 |
| hsa_circ_0073114 | 95 | 1 | 256 | 338 | 593 | 0 | 4.00E-112 |
| hsa_circ_0073114 | 95 | 1 | 256 | 338 | 593 | 0 | 3.00E-112 |
| hsa_circ_0073114 | 95 | 1 | 593 | 1 | 593 | 0 | 0 |
| hsa_circ_0073114 | 95 | 1 | 497 | 1 | 497 | 0 | 0 |
| hsa_circ_0073111 | 96 | 1 | 575 | 1 | 575 | 0 | 0 |
| hsa_circ_0073107 | 94 | 1 | 658 | 967 | 310 | 0 | 0 |
| hsa_circ_0002996 | 94 | 1 | 507 | 507 | 1 | 0 | 0 |
| hsa_circ_0073105 | 92 | 2 | 627 | 626 | 1 | 0 | 0 |
| hsa_circ_0073105 | 92 | 11 | 444 | 434 | 1 | 0 | 2.00E-174 |
| hsa_circ_0073101 | 93 | 11 | 864 | 1674 | 821 | 0 | 0 |
| hsa_circ_0073101 | 91 | 11 | 1602 | 1674 | 74 | 0.01 | 0 |
| hsa_circ_0073105 | 94 | 1 | 288 | 288 | 1 | 0 | 7.00E-122 |
| hsa_circ_0073101 | 91 | 1 | 988 | 997 | 1 | 0.01 | 0 |
| hsa_circ_0073103 | 85 | 1 | 420 | 429 | 1 | 0.03 | 3.00E-117 |
| hsa_circ_0073101 | 85 | 1 | 493 | 502 | 1 | 0.02 | 2.00E-144 |
| hsa_circ_0073099 | 86 | 1 | 598 | 609 | 3 | 0.02 | 1.00E-177 |
| hsa_circ_0073090 | 93 | 1 | 554 | 554 | 1 | 0 | 0 |
| hsa_circ_0073082 | 91 | 1 | 472 | 646 | 175 | 0 | 1.00E-180 |
| hsa_circ_0073082 | 91 | 1 | 528 | 530 | 3 | 0 | 0 |
| hsa_circ_0073080 | 94 | 1 | 530 | 1574 | 1045 | 0 | 0 |
| hsa_circ_0073080 | 88 | 1 | 910 | 1574 | 657 | 0.02 | 0 |
| hsa_circ_0073075 | 94 | 329 | 843 | 1 | 515 | 0 | 0 |
| hsa_circ_0073075 | 94 | 329 | 760 | 1 | 432 | 0 | 0 |
| hsa_circ_0073075 | 92 | 1 | 164 | 1 | 164 | 0 | 7.00E-60 |
| hsa_circ_0006071 | 71 | 31174 | 32553 | 65966 | 64587 | 0.07 | 8.00E-53 |
| hsa_circ_0066637 | 94 | 113 | 423 | 1 | 311 | 0 | 8.00E-133 |
| hsa_circ_0066637 | 94 | 113 | 823 | 1 | 711 | 0 | 0 |
| hsa_circ_0066637 | 94 | 113 | 1039 | 1 | 927 | 0 | 0 |
| hsa_circ_0066637 | 94 | 113 | 1354 | 1 | 1242 | 0 | 0 |
| hsa_circ_0066637 | 94 | 1 | 311 | 1 | 311 | 0 | 6.00E-133 |
| hsa_circ_0066637 | 94 | 1 | 711 | 1 | 711 | 0 | 0 |
| hsa_circ_0066637 | 94 | 1 | 927 | 1 | 927 | 0 | 0 |
| hsa_circ_0066637 | 94 | 1 | 1388 | 1 | 1385 | 0 | 0 |
| hsa_circ_0066637 | 94 | 1 | 1436 | 1 | 1433 | 0 | 0 |
| hsa_circ_0066640 | 93 | 1 | 400 | 206 | 605 | 0 | 3.00E-167 |
| hsa_circ_0066642 | 93 | 1 | 276 | 1 | 276 | 0 | 1.00E-113 |
| hsa_circ_0066642 | 94 | 1 | 492 | 1 | 492 | 0 | 0 |
| hsa_circ_0066637 | 95 | 1 | 531 | 712 | 1242 | 0 | 0 |
| hsa_circ_0066637 | 94 | 1 | 677 | 712 | 1385 | 0 | 0 |
| hsa_circ_0066637 | 95 | 1 | 517 | 1142 | 1655 | 0.01 | 0 |
| hsa_circ_0066637 | 96 | 1 | 185 | 1249 | 1433 | 0 | 8.00E-80 |
| hsa_circ_0066646 | 96 | 1 | 331 | 90 | 420 | 0 | 1.00E-150 |
| hsa_circ_0066655 | 93 | 1 | 581 | 2840 | 2260 | 0 | 0 |

novel_circ_005324 novel_circ_005325 novel_circ_005326 novel_circ_005328 novel_circ_005329 novel_circ_005330 novel_circ_005331 novel_circ_005332 novel_circ_005333 novel_circ_005334 novel_circ_005335 novel_circ_005336 novel_circ_005337 novel_circ_005338 novel_circ_005339 novel_circ_005340 novel_circ_005341 novel_circ_005342 novel_circ_005343 novel_circ_005344 novel_circ_005345 novel_circ_005346 novel_circ_005348 novel_circ_005349 novel_circ_005350 novel_circ_005351 novel_circ_005352 novel_circ_005353 novel_circ_005354 novel_circ_005355 novel_circ_005356 novel_circ_005357 novel_circ_005358 novel_circ_005359 novel_circ_005362 novel_circ_005363 novel_circ_005364 novel_circ_005365 novel_circ_005366 novel_circ_005367 novel_circ_005370 novel_circ_005371 novel_circ_005372 novel_circ_005373 novel_circ_005374 novel_circ_005375 novel_circ_005376 novel_circ_005377 novel_circ_005378 novel_circ_005379 novel_circ_005380 novel_circ_005381 novel_circ_005382 novel_circ_005383

| hsa_circ_0066656 | 95 | 1 | 903 | 1128 | 226 | 0 | 0 |
| --- | --- | --- | --- | --- | --- | --- | --- |
| hsa_circ_0066656 | 95 | 1 | 768 | 1128 | 361 | 0 | 0 |
| hsa_circ_0066656 | 95 | 1 | 560 | 1128 | 569 | 0 | 0 |
| hsa_circ_0066663 | 96 | 1 | 627 | 1 | 623 | 0.01 | 0 |
| hsa_circ_0066669 | 86 | 200 | 490 | 3180 | 2887 | 0.02 | 8.00E-84 |
| hsa_circ_0066671 | 93 | 1 | 820 | 3165 | 2343 | 0.01 | 0 |
| hsa_circ_0066671 | 92 | 520 | 1675 | 3501 | 2343 | 0 | 0 |
| hsa_circ_0066671 | 92 | 834 | 1989 | 3501 | 2343 | 0 | 0 |
| hsa_circ_0066671 | 92 | 1 | 697 | 3165 | 2466 | 0.01 | 0 |
| hsa_circ_0066671 | 91 | 520 | 1552 | 3501 | 2466 | 0 | 0 |
| hsa_circ_0066673 | 82 | 5 | 493 | 494 | 6 | 0 | 4.00E-118 |
| hsa_circ_0066673 | 89 | 1 | 314 | 994 | 681 | 0 | 1.00E-109 |
| hsa_circ_0066673 | 89 | 199 | 512 | 994 | 681 | 0 | 2.00E-109 |
| hsa_circ_0066675 | 93 | 1 | 296 | 296 | 1 | 0 | 3.00E-119 |
| hsa_circ_0066675 | 93 | 159 | 454 | 296 | 1 | 0 | 3.00E-119 |
| hsa_circ_0066675 | 93 | 18529 | 18824 | 296 | 1 | 0 | 9.00E-118 |
| hsa_circ_0066675 | 97 | 1 | 198 | 198 | 1 | 0 | 5.00E-90 |
| hsa_circ_0066675 | 93 | 1 | 296 | 296 | 1 | 0 | 2.00E-119 |
| hsa_circ_0066675 | 93 | 159 | 454 | 296 | 1 | 0 | 3.00E-119 |
| hsa_circ_0066675 | 93 | 18529 | 18824 | 296 | 1 | 0 | 8.00E-118 |
| hsa_circ_0066675 | 93 | 159 | 454 | 296 | 1 | 0 | 2.00E-119 |
| hsa_circ_0066675 | 93 | 18529 | 18824 | 296 | 1 | 0 | 8.00E-118 |
| hsa_circ_0066687 | 92 | 1 | 333 | 2580 | 2248 | 0 | 2.00E-133 |
| hsa_circ_0066691 | 92 | 1 | 509 | 509 | 1 | 0 | 0 |
| hsa_circ_0066691 | 90 | 1 | 2002 | 1999 | 1 | 0 | 0 |
| hsa_circ_0066691 | 92 | 1 | 391 | 509 | 119 | 0 | 1.00E-155 |
| hsa_circ_0066691 | 87 | 1 | 1416 | 1531 | 119 | 0 | 0 |
| hsa_circ_0066691 | 89 | 1 | 1884 | 1999 | 119 | 0 | 0 |
| hsa_circ_0066691 | 89 | 1 | 1492 | 1999 | 511 | 0 | 0 |
| hsa_circ_0003179 | 95 | 1 | 466 | 468 | 3 | 0 | 0 |
| hsa_circ_0066700 | 91 | 144 | 302 | 1 | 159 | 0 | 2.00E-53 |
| hsa_circ_0066700 | 92 | 144 | 685 | 1 | 542 | 0 | 0 |
| hsa_circ_0066700 | 92 | 144 | 685 | 1 | 542 | 0 | 0 |
| hsa_circ_0066702 | 92 | 1 | 299 | 1 | 299 | 0 | 5.00E-114 |
| hsa_circ_0004065 | 92 | 1 | 829 | 1 | 829 | 0 | 0 |
| hsa_circ_0004065 | 92 | 1 | 829 | 1 | 829 | 0 | 0 |
| hsa_circ_0004385 | 92 | 75 | 628 | 1 | 554 | 0 | 0 |
| hsa_circ_0004385 | 92 | 1 | 554 | 1 | 554 | 0 | 0 |
| hsa_circ_0079214 | 91 | 1 | 415 | 1 | 415 | 0 | 1.00E-160 |
| hsa_circ_0079215 | 92 | 1 | 255 | 1 | 255 | 0.01 | 1.00E-98 |
| hsa_circ_0007319 | 100 | 1 | 83 | 141798 | 141880 | 0 | 6.00E-36 |
| hsa_circ_0079256 | 90 | 1 | 517 | 1668 | 1149 | 0.02 | 0 |
| hsa_circ_0079255 | 92 | 314 | 1492 | 2495 | 1314 | 0.01 | 0 |
| hsa_circ_0079260 | 86 | 457 | 2076 | 2053 | 443 | 0.01 | 0 |
| hsa_circ_0007319 | 87 | 218 | 597 | 330273 | 329894 | 0.01 | 1.00E-117 |
| hsa_circ_0079261 | 88 | 11 | 1979 | 1969 | 1 | 0 | 0 |
| hsa_circ_0079289 | 93 | 1 | 940 | 940 | 1 | 0 | 0 |
| hsa_circ_0079289 | 91 | 1 | 1704 | 1689 | 1 | 0.01 | 0 |
| hsa_circ_0079289 | 91 | 1 | 1875 | 1860 | 1 | 0.01 | 0 |
| hsa_circ_0079289 | 93 | 1 | 861 | 940 | 80 | 0 | 0 |
| hsa_circ_0079289 | 94 | 1 | 712 | 940 | 229 | 0 | 0 |
| hsa_circ_0079289 | 93 | 1 | 523 | 940 | 418 | 0 | 0 |
| hsa_circ_0079289 | 91 | 1 | 1458 | 1860 | 418 | 0.01 | 0 |
| hsa_circ_0079289 | 90 | 1 | 1203 | 1860 | 673 | 0.02 | 0 |

novel_circ_005384 novel_circ_005385 novel_circ_005386 novel_circ_005387 novel_circ_005388 novel_circ_005389 novel_circ_005390 novel_circ_005391 novel_circ_005392 novel_circ_005393 novel_circ_005394 novel_circ_005400 novel_circ_005401 novel_circ_005404 novel_circ_005405 novel_circ_005406 novel_circ_005407 novel_circ_005408 novel_circ_005409 novel_circ_005410 novel_circ_005411 novel_circ_005412 novel_circ_005413 novel_circ_005414 novel_circ_005415 novel_circ_005416 novel_circ_005417 novel_circ_005418 novel_circ_005419 novel_circ_005420 novel_circ_005425 novel_circ_005426 novel_circ_005427 novel_circ_005428 novel_circ_005429 novel_circ_005430 novel_circ_005434 novel_circ_005435 novel_circ_005436 novel_circ_005437 novel_circ_005438 novel_circ_005439 novel_circ_005441 novel_circ_005442 novel_circ_005444 novel_circ_005445 novel_circ_005446 novel_circ_005447 novel_circ_005448 novel_circ_005449 novel_circ_005450 novel_circ_005451 novel_circ_005452 novel_circ_005453

| hsa_circ_0007501 | 90 | 1 | 935 | 920 | 1 | 0.02 | 0 |
| --- | --- | --- | --- | --- | --- | --- | --- |
| hsa_circ_0079296 | 90 | 4 | 270 | 267 | 1 | 0 | 1.00E-93 |
| hsa_circ_0007140 | 88 | 6 | 204 | 218 | 20 | 0 | 7.00E-61 |
| hsa_circ_0007140 | 94 | 1 | 220 | 223 | 1 | 0.01 | 9.00E-90 |
| hsa_circ_0079395 | 96 | 1 | 168 | 2924 | 3091 | 0 | 4.00E-72 |
| hsa_circ_0007319 | 82 | 195 | 896 | 2E+06 | 1581224 | 0.04 | 6.00E-167 |
| hsa_circ_0079364 | 87 | 1 | 378 | 378 | 1 | 0.01 | 4.00E-120 |
| hsa_circ_0079359 | 87 | 1 | 540 | 540 | 1 | 0 | 1.00E-171 |
| hsa_circ_0079353 | 87 | 1 | 621 | 621 | 1 | 0 | 0 |
| hsa_circ_0079359 | 87 | 1 | 289 | 289 | 1 | 0 | 1.00E-85 |
| hsa_circ_0079353 | 87 | 1 | 370 | 370 | 1 | 0 | 2.00E-115 |
| hsa_circ_0059507 | 93 | 1 | 524 | 524 | 1 | 0 | 0 |
| hsa_circ_0059498 | 92 | 1 | 651 | 1459 | 809 | 0 | 0 |
| hsa_circ_0059489 | 96 | 523 | 828 | 825 | 520 | 0 | 1.00E-139 |
| hsa_circ_0059489 | 95 | 172 | 299 | 825 | 698 | 0 | 3.00E-51 |
| hsa_circ_0059489 | 96 | 172 | 603 | 825 | 394 | 0 | 0 |
| hsa_circ_0059489 | 92 | 1 | 310 | 393 | 84 | 0 | 5.00E-119 |
| hsa_circ_0059486 | 94 | 3 | 172 | 170 | 1 | 0 | 2.00E-66 |
| hsa_circ_0059486 | 94 | 3 | 172 | 170 | 1 | 0 | 8.00E-65 |
| hsa_circ_0008147 | 94 | 18595 | 18661 | 956 | 1022 | 0 | 8.00E-19 |
| hsa_circ_0006643 | 92 | 3037 | 3124 | 817714 | 817801 | 0 | 1.00E-25 |
| hsa_circ_0059478 | 87 | 1 | 497 | 1029 | 518 | 0.03 | 6.00E-155 |
| hsa_circ_0059478 | 89 | 1 | 751 | 1029 | 264 | 0.02 | 0 |
| hsa_circ_0059478 | 90 | 1 | 1014 | 1029 | 1 | 0.02 | 0 |
| hsa_circ_0059476 | 92 | 1 | 356 | 356 | 1 | 0.01 | 1.00E-139 |
| hsa_circ_0059473 | 94 | 1 | 642 | 749 | 108 | 0 | 0 |
| hsa_circ_0059473 | 94 | 1 | 749 | 749 | 1 | 0 | 0 |
| hsa_circ_0059473 | 97 | 1 | 286 | 393 | 108 | 0 | 4.00E-134 |
| hsa_circ_0059473 | 96 | 1 | 393 | 393 | 1 | 0 | 0 |
| hsa_circ_0059459 | 94 | 1 | 296 | 296 | 1 | 0 | 1.00E-124 |
| hsa_circ_0059454 | 94 | 1 | 490 | 127 | 604 | 0.02 | 0 |
| hsa_circ_0069749 | 87 | 56 | 211 | 590550 | 590395 | 0 | 6.00E-42 |
| hsa_circ_0069763 | 84 | 1 | 855 | 1 | 858 | 0.01 | 0 |
| hsa_circ_0069763 | 84 | 1 | 1040 | 1 | 1046 | 0.01 | 0 |
| hsa_circ_0069763 | 84 | 1 | 1156 | 1 | 1162 | 0.01 | 0 |
| hsa_circ_0006643 | 95 | 1183 | 1219 | 940029 | 940065 | 0 | 3.00E-06 |
| hsa_circ_0087716 | 94 | 299 | 894 | 932 | 337 | 0 | 0 |
| hsa_circ_0087716 | 94 | 62 | 425 | 932 | 569 | 0 | 3.00E-157 |
| hsa_circ_0087716 | 94 | 62 | 534 | 932 | 460 | 0 | 0 |
| hsa_circ_0087716 | 94 | 62 | 657 | 932 | 337 | 0 | 0 |
| hsa_circ_0087716 | 94 | 1 | 473 | 932 | 460 | 0 | 0 |
| hsa_circ_0087716 | 94 | 1 | 596 | 932 | 337 | 0 | 0 |
| hsa_circ_0087708 | 92 | 106 | 202 | 301 | 205 | 0 | 7.00E-31 |
| hsa_circ_0002900 | 92 | 215 | 470 | 1347 | 1602 | 0 | 7.00E-99 |
| hsa_circ_0002718 | 90 | 4159 | 4377 | 219 | 1 | 0.02 | 1.00E-75 |
| hsa_circ_0087695 | 92 | 1 | 358 | 667 | 1024 | 0 | 5.00E-144 |
| hsa_circ_0087692 | 93 | 1060 | 1172 | 63 | 175 | 0 | 6.00E-39 |
| hsa_circ_0087681 | 94 | 1 | 771 | 1 | 771 | 0 | 0 |
| hsa_circ_0087681 | 94 | 1 | 673 | 1 | 673 | 0 | 0 |
| hsa_circ_0087671 | 97 | 1 | 190 | 1 | 190 | 0 | 2.00E-87 |
| hsa_circ_0087671 | 97 | 1 | 190 | 1 | 190 | 0 | 1.00E-87 |
| hsa_circ_0087666 | 93 | 1 | 770 | 770 | 1 | 0 | 0 |
| hsa_circ_0087667 | 92 | 2 | 232 | 231 | 1 | 0 | 1.00E-88 |
| hsa_circ_0087666 | 94 | 2 | 649 | 648 | 1 | 0 | 0 |

novel_circ_005454 novel_circ_005457 novel_circ_005458 novel_circ_005459 novel_circ_005460 novel_circ_005461 novel_circ_005462 novel_circ_005463 novel_circ_005464 novel_circ_005465 novel_circ_005466 novel_circ_005467 novel_circ_005468 novel_circ_005469 novel_circ_005470 novel_circ_005471 novel_circ_005472 novel_circ_005473 novel_circ_005474 novel_circ_005475 novel_circ_005476 novel_circ_005477 novel_circ_005478 novel_circ_005479 novel_circ_005480 novel_circ_005481 novel_circ_005482 novel_circ_005483 novel_circ_005484 novel_circ_005485 novel_circ_005486 novel_circ_005487 novel_circ_005490 novel_circ_005491 novel_circ_005492 novel_circ_005493 novel_circ_005494 novel_circ_005495 novel_circ_005496 novel_circ_005497 novel_circ_005498 novel_circ_005499 novel_circ_005500 novel_circ_005507 novel_circ_005508 novel_circ_005512 novel_circ_005513 novel_circ_005514 novel_circ_005515 novel_circ_005516 novel_circ_005517 novel_circ_005518 novel_circ_005519 novel_circ_005520

| hsa_circ_0004167 | 92 | 3 | 291 | 290 | 2 | 0 | 4.00E-114 |
| --- | --- | --- | --- | --- | --- | --- | --- |
| hsa_circ_0087086 | 90 | 1 | 254 | 204 | 454 | 0.01 | 1.00E-88 |
| hsa_circ_0087086 | 92 | 1 | 457 | 1 | 454 | 0.01 | 0 |
| hsa_circ_0009083 | 94 | 2 | 655 | 2 | 652 | 0 | 0 |
| hsa_circ_0087085 | 95 | 1 | 629 | 1 | 626 | 0 | 0 |
| hsa_circ_0009083 | 95 | 2 | 515 | 2 | 512 | 0.01 | 0 |
| hsa_circ_0087085 | 97 | 1 | 312 | 1 | 312 | 0 | 3.00E-150 |
| hsa_circ_0087083 | 97 | 151 | 302 | 152 | 1 | 0 | 3.00E-66 |
| hsa_circ_0087082 | 93 | 126 | 277 | 1 | 152 | 0 | 6.00E-58 |
| hsa_circ_0087082 | 93 | 269 | 420 | 1 | 152 | 0 | 9.00E-58 |
| hsa_circ_0087082 | 93 | 449 | 600 | 1 | 152 | 0 | 1.00E-57 |
| hsa_circ_0087079 | 93 | 1 | 358 | 511 | 154 | 0 | 5.00E-149 |
| hsa_circ_0087079 | 93 | 1 | 509 | 511 | 3 | 0 | 0 |
| hsa_circ_0087079 | 93 | 1 | 359 | 361 | 3 | 0 | 3.00E-146 |
| hsa_circ_0087078 | 92 | 1 | 631 | 631 | 1 | 0 | 0 |
| hsa_circ_0087069 | 92 | 1 | 201 | 376 | 576 | 0 | 2.00E-75 |
| hsa_circ_0087053 | 82 | 1 | 335 | 1 | 335 | 0.01 | 7.00E-73 |
| hsa_circ_0005833 | 86 | 4 | 933 | 4 | 944 | 0.02 | 0 |
| hsa_circ_0087048 | 85 | 4 | 620 | 4 | 631 | 0.02 | 2.00E-180 |
| hsa_circ_0087028 | 99 | 402 | 553 | 152 | 1 | 0 | 5.00E-71 |
| hsa_circ_0087027 | 97 | 1 | 347 | 344 | 1 | 0.01 | 8.00E-167 |
| hsa_circ_0087025 | 98 | 1 | 561 | 558 | 1 | 0.01 | 0 |
| hsa_circ_0087025 | 98 | 1 | 411 | 411 | 1 | 0 | 0 |
| hsa_circ_0087025 | 98 | 1 | 408 | 408 | 1 | 0 | 0 |
| hsa_circ_0087016 | 96 | 1 | 1101 | 1101 | 1 | 0 | 0 |
| hsa_circ_0087013 | 96 | 1 | 1223 | 1323 | 101 | 0 | 0 |
| hsa_circ_0087013 | 95 | 4 | 815 | 912 | 101 | 0 | 0 |
| hsa_circ_0087014 | 96 | 1 | 476 | 476 | 1 | 0 | 0 |
| hsa_circ_0087014 | 94 | 1 | 207 | 207 | 1 | 0 | 2.00E-85 |
| hsa_circ_0087006 | 94 | 1 | 1024 | 1112 | 89 | 0 | 0 |
| hsa_circ_0087006 | 94 | 1 | 1112 | 1112 | 1 | 0 | 0 |
| hsa_circ_0087006 | 94 | 1 | 1112 | 1112 | 1 | 0 | 0 |
| hsa_circ_0087000 | 95 | 1 | 268 | 1 | 268 | 0 | 2.00E-117 |
| hsa_circ_0087000 | 94 | 1 | 156 | 1 | 156 | 0 | 2.00E-60 |
| hsa_circ_0086996 | 94 | 1 | 350 | 14517 | 14866 | 0 | 6.00E-148 |
| hsa_circ_0086996 | 94 | 1 | 996 | 13871 | 14866 | 0 | 0 |
| hsa_circ_0086996 | 78 | 1 | 714 | 5971 | 5228 | 0.05 | 2.00E-121 |
| hsa_circ_0086994 | 97 | 1 | 134 | 113 | 246 | 0 | 3.00E-57 |
| hsa_circ_0086994 | 96 | 1 | 246 | 1 | 246 | 0 | 4.00E-111 |
| hsa_circ_0039719 | 95 | 584 | 1414 | 1083 | 253 | 0 | 0 |
| hsa_circ_0038442 | 88 | 8007 | 8230 | 559 | 329 | 0.04 | 5.00E-69 |
| hsa_circ_0038442 | 88 | 73640 | 73863 | 559 | 329 | 0.04 | 8.00E-69 |
| hsa_circ_0038442 | 88 | 82114 | 82337 | 559 | 329 | 0.04 | 9.00E-69 |
| hsa_circ_0005447 | 90 | 33966 | 34086 | 7474 | 7353 | 0.01 | 5.00E-35 |
| hsa_circ_0005447 | 90 | 33966 | 34086 | 7474 | 7353 | 0.01 | 7.00E-35 |
| hsa_circ_0039718 | 96 | 126 | 288 | 605 | 443 | 0 | 1.00E-70 |
| hsa_circ_0039718 | 93 | 126 | 730 | 605 | 1 | 0 | 0 |
| hsa_circ_0039718 | 93 | 126 | 730 | 605 | 1 | 0 | 0 |
| hsa_circ_0039718 | 93 | 6 | 355 | 605 | 256 | 0 | 1.00E-144 |
| hsa_circ_0039718 | 93 | 6 | 610 | 605 | 1 | 0 | 0 |
| hsa_circ_0039718 | 93 | 6 | 610 | 605 | 1 | 0 | 0 |
| hsa_circ_0039717 | 96 | 138 | 377 | 240 | 1 | 0 | 2.00E-108 |
| hsa_circ_0039717 | 96 | 1 | 240 | 240 | 1 | 0 | 1.00E-108 |
| hsa_circ_0077783 | 89 | 402 | 680 | 1 | 279 | 0 | 2.00E-91 |

novel_circ_005521 novel_circ_005522 novel_circ_005523 novel_circ_005524 novel_circ_005525 novel_circ_005526 novel_circ_005527 novel_circ_005528 novel_circ_005529 novel_circ_005530 novel_circ_005531 novel_circ_005532 novel_circ_005533 novel_circ_005534 novel_circ_005535 novel_circ_005536 novel_circ_005537 novel_circ_005538 novel_circ_005539 novel_circ_005558 novel_circ_005559 novel_circ_005560 novel_circ_005561 novel_circ_005562 novel_circ_005563 novel_circ_005564 novel_circ_005565 novel_circ_005567 novel_circ_005568 novel_circ_005569 novel_circ_005570 novel_circ_005571 novel_circ_005572 novel_circ_005573 novel_circ_005574 novel_circ_005575 novel_circ_005577 novel_circ_005578 novel_circ_005579 novel_circ_005581 novel_circ_005582 novel_circ_005583 novel_circ_005585 novel_circ_005586 novel_circ_005587 novel_circ_005588 novel_circ_005589 novel_circ_005590 novel_circ_005591 novel_circ_005592 novel_circ_005593 novel_circ_005594 novel_circ_005595 novel_circ_005596

| hsa_circ_0077780 | 85 | 345 | 1348 | 1 | 1010 | 0.01 | 0 |
| --- | --- | --- | --- | --- | --- | --- | --- |
| hsa_circ_0077780 | 85 | 34 | 1037 | 1 | 1010 | 0.01 | 0 |
| hsa_circ_0077780 | 85 | 1 | 1004 | 1 | 1010 | 0.01 | 0 |
| hsa_circ_0077780 | 85 | 34 | 1037 | 1 | 1010 | 0.01 | 0 |
| hsa_circ_0077780 | 85 | 345 | 1348 | 1 | 1010 | 0.01 | 0 |
| hsa_circ_0077780 | 85 | 567 | 1570 | 1 | 1010 | 0.01 | 0 |
| hsa_circ_0077777 | 92 | 1 | 948 | 1 | 948 | 0 | 0 |
| hsa_circ_0077778 | 93 | 1 | 311 | 524 | 834 | 0 | 1.00E-124 |
| hsa_circ_0077778 | 94 | 1 | 533 | 302 | 834 | 0 | 0 |
| hsa_circ_0077778 | 92 | 3 | 834 | 3 | 834 | 0 | 0 |
| hsa_circ_0077777 | 92 | 1 | 948 | 1 | 948 | 0 | 0 |
| hsa_circ_0077778 | 97 | 1 | 302 | 222 | 523 | 0 | 3.00E-141 |
| hsa_circ_0077777 | 92 | 1 | 637 | 1 | 637 | 0 | 0 |
| hsa_circ_0077777 | 90 | 1 | 415 | 1 | 415 | 0 | 6.00E-149 |
| hsa_circ_0077777 | 88 | 1 | 335 | 1 | 335 | 0 | 1.00E-109 |
| hsa_circ_0077777 | 92 | 1 | 114 | 1 | 114 | 0 | 5.00E-37 |
| hsa_circ_0077765 | 96 | 1 | 399 | 1 | 399 | 0 | 0 |
| hsa_circ_0077765 | 96 | 51354 | 51752 | 1 | 399 | 0 | 0 |
| hsa_circ_0077765 | 96 | 1 | 234 | 1 | 234 | 0 | 2.00E-104 |
| hsa_circ_0083082 | 90 | 1 | 392 | 1 | 392 | 0 | 3.00E-141 |
| hsa_circ_0001771 | 91 | 831 | 956 | 3 | 128 | 0 | 3.00E-41 |
| hsa_circ_0001771 | 91 | 3 | 128 | 3 | 128 | 0 | 3.00E-42 |
| hsa_circ_0001771 | 91 | 3 | 128 | 3 | 128 | 0 | 1.00E-40 |
| hsa_circ_0001771 | 89 | 3 | 518 | 3 | 524 | 0.01 | 0 |
| hsa_circ_0001771 | 89 | 3 | 518 | 3 | 524 | 0.01 | 0 |
| hsa_circ_0001771 | 89 | 3 | 518 | 3 | 524 | 0.01 | 0 |
| hsa_circ_0001771 | 89 | 3 | 518 | 3 | 524 | 0.01 | 0 |
| hsa_circ_0083096 | 87 | 1 | 313 | 1 | 319 | 0.02 | 2.00E-97 |
| hsa_circ_0083095 | 87 | 1 | 314 | 78 | 397 | 0.02 | 9.00E-98 |
| hsa_circ_0083095 | 87 | 1 | 314 | 78 | 397 | 0.02 | 1.00E-97 |
| hsa_circ_0083097 | 88 | 434 | 814 | 1 | 381 | 0 | 1.00E-124 |
| hsa_circ_0083097 | 88 | 434 | 819 | 1 | 386 | 0 | 1.00E-125 |
| hsa_circ_0083097 | 88 | 434 | 819 | 1 | 386 | 0 | 1.00E-125 |
| hsa_circ_0083097 | 88 | 1 | 381 | 1 | 381 | 0 | 4.00E-125 |
| hsa_circ_0083097 | 88 | 1 | 386 | 1 | 386 | 0 | 6.00E-126 |
| hsa_circ_0083097 | 88 | 1 | 386 | 1 | 386 | 0 | 7.00E-126 |
| hsa_circ_0083108 | 85 | 605 | 786 | 138 | 312 | 0.05 | 1.00E-43 |
| hsa_circ_0083107 | 81 | 605 | 1710 | 615 | 1719 | 0.03 | 0 |
| hsa_circ_0083108 | 85 | 472 | 653 | 138 | 312 | 0.05 | 1.00E-43 |
| hsa_circ_0083114 | 92 | 4 | 394 | 391 | 1 | 0 | 3.00E-152 |
| hsa_circ_0083114 | 90 | 6 | 630 | 625 | 1 | 0 | 0 |
| hsa_circ_0083114 | 90 | 1 | 703 | 703 | 1 | 0 | 0 |
| hsa_circ_0004673 | 88 | 114 | 1159 | 1046 | 1 | 0 | 0 |
| hsa_circ_0083119 | 88 | 114 | 1092 | 979 | 1 | 0 | 0 |
| hsa_circ_0083119 | 88 | 6 | 236 | 396 | 166 | 0 | 1.00E-73 |
| hsa_circ_0083119 | 89 | 1 | 309 | 474 | 166 | 0 | 2.00E-103 |
| hsa_circ_0083119 | 87 | 114 | 927 | 979 | 166 | 0 | 0 |
| hsa_circ_0083119 | 87 | 1 | 659 | 979 | 321 | 0 | 0 |
| hsa_circ_0083121 | 89 | 1 | 691 | 691 | 1 | 0 | 0 |
| hsa_circ_0083121 | 85 | 4 | 438 | 435 | 1 | 0 | 1.00E-121 |
| hsa_circ_0083121 | 87 | 1 | 578 | 578 | 1 | 0 | 0 |
| hsa_circ_0083121 | 89 | 1 | 691 | 691 | 1 | 0 | 0 |
| hsa_circ_0004644 | 84 | 4 | 362 | 362 | 4 | 0 | 3.00E-91 |
| hsa_circ_0083121 | 88 | 1 | 615 | 691 | 77 | 0 | 0 |

novel_circ_005597 novel_circ_005598 novel_circ_005599 novel_circ_005600 novel_circ_005601 novel_circ_005602 novel_circ_005604 novel_circ_005605 novel_circ_005606 novel_circ_005607 novel_circ_005608 novel_circ_005609 novel_circ_005610 novel_circ_005611 novel_circ_005613 novel_circ_005614 novel_circ_005615 novel_circ_005617 novel_circ_005618 novel_circ_005619 novel_circ_005621 novel_circ_005622 novel_circ_005623 novel_circ_005624 novel_circ_005625 novel_circ_005626 novel_circ_005627 novel_circ_005628 novel_circ_005629 novel_circ_005630 novel_circ_005631 novel_circ_005635 novel_circ_005639 novel_circ_005640 novel_circ_005641 novel_circ_005642 novel_circ_005643 novel_circ_005644 novel_circ_005645 novel_circ_005647 novel_circ_005648 novel_circ_005649 novel_circ_005650 novel_circ_005651 novel_circ_005652 novel_circ_005653 novel_circ_005654 novel_circ_005655 novel_circ_005656 novel_circ_005657 novel_circ_005658 novel_circ_005659 novel_circ_005660 novel_circ_005661

| hsa_circ_0083121 | 89 | 1 | 539 | 691 | 153 | 0 | 0 |
| --- | --- | --- | --- | --- | --- | --- | --- |
| hsa_circ_0083121 | 95 | 1 | 358 | 691 | 334 | 0 | 7.00E-156 |
| hsa_circ_0083125 | 94 | 1 | 244 | 244 | 1 | 0 | 2.00E-102 |
| hsa_circ_0083121 | 95 | 1 | 357 | 691 | 335 | 0 | 8.00E-157 |
| hsa_circ_0083121 | 95 | 1 | 259 | 691 | 433 | 0 | 1.00E-111 |
| hsa_circ_0005939 | 95 | 1 | 253 | 253 | 1 | 0 | 8.00E-111 |
| hsa_circ_0083146 | 96 | 1 | 55 | 1 | 55 | 0 | 3.00E-16 |
| hsa_circ_0083146 | 96 | 1 | 55 | 1 | 55 | 0 | 3.00E-16 |
| hsa_circ_0083150 | 85 | 1 | 563 | 1 | 563 | 0.01 | 2.00E-164 |
| hsa_circ_0083152 | 87 | 3 | 533 | 3 | 533 | 0 | 1.00E-166 |
| hsa_circ_0083152 | 85 | 3 | 1336 | 3 | 1342 | 0.01 | 0 |
| hsa_circ_0083154 | 83 | 1 | 1024 | 1 | 1030 | 0.01 | 0 |
| hsa_circ_0083156 | 86 | 1 | 1236 | 1 | 1242 | 0 | 0 |
| hsa_circ_0083156 | 87 | 1 | 1697 | 1 | 1703 | 0 | 0 |
| hsa_circ_0083158 | 86 | 1 | 1084 | 1 | 1090 | 0.01 | 0 |
| hsa_circ_0083158 | 87 | 1 | 1332 | 1 | 1338 | 0 | 0 |
| hsa_circ_0083156 | 87 | 1 | 1545 | 153 | 1703 | 0 | 0 |
| hsa_circ_0083160 | 86 | 1 | 896 | 1 | 902 | 0.01 | 0 |
| hsa_circ_0083160 | 88 | 1 | 1144 | 1 | 1150 | 0.01 | 0 |
| hsa_circ_0083160 | 88 | 1 | 1357 | 1 | 1363 | 0 | 0 |
| hsa_circ_0083160 | 86 | 1 | 809 | 88 | 902 | 0.01 | 0 |
| hsa_circ_0083160 | 88 | 1 | 1057 | 88 | 1150 | 0.01 | 0 |
| hsa_circ_0083160 | 88 | 1 | 1657 | 88 | 1750 | 0 | 0 |
| hsa_circ_0001774 | 90 | 1 | 424 | 1 | 424 | 0 | 3.00E-152 |
| hsa_circ_0083160 | 91 | 1 | 672 | 479 | 1150 | 0 | 0 |
| hsa_circ_0083160 | 91 | 1 | 885 | 479 | 1363 | 0 | 0 |
| hsa_circ_0083160 | 90 | 1 | 1141 | 479 | 1619 | 0 | 0 |
| hsa_circ_0083160 | 90 | 1 | 1272 | 479 | 1750 | 0 | 0 |
| hsa_circ_0083164 | 91 | 1 | 231 | 1 | 231 | 0 | 1.00E-83 |
| hsa_circ_0083164 | 91 | 1 | 692 | 1 | 692 | 0 | 0 |
| hsa_circ_0083166 | 91 | 1 | 461 | 1 | 461 | 0 | 2.00E-179 |
| hsa_circ_0083179 | 90 | 1 | 406 | 406 | 1 | 0 | 1.00E-145 |
| hsa_circ_0083213 | 83 | 8 | 616 | 609 | 1 | 0 | 2.00E-151 |
| hsa_circ_0083213 | 82 | 1 | 672 | 672 | 1 | 0 | 2.00E-166 |
| hsa_circ_0083213 | 85 | 1 | 1089 | 1089 | 1 | 0 | 0 |
| hsa_circ_0083213 | 82 | 1 | 672 | 672 | 1 | 0 | 2.00E-166 |
| hsa_circ_0083215 | 85 | 1 | 600 | 649 | 50 | 0 | 1.00E-171 |
| hsa_circ_0001777 | 90 | 1 | 417 | 417 | 1 | 0 | 1.00E-151 |
| hsa_circ_0083231 | 76 | 888 | 1277 | 124 | 525 | 0.04 | 8.00E-48 |
| hsa_circ_0057260 | 93 | 1 | 227 | 227 | 1 | 0 | 2.00E-91 |
| hsa_circ_0057260 | 95 | 1 | 677 | 677 | 1 | 0 | 0 |
| hsa_circ_0057259 | 95 | 1 | 2176 | 2266 | 91 | 0 | 0 |
| hsa_circ_0057259 | 95 | 1 | 2390 | 2480 | 91 | 0 | 0 |
| hsa_circ_0057260 | 97 | 1 | 256 | 485 | 230 | 0 | 4.00E-119 |
| hsa_circ_0057266 | 95 | 1 | 542 | 542 | 1 | 0 | 0 |
| hsa_circ_0057266 | 94 | 1 | 814 | 814 | 1 | 0 | 0 |
| hsa_circ_0057266 | 95 | 1 | 1691 | 1691 | 1 | 0 | 0 |
| hsa_circ_0057266 | 95 | 1 | 1905 | 1905 | 1 | 0 | 0 |
| hsa_circ_0057269 | 95 | 1 | 448 | 448 | 1 | 0 | 0 |
| hsa_circ_0057269 | 94 | 1 | 720 | 720 | 1 | 0 | 0 |
| hsa_circ_0057269 | 95 | 4 | 840 | 837 | 1 | 0 | 0 |
| hsa_circ_0057269 | 95 | 1 | 1119 | 1119 | 1 | 0 | 0 |
| hsa_circ_0057269 | 96 | 1 | 1597 | 1597 | 1 | 0 | 0 |
| hsa_circ_0057269 | 96 | 1 | 1811 | 1811 | 1 | 0 | 0 |

novel_circ_005662 novel_circ_005663 novel_circ_005664 novel_circ_005665 novel_circ_005666 novel_circ_005667 novel_circ_005668 novel_circ_005669 novel_circ_005670 novel_circ_005671 novel_circ_005672 novel_circ_005673 novel_circ_005674 novel_circ_005675 novel_circ_005676 novel_circ_005677 novel_circ_005678 novel_circ_005679 novel_circ_005680 novel_circ_005681 novel_circ_005682 novel_circ_005683 novel_circ_005684 novel_circ_005685 novel_circ_005686 novel_circ_005688 novel_circ_005689 novel_circ_005690 novel_circ_005691 novel_circ_005692 novel_circ_005693 novel_circ_005694 novel_circ_005695 novel_circ_005696 novel_circ_005697 novel_circ_005698 novel_circ_005699 novel_circ_005700 novel_circ_005701 novel_circ_005705 novel_circ_005706 novel_circ_005707 novel_circ_005709 novel_circ_005710 novel_circ_005711 novel_circ_005713 novel_circ_005714 novel_circ_005715 novel_circ_005716 novel_circ_005717 novel_circ_005718 novel_circ_005719 novel_circ_005720 novel_circ_005721

| hsa_circ_0057269 | 96 | 1 | 1713 | 1811 | 99 | 0 | 0 |
| --- | --- | --- | --- | --- | --- | --- | --- |
| hsa_circ_0057279 | 97 | 1 | 877 | 877 | 1 | 0 | 0 |
| hsa_circ_0057279 | 97 | 2 | 934 | 933 | 1 | 0 | 0 |
| hsa_circ_0057279 | 97 | 1 | 1091 | 1091 | 1 | 0 | 0 |
| hsa_circ_0057279 | 97 | 1 | 338 | 458 | 121 | 0 | 6.00E-163 |
| hsa_circ_0057279 | 97 | 1 | 553 | 673 | 121 | 0 | 0 |
| hsa_circ_0057279 | 97 | 1 | 757 | 877 | 121 | 0 | 0 |
| hsa_circ_0057279 | 97 | 2 | 814 | 933 | 121 | 0 | 0 |
| hsa_circ_0057279 | 97 | 1 | 971 | 1091 | 121 | 0 | 0 |
| hsa_circ_0057279 | 97 | 1 | 287 | 540 | 254 | 0 | 5.00E-133 |
| hsa_circ_0057279 | 97 | 1 | 624 | 877 | 254 | 0 | 0 |
| hsa_circ_0057279 | 97 | 2 | 681 | 933 | 254 | 0 | 0 |
| hsa_circ_0057279 | 97 | 1 | 838 | 1091 | 254 | 0 | 0 |
| hsa_circ_0057283 | 97 | 1 | 478 | 478 | 1 | 0 | 0 |
| hsa_circ_0057283 | 97 | 1 | 692 | 692 | 1 | 0 | 0 |
| hsa_circ_0057284 | 96 | 2 | 476 | 475 | 1 | 0 | 0 |
| hsa_circ_0057284 | 97 | 1 | 337 | 419 | 83 | 0 | 2.00E-162 |
| hsa_circ_0057284 | 97 | 2 | 394 | 475 | 83 | 0 | 0 |
| hsa_circ_0057284 | 97 | 1 | 551 | 633 | 83 | 0 | 0 |
| hsa_circ_0057284 | 96 | 1 | 738 | 820 | 83 | 0 | 0 |
| hsa_circ_0057288 | 96 | 1 | 344 | 344 | 1 | 0 | 3.00E-156 |
| hsa_circ_0057298 | 95 | 1 | 360 | 1 | 357 | 0.01 | 1.00E-160 |
| hsa_circ_0057296 | 95 | 1 | 701 | 144 | 841 | 0 | 0 |
| hsa_circ_0068801 | 88 | 89 | 507 | 32 | 451 | 0.01 | 4.00E-141 |
| hsa_circ_0001385 | 91 | 1 | 200 | 1 | 200 | 0 | 8.00E-70 |
| hsa_circ_0068816 | 87 | 5837 | 6392 | 556 | 1 | 0 | 0 |
| hsa_circ_0068816 | 89 | 1 | 680 | 680 | 1 | 0 | 0 |
| hsa_circ_0068816 | 88 | 1 | 823 | 823 | 1 | 0 | 0 |
| hsa_circ_0068816 | 89 | 1 | 485 | 823 | 339 | 0 | 9.00E-173 |
| hsa_circ_0068820 | 87 | 1153 | 1466 | 314 | 1 | 0 | 1.00E-95 |
| hsa_circ_0068820 | 88 | 1 | 380 | 380 | 1 | 0 | 7.00E-123 |
| hsa_circ_0005830 | 98 | 1343 | 1398 | 171 | 116 | 0 | 7.00E-19 |
| hsa_circ_0005830 | 94 | 191 | 312 | 237 | 116 | 0 | 3.00E-46 |
| hsa_circ_0068824 | 88 | 88 | 363 | 52 | 327 | 0.01 | 2.00E-88 |
| hsa_circ_0004462 | 86 | 19132 | 19803 | 805 | 142 | 0.02 | 0 |
| hsa_circ_0004462 | 86 | 24736 | 25407 | 805 | 142 | 0.02 | 0 |
| hsa_circ_0015678 | 96 | 441 | 904 | 2918 | 2455 | 0 | 0 |
| hsa_circ_0015678 | 95 | 1 | 585 | 2917 | 2333 | 0 | 0 |
| hsa_circ_0015678 | 90 | 1 | 2590 | 2917 | 346 | 0.03 | 0 |
| hsa_circ_0004689 | 89 | 2 | 466 | 2 | 466 | 0 | 1.00E-161 |
| hsa_circ_0015677 | 89 | 1 | 915 | 386 | 1300 | 0 | 0 |
| hsa_circ_0015677 | 90 | 1 | 1300 | 1 | 1300 | 0 | 0 |
| hsa_circ_0004689 | 89 | 2 | 466 | 2 | 466 | 0 | 1.00E-161 |
| hsa_circ_0015677 | 89 | 1 | 605 | 696 | 1300 | 0 | 0 |
| hsa_circ_0015677 | 89 | 1 | 915 | 386 | 1300 | 0 | 0 |
| hsa_circ_0015677 | 89 | 1 | 605 | 696 | 1300 | 0 | 0 |
| hsa_circ_0015677 | 89 | 1 | 915 | 386 | 1300 | 0 | 0 |
| hsa_circ_0015677 | 90 | 1 | 476 | 696 | 1171 | 0 | 4.00E-176 |
| hsa_circ_0015677 | 89 | 1 | 786 | 386 | 1171 | 0 | 0 |
| hsa_circ_0015677 | 90 | 1 | 1171 | 1 | 1171 | 0 | 0 |
| hsa_circ_0015677 | 92 | 3 | 283 | 105 | 385 | 0 | 1.00E-109 |
| hsa_circ_0015674 | 80 | 7 | 730 | 75 | 801 | 0.03 | 7.00E-146 |
| hsa_circ_0015664 | 92 | 150 | 565 | 1522 | 1107 | 0 | 2.00E-164 |
| hsa_circ_0015664 | 91 | 150 | 1662 | 1522 | 1 | 0.01 | 0 |

novel_circ_005722 novel_circ_005723 novel_circ_005724 novel_circ_005725 novel_circ_005726 novel_circ_005727 novel_circ_005728 novel_circ_005729 novel_circ_005730 novel_circ_005731 novel_circ_005732 novel_circ_005733 novel_circ_005734 novel_circ_005735 novel_circ_005736 novel_circ_005737 novel_circ_005738 novel_circ_005739 novel_circ_005740 novel_circ_005741 novel_circ_005743 novel_circ_005744 novel_circ_005745 novel_circ_005746 novel_circ_005747 novel_circ_005748 novel_circ_005749 novel_circ_005750 novel_circ_005751 novel_circ_005752 novel_circ_005753 novel_circ_005754 novel_circ_005755 novel_circ_005758 novel_circ_005759 novel_circ_005760 novel_circ_005762 novel_circ_005763 novel_circ_005764 novel_circ_005765 novel_circ_005766 novel_circ_005767 novel_circ_005770 novel_circ_005771 novel_circ_005772 novel_circ_005773 novel_circ_005774 novel_circ_005775 novel_circ_005776 novel_circ_005777 novel_circ_005778 novel_circ_005779 novel_circ_005780 novel_circ_005781

| hsa_circ_0015664 | 92 | 2412 | 2827 | 1522 | 1107 | 0 | 1.00E-163 |
| --- | --- | --- | --- | --- | --- | --- | --- |
| hsa_circ_0015664 | 91 | 2412 | 3924 | 1522 | 1 | 0.01 | 0 |
| hsa_circ_0015669 | 91 | 3 | 225 | 223 | 1 | 0 | 3.00E-79 |
| hsa_circ_0015668 | 91 | 3 | 290 | 288 | 1 | 0 | 2.00E-108 |
| hsa_circ_0015664 | 92 | 3 | 418 | 1522 | 1107 | 0 | 2.00E-164 |
| hsa_circ_0015664 | 92 | 3 | 544 | 1522 | 981 | 0 | 0 |
| hsa_circ_0015664 | 93 | 3 | 624 | 1522 | 901 | 0 | 0 |
| hsa_circ_0015664 | 91 | 3 | 1078 | 1522 | 438 | 0.01 | 0 |
| hsa_circ_0015664 | 91 | 3 | 1269 | 1522 | 247 | 0.01 | 0 |
| hsa_circ_0015664 | 91 | 3 | 1348 | 1522 | 168 | 0.01 | 0 |
| hsa_circ_0015664 | 91 | 3 | 1515 | 1522 | 1 | 0.01 | 0 |
| hsa_circ_0015664 | 91 | 3 | 1515 | 1522 | 1 | 0.01 | 0 |
| hsa_circ_0015664 | 91 | 3 | 1515 | 1522 | 1 | 0.01 | 0 |
| hsa_circ_0015664 | 91 | 1 | 1123 | 1299 | 168 | 0.01 | 0 |
| hsa_circ_0015664 | 91 | 1 | 1290 | 1299 | 1 | 0.01 | 0 |
| hsa_circ_0015664 | 91 | 1 | 658 | 1104 | 438 | 0.01 | 0 |
| hsa_circ_0015664 | 91 | 1 | 849 | 1104 | 247 | 0.01 | 0 |
| hsa_circ_0015664 | 91 | 1 | 928 | 1104 | 168 | 0.01 | 0 |
| hsa_circ_0015664 | 91 | 1 | 1095 | 1104 | 1 | 0.01 | 0 |
| hsa_circ_0015664 | 91 | 1 | 971 | 980 | 1 | 0.01 | 0 |
| hsa_circ_0015661 | 95 | 1 | 466 | 1 | 466 | 0 | 0 |
| hsa_circ_0015650 | 94 | 1 | 649 | 2185 | 1537 | 0 | 0 |
| hsa_circ_0015650 | 93 | 1 | 957 | 2185 | 1229 | 0 | 0 |
| hsa_circ_0015650 | 93 | 1 | 2186 | 2185 | 3 | 0 | 0 |
| hsa_circ_0015650 | 93 | 4 | 816 | 2041 | 1229 | 0 | 0 |
| hsa_circ_0015650 | 95 | 4 | 1191 | 2041 | 854 | 0 | 0 |
| hsa_circ_0015650 | 95 | 4 | 1503 | 2041 | 545 | 0 | 0 |
| hsa_circ_0015650 | 95 | 4 | 1504 | 2041 | 544 | 0 | 0 |
| hsa_circ_0015650 | 92 | 1 | 414 | 1642 | 1229 | 0 | 5.00E-165 |
| hsa_circ_0015650 | 95 | 3 | 995 | 1534 | 545 | 0 | 0 |
| hsa_circ_0015650 | 95 | 3 | 996 | 1534 | 544 | 0 | 0 |
| hsa_circ_0015653 | 98 | 1 | 209 | 209 | 1 | 0 | 8.00E-100 |
| hsa_circ_0015650 | 96 | 1 | 478 | 1019 | 545 | 0.01 | 0 |
| hsa_circ_0015646 | 93 | 1 | 142 | 336 | 477 | 0 | 2.00E-52 |
| hsa_circ_0015643 | 93 | 1 | 689 | 873 | 185 | 0 | 0 |
| hsa_circ_0015643 | 93 | 1 | 509 | 509 | 1 | 0 | 0 |
| hsa_circ_0015641 | 87 | 1 | 678 | 1 | 654 | 0.04 | 0 |
| hsa_circ_0015641 | 87 | 34 | 711 | 1 | 654 | 0.04 | 0 |
| hsa_circ_0015641 | 84 | 1 | 423 | 1 | 399 | 0.06 | 2.00E-108 |
| hsa_circ_0015640 | 86 | 1 | 456 | 178 | 609 | 0.05 | 1.00E-126 |
| hsa_circ_0015639 | 94 | 527 | 805 | 1 | 279 | 0 | 5.00E-118 |
| hsa_circ_0015639 | 94 | 527 | 715 | 1 | 189 | 0 | 2.00E-76 |
| hsa_circ_0015627 | 94 | 1 | 1080 | 384 | 1463 | 0 | 0 |
| hsa_circ_0015619 | 93 | 1 | 2604 | 665 | 3271 | 0 | 0 |
| hsa_circ_0015627 | 94 | 1 | 763 | 384 | 1146 | 0 | 0 |
| hsa_circ_0015624 | 95 | 1 | 528 | 428 | 955 | 0 | 0 |
| hsa_circ_0015619 | 94 | 1 | 1811 | 378 | 2191 | 0 | 0 |
| hsa_circ_0015619 | 91 | 1 | 858 | 665 | 1525 | 0 | 0 |
| hsa_circ_0015619 | 91 | 1 | 452 | 665 | 1116 | 0 | 8.00E-173 |
| hsa_circ_0015619 | 94 | 1 | 739 | 378 | 1116 | 0 | 0 |
| hsa_circ_0015619 | 91 | 1 | 320 | 665 | 984 | 0 | 6.00E-118 |
| hsa_circ_0015619 | 95 | 1 | 984 | 1 | 984 | 0 | 0 |
| hsa_circ_0015612 | 96 | 1 | 283 | 1 | 283 | 0 | 4.00E-129 |
| hsa_circ_0015611 | 96 | 169 | 457 | 10578 | 10867 | 0 | 1.00E-130 |

novel_circ_005782 novel_circ_005785 novel_circ_005786 novel_circ_005794 novel_circ_005795 novel_circ_005796 novel_circ_005797 novel_circ_005798 novel_circ_005800 novel_circ_005801 novel_circ_005803 novel_circ_005804 novel_circ_005805 novel_circ_005806 novel_circ_005807 novel_circ_005808 novel_circ_005809 novel_circ_005810 novel_circ_005811 novel_circ_005812 novel_circ_005813 novel_circ_005814 novel_circ_005815 novel_circ_005816 novel_circ_005817 novel_circ_005818 novel_circ_005819 novel_circ_005820 novel_circ_005821 novel_circ_005822 novel_circ_005823 novel_circ_005824 novel_circ_005825 novel_circ_005826 novel_circ_005827 novel_circ_005828 novel_circ_005829 novel_circ_005830 novel_circ_005831 novel_circ_005832 novel_circ_005833 novel_circ_005834 novel_circ_005835 novel_circ_005836 novel_circ_005837 novel_circ_005838 novel_circ_005840 novel_circ_005841 novel_circ_005842 novel_circ_005843 novel_circ_005844 novel_circ_005845 novel_circ_005846 novel_circ_005847

| hsa_circ_0015611 | 96 | 169 | 324 | 10578 | 10734 | 0.01 | 4.00E-65 |
| --- | --- | --- | --- | --- | --- | --- | --- |
| hsa_circ_0015581 | 90 | 1 | 600 | 1 | 600 | 0 | 0 |
| hsa_circ_0015581 | 90 | 1 | 436 | 1 | 436 | 0 | 3.00E-162 |
| hsa_circ_0020371 | 92 | 723 | 1600 | 1217 | 343 | 0.01 | 0 |
| hsa_circ_0020371 | 92 | 723 | 1792 | 1217 | 151 | 0 | 0 |
| hsa_circ_0020371 | 92 | 195 | 1072 | 1217 | 343 | 0.01 | 0 |
| hsa_circ_0020371 | 92 | 1 | 878 | 1217 | 343 | 0.01 | 0 |
| hsa_circ_0020371 | 90 | 1 | 505 | 844 | 343 | 0.01 | 0 |
| hsa_circ_0020368 | 92 | 1 | 420 | 686 | 267 | 0 | 1.00E-166 |
| hsa_circ_0020358 | 91 | 542 | 984 | 1 | 443 | 0 | 2.00E-167 |
| hsa_circ_0020356 | 95 | 204 | 648 | 300 | 744 | 0 | 0 |
| hsa_circ_0020356 | 92 | 1 | 298 | 1 | 298 | 0 | 3.00E-117 |
| hsa_circ_0002545 | 95 | 1 | 264 | 263 | 1 | 0 | 5.00E-113 |
| hsa_circ_0020352 | 95 | 1 | 267 | 418 | 153 | 0 | 2.00E-113 |
| hsa_circ_0003462 | 83 | 10713 | 11133 | 429 | 1 | 0.03 | 1.00E-104 |
| hsa_circ_0020352 | 96 | 1 | 163 | 314 | 153 | 0.01 | 5.00E-68 |
| hsa_circ_0020352 | 96 | 1 | 163 | 314 | 153 | 0.01 | 7.00E-68 |
| hsa_circ_0020352 | 96 | 1 | 163 | 314 | 153 | 0.01 | 8.00E-68 |
| hsa_circ_0020352 | 96 | 1 | 163 | 314 | 153 | 0.01 | 3.00E-67 |
| hsa_circ_0020342 | 95 | 1 | 481 | 272 | 752 | 0 | 0 |
| hsa_circ_0020342 | 95 | 3 | 680 | 75 | 752 | 0 | 0 |
| hsa_circ_0020342 | 95 | 155 | 906 | 1 | 752 | 0 | 0 |
| hsa_circ_0020342 | 96 | 3 | 320 | 75 | 392 | 0 | 4.00E-145 |
| hsa_circ_0020338 | 97 | 1 | 546 | 189 | 734 | 0 | 0 |
| hsa_circ_0020339 | 96 | 1 | 425 | 1 | 425 | 0 | 0 |
| hsa_circ_0020336 | 95 | 1 | 847 | 1 | 847 | 0 | 0 |
| hsa_circ_0020336 | 95 | 1 | 847 | 1 | 847 | 0 | 0 |
| hsa_circ_0004451 | 98 | 1 | 122 | 19831 | 19952 | 0 | 2.00E-51 |
| hsa_circ_0020336 | 95 | 1 | 847 | 1 | 847 | 0 | 0 |
| hsa_circ_0006545 | 90 | 1376 | 2583 | 1725 | 2930 | 0.02 | 0 |
| hsa_circ_0006545 | 90 | 5241 | 6448 | 1725 | 2930 | 0.02 | 0 |
| hsa_circ_0006545 | 90 | 1376 | 2587 | 1725 | 2934 | 0.02 | 0 |
| hsa_circ_0006545 | 90 | 1394 | 2605 | 1725 | 2934 | 0.02 | 0 |
| hsa_circ_0006545 | 90 | 5241 | 6452 | 1725 | 2934 | 0.02 | 0 |
| hsa_circ_0006545 | 90 | 5259 | 6470 | 1725 | 2934 | 0.02 | 0 |
| hsa_circ_0020329 | 94 | 1 | 326 | 584 | 259 | 0 | 4.00E-141 |
| hsa_circ_0020328 | 84 | 417 | 1467 | 1661 | 641 | 0.06 | 0 |
| hsa_circ_0002282 | 89 | 1 | 779 | 773 | 1 | 0.01 | 0 |
| hsa_circ_0020309 | 87 | 1 | 591 | 217 | 807 | 0 | 0 |
| hsa_circ_0020311 | 85 | 1 | 336 | 1 | 336 | 0 | 4.00E-95 |
| hsa_circ_0008898 | 90 | 1 | 549 | 549 | 1 | 0 | 0 |
| hsa_circ_0008898 | 90 | 1 | 321 | 321 | 1 | 0 | 8.00E-117 |
| hsa_circ_0008898 | 90 | 1 | 321 | 321 | 1 | 0 | 2.00E-116 |
| hsa_circ_0020307 | 86 | 5 | 1698 | 1698 | 1 | 0.01 | 0 |
| hsa_circ_0020304 | 87 | 5 | 1855 | 1855 | 1 | 0.01 | 0 |
| hsa_circ_0020293 | 95 | 154 | 515 | 1400 | 1044 | 0.01 | 2.00E-159 |
| hsa_circ_0020271 | 91 | 1 | 702 | 1 | 702 | 0 | 0 |
| hsa_circ_0020271 | 92 | 1 | 606 | 1 | 606 | 0 | 0 |
| hsa_circ_0020270 | 88 | 1 | 305 | 1 | 305 | 0 | 6.00E-98 |
| hsa_circ_0020269 | 88 | 16922 | 17228 | 599 | 905 | 0 | 3.00E-97 |
| hsa_circ_0020266 | 95 | 1 | 213 | 127 | 339 | 0 | 2.00E-90 |
| hsa_circ_0020264 | 93 | 1 | 540 | 232 | 771 | 0 | 0 |
| hsa_circ_0020264 | 93 | 1 | 701 | 71 | 771 | 0 | 0 |
| hsa_circ_0020264 | 92 | 1 | 327 | 232 | 558 | 0 | 8.00E-127 |

novel_circ_005848 novel_circ_005849 novel_circ_005850 novel_circ_005851 novel_circ_005852 novel_circ_005853 novel_circ_005854 novel_circ_005855 novel_circ_005856 novel_circ_005857 novel_circ_005858 novel_circ_005859 novel_circ_005860 novel_circ_005861 novel_circ_005862 novel_circ_005863 novel_circ_005864 novel_circ_005865 novel_circ_005866 novel_circ_005867 novel_circ_005868 novel_circ_005869 novel_circ_005870 novel_circ_005871 novel_circ_005872 novel_circ_005873 novel_circ_005874 novel_circ_005875 novel_circ_005876 novel_circ_005877 novel_circ_005878 novel_circ_005879 novel_circ_005880 novel_circ_005881 novel_circ_005882 novel_circ_005883 novel_circ_005884 novel_circ_005885 novel_circ_005886 novel_circ_005887 novel_circ_005888 novel_circ_005889 novel_circ_005890 novel_circ_005891 novel_circ_005892 novel_circ_005893 novel_circ_005894 novel_circ_005895 novel_circ_005896 novel_circ_005897 novel_circ_005898 novel_circ_005899 novel_circ_005900 novel_circ_005901

| hsa_circ_0020264 | 93 | 1 | 488 | 71 | 558 | 0 | 0 |
| --- | --- | --- | --- | --- | --- | --- | --- |
| hsa_circ_0020264 | 94 | 1 | 264 | 71 | 334 | 0 | 1.00E-108 |
| hsa_circ_0003419 | 90 | 1 | 483 | 477 | 1 | 0.02 | 5.00E-175 |
| hsa_circ_0008742 | 92 | 1 | 719 | 836 | 124 | 0.02 | 0 |
| hsa_circ_0008742 | 92 | 1 | 719 | 836 | 124 | 0.02 | 0 |
| hsa_circ_0008742 | 92 | 1 | 719 | 836 | 124 | 0.02 | 0 |
| hsa_circ_0008742 | 92 | 1 | 719 | 836 | 124 | 0.02 | 0 |
| hsa_circ_0008742 | 91 | 1 | 655 | 772 | 124 | 0.02 | 0 |
| hsa_circ_0008742 | 90 | 1 | 592 | 709 | 124 | 0.02 | 0 |
| hsa_circ_0008742 | 91 | 1 | 488 | 605 | 124 | 0.02 | 0 |
| hsa_circ_0008742 | 91 | 1 | 488 | 605 | 124 | 0.02 | 0 |
| hsa_circ_0008742 | 91 | 1 | 488 | 605 | 124 | 0.02 | 0 |
| hsa_circ_0008742 | 91 | 1 | 488 | 605 | 124 | 0.02 | 0 |
| hsa_circ_0007062 | 94 | 610 | 1055 | 567 | 122 | 0 | 0 |
| hsa_circ_0008742 | 95 | 1 | 236 | 359 | 124 | 0 | 5.00E-101 |
| hsa_circ_0007062 | 94 | 358 | 803 | 567 | 122 | 0 | 0 |
| hsa_circ_0007062 | 93 | 358 | 924 | 567 | 1 | 0 | 0 |
| hsa_circ_0007062 | 94 | 1 | 316 | 436 | 121 | 0 | 5.00E-131 |
| hsa_circ_0007062 | 93 | 1 | 436 | 436 | 1 | 0 | 1.00E-180 |
| hsa_circ_0020238 | 95 | 1 | 345 | 345 | 1 | 0 | 7.00E-155 |
| hsa_circ_0020238 | 95 | 1 | 345 | 345 | 1 | 0 | 1.00E-154 |
| hsa_circ_0020238 | 95 | 1 | 345 | 345 | 1 | 0 | 2.00E-154 |
| hsa_circ_0020238 | 95 | 1 | 345 | 345 | 1 | 0 | 2.00E-154 |
| hsa_circ_0020233 | 94 | 1327 | 1901 | 1339 | 765 | 0 | 0 |
| hsa_circ_0020237 | 91 | 79 | 370 | 347 | 56 | 0 | 3.00E-107 |
| hsa_circ_0020237 | 91 | 1 | 292 | 347 | 56 | 0 | 2.00E-107 |
| hsa_circ_0020235 | 93 | 630 | 975 | 346 | 1 | 0 | 3.00E-140 |
| hsa_circ_0020235 | 93 | 630 | 975 | 346 | 1 | 0 | 4.00E-140 |
| hsa_circ_0020233 | 94 | 982 | 1556 | 1339 | 765 | 0 | 0 |
| hsa_circ_0020235 | 93 | 460 | 805 | 346 | 1 | 0 | 3.00E-140 |
| hsa_circ_0020233 | 94 | 812 | 1386 | 1339 | 765 | 0 | 0 |
| hsa_circ_0020235 | 93 | 336 | 681 | 346 | 1 | 0 | 2.00E-140 |
| hsa_circ_0020235 | 93 | 336 | 681 | 346 | 1 | 0 | 3.00E-140 |
| hsa_circ_0020233 | 94 | 688 | 1262 | 1339 | 765 | 0 | 0 |
| hsa_circ_0020233 | 94 | 1 | 575 | 1339 | 765 | 0 | 0 |
| hsa_circ_0020225 | 92 | 1 | 565 | 1031 | 1595 | 0 | 0 |
| hsa_circ_0020225 | 92 | 1419 | 1983 | 1031 | 1595 | 0 | 0 |
| hsa_circ_0020222 | 92 | 1 | 1769 | 1 | 1769 | 0 | 0 |
| hsa_circ_0020222 | 92 | 1 | 1387 | 1 | 1387 | 0 | 0 |
| hsa_circ_0020227 | 91 | 1 | 369 | 416 | 784 | 0 | 4.00E-140 |
| hsa_circ_0020226 | 93 | 1 | 950 | 1 | 950 | 0 | 0 |
| hsa_circ_0020222 | 92 | 1 | 1119 | 1 | 1119 | 0 | 0 |
| hsa_circ_0020227 | 96 | 3 | 300 | 118 | 415 | 0 | 9.00E-136 |
| hsa_circ_0020222 | 93 | 1 | 942 | 1 | 942 | 0 | 0 |
| hsa_circ_0020226 | 92 | 1 | 281 | 1 | 281 | 0 | 5.00E-108 |
| hsa_circ_0020208 | 96 | 1 | 560 | 1 | 560 | 0 | 0 |
| hsa_circ_0020210 | 97 | 1 | 280 | 1 | 280 | 0 | 1.00E-130 |
| hsa_circ_0020208 | 97 | 1 | 389 | 1 | 389 | 0 | 0 |
| hsa_circ_0020210 | 97 | 1 | 280 | 1 | 280 | 0 | 2.00E-132 |
| hsa_circ_0020208 | 97 | 1 | 389 | 1 | 389 | 0 | 0 |
| hsa_circ_0020210 | 99 | 1 | 91 | 1 | 91 | 0 | 2.00E-38 |
| hsa_circ_0020208 | 98 | 1 | 200 | 1 | 200 | 0 | 8.00E-95 |
| hsa_circ_0020202 | 92 | 2 | 906 | 245 | 1149 | 0 | 0 |
| hsa_circ_0020201 | 90 | 91 | 1007 | 1 | 911 | 0.01 | 0 |

novel_circ_005902 novel_circ_005903 novel_circ_005904 novel_circ_005905 novel_circ_005906 novel_circ_005907 novel_circ_005908 novel_circ_005909 novel_circ_005910 novel_circ_005911 novel_circ_005912 novel_circ_005913 novel_circ_005914 novel_circ_005915 novel_circ_005916 novel_circ_005917 novel_circ_005918 novel_circ_005919 novel_circ_005920 novel_circ_005921 novel_circ_005922 novel_circ_005923 novel_circ_005924 novel_circ_005925 novel_circ_005926 novel_circ_005927 novel_circ_005928 novel_circ_005929 novel_circ_005930 novel_circ_005931 novel_circ_005932 novel_circ_005933 novel_circ_005935 novel_circ_005937 novel_circ_005938 novel_circ_005939 novel_circ_005940 novel_circ_005941 novel_circ_005942 novel_circ_005943 novel_circ_005944 novel_circ_005945 novel_circ_005946 novel_circ_005947 novel_circ_005948 novel_circ_005949 novel_circ_005950 novel_circ_005951 novel_circ_005952 novel_circ_005953 novel_circ_005954 novel_circ_005955 novel_circ_005956 novel_circ_005957

| hsa_circ_0020201 | 90 | 496 | 1412 | 1 | 911 | 0.01 | 0 |
| --- | --- | --- | --- | --- | --- | --- | --- |
| hsa_circ_0020196 | 89 | 1 | 1602 | 1 | 1590 | 0.01 | 0 |
| hsa_circ_0020200 | 91 | 285 | 1118 | 1 | 834 | 0 | 0 |
| hsa_circ_0020196 | 89 | 1 | 1602 | 1 | 1590 | 0.01 | 0 |
| hsa_circ_0020201 | 92 | 1 | 481 | 143 | 623 | 0 | 0 |
| hsa_circ_0020201 | 91 | 1 | 623 | 1 | 623 | 0 | 0 |
| hsa_circ_0020200 | 91 | 3 | 713 | 124 | 834 | 0 | 0 |
| hsa_circ_0020200 | 91 | 285 | 1118 | 1 | 834 | 0 | 0 |
| hsa_circ_0020196 | 89 | 1 | 1602 | 1 | 1590 | 0.01 | 0 |
| hsa_circ_0020196 | 90 | 3 | 846 | 747 | 1590 | 0 | 0 |
| hsa_circ_0020196 | 89 | 3 | 631 | 747 | 1375 | 0 | 0 |
| hsa_circ_0020196 | 88 | 3 | 495 | 747 | 1239 | 0 | 7.00E-169 |
| hsa_circ_0020196 | 88 | 1 | 1251 | 1 | 1239 | 0.01 | 0 |
| hsa_circ_0020198 | 89 | 1 | 395 | 6 | 401 | 0 | 8.00E-138 |
| hsa_circ_0020198 | 89 | 3 | 400 | 3 | 401 | 0 | 2.00E-139 |
| hsa_circ_0020198 | 88 | 3 | 284 | 3 | 284 | 0 | 3.00E-90 |
| hsa_circ_0020196 | 87 | 2 | 495 | 535 | 1028 | 0 | 4.00E-161 |
| hsa_circ_0020196 | 88 | 1 | 1040 | 1 | 1028 | 0.01 | 0 |
| hsa_circ_0020196 | 88 | 1 | 756 | 1 | 744 | 0.02 | 0 |
| hsa_circ_0020194 | 94 | 1 | 516 | 516 | 1 | 0 | 0 |
| hsa_circ_0020194 | 94 | 1 | 247 | 247 | 1 | 0 | 5.00E-102 |
| hsa_circ_0020188 | 89 | 156 | 550 | 819 | 419 | 0.01 | 5.00E-136 |
| hsa_circ_0020188 | 92 | 1 | 300 | 300 | 1 | 0 | 5.00E-115 |
| hsa_circ_0020187 | 94 | 135 | 299 | 165 | 1 | 0 | 4.00E-65 |
| hsa_circ_0059848 | 92 | 322 | 767 | 871 | 425 | 0 | 1.00E-177 |
| hsa_circ_0059848 | 92 | 2484 | 2929 | 871 | 425 | 0 | 6.00E-177 |
| hsa_circ_0059853 | 89 | 1 | 332 | 2502 | 2171 | 0 | 1.00E-114 |
| hsa_circ_0059856 | 87 | 1 | 459 | 456 | 1 | 0.01 | 4.00E-142 |
| hsa_circ_0059858 | 92 | 1 | 386 | 1 | 386 | 0 | 3.00E-151 |
| hsa_circ_0059858 | 92 | 1 | 386 | 1 | 386 | 0 | 4.00E-151 |
| hsa_circ_0059864 | 90 | 387 | 912 | 1 | 526 | 0 | 0 |
| hsa_circ_0059864 | 90 | 1 | 526 | 1 | 526 | 0 | 0 |
| hsa_circ_0059887 | 96 | 1 | 183 | 134 | 316 | 0 | 2.00E-80 |
| hsa_circ_0059905 | 92 | 1 | 538 | 1740 | 1208 | 0.02 | 0 |
| hsa_circ_0059934 | 95 | 1 | 405 | 1 | 405 | 0 | 2.00E-178 |
| hsa_circ_0059933 | 94 | 1 | 451 | 92 | 542 | 0 | 0 |
| hsa_circ_0059932 | 92 | 1 | 651 | 311 | 967 | 0.01 | 0 |
| hsa_circ_0059932 | 93 | 1 | 747 | 311 | 1063 | 0.01 | 0 |
| hsa_circ_0059932 | 93 | 1 | 1077 | 311 | 1393 | 0.01 | 0 |
| hsa_circ_0059932 | 93 | 1 | 1206 | 311 | 1522 | 0 | 0 |
| hsa_circ_0059932 | 94 | 1 | 1734 | 311 | 2050 | 0 | 0 |
| hsa_circ_0059932 | 94 | 1 | 1734 | 311 | 2050 | 0 | 0 |
| hsa_circ_0059939 | 91 | 1 | 526 | 1 | 532 | 0.01 | 0 |
| hsa_circ_0059939 | 92 | 1 | 622 | 1 | 628 | 0.01 | 0 |
| hsa_circ_0059939 | 92 | 1 | 797 | 1 | 803 | 0.01 | 0 |
| hsa_circ_0059939 | 92 | 1 | 867 | 1 | 873 | 0.01 | 0 |
| hsa_circ_0059939 | 92 | 1 | 952 | 1 | 958 | 0.01 | 0 |
| hsa_circ_0059939 | 92 | 1 | 1081 | 1 | 1087 | 0.01 | 0 |
| hsa_circ_0059932 | 94 | 1 | 1609 | 436 | 2050 | 0 | 0 |
| hsa_circ_0059932 | 94 | 1 | 1609 | 436 | 2050 | 0 | 0 |
| hsa_circ_0059932 | 94 | 1 | 1609 | 436 | 2050 | 0 | 0 |
| hsa_circ_0059932 | 94 | 1 | 1609 | 436 | 2050 | 0 | 0 |
| hsa_circ_0059932 | 94 | 1 | 1609 | 436 | 2050 | 0 | 0 |
| hsa_circ_0059941 | 90 | 1 | 388 | 1 | 394 | 0.02 | 6.00E-144 |

novel_circ_005958 novel_circ_005959 novel_circ_005960 novel_circ_005961 novel_circ_005962 novel_circ_005963 novel_circ_005964 novel_circ_005965 novel_circ_005966 novel_circ_005967 novel_circ_005968 novel_circ_005969 novel_circ_005970 novel_circ_005971 novel_circ_005972 novel_circ_005973 novel_circ_005974 novel_circ_005975 novel_circ_005976 novel_circ_005977 novel_circ_005978 novel_circ_005979 novel_circ_005980 novel_circ_005981 novel_circ_005982 novel_circ_005983 novel_circ_005984 novel_circ_005985 novel_circ_005986 novel_circ_005987 novel_circ_005988 novel_circ_005990 novel_circ_005991 novel_circ_005992 novel_circ_005993 novel_circ_005994 novel_circ_005995 novel_circ_005997 novel_circ_005998 novel_circ_005999 novel_circ_006000 novel_circ_006001 novel_circ_006002 novel_circ_006003 novel_circ_006004 novel_circ_006005 novel_circ_006006 novel_circ_006007 novel_circ_006008 novel_circ_006009 novel_circ_006010 novel_circ_006011 novel_circ_006012 novel_circ_006013

| hsa_circ_0059939 | 92 | 1 | 814 | 139 | 958 | 0.01 | 0 |
| --- | --- | --- | --- | --- | --- | --- | --- |
| hsa_circ_0059939 | 92 | 1 | 943 | 139 | 1087 | 0.01 | 0 |
| hsa_circ_0059932 | 94 | 1 | 1471 | 574 | 2050 | 0 | 0 |
| hsa_circ_0059942 | 91 | 1 | 342 | 1 | 348 | 0.02 | 8.00E-127 |
| hsa_circ_0059942 | 92 | 1 | 683 | 1 | 689 | 0.01 | 0 |
| hsa_circ_0059942 | 92 | 1 | 897 | 1 | 903 | 0.01 | 0 |
| hsa_circ_0059947 | 92 | 1 | 280 | 1 | 286 | 0.02 | 2.00E-107 |
| hsa_circ_0059947 | 93 | 2 | 341 | 192 | 531 | 0 | 5.00E-139 |
| hsa_circ_0059952 | 96 | 1 | 742 | 1 | 742 | 0 | 0 |
| hsa_circ_0059961 | 96 | 1 | 524 | 1 | 524 | 0 | 0 |
| hsa_circ_0059961 | 96 | 1 | 750 | 1 | 750 | 0 | 0 |
| hsa_circ_0059961 | 96 | 1 | 917 | 1 | 917 | 0 | 0 |
| hsa_circ_0059961 | 96 | 1 | 294 | 90 | 383 | 0 | 3.00E-135 |
| hsa_circ_0059961 | 96 | 1 | 435 | 90 | 524 | 0 | 0 |
| hsa_circ_0059961 | 95 | 1 | 661 | 90 | 750 | 0 | 0 |
| hsa_circ_0059961 | 95 | 1 | 828 | 90 | 917 | 0 | 0 |
| hsa_circ_0059962 | 95 | 2 | 534 | 136 | 668 | 0 | 0 |
| hsa_circ_0005677 | 95 | 1 | 226 | 1 | 226 | 0 | 2.00E-97 |
| hsa_circ_0005677 | 95 | 1 | 393 | 1 | 393 | 0 | 4.00E-175 |
| hsa_circ_0001142 | 95 | 1 | 665 | 1045 | 381 | 0 | 0 |
| hsa_circ_0059979 | 95 | 1 | 522 | 522 | 1 | 0 | 0 |
| hsa_circ_0001142 | 96 | 1 | 399 | 1444 | 1046 | 0 | 0 |
| hsa_circ_0059990 | 91 | 9 | 255 | 247 | 1 | 0 | 2.00E-92 |
| hsa_circ_0059997 | 94 | 389 | 1073 | 5935 | 5251 | 0 | 0 |
| hsa_circ_0059997 | 95 | 389 | 944 | 5935 | 5380 | 0 | 0 |
| hsa_circ_0059997 | 95 | 608 | 1163 | 5935 | 5380 | 0 | 0 |
| hsa_circ_0059999 | 95 | 389 | 821 | 433 | 1 | 0 | 0 |
| hsa_circ_0059999 | 95 | 678 | 1110 | 433 | 1 | 0 | 0 |
| hsa_circ_0060016 | 91 | 1 | 674 | 1053 | 380 | 0 | 0 |
| hsa_circ_0060015 | 93 | 3 | 246 | 3 | 246 | 0 | 4.00E-99 |
| hsa_circ_0060016 | 90 | 1 | 534 | 1053 | 520 | 0 | 0 |
| hsa_circ_0060037 | 93 | 1 | 394 | 394 | 1 | 0 | 6.00E-164 |
| hsa_circ_0060037 | 93 | 1480 | 1875 | 396 | 1 | 0 | 2.00E-164 |
| hsa_circ_0060037 | 94 | 7 | 523 | 517 | 1 | 0 | 0 |
| hsa_circ_0060037 | 94 | 1 | 637 | 637 | 1 | 0 | 0 |
| hsa_circ_0060033 | 94 | 1 | 883 | 1241 | 359 | 0 | 0 |
| hsa_circ_0060033 | 95 | 1 | 489 | 1241 | 753 | 0 | 0 |
| hsa_circ_0060049 | 93 | 1 | 569 | 1 | 569 | 0 | 0 |
| hsa_circ_0060049 | 94 | 1 | 731 | 1 | 731 | 0 | 0 |
| hsa_circ_0060050 | 94 | 150 | 1354 | 1 | 1205 | 0 | 0 |
| hsa_circ_0060050 | 94 | 1 | 420 | 1 | 420 | 0 | 5.00E-180 |
| hsa_circ_0060050 | 94 | 1 | 1205 | 1 | 1205 | 0 | 0 |
| hsa_circ_0060060 | 94 | 1 | 535 | 535 | 1 | 0 | 0 |
| hsa_circ_0060066 | 98 | 1 | 131 | 131 | 1 | 0 | 5.00E-58 |
| hsa_circ_0060066 | 95 | 2731 | 2971 | 241 | 1 | 0 | 1.00E-104 |
| hsa_circ_0060066 | 93 | 1 | 335 | 335 | 1 | 0 | 2.00E-134 |
| hsa_circ_0060067 | 96 | 2731 | 2913 | 183 | 1 | 0 | 2.00E-77 |
| hsa_circ_0060067 | 92 | 1 | 277 | 277 | 1 | 0 | 2.00E-107 |
| hsa_circ_0060066 | 91 | 1 | 382 | 440 | 59 | 0.01 | 1.00E-145 |
| hsa_circ_0060068 | 89 | 1 | 309 | 309 | 1 | 0.01 | 4.00E-105 |
| hsa_circ_0060075 | 89 | 1 | 1149 | 457 | 1602 | 0 | 0 |
| hsa_circ_0060075 | 88 | 1 | 706 | 897 | 1602 | 0 | 0 |
| hsa_circ_0060075 | 89 | 1 | 523 | 1080 | 1602 | 0 | 0 |
| hsa_circ_0060075 | 89 | 1 | 1596 | 1080 | 2675 | 0 | 0 |

novel_circ_006014 novel_circ_006015 novel_circ_006016 novel_circ_006017 novel_circ_006018 novel_circ_006019 novel_circ_006020 novel_circ_006021 novel_circ_006022 novel_circ_006023 novel_circ_006024 novel_circ_006025 novel_circ_006026 novel_circ_006027 novel_circ_006028 novel_circ_006029 novel_circ_006030 novel_circ_006031 novel_circ_006033 novel_circ_006034 novel_circ_006035 novel_circ_006036 novel_circ_006037 novel_circ_006038 novel_circ_006039 novel_circ_006040 novel_circ_006041 novel_circ_006042 novel_circ_006043 novel_circ_006044 novel_circ_006045 novel_circ_006046 novel_circ_006047 novel_circ_006048 novel_circ_006050 novel_circ_006051 novel_circ_006052 novel_circ_006053 novel_circ_006055 novel_circ_006056 novel_circ_006057 novel_circ_006058 novel_circ_006059 novel_circ_006060 novel_circ_006061 novel_circ_006062 novel_circ_006064 novel_circ_006065 novel_circ_006066 novel_circ_006067 novel_circ_006068 novel_circ_006069 novel_circ_006070 novel_circ_006071

| hsa_circ_0060075 | 89 | 1 | 1596 | 1080 | 2675 | 0 | 0 |
| --- | --- | --- | --- | --- | --- | --- | --- |
| hsa_circ_0060075 | 89 | 1 | 2313 | 1080 | 3398 | 0 | 0 |
| hsa_circ_0060079 | 89 | 1 | 717 | 135 | 857 | 0.01 | 0 |
| hsa_circ_0060120 | 91 | 1 | 1102 | 1102 | 1 | 0 | 0 |
| hsa_circ_0060120 | 91 | 1 | 1102 | 1102 | 1 | 0 | 0 |
| hsa_circ_0060120 | 90 | 1 | 1050 | 1102 | 53 | 0 | 0 |
| hsa_circ_0001146 | 95 | 83 | 1902 | 1820 | 1 | 0 | 0 |
| hsa_circ_0001146 | 95 | 184 | 2003 | 1820 | 1 | 0 | 0 |
| hsa_circ_0001146 | 95 | 83 | 1632 | 1820 | 271 | 0 | 0 |
| hsa_circ_0060136 | 94 | 1 | 691 | 691 | 1 | 0 | 0 |
| hsa_circ_0008143 | 94 | 1 | 640 | 640 | 1 | 0 | 0 |
| hsa_circ_0004870 | 94 | 1 | 560 | 562 | 3 | 0 | 0 |
| hsa_circ_0004870 | 94 | 2 | 678 | 679 | 3 | 0 | 0 |
| hsa_circ_0001147 | 94 | 1 | 203 | 204 | 2 | 0 | 2.00E-81 |
| hsa_circ_0001147 | 93 | 1 | 356 | 357 | 2 | 0 | 7.00E-148 |
| hsa_circ_0001147 | 93 | 2 | 474 | 474 | 2 | 0 | 0 |
| hsa_circ_0005848 | 94 | 1 | 290 | 291 | 2 | 0 | 5.00E-123 |
| hsa_circ_0005848 | 94 | 2 | 408 | 408 | 2 | 0 | 2.00E-174 |
| hsa_circ_0060147 | 98 | 1 | 372 | 1 | 372 | 0 | 0 |
| hsa_circ_0060151 | 97 | 1 | 257 | 1 | 257 | 0 | 1.00E-119 |
| hsa_circ_0060151 | 92 | 1 | 1211 | 1 | 1199 | 0.01 | 0 |
| hsa_circ_0060157 | 91 | 1 | 954 | 1 | 942 | 0.02 | 0 |
| hsa_circ_0060156 | 91 | 1 | 1676 | 86 | 1749 | 0.01 | 0 |
| hsa_circ_0060159 | 90 | 1 | 874 | 1 | 862 | 0.02 | 0 |
| hsa_circ_0060156 | 91 | 1 | 1596 | 166 | 1749 | 0.01 | 0 |
| hsa_circ_0060156 | 91 | 1 | 1596 | 166 | 1749 | 0.01 | 0 |
| hsa_circ_0060161 | 93 | 1 | 722 | 1 | 722 | 0 | 0 |
| hsa_circ_0060161 | 93 | 1 | 722 | 1 | 722 | 0 | 0 |
| hsa_circ_0060161 | 93 | 1 | 722 | 1 | 722 | 0 | 0 |
| hsa_circ_0060162 | 95 | 280 | 1018 | 1 | 739 | 0 | 0 |
| hsa_circ_0060164 | 89 | 1 | 400 | 197 | 608 | 0.03 | 4.00E-137 |
| hsa_circ_0060169 | 91 | 1 | 461 | 1 | 461 | 0 | 0 |
| hsa_circ_0060175 | 89 | 14 | 1042 | 9 | 1035 | 0 | 0 |
| hsa_circ_0060179 | 92 | 1542 | 1780 | 1 | 239 | 0 | 2.00E-90 |
| hsa_circ_0060201 | 91 | 4 | 717 | 714 | 1 | 0 | 0 |
| hsa_circ_0060201 | 91 | 1 | 389 | 611 | 223 | 0.01 | 2.00E-149 |
| hsa_circ_0060201 | 91 | 4 | 495 | 714 | 223 | 0 | 0 |
| hsa_circ_0060203 | 91 | 4 | 438 | 435 | 1 | 0 | 5.00E-165 |
| hsa_circ_0060222 | 88 | 418 | 1202 | 785 | 1 | 0 | 0 |
| hsa_circ_0060222 | 87 | 1 | 256 | 256 | 1 | 0 | 3.00E-77 |
| hsa_circ_0060222 | 88 | 418 | 1202 | 785 | 1 | 0 | 0 |
| hsa_circ_0060222 | 88 | 351 | 1135 | 785 | 1 | 0 | 0 |
| hsa_circ_0060222 | 88 | 418 | 1202 | 785 | 1 | 0 | 0 |
| hsa_circ_0060222 | 88 | 351 | 995 | 785 | 141 | 0 | 0 |
| hsa_circ_0060224 | 87 | 68 | 644 | 577 | 1 | 0 | 0 |
| hsa_circ_0060224 | 89 | 1 | 275 | 504 | 230 | 0 | 3.00E-91 |
| hsa_circ_0060226 | 91 | 406 | 2735 | 3295 | 966 | 0 | 0 |
| hsa_circ_0060226 | 91 | 1 | 1255 | 2724 | 1470 | 0 | 0 |
| hsa_circ_0004855 | 90 | 1 | 915 | 1092 | 178 | 0 | 0 |
| hsa_circ_0060226 | 91 | 1 | 1132 | 2941 | 1810 | 0 | 0 |
| hsa_circ_0060229 | 93 | 1 | 303 | 436 | 134 | 0 | 3.00E-125 |
| hsa_circ_0060226 | 93 | 1 | 520 | 2941 | 2422 | 0 | 0 |
| hsa_circ_0060226 | 93 | 7 | 687 | 3102 | 2422 | 0 | 0 |
| hsa_circ_0060234 | 93 | 7 | 522 | 516 | 1 | 0 | 0 |

novel_circ_006072 novel_circ_006073 novel_circ_006074 novel_circ_006075 novel_circ_006076 novel_circ_006077 novel_circ_006081 novel_circ_006082 novel_circ_006083 novel_circ_006084 novel_circ_006085 novel_circ_006086 novel_circ_006087 novel_circ_006088 novel_circ_006089 novel_circ_006090 novel_circ_006091 novel_circ_006092 novel_circ_006093 novel_circ_006094 novel_circ_006095 novel_circ_006096 novel_circ_006097 novel_circ_006098 novel_circ_006099 novel_circ_006100 novel_circ_006101 novel_circ_006102 novel_circ_006103 novel_circ_006104 novel_circ_006105 novel_circ_006106 novel_circ_006107 novel_circ_006108 novel_circ_006109 novel_circ_006110 novel_circ_006111 novel_circ_006112 novel_circ_006113 novel_circ_006114 novel_circ_006115 novel_circ_006116 novel_circ_006117 novel_circ_006118 novel_circ_006119 novel_circ_006120 novel_circ_006121 novel_circ_006122 novel_circ_006123 novel_circ_006124 novel_circ_006125 novel_circ_006126 novel_circ_006127 novel_circ_006128

| hsa_circ_0060234 | 92 | 1 | 217 | 355 | 139 | 0 | 2.00E-82 |
| --- | --- | --- | --- | --- | --- | --- | --- |
| hsa_circ_0060237 | 94 | 4 | 404 | 401 | 1 | 0 | 2.00E-169 |
| hsa_circ_0060234 | 94 | 1 | 960 | 1315 | 356 | 0 | 0 |
| hsa_circ_0060234 | 94 | 1 | 1094 | 1449 | 356 | 0 | 0 |
| hsa_circ_0060238 | 94 | 1 | 556 | 556 | 1 | 0 | 0 |
| hsa_circ_0060234 | 94 | 1 | 690 | 1449 | 760 | 0 | 0 |
| hsa_circ_0060251 | 91 | 1 | 1079 | 1 | 1079 | 0 | 0 |
| hsa_circ_0060256 | 92 | 1 | 483 | 1 | 483 | 0 | 0 |
| hsa_circ_0060256 | 91 | 1 | 779 | 1 | 779 | 0 | 0 |
| hsa_circ_0060256 | 90 | 1 | 885 | 1 | 885 | 0 | 0 |
| hsa_circ_0060251 | 92 | 1 | 1470 | 195 | 1664 | 0 | 0 |
| hsa_circ_0060267 | 90 | 1 | 613 | 1 | 613 | 0 | 0 |
| hsa_circ_0060278 | 90 | 1 | 296 | 1 | 296 | 0.01 | 3.00E-106 |
| hsa_circ_0060307 | 94 | 1 | 467 | 1 | 467 | 0 | 0 |
| hsa_circ_0060307 | 94 | 1 | 649 | 1 | 649 | 0 | 0 |
| hsa_circ_0060310 | 95 | 1 | 361 | 150 | 510 | 0 | 1.00E-160 |
| hsa_circ_0060310 | 94 | 1 | 572 | 150 | 721 | 0 | 0 |
| hsa_circ_0060322 | 87 | 1 | 485 | 491 | 1 | 0.02 | 4.00E-151 |
| hsa_circ_0060322 | 90 | 35 | 2536 | 2798 | 291 | 0 | 0 |
| hsa_circ_0007365 | 93 | 1 | 415 | 1 | 415 | 0 | 3.00E-172 |
| hsa_circ_0060336 | 93 | 10313 | 10899 | 224 | 810 | 0 | 0 |
| hsa_circ_0060336 | 93 | 10309 | 10731 | 224 | 646 | 0 | 3.00E-175 |
| hsa_circ_0060337 | 93 | 1 | 419 | 1 | 419 | 0 | 2.00E-174 |
| hsa_circ_0060337 | 93 | 1 | 583 | 1 | 583 | 0 | 0 |
| hsa_circ_0060337 | 93 | 1 | 770 | 1 | 770 | 0 | 0 |
| hsa_circ_0060337 | 93 | 1 | 902 | 1 | 902 | 0 | 0 |
| hsa_circ_0060336 | 94 | 1 | 1081 | 228 | 1308 | 0 | 0 |
| hsa_circ_0060336 | 95 | 1 | 1649 | 228 | 1876 | 0 | 0 |
| hsa_circ_0060336 | 96 | 1 | 2080 | 228 | 2307 | 0 | 0 |
| hsa_circ_0060336 | 96 | 1 | 2081 | 228 | 2308 | 0 | 0 |
| hsa_circ_0060336 | 96 | 1 | 2145 | 228 | 2372 | 0 | 0 |
| hsa_circ_0060336 | 94 | 1 | 865 | 444 | 1308 | 0 | 0 |
| hsa_circ_0060336 | 95 | 1 | 1433 | 444 | 1876 | 0 | 0 |
| hsa_circ_0060340 | 94 | 1 | 351 | 1 | 351 | 0 | 2.00E-148 |
| hsa_circ_0060340 | 94 | 1 | 483 | 1 | 483 | 0 | 0 |
| hsa_circ_0060340 | 96 | 1 | 1230 | 1 | 1230 | 0 | 0 |
| hsa_circ_0060340 | 96 | 1 | 1990 | 1 | 1990 | 0 | 0 |
| hsa_circ_0060340 | 96 | 1 | 1066 | 165 | 1230 | 0 | 0 |
| hsa_circ_0060340 | 97 | 1 | 1178 | 484 | 1661 | 0 | 0 |
| hsa_circ_0060340 | 97 | 1 | 1179 | 484 | 1662 | 0 | 0 |
| hsa_circ_0060340 | 97 | 1 | 1243 | 484 | 1726 | 0 | 0 |
| hsa_circ_0060340 | 97 | 1 | 1373 | 484 | 1856 | 0 | 0 |
| hsa_circ_0060340 | 97 | 1 | 1842 | 484 | 2325 | 0 | 0 |
| hsa_circ_0060336 | 96 | 1 | 3085 | 1130 | 4211 | 0 | 0 |
| hsa_circ_0060340 | 98 | 1 | 1045 | 812 | 1856 | 0 | 0 |
| hsa_circ_0060341 | 98 | 1 | 828 | 1 | 828 | 0 | 0 |
| hsa_circ_0060341 | 96 | 1 | 664 | 634 | 1297 | 0 | 0 |
| hsa_circ_0060344 | 97 | 1 | 264 | 1 | 264 | 0 | 6.00E-122 |
| hsa_circ_0060344 | 95 | 1 | 447 | 1 | 447 | 0 | 0 |
| hsa_circ_0060345 | 95 | 1 | 993 | 1 | 993 | 0 | 0 |
| hsa_circ_0060345 | 95 | 1 | 1318 | 1 | 1318 | 0 | 0 |
| hsa_circ_0060345 | 96 | 1 | 969 | 184 | 1152 | 0 | 0 |
| hsa_circ_0060348 | 92 | 1 | 801 | 401 | 1201 | 0 | 0 |
| hsa_circ_0060358 | 94 | 2 | 226 | 2 | 226 | 0 | 1.00E-93 |

novel_circ_006129 novel_circ_006130 novel_circ_006131 novel_circ_006132 novel_circ_006133 novel_circ_006134 novel_circ_006135 novel_circ_006136 novel_circ_006137 novel_circ_006138 novel_circ_006140 novel_circ_006141 novel_circ_006142 novel_circ_006145 novel_circ_006146 novel_circ_006147 novel_circ_006148 novel_circ_006149 novel_circ_006150 novel_circ_006151 novel_circ_006152 novel_circ_006153 novel_circ_006154 novel_circ_006155 novel_circ_006156 novel_circ_006157 novel_circ_006158 novel_circ_006159 novel_circ_006160 novel_circ_006161 novel_circ_006162 novel_circ_006163 novel_circ_006164 novel_circ_006165 novel_circ_006166 novel_circ_006167 novel_circ_006168 novel_circ_006169 novel_circ_006170 novel_circ_006171 novel_circ_006172 novel_circ_006173 novel_circ_006174 novel_circ_006175 novel_circ_006176 novel_circ_006177 novel_circ_006178 novel_circ_006179 novel_circ_006180 novel_circ_006181 novel_circ_006182 novel_circ_006183 novel_circ_006184 novel_circ_006185

| hsa_circ_0060358 | 95 | 2 | 355 | 2 | 355 | 0 | 4.00E-155 |
| --- | --- | --- | --- | --- | --- | --- | --- |
| hsa_circ_0060366 | 93 | 1 | 507 | 99 | 605 | 0 | 0 |
| hsa_circ_0060376 | 92 | 1 | 208 | 1 | 208 | 0 | 1.00E-77 |
| hsa_circ_0060377 | 97 | 353 | 575 | 1 | 223 | 0 | 9.00E-104 |
| hsa_circ_0060377 | 95 | 1 | 694 | 108 | 801 | 0 | 0 |
| hsa_circ_0060377 | 95 | 1 | 695 | 108 | 802 | 0 | 0 |
| hsa_circ_0060377 | 94 | 1 | 1336 | 108 | 1443 | 0 | 0 |
| hsa_circ_0060377 | 93 | 1 | 477 | 469 | 945 | 0 | 0 |
| hsa_circ_0060377 | 93 | 1 | 663 | 469 | 1131 | 0 | 0 |
| hsa_circ_0060377 | 93 | 1 | 642 | 802 | 1443 | 0 | 0 |
| hsa_circ_0060396 | 87 | 13 | 2987 | 2983 | 3 | 0.01 | 0 |
| hsa_circ_0060396 | 87 | 130 | 3104 | 2983 | 3 | 0.01 | 0 |
| hsa_circ_0060397 | 83 | 17515 | 18828 | 35929 | 34593 | 0.05 | 0 |
| hsa_circ_0060406 | 90 | 1 | 252 | 252 | 1 | 0 | 8.00E-90 |
| hsa_circ_0060406 | 92 | 1 | 1300 | 1300 | 1 | 0 | 0 |
| hsa_circ_0060406 | 93 | 1 | 854 | 1300 | 447 | 0 | 0 |
| hsa_circ_0060406 | 93 | 1 | 1019 | 1465 | 447 | 0 | 0 |
| hsa_circ_0060406 | 94 | 11716 | 12932 | 1663 | 447 | 0 | 0 |
| hsa_circ_0060406 | 93 | 1 | 1682 | 2128 | 447 | 0 | 0 |
| hsa_circ_0060414 | 99 | 11716 | 11913 | 198 | 1 | 0 | 7.00E-97 |
| hsa_circ_0060414 | 95 | 1 | 663 | 663 | 1 | 0 | 0 |
| hsa_circ_0060414 | 95 | 748 | 1410 | 663 | 1 | 0 | 0 |
| hsa_circ_0060417 | 94 | 1 | 843 | 1376 | 534 | 0 | 0 |
| hsa_circ_0060417 | 95 | 1 | 344 | 1376 | 1033 | 0 | 3.00E-151 |
| hsa_circ_0060421 | 93 | 1 | 819 | 819 | 1 | 0 | 0 |
| hsa_circ_0001159 | 93 | 1 | 521 | 521 | 1 | 0 | 0 |
| hsa_circ_0076176 | 93 | 1 | 677 | 677 | 1 | 0 | 0 |
| hsa_circ_0076172 | 94 | 1 | 917 | 1546 | 630 | 0 | 0 |
| hsa_circ_0076172 | 94 | 1 | 917 | 1546 | 630 | 0 | 0 |
| hsa_circ_0076176 | 92 | 11 | 281 | 271 | 1 | 0 | 2.00E-102 |
| hsa_circ_0076167 | 92 | 1 | 696 | 699 | 1 | 0 | 0 |
| hsa_circ_0076163 | 92 | 1 | 706 | 792 | 84 | 0 | 0 |
| hsa_circ_0001600 | 92 | 1 | 258 | 258 | 1 | 0 | 1.00E-98 |
| hsa_circ_0076149 | 92 | 1 | 591 | 776 | 186 | 0 | 0 |
| hsa_circ_0076102 | 90 | 1 | 528 | 1 | 528 | 0 | 0 |
| hsa_circ_0076084 | 92 | 1 | 764 | 1 | 761 | 0.01 | 0 |
| hsa_circ_0076082 | 89 | 12 | 711 | 582 | 1278 | 0.01 | 0 |
| hsa_circ_0076081 | 89 | 1 | 967 | 1 | 967 | 0 | 0 |
| hsa_circ_0076081 | 90 | 1 | 774 | 1 | 774 | 0 | 0 |
| hsa_circ_0076081 | 90 | 1 | 774 | 1 | 774 | 0 | 0 |
| hsa_circ_0076076 | 80 | 1 | 452 | 943 | 489 | 0.07 | 1.00E-86 |
| hsa_circ_0076069 | 91 | 1 | 479 | 2288 | 2766 | 0 | 0 |
| hsa_circ_0076069 | 90 | 1 | 785 | 1982 | 2766 | 0 | 0 |
| hsa_circ_0076069 | 88 | 1 | 1448 | 337 | 1784 | 0 | 0 |
| hsa_circ_0076061 | 92 | 1 | 695 | 1 | 695 | 0 | 0 |
| hsa_circ_0076061 | 91 | 1 | 502 | 1 | 502 | 0 | 0 |
| hsa_circ_0076058 | 95 | 1 | 294 | 1 | 294 | 0 | 2.00E-128 |
| hsa_circ_0076052 | 99 | 1 | 295 | 703 | 409 | 0 | 2.00E-147 |
| hsa_circ_0076052 | 99 | 1 | 295 | 703 | 409 | 0 | 3.00E-147 |
| hsa_circ_0076052 | 92 | 326 | 700 | 396 | 1 | 0.05 | 1.00E-153 |
| hsa_circ_0076052 | 92 | 1 | 329 | 350 | 1 | 0.06 | 4.00E-130 |
| hsa_circ_0076044 | 93 | 8537 | 8647 | 111 | 1 | 0 | 6.00E-37 |
| hsa_circ_0076043 | 93 | 1 | 241 | 241 | 1 | 0 | 2.00E-97 |
| hsa_circ_0076043 | 93 | 1 | 241 | 241 | 1 | 0 | 4.00E-97 |

novel_circ_006186 novel_circ_006187 novel_circ_006188 novel_circ_006189 novel_circ_006190 novel_circ_006191 novel_circ_006192 novel_circ_006193 novel_circ_006194 novel_circ_006195 novel_circ_006196 novel_circ_006197 novel_circ_006201 novel_circ_006202 novel_circ_006203 novel_circ_006204 novel_circ_006205 novel_circ_006206 novel_circ_006207 novel_circ_006208 novel_circ_006209 novel_circ_006210 novel_circ_006211 novel_circ_006212 novel_circ_006213 novel_circ_006214 novel_circ_006215 novel_circ_006216 novel_circ_006217 novel_circ_006218 novel_circ_006223 novel_circ_006224 novel_circ_006226 novel_circ_006227 novel_circ_006228 novel_circ_006229 novel_circ_006232 novel_circ_006233 novel_circ_006234 novel_circ_006235 novel_circ_006236 novel_circ_006237 novel_circ_006238 novel_circ_006240 novel_circ_006241 novel_circ_006242 novel_circ_006243 novel_circ_006244 novel_circ_006246 novel_circ_006247 novel_circ_006248 novel_circ_006249 novel_circ_006250 novel_circ_006252

| hsa_circ_0076041 | 92 | 982 | 1599 | 621 | 1 | 0 | 0 |
| --- | --- | --- | --- | --- | --- | --- | --- |
| hsa_circ_0076040 | 93 | 982 | 1816 | 838 | 1 | 0 | 0 |
| hsa_circ_0076039 | 93 | 982 | 1817 | 1270 | 432 | 0 | 0 |
| hsa_circ_0076039 | 93 | 982 | 2248 | 1270 | 1 | 0 | 0 |
| hsa_circ_0076039 | 95 | 4 | 218 | 646 | 432 | 0 | 1.00E-89 |
| hsa_circ_0076039 | 95 | 4 | 218 | 646 | 432 | 0 | 1.00E-89 |
| hsa_circ_0076039 | 93 | 4 | 649 | 646 | 1 | 0 | 0 |
| hsa_circ_0076039 | 93 | 4 | 649 | 646 | 1 | 0 | 0 |
| hsa_circ_0076039 | 93 | 1 | 432 | 432 | 1 | 0 | 2.00E-178 |
| hsa_circ_0076038 | 91 | 1 | 333 | 338 | 6 | 0 | 9.00E-125 |
| hsa_circ_0003625 | 96 | 1 | 339 | 1 | 339 | 0 | 8.00E-157 |
| hsa_circ_0003625 | 95 | 1 | 537 | 1 | 537 | 0 | 0 |
| hsa_circ_0004026 | 94 | 1 | 373 | 373 | 1 | 0 | 5.00E-159 |
| hsa_circ_0077817 | 96 | 1 | 272 | 272 | 1 | 0 | 5.00E-123 |
| hsa_circ_0077819 | 90 | 1 | 1195 | 3139 | 4333 | 0 | 0 |
| hsa_circ_0077819 | 93 | 1 | 433 | 4772 | 5204 | 0 | 0 |
| hsa_circ_0077824 | 91 | 3 | 496 | 494 | 1 | 0 | 0 |
| hsa_circ_0077824 | 91 | 3 | 496 | 494 | 1 | 0 | 0 |
| hsa_circ_0077829 | 94 | 53 | 531 | 62 | 540 | 0 | 0 |
| hsa_circ_0077829 | 92 | 53 | 1971 | 62 | 1980 | 0 | 0 |
| hsa_circ_0077829 | 94 | 2 | 597 | 81 | 676 | 0 | 0 |
| hsa_circ_0077829 | 93 | 2 | 1259 | 81 | 1338 | 0 | 0 |
| hsa_circ_0077829 | 93 | 1 | 293 | 384 | 676 | 0 | 1.00E-119 |
| hsa_circ_0077829 | 88 | 1 | 282 | 677 | 958 | 0 | 7.00E-92 |
| hsa_circ_0077829 | 91 | 1 | 642 | 1339 | 1980 | 0 | 0 |
| hsa_circ_0077834 | 93 | 1 | 289 | 293 | 5 | 0 | 2.00E-117 |
| hsa_circ_0077834 | 99 | 619 | 741 | 416 | 294 | 0 | 2.00E-56 |
| hsa_circ_0077834 | 99 | 787 | 909 | 416 | 294 | 0 | 3.00E-56 |
| hsa_circ_0077837 | 85 | 1 | 782 | 824 | 1 | 0.06 | 0 |
| hsa_circ_0001640 | 84 | 1 | 677 | 719 | 1 | 0.07 | 3.00E-179 |
| hsa_circ_0076984 | 90 | 34518 | 34632 | 1877 | 1766 | 0.03 | 2.00E-32 |
| hsa_circ_0076984 | 90 | 34518 | 34632 | 1877 | 1766 | 0.03 | 3.00E-32 |
| hsa_circ_0076984 | 90 | 30597 | 30711 | 1877 | 1766 | 0.03 | 2.00E-32 |
| hsa_circ_0076984 | 90 | 30597 | 30711 | 1877 | 1766 | 0.03 | 2.00E-32 |
| hsa_circ_0076984 | 90 | 30593 | 30707 | 1877 | 1766 | 0.03 | 2.00E-32 |
| hsa_circ_0076984 | 90 | 30593 | 30707 | 1877 | 1766 | 0.03 | 2.00E-32 |
| hsa_circ_0007636 | 79 | 45368 | 45603 | 1E+06 | 1430328 | 0.02 | 3.00E-37 |
| hsa_circ_0077842 | 92 | 1 | 1930 | 1930 | 1 | 0 | 0 |
| hsa_circ_0077842 | 92 | 144 | 2073 | 1930 | 1 | 0 | 0 |
| hsa_circ_0077842 | 92 | 390 | 2319 | 1930 | 1 | 0 | 0 |
| hsa_circ_0077842 | 93 | 1 | 1239 | 1930 | 692 | 0 | 0 |
| hsa_circ_0077842 | 93 | 144 | 1382 | 1930 | 692 | 0 | 0 |
| hsa_circ_0077845 | 94 | 1 | 607 | 607 | 1 | 0 | 0 |
| hsa_circ_0077847 | 93 | 1 | 728 | 728 | 1 | 0 | 0 |
| hsa_circ_0077847 | 92 | 3 | 392 | 872 | 483 | 0 | 9.00E-157 |
| hsa_circ_0077858 | 93 | 305 | 481 | 1 | 177 | 0 | 3.00E-68 |
| hsa_circ_0077858 | 93 | 1 | 630 | 299 | 928 | 0 | 0 |
| hsa_circ_0077858 | 93 | 1 | 630 | 299 | 928 | 0 | 0 |
| hsa_circ_0077866 | 92 | 1 | 845 | 2277 | 1433 | 0 | 0 |
| hsa_circ_0077866 | 92 | 1 | 1244 | 2676 | 1433 | 0 | 0 |
| hsa_circ_0077866 | 92 | 1 | 642 | 2277 | 1636 | 0 | 0 |
| hsa_circ_0077867 | 89 | 1 | 283 | 283 | 1 | 0 | 9.00E-96 |
| hsa_circ_0077866 | 92 | 1 | 399 | 2676 | 2278 | 0 | 9.00E-157 |
| hsa_circ_0077885 | 92 | 1 | 932 | 195 | 1108 | 0.02 | 0 |

novel_circ_006253 novel_circ_006254 novel_circ_006255 novel_circ_006256 novel_circ_006257 novel_circ_006258 novel_circ_006261 novel_circ_006262 novel_circ_006263 novel_circ_006264 novel_circ_006265 novel_circ_006266 novel_circ_006267 novel_circ_006268 novel_circ_006269 novel_circ_006270 novel_circ_006271 novel_circ_006272 novel_circ_006273 novel_circ_006274 novel_circ_006275 novel_circ_006276 novel_circ_006277 novel_circ_006278 novel_circ_006279 novel_circ_006280 novel_circ_006281 novel_circ_006282 novel_circ_006283 novel_circ_006284 novel_circ_006285 novel_circ_006286 novel_circ_006288 novel_circ_006289 novel_circ_006290 novel_circ_006291 novel_circ_006294 novel_circ_006296 novel_circ_006297 novel_circ_006300 novel_circ_006301 novel_circ_006302 novel_circ_006303 novel_circ_006304 novel_circ_006305 novel_circ_006306 novel_circ_006307 novel_circ_006308 novel_circ_006309 novel_circ_006310 novel_circ_006311 novel_circ_006312 novel_circ_006313 novel_circ_006314

| hsa_circ_0077885 | 92 | 1 | 932 | 195 | 1108 | 0.02 | 0 |
| --- | --- | --- | --- | --- | --- | --- | --- |
| hsa_circ_0077888 | 93 | 1 | 600 | 1 | 600 | 0 | 0 |
| hsa_circ_0077888 | 91 | 1 | 839 | 1 | 821 | 0.02 | 0 |
| hsa_circ_0077888 | 91 | 1 | 929 | 1 | 911 | 0.02 | 0 |
| hsa_circ_0077892 | 95 | 1 | 525 | 1 | 525 | 0 | 0 |
| hsa_circ_0077890 | 94 | 1 | 734 | 562 | 1294 | 0 | 0 |
| hsa_circ_0077900 | 94 | 1 | 668 | 668 | 1 | 0 | 0 |
| hsa_circ_0077900 | 94 | 4 | 815 | 812 | 1 | 0 | 0 |
| hsa_circ_0077900 | 92 | 1 | 1462 | 1468 | 1 | 0.01 | 0 |
| hsa_circ_0077900 | 94 | 1 | 668 | 668 | 1 | 0 | 0 |
| hsa_circ_0077900 | 94 | 4 | 815 | 812 | 1 | 0 | 0 |
| hsa_circ_0077901 | 94 | 4 | 714 | 711 | 1 | 0 | 0 |
| hsa_circ_0077901 | 92 | 1 | 1361 | 1367 | 1 | 0.01 | 0 |
| hsa_circ_0077901 | 91 | 1 | 1056 | 1367 | 306 | 0.01 | 0 |
| hsa_circ_0077901 | 90 | 1 | 794 | 1367 | 568 | 0.01 | 0 |
| hsa_circ_0077901 | 90 | 9 | 538 | 1250 | 715 | 0.02 | 0 |
| hsa_circ_0077912 | 94 | 1 | 246 | 261 | 16 | 0 | 2.00E-100 |
| hsa_circ_0077912 | 94 | 1 | 246 | 261 | 16 | 0 | 1.00E-101 |
| hsa_circ_0077912 | 86 | 1 | 444 | 438 | 16 | 0.05 | 9.00E-129 |
| hsa_circ_0077912 | 94 | 1 | 246 | 261 | 16 | 0 | 6.00E-102 |
| hsa_circ_0077912 | 86 | 1 | 444 | 438 | 16 | 0.05 | 7.00E-129 |
| hsa_circ_0077924 | 91 | 1 | 615 | 617 | 3 | 0 | 0 |
| hsa_circ_0077924 | 92 | 1 | 734 | 736 | 3 | 0 | 0 |
| hsa_circ_0077919 | 89 | 198 | 3114 | 2972 | 59 | 0 | 0 |
| hsa_circ_0077931 | 92 | 1 | 498 | 498 | 1 | 0 | 0 |
| hsa_circ_0077931 | 92 | 1 | 1279 | 1420 | 142 | 0 | 0 |
| hsa_circ_0077931 | 92 | 1 | 226 | 498 | 273 | 0 | 7.00E-86 |
| hsa_circ_0077931 | 92 | 1 | 1148 | 1420 | 273 | 0 | 0 |
| hsa_circ_0077931 | 89 | 198 | 2499 | 2571 | 273 | 0 | 0 |
| hsa_circ_0077935 | 92 | 1 | 692 | 692 | 1 | 0 | 0 |
| hsa_circ_0077932 | 88 | 5 | 1850 | 2180 | 338 | 0 | 0 |
| hsa_circ_0077932 | 88 | 198 | 2043 | 2180 | 338 | 0 | 0 |
| hsa_circ_0077932 | 87 | 198 | 1786 | 2180 | 595 | 0 | 0 |
| hsa_circ_0077939 | 86 | 5 | 1440 | 1433 | 1 | 0 | 0 |
| hsa_circ_0077939 | 86 | 5 | 1254 | 1433 | 187 | 0.01 | 0 |
| hsa_circ_0077943 | 94 | 65 | 485 | 1 | 421 | 0 | 1.00E-180 |
| hsa_circ_0077954 | 88 | 1 | 239 | 263 | 1 | 0.09 | 3.00E-77 |
| hsa_circ_0077954 | 88 | 1 | 239 | 263 | 1 | 0.09 | 3.00E-77 |
| hsa_circ_0077954 | 88 | 1 | 239 | 263 | 1 | 0.09 | 3.00E-77 |
| hsa_circ_0077956 | 94 | 70 | 474 | 405 | 1 | 0 | 1.00E-174 |
| hsa_circ_0077954 | 88 | 1 | 239 | 263 | 1 | 0.09 | 5.00E-78 |
| hsa_circ_0077956 | 94 | 70 | 474 | 405 | 1 | 0 | 7.00E-175 |
| hsa_circ_0077956 | 94 | 6993 | 7397 | 405 | 1 | 0 | 9.00E-174 |
| hsa_circ_0077956 | 94 | 70 | 474 | 405 | 1 | 0 | 5.00E-175 |
| hsa_circ_0077956 | 94 | 6993 | 7397 | 405 | 1 | 0 | 8.00E-174 |
| hsa_circ_0077957 | 92 | 70 | 360 | 291 | 1 | 0 | 4.00E-115 |
| hsa_circ_0077957 | 92 | 247 | 537 | 291 | 1 | 0 | 6.00E-115 |
| hsa_circ_0077957 | 92 | 6993 | 7283 | 291 | 1 | 0 | 9.00E-114 |
| hsa_circ_0077962 | 90 | 1 | 544 | 547 | 1 | 0.01 | 0 |
| hsa_circ_0077962 | 90 | 1 | 650 | 653 | 1 | 0 | 0 |
| hsa_circ_0077962 | 91 | 3 | 1183 | 1184 | 1 | 0 | 0 |
| hsa_circ_0077962 | 91 | 1 | 1382 | 1385 | 1 | 0 | 0 |
| hsa_circ_0077965 | 91 | 3 | 880 | 881 | 1 | 0 | 0 |
| hsa_circ_0077968 | 91 | 3 | 639 | 637 | 1 | 0 | 0 |

novel_circ_006315 novel_circ_006316 novel_circ_006317 novel_circ_006318 novel_circ_006319 novel_circ_006320 novel_circ_006321 novel_circ_006322 novel_circ_006323 novel_circ_006324 novel_circ_006325 novel_circ_006326 novel_circ_006327 novel_circ_006328 novel_circ_006329 novel_circ_006330 novel_circ_006331 novel_circ_006332 novel_circ_006333 novel_circ_006334 novel_circ_006335 novel_circ_006336 novel_circ_006340 novel_circ_006344 novel_circ_006345 novel_circ_006346 novel_circ_006347 novel_circ_006348 novel_circ_006349 novel_circ_006350 novel_circ_006351 novel_circ_006352 novel_circ_006355 novel_circ_006356 novel_circ_006357 novel_circ_006358 novel_circ_006359 novel_circ_006360 novel_circ_006361 novel_circ_006362 novel_circ_006363 novel_circ_006364 novel_circ_006365 novel_circ_006366 novel_circ_006367 novel_circ_006368 novel_circ_006369 novel_circ_006370 novel_circ_006371 novel_circ_006372 novel_circ_006373 novel_circ_006374 novel_circ_006375 novel_circ_006376

| hsa_circ_0077968 | 91 | 6 | 642 | 637 | 1 | 0 | 0 |
| --- | --- | --- | --- | --- | --- | --- | --- |
| hsa_circ_0077968 | 92 | 1 | 838 | 838 | 1 | 0 | 0 |
| hsa_circ_0077968 | 91 | 3 | 347 | 637 | 293 | 0 | 4.00E-130 |
| hsa_circ_0077968 | 91 | 6 | 350 | 637 | 293 | 0 | 4.00E-130 |
| hsa_circ_0077968 | 92 | 1 | 546 | 838 | 293 | 0 | 0 |
| hsa_circ_0077971 | 94 | 1 | 505 | 663 | 159 | 0 | 0 |
| hsa_circ_0077975 | 94 | 1 | 470 | 470 | 1 | 0 | 0 |
| hsa_circ_0077975 | 94 | 1 | 336 | 470 | 135 | 0 | 8.00E-142 |
| hsa_circ_0077975 | 94 | 1 | 1404 | 1538 | 135 | 0 | 0 |
| hsa_circ_0077975 | 94 | 1 | 254 | 470 | 217 | 0 | 5.00E-108 |
| hsa_circ_0077975 | 95 | 1 | 681 | 897 | 217 | 0 | 0 |
| hsa_circ_0077975 | 95 | 1 | 959 | 1175 | 217 | 0 | 0 |
| hsa_circ_0077976 | 95 | 1 | 1176 | 1176 | 1 | 0 | 0 |
| hsa_circ_0077977 | 95 | 1 | 427 | 427 | 1 | 0 | 0 |
| hsa_circ_0077977 | 95 | 1 | 1068 | 1068 | 1 | 0 | 0 |
| hsa_circ_0077977 | 95 | 1 | 1232 | 1232 | 1 | 0 | 0 |
| hsa_circ_0077980 | 95 | 1 | 754 | 754 | 1 | 0 | 0 |
| hsa_circ_0007798 | 95 | 1 | 278 | 278 | 1 | 0 | 5.00E-123 |
| hsa_circ_0007798 | 94 | 1 | 641 | 641 | 1 | 0 | 0 |
| hsa_circ_0007798 | 94 | 1 | 805 | 805 | 1 | 0 | 0 |
| hsa_circ_0077992 | 92 | 1 | 564 | 1 | 564 | 0 | 0 |
| hsa_circ_0077992 | 90 | 1 | 377 | 188 | 564 | 0 | 2.00E-139 |
| hsa_circ_0018413 | 95 | 1 | 327 | 142 | 468 | 0 | 4.00E-145 |
| hsa_circ_0008028 | 75 | 3612 | 6069 | 414651 | 417089 | 0.05 | 0 |
| hsa_circ_0047481 | 95 | 131 | 582 | 1 | 452 | 0 | 0 |
| hsa_circ_0047481 | 94 | 131 | 769 | 1 | 639 | 0 | 0 |
| hsa_circ_0047484 | 92 | 314 | 1083 | 1 | 770 | 0 | 0 |
| hsa_circ_0047487 | 93 | 386 | 1787 | 1 | 1402 | 0 | 0 |
| hsa_circ_0047481 | 96 | 1 | 255 | 1 | 255 | 0 | 1.00E-113 |
| hsa_circ_0047481 | 95 | 1 | 452 | 1 | 452 | 0 | 0 |
| hsa_circ_0047484 | 92 | 184 | 953 | 1 | 770 | 0 | 0 |
| hsa_circ_0047498 | 91 | 1 | 546 | 1 | 546 | 0 | 0 |
| hsa_circ_0085301 | 90 | 41767 | 41825 | 99900 | 99841 | 0.02 | 1.00E-10 |
| hsa_circ_0047503 | 90 | 149 | 795 | 123 | 782 | 0.03 | 0 |
| hsa_circ_0000845 | 90 | 151 | 840 | 1 | 705 | 0.03 | 0 |
| hsa_circ_0047504 | 90 | 1 | 645 | 1 | 658 | 0.03 | 0 |
| hsa_circ_0047504 | 94 | 1 | 3460 | 713 | 4172 | 0 | 0 |
| hsa_circ_0047524 | 90 | 1 | 1133 | 1416 | 284 | 0 | 0 |
| hsa_circ_0047524 | 90 | 3 | 1468 | 1749 | 284 | 0 | 0 |
| hsa_circ_0047524 | 90 | 1 | 1613 | 1896 | 284 | 0 | 0 |
| hsa_circ_0047525 | 90 | 1665 | 2029 | 365 | 1 | 0 | 9.00E-129 |
| hsa_circ_0047525 | 90 | 4 | 558 | 555 | 1 | 0 | 0 |
| hsa_circ_0047525 | 89 | 1 | 858 | 858 | 1 | 0 | 0 |
| hsa_circ_0047525 | 89 | 1 | 300 | 858 | 559 | 0 | 8.00E-102 |
| hsa_circ_0047525 | 89 | 1 | 463 | 1021 | 559 | 0 | 1.00E-160 |
| hsa_circ_0047525 | 90 | 1 | 904 | 1462 | 559 | 0 | 0 |
| hsa_circ_0047525 | 91 | 1 | 1416 | 1974 | 559 | 0 | 0 |
| hsa_circ_0047527 | 89 | 3 | 335 | 333 | 1 | 0 | 8.00E-117 |
| hsa_circ_0047527 | 89 | 1 | 480 | 480 | 1 | 0 | 5.00E-170 |
| hsa_circ_0047527 | 92 | 1 | 491 | 826 | 336 | 0 | 0 |
| hsa_circ_0047527 | 92 | 1 | 781 | 1116 | 336 | 0 | 0 |
| hsa_circ_0047524 | 92 | 1 | 1921 | 3672 | 1752 | 0 | 0 |
| hsa_circ_0047524 | 92 | 1 | 2531 | 4282 | 1752 | 0 | 0 |
| hsa_circ_0047527 | 94 | 1 | 346 | 826 | 481 | 0.01 | 2.00E-147 |

novel_circ_006377 novel_circ_006378 novel_circ_006379 novel_circ_006380 novel_circ_006381 novel_circ_006382 novel_circ_006383 novel_circ_006384 novel_circ_006385 novel_circ_006386 novel_circ_006387 novel_circ_006388 novel_circ_006389 novel_circ_006390 novel_circ_006398 novel_circ_006399 novel_circ_006400 novel_circ_006401 novel_circ_006402 novel_circ_006403 novel_circ_006404 novel_circ_006405 novel_circ_006406 novel_circ_006407 novel_circ_006408 novel_circ_006409 novel_circ_006410 novel_circ_006411 novel_circ_006412 novel_circ_006413 novel_circ_006415 novel_circ_006416 novel_circ_006417 novel_circ_006419 novel_circ_006420 novel_circ_006421 novel_circ_006422 novel_circ_006423 novel_circ_006424 novel_circ_006425 novel_circ_006426 novel_circ_006428 novel_circ_006429 novel_circ_006430 novel_circ_006431 novel_circ_006432 novel_circ_006434 novel_circ_006435 novel_circ_006436 novel_circ_006437 novel_circ_006438 novel_circ_006439 novel_circ_006440 novel_circ_006441

| hsa_circ_0047524 | 92 | 1 | 2386 | 4282 | 1897 | 0 | 0 |
| --- | --- | --- | --- | --- | --- | --- | --- |
| hsa_circ_0047524 | 91 | 1 | 1750 | 4282 | 2533 | 0 | 0 |
| hsa_circ_0047544 | 90 | 1 | 610 | 895 | 286 | 0 | 0 |
| hsa_circ_0047550 | 93 | 6 | 688 | 686 | 1 | 0 | 0 |
| hsa_circ_0047550 | 93 | 6 | 319 | 686 | 373 | 0 | 3.00E-126 |
| hsa_circ_0047550 | 93 | 6 | 245 | 686 | 447 | 0 | 1.00E-93 |
| hsa_circ_0047574 | 94 | 1 | 341 | 341 | 1 | 0 | 3.00E-146 |
| hsa_circ_0047574 | 93 | 1 | 590 | 590 | 1 | 0 | 0 |
| hsa_circ_0007699 | 95 | 7 | 785 | 7 | 785 | 0 | 0 |
| hsa_circ_0007699 | 95 | 7 | 785 | 7 | 785 | 0 | 0 |
| hsa_circ_0007699 | 95 | 7 | 785 | 7 | 785 | 0 | 0 |
| hsa_circ_0047584 | 92 | 184 | 310 | 1 | 124 | 0.02 | 7.00E-43 |
| hsa_circ_0047584 | 94 | 184 | 481 | 1 | 295 | 0.01 | 2.00E-124 |
| hsa_circ_0047584 | 94 | 1 | 298 | 1 | 295 | 0.01 | 1.00E-124 |
| hsa_circ_0047591 | 99 | 1 | 178 | 178 | 1 | 0 | 2.00E-86 |
| hsa_circ_0047591 | 97 | 1 | 484 | 484 | 1 | 0 | 0 |
| hsa_circ_0047591 | 96 | 1 | 1662 | 1662 | 1 | 0 | 0 |
| hsa_circ_0047591 | 97 | 1 | 607 | 645 | 39 | 0 | 0 |
| hsa_circ_0047591 | 95 | 1 | 1484 | 1662 | 179 | 0 | 0 |
| hsa_circ_0047591 | 95 | 5287 | 6771 | 1663 | 179 | 0 | 0 |
| hsa_circ_0047591 | 95 | 1 | 341 | 825 | 485 | 0 | 6.00E-153 |
| hsa_circ_0047591 | 95 | 1 | 1178 | 1662 | 485 | 0 | 0 |
| hsa_circ_0047591 | 95 | 1 | 1017 | 1662 | 646 | 0 | 0 |
| hsa_circ_0047591 | 94 | 1 | 362 | 1187 | 826 | 0 | 3.00E-156 |
| hsa_circ_0047591 | 95 | 1 | 837 | 1662 | 826 | 0 | 0 |
| hsa_circ_0047591 | 95 | 109 | 946 | 1663 | 826 | 0 | 0 |
| hsa_circ_0047591 | 95 | 1 | 702 | 1662 | 961 | 0 | 0 |
| hsa_circ_0047591 | 95 | 109 | 811 | 1663 | 961 | 0 | 0 |
| hsa_circ_0047596 | 96 | 1 | 475 | 475 | 1 | 0 | 0 |
| hsa_circ_0047591 | 96 | 109 | 584 | 1663 | 1188 | 0 | 0 |
| hsa_circ_0047597 | 95 | 428 | 886 | 1 | 459 | 0 | 0 |
| hsa_circ_0047597 | 95 | 237 | 695 | 1 | 459 | 0 | 0 |
| hsa_circ_0047597 | 95 | 218 | 676 | 1 | 459 | 0 | 0 |
| hsa_circ_0006242 | 93 | 1 | 294 | 294 | 1 | 0 | 7.00E-117 |
| hsa_circ_0006242 | 94 | 1 | 405 | 405 | 1 | 0 | 9.00E-172 |
| hsa_circ_0047602 | 93 | 1 | 320 | 320 | 1 | 0 | 1.00E-129 |
| hsa_circ_0047608 | 93 | 1 | 342 | 342 | 1 | 0 | 4.00E-140 |
| hsa_circ_0000847 | 93 | 1 | 404 | 404 | 1 | 0 | 2.00E-164 |
| hsa_circ_0000847 | 94 | 1 | 494 | 494 | 1 | 0 | 0 |
| hsa_circ_0000847 | 93 | 1 | 783 | 783 | 1 | 0.01 | 0 |
| hsa_circ_0047612 | 93 | 1 | 708 | 708 | 1 | 0.01 | 0 |
| hsa_circ_0018067 | 82 | 62490 | 62702 | 44685 | 44904 | 0.05 | 5.00E-41 |
| hsa_circ_0047624 | 93 | 1 | 255 | 1 | 255 | 0 | 3.00E-100 |
| hsa_circ_0047628 | 92 | 2 | 793 | 2 | 793 | 0 | 0 |
| hsa_circ_0047633 | 93 | 1 | 400 | 803 | 404 | 0 | 3.00E-162 |
| hsa_circ_0047636 | 94 | 1 | 286 | 286 | 1 | 0 | 2.00E-122 |
| hsa_circ_0047645 | 92 | 1 | 514 | 514 | 1 | 0 | 0 |
| hsa_circ_0047645 | 92 | 3 | 697 | 695 | 1 | 0 | 0 |
| hsa_circ_0005902 | 92 | 1 | 305 | 305 | 1 | 0 | 3.00E-121 |
| hsa_circ_0047655 | 94 | 8 | 351 | 347 | 4 | 0 | 1.00E-144 |
| hsa_circ_0047655 | 94 | 8 | 280 | 347 | 75 | 0 | 1.00E-113 |
| hsa_circ_0047653 | 91 | 1 | 472 | 816 | 344 | 0 | 0 |
| hsa_circ_0047671 | 92 | 168 | 867 | 771 | 72 | 0 | 0 |
| hsa_circ_0047677 | 92 | 3 | 428 | 2061 | 1636 | 0 | 5.00E-170 |

novel_circ_006442 novel_circ_006443 novel_circ_006444 novel_circ_006445 novel_circ_006448 novel_circ_006449 novel_circ_006450 novel_circ_006451 novel_circ_006452 novel_circ_006453 novel_circ_006454 novel_circ_006455 novel_circ_006456 novel_circ_006457 novel_circ_006458 novel_circ_006459 novel_circ_006460 novel_circ_006461 novel_circ_006462 novel_circ_006463 novel_circ_006464 novel_circ_006465 novel_circ_006466 novel_circ_006467 novel_circ_006468 novel_circ_006469 novel_circ_006470 novel_circ_006471 novel_circ_006472 novel_circ_006473 novel_circ_006474 novel_circ_006475 novel_circ_006476 novel_circ_006477 novel_circ_006478 novel_circ_006481 novel_circ_006482 novel_circ_006483 novel_circ_006484 novel_circ_006485 novel_circ_006486 novel_circ_006487 novel_circ_006488 novel_circ_006489 novel_circ_006490 novel_circ_006491 novel_circ_006492 novel_circ_006494 novel_circ_006495 novel_circ_006498 novel_circ_006499 novel_circ_006500 novel_circ_006501 novel_circ_006502

| hsa_circ_0047688 | 81 | 75609 | 77025 | 1 | 1416 | 0.02 | 0 |
| --- | --- | --- | --- | --- | --- | --- | --- |
| hsa_circ_0047688 | 81 | 183 | 1599 | 1 | 1416 | 0.02 | 0 |
| hsa_circ_0047688 | 81 | 1 | 1417 | 1 | 1416 | 0.02 | 0 |
| hsa_circ_0047688 | 81 | 1 | 1417 | 1 | 1416 | 0.02 | 0 |
| hsa_circ_0086342 | 93 | 1 | 242 | 1 | 242 | 0 | 5.00E-98 |
| hsa_circ_0086340 | 90 | 3 | 638 | 3 | 638 | 0 | 0 |
| hsa_circ_0086339 | 90 | 10990 | 11629 | 108 | 747 | 0 | 0 |
| hsa_circ_0086339 | 90 | 6 | 747 | 6 | 747 | 0 | 0 |
| hsa_circ_0086336 | 85 | 1 | 1494 | 243 | 1745 | 0.01 | 0 |
| hsa_circ_0086336 | 84 | 1 | 1328 | 243 | 1579 | 0.02 | 0 |
| hsa_circ_0086339 | 88 | 6 | 505 | 6 | 505 | 0 | 9.00E-168 |
| hsa_circ_0086338 | 81 | 1 | 856 | 1 | 865 | 0.02 | 0 |
| hsa_circ_0086336 | 85 | 1 | 1098 | 1 | 1107 | 0.01 | 0 |
| hsa_circ_0086336 | 85 | 1 | 989 | 1 | 998 | 0.01 | 0 |
| hsa_circ_0086336 | 96 | 1 | 242 | 1 | 242 | 0 | 1.00E-109 |
| hsa_circ_0086334 | 97 | 1 | 292 | 1 | 292 | 0 | 2.00E-137 |
| hsa_circ_0086332 | 95 | 1 | 486 | 1 | 486 | 0 | 0 |
| hsa_circ_0086326 | 95 | 1 | 777 | 162 | 938 | 0 | 0 |
| hsa_circ_0086326 | 94 | 3 | 938 | 3 | 938 | 0 | 0 |
| hsa_circ_0086326 | 93 | 1 | 291 | 162 | 452 | 0 | 3.00E-120 |
| hsa_circ_0086322 | 95 | 1 | 221 | 446 | 226 | 0 | 9.00E-95 |
| hsa_circ_0086321 | 91 | 1 | 589 | 589 | 1 | 0 | 0 |
| hsa_circ_0086318 | 92 | 1 | 941 | 1308 | 368 | 0 | 0 |
| hsa_circ_0086318 | 93 | 4 | 276 | 640 | 368 | 0 | 7.00E-112 |
| hsa_circ_0086315 | 90 | 1 | 613 | 1564 | 952 | 0 | 0 |
| hsa_circ_0086307 | 93 | 1 | 1605 | 1 | 1607 | 0 | 0 |
| hsa_circ_0086310 | 96 | 1 | 516 | 420 | 935 | 0 | 0 |
| hsa_circ_0086307 | 95 | 1 | 1045 | 1 | 1045 | 0 | 0 |
| hsa_circ_0086305 | 94 | 1 | 960 | 1 | 960 | 0 | 0 |
| hsa_circ_0086310 | 96 | 1 | 336 | 188 | 523 | 0 | 8.00E-157 |
| hsa_circ_0086310 | 97 | 1 | 232 | 188 | 419 | 0 | 3.00E-109 |
| hsa_circ_0086307 | 94 | 1 | 529 | 1 | 529 | 0 | 0 |
| hsa_circ_0086307 | 91 | 1 | 297 | 1 | 297 | 0.01 | 3.00E-110 |
| hsa_circ_0086305 | 93 | 1 | 516 | 1 | 516 | 0 | 0 |
| hsa_circ_0086303 | 91 | 1 | 494 | 1 | 491 | 0.02 | 0 |
| hsa_circ_0031217 | 88 | 1 | 946 | 1 | 934 | 0.01 | 0 |
| hsa_circ_0031237 | 94 | 4 | 463 | 526 | 67 | 0 | 0 |
| hsa_circ_0005663 | 90 | 1 | 410 | 507 | 98 | 0 | 2.00E-149 |
| hsa_circ_0031278 | 88 | 1 | 1604 | 1598 | 1 | 0.01 | 0 |
| hsa_circ_0031278 | 88 | 1 | 1690 | 1687 | 1 | 0.01 | 0 |
| hsa_circ_0031275 | 89 | 1 | 1922 | 2787 | 869 | 0.01 | 0 |
| hsa_circ_0031279 | 88 | 1 | 1488 | 1482 | 1 | 0.01 | 0 |
| hsa_circ_0031279 | 88 | 1 | 1574 | 1571 | 1 | 0.01 | 0 |
| hsa_circ_0031281 | 91 | 127 | 280 | 154 | 1 | 0 | 4.00E-52 |
| hsa_circ_0031283 | 95 | 1 | 357 | 357 | 1 | 0 | 2.00E-158 |
| hsa_circ_0031283 | 95 | 9530 | 9894 | 366 | 1 | 0 | 4.00E-158 |
| hsa_circ_0031288 | 96 | 1 | 152 | 1 | 152 | 0 | 6.00E-65 |
| hsa_circ_0031378 | 90 | 4 | 655 | 652 | 1 | 0 | 0 |
| hsa_circ_0031378 | 90 | 382 | 1307 | 926 | 1 | 0 | 0 |
| hsa_circ_0074029 | 75 | 59127 | 59446 | 207611 | 207936 | 0.04 | 2.00E-30 |
| hsa_circ_0070970 | 94 | 1 | 653 | 8578 | 7925 | 0 | 0 |
| hsa_circ_0070981 | 95 | 1 | 387 | 387 | 1 | 0 | 9.00E-172 |
| hsa_circ_0070981 | 93 | 1 | 514 | 514 | 1 | 0 | 0 |
| hsa_circ_0070981 | 92 | 2 | 803 | 802 | 1 | 0 | 0 |

novel_circ_006503 novel_circ_006504 novel_circ_006505 novel_circ_006506 novel_circ_006508 novel_circ_006509 novel_circ_006512 novel_circ_006513 novel_circ_006514 novel_circ_006515 novel_circ_006516 novel_circ_006517 novel_circ_006518 novel_circ_006519 novel_circ_006520 novel_circ_006521 novel_circ_006522 novel_circ_006523 novel_circ_006524 novel_circ_006525 novel_circ_006526 novel_circ_006527 novel_circ_006528 novel_circ_006529 novel_circ_006530 novel_circ_006531 novel_circ_006532 novel_circ_006533 novel_circ_006534 novel_circ_006535 novel_circ_006537 novel_circ_006538 novel_circ_006539 novel_circ_006540 novel_circ_006541 novel_circ_006542 novel_circ_006543 novel_circ_006544 novel_circ_006546 novel_circ_006547 novel_circ_006548 novel_circ_006549 novel_circ_006550 novel_circ_006551 novel_circ_006552 novel_circ_006553 novel_circ_006554 novel_circ_006555 novel_circ_006556 novel_circ_006557 novel_circ_006558 novel_circ_006560 novel_circ_006561 novel_circ_006562

| hsa_circ_0070981 | 94 | 1 | 342 | 387 | 46 | 0 | 2.00E-148 |
| --- | --- | --- | --- | --- | --- | --- | --- |
| hsa_circ_0070981 | 93 | 1 | 469 | 514 | 46 | 0 | 0 |
| hsa_circ_0070981 | 92 | 2 | 530 | 802 | 274 | 0 | 0 |
| hsa_circ_0036055 | 83 | 10209 | 10442 | 26451 | 26684 | 0.05 | 4.00E-51 |
| hsa_circ_0066635 | 78 | 15286 | 15444 | 94317 | 94477 | 0.01 | 1.00E-18 |
| hsa_circ_0066635 | 78 | 15873 | 16031 | 94317 | 94477 | 0.01 | 1.00E-18 |
| hsa_circ_0070982 | 93 | 1 | 1484 | 2435 | 953 | 0 | 0 |
| hsa_circ_0070982 | 93 | 146 | 1859 | 2665 | 953 | 0 | 0 |
| hsa_circ_0008825 | 89 | 2 | 327 | 491 | 166 | 0.01 | 1.00E-111 |
| hsa_circ_0007710 | 95 | 1 | 167 | 8681 | 8515 | 0 | 1.00E-67 |
| hsa_circ_0008825 | 88 | 1 | 240 | 404 | 166 | 0 | 1.00E-75 |
| hsa_circ_0008825 | 89 | 1 | 324 | 489 | 166 | 0.01 | 3.00E-111 |
| hsa_circ_0008825 | 89 | 2 | 327 | 491 | 166 | 0.01 | 2.00E-112 |
| hsa_circ_0008825 | 88 | 1 | 240 | 404 | 166 | 0 | 8.00E-76 |
| hsa_circ_0008825 | 89 | 1 | 324 | 489 | 166 | 0.01 | 2.00E-111 |
| hsa_circ_0008825 | 89 | 2 | 327 | 491 | 166 | 0.01 | 2.00E-112 |
| hsa_circ_0008825 | 84 | 23176 | 23650 | 639 | 166 | 0.02 | 1.00E-126 |
| hsa_circ_0008825 | 89 | 2 | 327 | 491 | 166 | 0.01 | 1.00E-112 |
| hsa_circ_0007466 | 89 | 1 | 323 | 323 | 1 | 0.01 | 3.00E-111 |
| hsa_circ_0008825 | 89 | 2 | 326 | 491 | 167 | 0.01 | 2.00E-112 |
| hsa_circ_0008825 | 84 | 23176 | 23649 | 639 | 167 | 0.02 | 5.00E-126 |
| hsa_circ_0070991 | 95 | 1 | 757 | 1 | 757 | 0 | 0 |
| hsa_circ_0070991 | 94 | 1 | 446 | 758 | 1203 | 0 | 0 |
| hsa_circ_0070994 | 96 | 1 | 402 | 344 | 745 | 0 | 0 |
| hsa_circ_0070994 | 96 | 1 | 549 | 344 | 892 | 0 | 0 |
| hsa_circ_0001442 | 89 | 203 | 474 | 272 | 1 | 0 | 2.00E-92 |
| hsa_circ_0001442 | 89 | 1 | 272 | 272 | 1 | 0 | 8.00E-93 |
| hsa_circ_0001442 | 89 | 203 | 474 | 272 | 1 | 0 | 1.00E-92 |
| hsa_circ_0001442 | 89 | 203 | 392 | 272 | 83 | 0 | 1.00E-60 |
| hsa_circ_0071001 | 92 | 1 | 1587 | 1851 | 253 | 0.01 | 0 |
| hsa_circ_0002586 | 92 | 368 | 927 | 1 | 560 | 0 | 0 |
| hsa_circ_0002586 | 90 | 1 | 228 | 1 | 228 | 0 | 3.00E-80 |
| hsa_circ_0002586 | 92 | 1 | 560 | 1 | 560 | 0 | 0 |
| hsa_circ_0002586 | 94 | 1 | 332 | 229 | 560 | 0 | 1.00E-139 |
| hsa_circ_0071009 | 92 | 1 | 266 | 2402 | 2137 | 0 | 1.00E-104 |
| hsa_circ_0071009 | 92 | 1 | 2227 | 4363 | 2137 | 0 | 0 |
| hsa_circ_0071009 | 92 | 1 | 562 | 2877 | 2316 | 0 | 0 |
| hsa_circ_0071009 | 90 | 1 | 409 | 2877 | 2469 | 0 | 1.00E-150 |
| hsa_circ_0071009 | 92 | 1 | 1071 | 3948 | 2878 | 0 | 0 |
| hsa_circ_0071009 | 92 | 1 | 1278 | 4155 | 2878 | 0 | 0 |
| hsa_circ_0071009 | 93 | 1 | 1486 | 4363 | 2878 | 0 | 0 |
| hsa_circ_0071009 | 92 | 6 | 1748 | 4620 | 2878 | 0 | 0 |
| hsa_circ_0071009 | 93 | 1 | 1068 | 4363 | 3296 | 0 | 0 |
| hsa_circ_0071010 | 91 | 1 | 374 | 744 | 371 | 0 | 2.00E-139 |
| hsa_circ_0071010 | 92 | 1 | 582 | 952 | 371 | 0 | 0 |
| hsa_circ_0071010 | 91 | 6 | 472 | 1209 | 743 | 0 | 2.00E-180 |
| hsa_circ_0071009 | 92 | 1 | 699 | 4854 | 4156 | 0 | 0 |
| hsa_circ_0071009 | 91 | 1 | 818 | 4973 | 4156 | 0 | 0 |
| hsa_circ_0071009 | 90 | 1 | 491 | 4854 | 4364 | 0 | 0 |
| hsa_circ_0071009 | 90 | 1 | 610 | 4973 | 4364 | 0 | 0 |
| hsa_circ_0071009 | 90 | 1 | 721 | 5084 | 4364 | 0 | 0 |
| hsa_circ_0071011 | 98 | 1 | 97 | 97 | 1 | 0 | 1.00E-40 |
| hsa_circ_0071011 | 94 | 1 | 503 | 503 | 1 | 0 | 0 |
| hsa_circ_0071013 | 93 | 1 | 406 | 406 | 1 | 0 | 3.00E-167 |

novel_circ_006563 novel_circ_006564 novel_circ_006566 novel_circ_006567 novel_circ_006568 novel_circ_006569 novel_circ_006570 novel_circ_006571 novel_circ_006572 novel_circ_006573 novel_circ_006574 novel_circ_006575 novel_circ_006576 novel_circ_006577 novel_circ_006578 novel_circ_006579 novel_circ_006580 novel_circ_006581 novel_circ_006582 novel_circ_006587 novel_circ_006588 novel_circ_006589 novel_circ_006590 novel_circ_006591 novel_circ_006592 novel_circ_006593 novel_circ_006595 novel_circ_006596 novel_circ_006597 novel_circ_006599 novel_circ_006600 novel_circ_006606 novel_circ_006607 novel_circ_006608 novel_circ_006609 novel_circ_006611 novel_circ_006612 novel_circ_006613 novel_circ_006614 novel_circ_006615 novel_circ_006616 novel_circ_006617 novel_circ_006618 novel_circ_006619 novel_circ_006620 novel_circ_006622 novel_circ_006623 novel_circ_006624 novel_circ_006625 novel_circ_006626 novel_circ_006627 novel_circ_006628 novel_circ_006629 novel_circ_006630

| hsa_circ_0071014 | 95 | 11 | 303 | 5 | 297 | 0 | 1.00E-129 |
| --- | --- | --- | --- | --- | --- | --- | --- |
| hsa_circ_0071014 | 95 | 11 | 430 | 5 | 424 | 0 | 0 |
| hsa_circ_0071026 | 90 | 2 | 507 | 506 | 1 | 0 | 0 |
| hsa_circ_0071026 | 91 | 1 | 922 | 922 | 1 | 0 | 0 |
| hsa_circ_0071026 | 89 | 4 | 1079 | 1076 | 1 | 0 | 0 |
| hsa_circ_0071026 | 89 | 1 | 1280 | 1283 | 1 | 0 | 0 |
| hsa_circ_0071026 | 90 | 1 | 1458 | 1461 | 1 | 0 | 0 |
| hsa_circ_0071028 | 92 | 2 | 237 | 238 | 3 | 0 | 5.00E-88 |
| hsa_circ_0071028 | 91 | 1 | 652 | 654 | 3 | 0 | 0 |
| hsa_circ_0071028 | 89 | 1 | 1010 | 1015 | 3 | 0 | 0 |
| hsa_circ_0071028 | 90 | 1 | 1188 | 1193 | 3 | 0 | 0 |
| hsa_circ_0071028 | 88 | 4 | 572 | 808 | 240 | 0 | 0 |
| hsa_circ_0071028 | 90 | 1 | 951 | 1193 | 240 | 0.01 | 0 |
| hsa_circ_0071030 | 91 | 1 | 502 | 539 | 35 | 0.01 | 0 |
| hsa_circ_0071029 | 91 | 1 | 847 | 1057 | 208 | 0.01 | 0 |
| hsa_circ_0071032 | 93 | 1 | 493 | 875 | 383 | 0 | 0 |
| hsa_circ_0071032 | 92 | 1 | 678 | 1060 | 383 | 0 | 0 |
| hsa_circ_0071037 | 95 | 286 | 522 | 238 | 2 | 0 | 1.00E-101 |
| hsa_circ_0071037 | 95 | 350 | 586 | 238 | 2 | 0 | 2.00E-101 |
| hsa_circ_0052104 | 72 | 791 | 1690 | 1199 | 2117 | 0.07 | 6.00E-61 |
| hsa_circ_0052097 | 73 | 60954 | 61489 | 1204 | 666 | 0.05 | 9.00E-38 |
| hsa_circ_0052110 | 79 | 19543 | 20010 | 1612 | 1145 | 0.02 | 6.00E-80 |
| hsa_circ_0010789 | 90 | 76987 | 77636 | 725 | 68 | 0.02 | 0 |
| hsa_circ_0052095 | 89 | 3 | 122 | 3 | 122 | 0 | 4.00E-35 |
| hsa_circ_0052115 | 72 | 564 | 2803 | 4054 | 1813 | 0.04 | 7.00E-171 |
| hsa_circ_0052115 | 72 | 564 | 2803 | 4054 | 1813 | 0.04 | 1.00E-170 |
| hsa_circ_0078674 | 84 | 1 | 637 | 1 | 634 | 0 | 1.00E-172 |
| hsa_circ_0078674 | 85 | 1 | 795 | 1 | 792 | 0 | 0 |
| hsa_circ_0078679 | 89 | 1 | 431 | 1 | 431 | 0 | 2.00E-149 |
| hsa_circ_0078771 | 83 | 11 | 1095 | 15 | 1100 | 0.01 | 0 |
| hsa_circ_0078771 | 83 | 11 | 1095 | 15 | 1100 | 0.01 | 0 |
| hsa_circ_0078787 | 92 | 684 | 946 | 1 | 263 | 0 | 1.00E-100 |
| hsa_circ_0078787 | 92 | 673 | 935 | 1 | 263 | 0 | 1.00E-100 |
| hsa_circ_0078787 | 92 | 670 | 932 | 1 | 263 | 0 | 1.00E-100 |
| hsa_circ_0078787 | 92 | 181 | 443 | 1 | 263 | 0 | 7.00E-101 |
| hsa_circ_0078787 | 92 | 93 | 355 | 1 | 263 | 0 | 6.00E-101 |
| hsa_circ_0078787 | 96 | 1 | 95 | 169 | 263 | 0 | 4.00E-36 |
| hsa_circ_0078790 | 92 | 106 | 218 | 488 | 376 | 0 | 4.00E-38 |
| hsa_circ_0078790 | 92 | 232 | 344 | 488 | 376 | 0 | 7.00E-38 |
| hsa_circ_0078790 | 92 | 475 | 587 | 488 | 376 | 0 | 1.00E-37 |
| hsa_circ_0074380 | 91 | 4 | 1197 | 1194 | 1 | 0 | 0 |
| hsa_circ_0074380 | 91 | 4 | 1197 | 1194 | 1 | 0 | 0 |
| hsa_circ_0074379 | 91 | 4 | 1481 | 1760 | 280 | 0 | 0 |
| hsa_circ_0005968 | 73 | 24558 | 25109 | 17896 | 17363 | 0.07 | 1.00E-34 |
| hsa_circ_0002739 | 91 | 46372 | 47203 | 835 | 1 | 0 | 0 |
| hsa_circ_0002739 | 91 | 8 | 708 | 835 | 132 | 0 | 0 |
| hsa_circ_0002739 | 91 | 8 | 839 | 835 | 1 | 0 | 0 |
| hsa_circ_0002739 | 92 | 3 | 541 | 670 | 132 | 0 | 0 |
| hsa_circ_0074374 | 99 | 1 | 276 | 704 | 979 | 0 | 5.00E-136 |
| hsa_circ_0074377 | 95 | 174 | 322 | 142 | 290 | 0 | 3.00E-61 |
| hsa_circ_0074374 | 95 | 3 | 413 | 3 | 413 | 0 | 0 |
| hsa_circ_0074370 | 94 | 67 | 554 | 1 | 488 | 0 | 0 |
| hsa_circ_0074375 | 96 | 1 | 165 | 1 | 165 | 0 | 2.00E-70 |
| hsa_circ_0074374 | 96 | 3 | 253 | 3 | 253 | 0 | 2.00E-111 |

novel_circ_006631 novel_circ_006632 novel_circ_006633 novel_circ_006634 novel_circ_006635 novel_circ_006636 novel_circ_006637 novel_circ_006638 novel_circ_006639 novel_circ_006640 novel_circ_006641 novel_circ_006642 novel_circ_006652 novel_circ_006653 novel_circ_006654 novel_circ_006655 novel_circ_006656 novel_circ_006657 novel_circ_006658 novel_circ_006659 novel_circ_006660 novel_circ_006667 novel_circ_006668 novel_circ_006674 novel_circ_006675 novel_circ_006676 novel_circ_006678 novel_circ_006679 novel_circ_006680 novel_circ_006682 novel_circ_006685 novel_circ_006686 novel_circ_006687 novel_circ_006688 novel_circ_006690 novel_circ_006691 novel_circ_006692 novel_circ_006693 novel_circ_006694 novel_circ_006695 novel_circ_006696 novel_circ_006699 novel_circ_006700 novel_circ_006701 novel_circ_006702 novel_circ_006703 novel_circ_006704 novel_circ_006705 novel_circ_006708 novel_circ_006712 novel_circ_006713 novel_circ_006714 novel_circ_006715 novel_circ_006716

| hsa_circ_0074370 | 95 | 1 | 328 | 1 | 328 | 0 | 2.00E-142 |
| --- | --- | --- | --- | --- | --- | --- | --- |
| hsa_circ_0074368 | 94 | 1 | 394 | 1 | 394 | 0 | 3.00E-172 |
| hsa_circ_0074361 | 94 | 1 | 431 | 796 | 1226 | 0 | 0 |
| hsa_circ_0074365 | 95 | 1 | 275 | 236 | 510 | 0 | 5.00E-118 |
| hsa_circ_0074365 | 93 | 1 | 510 | 1 | 510 | 0 | 0 |
| hsa_circ_0074361 | 94 | 1 | 795 | 1 | 795 | 0 | 0 |
| hsa_circ_0074365 | 93 | 1 | 431 | 1 | 431 | 0 | 4.00E-176 |
| hsa_circ_0074363 | 93 | 1 | 644 | 1 | 644 | 0 | 0 |
| hsa_circ_0074365 | 92 | 1 | 235 | 1 | 235 | 0 | 4.00E-89 |
| hsa_circ_0074361 | 94 | 1 | 285 | 1 | 285 | 0 | 7.00E-122 |
| hsa_circ_0074361 | 94 | 159 | 443 | 1 | 285 | 0 | 1.00E-121 |
| hsa_circ_0055857 | 84 | 1552 | 1662 | 54170 | 54060 | 0.02 | 2.00E-20 |
| hsa_circ_0032110 | 98 | 1 | 854 | 1 | 854 | 0 | 0 |
| hsa_circ_0000544 | 87 | 1 | 1325 | 586 | 1910 | 0 | 0 |
| hsa_circ_0000544 | 88 | 1 | 1686 | 222 | 1910 | 0.01 | 0 |
| hsa_circ_0000544 | 88 | 1446 | 3353 | 3 | 1910 | 0.01 | 0 |
| hsa_circ_0000544 | 86 | 1 | 1034 | 690 | 1723 | 0.01 | 0 |
| hsa_circ_0000544 | 86 | 1 | 1138 | 586 | 1723 | 0.01 | 0 |
| hsa_circ_0000544 | 88 | 1446 | 3166 | 3 | 1723 | 0.01 | 0 |
| hsa_circ_0032104 | 86 | 8 | 925 | 8 | 889 | 0.04 | 0 |
| hsa_circ_0032104 | 86 | 750 | 1667 | 8 | 889 | 0.04 | 0 |
| hsa_circ_0032102 | 92 | 93 | 636 | 1 | 544 | 0 | 0 |
| hsa_circ_0032099 | 92 | 1324 | 1587 | 264 | 1 | 0 | 2.00E-99 |
| hsa_circ_0032097 | 94 | 3 | 198 | 403 | 598 | 0 | 1.00E-78 |
| hsa_circ_0032096 | 93 | 2 | 310 | 39 | 347 | 0 | 4.00E-125 |
| hsa_circ_0032096 | 93 | 602 | 910 | 39 | 347 | 0 | 1.00E-124 |
| hsa_circ_0032095 | 83 | 1 | 264 | 179 | 439 | 0.02 | 4.00E-59 |
| hsa_circ_0032092 | 84 | 1 | 545 | 838 | 1379 | 0.02 | 5.00E-143 |
| hsa_circ_0032092 | 83 | 2 | 796 | 585 | 1379 | 0.02 | 0 |
| hsa_circ_0032092 | 82 | 1 | 1037 | 1 | 1046 | 0.03 | 0 |
| hsa_circ_0032092 | 82 | 1 | 637 | 201 | 837 | 0.02 | 4.00E-152 |
| hsa_circ_0032092 | 81 | 1 | 574 | 1 | 583 | 0.04 | 1.00E-127 |
| hsa_circ_0032089 | 83 | 1 | 887 | 381 | 1273 | 0.03 | 0 |
| hsa_circ_0032088 | 83 | 1 | 421 | 398 | 843 | 0.06 | 1.00E-105 |
| hsa_circ_0032085 | 92 | 1 | 1667 | 582 | 2242 | 0 | 0 |
| hsa_circ_0032085 | 94 | 1 | 232 | 99 | 330 | 0 | 3.00E-94 |
| hsa_circ_0032084 | 96 | 1 | 498 | 458 | 955 | 0 | 0 |
| hsa_circ_0032084 | 97 | 17712 | 17880 | 457 | 625 | 0 | 7.00E-74 |
| hsa_circ_0032084 | 95 | 1 | 335 | 291 | 625 | 0 | 3.00E-146 |
| hsa_circ_0000542 | 92 | 1 | 308 | 1 | 308 | 0 | 3.00E-121 |
| hsa_circ_0000542 | 94 | 1 | 173 | 1 | 173 | 0 | 3.00E-68 |
| hsa_circ_0032061 | 93 | 1 | 365 | 337 | 701 | 0 | 7.00E-153 |
| hsa_circ_0032061 | 94 | 1 | 284 | 337 | 620 | 0 | 1.00E-119 |
| hsa_circ_0032061 | 96 | 1 | 156 | 181 | 336 | 0 | 2.00E-65 |
| hsa_circ_0032060 | 81 | 4 | 1414 | 1884 | 463 | 0.01 | 0 |
| hsa_circ_0032060 | 81 | 4 | 1640 | 1884 | 222 | 0.02 | 0 |
| hsa_circ_0032060 | 81 | 4 | 1858 | 1884 | 1 | 0.02 | 0 |
| hsa_circ_0032060 | 81 | 4 | 1858 | 1884 | 1 | 0.02 | 0 |
| hsa_circ_0013905 | 92 | 1 | 647 | 647 | 1 | 0 | 0 |
| hsa_circ_0032055 | 98 | 1 | 180 | 1 | 180 | 0 | 2.00E-85 |
| hsa_circ_0032053 | 93 | 1926 | 2277 | 868 | 1219 | 0 | 2.00E-141 |
| hsa_circ_0032053 | 91 | 2 | 1219 | 2 | 1219 | 0 | 0 |
| hsa_circ_0032053 | 90 | 2 | 646 | 2 | 646 | 0 | 0 |
| hsa_circ_0032049 | 96 | 1 | 642 | 642 | 1 | 0 | 0 |

novel_circ_006717 novel_circ_006718 novel_circ_006719 novel_circ_006720 novel_circ_006721 novel_circ_006722 novel_circ_006723 novel_circ_006724 novel_circ_006725 novel_circ_006726 novel_circ_006727 novel_circ_006728 novel_circ_006729 novel_circ_006730 novel_circ_006731 novel_circ_006732 novel_circ_006733 novel_circ_006734 novel_circ_006735 novel_circ_006737 novel_circ_006738 novel_circ_006739 novel_circ_006740 novel_circ_006741 novel_circ_006742 novel_circ_006743 novel_circ_006744 novel_circ_006745 novel_circ_006746 novel_circ_006747 novel_circ_006748 novel_circ_006749 novel_circ_006750 novel_circ_006751 novel_circ_006752 novel_circ_006753 novel_circ_006758 novel_circ_006759 novel_circ_006760 novel_circ_006761 novel_circ_006762 novel_circ_006763 novel_circ_006764 novel_circ_006769 novel_circ_006770 novel_circ_006771 novel_circ_006772 novel_circ_006773 novel_circ_006774 novel_circ_006775 novel_circ_006776 novel_circ_006777 novel_circ_006778 novel_circ_006779

| hsa_circ_0032047 | 96 | 1 | 1269 | 1269 | 1 | 0 | 0 |
| --- | --- | --- | --- | --- | --- | --- | --- |
| hsa_circ_0032047 | 96 | 1 | 1269 | 1269 | 1 | 0 | 0 |
| hsa_circ_0032047 | 96 | 1 | 1269 | 1269 | 1 | 0 | 0 |
| hsa_circ_0032049 | 96 | 1 | 547 | 547 | 1 | 0 | 0 |
| hsa_circ_0032047 | 96 | 1 | 548 | 1174 | 627 | 0 | 0 |
| hsa_circ_0032047 | 96 | 1 | 816 | 1174 | 359 | 0 | 0 |
| hsa_circ_0032047 | 96 | 1 | 1026 | 1174 | 149 | 0 | 0 |
| hsa_circ_0032047 | 96 | 1 | 1174 | 1174 | 1 | 0 | 0 |
| hsa_circ_0032047 | 96 | 1 | 1174 | 1174 | 1 | 0 | 0 |
| hsa_circ_0032047 | 96 | 1 | 1174 | 1174 | 1 | 0 | 0 |
| hsa_circ_0032047 | 96 | 1 | 1174 | 1174 | 1 | 0 | 0 |
| hsa_circ_0032047 | 96 | 1 | 1174 | 1174 | 1 | 0 | 0 |
| hsa_circ_0032047 | 96 | 1 | 831 | 831 | 1 | 0 | 0 |
| hsa_circ_0032047 | 96 | 1 | 831 | 831 | 1 | 0 | 0 |
| hsa_circ_0032047 | 96 | 1 | 358 | 358 | 1 | 0 | 9.00E-167 |
| hsa_circ_0032045 | 96 | 212 | 675 | 464 | 1 | 0 | 0 |
| hsa_circ_0032045 | 96 | 212 | 675 | 464 | 1 | 0 | 0 |
| hsa_circ_0032045 | 97 | 1 | 317 | 317 | 1 | 0 | 6.00E-148 |
| hsa_circ_0032045 | 96 | 1 | 208 | 208 | 1 | 0 | 1.00E-92 |
| hsa_circ_0032043 | 92 | 3 | 321 | 372 | 690 | 0 | 2.00E-127 |
| hsa_circ_0032043 | 91 | 1 | 690 | 1 | 690 | 0 | 0 |
| hsa_circ_0032040 | 92 | 1 | 879 | 185 | 1063 | 0 | 0 |
| hsa_circ_0032030 | 95 | 1 | 951 | 1 | 951 | 0 | 0 |
| hsa_circ_0032030 | 97 | 1 | 324 | 241 | 564 | 0 | 4.00E-155 |
| hsa_circ_0032030 | 96 | 1 | 453 | 112 | 564 | 0 | 0 |
| hsa_circ_0032030 | 96 | 1 | 457 | 108 | 564 | 0 | 0 |
| hsa_circ_0032030 | 96 | 1 | 564 | 1 | 564 | 0 | 0 |
| hsa_circ_0032030 | 96 | 561 | 1124 | 1 | 564 | 0 | 0 |
| hsa_circ_0032029 | 92 | 1 | 302 | 692 | 993 | 0 | 3.00E-119 |
| hsa_circ_0032029 | 92 | 2 | 998 | 2 | 993 | 0.01 | 0 |
| hsa_circ_0032029 | 92 | 1 | 302 | 692 | 993 | 0 | 2.00E-119 |
| hsa_circ_0032029 | 91 | 1 | 878 | 122 | 993 | 0.01 | 0 |
| hsa_circ_0032029 | 92 | 2 | 998 | 2 | 993 | 0.01 | 0 |
| hsa_circ_0032029 | 92 | 1 | 302 | 692 | 993 | 0 | 2.00E-119 |
| hsa_circ_0032029 | 91 | 1 | 878 | 122 | 993 | 0.01 | 0 |
| hsa_circ_0032029 | 92 | 2 | 998 | 2 | 993 | 0.01 | 0 |
| hsa_circ_0069238 | 96 | 135 | 715 | 664 | 84 | 0 | 0 |
| hsa_circ_0069238 | 96 | 135 | 798 | 664 | 1 | 0 | 0 |
| hsa_circ_0069238 | 96 | 135 | 798 | 664 | 1 | 0 | 0 |
| hsa_circ_0069238 | 96 | 4 | 584 | 664 | 84 | 0 | 0 |
| hsa_circ_0069238 | 96 | 4 | 667 | 664 | 1 | 0 | 0 |
| hsa_circ_0069238 | 97 | 1 | 465 | 548 | 84 | 0 | 0 |
| hsa_circ_0006377 | 92 | 82 | 320 | 239 | 1 | 0 | 4.00E-91 |
| hsa_circ_0069230 | 89 | 884 | 1544 | 1 | 660 | 0 | 0 |
| hsa_circ_0069230 | 89 | 884 | 1544 | 1 | 660 | 0 | 0 |
| hsa_circ_0069230 | 90 | 884 | 1395 | 1 | 512 | 0 | 0 |
| hsa_circ_0069228 | 96 | 52 | 682 | 631 | 1 | 0 | 0 |
| hsa_circ_0069228 | 96 | 52 | 682 | 631 | 1 | 0 | 0 |
| hsa_circ_0069227 | 94 | 213 | 1766 | 1554 | 1 | 0 | 0 |
| hsa_circ_0069226 | 94 | 213 | 1767 | 1703 | 149 | 0 | 0 |
| hsa_circ_0069226 | 94 | 213 | 1915 | 1703 | 1 | 0 | 0 |
| hsa_circ_0069227 | 94 | 1 | 1550 | 1550 | 1 | 0 | 0 |
| hsa_circ_0069226 | 94 | 1 | 1551 | 1699 | 149 | 0 | 0 |
| hsa_circ_0069226 | 94 | 1 | 1699 | 1699 | 1 | 0 | 0 |

novel_circ_006780 novel_circ_006781 novel_circ_006782 novel_circ_006783 novel_circ_006789 novel_circ_006790 novel_circ_006791 novel_circ_006792 novel_circ_006793 novel_circ_006795 novel_circ_006796 novel_circ_006797 novel_circ_006798 novel_circ_006799 novel_circ_006800 novel_circ_006801 novel_circ_006809 novel_circ_006810 novel_circ_006811 novel_circ_006812 novel_circ_006813 novel_circ_006815 novel_circ_006816 novel_circ_006817 novel_circ_006819 novel_circ_006820 novel_circ_006821 novel_circ_006822 novel_circ_006823 novel_circ_006824 novel_circ_006825 novel_circ_006826 novel_circ_006827 novel_circ_006828 novel_circ_006829 novel_circ_006830 novel_circ_006831 novel_circ_006832 novel_circ_006833 novel_circ_006834 novel_circ_006835 novel_circ_006836 novel_circ_006837 novel_circ_006838 novel_circ_006839 novel_circ_006840 novel_circ_006841 novel_circ_006842 novel_circ_006843 novel_circ_006844 novel_circ_006845 novel_circ_006846 novel_circ_006847 novel_circ_006848

| hsa_circ_0069227 | 93 | 1 | 1084 | 1084 | 1 | 0 | 0 |
| --- | --- | --- | --- | --- | --- | --- | --- |
| hsa_circ_0069226 | 93 | 1 | 1085 | 1233 | 149 | 0 | 0 |
| hsa_circ_0069227 | 93 | 3 | 958 | 956 | 1 | 0 | 0 |
| hsa_circ_0069226 | 93 | 3 | 959 | 1105 | 149 | 0 | 0 |
| hsa_circ_0069225 | 94 | 169 | 427 | 3 | 261 | 0 | 1.00E-108 |
| hsa_circ_0069225 | 94 | 3 | 261 | 3 | 261 | 0 | 8.00E-109 |
| hsa_circ_0069225 | 94 | 169 | 427 | 3 | 261 | 0 | 1.00E-108 |
| hsa_circ_0069225 | 94 | 169 | 427 | 3 | 261 | 0 | 7.00E-109 |
| hsa_circ_0069224 | 96 | 1 | 514 | 1 | 514 | 0 | 0 |
| hsa_circ_0069223 | 87 | 1 | 1078 | 8795 | 7715 | 0 | 0 |
| hsa_circ_0004731 | 93 | 1 | 316 | 316 | 1 | 0 | 2.00E-127 |
| hsa_circ_0004731 | 92 | 1 | 219 | 219 | 1 | 0 | 4.00E-83 |
| hsa_circ_0004731 | 92 | 1 | 130 | 131 | 1 | 0.01 | 3.00E-44 |
| hsa_circ_0004731 | 92 | 1 | 130 | 131 | 1 | 0.01 | 1.00E-43 |
| hsa_circ_0004731 | 92 | 1 | 130 | 131 | 1 | 0.01 | 8.00E-44 |
| hsa_circ_0002389 | 93 | 367 | 548 | 182 | 1 | 0 | 5.00E-71 |
| hsa_circ_0023975 | 93 | 1 | 692 | 1 | 692 | 0.01 | 0 |
| hsa_circ_0023969 | 95 | 1 | 256 | 344 | 599 | 0 | 9.00E-109 |
| hsa_circ_0008359 | 95 | 1 | 255 | 1 | 255 | 0 | 1.00E-108 |
| hsa_circ_0008359 | 94 | 1 | 221 | 1 | 221 | 0 | 2.00E-91 |
| hsa_circ_0023969 | 94 | 2964 | 3185 | 343 | 564 | 0 | 9.00E-91 |
| hsa_circ_0023964 | 91 | 4 | 607 | 623 | 19 | 0 | 0 |
| hsa_circ_0023964 | 92 | 1 | 323 | 342 | 19 | 0 | 2.00E-124 |
| hsa_circ_0023964 | 93 | 4 | 247 | 263 | 19 | 0 | 1.00E-95 |
| hsa_circ_0009016 | 93 | 1 | 257 | 908405 | 908661 | 0 | 3.00E-104 |
| hsa_circ_0009016 | 93 | 157 | 414 | 908404 | 908661 | 0 | 5.00E-105 |
| hsa_circ_0023953 | 98 | 1 | 233 | 415 | 647 | 0 | 4.00E-113 |
| hsa_circ_0023949 | 96 | 1 | 699 | 154 | 852 | 0 | 0 |
| hsa_circ_0023949 | 96 | 3312 | 4012 | 152 | 852 | 0 | 0 |
| hsa_circ_0023949 | 96 | 1 | 852 | 1 | 852 | 0 | 0 |
| hsa_circ_0023949 | 96 | 1 | 500 | 247 | 746 | 0 | 0 |
| hsa_circ_0023949 | 96 | 1 | 593 | 154 | 746 | 0 | 0 |
| hsa_circ_0023949 | 96 | 3312 | 3906 | 152 | 746 | 0 | 0 |
| hsa_circ_0023949 | 96 | 1 | 746 | 1 | 746 | 0 | 0 |
| hsa_circ_0023949 | 96 | 1 | 366 | 247 | 612 | 0 | 4.00E-170 |
| hsa_circ_0023949 | 97 | 3312 | 3772 | 152 | 612 | 0 | 0 |
| hsa_circ_0023949 | 97 | 1 | 612 | 1 | 612 | 0 | 0 |
| hsa_circ_0023951 | 96 | 1 | 274 | 1 | 274 | 0 | 2.00E-122 |
| hsa_circ_0023949 | 96 | 1 | 367 | 154 | 520 | 0 | 1.00E-170 |
| hsa_circ_0023949 | 96 | 3312 | 3680 | 152 | 520 | 0 | 9.00E-171 |
| hsa_circ_0023949 | 96 | 1 | 520 | 1 | 520 | 0 | 0 |
| hsa_circ_0023949 | 96 | 1 | 285 | 154 | 438 | 0 | 7.00E-132 |
| hsa_circ_0023949 | 97 | 1 | 438 | 1 | 438 | 0 | 0 |
| hsa_circ_0023939 | 97 | 13536 | 13858 | 636 | 314 | 0 | 1.00E-149 |
| hsa_circ_0023939 | 97 | 13536 | 13952 | 636 | 220 | 0 | 0 |
| hsa_circ_0023939 | 97 | 13536 | 14071 | 636 | 101 | 0 | 0 |
| hsa_circ_0023911 | 95 | 13536 | 14661 | 1400 | 272 | 0 | 0 |
| hsa_circ_0023942 | 97 | 1 | 322 | 322 | 1 | 0 | 1.00E-150 |
| hsa_circ_0023941 | 97 | 1 | 416 | 416 | 1 | 0 | 0 |
| hsa_circ_0023939 | 97 | 1 | 535 | 635 | 101 | 0 | 0 |
| hsa_circ_0023939 | 96 | 1 | 635 | 635 | 1 | 0 | 0 |
| hsa_circ_0023934 | 96 | 1 | 763 | 763 | 1 | 0 | 0 |
| hsa_circ_0023928 | 96 | 1 | 1016 | 1024 | 9 | 0 | 0 |
| hsa_circ_0023924 | 95 | 1 | 1125 | 1128 | 1 | 0 | 0 |

novel_circ_006849 novel_circ_006850 novel_circ_006851 novel_circ_006852 novel_circ_006853 novel_circ_006854 novel_circ_006855 novel_circ_006856 novel_circ_006857 novel_circ_006858 novel_circ_006859 novel_circ_006860 novel_circ_006861 novel_circ_006862 novel_circ_006863 novel_circ_006864 novel_circ_006865 novel_circ_006866 novel_circ_006867 novel_circ_006868 novel_circ_006869 novel_circ_006870 novel_circ_006872 novel_circ_006873 novel_circ_006874 novel_circ_006875 novel_circ_006876 novel_circ_006877 novel_circ_006878 novel_circ_006880 novel_circ_006881 novel_circ_006882 novel_circ_006883 novel_circ_006884 novel_circ_006885 novel_circ_006886 novel_circ_006887 novel_circ_006888 novel_circ_006889 novel_circ_006890 novel_circ_006891 novel_circ_006892 novel_circ_006893 novel_circ_006894 novel_circ_006895 novel_circ_006896 novel_circ_006897 novel_circ_006898 novel_circ_006899 novel_circ_006900 novel_circ_006901 novel_circ_006902 novel_circ_006903 novel_circ_006904

| hsa_circ_0023918 | 96 | 1 | 1275 | 1278 | 1 | 0 | 0 |
| --- | --- | --- | --- | --- | --- | --- | --- |
| hsa_circ_0023910 | 96 | 1 | 1381 | 1549 | 166 | 0 | 0 |
| hsa_circ_0023891 | 94 | 1 | 1835 | 1814 | 1 | 0.01 | 0 |
| hsa_circ_0023939 | 97 | 1 | 316 | 416 | 101 | 0 | 3.00E-148 |
| hsa_circ_0023939 | 96 | 1 | 416 | 416 | 1 | 0 | 0 |
| hsa_circ_0023928 | 96 | 1 | 797 | 805 | 9 | 0 | 0 |
| hsa_circ_0023924 | 95 | 1 | 906 | 909 | 1 | 0 | 0 |
| hsa_circ_0023918 | 96 | 1 | 1056 | 1059 | 1 | 0 | 0 |
| hsa_circ_0023928 | 95 | 1 | 694 | 702 | 9 | 0 | 0 |
| hsa_circ_0023924 | 95 | 1 | 803 | 806 | 1 | 0 | 0 |
| hsa_circ_0023918 | 95 | 1 | 859 | 862 | 1 | 0 | 0 |
| hsa_circ_0023915 | 95 | 1 | 965 | 970 | 3 | 0 | 0 |
| hsa_circ_0023903 | 95 | 1 | 1230 | 1233 | 1 | 0 | 0 |
| hsa_circ_0023903 | 95 | 1 | 883 | 886 | 1 | 0 | 0 |
| hsa_circ_0023903 | 96 | 1 | 625 | 625 | 1 | 0 | 0 |
| hsa_circ_0023903 | 96 | 1 | 521 | 521 | 1 | 0 | 0 |
| hsa_circ_0023891 | 92 | 1 | 710 | 686 | 1 | 0.03 | 0 |
| hsa_circ_0023903 | 95 | 3 | 371 | 369 | 1 | 0 | 9.00E-167 |
| hsa_circ_0006629 | 95 | 3 | 560 | 558 | 1 | 0 | 0 |
| hsa_circ_0009016 | 93 | 216 | 392 | 225365 | 225189 | 0 | 2.00E-66 |
| hsa_circ_0009016 | 94 | 79 | 179 | 213944 | 213844 | 0 | 4.00E-36 |
| hsa_circ_0057585 | 81 | 58071 | 58223 | 15975 | 16126 | 0.04 | 9.00E-24 |
| hsa_circ_0078031 | 82 | 4236 | 4374 | 167091 | 167234 | 0.05 | 1.00E-23 |
| hsa_circ_0023871 | 94 | 1 | 162 | 216 | 55 | 0 | 2.00E-65 |
| hsa_circ_0023870 | 95 | 1 | 346 | 483 | 138 | 0 | 1.00E-155 |
| hsa_circ_0023869 | 96 | 1 | 421 | 577 | 157 | 0 | 0 |
| hsa_circ_0023868 | 93 | 1 | 225 | 600 | 376 | 0 | 3.00E-90 |
| hsa_circ_0023868 | 90 | 1 | 329 | 444 | 116 | 0 | 3.00E-116 |
| hsa_circ_0023868 | 91 | 2 | 260 | 374 | 116 | 0 | 8.00E-96 |
| hsa_circ_0023866 | 92 | 1 | 253 | 881 | 629 | 0 | 2.00E-97 |
| hsa_circ_0023866 | 93 | 1 | 153 | 528 | 376 | 0 | 4.00E-57 |
| hsa_circ_0023866 | 94 | 1 | 283 | 375 | 93 | 0 | 4.00E-119 |
| hsa_circ_0023866 | 93 | 1 | 375 | 375 | 1 | 0 | 9.00E-157 |
| hsa_circ_0023859 | 91 | 1 | 862 | 109 | 973 | 0 | 0 |
| hsa_circ_0023861 | 94 | 1 | 279 | 328 | 609 | 0.01 | 4.00E-117 |
| hsa_circ_0023861 | 94 | 1 | 405 | 202 | 609 | 0.01 | 1.00E-173 |
| hsa_circ_0023859 | 92 | 1 | 742 | 229 | 973 | 0 | 0 |
| hsa_circ_0023859 | 91 | 1 | 862 | 109 | 973 | 0 | 0 |
| hsa_circ_0023859 | 91 | 321 | 1182 | 109 | 973 | 0 | 0 |
| hsa_circ_0023859 | 91 | 1 | 689 | 109 | 797 | 0 | 0 |
| hsa_circ_0023859 | 91 | 157 | 845 | 109 | 797 | 0 | 0 |
| hsa_circ_0023859 | 91 | 321 | 1009 | 109 | 797 | 0 | 0 |
| hsa_circ_0023860 | 92 | 1 | 256 | 1 | 256 | 0 | 2.00E-97 |
| hsa_circ_0023854 | 93 | 543 | 3800 | 21 | 3281 | 0 | 0 |
| hsa_circ_0023854 | 93 | 543 | 3800 | 21 | 3281 | 0 | 0 |
| hsa_circ_0023853 | 93 | 316 | 3474 | 1 | 3153 | 0.01 | 0 |
| hsa_circ_0023853 | 93 | 190 | 3348 | 1 | 3153 | 0.01 | 0 |
| hsa_circ_0023853 | 93 | 316 | 3474 | 1 | 3153 | 0.01 | 0 |
| hsa_circ_0023850 | 97 | 1 | 507 | 1 | 507 | 0 | 0 |
| hsa_circ_0023842 | 92 | 1 | 753 | 1862 | 1110 | 0 | 0 |
| hsa_circ_0023843 | 92 | 4 | 612 | 962 | 354 | 0 | 0 |
| hsa_circ_0023843 | 92 | 4 | 965 | 962 | 1 | 0 | 0 |
| hsa_circ_0023843 | 94 | 1 | 338 | 863 | 526 | 0 | 6.00E-143 |
| hsa_circ_0023843 | 92 | 1 | 510 | 863 | 354 | 0 | 0 |

novel_circ_006905 novel_circ_006906 novel_circ_006908 novel_circ_006909 novel_circ_006911 novel_circ_006912 novel_circ_006913 novel_circ_006914 novel_circ_006915 novel_circ_006916 novel_circ_006917 novel_circ_006918 novel_circ_006919 novel_circ_006920 novel_circ_006921 novel_circ_006922 novel_circ_006923 novel_circ_006924 novel_circ_006925 novel_circ_006926 novel_circ_006927 novel_circ_006928 novel_circ_006929 novel_circ_006930 novel_circ_006931 novel_circ_006932 novel_circ_006933 novel_circ_006934 novel_circ_006935 novel_circ_006936 novel_circ_006937 novel_circ_006938 novel_circ_006939 novel_circ_006940 novel_circ_006941 novel_circ_006942 novel_circ_006943 novel_circ_006944 novel_circ_006945 novel_circ_006946 novel_circ_006947 novel_circ_006948 novel_circ_006949 novel_circ_006950 novel_circ_006951 novel_circ_006952 novel_circ_006953 novel_circ_006954 novel_circ_006955 novel_circ_006956 novel_circ_006957 novel_circ_006958 novel_circ_006959 novel_circ_006960

| hsa_circ_0023843 | 92 | 4 | 188 | 185 | 1 | 0 | 4.00E-69 |
| --- | --- | --- | --- | --- | --- | --- | --- |
| hsa_circ_0028415 | 88 | 7310 | 7877 | 1 | 565 | 0.01 | 0 |
| hsa_circ_0082434 | 91 | 1 | 200 | 200 | 1 | 0 | 8.00E-70 |
| hsa_circ_0082442 | 90 | 1 | 710 | 1 | 707 | 0 | 0 |
| hsa_circ_0082450 | 92 | 1 | 235 | 1 | 235 | 0 | 8.00E-91 |
| hsa_circ_0082450 | 95 | 4607 | 4706 | 233 | 332 | 0 | 4.00E-36 |
| hsa_circ_0082452 | 90 | 1 | 220 | 1 | 220 | 0 | 3.00E-74 |
| hsa_circ_0082454 | 94 | 1 | 472 | 1 | 472 | 0 | 0 |
| hsa_circ_0082483 | 92 | 389 | 768 | 979 | 600 | 0 | 7.00E-151 |
| hsa_circ_0082489 | 97 | 1 | 1255 | 1468 | 214 | 0 | 0 |
| hsa_circ_0082490 | 97 | 1 | 308 | 806 | 499 | 0 | 1.00E-144 |
| hsa_circ_0082489 | 97 | 1 | 449 | 1468 | 1020 | 0 | 0 |
| hsa_circ_0082489 | 95 | 1 | 251 | 1279 | 1029 | 0 | 1.00E-109 |
| hsa_circ_0082489 | 97 | 1 | 440 | 1468 | 1029 | 0 | 0 |
| hsa_circ_0082526 | 92 | 1 | 637 | 1 | 637 | 0 | 0 |
| hsa_circ_0082526 | 92 | 1 | 555 | 83 | 637 | 0 | 0 |
| hsa_circ_0082530 | 91 | 1 | 265 | 1 | 265 | 0 | 4.00E-99 |
| hsa_circ_0082534 | 91 | 1 | 2105 | 116 | 2223 | 0 | 0 |
| hsa_circ_0082539 | 90 | 3 | 461 | 3 | 461 | 0 | 5.00E-170 |
| hsa_circ_0082539 | 92 | 3 | 1062 | 3 | 1062 | 0 | 0 |
| hsa_circ_0082539 | 91 | 1 | 657 | 150 | 806 | 0 | 0 |
| hsa_circ_0082539 | 91 | 1 | 913 | 150 | 1062 | 0 | 0 |
| hsa_circ_0082540 | 93 | 1 | 460 | 140 | 599 | 0 | 0 |
| hsa_circ_0082542 | 93 | 3 | 333 | 3 | 333 | 0 | 2.00E-137 |
| hsa_circ_0003949 | 93 | 2 | 453 | 452 | 1 | 0 | 0 |
| hsa_circ_0082552 | 94 | 112 | 388 | 277 | 1 | 0 | 2.00E-115 |
| hsa_circ_0082552 | 93 | 112 | 362 | 277 | 27 | 0 | 3.00E-101 |
| hsa_circ_0082552 | 93 | 237 | 487 | 277 | 27 | 0 | 5.00E-101 |
| hsa_circ_0082552 | 93 | 338 | 588 | 277 | 27 | 0 | 6.00E-101 |
| hsa_circ_0082552 | 93 | 401 | 651 | 277 | 27 | 0 | 6.00E-101 |
| hsa_circ_0082553 | 95 | 104 | 294 | 332 | 142 | 0 | 4.00E-81 |
| hsa_circ_0082557 | 96 | 264 | 362 | 101 | 3 | 0 | 2.00E-38 |
| hsa_circ_0082557 | 93 | 1 | 837 | 937 | 101 | 0 | 0 |
| hsa_circ_0082557 | 92 | 1 | 505 | 606 | 102 | 0 | 0 |
| hsa_circ_0082557 | 93 | 1 | 836 | 937 | 102 | 0 | 0 |
| hsa_circ_0082557 | 93 | 1 | 305 | 606 | 302 | 0 | 6.00E-123 |
| hsa_circ_0082557 | 95 | 1 | 524 | 937 | 414 | 0 | 0 |
| hsa_circ_0082557 | 95 | 1 | 331 | 937 | 607 | 0 | 6.00E-144 |
| hsa_circ_0082557 | 95 | 1 | 331 | 937 | 607 | 0 | 5.00E-144 |
| hsa_circ_0082555 | 90 | 1 | 462 | 1506 | 1072 | 0.06 | 1.00E-160 |
| hsa_circ_0082555 | 90 | 1 | 401 | 1506 | 1133 | 0.07 | 5.00E-135 |
| hsa_circ_0082555 | 87 | 1 | 318 | 1506 | 1216 | 0.08 | 1.00E-90 |
| hsa_circ_0008013 | 97 | 1 | 387 | 387 | 1 | 0 | 0 |
| hsa_circ_0005047 | 91 | 1 | 548 | 548 | 1 | 0 | 0 |
| hsa_circ_0005047 | 90 | 1 | 724 | 724 | 1 | 0 | 0 |
| hsa_circ_0082577 | 94 | 1 | 267 | 1 | 267 | 0 | 3.00E-110 |
| hsa_circ_0082577 | 93 | 1 | 400 | 1 | 400 | 0 | 6.00E-164 |
| hsa_circ_0082577 | 93 | 1 | 517 | 1 | 517 | 0 | 0 |
| hsa_circ_0082577 | 93 | 1 | 626 | 1 | 626 | 0 | 0 |
| hsa_circ_0082577 | 93 | 1 | 779 | 1 | 779 | 0 | 0 |
| hsa_circ_0082577 | 93 | 1 | 897 | 1 | 897 | 0 | 0 |
| hsa_circ_0082577 | 93 | 1 | 1340 | 1 | 1340 | 0 | 0 |
| hsa_circ_0082588 | 92 | 1 | 497 | 1 | 497 | 0 | 0 |
| hsa_circ_0082595 | 90 | 1 | 732 | 1 | 735 | 0 | 0 |

novel_circ_006961 novel_circ_006962 novel_circ_006963 novel_circ_006964 novel_circ_006965 novel_circ_006966 novel_circ_006967 novel_circ_006968 novel_circ_006969 novel_circ_006970 novel_circ_006971 novel_circ_006972 novel_circ_006973 novel_circ_006974 novel_circ_006975 novel_circ_006976 novel_circ_006977 novel_circ_006978 novel_circ_006979 novel_circ_006980 novel_circ_006981 novel_circ_006982 novel_circ_006983 novel_circ_006984 novel_circ_006985 novel_circ_006986 novel_circ_006987 novel_circ_006988 novel_circ_006989 novel_circ_006990 novel_circ_006991 novel_circ_006992 novel_circ_006993 novel_circ_006994 novel_circ_006995 novel_circ_006996 novel_circ_006997 novel_circ_006998 novel_circ_006999 novel_circ_007000 novel_circ_007001 novel_circ_007002 novel_circ_007005 novel_circ_007006 novel_circ_007007 novel_circ_007008 novel_circ_007009 novel_circ_007010 novel_circ_007012 novel_circ_007013 novel_circ_007014 novel_circ_007015 novel_circ_007016 novel_circ_007017

| hsa_circ_0082598 | 88 | 1 | 1008 | 175 | 1185 | 0 | 0 |
| --- | --- | --- | --- | --- | --- | --- | --- |
| hsa_circ_0082598 | 89 | 1 | 837 | 349 | 1185 | 0 | 0 |
| hsa_circ_0082598 | 89 | 1 | 628 | 558 | 1185 | 0 | 0 |
| hsa_circ_0082598 | 88 | 2 | 459 | 728 | 1185 | 0 | 2.00E-154 |
| hsa_circ_0082615 | 90 | 3 | 315 | 313 | 1 | 0 | 5.00E-114 |
| hsa_circ_0082615 | 90 | 1 | 582 | 582 | 1 | 0 | 0 |
| hsa_circ_0082635 | 92 | 1 | 208 | 1 | 208 | 0 | 3.00E-79 |
| hsa_circ_0082635 | 93 | 1 | 576 | 1 | 576 | 0 | 0 |
| hsa_circ_0082638 | 93 | 1 | 927 | 1 | 927 | 0 | 0 |
| hsa_circ_0082638 | 94 | 1 | 998 | 1 | 998 | 0 | 0 |
| hsa_circ_0082638 | 93 | 1 | 1128 | 1 | 1128 | 0 | 0 |
| hsa_circ_0082639 | 94 | 94 | 3198 | 1 | 3108 | 0 | 0 |
| hsa_circ_0082639 | 94 | 94 | 3698 | 1 | 3611 | 0 | 0 |
| hsa_circ_0082639 | 94 | 94 | 3742 | 1 | 3655 | 0 | 0 |
| hsa_circ_0082639 | 96 | 1 | 239 | 1 | 239 | 0 | 2.00E-106 |
| hsa_circ_0082639 | 93 | 1 | 834 | 1 | 834 | 0 | 0 |
| hsa_circ_0082642 | 93 | 1 | 732 | 1 | 732 | 0 | 0 |
| hsa_circ_0082642 | 93 | 1 | 1052 | 1 | 1052 | 0 | 0 |
| hsa_circ_0082642 | 93 | 1 | 1308 | 1 | 1308 | 0 | 0 |
| hsa_circ_0082643 | 93 | 4 | 594 | 4 | 594 | 0 | 0 |
| hsa_circ_0082643 | 93 | 4 | 1170 | 4 | 1170 | 0 | 0 |
| hsa_circ_0082645 | 92 | 1 | 490 | 1 | 490 | 0 | 0 |
| hsa_circ_0082645 | 93 | 1 | 915 | 1 | 915 | 0 | 0 |
| hsa_circ_0082651 | 94 | 2 | 1951 | 2 | 1954 | 0 | 0 |
| hsa_circ_0082658 | 93 | 1 | 1548 | 1 | 1551 | 0 | 0 |
| hsa_circ_0082656 | 95 | 1 | 322 | 1603 | 1927 | 0.02 | 3.00E-141 |
| hsa_circ_0082660 | 95 | 1 | 746 | 1662 | 2407 | 0 | 0 |
| hsa_circ_0082660 | 95 | 1 | 531 | 1757 | 2287 | 0 | 0 |
| hsa_circ_0082662 | 94 | 1 | 413 | 112 | 524 | 0 | 3.00E-176 |
| hsa_circ_0082674 | 92 | 1 | 678 | 678 | 1 | 0 | 0 |
| hsa_circ_0082681 | 94 | 1 | 1084 | 1084 | 1 | 0 | 0 |
| hsa_circ_0082685 | 86 | 1 | 1004 | 998 | 1 | 0.01 | 0 |
| hsa_circ_0004684 | 87 | 1 | 861 | 856 | 2 | 0.01 | 0 |
| hsa_circ_0082688 | 88 | 1 | 659 | 660 | 8 | 0.01 | 0 |
| hsa_circ_0082689 | 87 | 1 | 542 | 536 | 1 | 0.01 | 5.00E-176 |
| hsa_circ_0082695 | 94 | 4 | 579 | 577 | 2 | 0 | 0 |
| hsa_circ_0082694 | 95 | 1 | 358 | 1277 | 920 | 0 | 1.00E-160 |
| hsa_circ_0082694 | 95 | 1 | 966 | 1885 | 920 | 0 | 0 |
| hsa_circ_0082694 | 95 | 1 | 1052 | 1971 | 920 | 0 | 0 |
| hsa_circ_0082699 | 95 | 1 | 653 | 741 | 89 | 0 | 0 |
| hsa_circ_0082706 | 91 | 2268 | 2410 | 143 | 1 | 0 | 5.00E-47 |
| hsa_circ_0082706 | 88 | 1 | 321 | 321 | 1 | 0 | 8.00E-107 |
| hsa_circ_0082720 | 92 | 2 | 1332 | 1331 | 1 | 0 | 0 |
| hsa_circ_0082720 | 91 | 2 | 1042 | 1331 | 292 | 0 | 0 |
| hsa_circ_0082722 | 92 | 1 | 333 | 513 | 181 | 0 | 2.00E-133 |
| hsa_circ_0082722 | 93 | 4 | 534 | 711 | 181 | 0 | 0 |
| hsa_circ_0082722 | 91 | 2 | 784 | 963 | 181 | 0 | 0 |
| hsa_circ_0082722 | 91 | 2 | 451 | 963 | 514 | 0 | 5.00E-170 |
| hsa_circ_0006013 | 97 | 1 | 421 | 421 | 1 | 0 | 0 |
| hsa_circ_0006013 | 97 | 1 | 587 | 587 | 1 | 0 | 0 |
| hsa_circ_0006013 | 97 | 1 | 1104 | 1104 | 1 | 0 | 0 |
| hsa_circ_0006013 | 97 | 351 | 1457 | 1107 | 1 | 0 | 0 |
| hsa_circ_0082746 | 96 | 1 | 298 | 298 | 1 | 0 | 9.00E-136 |
| hsa_circ_0082747 | 96 | 1 | 428 | 428 | 1 | 0 | 0 |

novel_circ_007018 novel_circ_007019 novel_circ_007020 novel_circ_007021 novel_circ_007022 novel_circ_007023 novel_circ_007024 novel_circ_007025 novel_circ_007026 novel_circ_007027 novel_circ_007028 novel_circ_007029 novel_circ_007030 novel_circ_007031 novel_circ_007032 novel_circ_007033 novel_circ_007034 novel_circ_007035 novel_circ_007038 novel_circ_007039 novel_circ_007040 novel_circ_007041 novel_circ_007042 novel_circ_007043 novel_circ_007046 novel_circ_007047 novel_circ_007048 novel_circ_007049 novel_circ_007050 novel_circ_007052 novel_circ_007071 novel_circ_007072 novel_circ_007076 novel_circ_007077 novel_circ_007078 novel_circ_007079 novel_circ_007080 novel_circ_007081 novel_circ_007082 novel_circ_007083 novel_circ_007084 novel_circ_007086 novel_circ_007087 novel_circ_007088 novel_circ_007089 novel_circ_007090 novel_circ_007091 novel_circ_007092 novel_circ_007093 novel_circ_007094 novel_circ_007095 novel_circ_007096 novel_circ_007097 novel_circ_007098

| hsa_circ_0082747 | 96 | 351 | 1036 | 686 | 1 | 0 | 0 |
| --- | --- | --- | --- | --- | --- | --- | --- |
| hsa_circ_0082755 | 96 | 351 | 870 | 520 | 1 | 0 | 0 |
| hsa_circ_0082755 | 96 | 620 | 1139 | 520 | 1 | 0 | 0 |
| hsa_circ_0006961 | 96 | 1 | 635 | 636 | 2 | 0 | 0 |
| hsa_circ_0082755 | 96 | 1 | 902 | 1454 | 553 | 0 | 0 |
| hsa_circ_0082759 | 97 | 351 | 608 | 258 | 1 | 0 | 7.00E-120 |
| hsa_circ_0006961 | 95 | 1 | 279 | 280 | 2 | 0 | 2.00E-121 |
| hsa_circ_0006961 | 96 | 1 | 428 | 429 | 2 | 0 | 0 |
| hsa_circ_0006961 | 96 | 1 | 635 | 636 | 2 | 0 | 0 |
| hsa_circ_0006961 | 96 | 1 | 635 | 636 | 2 | 0 | 0 |
| hsa_circ_0082755 | 96 | 1 | 730 | 1285 | 556 | 0 | 0 |
| hsa_circ_0082763 | 95 | 1 | 476 | 476 | 1 | 0 | 0 |
| hsa_circ_0082764 | 96 | 1 | 207 | 356 | 150 | 0 | 5.00E-92 |
| hsa_circ_0082763 | 96 | 1 | 471 | 740 | 270 | 0 | 0 |
| hsa_circ_0082755 | 97 | 1 | 573 | 1556 | 984 | 0 | 0 |
| hsa_circ_0082768 | 97 | 1 | 368 | 470 | 103 | 0 | 3.00E-175 |
| hsa_circ_0082770 | 97 | 1 | 366 | 366 | 1 | 0 | 4.00E-175 |
| hsa_circ_0035409 | 83 | 13282 | 13424 | 129026 | 128888 | 0.04 | 1.00E-26 |
| hsa_circ_0082772 | 94 | 21 | 613 | 10 | 602 | 0 | 0 |
| hsa_circ_0082790 | 92 | 1 | 413 | 552 | 140 | 0 | 3.00E-166 |
| hsa_circ_0082790 | 93 | 1 | 259 | 552 | 294 | 0 | 4.00E-104 |
| hsa_circ_0075808 | 96 | 289 | 373 | 1 | 85 | 0 | 3.00E-32 |
| hsa_circ_0075808 | 91 | 1 | 352 | 1 | 352 | 0.01 | 5.00E-134 |
| hsa_circ_0075809 | 91 | 1 | 170 | 1 | 170 | 0.01 | 7.00E-60 |
| hsa_circ_0075813 | 93 | 1 | 327 | 1 | 327 | 0 | 2.00E-133 |
| hsa_circ_0075813 | 91 | 1 | 390 | 1 | 390 | 0 | 4.00E-150 |
| hsa_circ_0005234 | 76 | 17434 | 17581 | 323777 | 323924 | 0.03 | 3.00E-10 |
| hsa_circ_0005234 | 76 | 6847 | 6994 | 323777 | 323924 | 0.03 | 2.00E-10 |
| hsa_circ_0005234 | 76 | 4202 | 4349 | 323777 | 323924 | 0.03 | 2.00E-10 |
| hsa_circ_0075817 | 77 | 67792 | 70231 | 5646 | 8125 | 0.06 | 0 |
| hsa_circ_0075846 | 92 | 1 | 117 | 256 | 372 | 0 | 7.00E-40 |
| hsa_circ_0075846 | 92 | 1 | 117 | 256 | 372 | 0 | 2.00E-39 |
| hsa_circ_0083906 | 86 | 141 | 308 | 2808 | 2641 | 0 | 6.00E-45 |
| hsa_circ_0083914 | 88 | 1 | 363 | 1 | 363 | 0 | 9.00E-117 |
| hsa_circ_0083914 | 88 | 1 | 467 | 1 | 467 | 0 | 2.00E-154 |
| hsa_circ_0083912 | 94 | 27760 | 28275 | 5310 | 5825 | 0 | 0 |
| hsa_circ_0083912 | 94 | 1 | 514 | 5312 | 5825 | 0 | 0 |
| hsa_circ_0083925 | 95 | 1 | 344 | 1 | 344 | 0 | 6.00E-153 |
| hsa_circ_0010864 | 93 | 1 | 471 | 471 | 1 | 0 | 0 |
| hsa_circ_0010864 | 93 | 1 | 273 | 273 | 1 | 0 | 3.00E-110 |
| hsa_circ_0010848 | 97 | 210 | 356 | 147 | 1 | 0 | 5.00E-62 |
| hsa_circ_0010832 | 93 | 1 | 991 | 1 | 991 | 0 | 0 |
| hsa_circ_0010834 | 94 | 1 | 226 | 1 | 226 | 0 | 3.00E-94 |
| hsa_circ_0010832 | 94 | 1 | 369 | 1 | 369 | 0 | 2.00E-158 |
| hsa_circ_0010814 | 96 | 2 | 837 | 2 | 837 | 0 | 0 |
| hsa_circ_0010826 | 95 | 1 | 332 | 1 | 332 | 0 | 6.00E-148 |
| hsa_circ_0010821 | 96 | 1 | 439 | 1 | 439 | 0 | 0 |
| hsa_circ_0010814 | 96 | 2 | 611 | 2 | 611 | 0 | 0 |
| hsa_circ_0010810 | 96 | 1 | 495 | 1 | 495 | 0 | 0 |
| hsa_circ_0010814 | 97 | 2 | 172 | 2 | 172 | 0 | 4.00E-77 |
| hsa_circ_0010810 | 93 | 1 | 134 | 1 | 134 | 0 | 7.00E-49 |
| hsa_circ_0010802 | 94 | 1 | 360 | 1 | 360 | 0 | 9.00E-157 |
| hsa_circ_0002720 | 93 | 1 | 492 | 1 | 492 | 0 | 0 |
| hsa_circ_0010793 | 92 | 1222 | 1698 | 1 | 477 | 0 | 0 |

novel_circ_007100 novel_circ_007101 novel_circ_007102 novel_circ_007103 novel_circ_007104 novel_circ_007105 novel_circ_007106 novel_circ_007107 novel_circ_007108 novel_circ_007109 novel_circ_007110 novel_circ_007111 novel_circ_007112 novel_circ_007113 novel_circ_007114 novel_circ_007115 novel_circ_007116 novel_circ_007117 novel_circ_007118 novel_circ_007119 novel_circ_007120 novel_circ_007121 novel_circ_007122 novel_circ_007123 novel_circ_007124 novel_circ_007125 novel_circ_007126 novel_circ_007127 novel_circ_007128 novel_circ_007129 novel_circ_007130 novel_circ_007131 novel_circ_007132 novel_circ_007133 novel_circ_007134 novel_circ_007135 novel_circ_007136 novel_circ_007137 novel_circ_007138 novel_circ_007139 novel_circ_007140 novel_circ_007141 novel_circ_007142 novel_circ_007143 novel_circ_007144 novel_circ_007145 novel_circ_007146 novel_circ_007147 novel_circ_007148 novel_circ_007149 novel_circ_007150 novel_circ_007151 novel_circ_007152 novel_circ_007153

| hsa_circ_0007418 | 84 | 33 | 899 | 34 | 900 | 0 | 0 |
| --- | --- | --- | --- | --- | --- | --- | --- |
| hsa_circ_0010785 | 96 | 1 | 536 | 1 | 536 | 0 | 0 |
| hsa_circ_0010539 | 92 | 1 | 278 | 857 | 580 | 0 | 1.00E-109 |
| hsa_circ_0010539 | 92 | 1 | 406 | 857 | 452 | 0 | 6.00E-159 |
| hsa_circ_0010539 | 92 | 1 | 641 | 857 | 217 | 0 | 0 |
| hsa_circ_0010539 | 92 | 1 | 857 | 857 | 1 | 0 | 0 |
| hsa_circ_0010539 | 91 | 1 | 520 | 736 | 217 | 0 | 0 |
| hsa_circ_0010539 | 91 | 1 | 736 | 736 | 1 | 0 | 0 |
| hsa_circ_0010525 | 92 | 160 | 2261 | 2671 | 573 | 0 | 0 |
| hsa_circ_0010535 | 92 | 1 | 903 | 1282 | 380 | 0 | 0 |
| hsa_circ_0010525 | 92 | 1 | 1525 | 2094 | 573 | 0 | 0 |
| hsa_circ_0010535 | 92 | 1 | 723 | 1102 | 380 | 0 | 0 |
| hsa_circ_0010535 | 92 | 1 | 795 | 1102 | 311 | 0 | 0 |
| hsa_circ_0010532 | 92 | 1 | 1345 | 1838 | 497 | 0 | 0 |
| hsa_circ_0010535 | 91 | 1 | 516 | 823 | 311 | 0.01 | 0 |
| hsa_circ_0010532 | 92 | 1 | 937 | 1559 | 626 | 0 | 0 |
| hsa_circ_0010532 | 92 | 1 | 1436 | 1559 | 127 | 0 | 0 |
| hsa_circ_0010532 | 91 | 1 | 868 | 1361 | 497 | 0 | 0 |
| hsa_circ_0010532 | 92 | 1 | 977 | 1361 | 388 | 0 | 0 |
| hsa_circ_0010532 | 92 | 1 | 622 | 1115 | 497 | 0 | 0 |
| hsa_circ_0010532 | 93 | 3 | 550 | 1044 | 497 | 0 | 0 |
| hsa_circ_0010532 | 93 | 3 | 659 | 1044 | 388 | 0 | 0 |
| hsa_circ_0010532 | 92 | 1 | 342 | 625 | 284 | 0 | 2.00E-130 |
| hsa_circ_0010521 | 92 | 157 | 608 | 1081 | 630 | 0 | 1.00E-177 |
| hsa_circ_0002402 | 93 | 1 | 440 | 442 | 3 | 0 | 0 |
| hsa_circ_0010505 | 91 | 1 | 333 | 1109 | 777 | 0 | 2.00E-123 |
| hsa_circ_0010500 | 92 | 151 | 951 | 1371 | 571 | 0 | 0 |
| hsa_circ_0010496 | 94 | 135 | 264 | 130 | 1 | 0 | 4.00E-49 |
| hsa_circ_0007227 | 95 | 135 | 360 | 226 | 1 | 0 | 3.00E-97 |
| hsa_circ_0007227 | 95 | 135 | 360 | 226 | 1 | 0 | 3.00E-97 |
| hsa_circ_0007227 | 95 | 1 | 225 | 225 | 1 | 0 | 9.00E-97 |
| hsa_circ_0010471 | 93 | 243 | 556 | 2074 | 1758 | 0.01 | 1.00E-126 |
| hsa_circ_0010471 | 94 | 243 | 777 | 2074 | 1537 | 0.01 | 0 |
| hsa_circ_0010471 | 91 | 243 | 1723 | 2074 | 591 | 0.01 | 0 |
| hsa_circ_0010481 | 94 | 1 | 513 | 516 | 1 | 0.01 | 0 |
| hsa_circ_0010475 | 92 | 1 | 1459 | 1465 | 1 | 0 | 0 |
| hsa_circ_0010481 | 95 | 1 | 221 | 221 | 1 | 0 | 2.00E-96 |
| hsa_circ_0010475 | 91 | 4 | 1069 | 1069 | 1 | 0 | 0 |
| hsa_circ_0010475 | 90 | 46 | 997 | 956 | 1 | 0 | 0 |
| hsa_circ_0010456 | 90 | 46 | 1086 | 3562 | 2518 | 0 | 0 |
| hsa_circ_0010475 | 90 | 1 | 946 | 949 | 1 | 0 | 0 |
| hsa_circ_0010475 | 90 | 1 | 943 | 946 | 1 | 0 | 0 |
| hsa_circ_0010475 | 89 | 3 | 837 | 838 | 1 | 0 | 0 |
| hsa_circ_0010471 | 90 | 3 | 926 | 1428 | 502 | 0 | 0 |
| hsa_circ_0010471 | 91 | 3 | 1427 | 1428 | 1 | 0 | 0 |
| hsa_circ_0010465 | 92 | 1 | 872 | 1070 | 199 | 0 | 0 |
| hsa_circ_0010465 | 93 | 1 | 1070 | 1070 | 1 | 0 | 0 |
| hsa_circ_0010465 | 93 | 4 | 783 | 978 | 199 | 0 | 0 |
| hsa_circ_0010465 | 93 | 4 | 981 | 978 | 1 | 0 | 0 |
| hsa_circ_0010465 | 93 | 1 | 536 | 734 | 199 | 0 | 0 |
| hsa_circ_0010465 | 94 | 1 | 734 | 734 | 1 | 0 | 0 |
| hsa_circ_0010465 | 90 | 1 | 171 | 369 | 199 | 0 | 4.00E-57 |
| hsa_circ_0010465 | 93 | 1 | 369 | 369 | 1 | 0 | 9.00E-152 |
| hsa_circ_0010462 | 94 | 1 | 858 | 858 | 1 | 0 | 0 |

novel_circ_007154 novel_circ_007155 novel_circ_007156 novel_circ_007157 novel_circ_007158 novel_circ_007159 novel_circ_007160 novel_circ_007161 novel_circ_007162 novel_circ_007163 novel_circ_007164 novel_circ_007165 novel_circ_007166 novel_circ_007167 novel_circ_007168 novel_circ_007169 novel_circ_007170 novel_circ_007171 novel_circ_007172 novel_circ_007173 novel_circ_007174 novel_circ_007175 novel_circ_007176 novel_circ_007177 novel_circ_007178 novel_circ_007179 novel_circ_007180 novel_circ_007181 novel_circ_007182 novel_circ_007183 novel_circ_007184 novel_circ_007185 novel_circ_007186 novel_circ_007187 novel_circ_007188 novel_circ_007189 novel_circ_007190 novel_circ_007192 novel_circ_007193 novel_circ_007194 novel_circ_007195 novel_circ_007196 novel_circ_007197 novel_circ_007198 novel_circ_007199 novel_circ_007200 novel_circ_007201 novel_circ_007202 novel_circ_007203 novel_circ_007204 novel_circ_007205 novel_circ_007206 novel_circ_007207 novel_circ_007208

| hsa_circ_0010461 | 94 | 1 | 1086 | 1086 | 1 | 0 | 0 |
| --- | --- | --- | --- | --- | --- | --- | --- |
| hsa_circ_0010459 | 91 | 1 | 1635 | 1808 | 174 | 0 | 0 |
| hsa_circ_0010461 | 94 | 4 | 915 | 912 | 1 | 0 | 0 |
| hsa_circ_0010462 | 94 | 1 | 489 | 489 | 1 | 0 | 0 |
| hsa_circ_0010461 | 94 | 1 | 717 | 717 | 1 | 0 | 0 |
| hsa_circ_0010459 | 94 | 1 | 809 | 1439 | 631 | 0 | 0 |
| hsa_circ_0010459 | 93 | 1 | 929 | 1439 | 511 | 0 | 0 |
| hsa_circ_0010459 | 91 | 1 | 1266 | 1439 | 174 | 0 | 0 |
| hsa_circ_0010459 | 91 | 1 | 1439 | 1439 | 1 | 0 | 0 |
| hsa_circ_0010459 | 91 | 1 | 1439 | 1439 | 1 | 0 | 0 |
| hsa_circ_0010453 | 92 | 1 | 1746 | 1975 | 230 | 0 | 0 |
| hsa_circ_0010453 | 91 | 1 | 1893 | 1975 | 83 | 0 | 0 |
| hsa_circ_0010460 | 92 | 4 | 440 | 437 | 1 | 0 | 8.00E-173 |
| hsa_circ_0010459 | 89 | 4 | 777 | 947 | 174 | 0 | 0 |
| hsa_circ_0010460 | 90 | 3 | 307 | 305 | 1 | 0 | 6.00E-108 |
| hsa_circ_0010459 | 88 | 3 | 644 | 815 | 174 | 0 | 0 |
| hsa_circ_0010460 | 90 | 7 | 212 | 206 | 1 | 0 | 9.00E-70 |
| hsa_circ_0010459 | 86 | 1 | 455 | 628 | 174 | 0 | 9.00E-138 |
| hsa_circ_0010446 | 97 | 1 | 754 | 990 | 237 | 0 | 0 |
| hsa_circ_0010446 | 96 | 1 | 990 | 990 | 1 | 0 | 0 |
| hsa_circ_0002437 | 96 | 1 | 1081 | 1081 | 1 | 0 | 0 |
| hsa_circ_0002437 | 96 | 1 | 1081 | 1081 | 1 | 0 | 0 |
| hsa_circ_0002437 | 96 | 1 | 885 | 885 | 1 | 0 | 0 |
| hsa_circ_0010446 | 96 | 1 | 694 | 694 | 1 | 0 | 0 |
| hsa_circ_0002437 | 96 | 1 | 785 | 785 | 1 | 0 | 0 |
| hsa_circ_0000023 | 96 | 155 | 945 | 791 | 1 | 0 | 0 |
| hsa_circ_0010446 | 96 | 1 | 540 | 540 | 1 | 0 | 0 |
| hsa_circ_0002437 | 96 | 1 | 631 | 631 | 1 | 0 | 0 |
| hsa_circ_0000023 | 96 | 1 | 791 | 791 | 1 | 0 | 0 |
| hsa_circ_0010439 | 96 | 1 | 903 | 903 | 1 | 0 | 0 |
| hsa_circ_0010446 | 96 | 1 | 380 | 380 | 1 | 0 | 3.00E-176 |
| hsa_circ_0002437 | 96 | 1 | 471 | 471 | 1 | 0 | 0 |
| hsa_circ_0010446 | 96 | 4 | 236 | 233 | 1 | 0 | 1.00E-104 |
| hsa_circ_0002437 | 96 | 4 | 327 | 324 | 1 | 0 | 2.00E-148 |
| hsa_circ_0000023 | 96 | 4 | 487 | 484 | 1 | 0 | 0 |
| hsa_circ_0007296 | 90 | 1 | 749 | 734 | 1 | 0.03 | 0 |
| hsa_circ_0010427 | 91 | 1 | 293 | 442 | 150 | 0 | 3.00E-111 |
| hsa_circ_0010417 | 89 | 2 | 1291 | 2 | 1310 | 0.02 | 0 |
| hsa_circ_0010417 | 89 | 1 | 773 | 263 | 1042 | 0.01 | 0 |
| hsa_circ_0010417 | 91 | 2 | 1035 | 2 | 1042 | 0.01 | 0 |
| hsa_circ_0010417 | 94 | 2 | 614 | 2 | 614 | 0 | 0 |
| hsa_circ_0010417 | 95 | 2 | 385 | 2 | 385 | 0 | 4.00E-170 |
| hsa_circ_0010385 | 94 | 3 | 123 | 471 | 351 | 0 | 4.00E-46 |
| hsa_circ_0010371 | 93 | 1 | 468 | 2178 | 1711 | 0 | 0 |
| hsa_circ_0010371 | 93 | 1 | 842 | 2178 | 1337 | 0 | 0 |
| hsa_circ_0010371 | 93 | 1 | 1218 | 2178 | 961 | 0 | 0 |
| hsa_circ_0010373 | 93 | 1 | 376 | 920 | 545 | 0 | 2.00E-157 |
| hsa_circ_0010373 | 90 | 1 | 356 | 544 | 189 | 0 | 3.00E-131 |
| hsa_circ_0010358 | 88 | 1 | 665 | 668 | 1 | 0 | 0 |
| hsa_circ_0010353 | 88 | 1 | 885 | 888 | 1 | 0 | 0 |
| hsa_circ_0010339 | 91 | 1 | 2319 | 2553 | 232 | 0 | 0 |
| hsa_circ_0010318 | 91 | 1 | 3973 | 4577 | 599 | 0 | 0 |
| hsa_circ_0010345 | 92 | 1 | 959 | 959 | 1 | 0 | 0 |
| hsa_circ_0010340 | 93 | 1 | 411 | 411 | 1 | 0 | 4.00E-170 |

novel_circ_007209 novel_circ_007210 novel_circ_007211 novel_circ_007212 novel_circ_007213 novel_circ_007214 novel_circ_007215 novel_circ_007216 novel_circ_007217 novel_circ_007218 novel_circ_007219 novel_circ_007220 novel_circ_007222 novel_circ_007223 novel_circ_007224 novel_circ_007225 novel_circ_007226 novel_circ_007227 novel_circ_007228 novel_circ_007229 novel_circ_007230 novel_circ_007231 novel_circ_007232 novel_circ_007233 novel_circ_007234 novel_circ_007235 novel_circ_007236 novel_circ_007237 novel_circ_007238 novel_circ_007239 novel_circ_007240 novel_circ_007241 novel_circ_007242 novel_circ_007243 novel_circ_007244 novel_circ_007245 novel_circ_007246 novel_circ_007247 novel_circ_007248 novel_circ_007251 novel_circ_007252 novel_circ_007253 novel_circ_007254 novel_circ_007255 novel_circ_007256 novel_circ_007257 novel_circ_007258 novel_circ_007259 novel_circ_007260 novel_circ_007261 novel_circ_007262 novel_circ_007263 novel_circ_007264 novel_circ_007265

| hsa_circ_0010320 | 91 | 1 | 2065 | 2068 | 1 | 0 | 0 |
| --- | --- | --- | --- | --- | --- | --- | --- |
| hsa_circ_0010333 | 90 | 4 | 582 | 582 | 1 | 0.01 | 0 |
| hsa_circ_0010320 | 92 | 1 | 1072 | 1072 | 1 | 0 | 0 |
| hsa_circ_0010305 | 93 | 1 | 1347 | 1347 | 1 | 0 | 0 |
| hsa_circ_0010315 | 93 | 1 | 808 | 808 | 1 | 0 | 0 |
| hsa_circ_0010315 | 93 | 1 | 591 | 591 | 1 | 0 | 0 |
| hsa_circ_0010305 | 93 | 1 | 966 | 966 | 1 | 0 | 0 |
| hsa_circ_0010300 | 98 | 1 | 414 | 414 | 1 | 0 | 0 |
| hsa_circ_0010261 | 95 | 1 | 401 | 1036 | 636 | 0 | 3.00E-176 |
| hsa_circ_0010261 | 94 | 1 | 1036 | 1036 | 1 | 0 | 0 |
| hsa_circ_0010255 | 92 | 2 | 876 | 875 | 1 | 0 | 0 |
| hsa_circ_0010242 | 93 | 1 | 354 | 354 | 1 | 0 | 3.00E-143 |
| hsa_circ_0010182 | 89 | 1 | 532 | 1835 | 1289 | 0.03 | 0 |
| hsa_circ_0010150 | 90 | 3 | 474 | 104 | 575 | 0 | 3.00E-177 |
| hsa_circ_0010150 | 94 | 3 | 296 | 104 | 397 | 0 | 3.00E-121 |
| hsa_circ_0010150 | 93 | 1 | 397 | 1 | 397 | 0 | 1.00E-161 |
| hsa_circ_0010146 | 93 | 20 | 387 | 368 | 1 | 0 | 2.00E-154 |
| hsa_circ_0010144 | 94 | 20 | 522 | 503 | 1 | 0 | 0 |
| hsa_circ_0010144 | 94 | 20 | 522 | 503 | 1 | 0 | 0 |
| hsa_circ_0010141 | 96 | 112 | 265 | 271 | 118 | 0 | 9.00E-66 |
| hsa_circ_0010114 | 94 | 1 | 215 | 594 | 808 | 0 | 4.00E-88 |
| hsa_circ_0010114 | 93 | 1 | 455 | 354 | 808 | 0 | 0 |
| hsa_circ_0010112 | 94 | 1 | 969 | 1 | 969 | 0 | 0 |
| hsa_circ_0010114 | 93 | 1 | 354 | 354 | 707 | 0 | 2.00E-143 |
| hsa_circ_0010112 | 94 | 1 | 868 | 1 | 868 | 0 | 0 |
| hsa_circ_0010114 | 93 | 1 | 233 | 354 | 586 | 0 | 5.00E-93 |
| hsa_circ_0010114 | 95 | 1 | 385 | 202 | 586 | 0 | 3.00E-172 |
| hsa_circ_0010112 | 95 | 2 | 511 | 5 | 514 | 0 | 0 |
| hsa_circ_0010112 | 95 | 1 | 514 | 1 | 514 | 0 | 0 |
| hsa_circ_0002237 | 98 | 1 | 798 | 1 | 798 | 0 | 0 |
| hsa_circ_0002237 | 98 | 1 | 798 | 1 | 798 | 0 | 0 |
| hsa_circ_0010056 | 93 | 1 | 551 | 1 | 551 | 0 | 0 |
| hsa_circ_0010052 | 93 | 1 | 678 | 238 | 915 | 0 | 0 |
| hsa_circ_0010056 | 93 | 1 | 361 | 1 | 361 | 0 | 2.00E-147 |
| hsa_circ_0010052 | 93 | 1 | 488 | 238 | 725 | 0 | 0 |
| hsa_circ_0010056 | 93 | 1 | 257 | 1 | 257 | 0 | 2.00E-101 |
| hsa_circ_0010054 | 92 | 1 | 384 | 1 | 384 | 0 | 4.00E-155 |
| hsa_circ_0010050 | 93 | 1 | 751 | 1 | 751 | 0 | 0 |
| hsa_circ_0010030 | 95 | 193 | 500 | 1 | 308 | 0 | 2.00E-134 |
| hsa_circ_0088336 | 97 | 11047 | 11082 | 2119 | 2084 | 0 | 1.00E-06 |
| hsa_circ_0010029 | 89 | 1 | 4408 | 1 | 4414 | 0.01 | 0 |
| hsa_circ_0010029 | 89 | 1 | 4408 | 1 | 4414 | 0.01 | 0 |
| hsa_circ_0010029 | 89 | 112 | 4519 | 1 | 4414 | 0.01 | 0 |
| hsa_circ_0010029 | 89 | 612 | 5019 | 1 | 4414 | 0.01 | 0 |
| hsa_circ_0010028 | 94 | 1 | 298 | 1 | 298 | 0 | 2.00E-125 |
| hsa_circ_0010028 | 94 | 1 | 296 | 1 | 296 | 0 | 1.00E-124 |
| hsa_circ_0010025 | 93 | 1 | 503 | 503 | 1 | 0 | 0 |
| hsa_circ_0010012 | 94 | 1 | 329 | 1 | 329 | 0 | 1.00E-139 |
| hsa_circ_0010010 | 93 | 1 | 1028 | 1 | 1028 | 0 | 0 |
| hsa_circ_0010010 | 93 | 1 | 699 | 1 | 699 | 0 | 0 |
| hsa_circ_0010009 | 93 | 1 | 1146 | 1050 | 2192 | 0 | 0 |
| hsa_circ_0010009 | 93 | 2 | 1528 | 669 | 2192 | 0 | 0 |
| hsa_circ_0010009 | 92 | 1 | 2195 | 1 | 2192 | 0 | 0 |
| hsa_circ_0009994 | 93 | 1 | 3710 | 1079 | 4785 | 0 | 0 |

novel_circ_007266 novel_circ_007267 novel_circ_007268 novel_circ_007269 novel_circ_007270 novel_circ_007271 novel_circ_007272 novel_circ_007273 novel_circ_007274 novel_circ_007275 novel_circ_007276 novel_circ_007277 novel_circ_007278 novel_circ_007279 novel_circ_007280 novel_circ_007281 novel_circ_007282 novel_circ_007283 novel_circ_007284 novel_circ_007285 novel_circ_007286 novel_circ_007287 novel_circ_007288 novel_circ_007289 novel_circ_007290 novel_circ_007291 novel_circ_007292 novel_circ_007293 novel_circ_007294 novel_circ_007295 novel_circ_007296 novel_circ_007297 novel_circ_007298 novel_circ_007299 novel_circ_007300 novel_circ_007301 novel_circ_007302 novel_circ_007303 novel_circ_007304 novel_circ_007305 novel_circ_007306 novel_circ_007307 novel_circ_007308 novel_circ_007309 novel_circ_007310 novel_circ_007312 novel_circ_007313 novel_circ_007314 novel_circ_007315 novel_circ_007316 novel_circ_007317 novel_circ_007318 novel_circ_007319 novel_circ_007320

| hsa_circ_0010010 | 93 | 1 | 396 | 1 | 396 | 0 | 9.00E-167 |
| --- | --- | --- | --- | --- | --- | --- | --- |
| hsa_circ_0010009 | 93 | 1 | 843 | 1050 | 1889 | 0 | 0 |
| hsa_circ_0010009 | 93 | 2 | 1225 | 669 | 1889 | 0 | 0 |
| hsa_circ_0010009 | 92 | 1 | 447 | 1050 | 1493 | 0.01 | 3.00E-177 |
| hsa_circ_0010009 | 92 | 2 | 829 | 669 | 1493 | 0 | 0 |
| hsa_circ_0010009 | 92 | 1 | 1496 | 1 | 1493 | 0 | 0 |
| hsa_circ_0010000 | 93 | 1 | 2121 | 48 | 2169 | 0 | 0 |
| hsa_circ_0010009 | 92 | 1 | 776 | 1 | 776 | 0 | 0 |
| hsa_circ_0010002 | 94 | 1 | 1377 | 212 | 1588 | 0 | 0 |
| hsa_circ_0010000 | 93 | 1 | 1739 | 48 | 1787 | 0 | 0 |
| hsa_circ_0010000 | 93 | 1 | 1503 | 48 | 1551 | 0 | 0 |
| hsa_circ_0010009 | 93 | 1 | 307 | 1 | 307 | 0 | 1.00E-125 |
| hsa_circ_0010003 | 94 | 3 | 769 | 3 | 769 | 0 | 0 |
| hsa_circ_0010001 | 94 | 1 | 1379 | 1 | 1379 | 0 | 0 |
| hsa_circ_0010001 | 93 | 1 | 610 | 1 | 610 | 0 | 0 |
| hsa_circ_0009996 | 94 | 1 | 1106 | 212 | 1317 | 0 | 0 |
| hsa_circ_0009998 | 94 | 1 | 688 | 208 | 895 | 0 | 0 |
| hsa_circ_0009996 | 93 | 1 | 505 | 212 | 716 | 0 | 0 |
| hsa_circ_0009994 | 89 | 1 | 367 | 1 | 367 | 0 | 3.00E-127 |
| hsa_circ_0009990 | 91 | 1 | 764 | 1 | 767 | 0 | 0 |
| hsa_circ_0009969 | 91 | 2 | 2057 | 2 | 2063 | 0 | 0 |
| hsa_circ_0009980 | 91 | 1 | 709 | 407 | 1115 | 0 | 0 |
| hsa_circ_0009980 | 90 | 1 | 1112 | 1 | 1115 | 0.01 | 0 |
| hsa_circ_0009965 | 91 | 1 | 2695 | 1 | 2698 | 0 | 0 |
| hsa_circ_0009965 | 92 | 1 | 1614 | 153 | 1766 | 0 | 0 |
| hsa_circ_0009965 | 92 | 1 | 1766 | 1 | 1766 | 0 | 0 |
| hsa_circ_0009965 | 91 | 1 | 1431 | 153 | 1583 | 0 | 0 |
| hsa_circ_0009965 | 92 | 1 | 1583 | 1 | 1583 | 0 | 0 |
| hsa_circ_0009965 | 92 | 1 | 1123 | 153 | 1275 | 0 | 0 |
| hsa_circ_0009965 | 92 | 1 | 1032 | 153 | 1184 | 0 | 0 |
| hsa_circ_0009965 | 92 | 1 | 1184 | 1 | 1184 | 0 | 0 |
| hsa_circ_0009963 | 91 | 1 | 2499 | 1 | 2499 | 0 | 0 |
| hsa_circ_0009960 | 91 | 1 | 3492 | 442 | 3933 | 0 | 0 |
| hsa_circ_0009963 | 91 | 1 | 2347 | 1 | 2347 | 0 | 0 |
| hsa_circ_0009962 | 91 | 1 | 2478 | 379 | 2856 | 0 | 0 |
| hsa_circ_0009950 | 93 | 1 | 272 | 252 | 523 | 0 | 5.00E-108 |
| hsa_circ_0009950 | 94 | 7 | 525 | 5 | 523 | 0 | 0 |
| hsa_circ_0009950 | 96 | 7 | 253 | 5 | 251 | 0 | 8.00E-111 |
| hsa_circ_0009926 | 92 | 1 | 712 | 1 | 712 | 0 | 0 |
| hsa_circ_0009915 | 92 | 3 | 1697 | 182 | 1876 | 0 | 0 |
| hsa_circ_0009915 | 93 | 1 | 685 | 479 | 1163 | 0 | 0 |
| hsa_circ_0009915 | 93 | 3 | 984 | 182 | 1163 | 0 | 0 |
| hsa_circ_0009915 | 93 | 1 | 1163 | 1 | 1163 | 0 | 0 |
| hsa_circ_0009882 | 91 | 1 | 899 | 1 | 899 | 0 | 0 |
| hsa_circ_0009870 | 90 | 1 | 234 | 1 | 234 | 0 | 6.00E-82 |
| hsa_circ_0009823 | 94 | 7 | 1223 | 1217 | 1 | 0 | 0 |
| hsa_circ_0009807 | 91 | 4 | 681 | 678 | 1 | 0 | 0 |
| hsa_circ_0009807 | 92 | 8 | 565 | 558 | 1 | 0 | 0 |
| hsa_circ_0009800 | 96 | 1 | 715 | 851 | 137 | 0 | 0 |
| hsa_circ_0000017 | 93 | 1 | 267 | 281 | 15 | 0 | 1.00E-108 |
| hsa_circ_0000017 | 93 | 1 | 281 | 281 | 1 | 0 | 5.00E-113 |
| hsa_circ_0009765 | 90 | 1 | 1505 | 1680 | 176 | 0 | 0 |
| hsa_circ_0009772 | 89 | 1 | 331 | 331 | 1 | 0 | 2.00E-112 |
| hsa_circ_0009765 | 89 | 1 | 1224 | 1399 | 176 | 0 | 0 |

novel_circ_007321 novel_circ_007322 novel_circ_007323 novel_circ_007324 novel_circ_007325 novel_circ_007327 novel_circ_007328 novel_circ_007329 novel_circ_007330 novel_circ_007336 novel_circ_007338 novel_circ_007339 novel_circ_007340 novel_circ_007341 novel_circ_007342 novel_circ_007343 novel_circ_007344 novel_circ_007345 novel_circ_007346 novel_circ_007347 novel_circ_007348 novel_circ_007349 novel_circ_007350 novel_circ_007351 novel_circ_007352 novel_circ_007353 novel_circ_007354 novel_circ_007355 novel_circ_007356 novel_circ_007357 novel_circ_007358 novel_circ_007359 novel_circ_007360 novel_circ_007361 novel_circ_007362 novel_circ_007363 novel_circ_007364 novel_circ_007365 novel_circ_007366 novel_circ_007367 novel_circ_007368 novel_circ_007369 novel_circ_007370 novel_circ_007371 novel_circ_007372 novel_circ_007373 novel_circ_007374 novel_circ_007375 novel_circ_007376 novel_circ_007377 novel_circ_007378 novel_circ_007379 novel_circ_007380 novel_circ_007381

| hsa_circ_0009765 | 89 | 1 | 702 | 877 | 176 | 0.01 | 0 |
| --- | --- | --- | --- | --- | --- | --- | --- |
| hsa_circ_0008660 | 91 | 1 | 141 | 165 | 305 | 0 | 9.00E-47 |
| hsa_circ_0008660 | 94 | 1 | 305 | 1 | 305 | 0 | 1.00E-129 |
| hsa_circ_0008660 | 94 | 253 | 557 | 1 | 305 | 0 | 2.00E-129 |
| hsa_circ_0009725 | 91 | 1 | 1248 | 1 | 1248 | 0 | 0 |
| hsa_circ_0009717 | 78 | 1 | 455 | 4782 | 4338 | 0.06 | 3.00E-71 |
| hsa_circ_0009717 | 78 | 617 | 1057 | 4338 | 4768 | 0.07 | 4.00E-65 |
| hsa_circ_0009717 | 76 | 1 | 405 | 4732 | 4338 | 0.07 | 2.00E-48 |
| hsa_circ_0009717 | 76 | 1 | 405 | 4732 | 4338 | 0.07 | 2.00E-48 |
| hsa_circ_0009717 | 81 | 768 | 931 | 3945 | 3777 | 0.06 | 2.00E-28 |
| hsa_circ_0009717 | 93 | 3 | 580 | 65 | 642 | 0 | 0 |
| hsa_circ_0009712 | 93 | 88 | 1082 | 3 | 997 | 0 | 0 |
| hsa_circ_0009718 | 93 | 1 | 240 | 147 | 386 | 0 | 1.00E-92 |
| hsa_circ_0009712 | 93 | 88 | 648 | 3 | 563 | 0 | 0 |
| hsa_circ_0009712 | 94 | 88 | 431 | 3 | 346 | 0 | 2.00E-144 |
| hsa_circ_0009712 | 93 | 88 | 300 | 3 | 215 | 0 | 4.00E-85 |
| hsa_circ_0009712 | 93 | 264 | 476 | 3 | 215 | 0 | 6.00E-85 |
| hsa_circ_0009712 | 93 | 320 | 532 | 3 | 215 | 0 | 7.00E-85 |
| hsa_circ_0009708 | 94 | 1 | 284 | 2357 | 2640 | 0 | 1.00E-117 |
| hsa_circ_0009708 | 93 | 1 | 873 | 1768 | 2640 | 0 | 0 |
| hsa_circ_0009708 | 93 | 1 | 1685 | 959 | 2640 | 0 | 0 |
| hsa_circ_0009708 | 93 | 1 | 375 | 2266 | 2640 | 0 | 2.00E-153 |
| hsa_circ_0009708 | 93 | 1 | 1685 | 959 | 2640 | 0 | 0 |
| hsa_circ_0009709 | 93 | 1 | 1576 | 1 | 1573 | 0 | 0 |
| hsa_circ_0009709 | 94 | 1 | 812 | 1 | 809 | 0 | 0 |
| hsa_circ_0009709 | 92 | 1 | 314 | 244 | 557 | 0 | 6.00E-123 |
| hsa_circ_0009708 | 94 | 264 | 633 | 144 | 513 | 0 | 4.00E-157 |
| hsa_circ_0009703 | 96 | 78 | 257 | 1 | 180 | 0 | 1.00E-78 |
| hsa_circ_0009702 | 93 | 1 | 327 | 146 | 472 | 0 | 2.00E-133 |
| hsa_circ_0009702 | 93 | 1 | 472 | 1 | 472 | 0 | 0 |
| hsa_circ_0009684 | 94 | 1 | 599 | 3615 | 4213 | 0 | 0 |
| hsa_circ_0009684 | 94 | 1 | 835 | 3379 | 4213 | 0 | 0 |
| hsa_circ_0009684 | 95 | 1 | 934 | 3280 | 4213 | 0 | 0 |
| hsa_circ_0009684 | 94 | 1 | 1971 | 2243 | 4213 | 0 | 0 |
| hsa_circ_0009684 | 94 | 1 | 2329 | 1885 | 4213 | 0 | 0 |
| hsa_circ_0009702 | 94 | 1 | 280 | 1 | 280 | 0 | 2.00E-117 |
| hsa_circ_0009684 | 95 | 1 | 407 | 3615 | 4021 | 0 | 0 |
| hsa_circ_0009684 | 94 | 1 | 1644 | 2243 | 3886 | 0 | 0 |
| hsa_circ_0009690 | 94 | 1 | 1372 | 359 | 1730 | 0 | 0 |
| hsa_circ_0009690 | 94 | 1 | 1730 | 1 | 1730 | 0 | 0 |
| hsa_circ_0009698 | 95 | 1 | 367 | 314 | 680 | 0 | 1.00E-160 |
| hsa_circ_0009690 | 93 | 2 | 896 | 501 | 1395 | 0 | 0 |
| hsa_circ_0009690 | 93 | 1 | 1037 | 359 | 1395 | 0 | 0 |
| hsa_circ_0009690 | 94 | 1 | 1267 | 1 | 1267 | 0 | 0 |
| hsa_circ_0009697 | 93 | 2 | 668 | 2 | 668 | 0 | 0 |
| hsa_circ_0009695 | 93 | 1 | 809 | 1 | 809 | 0 | 0 |
| hsa_circ_0009690 | 94 | 1 | 1167 | 1 | 1167 | 0 | 0 |
| hsa_circ_0009687 | 94 | 226 | 1396 | 758 | 1928 | 0 | 0 |
| hsa_circ_0009697 | 92 | 2 | 529 | 2 | 529 | 0 | 0 |
| hsa_circ_0009695 | 92 | 1 | 670 | 1 | 670 | 0 | 0 |
| hsa_circ_0009690 | 93 | 1 | 1028 | 1 | 1028 | 0 | 0 |
| hsa_circ_0009690 | 95 | 1 | 358 | 1 | 358 | 0 | 5.00E-159 |
| hsa_circ_0009687 | 95 | 226 | 587 | 758 | 1119 | 0 | 5.00E-161 |
| hsa_circ_0009690 | 95 | 1 | 240 | 1 | 240 | 0 | 3.00E-105 |

novel_circ_007382 novel_circ_007383 novel_circ_007384 novel_circ_007385 novel_circ_007386 novel_circ_007387 novel_circ_007388 novel_circ_007389 novel_circ_007390 novel_circ_007391 novel_circ_007392 novel_circ_007394 novel_circ_007395 novel_circ_007396 novel_circ_007399 novel_circ_007400 novel_circ_007401 novel_circ_007402 novel_circ_007403 novel_circ_007404 novel_circ_007405 novel_circ_007406 novel_circ_007407 novel_circ_007408 novel_circ_007409 novel_circ_007410 novel_circ_007411 novel_circ_007412 novel_circ_007413 novel_circ_007414 novel_circ_007415 novel_circ_007416 novel_circ_007417 novel_circ_007418 novel_circ_007419 novel_circ_007420 novel_circ_007421 novel_circ_007422 novel_circ_007423 novel_circ_007427 novel_circ_007436 novel_circ_007437 novel_circ_007438 novel_circ_007439 novel_circ_007440 novel_circ_007441 novel_circ_007442 novel_circ_007443 novel_circ_007444 novel_circ_007445 novel_circ_007446 novel_circ_007448 novel_circ_007449 novel_circ_007450

| hsa_circ_0009687 | 95 | 226 | 469 | 758 | 1001 | 0 | 3.00E-107 |
| --- | --- | --- | --- | --- | --- | --- | --- |
| hsa_circ_0005199 | 97 | 1315 | 1551 | 133 | 369 | 0 | 2.00E-109 |
| hsa_circ_0005199 | 95 | 1 | 369 | 1 | 369 | 0 | 2.00E-163 |
| hsa_circ_0009682 | 92 | 1 | 281 | 1 | 281 | 0 | 7.00E-108 |
| hsa_circ_0009679 | 86 | 1 | 347 | 1 | 355 | 0.02 | 4.00E-100 |
| hsa_circ_0009675 | 95 | 4 | 297 | 293 | 1 | 0 | 3.00E-126 |
| hsa_circ_0009670 | 91 | 1 | 892 | 894 | 3 | 0 | 0 |
| hsa_circ_0009667 | 90 | 1 | 1428 | 1428 | 1 | 0 | 0 |
| hsa_circ_0009661 | 91 | 1 | 1332 | 4189 | 2858 | 0 | 0 |
| hsa_circ_0009670 | 91 | 1 | 543 | 545 | 3 | 0 | 0 |
| hsa_circ_0009667 | 90 | 1 | 1079 | 1079 | 1 | 0 | 0 |
| hsa_circ_0009667 | 88 | 1 | 534 | 534 | 1 | 0 | 0 |
| hsa_circ_0009665 | 89 | 1 | 364 | 364 | 1 | 0 | 5.00E-124 |
| hsa_circ_0009640 | 93 | 638 | 750 | 1 | 113 | 0 | 4.00E-39 |
| hsa_circ_0009638 | 85 | 3 | 260 | 258 | 1 | 0 | 3.00E-70 |
| hsa_circ_0009589 | 94 | 3295 | 3962 | 976 | 309 | 0 | 0 |
| hsa_circ_0009597 | 92 | 1 | 469 | 540 | 72 | 0 | 0 |
| hsa_circ_0009597 | 93 | 1 | 540 | 540 | 1 | 0 | 0 |
| hsa_circ_0005039 | 94 | 1 | 666 | 666 | 1 | 0 | 0 |
| hsa_circ_0005039 | 99 | 1 | 197 | 197 | 1 | 0 | 4.00E-98 |
| hsa_circ_0009592 | 97 | 1 | 398 | 400 | 3 | 0 | 0 |
| hsa_circ_0009589 | 97 | 1 | 505 | 505 | 1 | 0 | 0 |
| hsa_circ_0009592 | 97 | 1 | 327 | 329 | 3 | 0 | 8.00E-157 |
| hsa_circ_0009589 | 96 | 1 | 434 | 434 | 1 | 0 | 0 |
| hsa_circ_0009589 | 95 | 14 | 322 | 309 | 1 | 0 | 4.00E-135 |
| hsa_circ_0009584 | 94 | 14 | 596 | 583 | 1 | 0 | 0 |
| hsa_circ_0009589 | 95 | 1 | 308 | 308 | 1 | 0 | 1.00E-134 |
| hsa_circ_0009586 | 95 | 1 | 357 | 357 | 1 | 0 | 8.00E-157 |
| hsa_circ_0009584 | 94 | 1 | 582 | 582 | 1 | 0 | 0 |
| hsa_circ_0009577 | 93 | 1 | 681 | 681 | 1 | 0 | 0 |
| hsa_circ_0009584 | 93 | 1 | 379 | 379 | 1 | 0 | 2.00E-157 |
| hsa_circ_0009584 | 93 | 1 | 274 | 274 | 1 | 0 | 4.00E-109 |
| hsa_circ_0009577 | 92 | 1 | 373 | 373 | 1 | 0 | 1.00E-145 |
| hsa_circ_0009572 | 93 | 1 | 454 | 617 | 164 | 0 | 0 |
| hsa_circ_0009584 | 92 | 1 | 225 | 225 | 1 | 0 | 6.00E-87 |
| hsa_circ_0009577 | 91 | 1 | 324 | 324 | 1 | 0 | 2.00E-123 |
| hsa_circ_0009572 | 93 | 1 | 405 | 568 | 164 | 0 | 4.00E-165 |
| hsa_circ_0009572 | 92 | 1 | 368 | 531 | 164 | 0 | 3.00E-146 |
| hsa_circ_0009569 | 94 | 1 | 456 | 456 | 1 | 0 | 0 |
| hsa_circ_0009551 | 91 | 389 | 758 | 1 | 370 | 0 | 2.00E-136 |
| hsa_circ_0009551 | 92 | 1 | 259 | 6 | 264 | 0 | 2.00E-97 |
| hsa_circ_0009551 | 92 | 1 | 264 | 1 | 264 | 0 | 3.00E-100 |
| hsa_circ_0009543 | 91 | 1 | 276 | 3022 | 3297 | 0 | 1.00E-103 |
| hsa_circ_0009543 | 99 | 1 | 136 | 258 | 393 | 0 | 7.00E-62 |
| hsa_circ_0002203 | 98 | 1 | 85 | 18229 | 18313 | 0 | 1.00E-32 |
| hsa_circ_0009543 | 99 | 1 | 190 | 1 | 190 | 0 | 1.00E-91 |
| hsa_circ_0009543 | 99 | 229 | 418 | 1 | 190 | 0 | 1.00E-91 |
| hsa_circ_0009543 | 99 | 1 | 190 | 1 | 190 | 0 | 1.00E-91 |
| hsa_circ_0008737 | 99 | 228 | 417 | 13198 | 13387 | 0 | 7.00E-94 |
| hsa_circ_0009540 | 95 | 1 | 204 | 204 | 1 | 0 | 5.00E-87 |
| hsa_circ_0008389 | 94 | 1 | 306 | 306 | 1 | 0 | 2.00E-128 |
| hsa_circ_0009536 | 93 | 59 | 331 | 273 | 1 | 0 | 8.00E-112 |
| hsa_circ_0009533 | 92 | 1 | 165 | 1 | 165 | 0 | 9.00E-59 |
| hsa_circ_0009522 | 85 | 3 | 459 | 1341 | 885 | 0 | 7.00E-129 |

novel_circ_007451 novel_circ_007452 novel_circ_007454 novel_circ_007455 novel_circ_007457 novel_circ_007458 novel_circ_007459 novel_circ_007460 novel_circ_007461 novel_circ_007462 novel_circ_007463 novel_circ_007464 novel_circ_007465 novel_circ_007466 novel_circ_007467 novel_circ_007468 novel_circ_007469 novel_circ_007470 novel_circ_007471 novel_circ_007472 novel_circ_007473 novel_circ_007475 novel_circ_007476 novel_circ_007477 novel_circ_007478 novel_circ_007479 novel_circ_007480 novel_circ_007481 novel_circ_007482 novel_circ_007483 novel_circ_007484 novel_circ_007485 novel_circ_007486 novel_circ_007487 novel_circ_007488 novel_circ_007490 novel_circ_007491 novel_circ_007492 novel_circ_007493 novel_circ_007494 novel_circ_007495 novel_circ_007496 novel_circ_007497 novel_circ_007499 novel_circ_007502 novel_circ_007503 novel_circ_007505 novel_circ_007506 novel_circ_007507 novel_circ_007508 novel_circ_007509 novel_circ_007511 novel_circ_007512 novel_circ_007513

| hsa_circ_0009519 | 76 | 4 | 900 | 4966 | 4093 | 0.07 | 9.00E-119 |
| --- | --- | --- | --- | --- | --- | --- | --- |
| hsa_circ_0009513 | 94 | 1 | 133 | 133 | 1 | 0 | 2.00E-50 |
| hsa_circ_0009504 | 91 | 1 | 871 | 1295 | 425 | 0 | 0 |
| hsa_circ_0009504 | 92 | 1 | 504 | 928 | 425 | 0 | 0 |
| hsa_circ_0005719 | 86 | 4 | 716 | 708 | 5 | 0.01 | 0 |
| hsa_circ_0009464 | 84 | 4 | 1166 | 1154 | 1 | 0.01 | 0 |
| hsa_circ_0009457 | 83 | 4 | 1504 | 1476 | 3 | 0.02 | 0 |
| hsa_circ_0009464 | 84 | 5 | 993 | 980 | 1 | 0.01 | 0 |
| hsa_circ_0009457 | 83 | 5 | 1331 | 1302 | 3 | 0.02 | 0 |
| hsa_circ_0005719 | 87 | 1 | 390 | 394 | 5 | 0 | 5.00E-125 |
| hsa_circ_0009467 | 85 | 1 | 531 | 531 | 1 | 0 | 6.00E-155 |
| hsa_circ_0009464 | 84 | 1 | 840 | 840 | 1 | 0 | 0 |
| hsa_circ_0009457 | 83 | 1 | 1178 | 1162 | 3 | 0.02 | 0 |
| hsa_circ_0005661 | 83 | 1 | 1880 | 1864 | 6 | 0.01 | 0 |
| hsa_circ_0009464 | 83 | 1 | 602 | 602 | 1 | 0 | 1.00E-156 |
| hsa_circ_0009458 | 82 | 1 | 803 | 785 | 1 | 0.02 | 0 |
| hsa_circ_0009457 | 82 | 1 | 940 | 924 | 3 | 0.02 | 0 |
| hsa_circ_0009464 | 82 | 10 | 446 | 437 | 1 | 0 | 2.00E-108 |
| hsa_circ_0009457 | 82 | 10 | 784 | 759 | 3 | 0.02 | 0 |
| hsa_circ_0005661 | 82 | 10 | 1486 | 1461 | 6 | 0.02 | 0 |
| hsa_circ_0009464 | 84 | 13 | 309 | 297 | 1 | 0 | 5.00E-79 |
| hsa_circ_0005661 | 84 | 1 | 839 | 841 | 6 | 0.01 | 0 |
| hsa_circ_0009445 | 83 | 1 | 1496 | 2013 | 526 | 0.01 | 0 |
| hsa_circ_0009452 | 84 | 8 | 455 | 445 | 1 | 0.02 | 2.00E-114 |
| hsa_circ_0009446 | 84 | 8 | 1109 | 1101 | 3 | 0.01 | 0 |
| hsa_circ_0087361 | 91 | 1 | 386 | 385 | 1 | 0 | 2.00E-145 |
| hsa_circ_0087360 | 91 | 387 | 1690 | 3059 | 1756 | 0 | 0 |
| hsa_circ_0087360 | 91 | 1 | 1304 | 3059 | 1756 | 0 | 0 |
| hsa_circ_0087360 | 91 | 1 | 1511 | 3059 | 1549 | 0 | 0 |
| hsa_circ_0028415 | 87 | 16697 | 17228 | 6 | 533 | 0.01 | 2.00E-169 |
| hsa_circ_0087359 | 92 | 2 | 403 | 780 | 379 | 0 | 4.00E-160 |
| hsa_circ_0087359 | 93 | 2 | 628 | 780 | 154 | 0 | 0 |
| hsa_circ_0087359 | 94 | 2 | 781 | 780 | 1 | 0 | 0 |
| hsa_circ_0087359 | 96 | 2 | 378 | 377 | 1 | 0 | 2.00E-174 |
| hsa_circ_0087359 | 98 | 1 | 153 | 153 | 1 | 0 | 3.00E-70 |
| hsa_circ_0005142 | 93 | 4 | 271 | 265 | 1 | 0.01 | 5.00E-108 |
| hsa_circ_0003715 | 93 | 4 | 928 | 922 | 1 | 0 | 0 |
| hsa_circ_0087353 | 93 | 4 | 1071 | 1065 | 1 | 0 | 0 |
| hsa_circ_0087346 | 93 | 4 | 1440 | 3411 | 1978 | 0 | 0 |
| hsa_circ_0087346 | 94 | 4 | 1568 | 3411 | 1850 | 0 | 0 |
| hsa_circ_0003715 | 92 | 1 | 776 | 773 | 1 | 0 | 0 |
| hsa_circ_0003715 | 92 | 1 | 657 | 657 | 1 | 0 | 0 |
| hsa_circ_0087348 | 95 | 1 | 512 | 512 | 1 | 0 | 0 |
| hsa_circ_0003163 | 76 | 6856 | 7081 | 1E+06 | 1169813 | 0.05 | 2.00E-23 |
| hsa_circ_0003163 | 76 | 6555 | 6780 | 1E+06 | 1169813 | 0.05 | 2.00E-23 |
| hsa_circ_0003163 | 76 | 6225 | 6450 | 1E+06 | 1169813 | 0.05 | 2.00E-23 |
| hsa_circ_0087319 | 91 | 1 | 737 | 737 | 1 | 0 | 0 |
| hsa_circ_0087270 | 90 | 1 | 510 | 1 | 510 | 0 | 0 |
| hsa_circ_0087267 | 91 | 1 | 311 | 181 | 491 | 0 | 1.00E-114 |
| hsa_circ_0087267 | 91 | 115 | 602 | 1 | 491 | 0.01 | 0 |
| hsa_circ_0087267 | 91 | 290 | 777 | 1 | 491 | 0.01 | 0 |
| hsa_circ_0087267 | 93 | 290 | 466 | 1 | 177 | 0 | 3.00E-68 |
| hsa_circ_0087267 | 93 | 394 | 570 | 1 | 177 | 0 | 3.00E-68 |
| hsa_circ_0087265 | 98 | 21490 | 21905 | 416 | 1 | 0 | 0 |

novel_circ_007514 novel_circ_007515 novel_circ_007516 novel_circ_007517 novel_circ_007518 novel_circ_007519 novel_circ_007520 novel_circ_007521 novel_circ_007522 novel_circ_007523 novel_circ_007524 novel_circ_007525 novel_circ_007526 novel_circ_007527 novel_circ_007528 novel_circ_007529 novel_circ_007530 novel_circ_007531 novel_circ_007532 novel_circ_007533 novel_circ_007534 novel_circ_007535 novel_circ_007536 novel_circ_007537 novel_circ_007538 novel_circ_007539 novel_circ_007540 novel_circ_007541 novel_circ_007542 novel_circ_007543 novel_circ_007544 novel_circ_007545 novel_circ_007546 novel_circ_007547 novel_circ_007548 novel_circ_007549 novel_circ_007550 novel_circ_007551 novel_circ_007552 novel_circ_007553 novel_circ_007554 novel_circ_007555 novel_circ_007556 novel_circ_007557 novel_circ_007558 novel_circ_007559 novel_circ_007560 novel_circ_007561 novel_circ_007562 novel_circ_007563 novel_circ_007564 novel_circ_007565 novel_circ_007566 novel_circ_007567

| hsa_circ_0087265 | 98 | 1 | 414 | 414 | 1 | 0 | 0 |
| --- | --- | --- | --- | --- | --- | --- | --- |
| hsa_circ_0087265 | 98 | 1 | 259 | 259 | 1 | 0 | 2.00E-127 |
| hsa_circ_0087262 | 91 | 1 | 284 | 1659 | 1376 | 0 | 3.00E-106 |
| hsa_circ_0057916 | 87 | 7282 | 8525 | 16 | 1266 | 0.02 | 0 |
| hsa_circ_0087255 | 92 | 2 | 1422 | 1559 | 2979 | 0 | 0 |
| hsa_circ_0087255 | 92 | 1 | 331 | 2649 | 2979 | 0 | 3.00E-132 |
| hsa_circ_0087255 | 92 | 1 | 1655 | 1325 | 2979 | 0 | 0 |
| hsa_circ_0087255 | 92 | 1 | 1655 | 1325 | 2979 | 0 | 0 |
| hsa_circ_0087255 | 92 | 1 | 387 | 1325 | 1711 | 0 | 4.00E-155 |
| hsa_circ_0087255 | 91 | 1 | 1428 | 284 | 1711 | 0 | 0 |
| hsa_circ_0087255 | 91 | 1 | 626 | 932 | 1557 | 0 | 0 |
| hsa_circ_0087257 | 90 | 1 | 1040 | 1 | 1040 | 0 | 0 |
| hsa_circ_0087255 | 90 | 1 | 1323 | 1 | 1323 | 0 | 0 |
| hsa_circ_0087253 | 90 | 1 | 1427 | 4207 | 5633 | 0 | 0 |
| hsa_circ_0087253 | 89 | 2 | 3603 | 2029 | 5633 | 0 | 0 |
| hsa_circ_0087257 | 90 | 1 | 911 | 1 | 911 | 0 | 0 |
| hsa_circ_0087257 | 91 | 1 | 648 | 1 | 648 | 0 | 0 |
| hsa_circ_0087255 | 90 | 1 | 931 | 1 | 931 | 0 | 0 |
| hsa_circ_0087253 | 90 | 1 | 1035 | 4207 | 5241 | 0 | 0 |
| hsa_circ_0087253 | 90 | 1 | 1611 | 3631 | 5241 | 0 | 0 |
| hsa_circ_0087253 | 90 | 1 | 2912 | 2330 | 5241 | 0 | 0 |
| hsa_circ_0087253 | 88 | 1 | 3857 | 728 | 4587 | 0 | 0 |
| hsa_circ_0087253 | 91 | 1 | 680 | 3631 | 4310 | 0 | 0 |
| hsa_circ_0087253 | 90 | 1 | 1232 | 3079 | 4310 | 0 | 0 |
| hsa_circ_0087253 | 89 | 1 | 1981 | 2330 | 4310 | 0 | 0 |
| hsa_circ_0087253 | 88 | 2 | 2280 | 2029 | 4310 | 0 | 0 |
| hsa_circ_0087253 | 88 | 1 | 3580 | 728 | 4310 | 0 | 0 |
| hsa_circ_0087253 | 91 | 1 | 576 | 3631 | 4206 | 0 | 0 |
| hsa_circ_0087253 | 90 | 1 | 1128 | 3079 | 4206 | 0.01 | 0 |
| hsa_circ_0087253 | 90 | 21 | 1361 | 2866 | 4206 | 0 | 0 |
| hsa_circ_0087253 | 90 | 2 | 1749 | 2459 | 4206 | 0 | 0 |
| hsa_circ_0087253 | 90 | 1 | 1877 | 2330 | 4206 | 0 | 0 |
| hsa_circ_0087253 | 88 | 2 | 2176 | 2029 | 4206 | 0 | 0 |
| hsa_circ_0087253 | 89 | 1 | 2481 | 1723 | 4206 | 0 | 0 |
| hsa_circ_0087252 | 89 | 144 | 4533 | 1 | 4396 | 0 | 0 |
| hsa_circ_0087253 | 91 | 1 | 415 | 3631 | 4045 | 0 | 6.00E-159 |
| hsa_circ_0087253 | 88 | 1 | 1003 | 2628 | 3630 | 0 | 0 |
| hsa_circ_0087253 | 88 | 1 | 897 | 2628 | 3524 | 0.01 | 0 |
| hsa_circ_0087253 | 88 | 1 | 2348 | 728 | 3078 | 0.01 | 0 |
| hsa_circ_0087253 | 88 | 1 | 2115 | 728 | 2845 | 0.01 | 0 |
| hsa_circ_0087253 | 87 | 1 | 1721 | 728 | 2451 | 0.01 | 0 |
| hsa_circ_0087253 | 88 | 1 | 1553 | 728 | 2280 | 0 | 0 |
| hsa_circ_0087252 | 89 | 432 | 2898 | 1 | 2470 | 0 | 0 |
| hsa_circ_0087253 | 90 | 1 | 683 | 1040 | 1722 | 0 | 0 |
| hsa_circ_0087253 | 88 | 1 | 995 | 728 | 1722 | 0 | 0 |
| hsa_circ_0087253 | 88 | 5053 | 6387 | 385 | 1722 | 0 | 0 |
| hsa_circ_0087253 | 88 | 1 | 1467 | 253 | 1722 | 0 | 0 |
| hsa_circ_0087252 | 89 | 144 | 2052 | 1 | 1912 | 0 | 0 |
| hsa_circ_0001863 | 87 | 1 | 827 | 1 | 827 | 0 | 0 |
| hsa_circ_0087252 | 88 | 369 | 1888 | 1 | 1523 | 0.01 | 0 |
| hsa_circ_0001863 | 84 | 1 | 392 | 1 | 392 | 0 | 2.00E-104 |
| hsa_circ_0087253 | 86 | 1 | 613 | 504 | 1119 | 0 | 0 |
| hsa_circ_0087252 | 88 | 144 | 1449 | 1 | 1309 | 0.01 | 0 |
| hsa_circ_0087252 | 88 | 369 | 1591 | 1 | 1226 | 0.01 | 0 |

novel_circ_007568 novel_circ_007569 novel_circ_007570 novel_circ_007571 novel_circ_007572 novel_circ_007573 novel_circ_007574 novel_circ_007575 novel_circ_007576 novel_circ_007577 novel_circ_007578 novel_circ_007579 novel_circ_007580 novel_circ_007581 novel_circ_007582 novel_circ_007583 novel_circ_007584 novel_circ_007585 novel_circ_007586 novel_circ_007587 novel_circ_007588 novel_circ_007589 novel_circ_007590 novel_circ_007591 novel_circ_007592 novel_circ_007593 novel_circ_007594 novel_circ_007595 novel_circ_007596 novel_circ_007597 novel_circ_007598 novel_circ_007599 novel_circ_007600 novel_circ_007601 novel_circ_007602 novel_circ_007603 novel_circ_007604 novel_circ_007605 novel_circ_007606 novel_circ_007607 novel_circ_007608 novel_circ_007609 novel_circ_007610 novel_circ_007611 novel_circ_007612 novel_circ_007614 novel_circ_007615 novel_circ_007616 novel_circ_007617 novel_circ_007618 novel_circ_007619 novel_circ_007620 novel_circ_007621 novel_circ_007622

| hsa_circ_0087246 | 90 | 1 | 1293 | 1 | 1296 | 0.01 | 0 |
| --- | --- | --- | --- | --- | --- | --- | --- |
| hsa_circ_0087252 | 90 | 1 | 912 | 1 | 915 | 0.01 | 0 |
| hsa_circ_0087246 | 90 | 1 | 1293 | 1 | 1296 | 0.01 | 0 |
| hsa_circ_0087253 | 91 | 1 | 513 | 116 | 628 | 0 | 0 |
| hsa_circ_0087252 | 91 | 1 | 818 | 1 | 818 | 0 | 0 |
| hsa_circ_0087252 | 91 | 144 | 961 | 1 | 818 | 0 | 0 |
| hsa_circ_0087246 | 89 | 1 | 1058 | 236 | 1296 | 0 | 0 |
| hsa_circ_0087246 | 90 | 1 | 1121 | 173 | 1296 | 0.01 | 0 |
| hsa_circ_0087246 | 90 | 1 | 1293 | 1 | 1296 | 0.01 | 0 |
| hsa_circ_0087241 | 90 | 1 | 1528 | 568 | 2098 | 0.01 | 0 |
| hsa_circ_0087241 | 91 | 88 | 2182 | 1 | 2098 | 0 | 0 |
| hsa_circ_0087252 | 91 | 1 | 690 | 1 | 690 | 0 | 0 |
| hsa_circ_0087246 | 89 | 1 | 1058 | 236 | 1296 | 0 | 0 |
| hsa_circ_0087246 | 90 | 1 | 1121 | 173 | 1296 | 0.01 | 0 |
| hsa_circ_0087246 | 90 | 1 | 1293 | 1 | 1296 | 0.01 | 0 |
| hsa_circ_0087252 | 91 | 1 | 442 | 1 | 442 | 0 | 3.00E-172 |
| hsa_circ_0087246 | 90 | 1 | 585 | 464 | 1048 | 0 | 0 |
| hsa_circ_0087246 | 90 | 2 | 680 | 370 | 1048 | 0 | 0 |
| hsa_circ_0087246 | 89 | 1 | 810 | 236 | 1048 | 0.01 | 0 |
| hsa_circ_0087246 | 90 | 1 | 1045 | 1 | 1048 | 0.01 | 0 |
| hsa_circ_0087244 | 91 | 1 | 1280 | 59 | 1341 | 0.01 | 0 |
| hsa_circ_0087241 | 91 | 1 | 1479 | 369 | 1850 | 0.01 | 0 |
| hsa_circ_0087246 | 89 | 2 | 543 | 370 | 911 | 0 | 0 |
| hsa_circ_0087246 | 88 | 1 | 673 | 236 | 911 | 0.01 | 0 |
| hsa_circ_0087246 | 89 | 1 | 908 | 1 | 911 | 0.01 | 0 |
| hsa_circ_0087246 | 87 | 1 | 368 | 236 | 606 | 0.01 | 2.00E-113 |
| hsa_circ_0087246 | 89 | 1 | 603 | 1 | 606 | 0.01 | 0 |
| hsa_circ_0087246 | 89 | 1 | 225 | 236 | 463 | 0.01 | 2.00E-72 |
| hsa_circ_0087246 | 91 | 1 | 460 | 1 | 463 | 0.02 | 2.00E-174 |
| hsa_circ_0087246 | 90 | 1 | 362 | 1 | 365 | 0.02 | 2.00E-133 |
| hsa_circ_0087244 | 91 | 1 | 597 | 59 | 658 | 0.01 | 0 |
| hsa_circ_0087241 | 91 | 1 | 434 | 369 | 802 | 0 | 2.00E-169 |
| hsa_circ_0087241 | 91 | 1 | 604 | 199 | 802 | 0 | 0 |
| hsa_circ_0087240 | 92 | 1 | 889 | 1 | 889 | 0 | 0 |
| hsa_circ_0087240 | 91 | 1 | 654 | 1 | 654 | 0 | 0 |
| hsa_circ_0087240 | 92 | 1 | 455 | 1 | 455 | 0 | 0 |
| hsa_circ_0087239 | 94 | 1 | 322 | 322 | 1 | 0 | 5.00E-139 |
| hsa_circ_0003654 | 84 | 95 | 1373 | 91 | 1368 | 0.02 | 0 |
| hsa_circ_0003654 | 84 | 95 | 1184 | 91 | 1179 | 0.02 | 0 |
| hsa_circ_0087234 | 92 | 1 | 576 | 1 | 576 | 0 | 0 |
| hsa_circ_0087234 | 92 | 1 | 474 | 1 | 474 | 0 | 0 |
| hsa_circ_0087234 | 90 | 1 | 262 | 1 | 262 | 0 | 9.00E-91 |
| hsa_circ_0087231 | 90 | 1 | 483 | 1 | 483 | 0 | 5.00E-180 |
| hsa_circ_0087227 | 91 | 1 | 597 | 106 | 702 | 0 | 0 |
| hsa_circ_0087229 | 92 | 1 | 335 | 1 | 335 | 0 | 3.00E-131 |
| hsa_circ_0027681 | 94 | 1 | 571 | 1 | 571 | 0 | 0 |
| hsa_circ_0027681 | 92 | 1 | 1400 | 1 | 1400 | 0 | 0 |
| hsa_circ_0027681 | 91 | 1 | 829 | 572 | 1400 | 0 | 0 |
| hsa_circ_0027681 | 91 | 1 | 829 | 572 | 1400 | 0 | 0 |
| hsa_circ_0027681 | 91 | 1 | 829 | 572 | 1400 | 0 | 0 |
| hsa_circ_0027681 | 91 | 1 | 829 | 572 | 1400 | 0 | 0 |
| hsa_circ_0027682 | 95 | 1 | 465 | 1 | 465 | 0 | 0 |
| hsa_circ_0027682 | 95 | 1 | 587 | 1 | 587 | 0 | 0 |
| hsa_circ_0027682 | 95 | 1 | 669 | 1 | 669 | 0 | 0 |

novel_circ_007623 novel_circ_007624 novel_circ_007625 novel_circ_007626 novel_circ_007627 novel_circ_007628 novel_circ_007632 novel_circ_007633 novel_circ_007634 novel_circ_007635 novel_circ_007636 novel_circ_007637 novel_circ_007638 novel_circ_007640 novel_circ_007641 novel_circ_007642 novel_circ_007643 novel_circ_007644 novel_circ_007648 novel_circ_007649 novel_circ_007650 novel_circ_007651 novel_circ_007652 novel_circ_007653 novel_circ_007654 novel_circ_007657 novel_circ_007658 novel_circ_007659 novel_circ_007660 novel_circ_007661 novel_circ_007662 novel_circ_007667 novel_circ_007668 novel_circ_007669 novel_circ_007670 novel_circ_007671 novel_circ_007672 novel_circ_007673 novel_circ_007674 novel_circ_007675 novel_circ_007676 novel_circ_007677 novel_circ_007678 novel_circ_007679 novel_circ_007680 novel_circ_007681 novel_circ_007682 novel_circ_007683 novel_circ_007684 novel_circ_007685 novel_circ_007686 novel_circ_007687 novel_circ_007688 novel_circ_007689

| hsa_circ_0027683 | 93 | 8178 | 8649 | 1 | 472 | 0 | 0 |
| --- | --- | --- | --- | --- | --- | --- | --- |
| hsa_circ_0027683 | 93 | 1 | 350 | 1 | 350 | 0 | 7.00E-143 |
| hsa_circ_0027683 | 93 | 1 | 472 | 1 | 472 | 0 | 0 |
| hsa_circ_0027682 | 93 | 1 | 473 | 116 | 588 | 0 | 0 |
| hsa_circ_0027682 | 94 | 1 | 554 | 116 | 669 | 0 | 0 |
| hsa_circ_0027682 | 93 | 1 | 669 | 116 | 784 | 0 | 0 |
| hsa_circ_0073741 | 87 | 1 | 614 | 1 | 614 | 0 | 0 |
| hsa_circ_0073741 | 88 | 1 | 735 | 1 | 735 | 0 | 0 |
| hsa_circ_0073748 | 92 | 2 | 580 | 2 | 580 | 0 | 0 |
| hsa_circ_0073748 | 92 | 2 | 580 | 2 | 580 | 0 | 0 |
| hsa_circ_0073748 | 91 | 2 | 1252 | 2 | 1252 | 0 | 0 |
| hsa_circ_0073748 | 91 | 2 | 1360 | 2 | 1360 | 0 | 0 |
| hsa_circ_0073750 | 98 | 1 | 244 | 244 | 1 | 0 | 3.00E-117 |
| hsa_circ_0073752 | 83 | 151 | 794 | 1 | 674 | 0.04 | 3.00E-169 |
| hsa_circ_0073752 | 83 | 151 | 794 | 1 | 674 | 0.04 | 5.00E-169 |
| hsa_circ_0073752 | 83 | 1 | 644 | 1 | 674 | 0.04 | 3.00E-169 |
| hsa_circ_0073752 | 83 | 1 | 644 | 1 | 674 | 0.04 | 4.00E-169 |
| hsa_circ_0073753 | 85 | 88 | 624 | 112 | 654 | 0.01 | 2.00E-156 |
| hsa_circ_0087783 | 92 | 9 | 654 | 73 | 718 | 0 | 0 |
| hsa_circ_0087784 | 93 | 1 | 499 | 1 | 499 | 0 | 0 |
| hsa_circ_0087789 | 93 | 1 | 489 | 1 | 489 | 0 | 0 |
| hsa_circ_0085310 | 94 | 3 | 197 | 164 | 358 | 0 | 1.00E-78 |
| hsa_circ_0085310 | 93 | 82578 | 82775 | 161 | 358 | 0 | 5.00E-76 |
| hsa_circ_0061760 | 89 | 47375 | 47685 | 703 | 1015 | 0.01 | 4.00E-106 |
| hsa_circ_0061760 | 89 | 57024 | 57334 | 703 | 1015 | 0.01 | 4.00E-106 |
| hsa_circ_0034300 | 73 | 2507 | 2778 | 4948 | 5213 | 0.05 | 5.00E-13 |
| hsa_circ_0085308 | 96 | 1 | 432 | 113 | 544 | 0 | 0 |
| hsa_circ_0085307 | 98 | 19337 | 19449 | 221 | 333 | 0 | 1.00E-47 |
| hsa_circ_0085307 | 96 | 1 | 231 | 103 | 333 | 0 | 1.00E-103 |
| hsa_circ_0085306 | 95 | 1 | 261 | 1 | 261 | 0 | 6.00E-112 |
| hsa_circ_0061589 | 87 | 2694 | 2773 | 73118 | 73037 | 0.02 | 6.00E-15 |
| hsa_circ_0085303 | 92 | 1 | 242 | 1 | 242 | 0 | 2.00E-91 |
| hsa_circ_0085302 | 92 | 52 | 296 | 55134 | 55378 | 0 | 6.00E-93 |
| hsa_circ_0085302 | 92 | 55 | 299 | 55134 | 55378 | 0 | 6.00E-93 |
| hsa_circ_0085302 | 92 | 171 | 415 | 55134 | 55378 | 0 | 8.00E-93 |
| hsa_circ_0085302 | 92 | 272 | 516 | 55134 | 55378 | 0 | 1.00E-92 |
| hsa_circ_0085302 | 92 | 22062 | 22306 | 55134 | 55378 | 0 | 5.00E-91 |
| hsa_circ_0085302 | 92 | 22065 | 22309 | 55134 | 55378 | 0 | 5.00E-91 |
| hsa_circ_0085302 | 80 | 36104 | 37215 | 605 | 1716 | 0.04 | 0 |
| hsa_circ_0085302 | 97 | 1 | 173 | 1 | 173 | 0 | 3.00E-78 |
| hsa_circ_0085301 | 97 | 100 | 274 | 24958 | 25132 | 0 | 4.00E-79 |
| hsa_circ_0085301 | 97 | 34873 | 35047 | 24958 | 25132 | 0 | 6.00E-77 |
| hsa_circ_0085301 | 97 | 210 | 384 | 24958 | 25132 | 0 | 6.00E-79 |
| hsa_circ_0085299 | 98 | 1 | 290 | 1 | 290 | 0 | 2.00E-139 |
| hsa_circ_0085296 | 98 | 10409 | 10700 | 1437 | 1728 | 0 | 4.00E-139 |
| hsa_circ_0085296 | 98 | 11449 | 11740 | 1437 | 1728 | 0 | 4.00E-139 |
| hsa_circ_0085299 | 95 | 1 | 110 | 181 | 290 | 0 | 2.00E-39 |
| hsa_circ_0085296 | 98 | 10409 | 10700 | 1437 | 1728 | 0 | 9.00E-139 |
| hsa_circ_0085296 | 94 | 1 | 340 | 1099 | 1438 | 0 | 5.00E-144 |
| hsa_circ_0085296 | 91 | 4392 | 5155 | 702 | 1438 | 0.04 | 0 |
| hsa_circ_0085296 | 92 | 1 | 893 | 573 | 1438 | 0.03 | 0 |
| hsa_circ_0085296 | 92 | 757 | 1654 | 569 | 1438 | 0.03 | 0 |
| hsa_circ_0085296 | 92 | 954 | 1851 | 569 | 1438 | 0.03 | 0 |
| hsa_circ_0085296 | 94 | 1 | 343 | 1030 | 1372 | 0 | 1.00E-145 |

novel_circ_007690 novel_circ_007691 novel_circ_007692 novel_circ_007693 novel_circ_007694 novel_circ_007695 novel_circ_007696 novel_circ_007697 novel_circ_007698 novel_circ_007699 novel_circ_007700 novel_circ_007701 novel_circ_007702 novel_circ_007703 novel_circ_007704 novel_circ_007705 novel_circ_007706 novel_circ_007707 novel_circ_007708 novel_circ_007709 novel_circ_007710 novel_circ_007711 novel_circ_007712 novel_circ_007713 novel_circ_007714 novel_circ_007715 novel_circ_007716 novel_circ_007717 novel_circ_007718 novel_circ_007724 novel_circ_007725 novel_circ_007726 novel_circ_007729 novel_circ_007730 novel_circ_007731 novel_circ_007732 novel_circ_007733 novel_circ_007734 novel_circ_007735 novel_circ_007736 novel_circ_007737 novel_circ_007738 novel_circ_007739 novel_circ_007740 novel_circ_007741 novel_circ_007742 novel_circ_007743 novel_circ_007744 novel_circ_007745 novel_circ_007746 novel_circ_007747 novel_circ_007748 novel_circ_007749 novel_circ_007750

| hsa_circ_0085296 | 90 | 4392 | 5089 | 702 | 1372 | 0.04 | 0 |
| --- | --- | --- | --- | --- | --- | --- | --- |
| hsa_circ_0085296 | 92 | 1 | 827 | 573 | 1372 | 0.03 | 0 |
| hsa_circ_0085296 | 92 | 757 | 1588 | 569 | 1372 | 0.03 | 0 |
| hsa_circ_0085296 | 92 | 954 | 1785 | 569 | 1372 | 0.03 | 0 |
| hsa_circ_0085296 | 92 | 1680 | 2511 | 569 | 1372 | 0.03 | 0 |
| hsa_circ_0085296 | 95 | 1 | 226 | 1030 | 1255 | 0 | 2.00E-97 |
| hsa_circ_0085296 | 90 | 4392 | 4972 | 702 | 1255 | 0.05 | 0 |
| hsa_circ_0085296 | 92 | 1 | 710 | 573 | 1255 | 0.04 | 0 |
| hsa_circ_0085296 | 92 | 157 | 871 | 569 | 1255 | 0.04 | 0 |
| hsa_circ_0085296 | 96 | 4392 | 4719 | 702 | 1029 | 0 | 7.00E-148 |
| hsa_circ_0085296 | 96 | 1 | 457 | 573 | 1029 | 0 | 0 |
| hsa_circ_0085296 | 96 | 157 | 618 | 569 | 1029 | 0 | 0 |
| hsa_circ_0085296 | 96 | 328 | 789 | 569 | 1029 | 0 | 0 |
| hsa_circ_0085296 | 94 | 209 | 714 | 68 | 573 | 0 | 0 |
| hsa_circ_0085296 | 96 | 4392 | 4719 | 702 | 1029 | 0 | 7.00E-148 |
| hsa_circ_0085296 | 96 | 1 | 457 | 573 | 1029 | 0 | 0 |
| hsa_circ_0085296 | 94 | 406 | 911 | 68 | 573 | 0 | 0 |
| hsa_circ_0085296 | 94 | 1132 | 1637 | 68 | 573 | 0 | 0 |
| hsa_circ_0085296 | 97 | 4392 | 4565 | 702 | 875 | 0 | 1.00E-75 |
| hsa_circ_0085296 | 97 | 1 | 303 | 573 | 875 | 0 | 3.00E-145 |
| hsa_circ_0085296 | 97 | 328 | 635 | 569 | 875 | 0 | 2.00E-145 |
| hsa_circ_0085296 | 94 | 406 | 911 | 68 | 573 | 0 | 0 |
| hsa_circ_0085296 | 94 | 1132 | 1637 | 68 | 573 | 0 | 0 |
| hsa_circ_0085296 | 92 | 1 | 284 | 289 | 572 | 0 | 3.00E-111 |
| hsa_circ_0085296 | 94 | 406 | 910 | 68 | 572 | 0 | 0 |
| hsa_circ_0085296 | 94 | 1132 | 1636 | 68 | 572 | 0 | 0 |
| hsa_circ_0085296 | 93 | 1 | 224 | 189 | 412 | 0 | 9.00E-90 |
| hsa_circ_0085296 | 94 | 1132 | 1476 | 68 | 412 | 0 | 2.00E-149 |
| hsa_circ_0085296 | 97 | 1132 | 1252 | 68 | 188 | 0 | 5.00E-50 |
| hsa_circ_0008249 | 95 | 70 | 354 | 1 | 285 | 0 | 2.00E-126 |
| hsa_circ_0008249 | 95 | 261 | 545 | 1 | 285 | 0 | 2.00E-126 |
| hsa_circ_0008249 | 96 | 1 | 187 | 1 | 187 | 0 | 1.00E-82 |
| hsa_circ_0085287 | 93 | 3 | 235 | 7 | 239 | 0 | 4.00E-91 |
| hsa_circ_0085285 | 99 | 1 | 481 | 481 | 1 | 0 | 0 |
| hsa_circ_0085283 | 97 | 1 | 828 | 828 | 1 | 0 | 0 |
| hsa_circ_0085275 | 96 | 1 | 1283 | 1614 | 332 | 0 | 0 |
| hsa_circ_0085283 | 97 | 1 | 544 | 544 | 1 | 0 | 0 |
| hsa_circ_0085276 | 96 | 1 | 999 | 1115 | 117 | 0 | 0 |
| hsa_circ_0085276 | 95 | 1 | 802 | 918 | 117 | 0 | 0 |
| hsa_circ_0085275 | 98 | 1 | 331 | 331 | 1 | 0 | 1.00E-160 |
| hsa_circ_0001819 | 95 | 1 | 324 | 324 | 1 | 0 | 2.00E-143 |
| hsa_circ_0085258 | 96 | 1 | 1036 | 1036 | 1 | 0 | 0 |
| hsa_circ_0085236 | 95 | 1 | 1722 | 4126 | 2405 | 0 | 0 |
| hsa_circ_0001819 | 95 | 1 | 279 | 279 | 1 | 0 | 1.00E-123 |
| hsa_circ_0001819 | 93 | 1 | 197 | 197 | 1 | 0 | 4.00E-78 |
| hsa_circ_0085258 | 97 | 1 | 712 | 712 | 1 | 0 | 0 |
| hsa_circ_0008217 | 96 | 1 | 1398 | 1398 | 1 | 0 | 0 |
| hsa_circ_0085255 | 94 | 1 | 383 | 383 | 1 | 0 | 1.00E-164 |
| hsa_circ_0008217 | 94 | 1 | 501 | 686 | 186 | 0 | 0 |
| hsa_circ_0008217 | 94 | 1 | 686 | 686 | 1 | 0 | 0 |
| hsa_circ_0085251 | 93 | 1 | 936 | 936 | 1 | 0 | 0 |
| hsa_circ_0085246 | 94 | 1 | 1610 | 1739 | 130 | 0 | 0 |
| hsa_circ_0085252 | 93 | 1 | 411 | 413 | 3 | 0 | 4.00E-170 |
| hsa_circ_0085252 | 93 | 1 | 293 | 295 | 3 | 0 | 6.00E-118 |

novel_circ_007751 novel_circ_007752 novel_circ_007753 novel_circ_007754 novel_circ_007756 novel_circ_007757 novel_circ_007758 novel_circ_007759 novel_circ_007760 novel_circ_007761 novel_circ_007762 novel_circ_007763 novel_circ_007764 novel_circ_007765 novel_circ_007766 novel_circ_007767 novel_circ_007768 novel_circ_007769 novel_circ_007770 novel_circ_007772 novel_circ_007773 novel_circ_007774 novel_circ_007775 novel_circ_007776 novel_circ_007777 novel_circ_007778 novel_circ_007779 novel_circ_007780 novel_circ_007781 novel_circ_007782 novel_circ_007783 novel_circ_007784 novel_circ_007785 novel_circ_007786 novel_circ_007787 novel_circ_007788 novel_circ_007789 novel_circ_007790 novel_circ_007791 novel_circ_007792 novel_circ_007793 novel_circ_007794 novel_circ_007795 novel_circ_007796 novel_circ_007797 novel_circ_007798 novel_circ_007799 novel_circ_007800 novel_circ_007801 novel_circ_007802 novel_circ_007803 novel_circ_007804 novel_circ_007805 novel_circ_007806

| hsa_circ_0085249 | 93 | 4 | 411 | 606 | 199 | 0 | 1.00E-171 |
| --- | --- | --- | --- | --- | --- | --- | --- |
| hsa_circ_0085249 | 94 | 4 | 609 | 606 | 1 | 0 | 0 |
| hsa_circ_0085246 | 95 | 1 | 691 | 691 | 1 | 0 | 0 |
| hsa_circ_0085246 | 95 | 1 | 407 | 536 | 130 | 0 | 0 |
| hsa_circ_0085236 | 93 | 1 | 257 | 257 | 1 | 0 | 2.00E-101 |
| hsa_circ_0085234 | 93 | 1 | 401 | 401 | 1 | 0 | 7.00E-168 |
| hsa_circ_0085232 | 94 | 1 | 525 | 525 | 1 | 0 | 0 |
| hsa_circ_0085201 | 95 | 1 | 2100 | 2100 | 1 | 0 | 0 |
| hsa_circ_0085201 | 95 | 1 | 1575 | 1575 | 1 | 0 | 0 |
| hsa_circ_0085200 | 94 | 1 | 1552 | 1772 | 221 | 0 | 0 |
| hsa_circ_0085201 | 95 | 1 | 1073 | 1073 | 1 | 0 | 0 |
| hsa_circ_0085201 | 96 | 1 | 592 | 592 | 1 | 0 | 0 |
| hsa_circ_0085200 | 95 | 1 | 508 | 728 | 221 | 0 | 0 |
| hsa_circ_0085197 | 94 | 1 | 1027 | 1084 | 64 | 0.01 | 0 |
| hsa_circ_0085198 | 93 | 1 | 691 | 685 | 1 | 0.01 | 0 |
| hsa_circ_0085196 | 89 | 1 | 601 | 632 | 1 | 0.05 | 0 |
| hsa_circ_0085193 | 89 | 1 | 662 | 693 | 1 | 0.04 | 0 |
| hsa_circ_0085191 | 90 | 1 | 923 | 957 | 1 | 0.04 | 0 |
| hsa_circ_0085183 | 91 | 1 | 1192 | 1372 | 147 | 0.03 | 0 |
| hsa_circ_0085191 | 92 | 50 | 679 | 633 | 1 | 0 | 0 |
| hsa_circ_0085183 | 94 | 50 | 1094 | 1048 | 1 | 0 | 0 |
| hsa_circ_0085191 | 92 | 1 | 322 | 325 | 1 | 0.01 | 3.00E-126 |
| hsa_circ_0085186 | 94 | 1 | 591 | 595 | 2 | 0.01 | 0 |
| hsa_circ_0085181 | 94 | 1 | 823 | 826 | 1 | 0 | 0 |
| hsa_circ_0085191 | 93 | 1 | 261 | 261 | 1 | 0 | 1.00E-103 |
| hsa_circ_0085178 | 92 | 1 | 502 | 502 | 1 | 0 | 0 |
| hsa_circ_0085178 | 92 | 1 | 502 | 502 | 1 | 0 | 0 |
| hsa_circ_0085178 | 94 | 1 | 346 | 346 | 1 | 0.01 | 7.00E-149 |
| hsa_circ_0085177 | 93 | 252 | 701 | 4233 | 3784 | 0 | 0 |
| hsa_circ_0085178 | 94 | 1 | 229 | 229 | 1 | 0 | 6.00E-94 |
| hsa_circ_0085177 | 93 | 135 | 582 | 4233 | 3786 | 0 | 0 |
| hsa_circ_0085177 | 93 | 135 | 584 | 4233 | 3784 | 0 | 0 |
| hsa_circ_0006425 | 85 | 1 | 610 | 1 | 610 | 0 | 9.00E-179 |
| hsa_circ_0006425 | 90 | 1 | 406 | 1 | 406 | 0 | 1.00E-150 |
| hsa_circ_0006425 | 89 | 1 | 318 | 1 | 318 | 0 | 2.00E-108 |
| hsa_circ_0073525 | 92 | 2 | 576 | 186 | 760 | 0 | 0 |
| hsa_circ_0006425 | 89 | 1 | 313 | 1 | 313 | 0 | 5.00E-109 |
| hsa_circ_0073525 | 91 | 1 | 433 | 323 | 755 | 0 | 1.00E-165 |
| hsa_circ_0073525 | 92 | 2 | 571 | 186 | 755 | 0 | 0 |
| hsa_circ_0073523 | 90 | 1 | 1029 | 267 | 1295 | 0 | 0 |
| hsa_circ_0073523 | 92 | 1 | 1296 | 1 | 1295 | 0 | 0 |
| hsa_circ_0073522 | 90 | 1 | 1440 | 1 | 1441 | 0 | 0 |
| hsa_circ_0073522 | 90 | 36 | 1475 | 1 | 1441 | 0 | 0 |
| hsa_circ_0073522 | 90 | 36 | 1285 | 1 | 1251 | 0.01 | 0 |
| hsa_circ_0073525 | 95 | 2 | 258 | 186 | 442 | 0 | 1.00E-109 |
| hsa_circ_0073522 | 91 | 1 | 1127 | 1 | 1128 | 0.01 | 0 |
| hsa_circ_0073522 | 91 | 36 | 1162 | 1 | 1128 | 0.01 | 0 |
| hsa_circ_0073523 | 94 | 1 | 541 | 1 | 540 | 0 | 0 |
| hsa_circ_0073522 | 91 | 1 | 685 | 1 | 686 | 0.01 | 0 |
| hsa_circ_0073522 | 91 | 36 | 720 | 1 | 686 | 0.01 | 0 |
| hsa_circ_0073522 | 90 | 36 | 446 | 1 | 412 | 0.02 | 5.00E-150 |
| hsa_circ_0073520 | 97 | 1723 | 1982 | 392 | 133 | 0 | 2.00E-120 |
| hsa_circ_0073520 | 97 | 1723 | 1982 | 392 | 133 | 0 | 3.00E-119 |
| hsa_circ_0001517 | 77 | 32920 | 34070 | 6604 | 5458 | 0.06 | 4.00E-171 |

novel_circ_007811 novel_circ_007815 novel_circ_007818 novel_circ_007819 novel_circ_007820 novel_circ_007822 novel_circ_007823 novel_circ_007824 novel_circ_007825 novel_circ_007826 novel_circ_007827 novel_circ_007828 novel_circ_007829 novel_circ_007830 novel_circ_007831 novel_circ_007832 novel_circ_007834 novel_circ_007836 novel_circ_007837 novel_circ_007838 novel_circ_007839 novel_circ_007840 novel_circ_007841 novel_circ_007842 novel_circ_007843 novel_circ_007844 novel_circ_007845 novel_circ_007846 novel_circ_007847 novel_circ_007848 novel_circ_007849 novel_circ_007850 novel_circ_007852 novel_circ_007855 novel_circ_007856 novel_circ_007857 novel_circ_007858 novel_circ_007859 novel_circ_007860 novel_circ_007861 novel_circ_007862 novel_circ_007863 novel_circ_007864 novel_circ_007865 novel_circ_007866 novel_circ_007869 novel_circ_007870 novel_circ_007871 novel_circ_007872 novel_circ_007873 novel_circ_007876 novel_circ_007878 novel_circ_007883 novel_circ_007890

| hsa_circ_0046430 | 85 | 1 | 1362 | 1 | 1365 | 0 | 0 |
| --- | --- | --- | --- | --- | --- | --- | --- |
| hsa_circ_0046395 | 89 | 1 | 1121 | 1121 | 1 | 0 | 0 |
| hsa_circ_0046294 | 83 | 30 | 190 | 350 | 509 | 0.02 | 3.00E-34 |
| hsa_circ_0046291 | 86 | 1 | 230 | 57 | 286 | 0.01 | 2.00E-64 |
| hsa_circ_0046278 | 89 | 1 | 414 | 425 | 12 | 0 | 1.00E-141 |
| hsa_circ_0046204 | 88 | 372 | 816 | 445 | 1 | 0 | 3.00E-145 |
| hsa_circ_0046204 | 88 | 291 | 735 | 445 | 1 | 0 | 2.00E-145 |
| hsa_circ_0046204 | 91 | 1 | 219 | 219 | 1 | 0 | 3.00E-80 |
| hsa_circ_0046195 | 89 | 4 | 466 | 463 | 1 | 0 | 7.00E-164 |
| hsa_circ_0046191 | 89 | 7 | 447 | 441 | 1 | 0 | 5.00E-155 |
| hsa_circ_0046065 | 91 | 1 | 373 | 1 | 373 | 0 | 1.00E-140 |
| hsa_circ_0046058 | 89 | 1 | 246 | 543 | 788 | 0 | 3.00E-80 |
| hsa_circ_0046062 | 91 | 1 | 383 | 1 | 383 | 0 | 7.00E-143 |
| hsa_circ_0046058 | 90 | 1 | 306 | 1 | 306 | 0 | 2.00E-108 |
| hsa_circ_0046055 | 91 | 1 | 389 | 1 | 389 | 0 | 7.00E-148 |
| hsa_circ_0046053 | 90 | 1 | 492 | 1 | 492 | 0 | 0 |
| hsa_circ_0045947 | 92 | 1 | 335 | 335 | 1 | 0 | 3.00E-131 |
| hsa_circ_0045913 | 91 | 1 | 1096 | 1096 | 1 | 0 | 0 |
| hsa_circ_0045913 | 92 | 6 | 568 | 563 | 1 | 0 | 0 |
| hsa_circ_0045903 | 90 | 3 | 522 | 3 | 522 | 0 | 0 |
| hsa_circ_0045894 | 90 | 1 | 469 | 1 | 469 | 0 | 1.00E-175 |
| hsa_circ_0045863 | 91 | 1 | 496 | 1 | 493 | 0.01 | 0 |
| hsa_circ_0045863 | 95 | 1 | 328 | 1 | 325 | 0.01 | 3.00E-141 |
| hsa_circ_0045861 | 97 | 151 | 417 | 1 | 267 | 0 | 1.00E-126 |
| hsa_circ_0045859 | 91 | 1 | 248 | 2830 | 3077 | 0 | 2.00E-91 |
| hsa_circ_0045859 | 85 | 1 | 3002 | 1 | 2996 | 0.02 | 0 |
| hsa_circ_0008622 | 94 | 1 | 449 | 1 | 449 | 0 | 0 |
| hsa_circ_0008622 | 94 | 48320 | 48768 | 1 | 449 | 0 | 0 |
| hsa_circ_0045858 | 94 | 193 | 755 | 1 | 563 | 0 | 0 |
| hsa_circ_0045856 | 94 | 2 | 307 | 352 | 657 | 0 | 3.00E-130 |
| hsa_circ_0045856 | 90 | 1 | 657 | 1 | 657 | 0 | 0 |
| hsa_circ_0045856 | 87 | 1 | 350 | 1 | 350 | 0 | 7.00E-108 |
| hsa_circ_0045842 | 89 | 1 | 503 | 1 | 503 | 0 | 0 |
| hsa_circ_0045834 | 93 | 1 | 948 | 1 | 945 | 0.01 | 0 |
| hsa_circ_0045791 | 90 | 121 | 611 | 709 | 219 | 0 | 2.00E-179 |
| hsa_circ_0045791 | 90 | 68 | 558 | 709 | 219 | 0 | 2.00E-179 |
| hsa_circ_0045791 | 88 | 2 | 274 | 418 | 147 | 0 | 3.00E-86 |
| hsa_circ_0045778 | 90 | 2 | 376 | 423 | 49 | 0 | 4.00E-135 |
| hsa_circ_0045763 | 90 | 1 | 586 | 1143 | 558 | 0 | 0 |
| hsa_circ_0045763 | 90 | 1 | 727 | 1143 | 417 | 0 | 0 |
| hsa_circ_0045766 | 91 | 5 | 613 | 609 | 1 | 0 | 0 |
| hsa_circ_0000805 | 91 | 1 | 193 | 193 | 1 | 0 | 3.00E-69 |
| hsa_circ_0045764 | 89 | 1 | 498 | 498 | 1 | 0 | 3.00E-178 |
| hsa_circ_0045747 | 93 | 1 | 406 | 406 | 1 | 0 | 1.00E-165 |
| hsa_circ_0045746 | 90 | 1 | 784 | 784 | 1 | 0 | 0 |
| hsa_circ_0045714 | 92 | 211 | 772 | 1 | 562 | 0 | 0 |
| hsa_circ_0045679 | 92 | 7 | 378 | 941 | 570 | 0 | 2.00E-148 |
| hsa_circ_0045679 | 92 | 7 | 458 | 941 | 490 | 0 | 0 |
| hsa_circ_0045640 | 93 | 1 | 292 | 292 | 1 | 0 | 2.00E-117 |
| hsa_circ_0045633 | 88 | 1 | 546 | 1579 | 1034 | 0 | 0 |
| hsa_circ_0045525 | 83 | 1 | 803 | 1975 | 1169 | 0.05 | 0 |
| hsa_circ_0055813 | 77 | 13159 | 13603 | 70823 | 70375 | 0.03 | 3.00E-63 |
| hsa_circ_0017174 | 80 | 1 | 1198 | 2333 | 3530 | 0 | 0 |
| hsa_circ_0017172 | 83 | 2 | 510 | 1625 | 2133 | 0 | 5.00E-126 |

novel_circ_007895 novel_circ_007899 novel_circ_007904 novel_circ_007905 novel_circ_007906 novel_circ_007914 novel_circ_007915 novel_circ_007916 novel_circ_007917 novel_circ_007918 novel_circ_007919 novel_circ_007920 novel_circ_007921 novel_circ_007922 novel_circ_007923 novel_circ_007924 novel_circ_007925 novel_circ_007926 novel_circ_007927 novel_circ_007928 novel_circ_007929 novel_circ_007930 novel_circ_007931 novel_circ_007932 novel_circ_007933 novel_circ_007934 novel_circ_007935 novel_circ_007936 novel_circ_007937 novel_circ_007938 novel_circ_007939 novel_circ_007940 novel_circ_007941 novel_circ_007943 novel_circ_007944 novel_circ_007945 novel_circ_007946 novel_circ_007947 novel_circ_007948 novel_circ_007953 novel_circ_007954 novel_circ_007955 novel_circ_007956 novel_circ_007957 novel_circ_007958 novel_circ_007959 novel_circ_007960 novel_circ_007961 novel_circ_007963 novel_circ_007964 novel_circ_007965 novel_circ_007966 novel_circ_007967 novel_circ_007969

| hsa_circ_0017159 | 84 | 1 | 867 | 1 | 867 | 0 | 0 |
| --- | --- | --- | --- | --- | --- | --- | --- |
| hsa_circ_0032327 | 87 | 4 | 322 | 319 | 1 | 0 | 2.00E-97 |
| hsa_circ_0017075 | 83 | 7 | 241 | 437 | 203 | 0 | 4.00E-54 |
| hsa_circ_0017075 | 81 | 7 | 443 | 437 | 1 | 0 | 4.00E-91 |
| hsa_circ_0017070 | 83 | 7 | 646 | 644 | 5 | 0 | 9.00E-164 |
| hsa_circ_0017050 | 91 | 72 | 803 | 1 | 732 | 0 | 0 |
| hsa_circ_0017050 | 91 | 165 | 896 | 1 | 732 | 0 | 0 |
| hsa_circ_0008531 | 93 | 4 | 268 | 345 | 81 | 0 | 9.00E-106 |
| hsa_circ_0008531 | 92 | 4 | 348 | 345 | 1 | 0 | 8.00E-137 |
| hsa_circ_0008531 | 92 | 4 | 348 | 345 | 1 | 0 | 1.00E-136 |
| hsa_circ_0008531 | 92 | 4 | 348 | 345 | 1 | 0 | 1.00E-136 |
| hsa_circ_0017045 | 94 | 271 | 659 | 3388 | 3000 | 0 | 3.00E-169 |
| hsa_circ_0017045 | 93 | 271 | 890 | 3388 | 2769 | 0 | 0 |
| hsa_circ_0017045 | 93 | 271 | 1157 | 3388 | 2502 | 0 | 0 |
| hsa_circ_0017045 | 91 | 271 | 1577 | 3388 | 2082 | 0 | 0 |
| hsa_circ_0017045 | 92 | 271 | 2107 | 3388 | 1540 | 0.01 | 0 |
| hsa_circ_0008531 | 93 | 1 | 237 | 237 | 1 | 0 | 1.00E-93 |
| hsa_circ_0008531 | 93 | 1 | 237 | 237 | 1 | 0 | 2.00E-93 |
| hsa_circ_0017045 | 95 | 160 | 468 | 3388 | 3080 | 0 | 5.00E-135 |
| hsa_circ_0017045 | 94 | 160 | 548 | 3388 | 3000 | 0 | 2.00E-169 |
| hsa_circ_0017045 | 95 | 94 | 402 | 3388 | 3080 | 0 | 5.00E-135 |
| hsa_circ_0017047 | 95 | 1 | 311 | 2980 | 2670 | 0 | 6.00E-138 |
| hsa_circ_0017047 | 91 | 1 | 311 | 2749 | 2439 | 0 | 1.00E-114 |
| hsa_circ_0017047 | 91 | 1 | 480 | 2749 | 2270 | 0 | 0 |
| hsa_circ_0017047 | 92 | 1 | 578 | 2749 | 2172 | 0 | 0 |
| hsa_circ_0017047 | 90 | 1 | 998 | 2749 | 1752 | 0 | 0 |
| hsa_circ_0017047 | 91 | 1 | 1528 | 2749 | 1210 | 0.01 | 0 |
| hsa_circ_0017048 | 90 | 1 | 231 | 232 | 2 | 0 | 3.00E-80 |
| hsa_circ_0017047 | 91 | 1 | 400 | 2669 | 2270 | 0 | 6.00E-154 |
| hsa_circ_0017047 | 92 | 1 | 498 | 2669 | 2172 | 0 | 0 |
| hsa_circ_0017047 | 90 | 1 | 278 | 1493 | 1210 | 0.02 | 1.00E-99 |
| hsa_circ_0017045 | 81 | 1 | 1539 | 1539 | 1 | 0.01 | 0 |
| hsa_circ_0017045 | 86 | 1 | 330 | 330 | 1 | 0.01 | 6.00E-95 |
| hsa_circ_0017017 | 83 | 3 | 317 | 2390 | 2076 | 0 | 2.00E-78 |
| hsa_circ_0017017 | 84 | 3 | 468 | 2390 | 1925 | 0.01 | 3.00E-127 |
| hsa_circ_0017017 | 83 | 3 | 604 | 2390 | 1789 | 0.01 | 2.00E-149 |
| hsa_circ_0017009 | 87 | 4 | 260 | 431 | 175 | 0.01 | 4.00E-76 |
| hsa_circ_0017004 | 77 | 4 | 1005 | 1002 | 1 | 0.02 | 5.00E-153 |
| hsa_circ_0017001 | 77 | 4 | 1007 | 1572 | 569 | 0.02 | 1.00E-153 |
| hsa_circ_0016995 | 75 | 3 | 879 | 1609 | 712 | 0.04 | 8.00E-104 |
| hsa_circ_0016995 | 75 | 3 | 879 | 1609 | 712 | 0.04 | 9.00E-104 |
| hsa_circ_0016972 | 89 | 2137 | 2600 | 464 | 1 | 0 | 2.00E-165 |
| hsa_circ_0016965 | 89 | 2137 | 2601 | 671 | 207 | 0 | 1.00E-165 |
| hsa_circ_0016965 | 87 | 2137 | 2807 | 671 | 1 | 0 | 0 |
| hsa_circ_0016963 | 85 | 2137 | 3051 | 915 | 1 | 0 | 0 |
| hsa_circ_0016972 | 90 | 1 | 375 | 375 | 1 | 0 | 2.00E-133 |
| hsa_circ_0016966 | 90 | 1 | 376 | 485 | 110 | 0 | 4.00E-133 |
| hsa_circ_0016965 | 87 | 1 | 582 | 582 | 1 | 0 | 0 |
| hsa_circ_0016949 | 84 | 1 | 1213 | 1213 | 1 | 0 | 0 |
| hsa_circ_0016949 | 83 | 1 | 1010 | 1010 | 1 | 0 | 0 |
| hsa_circ_0016949 | 86 | 12 | 559 | 548 | 1 | 0 | 1.00E-162 |
| hsa_circ_0016941 | 87 | 12 | 834 | 823 | 1 | 0 | 0 |
| hsa_circ_0016949 | 84 | 1 | 417 | 417 | 1 | 0 | 1.00E-111 |
| hsa_circ_0016917 | 85 | 1725 | 2047 | 323 | 1 | 0 | 4.00E-87 |

novel_circ_007970 novel_circ_007971 novel_circ_007974 novel_circ_007975 novel_circ_007976 novel_circ_007977 novel_circ_007978 novel_circ_007979 novel_circ_007980 novel_circ_007981 novel_circ_007985 novel_circ_007986 novel_circ_007987 novel_circ_007988 novel_circ_007989 novel_circ_007990 novel_circ_007991 novel_circ_007992 novel_circ_007994 novel_circ_007995 novel_circ_007997 novel_circ_007998 novel_circ_007999 novel_circ_008000 novel_circ_008001 novel_circ_008002 novel_circ_008003 novel_circ_008004 novel_circ_008005 novel_circ_008006 novel_circ_008007 novel_circ_008008 novel_circ_008012 novel_circ_008013 novel_circ_008014 novel_circ_008015 novel_circ_008016 novel_circ_008017 novel_circ_008018 novel_circ_008019 novel_circ_008020 novel_circ_008021 novel_circ_008022 novel_circ_008023 novel_circ_008024 novel_circ_008033 novel_circ_008034 novel_circ_008035 novel_circ_008036 novel_circ_008037 novel_circ_008045 novel_circ_008046 novel_circ_008047 novel_circ_008048

| hsa_circ_0016916 | 87 | 135 | 602 | 626 | 159 | 0 | 1.00E-146 |
| --- | --- | --- | --- | --- | --- | --- | --- |
| hsa_circ_0016916 | 90 | 1 | 279 | 437 | 159 | 0 | 3.00E-100 |
| hsa_circ_0016902 | 82 | 14 | 524 | 1852 | 2362 | 0.01 | 5.00E-117 |
| hsa_circ_0016902 | 80 | 1 | 675 | 1688 | 2362 | 0.01 | 2.00E-136 |
| hsa_circ_0016902 | 80 | 1 | 745 | 1618 | 2362 | 0.01 | 3.00E-160 |
| hsa_circ_0016902 | 78 | 576 | 1725 | 1186 | 2362 | 0.03 | 0 |
| hsa_circ_0016902 | 80 | 1 | 517 | 1688 | 2204 | 0.01 | 8.00E-104 |
| hsa_circ_0016902 | 81 | 1 | 587 | 1618 | 2204 | 0.01 | 1.00E-127 |
| hsa_circ_0016900 | 93 | 1 | 257 | 257 | 1 | 0 | 2.00E-101 |
| hsa_circ_0016895 | 88 | 1235 | 1655 | 34 | 454 | 0 | 1.00E-141 |
| hsa_circ_0016862 | 86 | 1 | 625 | 1178 | 1802 | 0 | 0 |
| hsa_circ_0016862 | 90 | 1 | 267 | 1051 | 1317 | 0 | 1.00E-93 |
| hsa_circ_0016868 | 83 | 1 | 432 | 295 | 726 | 0 | 2.00E-108 |
| hsa_circ_0016868 | 96 | 1 | 293 | 1 | 293 | 0 | 3.00E-131 |
| hsa_circ_0016846 | 91 | 1 | 248 | 1 | 248 | 0 | 2.00E-91 |
| hsa_circ_0016833 | 89 | 3 | 969 | 2949 | 1977 | 0.01 | 0 |
| hsa_circ_0016833 | 90 | 1 | 719 | 2701 | 1977 | 0.01 | 0 |
| hsa_circ_0008717 | 89 | 1 | 283 | 589 | 307 | 0 | 2.00E-97 |
| hsa_circ_0016815 | 85 | 1 | 546 | 546 | 1 | 0 | 6.00E-160 |
| hsa_circ_0016792 | 89 | 1 | 401 | 1234 | 834 | 0 | 3.00E-141 |
| hsa_circ_0015733 | 94 | 171 | 553 | 383 | 1 | 0 | 1.00E-165 |
| hsa_circ_0015733 | 94 | 171 | 553 | 383 | 1 | 0 | 9.00E-166 |
| hsa_circ_0015733 | 94 | 1 | 383 | 383 | 1 | 0 | 6.00E-166 |
| hsa_circ_0015733 | 94 | 189 | 571 | 383 | 1 | 0 | 8.00E-166 |
| hsa_circ_0015733 | 94 | 1 | 383 | 383 | 1 | 0 | 3.00E-166 |
| hsa_circ_0015733 | 94 | 1 | 319 | 383 | 65 | 0 | 1.00E-135 |
| hsa_circ_0015733 | 94 | 171 | 489 | 383 | 65 | 0 | 2.00E-135 |
| hsa_circ_0015735 | 87 | 1 | 600 | 1 | 601 | 0 | 0 |
| hsa_circ_0015735 | 87 | 1 | 600 | 1 | 601 | 0 | 0 |
| hsa_circ_0015735 | 87 | 1 | 600 | 1 | 601 | 0 | 0 |
| hsa_circ_0015735 | 87 | 1 | 600 | 1 | 601 | 0 | 0 |
| hsa_circ_0015735 | 87 | 1 | 600 | 1 | 601 | 0 | 0 |
| hsa_circ_0015738 | 93 | 2 | 841 | 2 | 841 | 0 | 0 |
| hsa_circ_0015738 | 94 | 1 | 492 | 107 | 598 | 0 | 0 |
| hsa_circ_0015738 | 94 | 1 | 492 | 107 | 598 | 0 | 0 |
| hsa_circ_0015739 | 92 | 1 | 199 | 1 | 199 | 0 | 7.00E-76 |
| hsa_circ_0015739 | 94 | 1 | 422 | 1 | 422 | 0 | 0 |
| hsa_circ_0015739 | 94 | 1 | 422 | 1 | 422 | 0 | 2.00E-180 |
| hsa_circ_0015749 | 93 | 1 | 182 | 1 | 182 | 0 | 8.00E-70 |
| hsa_circ_0015749 | 91 | 1 | 344 | 1 | 344 | 0 | 1.00E-129 |
| hsa_circ_0015749 | 91 | 1 | 445 | 1 | 445 | 0 | 6.00E-174 |
| hsa_circ_0015742 | 93 | 1 | 587 | 550 | 1136 | 0 | 0 |
| hsa_circ_0056157 | 79 | 2720 | 4377 | 1666 | 10 | 0.04 | 0 |
| hsa_circ_0029815 | 90 | 3954 | 4328 | 518 | 138 | 0.02 | 4.00E-135 |
| hsa_circ_0007554 | 89 | 43876 | 44298 | 427 | 3 | 0 | 3.00E-143 |
| hsa_circ_0015753 | 93 | 1 | 547 | 547 | 1 | 0 | 0 |
| hsa_circ_0015753 | 93 | 1 | 547 | 547 | 1 | 0 | 0 |
| hsa_circ_0015753 | 95 | 1 | 543 | 1090 | 548 | 0 | 0 |
| hsa_circ_0015753 | 95 | 52432 | 52974 | 1090 | 548 | 0 | 0 |
| hsa_circ_0015753 | 95 | 1 | 448 | 1090 | 643 | 0 | 0 |
| hsa_circ_0083793 | 100 | 20548 | 20589 | 63079 | 63038 | 0 | 2.00E-11 |
| hsa_circ_0012960 | 89 | 1 | 444 | 444 | 1 | 0 | 5.00E-158 |
| hsa_circ_0012960 | 89 | 101 | 544 | 444 | 1 | 0 | 6.00E-158 |
| hsa_circ_0012960 | 89 | 554 | 997 | 444 | 1 | 0 | 6.00E-158 |

novel_circ_008049 novel_circ_008059 novel_circ_008062 novel_circ_008063 novel_circ_008064 novel_circ_008065 novel_circ_008066 novel_circ_008067 novel_circ_008068 novel_circ_008069 novel_circ_008070 novel_circ_008071 novel_circ_008072 novel_circ_008073 novel_circ_008077 novel_circ_008078 novel_circ_008079 novel_circ_008080 novel_circ_008081 novel_circ_008082 novel_circ_008083 novel_circ_008084 novel_circ_008085 novel_circ_008086 novel_circ_008087 novel_circ_008088 novel_circ_008089 novel_circ_008090 novel_circ_008091 novel_circ_008092 novel_circ_008093 novel_circ_008094 novel_circ_008095 novel_circ_008096 novel_circ_008097 novel_circ_008098 novel_circ_008099 novel_circ_008100 novel_circ_008101 novel_circ_008102 novel_circ_008103 novel_circ_008104 novel_circ_008105 novel_circ_008106 novel_circ_008107 novel_circ_008108 novel_circ_008109 novel_circ_008110 novel_circ_008111 novel_circ_008112 novel_circ_008113 novel_circ_008114 novel_circ_008115 novel_circ_008116

| hsa_circ_0012960 | 89 | 851 | 941 | 444 | 354 | 0.02 | 2.00E-23 |
| --- | --- | --- | --- | --- | --- | --- | --- |
| hsa_circ_0008129 | 93 | 439 | 915 | 1 | 477 | 0 | 0 |
| hsa_circ_0012974 | 94 | 1 | 751 | 1660 | 910 | 0 | 0 |
| hsa_circ_0012975 | 95 | 1 | 402 | 402 | 1 | 0 | 4.00E-180 |
| hsa_circ_0012975 | 94 | 1 | 724 | 724 | 1 | 0 | 0 |
| hsa_circ_0012975 | 94 | 1 | 747 | 747 | 1 | 0 | 0 |
| hsa_circ_0012975 | 94 | 1 | 893 | 893 | 1 | 0 | 0 |
| hsa_circ_0012976 | 95 | 1 | 574 | 574 | 1 | 0 | 0 |
| hsa_circ_0012975 | 94 | 1 | 720 | 893 | 174 | 0 | 0 |
| hsa_circ_0012975 | 93 | 1 | 491 | 893 | 403 | 0 | 0 |
| hsa_circ_0012978 | 98 | 526 | 639 | 1 | 114 | 0 | 8.00E-50 |
| hsa_circ_0012978 | 92 | 526 | 831 | 1 | 306 | 0 | 1.00E-119 |
| hsa_circ_0012978 | 92 | 339 | 644 | 1 | 306 | 0 | 8.00E-120 |
| hsa_circ_0012978 | 92 | 171 | 476 | 1 | 306 | 0 | 6.00E-120 |
| hsa_circ_0012984 | 94 | 1 | 403 | 577 | 175 | 0 | 6.00E-174 |
| hsa_circ_0012986 | 95 | 201 | 1846 | 1646 | 1 | 0 | 0 |
| hsa_circ_0012987 | 95 | 1 | 1556 | 1556 | 1 | 0 | 0 |
| hsa_circ_0012986 | 95 | 201 | 1831 | 1646 | 16 | 0 | 0 |
| hsa_circ_0012986 | 95 | 2889 | 4519 | 1646 | 16 | 0 | 0 |
| hsa_circ_0006395 | 95 | 201 | 275 | 366 | 292 | 0 | 4.00E-25 |
| hsa_circ_0006395 | 95 | 2231 | 2305 | 366 | 292 | 0 | 3.00E-24 |
| hsa_circ_0012995 | 95 | 1 | 781 | 783 | 3 | 0 | 0 |
| hsa_circ_0012995 | 95 | 1 | 781 | 783 | 3 | 0 | 0 |
| hsa_circ_0012996 | 95 | 1 | 254 | 522 | 269 | 0 | 1.00E-109 |
| hsa_circ_0012996 | 94 | 1 | 865 | 1133 | 269 | 0 | 0 |
| hsa_circ_0012996 | 93 | 1 | 1038 | 1306 | 269 | 0 | 0 |
| hsa_circ_0012996 | 94 | 4 | 1441 | 1705 | 269 | 0 | 0 |
| hsa_circ_0012998 | 92 | 1 | 559 | 559 | 1 | 0 | 0 |
| hsa_circ_0012998 | 93 | 1 | 752 | 752 | 1 | 0 | 0 |
| hsa_circ_0012996 | 94 | 4 | 576 | 1705 | 1134 | 0.01 | 0 |
| hsa_circ_0012996 | 95 | 4 | 403 | 1705 | 1307 | 0.01 | 9.00E-177 |
| hsa_circ_0013000 | 92 | 4 | 250 | 246 | 1 | 0.01 | 3.00E-95 |
| hsa_circ_0013004 | 93 | 1 | 551 | 6 | 556 | 0 | 0 |
| hsa_circ_0013004 | 93 | 1 | 685 | 6 | 690 | 0 | 0 |
| hsa_circ_0013004 | 93 | 1 | 809 | 6 | 814 | 0 | 0 |
| hsa_circ_0013004 | 92 | 1 | 910 | 6 | 915 | 0 | 0 |
| hsa_circ_0013004 | 93 | 1 | 1288 | 6 | 1293 | 0 | 0 |
| hsa_circ_0013004 | 93 | 1 | 1288 | 6 | 1293 | 0 | 0 |
| hsa_circ_0013004 | 93 | 3 | 806 | 11 | 814 | 0 | 0 |
| hsa_circ_0013006 | 93 | 1 | 442 | 1 | 442 | 0 | 0 |
| hsa_circ_0013006 | 93 | 1 | 700 | 1 | 700 | 0 | 0 |
| hsa_circ_0013009 | 93 | 1 | 261 | 138 | 398 | 0 | 6.00E-107 |
| hsa_circ_0013009 | 94 | 1 | 385 | 138 | 522 | 0 | 5.00E-164 |
| hsa_circ_0013009 | 93 | 1 | 393 | 138 | 531 | 0 | 5.00E-164 |
| hsa_circ_0013009 | 92 | 1 | 486 | 138 | 623 | 0 | 0 |
| hsa_circ_0013006 | 93 | 1 | 765 | 316 | 1080 | 0 | 0 |
| hsa_circ_0013006 | 93 | 1 | 864 | 316 | 1179 | 0 | 0 |
| hsa_circ_0013012 | 94 | 102 | 613 | 1 | 512 | 0 | 0 |
| hsa_circ_0013012 | 93 | 1 | 279 | 1 | 279 | 0 | 1.00E-113 |
| hsa_circ_0013012 | 94 | 1 | 378 | 1 | 378 | 0 | 2.00E-163 |
| hsa_circ_0013012 | 94 | 1 | 512 | 1 | 512 | 0 | 0 |
| hsa_circ_0013012 | 94 | 1 | 567 | 1 | 567 | 0 | 0 |
| hsa_circ_0013012 | 94 | 1 | 320 | 193 | 512 | 0 | 3.00E-136 |
| hsa_circ_0013012 | 94 | 1 | 288 | 280 | 567 | 0 | 2.00E-123 |

novel_circ_008117 novel_circ_008118 novel_circ_008119 novel_circ_008120 novel_circ_008121 novel_circ_008122 novel_circ_008145 novel_circ_008146 novel_circ_008147 novel_circ_008148 novel_circ_008149 novel_circ_008150 novel_circ_008152 novel_circ_008154 novel_circ_008155 novel_circ_008156 novel_circ_008157 novel_circ_008158 novel_circ_008159 novel_circ_008160 novel_circ_008161 novel_circ_008162 novel_circ_008163 novel_circ_008164 novel_circ_008165 novel_circ_008166 novel_circ_008167 novel_circ_008168 novel_circ_008175 novel_circ_008176 novel_circ_008177 novel_circ_008178 novel_circ_008179 novel_circ_008180 novel_circ_008181 novel_circ_008182 novel_circ_008183 novel_circ_008184 novel_circ_008185 novel_circ_008186 novel_circ_008187 novel_circ_008188 novel_circ_008189 novel_circ_008190 novel_circ_008196 novel_circ_008197 novel_circ_008198 novel_circ_008199 novel_circ_008200 novel_circ_008201 novel_circ_008202 novel_circ_008203 novel_circ_008204 novel_circ_008205

| hsa_circ_0013024 | 94 | 1 | 743 | 743 | 1 | 0 | 0 |
| --- | --- | --- | --- | --- | --- | --- | --- |
| hsa_circ_0013024 | 95 | 1 | 885 | 885 | 1 | 0 | 0 |
| hsa_circ_0013026 | 95 | 1 | 597 | 597 | 1 | 0 | 0 |
| hsa_circ_0013026 | 95 | 1 | 739 | 739 | 1 | 0 | 0 |
| hsa_circ_0013031 | 95 | 1 | 522 | 522 | 1 | 0 | 0 |
| hsa_circ_0013035 | 100 | 62 | 102 | 1135 | 1095 | 0 | 2.00E-13 |
| hsa_circ_0009049 | 93 | 1 | 673 | 671 | 2 | 0 | 0 |
| hsa_circ_0012709 | 94 | 1 | 501 | 2426 | 1926 | 0 | 0 |
| hsa_circ_0012709 | 94 | 1 | 498 | 2423 | 1926 | 0 | 0 |
| hsa_circ_0012709 | 94 | 1 | 267 | 2192 | 1926 | 0 | 1.00E-113 |
| hsa_circ_0012709 | 94 | 1 | 435 | 2192 | 1758 | 0 | 0 |
| hsa_circ_0012687 | 95 | 1 | 432 | 628 | 197 | 0 | 0 |
| hsa_circ_0090964 | 95 | 3 | 368 | 714 | 1079 | 0 | 5.00E-163 |
| hsa_circ_0048002 | 85 | 1 | 548 | 1 | 548 | 0 | 5.00E-161 |
| hsa_circ_0048002 | 86 | 1 | 607 | 1 | 607 | 0 | 2.00E-180 |
| hsa_circ_0048002 | 86 | 1 | 754 | 1 | 754 | 0 | 0 |
| hsa_circ_0048002 | 86 | 1 | 836 | 1 | 836 | 0 | 0 |
| hsa_circ_0048002 | 86 | 1 | 908 | 1 | 911 | 0 | 0 |
| hsa_circ_0048003 | 88 | 1 | 580 | 1 | 580 | 0 | 0 |
| hsa_circ_0048003 | 89 | 1 | 429 | 152 | 580 | 0 | 2.00E-148 |
| hsa_circ_0048009 | 89 | 1 | 168 | 1 | 168 | 0 | 4.00E-52 |
| hsa_circ_0048009 | 90 | 1 | 220 | 1 | 220 | 0 | 2.00E-77 |
| hsa_circ_0048009 | 88 | 1 | 315 | 1 | 315 | 0 | 2.00E-103 |
| hsa_circ_0048009 | 88 | 1 | 396 | 1 | 396 | 0 | 5.00E-130 |
| hsa_circ_0048009 | 88 | 1 | 397 | 1 | 397 | 0 | 3.00E-129 |
| hsa_circ_0048009 | 87 | 1 | 469 | 1 | 472 | 0.01 | 2.00E-149 |
| hsa_circ_0048009 | 88 | 1 | 540 | 1 | 543 | 0.01 | 0 |
| hsa_circ_0048009 | 88 | 1 | 548 | 1 | 551 | 0.01 | 0 |
| hsa_circ_0027517 | 95 | 1 | 835 | 62 | 897 | 0 | 0 |
| hsa_circ_0027518 | 97 | 1 | 533 | 1 | 533 | 0 | 0 |
| hsa_circ_0027518 | 96 | 1 | 697 | 1 | 697 | 0 | 0 |
| hsa_circ_0027518 | 98 | 1 | 279 | 96 | 374 | 0 | 3.00E-135 |
| hsa_circ_0027518 | 97 | 1 | 438 | 96 | 533 | 0 | 0 |
| hsa_circ_0027518 | 97 | 1 | 346 | 188 | 533 | 0 | 1.00E-165 |
| hsa_circ_0027527 | 92 | 1 | 536 | 1 | 536 | 0 | 0 |
| hsa_circ_0027527 | 92 | 1 | 984 | 1 | 984 | 0 | 0 |
| hsa_circ_0027529 | 92 | 1 | 448 | 1 | 448 | 0 | 1.00E-180 |
| hsa_circ_0027527 | 91 | 1 | 472 | 657 | 1128 | 0 | 1.00E-180 |
| hsa_circ_0027533 | 91 | 1 | 537 | 1 | 537 | 0 | 0 |
| hsa_circ_0027534 | 92 | 3 | 261 | 3 | 261 | 0.01 | 3.00E-97 |
| hsa_circ_0027535 | 95 | 1 | 411 | 1 | 411 | 0 | 0 |
| hsa_circ_0027540 | 93 | 130 | 694 | 1 | 565 | 0.01 | 0 |
| hsa_circ_0027540 | 97 | 1 | 281 | 1 | 281 | 0 | 5.00E-133 |
| hsa_circ_0027540 | 93 | 1 | 452 | 114 | 565 | 0.01 | 0 |
| hsa_circ_0003427 | 72 | 5644 | 6012 | 574163 | 573804 | 0.07 | 1.00E-15 |
| hsa_circ_0027548 | 99 | 136 | 324 | 303 | 490 | 0.01 | 3.00E-91 |
| hsa_circ_0027548 | 99 | 136 | 391 | 303 | 557 | 0 | 2.00E-128 |
| hsa_circ_0027548 | 99 | 66 | 254 | 303 | 490 | 0.01 | 2.00E-91 |
| hsa_circ_0027555 | 98 | 124 | 1488 | 1 | 1365 | 0 | 0 |
| hsa_circ_0027555 | 98 | 1 | 398 | 1 | 398 | 0 | 0 |
| hsa_circ_0027558 | 98 | 1 | 411 | 1 | 411 | 0 | 0 |
| hsa_circ_0027558 | 97 | 1 | 1153 | 1 | 1153 | 0 | 0 |
| hsa_circ_0027558 | 97 | 1 | 1298 | 1 | 1298 | 0 | 0 |
| hsa_circ_0027558 | 97 | 1 | 967 | 332 | 1298 | 0 | 0 |

novel_circ_008206 novel_circ_008207 novel_circ_008208 novel_circ_008209 novel_circ_008210 novel_circ_008211 novel_circ_008212 novel_circ_008213 novel_circ_008214 novel_circ_008215 novel_circ_008216 novel_circ_008217 novel_circ_008221 novel_circ_008222 novel_circ_008223 novel_circ_008224 novel_circ_008225 novel_circ_008226 novel_circ_008227 novel_circ_008228 novel_circ_008229 novel_circ_008230 novel_circ_008231 novel_circ_008232 novel_circ_008233 novel_circ_008234 novel_circ_008235 novel_circ_008236 novel_circ_008237 novel_circ_008238 novel_circ_008239 novel_circ_008240 novel_circ_008241 novel_circ_008242 novel_circ_008243 novel_circ_008248 novel_circ_008249 novel_circ_008250 novel_circ_008251 novel_circ_008252 novel_circ_008260 novel_circ_008261 novel_circ_008262 novel_circ_008263 novel_circ_008264 novel_circ_008265 novel_circ_008266 novel_circ_008267 novel_circ_008268 novel_circ_008269 novel_circ_008270 novel_circ_008271 novel_circ_008272 novel_circ_008273

| hsa_circ_0027558 | 97 | 3 | 887 | 414 | 1298 | 0 | 0 |
| --- | --- | --- | --- | --- | --- | --- | --- |
| hsa_circ_0027564 | 95 | 1 | 128 | 1 | 128 | 0 | 1.00E-47 |
| hsa_circ_0027566 | 92 | 1 | 1239 | 1483 | 245 | 0 | 0 |
| hsa_circ_0027569 | 87 | 258 | 1917 | 2188 | 532 | 0 | 0 |
| hsa_circ_0027570 | 93 | 3 | 865 | 871 | 9 | 0 | 0 |
| hsa_circ_0027570 | 93 | 9227 | 9713 | 601 | 115 | 0 | 0 |
| hsa_circ_0027570 | 93 | 1 | 572 | 686 | 115 | 0 | 0 |
| hsa_circ_0027570 | 93 | 3 | 759 | 871 | 115 | 0 | 0 |
| hsa_circ_0027570 | 93 | 9227 | 9444 | 601 | 384 | 0 | 4.00E-83 |
| hsa_circ_0027570 | 93 | 1 | 303 | 686 | 384 | 0 | 7.00E-122 |
| hsa_circ_0027570 | 93 | 3 | 490 | 871 | 384 | 0 | 0 |
| hsa_circ_0027570 | 93 | 383 | 870 | 871 | 384 | 0 | 0 |
| hsa_circ_0027572 | 96 | 1 | 483 | 625 | 143 | 0 | 0 |
| hsa_circ_0027572 | 96 | 1175 | 1754 | 722 | 143 | 0 | 0 |
| hsa_circ_0027572 | 94 | 1 | 883 | 1025 | 143 | 0 | 0 |
| hsa_circ_0027572 | 93 | 1 | 1310 | 1452 | 143 | 0 | 0 |
| hsa_circ_0027572 | 93 | 3 | 1801 | 1941 | 143 | 0 | 0 |
| hsa_circ_0027573 | 93 | 1 | 1674 | 1785 | 112 | 0 | 0 |
| hsa_circ_0027575 | 93 | 1 | 1274 | 1274 | 1 | 0 | 0 |
| hsa_circ_0027575 | 92 | 3 | 680 | 916 | 239 | 0 | 0 |
| hsa_circ_0027575 | 94 | 1 | 593 | 1020 | 428 | 0 | 0 |
| hsa_circ_0027575 | 93 | 1 | 847 | 1274 | 428 | 0 | 0 |
| hsa_circ_0027573 | 93 | 1 | 2217 | 3155 | 939 | 0 | 0 |
| hsa_circ_0008912 | 93 | 3 | 304 | 489 | 188 | 0 | 1.00E-124 |
| hsa_circ_0027575 | 93 | 1 | 770 | 1384 | 615 | 0 | 0 |
| hsa_circ_0027573 | 94 | 7 | 785 | 3524 | 2746 | 0 | 0 |
| hsa_circ_0027573 | 93 | 1 | 883 | 3628 | 2746 | 0 | 0 |
| hsa_circ_0027573 | 93 | 4322 | 5204 | 3628 | 2746 | 0 | 0 |
| hsa_circ_0027573 | 93 | 125 | 1007 | 3628 | 2746 | 0 | 0 |
| hsa_circ_0027576 | 93 | 1 | 611 | 611 | 1 | 0 | 0 |
| hsa_circ_0027576 | 93 | 4319 | 4932 | 614 | 1 | 0 | 0 |
| hsa_circ_0027576 | 93 | 1 | 735 | 735 | 1 | 0 | 0 |
| hsa_circ_0027576 | 93 | 4319 | 4794 | 614 | 139 | 0 | 0 |
| hsa_circ_0027576 | 93 | 1 | 597 | 735 | 139 | 0 | 0 |
| hsa_circ_0027579 | 92 | 152 | 658 | 1 | 507 | 0 | 0 |
| hsa_circ_0027580 | 98 | 314 | 524 | 1 | 211 | 0 | 2.00E-100 |
| hsa_circ_0027580 | 98 | 314 | 524 | 1 | 211 | 0 | 2.00E-100 |
| hsa_circ_0027580 | 98 | 314 | 524 | 1 | 211 | 0 | 5.00E-100 |
| hsa_circ_0027580 | 98 | 1 | 211 | 1 | 211 | 0 | 3.00E-100 |
| hsa_circ_0027580 | 98 | 1 | 211 | 1 | 211 | 0 | 4.00E-100 |
| hsa_circ_0027583 | 97 | 1 | 396 | 1 | 396 | 0 | 0 |
| hsa_circ_0027584 | 98 | 1 | 269 | 1 | 269 | 0 | 1.00E-129 |
| hsa_circ_0027584 | 96 | 1 | 407 | 1 | 407 | 0 | 0 |
| hsa_circ_0027584 | 96 | 1 | 473 | 1 | 473 | 0 | 0 |
| hsa_circ_0027584 | 95 | 1 | 458 | 270 | 727 | 0 | 0 |
| hsa_circ_0027584 | 96 | 1 | 737 | 270 | 1006 | 0 | 0 |
| hsa_circ_0027584 | 97 | 1 | 409 | 408 | 816 | 0 | 0 |
| hsa_circ_0027584 | 96 | 1 | 599 | 408 | 1006 | 0 | 0 |
| hsa_circ_0027584 | 95 | 1 | 950 | 408 | 1357 | 0 | 0 |
| hsa_circ_0027584 | 96 | 1 | 533 | 474 | 1006 | 0 | 0 |
| hsa_circ_0027584 | 96 | 1 | 534 | 474 | 1007 | 0 | 0 |
| hsa_circ_0027587 | 94 | 1 | 541 | 278 | 818 | 0 | 0 |
| hsa_circ_0027584 | 95 | 1 | 853 | 817 | 1669 | 0 | 0 |
| hsa_circ_0027587 | 93 | 1 | 351 | 468 | 818 | 0 | 8.00E-147 |

novel_circ_008274 novel_circ_008275 novel_circ_008276 novel_circ_008281 novel_circ_008282 novel_circ_008283 novel_circ_008290 novel_circ_008291 novel_circ_008292 novel_circ_008293 novel_circ_008295 novel_circ_008296 novel_circ_008297 novel_circ_008298 novel_circ_008299 novel_circ_008300 novel_circ_008301 novel_circ_008302 novel_circ_008303 novel_circ_008304 novel_circ_008305 novel_circ_008306 novel_circ_008307 novel_circ_008308 novel_circ_008309 novel_circ_008310 novel_circ_008311 novel_circ_008312 novel_circ_008313 novel_circ_008314 novel_circ_008316 novel_circ_008317 novel_circ_008318 novel_circ_008319 novel_circ_008320 novel_circ_008321 novel_circ_008322 novel_circ_008323 novel_circ_008324 novel_circ_008326 novel_circ_008329 novel_circ_008331 novel_circ_008332 novel_circ_008333 novel_circ_008334 novel_circ_008335 novel_circ_008336 novel_circ_008337 novel_circ_008338 novel_circ_008339 novel_circ_008340 novel_circ_008341 novel_circ_008342 novel_circ_008343

| hsa_circ_0027584 | 94 | 1 | 745 | 1007 | 1751 | 0 | 0 |
| --- | --- | --- | --- | --- | --- | --- | --- |
| hsa_circ_0027587 | 92 | 1 | 203 | 616 | 818 | 0 | 2.00E-76 |
| hsa_circ_0027584 | 94 | 1 | 597 | 1155 | 1751 | 0 | 0 |
| hsa_circ_0079678 | 84 | 188 | 559 | 373 | 1 | 0.01 | 3.00E-96 |
| hsa_circ_0079678 | 84 | 2 | 373 | 373 | 1 | 0.01 | 1.00E-96 |
| hsa_circ_0079678 | 84 | 188 | 559 | 373 | 1 | 0.01 | 2.00E-96 |
| hsa_circ_0079682 | 83 | 441 | 1045 | 678 | 1261 | 0.04 | 3.00E-151 |
| hsa_circ_0079683 | 88 | 1 | 564 | 564 | 1 | 0 | 0 |
| hsa_circ_0079683 | 90 | 1 | 746 | 746 | 1 | 0 | 0 |
| hsa_circ_0079683 | 88 | 1 | 564 | 564 | 1 | 0 | 0 |
| hsa_circ_0079683 | 88 | 1 | 564 | 564 | 1 | 0 | 0 |
| hsa_circ_0079683 | 90 | 1 | 746 | 746 | 1 | 0 | 0 |
| hsa_circ_0079683 | 90 | 161 | 906 | 746 | 1 | 0 | 0 |
| hsa_circ_0079689 | 95 | 1 | 398 | 1 | 398 | 0 | 2.00E-179 |
| hsa_circ_0079689 | 95 | 1 | 557 | 1 | 557 | 0 | 0 |
| hsa_circ_0079689 | 94 | 1 | 598 | 1 | 598 | 0 | 0 |
| hsa_circ_0079689 | 94 | 1 | 756 | 1 | 756 | 0 | 0 |
| hsa_circ_0079689 | 93 | 1 | 1002 | 1 | 999 | 0 | 0 |
| hsa_circ_0079689 | 92 | 1 | 1192 | 1 | 1189 | 0 | 0 |
| hsa_circ_0079689 | 92 | 1 | 1193 | 1 | 1190 | 0 | 0 |
| hsa_circ_0079689 | 92 | 1 | 1193 | 1 | 1190 | 0 | 0 |
| hsa_circ_0079689 | 92 | 1 | 1193 | 1 | 1190 | 0 | 0 |
| hsa_circ_0079689 | 92 | 1 | 920 | 274 | 1190 | 0 | 0 |
| hsa_circ_0079689 | 92 | 1 | 920 | 274 | 1190 | 0 | 0 |
| hsa_circ_0079689 | 90 | 1 | 445 | 558 | 999 | 0.01 | 2.00E-159 |
| hsa_circ_0079689 | 90 | 1 | 635 | 558 | 1189 | 0 | 0 |
| hsa_circ_0079690 | 90 | 1 | 404 | 1 | 401 | 0.01 | 1.00E-146 |
| hsa_circ_0079689 | 91 | 1 | 463 | 599 | 1058 | 0.01 | 2.00E-174 |
| hsa_circ_0079689 | 90 | 1 | 594 | 599 | 1189 | 0.01 | 0 |
| hsa_circ_0079689 | 89 | 1 | 436 | 757 | 1189 | 0.01 | 8.00E-153 |
| hsa_circ_0079702 | 81 | 12367 | 12771 | 587 | 176 | 0.06 | 8.00E-82 |
| hsa_circ_0079713 | 93 | 1 | 436 | 1 | 436 | 0 | 0 |
| hsa_circ_0079712 | 92 | 1 | 809 | 580 | 1388 | 0 | 0 |
| hsa_circ_0079719 | 92 | 1 | 454 | 1 | 454 | 0 | 0 |
| hsa_circ_0079719 | 92 | 1 | 604 | 1 | 604 | 0 | 0 |
| hsa_circ_0079722 | 91 | 1 | 296 | 1 | 296 | 0 | 3.00E-111 |
| hsa_circ_0079722 | 91 | 1 | 878 | 1 | 878 | 0 | 0 |
| hsa_circ_0079722 | 93 | 1 | 327 | 297 | 623 | 0 | 4.00E-135 |
| hsa_circ_0079722 | 92 | 1 | 437 | 297 | 733 | 0 | 9.00E-178 |
| hsa_circ_0079729 | 88 | 5 | 688 | 120 | 803 | 0 | 0 |
| hsa_circ_0069858 | 79 | 32048 | 34278 | 133373 | 135622 | 0.05 | 0 |
| hsa_circ_0007319 | 83 | 260 | 456 | 2E+06 | 1532697 | 0.03 | 1.00E-42 |
| hsa_circ_0069858 | 90 | 453 | 670 | 296090 | 296307 | 0 | 7.00E-76 |
| hsa_circ_0069858 | 90 | 201 | 418 | 296090 | 296307 | 0 | 4.00E-76 |
| hsa_circ_0069860 | 92 | 126 | 926 | 1 | 801 | 0 | 0 |
| hsa_circ_0069860 | 93 | 3 | 407 | 885 | 1289 | 0 | 4.00E-165 |
| hsa_circ_0069860 | 91 | 3 | 593 | 885 | 1475 | 0 | 0 |
| hsa_circ_0069860 | 92 | 3 | 632 | 885 | 1514 | 0 | 0 |
| hsa_circ_0069860 | 92 | 3 | 1325 | 885 | 2207 | 0 | 0 |
| hsa_circ_0069862 | 91 | 1 | 532 | 1 | 532 | 0 | 0 |
| hsa_circ_0069862 | 92 | 1 | 918 | 1 | 918 | 0 | 0 |
| hsa_circ_0069865 | 92 | 1 | 307 | 1 | 307 | 0 | 5.00E-119 |
| hsa_circ_0069865 | 92 | 1 | 693 | 1 | 693 | 0 | 0 |
| hsa_circ_0069865 | 93 | 1 | 1124 | 1 | 1124 | 0 | 0 |

novel_circ_008344 novel_circ_008345 novel_circ_008346 novel_circ_008347 novel_circ_008348 novel_circ_008349 novel_circ_008350 novel_circ_008351 novel_circ_008352 novel_circ_008353 novel_circ_008354 novel_circ_008355 novel_circ_008356 novel_circ_008357 novel_circ_008358 novel_circ_008359 novel_circ_008360 novel_circ_008361 novel_circ_008362 novel_circ_008363 novel_circ_008364 novel_circ_008365 novel_circ_008366 novel_circ_008367 novel_circ_008368 novel_circ_008369 novel_circ_008370 novel_circ_008371 novel_circ_008372 novel_circ_008373 novel_circ_008374 novel_circ_008376 novel_circ_008377 novel_circ_008378 novel_circ_008379 novel_circ_008380 novel_circ_008381 novel_circ_008382 novel_circ_008383 novel_circ_008384 novel_circ_008385 novel_circ_008386 novel_circ_008387 novel_circ_008388 novel_circ_008389 novel_circ_008390 novel_circ_008391 novel_circ_008392 novel_circ_008393 novel_circ_008394 novel_circ_008395 novel_circ_008399 novel_circ_008400 novel_circ_008401

| hsa_circ_0069865 | 93 | 1 | 1283 | 1 | 1283 | 0 | 0 |
| --- | --- | --- | --- | --- | --- | --- | --- |
| hsa_circ_0069865 | 94 | 1 | 431 | 694 | 1124 | 0 | 0 |
| hsa_circ_0069865 | 92 | 1 | 982 | 694 | 1702 | 0.03 | 0 |
| hsa_circ_0069865 | 95 | 1 | 392 | 1311 | 1702 | 0 | 2.00E-174 |
| hsa_circ_0066710 | 94 | 102 | 321 | 1 | 220 | 0 | 1.00E-90 |
| hsa_circ_0066710 | 91 | 102 | 1301 | 1 | 1200 | 0 | 0 |
| hsa_circ_0066710 | 91 | 102 | 1301 | 1 | 1200 | 0 | 0 |
| hsa_circ_0066710 | 90 | 1 | 556 | 1 | 556 | 0 | 0 |
| hsa_circ_0066710 | 90 | 1 | 930 | 1 | 930 | 0 | 0 |
| hsa_circ_0066710 | 91 | 1 | 1200 | 1 | 1200 | 0 | 0 |
| hsa_circ_0066710 | 89 | 3 | 597 | 223 | 817 | 0 | 0 |
| hsa_circ_0066710 | 89 | 3 | 710 | 223 | 930 | 0 | 0 |
| hsa_circ_0066710 | 90 | 3 | 980 | 223 | 1200 | 0 | 0 |
| hsa_circ_0066712 | 95 | 252 | 673 | 3 | 424 | 0 | 0 |
| hsa_circ_0066717 | 90 | 1 | 635 | 635 | 1 | 0.01 | 0 |
| hsa_circ_0066717 | 90 | 4 | 1267 | 1279 | 1 | 0.02 | 0 |
| hsa_circ_0066717 | 90 | 1 | 1471 | 1486 | 1 | 0.01 | 0 |
| hsa_circ_0066719 | 91 | 1 | 515 | 515 | 1 | 0.01 | 0 |
| hsa_circ_0066719 | 90 | 1 | 961 | 976 | 1 | 0.02 | 0 |
| hsa_circ_0066719 | 90 | 4 | 1147 | 1159 | 1 | 0.02 | 0 |
| hsa_circ_0066719 | 90 | 1 | 820 | 976 | 142 | 0.03 | 0 |
| hsa_circ_0066720 | 89 | 1 | 446 | 461 | 1 | 0.03 | 5.00E-160 |
| hsa_circ_0066720 | 89 | 4 | 632 | 644 | 1 | 0.02 | 0 |
| hsa_circ_0066720 | 90 | 1 | 836 | 851 | 1 | 0.02 | 0 |
| hsa_circ_0066720 | 90 | 1 | 1056 | 1071 | 1 | 0.01 | 0 |
| hsa_circ_0066722 | 89 | 4 | 537 | 549 | 1 | 0.03 | 0 |
| hsa_circ_0066722 | 89 | 1 | 741 | 756 | 1 | 0.02 | 0 |
| hsa_circ_0066723 | 92 | 1 | 841 | 841 | 1 | 0 | 0 |
| hsa_circ_0066725 | 93 | 1 | 505 | 637 | 133 | 0 | 0 |
| hsa_circ_0061592 | 89 | 107 | 759 | 653 | 1 | 0 | 0 |
| hsa_circ_0061596 | 87 | 1 | 2131 | 1 | 2131 | 0 | 0 |
| hsa_circ_0061598 | 91 | 1 | 470 | 100 | 569 | 0 | 0 |
| hsa_circ_0061598 | 89 | 1 | 885 | 100 | 984 | 0 | 0 |
| hsa_circ_0061598 | 91 | 1 | 346 | 224 | 569 | 0 | 1.00E-130 |
| hsa_circ_0061598 | 89 | 1 | 569 | 224 | 792 | 0.01 | 0 |
| hsa_circ_0061598 | 87 | 1 | 1718 | 224 | 1941 | 0 | 0 |
| hsa_circ_0061600 | 87 | 1 | 1493 | 116 | 1608 | 0 | 0 |
| hsa_circ_0061600 | 86 | 3 | 1372 | 239 | 1608 | 0 | 0 |
| hsa_circ_0061603 | 85 | 1 | 685 | 296 | 980 | 0.01 | 0 |
| hsa_circ_0061603 | 89 | 1 | 440 | 1253 | 1692 | 0 | 2.00E-154 |
| hsa_circ_0008078 | 85 | 1 | 289 | 1 | 301 | 0.04 | 2.00E-77 |
| hsa_circ_0008078 | 87 | 1 | 412 | 1 | 424 | 0.03 | 1.00E-130 |
| hsa_circ_0008078 | 87 | 1 | 603 | 1 | 618 | 0.02 | 0 |
| hsa_circ_0061598 | 88 | 1 | 909 | 4017 | 4940 | 0.02 | 0 |
| hsa_circ_0061596 | 90 | 1 | 1644 | 4207 | 5862 | 0.01 | 0 |
| hsa_circ_0061598 | 92 | 1 | 531 | 4949 | 5479 | 0 | 0 |
| hsa_circ_0061596 | 97 | 1 | 328 | 5479 | 5806 | 0 | 5.00E-154 |
| hsa_circ_0061614 | 94 | 1 | 966 | 1 | 966 | 0 | 0 |
| hsa_circ_0061614 | 93 | 1 | 1169 | 1 | 1169 | 0 | 0 |
| hsa_circ_0061617 | 93 | 1 | 858 | 134 | 991 | 0 | 0 |
| hsa_circ_0061617 | 93 | 6 | 431 | 348 | 773 | 0 | 5.00E-175 |
| hsa_circ_0061625 | 93 | 1 | 671 | 78 | 748 | 0 | 0 |
| hsa_circ_0061625 | 92 | 1 | 1082 | 78 | 1159 | 0 | 0 |
| hsa_circ_0061625 | 94 | 2 | 495 | 150 | 643 | 0 | 0 |

novel_circ_008402 novel_circ_008403 novel_circ_008404 novel_circ_008405 novel_circ_008406 novel_circ_008407 novel_circ_008408 novel_circ_008409 novel_circ_008410 novel_circ_008411 novel_circ_008412 novel_circ_008414 novel_circ_008415 novel_circ_008416 novel_circ_008417 novel_circ_008418 novel_circ_008419 novel_circ_008420 novel_circ_008421 novel_circ_008422 novel_circ_008423 novel_circ_008424 novel_circ_008425 novel_circ_008426 novel_circ_008427 novel_circ_008428 novel_circ_008429 novel_circ_008430 novel_circ_008431 novel_circ_008432 novel_circ_008433 novel_circ_008434 novel_circ_008435 novel_circ_008436 novel_circ_008437 novel_circ_008438 novel_circ_008439 novel_circ_008440 novel_circ_008441 novel_circ_008442 novel_circ_008443 novel_circ_008444 novel_circ_008445 novel_circ_008446 novel_circ_008447 novel_circ_008448 novel_circ_008449 novel_circ_008450 novel_circ_008451 novel_circ_008454 novel_circ_008455 novel_circ_008456 novel_circ_008457 novel_circ_008458

| hsa_circ_0061625 | 93 | 2 | 600 | 150 | 748 | 0 | 0 |
| --- | --- | --- | --- | --- | --- | --- | --- |
| hsa_circ_0061625 | 95 | 1 | 347 | 297 | 643 | 0 | 1.00E-154 |
| hsa_circ_0061625 | 94 | 1 | 452 | 297 | 748 | 0 | 0 |
| hsa_circ_0061625 | 92 | 1 | 863 | 297 | 1159 | 0 | 0 |
| hsa_circ_0061625 | 91 | 1 | 614 | 546 | 1159 | 0 | 0 |
| hsa_circ_0061625 | 90 | 2 | 516 | 645 | 1159 | 0 | 0 |
| hsa_circ_0061627 | 90 | 1 | 411 | 1 | 411 | 0 | 1.00E-146 |
| hsa_circ_0061628 | 93 | 1 | 353 | 265 | 617 | 0 | 7.00E-148 |
| hsa_circ_0061628 | 92 | 1 | 631 | 265 | 895 | 0 | 0 |
| hsa_circ_0061628 | 91 | 1 | 701 | 265 | 965 | 0 | 0 |
| hsa_circ_0061628 | 91 | 1 | 701 | 265 | 965 | 0 | 0 |
| hsa_circ_0002763 | 85 | 7 | 1276 | 1284 | 12 | 0.01 | 0 |
| hsa_circ_0002763 | 85 | 145 | 1414 | 1284 | 12 | 0.01 | 0 |
| hsa_circ_0061655 | 84 | 8 | 941 | 937 | 1 | 0.02 | 0 |
| hsa_circ_0061655 | 85 | 7 | 1104 | 1101 | 1 | 0.02 | 0 |
| hsa_circ_0002763 | 91 | 1378 | 1515 | 2380 | 2246 | 0.02 | 6.00E-45 |
| hsa_circ_0061658 | 92 | 440 | 1230 | 3 | 793 | 0 | 0 |
| hsa_circ_0061658 | 92 | 3 | 793 | 3 | 793 | 0 | 0 |
| hsa_circ_0005304 | 82 | 3819 | 4367 | 579 | 4 | 0.05 | 4.00E-124 |
| hsa_circ_0061666 | 94 | 11986 | 12592 | 2 | 608 | 0 | 0 |
| hsa_circ_0061672 | 92 | 1 | 2490 | 1 | 2490 | 0 | 0 |
| hsa_circ_0061673 | 95 | 1 | 333 | 1 | 333 | 0 | 4.00E-145 |
| hsa_circ_0061673 | 94 | 1 | 395 | 1 | 395 | 0 | 7.00E-168 |
| hsa_circ_0061672 | 92 | 1 | 759 | 56 | 814 | 0 | 0 |
| hsa_circ_0061672 | 93 | 1 | 1218 | 56 | 1273 | 0 | 0 |
| hsa_circ_0061675 | 91 | 1 | 334 | 1 | 334 | 0 | 2.00E-127 |
| hsa_circ_0061675 | 91 | 1 | 469 | 1 | 469 | 0 | 7.00E-179 |
| hsa_circ_0061675 | 92 | 1 | 1009 | 1 | 1009 | 0 | 0 |
| hsa_circ_0061675 | 92 | 1 | 1631 | 1 | 1631 | 0 | 0 |
| hsa_circ_0061675 | 93 | 1 | 504 | 664 | 1167 | 0 | 0 |
| hsa_circ_0061675 | 93 | 1 | 225 | 785 | 1009 | 0 | 1.00E-88 |
| hsa_circ_0061675 | 94 | 1 | 383 | 785 | 1167 | 0 | 3.00E-166 |
| hsa_circ_0061683 | 85 | 5 | 1294 | 183 | 1475 | 0 | 0 |
| hsa_circ_0061683 | 85 | 1 | 1129 | 344 | 1475 | 0 | 0 |
| hsa_circ_0061683 | 85 | 1 | 1419 | 344 | 1765 | 0 | 0 |
| hsa_circ_0061686 | 89 | 1 | 302 | 304 | 3 | 0 | 1.00E-104 |
| hsa_circ_0001191 | 77 | 1 | 920 | 1 | 905 | 0.08 | 9.00E-128 |
| hsa_circ_0061695 | 99 | 1 | 283 | 1 | 283 | 0 | 4.00E-144 |
| hsa_circ_0061695 | 98 | 1 | 592 | 1 | 592 | 0 | 0 |
| hsa_circ_0061695 | 98 | 1 | 740 | 1 | 740 | 0 | 0 |
| hsa_circ_0061695 | 97 | 1 | 1315 | 1 | 1315 | 0 | 0 |
| hsa_circ_0061698 | 98 | 1 | 506 | 1 | 506 | 0 | 0 |
| hsa_circ_0061698 | 98 | 1 | 654 | 1 | 654 | 0 | 0 |
| hsa_circ_0061698 | 97 | 1 | 1229 | 1 | 1229 | 0 | 0 |
| hsa_circ_0061699 | 96 | 1 | 582 | 190 | 771 | 0 | 0 |
| hsa_circ_0061699 | 97 | 1 | 723 | 190 | 912 | 0 | 0 |
| hsa_circ_0061699 | 95 | 1 | 1155 | 190 | 1344 | 0 | 0 |
| hsa_circ_0061700 | 97 | 1 | 575 | 1 | 575 | 0 | 0 |
| hsa_circ_0061703 | 93 | 1 | 921 | 921 | 1 | 0 | 0 |
| hsa_circ_0061713 | 89 | 655 | 3307 | 3571 | 919 | 0.01 | 0 |
| hsa_circ_0061717 | 89 | 1 | 694 | 685 | 1 | 0.01 | 0 |
| hsa_circ_0061717 | 89 | 1 | 806 | 800 | 1 | 0.01 | 0 |
| hsa_circ_0061719 | 91 | 5 | 1851 | 1844 | 1 | 0 | 0 |
| hsa_circ_0061720 | 95 | 1 | 403 | 662 | 260 | 0 | 3.00E-177 |

novel_circ_008459 novel_circ_008460 novel_circ_008461 novel_circ_008462 novel_circ_008463 novel_circ_008464 novel_circ_008465 novel_circ_008466 novel_circ_008467 novel_circ_008468 novel_circ_008469 novel_circ_008470 novel_circ_008471 novel_circ_008472 novel_circ_008473 novel_circ_008474 novel_circ_008475 novel_circ_008476 novel_circ_008477 novel_circ_008478 novel_circ_008479 novel_circ_008480 novel_circ_008481 novel_circ_008482 novel_circ_008483 novel_circ_008484 novel_circ_008485 novel_circ_008486 novel_circ_008487 novel_circ_008488 novel_circ_008489 novel_circ_008490 novel_circ_008491 novel_circ_008496 novel_circ_008497 novel_circ_008498 novel_circ_008499 novel_circ_008500 novel_circ_008503 novel_circ_008504 novel_circ_008506 novel_circ_008508 novel_circ_008509 novel_circ_008510 novel_circ_008511 novel_circ_008512 novel_circ_008513 novel_circ_008521 novel_circ_008522 novel_circ_008523 novel_circ_008524 novel_circ_008525 novel_circ_008529 novel_circ_008530

| hsa_circ_0061720 | 95 | 1 | 785 | 1044 | 260 | 0 | 0 |
| --- | --- | --- | --- | --- | --- | --- | --- |
| hsa_circ_0061719 | 93 | 1 | 1209 | 1621 | 413 | 0 | 0 |
| hsa_circ_0061719 | 92 | 5 | 1439 | 1844 | 413 | 0 | 0 |
| hsa_circ_0061719 | 91 | 1 | 1533 | 1942 | 413 | 0 | 0 |
| hsa_circ_0061724 | 95 | 1 | 659 | 659 | 1 | 0 | 0 |
| hsa_circ_0061738 | 89 | 5 | 779 | 772 | 1 | 0 | 0 |
| hsa_circ_0061738 | 89 | 1 | 873 | 870 | 1 | 0 | 0 |
| hsa_circ_0061738 | 91 | 1 | 1983 | 1980 | 1 | 0 | 0 |
| hsa_circ_0061738 | 88 | 1 | 519 | 644 | 126 | 0 | 3.00E-178 |
| hsa_circ_0061738 | 88 | 5 | 654 | 772 | 126 | 0 | 0 |
| hsa_circ_0061738 | 88 | 1 | 748 | 870 | 126 | 0 | 0 |
| hsa_circ_0061741 | 87 | 5 | 420 | 413 | 1 | 0.01 | 5.00E-130 |
| hsa_circ_0061741 | 88 | 1 | 514 | 511 | 1 | 0.01 | 9.00E-168 |
| hsa_circ_0061741 | 89 | 1 | 698 | 695 | 1 | 0 | 0 |
| hsa_circ_0061741 | 87 | 1 | 324 | 511 | 191 | 0.01 | 5.00E-99 |
| hsa_circ_0061742 | 92 | 1 | 464 | 596 | 133 | 0 | 0 |
| hsa_circ_0061742 | 93 | 1 | 1346 | 1478 | 133 | 0 | 0 |
| hsa_circ_0061742 | 94 | 1 | 1901 | 2033 | 133 | 0 | 0 |
| hsa_circ_0061742 | 94 | 4 | 2151 | 2280 | 133 | 0 | 0 |
| hsa_circ_0061744 | 94 | 1 | 827 | 827 | 1 | 0 | 0 |
| hsa_circ_0061744 | 93 | 1 | 926 | 926 | 1 | 0 | 0 |
| hsa_circ_0061743 | 93 | 1 | 1068 | 1252 | 185 | 0 | 0 |
| hsa_circ_0061742 | 94 | 1 | 1623 | 2033 | 411 | 0 | 0 |
| hsa_circ_0061742 | 94 | 4 | 1873 | 2280 | 411 | 0 | 0 |
| hsa_circ_0061745 | 94 | 1 | 641 | 641 | 1 | 0 | 0 |
| hsa_circ_0061745 | 93 | 1 | 882 | 882 | 1 | 0 | 0 |
| hsa_circ_0061742 | 93 | 1 | 1054 | 1650 | 597 | 0 | 0 |
| hsa_circ_0061742 | 95 | 4 | 1687 | 2280 | 597 | 0 | 0 |
| hsa_circ_0061742 | 94 | 1 | 947 | 2033 | 1087 | 0 | 0 |
| hsa_circ_0061742 | 93 | 4 | 635 | 1869 | 1238 | 0 | 0 |
| hsa_circ_0061742 | 95 | 4 | 1046 | 2280 | 1238 | 0 | 0 |
| hsa_circ_0061756 | 96 | 4 | 734 | 731 | 1 | 0 | 0 |
| hsa_circ_0061757 | 97 | 4 | 633 | 630 | 1 | 0 | 0 |
| hsa_circ_0006663 | 88 | 1 | 531 | 531 | 1 | 0 | 6.00E-175 |
| hsa_circ_0037144 | 89 | 4 | 255 | 252 | 1 | 0 | 1.00E-83 |
| hsa_circ_0037144 | 89 | 1 | 354 | 354 | 1 | 0 | 2.00E-123 |
| hsa_circ_0037144 | 91 | 1 | 449 | 449 | 1 | 0 | 8.00E-173 |
| hsa_circ_0037144 | 88 | 1 | 210 | 354 | 145 | 0 | 1.00E-63 |
| hsa_circ_0003617 | 87 | 1 | 376 | 376 | 1 | 0 | 1.00E-115 |
| hsa_circ_0037182 | 83 | 1 | 548 | 1054 | 1604 | 0.01 | 1.00E-136 |
| hsa_circ_0037239 | 93 | 310 | 428 | 497 | 615 | 0 | 9.00E-43 |
| hsa_circ_0002445 | 88 | 1 | 447 | 565 | 119 | 0 | 5.00E-150 |
| hsa_circ_0002445 | 88 | 1 | 311 | 565 | 255 | 0 | 6.00E-103 |
| hsa_circ_0007865 | 85 | 30 | 674 | 645 | 1 | 0.01 | 0 |
| hsa_circ_0037446 | 86 | 63 | 230 | 375 | 205 | 0.02 | 6.00E-44 |
| hsa_circ_0037446 | 85 | 3 | 877 | 1100 | 205 | 0.03 | 0 |
| hsa_circ_0037449 | 87 | 3 | 647 | 768 | 118 | 0.01 | 0 |
| hsa_circ_0004886 | 85 | 1 | 266 | 8751 | 8492 | 0.02 | 2.00E-71 |
| hsa_circ_0037551 | 88 | 1 | 691 | 1 | 685 | 0.01 | 0 |
| hsa_circ_0004659 | 84 | 61703 | 61861 | 164811 | 164653 | 0 | 1.00E-32 |
| hsa_circ_0004659 | 84 | 45240 | 45398 | 164811 | 164653 | 0 | 1.00E-32 |
| hsa_circ_0054436 | 74 | 17656 | 18044 | 26817 | 26435 | 0.06 | 7.00E-31 |
| hsa_circ_0071449 | 96 | 58787 | 59247 | 1 | 461 | 0 | 0 |
| hsa_circ_0071449 | 96 | 1264 | 1724 | 1 | 461 | 0 | 0 |

novel_circ_008531 novel_circ_008532 novel_circ_008533 novel_circ_008534 novel_circ_008535 novel_circ_008540 novel_circ_008541 novel_circ_008542 novel_circ_008543 novel_circ_008544 novel_circ_008545 novel_circ_008546 novel_circ_008547 novel_circ_008548 novel_circ_008549 novel_circ_008550 novel_circ_008551 novel_circ_008552 novel_circ_008553 novel_circ_008554 novel_circ_008555 novel_circ_008556 novel_circ_008557 novel_circ_008559 novel_circ_008560 novel_circ_008565 novel_circ_008566 novel_circ_008567 novel_circ_008568 novel_circ_008569 novel_circ_008573 novel_circ_008577 novel_circ_008578 novel_circ_008579 novel_circ_008581 novel_circ_008582 novel_circ_008583 novel_circ_008584 novel_circ_008585 novel_circ_008586 novel_circ_008587 novel_circ_008588 novel_circ_008589 novel_circ_008590 novel_circ_008591 novel_circ_008592 novel_circ_008602 novel_circ_008603 novel_circ_008604 novel_circ_008605 novel_circ_008607 novel_circ_008608 novel_circ_008609 novel_circ_008610

| hsa_circ_0071449 | 96 | 1 | 461 | 1 | 461 | 0 | 0 |
| --- | --- | --- | --- | --- | --- | --- | --- |
| hsa_circ_0071449 | 96 | 1 | 461 | 1 | 461 | 0 | 0 |
| hsa_circ_0071449 | 96 | 1 | 457 | 5 | 461 | 0 | 0 |
| hsa_circ_0071449 | 96 | 1 | 679 | 462 | 1140 | 0 | 0 |
| hsa_circ_0071449 | 95 | 1 | 802 | 462 | 1263 | 0 | 0 |
| hsa_circ_0071453 | 89 | 11220 | 11593 | 697 | 326 | 0.03 | 2.00E-125 |
| hsa_circ_0071453 | 89 | 11220 | 11593 | 697 | 326 | 0.03 | 4.00E-125 |
| hsa_circ_0071458 | 88 | 1 | 1136 | 1 | 1136 | 0 | 0 |
| hsa_circ_0071458 | 88 | 2 | 440 | 289 | 727 | 0 | 6.00E-149 |
| hsa_circ_0046703 | 90 | 1 | 874 | 877 | 1 | 0 | 0 |
| hsa_circ_0046703 | 90 | 415 | 1288 | 877 | 1 | 0 | 0 |
| hsa_circ_0046719 | 93 | 1 | 704 | 1 | 704 | 0 | 0 |
| hsa_circ_0046719 | 94 | 1 | 1204 | 1 | 1204 | 0 | 0 |
| hsa_circ_0046719 | 94 | 1 | 1620 | 1 | 1620 | 0 | 0 |
| hsa_circ_0046727 | 94 | 1 | 710 | 222 | 931 | 0 | 0 |
| hsa_circ_0046731 | 94 | 1 | 445 | 146 | 590 | 0 | 0 |
| hsa_circ_0046719 | 93 | 1 | 2114 | 1966 | 4079 | 0 | 0 |
| hsa_circ_0046731 | 94 | 6 | 275 | 321 | 590 | 0 | 3.00E-115 |
| hsa_circ_0046731 | 94 | 6 | 1028 | 321 | 1343 | 0 | 0 |
| hsa_circ_0046735 | 93 | 1 | 376 | 378 | 753 | 0 | 5.00E-154 |
| hsa_circ_0046735 | 92 | 1 | 1292 | 378 | 1669 | 0 | 0 |
| hsa_circ_0046744 | 96 | 3 | 181 | 935 | 1113 | 0 | 2.00E-76 |
| hsa_circ_0046744 | 92 | 3 | 627 | 935 | 1559 | 0 | 0 |
| hsa_circ_0007238 | 90 | 1 | 597 | 599 | 3 | 0 | 0 |
| hsa_circ_0030237 | 87 | 4184 | 4291 | 11107 | 11214 | 0 | 3.00E-25 |
| hsa_circ_0046754 | 98 | 1 | 178 | 178 | 1 | 0 | 3.00E-82 |
| hsa_circ_0046754 | 98 | 216 | 393 | 178 | 1 | 0 | 5.00E-82 |
| hsa_circ_0046754 | 98 | 216 | 393 | 178 | 1 | 0 | 3.00E-82 |
| hsa_circ_0018265 | 85 | 5415 | 6275 | 187 | 1082 | 0.04 | 0 |
| hsa_circ_0057231 | 88 | 16279 | 16894 | 618 | 1 | 0.01 | 0 |
| hsa_circ_0036055 | 92 | 1596 | 1908 | 25658 | 25969 | 0 | 7.00E-119 |
| hsa_circ_0005102 | 81 | 26492 | 27159 | 2674 | 3327 | 0.07 | 1.00E-136 |
| hsa_circ_0024509 | 83 | 8454 | 8641 | 452 | 258 | 0.04 | 1.00E-39 |
| hsa_circ_0024509 | 83 | 8452 | 8639 | 452 | 258 | 0.04 | 1.00E-39 |
| hsa_circ_0046755 | 91 | 4 | 132 | 129 | 1 | 0 | 6.00E-42 |
| hsa_circ_0046755 | 91 | 1 | 307 | 2368 | 2062 | 0 | 5.00E-114 |
| hsa_circ_0046755 | 88 | 34 | 391 | 2951 | 2594 | 0.02 | 6.00E-119 |
| hsa_circ_0004611 | 93 | 303 | 568 | 266 | 1 | 0 | 1.00E-107 |
| hsa_circ_0004611 | 92 | 303 | 433 | 266 | 136 | 0 | 2.00E-44 |
| hsa_circ_0005152 | 94 | 1 | 358 | 358 | 1 | 0 | 2.00E-150 |
| hsa_circ_0005152 | 94 | 110 | 467 | 358 | 1 | 0 | 2.00E-150 |
| hsa_circ_0005152 | 94 | 110 | 467 | 358 | 1 | 0 | 2.00E-150 |
| hsa_circ_0008638 | 96 | 1 | 427 | 427 | 1 | 0 | 0 |
| hsa_circ_0008638 | 94 | 3 | 532 | 530 | 1 | 0 | 0 |
| hsa_circ_0008638 | 94 | 3 | 532 | 530 | 1 | 0 | 0 |
| hsa_circ_0004344 | 84 | 391 | 1005 | 618 | 1 | 0.05 | 1.00E-158 |
| hsa_circ_0037806 | 79 | 1 | 988 | 150 | 1119 | 0.07 | 7.00E-172 |
| hsa_circ_0037806 | 80 | 1 | 1222 | 150 | 1353 | 0.05 | 0 |
| hsa_circ_0037806 | 80 | 1 | 1222 | 150 | 1353 | 0.05 | 0 |
| hsa_circ_0037806 | 87 | 1 | 359 | 995 | 1353 | 0 | 5.00E-111 |
| hsa_circ_0037824 | 92 | 1 | 862 | 130 | 991 | 0 | 0 |
| hsa_circ_0037827 | 92 | 1 | 728 | 1 | 728 | 0 | 0 |
| hsa_circ_0037827 | 92 | 1 | 960 | 1 | 960 | 0 | 0 |
| hsa_circ_0037835 | 92 | 1 | 343 | 1 | 343 | 0 | 5.00E-134 |

novel_circ_008611 novel_circ_008612 novel_circ_008613 novel_circ_008614 novel_circ_008615 novel_circ_008616 novel_circ_008617 novel_circ_008618 novel_circ_008619 novel_circ_008620 novel_circ_008621 novel_circ_008622 novel_circ_008623 novel_circ_008624 novel_circ_008625 novel_circ_008626 novel_circ_008627 novel_circ_008628 novel_circ_008629 novel_circ_008630 novel_circ_008631 novel_circ_008632 novel_circ_008633 novel_circ_008634 novel_circ_008635 novel_circ_008636 novel_circ_008637 novel_circ_008638 novel_circ_008639 novel_circ_008641 novel_circ_008642 novel_circ_008643 novel_circ_008644 novel_circ_008645 novel_circ_008647 novel_circ_008648 novel_circ_008649 novel_circ_008650 novel_circ_008651 novel_circ_008652 novel_circ_008657 novel_circ_008658 novel_circ_008659 novel_circ_008660 novel_circ_008661 novel_circ_008662 novel_circ_008663 novel_circ_008664 novel_circ_008665 novel_circ_008666 novel_circ_008667 novel_circ_008668 novel_circ_008669 novel_circ_008670

| hsa_circ_0037835 | 92 | 1 | 575 | 1 | 575 | 0 | 0 |
| --- | --- | --- | --- | --- | --- | --- | --- |
| hsa_circ_0007846 | 89 | 1 | 692 | 1 | 689 | 0 | 0 |
| hsa_circ_0007846 | 89 | 1 | 696 | 1 | 693 | 0 | 0 |
| hsa_circ_0037868 | 87 | 1 | 784 | 1046 | 254 | 0.01 | 0 |
| hsa_circ_0037868 | 87 | 1 | 997 | 1247 | 254 | 0.02 | 0 |
| hsa_circ_0037868 | 91 | 1 | 1649 | 1899 | 254 | 0.01 | 0 |
| hsa_circ_0037870 | 94 | 1 | 550 | 538 | 1 | 0.02 | 0 |
| hsa_circ_0037870 | 94 | 1 | 865 | 853 | 1 | 0.01 | 0 |
| hsa_circ_0009128 | 99 | 1 | 224 | 224 | 1 | 0 | 2.00E-111 |
| hsa_circ_0009128 | 98 | 1 | 322 | 322 | 1 | 0 | 2.00E-157 |
| hsa_circ_0009128 | 96 | 1 | 539 | 539 | 1 | 0 | 0 |
| hsa_circ_0006559 | 95 | 1 | 445 | 445 | 1 | 0 | 0 |
| hsa_circ_0037875 | 94 | 1 | 315 | 315 | 1 | 0 | 8.00E-132 |
| hsa_circ_0037877 | 93 | 1 | 407 | 407 | 1 | 0 | 7.00E-168 |
| hsa_circ_0037877 | 93 | 1 | 906 | 906 | 1 | 0 | 0 |
| hsa_circ_0037877 | 93 | 1 | 1366 | 1366 | 1 | 0 | 0 |
| hsa_circ_0037877 | 93 | 1 | 741 | 906 | 166 | 0 | 0 |
| hsa_circ_0037877 | 93 | 1 | 1201 | 1366 | 166 | 0 | 0 |
| hsa_circ_0037879 | 91 | 358 | 2211 | 1854 | 1 | 0 | 0 |
| hsa_circ_0037877 | 94 | 1 | 709 | 1007 | 299 | 0 | 0 |
| hsa_circ_0037879 | 91 | 358 | 2211 | 1854 | 1 | 0 | 0 |
| hsa_circ_0037879 | 91 | 500 | 2353 | 1854 | 1 | 0 | 0 |
| hsa_circ_0037879 | 91 | 600 | 2453 | 1854 | 1 | 0 | 0 |
| hsa_circ_0037879 | 92 | 1 | 259 | 499 | 241 | 0 | 8.00E-101 |
| hsa_circ_0037879 | 94 | 1 | 360 | 600 | 241 | 0 | 9.00E-157 |
| hsa_circ_0037879 | 94 | 1 | 719 | 959 | 241 | 0 | 0 |
| hsa_circ_0037883 | 90 | 1 | 1414 | 1414 | 1 | 0 | 0 |
| hsa_circ_0037883 | 89 | 8 | 741 | 896 | 163 | 0 | 0 |
| hsa_circ_0037883 | 90 | 1 | 1252 | 1414 | 163 | 0 | 0 |
| hsa_circ_0005394 | 89 | 1 | 776 | 795 | 20 | 0 | 0 |
| hsa_circ_0005394 | 89 | 4 | 918 | 934 | 20 | 0 | 0 |
| hsa_circ_0005394 | 89 | 104 | 1018 | 934 | 20 | 0 | 0 |
| hsa_circ_0005420 | 92 | 4 | 340 | 337 | 1 | 0 | 5.00E-134 |
| hsa_circ_0005420 | 92 | 104 | 440 | 337 | 1 | 0 | 7.00E-134 |
| hsa_circ_0037895 | 95 | 3677 | 3938 | 262 | 1 | 0 | 3.00E-111 |
| hsa_circ_0037895 | 94 | 1 | 514 | 514 | 1 | 0 | 0 |
| hsa_circ_0037895 | 95 | 1 | 904 | 904 | 1 | 0 | 0 |
| hsa_circ_0037923 | 88 | 32212 | 32559 | 29 | 376 | 0 | 9.00E-110 |
| hsa_circ_0037923 | 88 | 5737 | 6084 | 29 | 376 | 0 | 2.00E-110 |
| hsa_circ_0037919 | 88 | 5737 | 6088 | 655 | 1006 | 0 | 2.00E-112 |
| hsa_circ_0037936 | 84 | 1 | 275 | 1 | 282 | 0.02 | 1.00E-69 |
| hsa_circ_0037941 | 95 | 1 | 539 | 1 | 539 | 0 | 0 |
| hsa_circ_0037940 | 94 | 1 | 677 | 218 | 894 | 0 | 0 |
| hsa_circ_0037942 | 96 | 1 | 473 | 1 | 473 | 0 | 0 |
| hsa_circ_0037942 | 95 | 1 | 341 | 133 | 473 | 0 | 6.00E-153 |
| hsa_circ_0037940 | 92 | 1 | 854 | 416 | 1275 | 0.01 | 0 |
| hsa_circ_0037951 | 89 | 3 | 602 | 600 | 1 | 0 | 0 |
| hsa_circ_0037953 | 90 | 3 | 408 | 406 | 1 | 0 | 6.00E-149 |
| hsa_circ_0037958 | 94 | 3 | 218 | 216 | 1 | 0 | 2.00E-90 |
| hsa_circ_0037958 | 94 | 5797 | 6012 | 216 | 1 | 0 | 8.00E-89 |
| hsa_circ_0037958 | 94 | 10 | 562 | 553 | 1 | 0 | 0 |
| hsa_circ_0037958 | 94 | 1 | 640 | 640 | 1 | 0 | 0 |
| hsa_circ_0037958 | 94 | 1 | 778 | 778 | 1 | 0 | 0 |
| hsa_circ_0037958 | 94 | 1 | 616 | 778 | 163 | 0 | 0 |

novel_circ_008671 novel_circ_008672 novel_circ_008673 novel_circ_008674 novel_circ_008675 novel_circ_008676 novel_circ_008677 novel_circ_008678 novel_circ_008679 novel_circ_008680 novel_circ_008681 novel_circ_008683 novel_circ_008685 novel_circ_008686 novel_circ_008687 novel_circ_008690 novel_circ_008691 novel_circ_008692 novel_circ_008693 novel_circ_008694 novel_circ_008695 novel_circ_008696 novel_circ_008697 novel_circ_008698 novel_circ_008699 novel_circ_008700 novel_circ_008702 novel_circ_008703 novel_circ_008704 novel_circ_008705 novel_circ_008706 novel_circ_008707 novel_circ_008708 novel_circ_008709 novel_circ_008710 novel_circ_008711 novel_circ_008712 novel_circ_008713 novel_circ_008714 novel_circ_008715 novel_circ_008716 novel_circ_008717 novel_circ_008718 novel_circ_008719 novel_circ_008720 novel_circ_008721 novel_circ_008722 novel_circ_008723 novel_circ_008724 novel_circ_008725 novel_circ_008726 novel_circ_008727 novel_circ_008728 novel_circ_008729

| hsa_circ_0037961 | 94 | 1 | 303 | 560 | 258 | 0 | 7.00E-127 |
| --- | --- | --- | --- | --- | --- | --- | --- |
| hsa_circ_0037969 | 93 | 1 | 260 | 260 | 1 | 0 | 5.00E-103 |
| hsa_circ_0037971 | 92 | 1 | 535 | 899 | 365 | 0 | 0 |
| hsa_circ_0038024 | 90 | 1 | 192 | 331 | 140 | 0 | 1.00E-63 |
| hsa_circ_0038024 | 89 | 1 | 331 | 331 | 1 | 0 | 1.00E-115 |
| hsa_circ_0038022 | 91 | 1 | 887 | 887 | 1 | 0 | 0 |
| hsa_circ_0038022 | 92 | 1 | 556 | 556 | 1 | 0 | 0 |
| hsa_circ_0038017 | 89 | 263 | 963 | 2419 | 1723 | 0.01 | 0 |
| hsa_circ_0003427 | 87 | 103 | 281 | 135618 | 135796 | 0 | 4.00E-51 |
| hsa_circ_0040118 | 88 | 1 | 343 | 429 | 90 | 0.01 | 2.00E-112 |
| hsa_circ_0040117 | 90 | 1 | 625 | 817 | 196 | 0 | 0 |
| hsa_circ_0040118 | 88 | 1 | 343 | 429 | 90 | 0.01 | 2.00E-112 |
| hsa_circ_0040117 | 90 | 1 | 561 | 817 | 260 | 0.01 | 0 |
| hsa_circ_0040114 | 89 | 1 | 847 | 1391 | 548 | 0 | 0 |
| hsa_circ_0040114 | 90 | 75 | 1068 | 1538 | 548 | 0 | 0 |
| hsa_circ_0038006 | 91 | 1 | 627 | 1 | 627 | 0 | 0 |
| hsa_circ_0038044 | 90 | 1 | 1481 | 2417 | 937 | 0 | 0 |
| hsa_circ_0038044 | 89 | 1 | 1204 | 2417 | 1214 | 0 | 0 |
| hsa_circ_0038044 | 90 | 1 | 986 | 2420 | 1435 | 0 | 0 |
| hsa_circ_0038048 | 88 | 1 | 369 | 450 | 82 | 0 | 4.00E-120 |
| hsa_circ_0038044 | 88 | 1 | 372 | 2420 | 2049 | 0 | 9.00E-122 |
| hsa_circ_0038049 | 88 | 174 | 917 | 746 | 3 | 0 | 0 |
| hsa_circ_0003427 | 95 | 1 | 690 | 740229 | 739543 | 0 | 0 |
| hsa_circ_0003427 | 95 | 242 | 934 | 740232 | 739543 | 0 | 0 |
| hsa_circ_0038080 | 90 | 1 | 566 | 1 | 566 | 0 | 0 |
| hsa_circ_0038080 | 89 | 1 | 761 | 1 | 761 | 0 | 0 |
| hsa_circ_0038088 | 87 | 1 | 458 | 1 | 458 | 0 | 9.00E-143 |
| hsa_circ_0038093 | 86 | 4 | 190 | 4 | 190 | 0 | 7.00E-51 |
| hsa_circ_0038093 | 86 | 4 | 320 | 4 | 320 | 0.01 | 1.00E-94 |
| hsa_circ_0038096 | 88 | 1 | 194 | 1 | 194 | 0.01 | 8.00E-60 |
| hsa_circ_0038112 | 90 | 1 | 294 | 1 | 295 | 0 | 2.00E-103 |
| hsa_circ_0038169 | 90 | 1 | 993 | 102 | 1094 | 0 | 0 |
| hsa_circ_0038212 | 92 | 1 | 979 | 1907 | 929 | 0 | 0 |
| hsa_circ_0038213 | 93 | 1 | 242 | 242 | 1 | 0 | 2.00E-96 |
| hsa_circ_0038212 | 90 | 1 | 384 | 801 | 418 | 0 | 9.00E-137 |
| hsa_circ_0038199 | 88 | 1 | 511 | 511 | 1 | 0 | 3.00E-172 |
| hsa_circ_0038191 | 94 | 2 | 196 | 195 | 1 | 0 | 3.00E-78 |
| hsa_circ_0002813 | 84 | 38320 | 38460 | 9264 | 9124 | 0 | 1.00E-29 |
| hsa_circ_0038239 | 94 | 1 | 606 | 606 | 1 | 0 | 0 |
| hsa_circ_0038243 | 95 | 1 | 319 | 319 | 1 | 0 | 1.00E-140 |
| hsa_circ_0038240 | 93 | 1 | 2564 | 2744 | 181 | 0 | 0 |
| hsa_circ_0038243 | 94 | 1 | 561 | 880 | 320 | 0 | 0 |
| hsa_circ_0038243 | 93 | 1 | 699 | 1018 | 320 | 0 | 0 |
| hsa_circ_0038240 | 93 | 1 | 2245 | 2744 | 500 | 0 | 0 |
| hsa_circ_0038252 | 94 | 8 | 811 | 804 | 1 | 0 | 0 |
| hsa_circ_0038254 | 92 | 1 | 1076 | 1076 | 1 | 0 | 0 |
| hsa_circ_0038256 | 92 | 1 | 507 | 735 | 229 | 0 | 0 |
| hsa_circ_0038252 | 93 | 4 | 1868 | 2904 | 1040 | 0 | 0 |
| hsa_circ_0038268 | 93 | 1 | 2434 | 2434 | 1 | 0 | 0 |
| hsa_circ_0038268 | 94 | 1 | 1385 | 1838 | 454 | 0 | 0 |
| hsa_circ_0038268 | 94 | 1 | 1981 | 2434 | 454 | 0 | 0 |
| hsa_circ_0038268 | 94 | 1 | 1194 | 1838 | 645 | 0 | 0 |
| hsa_circ_0038268 | 94 | 1 | 1790 | 2434 | 645 | 0 | 0 |
| hsa_circ_0038272 | 95 | 1 | 479 | 479 | 1 | 0 | 0 |

novel_circ_008730 novel_circ_008731 novel_circ_008732 novel_circ_008733 novel_circ_008734 novel_circ_008735 novel_circ_008736 novel_circ_008737 novel_circ_008738 novel_circ_008739 novel_circ_008740 novel_circ_008741 novel_circ_008742 novel_circ_008743 novel_circ_008744 novel_circ_008745 novel_circ_008746 novel_circ_008747 novel_circ_008748 novel_circ_008749 novel_circ_008750 novel_circ_008751 novel_circ_008752 novel_circ_008753 novel_circ_008754 novel_circ_008755 novel_circ_008756 novel_circ_008757 novel_circ_008758 novel_circ_008759 novel_circ_008760 novel_circ_008761 novel_circ_008762 novel_circ_008763 novel_circ_008764 novel_circ_008765 novel_circ_008766 novel_circ_008767 novel_circ_008768 novel_circ_008769 novel_circ_008773 novel_circ_008774 novel_circ_008775 novel_circ_008776 novel_circ_008777 novel_circ_008778 novel_circ_008779 novel_circ_008780 novel_circ_008781 novel_circ_008782 novel_circ_008783 novel_circ_008784 novel_circ_008785 novel_circ_008786

| hsa_circ_0038272 | 95 | 1 | 726 | 726 | 1 | 0 | 0 |
| --- | --- | --- | --- | --- | --- | --- | --- |
| hsa_circ_0038272 | 94 | 1 | 949 | 949 | 1 | 0 | 0 |
| hsa_circ_0038272 | 94 | 1 | 1322 | 1322 | 1 | 0 | 0 |
| hsa_circ_0038274 | 96 | 1 | 477 | 477 | 1 | 0 | 0 |
| hsa_circ_0038274 | 94 | 1 | 700 | 700 | 1 | 0 | 0 |
| hsa_circ_0038274 | 94 | 1 | 1073 | 1073 | 1 | 0 | 0 |
| hsa_circ_0038276 | 93 | 1 | 596 | 596 | 1 | 0 | 0 |
| hsa_circ_0038279 | 94 | 1 | 373 | 373 | 1 | 0 | 2.00E-162 |
| hsa_circ_0038280 | 89 | 1 | 686 | 928 | 219 | 0.03 | 0 |
| hsa_circ_0038279 | 93 | 1 | 4774 | 5174 | 374 | 0.01 | 0 |
| hsa_circ_0038280 | 92 | 1 | 1511 | 1809 | 275 | 0.02 | 0 |
| hsa_circ_0038279 | 92 | 3 | 1648 | 3692 | 2044 | 0 | 0 |
| hsa_circ_0038279 | 92 | 1 | 2432 | 4478 | 2044 | 0 | 0 |
| hsa_circ_0038288 | 92 | 3 | 1045 | 1046 | 1 | 0 | 0 |
| hsa_circ_0038279 | 94 | 1 | 2525 | 5174 | 2647 | 0 | 0 |
| hsa_circ_0038288 | 92 | 3 | 879 | 1046 | 170 | 0 | 0 |
| hsa_circ_0038279 | 95 | 1 | 1670 | 5174 | 3505 | 0 | 0 |
| hsa_circ_0038268 | 95 | 1 | 1884 | 7449 | 5566 | 0 | 0 |
| hsa_circ_0038291 | 94 | 1 | 412 | 1140 | 729 | 0 | 6.00E-179 |
| hsa_circ_0038279 | 95 | 1 | 1480 | 5174 | 3695 | 0 | 0 |
| hsa_circ_0038298 | 94 | 1 | 624 | 624 | 1 | 0 | 0 |
| hsa_circ_0038298 | 96 | 1 | 1282 | 1413 | 132 | 0 | 0 |
| hsa_circ_0038268 | 96 | 1 | 1798 | 7965 | 6168 | 0 | 0 |
| hsa_circ_0038298 | 97 | 1 | 798 | 1199 | 402 | 0 | 0 |
| hsa_circ_0038298 | 97 | 1 | 1012 | 1413 | 402 | 0 | 0 |
| hsa_circ_0038301 | 97 | 1 | 692 | 696 | 5 | 0 | 0 |
| hsa_circ_0038268 | 97 | 1 | 1422 | 7965 | 6544 | 0 | 0 |
| hsa_circ_0038303 | 97 | 1 | 1201 | 1201 | 1 | 0 | 0 |
| hsa_circ_0038305 | 97 | 1 | 297 | 297 | 1 | 0 | 3.00E-140 |
| hsa_circ_0038305 | 98 | 1 | 511 | 511 | 1 | 0 | 0 |
| hsa_circ_0038303 | 97 | 4 | 707 | 878 | 175 | 0 | 0 |
| hsa_circ_0038303 | 97 | 1 | 1027 | 1201 | 175 | 0 | 0 |
| hsa_circ_0038303 | 98 | 1 | 730 | 1201 | 472 | 0 | 0 |
| hsa_circ_0038303 | 98 | 59 | 788 | 1201 | 472 | 0 | 0 |
| hsa_circ_0038303 | 97 | 1 | 516 | 1201 | 686 | 0 | 0 |
| hsa_circ_0038303 | 98 | 1 | 320 | 1201 | 882 | 0 | 1.00E-154 |
| hsa_circ_0038319 | 91 | 150 | 769 | 1 | 620 | 0 | 0 |
| hsa_circ_0038316 | 87 | 118 | 403 | 3 | 288 | 0.01 | 6.00E-89 |
| hsa_circ_0038316 | 87 | 297 | 582 | 3 | 288 | 0.01 | 9.00E-89 |
| hsa_circ_0038316 | 89 | 297 | 503 | 3 | 209 | 0.01 | 3.00E-68 |
| hsa_circ_0038332 | 91 | 9 | 291 | 9 | 291 | 0 | 9.00E-106 |
| hsa_circ_0038336 | 94 | 1 | 440 | 1 | 440 | 0 | 0 |
| hsa_circ_0038336 | 94 | 1 | 1437 | 1 | 1437 | 0 | 0 |
| hsa_circ_0038351 | 92 | 1 | 477 | 1 | 477 | 0 | 0 |
| hsa_circ_0038351 | 94 | 1 | 997 | 202 | 1198 | 0 | 0 |
| hsa_circ_0038351 | 94 | 1 | 1137 | 202 | 1338 | 0 | 0 |
| hsa_circ_0038358 | 95 | 1 | 586 | 253 | 838 | 0 | 0 |
| hsa_circ_0038351 | 95 | 1 | 726 | 613 | 1338 | 0 | 0 |
| hsa_circ_0038358 | 95 | 1 | 523 | 316 | 838 | 0 | 0 |
| hsa_circ_0038365 | 95 | 1 | 356 | 1 | 356 | 0 | 1.00E-159 |
| hsa_circ_0038365 | 95 | 1 | 496 | 1 | 496 | 0 | 0 |
| hsa_circ_0038365 | 94 | 1 | 662 | 1 | 662 | 0 | 0 |
| hsa_circ_0025407 | 89 | 1 | 472 | 1438 | 1909 | 0 | 3.00E-162 |
| hsa_circ_0025374 | 88 | 2 | 3503 | 2 | 3462 | 0.03 | 0 |

novel_circ_008787 novel_circ_008788 novel_circ_008789 novel_circ_008790 novel_circ_008792 novel_circ_008793 novel_circ_008798 novel_circ_008799 novel_circ_008800 novel_circ_008801 novel_circ_008802 novel_circ_008803 novel_circ_008804 novel_circ_008805 novel_circ_008806 novel_circ_008807 novel_circ_008808 novel_circ_008809 novel_circ_008810 novel_circ_008811 novel_circ_008812 novel_circ_008813 novel_circ_008814 novel_circ_008815 novel_circ_008816 novel_circ_008817 novel_circ_008818 novel_circ_008819 novel_circ_008820 novel_circ_008821 novel_circ_008822 novel_circ_008825 novel_circ_008826 novel_circ_008827 novel_circ_008828 novel_circ_008829 novel_circ_008830 novel_circ_008831 novel_circ_008832 novel_circ_008833 novel_circ_008834 novel_circ_008835 novel_circ_008836 novel_circ_008837 novel_circ_008838 novel_circ_008841 novel_circ_008842 novel_circ_008843 novel_circ_008845 novel_circ_008846 novel_circ_008856 novel_circ_008857 novel_circ_008858 novel_circ_008860

| hsa_circ_0025374 | 93 | 2 | 900 | 2 | 899 | 0 | 0 |
| --- | --- | --- | --- | --- | --- | --- | --- |
| hsa_circ_0025374 | 92 | 2 | 754 | 2 | 753 | 0 | 0 |
| hsa_circ_0025371 | 88 | 136 | 1433 | 282 | 1583 | 0.02 | 0 |
| hsa_circ_0025374 | 91 | 2 | 463 | 2 | 462 | 0 | 8.00E-178 |
| hsa_circ_0025355 | 85 | 2 | 591 | 3406 | 2817 | 0 | 5.00E-171 |
| hsa_circ_0025355 | 86 | 2 | 404 | 3406 | 3004 | 0 | 6.00E-119 |
| hsa_circ_0090041 | 94 | 1 | 239 | 239 | 1 | 0 | 7.00E-98 |
| hsa_circ_0090042 | 96 | 1 | 508 | 598 | 91 | 0 | 0 |
| hsa_circ_0090041 | 96 | 1 | 1110 | 1598 | 489 | 0 | 0 |
| hsa_circ_0090041 | 96 | 1 | 1227 | 1715 | 489 | 0 | 0 |
| hsa_circ_0090042 | 96 | 1 | 382 | 598 | 217 | 0 | 1.00E-175 |
| hsa_circ_0090041 | 96 | 1 | 984 | 1598 | 615 | 0 | 0 |
| hsa_circ_0090041 | 96 | 1 | 1101 | 1715 | 615 | 0 | 0 |
| hsa_circ_0090041 | 97 | 1 | 520 | 1516 | 997 | 0 | 0 |
| hsa_circ_0090041 | 97 | 1 | 719 | 1715 | 997 | 0 | 0 |
| hsa_circ_0090041 | 97 | 7243 | 8018 | 1772 | 997 | 0 | 0 |
| hsa_circ_0090041 | 97 | 1 | 648 | 1715 | 1068 | 0 | 0 |
| hsa_circ_0090041 | 97 | 1 | 505 | 1715 | 1211 | 0 | 0 |
| hsa_circ_0090045 | 97 | 1 | 268 | 306 | 39 | 0 | 8.00E-126 |
| hsa_circ_0090041 | 97 | 1 | 467 | 1715 | 1249 | 0 | 0 |
| hsa_circ_0090041 | 97 | 7243 | 7766 | 1772 | 1249 | 0 | 0 |
| hsa_circ_0090041 | 97 | 6228 | 6644 | 1772 | 1356 | 0 | 0 |
| hsa_circ_0074829 | 92 | 76616 | 77342 | 734 | 8 | 0 | 0 |
| hsa_circ_0074829 | 92 | 77692 | 78418 | 734 | 8 | 0 | 0 |
| hsa_circ_0090046 | 96 | 6731 | 7185 | 1 | 455 | 0 | 0 |
| hsa_circ_0090046 | 96 | 16985 | 17439 | 1 | 455 | 0 | 0 |
| hsa_circ_0090046 | 96 | 1 | 367 | 1 | 367 | 0 | 1.00E-170 |
| hsa_circ_0090046 | 96 | 1 | 455 | 1 | 455 | 0 | 0 |
| hsa_circ_0090046 | 96 | 1 | 455 | 1 | 455 | 0 | 0 |
| hsa_circ_0090046 | 95 | 1 | 291 | 165 | 455 | 0 | 2.00E-128 |
| hsa_circ_0065103 | 83 | 589 | 689 | 7355 | 7453 | 0.02 | 3.00E-15 |
| hsa_circ_0090047 | 96 | 394 | 739 | 1 | 346 | 0 | 5.00E-157 |
| hsa_circ_0090047 | 96 | 394 | 1044 | 1 | 651 | 0 | 0 |
| hsa_circ_0090047 | 96 | 148 | 493 | 1 | 346 | 0 | 3.00E-157 |
| hsa_circ_0090047 | 96 | 148 | 798 | 1 | 651 | 0 | 0 |
| hsa_circ_0090047 | 96 | 148 | 798 | 1 | 651 | 0 | 0 |
| hsa_circ_0090047 | 96 | 148 | 798 | 1 | 651 | 0 | 0 |
| hsa_circ_0090047 | 96 | 1 | 346 | 1 | 346 | 0 | 2.00E-157 |
| hsa_circ_0090047 | 96 | 1 | 651 | 1 | 651 | 0 | 0 |
| hsa_circ_0090047 | 96 | 1 | 651 | 1 | 651 | 0 | 0 |
| hsa_circ_0090047 | 96 | 1 | 651 | 1 | 651 | 0 | 0 |
| hsa_circ_0090047 | 96 | 1 | 651 | 1 | 651 | 0 | 0 |
| hsa_circ_0090047 | 96 | 1 | 517 | 135 | 651 | 0 | 0 |
| hsa_circ_0090047 | 96 | 1 | 517 | 135 | 651 | 0 | 0 |
| hsa_circ_0090047 | 97 | 1 | 305 | 347 | 651 | 0 | 6.00E-143 |
| hsa_circ_0090048 | 93 | 1 | 1043 | 1 | 1037 | 0.01 | 0 |
| hsa_circ_0090053 | 94 | 1 | 472 | 1 | 472 | 0 | 0 |
| hsa_circ_0090053 | 94 | 1 | 548 | 1 | 548 | 0 | 0 |
| hsa_circ_0008619 | 90 | 1 | 568 | 568 | 1 | 0 | 0 |
| hsa_circ_0090096 | 89 | 1 | 342 | 380 | 39 | 0 | 9.00E-117 |
| hsa_circ_0001684 | 89 | 1 | 373 | 1 | 383 | 0.04 | 4.00E-130 |
| hsa_circ_0079611 | 89 | 2809 | 3212 | 12980 | 13393 | 0.04 | 2.00E-141 |
| hsa_circ_0079611 | 89 | 10347 | 10750 | 12980 | 13393 | 0.04 | 8.00E-141 |
| hsa_circ_0079573 | 93 | 1 | 443 | 495 | 937 | 0 | 0 |

novel_circ_008861 novel_circ_008862 novel_circ_008863 novel_circ_008864 novel_circ_008865 novel_circ_008866 novel_circ_008867 novel_circ_008868 novel_circ_008869 novel_circ_008870 novel_circ_008871 novel_circ_008872 novel_circ_008873 novel_circ_008874 novel_circ_008875 novel_circ_008876 novel_circ_008877 novel_circ_008878 novel_circ_008879 novel_circ_008898 novel_circ_008899 novel_circ_008900 novel_circ_008901 novel_circ_008902 novel_circ_008909 novel_circ_008912 novel_circ_008915 novel_circ_008916 novel_circ_008917 novel_circ_008918 novel_circ_008919 novel_circ_008920 novel_circ_008921 novel_circ_008922 novel_circ_008923 novel_circ_008924 novel_circ_008925 novel_circ_008926 novel_circ_008927 novel_circ_008928 novel_circ_008929 novel_circ_008930 novel_circ_008931 novel_circ_008932 novel_circ_008933 novel_circ_008934 novel_circ_008935 novel_circ_008936 novel_circ_008937 novel_circ_008938 novel_circ_008939 novel_circ_008940 novel_circ_008941 novel_circ_008942

| hsa_circ_0079573 | 94 | 325 | 1259 | 3 | 937 | 0 | 0 |
| --- | --- | --- | --- | --- | --- | --- | --- |
| hsa_circ_0079573 | 94 | 1 | 761 | 177 | 937 | 0 | 0 |
| hsa_circ_0079573 | 94 | 3 | 937 | 3 | 937 | 0 | 0 |
| hsa_circ_0079573 | 94 | 325 | 1259 | 3 | 937 | 0 | 0 |
| hsa_circ_0079573 | 94 | 404 | 1338 | 3 | 937 | 0 | 0 |
| hsa_circ_0079573 | 94 | 1685 | 1827 | 352 | 494 | 0 | 9.00E-54 |
| hsa_circ_0079573 | 97 | 1 | 318 | 177 | 494 | 0 | 3.00E-150 |
| hsa_circ_0079573 | 96 | 3 | 494 | 3 | 494 | 0 | 0 |
| hsa_circ_0079570 | 95 | 1 | 713 | 104 | 816 | 0 | 0 |
| hsa_circ_0079570 | 95 | 1 | 816 | 1 | 816 | 0 | 0 |
| hsa_circ_0079570 | 95 | 80 | 895 | 1 | 816 | 0 | 0 |
| hsa_circ_0079570 | 96 | 1 | 570 | 104 | 673 | 0 | 0 |
| hsa_circ_0079570 | 96 | 1 | 673 | 1 | 673 | 0 | 0 |
| hsa_circ_0079570 | 94 | 1 | 395 | 104 | 498 | 0 | 3.00E-171 |
| hsa_circ_0079570 | 94 | 1 | 498 | 1 | 498 | 0 | 0 |
| hsa_circ_0079567 | 94 | 79 | 577 | 544 | 1042 | 0 | 0 |
| hsa_circ_0079570 | 95 | 1 | 322 | 1 | 322 | 0 | 5.00E-144 |
| hsa_circ_0079570 | 95 | 1 | 197 | 1 | 197 | 0 | 4.00E-83 |
| hsa_circ_0005251 | 94 | 4 | 654 | 651 | 1 | 0 | 0 |
| hsa_circ_0066552 | 91 | 1 | 336 | 498 | 163 | 0 | 2.00E-128 |
| hsa_circ_0066552 | 92 | 1 | 498 | 498 | 1 | 0 | 0 |
| hsa_circ_0066552 | 94 | 1 | 162 | 162 | 1 | 0 | 2.00E-63 |
| hsa_circ_0066944 | 92 | 3 | 1054 | 5073 | 4022 | 0 | 0 |
| hsa_circ_0066939 | 89 | 1 | 249 | 249 | 1 | 0.02 | 6.00E-82 |
| hsa_circ_0074783 | 75 | 4893 | 5197 | 18663 | 18370 | 0.1 | 2.00E-24 |
| hsa_circ_0007133 | 75 | 1789 | 2372 | 372413 | 373010 | 0.07 | 2.00E-58 |
| hsa_circ_0066930 | 94 | 1 | 296 | 1373 | 1671 | 0.01 | 3.00E-125 |
| hsa_circ_0066930 | 94 | 1 | 514 | 1155 | 1671 | 0.01 | 0 |
| hsa_circ_0066930 | 95 | 1 | 844 | 825 | 1671 | 0 | 0 |
| hsa_circ_0066930 | 95 | 1 | 1202 | 467 | 1671 | 0 | 0 |
| hsa_circ_0066930 | 95 | 1 | 1350 | 319 | 1671 | 0 | 0 |
| hsa_circ_0066930 | 96 | 1 | 1485 | 184 | 1671 | 0 | 0 |
| hsa_circ_0066930 | 95 | 1 | 718 | 825 | 1545 | 0 | 0 |
| hsa_circ_0066930 | 95 | 1 | 548 | 825 | 1372 | 0 | 0 |
| hsa_circ_0066930 | 96 | 1 | 1054 | 319 | 1372 | 0 | 0 |
| hsa_circ_0066930 | 96 | 2 | 444 | 671 | 1113 | 0 | 0 |
| hsa_circ_0066930 | 96 | 1 | 647 | 467 | 1113 | 0 | 0 |
| hsa_circ_0066930 | 96 | 1 | 731 | 383 | 1113 | 0 | 0 |
| hsa_circ_0066930 | 96 | 1 | 795 | 319 | 1113 | 0 | 0 |
| hsa_circ_0066930 | 96 | 1 | 930 | 184 | 1113 | 0 | 0 |
| hsa_circ_0066930 | 96 | 1 | 663 | 383 | 1045 | 0 | 0 |
| hsa_circ_0066930 | 96 | 1 | 727 | 319 | 1045 | 0 | 0 |
| hsa_circ_0066930 | 96 | 1 | 862 | 184 | 1045 | 0 | 0 |
| hsa_circ_0066930 | 96 | 1 | 506 | 319 | 824 | 0 | 0 |
| hsa_circ_0066930 | 97 | 1 | 641 | 184 | 824 | 0 | 0 |
| hsa_circ_0066930 | 97 | 1 | 442 | 383 | 824 | 0 | 0 |
| hsa_circ_0066930 | 96 | 1 | 506 | 319 | 824 | 0 | 0 |
| hsa_circ_0066930 | 97 | 1 | 641 | 184 | 824 | 0 | 0 |
| hsa_circ_0066930 | 96 | 1 | 272 | 319 | 590 | 0 | 2.00E-121 |
| hsa_circ_0066930 | 97 | 1 | 407 | 184 | 590 | 0 | 0 |
| hsa_circ_0066930 | 96 | 1 | 508 | 83 | 590 | 0 | 0 |
| hsa_circ_0066930 | 96 | 1 | 590 | 1 | 590 | 0 | 0 |
| hsa_circ_0066930 | 96 | 99 | 688 | 1 | 590 | 0 | 0 |
| hsa_circ_0066930 | 95 | 1 | 466 | 1 | 466 | 0 | 0 |

novel_circ_008943 novel_circ_008944 novel_circ_008945 novel_circ_008946 novel_circ_008947 novel_circ_008948 novel_circ_008949 novel_circ_008950 novel_circ_008951 novel_circ_008952 novel_circ_008953 novel_circ_008954 novel_circ_008955 novel_circ_008956 novel_circ_008957 novel_circ_008958 novel_circ_008959 novel_circ_008960 novel_circ_008961 novel_circ_008962 novel_circ_008963 novel_circ_008964 novel_circ_008965 novel_circ_008966 novel_circ_008967 novel_circ_008968 novel_circ_008969 novel_circ_008970 novel_circ_008971 novel_circ_008972 novel_circ_008973 novel_circ_008974 novel_circ_008975 novel_circ_008976 novel_circ_008977 novel_circ_008978 novel_circ_008979 novel_circ_008980 novel_circ_008981 novel_circ_008982 novel_circ_008983 novel_circ_008984 novel_circ_008985 novel_circ_008986 novel_circ_008987 novel_circ_008988 novel_circ_008989 novel_circ_008990 novel_circ_008991 novel_circ_008992 novel_circ_008993 novel_circ_008994 novel_circ_008995 novel_circ_008996

| hsa_circ_0066930 | 95 | 99 | 480 | 1 | 382 | 0 | 3.00E-172 |
| --- | --- | --- | --- | --- | --- | --- | --- |
| hsa_circ_0066930 | 96 | 99 | 416 | 1 | 318 | 0 | 2.00E-143 |
| hsa_circ_0066924 | 95 | 1 | 337 | 3807 | 3471 | 0 | 2.00E-147 |
| hsa_circ_0066924 | 93 | 1 | 266 | 3585 | 3320 | 0.01 | 2.00E-106 |
| hsa_circ_0066905 | 93 | 1 | 407 | 407 | 1 | 0 | 3.00E-166 |
| hsa_circ_0066905 | 93 | 1 | 402 | 402 | 1 | 0 | 2.00E-163 |
| hsa_circ_0029639 | 95 | 1 | 252 | 437 | 688 | 0 | 4.00E-110 |
| hsa_circ_0029638 | 95 | 1 | 531 | 1 | 531 | 0 | 0 |
| hsa_circ_0029633 | 94 | 1 | 1158 | 1 | 1161 | 0 | 0 |
| hsa_circ_0029638 | 95 | 1 | 416 | 1 | 416 | 0 | 0 |
| hsa_circ_0029636 | 95 | 1 | 957 | 1 | 960 | 0 | 0 |
| hsa_circ_0029633 | 94 | 1 | 1158 | 1 | 1161 | 0 | 0 |
| hsa_circ_0029623 | 94 | 1 | 1331 | 2177 | 3510 | 0 | 0 |
| hsa_circ_0029623 | 95 | 2808 | 4291 | 2024 | 3510 | 0 | 0 |
| hsa_circ_0029623 | 96 | 1 | 1866 | 1642 | 3510 | 0 | 0 |
| hsa_circ_0029623 | 95 | 1 | 2600 | 905 | 3510 | 0 | 0 |
| hsa_circ_0029623 | 95 | 2 | 3457 | 49 | 3510 | 0 | 0 |
| hsa_circ_0029633 | 94 | 1 | 742 | 1 | 745 | 0 | 0 |
| hsa_circ_0029623 | 94 | 1 | 915 | 2177 | 3094 | 0 | 0 |
| hsa_circ_0029633 | 94 | 1 | 616 | 1 | 619 | 0 | 0 |
| hsa_circ_0029633 | 93 | 1 | 200 | 1 | 200 | 0 | 2.00E-76 |
| hsa_circ_0029628 | 98 | 1 | 778 | 384 | 1161 | 0 | 0 |
| hsa_circ_0029623 | 95 | 2 | 1858 | 49 | 1908 | 0 | 0 |
| hsa_circ_0029626 | 95 | 1 | 734 | 95 | 831 | 0 | 0 |
| hsa_circ_0029623 | 95 | 2 | 1591 | 49 | 1641 | 0 | 0 |
| hsa_circ_0029628 | 95 | 1 | 376 | 1 | 379 | 0.01 | 7.00E-168 |
| hsa_circ_0005966 | 95 | 1 | 662 | 1 | 665 | 0 | 0 |
| hsa_circ_0029626 | 95 | 451 | 1113 | 94 | 759 | 0 | 0 |
| hsa_circ_0029626 | 95 | 1 | 756 | 1 | 759 | 0 | 0 |
| hsa_circ_0029625 | 94 | 2 | 1519 | 2 | 1522 | 0 | 0 |
| hsa_circ_0029625 | 94 | 28756 | 30273 | 2 | 1522 | 0 | 0 |
| hsa_circ_0029625 | 94 | 71 | 1588 | 2 | 1522 | 0 | 0 |
| hsa_circ_0005966 | 95 | 1 | 449 | 1 | 452 | 0.01 | 0 |
| hsa_circ_0029625 | 94 | 2 | 857 | 2 | 857 | 0 | 0 |
| hsa_circ_0005966 | 81 | 1 | 662 | 1 | 665 | 0.01 | 2.00E-146 |
| hsa_circ_0005966 | 81 | 1 | 662 | 1 | 665 | 0.01 | 3.00E-146 |
| hsa_circ_0006778 | 91 | 1 | 302 | 302 | 1 | 0 | 6.00E-113 |
| hsa_circ_0029616 | 92 | 1 | 398 | 398 | 1 | 0 | 3.00E-156 |
| hsa_circ_0000464 | 90 | 1 | 579 | 680 | 86 | 0.03 | 0 |
| hsa_circ_0000464 | 90 | 1 | 664 | 680 | 1 | 0.02 | 0 |
| hsa_circ_0029614 | 90 | 1 | 770 | 786 | 1 | 0.02 | 0 |
| hsa_circ_0029611 | 98 | 1 | 228 | 228 | 1 | 0 | 3.00E-110 |
| hsa_circ_0006833 | 84 | 228 | 1413 | 463413 | 462214 | 0.05 | 0 |
| hsa_circ_0006833 | 93 | 12554 | 12726 | 428597 | 428425 | 0 | 2.00E-64 |
| hsa_circ_0029605 | 93 | 1 | 283 | 384 | 666 | 0 | 2.00E-112 |
| hsa_circ_0029605 | 92 | 1 | 525 | 142 | 666 | 0 | 0 |
| hsa_circ_0029605 | 91 | 1 | 666 | 1 | 666 | 0 | 0 |
| hsa_circ_0029605 | 91 | 1 | 538 | 1 | 538 | 0 | 0 |
| hsa_circ_0029605 | 90 | 1 | 383 | 1 | 383 | 0 | 7.00E-138 |
| hsa_circ_0029603 | 91 | 1 | 598 | 1678 | 2275 | 0 | 0 |
| hsa_circ_0029738 | 87 | 1 | 274 | 1380 | 1104 | 0.01 | 3.00E-81 |
| hsa_circ_0029738 | 87 | 1 | 526 | 1632 | 1104 | 0.01 | 1.00E-167 |
| hsa_circ_0029738 | 87 | 1 | 626 | 1732 | 1104 | 0.01 | 0 |
| hsa_circ_0029738 | 87 | 2 | 1216 | 2321 | 1104 | 0.01 | 0 |

novel_circ_008997 novel_circ_008998 novel_circ_008999 novel_circ_009000 novel_circ_009001 novel_circ_009002 novel_circ_009003 novel_circ_009004 novel_circ_009005 novel_circ_009006 novel_circ_009007 novel_circ_009008 novel_circ_009009 novel_circ_009010 novel_circ_009011 novel_circ_009012 novel_circ_009013 novel_circ_009014 novel_circ_009015 novel_circ_009016 novel_circ_009017 novel_circ_009018 novel_circ_009019 novel_circ_009020 novel_circ_009021 novel_circ_009022 novel_circ_009023 novel_circ_009024 novel_circ_009025 novel_circ_009026 novel_circ_009027 novel_circ_009028 novel_circ_009029 novel_circ_009030 novel_circ_009036 novel_circ_009037 novel_circ_009038 novel_circ_009039 novel_circ_009040 novel_circ_009041 novel_circ_009042 novel_circ_009043 novel_circ_009044 novel_circ_009045 novel_circ_009046 novel_circ_009047 novel_circ_009048 novel_circ_009049 novel_circ_009050 novel_circ_009051 novel_circ_009052 novel_circ_009053 novel_circ_009054 novel_circ_009055

| hsa_circ_0029738 | 88 | 4 | 1507 | 2610 | 1104 | 0.01 | 0 |
| --- | --- | --- | --- | --- | --- | --- | --- |
| hsa_circ_0029739 | 88 | 1 | 2233 | 2233 | 1 | 0 | 0 |
| hsa_circ_0029739 | 87 | 2 | 1023 | 1022 | 1 | 0.01 | 0 |
| hsa_circ_0029739 | 87 | 2 | 941 | 1022 | 83 | 0.01 | 0 |
| hsa_circ_0029739 | 88 | 4 | 1232 | 1311 | 83 | 0.01 | 0 |
| hsa_circ_0029739 | 88 | 1 | 1917 | 1999 | 83 | 0.01 | 0 |
| hsa_circ_0029739 | 88 | 1 | 2151 | 2233 | 83 | 0 | 0 |
| hsa_circ_0029740 | 88 | 4 | 981 | 1149 | 172 | 0.01 | 0 |
| hsa_circ_0029741 | 89 | 4 | 693 | 878 | 189 | 0.01 | 0 |
| hsa_circ_0029741 | 89 | 1 | 1378 | 1566 | 189 | 0 | 0 |
| hsa_circ_0029741 | 88 | 12 | 2425 | 2602 | 189 | 0 | 0 |
| hsa_circ_0029755 | 89 | 1 | 976 | 976 | 1 | 0 | 0 |
| hsa_circ_0029755 | 89 | 1 | 897 | 976 | 80 | 0 | 0 |
| hsa_circ_0029760 | 88 | 1 | 685 | 685 | 1 | 0 | 0 |
| hsa_circ_0029758 | 88 | 1 | 919 | 1084 | 166 | 0 | 0 |
| hsa_circ_0029764 | 88 | 1 | 339 | 341 | 3 | 0 | 4.00E-110 |
| hsa_circ_0029764 | 88 | 1 | 573 | 575 | 3 | 0 | 0 |
| hsa_circ_0029764 | 88 | 12 | 1386 | 1377 | 3 | 0 | 0 |
| hsa_circ_0029768 | 91 | 1 | 603 | 611 | 9 | 0 | 0 |
| hsa_circ_0029768 | 88 | 12 | 943 | 940 | 9 | 0 | 0 |
| hsa_circ_0029768 | 87 | 1 | 1010 | 1018 | 9 | 0 | 0 |
| hsa_circ_0029767 | 87 | 9 | 1345 | 1441 | 105 | 0 | 0 |
| hsa_circ_0029768 | 90 | 1 | 473 | 611 | 139 | 0 | 4.00E-171 |
| hsa_circ_0029767 | 86 | 9 | 1215 | 1441 | 235 | 0 | 0 |
| hsa_circ_0029768 | 87 | 1 | 312 | 611 | 300 | 0 | 8.00E-97 |
| hsa_circ_0029768 | 85 | 12 | 652 | 940 | 300 | 0 | 0 |
| hsa_circ_0029768 | 85 | 4 | 921 | 1217 | 300 | 0 | 0 |
| hsa_circ_0029767 | 85 | 9 | 1054 | 1441 | 396 | 0 | 0 |
| hsa_circ_0029768 | 84 | 12 | 477 | 940 | 475 | 0 | 3.00E-127 |
| hsa_circ_0029768 | 83 | 12 | 340 | 940 | 612 | 0.01 | 3.00E-81 |
| hsa_circ_0005198 | 83 | 9 | 592 | 584 | 1 | 0.01 | 2.00E-144 |
| hsa_circ_0005198 | 85 | 9 | 478 | 584 | 114 | 0 | 9.00E-133 |
| hsa_circ_0029772 | 87 | 9 | 335 | 470 | 144 | 0 | 9.00E-102 |
| hsa_circ_0029775 | 91 | 1 | 133 | 133 | 1 | 0 | 1.00E-43 |
| hsa_circ_0045519 | 93 | 804 | 1134 | 2851 | 2521 | 0 | 4.00E-135 |
| hsa_circ_0045519 | 93 | 482 | 812 | 2851 | 2521 | 0 | 3.00E-135 |
| hsa_circ_0045519 | 93 | 482 | 1188 | 2851 | 2145 | 0 | 0 |
| hsa_circ_0045519 | 93 | 482 | 1299 | 2851 | 2034 | 0 | 0 |
| hsa_circ_0045519 | 93 | 482 | 2287 | 2851 | 1046 | 0 | 0 |
| hsa_circ_0045519 | 93 | 482 | 2457 | 2851 | 876 | 0 | 0 |
| hsa_circ_0045519 | 93 | 482 | 2723 | 2851 | 610 | 0 | 0 |
| hsa_circ_0045519 | 93 | 482 | 3008 | 2851 | 325 | 0 | 0 |
| hsa_circ_0045519 | 93 | 231 | 2472 | 2851 | 610 | 0 | 0 |
| hsa_circ_0045519 | 94 | 1 | 1114 | 2851 | 1738 | 0 | 0 |
| hsa_circ_0045519 | 94 | 1 | 972 | 2709 | 1738 | 0 | 0 |
| hsa_circ_0045519 | 93 | 1 | 2634 | 2709 | 76 | 0 | 0 |
| hsa_circ_0045519 | 93 | 7 | 1594 | 2197 | 610 | 0 | 0 |
| hsa_circ_0045519 | 95 | 1 | 287 | 2144 | 1858 | 0 | 2.00E-126 |
| hsa_circ_0045519 | 96 | 1 | 407 | 2144 | 1738 | 0 | 0 |
| hsa_circ_0045519 | 93 | 1 | 1535 | 2144 | 610 | 0 | 0 |
| hsa_circ_0045519 | 93 | 1 | 2069 | 2144 | 76 | 0 | 0 |
| hsa_circ_0045519 | 93 | 1 | 812 | 1857 | 1046 | 0 | 0 |
| hsa_circ_0045519 | 93 | 1 | 982 | 1857 | 876 | 0 | 0 |
| hsa_circ_0045519 | 93 | 1 | 982 | 1857 | 876 | 0 | 0 |

novel_circ_009056 novel_circ_009057 novel_circ_009058 novel_circ_009059 novel_circ_009060 novel_circ_009061 novel_circ_009062 novel_circ_009063 novel_circ_009064 novel_circ_009065 novel_circ_009069 novel_circ_009070 novel_circ_009071 novel_circ_009072 novel_circ_009074 novel_circ_009075 novel_circ_009076 novel_circ_009077 novel_circ_009078 novel_circ_009079 novel_circ_009080 novel_circ_009081 novel_circ_009082 novel_circ_009083 novel_circ_009084 novel_circ_009085 novel_circ_009086 novel_circ_009087 novel_circ_009088 novel_circ_009089 novel_circ_009090 novel_circ_009091 novel_circ_009093 novel_circ_009094 novel_circ_009095 novel_circ_009096 novel_circ_009097 novel_circ_009098 novel_circ_009099 novel_circ_009100 novel_circ_009101 novel_circ_009102 novel_circ_009103 novel_circ_009104 novel_circ_009105 novel_circ_009106 novel_circ_009107 novel_circ_009108 novel_circ_009109 novel_circ_009110 novel_circ_009111 novel_circ_009112 novel_circ_009113 novel_circ_009114

| hsa_circ_0045519 | 92 | 1 | 1248 | 1857 | 610 | 0 | 0 |
| --- | --- | --- | --- | --- | --- | --- | --- |
| hsa_circ_0045519 | 92 | 1 | 1533 | 1857 | 325 | 0 | 0 |
| hsa_circ_0045519 | 93 | 8 | 692 | 1730 | 1046 | 0 | 0 |
| hsa_circ_0045519 | 93 | 8 | 862 | 1730 | 876 | 0 | 0 |
| hsa_circ_0045519 | 93 | 8 | 862 | 1730 | 876 | 0 | 0 |
| hsa_circ_0045519 | 92 | 8 | 1128 | 1730 | 610 | 0 | 0 |
| hsa_circ_0045519 | 92 | 8 | 1413 | 1730 | 325 | 0 | 0 |
| hsa_circ_0045519 | 91 | 1 | 638 | 1247 | 610 | 0 | 0 |
| hsa_circ_0045519 | 91 | 1 | 436 | 1045 | 610 | 0 | 6.00E-164 |
| hsa_circ_0045519 | 91 | 1 | 875 | 875 | 1 | 0 | 0 |
| hsa_circ_0045512 | 88 | 3 | 424 | 542 | 121 | 0 | 8.00E-143 |
| hsa_circ_0045513 | 77 | 371 | 1595 | 3789 | 2565 | 0.03 | 0 |
| hsa_circ_0045513 | 77 | 38 | 1257 | 3540 | 2321 | 0.03 | 0 |
| hsa_circ_0045513 | 79 | 18 | 865 | 3412 | 2565 | 0.02 | 8.00E-161 |
| hsa_circ_0045508 | 93 | 1 | 109 | 264 | 372 | 0 | 4.00E-37 |
| hsa_circ_0045508 | 93 | 184 | 446 | 1 | 263 | 0 | 2.00E-104 |
| hsa_circ_0045494 | 94 | 2052 | 2175 | 640 | 763 | 0 | 8.00E-46 |
| hsa_circ_0045492 | 86 | 262 | 537 | 3 | 278 | 0.01 | 1.00E-76 |
| hsa_circ_0004560 | 88 | 462 | 685 | 546 | 769 | 0.02 | 1.00E-67 |
| hsa_circ_0004560 | 88 | 589 | 812 | 546 | 769 | 0.02 | 2.00E-67 |
| hsa_circ_0004560 | 88 | 981 | 1204 | 546 | 769 | 0.02 | 3.00E-67 |
| hsa_circ_0045345 | 89 | 284 | 586 | 303 | 1 | 0 | 4.00E-103 |
| hsa_circ_0045350 | 90 | 1 | 1078 | 1072 | 1 | 0.01 | 0 |
| hsa_circ_0045350 | 90 | 1 | 937 | 1072 | 142 | 0.01 | 0 |
| hsa_circ_0045351 | 95 | 540 | 755 | 216 | 1 | 0 | 2.00E-91 |
| hsa_circ_0045354 | 92 | 1 | 427 | 427 | 1 | 0 | 3.00E-172 |
| hsa_circ_0045354 | 92 | 4 | 518 | 942 | 428 | 0 | 0 |
| hsa_circ_0045354 | 92 | 168 | 1134 | 1394 | 428 | 0 | 0 |
| hsa_circ_0045354 | 92 | 1156 | 2122 | 1394 | 428 | 0 | 0 |
| hsa_circ_0045354 | 92 | 2 | 450 | 1394 | 946 | 0 | 8.00E-178 |
| hsa_circ_0045354 | 92 | 168 | 616 | 1394 | 946 | 0 | 1.00E-177 |
| hsa_circ_0045354 | 92 | 249 | 697 | 1394 | 946 | 0 | 1.00E-177 |
| hsa_circ_0005547 | 90 | 1 | 644 | 650 | 1 | 0.01 | 0 |
| hsa_circ_0045358 | 91 | 1 | 478 | 478 | 1 | 0 | 0 |
| hsa_circ_0045358 | 90 | 1 | 305 | 478 | 174 | 0 | 1.00E-109 |
| hsa_circ_0045365 | 94 | 17956 | 18039 | 31 | 114 | 0 | 1.00E-26 |
| hsa_circ_0045365 | 94 | 4732 | 4815 | 31 | 114 | 0 | 3.00E-27 |
| hsa_circ_0045373 | 90 | 1 | 241 | 1 | 241 | 0 | 4.00E-84 |
| hsa_circ_0045373 | 92 | 1 | 630 | 1 | 630 | 0 | 0 |
| hsa_circ_0045376 | 93 | 1 | 389 | 1 | 389 | 0 | 2.00E-159 |
| hsa_circ_0045392 | 92 | 1 | 1403 | 1403 | 1 | 0 | 0 |
| hsa_circ_0045392 | 93 | 1 | 3064 | 3064 | 1 | 0 | 0 |
| hsa_circ_0045392 | 93 | 1020 | 4187 | 3168 | 1 | 0 | 0 |
| hsa_circ_0045397 | 94 | 1 | 451 | 451 | 1 | 0 | 0 |
| hsa_circ_0045397 | 93 | 4 | 1069 | 1066 | 1 | 0 | 0 |
| hsa_circ_0045397 | 94 | 1 | 1661 | 1661 | 1 | 0 | 0 |
| hsa_circ_0045397 | 94 | 287 | 2051 | 1765 | 1 | 0 | 0 |
| hsa_circ_0045397 | 94 | 1020 | 2784 | 1765 | 1 | 0 | 0 |
| hsa_circ_0045398 | 93 | 1 | 1210 | 1453 | 244 | 0 | 0 |
| hsa_circ_0045398 | 94 | 287 | 1600 | 1557 | 244 | 0 | 0 |
| hsa_circ_0045398 | 94 | 1 | 1005 | 1453 | 449 | 0 | 0 |
| hsa_circ_0045398 | 94 | 1020 | 2128 | 1557 | 449 | 0 | 0 |
| hsa_circ_0045398 | 95 | 1 | 592 | 1453 | 862 | 0 | 0 |
| hsa_circ_0045408 | 94 | 1 | 1018 | 1015 | 1 | 0 | 0 |

novel_circ_009115 novel_circ_009116 novel_circ_009117 novel_circ_009118 novel_circ_009119 novel_circ_009120 novel_circ_009121 novel_circ_009122 novel_circ_009123 novel_circ_009124 novel_circ_009130 novel_circ_009133 novel_circ_009134 novel_circ_009135 novel_circ_009136 novel_circ_009137 novel_circ_009138 novel_circ_009139 novel_circ_009140 novel_circ_009141 novel_circ_009142 novel_circ_009143 novel_circ_009144 novel_circ_009145 novel_circ_009146 novel_circ_009147 novel_circ_009148 novel_circ_009149 novel_circ_009150 novel_circ_009151 novel_circ_009152 novel_circ_009153 novel_circ_009154 novel_circ_009155 novel_circ_009156 novel_circ_009157 novel_circ_009158 novel_circ_009159 novel_circ_009160 novel_circ_009161 novel_circ_009162 novel_circ_009163 novel_circ_009164 novel_circ_009165 novel_circ_009166 novel_circ_009167 novel_circ_009168 novel_circ_009169 novel_circ_009170 novel_circ_009171 novel_circ_009173 novel_circ_009177 novel_circ_009178 novel_circ_009179

| hsa_circ_0045411 | 94 | 1 | 733 | 733 | 1 | 0 | 0 |
| --- | --- | --- | --- | --- | --- | --- | --- |
| hsa_circ_0045411 | 94 | 3286 | 4020 | 735 | 1 | 0 | 0 |
| hsa_circ_0045411 | 97 | 1 | 873 | 1338 | 466 | 0 | 0 |
| hsa_circ_0045412 | 97 | 1 | 915 | 915 | 1 | 0 | 0 |
| hsa_circ_0045416 | 95 | 1 | 316 | 1 | 316 | 0 | 2.00E-137 |
| hsa_circ_0045423 | 88 | 1 | 913 | 1 | 913 | 0 | 0 |
| hsa_circ_0045426 | 90 | 2 | 203 | 2 | 203 | 0 | 1.00E-67 |
| hsa_circ_0045426 | 85 | 2 | 593 | 2 | 593 | 0 | 2.00E-175 |
| hsa_circ_0045426 | 83 | 3 | 477 | 206 | 680 | 0 | 3.00E-122 |
| hsa_circ_0045427 | 86 | 1 | 676 | 1 | 676 | 0 | 0 |
| hsa_circ_0045426 | 85 | 1 | 1054 | 758 | 1817 | 0.01 | 0 |
| hsa_circ_0045437 | 94 | 1 | 823 | 1 | 823 | 0 | 0 |
| hsa_circ_0045437 | 94 | 1 | 1045 | 1 | 1045 | 0 | 0 |
| hsa_circ_0045437 | 93 | 1 | 1443 | 1 | 1440 | 0 | 0 |
| hsa_circ_0045446 | 93 | 1 | 673 | 4 | 676 | 0 | 0 |
| hsa_circ_0045446 | 92 | 1 | 1131 | 4 | 1128 | 0.01 | 0 |
| hsa_circ_0045450 | 95 | 1 | 485 | 1 | 485 | 0 | 0 |
| hsa_circ_0045457 | 94 | 6 | 254 | 6 | 254 | 0 | 1.00E-103 |
| hsa_circ_0045468 | 90 | 1 | 1643 | 110 | 1776 | 0.01 | 0 |
| hsa_circ_0000799 | 94 | 1 | 309 | 918 | 1226 | 0 | 2.00E-133 |
| hsa_circ_0000799 | 92 | 1 | 1023 | 1001 | 2026 | 0.02 | 0 |
| hsa_circ_0045320 | 94 | 50944 | 51663 | 805 | 86 | 0 | 0 |
| hsa_circ_0045320 | 94 | 1 | 720 | 805 | 86 | 0 | 0 |
| hsa_circ_0008633 | 94 | 1 | 616 | 616 | 1 | 0 | 0 |
| hsa_circ_0008633 | 94 | 1 | 531 | 531 | 1 | 0 | 0 |
| hsa_circ_0008633 | 95 | 1 | 244 | 244 | 1 | 0 | 8.00E-106 |
| hsa_circ_0045316 | 93 | 1 | 440 | 440 | 1 | 0 | 0 |
| hsa_circ_0009120 | 94 | 1 | 544 | 544 | 1 | 0 | 0 |
| hsa_circ_0045313 | 94 | 1 | 659 | 1299 | 641 | 0 | 0 |
| hsa_circ_0045313 | 94 | 1 | 1097 | 1299 | 203 | 0 | 0 |
| hsa_circ_0045313 | 94 | 1 | 654 | 1294 | 641 | 0 | 0 |
| hsa_circ_0045313 | 95 | 2 | 574 | 1213 | 641 | 0 | 0 |
| hsa_circ_0045313 | 95 | 2 | 891 | 1213 | 324 | 0 | 0 |
| hsa_circ_0045313 | 94 | 2 | 1012 | 1213 | 203 | 0 | 0 |
| hsa_circ_0045314 | 95 | 1 | 317 | 438 | 122 | 0 | 6.00E-138 |
| hsa_circ_0045311 | 88 | 1 | 762 | 592 | 1355 | 0.02 | 0 |
| hsa_circ_0045311 | 84 | 1 | 722 | 1 | 728 | 0.01 | 0 |
| hsa_circ_0045303 | 85 | 1 | 1287 | 78 | 1364 | 0.01 | 0 |
| hsa_circ_0045303 | 87 | 1 | 702 | 78 | 773 | 0.01 | 0 |
| hsa_circ_0045303 | 89 | 1 | 570 | 78 | 647 | 0 | 0 |
| hsa_circ_0045303 | 90 | 1 | 454 | 78 | 531 | 0 | 3.00E-167 |
| hsa_circ_0045303 | 90 | 1 | 348 | 78 | 425 | 0 | 9.00E-127 |
| hsa_circ_0045303 | 89 | 1 | 237 | 78 | 314 | 0 | 1.00E-78 |
| hsa_circ_0045292 | 95 | 1 | 773 | 1006 | 234 | 0 | 0 |
| hsa_circ_0045288 | 88 | 1 | 233 | 233 | 1 | 0 | 8.00E-73 |
| hsa_circ_0045288 | 88 | 1 | 233 | 233 | 1 | 0 | 1.00E-72 |
| hsa_circ_0045288 | 88 | 1 | 233 | 233 | 1 | 0 | 2.00E-72 |
| hsa_circ_0045288 | 88 | 1 | 233 | 233 | 1 | 0 | 2.00E-72 |
| hsa_circ_0045287 | 93 | 316 | 396 | 182 | 102 | 0 | 5.00E-25 |
| hsa_circ_0045287 | 92 | 316 | 492 | 182 | 6 | 0 | 3.00E-63 |
| hsa_circ_0006479 | 92 | 11 | 1665 | 1655 | 1 | 0 | 0 |
| hsa_circ_0045236 | 95 | 1 | 277 | 147 | 423 | 0 | 6.00E-119 |
| hsa_circ_0045236 | 94 | 1 | 375 | 49 | 423 | 0 | 1.00E-161 |
| hsa_circ_0045232 | 94 | 1 | 949 | 256 | 1204 | 0 | 0 |

novel_circ_009181 novel_circ_009182 novel_circ_009183 novel_circ_009184 novel_circ_009185 novel_circ_009186 novel_circ_009188 novel_circ_009189 novel_circ_009190 novel_circ_009191 novel_circ_009192 novel_circ_009193 novel_circ_009194 novel_circ_009195 novel_circ_009196 novel_circ_009197 novel_circ_009198 novel_circ_009199 novel_circ_009200 novel_circ_009201 novel_circ_009202 novel_circ_009203 novel_circ_009204 novel_circ_009205 novel_circ_009206 novel_circ_009207 novel_circ_009208 novel_circ_009209 novel_circ_009210 novel_circ_009211 novel_circ_009212 novel_circ_009213 novel_circ_009214 novel_circ_009215 novel_circ_009216 novel_circ_009217 novel_circ_009218 novel_circ_009219 novel_circ_009220 novel_circ_009221 novel_circ_009222 novel_circ_009225 novel_circ_009226 novel_circ_009227 novel_circ_009228 novel_circ_009229 novel_circ_009232 novel_circ_009233 novel_circ_009234 novel_circ_009235 novel_circ_009236 novel_circ_009237 novel_circ_009238 novel_circ_009239

| hsa_circ_0045222 | 93 | 5 | 634 | 246 | 878 | 0.01 | 0 |
| --- | --- | --- | --- | --- | --- | --- | --- |
| hsa_circ_0045218 | 95 | 1 | 573 | 684 | 112 | 0 | 0 |
| hsa_circ_0045218 | 96 | 1 | 111 | 111 | 1 | 0 | 5.00E-45 |
| hsa_circ_0045216 | 92 | 169 | 393 | 225 | 1 | 0 | 2.00E-83 |
| hsa_circ_0045215 | 92 | 169 | 626 | 458 | 1 | 0 | 0 |
| hsa_circ_0045216 | 92 | 1 | 225 | 225 | 1 | 0 | 1.00E-83 |
| hsa_circ_0045212 | 93 | 3 | 591 | 326 | 914 | 0 | 0 |
| hsa_circ_0045212 | 93 | 3 | 376 | 541 | 914 | 0 | 3.00E-156 |
| hsa_circ_0045212 | 93 | 3 | 591 | 326 | 914 | 0 | 0 |
| hsa_circ_0045212 | 94 | 3 | 469 | 326 | 792 | 0 | 0 |
| hsa_circ_0045212 | 93 | 3 | 356 | 326 | 679 | 0 | 4.00E-145 |
| hsa_circ_0045212 | 94 | 3 | 215 | 326 | 538 | 0 | 5.00E-87 |
| hsa_circ_0045203 | 94 | 1 | 718 | 1 | 719 | 0 | 0 |
| hsa_circ_0045207 | 96 | 1 | 328 | 1 | 329 | 0 | 5.00E-149 |
| hsa_circ_0045204 | 95 | 1 | 559 | 1 | 560 | 0 | 0 |
| hsa_circ_0045203 | 94 | 1 | 718 | 1 | 719 | 0 | 0 |
| hsa_circ_0045204 | 93 | 1 | 231 | 1 | 231 | 0 | 6.00E-92 |
| hsa_circ_0045203 | 93 | 1 | 390 | 1 | 390 | 0 | 9.00E-162 |
| hsa_circ_0045193 | 95 | 1 | 528 | 2079 | 2606 | 0 | 0 |
| hsa_circ_0045197 | 94 | 1 | 352 | 1264 | 1615 | 0 | 2.00E-152 |
| hsa_circ_0045195 | 96 | 1 | 1116 | 1 | 1116 | 0 | 0 |
| hsa_circ_0045195 | 96 | 1 | 1004 | 1 | 1004 | 0 | 0 |
| hsa_circ_0045193 | 96 | 2731 | 3868 | 283 | 1420 | 0 | 0 |
| hsa_circ_0045195 | 91 | 1 | 232 | 1 | 232 | 0 | 2.00E-82 |
| hsa_circ_0045185 | 95 | 1 | 682 | 1 | 682 | 0 | 0 |
| hsa_circ_0045170 | 95 | 27228 | 27911 | 71 | 754 | 0 | 0 |
| hsa_circ_0045185 | 95 | 1 | 600 | 1 | 600 | 0 | 0 |
| hsa_circ_0045170 | 95 | 27228 | 27829 | 71 | 672 | 0 | 0 |
| hsa_circ_0045170 | 95 | 27228 | 27378 | 71 | 221 | 0 | 2.00E-60 |
| hsa_circ_0045151 | 94 | 1 | 935 | 145 | 1079 | 0 | 0 |
| hsa_circ_0045143 | 96 | 1 | 711 | 1 | 711 | 0 | 0 |
| hsa_circ_0045139 | 96 | 1 | 793 | 166 | 958 | 0 | 0 |
| hsa_circ_0045139 | 98 | 1 | 573 | 166 | 738 | 0 | 0 |
| hsa_circ_0082183 | 96 | 1 | 226 | 427 | 652 | 0 | 7.00E-101 |
| hsa_circ_0082182 | 84 | 188 | 752 | 3 | 570 | 0.01 | 2.00E-152 |
| hsa_circ_0082158 | 95 | 13476 | 13619 | 216 | 73 | 0 | 9.00E-57 |
| hsa_circ_0082115 | 93 | 1 | 724 | 1 | 724 | 0 | 0 |
| hsa_circ_0082115 | 94 | 1 | 610 | 1 | 610 | 0 | 0 |
| hsa_circ_0082098 | 97 | 1 | 1933 | 1933 | 1 | 0 | 0 |
| hsa_circ_0082098 | 97 | 1 | 1837 | 1837 | 1 | 0 | 0 |
| hsa_circ_0082095 | 97 | 1 | 1892 | 2352 | 460 | 0 | 0 |
| hsa_circ_0073579 | 87 | 20 | 532 | 516 | 1 | 0.01 | 2.00E-160 |
| hsa_circ_0073579 | 82 | 1 | 408 | 657 | 247 | 0.01 | 3.00E-97 |
| hsa_circ_0073579 | 84 | 16 | 578 | 812 | 247 | 0.01 | 1.00E-156 |
| hsa_circ_0073580 | 88 | 16 | 267 | 452 | 201 | 0 | 8.00E-82 |
| hsa_circ_0073580 | 90 | 15 | 411 | 694 | 298 | 0 | 3.00E-147 |
| hsa_circ_0073586 | 95 | 1 | 437 | 1 | 437 | 0 | 0 |
| hsa_circ_0073586 | 94 | 1 | 549 | 1 | 549 | 0 | 0 |
| hsa_circ_0073586 | 94 | 1 | 663 | 1 | 663 | 0 | 0 |
| hsa_circ_0073586 | 93 | 1 | 747 | 1 | 747 | 0 | 0 |
| hsa_circ_0073586 | 92 | 1 | 852 | 1 | 852 | 0 | 0 |
| hsa_circ_0073588 | 93 | 1 | 510 | 1 | 510 | 0 | 0 |
| hsa_circ_0073588 | 93 | 1 | 594 | 1 | 594 | 0 | 0 |
| hsa_circ_0073588 | 92 | 1 | 699 | 1 | 699 | 0 | 0 |

novel_circ_009240 novel_circ_009242 novel_circ_009245 novel_circ_009246 novel_circ_009247 novel_circ_009248 novel_circ_009249 novel_circ_009250 novel_circ_009251 novel_circ_009252 novel_circ_009253 novel_circ_009254 novel_circ_009255 novel_circ_009256 novel_circ_009257 novel_circ_009258 novel_circ_009260 novel_circ_009261 novel_circ_009262 novel_circ_009263 novel_circ_009264 novel_circ_009265 novel_circ_009266 novel_circ_009267 novel_circ_009268 novel_circ_009269 novel_circ_009270 novel_circ_009271 novel_circ_009272 novel_circ_009273 novel_circ_009274 novel_circ_009275 novel_circ_009276 novel_circ_009277 novel_circ_009278 novel_circ_009279 novel_circ_009280 novel_circ_009281 novel_circ_009282 novel_circ_009284 novel_circ_009285 novel_circ_009286 novel_circ_009287 novel_circ_009288 novel_circ_009289 novel_circ_009290 novel_circ_009291 novel_circ_009292 novel_circ_009293 novel_circ_009294 novel_circ_009295 novel_circ_009296 novel_circ_009297 novel_circ_009298

| hsa_circ_0073593 | 91 | 2 | 412 | 2 | 412 | 0 | 2.00E-153 |
| --- | --- | --- | --- | --- | --- | --- | --- |
| hsa_circ_0073594 | 89 | 1 | 303 | 1 | 303 | 0 | 4.00E-105 |
| hsa_circ_0001520 | 94 | 1 | 379 | 1 | 379 | 0 | 5.00E-164 |
| hsa_circ_0001520 | 94 | 1 | 753 | 1 | 753 | 0 | 0 |
| hsa_circ_0001520 | 92 | 1 | 886 | 1 | 889 | 0 | 0 |
| hsa_circ_0001520 | 92 | 1 | 886 | 1 | 889 | 0 | 0 |
| hsa_circ_0073610 | 85 | 153 | 1417 | 1 | 1272 | 0.03 | 0 |
| hsa_circ_0073610 | 91 | 1 | 606 | 129 | 737 | 0 | 0 |
| hsa_circ_0073610 | 91 | 1 | 681 | 129 | 809 | 0.01 | 0 |
| hsa_circ_0073611 | 95 | 1 | 374 | 1 | 374 | 0 | 1.00E-164 |
| hsa_circ_0073611 | 91 | 1 | 507 | 1 | 510 | 0.01 | 0 |
| hsa_circ_0073610 | 90 | 1 | 582 | 228 | 809 | 0.01 | 0 |
| hsa_circ_0073610 | 83 | 1 | 208 | 602 | 809 | 0.03 | 9.00E-46 |
| hsa_circ_0003044 | 91 | 5 | 164 | 367 | 208 | 0 | 9.00E-55 |
| hsa_circ_0003044 | 91 | 35644 | 35803 | 367 | 208 | 0 | 1.00E-53 |
| hsa_circ_0003044 | 91 | 50455 | 50614 | 367 | 208 | 0 | 2.00E-53 |
| hsa_circ_0073617 | 93 | 1 | 1078 | 92 | 1169 | 0 | 0 |
| hsa_circ_0073617 | 92 | 1 | 1217 | 92 | 1308 | 0 | 0 |
| hsa_circ_0073617 | 92 | 1 | 881 | 289 | 1169 | 0 | 0 |
| hsa_circ_0073617 | 92 | 1 | 427 | 489 | 915 | 0 | 1.00E-170 |
| hsa_circ_0073617 | 92 | 1 | 532 | 489 | 1020 | 0 | 0 |
| hsa_circ_0073617 | 92 | 1 | 681 | 489 | 1169 | 0 | 0 |
| hsa_circ_0073617 | 91 | 1 | 820 | 489 | 1308 | 0 | 0 |
| hsa_circ_0073617 | 93 | 1 | 514 | 656 | 1169 | 0 | 0 |
| hsa_circ_0073617 | 92 | 1 | 653 | 656 | 1308 | 0 | 0 |
| hsa_circ_0073617 | 91 | 1 | 1291 | 656 | 1946 | 0 | 0 |
| hsa_circ_0073617 | 91 | 1 | 1374 | 656 | 2029 | 0 | 0 |
| hsa_circ_0073617 | 90 | 1 | 1873 | 656 | 2528 | 0 | 0 |
| hsa_circ_0073617 | 92 | 1 | 550 | 759 | 1308 | 0 | 0 |
| hsa_circ_0073617 | 91 | 1 | 1188 | 759 | 1946 | 0 | 0 |
| hsa_circ_0073617 | 91 | 1 | 1271 | 759 | 2029 | 0 | 0 |
| hsa_circ_0073617 | 91 | 1 | 2299 | 759 | 3057 | 0 | 0 |
| hsa_circ_0073618 | 91 | 1 | 393 | 1 | 393 | 0 | 2.00E-148 |
| hsa_circ_0073618 | 90 | 1 | 586 | 1 | 586 | 0 | 0 |
| hsa_circ_0073617 | 91 | 1 | 1114 | 916 | 2029 | 0 | 0 |
| hsa_circ_0073617 | 90 | 1 | 2142 | 916 | 3057 | 0 | 0 |
| hsa_circ_0073620 | 89 | 3 | 1220 | 139 | 1356 | 0 | 0 |
| hsa_circ_0073620 | 90 | 3 | 1749 | 139 | 1885 | 0 | 0 |
| hsa_circ_0073622 | 91 | 1 | 445 | 1 | 445 | 0 | 1.00E-170 |
| hsa_circ_0073625 | 92 | 1 | 252 | 1 | 252 | 0 | 1.00E-98 |
| hsa_circ_0073625 | 88 | 1 | 751 | 1 | 751 | 0 | 0 |
| hsa_circ_0073625 | 89 | 1 | 878 | 1 | 878 | 0 | 0 |
| hsa_circ_0073625 | 90 | 1 | 1683 | 1 | 1683 | 0 | 0 |
| hsa_circ_0073625 | 90 | 1 | 1111 | 170 | 1280 | 0 | 0 |
| hsa_circ_0073625 | 90 | 1 | 1514 | 170 | 1683 | 0 | 0 |
| hsa_circ_0073625 | 90 | 1 | 1028 | 253 | 1280 | 0 | 0 |
| hsa_circ_0073625 | 90 | 1 | 1431 | 253 | 1683 | 0 | 0 |
| hsa_circ_0073625 | 90 | 1 | 1996 | 253 | 2248 | 0 | 0 |
| hsa_circ_0073625 | 89 | 1 | 922 | 359 | 1280 | 0 | 0 |
| hsa_circ_0073625 | 93 | 1 | 529 | 752 | 1280 | 0 | 0 |
| hsa_circ_0073625 | 92 | 1 | 932 | 752 | 1683 | 0 | 0 |
| hsa_circ_0073625 | 92 | 1 | 1314 | 752 | 2065 | 0 | 0 |
| hsa_circ_0073625 | 92 | 2 | 785 | 1282 | 2065 | 0 | 0 |
| hsa_circ_0073626 | 92 | 1 | 293 | 307 | 599 | 0 | 1.00E-114 |

novel_circ_009299 novel_circ_009300 novel_circ_009304 novel_circ_009305 novel_circ_009306 novel_circ_009307 novel_circ_009308 novel_circ_009309 novel_circ_009310 novel_circ_009311 novel_circ_009312 novel_circ_009313 novel_circ_009314 novel_circ_009315 novel_circ_009316 novel_circ_009317 novel_circ_009318 novel_circ_009319 novel_circ_009320 novel_circ_009321 novel_circ_009322 novel_circ_009323 novel_circ_009324 novel_circ_009331 novel_circ_009332 novel_circ_009333 novel_circ_009334 novel_circ_009335 novel_circ_009336 novel_circ_009337 novel_circ_009338 novel_circ_009339 novel_circ_009340 novel_circ_009341 novel_circ_009342 novel_circ_009343 novel_circ_009344 novel_circ_009345 novel_circ_009346 novel_circ_009347 novel_circ_009348 novel_circ_009349 novel_circ_009350 novel_circ_009351 novel_circ_009352 novel_circ_009353 novel_circ_009354 novel_circ_009356 novel_circ_009357 novel_circ_009358 novel_circ_009359 novel_circ_009360 novel_circ_009361 novel_circ_009362

| hsa_circ_0073625 | 93 | 1 | 675 | 1391 | 2065 | 0 | 0 |
| --- | --- | --- | --- | --- | --- | --- | --- |
| hsa_circ_0073625 | 92 | 1 | 387 | 1862 | 2248 | 0 | 2.00E-153 |
| hsa_circ_0021486 | 93 | 1 | 332 | 54 | 385 | 0 | 2.00E-134 |
| hsa_circ_0006412 | 93 | 3 | 259 | 3 | 259 | 0 | 4.00E-101 |
| hsa_circ_0021487 | 88 | 2 | 1995 | 71 | 2067 | 0.01 | 0 |
| hsa_circ_0021490 | 91 | 1 | 499 | 1 | 499 | 0 | 0 |
| hsa_circ_0021494 | 90 | 3 | 667 | 3 | 676 | 0.01 | 0 |
| hsa_circ_0021496 | 92 | 1 | 660 | 1 | 660 | 0 | 0 |
| hsa_circ_0021496 | 93 | 1 | 424 | 237 | 660 | 0 | 1.00E-175 |
| hsa_circ_0021499 | 91 | 1 | 371 | 373 | 743 | 0 | 4.00E-141 |
| hsa_circ_0021499 | 91 | 1 | 371 | 373 | 743 | 0 | 5.00E-141 |
| hsa_circ_0021503 | 93 | 306 | 667 | 1 | 362 | 0 | 6.00E-151 |
| hsa_circ_0021503 | 90 | 306 | 947 | 1 | 642 | 0 | 0 |
| hsa_circ_0021503 | 93 | 146 | 433 | 1 | 288 | 0 | 1.00E-116 |
| hsa_circ_0021503 | 93 | 146 | 507 | 1 | 362 | 0 | 4.00E-151 |
| hsa_circ_0021503 | 93 | 1 | 288 | 1 | 288 | 0 | 7.00E-117 |
| hsa_circ_0021503 | 93 | 1 | 362 | 1 | 362 | 0 | 3.00E-151 |
| hsa_circ_0021503 | 90 | 1 | 642 | 1 | 642 | 0 | 0 |
| hsa_circ_0021503 | 94 | 1 | 222 | 67 | 288 | 0 | 5.00E-92 |
| hsa_circ_0021503 | 94 | 1 | 296 | 67 | 362 | 0 | 3.00E-126 |
| hsa_circ_0021503 | 90 | 1 | 489 | 67 | 555 | 0 | 2.00E-178 |
| hsa_circ_0021503 | 90 | 1 | 576 | 67 | 642 | 0 | 0 |
| hsa_circ_0021503 | 90 | 1 | 576 | 67 | 642 | 0 | 0 |
| hsa_circ_0021510 | 88 | 1 | 2340 | 1 | 2340 | 0 | 0 |
| hsa_circ_0021510 | 88 | 1 | 1336 | 115 | 1450 | 0 | 0 |
| hsa_circ_0021510 | 89 | 1 | 1941 | 115 | 2055 | 0 | 0 |
| hsa_circ_0021510 | 88 | 988 | 2368 | 469 | 1849 | 0 | 0 |
| hsa_circ_0021510 | 89 | 988 | 2859 | 469 | 2340 | 0 | 0 |
| hsa_circ_0021514 | 90 | 1 | 230 | 1 | 230 | 0 | 4.00E-78 |
| hsa_circ_0021514 | 89 | 1 | 532 | 1 | 532 | 0 | 0 |
| hsa_circ_0021514 | 89 | 1 | 684 | 1 | 684 | 0 | 0 |
| hsa_circ_0021510 | 88 | 1 | 982 | 469 | 1450 | 0 | 0 |
| hsa_circ_0021510 | 89 | 1 | 1152 | 469 | 1620 | 0 | 0 |
| hsa_circ_0021510 | 89 | 1 | 1152 | 469 | 1620 | 0 | 0 |
| hsa_circ_0021510 | 89 | 1 | 1250 | 469 | 1718 | 0 | 0 |
| hsa_circ_0021510 | 88 | 1 | 1381 | 469 | 1849 | 0 | 0 |
| hsa_circ_0021510 | 89 | 1 | 1872 | 469 | 2340 | 0 | 0 |
| hsa_circ_0021510 | 89 | 3 | 1357 | 701 | 2055 | 0 | 0 |
| hsa_circ_0021510 | 89 | 1 | 620 | 1001 | 1620 | 0 | 0 |
| hsa_circ_0021510 | 89 | 1 | 620 | 1001 | 1620 | 0 | 0 |
| hsa_circ_0021510 | 88 | 1 | 718 | 1001 | 1718 | 0 | 0 |
| hsa_circ_0021510 | 88 | 1 | 849 | 1001 | 1849 | 0 | 0 |
| hsa_circ_0021510 | 88 | 1 | 1340 | 1001 | 2340 | 0 | 0 |
| hsa_circ_0021515 | 90 | 1 | 435 | 1 | 435 | 0 | 1.00E-156 |
| hsa_circ_0021517 | 95 | 4322 | 4608 | 1 | 287 | 0 | 9.00E-127 |
| hsa_circ_0021517 | 93 | 4322 | 4750 | 1 | 429 | 0 | 1.00E-175 |
| hsa_circ_0021517 | 93 | 1 | 429 | 1 | 429 | 0 | 2.00E-176 |
| hsa_circ_0003427 | 98 | 33478 | 33519 | 873458 | 873417 | 0 | 2.00E-09 |
| hsa_circ_0003427 | 98 | 23930 | 23971 | 873458 | 873417 | 0 | 2.00E-09 |
| hsa_circ_0020417 | 92 | 1 | 278 | 1 | 278 | 0 | 1.00E-109 |
| hsa_circ_0020417 | 93 | 1 | 937 | 1 | 937 | 0 | 0 |
| hsa_circ_0020428 | 94 | 1 | 510 | 1 | 510 | 0 | 0 |
| hsa_circ_0020453 | 89 | 1 | 386 | 1 | 386 | 0.01 | 3.00E-131 |
| hsa_circ_0020453 | 89 | 1 | 712 | 1 | 712 | 0 | 0 |

novel_circ_009363 novel_circ_009364 novel_circ_009365 novel_circ_009366 novel_circ_009367 novel_circ_009369 novel_circ_009370 novel_circ_009371 novel_circ_009372 novel_circ_009374 novel_circ_009376 novel_circ_009377 novel_circ_009378 novel_circ_009379 novel_circ_009380 novel_circ_009381 novel_circ_009387 novel_circ_009388 novel_circ_009391 novel_circ_009392 novel_circ_009393 novel_circ_009394 novel_circ_009395 novel_circ_009398 novel_circ_009399 novel_circ_009400 novel_circ_009401 novel_circ_009406 novel_circ_009407 novel_circ_009408 novel_circ_009409 novel_circ_009410 novel_circ_009411 novel_circ_009412 novel_circ_009413 novel_circ_009415 novel_circ_009416 novel_circ_009417 novel_circ_009418 novel_circ_009419 novel_circ_009420 novel_circ_009421 novel_circ_009422 novel_circ_009423 novel_circ_009424 novel_circ_009425 novel_circ_009427 novel_circ_009428 novel_circ_009429 novel_circ_009430 novel_circ_009431 novel_circ_009432 novel_circ_009433 novel_circ_009434

| hsa_circ_0020453 | 89 | 1 | 812 | 1 | 812 | 0 | 0 |
| --- | --- | --- | --- | --- | --- | --- | --- |
| hsa_circ_0020453 | 89 | 1 | 822 | 1 | 822 | 0 | 0 |
| hsa_circ_0020453 | 90 | 1 | 999 | 1 | 999 | 0 | 0 |
| hsa_circ_0020458 | 90 | 1 | 613 | 1 | 613 | 0 | 0 |
| hsa_circ_0020474 | 93 | 1 | 330 | 1 | 330 | 0 | 8.00E-137 |
| hsa_circ_0020478 | 93 | 1 | 197 | 1 | 197 | 0 | 2.00E-76 |
| hsa_circ_0020480 | 92 | 1 | 690 | 209 | 898 | 0 | 0 |
| hsa_circ_0020485 | 95 | 1 | 205 | 347 | 551 | 0 | 1.00E-85 |
| hsa_circ_0000269 | 94 | 95 | 273 | 15 | 193 | 0 | 3.00E-71 |
| hsa_circ_0020507 | 90 | 12480 | 13133 | 1 | 667 | 0.02 | 0 |
| hsa_circ_0020520 | 95 | 3 | 377 | 5 | 379 | 0 | 4.00E-165 |
| hsa_circ_0020520 | 93 | 3 | 982 | 5 | 984 | 0 | 0 |
| hsa_circ_0020520 | 93 | 3 | 982 | 5 | 984 | 0 | 0 |
| hsa_circ_0020520 | 93 | 3 | 982 | 5 | 984 | 0 | 0 |
| hsa_circ_0020520 | 93 | 3 | 982 | 5 | 984 | 0 | 0 |
| hsa_circ_0005972 | 90 | 4 | 257 | 254 | 1 | 0 | 5.00E-88 |
| hsa_circ_0020546 | 89 | 1 | 341 | 1 | 341 | 0 | 7.00E-118 |
| hsa_circ_0020546 | 89 | 1 | 426 | 1 | 426 | 0 | 1.00E-151 |
| hsa_circ_0020556 | 87 | 3 | 319 | 3 | 313 | 0.02 | 3.00E-96 |
| hsa_circ_0020557 | 90 | 2 | 883 | 217 | 1098 | 0 | 0 |
| hsa_circ_0020557 | 92 | 1 | 234 | 865 | 1098 | 0 | 3.00E-90 |
| hsa_circ_0020561 | 85 | 1 | 273 | 428 | 165 | 0.03 | 2.00E-72 |
| hsa_circ_0020564 | 89 | 1 | 362 | 664 | 303 | 0 | 3.00E-126 |
| hsa_circ_0020584 | 90 | 423 | 756 | 1 | 334 | 0 | 5.00E-122 |
| hsa_circ_0016761 | 81 | 40 | 1038 | 38 | 1033 | 0.01 | 0 |
| hsa_circ_0016761 | 80 | 40 | 1175 | 38 | 1158 | 0.02 | 0 |
| hsa_circ_0016761 | 80 | 40 | 1175 | 38 | 1158 | 0.02 | 0 |
| hsa_circ_0015095 | 96 | 21147 | 21313 | 1 | 167 | 0 | 2.00E-69 |
| hsa_circ_0015095 | 96 | 19301 | 19467 | 1 | 167 | 0 | 2.00E-69 |
| hsa_circ_0015097 | 92 | 1 | 853 | 156 | 1002 | 0.01 | 0 |
| hsa_circ_0015097 | 96 | 1 | 396 | 465 | 860 | 0 | 0 |
| hsa_circ_0015097 | 94 | 1 | 843 | 592 | 1428 | 0.01 | 0 |
| hsa_circ_0015097 | 94 | 1 | 844 | 592 | 1429 | 0.01 | 0 |
| hsa_circ_0015101 | 91 | 1 | 432 | 143 | 568 | 0.01 | 5.00E-160 |
| hsa_circ_0015102 | 93 | 1 | 435 | 1 | 435 | 0 | 5.00E-180 |
| hsa_circ_0015118 | 89 | 14321 | 15537 | 1 | 1217 | 0 | 0 |
| hsa_circ_0015121 | 90 | 1 | 651 | 1 | 651 | 0 | 0 |
| hsa_circ_0015122 | 90 | 1 | 465 | 1 | 465 | 0 | 5.00E-170 |
| hsa_circ_0015123 | 89 | 1 | 824 | 1 | 824 | 0 | 0 |
| hsa_circ_0015123 | 89 | 1 | 688 | 137 | 824 | 0 | 0 |
| hsa_circ_0015126 | 89 | 5 | 473 | 5 | 473 | 0 | 3.00E-162 |
| hsa_circ_0015118 | 84 | 5 | 1322 | 749 | 2099 | 0.03 | 0 |
| hsa_circ_0015129 | 84 | 1 | 477 | 1 | 492 | 0.03 | 2.00E-125 |
| hsa_circ_0015118 | 87 | 1 | 711 | 959 | 1684 | 0.02 | 0 |
| hsa_circ_0015131 | 83 | 1 | 691 | 1 | 709 | 0.03 | 0 |
| hsa_circ_0015132 | 82 | 1 | 631 | 1 | 649 | 0.03 | 9.00E-149 |
| hsa_circ_0015135 | 82 | 1 | 930 | 1 | 948 | 0.02 | 0 |
| hsa_circ_0007277 | 87 | 1 | 491 | 1 | 491 | 0 | 9.00E-153 |
| hsa_circ_0007277 | 87 | 1 | 352 | 140 | 491 | 0 | 1.00E-110 |
| hsa_circ_0015140 | 94 | 2 | 412 | 2 | 412 | 0 | 4.00E-175 |
| hsa_circ_0015138 | 94 | 2 | 508 | 251 | 757 | 0 | 0 |
| hsa_circ_0015138 | 91 | 2 | 766 | 251 | 1018 | 0 | 0 |
| hsa_circ_0015138 | 95 | 1 | 228 | 530 | 757 | 0 | 1.00E-98 |
| hsa_circ_0015138 | 90 | 1 | 486 | 530 | 1018 | 0.01 | 3.00E-177 |

novel_circ_009435 novel_circ_009436 novel_circ_009438 novel_circ_009440 novel_circ_009441 novel_circ_009448 novel_circ_009449 novel_circ_009450 novel_circ_009451 novel_circ_009452 novel_circ_009453 novel_circ_009454 novel_circ_009455 novel_circ_009456 novel_circ_009457 novel_circ_009458 novel_circ_009459 novel_circ_009460 novel_circ_009461 novel_circ_009462 novel_circ_009463 novel_circ_009464 novel_circ_009465 novel_circ_009466 novel_circ_009467 novel_circ_009468 novel_circ_009469 novel_circ_009471 novel_circ_009472 novel_circ_009473 novel_circ_009474 novel_circ_009475 novel_circ_009476 novel_circ_009482 novel_circ_009483 novel_circ_009484 novel_circ_009485 novel_circ_009486 novel_circ_009488 novel_circ_009489 novel_circ_009490 novel_circ_009491 novel_circ_009492 novel_circ_009493 novel_circ_009499 novel_circ_009500 novel_circ_009501 novel_circ_009502 novel_circ_009503 novel_circ_009504 novel_circ_009505 novel_circ_009506 novel_circ_009507 novel_circ_009508

| hsa_circ_0004255 | 92 | 1 | 365 | 356 | 1 | 0.02 | 2.00E-143 |
| --- | --- | --- | --- | --- | --- | --- | --- |
| hsa_circ_0004255 | 90 | 4864 | 5055 | 356 | 174 | 0.05 | 2.00E-59 |
| hsa_circ_0007636 | 89 | 11522 | 11585 | 1E+06 | 1014980 | 0.02 | 1.00E-11 |
| hsa_circ_0018420 | 92 | 2186 | 2512 | 81 | 407 | 0 | 3.00E-129 |
| hsa_circ_0018420 | 92 | 1 | 327 | 81 | 407 | 0 | 4.00E-130 |
| hsa_circ_0018433 | 91 | 7 | 473 | 3568 | 3103 | 0 | 1.00E-175 |
| hsa_circ_0006246 | 93 | 1 | 419 | 419 | 1 | 0 | 3.00E-176 |
| hsa_circ_0006246 | 94 | 1 | 523 | 523 | 1 | 0 | 0 |
| hsa_circ_0018435 | 94 | 1 | 746 | 746 | 1 | 0.01 | 0 |
| hsa_circ_0018435 | 99 | 1 | 351 | 821 | 471 | 0 | 4.00E-180 |
| hsa_circ_0018436 | 94 | 1 | 1150 | 9278 | 8129 | 0 | 0 |
| hsa_circ_0018436 | 95 | 2 | 355 | 8605 | 8252 | 0 | 8.00E-157 |
| hsa_circ_0018436 | 93 | 1 | 872 | 9123 | 8252 | 0 | 0 |
| hsa_circ_0018436 | 92 | 1 | 954 | 10564 | 9611 | 0 | 0 |
| hsa_circ_0018436 | 90 | 1 | 367 | 10267 | 9901 | 0 | 1.00E-135 |
| hsa_circ_0018436 | 92 | 1 | 664 | 10564 | 9901 | 0 | 0 |
| hsa_circ_0018436 | 92 | 694 | 1357 | 10564 | 9901 | 0 | 0 |
| hsa_circ_0018439 | 92 | 7 | 792 | 885 | 100 | 0 | 0 |
| hsa_circ_0018439 | 93 | 7 | 396 | 885 | 496 | 0 | 9.00E-162 |
| hsa_circ_0018439 | 91 | 7 | 297 | 885 | 595 | 0 | 3.00E-110 |
| hsa_circ_0018440 | 90 | 4 | 708 | 681 | 1 | 0.03 | 0 |
| hsa_circ_0018441 | 94 | 4 | 399 | 396 | 1 | 0 | 9.00E-172 |
| hsa_circ_0018442 | 90 | 1 | 1247 | 3651 | 2408 | 0 | 0 |
| hsa_circ_0018445 | 89 | 1 | 975 | 1093 | 122 | 0.01 | 0 |
| hsa_circ_0018445 | 90 | 4 | 1467 | 1583 | 122 | 0 | 0 |
| hsa_circ_0018445 | 91 | 913 | 1269 | 1636 | 1280 | 0 | 3.00E-136 |
| hsa_circ_0018452 | 95 | 1 | 454 | 1 | 457 | 0.01 | 0 |
| hsa_circ_0050250 | 87 | 1 | 566 | 587 | 22 | 0 | 0 |
| hsa_circ_0050235 | 88 | 1 | 258 | 1 | 264 | 0.02 | 1.00E-83 |
| hsa_circ_0050232 | 88 | 86 | 346 | 309 | 575 | 0.02 | 4.00E-85 |
| hsa_circ_0050220 | 91 | 454 | 678 | 581 | 357 | 0 | 9.00E-80 |
| hsa_circ_0050220 | 87 | 1 | 581 | 581 | 1 | 0 | 0 |
| hsa_circ_0050219 | 90 | 1 | 748 | 1186 | 439 | 0 | 0 |
| hsa_circ_0050190 | 84 | 1 | 520 | 828 | 1340 | 0.02 | 6.00E-140 |
| hsa_circ_0005063 | 82 | 1 | 1584 | 1608 | 1 | 0.02 | 0 |
| hsa_circ_0050187 | 82 | 1 | 1705 | 1729 | 1 | 0.02 | 0 |
| hsa_circ_0050186 | 82 | 1 | 1707 | 2808 | 1078 | 0.02 | 0 |
| hsa_circ_0050186 | 81 | 1 | 2785 | 2808 | 6 | 0.03 | 0 |
| hsa_circ_0050183 | 90 | 1 | 240 | 240 | 1 | 0 | 3.00E-85 |
| hsa_circ_0050166 | 86 | 163 | 420 | 258 | 1 | 0.02 | 2.00E-73 |
| hsa_circ_0050166 | 86 | 163 | 420 | 258 | 1 | 0.02 | 2.00E-72 |
| hsa_circ_0050146 | 97 | 1 | 86 | 96 | 181 | 0 | 8.00E-33 |
| hsa_circ_0050144 | 91 | 118 | 498 | 1 | 381 | 0 | 2.00E-141 |
| hsa_circ_0050146 | 92 | 1 | 181 | 1 | 181 | 0 | 6.00E-66 |
| hsa_circ_0050029 | 92 | 1 | 445 | 792 | 348 | 0 | 1.00E-180 |
| hsa_circ_0050029 | 92 | 1 | 579 | 792 | 214 | 0 | 0 |
| hsa_circ_0050029 | 92 | 1 | 269 | 482 | 214 | 0 | 5.00E-103 |
| hsa_circ_0050029 | 91 | 1 | 482 | 482 | 1 | 0 | 0 |
| hsa_circ_0050027 | 92 | 1 | 858 | 1027 | 170 | 0 | 0 |
| hsa_circ_0050023 | 92 | 4 | 539 | 5563 | 5028 | 0 | 0 |
| hsa_circ_0050016 | 90 | 1 | 442 | 1 | 442 | 0 | 6.00E-164 |
| hsa_circ_0050016 | 90 | 1 | 317 | 1 | 317 | 0.01 | 1.00E-114 |
| hsa_circ_0050011 | 93 | 1 | 340 | 1 | 340 | 0 | 2.00E-137 |
| hsa_circ_0050006 | 88 | 1 | 229 | 1 | 229 | 0 | 8.00E-71 |

novel_circ_009509 novel_circ_009510 novel_circ_009511 novel_circ_009514 novel_circ_009515 novel_circ_009516 novel_circ_009517 novel_circ_009518 novel_circ_009519 novel_circ_009520 novel_circ_009521 novel_circ_009522 novel_circ_009523 novel_circ_009525 novel_circ_009526 novel_circ_009527 novel_circ_009528 novel_circ_009529 novel_circ_009530 novel_circ_009533 novel_circ_009534 novel_circ_009535 novel_circ_009536 novel_circ_009537 novel_circ_009538 novel_circ_009539 novel_circ_009542 novel_circ_009543 novel_circ_009544 novel_circ_009545 novel_circ_009546 novel_circ_009547 novel_circ_009548 novel_circ_009549 novel_circ_009550 novel_circ_009551 novel_circ_009552 novel_circ_009554 novel_circ_009555 novel_circ_009558 novel_circ_009559 novel_circ_009560 novel_circ_009561 novel_circ_009562 novel_circ_009563 novel_circ_009564 novel_circ_009565 novel_circ_009566 novel_circ_009567 novel_circ_009568 novel_circ_009569 novel_circ_009570 novel_circ_009571 novel_circ_009572

| hsa_circ_0049990 | 89 | 1 | 118 | 98 | 215 | 0 | 6.00E-34 |
| --- | --- | --- | --- | --- | --- | --- | --- |
| hsa_circ_0049990 | 89 | 929 | 1050 | 95 | 215 | 0.01 | 2.00E-33 |
| hsa_circ_0049978 | 91 | 3 | 435 | 1103 | 671 | 0 | 3.00E-162 |
| hsa_circ_0049887 | 88 | 403 | 636 | 772 | 539 | 0 | 2.00E-71 |
| hsa_circ_0049887 | 88 | 245 | 1004 | 772 | 1 | 0.02 | 0 |
| hsa_circ_0049885 | 91 | 4 | 312 | 309 | 1 | 0 | 4.00E-115 |
| hsa_circ_0049885 | 91 | 4 | 312 | 309 | 1 | 0 | 5.00E-115 |
| hsa_circ_0049885 | 91 | 4 | 312 | 309 | 1 | 0 | 1.00E-114 |
| hsa_circ_0049863 | 94 | 1 | 265 | 1 | 265 | 0 | 4.00E-109 |
| hsa_circ_0049862 | 93 | 1 | 399 | 1 | 399 | 0 | 2.00E-163 |
| hsa_circ_0049511 | 77 | 1134 | 1584 | 23801 | 23351 | 0.01 | 1.00E-66 |
| hsa_circ_0049519 | 85 | 3 | 121 | 2461 | 2343 | 0 | 2.00E-26 |
| hsa_circ_0049503 | 72 | 1466 | 2618 | 465 | 1635 | 0.05 | 3.00E-76 |
| hsa_circ_0049521 | 82 | 1 | 168 | 1 | 167 | 0.02 | 5.00E-31 |
| hsa_circ_0049521 | 82 | 1 | 168 | 1 | 167 | 0.02 | 2.00E-31 |
| hsa_circ_0049503 | 72 | 1131 | 1858 | 886 | 1635 | 0.08 | 2.00E-36 |
| hsa_circ_0047913 | 90 | 1 | 368 | 368 | 1 | 0 | 1.00E-134 |
| hsa_circ_0047916 | 94 | 1 | 653 | 653 | 1 | 0 | 0 |
| hsa_circ_0047918 | 93 | 1 | 429 | 429 | 1 | 0 | 2.00E-178 |
| hsa_circ_0047925 | 85 | 1 | 670 | 1500 | 831 | 0.01 | 0 |
| hsa_circ_0047929 | 92 | 1 | 532 | 621 | 90 | 0 | 0 |
| hsa_circ_0047945 | 90 | 1 | 989 | 986 | 1 | 0 | 0 |
| hsa_circ_0047945 | 89 | 1 | 1947 | 1947 | 1 | 0 | 0 |
| hsa_circ_0047948 | 90 | 1 | 782 | 779 | 1 | 0 | 0 |
| hsa_circ_0047949 | 89 | 1 | 253 | 370 | 118 | 0 | 8.00E-86 |
| hsa_circ_0047950 | 86 | 1 | 345 | 1088 | 741 | 0.01 | 5.00E-104 |
| hsa_circ_0052990 | 94 | 264 | 528 | 1 | 265 | 0 | 4.00E-112 |
| hsa_circ_0052990 | 94 | 107 | 371 | 1 | 265 | 0 | 3.00E-112 |
| hsa_circ_0052990 | 95 | 107 | 549 | 1 | 443 | 0 | 0 |
| hsa_circ_0052990 | 95 | 107 | 966 | 1 | 860 | 0 | 0 |
| hsa_circ_0052990 | 95 | 1 | 595 | 266 | 860 | 0 | 0 |
| hsa_circ_0052992 | 97 | 1 | 237 | 1 | 237 | 0 | 1.00E-108 |
| hsa_circ_0052990 | 94 | 1 | 1645 | 861 | 2505 | 0 | 0 |
| hsa_circ_0052993 | 92 | 1 | 885 | 1 | 885 | 0 | 0 |
| hsa_circ_0052993 | 92 | 1 | 1288 | 1 | 1288 | 0 | 0 |
| hsa_circ_0052993 | 92 | 1 | 1282 | 182 | 1463 | 0 | 0 |
| hsa_circ_0052993 | 94 | 1 | 505 | 784 | 1288 | 0 | 0 |
| hsa_circ_0052998 | 84 | 33 | 879 | 197 | 1043 | 0.01 | 0 |
| hsa_circ_0053007 | 92 | 1 | 639 | 1203 | 565 | 0 | 0 |
| hsa_circ_0012447 | 94 | 13399 | 13545 | 304 | 158 | 0 | 4.00E-55 |
| hsa_circ_0012447 | 92 | 201 | 504 | 304 | 1 | 0 | 8.00E-119 |
| hsa_circ_0012445 | 93 | 201 | 590 | 390 | 1 | 0 | 1.00E-161 |
| hsa_circ_0012445 | 93 | 201 | 590 | 390 | 1 | 0 | 2.00E-161 |
| hsa_circ_0012424 | 93 | 591 | 1624 | 1268 | 238 | 0 | 0 |
| hsa_circ_0012431 | 91 | 1 | 945 | 939 | 1 | 0.01 | 0 |
| hsa_circ_0012426 | 91 | 1 | 1015 | 1006 | 1 | 0.01 | 0 |
| hsa_circ_0012440 | 89 | 1 | 321 | 318 | 1 | 0.01 | 2.00E-107 |
| hsa_circ_0012438 | 90 | 1 | 412 | 409 | 1 | 0.01 | 8.00E-148 |
| hsa_circ_0012431 | 90 | 1 | 585 | 579 | 1 | 0.01 | 0 |
| hsa_circ_0012416 | 93 | 10 | 310 | 483 | 186 | 0.01 | 3.00E-121 |
| hsa_circ_0012412 | 94 | 1 | 319 | 322 | 1 | 0.01 | 6.00E-138 |
| hsa_circ_0012410 | 95 | 1 | 387 | 390 | 1 | 0.01 | 1.00E-170 |
| hsa_circ_0000070 | 96 | 1 | 493 | 496 | 1 | 0.01 | 0 |
| hsa_circ_0000070 | 98 | 1 | 290 | 290 | 1 | 0 | 1.00E-139 |

novel_circ_009573 novel_circ_009574 novel_circ_009575 novel_circ_009576 novel_circ_009577 novel_circ_009578 novel_circ_009579 novel_circ_009580 novel_circ_009581 novel_circ_009582 novel_circ_009583 novel_circ_009584 novel_circ_009588 novel_circ_009589 novel_circ_009590 novel_circ_009591 novel_circ_009592 novel_circ_009593 novel_circ_009594 novel_circ_009597 novel_circ_009598 novel_circ_009599 novel_circ_009600 novel_circ_009601 novel_circ_009602 novel_circ_009603 novel_circ_009604 novel_circ_009605 novel_circ_009606 novel_circ_009608 novel_circ_009609 novel_circ_009610 novel_circ_009612 novel_circ_009613 novel_circ_009614 novel_circ_009615 novel_circ_009616 novel_circ_009618 novel_circ_009619 novel_circ_009620 novel_circ_009621 novel_circ_009622 novel_circ_009623 novel_circ_009624 novel_circ_009625 novel_circ_009626 novel_circ_009627 novel_circ_009628 novel_circ_009629 novel_circ_009630 novel_circ_009631 novel_circ_009632 novel_circ_009633 novel_circ_009634

| hsa_circ_0012407 | 97 | 1 | 376 | 377 | 2 | 0 | 2.00E-177 |
| --- | --- | --- | --- | --- | --- | --- | --- |
| hsa_circ_0012401 | 96 | 1 | 611 | 611 | 1 | 0 | 0 |
| hsa_circ_0012401 | 96 | 1 | 524 | 524 | 1 | 0 | 0 |
| hsa_circ_0012402 | 97 | 1 | 273 | 273 | 1 | 0 | 3.00E-130 |
| hsa_circ_0012401 | 97 | 1 | 428 | 428 | 1 | 0 | 0 |
| hsa_circ_0012400 | 96 | 1 | 565 | 565 | 1 | 0 | 0 |
| hsa_circ_0012398 | 96 | 1 | 654 | 654 | 1 | 0 | 0 |
| hsa_circ_0012394 | 96 | 1 | 655 | 813 | 159 | 0 | 0 |
| hsa_circ_0012394 | 96 | 1 | 813 | 813 | 1 | 0 | 0 |
| hsa_circ_0012391 | 95 | 1 | 601 | 601 | 1 | 0 | 0 |
| hsa_circ_0012391 | 96 | 1 | 375 | 375 | 1 | 0 | 2.00E-173 |
| hsa_circ_0046937 | 74 | 10 | 355 | 3912 | 3579 | 0.07 | 9.00E-26 |
| hsa_circ_0012388 | 95 | 1 | 382 | 608 | 227 | 0 | 1.00E-170 |
| hsa_circ_0012387 | 94 | 1 | 708 | 708 | 1 | 0 | 0 |
| hsa_circ_0012387 | 94 | 1 | 661 | 708 | 48 | 0 | 0 |
| hsa_circ_0012387 | 93 | 1 | 414 | 414 | 1 | 0 | 2.00E-173 |
| hsa_circ_0012387 | 93 | 1 | 285 | 285 | 1 | 0 | 3.00E-115 |
| hsa_circ_0027443 | 97 | 1 | 311 | 1 | 311 | 0 | 6.00E-148 |
| hsa_circ_0027443 | 97 | 1 | 343 | 1 | 343 | 0 | 5.00E-164 |
| hsa_circ_0005505 | 89 | 3 | 303 | 3 | 303 | 0 | 1.00E-100 |
| hsa_circ_0005505 | 89 | 3 | 455 | 3 | 455 | 0 | 5.00E-160 |
| hsa_circ_0005505 | 89 | 3 | 456 | 3 | 456 | 0 | 3.00E-160 |
| hsa_circ_0005505 | 89 | 3 | 517 | 3 | 517 | 0 | 0 |
| hsa_circ_0005505 | 89 | 3 | 751 | 3 | 751 | 0 | 0 |
| hsa_circ_0006550 | 87 | 1 | 568 | 181 | 748 | 0 | 0 |
| hsa_circ_0003753 | 90 | 1 | 214 | 121 | 334 | 0 | 2.00E-72 |
| hsa_circ_0006550 | 88 | 1 | 448 | 301 | 748 | 0 | 6.00E-152 |
| hsa_circ_0006550 | 88 | 1 | 296 | 453 | 748 | 0 | 6.00E-93 |
| hsa_circ_0027449 | 84 | 1 | 1348 | 171 | 1548 | 0.03 | 0 |
| hsa_circ_0027451 | 93 | 1 | 306 | 306 | 1 | 0 | 2.00E-123 |
| hsa_circ_0027451 | 93 | 1 | 588 | 588 | 1 | 0 | 0 |
| hsa_circ_0027451 | 94 | 1 | 669 | 669 | 1 | 0 | 0 |
| hsa_circ_0027455 | 96 | 424 | 932 | 1 | 509 | 0 | 0 |
| hsa_circ_0027455 | 96 | 280 | 788 | 1 | 509 | 0 | 0 |
| hsa_circ_0027455 | 96 | 280 | 1081 | 1 | 802 | 0 | 0 |
| hsa_circ_0027455 | 95 | 1 | 2025 | 510 | 2534 | 0 | 0 |
| hsa_circ_0027455 | 94 | 1 | 1732 | 803 | 2534 | 0 | 0 |
| hsa_circ_0027467 | 87 | 1 | 449 | 1 | 452 | 0.01 | 5.00E-140 |
| hsa_circ_0027469 | 89 | 101 | 787 | 14 | 703 | 0 | 0 |
| hsa_circ_0027469 | 89 | 101 | 980 | 14 | 896 | 0 | 0 |
| hsa_circ_0027469 | 91 | 101 | 1591 | 14 | 1507 | 0 | 0 |
| hsa_circ_0027480 | 93 | 867 | 2561 | 1 | 1701 | 0 | 0 |
| hsa_circ_0027473 | 92 | 1 | 354 | 50 | 403 | 0 | 2.00E-138 |
| hsa_circ_0027473 | 92 | 1 | 965 | 50 | 1014 | 0 | 0 |
| hsa_circ_0027480 | 93 | 241 | 1767 | 1 | 1533 | 0 | 0 |
| hsa_circ_0027480 | 93 | 241 | 1935 | 1 | 1701 | 0 | 0 |
| hsa_circ_0027483 | 90 | 1 | 383 | 138 | 520 | 0 | 3.00E-141 |
| hsa_circ_0000415 | 96 | 1 | 240 | 1 | 240 | 0 | 1.00E-108 |
| hsa_circ_0000415 | 95 | 1 | 408 | 1 | 408 | 0 | 4.00E-180 |
| hsa_circ_0002770 | 94 | 2 | 389 | 2 | 389 | 0.01 | 1.00E-165 |
| hsa_circ_0027496 | 90 | 1 | 682 | 682 | 1 | 0 | 0 |
| hsa_circ_0027496 | 90 | 99 | 780 | 682 | 1 | 0 | 0 |
| hsa_circ_0027497 | 89 | 1 | 529 | 529 | 1 | 0 | 0 |
| hsa_circ_0000417 | 95 | 744 | 1710 | 633 | 1599 | 0 | 0 |

novel_circ_009635 novel_circ_009636 novel_circ_009637 novel_circ_009638 novel_circ_009639 novel_circ_009640 novel_circ_009641 novel_circ_009642 novel_circ_009643 novel_circ_009644 novel_circ_009645 novel_circ_009647 novel_circ_009648 novel_circ_009649 novel_circ_009650 novel_circ_009651 novel_circ_009652 novel_circ_009653 novel_circ_009655 novel_circ_009656 novel_circ_009657 novel_circ_009658 novel_circ_009659 novel_circ_009660 novel_circ_009661 novel_circ_009662 novel_circ_009664 novel_circ_009665 novel_circ_009666 novel_circ_009667 novel_circ_009668 novel_circ_009669 novel_circ_009670 novel_circ_009671 novel_circ_009672 novel_circ_009673 novel_circ_009674 novel_circ_009675 novel_circ_009677 novel_circ_009678 novel_circ_009679 novel_circ_009681 novel_circ_009683 novel_circ_009684 novel_circ_009685 novel_circ_009694 novel_circ_009695 novel_circ_009706 novel_circ_009709 novel_circ_009710 novel_circ_009711 novel_circ_009712 novel_circ_009713 novel_circ_009714

| hsa_circ_0027504 | 93 | 1 | 556 | 410 | 965 | 0 | 0 |
| --- | --- | --- | --- | --- | --- | --- | --- |
| hsa_circ_0050766 | 70 | 508 | 1221 | 6871 | 6158 | 0.06 | 5.00E-21 |
| hsa_circ_0050766 | 70 | 604 | 1317 | 6871 | 6158 | 0.06 | 6.00E-21 |
| hsa_circ_0050766 | 70 | 827 | 1540 | 6871 | 6158 | 0.06 | 6.00E-21 |
| hsa_circ_0050766 | 70 | 1019 | 1732 | 6871 | 6158 | 0.06 | 7.00E-21 |
| hsa_circ_0029594 | 84 | 9499 | 11190 | 90 | 1824 | 0.05 | 0 |
| hsa_circ_0029594 | 84 | 10779 | 12470 | 90 | 1824 | 0.05 | 0 |
| hsa_circ_0002866 | 80 | 301 | 391 | 208 | 118 | 0 | 2.00E-10 |
| hsa_circ_0002866 | 80 | 315 | 405 | 208 | 118 | 0 | 2.00E-10 |
| hsa_circ_0002866 | 80 | 787 | 877 | 208 | 118 | 0 | 3.00E-10 |
| hsa_circ_0002866 | 80 | 801 | 891 | 208 | 118 | 0 | 4.00E-10 |
| hsa_circ_0029593 | 83 | 4 | 1938 | 61 | 2008 | 0.06 | 0 |
| hsa_circ_0029593 | 89 | 4 | 226 | 61 | 280 | 0.01 | 2.00E-71 |
| hsa_circ_0029593 | 87 | 3954 | 4196 | 39 | 280 | 0.02 | 9.00E-72 |
| hsa_circ_0074573 | 80 | 10 | 188 | 214 | 36 | 0.02 | 5.00E-28 |
| hsa_circ_0074573 | 78 | 171 | 381 | 246 | 36 | 0.03 | 7.00E-29 |
| hsa_circ_0074573 | 78 | 2699 | 2909 | 246 | 36 | 0.03 | 5.00E-28 |
| hsa_circ_0007133 | 74 | 429 | 670 | 1E+06 | 1052162 | 0.02 | 2.00E-16 |
| hsa_circ_0007133 | 74 | 429 | 670 | 1E+06 | 1052162 | 0.02 | 8.00E-17 |
| hsa_circ_0050774 | 97 | 701 | 733 | 82 | 114 | 0 | 3.00E-06 |
| hsa_circ_0029590 | 87 | 8 | 646 | 902 | 264 | 0.01 | 0 |
| hsa_circ_0029582 | 86 | 8 | 1152 | 2571 | 1427 | 0.02 | 0 |
| hsa_circ_0029582 | 84 | 8 | 1359 | 2571 | 1246 | 0.03 | 0 |
| hsa_circ_0029590 | 88 | 1 | 478 | 741 | 264 | 0.01 | 3.00E-162 |
| hsa_circ_0029586 | 88 | 119 | 484 | 366 | 1 | 0.01 | 1.00E-116 |
| hsa_circ_0029586 | 88 | 7 | 243 | 237 | 1 | 0.02 | 1.00E-73 |
| hsa_circ_0029582 | 87 | 114 | 295 | 1427 | 1246 | 0 | 1.00E-52 |
| hsa_circ_0029563 | 83 | 48 | 3252 | 3595 | 376 | 0.01 | 0 |
| hsa_circ_0029573 | 86 | 1 | 622 | 622 | 1 | 0 | 0 |
| hsa_circ_0029566 | 86 | 1 | 1800 | 1833 | 34 | 0 | 0 |
| hsa_circ_0029561 | 91 | 12 | 459 | 448 | 1 | 0 | 3.00E-172 |
| hsa_circ_0008567 | 84 | 10 | 596 | 581 | 1 | 0.01 | 2.00E-159 |
| hsa_circ_0029551 | 84 | 10 | 597 | 704 | 123 | 0.01 | 5.00E-159 |
| hsa_circ_0029551 | 85 | 10 | 719 | 704 | 1 | 0.01 | 0 |
| hsa_circ_0029551 | 85 | 10 | 719 | 704 | 1 | 0.01 | 0 |
| hsa_circ_0029546 | 85 | 214 | 1240 | 3544 | 2515 | 0 | 0 |
| hsa_circ_0029546 | 86 | 1 | 467 | 3161 | 2692 | 0.01 | 1.00E-141 |
| hsa_circ_0029546 | 86 | 1 | 644 | 3161 | 2515 | 0 | 0 |
| hsa_circ_0029547 | 85 | 1 | 521 | 1262 | 739 | 0.01 | 1.00E-146 |
| hsa_circ_0029547 | 74 | 5 | 942 | 911 | 1 | 0.09 | 3.00E-81 |
| hsa_circ_0029540 | 87 | 1 | 516 | 516 | 1 | 0 | 6.00E-165 |
| hsa_circ_0004021 | 81 | 251 | 1314 | 217 | 1283 | 0.01 | 0 |
| hsa_circ_0029444 | 87 | 1 | 195 | 1 | 192 | 0.02 | 2.00E-55 |
| hsa_circ_0029421 | 85 | 79 | 431 | 355 | 3 | 0 | 3.00E-99 |
| hsa_circ_0029421 | 85 | 4 | 356 | 355 | 3 | 0 | 3.00E-99 |
| hsa_circ_0029416 | 80 | 29 | 657 | 1192 | 564 | 0.02 | 4.00E-129 |
| hsa_circ_0029384 | 87 | 2 | 1039 | 2 | 1039 | 0 | 0 |
| hsa_circ_0013829 | 74 | 41811 | 42671 | 152574 | 151713 | 0.04 | 2.00E-79 |
| hsa_circ_0068138 | 93 | 1 | 288 | 1 | 288 | 0 | 3.00E-115 |
| hsa_circ_0068138 | 89 | 1 | 629 | 1 | 629 | 0 | 0 |
| hsa_circ_0068138 | 89 | 1 | 1661 | 1 | 1661 | 0 | 0 |
| hsa_circ_0068138 | 89 | 1 | 2955 | 1 | 2955 | 0 | 0 |
| hsa_circ_0068138 | 92 | 1 | 408 | 118 | 525 | 0 | 1.00E-161 |
| hsa_circ_0068138 | 90 | 1 | 707 | 118 | 824 | 0 | 0 |

novel_circ_009715 novel_circ_009716 novel_circ_009717 novel_circ_009718 novel_circ_009719 novel_circ_009720 novel_circ_009721 novel_circ_009722 novel_circ_009723 novel_circ_009724 novel_circ_009725 novel_circ_009726 novel_circ_009727 novel_circ_009728 novel_circ_009729 novel_circ_009730 novel_circ_009731 novel_circ_009733 novel_circ_009734 novel_circ_009735 novel_circ_009736 novel_circ_009737 novel_circ_009738 novel_circ_009740 novel_circ_009741 novel_circ_009743 novel_circ_009744 novel_circ_009745 novel_circ_009746 novel_circ_009747 novel_circ_009749 novel_circ_009750 novel_circ_009751 novel_circ_009752 novel_circ_009755 novel_circ_009756 novel_circ_009757 novel_circ_009758 novel_circ_009759 novel_circ_009760 novel_circ_009761 novel_circ_009762 novel_circ_009763 novel_circ_009764 novel_circ_009765 novel_circ_009766 novel_circ_009767 novel_circ_009769 novel_circ_009770 novel_circ_009771 novel_circ_009772 novel_circ_009773 novel_circ_009774 novel_circ_009775

| hsa_circ_0068138 | 88 | 1 | 1544 | 118 | 1661 | 0 | 0 |
| --- | --- | --- | --- | --- | --- | --- | --- |
| hsa_circ_0068139 | 87 | 1 | 837 | 537 | 1373 | 0 | 0 |
| hsa_circ_0068140 | 89 | 1 | 1294 | 489 | 1782 | 0 | 0 |
| hsa_circ_0068140 | 90 | 1 | 1746 | 489 | 2234 | 0 | 0 |
| hsa_circ_0068140 | 89 | 1 | 971 | 667 | 1637 | 0 | 0 |
| hsa_circ_0068140 | 89 | 1 | 1116 | 667 | 1782 | 0 | 0 |
| hsa_circ_0068140 | 90 | 1 | 934 | 849 | 1782 | 0 | 0 |
| hsa_circ_0068140 | 89 | 1 | 403 | 1053 | 1455 | 0 | 6.00E-139 |
| hsa_circ_0068140 | 90 | 1 | 463 | 1053 | 1515 | 0 | 1.00E-170 |
| hsa_circ_0068140 | 90 | 1 | 730 | 1053 | 1782 | 0 | 0 |
| hsa_circ_0068140 | 91 | 1 | 900 | 1053 | 1952 | 0 | 0 |
| hsa_circ_0068140 | 91 | 1 | 1066 | 1053 | 2118 | 0 | 0 |
| hsa_circ_0068140 | 91 | 1 | 1066 | 1053 | 2118 | 0 | 0 |
| hsa_circ_0068140 | 91 | 1 | 1182 | 1053 | 2234 | 0 | 0 |
| hsa_circ_0068140 | 92 | 1 | 809 | 1310 | 2118 | 0 | 0 |
| hsa_circ_0068144 | 92 | 12008 | 12345 | 138 | 475 | 0 | 1.00E-129 |
| hsa_circ_0068144 | 92 | 12008 | 12345 | 138 | 475 | 0 | 1.00E-129 |
| hsa_circ_0068146 | 84 | 1 | 596 | 786 | 191 | 0.01 | 1.00E-162 |
| hsa_circ_0068146 | 84 | 6 | 724 | 909 | 191 | 0.01 | 0 |
| hsa_circ_0068146 | 86 | 1 | 918 | 1108 | 191 | 0 | 0 |
| hsa_circ_0068146 | 86 | 123 | 1310 | 1378 | 191 | 0 | 0 |
| hsa_circ_0068146 | 84 | 1 | 507 | 786 | 280 | 0 | 6.00E-135 |
| hsa_circ_0068146 | 86 | 1 | 829 | 1108 | 280 | 0 | 0 |
| hsa_circ_0068146 | 88 | 1 | 506 | 1108 | 603 | 0 | 9.00E-173 |
| hsa_circ_0068146 | 88 | 123 | 898 | 1378 | 603 | 0 | 0 |
| hsa_circ_0068146 | 89 | 1 | 321 | 1108 | 788 | 0 | 4.00E-110 |
| hsa_circ_0068146 | 91 | 1 | 194 | 1108 | 915 | 0 | 8.00E-70 |
| hsa_circ_0003085 | 88 | 123 | 504 | 382 | 1 | 0 | 2.00E-125 |
| hsa_circ_0008550 | 86 | 1 | 270 | 270 | 1 | 0 | 7.00E-77 |
| hsa_circ_0008550 | 86 | 123 | 392 | 270 | 1 | 0 | 1.00E-76 |
| hsa_circ_0068152 | 81 | 1 | 278 | 1387 | 1101 | 0.03 | 5.00E-55 |
| hsa_circ_0068152 | 81 | 155 | 432 | 1387 | 1101 | 0.03 | 7.00E-55 |
| hsa_circ_0068158 | 84 | 1 | 698 | 1950 | 1253 | 0.01 | 0 |
| hsa_circ_0068158 | 85 | 5417 | 5944 | 1971 | 1443 | 0 | 3.00E-147 |
| hsa_circ_0068164 | 92 | 91 | 1131 | 168 | 1215 | 0.01 | 0 |
| hsa_circ_0068166 | 92 | 96 | 1435 | 1 | 1348 | 0.01 | 0 |
| hsa_circ_0068166 | 92 | 96 | 1813 | 1 | 1726 | 0 | 0 |
| hsa_circ_0068166 | 92 | 1 | 1036 | 1 | 1044 | 0.01 | 0 |
| hsa_circ_0068166 | 92 | 1 | 1718 | 1 | 1726 | 0 | 0 |
| hsa_circ_0068169 | 93 | 1 | 981 | 1 | 981 | 0 | 0 |
| hsa_circ_0068169 | 93 | 1 | 1166 | 1 | 1166 | 0 | 0 |
| hsa_circ_0068169 | 93 | 1 | 1285 | 1 | 1285 | 0 | 0 |
| hsa_circ_0068169 | 92 | 1 | 1663 | 1 | 1663 | 0 | 0 |
| hsa_circ_0068169 | 92 | 1 | 1827 | 1 | 1827 | 0 | 0 |
| hsa_circ_0068169 | 92 | 1 | 304 | 982 | 1285 | 0 | 1.00E-120 |
| hsa_circ_0068169 | 91 | 1 | 682 | 982 | 1663 | 0 | 0 |
| hsa_circ_0068170 | 89 | 1 | 378 | 120 | 497 | 0 | 9.00E-132 |
| hsa_circ_0068173 | 89 | 3 | 672 | 3 | 669 | 0 | 0 |
| hsa_circ_0068173 | 89 | 3 | 834 | 3 | 831 | 0 | 0 |
| hsa_circ_0068178 | 88 | 1 | 437 | 1 | 437 | 0 | 2.00E-144 |
| hsa_circ_0068178 | 88 | 1 | 442 | 1 | 441 | 0 | 2.00E-144 |
| hsa_circ_0068184 | 93 | 1 | 390 | 1 | 387 | 0.01 | 3.00E-157 |
| hsa_circ_0068195 | 85 | 1 | 1020 | 1 | 1023 | 0.01 | 0 |
| hsa_circ_0068209 | 86 | 1 | 551 | 1 | 557 | 0.01 | 2.00E-164 |

novel_circ_009776 novel_circ_009777 novel_circ_009778 novel_circ_009779 novel_circ_009780 novel_circ_009781 novel_circ_009782 novel_circ_009783 novel_circ_009784 novel_circ_009785 novel_circ_009786 novel_circ_009787 novel_circ_009788 novel_circ_009789 novel_circ_009790 novel_circ_009791 novel_circ_009792 novel_circ_009793 novel_circ_009794 novel_circ_009797 novel_circ_009798 novel_circ_009799 novel_circ_009800 novel_circ_009801 novel_circ_009802 novel_circ_009804 novel_circ_009806 novel_circ_009807 novel_circ_009808 novel_circ_009809 novel_circ_009811 novel_circ_009813 novel_circ_009814 novel_circ_009815 novel_circ_009816 novel_circ_009817 novel_circ_009818 novel_circ_009819 novel_circ_009820 novel_circ_009821 novel_circ_009822 novel_circ_009823 novel_circ_009824 novel_circ_009825 novel_circ_009826 novel_circ_009827 novel_circ_009828 novel_circ_009829 novel_circ_009830 novel_circ_009831 novel_circ_009835 novel_circ_009836 novel_circ_009837 novel_circ_009838

| hsa_circ_0068213 | 84 | 1 | 762 | 1 | 789 | 0.03 | 0 |
| --- | --- | --- | --- | --- | --- | --- | --- |
| hsa_circ_0068213 | 84 | 1 | 939 | 1 | 966 | 0.03 | 0 |
| hsa_circ_0068250 | 89 | 1 | 517 | 517 | 1 | 0 | 0 |
| hsa_circ_0068253 | 91 | 1 | 317 | 317 | 1 | 0 | 2.00E-118 |
| hsa_circ_0068253 | 91 | 1 | 317 | 317 | 1 | 0 | 1.00E-119 |
| hsa_circ_0068253 | 91 | 1 | 700 | 703 | 1 | 0 | 0 |
| hsa_circ_0068253 | 91 | 1 | 629 | 703 | 72 | 0 | 0 |
| hsa_circ_0022196 | 95 | 7 | 471 | 561 | 97 | 0 | 0 |
| hsa_circ_0022204 | 93 | 10 | 309 | 300 | 1 | 0 | 2.00E-123 |
| hsa_circ_0022204 | 93 | 1 | 467 | 467 | 1 | 0 | 0 |
| hsa_circ_0022204 | 94 | 1 | 1763 | 1766 | 1 | 0 | 0 |
| hsa_circ_0022204 | 93 | 1 | 302 | 772 | 468 | 0.01 | 2.00E-123 |
| hsa_circ_0022204 | 94 | 1 | 1296 | 1766 | 468 | 0 | 0 |
| hsa_circ_0022204 | 94 | 1 | 1172 | 1766 | 592 | 0 | 0 |
| hsa_circ_0022204 | 94 | 1 | 775 | 1548 | 774 | 0 | 0 |
| hsa_circ_0022204 | 94 | 1 | 993 | 1766 | 774 | 0 | 0 |
| hsa_circ_0075358 | 83 | 1 | 1448 | 1 | 1463 | 0.01 | 0 |
| hsa_circ_0075358 | 87 | 1 | 301 | 1 | 301 | 0 | 1.00E-90 |
| hsa_circ_0075358 | 89 | 1 | 115 | 1 | 115 | 0 | 6.00E-32 |
| hsa_circ_0075336 | 85 | 1 | 903 | 997 | 95 | 0 | 0 |
| hsa_circ_0075336 | 85 | 1 | 708 | 802 | 95 | 0 | 0 |
| hsa_circ_0075338 | 87 | 1 | 457 | 457 | 1 | 0 | 7.00E-144 |
| hsa_circ_0075302 | 84 | 1 | 271 | 384 | 654 | 0 | 4.00E-69 |
| hsa_circ_0075302 | 86 | 1 | 461 | 194 | 654 | 0 | 4.00E-136 |
| hsa_circ_0075280 | 96 | 1 | 804 | 1097 | 294 | 0 | 0 |
| hsa_circ_0075274 | 86 | 1 | 555 | 1 | 555 | 0 | 6.00E-165 |
| hsa_circ_0059524 | 79 | 8 | 177 | 179 | 347 | 0.01 | 6.00E-25 |
| hsa_circ_0047050 | 73 | 794 | 1601 | 20825 | 20018 | 0.05 | 2.00E-59 |
| hsa_circ_0059524 | 79 | 8 | 177 | 179 | 347 | 0.01 | 2.00E-25 |
| hsa_circ_0047050 | 73 | 794 | 1601 | 20825 | 20018 | 0.05 | 1.00E-59 |
| hsa_circ_0012752 | 97 | 1 | 532 | 1 | 532 | 0 | 0 |
| hsa_circ_0012757 | 98 | 1 | 256 | 1 | 256 | 0 | 8.00E-126 |
| hsa_circ_0012757 | 97 | 1 | 385 | 1 | 385 | 0 | 0 |
| hsa_circ_0012759 | 96 | 1 | 345 | 1 | 345 | 0 | 2.00E-158 |
| hsa_circ_0012767 | 88 | 69 | 894 | 1 | 826 | 0 | 0 |
| hsa_circ_0012767 | 88 | 1 | 826 | 1 | 826 | 0 | 0 |
| hsa_circ_0012768 | 88 | 1 | 1920 | 1 | 1923 | 0 | 0 |
| hsa_circ_0012769 | 87 | 1 | 697 | 326 | 1025 | 0.01 | 0 |
| hsa_circ_0012769 | 88 | 1 | 1260 | 326 | 1588 | 0.01 | 0 |
| hsa_circ_0012772 | 88 | 1 | 650 | 1 | 650 | 0.01 | 0 |
| hsa_circ_0012772 | 88 | 1 | 944 | 1 | 944 | 0.01 | 0 |
| hsa_circ_0012772 | 88 | 1 | 852 | 93 | 944 | 0 | 0 |
| hsa_circ_0012772 | 89 | 1 | 563 | 382 | 944 | 0 | 0 |
| hsa_circ_0012776 | 88 | 1 | 429 | 1 | 429 | 0 | 5.00E-145 |
| hsa_circ_0012781 | 86 | 1 | 549 | 724 | 1269 | 0.01 | 4.00E-167 |
| hsa_circ_0012781 | 87 | 6 | 713 | 850 | 1557 | 0 | 0 |
| hsa_circ_0012781 | 87 | 6 | 713 | 850 | 1557 | 0 | 0 |
| hsa_circ_0012781 | 88 | 1 | 538 | 1020 | 1557 | 0 | 2.00E-178 |
| hsa_circ_0012781 | 90 | 1 | 286 | 1092 | 1377 | 0 | 2.00E-102 |
| hsa_circ_0012781 | 88 | 1 | 466 | 1092 | 1557 | 0 | 7.00E-154 |
| hsa_circ_0012783 | 91 | 248 | 505 | 551 | 807 | 0 | 2.00E-94 |
| hsa_circ_0012783 | 93 | 1 | 412 | 674 | 1085 | 0 | 6.00E-169 |
| hsa_circ_0012772 | 92 | 1 | 1028 | 3076 | 4103 | 0 | 0 |
| hsa_circ_0012772 | 93 | 1 | 429 | 3294 | 3722 | 0 | 5.00E-175 |

novel_circ_009839 novel_circ_009840 novel_circ_009841 novel_circ_009842 novel_circ_009843 novel_circ_009845 novel_circ_009846 novel_circ_009847 novel_circ_009848 novel_circ_009849 novel_circ_009850 novel_circ_009851 novel_circ_009852 novel_circ_009853 novel_circ_009854 novel_circ_009855 novel_circ_009856 novel_circ_009857 novel_circ_009858 novel_circ_009859 novel_circ_009860 novel_circ_009861 novel_circ_009862 novel_circ_009863 novel_circ_009864 novel_circ_009865 novel_circ_009866 novel_circ_009867 novel_circ_009868 novel_circ_009869 novel_circ_009870 novel_circ_009871 novel_circ_009872 novel_circ_009873 novel_circ_009874 novel_circ_009875 novel_circ_009876 novel_circ_009877 novel_circ_009878 novel_circ_009879 novel_circ_009880 novel_circ_009881 novel_circ_009882 novel_circ_009883 novel_circ_009884 novel_circ_009885 novel_circ_009886 novel_circ_009887 novel_circ_009888 novel_circ_009889 novel_circ_009890 novel_circ_009891 novel_circ_009893 novel_circ_009894

| hsa_circ_0012772 | 92 | 1 | 810 | 3294 | 4103 | 0 | 0 |
| --- | --- | --- | --- | --- | --- | --- | --- |
| hsa_circ_0012786 | 91 | 1 | 235 | 1 | 235 | 0 | 8.00E-86 |
| hsa_circ_0012786 | 89 | 1 | 350 | 1 | 350 | 0 | 1.00E-119 |
| hsa_circ_0012786 | 89 | 1 | 388 | 1 | 388 | 0 | 3.00E-132 |
| hsa_circ_0012786 | 91 | 1 | 616 | 1 | 616 | 0 | 0 |
| hsa_circ_0012787 | 92 | 7 | 381 | 7 | 381 | 0 | 9.00E-147 |
| hsa_circ_0008657 | 95 | 1 | 387 | 1 | 387 | 0 | 4.00E-175 |
| hsa_circ_0000080 | 92 | 1 | 1065 | 1 | 1065 | 0 | 0 |
| hsa_circ_0012799 | 95 | 1 | 713 | 713 | 1 | 0 | 0 |
| hsa_circ_0012799 | 95 | 1 | 713 | 713 | 1 | 0 | 0 |
| hsa_circ_0012799 | 95 | 1 | 713 | 713 | 1 | 0 | 0 |
| hsa_circ_0012799 | 95 | 1 | 606 | 713 | 108 | 0 | 0 |
| hsa_circ_0012801 | 93 | 4 | 750 | 747 | 1 | 0 | 0 |
| hsa_circ_0012801 | 94 | 1 | 597 | 597 | 1 | 0 | 0 |
| hsa_circ_0012804 | 93 | 1 | 453 | 453 | 1 | 0 | 0 |
| hsa_circ_0012804 | 92 | 4 | 413 | 603 | 194 | 0 | 8.00E-163 |
| hsa_circ_0012804 | 91 | 2 | 645 | 837 | 194 | 0 | 0 |
| hsa_circ_0012810 | 94 | 1 | 890 | 891 | 2 | 0 | 0 |
| hsa_circ_0012810 | 94 | 1 | 1076 | 1077 | 2 | 0 | 0 |
| hsa_circ_0012810 | 94 | 1 | 736 | 891 | 156 | 0 | 0 |
| hsa_circ_0012811 | 93 | 7 | 947 | 1081 | 141 | 0 | 0 |
| hsa_circ_0012811 | 94 | 1 | 1696 | 1836 | 141 | 0 | 0 |
| hsa_circ_0012811 | 93 | 1 | 654 | 1406 | 753 | 0 | 0 |
| hsa_circ_0012813 | 95 | 1 | 319 | 319 | 1 | 0 | 1.00E-140 |
| hsa_circ_0012819 | 94 | 1 | 647 | 647 | 1 | 0 | 0 |
| hsa_circ_0012819 | 95 | 2 | 1293 | 1292 | 1 | 0 | 0 |
| hsa_circ_0012819 | 95 | 1 | 1538 | 1538 | 1 | 0 | 0 |
| hsa_circ_0012819 | 95 | 1 | 1644 | 1644 | 1 | 0 | 0 |
| hsa_circ_0012821 | 95 | 1 | 972 | 1281 | 310 | 0 | 0 |
| hsa_circ_0012821 | 95 | 2 | 646 | 1035 | 391 | 0 | 0 |
| hsa_circ_0012821 | 95 | 1 | 891 | 1281 | 391 | 0 | 0 |
| hsa_circ_0012820 | 95 | 1 | 997 | 1481 | 485 | 0 | 0 |
| hsa_circ_0012821 | 94 | 1 | 674 | 1281 | 608 | 0 | 0 |
| hsa_circ_0012820 | 94 | 1 | 780 | 1481 | 702 | 0 | 0 |
| hsa_circ_0012821 | 95 | 1 | 588 | 1281 | 694 | 0 | 0 |
| hsa_circ_0001611 | 86 | 1722 | 1791 | 24078 | 24145 | 0.03 | 7.00E-11 |
| hsa_circ_0001611 | 86 | 9035 | 9104 | 24078 | 24145 | 0.03 | 3.00E-10 |
| hsa_circ_0012822 | 95 | 158 | 386 | 168 | 396 | 0 | 4.00E-98 |
| hsa_circ_0012822 | 93 | 158 | 620 | 168 | 630 | 0 | 0 |
| hsa_circ_0012822 | 94 | 158 | 1022 | 168 | 1032 | 0 | 0 |
| hsa_circ_0012822 | 93 | 158 | 1159 | 168 | 1169 | 0 | 0 |
| hsa_circ_0012822 | 94 | 158 | 1435 | 168 | 1445 | 0 | 0 |
| hsa_circ_0012822 | 94 | 158 | 1435 | 168 | 1445 | 0 | 0 |
| hsa_circ_0012824 | 95 | 1 | 228 | 1 | 228 | 0 | 1.00E-98 |
| hsa_circ_0012824 | 93 | 1 | 462 | 1 | 462 | 0 | 0 |
| hsa_circ_0012824 | 94 | 1 | 864 | 1 | 864 | 0 | 0 |
| hsa_circ_0012824 | 93 | 1 | 1001 | 1 | 1001 | 0 | 0 |
| hsa_circ_0012824 | 94 | 1 | 1157 | 1 | 1157 | 0 | 0 |
| hsa_circ_0012824 | 94 | 1 | 1277 | 1 | 1277 | 0 | 0 |
| hsa_circ_0012824 | 94 | 1 | 1277 | 1 | 1277 | 0 | 0 |
| hsa_circ_0012827 | 94 | 1 | 1049 | 1 | 1049 | 0 | 0 |
| hsa_circ_0012828 | 94 | 3 | 413 | 3 | 413 | 0 | 2.00E-178 |
| hsa_circ_0012831 | 91 | 538 | 1183 | 1 | 646 | 0 | 0 |
| hsa_circ_0012831 | 91 | 359 | 1004 | 1 | 646 | 0 | 0 |

novel_circ_009895 novel_circ_009896 novel_circ_009897 novel_circ_009900 novel_circ_009901 novel_circ_009902 novel_circ_009904 novel_circ_009905 novel_circ_009906 novel_circ_009907 novel_circ_009908 novel_circ_009909 novel_circ_009912 novel_circ_009913 novel_circ_009914 novel_circ_009915 novel_circ_009916 novel_circ_009917 novel_circ_009918 novel_circ_009919 novel_circ_009920 novel_circ_009921 novel_circ_009922 novel_circ_009923 novel_circ_009924 novel_circ_009925 novel_circ_009926 novel_circ_009927 novel_circ_009928 novel_circ_009929 novel_circ_009930 novel_circ_009931 novel_circ_009932 novel_circ_009933 novel_circ_009934 novel_circ_009935 novel_circ_009936 novel_circ_009938 novel_circ_009939 novel_circ_009940 novel_circ_009941 novel_circ_009942 novel_circ_009943 novel_circ_009944 novel_circ_009945 novel_circ_009946 novel_circ_009947 novel_circ_009948 novel_circ_009949 novel_circ_009950 novel_circ_009951 novel_circ_009952 novel_circ_009953 novel_circ_009954

| hsa_circ_0012831 | 91 | 1 | 646 | 1 | 646 | 0 | 0 |
| --- | --- | --- | --- | --- | --- | --- | --- |
| hsa_circ_0012831 | 91 | 1 | 646 | 1 | 646 | 0 | 0 |
| hsa_circ_0012831 | 92 | 1 | 510 | 137 | 646 | 0 | 0 |
| hsa_circ_0012833 | 97 | 1028 | 1161 | 1 | 134 | 0 | 3.00E-57 |
| hsa_circ_0012833 | 97 | 1028 | 1161 | 1 | 134 | 0 | 1.00E-56 |
| hsa_circ_0012833 | 95 | 1028 | 1310 | 1 | 283 | 0 | 4.00E-125 |
| hsa_circ_0012833 | 95 | 159 | 441 | 1 | 283 | 0 | 1.00E-125 |
| hsa_circ_0012833 | 93 | 159 | 759 | 1 | 601 | 0 | 0 |
| hsa_circ_0012833 | 95 | 91 | 373 | 1 | 283 | 0 | 1.00E-125 |
| hsa_circ_0012837 | 94 | 1 | 436 | 1 | 436 | 0 | 0 |
| hsa_circ_0012837 | 94 | 1 | 627 | 1 | 627 | 0 | 0 |
| hsa_circ_0012837 | 90 | 1 | 455 | 899 | 1353 | 0 | 2.00E-164 |
| hsa_circ_0012844 | 94 | 1 | 212 | 1 | 212 | 0 | 2.00E-86 |
| hsa_circ_0012844 | 95 | 1 | 319 | 1 | 319 | 0 | 5.00E-139 |
| hsa_circ_0012845 | 93 | 108 | 596 | 1 | 489 | 0 | 0 |
| hsa_circ_0012846 | 92 | 235 | 980 | 1 | 746 | 0 | 0 |
| hsa_circ_0012846 | 92 | 128 | 639 | 1 | 512 | 0 | 0 |
| hsa_circ_0012846 | 92 | 128 | 873 | 1 | 746 | 0 | 0 |
| hsa_circ_0012847 | 94 | 640 | 1870 | 1 | 1231 | 0 | 0 |
| hsa_circ_0012846 | 92 | 1 | 512 | 1 | 512 | 0 | 0 |
| hsa_circ_0012847 | 94 | 513 | 1743 | 1 | 1231 | 0 | 0 |
| hsa_circ_0012846 | 92 | 1 | 384 | 363 | 746 | 0 | 3.00E-153 |
| hsa_circ_0012847 | 92 | 151 | 776 | 1 | 626 | 0 | 0 |
| hsa_circ_0012847 | 92 | 151 | 940 | 1 | 790 | 0 | 0 |
| hsa_circ_0012847 | 94 | 151 | 1381 | 1 | 1231 | 0 | 0 |
| hsa_circ_0012847 | 93 | 1 | 392 | 235 | 626 | 0 | 2.00E-164 |
| hsa_circ_0012847 | 95 | 3 | 847 | 387 | 1231 | 0 | 0 |
| hsa_circ_0012849 | 96 | 3 | 436 | 3 | 436 | 0 | 0 |
| hsa_circ_0007009 | 93 | 1 | 586 | 112 | 697 | 0 | 0 |
| hsa_circ_0012852 | 93 | 224 | 1019 | 1 | 796 | 0 | 0 |
| hsa_circ_0012857 | 92 | 70 | 729 | 3 | 662 | 0 | 0 |
| hsa_circ_0012857 | 92 | 70 | 729 | 3 | 662 | 0 | 0 |
| hsa_circ_0012857 | 92 | 70 | 856 | 3 | 789 | 0 | 0 |
| hsa_circ_0012857 | 91 | 70 | 940 | 3 | 876 | 0 | 0 |
| hsa_circ_0012857 | 93 | 3 | 470 | 3 | 470 | 0 | 0 |
| hsa_circ_0012857 | 92 | 3 | 662 | 3 | 662 | 0 | 0 |
| hsa_circ_0012857 | 92 | 3 | 662 | 3 | 662 | 0 | 0 |
| hsa_circ_0012859 | 89 | 231 | 397 | 482 | 648 | 0.01 | 4.00E-51 |
| hsa_circ_0012860 | 89 | 1 | 249 | 1 | 249 | 0 | 1.00E-83 |
| hsa_circ_0012880 | 91 | 1 | 293 | 276 | 568 | 0 | 1.00E-109 |
| hsa_circ_0004659 | 92 | 1 | 570 | 569368 | 569937 | 0 | 0 |
| hsa_circ_0004659 | 92 | 1 | 570 | 569368 | 569937 | 0 | 0 |
| hsa_circ_0004659 | 93 | 1 | 450 | 569488 | 569937 | 0 | 0 |
| hsa_circ_0004659 | 93 | 1 | 450 | 569488 | 569937 | 0 | 0 |
| hsa_circ_0012885 | 96 | 1746 | 2095 | 1 | 350 | 0 | 9.00E-159 |
| hsa_circ_0012885 | 96 | 1 | 350 | 1 | 350 | 0 | 1.00E-159 |
| hsa_circ_0012885 | 95 | 1 | 473 | 1 | 473 | 0 | 0 |
| hsa_circ_0012885 | 92 | 1 | 1845 | 1 | 1845 | 0 | 0 |
| hsa_circ_0012885 | 92 | 1 | 1914 | 1 | 1914 | 0 | 0 |
| hsa_circ_0012885 | 92 | 1 | 1914 | 1 | 1914 | 0 | 0 |
| hsa_circ_0012885 | 90 | 1 | 914 | 1001 | 1914 | 0 | 0 |
| hsa_circ_0012892 | 84 | 175 | 679 | 1 | 514 | 0.02 | 6.00E-136 |
| hsa_circ_0012892 | 89 | 175 | 2858 | 1 | 2693 | 0 | 0 |
| hsa_circ_0005822 | 94 | 66655 | 66893 | 113 | 351 | 0 | 3.00E-97 |

novel_circ_009955 novel_circ_009956 novel_circ_009957 novel_circ_009958 novel_circ_009959 novel_circ_009960 novel_circ_009961 novel_circ_009963 novel_circ_009964 novel_circ_009965 novel_circ_009967 novel_circ_009968 novel_circ_009969 novel_circ_009970 novel_circ_009971 novel_circ_009972 novel_circ_009977 novel_circ_009978 novel_circ_009986 novel_circ_009987 novel_circ_009989 novel_circ_009993 novel_circ_009994 novel_circ_009995 novel_circ_009996 novel_circ_009997 novel_circ_009998 novel_circ_010001 novel_circ_010002 novel_circ_010003 novel_circ_010004 novel_circ_010005 novel_circ_010006 novel_circ_010007 novel_circ_010008 novel_circ_010009 novel_circ_010010 novel_circ_010011 novel_circ_010012 novel_circ_010013 novel_circ_010014 novel_circ_010015 novel_circ_010016 novel_circ_010017 novel_circ_010018 novel_circ_010019 novel_circ_010020 novel_circ_010021 novel_circ_010022 novel_circ_010023 novel_circ_010024 novel_circ_010025 novel_circ_010026 novel_circ_010027

| hsa_circ_0005822 | 94 | 25620 | 25858 | 113 | 351 | 0 | 1.00E-97 |
| --- | --- | --- | --- | --- | --- | --- | --- |
| hsa_circ_0005822 | 94 | 4799 | 5037 | 113 | 351 | 0 | 2.00E-98 |
| hsa_circ_0060526 | 93 | 1 | 1189 | 1 | 1189 | 0 | 0 |
| hsa_circ_0060526 | 93 | 82 | 1270 | 1 | 1189 | 0 | 0 |
| hsa_circ_0060526 | 93 | 1 | 1031 | 1 | 1031 | 0 | 0 |
| hsa_circ_0060526 | 93 | 82 | 1112 | 1 | 1031 | 0 | 0 |
| hsa_circ_0060526 | 93 | 1 | 715 | 1 | 715 | 0 | 0 |
| hsa_circ_0060488 | 88 | 1 | 744 | 744 | 1 | 0.01 | 0 |
| hsa_circ_0060490 | 88 | 1 | 412 | 412 | 1 | 0 | 6.00E-134 |
| hsa_circ_0060488 | 88 | 1 | 582 | 582 | 1 | 0.01 | 0 |
| hsa_circ_0060488 | 90 | 1 | 388 | 388 | 1 | 0.01 | 6.00E-139 |
| hsa_circ_0060488 | 90 | 1 | 308 | 308 | 1 | 0.01 | 6.00E-108 |
| hsa_circ_0006484 | 86 | 1 | 272 | 272 | 1 | 0 | 5.00E-78 |
| hsa_circ_0060475 | 93 | 368 | 580 | 1 | 213 | 0 | 3.00E-83 |
| hsa_circ_0060440 | 94 | 1 | 705 | 112 | 816 | 0 | 0 |
| hsa_circ_0060440 | 94 | 1 | 618 | 112 | 729 | 0 | 0 |
| hsa_circ_0002595 | 75 | 7207 | 7653 | 314260 | 314708 | 0.02 | 7.00E-45 |
| hsa_circ_0077585 | 75 | 21424 | 22050 | 8361 | 8981 | 0.04 | 2.00E-69 |
| hsa_circ_0019061 | 89 | 1 | 792 | 302 | 1093 | 0 | 0 |
| hsa_circ_0019061 | 91 | 1 | 453 | 378 | 830 | 0 | 1.00E-171 |
| hsa_circ_0019068 | 90 | 1 | 219 | 291 | 73 | 0 | 6.00E-77 |
| hsa_circ_0019071 | 96 | 1 | 427 | 427 | 1 | 0 | 0 |
| hsa_circ_0019071 | 96 | 4 | 681 | 678 | 1 | 0 | 0 |
| hsa_circ_0019072 | 94 | 3 | 607 | 906 | 302 | 0 | 0 |
| hsa_circ_0019072 | 93 | 3 | 351 | 906 | 558 | 0 | 2.00E-142 |
| hsa_circ_0019075 | 92 | 1 | 710 | 1 | 710 | 0 | 0 |
| hsa_circ_0019077 | 90 | 1 | 1057 | 1 | 1066 | 0.01 | 0 |
| hsa_circ_0019092 | 89 | 286 | 567 | 3 | 290 | 0.02 | 7.00E-95 |
| hsa_circ_0019092 | 91 | 135 | 783 | 3 | 657 | 0.01 | 0 |
| hsa_circ_0019092 | 93 | 135 | 972 | 3 | 846 | 0.01 | 0 |
| hsa_circ_0019092 | 91 | 3 | 651 | 3 | 657 | 0.01 | 0 |
| hsa_circ_0019092 | 93 | 3 | 900 | 3 | 906 | 0.01 | 0 |
| hsa_circ_0019095 | 93 | 1 | 362 | 1 | 362 | 0 | 1.00E-149 |
| hsa_circ_0019095 | 95 | 1 | 461 | 1 | 461 | 0 | 0 |
| hsa_circ_0019095 | 95 | 1 | 551 | 1 | 551 | 0 | 0 |
| hsa_circ_0019095 | 95 | 1 | 611 | 1 | 611 | 0 | 0 |
| hsa_circ_0019095 | 95 | 1 | 364 | 98 | 461 | 0 | 5.00E-164 |
| hsa_circ_0019099 | 93 | 1 | 683 | 1 | 683 | 0 | 0 |
| hsa_circ_0019099 | 93 | 1 | 553 | 131 | 683 | 0 | 0 |
| hsa_circ_0019102 | 93 | 222 | 494 | 1 | 273 | 0 | 3.00E-108 |
| hsa_circ_0019102 | 93 | 157 | 429 | 1 | 273 | 0 | 2.00E-108 |
| hsa_circ_0019102 | 93 | 1 | 273 | 1 | 273 | 0 | 1.00E-108 |
| hsa_circ_0019102 | 93 | 1 | 478 | 1 | 478 | 0 | 0 |
| hsa_circ_0019104 | 94 | 1 | 225 | 497 | 721 | 0 | 1.00E-93 |
| hsa_circ_0019105 | 94 | 1 | 349 | 210 | 558 | 0 | 2.00E-147 |
| hsa_circ_0019105 | 94 | 1 | 471 | 210 | 680 | 0 | 0 |
| hsa_circ_0019105 | 95 | 1 | 642 | 210 | 851 | 0 | 0 |
| hsa_circ_0019105 | 93 | 1 | 814 | 210 | 1023 | 0 | 0 |
| hsa_circ_0019105 | 93 | 1 | 814 | 210 | 1023 | 0 | 0 |
| hsa_circ_0008170 | 92 | 1 | 343 | 1 | 343 | 0 | 5.00E-134 |
| hsa_circ_0008170 | 92 | 1 | 343 | 1 | 343 | 0 | 3.00E-133 |
| hsa_circ_0008170 | 93 | 1 | 735 | 1 | 735 | 0 | 0 |
| hsa_circ_0008170 | 93 | 1 | 955 | 1 | 955 | 0 | 0 |
| hsa_circ_0008170 | 94 | 1 | 386 | 570 | 955 | 0 | 3.00E-166 |

novel_circ_010028 novel_circ_010029 novel_circ_010030 novel_circ_010031 novel_circ_010032 novel_circ_010033 novel_circ_010034 novel_circ_010035 novel_circ_010036 novel_circ_010037 novel_circ_010038 novel_circ_010039 novel_circ_010040 novel_circ_010041 novel_circ_010042 novel_circ_010043 novel_circ_010044 novel_circ_010045 novel_circ_010046 novel_circ_010047 novel_circ_010048 novel_circ_010049 novel_circ_010050 novel_circ_010051 novel_circ_010052 novel_circ_010053 novel_circ_010054 novel_circ_010055 novel_circ_010056 novel_circ_010057 novel_circ_010058 novel_circ_010059 novel_circ_010060 novel_circ_010061 novel_circ_010062 novel_circ_010063 novel_circ_010064 novel_circ_010065 novel_circ_010066 novel_circ_010067 novel_circ_010068 novel_circ_010070 novel_circ_010071 novel_circ_010072 novel_circ_010073 novel_circ_010074 novel_circ_010075 novel_circ_010076 novel_circ_010077 novel_circ_010078 novel_circ_010079 novel_circ_010080 novel_circ_010081 novel_circ_010082

| hsa_circ_0019110 | 93 | 1 | 685 | 692 | 1376 | 0 | 0 |
| --- | --- | --- | --- | --- | --- | --- | --- |
| hsa_circ_0019110 | 94 | 1 | 1315 | 692 | 2006 | 0 | 0 |
| hsa_circ_0019118 | 94 | 1 | 736 | 1 | 736 | 0 | 0 |
| hsa_circ_0019118 | 94 | 1 | 923 | 1 | 923 | 0 | 0 |
| hsa_circ_0019118 | 95 | 3 | 302 | 256 | 555 | 0 | 7.00E-132 |
| hsa_circ_0019118 | 95 | 1 | 368 | 556 | 923 | 0 | 7.00E-163 |
| hsa_circ_0019126 | 95 | 8941 | 9851 | 55 | 965 | 0 | 0 |
| hsa_circ_0019126 | 94 | 8941 | 11521 | 55 | 2635 | 0 | 0 |
| hsa_circ_0019126 | 96 | 6950 | 7594 | 55 | 699 | 0 | 0 |
| hsa_circ_0019126 | 95 | 6950 | 7860 | 55 | 965 | 0 | 0 |
| hsa_circ_0019126 | 95 | 6950 | 8236 | 55 | 1341 | 0 | 0 |
| hsa_circ_0019126 | 95 | 6950 | 8237 | 55 | 1342 | 0 | 0 |
| hsa_circ_0019128 | 94 | 1 | 504 | 1 | 504 | 0 | 0 |
| hsa_circ_0019128 | 94 | 1 | 1006 | 1 | 1006 | 0 | 0 |
| hsa_circ_0019129 | 94 | 1 | 785 | 274 | 1058 | 0 | 0 |
| hsa_circ_0019129 | 93 | 1 | 1013 | 415 | 1427 | 0 | 0 |
| hsa_circ_0019129 | 93 | 1 | 1795 | 415 | 2209 | 0 | 0 |
| hsa_circ_0019129 | 93 | 1 | 1670 | 540 | 2209 | 0 | 0 |
| hsa_circ_0019129 | 93 | 1 | 2131 | 540 | 2670 | 0 | 0 |
| hsa_circ_0019126 | 93 | 1 | 3334 | 966 | 4299 | 0 | 0 |
| hsa_circ_0019130 | 93 | 1 | 427 | 673 | 1099 | 0 | 1.00E-175 |
| hsa_circ_0019132 | 94 | 1 | 345 | 1 | 345 | 0 | 4.00E-150 |
| hsa_circ_0019136 | 92 | 1 | 461 | 1 | 461 | 0 | 0 |
| hsa_circ_0019139 | 93 | 1 | 443 | 150 | 592 | 0 | 0 |
| hsa_circ_0019136 | 92 | 1 | 279 | 1386 | 1664 | 0 | 3.00E-110 |
| hsa_circ_0019125 | 92 | 1 | 822 | 4185 | 5006 | 0 | 0 |
| hsa_circ_0019143 | 96 | 1 | 416 | 416 | 1 | 0 | 0 |
| hsa_circ_0019143 | 94 | 28 | 674 | 647 | 1 | 0 | 0 |
| hsa_circ_0019143 | 94 | 52 | 698 | 647 | 1 | 0 | 0 |
| hsa_circ_0019143 | 94 | 109 | 755 | 647 | 1 | 0 | 0 |
| hsa_circ_0019144 | 94 | 1 | 492 | 492 | 1 | 0 | 0 |
| hsa_circ_0019144 | 94 | 82 | 573 | 492 | 1 | 0 | 0 |
| hsa_circ_0019145 | 93 | 1 | 349 | 350 | 2 | 0 | 2.00E-142 |
| hsa_circ_0019145 | 93 | 1 | 376 | 377 | 2 | 0 | 1.00E-155 |
| hsa_circ_0019145 | 93 | 82 | 457 | 377 | 2 | 0 | 1.00E-155 |
| hsa_circ_0019145 | 93 | 173 | 548 | 377 | 2 | 0 | 2.00E-155 |
| hsa_circ_0019146 | 93 | 1 | 258 | 258 | 1 | 0 | 6.00E-102 |
| hsa_circ_0019146 | 93 | 82 | 339 | 258 | 1 | 0 | 9.00E-102 |
| hsa_circ_0019146 | 93 | 173 | 430 | 258 | 1 | 0 | 1.00E-101 |
| hsa_circ_0019148 | 97 | 4 | 92 | 214 | 126 | 0 | 5.00E-34 |
| hsa_circ_0019141 | 91 | 1 | 505 | 5680 | 5176 | 0.02 | 0 |
| hsa_circ_0019154 | 95 | 1 | 639 | 1243 | 605 | 0 | 0 |
| hsa_circ_0019155 | 91 | 1 | 204 | 204 | 1 | 0 | 1.00E-73 |
| hsa_circ_0019154 | 93 | 1 | 309 | 1058 | 750 | 0 | 8.00E-127 |
| hsa_circ_0019156 | 95 | 3 | 769 | 767 | 1 | 0 | 0 |
| hsa_circ_0019153 | 94 | 186 | 1332 | 2613 | 1467 | 0 | 0 |
| hsa_circ_0019153 | 94 | 186 | 1228 | 2613 | 1571 | 0 | 0 |
| hsa_circ_0019156 | 94 | 3 | 399 | 767 | 371 | 0 | 6.00E-169 |
| hsa_circ_0000251 | 92 | 1 | 563 | 563 | 1 | 0 | 0 |
| hsa_circ_0000251 | 92 | 1 | 393 | 563 | 171 | 0 | 2.00E-158 |
| hsa_circ_0019162 | 91 | 1 | 462 | 244 | 705 | 0 | 1.00E-176 |
| hsa_circ_0019162 | 90 | 1 | 366 | 340 | 705 | 0 | 4.00E-130 |
| hsa_circ_0019175 | 93 | 1 | 562 | 1 | 562 | 0 | 0 |
| hsa_circ_0019175 | 93 | 1 | 1852 | 1 | 1852 | 0 | 0 |

novel_circ_010083 novel_circ_010084 novel_circ_010085 novel_circ_010086 novel_circ_010087 novel_circ_010088 novel_circ_010089 novel_circ_010090 novel_circ_010091 novel_circ_010093 novel_circ_010095 novel_circ_010096 novel_circ_010097 novel_circ_010098 novel_circ_010099 novel_circ_010100 novel_circ_010101 novel_circ_010102 novel_circ_010103 novel_circ_010105 novel_circ_010106 novel_circ_010107 novel_circ_010108 novel_circ_010109 novel_circ_010110 novel_circ_010111 novel_circ_010112 novel_circ_010113 novel_circ_010115 novel_circ_010116 novel_circ_010117 novel_circ_010118 novel_circ_010119 novel_circ_010120 novel_circ_010121 novel_circ_010122 novel_circ_010123 novel_circ_010124 novel_circ_010125 novel_circ_010126 novel_circ_010127 novel_circ_010128 novel_circ_010129 novel_circ_010130 novel_circ_010131 novel_circ_010132 novel_circ_010133 novel_circ_010134 novel_circ_010135 novel_circ_010136 novel_circ_010137 novel_circ_010138 novel_circ_010139 novel_circ_010140

| hsa_circ_0019175 | 93 | 1 | 1541 | 312 | 1852 | 0 | 0 |
| --- | --- | --- | --- | --- | --- | --- | --- |
| hsa_circ_0019176 | 93 | 1 | 682 | 1 | 682 | 0 | 0 |
| hsa_circ_0019176 | 93 | 1 | 1315 | 1 | 1315 | 0 | 0 |
| hsa_circ_0019175 | 93 | 1 | 1495 | 358 | 1852 | 0 | 0 |
| hsa_circ_0019175 | 93 | 1 | 1635 | 358 | 1992 | 0 | 0 |
| hsa_circ_0019175 | 93 | 1 | 1711 | 358 | 2068 | 0 | 0 |
| hsa_circ_0019175 | 93 | 1 | 1290 | 563 | 1852 | 0 | 0 |
| hsa_circ_0019179 | 93 | 1 | 643 | 339 | 981 | 0 | 0 |
| hsa_circ_0019179 | 92 | 1 | 783 | 339 | 1121 | 0 | 0 |
| hsa_circ_0019219 | 87 | 86 | 721 | 2426 | 1768 | 0.03 | 0 |
| hsa_circ_0019219 | 92 | 86 | 602 | 2426 | 1910 | 0 | 0 |
| hsa_circ_0019223 | 84 | 1 | 1479 | 1 | 1486 | 0.02 | 0 |
| hsa_circ_0019222 | 84 | 1 | 1831 | 271 | 2126 | 0.03 | 0 |
| hsa_circ_0019222 | 88 | 1 | 289 | 1841 | 2126 | 0.01 | 6.00E-93 |
| hsa_circ_0019222 | 88 | 1 | 606 | 1841 | 2443 | 0 | 0 |
| hsa_circ_0019228 | 91 | 1 | 527 | 860 | 1392 | 0.01 | 0 |
| hsa_circ_0019224 | 90 | 1 | 720 | 3409 | 4128 | 0 | 0 |
| hsa_circ_0008528 | 95 | 15363 | 15479 | 27033 | 27149 | 0 | 2.00E-43 |
| hsa_circ_0008528 | 95 | 5785 | 5901 | 27033 | 27149 | 0 | 8.00E-44 |
| hsa_circ_0008528 | 95 | 1928 | 2044 | 27033 | 27149 | 0 | 3.00E-44 |
| hsa_circ_0006650 | 95 | 1928 | 2127 | 124 | 323 | 0 | 5.00E-82 |
| hsa_circ_0006650 | 96 | 1928 | 2350 | 124 | 546 | 0 | 0 |
| hsa_circ_0006650 | 96 | 1928 | 2433 | 124 | 629 | 0 | 0 |
| hsa_circ_0003155 | 95 | 1929 | 2833 | 1 | 905 | 0 | 0 |
| hsa_circ_0003155 | 96 | 1 | 422 | 1 | 422 | 0 | 0 |
| hsa_circ_0003155 | 96 | 1 | 505 | 1 | 505 | 0 | 0 |
| hsa_circ_0003155 | 95 | 1 | 905 | 1 | 905 | 0 | 0 |
| hsa_circ_0019242 | 94 | 1 | 481 | 309 | 789 | 0 | 0 |
| hsa_circ_0019243 | 97 | 1 | 123 | 290 | 412 | 0 | 5.00E-52 |
| hsa_circ_0019244 | 96 | 849 | 1304 | 1 | 456 | 0 | 0 |
| hsa_circ_0019244 | 94 | 849 | 1583 | 1 | 735 | 0 | 0 |
| hsa_circ_0019244 | 94 | 1 | 735 | 1 | 735 | 0 | 0 |
| hsa_circ_0019244 | 93 | 1 | 938 | 1 | 938 | 0 | 0 |
| hsa_circ_0019244 | 93 | 1 | 938 | 1 | 938 | 0 | 0 |
| hsa_circ_0019244 | 92 | 1 | 538 | 198 | 735 | 0 | 0 |
| hsa_circ_0000252 | 92 | 1 | 441 | 1 | 441 | 0 | 5.00E-175 |
| hsa_circ_0000252 | 92 | 1 | 644 | 1 | 644 | 0 | 0 |
| hsa_circ_0000252 | 92 | 1 | 762 | 1 | 762 | 0 | 0 |
| hsa_circ_0000252 | 92 | 1 | 922 | 1 | 922 | 0 | 0 |
| hsa_circ_0000252 | 92 | 1 | 922 | 1 | 922 | 0 | 0 |
| hsa_circ_0000252 | 90 | 1 | 279 | 163 | 441 | 0 | 3.00E-100 |
| hsa_circ_0000252 | 92 | 1 | 760 | 163 | 922 | 0 | 0 |
| hsa_circ_0008317 | 92 | 1 | 397 | 85 | 481 | 0 | 7.00E-159 |
| hsa_circ_0008317 | 93 | 1 | 277 | 205 | 481 | 0 | 2.00E-112 |
| hsa_circ_0008317 | 93 | 1 | 277 | 205 | 481 | 0 | 3.00E-112 |
| hsa_circ_0008317 | 94 | 1 | 160 | 322 | 481 | 0 | 2.00E-62 |
| hsa_circ_0000236 | 90 | 1 | 1144 | 1 | 1131 | 0.02 | 0 |
| hsa_circ_0000236 | 90 | 1 | 1144 | 1 | 1131 | 0.02 | 0 |
| hsa_circ_0000236 | 90 | 1 | 1144 | 1 | 1131 | 0.02 | 0 |
| hsa_circ_0009016 | 83 | 9364 | 9446 | 848879 | 848958 | 0.06 | 4.00E-10 |
| hsa_circ_0009016 | 83 | 4330 | 4412 | 848879 | 848958 | 0.06 | 2.00E-10 |
| hsa_circ_0009016 | 83 | 3854 | 3936 | 848879 | 848958 | 0.06 | 2.00E-10 |
| hsa_circ_0018274 | 91 | 927 | 1261 | 335 | 1 | 0 | 1.00E-125 |
| hsa_circ_0018274 | 91 | 978 | 1312 | 335 | 1 | 0 | 1.00E-125 |

novel_circ_010141 novel_circ_010142 novel_circ_010143 novel_circ_010144 novel_circ_010145 novel_circ_010146 novel_circ_010147 novel_circ_010148 novel_circ_010149 novel_circ_010150 novel_circ_010151 novel_circ_010152 novel_circ_010153 novel_circ_010163 novel_circ_010164 novel_circ_010165 novel_circ_010167 novel_circ_010168 novel_circ_010169 novel_circ_010170 novel_circ_010171 novel_circ_010172 novel_circ_010173 novel_circ_010174 novel_circ_010175 novel_circ_010176 novel_circ_010177 novel_circ_010178 novel_circ_010179 novel_circ_010180 novel_circ_010181 novel_circ_010182 novel_circ_010184 novel_circ_010185 novel_circ_010186 novel_circ_010189 novel_circ_010192 novel_circ_010193 novel_circ_010194 novel_circ_010198 novel_circ_010207 novel_circ_010208 novel_circ_010212 novel_circ_010213 novel_circ_010214 novel_circ_010215 novel_circ_010217 novel_circ_010218 novel_circ_010219 novel_circ_010220 novel_circ_010221 novel_circ_010222 novel_circ_010223 novel_circ_010224

| hsa_circ_0018274 | 91 | 1155 | 1489 | 335 | 1 | 0 | 1.00E-125 |
| --- | --- | --- | --- | --- | --- | --- | --- |
| hsa_circ_0018277 | 91 | 3 | 311 | 318 | 1 | 0.03 | 1.00E-116 |
| hsa_circ_0018280 | 83 | 1 | 1171 | 1210 | 57 | 0.02 | 0 |
| hsa_circ_0018280 | 83 | 918 | 2088 | 1210 | 57 | 0.02 | 0 |
| hsa_circ_0018281 | 90 | 1 | 300 | 301 | 1 | 0 | 6.00E-108 |
| hsa_circ_0018281 | 90 | 1 | 300 | 301 | 1 | 0 | 5.00E-108 |
| hsa_circ_0018281 | 90 | 1 | 300 | 301 | 1 | 0 | 4.00E-108 |
| hsa_circ_0018281 | 90 | 1 | 300 | 301 | 1 | 0 | 3.00E-108 |
| hsa_circ_0018281 | 90 | 1 | 300 | 301 | 1 | 0 | 2.00E-108 |
| hsa_circ_0075343 | 76 | 3972 | 4469 | 514 | 17 | 0.02 | 3.00E-67 |
| hsa_circ_0070350 | 87 | 4 | 238 | 543 | 309 | 0 | 3.00E-70 |
| hsa_circ_0002968 | 97 | 2 | 499 | 2 | 499 | 0 | 0 |
| hsa_circ_0002968 | 97 | 2 | 499 | 2 | 499 | 0 | 0 |
| hsa_circ_0019034 | 91 | 7 | 661 | 655 | 1 | 0 | 0 |
| hsa_circ_0019017 | 89 | 3 | 110 | 3 | 110 | 0 | 6.00E-29 |
| hsa_circ_0019016 | 88 | 119 | 230 | 280 | 391 | 0 | 8.00E-31 |
| hsa_circ_0090327 | 89 | 1 | 773 | 365 | 1137 | 0 | 0 |
| hsa_circ_0090332 | 89 | 777 | 1504 | 1 | 731 | 0 | 0 |
| hsa_circ_0090332 | 90 | 777 | 1619 | 1 | 846 | 0 | 0 |
| hsa_circ_0090332 | 90 | 777 | 1619 | 1 | 846 | 0 | 0 |
| hsa_circ_0090332 | 90 | 239 | 1081 | 1 | 846 | 0 | 0 |
| hsa_circ_0090332 | 93 | 1 | 442 | 1 | 442 | 0 | 0 |
| hsa_circ_0090332 | 89 | 1 | 728 | 1 | 731 | 0 | 0 |
| hsa_circ_0090332 | 90 | 1 | 843 | 1 | 846 | 0 | 0 |
| hsa_circ_0090332 | 90 | 1 | 843 | 1 | 846 | 0 | 0 |
| hsa_circ_0090332 | 90 | 1 | 843 | 1 | 846 | 0 | 0 |
| hsa_circ_0090332 | 93 | 1 | 312 | 131 | 442 | 0 | 8.00E-127 |
| hsa_circ_0090332 | 89 | 1 | 598 | 131 | 731 | 0 | 0 |
| hsa_circ_0090332 | 90 | 1 | 713 | 131 | 846 | 0 | 0 |
| hsa_circ_0090332 | 90 | 1 | 713 | 131 | 846 | 0 | 0 |
| hsa_circ_0090332 | 90 | 1 | 713 | 131 | 846 | 0 | 0 |
| hsa_circ_0090332 | 87 | 1 | 401 | 443 | 846 | 0.01 | 5.00E-131 |
| hsa_circ_0089861 | 87 | 1 | 709 | 3139 | 3862 | 0.02 | 0 |
| hsa_circ_0089861 | 82 | 135 | 742 | 3 | 607 | 0.04 | 1.00E-142 |
| hsa_circ_0089856 | 91 | 2 | 365 | 540 | 903 | 0 | 5.00E-139 |
| hsa_circ_0029815 | 89 | 23049 | 23574 | 538 | 5 | 0.02 | 0 |
| hsa_circ_0089851 | 81 | 9 | 267 | 480 | 222 | 0.01 | 9.00E-51 |
| hsa_circ_0089851 | 84 | 9 | 487 | 480 | 2 | 0 | 3.00E-129 |
| hsa_circ_0089851 | 88 | 1 | 220 | 221 | 2 | 0 | 6.00E-69 |
| hsa_circ_0061589 | 83 | 3584 | 3654 | 553840 | 553909 | 0.01 | 6.00E-07 |
| hsa_circ_0066697 | 85 | 57253 | 57693 | 468 | 17 | 0.02 | 4.00E-122 |
| hsa_circ_0066697 | 85 | 57253 | 57693 | 468 | 17 | 0.02 | 4.00E-122 |
| hsa_circ_0006368 | 93 | 23966 | 24022 | 66138 | 66194 | 0 | 5.00E-13 |
| hsa_circ_0006368 | 93 | 23906 | 23962 | 66138 | 66194 | 0 | 5.00E-13 |
| hsa_circ_0092268 | 89 | 1 | 973 | 1 | 973 | 0 | 0 |
| hsa_circ_0092268 | 89 | 1 | 787 | 187 | 973 | 0 | 0 |
| hsa_circ_0057546 | 94 | 1 | 603 | 776 | 1378 | 0 | 0 |
| hsa_circ_0008730 | 93 | 1 | 470 | 1 | 470 | 0 | 0 |
| hsa_circ_0008730 | 93 | 1 | 470 | 1 | 470 | 0 | 0 |
| hsa_circ_0057529 | 94 | 1 | 203 | 1 | 203 | 0 | 2.00E-81 |
| hsa_circ_0057523 | 95 | 1 | 1045 | 1 | 1045 | 0 | 0 |
| hsa_circ_0057519 | 95 | 628 | 1744 | 148 | 1264 | 0 | 0 |
| hsa_circ_0057523 | 95 | 1 | 1045 | 1 | 1045 | 0 | 0 |
| hsa_circ_0057519 | 95 | 628 | 1744 | 148 | 1264 | 0 | 0 |

novel_circ_010225 novel_circ_010226 novel_circ_010227 novel_circ_010228 novel_circ_010229 novel_circ_010230 novel_circ_010231 novel_circ_010232 novel_circ_010233 novel_circ_010234 novel_circ_010235 novel_circ_010236 novel_circ_010237 novel_circ_010238 novel_circ_010239 novel_circ_010240 novel_circ_010241 novel_circ_010243 novel_circ_010244 novel_circ_010247 novel_circ_010248 novel_circ_010250 novel_circ_010251 novel_circ_010252 novel_circ_010253 novel_circ_010254 novel_circ_010255 novel_circ_010256 novel_circ_010257 novel_circ_010258 novel_circ_010259 novel_circ_010260 novel_circ_010261 novel_circ_010262 novel_circ_010263 novel_circ_010264 novel_circ_010265 novel_circ_010270 novel_circ_010271 novel_circ_010272 novel_circ_010273 novel_circ_010274 novel_circ_010275 novel_circ_010276 novel_circ_010278 novel_circ_010280 novel_circ_010281 novel_circ_010282 novel_circ_010283 novel_circ_010284 novel_circ_010285 novel_circ_010286 novel_circ_010287 novel_circ_010288

| hsa_circ_0057524 | 94 | 1 | 520 | 396 | 915 | 0 | 0 |
| --- | --- | --- | --- | --- | --- | --- | --- |
| hsa_circ_0057524 | 95 | 5130 | 5743 | 302 | 915 | 0 | 0 |
| hsa_circ_0057524 | 95 | 1 | 671 | 245 | 915 | 0 | 0 |
| hsa_circ_0057523 | 95 | 1 | 1045 | 1 | 1045 | 0 | 0 |
| hsa_circ_0057519 | 95 | 628 | 1744 | 148 | 1264 | 0 | 0 |
| hsa_circ_0057523 | 95 | 1 | 820 | 1 | 820 | 0 | 0 |
| hsa_circ_0057523 | 95 | 1 | 643 | 1 | 643 | 0 | 0 |
| hsa_circ_0057523 | 95 | 1 | 525 | 1 | 525 | 0 | 0 |
| hsa_circ_0057523 | 94 | 1 | 433 | 1 | 433 | 0 | 0 |
| hsa_circ_0057523 | 94 | 1 | 433 | 1 | 433 | 0 | 0 |
| hsa_circ_0057520 | 95 | 628 | 1132 | 51 | 555 | 0 | 0 |
| hsa_circ_0057523 | 94 | 1 | 374 | 1 | 374 | 0 | 1.00E-159 |
| hsa_circ_0057520 | 95 | 628 | 1073 | 51 | 496 | 0 | 0 |
| hsa_circ_0057511 | 94 | 1 | 276 | 1 | 276 | 0 | 3.00E-115 |
| hsa_circ_0057509 | 94 | 1 | 1114 | 100 | 1213 | 0 | 0 |
| hsa_circ_0002024 | 94 | 1 | 1024 | 1 | 1024 | 0 | 0 |
| hsa_circ_0002024 | 94 | 1 | 838 | 1 | 838 | 0 | 0 |
| hsa_circ_0057504 | 87 | 18 | 853 | 839 | 1 | 0.01 | 0 |
| hsa_circ_0057499 | 86 | 18 | 1026 | 3436 | 2425 | 0 | 0 |
| hsa_circ_0057487 | 90 | 3 | 587 | 739 | 155 | 0 | 0 |
| hsa_circ_0057487 | 92 | 1 | 453 | 607 | 155 | 0 | 2.00E-178 |
| hsa_circ_0057471 | 96 | 1 | 182 | 1 | 182 | 0 | 6.00E-79 |
| hsa_circ_0057471 | 96 | 1 | 182 | 1 | 182 | 0 | 7.00E-80 |
| hsa_circ_0057464 | 92 | 293 | 603 | 6 | 316 | 0 | 6.00E-121 |
| hsa_circ_0057331 | 91 | 11536 | 12056 | 930 | 410 | 0 | 0 |
| hsa_circ_0057331 | 91 | 548 | 1068 | 930 | 410 | 0 | 0 |
| hsa_circ_0057331 | 91 | 14 | 442 | 930 | 502 | 0 | 5.00E-165 |
| hsa_circ_0057331 | 92 | 2 | 357 | 857 | 502 | 0 | 1.00E-139 |
| hsa_circ_0028149 | 88 | 1 | 272 | 272 | 1 | 0 | 2.00E-86 |
| hsa_circ_0028149 | 92 | 1137 | 1747 | 611 | 1 | 0 | 0 |
| hsa_circ_0028149 | 95 | 882 | 1130 | 611 | 363 | 0 | 6.00E-108 |
| hsa_circ_0028151 | 93 | 1 | 405 | 878 | 474 | 0 | 4.00E-170 |
| hsa_circ_0028158 | 93 | 1 | 313 | 313 | 1 | 0 | 1.00E-125 |
| hsa_circ_0028158 | 93 | 1 | 532 | 532 | 1 | 0 | 0 |
| hsa_circ_0028159 | 93 | 1 | 218 | 218 | 1 | 0 | 2.00E-86 |
| hsa_circ_0028158 | 94 | 1 | 437 | 532 | 96 | 0 | 0 |
| hsa_circ_0028164 | 94 | 1 | 948 | 1 | 948 | 0 | 0 |
| hsa_circ_0062647 | 96 | 1722 | 1872 | 1 | 151 | 0 | 3.00E-63 |
| hsa_circ_0062648 | 87 | 4 | 785 | 3 | 785 | 0 | 0 |
| hsa_circ_0062650 | 88 | 1 | 591 | 1 | 591 | 0 | 0 |
| hsa_circ_0062655 | 88 | 1 | 402 | 1 | 402 | 0 | 5.00E-135 |
| hsa_circ_0062662 | 89 | 1 | 302 | 1 | 302 | 0 | 1.00E-104 |
| hsa_circ_0062662 | 94 | 1 | 602 | 306 | 907 | 0 | 0 |
| hsa_circ_0062662 | 93 | 1 | 494 | 414 | 907 | 0 | 0 |
| hsa_circ_0062676 | 91 | 124 | 786 | 1343 | 682 | 0 | 0 |
| hsa_circ_0008799 | 75 | 16854 | 17216 | 122965 | 122597 | 0.06 | 3.00E-33 |
| hsa_circ_0062705 | 91 | 1 | 506 | 508 | 3 | 0 | 0 |
| hsa_circ_0062707 | 96 | 1 | 404 | 404 | 1 | 0 | 0 |
| hsa_circ_0062707 | 96 | 1 | 552 | 552 | 1 | 0 | 0 |
| hsa_circ_0022629 | 88 | 58734 | 60010 | 145 | 1438 | 0.01 | 0 |
| hsa_circ_0062717 | 82 | 1117 | 2457 | 2354 | 3699 | 0.06 | 0 |
| hsa_circ_0062729 | 91 | 1 | 255 | 1 | 255 | 0 | 1.00E-93 |
| hsa_circ_0062729 | 93 | 1 | 532 | 1 | 532 | 0 | 0 |
| hsa_circ_0062770 | 93 | 1 | 506 | 813 | 308 | 0 | 0 |

novel_circ_010289 novel_circ_010290 novel_circ_010291 novel_circ_010293 novel_circ_010294 novel_circ_010295 novel_circ_010296 novel_circ_010297 novel_circ_010302 novel_circ_010303 novel_circ_010305 novel_circ_010307 novel_circ_010308 novel_circ_010309 novel_circ_010310 novel_circ_010311 novel_circ_010312 novel_circ_010313 novel_circ_010314 novel_circ_010315 novel_circ_010316 novel_circ_010319 novel_circ_010320 novel_circ_010322 novel_circ_010323 novel_circ_010324 novel_circ_010325 novel_circ_010326 novel_circ_010327 novel_circ_010328 novel_circ_010329 novel_circ_010330 novel_circ_010331 novel_circ_010332 novel_circ_010333 novel_circ_010334 novel_circ_010335 novel_circ_010336 novel_circ_010337 novel_circ_010338 novel_circ_010339 novel_circ_010340 novel_circ_010341 novel_circ_010342 novel_circ_010343 novel_circ_010344 novel_circ_010345 novel_circ_010346 novel_circ_010347 novel_circ_010348 novel_circ_010349 novel_circ_010350 novel_circ_010351 novel_circ_010352

| hsa_circ_0062775 | 91 | 1 | 133 | 211 | 79 | 0 | 4.00E-44 |
| --- | --- | --- | --- | --- | --- | --- | --- |
| hsa_circ_0062770 | 94 | 276 | 487 | 1748 | 1537 | 0 | 5.00E-86 |
| hsa_circ_0005386 | 96 | 1 | 207 | 127 | 333 | 0 | 4.00E-90 |
| hsa_circ_0062798 | 92 | 4 | 593 | 590 | 1 | 0 | 0 |
| hsa_circ_0062805 | 92 | 3 | 595 | 3 | 597 | 0.01 | 0 |
| hsa_circ_0062807 | 93 | 4 | 542 | 4 | 544 | 0 | 0 |
| hsa_circ_0062811 | 94 | 1 | 290 | 1 | 290 | 0 | 5.00E-123 |
| hsa_circ_0062814 | 96 | 1 | 250 | 1 | 250 | 0 | 2.00E-112 |
| hsa_circ_0062832 | 92 | 1 | 724 | 724 | 1 | 0 | 0 |
| hsa_circ_0062835 | 92 | 1 | 249 | 1 | 249 | 0 | 3.00E-95 |
| hsa_circ_0031269 | 78 | 12828 | 13171 | 370 | 11 | 0.06 | 1.00E-53 |
| hsa_circ_0062894 | 94 | 1 | 370 | 1 | 370 | 0 | 1.00E-155 |
| hsa_circ_0062906 | 90 | 3 | 507 | 1271 | 764 | 0.01 | 0 |
| hsa_circ_0062906 | 92 | 4 | 2128 | 2891 | 764 | 0 | 0 |
| hsa_circ_0062907 | 94 | 1 | 218 | 563 | 346 | 0 | 2.00E-91 |
| hsa_circ_0062908 | 92 | 1 | 709 | 709 | 1 | 0 | 0 |
| hsa_circ_0062909 | 95 | 1 | 256 | 256 | 1 | 0 | 4.00E-109 |
| hsa_circ_0062910 | 93 | 1 | 1213 | 1214 | 2 | 0 | 0 |
| hsa_circ_0062910 | 93 | 1 | 665 | 1214 | 550 | 0 | 0 |
| hsa_circ_0062910 | 92 | 1 | 793 | 1342 | 550 | 0 | 0 |
| hsa_circ_0062910 | 92 | 7 | 867 | 1410 | 550 | 0 | 0 |
| hsa_circ_0062937 | 87 | 1 | 604 | 5807 | 5210 | 0.03 | 0 |
| hsa_circ_0062937 | 87 | 1 | 534 | 5807 | 5283 | 0.02 | 8.00E-169 |
| hsa_circ_0062941 | 92 | 3 | 305 | 3 | 305 | 0 | 3.00E-120 |
| hsa_circ_0062941 | 93 | 3 | 425 | 3 | 425 | 0 | 5.00E-175 |
| hsa_circ_0062941 | 95 | 3 | 813 | 3 | 813 | 0 | 0 |
| hsa_circ_0062941 | 95 | 1 | 431 | 136 | 566 | 0 | 0 |
| hsa_circ_0062941 | 96 | 1 | 574 | 136 | 709 | 0 | 0 |
| hsa_circ_0062941 | 94 | 1 | 950 | 136 | 1085 | 0 | 0 |
| hsa_circ_0062942 | 98 | 1 | 141 | 205 | 345 | 0 | 7.00E-64 |
| hsa_circ_0062943 | 94 | 1 | 581 | 1 | 581 | 0 | 0 |
| hsa_circ_0062958 | 90 | 1 | 354 | 287 | 640 | 0 | 4.00E-130 |
| hsa_circ_0028428 | 92 | 1 | 434 | 1048 | 1482 | 0 | 4.00E-176 |
| hsa_circ_0028428 | 91 | 1 | 972 | 522 | 1482 | 0.01 | 0 |
| hsa_circ_0028428 | 91 | 1 | 855 | 425 | 1267 | 0.01 | 0 |
| hsa_circ_0028428 | 89 | 1 | 606 | 522 | 1115 | 0.02 | 0 |
| hsa_circ_0028429 | 89 | 1 | 538 | 329 | 854 | 0.02 | 0 |
| hsa_circ_0028419 | 93 | 1 | 742 | 1 | 742 | 0 | 0 |
| hsa_circ_0002584 | 95 | 1 | 518 | 518 | 1 | 0 | 0 |
| hsa_circ_0028412 | 93 | 1 | 1158 | 1158 | 1 | 0 | 0 |
| hsa_circ_0028412 | 92 | 1 | 640 | 640 | 1 | 0 | 0 |
| hsa_circ_0028412 | 93 | 1 | 319 | 550 | 232 | 0 | 5.00E-129 |
| hsa_circ_0028412 | 93 | 1 | 379 | 550 | 172 | 0 | 1.00E-155 |
| hsa_circ_0028412 | 92 | 1 | 550 | 550 | 1 | 0 | 0 |
| hsa_circ_0028409 | 92 | 1 | 902 | 1834 | 933 | 0 | 0 |
| hsa_circ_0028412 | 92 | 1 | 419 | 419 | 1 | 0 | 8.00E-168 |
| hsa_circ_0028409 | 91 | 1 | 771 | 1703 | 933 | 0 | 0 |
| hsa_circ_0028409 | 92 | 1 | 1026 | 1703 | 678 | 0 | 0 |
| hsa_circ_0028412 | 90 | 1 | 310 | 310 | 1 | 0 | 2.00E-112 |
| hsa_circ_0028409 | 90 | 1 | 503 | 1594 | 1092 | 0 | 0 |
| hsa_circ_0028409 | 90 | 1 | 662 | 1594 | 933 | 0 | 0 |
| hsa_circ_0028409 | 91 | 1 | 917 | 1594 | 678 | 0 | 0 |
| hsa_circ_0028409 | 92 | 1 | 1473 | 1594 | 122 | 0 | 0 |
| hsa_circ_0028404 | 92 | 1 | 2296 | 3148 | 847 | 0 | 0 |

novel_circ_010354 novel_circ_010355 novel_circ_010357 novel_circ_010358 novel_circ_010359 novel_circ_010360 novel_circ_010361 novel_circ_010362 novel_circ_010363 novel_circ_010364 novel_circ_010365 novel_circ_010366 novel_circ_010367 novel_circ_010368 novel_circ_010369 novel_circ_010370 novel_circ_010371 novel_circ_010372 novel_circ_010373 novel_circ_010374 novel_circ_010375 novel_circ_010376 novel_circ_010377 novel_circ_010378 novel_circ_010379 novel_circ_010380 novel_circ_010381 novel_circ_010382 novel_circ_010383 novel_circ_010384 novel_circ_010385 novel_circ_010386 novel_circ_010387 novel_circ_010388 novel_circ_010389 novel_circ_010390 novel_circ_010391 novel_circ_010392 novel_circ_010393 novel_circ_010394 novel_circ_010395 novel_circ_010396 novel_circ_010397 novel_circ_010398 novel_circ_010399 novel_circ_010400 novel_circ_010402 novel_circ_010403 novel_circ_010404 novel_circ_010405 novel_circ_010406 novel_circ_010407 novel_circ_010408 novel_circ_010409

| hsa_circ_0028409 | 92 | 1 | 811 | 932 | 122 | 0 | 0 |
| --- | --- | --- | --- | --- | --- | --- | --- |
| hsa_circ_0028410 | 95 | 3 | 379 | 377 | 1 | 0 | 3.00E-166 |
| hsa_circ_0028404 | 94 | 1 | 823 | 1675 | 847 | 0.01 | 0 |
| hsa_circ_0028393 | 94 | 820 | 4059 | 3240 | 1 | 0 | 0 |
| hsa_circ_0028399 | 95 | 317 | 2162 | 1846 | 1 | 0 | 0 |
| hsa_circ_0028393 | 93 | 1 | 2968 | 2968 | 1 | 0 | 0 |
| hsa_circ_0028393 | 93 | 1 | 2734 | 2734 | 1 | 0 | 0 |
| hsa_circ_0028395 | 93 | 1 | 892 | 892 | 1 | 0 | 0 |
| hsa_circ_0028395 | 92 | 1 | 421 | 421 | 1 | 0 | 1.00E-165 |
| hsa_circ_0028382 | 91 | 1 | 897 | 1161 | 265 | 0 | 0 |
| hsa_circ_0028372 | 89 | 1 | 1512 | 1515 | 1 | 0 | 0 |
| hsa_circ_0028359 | 93 | 4 | 815 | 1150 | 339 | 0 | 0 |
| hsa_circ_0028357 | 92 | 4 | 1755 | 1919 | 168 | 0 | 0 |
| hsa_circ_0028352 | 88 | 1 | 281 | 1 | 284 | 0.02 | 3.00E-90 |
| hsa_circ_0028352 | 88 | 1 | 180 | 1 | 183 | 0.03 | 3.00E-54 |
| hsa_circ_0028351 | 89 | 1 | 586 | 1 | 589 | 0.01 | 0 |
| hsa_circ_0028341 | 95 | 1 | 344 | 344 | 1 | 0 | 1.00E-154 |
| hsa_circ_0028335 | 93 | 1 | 962 | 978 | 1 | 0.02 | 0 |
| hsa_circ_0028330 | 93 | 420 | 1554 | 1251 | 101 | 0.01 | 0 |
| hsa_circ_0028335 | 92 | 1 | 618 | 634 | 1 | 0.03 | 0 |
| hsa_circ_0028332 | 92 | 1 | 954 | 970 | 1 | 0.02 | 0 |
| hsa_circ_0028330 | 93 | 1 | 1235 | 1251 | 1 | 0.01 | 0 |
| hsa_circ_0028332 | 94 | 1 | 581 | 581 | 1 | 0 | 0 |
| hsa_circ_0028331 | 94 | 1 | 762 | 762 | 1 | 0 | 0 |
| hsa_circ_0028331 | 94 | 1 | 762 | 762 | 1 | 0 | 0 |
| hsa_circ_0028330 | 94 | 1 | 862 | 862 | 1 | 0 | 0 |
| hsa_circ_0028330 | 94 | 1 | 862 | 862 | 1 | 0 | 0 |
| hsa_circ_0028330 | 94 | 1 | 862 | 862 | 1 | 0 | 0 |
| hsa_circ_0028332 | 93 | 1 | 411 | 411 | 1 | 0 | 2.00E-168 |
| hsa_circ_0028331 | 93 | 1 | 592 | 592 | 1 | 0 | 0 |
| hsa_circ_0028331 | 93 | 1 | 592 | 592 | 1 | 0 | 0 |
| hsa_circ_0028330 | 94 | 1 | 692 | 692 | 1 | 0 | 0 |
| hsa_circ_0028330 | 94 | 1 | 692 | 692 | 1 | 0 | 0 |
| hsa_circ_0028330 | 94 | 1 | 692 | 692 | 1 | 0 | 0 |
| hsa_circ_0028328 | 94 | 593 | 1338 | 812 | 67 | 0 | 0 |
| hsa_circ_0028332 | 92 | 1 | 298 | 298 | 1 | 0 | 2.00E-117 |
| hsa_circ_0028331 | 93 | 1 | 479 | 479 | 1 | 0 | 0 |
| hsa_circ_0028330 | 94 | 1 | 579 | 579 | 1 | 0 | 0 |
| hsa_circ_0028328 | 96 | 182 | 559 | 812 | 435 | 0 | 3.00E-173 |
| hsa_circ_0028328 | 96 | 1 | 252 | 812 | 561 | 0 | 1.00E-113 |
| hsa_circ_0028328 | 96 | 1 | 378 | 812 | 435 | 0 | 2.00E-173 |
| hsa_circ_0028328 | 93 | 1 | 622 | 812 | 191 | 0 | 0 |
| hsa_circ_0028328 | 96 | 1343 | 1620 | 712 | 435 | 0 | 7.00E-124 |
| hsa_circ_0028321 | 89 | 175 | 772 | 598 | 1 | 0 | 0 |
| hsa_circ_0028315 | 94 | 2 | 479 | 367 | 844 | 0 | 0 |
| hsa_circ_0028309 | 92 | 1 | 639 | 1 | 624 | 0.02 | 0 |
| hsa_circ_0028300 | 89 | 1 | 529 | 576 | 1104 | 0 | 0 |
| hsa_circ_0028286 | 88 | 344 | 845 | 202 | 703 | 0 | 3.00E-170 |
| hsa_circ_0028286 | 88 | 344 | 845 | 202 | 703 | 0 | 3.00E-170 |
| hsa_circ_0028281 | 92 | 1 | 303 | 304 | 2 | 0 | 3.00E-120 |
| hsa_circ_0028275 | 92 | 1 | 668 | 668 | 1 | 0 | 0 |
| hsa_circ_0028272 | 94 | 1 | 972 | 3389 | 2418 | 0 | 0 |
| hsa_circ_0003884 | 93 | 4 | 474 | 471 | 1 | 0 | 0 |
| hsa_circ_0028273 | 94 | 4 | 564 | 561 | 1 | 0 | 0 |

novel_circ_010410 novel_circ_010411 novel_circ_010412 novel_circ_010413 novel_circ_010414 novel_circ_010415 novel_circ_010416 novel_circ_010417 novel_circ_010418 novel_circ_010419 novel_circ_010420 novel_circ_010421 novel_circ_010422 novel_circ_010426 novel_circ_010427 novel_circ_010428 novel_circ_010430 novel_circ_010431 novel_circ_010432 novel_circ_010433 novel_circ_010434 novel_circ_010435 novel_circ_010436 novel_circ_010437 novel_circ_010440 novel_circ_010441 novel_circ_010442 novel_circ_010443 novel_circ_010444 novel_circ_010445 novel_circ_010446 novel_circ_010447 novel_circ_010448 novel_circ_010449 novel_circ_010450 novel_circ_010451 novel_circ_010452 novel_circ_010453 novel_circ_010454 novel_circ_010455 novel_circ_010456 novel_circ_010457 novel_circ_010463 novel_circ_010464 novel_circ_010465 novel_circ_010466 novel_circ_010467 novel_circ_010468 novel_circ_010469 novel_circ_010470 novel_circ_010471 novel_circ_010472 novel_circ_010473 novel_circ_010474

| hsa_circ_0028272 | 94 | 4 | 668 | 3082 | 2418 | 0 | 0 |
| --- | --- | --- | --- | --- | --- | --- | --- |
| hsa_circ_0028275 | 95 | 1 | 215 | 215 | 1 | 0 | 4.00E-93 |
| hsa_circ_0028272 | 96 | 1 | 519 | 2936 | 2418 | 0 | 0 |
| hsa_circ_0003884 | 95 | 7 | 249 | 243 | 1 | 0 | 6.00E-107 |
| hsa_circ_0028272 | 96 | 7 | 443 | 2854 | 2418 | 0 | 0 |
| hsa_circ_0025598 | 89 | 10965 | 12040 | 1272 | 182 | 0.02 | 0 |
| hsa_circ_0028271 | 95 | 19496 | 19818 | 323 | 1 | 0 | 9.00E-143 |
| hsa_circ_0028271 | 95 | 1 | 320 | 320 | 1 | 0 | 6.00E-143 |
| hsa_circ_0028250 | 92 | 1 | 1467 | 1461 | 1 | 0 | 0 |
| hsa_circ_0028259 | 94 | 1 | 583 | 583 | 1 | 0 | 0 |
| hsa_circ_0028231 | 92 | 1 | 459 | 2705 | 3163 | 0 | 0 |
| hsa_circ_0028230 | 95 | 112 | 238 | 1 | 127 | 0 | 8.00E-51 |
| hsa_circ_0028230 | 98 | 112 | 159 | 1 | 48 | 0 | 2.00E-15 |
| hsa_circ_0085521 | 94 | 761 | 1003 | 243 | 1 | 0 | 3.00E-101 |
| hsa_circ_0085521 | 94 | 761 | 1003 | 243 | 1 | 0 | 6.00E-101 |
| hsa_circ_0085518 | 93 | 1358 | 2149 | 1961 | 1170 | 0 | 0 |
| hsa_circ_0085518 | 94 | 1 | 938 | 1820 | 883 | 0 | 0 |
| hsa_circ_0085518 | 95 | 1 | 287 | 1169 | 883 | 0 | 2.00E-126 |
| hsa_circ_0085518 | 95 | 1 | 225 | 882 | 658 | 0 | 6.00E-97 |
| hsa_circ_0085511 | 91 | 184 | 898 | 3 | 717 | 0 | 0 |
| hsa_circ_0085512 | 92 | 1 | 286 | 1 | 286 | 0 | 4.00E-109 |
| hsa_circ_0085510 | 91 | 1 | 392 | 1 | 392 | 0 | 3.00E-151 |
| hsa_circ_0085510 | 91 | 1 | 278 | 1 | 278 | 0 | 1.00E-104 |
| hsa_circ_0085507 | 91 | 1 | 158 | 158 | 1 | 0 | 8.00E-53 |
| hsa_circ_0074404 | 91 | 1 | 352 | 410 | 59 | 0 | 5.00E-134 |
| hsa_circ_0074400 | 92 | 4 | 1796 | 2099 | 307 | 0 | 0 |
| hsa_circ_0074408 | 93 | 1 | 831 | 1144 | 314 | 0 | 0 |
| hsa_circ_0074412 | 93 | 1 | 471 | 636 | 166 | 0 | 0 |
| hsa_circ_0074414 | 93 | 1 | 469 | 469 | 1 | 0 | 0 |
| hsa_circ_0074419 | 95 | 1 | 1223 | 1 | 1220 | 0 | 0 |
| hsa_circ_0074429 | 94 | 1 | 429 | 1 | 429 | 0 | 0 |
| hsa_circ_0074434 | 93 | 1 | 1187 | 1 | 1187 | 0 | 0 |
| hsa_circ_0074443 | 91 | 1 | 558 | 155 | 712 | 0 | 0 |
| hsa_circ_0074449 | 91 | 1 | 1064 | 100 | 1175 | 0.01 | 0 |
| hsa_circ_0074449 | 92 | 1 | 1530 | 100 | 1641 | 0.01 | 0 |
| hsa_circ_0074457 | 95 | 1 | 511 | 404 | 914 | 0 | 0 |
| hsa_circ_0074457 | 95 | 1 | 630 | 404 | 1033 | 0 | 0 |
| hsa_circ_0074459 | 95 | 1 | 888 | 1 | 888 | 0 | 0 |
| hsa_circ_0074461 | 95 | 1 | 348 | 1 | 348 | 0 | 2.00E-153 |
| hsa_circ_0074462 | 95 | 1 | 264 | 1 | 264 | 0 | 1.00E-113 |
| hsa_circ_0074463 | 95 | 1 | 343 | 343 | 1 | 0 | 5.00E-154 |
| hsa_circ_0074463 | 97 | 118 | 295 | 343 | 166 | 0 | 5.00E-79 |
| hsa_circ_0074472 | 87 | 1 | 324 | 1 | 325 | 0.01 | 8.00E-102 |
| hsa_circ_0074472 | 91 | 1 | 654 | 1 | 655 | 0 | 0 |
| hsa_circ_0074472 | 93 | 1 | 930 | 1 | 931 | 0 | 0 |
| hsa_circ_0074472 | 93 | 1 | 1150 | 1 | 1151 | 0 | 0 |
| hsa_circ_0074472 | 93 | 1 | 1469 | 1 | 1470 | 0 | 0 |
| hsa_circ_0074472 | 93 | 1 | 1803 | 1 | 1801 | 0 | 0 |
| hsa_circ_0074472 | 95 | 1 | 960 | 192 | 1151 | 0 | 0 |
| hsa_circ_0074475 | 95 | 1 | 815 | 1 | 815 | 0 | 0 |
| hsa_circ_0074475 | 94 | 1 | 1149 | 1 | 1146 | 0 | 0 |
| hsa_circ_0074477 | 97 | 1 | 232 | 1 | 232 | 0 | 7.00E-106 |
| hsa_circ_0074477 | 95 | 1 | 677 | 1 | 677 | 0 | 0 |
| hsa_circ_0074477 | 93 | 1 | 1011 | 1 | 1008 | 0 | 0 |

novel_circ_010475 novel_circ_010476 novel_circ_010477 novel_circ_010478 novel_circ_010479 novel_circ_010480 novel_circ_010481 novel_circ_010482 novel_circ_010483 novel_circ_010484 novel_circ_010486 novel_circ_010487 novel_circ_010488 novel_circ_010489 novel_circ_010490 novel_circ_010491 novel_circ_010493 novel_circ_010495 novel_circ_010500 novel_circ_010501 novel_circ_010503 novel_circ_010504 novel_circ_010505 novel_circ_010506 novel_circ_010508 novel_circ_010509 novel_circ_010510 novel_circ_010511 novel_circ_010512 novel_circ_010513 novel_circ_010514 novel_circ_010515 novel_circ_010516 novel_circ_010517 novel_circ_010518 novel_circ_010519 novel_circ_010520 novel_circ_010521 novel_circ_010522 novel_circ_010523 novel_circ_010524 novel_circ_010525 novel_circ_010526 novel_circ_010527 novel_circ_010528 novel_circ_010529 novel_circ_010530 novel_circ_010531 novel_circ_010532 novel_circ_010533 novel_circ_010534 novel_circ_010535 novel_circ_010536 novel_circ_010537

| hsa_circ_0074477 | 94 | 1 | 2544 | 1 | 2544 | 0 | 0 |
| --- | --- | --- | --- | --- | --- | --- | --- |
| hsa_circ_0074478 | 95 | 1 | 314 | 226 | 539 | 0 | 3.00E-136 |
| hsa_circ_0074477 | 94 | 1 | 1906 | 535 | 2440 | 0 | 0 |
| hsa_circ_0074479 | 96 | 1 | 1116 | 121 | 1239 | 0 | 0 |
| hsa_circ_0074481 | 93 | 1 | 735 | 1 | 735 | 0 | 0 |
| hsa_circ_0074483 | 96 | 1 | 569 | 569 | 1 | 0 | 0 |
| hsa_circ_0074483 | 95 | 1 | 723 | 723 | 1 | 0 | 0 |
| hsa_circ_0074483 | 95 | 328 | 1050 | 723 | 1 | 0 | 0 |
| hsa_circ_0074483 | 95 | 328 | 481 | 723 | 570 | 0 | 4.00E-62 |
| hsa_circ_0074483 | 95 | 7102 | 7255 | 723 | 570 | 0 | 6.00E-61 |
| hsa_circ_0080267 | 95 | 1 | 632 | 1 | 632 | 0 | 0 |
| hsa_circ_0080266 | 95 | 1 | 524 | 65 | 588 | 0 | 0 |
| hsa_circ_0080256 | 95 | 160 | 438 | 281 | 3 | 0 | 7.00E-120 |
| hsa_circ_0080249 | 91 | 1 | 211 | 308 | 518 | 0 | 1.00E-77 |
| hsa_circ_0080244 | 92 | 1 | 525 | 1 | 525 | 0 | 0 |
| hsa_circ_0080244 | 94 | 1 | 349 | 1 | 349 | 0 | 1.00E-150 |
| hsa_circ_0080238 | 84 | 92 | 309 | 3 | 220 | 0 | 3.00E-52 |
| hsa_circ_0080238 | 84 | 92 | 309 | 3 | 220 | 0 | 3.00E-52 |
| hsa_circ_0039116 | 93 | 1 | 141 | 1474 | 1614 | 0 | 6.00E-52 |
| hsa_circ_0039116 | 89 | 1 | 1157 | 317 | 1473 | 0 | 0 |
| hsa_circ_0039108 | 94 | 3 | 482 | 3 | 482 | 0 | 0 |
| hsa_circ_0004236 | 96 | 1 | 951 | 1 | 951 | 0 | 0 |
| hsa_circ_0004236 | 98 | 1 | 469 | 1 | 469 | 0 | 0 |
| hsa_circ_0039105 | 90 | 1 | 2835 | 325 | 3165 | 0 | 0 |
| hsa_circ_0039099 | 94 | 1 | 824 | 1 | 824 | 0 | 0 |
| hsa_circ_0039095 | 92 | 99 | 1532 | 69 | 1499 | 0 | 0 |
| hsa_circ_0039099 | 94 | 1 | 500 | 1 | 500 | 0 | 0 |
| hsa_circ_0039096 | 93 | 1 | 1004 | 1 | 1001 | 0 | 0 |
| hsa_circ_0039095 | 92 | 99 | 1208 | 69 | 1175 | 0 | 0 |
| hsa_circ_0039093 | 88 | 1 | 798 | 1 | 765 | 0.04 | 0 |
| hsa_circ_0039092 | 89 | 4 | 889 | 4 | 859 | 0.04 | 0 |
| hsa_circ_0039088 | 90 | 1 | 1966 | 1 | 1939 | 0.02 | 0 |
| hsa_circ_0039092 | 94 | 4 | 181 | 4 | 184 | 0.02 | 2.00E-71 |
| hsa_circ_0039088 | 92 | 1 | 1258 | 1 | 1264 | 0 | 0 |
| hsa_circ_0039088 | 92 | 1 | 579 | 1 | 579 | 0 | 0 |
| hsa_circ_0039088 | 93 | 1 | 252 | 1 | 252 | 0 | 3.00E-100 |
| hsa_circ_0005806 | 99 | 121 | 632 | 237 | 748 | 0 | 0 |
| hsa_circ_0038961 | 97 | 1 | 339 | 339 | 1 | 0 | 2.00E-163 |
| hsa_circ_0038853 | 94 | 2 | 198 | 284 | 88 | 0 | 4.00E-79 |
| hsa_circ_0005894 | 91 | 1 | 580 | 1 | 580 | 0 | 0 |
| hsa_circ_0038823 | 97 | 1 | 402 | 402 | 1 | 0 | 0 |
| hsa_circ_0038820 | 95 | 1 | 637 | 640 | 1 | 0 | 0 |
| hsa_circ_0038813 | 95 | 1 | 1218 | 1533 | 313 | 0 | 0 |
| hsa_circ_0038823 | 97 | 1 | 311 | 311 | 1 | 0 | 1.00E-149 |
| hsa_circ_0038820 | 95 | 1 | 546 | 549 | 1 | 0.01 | 0 |
| hsa_circ_0007807 | 95 | 1 | 581 | 581 | 1 | 0 | 0 |
| hsa_circ_0038817 | 96 | 1 | 691 | 691 | 1 | 0 | 0 |
| hsa_circ_0038813 | 95 | 1 | 893 | 893 | 1 | 0 | 0 |
| hsa_circ_0038810 | 95 | 1 | 963 | 963 | 1 | 0 | 0 |
| hsa_circ_0038808 | 95 | 1 | 1123 | 1123 | 1 | 0 | 0 |
| hsa_circ_0038780 | 93 | 1 | 2633 | 2633 | 1 | 0 | 0 |
| hsa_circ_0038813 | 95 | 1 | 312 | 312 | 1 | 0 | 7.00E-137 |
| hsa_circ_0038813 | 94 | 1 | 202 | 202 | 1 | 0 | 6.00E-81 |
| hsa_circ_0038813 | 94 | 1 | 202 | 202 | 1 | 0 | 7.00E-81 |

novel_circ_010538 novel_circ_010539 novel_circ_010540 novel_circ_010541 novel_circ_010542 novel_circ_010543 novel_circ_010544 novel_circ_010545 novel_circ_010546 novel_circ_010548 novel_circ_010549 novel_circ_010550 novel_circ_010552 novel_circ_010553 novel_circ_010554 novel_circ_010555 novel_circ_010556 novel_circ_010557 novel_circ_010558 novel_circ_010559 novel_circ_010562 novel_circ_010563 novel_circ_010564 novel_circ_010565 novel_circ_010566 novel_circ_010567 novel_circ_010568 novel_circ_010569 novel_circ_010570 novel_circ_010571 novel_circ_010572 novel_circ_010573 novel_circ_010575 novel_circ_010576 novel_circ_010577 novel_circ_010578 novel_circ_010579 novel_circ_010580 novel_circ_010584 novel_circ_010585 novel_circ_010586 novel_circ_010587 novel_circ_010588 novel_circ_010589 novel_circ_010590 novel_circ_010591 novel_circ_010592 novel_circ_010593 novel_circ_010594 novel_circ_010595 novel_circ_010596 novel_circ_010597 novel_circ_010598 novel_circ_010599

| hsa_circ_0038810 | 93 | 1 | 272 | 272 | 1 | 0 | 2.00E-111 |
| --- | --- | --- | --- | --- | --- | --- | --- |
| hsa_circ_0038808 | 93 | 1 | 432 | 432 | 1 | 0 | 2.00E-178 |
| hsa_circ_0038806 | 94 | 1 | 525 | 525 | 1 | 0 | 0 |
| hsa_circ_0038794 | 93 | 1 | 1007 | 1007 | 1 | 0 | 0 |
| hsa_circ_0038794 | 93 | 1 | 1007 | 1007 | 1 | 0 | 0 |
| hsa_circ_0038780 | 93 | 1 | 1942 | 1942 | 1 | 0 | 0 |
| hsa_circ_0038802 | 93 | 7 | 468 | 462 | 1 | 0 | 0 |
| hsa_circ_0038780 | 93 | 1 | 1670 | 1670 | 1 | 0 | 0 |
| hsa_circ_0038780 | 92 | 1 | 779 | 779 | 1 | 0 | 0 |
| hsa_circ_0038762 | 86 | 129 | 518 | 132 | 521 | 0 | 1.00E-116 |
| hsa_circ_0038762 | 86 | 261 | 650 | 132 | 521 | 0 | 2.00E-116 |
| hsa_circ_0038762 | 86 | 526 | 915 | 132 | 521 | 0 | 2.00E-116 |
| hsa_circ_0038761 | 94 | 1 | 98 | 335 | 432 | 0 | 2.00E-34 |
| hsa_circ_0038761 | 89 | 1 | 265 | 165 | 432 | 0.01 | 1.00E-87 |
| hsa_circ_0038761 | 91 | 1 | 342 | 88 | 432 | 0.01 | 4.00E-127 |
| hsa_circ_0038761 | 89 | 1 | 265 | 165 | 432 | 0.01 | 8.00E-88 |
| hsa_circ_0038753 | 82 | 1 | 999 | 1008 | 1 | 0.02 | 0 |
| hsa_circ_0038746 | 83 | 1 | 1097 | 1741 | 636 | 0.02 | 0 |
| hsa_circ_0038746 | 85 | 1 | 1372 | 1741 | 361 | 0.02 | 0 |
| hsa_circ_0038739 | 84 | 1 | 897 | 2062 | 1160 | 0.01 | 0 |
| hsa_circ_0038713 | 88 | 1 | 464 | 828 | 365 | 0 | 4.00E-156 |
| hsa_circ_0038699 | 86 | 1 | 397 | 1 | 396 | 0.01 | 5.00E-115 |
| hsa_circ_0038698 | 86 | 1 | 518 | 1 | 517 | 0.01 | 8.00E-159 |
| hsa_circ_0038699 | 86 | 1 | 397 | 1 | 396 | 0.01 | 5.00E-115 |
| hsa_circ_0038693 | 88 | 1 | 557 | 215 | 771 | 0 | 0 |
| hsa_circ_0038685 | 91 | 1 | 263 | 263 | 1 | 0.01 | 2.00E-94 |
| hsa_circ_0038684 | 93 | 152 | 668 | 609 | 93 | 0 | 0 |
| hsa_circ_0038678 | 97 | 1 | 343 | 769 | 1111 | 0 | 2.00E-162 |
| hsa_circ_0038678 | 97 | 1 | 550 | 562 | 1111 | 0 | 0 |
| hsa_circ_0038678 | 96 | 1 | 289 | 691 | 979 | 0 | 2.00E-132 |
| hsa_circ_0038678 | 97 | 1 | 418 | 562 | 979 | 0 | 0 |
| hsa_circ_0038677 | 99 | 177 | 899 | 1 | 723 | 0 | 0 |
| hsa_circ_0038677 | 98 | 177 | 770 | 1 | 594 | 0 | 0 |
| hsa_circ_0005969 | 82 | 1 | 1503 | 1 | 1511 | 0.06 | 0 |
| hsa_circ_0038669 | 91 | 215 | 785 | 103 | 673 | 0 | 0 |
| hsa_circ_0038669 | 92 | 1 | 673 | 1 | 673 | 0 | 0 |
| hsa_circ_0038668 | 94 | 1 | 983 | 1 | 983 | 0 | 0 |
| hsa_circ_0038668 | 95 | 138 | 681 | 1 | 544 | 0 | 0 |
| hsa_circ_0038661 | 94 | 1 | 469 | 1 | 469 | 0 | 0 |
| hsa_circ_0038656 | 93 | 1 | 624 | 419 | 1042 | 0 | 0 |
| hsa_circ_0038656 | 93 | 1 | 798 | 245 | 1042 | 0 | 0 |
| hsa_circ_0038656 | 93 | 1 | 418 | 1 | 418 | 0 | 1.00E-175 |
| hsa_circ_0038650 | 94 | 1 | 233 | 1 | 233 | 0 | 5.00E-98 |
| hsa_circ_0038647 | 94 | 1 | 316 | 1 | 316 | 0 | 1.00E-135 |
| hsa_circ_0038630 | 93 | 68 | 403 | 100 | 435 | 0 | 2.00E-138 |
| hsa_circ_0038630 | 93 | 68 | 300 | 100 | 332 | 0 | 3.00E-91 |
| hsa_circ_0038606 | 81 | 14 | 882 | 1352 | 485 | 0.02 | 0 |
| hsa_circ_0038598 | 93 | 1 | 622 | 622 | 1 | 0 | 0 |
| hsa_circ_0038573 | 91 | 1 | 653 | 993 | 341 | 0 | 0 |
| hsa_circ_0038572 | 91 | 1 | 1137 | 1137 | 1 | 0 | 0 |
| hsa_circ_0038561 | 91 | 1 | 1289 | 1731 | 443 | 0 | 0 |
| hsa_circ_0003672 | 93 | 3 | 456 | 599 | 146 | 0 | 0 |
| hsa_circ_0003672 | 94 | 3 | 601 | 599 | 1 | 0 | 0 |
| hsa_circ_0038555 | 93 | 139 | 1194 | 1564 | 506 | 0 | 0 |

novel_circ_010600 novel_circ_010601 novel_circ_010603 novel_circ_010604 novel_circ_010605 novel_circ_010606 novel_circ_010607 novel_circ_010608 novel_circ_010609 novel_circ_010610 novel_circ_010611 novel_circ_010612 novel_circ_010613 novel_circ_010614 novel_circ_010615 novel_circ_010616 novel_circ_010617 novel_circ_010618 novel_circ_010619 novel_circ_010620 novel_circ_010621 novel_circ_010622 novel_circ_010623 novel_circ_010624 novel_circ_010625 novel_circ_010626 novel_circ_010627 novel_circ_010628 novel_circ_010629 novel_circ_010630 novel_circ_010631 novel_circ_010632 novel_circ_010633 novel_circ_010634 novel_circ_010635 novel_circ_010637 novel_circ_010638 novel_circ_010642 novel_circ_010643 novel_circ_010647 novel_circ_010648 novel_circ_010649 novel_circ_010650 novel_circ_010651 novel_circ_010652 novel_circ_010653 novel_circ_010654 novel_circ_010655 novel_circ_010656 novel_circ_010657 novel_circ_010658 novel_circ_010659 novel_circ_010660 novel_circ_010661

| hsa_circ_0038555 | 92 | 1 | 593 | 1101 | 506 | 0.01 | 0 |
| --- | --- | --- | --- | --- | --- | --- | --- |
| hsa_circ_0038555 | 91 | 1 | 713 | 1101 | 386 | 0 | 0 |
| hsa_circ_0005448 | 81 | 27833 | 27941 | 279675 | 279564 | 0.06 | 3.00E-12 |
| hsa_circ_0072646 | 94 | 1 | 570 | 117 | 686 | 0 | 0 |
| hsa_circ_0002107 | 94 | 1 | 332 | 1 | 332 | 0 | 3.00E-141 |
| hsa_circ_0072646 | 94 | 1 | 570 | 117 | 686 | 0 | 0 |
| hsa_circ_0072637 | 95 | 1 | 800 | 565 | 1364 | 0 | 0 |
| hsa_circ_0072637 | 94 | 1 | 678 | 565 | 1242 | 0 | 0 |
| hsa_circ_0072643 | 95 | 1 | 468 | 193 | 660 | 0 | 0 |
| hsa_circ_0072634 | 94 | 1 | 416 | 1 | 416 | 0 | 3.00E-176 |
| hsa_circ_0072618 | 94 | 1 | 410 | 1 | 410 | 0 | 3.00E-176 |
| hsa_circ_0072610 | 94 | 1 | 511 | 1 | 511 | 0 | 0 |
| hsa_circ_0072618 | 95 | 1 | 277 | 1 | 277 | 0 | 4.00E-119 |
| hsa_circ_0072610 | 95 | 1 | 378 | 1 | 378 | 0 | 2.00E-168 |
| hsa_circ_0072597 | 97 | 1 | 649 | 144 | 792 | 0 | 0 |
| hsa_circ_0072597 | 96 | 1 | 792 | 1 | 792 | 0 | 0 |
| hsa_circ_0072597 | 96 | 1 | 524 | 1 | 524 | 0 | 0 |
| hsa_circ_0072592 | 97 | 1 | 1309 | 474 | 1782 | 0 | 0 |
| hsa_circ_0072597 | 97 | 1 | 316 | 144 | 459 | 0 | 2.00E-147 |
| hsa_circ_0072597 | 96 | 1 | 459 | 1 | 459 | 0 | 0 |
| hsa_circ_0072592 | 97 | 1 | 928 | 474 | 1401 | 0 | 0 |
| hsa_circ_0072592 | 98 | 1 | 247 | 1012 | 1258 | 0 | 4.00E-119 |
| hsa_circ_0001490 | 98 | 1 | 218 | 1 | 218 | 0 | 9.00E-105 |
| hsa_circ_0072592 | 96 | 214 | 1022 | 203 | 1011 | 0 | 0 |
| hsa_circ_0072586 | 98 | 3 | 719 | 1311 | 2027 | 0 | 0 |
| hsa_circ_0072586 | 97 | 1 | 1048 | 658 | 1705 | 0 | 0 |
| hsa_circ_0072586 | 96 | 1 | 912 | 658 | 1569 | 0 | 0 |
| hsa_circ_0072586 | 97 | 1 | 1569 | 1 | 1569 | 0 | 0 |
| hsa_circ_0072590 | 96 | 1 | 504 | 1 | 504 | 0 | 0 |
| hsa_circ_0072586 | 97 | 1 | 1161 | 1 | 1161 | 0 | 0 |
| hsa_circ_0072590 | 97 | 1 | 357 | 1 | 357 | 0 | 2.00E-168 |
| hsa_circ_0072586 | 97 | 1 | 837 | 1 | 837 | 0 | 0 |
| hsa_circ_0072586 | 98 | 1 | 657 | 1 | 657 | 0 | 0 |
| hsa_circ_0072584 | 98 | 21666 | 22026 | 1036 | 676 | 0 | 7.00E-179 |
| hsa_circ_0072586 | 98 | 1 | 357 | 1 | 357 | 0 | 2.00E-178 |
| hsa_circ_0072581 | 92 | 1 | 308 | 308 | 1 | 0 | 3.00E-121 |
| hsa_circ_0072581 | 92 | 1 | 308 | 308 | 1 | 0 | 4.00E-121 |
| hsa_circ_0015868 | 79 | 1 | 861 | 952 | 92 | 0.03 | 1.00E-164 |
| hsa_circ_0072578 | 93 | 1 | 397 | 3288 | 2892 | 0 | 3.00E-162 |
| hsa_circ_0003792 | 88 | 1 | 633 | 1 | 633 | 0 | 0 |
| hsa_circ_0003792 | 93 | 1 | 356 | 1 | 356 | 0 | 7.00E-148 |
| hsa_circ_0075033 | 92 | 1 | 278 | 416 | 139 | 0 | 1.00E-109 |
| hsa_circ_0075027 | 92 | 1 | 1281 | 1281 | 1 | 0 | 0 |
| hsa_circ_0075027 | 92 | 1 | 1003 | 1003 | 1 | 0 | 0 |
| hsa_circ_0075032 | 92 | 4 | 369 | 366 | 1 | 0 | 2.00E-143 |
| hsa_circ_0075027 | 92 | 4 | 865 | 862 | 1 | 0 | 0 |
| hsa_circ_0075029 | 91 | 1 | 369 | 369 | 1 | 0 | 4.00E-140 |
| hsa_circ_0074976 | 97 | 1 | 99 | 2926 | 3024 | 0 | 3.00E-40 |
| hsa_circ_0004738 | 93 | 1 | 354 | 1 | 354 | 0 | 4.00E-145 |
| hsa_circ_0074992 | 94 | 3 | 738 | 112 | 847 | 0 | 0 |
| hsa_circ_0074994 | 95 | 3 | 384 | 3 | 384 | 0 | 2.00E-167 |
| hsa_circ_0074992 | 93 | 1 | 493 | 1 | 493 | 0 | 0 |
| hsa_circ_0074994 | 96 | 11017 | 11207 | 3 | 193 | 0 | 5.00E-83 |
| hsa_circ_0002713 | 93 | 1 | 334 | 1 | 334 | 0 | 5.00E-139 |

novel_circ_010662 novel_circ_010663 novel_circ_010664 novel_circ_010665 novel_circ_010666 novel_circ_010667 novel_circ_010668 novel_circ_010669 novel_circ_010670 novel_circ_010671 novel_circ_010672 novel_circ_010673 novel_circ_010674 novel_circ_010675 novel_circ_010676 novel_circ_010677 novel_circ_010678 novel_circ_010679 novel_circ_010680 novel_circ_010681 novel_circ_010682 novel_circ_010683 novel_circ_010684 novel_circ_010685 novel_circ_010686 novel_circ_010687 novel_circ_010688 novel_circ_010689 novel_circ_010690 novel_circ_010691 novel_circ_010692 novel_circ_010693 novel_circ_010694 novel_circ_010695 novel_circ_010696 novel_circ_010697 novel_circ_010698 novel_circ_010699 novel_circ_010700 novel_circ_010701 novel_circ_010704 novel_circ_010705 novel_circ_010706 novel_circ_010707 novel_circ_010708 novel_circ_010709 novel_circ_010710 novel_circ_010711 novel_circ_010712 novel_circ_010713 novel_circ_010714 novel_circ_010715 novel_circ_010716 novel_circ_010717

| hsa_circ_0074987 | 79 | 14605 | 17762 | 21552 | 24656 | 0.07 | 0 |
| --- | --- | --- | --- | --- | --- | --- | --- |
| hsa_circ_0074987 | 79 | 11408 | 14565 | 21552 | 24656 | 0.07 | 0 |
| hsa_circ_0074976 | 93 | 1 | 1695 | 1 | 1695 | 0 | 0 |
| hsa_circ_0074976 | 93 | 1 | 1695 | 1 | 1695 | 0 | 0 |
| hsa_circ_0074988 | 90 | 1 | 436 | 321 | 756 | 0 | 1.00E-160 |
| hsa_circ_0074983 | 92 | 1 | 1221 | 1 | 1221 | 0 | 0 |
| hsa_circ_0074976 | 93 | 1 | 1690 | 3 | 1692 | 0 | 0 |
| hsa_circ_0074976 | 93 | 1 | 1692 | 1 | 1692 | 0 | 0 |
| hsa_circ_0074983 | 92 | 1 | 979 | 1 | 979 | 0 | 0 |
| hsa_circ_0074980 | 92 | 1 | 1045 | 168 | 1212 | 0 | 0 |
| hsa_circ_0074980 | 92 | 1 | 1212 | 1 | 1212 | 0 | 0 |
| hsa_circ_0074976 | 92 | 1 | 1450 | 1 | 1450 | 0 | 0 |
| hsa_circ_0074976 | 93 | 1 | 1256 | 1 | 1256 | 0 | 0 |
| hsa_circ_0074976 | 93 | 1 | 816 | 1 | 816 | 0 | 0 |
| hsa_circ_0074980 | 94 | 1 | 504 | 1 | 504 | 0 | 0 |
| hsa_circ_0074976 | 93 | 1 | 742 | 1 | 742 | 0 | 0 |
| hsa_circ_0074976 | 94 | 1 | 576 | 1 | 576 | 0 | 0 |
| hsa_circ_0074976 | 93 | 1 | 471 | 1 | 471 | 0 | 0 |
| hsa_circ_0074976 | 93 | 1 | 403 | 3 | 405 | 0 | 6.00E-164 |
| hsa_circ_0074976 | 93 | 1 | 405 | 1 | 405 | 0 | 4.00E-165 |
| hsa_circ_0074919 | 92 | 1 | 585 | 798 | 214 | 0 | 0 |
| hsa_circ_0074919 | 91 | 1 | 796 | 798 | 3 | 0 | 0 |
| hsa_circ_0074900 | 89 | 2 | 641 | 649 | 1 | 0.01 | 0 |
| hsa_circ_0074897 | 95 | 1 | 1034 | 1034 | 1 | 0 | 0 |
| hsa_circ_0074898 | 96 | 1 | 301 | 301 | 1 | 0 | 4.00E-139 |
| hsa_circ_0074897 | 96 | 1 | 427 | 427 | 1 | 0 | 0 |
| hsa_circ_0074880 | 92 | 1 | 1172 | 123 | 1294 | 0 | 0 |
| hsa_circ_0074894 | 90 | 1 | 279 | 1 | 279 | 0 | 7.00E-97 |
| hsa_circ_0074889 | 92 | 1 | 568 | 1 | 568 | 0 | 0 |
| hsa_circ_0074884 | 92 | 1 | 803 | 1 | 803 | 0 | 0 |
| hsa_circ_0074880 | 92 | 1 | 924 | 123 | 1046 | 0 | 0 |
| hsa_circ_0074877 | 92 | 1 | 1256 | 325 | 1580 | 0 | 0 |
| hsa_circ_0074884 | 92 | 1 | 630 | 1 | 630 | 0 | 0 |
| hsa_circ_0074877 | 92 | 1 | 1083 | 325 | 1407 | 0 | 0 |
| hsa_circ_0074889 | 94 | 1 | 289 | 1 | 289 | 0 | 4.00E-124 |
| hsa_circ_0074877 | 92 | 1 | 867 | 325 | 1191 | 0 | 0 |
| hsa_circ_0074884 | 91 | 1 | 232 | 1 | 232 | 0 | 8.00E-86 |
| hsa_circ_0074877 | 92 | 1 | 453 | 325 | 777 | 0 | 0 |
| hsa_circ_0074877 | 91 | 1 | 642 | 136 | 777 | 0 | 0 |
| hsa_circ_0074864 | 95 | 1 | 314 | 1 | 314 | 0 | 6.00E-138 |
| hsa_circ_0074859 | 92 | 1 | 472 | 1 | 472 | 0 | 0 |
| hsa_circ_0000701 | 93 | 32006 | 33643 | 1461 | 3095 | 0.01 | 0 |
| hsa_circ_0039354 | 93 | 1 | 1619 | 1 | 1616 | 0.01 | 0 |
| hsa_circ_0039354 | 94 | 1 | 1951 | 1 | 1948 | 0.01 | 0 |
| hsa_circ_0039358 | 95 | 1 | 1923 | 1 | 1923 | 0 | 0 |
| hsa_circ_0039358 | 95 | 1 | 2034 | 1 | 2034 | 0 | 0 |
| hsa_circ_0039358 | 95 | 1 | 2231 | 1 | 2231 | 0 | 0 |
| hsa_circ_0039359 | 97 | 1 | 366 | 1 | 366 | 0 | 4.00E-175 |
| hsa_circ_0039358 | 95 | 1 | 1154 | 226 | 1379 | 0 | 0 |
| hsa_circ_0039358 | 95 | 1 | 1698 | 226 | 1923 | 0 | 0 |
| hsa_circ_0039360 | 93 | 1 | 788 | 1 | 788 | 0 | 0 |
| hsa_circ_0039358 | 94 | 1 | 1443 | 592 | 2034 | 0 | 0 |
| hsa_circ_0039358 | 94 | 1 | 1640 | 592 | 2231 | 0 | 0 |
| hsa_circ_0039358 | 94 | 1 | 1640 | 592 | 2231 | 0 | 0 |

novel_circ_010718 novel_circ_010719 novel_circ_010720 novel_circ_010721 novel_circ_010722 novel_circ_010723 novel_circ_010724 novel_circ_010725 novel_circ_010726 novel_circ_010727 novel_circ_010728 novel_circ_010729 novel_circ_010730 novel_circ_010731 novel_circ_010732 novel_circ_010733 novel_circ_010734 novel_circ_010735 novel_circ_010736 novel_circ_010737 novel_circ_010738 novel_circ_010739 novel_circ_010740 novel_circ_010741 novel_circ_010742 novel_circ_010743 novel_circ_010744 novel_circ_010745 novel_circ_010746 novel_circ_010747 novel_circ_010748 novel_circ_010749 novel_circ_010750 novel_circ_010751 novel_circ_010752 novel_circ_010753 novel_circ_010754 novel_circ_010755 novel_circ_010756 novel_circ_010757 novel_circ_010758 novel_circ_010759 novel_circ_010760 novel_circ_010761 novel_circ_010762 novel_circ_010763 novel_circ_010764 novel_circ_010765 novel_circ_010766 novel_circ_010767 novel_circ_010768 novel_circ_010769 novel_circ_010770 novel_circ_010771

| hsa_circ_0039362 | 94 | 578 | 2606 | 1 | 2050 | 0.01 | 0 |
| --- | --- | --- | --- | --- | --- | --- | --- |
| hsa_circ_0039358 | 94 | 1 | 975 | 769 | 1743 | 0 | 0 |
| hsa_circ_0039358 | 94 | 1 | 1155 | 769 | 1923 | 0 | 0 |
| hsa_circ_0039367 | 94 | 1 | 758 | 181 | 938 | 0 | 0 |
| hsa_circ_0008031 | 94 | 1 | 397 | 1 | 397 | 0 | 6.00E-169 |
| hsa_circ_0039367 | 94 | 1 | 398 | 292 | 689 | 0 | 7.00E-169 |
| hsa_circ_0039367 | 94 | 1 | 250 | 689 | 938 | 0 | 8.00E-106 |
| hsa_circ_0039370 | 94 | 3 | 630 | 3 | 642 | 0.02 | 0 |
| hsa_circ_0039370 | 94 | 3 | 1976 | 3 | 1982 | 0.01 | 0 |
| hsa_circ_0039371 | 97 | 1 | 257 | 1 | 257 | 0 | 1.00E-119 |
| hsa_circ_0039371 | 96 | 1 | 466 | 1 | 466 | 0 | 0 |
| hsa_circ_0039371 | 96 | 1 | 423 | 44 | 466 | 0 | 0 |
| hsa_circ_0039371 | 94 | 1 | 1335 | 44 | 1372 | 0 | 0 |
| hsa_circ_0039371 | 94 | 1 | 1496 | 44 | 1533 | 0.01 | 0 |
| hsa_circ_0039370 | 94 | 1 | 1769 | 220 | 1982 | 0.01 | 0 |
| hsa_circ_0039374 | 94 | 1 | 912 | 1 | 906 | 0.01 | 0 |
| hsa_circ_0039374 | 93 | 1 | 1073 | 1 | 1067 | 0.01 | 0 |
| hsa_circ_0039370 | 93 | 1 | 1346 | 643 | 1982 | 0.01 | 0 |
| hsa_circ_0039370 | 94 | 21 | 437 | 1566 | 1982 | 0 | 0 |
| hsa_circ_0039370 | 94 | 18 | 434 | 1566 | 1982 | 0 | 0 |
| hsa_circ_0039370 | 96 | 1 | 273 | 1710 | 1982 | 0 | 5.00E-125 |
| hsa_circ_0039376 | 93 | 141 | 362 | 1 | 222 | 0 | 9.00E-87 |
| hsa_circ_0039376 | 92 | 141 | 491 | 1 | 351 | 0 | 6.00E-140 |
| hsa_circ_0039376 | 92 | 141 | 493 | 1 | 353 | 0 | 1.00E-140 |
| hsa_circ_0039378 | 93 | 1 | 329 | 1 | 329 | 0 | 3.00E-136 |
| hsa_circ_0039378 | 94 | 1 | 523 | 1 | 523 | 0 | 0 |
| hsa_circ_0039379 | 93 | 333 | 2460 | 1 | 2131 | 0 | 0 |
| hsa_circ_0039378 | 94 | 1 | 392 | 132 | 523 | 0 | 7.00E-168 |
| hsa_circ_0039378 | 93 | 1 | 621 | 132 | 752 | 0 | 0 |
| hsa_circ_0039378 | 94 | 1 | 808 | 132 | 939 | 0 | 0 |
| hsa_circ_0039378 | 93 | 1 | 1189 | 132 | 1320 | 0 | 0 |
| hsa_circ_0039379 | 94 | 1 | 774 | 1 | 774 | 0 | 0 |
| hsa_circ_0039380 | 93 | 1 | 709 | 1 | 709 | 0 | 0 |
| hsa_circ_0039380 | 93 | 1 | 817 | 1 | 817 | 0 | 0 |
| hsa_circ_0039380 | 93 | 1 | 1336 | 1 | 1336 | 0 | 0 |
| hsa_circ_0039380 | 95 | 6 | 413 | 135 | 542 | 0 | 0 |
| hsa_circ_0039380 | 94 | 6 | 580 | 135 | 709 | 0 | 0 |
| hsa_circ_0039380 | 94 | 6 | 794 | 135 | 923 | 0 | 0 |
| hsa_circ_0039380 | 93 | 6 | 1207 | 135 | 1336 | 0 | 0 |
| hsa_circ_0039380 | 94 | 1 | 354 | 356 | 709 | 0 | 8.00E-152 |
| hsa_circ_0039380 | 93 | 1 | 462 | 356 | 817 | 0 | 0 |
| hsa_circ_0039380 | 94 | 1 | 568 | 356 | 923 | 0 | 0 |
| hsa_circ_0039380 | 93 | 1 | 706 | 356 | 1061 | 0 | 0 |
| hsa_circ_0039380 | 93 | 1 | 981 | 356 | 1336 | 0 | 0 |
| hsa_circ_0039383 | 93 | 1 | 413 | 105 | 517 | 0 | 2.00E-169 |
| hsa_circ_0039383 | 92 | 1 | 1521 | 105 | 1628 | 0.01 | 0 |
| hsa_circ_0039383 | 92 | 1 | 1521 | 105 | 1628 | 0.01 | 0 |
| hsa_circ_0039385 | 94 | 1 | 275 | 1 | 275 | 0 | 1.00E-114 |
| hsa_circ_0039385 | 92 | 1 | 1383 | 1 | 1386 | 0.01 | 0 |
| hsa_circ_0039392 | 87 | 1 | 608 | 749 | 139 | 0.01 | 0 |
| hsa_circ_0039392 | 87 | 1 | 336 | 474 | 139 | 0.01 | 4.00E-105 |
| hsa_circ_0039392 | 92 | 1 | 1710 | 1851 | 139 | 0 | 0 |
| hsa_circ_0039392 | 87 | 1 | 534 | 749 | 213 | 0.01 | 4.00E-172 |
| hsa_circ_0039392 | 93 | 1 | 1370 | 1851 | 479 | 0 | 0 |

novel_circ_010772 novel_circ_010773 novel_circ_010774 novel_circ_010775 novel_circ_010776 novel_circ_010777 novel_circ_010778 novel_circ_010779 novel_circ_010780 novel_circ_010781 novel_circ_010782 novel_circ_010784 novel_circ_010785 novel_circ_010786 novel_circ_010787 novel_circ_010788 novel_circ_010789 novel_circ_010790 novel_circ_010791 novel_circ_010792 novel_circ_010793 novel_circ_010794 novel_circ_010795 novel_circ_010796 novel_circ_010797 novel_circ_010798 novel_circ_010799 novel_circ_010800 novel_circ_010802 novel_circ_010803 novel_circ_010804 novel_circ_010805 novel_circ_010806 novel_circ_010808 novel_circ_010809 novel_circ_010810 novel_circ_010811 novel_circ_010812 novel_circ_010813 novel_circ_010814 novel_circ_010815 novel_circ_010816 novel_circ_010817 novel_circ_010818 novel_circ_010819 novel_circ_010820 novel_circ_010821 novel_circ_010822 novel_circ_010823 novel_circ_010824 novel_circ_010825 novel_circ_010826 novel_circ_010827 novel_circ_010828

| hsa_circ_0039392 | 93 | 1 | 1290 | 1851 | 562 | 0 | 0 |
| --- | --- | --- | --- | --- | --- | --- | --- |
| hsa_circ_0039392 | 94 | 1 | 1102 | 1851 | 750 | 0 | 0 |
| hsa_circ_0039392 | 92 | 1 | 478 | 2329 | 1852 | 0 | 0 |
| hsa_circ_0039391 | 91 | 1 | 247 | 3392 | 3146 | 0 | 2.00E-92 |
| hsa_circ_0039399 | 93 | 4 | 368 | 4 | 368 | 0 | 7.00E-148 |
| hsa_circ_0039399 | 92 | 4 | 488 | 4 | 488 | 0 | 0 |
| hsa_circ_0039401 | 92 | 1 | 224 | 1 | 224 | 0 | 4.00E-83 |
| hsa_circ_0039401 | 92 | 1 | 344 | 1 | 344 | 0 | 6.00E-133 |
| hsa_circ_0039401 | 91 | 1 | 469 | 1 | 469 | 0 | 0 |
| hsa_circ_0039404 | 91 | 1 | 245 | 1 | 245 | 0 | 2.00E-91 |
| hsa_circ_0007068 | 91 | 1797 | 3051 | 2071 | 3310 | 0.02 | 0 |
| hsa_circ_0039422 | 95 | 143 | 562 | 1 | 420 | 0 | 0 |
| hsa_circ_0039429 | 90 | 1 | 219 | 219 | 1 | 0 | 3.00E-75 |
| hsa_circ_0008464 | 93 | 1 | 830 | 830 | 1 | 0 | 0 |
| hsa_circ_0008464 | 92 | 1 | 719 | 830 | 112 | 0 | 0 |
| hsa_circ_0008464 | 92 | 1 | 585 | 830 | 246 | 0 | 0 |
| hsa_circ_0008464 | 91 | 1 | 411 | 830 | 420 | 0 | 2.00E-153 |
| hsa_circ_0039442 | 97 | 1 | 265 | 4144 | 3880 | 0 | 2.00E-122 |
| hsa_circ_0039443 | 92 | 1 | 294 | 1 | 294 | 0 | 3.00E-115 |
| hsa_circ_0039446 | 92 | 147 | 632 | 1 | 486 | 0 | 0 |
| hsa_circ_0039446 | 90 | 147 | 1137 | 1 | 985 | 0.01 | 0 |
| hsa_circ_0039446 | 89 | 147 | 1260 | 1 | 1108 | 0.01 | 0 |
| hsa_circ_0039446 | 90 | 147 | 1489 | 1 | 1337 | 0.01 | 0 |
| hsa_circ_0039446 | 89 | 1 | 1067 | 48 | 1108 | 0.01 | 0 |
| hsa_circ_0039446 | 93 | 1 | 452 | 149 | 600 | 0 | 0 |
| hsa_circ_0039446 | 89 | 1 | 966 | 149 | 1108 | 0.01 | 0 |
| hsa_circ_0039446 | 90 | 1 | 1195 | 149 | 1337 | 0.01 | 0 |
| hsa_circ_0039446 | 90 | 1 | 1195 | 149 | 1337 | 0.01 | 0 |
| hsa_circ_0039452 | 88 | 1 | 743 | 1 | 737 | 0.01 | 0 |
| hsa_circ_0039452 | 88 | 1 | 743 | 1 | 737 | 0.01 | 0 |
| hsa_circ_0039452 | 91 | 1 | 352 | 386 | 737 | 0 | 3.00E-132 |
| hsa_circ_0039456 | 89 | 496 | 1410 | 915 | 1 | 0 | 0 |
| hsa_circ_0039456 | 89 | 496 | 1410 | 915 | 1 | 0 | 0 |
| hsa_circ_0039456 | 89 | 496 | 1410 | 915 | 1 | 0 | 0 |
| hsa_circ_0039456 | 90 | 79 | 863 | 915 | 131 | 0 | 0 |
| hsa_circ_0039456 | 90 | 268 | 1052 | 915 | 131 | 0 | 0 |
| hsa_circ_0039456 | 90 | 496 | 1280 | 915 | 131 | 0 | 0 |
| hsa_circ_0039456 | 93 | 1 | 468 | 915 | 448 | 0 | 0 |
| hsa_circ_0039457 | 93 | 4 | 687 | 684 | 1 | 0 | 0 |
| hsa_circ_0039457 | 92 | 4 | 495 | 684 | 193 | 0 | 0 |
| hsa_circ_0039476 | 94 | 1 | 192 | 1 | 192 | 0 | 5.00E-77 |
| hsa_circ_0039475 | 95 | 1 | 266 | 119 | 384 | 0 | 1.00E-114 |
| hsa_circ_0039475 | 92 | 1 | 497 | 119 | 615 | 0 | 0 |
| hsa_circ_0039475 | 92 | 1 | 630 | 119 | 748 | 0 | 0 |
| hsa_circ_0039479 | 91 | 1 | 434 | 1 | 434 | 0 | 2.00E-169 |
| hsa_circ_0039482 | 92 | 2 | 814 | 2 | 814 | 0 | 0 |
| hsa_circ_0039482 | 92 | 2 | 933 | 2 | 933 | 0 | 0 |
| hsa_circ_0006886 | 93 | 6 | 557 | 552 | 1 | 0 | 0 |
| hsa_circ_0006886 | 92 | 6 | 492 | 552 | 66 | 0 | 0 |
| hsa_circ_0039552 | 93 | 1 | 671 | 1 | 671 | 0 | 0 |
| hsa_circ_0039552 | 93 | 1 | 857 | 1 | 857 | 0 | 0 |
| hsa_circ_0039555 | 96 | 1 | 385 | 1 | 385 | 0 | 1.00E-175 |
| hsa_circ_0039554 | 95 | 1 | 645 | 167 | 811 | 0 | 0 |
| hsa_circ_0039559 | 94 | 1 | 504 | 1 | 504 | 0 | 0 |

novel_circ_010834 novel_circ_010835 novel_circ_010838 novel_circ_010839 novel_circ_010840 novel_circ_010841 novel_circ_010842 novel_circ_010843 novel_circ_010844 novel_circ_010845 novel_circ_010846 novel_circ_010847 novel_circ_010848 novel_circ_010849 novel_circ_010850 novel_circ_010851 novel_circ_010852 novel_circ_010853 novel_circ_010854 novel_circ_010855 novel_circ_010856 novel_circ_010857 novel_circ_010858 novel_circ_010859 novel_circ_010860 novel_circ_010861 novel_circ_010862 novel_circ_010863 novel_circ_010864 novel_circ_010865 novel_circ_010866 novel_circ_010867 novel_circ_010868 novel_circ_010869 novel_circ_010871 novel_circ_010872 novel_circ_010873 novel_circ_010874 novel_circ_010875 novel_circ_010876 novel_circ_010877 novel_circ_010878 novel_circ_010879 novel_circ_010880 novel_circ_010881 novel_circ_010882 novel_circ_010883 novel_circ_010884 novel_circ_010891 novel_circ_010892 novel_circ_010893 novel_circ_010894 novel_circ_010895 novel_circ_010896

| hsa_circ_0039613 | 95 | 1 | 463 | 557 | 95 | 0 | 0 |
| --- | --- | --- | --- | --- | --- | --- | --- |
| hsa_circ_0039614 | 89 | 157 | 393 | 1 | 237 | 0 | 2.00E-78 |
| hsa_circ_0039621 | 95 | 1 | 1136 | 1138 | 3 | 0 | 0 |
| hsa_circ_0039626 | 96 | 1 | 807 | 807 | 1 | 0 | 0 |
| hsa_circ_0039633 | 96 | 1 | 531 | 531 | 1 | 0 | 0 |
| hsa_circ_0039639 | 94 | 1 | 2124 | 2124 | 1 | 0 | 0 |
| hsa_circ_0039647 | 93 | 1 | 366 | 366 | 1 | 0 | 9.00E-152 |
| hsa_circ_0039647 | 94 | 1 | 663 | 663 | 1 | 0 | 0 |
| hsa_circ_0039647 | 94 | 1 | 794 | 794 | 1 | 0 | 0 |
| hsa_circ_0039647 | 94 | 1 | 1278 | 1278 | 1 | 0 | 0 |
| hsa_circ_0039647 | 95 | 1 | 2321 | 2321 | 1 | 0 | 0 |
| hsa_circ_0039653 | 94 | 1 | 912 | 912 | 1 | 0 | 0 |
| hsa_circ_0039662 | 96 | 1 | 1146 | 1146 | 1 | 0 | 0 |
| hsa_circ_0039664 | 96 | 1 | 552 | 552 | 1 | 0 | 0 |
| hsa_circ_0039664 | 96 | 1 | 631 | 631 | 1 | 0 | 0 |
| hsa_circ_0039664 | 96 | 1 | 918 | 918 | 1 | 0 | 0 |
| hsa_circ_0039664 | 96 | 1 | 1043 | 1043 | 1 | 0 | 0 |
| hsa_circ_0039664 | 96 | 1 | 1543 | 1543 | 1 | 0 | 0 |
| hsa_circ_0039664 | 96 | 1 | 1938 | 1938 | 1 | 0 | 0 |
| hsa_circ_0039667 | 97 | 1 | 491 | 491 | 1 | 0 | 0 |
| hsa_circ_0039667 | 96 | 1 | 991 | 991 | 1 | 0 | 0 |
| hsa_circ_0039670 | 96 | 1 | 912 | 912 | 1 | 0 | 0 |
| hsa_circ_0039670 | 95 | 1 | 1307 | 1307 | 1 | 0 | 0 |
| hsa_circ_0039670 | 95 | 1 | 895 | 1307 | 413 | 0 | 0 |
| hsa_circ_0039670 | 95 | 1 | 880 | 1307 | 428 | 0 | 0 |
| hsa_circ_0039692 | 93 | 1 | 275 | 275 | 1 | 0 | 2.00E-111 |
| hsa_circ_0039692 | 94 | 1 | 395 | 395 | 1 | 0 | 7.00E-173 |
| hsa_circ_0039692 | 95 | 1 | 1173 | 1173 | 1 | 0 | 0 |
| hsa_circ_0039695 | 96 | 1 | 778 | 898 | 121 | 0 | 0 |
| hsa_circ_0039692 | 97 | 1 | 1482 | 1877 | 396 | 0 | 0 |
| hsa_circ_0039696 | 97 | 1 | 537 | 537 | 1 | 0 | 0 |
| hsa_circ_0039695 | 97 | 1 | 706 | 1067 | 362 | 0 | 0 |
| hsa_circ_0039695 | 96 | 1 | 407 | 1067 | 661 | 0 | 0 |
| hsa_circ_0039704 | 93 | 4 | 443 | 437 | 1 | 0.01 | 5.00E-180 |
| hsa_circ_0029815 | 91 | 67162 | 67646 | 539 | 49 | 0.01 | 0 |
| hsa_circ_0072033 | 93 | 772 | 1162 | 1 | 391 | 0 | 4.00E-160 |
| hsa_circ_0072035 | 93 | 1 | 469 | 469 | 1 | 0 | 0 |
| hsa_circ_0072036 | 94 | 1 | 329 | 329 | 1 | 0 | 6.00E-138 |
| hsa_circ_0072044 | 96 | 1 | 309 | 309 | 1 | 0 | 3.00E-140 |
| hsa_circ_0072044 | 96 | 1 | 643 | 643 | 1 | 0 | 0 |
| hsa_circ_0072044 | 95 | 1 | 951 | 951 | 1 | 0 | 0 |
| hsa_circ_0072054 | 92 | 1 | 951 | 1652 | 702 | 0 | 0 |
| hsa_circ_0072062 | 96 | 1 | 229 | 229 | 1 | 0 | 2.00E-102 |
| hsa_circ_0072062 | 94 | 1 | 639 | 639 | 1 | 0 | 0 |
| hsa_circ_0072063 | 93 | 1 | 410 | 410 | 1 | 0 | 2.00E-169 |
| hsa_circ_0072063 | 91 | 1 | 552 | 552 | 1 | 0 | 0 |
| hsa_circ_0072065 | 91 | 1 | 378 | 378 | 1 | 0 | 2.00E-143 |
| hsa_circ_0072064 | 90 | 94 | 622 | 697 | 169 | 0 | 0 |
| hsa_circ_0072070 | 89 | 1 | 860 | 1919 | 1076 | 0.04 | 0 |
| hsa_circ_0072070 | 90 | 1 | 1107 | 2166 | 1076 | 0.03 | 0 |
| hsa_circ_0001470 | 95 | 1 | 247 | 247 | 1 | 0 | 8.00E-106 |
| hsa_circ_0072074 | 93 | 108 | 555 | 448 | 1 | 0 | 0 |
| hsa_circ_0072074 | 93 | 539 | 986 | 448 | 1 | 0 | 0 |
| hsa_circ_0072078 | 92 | 1 | 355 | 355 | 1 | 0.01 | 4.00E-137 |

novel_circ_010897 novel_circ_010898 novel_circ_010899 novel_circ_010900 novel_circ_010901 novel_circ_010902 novel_circ_010903 novel_circ_010904 novel_circ_010905 novel_circ_010906 novel_circ_010907 novel_circ_010908 novel_circ_010909 novel_circ_010910 novel_circ_010911 novel_circ_010912 novel_circ_010913 novel_circ_010914 novel_circ_010915 novel_circ_010916 novel_circ_010917 novel_circ_010919 novel_circ_010920 novel_circ_010921 novel_circ_010923 novel_circ_010924 novel_circ_010925 novel_circ_010926 novel_circ_010927 novel_circ_010928 novel_circ_010929 novel_circ_010930 novel_circ_010931 novel_circ_010932 novel_circ_010933 novel_circ_010934 novel_circ_010935 novel_circ_010936 novel_circ_010937 novel_circ_010939 novel_circ_010940 novel_circ_010941 novel_circ_010942 novel_circ_010943 novel_circ_010944 novel_circ_010945 novel_circ_010946 novel_circ_010947 novel_circ_010948 novel_circ_010949 novel_circ_010950 novel_circ_010951 novel_circ_010952 novel_circ_010953

| hsa_circ_0072078 | 92 | 1 | 428 | 428 | 1 | 0.01 | 2.00E-169 |
| --- | --- | --- | --- | --- | --- | --- | --- |
| hsa_circ_0072078 | 92 | 1 | 355 | 355 | 1 | 0.01 | 3.00E-137 |
| hsa_circ_0072078 | 92 | 1 | 638 | 632 | 1 | 0.02 | 0 |
| hsa_circ_0072088 | 97 | 1 | 336 | 336 | 1 | 0 | 8.00E-162 |
| hsa_circ_0072089 | 97 | 4 | 357 | 354 | 1 | 0 | 2.00E-168 |
| hsa_circ_0072089 | 96 | 1 | 520 | 520 | 1 | 0 | 0 |
| hsa_circ_0072089 | 95 | 1 | 1467 | 1467 | 1 | 0 | 0 |
| hsa_circ_0072096 | 95 | 1 | 429 | 429 | 1 | 0 | 0 |
| hsa_circ_0072096 | 94 | 7 | 1743 | 1716 | 1 | 0.01 | 0 |
| hsa_circ_0072096 | 94 | 1 | 681 | 1110 | 430 | 0 | 0 |
| hsa_circ_0072083 | 94 | 1 | 1597 | 4498 | 2923 | 0.01 | 0 |
| hsa_circ_0072101 | 97 | 7 | 364 | 358 | 1 | 0 | 5.00E-169 |
| hsa_circ_0072101 | 96 | 1 | 647 | 647 | 1 | 0 | 0 |
| hsa_circ_0007237 | 76 | 753 | 985 | 90265 | 90045 | 0.06 | 7.00E-23 |
| hsa_circ_0072113 | 92 | 1 | 1071 | 385 | 1455 | 0 | 0 |
| hsa_circ_0072117 | 87 | 1 | 479 | 666 | 188 | 0.01 | 9.00E-153 |
| hsa_circ_0072121 | 85 | 1 | 2280 | 2280 | 1 | 0.02 | 0 |
| hsa_circ_0072125 | 84 | 1 | 2103 | 2103 | 1 | 0.02 | 0 |
| hsa_circ_0072125 | 84 | 1 | 1950 | 2103 | 154 | 0.02 | 0 |
| hsa_circ_0072125 | 90 | 1 | 843 | 2103 | 1261 | 0 | 0 |
| hsa_circ_0072125 | 91 | 1 | 632 | 2103 | 1472 | 0 | 0 |
| hsa_circ_0076598 | 93 | 1 | 224 | 1 | 224 | 0 | 4.00E-88 |
| hsa_circ_0076595 | 92 | 1 | 218 | 1 | 218 | 0 | 8.00E-83 |
| hsa_circ_0076592 | 93 | 1 | 494 | 301 | 794 | 0 | 0 |
| hsa_circ_0076574 | 89 | 1 | 1244 | 1422 | 182 | 0 | 0 |
| hsa_circ_0076581 | 91 | 4 | 664 | 661 | 1 | 0 | 0 |
| hsa_circ_0076576 | 89 | 4 | 1034 | 1077 | 50 | 0 | 0 |
| hsa_circ_0076581 | 92 | 1 | 301 | 301 | 1 | 0 | 4.00E-119 |
| hsa_circ_0076580 | 91 | 1 | 422 | 430 | 9 | 0 | 8.00E-163 |
| hsa_circ_0076579 | 90 | 1 | 564 | 561 | 1 | 0.01 | 0 |
| hsa_circ_0076563 | 91 | 1 | 482 | 482 | 1 | 0 | 0 |
| hsa_circ_0076559 | 92 | 1 | 583 | 583 | 1 | 0 | 0 |
| hsa_circ_0076540 | 94 | 1 | 411 | 411 | 1 | 0 | 4.00E-175 |
| hsa_circ_0076515 | 91 | 7 | 792 | 7523 | 6741 | 0.01 | 0 |
| hsa_circ_0076515 | 92 | 7 | 1526 | 7523 | 6007 | 0.01 | 0 |
| hsa_circ_0076515 | 92 | 7 | 1526 | 7523 | 6007 | 0.01 | 0 |
| hsa_circ_0076515 | 92 | 7 | 1526 | 7523 | 6007 | 0.01 | 0 |
| hsa_circ_0076515 | 87 | 1570 | 7487 | 5963 | 1 | 0.03 | 0 |
| hsa_circ_0076515 | 91 | 1 | 640 | 7380 | 6741 | 0.01 | 0 |
| hsa_circ_0076438 | 93 | 1 | 214 | 579 | 792 | 0 | 3.00E-84 |
| hsa_circ_0076431 | 90 | 1 | 248 | 248 | 1 | 0 | 5.00E-88 |
| hsa_circ_0008801 | 90 | 1 | 124 | 1 | 124 | 0 | 5.00E-39 |
| hsa_circ_0076414 | 91 | 6 | 312 | 3 | 309 | 0 | 2.00E-115 |
| hsa_circ_0076411 | 91 | 1 | 1786 | 119 | 1904 | 0 | 0 |
| hsa_circ_0076412 | 91 | 1 | 1680 | 1 | 1680 | 0 | 0 |
| hsa_circ_0076395 | 90 | 1 | 1599 | 371 | 1981 | 0.01 | 0 |
| hsa_circ_0076395 | 90 | 1 | 1464 | 371 | 1846 | 0.01 | 0 |
| hsa_circ_0076395 | 90 | 1 | 1834 | 1 | 1846 | 0.01 | 0 |
| hsa_circ_0001604 | 86 | 1 | 392 | 1 | 392 | 0 | 4.00E-116 |
| hsa_circ_0076399 | 87 | 1 | 448 | 495 | 942 | 0 | 1.00E-140 |
| hsa_circ_0076395 | 90 | 1 | 1233 | 371 | 1606 | 0 | 0 |
| hsa_circ_0076395 | 90 | 1 | 1603 | 1 | 1606 | 0 | 0 |
| hsa_circ_0076399 | 87 | 1 | 357 | 495 | 851 | 0 | 9.00E-112 |
| hsa_circ_0076395 | 90 | 1 | 1512 | 1 | 1515 | 0 | 0 |

novel_circ_010954 novel_circ_010955 novel_circ_010956 novel_circ_010957 novel_circ_010958 novel_circ_010959 novel_circ_010960 novel_circ_010961 novel_circ_010962 novel_circ_010963 novel_circ_010964 novel_circ_010965 novel_circ_010966 novel_circ_010967 novel_circ_010968 novel_circ_010969 novel_circ_010970 novel_circ_010971 novel_circ_010972 novel_circ_010973 novel_circ_010974 novel_circ_010975 novel_circ_010976 novel_circ_010977 novel_circ_010978 novel_circ_010979 novel_circ_010980 novel_circ_010981 novel_circ_010982 novel_circ_010983 novel_circ_010984 novel_circ_010985 novel_circ_010986 novel_circ_010987 novel_circ_010988 novel_circ_010989 novel_circ_010990 novel_circ_010991 novel_circ_010992 novel_circ_010993 novel_circ_010994 novel_circ_010995 novel_circ_010996 novel_circ_010997 novel_circ_010998 novel_circ_010999 novel_circ_011001 novel_circ_011003 novel_circ_011004 novel_circ_011006 novel_circ_011007 novel_circ_011008 novel_circ_011009 novel_circ_011010

| hsa_circ_0076395 | 91 | 1 | 1006 | 371 | 1379 | 0 | 0 |
| --- | --- | --- | --- | --- | --- | --- | --- |
| hsa_circ_0076395 | 91 | 1 | 1376 | 1 | 1379 | 0 | 0 |
| hsa_circ_0076399 | 90 | 1 | 644 | 1 | 647 | 0 | 0 |
| hsa_circ_0076395 | 91 | 1 | 1211 | 1 | 1214 | 0 | 0 |
| hsa_circ_0076389 | 91 | 1 | 1469 | 50 | 1521 | 0 | 0 |
| hsa_circ_0076397 | 92 | 1 | 450 | 1 | 450 | 0 | 0 |
| hsa_circ_0076395 | 91 | 1 | 820 | 1 | 820 | 0 | 0 |
| hsa_circ_0076389 | 91 | 1 | 1078 | 50 | 1127 | 0 | 0 |
| hsa_circ_0076389 | 90 | 1 | 929 | 50 | 978 | 0 | 0 |
| hsa_circ_0076389 | 90 | 1 | 922 | 50 | 971 | 0 | 0 |
| hsa_circ_0076389 | 90 | 1 | 619 | 50 | 668 | 0 | 0 |
| hsa_circ_0076384 | 92 | 1 | 824 | 313 | 1136 | 0 | 0 |
| hsa_circ_0076384 | 92 | 1 | 1132 | 5 | 1136 | 0 | 0 |
| hsa_circ_0076384 | 92 | 1 | 1136 | 1 | 1136 | 0 | 0 |
| hsa_circ_0076383 | 92 | 1 | 1313 | 1 | 1313 | 0 | 0 |
| hsa_circ_0076379 | 93 | 1 | 1559 | 1 | 1559 | 0 | 0 |
| hsa_circ_0076371 | 93 | 1 | 1952 | 100 | 2051 | 0 | 0 |
| hsa_circ_0076370 | 92 | 1 | 2248 | 1 | 2248 | 0 | 0 |
| hsa_circ_0076390 | 91 | 1 | 258 | 1 | 258 | 0 | 1.00E-93 |
| hsa_circ_0076371 | 94 | 1 | 1591 | 100 | 1690 | 0 | 0 |
| hsa_circ_0076371 | 94 | 1 | 1488 | 100 | 1587 | 0 | 0 |
| hsa_circ_0076384 | 94 | 1 | 464 | 5 | 468 | 0 | 0 |
| hsa_circ_0076384 | 94 | 1 | 468 | 1 | 468 | 0 | 0 |
| hsa_circ_0076383 | 94 | 1 | 645 | 1 | 645 | 0 | 0 |
| hsa_circ_0076376 | 95 | 1 | 1020 | 1 | 1020 | 0 | 0 |
| hsa_circ_0076371 | 95 | 1 | 1284 | 100 | 1383 | 0 | 0 |
| hsa_circ_0076370 | 93 | 1 | 1580 | 1 | 1580 | 0 | 0 |
| hsa_circ_0076371 | 95 | 1 | 1181 | 100 | 1280 | 0 | 0 |
| hsa_circ_0076384 | 94 | 1 | 308 | 5 | 312 | 0 | 1.00E-129 |
| hsa_circ_0076384 | 94 | 1 | 312 | 1 | 312 | 0 | 8.00E-132 |
| hsa_circ_0076383 | 94 | 1 | 489 | 1 | 489 | 0 | 0 |
| hsa_circ_0076381 | 94 | 1 | 551 | 1 | 551 | 0 | 0 |
| hsa_circ_0076379 | 94 | 1 | 735 | 1 | 735 | 0 | 0 |
| hsa_circ_0076376 | 95 | 1 | 864 | 1 | 864 | 0 | 0 |
| hsa_circ_0076371 | 95 | 1 | 1128 | 100 | 1227 | 0 | 0 |
| hsa_circ_0076370 | 93 | 1 | 1424 | 1 | 1424 | 0 | 0 |
| hsa_circ_0076371 | 95 | 1 | 1003 | 100 | 1102 | 0 | 0 |
| hsa_circ_0076371 | 95 | 1 | 816 | 100 | 915 | 0 | 0 |
| hsa_circ_0076371 | 95 | 1 | 750 | 100 | 849 | 0 | 0 |
| hsa_circ_0076371 | 95 | 1 | 639 | 100 | 738 | 0 | 0 |
| hsa_circ_0076373 | 95 | 1 | 577 | 1 | 577 | 0 | 0 |
| hsa_circ_0076373 | 95 | 1 | 393 | 1 | 393 | 0 | 2.00E-178 |
| hsa_circ_0076373 | 94 | 1 | 264 | 1 | 264 | 0 | 3.00E-110 |
| hsa_circ_0076340 | 95 | 1 | 203 | 203 | 1 | 0 | 2.00E-86 |
| hsa_circ_0076328 | 93 | 1 | 289 | 1 | 289 | 0 | 2.00E-117 |
| hsa_circ_0076318 | 94 | 1 | 264 | 274 | 537 | 0 | 1.00E-108 |
| hsa_circ_0076286 | 94 | 46 | 381 | 34 | 369 | 0 | 9.00E-142 |
| hsa_circ_0076279 | 93 | 341 | 481 | 141 | 1 | 0 | 2.00E-51 |
| hsa_circ_0076279 | 93 | 341 | 481 | 141 | 1 | 0 | 2.00E-51 |
| hsa_circ_0076255 | 96 | 6 | 576 | 836 | 266 | 0 | 0 |
| hsa_circ_0076255 | 95 | 6 | 841 | 836 | 1 | 0 | 0 |
| hsa_circ_0076254 | 94 | 6 | 1181 | 1176 | 1 | 0 | 0 |
| hsa_circ_0076254 | 94 | 1 | 1117 | 1117 | 1 | 0 | 0 |
| hsa_circ_0076254 | 92 | 1 | 340 | 340 | 1 | 0 | 2.00E-132 |

novel_circ_011011 novel_circ_011012 novel_circ_011013 novel_circ_011014 novel_circ_011015 novel_circ_011016 novel_circ_011017 novel_circ_011018 novel_circ_011019 novel_circ_011020 novel_circ_011021 novel_circ_011022 novel_circ_011023 novel_circ_011024 novel_circ_011025 novel_circ_011028 novel_circ_011029 novel_circ_011031 novel_circ_011032 novel_circ_011033 novel_circ_011034 novel_circ_011035 novel_circ_011036 novel_circ_011037 novel_circ_011038 novel_circ_011039 novel_circ_011040 novel_circ_011041 novel_circ_011042 novel_circ_011043 novel_circ_011044 novel_circ_011045 novel_circ_011046 novel_circ_011047 novel_circ_011048 novel_circ_011049 novel_circ_011050 novel_circ_011051 novel_circ_011052 novel_circ_011053 novel_circ_011054 novel_circ_011055 novel_circ_011056 novel_circ_011057 novel_circ_011058 novel_circ_011059 novel_circ_011060 novel_circ_011061 novel_circ_011062 novel_circ_011063 novel_circ_011064 novel_circ_011065 novel_circ_011067 novel_circ_011069

| hsa_circ_0076253 | 93 | 111 | 408 | 298 | 1 | 0 | 3.00E-122 |
| --- | --- | --- | --- | --- | --- | --- | --- |
| hsa_circ_0076253 | 93 | 1 | 190 | 298 | 109 | 0 | 1.00E-72 |
| hsa_circ_0076253 | 93 | 1 | 298 | 298 | 1 | 0 | 2.00E-122 |
| hsa_circ_0076249 | 95 | 1 | 417 | 42 | 458 | 0 | 0 |
| hsa_circ_0076233 | 94 | 1 | 670 | 59 | 728 | 0 | 0 |
| hsa_circ_0076233 | 94 | 1 | 728 | 1 | 728 | 0 | 0 |
| hsa_circ_0076222 | 96 | 107 | 331 | 183 | 407 | 0 | 2.00E-98 |
| hsa_circ_0076218 | 96 | 560 | 754 | 1 | 195 | 0 | 2.00E-86 |
| hsa_circ_0076218 | 96 | 560 | 754 | 1 | 195 | 0 | 2.00E-86 |
| hsa_circ_0076218 | 95 | 560 | 688 | 1 | 129 | 0 | 2.00E-51 |
| hsa_circ_0076201 | 88 | 1 | 544 | 215 | 755 | 0.01 | 0 |
| hsa_circ_0076201 | 89 | 306 | 1052 | 9 | 755 | 0.01 | 0 |
| hsa_circ_0076201 | 88 | 1 | 425 | 215 | 636 | 0.01 | 1.00E-136 |
| hsa_circ_0076201 | 89 | 1 | 292 | 215 | 503 | 0.01 | 3.00E-96 |
| hsa_circ_0076201 | 90 | 306 | 800 | 9 | 503 | 0.01 | 0 |
| hsa_circ_0076190 | 97 | 85 | 466 | 444 | 63 | 0 | 0 |
| hsa_circ_0009139 | 92 | 1 | 601 | 1 | 601 | 0 | 0 |
| hsa_circ_0076180 | 95 | 1 | 494 | 1 | 494 | 0 | 0 |
| hsa_circ_0076180 | 92 | 1 | 186 | 1 | 186 | 0 | 2.00E-70 |
| hsa_circ_0011669 | 91 | 1 | 690 | 1 | 693 | 0 | 0 |
| hsa_circ_0011656 | 92 | 1 | 437 | 1 | 437 | 0 | 4.00E-176 |
| hsa_circ_0011656 | 92 | 1 | 237 | 1 | 237 | 0 | 3.00E-90 |
| hsa_circ_0011649 | 94 | 394 | 1116 | 146 | 868 | 0 | 0 |
| hsa_circ_0011632 | 95 | 1 | 1095 | 1 | 1095 | 0 | 0 |
| hsa_circ_0011634 | 95 | 2 | 886 | 2 | 886 | 0 | 0 |
| hsa_circ_0011632 | 95 | 1 | 1095 | 1 | 1095 | 0 | 0 |
| hsa_circ_0011650 | 95 | 1 | 446 | 1 | 446 | 0 | 0 |
| hsa_circ_0011649 | 94 | 394 | 879 | 146 | 631 | 0 | 0 |
| hsa_circ_0011646 | 95 | 1 | 378 | 1 | 378 | 0.01 | 4.00E-168 |
| hsa_circ_0011646 | 95 | 1 | 378 | 1 | 378 | 0.01 | 3.00E-168 |
| hsa_circ_0011640 | 96 | 1 | 749 | 1 | 749 | 0 | 0 |
| hsa_circ_0011634 | 95 | 2 | 886 | 2 | 886 | 0 | 0 |
| hsa_circ_0011632 | 95 | 1 | 1095 | 1 | 1095 | 0 | 0 |
| hsa_circ_0011646 | 95 | 1 | 378 | 1 | 378 | 0.01 | 2.00E-168 |
| hsa_circ_0011646 | 95 | 1 | 377 | 1 | 377 | 0.01 | 7.00E-168 |
| hsa_circ_0011640 | 96 | 1 | 491 | 1 | 491 | 0 | 0 |
| hsa_circ_0011634 | 95 | 2 | 508 | 2 | 508 | 0 | 0 |
| hsa_circ_0011632 | 95 | 1 | 717 | 1 | 717 | 0 | 0 |
| hsa_circ_0011634 | 93 | 2 | 137 | 2 | 137 | 0 | 5.00E-49 |
| hsa_circ_0011632 | 93 | 1 | 346 | 1 | 346 | 0 | 2.00E-142 |
| hsa_circ_0002828 | 93 | 1 | 467 | 1 | 467 | 0 | 0 |
| hsa_circ_0003435 | 93 | 14015 | 14483 | 171 | 639 | 0 | 0 |
| hsa_circ_0003435 | 95 | 1 | 639 | 1 | 639 | 0 | 0 |
| hsa_circ_0011623 | 95 | 1 | 437 | 1245 | 1681 | 0 | 0 |
| hsa_circ_0011617 | 94 | 1 | 811 | 185 | 995 | 0 | 0 |
| hsa_circ_0011617 | 95 | 1 | 995 | 1 | 995 | 0 | 0 |
| hsa_circ_0011599 | 91 | 1 | 2180 | 1 | 2156 | 0.01 | 0 |
| hsa_circ_0011604 | 94 | 1 | 371 | 1 | 371 | 0 | 1.00E-159 |
| hsa_circ_0011599 | 94 | 1 | 360 | 470 | 829 | 0 | 9.00E-152 |
| hsa_circ_0011599 | 92 | 1 | 663 | 167 | 829 | 0 | 0 |
| hsa_circ_0011599 | 92 | 1 | 829 | 1 | 829 | 0 | 0 |
| hsa_circ_0011599 | 93 | 1 | 272 | 470 | 741 | 0 | 5.00E-108 |
| hsa_circ_0011581 | 94 | 1 | 194 | 194 | 1 | 0 | 8.00E-80 |
| hsa_circ_0011573 | 85 | 14 | 332 | 336 | 5 | 0.04 | 1.00E-85 |

novel_circ_011070 novel_circ_011071 novel_circ_011072 novel_circ_011073 novel_circ_011074 novel_circ_011075 novel_circ_011076 novel_circ_011077 novel_circ_011078 novel_circ_011079 novel_circ_011080 novel_circ_011081 novel_circ_011082 novel_circ_011083 novel_circ_011084 novel_circ_011085 novel_circ_011086 novel_circ_011087 novel_circ_011088 novel_circ_011089 novel_circ_011090 novel_circ_011091 novel_circ_011092 novel_circ_011093 novel_circ_011094 novel_circ_011095 novel_circ_011096 novel_circ_011097 novel_circ_011098 novel_circ_011099 novel_circ_011100 novel_circ_011101 novel_circ_011102 novel_circ_011103 novel_circ_011104 novel_circ_011105 novel_circ_011106 novel_circ_011107 novel_circ_011108 novel_circ_011109 novel_circ_011110 novel_circ_011111 novel_circ_011112 novel_circ_011113 novel_circ_011114 novel_circ_011115 novel_circ_011116 novel_circ_011117 novel_circ_011118 novel_circ_011119 novel_circ_011120 novel_circ_011121 novel_circ_011122 novel_circ_011123

| hsa_circ_0011566 | 91 | 1 | 619 | 619 | 1 | 0 | 0 |
| --- | --- | --- | --- | --- | --- | --- | --- |
| hsa_circ_0011566 | 90 | 5231 | 5718 | 488 | 1 | 0 | 5.00E-180 |
| hsa_circ_0011566 | 90 | 3 | 486 | 484 | 1 | 0 | 1.00E-180 |
| hsa_circ_0011556 | 96 | 1 | 513 | 1534 | 2046 | 0 | 0 |
| hsa_circ_0011556 | 95 | 1 | 781 | 1266 | 2046 | 0 | 0 |
| hsa_circ_0011556 | 94 | 1 | 1282 | 768 | 2046 | 0 | 0 |
| hsa_circ_0011556 | 96 | 1 | 513 | 1534 | 2046 | 0 | 0 |
| hsa_circ_0011556 | 95 | 1 | 781 | 1266 | 2046 | 0 | 0 |
| hsa_circ_0011556 | 94 | 1 | 1282 | 768 | 2046 | 0 | 0 |
| hsa_circ_0011556 | 95 | 1 | 532 | 1266 | 1797 | 0 | 0 |
| hsa_circ_0011556 | 94 | 1 | 420 | 1266 | 1685 | 0 | 4.00E-180 |
| hsa_circ_0011548 | 94 | 2 | 2770 | 2 | 2767 | 0 | 0 |
| hsa_circ_0011556 | 92 | 1 | 268 | 1266 | 1533 | 0 | 4.00E-104 |
| hsa_circ_0011556 | 94 | 1 | 1269 | 1 | 1266 | 0 | 0 |
| hsa_circ_0011556 | 94 | 1 | 1268 | 1 | 1265 | 0 | 0 |
| hsa_circ_0011553 | 94 | 1 | 1419 | 1 | 1416 | 0 | 0 |
| hsa_circ_0011548 | 94 | 2 | 2350 | 2 | 2347 | 0 | 0 |
| hsa_circ_0011540 | 94 | 1 | 2691 | 756 | 3443 | 0 | 0 |
| hsa_circ_0011556 | 93 | 1 | 406 | 768 | 1170 | 0.01 | 2.00E-169 |
| hsa_circ_0011556 | 93 | 1 | 436 | 504 | 936 | 0.01 | 2.00E-179 |
| hsa_circ_0011556 | 93 | 1 | 939 | 1 | 936 | 0.01 | 0 |
| hsa_circ_0011553 | 93 | 8314 | 9254 | 150 | 1087 | 0.01 | 0 |
| hsa_circ_0011553 | 94 | 1 | 1090 | 1 | 1087 | 0 | 0 |
| hsa_circ_0011548 | 94 | 1 | 1633 | 389 | 2018 | 0 | 0 |
| hsa_circ_0011548 | 94 | 2 | 2021 | 2 | 2018 | 0 | 0 |
| hsa_circ_0011556 | 93 | 1 | 767 | 1 | 767 | 0 | 0 |
| hsa_circ_0011553 | 94 | 1 | 918 | 1 | 918 | 0 | 0 |
| hsa_circ_0011548 | 94 | 2 | 1849 | 2 | 1849 | 0 | 0 |
| hsa_circ_0011553 | 95 | 1 | 799 | 1 | 799 | 0 | 0 |
| hsa_circ_0011548 | 94 | 2 | 1730 | 2 | 1730 | 0 | 0 |
| hsa_circ_0011556 | 94 | 1 | 503 | 1 | 503 | 0 | 0 |
| hsa_circ_0011553 | 95 | 1 | 654 | 1 | 654 | 0 | 0 |
| hsa_circ_0011548 | 94 | 2 | 1585 | 2 | 1585 | 0 | 0 |
| hsa_circ_0011556 | 95 | 1 | 324 | 1 | 324 | 0.01 | 8.00E-142 |
| hsa_circ_0011553 | 95 | 8314 | 8639 | 150 | 475 | 0.01 | 2.00E-141 |
| hsa_circ_0011553 | 95 | 1 | 475 | 1 | 475 | 0 | 0 |
| hsa_circ_0011549 | 95 | 1 | 1018 | 1 | 1018 | 0 | 0 |
| hsa_circ_0011548 | 94 | 2 | 1406 | 2 | 1406 | 0 | 0 |
| hsa_circ_0011540 | 95 | 1 | 1747 | 756 | 2502 | 0 | 0 |
| hsa_circ_0011540 | 95 | 1 | 2505 | 1 | 2502 | 0 | 0 |
| hsa_circ_0011553 | 96 | 1 | 324 | 1 | 324 | 0.01 | 4.00E-150 |
| hsa_circ_0011548 | 95 | 2 | 1255 | 2 | 1255 | 0 | 0 |
| hsa_circ_0011550 | 94 | 1 | 427 | 117 | 543 | 0 | 0 |
| hsa_circ_0011549 | 94 | 1 | 694 | 1 | 694 | 0 | 0 |
| hsa_circ_0011540 | 95 | 1 | 2030 | 1 | 2027 | 0 | 0 |
| hsa_circ_0011548 | 94 | 2 | 388 | 2 | 388 | 0 | 2.00E-168 |
| hsa_circ_0011540 | 95 | 1 | 729 | 756 | 1484 | 0 | 0 |
| hsa_circ_0011540 | 95 | 1 | 1487 | 1 | 1484 | 0 | 0 |
| hsa_circ_0011540 | 95 | 190 | 1676 | 1 | 1484 | 0 | 0 |
| hsa_circ_0011540 | 96 | 3 | 577 | 522 | 1096 | 0 | 0 |
| hsa_circ_0011540 | 96 | 1 | 1099 | 1 | 1096 | 0 | 0 |
| hsa_circ_0011540 | 95 | 1 | 757 | 1 | 754 | 0 | 0 |
| hsa_circ_0011540 | 95 | 190 | 946 | 1 | 754 | 0 | 0 |
| hsa_circ_0011540 | 96 | 1 | 522 | 1 | 522 | 0 | 0 |

novel_circ_011124 novel_circ_011126 novel_circ_011127 novel_circ_011128 novel_circ_011129 novel_circ_011130 novel_circ_011131 novel_circ_011132 novel_circ_011133 novel_circ_011134 novel_circ_011135 novel_circ_011137 novel_circ_011138 novel_circ_011139 novel_circ_011140 novel_circ_011141 novel_circ_011142 novel_circ_011143 novel_circ_011144 novel_circ_011145 novel_circ_011146 novel_circ_011147 novel_circ_011148 novel_circ_011150 novel_circ_011151 novel_circ_011152 novel_circ_011153 novel_circ_011154 novel_circ_011155 novel_circ_011156 novel_circ_011157 novel_circ_011158 novel_circ_011159 novel_circ_011160 novel_circ_011161 novel_circ_011162 novel_circ_011163 novel_circ_011164 novel_circ_011165 novel_circ_011166 novel_circ_011167 novel_circ_011168 novel_circ_011169 novel_circ_011170 novel_circ_011171 novel_circ_011172 novel_circ_011173 novel_circ_011174 novel_circ_011175 novel_circ_011176 novel_circ_011177 novel_circ_011178 novel_circ_011179 novel_circ_011180

| hsa_circ_0011540 | 96 | 190 | 711 | 1 | 522 | 0 | 0 |
| --- | --- | --- | --- | --- | --- | --- | --- |
| hsa_circ_0011531 | 95 | 4 | 680 | 1960 | 1284 | 0 | 0 |
| hsa_circ_0011524 | 79 | 1 | 677 | 1 | 683 | 0.01 | 1.00E-122 |
| hsa_circ_0011524 | 79 | 1 | 677 | 1 | 683 | 0.01 | 1.00E-122 |
| hsa_circ_0011524 | 80 | 1 | 515 | 1 | 515 | 0.01 | 5.00E-106 |
| hsa_circ_0011524 | 85 | 1 | 309 | 1 | 309 | 0 | 2.00E-83 |
| hsa_circ_0011517 | 86 | 1 | 367 | 1064 | 698 | 0 | 3.00E-107 |
| hsa_circ_0011516 | 88 | 1 | 1382 | 1564 | 180 | 0 | 0 |
| hsa_circ_0011516 | 87 | 1 | 1558 | 1564 | 1 | 0.01 | 0 |
| hsa_circ_0011516 | 89 | 7 | 1207 | 1383 | 180 | 0 | 0 |
| hsa_circ_0011516 | 89 | 1 | 497 | 500 | 1 | 0.01 | 3.00E-178 |
| hsa_circ_0018987 | 93 | 1 | 209 | 169 | 377 | 0 | 4.00E-81 |
| hsa_circ_0018993 | 90 | 1 | 1658 | 1 | 1652 | 0 | 0 |
| hsa_circ_0018993 | 90 | 1 | 1658 | 1 | 1652 | 0 | 0 |
| hsa_circ_0018993 | 90 | 1 | 1658 | 1 | 1652 | 0 | 0 |
| hsa_circ_0018996 | 91 | 1 | 254 | 1 | 254 | 0 | 2.00E-91 |
| hsa_circ_0018996 | 92 | 1 | 450 | 1 | 450 | 0 | 1.00E-176 |
| hsa_circ_0018996 | 92 | 1 | 515 | 1 | 515 | 0 | 0 |
| hsa_circ_0018996 | 91 | 1 | 617 | 1 | 617 | 0 | 0 |
| hsa_circ_0018996 | 90 | 1 | 704 | 1 | 704 | 0 | 0 |
| hsa_circ_0018998 | 91 | 1 | 363 | 1 | 363 | 0 | 4.00E-140 |
| hsa_circ_0018998 | 90 | 1 | 450 | 1 | 450 | 0 | 2.00E-168 |
| hsa_circ_0051260 | 95 | 522 | 561 | 368 | 329 | 0 | 1.00E-08 |
| hsa_circ_0018999 | 93 | 1 | 171 | 171 | 1 | 0 | 6.00E-65 |
| hsa_circ_0091090 | 95 | 1 | 373 | 373 | 1 | 0 | 5.00E-169 |
| hsa_circ_0091090 | 95 | 19863 | 20237 | 375 | 1 | 0 | 2.00E-168 |
| hsa_circ_0091090 | 95 | 1 | 482 | 482 | 1 | 0 | 0 |
| hsa_circ_0091090 | 94 | 1 | 589 | 589 | 1 | 0 | 0 |
| hsa_circ_0091090 | 95 | 1 | 743 | 743 | 1 | 0 | 0 |
| hsa_circ_0091090 | 95 | 263 | 1005 | 743 | 1 | 0 | 0 |
| hsa_circ_0091090 | 95 | 394 | 1136 | 743 | 1 | 0 | 0 |
| hsa_circ_0091091 | 95 | 1 | 1195 | 1192 | 1 | 0 | 0 |
| hsa_circ_0091093 | 95 | 1 | 434 | 434 | 1 | 0 | 0 |
| hsa_circ_0091093 | 95 | 1 | 523 | 521 | 1 | 0.01 | 0 |
| hsa_circ_0091093 | 94 | 1 | 654 | 651 | 1 | 0 | 0 |
| hsa_circ_0091093 | 95 | 1 | 1086 | 1083 | 1 | 0 | 0 |
| hsa_circ_0091093 | 96 | 1 | 979 | 1083 | 108 | 0 | 0 |
| hsa_circ_0001931 | 94 | 1 | 393 | 390 | 1 | 0.01 | 1.00E-165 |
| hsa_circ_0091093 | 95 | 1 | 825 | 1083 | 262 | 0 | 0 |
| hsa_circ_0091096 | 97 | 1 | 432 | 432 | 1 | 0 | 0 |
| hsa_circ_0091096 | 97 | 4 | 610 | 607 | 1 | 0 | 0 |
| hsa_circ_0091096 | 97 | 4 | 1009 | 1006 | 1 | 0 | 0 |
| hsa_circ_0091096 | 95 | 1 | 1337 | 1352 | 1 | 0.01 | 0 |
| hsa_circ_0091096 | 95 | 1 | 1431 | 1446 | 1 | 0.01 | 0 |
| hsa_circ_0091093 | 93 | 1 | 1824 | 2481 | 652 | 0.01 | 0 |
| hsa_circ_0091100 | 96 | 4 | 577 | 574 | 1 | 0 | 0 |
| hsa_circ_0091100 | 92 | 1 | 1392 | 1398 | 1 | 0.02 | 0 |
| hsa_circ_0091101 | 90 | 1 | 487 | 1073 | 596 | 0.02 | 9.00E-173 |
| hsa_circ_0091101 | 88 | 1 | 393 | 1073 | 690 | 0.03 | 3.00E-127 |
| hsa_circ_0091126 | 89 | 1 | 1216 | 1 | 1216 | 0 | 0 |
| hsa_circ_0091128 | 93 | 1 | 3395 | 1 | 3395 | 0 | 0 |
| hsa_circ_0091128 | 93 | 5 | 1580 | 731 | 2306 | 0 | 0 |
| hsa_circ_0091128 | 93 | 5 | 2175 | 731 | 2901 | 0 | 0 |
| hsa_circ_0091128 | 94 | 5 | 2669 | 731 | 3395 | 0 | 0 |

novel_circ_011181 novel_circ_011182 novel_circ_011183 novel_circ_011184 novel_circ_011185 novel_circ_011186 novel_circ_011187 novel_circ_011188 novel_circ_011189 novel_circ_011190 novel_circ_011191 novel_circ_011192 novel_circ_011193 novel_circ_011194 novel_circ_011195 novel_circ_011196 novel_circ_011197 novel_circ_011198 novel_circ_011199 novel_circ_011200 novel_circ_011201 novel_circ_011202 novel_circ_011203 novel_circ_011204 novel_circ_011205 novel_circ_011206 novel_circ_011207 novel_circ_011208 novel_circ_011209 novel_circ_011210 novel_circ_011211 novel_circ_011212 novel_circ_011213 novel_circ_011214 novel_circ_011215 novel_circ_011216 novel_circ_011217 novel_circ_011218 novel_circ_011219 novel_circ_011220 novel_circ_011221 novel_circ_011222 novel_circ_011225 novel_circ_011226 novel_circ_011227 novel_circ_011228 novel_circ_011229 novel_circ_011230 novel_circ_011231 novel_circ_011232 novel_circ_011233 novel_circ_011234 novel_circ_011235 novel_circ_011237

| hsa_circ_0091138 | 94 | 1 | 744 | 1 | 744 | 0 | 0 |
| --- | --- | --- | --- | --- | --- | --- | --- |
| hsa_circ_0091138 | 94 | 1 | 1486 | 1 | 1486 | 0 | 0 |
| hsa_circ_0091138 | 94 | 1 | 1629 | 1 | 1629 | 0 | 0 |
| hsa_circ_0091139 | 94 | 1 | 375 | 1 | 375 | 0 | 2.00E-158 |
| hsa_circ_0091140 | 94 | 1 | 283 | 1 | 283 | 0 | 2.00E-117 |
| hsa_circ_0091139 | 93 | 1 | 796 | 93 | 888 | 0 | 0 |
| hsa_circ_0091139 | 94 | 1 | 1303 | 93 | 1395 | 0 | 0 |
| hsa_circ_0091143 | 94 | 1 | 400 | 196 | 595 | 0 | 6.00E-169 |
| hsa_circ_0091143 | 93 | 1 | 547 | 196 | 742 | 0 | 0 |
| hsa_circ_0091143 | 94 | 1 | 894 | 196 | 1089 | 0 | 0 |
| hsa_circ_0091143 | 95 | 1 | 1115 | 196 | 1310 | 0 | 0 |
| hsa_circ_0091153 | 95 | 1 | 224 | 1 | 224 | 0 | 9.00E-95 |
| hsa_circ_0072783 | 84 | 1 | 348 | 862 | 509 | 0.02 | 9.00E-92 |
| hsa_circ_0077477 | 91 | 1 | 1418 | 4163 | 2749 | 0.01 | 0 |
| hsa_circ_0077497 | 91 | 1 | 560 | 560 | 1 | 0 | 0 |
| hsa_circ_0077496 | 91 | 1 | 1136 | 1154 | 22 | 0.01 | 0 |
| hsa_circ_0077496 | 93 | 4 | 468 | 591 | 127 | 0.01 | 0 |
| hsa_circ_0077496 | 92 | 4 | 576 | 591 | 22 | 0.02 | 0 |
| hsa_circ_0077489 | 91 | 7 | 768 | 750 | 1 | 0.02 | 0 |
| hsa_circ_0077490 | 93 | 55 | 177 | 123 | 1 | 0 | 1.00E-43 |
| hsa_circ_0077480 | 94 | 4 | 1101 | 1479 | 382 | 0 | 0 |
| hsa_circ_0077460 | 93 | 4 | 2634 | 2631 | 1 | 0 | 0 |
| hsa_circ_0077480 | 93 | 1 | 645 | 645 | 1 | 0 | 0 |
| hsa_circ_0077465 | 93 | 4 | 518 | 517 | 3 | 0 | 0 |
| hsa_circ_0077460 | 93 | 4 | 663 | 660 | 1 | 0 | 0 |
| hsa_circ_0077454 | 93 | 4 | 1428 | 1425 | 1 | 0 | 0 |
| hsa_circ_0077455 | 93 | 1 | 1177 | 1289 | 113 | 0 | 0 |
| hsa_circ_0077465 | 94 | 1 | 265 | 267 | 3 | 0 | 4.00E-109 |
| hsa_circ_0077455 | 95 | 1 | 259 | 630 | 372 | 0 | 8.00E-111 |
| hsa_circ_0077455 | 94 | 1 | 392 | 630 | 239 | 0 | 3.00E-166 |
| hsa_circ_0077454 | 93 | 1 | 765 | 765 | 1 | 0 | 0 |
| hsa_circ_0077453 | 93 | 1 | 990 | 990 | 1 | 0 | 0 |
| hsa_circ_0070447 | 94 | 1 | 491 | 1 | 491 | 0 | 0 |
| hsa_circ_0070449 | 95 | 1 | 510 | 1 | 510 | 0 | 0 |
| hsa_circ_0024618 | 92 | 1 | 718 | 1 | 718 | 0 | 0 |
| hsa_circ_0024620 | 90 | 1 | 511 | 1 | 511 | 0 | 0 |
| hsa_circ_0024620 | 90 | 1 | 332 | 180 | 511 | 0.01 | 1.00E-119 |
| hsa_circ_0024635 | 88 | 205 | 639 | 1 | 435 | 0 | 3.00E-143 |
| hsa_circ_0024641 | 87 | 3 | 324 | 3 | 324 | 0 | 1.00E-100 |
| hsa_circ_0024643 | 89 | 1 | 302 | 37 | 338 | 0 | 3.00E-101 |
| hsa_circ_0024652 | 82 | 8081 | 8718 | 3234 | 3871 | 0.02 | 1.00E-149 |
| hsa_circ_0024652 | 82 | 735 | 1372 | 3234 | 3871 | 0.02 | 3.00E-150 |
| hsa_circ_0051259 | 78 | 669 | 874 | 1602 | 1397 | 0 | 5.00E-29 |
| hsa_circ_0003302 | 93 | 11145 | 12116 | 2 | 973 | 0 | 0 |
| hsa_circ_0003302 | 93 | 11124 | 12095 | 2 | 973 | 0 | 0 |
| hsa_circ_0003302 | 93 | 11077 | 12048 | 2 | 973 | 0 | 0 |
| hsa_circ_0003302 | 88 | 2 | 285 | 2 | 285 | 0 | 6.00E-93 |
| hsa_circ_0003302 | 93 | 2 | 973 | 2 | 973 | 0 | 0 |
| hsa_circ_0024658 | 95 | 1 | 683 | 141 | 823 | 0 | 0 |
| hsa_circ_0024658 | 95 | 1 | 604 | 220 | 823 | 0 | 0 |
| hsa_circ_0024658 | 95 | 1 | 501 | 323 | 823 | 0 | 0 |
| hsa_circ_0024661 | 88 | 1 | 470 | 1 | 470 | 0 | 9.00E-158 |
| hsa_circ_0024661 | 89 | 1 | 654 | 1 | 654 | 0 | 0 |
| hsa_circ_0024661 | 88 | 1 | 537 | 118 | 654 | 0 | 0 |

novel_circ_011238 novel_circ_011240 novel_circ_011241 novel_circ_011242 novel_circ_011243 novel_circ_011244 novel_circ_011245 novel_circ_011246 novel_circ_011247 novel_circ_011248 novel_circ_011249 novel_circ_011250 novel_circ_011251 novel_circ_011252 novel_circ_011253 novel_circ_011254 novel_circ_011255 novel_circ_011256 novel_circ_011257 novel_circ_011259 novel_circ_011266 novel_circ_011267 novel_circ_011268 novel_circ_011270 novel_circ_011271 novel_circ_011272 novel_circ_011273 novel_circ_011274 novel_circ_011275 novel_circ_011277 novel_circ_011278 novel_circ_011280 novel_circ_011282 novel_circ_011283 novel_circ_011289 novel_circ_011290 novel_circ_011291 novel_circ_011292 novel_circ_011293 novel_circ_011294 novel_circ_011295 novel_circ_011296 novel_circ_011297 novel_circ_011298 novel_circ_011299 novel_circ_011300 novel_circ_011301 novel_circ_011302 novel_circ_011303 novel_circ_011304 novel_circ_011305 novel_circ_011306 novel_circ_011307 novel_circ_011308

| hsa_circ_0024661 | 90 | 1057 | 1471 | 240 | 654 | 0 | 5.00E-150 |
| --- | --- | --- | --- | --- | --- | --- | --- |
| hsa_circ_0024666 | 90 | 1 | 411 | 1 | 411 | 0 | 5.00E-150 |
| hsa_circ_0024666 | 90 | 1 | 683 | 1 | 683 | 0 | 0 |
| hsa_circ_0024666 | 91 | 1 | 1154 | 1 | 1154 | 0 | 0 |
| hsa_circ_0024666 | 91 | 1 | 1166 | 1 | 1167 | 0 | 0 |
| hsa_circ_0024668 | 92 | 1 | 249 | 1 | 249 | 0 | 1.00E-93 |
| hsa_circ_0024668 | 90 | 1 | 521 | 1 | 521 | 0 | 0 |
| hsa_circ_0024668 | 91 | 1 | 714 | 1 | 714 | 0 | 0 |
| hsa_circ_0024668 | 92 | 2063 | 2255 | 60 | 249 | 0.02 | 1.00E-68 |
| hsa_circ_0024669 | 91 | 1 | 453 | 1 | 453 | 0 | 5.00E-170 |
| hsa_circ_0024669 | 91 | 1 | 924 | 1 | 924 | 0 | 0 |
| hsa_circ_0024668 | 91 | 1 | 936 | 68 | 1005 | 0 | 0 |
| hsa_circ_0024669 | 89 | 1 | 272 | 182 | 453 | 0 | 2.00E-91 |
| hsa_circ_0024669 | 91 | 1 | 465 | 182 | 646 | 0 | 2.00E-178 |
| hsa_circ_0024669 | 91 | 1 | 657 | 182 | 838 | 0 | 0 |
| hsa_circ_0024669 | 91 | 1 | 1106 | 182 | 1287 | 0 | 0 |
| hsa_circ_0024673 | 93 | 1 | 385 | 1 | 385 | 0 | 5.00E-159 |
| hsa_circ_0024673 | 92 | 1 | 471 | 1 | 471 | 0 | 0 |
| hsa_circ_0024673 | 92 | 1 | 483 | 1 | 484 | 0 | 0 |
| hsa_circ_0024674 | 91 | 1 | 641 | 1 | 641 | 0 | 0 |
| hsa_circ_0024675 | 91 | 2 | 360 | 165 | 523 | 0 | 7.00E-138 |
| hsa_circ_0024675 | 91 | 2 | 581 | 165 | 744 | 0 | 0 |
| hsa_circ_0024675 | 89 | 2 | 1364 | 165 | 1527 | 0 | 0 |
| hsa_circ_0024678 | 89 | 1 | 1021 | 1 | 1021 | 0 | 0 |
| hsa_circ_0024681 | 90 | 1 | 386 | 93 | 478 | 0 | 3.00E-141 |
| hsa_circ_0024687 | 88 | 1 | 939 | 1 | 939 | 0 | 0 |
| hsa_circ_0024685 | 88 | 1 | 1839 | 121 | 1959 | 0 | 0 |
| hsa_circ_0024687 | 87 | 1 | 813 | 127 | 939 | 0 | 0 |
| hsa_circ_0024691 | 91 | 1 | 289 | 265 | 553 | 0 | 9.00E-106 |
| hsa_circ_0024692 | 89 | 1 | 900 | 307 | 1206 | 0 | 0 |
| hsa_circ_0024685 | 86 | 1 | 475 | 2430 | 2904 | 0 | 7.00E-144 |
| hsa_circ_0001676 | 85 | 27096 | 27200 | 5924 | 6029 | 0.03 | 2.00E-19 |
| hsa_circ_0024704 | 84 | 1 | 342 | 557 | 898 | 0 | 2.00E-88 |
| hsa_circ_0024706 | 91 | 1 | 217 | 362 | 578 | 0 | 3.00E-79 |
| hsa_circ_0024728 | 90 | 1 | 605 | 367 | 971 | 0 | 0 |
| hsa_circ_0024728 | 90 | 1 | 869 | 367 | 1235 | 0 | 0 |
| hsa_circ_0024728 | 92 | 3 | 510 | 832 | 1339 | 0 | 0 |
| hsa_circ_0024728 | 91 | 3 | 366 | 976 | 1339 | 0 | 1.00E-135 |
| hsa_circ_0024459 | 91 | 1 | 675 | 5392 | 6066 | 0 | 0 |
| hsa_circ_0024462 | 90 | 1 | 2946 | 495 | 3446 | 0 | 0 |
| hsa_circ_0024466 | 89 | 1 | 311 | 1 | 311 | 0 | 6.00E-108 |
| hsa_circ_0024462 | 90 | 1 | 2946 | 495 | 3446 | 0 | 0 |
| hsa_circ_0024462 | 90 | 1 | 2946 | 495 | 3446 | 0 | 0 |
| hsa_circ_0024462 | 93 | 1 | 491 | 1 | 491 | 0 | 0 |
| hsa_circ_0024459 | 94 | 1 | 792 | 1 | 792 | 0 | 0 |
| hsa_circ_0024459 | 95 | 1 | 597 | 1 | 597 | 0 | 0 |
| hsa_circ_0024459 | 96 | 1 | 439 | 1 | 439 | 0 | 0 |
| hsa_circ_0024453 | 95 | 1 | 968 | 122 | 1089 | 0 | 0 |
| hsa_circ_0024452 | 93 | 1 | 217 | 1 | 217 | 0 | 3.00E-84 |
| hsa_circ_0024443 | 93 | 1 | 364 | 247 | 610 | 0 | 2.00E-147 |
| hsa_circ_0024439 | 95 | 1 | 413 | 1 | 413 | 0 | 0 |
| hsa_circ_0024435 | 91 | 1 | 306 | 1 | 306 | 0 | 4.00E-115 |
| hsa_circ_0024433 | 92 | 1 | 662 | 963 | 1624 | 0 | 0 |
| hsa_circ_0024433 | 95 | 1 | 329 | 1138 | 1466 | 0 | 6.00E-143 |

novel_circ_011309 novel_circ_011310 novel_circ_011311 novel_circ_011312 novel_circ_011313 novel_circ_011316 novel_circ_011317 novel_circ_011318 novel_circ_011320 novel_circ_011321 novel_circ_011322 novel_circ_011323 novel_circ_011324 novel_circ_011325 novel_circ_011326 novel_circ_011328 novel_circ_011329 novel_circ_011330 novel_circ_011331 novel_circ_011332 novel_circ_011333 novel_circ_011334 novel_circ_011335 novel_circ_011336 novel_circ_011337 novel_circ_011338 novel_circ_011339 novel_circ_011340 novel_circ_011341 novel_circ_011342 novel_circ_011343 novel_circ_011344 novel_circ_011345 novel_circ_011346 novel_circ_011347 novel_circ_011348 novel_circ_011349 novel_circ_011350 novel_circ_011351 novel_circ_011352 novel_circ_011353 novel_circ_011354 novel_circ_011355 novel_circ_011356 novel_circ_011357 novel_circ_011358 novel_circ_011359 novel_circ_011360 novel_circ_011361 novel_circ_011362 novel_circ_011363 novel_circ_011364 novel_circ_011365 novel_circ_011366

| hsa_circ_0024433 | 92 | 1 | 504 | 963 | 1466 | 0 | 0 |
| --- | --- | --- | --- | --- | --- | --- | --- |
| hsa_circ_0024434 | 96 | 1 | 215 | 189 | 403 | 0 | 9.00E-95 |
| hsa_circ_0024434 | 94 | 1 | 403 | 1 | 403 | 0 | 1.00E-170 |
| hsa_circ_0024427 | 89 | 1 | 1181 | 114 | 1297 | 0.03 | 0 |
| hsa_circ_0024428 | 91 | 1 | 747 | 1 | 729 | 0.02 | 0 |
| hsa_circ_0090332 | 96 | 1 | 846 | 1 | 846 | 0 | 0 |
| hsa_circ_0090332 | 94 | 1 | 452 | 131 | 582 | 0 | 0 |
| hsa_circ_0090322 | 99 | 1 | 339 | 601 | 939 | 0 | 2.00E-173 |
| hsa_circ_0090317 | 87 | 1 | 467 | 1 | 467 | 0 | 9.00E-148 |
| hsa_circ_0090314 | 88 | 1 | 283 | 198 | 480 | 0.01 | 4.00E-89 |
| hsa_circ_0090307 | 93 | 1 | 387 | 402 | 1 | 0.04 | 3.00E-162 |
| hsa_circ_0090306 | 94 | 1 | 471 | 486 | 1 | 0.03 | 0 |
| hsa_circ_0090305 | 95 | 1 | 711 | 726 | 1 | 0.02 | 0 |
| hsa_circ_0090304 | 96 | 1 | 281 | 704 | 424 | 0 | 5.00E-128 |
| hsa_circ_0090304 | 97 | 1 | 470 | 704 | 235 | 0 | 0 |
| hsa_circ_0092257 | 92 | 1 | 309 | 784 | 1092 | 0 | 4.00E-120 |
| hsa_circ_0092257 | 91 | 1 | 466 | 627 | 1092 | 0 | 0 |
| hsa_circ_0090279 | 95 | 1 | 1385 | 848 | 2232 | 0 | 0 |
| hsa_circ_0090267 | 94 | 1 | 678 | 1 | 678 | 0 | 0 |
| hsa_circ_0090261 | 93 | 1 | 820 | 1 | 820 | 0 | 0 |
| hsa_circ_0090261 | 93 | 1 | 549 | 1 | 549 | 0 | 0 |
| hsa_circ_0090258 | 94 | 1 | 980 | 1 | 980 | 0 | 0 |
| hsa_circ_0090227 | 96 | 1 | 1801 | 255 | 2055 | 0 | 0 |
| hsa_circ_0090227 | 96 | 1 | 2055 | 1 | 2055 | 0 | 0 |
| hsa_circ_0090227 | 97 | 1 | 1921 | 1 | 1921 | 0 | 0 |
| hsa_circ_0090227 | 97 | 1 | 1784 | 1 | 1784 | 0 | 0 |
| hsa_circ_0090227 | 97 | 1 | 1577 | 1 | 1577 | 0 | 0 |
| hsa_circ_0090232 | 96 | 1 | 557 | 1 | 557 | 0 | 0 |
| hsa_circ_0090227 | 97 | 1 | 811 | 1 | 811 | 0 | 0 |
| hsa_circ_0090227 | 97 | 1 | 593 | 1 | 593 | 0 | 0 |
| hsa_circ_0090227 | 98 | 1 | 478 | 1 | 478 | 0 | 0 |
| hsa_circ_0090227 | 98 | 1 | 397 | 1 | 397 | 0 | 0 |
| hsa_circ_0090227 | 99 | 1 | 254 | 1 | 254 | 0 | 5.00E-128 |
| hsa_circ_0090216 | 96 | 1 | 437 | 437 | 1 | 0 | 0 |
| hsa_circ_0090212 | 95 | 134 | 1435 | 1709 | 408 | 0 | 0 |
| hsa_circ_0090212 | 96 | 107 | 1043 | 1709 | 773 | 0 | 0 |
| hsa_circ_0090212 | 96 | 1 | 937 | 1709 | 773 | 0 | 0 |
| hsa_circ_0090212 | 96 | 1 | 1063 | 1709 | 647 | 0 | 0 |
| hsa_circ_0090212 | 95 | 1 | 1142 | 1709 | 568 | 0 | 0 |
| hsa_circ_0090212 | 95 | 1 | 1302 | 1709 | 408 | 0 | 0 |
| hsa_circ_0090212 | 94 | 1 | 1709 | 1709 | 1 | 0 | 0 |
| hsa_circ_0090206 | 94 | 542 | 2673 | 2795 | 664 | 0 | 0 |
| hsa_circ_0090206 | 93 | 238 | 1713 | 2795 | 1320 | 0 | 0 |
| hsa_circ_0090212 | 94 | 1 | 317 | 884 | 568 | 0 | 3.00E-136 |
| hsa_circ_0090212 | 94 | 1 | 477 | 884 | 408 | 0 | 0 |
| hsa_circ_0090212 | 92 | 1 | 807 | 884 | 78 | 0 | 0 |
| hsa_circ_0090212 | 92 | 1 | 772 | 772 | 1 | 0 | 0 |
| hsa_circ_0090206 | 94 | 1 | 1848 | 2399 | 552 | 0 | 0 |
| hsa_circ_0090208 | 94 | 1 | 612 | 1035 | 424 | 0 | 0 |
| hsa_circ_0090209 | 94 | 1 | 385 | 385 | 1 | 0 | 3.00E-167 |
| hsa_circ_0090209 | 94 | 1 | 308 | 308 | 1 | 0 | 6.00E-128 |
| hsa_circ_0090208 | 95 | 1 | 823 | 823 | 1 | 0 | 0 |
| hsa_circ_0090207 | 95 | 1 | 964 | 1076 | 113 | 0 | 0 |
| hsa_circ_0090197 | 92 | 97 | 438 | 1 | 342 | 0 | 1.00E-131 |

novel_circ_011376 novel_circ_011377 novel_circ_011378 novel_circ_011379 novel_circ_011380 novel_circ_011381 novel_circ_011382 novel_circ_011383 novel_circ_011384 novel_circ_011385 novel_circ_011386 novel_circ_011387 novel_circ_011388 novel_circ_011389 novel_circ_011390 novel_circ_011391 novel_circ_011392 novel_circ_011394 novel_circ_011396 novel_circ_011397 novel_circ_011401 novel_circ_011402 novel_circ_011403 novel_circ_011404 novel_circ_011405 novel_circ_011406 novel_circ_011407 novel_circ_011411 novel_circ_011412 novel_circ_011413 novel_circ_011414 novel_circ_011415 novel_circ_011416 novel_circ_011417 novel_circ_011419 novel_circ_011420 novel_circ_011421 novel_circ_011422 novel_circ_011423 novel_circ_011426 novel_circ_011427 novel_circ_011428 novel_circ_011429 novel_circ_011430 novel_circ_011431 novel_circ_011432 novel_circ_011433 novel_circ_011434 novel_circ_011435 novel_circ_011436 novel_circ_011437 novel_circ_011438 novel_circ_011439 novel_circ_011440

| hsa_circ_0079953 | 92 | 1 | 323 | 1 | 323 | 0 | 6.00E-128 |
| --- | --- | --- | --- | --- | --- | --- | --- |
| hsa_circ_0079953 | 92 | 1 | 433 | 1 | 433 | 0 | 3.00E-172 |
| hsa_circ_0079954 | 94 | 1 | 515 | 1939 | 2453 | 0 | 0 |
| hsa_circ_0079954 | 95 | 1 | 836 | 1939 | 2774 | 0 | 0 |
| hsa_circ_0079954 | 94 | 1 | 1233 | 1939 | 3171 | 0 | 0 |
| hsa_circ_0079954 | 94 | 1 | 1618 | 1939 | 3556 | 0 | 0 |
| hsa_circ_0079955 | 94 | 1 | 734 | 1 | 734 | 0 | 0 |
| hsa_circ_0079955 | 94 | 1 | 1131 | 1 | 1131 | 0 | 0 |
| hsa_circ_0079954 | 94 | 1 | 1516 | 2041 | 3556 | 0 | 0 |
| hsa_circ_0079956 | 94 | 1 | 583 | 1 | 583 | 0 | 0 |
| hsa_circ_0079956 | 94 | 1 | 980 | 1 | 980 | 0 | 0 |
| hsa_circ_0079954 | 94 | 1 | 1365 | 2192 | 3556 | 0 | 0 |
| hsa_circ_0079956 | 95 | 1 | 646 | 141 | 786 | 0 | 0 |
| hsa_circ_0079958 | 95 | 1 | 524 | 1 | 524 | 0 | 0 |
| hsa_circ_0005017 | 93 | 1 | 203 | 1 | 203 | 0 | 4.00E-78 |
| hsa_circ_0079954 | 93 | 1 | 782 | 2775 | 3556 | 0 | 0 |
| hsa_circ_0079969 | 91 | 1 | 278 | 1 | 278 | 0 | 5.00E-103 |
| hsa_circ_0080225 | 87 | 1 | 988 | 1 | 988 | 0 | 0 |
| hsa_circ_0072993 | 85 | 1 | 437 | 1 | 437 | 0 | 4.00E-126 |
| hsa_circ_0072993 | 86 | 1 | 880 | 1 | 877 | 0.01 | 0 |
| hsa_circ_0073000 | 86 | 1 | 723 | 1 | 723 | 0 | 0 |
| hsa_circ_0073000 | 86 | 1 | 763 | 1 | 763 | 0 | 0 |
| hsa_circ_0073000 | 88 | 1 | 1774 | 1 | 1790 | 0.01 | 0 |
| hsa_circ_0073000 | 90 | 1 | 2801 | 1 | 2817 | 0.01 | 0 |
| hsa_circ_0073006 | 92 | 6 | 916 | 958 | 1868 | 0 | 0 |
| hsa_circ_0073006 | 91 | 1 | 687 | 1285 | 1971 | 0 | 0 |
| hsa_circ_0073006 | 91 | 1 | 687 | 1285 | 1971 | 0 | 0 |
| hsa_circ_0002758 | 92 | 1 | 261 | 1 | 261 | 0 | 8.00E-100 |
| hsa_circ_0018625 | 94 | 1910 | 2067 | 183 | 340 | 0 | 5.00E-62 |
| hsa_circ_0018618 | 91 | 1 | 284 | 97 | 380 | 0 | 3.00E-106 |
| hsa_circ_0018618 | 92 | 1 | 380 | 1 | 380 | 0 | 2.00E-149 |
| hsa_circ_0018616 | 92 | 1 | 500 | 1 | 500 | 0 | 0 |
| hsa_circ_0018614 | 93 | 1 | 649 | 1 | 649 | 0 | 0 |
| hsa_circ_0018613 | 94 | 5379 | 5541 | 1 | 163 | 0 | 2.00E-64 |
| hsa_circ_0018605 | 91 | 1 | 1052 | 282 | 1353 | 0.02 | 0 |
| hsa_circ_0018611 | 93 | 1 | 753 | 1 | 753 | 0 | 0 |
| hsa_circ_0018608 | 93 | 1 | 923 | 1 | 923 | 0 | 0 |
| hsa_circ_0018605 | 92 | 1 | 463 | 170 | 632 | 0 | 0 |
| hsa_circ_0018605 | 92 | 1 | 282 | 170 | 451 | 0 | 2.00E-108 |
| hsa_circ_0018561 | 96 | 1 | 312 | 630 | 941 | 0 | 7.00E-142 |
| hsa_circ_0018561 | 96 | 1 | 458 | 484 | 941 | 0 | 0 |
| hsa_circ_0018561 | 95 | 1 | 762 | 180 | 941 | 0 | 0 |
| hsa_circ_0018561 | 95 | 77 | 855 | 1 | 779 | 0 | 0 |
| hsa_circ_0018561 | 95 | 371 | 1149 | 1 | 779 | 0 | 0 |
| hsa_circ_0018561 | 95 | 77 | 705 | 1 | 629 | 0 | 0 |
| hsa_circ_0018561 | 95 | 371 | 999 | 1 | 629 | 0 | 0 |
| hsa_circ_0018561 | 95 | 371 | 549 | 1 | 179 | 0 | 2.00E-74 |
| hsa_circ_0002371 | 92 | 6 | 230 | 6 | 230 | 0 | 3.00E-85 |
| hsa_circ_0018549 | 95 | 1 | 1536 | 1099 | 2634 | 0 | 0 |
| hsa_circ_0000240 | 94 | 1 | 730 | 803 | 1532 | 0 | 0 |
| hsa_circ_0000240 | 96 | 989 | 2296 | 225 | 1532 | 0 | 0 |
| hsa_circ_0000240 | 96 | 1 | 1532 | 1 | 1532 | 0 | 0 |
| hsa_circ_0000240 | 96 | 989 | 2184 | 225 | 1420 | 0 | 0 |
| hsa_circ_0000240 | 96 | 1 | 1420 | 1 | 1420 | 0 | 0 |

novel_circ_011441 novel_circ_011442 novel_circ_011443 novel_circ_011444 novel_circ_011445 novel_circ_011446 novel_circ_011447 novel_circ_011448 novel_circ_011449 novel_circ_011450 novel_circ_011451 novel_circ_011452 novel_circ_011453 novel_circ_011454 novel_circ_011455 novel_circ_011456 novel_circ_011457 novel_circ_011458 novel_circ_011459 novel_circ_011460 novel_circ_011461 novel_circ_011462 novel_circ_011463 novel_circ_011464 novel_circ_011465 novel_circ_011466 novel_circ_011467 novel_circ_011468 novel_circ_011469 novel_circ_011470 novel_circ_011471 novel_circ_011472 novel_circ_011473 novel_circ_011474 novel_circ_011475 novel_circ_011476 novel_circ_011477 novel_circ_011478 novel_circ_011479 novel_circ_011480 novel_circ_011481 novel_circ_011482 novel_circ_011483 novel_circ_011484 novel_circ_011485 novel_circ_011486 novel_circ_011487 novel_circ_011488 novel_circ_011489 novel_circ_011490 novel_circ_011491 novel_circ_011492 novel_circ_011493 novel_circ_011494

| hsa_circ_0000240 | 96 | 1 | 954 | 227 | 1180 | 0 | 0 |
| --- | --- | --- | --- | --- | --- | --- | --- |
| hsa_circ_0000240 | 96 | 989 | 1944 | 225 | 1180 | 0 | 0 |
| hsa_circ_0000240 | 96 | 1 | 1180 | 1 | 1180 | 0 | 0 |
| hsa_circ_0018557 | 98 | 1 | 576 | 1 | 576 | 0 | 0 |
| hsa_circ_0000240 | 98 | 34 | 611 | 225 | 802 | 0 | 0 |
| hsa_circ_0000240 | 98 | 989 | 1566 | 225 | 802 | 0 | 0 |
| hsa_circ_0000240 | 98 | 1 | 802 | 1 | 802 | 0 | 0 |
| hsa_circ_0018549 | 95 | 1 | 1596 | 79 | 1674 | 0 | 0 |
| hsa_circ_0018549 | 93 | 1 | 791 | 79 | 869 | 0 | 0 |
| hsa_circ_0018551 | 93 | 1 | 502 | 34 | 535 | 0 | 0 |
| hsa_circ_0018544 | 94 | 3 | 753 | 3 | 753 | 0 | 0 |
| hsa_circ_0018537 | 90 | 1 | 462 | 3001 | 3462 | 0 | 5.00E-170 |
| hsa_circ_0018537 | 90 | 1 | 552 | 2911 | 3462 | 0 | 0 |
| hsa_circ_0018537 | 91 | 1 | 703 | 2760 | 3462 | 0 | 0 |
| hsa_circ_0018537 | 85 | 3 | 3408 | 57 | 3462 | 0.01 | 0 |
| hsa_circ_0018538 | 94 | 1 | 379 | 213 | 591 | 0 | 1.00E-165 |
| hsa_circ_0018538 | 94 | 1 | 241 | 213 | 453 | 0 | 8.00E-101 |
| hsa_circ_0006753 | 89 | 1 | 397 | 1 | 397 | 0 | 3.00E-137 |
| hsa_circ_0018537 | 83 | 3 | 2705 | 57 | 2759 | 0.01 | 0 |
| hsa_circ_0018537 | 83 | 3 | 2493 | 57 | 2547 | 0.02 | 0 |
| hsa_circ_0018537 | 82 | 3 | 2291 | 57 | 2345 | 0.02 | 0 |
| hsa_circ_0018536 | 84 | 1 | 2060 | 384 | 2473 | 0.02 | 0 |
| hsa_circ_0018532 | 90 | 1 | 381 | 619 | 233 | 0.02 | 4.00E-140 |
| hsa_circ_0018535 | 93 | 1 | 189 | 189 | 1 | 0 | 5.00E-72 |
| hsa_circ_0018533 | 95 | 1 | 352 | 352 | 1 | 0 | 5.00E-154 |
| hsa_circ_0018532 | 95 | 1 | 421 | 421 | 1 | 0 | 0 |
| hsa_circ_0018532 | 96 | 1 | 232 | 232 | 1 | 0 | 3.00E-104 |
| hsa_circ_0009172 | 87 | 6 | 642 | 637 | 1 | 0 | 0 |
| hsa_circ_0018522 | 89 | 2 | 227 | 226 | 2 | 0 | 1.00E-73 |
| hsa_circ_0018505 | 94 | 477 | 878 | 402 | 1 | 0 | 1.00E-174 |
| hsa_circ_0018505 | 94 | 179 | 580 | 402 | 1 | 0 | 1.00E-174 |
| hsa_circ_0018505 | 94 | 179 | 580 | 402 | 1 | 0 | 1.00E-174 |
| hsa_circ_0018505 | 94 | 179 | 580 | 402 | 1 | 0 | 2.00E-174 |
| hsa_circ_0018505 | 94 | 5 | 220 | 402 | 187 | 0 | 5.00E-87 |
| hsa_circ_0018505 | 94 | 5 | 406 | 402 | 1 | 0 | 5.00E-175 |
| hsa_circ_0018505 | 94 | 5 | 406 | 402 | 1 | 0 | 1.00E-174 |
| hsa_circ_0018505 | 94 | 5 | 406 | 402 | 1 | 0 | 6.00E-175 |
| hsa_circ_0018505 | 94 | 5 | 406 | 402 | 1 | 0 | 1.00E-174 |
| hsa_circ_0000239 | 96 | 1 | 252 | 252 | 1 | 0 | 1.00E-113 |
| hsa_circ_0018504 | 94 | 253 | 541 | 289 | 1 | 0 | 4.00E-122 |
| hsa_circ_0018504 | 94 | 67 | 355 | 289 | 1 | 0 | 4.00E-122 |
| hsa_circ_0018504 | 94 | 1 | 289 | 289 | 1 | 0 | 2.00E-122 |
| hsa_circ_0018504 | 94 | 1 | 289 | 289 | 1 | 0 | 3.00E-122 |
| hsa_circ_0018504 | 94 | 1 | 289 | 289 | 1 | 0 | 5.00E-122 |
| hsa_circ_0018503 | 97 | 140 | 261 | 347 | 226 | 0 | 2.00E-51 |
| hsa_circ_0018503 | 94 | 42 | 388 | 347 | 1 | 0 | 3.00E-149 |
| hsa_circ_0006031 | 92 | 1 | 144 | 144 | 1 | 0 | 1.00E-51 |
| hsa_circ_0006031 | 92 | 1 | 144 | 144 | 1 | 0 | 3.00E-51 |
| hsa_circ_0018501 | 92 | 1 | 2217 | 1 | 2206 | 0.03 | 0 |
| hsa_circ_0018501 | 98 | 1 | 620 | 275 | 894 | 0 | 0 |
| hsa_circ_0018501 | 98 | 1 | 894 | 1 | 894 | 0 | 0 |
| hsa_circ_0006263 | 99 | 1 | 133 | 22891 | 22759 | 0 | 4.00E-62 |
| hsa_circ_0003259 | 94 | 381 | 1066 | 686 | 1 | 0 | 0 |
| hsa_circ_0006263 | 93 | 1 | 160 | 237 | 78 | 0 | 5.00E-59 |

novel_circ_011495 novel_circ_011496 novel_circ_011497 novel_circ_011498 novel_circ_011499 novel_circ_011500 novel_circ_011501 novel_circ_011502 novel_circ_011503 novel_circ_011504 novel_circ_011505 novel_circ_011506 novel_circ_011507 novel_circ_011508 novel_circ_011509 novel_circ_011510 novel_circ_011511 novel_circ_011512 novel_circ_011513 novel_circ_011514 novel_circ_011515 novel_circ_011516 novel_circ_011517 novel_circ_011518 novel_circ_011519 novel_circ_011520 novel_circ_011522 novel_circ_011523 novel_circ_011524 novel_circ_011526 novel_circ_011527 novel_circ_011528 novel_circ_011529 novel_circ_011532 novel_circ_011533 novel_circ_011534 novel_circ_011535 novel_circ_011536 novel_circ_011537 novel_circ_011538 novel_circ_011539 novel_circ_011540 novel_circ_011541 novel_circ_011542 novel_circ_011543 novel_circ_011544 novel_circ_011545 novel_circ_011546 novel_circ_011547 novel_circ_011548 novel_circ_011549 novel_circ_011550 novel_circ_011551 novel_circ_011552

| hsa_circ_0018489 | 95 | 248 | 549 | 394 | 93 | 0 | 2.00E-134 |
| --- | --- | --- | --- | --- | --- | --- | --- |
| hsa_circ_0007113 | 94 | 248 | 770 | 525 | 3 | 0 | 0 |
| hsa_circ_0003259 | 94 | 248 | 933 | 686 | 1 | 0 | 0 |
| hsa_circ_0018486 | 94 | 248 | 1135 | 1060 | 173 | 0 | 0 |
| hsa_circ_0009151 | 95 | 248 | 1495 | 1423 | 176 | 0 | 0 |
| hsa_circ_0018483 | 95 | 248 | 1790 | 1543 | 1 | 0 | 0 |
| hsa_circ_0018482 | 95 | 248 | 1913 | 1666 | 1 | 0 | 0 |
| hsa_circ_0007113 | 94 | 243 | 765 | 525 | 3 | 0 | 0 |
| hsa_circ_0003259 | 94 | 2 | 683 | 682 | 1 | 0 | 0 |
| hsa_circ_0003259 | 92 | 1 | 292 | 292 | 1 | 0 | 9.00E-116 |
| hsa_circ_0006196 | 94 | 1 | 363 | 363 | 1 | 0 | 4.00E-155 |
| hsa_circ_0018483 | 95 | 1 | 1018 | 1018 | 1 | 0 | 0 |
| hsa_circ_0018482 | 95 | 1 | 1141 | 1141 | 1 | 0 | 0 |
| hsa_circ_0018480 | 95 | 1 | 1292 | 1292 | 1 | 0 | 0 |
| hsa_circ_0018480 | 95 | 1 | 728 | 728 | 1 | 0 | 0 |
| hsa_circ_0018480 | 95 | 1 | 555 | 555 | 1 | 0 | 0 |
| hsa_circ_0018480 | 94 | 1 | 411 | 411 | 1 | 0 | 4.00E-180 |
| hsa_circ_0018475 | 96 | 1 | 728 | 912 | 185 | 0 | 0 |
| hsa_circ_0018480 | 95 | 1 | 312 | 312 | 1 | 0 | 3.00E-135 |
| hsa_circ_0018472 | 97 | 81 | 267 | 1 | 187 | 0 | 1.00E-83 |
| hsa_circ_0018472 | 97 | 81 | 267 | 1 | 187 | 0 | 4.00E-84 |
| hsa_circ_0018471 | 96 | 1 | 623 | 1 | 623 | 0 | 0 |
| hsa_circ_0018471 | 95 | 1 | 395 | 1 | 395 | 0 | 2.00E-179 |
| hsa_circ_0018471 | 95 | 118 | 512 | 1 | 395 | 0 | 2.00E-179 |
| hsa_circ_0018471 | 95 | 118 | 359 | 1 | 242 | 0 | 3.00E-106 |
| hsa_circ_0018470 | 96 | 1 | 79 | 79 | 1 | 0 | 4.00E-29 |
| hsa_circ_0072543 | 96 | 475 | 1157 | 556 | 1238 | 0 | 0 |
| hsa_circ_0072536 | 95 | 1 | 281 | 281 | 1 | 0 | 1.00E-122 |
| hsa_circ_0072536 | 95 | 1 | 281 | 281 | 1 | 0 | 2.00E-122 |
| hsa_circ_0072534 | 93 | 125 | 346 | 1 | 222 | 0 | 7.00E-88 |
| hsa_circ_0072534 | 93 | 125 | 346 | 1 | 222 | 0 | 2.00E-88 |
| hsa_circ_0072529 | 94 | 1 | 493 | 853 | 1345 | 0 | 0 |
| hsa_circ_0072529 | 94 | 1 | 401 | 853 | 1253 | 0 | 2.00E-169 |
| hsa_circ_0072509 | 88 | 16 | 1567 | 1552 | 1 | 0.01 | 0 |
| hsa_circ_0072515 | 88 | 2 | 306 | 305 | 1 | 0.01 | 1.00E-99 |
| hsa_circ_0072514 | 87 | 2 | 427 | 426 | 1 | 0.01 | 2.00E-133 |
| hsa_circ_0072511 | 87 | 2 | 594 | 1385 | 793 | 0.01 | 0 |
| hsa_circ_0072511 | 87 | 2 | 749 | 1385 | 638 | 0.01 | 0 |
| hsa_circ_0072511 | 87 | 2 | 909 | 1385 | 478 | 0 | 0 |
| hsa_circ_0072511 | 88 | 2 | 1389 | 1385 | 1 | 0.01 | 0 |
| hsa_circ_0072509 | 88 | 2 | 1491 | 1487 | 1 | 0 | 0 |
| hsa_circ_0072509 | 89 | 3 | 1185 | 1180 | 1 | 0 | 0 |
| hsa_circ_0072513 | 87 | 1 | 322 | 322 | 1 | 0 | 2.00E-97 |
| hsa_circ_0072506 | 90 | 1 | 1352 | 1446 | 98 | 0 | 0 |
| hsa_circ_0072506 | 91 | 9 | 1030 | 1116 | 98 | 0 | 0 |
| hsa_circ_0072509 | 90 | 1 | 582 | 579 | 1 | 0.01 | 0 |
| hsa_circ_0072506 | 94 | 10 | 485 | 478 | 3 | 0 | 0 |
| hsa_circ_0072506 | 94 | 1 | 288 | 385 | 98 | 0 | 3.00E-120 |
| hsa_circ_0072506 | 93 | 1 | 383 | 385 | 3 | 0 | 3.00E-161 |
| hsa_circ_0072498 | 94 | 128 | 771 | 644 | 1 | 0 | 0 |
| hsa_circ_0072498 | 94 | 1 | 644 | 644 | 1 | 0 | 0 |
| hsa_circ_0072498 | 94 | 1 | 644 | 644 | 1 | 0 | 0 |
| hsa_circ_0072496 | 93 | 419 | 1317 | 899 | 1 | 0 | 0 |
| hsa_circ_0072496 | 93 | 286 | 1184 | 899 | 1 | 0 | 0 |

novel_circ_011553 novel_circ_011554 novel_circ_011555 novel_circ_011556 novel_circ_011557 novel_circ_011558 novel_circ_011559 novel_circ_011560 novel_circ_011561 novel_circ_011562 novel_circ_011563 novel_circ_011564 novel_circ_011565 novel_circ_011566 novel_circ_011567 novel_circ_011568 novel_circ_011569 novel_circ_011570 novel_circ_011571 novel_circ_011572 novel_circ_011573 novel_circ_011574 novel_circ_011575 novel_circ_011576 novel_circ_011577 novel_circ_011578 novel_circ_011579 novel_circ_011580 novel_circ_011581 novel_circ_011582 novel_circ_011583 novel_circ_011584 novel_circ_011585 novel_circ_011586 novel_circ_011587 novel_circ_011588 novel_circ_011589 novel_circ_011590 novel_circ_011591 novel_circ_011592 novel_circ_011593 novel_circ_011596 novel_circ_011597 novel_circ_011598 novel_circ_011599 novel_circ_011600 novel_circ_011601 novel_circ_011602 novel_circ_011603 novel_circ_011605 novel_circ_011606 novel_circ_011607 novel_circ_011609 novel_circ_011610

| hsa_circ_0008731 | 91 | 1003 | 1531 | 636 | 108 | 0 | 0 |
| --- | --- | --- | --- | --- | --- | --- | --- |
| hsa_circ_0008731 | 91 | 6 | 534 | 636 | 108 | 0 | 0 |
| hsa_circ_0008731 | 91 | 6 | 641 | 636 | 1 | 0 | 0 |
| hsa_circ_0072496 | 91 | 6 | 646 | 899 | 259 | 0 | 0 |
| hsa_circ_0072496 | 92 | 6 | 755 | 899 | 150 | 0 | 0 |
| hsa_circ_0072496 | 93 | 6 | 904 | 899 | 1 | 0 | 0 |
| hsa_circ_0008731 | 90 | 1 | 363 | 470 | 108 | 0 | 4.00E-130 |
| hsa_circ_0072490 | 96 | 33358 | 33847 | 491 | 2 | 0 | 0 |
| hsa_circ_0072492 | 95 | 1 | 433 | 433 | 1 | 0 | 0 |
| hsa_circ_0072490 | 96 | 1 | 490 | 491 | 2 | 0 | 0 |
| hsa_circ_0072491 | 95 | 4 | 338 | 336 | 2 | 0 | 6.00E-148 |
| hsa_circ_0072489 | 94 | 4 | 516 | 513 | 1 | 0 | 0 |
| hsa_circ_0072487 | 95 | 1 | 1313 | 754 | 2066 | 0 | 0 |
| hsa_circ_0072487 | 95 | 1 | 1149 | 754 | 1902 | 0 | 0 |
| hsa_circ_0072487 | 95 | 1 | 1092 | 754 | 1845 | 0 | 0 |
| hsa_circ_0072487 | 95 | 1 | 1218 | 628 | 1845 | 0 | 0 |
| hsa_circ_0072487 | 95 | 1 | 464 | 1276 | 1739 | 0 | 0 |
| hsa_circ_0072487 | 95 | 1 | 986 | 754 | 1739 | 0 | 0 |
| hsa_circ_0072487 | 95 | 1 | 1112 | 628 | 1739 | 0 | 0 |
| hsa_circ_0072487 | 95 | 1 | 822 | 754 | 1575 | 0 | 0 |
| hsa_circ_0072487 | 95 | 1 | 948 | 628 | 1575 | 0 | 0 |
| hsa_circ_0072488 | 95 | 1 | 633 | 340 | 972 | 0 | 0 |
| hsa_circ_0072488 | 95 | 1 | 759 | 214 | 972 | 0 | 0 |
| hsa_circ_0072488 | 95 | 1 | 522 | 340 | 861 | 0 | 0 |
| hsa_circ_0072488 | 95 | 1 | 648 | 214 | 861 | 0 | 0 |
| hsa_circ_0072488 | 93 | 1 | 228 | 112 | 339 | 0 | 1.00E-88 |
| hsa_circ_0072487 | 94 | 1 | 551 | 203 | 753 | 0 | 0 |
| hsa_circ_0072487 | 94 | 1 | 654 | 100 | 753 | 0 | 0 |
| hsa_circ_0072487 | 94 | 1 | 753 | 1 | 753 | 0 | 0 |
| hsa_circ_0072477 | 94 | 160 | 1303 | 1 | 1144 | 0 | 0 |
| hsa_circ_0072477 | 94 | 1 | 916 | 1 | 916 | 0 | 0 |
| hsa_circ_0072484 | 95 | 3 | 459 | 3 | 459 | 0 | 0 |
| hsa_circ_0072474 | 91 | 1 | 617 | 539 | 1172 | 0.03 | 0 |
| hsa_circ_0072467 | 94 | 1 | 325 | 3446 | 3122 | 0 | 3.00E-137 |
| hsa_circ_0072471 | 92 | 1 | 728 | 877 | 147 | 0 | 0 |
| hsa_circ_0072471 | 92 | 1 | 874 | 877 | 1 | 0 | 0 |
| hsa_circ_0072471 | 92 | 1 | 750 | 753 | 1 | 0 | 0 |
| hsa_circ_0072469 | 96 | 1 | 362 | 456 | 95 | 0 | 7.00E-168 |
| hsa_circ_0072467 | 94 | 1 | 988 | 1284 | 297 | 0 | 0 |
| hsa_circ_0072469 | 94 | 1 | 231 | 231 | 1 | 0 | 6.00E-97 |
| hsa_circ_0072467 | 94 | 1 | 763 | 1059 | 297 | 0 | 0 |
| hsa_circ_0064026 | 80 | 15058 | 15263 | 36487 | 36684 | 0.08 | 9.00E-29 |
| hsa_circ_0007389 | 78 | 14432 | 14688 | 1E+06 | 1421171 | 0.03 | 3.00E-33 |
| hsa_circ_0007389 | 78 | 39252 | 39508 | 1E+06 | 1421171 | 0.03 | 4.00E-33 |
| hsa_circ_0007389 | 78 | 39252 | 39508 | 1E+06 | 1421171 | 0.03 | 3.00E-33 |
| hsa_circ_0007389 | 78 | 14432 | 14688 | 1E+06 | 1421171 | 0.03 | 1.00E-33 |
| hsa_circ_0007389 | 78 | 39252 | 39508 | 1E+06 | 1421171 | 0.03 | 2.00E-33 |
| hsa_circ_0032941 | 83 | 27917 | 27997 | 26282 | 26204 | 0.02 | 4.00E-09 |
| hsa_circ_0045877 | 93 | 17891 | 17934 | 475 | 432 | 0 | 1.00E-07 |
| hsa_circ_0026804 | 84 | 50882 | 51303 | 257 | 682 | 0.04 | 5.00E-105 |
| hsa_circ_0072464 | 97 | 146 | 414 | 269 | 1 | 0 | 4.00E-126 |
| hsa_circ_0072463 | 80 | 1 | 1978 | 1930 | 2 | 0.06 | 0 |
| hsa_circ_0072445 | 91 | 1 | 1535 | 1 | 1538 | 0 | 0 |
| hsa_circ_0072445 | 92 | 1 | 1248 | 1 | 1248 | 0 | 0 |

novel_circ_011611 novel_circ_011612 novel_circ_011613 novel_circ_011614 novel_circ_011615 novel_circ_011616 novel_circ_011617 novel_circ_011618 novel_circ_011619 novel_circ_011620 novel_circ_011621 novel_circ_011623 novel_circ_011624 novel_circ_011625 novel_circ_011626 novel_circ_011627 novel_circ_011628 novel_circ_011629 novel_circ_011630 novel_circ_011631 novel_circ_011632 novel_circ_011640 novel_circ_011644 novel_circ_011645 novel_circ_011646 novel_circ_011647 novel_circ_011648 novel_circ_011649 novel_circ_011650 novel_circ_011651 novel_circ_011652 novel_circ_011653 novel_circ_011654 novel_circ_011655 novel_circ_011656 novel_circ_011657 novel_circ_011658 novel_circ_011659 novel_circ_011660 novel_circ_011661 novel_circ_011662 novel_circ_011663 novel_circ_011664 novel_circ_011665 novel_circ_011666 novel_circ_011667 novel_circ_011668 novel_circ_011669 novel_circ_011670 novel_circ_011671 novel_circ_011672 novel_circ_011673 novel_circ_011674 novel_circ_011675

| hsa_circ_0072445 | 93 | 1 | 1029 | 1 | 1029 | 0 | 0 |
| --- | --- | --- | --- | --- | --- | --- | --- |
| hsa_circ_0072445 | 93 | 1 | 563 | 1 | 563 | 0 | 0 |
| hsa_circ_0072432 | 95 | 1 | 515 | 1430 | 1944 | 0 | 0 |
| hsa_circ_0072432 | 92 | 1 | 1108 | 718 | 1831 | 0.01 | 0 |
| hsa_circ_0072440 | 92 | 1 | 899 | 1 | 905 | 0.01 | 0 |
| hsa_circ_0072439 | 93 | 1 | 1051 | 1 | 1057 | 0.01 | 0 |
| hsa_circ_0072433 | 93 | 1 | 1385 | 1 | 1391 | 0 | 0 |
| hsa_circ_0072433 | 95 | 1 | 679 | 1 | 679 | 0 | 0 |
| hsa_circ_0072433 | 95 | 1 | 553 | 1 | 553 | 0 | 0 |
| hsa_circ_0072433 | 95 | 1 | 486 | 1 | 486 | 0 | 0 |
| hsa_circ_0072433 | 94 | 1 | 334 | 1 | 334 | 0 | 2.00E-142 |
| hsa_circ_0076676 | 92 | 1 | 314 | 314 | 1 | 0 | 3.00E-121 |
| hsa_circ_0004346 | 94 | 1 | 334 | 220 | 553 | 0 | 2.00E-140 |
| hsa_circ_0076678 | 92 | 1 | 666 | 1 | 666 | 0 | 0 |
| hsa_circ_0076678 | 92 | 1 | 477 | 190 | 666 | 0 | 0 |
| hsa_circ_0003724 | 95 | 2 | 221 | 331 | 112 | 0 | 2.00E-95 |
| hsa_circ_0003724 | 96 | 4 | 297 | 405 | 112 | 0 | 1.00E-131 |
| hsa_circ_0003724 | 95 | 1 | 437 | 548 | 112 | 0 | 0 |
| hsa_circ_0005112 | 93 | 1 | 318 | 318 | 1 | 0 | 4.00E-130 |
| hsa_circ_0005112 | 94 | 4 | 403 | 400 | 1 | 0 | 6.00E-169 |
| hsa_circ_0076687 | 97 | 1 | 262 | 1 | 262 | 0 | 8.00E-121 |
| hsa_circ_0003890 | 93 | 3778 | 3819 | 47588 | 47547 | 0 | 1.00E-06 |
| hsa_circ_0005897 | 89 | 1 | 266 | 266 | 1 | 0 | 2.00E-91 |
| hsa_circ_0001610 | 94 | 1 | 1144 | 1147 | 1 | 0 | 0 |
| hsa_circ_0076707 | 89 | 1 | 458 | 538 | 1004 | 0.03 | 1.00E-156 |
| hsa_circ_0076707 | 89 | 1 | 495 | 538 | 1041 | 0.03 | 7.00E-174 |
| hsa_circ_0076707 | 89 | 1 | 556 | 538 | 1102 | 0.03 | 0 |
| hsa_circ_0076707 | 90 | 1 | 864 | 538 | 1413 | 0.02 | 0 |
| hsa_circ_0076707 | 90 | 1 | 673 | 726 | 1413 | 0.02 | 0 |
| hsa_circ_0076708 | 95 | 6 | 200 | 6 | 200 | 0 | 5.00E-82 |
| hsa_circ_0076708 | 95 | 6 | 237 | 6 | 237 | 0 | 3.00E-99 |
| hsa_circ_0076708 | 94 | 6 | 298 | 6 | 298 | 0 | 1.00E-124 |
| hsa_circ_0076708 | 92 | 6 | 606 | 6 | 609 | 0 | 0 |
| hsa_circ_0076708 | 91 | 6 | 1000 | 6 | 1003 | 0 | 0 |
| hsa_circ_0076708 | 90 | 1 | 394 | 610 | 1003 | 0 | 6.00E-144 |
| hsa_circ_0076710 | 97 | 1 | 481 | 481 | 1 | 0 | 0 |
| hsa_circ_0076713 | 93 | 4019 | 5388 | 2867 | 1498 | 0 | 0 |
| hsa_circ_0076713 | 93 | 1 | 1739 | 3236 | 1498 | 0 | 0 |
| hsa_circ_0076713 | 94 | 425 | 1995 | 3236 | 1666 | 0 | 0 |
| hsa_circ_0076713 | 94 | 4 | 344 | 2286 | 1946 | 0 | 6.00E-148 |
| hsa_circ_0076713 | 93 | 4019 | 4940 | 2867 | 1946 | 0 | 0 |
| hsa_circ_0076713 | 94 | 1 | 1291 | 3236 | 1946 | 0 | 0 |
| hsa_circ_0076713 | 97 | 4 | 228 | 2286 | 2062 | 0 | 1.00E-103 |
| hsa_circ_0076713 | 94 | 4019 | 4824 | 2867 | 2062 | 0 | 0 |
| hsa_circ_0076713 | 94 | 1 | 1175 | 3236 | 2062 | 0 | 0 |
| hsa_circ_0076713 | 94 | 425 | 1599 | 3236 | 2062 | 0 | 0 |
| hsa_circ_0076713 | 93 | 4019 | 4708 | 2867 | 2178 | 0 | 0 |
| hsa_circ_0076713 | 94 | 425 | 1483 | 3236 | 2178 | 0 | 0 |
| hsa_circ_0076713 | 93 | 4019 | 4596 | 2867 | 2290 | 0 | 0 |
| hsa_circ_0076713 | 93 | 1 | 947 | 3236 | 2290 | 0 | 0 |
| hsa_circ_0076713 | 93 | 425 | 1371 | 3236 | 2290 | 0 | 0 |
| hsa_circ_0076713 | 95 | 1 | 368 | 3236 | 2869 | 0 | 2.00E-163 |
| hsa_circ_0076713 | 94 | 1 | 698 | 3236 | 2539 | 0 | 0 |
| hsa_circ_0076713 | 95 | 1 | 368 | 3236 | 2869 | 0 | 2.00E-163 |

novel_circ_011676 novel_circ_011677 novel_circ_011680 novel_circ_011681 novel_circ_011682 novel_circ_011683 novel_circ_011684 novel_circ_011686 novel_circ_011687 novel_circ_011688 novel_circ_011689 novel_circ_011690 novel_circ_011691 novel_circ_011692 novel_circ_011694 novel_circ_011695 novel_circ_011696 novel_circ_011697 novel_circ_011698 novel_circ_011699 novel_circ_011700 novel_circ_011701 novel_circ_011702 novel_circ_011703 novel_circ_011704 novel_circ_011705 novel_circ_011706 novel_circ_011707 novel_circ_011708 novel_circ_011709 novel_circ_011710 novel_circ_011711 novel_circ_011712 novel_circ_011713 novel_circ_011714 novel_circ_011715 novel_circ_011716 novel_circ_011717 novel_circ_011718 novel_circ_011719 novel_circ_011720 novel_circ_011721 novel_circ_011722 novel_circ_011723 novel_circ_011724 novel_circ_011725 novel_circ_011726 novel_circ_011727 novel_circ_011728 novel_circ_011729 novel_circ_011730 novel_circ_011731 novel_circ_011732 novel_circ_011733

| hsa_circ_0076713 | 94 | 1 | 524 | 3236 | 2713 | 0 | 0 |
| --- | --- | --- | --- | --- | --- | --- | --- |
| hsa_circ_0076713 | 94 | 425 | 948 | 3236 | 2713 | 0 | 0 |
| hsa_circ_0055385 | 92 | 1 | 507 | 696 | 190 | 0 | 0 |
| hsa_circ_0055385 | 93 | 4 | 624 | 810 | 190 | 0 | 0 |
| hsa_circ_0055385 | 92 | 6 | 728 | 912 | 190 | 0 | 0 |
| hsa_circ_0055385 | 92 | 1 | 355 | 696 | 342 | 0 | 2.00E-142 |
| hsa_circ_0055385 | 93 | 4 | 472 | 810 | 342 | 0 | 0 |
| hsa_circ_0055391 | 98 | 169 | 308 | 1 | 140 | 0 | 6.00E-63 |
| hsa_circ_0055391 | 99 | 169 | 585 | 1 | 417 | 0 | 0 |
| hsa_circ_0055391 | 96 | 169 | 868 | 1 | 700 | 0 | 0 |
| hsa_circ_0055391 | 96 | 169 | 868 | 1 | 700 | 0 | 0 |
| hsa_circ_0002088 | 95 | 1 | 201 | 1 | 201 | 0 | 1.00E-83 |
| hsa_circ_0002088 | 94 | 1 | 458 | 1 | 458 | 0 | 0 |
| hsa_circ_0055417 | 95 | 79 | 994 | 1 | 916 | 0 | 0 |
| hsa_circ_0081811 | 98 | 345 | 530 | 74 | 259 | 0 | 3.00E-88 |
| hsa_circ_0081811 | 98 | 345 | 956 | 74 | 685 | 0 | 0 |
| hsa_circ_0081811 | 97 | 345 | 1187 | 74 | 916 | 0 | 0 |
| hsa_circ_0081811 | 98 | 143 | 328 | 74 | 259 | 0 | 2.00E-88 |
| hsa_circ_0081811 | 98 | 143 | 754 | 74 | 685 | 0 | 0 |
| hsa_circ_0081811 | 97 | 143 | 985 | 74 | 916 | 0 | 0 |
| hsa_circ_0081811 | 97 | 143 | 1387 | 74 | 1318 | 0 | 0 |
| hsa_circ_0081811 | 98 | 1 | 300 | 75 | 374 | 0 | 2.00E-148 |
| hsa_circ_0081811 | 98 | 1 | 530 | 75 | 604 | 0 | 0 |
| hsa_circ_0081811 | 98 | 1 | 611 | 75 | 685 | 0 | 0 |
| hsa_circ_0081811 | 97 | 1 | 670 | 75 | 744 | 0 | 0 |
| hsa_circ_0081811 | 97 | 1 | 842 | 75 | 916 | 0 | 0 |
| hsa_circ_0081811 | 97 | 1 | 882 | 75 | 956 | 0 | 0 |
| hsa_circ_0081811 | 97 | 1 | 1244 | 75 | 1318 | 0 | 0 |
| hsa_circ_0081811 | 96 | 1 | 1472 | 75 | 1546 | 0 | 0 |
| hsa_circ_0081811 | 96 | 1 | 1836 | 75 | 1910 | 0 | 0 |
| hsa_circ_0081815 | 97 | 1 | 426 | 1 | 426 | 0 | 0 |
| hsa_circ_0081815 | 97 | 1 | 657 | 1 | 657 | 0 | 0 |
| hsa_circ_0081815 | 96 | 1 | 1059 | 1 | 1059 | 0 | 0 |
| hsa_circ_0081816 | 97 | 1 | 230 | 1 | 230 | 0 | 2.00E-107 |
| hsa_circ_0081816 | 97 | 1 | 311 | 1 | 311 | 0 | 6.00E-148 |
| hsa_circ_0081815 | 96 | 1 | 542 | 116 | 657 | 0 | 0 |
| hsa_circ_0081815 | 95 | 1 | 1437 | 116 | 1552 | 0 | 0 |
| hsa_circ_0081818 | 96 | 2 | 312 | 2 | 312 | 0 | 3.00E-141 |
| hsa_circ_0081818 | 96 | 2 | 714 | 2 | 714 | 0 | 0 |
| hsa_circ_0081818 | 96 | 2 | 942 | 2 | 942 | 0 | 0 |
| hsa_circ_0081820 | 95 | 1 | 231 | 1 | 231 | 0 | 3.00E-100 |
| hsa_circ_0081820 | 96 | 1 | 633 | 1 | 633 | 0 | 0 |
| hsa_circ_0081820 | 96 | 1 | 861 | 1 | 861 | 0 | 0 |
| hsa_circ_0081821 | 96 | 1 | 228 | 1 | 228 | 0 | 3.00E-100 |
| hsa_circ_0081818 | 94 | 1 | 493 | 715 | 1207 | 0 | 0 |
| hsa_circ_0081818 | 94 | 1 | 592 | 715 | 1306 | 0 | 0 |
| hsa_circ_0081822 | 93 | 1 | 364 | 1 | 364 | 0 | 1.00E-150 |
| hsa_circ_0081818 | 96 | 1 | 400 | 1781 | 2180 | 0 | 0 |
| hsa_circ_0081815 | 92 | 1 | 1475 | 2526 | 3997 | 0 | 0 |
| hsa_circ_0081831 | 90 | 1 | 482 | 483 | 2 | 0 | 2.00E-174 |
| hsa_circ_0081831 | 91 | 1 | 1313 | 1314 | 2 | 0 | 0 |
| hsa_circ_0081833 | 93 | 1 | 392 | 392 | 1 | 0 | 3.00E-161 |
| hsa_circ_0081827 | 93 | 1 | 547 | 1681 | 1132 | 0.01 | 0 |
| hsa_circ_0050134 | 83 | 8280 | 8745 | 478 | 3 | 0.03 | 4.00E-118 |

novel_circ_011734 novel_circ_011735 novel_circ_011736 novel_circ_011737 novel_circ_011738 novel_circ_011739 novel_circ_011740 novel_circ_011741 novel_circ_011742 novel_circ_011743 novel_circ_011744 novel_circ_011745 novel_circ_011746 novel_circ_011747 novel_circ_011748 novel_circ_011749 novel_circ_011750 novel_circ_011751 novel_circ_011752 novel_circ_011753 novel_circ_011759 novel_circ_011760 novel_circ_011761 novel_circ_011762 novel_circ_011763 novel_circ_011764 novel_circ_011765 novel_circ_011766 novel_circ_011767 novel_circ_011768 novel_circ_011769 novel_circ_011770 novel_circ_011771 novel_circ_011772 novel_circ_011773 novel_circ_011774 novel_circ_011775 novel_circ_011789 novel_circ_011790 novel_circ_011791 novel_circ_011792 novel_circ_011793 novel_circ_011794 novel_circ_011795 novel_circ_011796 novel_circ_011797 novel_circ_011798 novel_circ_011799 novel_circ_011800 novel_circ_011801 novel_circ_011802 novel_circ_011803 novel_circ_011804 novel_circ_011805

| hsa_circ_0081835 | 88 | 1 | 1167 | 2026 | 880 | 0.02 | 0 |
| --- | --- | --- | --- | --- | --- | --- | --- |
| hsa_circ_0081842 | 94 | 1 | 929 | 929 | 1 | 0 | 0 |
| hsa_circ_0081847 | 94 | 1 | 232 | 232 | 1 | 0 | 2.00E-97 |
| hsa_circ_0081847 | 95 | 1 | 520 | 520 | 1 | 0 | 0 |
| hsa_circ_0081847 | 94 | 1 | 837 | 837 | 1 | 0 | 0 |
| hsa_circ_0081847 | 93 | 1 | 940 | 1172 | 233 | 0 | 0 |
| hsa_circ_0081852 | 92 | 1 | 652 | 652 | 1 | 0 | 0 |
| hsa_circ_0081856 | 88 | 1 | 952 | 1207 | 256 | 0.01 | 0 |
| hsa_circ_0081856 | 87 | 1 | 762 | 1207 | 446 | 0.01 | 0 |
| hsa_circ_0005705 | 86 | 1 | 617 | 617 | 1 | 0.01 | 0 |
| hsa_circ_0081859 | 92 | 24 | 424 | 27 | 427 | 0 | 4.00E-161 |
| hsa_circ_0081858 | 93 | 24 | 1580 | 73 | 1629 | 0 | 0 |
| hsa_circ_0081860 | 92 | 1 | 481 | 1 | 481 | 0 | 0 |
| hsa_circ_0081862 | 93 | 1 | 415 | 139 | 553 | 0 | 1.00E-175 |
| hsa_circ_0081863 | 92 | 2 | 300 | 2 | 300 | 0 | 6.00E-118 |
| hsa_circ_0081864 | 99 | 1071 | 1145 | 75 | 1 | 0 | 2.00E-29 |
| hsa_circ_0081866 | 94 | 2 | 939 | 938 | 1 | 0 | 0 |
| hsa_circ_0081867 | 94 | 2 | 624 | 623 | 1 | 0 | 0 |
| hsa_circ_0081867 | 94 | 1 | 847 | 847 | 1 | 0 | 0 |
| hsa_circ_0081865 | 87 | 2487 | 3744 | 1589 | 316 | 0.02 | 0 |
| hsa_circ_0076906 | 92 | 1 | 327 | 1 | 327 | 0 | 4.00E-130 |
| hsa_circ_0076900 | 90 | 1 | 233 | 1 | 233 | 0.01 | 2.00E-81 |
| hsa_circ_0076893 | 89 | 146 | 679 | 9 | 546 | 0.01 | 0 |
| hsa_circ_0004119 | 91 | 1 | 456 | 639 | 177 | 0.02 | 1.00E-176 |
| hsa_circ_0004119 | 91 | 1 | 456 | 639 | 177 | 0.02 | 1.00E-176 |
| hsa_circ_0004119 | 92 | 1 | 632 | 639 | 1 | 0.01 | 0 |
| hsa_circ_0004119 | 93 | 20 | 449 | 606 | 177 | 0 | 1.00E-175 |
| hsa_circ_0076889 | 83 | 1 | 1041 | 8597 | 9652 | 0.06 | 0 |
| hsa_circ_0076887 | 95 | 1 | 531 | 1753 | 2283 | 0 | 0 |
| hsa_circ_0076887 | 90 | 1 | 2135 | 149 | 2283 | 0.01 | 0 |
| hsa_circ_0076886 | 90 | 517 | 2953 | 1 | 2437 | 0 | 0 |
| hsa_circ_0076886 | 90 | 517 | 2884 | 1 | 2368 | 0.01 | 0 |
| hsa_circ_0076887 | 95 | 1 | 275 | 1753 | 2027 | 0 | 1.00E-119 |
| hsa_circ_0076887 | 88 | 1 | 1604 | 149 | 1752 | 0.01 | 0 |
| hsa_circ_0076886 | 88 | 517 | 2422 | 1 | 1906 | 0.01 | 0 |
| hsa_circ_0007587 | 91 | 1 | 516 | 1 | 516 | 0 | 0 |
| hsa_circ_0007587 | 91 | 1 | 387 | 1 | 387 | 0 | 4.00E-150 |
| hsa_circ_0076873 | 91 | 1 | 201 | 201 | 1 | 0 | 5.00E-72 |
| hsa_circ_0002132 | 95 | 202 | 409 | 208 | 1 | 0 | 3.00E-87 |
| hsa_circ_0006880 | 94 | 1 | 90 | 4717 | 4628 | 0 | 4.00E-32 |
| hsa_circ_0006880 | 98 | 166 | 261 | 96 | 1 | 0 | 3.00E-40 |
| hsa_circ_0076872 | 92 | 260 | 428 | 169 | 1 | 0 | 2.00E-60 |
| hsa_circ_0076868 | 92 | 92 | 261 | 19678 | 19509 | 0 | 6.00E-61 |
| hsa_circ_0076872 | 92 | 1 | 167 | 167 | 1 | 0 | 7.00E-60 |
| hsa_circ_0076868 | 92 | 1 | 168 | 19676 | 19509 | 0 | 5.00E-60 |
| hsa_circ_0076868 | 92 | 1 | 168 | 19676 | 19509 | 0 | 7.00E-60 |
| hsa_circ_0076868 | 92 | 1 | 168 | 19676 | 19509 | 0 | 1.00E-59 |
| hsa_circ_0076867 | 92 | 730 | 980 | 251 | 1 | 0 | 9.00E-96 |
| hsa_circ_0076867 | 98 | 221 | 340 | 6317 | 6198 | 0 | 2.00E-53 |
| hsa_circ_0076867 | 98 | 1 | 121 | 6317 | 6197 | 0 | 6.00E-54 |
| hsa_circ_0076867 | 92 | 343 | 593 | 251 | 1 | 0 | 6.00E-96 |
| hsa_circ_0076862 | 92 | 343 | 1626 | 2946 | 1663 | 0 | 0 |
| hsa_circ_0076863 | 93 | 1 | 1778 | 2115 | 338 | 0 | 0 |
| hsa_circ_0076863 | 93 | 1 | 1936 | 2115 | 180 | 0 | 0 |

novel_circ_011806 novel_circ_011807 novel_circ_011808 novel_circ_011809 novel_circ_011810 novel_circ_011811 novel_circ_011812 novel_circ_011813 novel_circ_011814 novel_circ_011815 novel_circ_011816 novel_circ_011817 novel_circ_011818 novel_circ_011819 novel_circ_011820 novel_circ_011821 novel_circ_011822 novel_circ_011823 novel_circ_011824 novel_circ_011825 novel_circ_011826 novel_circ_011827 novel_circ_011828 novel_circ_011829 novel_circ_011830 novel_circ_011831 novel_circ_011832 novel_circ_011833 novel_circ_011834 novel_circ_011835 novel_circ_011836 novel_circ_011847 novel_circ_011848 novel_circ_011849 novel_circ_011850 novel_circ_011851 novel_circ_011852 novel_circ_011853 novel_circ_011854 novel_circ_011857 novel_circ_011858 novel_circ_011859 novel_circ_011860 novel_circ_011861 novel_circ_011862 novel_circ_011863 novel_circ_011864 novel_circ_011867 novel_circ_011869 novel_circ_011870 novel_circ_011871 novel_circ_011872 novel_circ_011873 novel_circ_011874

| hsa_circ_0076862 | 93 | 1 | 2254 | 2695 | 442 | 0 | 0 |
| --- | --- | --- | --- | --- | --- | --- | --- |
| hsa_circ_0076863 | 94 | 1 | 745 | 1082 | 338 | 0 | 0 |
| hsa_circ_0076862 | 94 | 1 | 1221 | 1662 | 442 | 0 | 0 |
| hsa_circ_0076862 | 94 | 1 | 1415 | 1662 | 248 | 0 | 0 |
| hsa_circ_0006287 | 93 | 1 | 434 | 435 | 2 | 0 | 0 |
| hsa_circ_0076863 | 94 | 1 | 619 | 956 | 338 | 0 | 0 |
| hsa_circ_0076863 | 94 | 1 | 777 | 956 | 180 | 0 | 0 |
| hsa_circ_0076862 | 94 | 1 | 1095 | 1536 | 442 | 0 | 0 |
| hsa_circ_0076862 | 94 | 1 | 1289 | 1536 | 248 | 0 | 0 |
| hsa_circ_0076862 | 94 | 1 | 476 | 917 | 442 | 0 | 0 |
| hsa_circ_0076838 | 91 | 1 | 1501 | 3595 | 2110 | 0.01 | 0 |
| hsa_circ_0076838 | 91 | 1 | 1348 | 3442 | 2110 | 0.01 | 0 |
| hsa_circ_0076838 | 91 | 4 | 1114 | 3205 | 2110 | 0.01 | 0 |
| hsa_circ_0076840 | 89 | 1 | 546 | 708 | 178 | 0.03 | 0 |
| hsa_circ_0076838 | 91 | 1 | 847 | 2941 | 2110 | 0.02 | 0 |
| hsa_circ_0076838 | 93 | 1 | 981 | 2109 | 1132 | 0 | 0 |
| hsa_circ_0076837 | 89 | 1 | 1781 | 3440 | 1636 | 0.01 | 0 |
| hsa_circ_0076837 | 93 | 4 | 1521 | 1965 | 448 | 0 | 0 |
| hsa_circ_0076833 | 92 | 1 | 990 | 990 | 1 | 0 | 0 |
| hsa_circ_0076829 | 95 | 1 | 327 | 327 | 1 | 0 | 8.00E-147 |
| hsa_circ_0076821 | 94 | 1 | 981 | 1635 | 655 | 0 | 0 |
| hsa_circ_0076822 | 94 | 1 | 534 | 534 | 1 | 0 | 0 |
| hsa_circ_0076822 | 94 | 1 | 534 | 534 | 1 | 0 | 0 |
| hsa_circ_0076821 | 94 | 2 | 813 | 1136 | 325 | 0 | 0 |
| hsa_circ_0076821 | 95 | 1 | 330 | 654 | 325 | 0 | 2.00E-143 |
| hsa_circ_0076821 | 95 | 1 | 324 | 324 | 1 | 0 | 4.00E-145 |
| hsa_circ_0076816 | 92 | 4 | 591 | 1146 | 559 | 0 | 0 |
| hsa_circ_0076814 | 92 | 1 | 823 | 1023 | 201 | 0 | 0 |
| hsa_circ_0076814 | 92 | 1 | 366 | 566 | 201 | 0 | 4.00E-145 |
| hsa_circ_0076812 | 93 | 1 | 801 | 801 | 1 | 0 | 0 |
| hsa_circ_0076810 | 93 | 1 | 914 | 1834 | 921 | 0 | 0 |
| hsa_circ_0082094 | 91 | 260 | 681 | 422 | 1 | 0 | 3.00E-164 |
| hsa_circ_0082093 | 91 | 260 | 837 | 578 | 1 | 0 | 0 |
| hsa_circ_0082093 | 91 | 163 | 740 | 578 | 1 | 0 | 0 |
| hsa_circ_0082094 | 91 | 1 | 422 | 422 | 1 | 0 | 2.00E-164 |
| hsa_circ_0082093 | 91 | 1 | 578 | 578 | 1 | 0 | 0 |
| hsa_circ_0082092 | 91 | 1 | 748 | 1159 | 415 | 0 | 0 |
| hsa_circ_0082091 | 87 | 260 | 815 | 556 | 1 | 0 | 1.00E-178 |
| hsa_circ_0082091 | 87 | 260 | 815 | 556 | 1 | 0 | 1.00E-178 |
| hsa_circ_0002765 | 87 | 17022 | 17097 | 13939 | 14014 | 0 | 7.00E-14 |
| hsa_circ_0082088 | 96 | 1 | 319 | 709 | 391 | 0 | 1.00E-145 |
| hsa_circ_0082088 | 96 | 1 | 332 | 709 | 378 | 0 | 3.00E-151 |
| hsa_circ_0082088 | 95 | 1 | 512 | 709 | 198 | 0 | 0 |
| hsa_circ_0082088 | 95 | 1 | 709 | 709 | 1 | 0 | 0 |
| hsa_circ_0082088 | 95 | 1 | 709 | 709 | 1 | 0 | 0 |
| hsa_circ_0082088 | 95 | 1 | 709 | 709 | 1 | 0 | 0 |
| hsa_circ_0082088 | 95 | 1 | 709 | 709 | 1 | 0 | 0 |
| hsa_circ_0029815 | 86 | 6314 | 6842 | 3 | 543 | 0.02 | 4.00E-159 |
| hsa_circ_0082081 | 93 | 4 | 414 | 10625 | 10215 | 0 | 1.00E-171 |
| hsa_circ_0082081 | 94 | 4 | 651 | 10625 | 9978 | 0 | 0 |
| hsa_circ_0082081 | 94 | 4 | 773 | 10625 | 9859 | 0 | 0 |
| hsa_circ_0082081 | 94 | 4 | 885 | 10625 | 9747 | 0 | 0 |
| hsa_circ_0082081 | 95 | 1 | 440 | 10295 | 9859 | 0.01 | 0 |
| hsa_circ_0082081 | 94 | 1 | 552 | 10295 | 9747 | 0.01 | 0 |

novel_circ_011875 novel_circ_011876 novel_circ_011877 novel_circ_011878 novel_circ_011879 novel_circ_011880 novel_circ_011881 novel_circ_011882 novel_circ_011883 novel_circ_011884 novel_circ_011885 novel_circ_011886 novel_circ_011888 novel_circ_011889 novel_circ_011890 novel_circ_011891 novel_circ_011892 novel_circ_011893 novel_circ_011894 novel_circ_011895 novel_circ_011896 novel_circ_011897 novel_circ_011898 novel_circ_011899 novel_circ_011900 novel_circ_011901 novel_circ_011902 novel_circ_011903 novel_circ_011904 novel_circ_011905 novel_circ_011906 novel_circ_011907 novel_circ_011908 novel_circ_011909 novel_circ_011910 novel_circ_011911 novel_circ_011912 novel_circ_011913 novel_circ_011916 novel_circ_011917 novel_circ_011918 novel_circ_011919 novel_circ_011920 novel_circ_011921 novel_circ_011922 novel_circ_011923 novel_circ_011924 novel_circ_011925 novel_circ_011929 novel_circ_011930 novel_circ_011931 novel_circ_011937 novel_circ_011938 novel_circ_011939

| hsa_circ_0082081 | 94 | 1 | 692 | 10295 | 9607 | 0 | 0 |
| --- | --- | --- | --- | --- | --- | --- | --- |
| hsa_circ_0082081 | 95 | 1 | 359 | 10214 | 9859 | 0.01 | 8.00E-157 |
| hsa_circ_0082081 | 95 | 1 | 359 | 10214 | 9859 | 0.01 | 2.00E-155 |
| hsa_circ_0082081 | 94 | 1 | 471 | 10214 | 9747 | 0.01 | 0 |
| hsa_circ_0082081 | 94 | 1 | 611 | 10214 | 9607 | 0 | 0 |
| hsa_circ_0082081 | 92 | 1 | 374 | 9977 | 9607 | 0.01 | 2.00E-148 |
| hsa_circ_0082081 | 92 | 1 | 252 | 9858 | 9607 | 0 | 1.00E-98 |
| hsa_circ_0082081 | 93 | 1 | 319 | 9858 | 9540 | 0 | 1.00E-130 |
| hsa_circ_0082081 | 92 | 4 | 515 | 9743 | 9232 | 0 | 0 |
| hsa_circ_0082081 | 92 | 4 | 515 | 9743 | 9232 | 0 | 0 |
| hsa_circ_0082081 | 92 | 4 | 515 | 9743 | 9232 | 0 | 0 |
| hsa_circ_0082081 | 92 | 1 | 375 | 9606 | 9232 | 0 | 4.00E-148 |
| hsa_circ_0082080 | 97 | 460 | 644 | 1398 | 1214 | 0 | 1.00E-82 |
| hsa_circ_0082080 | 96 | 460 | 746 | 1398 | 1112 | 0 | 7.00E-131 |
| hsa_circ_0082080 | 95 | 460 | 1225 | 1398 | 642 | 0.01 | 0 |
| hsa_circ_0082080 | 96 | 351 | 637 | 1398 | 1112 | 0 | 6.00E-131 |
| hsa_circ_0082080 | 95 | 20661 | 21244 | 1216 | 642 | 0.02 | 0 |
| hsa_circ_0082080 | 95 | 1 | 581 | 1213 | 642 | 0.02 | 0 |
| hsa_circ_0082080 | 94 | 1 | 1147 | 1213 | 76 | 0.01 | 0 |
| hsa_circ_0082080 | 93 | 1 | 1237 | 1213 | 1 | 0.02 | 0 |
| hsa_circ_0082080 | 98 | 1 | 313 | 1111 | 799 | 0 | 4.00E-154 |
| hsa_circ_0082080 | 92 | 1 | 270 | 902 | 642 | 0.03 | 9.00E-101 |
| hsa_circ_0082080 | 93 | 1 | 412 | 902 | 500 | 0.02 | 7.00E-168 |
| hsa_circ_0082080 | 93 | 1 | 836 | 902 | 76 | 0.01 | 0 |
| hsa_circ_0082080 | 91 | 1 | 926 | 902 | 1 | 0.03 | 0 |
| hsa_circ_0082080 | 91 | 1 | 926 | 902 | 1 | 0.03 | 0 |
| hsa_circ_0082080 | 91 | 1 | 926 | 902 | 1 | 0.03 | 0 |
| hsa_circ_0082080 | 91 | 1 | 926 | 902 | 1 | 0.03 | 0 |
| hsa_circ_0082080 | 95 | 4 | 419 | 795 | 380 | 0 | 0 |
| hsa_circ_0082080 | 94 | 4 | 723 | 795 | 76 | 0 | 0 |
| hsa_circ_0082080 | 92 | 4 | 813 | 795 | 1 | 0.02 | 0 |
| hsa_circ_0082080 | 94 | 1 | 566 | 641 | 76 | 0 | 0 |
| hsa_circ_0082080 | 91 | 1 | 656 | 641 | 1 | 0.02 | 0 |
| hsa_circ_0082080 | 91 | 1 | 656 | 641 | 1 | 0.02 | 0 |
| hsa_circ_0082080 | 88 | 1 | 394 | 379 | 1 | 0.04 | 4.00E-126 |
| hsa_circ_0082080 | 88 | 1 | 394 | 379 | 1 | 0.04 | 5.00E-126 |
| hsa_circ_0082080 | 88 | 1 | 394 | 379 | 1 | 0.04 | 6.00E-126 |
| hsa_circ_0082080 | 88 | 1 | 394 | 379 | 1 | 0.04 | 7.00E-126 |
| hsa_circ_0082076 | 92 | 14 | 1293 | 1280 | 1 | 0 | 0 |
| hsa_circ_0082061 | 93 | 1 | 555 | 5140 | 5694 | 0 | 0 |
| hsa_circ_0082061 | 92 | 1 | 591 | 4825 | 5415 | 0 | 0 |
| hsa_circ_0082061 | 93 | 1 | 1540 | 3876 | 5415 | 0 | 0 |
| hsa_circ_0082061 | 93 | 1 | 1096 | 3876 | 4971 | 0 | 0 |
| hsa_circ_0082061 | 93 | 1 | 949 | 3876 | 4824 | 0 | 0 |
| hsa_circ_0082061 | 96 | 1 | 379 | 3876 | 4254 | 0 | 2.00E-172 |
| hsa_circ_0082061 | 82 | 1 | 1023 | 128 | 1150 | 0.05 | 0 |
| hsa_circ_0082061 | 83 | 1077 | 2226 | 1 | 1150 | 0.04 | 0 |
| hsa_circ_0082061 | 91 | 679 | 805 | 1 | 127 | 0 | 6.00E-42 |
| hsa_circ_0082058 | 96 | 1 | 314 | 313 | 1 | 0 | 1.00E-140 |
| hsa_circ_0082056 | 96 | 1 | 509 | 508 | 1 | 0 | 0 |
| hsa_circ_0082046 | 87 | 1 | 1131 | 1 | 1137 | 0.01 | 0 |
| hsa_circ_0029310 | 94 | 1 | 667 | 667 | 1 | 0 | 0 |
| hsa_circ_0029306 | 90 | 1 | 1040 | 1061 | 1 | 0.02 | 0 |
| hsa_circ_0029303 | 90 | 1 | 1042 | 1352 | 290 | 0.02 | 0 |

novel_circ_011940 novel_circ_011941 novel_circ_011942 novel_circ_011943 novel_circ_011948 novel_circ_011949 novel_circ_011950 novel_circ_011951 novel_circ_011953 novel_circ_011955 novel_circ_011956 novel_circ_011957 novel_circ_011958 novel_circ_011959 novel_circ_011960 novel_circ_011961 novel_circ_011962 novel_circ_011963 novel_circ_011964 novel_circ_011965 novel_circ_011967 novel_circ_011968 novel_circ_011969 novel_circ_011970 novel_circ_011971 novel_circ_011974 novel_circ_011975 novel_circ_011976 novel_circ_011977 novel_circ_011978 novel_circ_011979 novel_circ_011980 novel_circ_011982 novel_circ_011983 novel_circ_011984 novel_circ_011985 novel_circ_011986 novel_circ_011987 novel_circ_011988 novel_circ_011991 novel_circ_011992 novel_circ_011993 novel_circ_011994 novel_circ_011995 novel_circ_011997 novel_circ_011998 novel_circ_011999 novel_circ_012000 novel_circ_012001 novel_circ_012003 novel_circ_012005 novel_circ_012006 novel_circ_012007 novel_circ_012008

| hsa_circ_0029303 | 90 | 1 | 1183 | 1352 | 149 | 0.02 | 0 |
| --- | --- | --- | --- | --- | --- | --- | --- |
| hsa_circ_0029310 | 94 | 1 | 600 | 600 | 1 | 0 | 0 |
| hsa_circ_0029300 | 82 | 3 | 954 | 2481 | 1557 | 0.05 | 0 |
| hsa_circ_0002783 | 80 | 1 | 811 | 788 | 5 | 0.05 | 2.00E-162 |
| hsa_circ_0029272 | 79 | 9 | 575 | 278 | 844 | 0 | 7.00E-110 |
| hsa_circ_0029272 | 79 | 1 | 844 | 1 | 844 | 0 | 6.00E-167 |
| hsa_circ_0029266 | 80 | 264 | 1407 | 195 | 1341 | 0.01 | 0 |
| hsa_circ_0029267 | 87 | 4 | 196 | 1 | 193 | 0 | 3.00E-55 |
| hsa_circ_0029238 | 90 | 2 | 340 | 157 | 495 | 0 | 2.00E-123 |
| hsa_circ_0029225 | 81 | 1 | 492 | 799 | 292 | 0.04 | 3.00E-109 |
| hsa_circ_0002510 | 88 | 1 | 562 | 550 | 3 | 0.02 | 0 |
| hsa_circ_0029218 | 90 | 1 | 762 | 1875 | 1128 | 0.02 | 0 |
| hsa_circ_0029218 | 91 | 1 | 508 | 1635 | 1128 | 0 | 0 |
| hsa_circ_0029218 | 94 | 1 | 386 | 1127 | 742 | 0 | 2.00E-164 |
| hsa_circ_0029210 | 93 | 1 | 1225 | 2472 | 1248 | 0 | 0 |
| hsa_circ_0029210 | 92 | 1 | 1743 | 2472 | 730 | 0 | 0 |
| hsa_circ_0029219 | 93 | 1 | 558 | 558 | 1 | 0 | 0 |
| hsa_circ_0029215 | 92 | 1 | 839 | 991 | 153 | 0 | 0 |
| hsa_circ_0029210 | 91 | 1 | 826 | 1247 | 422 | 0 | 0 |
| hsa_circ_0029210 | 92 | 1 | 1247 | 1247 | 1 | 0 | 0 |
| hsa_circ_0029212 | 91 | 1 | 444 | 629 | 186 | 0 | 1.00E-171 |
| hsa_circ_0029210 | 92 | 1 | 729 | 729 | 1 | 0 | 0 |
| hsa_circ_0029204 | 92 | 1 | 1663 | 8314 | 6650 | 0 | 0 |
| hsa_circ_0029209 | 93 | 1 | 496 | 831 | 336 | 0 | 0 |
| hsa_circ_0029209 | 95 | 6 | 334 | 330 | 2 | 0 | 6.00E-143 |
| hsa_circ_0029192 | 90 | 5 | 459 | 456 | 2 | 0 | 8.00E-168 |
| hsa_circ_0029183 | 87 | 5 | 986 | 1123 | 142 | 0.01 | 0 |
| hsa_circ_0029183 | 87 | 5 | 990 | 1123 | 139 | 0.01 | 0 |
| hsa_circ_0029169 | 82 | 6 | 312 | 400 | 97 | 0.03 | 6.00E-68 |
| hsa_circ_0029161 | 94 | 2 | 327 | 326 | 1 | 0 | 7.00E-136 |
| hsa_circ_0029160 | 95 | 1 | 189 | 523 | 335 | 0 | 5.00E-80 |
| hsa_circ_0029160 | 95 | 1 | 189 | 523 | 335 | 0 | 5.00E-80 |
| hsa_circ_0029073 | 88 | 293 | 611 | 321 | 3 | 0.01 | 2.00E-105 |
| hsa_circ_0029073 | 94 | 150 | 352 | 321 | 119 | 0 | 7.00E-83 |
| hsa_circ_0005465 | 84 | 1 | 746 | 763 | 5 | 0.03 | 0 |
| hsa_circ_0029063 | 85 | 1 | 1137 | 4451 | 3315 | 0 | 0 |
| hsa_circ_0029065 | 84 | 1 | 2088 | 2141 | 54 | 0 | 0 |
| hsa_circ_0029061 | 89 | 4 | 432 | 604 | 176 | 0 | 5.00E-150 |
| hsa_circ_0029049 | 92 | 356 | 607 | 1 | 252 | 0 | 8.00E-95 |
| hsa_circ_0029045 | 83 | 1 | 1226 | 1 | 1229 | 0 | 0 |
| hsa_circ_0029045 | 83 | 1 | 1226 | 1 | 1229 | 0 | 0 |
| hsa_circ_0029045 | 83 | 1 | 954 | 1 | 957 | 0 | 0 |
| hsa_circ_0029045 | 84 | 1 | 588 | 1 | 591 | 0.01 | 4.00E-162 |
| hsa_circ_0008670 | 91 | 1 | 314 | 104 | 417 | 0 | 6.00E-118 |
| hsa_circ_0029010 | 94 | 1 | 311 | 1 | 311 | 0 | 1.00E-129 |
| hsa_circ_0029003 | 88 | 1 | 233 | 1 | 233 | 0.01 | 5.00E-73 |
| hsa_circ_0028990 | 95 | 1 | 248 | 248 | 1 | 0 | 2.00E-106 |
| hsa_circ_0004513 | 93 | 1 | 408 | 220 | 627 | 0 | 2.00E-166 |
| hsa_circ_0004513 | 93 | 1 | 627 | 1 | 627 | 0 | 0 |
| hsa_circ_0028946 | 95 | 1 | 505 | 3956 | 3452 | 0 | 0 |
| hsa_circ_0028169 | 94 | 270 | 450 | 1 | 181 | 0 | 3.00E-72 |
| hsa_circ_0028169 | 94 | 270 | 538 | 1 | 269 | 0 | 2.00E-114 |
| hsa_circ_0028169 | 94 | 270 | 802 | 1 | 533 | 0 | 0 |
| hsa_circ_0028169 | 94 | 1 | 448 | 1 | 448 | 0 | 0 |

novel_circ_012009 novel_circ_012010 novel_circ_012011 novel_circ_012012 novel_circ_012013 novel_circ_012014 novel_circ_012015 novel_circ_012016 novel_circ_012017 novel_circ_012018 novel_circ_012019 novel_circ_012020 novel_circ_012021 novel_circ_012023 novel_circ_012024 novel_circ_012025 novel_circ_012026 novel_circ_012027 novel_circ_012028 novel_circ_012029 novel_circ_012030 novel_circ_012031 novel_circ_012032 novel_circ_012033 novel_circ_012034 novel_circ_012035 novel_circ_012036 novel_circ_012037 novel_circ_012038 novel_circ_012039 novel_circ_012040 novel_circ_012041 novel_circ_012042 novel_circ_012043 novel_circ_012044 novel_circ_012045 novel_circ_012046 novel_circ_012047 novel_circ_012048 novel_circ_012049 novel_circ_012050 novel_circ_012051 novel_circ_012052 novel_circ_012053 novel_circ_012054 novel_circ_012055 novel_circ_012056 novel_circ_012057 novel_circ_012058 novel_circ_012059 novel_circ_012060 novel_circ_012061 novel_circ_012062 novel_circ_012063

| hsa_circ_0004427 | 93 | 151 | 369 | 1 | 219 | 0 | 1.00E-84 |
| --- | --- | --- | --- | --- | --- | --- | --- |
| hsa_circ_0004427 | 93 | 1 | 219 | 1 | 219 | 0 | 3.00E-85 |
| hsa_circ_0028174 | 93 | 1 | 554 | 87 | 640 | 0 | 0 |
| hsa_circ_0028174 | 92 | 1 | 788 | 87 | 874 | 0 | 0 |
| hsa_circ_0028175 | 91 | 1 | 678 | 1 | 678 | 0 | 0 |
| hsa_circ_0028182 | 95 | 1 | 1407 | 1407 | 1 | 0 | 0 |
| hsa_circ_0005785 | 94 | 1 | 1031 | 1031 | 1 | 0 | 0 |
| hsa_circ_0028190 | 93 | 1 | 527 | 527 | 1 | 0 | 0 |
| hsa_circ_0028190 | 94 | 1 | 647 | 647 | 1 | 0 | 0 |
| hsa_circ_0028190 | 94 | 1 | 834 | 834 | 1 | 0 | 0 |
| hsa_circ_0028191 | 95 | 1 | 716 | 716 | 1 | 0 | 0 |
| hsa_circ_0028194 | 95 | 1 | 573 | 573 | 1 | 0 | 0 |
| hsa_circ_0028199 | 95 | 1 | 307 | 307 | 1 | 0 | 2.00E-137 |
| hsa_circ_0004839 | 94 | 1 | 235 | 317 | 83 | 0 | 3.00E-99 |
| hsa_circ_0012625 | 94 | 1 | 218 | 540 | 321 | 0.01 | 2.00E-90 |
| hsa_circ_0012625 | 93 | 1 | 287 | 540 | 252 | 0.01 | 3.00E-115 |
| hsa_circ_0012623 | 94 | 1 | 169 | 169 | 1 | 0 | 1.00E-67 |
| hsa_circ_0012622 | 95 | 1 | 286 | 636 | 351 | 0 | 4.00E-124 |
| hsa_circ_0012622 | 92 | 1 | 636 | 636 | 1 | 0 | 0 |
| hsa_circ_0012616 | 88 | 1 | 644 | 834 | 191 | 0 | 0 |
| hsa_circ_0012615 | 88 | 1 | 927 | 927 | 1 | 0 | 0 |
| hsa_circ_0012615 | 89 | 1 | 806 | 806 | 1 | 0 | 0 |
| hsa_circ_0012605 | 91 | 1 | 816 | 816 | 1 | 0 | 0 |
| hsa_circ_0012605 | 91 | 1 | 734 | 734 | 1 | 0 | 0 |
| hsa_circ_0012605 | 91 | 1 | 578 | 578 | 1 | 0 | 0 |
| hsa_circ_0002079 | 87 | 1 | 1067 | 1061 | 1 | 0.01 | 0 |
| hsa_circ_0012591 | 90 | 121 | 908 | 788 | 1 | 0 | 0 |
| hsa_circ_0012591 | 91 | 1 | 662 | 662 | 1 | 0 | 0 |
| hsa_circ_0012590 | 92 | 1 | 804 | 804 | 1 | 0 | 0 |
| hsa_circ_0012588 | 89 | 1 | 292 | 294 | 3 | 0 | 2.00E-97 |
| hsa_circ_0012585 | 92 | 279 | 578 | 1 | 300 | 0 | 3.00E-116 |
| hsa_circ_0012585 | 92 | 279 | 578 | 1 | 300 | 0 | 2.00E-116 |
| hsa_circ_0012562 | 95 | 1 | 297 | 513 | 809 | 0 | 2.00E-128 |
| hsa_circ_0012562 | 96 | 1 | 349 | 262 | 610 | 0 | 5.00E-159 |
| hsa_circ_0012557 | 93 | 281 | 1094 | 257 | 1073 | 0 | 0 |
| hsa_circ_0012557 | 93 | 447 | 1260 | 257 | 1073 | 0 | 0 |
| hsa_circ_0012557 | 94 | 281 | 779 | 257 | 755 | 0 | 0 |
| hsa_circ_0012553 | 87 | 147 | 650 | 941 | 447 | 0.02 | 1.00E-158 |
| hsa_circ_0012553 | 89 | 671 | 1159 | 487 | 1 | 0.02 | 2.00E-172 |
| hsa_circ_0012551 | 90 | 671 | 1276 | 1120 | 517 | 0.02 | 0 |
| hsa_circ_0012551 | 91 | 671 | 1454 | 1120 | 339 | 0.01 | 0 |
| hsa_circ_0012551 | 92 | 671 | 1592 | 1120 | 200 | 0.01 | 0 |
| hsa_circ_0012553 | 87 | 25 | 528 | 941 | 447 | 0.02 | 1.00E-158 |
| hsa_circ_0012553 | 89 | 549 | 1037 | 487 | 1 | 0.02 | 2.00E-172 |
| hsa_circ_0012551 | 90 | 549 | 1154 | 1120 | 517 | 0.02 | 0 |
| hsa_circ_0012551 | 91 | 549 | 1332 | 1120 | 339 | 0.01 | 0 |
| hsa_circ_0012551 | 92 | 549 | 1421 | 1120 | 250 | 0.01 | 0 |
| hsa_circ_0012551 | 92 | 549 | 1470 | 1120 | 200 | 0.01 | 0 |
| hsa_circ_0012551 | 95 | 1 | 459 | 797 | 339 | 0 | 0 |
| hsa_circ_0012539 | 95 | 1 | 917 | 2513 | 1597 | 0 | 0 |
| hsa_circ_0012551 | 95 | 1 | 516 | 516 | 1 | 0 | 0 |
| hsa_circ_0012541 | 96 | 1 | 849 | 4946 | 4098 | 0 | 0 |
| hsa_circ_0012541 | 96 | 1 | 1024 | 4946 | 3923 | 0 | 0 |
| hsa_circ_0012541 | 92 | 1 | 1888 | 4946 | 3068 | 0.01 | 0 |

novel_circ_012064 novel_circ_012065 novel_circ_012066 novel_circ_012067 novel_circ_012068 novel_circ_012069 novel_circ_012070 novel_circ_012071 novel_circ_012072 novel_circ_012073 novel_circ_012074 novel_circ_012075 novel_circ_012076 novel_circ_012077 novel_circ_012078 novel_circ_012079 novel_circ_012080 novel_circ_012081 novel_circ_012082 novel_circ_012083 novel_circ_012084 novel_circ_012085 novel_circ_012086 novel_circ_012087 novel_circ_012088 novel_circ_012089 novel_circ_012090 novel_circ_012091 novel_circ_012092 novel_circ_012093 novel_circ_012094 novel_circ_012095 novel_circ_012096 novel_circ_012097 novel_circ_012098 novel_circ_012099 novel_circ_012100 novel_circ_012101 novel_circ_012102 novel_circ_012103 novel_circ_012104 novel_circ_012105 novel_circ_012106 novel_circ_012107 novel_circ_012108 novel_circ_012109 novel_circ_012110 novel_circ_012111 novel_circ_012112 novel_circ_012113 novel_circ_012114 novel_circ_012115 novel_circ_012116 novel_circ_012117

| hsa_circ_0012541 | 92 | 3 | 1710 | 4766 | 3068 | 0.01 | 0 |
| --- | --- | --- | --- | --- | --- | --- | --- |
| hsa_circ_0012541 | 92 | 1 | 1621 | 4679 | 3068 | 0.01 | 0 |
| hsa_circ_0012541 | 89 | 1 | 1039 | 4097 | 3068 | 0.02 | 0 |
| hsa_circ_0012532 | 96 | 1 | 1211 | 2143 | 933 | 0 | 0 |
| hsa_circ_0012539 | 96 | 1 | 353 | 353 | 1 | 0 | 3.00E-161 |
| hsa_circ_0012537 | 96 | 1 | 443 | 557 | 115 | 0 | 0 |
| hsa_circ_0012537 | 96 | 1 | 557 | 557 | 1 | 0 | 0 |
| hsa_circ_0012532 | 96 | 1 | 944 | 1992 | 1049 | 0 | 0 |
| hsa_circ_0012532 | 96 | 1 | 1060 | 1992 | 933 | 0 | 0 |
| hsa_circ_0012532 | 95 | 1 | 1284 | 1992 | 709 | 0 | 0 |
| hsa_circ_0012532 | 95 | 1 | 1385 | 1992 | 608 | 0 | 0 |
| hsa_circ_0012532 | 94 | 1 | 1833 | 1992 | 160 | 0 | 0 |
| hsa_circ_0012532 | 94 | 1 | 1992 | 1992 | 1 | 0 | 0 |
| hsa_circ_0012532 | 94 | 1 | 1992 | 1992 | 1 | 0 | 0 |
| hsa_circ_0012532 | 94 | 1 | 1992 | 1992 | 1 | 0 | 0 |
| hsa_circ_0012537 | 96 | 1 | 356 | 470 | 115 | 0 | 3.00E-161 |
| hsa_circ_0012535 | 96 | 1 | 387 | 503 | 117 | 0 | 9.00E-177 |
| hsa_circ_0012535 | 96 | 1 | 503 | 503 | 1 | 0 | 0 |
| hsa_circ_0012532 | 94 | 1 | 828 | 1435 | 608 | 0 | 0 |
| hsa_circ_0012535 | 96 | 1 | 425 | 425 | 1 | 0 | 0 |
| hsa_circ_0012532 | 93 | 1 | 441 | 1048 | 608 | 0 | 0 |
| hsa_circ_0012532 | 92 | 1 | 889 | 1048 | 160 | 0 | 0 |
| hsa_circ_0012532 | 93 | 1 | 607 | 607 | 1 | 0 | 0 |
| hsa_circ_0012528 | 95 | 253 | 398 | 2 | 147 | 0 | 2.00E-59 |
| hsa_circ_0012528 | 95 | 339 | 484 | 2 | 147 | 0 | 3.00E-59 |
| hsa_circ_0012500 | 92 | 1 | 446 | 338 | 783 | 0 | 2.00E-179 |
| hsa_circ_0012502 | 94 | 1 | 501 | 1 | 501 | 0 | 0 |
| hsa_circ_0012502 | 95 | 1 | 395 | 1 | 395 | 0 | 2.00E-174 |
| hsa_circ_0012491 | 95 | 1 | 808 | 3168 | 3975 | 0 | 0 |
| hsa_circ_0012491 | 95 | 1 | 1378 | 2598 | 3975 | 0 | 0 |
| hsa_circ_0012491 | 96 | 1 | 801 | 3012 | 3812 | 0 | 0 |
| hsa_circ_0012491 | 95 | 1 | 924 | 2889 | 3812 | 0 | 0 |
| hsa_circ_0012491 | 95 | 1 | 720 | 3012 | 3731 | 0 | 0 |
| hsa_circ_0012499 | 95 | 1 | 188 | 226 | 413 | 0 | 8.00E-80 |
| hsa_circ_0012499 | 94 | 1 | 413 | 1 | 413 | 0 | 0 |
| hsa_circ_0012498 | 95 | 1 | 569 | 1 | 569 | 0 | 0 |
| hsa_circ_0012496 | 95 | 1 | 692 | 292 | 983 | 0 | 0 |
| hsa_circ_0012496 | 95 | 3 | 813 | 173 | 983 | 0 | 0 |
| hsa_circ_0012491 | 95 | 2561 | 3721 | 2420 | 3580 | 0 | 0 |
| hsa_circ_0012491 | 95 | 1 | 1260 | 2321 | 3580 | 0 | 0 |
| hsa_circ_0012491 | 92 | 1 | 3570 | 1 | 3580 | 0 | 0 |
| hsa_circ_0012499 | 95 | 1 | 307 | 1 | 307 | 0 | 4.00E-134 |
| hsa_circ_0012496 | 95 | 3 | 625 | 173 | 795 | 0 | 0 |
| hsa_circ_0012494 | 96 | 2561 | 3308 | 100 | 847 | 0 | 0 |
| hsa_circ_0012494 | 96 | 1 | 847 | 1 | 847 | 0 | 0 |
| hsa_circ_0012491 | 92 | 1 | 3157 | 1 | 3167 | 0.01 | 0 |
| hsa_circ_0012494 | 96 | 2561 | 3029 | 100 | 568 | 0 | 0 |
| hsa_circ_0012494 | 96 | 1 | 568 | 1 | 568 | 0 | 0 |
| hsa_circ_0012493 | 91 | 1 | 2773 | 1 | 2782 | 0 | 0 |
| hsa_circ_0012491 | 91 | 1 | 2878 | 1 | 2888 | 0.01 | 0 |
| hsa_circ_0012494 | 97 | 2561 | 2908 | 100 | 447 | 0 | 1.00E-163 |
| hsa_circ_0012494 | 96 | 1 | 447 | 1 | 447 | 0 | 0 |
| hsa_circ_0012493 | 90 | 1 | 2099 | 107 | 2214 | 0.01 | 0 |
| hsa_circ_0012493 | 90 | 1 | 2205 | 1 | 2214 | 0.01 | 0 |

novel_circ_012118 novel_circ_012119 novel_circ_012120 novel_circ_012121 novel_circ_012122 novel_circ_012123 novel_circ_012124 novel_circ_012125 novel_circ_012126 novel_circ_012127 novel_circ_012128 novel_circ_012129 novel_circ_012130 novel_circ_012131 novel_circ_012132 novel_circ_012133 novel_circ_012134 novel_circ_012135 novel_circ_012136 novel_circ_012137 novel_circ_012141 novel_circ_012142 novel_circ_012143 novel_circ_012144 novel_circ_012149 novel_circ_012150 novel_circ_012151 novel_circ_012152 novel_circ_012153 novel_circ_012154 novel_circ_012155 novel_circ_012156 novel_circ_012157 novel_circ_012158 novel_circ_012159 novel_circ_012160 novel_circ_012161 novel_circ_012162 novel_circ_012163 novel_circ_012165 novel_circ_012166 novel_circ_012167 novel_circ_012168 novel_circ_012169 novel_circ_012170 novel_circ_012171 novel_circ_012172 novel_circ_012173 novel_circ_012174 novel_circ_012175 novel_circ_012176 novel_circ_012177 novel_circ_012178 novel_circ_012179

| hsa_circ_0012491 | 90 | 1 | 2310 | 1 | 2320 | 0.01 | 0 |
| --- | --- | --- | --- | --- | --- | --- | --- |
| hsa_circ_0012481 | 94 | 1 | 738 | 803 | 75 | 0.01 | 0 |
| hsa_circ_0012481 | 94 | 1 | 738 | 803 | 75 | 0.01 | 0 |
| hsa_circ_0012481 | 94 | 1 | 812 | 803 | 1 | 0.01 | 0 |
| hsa_circ_0012481 | 94 | 1 | 812 | 803 | 1 | 0.01 | 0 |
| hsa_circ_0012473 | 95 | 1 | 328 | 488 | 161 | 0 | 5.00E-144 |
| hsa_circ_0012473 | 95 | 1 | 380 | 488 | 109 | 0 | 1.00E-169 |
| hsa_circ_0012470 | 94 | 1 | 1022 | 1585 | 564 | 0 | 0 |
| hsa_circ_0012470 | 93 | 1 | 1484 | 1585 | 102 | 0 | 0 |
| hsa_circ_0012470 | 93 | 1 | 985 | 1257 | 273 | 0 | 0 |
| hsa_circ_0012470 | 93 | 1 | 639 | 1202 | 564 | 0 | 0 |
| hsa_circ_0012470 | 93 | 1 | 767 | 1202 | 436 | 0 | 0 |
| hsa_circ_0012470 | 93 | 1 | 930 | 1202 | 273 | 0 | 0 |
| hsa_circ_0012470 | 93 | 1 | 1101 | 1202 | 102 | 0 | 0 |
| hsa_circ_0012470 | 93 | 1 | 662 | 1097 | 436 | 0 | 0 |
| hsa_circ_0012470 | 92 | 2 | 613 | 713 | 102 | 0 | 0 |
| hsa_circ_0012463 | 91 | 1 | 588 | 1 | 588 | 0 | 0 |
| hsa_circ_0012458 | 92 | 1 | 852 | 1 | 852 | 0 | 0 |
| hsa_circ_0012463 | 92 | 1 | 196 | 1 | 196 | 0 | 1.00E-72 |
| hsa_circ_0081991 | 92 | 1 | 360 | 498 | 857 | 0 | 2.00E-143 |
| hsa_circ_0081985 | 94 | 1 | 466 | 1 | 466 | 0 | 0 |
| hsa_circ_0081985 | 95 | 1 | 374 | 1 | 374 | 0 | 1.00E-169 |
| hsa_circ_0081985 | 95 | 4919 | 5292 | 1 | 374 | 0 | 2.00E-168 |
| hsa_circ_0081985 | 96 | 1 | 202 | 1 | 202 | 0 | 3.00E-89 |
| hsa_circ_0081983 | 97 | 1 | 278 | 427 | 150 | 0 | 2.00E-131 |
| hsa_circ_0081969 | 94 | 1 | 512 | 5355 | 4844 | 0 | 0 |
| hsa_circ_0081969 | 94 | 1 | 664 | 5355 | 4692 | 0 | 0 |
| hsa_circ_0081969 | 93 | 1 | 940 | 5355 | 4416 | 0 | 0 |
| hsa_circ_0081969 | 93 | 1 | 1029 | 5355 | 4327 | 0 | 0 |
| hsa_circ_0081969 | 92 | 1 | 1143 | 5355 | 4201 | 0.01 | 0 |
| hsa_circ_0081969 | 94 | 1 | 580 | 5271 | 4692 | 0 | 0 |
| hsa_circ_0081969 | 93 | 1 | 387 | 5230 | 4844 | 0 | 2.00E-158 |
| hsa_circ_0081969 | 94 | 1 | 539 | 5230 | 4692 | 0 | 0 |
| hsa_circ_0081969 | 93 | 1 | 483 | 5174 | 4692 | 0 | 0 |
| hsa_circ_0081969 | 92 | 1 | 848 | 5174 | 4327 | 0 | 0 |
| hsa_circ_0081982 | 93 | 7 | 386 | 380 | 1 | 0 | 7.00E-158 |
| hsa_circ_0081975 | 92 | 1 | 602 | 2371 | 1770 | 0 | 0 |
| hsa_circ_0005937 | 91 | 1 | 365 | 365 | 1 | 0 | 3.00E-136 |
| hsa_circ_0081980 | 88 | 1 | 479 | 491 | 1 | 0.02 | 2.00E-164 |
| hsa_circ_0081977 | 91 | 1 | 253 | 446 | 194 | 0 | 4.00E-94 |
| hsa_circ_0081976 | 92 | 1 | 456 | 456 | 1 | 0 | 0 |
| hsa_circ_0081972 | 94 | 1 | 1121 | 2370 | 1250 | 0 | 0 |
| hsa_circ_0081975 | 93 | 1 | 362 | 362 | 1 | 0 | 3.00E-151 |
| hsa_circ_0081974 | 95 | 7 | 194 | 188 | 1 | 0 | 8.00E-80 |
| hsa_circ_0081972 | 95 | 7 | 665 | 1908 | 1250 | 0 | 0 |
| hsa_circ_0081972 | 95 | 7 | 665 | 1908 | 1250 | 0 | 0 |
| hsa_circ_0081972 | 96 | 1 | 566 | 1815 | 1250 | 0 | 0 |
| hsa_circ_0081972 | 96 | 1 | 566 | 1815 | 1250 | 0 | 0 |
| hsa_circ_0081972 | 96 | 1 | 566 | 1815 | 1250 | 0 | 0 |
| hsa_circ_0081972 | 96 | 1 | 566 | 1815 | 1250 | 0 | 0 |
| hsa_circ_0081972 | 94 | 594 | 1185 | 1249 | 658 | 0 | 0 |
| hsa_circ_0081972 | 94 | 594 | 1581 | 1249 | 262 | 0 | 0 |
| hsa_circ_0081972 | 95 | 1 | 471 | 1720 | 1250 | 0 | 0 |
| hsa_circ_0081972 | 95 | 1 | 395 | 1644 | 1250 | 0 | 1.00E-177 |

novel_circ_012180 novel_circ_012181 novel_circ_012182 novel_circ_012183 novel_circ_012184 novel_circ_012185 novel_circ_012186 novel_circ_012187 novel_circ_012191 novel_circ_012192 novel_circ_012193 novel_circ_012194 novel_circ_012195 novel_circ_012196 novel_circ_012197 novel_circ_012198 novel_circ_012199 novel_circ_012200 novel_circ_012201 novel_circ_012202 novel_circ_012203 novel_circ_012204 novel_circ_012226 novel_circ_012227 novel_circ_012228 novel_circ_012229 novel_circ_012230 novel_circ_012231 novel_circ_012232 novel_circ_012233 novel_circ_012234 novel_circ_012235 novel_circ_012236 novel_circ_012237 novel_circ_012238 novel_circ_012239 novel_circ_012240 novel_circ_012241 novel_circ_012242 novel_circ_012243 novel_circ_012244 novel_circ_012245 novel_circ_012246 novel_circ_012247 novel_circ_012248 novel_circ_012249 novel_circ_012250 novel_circ_012251 novel_circ_012252 novel_circ_012253 novel_circ_012254 novel_circ_012255 novel_circ_012256 novel_circ_012257

| hsa_circ_0081972 | 96 | 263 | 513 | 1249 | 999 | 0 | 5.00E-111 |
| --- | --- | --- | --- | --- | --- | --- | --- |
| hsa_circ_0081972 | 94 | 263 | 854 | 1249 | 658 | 0 | 0 |
| hsa_circ_0081972 | 94 | 1 | 396 | 657 | 262 | 0 | 2.00E-173 |
| hsa_circ_0013075 | 88 | 1 | 1371 | 1478 | 114 | 0.01 | 0 |
| hsa_circ_0013073 | 96 | 1 | 254 | 254 | 1 | 0 | 1.00E-112 |
| hsa_circ_0013073 | 96 | 1 | 254 | 254 | 1 | 0 | 1.00E-112 |
| hsa_circ_0013071 | 84 | 1 | 1509 | 3422 | 1922 | 0.05 | 0 |
| hsa_circ_0013071 | 82 | 1 | 1315 | 3228 | 1922 | 0.05 | 0 |
| hsa_circ_0013062 | 85 | 1 | 488 | 488 | 1 | 0 | 4.00E-141 |
| hsa_circ_0013059 | 90 | 1 | 757 | 754 | 1 | 0 | 0 |
| hsa_circ_0013057 | 91 | 1 | 1176 | 1291 | 116 | 0 | 0 |
| hsa_circ_0013057 | 92 | 1 | 1291 | 1291 | 1 | 0 | 0 |
| hsa_circ_0013057 | 91 | 4 | 852 | 964 | 116 | 0 | 0 |
| hsa_circ_0013057 | 91 | 4 | 967 | 964 | 1 | 0 | 0 |
| hsa_circ_0004083 | 95 | 1 | 884 | 1 | 884 | 0 | 0 |
| hsa_circ_0004083 | 95 | 1 | 884 | 1 | 884 | 0 | 0 |
| hsa_circ_0004083 | 95 | 1 | 884 | 1 | 884 | 0 | 0 |
| hsa_circ_0004083 | 96 | 1 | 346 | 374 | 719 | 0 | 5.00E-159 |
| hsa_circ_0004083 | 96 | 1 | 719 | 1 | 719 | 0 | 0 |
| hsa_circ_0004083 | 96 | 1 | 596 | 1 | 596 | 0 | 0 |
| hsa_circ_0004083 | 94 | 1 | 290 | 1 | 290 | 0 | 5.00E-123 |
| hsa_circ_0004083 | 93 | 1 | 191 | 1 | 191 | 0 | 8.00E-75 |
| hsa_circ_0047331 | 94 | 1 | 721 | 1329 | 609 | 0 | 0 |
| hsa_circ_0047331 | 93 | 1 | 1569 | 2177 | 609 | 0 | 0 |
| hsa_circ_0047331 | 93 | 1 | 642 | 1647 | 1006 | 0 | 0 |
| hsa_circ_0047331 | 93 | 1 | 986 | 2177 | 1192 | 0 | 0 |
| hsa_circ_0047331 | 93 | 1 | 675 | 2177 | 1503 | 0.01 | 0 |
| hsa_circ_0047331 | 92 | 1 | 530 | 2177 | 1648 | 0.01 | 0 |
| hsa_circ_0035370 | 94 | 1 | 805 | 242 | 1046 | 0 | 0 |
| hsa_circ_0035368 | 94 | 1 | 1173 | 87 | 1259 | 0 | 0 |
| hsa_circ_0035370 | 95 | 6207 | 6620 | 485 | 898 | 0 | 0 |
| hsa_circ_0035370 | 94 | 1 | 501 | 398 | 898 | 0 | 0 |
| hsa_circ_0035370 | 94 | 1 | 579 | 320 | 898 | 0 | 0 |
| hsa_circ_0035368 | 94 | 1 | 1025 | 87 | 1111 | 0 | 0 |
| hsa_circ_0035370 | 94 | 1 | 498 | 320 | 817 | 0 | 0 |
| hsa_circ_0035370 | 93 | 1 | 441 | 320 | 760 | 0 | 0 |
| hsa_circ_0035369 | 94 | 1 | 519 | 1 | 519 | 0 | 0 |
| hsa_circ_0035366 | 94 | 1 | 349 | 317 | 665 | 0 | 5.00E-149 |
| hsa_circ_0035366 | 94 | 1 | 263 | 317 | 579 | 0 | 5.00E-108 |
| hsa_circ_0035366 | 92 | 1 | 579 | 1 | 579 | 0 | 0 |
| hsa_circ_0035366 | 92 | 1 | 288 | 158 | 445 | 0 | 7.00E-112 |
| hsa_circ_0035366 | 92 | 1 | 445 | 1 | 445 | 0 | 1.00E-175 |
| hsa_circ_0035363 | 93 | 1 | 895 | 143 | 1037 | 0 | 0 |
| hsa_circ_0035359 | 92 | 1 | 1265 | 443 | 1707 | 0 | 0 |
| hsa_circ_0035363 | 93 | 1 | 766 | 143 | 908 | 0 | 0 |
| hsa_circ_0035366 | 91 | 1 | 157 | 1 | 157 | 0 | 2.00E-54 |
| hsa_circ_0035364 | 93 | 1 | 437 | 171 | 607 | 0 | 0 |
| hsa_circ_0035364 | 93 | 1 | 607 | 1 | 607 | 0 | 0 |
| hsa_circ_0035361 | 92 | 1 | 977 | 221 | 1197 | 0 | 0 |
| hsa_circ_0035361 | 92 | 1 | 1197 | 1 | 1197 | 0 | 0 |
| hsa_circ_0035359 | 92 | 1 | 1354 | 66 | 1419 | 0 | 0 |
| hsa_circ_0035364 | 94 | 1 | 280 | 171 | 450 | 0 | 4.00E-119 |
| hsa_circ_0035364 | 94 | 1 | 450 | 1 | 450 | 0 | 0 |
| hsa_circ_0035361 | 92 | 1 | 820 | 221 | 1040 | 0 | 0 |

novel_circ_012258 novel_circ_012259 novel_circ_012260 novel_circ_012261 novel_circ_012262 novel_circ_012263 novel_circ_012264 novel_circ_012265 novel_circ_012269 novel_circ_012270 novel_circ_012271 novel_circ_012272 novel_circ_012274 novel_circ_012275 novel_circ_012276 novel_circ_012277 novel_circ_012280 novel_circ_012283 novel_circ_012284 novel_circ_012285 novel_circ_012286 novel_circ_012287 novel_circ_012288 novel_circ_012289 novel_circ_012290 novel_circ_012291 novel_circ_012292 novel_circ_012293 novel_circ_012294 novel_circ_012295 novel_circ_012296 novel_circ_012297 novel_circ_012298 novel_circ_012299 novel_circ_012300 novel_circ_012301 novel_circ_012302 novel_circ_012303 novel_circ_012304 novel_circ_012305 novel_circ_012306 novel_circ_012307 novel_circ_012308 novel_circ_012309 novel_circ_012310 novel_circ_012311 novel_circ_012312 novel_circ_012313 novel_circ_012314 novel_circ_012315 novel_circ_012316 novel_circ_012317 novel_circ_012318 novel_circ_012321

| hsa_circ_0035361 | 92 | 1 | 1040 | 1 | 1040 | 0 | 0 |
| --- | --- | --- | --- | --- | --- | --- | --- |
| hsa_circ_0035359 | 92 | 1 | 1197 | 66 | 1262 | 0 | 0 |
| hsa_circ_0035361 | 92 | 1 | 760 | 1 | 760 | 0 | 0 |
| hsa_circ_0035359 | 91 | 1 | 917 | 66 | 982 | 0 | 0 |
| hsa_circ_0035361 | 92 | 1 | 590 | 1 | 590 | 0 | 0 |
| hsa_circ_0035359 | 91 | 1 | 747 | 66 | 812 | 0 | 0 |
| hsa_circ_0035360 | 91 | 1 | 377 | 1 | 377 | 0.01 | 3.00E-141 |
| hsa_circ_0035359 | 95 | 24 | 88 | 1 | 65 | 0 | 7.00E-22 |
| hsa_circ_0013363 | 92 | 1 | 251 | 308 | 558 | 0 | 1.00E-94 |
| hsa_circ_0013363 | 93 | 1 | 1302 | 308 | 1609 | 0 | 0 |
| hsa_circ_0013363 | 93 | 1 | 408 | 475 | 882 | 0 | 1.00E-166 |
| hsa_circ_0013363 | 94 | 2 | 432 | 560 | 990 | 0 | 0 |
| hsa_circ_0013365 | 95 | 1 | 641 | 1 | 641 | 0 | 0 |
| hsa_circ_0013367 | 92 | 5 | 412 | 3209 | 2802 | 0 | 5.00E-160 |
| hsa_circ_0013367 | 93 | 5 | 275 | 3209 | 2939 | 0 | 4.00E-109 |
| hsa_circ_0013367 | 94 | 1 | 727 | 4269 | 3543 | 0 | 0 |
| hsa_circ_0013382 | 94 | 3 | 389 | 3 | 389 | 0 | 2.00E-163 |
| hsa_circ_0000100 | 94 | 155 | 440 | 1 | 286 | 0 | 7.00E-119 |
| hsa_circ_0000100 | 95 | 155 | 547 | 1 | 393 | 0 | 3.00E-173 |
| hsa_circ_0000100 | 95 | 155 | 547 | 1 | 393 | 0 | 4.00E-173 |
| hsa_circ_0013391 | 91 | 1 | 773 | 1 | 773 | 0 | 0 |
| hsa_circ_0013391 | 91 | 1 | 1025 | 1 | 1025 | 0 | 0 |
| hsa_circ_0013398 | 90 | 345 | 1046 | 702 | 1 | 0 | 0 |
| hsa_circ_0013399 | 91 | 345 | 707 | 363 | 1 | 0 | 8.00E-135 |
| hsa_circ_0013402 | 95 | 1 | 522 | 1879 | 1358 | 0 | 0 |
| hsa_circ_0013402 | 95 | 1 | 698 | 2049 | 1358 | 0.01 | 0 |
| hsa_circ_0013402 | 93 | 807 | 2299 | 2844 | 1358 | 0 | 0 |
| hsa_circ_0013403 | 94 | 1 | 479 | 473 | 1 | 0.01 | 0 |
| hsa_circ_0013403 | 93 | 1 | 713 | 707 | 1 | 0.01 | 0 |
| hsa_circ_0013403 | 93 | 1 | 1150 | 1144 | 1 | 0.01 | 0 |
| hsa_circ_0013405 | 94 | 337 | 1779 | 1443 | 1 | 0 | 0 |
| hsa_circ_0013405 | 93 | 1 | 984 | 984 | 1 | 0 | 0 |
| hsa_circ_0013403 | 92 | 1 | 671 | 1144 | 474 | 0 | 0 |
| hsa_circ_0013403 | 92 | 1 | 798 | 1271 | 474 | 0 | 0 |
| hsa_circ_0013405 | 94 | 1 | 1443 | 1443 | 1 | 0 | 0 |
| hsa_circ_0013405 | 94 | 86 | 1528 | 1443 | 1 | 0 | 0 |
| hsa_circ_0013405 | 94 | 337 | 1779 | 1443 | 1 | 0 | 0 |
| hsa_circ_0013405 | 92 | 1 | 437 | 513 | 77 | 0 | 4.00E-176 |
| hsa_circ_0013405 | 94 | 1 | 1367 | 1443 | 77 | 0 | 0 |
| hsa_circ_0013406 | 95 | 1 | 1109 | 1109 | 1 | 0 | 0 |
| hsa_circ_0013406 | 95 | 86 | 1194 | 1109 | 1 | 0 | 0 |
| hsa_circ_0013406 | 95 | 337 | 1445 | 1109 | 1 | 0 | 0 |
| hsa_circ_0013406 | 95 | 1 | 930 | 1109 | 180 | 0 | 0 |
| hsa_circ_0013406 | 95 | 86 | 1015 | 1109 | 180 | 0 | 0 |
| hsa_circ_0013406 | 95 | 337 | 1266 | 1109 | 180 | 0 | 0 |
| hsa_circ_0013408 | 95 | 1 | 803 | 803 | 1 | 0 | 0 |
| hsa_circ_0013408 | 95 | 170 | 972 | 803 | 1 | 0 | 0 |
| hsa_circ_0013437 | 93 | 1 | 653 | 653 | 1 | 0 | 0 |
| hsa_circ_0013437 | 93 | 1 | 653 | 653 | 1 | 0 | 0 |
| hsa_circ_0013442 | 94 | 1 | 660 | 663 | 1 | 0 | 0 |
| hsa_circ_0013455 | 96 | 1 | 338 | 425 | 88 | 0 | 6.00E-153 |
| hsa_circ_0013469 | 97 | 1 | 256 | 1 | 256 | 0 | 8.00E-121 |
| hsa_circ_0013470 | 95 | 1 | 95 | 225 | 319 | 0 | 1.00E-34 |
| hsa_circ_0013472 | 96 | 1 | 165 | 1208 | 1372 | 0 | 3.00E-70 |

novel_circ_012324 novel_circ_012325 novel_circ_012326 novel_circ_012327 novel_circ_012328 novel_circ_012329 novel_circ_012330 novel_circ_012331 novel_circ_012332 novel_circ_012334 novel_circ_012335 novel_circ_012336 novel_circ_012337 novel_circ_012338 novel_circ_012339 novel_circ_012340 novel_circ_012341 novel_circ_012342 novel_circ_012343 novel_circ_012344 novel_circ_012345 novel_circ_012346 novel_circ_012347 novel_circ_012348 novel_circ_012349 novel_circ_012350 novel_circ_012351 novel_circ_012352 novel_circ_012353 novel_circ_012354 novel_circ_012355 novel_circ_012356 novel_circ_012357 novel_circ_012358 novel_circ_012359 novel_circ_012360 novel_circ_012361 novel_circ_012362 novel_circ_012363 novel_circ_012364 novel_circ_012365 novel_circ_012366 novel_circ_012367 novel_circ_012368 novel_circ_012369 novel_circ_012370 novel_circ_012371 novel_circ_012374 novel_circ_012375 novel_circ_012376 novel_circ_012377 novel_circ_012378 novel_circ_012379 novel_circ_012380

| hsa_circ_0013487 | 91 | 1 | 1704 | 1801 | 80 | 0.02 | 0 |
| --- | --- | --- | --- | --- | --- | --- | --- |
| hsa_circ_0013489 | 90 | 6 | 491 | 489 | 1 | 0.01 | 9.00E-178 |
| hsa_circ_0013489 | 90 | 6 | 491 | 489 | 1 | 0.01 | 7.00E-179 |
| hsa_circ_0013489 | 90 | 146 | 704 | 562 | 1 | 0.01 | 0 |
| hsa_circ_0013497 | 94 | 16 | 697 | 19 | 702 | 0 | 0 |
| hsa_circ_0013497 | 94 | 16 | 706 | 19 | 711 | 0 | 0 |
| hsa_circ_0013497 | 94 | 16 | 706 | 19 | 711 | 0 | 0 |
| hsa_circ_0013497 | 96 | 1 | 290 | 413 | 702 | 0 | 3.00E-131 |
| hsa_circ_0013497 | 95 | 1 | 299 | 413 | 711 | 0 | 9.00E-133 |
| hsa_circ_0013518 | 97 | 95 | 705 | 1 | 611 | 0 | 0 |
| hsa_circ_0013518 | 96 | 1 | 351 | 1 | 351 | 0 | 8.00E-162 |
| hsa_circ_0013518 | 97 | 1 | 495 | 1 | 495 | 0 | 0 |
| hsa_circ_0013518 | 97 | 1 | 611 | 1 | 611 | 0 | 0 |
| hsa_circ_0013518 | 96 | 1 | 458 | 154 | 611 | 0 | 0 |
| hsa_circ_0013520 | 95 | 1 | 418 | 420 | 3 | 0 | 0 |
| hsa_circ_0013520 | 96 | 1 | 631 | 633 | 3 | 0 | 0 |
| hsa_circ_0013520 | 96 | 1 | 786 | 788 | 3 | 0 | 0 |
| hsa_circ_0013520 | 96 | 1 | 981 | 983 | 3 | 0 | 0 |
| hsa_circ_0013520 | 96 | 1 | 1036 | 1038 | 3 | 0 | 0 |
| hsa_circ_0013520 | 95 | 2 | 1196 | 1200 | 3 | 0 | 0 |
| hsa_circ_0013520 | 96 | 7 | 1279 | 1278 | 3 | 0 | 0 |
| hsa_circ_0000107 | 96 | 1 | 442 | 442 | 1 | 0 | 0 |
| hsa_circ_0000107 | 96 | 1 | 695 | 695 | 1 | 0 | 0 |
| hsa_circ_0000107 | 96 | 1 | 890 | 890 | 1 | 0 | 0 |
| hsa_circ_0000107 | 96 | 2 | 1105 | 1107 | 1 | 0 | 0 |
| hsa_circ_0000107 | 96 | 7 | 1188 | 1185 | 1 | 0 | 0 |
| hsa_circ_0013521 | 96 | 1 | 544 | 544 | 1 | 0 | 0 |
| hsa_circ_0013521 | 96 | 1 | 739 | 739 | 1 | 0 | 0 |
| hsa_circ_0000107 | 96 | 2 | 954 | 1107 | 152 | 0 | 0 |
| hsa_circ_0000107 | 96 | 7 | 1037 | 1185 | 152 | 0 | 0 |
| hsa_circ_0013521 | 96 | 1 | 368 | 544 | 177 | 0 | 3.00E-171 |
| hsa_circ_0013521 | 96 | 1 | 563 | 739 | 177 | 0 | 0 |
| hsa_circ_0013521 | 96 | 1 | 618 | 794 | 177 | 0 | 0 |
| hsa_circ_0000107 | 96 | 2 | 778 | 1107 | 328 | 0 | 0 |
| hsa_circ_0000107 | 96 | 7 | 861 | 1185 | 328 | 0 | 0 |
| hsa_circ_0013521 | 96 | 1 | 253 | 544 | 292 | 0 | 4.00E-114 |
| hsa_circ_0000107 | 95 | 2 | 663 | 1107 | 443 | 0 | 0 |
| hsa_circ_0000107 | 95 | 7 | 746 | 1185 | 443 | 0 | 0 |
| hsa_circ_0000107 | 95 | 2 | 565 | 1107 | 541 | 0.01 | 0 |
| hsa_circ_0000107 | 95 | 7 | 648 | 1185 | 541 | 0 | 0 |
| hsa_circ_0000107 | 95 | 7 | 493 | 1185 | 696 | 0.01 | 0 |
| hsa_circ_0000107 | 95 | 2 | 410 | 1107 | 696 | 0.01 | 0 |
| hsa_circ_0000107 | 95 | 7 | 493 | 1185 | 696 | 0.01 | 0 |
| hsa_circ_0013525 | 94 | 2 | 331 | 333 | 1 | 0.01 | 1.00E-140 |
| hsa_circ_0013525 | 94 | 7 | 414 | 411 | 1 | 0.01 | 6.00E-179 |
| hsa_circ_0013525 | 94 | 7 | 298 | 411 | 117 | 0.01 | 6.00E-123 |
| hsa_circ_0003277 | 94 | 7 | 243 | 240 | 1 | 0.01 | 2.00E-97 |
| hsa_circ_0013562 | 89 | 1 | 1272 | 1272 | 1 | 0 | 0 |
| hsa_circ_0013562 | 89 | 1 | 1272 | 1272 | 1 | 0 | 0 |
| hsa_circ_0013562 | 89 | 90 | 1361 | 1272 | 1 | 0 | 0 |
| hsa_circ_0013563 | 89 | 1 | 405 | 405 | 1 | 0 | 2.00E-143 |
| hsa_circ_0013573 | 92 | 1 | 849 | 1 | 849 | 0 | 0 |
| hsa_circ_0013573 | 93 | 1 | 1074 | 1 | 1074 | 0 | 0 |
| hsa_circ_0013573 | 93 | 1 | 1238 | 1 | 1238 | 0 | 0 |

novel_circ_012381 novel_circ_012382 novel_circ_012383 novel_circ_012384 novel_circ_012385 novel_circ_012386 novel_circ_012387 novel_circ_012388 novel_circ_012389 novel_circ_012390 novel_circ_012391 novel_circ_012392 novel_circ_012393 novel_circ_012394 novel_circ_012395 novel_circ_012396 novel_circ_012397 novel_circ_012398 novel_circ_012399 novel_circ_012400 novel_circ_012401 novel_circ_012402 novel_circ_012403 novel_circ_012404 novel_circ_012405 novel_circ_012406 novel_circ_012407 novel_circ_012408 novel_circ_012409 novel_circ_012410 novel_circ_012411 novel_circ_012412 novel_circ_012413 novel_circ_012414 novel_circ_012415 novel_circ_012416 novel_circ_012417 novel_circ_012418 novel_circ_012419 novel_circ_012420 novel_circ_012421 novel_circ_012422 novel_circ_012423 novel_circ_012424 novel_circ_012425 novel_circ_012426 novel_circ_012427 novel_circ_012428 novel_circ_012429 novel_circ_012430 novel_circ_012431 novel_circ_012432 novel_circ_012433 novel_circ_012434

| hsa_circ_0013573 | 93 | 1 | 1841 | 1 | 1841 | 0 | 0 |
| --- | --- | --- | --- | --- | --- | --- | --- |
| hsa_circ_0013573 | 93 | 1 | 2732 | 1 | 2732 | 0 | 0 |
| hsa_circ_0013576 | 93 | 1 | 498 | 1 | 498 | 0 | 0 |
| hsa_circ_0013576 | 92 | 1 | 783 | 1 | 783 | 0 | 0 |
| hsa_circ_0013576 | 93 | 1 | 1172 | 1 | 1172 | 0 | 0 |
| hsa_circ_0013576 | 93 | 1 | 2666 | 1 | 2666 | 0 | 0 |
| hsa_circ_0013578 | 92 | 1 | 573 | 136 | 708 | 0 | 0 |
| hsa_circ_0013582 | 94 | 1 | 603 | 165 | 767 | 0 | 0 |
| hsa_circ_0013579 | 92 | 1 | 1494 | 675 | 2168 | 0 | 0 |
| hsa_circ_0013589 | 95 | 1 | 237 | 1 | 237 | 0 | 3.00E-100 |
| hsa_circ_0013589 | 94 | 1 | 442 | 1 | 442 | 0 | 0 |
| hsa_circ_0013589 | 94 | 1 | 622 | 1 | 622 | 0 | 0 |
| hsa_circ_0013589 | 95 | 1 | 702 | 1 | 702 | 0 | 0 |
| hsa_circ_0013589 | 95 | 1 | 760 | 1 | 760 | 0 | 0 |
| hsa_circ_0013593 | 94 | 1 | 948 | 81 | 1028 | 0 | 0 |
| hsa_circ_0013595 | 93 | 1 | 568 | 1172 | 1739 | 0 | 0 |
| hsa_circ_0013595 | 93 | 1 | 942 | 1172 | 2113 | 0 | 0 |
| hsa_circ_0013599 | 93 | 1 | 375 | 375 | 1 | 0 | 5.00E-153 |
| hsa_circ_0013599 | 93 | 1 | 156 | 156 | 1 | 0 | 3.00E-58 |
| hsa_circ_0013599 | 93 | 1 | 375 | 375 | 1 | 0 | 4.00E-153 |
| hsa_circ_0013599 | 93 | 1 | 156 | 156 | 1 | 0 | 2.00E-58 |
| hsa_circ_0013599 | 93 | 1 | 375 | 375 | 1 | 0 | 3.00E-153 |
| hsa_circ_0013599 | 92 | 1 | 1343 | 1715 | 376 | 0 | 0 |
| hsa_circ_0003198 | 95 | 7 | 229 | 223 | 1 | 0 | 3.00E-94 |
| hsa_circ_0013606 | 95 | 187 | 585 | 399 | 1 | 0 | 0 |
| hsa_circ_0013605 | 99 | 1 | 676 | 5854 | 5179 | 0 | 0 |
| hsa_circ_0013605 | 99 | 1 | 676 | 5854 | 5179 | 0 | 0 |
| hsa_circ_0013605 | 99 | 7345 | 8022 | 5856 | 5179 | 0 | 0 |
| hsa_circ_0013605 | 99 | 1 | 674 | 5854 | 5181 | 0 | 0 |
| hsa_circ_0013605 | 99 | 7345 | 8020 | 5856 | 5181 | 0 | 0 |
| hsa_circ_0013618 | 96 | 1 | 1199 | 1 | 1202 | 0 | 0 |
| hsa_circ_0013618 | 95 | 1 | 2380 | 1 | 2383 | 0 | 0 |
| hsa_circ_0013619 | 95 | 1 | 679 | 1 | 679 | 0 | 0 |
| hsa_circ_0013619 | 95 | 1 | 905 | 1 | 905 | 0 | 0 |
| hsa_circ_0013620 | 95 | 1 | 1454 | 121 | 1571 | 0 | 0 |
| hsa_circ_0013621 | 94 | 1 | 793 | 390 | 1179 | 0 | 0 |
| hsa_circ_0013621 | 92 | 1 | 778 | 647 | 1421 | 0 | 0 |
| hsa_circ_0013621 | 92 | 1 | 775 | 650 | 1421 | 0 | 0 |
| hsa_circ_0013621 | 92 | 2 | 449 | 974 | 1421 | 0 | 1.00E-180 |
| hsa_circ_0013628 | 95 | 1 | 862 | 862 | 1 | 0 | 0 |
| hsa_circ_0013628 | 95 | 1 | 510 | 862 | 353 | 0 | 0 |
| hsa_circ_0013628 | 95 | 1 | 707 | 1059 | 353 | 0 | 0 |
| hsa_circ_0013628 | 95 | 1 | 908 | 1260 | 353 | 0 | 0 |
| hsa_circ_0013631 | 95 | 1 | 1138 | 1138 | 1 | 0 | 0 |
| hsa_circ_0013631 | 96 | 1 | 641 | 641 | 1 | 0 | 0 |
| hsa_circ_0013631 | 95 | 1 | 1138 | 1138 | 1 | 0 | 0 |
| hsa_circ_0013631 | 95 | 1 | 1138 | 1138 | 1 | 0 | 0 |
| hsa_circ_0013631 | 96 | 1 | 1535 | 1535 | 1 | 0 | 0 |
| hsa_circ_0013632 | 96 | 1 | 937 | 937 | 1 | 0 | 0 |
| hsa_circ_0013635 | 96 | 1 | 250 | 250 | 1 | 0 | 4.00E-114 |
| hsa_circ_0004127 | 97 | 1 | 278 | 278 | 1 | 0 | 5.00E-133 |
| hsa_circ_0004127 | 97 | 1 | 397 | 397 | 1 | 0 | 0 |
| hsa_circ_0013633 | 97 | 4082 | 4480 | 1171 | 773 | 0 | 0 |
| hsa_circ_0004127 | 97 | 1 | 264 | 397 | 134 | 0 | 1.00E-123 |

novel_circ_012436 novel_circ_012437 novel_circ_012438 novel_circ_012439 novel_circ_012440 novel_circ_012441 novel_circ_012442 novel_circ_012443 novel_circ_012444 novel_circ_012448 novel_circ_012449 novel_circ_012450 novel_circ_012451 novel_circ_012452 novel_circ_012453 novel_circ_012454 novel_circ_012455 novel_circ_012456 novel_circ_012457 novel_circ_012458 novel_circ_012459 novel_circ_012460 novel_circ_012461 novel_circ_012462 novel_circ_012463 novel_circ_012464 novel_circ_012465 novel_circ_012466 novel_circ_012467 novel_circ_012468 novel_circ_012469 novel_circ_012470 novel_circ_012471 novel_circ_012472 novel_circ_012473 novel_circ_012474 novel_circ_012475 novel_circ_012476 novel_circ_012477 novel_circ_012478 novel_circ_012479 novel_circ_012480 novel_circ_012481 novel_circ_012482 novel_circ_012483 novel_circ_012484 novel_circ_012485 novel_circ_012486 novel_circ_012489 novel_circ_012490 novel_circ_012491 novel_circ_012494 novel_circ_012495 novel_circ_012496

| hsa_circ_0013652 | 96 | 1 | 289 | 289 | 1 | 0 | 4.00E-129 |
| --- | --- | --- | --- | --- | --- | --- | --- |
| hsa_circ_0013652 | 96 | 1 | 1554 | 1554 | 1 | 0 | 0 |
| hsa_circ_0013652 | 96 | 1 | 856 | 1253 | 398 | 0 | 0 |
| hsa_circ_0013652 | 96 | 1 | 550 | 1253 | 704 | 0 | 0 |
| hsa_circ_0013651 | 96 | 1 | 1050 | 1873 | 824 | 0 | 0 |
| hsa_circ_0000115 | 96 | 1 | 337 | 337 | 1 | 0 | 1.00E-155 |
| hsa_circ_0013656 | 96 | 1 | 301 | 383 | 83 | 0 | 2.00E-137 |
| hsa_circ_0013656 | 96 | 1 | 500 | 582 | 83 | 0 | 0 |
| hsa_circ_0013651 | 99 | 1 | 295 | 2407 | 2113 | 0 | 6.00E-149 |
| hsa_circ_0083449 | 87 | 1 | 333 | 5602 | 5931 | 0.01 | 8.00E-104 |
| hsa_circ_0083449 | 91 | 1 | 1440 | 4501 | 5931 | 0.02 | 0 |
| hsa_circ_0083449 | 91 | 220 | 1967 | 4196 | 5931 | 0.01 | 0 |
| hsa_circ_0083462 | 92 | 1 | 1101 | 1556 | 2650 | 0.02 | 0 |
| hsa_circ_0083462 | 92 | 1 | 1052 | 1254 | 2305 | 0.01 | 0 |
| hsa_circ_0083462 | 92 | 1 | 854 | 1254 | 2107 | 0.02 | 0 |
| hsa_circ_0002887 | 91 | 1 | 414 | 1 | 417 | 0.03 | 1.00E-155 |
| hsa_circ_0083464 | 92 | 1 | 719 | 697 | 1415 | 0.02 | 0 |
| hsa_circ_0083464 | 92 | 220 | 941 | 694 | 1415 | 0.02 | 0 |
| hsa_circ_0083459 | 91 | 2 | 1645 | 2 | 1645 | 0 | 0 |
| hsa_circ_0083464 | 93 | 1 | 305 | 697 | 998 | 0.01 | 2.00E-123 |
| hsa_circ_0083464 | 93 | 220 | 527 | 694 | 998 | 0.01 | 6.00E-125 |
| hsa_circ_0083449 | 90 | 1 | 2292 | 1737 | 4028 | 0.01 | 0 |
| hsa_circ_0083449 | 90 | 1 | 2466 | 1563 | 4028 | 0.01 | 0 |
| hsa_circ_0083459 | 91 | 2 | 1121 | 2 | 1121 | 0.01 | 0 |
| hsa_circ_0083449 | 90 | 1 | 1262 | 2413 | 3674 | 0 | 0 |
| hsa_circ_0083449 | 90 | 1 | 1181 | 2131 | 3311 | 0.01 | 0 |
| hsa_circ_0083449 | 88 | 1 | 533 | 2413 | 2945 | 0.01 | 1.00E-177 |
| hsa_circ_0083449 | 89 | 1 | 815 | 2131 | 2945 | 0.01 | 0 |
| hsa_circ_0083449 | 89 | 1 | 1209 | 1737 | 2945 | 0.01 | 0 |
| hsa_circ_0083449 | 90 | 1 | 1383 | 1563 | 2945 | 0.01 | 0 |
| hsa_circ_0083457 | 89 | 1 | 423 | 1 | 423 | 0 | 5.00E-145 |
| hsa_circ_0083456 | 89 | 1 | 817 | 1 | 817 | 0.01 | 0 |
| hsa_circ_0083451 | 89 | 1 | 991 | 1377 | 2367 | 0.01 | 0 |
| hsa_circ_0083451 | 89 | 8 | 922 | 1185 | 2096 | 0.01 | 0 |
| hsa_circ_0083451 | 90 | 1 | 568 | 1377 | 1944 | 0.02 | 0 |
| hsa_circ_0083451 | 89 | 1 | 987 | 976 | 1944 | 0.03 | 0 |
| hsa_circ_0083454 | 89 | 556 | 1259 | 178 | 863 | 0.03 | 0 |
| hsa_circ_0083450 | 90 | 4 | 809 | 8 | 813 | 0 | 0 |
| hsa_circ_0083454 | 88 | 1 | 529 | 179 | 689 | 0.03 | 3.00E-177 |
| hsa_circ_0083454 | 88 | 268 | 797 | 178 | 689 | 0.03 | 1.00E-177 |
| hsa_circ_0083454 | 88 | 556 | 1085 | 178 | 689 | 0.03 | 2.00E-177 |
| hsa_circ_0083450 | 90 | 4 | 809 | 8 | 813 | 0 | 0 |
| hsa_circ_0083454 | 88 | 1 | 173 | 1 | 173 | 0 | 8.00E-53 |
| hsa_circ_0083451 | 93 | 1 | 179 | 517 | 695 | 0 | 3.00E-69 |
| hsa_circ_0083450 | 90 | 4 | 809 | 8 | 813 | 0 | 0 |
| hsa_circ_0083450 | 90 | 4 | 809 | 8 | 813 | 0 | 0 |
| hsa_circ_0083450 | 90 | 4 | 801 | 8 | 805 | 0.01 | 0 |
| hsa_circ_0083450 | 89 | 4 | 630 | 8 | 634 | 0.01 | 0 |
| hsa_circ_0083444 | 77 | 2 | 2436 | 2440 | 1 | 0.02 | 0 |
| hsa_circ_0083444 | 77 | 2 | 2436 | 2440 | 1 | 0.02 | 0 |
| hsa_circ_0083444 | 77 | 2 | 2436 | 2440 | 1 | 0.02 | 0 |
| hsa_circ_0083441 | 86 | 535 | 752 | 285 | 68 | 0 | 8.00E-61 |
| hsa_circ_0083441 | 86 | 1 | 215 | 282 | 68 | 0 | 9.00E-60 |
| hsa_circ_0083441 | 88 | 1 | 282 | 282 | 1 | 0 | 3.00E-90 |

novel_circ_012506 novel_circ_012507 novel_circ_012508 novel_circ_012509 novel_circ_012510 novel_circ_012511 novel_circ_012512 novel_circ_012513 novel_circ_012514 novel_circ_012515 novel_circ_012516 novel_circ_012517 novel_circ_012518 novel_circ_012519 novel_circ_012520 novel_circ_012521 novel_circ_012522 novel_circ_012523 novel_circ_012524 novel_circ_012525 novel_circ_012526 novel_circ_012527 novel_circ_012528 novel_circ_012529 novel_circ_012530 novel_circ_012531 novel_circ_012532 novel_circ_012533 novel_circ_012534 novel_circ_012535 novel_circ_012536 novel_circ_012537 novel_circ_012538 novel_circ_012539 novel_circ_012540 novel_circ_012541 novel_circ_012542 novel_circ_012545 novel_circ_012546 novel_circ_012547 novel_circ_012548 novel_circ_012549 novel_circ_012550 novel_circ_012551 novel_circ_012552 novel_circ_012553 novel_circ_012556 novel_circ_012557 novel_circ_012558 novel_circ_012559 novel_circ_012560 novel_circ_012561 novel_circ_012562 novel_circ_012563

| hsa_circ_0008555 | 91 | 2 | 297 | 218 | 513 | 0 | 6.00E-108 |
| --- | --- | --- | --- | --- | --- | --- | --- |
| hsa_circ_0008555 | 92 | 1 | 398 | 116 | 513 | 0 | 2.00E-159 |
| hsa_circ_0083420 | 97 | 1 | 288 | 1 | 288 | 0 | 2.00E-133 |
| hsa_circ_0083418 | 96 | 1 | 567 | 2442 | 1875 | 0 | 0 |
| hsa_circ_0083418 | 96 | 1 | 712 | 2442 | 1730 | 0 | 0 |
| hsa_circ_0083418 | 96 | 1 | 823 | 2442 | 1619 | 0 | 0 |
| hsa_circ_0083418 | 96 | 1 | 356 | 2230 | 1875 | 0 | 3.00E-161 |
| hsa_circ_0083418 | 96 | 1 | 501 | 2230 | 1730 | 0 | 0 |
| hsa_circ_0083418 | 96 | 1 | 612 | 2230 | 1619 | 0 | 0 |
| hsa_circ_0083410 | 95 | 1 | 474 | 1 | 474 | 0 | 0 |
| hsa_circ_0083406 | 94 | 1 | 698 | 96 | 793 | 0 | 0 |
| hsa_circ_0083410 | 98 | 1 | 254 | 1 | 254 | 0 | 2.00E-121 |
| hsa_circ_0083406 | 95 | 1 | 478 | 96 | 573 | 0 | 0 |
| hsa_circ_0083406 | 92 | 1 | 345 | 96 | 440 | 0 | 2.00E-138 |
| hsa_circ_0083400 | 90 | 1 | 704 | 1 | 704 | 0 | 0 |
| hsa_circ_0083400 | 89 | 9 | 197 | 516 | 704 | 0 | 2.00E-61 |
| hsa_circ_0083400 | 90 | 1 | 704 | 1 | 704 | 0 | 0 |
| hsa_circ_0083400 | 90 | 1 | 704 | 1 | 704 | 0 | 0 |
| hsa_circ_0083400 | 89 | 1 | 603 | 1 | 603 | 0 | 0 |
| hsa_circ_0083400 | 91 | 1 | 507 | 1 | 507 | 0 | 0 |
| hsa_circ_0083400 | 90 | 1 | 468 | 1 | 468 | 0 | 5.00E-175 |
| hsa_circ_0083400 | 91 | 1 | 396 | 1 | 396 | 0 | 4.00E-150 |
| hsa_circ_0083400 | 90 | 1 | 313 | 1 | 313 | 0 | 1.00E-110 |
| hsa_circ_0067689 | 93 | 2 | 338 | 138 | 474 | 0 | 5.00E-139 |
| hsa_circ_0067689 | 94 | 2 | 464 | 138 | 600 | 0 | 0 |
| hsa_circ_0067689 | 94 | 2 | 464 | 138 | 600 | 0 | 0 |
| hsa_circ_0067689 | 95 | 1 | 126 | 475 | 600 | 0 | 4.00E-50 |
| hsa_circ_0067692 | 90 | 1 | 123 | 126 | 1 | 0.02 | 8.00E-39 |
| hsa_circ_0067692 | 92 | 1 | 1038 | 1029 | 1 | 0.01 | 0 |
| hsa_circ_0067692 | 92 | 1 | 894 | 885 | 1 | 0.02 | 0 |
| hsa_circ_0067692 | 92 | 1 | 755 | 746 | 1 | 0.02 | 0 |
| hsa_circ_0067692 | 92 | 1 | 755 | 746 | 1 | 0.02 | 0 |
| hsa_circ_0067692 | 92 | 1 | 1013 | 1127 | 127 | 0.01 | 0 |
| hsa_circ_0067698 | 88 | 1 | 859 | 1303 | 442 | 0.01 | 0 |
| hsa_circ_0067698 | 90 | 168 | 1525 | 1802 | 442 | 0.01 | 0 |
| hsa_circ_0067698 | 89 | 168 | 1242 | 1802 | 725 | 0.01 | 0 |
| hsa_circ_0067698 | 92 | 168 | 832 | 1802 | 1138 | 0 | 0 |
| hsa_circ_0067700 | 90 | 259 | 533 | 1 | 275 | 0 | 7.00E-96 |
| hsa_circ_0067702 | 89 | 980 | 1580 | 1 | 601 | 0.01 | 0 |
| hsa_circ_0067702 | 90 | 529 | 1633 | 1 | 1105 | 0 | 0 |
| hsa_circ_0067701 | 89 | 1 | 446 | 83 | 528 | 0 | 8.00E-158 |
| hsa_circ_0067701 | 89 | 1 | 446 | 83 | 528 | 0 | 2.00E-157 |
| hsa_circ_0067702 | 89 | 447 | 1047 | 1 | 601 | 0.01 | 0 |
| hsa_circ_0067702 | 90 | 447 | 1344 | 1 | 898 | 0 | 0 |
| hsa_circ_0067702 | 90 | 447 | 1551 | 1 | 1105 | 0 | 0 |
| hsa_circ_0067702 | 90 | 1 | 690 | 416 | 1105 | 0.01 | 0 |
| hsa_circ_0067718 | 94 | 24561 | 25273 | 1 | 716 | 0 | 0 |
| hsa_circ_0067718 | 94 | 24558 | 25270 | 1 | 716 | 0 | 0 |
| hsa_circ_0067718 | 94 | 1 | 334 | 1 | 337 | 0.01 | 1.00E-144 |
| hsa_circ_0067718 | 94 | 1 | 713 | 1 | 716 | 0 | 0 |
| hsa_circ_0006801 | 94 | 1 | 379 | 1 | 379 | 0 | 2.00E-162 |
| hsa_circ_0001347 | 91 | 337 | 908 | 1 | 572 | 0 | 0 |
| hsa_circ_0001347 | 91 | 1 | 572 | 1 | 572 | 0 | 0 |
| hsa_circ_0067736 | 95 | 1481 | 1937 | 1 | 457 | 0 | 0 |

novel_circ_012564 novel_circ_012565 novel_circ_012566 novel_circ_012567 novel_circ_012568 novel_circ_012569 novel_circ_012570 novel_circ_012571 novel_circ_012572 novel_circ_012573 novel_circ_012574 novel_circ_012575 novel_circ_012576 novel_circ_012577 novel_circ_012578 novel_circ_012579 novel_circ_012580 novel_circ_012581 novel_circ_012582 novel_circ_012583 novel_circ_012584 novel_circ_012585 novel_circ_012586 novel_circ_012593 novel_circ_012594 novel_circ_012596 novel_circ_012597 novel_circ_012598 novel_circ_012599 novel_circ_012600 novel_circ_012601 novel_circ_012602 novel_circ_012603 novel_circ_012604 novel_circ_012605 novel_circ_012606 novel_circ_012607 novel_circ_012608 novel_circ_012609 novel_circ_012610 novel_circ_012611 novel_circ_012612 novel_circ_012613 novel_circ_012614 novel_circ_012615 novel_circ_012616 novel_circ_012617 novel_circ_012618 novel_circ_012619 novel_circ_012620 novel_circ_012621 novel_circ_012622 novel_circ_012623 novel_circ_012624

| hsa_circ_0067736 | 94 | 1481 | 2875 | 1 | 1395 | 0 | 0 |
| --- | --- | --- | --- | --- | --- | --- | --- |
| hsa_circ_0067736 | 94 | 1481 | 2877 | 1 | 1397 | 0 | 0 |
| hsa_circ_0067736 | 95 | 1 | 457 | 1 | 457 | 0 | 0 |
| hsa_circ_0067736 | 94 | 1 | 1395 | 1 | 1395 | 0 | 0 |
| hsa_circ_0067736 | 94 | 1 | 1397 | 1 | 1397 | 0 | 0 |
| hsa_circ_0067736 | 93 | 1 | 1869 | 1 | 1869 | 0 | 0 |
| hsa_circ_0067736 | 95 | 1 | 352 | 106 | 457 | 0 | 1.00E-155 |
| hsa_circ_0067736 | 94 | 1 | 1290 | 106 | 1395 | 0 | 0 |
| hsa_circ_0067736 | 94 | 1 | 1292 | 106 | 1397 | 0 | 0 |
| hsa_circ_0067736 | 93 | 1 | 1764 | 106 | 1869 | 0 | 0 |
| hsa_circ_0067736 | 94 | 1 | 551 | 458 | 1008 | 0 | 0 |
| hsa_circ_0067736 | 94 | 1 | 938 | 458 | 1395 | 0 | 0 |
| hsa_circ_0067736 | 94 | 1 | 940 | 458 | 1397 | 0 | 0 |
| hsa_circ_0067736 | 94 | 1 | 381 | 628 | 1008 | 0 | 9.00E-162 |
| hsa_circ_0067736 | 94 | 1 | 770 | 628 | 1397 | 0 | 0 |
| hsa_circ_0067736 | 91 | 1 | 474 | 1396 | 1869 | 0 | 5.00E-180 |
| hsa_circ_0067736 | 91 | 1 | 474 | 1396 | 1869 | 0 | 8.00E-180 |
| hsa_circ_0067737 | 90 | 1 | 427 | 149 | 575 | 0 | 6.00E-154 |
| hsa_circ_0067737 | 91 | 1 | 440 | 268 | 707 | 0 | 4.00E-171 |
| hsa_circ_0067737 | 93 | 1 | 1003 | 412 | 1414 | 0 | 0 |
| hsa_circ_0067738 | 95 | 1 | 502 | 1 | 502 | 0 | 0 |
| hsa_circ_0067738 | 94 | 1 | 839 | 1 | 839 | 0 | 0 |
| hsa_circ_0067738 | 93 | 1 | 707 | 133 | 839 | 0 | 0 |
| hsa_circ_0001349 | 94 | 1 | 372 | 1 | 375 | 0.01 | 1.00E-160 |
| hsa_circ_0007630 | 98 | 1 | 466 | 1 | 466 | 0 | 0 |
| hsa_circ_0067744 | 94 | 2 | 358 | 2 | 358 | 0 | 7.00E-155 |
| hsa_circ_0067745 | 94 | 206 | 660 | 1 | 455 | 0 | 0 |
| hsa_circ_0067745 | 94 | 76 | 530 | 1 | 455 | 0 | 0 |
| hsa_circ_0067745 | 94 | 1 | 455 | 1 | 455 | 0 | 0 |
| hsa_circ_0067746 | 95 | 4 | 810 | 1496 | 690 | 0 | 0 |
| hsa_circ_0067749 | 94 | 4 | 345 | 342 | 1 | 0 | 8.00E-147 |
| hsa_circ_0067753 | 93 | 2 | 600 | 987 | 392 | 0.01 | 0 |
| hsa_circ_0067758 | 91 | 1 | 445 | 990 | 546 | 0 | 3.00E-172 |
| hsa_circ_0002626 | 88 | 3 | 524 | 3 | 524 | 0 | 3.00E-178 |
| hsa_circ_0067762 | 94 | 693 | 1872 | 2263 | 1084 | 0 | 0 |
| hsa_circ_0067762 | 94 | 188 | 853 | 1081 | 416 | 0 | 0 |
| hsa_circ_0067762 | 94 | 693 | 1872 | 2263 | 1084 | 0 | 0 |
| hsa_circ_0067762 | 93 | 1 | 761 | 1844 | 1084 | 0 | 0 |
| hsa_circ_0067762 | 94 | 546 | 1725 | 2263 | 1084 | 0 | 0 |
| hsa_circ_0067762 | 94 | 693 | 1872 | 2263 | 1084 | 0 | 0 |
| hsa_circ_0067762 | 93 | 1 | 247 | 1330 | 1084 | 0 | 8.00E-99 |
| hsa_circ_0067762 | 92 | 1 | 606 | 1844 | 1239 | 0 | 0 |
| hsa_circ_0067762 | 93 | 693 | 1717 | 2263 | 1239 | 0 | 0 |
| hsa_circ_0067762 | 93 | 1 | 514 | 1844 | 1331 | 0 | 0 |
| hsa_circ_0067762 | 93 | 1 | 442 | 1844 | 1403 | 0 | 0 |
| hsa_circ_0067762 | 94 | 546 | 1406 | 2263 | 1403 | 0 | 0 |
| hsa_circ_0067766 | 94 | 1 | 990 | 990 | 1 | 0 | 0 |
| hsa_circ_0067766 | 94 | 1 | 990 | 990 | 1 | 0 | 0 |
| hsa_circ_0067766 | 94 | 1 | 990 | 990 | 1 | 0 | 0 |
| hsa_circ_0067766 | 94 | 1 | 990 | 990 | 1 | 0 | 0 |
| hsa_circ_0067767 | 94 | 1 | 692 | 692 | 1 | 0 | 0 |
| hsa_circ_0067770 | 92 | 3 | 208 | 206 | 1 | 0 | 4.00E-76 |
| hsa_circ_0067770 | 92 | 3 | 208 | 206 | 1 | 0 | 2.00E-76 |
| hsa_circ_0067772 | 93 | 1 | 703 | 707 | 2 | 0.01 | 0 |

novel_circ_012625 novel_circ_012626 novel_circ_012627 novel_circ_012628 novel_circ_012629 novel_circ_012630 novel_circ_012631 novel_circ_012632 novel_circ_012633 novel_circ_012634 novel_circ_012635 novel_circ_012636 novel_circ_012637 novel_circ_012640 novel_circ_012641 novel_circ_012642 novel_circ_012644 novel_circ_012645 novel_circ_012647 novel_circ_012648 novel_circ_012649 novel_circ_012650 novel_circ_012651 novel_circ_012652 novel_circ_012653 novel_circ_012655 novel_circ_012661 novel_circ_012662 novel_circ_012663 novel_circ_012664 novel_circ_012666 novel_circ_012667 novel_circ_012668 novel_circ_012669 novel_circ_012670 novel_circ_012671 novel_circ_012672 novel_circ_012673 novel_circ_012674 novel_circ_012683 novel_circ_012684 novel_circ_012685 novel_circ_012686 novel_circ_012687 novel_circ_012688 novel_circ_012689 novel_circ_012690 novel_circ_012691 novel_circ_012692 novel_circ_012693 novel_circ_012694 novel_circ_012695 novel_circ_012696 novel_circ_012697

| hsa_circ_0067774 | 96 | 1 | 303 | 303 | 1 | 0 | 7.00E-137 |
| --- | --- | --- | --- | --- | --- | --- | --- |
| hsa_circ_0067774 | 94 | 1 | 494 | 491 | 1 | 0.01 | 0 |
| hsa_circ_0008929 | 94 | 2 | 297 | 2 | 297 | 0 | 1.00E-124 |
| hsa_circ_0001351 | 94 | 2 | 1533 | 6221 | 7752 | 0 | 0 |
| hsa_circ_0001351 | 94 | 2 | 1533 | 6221 | 7752 | 0 | 0 |
| hsa_circ_0001351 | 94 | 2 | 1533 | 6221 | 7752 | 0 | 0 |
| hsa_circ_0001352 | 93 | 1 | 317 | 1 | 317 | 0 | 3.00E-131 |
| hsa_circ_0001352 | 94 | 1 | 677 | 1 | 677 | 0 | 0 |
| hsa_circ_0001352 | 94 | 1 | 1003 | 1 | 1003 | 0 | 0 |
| hsa_circ_0067781 | 94 | 1 | 562 | 1 | 562 | 0 | 0 |
| hsa_circ_0067781 | 94 | 1 | 714 | 1 | 714 | 0 | 0 |
| hsa_circ_0067781 | 94 | 1 | 888 | 1 | 888 | 0 | 0 |
| hsa_circ_0007389 | 80 | 7086 | 7263 | 155529 | 155349 | 0.02 | 3.00E-26 |
| hsa_circ_0006180 | 91 | 1 | 961 | 1 | 958 | 0 | 0 |
| hsa_circ_0006999 | 92 | 2 | 330 | 2 | 330 | 0 | 6.00E-128 |
| hsa_circ_0006999 | 92 | 2 | 330 | 2 | 330 | 0 | 1.00E-127 |
| hsa_circ_0067799 | 96 | 1 | 558 | 2640 | 2083 | 0 | 0 |
| hsa_circ_0067799 | 96 | 1 | 459 | 2640 | 2182 | 0 | 0 |
| hsa_circ_0022597 | 92 | 1 | 150 | 150 | 1 | 0 | 2.00E-53 |
| hsa_circ_0003843 | 94 | 2 | 515 | 514 | 1 | 0 | 0 |
| hsa_circ_0022602 | 83 | 8102 | 10073 | 378 | 2331 | 0.03 | 0 |
| hsa_circ_0022603 | 97 | 1 | 208 | 1 | 208 | 0 | 7.00E-96 |
| hsa_circ_0022604 | 95 | 209 | 464 | 1 | 256 | 0 | 8.00E-109 |
| hsa_circ_0022611 | 86 | 1 | 327 | 1 | 327 | 0 | 2.00E-93 |
| hsa_circ_0022613 | 94 | 1 | 1457 | 451 | 1907 | 0 | 0 |
| hsa_circ_0022702 | 95 | 1 | 354 | 444 | 91 | 0 | 8.00E-157 |
| hsa_circ_0022705 | 93 | 1 | 543 | 543 | 1 | 0 | 0 |
| hsa_circ_0022801 | 88 | 1 | 573 | 412 | 987 | 0.01 | 0 |
| hsa_circ_0022812 | 92 | 3 | 347 | 3 | 347 | 0 | 2.00E-133 |
| hsa_circ_0022819 | 93 | 1 | 269 | 1 | 269 | 0 | 1.00E-109 |
| hsa_circ_0004287 | 94 | 749 | 820 | 2 | 73 | 0 | 5.00E-23 |
| hsa_circ_0004287 | 82 | 1 | 919 | 797 | 1717 | 0.09 | 0 |
| hsa_circ_0004287 | 81 | 1 | 915 | 801 | 1717 | 0.09 | 0 |
| hsa_circ_0004287 | 81 | 1 | 915 | 801 | 1717 | 0.09 | 0 |
| hsa_circ_0002082 | 89 | 7 | 448 | 408 | 855 | 0.06 | 1.00E-155 |
| hsa_circ_0002082 | 90 | 4 | 409 | 430 | 843 | 0.06 | 4.00E-146 |
| hsa_circ_0002082 | 90 | 1 | 401 | 435 | 843 | 0.06 | 2.00E-143 |
| hsa_circ_0005915 | 92 | 1 | 203 | 898 | 1097 | 0.02 | 1.00E-74 |
| hsa_circ_0005915 | 97 | 1 | 68 | 1030 | 1097 | 0 | 3.00E-24 |
| hsa_circ_0024922 | 92 | 1 | 341 | 1 | 341 | 0 | 3.00E-136 |
| hsa_circ_0024925 | 88 | 1 | 170 | 102 | 271 | 0 | 2.00E-49 |
| hsa_circ_0003320 | 93 | 15253 | 15478 | 52 | 277 | 0 | 3.00E-87 |
| hsa_circ_0000342 | 94 | 1 | 435 | 435 | 1 | 0 | 0 |
| hsa_circ_0000342 | 95 | 1 | 666 | 666 | 1 | 0 | 0 |
| hsa_circ_0023677 | 95 | 1 | 415 | 415 | 1 | 0 | 0 |
| hsa_circ_0023679 | 95 | 1 | 280 | 380 | 101 | 0 | 4.00E-124 |
| hsa_circ_0023679 | 93 | 4 | 636 | 736 | 101 | 0 | 0 |
| hsa_circ_0023679 | 92 | 4 | 518 | 736 | 219 | 0.01 | 0 |
| hsa_circ_0023679 | 96 | 3 | 113 | 442 | 332 | 0 | 1.00E-45 |
| hsa_circ_0023679 | 90 | 4 | 356 | 736 | 381 | 0.01 | 4.00E-130 |
| hsa_circ_0023680 | 90 | 1 | 579 | 582 | 1 | 0.01 | 0 |
| hsa_circ_0008992 | 91 | 1 | 249 | 249 | 1 | 0 | 6.00E-92 |
| hsa_circ_0023685 | 93 | 4 | 460 | 495 | 39 | 0 | 0 |
| hsa_circ_0023685 | 93 | 349 | 805 | 495 | 39 | 0 | 0 |

novel_circ_012698 novel_circ_012699 novel_circ_012700 novel_circ_012701 novel_circ_012702 novel_circ_012703 novel_circ_012704 novel_circ_012705 novel_circ_012706 novel_circ_012707 novel_circ_012709 novel_circ_012710 novel_circ_012711 novel_circ_012712 novel_circ_012713 novel_circ_012716 novel_circ_012717 novel_circ_012718 novel_circ_012719 novel_circ_012720 novel_circ_012721 novel_circ_012722 novel_circ_012723 novel_circ_012724 novel_circ_012725 novel_circ_012726 novel_circ_012728 novel_circ_012730 novel_circ_012731 novel_circ_012732 novel_circ_012733 novel_circ_012735 novel_circ_012736 novel_circ_012737 novel_circ_012740 novel_circ_012743 novel_circ_012744 novel_circ_012745 novel_circ_012746 novel_circ_012747 novel_circ_012748 novel_circ_012749 novel_circ_012750 novel_circ_012751 novel_circ_012752 novel_circ_012753 novel_circ_012754 novel_circ_012755 novel_circ_012756 novel_circ_012757 novel_circ_012758 novel_circ_012759 novel_circ_012760 novel_circ_012761

| hsa_circ_0024372 | 94 | 1 | 330 | 1 | 330 | 0 | 8.00E-142 |
| --- | --- | --- | --- | --- | --- | --- | --- |
| hsa_circ_0024371 | 95 | 1 | 418 | 1 | 418 | 0 | 0 |
| hsa_circ_0024371 | 95 | 1 | 295 | 1 | 295 | 0 | 2.00E-127 |
| hsa_circ_0024363 | 96 | 2927 | 3109 | 1688 | 1506 | 0 | 2.00E-77 |
| hsa_circ_0024366 | 96 | 4 | 181 | 340 | 163 | 0 | 1.00E-77 |
| hsa_circ_0024366 | 94 | 4 | 343 | 340 | 1 | 0 | 2.00E-147 |
| hsa_circ_0024365 | 93 | 4 | 592 | 589 | 1 | 0 | 0 |
| hsa_circ_0024365 | 93 | 4 | 592 | 589 | 1 | 0 | 0 |
| hsa_circ_0024365 | 91 | 1 | 411 | 411 | 1 | 0 | 2.00E-158 |
| hsa_circ_0024365 | 91 | 1 | 411 | 411 | 1 | 0 | 1.00E-157 |
| hsa_circ_0084555 | 77 | 3377 | 3790 | 248 | 694 | 0.08 | 7.00E-53 |
| hsa_circ_0007636 | 74 | 20372 | 20937 | 1E+06 | 1206155 | 0.05 | 7.00E-51 |
| hsa_circ_0007636 | 74 | 61905 | 62470 | 1E+06 | 1206155 | 0.05 | 2.00E-50 |
| hsa_circ_0007636 | 74 | 20372 | 20937 | 1E+06 | 1206155 | 0.05 | 7.00E-51 |
| hsa_circ_0007636 | 74 | 20372 | 20937 | 1E+06 | 1206155 | 0.05 | 6.00E-51 |
| hsa_circ_0013763 | 80 | 6977 | 7241 | 17605 | 17337 | 0.04 | 8.00E-45 |
| hsa_circ_0013763 | 80 | 23903 | 24167 | 17605 | 17337 | 0.04 | 1.00E-44 |
| hsa_circ_0017796 | 84 | 189 | 567 | 380 | 2 | 0 | 4.00E-102 |
| hsa_circ_0017796 | 84 | 189 | 567 | 380 | 2 | 0 | 5.00E-102 |
| hsa_circ_0017796 | 84 | 189 | 567 | 380 | 2 | 0 | 6.00E-102 |
| hsa_circ_0007242 | 90 | 245 | 775 | 1 | 523 | 0.02 | 0 |
| hsa_circ_0007242 | 90 | 361 | 891 | 1 | 523 | 0.02 | 0 |
| hsa_circ_0007242 | 90 | 455 | 985 | 1 | 523 | 0.02 | 0 |
| hsa_circ_0007242 | 90 | 590 | 1120 | 1 | 523 | 0.02 | 0 |
| hsa_circ_0007242 | 90 | 1227 | 1757 | 1 | 523 | 0.02 | 0 |
| hsa_circ_0007242 | 91 | 455 | 827 | 1 | 370 | 0.01 | 7.00E-141 |
| hsa_circ_0017784 | 84 | 178 | 772 | 1 | 613 | 0.03 | 3.00E-160 |
| hsa_circ_0017784 | 87 | 1 | 383 | 1 | 383 | 0 | 3.00E-121 |
| hsa_circ_0017784 | 87 | 178 | 560 | 1 | 383 | 0 | 5.00E-121 |
| hsa_circ_0017782 | 91 | 2 | 342 | 2 | 342 | 0 | 3.00E-131 |
| hsa_circ_0017782 | 92 | 2 | 261 | 2 | 261 | 0 | 5.00E-98 |
| hsa_circ_0017776 | 90 | 1 | 495 | 416 | 910 | 0 | 0 |
| hsa_circ_0017776 | 91 | 1 | 747 | 164 | 910 | 0 | 0 |
| hsa_circ_0017776 | 93 | 2 | 291 | 74 | 363 | 0 | 6.00E-118 |
| hsa_circ_0017768 | 88 | 5 | 334 | 587 | 262 | 0.01 | 2.00E-107 |
| hsa_circ_0017757 | 91 | 1 | 551 | 1 | 551 | 0 | 0 |
| hsa_circ_0017757 | 91 | 155 | 705 | 1 | 551 | 0 | 0 |
| hsa_circ_0017757 | 91 | 155 | 705 | 1 | 551 | 0 | 0 |
| hsa_circ_0008826 | 89 | 1 | 888 | 1 | 888 | 0 | 0 |
| hsa_circ_0017738 | 90 | 2 | 641 | 2 | 641 | 0 | 0 |
| hsa_circ_0007813 | 89 | 1 | 1048 | 157 | 1204 | 0 | 0 |
| hsa_circ_0017717 | 95 | 1 | 356 | 699 | 341 | 0.01 | 4.00E-160 |
| hsa_circ_0017717 | 95 | 1 | 506 | 699 | 191 | 0.01 | 0 |
| hsa_circ_0017717 | 95 | 1 | 610 | 699 | 87 | 0 | 0 |
| hsa_circ_0017717 | 95 | 1 | 696 | 699 | 1 | 0 | 0 |
| hsa_circ_0017713 | 96 | 1 | 805 | 808 | 1 | 0 | 0 |
| hsa_circ_0017711 | 95 | 1 | 919 | 922 | 1 | 0 | 0 |
| hsa_circ_0017704 | 95 | 1 | 1036 | 2664 | 1626 | 0 | 0 |
| hsa_circ_0017704 | 95 | 1 | 1222 | 2664 | 1440 | 0 | 0 |
| hsa_circ_0017704 | 95 | 1 | 1426 | 2664 | 1236 | 0 | 0 |
| hsa_circ_0017711 | 96 | 1 | 309 | 309 | 1 | 0 | 7.00E-142 |
| hsa_circ_0017708 | 94 | 1 | 850 | 850 | 1 | 0 | 0 |
| hsa_circ_0017706 | 93 | 1 | 702 | 972 | 268 | 0.01 | 0 |
| hsa_circ_0017704 | 94 | 1 | 431 | 431 | 1 | 0 | 0 |

novel_circ_012762 novel_circ_012763 novel_circ_012764 novel_circ_012765 novel_circ_012766 novel_circ_012767 novel_circ_012768 novel_circ_012769 novel_circ_012770 novel_circ_012771 novel_circ_012772 novel_circ_012773 novel_circ_012774 novel_circ_012775 novel_circ_012778 novel_circ_012779 novel_circ_012780 novel_circ_012781 novel_circ_012782 novel_circ_012783 novel_circ_012784 novel_circ_012785 novel_circ_012786 novel_circ_012787 novel_circ_012788 novel_circ_012789 novel_circ_012790 novel_circ_012791 novel_circ_012792 novel_circ_012793 novel_circ_012795 novel_circ_012796 novel_circ_012799 novel_circ_012800 novel_circ_012801 novel_circ_012802 novel_circ_012805 novel_circ_012806 novel_circ_012807 novel_circ_012808 novel_circ_012809 novel_circ_012810 novel_circ_012811 novel_circ_012812 novel_circ_012813 novel_circ_012817 novel_circ_012818 novel_circ_012819 novel_circ_012820 novel_circ_012825 novel_circ_012826 novel_circ_012827 novel_circ_012828 novel_circ_012833

| hsa_circ_0017700 | 94 | 1 | 432 | 1717 | 1286 | 0 | 0 |
| --- | --- | --- | --- | --- | --- | --- | --- |
| hsa_circ_0017691 | 81 | 5384 | 5769 | 31590 | 31207 | 0.04 | 5.00E-81 |
| hsa_circ_0017690 | 96 | 1 | 151 | 151 | 1 | 0 | 2.00E-64 |
| hsa_circ_0017687 | 95 | 1 | 380 | 380 | 1 | 0 | 1.00E-169 |
| hsa_circ_0017685 | 95 | 1 | 921 | 921 | 1 | 0 | 0 |
| hsa_circ_0017685 | 92 | 1 | 388 | 649 | 262 | 0 | 1.00E-155 |
| hsa_circ_0017685 | 94 | 1 | 649 | 649 | 1 | 0 | 0 |
| hsa_circ_0017679 | 97 | 1 | 496 | 1041 | 1536 | 0 | 0 |
| hsa_circ_0017679 | 97 | 1 | 514 | 1023 | 1536 | 0 | 0 |
| hsa_circ_0017679 | 95 | 1 | 438 | 465 | 902 | 0 | 0 |
| hsa_circ_0017679 | 99 | 1 | 280 | 1 | 280 | 0 | 4.00E-139 |
| hsa_circ_0017679 | 98 | 1 | 199 | 1 | 199 | 0 | 1.00E-92 |
| hsa_circ_0017679 | 98 | 1 | 197 | 1 | 197 | 0 | 4.00E-93 |
| hsa_circ_0017679 | 98 | 275 | 471 | 1 | 197 | 0 | 1.00E-92 |
| hsa_circ_0017661 | 87 | 3 | 673 | 415 | 1085 | 0 | 0 |
| hsa_circ_0017648 | 90 | 1 | 486 | 487 | 3 | 0 | 2.00E-179 |
| hsa_circ_0017648 | 91 | 3 | 334 | 334 | 3 | 0 | 3.00E-126 |
| hsa_circ_0017642 | 97 | 1 | 434 | 434 | 1 | 0 | 0 |
| hsa_circ_0017637 | 95 | 1 | 684 | 684 | 1 | 0 | 0 |
| hsa_circ_0017632 | 95 | 1 | 767 | 767 | 1 | 0 | 0 |
| hsa_circ_0017630 | 94 | 1 | 894 | 894 | 1 | 0 | 0 |
| hsa_circ_0017620 | 94 | 1 | 1262 | 1262 | 1 | 0 | 0 |
| hsa_circ_0017614 | 94 | 1 | 1115 | 1399 | 285 | 0 | 0 |
| hsa_circ_0017620 | 92 | 1 | 578 | 578 | 1 | 0 | 0 |
| hsa_circ_0017620 | 92 | 1 | 495 | 495 | 1 | 0 | 0 |
| hsa_circ_0017616 | 92 | 1 | 605 | 605 | 1 | 0 | 0 |
| hsa_circ_0017620 | 93 | 3 | 368 | 366 | 1 | 0 | 2.00E-153 |
| hsa_circ_0017614 | 92 | 3 | 654 | 652 | 1 | 0 | 0 |
| hsa_circ_0017613 | 88 | 3 | 1085 | 1083 | 1 | 0 | 0 |
| hsa_circ_0017620 | 94 | 1 | 211 | 211 | 1 | 0 | 1.00E-87 |
| hsa_circ_0017609 | 90 | 1 | 986 | 1586 | 613 | 0.01 | 0 |
| hsa_circ_0017609 | 90 | 1 | 705 | 1305 | 613 | 0.02 | 0 |
| hsa_circ_0017605 | 93 | 10 | 255 | 13 | 258 | 0 | 3.00E-100 |
| hsa_circ_0060046 | 75 | 12349 | 12632 | 39362 | 39065 | 0.05 | 7.00E-25 |
| hsa_circ_0060046 | 75 | 12365 | 12648 | 39362 | 39065 | 0.05 | 7.00E-25 |
| hsa_circ_0060046 | 75 | 12349 | 12632 | 39362 | 39065 | 0.05 | 6.00E-25 |
| hsa_circ_0017595 | 89 | 1 | 2831 | 1 | 2828 | 0 | 0 |
| hsa_circ_0017602 | 92 | 1 | 629 | 238 | 866 | 0 | 0 |
| hsa_circ_0017598 | 91 | 1 | 1433 | 1240 | 2672 | 0 | 0 |
| hsa_circ_0017598 | 91 | 1 | 1618 | 1055 | 2672 | 0 | 0 |
| hsa_circ_0017595 | 89 | 1 | 2831 | 1 | 2828 | 0 | 0 |
| hsa_circ_0017602 | 88 | 1 | 359 | 1 | 359 | 0 | 1.00E-119 |
| hsa_circ_0017598 | 90 | 1 | 1111 | 1055 | 2165 | 0 | 0 |
| hsa_circ_0017595 | 85 | 1 | 1022 | 1 | 1019 | 0.01 | 0 |
| hsa_circ_0017595 | 84 | 1 | 755 | 1 | 752 | 0.01 | 0 |
| hsa_circ_0017572 | 90 | 1 | 260 | 5286 | 5545 | 0 | 2.00E-91 |
| hsa_circ_0017572 | 80 | 9 | 880 | 9 | 880 | 0.02 | 0 |
| hsa_circ_0017564 | 86 | 137 | 1683 | 91 | 1634 | 0.01 | 0 |
| hsa_circ_0017564 | 84 | 137 | 1444 | 91 | 1395 | 0.01 | 0 |
| hsa_circ_0003755 | 95 | 135 | 280 | 2865 | 3010 | 0 | 2.00E-57 |
| hsa_circ_0003755 | 95 | 1 | 146 | 2865 | 3010 | 0 | 7.00E-58 |
| hsa_circ_0003755 | 95 | 135 | 280 | 2865 | 3010 | 0 | 1.00E-57 |
| hsa_circ_0005150 | 93 | 12844 | 12959 | 120 | 5 | 0 | 5.00E-39 |
| hsa_circ_0024145 | 92 | 1 | 917 | 157 | 1073 | 0 | 0 |

novel_circ_012834 novel_circ_012835 novel_circ_012836 novel_circ_012837 novel_circ_012838 novel_circ_012839 novel_circ_012840 novel_circ_012841 novel_circ_012842 novel_circ_012843 novel_circ_012844 novel_circ_012845 novel_circ_012846 novel_circ_012847 novel_circ_012848 novel_circ_012849 novel_circ_012850 novel_circ_012851 novel_circ_012852 novel_circ_012853 novel_circ_012854 novel_circ_012855 novel_circ_012856 novel_circ_012857 novel_circ_012858 novel_circ_012859 novel_circ_012860 novel_circ_012861 novel_circ_012862 novel_circ_012863 novel_circ_012864 novel_circ_012865 novel_circ_012866 novel_circ_012867 novel_circ_012868 novel_circ_012869 novel_circ_012870 novel_circ_012871 novel_circ_012872 novel_circ_012873 novel_circ_012874 novel_circ_012875 novel_circ_012876 novel_circ_012877 novel_circ_012878 novel_circ_012879 novel_circ_012880 novel_circ_012881 novel_circ_012882 novel_circ_012883 novel_circ_012884 novel_circ_012891 novel_circ_012893 novel_circ_012894

| hsa_circ_0024131 | 92 | 1 | 1310 | 2584 | 3893 | 0 | 0 |
| --- | --- | --- | --- | --- | --- | --- | --- |
| hsa_circ_0024146 | 91 | 1 | 300 | 1 | 300 | 0 | 3.00E-110 |
| hsa_circ_0024145 | 90 | 1 | 417 | 547 | 963 | 0 | 5.00E-155 |
| hsa_circ_0024145 | 91 | 1 | 807 | 157 | 963 | 0 | 0 |
| hsa_circ_0024131 | 92 | 1 | 1200 | 2584 | 3783 | 0 | 0 |
| hsa_circ_0024131 | 92 | 3 | 1361 | 2425 | 3783 | 0 | 0 |
| hsa_circ_0024131 | 92 | 1 | 2414 | 1370 | 3783 | 0 | 0 |
| hsa_circ_0024131 | 92 | 1 | 2324 | 1370 | 3693 | 0 | 0 |
| hsa_circ_0024144 | 92 | 3 | 1061 | 3 | 1061 | 0 | 0 |
| hsa_circ_0024135 | 93 | 1 | 2114 | 224 | 2337 | 0 | 0 |
| hsa_circ_0024144 | 93 | 1 | 657 | 288 | 944 | 0 | 0 |
| hsa_circ_0024144 | 92 | 3 | 944 | 3 | 944 | 0 | 0 |
| hsa_circ_0024139 | 92 | 1 | 1149 | 196 | 1344 | 0 | 0 |
| hsa_circ_0024135 | 93 | 1 | 1997 | 224 | 2220 | 0 | 0 |
| hsa_circ_0024139 | 92 | 1 | 1044 | 196 | 1239 | 0 | 0 |
| hsa_circ_0024144 | 92 | 3 | 554 | 3 | 554 | 0 | 0 |
| hsa_circ_0024144 | 95 | 1 | 237 | 162 | 398 | 0 | 3.00E-100 |
| hsa_circ_0024135 | 93 | 1 | 1176 | 388 | 1563 | 0 | 0 |
| hsa_circ_0024135 | 93 | 1 | 1340 | 224 | 1563 | 0 | 0 |
| hsa_circ_0024135 | 93 | 1 | 1053 | 224 | 1276 | 0 | 0 |
| hsa_circ_0024136 | 93 | 1 | 369 | 285 | 653 | 0 | 2.00E-153 |
| hsa_circ_0024136 | 93 | 1 | 489 | 165 | 653 | 0 | 0 |
| hsa_circ_0024136 | 94 | 1 | 653 | 1 | 653 | 0 | 0 |
| hsa_circ_0024136 | 95 | 1 | 419 | 1 | 419 | 0 | 0 |
| hsa_circ_0024132 | 94 | 1 | 252 | 461 | 712 | 0 | 1.00E-103 |
| hsa_circ_0024132 | 94 | 1 | 588 | 125 | 712 | 0 | 0 |
| hsa_circ_0024131 | 94 | 1 | 1125 | 1 | 1125 | 0 | 0 |
| hsa_circ_0024121 | 93 | 1 | 3726 | 1511 | 5236 | 0 | 0 |
| hsa_circ_0024121 | 93 | 1 | 603 | 2417 | 3019 | 0 | 0 |
| hsa_circ_0024121 | 93 | 1 | 1761 | 921 | 2681 | 0 | 0 |
| hsa_circ_0024121 | 92 | 1 | 906 | 1511 | 2416 | 0 | 0 |
| hsa_circ_0024121 | 93 | 1 | 590 | 921 | 1510 | 0 | 0 |
| hsa_circ_0024120 | 94 | 1 | 2150 | 614 | 2763 | 0 | 0 |
| hsa_circ_0024120 | 94 | 1 | 2394 | 370 | 2763 | 0 | 0 |
| hsa_circ_0024120 | 93 | 1 | 1076 | 1098 | 2173 | 0 | 0 |
| hsa_circ_0024120 | 94 | 3 | 1676 | 500 | 2173 | 0 | 0 |
| hsa_circ_0024120 | 94 | 1 | 1553 | 370 | 1922 | 0 | 0 |
| hsa_circ_0024120 | 93 | 1 | 648 | 892 | 1539 | 0 | 0 |
| hsa_circ_0024120 | 94 | 1 | 798 | 742 | 1539 | 0 | 0 |
| hsa_circ_0024120 | 93 | 1 | 926 | 614 | 1539 | 0 | 0 |
| hsa_circ_0024120 | 94 | 1 | 1539 | 1 | 1539 | 0 | 0 |
| hsa_circ_0024120 | 95 | 1 | 728 | 370 | 1097 | 0 | 0 |
| hsa_circ_0024120 | 94 | 1 | 741 | 1 | 741 | 0 | 0 |
| hsa_circ_0024117 | 93 | 146 | 1040 | 1 | 895 | 0 | 0 |
| hsa_circ_0024117 | 93 | 146 | 627 | 1 | 482 | 0 | 0 |
| hsa_circ_0024115 | 98 | 1 | 255 | 469 | 215 | 0 | 3.00E-125 |
| hsa_circ_0024115 | 98 | 1 | 469 | 469 | 1 | 0 | 0 |
| hsa_circ_0024115 | 97 | 2 | 214 | 213 | 1 | 0 | 2.00E-98 |
| hsa_circ_0000353 | 79 | 889 | 1687 | 14516 | 13727 | 0.02 | 3.00E-149 |
| hsa_circ_0024101 | 96 | 1 | 244 | 1 | 244 | 0 | 4.00E-109 |
| hsa_circ_0008074 | 95 | 1 | 474 | 1 | 474 | 0 | 0 |
| hsa_circ_0003427 | 83 | 1738 | 1825 | 382852 | 382767 | 0.02 | 6.00E-11 |
| hsa_circ_0003427 | 83 | 1738 | 1825 | 382852 | 382767 | 0.02 | 5.00E-11 |
| hsa_circ_0028415 | 86 | 1295 | 1854 | 3 | 565 | 0.02 | 1.00E-167 |

novel_circ_012895 novel_circ_012896 novel_circ_012897 novel_circ_012898 novel_circ_012899 novel_circ_012900 novel_circ_012901 novel_circ_012902 novel_circ_012903 novel_circ_012920 novel_circ_012921 novel_circ_012922 novel_circ_012923 novel_circ_012924 novel_circ_012925 novel_circ_012926 novel_circ_012927 novel_circ_012928 novel_circ_012929 novel_circ_012930 novel_circ_012931 novel_circ_012932 novel_circ_012933 novel_circ_012934 novel_circ_012935 novel_circ_012936 novel_circ_012937 novel_circ_012938 novel_circ_012939 novel_circ_012940 novel_circ_012941 novel_circ_012942 novel_circ_012943 novel_circ_012944 novel_circ_012945 novel_circ_012946 novel_circ_012947 novel_circ_012948 novel_circ_012958 novel_circ_012961 novel_circ_012962 novel_circ_012966 novel_circ_012967 novel_circ_012968 novel_circ_012969 novel_circ_012970 novel_circ_012971 novel_circ_012972 novel_circ_012973 novel_circ_012974 novel_circ_012975 novel_circ_012976 novel_circ_012977 novel_circ_012978

| hsa_circ_0003427 | 83 | 1738 | 1825 | 382852 | 382767 | 0.02 | 8.00E-12 |
| --- | --- | --- | --- | --- | --- | --- | --- |
| hsa_circ_0028415 | 86 | 1295 | 1854 | 3 | 565 | 0.02 | 5.00E-168 |
| hsa_circ_0049157 | 98 | 4636 | 4680 | 3019 | 2975 | 0 | 8.00E-12 |
| hsa_circ_0024090 | 93 | 1 | 266 | 1 | 266 | 0 | 1.00E-105 |
| hsa_circ_0024090 | 93 | 1 | 160 | 107 | 266 | 0 | 2.00E-60 |
| hsa_circ_0024090 | 93 | 1 | 266 | 1 | 266 | 0 | 2.00E-106 |
| hsa_circ_0024090 | 93 | 145 | 410 | 1 | 266 | 0 | 4.00E-106 |
| hsa_circ_0024090 | 93 | 402 | 667 | 1 | 266 | 0 | 6.00E-106 |
| hsa_circ_0024089 | 94 | 1 | 144 | 76 | 219 | 0 | 7.00E-57 |
| hsa_circ_0069858 | 92 | 27456 | 27518 | 118891 | 118953 | 0 | 2.00E-14 |
| hsa_circ_0007745 | 93 | 1 | 430 | 1 | 430 | 0 | 1.00E-180 |
| hsa_circ_0007745 | 92 | 1 | 358 | 1 | 358 | 0 | 1.00E-140 |
| hsa_circ_0000159 | 84 | 1 | 562 | 1 | 593 | 0.06 | 5.00E-153 |
| hsa_circ_0000159 | 84 | 1 | 562 | 1 | 593 | 0.06 | 8.00E-154 |
| hsa_circ_0000159 | 81 | 1 | 433 | 1 | 464 | 0.07 | 7.00E-94 |
| hsa_circ_0000159 | 80 | 1 | 396 | 1 | 427 | 0.08 | 1.00E-76 |
| hsa_circ_0015183 | 94 | 1 | 287 | 2858 | 2572 | 0 | 5.00E-123 |
| hsa_circ_0015183 | 94 | 1 | 288 | 2858 | 2571 | 0 | 3.00E-123 |
| hsa_circ_0015190 | 93 | 285 | 585 | 867 | 567 | 0 | 9.00E-124 |
| hsa_circ_0015190 | 94 | 285 | 809 | 867 | 343 | 0 | 0 |
| hsa_circ_0015190 | 93 | 153 | 453 | 867 | 567 | 0 | 7.00E-124 |
| hsa_circ_0015190 | 94 | 153 | 677 | 867 | 343 | 0 | 0 |
| hsa_circ_0015190 | 94 | 153 | 1019 | 867 | 1 | 0 | 0 |
| hsa_circ_0015190 | 94 | 3 | 701 | 862 | 164 | 0 | 0 |
| hsa_circ_0015190 | 94 | 3 | 864 | 862 | 1 | 0 | 0 |
| hsa_circ_0015190 | 94 | 1 | 503 | 666 | 164 | 0 | 0 |
| hsa_circ_0015190 | 94 | 1 | 666 | 666 | 1 | 0 | 0 |
| hsa_circ_0015188 | 94 | 1 | 443 | 443 | 1 | 0 | 0 |
| hsa_circ_0015186 | 94 | 1 | 957 | 1056 | 100 | 0 | 0 |
| hsa_circ_0015186 | 94 | 1 | 960 | 1056 | 97 | 0 | 0 |
| hsa_circ_0015186 | 94 | 1 | 781 | 877 | 97 | 0 | 0 |
| hsa_circ_0015185 | 95 | 199 | 374 | 176 | 1 | 0 | 2.00E-74 |
| hsa_circ_0015184 | 94 | 4 | 290 | 287 | 1 | 0 | 5.00E-123 |
| hsa_circ_0015182 | 86 | 1 | 400 | 401 | 2 | 0 | 1.00E-120 |
| hsa_circ_0015180 | 89 | 1 | 274 | 604 | 331 | 0 | 2.00E-92 |
| hsa_circ_0015180 | 89 | 1 | 386 | 604 | 219 | 0 | 3.00E-136 |
| hsa_circ_0015178 | 86 | 1 | 406 | 403 | 1 | 0.01 | 5.00E-121 |
| hsa_circ_0015174 | 90 | 1 | 471 | 1 | 471 | 0 | 1.00E-171 |
| hsa_circ_0081384 | 86 | 1 | 676 | 49 | 729 | 0.02 | 0 |
| hsa_circ_0081378 | 88 | 1 | 867 | 4189 | 5058 | 0.01 | 0 |
| hsa_circ_0001727 | 88 | 1 | 660 | 1 | 663 | 0.02 | 0 |
| hsa_circ_0004074 | 84 | 521 | 1219 | 623 | 1321 | 0.03 | 0 |
| hsa_circ_0081365 | 88 | 4 | 358 | 85 | 439 | 0 | 2.00E-117 |
| hsa_circ_0081365 | 86 | 4 | 437 | 6 | 439 | 0 | 8.00E-133 |
| hsa_circ_0081365 | 86 | 438 | 875 | 3 | 439 | 0 | 2.00E-132 |
| hsa_circ_0081334 | 96 | 1 | 541 | 1 | 541 | 0 | 0 |
| hsa_circ_0081331 | 96 | 1 | 646 | 1 | 646 | 0 | 0 |
| hsa_circ_0081329 | 95 | 1 | 739 | 1 | 739 | 0 | 0 |
| hsa_circ_0081331 | 96 | 1 | 436 | 1 | 436 | 0 | 0 |
| hsa_circ_0081331 | 96 | 1 | 328 | 1 | 328 | 0 | 2.00E-152 |
| hsa_circ_0081329 | 95 | 1 | 421 | 1 | 421 | 0 | 0 |
| hsa_circ_0081322 | 93 | 6 | 712 | 707 | 1 | 0 | 0 |
| hsa_circ_0081318 | 91 | 1 | 303 | 4392 | 4090 | 0 | 8.00E-112 |
| hsa_circ_0081318 | 90 | 1 | 417 | 4392 | 3976 | 0 | 5.00E-155 |

novel_circ_012979 novel_circ_012980 novel_circ_012981 novel_circ_012982 novel_circ_012984 novel_circ_012985 novel_circ_012986 novel_circ_012987 novel_circ_012988 novel_circ_012989 novel_circ_012990 novel_circ_012991 novel_circ_012992 novel_circ_012994 novel_circ_012995 novel_circ_012996 novel_circ_012997 novel_circ_012998 novel_circ_012999 novel_circ_013000 novel_circ_013001 novel_circ_013002 novel_circ_013003 novel_circ_013004 novel_circ_013005 novel_circ_013006 novel_circ_013007 novel_circ_013008 novel_circ_013009 novel_circ_013010 novel_circ_013011 novel_circ_013012 novel_circ_013013 novel_circ_013014 novel_circ_013015 novel_circ_013016 novel_circ_013017 novel_circ_013018 novel_circ_013019 novel_circ_013020 novel_circ_013021 novel_circ_013022 novel_circ_013023 novel_circ_013024 novel_circ_013025 novel_circ_013026 novel_circ_013027 novel_circ_013028 novel_circ_013029 novel_circ_013030 novel_circ_013031 novel_circ_013032 novel_circ_013033 novel_circ_013034

| hsa_circ_0081318 | 89 | 2 | 755 | 4088 | 3335 | 0 | 0 |
| --- | --- | --- | --- | --- | --- | --- | --- |
| hsa_circ_0081318 | 89 | 2 | 840 | 4088 | 3250 | 0 | 0 |
| hsa_circ_0081318 | 89 | 1 | 725 | 3974 | 3250 | 0 | 0 |
| hsa_circ_0081305 | 90 | 3 | 809 | 3 | 809 | 0 | 0 |
| hsa_circ_0081297 | 90 | 1 | 612 | 1 | 612 | 0 | 0 |
| hsa_circ_0081289 | 90 | 1 | 942 | 657 | 1598 | 0 | 0 |
| hsa_circ_0081289 | 91 | 1 | 675 | 657 | 1331 | 0 | 0 |
| hsa_circ_0081289 | 90 | 1 | 915 | 417 | 1331 | 0 | 0 |
| hsa_circ_0081277 | 91 | 1 | 552 | 1 | 552 | 0 | 0 |
| hsa_circ_0081273 | 91 | 3 | 1229 | 3 | 1229 | 0 | 0 |
| hsa_circ_0081264 | 86 | 3 | 458 | 3 | 458 | 0 | 1.00E-136 |
| hsa_circ_0081252 | 89 | 1 | 1926 | 1 | 1926 | 0 | 0 |
| hsa_circ_0081230 | 94 | 7 | 687 | 7 | 687 | 0 | 0 |
| hsa_circ_0081215 | 86 | 2 | 422 | 2 | 417 | 0.04 | 1.00E-117 |
| hsa_circ_0081215 | 85 | 2 | 379 | 2 | 374 | 0.05 | 5.00E-100 |
| hsa_circ_0081207 | 82 | 1 | 2953 | 1 | 2959 | 0.01 | 0 |
| hsa_circ_0001725 | 83 | 1 | 3103 | 1 | 3109 | 0.01 | 0 |
| hsa_circ_0081205 | 91 | 7 | 426 | 6 | 426 | 0 | 5.00E-160 |
| hsa_circ_0081204 | 91 | 3 | 415 | 3 | 415 | 0 | 4.00E-156 |
| hsa_circ_0081204 | 91 | 3 | 282 | 3 | 282 | 0 | 3.00E-102 |
| hsa_circ_0081204 | 91 | 3 | 281 | 3 | 281 | 0 | 2.00E-103 |
| hsa_circ_0079406 | 92 | 1 | 584 | 584 | 1 | 0 | 0 |
| hsa_circ_0079406 | 92 | 1 | 584 | 584 | 1 | 0 | 0 |
| hsa_circ_0007319 | 91 | 120 | 311 | 890634 | 890443 | 0 | 9.00E-68 |
| hsa_circ_0007319 | 91 | 4 | 189 | 890628 | 890443 | 0 | 3.00E-66 |
| hsa_circ_0007319 | 91 | 120 | 311 | 890634 | 890443 | 0 | 7.00E-68 |
| hsa_circ_0007319 | 91 | 460 | 651 | 890634 | 890443 | 0 | 9.00E-68 |
| hsa_circ_0007319 | 91 | 986 | 1177 | 890634 | 890443 | 0 | 1.00E-67 |
| hsa_circ_0007319 | 91 | 4 | 189 | 890628 | 890443 | 0 | 3.00E-66 |
| hsa_circ_0007319 | 91 | 120 | 311 | 890634 | 890443 | 0 | 6.00E-68 |
| hsa_circ_0007319 | 91 | 986 | 1177 | 890634 | 890443 | 0 | 1.00E-67 |
| hsa_circ_0007319 | 91 | 1336 | 1527 | 890634 | 890443 | 0 | 1.00E-67 |
| hsa_circ_0007319 | 91 | 120 | 311 | 890634 | 890443 | 0 | 5.00E-68 |
| hsa_circ_0007319 | 91 | 120 | 311 | 890634 | 890443 | 0 | 2.00E-68 |
| hsa_circ_0079310 | 83 | 551 | 1928 | 1372 | 1 | 0.01 | 0 |
| hsa_circ_0079312 | 79 | 1 | 1036 | 1030 | 1 | 0.02 | 0 |
| hsa_circ_0079312 | 82 | 642 | 2017 | 1370 | 1 | 0.01 | 0 |
| hsa_circ_0079312 | 82 | 782 | 2157 | 1370 | 1 | 0.01 | 0 |
| hsa_circ_0008656 | 80 | 1 | 1109 | 1103 | 1 | 0.01 | 0 |
| hsa_circ_0079312 | 81 | 452 | 1659 | 1370 | 169 | 0.01 | 0 |
| hsa_circ_0079314 | 91 | 185 | 791 | 607 | 1 | 0 | 0 |
| hsa_circ_0079314 | 91 | 185 | 791 | 607 | 1 | 0 | 0 |
| hsa_circ_0079314 | 92 | 1 | 241 | 241 | 1 | 0 | 8.00E-91 |
| hsa_circ_0079314 | 91 | 1 | 607 | 607 | 1 | 0 | 0 |
| hsa_circ_0079314 | 91 | 185 | 791 | 607 | 1 | 0 | 0 |
| hsa_circ_0079314 | 91 | 375 | 981 | 607 | 1 | 0 | 0 |
| hsa_circ_0079314 | 91 | 510 | 1116 | 607 | 1 | 0 | 0 |
| hsa_circ_0079314 | 91 | 515 | 1121 | 607 | 1 | 0 | 0 |
| hsa_circ_0079316 | 91 | 201 | 740 | 540 | 1 | 0 | 0 |
| hsa_circ_0079316 | 92 | 11 | 448 | 540 | 103 | 0 | 5.00E-175 |
| hsa_circ_0007319 | 89 | 197 | 432 | 969185 | 968950 | 0 | 2.00E-79 |
| hsa_circ_0007319 | 89 | 298 | 533 | 969185 | 968950 | 0 | 2.00E-79 |
| hsa_circ_0079323 | 85 | 5 | 541 | 537 | 1 | 0.01 | 1.00E-149 |
| hsa_circ_0079323 | 85 | 166 | 702 | 537 | 1 | 0.01 | 1.00E-149 |

novel_circ_013035 novel_circ_013036 novel_circ_013038 novel_circ_013039 novel_circ_013040 novel_circ_013041 novel_circ_013042 novel_circ_013043 novel_circ_013044 novel_circ_013045 novel_circ_013046 novel_circ_013047 novel_circ_013049 novel_circ_013054 novel_circ_013055 novel_circ_013056 novel_circ_013057 novel_circ_013059 novel_circ_013060 novel_circ_013061 novel_circ_013062 novel_circ_013063 novel_circ_013064 novel_circ_013065 novel_circ_013067 novel_circ_013069 novel_circ_013070 novel_circ_013071 novel_circ_013072 novel_circ_013073 novel_circ_013074 novel_circ_013075 novel_circ_013076 novel_circ_013077 novel_circ_013078 novel_circ_013079 novel_circ_013080 novel_circ_013081 novel_circ_013085 novel_circ_013086 novel_circ_013087 novel_circ_013088 novel_circ_013089 novel_circ_013091 novel_circ_013092 novel_circ_013093 novel_circ_013094 novel_circ_013095 novel_circ_013096 novel_circ_013097 novel_circ_013098 novel_circ_013099 novel_circ_013100 novel_circ_013101

| hsa_circ_0079323 | 85 | 247 | 783 | 537 | 1 | 0.01 | 1.00E-149 |
| --- | --- | --- | --- | --- | --- | --- | --- |
| hsa_circ_0079323 | 85 | 347 | 883 | 537 | 1 | 0.01 | 1.00E-149 |
| hsa_circ_0079327 | 90 | 1 | 242 | 242 | 1 | 0 | 1.00E-82 |
| hsa_circ_0079327 | 93 | 1 | 181 | 242 | 62 | 0 | 3.00E-69 |
| hsa_circ_0079332 | 96 | 1 | 451 | 1 | 451 | 0 | 0 |
| hsa_circ_0079332 | 96 | 1 | 562 | 1 | 562 | 0 | 0 |
| hsa_circ_0079335 | 98 | 1 | 242 | 1 | 242 | 0 | 1.00E-114 |
| hsa_circ_0079335 | 95 | 1 | 833 | 1 | 833 | 0 | 0 |
| hsa_circ_0079338 | 93 | 1 | 508 | 84 | 591 | 0 | 0 |
| hsa_circ_0079338 | 88 | 1 | 1316 | 84 | 1402 | 0.01 | 0 |
| hsa_circ_0082931 | 96 | 1 | 274 | 1 | 274 | 0 | 2.00E-122 |
| hsa_circ_0082936 | 93 | 569 | 664 | 199 | 104 | 0 | 2.00E-31 |
| hsa_circ_0008095 | 93 | 1 | 410 | 410 | 1 | 0 | 2.00E-169 |
| hsa_circ_0082953 | 87 | 706 | 1081 | 1 | 376 | 0.01 | 2.00E-118 |
| hsa_circ_0082953 | 89 | 706 | 1199 | 1 | 494 | 0 | 2.00E-172 |
| hsa_circ_0082953 | 90 | 706 | 1352 | 1 | 647 | 0 | 0 |
| hsa_circ_0082953 | 89 | 168 | 293 | 1 | 126 | 0 | 8.00E-37 |
| hsa_circ_0082964 | 88 | 1 | 1817 | 1817 | 1 | 0 | 0 |
| hsa_circ_0082971 | 88 | 1 | 2221 | 2224 | 1 | 0 | 0 |
| hsa_circ_0082987 | 79 | 1 | 1913 | 2153 | 211 | 0.02 | 0 |
| hsa_circ_0082987 | 80 | 1 | 2053 | 2296 | 211 | 0.03 | 0 |
| hsa_circ_0082987 | 81 | 1 | 2165 | 2408 | 211 | 0.02 | 0 |
| hsa_circ_0082989 | 89 | 1 | 373 | 376 | 1 | 0.02 | 3.00E-131 |
| hsa_circ_0082989 | 90 | 1 | 485 | 488 | 1 | 0.01 | 0 |
| hsa_circ_0083002 | 89 | 1 | 1167 | 1167 | 1 | 0 | 0 |
| hsa_circ_0083004 | 91 | 1 | 945 | 948 | 4 | 0 | 0 |
| hsa_circ_0083003 | 92 | 1 | 1334 | 1470 | 137 | 0 | 0 |
| hsa_circ_0083010 | 92 | 1 | 666 | 666 | 1 | 0 | 0 |
| hsa_circ_0083011 | 93 | 1 | 948 | 950 | 3 | 0 | 0 |
| hsa_circ_0083011 | 91 | 1 | 1739 | 1741 | 3 | 0 | 0 |
| hsa_circ_0083023 | 89 | 1 | 347 | 347 | 1 | 0 | 7.00E-118 |
| hsa_circ_0083023 | 89 | 28 | 554 | 527 | 1 | 0 | 0 |
| hsa_circ_0083023 | 89 | 1 | 791 | 791 | 1 | 0 | 0 |
| hsa_circ_0083021 | 85 | 3 | 1510 | 1618 | 111 | 0.01 | 0 |
| hsa_circ_0083021 | 84 | 4 | 1585 | 1692 | 111 | 0.01 | 0 |
| hsa_circ_0083028 | 84 | 3 | 1163 | 1161 | 1 | 0.01 | 0 |
| hsa_circ_0083031 | 84 | 3 | 956 | 954 | 1 | 0.01 | 0 |
| hsa_circ_0083028 | 84 | 1 | 1467 | 1677 | 207 | 0.01 | 0 |
| hsa_circ_0083036 | 82 | 1 | 1230 | 1233 | 1 | 0.02 | 0 |
| hsa_circ_0083036 | 84 | 6 | 1517 | 1515 | 1 | 0.01 | 0 |
| hsa_circ_0083036 | 84 | 1 | 1680 | 1683 | 1 | 0.01 | 0 |
| hsa_circ_0083036 | 84 | 1 | 1939 | 1942 | 1 | 0.01 | 0 |
| hsa_circ_0083036 | 85 | 1 | 2140 | 2143 | 1 | 0.01 | 0 |
| hsa_circ_0083042 | 87 | 1 | 434 | 436 | 3 | 0 | 8.00E-138 |
| hsa_circ_0083042 | 89 | 6 | 721 | 718 | 3 | 0 | 0 |
| hsa_circ_0006784 | 90 | 1 | 450 | 450 | 1 | 0 | 5.00E-165 |
| hsa_circ_0006784 | 89 | 1 | 910 | 910 | 1 | 0 | 0 |
| hsa_circ_0083044 | 90 | 93 | 1141 | 1371 | 323 | 0 | 0 |
| hsa_circ_0083049 | 88 | 2 | 1026 | 1022 | 1 | 0 | 0 |
| hsa_circ_0083052 | 89 | 1 | 622 | 623 | 2 | 0 | 0 |
| hsa_circ_0083049 | 89 | 2 | 853 | 1022 | 174 | 0 | 0 |
| hsa_circ_0083056 | 88 | 1 | 458 | 460 | 3 | 0 | 4.00E-156 |
| hsa_circ_0083056 | 89 | 1 | 597 | 599 | 3 | 0 | 0 |
| hsa_circ_0083056 | 89 | 2 | 689 | 687 | 3 | 0 | 0 |

novel_circ_013102 novel_circ_013103 novel_circ_013104 novel_circ_013105 novel_circ_013106 novel_circ_013107 novel_circ_013109 novel_circ_013110 novel_circ_013111 novel_circ_013112 novel_circ_013113 novel_circ_013114 novel_circ_013115 novel_circ_013116 novel_circ_013117 novel_circ_013118 novel_circ_013135 novel_circ_013136 novel_circ_013137 novel_circ_013138 novel_circ_013139 novel_circ_013140 novel_circ_013142 novel_circ_013143 novel_circ_013145 novel_circ_013146 novel_circ_013147 novel_circ_013150 novel_circ_013151 novel_circ_013152 novel_circ_013162 novel_circ_013167 novel_circ_013171 novel_circ_013172 novel_circ_013173 novel_circ_013174 novel_circ_013175 novel_circ_013176 novel_circ_013177 novel_circ_013178 novel_circ_013179 novel_circ_013180 novel_circ_013181 novel_circ_013182 novel_circ_013183 novel_circ_013184 novel_circ_013185 novel_circ_013186 novel_circ_013187 novel_circ_013188 novel_circ_013189 novel_circ_013190 novel_circ_013191 novel_circ_013194

| hsa_circ_0083056 | 91 | 2 | 432 | 687 | 260 | 0.01 | 2.00E-163 |
| --- | --- | --- | --- | --- | --- | --- | --- |
| hsa_circ_0001770 | 91 | 2 | 231 | 227 | 1 | 0.01 | 6.00E-82 |
| hsa_circ_0035037 | 84 | 2232 | 2587 | 744 | 390 | 0.06 | 2.00E-87 |
| hsa_circ_0005181 | 98 | 1 | 59 | 1 | 59 | 0 | 1.00E-20 |
| hsa_circ_0002285 | 95 | 1 | 181 | 292 | 112 | 0 | 1.00E-75 |
| hsa_circ_0083062 | 94 | 282 | 1008 | 125 | 851 | 0 | 0 |
| hsa_circ_0083062 | 95 | 226 | 337 | 125 | 236 | 0 | 3.00E-42 |
| hsa_circ_0083062 | 94 | 226 | 859 | 125 | 758 | 0 | 0 |
| hsa_circ_0083062 | 94 | 226 | 952 | 125 | 851 | 0 | 0 |
| hsa_circ_0083062 | 94 | 1 | 633 | 126 | 758 | 0 | 0 |
| hsa_circ_0083062 | 94 | 1 | 726 | 126 | 851 | 0 | 0 |
| hsa_circ_0083063 | 93 | 1 | 204 | 1 | 204 | 0 | 2.00E-80 |
| hsa_circ_0083063 | 94 | 1 | 522 | 1 | 522 | 0 | 0 |
| hsa_circ_0083063 | 93 | 1 | 615 | 1 | 615 | 0 | 0 |
| hsa_circ_0083065 | 93 | 1 | 411 | 1 | 411 | 0 | 2.00E-173 |
| hsa_circ_0083065 | 93 | 1 | 411 | 1 | 411 | 0 | 1.00E-172 |
| hsa_circ_0067876 | 85 | 178 | 389 | 909 | 1111 | 0.04 | 2.00E-53 |
| hsa_circ_0067876 | 85 | 532 | 743 | 909 | 1111 | 0.04 | 5.00E-53 |
| hsa_circ_0067876 | 85 | 1000 | 1211 | 909 | 1111 | 0.04 | 8.00E-53 |
| hsa_circ_0067876 | 85 | 655 | 866 | 909 | 1111 | 0.04 | 5.00E-53 |
| hsa_circ_0067875 | 81 | 3 | 514 | 249 | 785 | 0.05 | 3.00E-112 |
| hsa_circ_0067875 | 82 | 1 | 637 | 124 | 785 | 0.04 | 4.00E-155 |
| hsa_circ_0025468 | 88 | 11535 | 11603 | 30062 | 29994 | 0 | 2.00E-13 |
| hsa_circ_0047474 | 73 | 16375 | 16856 | 12271 | 11776 | 0.04 | 6.00E-35 |
| hsa_circ_0056968 | 88 | 1682 | 1870 | 344 | 158 | 0.01 | 4.00E-56 |
| hsa_circ_0056968 | 88 | 19146 | 19334 | 344 | 158 | 0.01 | 8.00E-56 |
| hsa_circ_0056968 | 88 | 30743 | 30931 | 344 | 158 | 0.01 | 1.00E-55 |
| hsa_circ_0001357 | 83 | 1195 | 2366 | 1198 | 39 | 0.02 | 0 |
| hsa_circ_0001357 | 83 | 127 | 1298 | 1198 | 39 | 0.02 | 0 |
| hsa_circ_0001357 | 83 | 11 | 1182 | 1198 | 39 | 0.02 | 0 |
| hsa_circ_0034327 | 79 | 31805 | 31976 | 36914 | 36740 | 0.05 | 5.00E-22 |
| hsa_circ_0003760 | 81 | 15899 | 16039 | 2341 | 2199 | 0.04 | 6.00E-20 |
| hsa_circ_0003760 | 81 | 15899 | 16039 | 2341 | 2199 | 0.04 | 4.00E-20 |
| hsa_circ_0071269 | 92 | 1 | 692 | 692 | 1 | 0 | 0 |
| hsa_circ_0071270 | 93 | 1 | 510 | 510 | 1 | 0 | 0 |
| hsa_circ_0001451 | 89 | 50 | 647 | 571 | 1 | 0.05 | 0 |
| hsa_circ_0001451 | 89 | 140 | 737 | 571 | 1 | 0.05 | 0 |
| hsa_circ_0001451 | 89 | 206 | 803 | 571 | 1 | 0.05 | 0 |
| hsa_circ_0071280 | 91 | 1 | 420 | 1 | 420 | 0 | 2.00E-158 |
| hsa_circ_0071280 | 91 | 1 | 642 | 1 | 642 | 0 | 0 |
| hsa_circ_0071280 | 90 | 1 | 800 | 1 | 800 | 0 | 0 |
| hsa_circ_0071280 | 88 | 1 | 975 | 1 | 975 | 0 | 0 |
| hsa_circ_0071280 | 88 | 1 | 977 | 1 | 977 | 0 | 0 |
| hsa_circ_0071280 | 93 | 2522 | 2634 | 99 | 211 | 0 | 1.00E-38 |
| hsa_circ_0071281 | 91 | 1 | 540 | 1 | 540 | 0 | 0 |
| hsa_circ_0071281 | 90 | 1 | 698 | 1 | 698 | 0 | 0 |
| hsa_circ_0071281 | 88 | 1 | 873 | 1 | 873 | 0 | 0 |
| hsa_circ_0071280 | 88 | 1 | 875 | 103 | 977 | 0 | 0 |
| hsa_circ_0071283 | 87 | 1 | 668 | 1 | 668 | 0 | 0 |
| hsa_circ_0071283 | 87 | 1 | 670 | 1 | 670 | 0 | 0 |
| hsa_circ_0071283 | 90 | 1 | 380 | 114 | 493 | 0 | 7.00E-138 |
| hsa_circ_0071283 | 83 | 1 | 333 | 336 | 668 | 0 | 2.00E-83 |
| hsa_circ_0071283 | 84 | 1 | 335 | 336 | 670 | 0 | 1.00E-83 |
| hsa_circ_0071286 | 89 | 1 | 1480 | 1 | 1480 | 0 | 0 |

novel_circ_013195 novel_circ_013196 novel_circ_013198 novel_circ_013199 novel_circ_013200 novel_circ_013203 novel_circ_013204 novel_circ_013205 novel_circ_013208 novel_circ_013209 novel_circ_013210 novel_circ_013211 novel_circ_013212 novel_circ_013213 novel_circ_013214 novel_circ_013215 novel_circ_013216 novel_circ_013219 novel_circ_013220 novel_circ_013225 novel_circ_013228 novel_circ_013229 novel_circ_013230 novel_circ_013231 novel_circ_013232 novel_circ_013233 novel_circ_013234 novel_circ_013235 novel_circ_013236 novel_circ_013237 novel_circ_013241 novel_circ_013242 novel_circ_013243 novel_circ_013244 novel_circ_013245 novel_circ_013246 novel_circ_013247 novel_circ_013248 novel_circ_013249 novel_circ_013250 novel_circ_013251 novel_circ_013252 novel_circ_013253 novel_circ_013254 novel_circ_013255 novel_circ_013256 novel_circ_013257 novel_circ_013258 novel_circ_013262 novel_circ_013263 novel_circ_013264 novel_circ_013265 novel_circ_013266 novel_circ_013267

| hsa_circ_0071287 | 91 | 1 | 416 | 1 | 416 | 0 | 2.00E-159 |
| --- | --- | --- | --- | --- | --- | --- | --- |
| hsa_circ_0071287 | 89 | 1 | 234 | 183 | 416 | 0 | 1.00E-78 |
| hsa_circ_0071288 | 89 | 1 | 1057 | 1 | 1057 | 0 | 0 |
| hsa_circ_0071291 | 88 | 3 | 272 | 3 | 272 | 0 | 7.00E-87 |
| hsa_circ_0071290 | 87 | 1 | 408 | 829 | 1236 | 0 | 6.00E-130 |
| hsa_circ_0071298 | 86 | 1 | 416 | 1 | 416 | 0 | 4.00E-126 |
| hsa_circ_0071298 | 84 | 1 | 347 | 70 | 416 | 0 | 7.00E-93 |
| hsa_circ_0071298 | 84 | 1 | 347 | 70 | 416 | 0 | 3.00E-92 |
| hsa_circ_0008409 | 94 | 1 | 295 | 295 | 1 | 0 | 4.00E-124 |
| hsa_circ_0017818 | 92 | 1 | 238 | 238 | 1 | 0 | 8.00E-91 |
| hsa_circ_0052208 | 88 | 1 | 231 | 237 | 1 | 0.03 | 6.00E-72 |
| hsa_circ_0052245 | 92 | 1 | 468 | 2293 | 1826 | 0 | 0 |
| hsa_circ_0062302 | 90 | 1 | 295 | 165 | 459 | 0 | 1.00E-105 |
| hsa_circ_0062288 | 87 | 1 | 445 | 121 | 565 | 0 | 7.00E-139 |
| hsa_circ_0062252 | 98 | 1 | 360 | 360 | 1 | 0 | 4.00E-180 |
| hsa_circ_0062246 | 95 | 1 | 899 | 970 | 72 | 0 | 0 |
| hsa_circ_0062248 | 93 | 1 | 539 | 539 | 1 | 0 | 0 |
| hsa_circ_0062199 | 92 | 1 | 381 | 381 | 1 | 0 | 4.00E-150 |
| hsa_circ_0062324 | 93 | 89 | 168 | 1 | 80 | 0 | 2.00E-24 |
| hsa_circ_0070661 | 78 | 13523 | 14023 | 2 | 507 | 0.01 | 1.00E-85 |
| hsa_circ_0084622 | 92 | 9 | 313 | 305 | 1 | 0 | 5.00E-121 |
| hsa_circ_0084622 | 92 | 9 | 313 | 305 | 1 | 0 | 3.00E-121 |
| hsa_circ_0084623 | 92 | 9 | 220 | 213 | 2 | 0 | 2.00E-81 |
| hsa_circ_0084628 | 96 | 1 | 144 | 1 | 147 | 0.02 | 9.00E-61 |
| hsa_circ_0084628 | 86 | 176 | 1058 | 146 | 1013 | 0.03 | 0 |
| hsa_circ_0084628 | 86 | 2 | 783 | 247 | 1013 | 0.03 | 0 |
| hsa_circ_0084629 | 85 | 1 | 667 | 1 | 652 | 0.04 | 0 |
| hsa_circ_0084633 | 95 | 62 | 558 | 721 | 225 | 0 | 0 |
| hsa_circ_0084637 | 94 | 4 | 145 | 142 | 1 | 0 | 5.00E-56 |
| hsa_circ_0000143 | 87 | 4363 | 4422 | 48556 | 48498 | 0.02 | 8.00E-08 |
| hsa_circ_0084644 | 92 | 1 | 1447 | 1450 | 1 | 0 | 0 |
| hsa_circ_0084642 | 97 | 1 | 258 | 258 | 1 | 0 | 3.00E-120 |
| hsa_circ_0084642 | 91 | 1 | 627 | 627 | 1 | 0 | 0 |
| hsa_circ_0084642 | 91 | 1 | 861 | 861 | 1 | 0 | 0 |
| hsa_circ_0084642 | 91 | 1 | 966 | 966 | 1 | 0 | 0 |
| hsa_circ_0084644 | 92 | 1 | 1447 | 1450 | 1 | 0 | 0 |
| hsa_circ_0084644 | 89 | 1 | 780 | 783 | 1 | 0 | 0 |
| hsa_circ_0084644 | 92 | 1 | 1447 | 1450 | 1 | 0 | 0 |
| hsa_circ_0084644 | 94 | 1 | 1078 | 1450 | 370 | 0 | 0 |
| hsa_circ_0005883 | 91 | 1 | 176 | 180 | 2 | 0.02 | 2.00E-60 |
| hsa_circ_0005883 | 94 | 1 | 351 | 355 | 2 | 0.01 | 2.00E-147 |
| hsa_circ_0005883 | 95 | 1 | 843 | 847 | 2 | 0 | 0 |
| hsa_circ_0084646 | 96 | 1 | 739 | 742 | 1 | 0 | 0 |
| hsa_circ_0007077 | 96 | 1 | 271 | 271 | 1 | 0 | 4.00E-124 |
| hsa_circ_0004863 | 96 | 1 | 178 | 178 | 1 | 0 | 1.00E-77 |
| hsa_circ_0084648 | 93 | 501 | 649 | 1 | 149 | 0 | 3.00E-54 |
| hsa_circ_0084648 | 93 | 501 | 737 | 1 | 237 | 0 | 9.00E-95 |
| hsa_circ_0005144 | 78 | 17195 | 17415 | 452 | 238 | 0.04 | 2.00E-29 |
| hsa_circ_0084660 | 90 | 2 | 209 | 2 | 209 | 0.01 | 1.00E-72 |
| hsa_circ_0084660 | 88 | 1 | 1268 | 314 | 1584 | 0.01 | 0 |
| hsa_circ_0084660 | 88 | 1 | 1398 | 314 | 1714 | 0 | 0 |
| hsa_circ_0084660 | 89 | 1 | 1546 | 314 | 1862 | 0 | 0 |
| hsa_circ_0084660 | 89 | 1 | 1546 | 314 | 1862 | 0 | 0 |
| hsa_circ_0084660 | 88 | 3 | 1299 | 415 | 1714 | 0.01 | 0 |

novel_circ_013268 novel_circ_013269 novel_circ_013270 novel_circ_013271 novel_circ_013272 novel_circ_013273 novel_circ_013274 novel_circ_013275 novel_circ_013276 novel_circ_013277 novel_circ_013278 novel_circ_013279 novel_circ_013280 novel_circ_013281 novel_circ_013282 novel_circ_013283 novel_circ_013284 novel_circ_013285 novel_circ_013286 novel_circ_013287 novel_circ_013289 novel_circ_013290 novel_circ_013291 novel_circ_013292 novel_circ_013293 novel_circ_013294 novel_circ_013295 novel_circ_013296 novel_circ_013297 novel_circ_013298 novel_circ_013299 novel_circ_013300 novel_circ_013301 novel_circ_013302 novel_circ_013303 novel_circ_013304 novel_circ_013305 novel_circ_013306 novel_circ_013307 novel_circ_013308 novel_circ_013309 novel_circ_013310 novel_circ_013311 novel_circ_013312 novel_circ_013313 novel_circ_013314 novel_circ_013315 novel_circ_013316 novel_circ_013317 novel_circ_013318 novel_circ_013319 novel_circ_013320 novel_circ_013321 novel_circ_013322

| hsa_circ_0084672 | 88 | 9 | 528 | 9 | 531 | 0.01 | 1.00E-172 |
| --- | --- | --- | --- | --- | --- | --- | --- |
| hsa_circ_0084672 | 89 | 9 | 859 | 9 | 862 | 0.01 | 0 |
| hsa_circ_0084672 | 90 | 9 | 1007 | 9 | 1010 | 0 | 0 |
| hsa_circ_0084672 | 92 | 1 | 563 | 171 | 733 | 0 | 0 |
| hsa_circ_0084672 | 92 | 1 | 692 | 171 | 862 | 0 | 0 |
| hsa_circ_0084672 | 92 | 1 | 840 | 171 | 1010 | 0 | 0 |
| hsa_circ_0084672 | 92 | 1 | 840 | 171 | 1010 | 0 | 0 |
| hsa_circ_0084672 | 92 | 1 | 688 | 323 | 1010 | 0 | 0 |
| hsa_circ_0084683 | 92 | 1 | 331 | 1 | 331 | 0 | 5.00E-129 |
| hsa_circ_0084683 | 92 | 1 | 479 | 1 | 479 | 0 | 0 |
| hsa_circ_0084683 | 92 | 1 | 479 | 1 | 479 | 0 | 0 |
| hsa_circ_0084683 | 93 | 1 | 1472 | 1 | 1472 | 0 | 0 |
| hsa_circ_0084683 | 94 | 1 | 993 | 480 | 1472 | 0 | 0 |
| hsa_circ_0084683 | 92 | 1 | 577 | 896 | 1472 | 0 | 0 |
| hsa_circ_0084683 | 92 | 1 | 572 | 901 | 1472 | 0 | 0 |
| hsa_circ_0084683 | 93 | 1 | 430 | 1043 | 1472 | 0 | 1.00E-175 |
| hsa_circ_0084672 | 90 | 1 | 826 | 1679 | 2504 | 0 | 0 |
| hsa_circ_0084672 | 90 | 1 | 759 | 1746 | 2504 | 0 | 0 |
| hsa_circ_0084686 | 91 | 1 | 188 | 1 | 188 | 0 | 2.00E-66 |
| hsa_circ_0084672 | 88 | 1 | 501 | 2004 | 2504 | 0 | 5.00E-170 |
| hsa_circ_0084690 | 93 | 1 | 920 | 1059 | 140 | 0 | 0 |
| hsa_circ_0084690 | 92 | 1 | 611 | 1059 | 449 | 0 | 0 |
| hsa_circ_0084690 | 94 | 1 | 478 | 1059 | 582 | 0 | 0 |
| hsa_circ_0084695 | 95 | 1 | 405 | 405 | 1 | 0 | 0 |
| hsa_circ_0084695 | 96 | 1 | 595 | 870 | 276 | 0 | 0 |
| hsa_circ_0084697 | 97 | 1 | 465 | 465 | 1 | 0 | 0 |
| hsa_circ_0084697 | 96 | 1 | 536 | 828 | 293 | 0 | 0 |
| hsa_circ_0084698 | 96 | 1 | 363 | 363 | 1 | 0 | 4.00E-170 |
| hsa_circ_0084699 | 97 | 1 | 401 | 612 | 212 | 0 | 0 |
| hsa_circ_0084701 | 96 | 1 | 310 | 310 | 1 | 0 | 4.00E-144 |
| hsa_circ_0084701 | 95 | 1 | 1054 | 1054 | 1 | 0 | 0 |
| hsa_circ_0084701 | 95 | 1 | 1166 | 1166 | 1 | 0 | 0 |
| hsa_circ_0084701 | 95 | 1 | 744 | 1054 | 311 | 0 | 0 |
| hsa_circ_0084702 | 95 | 1 | 615 | 615 | 1 | 0 | 0 |
| hsa_circ_0084702 | 95 | 1 | 727 | 727 | 1 | 0 | 0 |
| hsa_circ_0084699 | 95 | 1 | 1647 | 2388 | 742 | 0 | 0 |
| hsa_circ_0084699 | 95 | 1 | 1189 | 2254 | 1066 | 0 | 0 |
| hsa_circ_0084699 | 95 | 4 | 551 | 2016 | 1469 | 0 | 0 |
| hsa_circ_0084699 | 95 | 1 | 894 | 2564 | 1671 | 0 | 0 |
| hsa_circ_0084705 | 96 | 1 | 545 | 645 | 101 | 0 | 0 |
| hsa_circ_0084705 | 95 | 1 | 656 | 756 | 101 | 0 | 0 |
| hsa_circ_0084705 | 95 | 305 | 960 | 756 | 101 | 0 | 0 |
| hsa_circ_0084705 | 95 | 485 | 1140 | 756 | 101 | 0 | 0 |
| hsa_circ_0084705 | 97 | 1 | 310 | 645 | 336 | 0 | 9.00E-146 |
| hsa_circ_0084705 | 96 | 1 | 421 | 756 | 336 | 0 | 0 |
| hsa_circ_0084705 | 96 | 305 | 725 | 756 | 336 | 0 | 0 |
| hsa_circ_0084705 | 94 | 1 | 287 | 756 | 470 | 0 | 5.00E-123 |
| hsa_circ_0084706 | 96 | 26754 | 27268 | 515 | 1 | 0 | 0 |
| hsa_circ_0084708 | 95 | 46011 | 46574 | 1 | 564 | 0 | 0 |
| hsa_circ_0084710 | 94 | 46083 | 46807 | 1 | 725 | 0 | 0 |
| hsa_circ_0084708 | 95 | 1 | 564 | 1 | 564 | 0 | 0 |
| hsa_circ_0084710 | 94 | 73 | 797 | 1 | 725 | 0 | 0 |
| hsa_circ_0084710 | 96 | 1 | 503 | 124 | 626 | 0 | 0 |
| hsa_circ_0084710 | 95 | 1 | 602 | 124 | 725 | 0 | 0 |

novel_circ_013323 novel_circ_013324 novel_circ_013325 novel_circ_013326 novel_circ_013327 novel_circ_013328 novel_circ_013329 novel_circ_013330 novel_circ_013331 novel_circ_013332 novel_circ_013333 novel_circ_013334 novel_circ_013335 novel_circ_013336 novel_circ_013337 novel_circ_013338 novel_circ_013339 novel_circ_013340 novel_circ_013341 novel_circ_013342 novel_circ_013343 novel_circ_013344 novel_circ_013345 novel_circ_013346 novel_circ_013347 novel_circ_013348 novel_circ_013349 novel_circ_013350 novel_circ_013351 novel_circ_013352 novel_circ_013353 novel_circ_013354 novel_circ_013355 novel_circ_013356 novel_circ_013357 novel_circ_013358 novel_circ_013359 novel_circ_013366 novel_circ_013367 novel_circ_013368 novel_circ_013369 novel_circ_013370 novel_circ_013371 novel_circ_013372 novel_circ_013373 novel_circ_013377 novel_circ_013378 novel_circ_013379 novel_circ_013380 novel_circ_013381 novel_circ_013382 novel_circ_013383 novel_circ_013384 novel_circ_013385

| hsa_circ_0084710 | 94 | 1 | 1003 | 124 | 1126 | 0 | 0 |
| --- | --- | --- | --- | --- | --- | --- | --- |
| hsa_circ_0084710 | 94 | 1 | 1449 | 124 | 1572 | 0 | 0 |
| hsa_circ_0084710 | 94 | 1 | 1688 | 124 | 1811 | 0 | 0 |
| hsa_circ_0084710 | 95 | 1 | 497 | 229 | 725 | 0 | 0 |
| hsa_circ_0084710 | 95 | 1 | 395 | 331 | 725 | 0 | 3.00E-176 |
| hsa_circ_0084710 | 93 | 1 | 533 | 493 | 1025 | 0 | 0 |
| hsa_circ_0084710 | 93 | 1 | 634 | 493 | 1126 | 0 | 0 |
| hsa_circ_0084710 | 93 | 1 | 500 | 627 | 1126 | 0 | 0 |
| hsa_circ_0084710 | 93 | 1 | 396 | 731 | 1126 | 0 | 9.00E-167 |
| hsa_circ_0084710 | 93 | 1 | 626 | 731 | 1356 | 0 | 0 |
| hsa_circ_0084710 | 94 | 1 | 1081 | 731 | 1811 | 0 | 0 |
| hsa_circ_0084711 | 93 | 1 | 230 | 247 | 476 | 0 | 4.00E-93 |
| hsa_circ_0084711 | 92 | 1158 | 2406 | 1380 | 2631 | 0 | 0 |
| hsa_circ_0084711 | 96 | 1 | 531 | 401 | 931 | 0 | 0 |
| hsa_circ_0084713 | 95 | 1 | 382 | 74 | 455 | 0 | 1.00E-170 |
| hsa_circ_0084710 | 92 | 470 | 2337 | 2260 | 4130 | 0 | 0 |
| hsa_circ_0084710 | 92 | 247 | 1858 | 2260 | 3874 | 0 | 0 |
| hsa_circ_0084710 | 92 | 247 | 2114 | 2260 | 4130 | 0 | 0 |
| hsa_circ_0084715 | 91 | 129 | 802 | 223 | 896 | 0 | 0 |
| hsa_circ_0084710 | 92 | 129 | 1996 | 2260 | 4130 | 0 | 0 |
| hsa_circ_0084715 | 91 | 1 | 431 | 466 | 896 | 0 | 8.00E-168 |
| hsa_circ_0084711 | 92 | 1 | 1006 | 1623 | 2631 | 0 | 0 |
| hsa_circ_0084710 | 92 | 1 | 1625 | 2503 | 4130 | 0 | 0 |
| hsa_circ_0084710 | 92 | 1 | 1883 | 2503 | 4388 | 0 | 0 |
| hsa_circ_0084710 | 92 | 1 | 938 | 2934 | 3874 | 0 | 0 |
| hsa_circ_0084710 | 93 | 1 | 1194 | 2934 | 4130 | 0 | 0 |
| hsa_circ_0084710 | 93 | 1 | 922 | 3209 | 4130 | 0 | 0 |
| hsa_circ_0084710 | 93 | 1 | 389 | 3383 | 3771 | 0 | 2.00E-159 |
| hsa_circ_0084710 | 93 | 1 | 492 | 3383 | 3874 | 0 | 0 |
| hsa_circ_0084710 | 93 | 1 | 748 | 3383 | 4130 | 0 | 0 |
| hsa_circ_0084710 | 92 | 3 | 321 | 3556 | 3874 | 0 | 1.00E-125 |
| hsa_circ_0084710 | 93 | 3 | 465 | 3556 | 4018 | 0 | 0 |
| hsa_circ_0084710 | 93 | 3 | 577 | 3556 | 4130 | 0 | 0 |
| hsa_circ_0084710 | 94 | 1 | 256 | 3875 | 4130 | 0 | 8.00E-106 |
| hsa_circ_0084710 | 92 | 3 | 370 | 4021 | 4388 | 0 | 3.00E-146 |
| hsa_circ_0068512 | 76 | 17929 | 18317 | 28967 | 28581 | 0.02 | 2.00E-43 |
| hsa_circ_0068512 | 76 | 17929 | 18317 | 28967 | 28581 | 0.02 | 2.00E-43 |
| hsa_circ_0008658 | 95 | 1 | 365 | 1 | 365 | 0 | 1.00E-159 |
| hsa_circ_0088361 | 90 | 2 | 577 | 2 | 577 | 0.01 | 0 |
| hsa_circ_0088357 | 92 | 1 | 330 | 650 | 321 | 0 | 2.00E-128 |
| hsa_circ_0088353 | 93 | 135 | 922 | 788 | 1 | 0 | 0 |
| hsa_circ_0088354 | 92 | 1534 | 2109 | 576 | 1 | 0 | 0 |
| hsa_circ_0088354 | 92 | 1 | 576 | 576 | 1 | 0 | 0 |
| hsa_circ_0088353 | 93 | 1 | 788 | 788 | 1 | 0 | 0 |
| hsa_circ_0088352 | 95 | 1 | 154 | 1798 | 1951 | 0 | 1.00E-63 |
| hsa_circ_0088326 | 89 | 1 | 639 | 4204 | 4842 | 0 | 0 |
| hsa_circ_0088326 | 89 | 1 | 915 | 3928 | 4842 | 0 | 0 |
| hsa_circ_0088333 | 91 | 1 | 376 | 117 | 492 | 0 | 1.00E-140 |
| hsa_circ_0088333 | 89 | 1 | 334 | 1 | 334 | 0 | 5.00E-114 |
| hsa_circ_0088332 | 90 | 1 | 441 | 400 | 840 | 0 | 1.00E-161 |
| hsa_circ_0001884 | 91 | 1 | 801 | 1 | 801 | 0 | 0 |
| hsa_circ_0088326 | 91 | 1 | 552 | 649 | 1200 | 0 | 0 |
| hsa_circ_0088325 | 91 | 251 | 2241 | 1 | 1985 | 0 | 0 |
| hsa_circ_0088326 | 92 | 1 | 375 | 649 | 1023 | 0 | 4.00E-150 |

novel_circ_013386 novel_circ_013387 novel_circ_013388 novel_circ_013389 novel_circ_013390 novel_circ_013391 novel_circ_013392 novel_circ_013393 novel_circ_013394 novel_circ_013395 novel_circ_013396 novel_circ_013397 novel_circ_013398 novel_circ_013399 novel_circ_013400 novel_circ_013401 novel_circ_013402 novel_circ_013403 novel_circ_013404 novel_circ_013405 novel_circ_013406 novel_circ_013408 novel_circ_013411 novel_circ_013412 novel_circ_013413 novel_circ_013414 novel_circ_013431 novel_circ_013432 novel_circ_013433 novel_circ_013441 novel_circ_013442 novel_circ_013445 novel_circ_013449 novel_circ_013450 novel_circ_013451 novel_circ_013452 novel_circ_013453 novel_circ_013455 novel_circ_013456 novel_circ_013457 novel_circ_013458 novel_circ_013459 novel_circ_013460 novel_circ_013461 novel_circ_013462 novel_circ_013463 novel_circ_013464 novel_circ_013465 novel_circ_013466 novel_circ_013467 novel_circ_013468 novel_circ_013469 novel_circ_013470 novel_circ_013471

| hsa_circ_0088325 | 91 | 251 | 1689 | 1 | 1433 | 0.01 | 0 |
| --- | --- | --- | --- | --- | --- | --- | --- |
| hsa_circ_0088325 | 91 | 251 | 1494 | 1 | 1238 | 0 | 0 |
| hsa_circ_0088325 | 91 | 251 | 1384 | 1 | 1128 | 0.01 | 0 |
| hsa_circ_0088325 | 92 | 1 | 654 | 132 | 785 | 0 | 0 |
| hsa_circ_0088325 | 92 | 251 | 1035 | 1 | 785 | 0 | 0 |
| hsa_circ_0088325 | 93 | 1 | 585 | 1 | 585 | 0 | 0 |
| hsa_circ_0088325 | 93 | 251 | 835 | 1 | 585 | 0 | 0 |
| hsa_circ_0088325 | 93 | 251 | 654 | 1 | 404 | 0 | 3.00E-169 |
| hsa_circ_0047460 | 85 | 1 | 266 | 136 | 401 | 0 | 5.00E-73 |
| hsa_circ_0047460 | 85 | 4677 | 4947 | 130 | 401 | 0 | 8.00E-73 |
| hsa_circ_0047460 | 87 | 1 | 401 | 1 | 401 | 0 | 8.00E-123 |
| hsa_circ_0047459 | 86 | 1 | 515 | 686 | 1200 | 0 | 2.00E-159 |
| hsa_circ_0047437 | 87 | 123 | 616 | 1 | 489 | 0.01 | 5.00E-154 |
| hsa_circ_0047435 | 92 | 1 | 449 | 1 | 449 | 0 | 1.00E-180 |
| hsa_circ_0047420 | 93 | 1 | 784 | 2190 | 1407 | 0 | 0 |
| hsa_circ_0047422 | 93 | 4 | 376 | 375 | 3 | 0 | 3.00E-152 |
| hsa_circ_0047420 | 93 | 4 | 459 | 1862 | 1407 | 0 | 0 |
| hsa_circ_0047420 | 93 | 4 | 650 | 1862 | 1216 | 0 | 0 |
| hsa_circ_0000842 | 96 | 1 | 638 | 638 | 1 | 0 | 0 |
| hsa_circ_0002659 | 82 | 12603 | 12690 | 181272 | 181357 | 0.04 | 6.00E-10 |
| hsa_circ_0002659 | 82 | 12512 | 12599 | 181272 | 181357 | 0.04 | 6.00E-10 |
| hsa_circ_0047402 | 95 | 1 | 610 | 1 | 610 | 0 | 0 |
| hsa_circ_0047388 | 92 | 1 | 299 | 551 | 849 | 0 | 6.00E-118 |
| hsa_circ_0047388 | 95 | 1 | 787 | 63 | 849 | 0 | 0 |
| hsa_circ_0047391 | 96 | 1 | 360 | 129 | 488 | 0 | 2.00E-168 |
| hsa_circ_0047391 | 97 | 1 | 488 | 1 | 488 | 0 | 0 |
| hsa_circ_0047386 | 93 | 395 | 527 | 133 | 1 | 0 | 9.00E-49 |
| hsa_circ_0047386 | 93 | 2379 | 2511 | 133 | 1 | 0 | 4.00E-48 |
| hsa_circ_0047386 | 93 | 226 | 358 | 133 | 1 | 0 | 6.00E-49 |
| hsa_circ_0063052 | 80 | 7433 | 7541 | 2177 | 2070 | 0.06 | 3.00E-11 |
| hsa_circ_0063052 | 80 | 9711 | 9819 | 2177 | 2070 | 0.06 | 3.00E-11 |
| hsa_circ_0063052 | 80 | 7433 | 7541 | 2177 | 2070 | 0.06 | 3.00E-11 |
| hsa_circ_0007319 | 86 | 3449 | 3516 | 357037 | 357104 | 0.06 | 2.00E-09 |
| hsa_circ_0047385 | 93 | 1 | 1173 | 1173 | 1 | 0 | 0 |
| hsa_circ_0005122 | 96 | 1 | 451 | 1 | 451 | 0 | 0 |
| hsa_circ_0005122 | 96 | 1 | 282 | 1 | 282 | 0 | 3.00E-129 |
| hsa_circ_0005122 | 96 | 1 | 282 | 1 | 282 | 0 | 3.00E-130 |
| hsa_circ_0047374 | 97 | 282 | 698 | 1242 | 826 | 0 | 0 |
| hsa_circ_0047374 | 97 | 87 | 503 | 1242 | 826 | 0 | 0 |
| hsa_circ_0047374 | 96 | 87 | 597 | 1242 | 732 | 0 | 0 |
| hsa_circ_0047374 | 93 | 87 | 1331 | 1242 | 1 | 0 | 0 |
| hsa_circ_0047375 | 92 | 498 | 585 | 263 | 176 | 0 | 5.00E-27 |
| hsa_circ_0047374 | 97 | 3 | 419 | 1242 | 826 | 0 | 0 |
| hsa_circ_0047374 | 93 | 3 | 1040 | 1242 | 208 | 0 | 0 |
| hsa_circ_0047374 | 93 | 3 | 1247 | 1242 | 1 | 0 | 0 |
| hsa_circ_0047366 | 93 | 3 | 2250 | 2391 | 156 | 0.01 | 0 |
| hsa_circ_0047366 | 93 | 3 | 2405 | 2391 | 1 | 0.01 | 0 |
| hsa_circ_0047374 | 99 | 1 | 158 | 979 | 822 | 0 | 3.00E-74 |
| hsa_circ_0047374 | 98 | 1 | 248 | 979 | 732 | 0 | 5.00E-118 |
| hsa_circ_0047374 | 92 | 1 | 775 | 979 | 208 | 0.01 | 0 |
| hsa_circ_0047374 | 92 | 1 | 876 | 979 | 107 | 0.01 | 0 |
| hsa_circ_0047366 | 93 | 1 | 1771 | 2128 | 358 | 0 | 0 |
| hsa_circ_0047366 | 93 | 1 | 1773 | 2128 | 356 | 0 | 0 |
| hsa_circ_0047354 | 93 | 1 | 2731 | 3456 | 738 | 0.01 | 0 |

novel_circ_013472 novel_circ_013473 novel_circ_013474 novel_circ_013475 novel_circ_013476 novel_circ_013477 novel_circ_013478 novel_circ_013479 novel_circ_013480 novel_circ_013481 novel_circ_013482 novel_circ_013483 novel_circ_013484 novel_circ_013485 novel_circ_013486 novel_circ_013487 novel_circ_013488 novel_circ_013489 novel_circ_013490 novel_circ_013491 novel_circ_013492 novel_circ_013493 novel_circ_013494 novel_circ_013495 novel_circ_013496 novel_circ_013497 novel_circ_013498 novel_circ_013499 novel_circ_013500 novel_circ_013501 novel_circ_013502 novel_circ_013503 novel_circ_013504 novel_circ_013507 novel_circ_013508 novel_circ_013509 novel_circ_013510 novel_circ_013511 novel_circ_013512 novel_circ_013513 novel_circ_013514 novel_circ_013515 novel_circ_013516 novel_circ_013517 novel_circ_013518 novel_circ_013519 novel_circ_013520 novel_circ_013521 novel_circ_013522 novel_circ_013523 novel_circ_013524 novel_circ_013525 novel_circ_013526 novel_circ_013527

| hsa_circ_0047354 | 93 | 1 | 2828 | 3456 | 641 | 0 | 0 |
| --- | --- | --- | --- | --- | --- | --- | --- |
| hsa_circ_0047374 | 90 | 1 | 621 | 825 | 208 | 0.01 | 0 |
| hsa_circ_0047366 | 92 | 1 | 1489 | 1974 | 489 | 0 | 0 |
| hsa_circ_0047366 | 92 | 1 | 1617 | 1974 | 358 | 0 | 0 |
| hsa_circ_0047366 | 92 | 2 | 1200 | 1882 | 684 | 0 | 0 |
| hsa_circ_0047366 | 92 | 2 | 1892 | 1882 | 1 | 0.01 | 0 |
| hsa_circ_0047367 | 93 | 1 | 673 | 673 | 1 | 0 | 0 |
| hsa_circ_0047366 | 93 | 1 | 868 | 1356 | 489 | 0 | 0 |
| hsa_circ_0047366 | 93 | 1 | 996 | 1356 | 358 | 0 | 0 |
| hsa_circ_0047366 | 93 | 1 | 1365 | 1356 | 1 | 0.01 | 0 |
| hsa_circ_0047361 | 93 | 1 | 1956 | 2113 | 167 | 0 | 0 |
| hsa_circ_0047367 | 94 | 1 | 572 | 572 | 1 | 0 | 0 |
| hsa_circ_0047367 | 93 | 1 | 466 | 466 | 1 | 0 | 0 |
| hsa_circ_0047366 | 93 | 1 | 789 | 1149 | 358 | 0 | 0 |
| hsa_circ_0047366 | 92 | 1 | 1158 | 1149 | 1 | 0.01 | 0 |
| hsa_circ_0047361 | 93 | 1 | 1749 | 1906 | 167 | 0.01 | 0 |
| hsa_circ_0047367 | 93 | 1 | 334 | 334 | 1 | 0 | 5.00E-134 |
| hsa_circ_0047366 | 92 | 1 | 692 | 683 | 1 | 0.01 | 0 |
| hsa_circ_0047361 | 93 | 1 | 1283 | 1440 | 167 | 0.01 | 0 |
| hsa_circ_0047361 | 93 | 1 | 746 | 912 | 167 | 0 | 0 |
| hsa_circ_0047361 | 94 | 1 | 688 | 757 | 70 | 0 | 0 |
| hsa_circ_0047358 | 93 | 2 | 373 | 648 | 277 | 0 | 9.00E-152 |
| hsa_circ_0047358 | 92 | 2 | 547 | 648 | 103 | 0 | 0 |
| hsa_circ_0047358 | 91 | 4 | 302 | 401 | 103 | 0 | 3.00E-111 |
| hsa_circ_0047350 | 93 | 1 | 356 | 356 | 1 | 0 | 1.00E-144 |
| hsa_circ_0047348 | 93 | 1 | 473 | 661 | 189 | 0 | 0 |
| hsa_circ_0047348 | 93 | 1 | 661 | 661 | 1 | 0 | 0 |
| hsa_circ_0047348 | 93 | 1 | 661 | 661 | 1 | 0 | 0 |
| hsa_circ_0047348 | 93 | 1 | 544 | 544 | 1 | 0 | 0 |
| hsa_circ_0047349 | 95 | 3 | 242 | 240 | 1 | 0 | 6.00E-102 |
| hsa_circ_0047348 | 94 | 3 | 430 | 428 | 1 | 0 | 0 |
| hsa_circ_0047348 | 94 | 3 | 430 | 428 | 1 | 0 | 0 |
| hsa_circ_0047348 | 93 | 1 | 305 | 305 | 1 | 0 | 5.00E-126 |
| hsa_circ_0087801 | 94 | 1 | 506 | 3790 | 3284 | 0 | 0 |
| hsa_circ_0087806 | 92 | 1 | 385 | 515 | 131 | 0 | 3.00E-152 |
| hsa_circ_0087810 | 91 | 3 | 890 | 4041 | 3154 | 0 | 0 |
| hsa_circ_0087810 | 89 | 3 | 392 | 4041 | 3652 | 0 | 4.00E-135 |
| hsa_circ_0087810 | 90 | 3 | 299 | 4041 | 3745 | 0 | 3.00E-105 |
| hsa_circ_0005443 | 92 | 1 | 350 | 355 | 6 | 0 | 1.00E-137 |
| hsa_circ_0005443 | 92 | 1 | 350 | 355 | 6 | 0 | 7.00E-138 |
| hsa_circ_0005443 | 92 | 152 | 501 | 355 | 6 | 0 | 1.00E-137 |
| hsa_circ_0087820 | 95 | 1 | 233 | 258 | 490 | 0 | 2.00E-101 |
| hsa_circ_0087820 | 95 | 1 | 370 | 258 | 627 | 0 | 1.00E-165 |
| hsa_circ_0087820 | 94 | 1 | 464 | 258 | 721 | 0 | 0 |
| hsa_circ_0087820 | 96 | 1 | 724 | 258 | 981 | 0 | 0 |
| hsa_circ_0087820 | 95 | 1 | 1458 | 258 | 1715 | 0 | 0 |
| hsa_circ_0087820 | 95 | 1 | 1914 | 258 | 2171 | 0 | 0 |
| hsa_circ_0087820 | 95 | 1 | 280 | 348 | 627 | 0 | 2.00E-122 |
| hsa_circ_0087823 | 96 | 1 | 491 | 1 | 491 | 0 | 0 |
| hsa_circ_0087824 | 95 | 3 | 494 | 3 | 494 | 0 | 0 |
| hsa_circ_0087824 | 95 | 3 | 498 | 3 | 498 | 0 | 0 |
| hsa_circ_0087824 | 95 | 3 | 1088 | 3 | 1088 | 0 | 0 |
| hsa_circ_0087824 | 98 | 1 | 260 | 95 | 354 | 0 | 1.00E-124 |
| hsa_circ_0087824 | 96 | 1 | 400 | 95 | 494 | 0 | 0 |

novel_circ_013528 novel_circ_013529 novel_circ_013530 novel_circ_013531 novel_circ_013532 novel_circ_013533 novel_circ_013534 novel_circ_013535 novel_circ_013536 novel_circ_013537 novel_circ_013538 novel_circ_013539 novel_circ_013540 novel_circ_013541 novel_circ_013542 novel_circ_013543 novel_circ_013544 novel_circ_013545 novel_circ_013546 novel_circ_013547 novel_circ_013548 novel_circ_013549 novel_circ_013550 novel_circ_013551 novel_circ_013552 novel_circ_013553 novel_circ_013554 novel_circ_013555 novel_circ_013556 novel_circ_013557 novel_circ_013558 novel_circ_013559 novel_circ_013560 novel_circ_013561 novel_circ_013562 novel_circ_013563 novel_circ_013564 novel_circ_013565 novel_circ_013566 novel_circ_013567 novel_circ_013568 novel_circ_013569 novel_circ_013570 novel_circ_013571 novel_circ_013577 novel_circ_013578 novel_circ_013579 novel_circ_013580 novel_circ_013582 novel_circ_013584 novel_circ_013585 novel_circ_013586 novel_circ_013587 novel_circ_013588

| hsa_circ_0087824 | 96 | 1 | 404 | 95 | 498 | 0 | 0 |
| --- | --- | --- | --- | --- | --- | --- | --- |
| hsa_circ_0087824 | 95 | 1 | 994 | 95 | 1088 | 0 | 0 |
| hsa_circ_0087824 | 95 | 1 | 1450 | 95 | 1544 | 0 | 0 |
| hsa_circ_0087825 | 94 | 1 | 740 | 1 | 740 | 0 | 0 |
| hsa_circ_0087825 | 94 | 1 | 824 | 1 | 824 | 0 | 0 |
| hsa_circ_0087825 | 95 | 1 | 1280 | 1 | 1280 | 0 | 0 |
| hsa_circ_0087829 | 95 | 1 | 594 | 1 | 594 | 0 | 0 |
| hsa_circ_0087838 | 92 | 1 | 228 | 1 | 228 | 0 | 6.00E-87 |
| hsa_circ_0087840 | 94 | 1 | 431 | 1 | 431 | 0 | 0 |
| hsa_circ_0087840 | 94 | 1 | 431 | 1 | 431 | 0 | 0 |
| hsa_circ_0003939 | 96 | 1 | 353 | 1 | 353 | 0 | 6.00E-163 |
| hsa_circ_0003939 | 95 | 1 | 442 | 1 | 442 | 0 | 0 |
| hsa_circ_0005171 | 93 | 1516 | 1942 | 1 | 427 | 0 | 1.00E-176 |
| hsa_circ_0003842 | 89 | 1673 | 2221 | 1 | 549 | 0 | 0 |
| hsa_circ_0003842 | 89 | 158 | 706 | 1 | 549 | 0 | 0 |
| hsa_circ_0003842 | 89 | 158 | 706 | 1 | 549 | 0 | 0 |
| hsa_circ_0003842 | 91 | 1 | 391 | 1 | 391 | 0 | 6.00E-149 |
| hsa_circ_0003842 | 89 | 1 | 549 | 1 | 549 | 0 | 0 |
| hsa_circ_0003842 | 89 | 1 | 549 | 1 | 549 | 0 | 0 |
| hsa_circ_0003842 | 84 | 1 | 276 | 274 | 549 | 0 | 3.00E-69 |
| hsa_circ_0003842 | 84 | 1 | 276 | 274 | 549 | 0 | 6.00E-68 |
| hsa_circ_0087849 | 94 | 1 | 6272 | 1 | 6265 | 0 | 0 |
| hsa_circ_0087849 | 93 | 1 | 6464 | 1 | 6457 | 0 | 0 |
| hsa_circ_0087849 | 93 | 1 | 6468 | 1 | 6460 | 0 | 0 |
| hsa_circ_0087849 | 93 | 1 | 6468 | 1 | 6460 | 0 | 0 |
| hsa_circ_0087849 | 95 | 278 | 911 | 6452 | 7086 | 0 | 0 |
| hsa_circ_0087849 | 94 | 7398 | 7807 | 6452 | 6862 | 0 | 7.00E-175 |
| hsa_circ_0087849 | 94 | 86 | 495 | 6452 | 6862 | 0 | 4.00E-176 |
| hsa_circ_0087849 | 95 | 86 | 719 | 6452 | 7086 | 0 | 0 |
| hsa_circ_0002064 | 95 | 91 | 852 | 1 | 762 | 0 | 0 |
| hsa_circ_0002064 | 94 | 1 | 405 | 1 | 405 | 0 | 4.00E-175 |
| hsa_circ_0002064 | 95 | 1 | 629 | 1 | 629 | 0 | 0 |
| hsa_circ_0002064 | 95 | 1 | 762 | 1 | 762 | 0 | 0 |
| hsa_circ_0007271 | 90 | 1 | 431 | 1 | 431 | 0.01 | 2.00E-159 |
| hsa_circ_0007271 | 91 | 1 | 487 | 1 | 487 | 0.01 | 0 |
| hsa_circ_0007271 | 92 | 1 | 615 | 1 | 615 | 0.01 | 0 |
| hsa_circ_0007271 | 91 | 1 | 1050 | 1 | 1050 | 0.01 | 0 |
| hsa_circ_0087859 | 90 | 3 | 405 | 3 | 405 | 0.01 | 1.00E-145 |
| hsa_circ_0087859 | 91 | 3 | 533 | 3 | 533 | 0.01 | 0 |
| hsa_circ_0087859 | 90 | 3 | 968 | 3 | 968 | 0.01 | 0 |
| hsa_circ_0087862 | 90 | 1 | 453 | 1 | 453 | 0.01 | 5.00E-165 |
| hsa_circ_0087863 | 91 | 1 | 619 | 1 | 619 | 0 | 0 |
| hsa_circ_0087863 | 90 | 1 | 563 | 57 | 619 | 0 | 0 |
| hsa_circ_0001880 | 89 | 1 | 435 | 1 | 435 | 0 | 2.00E-154 |
| hsa_circ_0087868 | 89 | 13 | 1093 | 1303 | 223 | 0 | 0 |
| hsa_circ_0087868 | 89 | 439 | 2433 | 2217 | 223 | 0 | 0 |
| hsa_circ_0087870 | 89 | 13 | 561 | 932 | 384 | 0 | 0 |
| hsa_circ_0087870 | 89 | 439 | 1901 | 1846 | 384 | 0 | 0 |
| hsa_circ_0087870 | 89 | 190 | 1302 | 1846 | 734 | 0 | 0 |
| hsa_circ_0087872 | 88 | 3 | 685 | 790 | 108 | 0 | 0 |
| hsa_circ_0087872 | 89 | 190 | 1091 | 1009 | 108 | 0 | 0 |
| hsa_circ_0087874 | 91 | 1 | 1057 | 1057 | 1 | 0 | 0 |
| hsa_circ_0087872 | 91 | 2 | 719 | 1009 | 292 | 0 | 0 |
| hsa_circ_0087875 | 91 | 2 | 967 | 1201 | 236 | 0 | 0 |

novel_circ_013589 novel_circ_013590 novel_circ_013591 novel_circ_013592 novel_circ_013593 novel_circ_013594 novel_circ_013598 novel_circ_013603 novel_circ_013604 novel_circ_013605 novel_circ_013608 novel_circ_013609 novel_circ_013610 novel_circ_013611 novel_circ_013612 novel_circ_013613 novel_circ_013620 novel_circ_013621 novel_circ_013622 novel_circ_013625 novel_circ_013626 novel_circ_013627 novel_circ_013628 novel_circ_013629 novel_circ_013630 novel_circ_013631 novel_circ_013633 novel_circ_013634 novel_circ_013635 novel_circ_013637 novel_circ_013638 novel_circ_013639 novel_circ_013641 novel_circ_013642 novel_circ_013644 novel_circ_013645 novel_circ_013646 novel_circ_013647 novel_circ_013648 novel_circ_013650 novel_circ_013651 novel_circ_013652 novel_circ_013653 novel_circ_013654 novel_circ_013655 novel_circ_013656 novel_circ_013657 novel_circ_013658 novel_circ_013659 novel_circ_013660 novel_circ_013661 novel_circ_013662 novel_circ_013663 novel_circ_013664

| hsa_circ_0087875 | 95 | 4 | 312 | 634 | 326 | 0 | 7.00E-137 |
| --- | --- | --- | --- | --- | --- | --- | --- |
| hsa_circ_0087875 | 92 | 1 | 561 | 886 | 326 | 0 | 0 |
| hsa_circ_0087879 | 90 | 2 | 316 | 564 | 250 | 0 | 4.00E-115 |
| hsa_circ_0007483 | 87 | 15 | 233 | 15 | 233 | 0 | 3.00E-65 |
| hsa_circ_0007483 | 88 | 15 | 238 | 15 | 238 | 0 | 1.00E-66 |
| hsa_circ_0006833 | 75 | 143 | 326 | 221600 | 221787 | 0.02 | 2.00E-13 |
| hsa_circ_0007483 | 94 | 1 | 233 | 1 | 233 | 0 | 3.00E-96 |
| hsa_circ_0024897 | 90 | 1 | 625 | 760 | 136 | 0 | 0 |
| hsa_circ_0024897 | 90 | 1 | 625 | 760 | 136 | 0 | 0 |
| hsa_circ_0024897 | 90 | 1 | 625 | 760 | 136 | 0 | 0 |
| hsa_circ_0047094 | 89 | 1170 | 1223 | 30042 | 29989 | 0 | 8.00E-09 |
| hsa_circ_0024884 | 92 | 1 | 333 | 550 | 218 | 0 | 2.00E-133 |
| hsa_circ_0024884 | 95 | 1 | 550 | 550 | 1 | 0 | 0 |
| hsa_circ_0024884 | 95 | 1 | 550 | 550 | 1 | 0 | 0 |
| hsa_circ_0024870 | 84 | 412 | 1098 | 1 | 705 | 0.03 | 0 |
| hsa_circ_0024870 | 84 | 2017 | 2703 | 1 | 705 | 0.03 | 0 |
| hsa_circ_0085023 | 82 | 15700 | 16060 | 105 | 471 | 0.02 | 3.00E-80 |
| hsa_circ_0085023 | 82 | 15700 | 16060 | 105 | 471 | 0.02 | 5.00E-80 |
| hsa_circ_0085023 | 82 | 15700 | 16060 | 105 | 471 | 0.02 | 5.00E-80 |
| hsa_circ_0024843 | 88 | 23118 | 23289 | 172 | 1 | 0 | 2.00E-50 |
| hsa_circ_0024842 | 90 | 23118 | 23427 | 310 | 1 | 0 | 8.00E-109 |
| hsa_circ_0005790 | 89 | 23118 | 23721 | 604 | 1 | 0 | 0 |
| hsa_circ_0024838 | 90 | 23118 | 23803 | 686 | 1 | 0 | 0 |
| hsa_circ_0024838 | 90 | 23118 | 23803 | 686 | 1 | 0 | 0 |
| hsa_circ_0024838 | 90 | 4 | 601 | 598 | 1 | 0 | 0 |
| hsa_circ_0024838 | 90 | 4 | 601 | 598 | 1 | 0 | 0 |
| hsa_circ_0024838 | 91 | 4 | 514 | 511 | 1 | 0 | 0 |
| hsa_circ_0024838 | 94 | 1 | 160 | 160 | 1 | 0 | 3.00E-62 |
| hsa_circ_0024838 | 94 | 1 | 160 | 160 | 1 | 0 | 4.00E-62 |
| hsa_circ_0024830 | 90 | 3 | 909 | 907 | 1 | 0 | 0 |
| hsa_circ_0063701 | 88 | 2 | 716 | 2 | 716 | 0 | 0 |
| hsa_circ_0063700 | 89 | 239 | 1042 | 1 | 804 | 0 | 0 |
| hsa_circ_0063682 | 90 | 210 | 525 | 569 | 254 | 0 | 8.00E-114 |
| hsa_circ_0063682 | 90 | 210 | 634 | 569 | 145 | 0 | 1.00E-152 |
| hsa_circ_0063654 | 90 | 1 | 574 | 574 | 1 | 0 | 0 |
| hsa_circ_0063651 | 91 | 1 | 832 | 3277 | 2446 | 0 | 0 |
| hsa_circ_0063651 | 91 | 1 | 962 | 3277 | 2316 | 0 | 0 |
| hsa_circ_0063651 | 91 | 1 | 388 | 2703 | 2316 | 0 | 1.00E-150 |
| hsa_circ_0008561 | 90 | 1020 | 5724 | 4720 | 1 | 0.01 | 0 |
| hsa_circ_0063631 | 95 | 1 | 563 | 193 | 755 | 0 | 0 |
| hsa_circ_0063613 | 92 | 1 | 1488 | 1 | 1488 | 0 | 0 |
| hsa_circ_0063613 | 92 | 1 | 1318 | 1 | 1318 | 0 | 0 |
| hsa_circ_0063590 | 87 | 3 | 535 | 3 | 535 | 0 | 5.00E-171 |
| hsa_circ_0063496 | 94 | 1 | 664 | 1012 | 1675 | 0 | 0 |
| hsa_circ_0008175 | 94 | 1 | 529 | 1 | 529 | 0 | 0 |
| hsa_circ_0063496 | 91 | 1 | 684 | 687 | 1370 | 0 | 0 |
| hsa_circ_0063492 | 90 | 2 | 1382 | 480 | 1860 | 0 | 0 |
| hsa_circ_0063492 | 91 | 1 | 713 | 247 | 959 | 0 | 0 |
| hsa_circ_0063492 | 91 | 2 | 293 | 480 | 771 | 0 | 9.00E-111 |
| hsa_circ_0063492 | 92 | 1 | 525 | 247 | 771 | 0 | 0 |
| hsa_circ_0003184 | 94 | 1 | 232 | 1 | 232 | 0 | 7.00E-96 |
| hsa_circ_0063479 | 93 | 1 | 815 | 490 | 1301 | 0 | 0 |
| hsa_circ_0007297 | 93 | 1 | 638 | 1 | 635 | 0 | 0 |
| hsa_circ_0004469 | 94 | 1 | 157 | 80 | 236 | 0 | 5.00E-61 |

novel_circ_013665 novel_circ_013666 novel_circ_013667 novel_circ_013668 novel_circ_013670 novel_circ_013671 novel_circ_013672 novel_circ_013673 novel_circ_013674 novel_circ_013675 novel_circ_013676 novel_circ_013677 novel_circ_013678 novel_circ_013679 novel_circ_013680 novel_circ_013681 novel_circ_013682 novel_circ_013683 novel_circ_013684 novel_circ_013685 novel_circ_013686 novel_circ_013687 novel_circ_013688 novel_circ_013689 novel_circ_013690 novel_circ_013691 novel_circ_013692 novel_circ_013693 novel_circ_013694 novel_circ_013695 novel_circ_013703 novel_circ_013704 novel_circ_013705 novel_circ_013706 novel_circ_013707 novel_circ_013708 novel_circ_013709 novel_circ_013710 novel_circ_013711 novel_circ_013712 novel_circ_013713 novel_circ_013714 novel_circ_013715 novel_circ_013716 novel_circ_013717 novel_circ_013718 novel_circ_013719 novel_circ_013720 novel_circ_013721 novel_circ_013722 novel_circ_013723 novel_circ_013724 novel_circ_013725 novel_circ_013726

| hsa_circ_0063471 | 90 | 1 | 374 | 1 | 380 | 0.02 | 7.00E-138 |
| --- | --- | --- | --- | --- | --- | --- | --- |
| hsa_circ_0063469 | 89 | 1 | 782 | 1 | 788 | 0.01 | 0 |
| hsa_circ_0063467 | 88 | 1 | 899 | 1 | 905 | 0.01 | 0 |
| hsa_circ_0063467 | 87 | 1 | 508 | 1 | 508 | 0 | 4.00E-162 |
| hsa_circ_0063459 | 94 | 1 | 280 | 639 | 360 | 0.01 | 2.00E-117 |
| hsa_circ_0063459 | 93 | 3 | 511 | 509 | 1 | 0 | 0 |
| hsa_circ_0063455 | 92 | 3 | 442 | 440 | 1 | 0 | 3.00E-174 |
| hsa_circ_0002877 | 96 | 2 | 262 | 261 | 1 | 0 | 1.00E-118 |
| hsa_circ_0063422 | 92 | 209 | 1330 | 5 | 1126 | 0 | 0 |
| hsa_circ_0063422 | 92 | 209 | 1215 | 5 | 1011 | 0 | 0 |
| hsa_circ_0063430 | 91 | 1 | 310 | 1 | 310 | 0 | 2.00E-117 |
| hsa_circ_0063422 | 91 | 209 | 639 | 5 | 435 | 0 | 6.00E-166 |
| hsa_circ_0063422 | 92 | 209 | 548 | 5 | 344 | 0 | 2.00E-135 |
| hsa_circ_0002811 | 91 | 1 | 326 | 1 | 326 | 0 | 3.00E-121 |
| hsa_circ_0063416 | 97 | 1 | 436 | 244 | 679 | 0 | 0 |
| hsa_circ_0063409 | 95 | 1 | 1323 | 1 | 1314 | 0.01 | 0 |
| hsa_circ_0063416 | 98 | 1 | 539 | 1 | 539 | 0 | 0 |
| hsa_circ_0063416 | 98 | 1 | 243 | 1 | 243 | 0 | 6.00E-117 |
| hsa_circ_0063414 | 97 | 1 | 558 | 1 | 558 | 0 | 0 |
| hsa_circ_0063412 | 94 | 1 | 421 | 744 | 1155 | 0.02 | 4.00E-176 |
| hsa_circ_0063412 | 96 | 1 | 1164 | 1 | 1155 | 0.01 | 0 |
| hsa_circ_0063412 | 97 | 1 | 875 | 1 | 875 | 0 | 0 |
| hsa_circ_0005206 | 97 | 1 | 492 | 76 | 567 | 0 | 0 |
| hsa_circ_0005206 | 97 | 1 | 567 | 1 | 567 | 0 | 0 |
| hsa_circ_0063412 | 97 | 1 | 743 | 1 | 743 | 0 | 0 |
| hsa_circ_0063409 | 96 | 1 | 902 | 1 | 902 | 0 | 0 |
| hsa_circ_0005206 | 99 | 1 | 320 | 122 | 441 | 0 | 6.00E-163 |
| hsa_circ_0063412 | 98 | 1 | 617 | 1 | 617 | 0 | 0 |
| hsa_circ_0063409 | 97 | 1 | 776 | 1 | 776 | 0 | 0 |
| hsa_circ_0063412 | 97 | 1 | 446 | 1 | 446 | 0 | 0 |
| hsa_circ_0063388 | 95 | 2 | 384 | 2 | 384 | 0 | 7.00E-168 |
| hsa_circ_0056199 | 96 | 1 | 537 | 1 | 537 | 0 | 0 |
| hsa_circ_0056198 | 96 | 1 | 645 | 1 | 645 | 0 | 0 |
| hsa_circ_0056510 | 86 | 6 | 377 | 447 | 76 | 0 | 1.00E-111 |
| hsa_circ_0056510 | 85 | 4 | 4021 | 4485 | 453 | 0.01 | 0 |
| hsa_circ_0056510 | 85 | 34 | 4210 | 4647 | 453 | 0.01 | 0 |
| hsa_circ_0086351 | 96 | 1 | 718 | 1009 | 292 | 0 | 0 |
| hsa_circ_0086351 | 96 | 1 | 592 | 1009 | 418 | 0 | 0 |
| hsa_circ_0086351 | 94 | 1 | 275 | 1284 | 1010 | 0 | 1.00E-114 |
| hsa_circ_0086351 | 93 | 1 | 575 | 1584 | 1010 | 0 | 0 |
| hsa_circ_0086352 | 89 | 1 | 288 | 372 | 97 | 0.04 | 5.00E-95 |
| hsa_circ_0086352 | 92 | 1 | 498 | 582 | 97 | 0.02 | 0 |
| hsa_circ_0086352 | 93 | 1 | 589 | 673 | 97 | 0.02 | 0 |
| hsa_circ_0086352 | 93 | 1 | 947 | 1031 | 97 | 0.01 | 0 |
| hsa_circ_0086352 | 93 | 2 | 950 | 1033 | 97 | 0.01 | 0 |
| hsa_circ_0086352 | 89 | 1 | 284 | 372 | 101 | 0.04 | 1.00E-94 |
| hsa_circ_0086352 | 94 | 1 | 585 | 673 | 101 | 0.02 | 0 |
| hsa_circ_0086352 | 94 | 1 | 3307 | 3395 | 101 | 0 | 0 |
| hsa_circ_0086353 | 94 | 1 | 1064 | 1155 | 92 | 0 | 0 |
| hsa_circ_0086356 | 94 | 1 | 1658 | 2364 | 707 | 0 | 0 |
| hsa_circ_0086360 | 93 | 1 | 1131 | 1631 | 501 | 0 | 0 |
| hsa_circ_0086360 | 93 | 1 | 1254 | 1754 | 501 | 0 | 0 |
| hsa_circ_0086360 | 94 | 3 | 1470 | 1968 | 501 | 0 | 0 |
| hsa_circ_0086361 | 93 | 1 | 582 | 582 | 1 | 0 | 0 |

novel_circ_013727 novel_circ_013728 novel_circ_013729 novel_circ_013730 novel_circ_013731 novel_circ_013735 novel_circ_013736 novel_circ_013737 novel_circ_013738 novel_circ_013739 novel_circ_013740 novel_circ_013741 novel_circ_013742 novel_circ_013743 novel_circ_013744 novel_circ_013745 novel_circ_013746 novel_circ_013747 novel_circ_013750 novel_circ_013751 novel_circ_013752 novel_circ_013753 novel_circ_013757 novel_circ_013758 novel_circ_013759 novel_circ_013760 novel_circ_013761 novel_circ_013762 novel_circ_013763 novel_circ_013764 novel_circ_013765 novel_circ_013766 novel_circ_013769 novel_circ_013771 novel_circ_013772 novel_circ_013773 novel_circ_013774 novel_circ_013775 novel_circ_013776 novel_circ_013777 novel_circ_013778 novel_circ_013783 novel_circ_013784 novel_circ_013787 novel_circ_013788 novel_circ_013789 novel_circ_013790 novel_circ_013791 novel_circ_013796 novel_circ_013799 novel_circ_013800 novel_circ_013801 novel_circ_013805 novel_circ_013806

| hsa_circ_0086361 | 94 | 1 | 852 | 852 | 1 | 0 | 0 |
| --- | --- | --- | --- | --- | --- | --- | --- |
| hsa_circ_0086360 | 95 | 3 | 293 | 2256 | 1968 | 0.01 | 1.00E-122 |
| hsa_circ_0086360 | 95 | 3 | 286 | 2256 | 1973 | 0 | 1.00E-124 |
| hsa_circ_0086360 | 95 | 76030 | 76313 | 2256 | 1973 | 0 | 3.00E-122 |
| hsa_circ_0005715 | 82 | 23365 | 23448 | 7821 | 7738 | 0.02 | 2.00E-09 |
| hsa_circ_0074674 | 94 | 3 | 749 | 350 | 1099 | 0 | 0 |
| hsa_circ_0074674 | 94 | 1 | 441 | 1 | 444 | 0.01 | 0 |
| hsa_circ_0074634 | 89 | 3 | 4889 | 10365 | 5485 | 0 | 0 |
| hsa_circ_0074634 | 89 | 3 | 5277 | 10365 | 5097 | 0 | 0 |
| hsa_circ_0074634 | 89 | 7 | 4903 | 10221 | 5331 | 0 | 0 |
| hsa_circ_0074634 | 89 | 3 | 4256 | 9732 | 5485 | 0 | 0 |
| hsa_circ_0074634 | 88 | 1 | 388 | 5484 | 5097 | 0 | 1.00E-125 |
| hsa_circ_0074635 | 86 | 1 | 1009 | 1009 | 1 | 0 | 0 |
| hsa_circ_0074635 | 86 | 3 | 811 | 809 | 1 | 0 | 0 |
| hsa_circ_0074634 | 86 | 3 | 957 | 4015 | 3061 | 0 | 0 |
| hsa_circ_0074634 | 86 | 1 | 606 | 2618 | 2007 | 0.01 | 0 |
| hsa_circ_0074634 | 87 | 1 | 976 | 2618 | 1634 | 0.01 | 0 |
| hsa_circ_0074630 | 87 | 1 | 593 | 1 | 593 | 0.01 | 0 |
| hsa_circ_0067444 | 83 | 1 | 626 | 398 | 1023 | 0.01 | 5.00E-156 |
| hsa_circ_0067444 | 86 | 1 | 825 | 512 | 1336 | 0 | 0 |
| hsa_circ_0067444 | 92 | 1 | 313 | 1024 | 1336 | 0.01 | 3.00E-122 |
| hsa_circ_0067444 | 92 | 1 | 313 | 1024 | 1336 | 0.01 | 6.00E-122 |
| hsa_circ_0067445 | 94 | 66 | 747 | 1 | 682 | 0 | 0 |
| hsa_circ_0067447 | 94 | 3 | 461 | 159 | 617 | 0 | 0 |
| hsa_circ_0067447 | 93 | 3 | 798 | 159 | 954 | 0 | 0 |
| hsa_circ_0067447 | 93 | 3 | 921 | 159 | 1077 | 0 | 0 |
| hsa_circ_0067447 | 93 | 3 | 1385 | 159 | 1541 | 0 | 0 |
| hsa_circ_0067447 | 93 | 1 | 1049 | 493 | 1541 | 0 | 0 |
| hsa_circ_0067449 | 94 | 1 | 297 | 289 | 585 | 0 | 2.00E-123 |
| hsa_circ_0067447 | 94 | 1 | 587 | 955 | 1541 | 0 | 0 |
| hsa_circ_0067447 | 94 | 3 | 464 | 1080 | 1541 | 0 | 0 |
| hsa_circ_0081520 | 83 | 4 | 179 | 191 | 16 | 0.01 | 8.00E-38 |
| hsa_circ_0003845 | 93 | 426 | 606 | 2 | 182 | 0 | 1.00E-68 |
| hsa_circ_0003845 | 93 | 129 | 309 | 2 | 182 | 0 | 5.00E-69 |
| hsa_circ_0003845 | 94 | 129 | 440 | 2 | 313 | 0 | 1.00E-131 |
| hsa_circ_0074485 | 93 | 1 | 150 | 60 | 209 | 0 | 4.00E-57 |
| hsa_circ_0074486 | 95 | 1 | 154 | 410 | 257 | 0 | 2.00E-64 |
| hsa_circ_0074486 | 97 | 1 | 294 | 550 | 257 | 0 | 2.00E-138 |
| hsa_circ_0074486 | 97 | 311 | 604 | 550 | 257 | 0 | 3.00E-138 |
| hsa_circ_0074486 | 97 | 7641 | 7934 | 550 | 257 | 0 | 4.00E-137 |
| hsa_circ_0074486 | 99 | 311 | 450 | 550 | 411 | 0 | 2.00E-64 |
| hsa_circ_0074505 | 92 | 1 | 1117 | 100 | 1216 | 0 | 0 |
| hsa_circ_0074508 | 91 | 1 | 317 | 129 | 445 | 0 | 1.00E-119 |
| hsa_circ_0074541 | 93 | 1 | 1053 | 1 | 1053 | 0 | 0 |
| hsa_circ_0074557 | 93 | 1 | 470 | 1953 | 1484 | 0 | 0 |
| hsa_circ_0074566 | 94 | 1 | 558 | 558 | 1 | 0 | 0 |
| hsa_circ_0074566 | 94 | 1 | 460 | 558 | 99 | 0 | 0 |
| hsa_circ_0074564 | 92 | 1132 | 1536 | 3652 | 3248 | 0 | 2.00E-162 |
| hsa_circ_0059428 | 95 | 19 | 178 | 18 | 177 | 0 | 3.00E-65 |
| hsa_circ_0059428 | 95 | 19 | 178 | 18 | 177 | 0 | 3.00E-65 |
| hsa_circ_0059428 | 95 | 19 | 178 | 18 | 177 | 0 | 8.00E-66 |
| hsa_circ_0055368 | 77 | 9580 | 9708 | 3445 | 3564 | 0.07 | 3.00E-07 |
| hsa_circ_0059426 | 91 | 1 | 473 | 1968 | 2440 | 0 | 0 |
| hsa_circ_0059426 | 93 | 1 | 877 | 1564 | 2440 | 0 | 0 |

novel_circ_013807 novel_circ_013808 novel_circ_013809 novel_circ_013810 novel_circ_013811 novel_circ_013812 novel_circ_013813 novel_circ_013814 novel_circ_013815 novel_circ_013816 novel_circ_013822 novel_circ_013823 novel_circ_013824 novel_circ_013825 novel_circ_013826 novel_circ_013840 novel_circ_013841 novel_circ_013856 novel_circ_013859 novel_circ_013860 novel_circ_013861 novel_circ_013862 novel_circ_013863 novel_circ_013864 novel_circ_013865 novel_circ_013866 novel_circ_013867 novel_circ_013868 novel_circ_013869 novel_circ_013870 novel_circ_013871 novel_circ_013872 novel_circ_013873 novel_circ_013874 novel_circ_013875 novel_circ_013876 novel_circ_013877 novel_circ_013878 novel_circ_013879 novel_circ_013880 novel_circ_013881 novel_circ_013883 novel_circ_013884 novel_circ_013885 novel_circ_013886 novel_circ_013887 novel_circ_013888 novel_circ_013889 novel_circ_013890 novel_circ_013891 novel_circ_013892 novel_circ_013893 novel_circ_013894 novel_circ_013895

| hsa_circ_0059426 | 93 | 541 | 1424 | 1557 | 2440 | 0 | 0 |
| --- | --- | --- | --- | --- | --- | --- | --- |
| hsa_circ_0059426 | 93 | 1 | 672 | 1564 | 2235 | 0 | 0 |
| hsa_circ_0059426 | 94 | 1 | 868 | 661 | 1528 | 0 | 0 |
| hsa_circ_0059426 | 94 | 1 | 654 | 770 | 1423 | 0 | 0 |
| hsa_circ_0059426 | 95 | 1 | 820 | 420 | 1239 | 0 | 0 |
| hsa_circ_0059426 | 93 | 1 | 305 | 770 | 1074 | 0 | 6.00E-123 |
| hsa_circ_0059426 | 96 | 1 | 695 | 286 | 980 | 0 | 0 |
| hsa_circ_0059426 | 97 | 1 | 484 | 286 | 769 | 0 | 0 |
| hsa_circ_0059426 | 97 | 1 | 241 | 420 | 660 | 0 | 2.00E-112 |
| hsa_circ_0059426 | 92 | 1 | 285 | 1 | 285 | 0 | 7.00E-112 |
| hsa_circ_0059421 | 94 | 3 | 305 | 271 | 573 | 0 | 4.00E-128 |
| hsa_circ_0059421 | 94 | 3 | 305 | 271 | 573 | 0 | 2.00E-128 |
| hsa_circ_0059421 | 94 | 3 | 305 | 271 | 573 | 0 | 2.00E-128 |
| hsa_circ_0059420 | 93 | 1 | 763 | 18446 | 19208 | 0 | 0 |
| hsa_circ_0059420 | 93 | 1 | 616 | 18446 | 19061 | 0 | 0 |
| hsa_circ_0056324 | 91 | 1 | 199 | 149 | 347 | 0 | 4.00E-72 |
| hsa_circ_0056324 | 91 | 1 | 347 | 1 | 347 | 0 | 1.00E-132 |
| hsa_circ_0005447 | 94 | 17454 | 17560 | 7469 | 7363 | 0 | 3.00E-37 |
| hsa_circ_0024152 | 93 | 1 | 719 | 719 | 1 | 0 | 0 |
| hsa_circ_0024152 | 93 | 123 | 841 | 719 | 1 | 0 | 0 |
| hsa_circ_0024152 | 93 | 185 | 903 | 719 | 1 | 0 | 0 |
| hsa_circ_0024153 | 93 | 30 | 797 | 768 | 1 | 0 | 0 |
| hsa_circ_0024153 | 92 | 4 | 330 | 327 | 1 | 0 | 2.00E-126 |
| hsa_circ_0024153 | 93 | 10 | 766 | 757 | 1 | 0 | 0 |
| hsa_circ_0024153 | 93 | 2 | 769 | 768 | 1 | 0 | 0 |
| hsa_circ_0024153 | 93 | 30 | 797 | 768 | 1 | 0 | 0 |
| hsa_circ_0024153 | 93 | 2 | 769 | 768 | 1 | 0 | 0 |
| hsa_circ_0024153 | 93 | 30 | 797 | 768 | 1 | 0 | 0 |
| hsa_circ_0024153 | 92 | 4 | 330 | 327 | 1 | 0 | 1.00E-126 |
| hsa_circ_0024153 | 93 | 2 | 769 | 768 | 1 | 0 | 0 |
| hsa_circ_0024153 | 93 | 30 | 797 | 768 | 1 | 0 | 0 |
| hsa_circ_0024153 | 93 | 2 | 769 | 768 | 1 | 0 | 0 |
| hsa_circ_0024153 | 93 | 30 | 797 | 768 | 1 | 0 | 0 |
| hsa_circ_0024153 | 93 | 10 | 649 | 757 | 118 | 0 | 0 |
| hsa_circ_0024153 | 93 | 2 | 652 | 768 | 118 | 0 | 0 |
| hsa_circ_0024153 | 93 | 30 | 680 | 768 | 118 | 0 | 0 |
| hsa_circ_0024154 | 93 | 2 | 439 | 438 | 1 | 0 | 0 |
| hsa_circ_0024154 | 93 | 30 | 467 | 438 | 1 | 0 | 0 |
| hsa_circ_0024154 | 93 | 2 | 301 | 438 | 139 | 0 | 3.00E-120 |
| hsa_circ_0024154 | 93 | 30 | 329 | 438 | 139 | 0 | 4.00E-120 |
| hsa_circ_0024154 | 93 | 30 | 212 | 438 | 256 | 0 | 5.00E-72 |
| hsa_circ_0024158 | 90 | 10 | 409 | 550 | 151 | 0 | 3.00E-142 |
| hsa_circ_0024158 | 90 | 2 | 516 | 665 | 151 | 0 | 0 |
| hsa_circ_0024158 | 90 | 161 | 947 | 937 | 151 | 0 | 0 |
| hsa_circ_0024158 | 90 | 399 | 1185 | 937 | 151 | 0 | 0 |
| hsa_circ_0024158 | 91 | 161 | 690 | 937 | 408 | 0 | 0 |
| hsa_circ_0024158 | 91 | 399 | 928 | 937 | 408 | 0 | 0 |
| hsa_circ_0024158 | 91 | 535 | 1064 | 937 | 408 | 0 | 0 |
| hsa_circ_0024158 | 91 | 2 | 379 | 937 | 560 | 0 | 2.00E-143 |
| hsa_circ_0024158 | 91 | 161 | 538 | 937 | 560 | 0 | 3.00E-143 |
| hsa_circ_0024158 | 91 | 399 | 776 | 937 | 560 | 0 | 4.00E-143 |
| hsa_circ_0024158 | 91 | 535 | 912 | 937 | 560 | 0 | 5.00E-143 |
| hsa_circ_0024158 | 91 | 399 | 669 | 937 | 667 | 0 | 2.00E-100 |
| hsa_circ_0024158 | 91 | 535 | 805 | 937 | 667 | 0 | 3.00E-100 |

novel_circ_013896 novel_circ_013897 novel_circ_013898 novel_circ_013899 novel_circ_013907 novel_circ_013908 novel_circ_013909 novel_circ_013910 novel_circ_013911 novel_circ_013912 novel_circ_013913 novel_circ_013914 novel_circ_013915 novel_circ_013916 novel_circ_013917 novel_circ_013918 novel_circ_013919 novel_circ_013920 novel_circ_013921 novel_circ_013922 novel_circ_013923 novel_circ_013924 novel_circ_013925 novel_circ_013926 novel_circ_013928 novel_circ_013929 novel_circ_013930 novel_circ_013931 novel_circ_013932 novel_circ_013933 novel_circ_013934 novel_circ_013935 novel_circ_013936 novel_circ_013937 novel_circ_013938 novel_circ_013939 novel_circ_013940 novel_circ_013941 novel_circ_013942 novel_circ_013943 novel_circ_013944 novel_circ_013945 novel_circ_013946 novel_circ_013947 novel_circ_013948 novel_circ_013949 novel_circ_013950 novel_circ_013951 novel_circ_013952 novel_circ_013953 novel_circ_013954 novel_circ_013955 novel_circ_013956 novel_circ_013957

| hsa_circ_0024158 | 92 | 161 | 360 | 937 | 738 | 0 | 7.00E-73 |
| --- | --- | --- | --- | --- | --- | --- | --- |
| hsa_circ_0024158 | 92 | 399 | 598 | 937 | 738 | 0 | 1.00E-72 |
| hsa_circ_0024158 | 92 | 535 | 734 | 937 | 738 | 0 | 2.00E-72 |
| hsa_circ_0024158 | 93 | 399 | 493 | 937 | 843 | 0 | 5.00E-31 |
| hsa_circ_0024166 | 91 | 1 | 672 | 674 | 3 | 0 | 0 |
| hsa_circ_0024172 | 95 | 1 | 419 | 1 | 419 | 0 | 0 |
| hsa_circ_0024172 | 96 | 1 | 565 | 1 | 565 | 0 | 0 |
| hsa_circ_0024172 | 96 | 1 | 646 | 1 | 646 | 0 | 0 |
| hsa_circ_0024172 | 96 | 1 | 646 | 1 | 646 | 0 | 0 |
| hsa_circ_0024172 | 96 | 1 | 646 | 1 | 646 | 0 | 0 |
| hsa_circ_0024172 | 96 | 1 | 740 | 1 | 740 | 0 | 0 |
| hsa_circ_0024172 | 95 | 1 | 979 | 1 | 979 | 0 | 0 |
| hsa_circ_0024167 | 96 | 1 | 1771 | 111 | 1881 | 0 | 0 |
| hsa_circ_0024176 | 95 | 1 | 702 | 1 | 702 | 0 | 0 |
| hsa_circ_0024177 | 95 | 9141 | 9671 | 1 | 531 | 0 | 0 |
| hsa_circ_0024176 | 96 | 9141 | 10265 | 370 | 1494 | 0 | 0 |
| hsa_circ_0024176 | 96 | 9141 | 10384 | 370 | 1613 | 0 | 0 |
| hsa_circ_0024177 | 95 | 1 | 531 | 1 | 531 | 0 | 0 |
| hsa_circ_0024178 | 93 | 1 | 239 | 1 | 239 | 0 | 1.00E-94 |
| hsa_circ_0024178 | 94 | 1 | 301 | 1 | 301 | 0 | 1.00E-125 |
| hsa_circ_0024178 | 95 | 1 | 437 | 1 | 437 | 0 | 0 |
| hsa_circ_0024177 | 96 | 1 | 693 | 95 | 787 | 0 | 0 |
| hsa_circ_0024177 | 97 | 1 | 454 | 334 | 787 | 0 | 0 |
| hsa_circ_0003897 | 92 | 49 | 507 | 1 | 459 | 0 | 0 |
| hsa_circ_0003897 | 93 | 1 | 144 | 316 | 459 | 0 | 3.00E-53 |
| hsa_circ_0003897 | 93 | 1 | 144 | 316 | 459 | 0 | 6.00E-53 |
| hsa_circ_0024206 | 90 | 1 | 569 | 1 | 569 | 0 | 0 |
| hsa_circ_0024208 | 91 | 1 | 1050 | 1 | 1059 | 0.01 | 0 |
| hsa_circ_0024209 | 90 | 1 | 558 | 1 | 567 | 0.02 | 0 |
| hsa_circ_0024209 | 90 | 1 | 880 | 1 | 889 | 0.01 | 0 |
| hsa_circ_0024209 | 90 | 1 | 1006 | 1 | 1015 | 0.01 | 0 |
| hsa_circ_0024213 | 90 | 1 | 448 | 1 | 448 | 0 | 1.00E-165 |
| hsa_circ_0024213 | 88 | 1 | 821 | 1 | 821 | 0 | 0 |
| hsa_circ_0024216 | 87 | 3 | 499 | 3 | 499 | 0 | 4.00E-161 |
| hsa_circ_0024216 | 88 | 3 | 714 | 3 | 714 | 0 | 0 |
| hsa_circ_0024216 | 88 | 3 | 953 | 3 | 953 | 0 | 0 |
| hsa_circ_0024216 | 89 | 3 | 1160 | 3 | 1160 | 0 | 0 |
| hsa_circ_0024216 | 90 | 3 | 2115 | 3 | 2112 | 0 | 0 |
| hsa_circ_0024216 | 88 | 1 | 827 | 127 | 953 | 0 | 0 |
| hsa_circ_0024220 | 91 | 1 | 446 | 373 | 818 | 0 | 4.00E-171 |
| hsa_circ_0005583 | 92 | 1 | 497 | 1 | 497 | 0 | 0 |
| hsa_circ_0024229 | 91 | 1 | 741 | 269 | 1009 | 0 | 0 |
| hsa_circ_0024229 | 90 | 1 | 599 | 411 | 1009 | 0 | 0 |
| hsa_circ_0024229 | 90 | 1 | 422 | 588 | 1009 | 0 | 8.00E-153 |
| hsa_circ_0024229 | 90 | 1 | 1076 | 588 | 1663 | 0 | 0 |
| hsa_circ_0024229 | 91 | 1 | 1477 | 588 | 2064 | 0 | 0 |
| hsa_circ_0024231 | 89 | 1 | 566 | 1 | 566 | 0 | 0 |
| hsa_circ_0024231 | 91 | 1 | 967 | 1 | 967 | 0 | 0 |
| hsa_circ_0024231 | 89 | 2 | 477 | 91 | 566 | 0 | 2.00E-169 |
| hsa_circ_0024231 | 90 | 1 | 374 | 193 | 566 | 0.01 | 7.00E-138 |
| hsa_circ_0024232 | 94 | 2 | 401 | 227 | 626 | 0 | 1.00E-170 |
| hsa_circ_0024231 | 93 | 2 | 735 | 568 | 1301 | 0 | 0 |
| hsa_circ_0024233 | 92 | 5 | 813 | 5 | 813 | 0 | 0 |
| hsa_circ_0024233 | 93 | 5 | 952 | 5 | 952 | 0 | 0 |

novel_circ_013958 novel_circ_013959 novel_circ_013960 novel_circ_013961 novel_circ_013962 novel_circ_013966 novel_circ_013967 novel_circ_013970 novel_circ_013973 novel_circ_013974 novel_circ_013975 novel_circ_013976 novel_circ_013977 novel_circ_013978 novel_circ_013979 novel_circ_013985 novel_circ_013986 novel_circ_013987 novel_circ_013988 novel_circ_013991 novel_circ_013992 novel_circ_013993 novel_circ_013994 novel_circ_013995 novel_circ_013996 novel_circ_013997 novel_circ_013998 novel_circ_013999 novel_circ_014000 novel_circ_014001 novel_circ_014002 novel_circ_014003 novel_circ_014004 novel_circ_014005 novel_circ_014006 novel_circ_014007 novel_circ_014008 novel_circ_014009 novel_circ_014012 novel_circ_014013 novel_circ_014014 novel_circ_014015 novel_circ_014016 novel_circ_014017 novel_circ_014018 novel_circ_014019 novel_circ_014020 novel_circ_014021 novel_circ_014022 novel_circ_014035 novel_circ_014039 novel_circ_014041 novel_circ_014042 novel_circ_014043

| hsa_circ_0024233 | 93 | 5 | 1035 | 5 | 1035 | 0 | 0 |
| --- | --- | --- | --- | --- | --- | --- | --- |
| hsa_circ_0024220 | 93 | 5 | 1443 | 4514 | 5952 | 0 | 0 |
| hsa_circ_0024220 | 93 | 1 | 1156 | 5050 | 6205 | 0 | 0 |
| hsa_circ_0024238 | 92 | 1 | 253 | 631 | 883 | 0 | 6.00E-97 |
| hsa_circ_0024240 | 94 | 312 | 490 | 30101 | 30279 | 0 | 5.00E-71 |
| hsa_circ_0006368 | 86 | 22068 | 22125 | 189842 | 189785 | 0 | 5.00E-07 |
| hsa_circ_0006368 | 86 | 22008 | 22065 | 189842 | 189785 | 0 | 5.00E-07 |
| hsa_circ_0024252 | 85 | 1 | 649 | 1 | 643 | 0.02 | 0 |
| hsa_circ_0024261 | 92 | 143 | 895 | 809 | 1561 | 0 | 0 |
| hsa_circ_0003541 | 92 | 1 | 752 | 1 | 752 | 0 | 0 |
| hsa_circ_0003541 | 92 | 1 | 892 | 1 | 892 | 0 | 0 |
| hsa_circ_0003541 | 92 | 1 | 1127 | 1 | 1127 | 0 | 0 |
| hsa_circ_0002773 | 95 | 1 | 371 | 371 | 1 | 0 | 6.00E-166 |
| hsa_circ_0002773 | 95 | 1 | 371 | 371 | 1 | 0 | 5.00E-166 |
| hsa_circ_0002773 | 95 | 161 | 531 | 371 | 1 | 0 | 5.00E-166 |
| hsa_circ_0024266 | 95 | 1 | 121 | 1 | 121 | 0 | 4.00E-46 |
| hsa_circ_0024266 | 95 | 1 | 121 | 1 | 121 | 0 | 4.00E-46 |
| hsa_circ_0024266 | 94 | 5171 | 5399 | 115 | 343 | 0 | 9.00E-93 |
| hsa_circ_0024266 | 94 | 3 | 162 | 184 | 343 | 0 | 2.00E-63 |
| hsa_circ_0024269 | 95 | 1 | 390 | 390 | 1 | 0 | 1.00E-171 |
| hsa_circ_0024269 | 94 | 1 | 711 | 711 | 1 | 0 | 0 |
| hsa_circ_0024269 | 94 | 1 | 1015 | 1015 | 1 | 0 | 0 |
| hsa_circ_0024270 | 95 | 1 | 370 | 370 | 1 | 0 | 2.00E-162 |
| hsa_circ_0024272 | 94 | 1 | 495 | 495 | 1 | 0 | 0 |
| hsa_circ_0024272 | 93 | 1 | 321 | 495 | 175 | 0 | 2.00E-133 |
| hsa_circ_0024277 | 92 | 1 | 1011 | 5358 | 4327 | 0.02 | 0 |
| hsa_circ_0024280 | 92 | 8065 | 8628 | 585 | 1 | 0.04 | 0 |
| hsa_circ_0024280 | 92 | 1 | 880 | 901 | 1 | 0.02 | 0 |
| hsa_circ_0024280 | 93 | 1 | 1016 | 1037 | 1 | 0.02 | 0 |
| hsa_circ_0024280 | 93 | 4 | 1311 | 1329 | 1 | 0.02 | 0 |
| hsa_circ_0024280 | 93 | 1 | 1450 | 1471 | 1 | 0.01 | 0 |
| hsa_circ_0024280 | 94 | 4 | 903 | 1329 | 430 | 0 | 0 |
| hsa_circ_0024280 | 95 | 4 | 625 | 1329 | 708 | 0 | 0 |
| hsa_circ_0024280 | 94 | 1 | 764 | 1471 | 708 | 0 | 0 |
| hsa_circ_0024280 | 96 | 4 | 431 | 1329 | 902 | 0 | 0 |
| hsa_circ_0024280 | 95 | 1 | 570 | 1471 | 902 | 0 | 0 |
| hsa_circ_0024280 | 94 | 4 | 295 | 1329 | 1038 | 0 | 4.00E-124 |
| hsa_circ_0024280 | 93 | 1 | 434 | 1471 | 1038 | 0 | 0 |
| hsa_circ_0024286 | 93 | 603 | 954 | 1 | 351 | 0 | 1.00E-144 |
| hsa_circ_0024288 | 93 | 955 | 1635 | 1 | 681 | 0 | 0 |
| hsa_circ_0024288 | 93 | 1 | 375 | 55 | 429 | 0 | 2.00E-153 |
| hsa_circ_0024288 | 92 | 1 | 465 | 55 | 519 | 0 | 0 |
| hsa_circ_0024289 | 92 | 3 | 319 | 327 | 643 | 0 | 1.00E-124 |
| hsa_circ_0024289 | 92 | 3 | 319 | 327 | 643 | 0 | 2.00E-124 |
| hsa_circ_0024289 | 93 | 1 | 229 | 415 | 643 | 0 | 2.00E-92 |
| hsa_circ_0024297 | 94 | 1 | 342 | 1 | 342 | 0.01 | 2.00E-143 |
| hsa_circ_0024293 | 93 | 1 | 890 | 407 | 1296 | 0 | 0 |
| hsa_circ_0024293 | 92 | 1 | 317 | 817 | 1133 | 0 | 3.00E-126 |
| hsa_circ_0024293 | 96 | 1 | 163 | 1134 | 1296 | 0 | 1.00E-68 |
| hsa_circ_0014873 | 86 | 10386 | 10455 | 15311 | 15242 | 0 | 3.00E-10 |
| hsa_circ_0024312 | 88 | 1 | 586 | 586 | 1 | 0 | 0 |
| hsa_circ_0024312 | 87 | 1 | 931 | 931 | 1 | 0 | 0 |
| hsa_circ_0024312 | 88 | 1 | 692 | 931 | 240 | 0 | 0 |
| hsa_circ_0024319 | 91 | 1 | 300 | 492 | 193 | 0 | 2.00E-113 |

novel_circ_014044 novel_circ_014045 novel_circ_014048 novel_circ_014049 novel_circ_014050 novel_circ_014051 novel_circ_014052 novel_circ_014053 novel_circ_014054 novel_circ_014055 novel_circ_014056 novel_circ_014057 novel_circ_014058 novel_circ_014059 novel_circ_014060 novel_circ_014061 novel_circ_014062 novel_circ_014063 novel_circ_014064 novel_circ_014065 novel_circ_014066 novel_circ_014067 novel_circ_014068 novel_circ_014069 novel_circ_014070 novel_circ_014071 novel_circ_014072 novel_circ_014075 novel_circ_014076 novel_circ_014077 novel_circ_014078 novel_circ_014079 novel_circ_014083 novel_circ_014084 novel_circ_014085 novel_circ_014086 novel_circ_014087 novel_circ_014088 novel_circ_014089 novel_circ_014090 novel_circ_014091 novel_circ_014092 novel_circ_014093 novel_circ_014094 novel_circ_014095 novel_circ_014096 novel_circ_014097 novel_circ_014098 novel_circ_014099 novel_circ_014100 novel_circ_014101 novel_circ_014107 novel_circ_014108 novel_circ_014109

| hsa_circ_0024324 | 92 | 4 | 317 | 314 | 1 | 0 | 1.00E-124 |
| --- | --- | --- | --- | --- | --- | --- | --- |
| hsa_circ_0024324 | 92 | 2 | 211 | 314 | 105 | 0 | 1.00E-78 |
| hsa_circ_0035126 | 98 | 978 | 1478 | 501 | 1 | 0 | 0 |
| hsa_circ_0035129 | 96 | 2 | 364 | 363 | 1 | 0 | 5.00E-168 |
| hsa_circ_0035129 | 96 | 2 | 364 | 363 | 1 | 0 | 4.00E-168 |
| hsa_circ_0078000 | 90 | 1 | 314 | 215 | 528 | 0.01 | 3.00E-111 |
| hsa_circ_0078000 | 91 | 1 | 245 | 284 | 528 | 0.01 | 5.00E-88 |
| hsa_circ_0078003 | 89 | 1 | 1463 | 1 | 1463 | 0 | 0 |
| hsa_circ_0078003 | 89 | 1 | 1464 | 1 | 1464 | 0 | 0 |
| hsa_circ_0078005 | 88 | 1 | 1024 | 335 | 1358 | 0 | 0 |
| hsa_circ_0078005 | 88 | 1 | 1025 | 335 | 1359 | 0 | 0 |
| hsa_circ_0078005 | 90 | 2732 | 4110 | 1354 | 2732 | 0 | 0 |
| hsa_circ_0078005 | 90 | 1 | 657 | 1359 | 2015 | 0 | 0 |
| hsa_circ_0078005 | 90 | 1 | 1374 | 1359 | 2732 | 0 | 0 |
| hsa_circ_0078005 | 90 | 1 | 1700 | 1359 | 3058 | 0 | 0 |
| hsa_circ_0078005 | 91 | 1 | 2215 | 1359 | 3573 | 0 | 0 |
| hsa_circ_0078005 | 91 | 1 | 752 | 2127 | 2878 | 0 | 0 |
| hsa_circ_0078005 | 91 | 1 | 1027 | 3327 | 4353 | 0 | 0 |
| hsa_circ_0078005 | 90 | 1 | 2249 | 3327 | 5572 | 0 | 0 |
| hsa_circ_0078005 | 90 | 1 | 778 | 3576 | 4353 | 0 | 0 |
| hsa_circ_0078005 | 89 | 1 | 2000 | 3576 | 5572 | 0 | 0 |
| hsa_circ_0078010 | 83 | 1 | 2132 | 3288 | 1144 | 0.01 | 0 |
| hsa_circ_0078012 | 89 | 166 | 472 | 309 | 3 | 0 | 7.00E-104 |
| hsa_circ_0006835 | 88 | 3 | 281 | 279 | 1 | 0 | 3.00E-90 |
| hsa_circ_0006835 | 88 | 296 | 574 | 279 | 1 | 0 | 7.00E-90 |
| hsa_circ_0006071 | 85 | 28 | 688 | 805 | 154 | 0.02 | 0 |
| hsa_circ_0001645 | 89 | 1 | 1041 | 1 | 1041 | 0.01 | 0 |
| hsa_circ_0007133 | 72 | 21776 | 22037 | 393122 | 393373 | 0.06 | 9.00E-09 |
| hsa_circ_0007133 | 72 | 5730 | 5991 | 393122 | 393373 | 0.06 | 7.00E-09 |
| hsa_circ_0078045 | 85 | 1 | 1328 | 154 | 1472 | 0.01 | 0 |
| hsa_circ_0009016 | 100 | 19071 | 19102 | 751252 | 751221 | 0 | 5.00E-06 |
| hsa_circ_0009016 | 100 | 19071 | 19102 | 751252 | 751221 | 0 | 5.00E-06 |
| hsa_circ_0066635 | 73 | 2391 | 2719 | 174320 | 174648 | 0.08 | 7.00E-20 |
| hsa_circ_0006449 | 95 | 1 | 203 | 378 | 580 | 0 | 8.00E-85 |
| hsa_circ_0006449 | 97 | 218 | 705 | 93 | 580 | 0 | 0 |
| hsa_circ_0057158 | 94 | 1 | 319 | 319 | 1 | 0 | 8.00E-134 |
| hsa_circ_0057146 | 97 | 1 | 415 | 946 | 532 | 0 | 0 |
| hsa_circ_0057146 | 96 | 1 | 709 | 946 | 238 | 0 | 0 |
| hsa_circ_0057145 | 96 | 71 | 1153 | 1083 | 1 | 0 | 0 |
| hsa_circ_0007967 | 96 | 1 | 639 | 726 | 88 | 0 | 0 |
| hsa_circ_0007967 | 96 | 1 | 726 | 726 | 1 | 0 | 0 |
| hsa_circ_0057145 | 96 | 1 | 1083 | 1083 | 1 | 0 | 0 |
| hsa_circ_0057144 | 96 | 1 | 1187 | 1189 | 3 | 0 | 0 |
| hsa_circ_0057153 | 96 | 1 | 248 | 427 | 180 | 0 | 1.00E-109 |
| hsa_circ_0057145 | 95 | 1 | 986 | 986 | 1 | 0 | 0 |
| hsa_circ_0057151 | 96 | 1 | 294 | 294 | 1 | 0 | 7.00E-132 |
| hsa_circ_0057146 | 96 | 4 | 237 | 234 | 1 | 0 | 3.00E-105 |
| hsa_circ_0057145 | 95 | 4 | 444 | 441 | 1 | 0 | 0 |
| hsa_circ_0057145 | 95 | 4292 | 4649 | 358 | 1 | 0 | 3.00E-156 |
| hsa_circ_0057138 | 92 | 88 | 475 | 1468 | 1081 | 0 | 7.00E-154 |
| hsa_circ_0057138 | 92 | 3 | 390 | 1468 | 1081 | 0 | 6.00E-154 |
| hsa_circ_0002642 | 97 | 1 | 1360 | 1360 | 1 | 0 | 0 |
| hsa_circ_0057128 | 97 | 1 | 1553 | 1553 | 1 | 0 | 0 |
| hsa_circ_0057127 | 97 | 1 | 1750 | 1750 | 1 | 0 | 0 |

novel_circ_014110 novel_circ_014111 novel_circ_014112 novel_circ_014113 novel_circ_014114 novel_circ_014115 novel_circ_014116 novel_circ_014117 novel_circ_014118 novel_circ_014119 novel_circ_014120 novel_circ_014121 novel_circ_014122 novel_circ_014123 novel_circ_014124 novel_circ_014125 novel_circ_014126 novel_circ_014127 novel_circ_014128 novel_circ_014129 novel_circ_014135 novel_circ_014136 novel_circ_014139 novel_circ_014140 novel_circ_014141 novel_circ_014142 novel_circ_014143 novel_circ_014146 novel_circ_014148 novel_circ_014149 novel_circ_014150 novel_circ_014151 novel_circ_014152 novel_circ_014153 novel_circ_014155 novel_circ_014156 novel_circ_014157 novel_circ_014158 novel_circ_014159 novel_circ_014160 novel_circ_014161 novel_circ_014163 novel_circ_014165 novel_circ_014166 novel_circ_014167 novel_circ_014168 novel_circ_014169 novel_circ_014170 novel_circ_014171 novel_circ_014172 novel_circ_014173 novel_circ_014174 novel_circ_014175 novel_circ_014177

| hsa_circ_0057127 | 97 | 1 | 1750 | 1750 | 1 | 0 | 0 |
| --- | --- | --- | --- | --- | --- | --- | --- |
| hsa_circ_0002642 | 97 | 1 | 1356 | 1356 | 1 | 0 | 0 |
| hsa_circ_0057116 | 95 | 1 | 295 | 980 | 1274 | 0 | 4.00E-129 |
| hsa_circ_0057115 | 94 | 1 | 1067 | 55 | 1121 | 0 | 0 |
| hsa_circ_0057115 | 95 | 1 | 1121 | 1 | 1121 | 0 | 0 |
| hsa_circ_0057115 | 95 | 1194 | 2314 | 1 | 1121 | 0 | 0 |
| hsa_circ_0057115 | 95 | 55 | 1169 | 7 | 1121 | 0 | 0 |
| hsa_circ_0057115 | 95 | 1 | 429 | 614 | 1042 | 0 | 0 |
| hsa_circ_0057115 | 94 | 1 | 575 | 468 | 1042 | 0 | 0 |
| hsa_circ_0057115 | 94 | 1 | 988 | 55 | 1042 | 0 | 0 |
| hsa_circ_0057115 | 95 | 1 | 1042 | 1 | 1042 | 0 | 0 |
| hsa_circ_0057115 | 94 | 55 | 1090 | 7 | 1042 | 0 | 0 |
| hsa_circ_0057115 | 94 | 1 | 777 | 55 | 831 | 0 | 0 |
| hsa_circ_0057115 | 94 | 1 | 413 | 55 | 467 | 0 | 2.00E-179 |
| hsa_circ_0057115 | 95 | 1 | 467 | 1 | 467 | 0 | 0 |
| hsa_circ_0057115 | 95 | 1194 | 1660 | 1 | 467 | 0 | 0 |
| hsa_circ_0057115 | 95 | 55 | 515 | 7 | 467 | 0 | 0 |
| hsa_circ_0057114 | 93 | 2 | 379 | 2 | 379 | 0 | 2.00E-153 |
| hsa_circ_0057114 | 91 | 2 | 232 | 2 | 232 | 0 | 3.00E-85 |
| hsa_circ_0075111 | 86 | 4 | 1405 | 1422 | 3 | 0.03 | 0 |
| hsa_circ_0070519 | 90 | 1 | 621 | 621 | 1 | 0.01 | 0 |
| hsa_circ_0002132 | 85 | 10359 | 10987 | 19760 | 20395 | 0.01 | 4.00E-176 |
| hsa_circ_0070527 | 88 | 1 | 271 | 1 | 265 | 0.02 | 9.00E-86 |
| hsa_circ_0070527 | 93 | 37 | 225 | 77 | 265 | 0 | 1.00E-73 |
| hsa_circ_0001428 | 81 | 7 | 552 | 546 | 1 | 0.02 | 3.00E-123 |
| hsa_circ_0070532 | 86 | 1 | 525 | 525 | 1 | 0.01 | 3.00E-158 |
| hsa_circ_0001428 | 87 | 4 | 636 | 1017 | 385 | 0.01 | 0 |
| hsa_circ_0070550 | 80 | 1 | 447 | 447 | 1 | 0.01 | 3.00E-91 |
| hsa_circ_0070550 | 80 | 1 | 447 | 447 | 1 | 0.01 | 2.00E-91 |
| hsa_circ_0070550 | 80 | 1 | 447 | 447 | 1 | 0.01 | 1.00E-91 |
| hsa_circ_0070551 | 90 | 1 | 176 | 176 | 1 | 0 | 3.00E-59 |
| hsa_circ_0070551 | 86 | 1 | 447 | 447 | 1 | 0 | 2.00E-131 |
| hsa_circ_0070552 | 85 | 314 | 755 | 442 | 1 | 0 | 3.00E-125 |
| hsa_circ_0070552 | 89 | 314 | 484 | 442 | 272 | 0 | 3.00E-53 |
| hsa_circ_0001434 | 87 | 1 | 388 | 388 | 1 | 0 | 6.00E-119 |
| hsa_circ_0001434 | 86 | 6 | 498 | 493 | 1 | 0 | 2.00E-150 |
| hsa_circ_0007668 | 86 | 166 | 666 | 501 | 1 | 0 | 4.00E-153 |
| hsa_circ_0007860 | 83 | 13 | 1258 | 1 | 1261 | 0.02 | 0 |
| hsa_circ_0007860 | 83 | 13 | 1258 | 1 | 1261 | 0.02 | 0 |
| hsa_circ_0007860 | 83 | 1 | 1246 | 1 | 1261 | 0.02 | 0 |
| hsa_circ_0007860 | 83 | 1 | 1246 | 1 | 1261 | 0.02 | 0 |
| hsa_circ_0070577 | 90 | 1 | 289 | 1 | 289 | 0 | 1.00E-103 |
| hsa_circ_0070583 | 89 | 1 | 551 | 711 | 161 | 0 | 0 |
| hsa_circ_0070583 | 89 | 1 | 637 | 797 | 161 | 0 | 0 |
| hsa_circ_0070587 | 90 | 3 | 338 | 336 | 1 | 0 | 2.00E-123 |
| hsa_circ_0070587 | 90 | 1 | 375 | 375 | 1 | 0 | 2.00E-138 |
| hsa_circ_0070587 | 90 | 2750 | 3124 | 375 | 1 | 0 | 2.00E-137 |
| hsa_circ_0070587 | 90 | 1 | 461 | 461 | 1 | 0 | 8.00E-168 |
| hsa_circ_0070587 | 90 | 1 | 545 | 545 | 1 | 0 | 0 |
| hsa_circ_0070587 | 90 | 1 | 1638 | 1638 | 1 | 0 | 0 |
| hsa_circ_0070596 | 89 | 1 | 170 | 170 | 1 | 0 | 7.00E-55 |
| hsa_circ_0070596 | 90 | 1 | 640 | 640 | 1 | 0 | 0 |
| hsa_circ_0070596 | 91 | 1 | 623 | 1263 | 641 | 0 | 0 |
| hsa_circ_0070596 | 89 | 1 | 334 | 1263 | 930 | 0 | 1.00E-115 |

novel_circ_014178 novel_circ_014181 novel_circ_014182 novel_circ_014183 novel_circ_014184 novel_circ_014185 novel_circ_014190 novel_circ_014191 novel_circ_014192 novel_circ_014193 novel_circ_014194 novel_circ_014195 novel_circ_014198 novel_circ_014199 novel_circ_014200 novel_circ_014201 novel_circ_014202 novel_circ_014203 novel_circ_014204 novel_circ_014205 novel_circ_014206 novel_circ_014207 novel_circ_014208 novel_circ_014209 novel_circ_014210 novel_circ_014211 novel_circ_014212 novel_circ_014213 novel_circ_014214 novel_circ_014215 novel_circ_014216 novel_circ_014217 novel_circ_014218 novel_circ_014219 novel_circ_014220 novel_circ_014221 novel_circ_014222 novel_circ_014223 novel_circ_014224 novel_circ_014226 novel_circ_014227 novel_circ_014228 novel_circ_014230 novel_circ_014231 novel_circ_014232 novel_circ_014233 novel_circ_014234 novel_circ_014235 novel_circ_014236 novel_circ_014237 novel_circ_014238 novel_circ_014239 novel_circ_014243 novel_circ_014244

| hsa_circ_0070587 | 86 | 9 | 738 | 2034 | 1305 | 0.01 | 0 |
| --- | --- | --- | --- | --- | --- | --- | --- |
| hsa_circ_0070605 | 88 | 1 | 410 | 1 | 416 | 0.02 | 2.00E-134 |
| hsa_circ_0070605 | 88 | 1 | 622 | 1 | 628 | 0.01 | 0 |
| hsa_circ_0070605 | 88 | 1 | 622 | 1 | 628 | 0.01 | 0 |
| hsa_circ_0070605 | 88 | 1 | 622 | 1 | 628 | 0.01 | 0 |
| hsa_circ_0070605 | 88 | 1 | 488 | 135 | 628 | 0.02 | 2.00E-164 |
| hsa_circ_0055373 | 88 | 3 | 303 | 971 | 671 | 0.01 | 5.00E-99 |
| hsa_circ_0055372 | 88 | 1 | 789 | 1083 | 292 | 0.01 | 0 |
| hsa_circ_0055373 | 88 | 1 | 543 | 546 | 1 | 0.01 | 0 |
| hsa_circ_0055372 | 89 | 1 | 665 | 959 | 292 | 0.01 | 0 |
| hsa_circ_0055355 | 91 | 1 | 518 | 153 | 670 | 0 | 0 |
| hsa_circ_0055355 | 91 | 121 | 790 | 1 | 670 | 0 | 0 |
| hsa_circ_0055279 | 92 | 1 | 569 | 279 | 847 | 0 | 0 |
| hsa_circ_0055279 | 93 | 1 | 461 | 279 | 739 | 0 | 0 |
| hsa_circ_0055278 | 94 | 2 | 261 | 260 | 1 | 0 | 5.00E-108 |
| hsa_circ_0055276 | 94 | 2 | 395 | 394 | 1 | 0 | 1.00E-170 |
| hsa_circ_0055274 | 94 | 2 | 784 | 1120 | 335 | 0 | 0 |
| hsa_circ_0055270 | 95 | 1 | 454 | 1 | 454 | 0 | 0 |
| hsa_circ_0006251 | 92 | 1 | 2137 | 1 | 2134 | 0 | 0 |
| hsa_circ_0055268 | 91 | 1 | 549 | 1 | 549 | 0 | 0 |
| hsa_circ_0055252 | 95 | 1 | 475 | 755 | 1229 | 0 | 0 |
| hsa_circ_0055252 | 95 | 2393 | 2867 | 755 | 1229 | 0 | 0 |
| hsa_circ_0055253 | 95 | 1 | 263 | 368 | 630 | 0 | 1.00E-114 |
| hsa_circ_0055253 | 95 | 1 | 263 | 368 | 630 | 0 | 1.00E-114 |
| hsa_circ_0055252 | 93 | 1 | 802 | 216 | 1017 | 0 | 0 |
| hsa_circ_0055252 | 94 | 1 | 1017 | 1 | 1017 | 0 | 0 |
| hsa_circ_0055249 | 90 | 1 | 364 | 1279 | 916 | 0 | 5.00E-134 |
| hsa_circ_0055244 | 87 | 1 | 812 | 1641 | 2455 | 0.01 | 0 |
| hsa_circ_0055234 | 85 | 1 | 2126 | 8891 | 11019 | 0.01 | 0 |
| hsa_circ_0055242 | 85 | 1 | 2005 | 1 | 2008 | 0.01 | 0 |
| hsa_circ_0055238 | 86 | 1 | 3840 | 6334 | 10206 | 0.01 | 0 |
| hsa_circ_0055242 | 83 | 1 | 844 | 1 | 841 | 0.01 | 0 |
| hsa_circ_0055238 | 86 | 1 | 2679 | 6334 | 9039 | 0.02 | 0 |
| hsa_circ_0055238 | 86 | 1 | 2810 | 6200 | 9039 | 0.02 | 0 |
| hsa_circ_0055238 | 86 | 1 | 2643 | 6200 | 8872 | 0.02 | 0 |
| hsa_circ_0055242 | 86 | 1 | 526 | 1 | 526 | 0.01 | 8.00E-159 |
| hsa_circ_0055238 | 87 | 1 | 2072 | 6334 | 8435 | 0.02 | 0 |
| hsa_circ_0055240 | 87 | 1 | 1835 | 1 | 1865 | 0.02 | 0 |
| hsa_circ_0055238 | 87 | 1 | 1966 | 6200 | 8198 | 0.02 | 0 |
| hsa_circ_0055234 | 83 | 3 | 622 | 3 | 628 | 0.04 | 9.00E-151 |
| hsa_circ_0055231 | 83 | 3 | 818 | 3 | 824 | 0.03 | 0 |
| hsa_circ_0055234 | 83 | 3 | 622 | 3 | 628 | 0.04 | 7.00E-151 |
| hsa_circ_0084772 | 82 | 107 | 777 | 713 | 47 | 0.04 | 3.00E-161 |
| hsa_circ_0055191 | 96 | 1 | 351 | 351 | 1 | 0 | 4.00E-160 |
| hsa_circ_0055188 | 96 | 1 | 556 | 556 | 1 | 0 | 0 |
| hsa_circ_0055188 | 98 | 4 | 390 | 387 | 1 | 0 | 0 |
| hsa_circ_0004643 | 93 | 1 | 246 | 246 | 1 | 0 | 3.00E-100 |
| hsa_circ_0055179 | 95 | 1 | 996 | 1131 | 136 | 0 | 0 |
| hsa_circ_0055176 | 95 | 6164 | 7229 | 1066 | 1 | 0 | 0 |
| hsa_circ_0055179 | 95 | 1 | 885 | 885 | 1 | 0 | 0 |
| hsa_circ_0055176 | 95 | 1 | 1065 | 1065 | 1 | 0 | 0 |
| hsa_circ_0055172 | 96 | 4 | 216 | 326 | 114 | 0 | 1.00E-93 |
| hsa_circ_0008576 | 95 | 2365 | 2420 | 493410 | 493355 | 0 | 9.00E-15 |
| hsa_circ_0008576 | 95 | 4405 | 4460 | 493410 | 493355 | 0 | 1.00E-14 |

novel_circ_014247 novel_circ_014250 novel_circ_014251 novel_circ_014252 novel_circ_014253 novel_circ_014254 novel_circ_014256 novel_circ_014257 novel_circ_014258 novel_circ_014259 novel_circ_014260 novel_circ_014261 novel_circ_014262 novel_circ_014263 novel_circ_014264 novel_circ_014265 novel_circ_014266 novel_circ_014267 novel_circ_014268 novel_circ_014269 novel_circ_014272 novel_circ_014273 novel_circ_014274 novel_circ_014275 novel_circ_014276 novel_circ_014277 novel_circ_014278 novel_circ_014279 novel_circ_014280 novel_circ_014281 novel_circ_014282 novel_circ_014283 novel_circ_014284 novel_circ_014285 novel_circ_014286 novel_circ_014287 novel_circ_014290 novel_circ_014291 novel_circ_014293 novel_circ_014294 novel_circ_014295 novel_circ_014296 novel_circ_014297 novel_circ_014298 novel_circ_014299 novel_circ_014300 novel_circ_014301 novel_circ_014302 novel_circ_014303 novel_circ_014304 novel_circ_014305 novel_circ_014306 novel_circ_014307 novel_circ_014308

| hsa_circ_0055147 | 90 | 1 | 3495 | 55600 | 59100 | 0 | 0 |
| --- | --- | --- | --- | --- | --- | --- | --- |
| hsa_circ_0055151 | 89 | 3 | 605 | 282 | 884 | 0 | 0 |
| hsa_circ_0055151 | 88 | 1 | 761 | 124 | 884 | 0 | 0 |
| hsa_circ_0055151 | 88 | 3 | 833 | 54 | 884 | 0 | 0 |
| hsa_circ_0055150 | 88 | 1 | 1879 | 63 | 1944 | 0 | 0 |
| hsa_circ_0055147 | 91 | 1 | 3457 | 55600 | 59062 | 0 | 0 |
| hsa_circ_0055151 | 89 | 1 | 438 | 321 | 758 | 0 | 2.00E-153 |
| hsa_circ_0055151 | 89 | 3 | 479 | 282 | 758 | 0 | 2.00E-168 |
| hsa_circ_0055151 | 88 | 1 | 635 | 124 | 758 | 0 | 0 |
| hsa_circ_0055151 | 87 | 3 | 173 | 282 | 452 | 0 | 9.00E-49 |
| hsa_circ_0055151 | 85 | 3 | 159 | 54 | 210 | 0 | 1.00E-37 |
| hsa_circ_0055150 | 89 | 1 | 956 | 102 | 1060 | 0.01 | 0 |
| hsa_circ_0001028 | 89 | 1 | 850 | 40 | 886 | 0 | 0 |
| hsa_circ_0001028 | 89 | 1 | 889 | 1 | 886 | 0 | 0 |
| hsa_circ_0055147 | 92 | 1 | 2467 | 55600 | 58066 | 0 | 0 |
| hsa_circ_0001028 | 88 | 1 | 727 | 40 | 763 | 0 | 0 |
| hsa_circ_0001028 | 88 | 1 | 766 | 1 | 763 | 0 | 0 |
| hsa_circ_0001028 | 88 | 1 | 580 | 40 | 616 | 0.01 | 0 |
| hsa_circ_0055147 | 93 | 1 | 1578 | 55600 | 57180 | 0 | 0 |
| hsa_circ_0055148 | 93 | 1 | 1516 | 1 | 1519 | 0 | 0 |
| hsa_circ_0055133 | 84 | 150 | 1273 | 1780 | 635 | 0.04 | 0 |
| hsa_circ_0055133 | 84 | 150 | 1293 | 1780 | 615 | 0.04 | 0 |
| hsa_circ_0055133 | 81 | 150 | 1885 | 1780 | 4 | 0.05 | 0 |
| hsa_circ_0006138 | 97 | 1 | 448 | 448 | 1 | 0 | 0 |
| hsa_circ_0055118 | 96 | 1 | 1008 | 1008 | 1 | 0 | 0 |
| hsa_circ_0055118 | 97 | 1 | 290 | 560 | 271 | 0 | 5.00E-138 |
| hsa_circ_0055118 | 96 | 1 | 560 | 560 | 1 | 0 | 0 |
| hsa_circ_0055118 | 94 | 1 | 270 | 270 | 1 | 0 | 3.00E-115 |
| hsa_circ_0004975 | 92 | 1 | 321 | 693 | 373 | 0 | 4.00E-125 |
| hsa_circ_0004975 | 94 | 3 | 213 | 475 | 265 | 0 | 7.00E-86 |
| hsa_circ_0004975 | 93 | 3 | 314 | 475 | 164 | 0 | 2.00E-128 |
| hsa_circ_0004975 | 92 | 3 | 477 | 475 | 1 | 0 | 0 |
| hsa_circ_0004975 | 91 | 1 | 372 | 372 | 1 | 0 | 2.00E-138 |
| hsa_circ_0055099 | 83 | 1 | 368 | 66 | 444 | 0.04 | 1.00E-90 |
| hsa_circ_0055099 | 85 | 1 | 433 | 1 | 444 | 0.03 | 1.00E-121 |
| hsa_circ_0055099 | 85 | 194 | 626 | 1 | 444 | 0.03 | 2.00E-121 |
| hsa_circ_0055097 | 90 | 1 | 208 | 243 | 450 | 0.02 | 8.00E-69 |
| hsa_circ_0055097 | 93 | 114 | 563 | 1 | 450 | 0.01 | 0 |
| hsa_circ_0055097 | 90 | 1 | 208 | 243 | 450 | 0.02 | 7.00E-69 |
| hsa_circ_0055097 | 93 | 114 | 563 | 1 | 450 | 0.01 | 0 |
| hsa_circ_0055097 | 98 | 1 | 162 | 67 | 228 | 0 | 9.00E-74 |
| hsa_circ_0055097 | 98 | 114 | 341 | 1 | 228 | 0 | 2.00E-108 |
| hsa_circ_0055085 | 95 | 1 | 371 | 493 | 123 | 0 | 7.00E-163 |
| hsa_circ_0055084 | 97 | 1 | 337 | 337 | 1 | 0 | 5.00E-159 |
| hsa_circ_0055082 | 97 | 1 | 521 | 1472 | 952 | 0 | 0 |
| hsa_circ_0055082 | 95 | 1 | 893 | 1472 | 580 | 0 | 0 |
| hsa_circ_0055082 | 95 | 1 | 893 | 1472 | 580 | 0 | 0 |
| hsa_circ_0055082 | 95 | 1 | 893 | 1472 | 580 | 0 | 0 |
| hsa_circ_0055082 | 97 | 1 | 399 | 1350 | 952 | 0 | 0 |
| hsa_circ_0055072 | 93 | 1 | 563 | 18754 | 18192 | 0 | 0 |
| hsa_circ_0055073 | 96 | 1 | 300 | 522 | 223 | 0 | 3.00E-135 |
| hsa_circ_0055073 | 95 | 1 | 411 | 522 | 112 | 0 | 0 |
| hsa_circ_0055073 | 96 | 1 | 315 | 426 | 112 | 0 | 8.00E-142 |
| hsa_circ_0055072 | 84 | 36 | 1366 | 6993 | 8351 | 0.03 | 0 |

novel_circ_014310 novel_circ_014311 novel_circ_014312 novel_circ_014313 novel_circ_014314 novel_circ_014315 novel_circ_014316 novel_circ_014317 novel_circ_014318 novel_circ_014319 novel_circ_014320 novel_circ_014321 novel_circ_014322 novel_circ_014323 novel_circ_014324 novel_circ_014325 novel_circ_014326 novel_circ_014327 novel_circ_014328 novel_circ_014329 novel_circ_014330 novel_circ_014331 novel_circ_014332 novel_circ_014333 novel_circ_014334 novel_circ_014335 novel_circ_014336 novel_circ_014340 novel_circ_014341 novel_circ_014342 novel_circ_014343 novel_circ_014344 novel_circ_014345 novel_circ_014346 novel_circ_014347 novel_circ_014348 novel_circ_014349 novel_circ_014350 novel_circ_014353 novel_circ_014354 novel_circ_014355 novel_circ_014356 novel_circ_014357 novel_circ_014358 novel_circ_014359 novel_circ_014360 novel_circ_014361 novel_circ_014362 novel_circ_014363 novel_circ_014364 novel_circ_014365 novel_circ_014366 novel_circ_014367 novel_circ_014368

| hsa_circ_0055066 | 91 | 2 | 240 | 657 | 419 | 0 | 2.00E-86 |
| --- | --- | --- | --- | --- | --- | --- | --- |
| hsa_circ_0055066 | 91 | 2 | 306 | 657 | 353 | 0 | 6.00E-113 |
| hsa_circ_0055066 | 92 | 2 | 422 | 657 | 237 | 0 | 1.00E-165 |
| hsa_circ_0055066 | 93 | 2 | 658 | 657 | 1 | 0 | 0 |
| hsa_circ_0055066 | 93 | 1 | 318 | 554 | 237 | 0 | 4.00E-130 |
| hsa_circ_0055066 | 94 | 1 | 351 | 351 | 1 | 0 | 8.00E-152 |
| hsa_circ_0055062 | 96 | 1 | 536 | 536 | 1 | 0 | 0 |
| hsa_circ_0055057 | 96 | 1 | 678 | 1002 | 325 | 0 | 0 |
| hsa_circ_0055048 | 93 | 677 | 1421 | 745 | 1 | 0 | 0 |
| hsa_circ_0055062 | 95 | 3 | 320 | 318 | 1 | 0 | 8.00E-142 |
| hsa_circ_0055061 | 96 | 3 | 382 | 380 | 1 | 0 | 7.00E-173 |
| hsa_circ_0055060 | 95 | 3 | 462 | 460 | 1 | 0 | 0 |
| hsa_circ_0055060 | 95 | 3 | 462 | 460 | 1 | 0 | 0 |
| hsa_circ_0055060 | 95 | 3 | 462 | 460 | 1 | 0 | 0 |
| hsa_circ_0055060 | 95 | 3 | 462 | 460 | 1 | 0 | 0 |
| hsa_circ_0055048 | 93 | 461 | 1205 | 745 | 1 | 0 | 0 |
| hsa_circ_0055048 | 93 | 1 | 473 | 473 | 1 | 0 | 0 |
| hsa_circ_0055048 | 93 | 1 | 377 | 377 | 1 | 0 | 3.00E-156 |
| hsa_circ_0055041 | 92 | 1 | 243 | 243 | 1 | 0 | 6.00E-92 |
| hsa_circ_0055040 | 92 | 1 | 411 | 411 | 1 | 0 | 1.00E-161 |
| hsa_circ_0055030 | 93 | 2 | 345 | 2 | 345 | 0 | 1.00E-139 |
| hsa_circ_0055030 | 93 | 2 | 196 | 2 | 196 | 0 | 5.00E-77 |
| hsa_circ_0001024 | 82 | 1 | 924 | 246 | 1187 | 0.03 | 0 |
| hsa_circ_0001024 | 82 | 1 | 1097 | 73 | 1187 | 0.02 | 0 |
| hsa_circ_0001024 | 83 | 1 | 1169 | 1 | 1187 | 0.02 | 0 |
| hsa_circ_0001024 | 82 | 1 | 974 | 73 | 1064 | 0.03 | 0 |
| hsa_circ_0001024 | 83 | 1 | 1046 | 1 | 1064 | 0.02 | 0 |
| hsa_circ_0055009 | 92 | 1 | 349 | 518 | 170 | 0.01 | 2.00E-137 |
| hsa_circ_0053961 | 92 | 1 | 174 | 1 | 174 | 0 | 9.00E-64 |
| hsa_circ_0053961 | 93 | 1 | 538 | 1 | 538 | 0.01 | 0 |
| hsa_circ_0053961 | 94 | 1 | 660 | 1 | 660 | 0.01 | 0 |
| hsa_circ_0053961 | 92 | 1 | 1041 | 1 | 1041 | 0 | 0 |
| hsa_circ_0053970 | 93 | 1 | 364 | 1 | 364 | 0.01 | 5.00E-149 |
| hsa_circ_0053970 | 94 | 1 | 486 | 1 | 486 | 0.01 | 0 |
| hsa_circ_0053970 | 93 | 1 | 867 | 1 | 867 | 0 | 0 |
| hsa_circ_0053979 | 92 | 1 | 243 | 1 | 243 | 0 | 6.00E-92 |
| hsa_circ_0054009 | 92 | 3 | 927 | 3 | 927 | 0 | 0 |
| hsa_circ_0054020 | 92 | 1 | 438 | 1 | 438 | 0 | 1.00E-176 |
| hsa_circ_0054029 | 92 | 1 | 309 | 309 | 1 | 0 | 4.00E-120 |
| hsa_circ_0054029 | 93 | 1 | 431 | 431 | 1 | 0 | 4.00E-176 |
| hsa_circ_0054029 | 93 | 1 | 655 | 655 | 1 | 0 | 0 |
| hsa_circ_0054029 | 94 | 1 | 792 | 792 | 1 | 0 | 0 |
| hsa_circ_0054029 | 95 | 1 | 1047 | 1047 | 1 | 0 | 0 |
| hsa_circ_0054029 | 95 | 1 | 1183 | 1183 | 1 | 0 | 0 |
| hsa_circ_0054029 | 95 | 137 | 1319 | 1183 | 1 | 0 | 0 |
| hsa_circ_0054031 | 94 | 1 | 645 | 651 | 7 | 0 | 0 |
| hsa_circ_0054032 | 95 | 1 | 344 | 346 | 3 | 0 | 6.00E-153 |
| hsa_circ_0054032 | 95 | 1 | 481 | 483 | 3 | 0 | 0 |
| hsa_circ_0054032 | 96 | 1 | 736 | 738 | 3 | 0 | 0 |
| hsa_circ_0054032 | 96 | 1 | 872 | 874 | 3 | 0 | 0 |
| hsa_circ_0054035 | 97 | 1 | 392 | 392 | 1 | 0 | 0 |
| hsa_circ_0054035 | 95 | 75 | 985 | 911 | 1 | 0 | 0 |
| hsa_circ_0054037 | 94 | 1 | 440 | 440 | 1 | 0 | 0 |
| hsa_circ_0054037 | 94 | 1 | 593 | 593 | 1 | 0 | 0 |

novel_circ_014369 novel_circ_014370 novel_circ_014371 novel_circ_014372 novel_circ_014373 novel_circ_014374 novel_circ_014375 novel_circ_014376 novel_circ_014377 novel_circ_014378 novel_circ_014379 novel_circ_014380 novel_circ_014381 novel_circ_014382 novel_circ_014383 novel_circ_014384 novel_circ_014385 novel_circ_014386 novel_circ_014387 novel_circ_014388 novel_circ_014389 novel_circ_014394 novel_circ_014395 novel_circ_014396 novel_circ_014397 novel_circ_014398 novel_circ_014399 novel_circ_014400 novel_circ_014401 novel_circ_014402 novel_circ_014403 novel_circ_014404 novel_circ_014405 novel_circ_014406 novel_circ_014408 novel_circ_014411 novel_circ_014412 novel_circ_014413 novel_circ_014414 novel_circ_014415 novel_circ_014416 novel_circ_014417 novel_circ_014418 novel_circ_014419 novel_circ_014420 novel_circ_014421 novel_circ_014422 novel_circ_014423 novel_circ_014424 novel_circ_014425 novel_circ_014426 novel_circ_014427 novel_circ_014428 novel_circ_014430

| hsa_circ_0054037 | 94 | 1 | 456 | 593 | 138 | 0 | 0 |
| --- | --- | --- | --- | --- | --- | --- | --- |
| hsa_circ_0054039 | 95 | 1 | 153 | 321 | 169 | 0 | 8.00E-64 |
| hsa_circ_0054037 | 95 | 1 | 257 | 697 | 441 | 0 | 2.00E-111 |
| hsa_circ_0054043 | 95 | 6 | 366 | 361 | 1 | 0 | 5.00E-159 |
| hsa_circ_0054043 | 92 | 1 | 858 | 861 | 1 | 0 | 0 |
| hsa_circ_0054043 | 91 | 1 | 1386 | 1389 | 1 | 0 | 0 |
| hsa_circ_0054043 | 91 | 1 | 644 | 861 | 215 | 0 | 0 |
| hsa_circ_0054051 | 91 | 588 | 2276 | 1692 | 1 | 0 | 0 |
| hsa_circ_0054054 | 92 | 588 | 1784 | 1464 | 268 | 0 | 0 |
| hsa_circ_0054063 | 92 | 1 | 2099 | 2186 | 88 | 0 | 0 |
| hsa_circ_0054076 | 92 | 1 | 351 | 351 | 1 | 0 | 4.00E-140 |
| hsa_circ_0054073 | 93 | 1 | 2409 | 2914 | 506 | 0 | 0 |
| hsa_circ_0054081 | 92 | 1 | 297 | 297 | 1 | 0 | 3.00E-115 |
| hsa_circ_0054081 | 93 | 1 | 847 | 847 | 1 | 0 | 0 |
| hsa_circ_0054092 | 94 | 3 | 340 | 338 | 1 | 0 | 6.00E-143 |
| hsa_circ_0054092 | 94 | 1 | 550 | 550 | 1 | 0 | 0 |
| hsa_circ_0054094 | 93 | 1 | 1402 | 1612 | 211 | 0 | 0 |
| hsa_circ_0054098 | 93 | 1 | 886 | 886 | 1 | 0 | 0 |
| hsa_circ_0054098 | 93 | 3 | 408 | 562 | 157 | 0 | 3.00E-167 |
| hsa_circ_0054098 | 93 | 1 | 730 | 886 | 157 | 0 | 0 |
| hsa_circ_0054098 | 93 | 1 | 949 | 1355 | 407 | 0 | 0 |
| hsa_circ_0054107 | 89 | 1 | 1918 | 2155 | 247 | 0 | 0 |
| hsa_circ_0054107 | 89 | 1000 | 2917 | 2155 | 247 | 0 | 0 |
| hsa_circ_0006444 | 91 | 82 | 894 | 1 | 813 | 0 | 0 |
| hsa_circ_0006444 | 88 | 1 | 1091 | 1 | 1093 | 0.01 | 0 |
| hsa_circ_0006444 | 90 | 1 | 702 | 112 | 813 | 0 | 0 |
| hsa_circ_0006444 | 90 | 1 | 429 | 385 | 813 | 0 | 1.00E-156 |
| hsa_circ_0006444 | 86 | 1 | 596 | 496 | 1093 | 0.01 | 3.00E-178 |
| hsa_circ_0054111 | 95 | 1 | 368 | 722 | 355 | 0 | 3.00E-161 |
| hsa_circ_0054119 | 87 | 118 | 1557 | 2193 | 742 | 0.03 | 0 |
| hsa_circ_0054127 | 93 | 1 | 429 | 429 | 1 | 0 | 0 |
| hsa_circ_0054119 | 86 | 118 | 1367 | 2193 | 935 | 0.03 | 0 |
| hsa_circ_0054119 | 84 | 118 | 1077 | 2193 | 1225 | 0.04 | 0 |
| hsa_circ_0000992 | 82 | 1 | 938 | 943 | 1 | 0.05 | 0 |
| hsa_circ_0054129 | 85 | 1330 | 1851 | 921 | 1451 | 0.02 | 6.00E-150 |
| hsa_circ_0002918 | 97 | 1 | 708 | 708 | 1 | 0 | 0 |
| hsa_circ_0002918 | 97 | 1 | 708 | 708 | 1 | 0 | 0 |
| hsa_circ_0002918 | 97 | 246 | 953 | 708 | 1 | 0 | 0 |
| hsa_circ_0054132 | 95 | 1 | 441 | 441 | 1 | 0 | 0 |
| hsa_circ_0000993 | 96 | 1 | 240 | 240 | 1 | 0 | 1.00E-108 |
| hsa_circ_0054132 | 96 | 1 | 485 | 686 | 202 | 0 | 0 |
| hsa_circ_0054136 | 94 | 2 | 357 | 358 | 3 | 0 | 3.00E-151 |
| hsa_circ_0054136 | 95 | 1 | 479 | 481 | 3 | 0 | 0 |
| hsa_circ_0054136 | 95 | 3 | 697 | 697 | 3 | 0 | 0 |
| hsa_circ_0054136 | 96 | 1 | 1025 | 1027 | 3 | 0 | 0 |
| hsa_circ_0054136 | 96 | 1 | 1382 | 1384 | 3 | 0 | 0 |
| hsa_circ_0054139 | 96 | 1 | 784 | 784 | 1 | 0 | 0 |
| hsa_circ_0054136 | 96 | 1 | 903 | 1384 | 482 | 0 | 0 |
| hsa_circ_0054136 | 96 | 1 | 685 | 1384 | 700 | 0 | 0 |
| hsa_circ_0054140 | 96 | 1 | 494 | 494 | 1 | 0 | 0 |
| hsa_circ_0054140 | 96 | 1 | 613 | 613 | 1 | 0 | 0 |
| hsa_circ_0054144 | 95 | 1 | 357 | 357 | 1 | 0 | 4.00E-160 |
| hsa_circ_0073233 | 82 | 5726 | 5898 | 11273 | 11454 | 0.05 | 2.00E-33 |
| hsa_circ_0054166 | 90 | 1 | 261 | 378 | 118 | 0 | 7.00E-92 |

novel_circ_014431 novel_circ_014432 novel_circ_014433 novel_circ_014434 novel_circ_014435 novel_circ_014436 novel_circ_014437 novel_circ_014438 novel_circ_014439 novel_circ_014440 novel_circ_014441 novel_circ_014442 novel_circ_014443 novel_circ_014444 novel_circ_014445 novel_circ_014446 novel_circ_014447 novel_circ_014449 novel_circ_014450 novel_circ_014451 novel_circ_014452 novel_circ_014453 novel_circ_014454 novel_circ_014455 novel_circ_014456 novel_circ_014457 novel_circ_014458 novel_circ_014459 novel_circ_014460 novel_circ_014461 novel_circ_014462 novel_circ_014463 novel_circ_014464 novel_circ_014465 novel_circ_014466 novel_circ_014469 novel_circ_014470 novel_circ_014471 novel_circ_014472 novel_circ_014473 novel_circ_014474 novel_circ_014475 novel_circ_014476 novel_circ_014477 novel_circ_014479 novel_circ_014480 novel_circ_014481 novel_circ_014482 novel_circ_014483 novel_circ_014485 novel_circ_014486 novel_circ_014487 novel_circ_014488 novel_circ_014489

| hsa_circ_0054162 | 91 | 1 | 455 | 1582 | 1128 | 0 | 4.00E-176 |
| --- | --- | --- | --- | --- | --- | --- | --- |
| hsa_circ_0054167 | 90 | 178 | 1010 | 839 | 1 | 0.01 | 0 |
| hsa_circ_0054169 | 92 | 1 | 834 | 1006 | 1838 | 0 | 0 |
| hsa_circ_0054169 | 92 | 1 | 834 | 1006 | 1838 | 0 | 0 |
| hsa_circ_0054170 | 94 | 24346 | 24611 | 45 | 310 | 0 | 4.00E-109 |
| hsa_circ_0054170 | 94 | 1 | 600 | 600 | 1 | 0 | 0 |
| hsa_circ_0054170 | 94 | 1 | 555 | 600 | 46 | 0 | 0 |
| hsa_circ_0054170 | 95 | 1 | 673 | 718 | 46 | 0 | 0 |
| hsa_circ_0054170 | 95 | 2 | 1283 | 1327 | 46 | 0 | 0 |
| hsa_circ_0054170 | 95 | 1221 | 2626 | 1451 | 46 | 0 | 0 |
| hsa_circ_0054170 | 95 | 1 | 290 | 600 | 311 | 0 | 3.00E-126 |
| hsa_circ_0054170 | 95 | 2 | 728 | 1327 | 601 | 0 | 0 |
| hsa_circ_0054170 | 95 | 1 | 851 | 1451 | 601 | 0 | 0 |
| hsa_circ_0054173 | 94 | 1 | 733 | 733 | 1 | 0 | 0 |
| hsa_circ_0054177 | 95 | 1 | 255 | 255 | 1 | 0 | 2.00E-108 |
| hsa_circ_0054177 | 96 | 1 | 888 | 888 | 1 | 0 | 0 |
| hsa_circ_0054178 | 96 | 1 | 777 | 777 | 1 | 0 | 0 |
| hsa_circ_0054187 | 93 | 1 | 162 | 162 | 1 | 0 | 4.00E-62 |
| hsa_circ_0054185 | 94 | 1 | 1940 | 2011 | 72 | 0 | 0 |
| hsa_circ_0054185 | 95 | 1 | 514 | 747 | 234 | 0 | 0 |
| hsa_circ_0054185 | 94 | 2 | 1037 | 1269 | 234 | 0 | 0 |
| hsa_circ_0054185 | 94 | 1 | 1778 | 2011 | 234 | 0 | 0 |
| hsa_circ_0054188 | 94 | 4 | 822 | 819 | 1 | 0 | 0 |
| hsa_circ_0054188 | 93 | 1 | 1017 | 1017 | 1 | 0 | 0 |
| hsa_circ_0054185 | 94 | 1 | 1218 | 1623 | 406 | 0 | 0 |
| hsa_circ_0054191 | 96 | 1 | 344 | 344 | 1 | 0 | 1.00E-159 |
| hsa_circ_0054191 | 96 | 1 | 386 | 386 | 1 | 0 | 1.00E-179 |
| hsa_circ_0054191 | 94 | 2 | 702 | 701 | 1 | 0 | 0 |
| hsa_circ_0054185 | 93 | 1 | 1443 | 2011 | 569 | 0 | 0 |
| hsa_circ_0054194 | 95 | 1 | 320 | 320 | 1 | 0 | 6.00E-143 |
| hsa_circ_0054194 | 93 | 4 | 480 | 477 | 1 | 0 | 0 |
| hsa_circ_0054192 | 93 | 1 | 876 | 937 | 62 | 0 | 0 |
| hsa_circ_0054185 | 93 | 1 | 1264 | 2011 | 748 | 0 | 0 |
| hsa_circ_0054195 | 91 | 4 | 315 | 397 | 86 | 0 | 2.00E-118 |
| hsa_circ_0054195 | 93 | 1 | 1099 | 1184 | 86 | 0 | 0 |
| hsa_circ_0054199 | 93 | 1 | 669 | 669 | 1 | 0 | 0 |
| hsa_circ_0054206 | 96 | 1 | 260 | 260 | 1 | 0 | 5.00E-118 |
| hsa_circ_0054195 | 94 | 1 | 648 | 1184 | 537 | 0 | 0 |
| hsa_circ_0054207 | 98 | 1 | 201 | 201 | 1 | 0 | 1.00E-93 |
| hsa_circ_0054209 | 93 | 1 | 467 | 467 | 1 | 0 | 0 |
| hsa_circ_0054209 | 93 | 1 | 388 | 467 | 80 | 0 | 3.00E-157 |
| hsa_circ_0054209 | 95 | 59 | 822 | 843 | 80 | 0 | 0 |
| hsa_circ_0054213 | 98 | 1 | 164 | 164 | 1 | 0 | 7.00E-75 |
| hsa_circ_0054213 | 99 | 1 | 91 | 164 | 74 | 0 | 1.00E-39 |
| hsa_circ_0070276 | 90 | 1 | 423 | 423 | 1 | 0 | 5.00E-155 |
| hsa_circ_0070276 | 90 | 1 | 423 | 423 | 1 | 0 | 6.00E-155 |
| hsa_circ_0070276 | 90 | 1 | 249 | 249 | 1 | 0 | 1.00E-88 |
| hsa_circ_0070276 | 90 | 1 | 249 | 249 | 1 | 0 | 2.00E-88 |
| hsa_circ_0070276 | 90 | 1 | 249 | 249 | 1 | 0 | 9.00E-88 |
| hsa_circ_0070272 | 91 | 6 | 342 | 526 | 190 | 0 | 5.00E-129 |
| hsa_circ_0070269 | 89 | 451 | 746 | 295 | 1 | 0 | 1.00E-98 |
| hsa_circ_0070269 | 89 | 83 | 378 | 295 | 1 | 0 | 6.00E-99 |
| hsa_circ_0070265 | 97 | 81 | 641 | 1 | 561 | 0 | 0 |
| hsa_circ_0007676 | 97 | 1 | 484 | 484 | 1 | 0 | 0 |

novel_circ_014490 novel_circ_014491 novel_circ_014492 novel_circ_014493 novel_circ_014494 novel_circ_014496 novel_circ_014497 novel_circ_014498 novel_circ_014499 novel_circ_014500 novel_circ_014501 novel_circ_014502 novel_circ_014503 novel_circ_014504 novel_circ_014505 novel_circ_014506 novel_circ_014507 novel_circ_014508 novel_circ_014509 novel_circ_014510 novel_circ_014511 novel_circ_014512 novel_circ_014513 novel_circ_014514 novel_circ_014515 novel_circ_014516 novel_circ_014517 novel_circ_014518 novel_circ_014519 novel_circ_014521 novel_circ_014522 novel_circ_014526 novel_circ_014527 novel_circ_014528 novel_circ_014530 novel_circ_014531 novel_circ_014532 novel_circ_014533 novel_circ_014534 novel_circ_014535 novel_circ_014536 novel_circ_014537 novel_circ_014538 novel_circ_014539 novel_circ_014540 novel_circ_014541 novel_circ_014542 novel_circ_014543 novel_circ_014544 novel_circ_014546 novel_circ_014547 novel_circ_014548 novel_circ_014549 novel_circ_014550

| hsa_circ_0007676 | 97 | 1 | 484 | 484 | 1 | 0 | 0 |
| --- | --- | --- | --- | --- | --- | --- | --- |
| hsa_circ_0007676 | 97 | 1 | 484 | 484 | 1 | 0 | 0 |
| hsa_circ_0007676 | 97 | 1 | 484 | 484 | 1 | 0 | 0 |
| hsa_circ_0007676 | 97 | 1 | 360 | 360 | 1 | 0 | 4.00E-170 |
| hsa_circ_0070256 | 95 | 582 | 894 | 313 | 1 | 0 | 3.00E-135 |
| hsa_circ_0006618 | 96 | 1 | 406 | 406 | 1 | 0 | 0 |
| hsa_circ_0070252 | 95 | 1 | 502 | 643 | 142 | 0 | 0 |
| hsa_circ_0070252 | 95 | 1 | 642 | 643 | 2 | 0 | 0 |
| hsa_circ_0070251 | 94 | 1 | 786 | 786 | 1 | 0 | 0 |
| hsa_circ_0006543 | 94 | 1 | 886 | 886 | 1 | 0 | 0 |
| hsa_circ_0070242 | 94 | 1 | 1201 | 2332 | 1132 | 0 | 0 |
| hsa_circ_0070242 | 93 | 1 | 1504 | 2332 | 820 | 0.01 | 0 |
| hsa_circ_0070242 | 93 | 1 | 1504 | 2332 | 820 | 0.01 | 0 |
| hsa_circ_0070242 | 93 | 1 | 1873 | 2332 | 448 | 0.01 | 0 |
| hsa_circ_0070242 | 92 | 1 | 2320 | 2332 | 1 | 0.01 | 0 |
| hsa_circ_0070232 | 92 | 1 | 2495 | 3487 | 981 | 0.01 | 0 |
| hsa_circ_0006618 | 96 | 1 | 403 | 403 | 1 | 0 | 0 |
| hsa_circ_0070252 | 95 | 1 | 639 | 640 | 2 | 0 | 0 |
| hsa_circ_0070232 | 92 | 1 | 2492 | 3484 | 981 | 0.01 | 0 |
| hsa_circ_0070252 | 94 | 1 | 559 | 560 | 2 | 0 | 0 |
| hsa_circ_0070251 | 93 | 1 | 380 | 380 | 1 | 0 | 2.00E-154 |
| hsa_circ_0070251 | 93 | 4 | 284 | 281 | 1 | 0 | 1.00E-114 |
| hsa_circ_0005635 | 94 | 1 | 315 | 315 | 1 | 0 | 8.00E-132 |
| hsa_circ_0070232 | 92 | 377 | 892 | 517 | 2 | 0 | 0 |
| hsa_circ_0070235 | 91 | 1 | 321 | 323 | 4 | 0 | 1.00E-119 |
| hsa_circ_0070232 | 92 | 1 | 514 | 515 | 2 | 0 | 0 |
| hsa_circ_0070232 | 91 | 1 | 455 | 456 | 2 | 0 | 8.00E-173 |
| hsa_circ_0070225 | 91 | 1 | 452 | 444 | 898 | 0.01 | 1.00E-176 |
| hsa_circ_0070225 | 92 | 1 | 554 | 342 | 898 | 0.01 | 0 |
| hsa_circ_0001420 | 99 | 1 | 463 | 808 | 346 | 0 | 0 |
| hsa_circ_0001420 | 99 | 1 | 710 | 808 | 99 | 0 | 0 |
| hsa_circ_0083652 | 82 | 37033 | 37138 | 2557 | 2454 | 0.02 | 2.00E-14 |
| hsa_circ_0088097 | 82 | 16193 | 17292 | 1117 | 1 | 0.03 | 0 |
| hsa_circ_0069858 | 80 | 34985 | 35117 | 258307 | 258437 | 0.02 | 6.00E-18 |
| hsa_circ_0070207 | 88 | 140 | 648 | 215 | 720 | 0.01 | 7.00E-170 |
| hsa_circ_0070207 | 88 | 301 | 809 | 215 | 720 | 0.01 | 9.00E-170 |
| hsa_circ_0070201 | 91 | 1 | 520 | 1 | 526 | 0.01 | 0 |
| hsa_circ_0070194 | 93 | 1 | 1225 | 1 | 1231 | 0 | 0 |
| hsa_circ_0070201 | 95 | 1 | 345 | 1 | 348 | 0.01 | 5.00E-154 |
| hsa_circ_0070194 | 95 | 1 | 1050 | 1 | 1053 | 0 | 0 |
| hsa_circ_0028415 | 87 | 4635 | 5083 | 6 | 452 | 0.01 | 4.00E-139 |
| hsa_circ_0070199 | 96 | 1 | 317 | 1 | 317 | 0 | 6.00E-143 |
| hsa_circ_0070198 | 96 | 2 | 399 | 2 | 399 | 0 | 0 |
| hsa_circ_0070194 | 95 | 1 | 889 | 1 | 889 | 0 | 0 |
| hsa_circ_0070198 | 95 | 2 | 319 | 2 | 319 | 0 | 8.00E-142 |
| hsa_circ_0070194 | 94 | 1 | 809 | 1 | 809 | 0 | 0 |
| hsa_circ_0070194 | 94 | 1 | 572 | 1 | 572 | 0 | 0 |
| hsa_circ_0070194 | 93 | 1 | 368 | 1 | 368 | 0 | 1.00E-154 |
| hsa_circ_0070182 | 93 | 7604 | 7881 | 189 | 466 | 0 | 2.00E-109 |
| hsa_circ_0070088 | 90 | 4822 | 5112 | 1 | 291 | 0 | 6.00E-104 |
| hsa_circ_0070088 | 90 | 1321 | 1611 | 1 | 291 | 0 | 2.00E-104 |
| hsa_circ_0006168 | 99 | 1 | 395 | 395 | 1 | 0 | 0 |
| hsa_circ_0070085 | 99 | 1 | 485 | 485 | 1 | 0 | 0 |
| hsa_circ_0070083 | 99 | 1 | 867 | 8707 | 7841 | 0 | 0 |

novel_circ_014551 novel_circ_014552 novel_circ_014554 novel_circ_014555 novel_circ_014556 novel_circ_014557 novel_circ_014558 novel_circ_014559 novel_circ_014560 novel_circ_014563 novel_circ_014564 novel_circ_014572 novel_circ_014573 novel_circ_014574 novel_circ_014575 novel_circ_014577 novel_circ_014578 novel_circ_014579 novel_circ_014583 novel_circ_014584 novel_circ_014586 novel_circ_014587 novel_circ_014588 novel_circ_014589 novel_circ_014590 novel_circ_014591 novel_circ_014592 novel_circ_014593 novel_circ_014594 novel_circ_014595 novel_circ_014596 novel_circ_014597 novel_circ_014599 novel_circ_014600 novel_circ_014601 novel_circ_014602 novel_circ_014603 novel_circ_014604 novel_circ_014605 novel_circ_014606 novel_circ_014607 novel_circ_014608 novel_circ_014609 novel_circ_014610 novel_circ_014611 novel_circ_014612 novel_circ_014615 novel_circ_014616 novel_circ_014617 novel_circ_014623 novel_circ_014624 novel_circ_014625 novel_circ_014626 novel_circ_014629

| hsa_circ_0070083 | 99 | 1 | 1724 | 8707 | 6983 | 0 | 0 |
| --- | --- | --- | --- | --- | --- | --- | --- |
| hsa_circ_0070083 | 98 | 1 | 583 | 7840 | 7258 | 0 | 0 |
| hsa_circ_0070075 | 92 | 1 | 286 | 361 | 76 | 0 | 9.00E-111 |
| hsa_circ_0003373 | 95 | 1 | 733 | 733 | 1 | 0 | 0 |
| hsa_circ_0008524 | 95 | 1 | 733 | 733 | 1 | 0 | 0 |
| hsa_circ_0003373 | 96 | 1 | 576 | 576 | 1 | 0 | 0 |
| hsa_circ_0003373 | 97 | 7 | 447 | 441 | 1 | 0 | 0 |
| hsa_circ_0028129 | 89 | 3 | 330 | 500 | 827 | 0 | 3.00E-110 |
| hsa_circ_0028121 | 90 | 1 | 314 | 1 | 314 | 0 | 1.00E-114 |
| hsa_circ_0028095 | 94 | 1 | 95 | 1 | 95 | 0 | 5.00E-33 |
| hsa_circ_0028093 | 91 | 3 | 344 | 5468 | 5809 | 0 | 4.00E-130 |
| hsa_circ_0028089 | 92 | 1 | 291 | 291 | 1 | 0 | 3.00E-115 |
| hsa_circ_0028089 | 91 | 1 | 187 | 187 | 1 | 0 | 1.00E-67 |
| hsa_circ_0028084 | 93 | 1 | 417 | 465 | 49 | 0 | 5.00E-170 |
| hsa_circ_0028083 | 92 | 1 | 194 | 194 | 1 | 0 | 4.00E-73 |
| hsa_circ_0028077 | 92 | 4 | 432 | 429 | 1 | 0 | 2.00E-173 |
| hsa_circ_0028068 | 93 | 4 | 683 | 680 | 1 | 0 | 0 |
| hsa_circ_0028057 | 91 | 1 | 1009 | 1808 | 800 | 0 | 0 |
| hsa_circ_0028936 | 96 | 1 | 479 | 479 | 1 | 0 | 0 |
| hsa_circ_0028936 | 96 | 1 | 401 | 401 | 1 | 0 | 0 |
| hsa_circ_0028914 | 96 | 1 | 217 | 258 | 474 | 0 | 1.00E-97 |
| hsa_circ_0028903 | 93 | 1 | 1708 | 1 | 1714 | 0 | 0 |
| hsa_circ_0028911 | 95 | 1 | 477 | 1 | 477 | 0 | 0 |
| hsa_circ_0028911 | 94 | 1 | 376 | 1 | 376 | 0 | 2.00E-162 |
| hsa_circ_0028895 | 93 | 1 | 1681 | 1 | 1687 | 0 | 0 |
| hsa_circ_0028908 | 89 | 1 | 558 | 1 | 564 | 0.01 | 0 |
| hsa_circ_0028903 | 92 | 1 | 880 | 1 | 886 | 0.01 | 0 |
| hsa_circ_0028895 | 97 | 1 | 774 | 1 | 774 | 0 | 0 |
| hsa_circ_0028903 | 98 | 1 | 322 | 1 | 322 | 0 | 2.00E-157 |
| hsa_circ_0028898 | 97 | 1 | 413 | 1 | 413 | 0 | 0 |
| hsa_circ_0028895 | 97 | 1 | 613 | 1 | 613 | 0 | 0 |
| hsa_circ_0028891 | 96 | 1 | 810 | 641 | 1450 | 0 | 0 |
| hsa_circ_0028706 | 91 | 1 | 590 | 595 | 6 | 0 | 0 |
| hsa_circ_0028702 | 92 | 4 | 542 | 539 | 1 | 0 | 0 |
| hsa_circ_0028692 | 98 | 1 | 250 | 3011 | 2762 | 0 | 4.00E-119 |
| hsa_circ_0003902 | 93 | 1 | 276 | 276 | 1 | 0 | 7.00E-112 |
| hsa_circ_0028697 | 94 | 1 | 955 | 1041 | 87 | 0 | 0 |
| hsa_circ_0028696 | 91 | 353 | 980 | 629 | 2 | 0 | 0 |
| hsa_circ_0028695 | 85 | 1 | 473 | 446 | 1 | 0.06 | 3.00E-128 |
| hsa_circ_0028695 | 84 | 4 | 419 | 389 | 1 | 0.06 | 5.00E-100 |
| hsa_circ_0028692 | 86 | 4 | 555 | 1345 | 821 | 0.05 | 8.00E-164 |
| hsa_circ_0028692 | 90 | 4 | 909 | 1345 | 467 | 0.03 | 0 |
| hsa_circ_0028692 | 93 | 1 | 576 | 1150 | 575 | 0 | 0 |
| hsa_circ_0028692 | 96 | 4 | 434 | 571 | 141 | 0 | 0 |
| hsa_circ_0028692 | 95 | 4 | 574 | 571 | 1 | 0 | 0 |
| hsa_circ_0028676 | 92 | 1 | 578 | 578 | 1 | 0 | 0 |
| hsa_circ_0028662 | 93 | 1 | 457 | 592 | 135 | 0 | 0 |
| hsa_circ_0028640 | 85 | 6 | 405 | 400 | 1 | 0 | 6.00E-114 |
| hsa_circ_0028633 | 94 | 174 | 392 | 219 | 1 | 0 | 2.00E-88 |
| hsa_circ_0028620 | 93 | 1 | 638 | 1321 | 684 | 0 | 0 |
| hsa_circ_0028620 | 92 | 1 | 1226 | 1226 | 1 | 0 | 0 |
| hsa_circ_0028622 | 93 | 5 | 313 | 309 | 1 | 0 | 2.00E-123 |
| hsa_circ_0028620 | 93 | 5 | 683 | 679 | 1 | 0 | 0 |
| hsa_circ_0028613 | 85 | 3 | 404 | 3 | 404 | 0 | 2.00E-113 |

novel_circ_014630 novel_circ_014631 novel_circ_014632 novel_circ_014633 novel_circ_014634 novel_circ_014635 novel_circ_014636 novel_circ_014637 novel_circ_014638 novel_circ_014639 novel_circ_014640 novel_circ_014641 novel_circ_014642 novel_circ_014643 novel_circ_014644 novel_circ_014645 novel_circ_014646 novel_circ_014647 novel_circ_014648 novel_circ_014649 novel_circ_014650 novel_circ_014651 novel_circ_014652 novel_circ_014653 novel_circ_014654 novel_circ_014655 novel_circ_014656 novel_circ_014657 novel_circ_014658 novel_circ_014659 novel_circ_014660 novel_circ_014661 novel_circ_014662 novel_circ_014663 novel_circ_014664 novel_circ_014665 novel_circ_014666 novel_circ_014667 novel_circ_014668 novel_circ_014669 novel_circ_014670 novel_circ_014671 novel_circ_014672 novel_circ_014673 novel_circ_014674 novel_circ_014675 novel_circ_014676 novel_circ_014677 novel_circ_014678 novel_circ_014679 novel_circ_014680 novel_circ_014681 novel_circ_014682 novel_circ_014683

| hsa_circ_0028609 | 88 | 1 | 816 | 1 | 816 | 0 | 0 |
| --- | --- | --- | --- | --- | --- | --- | --- |
| hsa_circ_0028607 | 92 | 1 | 356 | 1 | 356 | 0 | 1.00E-139 |
| hsa_circ_0028588 | 98 | 1 | 238 | 238 | 1 | 0 | 7.00E-116 |
| hsa_circ_0028586 | 99 | 1 | 169 | 169 | 1 | 0 | 1.00E-82 |
| hsa_circ_0028578 | 96 | 1 | 801 | 1759 | 959 | 0 | 0 |
| hsa_circ_0028578 | 96 | 1 | 802 | 1759 | 958 | 0 | 0 |
| hsa_circ_0028579 | 95 | 1 | 550 | 550 | 1 | 0 | 0 |
| hsa_circ_0028578 | 95 | 1 | 656 | 1613 | 958 | 0 | 0 |
| hsa_circ_0028575 | 95 | 1 | 1189 | 1289 | 101 | 0 | 0 |
| hsa_circ_0028573 | 95 | 1 | 427 | 427 | 1 | 0 | 0 |
| hsa_circ_0028573 | 95 | 1 | 424 | 424 | 1 | 0 | 0 |
| hsa_circ_0028571 | 93 | 1 | 938 | 1118 | 181 | 0 | 0 |
| hsa_circ_0028571 | 93 | 1 | 1116 | 1118 | 3 | 0 | 0 |
| hsa_circ_0028569 | 94 | 1 | 1342 | 1342 | 1 | 0 | 0 |
| hsa_circ_0028565 | 93 | 1 | 1064 | 1061 | 1 | 0.01 | 0 |
| hsa_circ_0028564 | 93 | 1 | 1253 | 1250 | 1 | 0.01 | 0 |
| hsa_circ_0028565 | 92 | 1 | 840 | 837 | 1 | 0.01 | 0 |
| hsa_circ_0047140 | 92 | 1 | 672 | 1 | 672 | 0 | 0 |
| hsa_circ_0047140 | 92 | 1 | 358 | 1 | 358 | 0 | 5.00E-144 |
| hsa_circ_0047139 | 94 | 1 | 436 | 94 | 529 | 0 | 0 |
| hsa_circ_0047128 | 88 | 1 | 1736 | 807 | 2552 | 0.01 | 0 |
| hsa_circ_0047130 | 92 | 1 | 452 | 44 | 495 | 0 | 0 |
| hsa_circ_0047130 | 92 | 1 | 495 | 1 | 495 | 0 | 0 |
| hsa_circ_0047128 | 91 | 1 | 459 | 1 | 459 | 0 | 2.00E-178 |
| hsa_circ_0047127 | 95 | 1 | 101 | 387 | 487 | 0 | 6.00E-37 |
| hsa_circ_0047127 | 93 | 1 | 215 | 273 | 487 | 0 | 1.00E-83 |
| hsa_circ_0047115 | 95 | 1 | 2244 | 1 | 2244 | 0 | 0 |
| hsa_circ_0047115 | 95 | 1 | 834 | 1194 | 2027 | 0 | 0 |
| hsa_circ_0047115 | 95 | 1 | 1426 | 602 | 2027 | 0 | 0 |
| hsa_circ_0047115 | 95 | 1 | 1571 | 457 | 2027 | 0 | 0 |
| hsa_circ_0047115 | 94 | 1 | 757 | 1194 | 1950 | 0 | 0 |
| hsa_circ_0047115 | 95 | 1 | 1494 | 457 | 1950 | 0 | 0 |
| hsa_circ_0047115 | 95 | 1 | 1301 | 457 | 1757 | 0 | 0 |
| hsa_circ_0047115 | 95 | 1 | 1690 | 68 | 1757 | 0 | 0 |
| hsa_circ_0047110 | 95 | 1 | 1862 | 1 | 1862 | 0 | 0 |
| hsa_circ_0047125 | 95 | 1 | 381 | 1 | 381 | 0 | 2.00E-168 |
| hsa_circ_0047118 | 95 | 1 | 973 | 146 | 1118 | 0 | 0 |
| hsa_circ_0047118 | 95 | 1 | 1118 | 1 | 1118 | 0 | 0 |
| hsa_circ_0047115 | 95 | 1 | 1507 | 68 | 1574 | 0 | 0 |
| hsa_circ_0047118 | 95 | 1 | 725 | 146 | 870 | 0 | 0 |
| hsa_circ_0047118 | 95 | 1 | 870 | 1 | 870 | 0 | 0 |
| hsa_circ_0047115 | 95 | 1 | 1054 | 273 | 1326 | 0 | 0 |
| hsa_circ_0047103 | 95 | 1 | 1733 | 1 | 1733 | 0 | 0 |
| hsa_circ_0047123 | 93 | 1 | 350 | 1 | 350 | 0 | 1.00E-144 |
| hsa_circ_0047121 | 94 | 1 | 458 | 1 | 458 | 0 | 0 |
| hsa_circ_0047119 | 95 | 1 | 588 | 5 | 592 | 0 | 0 |
| hsa_circ_0047119 | 95 | 1 | 592 | 1 | 592 | 0 | 0 |
| hsa_circ_0047118 | 95 | 1 | 737 | 1 | 737 | 0 | 0 |
| hsa_circ_0009149 | 95 | 1 | 921 | 1 | 921 | 0 | 0 |
| hsa_circ_0047115 | 95 | 1 | 1126 | 68 | 1193 | 0 | 0 |
| hsa_circ_0047115 | 95 | 1 | 1193 | 1 | 1193 | 0 | 0 |
| hsa_circ_0047110 | 95 | 1 | 1298 | 1 | 1298 | 0 | 0 |
| hsa_circ_0047107 | 95 | 1 | 1428 | 1 | 1428 | 0 | 0 |
| hsa_circ_0047103 | 95 | 1 | 1600 | 1 | 1600 | 0 | 0 |

novel_circ_014684 novel_circ_014685 novel_circ_014686 novel_circ_014687 novel_circ_014688 novel_circ_014689 novel_circ_014690 novel_circ_014691 novel_circ_014692 novel_circ_014693 novel_circ_014694 novel_circ_014695 novel_circ_014696 novel_circ_014697 novel_circ_014698 novel_circ_014699 novel_circ_014700 novel_circ_014701 novel_circ_014702 novel_circ_014703 novel_circ_014704 novel_circ_014705 novel_circ_014706 novel_circ_014707 novel_circ_014708 novel_circ_014709 novel_circ_014710 novel_circ_014711 novel_circ_014712 novel_circ_014713 novel_circ_014714 novel_circ_014715 novel_circ_014716 novel_circ_014717 novel_circ_014718 novel_circ_014719 novel_circ_014720 novel_circ_014721 novel_circ_014722 novel_circ_014723 novel_circ_014724 novel_circ_014725 novel_circ_014726 novel_circ_014727 novel_circ_014728 novel_circ_014729 novel_circ_014730 novel_circ_014731 novel_circ_014732 novel_circ_014733 novel_circ_014734 novel_circ_014735 novel_circ_014736 novel_circ_014737

| hsa_circ_0047118 | 96 | 1 | 585 | 1 | 585 | 0 | 0 |
| --- | --- | --- | --- | --- | --- | --- | --- |
| hsa_circ_0009149 | 95 | 1 | 769 | 1 | 769 | 0 | 0 |
| hsa_circ_0047115 | 95 | 1 | 1041 | 1 | 1041 | 0 | 0 |
| hsa_circ_0047110 | 95 | 1 | 1146 | 1 | 1146 | 0 | 0 |
| hsa_circ_0047107 | 95 | 1 | 1276 | 1 | 1276 | 0 | 0 |
| hsa_circ_0047103 | 95 | 1 | 1448 | 1 | 1448 | 0 | 0 |
| hsa_circ_0047118 | 97 | 1 | 387 | 1 | 387 | 0 | 0 |
| hsa_circ_0009149 | 96 | 1 | 571 | 1 | 571 | 0 | 0 |
| hsa_circ_0047115 | 96 | 1 | 776 | 68 | 843 | 0 | 0 |
| hsa_circ_0047115 | 95 | 1 | 843 | 1 | 843 | 0 | 0 |
| hsa_circ_0047110 | 95 | 1 | 948 | 1 | 948 | 0 | 0 |
| hsa_circ_0047107 | 95 | 1 | 1078 | 1 | 1078 | 0 | 0 |
| hsa_circ_0047103 | 95 | 1 | 1250 | 1 | 1250 | 0 | 0 |
| hsa_circ_0047107 | 95 | 1 | 970 | 1 | 970 | 0 | 0 |
| hsa_circ_0047115 | 95 | 1 | 388 | 68 | 455 | 0 | 3.00E-172 |
| hsa_circ_0047115 | 95 | 1 | 455 | 1 | 455 | 0 | 0 |
| hsa_circ_0047110 | 95 | 1 | 560 | 1 | 560 | 0 | 0 |
| hsa_circ_0047107 | 95 | 1 | 690 | 1 | 690 | 0 | 0 |
| hsa_circ_0047103 | 95 | 1 | 862 | 1 | 862 | 0 | 0 |
| hsa_circ_0047115 | 95 | 1 | 272 | 1 | 272 | 0 | 5.00E-118 |
| hsa_circ_0047110 | 95 | 1 | 377 | 1 | 377 | 0 | 7.00E-168 |
| hsa_circ_0047107 | 95 | 1 | 507 | 1 | 507 | 0 | 0 |
| hsa_circ_0047103 | 95 | 1 | 679 | 1 | 679 | 0 | 0 |
| hsa_circ_0047103 | 95 | 1 | 474 | 1 | 474 | 0 | 0 |
| hsa_circ_0047103 | 95 | 1 | 407 | 1 | 407 | 0 | 0 |
| hsa_circ_0047103 | 95 | 1 | 302 | 1 | 302 | 0 | 3.00E-131 |
| hsa_circ_0047089 | 91 | 1 | 175 | 223 | 49 | 0 | 9.00E-62 |
| hsa_circ_0047088 | 92 | 1 | 389 | 389 | 1 | 0 | 7.00E-153 |
| hsa_circ_0047080 | 87 | 1 | 2534 | 2514 | 1 | 0.02 | 0 |
| hsa_circ_0047078 | 87 | 1 | 2666 | 2646 | 1 | 0.02 | 0 |
| hsa_circ_0047080 | 90 | 1 | 291 | 291 | 1 | 0 | 2.00E-103 |
| hsa_circ_0047078 | 92 | 1 | 423 | 423 | 1 | 0 | 2.00E-168 |
| hsa_circ_0047076 | 93 | 1 | 513 | 513 | 1 | 0 | 0 |
| hsa_circ_0047074 | 92 | 1 | 657 | 657 | 1 | 0 | 0 |
| hsa_circ_0047074 | 92 | 1 | 657 | 657 | 1 | 0 | 0 |
| hsa_circ_0047074 | 92 | 1 | 657 | 657 | 1 | 0 | 0 |
| hsa_circ_0047070 | 91 | 514 | 2012 | 1522 | 17 | 0.02 | 0 |
| hsa_circ_0047070 | 91 | 514 | 2012 | 1522 | 17 | 0.02 | 0 |
| hsa_circ_0047070 | 91 | 514 | 2012 | 1522 | 17 | 0.02 | 0 |
| hsa_circ_0047080 | 93 | 1 | 176 | 176 | 1 | 0 | 3.00E-68 |
| hsa_circ_0047078 | 94 | 1 | 308 | 308 | 1 | 0 | 6.00E-133 |
| hsa_circ_0047076 | 95 | 1 | 398 | 398 | 1 | 0 | 7.00E-178 |
| hsa_circ_0047074 | 94 | 1 | 542 | 542 | 1 | 0 | 0 |
| hsa_circ_0047070 | 91 | 399 | 1897 | 1522 | 17 | 0.02 | 0 |
| hsa_circ_0047074 | 94 | 1 | 481 | 481 | 1 | 0 | 0 |
| hsa_circ_0047076 | 96 | 1 | 222 | 222 | 1 | 0 | 3.00E-100 |
| hsa_circ_0047074 | 94 | 1 | 366 | 366 | 1 | 0 | 3.00E-158 |
| hsa_circ_0047075 | 87 | 1 | 772 | 781 | 1 | 0.07 | 0 |
| hsa_circ_0047068 | 93 | 1 | 1034 | 3787 | 4817 | 0 | 0 |
| hsa_circ_0047068 | 94 | 3 | 361 | 2084 | 2442 | 0 | 7.00E-153 |
| hsa_circ_0047064 | 93 | 1 | 2559 | 952 | 3510 | 0 | 0 |
| hsa_circ_0047068 | 92 | 1 | 590 | 1608 | 2197 | 0 | 0 |
| hsa_circ_0047069 | 93 | 1 | 508 | 790 | 1297 | 0 | 0 |
| hsa_circ_0047069 | 93 | 1 | 841 | 457 | 1297 | 0 | 0 |

novel_circ_014738 novel_circ_014739 novel_circ_014740 novel_circ_014741 novel_circ_014742 novel_circ_014743 novel_circ_014744 novel_circ_014745 novel_circ_014746 novel_circ_014747 novel_circ_014748 novel_circ_014749 novel_circ_014750 novel_circ_014751 novel_circ_014752 novel_circ_014753 novel_circ_014754 novel_circ_014755 novel_circ_014756 novel_circ_014757 novel_circ_014758 novel_circ_014759 novel_circ_014760 novel_circ_014761 novel_circ_014762 novel_circ_014763 novel_circ_014764 novel_circ_014765 novel_circ_014766 novel_circ_014767 novel_circ_014768 novel_circ_014769 novel_circ_014770 novel_circ_014771 novel_circ_014772 novel_circ_014773 novel_circ_014774 novel_circ_014775 novel_circ_014776 novel_circ_014777 novel_circ_014778 novel_circ_014779 novel_circ_014780 novel_circ_014781 novel_circ_014782 novel_circ_014784 novel_circ_014785 novel_circ_014786 novel_circ_014797 novel_circ_014806 novel_circ_014807 novel_circ_014813 novel_circ_014814 novel_circ_014815

| hsa_circ_0047069 | 93 | 1 | 1297 | 1 | 1297 | 0 | 0 |
| --- | --- | --- | --- | --- | --- | --- | --- |
| hsa_circ_0047064 | 93 | 1 | 1858 | 952 | 2809 | 0 | 0 |
| hsa_circ_0047069 | 94 | 1 | 514 | 457 | 970 | 0 | 0 |
| hsa_circ_0047069 | 94 | 1 | 970 | 1 | 970 | 0 | 0 |
| hsa_circ_0047067 | 93 | 1 | 1531 | 1 | 1531 | 0 | 0 |
| hsa_circ_0047069 | 92 | 1 | 333 | 457 | 789 | 0 | 2.00E-133 |
| hsa_circ_0047069 | 94 | 1 | 789 | 1 | 789 | 0 | 0 |
| hsa_circ_0047068 | 93 | 1 | 975 | 259 | 1233 | 0 | 0 |
| hsa_circ_0047068 | 93 | 1 | 1113 | 121 | 1233 | 0 | 0 |
| hsa_circ_0047067 | 93 | 1 | 1350 | 1 | 1350 | 0 | 0 |
| hsa_circ_0047064 | 93 | 3513 | 4866 | 948 | 2301 | 0 | 0 |
| hsa_circ_0047064 | 93 | 3 | 2185 | 119 | 2301 | 0 | 0 |
| hsa_circ_0047064 | 93 | 3 | 1852 | 119 | 1968 | 0 | 0 |
| hsa_circ_0047065 | 92 | 3513 | 4077 | 672 | 1236 | 0 | 0 |
| hsa_circ_0047068 | 93 | 1 | 258 | 1 | 258 | 0 | 6.00E-102 |
| hsa_circ_0047067 | 92 | 1 | 375 | 1 | 375 | 0 | 9.00E-147 |
| hsa_circ_0047064 | 93 | 3 | 1210 | 119 | 1326 | 0 | 0 |
| hsa_circ_0047064 | 93 | 3 | 952 | 119 | 1068 | 0 | 0 |
| hsa_circ_0047066 | 94 | 1 | 477 | 1 | 477 | 0 | 0 |
| hsa_circ_0047064 | 94 | 3 | 835 | 119 | 951 | 0 | 0 |
| hsa_circ_0047064 | 93 | 1 | 944 | 1 | 951 | 0.01 | 0 |
| hsa_circ_0047064 | 94 | 3 | 712 | 119 | 828 | 0 | 0 |
| hsa_circ_0047064 | 93 | 3 | 535 | 119 | 651 | 0 | 0 |
| hsa_circ_0047064 | 92 | 1 | 644 | 1 | 651 | 0.01 | 0 |
| hsa_circ_0047064 | 94 | 3 | 358 | 119 | 474 | 0 | 7.00E-153 |
| hsa_circ_0047064 | 92 | 1 | 467 | 1 | 474 | 0.01 | 0 |
| hsa_circ_0047059 | 98 | 240 | 645 | 3098 | 2693 | 0 | 0 |
| hsa_circ_0047059 | 97 | 240 | 784 | 3098 | 2554 | 0 | 0 |
| hsa_circ_0047059 | 97 | 240 | 876 | 3098 | 2462 | 0 | 0 |
| hsa_circ_0047059 | 96 | 240 | 1036 | 3098 | 2302 | 0 | 0 |
| hsa_circ_0047059 | 98 | 1 | 406 | 3098 | 2693 | 0 | 0 |
| hsa_circ_0047059 | 97 | 1 | 545 | 3098 | 2554 | 0 | 0 |
| hsa_circ_0047059 | 96 | 1 | 621 | 2922 | 2302 | 0 | 0 |
| hsa_circ_0047059 | 95 | 1 | 536 | 2837 | 2302 | 0 | 0 |
| hsa_circ_0047059 | 95 | 1 | 597 | 2837 | 2241 | 0 | 0 |
| hsa_circ_0047059 | 95 | 1 | 686 | 2837 | 2152 | 0 | 0 |
| hsa_circ_0047059 | 96 | 1 | 871 | 2837 | 1967 | 0 | 0 |
| hsa_circ_0047059 | 92 | 1 | 532 | 692 | 161 | 0 | 0 |
| hsa_circ_0047059 | 93 | 1 | 692 | 692 | 1 | 0 | 0 |
| hsa_circ_0047054 | 95 | 1 | 869 | 2515 | 1647 | 0 | 0 |
| hsa_circ_0047054 | 95 | 1 | 638 | 2284 | 1647 | 0 | 0 |
| hsa_circ_0047055 | 95 | 1 | 549 | 549 | 1 | 0 | 0 |
| hsa_circ_0046659 | 95 | 1 | 388 | 1 | 388 | 0 | 1.00E-170 |
| hsa_circ_0005653 | 94 | 1 | 570 | 132 | 701 | 0 | 0 |
| hsa_circ_0005653 | 94 | 1 | 489 | 213 | 701 | 0 | 0 |
| hsa_circ_0046681 | 90 | 1 | 683 | 683 | 1 | 0 | 0 |
| hsa_circ_0046696 | 93 | 1 | 1059 | 1299 | 232 | 0.01 | 0 |
| hsa_circ_0046696 | 90 | 1 | 370 | 1299 | 921 | 0.03 | 3.00E-136 |
| hsa_circ_0057249 | 93 | 1447 | 1725 | 1 | 279 | 0 | 1.00E-112 |
| hsa_circ_0057239 | 91 | 306 | 1066 | 1 | 761 | 0 | 0 |
| hsa_circ_0057236 | 96 | 650 | 784 | 50 | 184 | 0 | 2.00E-56 |
| hsa_circ_0057230 | 90 | 190 | 595 | 417 | 1 | 0.03 | 2.00E-151 |
| hsa_circ_0057230 | 90 | 190 | 595 | 417 | 1 | 0.03 | 2.00E-151 |
| hsa_circ_0057230 | 94 | 1 | 212 | 212 | 1 | 0 | 1.00E-87 |

novel_circ_014816 novel_circ_014827 novel_circ_014828 novel_circ_014829 novel_circ_014830 novel_circ_014832 novel_circ_014833 novel_circ_014834 novel_circ_014835 novel_circ_014836 novel_circ_014837 novel_circ_014838 novel_circ_014839 novel_circ_014840 novel_circ_014841 novel_circ_014842 novel_circ_014843 novel_circ_014844 novel_circ_014845 novel_circ_014846 novel_circ_014847 novel_circ_014848 novel_circ_014849 novel_circ_014850 novel_circ_014851 novel_circ_014852 novel_circ_014853 novel_circ_014854 novel_circ_014855 novel_circ_014856 novel_circ_014857 novel_circ_014858 novel_circ_014859 novel_circ_014860 novel_circ_014861 novel_circ_014862 novel_circ_014863 novel_circ_014864 novel_circ_014865 novel_circ_014866 novel_circ_014867 novel_circ_014868 novel_circ_014869 novel_circ_014870 novel_circ_014871 novel_circ_014872 novel_circ_014873 novel_circ_014874 novel_circ_014875 novel_circ_014878 novel_circ_014879 novel_circ_014880 novel_circ_014881 novel_circ_014884

| hsa_circ_0057230 | 94 | 1 | 212 | 212 | 1 | 0 | 1.00E-87 |
| --- | --- | --- | --- | --- | --- | --- | --- |
| hsa_circ_0057229 | 91 | 540 | 902 | 1302 | 940 | 0 | 1.00E-139 |
| hsa_circ_0057229 | 90 | 540 | 1019 | 1302 | 823 | 0 | 1.00E-179 |
| hsa_circ_0057229 | 90 | 193 | 672 | 1302 | 823 | 0 | 7.00E-180 |
| hsa_circ_0057229 | 89 | 4 | 485 | 936 | 455 | 0 | 9.00E-173 |
| hsa_circ_0057206 | 94 | 3 | 618 | 120 | 735 | 0 | 0 |
| hsa_circ_0057206 | 94 | 3 | 735 | 3 | 735 | 0 | 0 |
| hsa_circ_0057206 | 94 | 3 | 458 | 120 | 575 | 0 | 0 |
| hsa_circ_0057203 | 93 | 84 | 368 | 466 | 176 | 0.02 | 4.00E-115 |
| hsa_circ_0057203 | 95 | 84 | 543 | 466 | 1 | 0.01 | 0 |
| hsa_circ_0057197 | 95 | 1 | 571 | 1756 | 2326 | 0 | 0 |
| hsa_circ_0057197 | 94 | 1 | 1483 | 868 | 2326 | 0.02 | 0 |
| hsa_circ_0057197 | 94 | 1 | 290 | 2037 | 2326 | 0 | 3.00E-121 |
| hsa_circ_0057197 | 94 | 1 | 1051 | 1276 | 2326 | 0 | 0 |
| hsa_circ_0057197 | 94 | 1 | 1483 | 868 | 2326 | 0.02 | 0 |
| hsa_circ_0057197 | 95 | 1 | 906 | 1276 | 2181 | 0 | 0 |
| hsa_circ_0057197 | 94 | 1 | 1338 | 868 | 2181 | 0.02 | 0 |
| hsa_circ_0057197 | 93 | 1 | 570 | 868 | 1413 | 0.04 | 0 |
| hsa_circ_0057197 | 94 | 1 | 842 | 596 | 1413 | 0.03 | 0 |
| hsa_circ_0057193 | 94 | 1 | 889 | 128 | 1010 | 0.01 | 0 |
| hsa_circ_0057192 | 94 | 1 | 1139 | 393 | 1525 | 0.01 | 0 |
| hsa_circ_0057192 | 94 | 1 | 938 | 393 | 1324 | 0.01 | 0 |
| hsa_circ_0057192 | 95 | 1 | 689 | 393 | 1075 | 0.01 | 0 |
| hsa_circ_0057192 | 94 | 1 | 559 | 393 | 945 | 0.01 | 0 |
| hsa_circ_0057192 | 96 | 1 | 373 | 393 | 765 | 0 | 1.00E-170 |
| hsa_circ_0057192 | 96 | 1 | 250 | 393 | 642 | 0 | 2.00E-112 |
| hsa_circ_0057187 | 85 | 1 | 2131 | 91 | 2207 | 0.05 | 0 |
| hsa_circ_0057182 | 95 | 1 | 412 | 1 | 412 | 0 | 0 |
| hsa_circ_0057178 | 95 | 1 | 464 | 364 | 827 | 0 | 0 |
| hsa_circ_0057178 | 95 | 1 | 592 | 236 | 827 | 0 | 0 |
| hsa_circ_0057176 | 95 | 1 | 908 | 81 | 988 | 0 | 0 |
| hsa_circ_0057172 | 96 | 3 | 468 | 78 | 543 | 0 | 0 |
| hsa_circ_0057170 | 96 | 1 | 555 | 191 | 745 | 0 | 0 |
| hsa_circ_0057170 | 95 | 1 | 646 | 100 | 745 | 0 | 0 |
| hsa_circ_0057173 | 96 | 3 | 359 | 3 | 359 | 0 | 8.00E-162 |
| hsa_circ_0057170 | 96 | 1 | 555 | 191 | 745 | 0 | 0 |
| hsa_circ_0057170 | 95 | 1 | 646 | 100 | 745 | 0 | 0 |
| hsa_circ_0057170 | 95 | 1 | 736 | 10 | 745 | 0 | 0 |
| hsa_circ_0057171 | 96 | 1 | 348 | 1 | 348 | 0 | 8.00E-162 |
| hsa_circ_0057170 | 96 | 1 | 439 | 100 | 538 | 0 | 0 |
| hsa_circ_0057170 | 95 | 1 | 529 | 10 | 538 | 0 | 0 |
| hsa_circ_0057170 | 95 | 1 | 287 | 100 | 386 | 0 | 5.00E-128 |
| hsa_circ_0013829 | 78 | 11490 | 11703 | 440080 | 440293 | 0.08 | 3.00E-25 |
| hsa_circ_0057169 | 96 | 1 | 357 | 357 | 1 | 0 | 8.00E-162 |
| hsa_circ_0057168 | 91 | 1 | 546 | 549 | 1 | 0.01 | 0 |
| hsa_circ_0028537 | 85 | 15 | 499 | 1971 | 1496 | 0.03 | 3.00E-133 |
| hsa_circ_0028537 | 87 | 15 | 726 | 1971 | 1269 | 0.02 | 0 |
| hsa_circ_0028501 | 84 | 10 | 837 | 1327 | 500 | 0 | 0 |
| hsa_circ_0028496 | 91 | 1 | 384 | 294 | 677 | 0 | 4.00E-145 |
| hsa_circ_0085528 | 92 | 1 | 429 | 1 | 430 | 0 | 2.00E-173 |
| hsa_circ_0042007 | 88 | 4 | 756 | 756 | 1 | 0 | 0 |
| hsa_circ_0042007 | 88 | 1 | 592 | 595 | 1 | 0.01 | 0 |
| hsa_circ_0041973 | 94 | 1 | 393 | 445 | 837 | 0 | 9.00E-167 |
| hsa_circ_0041944 | 94 | 1 | 579 | 579 | 1 | 0 | 0 |

novel_circ_014885 novel_circ_014887 novel_circ_014889 novel_circ_014892 novel_circ_014893 novel_circ_014894 novel_circ_014895 novel_circ_014896 novel_circ_014897 novel_circ_014898 novel_circ_014899 novel_circ_014900 novel_circ_014901 novel_circ_014902 novel_circ_014903 novel_circ_014904 novel_circ_014905 novel_circ_014906 novel_circ_014907 novel_circ_014908 novel_circ_014909 novel_circ_014910 novel_circ_014911 novel_circ_014912 novel_circ_014913 novel_circ_014914 novel_circ_014915 novel_circ_014917 novel_circ_014923 novel_circ_014927 novel_circ_014928 novel_circ_014929 novel_circ_014930 novel_circ_014931 novel_circ_014932 novel_circ_014933 novel_circ_014935 novel_circ_014936 novel_circ_014937 novel_circ_014938 novel_circ_014939 novel_circ_014940 novel_circ_014941 novel_circ_014942 novel_circ_014943 novel_circ_014944 novel_circ_014945 novel_circ_014947 novel_circ_014948 novel_circ_014949 novel_circ_014950 novel_circ_014951 novel_circ_014952 novel_circ_014954

| hsa_circ_0041944 | 94 | 1 | 432 | 432 | 1 | 0 | 0 |
| --- | --- | --- | --- | --- | --- | --- | --- |
| hsa_circ_0041901 | 91 | 1 | 548 | 1 | 548 | 0 | 0 |
| hsa_circ_0041796 | 85 | 3 | 298 | 3 | 298 | 0.01 | 2.00E-77 |
| hsa_circ_0041607 | 93 | 1 | 634 | 127 | 760 | 0 | 0 |
| hsa_circ_0041607 | 93 | 1 | 643 | 127 | 769 | 0 | 0 |
| hsa_circ_0041650 | 95 | 1 | 260 | 262 | 3 | 0 | 1.00E-114 |
| hsa_circ_0041673 | 94 | 130 | 415 | 48 | 333 | 0 | 3.00E-122 |
| hsa_circ_0041673 | 93 | 130 | 696 | 48 | 614 | 0 | 0 |
| hsa_circ_0041673 | 94 | 130 | 832 | 48 | 750 | 0 | 0 |
| hsa_circ_0041675 | 94 | 212 | 1011 | 1 | 800 | 0 | 0 |
| hsa_circ_0041675 | 94 | 212 | 1143 | 1 | 932 | 0 | 0 |
| hsa_circ_0041675 | 94 | 1 | 932 | 1 | 932 | 0 | 0 |
| hsa_circ_0041677 | 97 | 1 | 315 | 1 | 315 | 0 | 2.00E-148 |
| hsa_circ_0041677 | 96 | 1 | 447 | 1 | 447 | 0 | 0 |
| hsa_circ_0041677 | 95 | 1 | 1137 | 1 | 1137 | 0 | 0 |
| hsa_circ_0041682 | 95 | 1 | 311 | 1 | 311 | 0 | 3.00E-136 |
| hsa_circ_0041684 | 94 | 1 | 690 | 1 | 690 | 0 | 0 |
| hsa_circ_0041684 | 94 | 1 | 930 | 1 | 930 | 0 | 0 |
| hsa_circ_0041687 | 91 | 1 | 462 | 142 | 603 | 0 | 5.00E-175 |
| hsa_circ_0041691 | 93 | 1 | 559 | 559 | 1 | 0 | 0 |
| hsa_circ_0041691 | 92 | 1 | 432 | 559 | 128 | 0 | 1.00E-171 |
| hsa_circ_0041700 | 92 | 3 | 895 | 893 | 1 | 0 | 0 |
| hsa_circ_0041700 | 92 | 3 | 895 | 893 | 1 | 0 | 0 |
| hsa_circ_0041702 | 91 | 1 | 576 | 577 | 2 | 0 | 0 |
| hsa_circ_0041702 | 92 | 3 | 746 | 745 | 2 | 0 | 0 |
| hsa_circ_0041702 | 93 | 3 | 560 | 745 | 188 | 0 | 0 |
| hsa_circ_0041720 | 95 | 1 | 364 | 952 | 589 | 0 | 5.00E-164 |
| hsa_circ_0044203 | 74 | 66206 | 66825 | 2489 | 3111 | 0.08 | 9.00E-53 |
| hsa_circ_0058870 | 92 | 1 | 740 | 1015 | 276 | 0 | 0 |
| hsa_circ_0064280 | 93 | 5 | 544 | 687 | 148 | 0 | 0 |
| hsa_circ_0064280 | 92 | 5 | 691 | 687 | 1 | 0 | 0 |
| hsa_circ_0064280 | 92 | 1 | 405 | 405 | 1 | 0 | 1.00E-161 |
| hsa_circ_0064180 | 88 | 1 | 267 | 1 | 267 | 0 | 2.00E-83 |
| hsa_circ_0064175 | 93 | 1 | 267 | 1 | 267 | 0 | 1.00E-108 |
| hsa_circ_0064172 | 94 | 1 | 498 | 1 | 498 | 0 | 0 |
| hsa_circ_0064164 | 93 | 1 | 346 | 346 | 1 | 0 | 1.00E-140 |
| hsa_circ_0064136 | 94 | 1 | 694 | 1216 | 1909 | 0 | 0 |
| hsa_circ_0064136 | 93 | 1 | 952 | 958 | 1909 | 0 | 0 |
| hsa_circ_0064136 | 94 | 1 | 1663 | 244 | 1909 | 0 | 0 |
| hsa_circ_0064136 | 94 | 1 | 1906 | 1 | 1909 | 0 | 0 |
| hsa_circ_0064134 | 94 | 1467 | 3378 | 233 | 2147 | 0 | 0 |
| hsa_circ_0064136 | 94 | 1 | 1776 | 1 | 1779 | 0 | 0 |
| hsa_circ_0064136 | 94 | 1 | 969 | 244 | 1215 | 0 | 0 |
| hsa_circ_0064136 | 94 | 1 | 1212 | 1 | 1215 | 0 | 0 |
| hsa_circ_0064134 | 95 | 1467 | 2426 | 233 | 1195 | 0 | 0 |
| hsa_circ_0064128 | 99 | 1194 | 1433 | 208 | 447 | 0 | 2.00E-119 |
| hsa_circ_0064117 | 99 | 283 | 509 | 1063 | 1289 | 0 | 5.00E-114 |
| hsa_circ_0064108 | 92 | 1 | 315 | 2072 | 1758 | 0 | 4.00E-125 |
| hsa_circ_0064108 | 93 | 1 | 950 | 2072 | 1123 | 0 | 0 |
| hsa_circ_0064108 | 93 | 1 | 522 | 1757 | 1236 | 0 | 0 |
| hsa_circ_0064108 | 96 | 333 | 473 | 1021 | 881 | 0 | 3.00E-58 |
| hsa_circ_0064101 | 84 | 1 | 753 | 756 | 1 | 0.01 | 0 |
| hsa_circ_0064100 | 85 | 1 | 1026 | 1035 | 1 | 0.01 | 0 |
| hsa_circ_0064101 | 82 | 1 | 620 | 623 | 1 | 0.01 | 7.00E-150 |

novel_circ_014955 novel_circ_014956 novel_circ_014957 novel_circ_014958 novel_circ_014959 novel_circ_014960 novel_circ_014963 novel_circ_014965 novel_circ_014966 novel_circ_014967 novel_circ_014968 novel_circ_014971 novel_circ_014974 novel_circ_014975 novel_circ_014976 novel_circ_014977 novel_circ_014978 novel_circ_014979 novel_circ_014980 novel_circ_014981 novel_circ_014982 novel_circ_014983 novel_circ_014984 novel_circ_014985 novel_circ_014986 novel_circ_014987 novel_circ_014988 novel_circ_014989 novel_circ_014990 novel_circ_014991 novel_circ_014992 novel_circ_014993 novel_circ_014994 novel_circ_014995 novel_circ_014996 novel_circ_014997 novel_circ_014998 novel_circ_014999 novel_circ_015000 novel_circ_015001 novel_circ_015002 novel_circ_015003 novel_circ_015004 novel_circ_015005 novel_circ_015006 novel_circ_015007 novel_circ_015008 novel_circ_015009 novel_circ_015010 novel_circ_015011 novel_circ_015012 novel_circ_015013 novel_circ_015017 novel_circ_015018

| hsa_circ_0064100 | 84 | 1 | 758 | 902 | 142 | 0.01 | 0 |
| --- | --- | --- | --- | --- | --- | --- | --- |
| hsa_circ_0064098 | 82 | 1 | 1060 | 5383 | 4334 | 0.03 | 0 |
| hsa_circ_0064098 | 85 | 1 | 725 | 5045 | 4334 | 0.04 | 0 |
| hsa_circ_0064100 | 92 | 1 | 323 | 464 | 142 | 0 | 1.00E-124 |
| hsa_circ_0064098 | 84 | 1 | 625 | 4945 | 4334 | 0.04 | 3.00E-168 |
| hsa_circ_0064098 | 84 | 1 | 625 | 4945 | 4334 | 0.04 | 1.00E-166 |
| hsa_circ_0085943 | 89 | 1 | 305 | 373 | 69 | 0 | 1.00E-104 |
| hsa_circ_0085898 | 83 | 2518 | 3022 | 6526 | 6022 | 0.02 | 4.00E-126 |
| hsa_circ_0001831 | 93 | 133 | 255 | 121 | 1 | 0.02 | 6.00E-43 |
| hsa_circ_0001831 | 93 | 133 | 255 | 121 | 1 | 0.02 | 1.00E-42 |
| hsa_circ_0001829 | 84 | 11 | 634 | 631 | 1 | 0.02 | 2.00E-166 |
| hsa_circ_0005259 | 87 | 9449 | 9767 | 30987 | 30670 | 0.01 | 6.00E-97 |
| hsa_circ_0005259 | 87 | 284 | 602 | 30987 | 30670 | 0.01 | 5.00E-98 |
| hsa_circ_0005259 | 87 | 3537 | 3855 | 30987 | 30670 | 0.01 | 2.00E-97 |
| hsa_circ_0005259 | 87 | 205 | 523 | 30987 | 30670 | 0.01 | 4.00E-98 |
| hsa_circ_0085771 | 94 | 522 | 917 | 396 | 1 | 0 | 5.00E-173 |
| hsa_circ_0008778 | 94 | 522 | 1005 | 484 | 1 | 0 | 0 |
| hsa_circ_0005259 | 87 | 108 | 426 | 30987 | 30670 | 0.01 | 4.00E-98 |
| hsa_circ_0085771 | 94 | 425 | 820 | 396 | 1 | 0 | 4.00E-173 |
| hsa_circ_0008778 | 94 | 425 | 908 | 484 | 1 | 0 | 0 |
| hsa_circ_0005259 | 94 | 316 | 945 | 631 | 1 | 0 | 0 |
| hsa_circ_0002666 | 93 | 1 | 227 | 227 | 1 | 0 | 4.00E-88 |
| hsa_circ_0085771 | 94 | 1 | 394 | 394 | 1 | 0 | 3.00E-172 |
| hsa_circ_0008778 | 94 | 1 | 482 | 482 | 1 | 0 | 0 |
| hsa_circ_0005259 | 94 | 1 | 625 | 625 | 1 | 0 | 0 |
| hsa_circ_0007786 | 94 | 1 | 680 | 680 | 1 | 0 | 0 |
| hsa_circ_0085766 | 94 | 1 | 898 | 899 | 2 | 0 | 0 |
| hsa_circ_0085758 | 93 | 1 | 1209 | 1209 | 1 | 0 | 0 |
| hsa_circ_0005259 | 94 | 1 | 398 | 398 | 1 | 0 | 2.00E-174 |
| hsa_circ_0005259 | 93 | 5 | 231 | 227 | 1 | 0 | 2.00E-91 |
| hsa_circ_0085758 | 93 | 1 | 584 | 584 | 1 | 0 | 0 |
| hsa_circ_0085755 | 89 | 1 | 778 | 739 | 1 | 0.05 | 0 |
| hsa_circ_0085752 | 90 | 1 | 863 | 824 | 1 | 0.05 | 0 |
| hsa_circ_0085742 | 90 | 1 | 1476 | 1636 | 200 | 0.03 | 0 |
| hsa_circ_0085758 | 92 | 1 | 529 | 529 | 1 | 0 | 0 |
| hsa_circ_0085755 | 88 | 1 | 723 | 684 | 1 | 0.05 | 0 |
| hsa_circ_0085745 | 90 | 1 | 1421 | 1494 | 113 | 0.03 | 0 |
| hsa_circ_0085758 | 92 | 1 | 310 | 310 | 1 | 0 | 2.00E-122 |
| hsa_circ_0085755 | 86 | 1 | 504 | 465 | 1 | 0.08 | 2.00E-140 |
| hsa_circ_0085748 | 89 | 1 | 907 | 958 | 91 | 0.04 | 0 |
| hsa_circ_0085748 | 90 | 1 | 997 | 958 | 1 | 0.04 | 0 |
| hsa_circ_0085758 | 93 | 1 | 202 | 202 | 1 | 0 | 1.00E-77 |
| hsa_circ_0085755 | 84 | 1 | 396 | 357 | 1 | 0.1 | 1.00E-95 |
| hsa_circ_0085745 | 89 | 1 | 1094 | 1167 | 113 | 0.04 | 0 |
| hsa_circ_0085752 | 98 | 79 | 261 | 183 | 1 | 0 | 3.00E-85 |
| hsa_circ_0085748 | 94 | 79 | 669 | 591 | 1 | 0 | 0 |
| hsa_circ_0085745 | 92 | 79 | 874 | 908 | 113 | 0 | 0 |
| hsa_circ_0085748 | 94 | 1 | 493 | 493 | 1 | 0 | 0 |
| hsa_circ_0004238 | 92 | 1 | 698 | 698 | 1 | 0 | 0 |
| hsa_circ_0085748 | 92 | 1 | 318 | 408 | 91 | 0 | 2.00E-123 |
| hsa_circ_0085748 | 93 | 1 | 408 | 408 | 1 | 0 | 1.00E-166 |
| hsa_circ_0085745 | 91 | 1 | 725 | 725 | 1 | 0 | 0 |
| hsa_circ_0085721 | 91 | 9 | 636 | 760 | 136 | 0 | 0 |
| hsa_circ_0085710 | 92 | 9 | 2452 | 2441 | 1 | 0 | 0 |

novel_circ_015019 novel_circ_015020 novel_circ_015021 novel_circ_015027 novel_circ_015028 novel_circ_015029 novel_circ_015030 novel_circ_015031 novel_circ_015032 novel_circ_015033 novel_circ_015034 novel_circ_015035 novel_circ_015036 novel_circ_015037 novel_circ_015038 novel_circ_015039 novel_circ_015042 novel_circ_015043 novel_circ_015045 novel_circ_015046 novel_circ_015047 novel_circ_015048 novel_circ_015049 novel_circ_015050 novel_circ_015051 novel_circ_015052 novel_circ_015053 novel_circ_015054 novel_circ_015055 novel_circ_015056 novel_circ_015057 novel_circ_015058 novel_circ_015059 novel_circ_015060 novel_circ_015061 novel_circ_015062 novel_circ_015063 novel_circ_015064 novel_circ_015065 novel_circ_015066 novel_circ_015067 novel_circ_015068 novel_circ_015069 novel_circ_015070 novel_circ_015072 novel_circ_015073 novel_circ_015074 novel_circ_015075 novel_circ_015076 novel_circ_015077 novel_circ_015078 novel_circ_015079 novel_circ_015080 novel_circ_015081

| hsa_circ_0085708 | 84 | 1 | 213 | 1 | 210 | 0.01 | 4.00E-50 |
| --- | --- | --- | --- | --- | --- | --- | --- |
| hsa_circ_0085695 | 83 | 4 | 656 | 653 | 1 | 0 | 3.00E-169 |
| hsa_circ_0085681 | 93 | 1 | 245 | 245 | 1 | 0 | 3.00E-96 |
| hsa_circ_0006059 | 91 | 1 | 336 | 265 | 600 | 0 | 1.00E-126 |
| hsa_circ_0006059 | 91 | 1 | 483 | 118 | 600 | 0 | 0 |
| hsa_circ_0006059 | 91 | 1 | 600 | 1 | 600 | 0 | 0 |
| hsa_circ_0006059 | 91 | 120 | 719 | 1 | 600 | 0 | 0 |
| hsa_circ_0006059 | 91 | 1 | 336 | 265 | 600 | 0 | 8.00E-127 |
| hsa_circ_0006059 | 91 | 1 | 483 | 118 | 600 | 0 | 0 |
| hsa_circ_0006059 | 91 | 1 | 600 | 1 | 600 | 0 | 0 |
| hsa_circ_0006059 | 91 | 120 | 719 | 1 | 600 | 0 | 0 |
| hsa_circ_0006059 | 91 | 1 | 404 | 1 | 404 | 0 | 2.00E-154 |
| hsa_circ_0006059 | 91 | 120 | 523 | 1 | 404 | 0 | 2.00E-154 |
| hsa_circ_0006059 | 91 | 1 | 268 | 1 | 269 | 0 | 9.00E-99 |
| hsa_circ_0006059 | 91 | 1 | 264 | 1 | 264 | 0 | 1.00E-98 |
| hsa_circ_0006059 | 91 | 120 | 383 | 1 | 264 | 0 | 2.00E-98 |
| hsa_circ_0083285 | 87 | 1 | 388 | 1246 | 1633 | 0.01 | 1.00E-120 |
| hsa_circ_0083280 | 79 | 1 | 1720 | 120 | 1821 | 0.02 | 0 |
| hsa_circ_0002595 | 73 | 10676 | 11157 | 314709 | 314226 | 0.04 | 6.00E-38 |
| hsa_circ_0070506 | 93 | 1 | 318 | 2482 | 2165 | 0 | 4.00E-130 |
| hsa_circ_0070506 | 92 | 1 | 709 | 2482 | 1774 | 0 | 0 |
| hsa_circ_0070506 | 93 | 1 | 882 | 2482 | 1601 | 0 | 0 |
| hsa_circ_0070506 | 93 | 1 | 882 | 2482 | 1601 | 0 | 0 |
| hsa_circ_0070506 | 92 | 1 | 391 | 2164 | 1774 | 0 | 3.00E-152 |
| hsa_circ_0070506 | 92 | 1 | 564 | 2164 | 1601 | 0 | 0 |
| hsa_circ_0070506 | 92 | 1 | 564 | 2164 | 1601 | 0 | 0 |
| hsa_circ_0070506 | 95 | 1 | 378 | 1978 | 1601 | 0 | 9.00E-167 |
| hsa_circ_0070506 | 95 | 1 | 378 | 1978 | 1601 | 0 | 1.00E-165 |
| hsa_circ_0047659 | 82 | 1382 | 1761 | 408 | 23 | 0.02 | 9.00E-85 |
| hsa_circ_0070502 | 92 | 1 | 244 | 332 | 89 | 0 | 2.00E-92 |
| hsa_circ_0070500 | 92 | 1 | 813 | 949 | 137 | 0 | 0 |
| hsa_circ_0070500 | 92 | 1 | 949 | 949 | 1 | 0 | 0 |
| hsa_circ_0003640 | 92 | 1 | 271 | 85 | 355 | 0 | 2.00E-102 |
| hsa_circ_0003640 | 92 | 1 | 355 | 1 | 355 | 0 | 2.00E-137 |
| hsa_circ_0070498 | 92 | 1 | 447 | 1 | 447 | 0 | 0 |
| hsa_circ_0070497 | 93 | 1 | 508 | 1 | 508 | 0 | 0 |
| hsa_circ_0070495 | 93 | 1 | 621 | 1 | 621 | 0 | 0 |
| hsa_circ_0070493 | 93 | 1 | 673 | 1 | 673 | 0 | 0 |
| hsa_circ_0070493 | 94 | 1 | 226 | 1 | 226 | 0 | 3.00E-94 |
| hsa_circ_0070486 | 96 | 7 | 609 | 2824 | 2222 | 0 | 0 |
| hsa_circ_0070482 | 90 | 1 | 677 | 127 | 803 | 0 | 0 |
| hsa_circ_0070482 | 89 | 1 | 411 | 127 | 537 | 0 | 2.00E-143 |
| hsa_circ_0070479 | 94 | 1 | 231 | 4 | 234 | 0 | 2.00E-93 |
| hsa_circ_0070479 | 94 | 5437 | 5670 | 1 | 234 | 0 | 7.00E-94 |
| hsa_circ_0054959 | 97 | 1 | 477 | 512 | 36 | 0 | 0 |
| hsa_circ_0054959 | 97 | 1 | 512 | 512 | 1 | 0 | 0 |
| hsa_circ_0054958 | 98 | 1 | 1030 | 1030 | 1 | 0 | 0 |
| hsa_circ_0054957 | 97 | 1 | 1265 | 1759 | 495 | 0 | 0 |
| hsa_circ_0054957 | 97 | 1 | 1418 | 1759 | 342 | 0 | 0 |
| hsa_circ_0054957 | 97 | 1 | 1759 | 1759 | 1 | 0 | 0 |
| hsa_circ_0054951 | 96 | 1 | 2639 | 2645 | 1 | 0 | 0 |
| hsa_circ_0054951 | 96 | 1 | 2639 | 2645 | 1 | 0 | 0 |
| hsa_circ_0054958 | 98 | 2 | 632 | 631 | 1 | 0 | 0 |
| hsa_circ_0054958 | 98 | 1 | 518 | 518 | 1 | 0 | 0 |

novel_circ_015082 novel_circ_015083 novel_circ_015084 novel_circ_015085 novel_circ_015086 novel_circ_015087 novel_circ_015088 novel_circ_015089 novel_circ_015090 novel_circ_015091 novel_circ_015092 novel_circ_015093 novel_circ_015094 novel_circ_015095 novel_circ_015096 novel_circ_015097 novel_circ_015098 novel_circ_015099 novel_circ_015100 novel_circ_015101 novel_circ_015102 novel_circ_015103 novel_circ_015104 novel_circ_015105 novel_circ_015106 novel_circ_015107 novel_circ_015108 novel_circ_015109 novel_circ_015110 novel_circ_015111 novel_circ_015112 novel_circ_015113 novel_circ_015114 novel_circ_015115 novel_circ_015116 novel_circ_015117 novel_circ_015118 novel_circ_015119 novel_circ_015120 novel_circ_015121 novel_circ_015122 novel_circ_015123 novel_circ_015124 novel_circ_015126 novel_circ_015127 novel_circ_015128 novel_circ_015129 novel_circ_015130 novel_circ_015135 novel_circ_015136 novel_circ_015137 novel_circ_015138 novel_circ_015139 novel_circ_015140

| hsa_circ_0054957 | 97 | 1 | 753 | 1247 | 495 | 0 | 0 |
| --- | --- | --- | --- | --- | --- | --- | --- |
| hsa_circ_0054957 | 97 | 1 | 906 | 1247 | 342 | 0 | 0 |
| hsa_circ_0054957 | 96 | 1 | 1247 | 1247 | 1 | 0 | 0 |
| hsa_circ_0054951 | 96 | 1 | 1670 | 2133 | 464 | 0 | 0 |
| hsa_circ_0054951 | 95 | 1 | 1730 | 2133 | 398 | 0 | 0 |
| hsa_circ_0054951 | 95 | 1 | 1924 | 2133 | 204 | 0 | 0 |
| hsa_circ_0054949 | 96 | 1 | 2235 | 2241 | 1 | 0 | 0 |
| hsa_circ_0054958 | 98 | 1 | 386 | 386 | 1 | 0 | 0 |
| hsa_circ_0054957 | 96 | 1 | 1115 | 1115 | 1 | 0 | 0 |
| hsa_circ_0054957 | 95 | 1 | 388 | 729 | 342 | 0 | 1.00E-175 |
| hsa_circ_0054957 | 95 | 1 | 729 | 729 | 1 | 0 | 0 |
| hsa_circ_0054951 | 95 | 1 | 1152 | 1615 | 464 | 0 | 0 |
| hsa_circ_0054951 | 95 | 1 | 1609 | 1615 | 1 | 0 | 0 |
| hsa_circ_0054951 | 94 | 1 | 1018 | 1227 | 204 | 0.01 | 0 |
| hsa_circ_0054951 | 94 | 1 | 1221 | 1227 | 1 | 0 | 0 |
| hsa_circ_0054951 | 94 | 1 | 423 | 886 | 464 | 0 | 0 |
| hsa_circ_0054951 | 94 | 1 | 677 | 886 | 204 | 0.01 | 0 |
| hsa_circ_0054951 | 94 | 1 | 880 | 886 | 1 | 0.01 | 0 |
| hsa_circ_0054949 | 95 | 1 | 988 | 994 | 1 | 0.01 | 0 |
| hsa_circ_0054951 | 97 | 1 | 194 | 397 | 204 | 0 | 8.00E-90 |
| hsa_circ_0054947 | 94 | 1 | 441 | 1 | 441 | 0 | 0 |
| hsa_circ_0054943 | 94 | 186 | 1176 | 630 | 1620 | 0 | 0 |
| hsa_circ_0004227 | 94 | 1 | 235 | 1 | 235 | 0 | 2.00E-97 |
| hsa_circ_0054924 | 89 | 4419 | 6358 | 2272 | 316 | 0.02 | 0 |
| hsa_circ_0006759 | 94 | 7 | 309 | 303 | 1 | 0 | 8.00E-127 |
| hsa_circ_0006759 | 94 | 7 | 309 | 303 | 1 | 0 | 1.00E-125 |
| hsa_circ_0002370 | 93 | 7 | 750 | 744 | 1 | 0 | 0 |
| hsa_circ_0054924 | 91 | 7 | 1673 | 1667 | 1 | 0 | 0 |
| hsa_circ_0007387 | 90 | 1 | 608 | 610 | 3 | 0 | 0 |
| hsa_circ_0054927 | 89 | 1 | 799 | 799 | 1 | 0 | 0 |
| hsa_circ_0054924 | 90 | 1 | 923 | 923 | 1 | 0 | 0 |
| hsa_circ_0054920 | 90 | 1 | 987 | 1333 | 347 | 0 | 0 |
| hsa_circ_0054920 | 91 | 1 | 1085 | 1333 | 249 | 0 | 0 |
| hsa_circ_0054920 | 91 | 1 | 1085 | 1333 | 249 | 0 | 0 |
| hsa_circ_0054923 | 92 | 4 | 377 | 374 | 1 | 0 | 3.00E-146 |
| hsa_circ_0054922 | 92 | 4 | 475 | 640 | 169 | 0 | 0 |
| hsa_circ_0054922 | 92 | 4 | 475 | 640 | 169 | 0 | 0 |
| hsa_circ_0054918 | 95 | 1 | 495 | 1 | 495 | 0 | 0 |
| hsa_circ_0005552 | 95 | 1 | 751 | 1 | 751 | 0 | 0 |
| hsa_circ_0054913 | 94 | 1 | 1423 | 429 | 1851 | 0 | 0 |
| hsa_circ_0054914 | 93 | 1 | 1275 | 1 | 1275 | 0 | 0 |
| hsa_circ_0054914 | 94 | 1 | 1131 | 1 | 1131 | 0 | 0 |
| hsa_circ_0054914 | 96 | 1 | 228 | 1 | 228 | 0 | 3.00E-100 |
| hsa_circ_0054912 | 93 | 1 | 653 | 1117 | 1772 | 0.01 | 0 |
| hsa_circ_0054912 | 94 | 1 | 975 | 795 | 1772 | 0.01 | 0 |
| hsa_circ_0054912 | 95 | 1 | 788 | 795 | 1585 | 0 | 0 |
| hsa_circ_0006819 | 97 | 2 | 368 | 2 | 368 | 0 | 5.00E-174 |
| hsa_circ_0054912 | 91 | 2 | 399 | 189 | 586 | 0.02 | 2.00E-149 |
| hsa_circ_0054910 | 93 | 1 | 283 | 1 | 283 | 0 | 4.00E-110 |
| hsa_circ_0054910 | 93 | 1 | 283 | 1 | 283 | 0 | 4.00E-111 |
| hsa_circ_0054910 | 93 | 1 | 273 | 1 | 273 | 0 | 7.00E-112 |
| hsa_circ_0032798 | 84 | 6190 | 7331 | 1499 | 303 | 0.05 | 0 |
| hsa_circ_0007473 | 94 | 54 | 649 | 945 | 350 | 0 | 0 |
| hsa_circ_0054903 | 94 | 1 | 1076 | 1311 | 236 | 0 | 0 |

novel_circ_015141 novel_circ_015142 novel_circ_015143 novel_circ_015144 novel_circ_015145 novel_circ_015146 novel_circ_015147 novel_circ_015148 novel_circ_015149 novel_circ_015150 novel_circ_015151 novel_circ_015152 novel_circ_015153 novel_circ_015154 novel_circ_015155 novel_circ_015156 novel_circ_015157 novel_circ_015158 novel_circ_015159 novel_circ_015160 novel_circ_015161 novel_circ_015162 novel_circ_015163 novel_circ_015164 novel_circ_015165 novel_circ_015166 novel_circ_015167 novel_circ_015168 novel_circ_015169 novel_circ_015170 novel_circ_015171 novel_circ_015172 novel_circ_015173 novel_circ_015174 novel_circ_015175 novel_circ_015176 novel_circ_015177 novel_circ_015178 novel_circ_015179 novel_circ_015180 novel_circ_015181 novel_circ_015182 novel_circ_015183 novel_circ_015184 novel_circ_015185 novel_circ_015186 novel_circ_015187 novel_circ_015188 novel_circ_015189 novel_circ_015190 novel_circ_015191 novel_circ_015192 novel_circ_015193 novel_circ_015194

| hsa_circ_0005625 | 94 | 2 | 506 | 646 | 142 | 0 | 0 |
| --- | --- | --- | --- | --- | --- | --- | --- |
| hsa_circ_0005625 | 95 | 3 | 397 | 536 | 142 | 0 | 7.00E-178 |
| hsa_circ_0054899 | 96 | 1 | 307 | 307 | 1 | 0 | 4.00E-139 |
| hsa_circ_0054868 | 95 | 1 | 961 | 1068 | 108 | 0 | 0 |
| hsa_circ_0054852 | 95 | 1 | 1047 | 1047 | 1 | 0 | 0 |
| hsa_circ_0054852 | 95 | 4 | 756 | 753 | 1 | 0 | 0 |
| hsa_circ_0054852 | 95 | 1 | 561 | 561 | 1 | 0 | 0 |
| hsa_circ_0054842 | 95 | 12348 | 12768 | 1545 | 1123 | 0 | 0 |
| hsa_circ_0054842 | 96 | 12348 | 12969 | 1545 | 922 | 0 | 0 |
| hsa_circ_0054842 | 96 | 12348 | 13723 | 1545 | 168 | 0 | 0 |
| hsa_circ_0054845 | 95 | 1 | 419 | 1120 | 700 | 0 | 0 |
| hsa_circ_0054845 | 95 | 1 | 423 | 1120 | 695 | 0.01 | 0 |
| hsa_circ_0054845 | 96 | 1 | 620 | 1120 | 499 | 0 | 0 |
| hsa_circ_0054845 | 96 | 1 | 524 | 699 | 176 | 0 | 0 |
| hsa_circ_0054845 | 97 | 1 | 699 | 699 | 1 | 0 | 0 |
| hsa_circ_0054842 | 96 | 1 | 955 | 1122 | 168 | 0 | 0 |
| hsa_circ_0054837 | 95 | 1 | 1732 | 1948 | 217 | 0 | 0 |
| hsa_circ_0054837 | 95 | 1 | 1951 | 1948 | 1 | 0 | 0 |
| hsa_circ_0054833 | 95 | 1 | 2195 | 2192 | 1 | 0 | 0 |
| hsa_circ_0054827 | 95 | 1 | 2464 | 3117 | 657 | 0 | 0 |
| hsa_circ_0054774 | 95 | 1 | 3268 | 6561 | 3297 | 0 | 0 |
| hsa_circ_0054845 | 96 | 1 | 498 | 498 | 1 | 0 | 0 |
| hsa_circ_0054846 | 96 | 1 | 255 | 255 | 1 | 0 | 3.00E-115 |
| hsa_circ_0054842 | 96 | 1 | 493 | 660 | 168 | 0 | 0 |
| hsa_circ_0054837 | 95 | 1 | 1489 | 1486 | 1 | 0 | 0 |
| hsa_circ_0054833 | 94 | 1 | 1671 | 1668 | 1 | 0 | 0 |
| hsa_circ_0054843 | 95 | 1 | 256 | 256 | 1 | 0 | 2.00E-112 |
| hsa_circ_0054837 | 94 | 1 | 1252 | 1249 | 1 | 0 | 0 |
| hsa_circ_0054830 | 94 | 1 | 1765 | 2131 | 370 | 0 | 0 |
| hsa_circ_0054818 | 94 | 1 | 3007 | 3206 | 203 | 0 | 0 |
| hsa_circ_0054833 | 94 | 1 | 1073 | 1070 | 1 | 0 | 0 |
| hsa_circ_0054833 | 95 | 1 | 459 | 459 | 1 | 0 | 0 |
| hsa_circ_0054832 | 95 | 1 | 549 | 549 | 1 | 0 | 0 |
| hsa_circ_0054830 | 95 | 1 | 643 | 1097 | 455 | 0 | 0 |
| hsa_circ_0054830 | 95 | 1 | 728 | 1097 | 370 | 0 | 0 |
| hsa_circ_0054830 | 95 | 1 | 883 | 1097 | 215 | 0 | 0 |
| hsa_circ_0054830 | 95 | 1 | 997 | 1097 | 101 | 0 | 0 |
| hsa_circ_0054828 | 95 | 1 | 1257 | 1257 | 1 | 0 | 0 |
| hsa_circ_0054824 | 95 | 1 | 1532 | 1843 | 312 | 0 | 0 |
| hsa_circ_0054824 | 95 | 1 | 1633 | 1843 | 211 | 0 | 0 |
| hsa_circ_0054824 | 95 | 1 | 1843 | 1843 | 1 | 0 | 0 |
| hsa_circ_0054818 | 95 | 1 | 2262 | 2464 | 203 | 0 | 0 |
| hsa_circ_0054833 | 93 | 1 | 244 | 244 | 1 | 0 | 4.00E-99 |
| hsa_circ_0054832 | 93 | 1 | 334 | 334 | 1 | 0 | 5.00E-139 |
| hsa_circ_0054830 | 94 | 1 | 269 | 638 | 370 | 0 | 1.00E-114 |
| hsa_circ_0054830 | 95 | 1 | 424 | 638 | 215 | 0 | 0 |
| hsa_circ_0054830 | 95 | 1 | 538 | 638 | 101 | 0 | 0 |
| hsa_circ_0054824 | 95 | 1 | 1073 | 1384 | 312 | 0 | 0 |
| hsa_circ_0054821 | 95 | 1 | 1510 | 1510 | 1 | 0 | 0 |
| hsa_circ_0054830 | 95 | 1 | 269 | 369 | 101 | 0 | 5.00E-118 |
| hsa_circ_0054824 | 95 | 1 | 804 | 1115 | 312 | 0 | 0 |
| hsa_circ_0054828 | 96 | 1 | 260 | 260 | 1 | 0 | 2.00E-116 |
| hsa_circ_0054826 | 95 | 1 | 535 | 535 | 1 | 0 | 0 |
| hsa_circ_0054821 | 95 | 1 | 972 | 972 | 1 | 0 | 0 |

novel_circ_015195 novel_circ_015196 novel_circ_015197 novel_circ_015198 novel_circ_015199 novel_circ_015200 novel_circ_015201 novel_circ_015202 novel_circ_015203 novel_circ_015204 novel_circ_015205 novel_circ_015206 novel_circ_015207 novel_circ_015208 novel_circ_015209 novel_circ_015210 novel_circ_015211 novel_circ_015212 novel_circ_015213 novel_circ_015214 novel_circ_015215 novel_circ_015216 novel_circ_015217 novel_circ_015218 novel_circ_015219 novel_circ_015220 novel_circ_015221 novel_circ_015222 novel_circ_015223 novel_circ_015224 novel_circ_015225 novel_circ_015226 novel_circ_015227 novel_circ_015228 novel_circ_015229 novel_circ_015230 novel_circ_015231 novel_circ_015232 novel_circ_015233 novel_circ_015234 novel_circ_015235 novel_circ_015236 novel_circ_015237 novel_circ_015238 novel_circ_015240 novel_circ_015241 novel_circ_015242 novel_circ_015243 novel_circ_015244 novel_circ_015245 novel_circ_015246 novel_circ_015247 novel_circ_015248 novel_circ_015249

| hsa_circ_0054818 | 94 | 1 | 1005 | 1207 | 203 | 0 | 0 |
| --- | --- | --- | --- | --- | --- | --- | --- |
| hsa_circ_0054810 | 95 | 1 | 1714 | 1770 | 57 | 0 | 0 |
| hsa_circ_0054824 | 95 | 1 | 311 | 311 | 1 | 0 | 6.00E-138 |
| hsa_circ_0054818 | 94 | 1 | 730 | 932 | 203 | 0 | 0 |
| hsa_circ_0054824 | 95 | 1 | 210 | 210 | 1 | 0 | 2.00E-90 |
| hsa_circ_0054821 | 94 | 1 | 336 | 336 | 1 | 0 | 2.00E-143 |
| hsa_circ_0054818 | 94 | 1 | 629 | 831 | 203 | 0 | 0 |
| hsa_circ_0054810 | 95 | 1 | 1338 | 1394 | 57 | 0 | 0 |
| hsa_circ_0054818 | 94 | 4 | 419 | 618 | 203 | 0 | 8.00E-178 |
| hsa_circ_0054810 | 95 | 4 | 1128 | 1181 | 57 | 0 | 0 |
| hsa_circ_0054818 | 94 | 1 | 293 | 495 | 203 | 0 | 1.00E-124 |
| hsa_circ_0054810 | 96 | 1 | 708 | 764 | 57 | 0 | 0 |
| hsa_circ_0054745 | 95 | 1 | 3499 | 4292 | 794 | 0 | 0 |
| hsa_circ_0054810 | 95 | 1 | 507 | 563 | 57 | 0 | 0 |
| hsa_circ_0054807 | 95 | 1 | 868 | 868 | 1 | 0 | 0 |
| hsa_circ_0054801 | 95 | 1 | 1029 | 1095 | 67 | 0 | 0 |
| hsa_circ_0054795 | 95 | 1 | 1645 | 1645 | 1 | 0 | 0 |
| hsa_circ_0054803 | 94 | 1 | 345 | 345 | 1 | 0 | 4.00E-150 |
| hsa_circ_0054801 | 94 | 1 | 227 | 227 | 1 | 0 | 9.00E-95 |
| hsa_circ_0054792 | 94 | 1 | 406 | 406 | 1 | 0 | 6.00E-174 |
| hsa_circ_0054760 | 94 | 1 | 1049 | 1250 | 202 | 0 | 0 |
| hsa_circ_0054760 | 94 | 1 | 563 | 612 | 50 | 0 | 0 |
| hsa_circ_0054753 | 96 | 1 | 604 | 604 | 1 | 0 | 0 |
| hsa_circ_0054753 | 94 | 1 | 107 | 253 | 147 | 0 | 2.00E-39 |
| hsa_circ_0054737 | 97 | 1 | 1212 | 1212 | 1 | 0 | 0 |
| hsa_circ_0054737 | 95 | 1 | 577 | 577 | 1 | 0 | 0 |
| hsa_circ_0054722 | 96 | 1 | 1351 | 1351 | 1 | 0 | 0 |
| hsa_circ_0054725 | 97 | 1 | 827 | 827 | 1 | 0 | 0 |
| hsa_circ_0054722 | 97 | 1 | 987 | 987 | 1 | 0 | 0 |
| hsa_circ_0054709 | 92 | 1 | 846 | 1 | 849 | 0 | 0 |
| hsa_circ_0054709 | 92 | 1 | 745 | 1 | 748 | 0 | 0 |
| hsa_circ_0054708 | 95 | 1 | 657 | 1 | 657 | 0 | 0 |
| hsa_circ_0054700 | 92 | 1 | 695 | 1 | 695 | 0 | 0 |
| hsa_circ_0054691 | 94 | 1 | 524 | 93 | 616 | 0 | 0 |
| hsa_circ_0054690 | 94 | 150 | 908 | 7 | 765 | 0 | 0 |
| hsa_circ_0054691 | 94 | 1 | 524 | 93 | 616 | 0 | 0 |
| hsa_circ_0054690 | 94 | 150 | 908 | 7 | 765 | 0 | 0 |
| hsa_circ_0054690 | 94 | 150 | 843 | 7 | 700 | 0 | 0 |
| hsa_circ_0001011 | 93 | 6 | 382 | 6 | 382 | 0 | 3.00E-156 |
| hsa_circ_0001011 | 94 | 6 | 292 | 6 | 292 | 0 | 3.00E-121 |
| hsa_circ_0054686 | 91 | 1 | 904 | 140 | 1028 | 0.02 | 0 |
| hsa_circ_0054686 | 94 | 1 | 616 | 1 | 616 | 0 | 0 |
| hsa_circ_0054684 | 96 | 1 | 490 | 1 | 490 | 0 | 0 |
| hsa_circ_0004662 | 91 | 1 | 447 | 462 | 16 | 0 | 2.00E-173 |
| hsa_circ_0078610 | 90 | 40559 | 41104 | 1 | 546 | 0 | 0 |
| hsa_circ_0078610 | 91 | 1 | 428 | 1 | 428 | 0 | 8.00E-163 |
| hsa_circ_0078610 | 90 | 1 | 546 | 1 | 546 | 0 | 0 |
| hsa_circ_0078618 | 89 | 12196 | 14133 | 2 | 1939 | 0 | 0 |
| hsa_circ_0078618 | 90 | 2 | 1544 | 2 | 1544 | 0 | 0 |
| hsa_circ_0078619 | 91 | 1 | 1353 | 1 | 1353 | 0 | 0 |
| hsa_circ_0078618 | 89 | 1 | 1607 | 192 | 1798 | 0 | 0 |
| hsa_circ_0078618 | 89 | 1 | 1748 | 192 | 1939 | 0 | 0 |
| hsa_circ_0078618 | 90 | 1 | 2621 | 192 | 2821 | 0.01 | 0 |
| hsa_circ_0078620 | 90 | 244 | 1632 | 1 | 1398 | 0.01 | 0 |

novel_circ_015250 novel_circ_015251 novel_circ_015252 novel_circ_015253 novel_circ_015254 novel_circ_015255 novel_circ_015256 novel_circ_015260 novel_circ_015261 novel_circ_015262 novel_circ_015263 novel_circ_015264 novel_circ_015265 novel_circ_015268 novel_circ_015269 novel_circ_015270 novel_circ_015271 novel_circ_015272 novel_circ_015273 novel_circ_015274 novel_circ_015275 novel_circ_015276 novel_circ_015277 novel_circ_015278 novel_circ_015279 novel_circ_015280 novel_circ_015281 novel_circ_015283 novel_circ_015284 novel_circ_015286 novel_circ_015288 novel_circ_015294 novel_circ_015295 novel_circ_015296 novel_circ_015297 novel_circ_015298 novel_circ_015299 novel_circ_015300 novel_circ_015301 novel_circ_015302 novel_circ_015303 novel_circ_015304 novel_circ_015305 novel_circ_015306 novel_circ_015307 novel_circ_015309 novel_circ_015312 novel_circ_015313 novel_circ_015315 novel_circ_015316 novel_circ_015317 novel_circ_015318 novel_circ_015319 novel_circ_015320

| hsa_circ_0078622 | 89 | 3 | 607 | 3 | 619 | 0.02 | 0 |
| --- | --- | --- | --- | --- | --- | --- | --- |
| hsa_circ_0078622 | 91 | 3 | 271 | 351 | 619 | 0 | 5.00E-98 |
| hsa_circ_0078622 | 89 | 3 | 621 | 351 | 969 | 0 | 0 |
| hsa_circ_0078622 | 91 | 1 | 540 | 430 | 969 | 0 | 0 |
| hsa_circ_0078622 | 90 | 1 | 663 | 430 | 1092 | 0 | 0 |
| hsa_circ_0078623 | 90 | 1 | 230 | 1 | 230 | 0 | 1.00E-79 |
| hsa_circ_0078626 | 86 | 2 | 266 | 266 | 1 | 0.01 | 4.00E-74 |
| hsa_circ_0005328 | 97 | 24699 | 24958 | 1 | 260 | 0 | 2.00E-119 |
| hsa_circ_0005328 | 97 | 23133 | 23392 | 1 | 260 | 0 | 2.00E-119 |
| hsa_circ_0005328 | 97 | 1 | 260 | 1 | 260 | 0 | 2.00E-121 |
| hsa_circ_0005328 | 97 | 1 | 260 | 1 | 260 | 0 | 4.00E-121 |
| hsa_circ_0005328 | 97 | 1 | 260 | 1 | 260 | 0 | 9.00E-121 |
| hsa_circ_0005328 | 95 | 1 | 117 | 144 | 260 | 0 | 3.00E-45 |
| hsa_circ_0078629 | 97 | 89 | 388 | 1 | 300 | 0 | 2.00E-143 |
| hsa_circ_0078630 | 89 | 1 | 193 | 193 | 1 | 0 | 3.00E-60 |
| hsa_circ_0078634 | 86 | 1 | 707 | 707 | 1 | 0 | 0 |
| hsa_circ_0078634 | 88 | 1 | 564 | 564 | 1 | 0 | 0 |
| hsa_circ_0078634 | 86 | 1 | 707 | 707 | 1 | 0 | 0 |
| hsa_circ_0078634 | 87 | 2 | 894 | 893 | 1 | 0 | 0 |
| hsa_circ_0078634 | 87 | 1 | 987 | 987 | 1 | 0 | 0 |
| hsa_circ_0078634 | 88 | 4 | 1082 | 1079 | 1 | 0 | 0 |
| hsa_circ_0078634 | 89 | 1 | 1130 | 1130 | 1 | 0 | 0 |
| hsa_circ_0078634 | 89 | 1 | 1589 | 1589 | 1 | 0 | 0 |
| hsa_circ_0078634 | 89 | 1 | 1760 | 1760 | 1 | 0 | 0 |
| hsa_circ_0078634 | 87 | 1 | 814 | 987 | 174 | 0 | 0 |
| hsa_circ_0078634 | 89 | 1 | 957 | 1130 | 174 | 0 | 0 |
| hsa_circ_0078634 | 90 | 1 | 566 | 1130 | 565 | 0.01 | 0 |
| hsa_circ_0078637 | 92 | 1 | 423 | 423 | 1 | 0 | 1.00E-171 |
| hsa_circ_0078637 | 91 | 1 | 695 | 882 | 188 | 0 | 0 |
| hsa_circ_0078634 | 89 | 1 | 469 | 1918 | 1450 | 0 | 7.00E-164 |
| hsa_circ_0078639 | 91 | 1 | 329 | 329 | 1 | 0 | 1.00E-124 |
| hsa_circ_0007372 | 88 | 1 | 845 | 860 | 1 | 0.02 | 0 |
| hsa_circ_0008157 | 87 | 1 | 655 | 671 | 2 | 0.02 | 0 |
| hsa_circ_0024473 | 94 | 1 | 815 | 1 | 815 | 0 | 0 |
| hsa_circ_0024473 | 94 | 1 | 819 | 1 | 820 | 0 | 0 |
| hsa_circ_0024473 | 93 | 1 | 981 | 1 | 981 | 0 | 0 |
| hsa_circ_0024473 | 93 | 1 | 1129 | 1 | 1129 | 0 | 0 |
| hsa_circ_0024475 | 92 | 1 | 423 | 166 | 588 | 0 | 1.00E-166 |
| hsa_circ_0024475 | 94 | 1 | 257 | 332 | 588 | 0 | 2.00E-106 |
| hsa_circ_0024493 | 97 | 2959 | 3329 | 371 | 1 | 0 | 3.00E-175 |
| hsa_circ_0024493 | 97 | 1 | 464 | 464 | 1 | 0 | 0 |
| hsa_circ_0024492 | 97 | 1 | 611 | 777 | 167 | 0 | 0 |
| hsa_circ_0000360 | 97 | 2959 | 3211 | 254 | 2 | 0 | 2.00E-116 |
| hsa_circ_0000360 | 97 | 1 | 346 | 347 | 2 | 0 | 2.00E-162 |
| hsa_circ_0006588 | 95 | 6 | 634 | 627 | 1 | 0.01 | 0 |
| hsa_circ_0024529 | 95 | 1 | 354 | 1 | 354 | 0 | 8.00E-157 |
| hsa_circ_0061824 | 88 | 402 | 460 | 208 | 266 | 0 | 8.00E-11 |
| hsa_circ_0061824 | 88 | 20 | 78 | 208 | 266 | 0 | 4.00E-11 |
| hsa_circ_0024563 | 91 | 1 | 1260 | 334 | 1569 | 0.02 | 0 |
| hsa_circ_0024565 | 91 | 1 | 588 | 1 | 564 | 0.04 | 0 |
| hsa_circ_0024565 | 89 | 1 | 1012 | 1 | 988 | 0.02 | 0 |
| hsa_circ_0024569 | 91 | 42 | 865 | 18 | 841 | 0 | 0 |
| hsa_circ_0024569 | 90 | 42 | 1371 | 18 | 1347 | 0 | 0 |
| hsa_circ_0024572 | 88 | 1 | 424 | 1 | 424 | 0 | 3.00E-137 |

novel_circ_015321 novel_circ_015323 novel_circ_015324 novel_circ_015325 novel_circ_015326 novel_circ_015327 novel_circ_015328 novel_circ_015329 novel_circ_015330 novel_circ_015331 novel_circ_015332 novel_circ_015337 novel_circ_015338 novel_circ_015339 novel_circ_015340 novel_circ_015341 novel_circ_015342 novel_circ_015343 novel_circ_015344 novel_circ_015345 novel_circ_015346 novel_circ_015347 novel_circ_015348 novel_circ_015349 novel_circ_015350 novel_circ_015351 novel_circ_015352 novel_circ_015353 novel_circ_015354 novel_circ_015355 novel_circ_015356 novel_circ_015357 novel_circ_015358 novel_circ_015359 novel_circ_015360 novel_circ_015361 novel_circ_015363 novel_circ_015364 novel_circ_015365 novel_circ_015366 novel_circ_015367 novel_circ_015368 novel_circ_015369 novel_circ_015370 novel_circ_015371 novel_circ_015372 novel_circ_015373 novel_circ_015374 novel_circ_015375 novel_circ_015376 novel_circ_015377 novel_circ_015378 novel_circ_015379 novel_circ_015380

| hsa_circ_0024572 | 88 | 1 | 930 | 1 | 930 | 0 | 0 |
| --- | --- | --- | --- | --- | --- | --- | --- |
| hsa_circ_0024572 | 88 | 1 | 842 | 89 | 930 | 0 | 0 |
| hsa_circ_0024572 | 89 | 1 | 506 | 425 | 930 | 0 | 0 |
| hsa_circ_0024573 | 90 | 8 | 374 | 8 | 374 | 0 | 1.00E-135 |
| hsa_circ_0024573 | 77 | 1 | 237 | 474 | 686 | 0.11 | 3.00E-25 |
| hsa_circ_0024592 | 95 | 1 | 108 | 108 | 1 | 0 | 6.00E-42 |
| hsa_circ_0067816 | 90 | 1 | 425 | 206 | 630 | 0 | 8.00E-153 |
| hsa_circ_0067816 | 89 | 1 | 511 | 206 | 716 | 0 | 0 |
| hsa_circ_0067821 | 90 | 3 | 518 | 3 | 518 | 0 | 0 |
| hsa_circ_0067821 | 90 | 3 | 681 | 3 | 681 | 0 | 0 |
| hsa_circ_0067821 | 90 | 3 | 826 | 3 | 826 | 0 | 0 |
| hsa_circ_0083174 | 87 | 34034 | 34179 | 895 | 1040 | 0.05 | 1.00E-35 |
| hsa_circ_0005108 | 95 | 1 | 797 | 797 | 1 | 0 | 0 |
| hsa_circ_0005108 | 95 | 1 | 686 | 686 | 1 | 0 | 0 |
| hsa_circ_0031747 | 87 | 3 | 570 | 1003 | 1576 | 0.01 | 0 |
| hsa_circ_0031747 | 90 | 1 | 850 | 721 | 1576 | 0.01 | 0 |
| hsa_circ_0031747 | 91 | 1 | 1279 | 292 | 1576 | 0 | 0 |
| hsa_circ_0031747 | 92 | 1 | 559 | 721 | 1282 | 0.01 | 0 |
| hsa_circ_0031747 | 93 | 3 | 897 | 385 | 1282 | 0 | 0 |
| hsa_circ_0031749 | 93 | 1 | 355 | 268 | 625 | 0.01 | 7.00E-148 |
| hsa_circ_0031749 | 93 | 1 | 423 | 200 | 625 | 0.01 | 3.00E-177 |
| hsa_circ_0031749 | 94 | 1 | 459 | 164 | 625 | 0.01 | 0 |
| hsa_circ_0031749 | 94 | 1 | 622 | 1 | 625 | 0 | 0 |
| hsa_circ_0031747 | 93 | 1 | 852 | 292 | 1146 | 0 | 0 |
| hsa_circ_0031747 | 93 | 1 | 429 | 292 | 720 | 0 | 0 |
| hsa_circ_0031748 | 95 | 3 | 302 | 3 | 302 | 0 | 3.00E-130 |
| hsa_circ_0031746 | 91 | 11 | 1071 | 17 | 1065 | 0.01 | 0 |
| hsa_circ_0031747 | 92 | 1 | 230 | 292 | 521 | 0 | 2.00E-86 |
| hsa_circ_0031746 | 90 | 11 | 908 | 17 | 902 | 0.01 | 0 |
| hsa_circ_0031746 | 91 | 1 | 579 | 94 | 672 | 0 | 0 |
| hsa_circ_0031746 | 90 | 11 | 678 | 17 | 672 | 0.02 | 0 |
| hsa_circ_0031746 | 91 | 1 | 532 | 94 | 625 | 0 | 0 |
| hsa_circ_0031746 | 89 | 11 | 631 | 17 | 625 | 0.02 | 0 |
| hsa_circ_0031746 | 91 | 1 | 532 | 94 | 625 | 0 | 0 |
| hsa_circ_0031746 | 89 | 11 | 631 | 17 | 625 | 0.02 | 0 |
| hsa_circ_0031746 | 90 | 11 | 387 | 17 | 381 | 0.03 | 3.00E-131 |
| hsa_circ_0002395 | 91 | 1 | 198 | 186 | 1 | 0.06 | 5.00E-67 |
| hsa_circ_0031738 | 93 | 1 | 282 | 364 | 95 | 0.04 | 3.00E-110 |
| hsa_circ_0031738 | 93 | 1 | 374 | 364 | 3 | 0.03 | 7.00E-153 |
| hsa_circ_0031733 | 95 | 1 | 548 | 86 | 633 | 0 | 0 |
| hsa_circ_0031733 | 94 | 1 | 489 | 86 | 574 | 0 | 0 |
| hsa_circ_0031733 | 92 | 1 | 264 | 86 | 349 | 0 | 1.00E-103 |
| hsa_circ_0031723 | 94 | 1 | 240 | 321 | 82 | 0 | 3.00E-100 |
| hsa_circ_0031698 | 96 | 1 | 203 | 206 | 408 | 0 | 8.00E-90 |
| hsa_circ_0004204 | 93 | 1 | 443 | 1 | 443 | 0 | 0 |
| hsa_circ_0004204 | 92 | 1 | 332 | 1 | 332 | 0.01 | 1.00E-129 |
| hsa_circ_0031697 | 84 | 9 | 589 | 478 | 1058 | 0.02 | 5.00E-156 |
| hsa_circ_0031697 | 83 | 9 | 474 | 478 | 943 | 0.02 | 2.00E-115 |
| hsa_circ_0084772 | 88 | 8124 | 8687 | 3 | 565 | 0 | 0 |
| hsa_circ_0031669 | 96 | 1 | 557 | 5585 | 5029 | 0 | 0 |
| hsa_circ_0031669 | 96 | 1 | 696 | 5585 | 4890 | 0 | 0 |
| hsa_circ_0031669 | 96 | 1 | 905 | 5585 | 4681 | 0 | 0 |
| hsa_circ_0031669 | 95 | 1 | 1481 | 5585 | 4105 | 0 | 0 |
| hsa_circ_0031688 | 95 | 1 | 1080 | 2584 | 1505 | 0 | 0 |

novel_circ_015381 novel_circ_015382 novel_circ_015383 novel_circ_015384 novel_circ_015385 novel_circ_015386 novel_circ_015387 novel_circ_015388 novel_circ_015389 novel_circ_015390 novel_circ_015391 novel_circ_015392 novel_circ_015393 novel_circ_015394 novel_circ_015395 novel_circ_015396 novel_circ_015397 novel_circ_015398 novel_circ_015399 novel_circ_015400 novel_circ_015401 novel_circ_015402 novel_circ_015403 novel_circ_015404 novel_circ_015405 novel_circ_015406 novel_circ_015407 novel_circ_015408 novel_circ_015409 novel_circ_015410 novel_circ_015411 novel_circ_015412 novel_circ_015413 novel_circ_015414 novel_circ_015415 novel_circ_015416 novel_circ_015417 novel_circ_015418 novel_circ_015419 novel_circ_015420 novel_circ_015421 novel_circ_015422 novel_circ_015423 novel_circ_015424 novel_circ_015428 novel_circ_015429 novel_circ_015430 novel_circ_015431 novel_circ_015432 novel_circ_015433 novel_circ_015434 novel_circ_015442 novel_circ_015443 novel_circ_015444

| hsa_circ_0031688 | 95 | 1 | 1040 | 2406 | 1367 | 0 | 0 |
| --- | --- | --- | --- | --- | --- | --- | --- |
| hsa_circ_0031688 | 96 | 1 | 348 | 2290 | 1943 | 0 | 4.00E-160 |
| hsa_circ_0031688 | 96 | 3 | 449 | 2149 | 1703 | 0 | 0 |
| hsa_circ_0031688 | 95 | 3 | 647 | 2149 | 1505 | 0 | 0 |
| hsa_circ_0031688 | 95 | 3 | 785 | 2149 | 1367 | 0 | 0 |
| hsa_circ_0031689 | 95 | 1 | 1013 | 1525 | 513 | 0 | 0 |
| hsa_circ_0031689 | 95 | 1 | 528 | 1040 | 513 | 0 | 0 |
| hsa_circ_0031669 | 94 | 2206 | 4250 | 3259 | 1215 | 0 | 0 |
| hsa_circ_0031688 | 94 | 1 | 392 | 394 | 3 | 0 | 3.00E-166 |
| hsa_circ_0031682 | 94 | 1 | 1125 | 1125 | 1 | 0 | 0 |
| hsa_circ_0031677 | 94 | 1 | 1251 | 1251 | 1 | 0 | 0 |
| hsa_circ_0031673 | 94 | 1 | 1310 | 1599 | 290 | 0 | 0 |
| hsa_circ_0031673 | 94 | 1 | 1310 | 1599 | 290 | 0 | 0 |
| hsa_circ_0031673 | 94 | 1 | 1419 | 1599 | 181 | 0 | 0 |
| hsa_circ_0031673 | 94 | 1 | 1530 | 1599 | 70 | 0 | 0 |
| hsa_circ_0031669 | 94 | 1 | 1918 | 3132 | 1215 | 0 | 0 |
| hsa_circ_0031669 | 93 | 1 | 2852 | 3132 | 281 | 0 | 0 |
| hsa_circ_0031669 | 94 | 1 | 3132 | 3132 | 1 | 0 | 0 |
| hsa_circ_0031684 | 93 | 1 | 623 | 623 | 1 | 0 | 0 |
| hsa_circ_0031682 | 94 | 1 | 908 | 908 | 1 | 0 | 0 |
| hsa_circ_0031673 | 93 | 1 | 1202 | 1382 | 181 | 0 | 0 |
| hsa_circ_0031682 | 94 | 1 | 731 | 731 | 1 | 0 | 0 |
| hsa_circ_0031684 | 94 | 1 | 293 | 293 | 1 | 0 | 1.00E-124 |
| hsa_circ_0031682 | 95 | 1 | 578 | 578 | 1 | 0 | 0 |
| hsa_circ_0031677 | 95 | 1 | 704 | 704 | 1 | 0 | 0 |
| hsa_circ_0031673 | 94 | 1 | 763 | 1052 | 290 | 0 | 0 |
| hsa_circ_0031673 | 94 | 1 | 763 | 1052 | 290 | 0 | 0 |
| hsa_circ_0031673 | 94 | 1 | 872 | 1052 | 181 | 0 | 0 |
| hsa_circ_0031673 | 94 | 1 | 983 | 1052 | 70 | 0 | 0 |
| hsa_circ_0031673 | 94 | 1 | 1052 | 1052 | 1 | 0 | 0 |
| hsa_circ_0031671 | 94 | 1 | 1187 | 1371 | 185 | 0 | 0 |
| hsa_circ_0031671 | 94 | 1 | 1371 | 1371 | 1 | 0 | 0 |
| hsa_circ_0031669 | 94 | 1 | 2429 | 2585 | 157 | 0 | 0 |
| hsa_circ_0031669 | 94 | 1 | 2585 | 2585 | 1 | 0 | 0 |
| hsa_circ_0031665 | 94 | 1 | 2744 | 3028 | 285 | 0 | 0 |
| hsa_circ_0031665 | 94 | 1 | 2876 | 3028 | 153 | 0 | 0 |
| hsa_circ_0031673 | 93 | 1 | 405 | 474 | 70 | 0 | 4.00E-170 |
| hsa_circ_0031671 | 93 | 1 | 608 | 608 | 1 | 0 | 0 |
| hsa_circ_0031669 | 93 | 1 | 862 | 1214 | 353 | 0 | 0 |
| hsa_circ_0031669 | 93 | 1 | 934 | 1214 | 281 | 0 | 0 |
| hsa_circ_0031669 | 94 | 1 | 1058 | 1214 | 157 | 0 | 0 |
| hsa_circ_0031669 | 94 | 1 | 1214 | 1214 | 1 | 0 | 0 |
| hsa_circ_0031665 | 94 | 1 | 1505 | 1657 | 153 | 0 | 0 |
| hsa_circ_0031669 | 97 | 2 | 280 | 279 | 1 | 0 | 7.00E-132 |
| hsa_circ_0031647 | 94 | 1 | 183 | 1 | 183 | 0 | 1.00E-73 |
| hsa_circ_0031646 | 96 | 2 | 613 | 107 | 718 | 0 | 0 |
| hsa_circ_0031646 | 96 | 1 | 718 | 1 | 718 | 0 | 0 |
| hsa_circ_0031646 | 96 | 1 | 718 | 1 | 718 | 0 | 0 |
| hsa_circ_0031646 | 96 | 1 | 530 | 1 | 530 | 0 | 0 |
| hsa_circ_0031646 | 96 | 1 | 381 | 1 | 381 | 0 | 2.00E-173 |
| hsa_circ_0031646 | 95 | 1 | 230 | 1 | 230 | 0 | 9.00E-100 |
| hsa_circ_0031639 | 94 | 1 | 525 | 613 | 89 | 0 | 0 |
| hsa_circ_0031611 | 95 | 1 | 216 | 216 | 1 | 0 | 1.00E-93 |
| hsa_circ_0031610 | 96 | 1 | 462 | 462 | 1 | 0 | 0 |

novel_circ_015445 novel_circ_015446 novel_circ_015447 novel_circ_015448 novel_circ_015449 novel_circ_015450 novel_circ_015451 novel_circ_015452 novel_circ_015461 novel_circ_015463 novel_circ_015464 novel_circ_015465 novel_circ_015466 novel_circ_015467 novel_circ_015468 novel_circ_015469 novel_circ_015470 novel_circ_015471 novel_circ_015472 novel_circ_015474 novel_circ_015475 novel_circ_015476 novel_circ_015477 novel_circ_015478 novel_circ_015479 novel_circ_015480 novel_circ_015481 novel_circ_015482 novel_circ_015483 novel_circ_015484 novel_circ_015485 novel_circ_015486 novel_circ_015487 novel_circ_015488 novel_circ_015489 novel_circ_015490 novel_circ_015491 novel_circ_015492 novel_circ_015493 novel_circ_015494 novel_circ_015495 novel_circ_015496 novel_circ_015497 novel_circ_015498 novel_circ_015499 novel_circ_015500 novel_circ_015501 novel_circ_015502 novel_circ_015503 novel_circ_015504 novel_circ_015505 novel_circ_015506 novel_circ_015507 novel_circ_015508

| hsa_circ_0031608 | 96 | 1 | 558 | 740 | 183 | 0 | 0 |
| --- | --- | --- | --- | --- | --- | --- | --- |
| hsa_circ_0031608 | 96 | 1 | 664 | 740 | 77 | 0 | 0 |
| hsa_circ_0031607 | 96 | 1 | 867 | 867 | 1 | 0 | 0 |
| hsa_circ_0031603 | 96 | 1 | 1027 | 2877 | 1851 | 0 | 0 |
| hsa_circ_0031608 | 96 | 1 | 559 | 635 | 77 | 0 | 0 |
| hsa_circ_0031608 | 97 | 4 | 448 | 521 | 77 | 0 | 0 |
| hsa_circ_0031608 | 97 | 4 | 524 | 521 | 1 | 0 | 0 |
| hsa_circ_0031607 | 97 | 4 | 651 | 648 | 1 | 0 | 0 |
| hsa_circ_0008576 | 92 | 32708 | 32756 | 451041 | 451089 | 0.08 | 2.00E-08 |
| hsa_circ_0031591 | 99 | 1 | 147 | 532 | 678 | 0 | 3.00E-68 |
| hsa_circ_0031591 | 99 | 1 | 268 | 411 | 678 | 0 | 7.00E-134 |
| hsa_circ_0031591 | 98 | 1 | 270 | 262 | 531 | 0 | 6.00E-132 |
| hsa_circ_0031589 | 95 | 1 | 387 | 2023 | 2406 | 0.01 | 5.00E-174 |
| hsa_circ_0031589 | 88 | 1 | 1761 | 253 | 2022 | 0.01 | 0 |
| hsa_circ_0031589 | 88 | 1 | 2013 | 1 | 2022 | 0.01 | 0 |
| hsa_circ_0031588 | 95 | 1 | 358 | 1 | 358 | 0 | 5.00E-159 |
| hsa_circ_0031588 | 95 | 3505 | 3862 | 1 | 358 | 0 | 6.00E-158 |
| hsa_circ_0005236 | 96 | 1 | 464 | 1 | 464 | 0 | 0 |
| hsa_circ_0031585 | 96 | 1 | 1916 | 1 | 1916 | 0 | 0 |
| hsa_circ_0031578 | 85 | 1 | 561 | 2418 | 1855 | 0.02 | 4.00E-157 |
| hsa_circ_0031567 | 91 | 1 | 521 | 2485 | 1966 | 0 | 0 |
| hsa_circ_0031567 | 91 | 1 | 841 | 2485 | 1646 | 0 | 0 |
| hsa_circ_0031567 | 91 | 1 | 1264 | 2485 | 1223 | 0 | 0 |
| hsa_circ_0031567 | 91 | 1 | 1671 | 2485 | 816 | 0 | 0 |
| hsa_circ_0031567 | 91 | 1 | 1783 | 2485 | 704 | 0 | 0 |
| hsa_circ_0031567 | 91 | 1 | 2486 | 2485 | 1 | 0 | 0 |
| hsa_circ_0031567 | 91 | 1 | 1258 | 2073 | 816 | 0 | 0 |
| hsa_circ_0031567 | 92 | 1 | 1370 | 2073 | 704 | 0 | 0 |
| hsa_circ_0031574 | 92 | 1 | 742 | 898 | 157 | 0 | 0 |
| hsa_circ_0031567 | 91 | 1 | 1261 | 1964 | 704 | 0 | 0 |
| hsa_circ_0031565 | 91 | 1 | 1479 | 1775 | 297 | 0 | 0 |
| hsa_circ_0031567 | 91 | 1 | 450 | 450 | 1 | 0 | 1.00E-171 |
| hsa_circ_0031566 | 91 | 1 | 451 | 556 | 106 | 0 | 2.00E-171 |
| hsa_circ_0031566 | 92 | 1 | 556 | 556 | 1 | 0 | 0 |
| hsa_circ_0031565 | 90 | 1 | 747 | 747 | 1 | 0 | 0 |
| hsa_circ_0031558 | 91 | 1 | 1097 | 1097 | 1 | 0 | 0 |
| hsa_circ_0031555 | 92 | 1 | 1267 | 1267 | 1 | 0 | 0 |
| hsa_circ_0031536 | 92 | 1 | 2477 | 2480 | 1 | 0 | 0 |
| hsa_circ_0031555 | 95 | 1 | 360 | 360 | 1 | 0 | 8.00E-162 |
| hsa_circ_0031542 | 92 | 1 | 602 | 602 | 1 | 0 | 0 |
| hsa_circ_0031536 | 92 | 1 | 1071 | 1074 | 1 | 0 | 0 |
| hsa_circ_0031497 | 94 | 1 | 1480 | 1792 | 313 | 0 | 0 |
| hsa_circ_0031497 | 95 | 1 | 1792 | 1792 | 1 | 0 | 0 |
| hsa_circ_0031498 | 94 | 4 | 858 | 1244 | 390 | 0 | 0 |
| hsa_circ_0031498 | 94 | 4 | 957 | 1244 | 291 | 0 | 0 |
| hsa_circ_0031498 | 96 | 1 | 464 | 466 | 3 | 0 | 0 |
| hsa_circ_0031497 | 96 | 1 | 776 | 776 | 1 | 0 | 0 |
| hsa_circ_0031497 | 96 | 1 | 699 | 699 | 1 | 0 | 0 |
| hsa_circ_0031493 | 96 | 1 | 922 | 1881 | 960 | 0 | 0 |
| hsa_circ_0031493 | 95 | 1 | 1881 | 1881 | 1 | 0 | 0 |
| hsa_circ_0031494 | 96 | 1 | 874 | 1251 | 378 | 0 | 0 |
| hsa_circ_0031493 | 96 | 1 | 1357 | 1492 | 136 | 0 | 0 |
| hsa_circ_0031489 | 95 | 1 | 2300 | 2300 | 1 | 0 | 0 |
| hsa_circ_0031493 | 95 | 1 | 348 | 483 | 136 | 0 | 8.00E-152 |

novel_circ_015509 novel_circ_015510 novel_circ_015511 novel_circ_015512 novel_circ_015513 novel_circ_015514 novel_circ_015515 novel_circ_015516 novel_circ_015517 novel_circ_015518 novel_circ_015519 novel_circ_015520 novel_circ_015521 novel_circ_015522 novel_circ_015523 novel_circ_015524 novel_circ_015525 novel_circ_015526 novel_circ_015527 novel_circ_015528 novel_circ_015529 novel_circ_015530 novel_circ_015531 novel_circ_015532 novel_circ_015533 novel_circ_015534 novel_circ_015535 novel_circ_015536 novel_circ_015537 novel_circ_015538 novel_circ_015539 novel_circ_015540 novel_circ_015541 novel_circ_015542 novel_circ_015543 novel_circ_015544 novel_circ_015545 novel_circ_015546 novel_circ_015547 novel_circ_015548 novel_circ_015549 novel_circ_015550 novel_circ_015551 novel_circ_015552 novel_circ_015553 novel_circ_015554 novel_circ_015555 novel_circ_015557 novel_circ_015558 novel_circ_015559 novel_circ_015560 novel_circ_015561 novel_circ_015562 novel_circ_015563

| hsa_circ_0031493 | 94 | 1 | 483 | 483 | 1 | 0 | 0 |
| --- | --- | --- | --- | --- | --- | --- | --- |
| hsa_circ_0031493 | 94 | 1 | 336 | 336 | 1 | 0 | 8.00E-142 |
| hsa_circ_0031489 | 94 | 1 | 344 | 344 | 1 | 0 | 3.00E-146 |
| hsa_circ_0031482 | 97 | 1 | 1403 | 1403 | 1 | 0 | 0 |
| hsa_circ_0031469 | 96 | 1 | 2040 | 2040 | 1 | 0 | 0 |
| hsa_circ_0031460 | 96 | 1 | 2572 | 2572 | 1 | 0 | 0 |
| hsa_circ_0031482 | 98 | 1 | 1059 | 1059 | 1 | 0 | 0 |
| hsa_circ_0031473 | 94 | 1 | 414 | 414 | 1 | 0 | 1.00E-176 |
| hsa_circ_0031469 | 92 | 1 | 503 | 503 | 1 | 0 | 0 |
| hsa_circ_0031457 | 93 | 1 | 1502 | 2111 | 610 | 0 | 0 |
| hsa_circ_0031457 | 93 | 1 | 467 | 1076 | 610 | 0 | 0 |
| hsa_circ_0031457 | 95 | 1 | 1076 | 1076 | 1 | 0 | 0 |
| hsa_circ_0031454 | 95 | 1 | 1249 | 1545 | 297 | 0 | 0 |
| hsa_circ_0031454 | 95 | 1 | 1400 | 1545 | 146 | 0 | 0 |
| hsa_circ_0031449 | 94 | 1 | 364 | 1 | 365 | 0 | 1.00E-155 |
| hsa_circ_0031449 | 95 | 1 | 295 | 1 | 296 | 0 | 2.00E-127 |
| hsa_circ_0031447 | 94 | 1 | 255 | 434 | 180 | 0 | 3.00E-105 |
| hsa_circ_0031446 | 94 | 1 | 564 | 706 | 143 | 0 | 0 |
| hsa_circ_0031446 | 94 | 1 | 706 | 706 | 1 | 0 | 0 |
| hsa_circ_0031437 | 97 | 1 | 1178 | 1487 | 310 | 0 | 0 |
| hsa_circ_0031437 | 97 | 1 | 1487 | 1487 | 1 | 0 | 0 |
| hsa_circ_0031437 | 97 | 1 | 1487 | 1487 | 1 | 0 | 0 |
| hsa_circ_0031439 | 97 | 1 | 480 | 480 | 1 | 0 | 0 |
| hsa_circ_0031437 | 97 | 1 | 789 | 789 | 1 | 0 | 0 |
| hsa_circ_0031435 | 97 | 1 | 897 | 897 | 1 | 0 | 0 |
| hsa_circ_0031435 | 96 | 1 | 417 | 417 | 1 | 0 | 0 |
| hsa_circ_0000528 | 94 | 1 | 207 | 1 | 207 | 0 | 2.00E-85 |
| hsa_circ_0031425 | 95 | 1 | 495 | 141 | 635 | 0 | 0 |
| hsa_circ_0031425 | 95 | 1 | 635 | 1 | 635 | 0 | 0 |
| hsa_circ_0031423 | 94 | 1 | 735 | 321 | 1055 | 0 | 0 |
| hsa_circ_0031425 | 96 | 1 | 344 | 141 | 484 | 0 | 3.00E-156 |
| hsa_circ_0031425 | 95 | 1 | 484 | 1 | 484 | 0 | 0 |
| hsa_circ_0031423 | 95 | 1 | 584 | 321 | 904 | 0 | 0 |
| hsa_circ_0031425 | 95 | 1 | 387 | 1 | 387 | 0 | 4.00E-170 |
| hsa_circ_0031423 | 94 | 1 | 487 | 321 | 807 | 0 | 0 |
| hsa_circ_0031423 | 95 | 3 | 719 | 91 | 807 | 0 | 0 |
| hsa_circ_0031423 | 95 | 3 | 472 | 91 | 560 | 0 | 0 |
| hsa_circ_0031423 | 94 | 1 | 560 | 1 | 560 | 0 | 0 |
| hsa_circ_0031424 | 95 | 3 | 332 | 3 | 332 | 0 | 8.00E-147 |
| hsa_circ_0031422 | 94 | 2305 | 2484 | 213 | 392 | 0 | 7.00E-71 |
| hsa_circ_0031422 | 94 | 1 | 392 | 1 | 392 | 0 | 2.00E-169 |
| hsa_circ_0031420 | 94 | 1 | 552 | 1 | 552 | 0 | 0 |
| hsa_circ_0031414 | 89 | 1 | 560 | 391 | 956 | 0.01 | 0 |
| hsa_circ_0031414 | 89 | 1 | 560 | 391 | 956 | 0.01 | 0 |
| hsa_circ_0031414 | 90 | 1 | 784 | 167 | 956 | 0.01 | 0 |
| hsa_circ_0031414 | 91 | 126 | 1075 | 1 | 956 | 0.01 | 0 |
| hsa_circ_0031414 | 91 | 228 | 1177 | 1 | 956 | 0.01 | 0 |
| hsa_circ_0070477 | 88 | 1 | 1028 | 8417 | 7390 | 0 | 0 |
| hsa_circ_0070477 | 89 | 1 | 625 | 8417 | 7793 | 0 | 0 |
| hsa_circ_0070477 | 90 | 1 | 537 | 8417 | 7881 | 0 | 0 |
| hsa_circ_0070477 | 91 | 3 | 333 | 8748 | 8418 | 0 | 5.00E-124 |
| hsa_circ_0070477 | 91 | 1 | 514 | 8931 | 8418 | 0 | 0 |
| hsa_circ_0070477 | 92 | 1 | 618 | 9035 | 8418 | 0 | 0 |
| hsa_circ_0070477 | 94 | 223 | 469 | 9179 | 8933 | 0 | 2.00E-100 |

novel_circ_015564 novel_circ_015567 novel_circ_015568 novel_circ_015569 novel_circ_015570 novel_circ_015571 novel_circ_015572 novel_circ_015573 novel_circ_015574 novel_circ_015575 novel_circ_015576 novel_circ_015577 novel_circ_015578 novel_circ_015579 novel_circ_015580 novel_circ_015581 novel_circ_015582 novel_circ_015583 novel_circ_015584 novel_circ_015585 novel_circ_015586 novel_circ_015587 novel_circ_015588 novel_circ_015589 novel_circ_015590 novel_circ_015591 novel_circ_015592 novel_circ_015593 novel_circ_015594 novel_circ_015595 novel_circ_015596 novel_circ_015597 novel_circ_015598 novel_circ_015599 novel_circ_015600 novel_circ_015603 novel_circ_015604 novel_circ_015605 novel_circ_015606 novel_circ_015607 novel_circ_015608 novel_circ_015609 novel_circ_015610 novel_circ_015611 novel_circ_015612 novel_circ_015613 novel_circ_015614 novel_circ_015615 novel_circ_015616 novel_circ_015617 novel_circ_015618 novel_circ_015619 novel_circ_015620 novel_circ_015621

| hsa_circ_0070477 | 94 | 223 | 366 | 9179 | 9036 | 0 | 5.00E-55 |
| --- | --- | --- | --- | --- | --- | --- | --- |
| hsa_circ_0086289 | 93 | 1 | 559 | 1618 | 1060 | 0 | 0 |
| hsa_circ_0086289 | 92 | 1 | 776 | 1618 | 843 | 0 | 0 |
| hsa_circ_0086291 | 93 | 1 | 453 | 674 | 222 | 0 | 0 |
| hsa_circ_0086291 | 93 | 1 | 670 | 674 | 5 | 0 | 0 |
| hsa_circ_0086289 | 91 | 1 | 943 | 1059 | 117 | 0 | 0 |
| hsa_circ_0086289 | 91 | 1 | 722 | 838 | 117 | 0 | 0 |
| hsa_circ_0086289 | 91 | 1 | 836 | 838 | 3 | 0 | 0 |
| hsa_circ_0086289 | 91 | 1 | 661 | 663 | 3 | 0 | 0 |
| hsa_circ_0086289 | 92 | 1 | 470 | 472 | 3 | 0 | 0 |
| hsa_circ_0086288 | 94 | 1 | 479 | 1146 | 1624 | 0 | 0 |
| hsa_circ_0086288 | 94 | 1 | 775 | 850 | 1624 | 0 | 0 |
| hsa_circ_0086288 | 94 | 3 | 1621 | 3 | 1624 | 0 | 0 |
| hsa_circ_0086280 | 94 | 1 | 2161 | 1013 | 3176 | 0 | 0 |
| hsa_circ_0086288 | 94 | 1 | 296 | 850 | 1145 | 0 | 3.00E-126 |
| hsa_circ_0086280 | 94 | 1 | 2039 | 656 | 2697 | 0 | 0 |
| hsa_circ_0086288 | 94 | 3 | 846 | 3 | 849 | 0 | 0 |
| hsa_circ_0086280 | 94 | 1 | 300 | 1253 | 1552 | 0 | 3.00E-125 |
| hsa_circ_0086280 | 94 | 1 | 744 | 809 | 1552 | 0 | 0 |
| hsa_circ_0086280 | 93 | 1 | 897 | 656 | 1552 | 0 | 0 |
| hsa_circ_0086280 | 93 | 1 | 1272 | 281 | 1552 | 0 | 0 |
| hsa_circ_0086280 | 93 | 1 | 1552 | 1 | 1552 | 0 | 0 |
| hsa_circ_0086280 | 94 | 1 | 1413 | 1 | 1413 | 0 | 0 |
| hsa_circ_0086282 | 92 | 1 | 731 | 1 | 731 | 0 | 0 |
| hsa_circ_0086280 | 92 | 1 | 1011 | 1 | 1011 | 0 | 0 |
| hsa_circ_0086282 | 91 | 1 | 528 | 1 | 528 | 0 | 0 |
| hsa_circ_0086280 | 93 | 1 | 808 | 1 | 808 | 0 | 0 |
| hsa_circ_0086280 | 94 | 1 | 461 | 1 | 461 | 0 | 0 |
| hsa_circ_0086280 | 95 | 1 | 368 | 1 | 368 | 0 | 3.00E-161 |
| hsa_circ_0086277 | 94 | 1 | 556 | 1 | 556 | 0 | 0 |
| hsa_circ_0086276 | 94 | 1 | 576 | 1 | 576 | 0 | 0 |
| hsa_circ_0086280 | 95 | 1 | 280 | 1 | 280 | 0 | 2.00E-122 |
| hsa_circ_0086277 | 94 | 1 | 188 | 1 | 188 | 0 | 2.00E-76 |
| hsa_circ_0086276 | 93 | 1 | 296 | 1 | 296 | 0 | 1.00E-119 |
| hsa_circ_0086276 | 91 | 1 | 188 | 1 | 188 | 0 | 4.00E-68 |
| hsa_circ_0086268 | 94 | 3 | 298 | 1438 | 1733 | 0 | 3.00E-126 |
| hsa_circ_0086268 | 94 | 1 | 928 | 806 | 1733 | 0 | 0 |
| hsa_circ_0086268 | 95 | 1 | 327 | 1109 | 1435 | 0 | 2.00E-143 |
| hsa_circ_0086268 | 94 | 1 | 1435 | 1 | 1435 | 0 | 0 |
| hsa_circ_0086265 | 94 | 2 | 1547 | 1241 | 2786 | 0 | 0 |
| hsa_circ_0086265 | 94 | 1 | 2786 | 1 | 2786 | 0 | 0 |
| hsa_circ_0086268 | 94 | 1 | 440 | 806 | 1245 | 0 | 0 |
| hsa_circ_0086265 | 94 | 1 | 2596 | 1 | 2596 | 0 | 0 |
| hsa_circ_0086268 | 94 | 1 | 442 | 667 | 1108 | 0 | 0 |
| hsa_circ_0086268 | 94 | 1 | 805 | 1 | 805 | 0 | 0 |
| hsa_circ_0086265 | 94 | 1 | 1075 | 1082 | 2156 | 0 | 0 |
| hsa_circ_0086265 | 94 | 1 | 2156 | 1 | 2156 | 0 | 0 |
| hsa_circ_0086265 | 93 | 1 | 1801 | 1 | 1801 | 0 | 0 |
| hsa_circ_0086265 | 93 | 83 | 1883 | 1 | 1801 | 0 | 0 |
| hsa_circ_0086265 | 93 | 1 | 1351 | 1 | 1351 | 0 | 0 |
| hsa_circ_0086266 | 95 | 1 | 706 | 125 | 830 | 0 | 0 |
| hsa_circ_0086266 | 95 | 1 | 830 | 1 | 830 | 0 | 0 |
| hsa_circ_0086265 | 93 | 1 | 1081 | 1 | 1081 | 0 | 0 |
| hsa_circ_0086265 | 93 | 83 | 1163 | 1 | 1081 | 0 | 0 |

novel_circ_015622 novel_circ_015623 novel_circ_015624 novel_circ_015625 novel_circ_015626 novel_circ_015627 novel_circ_015628 novel_circ_015629 novel_circ_015630 novel_circ_015631 novel_circ_015632 novel_circ_015633 novel_circ_015635 novel_circ_015636 novel_circ_015637 novel_circ_015638 novel_circ_015639 novel_circ_015640 novel_circ_015641 novel_circ_015642 novel_circ_015643 novel_circ_015644 novel_circ_015645 novel_circ_015646 novel_circ_015647 novel_circ_015648 novel_circ_015649 novel_circ_015650 novel_circ_015651 novel_circ_015652 novel_circ_015653 novel_circ_015654 novel_circ_015655 novel_circ_015656 novel_circ_015657 novel_circ_015658 novel_circ_015659 novel_circ_015660 novel_circ_015661 novel_circ_015662 novel_circ_015663 novel_circ_015664 novel_circ_015665 novel_circ_015666 novel_circ_015667 novel_circ_015670 novel_circ_015673 novel_circ_015674 novel_circ_015675 novel_circ_015676 novel_circ_015677 novel_circ_015681 novel_circ_015682 novel_circ_015683

| hsa_circ_0086266 | 94 | 1 | 388 | 1 | 388 | 0 | 5.00E-169 |
| --- | --- | --- | --- | --- | --- | --- | --- |
| hsa_circ_0086265 | 91 | 1 | 639 | 1 | 639 | 0 | 0 |
| hsa_circ_0086265 | 91 | 83 | 721 | 1 | 639 | 0 | 0 |
| hsa_circ_0086265 | 90 | 1 | 375 | 1 | 375 | 0 | 2.00E-133 |
| hsa_circ_0086258 | 92 | 1727 | 1856 | 322 | 451 | 0 | 3.00E-44 |
| hsa_circ_0029409 | 89 | 332 | 540 | 18240 | 18033 | 0.01 | 4.00E-67 |
| hsa_circ_0086252 | 97 | 283 | 780 | 1 | 498 | 0 | 0 |
| hsa_circ_0086251 | 87 | 171 | 811 | 641 | 1 | 0.01 | 0 |
| hsa_circ_0086251 | 87 | 129 | 442 | 641 | 328 | 0 | 9.00E-98 |
| hsa_circ_0086251 | 87 | 129 | 769 | 641 | 1 | 0.01 | 0 |
| hsa_circ_0086249 | 92 | 1 | 318 | 109 | 426 | 0 | 4.00E-125 |
| hsa_circ_0086249 | 93 | 186 | 611 | 1 | 426 | 0 | 7.00E-175 |
| hsa_circ_0086248 | 92 | 1 | 486 | 486 | 1 | 0 | 0 |
| hsa_circ_0003860 | 88 | 1 | 1313 | 1322 | 1 | 0.01 | 0 |
| hsa_circ_0003860 | 86 | 1 | 1105 | 1114 | 1 | 0.02 | 0 |
| hsa_circ_0004221 | 95 | 1 | 273 | 587 | 315 | 0 | 1.00E-118 |
| hsa_circ_0004221 | 92 | 1 | 418 | 587 | 170 | 0 | 6.00E-169 |
| hsa_circ_0086245 | 97 | 1 | 128 | 223 | 96 | 0 | 3.00E-53 |
| hsa_circ_0086245 | 97 | 1 | 223 | 223 | 1 | 0 | 2.00E-102 |
| hsa_circ_0086244 | 95 | 1 | 482 | 482 | 1 | 0 | 0 |
| hsa_circ_0086240 | 96 | 1 | 557 | 557 | 1 | 0 | 0 |
| hsa_circ_0086244 | 94 | 1 | 357 | 357 | 1 | 0 | 4.00E-155 |
| hsa_circ_0086240 | 95 | 1 | 432 | 432 | 1 | 0 | 0 |
| hsa_circ_0086237 | 95 | 1 | 614 | 1085 | 472 | 0 | 0 |
| hsa_circ_0086237 | 96 | 1 | 734 | 1085 | 352 | 0 | 0 |
| hsa_circ_0086234 | 97 | 1 | 1488 | 1488 | 1 | 0 | 0 |
| hsa_circ_0086233 | 97 | 1 | 1697 | 1697 | 1 | 0 | 0 |
| hsa_circ_0086244 | 93 | 1 | 259 | 259 | 1 | 0 | 8.00E-106 |
| hsa_circ_0086238 | 95 | 1 | 636 | 636 | 1 | 0 | 0 |
| hsa_circ_0086234 | 97 | 1 | 1240 | 1390 | 151 | 0 | 0 |
| hsa_circ_0086234 | 97 | 1 | 1390 | 1390 | 1 | 0 | 0 |
| hsa_circ_0086233 | 97 | 1 | 1599 | 1599 | 1 | 0 | 0 |
| hsa_circ_0086238 | 97 | 1 | 377 | 377 | 1 | 0 | 1.00E-179 |
| hsa_circ_0086233 | 97 | 1 | 1340 | 1340 | 1 | 0 | 0 |
| hsa_circ_0086230 | 97 | 1 | 1494 | 1494 | 1 | 0 | 0 |
| hsa_circ_0086237 | 97 | 1 | 653 | 653 | 1 | 0 | 0 |
| hsa_circ_0086234 | 98 | 1 | 906 | 1056 | 151 | 0 | 0 |
| hsa_circ_0086234 | 97 | 1 | 1056 | 1056 | 1 | 0 | 0 |
| hsa_circ_0086233 | 97 | 1 | 1265 | 1265 | 1 | 0 | 0 |
| hsa_circ_0086230 | 97 | 1 | 1419 | 1419 | 1 | 0 | 0 |
| hsa_circ_0086234 | 98 | 1 | 724 | 874 | 151 | 0 | 0 |
| hsa_circ_0086234 | 98 | 1 | 874 | 874 | 1 | 0 | 0 |
| hsa_circ_0086233 | 98 | 1 | 1083 | 1083 | 1 | 0 | 0 |
| hsa_circ_0086234 | 98 | 1 | 754 | 754 | 1 | 0 | 0 |
| hsa_circ_0086233 | 98 | 1 | 963 | 963 | 1 | 0 | 0 |
| hsa_circ_0003163 | 77 | 11890 | 12234 | 210722 | 210368 | 0.04 | 2.00E-44 |
| hsa_circ_0086214 | 95 | 1 | 873 | 124 | 1002 | 0.01 | 0 |
| hsa_circ_0086214 | 93 | 1 | 243 | 124 | 366 | 0 | 1.00E-98 |
| hsa_circ_0088101 | 76 | 40897 | 41119 | 15535 | 15313 | 0.02 | 1.00E-21 |
| hsa_circ_0088101 | 76 | 40897 | 41119 | 15535 | 15313 | 0.02 | 1.00E-21 |
| hsa_circ_0088101 | 76 | 33584 | 33806 | 15535 | 15313 | 0.02 | 1.00E-21 |
| hsa_circ_0029815 | 89 | 26049 | 26564 | 543 | 3 | 0.05 | 0 |
| hsa_circ_0029815 | 89 | 26049 | 26564 | 543 | 3 | 0.05 | 0 |
| hsa_circ_0029815 | 89 | 26049 | 26564 | 543 | 3 | 0.05 | 0 |

novel_circ_015684 novel_circ_015685 novel_circ_015687 novel_circ_015688 novel_circ_015689 novel_circ_015690 novel_circ_015691 novel_circ_015692 novel_circ_015693 novel_circ_015694 novel_circ_015695 novel_circ_015696 novel_circ_015697 novel_circ_015698 novel_circ_015699 novel_circ_015701 novel_circ_015702 novel_circ_015703 novel_circ_015704 novel_circ_015705 novel_circ_015707 novel_circ_015708 novel_circ_015709 novel_circ_015710 novel_circ_015711 novel_circ_015712 novel_circ_015713 novel_circ_015714 novel_circ_015715 novel_circ_015716 novel_circ_015717 novel_circ_015718 novel_circ_015719 novel_circ_015720 novel_circ_015721 novel_circ_015722 novel_circ_015723 novel_circ_015724 novel_circ_015725 novel_circ_015726 novel_circ_015727 novel_circ_015728 novel_circ_015729 novel_circ_015730 novel_circ_015731 novel_circ_015732 novel_circ_015733 novel_circ_015734 novel_circ_015735 novel_circ_015736 novel_circ_015737 novel_circ_015739 novel_circ_015740 novel_circ_015741

| hsa_circ_0029815 | 89 | 26049 | 26564 | 543 | 3 | 0.05 | 0 |
| --- | --- | --- | --- | --- | --- | --- | --- |
| hsa_circ_0029815 | 89 | 26049 | 26564 | 543 | 3 | 0.05 | 0 |
| hsa_circ_0029815 | 89 | 12922 | 13437 | 543 | 3 | 0.05 | 0 |
| hsa_circ_0086208 | 86 | 1 | 546 | 332 | 856 | 0.04 | 2.00E-155 |
| hsa_circ_0086208 | 90 | 1 | 877 | 1 | 856 | 0.02 | 0 |
| hsa_circ_0086208 | 90 | 243 | 1119 | 1 | 856 | 0.02 | 0 |
| hsa_circ_0086208 | 96 | 1 | 331 | 1 | 331 | 0 | 2.00E-152 |
| hsa_circ_0086206 | 95 | 1 | 409 | 1138 | 1546 | 0 | 1.00E-180 |
| hsa_circ_0086207 | 94 | 1 | 955 | 102 | 1056 | 0 | 0 |
| hsa_circ_0086207 | 95 | 1 | 699 | 250 | 948 | 0 | 0 |
| hsa_circ_0086207 | 95 | 1 | 847 | 102 | 948 | 0 | 0 |
| hsa_circ_0086205 | 94 | 1 | 1138 | 692 | 1829 | 0 | 0 |
| hsa_circ_0086205 | 96 | 1 | 646 | 692 | 1337 | 0 | 0 |
| hsa_circ_0086202 | 92 | 4 | 1738 | 69 | 1791 | 0.01 | 0 |
| hsa_circ_0086202 | 90 | 4 | 1092 | 69 | 1145 | 0.02 | 0 |
| hsa_circ_0007075 | 90 | 1 | 451 | 1 | 451 | 0 | 9.00E-164 |
| hsa_circ_0086201 | 93 | 1 | 540 | 1 | 537 | 0.01 | 0 |
| hsa_circ_0086200 | 93 | 3 | 640 | 210 | 844 | 0 | 0 |
| hsa_circ_0086199 | 86 | 7 | 2877 | 167739 | 170591 | 0.01 | 0 |
| hsa_circ_0005062 | 86 | 7 | 2679 | 7 | 2661 | 0.01 | 0 |
| hsa_circ_0086190 | 91 | 672 | 1523 | 1 | 852 | 0 | 0 |
| hsa_circ_0086187 | 91 | 1 | 738 | 269 | 1006 | 0 | 0 |
| hsa_circ_0086189 | 92 | 1 | 248 | 54 | 301 | 0 | 2.00E-96 |
| hsa_circ_0086189 | 92 | 212 | 466 | 47 | 301 | 0 | 1.00E-96 |
| hsa_circ_0086185 | 91 | 185 | 920 | 743 | 8 | 0 | 0 |
| hsa_circ_0086186 | 94 | 1 | 189 | 189 | 1 | 0 | 2.00E-75 |
| hsa_circ_0086185 | 90 | 2 | 499 | 505 | 8 | 0 | 5.00E-180 |
| hsa_circ_0086185 | 90 | 1 | 367 | 374 | 8 | 0 | 1.00E-135 |
| hsa_circ_0086185 | 91 | 1 | 310 | 317 | 8 | 0 | 1.00E-115 |
| hsa_circ_0086185 | 90 | 1 | 258 | 265 | 8 | 0 | 3.00E-90 |
| hsa_circ_0087140 | 95 | 285 | 530 | 4 | 249 | 0 | 6.00E-105 |
| hsa_circ_0087141 | 94 | 414 | 983 | 2 | 571 | 0 | 0 |
| hsa_circ_0087141 | 93 | 1 | 453 | 119 | 571 | 0 | 0 |
| hsa_circ_0087143 | 91 | 1 | 731 | 1 | 731 | 0 | 0 |
| hsa_circ_0087147 | 90 | 18 | 317 | 18 | 317 | 0.01 | 3.00E-108 |
| hsa_circ_0087148 | 90 | 3 | 258 | 613 | 868 | 0 | 4.00E-89 |
| hsa_circ_0087148 | 90 | 3 | 719 | 613 | 1329 | 0 | 0 |
| hsa_circ_0087149 | 91 | 1 | 963 | 576 | 1538 | 0 | 0 |
| hsa_circ_0087153 | 92 | 181 | 513 | 333 | 1 | 0 | 4.00E-133 |
| hsa_circ_0087153 | 92 | 181 | 513 | 333 | 1 | 0 | 3.00E-133 |
| hsa_circ_0087153 | 92 | 633 | 965 | 333 | 1 | 0 | 5.00E-133 |
| hsa_circ_0004129 | 95 | 1 | 146 | 146 | 1 | 0 | 1.00E-57 |
| hsa_circ_0004129 | 92 | 1 | 282 | 282 | 1 | 0 | 7.00E-110 |
| hsa_circ_0004129 | 95 | 1 | 146 | 146 | 1 | 0 | 5.00E-58 |
| hsa_circ_0004129 | 92 | 1 | 282 | 282 | 1 | 0 | 3.00E-110 |
| hsa_circ_0005976 | 90 | 1 | 1262 | 1269 | 1 | 0.04 | 0 |
| hsa_circ_0005976 | 90 | 1 | 1262 | 1269 | 1 | 0.04 | 0 |
| hsa_circ_0087159 | 94 | 171 | 861 | 691 | 1 | 0 | 0 |
| hsa_circ_0087162 | 95 | 171 | 556 | 386 | 1 | 0 | 2.00E-169 |
| hsa_circ_0087163 | 95 | 1 | 237 | 342 | 106 | 0 | 6.00E-102 |
| hsa_circ_0087163 | 94 | 1 | 170 | 342 | 173 | 0 | 7.00E-67 |
| hsa_circ_0087166 | 93 | 23 | 656 | 1 | 634 | 0 | 0 |
| hsa_circ_0087166 | 93 | 1 | 634 | 1 | 634 | 0 | 0 |
| hsa_circ_0087174 | 91 | 1 | 375 | 1 | 375 | 0 | 9.00E-144 |

novel_circ_015742 novel_circ_015743 novel_circ_015744 novel_circ_015745 novel_circ_015746 novel_circ_015747 novel_circ_015748 novel_circ_015749 novel_circ_015750 novel_circ_015774 novel_circ_015775 novel_circ_015776 novel_circ_015777 novel_circ_015778 novel_circ_015779 novel_circ_015780 novel_circ_015781 novel_circ_015782 novel_circ_015786 novel_circ_015788 novel_circ_015789 novel_circ_015790 novel_circ_015791 novel_circ_015792 novel_circ_015793 novel_circ_015794 novel_circ_015795 novel_circ_015796 novel_circ_015797 novel_circ_015798 novel_circ_015799 novel_circ_015801 novel_circ_015802 novel_circ_015806 novel_circ_015807 novel_circ_015808 novel_circ_015809 novel_circ_015812 novel_circ_015813 novel_circ_015814 novel_circ_015815 novel_circ_015816 novel_circ_015817 novel_circ_015818 novel_circ_015820 novel_circ_015821 novel_circ_015824 novel_circ_015825 novel_circ_015826 novel_circ_015827 novel_circ_015828 novel_circ_015829 novel_circ_015830 novel_circ_015841

| hsa_circ_0087174 | 91 | 1 | 785 | 1 | 785 | 0 | 0 |
| --- | --- | --- | --- | --- | --- | --- | --- |
| hsa_circ_0087176 | 92 | 1 | 753 | 1 | 753 | 0 | 0 |
| hsa_circ_0087176 | 92 | 1 | 1097 | 1 | 1097 | 0 | 0 |
| hsa_circ_0087176 | 94 | 2 | 497 | 258 | 753 | 0 | 0 |
| hsa_circ_0087177 | 94 | 1 | 342 | 1 | 342 | 0 | 8.00E-147 |
| hsa_circ_0087178 | 94 | 1 | 228 | 1 | 228 | 0 | 1.00E-93 |
| hsa_circ_0087177 | 94 | 1 | 739 | 687 | 1425 | 0 | 0 |
| hsa_circ_0087177 | 94 | 1 | 492 | 934 | 1425 | 0 | 0 |
| hsa_circ_0087184 | 93 | 1 | 276 | 322 | 597 | 0 | 3.00E-110 |
| hsa_circ_0087193 | 93 | 1 | 502 | 718 | 217 | 0 | 0 |
| hsa_circ_0004492 | 91 | 3 | 703 | 701 | 1 | 0 | 0 |
| hsa_circ_0004492 | 90 | 8 | 1043 | 1039 | 1 | 0 | 0 |
| hsa_circ_0006643 | 97 | 30530 | 30568 | 734808 | 734770 | 0 | 5.00E-08 |
| hsa_circ_0006383 | 95 | 4 | 858 | 855 | 1 | 0 | 0 |
| hsa_circ_0001862 | 94 | 4 | 650 | 647 | 1 | 0 | 0 |
| hsa_circ_0002921 | 94 | 4 | 470 | 467 | 1 | 0 | 0 |
| hsa_circ_0087198 | 92 | 1 | 530 | 299 | 828 | 0 | 0 |
| hsa_circ_0087198 | 92 | 1 | 604 | 299 | 902 | 0 | 0 |
| hsa_circ_0041131 | 88 | 1 | 292 | 1 | 292 | 0 | 1.00E-95 |
| hsa_circ_0040988 | 90 | 1 | 294 | 1 | 294 | 0 | 3.00E-105 |
| hsa_circ_0040988 | 92 | 1 | 194 | 1 | 194 | 0 | 2.00E-71 |
| hsa_circ_0006945 | 89 | 2 | 305 | 2 | 305 | 0 | 2.00E-102 |
| hsa_circ_0040940 | 89 | 1 | 461 | 104 | 564 | 0 | 2.00E-164 |
| hsa_circ_0040830 | 85 | 1 | 796 | 1 | 781 | 0.02 | 0 |
| hsa_circ_0040830 | 89 | 1 | 397 | 1 | 397 | 0 | 6.00E-139 |
| hsa_circ_0040830 | 89 | 155 | 551 | 1 | 397 | 0 | 8.00E-139 |
| hsa_circ_0003098 | 90 | 1 | 293 | 1 | 293 | 0 | 1.00E-104 |
| hsa_circ_0040812 | 83 | 45 | 428 | 49 | 430 | 0.01 | 4.00E-91 |
| hsa_circ_0040801 | 92 | 1 | 626 | 3807 | 3182 | 0 | 0 |
| hsa_circ_0040787 | 85 | 8 | 327 | 322 | 3 | 0 | 1.00E-89 |
| hsa_circ_0040735 | 95 | 1 | 219 | 1 | 219 | 0 | 5.00E-92 |
| hsa_circ_0040705 | 92 | 1 | 462 | 1 | 462 | 0 | 0 |
| hsa_circ_0040700 | 83 | 1 | 1171 | 1 | 1171 | 0.01 | 0 |
| hsa_circ_0040679 | 89 | 12 | 729 | 932 | 214 | 0 | 0 |
| hsa_circ_0040678 | 92 | 481 | 1451 | 971 | 1 | 0 | 0 |
| hsa_circ_0040670 | 89 | 4 | 476 | 632 | 160 | 0 | 2.00E-169 |
| hsa_circ_0040670 | 89 | 4 | 635 | 632 | 1 | 0 | 0 |
| hsa_circ_0040594 | 88 | 1 | 1735 | 1 | 1731 | 0.01 | 0 |
| hsa_circ_0040597 | 88 | 1 | 529 | 341 | 869 | 0 | 2.00E-175 |
| hsa_circ_0040596 | 87 | 1 | 1220 | 1 | 1220 | 0 | 0 |
| hsa_circ_0040594 | 88 | 1 | 1335 | 1 | 1335 | 0 | 0 |
| hsa_circ_0040597 | 89 | 1 | 385 | 219 | 603 | 0 | 6.00E-134 |
| hsa_circ_0040594 | 89 | 1 | 806 | 1 | 806 | 0 | 0 |
| hsa_circ_0040594 | 89 | 1 | 466 | 1 | 466 | 0 | 7.00E-164 |
| hsa_circ_0040581 | 90 | 1 | 279 | 138 | 416 | 0 | 2.00E-98 |
| hsa_circ_0040574 | 94 | 392 | 746 | 355 | 1 | 0 | 3.00E-150 |
| hsa_circ_0040573 | 87 | 3 | 451 | 3 | 451 | 0 | 2.00E-144 |
| hsa_circ_0005109 | 94 | 1 | 355 | 490 | 844 | 0 | 5.00E-154 |
| hsa_circ_0005109 | 94 | 1 | 498 | 347 | 844 | 0 | 0 |
| hsa_circ_0005109 | 93 | 1 | 844 | 1 | 844 | 0 | 0 |
| hsa_circ_0005109 | 93 | 1 | 489 | 1 | 489 | 0 | 0 |
| hsa_circ_0005109 | 92 | 1 | 346 | 1 | 346 | 0 | 5.00E-139 |
| hsa_circ_0087096 | 81 | 5 | 979 | 985 | 11 | 0.01 | 0 |
| hsa_circ_0040551 | 85 | 742 | 1360 | 619 | 1 | 0 | 4.00E-175 |

novel_circ_015842 novel_circ_015844 novel_circ_015845 novel_circ_015846 novel_circ_015847 novel_circ_015848 novel_circ_015849 novel_circ_015852 novel_circ_015853 novel_circ_015854 novel_circ_015855 novel_circ_015856 novel_circ_015857 novel_circ_015858 novel_circ_015859 novel_circ_015860 novel_circ_015861 novel_circ_015862 novel_circ_015864 novel_circ_015865 novel_circ_015867 novel_circ_015868 novel_circ_015869 novel_circ_015870 novel_circ_015871 novel_circ_015872 novel_circ_015873 novel_circ_015874 novel_circ_015875 novel_circ_015876 novel_circ_015877 novel_circ_015878 novel_circ_015879 novel_circ_015880 novel_circ_015881 novel_circ_015882 novel_circ_015883 novel_circ_015884 novel_circ_015885 novel_circ_015892 novel_circ_015893 novel_circ_015894 novel_circ_015895 novel_circ_015896 novel_circ_015897 novel_circ_015898 novel_circ_015899 novel_circ_015900 novel_circ_015901 novel_circ_015902 novel_circ_015903 novel_circ_015904 novel_circ_015905 novel_circ_015906

| hsa_circ_0008913 | 85 | 742 | 1606 | 952 | 88 | 0 | 0 |
| --- | --- | --- | --- | --- | --- | --- | --- |
| hsa_circ_0040540 | 90 | 1 | 464 | 960 | 491 | 0.01 | 2.00E-171 |
| hsa_circ_0055876 | 90 | 10577 | 10927 | 433 | 83 | 0 | 2.00E-123 |
| hsa_circ_0040540 | 94 | 2 | 291 | 491 | 201 | 0 | 3.00E-121 |
| hsa_circ_0040537 | 97 | 1 | 198 | 97 | 292 | 0.01 | 8.00E-90 |
| hsa_circ_0040511 | 88 | 1 | 488 | 488 | 1 | 0 | 4.00E-161 |
| hsa_circ_0040505 | 90 | 6 | 266 | 265 | 4 | 0 | 1.00E-93 |
| hsa_circ_0040490 | 83 | 10 | 495 | 489 | 1 | 0.03 | 3.00E-122 |
| hsa_circ_0040471 | 96 | 1 | 336 | 1235 | 900 | 0 | 8.00E-152 |
| hsa_circ_0040471 | 92 | 1 | 891 | 1115 | 225 | 0 | 0 |
| hsa_circ_0040472 | 91 | 1 | 675 | 675 | 1 | 0 | 0 |
| hsa_circ_0040472 | 90 | 1 | 399 | 399 | 1 | 0 | 2.00E-143 |
| hsa_circ_0040467 | 88 | 1 | 256 | 1682 | 1427 | 0 | 2.00E-82 |
| hsa_circ_0040467 | 90 | 1 | 394 | 1682 | 1289 | 0 | 3.00E-142 |
| hsa_circ_0040467 | 91 | 1 | 414 | 1029 | 616 | 0 | 2.00E-158 |
| hsa_circ_0040467 | 92 | 1 | 676 | 1029 | 354 | 0 | 0 |
| hsa_circ_0040467 | 91 | 1 | 921 | 1029 | 109 | 0 | 0 |
| hsa_circ_0040467 | 91 | 4 | 600 | 705 | 109 | 0 | 0 |
| hsa_circ_0040128 | 92 | 1 | 380 | 1 | 380 | 0 | 2.00E-149 |
| hsa_circ_0040135 | 92 | 1 | 583 | 1 | 583 | 0 | 0 |
| hsa_circ_0040204 | 89 | 6 | 542 | 546 | 1 | 0.02 | 0 |
| hsa_circ_0040202 | 92 | 1 | 337 | 1013 | 677 | 0 | 5.00E-134 |
| hsa_circ_0040202 | 91 | 1 | 483 | 1159 | 677 | 0 | 0 |
| hsa_circ_0040207 | 91 | 95 | 595 | 1 | 501 | 0 | 0 |
| hsa_circ_0040207 | 91 | 1 | 501 | 1 | 501 | 0 | 0 |
| hsa_circ_0040208 | 90 | 1 | 332 | 1 | 332 | 0 | 3.00E-121 |
| hsa_circ_0040208 | 91 | 1 | 447 | 1 | 447 | 0 | 5.00E-175 |
| hsa_circ_0040217 | 91 | 339 | 556 | 1 | 218 | 0 | 1.00E-77 |
| hsa_circ_0040209 | 90 | 246 | 972 | 1 | 727 | 0 | 0 |
| hsa_circ_0040254 | 94 | 1 | 315 | 1 | 315 | 0 | 2.00E-133 |
| hsa_circ_0040285 | 94 | 1 | 308 | 1 | 308 | 0 | 1.00E-129 |
| hsa_circ_0040285 | 93 | 1 | 608 | 1 | 608 | 0 | 0 |
| hsa_circ_0040285 | 92 | 1 | 883 | 1 | 883 | 0 | 0 |
| hsa_circ_0040285 | 92 | 1 | 1061 | 1 | 1061 | 0 | 0 |
| hsa_circ_0040285 | 92 | 1 | 1424 | 1 | 1424 | 0 | 0 |
| hsa_circ_0040288 | 91 | 1 | 575 | 1 | 575 | 0 | 0 |
| hsa_circ_0040290 | 90 | 1 | 419 | 1 | 419 | 0 | 4.00E-151 |
| hsa_circ_0040290 | 89 | 1 | 275 | 145 | 419 | 0 | 1.00E-94 |
| hsa_circ_0040288 | 92 | 1 | 1225 | 579 | 1803 | 0 | 0 |
| hsa_circ_0040336 | 86 | 1 | 1087 | 2200 | 1102 | 0.01 | 0 |
| hsa_circ_0040338 | 92 | 1 | 832 | 832 | 1 | 0 | 0 |
| hsa_circ_0040338 | 91 | 1 | 1033 | 1033 | 1 | 0 | 0 |
| hsa_circ_0005638 | 90 | 1 | 495 | 495 | 1 | 0 | 5.00E-180 |
| hsa_circ_0005638 | 90 | 1 | 808 | 808 | 1 | 0 | 0 |
| hsa_circ_0040352 | 90 | 1 | 157 | 157 | 1 | 0 | 1.00E-52 |
| hsa_circ_0040352 | 90 | 1 | 893 | 893 | 1 | 0 | 0 |
| hsa_circ_0040357 | 89 | 3084 | 3664 | 581 | 1 | 0 | 0 |
| hsa_circ_0040357 | 90 | 1 | 736 | 736 | 1 | 0 | 0 |
| hsa_circ_0040352 | 91 | 1 | 1471 | 1634 | 158 | 0 | 0 |
| hsa_circ_0000715 | 91 | 3084 | 3461 | 378 | 1 | 0 | 4.00E-144 |
| hsa_circ_0000715 | 92 | 1 | 533 | 533 | 1 | 0 | 0 |
| hsa_circ_0040368 | 95 | 1 | 368 | 368 | 1 | 0 | 7.00E-163 |
| hsa_circ_0040368 | 96 | 1 | 771 | 870 | 100 | 0 | 0 |
| hsa_circ_0040375 | 97 | 1 | 374 | 374 | 1 | 0 | 3.00E-176 |

novel_circ_015907 novel_circ_015908 novel_circ_015909 novel_circ_015910 novel_circ_015911 novel_circ_015912 novel_circ_015913 novel_circ_015914 novel_circ_015915 novel_circ_015916 novel_circ_015917 novel_circ_015918 novel_circ_015919 novel_circ_015920 novel_circ_015921 novel_circ_015924 novel_circ_015925 novel_circ_015926 novel_circ_015927 novel_circ_015931 novel_circ_015932 novel_circ_015933 novel_circ_015934 novel_circ_015935 novel_circ_015936 novel_circ_015937 novel_circ_015938 novel_circ_015939 novel_circ_015943 novel_circ_015944 novel_circ_015945 novel_circ_015946 novel_circ_015947 novel_circ_015949 novel_circ_015950 novel_circ_015951 novel_circ_015952 novel_circ_015953 novel_circ_015954 novel_circ_015957 novel_circ_015958 novel_circ_015959 novel_circ_015960 novel_circ_015961 novel_circ_015962 novel_circ_015963 novel_circ_015964 novel_circ_015965 novel_circ_015966 novel_circ_015967 novel_circ_015968 novel_circ_015970 novel_circ_015971 novel_circ_015972

| hsa_circ_0040375 | 96 | 1 | 502 | 502 | 1 | 0 | 0 |
| --- | --- | --- | --- | --- | --- | --- | --- |
| hsa_circ_0040375 | 96 | 1 | 715 | 715 | 1 | 0 | 0 |
| hsa_circ_0040375 | 96 | 8514 | 9229 | 716 | 1 | 0 | 0 |
| hsa_circ_0040375 | 96 | 1 | 1025 | 1025 | 1 | 0 | 0 |
| hsa_circ_0040377 | 96 | 1 | 341 | 588 | 248 | 0 | 3.00E-156 |
| hsa_circ_0040377 | 96 | 5675 | 6016 | 589 | 248 | 0 | 2.00E-155 |
| hsa_circ_0040377 | 96 | 1 | 707 | 954 | 248 | 0 | 0 |
| hsa_circ_0040377 | 96 | 1 | 1308 | 1555 | 248 | 0 | 0 |
| hsa_circ_0040384 | 96 | 1 | 598 | 657 | 60 | 0 | 0 |
| hsa_circ_0040384 | 96 | 1 | 723 | 782 | 60 | 0 | 0 |
| hsa_circ_0040384 | 96 | 1 | 927 | 986 | 60 | 0 | 0 |
| hsa_circ_0040394 | 98 | 1 | 329 | 645 | 317 | 0 | 1.00E-159 |
| hsa_circ_0040402 | 92 | 1 | 284 | 1 | 284 | 0 | 1.00E-109 |
| hsa_circ_0040402 | 93 | 1 | 372 | 1 | 372 | 0 | 2.00E-153 |
| hsa_circ_0040397 | 89 | 1 | 1538 | 870 | 2401 | 0.02 | 0 |
| hsa_circ_0040435 | 96 | 7 | 3244 | 3256 | 1 | 0.01 | 0 |
| hsa_circ_0048748 | 88 | 1 | 486 | 623 | 138 | 0 | 5.00E-165 |
| hsa_circ_0048757 | 83 | 8 | 269 | 262 | 1 | 0.02 | 4.00E-59 |
| hsa_circ_0048758 | 84 | 1 | 272 | 1 | 272 | 0 | 1.00E-69 |
| hsa_circ_0003679 | 91 | 6 | 293 | 6 | 293 | 0 | 7.00E-107 |
| hsa_circ_0036687 | 93 | 1 | 342 | 1 | 342 | 0 | 8.00E-142 |
| hsa_circ_0036685 | 94 | 1 | 528 | 301 | 828 | 0 | 0 |
| hsa_circ_0036692 | 93 | 1 | 359 | 353 | 711 | 0 | 1.00E-149 |
| hsa_circ_0036692 | 93 | 2 | 266 | 447 | 711 | 0 | 8.00E-106 |
| hsa_circ_0036692 | 93 | 1753 | 1838 | 626 | 711 | 0 | 4.00E-27 |
| hsa_circ_0036697 | 90 | 6 | 522 | 6 | 522 | 0 | 0 |
| hsa_circ_0036706 | 90 | 1 | 357 | 1 | 357 | 0 | 9.00E-132 |
| hsa_circ_0036706 | 90 | 1 | 626 | 1 | 626 | 0 | 0 |
| hsa_circ_0036729 | 88 | 3 | 886 | 508 | 1391 | 0 | 0 |
| hsa_circ_0036748 | 79 | 4 | 794 | 5150 | 4329 | 0.06 | 5.00E-152 |
| hsa_circ_0036748 | 93 | 205 | 514 | 5225 | 4916 | 0 | 2.00E-125 |
| hsa_circ_0002080 | 95 | 1 | 204 | 204 | 1 | 0 | 2.00E-85 |
| hsa_circ_0002080 | 95 | 1 | 204 | 204 | 1 | 0 | 7.00E-87 |
| hsa_circ_0025824 | 81 | 5653 | 5782 | 74283 | 74153 | 0.05 | 3.00E-19 |
| hsa_circ_0036755 | 93 | 1 | 1491 | 1 | 1486 | 0.01 | 0 |
| hsa_circ_0036755 | 97 | 1 | 367 | 1487 | 1853 | 0 | 1.00E-175 |
| hsa_circ_0036759 | 92 | 3 | 594 | 592 | 1 | 0 | 0 |
| hsa_circ_0036759 | 92 | 4 | 760 | 757 | 1 | 0 | 0 |
| hsa_circ_0036759 | 92 | 1 | 852 | 852 | 1 | 0 | 0 |
| hsa_circ_0036871 | 91 | 1 | 330 | 197 | 535 | 0.03 | 6.00E-122 |
| hsa_circ_0036871 | 95 | 269 | 655 | 1 | 387 | 0 | 2.00E-171 |
| hsa_circ_0036870 | 92 | 237 | 749 | 4 | 516 | 0 | 0 |
| hsa_circ_0036870 | 92 | 237 | 749 | 4 | 516 | 0 | 0 |
| hsa_circ_0036867 | 92 | 666 | 1338 | 1 | 673 | 0 | 0 |
| hsa_circ_0036867 | 92 | 1 | 481 | 193 | 673 | 0 | 0 |
| hsa_circ_0036867 | 92 | 666 | 1338 | 1 | 673 | 0 | 0 |
| hsa_circ_0036867 | 92 | 927 | 1599 | 1 | 673 | 0 | 0 |
| hsa_circ_0036867 | 92 | 1884 | 2556 | 1 | 673 | 0 | 0 |
| hsa_circ_0036867 | 92 | 666 | 1189 | 1 | 524 | 0 | 0 |
| hsa_circ_0036867 | 90 | 666 | 976 | 1 | 311 | 0 | 2.00E-112 |
| hsa_circ_0036867 | 90 | 927 | 1237 | 1 | 311 | 0 | 3.00E-112 |
| hsa_circ_0036863 | 87 | 1 | 679 | 355 | 1033 | 0 | 0 |
| hsa_circ_0036863 | 87 | 6 | 1033 | 6 | 1033 | 0 | 0 |
| hsa_circ_0036863 | 89 | 6 | 349 | 6 | 349 | 0 | 3.00E-116 |

novel_circ_015973 novel_circ_015974 novel_circ_015975 novel_circ_015976 novel_circ_015977 novel_circ_015978 novel_circ_015979 novel_circ_015980 novel_circ_015982 novel_circ_015983 novel_circ_015986 novel_circ_015987 novel_circ_015988 novel_circ_015991 novel_circ_015992 novel_circ_015993 novel_circ_015994 novel_circ_015995 novel_circ_015996 novel_circ_015997 novel_circ_015998 novel_circ_016003 novel_circ_016004 novel_circ_016005 novel_circ_016006 novel_circ_016008 novel_circ_016009 novel_circ_016010 novel_circ_016011 novel_circ_016012 novel_circ_016013 novel_circ_016016 novel_circ_016017 novel_circ_016018 novel_circ_016019 novel_circ_016020 novel_circ_016021 novel_circ_016022 novel_circ_016023 novel_circ_016024 novel_circ_016025 novel_circ_016026 novel_circ_016027 novel_circ_016028 novel_circ_016029 novel_circ_016030 novel_circ_016031 novel_circ_016032 novel_circ_016033 novel_circ_016034 novel_circ_016035 novel_circ_016036 novel_circ_016037 novel_circ_016038

| hsa_circ_0036858 | 90 | 1 | 486 | 1 | 486 | 0 | 1.00E-176 |
| --- | --- | --- | --- | --- | --- | --- | --- |
| hsa_circ_0036848 | 94 | 1 | 218 | 1 | 218 | 0 | 2.00E-91 |
| hsa_circ_0036835 | 94 | 1 | 1756 | 1 | 1756 | 0 | 0 |
| hsa_circ_0036839 | 93 | 1 | 963 | 234 | 1196 | 0 | 0 |
| hsa_circ_0036839 | 93 | 1 | 840 | 234 | 1073 | 0 | 0 |
| hsa_circ_0036833 | 93 | 1 | 980 | 1 | 980 | 0 | 0 |
| hsa_circ_0036813 | 91 | 1 | 1498 | 165 | 1662 | 0 | 0 |
| hsa_circ_0036813 | 91 | 1 | 1498 | 165 | 1662 | 0 | 0 |
| hsa_circ_0036599 | 96 | 4 | 260 | 257 | 1 | 0 | 2.00E-116 |
| hsa_circ_0036600 | 89 | 284 | 2291 | 7 | 2005 | 0.01 | 0 |
| hsa_circ_0036619 | 94 | 1 | 389 | 1 | 392 | 0.01 | 4.00E-165 |
| hsa_circ_0036622 | 89 | 1 | 415 | 1 | 418 | 0.01 | 2.00E-144 |
| hsa_circ_0036576 | 95 | 700 | 836 | 2014 | 1878 | 0 | 4.00E-54 |
| hsa_circ_0008353 | 86 | 8 | 495 | 8 | 483 | 0.03 | 2.00E-139 |
| hsa_circ_0008353 | 86 | 8 | 495 | 8 | 483 | 0.03 | 3.00E-139 |
| hsa_circ_0008353 | 86 | 8 | 495 | 8 | 483 | 0.03 | 4.00E-139 |
| hsa_circ_0008353 | 89 | 34 | 321 | 196 | 483 | 0.01 | 4.00E-95 |
| hsa_circ_0008353 | 89 | 34 | 321 | 196 | 483 | 0.01 | 8.00E-95 |
| hsa_circ_0008353 | 89 | 34 | 321 | 196 | 483 | 0.01 | 2.00E-94 |
| hsa_circ_0036584 | 93 | 1 | 193 | 193 | 1 | 0 | 1.00E-75 |
| hsa_circ_0036584 | 92 | 1 | 654 | 654 | 1 | 0 | 0 |
| hsa_circ_0000646 | 96 | 1 | 445 | 445 | 1 | 0 | 0 |
| hsa_circ_0000646 | 97 | 1 | 522 | 522 | 1 | 0 | 0 |
| hsa_circ_0036588 | 97 | 1 | 375 | 375 | 1 | 0 | 2.00E-178 |
| hsa_circ_0068841 | 91 | 167 | 487 | 609 | 929 | 0 | 1.00E-121 |
| hsa_circ_0068851 | 90 | 1 | 535 | 520 | 1 | 0.03 | 0 |
| hsa_circ_0068851 | 93 | 1 | 453 | 520 | 68 | 0 | 0 |
| hsa_circ_0068882 | 91 | 1 | 342 | 4427 | 4086 | 0 | 4.00E-130 |
| hsa_circ_0068882 | 91 | 1 | 486 | 4571 | 4086 | 0 | 0 |
| hsa_circ_0068882 | 87 | 1 | 937 | 5022 | 4086 | 0.01 | 0 |
| hsa_circ_0068882 | 87 | 1550 | 2486 | 5022 | 4086 | 0.01 | 0 |
| hsa_circ_0068889 | 90 | 360 | 1151 | 173 | 964 | 0 | 0 |
| hsa_circ_0068889 | 90 | 360 | 1801 | 173 | 1614 | 0 | 0 |
| hsa_circ_0068889 | 90 | 126 | 917 | 173 | 964 | 0 | 0 |
| hsa_circ_0068893 | 90 | 1 | 789 | 1 | 789 | 0 | 0 |
| hsa_circ_0068893 | 91 | 1 | 956 | 1 | 956 | 0 | 0 |
| hsa_circ_0068893 | 90 | 1 | 1439 | 1 | 1439 | 0 | 0 |
| hsa_circ_0068893 | 89 | 1 | 1584 | 1 | 1584 | 0 | 0 |
| hsa_circ_0068893 | 90 | 1 | 1703 | 1 | 1703 | 0 | 0 |
| hsa_circ_0068893 | 90 | 1 | 1913 | 1 | 1910 | 0 | 0 |
| hsa_circ_0068892 | 91 | 1 | 2140 | 1 | 2137 | 0 | 0 |
| hsa_circ_0068892 | 86 | 1 | 3650 | 1 | 3639 | 0.01 | 0 |
| hsa_circ_0068892 | 86 | 1 | 3650 | 1 | 3639 | 0.01 | 0 |
| hsa_circ_0003112 | 91 | 1 | 813 | 1 | 813 | 0 | 0 |
| hsa_circ_0068895 | 91 | 1 | 650 | 1 | 650 | 0 | 0 |
| hsa_circ_0068896 | 89 | 1 | 483 | 1 | 483 | 0 | 1.00E-166 |
| hsa_circ_0068893 | 92 | 2679 | 4169 | 1911 | 3401 | 0 | 0 |
| hsa_circ_0068893 | 91 | 1 | 637 | 1911 | 2547 | 0 | 0 |
| hsa_circ_0068897 | 93 | 1 | 1034 | 326 | 1359 | 0 | 0 |
| hsa_circ_0068897 | 94 | 3 | 854 | 508 | 1359 | 0 | 0 |
| hsa_circ_0068897 | 92 | 1 | 491 | 869 | 1359 | 0 | 0 |
| hsa_circ_0068893 | 92 | 1 | 740 | 2911 | 3650 | 0 | 0 |
| hsa_circ_0068897 | 91 | 1 | 387 | 973 | 1359 | 0 | 2.00E-148 |
| hsa_circ_0007906 | 92 | 1 | 249 | 1 | 249 | 0 | 6.00E-97 |

novel_circ_016040 novel_circ_016041 novel_circ_016042 novel_circ_016043 novel_circ_016044 novel_circ_016045 novel_circ_016047 novel_circ_016048 novel_circ_016052 novel_circ_016053 novel_circ_016054 novel_circ_016055 novel_circ_016056 novel_circ_016057 novel_circ_016058 novel_circ_016059 novel_circ_016060 novel_circ_016061 novel_circ_016062 novel_circ_016063 novel_circ_016064 novel_circ_016065 novel_circ_016066 novel_circ_016067 novel_circ_016068 novel_circ_016069 novel_circ_016070 novel_circ_016071 novel_circ_016072 novel_circ_016073 novel_circ_016074 novel_circ_016075 novel_circ_016076 novel_circ_016077 novel_circ_016078 novel_circ_016079 novel_circ_016080 novel_circ_016081 novel_circ_016082 novel_circ_016083 novel_circ_016084 novel_circ_016085 novel_circ_016086 novel_circ_016088 novel_circ_016119 novel_circ_016120 novel_circ_016121 novel_circ_016122 novel_circ_016123 novel_circ_016124 novel_circ_016125 novel_circ_016126 novel_circ_016127 novel_circ_016128

| hsa_circ_0068912 | 90 | 5065 | 5293 | 229 | 1 | 0 | 2.00E-79 |
| --- | --- | --- | --- | --- | --- | --- | --- |
| hsa_circ_0068912 | 90 | 11897 | 12125 | 229 | 1 | 0 | 4.00E-79 |
| hsa_circ_0068916 | 93 | 7 | 623 | 617 | 1 | 0 | 0 |
| hsa_circ_0068916 | 93 | 148 | 764 | 617 | 1 | 0 | 0 |
| hsa_circ_0068916 | 92 | 7 | 521 | 617 | 103 | 0 | 0 |
| hsa_circ_0068916 | 92 | 7 | 341 | 617 | 283 | 0 | 3.00E-131 |
| hsa_circ_0004803 | 85 | 1 | 277 | 1 | 281 | 0.05 | 7.00E-72 |
| hsa_circ_0004803 | 86 | 1 | 357 | 1 | 361 | 0.04 | 3.00E-106 |
| hsa_circ_0068922 | 91 | 159 | 561 | 1 | 403 | 0 | 4.00E-152 |
| hsa_circ_0068922 | 92 | 159 | 686 | 1 | 528 | 0 | 0 |
| hsa_circ_0068922 | 91 | 159 | 912 | 1 | 754 | 0 | 0 |
| hsa_circ_0068922 | 90 | 159 | 1854 | 1 | 1696 | 0 | 0 |
| hsa_circ_0068924 | 92 | 1 | 360 | 1 | 360 | 0 | 4.00E-140 |
| hsa_circ_0068922 | 90 | 1 | 942 | 169 | 1110 | 0 | 0 |
| hsa_circ_0068924 | 90 | 1 | 273 | 236 | 508 | 0 | 3.00E-95 |
| hsa_circ_0068922 | 91 | 1 | 351 | 404 | 754 | 0 | 4.00E-130 |
| hsa_circ_0068922 | 89 | 15 | 932 | 527 | 1444 | 0 | 0 |
| hsa_circ_0068922 | 88 | 1 | 226 | 529 | 754 | 0 | 2.00E-72 |
| hsa_circ_0068922 | 89 | 1 | 582 | 529 | 1110 | 0 | 0 |
| hsa_circ_0068922 | 89 | 1 | 916 | 529 | 1444 | 0 | 0 |
| hsa_circ_0068922 | 89 | 1 | 1168 | 529 | 1696 | 0 | 0 |
| hsa_circ_0068922 | 89 | 1 | 1169 | 529 | 1697 | 0 | 0 |
| hsa_circ_0068922 | 90 | 1 | 2107 | 529 | 2641 | 0.01 | 0 |
| hsa_circ_0068920 | 89 | 1 | 3203 | 812 | 4020 | 0.01 | 0 |
| hsa_circ_0068927 | 90 | 1 | 434 | 1 | 434 | 0 | 4.00E-161 |
| hsa_circ_0068927 | 89 | 1 | 1021 | 1 | 1021 | 0 | 0 |
| hsa_circ_0068929 | 90 | 1 | 356 | 1 | 356 | 0 | 3.00E-126 |
| hsa_circ_0003869 | 88 | 1 | 334 | 1 | 334 | 0 | 5.00E-109 |
| hsa_circ_0003869 | 89 | 1 | 586 | 1 | 586 | 0 | 0 |
| hsa_circ_0068927 | 89 | 1 | 2621 | 435 | 3061 | 0.01 | 0 |
| hsa_circ_0068929 | 93 | 1 | 451 | 691 | 1141 | 0 | 0 |
| hsa_circ_0068927 | 88 | 1 | 1280 | 1782 | 3061 | 0 | 0 |
| hsa_circ_0068927 | 88 | 1 | 1380 | 1782 | 3143 | 0.02 | 0 |
| hsa_circ_0068927 | 88 | 1 | 1380 | 1782 | 3143 | 0.02 | 0 |
| hsa_circ_0068927 | 88 | 1 | 1380 | 1782 | 3143 | 0.02 | 0 |
| hsa_circ_0068927 | 88 | 1 | 1096 | 1966 | 3061 | 0 | 0 |
| hsa_circ_0068927 | 87 | 1 | 1196 | 1966 | 3143 | 0.02 | 0 |
| hsa_circ_0003758 | 87 | 1 | 865 | 1 | 865 | 0 | 0 |
| hsa_circ_0068927 | 86 | 1 | 965 | 2197 | 3143 | 0.02 | 0 |
| hsa_circ_0068927 | 95 | 1 | 82 | 3062 | 3143 | 0 | 6.00E-29 |
| hsa_circ_0068943 | 92 | 1 | 378 | 1 | 378 | 0 | 9.00E-147 |
| hsa_circ_0068943 | 92 | 1 | 530 | 1 | 530 | 0 | 0 |
| hsa_circ_0002630 | 95 | 1 | 315 | 1 | 315 | 0 | 2.00E-138 |
| hsa_circ_0068948 | 98 | 1 | 193 | 523 | 715 | 0 | 4.00E-92 |
| hsa_circ_0068969 | 95 | 1 | 345 | 1 | 345 | 0 | 8.00E-152 |
| hsa_circ_0068969 | 94 | 1 | 484 | 1 | 484 | 0 | 0 |
| hsa_circ_0068969 | 92 | 1 | 805 | 1 | 805 | 0 | 0 |
| hsa_circ_0068969 | 90 | 1 | 1010 | 1 | 1010 | 0 | 0 |
| hsa_circ_0068969 | 90 | 1 | 1134 | 1 | 1134 | 0 | 0 |
| hsa_circ_0068969 | 90 | 1 | 1480 | 1 | 1480 | 0 | 0 |
| hsa_circ_0068969 | 89 | 1 | 2526 | 1 | 2526 | 0 | 0 |
| hsa_circ_0068969 | 89 | 1 | 2682 | 1 | 2682 | 0 | 0 |
| hsa_circ_0068969 | 89 | 1 | 3032 | 1 | 3032 | 0 | 0 |
| hsa_circ_0068969 | 94 | 1 | 480 | 5 | 484 | 0 | 0 |

novel_circ_016129 novel_circ_016130 novel_circ_016131 novel_circ_016132 novel_circ_016133 novel_circ_016134 novel_circ_016135 novel_circ_016136 novel_circ_016137 novel_circ_016138 novel_circ_016139 novel_circ_016140 novel_circ_016141 novel_circ_016142 novel_circ_016143 novel_circ_016144 novel_circ_016145 novel_circ_016146 novel_circ_016147 novel_circ_016148 novel_circ_016149 novel_circ_016150 novel_circ_016151 novel_circ_016152 novel_circ_016153 novel_circ_016154 novel_circ_016155 novel_circ_016156 novel_circ_016157 novel_circ_016158 novel_circ_016159 novel_circ_016160 novel_circ_016161 novel_circ_016162 novel_circ_016163 novel_circ_016164 novel_circ_016165 novel_circ_016166 novel_circ_016167 novel_circ_016168 novel_circ_016169 novel_circ_016171 novel_circ_016172 novel_circ_016173 novel_circ_016174 novel_circ_016175 novel_circ_016176 novel_circ_016177 novel_circ_016178 novel_circ_016184 novel_circ_016185 novel_circ_016186 novel_circ_016187 novel_circ_016188

| hsa_circ_0068969 | 93 | 1 | 400 | 85 | 484 | 0 | 1.00E-165 |
| --- | --- | --- | --- | --- | --- | --- | --- |
| hsa_circ_0068971 | 91 | 1 | 279 | 1 | 279 | 0 | 3.00E-105 |
| hsa_circ_0068979 | 88 | 1 | 996 | 1 | 996 | 0 | 0 |
| hsa_circ_0068988 | 87 | 3 | 544 | 3 | 544 | 0 | 2.00E-174 |
| hsa_circ_0068988 | 87 | 3 | 544 | 3 | 544 | 0 | 2.00E-173 |
| hsa_circ_0068988 | 87 | 3 | 777 | 3 | 777 | 0 | 0 |
| hsa_circ_0068991 | 89 | 3192 | 4620 | 124 | 1552 | 0 | 0 |
| hsa_circ_0068991 | 89 | 3 | 626 | 127 | 750 | 0 | 0 |
| hsa_circ_0068991 | 89 | 3 | 922 | 127 | 1046 | 0 | 0 |
| hsa_circ_0068991 | 89 | 3 | 1428 | 127 | 1552 | 0 | 0 |
| hsa_circ_0068995 | 90 | 3 | 395 | 3 | 395 | 0 | 4.00E-140 |
| hsa_circ_0068995 | 89 | 3 | 532 | 3 | 532 | 0 | 0 |
| hsa_circ_0068995 | 89 | 3 | 691 | 3 | 691 | 0 | 0 |
| hsa_circ_0068995 | 90 | 3 | 1197 | 3 | 1197 | 0 | 0 |
| hsa_circ_0069005 | 90 | 1 | 822 | 1 | 822 | 0 | 0 |
| hsa_circ_0069008 | 92 | 1 | 909 | 204 | 1112 | 0 | 0 |
| hsa_circ_0069007 | 93 | 1 | 1251 | 356 | 1606 | 0 | 0 |
| hsa_circ_0069007 | 93 | 1 | 1368 | 356 | 1723 | 0 | 0 |
| hsa_circ_0069007 | 93 | 1 | 1727 | 356 | 2082 | 0 | 0 |
| hsa_circ_0069007 | 93 | 1 | 1870 | 356 | 2225 | 0 | 0 |
| hsa_circ_0069007 | 91 | 1 | 3497 | 356 | 3852 | 0 | 0 |
| hsa_circ_0069007 | 92 | 1 | 859 | 611 | 1469 | 0 | 0 |
| hsa_circ_0069007 | 93 | 1 | 996 | 611 | 1606 | 0 | 0 |
| hsa_circ_0069007 | 93 | 1 | 1615 | 611 | 2225 | 0 | 0 |
| hsa_circ_0069007 | 93 | 1 | 1504 | 722 | 2225 | 0 | 0 |
| hsa_circ_0069010 | 93 | 1 | 700 | 1 | 700 | 0 | 0 |
| hsa_circ_0069011 | 94 | 1 | 459 | 163 | 621 | 0 | 0 |
| hsa_circ_0069011 | 94 | 1 | 905 | 219 | 1123 | 0 | 0 |
| hsa_circ_0069013 | 94 | 1 | 756 | 1 | 756 | 0 | 0 |
| hsa_circ_0069013 | 92 | 1 | 962 | 1 | 961 | 0 | 0 |
| hsa_circ_0069013 | 93 | 1 | 476 | 138 | 613 | 0 | 0 |
| hsa_circ_0069013 | 93 | 1 | 619 | 138 | 756 | 0 | 0 |
| hsa_circ_0069013 | 92 | 1 | 825 | 138 | 961 | 0 | 0 |
| hsa_circ_0069016 | 93 | 1 | 502 | 1 | 502 | 0 | 0 |
| hsa_circ_0069017 | 87 | 1 | 206 | 144 | 348 | 0 | 1.00E-59 |
| hsa_circ_0069021 | 90 | 1 | 499 | 1 | 499 | 0 | 0 |
| hsa_circ_0069021 | 90 | 1 | 838 | 1 | 838 | 0 | 0 |
| hsa_circ_0069021 | 89 | 1 | 1419 | 1 | 1419 | 0 | 0 |
| hsa_circ_0069023 | 89 | 3 | 896 | 744 | 1637 | 0 | 0 |
| hsa_circ_0069023 | 90 | 1 | 632 | 843 | 1474 | 0 | 0 |
| hsa_circ_0006091 | 84 | 1 | 2085 | 1 | 2099 | 0.01 | 0 |
| hsa_circ_0057643 | 95 | 365 | 891 | 527 | 1 | 0 | 0 |
| hsa_circ_0057646 | 96 | 1 | 1776 | 1895 | 120 | 0 | 0 |
| hsa_circ_0057646 | 96 | 1 | 297 | 416 | 120 | 0 | 7.00E-135 |
| hsa_circ_0057646 | 96 | 1 | 1776 | 1895 | 120 | 0 | 0 |
| hsa_circ_0057647 | 96 | 1 | 1479 | 1662 | 184 | 0 | 0 |
| hsa_circ_0057653 | 96 | 1 | 635 | 815 | 181 | 0 | 0 |
| hsa_circ_0057653 | 96 | 1 | 335 | 815 | 481 | 0 | 3.00E-151 |
| hsa_circ_0057676 | 95 | 1 | 442 | 442 | 1 | 0 | 0 |
| hsa_circ_0009016 | 71 | 44278 | 45111 | 101223 | 100383 | 0.06 | 2.00E-29 |
| hsa_circ_0057684 | 93 | 1 | 2679 | 1 | 2679 | 0 | 0 |
| hsa_circ_0057686 | 96 | 1 | 567 | 567 | 1 | 0 | 0 |
| hsa_circ_0057686 | 95 | 1 | 1143 | 1143 | 1 | 0 | 0 |
| hsa_circ_0008265 | 97 | 1 | 369 | 369 | 1 | 0 | 8.00E-177 |

novel_circ_016189 novel_circ_016190 novel_circ_016191 novel_circ_016192 novel_circ_016193 novel_circ_016194 novel_circ_016195 novel_circ_016196 novel_circ_016197 novel_circ_016202 novel_circ_016203 novel_circ_016204 novel_circ_016205 novel_circ_016206 novel_circ_016207 novel_circ_016208 novel_circ_016209 novel_circ_016210 novel_circ_016211 novel_circ_016212 novel_circ_016213 novel_circ_016214 novel_circ_016217 novel_circ_016218 novel_circ_016219 novel_circ_016220 novel_circ_016221 novel_circ_016222 novel_circ_016223 novel_circ_016224 novel_circ_016225 novel_circ_016226 novel_circ_016227 novel_circ_016228 novel_circ_016229 novel_circ_016230 novel_circ_016231 novel_circ_016232 novel_circ_016233 novel_circ_016234 novel_circ_016235 novel_circ_016236 novel_circ_016237 novel_circ_016238 novel_circ_016239 novel_circ_016241 novel_circ_016242 novel_circ_016243 novel_circ_016244 novel_circ_016245 novel_circ_016246 novel_circ_016247 novel_circ_016248 novel_circ_016249

| hsa_circ_0008265 | 96 | 1 | 945 | 945 | 1 | 0 | 0 |
| --- | --- | --- | --- | --- | --- | --- | --- |
| hsa_circ_0008928 | 95 | 1 | 576 | 576 | 1 | 0.01 | 0 |
| hsa_circ_0008928 | 96 | 1 | 827 | 827 | 1 | 0 | 0 |
| hsa_circ_0003915 | 98 | 1 | 531 | 531 | 1 | 0 | 0 |
| hsa_circ_0002867 | 99 | 1 | 251 | 251 | 1 | 0 | 1.00E-124 |
| hsa_circ_0002867 | 97 | 1 | 428 | 428 | 1 | 0 | 0 |
| hsa_circ_0057693 | 94 | 1 | 177 | 177 | 1 | 0 | 4.00E-72 |
| hsa_circ_0057697 | 94 | 1 | 127 | 73 | 199 | 0 | 9.00E-48 |
| hsa_circ_0057697 | 94 | 1 | 127 | 73 | 199 | 0 | 2.00E-47 |
| hsa_circ_0057700 | 93 | 5 | 810 | 5 | 810 | 0 | 0 |
| hsa_circ_0057702 | 93 | 1 | 749 | 1 | 749 | 0 | 0 |
| hsa_circ_0057702 | 92 | 1 | 640 | 110 | 749 | 0 | 0 |
| hsa_circ_0057703 | 95 | 1 | 343 | 1 | 343 | 0 | 1.00E-150 |
| hsa_circ_0057704 | 92 | 1 | 392 | 392 | 1 | 0 | 2.00E-154 |
| hsa_circ_0057717 | 97 | 1 | 272 | 1 | 272 | 0 | 5.00E-128 |
| hsa_circ_0057720 | 96 | 3 | 297 | 3 | 297 | 0 | 9.00E-136 |
| hsa_circ_0057720 | 96 | 3 | 725 | 3 | 725 | 0 | 0 |
| hsa_circ_0057720 | 96 | 3 | 864 | 3 | 864 | 0 | 0 |
| hsa_circ_0057722 | 96 | 3 | 567 | 139 | 703 | 0 | 0 |
| hsa_circ_0057722 | 96 | 3 | 690 | 139 | 826 | 0 | 0 |
| hsa_circ_0057727 | 93 | 397 | 517 | 121 | 1 | 0 | 3.00E-43 |
| hsa_circ_0057727 | 93 | 397 | 517 | 121 | 1 | 0 | 9.00E-44 |
| hsa_circ_0057731 | 86 | 461 | 750 | 1 | 290 | 0 | 2.00E-82 |
| hsa_circ_0057731 | 87 | 461 | 973 | 1 | 513 | 0 | 2.00E-166 |
| hsa_circ_0057731 | 87 | 461 | 973 | 1 | 513 | 0 | 3.00E-166 |
| hsa_circ_0057736 | 93 | 1 | 439 | 758 | 320 | 0 | 0 |
| hsa_circ_0057737 | 93 | 7 | 293 | 397 | 111 | 0 | 3.00E-116 |
| hsa_circ_0057737 | 90 | 4 | 713 | 820 | 111 | 0 | 0 |
| hsa_circ_0057737 | 89 | 4 | 614 | 820 | 210 | 0 | 0 |
| hsa_circ_0057738 | 95 | 1 | 678 | 678 | 1 | 0 | 0 |
| hsa_circ_0057738 | 95 | 4 | 417 | 414 | 1 | 0 | 0 |
| hsa_circ_0057738 | 95 | 1 | 498 | 498 | 1 | 0 | 0 |
| hsa_circ_0057738 | 95 | 1 | 678 | 678 | 1 | 0 | 0 |
| hsa_circ_0057738 | 96 | 4 | 329 | 414 | 89 | 0 | 6.00E-148 |
| hsa_circ_0057738 | 95 | 1 | 590 | 678 | 89 | 0 | 0 |
| hsa_circ_0057739 | 94 | 1 | 293 | 293 | 1 | 0 | 1.00E-124 |
| hsa_circ_0057738 | 95 | 1 | 473 | 678 | 206 | 0 | 0 |
| hsa_circ_0057738 | 95 | 350 | 824 | 680 | 206 | 0 | 0 |
| hsa_circ_0057740 | 95 | 1 | 377 | 377 | 1 | 0 | 3.00E-171 |
| hsa_circ_0057740 | 96 | 446 | 824 | 379 | 1 | 0 | 6.00E-172 |
| hsa_circ_0057742 | 96 | 350 | 531 | 182 | 1 | 0 | 2.00E-79 |
| hsa_circ_0057742 | 93 | 1 | 277 | 459 | 181 | 0.01 | 1.00E-112 |
| hsa_circ_0002860 | 83 | 3 | 736 | 3 | 739 | 0.02 | 0 |
| hsa_circ_0002860 | 83 | 3 | 928 | 3 | 925 | 0.03 | 0 |
| hsa_circ_0007530 | 84 | 2 | 516 | 2 | 507 | 0.03 | 1.00E-131 |
| hsa_circ_0057759 | 89 | 1 | 1166 | 1169 | 1 | 0 | 0 |
| hsa_circ_0057759 | 92 | 1 | 131 | 1300 | 1170 | 0 | 4.00E-44 |
| hsa_circ_0057759 | 93 | 1 | 206 | 1300 | 1095 | 0 | 9.00E-80 |
| hsa_circ_0057756 | 94 | 1 | 689 | 5795 | 5107 | 0 | 0 |
| hsa_circ_0057756 | 94 | 603 | 1291 | 5795 | 5107 | 0 | 0 |
| hsa_circ_0057756 | 90 | 4 | 479 | 6277 | 5796 | 0.01 | 1.00E-177 |
| hsa_circ_0057756 | 90 | 4 | 479 | 6277 | 5796 | 0.01 | 9.00E-178 |
| hsa_circ_0057762 | 92 | 1 | 331 | 189 | 519 | 0.01 | 1.00E-130 |
| hsa_circ_0057771 | 78 | 1 | 183 | 195 | 1 | 0.08 | 4.00E-23 |

novel_circ_016250 novel_circ_016251 novel_circ_016252 novel_circ_016253 novel_circ_016254 novel_circ_016255 novel_circ_016256 novel_circ_016257 novel_circ_016258 novel_circ_016259 novel_circ_016260 novel_circ_016261 novel_circ_016262 novel_circ_016263 novel_circ_016264 novel_circ_016265 novel_circ_016266 novel_circ_016267 novel_circ_016268 novel_circ_016269 novel_circ_016270 novel_circ_016271 novel_circ_016272 novel_circ_016273 novel_circ_016274 novel_circ_016275 novel_circ_016276 novel_circ_016277 novel_circ_016278 novel_circ_016279 novel_circ_016280 novel_circ_016281 novel_circ_016282 novel_circ_016283 novel_circ_016284 novel_circ_016285 novel_circ_016286 novel_circ_016287 novel_circ_016288 novel_circ_016289 novel_circ_016290 novel_circ_016291 novel_circ_016292 novel_circ_016293 novel_circ_016294 novel_circ_016295 novel_circ_016296 novel_circ_016297 novel_circ_016298 novel_circ_016299 novel_circ_016300 novel_circ_016301 novel_circ_016303 novel_circ_016304

| hsa_circ_0057771 | 78 | 1 | 183 | 195 | 1 | 0.08 | 1.00E-23 |
| --- | --- | --- | --- | --- | --- | --- | --- |
| hsa_circ_0057777 | 92 | 1 | 878 | 878 | 1 | 0 | 0 |
| hsa_circ_0057777 | 92 | 1 | 1332 | 1332 | 1 | 0 | 0 |
| hsa_circ_0057777 | 91 | 1 | 576 | 576 | 1 | 0 | 0 |
| hsa_circ_0057777 | 92 | 1 | 300 | 300 | 1 | 0 | 7.00E-117 |
| hsa_circ_0057778 | 92 | 1 | 768 | 891 | 124 | 0 | 0 |
| hsa_circ_0057778 | 92 | 1 | 790 | 1491 | 702 | 0 | 0 |
| hsa_circ_0057778 | 93 | 2 | 1122 | 1822 | 702 | 0 | 0 |
| hsa_circ_0004852 | 92 | 2 | 277 | 354 | 79 | 0 | 1.00E-108 |
| hsa_circ_0057778 | 92 | 1 | 712 | 1491 | 780 | 0 | 0 |
| hsa_circ_0057778 | 93 | 2 | 1044 | 1822 | 780 | 0 | 0 |
| hsa_circ_0057778 | 93 | 1 | 264 | 1155 | 892 | 0 | 7.00E-107 |
| hsa_circ_0057778 | 92 | 1 | 435 | 1491 | 1057 | 0 | 2.00E-173 |
| hsa_circ_0057778 | 91 | 1 | 336 | 1491 | 1156 | 0 | 8.00E-127 |
| hsa_circ_0057778 | 90 | 1 | 2285 | 4463 | 2191 | 0.01 | 0 |
| hsa_circ_0057778 | 94 | 1 | 229 | 2052 | 1824 | 0 | 3.00E-94 |
| hsa_circ_0057778 | 94 | 247 | 603 | 2180 | 1824 | 0 | 1.00E-151 |
| hsa_circ_0057782 | 93 | 10 | 398 | 593 | 205 | 0.01 | 1.00E-162 |
| hsa_circ_0057778 | 92 | 1 | 476 | 2666 | 2191 | 0 | 0 |
| hsa_circ_0057778 | 92 | 1 | 573 | 2763 | 2191 | 0 | 0 |
| hsa_circ_0057778 | 92 | 1 | 742 | 2932 | 2191 | 0 | 0 |
| hsa_circ_0057778 | 92 | 1 | 1100 | 3290 | 2191 | 0 | 0 |
| hsa_circ_0057778 | 92 | 1916 | 3017 | 3292 | 2191 | 0 | 0 |
| hsa_circ_0057778 | 91 | 6 | 1259 | 3432 | 2191 | 0.01 | 0 |
| hsa_circ_0057778 | 90 | 1 | 2285 | 4463 | 2191 | 0.01 | 0 |
| hsa_circ_0057778 | 90 | 9055 | 11341 | 4465 | 2191 | 0.01 | 0 |
| hsa_circ_0057782 | 93 | 1 | 215 | 419 | 205 | 0.01 | 2.00E-84 |
| hsa_circ_0057778 | 92 | 1 | 573 | 2763 | 2191 | 0 | 0 |
| hsa_circ_0057778 | 92 | 1 | 742 | 2932 | 2191 | 0 | 0 |
| hsa_circ_0057778 | 92 | 1 | 1100 | 3290 | 2191 | 0 | 0 |
| hsa_circ_0057778 | 92 | 1916 | 3017 | 3292 | 2191 | 0 | 0 |
| hsa_circ_0057778 | 90 | 1 | 2285 | 4463 | 2191 | 0.01 | 0 |
| hsa_circ_0057778 | 90 | 9055 | 11341 | 4465 | 2191 | 0.01 | 0 |
| hsa_circ_0057778 | 92 | 1 | 530 | 2763 | 2234 | 0 | 0 |
| hsa_circ_0057778 | 92 | 1 | 699 | 2932 | 2234 | 0 | 0 |
| hsa_circ_0057778 | 92 | 1 | 1057 | 3290 | 2234 | 0 | 0 |
| hsa_circ_0057778 | 92 | 1916 | 2974 | 3292 | 2234 | 0 | 0 |
| hsa_circ_0057778 | 92 | 1 | 358 | 2763 | 2406 | 0 | 5.00E-139 |
| hsa_circ_0057778 | 89 | 1 | 2070 | 4463 | 2406 | 0.01 | 0 |
| hsa_circ_0057778 | 92 | 1 | 624 | 3290 | 2667 | 0 | 0 |
| hsa_circ_0057778 | 92 | 1916 | 2541 | 3292 | 2667 | 0 | 0 |
| hsa_circ_0057778 | 89 | 9055 | 10865 | 4465 | 2667 | 0.01 | 0 |
| hsa_circ_0057778 | 89 | 1 | 1712 | 4463 | 2764 | 0.01 | 0 |
| hsa_circ_0057778 | 89 | 9055 | 10768 | 4465 | 2764 | 0.01 | 0 |
| hsa_circ_0057778 | 88 | 1 | 1543 | 4463 | 2933 | 0.01 | 0 |
| hsa_circ_0057778 | 88 | 1 | 1158 | 4463 | 3306 | 0 | 0 |
| hsa_circ_0057778 | 88 | 1 | 1078 | 4383 | 3306 | 0 | 0 |
| hsa_circ_0057778 | 88 | 1 | 1158 | 4463 | 3306 | 0 | 0 |
| hsa_circ_0057778 | 90 | 1 | 235 | 4463 | 4229 | 0 | 2.00E-82 |
| hsa_circ_0057783 | 94 | 239 | 483 | 1 | 245 | 0 | 5.00E-101 |
| hsa_circ_0057783 | 94 | 1 | 245 | 1 | 245 | 0 | 2.00E-101 |
| hsa_circ_0057783 | 92 | 1 | 342 | 246 | 587 | 0 | 2.00E-133 |
| hsa_circ_0057789 | 94 | 1 | 259 | 1 | 259 | 0 | 2.00E-107 |
| hsa_circ_0057789 | 94 | 1 | 324 | 1 | 324 | 0 | 4.00E-140 |

novel_circ_016305 novel_circ_016306 novel_circ_016307 novel_circ_016308 novel_circ_016309 novel_circ_016310 novel_circ_016311 novel_circ_016312 novel_circ_016313 novel_circ_016314 novel_circ_016315 novel_circ_016316 novel_circ_016317 novel_circ_016318 novel_circ_016319 novel_circ_016320 novel_circ_016321 novel_circ_016322 novel_circ_016323 novel_circ_016324 novel_circ_016325 novel_circ_016326 novel_circ_016327 novel_circ_016328 novel_circ_016329 novel_circ_016332 novel_circ_016333 novel_circ_016334 novel_circ_016335 novel_circ_016336 novel_circ_016337 novel_circ_016338 novel_circ_016339 novel_circ_016340 novel_circ_016341 novel_circ_016342 novel_circ_016343 novel_circ_016344 novel_circ_016345 novel_circ_016346 novel_circ_016347 novel_circ_016348 novel_circ_016350 novel_circ_016351 novel_circ_016352 novel_circ_016353 novel_circ_016354 novel_circ_016355 novel_circ_016356 novel_circ_016357 novel_circ_016358 novel_circ_016359 novel_circ_016360 novel_circ_016361

| hsa_circ_0057789 | 95 | 1 | 732 | 1 | 732 | 0 | 0 |
| --- | --- | --- | --- | --- | --- | --- | --- |
| hsa_circ_0057793 | 93 | 1 | 903 | 1 | 903 | 0 | 0 |
| hsa_circ_0057794 | 91 | 1 | 495 | 1 | 495 | 0 | 0 |
| hsa_circ_0057801 | 95 | 1 | 342 | 1 | 342 | 0 | 2.00E-153 |
| hsa_circ_0057801 | 95 | 1 | 545 | 1 | 545 | 0 | 0 |
| hsa_circ_0057801 | 94 | 1 | 1200 | 1 | 1200 | 0 | 0 |
| hsa_circ_0057808 | 95 | 1 | 203 | 1 | 203 | 0 | 2.00E-86 |
| hsa_circ_0057808 | 94 | 1 | 434 | 1 | 434 | 0 | 0 |
| hsa_circ_0057808 | 93 | 1 | 549 | 1 | 549 | 0 | 0 |
| hsa_circ_0057808 | 94 | 1 | 858 | 1 | 858 | 0 | 0 |
| hsa_circ_0057818 | 93 | 1 | 1738 | 1 | 1738 | 0 | 0 |
| hsa_circ_0057818 | 93 | 1 | 1453 | 286 | 1738 | 0 | 0 |
| hsa_circ_0057821 | 94 | 208 | 351 | 1 | 144 | 0 | 1.00E-56 |
| hsa_circ_0057821 | 91 | 1 | 370 | 1 | 370 | 0 | 5.00E-139 |
| hsa_circ_0057821 | 93 | 1 | 491 | 1 | 491 | 0 | 0 |
| hsa_circ_0057822 | 87 | 127 | 1101 | 972 | 1 | 0.01 | 0 |
| hsa_circ_0057823 | 91 | 4 | 920 | 914 | 1 | 0 | 0 |
| hsa_circ_0057823 | 91 | 4 | 920 | 914 | 1 | 0 | 0 |
| hsa_circ_0057823 | 90 | 2 | 678 | 674 | 1 | 0 | 0 |
| hsa_circ_0057823 | 91 | 4 | 920 | 914 | 1 | 0 | 0 |
| hsa_circ_0057823 | 91 | 4 | 920 | 914 | 1 | 0 | 0 |
| hsa_circ_0057823 | 93 | 4 | 790 | 914 | 128 | 0 | 0 |
| hsa_circ_0057824 | 93 | 2 | 449 | 547 | 100 | 0 | 0 |
| hsa_circ_0057823 | 94 | 4 | 366 | 914 | 552 | 0 | 9.00E-157 |
| hsa_circ_0001094 | 94 | 4 | 242 | 239 | 1 | 0 | 1.00E-99 |
| hsa_circ_0003747 | 92 | 632 | 752 | 1 | 121 | 0 | 4.00E-40 |
| hsa_circ_0003747 | 92 | 632 | 752 | 1 | 121 | 0 | 4.00E-40 |
| hsa_circ_0057830 | 87 | 820 | 1215 | 1 | 405 | 0.04 | 9.00E-122 |
| hsa_circ_0003747 | 92 | 461 | 581 | 1 | 121 | 0 | 3.00E-40 |
| hsa_circ_0003747 | 92 | 461 | 581 | 1 | 121 | 0 | 3.00E-40 |
| hsa_circ_0057830 | 87 | 649 | 1044 | 1 | 405 | 0.04 | 8.00E-122 |
| hsa_circ_0057830 | 87 | 649 | 1044 | 1 | 405 | 0.04 | 8.00E-122 |
| hsa_circ_0003747 | 92 | 457 | 577 | 1 | 121 | 0 | 3.00E-40 |
| hsa_circ_0003747 | 92 | 349 | 469 | 1 | 121 | 0 | 3.00E-40 |
| hsa_circ_0003747 | 92 | 229 | 349 | 1 | 121 | 0 | 2.00E-40 |
| hsa_circ_0057830 | 87 | 417 | 812 | 1 | 405 | 0.04 | 8.00E-122 |
| hsa_circ_0057830 | 87 | 189 | 584 | 1 | 405 | 0.04 | 4.00E-122 |
| hsa_circ_0057830 | 87 | 189 | 584 | 1 | 405 | 0.04 | 5.00E-122 |
| hsa_circ_0057830 | 93 | 1 | 275 | 131 | 405 | 0 | 2.00E-111 |
| hsa_circ_0057830 | 93 | 1 | 275 | 131 | 405 | 0 | 5.00E-111 |
| hsa_circ_0057830 | 94 | 1 | 190 | 216 | 405 | 0 | 1.00E-75 |
| hsa_circ_0057831 | 93 | 1 | 1221 | 1 | 1221 | 0 | 0 |
| hsa_circ_0057855 | 93 | 2 | 557 | 2 | 557 | 0 | 0 |
| hsa_circ_0057855 | 92 | 2 | 1456 | 2 | 1456 | 0 | 0 |
| hsa_circ_0057855 | 91 | 1 | 1323 | 134 | 1456 | 0 | 0 |
| hsa_circ_0057855 | 91 | 1 | 899 | 558 | 1456 | 0 | 0 |
| hsa_circ_0057862 | 90 | 2 | 1035 | 2 | 1035 | 0 | 0 |
| hsa_circ_0057861 | 93 | 1 | 401 | 2058 | 2458 | 0 | 7.00E-163 |
| hsa_circ_0057861 | 93 | 1 | 535 | 2058 | 2592 | 0 | 0 |
| hsa_circ_0057861 | 93 | 1 | 858 | 2058 | 2918 | 0 | 0 |
| hsa_circ_0057860 | 92 | 1 | 533 | 3451 | 3983 | 0 | 0 |
| hsa_circ_0057860 | 92 | 1 | 643 | 3451 | 4093 | 0 | 0 |
| hsa_circ_0057860 | 92 | 1 | 643 | 3451 | 4093 | 0 | 0 |
| hsa_circ_0057860 | 92 | 1 | 643 | 3451 | 4093 | 0 | 0 |

novel_circ_016362 novel_circ_016363 novel_circ_016364 novel_circ_016365 novel_circ_016366 novel_circ_016367 novel_circ_016368 novel_circ_016369 novel_circ_016370 novel_circ_016371 novel_circ_016374 novel_circ_016376 novel_circ_016377 novel_circ_016378 novel_circ_016379 novel_circ_016380 novel_circ_016381 novel_circ_016382 novel_circ_016383 novel_circ_016384 novel_circ_016385 novel_circ_016386 novel_circ_016387 novel_circ_016388 novel_circ_016389 novel_circ_016391 novel_circ_016392 novel_circ_016393 novel_circ_016394 novel_circ_016396 novel_circ_016399 novel_circ_016400 novel_circ_016401 novel_circ_016402 novel_circ_016403 novel_circ_016404 novel_circ_016405 novel_circ_016406 novel_circ_016407 novel_circ_016408 novel_circ_016415 novel_circ_016416 novel_circ_016417 novel_circ_016418 novel_circ_016419 novel_circ_016422 novel_circ_016423 novel_circ_016425 novel_circ_016426 novel_circ_016427 novel_circ_016428 novel_circ_016429 novel_circ_016430 novel_circ_016431

| hsa_circ_0057866 | 92 | 1 | 423 | 1 | 423 | 0 | 2.00E-168 |
| --- | --- | --- | --- | --- | --- | --- | --- |
| hsa_circ_0057860 | 92 | 1 | 533 | 3561 | 4093 | 0 | 0 |
| hsa_circ_0057860 | 92 | 1 | 533 | 3561 | 4093 | 0 | 0 |
| hsa_circ_0057860 | 92 | 1 | 533 | 3561 | 4093 | 0 | 0 |
| hsa_circ_0057860 | 92 | 1 | 533 | 3561 | 4093 | 0 | 0 |
| hsa_circ_0057860 | 92 | 1 | 533 | 3561 | 4093 | 0 | 0 |
| hsa_circ_0057860 | 92 | 1 | 415 | 3679 | 4093 | 0 | 6.00E-164 |
| hsa_circ_0057868 | 92 | 1 | 279 | 1 | 279 | 0 | 1.00E-108 |
| hsa_circ_0057870 | 92 | 1 | 528 | 1 | 528 | 0 | 0 |
| hsa_circ_0057870 | 92 | 1 | 528 | 1 | 528 | 0 | 0 |
| hsa_circ_0005307 | 91 | 683 | 859 | 1 | 177 | 0 | 2.00E-61 |
| hsa_circ_0057875 | 94 | 155 | 733 | 4263 | 4823 | 0.03 | 0 |
| hsa_circ_0057875 | 94 | 155 | 733 | 4263 | 4823 | 0.03 | 0 |
| hsa_circ_0057875 | 94 | 1 | 565 | 4277 | 4823 | 0.03 | 0 |
| hsa_circ_0057877 | 98 | 1 | 370 | 1 | 370 | 0 | 0 |
| hsa_circ_0057876 | 97 | 120 | 305 | 1372 | 1557 | 0 | 3.00E-83 |
| hsa_circ_0007951 | 96 | 1 | 420 | 1 | 420 | 0 | 0 |
| hsa_circ_0007951 | 96 | 1 | 513 | 1 | 513 | 0 | 0 |
| hsa_circ_0057880 | 94 | 1 | 1569 | 1569 | 1 | 0 | 0 |
| hsa_circ_0057880 | 94 | 1 | 681 | 681 | 1 | 0 | 0 |
| hsa_circ_0057880 | 94 | 1 | 888 | 1569 | 682 | 0 | 0 |
| hsa_circ_0057882 | 93 | 1 | 813 | 813 | 1 | 0 | 0 |
| hsa_circ_0005613 | 91 | 1 | 506 | 506 | 1 | 0 | 0 |
| hsa_circ_0005613 | 92 | 4 | 612 | 609 | 1 | 0 | 0 |
| hsa_circ_0005613 | 93 | 1 | 732 | 732 | 1 | 0 | 0 |
| hsa_circ_0074029 | 75 | 37288 | 37613 | 36511 | 36834 | 0.02 | 1.00E-31 |
| hsa_circ_0074029 | 75 | 48844 | 49169 | 36511 | 36834 | 0.02 | 2.00E-31 |
| hsa_circ_0074029 | 75 | 37288 | 37613 | 36511 | 36834 | 0.02 | 1.00E-31 |
| hsa_circ_0074029 | 75 | 48844 | 49169 | 36511 | 36834 | 0.02 | 1.00E-31 |
| hsa_circ_0001106 | 78 | 8730 | 9113 | 61170 | 60781 | 0.04 | 2.00E-56 |
| hsa_circ_0074541 | 79 | 919 | 1356 | 277 | 714 | 0.02 | 2.00E-74 |
| hsa_circ_0074541 | 77 | 919 | 1566 | 277 | 924 | 0.01 | 4.00E-101 |
| hsa_circ_0074541 | 77 | 919 | 1691 | 277 | 1049 | 0.01 | 2.00E-115 |
| hsa_circ_0074541 | 77 | 919 | 1691 | 277 | 1049 | 0.01 | 5.00E-115 |
| hsa_circ_0074541 | 77 | 919 | 1691 | 277 | 1049 | 0.01 | 2.00E-114 |
| hsa_circ_0074541 | 77 | 919 | 1691 | 277 | 1049 | 0.01 | 4.00E-114 |
| hsa_circ_0074544 | 77 | 1 | 429 | 1 | 429 | 0.02 | 1.00E-61 |
| hsa_circ_0074544 | 76 | 1 | 554 | 1 | 554 | 0.01 | 5.00E-76 |
| hsa_circ_0074544 | 76 | 1 | 564 | 1 | 564 | 0.01 | 1.00E-74 |
| hsa_circ_0074544 | 77 | 41 | 466 | 129 | 554 | 0 | 6.00E-65 |
| hsa_circ_0070727 | 90 | 1 | 1537 | 1818 | 282 | 0 | 0 |
| hsa_circ_0070731 | 90 | 1 | 1129 | 1129 | 1 | 0 | 0 |
| hsa_circ_0070731 | 89 | 1 | 518 | 660 | 143 | 0 | 0 |
| hsa_circ_0070731 | 89 | 1 | 987 | 1129 | 143 | 0 | 0 |
| hsa_circ_0006472 | 89 | 1 | 469 | 469 | 1 | 0 | 1.00E-165 |
| hsa_circ_0070751 | 93 | 3 | 666 | 781 | 118 | 0 | 0 |
| hsa_circ_0007815 | 97 | 1877 | 1913 | 7396 | 7432 | 0 | 9.00E-07 |
| hsa_circ_0070775 | 93 | 1 | 827 | 1 | 827 | 0 | 0 |
| hsa_circ_0070775 | 93 | 1 | 827 | 1 | 827 | 0 | 0 |
| hsa_circ_0070775 | 92 | 1 | 1144 | 1 | 1144 | 0 | 0 |
| hsa_circ_0070784 | 87 | 2 | 877 | 1338 | 2213 | 0 | 0 |
| hsa_circ_0070784 | 86 | 2 | 944 | 1338 | 2280 | 0 | 0 |
| hsa_circ_0070784 | 85 | 1 | 797 | 1484 | 2280 | 0 | 0 |
| hsa_circ_0070805 | 93 | 1 | 1480 | 1480 | 1 | 0 | 0 |

novel_circ_016432 novel_circ_016433 novel_circ_016435 novel_circ_016436 novel_circ_016437 novel_circ_016438 novel_circ_016439 novel_circ_016440 novel_circ_016441 novel_circ_016442 novel_circ_016443 novel_circ_016444 novel_circ_016445 novel_circ_016446 novel_circ_016449 novel_circ_016450 novel_circ_016451 novel_circ_016452 novel_circ_016453 novel_circ_016454 novel_circ_016455 novel_circ_016456 novel_circ_016457 novel_circ_016458 novel_circ_016459 novel_circ_016460 novel_circ_016461 novel_circ_016462 novel_circ_016463 novel_circ_016464 novel_circ_016465 novel_circ_016466 novel_circ_016467 novel_circ_016468 novel_circ_016469 novel_circ_016470 novel_circ_016471 novel_circ_016472 novel_circ_016473 novel_circ_016474 novel_circ_016475 novel_circ_016476 novel_circ_016477 novel_circ_016478 novel_circ_016479 novel_circ_016480 novel_circ_016481 novel_circ_016482 novel_circ_016483 novel_circ_016484 novel_circ_016485 novel_circ_016486 novel_circ_016487 novel_circ_016488

| hsa_circ_0070805 | 93 | 4 | 831 | 888 | 61 | 0 | 0 |
| --- | --- | --- | --- | --- | --- | --- | --- |
| hsa_circ_0070805 | 94 | 1 | 979 | 1480 | 502 | 0 | 0 |
| hsa_circ_0002474 | 94 | 1 | 679 | 679 | 1 | 0 | 0 |
| hsa_circ_0070812 | 79 | 88635 | 89478 | 3 | 867 | 0.06 | 9.00E-159 |
| hsa_circ_0070812 | 79 | 1782 | 2625 | 3 | 867 | 0.06 | 1.00E-159 |
| hsa_circ_0070816 | 92 | 1 | 540 | 540 | 1 | 0 | 0 |
| hsa_circ_0070816 | 93 | 1 | 783 | 783 | 1 | 0 | 0 |
| hsa_circ_0070816 | 93 | 1 | 863 | 863 | 1 | 0 | 0 |
| hsa_circ_0070818 | 92 | 1 | 435 | 435 | 1 | 0 | 2.00E-173 |
| hsa_circ_0070818 | 93 | 1 | 678 | 678 | 1 | 0 | 0 |
| hsa_circ_0070818 | 93 | 1 | 758 | 758 | 1 | 0 | 0 |
| hsa_circ_0070819 | 93 | 1 | 672 | 672 | 1 | 0 | 0 |
| hsa_circ_0070818 | 91 | 1 | 888 | 1323 | 436 | 0 | 0 |
| hsa_circ_0070818 | 93 | 1 | 206 | 1530 | 1325 | 0 | 9.00E-80 |
| hsa_circ_0070825 | 93 | 224 | 663 | 1 | 440 | 0 | 0 |
| hsa_circ_0070825 | 93 | 224 | 663 | 1 | 440 | 0 | 0 |
| hsa_circ_0070825 | 93 | 1 | 440 | 1 | 440 | 0 | 0 |
| hsa_circ_0070825 | 93 | 1 | 440 | 1 | 440 | 0 | 0 |
| hsa_circ_0070831 | 93 | 1 | 165 | 165 | 1 | 0 | 4.00E-63 |
| hsa_circ_0070831 | 95 | 1 | 305 | 305 | 1 | 0 | 2.00E-132 |
| hsa_circ_0070831 | 92 | 1 | 961 | 958 | 1 | 0 | 0 |
| hsa_circ_0070831 | 91 | 1 | 1078 | 1075 | 1 | 0 | 0 |
| hsa_circ_0070831 | 91 | 188 | 1338 | 1148 | 1 | 0 | 0 |
| hsa_circ_0070831 | 91 | 493 | 1643 | 1148 | 1 | 0 | 0 |
| hsa_circ_0070831 | 93 | 1 | 165 | 165 | 1 | 0 | 3.00E-63 |
| hsa_circ_0070831 | 94 | 1 | 371 | 371 | 1 | 0 | 1.00E-157 |
| hsa_circ_0070831 | 92 | 4 | 642 | 636 | 1 | 0 | 0 |
| hsa_circ_0070831 | 92 | 1 | 961 | 958 | 1 | 0 | 0 |
| hsa_circ_0070831 | 91 | 188 | 1338 | 1148 | 1 | 0 | 0 |
| hsa_circ_0070833 | 91 | 4 | 477 | 471 | 1 | 0.01 | 0 |
| hsa_circ_0070833 | 91 | 528 | 1005 | 475 | 1 | 0.01 | 0 |
| hsa_circ_0070831 | 91 | 1 | 665 | 827 | 166 | 0 | 0 |
| hsa_circ_0070831 | 91 | 1 | 796 | 958 | 166 | 0 | 0 |
| hsa_circ_0070831 | 91 | 188 | 1173 | 1148 | 166 | 0 | 0 |
| hsa_circ_0070831 | 91 | 427 | 1412 | 1148 | 166 | 0 | 0 |
| hsa_circ_0070831 | 90 | 188 | 1033 | 1148 | 306 | 0 | 0 |
| hsa_circ_0070837 | 91 | 1 | 258 | 260 | 3 | 0 | 6.00E-95 |
| hsa_circ_0070837 | 91 | 1 | 258 | 260 | 3 | 0 | 5.00E-95 |
| hsa_circ_0070837 | 91 | 1 | 426 | 499 | 74 | 0 | 5.00E-165 |
| hsa_circ_0070837 | 91 | 1 | 239 | 499 | 261 | 0 | 2.00E-86 |
| hsa_circ_0070848 | 95 | 1 | 387 | 81 | 467 | 0 | 4.00E-175 |
| hsa_circ_0070848 | 95 | 1 | 848 | 81 | 928 | 0 | 0 |
| hsa_circ_0070848 | 94 | 1 | 961 | 81 | 1041 | 0 | 0 |
| hsa_circ_0070848 | 94 | 1 | 961 | 81 | 1041 | 0 | 0 |
| hsa_circ_0070849 | 95 | 1 | 446 | 1 | 446 | 0 | 0 |
| hsa_circ_0070849 | 94 | 1 | 810 | 1 | 810 | 0 | 0 |
| hsa_circ_0070849 | 94 | 1 | 810 | 1 | 810 | 0 | 0 |
| hsa_circ_0070852 | 93 | 841 | 1894 | 215 | 1268 | 0 | 0 |
| hsa_circ_0070852 | 93 | 841 | 2546 | 215 | 1920 | 0 | 0 |
| hsa_circ_0070849 | 94 | 1 | 210 | 237 | 446 | 0 | 5.00E-87 |
| hsa_circ_0070849 | 95 | 1 | 366 | 237 | 602 | 0 | 9.00E-162 |
| hsa_circ_0070849 | 94 | 1 | 574 | 237 | 810 | 0 | 0 |
| hsa_circ_0070852 | 93 | 141 | 1194 | 215 | 1268 | 0 | 0 |
| hsa_circ_0070856 | 94 | 1 | 366 | 1 | 366 | 0 | 4.00E-155 |

novel_circ_016489 novel_circ_016490 novel_circ_016491 novel_circ_016492 novel_circ_016493 novel_circ_016494 novel_circ_016495 novel_circ_016496 novel_circ_016497 novel_circ_016498 novel_circ_016499 novel_circ_016500 novel_circ_016501 novel_circ_016502 novel_circ_016503 novel_circ_016504 novel_circ_016505 novel_circ_016506 novel_circ_016507 novel_circ_016508 novel_circ_016509 novel_circ_016510 novel_circ_016511 novel_circ_016512 novel_circ_016513 novel_circ_016514 novel_circ_016515 novel_circ_016516 novel_circ_016517 novel_circ_016518 novel_circ_016519 novel_circ_016520 novel_circ_016521 novel_circ_016522 novel_circ_016523 novel_circ_016524 novel_circ_016525 novel_circ_016526 novel_circ_016527 novel_circ_016528 novel_circ_016529 novel_circ_016530 novel_circ_016531 novel_circ_016532 novel_circ_016533 novel_circ_016534 novel_circ_016535 novel_circ_016536 novel_circ_016537 novel_circ_016538 novel_circ_016539 novel_circ_016540 novel_circ_016541 novel_circ_016542

| hsa_circ_0070860 | 93 | 1 | 909 | 511 | 1419 | 0 | 0 |
| --- | --- | --- | --- | --- | --- | --- | --- |
| hsa_circ_0070860 | 91 | 1 | 570 | 1420 | 1989 | 0 | 0 |
| hsa_circ_0070861 | 91 | 1 | 456 | 611 | 1066 | 0 | 1.00E-176 |
| hsa_circ_0070856 | 93 | 1 | 3750 | 5247 | 8999 | 0 | 0 |
| hsa_circ_0070856 | 93 | 1 | 2052 | 5954 | 8005 | 0 | 0 |
| hsa_circ_0070865 | 95 | 1 | 559 | 692 | 1250 | 0 | 0 |
| hsa_circ_0070865 | 94 | 1 | 970 | 692 | 1661 | 0 | 0 |
| hsa_circ_0070863 | 94 | 1 | 2361 | 837 | 3200 | 0 | 0 |
| hsa_circ_0070866 | 94 | 1 | 567 | 1 | 567 | 0 | 0 |
| hsa_circ_0070866 | 94 | 1 | 766 | 1 | 766 | 0 | 0 |
| hsa_circ_0070866 | 94 | 1 | 826 | 1 | 826 | 0 | 0 |
| hsa_circ_0070863 | 94 | 1 | 1480 | 2225 | 3704 | 0 | 0 |
| hsa_circ_0070863 | 94 | 1 | 452 | 2349 | 2800 | 0 | 0 |
| hsa_circ_0070863 | 94 | 1 | 852 | 2349 | 3200 | 0 | 0 |
| hsa_circ_0070863 | 94 | 1 | 1356 | 2349 | 3704 | 0 | 0 |
| hsa_circ_0070867 | 94 | 1 | 1155 | 92 | 1246 | 0 | 0 |
| hsa_circ_0070867 | 95 | 1 | 685 | 562 | 1246 | 0 | 0 |
| hsa_circ_0070863 | 95 | 1 | 1116 | 4288 | 5403 | 0 | 0 |
| hsa_circ_0070872 | 89 | 1 | 1548 | 240 | 1787 | 0.01 | 0 |
| hsa_circ_0070872 | 89 | 1 | 1703 | 240 | 1942 | 0.01 | 0 |
| hsa_circ_0070875 | 89 | 123 | 1913 | 1 | 1791 | 0.01 | 0 |
| hsa_circ_0070887 | 90 | 1 | 437 | 131 | 567 | 0 | 8.00E-163 |
| hsa_circ_0070887 | 89 | 1 | 592 | 131 | 722 | 0 | 0 |
| hsa_circ_0005291 | 93 | 1 | 471 | 1 | 471 | 0 | 0 |
| hsa_circ_0070897 | 89 | 1 | 1855 | 5 | 1844 | 0.01 | 0 |
| hsa_circ_0070898 | 88 | 1 | 938 | 614 | 1536 | 0.03 | 0 |
| hsa_circ_0070902 | 92 | 1 | 1031 | 4596 | 5617 | 0.01 | 0 |
| hsa_circ_0070902 | 92 | 1 | 343 | 5275 | 5617 | 0 | 2.00E-137 |
| hsa_circ_0070898 | 90 | 1 | 605 | 7167 | 7771 | 0 | 0 |
| hsa_circ_0022368 | 83 | 96 | 1024 | 15 | 968 | 0.03 | 0 |
| hsa_circ_0070905 | 89 | 1 | 675 | 623 | 1303 | 0.01 | 0 |
| hsa_circ_0070909 | 90 | 1 | 796 | 1 | 796 | 0 | 0 |
| hsa_circ_0070909 | 90 | 3 | 480 | 225 | 702 | 0 | 3.00E-172 |
| hsa_circ_0070911 | 93 | 1 | 199 | 1 | 199 | 0 | 3.00E-79 |
| hsa_circ_0070911 | 93 | 1 | 321 | 1 | 321 | 0 | 2.00E-133 |
| hsa_circ_0070910 | 94 | 1 | 556 | 361 | 916 | 0 | 0 |
| hsa_circ_0070910 | 94 | 1 | 1030 | 361 | 1390 | 0 | 0 |
| hsa_circ_0070910 | 94 | 1 | 1030 | 361 | 1390 | 0 | 0 |
| hsa_circ_0070910 | 93 | 1 | 498 | 419 | 916 | 0 | 0 |
| hsa_circ_0070910 | 94 | 1 | 357 | 560 | 916 | 0 | 8.00E-152 |
| hsa_circ_0070910 | 94 | 1 | 708 | 683 | 1390 | 0 | 0 |
| hsa_circ_0070910 | 94 | 1 | 708 | 683 | 1390 | 0 | 0 |
| hsa_circ_0070910 | 94 | 1 | 322 | 917 | 1238 | 0 | 1.00E-135 |
| hsa_circ_0070910 | 94 | 1 | 474 | 917 | 1390 | 0 | 0 |
| hsa_circ_0070912 | 94 | 1 | 259 | 1 | 259 | 0 | 4.00E-109 |
| hsa_circ_0070912 | 95 | 1 | 393 | 1 | 393 | 0 | 2.00E-178 |
| hsa_circ_0070912 | 96 | 1 | 586 | 1 | 586 | 0 | 0 |
| hsa_circ_0070912 | 96 | 1 | 586 | 1 | 586 | 0 | 0 |
| hsa_circ_0070912 | 96 | 1 | 586 | 1 | 586 | 0 | 0 |
| hsa_circ_0070912 | 96 | 1 | 586 | 1 | 586 | 0 | 0 |
| hsa_circ_0070912 | 96 | 1 | 586 | 1 | 586 | 0 | 0 |
| hsa_circ_0070913 | 94 | 135 | 571 | 1 | 434 | 0.01 | 0 |
| hsa_circ_0070914 | 88 | 1 | 294 | 109 | 390 | 0.04 | 1.00E-90 |
| hsa_circ_0070923 | 87 | 1 | 442 | 445 | 1 | 0.02 | 2.00E-139 |

novel_circ_016543 novel_circ_016544 novel_circ_016545 novel_circ_016546 novel_circ_016547 novel_circ_016548 novel_circ_016549 novel_circ_016550 novel_circ_016551 novel_circ_016552 novel_circ_016553 novel_circ_016554 novel_circ_016555 novel_circ_016556 novel_circ_016557 novel_circ_016558 novel_circ_016559 novel_circ_016560 novel_circ_016561 novel_circ_016562 novel_circ_016563 novel_circ_016564 novel_circ_016565 novel_circ_016566 novel_circ_016573 novel_circ_016575 novel_circ_016579 novel_circ_016580 novel_circ_016581 novel_circ_016582 novel_circ_016583 novel_circ_016584 novel_circ_016585 novel_circ_016586 novel_circ_016587 novel_circ_016588 novel_circ_016589 novel_circ_016590 novel_circ_016591 novel_circ_016592 novel_circ_016593 novel_circ_016594 novel_circ_016595 novel_circ_016596 novel_circ_016597 novel_circ_016598 novel_circ_016599 novel_circ_016600 novel_circ_016601 novel_circ_016602 novel_circ_016603 novel_circ_016604 novel_circ_016605 novel_circ_016606

| hsa_circ_0070926 | 90 | 1 | 491 | 491 | 1 | 0 | 2.00E-179 |
| --- | --- | --- | --- | --- | --- | --- | --- |
| hsa_circ_0070928 | 95 | 1 | 194 | 1 | 194 | 0 | 6.00E-83 |
| hsa_circ_0070928 | 95 | 1 | 194 | 1 | 194 | 0 | 8.00E-83 |
| hsa_circ_0070929 | 95 | 195 | 459 | 1 | 265 | 0 | 1.00E-113 |
| hsa_circ_0070929 | 95 | 195 | 459 | 1 | 265 | 0 | 1.00E-113 |
| hsa_circ_0070929 | 95 | 195 | 459 | 1 | 265 | 0 | 1.00E-113 |
| hsa_circ_0070929 | 95 | 195 | 459 | 1 | 265 | 0 | 1.00E-113 |
| hsa_circ_0070930 | 89 | 820 | 1197 | 231 | 608 | 0 | 3.00E-131 |
| hsa_circ_0070930 | 89 | 820 | 1197 | 231 | 608 | 0 | 4.00E-131 |
| hsa_circ_0070929 | 96 | 1 | 164 | 102 | 265 | 0 | 2.00E-69 |
| hsa_circ_0070930 | 93 | 193 | 421 | 1 | 229 | 0 | 4.00E-92 |
| hsa_circ_0070930 | 89 | 194 | 571 | 231 | 608 | 0 | 2.00E-131 |
| hsa_circ_0070930 | 89 | 104 | 481 | 231 | 608 | 0 | 2.00E-131 |
| hsa_circ_0070930 | 89 | 2 | 379 | 231 | 608 | 0 | 1.00E-131 |
| hsa_circ_0070936 | 92 | 4 | 742 | 4 | 745 | 0 | 0 |
| hsa_circ_0070940 | 91 | 1 | 633 | 1 | 630 | 0.01 | 0 |
| hsa_circ_0070940 | 91 | 1 | 633 | 1 | 630 | 0.01 | 0 |
| hsa_circ_0070940 | 92 | 931 | 1467 | 633 | 1166 | 0.01 | 0 |
| hsa_circ_0070940 | 92 | 765 | 933 | 633 | 801 | 0 | 3.00E-60 |
| hsa_circ_0070940 | 92 | 3 | 539 | 633 | 1166 | 0.01 | 0 |
| hsa_circ_0070940 | 92 | 3 | 539 | 633 | 1166 | 0.01 | 0 |
| hsa_circ_0070942 | 94 | 30 | 366 | 30 | 363 | 0.01 | 3.00E-144 |
| hsa_circ_0070942 | 88 | 107 | 443 | 505 | 842 | 0 | 1.00E-110 |
| hsa_circ_0070949 | 95 | 1 | 322 | 1 | 322 | 0 | 2.00E-142 |
| hsa_circ_0059689 | 72 | 729 | 1337 | 2398 | 1787 | 0.05 | 2.00E-31 |
| hsa_circ_0042191 | 95 | 1 | 228 | 438 | 211 | 0 | 1.00E-98 |
| hsa_circ_0005471 | 92 | 1 | 312 | 312 | 1 | 0 | 4.00E-120 |
| hsa_circ_0005069 | 91 | 1 | 505 | 505 | 1 | 0 | 0 |
| hsa_circ_0042181 | 92 | 1 | 688 | 688 | 1 | 0 | 0 |
| hsa_circ_0042179 | 92 | 1 | 802 | 802 | 1 | 0 | 0 |
| hsa_circ_0042176 | 94 | 1 | 1243 | 1579 | 337 | 0 | 0 |
| hsa_circ_0042176 | 94 | 1 | 1579 | 1579 | 1 | 0 | 0 |
| hsa_circ_0005069 | 90 | 1 | 327 | 327 | 1 | 0 | 4.00E-115 |
| hsa_circ_0042181 | 91 | 1 | 510 | 510 | 1 | 0 | 0 |
| hsa_circ_0042181 | 92 | 1 | 376 | 376 | 1 | 0 | 1.00E-150 |
| hsa_circ_0042179 | 93 | 1 | 490 | 490 | 1 | 0 | 0 |
| hsa_circ_0042176 | 95 | 1 | 931 | 1267 | 337 | 0 | 0 |
| hsa_circ_0042176 | 95 | 1 | 1110 | 1267 | 158 | 0 | 0 |
| hsa_circ_0042176 | 95 | 1 | 1267 | 1267 | 1 | 0 | 0 |
| hsa_circ_0042176 | 94 | 1 | 336 | 336 | 1 | 0 | 4.00E-145 |
| hsa_circ_0042169 | 86 | 1 | 879 | 882 | 1 | 0 | 0 |
| hsa_circ_0042169 | 85 | 1 | 831 | 834 | 1 | 0 | 0 |
| hsa_circ_0042169 | 94 | 1 | 326 | 326 | 1 | 0 | 3.00E-136 |
| hsa_circ_0042169 | 94 | 1 | 326 | 326 | 1 | 0 | 6.00E-136 |
| hsa_circ_0042164 | 92 | 499 | 1154 | 656 | 1 | 0 | 0 |
| hsa_circ_0042161 | 92 | 499 | 1281 | 1937 | 1155 | 0 | 0 |
| hsa_circ_0042150 | 93 | 4 | 2199 | 2578 | 383 | 0 | 0 |
| hsa_circ_0042161 | 92 | 1 | 438 | 438 | 1 | 0 | 2.00E-173 |
| hsa_circ_0042150 | 95 | 1 | 789 | 1171 | 383 | 0 | 0 |
| hsa_circ_0042150 | 92 | 9 | 385 | 374 | 1 | 0.01 | 4.00E-150 |
| hsa_circ_0042136 | 93 | 1 | 569 | 112 | 680 | 0 | 0 |
| hsa_circ_0042136 | 94 | 1 | 259 | 112 | 371 | 0 | 4.00E-106 |
| hsa_circ_0042136 | 94 | 1 | 259 | 112 | 371 | 0 | 2.00E-106 |
| hsa_circ_0042136 | 94 | 1 | 253 | 112 | 364 | 0 | 2.00E-107 |

novel_circ_016607 novel_circ_016608 novel_circ_016609 novel_circ_016610 novel_circ_016611 novel_circ_016614 novel_circ_016615 novel_circ_016616 novel_circ_016617 novel_circ_016618 novel_circ_016619 novel_circ_016620 novel_circ_016621 novel_circ_016622 novel_circ_016623 novel_circ_016624 novel_circ_016625 novel_circ_016626 novel_circ_016627 novel_circ_016628 novel_circ_016629 novel_circ_016630 novel_circ_016631 novel_circ_016632 novel_circ_016633 novel_circ_016634 novel_circ_016635 novel_circ_016636 novel_circ_016637 novel_circ_016638 novel_circ_016639 novel_circ_016640 novel_circ_016641 novel_circ_016642 novel_circ_016643 novel_circ_016644 novel_circ_016645 novel_circ_016646 novel_circ_016647 novel_circ_016648 novel_circ_016649 novel_circ_016650 novel_circ_016651 novel_circ_016653 novel_circ_016654 novel_circ_016655 novel_circ_016656 novel_circ_016657 novel_circ_016658 novel_circ_016659 novel_circ_016660 novel_circ_016661 novel_circ_016662 novel_circ_016663

| hsa_circ_0042136 | 95 | 1 | 364 | 1 | 364 | 0 | 5.00E-159 |
| --- | --- | --- | --- | --- | --- | --- | --- |
| hsa_circ_0042137 | 96 | 1 | 96 | 1 | 96 | 0 | 2.00E-37 |
| hsa_circ_0042437 | 86 | 1 | 342 | 1 | 342 | 0 | 2.00E-98 |
| hsa_circ_0042429 | 88 | 1 | 461 | 1 | 461 | 0 | 9.00E-153 |
| hsa_circ_0042428 | 82 | 15 | 2035 | 15 | 2041 | 0 | 0 |
| hsa_circ_0052968 | 94 | 2199 | 2262 | 1584 | 1647 | 0 | 8.00E-17 |
| hsa_circ_0042421 | 92 | 49 | 789 | 840 | 100 | 0 | 0 |
| hsa_circ_0042419 | 94 | 49 | 1234 | 1186 | 1 | 0 | 0 |
| hsa_circ_0042415 | 93 | 49 | 1553 | 1505 | 1 | 0 | 0 |
| hsa_circ_0042410 | 93 | 49 | 1663 | 1615 | 1 | 0 | 0 |
| hsa_circ_0042409 | 93 | 49 | 1746 | 1698 | 1 | 0 | 0 |
| hsa_circ_0042421 | 92 | 1 | 741 | 840 | 100 | 0 | 0 |
| hsa_circ_0042421 | 93 | 1 | 840 | 840 | 1 | 0 | 0 |
| hsa_circ_0042419 | 94 | 1 | 1186 | 1186 | 1 | 0 | 0 |
| hsa_circ_0042415 | 94 | 1 | 1331 | 1505 | 175 | 0 | 0 |
| hsa_circ_0042415 | 93 | 1 | 1505 | 1505 | 1 | 0 | 0 |
| hsa_circ_0042409 | 93 | 1 | 1698 | 1698 | 1 | 0 | 0 |
| hsa_circ_0004719 | 91 | 3 | 558 | 556 | 1 | 0 | 0 |
| hsa_circ_0042419 | 93 | 3 | 1003 | 1001 | 1 | 0 | 0 |
| hsa_circ_0042421 | 91 | 1 | 557 | 653 | 97 | 0 | 0 |
| hsa_circ_0042419 | 97 | 1 | 445 | 445 | 1 | 0 | 0 |
| hsa_circ_0042415 | 94 | 1 | 764 | 764 | 1 | 0 | 0 |
| hsa_circ_0042410 | 94 | 1 | 874 | 874 | 1 | 0 | 0 |
| hsa_circ_0042409 | 94 | 1 | 957 | 957 | 1 | 0 | 0 |
| hsa_circ_0042410 | 91 | 1 | 429 | 429 | 1 | 0 | 2.00E-163 |
| hsa_circ_0042409 | 91 | 1 | 512 | 512 | 1 | 0 | 0 |
| hsa_circ_0042408 | 93 | 1 | 342 | 342 | 1 | 0 | 2.00E-138 |
| hsa_circ_0042405 | 89 | 3 | 350 | 348 | 1 | 0 | 9.00E-122 |
| hsa_circ_0042400 | 91 | 3 | 492 | 799 | 310 | 0 | 0 |
| hsa_circ_0042400 | 91 | 3 | 629 | 799 | 173 | 0 | 0 |
| hsa_circ_0042400 | 92 | 3 | 801 | 799 | 1 | 0 | 0 |
| hsa_circ_0042393 | 92 | 3 | 1000 | 1512 | 515 | 0 | 0 |
| hsa_circ_0042393 | 91 | 3 | 1227 | 1512 | 288 | 0 | 0 |
| hsa_circ_0042400 | 92 | 1 | 318 | 627 | 310 | 0 | 8.00E-127 |
| hsa_circ_0042400 | 91 | 1 | 381 | 553 | 173 | 0 | 9.00E-147 |
| hsa_circ_0042400 | 94 | 1 | 309 | 309 | 1 | 0 | 3.00E-130 |
| hsa_circ_0042394 | 92 | 1 | 508 | 508 | 1 | 0 | 0 |
| hsa_circ_0042393 | 91 | 1 | 227 | 514 | 288 | 0 | 2.00E-81 |
| hsa_circ_0042392 | 89 | 1 | 806 | 806 | 1 | 0 | 0 |
| hsa_circ_0042392 | 88 | 3 | 579 | 577 | 1 | 0 | 0 |
| hsa_circ_0042386 | 88 | 3 | 938 | 936 | 1 | 0 | 0 |
| hsa_circ_0042386 | 88 | 3 | 938 | 936 | 1 | 0 | 0 |
| hsa_circ_0042386 | 88 | 3 | 938 | 936 | 1 | 0 | 0 |
| hsa_circ_0042358 | 90 | 1 | 237 | 1 | 238 | 0 | 1.00E-83 |
| hsa_circ_0042358 | 89 | 1 | 655 | 1 | 656 | 0 | 0 |
| hsa_circ_0042358 | 89 | 1 | 711 | 1 | 712 | 0 | 0 |
| hsa_circ_0042358 | 89 | 1 | 268 | 97 | 364 | 0 | 4.00E-89 |
| hsa_circ_0042359 | 90 | 1 | 288 | 1 | 288 | 0 | 7.00E-102 |
| hsa_circ_0042364 | 91 | 1 | 149 | 1 | 149 | 0 | 2.00E-49 |
| hsa_circ_0083827 | 95 | 1 | 265 | 1 | 265 | 0 | 8.00E-116 |
| hsa_circ_0083825 | 96 | 1 | 351 | 1 | 351 | 0 | 2.00E-163 |
| hsa_circ_0083823 | 96 | 1 | 828 | 81 | 908 | 0 | 0 |
| hsa_circ_0083823 | 96 | 2 | 908 | 2 | 908 | 0 | 0 |
| hsa_circ_0006175 | 97 | 1 | 563 | 1 | 563 | 0 | 0 |

novel_circ_016664 novel_circ_016665 novel_circ_016666 novel_circ_016667 novel_circ_016668 novel_circ_016669 novel_circ_016671 novel_circ_016672 novel_circ_016673 novel_circ_016674 novel_circ_016676 novel_circ_016677 novel_circ_016678 novel_circ_016679 novel_circ_016680 novel_circ_016681 novel_circ_016682 novel_circ_016683 novel_circ_016684 novel_circ_016685 novel_circ_016688 novel_circ_016689 novel_circ_016691 novel_circ_016692 novel_circ_016693 novel_circ_016697 novel_circ_016698 novel_circ_016699 novel_circ_016700 novel_circ_016701 novel_circ_016702 novel_circ_016703 novel_circ_016704 novel_circ_016705 novel_circ_016709 novel_circ_016710 novel_circ_016711 novel_circ_016712 novel_circ_016713 novel_circ_016714 novel_circ_016715 novel_circ_016716 novel_circ_016717 novel_circ_016718 novel_circ_016719 novel_circ_016725 novel_circ_016726 novel_circ_016728 novel_circ_016729 novel_circ_016730 novel_circ_016735 novel_circ_016736 novel_circ_016737 novel_circ_016738

| hsa_circ_0083823 | 96 | 2 | 643 | 2 | 643 | 0 | 0 |
| --- | --- | --- | --- | --- | --- | --- | --- |
| hsa_circ_0083823 | 96 | 20298 | 20939 | 2 | 643 | 0 | 0 |
| hsa_circ_0006175 | 96 | 1 | 477 | 1 | 477 | 0 | 0 |
| hsa_circ_0083823 | 96 | 2 | 557 | 2 | 557 | 0 | 0 |
| hsa_circ_0083819 | 94 | 4 | 479 | 476 | 1 | 0 | 0 |
| hsa_circ_0083813 | 94 | 4 | 735 | 1025 | 294 | 0 | 0 |
| hsa_circ_0083803 | 89 | 1 | 2850 | 1 | 2842 | 0 | 0 |
| hsa_circ_0083803 | 89 | 109 | 2958 | 1 | 2842 | 0 | 0 |
| hsa_circ_0083804 | 89 | 1 | 2631 | 1 | 2623 | 0 | 0 |
| hsa_circ_0083803 | 89 | 1 | 2725 | 1 | 2717 | 0 | 0 |
| hsa_circ_0083797 | 95 | 1 | 1018 | 1 | 1018 | 0 | 0 |
| hsa_circ_0083797 | 95 | 1 | 1018 | 1 | 1018 | 0 | 0 |
| hsa_circ_0083796 | 95 | 1 | 1215 | 1 | 1215 | 0 | 0 |
| hsa_circ_0083795 | 90 | 1 | 535 | 1 | 533 | 0.01 | 0 |
| hsa_circ_0083793 | 91 | 322 | 447 | 97724 | 97599 | 0 | 1.00E-41 |
| hsa_circ_0083794 | 89 | 233 | 857 | 1291 | 688 | 0.03 | 0 |
| hsa_circ_0083794 | 88 | 233 | 1076 | 1291 | 469 | 0.03 | 0 |
| hsa_circ_0083794 | 88 | 233 | 1179 | 1291 | 363 | 0.03 | 0 |
| hsa_circ_0083794 | 92 | 23 | 544 | 1209 | 688 | 0 | 0 |
| hsa_circ_0083794 | 90 | 23 | 866 | 1209 | 363 | 0.01 | 0 |
| hsa_circ_0001785 | 92 | 2 | 467 | 2 | 467 | 0 | 0 |
| hsa_circ_0001784 | 91 | 1 | 483 | 1 | 483 | 0 | 0 |
| hsa_circ_0083761 | 94 | 1 | 347 | 1 | 347 | 0 | 6.00E-148 |
| hsa_circ_0083760 | 94 | 285 | 633 | 240 | 588 | 0 | 9.00E-149 |
| hsa_circ_0083760 | 93 | 2 | 241 | 2 | 241 | 0 | 1.00E-93 |
| hsa_circ_0083749 | 93 | 1 | 469 | 499 | 967 | 0 | 0 |
| hsa_circ_0083749 | 92 | 1 | 498 | 1 | 498 | 0 | 0 |
| hsa_circ_0005013 | 92 | 1 | 274 | 1 | 274 | 0 | 9.00E-106 |
| hsa_circ_0083743 | 87 | 1 | 2455 | 225 | 2674 | 0.03 | 0 |
| hsa_circ_0083743 | 93 | 1 | 642 | 225 | 866 | 0.01 | 0 |
| hsa_circ_0002131 | 94 | 1 | 330 | 185 | 511 | 0.01 | 2.00E-137 |
| hsa_circ_0083741 | 96 | 3 | 427 | 3 | 427 | 0 | 0 |
| hsa_circ_0083738 | 96 | 1 | 718 | 265 | 982 | 0 | 0 |
| hsa_circ_0083738 | 98 | 13311 | 13572 | 1 | 262 | 0 | 2.00E-127 |
| hsa_circ_0083734 | 97 | 393 | 577 | 353 | 169 | 0 | 1.00E-82 |
| hsa_circ_0083734 | 94 | 393 | 745 | 353 | 1 | 0 | 3.00E-149 |
| hsa_circ_0083734 | 94 | 393 | 745 | 353 | 1 | 0 | 4.00E-149 |
| hsa_circ_0083732 | 95 | 653 | 1135 | 2609 | 2127 | 0 | 0 |
| hsa_circ_0083732 | 94 | 653 | 1348 | 2609 | 1914 | 0 | 0 |
| hsa_circ_0083734 | 97 | 269 | 453 | 353 | 169 | 0 | 9.00E-83 |
| hsa_circ_0083709 | 92 | 1 | 1066 | 100 | 1165 | 0 | 0 |
| hsa_circ_0083708 | 91 | 3 | 711 | 146 | 854 | 0.01 | 0 |
| hsa_circ_0038457 | 90 | 1 | 1091 | 1 | 1091 | 0 | 0 |
| hsa_circ_0038457 | 90 | 1 | 933 | 1 | 933 | 0 | 0 |
| hsa_circ_0038457 | 90 | 1 | 579 | 1 | 579 | 0 | 0 |
| hsa_circ_0038400 | 96 | 1 | 252 | 233 | 484 | 0 | 9.00E-112 |
| hsa_circ_0038400 | 96 | 1 | 252 | 233 | 484 | 0 | 1.00E-113 |
| hsa_circ_0038398 | 95 | 1 | 536 | 536 | 1 | 0 | 0 |
| hsa_circ_0038391 | 88 | 1 | 446 | 241 | 683 | 0.01 | 2.00E-148 |
| hsa_circ_0038383 | 90 | 3 | 679 | 3867 | 3194 | 0 | 0 |
| hsa_circ_0064628 | 93 | 1 | 347 | 918 | 575 | 0.01 | 8.00E-142 |
| hsa_circ_0064628 | 94 | 1 | 596 | 918 | 326 | 0.01 | 0 |
| hsa_circ_0064624 | 90 | 1 | 529 | 718 | 190 | 0 | 0 |
| hsa_circ_0064624 | 91 | 1 | 718 | 718 | 1 | 0 | 0 |

novel_circ_016739 novel_circ_016740 novel_circ_016741 novel_circ_016742 novel_circ_016743 novel_circ_016744 novel_circ_016745 novel_circ_016747 novel_circ_016748 novel_circ_016749 novel_circ_016750 novel_circ_016751 novel_circ_016752 novel_circ_016753 novel_circ_016754 novel_circ_016755 novel_circ_016756 novel_circ_016757 novel_circ_016758 novel_circ_016759 novel_circ_016760 novel_circ_016761 novel_circ_016762 novel_circ_016763 novel_circ_016764 novel_circ_016765 novel_circ_016766 novel_circ_016767 novel_circ_016768 novel_circ_016769 novel_circ_016770 novel_circ_016771 novel_circ_016772 novel_circ_016773 novel_circ_016774 novel_circ_016775 novel_circ_016776 novel_circ_016777 novel_circ_016778 novel_circ_016779 novel_circ_016780 novel_circ_016781 novel_circ_016782 novel_circ_016783 novel_circ_016784 novel_circ_016785 novel_circ_016786 novel_circ_016787 novel_circ_016788 novel_circ_016789 novel_circ_016790 novel_circ_016791 novel_circ_016792 novel_circ_016793

| hsa_circ_0064622 | 91 | 1 | 722 | 1706 | 985 | 0 | 0 |
| --- | --- | --- | --- | --- | --- | --- | --- |
| hsa_circ_0064622 | 92 | 1 | 1204 | 1706 | 500 | 0 | 0 |
| hsa_circ_0064622 | 92 | 1 | 1204 | 1706 | 500 | 0 | 0 |
| hsa_circ_0064622 | 92 | 1 | 1204 | 1706 | 500 | 0 | 0 |
| hsa_circ_0064618 | 91 | 1270 | 3587 | 2318 | 1 | 0 | 0 |
| hsa_circ_0064618 | 91 | 1270 | 3587 | 2318 | 1 | 0 | 0 |
| hsa_circ_0064618 | 91 | 1270 | 3587 | 2318 | 1 | 0 | 0 |
| hsa_circ_0064623 | 97 | 1 | 117 | 512 | 396 | 0 | 2.00E-48 |
| hsa_circ_0064623 | 97 | 1 | 117 | 512 | 396 | 0 | 3.00E-48 |
| hsa_circ_0064622 | 90 | 183 | 674 | 494 | 3 | 0 | 0 |
| hsa_circ_0064621 | 92 | 183 | 851 | 669 | 1 | 0 | 0 |
| hsa_circ_0064620 | 92 | 183 | 1337 | 1431 | 277 | 0 | 0 |
| hsa_circ_0064619 | 91 | 183 | 2270 | 2088 | 1 | 0 | 0 |
| hsa_circ_0064618 | 91 | 183 | 2500 | 2318 | 1 | 0 | 0 |
| hsa_circ_0064618 | 91 | 183 | 2500 | 2318 | 1 | 0 | 0 |
| hsa_circ_0064618 | 91 | 183 | 2500 | 2318 | 1 | 0 | 0 |
| hsa_circ_0064619 | 91 | 4 | 1848 | 1846 | 1 | 0 | 0 |
| hsa_circ_0064619 | 89 | 1 | 690 | 933 | 244 | 0 | 0 |
| hsa_circ_0064618 | 90 | 1 | 1163 | 1163 | 1 | 0 | 0 |
| hsa_circ_0064618 | 90 | 1 | 1163 | 1163 | 1 | 0 | 0 |
| hsa_circ_0064619 | 89 | 1 | 243 | 243 | 1 | 0 | 6.00E-82 |
| hsa_circ_0064618 | 91 | 1 | 375 | 473 | 99 | 0 | 4.00E-140 |
| hsa_circ_0003925 | 92 | 1 | 402 | 399 | 1 | 0.01 | 1.00E-155 |
| hsa_circ_0064612 | 92 | 760 | 1348 | 589 | 1 | 0 | 0 |
| hsa_circ_0064610 | 90 | 439 | 569 | 86618 | 86489 | 0.01 | 2.00E-40 |
| hsa_circ_0064610 | 90 | 439 | 569 | 86618 | 86489 | 0.01 | 3.00E-40 |
| hsa_circ_0064610 | 90 | 214 | 344 | 86618 | 86489 | 0.01 | 2.00E-40 |
| hsa_circ_0064610 | 90 | 1 | 131 | 86618 | 86489 | 0.01 | 2.00E-40 |
| hsa_circ_0064608 | 91 | 10 | 223 | 214 | 1 | 0 | 2.00E-77 |
| hsa_circ_0064607 | 91 | 224 | 602 | 379 | 1 | 0 | 2.00E-145 |
| hsa_circ_0064592 | 89 | 1 | 247 | 5120 | 4874 | 0 | 4.00E-84 |
| hsa_circ_0064592 | 95 | 4 | 301 | 4961 | 4664 | 0 | 2.00E-132 |
| hsa_circ_0064592 | 95 | 4 | 612 | 4961 | 4353 | 0 | 0 |
| hsa_circ_0064592 | 95 | 4 | 786 | 4961 | 4179 | 0 | 0 |
| hsa_circ_0064592 | 95 | 1 | 935 | 4873 | 3939 | 0 | 0 |
| hsa_circ_0064592 | 94 | 1 | 1148 | 4663 | 3516 | 0 | 0 |
| hsa_circ_0064600 | 93 | 4 | 661 | 1296 | 639 | 0 | 0 |
| hsa_circ_0064600 | 92 | 1 | 806 | 1173 | 368 | 0 | 0 |
| hsa_circ_0064592 | 95 | 5 | 1096 | 2705 | 1614 | 0 | 0 |
| hsa_circ_0064592 | 95 | 1 | 864 | 2477 | 1614 | 0 | 0 |
| hsa_circ_0064593 | 91 | 1 | 700 | 703 | 1 | 0 | 0 |
| hsa_circ_0015346 | 94 | 1040 | 1568 | 3659 | 3131 | 0 | 0 |
| hsa_circ_0015346 | 92 | 1040 | 2026 | 3659 | 2673 | 0 | 0 |
| hsa_circ_0015346 | 94 | 478 | 1006 | 3659 | 3131 | 0 | 0 |
| hsa_circ_0015346 | 92 | 478 | 1464 | 3659 | 2673 | 0 | 0 |
| hsa_circ_0015346 | 89 | 1 | 456 | 3128 | 2673 | 0 | 1.00E-156 |
| hsa_circ_0015336 | 96 | 1 | 270 | 76 | 345 | 0 | 3.00E-120 |
| hsa_circ_0015335 | 95 | 1 | 459 | 1 | 459 | 0 | 0 |
| hsa_circ_0006029 | 95 | 1 | 245 | 413 | 657 | 0 | 2.00E-106 |
| hsa_circ_0006029 | 94 | 1 | 387 | 271 | 657 | 0 | 9.00E-167 |
| hsa_circ_0015324 | 95 | 1 | 746 | 995 | 1740 | 0 | 0 |
| hsa_circ_0015324 | 93 | 10 | 1501 | 245 | 1740 | 0 | 0 |
| hsa_circ_0015332 | 95 | 1 | 337 | 1 | 337 | 0 | 5.00E-149 |
| hsa_circ_0015324 | 95 | 1 | 511 | 1090 | 1600 | 0 | 0 |

novel_circ_016794 novel_circ_016795 novel_circ_016796 novel_circ_016797 novel_circ_016798 novel_circ_016799 novel_circ_016800 novel_circ_016801 novel_circ_016802 novel_circ_016803 novel_circ_016804 novel_circ_016805 novel_circ_016806 novel_circ_016807 novel_circ_016808 novel_circ_016809 novel_circ_016810 novel_circ_016811 novel_circ_016812 novel_circ_016813 novel_circ_016814 novel_circ_016815 novel_circ_016816 novel_circ_016817 novel_circ_016818 novel_circ_016819 novel_circ_016820 novel_circ_016821 novel_circ_016822 novel_circ_016823 novel_circ_016824 novel_circ_016825 novel_circ_016826 novel_circ_016827 novel_circ_016828 novel_circ_016830 novel_circ_016831 novel_circ_016832 novel_circ_016833 novel_circ_016834 novel_circ_016837 novel_circ_016838 novel_circ_016839 novel_circ_016840 novel_circ_016841 novel_circ_016842 novel_circ_016843 novel_circ_016844 novel_circ_016845 novel_circ_016847 novel_circ_016848 novel_circ_016849 novel_circ_016850 novel_circ_016851

| hsa_circ_0015324 | 95 | 1 | 606 | 995 | 1600 | 0 | 0 |
| --- | --- | --- | --- | --- | --- | --- | --- |
| hsa_circ_0015324 | 95 | 1 | 992 | 609 | 1600 | 0 | 0 |
| hsa_circ_0015324 | 95 | 413 | 1406 | 607 | 1600 | 0 | 0 |
| hsa_circ_0015324 | 93 | 10 | 1361 | 245 | 1600 | 0 | 0 |
| hsa_circ_0015324 | 94 | 1 | 655 | 609 | 1263 | 0 | 0 |
| hsa_circ_0015324 | 94 | 413 | 1069 | 607 | 1263 | 0 | 0 |
| hsa_circ_0015324 | 92 | 10 | 1024 | 245 | 1263 | 0.01 | 0 |
| hsa_circ_0015324 | 91 | 10 | 755 | 245 | 994 | 0.01 | 0 |
| hsa_circ_0015328 | 91 | 1 | 401 | 1 | 404 | 0.01 | 4.00E-150 |
| hsa_circ_0015326 | 89 | 10 | 369 | 1 | 364 | 0.02 | 2.00E-124 |
| hsa_circ_0004927 | 97 | 1 | 380 | 381 | 1 | 0.01 | 1.00E-179 |
| hsa_circ_0015322 | 96 | 381 | 1118 | 738 | 1 | 0 | 0 |
| hsa_circ_0015322 | 96 | 1 | 617 | 617 | 1 | 0 | 0 |
| hsa_circ_0015320 | 96 | 1 | 750 | 750 | 1 | 0 | 0 |
| hsa_circ_0015317 | 96 | 1 | 869 | 2018 | 1150 | 0 | 0 |
| hsa_circ_0015317 | 96 | 1 | 982 | 2018 | 1037 | 0 | 0 |
| hsa_circ_0015317 | 94 | 1 | 1479 | 2018 | 540 | 0 | 0 |
| hsa_circ_0015317 | 93 | 1 | 2010 | 2018 | 9 | 0 | 0 |
| hsa_circ_0015312 | 93 | 417 | 2609 | 10141 | 7949 | 0 | 0 |
| hsa_circ_0015317 | 96 | 1 | 566 | 1602 | 1037 | 0 | 0 |
| hsa_circ_0015319 | 96 | 1 | 365 | 647 | 283 | 0 | 3.00E-166 |
| hsa_circ_0015317 | 92 | 1 | 610 | 1149 | 540 | 0 | 0 |
| hsa_circ_0015312 | 92 | 1 | 1302 | 9688 | 8387 | 0 | 0 |
| hsa_circ_0015317 | 90 | 1 | 586 | 754 | 169 | 0 | 0 |
| hsa_circ_0015317 | 91 | 1 | 746 | 754 | 9 | 0 | 0 |
| hsa_circ_0015315 | 92 | 1 | 907 | 1345 | 439 | 0 | 0 |
| hsa_circ_0015315 | 92 | 1 | 1345 | 1345 | 1 | 0 | 0 |
| hsa_circ_0015312 | 92 | 1 | 1516 | 9293 | 7775 | 0 | 0 |
| hsa_circ_0015312 | 92 | 1 | 1632 | 9293 | 7659 | 0 | 0 |
| hsa_circ_0015312 | 92 | 1 | 1632 | 9293 | 7659 | 0 | 0 |
| hsa_circ_0015317 | 92 | 1 | 531 | 539 | 9 | 0 | 0 |
| hsa_circ_0015315 | 93 | 1 | 591 | 591 | 1 | 0 | 0 |
| hsa_circ_0015312 | 92 | 1 | 762 | 8539 | 7775 | 0 | 0 |
| hsa_circ_0015315 | 91 | 1 | 438 | 438 | 1 | 0 | 2.00E-168 |
| hsa_circ_0015312 | 91 | 1 | 609 | 8386 | 7775 | 0 | 0 |
| hsa_circ_0015282 | 90 | 101 | 1623 | 1 | 1523 | 0 | 0 |
| hsa_circ_0015285 | 86 | 1 | 471 | 1 | 472 | 0 | 1.00E-141 |
| hsa_circ_0015282 | 90 | 101 | 1535 | 1 | 1436 | 0 | 0 |
| hsa_circ_0015282 | 93 | 101 | 1001 | 1 | 901 | 0 | 0 |
| hsa_circ_0015283 | 95 | 1 | 215 | 1 | 215 | 0 | 4.00E-93 |
| hsa_circ_0015280 | 95 | 1 | 316 | 1 | 316 | 0 | 5.00E-139 |
| hsa_circ_0015275 | 95 | 1 | 450 | 445 | 894 | 0 | 0 |
| hsa_circ_0005794 | 94 | 1 | 343 | 1 | 343 | 0 | 2.00E-147 |
| hsa_circ_0015278 | 94 | 1 | 462 | 1 | 462 | 0 | 0 |
| hsa_circ_0015278 | 94 | 1 | 328 | 1 | 328 | 0 | 5.00E-139 |
| hsa_circ_0015273 | 95 | 1 | 698 | 575 | 1272 | 0 | 0 |
| hsa_circ_0015273 | 95 | 1 | 554 | 575 | 1128 | 0 | 0 |
| hsa_circ_0015273 | 96 | 1 | 370 | 575 | 944 | 0 | 1.00E-170 |
| hsa_circ_0015273 | 95 | 1 | 574 | 1 | 574 | 0 | 0 |
| hsa_circ_0036251 | 87 | 5148 | 5822 | 675 | 1 | 0 | 0 |
| hsa_circ_0036251 | 87 | 1 | 675 | 675 | 1 | 0 | 0 |
| hsa_circ_0036251 | 87 | 5148 | 5822 | 675 | 1 | 0 | 0 |
| hsa_circ_0036251 | 86 | 3 | 408 | 406 | 1 | 0 | 6.00E-119 |
| hsa_circ_0036251 | 87 | 1 | 675 | 675 | 1 | 0 | 0 |

novel_circ_016852 novel_circ_016853 novel_circ_016856 novel_circ_016857 novel_circ_016858 novel_circ_016859 novel_circ_016860 novel_circ_016861 novel_circ_016863 novel_circ_016864 novel_circ_016865 novel_circ_016866 novel_circ_016867 novel_circ_016868 novel_circ_016869 novel_circ_016870 novel_circ_016871 novel_circ_016872 novel_circ_016873 novel_circ_016874 novel_circ_016875 novel_circ_016876 novel_circ_016877 novel_circ_016878 novel_circ_016879 novel_circ_016880 novel_circ_016881 novel_circ_016882 novel_circ_016883 novel_circ_016884 novel_circ_016885 novel_circ_016886 novel_circ_016887 novel_circ_016888 novel_circ_016889 novel_circ_016890 novel_circ_016891 novel_circ_016892 novel_circ_016893 novel_circ_016894 novel_circ_016895 novel_circ_016896 novel_circ_016897 novel_circ_016898 novel_circ_016899 novel_circ_016900 novel_circ_016901 novel_circ_016902 novel_circ_016906 novel_circ_016908 novel_circ_016909 novel_circ_016910 novel_circ_016911 novel_circ_016912

| hsa_circ_0036251 | 89 | 1 | 401 | 675 | 275 | 0 | 3.00E-141 |
| --- | --- | --- | --- | --- | --- | --- | --- |
| hsa_circ_0036253 | 98 | 1 | 348 | 348 | 1 | 0 | 3.00E-172 |
| hsa_circ_0070963 | 86 | 1 | 325 | 325 | 1 | 0 | 2.00E-97 |
| hsa_circ_0070963 | 86 | 1 | 325 | 325 | 1 | 0 | 4.00E-97 |
| hsa_circ_0004160 | 95 | 1 | 405 | 405 | 1 | 0 | 4.00E-180 |
| hsa_circ_0005333 | 96 | 1 | 557 | 557 | 1 | 0 | 0 |
| hsa_circ_0004659 | 95 | 27159 | 27198 | 1E+06 | 1078581 | 0.02 | 8.00E-07 |
| hsa_circ_0014617 | 95 | 1 | 118 | 724 | 841 | 0 | 4.00E-45 |
| hsa_circ_0014592 | 95 | 26022 | 26535 | 515 | 2 | 0 | 0 |
| hsa_circ_0014592 | 95 | 9163 | 9676 | 515 | 2 | 0 | 0 |
| hsa_circ_0014592 | 95 | 7 | 520 | 515 | 2 | 0 | 0 |
| hsa_circ_0014591 | 95 | 7 | 5094 | 5079 | 1 | 0 | 0 |
| hsa_circ_0014589 | 94 | 1 | 4678 | 4666 | 1 | 0 | 0 |
| hsa_circ_0014586 | 91 | 2385 | 3412 | 1022 | 1 | 0.01 | 0 |
| hsa_circ_0014584 | 91 | 2385 | 3507 | 1117 | 1 | 0.01 | 0 |
| hsa_circ_0003247 | 90 | 3 | 850 | 842 | 1 | 0.01 | 0 |
| hsa_circ_0014586 | 91 | 3 | 1030 | 1022 | 1 | 0.01 | 0 |
| hsa_circ_0014584 | 91 | 3 | 1125 | 1117 | 1 | 0.01 | 0 |
| hsa_circ_0014579 | 91 | 3 | 1708 | 1700 | 1 | 0.01 | 0 |
| hsa_circ_0003247 | 90 | 3 | 745 | 740 | 1 | 0.01 | 0 |
| hsa_circ_0014584 | 95 | 1 | 275 | 275 | 1 | 0 | 5.00E-118 |
| hsa_circ_0014579 | 93 | 1 | 858 | 858 | 1 | 0 | 0 |
| hsa_circ_0014581 | 92 | 1 | 324 | 324 | 1 | 0 | 4.00E-125 |
| hsa_circ_0014579 | 92 | 1 | 678 | 678 | 1 | 0 | 0 |
| hsa_circ_0014581 | 92 | 1 | 229 | 229 | 1 | 0 | 7.00E-86 |
| hsa_circ_0014579 | 92 | 1 | 583 | 583 | 1 | 0 | 0 |
| hsa_circ_0014575 | 93 | 1 | 692 | 692 | 1 | 0 | 0 |
| hsa_circ_0014579 | 92 | 4 | 509 | 506 | 1 | 0 | 0 |
| hsa_circ_0014579 | 93 | 1 | 354 | 354 | 1 | 0 | 4.00E-145 |
| hsa_circ_0014575 | 93 | 1 | 463 | 463 | 1 | 0 | 0 |
| hsa_circ_0014573 | 92 | 1 | 723 | 723 | 1 | 0 | 0 |
| hsa_circ_0014571 | 92 | 1 | 879 | 881 | 3 | 0 | 0 |
| hsa_circ_0014568 | 93 | 1 | 1034 | 1034 | 1 | 0 | 0 |
| hsa_circ_0014562 | 93 | 1 | 1008 | 1008 | 1 | 0 | 0 |
| hsa_circ_0014568 | 93 | 1 | 311 | 311 | 1 | 0 | 3.00E-126 |
| hsa_circ_0014562 | 94 | 1 | 748 | 748 | 1 | 0 | 0 |
| hsa_circ_0014561 | 94 | 1 | 835 | 835 | 1 | 0 | 0 |
| hsa_circ_0014554 | 94 | 1 | 1238 | 1238 | 1 | 0 | 0 |
| hsa_circ_0014564 | 95 | 1 | 340 | 341 | 2 | 0 | 1.00E-150 |
| hsa_circ_0014562 | 94 | 1 | 590 | 590 | 1 | 0 | 0 |
| hsa_circ_0014561 | 94 | 1 | 677 | 677 | 1 | 0 | 0 |
| hsa_circ_0014562 | 94 | 1 | 437 | 437 | 1 | 0 | 0 |
| hsa_circ_0014549 | 94 | 1 | 905 | 947 | 43 | 0 | 0 |
| hsa_circ_0014554 | 94 | 1 | 403 | 403 | 1 | 0 | 6.00E-174 |
| hsa_circ_0014549 | 95 | 1 | 569 | 611 | 43 | 0 | 0 |
| hsa_circ_0014549 | 95 | 3 | 478 | 518 | 43 | 0 | 0 |
| hsa_circ_0014548 | 93 | 3 | 822 | 820 | 1 | 0 | 0 |
| hsa_circ_0014526 | 92 | 1 | 933 | 1143 | 214 | 0 | 0 |
| hsa_circ_0036064 | 93 | 34200 | 34681 | 484 | 3 | 0 | 0 |
| hsa_circ_0036047 | 94 | 1 | 698 | 134 | 831 | 0 | 0 |
| hsa_circ_0036047 | 94 | 1 | 831 | 1 | 831 | 0 | 0 |
| hsa_circ_0036047 | 94 | 3 | 341 | 362 | 700 | 0 | 4.00E-145 |
| hsa_circ_0036047 | 94 | 1 | 567 | 134 | 700 | 0 | 0 |
| hsa_circ_0036047 | 95 | 1 | 700 | 1 | 700 | 0 | 0 |

novel_circ_016913 novel_circ_016914 novel_circ_016915 novel_circ_016916 novel_circ_016917 novel_circ_016918 novel_circ_016919 novel_circ_016921 novel_circ_016922 novel_circ_016923 novel_circ_016924 novel_circ_016925 novel_circ_016926 novel_circ_016927 novel_circ_016928 novel_circ_016930 novel_circ_016931 novel_circ_016932 novel_circ_016933 novel_circ_016935 novel_circ_016936 novel_circ_016937 novel_circ_016938 novel_circ_016939 novel_circ_016940 novel_circ_016941 novel_circ_016942 novel_circ_016943 novel_circ_016944 novel_circ_016945 novel_circ_016946 novel_circ_016947 novel_circ_016950 novel_circ_016951 novel_circ_016952 novel_circ_016953 novel_circ_016954 novel_circ_016955 novel_circ_016956 novel_circ_016957 novel_circ_016958 novel_circ_016959 novel_circ_016960 novel_circ_016961 novel_circ_016962 novel_circ_016963 novel_circ_016964 novel_circ_016965 novel_circ_016966 novel_circ_016967 novel_circ_016968 novel_circ_016969 novel_circ_016970 novel_circ_016971

| hsa_circ_0036047 | 95 | 446 | 1145 | 1 | 700 | 0 | 0 |
| --- | --- | --- | --- | --- | --- | --- | --- |
| hsa_circ_0036047 | 94 | 1 | 406 | 134 | 539 | 0 | 1.00E-175 |
| hsa_circ_0036047 | 95 | 1 | 332 | 134 | 465 | 0 | 3.00E-146 |
| hsa_circ_0036047 | 95 | 1 | 465 | 1 | 465 | 0 | 0 |
| hsa_circ_0036047 | 95 | 446 | 910 | 1 | 465 | 0 | 0 |
| hsa_circ_0036047 | 96 | 1 | 359 | 1 | 359 | 0 | 7.00E-163 |
| hsa_circ_0036047 | 96 | 446 | 804 | 1 | 359 | 0 | 2.00E-162 |
| hsa_circ_0036033 | 95 | 1 | 308 | 257 | 564 | 0 | 3.00E-136 |
| hsa_circ_0036033 | 96 | 1 | 236 | 257 | 492 | 0 | 2.00E-106 |
| hsa_circ_0036029 | 94 | 1 | 484 | 69 | 552 | 0 | 0 |
| hsa_circ_0005498 | 94 | 1 | 293 | 1 | 293 | 0 | 1.00E-124 |
| hsa_circ_0003651 | 93 | 1 | 378 | 378 | 1 | 0 | 2.00E-153 |
| hsa_circ_0000620 | 93 | 1 | 462 | 462 | 1 | 0 | 0 |
| hsa_circ_0036017 | 87 | 4 | 357 | 351 | 1 | 0.01 | 5.00E-109 |
| hsa_circ_0003973 | 94 | 1 | 452 | 1 | 452 | 0 | 0 |
| hsa_circ_0035971 | 92 | 1 | 488 | 1 | 488 | 0 | 0 |
| hsa_circ_0035963 | 86 | 1 | 332 | 1 | 332 | 0 | 7.00E-98 |
| hsa_circ_0035963 | 86 | 535 | 866 | 1 | 332 | 0 | 2.00E-97 |
| hsa_circ_0035959 | 88 | 155 | 1516 | 1 | 1365 | 0 | 0 |
| hsa_circ_0035961 | 90 | 4 | 580 | 184 | 760 | 0 | 0 |
| hsa_circ_0035961 | 88 | 2 | 757 | 2 | 760 | 0 | 0 |
| hsa_circ_0035959 | 89 | 1 | 1013 | 266 | 1281 | 0 | 0 |
| hsa_circ_0035959 | 88 | 155 | 1432 | 1 | 1281 | 0 | 0 |
| hsa_circ_0035959 | 89 | 1 | 765 | 266 | 1033 | 0 | 0 |
| hsa_circ_0035959 | 88 | 155 | 1184 | 1 | 1033 | 0 | 0 |
| hsa_circ_0035961 | 88 | 2 | 347 | 2 | 350 | 0.01 | 7.00E-113 |
| hsa_circ_0035959 | 89 | 1 | 603 | 266 | 871 | 0 | 0 |
| hsa_circ_0035959 | 88 | 155 | 1022 | 1 | 871 | 0 | 0 |
| hsa_circ_0035959 | 87 | 155 | 852 | 1 | 701 | 0 | 0 |
| hsa_circ_0035959 | 88 | 155 | 675 | 1 | 521 | 0 | 1.00E-177 |
| hsa_circ_0035959 | 87 | 155 | 596 | 1 | 442 | 0 | 4.00E-142 |
| hsa_circ_0035959 | 85 | 155 | 419 | 1 | 265 | 0 | 1.00E-70 |
| hsa_circ_0035957 | 92 | 6 | 667 | 661 | 1 | 0 | 0 |
| hsa_circ_0035957 | 93 | 4 | 583 | 580 | 1 | 0 | 0 |
| hsa_circ_0035947 | 94 | 4 | 1512 | 1506 | 1 | 0 | 0 |
| hsa_circ_0035938 | 94 | 4 | 1978 | 1972 | 1 | 0 | 0 |
| hsa_circ_0035954 | 95 | 1 | 732 | 729 | 1 | 0 | 0 |
| hsa_circ_0035947 | 94 | 1 | 1179 | 1176 | 1 | 0 | 0 |
| hsa_circ_0035938 | 94 | 1 | 1645 | 1642 | 1 | 0 | 0 |
| hsa_circ_0035938 | 94 | 1 | 1395 | 1392 | 1 | 0 | 0 |
| hsa_circ_0035954 | 94 | 3 | 412 | 407 | 1 | 0.01 | 1.00E-176 |
| hsa_circ_0035947 | 94 | 3 | 859 | 854 | 1 | 0 | 0 |
| hsa_circ_0035938 | 94 | 3 | 1325 | 1320 | 1 | 0 | 0 |
| hsa_circ_0035930 | 93 | 4 | 1903 | 3980 | 2084 | 0 | 0 |
| hsa_circ_0035938 | 94 | 4 | 466 | 463 | 1 | 0 | 0 |
| hsa_circ_0035933 | 93 | 1 | 614 | 2089 | 1476 | 0.01 | 0 |
| hsa_circ_0035933 | 93 | 1 | 746 | 2089 | 1347 | 0.01 | 0 |
| hsa_circ_0035933 | 92 | 1 | 991 | 2089 | 1102 | 0.01 | 0 |
| hsa_circ_0035930 | 90 | 1 | 2282 | 2697 | 419 | 0.01 | 0 |
| hsa_circ_0035927 | 90 | 1 | 3154 | 3151 | 1 | 0 | 0 |
| hsa_circ_0035934 | 88 | 1 | 245 | 249 | 5 | 0 | 2.00E-76 |
| hsa_circ_0035933 | 89 | 1 | 1346 | 1346 | 1 | 0.01 | 0 |
| hsa_circ_0035931 | 89 | 1 | 1536 | 1807 | 272 | 0.01 | 0 |
| hsa_circ_0035931 | 89 | 1 | 1631 | 1807 | 177 | 0 | 0 |

novel_circ_016972 novel_circ_016973 novel_circ_016974 novel_circ_016975 novel_circ_016976 novel_circ_016977 novel_circ_016978 novel_circ_016979 novel_circ_016980 novel_circ_016981 novel_circ_016983 novel_circ_016984 novel_circ_016985 novel_circ_016986 novel_circ_016987 novel_circ_016988 novel_circ_016989 novel_circ_016990 novel_circ_016991 novel_circ_016992 novel_circ_016993 novel_circ_016994 novel_circ_016995 novel_circ_016996 novel_circ_016997 novel_circ_016998 novel_circ_016999 novel_circ_017000 novel_circ_017001 novel_circ_017002 novel_circ_017003 novel_circ_017004 novel_circ_017005 novel_circ_017006 novel_circ_017007 novel_circ_017008 novel_circ_017009 novel_circ_017010 novel_circ_017013 novel_circ_017016 novel_circ_017020 novel_circ_017021 novel_circ_017022 novel_circ_017027 novel_circ_017030 novel_circ_017033 novel_circ_017034 novel_circ_017035 novel_circ_017036 novel_circ_017037 novel_circ_017038 novel_circ_017040 novel_circ_017041 novel_circ_017042

| hsa_circ_0035930 | 89 | 1 | 1954 | 1954 | 1 | 0 | 0 |
| --- | --- | --- | --- | --- | --- | --- | --- |
| hsa_circ_0035927 | 89 | 1 | 2153 | 2408 | 256 | 0 | 0 |
| hsa_circ_0035927 | 89 | 1 | 2408 | 2408 | 1 | 0 | 0 |
| hsa_circ_0035933 | 90 | 1 | 1097 | 1097 | 1 | 0.01 | 0 |
| hsa_circ_0035931 | 89 | 1 | 1287 | 1558 | 272 | 0.01 | 0 |
| hsa_circ_0035930 | 89 | 1 | 323 | 323 | 1 | 0 | 6.00E-113 |
| hsa_circ_0035927 | 88 | 1 | 777 | 777 | 1 | 0 | 0 |
| hsa_circ_0035919 | 92 | 534 | 792 | 259 | 1 | 0 | 3.00E-100 |
| hsa_circ_0035919 | 92 | 534 | 792 | 259 | 1 | 0 | 4.00E-100 |
| hsa_circ_0035919 | 92 | 160 | 418 | 259 | 1 | 0 | 3.00E-100 |
| hsa_circ_0035912 | 96 | 1 | 270 | 270 | 1 | 0 | 3.00E-120 |
| hsa_circ_0006487 | 96 | 1 | 383 | 383 | 1 | 0 | 3.00E-176 |
| hsa_circ_0006807 | 96 | 1 | 556 | 557 | 2 | 0 | 0 |
| hsa_circ_0035911 | 94 | 1 | 726 | 726 | 1 | 0 | 0 |
| hsa_circ_0035905 | 94 | 1 | 837 | 966 | 130 | 0 | 0 |
| hsa_circ_0035905 | 94 | 1 | 966 | 966 | 1 | 0 | 0 |
| hsa_circ_0035902 | 94 | 1 | 1028 | 1129 | 102 | 0 | 0 |
| hsa_circ_0035900 | 94 | 1 | 1467 | 1547 | 81 | 0 | 0 |
| hsa_circ_0035900 | 94 | 1 | 1547 | 1547 | 1 | 0 | 0 |
| hsa_circ_0035900 | 94 | 1 | 1547 | 1547 | 1 | 0 | 0 |
| hsa_circ_0035894 | 94 | 558 | 2130 | 1980 | 408 | 0 | 0 |
| hsa_circ_0035905 | 92 | 1 | 409 | 409 | 1 | 0 | 1.00E-165 |
| hsa_circ_0035902 | 93 | 1 | 471 | 572 | 102 | 0 | 0 |
| hsa_circ_0035902 | 94 | 1 | 572 | 572 | 1 | 0 | 0 |
| hsa_circ_0035900 | 94 | 1 | 990 | 990 | 1 | 0 | 0 |
| hsa_circ_0035894 | 94 | 1 | 1573 | 1980 | 408 | 0 | 0 |
| hsa_circ_0035900 | 94 | 4 | 439 | 516 | 81 | 0 | 0 |
| hsa_circ_0035900 | 95 | 4 | 519 | 516 | 1 | 0 | 0 |
| hsa_circ_0035896 | 95 | 4 | 954 | 1098 | 148 | 0 | 0 |
| hsa_circ_0035894 | 95 | 4 | 1102 | 1506 | 408 | 0 | 0 |
| hsa_circ_0035894 | 95 | 4 | 1102 | 1506 | 408 | 0 | 0 |
| hsa_circ_0035900 | 94 | 2 | 418 | 417 | 1 | 0 | 4.00E-180 |
| hsa_circ_0035894 | 95 | 2 | 1001 | 1407 | 408 | 0 | 0 |
| hsa_circ_0035895 | 95 | 1 | 583 | 735 | 153 | 0 | 0 |
| hsa_circ_0035895 | 96 | 1 | 430 | 582 | 153 | 0 | 0 |
| hsa_circ_0035894 | 94 | 459 | 866 | 408 | 1 | 0 | 4.00E-178 |
| hsa_circ_0035895 | 95 | 1 | 148 | 300 | 153 | 0 | 8.00E-59 |
| hsa_circ_0035894 | 94 | 313 | 720 | 408 | 1 | 0 | 4.00E-178 |
| hsa_circ_0052473 | 87 | 1 | 253 | 1 | 253 | 0 | 4.00E-74 |
| hsa_circ_0052460 | 90 | 1 | 470 | 473 | 4 | 0 | 4.00E-171 |
| hsa_circ_0052455 | 91 | 557 | 1078 | 522 | 1 | 0 | 0 |
| hsa_circ_0052454 | 88 | 1 | 369 | 369 | 1 | 0 | 9.00E-122 |
| hsa_circ_0052454 | 87 | 1 | 306 | 306 | 1 | 0 | 2.00E-94 |
| hsa_circ_0067244 | 87 | 1 | 653 | 1 | 653 | 0 | 0 |
| hsa_circ_0005732 | 92 | 1 | 504 | 1 | 504 | 0 | 0 |
| hsa_circ_0005732 | 92 | 1 | 504 | 1 | 504 | 0 | 0 |
| hsa_circ_0005732 | 92 | 1 | 310 | 1 | 310 | 0 | 2.00E-122 |
| hsa_circ_0056756 | 96 | 1 | 387 | 1 | 387 | 0 | 9.00E-177 |
| hsa_circ_0056755 | 95 | 2 | 559 | 2 | 559 | 0 | 0 |
| hsa_circ_0056755 | 95 | 112 | 669 | 2 | 559 | 0 | 0 |
| hsa_circ_0056755 | 92 | 112 | 282 | 2 | 172 | 0 | 2.00E-63 |
| hsa_circ_0003891 | 93 | 3 | 258 | 156 | 411 | 0 | 2.00E-102 |
| hsa_circ_0056961 | 87 | 3 | 1189 | 1789 | 603 | 0 | 0 |
| hsa_circ_0056961 | 86 | 3 | 1789 | 1789 | 3 | 0 | 0 |

novel_circ_017043 novel_circ_017044 novel_circ_017045 novel_circ_017046 novel_circ_017047 novel_circ_017048 novel_circ_017049 novel_circ_017050 novel_circ_017051 novel_circ_017052 novel_circ_017059 novel_circ_017060 novel_circ_017061 novel_circ_017062 novel_circ_017063 novel_circ_017064 novel_circ_017065 novel_circ_017066 novel_circ_017067 novel_circ_017068 novel_circ_017069 novel_circ_017070 novel_circ_017071 novel_circ_017072 novel_circ_017073 novel_circ_017074 novel_circ_017075 novel_circ_017076 novel_circ_017077 novel_circ_017078 novel_circ_017079 novel_circ_017080 novel_circ_017081 novel_circ_017082 novel_circ_017084 novel_circ_017091 novel_circ_017092 novel_circ_017094 novel_circ_017095 novel_circ_017096 novel_circ_017097 novel_circ_017098 novel_circ_017099 novel_circ_017100 novel_circ_017101 novel_circ_017102 novel_circ_017103 novel_circ_017104 novel_circ_017105 novel_circ_017106 novel_circ_017107 novel_circ_017108 novel_circ_017109 novel_circ_017110

| hsa_circ_0056961 | 86 | 3 | 1789 | 1789 | 3 | 0 | 0 |
| --- | --- | --- | --- | --- | --- | --- | --- |
| hsa_circ_0056961 | 86 | 3 | 1789 | 1789 | 3 | 0 | 0 |
| hsa_circ_0056961 | 84 | 6 | 732 | 729 | 3 | 0.01 | 0 |
| hsa_circ_0056955 | 86 | 6 | 1109 | 1101 | 1 | 0.01 | 0 |
| hsa_circ_0056955 | 90 | 1 | 490 | 487 | 1 | 0.01 | 9.00E-178 |
| hsa_circ_0056951 | 88 | 1 | 915 | 915 | 1 | 0.02 | 0 |
| hsa_circ_0056951 | 88 | 1 | 915 | 915 | 1 | 0.02 | 0 |
| hsa_circ_0056951 | 88 | 1 | 915 | 915 | 1 | 0.02 | 0 |
| hsa_circ_0056951 | 88 | 1 | 915 | 915 | 1 | 0.02 | 0 |
| hsa_circ_0056951 | 88 | 1 | 915 | 915 | 1 | 0.02 | 0 |
| hsa_circ_0056945 | 86 | 1 | 236 | 1 | 236 | 0 | 5.00E-68 |
| hsa_circ_0056944 | 88 | 1 | 329 | 52 | 380 | 0 | 1.00E-109 |
| hsa_circ_0056944 | 89 | 1 | 380 | 1 | 380 | 0 | 7.00E-133 |
| hsa_circ_0056941 | 89 | 1 | 438 | 238 | 675 | 0 | 1.00E-151 |
| hsa_circ_0056944 | 94 | 1 | 144 | 1 | 144 | 0 | 2.00E-55 |
| hsa_circ_0056941 | 94 | 1 | 237 | 1 | 237 | 0 | 6.00E-97 |
| hsa_circ_0056941 | 94 | 15799 | 16035 | 1 | 237 | 0 | 4.00E-95 |
| hsa_circ_0047658 | 85 | 1481 | 1714 | 68 | 301 | 0 | 2.00E-58 |
| hsa_circ_0056933 | 96 | 58563 | 58983 | 1169 | 749 | 0 | 0 |
| hsa_circ_0056933 | 96 | 7907 | 8327 | 1169 | 749 | 0 | 0 |
| hsa_circ_0056933 | 95 | 7907 | 8673 | 1169 | 403 | 0 | 0 |
| hsa_circ_0056933 | 96 | 7907 | 8788 | 1169 | 288 | 0 | 0 |
| hsa_circ_0001079 | 96 | 1 | 420 | 420 | 1 | 0 | 0 |
| hsa_circ_0002029 | 95 | 1 | 766 | 766 | 1 | 0 | 0 |
| hsa_circ_0001078 | 96 | 1 | 881 | 881 | 1 | 0 | 0 |
| hsa_circ_0002029 | 95 | 1 | 653 | 653 | 1 | 0 | 0 |
| hsa_circ_0002029 | 95 | 3 | 544 | 542 | 1 | 0 | 0 |
| hsa_circ_0002029 | 95 | 1 | 346 | 346 | 1 | 0 | 2.00E-152 |
| hsa_circ_0001078 | 96 | 1 | 461 | 461 | 1 | 0 | 0 |
| hsa_circ_0056933 | 96 | 1 | 464 | 748 | 285 | 0 | 0 |
| hsa_circ_0056933 | 94 | 1 | 745 | 748 | 1 | 0 | 0 |
| hsa_circ_0056933 | 94 | 1 | 399 | 402 | 1 | 0.01 | 3.00E-172 |
| hsa_circ_0056933 | 92 | 1 | 284 | 287 | 1 | 0.01 | 3.00E-110 |
| hsa_circ_0056929 | 93 | 1 | 406 | 409 | 1 | 0.01 | 7.00E-168 |
| hsa_circ_0029815 | 89 | 36258 | 36799 | 543 | 2 | 0 | 0 |
| hsa_circ_0056922 | 80 | 1870 | 2869 | 1290 | 291 | 0.01 | 0 |
| hsa_circ_0056922 | 80 | 1870 | 2869 | 1290 | 291 | 0.01 | 0 |
| hsa_circ_0056922 | 80 | 91 | 1090 | 1290 | 291 | 0.01 | 0 |
| hsa_circ_0056922 | 81 | 1 | 770 | 1247 | 478 | 0 | 3.00E-179 |
| hsa_circ_0056922 | 82 | 1 | 772 | 1247 | 476 | 0 | 1.00E-179 |
| hsa_circ_0056922 | 82 | 3 | 718 | 1005 | 291 | 0.01 | 3.00E-170 |
| hsa_circ_0056919 | 89 | 242 | 692 | 448 | 1 | 0.01 | 6.00E-156 |
| hsa_circ_0056918 | 88 | 535 | 1069 | 535 | 1 | 0 | 0 |
| hsa_circ_0056916 | 86 | 1 | 622 | 1567 | 946 | 0.01 | 0 |
| hsa_circ_0056907 | 90 | 1 | 2369 | 4450 | 2082 | 0 | 0 |
| hsa_circ_0056916 | 84 | 1 | 429 | 1374 | 946 | 0.01 | 5.00E-115 |
| hsa_circ_0056915 | 90 | 4 | 1482 | 1561 | 83 | 0 | 0 |
| hsa_circ_0056907 | 90 | 4 | 1568 | 3955 | 2391 | 0 | 0 |
| hsa_circ_0056916 | 90 | 1 | 460 | 787 | 328 | 0 | 1.00E-165 |
| hsa_circ_0056916 | 90 | 1 | 787 | 787 | 1 | 0 | 0 |
| hsa_circ_0056915 | 89 | 1 | 894 | 1276 | 383 | 0 | 0 |
| hsa_circ_0056916 | 90 | 1 | 562 | 562 | 1 | 0 | 0 |
| hsa_circ_0056915 | 91 | 1 | 858 | 1051 | 194 | 0 | 0 |
| hsa_circ_0056909 | 92 | 1 | 1473 | 1473 | 1 | 0 | 0 |

novel_circ_017111 novel_circ_017112 novel_circ_017113 novel_circ_017114 novel_circ_017115 novel_circ_017116 novel_circ_017125 novel_circ_017126 novel_circ_017127 novel_circ_017128 novel_circ_017129 novel_circ_017130 novel_circ_017131 novel_circ_017132 novel_circ_017133 novel_circ_017134 novel_circ_017135 novel_circ_017136 novel_circ_017137 novel_circ_017138 novel_circ_017139 novel_circ_017142 novel_circ_017143 novel_circ_017144 novel_circ_017145 novel_circ_017146 novel_circ_017147 novel_circ_017148 novel_circ_017149 novel_circ_017150 novel_circ_017151 novel_circ_017152 novel_circ_017153 novel_circ_017154 novel_circ_017155 novel_circ_017156 novel_circ_017157 novel_circ_017158 novel_circ_017159 novel_circ_017160 novel_circ_017161 novel_circ_017162 novel_circ_017163 novel_circ_017164 novel_circ_017165 novel_circ_017166 novel_circ_017167 novel_circ_017168 novel_circ_017169 novel_circ_017170 novel_circ_017171 novel_circ_017172 novel_circ_017173 novel_circ_017174

| hsa_circ_0056907 | 91 | 1 | 1662 | 3133 | 1472 | 0 | 0 |
| --- | --- | --- | --- | --- | --- | --- | --- |
| hsa_circ_0056915 | 94 | 1 | 296 | 489 | 194 | 0 | 6.00E-123 |
| hsa_circ_0056910 | 93 | 1 | 313 | 313 | 1 | 0 | 2.00E-127 |
| hsa_circ_0056907 | 91 | 1 | 853 | 2394 | 1542 | 0 | 0 |
| hsa_circ_0061275 | 92 | 3 | 186 | 201 | 18 | 0.01 | 3.00E-69 |
| hsa_circ_0061273 | 91 | 4 | 3368 | 7289 | 3932 | 0.01 | 0 |
| hsa_circ_0066556 | 92 | 22897 | 23223 | 1 | 327 | 0 | 1.00E-126 |
| hsa_circ_0066556 | 92 | 1 | 327 | 1 | 327 | 0 | 2.00E-128 |
| hsa_circ_0066557 | 93 | 1 | 279 | 1 | 279 | 0 | 2.00E-113 |
| hsa_circ_0066557 | 93 | 1 | 279 | 1 | 279 | 0 | 3.00E-113 |
| hsa_circ_0066557 | 93 | 1 | 279 | 1 | 279 | 0 | 4.00E-113 |
| hsa_circ_0066558 | 93 | 588 | 1037 | 3 | 452 | 0 | 0 |
| hsa_circ_0066558 | 92 | 291 | 1450 | 3 | 1162 | 0 | 0 |
| hsa_circ_0066558 | 92 | 1 | 816 | 164 | 979 | 0 | 0 |
| hsa_circ_0066558 | 93 | 1 | 1454 | 164 | 1617 | 0 | 0 |
| hsa_circ_0066559 | 95 | 1 | 453 | 835 | 1287 | 0 | 0 |
| hsa_circ_0066560 | 94 | 579 | 1203 | 282 | 906 | 0 | 0 |
| hsa_circ_0066560 | 94 | 3 | 624 | 286 | 906 | 0 | 0 |
| hsa_circ_0066560 | 94 | 1 | 467 | 440 | 906 | 0 | 0 |
| hsa_circ_0047474 | 72 | 3232 | 3963 | 12268 | 11557 | 0.07 | 1.00E-45 |
| hsa_circ_0086616 | 88 | 44048 | 45529 | 1538 | 62 | 0.02 | 0 |
| hsa_circ_0044673 | 94 | 1 | 338 | 332 | 1 | 0.02 | 6.00E-143 |
| hsa_circ_0044667 | 96 | 416 | 1153 | 738 | 1 | 0 | 0 |
| hsa_circ_0044667 | 96 | 161 | 898 | 738 | 1 | 0 | 0 |
| hsa_circ_0044672 | 97 | 118 | 370 | 253 | 1 | 0 | 6.00E-119 |
| hsa_circ_0044667 | 95 | 1 | 485 | 485 | 1 | 0 | 0 |
| hsa_circ_0044667 | 95 | 1 | 396 | 396 | 1 | 0 | 9.00E-177 |
| hsa_circ_0002605 | 94 | 1 | 320 | 317 | 1 | 0.01 | 2.00E-133 |
| hsa_circ_0044652 | 94 | 1 | 321 | 567 | 250 | 0.01 | 9.00E-134 |
| hsa_circ_0044649 | 92 | 319 | 750 | 695 | 264 | 0 | 4.00E-173 |
| hsa_circ_0002605 | 93 | 1 | 249 | 246 | 1 | 0.01 | 2.00E-97 |
| hsa_circ_0044651 | 91 | 248 | 557 | 310 | 1 | 0 | 9.00E-114 |
| hsa_circ_0044649 | 92 | 248 | 679 | 695 | 264 | 0 | 3.00E-173 |
| hsa_circ_0044651 | 91 | 153 | 462 | 310 | 1 | 0 | 7.00E-114 |
| hsa_circ_0044651 | 91 | 150 | 459 | 310 | 1 | 0 | 7.00E-114 |
| hsa_circ_0044642 | 91 | 152 | 987 | 2466 | 1643 | 0.01 | 0 |
| hsa_circ_0044651 | 91 | 1 | 308 | 308 | 1 | 0 | 6.00E-113 |
| hsa_circ_0044650 | 92 | 1 | 430 | 430 | 1 | 0 | 3.00E-172 |
| hsa_circ_0044649 | 92 | 1 | 397 | 385 | 1 | 0.03 | 3.00E-152 |
| hsa_circ_0044642 | 92 | 1 | 528 | 2158 | 1643 | 0.02 | 0 |
| hsa_circ_0044642 | 91 | 1 | 889 | 2158 | 1270 | 0.03 | 0 |
| hsa_circ_0044642 | 92 | 1 | 1319 | 2158 | 840 | 0.02 | 0 |
| hsa_circ_0044642 | 92 | 1 | 1321 | 2158 | 838 | 0.02 | 0 |
| hsa_circ_0044642 | 92 | 1 | 2007 | 2158 | 152 | 0.01 | 0 |
| hsa_circ_0044648 | 93 | 1 | 734 | 953 | 220 | 0 | 0 |
| hsa_circ_0044648 | 93 | 1 | 736 | 953 | 218 | 0 | 0 |
| hsa_circ_0044642 | 92 | 1 | 1149 | 1573 | 425 | 0 | 0 |
| hsa_circ_0044643 | 91 | 3 | 541 | 688 | 150 | 0 | 0 |
| hsa_circ_0044643 | 91 | 3 | 622 | 688 | 69 | 0 | 0 |
| hsa_circ_0044643 | 91 | 3 | 688 | 688 | 3 | 0 | 0 |
| hsa_circ_0044634 | 92 | 3 | 1491 | 1489 | 1 | 0 | 0 |
| hsa_circ_0044635 | 93 | 1 | 286 | 502 | 217 | 0 | 4.00E-114 |
| hsa_circ_0044634 | 93 | 1 | 652 | 652 | 1 | 0 | 0 |
| hsa_circ_0044622 | 91 | 1 | 638 | 3780 | 3137 | 0.02 | 0 |

novel_circ_017176 novel_circ_017177 novel_circ_017178 novel_circ_017179 novel_circ_017180 novel_circ_017181 novel_circ_017182 novel_circ_017183 novel_circ_017184 novel_circ_017185 novel_circ_017186 novel_circ_017187 novel_circ_017188 novel_circ_017189 novel_circ_017190 novel_circ_017191 novel_circ_017192 novel_circ_017193 novel_circ_017194 novel_circ_017195 novel_circ_017196 novel_circ_017198 novel_circ_017199 novel_circ_017201 novel_circ_017202 novel_circ_017203 novel_circ_017204 novel_circ_017206 novel_circ_017207 novel_circ_017208 novel_circ_017209 novel_circ_017210 novel_circ_017211 novel_circ_017212 novel_circ_017213 novel_circ_017214 novel_circ_017215 novel_circ_017216 novel_circ_017217 novel_circ_017218 novel_circ_017219 novel_circ_017220 novel_circ_017221 novel_circ_017222 novel_circ_017223 novel_circ_017224 novel_circ_017225 novel_circ_017226 novel_circ_017227 novel_circ_017228 novel_circ_017229 novel_circ_017230 novel_circ_017231 novel_circ_017232

| hsa_circ_0002515 | 90 | 1 | 332 | 336 | 5 | 0 | 3.00E-121 |
| --- | --- | --- | --- | --- | --- | --- | --- |
| hsa_circ_0069149 | 91 | 1 | 548 | 548 | 1 | 0 | 0 |
| hsa_circ_0069146 | 91 | 1 | 728 | 728 | 1 | 0 | 0 |
| hsa_circ_0069146 | 91 | 1 | 392 | 392 | 1 | 0 | 3.00E-151 |
| hsa_circ_0069140 | 89 | 1 | 358 | 508 | 151 | 0 | 1.00E-125 |
| hsa_circ_0069140 | 89 | 1 | 508 | 508 | 1 | 0 | 2.00E-180 |
| hsa_circ_0069139 | 89 | 1 | 866 | 866 | 1 | 0 | 0 |
| hsa_circ_0069140 | 88 | 1 | 328 | 328 | 1 | 0 | 2.00E-107 |
| hsa_circ_0069139 | 88 | 1 | 540 | 686 | 147 | 0 | 0 |
| hsa_circ_0069139 | 89 | 1 | 358 | 358 | 1 | 0 | 1.00E-125 |
| hsa_circ_0069135 | 89 | 1 | 476 | 476 | 1 | 0 | 9.00E-168 |
| hsa_circ_0069120 | 93 | 1 | 311 | 96 | 406 | 0 | 6.00E-128 |
| hsa_circ_0069118 | 94 | 1 | 487 | 1 | 487 | 0 | 0 |
| hsa_circ_0008246 | 93 | 1 | 594 | 321 | 914 | 0 | 0 |
| hsa_circ_0008246 | 92 | 1 | 914 | 1 | 914 | 0 | 0 |
| hsa_circ_0069106 | 92 | 951 | 1865 | 860 | 1774 | 0 | 0 |
| hsa_circ_0069120 | 93 | 1 | 201 | 96 | 296 | 0 | 1.00E-78 |
| hsa_circ_0069106 | 86 | 1 | 851 | 1 | 860 | 0.01 | 0 |
| hsa_circ_0069099 | 98 | 1 | 149 | 1 | 149 | 0 | 3.00E-68 |
| hsa_circ_0069097 | 96 | 1 | 639 | 1 | 638 | 0 | 0 |
| hsa_circ_0069097 | 95 | 1 | 501 | 1 | 500 | 0 | 0 |
| hsa_circ_0069084 | 95 | 1764 | 1947 | 349 | 166 | 0 | 2.00E-76 |
| hsa_circ_0056467 | 78 | 1 | 1055 | 1 | 1058 | 0.02 | 1.00E-175 |
| hsa_circ_0056471 | 90 | 3 | 320 | 3 | 320 | 0 | 2.00E-113 |
| hsa_circ_0003524 | 96 | 4 | 165 | 162 | 1 | 0 | 9.00E-69 |
| hsa_circ_0056487 | 87 | 1 | 540 | 1 | 540 | 0 | 6.00E-170 |
| hsa_circ_0025580 | 74 | 18505 | 19004 | 12920 | 13401 | 0.07 | 3.00E-42 |
| hsa_circ_0056432 | 93 | 1 | 545 | 1259 | 1803 | 0 | 0 |
| hsa_circ_0056432 | 94 | 1 | 813 | 991 | 1803 | 0 | 0 |
| hsa_circ_0056432 | 96 | 1 | 276 | 1259 | 1534 | 0 | 1.00E-123 |
| hsa_circ_0056432 | 95 | 1 | 459 | 1076 | 1534 | 0 | 0 |
| hsa_circ_0056432 | 95 | 1 | 544 | 991 | 1534 | 0 | 0 |
| hsa_circ_0056432 | 92 | 810 | 2231 | 1 | 1419 | 0 | 0 |
| hsa_circ_0056432 | 91 | 132 | 1124 | 1 | 990 | 0 | 0 |
| hsa_circ_0056432 | 91 | 1 | 993 | 1 | 990 | 0 | 0 |
| hsa_circ_0056432 | 91 | 132 | 1124 | 1 | 990 | 0 | 0 |
| hsa_circ_0056425 | 92 | 1 | 836 | 435 | 1270 | 0 | 0 |
| hsa_circ_0056432 | 92 | 1 | 469 | 1 | 469 | 0 | 0 |
| hsa_circ_0056425 | 92 | 1 | 600 | 435 | 1034 | 0 | 0 |
| hsa_circ_0056419 | 92 | 1 | 1575 | 137 | 1711 | 0 | 0 |
| hsa_circ_0056419 | 93 | 1 | 1711 | 1 | 1711 | 0 | 0 |
| hsa_circ_0056425 | 92 | 1 | 330 | 435 | 764 | 0 | 8.00E-132 |
| hsa_circ_0056426 | 92 | 1 | 470 | 1 | 470 | 0 | 0 |
| hsa_circ_0056420 | 92 | 1 | 678 | 1 | 678 | 0 | 0 |
| hsa_circ_0056419 | 92 | 1 | 385 | 269 | 653 | 0 | 1.00E-150 |
| hsa_circ_0056419 | 93 | 1 | 517 | 137 | 653 | 0 | 0 |
| hsa_circ_0056419 | 93 | 1 | 653 | 1 | 653 | 0 | 0 |
| hsa_circ_0056405 | 92 | 1 | 1443 | 736 | 2178 | 0 | 0 |
| hsa_circ_0056419 | 94 | 1 | 433 | 1 | 433 | 0 | 0 |
| hsa_circ_0056415 | 92 | 1 | 882 | 1 | 882 | 0 | 0 |
| hsa_circ_0056413 | 92 | 1 | 1144 | 352 | 1495 | 0 | 0 |
| hsa_circ_0056413 | 92 | 1 | 1223 | 273 | 1495 | 0 | 0 |
| hsa_circ_0056413 | 92 | 1 | 1155 | 176 | 1330 | 0 | 0 |
| hsa_circ_0056405 | 92 | 1 | 1657 | 137 | 1793 | 0 | 0 |

novel_circ_017233 novel_circ_017234 novel_circ_017235 novel_circ_017236 novel_circ_017237 novel_circ_017238 novel_circ_017239 novel_circ_017240 novel_circ_017241 novel_circ_017242 novel_circ_017243 novel_circ_017244 novel_circ_017245 novel_circ_017246 novel_circ_017247 novel_circ_017248 novel_circ_017249 novel_circ_017250 novel_circ_017251 novel_circ_017252 novel_circ_017253 novel_circ_017254 novel_circ_017255 novel_circ_017256 novel_circ_017257 novel_circ_017258 novel_circ_017259 novel_circ_017260 novel_circ_017261 novel_circ_017262 novel_circ_017263 novel_circ_017264 novel_circ_017265 novel_circ_017272 novel_circ_017275 novel_circ_017280 novel_circ_017284 novel_circ_017285 novel_circ_017286 novel_circ_017287 novel_circ_017288 novel_circ_017289 novel_circ_017290 novel_circ_017291 novel_circ_017292 novel_circ_017293 novel_circ_017294 novel_circ_017295 novel_circ_017296 novel_circ_017297 novel_circ_017299 novel_circ_017300 novel_circ_017301 novel_circ_017302

| hsa_circ_0056415 | 91 | 1 | 443 | 1 | 443 | 0 | 2.00E-169 |
| --- | --- | --- | --- | --- | --- | --- | --- |
| hsa_circ_0056409 | 91 | 1 | 1100 | 1 | 1100 | 0 | 0 |
| hsa_circ_0056405 | 91 | 1 | 1183 | 137 | 1319 | 0 | 0 |
| hsa_circ_0056405 | 91 | 4 | 1319 | 4 | 1319 | 0 | 0 |
| hsa_circ_0056409 | 90 | 1 | 688 | 1 | 688 | 0 | 0 |
| hsa_circ_0056409 | 91 | 1 | 595 | 1 | 595 | 0 | 0 |
| hsa_circ_0056405 | 91 | 4 | 814 | 4 | 814 | 0 | 0 |
| hsa_circ_0056405 | 91 | 4 | 735 | 4 | 735 | 0 | 0 |
| hsa_circ_0056405 | 91 | 4 | 728 | 4 | 728 | 0 | 0 |
| hsa_circ_0056405 | 91 | 1 | 500 | 137 | 636 | 0 | 0 |
| hsa_circ_0056405 | 91 | 4 | 636 | 4 | 636 | 0 | 0 |
| hsa_circ_0056405 | 90 | 4 | 463 | 4 | 463 | 0 | 3.00E-172 |
| hsa_circ_0056405 | 91 | 1617 | 1834 | 133 | 350 | 0 | 9.00E-79 |
| hsa_circ_0056405 | 91 | 4 | 350 | 4 | 350 | 0 | 3.00E-131 |
| hsa_circ_0056393 | 92 | 3 | 486 | 484 | 1 | 0 | 0 |
| hsa_circ_0056378 | 90 | 1 | 827 | 839 | 1 | 0.02 | 0 |
| hsa_circ_0056371 | 96 | 1 | 762 | 762 | 1 | 0 | 0 |
| hsa_circ_0003192 | 96 | 60 | 800 | 741 | 1 | 0 | 0 |
| hsa_circ_0056362 | 95 | 1 | 393 | 393 | 1 | 0 | 9.00E-177 |
| hsa_circ_0003192 | 96 | 1 | 739 | 739 | 1 | 0 | 0 |
| hsa_circ_0003192 | 96 | 1 | 736 | 736 | 1 | 0 | 0 |
| hsa_circ_0003192 | 97 | 1 | 451 | 451 | 1 | 0 | 0 |
| hsa_circ_0056356 | 97 | 750 | 1110 | 361 | 1 | 0 | 7.00E-172 |
| hsa_circ_0056345 | 92 | 1 | 482 | 594 | 113 | 0 | 0 |
| hsa_circ_0056345 | 93 | 1 | 594 | 594 | 1 | 0 | 0 |
| hsa_circ_0056345 | 93 | 1 | 594 | 594 | 1 | 0 | 0 |
| hsa_circ_0056341 | 97 | 358 | 498 | 141 | 1 | 0 | 1.00E-61 |
| hsa_circ_0056341 | 97 | 358 | 498 | 141 | 1 | 0 | 4.00E-61 |
| hsa_circ_0056341 | 97 | 193 | 333 | 141 | 1 | 0 | 9.00E-62 |
| hsa_circ_0056341 | 97 | 193 | 333 | 141 | 1 | 0 | 3.00E-61 |
| hsa_circ_0056341 | 97 | 123 | 263 | 141 | 1 | 0 | 7.00E-62 |
| hsa_circ_0056341 | 97 | 123 | 263 | 141 | 1 | 0 | 3.00E-61 |
| hsa_circ_0056341 | 96 | 1 | 100 | 100 | 1 | 0 | 2.00E-38 |
| hsa_circ_0056329 | 90 | 4 | 539 | 1125 | 592 | 0 | 0 |
| hsa_circ_0011965 | 85 | 6 | 1413 | 14267 | 15692 | 0.02 | 0 |
| hsa_circ_0011992 | 93 | 4 | 175 | 396 | 225 | 0 | 6.00E-66 |
| hsa_circ_0012139 | 96 | 1 | 442 | 442 | 1 | 0 | 0 |
| hsa_circ_0012139 | 96 | 361 | 803 | 443 | 1 | 0 | 0 |
| hsa_circ_0077745 | 78 | 1446 | 1590 | 6017 | 6169 | 0.06 | 3.00E-15 |
| hsa_circ_0077745 | 78 | 1456 | 1600 | 6017 | 6169 | 0.06 | 3.00E-15 |
| hsa_circ_0012142 | 96 | 1 | 342 | 342 | 1 | 0 | 8.00E-157 |
| hsa_circ_0012142 | 96 | 361 | 703 | 343 | 1 | 0 | 5.00E-157 |
| hsa_circ_0012145 | 97 | 1 | 269 | 269 | 1 | 0 | 2.00E-126 |
| hsa_circ_0012145 | 97 | 5779 | 6050 | 272 | 1 | 0 | 1.00E-126 |
| hsa_circ_0012151 | 99 | 1 | 272 | 272 | 1 | 0 | 1.00E-137 |
| hsa_circ_0012151 | 99 | 1 | 278 | 278 | 1 | 0 | 6.00E-141 |
| hsa_circ_0012151 | 99 | 1 | 278 | 278 | 1 | 0 | 5.00E-141 |
| hsa_circ_0012151 | 99 | 1 | 278 | 278 | 1 | 0 | 4.00E-141 |
| hsa_circ_0012152 | 95 | 1 | 742 | 1 | 742 | 0 | 0 |
| hsa_circ_0012153 | 93 | 1 | 281 | 1 | 281 | 0 | 2.00E-111 |
| hsa_circ_0012183 | 88 | 1 | 730 | 1004 | 269 | 0.01 | 0 |
| hsa_circ_0012183 | 89 | 1 | 858 | 1132 | 269 | 0.01 | 0 |
| hsa_circ_0012183 | 89 | 1 | 644 | 1132 | 483 | 0.01 | 0 |
| hsa_circ_0012187 | 89 | 1 | 540 | 546 | 1 | 0.01 | 0 |

novel_circ_017303 novel_circ_017304 novel_circ_017305 novel_circ_017306 novel_circ_017307 novel_circ_017308 novel_circ_017309 novel_circ_017310 novel_circ_017311 novel_circ_017312 novel_circ_017313 novel_circ_017316 novel_circ_017317 novel_circ_017318 novel_circ_017322 novel_circ_017324 novel_circ_017325 novel_circ_017326 novel_circ_017327 novel_circ_017328 novel_circ_017330 novel_circ_017331 novel_circ_017332 novel_circ_017333 novel_circ_017334 novel_circ_017335 novel_circ_017336 novel_circ_017337 novel_circ_017339 novel_circ_017341 novel_circ_017342 novel_circ_017349 novel_circ_017350 novel_circ_017351 novel_circ_017352 novel_circ_017353 novel_circ_017354 novel_circ_017356 novel_circ_017357 novel_circ_017358 novel_circ_017359 novel_circ_017360 novel_circ_017361 novel_circ_017362 novel_circ_017363 novel_circ_017364 novel_circ_017365 novel_circ_017366 novel_circ_017367 novel_circ_017368 novel_circ_017369 novel_circ_017370 novel_circ_017371 novel_circ_017372

| hsa_circ_0012184 | 94 | 1 | 202 | 906 | 705 | 0 | 1.00E-82 |
| --- | --- | --- | --- | --- | --- | --- | --- |
| hsa_circ_0012184 | 92 | 1 | 508 | 1212 | 705 | 0 | 0 |
| hsa_circ_0012192 | 94 | 1 | 272 | 272 | 1 | 0 | 2.00E-116 |
| hsa_circ_0012192 | 93 | 1 | 418 | 418 | 1 | 0 | 6.00E-174 |
| hsa_circ_0012214 | 92 | 1 | 397 | 397 | 1 | 0 | 2.00E-155 |
| hsa_circ_0012214 | 92 | 534 | 1720 | 1187 | 1 | 0 | 0 |
| hsa_circ_0012214 | 92 | 1 | 397 | 397 | 1 | 0 | 1.00E-155 |
| hsa_circ_0012214 | 93 | 534 | 1038 | 1187 | 683 | 0 | 0 |
| hsa_circ_0012214 | 92 | 534 | 861 | 1187 | 860 | 0 | 1.00E-128 |
| hsa_circ_0012214 | 94 | 153 | 298 | 1187 | 1042 | 0 | 3.00E-56 |
| hsa_circ_0012214 | 94 | 534 | 679 | 1187 | 1042 | 0 | 7.00E-56 |
| hsa_circ_0012221 | 93 | 1 | 433 | 23683 | 23254 | 0.01 | 2.00E-179 |
| hsa_circ_0012221 | 93 | 1 | 311 | 23683 | 23376 | 0.01 | 8.00E-127 |
| hsa_circ_0012256 | 95 | 1 | 228 | 228 | 1 | 0 | 1.00E-98 |
| hsa_circ_0084810 | 86 | 441 | 1317 | 1 | 874 | 0.01 | 0 |
| hsa_circ_0084810 | 86 | 1 | 294 | 581 | 874 | 0 | 2.00E-83 |
| hsa_circ_0084810 | 88 | 1 | 838 | 581 | 1418 | 0 | 0 |
| hsa_circ_0084810 | 92 | 1 | 1008 | 1425 | 2432 | 0 | 0 |
| hsa_circ_0084810 | 92 | 1 | 1008 | 1425 | 2432 | 0 | 0 |
| hsa_circ_0084810 | 90 | 1 | 483 | 1950 | 2432 | 0 | 5.00E-180 |
| hsa_circ_0084811 | 92 | 1 | 649 | 123 | 771 | 0 | 0 |
| hsa_circ_0064567 | 99 | 112 | 274 | 163 | 1 | 0 | 4.00E-79 |
| hsa_circ_0061482 | 92 | 1 | 467 | 1 | 472 | 0.02 | 0 |
| hsa_circ_0061482 | 91 | 1 | 612 | 1 | 618 | 0.01 | 0 |
| hsa_circ_0061482 | 91 | 1 | 421 | 198 | 618 | 0 | 1.00E-160 |
| hsa_circ_0061483 | 92 | 145 | 315 | 1 | 171 | 0 | 2.00E-63 |
| hsa_circ_0061483 | 92 | 1 | 171 | 1 | 171 | 0 | 9.00E-64 |
| hsa_circ_0061483 | 87 | 1 | 739 | 1 | 743 | 0.01 | 0 |
| hsa_circ_0061483 | 86 | 1 | 568 | 172 | 743 | 0.01 | 4.00E-173 |
| hsa_circ_0061485 | 93 | 1262 | 1503 | 242 | 1 | 0 | 3.00E-97 |
| hsa_circ_0061485 | 93 | 1452 | 1693 | 242 | 1 | 0 | 4.00E-97 |
| hsa_circ_0008462 | 94 | 1 | 310 | 310 | 1 | 0 | 5.00E-129 |
| hsa_circ_0008462 | 93 | 1 | 665 | 665 | 1 | 0 | 0 |
| hsa_circ_0008462 | 94 | 1 | 494 | 578 | 85 | 0 | 0 |
| hsa_circ_0061490 | 94 | 3 | 827 | 1109 | 285 | 0 | 0 |
| hsa_circ_0061490 | 94 | 1 | 951 | 1235 | 285 | 0 | 0 |
| hsa_circ_0061490 | 95 | 1 | 1141 | 1425 | 285 | 0 | 0 |
| hsa_circ_0061499 | 77 | 405 | 883 | 16 | 485 | 0.03 | 2.00E-71 |
| hsa_circ_0061497 | 78 | 405 | 1052 | 108 | 746 | 0.03 | 8.00E-107 |
| hsa_circ_0061497 | 80 | 405 | 1183 | 108 | 877 | 0.02 | 6.00E-158 |
| hsa_circ_0061501 | 78 | 15 | 621 | 15 | 612 | 0.03 | 1.00E-102 |
| hsa_circ_0061509 | 84 | 7 | 479 | 7 | 473 | 0.03 | 5.00E-121 |
| hsa_circ_0061509 | 84 | 7 | 479 | 7 | 473 | 0.03 | 9.00E-121 |
| hsa_circ_0061509 | 84 | 7 | 479 | 7 | 473 | 0.03 | 1.00E-120 |
| hsa_circ_0061510 | 79 | 301 | 761 | 1 | 467 | 0.03 | 3.00E-79 |
| hsa_circ_0061510 | 79 | 301 | 761 | 1 | 467 | 0.03 | 5.00E-79 |
| hsa_circ_0061510 | 79 | 146 | 606 | 1 | 467 | 0.03 | 4.00E-79 |
| hsa_circ_0061523 | 95 | 1 | 332 | 2815 | 2484 | 0 | 1.00E-149 |
| hsa_circ_0061528 | 93 | 7 | 411 | 406 | 1 | 0 | 5.00E-170 |
| hsa_circ_0061528 | 95 | 1 | 274 | 274 | 1 | 0 | 9.00E-121 |
| hsa_circ_0061528 | 93 | 7 | 411 | 406 | 1 | 0 | 4.00E-170 |
| hsa_circ_0061536 | 92 | 1 | 3918 | 1 | 3966 | 0.01 | 0 |
| hsa_circ_0061539 | 97 | 1 | 497 | 1 | 497 | 0 | 0 |
| hsa_circ_0061537 | 97 | 1 | 605 | 5917 | 6521 | 0 | 0 |

novel_circ_017373 novel_circ_017374 novel_circ_017375 novel_circ_017376 novel_circ_017377 novel_circ_017378 novel_circ_017379 novel_circ_017380 novel_circ_017381 novel_circ_017382 novel_circ_017383 novel_circ_017384 novel_circ_017385 novel_circ_017386 novel_circ_017387 novel_circ_017388 novel_circ_017389 novel_circ_017390 novel_circ_017396 novel_circ_017397 novel_circ_017398 novel_circ_017399 novel_circ_017400 novel_circ_017401 novel_circ_017402 novel_circ_017403 novel_circ_017404 novel_circ_017405 novel_circ_017406 novel_circ_017407 novel_circ_017408 novel_circ_017409 novel_circ_017410 novel_circ_017411 novel_circ_017412 novel_circ_017413 novel_circ_017414 novel_circ_017415 novel_circ_017416 novel_circ_017417 novel_circ_017418 novel_circ_017419 novel_circ_017420 novel_circ_017421 novel_circ_017422 novel_circ_017423 novel_circ_017424 novel_circ_017425 novel_circ_017426 novel_circ_017427 novel_circ_017428 novel_circ_017429 novel_circ_017430 novel_circ_017431

| hsa_circ_0061542 | 96 | 1 | 336 | 1 | 336 | 0 | 8.00E-157 |
| --- | --- | --- | --- | --- | --- | --- | --- |
| hsa_circ_0061546 | 89 | 1 | 918 | 1318 | 395 | 0.01 | 0 |
| hsa_circ_0004339 | 91 | 5 | 945 | 944 | 1 | 0 | 0 |
| hsa_circ_0061550 | 90 | 2 | 820 | 1235 | 413 | 0 | 0 |
| hsa_circ_0061550 | 90 | 5 | 827 | 1238 | 413 | 0 | 0 |
| hsa_circ_0061553 | 90 | 4 | 211 | 1062 | 855 | 0 | 1.00E-72 |
| hsa_circ_0061555 | 97 | 152 | 265 | 360 | 247 | 0 | 2.00E-48 |
| hsa_circ_0061555 | 97 | 224 | 337 | 360 | 247 | 0 | 2.00E-48 |
| hsa_circ_0061558 | 97 | 194 | 571 | 1 | 378 | 0 | 0 |
| hsa_circ_0061558 | 97 | 106 | 322 | 1 | 217 | 0 | 1.00E-100 |
| hsa_circ_0061558 | 97 | 106 | 483 | 1 | 378 | 0 | 0 |
| hsa_circ_0061558 | 97 | 1 | 378 | 1 | 378 | 0 | 0 |
| hsa_circ_0061558 | 96 | 1 | 595 | 61 | 655 | 0 | 0 |
| hsa_circ_0061563 | 94 | 1 | 277 | 1 | 277 | 0 | 9.00E-116 |
| hsa_circ_0061560 | 93 | 1 | 696 | 162 | 857 | 0 | 0 |
| hsa_circ_0061569 | 92 | 249 | 697 | 1 | 449 | 0 | 0 |
| hsa_circ_0061570 | 94 | 1343 | 1854 | 1 | 512 | 0 | 0 |
| hsa_circ_0061570 | 94 | 433 | 944 | 1 | 512 | 0 | 0 |
| hsa_circ_0002533 | 88 | 1 | 184 | 1 | 163 | 0.11 | 4.00E-48 |
| hsa_circ_0020044 | 89 | 2 | 474 | 572 | 93 | 0.01 | 9.00E-163 |
| hsa_circ_0020044 | 88 | 2 | 478 | 572 | 89 | 0.01 | 7.00E-163 |
| hsa_circ_0020040 | 96 | 1 | 313 | 636 | 324 | 0 | 5.00E-142 |
| hsa_circ_0020040 | 93 | 253 | 577 | 325 | 1 | 0 | 9.00E-134 |
| hsa_circ_0020035 | 92 | 4 | 882 | 5945 | 5067 | 0 | 0 |
| hsa_circ_0020035 | 93 | 4 | 996 | 5945 | 4953 | 0 | 0 |
| hsa_circ_0089013 | 91 | 1 | 495 | 256 | 750 | 0 | 0 |
| hsa_circ_0089012 | 89 | 3 | 368 | 3 | 368 | 0 | 4.00E-125 |
| hsa_circ_0089012 | 89 | 214 | 579 | 3 | 368 | 0 | 7.00E-125 |
| hsa_circ_0089011 | 91 | 1 | 269 | 561 | 293 | 0 | 2.00E-101 |
| hsa_circ_0088950 | 90 | 1 | 556 | 1 | 556 | 0 | 0 |
| hsa_circ_0088934 | 92 | 1 | 1423 | 1 | 1423 | 0 | 0 |
| hsa_circ_0088950 | 90 | 1 | 403 | 1 | 403 | 0 | 6.00E-149 |
| hsa_circ_0088935 | 92 | 1 | 1144 | 1 | 1144 | 0 | 0 |
| hsa_circ_0088934 | 92 | 1 | 1270 | 1 | 1270 | 0 | 0 |
| hsa_circ_0088939 | 93 | 1 | 528 | 1 | 528 | 0 | 0 |
| hsa_circ_0088934 | 93 | 1 | 666 | 1 | 666 | 0 | 0 |
| hsa_circ_0088934 | 93 | 1 | 545 | 1 | 545 | 0 | 0 |
| hsa_circ_0088935 | 94 | 1 | 213 | 1 | 213 | 0 | 5.00E-87 |
| hsa_circ_0088934 | 93 | 1 | 339 | 1 | 339 | 0 | 4.00E-140 |
| hsa_circ_0088932 | 94 | 1 | 424 | 1 | 424 | 0 | 0 |
| hsa_circ_0088908 | 94 | 1 | 494 | 683 | 190 | 0 | 0 |
| hsa_circ_0088873 | 91 | 1 | 922 | 600 | 1521 | 0 | 0 |
| hsa_circ_0088843 | 91 | 1 | 1543 | 345 | 1887 | 0 | 0 |
| hsa_circ_0088843 | 91 | 1 | 1752 | 136 | 1887 | 0 | 0 |
| hsa_circ_0088872 | 95 | 1 | 147 | 1 | 147 | 0 | 8.00E-59 |
| hsa_circ_0088867 | 92 | 1 | 445 | 183 | 627 | 0 | 1.00E-175 |
| hsa_circ_0088845 | 95 | 1 | 60 | 106 | 165 | 0 | 2.00E-19 |
| hsa_circ_0088843 | 92 | 1 | 364 | 345 | 708 | 0 | 2.00E-142 |
| hsa_circ_0088832 | 91 | 1 | 434 | 289 | 722 | 0 | 2.00E-164 |
| hsa_circ_0088830 | 94 | 1 | 366 | 1 | 366 | 0 | 4.00E-155 |
| hsa_circ_0088814 | 86 | 1 | 786 | 1 | 789 | 0 | 0 |
| hsa_circ_0088814 | 84 | 1 | 330 | 1 | 333 | 0.01 | 2.00E-87 |
| hsa_circ_0088803 | 93 | 872 | 1744 | 1 | 873 | 0 | 0 |
| hsa_circ_0001893 | 94 | 1 | 594 | 1 | 594 | 0 | 0 |

novel_circ_017432 novel_circ_017433 novel_circ_017434 novel_circ_017435 novel_circ_017436 novel_circ_017437 novel_circ_017438 novel_circ_017439 novel_circ_017440 novel_circ_017441 novel_circ_017442 novel_circ_017443 novel_circ_017444 novel_circ_017445 novel_circ_017447 novel_circ_017448 novel_circ_017449 novel_circ_017450 novel_circ_017451 novel_circ_017452 novel_circ_017453 novel_circ_017454 novel_circ_017455 novel_circ_017456 novel_circ_017457 novel_circ_017458 novel_circ_017459 novel_circ_017460 novel_circ_017461 novel_circ_017462 novel_circ_017463 novel_circ_017464 novel_circ_017465 novel_circ_017466 novel_circ_017467 novel_circ_017468 novel_circ_017469 novel_circ_017470 novel_circ_017471 novel_circ_017472 novel_circ_017473 novel_circ_017475 novel_circ_017476 novel_circ_017477 novel_circ_017478 novel_circ_017479 novel_circ_017480 novel_circ_017481 novel_circ_017483 novel_circ_017484 novel_circ_017485 novel_circ_017486 novel_circ_017487 novel_circ_017488

| hsa_circ_0088737 | 80 | 26 | 1515 | 1514 | 1 | 0.04 | 0 |
| --- | --- | --- | --- | --- | --- | --- | --- |
| hsa_circ_0005931 | 93 | 252 | 522 | 216 | 486 | 0 | 4.00E-107 |
| hsa_circ_0088693 | 93 | 1 | 673 | 1 | 673 | 0 | 0 |
| hsa_circ_0088687 | 91 | 1 | 638 | 157 | 794 | 0 | 0 |
| hsa_circ_0088687 | 91 | 1 | 577 | 157 | 733 | 0 | 0 |
| hsa_circ_0088634 | 90 | 3 | 618 | 518 | 1133 | 0 | 0 |
| hsa_circ_0088636 | 90 | 3 | 551 | 3 | 551 | 0 | 0 |
| hsa_circ_0088634 | 92 | 1 | 843 | 224 | 1066 | 0 | 0 |
| hsa_circ_0088632 | 92 | 110 | 1221 | 1 | 1112 | 0 | 0 |
| hsa_circ_0088634 | 93 | 1 | 907 | 1 | 907 | 0 | 0 |
| hsa_circ_0088634 | 96 | 1 | 292 | 224 | 515 | 0 | 4.00E-134 |
| hsa_circ_0088634 | 96 | 1 | 408 | 108 | 515 | 0 | 0 |
| hsa_circ_0088634 | 95 | 1 | 515 | 1 | 515 | 0 | 0 |
| hsa_circ_0088629 | 94 | 1 | 670 | 655 | 1324 | 0 | 0 |
| hsa_circ_0088629 | 93 | 1 | 348 | 1 | 348 | 0 | 4.00E-145 |
| hsa_circ_0088624 | 94 | 1 | 310 | 85 | 394 | 0 | 2.00E-132 |
| hsa_circ_0088624 | 94 | 1 | 394 | 1 | 394 | 0 | 1.00E-170 |
| hsa_circ_0088622 | 94 | 1 | 445 | 1 | 445 | 0 | 0 |
| hsa_circ_0088622 | 94 | 230 | 674 | 1 | 445 | 0 | 0 |
| hsa_circ_0088622 | 94 | 230 | 547 | 1 | 318 | 0 | 6.00E-135 |
| hsa_circ_0088621 | 94 | 4 | 280 | 6 | 281 | 0 | 9.00E-117 |
| hsa_circ_0088621 | 94 | 4 | 280 | 6 | 281 | 0 | 9.00E-115 |
| hsa_circ_0088621 | 94 | 4 | 280 | 6 | 281 | 0 | 7.00E-117 |
| hsa_circ_0088621 | 95 | 4 | 229 | 6 | 230 | 0 | 3.00E-95 |
| hsa_circ_0088614 | 91 | 1 | 354 | 1 | 348 | 0.02 | 8.00E-132 |
| hsa_circ_0088614 | 91 | 1 | 354 | 1 | 348 | 0.02 | 2.00E-133 |
| hsa_circ_0088612 | 91 | 1 | 451 | 109 | 553 | 0.01 | 6.00E-174 |
| hsa_circ_0088612 | 93 | 1 | 559 | 1 | 553 | 0.01 | 0 |
| hsa_circ_0088612 | 91 | 1 | 356 | 109 | 458 | 0.02 | 3.00E-131 |
| hsa_circ_0088612 | 92 | 1 | 464 | 1 | 458 | 0.01 | 0 |
| hsa_circ_0088606 | 98 | 1 | 329 | 1 | 329 | 0 | 9.00E-158 |
| hsa_circ_0088606 | 98 | 1 | 327 | 1 | 327 | 0 | 2.00E-158 |
| hsa_circ_0003991 | 99 | 1 | 569 | 1 | 569 | 0 | 0 |
| hsa_circ_0027440 | 90 | 1 | 253 | 1 | 253 | 0 | 7.00E-89 |
| hsa_circ_0027439 | 91 | 1 | 697 | 1 | 697 | 0 | 0 |
| hsa_circ_0027439 | 91 | 1 | 388 | 170 | 557 | 0 | 1.00E-150 |
| hsa_circ_0027439 | 92 | 1 | 444 | 1 | 444 | 0 | 5.00E-175 |
| hsa_circ_0027439 | 92 | 379 | 822 | 1 | 444 | 0 | 9.00E-175 |
| hsa_circ_0027439 | 92 | 441 | 884 | 1 | 444 | 0 | 1.00E-174 |
| hsa_circ_0027439 | 91 | 441 | 778 | 1 | 338 | 0 | 7.00E-126 |
| hsa_circ_0027439 | 91 | 441 | 609 | 1 | 169 | 0 | 1.00E-58 |
| hsa_circ_0027434 | 94 | 1 | 1008 | 1227 | 220 | 0 | 0 |
| hsa_circ_0027438 | 94 | 1 | 372 | 540 | 169 | 0 | 4.00E-160 |
| hsa_circ_0027436 | 94 | 1 | 846 | 846 | 1 | 0 | 0 |
| hsa_circ_0027434 | 94 | 1 | 948 | 1167 | 220 | 0 | 0 |
| hsa_circ_0027423 | 98 | 3323 | 3503 | 640 | 820 | 0 | 7.00E-83 |
| hsa_circ_0027423 | 98 | 3323 | 3503 | 640 | 820 | 0 | 7.00E-83 |
| hsa_circ_0027423 | 96 | 1 | 273 | 548 | 820 | 0 | 8.00E-125 |
| hsa_circ_0027423 | 98 | 1 | 181 | 640 | 820 | 0 | 7.00E-84 |
| hsa_circ_0027423 | 94 | 1 | 529 | 292 | 820 | 0 | 0 |
| hsa_circ_0027423 | 94 | 1 | 529 | 292 | 820 | 0 | 0 |
| hsa_circ_0027422 | 94 | 3 | 182 | 520 | 699 | 0 | 1.00E-71 |
| hsa_circ_0027422 | 96 | 1 | 453 | 247 | 699 | 0 | 0 |
| hsa_circ_0027422 | 95 | 1 | 571 | 129 | 699 | 0 | 0 |

novel_circ_017489 novel_circ_017490 novel_circ_017491 novel_circ_017492 novel_circ_017493 novel_circ_017494 novel_circ_017495 novel_circ_017496 novel_circ_017497 novel_circ_017498 novel_circ_017499 novel_circ_017500 novel_circ_017503 novel_circ_017510 novel_circ_017511 novel_circ_017512 novel_circ_017513 novel_circ_017514 novel_circ_017515 novel_circ_017516 novel_circ_017517 novel_circ_017518 novel_circ_017519 novel_circ_017520 novel_circ_017521 novel_circ_017522 novel_circ_017523 novel_circ_017524 novel_circ_017525 novel_circ_017526 novel_circ_017527 novel_circ_017528 novel_circ_017529 novel_circ_017530 novel_circ_017531 novel_circ_017532 novel_circ_017533 novel_circ_017534 novel_circ_017535 novel_circ_017536 novel_circ_017537 novel_circ_017538 novel_circ_017539 novel_circ_017540 novel_circ_017541 novel_circ_017542 novel_circ_017543 novel_circ_017544 novel_circ_017545 novel_circ_017546 novel_circ_017547 novel_circ_017548 novel_circ_017549 novel_circ_017550

| hsa_circ_0006268 | 96 | 1 | 471 | 1 | 471 | 0 | 0 |
| --- | --- | --- | --- | --- | --- | --- | --- |
| hsa_circ_0027409 | 93 | 1 | 902 | 1525 | 2426 | 0 | 0 |
| hsa_circ_0027409 | 93 | 1 | 1609 | 701 | 2309 | 0 | 0 |
| hsa_circ_0027404 | 95 | 1 | 1738 | 1 | 1738 | 0 | 0 |
| hsa_circ_0027404 | 94 | 1 | 1154 | 1 | 1154 | 0 | 0 |
| hsa_circ_0027409 | 96 | 1 | 643 | 58 | 700 | 0 | 0 |
| hsa_circ_0027404 | 95 | 1 | 917 | 1 | 917 | 0 | 0 |
| hsa_circ_0027389 | 94 | 1 | 283 | 103 | 385 | 0 | 2.00E-117 |
| hsa_circ_0027387 | 94 | 1 | 607 | 1 | 607 | 0 | 0 |
| hsa_circ_0027383 | 93 | 1 | 919 | 1 | 919 | 0 | 0 |
| hsa_circ_0027387 | 95 | 1 | 324 | 1 | 324 | 0 | 8.00E-142 |
| hsa_circ_0027380 | 94 | 3 | 359 | 3 | 359 | 0 | 4.00E-155 |
| hsa_circ_0005890 | 85 | 5415 | 5488 | 20154 | 20227 | 0 | 5.00E-11 |
| hsa_circ_0027365 | 94 | 1 | 1495 | 2646 | 4134 | 0 | 0 |
| hsa_circ_0027374 | 96 | 1 | 376 | 292 | 667 | 0 | 5.00E-174 |
| hsa_circ_0027365 | 95 | 1 | 1296 | 2845 | 4134 | 0 | 0 |
| hsa_circ_0027365 | 94 | 1 | 1495 | 2646 | 4134 | 0 | 0 |
| hsa_circ_0027365 | 94 | 1 | 2128 | 2013 | 4134 | 0 | 0 |
| hsa_circ_0027374 | 96 | 1 | 252 | 292 | 543 | 0 | 6.00E-112 |
| hsa_circ_0027374 | 96 | 1 | 543 | 1 | 543 | 0 | 0 |
| hsa_circ_0027365 | 94 | 1 | 2004 | 2013 | 4010 | 0 | 0 |
| hsa_circ_0027374 | 96 | 1 | 482 | 1 | 482 | 0 | 0 |
| hsa_circ_0027365 | 94 | 1 | 1310 | 2646 | 3949 | 0 | 0 |
| hsa_circ_0027374 | 97 | 1 | 291 | 1 | 291 | 0 | 3.00E-135 |
| hsa_circ_0027372 | 93 | 1 | 1119 | 847 | 1959 | 0.01 | 0 |
| hsa_circ_0027374 | 96 | 1 | 162 | 1 | 162 | 0 | 2.00E-70 |
| hsa_circ_0027372 | 94 | 1 | 629 | 1046 | 1668 | 0.01 | 0 |
| hsa_circ_0027372 | 92 | 1 | 828 | 847 | 1668 | 0.01 | 0 |
| hsa_circ_0027371 | 94 | 1 | 566 | 1 | 566 | 0 | 0 |
| hsa_circ_0027367 | 88 | 1 | 384 | 138 | 521 | 0 | 2.00E-123 |
| hsa_circ_0027367 | 88 | 1 | 521 | 1 | 521 | 0 | 4.00E-171 |
| hsa_circ_0027365 | 91 | 1 | 841 | 225 | 1065 | 0 | 0 |
| hsa_circ_0027362 | 94 | 1 | 811 | 325 | 1135 | 0 | 0 |
| hsa_circ_0027361 | 97 | 1 | 712 | 117 | 828 | 0 | 0 |
| hsa_circ_0027361 | 97 | 1 | 712 | 117 | 828 | 0 | 0 |
| hsa_circ_0027361 | 97 | 1 | 610 | 117 | 726 | 0 | 0 |
| hsa_circ_0027358 | 96 | 1 | 558 | 1 | 558 | 0 | 0 |
| hsa_circ_0027357 | 96 | 6622 | 7182 | 230 | 790 | 0 | 0 |
| hsa_circ_0027357 | 96 | 4 | 703 | 91 | 790 | 0 | 0 |
| hsa_circ_0027350 | 96 | 1 | 1256 | 129 | 1384 | 0 | 0 |
| hsa_circ_0027357 | 96 | 4 | 319 | 91 | 406 | 0 | 1.00E-145 |
| hsa_circ_0027350 | 96 | 1 | 872 | 129 | 1000 | 0 | 0 |
| hsa_circ_0027351 | 96 | 1 | 698 | 1 | 698 | 0 | 0 |
| hsa_circ_0027351 | 96 | 1 | 698 | 1 | 698 | 0 | 0 |
| hsa_circ_0027350 | 96 | 1 | 826 | 1 | 826 | 0 | 0 |
| hsa_circ_0027354 | 96 | 1 | 149 | 1 | 149 | 0 | 3.00E-63 |
| hsa_circ_0027351 | 96 | 1 | 553 | 1 | 553 | 0 | 0 |
| hsa_circ_0027353 | 95 | 772 | 833 | 144 | 205 | 0 | 4.00E-19 |
| hsa_circ_0027351 | 96 | 1 | 463 | 1 | 463 | 0 | 0 |
| hsa_circ_0027350 | 96 | 1 | 532 | 1 | 532 | 0 | 0 |
| hsa_circ_0027350 | 96 | 1 | 386 | 1 | 386 | 0 | 0 |
| hsa_circ_0073485 | 92 | 3 | 987 | 985 | 1 | 0 | 0 |
| hsa_circ_0073485 | 92 | 3 | 987 | 985 | 1 | 0 | 0 |
| hsa_circ_0073485 | 92 | 3 | 808 | 985 | 180 | 0 | 0 |

novel_circ_017551 novel_circ_017552 novel_circ_017553 novel_circ_017554 novel_circ_017555 novel_circ_017556 novel_circ_017557 novel_circ_017558 novel_circ_017559 novel_circ_017560 novel_circ_017561 novel_circ_017562 novel_circ_017563 novel_circ_017564 novel_circ_017565 novel_circ_017566 novel_circ_017567 novel_circ_017569 novel_circ_017570 novel_circ_017571 novel_circ_017572 novel_circ_017573 novel_circ_017574 novel_circ_017575 novel_circ_017576 novel_circ_017577 novel_circ_017578 novel_circ_017579 novel_circ_017580 novel_circ_017581 novel_circ_017582 novel_circ_017583 novel_circ_017584 novel_circ_017585 novel_circ_017586 novel_circ_017587 novel_circ_017588 novel_circ_017589 novel_circ_017590 novel_circ_017591 novel_circ_017592 novel_circ_017593 novel_circ_017594 novel_circ_017595 novel_circ_017596 novel_circ_017597 novel_circ_017598 novel_circ_017599 novel_circ_017600 novel_circ_017601 novel_circ_017602 novel_circ_017603 novel_circ_017604 novel_circ_017605

| hsa_circ_0073485 | 94 | 3 | 516 | 985 | 472 | 0 | 0 |
| --- | --- | --- | --- | --- | --- | --- | --- |
| hsa_circ_0073485 | 95 | 3 | 421 | 985 | 567 | 0 | 0 |
| hsa_circ_0073485 | 95 | 1 | 994 | 1560 | 567 | 0 | 0 |
| hsa_circ_0073485 | 95 | 1 | 704 | 1560 | 857 | 0 | 0 |
| hsa_circ_0073485 | 95 | 1 | 492 | 1859 | 1368 | 0 | 0 |
| hsa_circ_0073485 | 96 | 2 | 880 | 2246 | 1368 | 0 | 0 |
| hsa_circ_0073485 | 95 | 1 | 299 | 1859 | 1561 | 0 | 6.00E-133 |
| hsa_circ_0073485 | 95 | 2 | 371 | 1930 | 1561 | 0 | 1.00E-165 |
| hsa_circ_0073485 | 96 | 2 | 687 | 2246 | 1561 | 0 | 0 |
| hsa_circ_0073485 | 95 | 1 | 1502 | 3062 | 1561 | 0 | 0 |
| hsa_circ_0073485 | 98 | 2 | 316 | 2246 | 1932 | 0 | 2.00E-153 |
| hsa_circ_0073485 | 95 | 1 | 1637 | 3568 | 1932 | 0 | 0 |
| hsa_circ_0073489 | 94 | 1 | 815 | 815 | 1 | 0 | 0 |
| hsa_circ_0073489 | 94 | 1 | 1321 | 1321 | 1 | 0 | 0 |
| hsa_circ_0073489 | 94 | 1 | 1132 | 1321 | 190 | 0 | 0 |
| hsa_circ_0073489 | 95 | 1 | 506 | 1321 | 816 | 0 | 0 |
| hsa_circ_0073489 | 95 | 1 | 487 | 1808 | 1322 | 0 | 0 |
| hsa_circ_0006759 | 76 | 15954 | 16235 | 34924 | 35212 | 0.04 | 3.00E-31 |
| hsa_circ_0018460 | 97 | 1 | 317 | 317 | 1 | 0 | 6.00E-148 |
| hsa_circ_0018459 | 94 | 1 | 1107 | 2149 | 1040 | 0 | 0 |
| hsa_circ_0018459 | 93 | 216 | 2122 | 2949 | 1040 | 0 | 0 |
| hsa_circ_0018459 | 93 | 418 | 2324 | 2949 | 1040 | 0 | 0 |
| hsa_circ_0018459 | 92 | 418 | 2147 | 2949 | 1217 | 0 | 0 |
| hsa_circ_0018462 | 97 | 5 | 254 | 250 | 1 | 0 | 8.00E-116 |
| hsa_circ_0018462 | 93 | 1 | 514 | 514 | 1 | 0 | 0 |
| hsa_circ_0018459 | 93 | 1 | 790 | 2149 | 1357 | 0.01 | 0 |
| hsa_circ_0018459 | 92 | 1 | 1437 | 2796 | 1357 | 0 | 0 |
| hsa_circ_0018459 | 92 | 1 | 1590 | 2949 | 1357 | 0 | 0 |
| hsa_circ_0018459 | 92 | 216 | 1805 | 2949 | 1357 | 0 | 0 |
| hsa_circ_0018459 | 92 | 418 | 2007 | 2949 | 1357 | 0 | 0 |
| hsa_circ_0018465 | 92 | 1 | 3171 | 3168 | 1 | 0 | 0 |
| hsa_circ_0018459 | 92 | 216 | 1682 | 2949 | 1480 | 0 | 0 |
| hsa_circ_0018459 | 92 | 418 | 1884 | 2949 | 1480 | 0 | 0 |
| hsa_circ_0018463 | 91 | 1 | 536 | 670 | 132 | 0.01 | 0 |
| hsa_circ_0018459 | 91 | 216 | 1551 | 2949 | 1611 | 0 | 0 |
| hsa_circ_0018463 | 92 | 1 | 445 | 670 | 223 | 0.01 | 8.00E-178 |
| hsa_circ_0018459 | 92 | 418 | 1662 | 2949 | 1702 | 0 | 0 |
| hsa_circ_0018463 | 91 | 1 | 923 | 1317 | 392 | 0 | 0 |
| hsa_circ_0018459 | 91 | 1 | 1076 | 2949 | 1871 | 0 | 0 |
| hsa_circ_0018459 | 91 | 216 | 1291 | 2949 | 1871 | 0 | 0 |
| hsa_circ_0018459 | 91 | 418 | 1493 | 2949 | 1871 | 0 | 0 |
| hsa_circ_0018465 | 92 | 1 | 3171 | 3168 | 1 | 0 | 0 |
| hsa_circ_0018459 | 91 | 216 | 1015 | 2949 | 2150 | 0 | 0 |
| hsa_circ_0018465 | 95 | 1 | 788 | 788 | 1 | 0 | 0 |
| hsa_circ_0018465 | 95 | 2 | 368 | 585 | 219 | 0 | 3.00E-165 |
| hsa_circ_0018465 | 95 | 2 | 368 | 585 | 219 | 0 | 1.00E-165 |
| hsa_circ_0018465 | 92 | 1 | 2953 | 3168 | 219 | 0 | 0 |
| hsa_circ_0018465 | 92 | 1 | 2800 | 3168 | 372 | 0 | 0 |
| hsa_circ_0018465 | 92 | 1 | 2585 | 3168 | 587 | 0 | 0 |
| hsa_circ_0018465 | 91 | 1 | 2383 | 3168 | 786 | 0 | 0 |
| hsa_circ_0018466 | 92 | 1 | 2253 | 2247 | 1 | 0 | 0 |
| hsa_circ_0018466 | 99 | 1 | 231 | 2247 | 2017 | 0 | 1.00E-113 |
| hsa_circ_0005144 | 95 | 1 | 165 | 165 | 1 | 0 | 7.00E-67 |
| hsa_circ_0018467 | 93 | 1 | 196 | 78719 | 78524 | 0 | 3.00E-77 |

novel_circ_017606 novel_circ_017607 novel_circ_017609 novel_circ_017610 novel_circ_017611 novel_circ_017612 novel_circ_017613 novel_circ_017614 novel_circ_017615 novel_circ_017616 novel_circ_017618 novel_circ_017619 novel_circ_017620 novel_circ_017621 novel_circ_017622 novel_circ_017623 novel_circ_017624 novel_circ_017627 novel_circ_017628 novel_circ_017629 novel_circ_017630 novel_circ_017631 novel_circ_017632 novel_circ_017633 novel_circ_017634 novel_circ_017635 novel_circ_017636 novel_circ_017637 novel_circ_017638 novel_circ_017639 novel_circ_017640 novel_circ_017641 novel_circ_017642 novel_circ_017643 novel_circ_017644 novel_circ_017645 novel_circ_017646 novel_circ_017647 novel_circ_017648 novel_circ_017649 novel_circ_017650 novel_circ_017651 novel_circ_017652 novel_circ_017653 novel_circ_017654 novel_circ_017655 novel_circ_017656 novel_circ_017657 novel_circ_017658 novel_circ_017660 novel_circ_017661 novel_circ_017664 novel_circ_017666 novel_circ_017667

| hsa_circ_0005144 | 95 | 51 | 215 | 165 | 1 | 0 | 5.00E-67 |
| --- | --- | --- | --- | --- | --- | --- | --- |
| hsa_circ_0005144 | 95 | 121 | 285 | 165 | 1 | 0 | 7.00E-67 |
| hsa_circ_0030001 | 94 | 1 | 569 | 569 | 1 | 0 | 0 |
| hsa_circ_0002923 | 89 | 1 | 1284 | 1290 | 1 | 0.02 | 0 |
| hsa_circ_0002923 | 90 | 1 | 992 | 1290 | 281 | 0.02 | 0 |
| hsa_circ_0030004 | 88 | 15 | 1203 | 1432 | 258 | 0.02 | 0 |
| hsa_circ_0030006 | 97 | 90 | 415 | 326 | 1 | 0 | 8.00E-153 |
| hsa_circ_0030006 | 97 | 94 | 419 | 326 | 1 | 0 | 8.00E-153 |
| hsa_circ_0030007 | 94 | 1 | 207 | 207 | 1 | 0 | 1.00E-83 |
| hsa_circ_0030007 | 93 | 220 | 495 | 276 | 1 | 0 | 1.00E-111 |
| hsa_circ_0030012 | 94 | 1 | 1683 | 1687 | 1 | 0 | 0 |
| hsa_circ_0030014 | 95 | 1 | 756 | 1267 | 512 | 0 | 0 |
| hsa_circ_0030014 | 95 | 1 | 759 | 1270 | 512 | 0 | 0 |
| hsa_circ_0000475 | 95 | 1 | 402 | 402 | 1 | 0 | 0 |
| hsa_circ_0000475 | 95 | 1 | 405 | 405 | 1 | 0 | 0 |
| hsa_circ_0000475 | 95 | 1 | 231 | 402 | 172 | 0 | 1.00E-98 |
| hsa_circ_0000475 | 96 | 1 | 396 | 567 | 172 | 0 | 0 |
| hsa_circ_0030019 | 93 | 6 | 140 | 135 | 1 | 0 | 5.00E-48 |
| hsa_circ_0030019 | 93 | 6 | 140 | 135 | 1 | 0 | 4.00E-48 |
| hsa_circ_0002265 | 94 | 1 | 519 | 519 | 1 | 0 | 0 |
| hsa_circ_0002265 | 94 | 406 | 924 | 519 | 1 | 0 | 0 |
| hsa_circ_0002265 | 94 | 10031 | 10549 | 519 | 1 | 0 | 0 |
| hsa_circ_0030022 | 87 | 148 | 756 | 1 | 609 | 0 | 0 |
| hsa_circ_0030022 | 89 | 4 | 378 | 235 | 609 | 0 | 9.00E-127 |
| hsa_circ_0030026 | 92 | 175 | 370 | 362 | 167 | 0 | 6.00E-74 |
| hsa_circ_0030032 | 95 | 145 | 272 | 1 | 128 | 0 | 4.00E-51 |
| hsa_circ_0030032 | 95 | 145 | 272 | 1 | 128 | 0 | 5.00E-51 |
| hsa_circ_0030033 | 94 | 637 | 921 | 2 | 286 | 0 | 5.00E-118 |
| hsa_circ_0030033 | 94 | 637 | 1673 | 2 | 1038 | 0 | 0 |
| hsa_circ_0030033 | 94 | 637 | 1673 | 2 | 1038 | 0 | 0 |
| hsa_circ_0030033 | 95 | 96 | 590 | 2 | 496 | 0 | 0 |
| hsa_circ_0030033 | 94 | 2 | 215 | 2 | 215 | 0 | 3.00E-89 |
| hsa_circ_0030033 | 94 | 2 | 286 | 2 | 286 | 0 | 2.00E-118 |
| hsa_circ_0030033 | 94 | 2 | 378 | 2 | 378 | 0 | 3.00E-161 |
| hsa_circ_0030033 | 95 | 2 | 496 | 2 | 496 | 0 | 0 |
| hsa_circ_0030033 | 94 | 2 | 796 | 2 | 796 | 0 | 0 |
| hsa_circ_0030033 | 94 | 2 | 904 | 2 | 904 | 0 | 0 |
| hsa_circ_0030033 | 94 | 2 | 1038 | 2 | 1038 | 0 | 0 |
| hsa_circ_0030033 | 94 | 2 | 1038 | 2 | 1038 | 0 | 0 |
| hsa_circ_0030033 | 94 | 2 | 1038 | 2 | 1038 | 0 | 0 |
| hsa_circ_0030033 | 94 | 2 | 1038 | 2 | 1038 | 0 | 0 |
| hsa_circ_0030033 | 94 | 1 | 817 | 222 | 1038 | 0 | 0 |
| hsa_circ_0030034 | 94 | 1 | 752 | 1 | 752 | 0 | 0 |
| hsa_circ_0030034 | 94 | 1 | 752 | 1 | 752 | 0 | 0 |
| hsa_circ_0030034 | 94 | 1 | 752 | 1 | 752 | 0 | 0 |
| hsa_circ_0030037 | 87 | 233 | 431 | 543 | 741 | 0.01 | 2.00E-55 |
| hsa_circ_0030037 | 76 | 1 | 812 | 543 | 1348 | 0.07 | 1.00E-105 |
| hsa_circ_0030348 | 96 | 1 | 432 | 678 | 247 | 0 | 0 |
| hsa_circ_0030329 | 89 | 1 | 172 | 174 | 1 | 0.01 | 3.00E-54 |
| hsa_circ_0030327 | 87 | 240 | 565 | 328 | 3 | 0 | 4.00E-99 |
| hsa_circ_0030327 | 87 | 1 | 326 | 328 | 3 | 0 | 3.00E-99 |
| hsa_circ_0030325 | 91 | 1 | 929 | 1 | 932 | 0 | 0 |
| hsa_circ_0030323 | 88 | 259 | 584 | 578 | 253 | 0 | 5.00E-106 |
| hsa_circ_0030323 | 92 | 1 | 414 | 414 | 1 | 0 | 2.00E-163 |

novel_circ_017668 novel_circ_017669 novel_circ_017670 novel_circ_017671 novel_circ_017672 novel_circ_017673 novel_circ_017674 novel_circ_017675 novel_circ_017676 novel_circ_017677 novel_circ_017678 novel_circ_017680 novel_circ_017683 novel_circ_017686 novel_circ_017689 novel_circ_017690 novel_circ_017691 novel_circ_017692 novel_circ_017693 novel_circ_017694 novel_circ_017695 novel_circ_017696 novel_circ_017697 novel_circ_017698 novel_circ_017699 novel_circ_017700 novel_circ_017705 novel_circ_017706 novel_circ_017707 novel_circ_017708 novel_circ_017709 novel_circ_017711 novel_circ_017712 novel_circ_017713 novel_circ_017714 novel_circ_017715 novel_circ_017716 novel_circ_017717 novel_circ_017718 novel_circ_017719 novel_circ_017720 novel_circ_017721 novel_circ_017722 novel_circ_017723 novel_circ_017724 novel_circ_017725 novel_circ_017726 novel_circ_017727 novel_circ_017728 novel_circ_017729 novel_circ_017730 novel_circ_017731 novel_circ_017732 novel_circ_017733

| hsa_circ_0030323 | 92 | 1 | 414 | 414 | 1 | 0 | 3.00E-163 |
| --- | --- | --- | --- | --- | --- | --- | --- |
| hsa_circ_0030323 | 92 | 1 | 414 | 414 | 1 | 0 | 4.00E-163 |
| hsa_circ_0030320 | 92 | 416 | 1353 | 938 | 1 | 0 | 0 |
| hsa_circ_0030313 | 96 | 1357 | 1700 | 232 | 575 | 0 | 7.00E-159 |
| hsa_circ_0030313 | 92 | 1 | 233 | 1 | 233 | 0 | 1.00E-89 |
| hsa_circ_0030306 | 94 | 1 | 552 | 143 | 694 | 0 | 0 |
| hsa_circ_0030312 | 94 | 1 | 240 | 1 | 240 | 0 | 3.00E-100 |
| hsa_circ_0030308 | 95 | 1 | 446 | 75 | 520 | 0 | 0 |
| hsa_circ_0084772 | 87 | 15072 | 15727 | 23 | 687 | 0.01 | 0 |
| hsa_circ_0079012 | 93 | 1 | 255 | 635 | 381 | 0 | 1.00E-103 |
| hsa_circ_0078938 | 95 | 81 | 210 | 512 | 383 | 0 | 5.00E-52 |
| hsa_circ_0078975 | 92 | 196 | 449 | 3666 | 3413 | 0 | 2.00E-97 |
| hsa_circ_0078947 | 91 | 1 | 132 | 132 | 1 | 0 | 2.00E-43 |
| hsa_circ_0001871 | 80 | 132 | 880 | 11 | 765 | 0.02 | 4.00E-159 |
| hsa_circ_0002493 | 89 | 1 | 290 | 290 | 1 | 0 | 2.00E-99 |
| hsa_circ_0003708 | 87 | 4 | 700 | 691 | 1 | 0.01 | 0 |
| hsa_circ_0003708 | 88 | 1 | 455 | 455 | 1 | 0 | 4.00E-151 |
| hsa_circ_0003708 | 88 | 1 | 455 | 455 | 1 | 0 | 4.00E-150 |
| hsa_circ_0087433 | 93 | 1 | 486 | 1 | 486 | 0 | 0 |
| hsa_circ_0087432 | 93 | 1 | 535 | 211 | 745 | 0 | 0 |
| hsa_circ_0087432 | 93 | 299 | 833 | 211 | 745 | 0 | 0 |
| hsa_circ_0087432 | 93 | 18039 | 18573 | 211 | 745 | 0 | 0 |
| hsa_circ_0087432 | 93 | 299 | 599 | 211 | 511 | 0 | 2.00E-120 |
| hsa_circ_0087432 | 86 | 2 | 204 | 2 | 210 | 0.03 | 2.00E-54 |
| hsa_circ_0087432 | 86 | 2 | 204 | 2 | 210 | 0.03 | 9.00E-55 |
| hsa_circ_0087428 | 85 | 17706 | 17944 | 7568 | 7814 | 0.03 | 1.00E-61 |
| hsa_circ_0080901 | 92 | 1 | 368 | 368 | 1 | 0 | 1.00E-144 |
| hsa_circ_0042931 | 96 | 1 | 1580 | 775 | 2354 | 0 | 0 |
| hsa_circ_0042932 | 95 | 1 | 370 | 139 | 508 | 0 | 1.00E-165 |
| hsa_circ_0042932 | 94 | 1 | 629 | 139 | 767 | 0 | 0 |
| hsa_circ_0042934 | 96 | 1 | 762 | 427 | 1188 | 0 | 0 |
| hsa_circ_0042949 | 87 | 1 | 596 | 669 | 74 | 0 | 0 |
| hsa_circ_0042949 | 86 | 1 | 529 | 669 | 141 | 0 | 4.00E-162 |
| hsa_circ_0042955 | 89 | 4 | 427 | 421 | 1 | 0.01 | 4.00E-146 |
| hsa_circ_0042971 | 97 | 3 | 643 | 3 | 643 | 0 | 0 |
| hsa_circ_0042971 | 97 | 3 | 927 | 3 | 927 | 0 | 0 |
| hsa_circ_0042974 | 97 | 1 | 462 | 1 | 462 | 0 | 0 |
| hsa_circ_0042979 | 96 | 1 | 95 | 1 | 95 | 0 | 2.00E-35 |
| hsa_circ_0042979 | 97 | 1 | 378 | 1 | 378 | 0 | 0 |
| hsa_circ_0042979 | 96 | 1 | 614 | 1 | 614 | 0 | 0 |
| hsa_circ_0042985 | 95 | 1 | 394 | 1 | 394 | 0 | 3.00E-177 |
| hsa_circ_0042988 | 95 | 1 | 581 | 1 | 581 | 0 | 0 |
| hsa_circ_0042988 | 96 | 1 | 437 | 145 | 581 | 0 | 0 |
| hsa_circ_0042859 | 92 | 1 | 943 | 943 | 1 | 0 | 0 |
| hsa_circ_0042859 | 91 | 1 | 469 | 469 | 1 | 0 | 2.00E-180 |
| hsa_circ_0042859 | 92 | 1 | 735 | 735 | 1 | 0 | 0 |
| hsa_circ_0042859 | 92 | 1 | 943 | 943 | 1 | 0 | 0 |
| hsa_circ_0042861 | 93 | 1 | 695 | 830 | 136 | 0 | 0 |
| hsa_circ_0042861 | 93 | 1 | 474 | 830 | 357 | 0 | 0 |
| hsa_circ_0042861 | 92 | 1 | 296 | 830 | 535 | 0 | 3.00E-116 |
| hsa_circ_0000756 | 86 | 1 | 712 | 1 | 715 | 0.01 | 0 |
| hsa_circ_0042869 | 89 | 3 | 599 | 948 | 1550 | 0.01 | 0 |
| hsa_circ_0042869 | 90 | 3 | 690 | 948 | 1641 | 0.01 | 0 |
| hsa_circ_0003422 | 91 | 3 | 936 | 224722 | 225661 | 0.01 | 0 |

novel_circ_017734 novel_circ_017735 novel_circ_017736 novel_circ_017737 novel_circ_017738 novel_circ_017739 novel_circ_017740 novel_circ_017741 novel_circ_017742 novel_circ_017743 novel_circ_017744 novel_circ_017746 novel_circ_017747 novel_circ_017748 novel_circ_017749 novel_circ_017750 novel_circ_017751 novel_circ_017753 novel_circ_017754 novel_circ_017755 novel_circ_017757 novel_circ_017758 novel_circ_017759 novel_circ_017760 novel_circ_017761 novel_circ_017762 novel_circ_017763 novel_circ_017764 novel_circ_017765 novel_circ_017766 novel_circ_017767 novel_circ_017768 novel_circ_017769 novel_circ_017770 novel_circ_017771 novel_circ_017772 novel_circ_017773 novel_circ_017774 novel_circ_017775 novel_circ_017776 novel_circ_017777 novel_circ_017778 novel_circ_017779 novel_circ_017780 novel_circ_017781 novel_circ_017782 novel_circ_017783 novel_circ_017784 novel_circ_017785 novel_circ_017786 novel_circ_017787 novel_circ_017788 novel_circ_017789 novel_circ_017790

| hsa_circ_0003422 | 88 | 3 | 1987 | 224722 | 226703 | 0.01 | 0 |
| --- | --- | --- | --- | --- | --- | --- | --- |
| hsa_circ_0003422 | 94 | 1 | 337 | 225325 | 225661 | 0 | 2.00E-142 |
| hsa_circ_0042999 | 96 | 1 | 503 | 1 | 503 | 0 | 0 |
| hsa_circ_0042999 | 95 | 1 | 1699 | 1 | 1699 | 0 | 0 |
| hsa_circ_0043004 | 96 | 1 | 444 | 1 | 444 | 0 | 0 |
| hsa_circ_0043005 | 96 | 1 | 362 | 1 | 362 | 0 | 1.00E-164 |
| hsa_circ_0043007 | 95 | 1 | 540 | 1 | 540 | 0 | 0 |
| hsa_circ_0043007 | 94 | 1 | 1003 | 1 | 1003 | 0 | 0 |
| hsa_circ_0043007 | 94 | 1 | 1087 | 1 | 1087 | 0 | 0 |
| hsa_circ_0043007 | 94 | 1 | 661 | 212 | 872 | 0 | 0 |
| hsa_circ_0043007 | 94 | 1 | 792 | 212 | 1003 | 0 | 0 |
| hsa_circ_0043009 | 93 | 1 | 332 | 1 | 332 | 0 | 6.00E-138 |
| hsa_circ_0043013 | 92 | 1 | 363 | 1 | 363 | 0 | 9.00E-142 |
| hsa_circ_0006129 | 97 | 1 | 550 | 1 | 550 | 0 | 0 |
| hsa_circ_0043039 | 97 | 1 | 323 | 1 | 323 | 0 | 3.00E-151 |
| hsa_circ_0043049 | 93 | 1 | 963 | 963 | 1 | 0 | 0 |
| hsa_circ_0043058 | 93 | 1 | 375 | 375 | 1 | 0 | 4.00E-155 |
| hsa_circ_0043082 | 91 | 1 | 548 | 1 | 551 | 0.01 | 0 |
| hsa_circ_0043082 | 90 | 1 | 692 | 1 | 695 | 0 | 0 |
| hsa_circ_0043084 | 93 | 1 | 350 | 1 | 350 | 0 | 3.00E-141 |
| hsa_circ_0029815 | 90 | 9550 | 10063 | 519 | 6 | 0 | 0 |
| hsa_circ_0029815 | 90 | 9550 | 10063 | 519 | 6 | 0 | 0 |
| hsa_circ_0035310 | 88 | 7473 | 7677 | 2333 | 2132 | 0.05 | 8.00E-58 |
| hsa_circ_0035310 | 88 | 9728 | 9932 | 2333 | 2132 | 0.05 | 9.00E-58 |
| hsa_circ_0043131 | 95 | 1 | 465 | 194 | 658 | 0 | 0 |
| hsa_circ_0043127 | 95 | 1 | 1299 | 440 | 1738 | 0 | 0 |
| hsa_circ_0043127 | 95 | 1 | 1169 | 440 | 1608 | 0 | 0 |
| hsa_circ_0043127 | 95 | 1 | 1023 | 440 | 1462 | 0 | 0 |
| hsa_circ_0043127 | 95 | 1 | 834 | 440 | 1273 | 0 | 0 |
| hsa_circ_0043124 | 94 | 1 | 1464 | 1 | 1464 | 0 | 0 |
| hsa_circ_0043127 | 96 | 1 | 381 | 440 | 820 | 0 | 9.00E-177 |
| hsa_circ_0044914 | 88 | 10 | 536 | 530 | 1 | 0.01 | 6.00E-180 |
| hsa_circ_0044913 | 88 | 10 | 538 | 915 | 384 | 0.01 | 0 |
| hsa_circ_0044911 | 91 | 4 | 307 | 1350 | 1047 | 0 | 5.00E-114 |
| hsa_circ_0043293 | 91 | 512 | 2540 | 3088 | 1059 | 0.01 | 0 |
| hsa_circ_0043293 | 91 | 1 | 2019 | 3077 | 1059 | 0.01 | 0 |
| hsa_circ_0043293 | 91 | 1 | 2019 | 3077 | 1059 | 0.01 | 0 |
| hsa_circ_0043293 | 91 | 1 | 2019 | 3077 | 1059 | 0.01 | 0 |
| hsa_circ_0043293 | 91 | 1 | 2019 | 3077 | 1059 | 0.01 | 0 |
| hsa_circ_0043293 | 91 | 4 | 1785 | 2840 | 1059 | 0.01 | 0 |
| hsa_circ_0043293 | 91 | 4 | 1785 | 2840 | 1059 | 0.01 | 0 |
| hsa_circ_0043295 | 92 | 1 | 577 | 1707 | 1128 | 0.01 | 0 |
| hsa_circ_0043295 | 90 | 6 | 316 | 1256 | 946 | 0.02 | 6.00E-113 |
| hsa_circ_0043295 | 91 | 1 | 1130 | 1127 | 1 | 0.01 | 0 |
| hsa_circ_0043295 | 91 | 1 | 1130 | 1127 | 1 | 0.01 | 0 |
| hsa_circ_0043293 | 92 | 1 | 1052 | 1058 | 1 | 0.01 | 0 |
| hsa_circ_0043293 | 92 | 6 | 917 | 915 | 1 | 0.01 | 0 |
| hsa_circ_0043293 | 94 | 1 | 464 | 464 | 1 | 0 | 0 |
| hsa_circ_0043288 | 93 | 1 | 251 | 292 | 542 | 0 | 2.00E-101 |
| hsa_circ_0043288 | 95 | 1 | 219 | 1 | 219 | 0 | 5.00E-92 |
| hsa_circ_0043281 | 93 | 1 | 515 | 1 | 518 | 0.01 | 0 |
| hsa_circ_0008283 | 92 | 1 | 317 | 1 | 320 | 0.01 | 2.00E-123 |
| hsa_circ_0043281 | 92 | 1 | 409 | 1 | 412 | 0.01 | 3.00E-166 |
| hsa_circ_0008283 | 91 | 1 | 244 | 1 | 247 | 0.01 | 5.00E-88 |

novel_circ_017791 novel_circ_017792 novel_circ_017793 novel_circ_017794 novel_circ_017795 novel_circ_017796 novel_circ_017797 novel_circ_017798 novel_circ_017799 novel_circ_017800 novel_circ_017801 novel_circ_017802 novel_circ_017803 novel_circ_017804 novel_circ_017805 novel_circ_017806 novel_circ_017807 novel_circ_017808 novel_circ_017809 novel_circ_017810 novel_circ_017811 novel_circ_017812 novel_circ_017813 novel_circ_017814 novel_circ_017815 novel_circ_017816 novel_circ_017817 novel_circ_017818 novel_circ_017819 novel_circ_017820 novel_circ_017821 novel_circ_017822 novel_circ_017823 novel_circ_017824 novel_circ_017826 novel_circ_017827 novel_circ_017828 novel_circ_017829 novel_circ_017830 novel_circ_017831 novel_circ_017832 novel_circ_017834 novel_circ_017835 novel_circ_017836 novel_circ_017837 novel_circ_017838 novel_circ_017839 novel_circ_017840 novel_circ_017841 novel_circ_017842 novel_circ_017843 novel_circ_017844 novel_circ_017845 novel_circ_017846

| hsa_circ_0043281 | 92 | 1 | 336 | 1 | 339 | 0.01 | 1.00E-130 |
| --- | --- | --- | --- | --- | --- | --- | --- |
| hsa_circ_0043265 | 87 | 2 | 300 | 1290 | 992 | 0.01 | 3.00E-91 |
| hsa_circ_0043265 | 91 | 2 | 572 | 1290 | 720 | 0 | 0 |
| hsa_circ_0043265 | 93 | 2 | 1081 | 1290 | 211 | 0 | 0 |
| hsa_circ_0000759 | 95 | 1 | 272 | 272 | 1 | 0 | 5.00E-118 |
| hsa_circ_0043265 | 95 | 1 | 781 | 991 | 211 | 0 | 0 |
| hsa_circ_0043259 | 94 | 1 | 1971 | 1971 | 1 | 0 | 0 |
| hsa_circ_0043242 | 94 | 1 | 2908 | 3369 | 462 | 0 | 0 |
| hsa_circ_0043265 | 94 | 1 | 317 | 527 | 211 | 0 | 6.00E-133 |
| hsa_circ_0043254 | 94 | 1 | 1793 | 1793 | 1 | 0 | 0 |
| hsa_circ_0043265 | 93 | 1 | 218 | 428 | 211 | 0 | 9.00E-85 |
| hsa_circ_0043259 | 94 | 1 | 1190 | 1190 | 1 | 0 | 0 |
| hsa_circ_0043259 | 95 | 1 | 326 | 809 | 484 | 0 | 1.00E-144 |
| hsa_circ_0043260 | 94 | 1 | 255 | 255 | 1 | 0 | 6.00E-107 |
| hsa_circ_0043248 | 93 | 1 | 1420 | 1534 | 115 | 0 | 0 |
| hsa_circ_0043242 | 94 | 1 | 1857 | 1881 | 25 | 0 | 0 |
| hsa_circ_0043247 | 95 | 1 | 437 | 437 | 1 | 0 | 0 |
| hsa_circ_0043232 | 95 | 1 | 618 | 618 | 1 | 0 | 0 |
| hsa_circ_0043228 | 95 | 1 | 777 | 1107 | 331 | 0 | 0 |
| hsa_circ_0043228 | 95 | 1 | 573 | 903 | 331 | 0 | 0 |
| hsa_circ_0043228 | 95 | 1 | 671 | 903 | 233 | 0 | 0 |
| hsa_circ_0043228 | 94 | 1 | 903 | 903 | 1 | 0 | 0 |
| hsa_circ_0043222 | 95 | 1 | 1498 | 1676 | 179 | 0 | 0 |
| hsa_circ_0043228 | 94 | 1 | 515 | 747 | 233 | 0 | 0 |
| hsa_circ_0043228 | 94 | 1 | 600 | 600 | 1 | 0 | 0 |
| hsa_circ_0043228 | 94 | 1 | 330 | 330 | 1 | 0 | 8.00E-142 |
| hsa_circ_0043222 | 95 | 1 | 595 | 773 | 179 | 0 | 0 |
| hsa_circ_0043221 | 94 | 1 | 886 | 1349 | 464 | 0 | 0 |
| hsa_circ_0043221 | 94 | 3 | 326 | 461 | 138 | 0 | 4.00E-140 |
| hsa_circ_0043209 | 92 | 3860 | 4527 | 114 | 787 | 0.01 | 0 |
| hsa_circ_0043209 | 91 | 3860 | 4306 | 114 | 566 | 0.01 | 1.00E-170 |
| hsa_circ_0043209 | 91 | 1 | 560 | 1 | 566 | 0.01 | 0 |
| hsa_circ_0043206 | 87 | 5 | 566 | 5 | 551 | 0.03 | 3.00E-172 |
| hsa_circ_0043206 | 87 | 5 | 564 | 5 | 549 | 0.03 | 1.00E-171 |
| hsa_circ_0043195 | 94 | 1 | 867 | 176 | 1045 | 0 | 0 |
| hsa_circ_0043195 | 95 | 1 | 370 | 1 | 370 | 0 | 2.00E-162 |
| hsa_circ_0043192 | 93 | 1 | 385 | 1 | 379 | 0.02 | 3.00E-157 |
| hsa_circ_0043189 | 93 | 225 | 724 | 353 | 846 | 0.01 | 0 |
| hsa_circ_0043186 | 95 | 1 | 434 | 1 | 434 | 0 | 0 |
| hsa_circ_0043189 | 95 | 1 | 353 | 1 | 353 | 0 | 6.00E-158 |
| hsa_circ_0043186 | 95 | 1 | 434 | 1 | 434 | 0 | 0 |
| hsa_circ_0044916 | 92 | 1 | 999 | 999 | 1 | 0 | 0 |
| hsa_circ_0044921 | 90 | 1 | 481 | 481 | 1 | 0 | 1.00E-180 |
| hsa_circ_0044922 | 94 | 1 | 1044 | 1044 | 1 | 0 | 0 |
| hsa_circ_0044922 | 95 | 1 | 1463 | 1463 | 1 | 0 | 0 |
| hsa_circ_0044931 | 94 | 1 | 185 | 185 | 1 | 0 | 8.00E-75 |
| hsa_circ_0044931 | 94 | 1 | 397 | 397 | 1 | 0 | 3.00E-172 |
| hsa_circ_0044931 | 93 | 3 | 509 | 506 | 1 | 0 | 0 |
| hsa_circ_0044931 | 93 | 3 | 661 | 659 | 1 | 0 | 0 |
| hsa_circ_0044931 | 94 | 1 | 836 | 836 | 1 | 0 | 0 |
| hsa_circ_0044931 | 95 | 1 | 1255 | 1255 | 1 | 0 | 0 |
| hsa_circ_0044931 | 95 | 7 | 1412 | 1406 | 1 | 0 | 0 |
| hsa_circ_0044931 | 95 | 1 | 1567 | 1567 | 1 | 0 | 0 |
| hsa_circ_0044931 | 95 | 1 | 1826 | 1826 | 1 | 0 | 0 |

novel_circ_017847 novel_circ_017848 novel_circ_017849 novel_circ_017850 novel_circ_017851 novel_circ_017852 novel_circ_017853 novel_circ_017854 novel_circ_017855 novel_circ_017856 novel_circ_017857 novel_circ_017858 novel_circ_017859 novel_circ_017860 novel_circ_017861 novel_circ_017862 novel_circ_017863 novel_circ_017864 novel_circ_017865 novel_circ_017866 novel_circ_017867 novel_circ_017868 novel_circ_017869 novel_circ_017870 novel_circ_017871 novel_circ_017872 novel_circ_017873 novel_circ_017874 novel_circ_017875 novel_circ_017876 novel_circ_017877 novel_circ_017878 novel_circ_017879 novel_circ_017880 novel_circ_017881 novel_circ_017882 novel_circ_017883 novel_circ_017884 novel_circ_017885 novel_circ_017886 novel_circ_017887 novel_circ_017888 novel_circ_017889 novel_circ_017890 novel_circ_017891 novel_circ_017892 novel_circ_017893 novel_circ_017894 novel_circ_017895 novel_circ_017896 novel_circ_017897 novel_circ_017898 novel_circ_017899 novel_circ_017900

| hsa_circ_0044931 | 94 | 4 | 2002 | 1999 | 1 | 0 | 0 |
| --- | --- | --- | --- | --- | --- | --- | --- |
| hsa_circ_0044931 | 94 | 1 | 2195 | 2195 | 1 | 0 | 0 |
| hsa_circ_0044931 | 93 | 3 | 324 | 506 | 186 | 0 | 8.00E-132 |
| hsa_circ_0044931 | 93 | 3 | 476 | 659 | 186 | 0 | 0 |
| hsa_circ_0044931 | 94 | 1 | 651 | 836 | 186 | 0 | 0 |
| hsa_circ_0044931 | 95 | 1 | 1070 | 1255 | 186 | 0 | 0 |
| hsa_circ_0044931 | 95 | 7 | 1227 | 1406 | 186 | 0 | 0 |
| hsa_circ_0044931 | 95 | 1 | 1382 | 1567 | 186 | 0 | 0 |
| hsa_circ_0044931 | 94 | 4 | 1817 | 1999 | 186 | 0 | 0 |
| hsa_circ_0044931 | 94 | 1 | 2010 | 2195 | 186 | 0 | 0 |
| hsa_circ_0044931 | 94 | 280 | 2957 | 2863 | 186 | 0 | 0 |
| hsa_circ_0044932 | 93 | 1 | 439 | 439 | 1 | 0 | 0 |
| hsa_circ_0044931 | 94 | 1 | 1798 | 2195 | 398 | 0 | 0 |
| hsa_circ_0044935 | 98 | 1 | 245 | 419 | 175 | 0 | 2.00E-116 |
| hsa_circ_0044935 | 94 | 1 | 1185 | 1359 | 175 | 0 | 0 |
| hsa_circ_0044936 | 93 | 1 | 940 | 1079 | 140 | 0 | 0 |
| hsa_circ_0044937 | 93 | 1 | 783 | 865 | 83 | 0 | 0 |
| hsa_circ_0044940 | 93 | 1 | 1457 | 1575 | 119 | 0 | 0 |
| hsa_circ_0044943 | 89 | 1 | 369 | 369 | 1 | 0 | 4.00E-130 |
| hsa_circ_0044940 | 93 | 1 | 1316 | 1575 | 260 | 0 | 0 |
| hsa_circ_0044940 | 94 | 1 | 1137 | 1575 | 439 | 0 | 0 |
| hsa_circ_0044945 | 94 | 1 | 668 | 668 | 1 | 0 | 0 |
| hsa_circ_0044940 | 95 | 1 | 947 | 1575 | 629 | 0 | 0 |
| hsa_circ_0044940 | 95 | 1 | 1053 | 1681 | 629 | 0 | 0 |
| hsa_circ_0044940 | 94 | 1 | 1181 | 1809 | 629 | 0 | 0 |
| hsa_circ_0044951 | 94 | 1 | 419 | 419 | 1 | 0 | 0 |
| hsa_circ_0044951 | 94 | 1 | 698 | 698 | 1 | 0 | 0 |
| hsa_circ_0044951 | 95 | 1 | 356 | 419 | 64 | 0 | 1.00E-159 |
| hsa_circ_0044951 | 95 | 1 | 516 | 579 | 64 | 0 | 0 |
| hsa_circ_0044951 | 95 | 1 | 635 | 698 | 64 | 0 | 0 |
| hsa_circ_0044951 | 95 | 1 | 869 | 932 | 64 | 0 | 0 |
| hsa_circ_0044952 | 97 | 1 | 240 | 240 | 1 | 0 | 3.00E-110 |
| hsa_circ_0044952 | 96 | 1 | 400 | 400 | 1 | 0 | 0 |
| hsa_circ_0044952 | 96 | 1 | 519 | 519 | 1 | 0 | 0 |
| hsa_circ_0044952 | 96 | 1 | 625 | 625 | 1 | 0 | 0 |
| hsa_circ_0044952 | 95 | 1 | 517 | 625 | 109 | 0 | 0 |
| hsa_circ_0044956 | 95 | 1 | 279 | 279 | 1 | 0 | 3.00E-120 |
| hsa_circ_0044956 | 95 | 1 | 385 | 385 | 1 | 0 | 1.00E-170 |
| hsa_circ_0044952 | 94 | 1 | 513 | 753 | 241 | 0 | 0 |
| hsa_circ_0044952 | 95 | 1 | 230 | 753 | 524 | 0 | 4.00E-98 |
| hsa_circ_0044966 | 97 | 1 | 533 | 923 | 391 | 0 | 0 |
| hsa_circ_0044966 | 98 | 1 | 365 | 923 | 559 | 0 | 1.00E-179 |
| hsa_circ_0044969 | 96 | 3 | 229 | 3 | 229 | 0 | 9.00E-100 |
| hsa_circ_0044969 | 95 | 3 | 545 | 3 | 545 | 0 | 0 |
| hsa_circ_0044969 | 95 | 1 | 542 | 4 | 545 | 0 | 0 |
| hsa_circ_0044971 | 95 | 1 | 316 | 1 | 316 | 0 | 1.00E-140 |
| hsa_circ_0044988 | 94 | 1 | 590 | 83 | 672 | 0 | 0 |
| hsa_circ_0044988 | 94 | 1 | 684 | 83 | 766 | 0 | 0 |
| hsa_circ_0044991 | 94 | 1 | 611 | 1 | 611 | 0 | 0 |
| hsa_circ_0044991 | 94 | 1 | 745 | 1 | 745 | 0 | 0 |
| hsa_circ_0044991 | 94 | 1 | 503 | 109 | 611 | 0 | 0 |
| hsa_circ_0044994 | 94 | 1 | 332 | 1 | 332 | 0 | 6.00E-143 |
| hsa_circ_0044994 | 95 | 1 | 426 | 1 | 426 | 0 | 0 |
| hsa_circ_0044994 | 95 | 1 | 560 | 1 | 560 | 0 | 0 |

novel_circ_017901 novel_circ_017902 novel_circ_017903 novel_circ_017904 novel_circ_017905 novel_circ_017906 novel_circ_017907 novel_circ_017908 novel_circ_017909 novel_circ_017910 novel_circ_017911 novel_circ_017912 novel_circ_017913 novel_circ_017914 novel_circ_017915 novel_circ_017916 novel_circ_017917 novel_circ_017918 novel_circ_017919 novel_circ_017920 novel_circ_017921 novel_circ_017922 novel_circ_017923 novel_circ_017924 novel_circ_017925 novel_circ_017926 novel_circ_017927 novel_circ_017928 novel_circ_017929 novel_circ_017930 novel_circ_017931 novel_circ_017932 novel_circ_017933 novel_circ_017934 novel_circ_017935 novel_circ_017936 novel_circ_017937 novel_circ_017941 novel_circ_017942 novel_circ_017943 novel_circ_017945 novel_circ_017946 novel_circ_017947 novel_circ_017948 novel_circ_017949 novel_circ_017950 novel_circ_017951 novel_circ_017952 novel_circ_017953 novel_circ_017954 novel_circ_017955 novel_circ_017956 novel_circ_017957 novel_circ_017958

| hsa_circ_0044996 | 94 | 1 | 265 | 85 | 349 | 0 | 4.00E-109 |
| --- | --- | --- | --- | --- | --- | --- | --- |
| hsa_circ_0045001 | 93 | 1 | 788 | 1 | 788 | 0 | 0 |
| hsa_circ_0044994 | 93 | 1 | 789 | 1022 | 1810 | 0 | 0 |
| hsa_circ_0045001 | 92 | 1 | 663 | 126 | 788 | 0 | 0 |
| hsa_circ_0045006 | 95 | 1 | 396 | 1 | 396 | 0 | 9.00E-177 |
| hsa_circ_0045004 | 95 | 1 | 397 | 102 | 498 | 0 | 3.00E-177 |
| hsa_circ_0045004 | 95 | 1 | 397 | 102 | 498 | 0 | 4.00E-177 |
| hsa_circ_0045004 | 95 | 1 | 397 | 102 | 498 | 0 | 3.00E-175 |
| hsa_circ_0087466 | 85 | 15769 | 15898 | 220 | 91 | 0.02 | 4.00E-27 |
| hsa_circ_0079526 | 96 | 1 | 642 | 1 | 642 | 0 | 0 |
| hsa_circ_0079526 | 98 | 1 | 400 | 243 | 642 | 0 | 0 |
| hsa_circ_0079526 | 98 | 1 | 532 | 243 | 774 | 0 | 0 |
| hsa_circ_0079526 | 95 | 1 | 1200 | 243 | 1445 | 0 | 0 |
| hsa_circ_0079526 | 95 | 1 | 1200 | 243 | 1445 | 0 | 0 |
| hsa_circ_0079526 | 98 | 1 | 391 | 252 | 642 | 0 | 0 |
| hsa_circ_0005331 | 94 | 135 | 1064 | 3 | 935 | 0 | 0 |
| hsa_circ_0005331 | 92 | 3 | 668 | 3 | 671 | 0 | 0 |
| hsa_circ_0005331 | 92 | 3 | 668 | 3 | 671 | 0 | 0 |
| hsa_circ_0005331 | 94 | 3 | 932 | 3 | 935 | 0 | 0 |
| hsa_circ_0079527 | 92 | 1 | 552 | 1 | 555 | 0.01 | 0 |
| hsa_circ_0079527 | 92 | 1 | 552 | 1 | 555 | 0.01 | 0 |
| hsa_circ_0079527 | 94 | 1 | 816 | 1 | 819 | 0 | 0 |
| hsa_circ_0007904 | 98 | 1 | 264 | 1 | 264 | 0 | 1.00E-128 |
| hsa_circ_0079528 | 92 | 9 | 486 | 6 | 483 | 0 | 0 |
| hsa_circ_0079528 | 93 | 9 | 594 | 6 | 591 | 0 | 0 |
| hsa_circ_0079528 | 96 | 3 | 305 | 181 | 483 | 0 | 2.00E-138 |
| hsa_circ_0079528 | 96 | 3 | 413 | 181 | 591 | 0 | 0 |
| hsa_circ_0079540 | 91 | 1 | 587 | 1 | 587 | 0 | 0 |
| hsa_circ_0079539 | 91 | 1 | 747 | 87 | 833 | 0 | 0 |
| hsa_circ_0079541 | 90 | 1 | 572 | 1 | 572 | 0 | 0 |
| hsa_circ_0079541 | 91 | 1 | 668 | 1 | 668 | 0 | 0 |
| hsa_circ_0079541 | 91 | 1 | 893 | 1 | 893 | 0 | 0 |
| hsa_circ_0079545 | 96 | 46704 | 47134 | 1670 | 2100 | 0 | 0 |
| hsa_circ_0079546 | 96 | 1 | 429 | 1 | 429 | 0 | 0 |
| hsa_circ_0079547 | 88 | 367 | 527 | 1 | 161 | 0 | 1.00E-47 |
| hsa_circ_0079547 | 92 | 1 | 913 | 465 | 1374 | 0 | 0 |
| hsa_circ_0079547 | 93 | 1 | 564 | 814 | 1374 | 0.01 | 0 |
| hsa_circ_0047325 | 98 | 1 | 324 | 324 | 1 | 0 | 4.00E-160 |
| hsa_circ_0047325 | 98 | 1 | 324 | 324 | 1 | 0 | 1.00E-159 |
| hsa_circ_0047325 | 98 | 1 | 145 | 145 | 1 | 0 | 4.00E-65 |
| hsa_circ_0047317 | 87 | 1 | 990 | 255 | 1247 | 0.01 | 0 |
| hsa_circ_0047323 | 94 | 1 | 484 | 1 | 484 | 0 | 0 |
| hsa_circ_0047317 | 87 | 1 | 990 | 255 | 1247 | 0.01 | 0 |
| hsa_circ_0047317 | 87 | 1 | 1250 | 1 | 1247 | 0.01 | 0 |
| hsa_circ_0047323 | 93 | 1 | 301 | 1 | 301 | 0 | 1.00E-120 |
| hsa_circ_0047317 | 87 | 1 | 990 | 255 | 1247 | 0.01 | 0 |
| hsa_circ_0047317 | 87 | 1 | 990 | 255 | 1247 | 0.01 | 0 |
| hsa_circ_0047317 | 87 | 1 | 1250 | 1 | 1247 | 0.01 | 0 |
| hsa_circ_0047301 | 97 | 1 | 180 | 3866 | 3687 | 0 | 5.00E-81 |
| hsa_circ_0047303 | 94 | 1 | 3353 | 3353 | 1 | 0 | 0 |
| hsa_circ_0047301 | 94 | 1 | 3438 | 3686 | 249 | 0 | 0 |
| hsa_circ_0047301 | 94 | 1 | 248 | 248 | 1 | 0 | 1.00E-104 |
| hsa_circ_0068717 | 92 | 1 | 313 | 718 | 406 | 0 | 2.00E-122 |
| hsa_circ_0068717 | 92 | 7 | 429 | 828 | 406 | 0 | 2.00E-168 |

novel_circ_017959 novel_circ_017960 novel_circ_017961 novel_circ_017962 novel_circ_017963 novel_circ_017964 novel_circ_017965 novel_circ_017966 novel_circ_017967 novel_circ_017968 novel_circ_017969 novel_circ_017970 novel_circ_017971 novel_circ_017972 novel_circ_017973 novel_circ_017974 novel_circ_017975 novel_circ_017976 novel_circ_017977 novel_circ_017980 novel_circ_017981 novel_circ_017982 novel_circ_017983 novel_circ_017984 novel_circ_017985 novel_circ_017986 novel_circ_017987 novel_circ_017988 novel_circ_017989 novel_circ_017990 novel_circ_017991 novel_circ_017992 novel_circ_017993 novel_circ_017994 novel_circ_017995 novel_circ_017996 novel_circ_018000 novel_circ_018001 novel_circ_018002 novel_circ_018003 novel_circ_018004 novel_circ_018005 novel_circ_018006 novel_circ_018007 novel_circ_018008 novel_circ_018009 novel_circ_018010 novel_circ_018011 novel_circ_018012 novel_circ_018013 novel_circ_018014 novel_circ_018015 novel_circ_018016 novel_circ_018017

| hsa_circ_0068716 | 90 | 1 | 522 | 1479 | 955 | 0.01 | 0 |
| --- | --- | --- | --- | --- | --- | --- | --- |
| hsa_circ_0068716 | 88 | 1 | 133 | 1524 | 1389 | 0.02 | 3.00E-38 |
| hsa_circ_0068729 | 94 | 1 | 350 | 128 | 477 | 0 | 1.00E-149 |
| hsa_circ_0068729 | 94 | 1 | 449 | 128 | 573 | 0.01 | 0 |
| hsa_circ_0068729 | 93 | 1 | 571 | 128 | 695 | 0.01 | 0 |
| hsa_circ_0068729 | 91 | 1 | 717 | 128 | 844 | 0.01 | 0 |
| hsa_circ_0068729 | 91 | 1 | 794 | 128 | 921 | 0.01 | 0 |
| hsa_circ_0068729 | 91 | 1 | 995 | 128 | 1122 | 0.01 | 0 |
| hsa_circ_0068729 | 90 | 1 | 1025 | 128 | 1152 | 0.01 | 0 |
| hsa_circ_0068729 | 90 | 1 | 1093 | 128 | 1232 | 0.02 | 0 |
| hsa_circ_0068732 | 89 | 107 | 1578 | 1 | 1490 | 0.02 | 0 |
| hsa_circ_0068732 | 90 | 1 | 688 | 1 | 688 | 0.01 | 0 |
| hsa_circ_0068732 | 90 | 1 | 889 | 1 | 889 | 0.01 | 0 |
| hsa_circ_0068741 | 89 | 1 | 344 | 1 | 347 | 0.01 | 4.00E-115 |
| hsa_circ_0068741 | 88 | 1 | 545 | 1 | 548 | 0.01 | 0 |
| hsa_circ_0068741 | 88 | 1 | 575 | 1 | 578 | 0.01 | 0 |
| hsa_circ_0068745 | 87 | 1 | 421 | 4 | 427 | 0.01 | 2.00E-134 |
| hsa_circ_0068761 | 89 | 1 | 480 | 1 | 486 | 0.01 | 2.00E-168 |
| hsa_circ_0008351 | 89 | 3 | 411 | 3 | 414 | 0.01 | 8.00E-143 |
| hsa_circ_0068772 | 90 | 331 | 825 | 1 | 498 | 0.01 | 0 |
| hsa_circ_0068772 | 90 | 331 | 1007 | 1 | 680 | 0 | 0 |
| hsa_circ_0068772 | 90 | 331 | 1007 | 1 | 680 | 0 | 0 |
| hsa_circ_0068772 | 90 | 331 | 1007 | 1 | 680 | 0 | 0 |
| hsa_circ_0068772 | 90 | 331 | 1007 | 1 | 680 | 0 | 0 |
| hsa_circ_0068772 | 89 | 1 | 374 | 1 | 377 | 0.01 | 9.00E-132 |
| hsa_circ_0005098 | 95 | 1 | 319 | 1 | 319 | 0 | 5.00E-139 |
| hsa_circ_0068772 | 93 | 1 | 501 | 180 | 680 | 0 | 0 |
| hsa_circ_0068772 | 93 | 1 | 501 | 180 | 680 | 0 | 0 |
| hsa_circ_0068772 | 93 | 1 | 501 | 180 | 680 | 0 | 0 |
| hsa_circ_0068772 | 92 | 1 | 395 | 286 | 680 | 0 | 2.00E-154 |
| hsa_circ_0068772 | 92 | 1 | 395 | 286 | 680 | 0 | 2.00E-154 |
| hsa_circ_0068772 | 92 | 1 | 395 | 286 | 680 | 0 | 4.00E-154 |
| hsa_circ_0068772 | 91 | 1 | 300 | 381 | 680 | 0 | 1.00E-111 |
| hsa_circ_0068772 | 91 | 1 | 300 | 381 | 680 | 0 | 2.00E-111 |
| hsa_circ_0068772 | 89 | 1 | 182 | 499 | 680 | 0 | 9.00E-58 |
| hsa_circ_0068772 | 89 | 1 | 182 | 499 | 680 | 0 | 2.00E-57 |
| hsa_circ_0087366 | 82 | 66 | 790 | 776 | 1 | 0.07 | 5.00E-171 |
| hsa_circ_0087366 | 82 | 66 | 790 | 776 | 1 | 0.07 | 5.00E-171 |
| hsa_circ_0006245 | 93 | 1 | 433 | 433 | 1 | 0 | 0 |
| hsa_circ_0067117 | 95 | 1 | 635 | 3668 | 3034 | 0 | 0 |
| hsa_circ_0067117 | 93 | 1 | 921 | 3668 | 2748 | 0 | 0 |
| hsa_circ_0067117 | 93 | 3 | 745 | 3490 | 2748 | 0 | 0 |
| hsa_circ_0067117 | 93 | 4 | 665 | 3409 | 2748 | 0 | 0 |
| hsa_circ_0067117 | 91 | 4 | 1352 | 3409 | 2061 | 0 | 0 |
| hsa_circ_0067117 | 93 | 1 | 488 | 3235 | 2748 | 0 | 0 |
| hsa_circ_0067117 | 91 | 1 | 1175 | 3235 | 2061 | 0 | 0 |
| hsa_circ_0067117 | 90 | 1 | 286 | 3033 | 2748 | 0 | 9.00E-101 |
| hsa_circ_0067117 | 89 | 1 | 973 | 3033 | 2061 | 0 | 0 |
| hsa_circ_0067117 | 86 | 2 | 1423 | 2059 | 621 | 0.04 | 0 |
| hsa_circ_0067117 | 86 | 1 | 1240 | 1877 | 621 | 0.04 | 0 |
| hsa_circ_0067112 | 92 | 3 | 456 | 645 | 192 | 0 | 0 |
| hsa_circ_0067108 | 91 | 1 | 451 | 451 | 1 | 0 | 6.00E-174 |
| hsa_circ_0067107 | 94 | 182 | 310 | 131 | 3 | 0 | 2.00E-48 |
| hsa_circ_0067101 | 97 | 372 | 722 | 601 | 251 | 0 | 8.00E-165 |

novel_circ_018018 novel_circ_018019 novel_circ_018020 novel_circ_018021 novel_circ_018022 novel_circ_018023 novel_circ_018024 novel_circ_018025 novel_circ_018026 novel_circ_018027 novel_circ_018028 novel_circ_018029 novel_circ_018031 novel_circ_018032 novel_circ_018033 novel_circ_018034 novel_circ_018035 novel_circ_018036 novel_circ_018037 novel_circ_018038 novel_circ_018039 novel_circ_018040 novel_circ_018041 novel_circ_018042 novel_circ_018043 novel_circ_018044 novel_circ_018045 novel_circ_018046 novel_circ_018047 novel_circ_018052 novel_circ_018053 novel_circ_018054 novel_circ_018055 novel_circ_018056 novel_circ_018057 novel_circ_018058 novel_circ_018059 novel_circ_018060 novel_circ_018061 novel_circ_018062 novel_circ_018067 novel_circ_018068 novel_circ_018069 novel_circ_018070 novel_circ_018072 novel_circ_018073 novel_circ_018075 novel_circ_018076 novel_circ_018077 novel_circ_018078 novel_circ_018079 novel_circ_018080 novel_circ_018081 novel_circ_018082

| hsa_circ_0067101 | 97 | 372 | 848 | 601 | 125 | 0 | 0 |
| --- | --- | --- | --- | --- | --- | --- | --- |
| hsa_circ_0067101 | 97 | 372 | 849 | 601 | 124 | 0 | 0 |
| hsa_circ_0067101 | 97 | 367 | 717 | 601 | 251 | 0 | 8.00E-165 |
| hsa_circ_0067101 | 97 | 196 | 546 | 601 | 251 | 0 | 6.00E-165 |
| hsa_circ_0067101 | 97 | 196 | 672 | 601 | 125 | 0 | 0 |
| hsa_circ_0067101 | 97 | 196 | 673 | 601 | 124 | 0 | 0 |
| hsa_circ_0067102 | 97 | 1 | 475 | 599 | 125 | 0 | 0 |
| hsa_circ_0067098 | 99 | 166 | 373 | 208 | 1 | 0 | 3.00E-102 |
| hsa_circ_0067098 | 99 | 1 | 208 | 208 | 1 | 0 | 1.00E-102 |
| hsa_circ_0067092 | 89 | 1 | 339 | 774 | 435 | 0 | 8.00E-117 |
| hsa_circ_0067092 | 90 | 1 | 571 | 774 | 203 | 0 | 0 |
| hsa_circ_0067092 | 90 | 1 | 685 | 774 | 89 | 0 | 0 |
| hsa_circ_0067084 | 80 | 386 | 1578 | 1733 | 541 | 0.01 | 0 |
| hsa_circ_0067081 | 86 | 1 | 291 | 710 | 420 | 0 | 3.00E-85 |
| hsa_circ_0067081 | 88 | 1 | 541 | 710 | 170 | 0 | 0 |
| hsa_circ_0067081 | 89 | 1 | 710 | 710 | 1 | 0 | 0 |
| hsa_circ_0067075 | 90 | 1 | 1193 | 1623 | 431 | 0 | 0 |
| hsa_circ_0067081 | 91 | 1167 | 1414 | 417 | 170 | 0 | 6.00E-89 |
| hsa_circ_0067076 | 92 | 1167 | 1931 | 765 | 1 | 0 | 0 |
| hsa_circ_0067075 | 91 | 3 | 1332 | 1330 | 1 | 0 | 0 |
| hsa_circ_0067075 | 91 | 3 | 1332 | 1330 | 1 | 0 | 0 |
| hsa_circ_0067076 | 92 | 1 | 517 | 517 | 1 | 0 | 0 |
| hsa_circ_0067075 | 91 | 1 | 1082 | 1082 | 1 | 0 | 0 |
| hsa_circ_0067076 | 92 | 1 | 348 | 348 | 1 | 0 | 4.00E-135 |
| hsa_circ_0067075 | 90 | 1 | 913 | 913 | 1 | 0 | 0 |
| hsa_circ_0067075 | 90 | 1 | 913 | 913 | 1 | 0 | 0 |
| hsa_circ_0067075 | 89 | 1 | 565 | 565 | 1 | 0 | 0 |
| hsa_circ_0067075 | 89 | 1 | 565 | 565 | 1 | 0 | 0 |
| hsa_circ_0006118 | 90 | 157 | 1002 | 3 | 848 | 0 | 0 |
| hsa_circ_0067071 | 94 | 1 | 642 | 1087 | 1737 | 0.01 | 0 |
| hsa_circ_0067071 | 94 | 1 | 895 | 834 | 1737 | 0.01 | 0 |
| hsa_circ_0067071 | 94 | 1 | 792 | 834 | 1634 | 0.01 | 0 |
| hsa_circ_0067071 | 94 | 1 | 456 | 538 | 993 | 0 | 0 |
| hsa_circ_0067071 | 93 | 1 | 296 | 538 | 833 | 0 | 3.00E-121 |
| hsa_circ_0067071 | 94 | 1 | 718 | 116 | 833 | 0 | 0 |
| hsa_circ_0067071 | 94 | 151 | 983 | 1 | 833 | 0 | 0 |
| hsa_circ_0067071 | 93 | 1 | 302 | 355 | 656 | 0 | 3.00E-121 |
| hsa_circ_0067071 | 93 | 1 | 541 | 116 | 656 | 0 | 0 |
| hsa_circ_0067071 | 94 | 151 | 806 | 1 | 656 | 0 | 0 |
| hsa_circ_0067071 | 94 | 1 | 422 | 116 | 537 | 0 | 0 |
| hsa_circ_0002236 | 96 | 1 | 541 | 1 | 541 | 0 | 0 |
| hsa_circ_0002236 | 96 | 202 | 742 | 1 | 541 | 0 | 0 |
| hsa_circ_0002236 | 96 | 202 | 676 | 1 | 475 | 0 | 0 |
| hsa_circ_0002236 | 98 | 202 | 560 | 1 | 359 | 0 | 5.00E-176 |
| hsa_circ_0067020 | 93 | 1 | 731 | 1 | 731 | 0 | 0 |
| hsa_circ_0067020 | 93 | 1 | 564 | 1 | 564 | 0 | 0 |
| hsa_circ_0067008 | 92 | 267 | 701 | 1 | 435 | 0 | 4.00E-173 |
| hsa_circ_0067008 | 90 | 1 | 238 | 1 | 238 | 0 | 4.00E-84 |
| hsa_circ_0067006 | 90 | 292 | 784 | 493 | 1 | 0 | 0 |
| hsa_circ_0067006 | 90 | 292 | 784 | 493 | 1 | 0 | 0 |
| hsa_circ_0044906 | 96 | 1 | 470 | 1 | 470 | 0 | 0 |
| hsa_circ_0044904 | 97 | 1 | 561 | 193 | 753 | 0 | 0 |
| hsa_circ_0044904 | 97 | 1 | 753 | 1 | 753 | 0 | 0 |
| hsa_circ_0044906 | 95 | 1 | 249 | 109 | 357 | 0 | 1.00E-108 |

novel_circ_018083 novel_circ_018084 novel_circ_018085 novel_circ_018086 novel_circ_018087 novel_circ_018088 novel_circ_018089 novel_circ_018090 novel_circ_018091 novel_circ_018092 novel_circ_018093 novel_circ_018094 novel_circ_018095 novel_circ_018096 novel_circ_018097 novel_circ_018098 novel_circ_018099 novel_circ_018100 novel_circ_018101 novel_circ_018102 novel_circ_018103 novel_circ_018104 novel_circ_018105 novel_circ_018106 novel_circ_018107 novel_circ_018108 novel_circ_018109 novel_circ_018110 novel_circ_018111 novel_circ_018112 novel_circ_018113 novel_circ_018114 novel_circ_018115 novel_circ_018116 novel_circ_018118 novel_circ_018119 novel_circ_018120 novel_circ_018121 novel_circ_018122 novel_circ_018123 novel_circ_018124 novel_circ_018125 novel_circ_018126 novel_circ_018129 novel_circ_018130 novel_circ_018131 novel_circ_018132 novel_circ_018133 novel_circ_018134 novel_circ_018135 novel_circ_018136 novel_circ_018137 novel_circ_018138 novel_circ_018139

| hsa_circ_0044906 | 96 | 1 | 357 | 1 | 357 | 0 | 4.00E-165 |
| --- | --- | --- | --- | --- | --- | --- | --- |
| hsa_circ_0044904 | 97 | 1 | 448 | 193 | 640 | 0 | 0 |
| hsa_circ_0044903 | 97 | 1 | 698 | 1 | 698 | 0 | 0 |
| hsa_circ_0044902 | 98 | 1 | 846 | 70 | 915 | 0 | 0 |
| hsa_circ_0044900 | 98 | 1 | 978 | 1 | 978 | 0 | 0 |
| hsa_circ_0044906 | 96 | 1 | 249 | 1 | 249 | 0 | 3.00E-110 |
| hsa_circ_0044903 | 98 | 1 | 449 | 1 | 449 | 0 | 0 |
| hsa_circ_0044902 | 99 | 1 | 217 | 1 | 217 | 0 | 1.00E-107 |
| hsa_circ_0044900 | 99 | 1 | 388 | 1 | 388 | 0 | 0 |
| hsa_circ_0044900 | 99 | 1 | 240 | 1 | 240 | 0 | 3.00E-120 |
| hsa_circ_0044891 | 91 | 218 | 1046 | 1667 | 839 | 0 | 0 |
| hsa_circ_0044878 | 93 | 21788 | 21957 | 111 | 280 | 0 | 4.00E-62 |
| hsa_circ_0044878 | 93 | 21788 | 21957 | 111 | 280 | 0 | 3.00E-62 |
| hsa_circ_0029815 | 94 | 2953 | 3395 | 101 | 543 | 0 | 0 |
| hsa_circ_0044882 | 93 | 1 | 169 | 1 | 169 | 0 | 1.00E-62 |
| hsa_circ_0044878 | 93 | 21788 | 21957 | 111 | 280 | 0 | 1.00E-62 |
| hsa_circ_0029815 | 94 | 2953 | 3395 | 101 | 543 | 0 | 0 |
| hsa_circ_0029815 | 94 | 2953 | 3395 | 101 | 543 | 0 | 0 |
| hsa_circ_0029815 | 94 | 2953 | 3395 | 101 | 543 | 0 | 0 |
| hsa_circ_0044858 | 95 | 1 | 504 | 434 | 937 | 0 | 0 |
| hsa_circ_0044847 | 94 | 1 | 2546 | 1 | 2546 | 0 | 0 |
| hsa_circ_0044855 | 94 | 1 | 607 | 1 | 607 | 0 | 0 |
| hsa_circ_0044851 | 94 | 1 | 1159 | 1 | 1159 | 0 | 0 |
| hsa_circ_0044848 | 94 | 1 | 1447 | 163 | 1609 | 0 | 0 |
| hsa_circ_0044845 | 94 | 1 | 639 | 1 | 639 | 0 | 0 |
| hsa_circ_0044841 | 94 | 1 | 833 | 548 | 1380 | 0 | 0 |
| hsa_circ_0044841 | 93 | 1 | 609 | 548 | 1156 | 0 | 0 |
| hsa_circ_0044842 | 93 | 1 | 370 | 428 | 797 | 0 | 5.00E-154 |
| hsa_circ_0044841 | 95 | 1 | 547 | 1 | 547 | 0 | 0 |
| hsa_circ_0044841 | 95 | 147 | 693 | 1 | 547 | 0 | 0 |
| hsa_circ_0044841 | 95 | 1 | 415 | 1 | 415 | 0 | 0 |
| hsa_circ_0044838 | 97 | 1 | 153 | 313 | 465 | 0 | 8.00E-69 |
| hsa_circ_0044838 | 93 | 1 | 314 | 1 | 312 | 0.01 | 7.00E-128 |
| hsa_circ_0044838 | 93 | 1 | 314 | 1 | 312 | 0.01 | 5.00E-129 |
| hsa_circ_0044836 | 91 | 1 | 134 | 210 | 343 | 0 | 2.00E-44 |
| hsa_circ_0044836 | 95 | 1 | 343 | 1 | 343 | 0 | 5.00E-152 |
| hsa_circ_0044836 | 95 | 47 | 389 | 1 | 343 | 0 | 5.00E-152 |
| hsa_circ_0044836 | 95 | 4371 | 4713 | 1 | 343 | 0 | 4.00E-151 |
| hsa_circ_0044836 | 95 | 183 | 525 | 1 | 343 | 0 | 6.00E-152 |
| hsa_circ_0044836 | 95 | 1 | 343 | 1 | 343 | 0 | 2.00E-152 |
| hsa_circ_0044836 | 95 | 4371 | 4713 | 1 | 343 | 0 | 3.00E-151 |
| hsa_circ_0044836 | 95 | 183 | 525 | 1 | 343 | 0 | 4.00E-152 |
| hsa_circ_0044836 | 98 | 183 | 391 | 1 | 209 | 0 | 8.00E-98 |
| hsa_circ_0044824 | 95 | 1 | 369 | 686 | 318 | 0 | 4.00E-165 |
| hsa_circ_0044824 | 95 | 1 | 686 | 686 | 1 | 0 | 0 |
| hsa_circ_0044824 | 95 | 1 | 528 | 528 | 1 | 0 | 0 |
| hsa_circ_0044824 | 95 | 1 | 315 | 440 | 126 | 0 | 8.00E-137 |
| hsa_circ_0044824 | 95 | 1 | 440 | 440 | 1 | 0 | 0 |
| hsa_circ_0044819 | 95 | 1 | 491 | 491 | 1 | 0 | 0 |
| hsa_circ_0044809 | 94 | 1 | 830 | 1384 | 555 | 0 | 0 |
| hsa_circ_0044806 | 92 | 1 | 1888 | 1888 | 1 | 0 | 0 |
| hsa_circ_0044806 | 92 | 1 | 1888 | 1888 | 1 | 0 | 0 |
| hsa_circ_0044824 | 95 | 1 | 192 | 317 | 126 | 0 | 5.00E-82 |
| hsa_circ_0044824 | 95 | 1 | 317 | 317 | 1 | 0 | 3.00E-141 |

novel_circ_018140 novel_circ_018141 novel_circ_018142 novel_circ_018143 novel_circ_018144 novel_circ_018145 novel_circ_018146 novel_circ_018147 novel_circ_018148 novel_circ_018149 novel_circ_018150 novel_circ_018151 novel_circ_018152 novel_circ_018153 novel_circ_018154 novel_circ_018155 novel_circ_018156 novel_circ_018157 novel_circ_018158 novel_circ_018159 novel_circ_018160 novel_circ_018161 novel_circ_018162 novel_circ_018163 novel_circ_018164 novel_circ_018165 novel_circ_018166 novel_circ_018167 novel_circ_018168 novel_circ_018169 novel_circ_018170 novel_circ_018171 novel_circ_018172 novel_circ_018173 novel_circ_018174 novel_circ_018175 novel_circ_018176 novel_circ_018177 novel_circ_018178 novel_circ_018179 novel_circ_018180 novel_circ_018181 novel_circ_018182 novel_circ_018183 novel_circ_018184 novel_circ_018185 novel_circ_018186 novel_circ_018187 novel_circ_018191 novel_circ_018193 novel_circ_018194 novel_circ_018195 novel_circ_018201 novel_circ_018202

| hsa_circ_0044817 | 95 | 1 | 450 | 450 | 1 | 0 | 0 |
| --- | --- | --- | --- | --- | --- | --- | --- |
| hsa_circ_0044806 | 92 | 1 | 1765 | 1765 | 1 | 0 | 0 |
| hsa_circ_0044809 | 94 | 1 | 583 | 1137 | 555 | 0 | 0 |
| hsa_circ_0044809 | 93 | 1 | 914 | 1137 | 224 | 0.01 | 0 |
| hsa_circ_0044806 | 92 | 1 | 1641 | 1641 | 1 | 0 | 0 |
| hsa_circ_0044806 | 92 | 1 | 1641 | 1641 | 1 | 0 | 0 |
| hsa_circ_0044806 | 92 | 45 | 1624 | 1579 | 1 | 0 | 0 |
| hsa_circ_0044806 | 92 | 45 | 1624 | 1579 | 1 | 0 | 0 |
| hsa_circ_0044817 | 95 | 2 | 258 | 257 | 1 | 0 | 5.00E-113 |
| hsa_circ_0044816 | 95 | 2 | 335 | 334 | 1 | 0 | 5.00E-149 |
| hsa_circ_0044812 | 94 | 2 | 515 | 982 | 469 | 0 | 0 |
| hsa_circ_0044812 | 95 | 2 | 630 | 982 | 354 | 0 | 0 |
| hsa_circ_0044812 | 93 | 2 | 846 | 982 | 138 | 0.01 | 0 |
| hsa_circ_0044812 | 92 | 2 | 983 | 982 | 1 | 0.01 | 0 |
| hsa_circ_0044809 | 92 | 2 | 1064 | 1068 | 6 | 0.01 | 0 |
| hsa_circ_0044806 | 92 | 2 | 1573 | 1572 | 1 | 0 | 0 |
| hsa_circ_0044806 | 92 | 2 | 1573 | 1572 | 1 | 0 | 0 |
| hsa_circ_0044806 | 92 | 2 | 1573 | 1572 | 1 | 0 | 0 |
| hsa_circ_0044806 | 92 | 2 | 1573 | 1572 | 1 | 0 | 0 |
| hsa_circ_0044813 | 92 | 1 | 511 | 511 | 1 | 0.01 | 0 |
| hsa_circ_0044806 | 91 | 1 | 1238 | 1238 | 1 | 0 | 0 |
| hsa_circ_0044809 | 91 | 1 | 549 | 554 | 6 | 0.01 | 0 |
| hsa_circ_0044806 | 91 | 1 | 1058 | 1058 | 1 | 0.01 | 0 |
| hsa_circ_0044806 | 90 | 1 | 943 | 943 | 1 | 0.01 | 0 |
| hsa_circ_0044806 | 91 | 4 | 727 | 724 | 1 | 0 | 0 |
| hsa_circ_0044804 | 91 | 138 | 856 | 719 | 1 | 0 | 0 |
| hsa_circ_0044803 | 92 | 421 | 1165 | 745 | 1 | 0 | 0 |
| hsa_circ_0044806 | 92 | 1 | 590 | 590 | 1 | 0 | 0 |
| hsa_circ_0044804 | 91 | 1 | 719 | 719 | 1 | 0 | 0 |
| hsa_circ_0044803 | 92 | 284 | 1028 | 745 | 1 | 0 | 0 |
| hsa_circ_0044803 | 92 | 284 | 1028 | 745 | 1 | 0 | 0 |
| hsa_circ_0044803 | 92 | 284 | 1028 | 745 | 1 | 0 | 0 |
| hsa_circ_0044806 | 92 | 1 | 504 | 504 | 1 | 0 | 0 |
| hsa_circ_0044803 | 92 | 198 | 823 | 745 | 120 | 0 | 0 |
| hsa_circ_0044803 | 92 | 198 | 942 | 745 | 1 | 0 | 0 |
| hsa_circ_0044806 | 92 | 1 | 500 | 500 | 1 | 0 | 0 |
| hsa_circ_0044803 | 93 | 1 | 438 | 438 | 1 | 0 | 0 |
| hsa_circ_0002809 | 90 | 1 | 628 | 634 | 1 | 0.01 | 0 |
| hsa_circ_0044801 | 84 | 1 | 196 | 1377 | 1176 | 0.04 | 3.00E-46 |
| hsa_circ_0044800 | 97 | 1 | 238 | 190 | 427 | 0 | 7.00E-111 |
| hsa_circ_0044800 | 95 | 1 | 427 | 1 | 427 | 0 | 0 |
| hsa_circ_0044800 | 95 | 1040 | 1466 | 1 | 427 | 0 | 0 |
| hsa_circ_0044800 | 95 | 201 | 627 | 1 | 427 | 0 | 0 |
| hsa_circ_0044800 | 95 | 320 | 746 | 1 | 427 | 0 | 0 |
| hsa_circ_0044800 | 93 | 320 | 508 | 1 | 189 | 0 | 3.00E-73 |
| hsa_circ_0044796 | 89 | 3861 | 3998 | 270 | 133 | 0 | 3.00E-40 |
| hsa_circ_0044795 | 90 | 1 | 394 | 168 | 561 | 0 | 1.00E-145 |
| hsa_circ_0044794 | 91 | 1 | 560 | 1 | 560 | 0 | 0 |
| hsa_circ_0044769 | 90 | 1 | 781 | 3666 | 2886 | 0 | 0 |
| hsa_circ_0044759 | 98 | 1 | 192 | 103 | 294 | 0 | 2.00E-90 |
| hsa_circ_0044731 | 99 | 1 | 95 | 210 | 304 | 0 | 2.00E-40 |
| hsa_circ_0044724 | 86 | 940 | 2583 | 1027 | 2661 | 0.01 | 0 |
| hsa_circ_0044712 | 93 | 1 | 633 | 145 | 777 | 0 | 0 |
| hsa_circ_0044710 | 93 | 1 | 897 | 161 | 1057 | 0 | 0 |

novel_circ_018203 novel_circ_018204 novel_circ_018205 novel_circ_018206 novel_circ_018208 novel_circ_018209 novel_circ_018210 novel_circ_018211 novel_circ_018212 novel_circ_018213 novel_circ_018214 novel_circ_018215 novel_circ_018216 novel_circ_018217 novel_circ_018218 novel_circ_018219 novel_circ_018220 novel_circ_018221 novel_circ_018222 novel_circ_018223 novel_circ_018224 novel_circ_018225 novel_circ_018226 novel_circ_018227 novel_circ_018228 novel_circ_018229 novel_circ_018230 novel_circ_018231 novel_circ_018232 novel_circ_018233 novel_circ_018234 novel_circ_018235 novel_circ_018236 novel_circ_018237 novel_circ_018238 novel_circ_018239 novel_circ_018242 novel_circ_018243 novel_circ_018244 novel_circ_018245 novel_circ_018246 novel_circ_018247 novel_circ_018248 novel_circ_018249 novel_circ_018250 novel_circ_018251 novel_circ_018252 novel_circ_018253 novel_circ_018254 novel_circ_018255 novel_circ_018256 novel_circ_018257 novel_circ_018258 novel_circ_018259

| hsa_circ_0003108 | 94 | 1 | 364 | 1 | 364 | 0 | 1.00E-155 |
| --- | --- | --- | --- | --- | --- | --- | --- |
| hsa_circ_0044710 | 93 | 1 | 749 | 1 | 749 | 0 | 0 |
| hsa_circ_0044713 | 93 | 1 | 324 | 1 | 324 | 0 | 2.00E-133 |
| hsa_circ_0044710 | 93 | 1 | 424 | 1 | 424 | 0 | 6.00E-174 |
| hsa_circ_0044707 | 84 | 1 | 308 | 119 | 426 | 0.02 | 1.00E-79 |
| hsa_circ_0044702 | 97 | 1 | 490 | 490 | 1 | 0 | 0 |
| hsa_circ_0044702 | 97 | 1 | 490 | 490 | 1 | 0 | 0 |
| hsa_circ_0044702 | 97 | 1 | 338 | 408 | 71 | 0 | 6.00E-163 |
| hsa_circ_0044702 | 97 | 1 | 408 | 408 | 1 | 0 | 0 |
| hsa_circ_0059170 | 94 | 1 | 322 | 322 | 1 | 0 | 2.00E-137 |
| hsa_circ_0059169 | 95 | 1 | 475 | 619 | 145 | 0 | 0 |
| hsa_circ_0059168 | 95 | 1 | 730 | 730 | 1 | 0 | 0 |
| hsa_circ_0059160 | 95 | 1 | 1318 | 2552 | 1235 | 0 | 0 |
| hsa_circ_0059169 | 97 | 1 | 265 | 409 | 145 | 0 | 2.00E-122 |
| hsa_circ_0059169 | 97 | 1 | 409 | 409 | 1 | 0 | 0 |
| hsa_circ_0059168 | 97 | 1 | 520 | 520 | 1 | 0 | 0 |
| hsa_circ_0059163 | 96 | 1 | 959 | 959 | 1 | 0 | 0 |
| hsa_circ_0059168 | 97 | 1 | 408 | 408 | 1 | 0 | 0 |
| hsa_circ_0059163 | 96 | 1 | 605 | 694 | 90 | 0 | 0 |
| hsa_circ_0059163 | 95 | 1 | 694 | 694 | 1 | 0 | 0 |
| hsa_circ_0059163 | 95 | 1 | 550 | 550 | 1 | 0 | 0 |
| hsa_circ_0059163 | 95 | 1 | 439 | 439 | 1 | 0 | 0 |
| hsa_circ_0059163 | 94 | 1 | 337 | 337 | 1 | 0 | 5.00E-144 |
| hsa_circ_0059154 | 91 | 1 | 267 | 267 | 1 | 0 | 7.00E-97 |
| hsa_circ_0059692 | 96 | 1 | 271 | 298 | 568 | 0 | 8.00E-121 |
| hsa_circ_0002302 | 95 | 1514 | 1857 | 1 | 344 | 0 | 2.00E-150 |
| hsa_circ_0059777 | 93 | 1739 | 2578 | 1 | 842 | 0 | 0 |
| hsa_circ_0059777 | 93 | 1739 | 2583 | 1 | 847 | 0 | 0 |
| hsa_circ_0002302 | 95 | 1 | 344 | 1 | 344 | 0 | 3.00E-151 |
| hsa_circ_0002302 | 95 | 1 | 344 | 1 | 344 | 0 | 5.00E-151 |
| hsa_circ_0059777 | 93 | 226 | 1065 | 1 | 842 | 0 | 0 |
| hsa_circ_0059777 | 93 | 226 | 1070 | 1 | 847 | 0 | 0 |
| hsa_circ_0059777 | 93 | 1 | 721 | 120 | 842 | 0.01 | 0 |
| hsa_circ_0059777 | 92 | 1 | 501 | 340 | 842 | 0.01 | 0 |
| hsa_circ_0059779 | 94 | 1 | 195 | 1 | 195 | 0 | 1.00E-78 |
| hsa_circ_0059785 | 91 | 1 | 210 | 110 | 319 | 0 | 6.00E-77 |
| hsa_circ_0007581 | 95 | 73 | 428 | 1 | 356 | 0 | 8.00E-158 |
| hsa_circ_0003183 | 94 | 1 | 176 | 1 | 176 | 0 | 7.00E-70 |
| hsa_circ_0003183 | 94 | 1 | 288 | 1 | 288 | 0 | 2.00E-123 |
| hsa_circ_0084591 | 92 | 1 | 2281 | 1 | 2270 | 0.01 | 0 |
| hsa_circ_0084591 | 94 | 1 | 777 | 1840 | 2616 | 0 | 0 |
| hsa_circ_0084591 | 94 | 1 | 833 | 1840 | 2672 | 0 | 0 |
| hsa_circ_0084591 | 95 | 1 | 1710 | 1840 | 3549 | 0 | 0 |
| hsa_circ_0084591 | 93 | 1 | 346 | 2271 | 2616 | 0 | 5.00E-144 |
| hsa_circ_0084591 | 94 | 1 | 402 | 2271 | 2672 | 0 | 2.00E-173 |
| hsa_circ_0084591 | 94 | 1 | 517 | 2271 | 2787 | 0 | 0 |
| hsa_circ_0084591 | 95 | 1 | 601 | 2271 | 2871 | 0 | 0 |
| hsa_circ_0084591 | 95 | 1 | 1279 | 2271 | 3549 | 0 | 0 |
| hsa_circ_0084591 | 95 | 1 | 1682 | 2271 | 3952 | 0 | 0 |
| hsa_circ_0084591 | 95 | 1 | 1540 | 2413 | 3952 | 0 | 0 |
| hsa_circ_0084591 | 96 | 1 | 877 | 2673 | 3549 | 0 | 0 |
| hsa_circ_0084591 | 95 | 1 | 1280 | 2673 | 3952 | 0 | 0 |
| hsa_circ_0084593 | 95 | 1 | 1081 | 1 | 1081 | 0 | 0 |
| hsa_circ_0084594 | 93 | 1 | 577 | 367 | 943 | 0 | 0 |

novel_circ_018260 novel_circ_018261 novel_circ_018268 novel_circ_018269 novel_circ_018270 novel_circ_018271 novel_circ_018272 novel_circ_018274 novel_circ_018275 novel_circ_018276 novel_circ_018277 novel_circ_018278 novel_circ_018279 novel_circ_018280 novel_circ_018281 novel_circ_018282 novel_circ_018287 novel_circ_018295 novel_circ_018296 novel_circ_018297 novel_circ_018298 novel_circ_018299 novel_circ_018300 novel_circ_018301 novel_circ_018306 novel_circ_018307 novel_circ_018311 novel_circ_018312 novel_circ_018313 novel_circ_018314 novel_circ_018315 novel_circ_018316 novel_circ_018317 novel_circ_018318 novel_circ_018321 novel_circ_018322 novel_circ_018323 novel_circ_018324 novel_circ_018325 novel_circ_018326 novel_circ_018327 novel_circ_018328 novel_circ_018329 novel_circ_018330 novel_circ_018331 novel_circ_018332 novel_circ_018333 novel_circ_018334 novel_circ_018335 novel_circ_018336 novel_circ_018337 novel_circ_018338 novel_circ_018339 novel_circ_018340

| hsa_circ_0084594 | 94 | 1 | 400 | 544 | 943 | 0 | 6.00E-174 |
| --- | --- | --- | --- | --- | --- | --- | --- |
| hsa_circ_0084594 | 95 | 1 | 317 | 1699 | 2015 | 0 | 6.00E-138 |
| hsa_circ_0084595 | 93 | 1 | 347 | 1 | 347 | 0 | 3.00E-141 |
| hsa_circ_0084599 | 90 | 4 | 689 | 686 | 1 | 0 | 0 |
| hsa_circ_0084605 | 88 | 4 | 327 | 324 | 1 | 0 | 2.00E-103 |
| hsa_circ_0084605 | 89 | 1 | 464 | 464 | 1 | 0 | 2.00E-159 |
| hsa_circ_0084605 | 88 | 1 | 615 | 615 | 1 | 0 | 0 |
| hsa_circ_0005335 | 97 | 1 | 249 | 249 | 1 | 0 | 2.00E-114 |
| hsa_circ_0005335 | 97 | 1 | 249 | 249 | 1 | 0 | 2.00E-114 |
| hsa_circ_0005335 | 93 | 1 | 444 | 444 | 1 | 0 | 0 |
| hsa_circ_0039316 | 94 | 2 | 444 | 4979 | 4537 | 0 | 0 |
| hsa_circ_0039316 | 92 | 1 | 2132 | 6671 | 4537 | 0 | 0 |
| hsa_circ_0039316 | 92 | 1 | 1916 | 6671 | 4753 | 0 | 0 |
| hsa_circ_0039316 | 92 | 1 | 1819 | 6671 | 4850 | 0 | 0 |
| hsa_circ_0039316 | 91 | 1 | 1688 | 6671 | 4981 | 0 | 0 |
| hsa_circ_0039316 | 91 | 1 | 1688 | 6671 | 4981 | 0 | 0 |
| hsa_circ_0039319 | 96 | 1 | 248 | 248 | 1 | 0 | 1.00E-109 |
| hsa_circ_0039343 | 91 | 1 | 385 | 629 | 245 | 0 | 3.00E-147 |
| hsa_circ_0039348 | 90 | 1 | 1030 | 1 | 1034 | 0.01 | 0 |
| hsa_circ_0039348 | 90 | 1 | 1030 | 1 | 1034 | 0.01 | 0 |
| hsa_circ_0039348 | 90 | 1 | 1030 | 1 | 1034 | 0.01 | 0 |
| hsa_circ_0039348 | 90 | 1 | 1030 | 1 | 1034 | 0.01 | 0 |
| hsa_circ_0039348 | 90 | 1 | 1030 | 1 | 1034 | 0.01 | 0 |
| hsa_circ_0039348 | 89 | 1 | 407 | 628 | 1034 | 0 | 3.00E-142 |
| hsa_circ_0039349 | 95 | 432 | 723 | 1 | 292 | 0 | 2.00E-125 |
| hsa_circ_0039349 | 94 | 1 | 361 | 160 | 520 | 0 | 4.00E-152 |
| hsa_circ_0027689 | 93 | 206 | 1283 | 1 | 1078 | 0 | 0 |
| hsa_circ_0027689 | 93 | 206 | 1665 | 1 | 1460 | 0 | 0 |
| hsa_circ_0027689 | 93 | 1 | 825 | 1 | 825 | 0 | 0 |
| hsa_circ_0027696 | 91 | 1 | 253 | 1 | 253 | 0.01 | 2.00E-92 |
| hsa_circ_0027696 | 92 | 1 | 635 | 1 | 635 | 0 | 0 |
| hsa_circ_0027698 | 94 | 1 | 397 | 217 | 613 | 0 | 6.00E-169 |
| hsa_circ_0002375 | 94 | 5 | 241 | 492 | 256 | 0 | 1.00E-98 |
| hsa_circ_0002375 | 91 | 4 | 412 | 664 | 256 | 0 | 3.00E-157 |
| hsa_circ_0027702 | 92 | 353 | 932 | 580 | 1 | 0 | 0 |
| hsa_circ_0027702 | 93 | 1 | 153 | 153 | 1 | 0 | 1.00E-58 |
| hsa_circ_0027702 | 92 | 181 | 760 | 580 | 1 | 0 | 0 |
| hsa_circ_0027702 | 92 | 353 | 932 | 580 | 1 | 0 | 0 |
| hsa_circ_0027702 | 94 | 954 | 1108 | 155 | 1 | 0 | 5.00E-59 |
| hsa_circ_0027702 | 92 | 1 | 222 | 223 | 1 | 0 | 3.00E-85 |
| hsa_circ_0027702 | 92 | 181 | 760 | 580 | 1 | 0 | 0 |
| hsa_circ_0027702 | 92 | 353 | 932 | 580 | 1 | 0 | 0 |
| hsa_circ_0027702 | 92 | 181 | 538 | 580 | 223 | 0 | 8.00E-144 |
| hsa_circ_0027703 | 93 | 1 | 352 | 352 | 1 | 0 | 2.00E-142 |
| hsa_circ_0027705 | 96 | 1 | 618 | 726 | 109 | 0 | 0 |
| hsa_circ_0027705 | 96 | 1 | 406 | 726 | 321 | 0 | 0 |
| hsa_circ_0027705 | 96 | 1 | 805 | 1339 | 535 | 0 | 0 |
| hsa_circ_0027707 | 96 | 629 | 2664 | 2036 | 1 | 0 | 0 |
| hsa_circ_0027707 | 96 | 1 | 855 | 855 | 1 | 0 | 0 |
| hsa_circ_0027707 | 97 | 1 | 1098 | 1098 | 1 | 0 | 0 |
| hsa_circ_0027707 | 97 | 1 | 660 | 855 | 196 | 0 | 0 |
| hsa_circ_0027707 | 96 | 1 | 1460 | 1655 | 196 | 0 | 0 |
| hsa_circ_0027707 | 96 | 629 | 2289 | 2036 | 376 | 0 | 0 |
| hsa_circ_0027707 | 98 | 1 | 485 | 1098 | 614 | 0 | 0 |

novel_circ_018341 novel_circ_018342 novel_circ_018343 novel_circ_018344 novel_circ_018345 novel_circ_018346 novel_circ_018347 novel_circ_018348 novel_circ_018349 novel_circ_018350 novel_circ_018351 novel_circ_018352 novel_circ_018353 novel_circ_018359 novel_circ_018360 novel_circ_018361 novel_circ_018362 novel_circ_018363 novel_circ_018364 novel_circ_018365 novel_circ_018366 novel_circ_018367 novel_circ_018368 novel_circ_018369 novel_circ_018370 novel_circ_018371 novel_circ_018373 novel_circ_018374 novel_circ_018375 novel_circ_018376 novel_circ_018377 novel_circ_018378 novel_circ_018379 novel_circ_018380 novel_circ_018381 novel_circ_018382 novel_circ_018383 novel_circ_018384 novel_circ_018385 novel_circ_018386 novel_circ_018387 novel_circ_018388 novel_circ_018389 novel_circ_018390 novel_circ_018391 novel_circ_018392 novel_circ_018393 novel_circ_018394 novel_circ_018395 novel_circ_018396 novel_circ_018397 novel_circ_018398 novel_circ_018399 novel_circ_018400

| hsa_circ_0027707 | 96 | 1 | 1042 | 1655 | 614 | 0 | 0 |
| --- | --- | --- | --- | --- | --- | --- | --- |
| hsa_circ_0027707 | 96 | 1 | 1423 | 2036 | 614 | 0 | 0 |
| hsa_circ_0027707 | 96 | 629 | 2051 | 2036 | 614 | 0 | 0 |
| hsa_circ_0027707 | 96 | 733 | 2155 | 2036 | 614 | 0 | 0 |
| hsa_circ_0027707 | 95 | 1 | 1181 | 2036 | 856 | 0 | 0 |
| hsa_circ_0027707 | 95 | 629 | 1809 | 2036 | 856 | 0 | 0 |
| hsa_circ_0027707 | 95 | 629 | 1565 | 2036 | 1100 | 0 | 0 |
| hsa_circ_0027707 | 96 | 1 | 522 | 2036 | 1515 | 0 | 0 |
| hsa_circ_0000426 | 96 | 4 | 628 | 624 | 1 | 0 | 0 |
| hsa_circ_0000426 | 96 | 4 | 628 | 624 | 1 | 0 | 0 |
| hsa_circ_0000426 | 96 | 108 | 732 | 624 | 1 | 0 | 0 |
| hsa_circ_0007621 | 94 | 4 | 430 | 426 | 1 | 0.01 | 0 |
| hsa_circ_0007621 | 94 | 108 | 534 | 426 | 1 | 0.01 | 0 |
| hsa_circ_0035543 | 81 | 4038 | 4221 | 19 | 201 | 0.01 | 3.00E-31 |
| hsa_circ_0027713 | 88 | 115 | 1026 | 924 | 1 | 0.01 | 0 |
| hsa_circ_0027713 | 88 | 115 | 1026 | 924 | 1 | 0.01 | 0 |
| hsa_circ_0027713 | 88 | 115 | 1026 | 924 | 1 | 0.01 | 0 |
| hsa_circ_0027715 | 93 | 250 | 1136 | 1038 | 152 | 0 | 0 |
| hsa_circ_0027725 | 95 | 1 | 377 | 1 | 377 | 0 | 3.00E-166 |
| hsa_circ_0027726 | 93 | 1 | 351 | 1 | 351 | 0 | 8.00E-142 |
| hsa_circ_0027726 | 91 | 1 | 496 | 1 | 496 | 0 | 0 |
| hsa_circ_0027726 | 90 | 1 | 692 | 1 | 692 | 0 | 0 |
| hsa_circ_0027725 | 88 | 10 | 496 | 1336 | 1819 | 0.01 | 2.00E-164 |
| hsa_circ_0027728 | 89 | 2 | 609 | 2314 | 1707 | 0 | 0 |
| hsa_circ_0027728 | 89 | 3 | 1034 | 2738 | 1707 | 0 | 0 |
| hsa_circ_0027728 | 89 | 3 | 650 | 2738 | 2091 | 0 | 0 |
| hsa_circ_0027737 | 94 | 2437 | 2683 | 2369 | 2123 | 0 | 4.00E-103 |
| hsa_circ_0027739 | 93 | 3 | 1173 | 1165 | 1 | 0.01 | 0 |
| hsa_circ_0027739 | 93 | 74 | 1559 | 1477 | 1 | 0.01 | 0 |
| hsa_circ_0027739 | 93 | 3 | 1173 | 1165 | 1 | 0.01 | 0 |
| hsa_circ_0027739 | 93 | 74 | 1559 | 1477 | 1 | 0.01 | 0 |
| hsa_circ_0027739 | 93 | 3 | 895 | 1165 | 279 | 0.01 | 0 |
| hsa_circ_0027739 | 93 | 5 | 977 | 1242 | 279 | 0.01 | 0 |
| hsa_circ_0027739 | 93 | 1 | 1208 | 1477 | 279 | 0.01 | 0 |
| hsa_circ_0027739 | 93 | 74 | 1281 | 1477 | 279 | 0.01 | 0 |
| hsa_circ_0027739 | 92 | 5 | 855 | 1242 | 401 | 0.01 | 0 |
| hsa_circ_0027739 | 92 | 1 | 1086 | 1477 | 401 | 0.01 | 0 |
| hsa_circ_0027739 | 92 | 74 | 1159 | 1477 | 401 | 0.01 | 0 |
| hsa_circ_0027739 | 92 | 1 | 920 | 1477 | 567 | 0.01 | 0 |
| hsa_circ_0027739 | 92 | 74 | 993 | 1477 | 567 | 0.01 | 0 |
| hsa_circ_0027739 | 93 | 3 | 419 | 1165 | 749 | 0 | 2.00E-173 |
| hsa_circ_0027739 | 93 | 5 | 501 | 1242 | 749 | 0.01 | 0 |
| hsa_circ_0027739 | 93 | 1 | 732 | 1477 | 749 | 0 | 0 |
| hsa_circ_0027739 | 93 | 74 | 805 | 1477 | 749 | 0 | 0 |
| hsa_circ_0027742 | 93 | 74 | 386 | 310 | 1 | 0.01 | 2.00E-124 |
| hsa_circ_0027742 | 93 | 74 | 304 | 310 | 80 | 0 | 6.00E-91 |
| hsa_circ_0027753 | 93 | 1 | 812 | 812 | 1 | 0 | 0 |
| hsa_circ_0027761 | 92 | 1 | 245 | 245 | 1 | 0 | 5.00E-93 |
| hsa_circ_0027761 | 92 | 1 | 397 | 397 | 1 | 0 | 1.00E-155 |
| hsa_circ_0027767 | 96 | 1 | 132 | 1 | 132 | 0 | 3.00E-55 |
| hsa_circ_0027767 | 92 | 138 | 674 | 211 | 747 | 0 | 0 |
| hsa_circ_0027770 | 92 | 1 | 574 | 1 | 574 | 0 | 0 |
| hsa_circ_0027770 | 92 | 1 | 900 | 1 | 906 | 0.01 | 0 |
| hsa_circ_0027768 | 93 | 1 | 1094 | 177 | 1276 | 0.01 | 0 |

novel_circ_018401 novel_circ_018402 novel_circ_018403 novel_circ_018404 novel_circ_018405 novel_circ_018406 novel_circ_018407 novel_circ_018408 novel_circ_018409 novel_circ_018410 novel_circ_018411 novel_circ_018412 novel_circ_018413 novel_circ_018414 novel_circ_018415 novel_circ_018416 novel_circ_018417 novel_circ_018418 novel_circ_018419 novel_circ_018420 novel_circ_018425 novel_circ_018426 novel_circ_018427 novel_circ_018428 novel_circ_018429 novel_circ_018430 novel_circ_018431 novel_circ_018432 novel_circ_018433 novel_circ_018434 novel_circ_018435 novel_circ_018436 novel_circ_018437 novel_circ_018438 novel_circ_018439 novel_circ_018440 novel_circ_018441 novel_circ_018442 novel_circ_018443 novel_circ_018444 novel_circ_018445 novel_circ_018446 novel_circ_018447 novel_circ_018448 novel_circ_018457 novel_circ_018458 novel_circ_018459 novel_circ_018460 novel_circ_018461 novel_circ_018462 novel_circ_018463 novel_circ_018464 novel_circ_018465 novel_circ_018466

| hsa_circ_0027770 | 93 | 1 | 413 | 488 | 906 | 0.01 | 2.00E-174 |
| --- | --- | --- | --- | --- | --- | --- | --- |
| hsa_circ_0027768 | 94 | 1 | 607 | 664 | 1276 | 0.01 | 0 |
| hsa_circ_0027768 | 94 | 1 | 520 | 751 | 1276 | 0.01 | 0 |
| hsa_circ_0027768 | 95 | 1 | 194 | 1083 | 1276 | 0 | 4.00E-83 |
| hsa_circ_0027774 | 96 | 1 | 542 | 1 | 542 | 0 | 0 |
| hsa_circ_0027774 | 96 | 1 | 639 | 1 | 639 | 0 | 0 |
| hsa_circ_0027782 | 82 | 1 | 1498 | 1498 | 1 | 0.01 | 0 |
| hsa_circ_0027810 | 96 | 4 | 572 | 569 | 1 | 0 | 0 |
| hsa_circ_0027806 | 93 | 96 | 403 | 555 | 248 | 0 | 4.00E-126 |
| hsa_circ_0027806 | 93 | 211 | 518 | 555 | 248 | 0 | 5.00E-126 |
| hsa_circ_0027806 | 93 | 268 | 575 | 555 | 248 | 0 | 5.00E-126 |
| hsa_circ_0027810 | 96 | 1 | 425 | 425 | 1 | 0 | 0 |
| hsa_circ_0027807 | 93 | 211 | 397 | 187 | 1 | 0 | 1.00E-70 |
| hsa_circ_0027810 | 96 | 1 | 425 | 425 | 1 | 0 | 0 |
| hsa_circ_0027810 | 96 | 4 | 572 | 569 | 1 | 0 | 0 |
| hsa_circ_0027810 | 96 | 1 | 425 | 425 | 1 | 0 | 0 |
| hsa_circ_0027810 | 96 | 4 | 572 | 569 | 1 | 0 | 0 |
| hsa_circ_0027810 | 98 | 1 | 299 | 425 | 127 | 0 | 1.00E-144 |
| hsa_circ_0027810 | 96 | 4 | 446 | 569 | 127 | 0 | 0 |
| hsa_circ_0002762 | 95 | 4 | 312 | 309 | 1 | 0 | 7.00E-137 |
| hsa_circ_0018920 | 96 | 1 | 748 | 881 | 134 | 0 | 0 |
| hsa_circ_0018920 | 97 | 1 | 636 | 881 | 246 | 0 | 0 |
| hsa_circ_0018920 | 97 | 48 | 683 | 881 | 246 | 0 | 0 |
| hsa_circ_0018920 | 95 | 1 | 443 | 881 | 439 | 0 | 0 |
| hsa_circ_0018920 | 99 | 1 | 343 | 1398 | 1056 | 0 | 5.00E-174 |
| hsa_circ_0018922 | 95 | 1 | 415 | 415 | 1 | 0 | 0 |
| hsa_circ_0018922 | 95 | 1 | 526 | 526 | 1 | 0 | 0 |
| hsa_circ_0018916 | 96 | 1 | 621 | 12775 | 12155 | 0 | 0 |
| hsa_circ_0018924 | 97 | 1 | 268 | 268 | 1 | 0 | 4.00E-124 |
| hsa_circ_0018924 | 95 | 15049 | 15210 | 430 | 269 | 0 | 4.00E-65 |
| hsa_circ_0018931 | 89 | 1 | 354 | 354 | 1 | 0 | 2.00E-123 |
| hsa_circ_0018934 | 93 | 1 | 942 | 1201 | 260 | 0 | 0 |
| hsa_circ_0018935 | 93 | 1 | 573 | 573 | 1 | 0 | 0 |
| hsa_circ_0018949 | 94 | 1 | 595 | 595 | 1 | 0 | 0 |
| hsa_circ_0018950 | 93 | 1 | 462 | 462 | 1 | 0 | 0 |
| hsa_circ_0018951 | 92 | 1 | 712 | 712 | 1 | 0 | 0 |
| hsa_circ_0018952 | 92 | 1 | 428 | 542 | 115 | 0 | 8.00E-168 |
| hsa_circ_0018953 | 93 | 1 | 257 | 257 | 1 | 0 | 5.00E-103 |
| hsa_circ_0018955 | 94 | 1 | 450 | 450 | 1 | 0 | 0 |
| hsa_circ_0018956 | 94 | 1 | 338 | 338 | 1 | 0 | 6.00E-143 |
| hsa_circ_0018960 | 92 | 1 | 1113 | 1452 | 340 | 0 | 0 |
| hsa_circ_0018960 | 92 | 1 | 971 | 1452 | 482 | 0 | 0 |
| hsa_circ_0029815 | 90 | 51038 | 51497 | 539 | 79 | 0 | 6.00E-170 |
| hsa_circ_0018968 | 96 | 1 | 329 | 1 | 329 | 0 | 1.00E-149 |
| hsa_circ_0012728 | 93 | 1 | 641 | 641 | 1 | 0 | 0 |
| hsa_circ_0012728 | 94 | 1 | 772 | 772 | 1 | 0 | 0 |
| hsa_circ_0012728 | 94 | 1 | 772 | 772 | 1 | 0 | 0 |
| hsa_circ_0012728 | 93 | 1 | 641 | 641 | 1 | 0 | 0 |
| hsa_circ_0012728 | 94 | 1 | 772 | 772 | 1 | 0 | 0 |
| hsa_circ_0012727 | 94 | 1 | 322 | 322 | 1 | 0 | 1.00E-135 |
| hsa_circ_0012727 | 93 | 1 | 592 | 592 | 1 | 0 | 0 |
| hsa_circ_0012728 | 93 | 1 | 636 | 636 | 1 | 0 | 0 |
| hsa_circ_0012728 | 93 | 1 | 641 | 641 | 1 | 0 | 0 |
| hsa_circ_0012729 | 89 | 1 | 1449 | 1443 | 1 | 0.01 | 0 |

novel_circ_018467 novel_circ_018468 novel_circ_018469 novel_circ_018470 novel_circ_018471 novel_circ_018473 novel_circ_018474 novel_circ_018475 novel_circ_018490 novel_circ_018496 novel_circ_018497 novel_circ_018498 novel_circ_018499 novel_circ_018500 novel_circ_018501 novel_circ_018502 novel_circ_018503 novel_circ_018504 novel_circ_018505 novel_circ_018506 novel_circ_018507 novel_circ_018508 novel_circ_018509 novel_circ_018510 novel_circ_018512 novel_circ_018514 novel_circ_018515 novel_circ_018516 novel_circ_018517 novel_circ_018518 novel_circ_018520 novel_circ_018521 novel_circ_018522 novel_circ_018523 novel_circ_018524 novel_circ_018525 novel_circ_018526 novel_circ_018527 novel_circ_018528 novel_circ_018529 novel_circ_018530 novel_circ_018533 novel_circ_018534 novel_circ_018535 novel_circ_018536 novel_circ_018537 novel_circ_018540 novel_circ_018541 novel_circ_018542 novel_circ_018543 novel_circ_018544 novel_circ_018545 novel_circ_018547 novel_circ_018548

| hsa_circ_0012732 | 93 | 1 | 271 | 271 | 1 | 0 | 4.00E-108 |
| --- | --- | --- | --- | --- | --- | --- | --- |
| hsa_circ_0012732 | 95 | 1 | 402 | 402 | 1 | 0 | 2.00E-178 |
| hsa_circ_0012742 | 85 | 1 | 866 | 860 | 1 | 0.02 | 0 |
| hsa_circ_0012740 | 85 | 352 | 1218 | 992 | 132 | 0.02 | 0 |
| hsa_circ_0012740 | 85 | 431 | 1297 | 992 | 132 | 0.02 | 0 |
| hsa_circ_0006269 | 93 | 42 | 476 | 45 | 479 | 0 | 5.00E-180 |
| hsa_circ_0006269 | 93 | 42 | 565 | 45 | 568 | 0 | 0 |
| hsa_circ_0006633 | 93 | 1 | 353 | 1 | 353 | 0 | 7.00E-143 |
| hsa_circ_0002518 | 93 | 1 | 630 | 630 | 1 | 0 | 0 |
| hsa_circ_0069332 | 87 | 1 | 593 | 593 | 1 | 0 | 0 |
| hsa_circ_0069332 | 88 | 1 | 448 | 593 | 146 | 0 | 1.00E-145 |
| hsa_circ_0005298 | 90 | 1 | 655 | 1 | 655 | 0 | 0 |
| hsa_circ_0069343 | 84 | 1 | 276 | 1 | 276 | 0 | 3.00E-70 |
| hsa_circ_0069343 | 85 | 1 | 357 | 1 | 357 | 0 | 9.00E-102 |
| hsa_circ_0069343 | 88 | 1 | 581 | 1 | 581 | 0 | 0 |
| hsa_circ_0007107 | 91 | 2 | 502 | 2 | 502 | 0 | 0 |
| hsa_circ_0069351 | 91 | 1279 | 1869 | 1 | 591 | 0 | 0 |
| hsa_circ_0069351 | 90 | 236 | 1035 | 1 | 800 | 0 | 0 |
| hsa_circ_0069351 | 90 | 103 | 902 | 1 | 800 | 0 | 0 |
| hsa_circ_0069351 | 90 | 1 | 755 | 46 | 800 | 0 | 0 |
| hsa_circ_0069354 | 91 | 122 | 584 | 2 | 464 | 0 | 8.00E-179 |
| hsa_circ_0069354 | 91 | 2 | 464 | 2 | 464 | 0 | 6.00E-179 |
| hsa_circ_0069354 | 92 | 1 | 370 | 465 | 834 | 0 | 3.00E-147 |
| hsa_circ_0069354 | 87 | 1 | 692 | 465 | 1138 | 0.03 | 0 |
| hsa_circ_0069358 | 89 | 1 | 1977 | 2833 | 856 | 0.01 | 0 |
| hsa_circ_0069360 | 91 | 1 | 587 | 590 | 1 | 0.01 | 0 |
| hsa_circ_0069358 | 90 | 1 | 1692 | 2833 | 1139 | 0 | 0 |
| hsa_circ_0069361 | 92 | 1 | 498 | 601 | 104 | 0 | 0 |
| hsa_circ_0069362 | 90 | 4 | 999 | 996 | 1 | 0 | 0 |
| hsa_circ_0069362 | 90 | 1 | 1391 | 1391 | 1 | 0 | 0 |
| hsa_circ_0069362 | 89 | 1 | 593 | 879 | 287 | 0 | 0 |
| hsa_circ_0069362 | 89 | 4 | 713 | 996 | 287 | 0 | 0 |
| hsa_circ_0069362 | 89 | 1 | 1105 | 1391 | 287 | 0 | 0 |
| hsa_circ_0069362 | 88 | 608 | 2413 | 2095 | 287 | 0 | 0 |
| hsa_circ_0069362 | 89 | 4 | 629 | 996 | 371 | 0 | 0 |
| hsa_circ_0069362 | 89 | 1 | 1021 | 1391 | 371 | 0 | 0 |
| hsa_circ_0069362 | 89 | 608 | 1961 | 2095 | 739 | 0 | 0 |
| hsa_circ_0069364 | 89 | 1 | 512 | 512 | 1 | 0 | 0 |
| hsa_circ_0069364 | 87 | 1 | 916 | 919 | 1 | 0.01 | 0 |
| hsa_circ_0069364 | 88 | 1 | 1213 | 1216 | 1 | 0 | 0 |
| hsa_circ_0069364 | 88 | 608 | 1820 | 1216 | 1 | 0 | 0 |
| hsa_circ_0069375 | 89 | 13 | 609 | 808 | 212 | 0 | 0 |
| hsa_circ_0069375 | 89 | 13 | 609 | 808 | 212 | 0 | 0 |
| hsa_circ_0069375 | 89 | 13 | 609 | 808 | 212 | 0 | 0 |
| hsa_circ_0069376 | 89 | 13 | 571 | 559 | 1 | 0 | 0 |
| hsa_circ_0069376 | 89 | 13 | 571 | 559 | 1 | 0 | 0 |
| hsa_circ_0069384 | 93 | 21 | 393 | 1 | 373 | 0 | 3.00E-152 |
| hsa_circ_0069384 | 93 | 21 | 393 | 1 | 373 | 0 | 2.00E-151 |
| hsa_circ_0069384 | 93 | 1 | 373 | 1 | 373 | 0 | 2.00E-152 |
| hsa_circ_0069384 | 93 | 1 | 373 | 1 | 373 | 0 | 2.00E-151 |
| hsa_circ_0069384 | 89 | 1 | 598 | 1 | 598 | 0 | 0 |
| hsa_circ_0069387 | 93 | 1 | 619 | 384 | 1002 | 0 | 0 |
| hsa_circ_0069387 | 92 | 1 | 481 | 522 | 1002 | 0 | 0 |
| hsa_circ_0069387 | 93 | 1 | 318 | 685 | 1002 | 0 | 8.00E-132 |

novel_circ_018550 novel_circ_018551 novel_circ_018552 novel_circ_018553 novel_circ_018554 novel_circ_018555 novel_circ_018556 novel_circ_018557 novel_circ_018558 novel_circ_018559 novel_circ_018560 novel_circ_018561 novel_circ_018562 novel_circ_018563 novel_circ_018564 novel_circ_018565 novel_circ_018566 novel_circ_018567 novel_circ_018568 novel_circ_018569 novel_circ_018570 novel_circ_018571 novel_circ_018572 novel_circ_018573 novel_circ_018574 novel_circ_018575 novel_circ_018576 novel_circ_018577 novel_circ_018578 novel_circ_018579 novel_circ_018580 novel_circ_018581 novel_circ_018582 novel_circ_018583 novel_circ_018584 novel_circ_018585 novel_circ_018586 novel_circ_018587 novel_circ_018588 novel_circ_018589 novel_circ_018590 novel_circ_018591 novel_circ_018592 novel_circ_018593 novel_circ_018594 novel_circ_018595 novel_circ_018596 novel_circ_018597 novel_circ_018598 novel_circ_018599 novel_circ_018600 novel_circ_018601 novel_circ_018602 novel_circ_018603

| hsa_circ_0001399 | 88 | 247 | 1338 | 1 | 1116 | 0.02 | 0 |
| --- | --- | --- | --- | --- | --- | --- | --- |
| hsa_circ_0001399 | 88 | 116 | 1207 | 1 | 1116 | 0.02 | 0 |
| hsa_circ_0001399 | 89 | 1 | 584 | 1 | 584 | 0 | 0 |
| hsa_circ_0001399 | 88 | 1 | 1092 | 1 | 1116 | 0.02 | 0 |
| hsa_circ_0069391 | 87 | 1 | 864 | 117 | 1004 | 0.03 | 0 |
| hsa_circ_0069391 | 87 | 1 | 864 | 117 | 1004 | 0.03 | 0 |
| hsa_circ_0069392 | 87 | 1 | 508 | 1 | 532 | 0.05 | 2.00E-165 |
| hsa_circ_0078192 | 92 | 1 | 302 | 143 | 444 | 0 | 3.00E-116 |
| hsa_circ_0078192 | 93 | 136 | 579 | 1 | 444 | 0 | 0 |
| hsa_circ_0078192 | 94 | 136 | 477 | 1 | 342 | 0 | 5.00E-145 |
| hsa_circ_0078192 | 97 | 136 | 233 | 1 | 98 | 0 | 1.00E-39 |
| hsa_circ_0078187 | 94 | 1 | 648 | 1 | 648 | 0 | 0 |
| hsa_circ_0078187 | 93 | 1 | 536 | 1 | 536 | 0 | 0 |
| hsa_circ_0078174 | 94 | 1 | 465 | 1451 | 1915 | 0 | 0 |
| hsa_circ_0078174 | 94 | 1 | 1143 | 773 | 1915 | 0 | 0 |
| hsa_circ_0078174 | 95 | 1 | 1628 | 288 | 1915 | 0 | 0 |
| hsa_circ_0078174 | 95 | 1 | 1229 | 288 | 1516 | 0 | 0 |
| hsa_circ_0078174 | 95 | 1 | 1432 | 85 | 1516 | 0 | 0 |
| hsa_circ_0078174 | 95 | 1 | 1516 | 1 | 1516 | 0 | 0 |
| hsa_circ_0078177 | 95 | 1 | 1163 | 80 | 1242 | 0 | 0 |
| hsa_circ_0078183 | 95 | 1 | 678 | 42 | 719 | 0 | 0 |
| hsa_circ_0078180 | 95 | 1 | 935 | 1 | 935 | 0 | 0 |
| hsa_circ_0078177 | 95 | 1 | 1163 | 80 | 1242 | 0 | 0 |
| hsa_circ_0078177 | 95 | 1 | 1242 | 1 | 1242 | 0 | 0 |
| hsa_circ_0078174 | 95 | 1 | 1366 | 85 | 1450 | 0 | 0 |
| hsa_circ_0078174 | 96 | 1 | 1450 | 1 | 1450 | 0 | 0 |
| hsa_circ_0078174 | 97 | 1 | 287 | 1 | 287 | 0 | 5.00E-133 |
| hsa_circ_0078166 | 97 | 1 | 486 | 1 | 486 | 0 | 0 |
| hsa_circ_0078156 | 97 | 398 | 1066 | 99 | 767 | 0 | 0 |
| hsa_circ_0078174 | 96 | 1 | 208 | 1 | 208 | 0 | 1.00E-92 |
| hsa_circ_0078170 | 96 | 1 | 272 | 1 | 272 | 0 | 2.00E-121 |
| hsa_circ_0078166 | 97 | 1 | 407 | 1 | 407 | 0 | 0 |
| hsa_circ_0078161 | 97 | 1 | 508 | 1 | 508 | 0 | 0 |
| hsa_circ_0078158 | 97 | 398 | 987 | 1 | 590 | 0 | 0 |
| hsa_circ_0078161 | 97 | 1 | 384 | 1 | 384 | 0 | 0 |
| hsa_circ_0078158 | 97 | 398 | 863 | 1 | 466 | 0 | 0 |
| hsa_circ_0078158 | 97 | 1 | 183 | 1 | 183 | 0 | 4.00E-82 |
| hsa_circ_0078158 | 97 | 398 | 580 | 1 | 183 | 0 | 2.00E-81 |
| hsa_circ_0078158 | 97 | 13867 | 14049 | 1 | 183 | 0 | 4.00E-80 |
| hsa_circ_0080684 | 88 | 1 | 403 | 4913 | 4499 | 0.03 | 3.00E-137 |
| hsa_circ_0080474 | 89 | 1 | 284 | 539 | 256 | 0 | 3.00E-97 |
| hsa_circ_0080482 | 97 | 1 | 361 | 361 | 1 | 0 | 2.00E-172 |
| hsa_circ_0080482 | 93 | 1 | 839 | 839 | 1 | 0 | 0 |
| hsa_circ_0080483 | 92 | 1 | 656 | 656 | 1 | 0 | 0 |
| hsa_circ_0080479 | 93 | 1 | 3025 | 3620 | 596 | 0 | 0 |
| hsa_circ_0080487 | 90 | 1 | 478 | 478 | 1 | 0 | 7.00E-179 |
| hsa_circ_0080490 | 92 | 1 | 2161 | 2295 | 135 | 0 | 0 |
| hsa_circ_0080492 | 94 | 1 | 667 | 667 | 1 | 0 | 0 |
| hsa_circ_0080492 | 94 | 1 | 469 | 667 | 199 | 0 | 0 |
| hsa_circ_0080496 | 92 | 1 | 268 | 268 | 1 | 0 | 8.00E-104 |
| hsa_circ_0008576 | 93 | 127 | 313 | 218539 | 218353 | 0 | 2.00E-70 |
| hsa_circ_0080607 | 94 | 1 | 664 | 1 | 664 | 0 | 0 |
| hsa_circ_0080608 | 94 | 1 | 319 | 1 | 319 | 0 | 2.00E-137 |
| hsa_circ_0080608 | 94 | 1 | 525 | 1 | 525 | 0 | 0 |

novel_circ_018604 novel_circ_018605 novel_circ_018606 novel_circ_018607 novel_circ_018608 novel_circ_018609 novel_circ_018610 novel_circ_018611 novel_circ_018612 novel_circ_018613 novel_circ_018614 novel_circ_018615 novel_circ_018616 novel_circ_018617 novel_circ_018618 novel_circ_018619 novel_circ_018620 novel_circ_018621 novel_circ_018622 novel_circ_018623 novel_circ_018624 novel_circ_018625 novel_circ_018626 novel_circ_018627 novel_circ_018628 novel_circ_018629 novel_circ_018630 novel_circ_018631 novel_circ_018632 novel_circ_018633 novel_circ_018634 novel_circ_018635 novel_circ_018636 novel_circ_018637 novel_circ_018638 novel_circ_018639 novel_circ_018640 novel_circ_018641 novel_circ_018642 novel_circ_018643 novel_circ_018644 novel_circ_018645 novel_circ_018646 novel_circ_018651 novel_circ_018652 novel_circ_018653 novel_circ_018654 novel_circ_018655 novel_circ_018656 novel_circ_018657 novel_circ_018659 novel_circ_018660 novel_circ_018661 novel_circ_018662

| hsa_circ_0080605 | 92 | 1 | 936 | 379 | 1309 | 0.01 | 0 |
| --- | --- | --- | --- | --- | --- | --- | --- |
| hsa_circ_0080612 | 92 | 3 | 723 | 3 | 718 | 0.01 | 0 |
| hsa_circ_0080612 | 92 | 1 | 624 | 100 | 718 | 0.01 | 0 |
| hsa_circ_0080613 | 94 | 1 | 185 | 79 | 258 | 0.03 | 2.00E-71 |
| hsa_circ_0080612 | 92 | 1 | 546 | 178 | 718 | 0.01 | 0 |
| hsa_circ_0080612 | 92 | 1 | 1176 | 178 | 1348 | 0 | 0 |
| hsa_circ_0080612 | 92 | 1 | 543 | 181 | 718 | 0.01 | 0 |
| hsa_circ_0080612 | 92 | 1 | 1173 | 181 | 1348 | 0 | 0 |
| hsa_circ_0080612 | 91 | 1 | 486 | 238 | 718 | 0.01 | 0 |
| hsa_circ_0080612 | 92 | 1 | 617 | 238 | 849 | 0.01 | 0 |
| hsa_circ_0080612 | 92 | 1 | 1116 | 238 | 1348 | 0 | 0 |
| hsa_circ_0080612 | 90 | 53 | 419 | 352 | 718 | 0 | 1.00E-135 |
| hsa_circ_0080612 | 92 | 53 | 1049 | 352 | 1348 | 0 | 0 |
| hsa_circ_0080614 | 91 | 1 | 361 | 1 | 361 | 0 | 5.00E-134 |
| hsa_circ_0080614 | 92 | 1 | 492 | 1 | 492 | 0 | 0 |
| hsa_circ_0080612 | 92 | 1 | 991 | 358 | 1348 | 0 | 0 |
| hsa_circ_0080614 | 93 | 1 | 250 | 112 | 361 | 0 | 8.00E-101 |
| hsa_circ_0080612 | 93 | 1 | 880 | 469 | 1348 | 0 | 0 |
| hsa_circ_0080612 | 93 | 1 | 630 | 719 | 1348 | 0 | 0 |
| hsa_circ_0080616 | 92 | 1 | 499 | 1 | 499 | 0 | 0 |
| hsa_circ_0080616 | 92 | 1 | 735 | 1 | 735 | 0 | 0 |
| hsa_circ_0002325 | 91 | 1 | 256 | 1 | 256 | 0 | 8.00E-96 |
| hsa_circ_0080661 | 95 | 1 | 489 | 870 | 382 | 0 | 0 |
| hsa_circ_0080417 | 94 | 1 | 213 | 1 | 213 | 0.01 | 1.00E-88 |
| hsa_circ_0080417 | 94 | 1 | 213 | 1 | 213 | 0.01 | 2.00E-86 |
| hsa_circ_0080420 | 91 | 1 | 707 | 1 | 726 | 0.03 | 0 |
| hsa_circ_0080420 | 92 | 1 | 928 | 1 | 947 | 0.02 | 0 |
| hsa_circ_0080420 | 93 | 1 | 973 | 1 | 992 | 0.02 | 0 |
| hsa_circ_0080420 | 93 | 1 | 1069 | 1 | 1088 | 0.02 | 0 |
| hsa_circ_0080420 | 93 | 1 | 1069 | 1 | 1088 | 0.02 | 0 |
| hsa_circ_0080420 | 93 | 1 | 1069 | 1 | 1088 | 0.02 | 0 |
| hsa_circ_0080420 | 93 | 1 | 1069 | 1 | 1088 | 0.02 | 0 |
| hsa_circ_0080420 | 93 | 1 | 1069 | 1 | 1088 | 0.02 | 0 |
| hsa_circ_0080420 | 93 | 1 | 1069 | 1 | 1088 | 0.02 | 0 |
| hsa_circ_0080424 | 91 | 3 | 351 | 3 | 351 | 0 | 1.00E-130 |
| hsa_circ_0080424 | 92 | 3 | 724 | 3 | 724 | 0 | 0 |
| hsa_circ_0080424 | 92 | 3 | 724 | 3 | 724 | 0 | 0 |
| hsa_circ_0035409 | 83 | 9914 | 10027 | 74850 | 74737 | 0.02 | 6.00E-20 |
| hsa_circ_0080424 | 92 | 2 | 540 | 186 | 724 | 0 | 0 |
| hsa_circ_0080424 | 92 | 2 | 540 | 186 | 724 | 0 | 0 |
| hsa_circ_0080425 | 93 | 1 | 373 | 1 | 373 | 0 | 2.00E-152 |
| hsa_circ_0080425 | 93 | 1 | 373 | 1 | 373 | 0 | 4.00E-152 |
| hsa_circ_0080427 | 92 | 1 | 538 | 1 | 538 | 0 | 0 |
| hsa_circ_0007603 | 89 | 1 | 312 | 314 | 2 | 0 | 8.00E-107 |
| hsa_circ_0080397 | 90 | 1 | 422 | 195 | 617 | 0 | 4.00E-151 |
| hsa_circ_0080397 | 90 | 1 | 541 | 76 | 617 | 0 | 0 |
| hsa_circ_0080396 | 90 | 1 | 594 | 1 | 595 | 0 | 0 |
| hsa_circ_0080392 | 91 | 28 | 700 | 454 | 1127 | 0 | 0 |
| hsa_circ_0080394 | 92 | 28 | 388 | 40 | 400 | 0 | 6.00E-144 |
| hsa_circ_0080394 | 92 | 28 | 278 | 40 | 290 | 0 | 5.00E-98 |
| hsa_circ_0080371 | 91 | 1 | 1038 | 178 | 1214 | 0 | 0 |
| hsa_circ_0080371 | 92 | 1 | 1215 | 1 | 1214 | 0 | 0 |
| hsa_circ_0080376 | 93 | 1 | 479 | 1 | 479 | 0 | 0 |
| hsa_circ_0080374 | 91 | 1 | 765 | 1 | 765 | 0 | 0 |

novel_circ_018663 novel_circ_018664 novel_circ_018665 novel_circ_018666 novel_circ_018667 novel_circ_018668 novel_circ_018669 novel_circ_018670 novel_circ_018671 novel_circ_018672 novel_circ_018673 novel_circ_018674 novel_circ_018675 novel_circ_018676 novel_circ_018677 novel_circ_018678 novel_circ_018680 novel_circ_018690 novel_circ_018691 novel_circ_018692 novel_circ_018693 novel_circ_018694 novel_circ_018695 novel_circ_018697 novel_circ_018698 novel_circ_018699 novel_circ_018700 novel_circ_018701 novel_circ_018702 novel_circ_018703 novel_circ_018704 novel_circ_018705 novel_circ_018706 novel_circ_018707 novel_circ_018708 novel_circ_018713 novel_circ_018714 novel_circ_018715 novel_circ_018716 novel_circ_018717 novel_circ_018718 novel_circ_018719 novel_circ_018720 novel_circ_018724 novel_circ_018725 novel_circ_018730 novel_circ_018731 novel_circ_018732 novel_circ_018735 novel_circ_018736 novel_circ_018737 novel_circ_018738 novel_circ_018739 novel_circ_018740

| hsa_circ_0080371 | 92 | 1 | 942 | 1 | 942 | 0 | 0 |
| --- | --- | --- | --- | --- | --- | --- | --- |
| hsa_circ_0080371 | 91 | 1 | 798 | 1 | 798 | 0 | 0 |
| hsa_circ_0080371 | 90 | 1 | 614 | 1 | 614 | 0 | 0 |
| hsa_circ_0080371 | 90 | 1 | 463 | 1 | 463 | 0 | 6.00E-174 |
| hsa_circ_0005610 | 92 | 1 | 644 | 1 | 641 | 0 | 0 |
| hsa_circ_0007989 | 92 | 1 | 830 | 4 | 830 | 0 | 0 |
| hsa_circ_0007989 | 92 | 1 | 833 | 1 | 830 | 0 | 0 |
| hsa_circ_0005610 | 93 | 1 | 337 | 1 | 334 | 0.01 | 1.00E-134 |
| hsa_circ_0007989 | 92 | 1 | 523 | 4 | 523 | 0.01 | 0 |
| hsa_circ_0007989 | 92 | 1 | 526 | 1 | 523 | 0.01 | 0 |
| hsa_circ_0007989 | 91 | 1 | 353 | 4 | 356 | 0 | 1.00E-134 |
| hsa_circ_0007989 | 91 | 1 | 356 | 1 | 356 | 0 | 3.00E-136 |
| hsa_circ_0006041 | 94 | 1 | 1145 | 1 | 1145 | 0 | 0 |
| hsa_circ_0006041 | 94 | 1 | 946 | 1 | 946 | 0 | 0 |
| hsa_circ_0068031 | 91 | 1 | 502 | 1 | 502 | 0 | 0 |
| hsa_circ_0068031 | 91 | 91 | 592 | 1 | 502 | 0 | 0 |
| hsa_circ_0009016 | 91 | 29311 | 29363 | 305853 | 305901 | 0.08 | 6.00E-08 |
| hsa_circ_0000209 | 80 | 3710 | 3901 | 2120 | 2308 | 0.03 | 6.00E-30 |
| hsa_circ_0003046 | 90 | 1 | 813 | 1 | 813 | 0 | 0 |
| hsa_circ_0003046 | 90 | 1 | 813 | 1 | 813 | 0 | 0 |
| hsa_circ_0003046 | 90 | 1 | 813 | 1 | 813 | 0 | 0 |
| hsa_circ_0068015 | 92 | 1 | 1683 | 2592 | 4274 | 0 | 0 |
| hsa_circ_0068015 | 92 | 1 | 1314 | 2592 | 3905 | 0 | 0 |
| hsa_circ_0067988 | 85 | 1 | 535 | 1 | 526 | 0.02 | 1.00E-152 |
| hsa_circ_0067987 | 86 | 1 | 612 | 1 | 603 | 0.01 | 2.00E-180 |
| hsa_circ_0067979 | 90 | 1 | 139 | 1 | 139 | 0 | 5.00E-44 |
| hsa_circ_0067966 | 90 | 97 | 237 | 143 | 283 | 0 | 5.00E-45 |
| hsa_circ_0067962 | 88 | 1 | 640 | 637 | 1 | 0 | 0 |
| hsa_circ_0067959 | 89 | 1 | 1261 | 1784 | 527 | 0 | 0 |
| hsa_circ_0067959 | 88 | 1 | 1787 | 1784 | 1 | 0 | 0 |
| hsa_circ_0067958 | 86 | 16 | 457 | 561 | 120 | 0 | 7.00E-129 |
| hsa_circ_0067948 | 94 | 20551 | 20616 | 716 | 651 | 0 | 3.00E-18 |
| hsa_circ_0067948 | 94 | 1 | 459 | 593 | 135 | 0 | 0 |
| hsa_circ_0002387 | 92 | 1 | 333 | 333 | 1 | 0 | 2.00E-133 |
| hsa_circ_0067942 | 93 | 1 | 865 | 865 | 1 | 0 | 0 |
| hsa_circ_0025614 | 96 | 1 | 224 | 224 | 1 | 0 | 4.00E-98 |
| hsa_circ_0025614 | 96 | 1 | 224 | 224 | 1 | 0 | 3.00E-97 |
| hsa_circ_0025610 | 95 | 1 | 699 | 1131 | 433 | 0 | 0 |
| hsa_circ_0025610 | 94 | 1 | 863 | 1131 | 269 | 0 | 0 |
| hsa_circ_0025608 | 93 | 1 | 1495 | 1495 | 1 | 0 | 0 |
| hsa_circ_0025610 | 94 | 1 | 351 | 619 | 269 | 0 | 4.00E-150 |
| hsa_circ_0025608 | 92 | 1 | 796 | 796 | 1 | 0 | 0 |
| hsa_circ_0025608 | 92 | 1 | 631 | 631 | 1 | 0 | 0 |
| hsa_circ_0025604 | 94 | 1 | 433 | 340 | 772 | 0 | 0 |
| hsa_circ_0025604 | 95 | 1 | 299 | 340 | 638 | 0 | 1.00E-129 |
| hsa_circ_0025577 | 94 | 919 | 1275 | 1 | 357 | 0 | 2.00E-154 |
| hsa_circ_0025577 | 93 | 658 | 853 | 1 | 196 | 0 | 7.00E-77 |
| hsa_circ_0025577 | 93 | 919 | 1114 | 1 | 196 | 0 | 9.00E-77 |
| hsa_circ_0025573 | 88 | 1 | 711 | 1 | 711 | 0 | 0 |
| hsa_circ_0025568 | 88 | 1 | 1249 | 1 | 1240 | 0.01 | 0 |
| hsa_circ_0025564 | 88 | 1295 | 2549 | 46 | 1291 | 0.01 | 0 |
| hsa_circ_0025573 | 87 | 1 | 599 | 1 | 599 | 0 | 0 |
| hsa_circ_0025568 | 87 | 1 | 1137 | 1 | 1128 | 0.01 | 0 |
| hsa_circ_0025564 | 87 | 1295 | 2437 | 46 | 1179 | 0.01 | 0 |

novel_circ_018741 novel_circ_018742 novel_circ_018743 novel_circ_018744 novel_circ_018745 novel_circ_018746 novel_circ_018747 novel_circ_018748 novel_circ_018749 novel_circ_018750 novel_circ_018751 novel_circ_018752 novel_circ_018758 novel_circ_018759 novel_circ_018760 novel_circ_018761 novel_circ_018762 novel_circ_018763 novel_circ_018764 novel_circ_018765 novel_circ_018766 novel_circ_018767 novel_circ_018768 novel_circ_018769 novel_circ_018770 novel_circ_018771 novel_circ_018772 novel_circ_018773 novel_circ_018774 novel_circ_018775 novel_circ_018781 novel_circ_018782 novel_circ_018783 novel_circ_018784 novel_circ_018786 novel_circ_018787 novel_circ_018788 novel_circ_018790 novel_circ_018791 novel_circ_018792 novel_circ_018793 novel_circ_018794 novel_circ_018795 novel_circ_018796 novel_circ_018797 novel_circ_018798 novel_circ_018799 novel_circ_018800 novel_circ_018803 novel_circ_018804 novel_circ_018805 novel_circ_018806 novel_circ_018807 novel_circ_018808

| hsa_circ_0025573 | 84 | 1 | 461 | 1 | 461 | 0 | 9.00E-123 |
| --- | --- | --- | --- | --- | --- | --- | --- |
| hsa_circ_0025568 | 86 | 1 | 999 | 1 | 990 | 0.01 | 0 |
| hsa_circ_0025564 | 86 | 1295 | 2299 | 46 | 1041 | 0.01 | 0 |
| hsa_circ_0025564 | 86 | 1 | 1050 | 1 | 1041 | 0.01 | 0 |
| hsa_circ_0025573 | 86 | 1 | 277 | 1 | 277 | 0 | 2.00E-77 |
| hsa_circ_0025571 | 86 | 1 | 500 | 1 | 491 | 0.02 | 6.00E-150 |
| hsa_circ_0025568 | 87 | 1 | 758 | 1 | 749 | 0.01 | 0 |
| hsa_circ_0025564 | 87 | 1295 | 2058 | 46 | 800 | 0.01 | 0 |
| hsa_circ_0025564 | 87 | 1 | 809 | 1 | 800 | 0.01 | 0 |
| hsa_circ_0025568 | 88 | 1 | 538 | 1 | 529 | 0.02 | 1.00E-177 |
| hsa_circ_0025564 | 88 | 1295 | 1838 | 46 | 580 | 0.02 | 7.00E-179 |
| hsa_circ_0025564 | 88 | 1 | 589 | 1 | 580 | 0.02 | 0 |
| hsa_circ_0025560 | 97 | 1 | 182 | 313 | 494 | 0 | 3.00E-83 |
| hsa_circ_0025560 | 96 | 1 | 494 | 1 | 494 | 0 | 0 |
| hsa_circ_0025558 | 96 | 29787 | 30282 | 107 | 602 | 0 | 0 |
| hsa_circ_0025558 | 96 | 1 | 602 | 1 | 602 | 0 | 0 |
| hsa_circ_0025554 | 96 | 3440 | 4042 | 208 | 810 | 0 | 0 |
| hsa_circ_0025554 | 97 | 1 | 810 | 1 | 810 | 0 | 0 |
| hsa_circ_0025558 | 96 | 29787 | 30168 | 107 | 488 | 0 | 1.00E-173 |
| hsa_circ_0025558 | 96 | 1 | 488 | 1 | 488 | 0 | 0 |
| hsa_circ_0025558 | 96 | 29787 | 30104 | 107 | 424 | 0 | 4.00E-143 |
| hsa_circ_0025558 | 96 | 1 | 424 | 1 | 424 | 0 | 0 |
| hsa_circ_0025554 | 97 | 1 | 632 | 1 | 632 | 0 | 0 |
| hsa_circ_0025558 | 96 | 29787 | 30100 | 107 | 420 | 0 | 6.00E-141 |
| hsa_circ_0025558 | 96 | 1 | 420 | 1 | 420 | 0 | 0 |
| hsa_circ_0025554 | 96 | 3440 | 3860 | 208 | 628 | 0 | 0 |
| hsa_circ_0025554 | 97 | 1 | 628 | 1 | 628 | 0 | 0 |
| hsa_circ_0025558 | 95 | 29787 | 29975 | 107 | 295 | 0 | 4.00E-78 |
| hsa_circ_0025558 | 95 | 1 | 295 | 1 | 295 | 0 | 9.00E-131 |
| hsa_circ_0025554 | 97 | 1 | 503 | 1 | 503 | 0 | 0 |
| hsa_circ_0025551 | 89 | 1 | 258 | 295 | 555 | 0.01 | 2.00E-83 |
| hsa_circ_0025548 | 90 | 1 | 1192 | 291 | 1482 | 0 | 0 |
| hsa_circ_0025551 | 89 | 1 | 258 | 295 | 555 | 0.01 | 2.00E-83 |
| hsa_circ_0025551 | 89 | 1 | 258 | 295 | 555 | 0.01 | 2.00E-83 |
| hsa_circ_0025551 | 89 | 1 | 258 | 295 | 555 | 0.01 | 1.00E-83 |
| hsa_circ_0025551 | 91 | 1 | 489 | 64 | 555 | 0.01 | 0 |
| hsa_circ_0025551 | 89 | 1 | 258 | 295 | 555 | 0.01 | 9.00E-84 |
| hsa_circ_0025551 | 91 | 1 | 439 | 64 | 505 | 0.01 | 4.00E-166 |
| hsa_circ_0025548 | 90 | 1 | 1192 | 291 | 1482 | 0 | 0 |
| hsa_circ_0025548 | 90 | 1 | 1192 | 291 | 1482 | 0 | 0 |
| hsa_circ_0025548 | 90 | 1 | 1192 | 291 | 1482 | 0 | 0 |
| hsa_circ_0025550 | 89 | 1 | 1018 | 117 | 1134 | 0 | 0 |
| hsa_circ_0025550 | 89 | 1 | 934 | 117 | 1050 | 0 | 0 |
| hsa_circ_0025548 | 90 | 1 | 1151 | 74 | 1224 | 0.01 | 0 |
| hsa_circ_0025550 | 87 | 1 | 468 | 117 | 584 | 0.01 | 2.00E-148 |
| hsa_circ_0025547 | 93 | 1 | 178 | 206 | 383 | 0 | 3.00E-69 |
| hsa_circ_0025547 | 93 | 3 | 299 | 87 | 383 | 0 | 7.00E-122 |
| hsa_circ_0025547 | 93 | 1 | 383 | 1 | 383 | 0 | 3.00E-156 |
| hsa_circ_0084765 | 95 | 1 | 432 | 432 | 1 | 0 | 0 |
| hsa_circ_0084764 | 95 | 1 | 702 | 702 | 1 | 0 | 0 |
| hsa_circ_0084764 | 95 | 1 | 408 | 408 | 1 | 0 | 4.00E-180 |
| hsa_circ_0084764 | 95 | 1 | 408 | 408 | 1 | 0 | 7.00E-180 |
| hsa_circ_0084764 | 94 | 1 | 270 | 270 | 1 | 0 | 7.00E-112 |
| hsa_circ_0084764 | 94 | 1 | 270 | 270 | 1 | 0 | 1.00E-111 |

novel_circ_018814 novel_circ_018815 novel_circ_018816 novel_circ_018817 novel_circ_018818 novel_circ_018819 novel_circ_018820 novel_circ_018821 novel_circ_018823 novel_circ_018824 novel_circ_018825 novel_circ_018826 novel_circ_018827 novel_circ_018828 novel_circ_018829 novel_circ_018830 novel_circ_018831 novel_circ_018832 novel_circ_018833 novel_circ_018834 novel_circ_018835 novel_circ_018836 novel_circ_018837 novel_circ_018838 novel_circ_018839 novel_circ_018840 novel_circ_018841 novel_circ_018842 novel_circ_018844 novel_circ_018845 novel_circ_018846 novel_circ_018847 novel_circ_018848 novel_circ_018849 novel_circ_018850 novel_circ_018852 novel_circ_018854 novel_circ_018855 novel_circ_018856 novel_circ_018857 novel_circ_018858 novel_circ_018859 novel_circ_018860 novel_circ_018861 novel_circ_018862 novel_circ_018863 novel_circ_018864 novel_circ_018865 novel_circ_018866 novel_circ_018868 novel_circ_018869 novel_circ_018870 novel_circ_018871 novel_circ_018872

| hsa_circ_0066808 | 87 | 12 | 547 | 184 | 715 | 0.02 | 6.00E-170 |
| --- | --- | --- | --- | --- | --- | --- | --- |
| hsa_circ_0066807 | 86 | 446 | 1115 | 4244 | 3566 | 0.01 | 0 |
| hsa_circ_0066807 | 86 | 1 | 670 | 4244 | 3566 | 0.01 | 0 |
| hsa_circ_0066806 | 95 | 1 | 131 | 1 | 131 | 0 | 7.00E-53 |
| hsa_circ_0066806 | 95 | 100 | 230 | 1 | 131 | 0 | 1.00E-52 |
| hsa_circ_0066806 | 95 | 100 | 230 | 1 | 131 | 0 | 7.00E-53 |
| hsa_circ_0066798 | 89 | 1 | 327 | 83 | 409 | 0 | 4.00E-113 |
| hsa_circ_0066798 | 89 | 1 | 327 | 83 | 409 | 0 | 2.00E-113 |
| hsa_circ_0066793 | 91 | 713 | 852 | 111 | 250 | 0 | 2.00E-47 |
| hsa_circ_0066793 | 92 | 1 | 250 | 1 | 250 | 0 | 8.00E-96 |
| hsa_circ_0005128 | 92 | 1 | 449 | 235 | 680 | 0.01 | 2.00E-178 |
| hsa_circ_0004125 | 91 | 1 | 188 | 1 | 188 | 0 | 4.00E-66 |
| hsa_circ_0066786 | 89 | 1 | 660 | 1 | 666 | 0.01 | 0 |
| hsa_circ_0007315 | 92 | 1 | 211 | 1 | 211 | 0 | 4.00E-79 |
| hsa_circ_0007315 | 92 | 1 | 211 | 1 | 211 | 0 | 3.00E-79 |
| hsa_circ_0066776 | 94 | 1 | 909 | 1 | 909 | 0 | 0 |
| hsa_circ_0066776 | 94 | 1 | 757 | 1 | 757 | 0 | 0 |
| hsa_circ_0005621 | 88 | 3 | 268 | 266 | 1 | 0 | 1.00E-84 |
| hsa_circ_0005621 | 89 | 4 | 413 | 410 | 1 | 0 | 4.00E-141 |
| hsa_circ_0014623 | 88 | 4492 | 5363 | 2835 | 1964 | 0 | 0 |
| hsa_circ_0014623 | 88 | 1 | 975 | 2938 | 1964 | 0 | 0 |
| hsa_circ_0005621 | 89 | 1 | 753 | 871 | 119 | 0 | 0 |
| hsa_circ_0014630 | 88 | 1 | 286 | 458 | 173 | 0 | 2.00E-92 |
| hsa_circ_0014632 | 89 | 8 | 482 | 579 | 105 | 0 | 7.00E-169 |
| hsa_circ_0014630 | 90 | 567 | 1141 | 1282 | 708 | 0 | 0 |
| hsa_circ_0014630 | 90 | 1098 | 1672 | 1282 | 708 | 0 | 0 |
| hsa_circ_0000139 | 83 | 47 | 576 | 530 | 1 | 0 | 4.00E-134 |
| hsa_circ_0000139 | 83 | 2 | 531 | 530 | 1 | 0 | 3.00E-134 |
| hsa_circ_0014633 | 92 | 1 | 263 | 263 | 1 | 0 | 1.00E-99 |
| hsa_circ_0014633 | 92 | 1 | 266 | 266 | 1 | 0 | 2.00E-101 |
| hsa_circ_0014641 | 95 | 1 | 802 | 802 | 1 | 0 | 0 |
| hsa_circ_0014641 | 94 | 1 | 437 | 749 | 313 | 0 | 0 |
| hsa_circ_0014642 | 94 | 1 | 376 | 376 | 1 | 0 | 2.00E-162 |
| hsa_circ_0014643 | 93 | 1 | 217 | 217 | 1 | 0 | 3.00E-84 |
| hsa_circ_0014696 | 90 | 1 | 404 | 404 | 1 | 0 | 8.00E-148 |
| hsa_circ_0014774 | 89 | 1 | 318 | 1 | 318 | 0 | 8.00E-107 |
| hsa_circ_0014824 | 91 | 1 | 550 | 550 | 1 | 0 | 0 |
| hsa_circ_0039278 | 95 | 1 | 493 | 1 | 493 | 0 | 0 |
| hsa_circ_0039279 | 93 | 1 | 404 | 137 | 540 | 0 | 7.00E-168 |
| hsa_circ_0039283 | 93 | 3 | 245 | 3 | 245 | 0 | 1.00E-98 |
| hsa_circ_0039283 | 93 | 3 | 1264 | 3 | 1264 | 0 | 0 |
| hsa_circ_0039288 | 93 | 1 | 656 | 1 | 656 | 0 | 0 |
| hsa_circ_0039298 | 93 | 1 | 747 | 1 | 747 | 0 | 0 |
| hsa_circ_0039298 | 92 | 1 | 1008 | 1 | 1008 | 0 | 0 |
| hsa_circ_0039298 | 93 | 5 | 1056 | 99 | 1150 | 0 | 0 |
| hsa_circ_0039298 | 94 | 1 | 512 | 236 | 747 | 0 | 0 |
| hsa_circ_0039298 | 93 | 1 | 915 | 236 | 1150 | 0 | 0 |
| hsa_circ_0039306 | 92 | 1 | 554 | 1 | 554 | 0 | 0 |
| hsa_circ_0039313 | 92 | 1 | 399 | 1 | 399 | 0 | 9.00E-162 |
| hsa_circ_0069595 | 91 | 1 | 160 | 1 | 160 | 0 | 2.00E-54 |
| hsa_circ_0069593 | 92 | 1 | 598 | 326 | 923 | 0 | 0 |
| hsa_circ_0069600 | 95 | 1 | 248 | 1 | 248 | 0 | 2.00E-106 |
| hsa_circ_0069600 | 92 | 1 | 403 | 1 | 403 | 0 | 4.00E-157 |
| hsa_circ_0069600 | 92 | 1 | 403 | 1 | 403 | 0 | 5.00E-157 |

novel_circ_018873 novel_circ_018874 novel_circ_018879 novel_circ_018880 novel_circ_018881 novel_circ_018882 novel_circ_018883 novel_circ_018885 novel_circ_018886 novel_circ_018887 novel_circ_018888 novel_circ_018889 novel_circ_018890 novel_circ_018891 novel_circ_018892 novel_circ_018893 novel_circ_018895 novel_circ_018896 novel_circ_018897 novel_circ_018898 novel_circ_018899 novel_circ_018900 novel_circ_018901 novel_circ_018902 novel_circ_018903 novel_circ_018904 novel_circ_018905 novel_circ_018906 novel_circ_018907 novel_circ_018908 novel_circ_018909 novel_circ_018910 novel_circ_018911 novel_circ_018919 novel_circ_018920 novel_circ_018921 novel_circ_018922 novel_circ_018923 novel_circ_018926 novel_circ_018927 novel_circ_018928 novel_circ_018929 novel_circ_018931 novel_circ_018932 novel_circ_018933 novel_circ_018934 novel_circ_018935 novel_circ_018936 novel_circ_018937 novel_circ_018938 novel_circ_018939 novel_circ_018940 novel_circ_018941 novel_circ_018942

| hsa_circ_0069600 | 92 | 1 | 403 | 1 | 403 | 0 | 1.00E-156 |
| --- | --- | --- | --- | --- | --- | --- | --- |
| hsa_circ_0069600 | 87 | 3 | 155 | 251 | 403 | 0 | 8.00E-42 |
| hsa_circ_0069603 | 89 | 1 | 888 | 888 | 1 | 0 | 0 |
| hsa_circ_0069603 | 92 | 1 | 504 | 593 | 90 | 0 | 0 |
| hsa_circ_0069603 | 90 | 1 | 799 | 888 | 90 | 0 | 0 |
| hsa_circ_0069604 | 90 | 1 | 634 | 691 | 58 | 0 | 0 |
| hsa_circ_0069604 | 90 | 1 | 572 | 691 | 120 | 0 | 0 |
| hsa_circ_0069611 | 93 | 4 | 374 | 374 | 4 | 0 | 7.00E-153 |
| hsa_circ_0069611 | 91 | 1 | 599 | 602 | 4 | 0 | 0 |
| hsa_circ_0069611 | 91 | 1 | 908 | 911 | 4 | 0 | 0 |
| hsa_circ_0069611 | 91 | 1 | 981 | 984 | 4 | 0 | 0 |
| hsa_circ_0069611 | 91 | 1 | 1321 | 1324 | 4 | 0 | 0 |
| hsa_circ_0069611 | 91 | 1 | 811 | 984 | 174 | 0 | 0 |
| hsa_circ_0069611 | 91 | 1 | 1151 | 1324 | 174 | 0 | 0 |
| hsa_circ_0069611 | 91 | 1 | 671 | 911 | 241 | 0 | 0 |
| hsa_circ_0069611 | 91 | 1 | 1084 | 1324 | 241 | 0 | 0 |
| hsa_circ_0069614 | 90 | 2 | 295 | 294 | 1 | 0 | 2.00E-103 |
| hsa_circ_0069614 | 90 | 1 | 345 | 345 | 1 | 0 | 2.00E-123 |
| hsa_circ_0069614 | 90 | 1 | 534 | 534 | 1 | 0 | 0 |
| hsa_circ_0069614 | 90 | 1 | 607 | 607 | 1 | 0 | 0 |
| hsa_circ_0069614 | 90 | 1 | 947 | 947 | 1 | 0 | 0 |
| hsa_circ_0069611 | 91 | 1 | 1497 | 1874 | 378 | 0 | 0 |
| hsa_circ_0069616 | 91 | 1 | 382 | 382 | 1 | 0 | 1.00E-145 |
| hsa_circ_0069616 | 91 | 1 | 722 | 722 | 1 | 0 | 0 |
| hsa_circ_0069616 | 92 | 1 | 1000 | 1000 | 1 | 0 | 0 |
| hsa_circ_0001408 | 91 | 1 | 412 | 519 | 108 | 0 | 1.00E-155 |
| hsa_circ_0001408 | 93 | 1 | 406 | 797 | 392 | 0 | 6.00E-169 |
| hsa_circ_0069617 | 92 | 1 | 534 | 1058 | 525 | 0 | 0 |
| hsa_circ_0069620 | 91 | 2 | 198 | 475 | 279 | 0 | 2.00E-71 |
| hsa_circ_0069620 | 92 | 113 | 387 | 553 | 279 | 0 | 2.00E-104 |
| hsa_circ_0069620 | 92 | 159 | 433 | 553 | 279 | 0 | 2.00E-104 |
| hsa_circ_0069621 | 93 | 1 | 313 | 314 | 2 | 0 | 5.00E-129 |
| hsa_circ_0069621 | 93 | 1 | 215 | 314 | 100 | 0 | 2.00E-86 |
| hsa_circ_0007207 | 96 | 1 | 744 | 747 | 1 | 0 | 0 |
| hsa_circ_0061427 | 93 | 1 | 1200 | 2266 | 1064 | 0.01 | 0 |
| hsa_circ_0061423 | 93 | 1 | 2452 | 2458 | 1 | 0 | 0 |
| hsa_circ_0061423 | 93 | 1 | 1885 | 1888 | 1 | 0 | 0 |
| hsa_circ_0061423 | 92 | 7 | 1708 | 1705 | 1 | 0 | 0 |
| hsa_circ_0061423 | 95 | 1 | 975 | 975 | 1 | 0 | 0 |
| hsa_circ_0061423 | 95 | 1 | 874 | 874 | 1 | 0 | 0 |
| hsa_circ_0018940 | 85 | 1454 | 1517 | 17958 | 18023 | 0.03 | 8.00E-09 |
| hsa_circ_0007400 | 82 | 20497 | 20636 | 19565 | 19425 | 0.01 | 4.00E-24 |
| hsa_circ_0061410 | 91 | 1 | 1938 | 2585 | 634 | 0.01 | 0 |
| hsa_circ_0061410 | 93 | 3 | 1143 | 2403 | 1255 | 0.01 | 0 |
| hsa_circ_0061410 | 93 | 3 | 1591 | 2403 | 807 | 0.01 | 0 |
| hsa_circ_0061410 | 93 | 3 | 1764 | 2403 | 634 | 0 | 0 |
| hsa_circ_0061414 | 94 | 1 | 1422 | 1820 | 399 | 0 | 0 |
| hsa_circ_0061414 | 94 | 1 | 1595 | 1820 | 226 | 0 | 0 |
| hsa_circ_0061414 | 93 | 1 | 448 | 846 | 399 | 0 | 0 |
| hsa_circ_0061414 | 93 | 1 | 621 | 846 | 226 | 0 | 0 |
| hsa_circ_0061412 | 92 | 1 | 1032 | 1179 | 148 | 0 | 0 |
| hsa_circ_0061412 | 92 | 1 | 1179 | 1179 | 1 | 0 | 0 |
| hsa_circ_0061410 | 92 | 1 | 1254 | 1254 | 1 | 0 | 0 |
| hsa_circ_0061408 | 92 | 1 | 1530 | 1924 | 395 | 0 | 0 |

novel_circ_018943 novel_circ_018944 novel_circ_018945 novel_circ_018946 novel_circ_018947 novel_circ_018948 novel_circ_018949 novel_circ_018950 novel_circ_018951 novel_circ_018952 novel_circ_018953 novel_circ_018954 novel_circ_018955 novel_circ_018956 novel_circ_018957 novel_circ_018958 novel_circ_018959 novel_circ_018960 novel_circ_018961 novel_circ_018962 novel_circ_018963 novel_circ_018964 novel_circ_018965 novel_circ_018966 novel_circ_018967 novel_circ_018968 novel_circ_018969 novel_circ_018970 novel_circ_018971 novel_circ_018972 novel_circ_018973 novel_circ_018979 novel_circ_018981 novel_circ_018982 novel_circ_018983 novel_circ_018984 novel_circ_018985 novel_circ_018986 novel_circ_018987 novel_circ_018990 novel_circ_018991 novel_circ_018992 novel_circ_018993 novel_circ_018994 novel_circ_018995 novel_circ_018996 novel_circ_018997 novel_circ_018998 novel_circ_018999 novel_circ_019000 novel_circ_019001 novel_circ_019002 novel_circ_019003 novel_circ_019004

| hsa_circ_0061408 | 92 | 1 | 1924 | 1924 | 1 | 0 | 0 |
| --- | --- | --- | --- | --- | --- | --- | --- |
| hsa_circ_0061408 | 92 | 1 | 1924 | 1924 | 1 | 0 | 0 |
| hsa_circ_0061408 | 92 | 1 | 1924 | 1924 | 1 | 0 | 0 |
| hsa_circ_0061414 | 91 | 1 | 398 | 398 | 1 | 0 | 2.00E-149 |
| hsa_circ_0061413 | 90 | 1 | 411 | 411 | 1 | 0 | 1.00E-151 |
| hsa_circ_0061412 | 91 | 1 | 558 | 558 | 1 | 0 | 0 |
| hsa_circ_0061410 | 91 | 1 | 633 | 633 | 1 | 0 | 0 |
| hsa_circ_0061408 | 91 | 1 | 909 | 1303 | 395 | 0 | 0 |
| hsa_circ_0061408 | 92 | 1 | 580 | 892 | 313 | 0 | 0 |
| hsa_circ_0061408 | 92 | 1 | 892 | 892 | 1 | 0 | 0 |
| hsa_circ_0061408 | 92 | 1 | 892 | 892 | 1 | 0 | 0 |
| hsa_circ_0061408 | 92 | 1 | 892 | 892 | 1 | 0 | 0 |
| hsa_circ_0061408 | 91 | 1 | 351 | 745 | 395 | 0 | 4.00E-135 |
| hsa_circ_0061408 | 92 | 1 | 745 | 745 | 1 | 0 | 0 |
| hsa_circ_0061408 | 91 | 1 | 443 | 670 | 228 | 0 | 4.00E-171 |
| hsa_circ_0061408 | 92 | 1 | 499 | 499 | 1 | 0 | 0 |
| hsa_circ_0061405 | 92 | 1 | 603 | 603 | 1 | 0 | 0 |
| hsa_circ_0061403 | 92 | 1 | 604 | 783 | 180 | 0 | 0 |
| hsa_circ_0061408 | 92 | 1 | 394 | 394 | 1 | 0 | 3.00E-157 |
| hsa_circ_0061405 | 92 | 1 | 498 | 498 | 1 | 0 | 0 |
| hsa_circ_0061403 | 92 | 1 | 499 | 678 | 180 | 0 | 0 |
| hsa_circ_0061403 | 91 | 1 | 417 | 596 | 180 | 0 | 3.00E-161 |
| hsa_circ_0061405 | 92 | 2065 | 2171 | 107 | 1 | 0 | 1.00E-33 |
| hsa_circ_0061403 | 93 | 478 | 658 | 181 | 1 | 0 | 3.00E-70 |
| hsa_circ_0061403 | 93 | 1 | 180 | 180 | 1 | 0 | 5.00E-70 |
| hsa_circ_0061403 | 93 | 1 | 180 | 180 | 1 | 0 | 6.00E-70 |
| hsa_circ_0061402 | 93 | 485 | 783 | 570 | 272 | 0 | 7.00E-121 |
| hsa_circ_0061402 | 92 | 485 | 890 | 570 | 165 | 0 | 1.00E-158 |
| hsa_circ_0061402 | 91 | 485 | 1054 | 570 | 1 | 0 | 0 |
| hsa_circ_0061402 | 93 | 305 | 603 | 570 | 272 | 0 | 6.00E-121 |
| hsa_circ_0011514 | 95 | 53028 | 53069 | 6623 | 6664 | 0 | 1.00E-07 |
| hsa_circ_0077500 | 81 | 322 | 1088 | 4 | 770 | 0 | 3.00E-172 |
| hsa_circ_0077498 | 80 | 1 | 547 | 1611 | 2160 | 0.01 | 2.00E-109 |
| hsa_circ_0077498 | 80 | 1 | 765 | 1611 | 2378 | 0.01 | 5.00E-157 |
| hsa_circ_0077500 | 81 | 208 | 974 | 4 | 770 | 0 | 2.00E-172 |
| hsa_circ_0006158 | 92 | 1 | 1849 | 1 | 1843 | 0.01 | 0 |
| hsa_circ_0061395 | 92 | 1 | 1548 | 1 | 1542 | 0.01 | 0 |
| hsa_circ_0006158 | 92 | 1 | 1842 | 1 | 1836 | 0.01 | 0 |
| hsa_circ_0006158 | 91 | 1 | 294 | 1 | 294 | 0 | 3.00E-110 |
| hsa_circ_0077306 | 96 | 300 | 754 | 455 | 1 | 0 | 0 |
| hsa_circ_0077306 | 96 | 300 | 754 | 455 | 1 | 0 | 0 |
| hsa_circ_0077306 | 96 | 4 | 458 | 455 | 1 | 0 | 0 |
| hsa_circ_0077306 | 96 | 4 | 458 | 455 | 1 | 0 | 0 |
| hsa_circ_0077304 | 93 | 1 | 527 | 527 | 1 | 0 | 0 |
| hsa_circ_0077303 | 92 | 1 | 647 | 3980 | 3334 | 0 | 0 |
| hsa_circ_0077303 | 92 | 1 | 573 | 3906 | 3334 | 0 | 0 |
| hsa_circ_0077303 | 93 | 1 | 441 | 3774 | 3334 | 0 | 5.00E-180 |
| hsa_circ_0077303 | 91 | 2 | 250 | 3582 | 3334 | 0 | 6.00E-92 |
| hsa_circ_0077297 | 88 | 156 | 655 | 1 | 491 | 0.03 | 2.00E-161 |
| hsa_circ_0077296 | 95 | 1 | 301 | 303 | 3 | 0 | 4.00E-134 |
| hsa_circ_0002752 | 94 | 1 | 620 | 620 | 1 | 0 | 0 |
| hsa_circ_0002752 | 93 | 1 | 317 | 317 | 1 | 0 | 3.00E-131 |
| hsa_circ_0003393 | 95 | 1 | 529 | 529 | 1 | 0 | 0 |
| hsa_circ_0077291 | 95 | 77 | 664 | 996 | 409 | 0 | 0 |

novel_circ_019005 novel_circ_019006 novel_circ_019007 novel_circ_019008 novel_circ_019009 novel_circ_019010 novel_circ_019011 novel_circ_019012 novel_circ_019013 novel_circ_019015 novel_circ_019016 novel_circ_019017 novel_circ_019018 novel_circ_019019 novel_circ_019020 novel_circ_019021 novel_circ_019022 novel_circ_019023 novel_circ_019024 novel_circ_019025 novel_circ_019026 novel_circ_019027 novel_circ_019028 novel_circ_019029 novel_circ_019033 novel_circ_019034 novel_circ_019035 novel_circ_019036 novel_circ_019037 novel_circ_019038 novel_circ_019039 novel_circ_019040 novel_circ_019041 novel_circ_019042 novel_circ_019043 novel_circ_019044 novel_circ_019054 novel_circ_019055 novel_circ_019056 novel_circ_019057 novel_circ_019058 novel_circ_019060 novel_circ_019061 novel_circ_019062 novel_circ_019063 novel_circ_019064 novel_circ_019065 novel_circ_019066 novel_circ_019067 novel_circ_019068 novel_circ_019069 novel_circ_019070 novel_circ_019071 novel_circ_019072

| hsa_circ_0077291 | 95 | 1 | 588 | 996 | 409 | 0 | 0 |
| --- | --- | --- | --- | --- | --- | --- | --- |
| hsa_circ_0003393 | 97 | 4 | 212 | 209 | 1 | 0 | 9.00E-95 |
| hsa_circ_0077291 | 97 | 4 | 215 | 752 | 541 | 0 | 2.00E-94 |
| hsa_circ_0077291 | 97 | 4 | 347 | 752 | 409 | 0 | 3.00E-161 |
| hsa_circ_0077291 | 96 | 4 | 420 | 752 | 336 | 0 | 0 |
| hsa_circ_0077291 | 95 | 4 | 585 | 752 | 171 | 0 | 0 |
| hsa_circ_0077291 | 95 | 4 | 654 | 752 | 102 | 0 | 0 |
| hsa_circ_0077292 | 94 | 5 | 373 | 369 | 1 | 0 | 2.00E-158 |
| hsa_circ_0077291 | 94 | 5 | 543 | 539 | 1 | 0 | 0 |
| hsa_circ_0011071 | 82 | 1 | 466 | 4909 | 4435 | 0.02 | 2.00E-104 |
| hsa_circ_0011051 | 94 | 1 | 347 | 1 | 347 | 0 | 3.00E-146 |
| hsa_circ_0007283 | 94 | 1 | 467 | 672 | 206 | 0 | 0 |
| hsa_circ_0087220 | 97 | 4723 | 5516 | 794 | 1 | 0 | 0 |
| hsa_circ_0087220 | 97 | 371 | 1164 | 794 | 1 | 0 | 0 |
| hsa_circ_0004189 | 97 | 1 | 360 | 360 | 1 | 0 | 4.00E-170 |
| hsa_circ_0087220 | 98 | 1 | 680 | 794 | 115 | 0 | 0 |
| hsa_circ_0087220 | 97 | 1 | 794 | 794 | 1 | 0 | 0 |
| hsa_circ_0087218 | 97 | 1 | 898 | 898 | 1 | 0 | 0 |
| hsa_circ_0087220 | 96 | 1 | 165 | 598 | 434 | 0 | 4.00E-71 |
| hsa_circ_0087220 | 98 | 1 | 484 | 598 | 115 | 0 | 0 |
| hsa_circ_0087220 | 98 | 1 | 598 | 598 | 1 | 0 | 0 |
| hsa_circ_0087220 | 98 | 4463 | 4897 | 435 | 1 | 0 | 0 |
| hsa_circ_0087220 | 98 | 1 | 434 | 434 | 1 | 0 | 0 |
| hsa_circ_0087220 | 97 | 1 | 293 | 293 | 1 | 0 | 1.00E-139 |
| hsa_circ_0066593 | 86 | 1 | 849 | 9629 | 8778 | 0.02 | 0 |
| hsa_circ_0007569 | 93 | 29 | 255 | 227 | 1 | 0 | 9.00E-88 |
| hsa_circ_0063325 | 94 | 1 | 507 | 507 | 1 | 0 | 0 |
| hsa_circ_0063325 | 93 | 1 | 1041 | 1100 | 63 | 0 | 0 |
| hsa_circ_0063328 | 93 | 7 | 208 | 202 | 1 | 0 | 3.00E-79 |
| hsa_circ_0063328 | 93 | 1 | 342 | 342 | 1 | 0 | 8.00E-142 |
| hsa_circ_0063328 | 93 | 1 | 442 | 442 | 1 | 0 | 0 |
| hsa_circ_0063328 | 93 | 1 | 596 | 593 | 1 | 0.01 | 0 |
| hsa_circ_0063332 | 94 | 1 | 453 | 451 | 2 | 0.01 | 0 |
| hsa_circ_0063333 | 94 | 1 | 388 | 385 | 1 | 0.01 | 3.00E-166 |
| hsa_circ_0001230 | 94 | 1 | 254 | 251 | 1 | 0.01 | 1.00E-103 |
| hsa_circ_0063345 | 91 | 2 | 213 | 2 | 213 | 0 | 2.00E-76 |
| hsa_circ_0004030 | 94 | 7 | 1129 | 7 | 1129 | 0 | 0 |
| hsa_circ_0004030 | 94 | 7 | 2406 | 7 | 2406 | 0 | 0 |
| hsa_circ_0064928 | 94 | 7 | 2409 | 227 | 2629 | 0 | 0 |
| hsa_circ_0064951 | 91 | 1 | 266 | 266 | 1 | 0 | 2.00E-96 |
| hsa_circ_0064951 | 91 | 1 | 372 | 372 | 1 | 0 | 4.00E-140 |
| hsa_circ_0064954 | 88 | 3 | 791 | 783 | 4 | 0.04 | 0 |
| hsa_circ_0064954 | 89 | 1 | 326 | 523 | 198 | 0.01 | 6.00E-113 |
| hsa_circ_0064954 | 91 | 3 | 588 | 783 | 198 | 0 | 0 |
| hsa_circ_0064956 | 91 | 3 | 128 | 491 | 366 | 0 | 5.00E-40 |
| hsa_circ_0064956 | 89 | 1 | 473 | 838 | 366 | 0 | 2.00E-169 |
| hsa_circ_0064956 | 91 | 1 | 557 | 922 | 366 | 0 | 0 |
| hsa_circ_0064956 | 92 | 4 | 665 | 1027 | 366 | 0 | 0 |
| hsa_circ_0064957 | 91 | 1 | 429 | 429 | 1 | 0 | 1.00E-161 |
| hsa_circ_0064957 | 92 | 4 | 537 | 534 | 1 | 0 | 0 |
| hsa_circ_0064957 | 92 | 1 | 433 | 616 | 184 | 0 | 1.00E-170 |
| hsa_circ_0064957 | 94 | 1 | 271 | 616 | 346 | 0 | 4.00E-114 |
| hsa_circ_0064956 | 90 | 10 | 949 | 1778 | 839 | 0 | 0 |
| hsa_circ_0064960 | 88 | 10 | 757 | 856 | 109 | 0 | 0 |

novel_circ_019073 novel_circ_019074 novel_circ_019075 novel_circ_019076 novel_circ_019077 novel_circ_019078 novel_circ_019079 novel_circ_019081 novel_circ_019082 novel_circ_019083 novel_circ_019084 novel_circ_019085 novel_circ_019088 novel_circ_019089 novel_circ_019091 novel_circ_019092 novel_circ_019093 novel_circ_019094 novel_circ_019095 novel_circ_019096 novel_circ_019097 novel_circ_019098 novel_circ_019099 novel_circ_019100 novel_circ_019103 novel_circ_019105 novel_circ_019106 novel_circ_019107 novel_circ_019108 novel_circ_019111 novel_circ_019112 novel_circ_019113 novel_circ_019114 novel_circ_019116 novel_circ_019117 novel_circ_019118 novel_circ_019119 novel_circ_019120 novel_circ_019121 novel_circ_019122 novel_circ_019123 novel_circ_019124 novel_circ_019125 novel_circ_019126 novel_circ_019127 novel_circ_019128 novel_circ_019129 novel_circ_019131 novel_circ_019132 novel_circ_019133 novel_circ_019134 novel_circ_019136 novel_circ_019137 novel_circ_019138

| hsa_circ_0064960 | 88 | 1005 | 1752 | 856 | 109 | 0 | 0 |
| --- | --- | --- | --- | --- | --- | --- | --- |
| hsa_circ_0064962 | 88 | 10 | 388 | 669 | 291 | 0 | 3.00E-127 |
| hsa_circ_0064962 | 90 | 284 | 772 | 1339 | 851 | 0 | 9.00E-180 |
| hsa_circ_0064962 | 94 | 284 | 423 | 1339 | 1200 | 0 | 2.00E-54 |
| hsa_circ_0064972 | 93 | 1 | 537 | 1 | 537 | 0 | 0 |
| hsa_circ_0064972 | 93 | 1 | 750 | 1 | 750 | 0 | 0 |
| hsa_circ_0064973 | 93 | 1 | 420 | 1 | 420 | 0 | 2.00E-173 |
| hsa_circ_0064973 | 92 | 1 | 527 | 421 | 947 | 0 | 0 |
| hsa_circ_0064987 | 94 | 46 | 309 | 1 | 264 | 0 | 4.00E-110 |
| hsa_circ_0064987 | 93 | 46 | 513 | 1 | 468 | 0 | 0 |
| hsa_circ_0064991 | 85 | 1 | 3114 | 157 | 3270 | 0.01 | 0 |
| hsa_circ_0064991 | 85 | 1 | 3110 | 161 | 3270 | 0.01 | 0 |
| hsa_circ_0015773 | 95 | 279 | 481 | 203 | 1 | 0 | 3.00E-84 |
| hsa_circ_0015773 | 95 | 557 | 759 | 203 | 1 | 0 | 4.00E-84 |
| hsa_circ_0015775 | 90 | 1 | 366 | 366 | 1 | 0 | 2.00E-133 |
| hsa_circ_0015781 | 93 | 1 | 330 | 330 | 1 | 0 | 2.00E-133 |
| hsa_circ_0015781 | 93 | 1 | 376 | 376 | 1 | 0 | 1.00E-155 |
| hsa_circ_0015781 | 92 | 35 | 477 | 445 | 1 | 0 | 5.00E-175 |
| hsa_circ_0008785 | 94 | 1 | 274 | 274 | 1 | 0 | 9.00E-116 |
| hsa_circ_0015803 | 95 | 3 | 226 | 3 | 226 | 0 | 9.00E-95 |
| hsa_circ_0015803 | 94 | 3 | 400 | 3 | 400 | 0 | 2.00E-174 |
| hsa_circ_0015803 | 93 | 3 | 617 | 3 | 617 | 0 | 0 |
| hsa_circ_0015803 | 94 | 3 | 826 | 3 | 826 | 0 | 0 |
| hsa_circ_0015805 | 93 | 1 | 532 | 1 | 532 | 0 | 0 |
| hsa_circ_0015832 | 90 | 1 | 792 | 792 | 1 | 0 | 0 |
| hsa_circ_0015843 | 95 | 1 | 260 | 1 | 260 | 0 | 2.00E-111 |
| hsa_circ_0015843 | 95 | 1 | 422 | 1 | 422 | 0 | 0 |
| hsa_circ_0015843 | 94 | 1 | 510 | 1 | 510 | 0 | 0 |
| hsa_circ_0006265 | 94 | 1 | 250 | 1 | 250 | 0 | 9.00E-104 |
| hsa_circ_0015849 | 91 | 1 | 2350 | 1 | 2356 | 0 | 0 |
| hsa_circ_0015850 | 91 | 1 | 2197 | 1 | 2203 | 0 | 0 |
| hsa_circ_0088278 | 81 | 1 | 653 | 659 | 1 | 0.03 | 8.00E-145 |
| hsa_circ_0088278 | 84 | 1 | 1897 | 1903 | 1 | 0.03 | 0 |
| hsa_circ_0088279 | 84 | 7 | 664 | 1513 | 868 | 0.03 | 2.00E-180 |
| hsa_circ_0088279 | 84 | 1 | 784 | 1651 | 868 | 0.04 | 0 |
| hsa_circ_0088279 | 85 | 1 | 960 | 1824 | 868 | 0.03 | 0 |
| hsa_circ_0088278 | 86 | 1 | 1035 | 2477 | 1449 | 0.01 | 0 |
| hsa_circ_0088283 | 86 | 1 | 808 | 807 | 3 | 0 | 0 |
| hsa_circ_0088283 | 86 | 395 | 1575 | 1171 | 3 | 0.01 | 0 |
| hsa_circ_0088283 | 86 | 1049 | 2229 | 1171 | 3 | 0.01 | 0 |
| hsa_circ_0088283 | 84 | 395 | 1338 | 1171 | 240 | 0.01 | 0 |
| hsa_circ_0088283 | 84 | 759 | 1702 | 1171 | 240 | 0.01 | 0 |
| hsa_circ_0088283 | 87 | 4 | 537 | 2225 | 1683 | 0.02 | 9.00E-169 |
| hsa_circ_0088284 | 89 | 9 | 755 | 787 | 38 | 0 | 0 |
| hsa_circ_0088284 | 89 | 9 | 315 | 787 | 481 | 0 | 1.00E-105 |
| hsa_circ_0004883 | 90 | 1 | 928 | 928 | 1 | 0 | 0 |
| hsa_circ_0088285 | 92 | 1 | 257 | 698 | 443 | 0 | 2.00E-97 |
| hsa_circ_0003992 | 96 | 3 | 391 | 389 | 1 | 0 | 0 |
| hsa_circ_0003992 | 96 | 1 | 586 | 586 | 1 | 0 | 0 |
| hsa_circ_0088289 | 99 | 1 | 510 | 705 | 196 | 0 | 0 |
| hsa_circ_0088298 | 90 | 1 | 577 | 574 | 1 | 0.01 | 0 |
| hsa_circ_0030138 | 87 | 1 | 4913 | 401 | 5322 | 0.01 | 0 |
| hsa_circ_0030138 | 87 | 1 | 5096 | 218 | 5322 | 0.01 | 0 |
| hsa_circ_0030138 | 87 | 1 | 5213 | 101 | 5322 | 0.01 | 0 |

novel_circ_019139 novel_circ_019140 novel_circ_019141 novel_circ_019142 novel_circ_019143 novel_circ_019144 novel_circ_019145 novel_circ_019146 novel_circ_019147 novel_circ_019148 novel_circ_019149 novel_circ_019150 novel_circ_019151 novel_circ_019152 novel_circ_019153 novel_circ_019154 novel_circ_019155 novel_circ_019156 novel_circ_019157 novel_circ_019158 novel_circ_019159 novel_circ_019160 novel_circ_019161 novel_circ_019162 novel_circ_019163 novel_circ_019164 novel_circ_019165 novel_circ_019167 novel_circ_019169 novel_circ_019170 novel_circ_019171 novel_circ_019172 novel_circ_019173 novel_circ_019174 novel_circ_019175 novel_circ_019176 novel_circ_019177 novel_circ_019178 novel_circ_019179 novel_circ_019180 novel_circ_019181 novel_circ_019182 novel_circ_019183 novel_circ_019184 novel_circ_019186 novel_circ_019187 novel_circ_019188 novel_circ_019189 novel_circ_019190 novel_circ_019191 novel_circ_019192 novel_circ_019193 novel_circ_019194 novel_circ_019195

| hsa_circ_0030138 | 87 | 67 | 5323 | 57 | 5322 | 0.01 | 0 |
| --- | --- | --- | --- | --- | --- | --- | --- |
| hsa_circ_0030140 | 87 | 1 | 4757 | 1 | 4766 | 0.01 | 0 |
| hsa_circ_0030139 | 93 | 1 | 183 | 1 | 183 | 0 | 2.00E-70 |
| hsa_circ_0030138 | 91 | 1 | 300 | 101 | 400 | 0 | 3.00E-110 |
| hsa_circ_0030138 | 91 | 67 | 410 | 57 | 400 | 0 | 8.00E-128 |
| hsa_circ_0030138 | 93 | 1 | 267 | 101 | 367 | 0 | 3.00E-105 |
| hsa_circ_0030138 | 92 | 67 | 377 | 57 | 367 | 0 | 7.00E-123 |
| hsa_circ_0030132 | 94 | 1 | 559 | 2160 | 2718 | 0 | 0 |
| hsa_circ_0030132 | 93 | 1 | 1661 | 1058 | 2718 | 0 | 0 |
| hsa_circ_0030132 | 93 | 1 | 428 | 2160 | 2587 | 0 | 2.00E-174 |
| hsa_circ_0030135 | 93 | 1 | 844 | 133 | 976 | 0 | 0 |
| hsa_circ_0030135 | 93 | 2 | 976 | 2 | 976 | 0 | 0 |
| hsa_circ_0030132 | 91 | 1 | 1893 | 264 | 2156 | 0 | 0 |
| hsa_circ_0030132 | 91 | 1 | 1732 | 264 | 1995 | 0 | 0 |
| hsa_circ_0030135 | 94 | 2 | 566 | 2 | 566 | 0 | 0 |
| hsa_circ_0030135 | 94 | 2 | 458 | 2 | 458 | 0 | 0 |
| hsa_circ_0030132 | 91 | 1 | 1375 | 264 | 1638 | 0 | 0 |
| hsa_circ_0030132 | 91 | 1 | 1638 | 1 | 1638 | 0 | 0 |
| hsa_circ_0030132 | 92 | 1 | 520 | 264 | 783 | 0 | 0 |
| hsa_circ_0030132 | 93 | 1 | 512 | 1 | 512 | 0 | 0 |
| hsa_circ_0003760 | 97 | 20657 | 20848 | 8369 | 8560 | 0 | 1.00E-86 |
| hsa_circ_0003760 | 97 | 21799 | 21990 | 8369 | 8560 | 0 | 1.00E-86 |
| hsa_circ_0030119 | 93 | 9915 | 10398 | 1262 | 779 | 0 | 0 |
| hsa_circ_0030119 | 93 | 1 | 484 | 1262 | 779 | 0 | 0 |
| hsa_circ_0030120 | 94 | 1 | 406 | 839 | 434 | 0 | 3.00E-172 |
| hsa_circ_0030120 | 91 | 1 | 428 | 429 | 2 | 0 | 2.00E-164 |
| hsa_circ_0030119 | 89 | 1 | 450 | 450 | 1 | 0 | 1.00E-156 |
| hsa_circ_0030112 | 91 | 1 | 983 | 983 | 1 | 0 | 0 |
| hsa_circ_0030112 | 92 | 1 | 638 | 638 | 1 | 0 | 0 |
| hsa_circ_0030102 | 91 | 1 | 1001 | 1001 | 1 | 0 | 0 |
| hsa_circ_0030112 | 92 | 1 | 477 | 477 | 1 | 0 | 0 |
| hsa_circ_0030112 | 92 | 1 | 363 | 363 | 1 | 0 | 2.00E-143 |
| hsa_circ_0030108 | 91 | 1 | 470 | 470 | 1 | 0 | 0 |
| hsa_circ_0030107 | 91 | 1 | 649 | 649 | 1 | 0 | 0 |
| hsa_circ_0030107 | 90 | 7 | 402 | 396 | 1 | 0 | 5.00E-145 |
| hsa_circ_0030095 | 94 | 1 | 767 | 876 | 110 | 0 | 0 |
| hsa_circ_0030095 | 93 | 1 | 723 | 723 | 1 | 0 | 0 |
| hsa_circ_0030093 | 92 | 1 | 1062 | 1474 | 413 | 0 | 0 |
| hsa_circ_0030093 | 90 | 1 | 1037 | 1338 | 302 | 0 | 0 |
| hsa_circ_0030093 | 90 | 1 | 600 | 1012 | 413 | 0 | 0 |
| hsa_circ_0030093 | 89 | 1 | 448 | 860 | 413 | 0 | 6.00E-154 |
| hsa_circ_0030090 | 89 | 1 | 1211 | 1211 | 1 | 0 | 0 |
| hsa_circ_0030088 | 90 | 1 | 1498 | 1495 | 1 | 0 | 0 |
| hsa_circ_0030090 | 92 | 1 | 652 | 652 | 1 | 0 | 0 |
| hsa_circ_0030075 | 90 | 1 | 660 | 660 | 1 | 0 | 0 |
| hsa_circ_0030068 | 92 | 1 | 396 | 961 | 1356 | 0 | 4.00E-160 |
| hsa_circ_0030068 | 94 | 1 | 599 | 758 | 1356 | 0 | 0 |
| hsa_circ_0030069 | 95 | 1 | 875 | 1 | 875 | 0 | 0 |
| hsa_circ_0030068 | 95 | 1 | 960 | 1 | 960 | 0 | 0 |
| hsa_circ_0030069 | 94 | 1 | 768 | 1 | 768 | 0 | 0 |
| hsa_circ_0030068 | 94 | 1 | 853 | 1 | 853 | 0 | 0 |
| hsa_circ_0030069 | 94 | 1 | 672 | 1 | 672 | 0 | 0 |
| hsa_circ_0030068 | 94 | 1 | 757 | 1 | 757 | 0 | 0 |
| hsa_circ_0030069 | 94 | 1 | 398 | 1 | 398 | 0 | 2.00E-174 |

novel_circ_019196 novel_circ_019197 novel_circ_019198 novel_circ_019199 novel_circ_019200 novel_circ_019201 novel_circ_019202 novel_circ_019204 novel_circ_019208 novel_circ_019209 novel_circ_019210 novel_circ_019211 novel_circ_019212 novel_circ_019213 novel_circ_019214 novel_circ_019215 novel_circ_019216 novel_circ_019217 novel_circ_019219 novel_circ_019220 novel_circ_019221 novel_circ_019222 novel_circ_019223 novel_circ_019224 novel_circ_019225 novel_circ_019226 novel_circ_019227 novel_circ_019228 novel_circ_019229 novel_circ_019230 novel_circ_019231 novel_circ_019232 novel_circ_019233 novel_circ_019234 novel_circ_019235 novel_circ_019236 novel_circ_019237 novel_circ_019238 novel_circ_019239 novel_circ_019240 novel_circ_019244 novel_circ_019245 novel_circ_019246 novel_circ_019247 novel_circ_019248 novel_circ_019249 novel_circ_019250 novel_circ_019251 novel_circ_019252 novel_circ_019253 novel_circ_019254 novel_circ_019255 novel_circ_019256 novel_circ_019257

| hsa_circ_0030068 | 94 | 1 | 483 | 1 | 483 | 0 | 0 |
| --- | --- | --- | --- | --- | --- | --- | --- |
| hsa_circ_0030063 | 87 | 1 | 708 | 705 | 1 | 0.01 | 0 |
| hsa_circ_0030059 | 88 | 1 | 999 | 996 | 1 | 0.01 | 0 |
| hsa_circ_0030059 | 90 | 1 | 399 | 399 | 1 | 0 | 3.00E-143 |
| hsa_circ_0030059 | 89 | 1 | 291 | 291 | 1 | 0 | 3.00E-98 |
| hsa_circ_0030059 | 90 | 4 | 118 | 115 | 1 | 0 | 1.00E-35 |
| hsa_circ_0030059 | 90 | 1 | 114 | 114 | 1 | 0 | 5.00E-35 |
| hsa_circ_0030364 | 94 | 2 | 141 | 2 | 141 | 0 | 8.00E-55 |
| hsa_circ_0036002 | 90 | 15780 | 15831 | 45648 | 45699 | 0 | 1.00E-08 |
| hsa_circ_0085667 | 88 | 1 | 429 | 3473 | 3045 | 0 | 2.00E-143 |
| hsa_circ_0007209 | 86 | 1 | 1785 | 2027 | 234 | 0.01 | 0 |
| hsa_circ_0007209 | 87 | 1 | 2013 | 2027 | 6 | 0.01 | 0 |
| hsa_circ_0085671 | 88 | 1 | 2104 | 2528 | 416 | 0.01 | 0 |
| hsa_circ_0085671 | 88 | 1 | 2428 | 2528 | 92 | 0.01 | 0 |
| hsa_circ_0085671 | 93 | 1 | 501 | 501 | 1 | 0 | 0 |
| hsa_circ_0085669 | 93 | 1 | 640 | 640 | 1 | 0 | 0 |
| hsa_circ_0085667 | 93 | 1 | 759 | 1017 | 259 | 0 | 0 |
| hsa_circ_0085667 | 92 | 1 | 1017 | 1017 | 1 | 0 | 0 |
| hsa_circ_0013828 | 73 | 54419 | 56817 | 24098 | 26496 | 0.04 | 0 |
| hsa_circ_0085636 | 92 | 1 | 2786 | 293 | 3073 | 0 | 0 |
| hsa_circ_0085636 | 94 | 1 | 1877 | 833 | 2709 | 0 | 0 |
| hsa_circ_0085636 | 93 | 1 | 1244 | 1173 | 2416 | 0 | 0 |
| hsa_circ_0085636 | 94 | 10 | 1674 | 752 | 2416 | 0 | 0 |
| hsa_circ_0085636 | 88 | 1 | 537 | 1698 | 2234 | 0 | 0 |
| hsa_circ_0085636 | 94 | 1 | 1072 | 833 | 1904 | 0 | 0 |
| hsa_circ_0085636 | 95 | 1 | 1239 | 666 | 1904 | 0 | 0 |
| hsa_circ_0085636 | 95 | 1 | 1496 | 409 | 1904 | 0 | 0 |
| hsa_circ_0085636 | 95 | 1 | 1611 | 293 | 1904 | 0 | 0 |
| hsa_circ_0085636 | 97 | 1 | 1404 | 293 | 1697 | 0 | 0 |
| hsa_circ_0085636 | 97 | 1 | 1408 | 289 | 1697 | 0 | 0 |
| hsa_circ_0085636 | 97 | 1 | 1219 | 289 | 1508 | 0 | 0 |
| hsa_circ_0085639 | 96 | 1 | 423 | 1 | 423 | 0 | 0 |
| hsa_circ_0085636 | 96 | 1 | 962 | 293 | 1255 | 0 | 0 |
| hsa_circ_0085636 | 96 | 1 | 966 | 289 | 1255 | 0 | 0 |
| hsa_circ_0085636 | 96 | 1 | 879 | 293 | 1172 | 0 | 0 |
| hsa_circ_0085636 | 96 | 1 | 883 | 289 | 1172 | 0 | 0 |
| hsa_circ_0085636 | 96 | 1 | 757 | 289 | 1046 | 0 | 0 |
| hsa_circ_0085636 | 96 | 1 | 461 | 293 | 754 | 0 | 0 |
| hsa_circ_0085636 | 96 | 1 | 465 | 289 | 754 | 0 | 0 |
| hsa_circ_0085636 | 96 | 1 | 376 | 289 | 665 | 0 | 2.00E-172 |
| hsa_circ_0085631 | 93 | 1 | 295 | 2056 | 2350 | 0 | 9.00E-121 |
| hsa_circ_0085631 | 91 | 3 | 1369 | 984 | 2350 | 0 | 0 |
| hsa_circ_0085635 | 93 | 1 | 411 | 1 | 411 | 0 | 2.00E-168 |
| hsa_circ_0085634 | 93 | 1 | 578 | 305 | 882 | 0 | 0 |
| hsa_circ_0085634 | 90 | 3 | 746 | 139 | 882 | 0 | 0 |
| hsa_circ_0085634 | 89 | 3 | 584 | 139 | 720 | 0 | 0 |
| hsa_circ_0085634 | 91 | 1 | 720 | 1 | 720 | 0 | 0 |
| hsa_circ_0007931 | 95 | 1 | 410 | 1 | 410 | 0 | 0 |
| hsa_circ_0085631 | 95 | 1 | 561 | 206 | 766 | 0 | 0 |
| hsa_circ_0085632 | 94 | 1 | 279 | 1 | 279 | 0 | 3.00E-115 |
| hsa_circ_0087796 | 96 | 1 | 200 | 2350 | 2549 | 0 | 5.00E-86 |
| hsa_circ_0081028 | 85 | 4 | 963 | 963 | 1 | 0 | 0 |
| hsa_circ_0081027 | 86 | 4 | 1311 | 1624 | 314 | 0 | 0 |
| hsa_circ_0081026 | 86 | 85 | 2134 | 2226 | 174 | 0 | 0 |

novel_circ_019258 novel_circ_019259 novel_circ_019260 novel_circ_019261 novel_circ_019262 novel_circ_019263 novel_circ_019264 novel_circ_019266 novel_circ_019267 novel_circ_019268 novel_circ_019269 novel_circ_019271 novel_circ_019272 novel_circ_019273 novel_circ_019274 novel_circ_019275 novel_circ_019276 novel_circ_019277 novel_circ_019278 novel_circ_019279 novel_circ_019280 novel_circ_019281 novel_circ_019282 novel_circ_019283 novel_circ_019284 novel_circ_019285 novel_circ_019286 novel_circ_019287 novel_circ_019288 novel_circ_019289 novel_circ_019290 novel_circ_019291 novel_circ_019292 novel_circ_019293 novel_circ_019294 novel_circ_019295 novel_circ_019296 novel_circ_019297 novel_circ_019298 novel_circ_019300 novel_circ_019303 novel_circ_019304 novel_circ_019305 novel_circ_019306 novel_circ_019307 novel_circ_019309 novel_circ_019310 novel_circ_019311 novel_circ_019312 novel_circ_019313 novel_circ_019314 novel_circ_019315 novel_circ_019316 novel_circ_019317

| hsa_circ_0081026 | 87 | 1 | 867 | 1224 | 358 | 0 | 0 |
| --- | --- | --- | --- | --- | --- | --- | --- |
| hsa_circ_0081026 | 86 | 1 | 401 | 913 | 513 | 0 | 8.00E-118 |
| hsa_circ_0081026 | 87 | 1 | 556 | 913 | 358 | 0 | 8.00E-174 |
| hsa_circ_0081026 | 86 | 1 | 740 | 913 | 174 | 0 | 0 |
| hsa_circ_0081025 | 88 | 1 | 1113 | 1966 | 854 | 0 | 0 |
| hsa_circ_0081025 | 90 | 1 | 1537 | 1966 | 430 | 0 | 0 |
| hsa_circ_0081025 | 90 | 1 | 1764 | 1966 | 203 | 0 | 0 |
| hsa_circ_0081025 | 91 | 4 | 981 | 1407 | 430 | 0 | 0 |
| hsa_circ_0081025 | 94 | 1 | 716 | 918 | 203 | 0 | 0 |
| hsa_circ_0081025 | 94 | 1 | 651 | 853 | 203 | 0 | 0 |
| hsa_circ_0081024 | 94 | 1 | 329 | 329 | 1 | 0 | 6.00E-138 |
| hsa_circ_0081017 | 94 | 1 | 1198 | 90 | 1287 | 0 | 0 |
| hsa_circ_0081017 | 94 | 1 | 821 | 90 | 910 | 0 | 0 |
| hsa_circ_0081017 | 93 | 1 | 532 | 90 | 621 | 0 | 0 |
| hsa_circ_0081013 | 94 | 1 | 1131 | 577 | 1707 | 0 | 0 |
| hsa_circ_0081013 | 94 | 322 | 1755 | 277 | 1707 | 0 | 0 |
| hsa_circ_0081013 | 94 | 377 | 1810 | 277 | 1707 | 0 | 0 |
| hsa_circ_0081017 | 92 | 1 | 399 | 90 | 488 | 0 | 9.00E-162 |
| hsa_circ_0007187 | 94 | 3 | 697 | 121 | 815 | 0 | 0 |
| hsa_circ_0081013 | 94 | 1 | 998 | 577 | 1574 | 0 | 0 |
| hsa_circ_0081013 | 94 | 1 | 1299 | 279 | 1574 | 0 | 0 |
| hsa_circ_0081013 | 94 | 377 | 1677 | 277 | 1574 | 0 | 0 |
| hsa_circ_0081017 | 93 | 1 | 401 | 1 | 401 | 0 | 2.00E-164 |
| hsa_circ_0009112 | 94 | 3 | 610 | 3 | 610 | 0 | 0 |
| hsa_circ_0081013 | 94 | 1 | 911 | 577 | 1487 | 0 | 0 |
| hsa_circ_0081013 | 94 | 1 | 1212 | 279 | 1487 | 0 | 0 |
| hsa_circ_0081013 | 94 | 377 | 1590 | 277 | 1487 | 0 | 0 |
| hsa_circ_0009112 | 95 | 3 | 443 | 3 | 443 | 0 | 0 |
| hsa_circ_0081013 | 94 | 377 | 1423 | 277 | 1320 | 0 | 0 |
| hsa_circ_0081013 | 95 | 1 | 510 | 577 | 1086 | 0 | 0 |
| hsa_circ_0081013 | 95 | 377 | 1189 | 277 | 1086 | 0 | 0 |
| hsa_circ_0081014 | 95 | 1 | 301 | 299 | 599 | 0 | 9.00E-131 |
| hsa_circ_0081014 | 95 | 1 | 602 | 1 | 599 | 0 | 0 |
| hsa_circ_0081013 | 95 | 322 | 925 | 277 | 877 | 0 | 0 |
| hsa_circ_0081013 | 95 | 377 | 980 | 277 | 877 | 0 | 0 |
| hsa_circ_0081013 | 94 | 377 | 862 | 277 | 759 | 0.01 | 0 |
| hsa_circ_0081014 | 94 | 1 | 301 | 1 | 298 | 0.01 | 6.00E-128 |
| hsa_circ_0081013 | 94 | 377 | 679 | 277 | 576 | 0.01 | 1.00E-128 |
| hsa_circ_0081013 | 93 | 3 | 275 | 3 | 278 | 0.01 | 2.00E-112 |
| hsa_circ_0081006 | 93 | 992 | 2027 | 1036 | 1 | 0 | 0 |
| hsa_circ_0081006 | 96 | 888 | 1035 | 1036 | 889 | 0 | 8.00E-62 |
| hsa_circ_0081006 | 93 | 888 | 1151 | 1036 | 773 | 0 | 1.00E-104 |
| hsa_circ_0081006 | 92 | 888 | 1461 | 1036 | 463 | 0 | 0 |
| hsa_circ_0081006 | 92 | 888 | 1628 | 1036 | 296 | 0 | 0 |
| hsa_circ_0081006 | 92 | 888 | 1716 | 1036 | 208 | 0 | 0 |
| hsa_circ_0081006 | 92 | 505 | 1078 | 1036 | 463 | 0 | 0 |
| hsa_circ_0081006 | 92 | 505 | 1245 | 1036 | 296 | 0 | 0 |
| hsa_circ_0081006 | 92 | 145 | 885 | 1036 | 296 | 0 | 0 |
| hsa_circ_0081006 | 92 | 1 | 741 | 1036 | 296 | 0 | 0 |
| hsa_circ_0081006 | 93 | 1 | 771 | 771 | 1 | 0 | 0 |
| hsa_circ_0081006 | 92 | 1 | 255 | 462 | 208 | 0 | 1.00E-98 |
| hsa_circ_0081006 | 94 | 1 | 462 | 462 | 1 | 0 | 0 |
| hsa_circ_0081002 | 93 | 6 | 273 | 573 | 306 | 0.01 | 2.00E-107 |
| hsa_circ_0081002 | 92 | 6 | 403 | 573 | 176 | 0.01 | 7.00E-158 |

novel_circ_019318 novel_circ_019319 novel_circ_019320 novel_circ_019321 novel_circ_019322 novel_circ_019323 novel_circ_019324 novel_circ_019325 novel_circ_019328 novel_circ_019329 novel_circ_019330 novel_circ_019331 novel_circ_019332 novel_circ_019333 novel_circ_019334 novel_circ_019337 novel_circ_019339 novel_circ_019342 novel_circ_019343 novel_circ_019345 novel_circ_019346 novel_circ_019347 novel_circ_019348 novel_circ_019349 novel_circ_019352 novel_circ_019353 novel_circ_019359 novel_circ_019375 novel_circ_019376 novel_circ_019377 novel_circ_019378 novel_circ_019383 novel_circ_019384 novel_circ_019385 novel_circ_019386 novel_circ_019387 novel_circ_019388 novel_circ_019389 novel_circ_019390 novel_circ_019391 novel_circ_019393 novel_circ_019394 novel_circ_019395 novel_circ_019396 novel_circ_019397 novel_circ_019405 novel_circ_019406 novel_circ_019407 novel_circ_019409 novel_circ_019410 novel_circ_019411 novel_circ_019412 novel_circ_019414 novel_circ_019415

| hsa_circ_0081002 | 92 | 6 | 578 | 573 | 1 | 0 | 0 |
| --- | --- | --- | --- | --- | --- | --- | --- |
| hsa_circ_0007299 | 94 | 1 | 265 | 265 | 1 | 0 | 2.00E-112 |
| hsa_circ_0080991 | 91 | 4 | 970 | 10669 | 11638 | 0 | 0 |
| hsa_circ_0080998 | 91 | 4 | 611 | 1359 | 1969 | 0 | 0 |
| hsa_circ_0080997 | 95 | 1 | 353 | 1640 | 1992 | 0 | 3.00E-156 |
| hsa_circ_0080997 | 93 | 3 | 233 | 1033 | 1263 | 0 | 3.00E-91 |
| hsa_circ_0080997 | 91 | 1 | 1036 | 1 | 1030 | 0.01 | 0 |
| hsa_circ_0080992 | 84 | 1 | 905 | 220 | 1139 | 0.03 | 0 |
| hsa_circ_0039747 | 94 | 4 | 400 | 397 | 1 | 0 | 6.00E-169 |
| hsa_circ_0039747 | 93 | 1 | 631 | 631 | 1 | 0 | 0 |
| hsa_circ_0039748 | 94 | 1 | 401 | 495 | 95 | 0 | 7.00E-173 |
| hsa_circ_0039752 | 96 | 1 | 819 | 1225 | 407 | 0 | 0 |
| hsa_circ_0039760 | 98 | 3 | 286 | 284 | 1 | 0 | 2.00E-136 |
| hsa_circ_0039760 | 96 | 1 | 127 | 413 | 287 | 0 | 8.00E-53 |
| hsa_circ_0000707 | 99 | 1 | 234 | 1 | 234 | 0 | 3.00E-115 |
| hsa_circ_0008422 | 86 | 1 | 404 | 555779 | 556176 | 0.03 | 2.00E-115 |
| hsa_circ_0081610 | 93 | 1 | 406 | 1 | 406 | 0 | 1.00E-165 |
| hsa_circ_0081479 | 90 | 4 | 376 | 375 | 3 | 0.01 | 3.00E-137 |
| hsa_circ_0081475 | 89 | 1 | 438 | 1 | 438 | 0 | 1.00E-151 |
| hsa_circ_0080943 | 85 | 1 | 1262 | 1645 | 381 | 0 | 0 |
| hsa_circ_0080942 | 88 | 1101 | 2899 | 1799 | 1 | 0 | 0 |
| hsa_circ_0080942 | 89 | 4 | 1407 | 1404 | 1 | 0.01 | 0 |
| hsa_circ_0080942 | 89 | 4 | 1407 | 1404 | 1 | 0.01 | 0 |
| hsa_circ_0080940 | 93 | 1 | 2010 | 2015 | 6 | 0 | 0 |
| hsa_circ_0091531 | 97 | 12464 | 12501 | 90646 | 90609 | 0 | 7.00E-08 |
| hsa_circ_0091531 | 97 | 10276 | 10313 | 90646 | 90609 | 0 | 6.00E-08 |
| hsa_circ_0091531 | 97 | 10139 | 10176 | 90646 | 90609 | 0 | 6.00E-08 |
| hsa_circ_0003159 | 95 | 1 | 353 | 353 | 1 | 0 | 6.00E-158 |
| hsa_circ_0003159 | 96 | 1 | 221 | 221 | 1 | 0 | 4.00E-98 |
| hsa_circ_0080937 | 94 | 1 | 364 | 364 | 1 | 0 | 1.00E-155 |
| hsa_circ_0080933 | 95 | 1 | 811 | 1094 | 284 | 0 | 0 |
| hsa_circ_0078489 | 88 | 1 | 483 | 1 | 483 | 0 | 1.00E-161 |
| hsa_circ_0078472 | 92 | 1 | 483 | 1 | 483 | 0 | 0 |
| hsa_circ_0078465 | 92 | 1 | 645 | 1 | 645 | 0 | 0 |
| hsa_circ_0078462 | 92 | 1 | 774 | 1 | 774 | 0 | 0 |
| hsa_circ_0078462 | 92 | 1 | 604 | 1 | 604 | 0 | 0 |
| hsa_circ_0078462 | 92 | 1 | 291 | 1 | 291 | 0 | 2.00E-113 |
| hsa_circ_0078457 | 84 | 606 | 2362 | 1 | 1738 | 0.04 | 0 |
| hsa_circ_0078457 | 84 | 606 | 2233 | 1 | 1609 | 0.05 | 0 |
| hsa_circ_0078457 | 84 | 55172 | 56799 | 1 | 1609 | 0.05 | 0 |
| hsa_circ_0078456 | 91 | 1 | 354 | 356 | 3 | 0 | 9.00E-132 |
| hsa_circ_0078455 | 86 | 130 | 739 | 610 | 1 | 0.01 | 0 |
| hsa_circ_0078455 | 86 | 1 | 610 | 610 | 1 | 0.01 | 0 |
| hsa_circ_0078455 | 86 | 1 | 610 | 610 | 1 | 0.01 | 0 |
| hsa_circ_0078455 | 85 | 1 | 473 | 474 | 1 | 0.01 | 4.00E-134 |
| hsa_circ_0078438 | 92 | 3 | 334 | 778 | 1109 | 0 | 1.00E-129 |
| hsa_circ_0003004 | 88 | 1 | 646 | 1 | 649 | 0.01 | 0 |
| hsa_circ_0078437 | 89 | 1 | 934 | 1 | 937 | 0.01 | 0 |
| hsa_circ_0078427 | 93 | 416 | 830 | 111 | 525 | 0 | 1.00E-168 |
| hsa_circ_0026804 | 83 | 10219 | 10660 | 237 | 682 | 0.03 | 1.00E-106 |
| hsa_circ_0078415 | 94 | 3 | 270 | 3 | 270 | 0 | 2.00E-112 |
| hsa_circ_0078413 | 91 | 1 | 514 | 1 | 514 | 0 | 0 |
| hsa_circ_0078400 | 92 | 1 | 264 | 1 | 261 | 0.01 | 2.00E-102 |
| hsa_circ_0078397 | 91 | 1 | 195 | 1 | 195 | 0 | 2.00E-68 |

novel_circ_019416 novel_circ_019417 novel_circ_019418 novel_circ_019419 novel_circ_019420 novel_circ_019422 novel_circ_019423 novel_circ_019424 novel_circ_019425 novel_circ_019426 novel_circ_019427 novel_circ_019428 novel_circ_019429 novel_circ_019430 novel_circ_019431 novel_circ_019432 novel_circ_019433 novel_circ_019434 novel_circ_019435 novel_circ_019436 novel_circ_019437 novel_circ_019438 novel_circ_019439 novel_circ_019440 novel_circ_019444 novel_circ_019445 novel_circ_019446 novel_circ_019447 novel_circ_019448 novel_circ_019449 novel_circ_019450 novel_circ_019451 novel_circ_019452 novel_circ_019453 novel_circ_019454 novel_circ_019456 novel_circ_019457 novel_circ_019458 novel_circ_019459 novel_circ_019460 novel_circ_019461 novel_circ_019462 novel_circ_019463 novel_circ_019464 novel_circ_019465 novel_circ_019470 novel_circ_019472 novel_circ_019473 novel_circ_019474 novel_circ_019475 novel_circ_019476 novel_circ_019477 novel_circ_019478 novel_circ_019479

| hsa_circ_0078397 | 91 | 1 | 195 | 1 | 195 | 0 | 1.00E-68 |
| --- | --- | --- | --- | --- | --- | --- | --- |
| hsa_circ_0032962 | 96 | 1 | 1479 | 1479 | 1 | 0 | 0 |
| hsa_circ_0032969 | 93 | 485 | 1035 | 554 | 4 | 0 | 0 |
| hsa_circ_0032970 | 94 | 127 | 484 | 358 | 1 | 0 | 2.00E-150 |
| hsa_circ_0032979 | 91 | 6 | 414 | 409 | 1 | 0 | 4.00E-157 |
| hsa_circ_0032979 | 91 | 1 | 499 | 1017 | 519 | 0 | 0 |
| hsa_circ_0032979 | 88 | 1 | 3855 | 4388 | 519 | 0 | 0 |
| hsa_circ_0032981 | 88 | 1 | 3356 | 3512 | 142 | 0.01 | 0 |
| hsa_circ_0032982 | 89 | 1 | 982 | 985 | 1 | 0.01 | 0 |
| hsa_circ_0032982 | 89 | 1 | 982 | 985 | 1 | 0.01 | 0 |
| hsa_circ_0032982 | 89 | 1 | 1044 | 1047 | 1 | 0.01 | 0 |
| hsa_circ_0032982 | 89 | 1 | 348 | 874 | 530 | 0.01 | 2.00E-117 |
| hsa_circ_0032986 | 93 | 1 | 377 | 547 | 171 | 0 | 4.00E-156 |
| hsa_circ_0032986 | 94 | 1 | 400 | 570 | 171 | 0 | 7.00E-169 |
| hsa_circ_0032986 | 94 | 449 | 848 | 570 | 171 | 0 | 1.00E-168 |
| hsa_circ_0032986 | 94 | 649 | 1048 | 570 | 171 | 0 | 2.00E-168 |
| hsa_circ_0032986 | 94 | 449 | 845 | 570 | 174 | 0 | 1.00E-168 |
| hsa_circ_0032986 | 94 | 1 | 397 | 570 | 174 | 0 | 6.00E-169 |
| hsa_circ_0032986 | 94 | 449 | 845 | 570 | 174 | 0 | 1.00E-168 |
| hsa_circ_0032986 | 94 | 649 | 1045 | 570 | 174 | 0 | 2.00E-168 |
| hsa_circ_0007959 | 95 | 1 | 360 | 363 | 1 | 0.01 | 6.00E-159 |
| hsa_circ_0032997 | 92 | 1 | 1050 | 237 | 1286 | 0 | 0 |
| hsa_circ_0033000 | 92 | 1 | 880 | 1 | 880 | 0 | 0 |
| hsa_circ_0033004 | 84 | 3 | 409 | 408 | 2 | 0 | 8.00E-108 |
| hsa_circ_0085302 | 80 | 21092 | 21263 | 3849 | 3678 | 0.01 | 1.00E-26 |
| hsa_circ_0016436 | 97 | 1 | 1900 | 1 | 1900 | 0 | 0 |
| hsa_circ_0016436 | 97 | 1 | 1900 | 1 | 1900 | 0 | 0 |
| hsa_circ_0016454 | 91 | 1 | 318 | 318 | 1 | 0 | 2.00E-118 |
| hsa_circ_0016454 | 93 | 1 | 325 | 643 | 319 | 0 | 1.00E-130 |
| hsa_circ_0016459 | 98 | 1 | 429 | 1 | 429 | 0 | 0 |
| hsa_circ_0007131 | 94 | 119 | 466 | 1 | 348 | 0 | 1.00E-146 |
| hsa_circ_0007131 | 94 | 119 | 606 | 1 | 488 | 0 | 0 |
| hsa_circ_0007131 | 94 | 119 | 606 | 1 | 488 | 0 | 0 |
| hsa_circ_0007131 | 94 | 1 | 348 | 1 | 348 | 0 | 8.00E-147 |
| hsa_circ_0007131 | 94 | 1 | 488 | 1 | 488 | 0 | 0 |
| hsa_circ_0016461 | 93 | 1 | 446 | 1 | 443 | 0.01 | 0 |
| hsa_circ_0016461 | 93 | 1 | 446 | 1 | 443 | 0.01 | 0 |
| hsa_circ_0016461 | 92 | 1 | 637 | 1 | 634 | 0 | 0 |
| hsa_circ_0016460 | 93 | 1 | 753 | 478 | 1227 | 0 | 0 |
| hsa_circ_0016462 | 93 | 1 | 563 | 1 | 560 | 0.01 | 0 |
| hsa_circ_0016465 | 93 | 1 | 307 | 1 | 307 | 0 | 2.00E-127 |
| hsa_circ_0006536 | 94 | 1 | 648 | 1 | 648 | 0 | 0 |
| hsa_circ_0006536 | 94 | 1 | 745 | 1 | 745 | 0 | 0 |
| hsa_circ_0016468 | 92 | 159 | 577 | 3 | 421 | 0 | 2.00E-164 |
| hsa_circ_0016468 | 91 | 1 | 324 | 98 | 421 | 0 | 8.00E-122 |
| hsa_circ_0043379 | 94 | 1 | 648 | 648 | 1 | 0 | 0 |
| hsa_circ_0043406 | 97 | 1 | 507 | 507 | 1 | 0 | 0 |
| hsa_circ_0043406 | 97 | 1 | 709 | 709 | 1 | 0 | 0 |
| hsa_circ_0043406 | 97 | 1 | 805 | 805 | 1 | 0 | 0 |
| hsa_circ_0043408 | 97 | 1 | 192 | 237 | 46 | 0 | 1.00E-88 |
| hsa_circ_0043408 | 97 | 1 | 394 | 439 | 46 | 0 | 0 |
| hsa_circ_0043408 | 97 | 1 | 490 | 535 | 46 | 0 | 0 |
| hsa_circ_0043410 | 95 | 4 | 335 | 534 | 203 | 0 | 6.00E-148 |
| hsa_circ_0043410 | 94 | 4 | 239 | 534 | 299 | 0 | 1.00E-99 |

novel_circ_019480 novel_circ_019481 novel_circ_019482 novel_circ_019483 novel_circ_019484 novel_circ_019485 novel_circ_019486 novel_circ_019487 novel_circ_019488 novel_circ_019489 novel_circ_019490 novel_circ_019491 novel_circ_019492 novel_circ_019493 novel_circ_019494 novel_circ_019498 novel_circ_019500 novel_circ_019501 novel_circ_019502 novel_circ_019503 novel_circ_019506 novel_circ_019507 novel_circ_019508 novel_circ_019509 novel_circ_019510 novel_circ_019511 novel_circ_019512 novel_circ_019513 novel_circ_019514 novel_circ_019515 novel_circ_019516 novel_circ_019517 novel_circ_019518 novel_circ_019519 novel_circ_019520 novel_circ_019521 novel_circ_019522 novel_circ_019523 novel_circ_019524 novel_circ_019525 novel_circ_019526 novel_circ_019527 novel_circ_019528 novel_circ_019529 novel_circ_019530 novel_circ_019531 novel_circ_019532 novel_circ_019533 novel_circ_019534 novel_circ_019535 novel_circ_019536 novel_circ_019537 novel_circ_019538 novel_circ_019539

| hsa_circ_0000761 | 97 | 4 | 170 | 167 | 1 | 0 | 7.00E-75 |
| --- | --- | --- | --- | --- | --- | --- | --- |
| hsa_circ_0043425 | 94 | 4 | 558 | 555 | 1 | 0 | 0 |
| hsa_circ_0043425 | 94 | 4360 | 4918 | 559 | 1 | 0 | 0 |
| hsa_circ_0043425 | 94 | 1 | 722 | 722 | 1 | 0 | 0 |
| hsa_circ_0043425 | 94 | 1 | 869 | 869 | 1 | 0 | 0 |
| hsa_circ_0043429 | 94 | 4 | 356 | 353 | 1 | 0 | 7.00E-153 |
| hsa_circ_0043429 | 94 | 3691 | 4047 | 357 | 1 | 0 | 2.00E-152 |
| hsa_circ_0043429 | 93 | 1 | 446 | 446 | 1 | 0 | 0 |
| hsa_circ_0043449 | 95 | 886 | 1373 | 1 | 488 | 0 | 0 |
| hsa_circ_0043449 | 95 | 886 | 1563 | 1 | 678 | 0 | 0 |
| hsa_circ_0043449 | 94 | 883 | 1199 | 1 | 317 | 0 | 2.00E-132 |
| hsa_circ_0043449 | 94 | 1 | 317 | 1 | 317 | 0 | 6.00E-133 |
| hsa_circ_0043449 | 95 | 1 | 488 | 1 | 488 | 0 | 0 |
| hsa_circ_0043449 | 95 | 1 | 678 | 1 | 678 | 0 | 0 |
| hsa_circ_0043450 | 96 | 1 | 349 | 312 | 660 | 0 | 5.00E-159 |
| hsa_circ_0006643 | 87 | 17857 | 17932 | 711826 | 711751 | 0 | 8.00E-14 |
| hsa_circ_0043500 | 99 | 1 | 383 | 321 | 703 | 0 | 0 |
| hsa_circ_0043521 | 95 | 1 | 265 | 1 | 265 | 0.01 | 6.00E-114 |
| hsa_circ_0043521 | 95 | 1 | 265 | 1 | 265 | 0.01 | 2.00E-113 |
| hsa_circ_0043521 | 95 | 1 | 265 | 1 | 265 | 0.01 | 2.00E-113 |
| hsa_circ_0043694 | 90 | 1 | 364 | 364 | 1 | 0 | 2.00E-132 |
| hsa_circ_0043748 | 95 | 1 | 273 | 530 | 258 | 0 | 3.00E-120 |
| hsa_circ_0043746 | 95 | 126 | 587 | 1093 | 632 | 0 | 0 |
| hsa_circ_0043749 | 94 | 1 | 314 | 314 | 1 | 0 | 6.00E-133 |
| hsa_circ_0043781 | 93 | 1 | 604 | 604 | 1 | 0 | 0 |
| hsa_circ_0043787 | 95 | 2 | 295 | 384 | 91 | 0 | 7.00E-127 |
| hsa_circ_0043816 | 93 | 1 | 395 | 395 | 1 | 0 | 7.00E-163 |
| hsa_circ_0043825 | 94 | 1 | 541 | 80 | 620 | 0 | 0 |
| hsa_circ_0043825 | 94 | 1 | 635 | 80 | 714 | 0 | 0 |
| hsa_circ_0043827 | 94 | 1 | 387 | 22 | 408 | 0 | 9.00E-167 |
| hsa_circ_0043829 | 95 | 1 | 177 | 1 | 177 | 0 | 9.00E-74 |
| hsa_circ_0043829 | 95 | 1 | 541 | 1 | 541 | 0 | 0 |
| hsa_circ_0043827 | 94 | 1 | 839 | 232 | 1067 | 0 | 0 |
| hsa_circ_0043827 | 93 | 1 | 929 | 232 | 1157 | 0 | 0 |
| hsa_circ_0043827 | 93 | 1 | 1049 | 232 | 1277 | 0 | 0 |
| hsa_circ_0043827 | 93 | 1 | 1266 | 232 | 1494 | 0 | 0 |
| hsa_circ_0043827 | 93 | 1 | 1374 | 232 | 1602 | 0 | 0 |
| hsa_circ_0043830 | 94 | 1 | 756 | 1 | 753 | 0 | 0 |
| hsa_circ_0043832 | 93 | 1 | 752 | 1 | 749 | 0 | 0 |
| hsa_circ_0043838 | 93 | 1 | 336 | 1 | 336 | 0 | 4.00E-135 |
| hsa_circ_0043852 | 95 | 1 | 704 | 1936 | 1233 | 0 | 0 |
| hsa_circ_0043854 | 95 | 1 | 571 | 571 | 1 | 0 | 0 |
| hsa_circ_0043855 | 95 | 1 | 350 | 488 | 139 | 0 | 6.00E-158 |
| hsa_circ_0043858 | 94 | 1 | 683 | 114 | 796 | 0 | 0 |
| hsa_circ_0043874 | 91 | 186 | 913 | 1 | 728 | 0 | 0 |
| hsa_circ_0043877 | 92 | 1 | 322 | 1 | 322 | 0 | 1.00E-125 |
| hsa_circ_0043877 | 92 | 1 | 272 | 51 | 322 | 0 | 5.00E-103 |
| hsa_circ_0043892 | 95 | 5 | 1087 | 1977 | 895 | 0 | 0 |
| hsa_circ_0043892 | 96 | 1 | 359 | 1434 | 1076 | 0 | 1.00E-164 |
| hsa_circ_0043892 | 95 | 1 | 536 | 1611 | 1076 | 0 | 0 |
| hsa_circ_0043892 | 95 | 5 | 906 | 1977 | 1076 | 0 | 0 |
| hsa_circ_0043892 | 95 | 3 | 1034 | 2107 | 1076 | 0 | 0 |
| hsa_circ_0043892 | 94 | 5 | 547 | 1977 | 1435 | 0 | 0 |
| hsa_circ_0043897 | 95 | 5 | 370 | 366 | 1 | 0 | 4.00E-165 |

novel_circ_019540 novel_circ_019541 novel_circ_019542 novel_circ_019543 novel_circ_019544 novel_circ_019545 novel_circ_019546 novel_circ_019547 novel_circ_019548 novel_circ_019555 novel_circ_019556 novel_circ_019560 novel_circ_019561 novel_circ_019563 novel_circ_019564 novel_circ_019565 novel_circ_019566 novel_circ_019567 novel_circ_019568 novel_circ_019569 novel_circ_019570 novel_circ_019571 novel_circ_019572 novel_circ_019573 novel_circ_019574 novel_circ_019575 novel_circ_019576 novel_circ_019577 novel_circ_019578 novel_circ_019579 novel_circ_019580 novel_circ_019581 novel_circ_019582 novel_circ_019583 novel_circ_019584 novel_circ_019585 novel_circ_019586 novel_circ_019587 novel_circ_019588 novel_circ_019589 novel_circ_019590 novel_circ_019591 novel_circ_019593 novel_circ_019594 novel_circ_019595 novel_circ_019596 novel_circ_019597 novel_circ_019598 novel_circ_019599 novel_circ_019600 novel_circ_019601 novel_circ_019603 novel_circ_019604 novel_circ_019605

| hsa_circ_0043897 | 95 | 3 | 498 | 496 | 1 | 0 | 0 |
| --- | --- | --- | --- | --- | --- | --- | --- |
| hsa_circ_0043897 | 95 | 3 | 377 | 496 | 122 | 0 | 4.00E-165 |
| hsa_circ_0043897 | 93 | 57 | 466 | 531 | 122 | 0.01 | 2.00E-169 |
| hsa_circ_0043898 | 92 | 57 | 346 | 290 | 1 | 0.02 | 7.00E-113 |
| hsa_circ_0043920 | 91 | 1 | 418 | 418 | 1 | 0 | 1.00E-160 |
| hsa_circ_0043920 | 91 | 1 | 578 | 578 | 1 | 0 | 0 |
| hsa_circ_0043924 | 94 | 1 | 358 | 511 | 868 | 0 | 1.00E-155 |
| hsa_circ_0043973 | 92 | 77 | 674 | 104 | 701 | 0 | 0 |
| hsa_circ_0027961 | 84 | 256 | 1143 | 1128 | 245 | 0.04 | 0 |
| hsa_circ_0005234 | 80 | 7184 | 7305 | 230425 | 230303 | 0.01 | 9.00E-15 |
| hsa_circ_0005234 | 80 | 7187 | 7308 | 230425 | 230303 | 0.01 | 9.00E-15 |
| hsa_circ_0077398 | 96 | 1 | 492 | 492 | 1 | 0 | 0 |
| hsa_circ_0077398 | 96 | 1 | 492 | 492 | 1 | 0 | 0 |
| hsa_circ_0077390 | 95 | 6 | 867 | 1008 | 147 | 0 | 0 |
| hsa_circ_0077390 | 95 | 6 | 598 | 869 | 277 | 0 | 0 |
| hsa_circ_0077389 | 96 | 4 | 168 | 165 | 1 | 0 | 4.00E-72 |
| hsa_circ_0077389 | 96 | 4 | 168 | 165 | 1 | 0 | 3.00E-71 |
| hsa_circ_0077388 | 97 | 4 | 284 | 281 | 1 | 0 | 5.00E-133 |
| hsa_circ_0004941 | 91 | 1 | 1314 | 1314 | 1 | 0 | 0 |
| hsa_circ_0077375 | 93 | 1 | 675 | 895 | 221 | 0 | 0 |
| hsa_circ_0004941 | 89 | 1 | 413 | 413 | 1 | 0 | 2.00E-144 |
| hsa_circ_0077371 | 91 | 1 | 590 | 853 | 265 | 0 | 0 |
| hsa_circ_0077368 | 90 | 1 | 914 | 1126 | 213 | 0 | 0 |
| hsa_circ_0077371 | 88 | 1 | 276 | 634 | 359 | 0 | 3.00E-90 |
| hsa_circ_0077371 | 90 | 1 | 371 | 634 | 265 | 0 | 1.00E-135 |
| hsa_circ_0077368 | 90 | 1 | 695 | 907 | 213 | 0 | 0 |
| hsa_circ_0077370 | 91 | 1 | 419 | 419 | 1 | 0 | 8.00E-163 |
| hsa_circ_0077361 | 93 | 1 | 331 | 687 | 357 | 0 | 1.00E-135 |
| hsa_circ_0077361 | 92 | 1 | 687 | 687 | 1 | 0 | 0 |
| hsa_circ_0077356 | 91 | 1 | 960 | 1294 | 335 | 0 | 0 |
| hsa_circ_0077355 | 91 | 1 | 1492 | 1789 | 298 | 0 | 0 |
| hsa_circ_0077356 | 89 | 1 | 275 | 1024 | 750 | 0 | 1.00E-94 |
| hsa_circ_0077356 | 91 | 1 | 690 | 1024 | 335 | 0 | 0 |
| hsa_circ_0077355 | 92 | 1 | 1519 | 1519 | 1 | 0 | 0 |
| hsa_circ_0077356 | 92 | 1 | 415 | 749 | 335 | 0 | 1.00E-165 |
| hsa_circ_0077355 | 93 | 1 | 827 | 827 | 1 | 0 | 0 |
| hsa_circ_0077350 | 87 | 2 | 475 | 907 | 434 | 0 | 1.00E-151 |
| hsa_circ_0077338 | 87 | 19 | 603 | 585 | 1 | 0 | 0 |
| hsa_circ_0077321 | 90 | 1 | 230 | 349 | 117 | 0.01 | 3.00E-80 |
| hsa_circ_0077311 | 88 | 2 | 921 | 1109 | 190 | 0.01 | 0 |
| hsa_circ_0079666 | 95 | 1 | 393 | 393 | 1 | 0 | 2.00E-178 |
| hsa_circ_0087649 | 91 | 333 | 379 | 58 | 12 | 0 | 2.00E-09 |
| hsa_circ_0074176 | 92 | 1 | 314 | 314 | 1 | 0 | 6.00E-123 |
| hsa_circ_0074176 | 92 | 1 | 414 | 414 | 1 | 0 | 5.00E-165 |
| hsa_circ_0074176 | 92 | 41 | 456 | 416 | 1 | 0 | 4.00E-166 |
| hsa_circ_0074176 | 89 | 3 | 535 | 521 | 1 | 0.02 | 0 |
| hsa_circ_0074178 | 93 | 1 | 292 | 292 | 1 | 0 | 2.00E-117 |
| hsa_circ_0074176 | 89 | 3 | 413 | 521 | 123 | 0.03 | 5.00E-140 |
| hsa_circ_0074197 | 93 | 115 | 1394 | 1549 | 2831 | 0 | 0 |
| hsa_circ_0001538 | 97 | 1 | 344 | 1 | 344 | 0 | 3.00E-161 |
| hsa_circ_0001538 | 97 | 1 | 339 | 6 | 344 | 0 | 4.00E-160 |
| hsa_circ_0004503 | 99 | 27 | 265 | 42 | 280 | 0 | 1.00E-119 |
| hsa_circ_0004503 | 98 | 27 | 359 | 42 | 374 | 0 | 8.00E-167 |
| hsa_circ_0074230 | 99 | 50 | 653 | 1 | 605 | 0 | 0 |

novel_circ_019608 novel_circ_019609 novel_circ_019610 novel_circ_019611 novel_circ_019612 novel_circ_019613 novel_circ_019614 novel_circ_019615 novel_circ_019616 novel_circ_019617 novel_circ_019618 novel_circ_019619 novel_circ_019620 novel_circ_019621 novel_circ_019622 novel_circ_019623 novel_circ_019624 novel_circ_019625 novel_circ_019626 novel_circ_019627 novel_circ_019628 novel_circ_019629 novel_circ_019630 novel_circ_019631 novel_circ_019632 novel_circ_019633 novel_circ_019634 novel_circ_019635 novel_circ_019636 novel_circ_019637 novel_circ_019649 novel_circ_019650 novel_circ_019651 novel_circ_019652 novel_circ_019653 novel_circ_019654 novel_circ_019655 novel_circ_019656 novel_circ_019657 novel_circ_019658 novel_circ_019659 novel_circ_019660 novel_circ_019661 novel_circ_019662 novel_circ_019663 novel_circ_019664 novel_circ_019665 novel_circ_019666 novel_circ_019667 novel_circ_019668 novel_circ_019669 novel_circ_019670 novel_circ_019671 novel_circ_019672

| hsa_circ_0074249 | 95 | 1 | 598 | 1 | 607 | 0.01 | 0 |
| --- | --- | --- | --- | --- | --- | --- | --- |
| hsa_circ_0074249 | 95 | 1 | 927 | 1 | 936 | 0.01 | 0 |
| hsa_circ_0074249 | 95 | 1 | 1357 | 1 | 1366 | 0.01 | 0 |
| hsa_circ_0074249 | 95 | 1 | 1467 | 1 | 1476 | 0.01 | 0 |
| hsa_circ_0074249 | 95 | 1 | 1469 | 1 | 1478 | 0.01 | 0 |
| hsa_circ_0074251 | 94 | 1 | 444 | 1 | 453 | 0.02 | 0 |
| hsa_circ_0074251 | 94 | 1 | 773 | 1 | 782 | 0.01 | 0 |
| hsa_circ_0074251 | 94 | 1 | 1313 | 1 | 1322 | 0.01 | 0 |
| hsa_circ_0074256 | 95 | 1 | 477 | 149 | 625 | 0 | 0 |
| hsa_circ_0074256 | 95 | 1 | 907 | 149 | 1055 | 0 | 0 |
| hsa_circ_0074257 | 94 | 1 | 329 | 1 | 329 | 0 | 6.00E-138 |
| hsa_circ_0074257 | 94 | 1 | 759 | 1 | 759 | 0 | 0 |
| hsa_circ_0074257 | 95 | 1 | 430 | 330 | 759 | 0 | 0 |
| hsa_circ_0074257 | 95 | 1 | 540 | 330 | 869 | 0 | 0 |
| hsa_circ_0074268 | 96 | 1 | 1520 | 104 | 1623 | 0 | 0 |
| hsa_circ_0074270 | 93 | 1 | 404 | 1 | 404 | 0 | 7.00E-168 |
| hsa_circ_0074268 | 94 | 1 | 761 | 863 | 1623 | 0 | 0 |
| hsa_circ_0074268 | 94 | 1 | 1100 | 863 | 1962 | 0 | 0 |
| hsa_circ_0074268 | 94 | 1 | 1312 | 863 | 2174 | 0 | 0 |
| hsa_circ_0074268 | 93 | 1 | 1945 | 863 | 2798 | 0 | 0 |
| hsa_circ_0074268 | 93 | 1 | 3855 | 863 | 4708 | 0 | 0 |
| hsa_circ_0074268 | 93 | 1 | 4032 | 863 | 4885 | 0 | 0 |
| hsa_circ_0074268 | 95 | 1 | 357 | 1267 | 1623 | 0 | 4.00E-160 |
| hsa_circ_0074268 | 95 | 1 | 696 | 1267 | 1962 | 0 | 0 |
| hsa_circ_0001542 | 94 | 1 | 339 | 1 | 339 | 0 | 8.00E-147 |
| hsa_circ_0074268 | 95 | 1 | 435 | 1624 | 2058 | 0 | 0 |
| hsa_circ_0074268 | 93 | 1 | 1331 | 1624 | 2945 | 0.01 | 0 |
| hsa_circ_0074268 | 93 | 1 | 2542 | 2176 | 4708 | 0 | 0 |
| hsa_circ_0074273 | 93 | 1 | 1910 | 449 | 2358 | 0 | 0 |
| hsa_circ_0074273 | 93 | 1 | 1763 | 596 | 2358 | 0 | 0 |
| hsa_circ_0007420 | 83 | 1402 | 1975 | 121 | 694 | 0 | 8.00E-145 |
| hsa_circ_0072352 | 97 | 256 | 714 | 459 | 1 | 0 | 0 |
| hsa_circ_0001478 | 96 | 256 | 1328 | 1073 | 1 | 0 | 0 |
| hsa_circ_0072352 | 97 | 1 | 458 | 458 | 1 | 0 | 0 |
| hsa_circ_0001478 | 96 | 1 | 1072 | 1072 | 1 | 0 | 0 |
| hsa_circ_0001478 | 95 | 1 | 614 | 614 | 1 | 0 | 0 |
| hsa_circ_0072335 | 95 | 27746 | 28469 | 792 | 69 | 0 | 0 |
| hsa_circ_0001476 | 80 | 3506 | 3965 | 643 | 198 | 0.05 | 5.00E-84 |
| hsa_circ_0001476 | 94 | 1 | 295 | 295 | 1 | 0 | 2.00E-122 |
| hsa_circ_0006308 | 95 | 1 | 486 | 486 | 1 | 0 | 0 |
| hsa_circ_0072335 | 95 | 1 | 656 | 792 | 137 | 0 | 0 |
| hsa_circ_0072335 | 95 | 1 | 724 | 792 | 69 | 0 | 0 |
| hsa_circ_0072335 | 95 | 1 | 792 | 792 | 1 | 0 | 0 |
| hsa_circ_0072325 | 95 | 99 | 1954 | 2148 | 293 | 0 | 0 |
| hsa_circ_0001476 | 95 | 1 | 197 | 197 | 1 | 0 | 4.00E-83 |
| hsa_circ_0006308 | 96 | 1 | 388 | 388 | 1 | 0 | 1.00E-180 |
| hsa_circ_0072336 | 96 | 1 | 558 | 558 | 1 | 0 | 0 |
| hsa_circ_0072335 | 96 | 1 | 626 | 694 | 69 | 0 | 0 |
| hsa_circ_0072335 | 95 | 1 | 694 | 694 | 1 | 0 | 0 |
| hsa_circ_0006308 | 96 | 1 | 323 | 323 | 1 | 0 | 1.00E-149 |
| hsa_circ_0072335 | 96 | 1 | 561 | 629 | 69 | 0 | 0 |
| hsa_circ_0072335 | 95 | 1 | 629 | 629 | 1 | 0 | 0 |
| hsa_circ_0072335 | 96 | 1 | 497 | 497 | 1 | 0 | 0 |
| hsa_circ_0072335 | 95 | 1 | 238 | 306 | 69 | 0 | 8.00E-101 |

novel_circ_019673 novel_circ_019674 novel_circ_019675 novel_circ_019676 novel_circ_019677 novel_circ_019678 novel_circ_019680 novel_circ_019681 novel_circ_019682 novel_circ_019683 novel_circ_019684 novel_circ_019685 novel_circ_019686 novel_circ_019687 novel_circ_019688 novel_circ_019689 novel_circ_019690 novel_circ_019691 novel_circ_019692 novel_circ_019693 novel_circ_019694 novel_circ_019695 novel_circ_019696 novel_circ_019697 novel_circ_019698 novel_circ_019699 novel_circ_019700 novel_circ_019701 novel_circ_019702 novel_circ_019703 novel_circ_019704 novel_circ_019705 novel_circ_019706 novel_circ_019707 novel_circ_019708 novel_circ_019709 novel_circ_019710 novel_circ_019712 novel_circ_019713 novel_circ_019714 novel_circ_019715 novel_circ_019716 novel_circ_019717 novel_circ_019718 novel_circ_019719 novel_circ_019720 novel_circ_019721 novel_circ_019722 novel_circ_019723 novel_circ_019724 novel_circ_019725 novel_circ_019726 novel_circ_019727 novel_circ_019728

| hsa_circ_0072335 | 94 | 1 | 306 | 306 | 1 | 0 | 7.00E-132 |
| --- | --- | --- | --- | --- | --- | --- | --- |
| hsa_circ_0072325 | 95 | 4 | 1298 | 1587 | 293 | 0 | 0 |
| hsa_circ_0072325 | 95 | 3 | 1079 | 1369 | 293 | 0 | 0 |
| hsa_circ_0072326 | 94 | 1 | 336 | 463 | 128 | 0 | 4.00E-145 |
| hsa_circ_0072323 | 93 | 1 | 782 | 1412 | 631 | 0 | 0 |
| hsa_circ_0072323 | 95 | 1 | 626 | 628 | 3 | 0 | 0 |
| hsa_circ_0072309 | 86 | 1 | 580 | 580 | 1 | 0 | 2.00E-180 |
| hsa_circ_0072307 | 85 | 1 | 755 | 755 | 1 | 0 | 0 |
| hsa_circ_0072306 | 87 | 1 | 1140 | 1456 | 317 | 0 | 0 |
| hsa_circ_0072306 | 87 | 1 | 1452 | 1456 | 5 | 0 | 0 |
| hsa_circ_0072305 | 87 | 1 | 1617 | 1904 | 288 | 0 | 0 |
| hsa_circ_0072305 | 87 | 1 | 1690 | 1904 | 215 | 0 | 0 |
| hsa_circ_0072305 | 87 | 1 | 1904 | 1904 | 1 | 0 | 0 |
| hsa_circ_0072304 | 87 | 1 | 2082 | 2084 | 3 | 0 | 0 |
| hsa_circ_0072300 | 87 | 1 | 2354 | 2354 | 1 | 0 | 0 |
| hsa_circ_0072298 | 88 | 1 | 2610 | 2689 | 80 | 0 | 0 |
| hsa_circ_0072306 | 87 | 1 | 979 | 1295 | 317 | 0 | 0 |
| hsa_circ_0072304 | 88 | 1 | 1666 | 1668 | 3 | 0 | 0 |
| hsa_circ_0072298 | 88 | 1 | 2145 | 2252 | 108 | 0 | 0 |
| hsa_circ_0072306 | 89 | 6 | 385 | 696 | 317 | 0.01 | 7.00E-133 |
| hsa_circ_0072305 | 88 | 1 | 550 | 764 | 215 | 0 | 0 |
| hsa_circ_0072305 | 89 | 1 | 764 | 764 | 1 | 0 | 0 |
| hsa_circ_0072298 | 89 | 1 | 1442 | 1549 | 108 | 0 | 0 |
| hsa_circ_0072298 | 89 | 1 | 1470 | 1549 | 80 | 0 | 0 |
| hsa_circ_0072298 | 89 | 3 | 1421 | 1526 | 108 | 0 | 0 |
| hsa_circ_0072305 | 90 | 1 | 448 | 448 | 1 | 0 | 1.00E-160 |
| hsa_circ_0072300 | 89 | 1 | 898 | 898 | 1 | 0 | 0 |
| hsa_circ_0072298 | 90 | 1 | 1154 | 1233 | 80 | 0 | 0 |
| hsa_circ_0029815 | 88 | 18151 | 18574 | 548 | 116 | 0.02 | 5.00E-139 |
| hsa_circ_0009297 | 92 | 17952 | 18549 | 394 | 991 | 0 | 0 |
| hsa_circ_0072295 | 95 | 1 | 461 | 500 | 960 | 0 | 0 |
| hsa_circ_0072295 | 93 | 1 | 785 | 176 | 960 | 0 | 0 |
| hsa_circ_0072295 | 93 | 1 | 960 | 1 | 960 | 0 | 0 |
| hsa_circ_0072295 | 93 | 1 | 797 | 1 | 797 | 0 | 0 |
| hsa_circ_0072295 | 91 | 1 | 324 | 176 | 499 | 0 | 8.00E-122 |
| hsa_circ_0072295 | 92 | 1 | 499 | 1 | 499 | 0 | 0 |
| hsa_circ_0072286 | 89 | 1 | 514 | 151 | 661 | 0.01 | 2.00E-179 |
| hsa_circ_0072280 | 93 | 2 | 399 | 398 | 1 | 0 | 3.00E-166 |
| hsa_circ_0072275 | 93 | 1 | 330 | 603 | 274 | 0 | 8.00E-137 |
| hsa_circ_0072259 | 93 | 1 | 634 | 1096 | 463 | 0 | 0 |
| hsa_circ_0072250 | 94 | 1 | 676 | 807 | 132 | 0 | 0 |
| hsa_circ_0072259 | 94 | 1 | 277 | 278 | 2 | 0 | 2.00E-117 |
| hsa_circ_0072253 | 95 | 1 | 394 | 394 | 1 | 0 | 5.00E-174 |
| hsa_circ_0072250 | 94 | 1 | 623 | 623 | 1 | 0 | 0 |
| hsa_circ_0072246 | 93 | 1 | 1110 | 1110 | 1 | 0 | 0 |
| hsa_circ_0072239 | 92 | 6 | 794 | 789 | 1 | 0 | 0 |
| hsa_circ_0072246 | 91 | 2 | 414 | 413 | 1 | 0 | 2.00E-159 |
| hsa_circ_0072244 | 92 | 2 | 543 | 543 | 2 | 0 | 0 |
| hsa_circ_0072232 | 93 | 2 | 779 | 778 | 1 | 0 | 0 |
| hsa_circ_0072231 | 93 | 2 | 890 | 889 | 1 | 0 | 0 |
| hsa_circ_0072232 | 95 | 1 | 365 | 365 | 1 | 0 | 7.00E-163 |
| hsa_circ_0072231 | 94 | 1 | 476 | 476 | 1 | 0 | 0 |
| hsa_circ_0072226 | 93 | 1 | 613 | 920 | 308 | 0 | 0 |
| hsa_circ_0072226 | 93 | 1 | 720 | 920 | 201 | 0 | 0 |

novel_circ_019730 novel_circ_019731 novel_circ_019732 novel_circ_019733 novel_circ_019734 novel_circ_019735 novel_circ_019736 novel_circ_019737 novel_circ_019738 novel_circ_019739 novel_circ_019740 novel_circ_019741 novel_circ_019742 novel_circ_019744 novel_circ_019745 novel_circ_019746 novel_circ_019747 novel_circ_019748 novel_circ_019755 novel_circ_019756 novel_circ_019757 novel_circ_019758 novel_circ_019759 novel_circ_019760 novel_circ_019761 novel_circ_019762 novel_circ_019763 novel_circ_019774 novel_circ_019775 novel_circ_019776 novel_circ_019777 novel_circ_019778 novel_circ_019779 novel_circ_019780 novel_circ_019781 novel_circ_019782 novel_circ_019783 novel_circ_019784 novel_circ_019785 novel_circ_019786 novel_circ_019787 novel_circ_019788 novel_circ_019789 novel_circ_019790 novel_circ_019791 novel_circ_019792 novel_circ_019793 novel_circ_019794 novel_circ_019795 novel_circ_019796 novel_circ_019797 novel_circ_019798 novel_circ_019799 novel_circ_019800

| hsa_circ_0008755 | 90 | 18 | 718 | 707 | 6 | 0 | 0 |
| --- | --- | --- | --- | --- | --- | --- | --- |
| hsa_circ_0072216 | 90 | 128 | 984 | 857 | 1 | 0 | 0 |
| hsa_circ_0072214 | 87 | 128 | 2538 | 2419 | 6 | 0.01 | 0 |
| hsa_circ_0072216 | 90 | 1 | 261 | 261 | 1 | 0 | 1.00E-93 |
| hsa_circ_0072211 | 86 | 1 | 2240 | 2243 | 1 | 0.01 | 0 |
| hsa_circ_0072211 | 86 | 1 | 2240 | 2243 | 1 | 0.01 | 0 |
| hsa_circ_0072209 | 86 | 445 | 3131 | 6279 | 3590 | 0.01 | 0 |
| hsa_circ_0072209 | 87 | 445 | 4375 | 6279 | 2354 | 0.01 | 0 |
| hsa_circ_0072214 | 86 | 1 | 1554 | 1562 | 6 | 0.01 | 0 |
| hsa_circ_0072211 | 86 | 2 | 629 | 628 | 1 | 0 | 0 |
| hsa_circ_0072209 | 87 | 2 | 858 | 5108 | 4252 | 0 | 0 |
| hsa_circ_0072211 | 90 | 9 | 238 | 412 | 183 | 0 | 5.00E-78 |
| hsa_circ_0072209 | 87 | 9 | 649 | 4892 | 4252 | 0 | 0 |
| hsa_circ_0072209 | 92 | 1 | 517 | 3728 | 3212 | 0.01 | 0 |
| hsa_circ_0072209 | 89 | 1 | 821 | 3728 | 2920 | 0.02 | 0 |
| hsa_circ_0072209 | 89 | 1 | 682 | 3589 | 2920 | 0.02 | 0 |
| hsa_circ_0072209 | 88 | 1 | 1244 | 3589 | 2354 | 0.01 | 0 |
| hsa_circ_0072209 | 88 | 1 | 1244 | 3589 | 2354 | 0.01 | 0 |
| hsa_circ_0072208 | 83 | 6 | 1864 | 2386 | 522 | 0.01 | 0 |
| hsa_circ_0072208 | 83 | 1 | 1627 | 2311 | 679 | 0.01 | 0 |
| hsa_circ_0072208 | 83 | 32 | 1549 | 2199 | 679 | 0.01 | 0 |
| hsa_circ_0072208 | 89 | 3 | 459 | 976 | 517 | 0.01 | 1.00E-157 |
| hsa_circ_0072208 | 89 | 3 | 459 | 976 | 517 | 0.01 | 2.00E-157 |
| hsa_circ_0072208 | 89 | 3 | 459 | 976 | 517 | 0.01 | 8.00E-157 |
| hsa_circ_0072208 | 93 | 1 | 107 | 623 | 517 | 0 | 2.00E-37 |
| hsa_circ_0072208 | 93 | 1 | 107 | 623 | 517 | 0 | 2.00E-37 |
| hsa_circ_0072208 | 93 | 1 | 107 | 623 | 517 | 0 | 2.00E-37 |
| hsa_circ_0072200 | 95 | 1 | 596 | 1 | 596 | 0 | 0 |
| hsa_circ_0072197 | 96 | 1 | 905 | 1 | 905 | 0 | 0 |
| hsa_circ_0072195 | 95 | 1 | 846 | 1 | 846 | 0 | 0 |
| hsa_circ_0072195 | 94 | 1 | 476 | 1 | 476 | 0 | 0 |
| hsa_circ_0072182 | 96 | 1 | 875 | 4215 | 5089 | 0 | 0 |
| hsa_circ_0072195 | 96 | 1 | 390 | 1 | 390 | 0 | 2.00E-178 |
| hsa_circ_0072191 | 97 | 1 | 636 | 289 | 924 | 0 | 0 |
| hsa_circ_0072190 | 97 | 1 | 780 | 319 | 1098 | 0 | 0 |
| hsa_circ_0072190 | 96 | 1 | 699 | 1 | 699 | 0 | 0 |
| hsa_circ_0072189 | 96 | 1 | 1066 | 84 | 1149 | 0 | 0 |
| hsa_circ_0072190 | 96 | 1 | 246 | 319 | 564 | 0 | 3.00E-110 |
| hsa_circ_0072190 | 97 | 1 | 318 | 1 | 318 | 0 | 2.00E-148 |
| hsa_circ_0072189 | 96 | 1 | 367 | 84 | 450 | 0 | 2.00E-172 |
| hsa_circ_0072184 | 95 | 1 | 556 | 320 | 875 | 0 | 0 |
| hsa_circ_0072184 | 96 | 1 | 875 | 1 | 875 | 0 | 0 |
| hsa_circ_0072184 | 96 | 1 | 334 | 320 | 653 | 0 | 1.00E-150 |
| hsa_circ_0072184 | 96 | 1 | 566 | 88 | 653 | 0 | 0 |
| hsa_circ_0072183 | 96 | 1 | 575 | 544 | 1118 | 0 | 0 |
| hsa_circ_0072183 | 97 | 1 | 360 | 184 | 543 | 0 | 4.00E-170 |
| hsa_circ_0072183 | 97 | 1 | 543 | 1 | 543 | 0 | 0 |
| hsa_circ_0072182 | 94 | 1 | 1626 | 1 | 1626 | 0 | 0 |
| hsa_circ_0072178 | 95 | 1 | 255 | 253 | 510 | 0.01 | 8.00E-111 |
| hsa_circ_0072174 | 96 | 1 | 801 | 1 | 804 | 0 | 0 |
| hsa_circ_0072172 | 96 | 1 | 944 | 1 | 947 | 0 | 0 |
| hsa_circ_0072178 | 98 | 1 | 252 | 1 | 252 | 0 | 3.00E-120 |
| hsa_circ_0072174 | 97 | 1 | 546 | 1 | 546 | 0 | 0 |
| hsa_circ_0072172 | 97 | 1 | 689 | 1 | 689 | 0 | 0 |

novel_circ_019801 novel_circ_019802 novel_circ_019806 novel_circ_019807 novel_circ_019808 novel_circ_019809 novel_circ_019810 novel_circ_019811 novel_circ_019812 novel_circ_019813 novel_circ_019814 novel_circ_019815 novel_circ_019816 novel_circ_019818 novel_circ_019819 novel_circ_019820 novel_circ_019821 novel_circ_019822 novel_circ_019823 novel_circ_019824 novel_circ_019825 novel_circ_019826 novel_circ_019827 novel_circ_019828 novel_circ_019831 novel_circ_019832 novel_circ_019833 novel_circ_019834 novel_circ_019835 novel_circ_019836 novel_circ_019838 novel_circ_019840 novel_circ_019841 novel_circ_019846 novel_circ_019848 novel_circ_019849 novel_circ_019850 novel_circ_019851 novel_circ_019852 novel_circ_019853 novel_circ_019854 novel_circ_019855 novel_circ_019856 novel_circ_019858 novel_circ_019859 novel_circ_019860 novel_circ_019861 novel_circ_019862 novel_circ_019863 novel_circ_019865 novel_circ_019866 novel_circ_019867 novel_circ_019868 novel_circ_019869

| hsa_circ_0072172 | 97 | 1 | 437 | 1 | 437 | 0 | 0 |
| --- | --- | --- | --- | --- | --- | --- | --- |
| hsa_circ_0072156 | 92 | 1 | 1464 | 796 | 2257 | 0 | 0 |
| hsa_circ_0072155 | 95 | 1 | 260 | 260 | 1 | 0 | 2.00E-111 |
| hsa_circ_0002292 | 93 | 1 | 481 | 560 | 80 | 0 | 0 |
| hsa_circ_0002292 | 93 | 1 | 560 | 560 | 1 | 0 | 0 |
| hsa_circ_0002292 | 93 | 1 | 560 | 560 | 1 | 0 | 0 |
| hsa_circ_0002292 | 93 | 1 | 560 | 560 | 1 | 0 | 0 |
| hsa_circ_0002292 | 92 | 1 | 392 | 471 | 80 | 0 | 7.00E-158 |
| hsa_circ_0002292 | 93 | 1 | 471 | 471 | 1 | 0 | 0 |
| hsa_circ_0002292 | 93 | 1 | 471 | 471 | 1 | 0 | 0 |
| hsa_circ_0002292 | 91 | 1 | 300 | 300 | 1 | 0 | 3.00E-113 |
| hsa_circ_0002292 | 91 | 1 | 300 | 300 | 1 | 0 | 4.00E-113 |
| hsa_circ_0002292 | 94 | 1 | 79 | 79 | 1 | 0 | 2.00E-25 |
| hsa_circ_0072149 | 90 | 1 | 621 | 1 | 621 | 0 | 0 |
| hsa_circ_0001471 | 91 | 7 | 430 | 804 | 380 | 0 | 6.00E-164 |
| hsa_circ_0001471 | 92 | 7 | 598 | 804 | 212 | 0 | 0 |
| hsa_circ_0001471 | 93 | 7 | 809 | 804 | 1 | 0 | 0 |
| hsa_circ_0001471 | 93 | 7 | 809 | 804 | 1 | 0 | 0 |
| hsa_circ_0001471 | 93 | 7 | 809 | 804 | 1 | 0 | 0 |
| hsa_circ_0001471 | 93 | 7 | 809 | 804 | 1 | 0 | 0 |
| hsa_circ_0001471 | 93 | 7 | 809 | 804 | 1 | 0 | 0 |
| hsa_circ_0001471 | 93 | 7 | 809 | 804 | 1 | 0 | 0 |
| hsa_circ_0001471 | 93 | 7 | 809 | 804 | 1 | 0 | 0 |
| hsa_circ_0001471 | 93 | 7 | 809 | 804 | 1 | 0 | 0 |
| hsa_circ_0006624 | 90 | 4209 | 4570 | 362 | 1 | 0.01 | 4.00E-130 |
| hsa_circ_0006624 | 90 | 4209 | 4570 | 362 | 1 | 0.01 | 6.00E-130 |
| hsa_circ_0006624 | 90 | 1 | 362 | 362 | 1 | 0.01 | 2.00E-130 |
| hsa_circ_0006833 | 78 | 17854 | 18784 | 217822 | 218775 | 0.06 | 4.00E-161 |
| hsa_circ_0072125 | 92 | 7 | 543 | 2640 | 2104 | 0 | 0 |
| hsa_circ_0061830 | 90 | 2 | 345 | 830 | 1173 | 0 | 2.00E-124 |
| hsa_circ_0061830 | 91 | 122 | 415 | 373 | 666 | 0 | 2.00E-108 |
| hsa_circ_0061811 | 94 | 971 | 1101 | 1 | 131 | 0 | 5.00E-49 |
| hsa_circ_0061811 | 94 | 13623 | 13753 | 1 | 131 | 0 | 7.00E-48 |
| hsa_circ_0086199 | 83 | 28998 | 29101 | 100281 | 100178 | 0.02 | 8.00E-16 |
| hsa_circ_0086199 | 83 | 12785 | 12888 | 100281 | 100178 | 0.02 | 4.00E-16 |
| hsa_circ_0061767 | 99 | 3 | 465 | 4843 | 4381 | 0 | 0 |
| hsa_circ_0061770 | 95 | 1 | 994 | 1848 | 855 | 0 | 0 |
| hsa_circ_0061770 | 95 | 1 | 994 | 1848 | 855 | 0 | 0 |
| hsa_circ_0061770 | 95 | 1 | 568 | 1422 | 855 | 0 | 0 |
| hsa_circ_0061770 | 91 | 408 | 981 | 574 | 1 | 0 | 0 |
| hsa_circ_0061770 | 91 | 1 | 573 | 573 | 1 | 0 | 0 |
| hsa_circ_0061767 | 95 | 1 | 662 | 2532 | 1871 | 0 | 0 |
| hsa_circ_0061767 | 92 | 1 | 535 | 1192 | 658 | 0 | 0 |
| hsa_circ_0005869 | 97 | 1 | 538 | 1 | 538 | 0 | 0 |
| hsa_circ_0005869 | 95 | 1 | 303 | 99 | 401 | 0 | 3.00E-135 |
| hsa_circ_0005869 | 96 | 1 | 858 | 99 | 956 | 0 | 0 |
| hsa_circ_0005869 | 95 | 1 | 460 | 253 | 712 | 0 | 0 |
| hsa_circ_0005869 | 95 | 1 | 704 | 253 | 956 | 0 | 0 |
| hsa_circ_0042030 | 92 | 1 | 838 | 1388 | 551 | 0 | 0 |
| hsa_circ_0042037 | 95 | 1 | 469 | 641 | 173 | 0 | 0 |
| hsa_circ_0042048 | 94 | 1 | 1138 | 1138 | 1 | 0 | 0 |
| hsa_circ_0042039 | 93 | 1 | 547 | 788 | 242 | 0 | 0 |
| hsa_circ_0042048 | 94 | 1 | 1138 | 1138 | 1 | 0 | 0 |
| hsa_circ_0042042 | 91 | 1 | 316 | 316 | 1 | 0 | 2.00E-117 |

novel_circ_019870 novel_circ_019871 novel_circ_019872 novel_circ_019873 novel_circ_019874 novel_circ_019875 novel_circ_019876 novel_circ_019877 novel_circ_019878 novel_circ_019879 novel_circ_019880 novel_circ_019881 novel_circ_019882 novel_circ_019883 novel_circ_019884 novel_circ_019885 novel_circ_019886 novel_circ_019887 novel_circ_019888 novel_circ_019896 novel_circ_019899 novel_circ_019900 novel_circ_019901 novel_circ_019902 novel_circ_019904 novel_circ_019905 novel_circ_019906 novel_circ_019907 novel_circ_019908 novel_circ_019909 novel_circ_019910 novel_circ_019911 novel_circ_019912 novel_circ_019913 novel_circ_019914 novel_circ_019915 novel_circ_019916 novel_circ_019917 novel_circ_019918 novel_circ_019919 novel_circ_019922 novel_circ_019923 novel_circ_019924 novel_circ_019927 novel_circ_019928 novel_circ_019929 novel_circ_019930 novel_circ_019931 novel_circ_019932 novel_circ_019933 novel_circ_019934 novel_circ_019935 novel_circ_019936 novel_circ_019942

| hsa_circ_0042048 | 93 | 4 | 289 | 286 | 1 | 0 | 4.00E-114 |
| --- | --- | --- | --- | --- | --- | --- | --- |
| hsa_circ_0042048 | 94 | 4 | 1231 | 1228 | 1 | 0 | 0 |
| hsa_circ_0042048 | 94 | 1 | 1334 | 1334 | 1 | 0 | 0 |
| hsa_circ_0042048 | 93 | 1 | 863 | 975 | 113 | 0 | 0 |
| hsa_circ_0042048 | 93 | 1 | 860 | 975 | 116 | 0 | 0 |
| hsa_circ_0042048 | 92 | 1 | 445 | 735 | 291 | 0 | 3.00E-177 |
| hsa_circ_0042048 | 93 | 1 | 685 | 975 | 291 | 0 | 0 |
| hsa_circ_0042048 | 94 | 1 | 848 | 1138 | 291 | 0 | 0 |
| hsa_circ_0042048 | 93 | 1 | 272 | 735 | 464 | 0 | 2.00E-111 |
| hsa_circ_0042048 | 97 | 1 | 403 | 1138 | 736 | 0 | 0 |
| hsa_circ_0042048 | 98 | 1 | 163 | 1138 | 976 | 0 | 2.00E-74 |
| hsa_circ_0042048 | 97 | 1 | 359 | 1334 | 976 | 0 | 1.00E-169 |
| hsa_circ_0042049 | 96 | 1 | 195 | 196 | 2 | 0 | 5.00E-87 |
| hsa_circ_0042048 | 95 | 2 | 561 | 1894 | 1335 | 0 | 0 |
| hsa_circ_0042048 | 95 | 2 | 533 | 1894 | 1363 | 0 | 0 |
| hsa_circ_0042048 | 95 | 2 | 375 | 1894 | 1521 | 0 | 1.00E-164 |
| hsa_circ_0042065 | 94 | 1 | 306 | 801 | 496 | 0 | 3.00E-130 |
| hsa_circ_0042085 | 89 | 2015 | 2749 | 1 | 735 | 0 | 0 |
| hsa_circ_0042085 | 89 | 1 | 735 | 1 | 735 | 0 | 0 |
| hsa_circ_0083499 | 88 | 1 | 352 | 695 | 1046 | 0 | 3.00E-117 |
| hsa_circ_0020103 | 77 | 36 | 313 | 317 | 43 | 0.04 | 1.00E-36 |
| hsa_circ_0083529 | 94 | 1 | 171 | 1 | 171 | 0 | 4.00E-67 |
| hsa_circ_0083615 | 87 | 671 | 1415 | 477 | 1227 | 0.01 | 0 |
| hsa_circ_0083617 | 89 | 1780 | 2637 | 1 | 858 | 0 | 0 |
| hsa_circ_0083623 | 89 | 1527 | 1848 | 2 | 323 | 0 | 1.00E-106 |
| hsa_circ_0083623 | 90 | 1527 | 1960 | 2 | 435 | 0 | 8.00E-159 |
| hsa_circ_0083623 | 90 | 1527 | 1962 | 2 | 437 | 0 | 1.00E-159 |
| hsa_circ_0083623 | 89 | 1527 | 2617 | 2 | 1092 | 0.01 | 0 |
| hsa_circ_0083623 | 87 | 1527 | 2795 | 2 | 1297 | 0.03 | 0 |
| hsa_circ_0083623 | 85 | 1527 | 3146 | 2 | 1660 | 0.03 | 0 |
| hsa_circ_0083623 | 89 | 402 | 723 | 2 | 323 | 0 | 5.00E-107 |
| hsa_circ_0083623 | 89 | 402 | 1492 | 2 | 1092 | 0.01 | 0 |
| hsa_circ_0083623 | 87 | 402 | 1670 | 2 | 1297 | 0.03 | 0 |
| hsa_circ_0083623 | 85 | 402 | 2021 | 2 | 1660 | 0.03 | 0 |
| hsa_circ_0083623 | 89 | 2 | 323 | 2 | 323 | 0 | 2.00E-107 |
| hsa_circ_0083623 | 90 | 2 | 581 | 2 | 581 | 0 | 0 |
| hsa_circ_0083623 | 89 | 2 | 1092 | 2 | 1092 | 0.01 | 0 |
| hsa_circ_0083623 | 87 | 2 | 1270 | 2 | 1297 | 0.03 | 0 |
| hsa_circ_0083623 | 85 | 2 | 1621 | 2 | 1660 | 0.03 | 0 |
| hsa_circ_0083624 | 92 | 3 | 383 | 3 | 383 | 0 | 9.00E-152 |
| hsa_circ_0071370 | 82 | 4 | 410 | 544 | 138 | 0 | 2.00E-94 |
| hsa_circ_0071370 | 82 | 4 | 547 | 544 | 1 | 0 | 4.00E-132 |
| hsa_circ_0071369 | 83 | 4 | 650 | 647 | 1 | 0 | 1.00E-167 |
| hsa_circ_0087133 | 91 | 15404 | 15449 | 1887 | 1932 | 0 | 8.00E-07 |
| hsa_circ_0071365 | 97 | 188 | 266 | 375 | 297 | 0 | 9.00E-31 |
| hsa_circ_0071365 | 94 | 188 | 510 | 375 | 53 | 0 | 1.00E-137 |
| hsa_circ_0071365 | 94 | 188 | 561 | 375 | 2 | 0 | 1.00E-162 |
| hsa_circ_0071365 | 94 | 188 | 561 | 375 | 2 | 0 | 2.00E-162 |
| hsa_circ_0071365 | 94 | 188 | 561 | 375 | 2 | 0 | 2.00E-162 |
| hsa_circ_0071365 | 94 | 188 | 561 | 375 | 2 | 0 | 2.00E-162 |
| hsa_circ_0071365 | 94 | 102 | 475 | 375 | 2 | 0 | 1.00E-162 |
| hsa_circ_0071365 | 94 | 102 | 475 | 375 | 2 | 0 | 1.00E-162 |
| hsa_circ_0071365 | 94 | 102 | 475 | 375 | 2 | 0 | 2.00E-162 |
| hsa_circ_0071363 | 84 | 291 | 585 | 295 | 1 | 0 | 2.00E-75 |

novel_circ_019943 novel_circ_019944 novel_circ_019945 novel_circ_019946 novel_circ_019947 novel_circ_019955 novel_circ_019957 novel_circ_019958 novel_circ_019959 novel_circ_019960 novel_circ_019961 novel_circ_019962 novel_circ_019963 novel_circ_019964 novel_circ_019965 novel_circ_019966 novel_circ_019967 novel_circ_019968 novel_circ_019969 novel_circ_019970 novel_circ_019971 novel_circ_019972 novel_circ_019973 novel_circ_019974 novel_circ_019975 novel_circ_019976 novel_circ_019986 novel_circ_019987 novel_circ_019988 novel_circ_019989 novel_circ_019990 novel_circ_019991 novel_circ_019992 novel_circ_019993 novel_circ_019997 novel_circ_019998 novel_circ_019999 novel_circ_020000 novel_circ_020001 novel_circ_020002 novel_circ_020003 novel_circ_020004 novel_circ_020005 novel_circ_020008 novel_circ_020009 novel_circ_020010 novel_circ_020011 novel_circ_020014 novel_circ_020015 novel_circ_020016 novel_circ_020017 novel_circ_020018 novel_circ_020019 novel_circ_020020

| hsa_circ_0071363 | 84 | 124 | 418 | 295 | 1 | 0 | 1.00E-75 |
| --- | --- | --- | --- | --- | --- | --- | --- |
| hsa_circ_0071363 | 84 | 3 | 297 | 295 | 1 | 0 | 1.00E-75 |
| hsa_circ_0071361 | 90 | 322 | 590 | 502 | 234 | 0 | 6.00E-96 |
| hsa_circ_0071361 | 90 | 25 | 293 | 502 | 234 | 0 | 3.00E-96 |
| hsa_circ_0071361 | 90 | 1 | 269 | 502 | 234 | 0 | 2.00E-96 |
| hsa_circ_0042106 | 90 | 112 | 221 | 32 | 141 | 0 | 1.00E-32 |
| hsa_circ_0042094 | 98 | 1 | 822 | 104 | 925 | 0 | 0 |
| hsa_circ_0042094 | 98 | 1 | 925 | 1 | 925 | 0 | 0 |
| hsa_circ_0042100 | 97 | 1 | 378 | 1 | 378 | 0 | 0 |
| hsa_circ_0042094 | 98 | 1 | 673 | 104 | 776 | 0 | 0 |
| hsa_circ_0042094 | 98 | 1 | 776 | 1 | 776 | 0 | 0 |
| hsa_circ_0042099 | 97 | 1 | 420 | 1 | 420 | 0 | 0 |
| hsa_circ_0007456 | 98 | 1 | 595 | 1 | 595 | 0 | 0 |
| hsa_circ_0042094 | 98 | 1 | 698 | 1 | 698 | 0 | 0 |
| hsa_circ_0007456 | 99 | 1 | 467 | 1 | 467 | 0 | 0 |
| hsa_circ_0042094 | 99 | 1 | 570 | 1 | 570 | 0 | 0 |
| hsa_circ_0042094 | 99 | 1 | 398 | 1 | 398 | 0 | 0 |
| hsa_circ_0042094 | 99 | 4090 | 4487 | 1 | 398 | 0 | 0 |
| hsa_circ_0042094 | 99 | 1 | 278 | 1 | 278 | 0 | 5.00E-143 |
| hsa_circ_0042094 | 99 | 34 | 311 | 1 | 278 | 0 | 6.00E-143 |
| hsa_circ_0042094 | 99 | 4087 | 4364 | 1 | 278 | 0 | 9.00E-142 |
| hsa_circ_0042094 | 99 | 4090 | 4367 | 1 | 278 | 0 | 9.00E-142 |
| hsa_circ_0042089 | 85 | 24 | 656 | 748 | 107 | 0.02 | 1.00E-178 |
| hsa_circ_0042089 | 85 | 24 | 656 | 748 | 107 | 0.02 | 1.00E-178 |
| hsa_circ_0042089 | 85 | 24 | 656 | 748 | 107 | 0.02 | 2.00E-178 |
| hsa_circ_0042089 | 85 | 24 | 656 | 748 | 107 | 0.02 | 4.00E-178 |
| hsa_circ_0063152 | 94 | 1 | 509 | 509 | 1 | 0 | 0 |
| hsa_circ_0063174 | 92 | 1 | 542 | 690 | 149 | 0 | 0 |
| hsa_circ_0063174 | 92 | 1 | 223 | 690 | 468 | 0 | 7.00E-86 |
| hsa_circ_0073278 | 92 | 1 | 263 | 4123 | 3858 | 0.01 | 1.00E-103 |
| hsa_circ_0073299 | 88 | 12 | 826 | 152 | 966 | 0 | 0 |
| hsa_circ_0073315 | 91 | 1 | 441 | 1 | 441 | 0 | 1.00E-166 |
| hsa_circ_0073324 | 90 | 1 | 538 | 1 | 538 | 0 | 0 |
| hsa_circ_0004758 | 97 | 9444 | 9479 | 1477 | 1442 | 0 | 1.00E-06 |
| hsa_circ_0072981 | 83 | 8980 | 9386 | 403 | 817 | 0.03 | 2.00E-101 |
| hsa_circ_0029734 | 81 | 1170 | 1275 | 137403 | 137296 | 0.02 | 9.00E-15 |
| hsa_circ_0006855 | 89 | 298 | 372 | 1 | 75 | 0 | 2.00E-18 |
| hsa_circ_0022510 | 94 | 1 | 225 | 226 | 2 | 0 | 1.00E-93 |
| hsa_circ_0008011 | 92 | 13 | 333 | 330 | 10 | 0 | 2.00E-123 |
| hsa_circ_0022481 | 92 | 1 | 1365 | 1365 | 1 | 0 | 0 |
| hsa_circ_0022481 | 92 | 1 | 346 | 346 | 1 | 0 | 1.00E-135 |
| hsa_circ_0022429 | 89 | 1 | 1152 | 9843 | 10994 | 0 | 0 |
| hsa_circ_0022429 | 89 | 104 | 4650 | 5218 | 9764 | 0 | 0 |
| hsa_circ_0030630 | 89 | 1 | 778 | 946 | 166 | 0 | 0 |
| hsa_circ_0030617 | 94 | 1 | 302 | 154 | 455 | 0 | 3.00E-126 |
| hsa_circ_0030617 | 94 | 3 | 455 | 4 | 455 | 0 | 0 |
| hsa_circ_0030610 | 94 | 1 | 300 | 667 | 368 | 0 | 7.00E-127 |
| hsa_circ_0030563 | 93 | 1 | 749 | 1 | 749 | 0 | 0 |
| hsa_circ_0030563 | 93 | 1 | 749 | 1 | 749 | 0 | 0 |
| hsa_circ_0030563 | 94 | 1 | 591 | 1 | 591 | 0 | 0 |
| hsa_circ_0030563 | 92 | 1 | 360 | 1 | 360 | 0 | 9.00E-142 |
| hsa_circ_0030560 | 96 | 1 | 303 | 303 | 1 | 0 | 2.00E-138 |
| hsa_circ_0030559 | 95 | 304 | 672 | 369 | 1 | 0 | 4.00E-163 |
| hsa_circ_0030559 | 95 | 235 | 603 | 369 | 1 | 0 | 4.00E-163 |

novel_circ_020021 novel_circ_020034 novel_circ_020035 novel_circ_020036 novel_circ_020037 novel_circ_020038 novel_circ_020039 novel_circ_020040 novel_circ_020041 novel_circ_020042 novel_circ_020043 novel_circ_020044 novel_circ_020045 novel_circ_020046 novel_circ_020047 novel_circ_020048 novel_circ_020049 novel_circ_020050 novel_circ_020051 novel_circ_020052 novel_circ_020053 novel_circ_020054 novel_circ_020055 novel_circ_020056 novel_circ_020057 novel_circ_020058 novel_circ_020059 novel_circ_020060 novel_circ_020061 novel_circ_020062 novel_circ_020063 novel_circ_020064 novel_circ_020065 novel_circ_020066 novel_circ_020067 novel_circ_020068 novel_circ_020069 novel_circ_020071 novel_circ_020072 novel_circ_020073 novel_circ_020074 novel_circ_020075 novel_circ_020076 novel_circ_020077 novel_circ_020078 novel_circ_020079 novel_circ_020080 novel_circ_020081 novel_circ_020082 novel_circ_020083 novel_circ_020084 novel_circ_020087 novel_circ_020088 novel_circ_020089

| hsa_circ_0030559 | 95 | 144 | 512 | 369 | 1 | 0 | 3.00E-163 |
| --- | --- | --- | --- | --- | --- | --- | --- |
| hsa_circ_0056044 | 88 | 1 | 511 | 1 | 544 | 0.06 | 9.00E-178 |
| hsa_circ_0056053 | 91 | 1 | 545 | 684 | 140 | 0 | 0 |
| hsa_circ_0056060 | 94 | 1 | 541 | 632 | 92 | 0 | 0 |
| hsa_circ_0056060 | 94 | 4 | 1382 | 1470 | 92 | 0 | 0 |
| hsa_circ_0056066 | 93 | 4 | 1151 | 1148 | 1 | 0 | 0 |
| hsa_circ_0056054 | 92 | 1 | 3112 | 4782 | 1671 | 0 | 0 |
| hsa_circ_0056074 | 92 | 1 | 1139 | 1354 | 216 | 0 | 0 |
| hsa_circ_0056054 | 92 | 1 | 2778 | 4782 | 2005 | 0 | 0 |
| hsa_circ_0056054 | 92 | 1 | 2599 | 4782 | 2184 | 0 | 0 |
| hsa_circ_0056087 | 92 | 1 | 585 | 585 | 1 | 0 | 0 |
| hsa_circ_0056087 | 92 | 1 | 1110 | 1110 | 1 | 0 | 0 |
| hsa_circ_0056087 | 92 | 1 | 1363 | 1363 | 1 | 0 | 0 |
| hsa_circ_0056080 | 92 | 1 | 2412 | 2528 | 117 | 0 | 0 |
| hsa_circ_0056096 | 92 | 1 | 344 | 344 | 1 | 0 | 3.00E-136 |
| hsa_circ_0056096 | 92 | 1 | 2171 | 2171 | 1 | 0 | 0 |
| hsa_circ_0056096 | 93 | 1 | 2408 | 2408 | 1 | 0 | 0 |
| hsa_circ_0056096 | 92 | 1 | 1827 | 2171 | 345 | 0 | 0 |
| hsa_circ_0056096 | 93 | 1 | 2064 | 2408 | 345 | 0 | 0 |
| hsa_circ_0056101 | 90 | 1 | 272 | 337 | 66 | 0 | 5.00E-98 |
| hsa_circ_0056101 | 92 | 1 | 525 | 590 | 66 | 0 | 0 |
| hsa_circ_0056101 | 92 | 1 | 1574 | 1639 | 66 | 0 | 0 |
| hsa_circ_0056101 | 92 | 1 | 1437 | 1639 | 203 | 0 | 0 |
| hsa_circ_0056101 | 93 | 1 | 1674 | 1876 | 203 | 0 | 0 |
| hsa_circ_0056101 | 93 | 1 | 1297 | 1634 | 338 | 0 | 0 |
| hsa_circ_0056101 | 93 | 1 | 1302 | 1639 | 338 | 0 | 0 |
| hsa_circ_0056101 | 93 | 1 | 1145 | 1639 | 495 | 0 | 0 |
| hsa_circ_0056101 | 93 | 1 | 1382 | 1876 | 495 | 0 | 0 |
| hsa_circ_0056101 | 93 | 1 | 1049 | 1639 | 591 | 0 | 0 |
| hsa_circ_0056114 | 93 | 1 | 472 | 472 | 1 | 0 | 0 |
| hsa_circ_0056114 | 94 | 1 | 635 | 709 | 75 | 0 | 0 |
| hsa_circ_0056120 | 89 | 23 | 666 | 50 | 696 | 0 | 0 |
| hsa_circ_0056121 | 92 | 1 | 275 | 1 | 275 | 0 | 5.00E-108 |
| hsa_circ_0056120 | 90 | 1 | 813 | 422 | 1234 | 0 | 0 |
| hsa_circ_0056120 | 89 | 3 | 538 | 699 | 1234 | 0 | 0 |
| hsa_circ_0056124 | 90 | 1 | 335 | 1 | 335 | 0 | 6.00E-123 |
| hsa_circ_0056124 | 90 | 1 | 783 | 337 | 1119 | 0 | 0 |
| hsa_circ_0056128 | 91 | 61 | 665 | 868 | 1470 | 0.01 | 0 |
| hsa_circ_0007133 | 92 | 133 | 258 | 2E+06 | 1573517 | 0.05 | 2.00E-41 |
| hsa_circ_0007133 | 96 | 180 | 304 | 2E+06 | 1642749 | 0 | 3.00E-51 |
| hsa_circ_0056132 | 93 | 182 | 946 | 1 | 765 | 0 | 0 |
| hsa_circ_0056132 | 93 | 182 | 1054 | 1 | 873 | 0 | 0 |
| hsa_circ_0056136 | 91 | 1 | 448 | 1 | 448 | 0 | 6.00E-169 |
| hsa_circ_0056136 | 92 | 1 | 862 | 1 | 862 | 0 | 0 |
| hsa_circ_0056138 | 90 | 1 | 369 | 1 | 369 | 0 | 9.00E-132 |
| hsa_circ_0056138 | 91 | 1 | 639 | 1 | 639 | 0 | 0 |
| hsa_circ_0056142 | 91 | 711 | 3510 | 1 | 2800 | 0 | 0 |
| hsa_circ_0056142 | 88 | 1 | 344 | 642 | 985 | 0.01 | 7.00E-113 |
| hsa_circ_0056142 | 89 | 1 | 626 | 642 | 1267 | 0.01 | 0 |
| hsa_circ_0056142 | 89 | 1 | 760 | 642 | 1401 | 0.01 | 0 |
| hsa_circ_0056153 | 91 | 1 | 1100 | 445 | 1544 | 0 | 0 |
| hsa_circ_0085541 | 76 | 2588 | 2797 | 1200 | 1411 | 0.06 | 2.00E-18 |
| hsa_circ_0009016 | 72 | 17141 | 17950 | 101077 | 100272 | 0.03 | 8.00E-57 |
| hsa_circ_0009016 | 72 | 17141 | 17950 | 101077 | 100272 | 0.03 | 1.00E-56 |

novel_circ_020093 novel_circ_020095 novel_circ_020096 novel_circ_020097 novel_circ_020098 novel_circ_020099 novel_circ_020100 novel_circ_020101 novel_circ_020102 novel_circ_020103 novel_circ_020104 novel_circ_020105 novel_circ_020106 novel_circ_020107 novel_circ_020108 novel_circ_020109 novel_circ_020110 novel_circ_020111 novel_circ_020112 novel_circ_020113 novel_circ_020114 novel_circ_020115 novel_circ_020116 novel_circ_020123 novel_circ_020126 novel_circ_020127 novel_circ_020129 novel_circ_020130 novel_circ_020131 novel_circ_020133 novel_circ_020134 novel_circ_020135 novel_circ_020136 novel_circ_020138 novel_circ_020141 novel_circ_020143 novel_circ_020144 novel_circ_020145 novel_circ_020146 novel_circ_020147 novel_circ_020148 novel_circ_020149 novel_circ_020150 novel_circ_020151 novel_circ_020152 novel_circ_020153 novel_circ_020154 novel_circ_020155 novel_circ_020156 novel_circ_020157 novel_circ_020158 novel_circ_020159 novel_circ_020160 novel_circ_020161

| hsa_circ_0002595 | 73 | 50500 | 50934 | 314709 | 314273 | 0.02 | 4.00E-33 |
| --- | --- | --- | --- | --- | --- | --- | --- |
| hsa_circ_0085562 | 95 | 1 | 645 | 645 | 1 | 0 | 0 |
| hsa_circ_0085562 | 95 | 1 | 767 | 767 | 1 | 0 | 0 |
| hsa_circ_0085562 | 95 | 1 | 850 | 850 | 1 | 0 | 0 |
| hsa_circ_0085562 | 96 | 1 | 933 | 933 | 1 | 0 | 0 |
| hsa_circ_0085562 | 96 | 235 | 1169 | 935 | 1 | 0 | 0 |
| hsa_circ_0085562 | 95 | 1 | 645 | 645 | 1 | 0 | 0 |
| hsa_circ_0085562 | 95 | 1 | 767 | 767 | 1 | 0 | 0 |
| hsa_circ_0085562 | 95 | 1 | 850 | 850 | 1 | 0 | 0 |
| hsa_circ_0085562 | 95 | 1 | 640 | 767 | 128 | 0 | 0 |
| hsa_circ_0085562 | 95 | 1 | 723 | 850 | 128 | 0 | 0 |
| hsa_circ_0085563 | 96 | 1 | 321 | 438 | 118 | 0 | 8.00E-147 |
| hsa_circ_0085563 | 95 | 1 | 443 | 560 | 118 | 0 | 0 |
| hsa_circ_0085563 | 97 | 1 | 243 | 438 | 196 | 0 | 6.00E-112 |
| hsa_circ_0085563 | 97 | 235 | 524 | 728 | 439 | 0 | 5.00E-136 |
| hsa_circ_0085568 | 91 | 1 | 923 | 3444 | 2531 | 0.01 | 0 |
| hsa_circ_0085568 | 92 | 1 | 1094 | 3615 | 2531 | 0.01 | 0 |
| hsa_circ_0085593 | 92 | 4 | 472 | 469 | 1 | 0 | 0 |
| hsa_circ_0085596 | 94 | 4 | 308 | 305 | 1 | 0 | 3.00E-131 |
| hsa_circ_0085596 | 95 | 4 | 259 | 305 | 50 | 0 | 4.00E-109 |
| hsa_circ_0085603 | 96 | 1 | 334 | 334 | 1 | 0 | 1.00E-155 |
| hsa_circ_0085603 | 97 | 1 | 420 | 420 | 1 | 0 | 0 |
| hsa_circ_0085603 | 95 | 1 | 550 | 550 | 1 | 0 | 0 |
| hsa_circ_0005343 | 82 | 31475 | 31587 | 108490 | 108379 | 0.04 | 3.00E-15 |
| hsa_circ_0068520 | 91 | 1 | 160 | 928 | 1087 | 0 | 7.00E-54 |
| hsa_circ_0068520 | 85 | 1 | 806 | 282 | 1087 | 0 | 0 |
| hsa_circ_0068520 | 85 | 1 | 806 | 282 | 1087 | 0 | 0 |
| hsa_circ_0068520 | 94 | 3 | 399 | 3 | 399 | 0 | 6.00E-174 |
| hsa_circ_0068520 | 95 | 3 | 281 | 3 | 281 | 0 | 1.00E-123 |
| hsa_circ_0068519 | 90 | 8 | 664 | 633 | 1289 | 0 | 0 |
| hsa_circ_0068519 | 90 | 1 | 987 | 153 | 1139 | 0 | 0 |
| hsa_circ_0068519 | 89 | 8 | 365 | 633 | 990 | 0 | 1.00E-125 |
| hsa_circ_0003347 | 91 | 1 | 471 | 1 | 471 | 0 | 0 |
| hsa_circ_0068510 | 89 | 1 | 684 | 1 | 684 | 0 | 0 |
| hsa_circ_0068482 | 87 | 1 | 774 | 1 | 787 | 0.04 | 0 |
| hsa_circ_0068557 | 85 | 1 | 894 | 2736 | 1840 | 0.02 | 0 |
| hsa_circ_0068570 | 93 | 1 | 1513 | 1492 | 1 | 0.01 | 0 |
| hsa_circ_0068570 | 93 | 183 | 1849 | 1708 | 63 | 0.01 | 0 |
| hsa_circ_0068571 | 93 | 1 | 370 | 668 | 299 | 0 | 2.00E-152 |
| hsa_circ_0068570 | 92 | 1 | 1042 | 1492 | 472 | 0.02 | 0 |
| hsa_circ_0002382 | 94 | 1 | 556 | 556 | 1 | 0 | 0 |
| hsa_circ_0068570 | 93 | 2 | 815 | 1378 | 565 | 0 | 0 |
| hsa_circ_0068570 | 92 | 1 | 949 | 1492 | 565 | 0.02 | 0 |
| hsa_circ_0068570 | 92 | 1 | 1120 | 1663 | 565 | 0.02 | 0 |
| hsa_circ_0068570 | 93 | 185 | 1349 | 1708 | 565 | 0.02 | 0 |
| hsa_circ_0068573 | 90 | 1 | 393 | 581 | 210 | 0.05 | 6.00E-134 |
| hsa_circ_0068573 | 91 | 1 | 564 | 752 | 210 | 0.04 | 0 |
| hsa_circ_0068573 | 92 | 1 | 852 | 1040 | 210 | 0.02 | 0 |
| hsa_circ_0068573 | 90 | 1 | 458 | 752 | 316 | 0.05 | 5.00E-160 |
| hsa_circ_0068575 | 95 | 1 | 284 | 284 | 1 | 0 | 2.00E-126 |
| hsa_circ_0068575 | 95 | 1 | 572 | 572 | 1 | 0 | 0 |
| hsa_circ_0068575 | 95 | 1 | 746 | 746 | 1 | 0 | 0 |
| hsa_circ_0068575 | 94 | 1 | 633 | 746 | 114 | 0 | 0 |
| hsa_circ_0007347 | 93 | 1 | 384 | 459 | 76 | 0 | 4.00E-160 |

novel_circ_020162 novel_circ_020163 novel_circ_020164 novel_circ_020166 novel_circ_020167 novel_circ_020168 novel_circ_020169 novel_circ_020170 novel_circ_020171 novel_circ_020172 novel_circ_020173 novel_circ_020174 novel_circ_020175 novel_circ_020176 novel_circ_020177 novel_circ_020178 novel_circ_020179 novel_circ_020180 novel_circ_020181 novel_circ_020182 novel_circ_020183 novel_circ_020184 novel_circ_020185 novel_circ_020186 novel_circ_020187 novel_circ_020188 novel_circ_020189 novel_circ_020190 novel_circ_020191 novel_circ_020192 novel_circ_020193 novel_circ_020194 novel_circ_020195 novel_circ_020196 novel_circ_020197 novel_circ_020198 novel_circ_020199 novel_circ_020200 novel_circ_020204 novel_circ_020205 novel_circ_020206 novel_circ_020207 novel_circ_020208 novel_circ_020209 novel_circ_020210 novel_circ_020212 novel_circ_020213 novel_circ_020214 novel_circ_020215 novel_circ_020216 novel_circ_020218 novel_circ_020224 novel_circ_020227 novel_circ_020228

| hsa_circ_0005400 | 93 | 1 | 243 | 243 | 1 | 0 | 3.00E-95 |
| --- | --- | --- | --- | --- | --- | --- | --- |
| hsa_circ_0068575 | 94 | 1 | 417 | 746 | 330 | 0 | 1.00E-176 |
| hsa_circ_0068575 | 94 | 1 | 475 | 804 | 330 | 0 | 0 |
| hsa_circ_0068708 | 94 | 1 | 167 | 167 | 1 | 0 | 1.00E-66 |
| hsa_circ_0068698 | 95 | 911 | 1693 | 904 | 122 | 0 | 0 |
| hsa_circ_0068698 | 95 | 1 | 537 | 803 | 267 | 0 | 0 |
| hsa_circ_0068698 | 95 | 1 | 682 | 803 | 122 | 0 | 0 |
| hsa_circ_0068695 | 95 | 1 | 960 | 960 | 1 | 0 | 0 |
| hsa_circ_0068699 | 96 | 4 | 483 | 480 | 1 | 0 | 0 |
| hsa_circ_0068698 | 96 | 4 | 628 | 746 | 122 | 0 | 0 |
| hsa_circ_0068695 | 95 | 4 | 906 | 903 | 1 | 0 | 0 |
| hsa_circ_0068698 | 95 | 1 | 577 | 698 | 122 | 0 | 0 |
| hsa_circ_0068698 | 95 | 1 | 403 | 403 | 1 | 0 | 3.00E-177 |
| hsa_circ_0068685 | 95 | 1 | 462 | 501 | 43 | 0.01 | 0 |
| hsa_circ_0068683 | 96 | 499 | 1010 | 512 | 1 | 0 | 0 |
| hsa_circ_0068683 | 96 | 1 | 275 | 385 | 111 | 0 | 2.00E-126 |
| hsa_circ_0068676 | 93 | 1 | 321 | 2002 | 1682 | 0 | 4.00E-130 |
| hsa_circ_0068670 | 96 | 1 | 800 | 1545 | 2344 | 0 | 0 |
| hsa_circ_0068670 | 96 | 1 | 644 | 1545 | 2188 | 0 | 0 |
| hsa_circ_0068670 | 96 | 1 | 593 | 1545 | 2137 | 0 | 0 |
| hsa_circ_0068670 | 95 | 1 | 509 | 1545 | 2053 | 0 | 0 |
| hsa_circ_0068673 | 95 | 1 | 371 | 1 | 371 | 0 | 7.00E-163 |
| hsa_circ_0068673 | 94 | 1 | 295 | 1 | 295 | 0 | 2.00E-122 |
| hsa_circ_0068673 | 94 | 1 | 293 | 1 | 293 | 0 | 3.00E-121 |
| hsa_circ_0068668 | 89 | 36 | 1578 | 217 | 1762 | 0 | 0 |
| hsa_circ_0068666 | 95 | 3 | 335 | 447 | 779 | 0 | 4.00E-150 |
| hsa_circ_0068666 | 92 | 1 | 715 | 65 | 779 | 0 | 0 |
| hsa_circ_0068664 | 91 | 8 | 1020 | 8 | 1020 | 0 | 0 |
| hsa_circ_0068666 | 91 | 1 | 577 | 65 | 641 | 0 | 0 |
| hsa_circ_0068666 | 91 | 1 | 641 | 1 | 641 | 0 | 0 |
| hsa_circ_0068664 | 90 | 8 | 882 | 8 | 882 | 0 | 0 |
| hsa_circ_0068666 | 89 | 1 | 375 | 65 | 439 | 0 | 9.00E-132 |
| hsa_circ_0068661 | 91 | 3 | 1074 | 304 | 1375 | 0 | 0 |
| hsa_circ_0068666 | 90 | 1 | 344 | 1 | 344 | 0 | 3.00E-121 |
| hsa_circ_0068664 | 89 | 8 | 585 | 8 | 585 | 0 | 0 |
| hsa_circ_0068664 | 89 | 8 | 467 | 8 | 467 | 0 | 7.00E-164 |
| hsa_circ_0068664 | 89 | 8 | 305 | 8 | 305 | 0 | 2.00E-102 |
| hsa_circ_0068662 | 94 | 3 | 457 | 3 | 457 | 0 | 0 |
| hsa_circ_0068645 | 95 | 1 | 395 | 1155 | 761 | 0 | 3.00E-176 |
| hsa_circ_0068645 | 96 | 1 | 761 | 1155 | 395 | 0 | 0 |
| hsa_circ_0001380 | 94 | 1 | 247 | 247 | 1 | 0 | 8.00E-101 |
| hsa_circ_0068645 | 95 | 1 | 522 | 522 | 1 | 0 | 0 |
| hsa_circ_0068645 | 94 | 1 | 394 | 394 | 1 | 0 | 1.00E-170 |
| hsa_circ_0068639 | 92 | 1 | 269 | 269 | 1 | 0 | 5.00E-103 |
| hsa_circ_0068617 | 85 | 1 | 1061 | 1491 | 431 | 0.01 | 0 |
| hsa_circ_0068624 | 83 | 1 | 662 | 662 | 1 | 0.02 | 3.00E-169 |
| hsa_circ_0068617 | 83 | 1 | 1080 | 1230 | 151 | 0.01 | 0 |
| hsa_circ_0068624 | 89 | 48 | 316 | 269 | 1 | 0 | 3.00E-91 |
| hsa_circ_0068618 | 86 | 1 | 631 | 717 | 87 | 0 | 0 |
| hsa_circ_0068618 | 83 | 1 | 418 | 504 | 87 | 0 | 3.00E-107 |
| hsa_circ_0068589 | 92 | 4 | 431 | 797 | 370 | 0 | 8.00E-173 |
| hsa_circ_0074854 | 96 | 1 | 268 | 1 | 268 | 0 | 4.00E-119 |
| hsa_circ_0020013 | 93 | 1 | 367 | 1 | 367 | 0 | 5.00E-154 |
| hsa_circ_0020016 | 95 | 1 | 453 | 180 | 632 | 0 | 0 |

novel_circ_020229 novel_circ_020230 novel_circ_020231 novel_circ_020232 novel_circ_020233 novel_circ_020234 novel_circ_020235 novel_circ_020236 novel_circ_020237 novel_circ_020238 novel_circ_020239 novel_circ_020240 novel_circ_020241 novel_circ_020242 novel_circ_020243 novel_circ_020244 novel_circ_020246 novel_circ_020247 novel_circ_020248 novel_circ_020249 novel_circ_020250 novel_circ_020251 novel_circ_020252 novel_circ_020253 novel_circ_020254 novel_circ_020255 novel_circ_020256 novel_circ_020257 novel_circ_020258 novel_circ_020259 novel_circ_020260 novel_circ_020261 novel_circ_020262 novel_circ_020263 novel_circ_020264 novel_circ_020265 novel_circ_020266 novel_circ_020267 novel_circ_020268 novel_circ_020269 novel_circ_020270 novel_circ_020271 novel_circ_020272 novel_circ_020273 novel_circ_020274 novel_circ_020275 novel_circ_020276 novel_circ_020277 novel_circ_020279 novel_circ_020280 novel_circ_020281 novel_circ_020282 novel_circ_020283 novel_circ_020284

| hsa_circ_0020019 | 95 | 1 | 294 | 1 | 294 | 0 | 7.00E-127 |
| --- | --- | --- | --- | --- | --- | --- | --- |
| hsa_circ_0020019 | 91 | 1 | 371 | 295 | 662 | 0.01 | 2.00E-138 |
| hsa_circ_0020019 | 93 | 1 | 948 | 295 | 1239 | 0.01 | 0 |
| hsa_circ_0020020 | 94 | 1 | 577 | 1 | 577 | 0 | 0 |
| hsa_circ_0020020 | 95 | 1 | 721 | 1 | 721 | 0 | 0 |
| hsa_circ_0020020 | 95 | 1 | 872 | 1 | 872 | 0 | 0 |
| hsa_circ_0020020 | 95 | 1 | 977 | 1 | 977 | 0 | 0 |
| hsa_circ_0020020 | 94 | 1 | 2014 | 1 | 2014 | 0 | 0 |
| hsa_circ_0020020 | 94 | 1 | 2384 | 1 | 2384 | 0 | 0 |
| hsa_circ_0020020 | 94 | 1 | 2388 | 1 | 2388 | 0 | 0 |
| hsa_circ_0020020 | 95 | 1 | 763 | 215 | 977 | 0 | 0 |
| hsa_circ_0020020 | 94 | 1 | 1339 | 215 | 1553 | 0 | 0 |
| hsa_circ_0020020 | 94 | 1 | 2174 | 215 | 2388 | 0 | 0 |
| hsa_circ_0020020 | 95 | 1 | 559 | 419 | 977 | 0 | 0 |
| hsa_circ_0020021 | 97 | 1 | 293 | 1 | 293 | 0 | 3.00E-136 |
| hsa_circ_0020020 | 96 | 1 | 398 | 580 | 977 | 0 | 0 |
| hsa_circ_0020030 | 94 | 139 | 1072 | 1 | 937 | 0.01 | 0 |
| hsa_circ_0020030 | 94 | 139 | 1210 | 1 | 1075 | 0 | 0 |
| hsa_circ_0020030 | 94 | 1 | 934 | 1 | 937 | 0.01 | 0 |
| hsa_circ_0020030 | 94 | 1 | 1072 | 1 | 1075 | 0 | 0 |
| hsa_circ_0020030 | 95 | 1 | 1203 | 1 | 1206 | 0 | 0 |
| hsa_circ_0020030 | 95 | 1 | 1392 | 1 | 1395 | 0 | 0 |
| hsa_circ_0020030 | 95 | 1 | 1515 | 1 | 1518 | 0 | 0 |
| hsa_circ_0020030 | 95 | 1 | 1771 | 1 | 1774 | 0 | 0 |
| hsa_circ_0020031 | 97 | 1 | 458 | 1 | 458 | 0 | 0 |
| hsa_circ_0020030 | 96 | 1 | 581 | 938 | 1518 | 0 | 0 |
| hsa_circ_0020030 | 94 | 1 | 312 | 1207 | 1518 | 0 | 2.00E-133 |
| hsa_circ_0020030 | 95 | 1 | 379 | 1396 | 1774 | 0 | 5.00E-169 |
| hsa_circ_0071173 | 91 | 84456 | 84624 | 169 | 1 | 0 | 7.00E-55 |
| hsa_circ_0071188 | 92 | 1 | 211 | 261 | 54 | 0.01 | 2.00E-77 |
| hsa_circ_0071188 | 92 | 7 | 290 | 334 | 54 | 0.01 | 1.00E-104 |
| hsa_circ_0071188 | 88 | 1 | 424 | 474 | 54 | 0.01 | 5.00E-139 |
| hsa_circ_0071188 | 92 | 1 | 211 | 261 | 54 | 0.01 | 1.00E-77 |
| hsa_circ_0071188 | 92 | 7 | 290 | 334 | 54 | 0.01 | 6.00E-105 |
| hsa_circ_0071188 | 88 | 1 | 424 | 474 | 54 | 0.01 | 2.00E-139 |
| hsa_circ_0071195 | 89 | 1 | 409 | 409 | 1 | 0 | 1.00E-140 |
| hsa_circ_0071218 | 79 | 1 | 1928 | 1963 | 21 | 0.03 | 0 |
| hsa_circ_0071203 | 88 | 1 | 347 | 347 | 1 | 0 | 3.00E-111 |
| hsa_circ_0071218 | 84 | 3 | 376 | 388 | 21 | 0.02 | 4.00E-94 |
| hsa_circ_0071218 | 84 | 3 | 376 | 388 | 21 | 0.02 | 2.00E-94 |
| hsa_circ_0071222 | 91 | 5 | 512 | 740 | 233 | 0 | 0 |
| hsa_circ_0071239 | 91 | 1 | 443 | 443 | 1 | 0 | 4.00E-166 |
| hsa_circ_0071245 | 89 | 1 | 1359 | 1359 | 1 | 0 | 0 |
| hsa_circ_0071246 | 90 | 1 | 612 | 1375 | 764 | 0 | 0 |
| hsa_circ_0071249 | 89 | 1 | 465 | 465 | 1 | 0 | 2.00E-163 |
| hsa_circ_0071246 | 90 | 2 | 428 | 1707 | 1281 | 0 | 6.00E-159 |
| hsa_circ_0071251 | 92 | 2 | 328 | 332 | 6 | 0 | 8.00E-127 |
| hsa_circ_0071256 | 84 | 1 | 839 | 2658 | 1820 | 0.01 | 0 |
| hsa_circ_0071256 | 85 | 1 | 439 | 2658 | 2220 | 0 | 3.00E-122 |
| hsa_circ_0071256 | 85 | 3 | 512 | 2729 | 2220 | 0 | 6.00E-145 |
| hsa_circ_0071256 | 85 | 1 | 389 | 2658 | 2270 | 0.01 | 4.00E-111 |
| hsa_circ_0071256 | 86 | 1 | 1112 | 3381 | 2270 | 0.01 | 0 |
| hsa_circ_0071265 | 84 | 1 | 849 | 1 | 854 | 0.02 | 0 |
| hsa_circ_0071265 | 85 | 1 | 1095 | 1 | 1100 | 0.02 | 0 |

novel_circ_020285 novel_circ_020286 novel_circ_020287 novel_circ_020288 novel_circ_020289 novel_circ_020290 novel_circ_020291 novel_circ_020292 novel_circ_020293 novel_circ_020294 novel_circ_020295 novel_circ_020296 novel_circ_020297 novel_circ_020298 novel_circ_020299 novel_circ_020300 novel_circ_020301 novel_circ_020302 novel_circ_020303 novel_circ_020304 novel_circ_020305 novel_circ_020306 novel_circ_020307 novel_circ_020308 novel_circ_020309 novel_circ_020310 novel_circ_020311 novel_circ_020314 novel_circ_020316 novel_circ_020317 novel_circ_020319 novel_circ_020320 novel_circ_020321 novel_circ_020322 novel_circ_020323 novel_circ_020324 novel_circ_020325 novel_circ_020326 novel_circ_020327 novel_circ_020328 novel_circ_020329 novel_circ_020330 novel_circ_020331 novel_circ_020332 novel_circ_020333 novel_circ_020334 novel_circ_020335 novel_circ_020336 novel_circ_020337 novel_circ_020338 novel_circ_020339 novel_circ_020340 novel_circ_020341 novel_circ_020342

| hsa_circ_0071265 | 86 | 1 | 627 | 228 | 854 | 0 | 0 |
| --- | --- | --- | --- | --- | --- | --- | --- |
| hsa_circ_0039875 | 90 | 1 | 2373 | 1 | 2361 | 0.01 | 0 |
| hsa_circ_0039880 | 90 | 1 | 1819 | 12 | 1818 | 0.01 | 0 |
| hsa_circ_0039923 | 93 | 1 | 393 | 1 | 393 | 0 | 9.00E-162 |
| hsa_circ_0039929 | 92 | 7393 | 8669 | 1 | 1298 | 0.02 | 0 |
| hsa_circ_0039929 | 92 | 7393 | 9183 | 1 | 1812 | 0.01 | 0 |
| hsa_circ_0039929 | 92 | 5669 | 6945 | 1 | 1298 | 0.02 | 0 |
| hsa_circ_0039929 | 92 | 5669 | 7145 | 1 | 1498 | 0.02 | 0 |
| hsa_circ_0039929 | 92 | 5669 | 7318 | 1 | 1671 | 0.01 | 0 |
| hsa_circ_0039929 | 92 | 5669 | 7459 | 1 | 1812 | 0.01 | 0 |
| hsa_circ_0039929 | 92 | 5669 | 8641 | 1 | 3003 | 0.01 | 0 |
| hsa_circ_0039929 | 91 | 1 | 1114 | 1 | 1135 | 0.02 | 0 |
| hsa_circ_0039929 | 92 | 1 | 1277 | 1 | 1298 | 0.02 | 0 |
| hsa_circ_0039929 | 92 | 1 | 1477 | 1 | 1498 | 0.02 | 0 |
| hsa_circ_0039929 | 92 | 1 | 1650 | 1 | 1671 | 0.01 | 0 |
| hsa_circ_0039929 | 92 | 1 | 1791 | 1 | 1812 | 0.01 | 0 |
| hsa_circ_0039929 | 92 | 1 | 2973 | 1 | 3003 | 0.01 | 0 |
| hsa_circ_0039929 | 92 | 1 | 2973 | 1 | 3003 | 0.01 | 0 |
| hsa_circ_0039931 | 95 | 1 | 536 | 1 | 536 | 0 | 0 |
| hsa_circ_0039932 | 94 | 1 | 373 | 1 | 373 | 0 | 1.00E-160 |
| hsa_circ_0039932 | 95 | 1 | 514 | 1 | 514 | 0 | 0 |
| hsa_circ_0039931 | 93 | 1 | 1696 | 164 | 1868 | 0.01 | 0 |
| hsa_circ_0039931 | 92 | 2 | 1496 | 365 | 1868 | 0.01 | 0 |
| hsa_circ_0039931 | 92 | 2 | 1496 | 365 | 1868 | 0.01 | 0 |
| hsa_circ_0000712 | 92 | 1 | 1323 | 1 | 1332 | 0.01 | 0 |
| hsa_circ_0039935 | 92 | 1 | 1182 | 1 | 1191 | 0.01 | 0 |
| hsa_circ_0039935 | 92 | 1 | 1182 | 1 | 1191 | 0.01 | 0 |
| hsa_circ_0039965 | 90 | 179 | 474 | 1 | 296 | 0 | 1.00E-102 |
| hsa_circ_0040006 | 89 | 8 | 331 | 2 | 325 | 0 | 2.00E-108 |
| hsa_circ_0040008 | 88 | 1 | 266 | 142 | 407 | 0.01 | 2.00E-86 |
| hsa_circ_0040018 | 91 | 1 | 298 | 1 | 298 | 0 | 2.00E-112 |
| hsa_circ_0040020 | 90 | 5 | 476 | 5 | 476 | 0 | 1.00E-175 |
| hsa_circ_0040020 | 90 | 5 | 573 | 5 | 573 | 0 | 0 |
| hsa_circ_0040024 | 91 | 1 | 813 | 1 | 813 | 0 | 0 |
| hsa_circ_0008635 | 90 | 1 | 280 | 1 | 280 | 0 | 2.00E-97 |
| hsa_circ_0040040 | 86 | 23531 | 23875 | 212 | 565 | 0.03 | 3.00E-108 |
| hsa_circ_0040040 | 86 | 10261 | 10605 | 212 | 565 | 0.03 | 1.00E-108 |
| hsa_circ_0040040 | 89 | 10261 | 10805 | 212 | 765 | 0.02 | 0 |
| hsa_circ_0040040 | 88 | 10261 | 10990 | 212 | 950 | 0.01 | 0 |
| hsa_circ_0040041 | 93 | 1 | 340 | 1 | 340 | 0 | 2.00E-137 |
| hsa_circ_0040041 | 90 | 1 | 525 | 1 | 525 | 0 | 0 |
| hsa_circ_0040042 | 91 | 1 | 382 | 1 | 382 | 0 | 3.00E-142 |
| hsa_circ_0040063 | 90 | 1 | 820 | 820 | 1 | 0 | 0 |
| hsa_circ_0040074 | 97 | 1 | 943 | 1 | 943 | 0 | 0 |
| hsa_circ_0040074 | 97 | 1 | 1116 | 1 | 1116 | 0 | 0 |
| hsa_circ_0040074 | 96 | 1 | 1304 | 1 | 1304 | 0 | 0 |
| hsa_circ_0040073 | 96 | 1 | 1521 | 189 | 1709 | 0 | 0 |
| hsa_circ_0040077 | 94 | 1 | 384 | 1 | 384 | 0 | 9.00E-167 |
| hsa_circ_0040077 | 94 | 1 | 557 | 1 | 557 | 0 | 0 |
| hsa_circ_0040077 | 94 | 1 | 745 | 1 | 745 | 0 | 0 |
| hsa_circ_0040077 | 94 | 1 | 878 | 1 | 878 | 0 | 0 |
| hsa_circ_0040077 | 94 | 1 | 962 | 1 | 962 | 0 | 0 |
| hsa_circ_0040077 | 93 | 1 | 364 | 194 | 557 | 0 | 1.00E-150 |
| hsa_circ_0040078 | 96 | 1 | 405 | 1 | 405 | 0 | 0 |

novel_circ_020343 novel_circ_020344 novel_circ_020345 novel_circ_020346 novel_circ_020347 novel_circ_020348 novel_circ_020349 novel_circ_020350 novel_circ_020351 novel_circ_020352 novel_circ_020353 novel_circ_020354 novel_circ_020355 novel_circ_020356 novel_circ_020357 novel_circ_020358 novel_circ_020359 novel_circ_020360 novel_circ_020361 novel_circ_020362 novel_circ_020363 novel_circ_020364 novel_circ_020365 novel_circ_020366 novel_circ_020367 novel_circ_020368 novel_circ_020369 novel_circ_020370 novel_circ_020371 novel_circ_020372 novel_circ_020373 novel_circ_020374 novel_circ_020375 novel_circ_020376 novel_circ_020377 novel_circ_020378 novel_circ_020379 novel_circ_020380 novel_circ_020381 novel_circ_020382 novel_circ_020383 novel_circ_020384 novel_circ_020385 novel_circ_020386 novel_circ_020387 novel_circ_020388 novel_circ_020389 novel_circ_020390 novel_circ_020391 novel_circ_020392 novel_circ_020393 novel_circ_020396 novel_circ_020397 novel_circ_020398

| hsa_circ_0040077 | 92 | 1 | 3107 | 746 | 3846 | 0 | 0 |
| --- | --- | --- | --- | --- | --- | --- | --- |
| hsa_circ_0077502 | 94 | 1 | 499 | 499 | 1 | 0 | 0 |
| hsa_circ_0077502 | 94 | 1 | 649 | 649 | 1 | 0 | 0 |
| hsa_circ_0077502 | 94 | 1 | 1439 | 1439 | 1 | 0 | 0 |
| hsa_circ_0077504 | 94 | 1 | 329 | 329 | 1 | 0 | 3.00E-141 |
| hsa_circ_0077504 | 94 | 1 | 479 | 479 | 1 | 0 | 0 |
| hsa_circ_0077504 | 94 | 1 | 1269 | 1269 | 1 | 0 | 0 |
| hsa_circ_0077506 | 95 | 1 | 1137 | 1137 | 1 | 0 | 0 |
| hsa_circ_0077506 | 95 | 1 | 790 | 1137 | 348 | 0 | 0 |
| hsa_circ_0077506 | 94 | 1 | 702 | 1137 | 436 | 0 | 0 |
| hsa_circ_0077509 | 94 | 1 | 492 | 492 | 1 | 0 | 0 |
| hsa_circ_0077509 | 95 | 1 | 643 | 643 | 1 | 0 | 0 |
| hsa_circ_0077509 | 94 | 1 | 949 | 949 | 1 | 0 | 0 |
| hsa_circ_0077509 | 95 | 1 | 1164 | 1164 | 1 | 0 | 0 |
| hsa_circ_0077512 | 95 | 1 | 540 | 540 | 1 | 0 | 0 |
| hsa_circ_0077512 | 95 | 1 | 672 | 672 | 1 | 0 | 0 |
| hsa_circ_0077504 | 95 | 1 | 748 | 2017 | 1270 | 0 | 0 |
| hsa_circ_0077512 | 93 | 1 | 306 | 457 | 152 | 0 | 3.00E-125 |
| hsa_circ_0077512 | 94 | 1 | 389 | 540 | 152 | 0 | 7.00E-168 |
| hsa_circ_0077512 | 94 | 1 | 521 | 672 | 152 | 0 | 0 |
| hsa_circ_0077521 | 95 | 1 | 208 | 208 | 1 | 0 | 3.00E-89 |
| hsa_circ_0077521 | 96 | 1 | 458 | 458 | 1 | 0 | 0 |
| hsa_circ_0077523 | 97 | 1 | 250 | 250 | 1 | 0 | 8.00E-116 |
| hsa_circ_0077528 | 93 | 4 | 607 | 604 | 1 | 0 | 0 |
| hsa_circ_0077528 | 91 | 1 | 963 | 969 | 1 | 0.01 | 0 |
| hsa_circ_0077528 | 91 | 1 | 653 | 969 | 311 | 0.01 | 0 |
| hsa_circ_0077528 | 88 | 1 | 356 | 969 | 608 | 0.02 | 1.00E-119 |
| hsa_circ_0077529 | 92 | 1 | 494 | 494 | 1 | 0 | 0 |
| hsa_circ_0077529 | 92 | 1 | 390 | 494 | 105 | 0 | 9.00E-152 |
| hsa_circ_0077531 | 95 | 268 | 597 | 330 | 1 | 0 | 3.00E-148 |
| hsa_circ_0077536 | 97 | 1 | 370 | 370 | 1 | 0 | 1.00E-175 |
| hsa_circ_0077536 | 97 | 167 | 536 | 370 | 1 | 0 | 2.00E-175 |
| hsa_circ_0077536 | 95 | 1 | 242 | 242 | 1 | 0 | 1.00E-104 |
| hsa_circ_0077536 | 97 | 1 | 370 | 370 | 1 | 0 | 1.00E-175 |
| hsa_circ_0077536 | 97 | 167 | 536 | 370 | 1 | 0 | 2.00E-175 |
| hsa_circ_0077536 | 98 | 1 | 207 | 370 | 164 | 0 | 5.00E-97 |
| hsa_circ_0077536 | 98 | 167 | 373 | 370 | 164 | 0 | 1.00E-96 |
| hsa_circ_0077539 | 90 | 1 | 413 | 414 | 3 | 0 | 4.00E-152 |
| hsa_circ_0077539 | 91 | 6 | 462 | 458 | 3 | 0 | 1.00E-171 |
| hsa_circ_0077539 | 92 | 1 | 808 | 809 | 3 | 0 | 0 |
| hsa_circ_0077541 | 93 | 1 | 346 | 346 | 1 | 0 | 5.00E-144 |
| hsa_circ_0077544 | 91 | 1 | 306 | 306 | 1 | 0 | 4.00E-115 |
| hsa_circ_0001633 | 96 | 19 | 343 | 1 | 325 | 0 | 2.00E-150 |
| hsa_circ_0001633 | 96 | 19 | 343 | 1 | 325 | 0 | 2.00E-150 |
| hsa_circ_0001633 | 96 | 1 | 325 | 1 | 325 | 0 | 1.00E-150 |
| hsa_circ_0001633 | 96 | 1 | 325 | 1 | 325 | 0 | 7.00E-150 |
| hsa_circ_0001633 | 96 | 1 | 325 | 1 | 325 | 0 | 1.00E-150 |
| hsa_circ_0001633 | 96 | 1 | 325 | 1 | 325 | 0 | 1.00E-148 |
| hsa_circ_0001633 | 96 | 1 | 325 | 1 | 325 | 0 | 2.00E-148 |
| hsa_circ_0001633 | 96 | 1 | 325 | 1 | 325 | 0 | 2.00E-150 |
| hsa_circ_0001633 | 96 | 1 | 325 | 1 | 325 | 0 | 2.00E-150 |
| hsa_circ_0077549 | 92 | 5 | 261 | 257 | 1 | 0 | 9.00E-98 |
| hsa_circ_0077549 | 92 | 5 | 261 | 257 | 1 | 0 | 5.00E-98 |
| hsa_circ_0077549 | 94 | 1 | 1708 | 1711 | 1 | 0 | 0 |

novel_circ_020399 novel_circ_020400 novel_circ_020401 novel_circ_020402 novel_circ_020403 novel_circ_020404 novel_circ_020405 novel_circ_020406 novel_circ_020409 novel_circ_020410 novel_circ_020413 novel_circ_020414 novel_circ_020415 novel_circ_020416 novel_circ_020417 novel_circ_020418 novel_circ_020419 novel_circ_020420 novel_circ_020421 novel_circ_020422 novel_circ_020423 novel_circ_020424 novel_circ_020427 novel_circ_020428 novel_circ_020429 novel_circ_020430 novel_circ_020434 novel_circ_020436 novel_circ_020437 novel_circ_020438 novel_circ_020439 novel_circ_020440 novel_circ_020443 novel_circ_020444 novel_circ_020445 novel_circ_020446 novel_circ_020447 novel_circ_020448 novel_circ_020449 novel_circ_020450 novel_circ_020451 novel_circ_020452 novel_circ_020453 novel_circ_020454 novel_circ_020455 novel_circ_020456 novel_circ_020457 novel_circ_020458 novel_circ_020459 novel_circ_020460 novel_circ_020461 novel_circ_020462 novel_circ_020463 novel_circ_020464

| hsa_circ_0077549 | 94 | 1 | 934 | 1202 | 266 | 0 | 0 |
| --- | --- | --- | --- | --- | --- | --- | --- |
| hsa_circ_0077549 | 95 | 1 | 1443 | 1711 | 266 | 0 | 0 |
| hsa_circ_0008925 | 95 | 1 | 1133 | 1133 | 1 | 0 | 0 |
| hsa_circ_0009144 | 96 | 1 | 830 | 830 | 1 | 0 | 0 |
| hsa_circ_0077556 | 96 | 1 | 737 | 737 | 1 | 0 | 0 |
| hsa_circ_0077559 | 95 | 1 | 400 | 509 | 110 | 0 | 6.00E-179 |
| hsa_circ_0005115 | 95 | 1 | 349 | 349 | 1 | 0 | 2.00E-152 |
| hsa_circ_0077561 | 95 | 1 | 290 | 290 | 1 | 0 | 5.00E-128 |
| hsa_circ_0077567 | 93 | 4 | 327 | 864 | 546 | 0.02 | 4.00E-130 |
| hsa_circ_0077567 | 94 | 1 | 423 | 963 | 546 | 0.01 | 5.00E-180 |
| hsa_circ_0006404 | 93 | 1 | 1436 | 1 | 1435 | 0 | 0 |
| hsa_circ_0077570 | 93 | 262 | 334 | 1 | 73 | 0 | 3.00E-22 |
| hsa_circ_0077570 | 89 | 262 | 498 | 1 | 237 | 0 | 1.00E-76 |
| hsa_circ_0077570 | 89 | 262 | 681 | 1 | 420 | 0 | 8.00E-145 |
| hsa_circ_0077579 | 95 | 1 | 662 | 693 | 32 | 0 | 0 |
| hsa_circ_0004131 | 89 | 2 | 343 | 2 | 343 | 0 | 9.00E-117 |
| hsa_circ_0004131 | 89 | 2 | 343 | 2 | 343 | 0 | 1.00E-116 |
| hsa_circ_0077585 | 87 | 1 | 457 | 14715 | 14259 | 0 | 9.00E-144 |
| hsa_circ_0077585 | 87 | 1 | 457 | 14715 | 14259 | 0 | 7.00E-144 |
| hsa_circ_0077605 | 93 | 163 | 248 | 86 | 1 | 0 | 5.00E-28 |
| hsa_circ_0077605 | 92 | 983 | 1234 | 336 | 85 | 0 | 7.00E-98 |
| hsa_circ_0077605 | 94 | 983 | 1150 | 336 | 169 | 0 | 3.00E-66 |
| hsa_circ_0077607 | 92 | 1 | 580 | 1 | 580 | 0 | 0 |
| hsa_circ_0077610 | 93 | 224 | 1684 | 1 | 1461 | 0 | 0 |
| hsa_circ_0077610 | 93 | 224 | 2310 | 1 | 2087 | 0 | 0 |
| hsa_circ_0077616 | 92 | 1 | 448 | 663 | 1110 | 0 | 6.00E-179 |
| hsa_circ_0077618 | 95 | 1 | 215 | 238 | 452 | 0 | 2.00E-91 |
| hsa_circ_0077625 | 95 | 1 | 346 | 517 | 172 | 0 | 1.00E-155 |
| hsa_circ_0077628 | 96 | 1 | 249 | 252 | 4 | 0 | 1.00E-113 |
| hsa_circ_0077628 | 97 | 1 | 325 | 328 | 4 | 0 | 5.00E-154 |
| hsa_circ_0077628 | 97 | 61 | 387 | 330 | 4 | 0 | 4.00E-155 |
| hsa_circ_0077631 | 99 | 1 | 214 | 1 | 214 | 0 | 1.00E-107 |
| hsa_circ_0077641 | 95 | 4 | 786 | 783 | 1 | 0 | 0 |
| hsa_circ_0077641 | 95 | 1 | 438 | 648 | 211 | 0 | 0 |
| hsa_circ_0077641 | 95 | 4 | 576 | 783 | 211 | 0 | 0 |
| hsa_circ_0077641 | 95 | 1 | 691 | 901 | 211 | 0 | 0 |
| hsa_circ_0077641 | 95 | 111 | 801 | 901 | 211 | 0 | 0 |
| hsa_circ_0077642 | 95 | 1 | 1115 | 1115 | 1 | 0 | 0 |
| hsa_circ_0077642 | 95 | 1 | 1257 | 1257 | 1 | 0 | 0 |
| hsa_circ_0077643 | 97 | 1 | 225 | 363 | 139 | 0 | 1.00E-103 |
| hsa_circ_0077643 | 95 | 1 | 928 | 1066 | 139 | 0 | 0 |
| hsa_circ_0077642 | 94 | 1 | 1587 | 1916 | 330 | 0 | 0 |
| hsa_circ_0077642 | 94 | 150 | 1738 | 1918 | 330 | 0 | 0 |
| hsa_circ_0077642 | 94 | 3267 | 4855 | 1918 | 330 | 0 | 0 |
| hsa_circ_0077644 | 94 | 1 | 813 | 813 | 1 | 0 | 0 |
| hsa_circ_0077646 | 94 | 1 | 702 | 703 | 2 | 0 | 0 |
| hsa_circ_0077646 | 94 | 1 | 1056 | 1057 | 2 | 0 | 0 |
| hsa_circ_0077644 | 94 | 1 | 1361 | 1472 | 112 | 0 | 0 |
| hsa_circ_0077644 | 94 | 150 | 1512 | 1474 | 112 | 0 | 0 |
| hsa_circ_0077644 | 94 | 3267 | 4629 | 1474 | 112 | 0 | 0 |
| hsa_circ_0077644 | 94 | 1267 | 2629 | 1474 | 112 | 0 | 0 |
| hsa_circ_0077646 | 95 | 1 | 387 | 703 | 317 | 0 | 9.00E-172 |
| hsa_circ_0077644 | 94 | 276 | 1078 | 1474 | 672 | 0 | 0 |
| hsa_circ_0077644 | 94 | 3267 | 4069 | 1474 | 672 | 0 | 0 |

novel_circ_020465 novel_circ_020466 novel_circ_020467 novel_circ_020468 novel_circ_020469 novel_circ_020470 novel_circ_020471 novel_circ_020472 novel_circ_020473 novel_circ_020474 novel_circ_020475 novel_circ_020476 novel_circ_020477 novel_circ_020478 novel_circ_020479 novel_circ_020480 novel_circ_020481 novel_circ_020482 novel_circ_020483 novel_circ_020484 novel_circ_020486 novel_circ_020487 novel_circ_020488 novel_circ_020489 novel_circ_020490 novel_circ_020491 novel_circ_020492 novel_circ_020493 novel_circ_020494 novel_circ_020496 novel_circ_020497 novel_circ_020498 novel_circ_020499 novel_circ_020500 novel_circ_020501 novel_circ_020502 novel_circ_020503 novel_circ_020504 novel_circ_020505 novel_circ_020506 novel_circ_020507 novel_circ_020508 novel_circ_020509 novel_circ_020510 novel_circ_020511 novel_circ_020512 novel_circ_020514 novel_circ_020516 novel_circ_020517 novel_circ_020518 novel_circ_020519 novel_circ_020520 novel_circ_020521 novel_circ_020522

| hsa_circ_0077647 | 94 | 276 | 936 | 661 | 1 | 0 | 0 |
| --- | --- | --- | --- | --- | --- | --- | --- |
| hsa_circ_0077652 | 90 | 1 | 908 | 914 | 1 | 0.01 | 0 |
| hsa_circ_0077649 | 91 | 1 | 1268 | 1313 | 43 | 0.01 | 0 |
| hsa_circ_0077647 | 94 | 150 | 456 | 661 | 355 | 0 | 1.00E-130 |
| hsa_circ_0077647 | 94 | 276 | 582 | 661 | 355 | 0 | 2.00E-130 |
| hsa_circ_0077652 | 90 | 1 | 908 | 914 | 1 | 0.01 | 0 |
| hsa_circ_0077649 | 91 | 1 | 1268 | 1313 | 43 | 0.01 | 0 |
| hsa_circ_0077652 | 90 | 1 | 908 | 914 | 1 | 0.01 | 0 |
| hsa_circ_0077649 | 91 | 1 | 1268 | 1313 | 43 | 0.01 | 0 |
| hsa_circ_0077652 | 90 | 1 | 908 | 914 | 1 | 0.01 | 0 |
| hsa_circ_0077653 | 90 | 3 | 355 | 354 | 1 | 0 | 7.00E-128 |
| hsa_circ_0077657 | 87 | 47305 | 48684 | 1578 | 149 | 0.04 | 0 |
| hsa_circ_0077657 | 95 | 1 | 541 | 692 | 149 | 0.01 | 0 |
| hsa_circ_0077657 | 87 | 47305 | 48684 | 1578 | 149 | 0.04 | 0 |
| hsa_circ_0077660 | 82 | 47305 | 48143 | 1047 | 162 | 0.07 | 0 |
| hsa_circ_0077666 | 96 | 1 | 518 | 518 | 1 | 0 | 0 |
| hsa_circ_0077665 | 96 | 4 | 873 | 1127 | 258 | 0 | 0 |
| hsa_circ_0077666 | 96 | 1 | 518 | 518 | 1 | 0 | 0 |
| hsa_circ_0077665 | 96 | 1 | 615 | 872 | 258 | 0 | 0 |
| hsa_circ_0077665 | 97 | 4 | 355 | 1127 | 776 | 0 | 2.00E-167 |
| hsa_circ_0077668 | 92 | 4 | 743 | 740 | 1 | 0 | 0 |
| hsa_circ_0077669 | 91 | 4 | 238 | 423 | 189 | 0 | 2.00E-87 |
| hsa_circ_0077687 | 91 | 1 | 361 | 1278 | 1644 | 0.05 | 3.00E-137 |
| hsa_circ_0077688 | 96 | 1 | 1326 | 1326 | 1 | 0 | 0 |
| hsa_circ_0077688 | 96 | 1 | 1170 | 1326 | 157 | 0 | 0 |
| hsa_circ_0077691 | 96 | 1 | 789 | 789 | 1 | 0 | 0 |
| hsa_circ_0077691 | 96 | 1 | 680 | 789 | 110 | 0 | 0 |
| hsa_circ_0077691 | 95 | 1 | 445 | 789 | 345 | 0 | 0 |
| hsa_circ_0077693 | 91 | 189 | 304 | 176572 | 176459 | 0.02 | 1.00E-35 |
| hsa_circ_0032686 | 90 | 1 | 224 | 1 | 224 | 0 | 4.00E-78 |
| hsa_circ_0032698 | 94 | 1 | 297 | 1 | 297 | 0 | 3.00E-125 |
| hsa_circ_0032698 | 94 | 1 | 428 | 1 | 428 | 0 | 0 |
| hsa_circ_0032698 | 94 | 1 | 511 | 1 | 511 | 0 | 0 |
| hsa_circ_0032699 | 94 | 1 | 557 | 1 | 557 | 0 | 0 |
| hsa_circ_0032702 | 94 | 1 | 236 | 1 | 236 | 0 | 2.00E-96 |
| hsa_circ_0032702 | 93 | 1 | 540 | 1 | 540 | 0 | 0 |
| hsa_circ_0032707 | 93 | 1 | 457 | 1 | 457 | 0 | 0 |
| hsa_circ_0032710 | 93 | 1 | 302 | 1 | 302 | 0 | 6.00E-123 |
| hsa_circ_0032718 | 95 | 1 | 206 | 1 | 206 | 0 | 2.00E-86 |
| hsa_circ_0032720 | 91 | 3 | 583 | 3 | 586 | 0.01 | 0 |
| hsa_circ_0032720 | 91 | 3 | 1060 | 3 | 1066 | 0.01 | 0 |
| hsa_circ_0032722 | 92 | 1 | 595 | 1 | 601 | 0.01 | 0 |
| hsa_circ_0032722 | 92 | 1 | 595 | 1 | 601 | 0.01 | 0 |
| hsa_circ_0032727 | 87 | 673 | 1555 | 47 | 920 | 0.01 | 0 |
| hsa_circ_0032727 | 87 | 555 | 1241 | 47 | 724 | 0.01 | 0 |
| hsa_circ_0032727 | 87 | 555 | 1437 | 47 | 920 | 0.01 | 0 |
| hsa_circ_0007946 | 88 | 8 | 414 | 8 | 414 | 0 | 8.00E-138 |
| hsa_circ_0032738 | 95 | 1 | 322 | 1 | 322 | 0 | 5.00E-144 |
| hsa_circ_0032738 | 95 | 1 | 390 | 1 | 390 | 0 | 1.00E-171 |
| hsa_circ_0032738 | 95 | 1 | 390 | 1 | 390 | 0 | 2.00E-171 |
| hsa_circ_0032742 | 94 | 3 | 325 | 3 | 325 | 0 | 2.00E-139 |
| hsa_circ_0032742 | 94 | 3 | 325 | 3 | 325 | 0 | 2.00E-139 |
| hsa_circ_0032747 | 96 | 109 | 249 | 1 | 141 | 0 | 1.00E-58 |
| hsa_circ_0032747 | 93 | 109 | 344 | 1 | 236 | 0.02 | 7.00E-93 |

novel_circ_020523 novel_circ_020524 novel_circ_020525 novel_circ_020526 novel_circ_020530 novel_circ_020531 novel_circ_020532 novel_circ_020533 novel_circ_020534 novel_circ_020536 novel_circ_020537 novel_circ_020538 novel_circ_020539 novel_circ_020540 novel_circ_020541 novel_circ_020542 novel_circ_020543 novel_circ_020545 novel_circ_020548 novel_circ_020549 novel_circ_020550 novel_circ_020551 novel_circ_020552 novel_circ_020553 novel_circ_020554 novel_circ_020555 novel_circ_020556 novel_circ_020557 novel_circ_020558 novel_circ_020559 novel_circ_020560 novel_circ_020561 novel_circ_020562 novel_circ_020563 novel_circ_020564 novel_circ_020565 novel_circ_020566 novel_circ_020567 novel_circ_020568 novel_circ_020569 novel_circ_020571 novel_circ_020572 novel_circ_020573 novel_circ_020574 novel_circ_020575 novel_circ_020577 novel_circ_020578 novel_circ_020579 novel_circ_020580 novel_circ_020582 novel_circ_020583 novel_circ_020584 novel_circ_020585 novel_circ_020586

| hsa_circ_0086198 | 95 | 6473 | 6513 | 16211 | 16252 | 0.02 | 9.00E-08 |
| --- | --- | --- | --- | --- | --- | --- | --- |
| hsa_circ_0086198 | 95 | 30880 | 30920 | 16211 | 16252 | 0.02 | 2.00E-07 |
| hsa_circ_0024151 | 93 | 14452 | 14518 | 48275 | 48209 | 0 | 1.00E-16 |
| hsa_circ_0006833 | 75 | 13544 | 13810 | 228654 | 228390 | 0.02 | 2.00E-25 |
| hsa_circ_0032755 | 94 | 1 | 540 | 3219 | 2680 | 0 | 0 |
| hsa_circ_0046155 | 100 | 2498 | 2529 | 5938 | 5969 | 0 | 1.00E-06 |
| hsa_circ_0032756 | 93 | 2 | 153 | 386 | 537 | 0 | 1.00E-56 |
| hsa_circ_0002849 | 91 | 1 | 485 | 678 | 194 | 0 | 0 |
| hsa_circ_0032767 | 87 | 83 | 207 | 130 | 6 | 0 | 7.00E-33 |
| hsa_circ_0032787 | 91 | 1 | 824 | 821 | 1 | 0 | 0 |
| hsa_circ_0032787 | 92 | 1 | 453 | 453 | 1 | 0 | 0 |
| hsa_circ_0032787 | 91 | 1 | 824 | 821 | 1 | 0 | 0 |
| hsa_circ_0032787 | 91 | 1 | 979 | 976 | 1 | 0 | 0 |
| hsa_circ_0032791 | 89 | 1 | 371 | 368 | 1 | 0.01 | 4.00E-130 |
| hsa_circ_0032792 | 93 | 92 | 285 | 194 | 1 | 0 | 3.00E-76 |
| hsa_circ_0032792 | 93 | 255 | 448 | 194 | 1 | 0 | 4.00E-76 |
| hsa_circ_0032806 | 81 | 1 | 1286 | 1 | 1277 | 0.03 | 0 |
| hsa_circ_0032809 | 94 | 3 | 464 | 3 | 464 | 0 | 0 |
| hsa_circ_0032810 | 92 | 1 | 182 | 1 | 182 | 0 | 4.00E-68 |
| hsa_circ_0032811 | 94 | 38073 | 38630 | 1 | 558 | 0 | 0 |
| hsa_circ_0032811 | 94 | 1 | 558 | 1 | 558 | 0 | 0 |
| hsa_circ_0032811 | 96 | 1 | 386 | 173 | 558 | 0 | 0 |
| hsa_circ_0032813 | 98 | 1 | 88 | 309 | 396 | 0 | 6.00E-35 |
| hsa_circ_0032823 | 93 | 10 | 402 | 586 | 194 | 0 | 4.00E-160 |
| hsa_circ_0032823 | 91 | 1 | 823 | 2572 | 1750 | 0 | 0 |
| hsa_circ_0032823 | 91 | 1 | 615 | 2572 | 1958 | 0 | 0 |
| hsa_circ_0002499 | 91 | 1 | 528 | 528 | 1 | 0 | 0 |
| hsa_circ_0032823 | 92 | 3 | 777 | 2819 | 2045 | 0 | 0 |
| hsa_circ_0032823 | 92 | 155 | 929 | 2819 | 2045 | 0 | 0 |
| hsa_circ_0032829 | 95 | 1 | 621 | 621 | 1 | 0 | 0 |
| hsa_circ_0032829 | 94 | 106 | 993 | 888 | 1 | 0 | 0 |
| hsa_circ_0032829 | 95 | 5609 | 5931 | 413 | 91 | 0 | 6.00E-140 |
| hsa_circ_0032829 | 95 | 1 | 531 | 621 | 91 | 0 | 0 |
| hsa_circ_0032829 | 94 | 4 | 801 | 888 | 91 | 0 | 0 |
| hsa_circ_0032829 | 94 | 106 | 903 | 888 | 91 | 0 | 0 |
| hsa_circ_0032829 | 92 | 4 | 270 | 888 | 622 | 0 | 1.00E-103 |
| hsa_circ_0032829 | 92 | 106 | 372 | 888 | 622 | 0 | 2.00E-103 |
| hsa_circ_0032834 | 87 | 3 | 462 | 481 | 1 | 0.05 | 2.00E-149 |
| hsa_circ_0032834 | 86 | 16 | 740 | 754 | 1 | 0.05 | 0 |
| hsa_circ_0032835 | 85 | 16 | 542 | 556 | 10 | 0.05 | 1.00E-146 |
| hsa_circ_0032839 | 92 | 1 | 403 | 403 | 1 | 0 | 1.00E-160 |
| hsa_circ_0032838 | 93 | 120 | 736 | 1096 | 480 | 0 | 0 |
| hsa_circ_0032838 | 94 | 120 | 470 | 1096 | 746 | 0 | 1.00E-151 |
| hsa_circ_0032838 | 94 | 288 | 638 | 1096 | 746 | 0 | 2.00E-151 |
| hsa_circ_0032838 | 94 | 120 | 315 | 1096 | 901 | 0 | 5.00E-79 |
| hsa_circ_0006643 | 93 | 1 | 168 | 797135 | 796969 | 0.01 | 1.00E-64 |
| hsa_circ_0003953 | 88 | 1 | 990 | 1 | 1004 | 0.02 | 0 |
| hsa_circ_0013962 | 94 | 1 | 782 | 233 | 1014 | 0 | 0 |
| hsa_circ_0013966 | 95 | 1 | 388 | 1 | 388 | 0 | 1.00E-170 |
| hsa_circ_0013962 | 93 | 1 | 521 | 126 | 646 | 0 | 0 |
| hsa_circ_0013962 | 93 | 1 | 411 | 126 | 536 | 0 | 2.00E-168 |
| hsa_circ_0013957 | 87 | 403 | 664 | 262 | 1 | 0 | 2.00E-80 |
| hsa_circ_0013957 | 87 | 323 | 584 | 262 | 1 | 0 | 2.00E-80 |
| hsa_circ_0007636 | 85 | 24 | 215 | 1E+06 | 1094616 | 0.02 | 3.00E-49 |

novel_circ_020588 novel_circ_020589 novel_circ_020590 novel_circ_020591 novel_circ_020592 novel_circ_020593 novel_circ_020594 novel_circ_020595 novel_circ_020596 novel_circ_020597 novel_circ_020598 novel_circ_020599 novel_circ_020600 novel_circ_020601 novel_circ_020602 novel_circ_020603 novel_circ_020604 novel_circ_020605 novel_circ_020606 novel_circ_020607 novel_circ_020608 novel_circ_020609 novel_circ_020610 novel_circ_020611 novel_circ_020612 novel_circ_020613 novel_circ_020614 novel_circ_020615 novel_circ_020616 novel_circ_020617 novel_circ_020623 novel_circ_020624 novel_circ_020625 novel_circ_020626 novel_circ_020627 novel_circ_020628 novel_circ_020629 novel_circ_020630 novel_circ_020631 novel_circ_020632 novel_circ_020633 novel_circ_020634 novel_circ_020635 novel_circ_020636 novel_circ_020637 novel_circ_020638 novel_circ_020639 novel_circ_020640 novel_circ_020641 novel_circ_020642 novel_circ_020643 novel_circ_020644 novel_circ_020647 novel_circ_020652

| hsa_circ_0062584 | 91 | 5 | 622 | 174 | 791 | 0 | 0 |
| --- | --- | --- | --- | --- | --- | --- | --- |
| hsa_circ_0062594 | 88 | 474 | 806 | 183 | 515 | 0 | 3.00E-105 |
| hsa_circ_0062610 | 91 | 1 | 260 | 1 | 260 | 0 | 1.00E-94 |
| hsa_circ_0062608 | 90 | 1 | 344 | 1 | 344 | 0 | 3.00E-121 |
| hsa_circ_0062604 | 90 | 1 | 435 | 2690 | 3124 | 0 | 5.00E-160 |
| hsa_circ_0062604 | 90 | 1 | 691 | 2434 | 3124 | 0 | 0 |
| hsa_circ_0062604 | 91 | 1 | 867 | 2258 | 3124 | 0 | 0 |
| hsa_circ_0062604 | 91 | 1 | 941 | 2184 | 3124 | 0 | 0 |
| hsa_circ_0062604 | 91 | 1 | 1149 | 1976 | 3124 | 0 | 0 |
| hsa_circ_0062607 | 92 | 1 | 597 | 209 | 805 | 0 | 0 |
| hsa_circ_0062607 | 92 | 1 | 506 | 209 | 714 | 0 | 0 |
| hsa_circ_0062607 | 92 | 1 | 340 | 283 | 622 | 0 | 5.00E-134 |
| hsa_circ_0062607 | 92 | 1 | 414 | 209 | 622 | 0 | 2.00E-163 |
| hsa_circ_0062604 | 89 | 1 | 2581 | 1 | 2597 | 0.01 | 0 |
| hsa_circ_0062604 | 89 | 1 | 2417 | 1 | 2433 | 0.01 | 0 |
| hsa_circ_0062605 | 89 | 1 | 1842 | 1 | 1839 | 0 | 0 |
| hsa_circ_0062604 | 88 | 1 | 1977 | 191 | 2183 | 0.01 | 0 |
| hsa_circ_0062604 | 89 | 1 | 2167 | 1 | 2183 | 0.01 | 0 |
| hsa_circ_0062604 | 89 | 10183 | 12349 | 1 | 2183 | 0.01 | 0 |
| hsa_circ_0062540 | 92 | 1 | 250 | 471 | 720 | 0 | 2.00E-97 |
| hsa_circ_0062540 | 93 | 1 | 310 | 411 | 720 | 0 | 5.00E-124 |
| hsa_circ_0062516 | 87 | 1 | 260 | 308 | 567 | 0 | 5.00E-78 |
| hsa_circ_0002514 | 93 | 429 | 1206 | 391 | 1171 | 0 | 0 |
| hsa_circ_0047836 | 87 | 102 | 428 | 73 | 398 | 0.02 | 4.00E-101 |
| hsa_circ_0047836 | 90 | 102 | 843 | 73 | 813 | 0.01 | 0 |
| hsa_circ_0047836 | 90 | 102 | 1199 | 73 | 1169 | 0.01 | 0 |
| hsa_circ_0047836 | 91 | 102 | 1453 | 73 | 1423 | 0.01 | 0 |
| hsa_circ_0047836 | 92 | 102 | 1712 | 73 | 1682 | 0 | 0 |
| hsa_circ_0047836 | 92 | 1 | 771 | 399 | 1169 | 0 | 0 |
| hsa_circ_0047836 | 92 | 1 | 1025 | 399 | 1423 | 0 | 0 |
| hsa_circ_0017197 | 99 | 1 | 170 | 281 | 112 | 0 | 7.00E-81 |
| hsa_circ_0017196 | 99 | 55149 | 55319 | 410 | 240 | 0 | 5.00E-81 |
| hsa_circ_0017196 | 99 | 55149 | 55318 | 410 | 241 | 0 | 1.00E-80 |
| hsa_circ_0017217 | 94 | 270 | 675 | 2717 | 2312 | 0 | 2.00E-175 |
| hsa_circ_0017218 | 99 | 1 | 148 | 319 | 172 | 0 | 2.00E-70 |
| hsa_circ_0017223 | 92 | 10 | 2198 | 2186 | 1 | 0 | 0 |
| hsa_circ_0017223 | 92 | 1 | 2806 | 2803 | 1 | 0 | 0 |
| hsa_circ_0017227 | 91 | 3 | 1836 | 1831 | 1 | 0 | 0 |
| hsa_circ_0017227 | 91 | 10 | 1963 | 1951 | 1 | 0 | 0 |
| hsa_circ_0017227 | 91 | 4 | 2113 | 2107 | 1 | 0 | 0 |
| hsa_circ_0017227 | 92 | 1 | 2407 | 2404 | 1 | 0 | 0 |
| hsa_circ_0017227 | 92 | 1 | 2571 | 2568 | 1 | 0 | 0 |
| hsa_circ_0017227 | 92 | 1 | 3048 | 3045 | 1 | 0 | 0 |
| hsa_circ_0017227 | 93 | 1 | 3720 | 3717 | 1 | 0 | 0 |
| hsa_circ_0017227 | 95 | 1 | 735 | 2568 | 1834 | 0 | 0 |
| hsa_circ_0017227 | 95 | 1 | 608 | 2568 | 1961 | 0 | 0 |
| hsa_circ_0017227 | 97 | 1 | 458 | 2568 | 2111 | 0 | 0 |
| hsa_circ_0017227 | 95 | 1 | 1607 | 3717 | 2111 | 0 | 0 |
| hsa_circ_0009111 | 92 | 1 | 298 | 298 | 1 | 0 | 9.00E-116 |
| hsa_circ_0009111 | 94 | 1 | 672 | 672 | 1 | 0 | 0 |
| hsa_circ_0009111 | 94 | 1 | 537 | 672 | 136 | 0 | 0 |
| hsa_circ_0017230 | 95 | 1 | 236 | 236 | 1 | 0 | 4.00E-103 |
| hsa_circ_0017233 | 86 | 167 | 1431 | 1 | 1253 | 0.01 | 0 |
| hsa_circ_0017233 | 85 | 154 | 1301 | 1 | 1136 | 0.01 | 0 |

novel_circ_020653 novel_circ_020654 novel_circ_020655 novel_circ_020656 novel_circ_020657 novel_circ_020658 novel_circ_020660 novel_circ_020661 novel_circ_020662 novel_circ_020663 novel_circ_020664 novel_circ_020665 novel_circ_020666 novel_circ_020667 novel_circ_020668 novel_circ_020669 novel_circ_020670 novel_circ_020671 novel_circ_020672 novel_circ_020674 novel_circ_020675 novel_circ_020677 novel_circ_020681 novel_circ_020682 novel_circ_020683 novel_circ_020686 novel_circ_020688 novel_circ_020689 novel_circ_020690 novel_circ_020691 novel_circ_020693 novel_circ_020695 novel_circ_020696 novel_circ_020697 novel_circ_020698 novel_circ_020699 novel_circ_020700 novel_circ_020701 novel_circ_020702 novel_circ_020703 novel_circ_020705 novel_circ_020707 novel_circ_020708 novel_circ_020709 novel_circ_020710 novel_circ_020711 novel_circ_020712 novel_circ_020713 novel_circ_020714 novel_circ_020715 novel_circ_020716 novel_circ_020717 novel_circ_020718 novel_circ_020719

| hsa_circ_0017235 | 84 | 1 | 390 | 1 | 378 | 0.04 | 2.00E-98 |
| --- | --- | --- | --- | --- | --- | --- | --- |
| hsa_circ_0017235 | 85 | 1 | 822 | 1 | 810 | 0.02 | 0 |
| hsa_circ_0017235 | 86 | 1 | 939 | 1 | 927 | 0.01 | 0 |
| hsa_circ_0017235 | 87 | 1 | 1082 | 1 | 1070 | 0.01 | 0 |
| hsa_circ_0017240 | 93 | 1 | 512 | 406 | 917 | 0 | 0 |
| hsa_circ_0017241 | 93 | 1 | 369 | 1 | 369 | 0 | 4.00E-150 |
| hsa_circ_0017242 | 96 | 1 | 303 | 303 | 1 | 0 | 7.00E-137 |
| hsa_circ_0017242 | 94 | 1 | 432 | 432 | 1 | 0 | 0 |
| hsa_circ_0017242 | 94 | 1091 | 1524 | 434 | 1 | 0 | 0 |
| hsa_circ_0017242 | 95 | 1 | 555 | 555 | 1 | 0 | 0 |
| hsa_circ_0017242 | 96 | 1 | 822 | 822 | 1 | 0 | 0 |
| hsa_circ_0017242 | 96 | 1 | 467 | 555 | 89 | 0 | 0 |
| hsa_circ_0017244 | 94 | 1 | 252 | 252 | 1 | 0 | 6.00E-107 |
| hsa_circ_0017252 | 98 | 3 | 524 | 522 | 1 | 0 | 0 |
| hsa_circ_0017252 | 97 | 1 | 650 | 650 | 1 | 0 | 0 |
| hsa_circ_0017253 | 97 | 1 | 515 | 515 | 1 | 0 | 0 |
| hsa_circ_0017253 | 97 | 121 | 636 | 516 | 1 | 0 | 0 |
| hsa_circ_0017254 | 97 | 1 | 383 | 383 | 1 | 0 | 0 |
| hsa_circ_0004649 | 96 | 240 | 366 | 127 | 1 | 0 | 3.00E-52 |
| hsa_circ_0017264 | 95 | 1 | 762 | 762 | 1 | 0 | 0 |
| hsa_circ_0017262 | 95 | 965 | 1727 | 2060 | 1298 | 0 | 0 |
| hsa_circ_0017268 | 97 | 95 | 236 | 1 | 142 | 0 | 2.00E-62 |
| hsa_circ_0017272 | 94 | 1 | 512 | 512 | 1 | 0 | 0 |
| hsa_circ_0017272 | 94 | 1 | 1307 | 1307 | 1 | 0 | 0 |
| hsa_circ_0017272 | 95 | 1 | 1547 | 1547 | 1 | 0 | 0 |
| hsa_circ_0005343 | 86 | 23700 | 23818 | 234842 | 234964 | 0.03 | 5.00E-27 |
| hsa_circ_0017278 | 88 | 86 | 348 | 1 | 263 | 0 | 2.00E-84 |
| hsa_circ_0017278 | 88 | 1 | 263 | 1 | 263 | 0 | 1.00E-84 |
| hsa_circ_0017280 | 91 | 1 | 534 | 1 | 534 | 0 | 0 |
| hsa_circ_0017280 | 90 | 1 | 701 | 1 | 701 | 0 | 0 |
| hsa_circ_0007853 | 91 | 1 | 908 | 1 | 908 | 0 | 0 |
| hsa_circ_0017294 | 94 | 1 | 263 | 263 | 1 | 0 | 5.00E-108 |
| hsa_circ_0017294 | 95 | 1 | 374 | 374 | 1 | 0 | 7.00E-168 |
| hsa_circ_0017294 | 95 | 1 | 477 | 477 | 1 | 0 | 0 |
| hsa_circ_0017294 | 94 | 1 | 545 | 545 | 1 | 0 | 0 |
| hsa_circ_0017302 | 95 | 1 | 282 | 282 | 1 | 0 | 3.00E-125 |
| hsa_circ_0017298 | 78 | 50647 | 51753 | 1110 | 1 | 0.06 | 0 |
| hsa_circ_0017298 | 78 | 50744 | 51850 | 1110 | 1 | 0.06 | 0 |
| hsa_circ_0005034 | 92 | 1 | 303 | 303 | 1 | 0 | 2.00E-118 |
| hsa_circ_0017308 | 85 | 157 | 456 | 300 | 1 | 0 | 3.00E-83 |
| hsa_circ_0017310 | 86 | 1 | 430 | 1 | 430 | 0 | 6.00E-129 |
| hsa_circ_0017313 | 90 | 1 | 505 | 538 | 1042 | 0 | 0 |
| hsa_circ_0017313 | 89 | 1 | 623 | 538 | 1160 | 0 | 0 |
| hsa_circ_0017313 | 92 | 9 | 211 | 659 | 861 | 0 | 2.00E-76 |
| hsa_circ_0017313 | 89 | 9 | 799 | 659 | 1449 | 0 | 0 |
| hsa_circ_0017314 | 88 | 1 | 705 | 1 | 705 | 0 | 0 |
| hsa_circ_0017314 | 88 | 1 | 718 | 1 | 718 | 0 | 0 |
| hsa_circ_0017314 | 89 | 1 | 295 | 312 | 606 | 0 | 4.00E-99 |
| hsa_circ_0017316 | 84 | 1 | 2132 | 2135 | 1 | 0.01 | 0 |
| hsa_circ_0017321 | 83 | 1 | 1890 | 1882 | 2 | 0.03 | 0 |
| hsa_circ_0017323 | 79 | 4 | 1026 | 1014 | 1 | 0.05 | 0 |
| hsa_circ_0017323 | 82 | 1 | 1605 | 1596 | 1 | 0.03 | 0 |
| hsa_circ_0017323 | 84 | 1 | 2121 | 2112 | 1 | 0.02 | 0 |
| hsa_circ_0017323 | 85 | 1 | 2215 | 2206 | 1 | 0.02 | 0 |

novel_circ_020721 novel_circ_020722 novel_circ_020723 novel_circ_020724 novel_circ_020725 novel_circ_020726 novel_circ_020727 novel_circ_020728 novel_circ_020729 novel_circ_020730 novel_circ_020731 novel_circ_020732 novel_circ_020733 novel_circ_020734 novel_circ_020735 novel_circ_020736 novel_circ_020737 novel_circ_020738 novel_circ_020739 novel_circ_020740 novel_circ_020741 novel_circ_020742 novel_circ_020743 novel_circ_020744 novel_circ_020745 novel_circ_020746 novel_circ_020747 novel_circ_020748 novel_circ_020749 novel_circ_020750 novel_circ_020751 novel_circ_020752 novel_circ_020753 novel_circ_020754 novel_circ_020755 novel_circ_020756 novel_circ_020757 novel_circ_020758 novel_circ_020759 novel_circ_020760 novel_circ_020761 novel_circ_020762 novel_circ_020763 novel_circ_020764 novel_circ_020765 novel_circ_020766 novel_circ_020767 novel_circ_020768 novel_circ_020769 novel_circ_020770 novel_circ_020771 novel_circ_020772 novel_circ_020773 novel_circ_020774

| hsa_circ_0017323 | 80 | 4 | 873 | 1014 | 172 | 0.04 | 1.00E-173 |
| --- | --- | --- | --- | --- | --- | --- | --- |
| hsa_circ_0017323 | 81 | 1 | 1024 | 1168 | 172 | 0.03 | 0 |
| hsa_circ_0017323 | 82 | 1 | 1175 | 1319 | 172 | 0.03 | 0 |
| hsa_circ_0017323 | 86 | 1 | 2062 | 2206 | 172 | 0.02 | 0 |
| hsa_circ_0017323 | 86 | 9 | 2308 | 2444 | 172 | 0.02 | 0 |
| hsa_circ_0017323 | 87 | 9 | 2995 | 3131 | 172 | 0.01 | 0 |
| hsa_circ_0017323 | 86 | 1 | 603 | 1319 | 717 | 0 | 0 |
| hsa_circ_0017323 | 87 | 1 | 880 | 1596 | 717 | 0 | 0 |
| hsa_circ_0017323 | 88 | 1 | 1068 | 1784 | 717 | 0 | 0 |
| hsa_circ_0017323 | 89 | 1 | 1917 | 2633 | 717 | 0 | 0 |
| hsa_circ_0017328 | 91 | 1 | 3226 | 3226 | 1 | 0 | 0 |
| hsa_circ_0017328 | 89 | 1 | 687 | 687 | 1 | 0 | 0 |
| hsa_circ_0017328 | 89 | 1 | 875 | 875 | 1 | 0 | 0 |
| hsa_circ_0017328 | 90 | 1 | 1203 | 1203 | 1 | 0 | 0 |
| hsa_circ_0017328 | 90 | 1 | 1724 | 1724 | 1 | 0 | 0 |
| hsa_circ_0017328 | 90 | 9 | 2230 | 2222 | 1 | 0 | 0 |
| hsa_circ_0017333 | 90 | 8309 | 8501 | 426 | 618 | 0 | 2.00E-62 |
| hsa_circ_0017330 | 89 | 1 | 767 | 767 | 1 | 0 | 0 |
| hsa_circ_0017330 | 90 | 1 | 1616 | 1616 | 1 | 0 | 0 |
| hsa_circ_0017330 | 90 | 9 | 2122 | 2114 | 1 | 0 | 0 |
| hsa_circ_0017330 | 91 | 1 | 3118 | 3118 | 1 | 0 | 0 |
| hsa_circ_0017333 | 90 | 1 | 612 | 616 | 5 | 0 | 0 |
| hsa_circ_0017330 | 91 | 1 | 2963 | 3118 | 156 | 0 | 0 |
| hsa_circ_0017333 | 90 | 1 | 1314 | 1465 | 152 | 0 | 0 |
| hsa_circ_0017333 | 90 | 1 | 1037 | 1465 | 429 | 0 | 0 |
| hsa_circ_0017330 | 91 | 9 | 1543 | 2114 | 580 | 0 | 0 |
| hsa_circ_0017330 | 91 | 10 | 1896 | 2466 | 580 | 0 | 0 |
| hsa_circ_0017330 | 91 | 1 | 2539 | 3118 | 580 | 0 | 0 |
| hsa_circ_0017330 | 91 | 1 | 2667 | 3246 | 580 | 0 | 0 |
| hsa_circ_0017335 | 91 | 1 | 328 | 516 | 189 | 0 | 5.00E-124 |
| hsa_circ_0017335 | 92 | 1 | 422 | 610 | 189 | 0 | 2.00E-169 |
| hsa_circ_0017335 | 91 | 9 | 668 | 848 | 189 | 0 | 0 |
| hsa_circ_0017337 | 92 | 1 | 275 | 275 | 1 | 0 | 5.00E-108 |
| hsa_circ_0017330 | 90 | 9 | 933 | 2114 | 1190 | 0 | 0 |
| hsa_circ_0017330 | 91 | 1 | 1169 | 2358 | 1190 | 0 | 0 |
| hsa_circ_0017330 | 91 | 1 | 1929 | 3118 | 1190 | 0 | 0 |
| hsa_circ_0017330 | 91 | 1 | 2057 | 3246 | 1190 | 0 | 0 |
| hsa_circ_0017344 | 91 | 1 | 1570 | 1570 | 1 | 0 | 0 |
| hsa_circ_0017344 | 91 | 1 | 1824 | 1824 | 1 | 0 | 0 |
| hsa_circ_0017344 | 91 | 9 | 687 | 820 | 142 | 0 | 0 |
| hsa_circ_0017344 | 92 | 1 | 923 | 1064 | 142 | 0 | 0 |
| hsa_circ_0017344 | 91 | 1 | 1683 | 1824 | 142 | 0 | 0 |
| hsa_circ_0017344 | 92 | 9 | 506 | 820 | 323 | 0 | 0 |
| hsa_circ_0017344 | 92 | 1 | 742 | 1064 | 323 | 0 | 0 |
| hsa_circ_0017344 | 92 | 10 | 859 | 1172 | 323 | 0 | 0 |
| hsa_circ_0017344 | 92 | 1 | 1248 | 1570 | 323 | 0 | 0 |
| hsa_circ_0017344 | 91 | 1 | 1502 | 1824 | 323 | 0 | 0 |
| hsa_circ_0017330 | 91 | 1 | 1630 | 3246 | 1617 | 0 | 0 |
| hsa_circ_0017344 | 92 | 1 | 466 | 979 | 514 | 0 | 0 |
| hsa_circ_0017344 | 92 | 1 | 1311 | 1824 | 514 | 0 | 0 |
| hsa_circ_0017344 | 92 | 1 | 742 | 1570 | 829 | 0 | 0 |
| hsa_circ_0017344 | 92 | 1 | 996 | 1824 | 829 | 0 | 0 |
| hsa_circ_0017330 | 91 | 1 | 1124 | 3246 | 2123 | 0 | 0 |
| hsa_circ_0017344 | 92 | 1 | 591 | 1570 | 980 | 0 | 0 |

novel_circ_020775 novel_circ_020776 novel_circ_020777 novel_circ_020778 novel_circ_020779 novel_circ_020780 novel_circ_020781 novel_circ_020782 novel_circ_020783 novel_circ_020786 novel_circ_020787 novel_circ_020788 novel_circ_020789 novel_circ_020790 novel_circ_020791 novel_circ_020792 novel_circ_020793 novel_circ_020794 novel_circ_020795 novel_circ_020796 novel_circ_020797 novel_circ_020798 novel_circ_020799 novel_circ_020800 novel_circ_020801 novel_circ_020802 novel_circ_020803 novel_circ_020804 novel_circ_020805 novel_circ_020806 novel_circ_020807 novel_circ_020808 novel_circ_020809 novel_circ_020810 novel_circ_020811 novel_circ_020812 novel_circ_020813 novel_circ_020814 novel_circ_020815 novel_circ_020816 novel_circ_020817 novel_circ_020822 novel_circ_020823 novel_circ_020824 novel_circ_020825 novel_circ_020826 novel_circ_020827 novel_circ_020828 novel_circ_020829 novel_circ_020830 novel_circ_020831 novel_circ_020832 novel_circ_020833 novel_circ_020834

| hsa_circ_0017344 | 91 | 1 | 845 | 1824 | 980 | 0 | 0 |
| --- | --- | --- | --- | --- | --- | --- | --- |
| hsa_circ_0017344 | 89 | 6 | 325 | 1384 | 1065 | 0 | 1.00E-109 |
| hsa_circ_0017344 | 91 | 1 | 506 | 1570 | 1065 | 0 | 0 |
| hsa_circ_0017344 | 91 | 1 | 760 | 1824 | 1065 | 0 | 0 |
| hsa_circ_0017330 | 91 | 1 | 888 | 3246 | 2359 | 0 | 0 |
| hsa_circ_0017344 | 90 | 1 | 389 | 1570 | 1182 | 0 | 2.00E-144 |
| hsa_circ_0017344 | 91 | 1 | 643 | 1824 | 1182 | 0 | 0 |
| hsa_circ_0017345 | 93 | 1 | 435 | 435 | 1 | 0 | 2.00E-178 |
| hsa_circ_0017330 | 92 | 1 | 563 | 3246 | 2684 | 0 | 0 |
| hsa_circ_0016754 | 97 | 205 | 625 | 421 | 1 | 0 | 0 |
| hsa_circ_0016754 | 98 | 1 | 272 | 421 | 150 | 0 | 2.00E-131 |
| hsa_circ_0016754 | 97 | 1 | 421 | 421 | 1 | 0 | 0 |
| hsa_circ_0016752 | 97 | 1 | 515 | 515 | 1 | 0 | 0 |
| hsa_circ_0016751 | 97 | 177 | 965 | 789 | 1 | 0 | 0 |
| hsa_circ_0016751 | 97 | 177 | 965 | 789 | 1 | 0 | 0 |
| hsa_circ_0016747 | 96 | 422 | 1335 | 2305 | 1392 | 0 | 0 |
| hsa_circ_0016747 | 96 | 422 | 1335 | 2305 | 1392 | 0 | 0 |
| hsa_circ_0016754 | 97 | 7909 | 8239 | 331 | 1 | 0 | 3.00E-154 |
| hsa_circ_0016752 | 97 | 7909 | 8333 | 425 | 1 | 0 | 0 |
| hsa_circ_0016754 | 97 | 1 | 329 | 329 | 1 | 0 | 1.00E-154 |
| hsa_circ_0016752 | 97 | 1 | 423 | 423 | 1 | 0 | 0 |
| hsa_circ_0016754 | 96 | 1 | 245 | 245 | 1 | 0 | 2.00E-111 |
| hsa_circ_0016747 | 96 | 1 | 791 | 2305 | 1515 | 0 | 0 |
| hsa_circ_0016751 | 96 | 4 | 450 | 447 | 1 | 0 | 0 |
| hsa_circ_0003659 | 96 | 4 | 697 | 694 | 1 | 0 | 0 |
| hsa_circ_0016747 | 96 | 4 | 820 | 2208 | 1392 | 0 | 0 |
| hsa_circ_0016747 | 96 | 4 | 820 | 2208 | 1392 | 0 | 0 |
| hsa_circ_0016743 | 96 | 165 | 269 | 105 | 1 | 0 | 1.00E-41 |
| hsa_circ_0016740 | 94 | 272 | 596 | 325 | 1 | 0 | 9.00E-139 |
| hsa_circ_0016737 | 93 | 272 | 1035 | 1421 | 658 | 0 | 0 |
| hsa_circ_0016736 | 95 | 108 | 1953 | 1964 | 119 | 0 | 0 |
| hsa_circ_0016740 | 94 | 3 | 327 | 325 | 1 | 0 | 5.00E-139 |
| hsa_circ_0016737 | 93 | 3 | 766 | 1421 | 658 | 0 | 0 |
| hsa_circ_0016736 | 95 | 3 | 1521 | 1964 | 446 | 0 | 0 |
| hsa_circ_0016736 | 95 | 3 | 1848 | 1964 | 119 | 0 | 0 |
| hsa_circ_0016737 | 93 | 1 | 682 | 1339 | 658 | 0 | 0 |
| hsa_circ_0016736 | 95 | 1 | 1437 | 1882 | 446 | 0 | 0 |
| hsa_circ_0016736 | 95 | 1 | 1082 | 1200 | 119 | 0 | 0 |
| hsa_circ_0016736 | 94 | 1 | 327 | 445 | 119 | 0 | 2.00E-138 |
| hsa_circ_0016736 | 94 | 1 | 327 | 445 | 119 | 0 | 3.00E-138 |
| hsa_circ_0016736 | 93 | 1 | 242 | 360 | 119 | 0 | 1.00E-97 |
| hsa_circ_0016713 | 90 | 1 | 196 | 196 | 1 | 0 | 6.00E-66 |
| hsa_circ_0016713 | 90 | 1 | 196 | 196 | 1 | 0 | 2.00E-65 |
| hsa_circ_0016711 | 90 | 533 | 913 | 1537 | 1157 | 0 | 2.00E-136 |
| hsa_circ_0016708 | 95 | 1 | 233 | 905 | 673 | 0 | 1.00E-99 |
| hsa_circ_0016708 | 96 | 1 | 393 | 777 | 385 | 0 | 0 |
| hsa_circ_0016708 | 96 | 1 | 393 | 777 | 385 | 0 | 0 |
| hsa_circ_0016706 | 95 | 1 | 356 | 437 | 82 | 0 | 6.00E-158 |
| hsa_circ_0016704 | 94 | 1 | 429 | 2154 | 1726 | 0 | 0 |
| hsa_circ_0016703 | 96 | 443 | 617 | 362 | 188 | 0 | 2.00E-75 |
| hsa_circ_0016703 | 96 | 160 | 334 | 362 | 188 | 0 | 1.00E-75 |
| hsa_circ_0016702 | 95 | 1 | 472 | 472 | 1 | 0 | 0 |
| hsa_circ_0016696 | 89 | 2 | 515 | 3835 | 3316 | 0.01 | 0 |
| hsa_circ_0016696 | 83 | 2 | 1008 | 3835 | 2823 | 0.02 | 0 |

novel_circ_020835 novel_circ_020836 novel_circ_020837 novel_circ_020838 novel_circ_020839 novel_circ_020840 novel_circ_020841 novel_circ_020842 novel_circ_020843 novel_circ_020844 novel_circ_020845 novel_circ_020846 novel_circ_020847 novel_circ_020848 novel_circ_020849 novel_circ_020850 novel_circ_020851 novel_circ_020852 novel_circ_020853 novel_circ_020854 novel_circ_020855 novel_circ_020856 novel_circ_020857 novel_circ_020858 novel_circ_020859 novel_circ_020860 novel_circ_020861 novel_circ_020862 novel_circ_020863 novel_circ_020864 novel_circ_020865 novel_circ_020866 novel_circ_020867 novel_circ_020868 novel_circ_020869 novel_circ_020870 novel_circ_020871 novel_circ_020872 novel_circ_020873 novel_circ_020874 novel_circ_020875 novel_circ_020876 novel_circ_020877 novel_circ_020878 novel_circ_020879 novel_circ_020880 novel_circ_020881 novel_circ_020882 novel_circ_020883 novel_circ_020884 novel_circ_020885 novel_circ_020886 novel_circ_020887 novel_circ_020888

| hsa_circ_0016677 | 91 | 1 | 678 | 680 | 3 | 0 | 0 |
| --- | --- | --- | --- | --- | --- | --- | --- |
| hsa_circ_0016667 | 92 | 1 | 369 | 1 | 369 | 0 | 9.00E-147 |
| hsa_circ_0016662 | 94 | 1 | 340 | 344 | 1 | 0.01 | 8.00E-147 |
| hsa_circ_0016662 | 94 | 1 | 340 | 344 | 1 | 0.01 | 1.00E-146 |
| hsa_circ_0016662 | 97 | 1 | 178 | 178 | 1 | 0 | 5.00E-79 |
| hsa_circ_0016657 | 95 | 1 | 520 | 11501 | 10982 | 0 | 0 |
| hsa_circ_0016596 | 97 | 65 | 457 | 5744 | 5352 | 0 | 0 |
| hsa_circ_0002322 | 97 | 1 | 392 | 392 | 1 | 0 | 0 |
| hsa_circ_0006962 | 96 | 1 | 531 | 672 | 142 | 0 | 0 |
| hsa_circ_0006962 | 97 | 1 | 672 | 672 | 1 | 0 | 0 |
| hsa_circ_0016588 | 96 | 1 | 792 | 1333 | 542 | 0 | 0 |
| hsa_circ_0016588 | 97 | 1 | 1147 | 1333 | 187 | 0 | 0 |
| hsa_circ_0016588 | 97 | 1 | 1147 | 1333 | 187 | 0 | 0 |
| hsa_circ_0016588 | 97 | 1 | 1333 | 1333 | 1 | 0 | 0 |
| hsa_circ_0016588 | 97 | 1 | 1333 | 1333 | 1 | 0 | 0 |
| hsa_circ_0006962 | 96 | 1 | 535 | 535 | 1 | 0 | 0 |
| hsa_circ_0006962 | 97 | 1 | 437 | 437 | 1 | 0 | 0 |
| hsa_circ_0006962 | 96 | 1 | 280 | 280 | 1 | 0 | 4.00E-129 |
| hsa_circ_0016592 | 96 | 1 | 400 | 546 | 147 | 0 | 0 |
| hsa_circ_0016588 | 97 | 1 | 755 | 941 | 187 | 0 | 0 |
| hsa_circ_0016588 | 97 | 1 | 755 | 941 | 187 | 0 | 0 |
| hsa_circ_0016588 | 97 | 1 | 941 | 941 | 1 | 0 | 0 |
| hsa_circ_0016588 | 97 | 1 | 941 | 941 | 1 | 0 | 0 |
| hsa_circ_0016588 | 97 | 1 | 616 | 802 | 187 | 0 | 0 |
| hsa_circ_0016588 | 97 | 1 | 616 | 802 | 187 | 0 | 0 |
| hsa_circ_0016588 | 97 | 1 | 802 | 802 | 1 | 0 | 0 |
| hsa_circ_0016588 | 97 | 1 | 802 | 802 | 1 | 0 | 0 |
| hsa_circ_0016588 | 97 | 4 | 475 | 658 | 187 | 0 | 0 |
| hsa_circ_0016588 | 97 | 4 | 475 | 658 | 187 | 0 | 0 |
| hsa_circ_0016584 | 87 | 5 | 581 | 118 | 706 | 0.03 | 0 |
| hsa_circ_0016584 | 88 | 1 | 694 | 1 | 706 | 0.02 | 0 |
| hsa_circ_0016584 | 89 | 5 | 505 | 118 | 619 | 0.01 | 3.00E-178 |
| hsa_circ_0016584 | 90 | 1 | 618 | 1 | 619 | 0.01 | 0 |
| hsa_circ_0016584 | 95 | 1 | 248 | 1 | 248 | 0 | 2.00E-106 |
| hsa_circ_0016582 | 90 | 1 | 328 | 331 | 1 | 0.01 | 3.00E-116 |
| hsa_circ_0016577 | 89 | 1 | 986 | 1620 | 638 | 0.01 | 0 |
| hsa_circ_0016579 | 87 | 1 | 453 | 565 | 119 | 0.01 | 2.00E-143 |
| hsa_circ_0016579 | 89 | 1 | 571 | 565 | 1 | 0.01 | 0 |
| hsa_circ_0016577 | 93 | 1 | 263 | 900 | 638 | 0 | 1.00E-104 |
| hsa_circ_0016577 | 91 | 1 | 837 | 900 | 64 | 0 | 0 |
| hsa_circ_0016577 | 90 | 3 | 614 | 780 | 169 | 0 | 0 |
| hsa_circ_0016577 | 90 | 3 | 719 | 780 | 64 | 0 | 0 |
| hsa_circ_0016576 | 91 | 3 | 902 | 900 | 1 | 0 | 0 |
| hsa_circ_0016572 | 90 | 3 | 1107 | 1107 | 3 | 0 | 0 |
| hsa_circ_0016578 | 90 | 1 | 469 | 574 | 106 | 0 | 7.00E-169 |
| hsa_circ_0016576 | 90 | 1 | 757 | 757 | 1 | 0 | 0 |
| hsa_circ_0016572 | 90 | 1 | 962 | 964 | 3 | 0 | 0 |
| hsa_circ_0016570 | 90 | 1 | 1041 | 1041 | 1 | 0 | 0 |
| hsa_circ_0016570 | 90 | 1 | 1041 | 1041 | 1 | 0 | 0 |
| hsa_circ_0016576 | 92 | 1 | 507 | 507 | 1 | 0 | 0 |
| hsa_circ_0016573 | 92 | 1 | 607 | 607 | 1 | 0 | 0 |
| hsa_circ_0016572 | 91 | 1 | 712 | 714 | 3 | 0 | 0 |
| hsa_circ_0016570 | 91 | 1 | 791 | 791 | 1 | 0 | 0 |
| hsa_circ_0016570 | 91 | 1 | 791 | 791 | 1 | 0 | 0 |

novel_circ_020892 novel_circ_020894 novel_circ_020895 novel_circ_020896 novel_circ_020897 novel_circ_020898 novel_circ_020899 novel_circ_020900 novel_circ_020901 novel_circ_020902 novel_circ_020903 novel_circ_020904 novel_circ_020905 novel_circ_020906 novel_circ_020908 novel_circ_020909 novel_circ_020910 novel_circ_020911 novel_circ_020912 novel_circ_020913 novel_circ_020915 novel_circ_020916 novel_circ_020917 novel_circ_020918 novel_circ_020919 novel_circ_020920 novel_circ_020921 novel_circ_020922 novel_circ_020923 novel_circ_020924 novel_circ_020925 novel_circ_020926 novel_circ_020927 novel_circ_020928 novel_circ_020929 novel_circ_020931 novel_circ_020934 novel_circ_020935 novel_circ_020936 novel_circ_020937 novel_circ_020938 novel_circ_020939 novel_circ_020940 novel_circ_020941 novel_circ_020942 novel_circ_020943 novel_circ_020944 novel_circ_020945 novel_circ_020946 novel_circ_020947 novel_circ_020948 novel_circ_020949 novel_circ_020950 novel_circ_020951

| hsa_circ_0013049 | 93 | 1 | 384 | 1 | 384 | 0 | 9.00E-162 |
| --- | --- | --- | --- | --- | --- | --- | --- |
| hsa_circ_0019298 | 97 | 1 | 226 | 226 | 1 | 0 | 3.00E-104 |
| hsa_circ_0019298 | 95 | 1 | 525 | 525 | 1 | 0 | 0 |
| hsa_circ_0019298 | 94 | 1 | 1083 | 1083 | 1 | 0 | 0 |
| hsa_circ_0019298 | 93 | 4 | 472 | 600 | 132 | 0 | 0 |
| hsa_circ_0019298 | 94 | 1 | 857 | 1083 | 227 | 0 | 0 |
| hsa_circ_0019299 | 94 | 1 | 554 | 690 | 137 | 0 | 0 |
| hsa_circ_0019300 | 93 | 1 | 319 | 319 | 1 | 0 | 1.00E-130 |
| hsa_circ_0019317 | 98 | 1 | 567 | 1 | 567 | 0 | 0 |
| hsa_circ_0008102 | 99 | 1 | 421 | 1 | 421 | 0 | 0 |
| hsa_circ_0008102 | 98 | 1 | 515 | 1 | 515 | 0 | 0 |
| hsa_circ_0019322 | 89 | 1 | 314 | 380 | 67 | 0 | 1.00E-109 |
| hsa_circ_0019321 | 91 | 1 | 871 | 5378 | 4508 | 0 | 0 |
| hsa_circ_0019323 | 92 | 1 | 557 | 557 | 1 | 0 | 0 |
| hsa_circ_0019377 | 92 | 202 | 487 | 1 | 286 | 0 | 8.00E-109 |
| hsa_circ_0019377 | 92 | 1 | 286 | 1 | 286 | 0 | 4.00E-109 |
| hsa_circ_0019389 | 80 | 231 | 2053 | 21632 | 23433 | 0.03 | 0 |
| hsa_circ_0019388 | 81 | 233 | 2226 | 198 | 2170 | 0.02 | 0 |
| hsa_circ_0019390 | 80 | 1 | 1820 | 1 | 1799 | 0.03 | 0 |
| hsa_circ_0019388 | 81 | 1 | 1993 | 199 | 2170 | 0.02 | 0 |
| hsa_circ_0004462 | 84 | 8558 | 9047 | 631 | 144 | 0.04 | 2.00E-128 |
| hsa_circ_0019422 | 93 | 1 | 354 | 1 | 354 | 0 | 9.00E-147 |
| hsa_circ_0019420 | 88 | 1 | 626 | 702 | 1327 | 0 | 0 |
| hsa_circ_0019424 | 93 | 1 | 342 | 210 | 551 | 0 | 2.00E-138 |
| hsa_circ_0019424 | 92 | 1 | 512 | 210 | 721 | 0 | 0 |
| hsa_circ_0019424 | 92 | 1 | 440 | 282 | 721 | 0 | 2.00E-174 |
| hsa_circ_0019426 | 92 | 1 | 380 | 1 | 380 | 0 | 2.00E-149 |
| hsa_circ_0019491 | 88 | 1 | 654 | 896 | 237 | 0.01 | 0 |
| hsa_circ_0008393 | 90 | 6 | 277 | 272 | 1 | 0 | 2.00E-95 |
| hsa_circ_0019502 | 94 | 1 | 498 | 595 | 98 | 0 | 0 |
| hsa_circ_0019502 | 95 | 488 | 1192 | 1024 | 320 | 0 | 0 |
| hsa_circ_0019503 | 96 | 463 | 1126 | 664 | 1 | 0 | 0 |
| hsa_circ_0019503 | 96 | 253 | 618 | 664 | 299 | 0 | 7.00E-170 |
| hsa_circ_0019503 | 96 | 368 | 733 | 664 | 299 | 0 | 8.00E-170 |
| hsa_circ_0019503 | 96 | 463 | 828 | 664 | 299 | 0 | 9.00E-170 |
| hsa_circ_0019505 | 91 | 1 | 330 | 330 | 1 | 0 | 1.00E-121 |
| hsa_circ_0073760 | 95 | 1 | 292 | 1 | 292 | 0 | 4.00E-129 |
| hsa_circ_0073760 | 95 | 1 | 432 | 1 | 432 | 0 | 0 |
| hsa_circ_0073760 | 95 | 1 | 651 | 1 | 651 | 0 | 0 |
| hsa_circ_0073760 | 95 | 1 | 865 | 1 | 865 | 0 | 0 |
| hsa_circ_0073760 | 95 | 1 | 1017 | 1 | 1017 | 0 | 0 |
| hsa_circ_0073764 | 95 | 293 | 1604 | 1 | 1312 | 0 | 0 |
| hsa_circ_0073764 | 95 | 293 | 1719 | 1 | 1427 | 0 | 0 |
| hsa_circ_0073764 | 95 | 293 | 1719 | 1 | 1427 | 0 | 0 |
| hsa_circ_0073764 | 95 | 1 | 725 | 1 | 725 | 0 | 0 |
| hsa_circ_0073767 | 95 | 1 | 365 | 1 | 365 | 0 | 3.00E-161 |
| hsa_circ_0073767 | 95 | 1 | 597 | 1 | 597 | 0 | 0 |
| hsa_circ_0073767 | 95 | 1 | 699 | 1 | 699 | 0 | 0 |
| hsa_circ_0073767 | 95 | 1 | 1067 | 1 | 1067 | 0 | 0 |
| hsa_circ_0073767 | 95 | 1 | 1208 | 1 | 1208 | 0 | 0 |
| hsa_circ_0073772 | 95 | 1 | 384 | 1 | 384 | 0 | 2.00E-168 |
| hsa_circ_0073772 | 95 | 1 | 486 | 1 | 486 | 0 | 0 |
| hsa_circ_0073774 | 96 | 1 | 232 | 1 | 232 | 0 | 3.00E-104 |
| hsa_circ_0073774 | 96 | 1 | 334 | 1 | 334 | 0 | 2.00E-152 |

novel_circ_020952 novel_circ_020953 novel_circ_020954 novel_circ_020955 novel_circ_020956 novel_circ_020957 novel_circ_020958 novel_circ_020959 novel_circ_020960 novel_circ_020961 novel_circ_020962 novel_circ_020963 novel_circ_020964 novel_circ_020965 novel_circ_020966 novel_circ_020969 novel_circ_020970 novel_circ_020971 novel_circ_020972 novel_circ_020974 novel_circ_020975 novel_circ_020976 novel_circ_020977 novel_circ_020978 novel_circ_020980 novel_circ_020981 novel_circ_020982 novel_circ_020988 novel_circ_020989 novel_circ_020992 novel_circ_020993 novel_circ_020994 novel_circ_020995 novel_circ_020996 novel_circ_020997 novel_circ_020998 novel_circ_020999 novel_circ_021000 novel_circ_021001 novel_circ_021002 novel_circ_021003 novel_circ_021004 novel_circ_021005 novel_circ_021006 novel_circ_021007 novel_circ_021008 novel_circ_021009 novel_circ_021010 novel_circ_021011 novel_circ_021012 novel_circ_021013 novel_circ_021014 novel_circ_021015 novel_circ_021016

| hsa_circ_0073774 | 96 | 1 | 587 | 1 | 587 | 0 | 0 |
| --- | --- | --- | --- | --- | --- | --- | --- |
| hsa_circ_0073772 | 95 | 1 | 702 | 153 | 854 | 0 | 0 |
| hsa_circ_0073772 | 95 | 1 | 843 | 153 | 995 | 0 | 0 |
| hsa_circ_0073772 | 95 | 1 | 843 | 153 | 995 | 0 | 0 |
| hsa_circ_0073772 | 95 | 1 | 843 | 153 | 995 | 0 | 0 |
| hsa_circ_0073774 | 95 | 1 | 355 | 233 | 587 | 0 | 5.00E-159 |
| hsa_circ_0073772 | 94 | 1 | 611 | 385 | 995 | 0 | 0 |
| hsa_circ_0073772 | 94 | 1 | 611 | 385 | 995 | 0 | 0 |
| hsa_circ_0073774 | 95 | 1 | 253 | 335 | 587 | 0 | 8.00E-111 |
| hsa_circ_0073772 | 94 | 1 | 253 | 743 | 995 | 0 | 7.00E-104 |
| hsa_circ_0073775 | 94 | 441 | 933 | 1 | 496 | 0.01 | 0 |
| hsa_circ_0073775 | 92 | 81 | 374 | 1 | 297 | 0.01 | 6.00E-114 |
| hsa_circ_0073775 | 96 | 1 | 199 | 298 | 496 | 0 | 3.00E-87 |
| hsa_circ_0073784 | 94 | 1 | 375 | 375 | 1 | 0 | 2.00E-163 |
| hsa_circ_0003173 | 91 | 3 | 489 | 487 | 1 | 0 | 0 |
| hsa_circ_0073810 | 95 | 1 | 415 | 1 | 415 | 0 | 0 |
| hsa_circ_0073816 | 92 | 1 | 445 | 1 | 445 | 0 | 1.00E-180 |
| hsa_circ_0073816 | 92 | 1 | 806 | 1 | 806 | 0 | 0 |
| hsa_circ_0073817 | 92 | 1 | 648 | 1 | 648 | 0 | 0 |
| hsa_circ_0028324 | 82 | 885 | 1062 | 2603 | 2775 | 0.06 | 4.00E-30 |
| hsa_circ_0058230 | 95 | 1 | 545 | 1 | 545 | 0 | 0 |
| hsa_circ_0058189 | 90 | 1233 | 1406 | 1 | 174 | 0.02 | 4.00E-56 |
| hsa_circ_0058186 | 89 | 82 | 214 | 106 | 238 | 0 | 3.00E-40 |
| hsa_circ_0058186 | 89 | 82 | 214 | 106 | 238 | 0 | 2.00E-40 |
| hsa_circ_0058168 | 88 | 1 | 875 | 308 | 1179 | 0.01 | 0 |
| hsa_circ_0058168 | 87 | 5 | 1182 | 5 | 1179 | 0.01 | 0 |
| hsa_circ_0058171 | 87 | 1 | 539 | 1 | 539 | 0 | 2.00E-169 |
| hsa_circ_0058159 | 89 | 7427 | 7600 | 2835 | 2663 | 0.02 | 2.00E-51 |
| hsa_circ_0058119 | 90 | 1 | 264 | 264 | 1 | 0 | 1.00E-93 |
| hsa_circ_0081169 | 94 | 1 | 339 | 372 | 710 | 0 | 4.00E-145 |
| hsa_circ_0081169 | 93 | 1 | 762 | 372 | 1133 | 0 | 0 |
| hsa_circ_0081169 | 94 | 1 | 852 | 372 | 1223 | 0 | 0 |
| hsa_circ_0081169 | 94 | 1 | 1311 | 372 | 1682 | 0 | 0 |
| hsa_circ_0081169 | 94 | 1 | 414 | 1581 | 1994 | 0 | 4.00E-178 |
| hsa_circ_0008347 | 79 | 23 | 823 | 116226 | 115421 | 0.04 | 4.00E-147 |
| hsa_circ_0081170 | 94 | 2 | 591 | 590 | 1 | 0 | 0 |
| hsa_circ_0081170 | 94 | 3 | 790 | 788 | 1 | 0 | 0 |
| hsa_circ_0081170 | 94 | 9 | 1144 | 1136 | 1 | 0 | 0 |
| hsa_circ_0081170 | 95 | 1 | 239 | 428 | 190 | 0 | 1.00E-104 |
| hsa_circ_0081170 | 93 | 9 | 716 | 1136 | 429 | 0 | 0 |
| hsa_circ_0081171 | 93 | 9 | 553 | 545 | 1 | 0 | 0 |
| hsa_circ_0081171 | 94 | 9 | 354 | 545 | 200 | 0 | 1.00E-145 |
| hsa_circ_0081174 | 96 | 1 | 254 | 1678 | 1931 | 0 | 1.00E-114 |
| hsa_circ_0081174 | 96 | 1 | 359 | 1678 | 2036 | 0 | 7.00E-163 |
| hsa_circ_0081174 | 95 | 1 | 495 | 1678 | 2172 | 0 | 0 |
| hsa_circ_0081174 | 95 | 1 | 495 | 1678 | 2172 | 0 | 0 |
| hsa_circ_0081174 | 94 | 566 | 1333 | 2180 | 2947 | 0 | 0 |
| hsa_circ_0081174 | 92 | 207 | 421 | 2180 | 2394 | 0 | 2.00E-79 |
| hsa_circ_0081174 | 94 | 207 | 705 | 2180 | 2678 | 0 | 0 |
| hsa_circ_0081174 | 95 | 207 | 1126 | 2180 | 3099 | 0 | 0 |
| hsa_circ_0081174 | 91 | 4911 | 5071 | 2234 | 2394 | 0 | 2.00E-53 |
| hsa_circ_0081174 | 94 | 1 | 709 | 2239 | 2947 | 0 | 0 |
| hsa_circ_0081174 | 95 | 1 | 553 | 2395 | 2947 | 0 | 0 |
| hsa_circ_0081174 | 89 | 1 | 524 | 3892 | 4391 | 0.05 | 4.00E-176 |

novel_circ_021023 novel_circ_021026 novel_circ_021040 novel_circ_021041 novel_circ_021042 novel_circ_021043 novel_circ_021044 novel_circ_021045 novel_circ_021046 novel_circ_021049 novel_circ_021050 novel_circ_021051 novel_circ_021052 novel_circ_021053 novel_circ_021054 novel_circ_021055 novel_circ_021056 novel_circ_021057 novel_circ_021058 novel_circ_021059 novel_circ_021060 novel_circ_021061 novel_circ_021062 novel_circ_021063 novel_circ_021064 novel_circ_021065 novel_circ_021067 novel_circ_021068 novel_circ_021069 novel_circ_021070 novel_circ_021072 novel_circ_021073 novel_circ_021074 novel_circ_021075 novel_circ_021076 novel_circ_021077 novel_circ_021078 novel_circ_021079 novel_circ_021080 novel_circ_021081 novel_circ_021082 novel_circ_021083 novel_circ_021084 novel_circ_021085 novel_circ_021086 novel_circ_021087 novel_circ_021088 novel_circ_021089 novel_circ_021090 novel_circ_021091 novel_circ_021092 novel_circ_021093 novel_circ_021099 novel_circ_021101

| hsa_circ_0071254 | 92 | 19580 | 19735 | 184 | 29 | 0 | 4.00E-53 |
| --- | --- | --- | --- | --- | --- | --- | --- |
| hsa_circ_0071254 | 92 | 19263 | 19418 | 184 | 29 | 0 | 5.00E-53 |
| hsa_circ_0081197 | 93 | 1 | 362 | 362 | 1 | 0 | 9.00E-148 |
| hsa_circ_0081197 | 92 | 212 | 677 | 466 | 1 | 0 | 0 |
| hsa_circ_0081197 | 93 | 9 | 675 | 1152 | 486 | 0 | 0 |
| hsa_circ_0081197 | 92 | 1 | 255 | 362 | 108 | 0 | 1.00E-98 |
| hsa_circ_0081197 | 92 | 212 | 570 | 466 | 108 | 0 | 5.00E-141 |
| hsa_circ_0081197 | 93 | 9 | 675 | 1152 | 486 | 0 | 0 |
| hsa_circ_0081197 | 93 | 9 | 675 | 1152 | 486 | 0 | 0 |
| hsa_circ_0085433 | 99 | 1 | 300 | 300 | 1 | 0 | 3.00E-150 |
| hsa_circ_0085434 | 99 | 1 | 204 | 204 | 1 | 0 | 2.00E-100 |
| hsa_circ_0085443 | 90 | 1 | 692 | 264 | 955 | 0 | 0 |
| hsa_circ_0085446 | 91 | 1 | 513 | 1 | 513 | 0 | 0 |
| hsa_circ_0085446 | 89 | 1 | 911 | 1 | 917 | 0.01 | 0 |
| hsa_circ_0085446 | 90 | 1 | 1045 | 1 | 1051 | 0.01 | 0 |
| hsa_circ_0085443 | 91 | 1 | 1744 | 443 | 2192 | 0.01 | 0 |
| hsa_circ_0085443 | 92 | 1 | 833 | 1360 | 2192 | 0 | 0 |
| hsa_circ_0085443 | 92 | 1 | 970 | 1360 | 2329 | 0 | 0 |
| hsa_circ_0085443 | 90 | 1 | 1522 | 1360 | 2881 | 0 | 0 |
| hsa_circ_0085443 | 92 | 1 | 699 | 1494 | 2192 | 0 | 0 |
| hsa_circ_0085443 | 87 | 1 | 370 | 2194 | 2563 | 0 | 1.00E-115 |
| hsa_circ_0085443 | 88 | 1 | 688 | 2194 | 2881 | 0 | 0 |
| hsa_circ_0085443 | 86 | 1 | 768 | 2194 | 2958 | 0 | 0 |
| hsa_circ_0085443 | 90 | 10 | 318 | 2573 | 2881 | 0 | 4.00E-110 |
| hsa_circ_0085452 | 85 | 1 | 836 | 1142 | 310 | 0 | 0 |
| hsa_circ_0002663 | 85 | 1 | 701 | 698 | 1 | 0 | 0 |
| hsa_circ_0085461 | 93 | 1 | 760 | 760 | 1 | 0 | 0 |
| hsa_circ_0085464 | 95 | 1 | 493 | 493 | 1 | 0 | 0 |
| hsa_circ_0085472 | 90 | 1 | 1442 | 1475 | 1 | 0.02 | 0 |
| hsa_circ_0085477 | 94 | 1 | 151 | 1 | 151 | 0 | 7.00E-59 |
| hsa_circ_0085483 | 92 | 278 | 483 | 1 | 205 | 0 | 2.00E-75 |
| hsa_circ_0085483 | 93 | 1 | 976 | 1 | 976 | 0 | 0 |
| hsa_circ_0085483 | 94 | 1 | 209 | 206 | 414 | 0 | 9.00E-85 |
| hsa_circ_0085483 | 92 | 1 | 1392 | 206 | 1597 | 0 | 0 |
| hsa_circ_0085484 | 93 | 1 | 708 | 1 | 708 | 0 | 0 |
| hsa_circ_0085489 | 92 | 1 | 754 | 201 | 954 | 0 | 0 |
| hsa_circ_0018412 | 95 | 1 | 173 | 1 | 173 | 0 | 3.00E-73 |
| hsa_circ_0018412 | 95 | 1 | 181 | 1 | 182 | 0.01 | 4.00E-74 |
| hsa_circ_0018412 | 95 | 1 | 181 | 1 | 182 | 0.01 | 1.00E-71 |
| hsa_circ_0064027 | 81 | 144 | 1138 | 114 | 1118 | 0.01 | 0 |
| hsa_circ_0084860 | 94 | 155 | 468 | 1 | 314 | 0 | 9.00E-133 |
| hsa_circ_0084860 | 94 | 155 | 680 | 1 | 526 | 0 | 0 |
| hsa_circ_0084860 | 94 | 155 | 766 | 1 | 612 | 0 | 0 |
| hsa_circ_0084860 | 92 | 155 | 947 | 1 | 796 | 0 | 0 |
| hsa_circ_0084860 | 89 | 155 | 1106 | 1 | 958 | 0.01 | 0 |
| hsa_circ_0084860 | 94 | 1 | 526 | 1 | 526 | 0 | 0 |
| hsa_circ_0084864 | 95 | 1 | 576 | 1 | 576 | 0 | 0 |
| hsa_circ_0084867 | 86 | 964 | 1305 | 339 | 1 | 0.01 | 5.00E-100 |
| hsa_circ_0084866 | 86 | 444 | 1360 | 1754 | 838 | 0.01 | 0 |
| hsa_circ_0084866 | 88 | 444 | 1087 | 1754 | 1111 | 0 | 0 |
| hsa_circ_0084866 | 89 | 310 | 823 | 1754 | 1241 | 0 | 1.00E-178 |
| hsa_circ_0084866 | 89 | 444 | 957 | 1754 | 1241 | 0 | 1.00E-178 |
| hsa_circ_0084871 | 92 | 1 | 496 | 1 | 496 | 0 | 0 |
| hsa_circ_0064004 | 86 | 1 | 321 | 562 | 242 | 0 | 4.00E-95 |

novel_circ_021102 novel_circ_021103 novel_circ_021104 novel_circ_021105 novel_circ_021106 novel_circ_021107 novel_circ_021108 novel_circ_021109 novel_circ_021110 novel_circ_021111 novel_circ_021112 novel_circ_021113 novel_circ_021114 novel_circ_021115 novel_circ_021116 novel_circ_021118 novel_circ_021120 novel_circ_021128 novel_circ_021129 novel_circ_021130 novel_circ_021131 novel_circ_021132 novel_circ_021133 novel_circ_021134 novel_circ_021135 novel_circ_021136 novel_circ_021138 novel_circ_021139 novel_circ_021140 novel_circ_021141 novel_circ_021142 novel_circ_021143 novel_circ_021144 novel_circ_021145 novel_circ_021146 novel_circ_021153 novel_circ_021156 novel_circ_021157 novel_circ_021158 novel_circ_021159 novel_circ_021160 novel_circ_021161 novel_circ_021162 novel_circ_021163 novel_circ_021164 novel_circ_021165 novel_circ_021166 novel_circ_021167 novel_circ_021168 novel_circ_021169 novel_circ_021170 novel_circ_021171 novel_circ_021172 novel_circ_021173

| hsa_circ_0063952 | 93 | 1 | 280 | 1 | 280 | 0 | 9.00E-111 |
| --- | --- | --- | --- | --- | --- | --- | --- |
| hsa_circ_0063948 | 92 | 1 | 507 | 635 | 1141 | 0 | 0 |
| hsa_circ_0063948 | 91 | 1 | 573 | 569 | 1141 | 0 | 0 |
| hsa_circ_0063948 | 91 | 1 | 711 | 431 | 1141 | 0 | 0 |
| hsa_circ_0063948 | 91 | 1 | 852 | 290 | 1141 | 0 | 0 |
| hsa_circ_0063948 | 91 | 1 | 898 | 244 | 1141 | 0 | 0 |
| hsa_circ_0063948 | 91 | 1 | 1141 | 1 | 1141 | 0 | 0 |
| hsa_circ_0063948 | 91 | 1 | 223 | 635 | 857 | 0 | 7.00E-81 |
| hsa_circ_0063948 | 91 | 1 | 614 | 244 | 857 | 0 | 0 |
| hsa_circ_0063949 | 90 | 1 | 504 | 1 | 504 | 0 | 0 |
| hsa_circ_0063949 | 91 | 1 | 391 | 1 | 391 | 0 | 1.00E-150 |
| hsa_circ_0063948 | 91 | 1 | 634 | 1 | 634 | 0 | 0 |
| hsa_circ_0063948 | 92 | 1 | 430 | 1 | 430 | 0 | 3.00E-172 |
| hsa_circ_0063944 | 87 | 1 | 581 | 1 | 561 | 0.04 | 0 |
| hsa_circ_0063944 | 85 | 1 | 394 | 1 | 374 | 0.06 | 1.00E-101 |
| hsa_circ_0063886 | 89 | 1 | 328 | 881 | 554 | 0 | 1.00E-110 |
| hsa_circ_0063883 | 87 | 1 | 774 | 1 | 789 | 0.02 | 0 |
| hsa_circ_0014416 | 87 | 5217 | 5467 | 255 | 2 | 0.02 | 1.00E-71 |
| hsa_circ_0056753 | 93 | 3365 | 3503 | 50 | 187 | 0.01 | 5.00E-49 |
| hsa_circ_0056750 | 92 | 1 | 671 | 795 | 110 | 0.02 | 0 |
| hsa_circ_0056749 | 93 | 1 | 845 | 1134 | 275 | 0.02 | 0 |
| hsa_circ_0056749 | 93 | 1 | 1010 | 1134 | 110 | 0.02 | 0 |
| hsa_circ_0056745 | 94 | 1 | 1268 | 1743 | 461 | 0.01 | 0 |
| hsa_circ_0056749 | 95 | 1 | 339 | 448 | 110 | 0 | 2.00E-148 |
| hsa_circ_0056745 | 95 | 1 | 285 | 609 | 325 | 0 | 1.00E-123 |
| hsa_circ_0056744 | 95 | 3 | 692 | 690 | 1 | 0 | 0 |
| hsa_circ_0056739 | 89 | 1 | 863 | 872 | 1722 | 0.03 | 0 |
| hsa_circ_0056739 | 94 | 1 | 433 | 1 | 433 | 0 | 0 |
| hsa_circ_0056737 | 93 | 1 | 517 | 78 | 594 | 0 | 0 |
| hsa_circ_0056736 | 94 | 1 | 675 | 1 | 675 | 0 | 0 |
| hsa_circ_0056736 | 94 | 85 | 759 | 1 | 675 | 0 | 0 |
| hsa_circ_0056736 | 94 | 1 | 581 | 1 | 581 | 0 | 0 |
| hsa_circ_0056736 | 94 | 1 | 395 | 1 | 395 | 0 | 7.00E-168 |
| hsa_circ_0056736 | 94 | 85 | 479 | 1 | 395 | 0 | 9.00E-168 |
| hsa_circ_0056736 | 93 | 85 | 326 | 1 | 242 | 0 | 6.00E-98 |
| hsa_circ_0056733 | 95 | 86 | 396 | 900 | 590 | 0 | 4.00E-136 |
| hsa_circ_0056731 | 96 | 612 | 969 | 544 | 187 | 0 | 1.00E-163 |
| hsa_circ_0056731 | 95 | 612 | 1155 | 544 | 1 | 0 | 0 |
| hsa_circ_0056731 | 96 | 388 | 745 | 544 | 187 | 0 | 1.00E-163 |
| hsa_circ_0056731 | 98 | 369 | 478 | 544 | 435 | 0 | 1.00E-47 |
| hsa_circ_0056731 | 96 | 369 | 630 | 544 | 283 | 0 | 1.00E-118 |
| hsa_circ_0056731 | 96 | 369 | 726 | 544 | 187 | 0 | 1.00E-163 |
| hsa_circ_0056731 | 96 | 1 | 262 | 544 | 283 | 0 | 4.00E-119 |
| hsa_circ_0056731 | 96 | 1 | 358 | 544 | 187 | 0 | 5.00E-164 |
| hsa_circ_0056731 | 95 | 1 | 544 | 544 | 1 | 0 | 0 |
| hsa_circ_0056731 | 95 | 1 | 248 | 434 | 187 | 0 | 2.00E-106 |
| hsa_circ_0056731 | 94 | 1 | 434 | 434 | 1 | 0 | 0 |
| hsa_circ_0056731 | 94 | 1 | 282 | 282 | 1 | 0 | 7.00E-117 |
| hsa_circ_0056730 | 96 | 213 | 694 | 13806 | 13325 | 0 | 0 |
| hsa_circ_0056730 | 85 | 213 | 1904 | 13806 | 12110 | 0.04 | 0 |
| hsa_circ_0056730 | 85 | 213 | 1904 | 13806 | 12110 | 0.04 | 0 |
| hsa_circ_0056730 | 85 | 213 | 1904 | 13806 | 12110 | 0.04 | 0 |
| hsa_circ_0056723 | 91 | 1 | 842 | 3992 | 4836 | 0 | 0 |
| hsa_circ_0056723 | 93 | 1 | 495 | 3992 | 4486 | 0 | 0 |

novel_circ_021174 novel_circ_021175 novel_circ_021176 novel_circ_021177 novel_circ_021178 novel_circ_021179 novel_circ_021180 novel_circ_021181 novel_circ_021182 novel_circ_021183 novel_circ_021184 novel_circ_021185 novel_circ_021186 novel_circ_021187 novel_circ_021188 novel_circ_021189 novel_circ_021190 novel_circ_021191 novel_circ_021192 novel_circ_021193 novel_circ_021195 novel_circ_021196 novel_circ_021198 novel_circ_021199 novel_circ_021214 novel_circ_021215 novel_circ_021216 novel_circ_021217 novel_circ_021218 novel_circ_021219 novel_circ_021220 novel_circ_021221 novel_circ_021222 novel_circ_021223 novel_circ_021224 novel_circ_021225 novel_circ_021226 novel_circ_021227 novel_circ_021228 novel_circ_021229 novel_circ_021230 novel_circ_021231 novel_circ_021232 novel_circ_021233 novel_circ_021234 novel_circ_021235 novel_circ_021236 novel_circ_021237 novel_circ_021238 novel_circ_021239 novel_circ_021240 novel_circ_021241 novel_circ_021242 novel_circ_021243

| hsa_circ_0056723 | 93 | 1 | 341 | 3992 | 4332 | 0 | 1.00E-139 |
| --- | --- | --- | --- | --- | --- | --- | --- |
| hsa_circ_0056722 | 90 | 1 | 609 | 611 | 1219 | 0 | 0 |
| hsa_circ_0056722 | 90 | 1 | 709 | 511 | 1219 | 0 | 0 |
| hsa_circ_0056722 | 90 | 1 | 967 | 253 | 1219 | 0 | 0 |
| hsa_circ_0056722 | 90 | 1 | 1148 | 72 | 1219 | 0 | 0 |
| hsa_circ_0056722 | 90 | 1 | 611 | 511 | 1121 | 0 | 0 |
| hsa_circ_0056722 | 90 | 1 | 1050 | 72 | 1121 | 0 | 0 |
| hsa_circ_0056722 | 90 | 1 | 512 | 1 | 509 | 0.01 | 0 |
| hsa_circ_0056714 | 90 | 1 | 1457 | 1 | 1457 | 0 | 0 |
| hsa_circ_0056711 | 91 | 1 | 1740 | 1 | 1740 | 0 | 0 |
| hsa_circ_0056714 | 91 | 1 | 1200 | 1 | 1200 | 0 | 0 |
| hsa_circ_0056711 | 91 | 1 | 1483 | 1 | 1483 | 0 | 0 |
| hsa_circ_0056708 | 91 | 129 | 1706 | 1 | 1578 | 0 | 0 |
| hsa_circ_0056714 | 91 | 1 | 1019 | 1 | 1019 | 0.01 | 0 |
| hsa_circ_0056711 | 91 | 1 | 1137 | 1 | 1140 | 0 | 0 |
| hsa_circ_0056711 | 92 | 1 | 692 | 1 | 692 | 0 | 0 |
| hsa_circ_0056711 | 94 | 1 | 574 | 1 | 574 | 0 | 0 |
| hsa_circ_0056711 | 92 | 1 | 422 | 1 | 422 | 0 | 8.00E-168 |
| hsa_circ_0056711 | 93 | 1 | 281 | 1 | 281 | 0 | 3.00E-111 |
| hsa_circ_0056705 | 90 | 1 | 504 | 98 | 601 | 0 | 0 |
| hsa_circ_0056688 | 80 | 511 | 2071 | 1742 | 3297 | 0.06 | 0 |
| hsa_circ_0056688 | 83 | 15 | 711 | 2951 | 2261 | 0.04 | 5.00E-172 |
| hsa_circ_0056686 | 92 | 1094 | 1648 | 1 | 555 | 0 | 0 |
| hsa_circ_0056686 | 94 | 188 | 422 | 1 | 235 | 0 | 3.00E-97 |
| hsa_circ_0056685 | 97 | 1 | 490 | 1 | 490 | 0 | 0 |
| hsa_circ_0056684 | 97 | 6 | 580 | 480 | 1054 | 0 | 0 |
| hsa_circ_0056684 | 96 | 1 | 1054 | 1 | 1054 | 0 | 0 |
| hsa_circ_0056682 | 97 | 21772 | 23178 | 161 | 1567 | 0 | 0 |
| hsa_circ_0056682 | 97 | 1 | 353 | 161 | 513 | 0 | 1.00E-169 |
| hsa_circ_0056678 | 93 | 1 | 287 | 5188 | 5474 | 0.01 | 3.00E-116 |
| hsa_circ_0056678 | 95 | 1026 | 2731 | 3769 | 5474 | 0 | 0 |
| hsa_circ_0056678 | 95 | 1 | 1283 | 3979 | 5261 | 0 | 0 |
| hsa_circ_0056678 | 95 | 1026 | 2444 | 3769 | 5187 | 0 | 0 |
| hsa_circ_0056679 | 98 | 3 | 328 | 3 | 328 | 0 | 1.00E-159 |
| hsa_circ_0056678 | 93 | 1 | 953 | 369 | 1321 | 0.02 | 0 |
| hsa_circ_0047282 | 94 | 1 | 440 | 264 | 703 | 0 | 0 |
| hsa_circ_0047282 | 93 | 1 | 436 | 170 | 605 | 0 | 6.00E-179 |
| hsa_circ_0047272 | 96 | 1 | 817 | 1 | 817 | 0 | 0 |
| hsa_circ_0047277 | 96 | 3 | 524 | 3 | 524 | 0 | 0 |
| hsa_circ_0047275 | 96 | 1 | 639 | 130 | 768 | 0 | 0 |
| hsa_circ_0047272 | 96 | 1 | 817 | 1 | 817 | 0 | 0 |
| hsa_circ_0047272 | 95 | 1 | 293 | 1 | 293 | 0 | 5.00E-128 |
| hsa_circ_0047181 | 89 | 4 | 695 | 1020 | 329 | 0.01 | 0 |
| hsa_circ_0047178 | 94 | 1 | 204 | 820 | 617 | 0 | 1.00E-83 |
| hsa_circ_0047152 | 95 | 1 | 637 | 1433 | 2069 | 0 | 0 |
| hsa_circ_0047145 | 91 | 1 | 382 | 382 | 1 | 0 | 6.00E-144 |
| hsa_circ_0047145 | 90 | 1 | 199 | 199 | 1 | 0 | 3.00E-69 |
| hsa_circ_0072030 | 88 | 1 | 356 | 1999 | 1646 | 0.01 | 1.00E-115 |
| hsa_circ_0072030 | 92 | 2 | 583 | 1644 | 1063 | 0 | 0 |
| hsa_circ_0072030 | 91 | 2 | 1025 | 1644 | 621 | 0 | 0 |
| hsa_circ_0072030 | 91 | 2 | 1160 | 1644 | 486 | 0 | 0 |
| hsa_circ_0072030 | 92 | 2 | 1284 | 1644 | 362 | 0 | 0 |
| hsa_circ_0072030 | 90 | 6 | 442 | 1057 | 621 | 0 | 8.00E-163 |
| hsa_circ_0072030 | 91 | 6 | 577 | 1057 | 486 | 0 | 0 |

novel_circ_021244 novel_circ_021245 novel_circ_021246 novel_circ_021247 novel_circ_021248 novel_circ_021249 novel_circ_021250 novel_circ_021251 novel_circ_021252 novel_circ_021253 novel_circ_021256 novel_circ_021285 novel_circ_021286 novel_circ_021287 novel_circ_021288 novel_circ_021289 novel_circ_021290 novel_circ_021291 novel_circ_021292 novel_circ_021293 novel_circ_021294 novel_circ_021295 novel_circ_021296 novel_circ_021297 novel_circ_021299 novel_circ_021300 novel_circ_021301 novel_circ_021302 novel_circ_021303 novel_circ_021304 novel_circ_021305 novel_circ_021306 novel_circ_021307 novel_circ_021312 novel_circ_021315 novel_circ_021316 novel_circ_021317 novel_circ_021318 novel_circ_021319 novel_circ_021320 novel_circ_021323 novel_circ_021327 novel_circ_021328 novel_circ_021329 novel_circ_021330 novel_circ_021331 novel_circ_021332 novel_circ_021333 novel_circ_021334 novel_circ_021335 novel_circ_021336 novel_circ_021337 novel_circ_021338 novel_circ_021339

| hsa_circ_0072030 | 92 | 6 | 701 | 1057 | 362 | 0 | 0 |
| --- | --- | --- | --- | --- | --- | --- | --- |
| hsa_circ_0072030 | 92 | 6 | 810 | 1057 | 253 | 0 | 0 |
| hsa_circ_0072030 | 92 | 6 | 1062 | 1057 | 1 | 0 | 0 |
| hsa_circ_0072030 | 93 | 7 | 874 | 868 | 1 | 0 | 0 |
| hsa_circ_0003163 | 86 | 13423 | 13493 | 1E+06 | 1049330 | 0.03 | 6.00E-11 |
| hsa_circ_0006061 | 81 | 11122 | 11206 | 64293 | 64211 | 0.02 | 4.00E-08 |
| hsa_circ_0006061 | 81 | 11122 | 11206 | 64293 | 64211 | 0.02 | 3.00E-08 |
| hsa_circ_0006061 | 81 | 11122 | 11206 | 64293 | 64211 | 0.02 | 2.00E-08 |
| hsa_circ_0008239 | 91 | 1 | 371 | 1 | 371 | 0 | 7.00E-143 |
| hsa_circ_0002943 | 89 | 3 | 508 | 3 | 508 | 0 | 2.00E-179 |
| hsa_circ_0021542 | 84 | 2565 | 3245 | 4002 | 4681 | 0.04 | 8.00E-176 |
| hsa_circ_0061328 | 94 | 1 | 579 | 1 | 579 | 0 | 0 |
| hsa_circ_0061328 | 94 | 1 | 774 | 1 | 774 | 0 | 0 |
| hsa_circ_0061328 | 94 | 1 | 1162 | 1 | 1162 | 0 | 0 |
| hsa_circ_0061329 | 95 | 1 | 476 | 1 | 476 | 0 | 0 |
| hsa_circ_0061333 | 93 | 1 | 248 | 248 | 1 | 0 | 5.00E-98 |
| hsa_circ_0061333 | 91 | 1 | 524 | 524 | 1 | 0 | 0 |
| hsa_circ_0061332 | 91 | 210 | 1118 | 2134 | 1223 | 0 | 0 |
| hsa_circ_0061332 | 90 | 76 | 682 | 2134 | 1525 | 0 | 0 |
| hsa_circ_0061339 | 92 | 1 | 594 | 597 | 1 | 0.01 | 0 |
| hsa_circ_0061339 | 93 | 1 | 819 | 822 | 1 | 0 | 0 |
| hsa_circ_0061339 | 90 | 1 | 226 | 229 | 1 | 0.01 | 2.00E-76 |
| hsa_circ_0061339 | 92 | 1 | 594 | 597 | 1 | 0.01 | 0 |
| hsa_circ_0061339 | 93 | 1 | 819 | 822 | 1 | 0 | 0 |
| hsa_circ_0061339 | 94 | 1 | 722 | 822 | 101 | 0 | 0 |
| hsa_circ_0061344 | 94 | 1 | 234 | 234 | 1 | 0 | 6.00E-97 |
| hsa_circ_0061344 | 94 | 1 | 368 | 368 | 1 | 0 | 7.00E-158 |
| hsa_circ_0061344 | 94 | 1 | 593 | 593 | 1 | 0 | 0 |
| hsa_circ_0061344 | 93 | 1 | 796 | 796 | 1 | 0 | 0 |
| hsa_circ_0061344 | 93 | 114 | 1103 | 990 | 1 | 0 | 0 |
| hsa_circ_0061346 | 91 | 1 | 510 | 678 | 169 | 0 | 0 |
| hsa_circ_0003038 | 92 | 1 | 307 | 307 | 1 | 0 | 5.00E-119 |
| hsa_circ_0003038 | 92 | 131 | 437 | 307 | 1 | 0 | 7.00E-119 |
| hsa_circ_0009010 | 73 | 6616 | 7019 | 69146 | 68723 | 0.1 | 7.00E-20 |
| hsa_circ_0084555 | 86 | 4184 | 4564 | 642 | 259 | 0.01 | 5.00E-111 |
| hsa_circ_0059524 | 84 | 237 | 346 | 190 | 299 | 0.04 | 5.00E-21 |
| hsa_circ_0059524 | 84 | 196 | 305 | 190 | 299 | 0.04 | 4.00E-21 |
| hsa_circ_0052055 | 89 | 1 | 1957 | 3504 | 1551 | 0 | 0 |
| hsa_circ_0052054 | 88 | 173 | 325 | 2124 | 1972 | 0 | 4.00E-45 |
| hsa_circ_0052054 | 83 | 1 | 1846 | 2098 | 240 | 0.02 | 0 |
| hsa_circ_0021801 | 85 | 1 | 234 | 197 | 430 | 0 | 5.00E-60 |
| hsa_circ_0021824 | 97 | 1 | 384 | 699 | 316 | 0 | 0 |
| hsa_circ_0021824 | 97 | 1 | 633 | 948 | 316 | 0 | 0 |
| hsa_circ_0021824 | 97 | 1 | 636 | 951 | 316 | 0 | 0 |
| hsa_circ_0021824 | 96 | 1 | 843 | 1158 | 316 | 0 | 0 |
| hsa_circ_0021824 | 95 | 1 | 459 | 1158 | 700 | 0 | 0 |
| hsa_circ_0000296 | 95 | 1 | 243 | 236 | 1 | 0.03 | 2.00E-101 |
| hsa_circ_0000296 | 95 | 153 | 396 | 237 | 1 | 0.03 | 1.00E-101 |
| hsa_circ_0000296 | 95 | 14268 | 14511 | 237 | 1 | 0.03 | 4.00E-100 |
| hsa_circ_0000296 | 95 | 27814 | 28057 | 237 | 1 | 0.03 | 8.00E-100 |
| hsa_circ_0000296 | 95 | 27845 | 28088 | 237 | 1 | 0.03 | 8.00E-100 |
| hsa_circ_0021847 | 97 | 3 | 344 | 342 | 1 | 0 | 4.00E-165 |
| hsa_circ_0021849 | 97 | 1 | 471 | 473 | 3 | 0 | 0 |
| hsa_circ_0021856 | 98 | 1 | 362 | 362 | 1 | 0 | 6.00E-178 |

novel_circ_021340 novel_circ_021341 novel_circ_021342 novel_circ_021343 novel_circ_021344 novel_circ_021345 novel_circ_021346 novel_circ_021347 novel_circ_021348 novel_circ_021349 novel_circ_021350 novel_circ_021351 novel_circ_021352 novel_circ_021353 novel_circ_021354 novel_circ_021355 novel_circ_021356 novel_circ_021357 novel_circ_021358 novel_circ_021359 novel_circ_021360 novel_circ_021361 novel_circ_021362 novel_circ_021363 novel_circ_021364 novel_circ_021365 novel_circ_021366 novel_circ_021367 novel_circ_021368 novel_circ_021369 novel_circ_021370 novel_circ_021371 novel_circ_021372 novel_circ_021373 novel_circ_021374 novel_circ_021375 novel_circ_021376 novel_circ_021377 novel_circ_021378 novel_circ_021379 novel_circ_021380 novel_circ_021381 novel_circ_021383 novel_circ_021411 novel_circ_021412 novel_circ_021413 novel_circ_021414 novel_circ_021415 novel_circ_021416 novel_circ_021417 novel_circ_021418 novel_circ_021419 novel_circ_021420 novel_circ_021421

| hsa_circ_0021856 | 98 | 1 | 449 | 449 | 1 | 0 | 0 |
| --- | --- | --- | --- | --- | --- | --- | --- |
| hsa_circ_0021856 | 95 | 997 | 2483 | 1487 | 1 | 0 | 0 |
| hsa_circ_0021859 | 99 | 1 | 267 | 267 | 1 | 0 | 1.00E-133 |
| hsa_circ_0005548 | 93 | 997 | 2034 | 1038 | 1 | 0 | 0 |
| hsa_circ_0005548 | 93 | 1080 | 2117 | 1038 | 1 | 0 | 0 |
| hsa_circ_0021865 | 93 | 1 | 736 | 738 | 1 | 0 | 0 |
| hsa_circ_0021867 | 93 | 1 | 669 | 671 | 1 | 0 | 0 |
| hsa_circ_0004534 | 93 | 1 | 495 | 498 | 2 | 0 | 0 |
| hsa_circ_0021869 | 95 | 1 | 283 | 57 | 339 | 0 | 2.00E-122 |
| hsa_circ_0021869 | 95 | 1 | 802 | 57 | 858 | 0 | 0 |
| hsa_circ_0021869 | 95 | 1 | 519 | 340 | 858 | 0 | 0 |
| hsa_circ_0021869 | 95 | 2 | 331 | 529 | 858 | 0 | 2.00E-143 |
| hsa_circ_0021872 | 93 | 1 | 193 | 1 | 193 | 0 | 6.00E-76 |
| hsa_circ_0021883 | 93 | 1 | 318 | 581 | 264 | 0 | 2.00E-128 |
| hsa_circ_0021900 | 95 | 652 | 916 | 265 | 1 | 0 | 1.00E-113 |
| hsa_circ_0021900 | 93 | 1 | 444 | 444 | 1 | 0 | 0 |
| hsa_circ_0021907 | 93 | 1 | 468 | 468 | 1 | 0 | 0 |
| hsa_circ_0021907 | 93 | 1 | 810 | 1278 | 469 | 0 | 0 |
| hsa_circ_0021907 | 91 | 4 | 939 | 1938 | 1003 | 0 | 0 |
| hsa_circ_0021909 | 92 | 1 | 675 | 675 | 1 | 0 | 0 |
| hsa_circ_0021910 | 90 | 4 | 663 | 660 | 1 | 0 | 0 |
| hsa_circ_0021910 | 90 | 1 | 838 | 838 | 1 | 0 | 0 |
| hsa_circ_0021912 | 91 | 4 | 574 | 571 | 1 | 0 | 0 |
| hsa_circ_0021912 | 91 | 1 | 749 | 749 | 1 | 0 | 0 |
| hsa_circ_0021916 | 91 | 4 | 243 | 240 | 1 | 0 | 6.00E-87 |
| hsa_circ_0021916 | 91 | 1 | 418 | 418 | 1 | 0 | 1.00E-160 |
| hsa_circ_0021916 | 91 | 1 | 558 | 558 | 1 | 0 | 0 |
| hsa_circ_0021916 | 92 | 1 | 1023 | 1023 | 1 | 0 | 0 |
| hsa_circ_0021924 | 91 | 1 | 885 | 885 | 1 | 0 | 0 |
| hsa_circ_0021926 | 93 | 1 | 625 | 1192 | 568 | 0 | 0 |
| hsa_circ_0021926 | 93 | 1 | 832 | 1399 | 568 | 0 | 0 |
| hsa_circ_0021926 | 93 | 1 | 1120 | 1687 | 568 | 0 | 0 |
| hsa_circ_0021927 | 93 | 1 | 406 | 406 | 1 | 0 | 3.00E-167 |
| hsa_circ_0021926 | 93 | 1 | 901 | 1687 | 787 | 0 | 0 |
| hsa_circ_0021929 | 93 | 1 | 495 | 495 | 1 | 0 | 0 |
| hsa_circ_0021964 | 91 | 1 | 243 | 1551 | 1793 | 0 | 3.00E-90 |
| hsa_circ_0021964 | 91 | 1 | 513 | 1794 | 2303 | 0.01 | 0 |
| hsa_circ_0003509 | 96 | 1 | 1514 | 1510 | 1 | 0 | 0 |
| hsa_circ_0000304 | 97 | 1 | 697 | 697 | 1 | 0 | 0 |
| hsa_circ_0022005 | 97 | 1 | 535 | 535 | 1 | 0 | 0 |
| hsa_circ_0004112 | 96 | 1 | 320 | 320 | 1 | 0 | 3.00E-146 |
| hsa_circ_0004112 | 96 | 1 | 271 | 320 | 50 | 0 | 8.00E-121 |
| hsa_circ_0022039 | 93 | 1 | 192 | 192 | 1 | 0 | 1.00E-73 |
| hsa_circ_0022041 | 89 | 1 | 967 | 1951 | 997 | 0.01 | 0 |
| hsa_circ_0022049 | 94 | 1 | 322 | 633 | 312 | 0 | 5.00E-139 |
| hsa_circ_0022049 | 95 | 1 | 427 | 738 | 312 | 0 | 0 |
| hsa_circ_0022049 | 93 | 1 | 552 | 860 | 312 | 0.01 | 0 |
| hsa_circ_0022049 | 93 | 4 | 555 | 860 | 312 | 0.01 | 0 |
| hsa_circ_0022049 | 92 | 1 | 1108 | 1413 | 312 | 0.01 | 0 |
| hsa_circ_0022049 | 91 | 1 | 1716 | 2006 | 312 | 0.01 | 0 |
| hsa_circ_0022052 | 90 | 1 | 949 | 1143 | 213 | 0.02 | 0 |
| hsa_circ_0022056 | 89 | 1 | 339 | 324 | 1 | 0.04 | 2.00E-112 |
| hsa_circ_0022067 | 92 | 1 | 235 | 235 | 1 | 0 | 4.00E-89 |
| hsa_circ_0022075 | 93 | 1 | 394 | 394 | 1 | 0 | 1.00E-165 |

novel_circ_021422 novel_circ_021423 novel_circ_021424 novel_circ_021425 novel_circ_021426 novel_circ_021427 novel_circ_021428 novel_circ_021429 novel_circ_021430 novel_circ_021435 novel_circ_021436 novel_circ_021437 novel_circ_021438 novel_circ_021448 novel_circ_021449 novel_circ_021450 novel_circ_021451 novel_circ_021452 novel_circ_021453 novel_circ_021454 novel_circ_021455 novel_circ_021456 novel_circ_021457 novel_circ_021458 novel_circ_021459 novel_circ_021460 novel_circ_021461 novel_circ_021462 novel_circ_021463 novel_circ_021464 novel_circ_021465 novel_circ_021466 novel_circ_021469 novel_circ_021470 novel_circ_021471 novel_circ_021472 novel_circ_021473 novel_circ_021474 novel_circ_021475 novel_circ_021476 novel_circ_021477 novel_circ_021478 novel_circ_021479 novel_circ_021480 novel_circ_021481 novel_circ_021482 novel_circ_021483 novel_circ_021486 novel_circ_021487 novel_circ_021488 novel_circ_021490 novel_circ_021491 novel_circ_021492 novel_circ_021493

| hsa_circ_0022074 | 92 | 1 | 1337 | 1498 | 162 | 0 | 0 |
| --- | --- | --- | --- | --- | --- | --- | --- |
| hsa_circ_0022079 | 92 | 1 | 1618 | 1809 | 192 | 0 | 0 |
| hsa_circ_0022088 | 90 | 1 | 494 | 494 | 1 | 0 | 2.00E-179 |
| hsa_circ_0022088 | 91 | 1 | 1169 | 1169 | 1 | 0 | 0 |
| hsa_circ_0022093 | 91 | 1 | 809 | 810 | 2 | 0 | 0 |
| hsa_circ_0022093 | 91 | 1 | 963 | 964 | 2 | 0 | 0 |
| hsa_circ_0022093 | 92 | 1 | 521 | 810 | 290 | 0 | 0 |
| hsa_circ_0022093 | 92 | 1 | 675 | 964 | 290 | 0 | 0 |
| hsa_circ_0022095 | 90 | 1 | 412 | 837 | 426 | 0 | 3.00E-152 |
| hsa_circ_0077741 | 95 | 3856 | 4068 | 213 | 1 | 0 | 5.00E-89 |
| hsa_circ_0077741 | 95 | 3856 | 4068 | 213 | 1 | 0 | 8.00E-89 |
| hsa_circ_0077741 | 95 | 3856 | 4068 | 213 | 1 | 0 | 5.00E-89 |
| hsa_circ_0077741 | 95 | 1 | 213 | 213 | 1 | 0 | 5.00E-90 |
| hsa_circ_0004285 | 93 | 4 | 811 | 808 | 1 | 0 | 0 |
| hsa_circ_0004285 | 93 | 4 | 811 | 808 | 1 | 0 | 0 |
| hsa_circ_0077739 | 91 | 1 | 287 | 287 | 1 | 0 | 3.00E-106 |
| hsa_circ_0077738 | 94 | 1 | 159 | 1441 | 1283 | 0 | 4.00E-62 |
| hsa_circ_0077738 | 91 | 1 | 947 | 1441 | 495 | 0 | 0 |
| hsa_circ_0077734 | 91 | 1 | 1069 | 1790 | 725 | 0 | 0 |
| hsa_circ_0077734 | 91 | 1 | 1187 | 1790 | 607 | 0 | 0 |
| hsa_circ_0077734 | 91 | 1 | 1793 | 1790 | 1 | 0 | 0 |
| hsa_circ_0077733 | 91 | 1 | 1844 | 2181 | 341 | 0 | 0 |
| hsa_circ_0077733 | 92 | 1 | 2184 | 2181 | 1 | 0 | 0 |
| hsa_circ_0077738 | 90 | 1 | 788 | 1282 | 495 | 0 | 0 |
| hsa_circ_0077734 | 91 | 1 | 1634 | 1631 | 1 | 0 | 0 |
| hsa_circ_0077733 | 91 | 1 | 1685 | 2022 | 341 | 0 | 0 |
| hsa_circ_0077734 | 91 | 1 | 573 | 843 | 274 | 0.01 | 0 |
| hsa_circ_0077734 | 91 | 1 | 728 | 843 | 119 | 0 | 0 |
| hsa_circ_0077734 | 92 | 1 | 846 | 843 | 1 | 0 | 0 |
| hsa_circ_0077733 | 92 | 1 | 897 | 1234 | 341 | 0 | 0 |
| hsa_circ_0077733 | 92 | 1 | 1237 | 1234 | 1 | 0 | 0 |
| hsa_circ_0077734 | 92 | 3 | 606 | 604 | 1 | 0 | 0 |
| hsa_circ_0077726 | 94 | 1 | 627 | 973 | 347 | 0 | 0 |
| hsa_circ_0006146 | 96 | 138 | 1249 | 4 | 1115 | 0 | 0 |
| hsa_circ_0077712 | 96 | 1 | 752 | 1 | 752 | 0 | 0 |
| hsa_circ_0077712 | 95 | 1 | 563 | 1 | 563 | 0 | 0 |
| hsa_circ_0006146 | 95 | 4 | 297 | 4 | 297 | 0 | 3.00E-130 |
| hsa_circ_0077712 | 95 | 1 | 431 | 1 | 431 | 0 | 0 |
| hsa_circ_0077710 | 86 | 12 | 675 | 964 | 292 | 0.02 | 0 |
| hsa_circ_0077710 | 87 | 12 | 964 | 964 | 3 | 0.02 | 0 |
| hsa_circ_0077710 | 90 | 1 | 400 | 402 | 3 | 0 | 3.00E-147 |
| hsa_circ_0077705 | 91 | 1 | 901 | 897 | 1 | 0 | 0 |
| hsa_circ_0064036 | 91 | 113 | 544 | 432 | 1 | 0 | 3.00E-163 |
| hsa_circ_0064032 | 91 | 113 | 602 | 492 | 3 | 0 | 0 |
| hsa_circ_0064036 | 91 | 4 | 435 | 432 | 1 | 0 | 2.00E-163 |
| hsa_circ_0064032 | 91 | 4 | 493 | 492 | 3 | 0 | 0 |
| hsa_circ_0064036 | 91 | 5 | 352 | 348 | 1 | 0 | 4.00E-130 |
| hsa_circ_0003400 | 95 | 10 | 576 | 567 | 1 | 0 | 0 |
| hsa_circ_0003400 | 96 | 1 | 373 | 373 | 1 | 0 | 1.00E-170 |
| hsa_circ_0089948 | 90 | 1 | 1228 | 1228 | 1 | 0 | 0 |
| hsa_circ_0006020 | 92 | 7408 | 7708 | 1 | 304 | 0.01 | 7.00E-115 |
| hsa_circ_0006020 | 93 | 7408 | 7800 | 1 | 396 | 0.01 | 3.00E-164 |
| hsa_circ_0006020 | 92 | 1 | 301 | 1 | 304 | 0.01 | 3.00E-116 |
| hsa_circ_0006020 | 93 | 1 | 393 | 1 | 396 | 0.01 | 1.00E-165 |

novel_circ_021494 novel_circ_021495 novel_circ_021496 novel_circ_021499 novel_circ_021500 novel_circ_021501 novel_circ_021502 novel_circ_021503 novel_circ_021504 novel_circ_021505 novel_circ_021506 novel_circ_021507 novel_circ_021508 novel_circ_021509 novel_circ_021510 novel_circ_021511 novel_circ_021512 novel_circ_021513 novel_circ_021514 novel_circ_021515 novel_circ_021516 novel_circ_021517 novel_circ_021518 novel_circ_021519 novel_circ_021520 novel_circ_021522 novel_circ_021523 novel_circ_021526 novel_circ_021528 novel_circ_021529 novel_circ_021531 novel_circ_021532 novel_circ_021533 novel_circ_021534 novel_circ_021535 novel_circ_021536 novel_circ_021537 novel_circ_021538 novel_circ_021539 novel_circ_021540 novel_circ_021541 novel_circ_021542 novel_circ_021543 novel_circ_021544 novel_circ_021545 novel_circ_021546 novel_circ_021547 novel_circ_021548 novel_circ_021549 novel_circ_021550 novel_circ_021551 novel_circ_021552 novel_circ_021553 novel_circ_021554

| hsa_circ_0006020 | 93 | 1 | 393 | 1 | 396 | 0.01 | 2.00E-165 |
| --- | --- | --- | --- | --- | --- | --- | --- |
| hsa_circ_0006020 | 93 | 1 | 393 | 1 | 396 | 0.01 | 2.00E-165 |
| hsa_circ_0092270 | 82 | 11 | 740 | 11 | 765 | 0.06 | 5.00E-167 |
| hsa_circ_0089961 | 93 | 1 | 340 | 340 | 1 | 0 | 3.00E-137 |
| hsa_circ_0089961 | 94 | 1 | 879 | 937 | 59 | 0 | 0 |
| hsa_circ_0005840 | 95 | 1 | 263 | 263 | 1 | 0 | 1.00E-114 |
| hsa_circ_0005840 | 96 | 1 | 290 | 290 | 1 | 0 | 1.00E-129 |
| hsa_circ_0089966 | 94 | 239 | 362 | 1 | 124 | 0 | 3.00E-47 |
| hsa_circ_0089966 | 91 | 509 | 878 | 126 | 498 | 0.01 | 4.00E-139 |
| hsa_circ_0089966 | 91 | 271 | 640 | 126 | 498 | 0.01 | 3.00E-139 |
| hsa_circ_0089966 | 90 | 271 | 779 | 126 | 634 | 0.01 | 0 |
| hsa_circ_0089966 | 90 | 271 | 986 | 126 | 841 | 0.01 | 0 |
| hsa_circ_0089966 | 90 | 271 | 1081 | 126 | 936 | 0.01 | 0 |
| hsa_circ_0089966 | 89 | 271 | 1151 | 126 | 1006 | 0.01 | 0 |
| hsa_circ_0089969 | 87 | 3 | 237 | 3 | 234 | 0.01 | 5.00E-68 |
| hsa_circ_0089969 | 88 | 3 | 444 | 3 | 441 | 0.01 | 8.00E-148 |
| hsa_circ_0089969 | 88 | 3 | 539 | 3 | 536 | 0.01 | 2.00E-175 |
| hsa_circ_0089966 | 91 | 3 | 846 | 403 | 1243 | 0 | 0 |
| hsa_circ_0089969 | 88 | 1 | 441 | 99 | 536 | 0.01 | 6.00E-149 |
| hsa_circ_0089970 | 97 | 1 | 199 | 171 | 369 | 0 | 2.00E-92 |
| hsa_circ_0089972 | 95 | 1 | 62 | 1 | 62 | 0 | 7.00E-20 |
| hsa_circ_0089972 | 95 | 1 | 62 | 1 | 62 | 0 | 2.00E-19 |
| hsa_circ_0089972 | 89 | 151 | 491 | 58 | 398 | 0 | 2.00E-114 |
| hsa_circ_0089973 | 88 | 1 | 336 | 1 | 336 | 0 | 8.00E-112 |
| hsa_circ_0089979 | 87 | 220 | 598 | 389 | 8 | 0.01 | 1.00E-117 |
| hsa_circ_0089980 | 88 | 1 | 266 | 1 | 261 | 0.04 | 2.00E-81 |
| hsa_circ_0001912 | 88 | 5 | 267 | 1 | 258 | 0.04 | 3.00E-81 |
| hsa_circ_0089981 | 91 | 600 | 2131 | 1 | 1532 | 0 | 0 |
| hsa_circ_0089981 | 94 | 282 | 514 | 1 | 233 | 0 | 1.00E-97 |
| hsa_circ_0005234 | 76 | 11331 | 11579 | 321020 | 321266 | 0.05 | 4.00E-24 |
| hsa_circ_0090002 | 88 | 4 | 1411 | 2391 | 975 | 0.01 | 0 |
| hsa_circ_0090012 | 88 | 1 | 528 | 673 | 146 | 0 | 3.00E-173 |
| hsa_circ_0090012 | 88 | 4 | 598 | 994 | 391 | 0.01 | 0 |
| hsa_circ_0090012 | 91 | 6 | 578 | 1255 | 674 | 0.02 | 0 |
| hsa_circ_0090019 | 92 | 1 | 1173 | 3255 | 2083 | 0 | 0 |
| hsa_circ_0090029 | 92 | 4 | 447 | 1715 | 1272 | 0 | 5.00E-180 |
| hsa_circ_0090029 | 92 | 1 | 641 | 1912 | 1272 | 0 | 0 |
| hsa_circ_0090031 | 92 | 1 | 470 | 470 | 1 | 0 | 0 |
| hsa_circ_0001913 | 95 | 262 | 468 | 207 | 1 | 0 | 1.00E-86 |
| hsa_circ_0090033 | 94 | 1 | 358 | 895 | 538 | 0 | 1.00E-155 |
| hsa_circ_0008270 | 89 | 2 | 147 | 363 | 508 | 0 | 2.00E-44 |
| hsa_circ_0032943 | 92 | 1 | 291 | 291 | 1 | 0 | 7.00E-112 |
| hsa_circ_0032942 | 94 | 1 | 515 | 515 | 1 | 0 | 0 |
| hsa_circ_0032943 | 93 | 1 | 219 | 219 | 1 | 0 | 5.00E-87 |
| hsa_circ_0032943 | 93 | 1 | 219 | 219 | 1 | 0 | 9.00E-85 |
| hsa_circ_0032942 | 95 | 1 | 443 | 443 | 1 | 0 | 0 |
| hsa_circ_0032937 | 95 | 1 | 631 | 631 | 1 | 0 | 0 |
| hsa_circ_0032936 | 93 | 220 | 1469 | 1250 | 1 | 0 | 0 |
| hsa_circ_0032942 | 96 | 1 | 224 | 224 | 1 | 0 | 2.00E-101 |
| hsa_circ_0032937 | 97 | 1 | 412 | 412 | 1 | 0 | 0 |
| hsa_circ_0032936 | 95 | 1 | 851 | 1250 | 400 | 0 | 0 |
| hsa_circ_0032936 | 94 | 1 | 985 | 1250 | 266 | 0 | 0 |
| hsa_circ_0032936 | 93 | 1 | 1250 | 1250 | 1 | 0 | 0 |
| hsa_circ_0032937 | 97 | 1 | 296 | 296 | 1 | 0 | 5.00E-138 |

novel_circ_021555 novel_circ_021556 novel_circ_021557 novel_circ_021558 novel_circ_021559 novel_circ_021560 novel_circ_021561 novel_circ_021562 novel_circ_021563 novel_circ_021564 novel_circ_021565 novel_circ_021567 novel_circ_021568 novel_circ_021569 novel_circ_021570 novel_circ_021571 novel_circ_021572 novel_circ_021573 novel_circ_021574 novel_circ_021576 novel_circ_021577 novel_circ_021578 novel_circ_021579 novel_circ_021580 novel_circ_021581 novel_circ_021582 novel_circ_021583 novel_circ_021584 novel_circ_021585 novel_circ_021586 novel_circ_021587 novel_circ_021588 novel_circ_021589 novel_circ_021590 novel_circ_021591 novel_circ_021592 novel_circ_021593 novel_circ_021594 novel_circ_021595 novel_circ_021596 novel_circ_021597 novel_circ_021598 novel_circ_021599 novel_circ_021600 novel_circ_021601 novel_circ_021602 novel_circ_021603 novel_circ_021604 novel_circ_021605 novel_circ_021606 novel_circ_021607 novel_circ_021608 novel_circ_021609 novel_circ_021610

| hsa_circ_0032936 | 93 | 1 | 1134 | 1134 | 1 | 0 | 0 |
| --- | --- | --- | --- | --- | --- | --- | --- |
| hsa_circ_0032936 | 92 | 38 | 1063 | 1026 | 1 | 0 | 0 |
| hsa_circ_0032937 | 97 | 1 | 188 | 188 | 1 | 0 | 8.00E-85 |
| hsa_circ_0032936 | 93 | 1 | 761 | 1026 | 266 | 0 | 0 |
| hsa_circ_0032936 | 92 | 1 | 1026 | 1026 | 1 | 0 | 0 |
| hsa_circ_0032936 | 92 | 1 | 1026 | 1026 | 1 | 0 | 0 |
| hsa_circ_0032936 | 91 | 1 | 838 | 838 | 1 | 0 | 0 |
| hsa_circ_0032936 | 91 | 2 | 265 | 264 | 1 | 0 | 3.00E-98 |
| hsa_circ_0032936 | 91 | 2 | 265 | 264 | 1 | 0 | 4.00E-98 |
| hsa_circ_0032936 | 90 | 1 | 171 | 171 | 1 | 0 | 1.00E-56 |
| hsa_circ_0032936 | 90 | 1 | 171 | 171 | 1 | 0 | 2.00E-56 |
| hsa_circ_0032934 | 97 | 1 | 253 | 332 | 80 | 0 | 4.00E-119 |
| hsa_circ_0005422 | 94 | 1 | 707 | 707 | 1 | 0 | 0 |
| hsa_circ_0005422 | 93 | 4 | 375 | 372 | 1 | 0 | 4.00E-155 |
| hsa_circ_0005422 | 93 | 1 | 202 | 202 | 1 | 0 | 1.00E-77 |
| hsa_circ_0032927 | 93 | 1 | 801 | 801 | 1 | 0 | 0 |
| hsa_circ_0032927 | 93 | 1 | 737 | 737 | 1 | 0 | 0 |
| hsa_circ_0032927 | 93 | 1 | 599 | 599 | 1 | 0 | 0 |
| hsa_circ_0032917 | 92 | 315 | 654 | 344 | 5 | 0 | 5.00E-132 |
| hsa_circ_0032910 | 86 | 1 | 1480 | 1631 | 140 | 0.02 | 0 |
| hsa_circ_0005987 | 95 | 117 | 673 | 557 | 1 | 0 | 0 |
| hsa_circ_0005987 | 95 | 1 | 557 | 557 | 1 | 0 | 0 |
| hsa_circ_0032889 | 94 | 1 | 694 | 759 | 66 | 0 | 0 |
| hsa_circ_0032889 | 94 | 1 | 694 | 759 | 66 | 0 | 0 |
| hsa_circ_0032884 | 93 | 3 | 644 | 124 | 765 | 0 | 0 |
| hsa_circ_0032884 | 93 | 1 | 765 | 1 | 765 | 0 | 0 |
| hsa_circ_0032884 | 92 | 1 | 480 | 1 | 480 | 0 | 0 |
| hsa_circ_0032875 | 94 | 7790 | 9249 | 1460 | 1 | 0 | 0 |
| hsa_circ_0032882 | 92 | 1 | 259 | 259 | 1 | 0 | 4.00E-99 |
| hsa_circ_0032880 | 95 | 1 | 510 | 852 | 343 | 0 | 0 |
| hsa_circ_0032880 | 95 | 1 | 650 | 852 | 203 | 0 | 0 |
| hsa_circ_0032880 | 94 | 1 | 852 | 852 | 1 | 0 | 0 |
| hsa_circ_0032877 | 94 | 1 | 853 | 990 | 138 | 0 | 0 |
| hsa_circ_0032877 | 95 | 1 | 990 | 990 | 1 | 0 | 0 |
| hsa_circ_0032875 | 94 | 1 | 1247 | 1460 | 214 | 0 | 0 |
| hsa_circ_0032875 | 94 | 1 | 1460 | 1460 | 1 | 0 | 0 |
| hsa_circ_0032871 | 94 | 1 | 2041 | 2041 | 1 | 0 | 0 |
| hsa_circ_0032880 | 96 | 1 | 692 | 692 | 1 | 0 | 0 |
| hsa_circ_0032877 | 96 | 1 | 830 | 830 | 1 | 0 | 0 |
| hsa_circ_0032875 | 95 | 1 | 1087 | 1300 | 214 | 0 | 0 |
| hsa_circ_0032875 | 95 | 1 | 1300 | 1300 | 1 | 0 | 0 |
| hsa_circ_0032871 | 95 | 1 | 1468 | 1881 | 414 | 0 | 0 |
| hsa_circ_0032872 | 95 | 1 | 1369 | 1369 | 1 | 0 | 0 |
| hsa_circ_0032880 | 95 | 1 | 524 | 524 | 1 | 0 | 0 |
| hsa_circ_0032875 | 94 | 1 | 1132 | 1132 | 1 | 0 | 0 |
| hsa_circ_0032871 | 95 | 1 | 1407 | 1713 | 307 | 0 | 0 |
| hsa_circ_0032880 | 95 | 1 | 338 | 338 | 1 | 0 | 6.00E-148 |
| hsa_circ_0032875 | 94 | 1 | 608 | 608 | 1 | 0 | 0 |
| hsa_circ_0032872 | 95 | 1 | 776 | 776 | 1 | 0 | 0 |
| hsa_circ_0032875 | 93 | 1 | 470 | 470 | 1 | 0 | 0 |
| hsa_circ_0032871 | 94 | 1 | 1051 | 1051 | 1 | 0 | 0 |
| hsa_circ_0032870 | 94 | 1 | 882 | 1467 | 586 | 0 | 0 |
| hsa_circ_0032870 | 95 | 1 | 1197 | 1467 | 271 | 0 | 0 |
| hsa_circ_0032862 | 94 | 1 | 2580 | 2874 | 295 | 0 | 0 |

novel_circ_021611 novel_circ_021612 novel_circ_021613 novel_circ_021614 novel_circ_021615 novel_circ_021616 novel_circ_021617 novel_circ_021618 novel_circ_021619 novel_circ_021620 novel_circ_021621 novel_circ_021622 novel_circ_021623 novel_circ_021624 novel_circ_021625 novel_circ_021626 novel_circ_021627 novel_circ_021628 novel_circ_021629 novel_circ_021630 novel_circ_021631 novel_circ_021632 novel_circ_021633 novel_circ_021634 novel_circ_021635 novel_circ_021636 novel_circ_021637 novel_circ_021638 novel_circ_021639 novel_circ_021640 novel_circ_021641 novel_circ_021642 novel_circ_021644 novel_circ_021645 novel_circ_021646 novel_circ_021648 novel_circ_021649 novel_circ_021650 novel_circ_021664 novel_circ_021666 novel_circ_021667 novel_circ_021668 novel_circ_021669 novel_circ_021671 novel_circ_021672 novel_circ_021673 novel_circ_021674 novel_circ_021675 novel_circ_021676 novel_circ_021677 novel_circ_021678 novel_circ_021681 novel_circ_021682 novel_circ_021683

| hsa_circ_0032870 | 94 | 1 | 714 | 1299 | 586 | 0 | 0 |
| --- | --- | --- | --- | --- | --- | --- | --- |
| hsa_circ_0032870 | 95 | 1 | 1157 | 1299 | 143 | 0 | 0 |
| hsa_circ_0032866 | 95 | 1 | 1566 | 2072 | 507 | 0 | 0 |
| hsa_circ_0032870 | 94 | 1 | 691 | 1073 | 383 | 0 | 0 |
| hsa_circ_0032870 | 95 | 4 | 744 | 883 | 143 | 0 | 0 |
| hsa_circ_0032870 | 97 | 1 | 544 | 686 | 143 | 0 | 0 |
| hsa_circ_0032870 | 96 | 1 | 443 | 585 | 143 | 0 | 0 |
| hsa_circ_0032862 | 95 | 1 | 1698 | 1992 | 295 | 0 | 0 |
| hsa_circ_0032870 | 95 | 1 | 329 | 471 | 143 | 0 | 3.00E-146 |
| hsa_circ_0032862 | 94 | 1 | 1584 | 1878 | 295 | 0 | 0 |
| hsa_circ_0032856 | 94 | 1 | 2684 | 2684 | 1 | 0 | 0 |
| hsa_circ_0032866 | 94 | 1 | 374 | 880 | 507 | 0 | 7.00E-163 |
| hsa_circ_0032866 | 94 | 1 | 880 | 880 | 1 | 0 | 0 |
| hsa_circ_0032866 | 93 | 1 | 595 | 595 | 1 | 0 | 0 |
| hsa_circ_0032863 | 94 | 1 | 828 | 828 | 1 | 0 | 0 |
| hsa_circ_0032863 | 95 | 1 | 360 | 360 | 1 | 0 | 4.00E-160 |
| hsa_circ_0032862 | 95 | 1 | 459 | 654 | 196 | 0 | 0 |
| hsa_circ_0032856 | 94 | 1 | 1661 | 1661 | 1 | 0 | 0 |
| hsa_circ_0032856 | 94 | 1 | 1661 | 1661 | 1 | 0 | 0 |
| hsa_circ_0032863 | 95 | 1 | 209 | 209 | 1 | 0 | 9.00E-90 |
| hsa_circ_0032862 | 95 | 1 | 304 | 503 | 200 | 0 | 2.00E-132 |
| hsa_circ_0032862 | 95 | 1 | 308 | 503 | 196 | 0 | 1.00E-134 |
| hsa_circ_0032857 | 93 | 1 | 870 | 1347 | 478 | 0 | 0 |
| hsa_circ_0032856 | 94 | 1 | 1510 | 1510 | 1 | 0 | 0 |
| hsa_circ_0032856 | 94 | 1 | 1510 | 1510 | 1 | 0 | 0 |
| hsa_circ_0032856 | 93 | 3 | 1181 | 1179 | 1 | 0 | 0 |
| hsa_circ_0032856 | 93 | 3 | 1181 | 1179 | 1 | 0 | 0 |
| hsa_circ_0032857 | 95 | 1 | 367 | 844 | 478 | 0 | 1.00E-165 |
| hsa_circ_0032857 | 95 | 1 | 482 | 844 | 363 | 0 | 0 |
| hsa_circ_0032856 | 94 | 1 | 640 | 640 | 1 | 0 | 0 |
| hsa_circ_0032856 | 94 | 1 | 640 | 640 | 1 | 0 | 0 |
| hsa_circ_0032856 | 94 | 1 | 163 | 163 | 1 | 0.01 | 7.00E-64 |
| hsa_circ_0003164 | 92 | 783 | 1200 | 1 | 418 | 0 | 2.00E-168 |
| hsa_circ_0003164 | 94 | 1 | 262 | 1 | 262 | 0 | 2.00E-107 |
| hsa_circ_0003164 | 94 | 783 | 1044 | 1 | 262 | 0 | 8.00E-107 |
| hsa_circ_0032851 | 94 | 1 | 582 | 582 | 1 | 0 | 0 |
| hsa_circ_0032851 | 94 | 2 | 345 | 344 | 1 | 0 | 3.00E-146 |
| hsa_circ_0032851 | 94 | 2 | 345 | 344 | 1 | 0 | 1.00E-145 |
| hsa_circ_0064026 | 90 | 7915 | 7966 | 231151 | 231100 | 0 | 7.00E-09 |
| hsa_circ_0066445 | 92 | 1 | 1299 | 2181 | 883 | 0 | 0 |
| hsa_circ_0066445 | 92 | 1 | 1085 | 1967 | 883 | 0 | 0 |
| hsa_circ_0066445 | 92 | 1 | 339 | 339 | 1 | 0 | 8.00E-132 |
| hsa_circ_0066443 | 92 | 1 | 681 | 681 | 1 | 0 | 0 |
| hsa_circ_0066438 | 97 | 1 | 329 | 329 | 1 | 0 | 6.00E-158 |
| hsa_circ_0066436 | 97 | 1 | 587 | 1 | 587 | 0 | 0 |
| hsa_circ_0066434 | 98 | 102 | 427 | 185 | 510 | 0 | 8.00E-158 |
| hsa_circ_0066434 | 97 | 102 | 322 | 185 | 405 | 0 | 3.00E-101 |
| hsa_circ_0066427 | 94 | 1 | 447 | 1312 | 866 | 0 | 0 |
| hsa_circ_0066427 | 93 | 1 | 333 | 1198 | 866 | 0 | 2.00E-138 |
| hsa_circ_0066428 | 94 | 1 | 356 | 356 | 1 | 0 | 7.00E-153 |
| hsa_circ_0066427 | 94 | 1 | 608 | 784 | 177 | 0 | 0 |
| hsa_circ_0066426 | 98 | 1364 | 1669 | 307 | 2 | 0 | 9.00E-148 |
| hsa_circ_0066426 | 98 | 1039 | 1344 | 307 | 2 | 0 | 7.00E-148 |
| hsa_circ_0066426 | 100 | 731 | 878 | 307 | 160 | 0 | 7.00E-72 |

novel_circ_021684 novel_circ_021685 novel_circ_021686 novel_circ_021687 novel_circ_021688 novel_circ_021689 novel_circ_021690 novel_circ_021691 novel_circ_021692 novel_circ_021693 novel_circ_021694 novel_circ_021695 novel_circ_021696 novel_circ_021698 novel_circ_021699 novel_circ_021700 novel_circ_021701 novel_circ_021702 novel_circ_021703 novel_circ_021704 novel_circ_021705 novel_circ_021706 novel_circ_021707 novel_circ_021709 novel_circ_021710 novel_circ_021711 novel_circ_021712 novel_circ_021713 novel_circ_021714 novel_circ_021715 novel_circ_021716 novel_circ_021717 novel_circ_021718 novel_circ_021719 novel_circ_021720 novel_circ_021721 novel_circ_021722 novel_circ_021723 novel_circ_021724 novel_circ_021725 novel_circ_021726 novel_circ_021727 novel_circ_021728 novel_circ_021729 novel_circ_021730 novel_circ_021731 novel_circ_021732 novel_circ_021733 novel_circ_021735 novel_circ_021736 novel_circ_021737 novel_circ_021738 novel_circ_021739 novel_circ_021740

| hsa_circ_0066426 | 98 | 731 | 1036 | 307 | 2 | 0 | 6.00E-148 |
| --- | --- | --- | --- | --- | --- | --- | --- |
| hsa_circ_0066426 | 98 | 731 | 1036 | 307 | 2 | 0 | 6.00E-148 |
| hsa_circ_0066426 | 100 | 603 | 750 | 307 | 160 | 0 | 6.00E-72 |
| hsa_circ_0066426 | 98 | 603 | 908 | 307 | 2 | 0 | 5.00E-148 |
| hsa_circ_0066426 | 98 | 603 | 908 | 307 | 2 | 0 | 6.00E-148 |
| hsa_circ_0066426 | 100 | 304 | 451 | 307 | 160 | 0 | 3.00E-72 |
| hsa_circ_0066426 | 99 | 304 | 526 | 307 | 85 | 0 | 2.00E-110 |
| hsa_circ_0066426 | 98 | 304 | 609 | 307 | 2 | 0 | 3.00E-148 |
| hsa_circ_0066426 | 98 | 304 | 609 | 307 | 2 | 0 | 4.00E-148 |
| hsa_circ_0066426 | 98 | 1471 | 1776 | 307 | 2 | 0 | 1.00E-147 |
| hsa_circ_0066426 | 99 | 157 | 379 | 307 | 85 | 0 | 1.00E-110 |
| hsa_circ_0066426 | 98 | 157 | 462 | 307 | 2 | 0 | 2.00E-148 |
| hsa_circ_0066426 | 98 | 157 | 462 | 307 | 2 | 0 | 3.00E-148 |
| hsa_circ_0066424 | 96 | 1 | 283 | 527 | 809 | 0 | 4.00E-129 |
| hsa_circ_0066424 | 96 | 2158 | 2445 | 522 | 809 | 0 | 6.00E-131 |
| hsa_circ_0066424 | 94 | 1 | 456 | 354 | 809 | 0 | 0 |
| hsa_circ_0066424 | 94 | 1 | 809 | 1 | 809 | 0 | 0 |
| hsa_circ_0066420 | 94 | 1 | 906 | 1527 | 2432 | 0 | 0 |
| hsa_circ_0066420 | 95 | 3 | 1310 | 1125 | 2432 | 0 | 0 |
| hsa_circ_0066424 | 94 | 1 | 353 | 1 | 353 | 0 | 6.00E-153 |
| hsa_circ_0066420 | 94 | 1 | 450 | 1527 | 1976 | 0 | 0 |
| hsa_circ_0066422 | 96 | 3 | 404 | 3 | 404 | 0 | 0 |
| hsa_circ_0066422 | 95 | 3 | 312 | 3 | 312 | 0 | 2.00E-137 |
| hsa_circ_0066403 | 97 | 30185 | 30285 | 804 | 904 | 0 | 3.00E-39 |
| hsa_circ_0066549 | 95 | 1 | 188 | 188 | 1 | 0 | 4.00E-78 |
| hsa_circ_0004340 | 91 | 1 | 349 | 343 | 1 | 0.02 | 5.00E-129 |
| hsa_circ_0002006 | 91 | 1 | 462 | 456 | 1 | 0.01 | 2.00E-173 |
| hsa_circ_0066543 | 88 | 66 | 590 | 728 | 210 | 0.01 | 3.00E-178 |
| hsa_circ_0066543 | 89 | 66 | 799 | 728 | 1 | 0.01 | 0 |
| hsa_circ_0066543 | 89 | 66 | 799 | 728 | 1 | 0.01 | 0 |
| hsa_circ_0066538 | 88 | 189 | 1044 | 2410 | 1561 | 0.01 | 0 |
| hsa_circ_0002006 | 90 | 1 | 397 | 391 | 1 | 0.02 | 3.00E-142 |
| hsa_circ_0002006 | 88 | 1 | 274 | 268 | 1 | 0.02 | 9.00E-86 |
| hsa_circ_0066543 | 87 | 1 | 402 | 605 | 210 | 0.01 | 1.00E-121 |
| hsa_circ_0066538 | 88 | 1 | 856 | 2410 | 1561 | 0.01 | 0 |
| hsa_circ_0066538 | 89 | 1 | 695 | 2255 | 1561 | 0 | 0 |
| hsa_circ_0066541 | 88 | 2 | 582 | 581 | 1 | 0 | 0 |
| hsa_circ_0066541 | 88 | 1 | 331 | 454 | 124 | 0.01 | 2.00E-107 |
| hsa_circ_0066541 | 90 | 1 | 454 | 454 | 1 | 0 | 6.00E-164 |
| hsa_circ_0066541 | 88 | 1 | 245 | 245 | 1 | 0.01 | 5.00E-78 |
| hsa_circ_0066536 | 95 | 13710 | 14161 | 452 | 1 | 0 | 0 |
| hsa_circ_0066536 | 95 | 6392 | 6843 | 452 | 1 | 0 | 0 |
| hsa_circ_0066536 | 95 | 149 | 600 | 452 | 1 | 0 | 0 |
| hsa_circ_0001322 | 96 | 149 | 758 | 609 | 1 | 0 | 0 |
| hsa_circ_0001322 | 96 | 149 | 758 | 609 | 1 | 0 | 0 |
| hsa_circ_0066536 | 95 | 1 | 452 | 452 | 1 | 0 | 0 |
| hsa_circ_0066536 | 96 | 1 | 379 | 379 | 1 | 0 | 5.00E-174 |
| hsa_circ_0001322 | 99 | 1 | 158 | 157 | 1 | 0.01 | 1.00E-74 |
| hsa_circ_0066533 | 96 | 156 | 247 | 153 | 62 | 0 | 1.00E-34 |
| hsa_circ_0008234 | 95 | 1 | 587 | 587 | 1 | 0 | 0 |
| hsa_circ_0066531 | 95 | 1 | 692 | 692 | 1 | 0 | 0 |
| hsa_circ_0008234 | 94 | 1 | 359 | 359 | 1 | 0 | 3.00E-156 |
| hsa_circ_0066524 | 96 | 1 | 468 | 468 | 1 | 0 | 0 |
| hsa_circ_0066524 | 96 | 2 | 384 | 383 | 1 | 0 | 8.00E-176 |

novel_circ_021741 novel_circ_021742 novel_circ_021743 novel_circ_021744 novel_circ_021745 novel_circ_021746 novel_circ_021747 novel_circ_021748 novel_circ_021749 novel_circ_021750 novel_circ_021751 novel_circ_021752 novel_circ_021753 novel_circ_021754 novel_circ_021755 novel_circ_021756 novel_circ_021757 novel_circ_021758 novel_circ_021759 novel_circ_021760 novel_circ_021761 novel_circ_021762 novel_circ_021763 novel_circ_021764 novel_circ_021765 novel_circ_021766 novel_circ_021767 novel_circ_021768 novel_circ_021769 novel_circ_021773 novel_circ_021774 novel_circ_021775 novel_circ_021776 novel_circ_021777 novel_circ_021778 novel_circ_021779 novel_circ_021780 novel_circ_021781 novel_circ_021782 novel_circ_021783 novel_circ_021784 novel_circ_021785 novel_circ_021786 novel_circ_021787 novel_circ_021788 novel_circ_021789 novel_circ_021790 novel_circ_021791 novel_circ_021792 novel_circ_021793 novel_circ_021795 novel_circ_021796 novel_circ_021798 novel_circ_021799

| hsa_circ_0091015 | 96 | 1 | 362 | 118 | 479 | 0 | 1.00E-164 |
| --- | --- | --- | --- | --- | --- | --- | --- |
| hsa_circ_0091015 | 95 | 1 | 581 | 118 | 698 | 0 | 0 |
| hsa_circ_0091015 | 95 | 1 | 1008 | 118 | 1125 | 0 | 0 |
| hsa_circ_0091015 | 95 | 1 | 427 | 699 | 1125 | 0 | 0 |
| hsa_circ_0091020 | 93 | 883 | 1871 | 432 | 1420 | 0 | 0 |
| hsa_circ_0091020 | 93 | 883 | 2497 | 432 | 2046 | 0 | 0 |
| hsa_circ_0091020 | 93 | 883 | 2877 | 432 | 2426 | 0 | 0 |
| hsa_circ_0091020 | 93 | 883 | 3089 | 432 | 2638 | 0 | 0 |
| hsa_circ_0091020 | 93 | 883 | 3844 | 432 | 3393 | 0 | 0 |
| hsa_circ_0091020 | 93 | 883 | 4155 | 432 | 3704 | 0 | 0 |
| hsa_circ_0091020 | 94 | 3 | 425 | 3 | 425 | 0 | 0 |
| hsa_circ_0091020 | 94 | 3 | 425 | 3 | 425 | 0 | 0 |
| hsa_circ_0091020 | 92 | 456 | 1091 | 432 | 1067 | 0 | 0 |
| hsa_circ_0091020 | 92 | 456 | 1233 | 432 | 1209 | 0 | 0 |
| hsa_circ_0091020 | 93 | 456 | 1444 | 432 | 1420 | 0 | 0 |
| hsa_circ_0091020 | 93 | 456 | 2070 | 432 | 2046 | 0 | 0 |
| hsa_circ_0091020 | 93 | 456 | 2450 | 432 | 2426 | 0 | 0 |
| hsa_circ_0091020 | 93 | 1 | 837 | 1210 | 2046 | 0 | 0 |
| hsa_circ_0091020 | 93 | 1 | 837 | 1210 | 2046 | 0 | 0 |
| hsa_circ_0007887 | 92 | 1 | 380 | 1 | 380 | 0 | 3.00E-151 |
| hsa_circ_0091020 | 94 | 1 | 1347 | 2047 | 3393 | 0 | 0 |
| hsa_circ_0091020 | 94 | 2 | 967 | 2428 | 3393 | 0 | 0 |
| hsa_circ_0091020 | 95 | 2 | 755 | 2640 | 3393 | 0 | 0 |
| hsa_circ_0091020 | 95 | 1 | 750 | 2644 | 3393 | 0 | 0 |
| hsa_circ_0091024 | 95 | 1 | 369 | 1 | 369 | 0 | 4.00E-165 |
| hsa_circ_0091037 | 96 | 118 | 2311 | 1 | 2194 | 0 | 0 |
| hsa_circ_0091039 | 95 | 1 | 436 | 1 | 436 | 0 | 0 |
| hsa_circ_0091039 | 95 | 1 | 685 | 1 | 685 | 0 | 0 |
| hsa_circ_0091039 | 95 | 1 | 1270 | 1 | 1270 | 0 | 0 |
| hsa_circ_0011509 | 92 | 1 | 218 | 330 | 113 | 0 | 8.00E-81 |
| hsa_circ_0011508 | 92 | 1 | 403 | 403 | 1 | 0 | 1.00E-160 |
| hsa_circ_0011500 | 93 | 1 | 1257 | 1257 | 1 | 0 | 0 |
| hsa_circ_0011503 | 94 | 1 | 464 | 464 | 1 | 0 | 0 |
| hsa_circ_0011500 | 93 | 1 | 647 | 1169 | 523 | 0 | 0 |
| hsa_circ_0011500 | 92 | 3 | 522 | 520 | 1 | 0 | 0 |
| hsa_circ_0011495 | 91 | 7 | 476 | 1192 | 723 | 0 | 0 |
| hsa_circ_0011480 | 83 | 684 | 1651 | 1847 | 868 | 0.02 | 0 |
| hsa_circ_0011480 | 96 | 1 | 448 | 867 | 420 | 0 | 0 |
| hsa_circ_0011469 | 93 | 108 | 660 | 432 | 996 | 0.02 | 0 |
| hsa_circ_0011469 | 93 | 234 | 786 | 432 | 996 | 0.02 | 0 |
| hsa_circ_0011461 | 95 | 1 | 332 | 2583 | 2252 | 0 | 6.00E-148 |
| hsa_circ_0011459 | 93 | 1 | 351 | 351 | 1 | 0 | 8.00E-147 |
| hsa_circ_0005810 | 93 | 1 | 629 | 770 | 142 | 0 | 0 |
| hsa_circ_0004013 | 92 | 1 | 978 | 978 | 1 | 0 | 0 |
| hsa_circ_0005057 | 92 | 1 | 980 | 1110 | 131 | 0 | 0 |
| hsa_circ_0011451 | 89 | 1 | 272 | 954 | 1225 | 0 | 3.00E-91 |
| hsa_circ_0011451 | 84 | 1 | 1237 | 1 | 1225 | 0.03 | 0 |
| hsa_circ_0011451 | 89 | 1 | 190 | 954 | 1143 | 0 | 6.00E-61 |
| hsa_circ_0011451 | 84 | 1 | 1155 | 1 | 1143 | 0.03 | 0 |
| hsa_circ_0011451 | 83 | 1 | 962 | 1 | 950 | 0.03 | 0 |
| hsa_circ_0011443 | 90 | 1 | 627 | 849 | 223 | 0 | 0 |
| hsa_circ_0011431 | 93 | 3 | 482 | 3 | 482 | 0 | 0 |
| hsa_circ_0011414 | 92 | 121 | 285 | 165 | 1 | 0 | 2.00E-58 |
| hsa_circ_0011403 | 91 | 3 | 374 | 3 | 374 | 0 | 2.00E-143 |

novel_circ_021800 novel_circ_021801 novel_circ_021802 novel_circ_021803 novel_circ_021804 novel_circ_021805 novel_circ_021806 novel_circ_021807 novel_circ_021808 novel_circ_021809 novel_circ_021810 novel_circ_021811 novel_circ_021812 novel_circ_021814 novel_circ_021815 novel_circ_021816 novel_circ_021817 novel_circ_021818 novel_circ_021819 novel_circ_021820 novel_circ_021821 novel_circ_021822 novel_circ_021823 novel_circ_021824 novel_circ_021825 novel_circ_021826 novel_circ_021827 novel_circ_021828 novel_circ_021829 novel_circ_021830 novel_circ_021831 novel_circ_021832 novel_circ_021833 novel_circ_021834 novel_circ_021835 novel_circ_021836 novel_circ_021837 novel_circ_021838 novel_circ_021839 novel_circ_021840 novel_circ_021841 novel_circ_021842 novel_circ_021843 novel_circ_021844 novel_circ_021845 novel_circ_021848 novel_circ_021849 novel_circ_021850 novel_circ_021851 novel_circ_021852 novel_circ_021853 novel_circ_021854 novel_circ_021855 novel_circ_021856

| hsa_circ_0011348 | 92 | 1 | 863 | 328 | 1190 | 0 | 0 |
| --- | --- | --- | --- | --- | --- | --- | --- |
| hsa_circ_0011350 | 93 | 1 | 432 | 1 | 432 | 0 | 5.00E-180 |
| hsa_circ_0011345 | 92 | 1 | 422 | 93 | 514 | 0 | 1.00E-167 |
| hsa_circ_0004289 | 92 | 1 | 388 | 1 | 388 | 0 | 6.00E-154 |
| hsa_circ_0004289 | 94 | 1 | 242 | 1 | 242 | 0 | 1.00E-99 |
| hsa_circ_0011338 | 92 | 1 | 697 | 689 | 1 | 0.03 | 0 |
| hsa_circ_0007364 | 93 | 1 | 790 | 782 | 1 | 0.03 | 0 |
| hsa_circ_0011336 | 94 | 1 | 996 | 988 | 1 | 0.02 | 0 |
| hsa_circ_0011334 | 94 | 1 | 1154 | 3528 | 2383 | 0.02 | 0 |
| hsa_circ_0011334 | 93 | 1 | 1309 | 3528 | 2228 | 0.02 | 0 |
| hsa_circ_0011334 | 93 | 1 | 1309 | 3528 | 2228 | 0.02 | 0 |
| hsa_circ_0011334 | 93 | 1 | 1309 | 3528 | 2228 | 0.02 | 0 |
| hsa_circ_0011336 | 99 | 1 | 206 | 206 | 1 | 0 | 8.00E-100 |
| hsa_circ_0011269 | 94 | 71 | 330 | 933 | 1193 | 0 | 6.00E-108 |
| hsa_circ_0011269 | 94 | 192 | 451 | 933 | 1193 | 0 | 9.00E-108 |
| hsa_circ_0007617 | 95 | 1 | 179 | 1 | 179 | 0 | 2.00E-74 |
| hsa_circ_0011262 | 92 | 4 | 390 | 880 | 494 | 0 | 9.00E-152 |
| hsa_circ_0011262 | 93 | 4 | 513 | 880 | 371 | 0 | 0 |
| hsa_circ_0011265 | 91 | 4 | 260 | 257 | 1 | 0 | 1.00E-94 |
| hsa_circ_0007391 | 93 | 4 | 383 | 584 | 205 | 0 | 3.00E-156 |
| hsa_circ_0007391 | 92 | 4 | 587 | 584 | 1 | 0 | 0 |
| hsa_circ_0011264 | 92 | 4 | 649 | 646 | 1 | 0 | 0 |
| hsa_circ_0007391 | 93 | 2 | 289 | 492 | 205 | 0 | 2.00E-118 |
| hsa_circ_0011264 | 92 | 2 | 555 | 554 | 1 | 0 | 0 |
| hsa_circ_0011260 | 88 | 713 | 829 | 185 | 70 | 0.01 | 1.00E-30 |
| hsa_circ_0011260 | 88 | 464 | 580 | 185 | 70 | 0.01 | 9.00E-31 |
| hsa_circ_0011255 | 98 | 1 | 726 | 726 | 1 | 0 | 0 |
| hsa_circ_0011255 | 98 | 1 | 723 | 723 | 1 | 0 | 0 |
| hsa_circ_0011255 | 96 | 1 | 438 | 438 | 1 | 0 | 0 |
| hsa_circ_0011251 | 93 | 1 | 532 | 634 | 103 | 0 | 0 |
| hsa_circ_0011251 | 93 | 1 | 634 | 634 | 1 | 0 | 0 |
| hsa_circ_0011243 | 92 | 1 | 786 | 1069 | 284 | 0 | 0 |
| hsa_circ_0011232 | 90 | 1 | 2133 | 2136 | 7 | 0 | 0 |
| hsa_circ_0011232 | 90 | 1 | 2139 | 2136 | 1 | 0 | 0 |
| hsa_circ_0011230 | 90 | 1 | 2271 | 2274 | 1 | 0 | 0 |
| hsa_circ_0011251 | 91 | 31 | 196 | 166 | 1 | 0 | 1.00E-57 |
| hsa_circ_0011245 | 88 | 31 | 348 | 318 | 1 | 0 | 2.00E-103 |
| hsa_circ_0011241 | 89 | 31 | 1168 | 1135 | 1 | 0 | 0 |
| hsa_circ_0011241 | 90 | 1 | 820 | 817 | 1 | 0 | 0 |
| hsa_circ_0011241 | 89 | 1 | 681 | 678 | 1 | 0 | 0 |
| hsa_circ_0011230 | 86 | 1 | 397 | 403 | 1 | 0.01 | 1.00E-120 |
| hsa_circ_0011220 | 91 | 4 | 248 | 245 | 1 | 0 | 1.00E-89 |
| hsa_circ_0011212 | 85 | 4 | 1012 | 4933 | 3913 | 0.02 | 0 |
| hsa_circ_0067553 | 92 | 1 | 396 | 2270 | 1875 | 0 | 2.00E-158 |
| hsa_circ_0067553 | 94 | 1 | 739 | 2270 | 1532 | 0 | 0 |
| hsa_circ_0067534 | 85 | 185 | 778 | 776 | 183 | 0 | 2.00E-171 |
| hsa_circ_0067534 | 86 | 185 | 909 | 776 | 52 | 0 | 0 |
| hsa_circ_0067534 | 87 | 185 | 960 | 776 | 1 | 0 | 0 |
| hsa_circ_0067523 | 87 | 185 | 1415 | 1867 | 637 | 0 | 0 |
| hsa_circ_0067523 | 87 | 185 | 1515 | 1867 | 537 | 0 | 0 |
| hsa_circ_0067523 | 88 | 185 | 1694 | 1867 | 358 | 0 | 0 |
| hsa_circ_0067523 | 88 | 185 | 1804 | 1867 | 248 | 0 | 0 |
| hsa_circ_0067523 | 88 | 185 | 1883 | 1867 | 169 | 0 | 0 |
| hsa_circ_0067523 | 88 | 185 | 2051 | 1867 | 1 | 0 | 0 |

novel_circ_021857 novel_circ_021859 novel_circ_021860 novel_circ_021861 novel_circ_021862 novel_circ_021863 novel_circ_021868 novel_circ_021871 novel_circ_021872 novel_circ_021873 novel_circ_021875 novel_circ_021876 novel_circ_021877 novel_circ_021878 novel_circ_021879 novel_circ_021880 novel_circ_021881 novel_circ_021882 novel_circ_021883 novel_circ_021884 novel_circ_021885 novel_circ_021886 novel_circ_021887 novel_circ_021888 novel_circ_021889 novel_circ_021890 novel_circ_021891 novel_circ_021892 novel_circ_021893 novel_circ_021894 novel_circ_021895 novel_circ_021896 novel_circ_021897 novel_circ_021898 novel_circ_021899 novel_circ_021900 novel_circ_021901 novel_circ_021902 novel_circ_021903 novel_circ_021904 novel_circ_021905 novel_circ_021906 novel_circ_021907 novel_circ_021908 novel_circ_021909 novel_circ_021910 novel_circ_021911 novel_circ_021912 novel_circ_021913 novel_circ_021914 novel_circ_021915 novel_circ_021916 novel_circ_021917 novel_circ_021918

| hsa_circ_0067518 | 89 | 682 | 2454 | 1773 | 1 | 0 | 0 |
| --- | --- | --- | --- | --- | --- | --- | --- |
| hsa_circ_0067534 | 86 | 5 | 729 | 776 | 52 | 0 | 0 |
| hsa_circ_0067523 | 87 | 5 | 1335 | 1867 | 537 | 0 | 0 |
| hsa_circ_0067523 | 88 | 1 | 555 | 1091 | 537 | 0 | 0 |
| hsa_circ_0067523 | 90 | 1 | 923 | 1091 | 169 | 0 | 0 |
| hsa_circ_0067518 | 90 | 1 | 939 | 939 | 1 | 0 | 0 |
| hsa_circ_0067514 | 87 | 157 | 1105 | 948 | 1 | 0 | 0 |
| hsa_circ_0067511 | 88 | 9151 | 9416 | 94 | 359 | 0 | 9.00E-85 |
| hsa_circ_0067508 | 92 | 2 | 523 | 250 | 771 | 0 | 0 |
| hsa_circ_0067508 | 93 | 559 | 1164 | 1 | 606 | 0 | 0 |
| hsa_circ_0067508 | 92 | 1 | 429 | 82 | 510 | 0 | 2.00E-168 |
| hsa_circ_0067508 | 93 | 1 | 510 | 1 | 510 | 0 | 0 |
| hsa_circ_0067508 | 97 | 1 | 248 | 1 | 248 | 0 | 2.00E-116 |
| hsa_circ_0067505 | 93 | 1 | 407 | 78 | 484 | 0 | 6.00E-166 |
| hsa_circ_0005404 | 93 | 1 | 406 | 1 | 406 | 0 | 1.00E-165 |
| hsa_circ_0067505 | 94 | 1 | 389 | 1 | 389 | 0 | 7.00E-168 |
| hsa_circ_0007133 | 84 | 2622 | 2732 | 542785 | 542897 | 0.05 | 3.00E-21 |
| hsa_circ_0088373 | 95 | 1 | 663 | 663 | 1 | 0 | 0 |
| hsa_circ_0088373 | 96 | 1 | 1480 | 1483 | 1 | 0 | 0 |
| hsa_circ_0088373 | 95 | 1 | 3181 | 3184 | 1 | 0 | 0 |
| hsa_circ_0088381 | 96 | 1 | 817 | 967 | 148 | 0 | 0 |
| hsa_circ_0088387 | 95 | 1 | 422 | 422 | 1 | 0 | 0 |
| hsa_circ_0088387 | 95 | 1 | 1403 | 1403 | 1 | 0 | 0 |
| hsa_circ_0088387 | 95 | 1 | 1701 | 1701 | 1 | 0 | 0 |
| hsa_circ_0088390 | 94 | 1 | 566 | 566 | 1 | 0 | 0 |
| hsa_circ_0088390 | 94 | 1 | 1094 | 1094 | 1 | 0 | 0 |
| hsa_circ_0088390 | 95 | 1 | 1392 | 1392 | 1 | 0 | 0 |
| hsa_circ_0088391 | 94 | 1 | 453 | 453 | 1 | 0 | 0 |
| hsa_circ_0088391 | 95 | 1 | 1279 | 1279 | 1 | 0 | 0 |
| hsa_circ_0088395 | 94 | 1 | 410 | 410 | 1 | 0 | 7.00E-178 |
| hsa_circ_0088393 | 95 | 1 | 528 | 729 | 202 | 0 | 0 |
| hsa_circ_0088393 | 96 | 1 | 826 | 1027 | 202 | 0 | 0 |
| hsa_circ_0088396 | 96 | 1 | 650 | 650 | 1 | 0 | 0 |
| hsa_circ_0088397 | 97 | 1 | 416 | 416 | 1 | 0 | 0 |
| hsa_circ_0088399 | 94 | 130 | 1550 | 1 | 1423 | 0 | 0 |
| hsa_circ_0088399 | 94 | 130 | 1970 | 1 | 1843 | 0 | 0 |
| hsa_circ_0088399 | 93 | 1 | 637 | 1 | 639 | 0.01 | 0 |
| hsa_circ_0088399 | 94 | 1 | 970 | 1 | 972 | 0 | 0 |
| hsa_circ_0088399 | 95 | 1 | 1251 | 1 | 1253 | 0 | 0 |
| hsa_circ_0088399 | 94 | 1 | 1421 | 1 | 1423 | 0 | 0 |
| hsa_circ_0088399 | 94 | 1 | 1596 | 1 | 1598 | 0 | 0 |
| hsa_circ_0088399 | 94 | 1 | 1841 | 1 | 1843 | 0 | 0 |
| hsa_circ_0088399 | 94 | 1 | 1842 | 1 | 1844 | 0 | 0 |
| hsa_circ_0088399 | 94 | 1 | 1282 | 139 | 1423 | 0 | 0 |
| hsa_circ_0088399 | 94 | 1 | 1702 | 139 | 1843 | 0 | 0 |
| hsa_circ_0088400 | 91 | 1 | 437 | 1 | 440 | 0.01 | 2.00E-168 |
| hsa_circ_0088400 | 94 | 1 | 770 | 1 | 773 | 0 | 0 |
| hsa_circ_0088400 | 94 | 1 | 1221 | 1 | 1224 | 0 | 0 |
| hsa_circ_0088399 | 94 | 1 | 1396 | 200 | 1598 | 0 | 0 |
| hsa_circ_0088399 | 94 | 1 | 1641 | 200 | 1843 | 0 | 0 |
| hsa_circ_0002672 | 97 | 1 | 333 | 1 | 333 | 0 | 8.00E-157 |
| hsa_circ_0088402 | 96 | 1 | 784 | 206 | 989 | 0 | 0 |
| hsa_circ_0088404 | 95 | 1 | 451 | 1 | 451 | 0 | 0 |
| hsa_circ_0088404 | 95 | 1 | 626 | 1 | 626 | 0 | 0 |

novel_circ_021919 novel_circ_021920 novel_circ_021921 novel_circ_021922 novel_circ_021923 novel_circ_021924 novel_circ_021925 novel_circ_021926 novel_circ_021927 novel_circ_021928 novel_circ_021929 novel_circ_021930 novel_circ_021931 novel_circ_021932 novel_circ_021933 novel_circ_021934 novel_circ_021935 novel_circ_021936 novel_circ_021937 novel_circ_021938 novel_circ_021939 novel_circ_021940 novel_circ_021941 novel_circ_021942 novel_circ_021943 novel_circ_021944 novel_circ_021945 novel_circ_021946 novel_circ_021947 novel_circ_021948 novel_circ_021949 novel_circ_021950 novel_circ_021951 novel_circ_021955 novel_circ_021956 novel_circ_021957 novel_circ_021958 novel_circ_021959 novel_circ_021960 novel_circ_021961 novel_circ_021962 novel_circ_021963 novel_circ_021964 novel_circ_021965 novel_circ_021967 novel_circ_021968 novel_circ_021969 novel_circ_021970 novel_circ_021971 novel_circ_021972 novel_circ_021973 novel_circ_021974 novel_circ_021975 novel_circ_021976

| hsa_circ_0088404 | 95 | 1 | 720 | 1 | 720 | 0 | 0 |
| --- | --- | --- | --- | --- | --- | --- | --- |
| hsa_circ_0088404 | 95 | 1 | 871 | 1 | 871 | 0 | 0 |
| hsa_circ_0088406 | 95 | 1 | 420 | 1 | 420 | 0 | 0 |
| hsa_circ_0088406 | 96 | 375 | 839 | 415 | 879 | 0 | 0 |
| hsa_circ_0088405 | 96 | 375 | 1010 | 688 | 1323 | 0 | 0 |
| hsa_circ_0088408 | 95 | 1 | 833 | 1 | 833 | 0 | 0 |
| hsa_circ_0007661 | 97 | 1 | 270 | 76 | 345 | 0 | 3.00E-125 |
| hsa_circ_0088410 | 95 | 1 | 203 | 172 | 374 | 0 | 8.00E-85 |
| hsa_circ_0088416 | 96 | 1 | 1407 | 1407 | 1 | 0 | 0 |
| hsa_circ_0088416 | 95 | 166 | 2108 | 1942 | 1 | 0 | 0 |
| hsa_circ_0088416 | 95 | 166 | 2108 | 1942 | 1 | 0 | 0 |
| hsa_circ_0088416 | 95 | 166 | 2063 | 1942 | 46 | 0 | 0 |
| hsa_circ_0088421 | 98 | 1 | 510 | 510 | 1 | 0 | 0 |
| hsa_circ_0088421 | 97 | 1 | 655 | 655 | 1 | 0 | 0 |
| hsa_circ_0088421 | 97 | 1 | 821 | 821 | 1 | 0 | 0 |
| hsa_circ_0088421 | 96 | 1 | 1042 | 1042 | 1 | 0 | 0 |
| hsa_circ_0088421 | 96 | 166 | 1211 | 1045 | 1 | 0 | 0 |
| hsa_circ_0088423 | 94 | 1 | 701 | 791 | 93 | 0 | 0 |
| hsa_circ_0088426 | 94 | 1 | 556 | 554 | 1 | 0 | 0 |
| hsa_circ_0088449 | 94 | 1 | 228 | 228 | 1 | 0 | 1.00E-93 |
| hsa_circ_0088449 | 95 | 1 | 274 | 274 | 1 | 0 | 2.00E-117 |
| hsa_circ_0088449 | 94 | 2 | 375 | 374 | 1 | 0 | 7.00E-163 |
| hsa_circ_0088469 | 95 | 1 | 316 | 316 | 1 | 0 | 2.00E-137 |
| hsa_circ_0088469 | 95 | 20772 | 21088 | 317 | 1 | 0 | 4.00E-136 |
| hsa_circ_0088476 | 98 | 13498 | 13620 | 123 | 1 | 0 | 6.00E-53 |
| hsa_circ_0088478 | 100 | 1 | 45 | 94 | 50 | 0 | 5.00E-13 |
| hsa_circ_0088476 | 98 | 13498 | 13620 | 123 | 1 | 0 | 2.00E-53 |
| hsa_circ_0088476 | 98 | 40340 | 40462 | 123 | 1 | 0 | 6.00E-53 |
| hsa_circ_0088506 | 94 | 1 | 913 | 913 | 1 | 0 | 0 |
| hsa_circ_0088510 | 94 | 1 | 638 | 638 | 1 | 0 | 0 |
| hsa_circ_0088510 | 94 | 1 | 694 | 694 | 1 | 0 | 0 |
| hsa_circ_0088510 | 95 | 1 | 937 | 937 | 1 | 0 | 0 |
| hsa_circ_0088514 | 99 | 1 | 299 | 299 | 1 | 0 | 3.00E-151 |
| hsa_circ_0088528 | 98 | 1 | 98 | 98 | 1 | 0 | 3.00E-41 |
| hsa_circ_0088528 | 95 | 1 | 264 | 264 | 1 | 0 | 1.00E-116 |
| hsa_circ_0088528 | 95 | 1 | 264 | 264 | 1 | 0 | 6.00E-117 |
| hsa_circ_0088532 | 91 | 1 | 511 | 530 | 1 | 0.04 | 0 |
| hsa_circ_0088536 | 90 | 4 | 519 | 535 | 1 | 0.04 | 0 |
| hsa_circ_0088539 | 93 | 4 | 383 | 380 | 1 | 0 | 2.00E-159 |
| hsa_circ_0088536 | 94 | 1 | 918 | 1073 | 156 | 0 | 0 |
| hsa_circ_0088545 | 95 | 2 | 414 | 510 | 98 | 0 | 0 |
| hsa_circ_0088551 | 97 | 1 | 290 | 290 | 1 | 0 | 2.00E-136 |
| hsa_circ_0088552 | 97 | 1 | 208 | 208 | 1 | 0 | 3.00E-94 |
| hsa_circ_0088552 | 96 | 1 | 304 | 304 | 1 | 0 | 2.00E-137 |
| hsa_circ_0088577 | 95 | 1 | 325 | 1 | 325 | 0 | 5.00E-144 |
| hsa_circ_0088577 | 94 | 1 | 486 | 1 | 486 | 0 | 0 |
| hsa_circ_0088578 | 92 | 1863 | 2030 | 185 | 351 | 0.01 | 1.00E-58 |
| hsa_circ_0088578 | 93 | 1 | 417 | 191 | 607 | 0 | 1.00E-171 |
| hsa_circ_0088578 | 94 | 1 | 591 | 191 | 781 | 0 | 0 |
| hsa_circ_0088578 | 95 | 1 | 985 | 191 | 1175 | 0 | 0 |
| hsa_circ_0088578 | 95 | 1 | 985 | 191 | 1175 | 0 | 0 |
| hsa_circ_0088578 | 95 | 1 | 985 | 191 | 1175 | 0 | 0 |
| hsa_circ_0088578 | 95 | 1 | 985 | 191 | 1175 | 0 | 0 |
| hsa_circ_0088580 | 94 | 1 | 430 | 1 | 430 | 0 | 0 |

novel_circ_021977 novel_circ_021978 novel_circ_021979 novel_circ_021980 novel_circ_021981 novel_circ_021982 novel_circ_021983 novel_circ_021984 novel_circ_021985 novel_circ_021986 novel_circ_021987 novel_circ_021988 novel_circ_021989 novel_circ_021998 novel_circ_021999 novel_circ_022000 novel_circ_022001 novel_circ_022002 novel_circ_022003 novel_circ_022004 novel_circ_022005 novel_circ_022006 novel_circ_022007 novel_circ_022008 novel_circ_022009 novel_circ_022010 novel_circ_022011 novel_circ_022012 novel_circ_022013 novel_circ_022014 novel_circ_022015 novel_circ_022016 novel_circ_022017 novel_circ_022018 novel_circ_022019 novel_circ_022020 novel_circ_022021 novel_circ_022022 novel_circ_022023 novel_circ_022024 novel_circ_022025 novel_circ_022026 novel_circ_022027 novel_circ_022030 novel_circ_022031 novel_circ_022032 novel_circ_022033 novel_circ_022034 novel_circ_022035 novel_circ_022036 novel_circ_022037 novel_circ_022038 novel_circ_022039 novel_circ_022040

| hsa_circ_0088580 | 92 | 1 | 300 | 131 | 430 | 0 | 2.00E-118 |
| --- | --- | --- | --- | --- | --- | --- | --- |
| hsa_circ_0088580 | 95 | 1 | 694 | 131 | 824 | 0 | 0 |
| hsa_circ_0088580 | 95 | 1 | 694 | 131 | 824 | 0 | 0 |
| hsa_circ_0088580 | 95 | 1 | 694 | 131 | 824 | 0 | 0 |
| hsa_circ_0088583 | 97 | 1 | 568 | 1 | 568 | 0 | 0 |
| hsa_circ_0088591 | 92 | 3 | 588 | 207 | 792 | 0 | 0 |
| hsa_circ_0003984 | 95 | 1 | 387 | 387 | 1 | 0 | 4.00E-170 |
| hsa_circ_0088596 | 96 | 1 | 460 | 16282 | 15823 | 0 | 0 |
| hsa_circ_0001890 | 97 | 1 | 567 | 567 | 1 | 0 | 0 |
| hsa_circ_0001890 | 97 | 1 | 567 | 567 | 1 | 0 | 0 |
| hsa_circ_0001890 | 97 | 1 | 563 | 563 | 1 | 0 | 0 |
| hsa_circ_0001890 | 97 | 1 | 567 | 567 | 1 | 0 | 0 |
| hsa_circ_0001890 | 97 | 74 | 640 | 567 | 1 | 0 | 0 |
| hsa_circ_0002765 | 77 | 1 | 911 | 17858 | 18779 | 0.08 | 1.00E-130 |
| hsa_circ_0004126 | 77 | 1 | 897 | 1 | 908 | 0.08 | 1.00E-124 |
| hsa_circ_0004126 | 78 | 13139 | 14310 | 13631 | 14819 | 0.08 | 0 |
| hsa_circ_0004126 | 78 | 13139 | 14310 | 13631 | 14819 | 0.08 | 0 |
| hsa_circ_0004126 | 78 | 13139 | 14310 | 13631 | 14819 | 0.08 | 0 |
| hsa_circ_0004126 | 77 | 1 | 890 | 8 | 908 | 0.08 | 1.00E-120 |
| hsa_circ_0018087 | 79 | 1 | 705 | 1 | 713 | 0.08 | 1.00E-129 |
| hsa_circ_0004907 | 92 | 1 | 423 | 1 | 426 | 0.02 | 2.00E-166 |
| hsa_circ_0018090 | 90 | 1 | 830 | 836 | 1 | 0.01 | 0 |
| hsa_circ_0018090 | 91 | 3 | 974 | 978 | 1 | 0.01 | 0 |
| hsa_circ_0018092 | 91 | 2 | 386 | 502 | 118 | 0 | 3.00E-147 |
| hsa_circ_0018092 | 94 | 2 | 184 | 502 | 320 | 0 | 1.00E-73 |
| hsa_circ_0018092 | 93 | 3 | 246 | 563 | 320 | 0 | 4.00E-99 |
| hsa_circ_0018095 | 90 | 1 | 345 | 360 | 1 | 0.04 | 1.00E-125 |
| hsa_circ_0018092 | 92 | 1 | 567 | 1003 | 422 | 0.03 | 0 |
| hsa_circ_0018092 | 92 | 7 | 831 | 1261 | 422 | 0.02 | 0 |
| hsa_circ_0018092 | 91 | 7 | 749 | 1261 | 504 | 0.02 | 0 |
| hsa_circ_0018098 | 94 | 1 | 423 | 438 | 16 | 0 | 0 |
| hsa_circ_0008251 | 94 | 1 | 348 | 348 | 1 | 0 | 8.00E-147 |
| hsa_circ_0008251 | 93 | 7 | 612 | 606 | 1 | 0 | 0 |
| hsa_circ_0018102 | 94 | 1 | 222 | 222 | 1 | 0 | 5.00E-92 |
| hsa_circ_0018102 | 93 | 7 | 486 | 480 | 1 | 0 | 0 |
| hsa_circ_0018102 | 93 | 6299 | 6785 | 487 | 1 | 0 | 0 |
| hsa_circ_0002813 | 92 | 3034 | 3790 | 757 | 1 | 0 | 0 |
| hsa_circ_0002813 | 92 | 7156 | 7912 | 757 | 1 | 0 | 0 |
| hsa_circ_0018120 | 94 | 1 | 368 | 368 | 1 | 0 | 3.00E-156 |
| hsa_circ_0018129 | 96 | 1 | 376 | 376 | 1 | 0 | 1.00E-175 |
| hsa_circ_0018130 | 97 | 1 | 372 | 497 | 126 | 0 | 4.00E-180 |
| hsa_circ_0018135 | 97 | 1 | 694 | 694 | 1 | 0 | 0 |
| hsa_circ_0018137 | 94 | 1 | 662 | 1552 | 891 | 0 | 0 |
| hsa_circ_0018148 | 85 | 1 | 1036 | 1036 | 1 | 0.01 | 0 |
| hsa_circ_0018148 | 85 | 1 | 1126 | 1126 | 1 | 0.01 | 0 |
| hsa_circ_0018148 | 86 | 4 | 1222 | 1219 | 1 | 0 | 0 |
| hsa_circ_0018148 | 87 | 1 | 2011 | 2011 | 1 | 0 | 0 |
| hsa_circ_0018156 | 88 | 1 | 788 | 885 | 98 | 0 | 0 |
| hsa_circ_0018160 | 88 | 4 | 633 | 971 | 342 | 0 | 0 |
| hsa_circ_0018160 | 89 | 4 | 301 | 971 | 674 | 0 | 1.00E-100 |
| hsa_circ_0002169 | 92 | 1 | 551 | 551 | 1 | 0 | 0 |
| hsa_circ_0002169 | 91 | 1 | 733 | 733 | 1 | 0 | 0 |
| hsa_circ_0018171 | 90 | 3 | 858 | 1199 | 344 | 0 | 0 |
| hsa_circ_0018171 | 90 | 3 | 847 | 1199 | 355 | 0 | 0 |

novel_circ_022041 novel_circ_022042 novel_circ_022043 novel_circ_022044 novel_circ_022045 novel_circ_022046 novel_circ_022047 novel_circ_022048 novel_circ_022049 novel_circ_022050 novel_circ_022051 novel_circ_022052 novel_circ_022053 novel_circ_022055 novel_circ_022056 novel_circ_022057 novel_circ_022058 novel_circ_022059 novel_circ_022060 novel_circ_022061 novel_circ_022062 novel_circ_022063 novel_circ_022064 novel_circ_022065 novel_circ_022068 novel_circ_022069 novel_circ_022071 novel_circ_022074 novel_circ_022079 novel_circ_022080 novel_circ_022081 novel_circ_022082 novel_circ_022083 novel_circ_022084 novel_circ_022085 novel_circ_022086 novel_circ_022087 novel_circ_022094 novel_circ_022095 novel_circ_022096 novel_circ_022098 novel_circ_022099 novel_circ_022100 novel_circ_022101 novel_circ_022104 novel_circ_022105 novel_circ_022106 novel_circ_022107 novel_circ_022108 novel_circ_022109 novel_circ_022110 novel_circ_022111 novel_circ_022112 novel_circ_022113

| hsa_circ_0018176 | 88 | 1 | 1326 | 1527 | 190 | 0.01 | 0 |
| --- | --- | --- | --- | --- | --- | --- | --- |
| hsa_circ_0018174 | 89 | 3 | 615 | 613 | 1 | 0 | 0 |
| hsa_circ_0018174 | 89 | 919 | 1534 | 617 | 1 | 0 | 0 |
| hsa_circ_0018176 | 88 | 1 | 1326 | 1527 | 190 | 0.01 | 0 |
| hsa_circ_0018178 | 88 | 1 | 1316 | 1328 | 1 | 0.01 | 0 |
| hsa_circ_0005486 | 86 | 1 | 355 | 360 | 6 | 0 | 3.00E-107 |
| hsa_circ_0005486 | 88 | 1 | 1160 | 1177 | 6 | 0.01 | 0 |
| hsa_circ_0018180 | 88 | 1 | 766 | 817 | 40 | 0.02 | 0 |
| hsa_circ_0018176 | 89 | 1 | 942 | 1703 | 750 | 0.01 | 0 |
| hsa_circ_0018181 | 87 | 1 | 673 | 825 | 141 | 0.02 | 0 |
| hsa_circ_0018181 | 91 | 1 | 176 | 825 | 650 | 0 | 2.00E-61 |
| hsa_circ_0018181 | 93 | 1 | 308 | 957 | 650 | 0 | 3.00E-126 |
| hsa_circ_0018183 | 92 | 1 | 224 | 224 | 1 | 0 | 9.00E-85 |
| hsa_circ_0027871 | 78 | 9808 | 10064 | 7840 | 8100 | 0.02 | 4.00E-35 |
| hsa_circ_0018190 | 93 | 1 | 293 | 293 | 1 | 0 | 3.00E-119 |
| hsa_circ_0018190 | 93 | 1 | 1192 | 1192 | 1 | 0 | 0 |
| hsa_circ_0018190 | 93 | 1 | 168 | 168 | 1 | 0 | 9.00E-64 |
| hsa_circ_0018191 | 94 | 1 | 369 | 604 | 236 | 0 | 9.00E-157 |
| hsa_circ_0018191 | 93 | 1 | 897 | 1132 | 236 | 0 | 0 |
| hsa_circ_0018191 | 94 | 1 | 736 | 1132 | 397 | 0 | 0 |
| hsa_circ_0018191 | 92 | 1 | 528 | 1132 | 605 | 0 | 0 |
| hsa_circ_0000234 | 90 | 1 | 339 | 339 | 1 | 0 | 2.00E-123 |
| hsa_circ_0000234 | 90 | 135 | 473 | 339 | 1 | 0 | 3.00E-123 |
| hsa_circ_0000234 | 90 | 348 | 686 | 339 | 1 | 0 | 4.00E-123 |
| hsa_circ_0018206 | 91 | 111 | 592 | 1 | 482 | 0 | 0 |
| hsa_circ_0018206 | 91 | 1 | 482 | 1 | 482 | 0 | 0 |
| hsa_circ_0062157 | 87 | 2 | 411 | 410 | 1 | 0 | 2.00E-129 |
| hsa_circ_0005343 | 90 | 2886 | 2934 | 137005 | 137053 | 0 | 1.00E-07 |
| hsa_circ_0006071 | 93 | 365 | 405 | 12483 | 12441 | 0.05 | 8.00E-08 |
| hsa_circ_0062178 | 89 | 1 | 364 | 364 | 1 | 0.02 | 5.00E-124 |
| hsa_circ_0062179 | 87 | 1 | 745 | 919 | 175 | 0 | 0 |
| hsa_circ_0062179 | 89 | 3 | 1376 | 1548 | 175 | 0 | 0 |
| hsa_circ_0062180 | 88 | 1 | 338 | 345 | 8 | 0 | 7.00E-113 |
| hsa_circ_0062180 | 88 | 1 | 562 | 569 | 8 | 0 | 0 |
| hsa_circ_0062179 | 89 | 1 | 596 | 1293 | 698 | 0 | 0 |
| hsa_circ_0013829 | 80 | 3557 | 3700 | 113011 | 113156 | 0.04 | 3.00E-21 |
| hsa_circ_0079428 | 93 | 1 | 939 | 1 | 933 | 0.01 | 0 |
| hsa_circ_0069397 | 86 | 1 | 582 | 582 | 1 | 0 | 3.00E-178 |
| hsa_circ_0069397 | 84 | 116 | 1624 | 1512 | 1 | 0.01 | 0 |
| hsa_circ_0069399 | 83 | 116 | 1042 | 930 | 1 | 0.02 | 0 |
| hsa_circ_0030218 | 90 | 1 | 730 | 44 | 775 | 0 | 0 |
| hsa_circ_0073636 | 89 | 1 | 3136 | 4707 | 1544 | 0.03 | 0 |
| hsa_circ_0073638 | 96 | 1 | 374 | 3484 | 3111 | 0 | 7.00E-173 |
| hsa_circ_0073645 | 86 | 119 | 862 | 1511 | 768 | 0 | 0 |
| hsa_circ_0047716 | 98 | 1 | 353 | 52 | 404 | 0 | 6.00E-173 |
| hsa_circ_0006952 | 98 | 34 | 650 | 238 | 854 | 0 | 0 |
| hsa_circ_0006952 | 96 | 4 | 860 | 7 | 854 | 0.01 | 0 |
| hsa_circ_0047709 | 94 | 1 | 1088 | 1 | 1082 | 0.02 | 0 |
| hsa_circ_0047709 | 93 | 1 | 791 | 1 | 794 | 0.01 | 0 |
| hsa_circ_0047709 | 93 | 1 | 578 | 1 | 578 | 0.01 | 0 |
| hsa_circ_0047701 | 92 | 1 | 683 | 105 | 787 | 0 | 0 |
| hsa_circ_0047693 | 92 | 124 | 1432 | 1 | 1309 | 0 | 0 |
| hsa_circ_0047701 | 92 | 1 | 580 | 105 | 684 | 0 | 0 |
| hsa_circ_0047701 | 93 | 1 | 684 | 1 | 684 | 0 | 0 |

novel_circ_022114 novel_circ_022115 novel_circ_022116 novel_circ_022117 novel_circ_022118 novel_circ_022119 novel_circ_022120 novel_circ_022121 novel_circ_022122 novel_circ_022123 novel_circ_022124 novel_circ_022125 novel_circ_022126 novel_circ_022127 novel_circ_022128 novel_circ_022129 novel_circ_022130 novel_circ_022131 novel_circ_022132 novel_circ_022134 novel_circ_022135 novel_circ_022136 novel_circ_022137 novel_circ_022138 novel_circ_022139 novel_circ_022140 novel_circ_022142 novel_circ_022143 novel_circ_022144 novel_circ_022145 novel_circ_022146 novel_circ_022147 novel_circ_022148 novel_circ_022149 novel_circ_022150 novel_circ_022151 novel_circ_022152 novel_circ_022153 novel_circ_022154 novel_circ_022155 novel_circ_022156 novel_circ_022157 novel_circ_022158 novel_circ_022159 novel_circ_022160 novel_circ_022161 novel_circ_022162 novel_circ_022163 novel_circ_022164 novel_circ_022165 novel_circ_022166 novel_circ_022167 novel_circ_022168 novel_circ_022169

| hsa_circ_0000850 | 92 | 1 | 1329 | 1 | 1326 | 0 | 0 |
| --- | --- | --- | --- | --- | --- | --- | --- |
| hsa_circ_0047701 | 94 | 1 | 426 | 1 | 426 | 0 | 0 |
| hsa_circ_0047693 | 92 | 1 | 284 | 1 | 284 | 0 | 1.00E-109 |
| hsa_circ_0000850 | 89 | 1 | 407 | 1 | 404 | 0.01 | 1.00E-141 |
| hsa_circ_0055813 | 76 | 49329 | 49886 | 70375 | 70936 | 0.04 | 2.00E-70 |
| hsa_circ_0092177 | 95 | 3382 | 3424 | 1110 | 1152 | 0 | 7.00E-09 |
| hsa_circ_0008068 | 95 | 4 | 1161 | 1158 | 1 | 0 | 0 |
| hsa_circ_0008068 | 96 | 1 | 655 | 655 | 1 | 0 | 0 |
| hsa_circ_0008068 | 96 | 7284 | 7940 | 657 | 1 | 0 | 0 |
| hsa_circ_0008068 | 96 | 1 | 824 | 824 | 1 | 0 | 0 |
| hsa_circ_0008068 | 96 | 1 | 985 | 985 | 1 | 0 | 0 |
| hsa_circ_0008068 | 95 | 4 | 1161 | 1158 | 1 | 0 | 0 |
| hsa_circ_0008068 | 95 | 275 | 1432 | 1158 | 1 | 0 | 0 |
| hsa_circ_0008068 | 96 | 1 | 520 | 655 | 136 | 0 | 0 |
| hsa_circ_0008068 | 95 | 4 | 1026 | 1158 | 136 | 0 | 0 |
| hsa_circ_0002310 | 95 | 4 | 740 | 737 | 1 | 0 | 0 |
| hsa_circ_0002310 | 95 | 1 | 335 | 564 | 230 | 0 | 1.00E-148 |
| hsa_circ_0002310 | 94 | 4 | 511 | 737 | 230 | 0 | 0 |
| hsa_circ_0008651 | 93 | 4 | 337 | 334 | 1 | 0 | 2.00E-137 |
| hsa_circ_0029905 | 93 | 1 | 849 | 849 | 1 | 0 | 0 |
| hsa_circ_0029905 | 93 | 1 | 1213 | 1213 | 1 | 0 | 0 |
| hsa_circ_0029905 | 94 | 1 | 608 | 849 | 242 | 0 | 0 |
| hsa_circ_0029905 | 94 | 1 | 972 | 1213 | 242 | 0 | 0 |
| hsa_circ_0029905 | 94 | 2 | 1030 | 1270 | 242 | 0 | 0 |
| hsa_circ_0029908 | 90 | 1 | 468 | 138 | 605 | 0 | 2.00E-168 |
| hsa_circ_0054279 | 75 | 11219 | 11588 | 19747 | 20107 | 0.06 | 6.00E-36 |
| hsa_circ_0029911 | 93 | 1 | 250 | 1 | 250 | 0 | 8.00E-101 |
| hsa_circ_0029911 | 94 | 1 | 394 | 1 | 394 | 0 | 1.00E-170 |
| hsa_circ_0029911 | 94 | 1 | 643 | 1 | 643 | 0 | 0 |
| hsa_circ_0029911 | 95 | 1 | 815 | 1 | 815 | 0 | 0 |
| hsa_circ_0029911 | 94 | 1 | 1109 | 1 | 1109 | 0 | 0 |
| hsa_circ_0029911 | 94 | 1 | 1322 | 1 | 1322 | 0 | 0 |
| hsa_circ_0029911 | 95 | 1 | 1822 | 1 | 1822 | 0 | 0 |
| hsa_circ_0029912 | 96 | 1 | 389 | 55 | 443 | 0 | 7.00E-178 |
| hsa_circ_0029911 | 95 | 1 | 855 | 255 | 1109 | 0 | 0 |
| hsa_circ_0029911 | 95 | 1 | 1068 | 255 | 1322 | 0 | 0 |
| hsa_circ_0029914 | 94 | 2 | 715 | 2 | 715 | 0 | 0 |
| hsa_circ_0029914 | 95 | 2 | 928 | 2 | 928 | 0 | 0 |
| hsa_circ_0029911 | 95 | 2 | 1428 | 396 | 1822 | 0 | 0 |
| hsa_circ_0029917 | 95 | 1 | 676 | 82 | 757 | 0 | 0 |
| hsa_circ_0029917 | 95 | 1 | 1176 | 82 | 1257 | 0 | 0 |
| hsa_circ_0029917 | 94 | 1 | 1618 | 82 | 1699 | 0 | 0 |
| hsa_circ_0029917 | 94 | 1 | 3030 | 82 | 3111 | 0 | 0 |
| hsa_circ_0029917 | 93 | 1 | 294 | 251 | 544 | 0 | 7.00E-117 |
| hsa_circ_0029917 | 96 | 1 | 713 | 545 | 1257 | 0 | 0 |
| hsa_circ_0029918 | 93 | 1 | 942 | 110 | 1051 | 0 | 0 |
| hsa_circ_0029918 | 95 | 1 | 413 | 197 | 609 | 0 | 0 |
| hsa_circ_0029918 | 93 | 1 | 770 | 369 | 1138 | 0 | 0 |
| hsa_circ_0029918 | 90 | 1 | 442 | 610 | 1051 | 0 | 1.00E-165 |
| hsa_circ_0029919 | 93 | 1 | 3123 | 1 | 3123 | 0 | 0 |
| hsa_circ_0029919 | 95 | 1 | 427 | 639 | 1065 | 0 | 0 |
| hsa_circ_0029919 | 93 | 1 | 699 | 639 | 1337 | 0 | 0 |
| hsa_circ_0029919 | 93 | 1 | 699 | 639 | 1337 | 0 | 0 |
| hsa_circ_0029919 | 92 | 1 | 1103 | 639 | 1741 | 0 | 0 |

novel_circ_022170 novel_circ_022171 novel_circ_022172 novel_circ_022173 novel_circ_022174 novel_circ_022175 novel_circ_022176 novel_circ_022177 novel_circ_022178 novel_circ_022179 novel_circ_022180 novel_circ_022181 novel_circ_022182 novel_circ_022183 novel_circ_022184 novel_circ_022185 novel_circ_022186 novel_circ_022187 novel_circ_022188 novel_circ_022189 novel_circ_022190 novel_circ_022191 novel_circ_022192 novel_circ_022193 novel_circ_022194 novel_circ_022195 novel_circ_022196 novel_circ_022197 novel_circ_022198 novel_circ_022199 novel_circ_022200 novel_circ_022201 novel_circ_022202 novel_circ_022203 novel_circ_022204 novel_circ_022205 novel_circ_022206 novel_circ_022207 novel_circ_022208 novel_circ_022209 novel_circ_022210 novel_circ_022211 novel_circ_022212 novel_circ_022213 novel_circ_022214 novel_circ_022215 novel_circ_022216 novel_circ_022217 novel_circ_022218 novel_circ_022219 novel_circ_022220 novel_circ_022221 novel_circ_022222 novel_circ_022223

| hsa_circ_0029921 | 90 | 1 | 404 | 428 | 831 | 0 | 2.00E-149 |
| --- | --- | --- | --- | --- | --- | --- | --- |
| hsa_circ_0029921 | 91 | 1 | 661 | 428 | 1088 | 0 | 0 |
| hsa_circ_0029919 | 92 | 1 | 1188 | 1338 | 2525 | 0 | 0 |
| hsa_circ_0029922 | 92 | 1 | 257 | 403 | 659 | 0 | 5.00E-98 |
| hsa_circ_0029922 | 93 | 1 | 598 | 403 | 1000 | 0 | 0 |
| hsa_circ_0029919 | 92 | 1 | 784 | 1742 | 2525 | 0 | 0 |
| hsa_circ_0029919 | 93 | 1 | 441 | 2085 | 2525 | 0 | 0 |
| hsa_circ_0029919 | 92 | 1 | 784 | 2340 | 3123 | 0 | 0 |
| hsa_circ_0029919 | 92 | 1 | 1282 | 2725 | 4006 | 0 | 0 |
| hsa_circ_0029919 | 93 | 3 | 277 | 2849 | 3123 | 0 | 3.00E-111 |
| hsa_circ_0029919 | 92 | 3 | 1160 | 2849 | 4006 | 0 | 0 |
| hsa_circ_0029919 | 92 | 3 | 1301 | 2849 | 4147 | 0 | 0 |
| hsa_circ_0029919 | 92 | 1 | 877 | 3130 | 4006 | 0 | 0 |
| hsa_circ_0029919 | 92 | 1 | 1018 | 3130 | 4147 | 0 | 0 |
| hsa_circ_0029919 | 92 | 1 | 1435 | 3130 | 4564 | 0 | 0 |
| hsa_circ_0029919 | 96 | 1 | 269 | 3738 | 4006 | 0 | 1.00E-119 |
| hsa_circ_0029919 | 94 | 1 | 410 | 3738 | 4147 | 0 | 2.00E-179 |
| hsa_circ_0029919 | 93 | 1 | 827 | 3738 | 4564 | 0 | 0 |
| hsa_circ_0029919 | 93 | 1 | 998 | 3738 | 4735 | 0 | 0 |
| hsa_circ_0029919 | 92 | 1 | 1525 | 3738 | 5262 | 0 | 0 |
| hsa_circ_0029919 | 92 | 1 | 1718 | 3738 | 5455 | 0 | 0 |
| hsa_circ_0029925 | 91 | 1 | 968 | 589 | 1556 | 0 | 0 |
| hsa_circ_0029925 | 92 | 1 | 1053 | 589 | 1641 | 0 | 0 |
| hsa_circ_0029925 | 94 | 1 | 340 | 1119 | 1458 | 0 | 2.00E-142 |
| hsa_circ_0029925 | 94 | 1 | 523 | 1119 | 1641 | 0 | 0 |
| hsa_circ_0029925 | 94 | 1 | 634 | 1119 | 1752 | 0 | 0 |
| hsa_circ_0005723 | 83 | 3 | 1008 | 37578 | 36576 | 0.06 | 0 |
| hsa_circ_0029937 | 96 | 3 | 166 | 164 | 1 | 0 | 7.00E-70 |
| hsa_circ_0029943 | 95 | 1 | 643 | 1 | 643 | 0 | 0 |
| hsa_circ_0029943 | 95 | 1 | 1076 | 1 | 1076 | 0 | 0 |
| hsa_circ_0029943 | 95 | 1 | 1488 | 1 | 1489 | 0 | 0 |
| hsa_circ_0029947 | 95 | 1 | 845 | 226 | 1071 | 0 | 0 |
| hsa_circ_0029949 | 95 | 1 | 1349 | 212 | 1560 | 0 | 0 |
| hsa_circ_0029949 | 95 | 1 | 1203 | 358 | 1560 | 0 | 0 |
| hsa_circ_0029953 | 94 | 1 | 271 | 1 | 271 | 0 | 9.00E-116 |
| hsa_circ_0029953 | 95 | 1 | 384 | 1 | 384 | 0 | 4.00E-170 |
| hsa_circ_0029953 | 95 | 1 | 654 | 1 | 654 | 0 | 0 |
| hsa_circ_0029953 | 96 | 1 | 937 | 1 | 937 | 0 | 0 |
| hsa_circ_0029948 | 95 | 1 | 1266 | 765 | 2030 | 0 | 0 |
| hsa_circ_0029948 | 94 | 1 | 1472 | 765 | 2236 | 0 | 0 |
| hsa_circ_0029953 | 95 | 3 | 523 | 134 | 654 | 0 | 0 |
| hsa_circ_0029953 | 96 | 3 | 806 | 134 | 937 | 0 | 0 |
| hsa_circ_0029956 | 96 | 1 | 666 | 1 | 666 | 0 | 0 |
| hsa_circ_0029956 | 95 | 1 | 1201 | 1 | 1201 | 0 | 0 |
| hsa_circ_0029957 | 96 | 1 | 550 | 1 | 550 | 0 | 0 |
| hsa_circ_0029957 | 97 | 1 | 444 | 107 | 550 | 0 | 0 |
| hsa_circ_0029956 | 94 | 1 | 979 | 223 | 1201 | 0 | 0 |
| hsa_circ_0029966 | 98 | 1 | 283 | 1 | 283 | 0 | 9.00E-136 |
| hsa_circ_0029966 | 94 | 1 | 818 | 1 | 818 | 0 | 0 |
| hsa_circ_0029966 | 93 | 1 | 535 | 284 | 818 | 0 | 0 |
| hsa_circ_0029966 | 93 | 1 | 650 | 284 | 933 | 0 | 0 |
| hsa_circ_0029980 | 97 | 65149 | 66093 | 1 | 945 | 0 | 0 |
| hsa_circ_0029980 | 97 | 1 | 429 | 1 | 429 | 0 | 0 |
| hsa_circ_0029980 | 97 | 1 | 678 | 1 | 678 | 0 | 0 |

novel_circ_022224 novel_circ_022225 novel_circ_022226 novel_circ_022227 novel_circ_022228 novel_circ_022229 novel_circ_022230 novel_circ_022231 novel_circ_022232 novel_circ_022233 novel_circ_022234 novel_circ_022235 novel_circ_022236 novel_circ_022237 novel_circ_022238 novel_circ_022239 novel_circ_022240 novel_circ_022241 novel_circ_022242 novel_circ_022243 novel_circ_022244 novel_circ_022245 novel_circ_022246 novel_circ_022247 novel_circ_022248 novel_circ_022249 novel_circ_022250 novel_circ_022251 novel_circ_022252 novel_circ_022253 novel_circ_022254 novel_circ_022255 novel_circ_022256 novel_circ_022257 novel_circ_022258 novel_circ_022259 novel_circ_022260 novel_circ_022261 novel_circ_022262 novel_circ_022263 novel_circ_022264 novel_circ_022265 novel_circ_022266 novel_circ_022267 novel_circ_022268 novel_circ_022269 novel_circ_022270 novel_circ_022271 novel_circ_022272 novel_circ_022273 novel_circ_022274 novel_circ_022275 novel_circ_022276 novel_circ_022277

| hsa_circ_0029980 | 97 | 1 | 798 | 1 | 798 | 0 | 0 |
| --- | --- | --- | --- | --- | --- | --- | --- |
| hsa_circ_0029980 | 97 | 1 | 945 | 1 | 945 | 0 | 0 |
| hsa_circ_0029980 | 96 | 1 | 1277 | 1 | 1277 | 0 | 0 |
| hsa_circ_0029980 | 96 | 1 | 1380 | 1 | 1386 | 0 | 0 |
| hsa_circ_0029980 | 95 | 1 | 1702 | 1 | 1708 | 0 | 0 |
| hsa_circ_0029980 | 95 | 1 | 2036 | 1 | 2042 | 0 | 0 |
| hsa_circ_0029980 | 95 | 1 | 2037 | 1 | 2043 | 0 | 0 |
| hsa_circ_0029980 | 95 | 47 | 1852 | 231 | 2042 | 0 | 0 |
| hsa_circ_0029980 | 95 | 47 | 1853 | 231 | 2043 | 0 | 0 |
| hsa_circ_0029982 | 97 | 1 | 446 | 1 | 446 | 0 | 0 |
| hsa_circ_0029982 | 97 | 1 | 713 | 1 | 713 | 0 | 0 |
| hsa_circ_0029982 | 96 | 1 | 1045 | 1 | 1045 | 0 | 0 |
| hsa_circ_0029982 | 95 | 1 | 1148 | 1 | 1154 | 0.01 | 0 |
| hsa_circ_0029982 | 95 | 1 | 1470 | 1 | 1476 | 0 | 0 |
| hsa_circ_0029980 | 95 | 1 | 1804 | 233 | 2042 | 0 | 0 |
| hsa_circ_0029980 | 95 | 1 | 1805 | 233 | 2043 | 0 | 0 |
| hsa_circ_0029984 | 97 | 1 | 516 | 1 | 516 | 0 | 0 |
| hsa_circ_0029984 | 95 | 1 | 848 | 1 | 848 | 0 | 0 |
| hsa_circ_0029982 | 95 | 1 | 951 | 198 | 1154 | 0.01 | 0 |
| hsa_circ_0029982 | 95 | 1 | 1273 | 198 | 1476 | 0 | 0 |
| hsa_circ_0029985 | 96 | 1 | 267 | 1 | 267 | 0 | 6.00E-122 |
| hsa_circ_0029985 | 95 | 1 | 599 | 1 | 599 | 0 | 0 |
| hsa_circ_0029982 | 94 | 1 | 702 | 447 | 1154 | 0.01 | 0 |
| hsa_circ_0029982 | 94 | 1 | 1024 | 447 | 1476 | 0.01 | 0 |
| hsa_circ_0029980 | 94 | 1 | 1358 | 679 | 2042 | 0 | 0 |
| hsa_circ_0029980 | 94 | 1 | 1359 | 679 | 2043 | 0 | 0 |
| hsa_circ_0029987 | 93 | 1 | 332 | 1 | 332 | 0 | 6.00E-138 |
| hsa_circ_0029987 | 93 | 1 | 435 | 1 | 441 | 0.01 | 5.00E-180 |
| hsa_circ_0029987 | 94 | 1 | 757 | 1 | 763 | 0.01 | 0 |
| hsa_circ_0029987 | 94 | 1 | 1091 | 1 | 1097 | 0.01 | 0 |
| hsa_circ_0029987 | 94 | 1 | 1092 | 1 | 1098 | 0.01 | 0 |
| hsa_circ_0029987 | 94 | 1 | 1092 | 1 | 1098 | 0.01 | 0 |
| hsa_circ_0029987 | 94 | 2283 | 2708 | 332 | 763 | 0.01 | 0 |
| hsa_circ_0029987 | 94 | 2283 | 3043 | 332 | 1098 | 0.01 | 0 |
| hsa_circ_0029989 | 95 | 1 | 322 | 110 | 431 | 0 | 1.00E-140 |
| hsa_circ_0029989 | 94 | 1 | 656 | 110 | 765 | 0 | 0 |
| hsa_circ_0029989 | 94 | 1 | 657 | 110 | 766 | 0 | 0 |
| hsa_circ_0029989 | 95 | 15936 | 16139 | 671 | 874 | 0 | 2.00E-83 |
| hsa_circ_0029989 | 94 | 1 | 504 | 263 | 766 | 0 | 0 |
| hsa_circ_0029989 | 94 | 1 | 334 | 432 | 765 | 0 | 1.00E-140 |
| hsa_circ_0029989 | 94 | 1 | 335 | 432 | 766 | 0 | 1.00E-140 |
| hsa_circ_0029989 | 95 | 5867 | 6070 | 671 | 874 | 0 | 8.00E-84 |
| hsa_circ_0029989 | 94 | 1 | 2326 | 766 | 3091 | 0 | 0 |
| hsa_circ_0029989 | 94 | 1 | 399 | 875 | 1273 | 0 | 4.00E-175 |
| hsa_circ_0029989 | 94 | 1 | 1788 | 875 | 2662 | 0 | 0 |
| hsa_circ_0029989 | 94 | 1 | 1706 | 957 | 2662 | 0 | 0 |
| hsa_circ_0029989 | 94 | 1 | 1235 | 1274 | 2508 | 0 | 0 |
| hsa_circ_0029989 | 94 | 1 | 1389 | 1274 | 2662 | 0 | 0 |
| hsa_circ_0029989 | 94 | 1 | 1818 | 1274 | 3091 | 0 | 0 |
| hsa_circ_0029989 | 95 | 1 | 475 | 2509 | 2983 | 0 | 0 |
| hsa_circ_0029989 | 95 | 1 | 583 | 2509 | 3091 | 0 | 0 |
| hsa_circ_0029989 | 94 | 1 | 1287 | 2509 | 3795 | 0 | 0 |
| hsa_circ_0029987 | 94 | 1 | 1569 | 2841 | 4409 | 0 | 0 |
| hsa_circ_0029987 | 95 | 1 | 1697 | 2841 | 4537 | 0 | 0 |

novel_circ_022278 novel_circ_022279 novel_circ_022280 novel_circ_022281 novel_circ_022282 novel_circ_022283 novel_circ_022284 novel_circ_022285 novel_circ_022286 novel_circ_022287 novel_circ_022288 novel_circ_022289 novel_circ_022290 novel_circ_022291 novel_circ_022292 novel_circ_022293 novel_circ_022294 novel_circ_022295 novel_circ_022296 novel_circ_022297 novel_circ_022298 novel_circ_022299 novel_circ_022300 novel_circ_022301 novel_circ_022302 novel_circ_022303 novel_circ_022304 novel_circ_022305 novel_circ_022306 novel_circ_022307 novel_circ_022308 novel_circ_022309 novel_circ_022310 novel_circ_022311 novel_circ_022312 novel_circ_022314 novel_circ_022315 novel_circ_022316 novel_circ_022317 novel_circ_022318 novel_circ_022319 novel_circ_022320 novel_circ_022321 novel_circ_022322 novel_circ_022323 novel_circ_022324 novel_circ_022325 novel_circ_022326 novel_circ_022327 novel_circ_022330 novel_circ_022332 novel_circ_022333 novel_circ_022334 novel_circ_022335

| hsa_circ_0029989 | 93 | 1 | 312 | 2672 | 2983 | 0 | 4.00E-125 |
| --- | --- | --- | --- | --- | --- | --- | --- |
| hsa_circ_0029989 | 93 | 1 | 420 | 2672 | 3091 | 0 | 1.00E-176 |
| hsa_circ_0029989 | 94 | 1 | 685 | 2672 | 3356 | 0 | 0 |
| hsa_circ_0029989 | 94 | 1 | 1124 | 2672 | 3795 | 0 | 0 |
| hsa_circ_0029987 | 94 | 1 | 1406 | 3004 | 4409 | 0 | 0 |
| hsa_circ_0029987 | 94 | 1 | 1534 | 3004 | 4537 | 0 | 0 |
| hsa_circ_0029989 | 95 | 3 | 504 | 2855 | 3356 | 0 | 0 |
| hsa_circ_0029989 | 94 | 3 | 943 | 2855 | 3795 | 0 | 0 |
| hsa_circ_0029987 | 94 | 3 | 1225 | 3187 | 4409 | 0 | 0 |
| hsa_circ_0029987 | 95 | 3 | 1353 | 3187 | 4537 | 0 | 0 |
| hsa_circ_0029990 | 96 | 1 | 265 | 1 | 265 | 0 | 8.00E-121 |
| hsa_circ_0029990 | 94 | 1 | 704 | 1 | 704 | 0 | 0 |
| hsa_circ_0029990 | 94 | 1 | 986 | 1 | 986 | 0 | 0 |
| hsa_circ_0029987 | 95 | 1 | 1114 | 3424 | 4537 | 0 | 0 |
| hsa_circ_0029991 | 92 | 1 | 439 | 97 | 535 | 0 | 3.00E-177 |
| hsa_circ_0029991 | 94 | 1 | 721 | 97 | 817 | 0 | 0 |
| hsa_circ_0029987 | 94 | 1 | 849 | 3689 | 4537 | 0 | 0 |
| hsa_circ_0029991 | 96 | 1 | 282 | 536 | 817 | 0 | 7.00E-127 |
| hsa_circ_0029987 | 97 | 1 | 410 | 4128 | 4537 | 0 | 0 |
| hsa_circ_0029992 | 95 | 1 | 403 | 256 | 658 | 0 | 3.00E-177 |
| hsa_circ_0029992 | 94 | 1 | 341 | 318 | 658 | 0 | 6.00E-148 |
| hsa_circ_0029992 | 94 | 1 | 276 | 383 | 658 | 0 | 3.00E-115 |
| hsa_circ_0029992 | 94 | 1 | 545 | 383 | 927 | 0 | 0 |
| hsa_circ_0029992 | 95 | 1 | 269 | 659 | 927 | 0 | 9.00E-118 |
| hsa_circ_0029992 | 98 | 176 | 314 | 926 | 1064 | 0 | 2.00E-62 |
| hsa_circ_0029992 | 95 | 1 | 450 | 1065 | 1514 | 0 | 0 |
| hsa_circ_0029992 | 95 | 1 | 864 | 1065 | 1928 | 0 | 0 |
| hsa_circ_0029992 | 95 | 1 | 1032 | 1065 | 2096 | 0 | 0 |
| hsa_circ_0029992 | 96 | 1 | 490 | 1286 | 1775 | 0 | 0 |
| hsa_circ_0029992 | 95 | 1 | 811 | 1286 | 2096 | 0 | 0 |
| hsa_circ_0029992 | 97 | 3 | 374 | 1404 | 1775 | 0 | 4.00E-175 |
| hsa_circ_0029992 | 96 | 3 | 527 | 1404 | 1928 | 0 | 0 |
| hsa_circ_0029992 | 95 | 3 | 695 | 1404 | 2096 | 0 | 0 |
| hsa_circ_0029992 | 96 | 1 | 414 | 1515 | 1928 | 0 | 0 |
| hsa_circ_0029992 | 95 | 1 | 582 | 1515 | 2096 | 0 | 0 |
| hsa_circ_0029995 | 95 | 1 | 743 | 824 | 82 | 0 | 0 |
| hsa_circ_0029995 | 95 | 1 | 325 | 715 | 391 | 0 | 5.00E-144 |
| hsa_circ_0029995 | 95 | 1 | 434 | 824 | 391 | 0 | 0 |
| hsa_circ_0029995 | 95 | 86 | 519 | 824 | 391 | 0 | 0 |
| hsa_circ_0029995 | 95 | 181 | 614 | 824 | 391 | 0 | 0 |
| hsa_circ_0029995 | 94 | 1 | 178 | 715 | 538 | 0 | 1.00E-72 |
| hsa_circ_0029995 | 95 | 1 | 287 | 824 | 538 | 0 | 1.00E-124 |
| hsa_circ_0029995 | 95 | 474 | 582 | 824 | 716 | 0 | 5.00E-42 |
| hsa_circ_0017553 | 92 | 1 | 663 | 1 | 663 | 0 | 0 |
| hsa_circ_0017523 | 84 | 298 | 486 | 355 | 543 | 0 | 6.00E-45 |
| hsa_circ_0090139 | 93 | 1 | 622 | 276 | 897 | 0 | 0 |
| hsa_circ_0090141 | 94 | 1 | 563 | 4982 | 4420 | 0 | 0 |
| hsa_circ_0090141 | 94 | 118 | 680 | 4982 | 4420 | 0 | 0 |
| hsa_circ_0090141 | 94 | 218 | 780 | 4982 | 4420 | 0 | 0 |
| hsa_circ_0090143 | 88 | 1 | 538 | 1 | 538 | 0.01 | 2.00E-180 |
| hsa_circ_0090148 | 89 | 26 | 968 | 26 | 980 | 0.01 | 0 |
| hsa_circ_0090148 | 91 | 26 | 1498 | 26 | 1510 | 0.01 | 0 |
| hsa_circ_0090148 | 92 | 8 | 599 | 389 | 980 | 0 | 0 |
| hsa_circ_0090148 | 93 | 8 | 953 | 389 | 1334 | 0 | 0 |

novel_circ_022336 novel_circ_022337 novel_circ_022338 novel_circ_022339 novel_circ_022340 novel_circ_022341 novel_circ_022342 novel_circ_022343 novel_circ_022344 novel_circ_022352 novel_circ_022353 novel_circ_022354 novel_circ_022355 novel_circ_022356 novel_circ_022358 novel_circ_022359 novel_circ_022360 novel_circ_022361 novel_circ_022362 novel_circ_022363 novel_circ_022364 novel_circ_022365 novel_circ_022379 novel_circ_022383 novel_circ_022384 novel_circ_022385 novel_circ_022386 novel_circ_022387 novel_circ_022388 novel_circ_022389 novel_circ_022390 novel_circ_022391 novel_circ_022392 novel_circ_022393 novel_circ_022394 novel_circ_022395 novel_circ_022396 novel_circ_022397 novel_circ_022398 novel_circ_022399 novel_circ_022400 novel_circ_022401 novel_circ_022402 novel_circ_022403 novel_circ_022404 novel_circ_022405 novel_circ_022406 novel_circ_022407 novel_circ_022408 novel_circ_022409 novel_circ_022410 novel_circ_022411 novel_circ_022412 novel_circ_022413

| hsa_circ_0090148 | 93 | 8 | 1259 | 389 | 1640 | 0 | 0 |
| --- | --- | --- | --- | --- | --- | --- | --- |
| hsa_circ_0090148 | 94 | 8 | 1604 | 389 | 1985 | 0 | 0 |
| hsa_circ_0090148 | 94 | 8 | 1877 | 389 | 2258 | 0 | 0 |
| hsa_circ_0090163 | 94 | 1 | 597 | 1 | 597 | 0 | 0 |
| hsa_circ_0090163 | 94 | 1 | 770 | 1 | 770 | 0 | 0 |
| hsa_circ_0090163 | 93 | 1 | 1083 | 1 | 1083 | 0 | 0 |
| hsa_circ_0090167 | 94 | 1 | 305 | 1 | 305 | 0 | 6.00E-128 |
| hsa_circ_0090167 | 93 | 1 | 618 | 1 | 618 | 0 | 0 |
| hsa_circ_0090168 | 92 | 1 | 522 | 1 | 525 | 0.01 | 0 |
| hsa_circ_0006518 | 96 | 1 | 259 | 1 | 259 | 0 | 2.00E-117 |
| hsa_circ_0090175 | 99 | 162 | 339 | 178 | 1 | 0 | 2.00E-87 |
| hsa_circ_0090176 | 94 | 1 | 1815 | 1800 | 1 | 0.01 | 0 |
| hsa_circ_0090176 | 94 | 1 | 1654 | 1800 | 162 | 0.01 | 0 |
| hsa_circ_0090176 | 85 | 1 | 502 | 2092 | 1609 | 0.05 | 7.00E-134 |
| hsa_circ_0090177 | 95 | 1 | 368 | 493 | 126 | 0 | 1.00E-164 |
| hsa_circ_0090177 | 98 | 1 | 746 | 1239 | 494 | 0 | 0 |
| hsa_circ_0090177 | 96 | 1 | 446 | 1685 | 1240 | 0 | 0 |
| hsa_circ_0090177 | 96 | 76 | 521 | 1685 | 1240 | 0 | 0 |
| hsa_circ_0090179 | 95 | 1 | 720 | 720 | 1 | 0 | 0 |
| hsa_circ_0090179 | 94 | 1 | 390 | 390 | 1 | 0 | 9.00E-167 |
| hsa_circ_0090179 | 95 | 1 | 720 | 720 | 1 | 0 | 0 |
| hsa_circ_0090179 | 95 | 1 | 330 | 720 | 391 | 0 | 2.00E-148 |
| hsa_circ_0090181 | 93 | 1 | 314 | 311 | 1 | 0.01 | 8.00E-125 |
| hsa_circ_0067376 | 90 | 1 | 387 | 1 | 387 | 0 | 4.00E-140 |
| hsa_circ_0067374 | 90 | 1 | 501 | 1463 | 1963 | 0 | 0 |
| hsa_circ_0067374 | 91 | 652 | 2604 | 2 | 1963 | 0 | 0 |
| hsa_circ_0067374 | 90 | 1 | 409 | 1463 | 1871 | 0 | 3.00E-147 |
| hsa_circ_0067374 | 90 | 1 | 1264 | 599 | 1871 | 0.01 | 0 |
| hsa_circ_0067374 | 89 | 1 | 647 | 920 | 1575 | 0.01 | 0 |
| hsa_circ_0067375 | 92 | 1 | 396 | 1 | 396 | 0 | 9.00E-157 |
| hsa_circ_0067358 | 92 | 5 | 558 | 3204 | 3757 | 0 | 0 |
| hsa_circ_0067358 | 92 | 1 | 951 | 2807 | 3757 | 0 | 0 |
| hsa_circ_0067358 | 91 | 1 | 1053 | 2705 | 3757 | 0 | 0 |
| hsa_circ_0067358 | 92 | 1 | 2283 | 1490 | 3757 | 0.01 | 0 |
| hsa_circ_0067358 | 92 | 1 | 749 | 2807 | 3555 | 0 | 0 |
| hsa_circ_0067358 | 91 | 1 | 851 | 2705 | 3555 | 0 | 0 |
| hsa_circ_0067364 | 90 | 1 | 594 | 811 | 1404 | 0 | 0 |
| hsa_circ_0067370 | 91 | 1 | 393 | 103 | 495 | 0 | 9.00E-152 |
| hsa_circ_0067363 | 92 | 43 | 1434 | 792 | 2168 | 0.01 | 0 |
| hsa_circ_0067366 | 97 | 1 | 264 | 1 | 264 | 0 | 1.00E-123 |
| hsa_circ_0067365 | 94 | 1 | 418 | 84 | 501 | 0 | 0 |
| hsa_circ_0067365 | 95 | 1 | 501 | 1 | 501 | 0 | 0 |
| hsa_circ_0067363 | 93 | 1 | 832 | 794 | 1610 | 0.02 | 0 |
| hsa_circ_0067363 | 93 | 43 | 876 | 792 | 1610 | 0.02 | 0 |
| hsa_circ_0067361 | 92 | 1 | 2187 | 202 | 2373 | 0.01 | 0 |
| hsa_circ_0067357 | 93 | 1 | 2737 | 1 | 2722 | 0.01 | 0 |
| hsa_circ_0067363 | 92 | 1 | 903 | 459 | 1346 | 0.02 | 0 |
| hsa_circ_0067361 | 92 | 1 | 2124 | 1 | 2109 | 0.01 | 0 |
| hsa_circ_0067357 | 92 | 1 | 2473 | 1 | 2458 | 0.01 | 0 |
| hsa_circ_0067363 | 94 | 1 | 759 | 351 | 1109 | 0 | 0 |
| hsa_circ_0067361 | 92 | 1 | 1671 | 202 | 1872 | 0 | 0 |
| hsa_circ_0067363 | 94 | 1 | 335 | 459 | 793 | 0 | 6.00E-143 |
| hsa_circ_0067363 | 94 | 1 | 443 | 351 | 793 | 0 | 0 |
| hsa_circ_0067357 | 92 | 1 | 1570 | 1 | 1570 | 0 | 0 |

novel_circ_022414 novel_circ_022415 novel_circ_022425 novel_circ_022426 novel_circ_022428 novel_circ_022429 novel_circ_022431 novel_circ_022432 novel_circ_022433 novel_circ_022434 novel_circ_022435 novel_circ_022436 novel_circ_022437 novel_circ_022438 novel_circ_022443 novel_circ_022444 novel_circ_022446 novel_circ_022447 novel_circ_022448 novel_circ_022449 novel_circ_022450 novel_circ_022451 novel_circ_022452 novel_circ_022453 novel_circ_022454 novel_circ_022455 novel_circ_022456 novel_circ_022457 novel_circ_022458 novel_circ_022459 novel_circ_022460 novel_circ_022461 novel_circ_022462 novel_circ_022463 novel_circ_022464 novel_circ_022465 novel_circ_022467 novel_circ_022468 novel_circ_022469 novel_circ_022470 novel_circ_022471 novel_circ_022475 novel_circ_022476 novel_circ_022477 novel_circ_022478 novel_circ_022479 novel_circ_022480 novel_circ_022481 novel_circ_022482 novel_circ_022483 novel_circ_022484 novel_circ_022485 novel_circ_022486 novel_circ_022487

| hsa_circ_0067362 | 90 | 1 | 517 | 1 | 517 | 0 | 0 |
| --- | --- | --- | --- | --- | --- | --- | --- |
| hsa_circ_0067358 | 94 | 2 | 469 | 2 | 469 | 0 | 0 |
| hsa_circ_0067343 | 88 | 1 | 251 | 582 | 832 | 0 | 2.00E-77 |
| hsa_circ_0067343 | 88 | 1 | 251 | 582 | 832 | 0 | 8.00E-78 |
| hsa_circ_0005797 | 90 | 1 | 525 | 1 | 525 | 0 | 0 |
| hsa_circ_0005797 | 91 | 1 | 354 | 1 | 354 | 0 | 4.00E-135 |
| hsa_circ_0067339 | 85 | 1 | 1071 | 1598 | 525 | 0 | 0 |
| hsa_circ_0067335 | 84 | 1 | 1663 | 2608 | 943 | 0 | 0 |
| hsa_circ_0067339 | 89 | 1 | 357 | 881 | 525 | 0 | 4.00E-125 |
| hsa_circ_0067335 | 84 | 1 | 1083 | 2025 | 943 | 0 | 0 |
| hsa_circ_0067335 | 85 | 1 | 1648 | 2025 | 378 | 0 | 0 |
| hsa_circ_0067335 | 85 | 1 | 599 | 1144 | 546 | 0 | 2.00E-174 |
| hsa_circ_0060562 | 93 | 127 | 744 | 1 | 618 | 0 | 0 |
| hsa_circ_0060562 | 92 | 1 | 332 | 287 | 618 | 0 | 3.00E-131 |
| hsa_circ_0060582 | 94 | 7 | 721 | 845 | 131 | 0 | 0 |
| hsa_circ_0060585 | 91 | 7 | 287 | 281 | 1 | 0 | 6.00E-103 |
| hsa_circ_0060599 | 94 | 1 | 319 | 319 | 1 | 0 | 1.00E-135 |
| hsa_circ_0060599 | 93 | 1 | 400 | 400 | 1 | 0 | 3.00E-167 |
| hsa_circ_0060605 | 90 | 1 | 1029 | 1044 | 1 | 0.01 | 0 |
| hsa_circ_0001163 | 89 | 1 | 510 | 510 | 1 | 0 | 1.00E-176 |
| hsa_circ_0001163 | 89 | 1 | 663 | 663 | 1 | 0 | 0 |
| hsa_circ_0060604 | 88 | 1 | 804 | 1238 | 435 | 0 | 0 |
| hsa_circ_0060604 | 90 | 1 | 1286 | 1720 | 435 | 0 | 0 |
| hsa_circ_0060617 | 91 | 1 | 623 | 623 | 1 | 0 | 0 |
| hsa_circ_0060617 | 93 | 7 | 961 | 955 | 1 | 0 | 0 |
| hsa_circ_0004212 | 93 | 1 | 482 | 482 | 1 | 0 | 0 |
| hsa_circ_0004212 | 94 | 1 | 732 | 732 | 1 | 0 | 0 |
| hsa_circ_0060617 | 94 | 7 | 820 | 955 | 142 | 0 | 0 |
| hsa_circ_0060618 | 95 | 1 | 431 | 431 | 1 | 0 | 0 |
| hsa_circ_0060618 | 95 | 1 | 545 | 545 | 1 | 0 | 0 |
| hsa_circ_0060623 | 97 | 4 | 439 | 436 | 1 | 0 | 0 |
| hsa_circ_0060623 | 97 | 1 | 514 | 514 | 1 | 0 | 0 |
| hsa_circ_0060623 | 96 | 216 | 659 | 444 | 1 | 0 | 0 |
| hsa_circ_0060623 | 98 | 4 | 351 | 436 | 89 | 0 | 4.00E-170 |
| hsa_circ_0060624 | 99 | 4 | 258 | 255 | 1 | 0 | 1.00E-128 |
| hsa_circ_0060624 | 99 | 1 | 333 | 333 | 1 | 0 | 4.00E-170 |
| hsa_circ_0011678 | 89 | 1 | 1084 | 1 | 1075 | 0.01 | 0 |
| hsa_circ_0011678 | 93 | 1 | 558 | 706 | 1263 | 0 | 0 |
| hsa_circ_0011697 | 94 | 813 | 881 | 69 | 1 | 0 | 4.00E-21 |
| hsa_circ_0011697 | 94 | 813 | 881 | 69 | 1 | 0 | 3.00E-21 |
| hsa_circ_0011697 | 87 | 8 | 249 | 242 | 1 | 0 | 1.00E-72 |
| hsa_circ_0011720 | 93 | 1 | 419 | 524 | 106 | 0 | 8.00E-173 |
| hsa_circ_0011720 | 94 | 1 | 1114 | 1222 | 106 | 0 | 0 |
| hsa_circ_0011720 | 94 | 2062 | 3175 | 1222 | 106 | 0 | 0 |
| hsa_circ_0011720 | 94 | 2062 | 2756 | 1222 | 525 | 0 | 0 |
| hsa_circ_0005807 | 89 | 15 | 531 | 517 | 1 | 0 | 0 |
| hsa_circ_0011728 | 89 | 2 | 646 | 972 | 328 | 0 | 0 |
| hsa_circ_0011729 | 89 | 1 | 274 | 274 | 1 | 0 | 2.00E-92 |
| hsa_circ_0011742 | 93 | 1 | 437 | 493 | 57 | 0 | 2.00E-179 |
| hsa_circ_0011744 | 92 | 1 | 276 | 276 | 1 | 0 | 1.00E-108 |
| hsa_circ_0011762 | 96 | 1 | 458 | 458 | 1 | 0 | 0 |
| hsa_circ_0011762 | 96 | 1 | 458 | 458 | 1 | 0 | 0 |
| hsa_circ_0011762 | 96 | 1 | 519 | 662 | 144 | 0 | 0 |
| hsa_circ_0011762 | 96 | 1 | 404 | 662 | 259 | 0 | 0 |

novel_circ_022488 novel_circ_022489 novel_circ_022490 novel_circ_022492 novel_circ_022493 novel_circ_022494 novel_circ_022495 novel_circ_022496 novel_circ_022497 novel_circ_022498 novel_circ_022499 novel_circ_022501 novel_circ_022502 novel_circ_022503 novel_circ_022504 novel_circ_022505 novel_circ_022506 novel_circ_022508 novel_circ_022509 novel_circ_022511 novel_circ_022512 novel_circ_022513 novel_circ_022514 novel_circ_022515 novel_circ_022516 novel_circ_022517 novel_circ_022518 novel_circ_022519 novel_circ_022520 novel_circ_022521 novel_circ_022522 novel_circ_022523 novel_circ_022524 novel_circ_022525 novel_circ_022526 novel_circ_022527 novel_circ_022528 novel_circ_022529 novel_circ_022530 novel_circ_022531 novel_circ_022532 novel_circ_022533 novel_circ_022534 novel_circ_022535 novel_circ_022536 novel_circ_022537 novel_circ_022538 novel_circ_022539 novel_circ_022540 novel_circ_022541 novel_circ_022542 novel_circ_022543 novel_circ_022544 novel_circ_022545

| hsa_circ_0003529 | 93 | 1 | 276 | 1 | 276 | 0 | 1.00E-113 |
| --- | --- | --- | --- | --- | --- | --- | --- |
| hsa_circ_0011773 | 91 | 1 | 3414 | 94 | 3510 | 0 | 0 |
| hsa_circ_0011781 | 91 | 1 | 2531 | 1 | 2534 | 0 | 0 |
| hsa_circ_0011797 | 91 | 1 | 480 | 1 | 480 | 0 | 0 |
| hsa_circ_0011797 | 89 | 1 | 1116 | 1 | 1116 | 0 | 0 |
| hsa_circ_0011803 | 89 | 1 | 1464 | 1 | 1464 | 0 | 0 |
| hsa_circ_0011815 | 90 | 1 | 449 | 1 | 449 | 0 | 2.00E-164 |
| hsa_circ_0011819 | 90 | 152 | 357 | 2 | 207 | 0 | 5.00E-71 |
| hsa_circ_0011814 | 86 | 1 | 1638 | 670 | 2307 | 0 | 0 |
| hsa_circ_0011814 | 88 | 1 | 4992 | 670 | 5670 | 0 | 0 |
| hsa_circ_0011828 | 88 | 1 | 958 | 125 | 1082 | 0 | 0 |
| hsa_circ_0011835 | 88 | 1 | 296 | 1 | 305 | 0.03 | 7.00E-97 |
| hsa_circ_0011836 | 93 | 1 | 500 | 2299 | 2798 | 0 | 0 |
| hsa_circ_0011841 | 93 | 1 | 229 | 208 | 436 | 0 | 4.00E-89 |
| hsa_circ_0005788 | 91 | 1 | 672 | 220 | 891 | 0 | 0 |
| hsa_circ_0011844 | 91 | 1 | 654 | 1869 | 2522 | 0 | 0 |
| hsa_circ_0011846 | 92 | 1 | 484 | 1 | 484 | 0 | 0 |
| hsa_circ_0034959 | 92 | 1 | 111 | 1 | 111 | 0 | 3.00E-37 |
| hsa_circ_0034956 | 91 | 1 | 363 | 1 | 363 | 0 | 4.00E-135 |
| hsa_circ_0034949 | 90 | 1 | 211 | 89 | 299 | 0 | 3.00E-72 |
| hsa_circ_0034948 | 91 | 2 | 356 | 133 | 487 | 0 | 9.00E-134 |
| hsa_circ_0034950 | 90 | 1 | 129 | 1 | 129 | 0 | 3.00E-40 |
| hsa_circ_0034949 | 90 | 1 | 211 | 89 | 299 | 0 | 2.00E-72 |
| hsa_circ_0034941 | 90 | 1 | 1129 | 1 | 1129 | 0 | 0 |
| hsa_circ_0034941 | 91 | 1 | 807 | 1 | 807 | 0 | 0 |
| hsa_circ_0034941 | 91 | 1 | 706 | 1 | 706 | 0 | 0 |
| hsa_circ_0034941 | 92 | 1 | 267 | 1 | 267 | 0 | 7.00E-102 |
| hsa_circ_0034930 | 93 | 1 | 447 | 780 | 334 | 0 | 0 |
| hsa_circ_0034926 | 95 | 258 | 1587 | 1573 | 244 | 0 | 0 |
| hsa_circ_0034907 | 94 | 258 | 2109 | 3880 | 2029 | 0 | 0 |
| hsa_circ_0034926 | 95 | 1 | 1251 | 1494 | 244 | 0 | 0 |
| hsa_circ_0034926 | 95 | 1 | 1140 | 1383 | 244 | 0 | 0 |
| hsa_circ_0034929 | 96 | 1 | 870 | 870 | 1 | 0 | 0 |
| hsa_circ_0034929 | 96 | 4 | 807 | 804 | 1 | 0 | 0 |
| hsa_circ_0034929 | 96 | 1 | 357 | 486 | 130 | 0 | 4.00E-165 |
| hsa_circ_0034929 | 95 | 1 | 486 | 486 | 1 | 0 | 0 |
| hsa_circ_0034923 | 94 | 1 | 840 | 1008 | 169 | 0 | 0 |
| hsa_circ_0034928 | 93 | 1 | 410 | 410 | 1 | 0 | 7.00E-168 |
| hsa_circ_0034924 | 92 | 1 | 173 | 239 | 67 | 0 | 7.00E-65 |
| hsa_circ_0034919 | 94 | 6 | 695 | 950 | 270 | 0.01 | 0 |
| hsa_circ_0034905 | 93 | 6 | 2127 | 2110 | 1 | 0.01 | 0 |
| hsa_circ_0034918 | 94 | 1 | 472 | 475 | 4 | 0 | 0 |
| hsa_circ_0034914 | 94 | 1 | 719 | 719 | 1 | 0 | 0 |
| hsa_circ_0034911 | 93 | 1 | 817 | 817 | 1 | 0 | 0 |
| hsa_circ_0034909 | 93 | 1 | 911 | 1113 | 206 | 0 | 0 |
| hsa_circ_0034909 | 92 | 1 | 1060 | 1113 | 57 | 0 | 0 |
| hsa_circ_0034905 | 93 | 1 | 1432 | 1429 | 1 | 0 | 0 |
| hsa_circ_0034919 | 96 | 1 | 182 | 182 | 1 | 0 | 2.00E-79 |
| hsa_circ_0034914 | 94 | 1 | 632 | 632 | 1 | 0 | 0 |
| hsa_circ_0034914 | 92 | 1 | 450 | 450 | 1 | 0 | 0 |
| hsa_circ_0034907 | 91 | 1 | 944 | 1009 | 69 | 0 | 0 |
| hsa_circ_0034905 | 92 | 1 | 1163 | 1160 | 1 | 0 | 0 |
| hsa_circ_0034905 | 92 | 1 | 713 | 710 | 1 | 0 | 0 |
| hsa_circ_0034899 | 91 | 1 | 801 | 798 | 1 | 0 | 0 |

novel_circ_022546 novel_circ_022547 novel_circ_022548 novel_circ_022549 novel_circ_022550 novel_circ_022551 novel_circ_022552 novel_circ_022553 novel_circ_022554 novel_circ_022555 novel_circ_022556 novel_circ_022557 novel_circ_022558 novel_circ_022559 novel_circ_022560 novel_circ_022561 novel_circ_022562 novel_circ_022563 novel_circ_022564 novel_circ_022565 novel_circ_022566 novel_circ_022567 novel_circ_022568 novel_circ_022569 novel_circ_022570 novel_circ_022571 novel_circ_022572 novel_circ_022573 novel_circ_022574 novel_circ_022576 novel_circ_022577 novel_circ_022578 novel_circ_022579 novel_circ_022580 novel_circ_022581 novel_circ_022582 novel_circ_022583 novel_circ_022584 novel_circ_022585 novel_circ_022586 novel_circ_022587 novel_circ_022588 novel_circ_022589 novel_circ_022590 novel_circ_022591 novel_circ_022592 novel_circ_022593 novel_circ_022594 novel_circ_022595 novel_circ_022596 novel_circ_022597 novel_circ_022598 novel_circ_022599 novel_circ_022600

| hsa_circ_0034896 | 91 | 1 | 935 | 1041 | 110 | 0 | 0 |
| --- | --- | --- | --- | --- | --- | --- | --- |
| hsa_circ_0034905 | 92 | 16 | 615 | 600 | 1 | 0 | 0 |
| hsa_circ_0034896 | 92 | 16 | 837 | 931 | 110 | 0 | 0 |
| hsa_circ_0034905 | 93 | 1 | 521 | 521 | 1 | 0 | 0 |
| hsa_circ_0034896 | 92 | 1 | 743 | 852 | 110 | 0 | 0 |
| hsa_circ_0034905 | 93 | 1 | 372 | 372 | 1 | 0 | 4.00E-155 |
| hsa_circ_0034896 | 91 | 1 | 222 | 331 | 110 | 0 | 1.00E-78 |
| hsa_circ_0034880 | 94 | 1 | 284 | 284 | 1 | 0 | 2.00E-121 |
| hsa_circ_0034879 | 94 | 1 | 288 | 358 | 71 | 0 | 4.00E-120 |
| hsa_circ_0034879 | 95 | 1 | 358 | 358 | 1 | 0 | 5.00E-159 |
| hsa_circ_0006297 | 94 | 1 | 603 | 604 | 2 | 0 | 0 |
| hsa_circ_0006297 | 94 | 1 | 467 | 468 | 2 | 0 | 0 |
| hsa_circ_0034869 | 94 | 1 | 534 | 753 | 220 | 0 | 0 |
| hsa_circ_0034869 | 95 | 1 | 753 | 753 | 1 | 0 | 0 |
| hsa_circ_0034868 | 94 | 1 | 911 | 911 | 1 | 0 | 0 |
| hsa_circ_0034864 | 94 | 1 | 1128 | 1929 | 802 | 0 | 0 |
| hsa_circ_0006297 | 94 | 1 | 319 | 320 | 2 | 0 | 1.00E-135 |
| hsa_circ_0034875 | 94 | 1 | 386 | 386 | 1 | 0 | 2.00E-164 |
| hsa_circ_0034869 | 94 | 1 | 605 | 605 | 1 | 0 | 0 |
| hsa_circ_0034864 | 94 | 1 | 1118 | 1707 | 590 | 0 | 0 |
| hsa_circ_0034869 | 95 | 1 | 285 | 285 | 1 | 0 | 3.00E-125 |
| hsa_circ_0034868 | 94 | 1 | 443 | 443 | 1 | 0 | 0 |
| hsa_circ_0034864 | 94 | 1 | 660 | 1461 | 802 | 0 | 0 |
| hsa_circ_0034864 | 94 | 1 | 594 | 1395 | 802 | 0 | 0 |
| hsa_circ_0034864 | 93 | 1 | 1395 | 1395 | 1 | 0 | 0 |
| hsa_circ_0034864 | 93 | 1 | 587 | 1176 | 590 | 0 | 0 |
| hsa_circ_0034864 | 92 | 1 | 1176 | 1176 | 1 | 0 | 0 |
| hsa_circ_0034864 | 93 | 1 | 429 | 1018 | 590 | 0 | 0 |
| hsa_circ_0034836 | 89 | 1 | 316 | 12334 | 12649 | 0 | 5.00E-109 |
| hsa_circ_0034829 | 93 | 71 | 187 | 1 | 117 | 0 | 5.00E-42 |
| hsa_circ_0034819 | 88 | 353 | 2530 | 2182 | 1 | 0.01 | 0 |
| hsa_circ_0034819 | 88 | 205 | 2382 | 2182 | 1 | 0.01 | 0 |
| hsa_circ_0034819 | 88 | 205 | 2382 | 2182 | 1 | 0.01 | 0 |
| hsa_circ_0034811 | 92 | 1 | 625 | 7067 | 6440 | 0 | 0 |
| hsa_circ_0034817 | 90 | 3 | 1117 | 1177 | 57 | 0.01 | 0 |
| hsa_circ_0034817 | 90 | 3 | 1173 | 1177 | 1 | 0.01 | 0 |
| hsa_circ_0034813 | 90 | 3 | 1454 | 1950 | 493 | 0 | 0 |
| hsa_circ_0034817 | 90 | 1 | 834 | 837 | 1 | 0 | 0 |
| hsa_circ_0034815 | 91 | 1 | 1115 | 1118 | 1 | 0 | 0 |
| hsa_circ_0034814 | 90 | 1 | 1225 | 1490 | 263 | 0 | 0 |
| hsa_circ_0034811 | 91 | 1 | 1745 | 6439 | 4686 | 0.01 | 0 |
| hsa_circ_0034811 | 84 | 1 | 3186 | 6439 | 3216 | 0.04 | 0 |
| hsa_circ_0034815 | 93 | 1 | 337 | 337 | 1 | 0 | 1.00E-135 |
| hsa_circ_0034811 | 96 | 1 | 327 | 5012 | 4686 | 0 | 2.00E-148 |
| hsa_circ_0034808 | 88 | 1 | 346 | 1184 | 1529 | 0 | 1.00E-115 |
| hsa_circ_0034808 | 91 | 3 | 684 | 502 | 1183 | 0 | 0 |
| hsa_circ_0034802 | 89 | 1 | 328 | 393 | 63 | 0.01 | 3.00E-111 |
| hsa_circ_0034799 | 93 | 1 | 558 | 558 | 1 | 0 | 0 |
| hsa_circ_0034799 | 93 | 1 | 558 | 558 | 1 | 0 | 0 |
| hsa_circ_0034799 | 94 | 1 | 378 | 378 | 1 | 0 | 9.00E-165 |
| hsa_circ_0034796 | 96 | 165 | 403 | 239 | 1 | 0 | 5.00E-106 |
| hsa_circ_0034796 | 95 | 3 | 157 | 155 | 1 | 0 | 1.00E-62 |
| hsa_circ_0034794 | 95 | 1 | 269 | 461 | 193 | 0 | 5.00E-118 |
| hsa_circ_0034794 | 94 | 1 | 395 | 395 | 1 | 0 | 7.00E-168 |

novel_circ_022601 novel_circ_022602 novel_circ_022603 novel_circ_022604 novel_circ_022605 novel_circ_022606 novel_circ_022607 novel_circ_022608 novel_circ_022610 novel_circ_022611 novel_circ_022612 novel_circ_022613 novel_circ_022614 novel_circ_022615 novel_circ_022616 novel_circ_022617 novel_circ_022618 novel_circ_022619 novel_circ_022620 novel_circ_022621 novel_circ_022622 novel_circ_022625 novel_circ_022627 novel_circ_022628 novel_circ_022629 novel_circ_022630 novel_circ_022631 novel_circ_022632 novel_circ_022633 novel_circ_022634 novel_circ_022635 novel_circ_022636 novel_circ_022637 novel_circ_022638 novel_circ_022639 novel_circ_022640 novel_circ_022641 novel_circ_022642 novel_circ_022643 novel_circ_022644 novel_circ_022645 novel_circ_022646 novel_circ_022647 novel_circ_022648 novel_circ_022649 novel_circ_022650 novel_circ_022651 novel_circ_022652 novel_circ_022654 novel_circ_022655 novel_circ_022656 novel_circ_022658 novel_circ_022659 novel_circ_022660

| hsa_circ_0034785 | 92 | 1 | 618 | 748 | 131 | 0 | 0 |
| --- | --- | --- | --- | --- | --- | --- | --- |
| hsa_circ_0034785 | 94 | 1 | 556 | 556 | 1 | 0 | 0 |
| hsa_circ_0034785 | 94 | 3 | 372 | 370 | 1 | 0 | 1.00E-155 |
| hsa_circ_0034784 | 94 | 3 | 515 | 513 | 1 | 0 | 0 |
| hsa_circ_0034742 | 92 | 1 | 904 | 5945 | 6851 | 0.01 | 0 |
| hsa_circ_0034753 | 95 | 1 | 553 | 2758 | 3310 | 0 | 0 |
| hsa_circ_0034753 | 93 | 1 | 733 | 2575 | 3310 | 0.01 | 0 |
| hsa_circ_0034753 | 91 | 1 | 499 | 2575 | 3076 | 0.01 | 0 |
| hsa_circ_0034756 | 89 | 1 | 1805 | 1 | 1796 | 0.01 | 0 |
| hsa_circ_0034753 | 90 | 1 | 2432 | 152 | 2574 | 0.01 | 0 |
| hsa_circ_0034733 | 92 | 1 | 4486 | 1 | 4501 | 0.01 | 0 |
| hsa_circ_0034743 | 92 | 1 | 412 | 1 | 412 | 0 | 3.00E-162 |
| hsa_circ_0034742 | 90 | 1 | 1361 | 1 | 1361 | 0 | 0 |
| hsa_circ_0034733 | 91 | 1 | 2489 | 1 | 2492 | 0 | 0 |
| hsa_circ_0034742 | 90 | 1 | 949 | 1 | 949 | 0 | 0 |
| hsa_circ_0034733 | 91 | 1 | 2077 | 1 | 2080 | 0 | 0 |
| hsa_circ_0034733 | 93 | 1 | 1126 | 1 | 1129 | 0 | 0 |
| hsa_circ_0034730 | 93 | 143 | 1269 | 114 | 1243 | 0 | 0 |
| hsa_circ_0034702 | 93 | 1 | 314 | 228 | 541 | 0 | 3.00E-126 |
| hsa_circ_0034702 | 93 | 1 | 426 | 116 | 541 | 0 | 1.00E-176 |
| hsa_circ_0034702 | 94 | 1 | 541 | 1 | 541 | 0 | 0 |
| hsa_circ_0034682 | 93 | 1 | 282 | 1 | 282 | 0 | 3.00E-115 |
| hsa_circ_0034676 | 93 | 1 | 1379 | 1648 | 279 | 0.01 | 0 |
| hsa_circ_0004279 | 94 | 1 | 336 | 336 | 1 | 0 | 8.00E-142 |
| hsa_circ_0034680 | 94 | 1 | 594 | 594 | 1 | 0 | 0 |
| hsa_circ_0034676 | 93 | 1 | 790 | 1068 | 279 | 0 | 0 |
| hsa_circ_0034676 | 93 | 1 | 858 | 1068 | 211 | 0 | 0 |
| hsa_circ_0034674 | 94 | 1 | 1245 | 1245 | 1 | 0 | 0 |
| hsa_circ_0034680 | 95 | 3 | 473 | 471 | 1 | 0 | 0 |
| hsa_circ_0034676 | 93 | 4 | 454 | 729 | 279 | 0 | 0 |
| hsa_circ_0034674 | 95 | 1 | 455 | 455 | 1 | 0 | 0 |
| hsa_circ_0034661 | 94 | 1 | 1583 | 1580 | 1 | 0 | 0 |
| hsa_circ_0034674 | 95 | 1 | 387 | 387 | 1 | 0 | 2.00E-173 |
| hsa_circ_0034665 | 94 | 1 | 590 | 1181 | 592 | 0 | 0 |
| hsa_circ_0034665 | 94 | 1 | 675 | 1181 | 507 | 0 | 0 |
| hsa_circ_0034665 | 94 | 1 | 793 | 1181 | 389 | 0 | 0 |
| hsa_circ_0034661 | 94 | 1 | 1515 | 1512 | 1 | 0 | 0 |
| hsa_circ_0034656 | 93 | 1 | 1650 | 2007 | 355 | 0.01 | 0 |
| hsa_circ_0034656 | 94 | 1 | 2004 | 2007 | 1 | 0 | 0 |
| hsa_circ_0034650 | 94 | 1 | 2172 | 2842 | 668 | 0 | 0 |
| hsa_circ_0034661 | 94 | 1 | 1068 | 1065 | 1 | 0 | 0 |
| hsa_circ_0034661 | 94 | 4 | 840 | 834 | 1 | 0 | 0 |
| hsa_circ_0034656 | 93 | 1 | 1125 | 1128 | 1 | 0.01 | 0 |
| hsa_circ_0034628 | 86 | 1 | 349 | 723 | 387 | 0.03 | 5.00E-99 |
| hsa_circ_0034628 | 88 | 1 | 738 | 723 | 1 | 0.02 | 0 |
| hsa_circ_0034628 | 89 | 1 | 389 | 386 | 1 | 0.01 | 1.00E-136 |
| hsa_circ_0034623 | 91 | 1 | 703 | 700 | 1 | 0.01 | 0 |
| hsa_circ_0034622 | 93 | 1 | 388 | 388 | 1 | 0 | 3.00E-157 |
| hsa_circ_0034592 | 91 | 1059 | 2060 | 1062 | 2066 | 0.01 | 0 |
| hsa_circ_0034588 | 92 | 1 | 176 | 1 | 176 | 0 | 7.00E-65 |
| hsa_circ_0034586 | 93 | 1 | 273 | 1 | 273 | 0 | 1.00E-108 |
| hsa_circ_0034572 | 91 | 1 | 404 | 1 | 404 | 0 | 8.00E-153 |
| hsa_circ_0034558 | 95 | 1 | 384 | 814 | 1197 | 0 | 2.00E-168 |
| hsa_circ_0034558 | 91 | 1 | 240 | 355 | 594 | 0 | 6.00E-87 |

novel_circ_022661 novel_circ_022662 novel_circ_022663 novel_circ_022664 novel_circ_022665 novel_circ_022666 novel_circ_022667 novel_circ_022668 novel_circ_022669 novel_circ_022670 novel_circ_022671 novel_circ_022672 novel_circ_022673 novel_circ_022674 novel_circ_022675 novel_circ_022676 novel_circ_022677 novel_circ_022678 novel_circ_022679 novel_circ_022680 novel_circ_022681 novel_circ_022682 novel_circ_022684 novel_circ_022685 novel_circ_022686 novel_circ_022687 novel_circ_022688 novel_circ_022689 novel_circ_022690 novel_circ_022691 novel_circ_022692 novel_circ_022693 novel_circ_022694 novel_circ_022695 novel_circ_022696 novel_circ_022697 novel_circ_022698 novel_circ_022699 novel_circ_022700 novel_circ_022701 novel_circ_022702 novel_circ_022703 novel_circ_022704 novel_circ_022705 novel_circ_022706 novel_circ_022707 novel_circ_022708 novel_circ_022709 novel_circ_022710 novel_circ_022711 novel_circ_022713 novel_circ_022714 novel_circ_022715 novel_circ_022716

| hsa_circ_0034556 | 95 | 2 | 286 | 487 | 771 | 0 | 1.00E-123 |
| --- | --- | --- | --- | --- | --- | --- | --- |
| hsa_circ_0034556 | 92 | 1 | 435 | 1 | 435 | 0 | 2.00E-173 |
| hsa_circ_0034555 | 92 | 367 | 957 | 1 | 591 | 0 | 0 |
| hsa_circ_0034555 | 93 | 1 | 393 | 1 | 393 | 0 | 2.00E-163 |
| hsa_circ_0034552 | 88 | 4872 | 6650 | 3 | 1769 | 0.01 | 0 |
| hsa_circ_0034552 | 88 | 502 | 2280 | 3 | 1769 | 0.01 | 0 |
| hsa_circ_0034551 | 91 | 1 | 602 | 308 | 909 | 0 | 0 |
| hsa_circ_0034551 | 92 | 1 | 599 | 195 | 793 | 0 | 0 |
| hsa_circ_0034546 | 84 | 658 | 1065 | 919 | 512 | 0 | 3.00E-106 |
| hsa_circ_0034546 | 84 | 225 | 494 | 919 | 650 | 0 | 3.00E-68 |
| hsa_circ_0034546 | 84 | 225 | 632 | 919 | 512 | 0 | 2.00E-106 |
| hsa_circ_0034546 | 86 | 1 | 502 | 511 | 9 | 0.02 | 3.00E-152 |
| hsa_circ_0034546 | 86 | 3 | 498 | 505 | 9 | 0.02 | 2.00E-150 |
| hsa_circ_0080955 | 88 | 126 | 862 | 1 | 737 | 0 | 0 |
| hsa_circ_0080957 | 85 | 103 | 424 | 1 | 322 | 0 | 7.00E-89 |
| hsa_circ_0080955 | 88 | 126 | 862 | 1 | 737 | 0 | 0 |
| hsa_circ_0080955 | 89 | 126 | 758 | 1 | 633 | 0 | 0 |
| hsa_circ_0080955 | 90 | 126 | 532 | 1 | 407 | 0 | 5.00E-146 |
| hsa_circ_0080955 | 90 | 262 | 668 | 1 | 407 | 0 | 6.00E-146 |
| hsa_circ_0080955 | 91 | 126 | 432 | 1 | 307 | 0 | 7.00E-114 |
| hsa_circ_0080955 | 91 | 262 | 568 | 1 | 307 | 0 | 9.00E-114 |
| hsa_circ_0080953 | 96 | 34 | 524 | 245 | 735 | 0 | 0 |
| hsa_circ_0080953 | 95 | 1 | 152 | 584 | 735 | 0 | 1.00E-60 |
| hsa_circ_0080953 | 96 | 896 | 1386 | 245 | 735 | 0 | 0 |
| hsa_circ_0080953 | 95 | 1 | 152 | 584 | 735 | 0 | 1.00E-60 |
| hsa_circ_0080953 | 95 | 1 | 381 | 355 | 735 | 0 | 2.00E-169 |
| hsa_circ_0080953 | 96 | 479 | 969 | 245 | 735 | 0 | 0 |
| hsa_circ_0080953 | 96 | 896 | 1386 | 245 | 735 | 0 | 0 |
| hsa_circ_0080953 | 95 | 1 | 381 | 355 | 735 | 0 | 1.00E-169 |
| hsa_circ_0080953 | 96 | 896 | 1386 | 245 | 735 | 0 | 0 |
| hsa_circ_0080953 | 95 | 1 | 381 | 355 | 735 | 0 | 8.00E-170 |
| hsa_circ_0080953 | 96 | 479 | 969 | 245 | 735 | 0 | 0 |
| hsa_circ_0080953 | 96 | 896 | 1386 | 245 | 735 | 0 | 0 |
| hsa_circ_0080953 | 96 | 896 | 1386 | 245 | 735 | 0 | 0 |
| hsa_circ_0080953 | 95 | 1 | 381 | 355 | 735 | 0 | 4.00E-170 |
| hsa_circ_0080953 | 96 | 479 | 969 | 245 | 735 | 0 | 0 |
| hsa_circ_0080953 | 96 | 896 | 1386 | 245 | 735 | 0 | 0 |
| hsa_circ_0080950 | 94 | 1 | 479 | 364 | 842 | 0 | 0 |
| hsa_circ_0080950 | 94 | 418 | 896 | 364 | 842 | 0 | 0 |
| hsa_circ_0080950 | 95 | 1 | 445 | 364 | 808 | 0 | 0 |
| hsa_circ_0080950 | 95 | 297 | 741 | 364 | 808 | 0 | 0 |
| hsa_circ_0080950 | 95 | 418 | 862 | 364 | 808 | 0 | 0 |
| hsa_circ_0080950 | 93 | 1 | 287 | 364 | 650 | 0.01 | 1.00E-114 |
| hsa_circ_0080950 | 94 | 1 | 361 | 3 | 363 | 0 | 5.00E-157 |
| hsa_circ_0080950 | 92 | 1 | 210 | 364 | 573 | 0.01 | 2.00E-80 |
| hsa_circ_0080950 | 92 | 180 | 389 | 364 | 573 | 0.01 | 5.00E-80 |
| hsa_circ_0080950 | 94 | 1 | 361 | 3 | 363 | 0 | 4.00E-157 |
| hsa_circ_0080950 | 94 | 1 | 361 | 3 | 363 | 0 | 3.00E-157 |
| hsa_circ_0080950 | 94 | 1 | 361 | 3 | 363 | 0 | 2.00E-157 |
| hsa_circ_0080950 | 92 | 1 | 238 | 3 | 240 | 0 | 2.00E-92 |
| hsa_circ_0080949 | 93 | 318 | 900 | 893 | 311 | 0 | 0 |
| hsa_circ_0080949 | 93 | 197 | 779 | 893 | 311 | 0 | 0 |
| hsa_circ_0080949 | 92 | 1 | 259 | 658 | 400 | 0 | 8.00E-101 |
| hsa_circ_0080949 | 92 | 1 | 348 | 658 | 311 | 0 | 8.00E-137 |

novel_circ_022717 novel_circ_022718 novel_circ_022719 novel_circ_022720 novel_circ_022721 novel_circ_022722 novel_circ_022723 novel_circ_022724 novel_circ_022725 novel_circ_022726 novel_circ_022727 novel_circ_022728 novel_circ_022729 novel_circ_022738 novel_circ_022739 novel_circ_022740 novel_circ_022741 novel_circ_022742 novel_circ_022743 novel_circ_022744 novel_circ_022745 novel_circ_022746 novel_circ_022747 novel_circ_022748 novel_circ_022749 novel_circ_022750 novel_circ_022751 novel_circ_022752 novel_circ_022753 novel_circ_022754 novel_circ_022755 novel_circ_022756 novel_circ_022757 novel_circ_022758 novel_circ_022759 novel_circ_022760 novel_circ_022761 novel_circ_022763 novel_circ_022764 novel_circ_022765 novel_circ_022767 novel_circ_022768 novel_circ_022769 novel_circ_022772 novel_circ_022773 novel_circ_022774 novel_circ_022775 novel_circ_022776 novel_circ_022777 novel_circ_022779 novel_circ_022780 novel_circ_022781 novel_circ_022782 novel_circ_022783

| hsa_circ_0080949 | 93 | 1 | 528 | 658 | 130 | 0 | 0 |
| --- | --- | --- | --- | --- | --- | --- | --- |
| hsa_circ_0080949 | 93 | 1 | 658 | 658 | 1 | 0 | 0 |
| hsa_circ_0080949 | 93 | 1 | 658 | 658 | 1 | 0 | 0 |
| hsa_circ_0080949 | 93 | 1 | 658 | 658 | 1 | 0 | 0 |
| hsa_circ_0080949 | 93 | 1 | 658 | 658 | 1 | 0 | 0 |
| hsa_circ_0080949 | 93 | 1 | 658 | 658 | 1 | 0 | 0 |
| hsa_circ_0080949 | 93 | 1 | 658 | 658 | 1 | 0 | 0 |
| hsa_circ_0080949 | 93 | 1 | 422 | 552 | 130 | 0 | 8.00E-173 |
| hsa_circ_0080949 | 94 | 1 | 310 | 310 | 1 | 0 | 1.00E-130 |
| hsa_circ_0080949 | 94 | 1 | 310 | 310 | 1 | 0 | 2.00E-130 |
| hsa_circ_0080949 | 94 | 1 | 310 | 310 | 1 | 0 | 5.00E-130 |
| hsa_circ_0080949 | 94 | 1 | 310 | 310 | 1 | 0 | 7.00E-130 |
| hsa_circ_0080949 | 94 | 1 | 310 | 310 | 1 | 0 | 8.00E-130 |
| hsa_circ_0075510 | 93 | 1 | 697 | 1 | 697 | 0 | 0 |
| hsa_circ_0075510 | 93 | 1 | 1225 | 1 | 1225 | 0 | 0 |
| hsa_circ_0075510 | 93 | 1 | 895 | 331 | 1225 | 0 | 0 |
| hsa_circ_0075510 | 92 | 1 | 377 | 698 | 1074 | 0 | 7.00E-148 |
| hsa_circ_0078195 | 94 | 32072 | 32461 | 1 | 390 | 0 | 4.00E-163 |
| hsa_circ_0078195 | 94 | 16086 | 16475 | 1 | 390 | 0 | 2.00E-163 |
| hsa_circ_0078195 | 94 | 1 | 390 | 1 | 390 | 0 | 4.00E-165 |
| hsa_circ_0078195 | 94 | 1 | 488 | 1 | 488 | 0 | 0 |
| hsa_circ_0078195 | 94 | 1 | 488 | 1 | 488 | 0 | 0 |
| hsa_circ_0078195 | 94 | 1 | 234 | 157 | 390 | 0 | 1.00E-98 |
| hsa_circ_0078197 | 95 | 81 | 490 | 1 | 410 | 0 | 2.00E-180 |
| hsa_circ_0078198 | 98 | 269 | 460 | 149 | 340 | 0 | 1.00E-91 |
| hsa_circ_0078198 | 93 | 269 | 1979 | 149 | 1841 | 0.01 | 0 |
| hsa_circ_0078198 | 93 | 120 | 1830 | 149 | 1841 | 0.01 | 0 |
| hsa_circ_0078200 | 93 | 1 | 1710 | 1 | 1692 | 0.01 | 0 |
| hsa_circ_0078201 | 93 | 1 | 1519 | 1 | 1501 | 0.01 | 0 |
| hsa_circ_0007392 | 96 | 1 | 451 | 451 | 1 | 0 | 0 |
| hsa_circ_0003485 | 96 | 1 | 665 | 665 | 1 | 0 | 0 |
| hsa_circ_0032332 | 96 | 1 | 732 | 732 | 1 | 0 | 0 |
| hsa_circ_0032330 | 95 | 1 | 860 | 860 | 1 | 0 | 0 |
| hsa_circ_0007392 | 95 | 3 | 307 | 305 | 1 | 0 | 1.00E-134 |
| hsa_circ_0003485 | 95 | 3 | 521 | 519 | 1 | 0 | 0 |
| hsa_circ_0032332 | 95 | 1 | 551 | 551 | 1 | 0 | 0 |
| hsa_circ_0032332 | 96 | 1 | 281 | 281 | 1 | 0 | 2.00E-126 |
| hsa_circ_0032302 | 91 | 25 | 780 | 77 | 832 | 0 | 0 |
| hsa_circ_0032294 | 84 | 2 | 744 | 1594 | 852 | 0.01 | 0 |
| hsa_circ_0032292 | 90 | 3 | 948 | 1110 | 165 | 0 | 0 |
| hsa_circ_0032278 | 90 | 3 | 505 | 503 | 1 | 0 | 0 |
| hsa_circ_0032278 | 91 | 1 | 400 | 400 | 1 | 0 | 6.00E-149 |
| hsa_circ_0032275 | 93 | 1 | 424 | 425 | 2 | 0 | 8.00E-174 |
| hsa_circ_0032272 | 94 | 749 | 855 | 153 | 259 | 0 | 4.00E-39 |
| hsa_circ_0032266 | 95 | 1 | 810 | 1 | 810 | 0 | 0 |
| hsa_circ_0032262 | 94 | 6 | 1484 | 609 | 2087 | 0 | 0 |
| hsa_circ_0032262 | 94 | 6 | 858 | 609 | 1461 | 0 | 0 |
| hsa_circ_0032263 | 94 | 6 | 674 | 6 | 674 | 0 | 0 |
| hsa_circ_0032262 | 95 | 14 | 612 | 10 | 603 | 0.01 | 0 |
| hsa_circ_0032258 | 94 | 2938 | 3095 | 463 | 620 | 0 | 7.00E-62 |
| hsa_circ_0032258 | 94 | 12921 | 13078 | 463 | 620 | 0 | 3.00E-61 |
| hsa_circ_0073022 | 88 | 13770 | 14580 | 53 | 866 | 0.01 | 0 |
| hsa_circ_0073022 | 88 | 49581 | 50391 | 53 | 866 | 0.01 | 0 |
| hsa_circ_0032258 | 98 | 35964 | 36101 | 1 | 138 | 0 | 2.00E-59 |

novel_circ_022784 novel_circ_022785 novel_circ_022786 novel_circ_022787 novel_circ_022788 novel_circ_022789 novel_circ_022790 novel_circ_022791 novel_circ_022792 novel_circ_022793 novel_circ_022794 novel_circ_022795 novel_circ_022796 novel_circ_022797 novel_circ_022798 novel_circ_022799 novel_circ_022800 novel_circ_022801 novel_circ_022802 novel_circ_022803 novel_circ_022804 novel_circ_022805 novel_circ_022806 novel_circ_022807 novel_circ_022808 novel_circ_022809 novel_circ_022810 novel_circ_022811 novel_circ_022812 novel_circ_022813 novel_circ_022814 novel_circ_022815 novel_circ_022816 novel_circ_022818 novel_circ_022819 novel_circ_022820 novel_circ_022821 novel_circ_022822 novel_circ_022823 novel_circ_022824 novel_circ_022825 novel_circ_022826 novel_circ_022827 novel_circ_022828 novel_circ_022829 novel_circ_022830 novel_circ_022831 novel_circ_022832 novel_circ_022834 novel_circ_022835 novel_circ_022836 novel_circ_022837 novel_circ_022838 novel_circ_022839

| hsa_circ_0032258 | 98 | 35964 | 36101 | 1 | 138 | 0 | 1.00E-59 |
| --- | --- | --- | --- | --- | --- | --- | --- |
| hsa_circ_0032251 | 95 | 1 | 273 | 68 | 340 | 0 | 1.00E-117 |
| hsa_circ_0032251 | 95 | 1 | 273 | 68 | 340 | 0 | 2.00E-118 |
| hsa_circ_0032251 | 95 | 6842 | 7117 | 65 | 340 | 0 | 6.00E-120 |
| hsa_circ_0011979 | 89 | 29911 | 31139 | 303 | 1545 | 0.02 | 0 |
| hsa_circ_0011979 | 89 | 38447 | 39675 | 303 | 1545 | 0.02 | 0 |
| hsa_circ_0011979 | 89 | 29911 | 31139 | 303 | 1545 | 0.02 | 0 |
| hsa_circ_0082241 | 93 | 4 | 2390 | 2387 | 1 | 0 | 0 |
| hsa_circ_0082241 | 93 | 1 | 2316 | 2316 | 1 | 0 | 0 |
| hsa_circ_0082241 | 93 | 4 | 2390 | 2387 | 1 | 0 | 0 |
| hsa_circ_0082241 | 93 | 4 | 2277 | 2387 | 114 | 0 | 0 |
| hsa_circ_0082248 | 92 | 1 | 212 | 212 | 1 | 0 | 4.00E-78 |
| hsa_circ_0082249 | 94 | 1 | 488 | 488 | 1 | 0 | 0 |
| hsa_circ_0082249 | 94 | 3 | 680 | 679 | 1 | 0 | 0 |
| hsa_circ_0082266 | 95 | 3 | 284 | 283 | 1 | 0 | 3.00E-125 |
| hsa_circ_0082266 | 94 | 1 | 624 | 624 | 1 | 0 | 0 |
| hsa_circ_0082260 | 94 | 1 | 1387 | 1666 | 280 | 0 | 0 |
| hsa_circ_0082266 | 94 | 1 | 532 | 624 | 93 | 0 | 0 |
| hsa_circ_0082260 | 94 | 4 | 1369 | 1737 | 372 | 0 | 0 |
| hsa_circ_0082260 | 93 | 1 | 1103 | 1666 | 564 | 0 | 0 |
| hsa_circ_0082260 | 94 | 4 | 1177 | 1737 | 564 | 0 | 0 |
| hsa_circ_0082270 | 93 | 1 | 763 | 871 | 109 | 0 | 0 |
| hsa_circ_0082270 | 94 | 1 | 1038 | 1146 | 109 | 0 | 0 |
| hsa_circ_0082272 | 92 | 1 | 315 | 315 | 1 | 0 | 4.00E-125 |
| hsa_circ_0082272 | 93 | 1 | 616 | 616 | 1 | 0 | 0 |
| hsa_circ_0082272 | 94 | 4 | 690 | 687 | 1 | 0 | 0 |
| hsa_circ_0082272 | 94 | 1 | 891 | 891 | 1 | 0 | 0 |
| hsa_circ_0082272 | 93 | 1 | 320 | 459 | 140 | 0 | 3.00E-131 |
| hsa_circ_0082273 | 94 | 1 | 301 | 301 | 1 | 0 | 4.00E-129 |
| hsa_circ_0082273 | 95 | 1 | 576 | 576 | 1 | 0 | 0 |
| hsa_circ_0082277 | 95 | 1 | 432 | 432 | 1 | 0 | 0 |
| hsa_circ_0082277 | 96 | 1 | 593 | 593 | 1 | 0 | 0 |
| hsa_circ_0082277 | 98 | 1 | 362 | 593 | 232 | 0 | 3.00E-176 |
| hsa_circ_0082290 | 95 | 4 | 460 | 1 | 457 | 0 | 0 |
| hsa_circ_0082290 | 94 | 1 | 350 | 1 | 350 | 0 | 1.00E-149 |
| hsa_circ_0082290 | 95 | 1 | 457 | 1 | 457 | 0 | 0 |
| hsa_circ_0082291 | 93 | 1 | 241 | 1 | 241 | 0 | 2.00E-97 |
| hsa_circ_0082290 | 94 | 1 | 348 | 110 | 457 | 0 | 5.00E-150 |
| hsa_circ_0082290 | 94 | 1 | 348 | 110 | 457 | 0 | 6.00E-150 |
| hsa_circ_0082292 | 99 | 378 | 448 | 1 | 71 | 0 | 1.00E-27 |
| hsa_circ_0082292 | 96 | 378 | 543 | 1 | 166 | 0 | 2.00E-70 |
| hsa_circ_0082292 | 94 | 1 | 95 | 72 | 166 | 0 | 4.00E-33 |
| hsa_circ_0082292 | 94 | 1 | 95 | 72 | 166 | 0 | 7.00E-33 |
| hsa_circ_0082307 | 94 | 6 | 615 | 6 | 615 | 0 | 0 |
| hsa_circ_0082307 | 94 | 6 | 774 | 6 | 774 | 0 | 0 |
| hsa_circ_0082307 | 93 | 6 | 1232 | 6 | 1232 | 0 | 0 |
| hsa_circ_0082307 | 93 | 6 | 1357 | 6 | 1357 | 0 | 0 |
| hsa_circ_0082311 | 93 | 1 | 742 | 142 | 883 | 0 | 0 |
| hsa_circ_0082316 | 100 | 1 | 93 | 93 | 1 | 0 | 3.00E-41 |
| hsa_circ_0082316 | 98 | 1 | 245 | 245 | 1 | 0 | 2.00E-116 |
| hsa_circ_0082327 | 92 | 1 | 212 | 212 | 1 | 0 | 9.00E-80 |
| hsa_circ_0007735 | 91 | 1 | 475 | 630 | 156 | 0 | 1.00E-180 |
| hsa_circ_0082335 | 93 | 1 | 377 | 1 | 377 | 0 | 7.00E-158 |
| hsa_circ_0082335 | 94 | 1 | 866 | 1 | 866 | 0 | 0 |

novel_circ_022840 novel_circ_022841 novel_circ_022842 novel_circ_022843 novel_circ_022844 novel_circ_022845 novel_circ_022846 novel_circ_022847 novel_circ_022848 novel_circ_022849 novel_circ_022850 novel_circ_022851 novel_circ_022852 novel_circ_022853 novel_circ_022854 novel_circ_022859 novel_circ_022861 novel_circ_022872 novel_circ_022873 novel_circ_022880 novel_circ_022881 novel_circ_022882 novel_circ_022885 novel_circ_022886 novel_circ_022887 novel_circ_022888 novel_circ_022889 novel_circ_022890 novel_circ_022891 novel_circ_022892 novel_circ_022893 novel_circ_022894 novel_circ_022895 novel_circ_022896 novel_circ_022897 novel_circ_022898 novel_circ_022899 novel_circ_022900 novel_circ_022901 novel_circ_022902 novel_circ_022904 novel_circ_022905 novel_circ_022906 novel_circ_022909 novel_circ_022910 novel_circ_022911 novel_circ_022912 novel_circ_022913 novel_circ_022914 novel_circ_022915 novel_circ_022916 novel_circ_022918 novel_circ_022919 novel_circ_022920

| hsa_circ_0082335 | 94 | 1 | 866 | 1 | 866 | 0 | 0 |
| --- | --- | --- | --- | --- | --- | --- | --- |
| hsa_circ_0082335 | 94 | 3 | 644 | 225 | 866 | 0 | 0 |
| hsa_circ_0082335 | 94 | 1 | 489 | 378 | 866 | 0 | 0 |
| hsa_circ_0082335 | 94 | 1 | 489 | 378 | 866 | 0 | 0 |
| hsa_circ_0082335 | 95 | 1 | 340 | 527 | 866 | 0 | 5.00E-149 |
| hsa_circ_0082342 | 94 | 1 | 385 | 385 | 1 | 0 | 5.00E-164 |
| hsa_circ_0082342 | 93 | 1 | 608 | 608 | 1 | 0 | 0 |
| hsa_circ_0082342 | 93 | 1 | 856 | 856 | 1 | 0 | 0 |
| hsa_circ_0082342 | 93 | 1 | 535 | 608 | 74 | 0 | 0 |
| hsa_circ_0082346 | 93 | 1 | 534 | 534 | 1 | 0 | 0 |
| hsa_circ_0082346 | 93 | 1 | 782 | 782 | 1 | 0 | 0 |
| hsa_circ_0082348 | 93 | 1 | 321 | 436 | 116 | 0 | 8.00E-132 |
| hsa_circ_0082346 | 92 | 1 | 569 | 782 | 214 | 0 | 0 |
| hsa_circ_0082348 | 93 | 1 | 223 | 436 | 214 | 0 | 2.00E-87 |
| hsa_circ_0082346 | 92 | 1 | 471 | 782 | 312 | 0 | 0 |
| hsa_circ_0082381 | 96 | 1 | 158 | 158 | 1 | 0 | 5.00E-66 |
| hsa_circ_0082384 | 95 | 1 | 412 | 412 | 1 | 0 | 0 |
| hsa_circ_0007239 | 81 | 21986 | 22364 | 361 | 1 | 0.06 | 8.00E-70 |
| hsa_circ_0007239 | 81 | 22377 | 22755 | 361 | 1 | 0.06 | 8.00E-70 |
| hsa_circ_0007239 | 81 | 22377 | 22755 | 361 | 1 | 0.06 | 4.00E-70 |
| hsa_circ_0082389 | 88 | 950 | 1449 | 502 | 5 | 0.04 | 1.00E-166 |
| hsa_circ_0007239 | 81 | 22377 | 22755 | 361 | 1 | 0.06 | 2.00E-70 |
| hsa_circ_0082400 | 94 | 1 | 605 | 1 | 605 | 0 | 0 |
| hsa_circ_0082404 | 93 | 1 | 535 | 1 | 535 | 0 | 0 |
| hsa_circ_0082400 | 95 | 1 | 1125 | 303 | 1427 | 0 | 0 |
| hsa_circ_0082400 | 94 | 1 | 1684 | 303 | 1986 | 0 | 0 |
| hsa_circ_0082400 | 95 | 2 | 1018 | 411 | 1427 | 0 | 0 |
| hsa_circ_0082400 | 95 | 1 | 1015 | 413 | 1427 | 0 | 0 |
| hsa_circ_0082410 | 96 | 1 | 470 | 304 | 773 | 0 | 0 |
| hsa_circ_0082400 | 96 | 1 | 822 | 606 | 1427 | 0 | 0 |
| hsa_circ_0082400 | 95 | 1 | 1328 | 606 | 1933 | 0 | 0 |
| hsa_circ_0003588 | 96 | 1 | 565 | 1 | 565 | 0 | 0 |
| hsa_circ_0082416 | 95 | 1 | 1071 | 114 | 1184 | 0 | 0 |
| hsa_circ_0082420 | 95 | 1 | 500 | 1 | 500 | 0 | 0 |
| hsa_circ_0082420 | 94 | 1 | 615 | 1 | 615 | 0 | 0 |
| hsa_circ_0082420 | 95 | 1 | 858 | 1 | 858 | 0 | 0 |
| hsa_circ_0082416 | 95 | 1 | 911 | 327 | 1237 | 0 | 0 |
| hsa_circ_0082420 | 93 | 1 | 263 | 353 | 615 | 0 | 1.00E-104 |
| hsa_circ_0082420 | 95 | 1 | 506 | 353 | 858 | 0 | 0 |
| hsa_circ_0082420 | 96 | 1 | 358 | 501 | 858 | 0 | 5.00E-164 |
| hsa_circ_0082428 | 93 | 1 | 716 | 1195 | 480 | 0 | 0 |
| hsa_circ_0082428 | 92 | 2 | 493 | 2748 | 2257 | 0 | 0 |
| hsa_circ_0042202 | 72 | 949 | 1989 | 3087 | 2045 | 0.05 | 4.00E-68 |
| hsa_circ_0075261 | 96 | 669 | 950 | 284 | 3 | 0 | 2.00E-126 |
| hsa_circ_0075261 | 96 | 669 | 950 | 284 | 3 | 0 | 3.00E-126 |
| hsa_circ_0075261 | 96 | 382 | 663 | 284 | 3 | 0 | 2.00E-126 |
| hsa_circ_0075261 | 96 | 68 | 349 | 284 | 3 | 0 | 1.00E-126 |
| hsa_circ_0075261 | 96 | 1 | 282 | 284 | 3 | 0 | 9.00E-127 |
| hsa_circ_0075261 | 96 | 1 | 282 | 284 | 3 | 0 | 1.00E-126 |
| hsa_circ_0075261 | 96 | 1 | 282 | 284 | 3 | 0 | 2.00E-126 |
| hsa_circ_0075261 | 96 | 1 | 282 | 284 | 3 | 0 | 2.00E-126 |
| hsa_circ_0002972 | 89 | 7 | 866 | 860 | 1 | 0.01 | 0 |
| hsa_circ_0075230 | 91 | 1 | 364 | 364 | 1 | 0 | 5.00E-139 |
| hsa_circ_0075167 | 91 | 1 | 476 | 1 | 476 | 0 | 0 |

novel_circ_022921 novel_circ_022922 novel_circ_022923 novel_circ_022924 novel_circ_022925 novel_circ_022926 novel_circ_022927 novel_circ_022928 novel_circ_022929 novel_circ_022930 novel_circ_022931 novel_circ_022932 novel_circ_022933 novel_circ_022934 novel_circ_022935 novel_circ_022936 novel_circ_022937 novel_circ_022938 novel_circ_022939 novel_circ_022940 novel_circ_022941 novel_circ_022942 novel_circ_022943 novel_circ_022944 novel_circ_022945 novel_circ_022946 novel_circ_022947 novel_circ_022948 novel_circ_022949 novel_circ_022950 novel_circ_022951 novel_circ_022952 novel_circ_022953 novel_circ_022954 novel_circ_022955 novel_circ_022956 novel_circ_022957 novel_circ_022958 novel_circ_022959 novel_circ_022960 novel_circ_022961 novel_circ_022962 novel_circ_022963 novel_circ_022964 novel_circ_022965 novel_circ_022966 novel_circ_022967 novel_circ_022968 novel_circ_022969 novel_circ_022970 novel_circ_022971 novel_circ_022972 novel_circ_022973 novel_circ_022974

| hsa_circ_0075163 | 91 | 1 | 857 | 970 | 1826 | 0 | 0 |
| --- | --- | --- | --- | --- | --- | --- | --- |
| hsa_circ_0075163 | 90 | 1 | 981 | 846 | 1826 | 0 | 0 |
| hsa_circ_0075163 | 90 | 1 | 1244 | 583 | 1826 | 0 | 0 |
| hsa_circ_0075163 | 91 | 1 | 1701 | 126 | 1826 | 0 | 0 |
| hsa_circ_0075163 | 91 | 1 | 1826 | 1 | 1826 | 0 | 0 |
| hsa_circ_0075163 | 90 | 1 | 744 | 970 | 1713 | 0 | 0 |
| hsa_circ_0075163 | 90 | 1 | 1588 | 126 | 1713 | 0 | 0 |
| hsa_circ_0075163 | 89 | 1 | 660 | 846 | 1505 | 0 | 0 |
| hsa_circ_0075163 | 90 | 1 | 1380 | 126 | 1505 | 0 | 0 |
| hsa_circ_0075165 | 90 | 1 | 381 | 269 | 649 | 0 | 9.00E-137 |
| hsa_circ_0075165 | 90 | 1 | 505 | 145 | 649 | 0 | 0 |
| hsa_circ_0075163 | 89 | 1 | 768 | 583 | 1350 | 0 | 0 |
| hsa_circ_0075163 | 90 | 1 | 1225 | 126 | 1350 | 0 | 0 |
| hsa_circ_0075164 | 88 | 1 | 387 | 458 | 844 | 0 | 4.00E-125 |
| hsa_circ_0075164 | 87 | 1 | 463 | 382 | 844 | 0 | 3.00E-147 |
| hsa_circ_0075164 | 90 | 1 | 844 | 1 | 844 | 0 | 0 |
| hsa_circ_0075163 | 91 | 1 | 969 | 1 | 969 | 0 | 0 |
| hsa_circ_0075162 | 86 | 1 | 3541 | 1 | 3529 | 0.01 | 0 |
| hsa_circ_0075159 | 87 | 1 | 3850 | 1 | 3838 | 0.01 | 0 |
| hsa_circ_0004473 | 86 | 1 | 339 | 1 | 339 | 0 | 9.00E-102 |
| hsa_circ_0075164 | 91 | 1 | 720 | 1 | 720 | 0 | 0 |
| hsa_circ_0075164 | 93 | 1 | 457 | 1 | 457 | 0 | 0 |
| hsa_circ_0075163 | 93 | 1 | 582 | 1 | 582 | 0 | 0 |
| hsa_circ_0075162 | 86 | 1 | 3154 | 1 | 3142 | 0.01 | 0 |
| hsa_circ_0075159 | 87 | 1 | 3327 | 137 | 3451 | 0.01 | 0 |
| hsa_circ_0075159 | 87 | 1 | 3463 | 1 | 3451 | 0.01 | 0 |
| hsa_circ_0075159 | 87 | 1071 | 4533 | 1 | 3451 | 0.01 | 0 |
| hsa_circ_0075164 | 94 | 1 | 381 | 1 | 381 | 0 | 9.00E-167 |
| hsa_circ_0075163 | 95 | 1 | 506 | 1 | 506 | 0 | 0 |
| hsa_circ_0075162 | 86 | 1 | 3078 | 1 | 3066 | 0.01 | 0 |
| hsa_circ_0075159 | 87 | 1 | 3387 | 1 | 3375 | 0.01 | 0 |
| hsa_circ_0075159 | 86 | 1 | 3006 | 1 | 2994 | 0.01 | 0 |
| hsa_circ_0075156 | 86 | 1068 | 4076 | 4756 | 7752 | 0.01 | 0 |
| hsa_circ_0075162 | 85 | 1 | 2572 | 1 | 2560 | 0.01 | 0 |
| hsa_circ_0075159 | 86 | 1 | 2881 | 1 | 2869 | 0.01 | 0 |
| hsa_circ_0075159 | 94 | 1 | 309 | 1 | 309 | 0 | 3.00E-130 |
| hsa_circ_0075153 | 95 | 1 | 938 | 1 | 944 | 0.01 | 0 |
| hsa_circ_0001559 | 96 | 1 | 343 | 1 | 343 | 0 | 8.00E-159 |
| hsa_circ_0008631 | 87 | 1 | 243 | 240 | 1 | 0.02 | 2.00E-72 |
| hsa_circ_0075138 | 91 | 1 | 1211 | 1605 | 398 | 0 | 0 |
| hsa_circ_0075138 | 89 | 1 | 1611 | 1605 | 1 | 0 | 0 |
| hsa_circ_0075138 | 92 | 2 | 1056 | 1452 | 398 | 0 | 0 |
| hsa_circ_0075138 | 92 | 1 | 1053 | 1450 | 398 | 0 | 0 |
| hsa_circ_0075138 | 92 | 1 | 968 | 1365 | 398 | 0 | 0 |
| hsa_circ_0075138 | 91 | 1 | 1110 | 1365 | 259 | 0 | 0 |
| hsa_circ_0075138 | 90 | 1 | 1214 | 1365 | 155 | 0 | 0 |
| hsa_circ_0075138 | 89 | 1 | 1368 | 1365 | 1 | 0 | 0 |
| hsa_circ_0075138 | 89 | 4 | 1243 | 1237 | 1 | 0 | 0 |
| hsa_circ_0075138 | 86 | 19 | 246 | 382 | 155 | 0 | 6.00E-62 |
| hsa_circ_0075138 | 84 | 19 | 400 | 382 | 1 | 0 | 1.00E-100 |
| hsa_circ_0075114 | 94 | 1 | 289 | 318 | 606 | 0 | 2.00E-120 |
| hsa_circ_0075112 | 95 | 1 | 392 | 205 | 596 | 0 | 7.00E-173 |
| hsa_circ_0001557 | 86 | 1 | 521 | 1 | 524 | 0.01 | 6.00E-155 |
| hsa_circ_0075095 | 87 | 71 | 746 | 1 | 679 | 0 | 0 |

novel_circ_022975 novel_circ_022976 novel_circ_022977 novel_circ_022978 novel_circ_022979 novel_circ_022980 novel_circ_022981 novel_circ_022986 novel_circ_022987 novel_circ_022988 novel_circ_022989 novel_circ_022990 novel_circ_022991 novel_circ_022992 novel_circ_022993 novel_circ_022994 novel_circ_022995 novel_circ_022996 novel_circ_023002 novel_circ_023003 novel_circ_023004 novel_circ_023005 novel_circ_023006 novel_circ_023007 novel_circ_023008 novel_circ_023009 novel_circ_023010 novel_circ_023011 novel_circ_023012 novel_circ_023013 novel_circ_023014 novel_circ_023015 novel_circ_023017 novel_circ_023018 novel_circ_023020 novel_circ_023021 novel_circ_023022 novel_circ_023023 novel_circ_023024 novel_circ_023025 novel_circ_023026 novel_circ_023027 novel_circ_023028 novel_circ_023029 novel_circ_023035 novel_circ_023041 novel_circ_023042 novel_circ_023043 novel_circ_023044 novel_circ_023046 novel_circ_023047 novel_circ_023051 novel_circ_023052 novel_circ_023053

| hsa_circ_0075095 | 87 | 304 | 979 | 1 | 679 | 0 | 0 |
| --- | --- | --- | --- | --- | --- | --- | --- |
| hsa_circ_0001557 | 86 | 1 | 436 | 1 | 439 | 0.01 | 2.00E-129 |
| hsa_circ_0075095 | 87 | 71 | 661 | 1 | 594 | 0.01 | 0 |
| hsa_circ_0001557 | 84 | 1 | 279 | 1 | 282 | 0.02 | 2.00E-72 |
| hsa_circ_0075095 | 86 | 1 | 434 | 1 | 437 | 0.01 | 2.00E-133 |
| hsa_circ_0002171 | 83 | 747 | 1398 | 1 | 685 | 0.05 | 5.00E-167 |
| hsa_circ_0075073 | 90 | 172 | 610 | 1 | 439 | 0 | 9.00E-159 |
| hsa_circ_0075069 | 96 | 107 | 498 | 25 | 416 | 0 | 2.00E-179 |
| hsa_circ_0075069 | 96 | 264 | 655 | 25 | 416 | 0 | 3.00E-179 |
| hsa_circ_0008042 | 96 | 1 | 106 | 1 | 106 | 0 | 1.00E-42 |
| hsa_circ_0008042 | 89 | 1 | 162 | 1 | 174 | 0.07 | 4.00E-49 |
| hsa_circ_0075061 | 94 | 1 | 385 | 1414 | 1798 | 0 | 5.00E-164 |
| hsa_circ_0075061 | 94 | 1 | 1213 | 201 | 1413 | 0 | 0 |
| hsa_circ_0075061 | 93 | 7 | 1402 | 11 | 1413 | 0.01 | 0 |
| hsa_circ_0064635 | 97 | 7365 | 7607 | 88 | 330 | 0 | 4.00E-112 |
| hsa_circ_0064654 | 90 | 76 | 435 | 1 | 360 | 0 | 5.00E-130 |
| hsa_circ_0064654 | 90 | 1 | 360 | 1 | 360 | 0 | 4.00E-130 |
| hsa_circ_0064654 | 91 | 1 | 1296 | 1 | 1302 | 0.01 | 0 |
| hsa_circ_0064662 | 96 | 1 | 397 | 1 | 397 | 0 | 0 |
| hsa_circ_0064662 | 96 | 1 | 1873 | 1 | 1873 | 0 | 0 |
| hsa_circ_0064662 | 96 | 1 | 904 | 110 | 1013 | 0 | 0 |
| hsa_circ_0064662 | 97 | 1 | 450 | 564 | 1013 | 0 | 0 |
| hsa_circ_0064662 | 95 | 1 | 1310 | 564 | 1873 | 0 | 0 |
| hsa_circ_0064662 | 95 | 1 | 1523 | 564 | 2086 | 0 | 0 |
| hsa_circ_0064662 | 95 | 1 | 1277 | 810 | 2086 | 0 | 0 |
| hsa_circ_0064662 | 95 | 1 | 1015 | 859 | 1873 | 0 | 0 |
| hsa_circ_0064662 | 93 | 1 | 400 | 1014 | 1413 | 0 | 3.00E-162 |
| hsa_circ_0064662 | 95 | 1 | 648 | 1226 | 1873 | 0 | 0 |
| hsa_circ_0064662 | 95 | 1 | 861 | 1226 | 2086 | 0 | 0 |
| hsa_circ_0064662 | 96 | 1 | 460 | 1414 | 1873 | 0 | 0 |
| hsa_circ_0064662 | 95 | 1 | 673 | 1414 | 2086 | 0 | 0 |
| hsa_circ_0064673 | 92 | 1 | 442 | 964 | 517 | 0.01 | 1.00E-175 |
| hsa_circ_0064675 | 90 | 179 | 571 | 1 | 393 | 0 | 1.00E-141 |
| hsa_circ_0064675 | 90 | 1 | 393 | 1 | 393 | 0 | 1.00E-141 |
| hsa_circ_0064687 | 96 | 1 | 342 | 342 | 1 | 0 | 2.00E-158 |
| hsa_circ_0064687 | 88 | 1 | 518 | 860 | 343 | 0 | 9.00E-178 |
| hsa_circ_0064687 | 89 | 1 | 347 | 860 | 514 | 0 | 3.00E-121 |
| hsa_circ_0064695 | 92 | 1 | 543 | 1 | 543 | 0 | 0 |
| hsa_circ_0064695 | 92 | 1 | 745 | 1 | 745 | 0 | 0 |
| hsa_circ_0064696 | 90 | 1 | 583 | 1 | 583 | 0 | 0 |
| hsa_circ_0064693 | 89 | 1 | 1235 | 258 | 1492 | 0 | 0 |
| hsa_circ_0064693 | 89 | 1 | 854 | 639 | 1492 | 0 | 0 |
| hsa_circ_0064693 | 88 | 5 | 502 | 995 | 1492 | 0 | 6.00E-165 |
| hsa_circ_0064698 | 92 | 1 | 380 | 1 | 380 | 0 | 2.00E-149 |
| hsa_circ_0030357 | 79 | 25136 | 26075 | 1820 | 837 | 0.05 | 6.00E-176 |
| hsa_circ_0083371 | 86 | 3 | 967 | 1124 | 160 | 0 | 0 |
| hsa_circ_0083371 | 87 | 3 | 1126 | 1124 | 1 | 0 | 0 |
| hsa_circ_0083371 | 87 | 3 | 1126 | 1124 | 1 | 0 | 0 |
| hsa_circ_0083371 | 87 | 3 | 1126 | 1124 | 1 | 0 | 0 |
| hsa_circ_0083371 | 87 | 1 | 884 | 884 | 1 | 0 | 0 |
| hsa_circ_0083371 | 87 | 1 | 884 | 884 | 1 | 0 | 0 |
| hsa_circ_0006762 | 93 | 1 | 659 | 659 | 1 | 0 | 0 |
| hsa_circ_0006762 | 93 | 353 | 1011 | 659 | 1 | 0 | 0 |
| hsa_circ_0006762 | 93 | 1 | 659 | 659 | 1 | 0 | 0 |

novel_circ_023054 novel_circ_023055 novel_circ_023056 novel_circ_023057 novel_circ_023058 novel_circ_023059 novel_circ_023060 novel_circ_023061 novel_circ_023062 novel_circ_023063 novel_circ_023064 novel_circ_023065 novel_circ_023066 novel_circ_023067 novel_circ_023068 novel_circ_023069 novel_circ_023070 novel_circ_023071 novel_circ_023073 novel_circ_023074 novel_circ_023075 novel_circ_023076 novel_circ_023077 novel_circ_023078 novel_circ_023079 novel_circ_023080 novel_circ_023081 novel_circ_023082 novel_circ_023083 novel_circ_023084 novel_circ_023085 novel_circ_023086 novel_circ_023088 novel_circ_023089 novel_circ_023090 novel_circ_023091 novel_circ_023092 novel_circ_023094 novel_circ_023095 novel_circ_023096 novel_circ_023097 novel_circ_023098 novel_circ_023099 novel_circ_023100 novel_circ_023101 novel_circ_023102 novel_circ_023103 novel_circ_023104 novel_circ_023105 novel_circ_023106 novel_circ_023107 novel_circ_023108 novel_circ_023109 novel_circ_023110

| hsa_circ_0030402 | 91 | 4 | 426 | 591 | 169 | 0 | 1.00E-161 |
| --- | --- | --- | --- | --- | --- | --- | --- |
| hsa_circ_0030402 | 90 | 4 | 352 | 591 | 243 | 0 | 2.00E-127 |
| hsa_circ_0008506 | 96 | 7 | 551 | 7 | 551 | 0 | 0 |
| hsa_circ_0008506 | 95 | 7 | 642 | 7 | 642 | 0 | 0 |
| hsa_circ_0008506 | 92 | 7 | 738 | 7 | 717 | 0.03 | 0 |
| hsa_circ_0054504 | 92 | 7 | 807 | 149 | 928 | 0.03 | 0 |
| hsa_circ_0008506 | 96 | 4 | 548 | 7 | 551 | 0 | 0 |
| hsa_circ_0054510 | 94 | 253 | 1028 | 104 | 879 | 0 | 0 |
| hsa_circ_0054512 | 96 | 1 | 249 | 1 | 249 | 0 | 1.00E-113 |
| hsa_circ_0054512 | 95 | 1 | 568 | 1 | 568 | 0 | 0 |
| hsa_circ_0054512 | 94 | 1 | 771 | 1 | 771 | 0 | 0 |
| hsa_circ_0054512 | 94 | 1 | 1099 | 1 | 1099 | 0 | 0 |
| hsa_circ_0054510 | 94 | 1 | 1192 | 109 | 1300 | 0 | 0 |
| hsa_circ_0054510 | 93 | 1 | 1808 | 109 | 1916 | 0 | 0 |
| hsa_circ_0054512 | 93 | 1 | 624 | 148 | 771 | 0 | 0 |
| hsa_circ_0054512 | 92 | 1 | 522 | 250 | 771 | 0 | 0 |
| hsa_circ_0054512 | 94 | 1 | 850 | 250 | 1099 | 0 | 0 |
| hsa_circ_0054512 | 92 | 2 | 298 | 475 | 771 | 0 | 3.00E-115 |
| hsa_circ_0054519 | 93 | 1 | 373 | 231 | 603 | 0 | 2.00E-152 |
| hsa_circ_0054522 | 88 | 23 | 2177 | 20 | 2180 | 0.01 | 0 |
| hsa_circ_0022702 | 89 | 1 | 90 | 90 | 1 | 0 | 3.00E-23 |
| hsa_circ_0022702 | 82 | 25 | 262 | 238 | 1 | 0.01 | 1.00E-48 |
| hsa_circ_0054524 | 94 | 1 | 571 | 573 | 3 | 0 | 0 |
| hsa_circ_0054524 | 94 | 1 | 694 | 696 | 3 | 0 | 0 |
| hsa_circ_0054524 | 94 | 1055 | 1748 | 696 | 3 | 0 | 0 |
| hsa_circ_0054524 | 94 | 1 | 571 | 573 | 3 | 0 | 0 |
| hsa_circ_0054524 | 94 | 1 | 694 | 696 | 3 | 0 | 0 |
| hsa_circ_0054524 | 94 | 232 | 925 | 696 | 3 | 0 | 0 |
| hsa_circ_0054524 | 94 | 1055 | 1748 | 696 | 3 | 0 | 0 |
| hsa_circ_0054524 | 94 | 1 | 571 | 573 | 3 | 0 | 0 |
| hsa_circ_0054524 | 94 | 1 | 694 | 696 | 3 | 0 | 0 |
| hsa_circ_0054524 | 94 | 232 | 925 | 696 | 3 | 0 | 0 |
| hsa_circ_0054525 | 99 | 1 | 162 | 162 | 1 | 0 | 2.00E-76 |
| hsa_circ_0054525 | 98 | 1 | 509 | 509 | 1 | 0 | 0 |
| hsa_circ_0054525 | 98 | 1 | 509 | 509 | 1 | 0 | 0 |
| hsa_circ_0001004 | 100 | 1 | 33 | 819 | 787 | 0 | 1.00E-09 |
| hsa_circ_0001004 | 98 | 1 | 51 | 837 | 787 | 0 | 1.00E-17 |
| hsa_circ_0001004 | 86 | 1 | 763 | 789 | 13 | 0.02 | 0 |
| hsa_circ_0002132 | 85 | 10364 | 10457 | 34463 | 34367 | 0.03 | 5.00E-16 |
| hsa_circ_0054527 | 89 | 1 | 765 | 765 | 1 | 0 | 0 |
| hsa_circ_0054527 | 87 | 1 | 389 | 389 | 1 | 0 | 3.00E-121 |
| hsa_circ_0054527 | 88 | 1 | 587 | 587 | 1 | 0 | 0 |
| hsa_circ_0054527 | 89 | 7 | 706 | 700 | 1 | 0 | 0 |
| hsa_circ_0054527 | 89 | 1 | 765 | 765 | 1 | 0 | 0 |
| hsa_circ_0054527 | 90 | 3 | 901 | 899 | 1 | 0 | 0 |
| hsa_circ_0054527 | 90 | 1 | 1014 | 1014 | 1 | 0 | 0 |
| hsa_circ_0054527 | 90 | 1 | 1173 | 1173 | 1 | 0 | 0 |
| hsa_circ_0054527 | 90 | 13 | 1382 | 1370 | 1 | 0 | 0 |
| hsa_circ_0054527 | 89 | 1 | 635 | 765 | 131 | 0 | 0 |
| hsa_circ_0054528 | 87 | 1 | 258 | 258 | 1 | 0 | 7.00E-77 |
| hsa_circ_0054528 | 89 | 1 | 634 | 634 | 1 | 0 | 0 |
| hsa_circ_0054527 | 90 | 1 | 883 | 1014 | 132 | 0 | 0 |
| hsa_circ_0054527 | 90 | 1 | 1042 | 1173 | 132 | 0 | 0 |
| hsa_circ_0054527 | 90 | 13 | 1251 | 1370 | 132 | 0 | 0 |

novel_circ_023111 novel_circ_023112 novel_circ_023113 novel_circ_023114 novel_circ_023115 novel_circ_023116 novel_circ_023117 novel_circ_023118 novel_circ_023119 novel_circ_023122 novel_circ_023123 novel_circ_023124 novel_circ_023126 novel_circ_023127 novel_circ_023128 novel_circ_023129 novel_circ_023130 novel_circ_023131 novel_circ_023132 novel_circ_023133 novel_circ_023134 novel_circ_023135 novel_circ_023136 novel_circ_023137 novel_circ_023138 novel_circ_023139 novel_circ_023140 novel_circ_023141 novel_circ_023142 novel_circ_023143 novel_circ_023144 novel_circ_023145 novel_circ_023146 novel_circ_023148 novel_circ_023149 novel_circ_023150 novel_circ_023151 novel_circ_023152 novel_circ_023153 novel_circ_023154 novel_circ_023155 novel_circ_023156 novel_circ_023157 novel_circ_023158 novel_circ_023159 novel_circ_023160 novel_circ_023161 novel_circ_023162 novel_circ_023163 novel_circ_023164 novel_circ_023165 novel_circ_023166 novel_circ_023167 novel_circ_023168

| hsa_circ_0054530 | 91 | 1 | 375 | 376 | 2 | 0 | 4.00E-145 |
| --- | --- | --- | --- | --- | --- | --- | --- |
| hsa_circ_0054530 | 92 | 1 | 624 | 625 | 2 | 0 | 0 |
| hsa_circ_0054530 | 91 | 1 | 783 | 784 | 2 | 0.01 | 0 |
| hsa_circ_0054530 | 92 | 13 | 992 | 981 | 2 | 0 | 0 |
| hsa_circ_0054530 | 92 | 13 | 615 | 981 | 379 | 0.01 | 0 |
| hsa_circ_0054530 | 92 | 13 | 481 | 981 | 513 | 0.01 | 0 |
| hsa_circ_0002416 | 91 | 13 | 368 | 356 | 1 | 0.01 | 7.00E-133 |
| hsa_circ_0054532 | 96 | 1 | 264 | 1 | 264 | 0 | 9.00E-120 |
| hsa_circ_0054535 | 94 | 612 | 736 | 1 | 125 | 0 | 8.00E-46 |
| hsa_circ_0054535 | 94 | 577 | 701 | 1 | 125 | 0 | 7.00E-46 |
| hsa_circ_0054535 | 94 | 577 | 855 | 1 | 279 | 0 | 1.00E-114 |
| hsa_circ_0054535 | 94 | 353 | 477 | 1 | 125 | 0 | 5.00E-46 |
| hsa_circ_0054535 | 94 | 223 | 501 | 1 | 279 | 0 | 6.00E-115 |
| hsa_circ_0054535 | 94 | 1 | 154 | 126 | 279 | 0 | 2.00E-59 |
| hsa_circ_0054542 | 95 | 1 | 590 | 592 | 3 | 0 | 0 |
| hsa_circ_0054542 | 95 | 1 | 719 | 721 | 3 | 0 | 0 |
| hsa_circ_0054542 | 93 | 1 | 1337 | 1345 | 3 | 0.01 | 0 |
| hsa_circ_0054542 | 93 | 1 | 1922 | 1930 | 3 | 0.01 | 0 |
| hsa_circ_0054542 | 95 | 1 | 297 | 436 | 140 | 0 | 7.00E-132 |
| hsa_circ_0005229 | 95 | 1 | 285 | 285 | 1 | 0 | 1.00E-123 |
| hsa_circ_0005229 | 95 | 1 | 371 | 371 | 1 | 0 | 1.00E-164 |
| hsa_circ_0054542 | 92 | 1 | 903 | 1345 | 437 | 0.01 | 0 |
| hsa_circ_0054542 | 92 | 1 | 747 | 1345 | 593 | 0.02 | 0 |
| hsa_circ_0054546 | 91 | 1 | 532 | 538 | 1 | 0.02 | 0 |
| hsa_circ_0054546 | 93 | 1 | 503 | 1041 | 539 | 0 | 0 |
| hsa_circ_0054546 | 93 | 1 | 585 | 1123 | 539 | 0 | 0 |
| hsa_circ_0054542 | 92 | 1 | 906 | 2251 | 1346 | 0 | 0 |
| hsa_circ_0054546 | 93 | 1 | 432 | 1123 | 692 | 0 | 1.00E-176 |
| hsa_circ_0054542 | 92 | 1 | 753 | 2251 | 1499 | 0 | 0 |
| hsa_circ_0054548 | 92 | 1 | 403 | 557 | 155 | 0 | 3.00E-157 |
| hsa_circ_0054548 | 91 | 1 | 613 | 767 | 155 | 0 | 0 |
| hsa_circ_0054549 | 93 | 1 | 364 | 1056 | 693 | 0 | 5.00E-149 |
| hsa_circ_0054557 | 95 | 2 | 258 | 591 | 335 | 0 | 5.00E-113 |
| hsa_circ_0054559 | 95 | 1 | 195 | 1 | 195 | 0 | 5.00E-82 |
| hsa_circ_0054560 | 84 | 41224 | 41722 | 167 | 646 | 0.06 | 5.00E-123 |
| hsa_circ_0054571 | 94 | 1 | 441 | 758 | 1198 | 0 | 0 |
| hsa_circ_0054587 | 96 | 1 | 650 | 248 | 897 | 0 | 0 |
| hsa_circ_0054587 | 95 | 1 | 1247 | 248 | 1494 | 0 | 0 |
| hsa_circ_0054587 | 95 | 1 | 1457 | 248 | 1704 | 0 | 0 |
| hsa_circ_0054587 | 96 | 1 | 490 | 408 | 897 | 0 | 0 |
| hsa_circ_0054587 | 95 | 1 | 1087 | 408 | 1494 | 0 | 0 |
| hsa_circ_0054587 | 97 | 1 | 391 | 507 | 897 | 0 | 0 |
| hsa_circ_0054589 | 94 | 1 | 807 | 1 | 807 | 0 | 0 |
| hsa_circ_0054590 | 93 | 1 | 395 | 1 | 395 | 0 | 7.00E-163 |
| hsa_circ_0054590 | 93 | 1 | 883 | 1 | 883 | 0 | 0 |
| hsa_circ_0054590 | 93 | 1 | 467 | 139 | 605 | 0 | 0 |
| hsa_circ_0054590 | 94 | 1 | 745 | 139 | 883 | 0 | 0 |
| hsa_circ_0054590 | 94 | 1 | 1953 | 1003 | 2955 | 0 | 0 |
| hsa_circ_0054590 | 95 | 3 | 398 | 1298 | 1693 | 0 | 2.00E-178 |
| hsa_circ_0054590 | 95 | 3 | 638 | 1298 | 1933 | 0 | 0 |
| hsa_circ_0054590 | 94 | 3 | 1065 | 1298 | 2360 | 0 | 0 |
| hsa_circ_0054590 | 94 | 3 | 1331 | 1298 | 2626 | 0 | 0 |
| hsa_circ_0054590 | 94 | 3 | 1660 | 1298 | 2955 | 0 | 0 |
| hsa_circ_0054591 | 93 | 1 | 693 | 241 | 933 | 0 | 0 |

novel_circ_023169 novel_circ_023170 novel_circ_023171 novel_circ_023172 novel_circ_023176 novel_circ_023177 novel_circ_023178 novel_circ_023179 novel_circ_023180 novel_circ_023181 novel_circ_023182 novel_circ_023183 novel_circ_023184 novel_circ_023185 novel_circ_023186 novel_circ_023187 novel_circ_023188 novel_circ_023190 novel_circ_023191 novel_circ_023192 novel_circ_023193 novel_circ_023194 novel_circ_023195 novel_circ_023196 novel_circ_023197 novel_circ_023198 novel_circ_023199 novel_circ_023200 novel_circ_023201 novel_circ_023202 novel_circ_023203 novel_circ_023204 novel_circ_023205 novel_circ_023206 novel_circ_023207 novel_circ_023208 novel_circ_023209 novel_circ_023210 novel_circ_023211 novel_circ_023212 novel_circ_023213 novel_circ_023215 novel_circ_023216 novel_circ_023217 novel_circ_023218 novel_circ_023220 novel_circ_023222 novel_circ_023223 novel_circ_023224 novel_circ_023226 novel_circ_023227 novel_circ_023228 novel_circ_023229 novel_circ_023230

| hsa_circ_0054591 | 92 | 1 | 506 | 757 | 1262 | 0 | 0 |
| --- | --- | --- | --- | --- | --- | --- | --- |
| hsa_circ_0054592 | 92 | 1 | 329 | 1 | 329 | 0 | 3.00E-131 |
| hsa_circ_0054595 | 95 | 1 | 347 | 347 | 1 | 0 | 6.00E-153 |
| hsa_circ_0054598 | 88 | 1 | 2451 | 2457 | 1 | 0.01 | 0 |
| hsa_circ_0054606 | 89 | 108 | 1229 | 1321 | 206 | 0.02 | 0 |
| hsa_circ_0054611 | 95 | 1 | 1423 | 2327 | 908 | 0 | 0 |
| hsa_circ_0054611 | 95 | 1 | 1063 | 2327 | 1265 | 0 | 0 |
| hsa_circ_0054614 | 94 | 3 | 333 | 1077 | 747 | 0 | 5.00E-139 |
| hsa_circ_0054611 | 94 | 1 | 755 | 2327 | 1573 | 0 | 0 |
| hsa_circ_0054611 | 93 | 1 | 609 | 2327 | 1719 | 0 | 0 |
| hsa_circ_0054611 | 94 | 1 | 422 | 2327 | 1906 | 0 | 0 |
| hsa_circ_0054616 | 95 | 1 | 597 | 597 | 1 | 0 | 0 |
| hsa_circ_0054617 | 95 | 1 | 435 | 435 | 1 | 0 | 0 |
| hsa_circ_0054617 | 95 | 127 | 928 | 802 | 1 | 0 | 0 |
| hsa_circ_0054617 | 93 | 1 | 306 | 2280 | 1975 | 0 | 7.00E-127 |
| hsa_circ_0054611 | 94 | 1 | 561 | 5021 | 4461 | 0 | 0 |
| hsa_circ_0054623 | 97 | 311 | 535 | 398 | 174 | 0 | 3.00E-103 |
| hsa_circ_0054628 | 91 | 1 | 723 | 689 | 1 | 0.05 | 0 |
| hsa_circ_0054628 | 92 | 103 | 1027 | 891 | 1 | 0.04 | 0 |
| hsa_circ_0054628 | 97 | 1 | 495 | 689 | 195 | 0 | 0 |
| hsa_circ_0054628 | 98 | 1 | 654 | 848 | 195 | 0 | 0 |
| hsa_circ_0054628 | 97 | 547 | 1243 | 891 | 195 | 0 | 0 |
| hsa_circ_0054629 | 97 | 103 | 474 | 512 | 141 | 0 | 2.00E-178 |
| hsa_circ_0054628 | 96 | 1 | 471 | 1455 | 985 | 0 | 0 |
| hsa_circ_0054628 | 97 | 1 | 549 | 1533 | 985 | 0 | 0 |
| hsa_circ_0054628 | 96 | 1 | 469 | 1455 | 987 | 0 | 0 |
| hsa_circ_0054628 | 97 | 1 | 547 | 1533 | 987 | 0 | 0 |
| hsa_circ_0054628 | 96 | 4 | 1171 | 2154 | 987 | 0 | 0 |
| hsa_circ_0001007 | 97 | 1 | 443 | 444 | 2 | 0 | 0 |
| hsa_circ_0054631 | 97 | 1 | 312 | 312 | 1 | 0 | 2.00E-148 |
| hsa_circ_0054628 | 97 | 2176 | 2490 | 1536 | 1222 | 0 | 3.00E-149 |
| hsa_circ_0054628 | 95 | 4 | 936 | 2154 | 1222 | 0 | 0 |
| hsa_circ_0054628 | 95 | 1 | 1035 | 2256 | 1222 | 0 | 0 |
| hsa_circ_0054628 | 95 | 4 | 624 | 2154 | 1534 | 0 | 0 |
| hsa_circ_0054628 | 94 | 1 | 723 | 2256 | 1534 | 0 | 0 |
| hsa_circ_0054628 | 94 | 57 | 779 | 2256 | 1534 | 0 | 0 |
| hsa_circ_0054633 | 83 | 3 | 1432 | 3029 | 1578 | 0.04 | 0 |
| hsa_circ_0054641 | 89 | 3 | 560 | 626 | 57 | 0.02 | 0 |
| hsa_circ_0054649 | 95 | 1 | 200 | 271 | 72 | 0 | 4.00E-83 |
| hsa_circ_0054649 | 93 | 1 | 497 | 568 | 72 | 0 | 0 |
| hsa_circ_0054654 | 96 | 1 | 320 | 320 | 1 | 0 | 1.00E-144 |
| hsa_circ_0054666 | 92 | 137 | 313 | 4 | 180 | 0 | 6.00E-65 |
| hsa_circ_0054666 | 92 | 137 | 313 | 4 | 180 | 0 | 8.00E-65 |
| hsa_circ_0004435 | 91 | 1 | 166 | 166 | 1 | 0 | 1.00E-57 |
| hsa_circ_0004435 | 94 | 181 | 507 | 327 | 1 | 0 | 3.00E-138 |
| hsa_circ_0054680 | 95 | 20 | 231 | 83 | 294 | 0 | 6.00E-89 |
| hsa_circ_0045116 | 98 | 1 | 282 | 1 | 282 | 0 | 3.00E-135 |
| hsa_circ_0045106 | 90 | 1 | 247 | 1 | 247 | 0 | 8.00E-86 |
| hsa_circ_0045103 | 86 | 1 | 687 | 93 | 806 | 0.04 | 0 |
| hsa_circ_0044229 | 95 | 1 | 1046 | 1542 | 482 | 0.02 | 0 |
| hsa_circ_0044232 | 97 | 1 | 854 | 1085 | 232 | 0 | 0 |
| hsa_circ_0044232 | 95 | 1 | 970 | 1085 | 101 | 0.02 | 0 |
| hsa_circ_0044232 | 95 | 2 | 822 | 936 | 101 | 0.03 | 0 |
| hsa_circ_0044232 | 95 | 2 | 922 | 936 | 1 | 0.02 | 0 |

novel_circ_023231 novel_circ_023232 novel_circ_023233 novel_circ_023234 novel_circ_023235 novel_circ_023236 novel_circ_023237 novel_circ_023238 novel_circ_023239 novel_circ_023240 novel_circ_023241 novel_circ_023242 novel_circ_023243 novel_circ_023244 novel_circ_023245 novel_circ_023246 novel_circ_023247 novel_circ_023248 novel_circ_023249 novel_circ_023250 novel_circ_023251 novel_circ_023252 novel_circ_023253 novel_circ_023254 novel_circ_023255 novel_circ_023256 novel_circ_023257 novel_circ_023258 novel_circ_023259 novel_circ_023260 novel_circ_023261 novel_circ_023262 novel_circ_023263 novel_circ_023264 novel_circ_023265 novel_circ_023266 novel_circ_023267 novel_circ_023268 novel_circ_023269 novel_circ_023270 novel_circ_023271 novel_circ_023272 novel_circ_023273 novel_circ_023274 novel_circ_023275 novel_circ_023276 novel_circ_023277 novel_circ_023278 novel_circ_023279 novel_circ_023280 novel_circ_023281 novel_circ_023282 novel_circ_023283 novel_circ_023285

| hsa_circ_0044233 | 97 | 1 | 465 | 465 | 1 | 0 | 0 |
| --- | --- | --- | --- | --- | --- | --- | --- |
| hsa_circ_0002502 | 97 | 1 | 580 | 580 | 1 | 0 | 0 |
| hsa_circ_0044232 | 94 | 1 | 696 | 811 | 101 | 0.03 | 0 |
| hsa_circ_0044227 | 96 | 1 | 843 | 843 | 1 | 0 | 0 |
| hsa_circ_0044229 | 97 | 1 | 381 | 381 | 1 | 0 | 0 |
| hsa_circ_0044227 | 96 | 1 | 743 | 743 | 1 | 0 | 0 |
| hsa_circ_0044222 | 96 | 1 | 688 | 3761 | 3074 | 0 | 0 |
| hsa_circ_0046624 | 96 | 1 | 1372 | 1 | 1378 | 0 | 0 |
| hsa_circ_0044191 | 94 | 1 | 1487 | 77673 | 76184 | 0.01 | 0 |
| hsa_circ_0046624 | 96 | 1 | 1514 | 1 | 1520 | 0 | 0 |
| hsa_circ_0046630 | 96 | 1 | 244 | 1 | 244 | 0 | 8.00E-111 |
| hsa_circ_0046630 | 94 | 1 | 556 | 1 | 559 | 0.01 | 0 |
| hsa_circ_0046630 | 94 | 1 | 728 | 1 | 731 | 0 | 0 |
| hsa_circ_0046630 | 94 | 1 | 728 | 1 | 731 | 0 | 0 |
| hsa_circ_0046633 | 94 | 1 | 414 | 1 | 417 | 0.01 | 1.00E-175 |
| hsa_circ_0046633 | 94 | 1 | 586 | 1 | 589 | 0.01 | 0 |
| hsa_circ_0046633 | 94 | 1 | 586 | 1 | 589 | 0.01 | 0 |
| hsa_circ_0046634 | 93 | 1 | 312 | 1 | 315 | 0.01 | 1.00E-125 |
| hsa_circ_0046633 | 93 | 1 | 484 | 103 | 589 | 0.01 | 0 |
| hsa_circ_0046633 | 93 | 1 | 484 | 103 | 589 | 0.01 | 0 |
| hsa_circ_0046637 | 93 | 1 | 520 | 1 | 520 | 0.01 | 0 |
| hsa_circ_0046630 | 93 | 1 | 637 | 732 | 1371 | 0.01 | 0 |
| hsa_circ_0006625 | 94 | 1 | 177 | 162177 | 162353 | 0 | 9.00E-72 |
| hsa_circ_0006625 | 94 | 1 | 177 | 162177 | 162353 | 0 | 1.00E-71 |
| hsa_circ_0006625 | 94 | 507 | 707 | 199214 | 199414 | 0 | 9.00E-80 |
| hsa_circ_0046611 | 93 | 708 | 1136 | 429 | 1 | 0 | 1.00E-179 |
| hsa_circ_0046611 | 93 | 708 | 1136 | 429 | 1 | 0 | 2.00E-179 |
| hsa_circ_0046611 | 93 | 708 | 1136 | 429 | 1 | 0 | 3.00E-179 |
| hsa_circ_0006625 | 94 | 181 | 340 | 167901 | 168060 | 0 | 5.00E-64 |
| hsa_circ_0006625 | 94 | 340 | 540 | 199214 | 199414 | 0 | 7.00E-80 |
| hsa_circ_0046611 | 93 | 201 | 629 | 429 | 1 | 0 | 7.00E-180 |
| hsa_circ_0046611 | 93 | 201 | 629 | 429 | 1 | 0 | 1.00E-179 |
| hsa_circ_0046611 | 93 | 201 | 629 | 429 | 1 | 0 | 3.00E-179 |
| hsa_circ_0046611 | 93 | 201 | 629 | 429 | 1 | 0 | 1.00E-179 |
| hsa_circ_0046611 | 93 | 1 | 429 | 429 | 1 | 0 | 5.00E-180 |
| hsa_circ_0046611 | 93 | 1 | 429 | 429 | 1 | 0 | 9.00E-180 |
| hsa_circ_0046611 | 93 | 1 | 429 | 429 | 1 | 0 | 3.00E-179 |
| hsa_circ_0003466 | 91 | 97 | 220 | 18061 | 18184 | 0 | 1.00E-40 |
| hsa_circ_0003466 | 86 | 1212 | 1793 | 20505 | 21084 | 0.04 | 7.00E-179 |
| hsa_circ_0044217 | 92 | 1 | 383 | 66 | 448 | 0 | 2.00E-149 |
| hsa_circ_0021765 | 93 | 95 | 624 | 1 | 530 | 0 | 0 |
| hsa_circ_0021765 | 93 | 95 | 1261 | 1 | 1167 | 0 | 0 |
| hsa_circ_0021765 | 93 | 1 | 530 | 1 | 530 | 0 | 0 |
| hsa_circ_0021767 | 94 | 1 | 333 | 196 | 528 | 0 | 8.00E-142 |
| hsa_circ_0021767 | 93 | 1 | 404 | 196 | 599 | 0 | 2.00E-169 |
| hsa_circ_0021769 | 94 | 1 | 260 | 1 | 260 | 0 | 2.00E-109 |
| hsa_circ_0021769 | 94 | 1 | 260 | 1 | 260 | 0 | 2.00E-109 |
| hsa_circ_0021771 | 92 | 505 | 2092 | 1 | 1588 | 0 | 0 |
| hsa_circ_0021771 | 92 | 505 | 2092 | 1 | 1588 | 0 | 0 |
| hsa_circ_0021779 | 89 | 172 | 585 | 188 | 601 | 0 | 3.00E-143 |
| hsa_circ_0021779 | 91 | 172 | 950 | 188 | 966 | 0 | 0 |
| hsa_circ_0021779 | 90 | 1 | 535 | 188 | 722 | 0 | 0 |
| hsa_circ_0021779 | 91 | 1 | 779 | 188 | 966 | 0 | 0 |
| hsa_circ_0091590 | 82 | 77 | 721 | 74 | 703 | 0.05 | 3.00E-142 |

novel_circ_023286 novel_circ_023287 novel_circ_023288 novel_circ_023289 novel_circ_023290 novel_circ_023291 novel_circ_023292 novel_circ_023293 novel_circ_023294 novel_circ_023295 novel_circ_023296 novel_circ_023298 novel_circ_023299 novel_circ_023300 novel_circ_023301 novel_circ_023302 novel_circ_023303 novel_circ_023304 novel_circ_023305 novel_circ_023306 novel_circ_023307 novel_circ_023308 novel_circ_023309 novel_circ_023310 novel_circ_023311 novel_circ_023312 novel_circ_023313 novel_circ_023314 novel_circ_023315 novel_circ_023316 novel_circ_023317 novel_circ_023318 novel_circ_023319 novel_circ_023320 novel_circ_023321 novel_circ_023324 novel_circ_023325 novel_circ_023326 novel_circ_023328 novel_circ_023329 novel_circ_023330 novel_circ_023331 novel_circ_023332 novel_circ_023333 novel_circ_023334 novel_circ_023335 novel_circ_023336 novel_circ_023337 novel_circ_023338 novel_circ_023339 novel_circ_023340 novel_circ_023341 novel_circ_023342 novel_circ_023346

| hsa_circ_0021788 | 86 | 12 | 726 | 393 | 1112 | 0.01 | 0 |
| --- | --- | --- | --- | --- | --- | --- | --- |
| hsa_circ_0015894 | 96 | 5132 | 5358 | 137 | 363 | 0 | 2.00E-98 |
| hsa_circ_0015894 | 92 | 1 | 437 | 367 | 803 | 0 | 8.00E-173 |
| hsa_circ_0015911 | 94 | 1 | 198 | 1 | 198 | 0 | 5.00E-82 |
| hsa_circ_0015920 | 95 | 3 | 351 | 3 | 351 | 0 | 1.00E-155 |
| hsa_circ_0015925 | 95 | 1 | 176 | 1 | 176 | 0 | 3.00E-73 |
| hsa_circ_0015931 | 94 | 1 | 885 | 161 | 1045 | 0 | 0 |
| hsa_circ_0015938 | 94 | 1 | 733 | 1 | 733 | 0 | 0 |
| hsa_circ_0015939 | 95 | 1 | 407 | 1 | 407 | 0 | 0 |
| hsa_circ_0015939 | 95 | 1 | 634 | 1 | 634 | 0 | 0 |
| hsa_circ_0015947 | 94 | 1 | 301 | 555 | 855 | 0 | 4.00E-129 |
| hsa_circ_0015981 | 94 | 1 | 630 | 1 | 630 | 0 | 0 |
| hsa_circ_0015986 | 94 | 1 | 424 | 1 | 424 | 0 | 1.00E-180 |
| hsa_circ_0015986 | 93 | 1 | 719 | 1 | 719 | 0 | 0 |
| hsa_circ_0015986 | 95 | 1 | 305 | 120 | 424 | 0 | 1.00E-134 |
| hsa_circ_0015988 | 92 | 1 | 440 | 1 | 440 | 0 | 4.00E-176 |
| hsa_circ_0015989 | 92 | 1 | 526 | 1 | 526 | 0 | 0 |
| hsa_circ_0015989 | 94 | 1 | 209 | 318 | 526 | 0 | 3.00E-84 |
| hsa_circ_0015988 | 91 | 391 | 666 | 965 | 1240 | 0 | 8.00E-100 |
| hsa_circ_0015989 | 94 | 1 | 126 | 401 | 526 | 0 | 1.00E-46 |
| hsa_circ_0015988 | 91 | 308 | 583 | 965 | 1240 | 0 | 7.00E-100 |
| hsa_circ_0015991 | 89 | 1 | 819 | 1 | 819 | 0 | 0 |
| hsa_circ_0015991 | 89 | 1 | 819 | 1 | 819 | 0 | 0 |
| hsa_circ_0015992 | 88 | 1 | 545 | 92 | 636 | 0 | 2.00E-179 |
| hsa_circ_0015992 | 88 | 1 | 545 | 92 | 636 | 0 | 2.00E-178 |
| hsa_circ_0016022 | 92 | 1 | 622 | 773 | 152 | 0 | 0 |
| hsa_circ_0016030 | 92 | 1 | 315 | 315 | 1 | 0 | 2.00E-123 |
| hsa_circ_0016030 | 91 | 1 | 478 | 478 | 1 | 0 | 0 |
| hsa_circ_0016043 | 92 | 1 | 621 | 780 | 160 | 0 | 0 |
| hsa_circ_0016042 | 93 | 7 | 915 | 1250 | 342 | 0 | 0 |
| hsa_circ_0016047 | 94 | 7 | 526 | 789 | 270 | 0 | 0 |
| hsa_circ_0003126 | 94 | 4 | 365 | 369 | 8 | 0 | 3.00E-156 |
| hsa_circ_0016051 | 93 | 116 | 418 | 303 | 1 | 0 | 1.00E-121 |
| hsa_circ_0016052 | 91 | 1 | 263 | 265 | 3 | 0 | 5.00E-98 |
| hsa_circ_0016053 | 94 | 3 | 218 | 216 | 1 | 0 | 1.00E-86 |
| hsa_circ_0016063 | 94 | 1 | 450 | 1 | 450 | 0 | 0 |
| hsa_circ_0016063 | 94 | 1 | 450 | 1 | 450 | 0 | 0 |
| hsa_circ_0016063 | 95 | 1 | 274 | 177 | 450 | 0 | 2.00E-117 |
| hsa_circ_0004290 | 87 | 1 | 665 | 1 | 657 | 0.02 | 0 |
| hsa_circ_0016095 | 89 | 1 | 607 | 1 | 607 | 0 | 0 |
| hsa_circ_0016108 | 92 | 1 | 313 | 1 | 319 | 0.02 | 1.00E-120 |
| hsa_circ_0016108 | 92 | 1 | 641 | 1 | 647 | 0.01 | 0 |
| hsa_circ_0016106 | 92 | 1 | 837 | 424 | 1266 | 0.01 | 0 |
| hsa_circ_0016106 | 91 | 1 | 950 | 424 | 1379 | 0.01 | 0 |
| hsa_circ_0016106 | 92 | 1 | 1010 | 424 | 1439 | 0.01 | 0 |
| hsa_circ_0016106 | 92 | 1 | 1010 | 424 | 1439 | 0.01 | 0 |
| hsa_circ_0016109 | 89 | 438 | 2093 | 1 | 1641 | 0.01 | 0 |
| hsa_circ_0007684 | 91 | 1 | 799 | 1 | 799 | 0 | 0 |
| hsa_circ_0016109 | 91 | 1 | 675 | 1 | 675 | 0 | 0 |
| hsa_circ_0016109 | 89 | 1 | 1656 | 1 | 1641 | 0.01 | 0 |
| hsa_circ_0016110 | 91 | 1 | 309 | 1 | 309 | 0 | 2.00E-113 |
| hsa_circ_0016109 | 90 | 1 | 471 | 205 | 675 | 0 | 1.00E-176 |
| hsa_circ_0016109 | 88 | 1 | 1452 | 205 | 1641 | 0.01 | 0 |
| hsa_circ_0016111 | 88 | 8 | 912 | 434 | 1320 | 0.02 | 0 |

novel_circ_023347 novel_circ_023348 novel_circ_023349 novel_circ_023350 novel_circ_023351 novel_circ_023352 novel_circ_023353 novel_circ_023354 novel_circ_023355 novel_circ_023356 novel_circ_023362 novel_circ_023363 novel_circ_023364 novel_circ_023365 novel_circ_023366 novel_circ_023367 novel_circ_023369 novel_circ_023370 novel_circ_023371 novel_circ_023372 novel_circ_023373 novel_circ_023374 novel_circ_023375 novel_circ_023376 novel_circ_023377 novel_circ_023378 novel_circ_023379 novel_circ_023380 novel_circ_023381 novel_circ_023382 novel_circ_023383 novel_circ_023384 novel_circ_023385 novel_circ_023386 novel_circ_023391 novel_circ_023395 novel_circ_023396 novel_circ_023398 novel_circ_023399 novel_circ_023400 novel_circ_023405 novel_circ_023406 novel_circ_023409 novel_circ_023411 novel_circ_023412 novel_circ_023413 novel_circ_023415 novel_circ_023420 novel_circ_023422 novel_circ_023424 novel_circ_023425 novel_circ_023427 novel_circ_023429 novel_circ_023430

| hsa_circ_0016111 | 88 | 1 | 331 | 1005 | 1320 | 0.05 | 6.00E-103 |
| --- | --- | --- | --- | --- | --- | --- | --- |
| hsa_circ_0009157 | 85 | 1 | 311 | 1 | 332 | 0.06 | 4.00E-85 |
| hsa_circ_0016147 | 94 | 4 | 437 | 591 | 158 | 0 | 0 |
| hsa_circ_0016160 | 85 | 1 | 397 | 1 | 378 | 0.05 | 2.00E-108 |
| hsa_circ_0016160 | 86 | 1 | 559 | 1 | 540 | 0.03 | 6.00E-165 |
| hsa_circ_0016160 | 90 | 15 | 446 | 109 | 540 | 0 | 5.00E-155 |
| hsa_circ_0016162 | 90 | 1 | 427 | 1 | 427 | 0 | 6.00E-154 |
| hsa_circ_0016162 | 90 | 1 | 591 | 1 | 594 | 0.01 | 0 |
| hsa_circ_0016164 | 89 | 1 | 352 | 1 | 352 | 0 | 5.00E-124 |
| hsa_circ_0016166 | 93 | 1 | 231 | 1 | 231 | 0 | 3.00E-90 |
| hsa_circ_0016178 | 92 | 1 | 188 | 188 | 1 | 0 | 8.00E-70 |
| hsa_circ_0003527 | 96 | 174 | 477 | 304 | 1 | 0 | 4.00E-137 |
| hsa_circ_0016191 | 92 | 3 | 781 | 912 | 134 | 0 | 0 |
| hsa_circ_0016195 | 93 | 3 | 317 | 315 | 1 | 0 | 2.00E-128 |
| hsa_circ_0016200 | 91 | 4 | 670 | 667 | 1 | 0 | 0 |
| hsa_circ_0016200 | 92 | 1 | 1059 | 1059 | 1 | 0 | 0 |
| hsa_circ_0016212 | 89 | 1 | 993 | 990 | 1 | 0.01 | 0 |
| hsa_circ_0016212 | 90 | 1 | 1209 | 1206 | 1 | 0 | 0 |
| hsa_circ_0013815 | 93 | 1 | 193 | 562 | 754 | 0 | 6.00E-76 |
| hsa_circ_0013815 | 93 | 1 | 193 | 562 | 754 | 0 | 1.00E-75 |
| hsa_circ_0006162 | 96 | 1 | 163 | 4786 | 4948 | 0 | 5.00E-71 |
| hsa_circ_0016225 | 94 | 164 | 571 | 1 | 408 | 0 | 6.00E-175 |
| hsa_circ_0016228 | 93 | 1 | 345 | 1 | 345 | 0 | 8.00E-142 |
| hsa_circ_0016228 | 93 | 1 | 345 | 1 | 345 | 0 | 4.00E-141 |
| hsa_circ_0016228 | 92 | 1 | 570 | 1 | 570 | 0 | 0 |
| hsa_circ_0016228 | 93 | 1 | 869 | 1 | 869 | 0 | 0 |
| hsa_circ_0016228 | 93 | 1 | 1008 | 1 | 1008 | 0 | 0 |
| hsa_circ_0016228 | 93 | 1 | 1069 | 1 | 1069 | 0 | 0 |
| hsa_circ_0016228 | 94 | 1 | 1282 | 1 | 1282 | 0 | 0 |
| hsa_circ_0016228 | 94 | 1 | 1388 | 1 | 1388 | 0 | 0 |
| hsa_circ_0016228 | 96 | 1 | 199 | 871 | 1069 | 0 | 3.00E-89 |
| hsa_circ_0016228 | 97 | 1 | 518 | 871 | 1388 | 0 | 0 |
| hsa_circ_0016242 | 92 | 1 | 522 | 123 | 647 | 0.01 | 0 |
| hsa_circ_0016250 | 90 | 1 | 969 | 1087 | 122 | 0.01 | 0 |
| hsa_circ_0065366 | 90 | 157 | 223 | 344 | 278 | 0 | 1.00E-15 |
| hsa_circ_0002472 | 92 | 4 | 1451 | 1448 | 1 | 0 | 0 |
| hsa_circ_0016322 | 88 | 504 | 729 | 1233 | 1007 | 0.01 | 3.00E-70 |
| hsa_circ_0016334 | 95 | 1 | 381 | 1 | 381 | 0 | 9.00E-172 |
| hsa_circ_0016334 | 95 | 1 | 381 | 1 | 381 | 0 | 3.00E-171 |
| hsa_circ_0016334 | 93 | 1 | 216 | 166 | 381 | 0 | 1.00E-84 |
| hsa_circ_0010890 | 96 | 1 | 214 | 123 | 336 | 0 | 3.00E-94 |
| hsa_circ_0003757 | 86 | 1 | 353 | 353 | 1 | 0.01 | 1.00E-104 |
| hsa_circ_0010909 | 92 | 1 | 241 | 1 | 241 | 0 | 2.00E-92 |
| hsa_circ_0010915 | 92 | 288 | 1392 | 1 | 1108 | 0.01 | 0 |
| hsa_circ_0010915 | 91 | 117 | 907 | 1 | 794 | 0.01 | 0 |
| hsa_circ_0010915 | 91 | 1 | 791 | 1 | 794 | 0.01 | 0 |
| hsa_circ_0005609 | 96 | 1 | 343 | 1 | 343 | 0 | 1.00E-155 |
| hsa_circ_0010928 | 91 | 1327 | 1767 | 1 | 441 | 0 | 2.00E-169 |
| hsa_circ_0010928 | 91 | 1 | 441 | 1 | 441 | 0 | 5.00E-170 |
| hsa_circ_0010935 | 94 | 1 | 1071 | 1 | 1071 | 0 | 0 |
| hsa_circ_0010942 | 92 | 257 | 371 | 1 | 115 | 0 | 6.00E-39 |
| hsa_circ_0010961 | 93 | 458 | 551 | 420 | 327 | 0 | 2.00E-30 |
| hsa_circ_0010973 | 90 | 15 | 919 | 21 | 925 | 0 | 0 |
| hsa_circ_0010973 | 90 | 15 | 1357 | 21 | 1363 | 0 | 0 |

novel_circ_023431 novel_circ_023432 novel_circ_023433 novel_circ_023434 novel_circ_023435 novel_circ_023436 novel_circ_023437 novel_circ_023438 novel_circ_023439 novel_circ_023440 novel_circ_023441 novel_circ_023442 novel_circ_023443 novel_circ_023444 novel_circ_023445 novel_circ_023446 novel_circ_023448 novel_circ_023449 novel_circ_023451 novel_circ_023452 novel_circ_023460 novel_circ_023461 novel_circ_023462 novel_circ_023463 novel_circ_023464 novel_circ_023467 novel_circ_023468 novel_circ_023470 novel_circ_023471 novel_circ_023472 novel_circ_023473 novel_circ_023474 novel_circ_023475 novel_circ_023476 novel_circ_023477 novel_circ_023478 novel_circ_023479 novel_circ_023480 novel_circ_023481 novel_circ_023482 novel_circ_023483 novel_circ_023484 novel_circ_023485 novel_circ_023486 novel_circ_023487 novel_circ_023488 novel_circ_023489 novel_circ_023490 novel_circ_023491 novel_circ_023492 novel_circ_023493 novel_circ_023494 novel_circ_023495 novel_circ_023496

| hsa_circ_0010976 | 91 | 1 | 848 | 1 | 848 | 0 | 0 |
| --- | --- | --- | --- | --- | --- | --- | --- |
| hsa_circ_0010978 | 90 | 1 | 695 | 1 | 695 | 0 | 0 |
| hsa_circ_0010978 | 90 | 1 | 1133 | 1 | 1133 | 0 | 0 |
| hsa_circ_0010981 | 91 | 1 | 438 | 1 | 438 | 0 | 1.00E-166 |
| hsa_circ_0010982 | 90 | 1 | 402 | 1 | 402 | 0 | 2.00E-148 |
| hsa_circ_0010996 | 94 | 3 | 479 | 216 | 692 | 0 | 0 |
| hsa_circ_0010996 | 94 | 3 | 594 | 216 | 807 | 0 | 0 |
| hsa_circ_0010996 | 93 | 3 | 702 | 216 | 915 | 0 | 0 |
| hsa_circ_0007537 | 91 | 1 | 223 | 1 | 223 | 0 | 2.00E-82 |
| hsa_circ_0011013 | 94 | 1 | 666 | 1 | 666 | 0 | 0 |
| hsa_circ_0011013 | 94 | 1 | 783 | 1 | 783 | 0 | 0 |
| hsa_circ_0011014 | 94 | 1 | 570 | 1 | 570 | 0 | 0 |
| hsa_circ_0011016 | 96 | 1 | 481 | 1 | 481 | 0 | 0 |
| hsa_circ_0011030 | 90 | 157 | 446 | 290 | 1 | 0 | 9.00E-103 |
| hsa_circ_0011032 | 91 | 9 | 421 | 417 | 2 | 0.01 | 2.00E-156 |
| hsa_circ_0011032 | 91 | 9 | 421 | 417 | 2 | 0.01 | 1.00E-156 |
| hsa_circ_0037057 | 89 | 271 | 734 | 1 | 464 | 0 | 3.00E-164 |
| hsa_circ_0037057 | 89 | 139 | 602 | 1 | 464 | 0 | 2.00E-164 |
| hsa_circ_0037057 | 89 | 1 | 464 | 1 | 464 | 0 | 2.00E-164 |
| hsa_circ_0037057 | 89 | 1 | 390 | 75 | 464 | 0 | 2.00E-138 |
| hsa_circ_0059418 | 93 | 1 | 333 | 337 | 1 | 0.01 | 9.00E-134 |
| hsa_circ_0059418 | 93 | 1 | 333 | 337 | 1 | 0.01 | 1.00E-134 |
| hsa_circ_0059418 | 93 | 1 | 291 | 337 | 47 | 0 | 7.00E-117 |
| hsa_circ_0059420 | 81 | 1378 | 2312 | 2751 | 1818 | 0.05 | 0 |
| hsa_circ_0059420 | 81 | 5033 | 5967 | 2751 | 1818 | 0.05 | 0 |
| hsa_circ_0029646 | 91 | 300 | 801 | 1 | 499 | 0.01 | 0 |
| hsa_circ_0029646 | 90 | 300 | 1287 | 1 | 985 | 0.01 | 0 |
| hsa_circ_0029646 | 90 | 104 | 521 | 1 | 415 | 0.02 | 1.00E-152 |
| hsa_circ_0029646 | 91 | 2461 | 3032 | 414 | 985 | 0 | 0 |
| hsa_circ_0029646 | 91 | 2390 | 2961 | 414 | 985 | 0 | 0 |
| hsa_circ_0029646 | 91 | 1 | 383 | 603 | 985 | 0 | 6.00E-146 |
| hsa_circ_0029648 | 90 | 1 | 410 | 564 | 155 | 0 | 8.00E-153 |
| hsa_circ_0029648 | 90 | 244 | 653 | 564 | 155 | 0 | 1.00E-152 |
| hsa_circ_0003852 | 88 | 1 | 364 | 364 | 1 | 0 | 1.00E-120 |
| hsa_circ_0029653 | 95 | 1 | 2085 | 2085 | 1 | 0 | 0 |
| hsa_circ_0029668 | 94 | 1 | 594 | 594 | 1 | 0 | 0 |
| hsa_circ_0029668 | 94 | 1 | 769 | 769 | 1 | 0 | 0 |
| hsa_circ_0029668 | 95 | 1 | 939 | 939 | 1 | 0 | 0 |
| hsa_circ_0029668 | 96 | 1 | 1743 | 1743 | 1 | 0 | 0 |
| hsa_circ_0029668 | 96 | 1 | 1622 | 1743 | 122 | 0 | 0 |
| hsa_circ_0029668 | 96 | 1 | 1955 | 2076 | 122 | 0 | 0 |
| hsa_circ_0029675 | 97 | 1 | 804 | 804 | 1 | 0 | 0 |
| hsa_circ_0029680 | 97 | 1 | 333 | 333 | 1 | 0 | 8.00E-157 |
| hsa_circ_0029680 | 97 | 1 | 717 | 717 | 1 | 0 | 0 |
| hsa_circ_0029680 | 97 | 1 | 856 | 856 | 1 | 0 | 0 |
| hsa_circ_0029680 | 97 | 1 | 523 | 856 | 334 | 0 | 0 |
| hsa_circ_0029680 | 97 | 155 | 820 | 999 | 334 | 0 | 0 |
| hsa_circ_0029680 | 97 | 155 | 436 | 999 | 718 | 0 | 1.00E-131 |
| hsa_circ_0029680 | 97 | 171 | 452 | 999 | 718 | 0 | 1.00E-131 |
| hsa_circ_0029680 | 97 | 277 | 558 | 999 | 718 | 0 | 1.00E-131 |
| hsa_circ_0007590 | 93 | 1 | 583 | 583 | 1 | 0 | 0 |
| hsa_circ_0029693 | 90 | 169 | 579 | 410 | 1 | 0 | 2.00E-149 |
| hsa_circ_0029704 | 95 | 192 | 695 | 617 | 114 | 0 | 0 |
| hsa_circ_0029704 | 95 | 296 | 799 | 617 | 114 | 0 | 0 |

novel_circ_023497 novel_circ_023498 novel_circ_023499 novel_circ_023500 novel_circ_023501 novel_circ_023502 novel_circ_023503 novel_circ_023504 novel_circ_023505 novel_circ_023506 novel_circ_023507 novel_circ_023508 novel_circ_023509 novel_circ_023510 novel_circ_023515 novel_circ_023516 novel_circ_023517 novel_circ_023518 novel_circ_023519 novel_circ_023520 novel_circ_023521 novel_circ_023522 novel_circ_023523 novel_circ_023525 novel_circ_023526 novel_circ_023527 novel_circ_023528 novel_circ_023529 novel_circ_023530 novel_circ_023531 novel_circ_023532 novel_circ_023533 novel_circ_023534 novel_circ_023535 novel_circ_023536 novel_circ_023537 novel_circ_023538 novel_circ_023539 novel_circ_023540 novel_circ_023541 novel_circ_023542 novel_circ_023543 novel_circ_023544 novel_circ_023545 novel_circ_023546 novel_circ_023547 novel_circ_023548 novel_circ_023549 novel_circ_023550 novel_circ_023551 novel_circ_023556 novel_circ_023557 novel_circ_023558 novel_circ_023559

| hsa_circ_0029704 | 95 | 71 | 484 | 617 | 204 | 0 | 0 |
| --- | --- | --- | --- | --- | --- | --- | --- |
| hsa_circ_0029704 | 95 | 192 | 605 | 617 | 204 | 0 | 0 |
| hsa_circ_0008763 | 95 | 1 | 449 | 449 | 1 | 0 | 0 |
| hsa_circ_0008763 | 95 | 1 | 476 | 476 | 1 | 0 | 0 |
| hsa_circ_0029704 | 96 | 71 | 357 | 617 | 331 | 0 | 3.00E-131 |
| hsa_circ_0008763 | 94 | 1 | 345 | 345 | 1 | 0 | 1.00E-146 |
| hsa_circ_0008763 | 95 | 1 | 449 | 449 | 1 | 0 | 0 |
| hsa_circ_0008763 | 95 | 1 | 476 | 476 | 1 | 0 | 0 |
| hsa_circ_0006732 | 95 | 1 | 225 | 225 | 1 | 0 | 3.00E-95 |
| hsa_circ_0029712 | 94 | 1 | 537 | 1202 | 666 | 0 | 0 |
| hsa_circ_0029712 | 94 | 1 | 270 | 1202 | 933 | 0 | 3.00E-115 |
| hsa_circ_0029712 | 92 | 1 | 572 | 1507 | 933 | 0.01 | 0 |
| hsa_circ_0029712 | 92 | 13525 | 14096 | 1507 | 933 | 0.01 | 0 |
| hsa_circ_0029712 | 92 | 143 | 714 | 1507 | 933 | 0.01 | 0 |
| hsa_circ_0003408 | 91 | 6 | 2014 | 2009 | 1 | 0 | 0 |
| hsa_circ_0029720 | 95 | 6 | 286 | 281 | 1 | 0 | 1.00E-124 |
| hsa_circ_0029722 | 92 | 265 | 529 | 215 | 479 | 0 | 8.00E-104 |
| hsa_circ_0029729 | 89 | 1 | 196 | 196 | 1 | 0 | 3.00E-64 |
| hsa_circ_0029729 | 93 | 1 | 742 | 938 | 197 | 0 | 0 |
| hsa_circ_0007891 | 92 | 1 | 547 | 547 | 1 | 0 | 0 |
| hsa_circ_0029729 | 92 | 1 | 600 | 991 | 392 | 0 | 0 |
| hsa_circ_0029732 | 90 | 1 | 503 | 503 | 1 | 0 | 0 |
| hsa_circ_0003040 | 84 | 8 | 507 | 8 | 510 | 0.02 | 6.00E-135 |
| hsa_circ_0029777 | 94 | 381 | 1066 | 686 | 1 | 0 | 0 |
| hsa_circ_0029777 | 94 | 948 | 1633 | 686 | 1 | 0 | 0 |
| hsa_circ_0029777 | 93 | 381 | 733 | 686 | 334 | 0 | 1.00E-147 |
| hsa_circ_0029777 | 92 | 1 | 236 | 619 | 384 | 0 | 2.00E-91 |
| hsa_circ_0029777 | 93 | 381 | 683 | 686 | 384 | 0 | 8.00E-125 |
| hsa_circ_0029777 | 92 | 1 | 433 | 1174 | 744 | 0 | 1.00E-174 |
| hsa_circ_0029777 | 93 | 1 | 596 | 1337 | 744 | 0 | 0 |
| hsa_circ_0029777 | 93 | 118 | 713 | 1337 | 744 | 0 | 0 |
| hsa_circ_0029777 | 96 | 249 | 382 | 753 | 620 | 0 | 4.00E-56 |
| hsa_circ_0029780 | 93 | 1 | 450 | 450 | 1 | 0 | 0 |
| hsa_circ_0029780 | 93 | 4 | 567 | 564 | 1 | 0 | 0 |
| hsa_circ_0029789 | 90 | 1 | 244 | 151 | 397 | 0.01 | 2.00E-86 |
| hsa_circ_0029789 | 92 | 1 | 711 | 151 | 864 | 0 | 0 |
| hsa_circ_0029790 | 94 | 1 | 440 | 1 | 440 | 0 | 0 |
| hsa_circ_0029791 | 94 | 1 | 199 | 1 | 199 | 0 | 1.00E-82 |
| hsa_circ_0029798 | 92 | 42 | 591 | 1 | 550 | 0 | 0 |
| hsa_circ_0029798 | 92 | 1 | 550 | 1 | 550 | 0 | 0 |
| hsa_circ_0029798 | 93 | 1 | 310 | 75 | 384 | 0 | 2.00E-127 |
| hsa_circ_0029798 | 93 | 1 | 476 | 75 | 550 | 0 | 0 |
| hsa_circ_0029798 | 92 | 1 | 682 | 75 | 756 | 0 | 0 |
| hsa_circ_0029798 | 93 | 1 | 406 | 145 | 550 | 0 | 1.00E-165 |
| hsa_circ_0029798 | 91 | 8 | 604 | 558 | 1154 | 0 | 0 |
| hsa_circ_0029798 | 93 | 15775 | 16072 | 1875 | 2172 | 0 | 3.00E-117 |
| hsa_circ_0029801 | 93 | 1 | 295 | 1 | 295 | 0 | 2.00E-117 |
| hsa_circ_0029802 | 94 | 1 | 429 | 76 | 504 | 0 | 0 |
| hsa_circ_0029802 | 93 | 9 | 119 | 286 | 396 | 0 | 6.00E-39 |
| hsa_circ_0029802 | 95 | 9 | 227 | 286 | 504 | 0 | 1.00E-93 |
| hsa_circ_0018902 | 93 | 1 | 948 | 1 | 949 | 0 | 0 |
| hsa_circ_0018903 | 93 | 1 | 878 | 1 | 879 | 0 | 0 |
| hsa_circ_0018903 | 97 | 1 | 2008 | 880 | 2887 | 0 | 0 |
| hsa_circ_0018903 | 97 | 3 | 1899 | 991 | 2887 | 0 | 0 |

novel_circ_023560 novel_circ_023562 novel_circ_023563 novel_circ_023564 novel_circ_023565 novel_circ_023566 novel_circ_023568 novel_circ_023570 novel_circ_023571 novel_circ_023572 novel_circ_023573 novel_circ_023574 novel_circ_023575 novel_circ_023576 novel_circ_023577 novel_circ_023578 novel_circ_023579 novel_circ_023582 novel_circ_023584 novel_circ_023585 novel_circ_023586 novel_circ_023587 novel_circ_023588 novel_circ_023591 novel_circ_023592 novel_circ_023593 novel_circ_023594 novel_circ_023595 novel_circ_023596 novel_circ_023597 novel_circ_023598 novel_circ_023599 novel_circ_023600 novel_circ_023601 novel_circ_023602 novel_circ_023603 novel_circ_023604 novel_circ_023605 novel_circ_023606 novel_circ_023607 novel_circ_023608 novel_circ_023609 novel_circ_023610 novel_circ_023611 novel_circ_023612 novel_circ_023613 novel_circ_023614 novel_circ_023615 novel_circ_023616 novel_circ_023617 novel_circ_023618 novel_circ_023619 novel_circ_023620 novel_circ_023621

| hsa_circ_0018903 | 97 | 1 | 731 | 2252 | 2982 | 0 | 0 |
| --- | --- | --- | --- | --- | --- | --- | --- |
| hsa_circ_0018905 | 96 | 1 | 593 | 1 | 593 | 0 | 0 |
| hsa_circ_0018905 | 96 | 1 | 807 | 1 | 807 | 0 | 0 |
| hsa_circ_0018905 | 96 | 1 | 807 | 1 | 807 | 0 | 0 |
| hsa_circ_0018905 | 96 | 1 | 807 | 1 | 807 | 0 | 0 |
| hsa_circ_0018906 | 97 | 2 | 214 | 2 | 214 | 0 | 2.00E-98 |
| hsa_circ_0084876 | 86 | 1 | 392 | 227 | 618 | 0 | 4.00E-116 |
| hsa_circ_0004394 | 94 | 3 | 260 | 258 | 1 | 0 | 1.00E-108 |
| hsa_circ_0084880 | 96 | 1 | 1191 | 1191 | 1 | 0 | 0 |
| hsa_circ_0084880 | 97 | 1 | 721 | 721 | 1 | 0 | 0 |
| hsa_circ_0084880 | 96 | 1 | 539 | 539 | 1 | 0 | 0 |
| hsa_circ_0084880 | 97 | 1 | 811 | 811 | 1 | 0 | 0 |
| hsa_circ_0084880 | 96 | 1 | 1191 | 1191 | 1 | 0 | 0 |
| hsa_circ_0084881 | 98 | 1 | 272 | 272 | 1 | 0 | 5.00E-133 |
| hsa_circ_0084881 | 96 | 1 | 652 | 652 | 1 | 0 | 0 |
| hsa_circ_0084881 | 96 | 74187 | 74839 | 653 | 1 | 0 | 0 |
| hsa_circ_0082816 | 90 | 215 | 452 | 829 | 592 | 0 | 7.00E-84 |
| hsa_circ_0056665 | 96 | 2484 | 2664 | 181 | 1 | 0 | 4.00E-78 |
| hsa_circ_0008208 | 92 | 2 | 606 | 608 | 1 | 0 | 0 |
| hsa_circ_0008208 | 96 | 61502 | 61845 | 344 | 1 | 0 | 6.00E-154 |
| hsa_circ_0008208 | 96 | 61499 | 61842 | 344 | 1 | 0 | 6.00E-154 |
| hsa_circ_0008208 | 96 | 61448 | 61791 | 344 | 1 | 0 | 6.00E-154 |
| hsa_circ_0008208 | 96 | 4 | 342 | 339 | 1 | 0 | 4.00E-155 |
| hsa_circ_0076937 | 97 | 1 | 438 | 1 | 438 | 0 | 0 |
| hsa_circ_0076937 | 95 | 1 | 768 | 1 | 768 | 0 | 0 |
| hsa_circ_0076937 | 95 | 1 | 977 | 1 | 977 | 0 | 0 |
| hsa_circ_0076937 | 95 | 1 | 977 | 1 | 977 | 0 | 0 |
| hsa_circ_0076937 | 95 | 1 | 1172 | 1 | 1172 | 0 | 0 |
| hsa_circ_0076940 | 92 | 1 | 495 | 1 | 495 | 0 | 0 |
| hsa_circ_0076940 | 93 | 1 | 704 | 1 | 704 | 0 | 0 |
| hsa_circ_0076937 | 95 | 1 | 1077 | 274 | 1350 | 0 | 0 |
| hsa_circ_0076937 | 95 | 1 | 912 | 439 | 1350 | 0 | 0 |
| hsa_circ_0076942 | 97 | 1 | 209 | 1 | 209 | 0 | 8.00E-95 |
| hsa_circ_0076942 | 97 | 1 | 404 | 1 | 404 | 0 | 0 |
| hsa_circ_0076942 | 97 | 1 | 578 | 1 | 578 | 0 | 0 |
| hsa_circ_0076942 | 97 | 1 | 582 | 1 | 582 | 0 | 0 |
| hsa_circ_0076942 | 97 | 1 | 808 | 1 | 808 | 0 | 0 |
| hsa_circ_0076945 | 98 | 10822 | 11194 | 1 | 373 | 0 | 0 |
| hsa_circ_0076945 | 96 | 10822 | 11522 | 1 | 701 | 0 | 0 |
| hsa_circ_0076945 | 97 | 1 | 195 | 1 | 195 | 0 | 2.00E-90 |
| hsa_circ_0076945 | 98 | 1 | 369 | 1 | 369 | 0 | 4.00E-180 |
| hsa_circ_0076945 | 98 | 1 | 373 | 1 | 373 | 0 | 0 |
| hsa_circ_0076945 | 97 | 1 | 599 | 1 | 599 | 0 | 0 |
| hsa_circ_0076945 | 96 | 1 | 701 | 1 | 701 | 0 | 0 |
| hsa_circ_0076949 | 96 | 1 | 404 | 1 | 404 | 0 | 0 |
| hsa_circ_0076949 | 96 | 1 | 506 | 1 | 506 | 0 | 0 |
| hsa_circ_0076949 | 95 | 1 | 437 | 70 | 506 | 0 | 0 |
| hsa_circ_0076949 | 94 | 8198 | 8573 | 176 | 551 | 0 | 3.00E-159 |
| hsa_circ_0076950 | 95 | 1 | 328 | 1 | 328 | 0 | 2.00E-142 |
| hsa_circ_0076950 | 94 | 1 | 373 | 1 | 373 | 0 | 5.00E-159 |
| hsa_circ_0076949 | 92 | 1 | 148 | 405 | 552 | 0 | 5.00E-50 |
| hsa_circ_0076952 | 98 | 24406 | 24739 | 1 | 334 | 0 | 8.00E-164 |
| hsa_circ_0076952 | 98 | 1 | 334 | 1 | 334 | 0 | 1.00E-165 |
| hsa_circ_0076945 | 95 | 1 | 374 | 1081 | 1454 | 0 | 3.00E-166 |

novel_circ_023622 novel_circ_023623 novel_circ_023624 novel_circ_023625 novel_circ_023626 novel_circ_023627 novel_circ_023628 novel_circ_023629 novel_circ_023630 novel_circ_023631 novel_circ_023632 novel_circ_023633 novel_circ_023634 novel_circ_023635 novel_circ_023636 novel_circ_023639 novel_circ_023641 novel_circ_023647 novel_circ_023648 novel_circ_023662 novel_circ_023663 novel_circ_023664 novel_circ_023665 novel_circ_023666 novel_circ_023667 novel_circ_023668 novel_circ_023669 novel_circ_023670 novel_circ_023671 novel_circ_023672 novel_circ_023675 novel_circ_023676 novel_circ_023677 novel_circ_023678 novel_circ_023679 novel_circ_023681 novel_circ_023682 novel_circ_023683 novel_circ_023684 novel_circ_023685 novel_circ_023686 novel_circ_023687 novel_circ_023688 novel_circ_023689 novel_circ_023691 novel_circ_023692 novel_circ_023693 novel_circ_023694 novel_circ_023695 novel_circ_023696 novel_circ_023697 novel_circ_023698 novel_circ_023699 novel_circ_023700

| hsa_circ_0076945 | 96 | 1 | 645 | 1081 | 1725 | 0 | 0 |
| --- | --- | --- | --- | --- | --- | --- | --- |
| hsa_circ_0076945 | 95 | 1 | 781 | 1081 | 1861 | 0 | 0 |
| hsa_circ_0076945 | 95 | 1 | 781 | 1081 | 1861 | 0 | 0 |
| hsa_circ_0076945 | 95 | 1 | 781 | 1081 | 1861 | 0 | 0 |
| hsa_circ_0076945 | 96 | 1 | 630 | 1232 | 1861 | 0 | 0 |
| hsa_circ_0076945 | 96 | 1 | 560 | 1302 | 1861 | 0 | 0 |
| hsa_circ_0076945 | 96 | 1 | 493 | 1369 | 1861 | 0 | 0 |
| hsa_circ_0076945 | 96 | 1 | 493 | 1369 | 1861 | 0 | 0 |
| hsa_circ_0076945 | 96 | 1 | 493 | 1369 | 1861 | 0 | 0 |
| hsa_circ_0076945 | 96 | 1 | 407 | 1455 | 1861 | 0 | 0 |
| hsa_circ_0076945 | 96 | 1 | 407 | 1455 | 1861 | 0 | 0 |
| hsa_circ_0076945 | 96 | 1 | 407 | 1455 | 1861 | 0 | 0 |
| hsa_circ_0076945 | 94 | 1 | 308 | 1554 | 1861 | 0 | 2.00E-132 |
| hsa_circ_0076945 | 95 | 1 | 136 | 1726 | 1861 | 0 | 1.00E-53 |
| hsa_circ_0076945 | 95 | 1 | 136 | 1726 | 1861 | 0 | 2.00E-53 |
| hsa_circ_0076953 | 95 | 239 | 404 | 166 | 1 | 0 | 7.00E-67 |
| hsa_circ_0018218 | 86 | 12 | 143 | 114 | 245 | 0 | 3.00E-31 |
| hsa_circ_0030832 | 88 | 265 | 473 | 1 | 209 | 0 | 2.00E-64 |
| hsa_circ_0030832 | 87 | 1 | 378 | 72 | 449 | 0 | 9.00E-117 |
| hsa_circ_0030915 | 88 | 1 | 418 | 1 | 418 | 0 | 3.00E-137 |
| hsa_circ_0030915 | 88 | 1 | 507 | 1 | 507 | 0 | 6.00E-170 |
| hsa_circ_0030918 | 86 | 1 | 333 | 1 | 333 | 0 | 4.00E-95 |
| hsa_circ_0030918 | 86 | 1 | 422 | 1 | 422 | 0 | 8.00E-128 |
| hsa_circ_0030919 | 88 | 1 | 291 | 1 | 291 | 0 | 3.00E-95 |
| hsa_circ_0030923 | 87 | 1 | 660 | 1 | 660 | 0 | 0 |
| hsa_circ_0030926 | 86 | 1 | 469 | 1 | 469 | 0 | 7.00E-144 |
| hsa_circ_0030926 | 87 | 1 | 556 | 1 | 556 | 0 | 0 |
| hsa_circ_0030926 | 87 | 1 | 765 | 1 | 765 | 0 | 0 |
| hsa_circ_0030912 | 89 | 1895 | 2046 | 12873 | 13024 | 0 | 2.00E-44 |
| hsa_circ_0030940 | 84 | 40 | 614 | 712 | 138 | 0 | 2.00E-159 |
| hsa_circ_0030990 | 85 | 1 | 463 | 328 | 790 | 0 | 3.00E-132 |
| hsa_circ_0004096 | 93 | 1 | 317 | 317 | 1 | 0 | 6.00E-128 |
| hsa_circ_0031082 | 88 | 103 | 658 | 2 | 557 | 0 | 0 |
| hsa_circ_0031082 | 88 | 2 | 557 | 2 | 557 | 0 | 0 |
| hsa_circ_0031087 | 91 | 1 | 506 | 1 | 506 | 0 | 0 |
| hsa_circ_0085129 | 93 | 108 | 599 | 492 | 1 | 0 | 0 |
| hsa_circ_0085129 | 94 | 6 | 338 | 492 | 160 | 0 | 4.00E-140 |
| hsa_circ_0085119 | 95 | 1 | 1008 | 1008 | 1 | 0 | 0 |
| hsa_circ_0085119 | 95 | 1 | 1008 | 1008 | 1 | 0 | 0 |
| hsa_circ_0085123 | 94 | 1 | 423 | 423 | 1 | 0 | 0 |
| hsa_circ_0085119 | 94 | 1 | 799 | 799 | 1 | 0 | 0 |
| hsa_circ_0085123 | 94 | 1 | 278 | 278 | 1 | 0 | 2.00E-116 |
| hsa_circ_0085119 | 95 | 1 | 491 | 491 | 1 | 0 | 0 |
| hsa_circ_0085119 | 95 | 1 | 376 | 376 | 1 | 0 | 1.00E-165 |
| hsa_circ_0085114 | 85 | 156 | 580 | 3 | 427 | 0.01 | 5.00E-121 |
| hsa_circ_0085112 | 85 | 1 | 288 | 1 | 288 | 0.01 | 4.00E-80 |
| hsa_circ_0085108 | 90 | 1 | 1150 | 348 | 1497 | 0 | 0 |
| hsa_circ_0085106 | 91 | 1 | 1845 | 1 | 1845 | 0 | 0 |
| hsa_circ_0085105 | 94 | 1 | 1242 | 1 | 1242 | 0 | 0 |
| hsa_circ_0085102 | 88 | 1 | 532 | 1 | 532 | 0 | 2.00E-175 |
| hsa_circ_0085101 | 92 | 1 | 596 | 1 | 596 | 0 | 0 |
| hsa_circ_0085097 | 93 | 1 | 685 | 1 | 685 | 0 | 0 |
| hsa_circ_0085097 | 92 | 1 | 826 | 1 | 826 | 0 | 0 |
| hsa_circ_0085094 | 93 | 1 | 475 | 113 | 587 | 0 | 0 |

novel_circ_023701 novel_circ_023702 novel_circ_023703 novel_circ_023704 novel_circ_023705 novel_circ_023706 novel_circ_023707 novel_circ_023708 novel_circ_023709 novel_circ_023710 novel_circ_023711 novel_circ_023712 novel_circ_023713 novel_circ_023714 novel_circ_023715 novel_circ_023716 novel_circ_023717 novel_circ_023718 novel_circ_023719 novel_circ_023720 novel_circ_023721 novel_circ_023722 novel_circ_023724 novel_circ_023725 novel_circ_023726 novel_circ_023727 novel_circ_023728 novel_circ_023729 novel_circ_023730 novel_circ_023731 novel_circ_023732 novel_circ_023733 novel_circ_023734 novel_circ_023735 novel_circ_023736 novel_circ_023737 novel_circ_023738 novel_circ_023739 novel_circ_023740 novel_circ_023741 novel_circ_023742 novel_circ_023743 novel_circ_023744 novel_circ_023745 novel_circ_023746 novel_circ_023747 novel_circ_023748 novel_circ_023749 novel_circ_023750 novel_circ_023751 novel_circ_023752 novel_circ_023753 novel_circ_023764 novel_circ_023765

| hsa_circ_0085094 | 92 | 3 | 591 | 3 | 587 | 0.01 | 0 |
| --- | --- | --- | --- | --- | --- | --- | --- |
| hsa_circ_0085093 | 92 | 1 | 1000 | 855 | 1850 | 0.01 | 0 |
| hsa_circ_0085093 | 93 | 356 | 1496 | 714 | 1850 | 0.01 | 0 |
| hsa_circ_0085093 | 92 | 1 | 521 | 855 | 1375 | 0.01 | 0 |
| hsa_circ_0085093 | 93 | 356 | 1017 | 714 | 1375 | 0.01 | 0 |
| hsa_circ_0085093 | 93 | 560 | 1221 | 714 | 1375 | 0.01 | 0 |
| hsa_circ_0085083 | 92 | 1 | 779 | 364 | 1142 | 0 | 0 |
| hsa_circ_0085093 | 94 | 356 | 905 | 714 | 1263 | 0.01 | 0 |
| hsa_circ_0085083 | 92 | 1 | 779 | 364 | 1142 | 0 | 0 |
| hsa_circ_0085093 | 89 | 1 | 425 | 1 | 425 | 0 | 4.00E-151 |
| hsa_circ_0085093 | 90 | 1 | 221 | 1 | 221 | 0 | 4.00E-78 |
| hsa_circ_0085079 | 89 | 1 | 842 | 1 | 842 | 0 | 0 |
| hsa_circ_0085074 | 92 | 1 | 932 | 183 | 1114 | 0 | 0 |
| hsa_circ_0085079 | 89 | 1 | 621 | 1 | 621 | 0 | 0 |
| hsa_circ_0085074 | 92 | 1 | 930 | 183 | 1112 | 0 | 0 |
| hsa_circ_0085079 | 88 | 1 | 386 | 1 | 386 | 0 | 2.00E-129 |
| hsa_circ_0085075 | 88 | 24185 | 24571 | 174 | 560 | 0 | 3.00E-128 |
| hsa_circ_0085074 | 92 | 1 | 695 | 183 | 877 | 0 | 0 |
| hsa_circ_0085074 | 94 | 1 | 567 | 183 | 749 | 0 | 0 |
| hsa_circ_0085074 | 95 | 1 | 419 | 183 | 601 | 0 | 0 |
| hsa_circ_0004366 | 97 | 1 | 307 | 1 | 307 | 0 | 4.00E-144 |
| hsa_circ_0085074 | 95 | 3 | 489 | 3 | 489 | 0 | 0 |
| hsa_circ_0085071 | 91 | 1 | 307 | 1 | 307 | 0 | 5.00E-114 |
| hsa_circ_0085066 | 92 | 1 | 672 | 1 | 672 | 0 | 0 |
| hsa_circ_0085064 | 92 | 7 | 864 | 95 | 952 | 0 | 0 |
| hsa_circ_0085064 | 91 | 1 | 952 | 1 | 952 | 0 | 0 |
| hsa_circ_0085063 | 92 | 1 | 1090 | 1 | 1090 | 0 | 0 |
| hsa_circ_0085059 | 92 | 1 | 1309 | 270 | 1578 | 0 | 0 |
| hsa_circ_0085059 | 91 | 1 | 1578 | 1 | 1578 | 0 | 0 |
| hsa_circ_0085058 | 91 | 1 | 1753 | 1 | 1753 | 0 | 0 |
| hsa_circ_0085057 | 92 | 1 | 2103 | 1 | 2103 | 0 | 0 |
| hsa_circ_0085064 | 92 | 7 | 682 | 95 | 770 | 0 | 0 |
| hsa_circ_0085063 | 92 | 1 | 908 | 1 | 908 | 0 | 0 |
| hsa_circ_0085059 | 91 | 1 | 1396 | 1 | 1396 | 0 | 0 |
| hsa_circ_0085058 | 91 | 1 | 1571 | 1 | 1571 | 0 | 0 |
| hsa_circ_0085051 | 92 | 1 | 2186 | 1 | 2186 | 0 | 0 |
| hsa_circ_0085063 | 92 | 1 | 783 | 1 | 783 | 0 | 0 |
| hsa_circ_0085062 | 92 | 1 | 906 | 1 | 906 | 0 | 0 |
| hsa_circ_0085058 | 91 | 1 | 1446 | 1 | 1446 | 0 | 0 |
| hsa_circ_0085050 | 92 | 3 | 2237 | 3 | 2237 | 0 | 0 |
| hsa_circ_0085058 | 91 | 1 | 889 | 1 | 889 | 0 | 0 |
| hsa_circ_0085055 | 90 | 1 | 915 | 1 | 915 | 0 | 0 |
| hsa_circ_0085051 | 91 | 1 | 1059 | 1 | 1059 | 0 | 0 |
| hsa_circ_0085050 | 91 | 3 | 1235 | 3 | 1235 | 0 | 0 |
| hsa_circ_0085050 | 93 | 3 | 791 | 3 | 791 | 0 | 0 |
| hsa_circ_0085050 | 91 | 3 | 441 | 3 | 441 | 0 | 6.00E-169 |
| hsa_circ_0085047 | 94 | 1 | 325 | 658 | 334 | 0 | 1.00E-140 |
| hsa_circ_0085047 | 96 | 1 | 333 | 333 | 1 | 0 | 2.00E-153 |
| hsa_circ_0085033 | 88 | 1 | 219 | 444 | 226 | 0.01 | 6.00E-67 |
| hsa_circ_0085027 | 86 | 1 | 276 | 1 | 276 | 0 | 3.00E-80 |
| hsa_circ_0017908 | 91 | 160 | 702 | 688 | 146 | 0 | 0 |
| hsa_circ_0017908 | 91 | 4 | 546 | 688 | 146 | 0 | 0 |
| hsa_circ_0017898 | 94 | 1 | 465 | 1 | 465 | 0 | 0 |
| hsa_circ_0017897 | 95 | 1 | 556 | 1 | 556 | 0 | 0 |

novel_circ_023766 novel_circ_023767 novel_circ_023775 novel_circ_023776 novel_circ_023777 novel_circ_023778 novel_circ_023779 novel_circ_023780 novel_circ_023781 novel_circ_023782 novel_circ_023783 novel_circ_023784 novel_circ_023785 novel_circ_023786 novel_circ_023787 novel_circ_023788 novel_circ_023790 novel_circ_023792 novel_circ_023793 novel_circ_023794 novel_circ_023797 novel_circ_023798 novel_circ_023799 novel_circ_023800 novel_circ_023814 novel_circ_023815 novel_circ_023816 novel_circ_023817 novel_circ_023818 novel_circ_023819 novel_circ_023820 novel_circ_023821 novel_circ_023822 novel_circ_023823 novel_circ_023824 novel_circ_023825 novel_circ_023826 novel_circ_023827 novel_circ_023828 novel_circ_023829 novel_circ_023830 novel_circ_023831 novel_circ_023832 novel_circ_023833 novel_circ_023835 novel_circ_023838 novel_circ_023839 novel_circ_023840 novel_circ_023841 novel_circ_023842 novel_circ_023844 novel_circ_023845 novel_circ_023847 novel_circ_023848

| hsa_circ_0017897 | 95 | 1 | 468 | 1 | 468 | 0 | 0 |
| --- | --- | --- | --- | --- | --- | --- | --- |
| hsa_circ_0002091 | 97 | 1614 | 2138 | 628 | 104 | 0 | 0 |
| hsa_circ_0017863 | 87 | 1000 | 1894 | 2058 | 1161 | 0 | 0 |
| hsa_circ_0017863 | 85 | 1 | 881 | 1029 | 152 | 0.01 | 0 |
| hsa_circ_0006577 | 94 | 3 | 489 | 487 | 1 | 0 | 0 |
| hsa_circ_0006577 | 94 | 3 | 438 | 436 | 1 | 0 | 0 |
| hsa_circ_0006577 | 94 | 3 | 438 | 436 | 1 | 0 | 0 |
| hsa_circ_0017853 | 97 | 1 | 315 | 315 | 1 | 0 | 7.00E-152 |
| hsa_circ_0017852 | 97 | 1 | 482 | 482 | 1 | 0 | 0 |
| hsa_circ_0006665 | 96 | 1 | 636 | 636 | 1 | 0 | 0 |
| hsa_circ_0000220 | 96 | 1 | 707 | 707 | 1 | 0 | 0 |
| hsa_circ_0000219 | 96 | 1 | 788 | 788 | 1 | 0 | 0 |
| hsa_circ_0000219 | 95 | 2 | 421 | 420 | 1 | 0 | 0 |
| hsa_circ_0017848 | 95 | 2 | 494 | 493 | 1 | 0 | 0 |
| hsa_circ_0000219 | 94 | 1 | 306 | 306 | 1 | 0 | 3.00E-130 |
| hsa_circ_0017848 | 94 | 1 | 379 | 379 | 1 | 0 | 5.00E-164 |
| hsa_circ_0017836 | 88 | 1 | 608 | 610 | 3 | 0 | 0 |
| hsa_circ_0017834 | 93 | 322 | 657 | 336 | 1 | 0 | 8.00E-140 |
| hsa_circ_0017834 | 93 | 1 | 336 | 336 | 1 | 0 | 4.00E-140 |
| hsa_circ_0005697 | 90 | 1 | 383 | 383 | 1 | 0 | 3.00E-136 |
| hsa_circ_0066590 | 95 | 294 | 462 | 3 | 171 | 0 | 2.00E-70 |
| hsa_circ_0066590 | 97 | 294 | 553 | 3 | 262 | 0 | 2.00E-119 |
| hsa_circ_0066590 | 95 | 294 | 732 | 3 | 441 | 0 | 0 |
| hsa_circ_0066590 | 95 | 3 | 441 | 3 | 441 | 0 | 0 |
| hsa_circ_0030393 | 96 | 1 | 102 | 2499 | 2398 | 0 | 7.00E-40 |
| hsa_circ_0014423 | 95 | 1 | 197 | 249 | 445 | 0 | 2.00E-82 |
| hsa_circ_0014423 | 93 | 1 | 445 | 1 | 445 | 0 | 0 |
| hsa_circ_0014410 | 97 | 1 | 198 | 529 | 726 | 0 | 3.00E-90 |
| hsa_circ_0014392 | 87 | 1 | 416 | 1 | 451 | 0.08 | 3.00E-132 |
| hsa_circ_0014386 | 91 | 18654 | 18853 | 172 | 371 | 0 | 9.00E-68 |
| hsa_circ_0014374 | 96 | 8581 | 8750 | 189 | 358 | 0 | 6.00E-71 |
| hsa_circ_0014374 | 96 | 8581 | 8750 | 189 | 358 | 0 | 3.00E-71 |
| hsa_circ_0014376 | 96 | 1 | 169 | 1 | 169 | 0 | 5.00E-72 |
| hsa_circ_0014371 | 99 | 1 | 131 | 1 | 131 | 0 | 4.00E-60 |
| hsa_circ_0014362 | 84 | 1 | 539 | 942 | 398 | 0.03 | 5.00E-141 |
| hsa_circ_0014362 | 80 | 1 | 978 | 942 | 3 | 0.07 | 0 |
| hsa_circ_0014318 | 94 | 337 | 598 | 565 | 304 | 0 | 9.00E-109 |
| hsa_circ_0014318 | 94 | 1 | 262 | 565 | 304 | 0 | 4.00E-109 |
| hsa_circ_0014318 | 95 | 1 | 303 | 303 | 1 | 0 | 7.00E-132 |
| hsa_circ_0014316 | 95 | 1 | 619 | 933 | 315 | 0 | 0 |
| hsa_circ_0014316 | 97 | 1 | 314 | 314 | 1 | 0 | 6.00E-148 |
| hsa_circ_0014306 | 94 | 8 | 681 | 8 | 681 | 0 | 0 |
| hsa_circ_0014296 | 95 | 1 | 1037 | 1 | 1037 | 0 | 0 |
| hsa_circ_0014294 | 93 | 1 | 373 | 1 | 373 | 0 | 2.00E-152 |
| hsa_circ_0014243 | 95 | 567 | 1247 | 596 | 1276 | 0.01 | 0 |
| hsa_circ_0054462 | 92 | 3 | 247 | 3 | 247 | 0 | 1.00E-94 |
| hsa_circ_0008101 | 92 | 8712 | 9128 | 164 | 580 | 0 | 1.00E-163 |
| hsa_circ_0008101 | 91 | 1 | 580 | 1 | 580 | 0 | 0 |
| hsa_circ_0008101 | 91 | 9582 | 10161 | 1 | 580 | 0 | 0 |
| hsa_circ_0008101 | 91 | 9582 | 9914 | 1 | 333 | 0 | 1.00E-123 |
| hsa_circ_0054459 | 93 | 9 | 321 | 307 | 1 | 0.02 | 1.00E-125 |
| hsa_circ_0062417 | 88 | 42118 | 42467 | 356 | 1 | 0.02 | 4.00E-112 |
| hsa_circ_0054456 | 93 | 15 | 458 | 444 | 1 | 0 | 0 |
| hsa_circ_0054444 | 92 | 1 | 432 | 86 | 517 | 0 | 2.00E-173 |

novel_circ_023849 novel_circ_023850 novel_circ_023851 novel_circ_023852 novel_circ_023853 novel_circ_023854 novel_circ_023855 novel_circ_023856 novel_circ_023857 novel_circ_023858 novel_circ_023859 novel_circ_023860 novel_circ_023861 novel_circ_023862 novel_circ_023863 novel_circ_023865 novel_circ_023867 novel_circ_023868 novel_circ_023869 novel_circ_023870 novel_circ_023871 novel_circ_023872 novel_circ_023873 novel_circ_023874 novel_circ_023875 novel_circ_023876 novel_circ_023877 novel_circ_023878 novel_circ_023879 novel_circ_023880 novel_circ_023881 novel_circ_023882 novel_circ_023883 novel_circ_023884 novel_circ_023885 novel_circ_023886 novel_circ_023887 novel_circ_023888 novel_circ_023889 novel_circ_023890 novel_circ_023891 novel_circ_023892 novel_circ_023893 novel_circ_023894 novel_circ_023898 novel_circ_023899 novel_circ_023900 novel_circ_023901 novel_circ_023902 novel_circ_023903 novel_circ_023904 novel_circ_023905 novel_circ_023906 novel_circ_023907

| hsa_circ_0054441 | 84 | 1 | 627 | 631 | 1255 | 0.04 | 1.00E-167 |
| --- | --- | --- | --- | --- | --- | --- | --- |
| hsa_circ_0054441 | 93 | 1 | 475 | 156 | 630 | 0 | 0 |
| hsa_circ_0054441 | 93 | 1 | 328 | 156 | 483 | 0 | 2.00E-132 |
| hsa_circ_0004447 | 94 | 1 | 374 | 241 | 614 | 0 | 1.00E-157 |
| hsa_circ_0004447 | 93 | 1 | 240 | 1 | 240 | 0 | 1.00E-93 |
| hsa_circ_0003882 | 92 | 1 | 411 | 1 | 411 | 0 | 1.00E-161 |
| hsa_circ_0054431 | 91 | 99 | 1589 | 1491 | 1 | 0 | 0 |
| hsa_circ_0054431 | 92 | 1 | 1226 | 1226 | 1 | 0 | 0 |
| hsa_circ_0054431 | 91 | 1 | 858 | 1059 | 202 | 0 | 0 |
| hsa_circ_0054431 | 93 | 1 | 802 | 802 | 1 | 0 | 0 |
| hsa_circ_0054431 | 93 | 2 | 705 | 704 | 1 | 0 | 0 |
| hsa_circ_0054431 | 94 | 1 | 569 | 569 | 1 | 0 | 0 |
| hsa_circ_0054431 | 95 | 1 | 465 | 465 | 1 | 0 | 0 |
| hsa_circ_0054414 | 95 | 1 | 639 | 639 | 1 | 0 | 0 |
| hsa_circ_0006530 | 95 | 1 | 247 | 1 | 247 | 0 | 2.00E-107 |
| hsa_circ_0054409 | 93 | 1 | 238 | 1 | 238 | 0 | 3.00E-92 |
| hsa_circ_0054409 | 93 | 1 | 238 | 1 | 238 | 0 | 3.00E-92 |
| hsa_circ_0054409 | 93 | 1 | 238 | 1 | 238 | 0 | 4.00E-94 |
| hsa_circ_0054408 | 93 | 4 | 397 | 1524 | 1131 | 0 | 6.00E-164 |
| hsa_circ_0054408 | 93 | 4 | 533 | 1524 | 995 | 0 | 0 |
| hsa_circ_0054408 | 93 | 4 | 936 | 1524 | 592 | 0 | 0 |
| hsa_circ_0054408 | 94 | 4 | 1134 | 1524 | 394 | 0 | 0 |
| hsa_circ_0054408 | 93 | 4 | 1310 | 1524 | 218 | 0 | 0 |
| hsa_circ_0054408 | 93 | 4 | 1527 | 1524 | 1 | 0 | 0 |
| hsa_circ_0054408 | 93 | 4 | 1527 | 1524 | 1 | 0 | 0 |
| hsa_circ_0054408 | 93 | 4 | 1527 | 1524 | 1 | 0 | 0 |
| hsa_circ_0054408 | 93 | 2 | 410 | 1403 | 995 | 0 | 1.00E-170 |
| hsa_circ_0054408 | 93 | 2 | 813 | 1403 | 592 | 0 | 0 |
| hsa_circ_0054408 | 94 | 2 | 1187 | 1403 | 218 | 0 | 0 |
| hsa_circ_0054408 | 93 | 1 | 746 | 1337 | 592 | 0 | 0 |
| hsa_circ_0054408 | 94 | 1 | 944 | 1337 | 394 | 0 | 0 |
| hsa_circ_0054408 | 93 | 1 | 1120 | 1337 | 218 | 0 | 0 |
| hsa_circ_0054408 | 93 | 1 | 1117 | 1334 | 218 | 0 | 0 |
| hsa_circ_0054408 | 93 | 1 | 1044 | 1261 | 218 | 0 | 0 |
| hsa_circ_0054408 | 93 | 1 | 539 | 1130 | 592 | 0 | 0 |
| hsa_circ_0054408 | 94 | 1 | 737 | 1130 | 394 | 0 | 0 |
| hsa_circ_0054408 | 93 | 1 | 913 | 1130 | 218 | 0 | 0 |
| hsa_circ_0054408 | 93 | 1 | 1130 | 1130 | 1 | 0 | 0 |
| hsa_circ_0054408 | 93 | 1 | 1130 | 1130 | 1 | 0 | 0 |
| hsa_circ_0054408 | 94 | 1 | 403 | 994 | 592 | 0 | 1.00E-170 |
| hsa_circ_0054408 | 95 | 1 | 601 | 994 | 394 | 0 | 0 |
| hsa_circ_0054408 | 94 | 1 | 777 | 994 | 218 | 0 | 0 |
| hsa_circ_0054408 | 94 | 1 | 994 | 994 | 1 | 0 | 0 |
| hsa_circ_0054408 | 94 | 4 | 374 | 588 | 218 | 0 | 1.00E-159 |
| hsa_circ_0054405 | 94 | 288 | 1349 | 1982 | 3042 | 0.02 | 0 |
| hsa_circ_0054405 | 94 | 288 | 1349 | 1982 | 3042 | 0.02 | 0 |
| hsa_circ_0009062 | 96 | 1 | 288 | 1 | 288 | 0 | 3.00E-130 |
| hsa_circ_0006882 | 96 | 996 | 1285 | 859 | 1148 | 0 | 1.00E-130 |
| hsa_circ_0006882 | 96 | 1 | 1142 | 1 | 1148 | 0.01 | 0 |
| hsa_circ_0006882 | 96 | 1 | 972 | 1 | 978 | 0.01 | 0 |
| hsa_circ_0006882 | 96 | 1 | 854 | 1 | 860 | 0.01 | 0 |
| hsa_circ_0054390 | 89 | 4 | 1113 | 1110 | 1 | 0 | 0 |
| hsa_circ_0054382 | 89 | 4 | 1138 | 1370 | 236 | 0 | 0 |
| hsa_circ_0054386 | 88 | 2 | 957 | 998 | 43 | 0 | 0 |

novel_circ_023908 novel_circ_023909 novel_circ_023910 novel_circ_023911 novel_circ_023912 novel_circ_023913 novel_circ_023914 novel_circ_023915 novel_circ_023917 novel_circ_023918 novel_circ_023919 novel_circ_023920 novel_circ_023921 novel_circ_023922 novel_circ_023923 novel_circ_023924 novel_circ_023925 novel_circ_023926 novel_circ_023927 novel_circ_023928 novel_circ_023929 novel_circ_023930 novel_circ_023931 novel_circ_023932 novel_circ_023933 novel_circ_023934 novel_circ_023935 novel_circ_023936 novel_circ_023937 novel_circ_023938 novel_circ_023939 novel_circ_023940 novel_circ_023941 novel_circ_023942 novel_circ_023943 novel_circ_023944 novel_circ_023945 novel_circ_023946 novel_circ_023947 novel_circ_023948 novel_circ_023949 novel_circ_023950 novel_circ_023951 novel_circ_023952 novel_circ_023953 novel_circ_023954 novel_circ_023955 novel_circ_023956 novel_circ_023957 novel_circ_023958 novel_circ_023959 novel_circ_023960 novel_circ_023962 novel_circ_023963

| hsa_circ_0054391 | 84 | 1 | 227 | 227 | 1 | 0 | 1.00E-54 |
| --- | --- | --- | --- | --- | --- | --- | --- |
| hsa_circ_0054372 | 92 | 1 | 533 | 533 | 1 | 0 | 0 |
| hsa_circ_0054355 | 91 | 5 | 390 | 388 | 3 | 0 | 2.00E-144 |
| hsa_circ_0054355 | 87 | 1 | 158 | 160 | 3 | 0 | 3.00E-43 |
| hsa_circ_0054329 | 91 | 1 | 305 | 1 | 305 | 0 | 5.00E-115 |
| hsa_circ_0054327 | 92 | 1 | 453 | 79 | 531 | 0 | 0 |
| hsa_circ_0054326 | 92 | 1 | 482 | 1 | 485 | 0.01 | 0 |
| hsa_circ_0054326 | 92 | 90 | 571 | 1 | 485 | 0.01 | 0 |
| hsa_circ_0007879 | 93 | 1800 | 2621 | 1 | 822 | 0 | 0 |
| hsa_circ_0054321 | 88 | 1 | 913 | 1248 | 336 | 0 | 0 |
| hsa_circ_0054321 | 89 | 1 | 692 | 1027 | 336 | 0.01 | 0 |
| hsa_circ_0054315 | 86 | 12 | 499 | 1052 | 565 | 0 | 8.00E-148 |
| hsa_circ_0054316 | 90 | 1 | 136 | 590 | 455 | 0 | 9.00E-44 |
| hsa_circ_0054316 | 91 | 5772 | 5931 | 160 | 1 | 0 | 1.00E-52 |
| hsa_circ_0054315 | 91 | 5772 | 5935 | 270 | 107 | 0 | 2.00E-54 |
| hsa_circ_0054315 | 91 | 5772 | 5935 | 270 | 107 | 0 | 5.00E-54 |
| hsa_circ_0054308 | 90 | 1 | 391 | 391 | 1 | 0 | 1.00E-140 |
| hsa_circ_0054293 | 86 | 7 | 1069 | 1063 | 1 | 0.01 | 0 |
| hsa_circ_0054293 | 85 | 1 | 858 | 858 | 1 | 0.01 | 0 |
| hsa_circ_0054275 | 93 | 1 | 823 | 1 | 823 | 0 | 0 |
| hsa_circ_0054261 | 94 | 1 | 512 | 95 | 606 | 0 | 0 |
| hsa_circ_0054261 | 95 | 1 | 412 | 95 | 506 | 0 | 0 |
| hsa_circ_0054261 | 94 | 1 | 309 | 95 | 403 | 0 | 2.00E-133 |
| hsa_circ_0054261 | 94 | 1 | 403 | 1 | 403 | 0 | 6.00E-174 |
| hsa_circ_0054261 | 95 | 1 | 191 | 95 | 285 | 0 | 8.00E-80 |
| hsa_circ_0057603 | 91 | 1 | 293 | 293 | 1 | 0 | 6.00E-108 |
| hsa_circ_0057603 | 92 | 1 | 480 | 480 | 1 | 0 | 0 |
| hsa_circ_0057603 | 91 | 1 | 678 | 678 | 1 | 0 | 0 |
| hsa_circ_0057603 | 92 | 1 | 769 | 769 | 1 | 0 | 0 |
| hsa_circ_0057603 | 91 | 1 | 863 | 863 | 1 | 0 | 0 |
| hsa_circ_0057604 | 92 | 1 | 334 | 334 | 1 | 0 | 2.00E-132 |
| hsa_circ_0057604 | 92 | 1 | 289 | 425 | 137 | 0 | 2.00E-112 |
| hsa_circ_0057604 | 91 | 1 | 383 | 519 | 137 | 0 | 3.00E-146 |
| hsa_circ_0057604 | 91 | 1 | 724 | 860 | 137 | 0 | 0 |
| hsa_circ_0057604 | 91 | 1 | 800 | 936 | 137 | 0 | 0 |
| hsa_circ_0057603 | 91 | 1 | 1223 | 1703 | 481 | 0 | 0 |
| hsa_circ_0057605 | 90 | 1 | 302 | 526 | 225 | 0 | 6.00E-108 |
| hsa_circ_0057606 | 93 | 1 | 543 | 543 | 1 | 0 | 0 |
| hsa_circ_0057607 | 94 | 1 | 366 | 366 | 1 | 0 | 9.00E-157 |
| hsa_circ_0057605 | 95 | 1 | 524 | 1303 | 780 | 0 | 0 |
| hsa_circ_0057605 | 95 | 1 | 872 | 1651 | 780 | 0 | 0 |
| hsa_circ_0057605 | 95 | 1 | 1026 | 1805 | 780 | 0 | 0 |
| hsa_circ_0057605 | 96 | 1 | 787 | 1651 | 865 | 0 | 0 |
| hsa_circ_0057605 | 96 | 1 | 626 | 1651 | 1026 | 0 | 0 |
| hsa_circ_0057605 | 96 | 1 | 506 | 1651 | 1146 | 0 | 0 |
| hsa_circ_0057605 | 96 | 1 | 660 | 1805 | 1146 | 0 | 0 |
| hsa_circ_0057616 | 93 | 1 | 623 | 625 | 3 | 0 | 0 |
| hsa_circ_0057616 | 93 | 1 | 807 | 809 | 3 | 0 | 0 |
| hsa_circ_0057621 | 95 | 1 | 550 | 550 | 1 | 0 | 0 |
| hsa_circ_0057621 | 94 | 1 | 1389 | 1389 | 1 | 0 | 0 |
| hsa_circ_0057621 | 93 | 1 | 839 | 1389 | 551 | 0 | 0 |
| hsa_circ_0057625 | 92 | 1 | 645 | 645 | 1 | 0 | 0 |
| hsa_circ_0015154 | 88 | 1 | 341 | 342 | 2 | 0 | 3.00E-111 |
| hsa_circ_0000156 | 94 | 1 | 386 | 386 | 1 | 0 | 7.00E-166 |

novel_circ_023964 novel_circ_023965 novel_circ_023966 novel_circ_023967 novel_circ_023968 novel_circ_023969 novel_circ_023972 novel_circ_023973 novel_circ_023974 novel_circ_023975 novel_circ_023976 novel_circ_023977 novel_circ_023978 novel_circ_023979 novel_circ_023980 novel_circ_023981 novel_circ_023982 novel_circ_023983 novel_circ_023984 novel_circ_023985 novel_circ_023986 novel_circ_023987 novel_circ_023988 novel_circ_023989 novel_circ_023990 novel_circ_023991 novel_circ_023992 novel_circ_023993 novel_circ_023994 novel_circ_023995 novel_circ_023996 novel_circ_023997 novel_circ_023998 novel_circ_023999 novel_circ_024000 novel_circ_024001 novel_circ_024002 novel_circ_024003 novel_circ_024004 novel_circ_024005 novel_circ_024006 novel_circ_024007 novel_circ_024008 novel_circ_024009 novel_circ_024010 novel_circ_024013 novel_circ_024014 novel_circ_024015 novel_circ_024016 novel_circ_024017 novel_circ_024018 novel_circ_024019 novel_circ_024020 novel_circ_024021

| hsa_circ_0000156 | 94 | 1 | 386 | 386 | 1 | 0 | 6.00E-166 |
| --- | --- | --- | --- | --- | --- | --- | --- |
| hsa_circ_0000156 | 94 | 1 | 386 | 386 | 1 | 0 | 3.00E-166 |
| hsa_circ_0000156 | 94 | 9442 | 9827 | 386 | 1 | 0 | 9.00E-165 |
| hsa_circ_0000156 | 94 | 18635 | 19020 | 386 | 1 | 0 | 2.00E-164 |
| hsa_circ_0015159 | 90 | 7 | 640 | 85 | 718 | 0 | 0 |
| hsa_circ_0084791 | 96 | 495 | 3630 | 1 | 3139 | 0 | 0 |
| hsa_circ_0049069 | 97 | 1 | 835 | 171 | 1005 | 0 | 0 |
| hsa_circ_0049072 | 96 | 1 | 559 | 1 | 559 | 0 | 0 |
| hsa_circ_0049077 | 95 | 836 | 1637 | 1 | 802 | 0 | 0 |
| hsa_circ_0049077 | 94 | 1 | 853 | 1 | 853 | 0 | 0 |
| hsa_circ_0049084 | 89 | 1 | 1181 | 1181 | 1 | 0 | 0 |
| hsa_circ_0067855 | 94 | 1 | 210 | 1 | 210 | 0 | 5.00E-87 |
| hsa_circ_0067855 | 95 | 1 | 301 | 1 | 301 | 0 | 2.00E-132 |
| hsa_circ_0067855 | 95 | 1 | 595 | 1 | 595 | 0 | 0 |
| hsa_circ_0067859 | 92 | 3 | 439 | 388 | 824 | 0 | 8.00E-178 |
| hsa_circ_0067859 | 93 | 3 | 510 | 388 | 895 | 0 | 0 |
| hsa_circ_0033102 | 93 | 1 | 348 | 519 | 172 | 0 | 2.00E-143 |
| hsa_circ_0033103 | 92 | 1 | 271 | 271 | 1 | 0 | 4.00E-104 |
| hsa_circ_0033108 | 91 | 1 | 1102 | 1102 | 1 | 0 | 0 |
| hsa_circ_0033108 | 91 | 1 | 2174 | 2174 | 1 | 0 | 0 |
| hsa_circ_0033108 | 92 | 1 | 821 | 1102 | 282 | 0 | 0 |
| hsa_circ_0033108 | 91 | 1 | 1198 | 1479 | 282 | 0 | 0 |
| hsa_circ_0033108 | 91 | 4 | 1480 | 1758 | 282 | 0 | 0 |
| hsa_circ_0033108 | 91 | 1 | 1893 | 2174 | 282 | 0 | 0 |
| hsa_circ_0033105 | 92 | 1 | 3033 | 3517 | 482 | 0 | 0 |
| hsa_circ_0033105 | 92 | 257 | 3285 | 3513 | 482 | 0 | 0 |
| hsa_circ_0033109 | 92 | 1 | 670 | 670 | 1 | 0 | 0 |
| hsa_circ_0033109 | 92 | 6 | 773 | 768 | 1 | 0 | 0 |
| hsa_circ_0033109 | 92 | 1 | 1047 | 1047 | 1 | 0 | 0 |
| hsa_circ_0033109 | 91 | 4 | 1329 | 1326 | 1 | 0 | 0 |
| hsa_circ_0033109 | 92 | 1 | 1742 | 1742 | 1 | 0 | 0 |
| hsa_circ_0033109 | 92 | 1 | 2882 | 2885 | 1 | 0 | 0 |
| hsa_circ_0033109 | 92 | 257 | 3134 | 2881 | 1 | 0 | 0 |
| hsa_circ_0033109 | 92 | 420 | 3297 | 2881 | 1 | 0 | 0 |
| hsa_circ_0033110 | 92 | 4 | 998 | 995 | 1 | 0 | 0 |
| hsa_circ_0033110 | 90 | 1 | 1330 | 1672 | 340 | 0 | 0 |
| hsa_circ_0033110 | 91 | 1 | 1613 | 1955 | 340 | 0 | 0 |
| hsa_circ_0033110 | 92 | 1 | 1506 | 1955 | 447 | 0 | 0 |
| hsa_circ_0033110 | 92 | 1 | 2105 | 2554 | 447 | 0 | 0 |
| hsa_circ_0033110 | 90 | 1 | 953 | 1672 | 717 | 0 | 0 |
| hsa_circ_0033110 | 94 | 1 | 882 | 2554 | 1673 | 0 | 0 |
| hsa_circ_0033110 | 94 | 257 | 1134 | 2550 | 1673 | 0 | 0 |
| hsa_circ_0002223 | 93 | 1 | 599 | 599 | 1 | 0 | 0 |
| hsa_circ_0002223 | 93 | 257 | 851 | 595 | 1 | 0 | 0 |
| hsa_circ_0002223 | 93 | 420 | 1014 | 595 | 1 | 0 | 0 |
| hsa_circ_0033116 | 98 | 1 | 241 | 1 | 241 | 0 | 3.00E-114 |
| hsa_circ_0033116 | 97 | 1 | 323 | 1 | 323 | 0 | 1.00E-154 |
| hsa_circ_0033116 | 97 | 1 | 487 | 1 | 487 | 0 | 0 |
| hsa_circ_0033116 | 96 | 1 | 597 | 1 | 597 | 0 | 0 |
| hsa_circ_0033116 | 98 | 1 | 1391 | 1 | 1391 | 0 | 0 |
| hsa_circ_0033116 | 98 | 1 | 1513 | 1 | 1513 | 0 | 0 |
| hsa_circ_0033116 | 98 | 1 | 1656 | 1 | 1656 | 0 | 0 |
| hsa_circ_0033116 | 96 | 1 | 2378 | 1 | 2394 | 0.01 | 0 |
| hsa_circ_0033116 | 97 | 1 | 149 | 175 | 323 | 0 | 1.00E-66 |

novel_circ_024022 novel_circ_024023 novel_circ_024024 novel_circ_024025 novel_circ_024026 novel_circ_024027 novel_circ_024028 novel_circ_024029 novel_circ_024030 novel_circ_024031 novel_circ_024032 novel_circ_024033 novel_circ_024034 novel_circ_024035 novel_circ_024036 novel_circ_024037 novel_circ_024038 novel_circ_024039 novel_circ_024040 novel_circ_024041 novel_circ_024042 novel_circ_024043 novel_circ_024044 novel_circ_024046 novel_circ_024047 novel_circ_024048 novel_circ_024049 novel_circ_024050 novel_circ_024055 novel_circ_024056 novel_circ_024057 novel_circ_024058 novel_circ_024059 novel_circ_024060 novel_circ_024061 novel_circ_024062 novel_circ_024064 novel_circ_024067 novel_circ_024068 novel_circ_024069 novel_circ_024070 novel_circ_024071 novel_circ_024073 novel_circ_024075 novel_circ_024076 novel_circ_024077 novel_circ_024078 novel_circ_024079 novel_circ_024080 novel_circ_024081 novel_circ_024082 novel_circ_024083 novel_circ_024084 novel_circ_024085

| hsa_circ_0033117 | 96 | 1 | 164 | 1 | 164 | 0 | 1.00E-71 |
| --- | --- | --- | --- | --- | --- | --- | --- |
| hsa_circ_0033122 | 95 | 1 | 1217 | 261 | 1493 | 0.02 | 0 |
| hsa_circ_0033122 | 96 | 1 | 697 | 381 | 1095 | 0.03 | 0 |
| hsa_circ_0033122 | 95 | 1 | 587 | 491 | 1095 | 0.03 | 0 |
| hsa_circ_0033122 | 95 | 1 | 725 | 491 | 1233 | 0.02 | 0 |
| hsa_circ_0033122 | 95 | 1 | 987 | 491 | 1493 | 0.02 | 0 |
| hsa_circ_0033122 | 94 | 1 | 465 | 613 | 1095 | 0.04 | 0 |
| hsa_circ_0033122 | 94 | 1 | 603 | 613 | 1233 | 0.03 | 0 |
| hsa_circ_0033122 | 94 | 1 | 865 | 613 | 1493 | 0.02 | 0 |
| hsa_circ_0033122 | 94 | 1 | 385 | 756 | 1158 | 0.04 | 1.00E-165 |
| hsa_circ_0033122 | 92 | 1 | 460 | 756 | 1233 | 0.04 | 0 |
| hsa_circ_0033122 | 94 | 1 | 722 | 756 | 1493 | 0.03 | 0 |
| hsa_circ_0033122 | 92 | 1 | 457 | 759 | 1233 | 0.04 | 0 |
| hsa_circ_0033122 | 95 | 1 | 400 | 1096 | 1493 | 0.01 | 4.00E-175 |
| hsa_circ_0033129 | 92 | 2 | 1166 | 103 | 1267 | 0 | 0 |
| hsa_circ_0033144 | 93 | 1 | 363 | 366 | 1 | 0.01 | 5.00E-149 |
| hsa_circ_0033144 | 93 | 1 | 366 | 369 | 1 | 0.01 | 1.00E-150 |
| hsa_circ_0033145 | 93 | 113 | 856 | 1072 | 329 | 0 | 0 |
| hsa_circ_0000567 | 93 | 1 | 479 | 479 | 1 | 0 | 0 |
| hsa_circ_0000567 | 93 | 1 | 682 | 683 | 1 | 0 | 0 |
| hsa_circ_0000567 | 93 | 1743 | 2424 | 683 | 1 | 0 | 0 |
| hsa_circ_0000567 | 93 | 3 | 544 | 570 | 29 | 0 | 0 |
| hsa_circ_0000567 | 93 | 1 | 654 | 683 | 29 | 0 | 0 |
| hsa_circ_0033156 | 86 | 1 | 792 | 538 | 1329 | 0.01 | 0 |
| hsa_circ_0033158 | 90 | 1 | 601 | 668 | 1268 | 0 | 0 |
| hsa_circ_0033165 | 94 | 1 | 347 | 1 | 347 | 0 | 6.00E-148 |
| hsa_circ_0033195 | 90 | 1514 | 1648 | 109 | 244 | 0.01 | 3.00E-42 |
| hsa_circ_0033195 | 90 | 1514 | 1648 | 109 | 244 | 0.01 | 4.00E-42 |
| hsa_circ_0005890 | 73 | 14669 | 15040 | 63749 | 63381 | 0.04 | 2.00E-20 |
| hsa_circ_0002571 | 73 | 30598 | 32511 | 6830 | 8745 | 0.07 | 1.00E-168 |
| hsa_circ_0002571 | 73 | 30598 | 32511 | 6830 | 8745 | 0.07 | 1.00E-168 |
| hsa_circ_0002571 | 73 | 30598 | 32511 | 6830 | 8745 | 0.07 | 1.00E-168 |
| hsa_circ_0002571 | 73 | 30598 | 32511 | 6830 | 8745 | 0.07 | 1.00E-168 |
| hsa_circ_0002571 | 73 | 30598 | 32511 | 6830 | 8745 | 0.07 | 2.00E-168 |
| hsa_circ_0002571 | 73 | 30598 | 32511 | 6830 | 8745 | 0.07 | 2.00E-168 |
| hsa_circ_0005890 | 73 | 11711 | 12082 | 63749 | 63381 | 0.04 | 2.00E-20 |
| hsa_circ_0002571 | 73 | 9437 | 11350 | 6830 | 8745 | 0.07 | 1.00E-168 |
| hsa_circ_0002571 | 73 | 8651 | 10564 | 6830 | 8745 | 0.07 | 5.00E-169 |
| hsa_circ_0002571 | 73 | 8651 | 10564 | 6830 | 8745 | 0.07 | 5.00E-169 |
| hsa_circ_0002571 | 73 | 8651 | 10564 | 6830 | 8745 | 0.07 | 6.00E-169 |
| hsa_circ_0002571 | 73 | 7187 | 9100 | 6830 | 8745 | 0.07 | 1.00E-168 |
| hsa_circ_0002571 | 73 | 4929 | 6842 | 6830 | 8745 | 0.07 | 1.00E-168 |
| hsa_circ_0002571 | 73 | 1 | 1332 | 7407 | 8745 | 0.06 | 3.00E-117 |
| hsa_circ_0004600 | 90 | 6438 | 6519 | 3293 | 3374 | 0 | 6.00E-21 |
| hsa_circ_0004600 | 90 | 6438 | 6519 | 3293 | 3374 | 0 | 8.00E-21 |
| hsa_circ_0004600 | 90 | 6438 | 6519 | 3293 | 3374 | 0 | 1.00E-20 |
| hsa_circ_0007636 | 78 | 18369 | 18599 | 1E+06 | 1301363 | 0.05 | 1.00E-27 |
| hsa_circ_0006541 | 73 | 20444 | 20996 | 6180 | 6742 | 0.08 | 8.00E-35 |
| hsa_circ_0006541 | 73 | 20444 | 20996 | 6180 | 6742 | 0.08 | 9.00E-35 |
| hsa_circ_0004600 | 75 | 37923 | 38429 | 3025 | 3524 | 0.09 | 1.00E-45 |
| hsa_circ_0004600 | 90 | 6308 | 6389 | 3293 | 3374 | 0 | 6.00E-21 |
| hsa_circ_0004600 | 90 | 6308 | 6389 | 3293 | 3374 | 0 | 7.00E-21 |
| hsa_circ_0004600 | 90 | 6308 | 6389 | 3293 | 3374 | 0 | 1.00E-20 |
| hsa_circ_0006541 | 73 | 20314 | 20866 | 6180 | 6742 | 0.08 | 8.00E-35 |

novel_circ_024086 novel_circ_024087 novel_circ_024088 novel_circ_024090 novel_circ_024091 novel_circ_024092 novel_circ_024093 novel_circ_024097 novel_circ_024098 novel_circ_024099 novel_circ_024100 novel_circ_024101 novel_circ_024102 novel_circ_024103 novel_circ_024104 novel_circ_024105 novel_circ_024112 novel_circ_024113 novel_circ_024114 novel_circ_024115 novel_circ_024116 novel_circ_024117 novel_circ_024118 novel_circ_024119 novel_circ_024120 novel_circ_024125 novel_circ_024127 novel_circ_024128 novel_circ_024129 novel_circ_024130 novel_circ_024132 novel_circ_024133 novel_circ_024137 novel_circ_024138 novel_circ_024139 novel_circ_024140 novel_circ_024141 novel_circ_024142 novel_circ_024143 novel_circ_024144 novel_circ_024145 novel_circ_024146 novel_circ_024148 novel_circ_024149 novel_circ_024150 novel_circ_024151 novel_circ_024152 novel_circ_024153 novel_circ_024154 novel_circ_024155 novel_circ_024156 novel_circ_024157 novel_circ_024159 novel_circ_024160

| hsa_circ_0006541 | 73 | 20314 | 20866 | 6180 | 6742 | 0.08 | 9.00E-35 |
| --- | --- | --- | --- | --- | --- | --- | --- |
| hsa_circ_0004600 | 90 | 3699 | 3780 | 3293 | 3374 | 0 | 4.00E-21 |
| hsa_circ_0004600 | 90 | 3699 | 3780 | 3293 | 3374 | 0 | 8.00E-21 |
| hsa_circ_0004600 | 90 | 2870 | 2951 | 3293 | 3374 | 0 | 3.00E-21 |
| hsa_circ_0006541 | 73 | 15860 | 16412 | 6180 | 6742 | 0.08 | 7.00E-35 |
| hsa_circ_0004600 | 90 | 391 | 472 | 3293 | 3374 | 0 | 6.00E-21 |
| hsa_circ_0004600 | 90 | 391 | 472 | 3293 | 3374 | 0 | 9.00E-21 |
| hsa_circ_0006541 | 73 | 8082 | 8634 | 6180 | 6742 | 0.08 | 5.00E-35 |
| hsa_circ_0006541 | 73 | 4335 | 4887 | 6180 | 6742 | 0.08 | 2.00E-35 |
| hsa_circ_0006541 | 73 | 4335 | 4887 | 6180 | 6742 | 0.08 | 3.00E-35 |
| hsa_circ_0006541 | 73 | 3115 | 3667 | 6180 | 6742 | 0.08 | 2.00E-35 |
| hsa_circ_0006541 | 73 | 3115 | 3667 | 6180 | 6742 | 0.08 | 4.00E-35 |
| hsa_circ_0009016 | 74 | 4433 | 4978 | 6758 | 7309 | 0.06 | 4.00E-45 |
| hsa_circ_0009016 | 74 | 4433 | 4978 | 6758 | 7309 | 0.06 | 7.00E-45 |
| hsa_circ_0055857 | 79 | 4857 | 4989 | 59269 | 59135 | 0.03 | 2.00E-14 |
| hsa_circ_0055857 | 79 | 4857 | 4989 | 59269 | 59135 | 0.03 | 2.00E-14 |
| hsa_circ_0033203 | 96 | 1 | 335 | 1 | 335 | 0 | 3.00E-151 |
| hsa_circ_0033203 | 95 | 1 | 551 | 1 | 551 | 0 | 0 |
| hsa_circ_0033203 | 93 | 1 | 953 | 1 | 953 | 0 | 0 |
| hsa_circ_0033205 | 93 | 1 | 216 | 1 | 216 | 0 | 3.00E-85 |
| hsa_circ_0033205 | 93 | 1 | 394 | 1 | 394 | 0 | 1.00E-160 |
| hsa_circ_0033205 | 92 | 1 | 522 | 1 | 522 | 0 | 0 |
| hsa_circ_0033205 | 92 | 1 | 618 | 1 | 618 | 0 | 0 |
| hsa_circ_0033205 | 93 | 1 | 334 | 61 | 394 | 0 | 1.00E-135 |
| hsa_circ_0033205 | 92 | 1 | 462 | 61 | 522 | 0 | 0 |
| hsa_circ_0033212 | 88 | 1 | 1205 | 1 | 1205 | 0 | 0 |
| hsa_circ_0033227 | 87 | 1 | 2843 | 1 | 2843 | 0 | 0 |
| hsa_circ_0033235 | 88 | 1 | 1554 | 1 | 1554 | 0 | 0 |
| hsa_circ_0033247 | 87 | 1 | 408 | 1 | 408 | 0 | 5.00E-130 |
| hsa_circ_0033267 | 89 | 1 | 738 | 1 | 738 | 0 | 0 |
| hsa_circ_0033304 | 88 | 1 | 392 | 1 | 395 | 0.01 | 1.00E-131 |
| hsa_circ_0033351 | 87 | 1 | 660 | 1 | 663 | 0.01 | 0 |
| hsa_circ_0004275 | 96 | 1 | 261 | 1 | 261 | 0 | 6.00E-117 |
| hsa_circ_0004275 | 90 | 1 | 727 | 1 | 727 | 0 | 0 |
| hsa_circ_0004275 | 90 | 1 | 727 | 1 | 727 | 0 | 0 |
| hsa_circ_0033408 | 87 | 1 | 1186 | 292 | 1489 | 0.01 | 0 |
| hsa_circ_0033408 | 84 | 1 | 3421 | 292 | 3712 | 0.02 | 0 |
| hsa_circ_0033408 | 84 | 1 | 3422 | 292 | 3713 | 0.02 | 0 |
| hsa_circ_0033408 | 84 | 1 | 3570 | 292 | 3861 | 0.02 | 0 |
| hsa_circ_0033409 | 87 | 5 | 466 | 5 | 466 | 0 | 2.00E-148 |
| hsa_circ_0033409 | 87 | 5 | 466 | 5 | 466 | 0 | 5.00E-148 |
| hsa_circ_0033409 | 89 | 1 | 714 | 2596 | 3309 | 0 | 0 |
| hsa_circ_0033416 | 94 | 300 | 692 | 1 | 393 | 0 | 2.00E-171 |
| hsa_circ_0033416 | 94 | 216 | 608 | 1 | 393 | 0 | 1.00E-171 |
| hsa_circ_0033416 | 94 | 216 | 744 | 1 | 529 | 0 | 0 |
| hsa_circ_0033416 | 94 | 216 | 744 | 1 | 529 | 0 | 0 |
| hsa_circ_0033416 | 94 | 163 | 555 | 1 | 393 | 0 | 1.00E-171 |
| hsa_circ_0006016 | 95 | 1 | 331 | 1 | 331 | 0 | 5.00E-144 |
| hsa_circ_0006016 | 95 | 1 | 331 | 1 | 331 | 0 | 8.00E-144 |
| hsa_circ_0006016 | 94 | 1 | 136 | 196 | 331 | 0 | 3.00E-52 |
| hsa_circ_0006016 | 94 | 1 | 136 | 196 | 331 | 0 | 4.00E-52 |
| hsa_circ_0033426 | 88 | 398 | 979 | 6 | 587 | 0 | 0 |
| hsa_circ_0033446 | 85 | 2 | 2232 | 2595 | 368 | 0 | 0 |
| hsa_circ_0033446 | 86 | 2 | 1539 | 2595 | 1061 | 0 | 0 |

novel_circ_024161 novel_circ_024162 novel_circ_024163 novel_circ_024164 novel_circ_024165 novel_circ_024166 novel_circ_024167 novel_circ_024168 novel_circ_024169 novel_circ_024170 novel_circ_024171 novel_circ_024172 novel_circ_024173 novel_circ_024174 novel_circ_024175 novel_circ_024176 novel_circ_024177 novel_circ_024178 novel_circ_024179 novel_circ_024180 novel_circ_024181 novel_circ_024182 novel_circ_024183 novel_circ_024184 novel_circ_024185 novel_circ_024186 novel_circ_024187 novel_circ_024188 novel_circ_024189 novel_circ_024190 novel_circ_024191 novel_circ_024192 novel_circ_024193 novel_circ_024194 novel_circ_024195 novel_circ_024197 novel_circ_024198 novel_circ_024199 novel_circ_024203 novel_circ_024204 novel_circ_024205 novel_circ_024206 novel_circ_024207 novel_circ_024210 novel_circ_024211 novel_circ_024212 novel_circ_024213 novel_circ_024214 novel_circ_024215 novel_circ_024216 novel_circ_024217 novel_circ_024218 novel_circ_024219 novel_circ_024220

| hsa_circ_0033451 | 84 | 2 | 582 | 1313 | 733 | 0 | 2.00E-154 |
| --- | --- | --- | --- | --- | --- | --- | --- |
| hsa_circ_0033451 | 85 | 1 | 693 | 1425 | 733 | 0 | 0 |
| hsa_circ_0033451 | 85 | 1 | 1357 | 2089 | 733 | 0 | 0 |
| hsa_circ_0033459 | 85 | 1 | 664 | 775 | 112 | 0.01 | 0 |
| hsa_circ_0033459 | 87 | 1 | 1437 | 1548 | 112 | 0 | 0 |
| hsa_circ_0033459 | 87 | 1 | 1709 | 1820 | 112 | 0 | 0 |
| hsa_circ_0005178 | 91 | 1 | 329 | 329 | 1 | 0 | 3.00E-121 |
| hsa_circ_0005178 | 91 | 5493 | 5823 | 331 | 1 | 0 | 4.00E-121 |
| hsa_circ_0005178 | 91 | 1 | 421 | 421 | 1 | 0 | 6.00E-159 |
| hsa_circ_0000571 | 91 | 5493 | 5674 | 182 | 1 | 0 | 1.00E-61 |
| hsa_circ_0000571 | 91 | 1 | 272 | 272 | 1 | 0 | 1.00E-99 |
| hsa_circ_0033474 | 94 | 32758 | 33189 | 1 | 432 | 0 | 0 |
| hsa_circ_0033474 | 93 | 1 | 192 | 1 | 192 | 0 | 1.00E-73 |
| hsa_circ_0033474 | 93 | 1 | 194 | 1 | 194 | 0 | 7.00E-74 |
| hsa_circ_0033474 | 93 | 1 | 194 | 1 | 194 | 0 | 8.00E-74 |
| hsa_circ_0033474 | 93 | 1 | 246 | 1 | 246 | 0 | 3.00E-100 |
| hsa_circ_0033474 | 93 | 1 | 246 | 1 | 246 | 0 | 4.00E-99 |
| hsa_circ_0033474 | 94 | 1 | 295 | 1 | 295 | 0 | 4.00E-124 |
| hsa_circ_0033474 | 94 | 1 | 361 | 1 | 361 | 0 | 2.00E-157 |
| hsa_circ_0033474 | 94 | 1 | 432 | 1 | 432 | 0 | 0 |
| hsa_circ_0033474 | 94 | 1 | 432 | 1 | 432 | 0 | 0 |
| hsa_circ_0033478 | 92 | 296 | 1213 | 1 | 918 | 0 | 0 |
| hsa_circ_0033478 | 93 | 296 | 1431 | 1 | 1136 | 0 | 0 |
| hsa_circ_0033478 | 93 | 296 | 1535 | 1 | 1240 | 0 | 0 |
| hsa_circ_0033478 | 92 | 1 | 852 | 67 | 918 | 0 | 0 |
| hsa_circ_0033478 | 93 | 1 | 1174 | 67 | 1240 | 0 | 0 |
| hsa_circ_0033482 | 92 | 1 | 781 | 1 | 781 | 0 | 0 |
| hsa_circ_0033482 | 95 | 1 | 220 | 295 | 514 | 0 | 2.00E-92 |
| hsa_circ_0033482 | 94 | 1 | 487 | 295 | 781 | 0 | 0 |
| hsa_circ_0033482 | 94 | 1 | 705 | 295 | 999 | 0 | 0 |
| hsa_circ_0033482 | 95 | 1 | 809 | 295 | 1103 | 0 | 0 |
| hsa_circ_0033484 | 96 | 1 | 218 | 1 | 218 | 0 | 2.00E-96 |
| hsa_circ_0033485 | 92 | 2 | 255 | 2 | 255 | 0 | 5.00E-98 |
| hsa_circ_0033492 | 90 | 1 | 198 | 1 | 198 | 0 | 7.00E-67 |
| hsa_circ_0033492 | 90 | 1 | 198 | 1 | 198 | 0 | 1.00E-66 |
| hsa_circ_0033509 | 87 | 494 | 1260 | 1 | 767 | 0 | 0 |
| hsa_circ_0033509 | 86 | 494 | 1489 | 1 | 996 | 0 | 0 |
| hsa_circ_0033509 | 87 | 1 | 1052 | 80 | 1131 | 0 | 0 |
| hsa_circ_0033506 | 84 | 2 | 457 | 889 | 1372 | 0.06 | 8.00E-121 |
| hsa_circ_0033522 | 90 | 1 | 320 | 322 | 3 | 0 | 1.00E-112 |
| hsa_circ_0033522 | 90 | 1 | 320 | 322 | 3 | 0 | 6.00E-113 |
| hsa_circ_0005791 | 91 | 1 | 345 | 345 | 1 | 0 | 8.00E-132 |
| hsa_circ_0005791 | 90 | 1 | 268 | 345 | 78 | 0 | 9.00E-96 |
| hsa_circ_0033552 | 89 | 1 | 344 | 535 | 192 | 0 | 3.00E-116 |
| hsa_circ_0074053 | 94 | 3 | 572 | 3 | 572 | 0 | 0 |
| hsa_circ_0074053 | 94 | 3 | 942 | 3 | 942 | 0 | 0 |
| hsa_circ_0074053 | 95 | 3 | 1423 | 3 | 1423 | 0 | 0 |
| hsa_circ_0074051 | 89 | 3 | 2833 | 87 | 2937 | 0.03 | 0 |
| hsa_circ_0074054 | 96 | 1 | 479 | 121 | 599 | 0 | 0 |
| hsa_circ_0074055 | 97 | 1 | 257 | 1 | 257 | 0 | 1.00E-119 |
| hsa_circ_0074059 | 92 | 3 | 357 | 355 | 1 | 0 | 5.00E-139 |
| hsa_circ_0074061 | 95 | 3 | 242 | 240 | 1 | 0 | 1.00E-103 |
| hsa_circ_0074064 | 93 | 1 | 808 | 814 | 1 | 0.01 | 0 |
| hsa_circ_0074064 | 93 | 1 | 541 | 547 | 1 | 0.01 | 0 |

novel_circ_024221 novel_circ_024222 novel_circ_024223 novel_circ_024224 novel_circ_024225 novel_circ_024226 novel_circ_024227 novel_circ_024228 novel_circ_024229 novel_circ_024230 novel_circ_024231 novel_circ_024232 novel_circ_024233 novel_circ_024234 novel_circ_024235 novel_circ_024236 novel_circ_024237 novel_circ_024238 novel_circ_024239 novel_circ_024240 novel_circ_024241 novel_circ_024242 novel_circ_024243 novel_circ_024244 novel_circ_024245 novel_circ_024246 novel_circ_024248 novel_circ_024252 novel_circ_024253 novel_circ_024254 novel_circ_024255 novel_circ_024257 novel_circ_024258 novel_circ_024259 novel_circ_024260 novel_circ_024261 novel_circ_024262 novel_circ_024263 novel_circ_024265 novel_circ_024266 novel_circ_024267 novel_circ_024268 novel_circ_024269 novel_circ_024270 novel_circ_024277 novel_circ_024278 novel_circ_024279 novel_circ_024280 novel_circ_024308 novel_circ_024309 novel_circ_024312 novel_circ_024313 novel_circ_024314 novel_circ_024315

| hsa_circ_0074064 | 95 | 1 | 385 | 814 | 430 | 0 | 5.00E-174 |
| --- | --- | --- | --- | --- | --- | --- | --- |
| hsa_circ_0074064 | 95 | 16867 | 17753 | 1316 | 430 | 0 | 0 |
| hsa_circ_0002927 | 94 | 1 | 267 | 267 | 1 | 0 | 7.00E-112 |
| hsa_circ_0002927 | 94 | 1 | 377 | 377 | 1 | 0 | 7.00E-163 |
| hsa_circ_0002927 | 94 | 1 | 540 | 540 | 1 | 0 | 0 |
| hsa_circ_0002927 | 94 | 1 | 662 | 662 | 1 | 0 | 0 |
| hsa_circ_0002927 | 94 | 1 | 769 | 769 | 1 | 0 | 0 |
| hsa_circ_0002927 | 94 | 4602 | 5370 | 769 | 1 | 0 | 0 |
| hsa_circ_0002927 | 94 | 16867 | 17635 | 769 | 1 | 0 | 0 |
| hsa_circ_0074065 | 94 | 1 | 395 | 395 | 1 | 0 | 3.00E-171 |
| hsa_circ_0002927 | 94 | 1 | 502 | 769 | 268 | 0 | 0 |
| hsa_circ_0002927 | 94 | 4602 | 5103 | 769 | 268 | 0 | 0 |
| hsa_circ_0002927 | 94 | 16867 | 17368 | 769 | 268 | 0 | 0 |
| hsa_circ_0074066 | 94 | 1 | 285 | 285 | 1 | 0 | 3.00E-120 |
| hsa_circ_0074066 | 94 | 1 | 392 | 392 | 1 | 0 | 7.00E-168 |
| hsa_circ_0074066 | 94 | 4602 | 4993 | 392 | 1 | 0 | 1.00E-166 |
| hsa_circ_0074066 | 94 | 16867 | 17258 | 392 | 1 | 0 | 3.00E-166 |
| hsa_circ_0074066 | 93 | 1 | 229 | 392 | 164 | 0 | 7.00E-91 |
| hsa_circ_0074066 | 93 | 4602 | 4830 | 392 | 164 | 0 | 2.00E-89 |
| hsa_circ_0074066 | 94 | 534 | 642 | 392 | 284 | 0 | 3.00E-37 |
| hsa_circ_0052003 | 90 | 1 | 507 | 3316 | 3822 | 0 | 0 |
| hsa_circ_0003395 | 93 | 1 | 403 | 1 | 403 | 0 | 6.00E-169 |
| hsa_circ_0051958 | 90 | 608 | 805 | 510 | 707 | 0 | 2.00E-66 |
| hsa_circ_0051958 | 89 | 1 | 266 | 241 | 506 | 0 | 5.00E-88 |
| hsa_circ_0051922 | 85 | 1 | 730 | 4774 | 5497 | 0.02 | 0 |
| hsa_circ_0051827 | 91 | 1 | 339 | 1 | 339 | 0 | 8.00E-127 |
| hsa_circ_0056900 | 91 | 1 | 557 | 1 | 557 | 0 | 0 |
| hsa_circ_0045264 | 77 | 34 | 512 | 3921 | 3443 | 0 | 1.00E-72 |
| hsa_circ_0045264 | 76 | 1 | 566 | 4008 | 3443 | 0.01 | 6.00E-81 |
| hsa_circ_0045264 | 74 | 20 | 848 | 4268 | 3443 | 0.02 | 2.00E-92 |
| hsa_circ_0045264 | 74 | 482 | 1421 | 4379 | 3443 | 0.01 | 1.00E-105 |
| hsa_circ_0056922 | 84 | 229 | 452 | 2715 | 2492 | 0 | 2.00E-55 |
| hsa_circ_0056922 | 84 | 229 | 452 | 2715 | 2492 | 0 | 2.00E-55 |
| hsa_circ_0056922 | 84 | 229 | 452 | 2715 | 2492 | 0 | 1.00E-55 |
| hsa_circ_0056922 | 84 | 229 | 452 | 2715 | 2492 | 0 | 1.00E-55 |
| hsa_circ_0026327 | 81 | 1 | 430 | 1 | 430 | 0.01 | 4.00E-90 |
| hsa_circ_0056922 | 84 | 229 | 452 | 2715 | 2492 | 0 | 6.00E-56 |
| hsa_circ_0026327 | 81 | 1 | 430 | 1 | 430 | 0.01 | 3.00E-90 |
| hsa_circ_0026327 | 82 | 1 | 206 | 1 | 206 | 0.01 | 2.00E-41 |
| hsa_circ_0026327 | 79 | 1 | 337 | 1 | 337 | 0.01 | 3.00E-57 |
| hsa_circ_0056922 | 84 | 137 | 358 | 2715 | 2494 | 0 | 3.00E-53 |
| hsa_circ_0056922 | 84 | 137 | 358 | 2715 | 2494 | 0 | 1.00E-52 |
| hsa_circ_0056923 | 89 | 4 | 137 | 274 | 141 | 0 | 3.00E-39 |
| hsa_circ_0056923 | 89 | 4 | 137 | 274 | 141 | 0 | 8.00E-39 |
| hsa_circ_0056899 | 88 | 202 | 734 | 533 | 1 | 0 | 0 |
| hsa_circ_0056894 | 91 | 1 | 620 | 955 | 336 | 0 | 0 |
| hsa_circ_0056894 | 92 | 1 | 431 | 766 | 336 | 0 | 1.00E-172 |
| hsa_circ_0056892 | 82 | 7 | 1745 | 1736 | 1 | 0.01 | 0 |
| hsa_circ_0090999 | 86 | 7411 | 9116 | 7 | 1742 | 0.03 | 0 |
| hsa_circ_0090999 | 86 | 24144 | 25849 | 7 | 1742 | 0.03 | 0 |
| hsa_circ_0090999 | 86 | 7411 | 9116 | 7 | 1742 | 0.03 | 0 |
| hsa_circ_0090999 | 86 | 24144 | 25849 | 7 | 1742 | 0.03 | 0 |
| hsa_circ_0054669 | 87 | 7316 | 7924 | 635 | 1 | 0.04 | 0 |
| hsa_circ_0056878 | 98 | 1 | 309 | 1 | 309 | 0 | 2.00E-153 |

novel_circ_024316 novel_circ_024317 novel_circ_024318 novel_circ_024320 novel_circ_024321 novel_circ_024324 novel_circ_024325 novel_circ_024326 novel_circ_024327 novel_circ_024328 novel_circ_024329 novel_circ_024330 novel_circ_024335 novel_circ_024336 novel_circ_024337 novel_circ_024338 novel_circ_024339 novel_circ_024344 novel_circ_024345 novel_circ_024346 novel_circ_024347 novel_circ_024348 novel_circ_024349 novel_circ_024350 novel_circ_024351 novel_circ_024352 novel_circ_024353 novel_circ_024354 novel_circ_024355 novel_circ_024356 novel_circ_024357 novel_circ_024358 novel_circ_024359 novel_circ_024360 novel_circ_024361 novel_circ_024362 novel_circ_024363 novel_circ_024364 novel_circ_024365 novel_circ_024366 novel_circ_024367 novel_circ_024368 novel_circ_024369 novel_circ_024370 novel_circ_024373 novel_circ_024374 novel_circ_024375 novel_circ_024376 novel_circ_024377 novel_circ_024378 novel_circ_024379 novel_circ_024381 novel_circ_024382 novel_circ_024383

| hsa_circ_0056876 | 98 | 1 | 723 | 1 | 723 | 0 | 0 |
| --- | --- | --- | --- | --- | --- | --- | --- |
| hsa_circ_0005227 | 91 | 3 | 379 | 3 | 376 | 0.01 | 4.00E-140 |
| hsa_circ_0005227 | 92 | 3 | 254 | 3 | 251 | 0.01 | 2.00E-97 |
| hsa_circ_0056852 | 88 | 1 | 636 | 636 | 1 | 0 | 0 |
| hsa_circ_0056852 | 88 | 1 | 467 | 467 | 1 | 0.01 | 2.00E-155 |
| hsa_circ_0056837 | 92 | 7 | 2193 | 117 | 2297 | 0.03 | 0 |
| hsa_circ_0056844 | 91 | 1 | 1369 | 1 | 1388 | 0.02 | 0 |
| hsa_circ_0056837 | 92 | 7 | 1890 | 117 | 2015 | 0.02 | 0 |
| hsa_circ_0056838 | 92 | 7 | 1780 | 9 | 1797 | 0.02 | 0 |
| hsa_circ_0056838 | 92 | 7 | 1610 | 9 | 1627 | 0.02 | 0 |
| hsa_circ_0056841 | 93 | 1 | 364 | 1 | 361 | 0.01 | 3.00E-151 |
| hsa_circ_0056838 | 94 | 7 | 529 | 9 | 528 | 0.01 | 0 |
| hsa_circ_0023981 | 90 | 1 | 510 | 481 | 1 | 0.06 | 1.00E-176 |
| hsa_circ_0023981 | 91 | 1 | 799 | 770 | 1 | 0.04 | 0 |
| hsa_circ_0023981 | 90 | 1 | 1016 | 987 | 1 | 0.03 | 0 |
| hsa_circ_0023982 | 91 | 1 | 588 | 588 | 1 | 0 | 0 |
| hsa_circ_0023988 | 93 | 1 | 476 | 476 | 1 | 0 | 0 |
| hsa_circ_0023992 | 94 | 61 | 319 | 318 | 60 | 0 | 2.00E-107 |
| hsa_circ_0024003 | 92 | 16916 | 20230 | 1 | 3309 | 0 | 0 |
| hsa_circ_0024003 | 92 | 1 | 3315 | 1 | 3309 | 0 | 0 |
| hsa_circ_0024003 | 92 | 69325 | 69640 | 3309 | 3624 | 0 | 5.00E-120 |
| hsa_circ_0024003 | 92 | 1 | 315 | 3310 | 3624 | 0 | 8.00E-122 |
| hsa_circ_0024003 | 95 | 169 | 339 | 3831 | 4001 | 0 | 4.00E-70 |
| hsa_circ_0024003 | 94 | 169 | 551 | 3831 | 4213 | 0 | 9.00E-166 |
| hsa_circ_0024003 | 94 | 169 | 690 | 3831 | 4352 | 0 | 0 |
| hsa_circ_0024005 | 92 | 2 | 848 | 847 | 1 | 0 | 0 |
| hsa_circ_0024005 | 93 | 125 | 1152 | 1028 | 1 | 0 | 0 |
| hsa_circ_0024005 | 92 | 125 | 1139 | 1028 | 14 | 0 | 0 |
| hsa_circ_0024005 | 92 | 2 | 678 | 847 | 171 | 0 | 0 |
| hsa_circ_0024005 | 93 | 125 | 982 | 1028 | 171 | 0 | 0 |
| hsa_circ_0024005 | 94 | 1 | 360 | 1028 | 669 | 0 | 9.00E-157 |
| hsa_circ_0024005 | 94 | 125 | 484 | 1028 | 669 | 0 | 1.00E-156 |
| hsa_circ_0066513 | 92 | 502 | 706 | 205 | 1 | 0 | 5.00E-77 |
| hsa_circ_0066513 | 92 | 230 | 434 | 205 | 1 | 0 | 3.00E-77 |
| hsa_circ_0066508 | 96 | 122 | 285 | 1916 | 1753 | 0 | 1.00E-69 |
| hsa_circ_0066505 | 93 | 232 | 647 | 666 | 251 | 0 | 3.00E-169 |
| hsa_circ_0066505 | 92 | 232 | 897 | 666 | 1 | 0 | 0 |
| hsa_circ_0066505 | 93 | 128 | 543 | 666 | 251 | 0 | 2.00E-169 |
| hsa_circ_0066503 | 92 | 413 | 719 | 307 | 1 | 0 | 2.00E-120 |
| hsa_circ_0066500 | 92 | 413 | 823 | 411 | 1 | 0 | 6.00E-163 |
| hsa_circ_0066500 | 92 | 1 | 411 | 411 | 1 | 0 | 3.00E-163 |
| hsa_circ_0066500 | 92 | 1 | 411 | 411 | 1 | 0 | 4.00E-163 |
| hsa_circ_0066500 | 91 | 1 | 104 | 104 | 1 | 0 | 9.00E-33 |
| hsa_circ_0066496 | 95 | 1 | 130 | 131 | 2 | 0 | 9.00E-51 |
| hsa_circ_0066488 | 94 | 1 | 836 | 836 | 1 | 0 | 0 |
| hsa_circ_0066488 | 94 | 1 | 645 | 645 | 1 | 0 | 0 |
| hsa_circ_0066488 | 96 | 1 | 402 | 402 | 1 | 0 | 0 |
| hsa_circ_0066488 | 95 | 1 | 305 | 305 | 1 | 0 | 1.00E-134 |
| hsa_circ_0066485 | 95 | 1 | 307 | 426 | 120 | 0 | 5.00E-134 |
| hsa_circ_0066478 | 88 | 1 | 282 | 282 | 1 | 0 | 3.00E-90 |
| hsa_circ_0066475 | 90 | 1 | 795 | 795 | 1 | 0 | 0 |
| hsa_circ_0003101 | 93 | 1 | 420 | 1 | 420 | 0 | 1.00E-171 |
| hsa_circ_0066451 | 93 | 1 | 1737 | 2355 | 619 | 0 | 0 |
| hsa_circ_0066454 | 92 | 1 | 404 | 1208 | 805 | 0.01 | 7.00E-163 |

novel_circ_024384 novel_circ_024385 novel_circ_024386 novel_circ_024387 novel_circ_024388 novel_circ_024389 novel_circ_024390 novel_circ_024391 novel_circ_024392 novel_circ_024393 novel_circ_024394 novel_circ_024395 novel_circ_024396 novel_circ_024397 novel_circ_024398 novel_circ_024399 novel_circ_024400 novel_circ_024401 novel_circ_024402 novel_circ_024404 novel_circ_024405 novel_circ_024406 novel_circ_024407 novel_circ_024408 novel_circ_024411 novel_circ_024412 novel_circ_024413 novel_circ_024414 novel_circ_024415 novel_circ_024416 novel_circ_024417 novel_circ_024418 novel_circ_024419 novel_circ_024420 novel_circ_024421 novel_circ_024422 novel_circ_024423 novel_circ_024424 novel_circ_024425 novel_circ_024426 novel_circ_024427 novel_circ_024428 novel_circ_024429 novel_circ_024430 novel_circ_024431 novel_circ_024433 novel_circ_024434 novel_circ_024435 novel_circ_024436 novel_circ_024437 novel_circ_024438 novel_circ_024439 novel_circ_024440 novel_circ_024441

| hsa_circ_0066454 | 92 | 1 | 1208 | 1208 | 1 | 0 | 0 |
| --- | --- | --- | --- | --- | --- | --- | --- |
| hsa_circ_0066454 | 92 | 1 | 1122 | 1125 | 1 | 0 | 0 |
| hsa_circ_0066454 | 92 | 1 | 1086 | 1089 | 1 | 0 | 0 |
| hsa_circ_0066454 | 92 | 1 | 897 | 897 | 1 | 0 | 0 |
| hsa_circ_0066454 | 92 | 1 | 804 | 804 | 1 | 0 | 0 |
| hsa_circ_0066448 | 95 | 1 | 783 | 1082 | 300 | 0 | 0 |
| hsa_circ_0020185 | 95 | 1 | 444 | 997 | 1439 | 0 | 0 |
| hsa_circ_0020185 | 93 | 1 | 768 | 673 | 1439 | 0 | 0 |
| hsa_circ_0020185 | 93 | 1 | 1215 | 226 | 1439 | 0 | 0 |
| hsa_circ_0020185 | 93 | 148 | 1362 | 226 | 1439 | 0 | 0 |
| hsa_circ_0020185 | 93 | 3017 | 4231 | 226 | 1439 | 0 | 0 |
| hsa_circ_0020185 | 93 | 130 | 1569 | 1 | 1439 | 0 | 0 |
| hsa_circ_0020185 | 93 | 267 | 1706 | 1 | 1439 | 0 | 0 |
| hsa_circ_0020185 | 92 | 1 | 647 | 226 | 872 | 0 | 0 |
| hsa_circ_0020185 | 92 | 1 | 572 | 226 | 797 | 0 | 0 |
| hsa_circ_0020185 | 92 | 3017 | 3588 | 226 | 797 | 0 | 0 |
| hsa_circ_0020185 | 93 | 130 | 926 | 1 | 797 | 0 | 0 |
| hsa_circ_0020185 | 93 | 1 | 376 | 226 | 601 | 0 | 5.00E-154 |
| hsa_circ_0020185 | 93 | 148 | 523 | 226 | 601 | 0 | 8.00E-154 |
| hsa_circ_0020178 | 97 | 1 | 1020 | 3346 | 2327 | 0 | 0 |
| hsa_circ_0004498 | 97 | 1 | 1017 | 1259 | 242 | 0 | 0 |
| hsa_circ_0020180 | 96 | 1 | 490 | 490 | 1 | 0 | 0 |
| hsa_circ_0020179 | 95 | 1 | 554 | 554 | 1 | 0 | 0 |
| hsa_circ_0005516 | 93 | 1 | 350 | 350 | 1 | 0 | 7.00E-143 |
| hsa_circ_0020147 | 94 | 1 | 785 | 785 | 1 | 0 | 0 |
| hsa_circ_0020147 | 93 | 1 | 376 | 376 | 1 | 0 | 2.00E-157 |
| hsa_circ_0020138 | 89 | 1 | 1007 | 1409 | 403 | 0.01 | 0 |
| hsa_circ_0020138 | 88 | 1 | 668 | 1070 | 403 | 0.02 | 0 |
| hsa_circ_0020134 | 98 | 5829 | 6058 | 230 | 1 | 0 | 6.00E-110 |
| hsa_circ_0002028 | 99 | 1 | 429 | 429 | 1 | 0 | 0 |
| hsa_circ_0020130 | 97 | 1 | 887 | 12749 | 11863 | 0 | 0 |
| hsa_circ_0020130 | 82 | 1 | 3051 | 12749 | 9720 | 0.07 | 0 |
| hsa_circ_0008910 | 91 | 1 | 251 | 251 | 1 | 0 | 2.00E-91 |
| hsa_circ_0003461 | 92 | 1 | 281 | 1 | 281 | 0 | 5.00E-108 |
| hsa_circ_0071500 | 87 | 1 | 1186 | 1 | 1177 | 0.01 | 0 |
| hsa_circ_0071502 | 91 | 1 | 504 | 507 | 1 | 0.01 | 0 |
| hsa_circ_0071505 | 92 | 1 | 241 | 226 | 466 | 0 | 2.00E-92 |
| hsa_circ_0071505 | 93 | 1 | 456 | 226 | 681 | 0 | 0 |
| hsa_circ_0071508 | 93 | 1 | 1218 | 98 | 1315 | 0 | 0 |
| hsa_circ_0071508 | 92 | 1 | 1797 | 98 | 1894 | 0 | 0 |
| hsa_circ_0071508 | 91 | 1 | 2015 | 98 | 2112 | 0 | 0 |
| hsa_circ_0071509 | 92 | 1 | 649 | 1 | 649 | 0 | 0 |
| hsa_circ_0071512 | 91 | 1 | 1421 | 1 | 1421 | 0 | 0 |
| hsa_circ_0071512 | 90 | 1 | 1639 | 1 | 1639 | 0 | 0 |
| hsa_circ_0071513 | 91 | 1 | 396 | 1 | 396 | 0 | 4.00E-150 |
| hsa_circ_0071513 | 93 | 1 | 326 | 202 | 527 | 0 | 3.00E-131 |
| hsa_circ_0071513 | 91 | 1 | 819 | 202 | 1020 | 0 | 0 |
| hsa_circ_0071513 | 91 | 1 | 1061 | 202 | 1262 | 0 | 0 |
| hsa_circ_0071513 | 90 | 1 | 1279 | 202 | 1480 | 0 | 0 |
| hsa_circ_0071513 | 90 | 1 | 1279 | 202 | 1480 | 0 | 0 |
| hsa_circ_0071514 | 94 | 3 | 287 | 3 | 287 | 0 | 3.00E-120 |
| hsa_circ_0071514 | 91 | 3 | 624 | 3 | 624 | 0 | 0 |
| hsa_circ_0071514 | 91 | 3 | 866 | 3 | 866 | 0 | 0 |
| hsa_circ_0071514 | 90 | 1 | 493 | 132 | 624 | 0 | 0 |

novel_circ_024442 novel_circ_024443 novel_circ_024444 novel_circ_024445 novel_circ_024446 novel_circ_024447 novel_circ_024448 novel_circ_024449 novel_circ_024450 novel_circ_024451 novel_circ_024452 novel_circ_024453 novel_circ_024454 novel_circ_024455 novel_circ_024456 novel_circ_024457 novel_circ_024458 novel_circ_024459 novel_circ_024460 novel_circ_024461 novel_circ_024462 novel_circ_024464 novel_circ_024465 novel_circ_024466 novel_circ_024467 novel_circ_024470 novel_circ_024471 novel_circ_024479 novel_circ_024480 novel_circ_024481 novel_circ_024482 novel_circ_024483 novel_circ_024484 novel_circ_024485 novel_circ_024486 novel_circ_024487 novel_circ_024488 novel_circ_024489 novel_circ_024490 novel_circ_024491 novel_circ_024492 novel_circ_024496 novel_circ_024499 novel_circ_024503 novel_circ_024510 novel_circ_024511 novel_circ_024512 novel_circ_024513 novel_circ_024514 novel_circ_024515 novel_circ_024516 novel_circ_024517 novel_circ_024518 novel_circ_024519

| hsa_circ_0071514 | 90 | 1 | 735 | 132 | 866 | 0 | 0 |
| --- | --- | --- | --- | --- | --- | --- | --- |
| hsa_circ_0071513 | 88 | 1 | 797 | 684 | 1480 | 0 | 0 |
| hsa_circ_0071513 | 88 | 1 | 797 | 684 | 1480 | 0 | 0 |
| hsa_circ_0071513 | 88 | 1 | 797 | 684 | 1480 | 0 | 0 |
| hsa_circ_0071513 | 88 | 1 | 797 | 684 | 1480 | 0 | 0 |
| hsa_circ_0071527 | 94 | 4 | 535 | 532 | 1 | 0 | 0 |
| hsa_circ_0071527 | 94 | 4 | 535 | 532 | 1 | 0 | 0 |
| hsa_circ_0071527 | 94 | 1 | 442 | 442 | 1 | 0 | 0 |
| hsa_circ_0071527 | 94 | 4 | 535 | 532 | 1 | 0 | 0 |
| hsa_circ_0071527 | 93 | 86 | 621 | 536 | 1 | 0 | 0 |
| hsa_circ_0071532 | 88 | 1 | 426 | 2232 | 1807 | 0 | 2.00E-143 |
| hsa_circ_0071532 | 89 | 1 | 551 | 2357 | 1807 | 0 | 0 |
| hsa_circ_0071532 | 89 | 382 | 932 | 2357 | 1807 | 0 | 0 |
| hsa_circ_0071535 | 83 | 242 | 972 | 97 | 827 | 0.01 | 0 |
| hsa_circ_0071535 | 90 | 1 | 529 | 665 | 1196 | 0.01 | 0 |
| hsa_circ_0071540 | 90 | 1 | 435 | 435 | 1 | 0 | 5.00E-160 |
| hsa_circ_0071540 | 90 | 1 | 524 | 524 | 1 | 0 | 0 |
| hsa_circ_0071548 | 87 | 1 | 269 | 266 | 1 | 0.01 | 3.00E-80 |
| hsa_circ_0071549 | 84 | 1 | 1058 | 1070 | 1 | 0.02 | 0 |
| hsa_circ_0071558 | 91 | 1 | 302 | 1 | 302 | 0 | 6.00E-113 |
| hsa_circ_0071558 | 90 | 1 | 662 | 1 | 662 | 0 | 0 |
| hsa_circ_0071566 | 91 | 2 | 429 | 2 | 429 | 0 | 4.00E-161 |
| hsa_circ_0071569 | 92 | 1 | 398 | 1 | 398 | 0 | 3.00E-161 |
| hsa_circ_0071568 | 94 | 12 | 1084 | 670 | 1742 | 0 | 0 |
| hsa_circ_0071568 | 93 | 1 | 255 | 956 | 1210 | 0 | 1.00E-103 |
| hsa_circ_0071584 | 91 | 544 | 961 | 797 | 380 | 0 | 1.00E-158 |
| hsa_circ_0071584 | 89 | 1 | 312 | 1108 | 797 | 0 | 2.00E-106 |
| hsa_circ_0071600 | 83 | 7 | 346 | 347 | 2 | 0.02 | 5.00E-84 |
| hsa_circ_0071611 | 88 | 1 | 487 | 487 | 1 | 0 | 7.00E-164 |
| hsa_circ_0071612 | 88 | 4 | 675 | 678 | 1 | 0.01 | 0 |
| hsa_circ_0071616 | 89 | 1 | 315 | 315 | 1 | 0 | 2.00E-108 |
| hsa_circ_0071616 | 88 | 11 | 3601 | 3588 | 1 | 0 | 0 |
| hsa_circ_0071618 | 88 | 11 | 3286 | 3273 | 1 | 0 | 0 |
| hsa_circ_0030378 | 95 | 1 | 312 | 1 | 312 | 0 | 7.00E-137 |
| hsa_circ_0030378 | 93 | 1 | 454 | 1 | 454 | 0 | 0 |
| hsa_circ_0030378 | 94 | 1 | 1100 | 1 | 1100 | 0 | 0 |
| hsa_circ_0030386 | 90 | 1 | 851 | 1 | 851 | 0 | 0 |
| hsa_circ_0030386 | 91 | 1 | 977 | 1 | 977 | 0 | 0 |
| hsa_circ_0077745 | 85 | 21687 | 21747 | 11282 | 11342 | 0 | 9.00E-07 |
| hsa_circ_0077745 | 85 | 21687 | 21747 | 11282 | 11342 | 0 | 8.00E-07 |
| hsa_circ_0077745 | 85 | 21687 | 21747 | 11282 | 11342 | 0 | 4.00E-07 |
| hsa_circ_0079763 | 76 | 20695 | 20879 | 221257 | 221444 | 0.07 | 2.00E-14 |
| hsa_circ_0079763 | 76 | 20695 | 20879 | 221257 | 221444 | 0.07 | 2.00E-14 |
| hsa_circ_0068765 | 90 | 10316 | 10745 | 442 | 13 | 0 | 5.00E-155 |
| hsa_circ_0031778 | 91 | 112 | 821 | 13 | 722 | 0 | 0 |
| hsa_circ_0031782 | 92 | 1 | 814 | 883 | 70 | 0 | 0 |
| hsa_circ_0007999 | 97 | 7938 | 8056 | 122920 | 123038 | 0 | 4.00E-48 |
| hsa_circ_0007999 | 97 | 71 | 189 | 122920 | 123038 | 0 | 1.00E-49 |
| hsa_circ_0007999 | 97 | 71 | 189 | 122920 | 123038 | 0 | 2.00E-49 |
| hsa_circ_0004984 | 98 | 311 | 471 | 3 | 163 | 0 | 1.00E-72 |
| hsa_circ_0031790 | 92 | 388 | 727 | 1 | 340 | 0 | 5.00E-132 |
| hsa_circ_0031790 | 93 | 388 | 798 | 1 | 411 | 0 | 4.00E-168 |
| hsa_circ_0031790 | 93 | 388 | 798 | 1 | 411 | 0 | 5.00E-168 |
| hsa_circ_0004984 | 98 | 240 | 400 | 3 | 163 | 0 | 8.00E-73 |

novel_circ_024520 novel_circ_024521 novel_circ_024522 novel_circ_024523 novel_circ_024524 novel_circ_024525 novel_circ_024526 novel_circ_024527 novel_circ_024528 novel_circ_024532 novel_circ_024533 novel_circ_024534 novel_circ_024535 novel_circ_024536 novel_circ_024537 novel_circ_024539 novel_circ_024540 novel_circ_024541 novel_circ_024542 novel_circ_024543 novel_circ_024544 novel_circ_024545 novel_circ_024546 novel_circ_024547 novel_circ_024548 novel_circ_024549 novel_circ_024550 novel_circ_024551 novel_circ_024552 novel_circ_024553 novel_circ_024554 novel_circ_024555 novel_circ_024556 novel_circ_024557 novel_circ_024558 novel_circ_024559 novel_circ_024560 novel_circ_024561 novel_circ_024562 novel_circ_024563 novel_circ_024564 novel_circ_024565 novel_circ_024566 novel_circ_024567 novel_circ_024568 novel_circ_024569 novel_circ_024570 novel_circ_024571 novel_circ_024572 novel_circ_024573 novel_circ_024574 novel_circ_024575 novel_circ_024576 novel_circ_024577

| hsa_circ_0031790 | 93 | 317 | 727 | 1 | 411 | 0 | 5.00E-168 |
| --- | --- | --- | --- | --- | --- | --- | --- |
| hsa_circ_0031790 | 92 | 80 | 419 | 1 | 340 | 0 | 3.00E-132 |
| hsa_circ_0031790 | 92 | 1 | 327 | 85 | 411 | 0 | 1.00E-126 |
| hsa_circ_0031790 | 94 | 1 | 184 | 228 | 411 | 0 | 5.00E-74 |
| hsa_circ_0031793 | 95 | 1 | 400 | 81 | 480 | 0 | 1.00E-175 |
| hsa_circ_0031794 | 95 | 1 | 282 | 1 | 282 | 0 | 7.00E-122 |
| hsa_circ_0006066 | 95 | 1 | 369 | 198174 | 197806 | 0 | 2.00E-163 |
| hsa_circ_0006066 | 95 | 1 | 402 | 198207 | 197806 | 0 | 2.00E-178 |
| hsa_circ_0006066 | 94 | 1 | 678 | 198483 | 197806 | 0 | 0 |
| hsa_circ_0006066 | 90 | 1 | 1136 | 199358 | 198208 | 0.02 | 0 |
| hsa_circ_0006066 | 92 | 1 | 1783 | 200528 | 198731 | 0.01 | 0 |
| hsa_circ_0031809 | 95 | 4 | 238 | 235 | 1 | 0 | 8.00E-101 |
| hsa_circ_0006066 | 94 | 1 | 1521 | 200528 | 199008 | 0 | 0 |
| hsa_circ_0031812 | 95 | 1 | 256 | 256 | 1 | 0 | 2.00E-112 |
| hsa_circ_0006066 | 94 | 1 | 1170 | 200528 | 199359 | 0 | 0 |
| hsa_circ_0031824 | 95 | 259 | 3292 | 3034 | 1 | 0 | 0 |
| hsa_circ_0031824 | 96 | 1 | 670 | 712 | 43 | 0 | 0 |
| hsa_circ_0031824 | 95 | 1 | 1485 | 1527 | 43 | 0 | 0 |
| hsa_circ_0031824 | 96 | 1 | 914 | 1218 | 305 | 0 | 0 |
| hsa_circ_0031826 | 95 | 1 | 624 | 797 | 174 | 0 | 0 |
| hsa_circ_0004040 | 95 | 1 | 506 | 506 | 1 | 0 | 0 |
| hsa_circ_0031826 | 95 | 1 | 610 | 901 | 292 | 0 | 0 |
| hsa_circ_0031826 | 95 | 2 | 733 | 1023 | 292 | 0 | 0 |
| hsa_circ_0031826 | 95 | 1 | 815 | 1106 | 292 | 0 | 0 |
| hsa_circ_0031824 | 94 | 259 | 2580 | 3034 | 713 | 0 | 0 |
| hsa_circ_0004040 | 94 | 1 | 343 | 506 | 164 | 0 | 1.00E-145 |
| hsa_circ_0031826 | 94 | 1 | 447 | 901 | 455 | 0 | 0 |
| hsa_circ_0031826 | 94 | 2 | 570 | 1023 | 455 | 0 | 0 |
| hsa_circ_0031826 | 94 | 1 | 652 | 1106 | 455 | 0 | 0 |
| hsa_circ_0031824 | 94 | 259 | 2417 | 3034 | 876 | 0 | 0 |
| hsa_circ_0031826 | 93 | 1 | 327 | 901 | 575 | 0 | 4.00E-135 |
| hsa_circ_0031826 | 94 | 1 | 532 | 1106 | 575 | 0 | 0 |
| hsa_circ_0031826 | 94 | 2 | 1188 | 1761 | 575 | 0 | 0 |
| hsa_circ_0031824 | 94 | 259 | 2297 | 3034 | 996 | 0 | 0 |
| hsa_circ_0031826 | 94 | 2 | 861 | 1761 | 902 | 0 | 0 |
| hsa_circ_0031824 | 94 | 1 | 989 | 2311 | 1323 | 0 | 0 |
| hsa_circ_0031824 | 94 | 259 | 1970 | 3034 | 1323 | 0 | 0 |
| hsa_circ_0031828 | 94 | 1 | 558 | 558 | 1 | 0 | 0 |
| hsa_circ_0031831 | 94 | 4 | 756 | 753 | 1 | 0 | 0 |
| hsa_circ_0031831 | 94 | 1 | 882 | 882 | 1 | 0 | 0 |
| hsa_circ_0031832 | 94 | 4 | 645 | 642 | 1 | 0 | 0 |
| hsa_circ_0031831 | 94 | 1 | 771 | 882 | 112 | 0 | 0 |
| hsa_circ_0031831 | 93 | 1 | 627 | 882 | 256 | 0 | 0 |
| hsa_circ_0031836 | 91 | 1 | 258 | 258 | 1 | 0 | 6.00E-97 |
| hsa_circ_0031842 | 93 | 1 | 290 | 290 | 1 | 0 | 5.00E-118 |
| hsa_circ_0031842 | 93 | 1 | 656 | 656 | 1 | 0 | 0 |
| hsa_circ_0031842 | 93 | 1 | 788 | 788 | 1 | 0 | 0 |
| hsa_circ_0031843 | 93 | 1 | 924 | 924 | 1 | 0 | 0 |
| hsa_circ_0031843 | 93 | 245 | 1168 | 924 | 1 | 0 | 0 |
| hsa_circ_0031843 | 94 | 1 | 524 | 524 | 1 | 0 | 0 |
| hsa_circ_0031843 | 94 | 1 | 366 | 524 | 159 | 0 | 4.00E-155 |
| hsa_circ_0031843 | 92 | 1 | 598 | 924 | 327 | 0 | 0 |
| hsa_circ_0031843 | 93 | 1 | 563 | 924 | 362 | 0 | 0 |
| hsa_circ_0031843 | 92 | 1 | 400 | 924 | 525 | 0 | 3.00E-157 |

novel_circ_024578 novel_circ_024579 novel_circ_024580 novel_circ_024581 novel_circ_024582 novel_circ_024583 novel_circ_024584 novel_circ_024585 novel_circ_024586 novel_circ_024587 novel_circ_024588 novel_circ_024589 novel_circ_024590 novel_circ_024591 novel_circ_024592 novel_circ_024593 novel_circ_024594 novel_circ_024595 novel_circ_024596 novel_circ_024597 novel_circ_024598 novel_circ_024599 novel_circ_024600 novel_circ_024601 novel_circ_024602 novel_circ_024604 novel_circ_024605 novel_circ_024606 novel_circ_024607 novel_circ_024608 novel_circ_024609 novel_circ_024612 novel_circ_024614 novel_circ_024616 novel_circ_024617 novel_circ_024619 novel_circ_024620 novel_circ_024621 novel_circ_024622 novel_circ_024623 novel_circ_024624 novel_circ_024625 novel_circ_024626 novel_circ_024627 novel_circ_024628 novel_circ_024629 novel_circ_024630 novel_circ_024631 novel_circ_024632 novel_circ_024633 novel_circ_024634 novel_circ_024635 novel_circ_024636 novel_circ_024637

| hsa_circ_0031845 | 92 | 1 | 268 | 268 | 1 | 0 | 4.00E-104 |
| --- | --- | --- | --- | --- | --- | --- | --- |
| hsa_circ_0031845 | 92 | 245 | 512 | 268 | 1 | 0 | 8.00E-104 |
| hsa_circ_0031854 | 95 | 11 | 501 | 493 | 3 | 0 | 0 |
| hsa_circ_0031855 | 96 | 11 | 341 | 331 | 1 | 0 | 2.00E-152 |
| hsa_circ_0031855 | 95 | 381 | 1206 | 826 | 1 | 0 | 0 |
| hsa_circ_0031855 | 94 | 1 | 259 | 600 | 342 | 0 | 4.00E-109 |
| hsa_circ_0031855 | 96 | 1 | 484 | 825 | 342 | 0 | 0 |
| hsa_circ_0031856 | 95 | 157 | 849 | 863 | 171 | 0 | 0 |
| hsa_circ_0002949 | 95 | 157 | 770 | 614 | 1 | 0 | 0 |
| hsa_circ_0002949 | 95 | 157 | 571 | 614 | 200 | 0 | 0 |
| hsa_circ_0031865 | 95 | 1 | 376 | 376 | 1 | 0 | 1.00E-170 |
| hsa_circ_0031869 | 95 | 1 | 212 | 212 | 1 | 0 | 2.00E-91 |
| hsa_circ_0031870 | 94 | 1 | 156 | 156 | 1 | 0 | 4.00E-62 |
| hsa_circ_0006342 | 90 | 1 | 248 | 1 | 248 | 0 | 5.00E-88 |
| hsa_circ_0006342 | 92 | 1 | 488 | 1 | 488 | 0 | 0 |
| hsa_circ_0006342 | 93 | 1 | 539 | 1 | 539 | 0 | 0 |
| hsa_circ_0006342 | 93 | 1 | 596 | 1 | 596 | 0 | 0 |
| hsa_circ_0031872 | 96 | 1 | 415 | 415 | 1 | 0 | 0 |
| hsa_circ_0031872 | 95 | 6 | 859 | 851 | 1 | 0 | 0 |
| hsa_circ_0031876 | 94 | 2 | 417 | 541 | 126 | 0 | 2.00E-179 |
| hsa_circ_0031876 | 92 | 442 | 1002 | 683 | 126 | 0.01 | 0 |
| hsa_circ_0008945 | 88 | 1 | 250 | 261 | 6 | 0.02 | 7.00E-79 |
| hsa_circ_0008945 | 90 | 529 | 899 | 382 | 6 | 0.02 | 2.00E-132 |
| hsa_circ_0008945 | 90 | 3281 | 3651 | 382 | 6 | 0.02 | 7.00E-132 |
| hsa_circ_0028324 | 91 | 155 | 211 | 2860 | 2804 | 0 | 3.00E-13 |
| hsa_circ_0031877 | 91 | 457 | 1137 | 1339 | 659 | 0 | 0 |
| hsa_circ_0031880 | 91 | 1 | 252 | 252 | 1 | 0 | 6.00E-92 |
| hsa_circ_0031880 | 93 | 1 | 456 | 456 | 1 | 0 | 0 |
| hsa_circ_0031891 | 92 | 103 | 285 | 427 | 245 | 0 | 2.00E-68 |
| hsa_circ_0031892 | 94 | 1 | 304 | 304 | 1 | 0 | 7.00E-129 |
| hsa_circ_0031892 | 94 | 256 | 559 | 304 | 1 | 0 | 1.00E-128 |
| hsa_circ_0031893 | 95 | 1 | 388 | 685 | 298 | 0 | 1.00E-175 |
| hsa_circ_0031914 | 90 | 3 | 468 | 733 | 1198 | 0 | 3.00E-172 |
| hsa_circ_0031934 | 86 | 4 | 813 | 2347 | 1547 | 0.02 | 0 |
| hsa_circ_0031934 | 89 | 4 | 565 | 2347 | 1786 | 0 | 0 |
| hsa_circ_0031938 | 93 | 6 | 298 | 295 | 3 | 0 | 1.00E-119 |
| hsa_circ_0031938 | 93 | 1 | 350 | 352 | 3 | 0 | 1.00E-144 |
| hsa_circ_0031934 | 92 | 1 | 714 | 3800 | 3087 | 0 | 0 |
| hsa_circ_0031934 | 92 | 1 | 863 | 3949 | 3087 | 0 | 0 |
| hsa_circ_0031934 | 90 | 4 | 1174 | 4262 | 3087 | 0.01 | 0 |
| hsa_circ_0031938 | 93 | 1 | 228 | 352 | 125 | 0 | 6.00E-92 |
| hsa_circ_0031934 | 92 | 1 | 592 | 3800 | 3209 | 0 | 0 |
| hsa_circ_0031934 | 92 | 1 | 741 | 3949 | 3209 | 0 | 0 |
| hsa_circ_0031939 | 91 | 1 | 513 | 513 | 1 | 0 | 0 |
| hsa_circ_0031939 | 89 | 4 | 824 | 826 | 1 | 0.01 | 0 |
| hsa_circ_0031939 | 89 | 4 | 673 | 826 | 152 | 0.01 | 0 |
| hsa_circ_0031939 | 88 | 4 | 576 | 826 | 249 | 0.02 | 0 |
| hsa_circ_0031940 | 88 | 4 | 460 | 462 | 1 | 0.02 | 2.00E-148 |
| hsa_circ_0031941 | 94 | 1 | 273 | 1 | 273 | 0 | 3.00E-115 |
| hsa_circ_0031942 | 95 | 230 | 624 | 1 | 395 | 0 | 5.00E-176 |
| hsa_circ_0031945 | 94 | 3 | 317 | 315 | 1 | 0 | 8.00E-132 |
| hsa_circ_0031946 | 94 | 121 | 941 | 821 | 1 | 0 | 0 |
| hsa_circ_0031947 | 95 | 121 | 562 | 442 | 1 | 0 | 0 |
| hsa_circ_0031948 | 94 | 121 | 515 | 395 | 1 | 0 | 9.00E-173 |

novel_circ_024638 novel_circ_024639 novel_circ_024640 novel_circ_024641 novel_circ_024642 novel_circ_024643 novel_circ_024645 novel_circ_024646 novel_circ_024647 novel_circ_024648 novel_circ_024649 novel_circ_024650 novel_circ_024651 novel_circ_024652 novel_circ_024653 novel_circ_024654 novel_circ_024655 novel_circ_024656 novel_circ_024657 novel_circ_024658 novel_circ_024659 novel_circ_024660 novel_circ_024664 novel_circ_024665 novel_circ_024666 novel_circ_024667 novel_circ_024668 novel_circ_024669 novel_circ_024670 novel_circ_024671 novel_circ_024672 novel_circ_024676 novel_circ_024678 novel_circ_024679 novel_circ_024680 novel_circ_024681 novel_circ_024682 novel_circ_024683 novel_circ_024684 novel_circ_024685 novel_circ_024686 novel_circ_024687 novel_circ_024688 novel_circ_024689 novel_circ_024690 novel_circ_024691 novel_circ_024692 novel_circ_024693 novel_circ_024694 novel_circ_024695 novel_circ_024696 novel_circ_024698 novel_circ_024699 novel_circ_024700

| hsa_circ_0031948 | 96 | 121 | 394 | 395 | 122 | 0 | 3.00E-122 |
| --- | --- | --- | --- | --- | --- | --- | --- |
| hsa_circ_0031970 | 95 | 1 | 308 | 310 | 3 | 0 | 1.00E-134 |
| hsa_circ_0031970 | 94 | 1 | 416 | 418 | 3 | 0 | 7.00E-178 |
| hsa_circ_0031970 | 95 | 1 | 745 | 747 | 3 | 0 | 0 |
| hsa_circ_0031970 | 95 | 169 | 605 | 747 | 311 | 0 | 0 |
| hsa_circ_0031970 | 96 | 403 | 731 | 747 | 419 | 0 | 1.00E-152 |
| hsa_circ_0031973 | 95 | 1 | 1025 | 1025 | 1 | 0 | 0 |
| hsa_circ_0031973 | 95 | 1 | 1025 | 1025 | 1 | 0 | 0 |
| hsa_circ_0031973 | 95 | 4 | 830 | 827 | 1 | 0 | 0 |
| hsa_circ_0031973 | 95 | 1 | 1025 | 1025 | 1 | 0 | 0 |
| hsa_circ_0031973 | 95 | 1195 | 1829 | 711 | 77 | 0 | 0 |
| hsa_circ_0031973 | 95 | 4 | 754 | 827 | 77 | 0 | 0 |
| hsa_circ_0031973 | 95 | 1 | 949 | 1025 | 77 | 0 | 0 |
| hsa_circ_0031976 | 96 | 1195 | 1566 | 372 | 1 | 0 | 2.00E-169 |
| hsa_circ_0031976 | 96 | 4 | 491 | 488 | 1 | 0 | 0 |
| hsa_circ_0031976 | 95 | 1 | 686 | 686 | 1 | 0 | 0 |
| hsa_circ_0031976 | 96 | 1195 | 1459 | 372 | 108 | 0 | 2.00E-118 |
| hsa_circ_0031976 | 96 | 4 | 384 | 488 | 108 | 0 | 4.00E-175 |
| hsa_circ_0031976 | 95 | 1 | 579 | 686 | 108 | 0 | 0 |
| hsa_circ_0031977 | 96 | 1195 | 1352 | 158 | 1 | 0 | 3.00E-67 |
| hsa_circ_0031977 | 96 | 4 | 277 | 274 | 1 | 0 | 4.00E-124 |
| hsa_circ_0031977 | 95 | 1 | 472 | 472 | 1 | 0 | 0 |
| hsa_circ_0031989 | 96 | 1 | 519 | 1 | 519 | 0 | 0 |
| hsa_circ_0031989 | 97 | 1 | 783 | 1 | 783 | 0 | 0 |
| hsa_circ_0032003 | 92 | 7 | 1500 | 1757 | 270 | 0.01 | 0 |
| hsa_circ_0032008 | 92 | 7 | 910 | 904 | 1 | 0 | 0 |
| hsa_circ_0032012 | 90 | 3 | 584 | 824 | 243 | 0 | 0 |
| hsa_circ_0032012 | 91 | 1 | 1022 | 1264 | 243 | 0 | 0 |
| hsa_circ_0032013 | 91 | 1 | 649 | 837 | 189 | 0 | 0 |
| hsa_circ_0032013 | 93 | 1 | 438 | 837 | 400 | 0 | 5.00E-180 |
| hsa_circ_0032026 | 93 | 1 | 468 | 4298 | 3831 | 0 | 0 |
| hsa_circ_0003163 | 80 | 2934 | 3289 | 1E+06 | 1178949 | 0.06 | 2.00E-61 |
| hsa_circ_0031752 | 95 | 1 | 653 | 5521 | 4869 | 0 | 0 |
| hsa_circ_0031754 | 96 | 1 | 899 | 899 | 1 | 0 | 0 |
| hsa_circ_0031756 | 91 | 599 | 1192 | 1 | 594 | 0 | 0 |
| hsa_circ_0031756 | 90 | 599 | 1618 | 1 | 1014 | 0.01 | 0 |
| hsa_circ_0031756 | 90 | 599 | 1772 | 1 | 1168 | 0.01 | 0 |
| hsa_circ_0031756 | 91 | 307 | 900 | 1 | 594 | 0 | 0 |
| hsa_circ_0031756 | 91 | 1 | 594 | 1 | 594 | 0 | 0 |
| hsa_circ_0031760 | 85 | 1 | 197 | 1 | 191 | 0.03 | 8.00E-50 |
| hsa_circ_0031760 | 90 | 1 | 1023 | 1 | 1017 | 0.01 | 0 |
| hsa_circ_0031760 | 90 | 1 | 1023 | 1 | 1017 | 0.01 | 0 |
| hsa_circ_0031760 | 90 | 1 | 1023 | 1 | 1017 | 0.01 | 0 |
| hsa_circ_0031765 | 91 | 1 | 246 | 827 | 1072 | 0 | 6.00E-92 |
| hsa_circ_0031766 | 94 | 29 | 481 | 19 | 471 | 0 | 0 |
| hsa_circ_0031766 | 95 | 711 | 1164 | 469 | 922 | 0 | 0 |
| hsa_circ_0031766 | 95 | 711 | 1272 | 469 | 1030 | 0 | 0 |
| hsa_circ_0031766 | 95 | 711 | 2213 | 469 | 1971 | 0 | 0 |
| hsa_circ_0031766 | 93 | 1 | 334 | 138 | 471 | 0.01 | 1.00E-138 |
| hsa_circ_0031766 | 95 | 1 | 726 | 470 | 1195 | 0 | 0 |
| hsa_circ_0031767 | 95 | 1 | 442 | 1 | 442 | 0 | 0 |
| hsa_circ_0069283 | 96 | 277 | 622 | 346 | 1 | 0 | 5.00E-156 |
| hsa_circ_0069283 | 96 | 1 | 346 | 346 | 1 | 0 | 2.00E-157 |
| hsa_circ_0069283 | 96 | 277 | 622 | 346 | 1 | 0 | 4.00E-157 |

novel_circ_024701 novel_circ_024702 novel_circ_024703 novel_circ_024704 novel_circ_024705 novel_circ_024706 novel_circ_024707 novel_circ_024708 novel_circ_024709 novel_circ_024710 novel_circ_024711 novel_circ_024712 novel_circ_024713 novel_circ_024714 novel_circ_024715 novel_circ_024716 novel_circ_024718 novel_circ_024728 novel_circ_024729 novel_circ_024731 novel_circ_024732 novel_circ_024733 novel_circ_024734 novel_circ_024735 novel_circ_024736 novel_circ_024737 novel_circ_024738 novel_circ_024739 novel_circ_024740 novel_circ_024741 novel_circ_024742 novel_circ_024747 novel_circ_024748 novel_circ_024749 novel_circ_024750 novel_circ_024751 novel_circ_024752 novel_circ_024753 novel_circ_024754 novel_circ_024755 novel_circ_024756 novel_circ_024757 novel_circ_024758 novel_circ_024759 novel_circ_024760 novel_circ_024761 novel_circ_024762 novel_circ_024763 novel_circ_024764 novel_circ_024765 novel_circ_024766 novel_circ_024767 novel_circ_024768 novel_circ_024769

| hsa_circ_0069283 | 96 | 25899 | 26244 | 346 | 1 | 0 | 2.00E-155 |
| --- | --- | --- | --- | --- | --- | --- | --- |
| hsa_circ_0069283 | 94 | 1 | 253 | 346 | 94 | 0 | 8.00E-107 |
| hsa_circ_0069285 | 99 | 3 | 276 | 274 | 1 | 0 | 4.00E-135 |
| hsa_circ_0069283 | 94 | 1 | 252 | 346 | 95 | 0 | 6.00E-107 |
| hsa_circ_0069283 | 94 | 211 | 462 | 346 | 95 | 0 | 1.00E-106 |
| hsa_circ_0069285 | 99 | 3 | 276 | 274 | 1 | 0 | 2.00E-135 |
| hsa_circ_0069285 | 99 | 173 | 446 | 274 | 1 | 0 | 2.00E-135 |
| hsa_circ_0069285 | 99 | 576 | 849 | 274 | 1 | 0 | 4.00E-135 |
| hsa_circ_0069285 | 99 | 25615 | 25888 | 274 | 1 | 0 | 9.00E-134 |
| hsa_circ_0069285 | 99 | 25625 | 25898 | 274 | 1 | 0 | 9.00E-134 |
| hsa_circ_0069285 | 98 | 1 | 210 | 210 | 1 | 0 | 2.00E-100 |
| hsa_circ_0069285 | 99 | 3 | 276 | 274 | 1 | 0 | 8.00E-136 |
| hsa_circ_0069285 | 99 | 173 | 446 | 274 | 1 | 0 | 1.00E-135 |
| hsa_circ_0069285 | 99 | 25615 | 25888 | 274 | 1 | 0 | 9.00E-134 |
| hsa_circ_0069285 | 99 | 25625 | 25898 | 274 | 1 | 0 | 9.00E-134 |
| hsa_circ_0069306 | 95 | 1 | 75 | 1 | 75 | 0 | 5.00E-25 |
| hsa_circ_0069294 | 85 | 1 | 665 | 2572 | 3236 | 0 | 0 |
| hsa_circ_0002865 | 90 | 1 | 396 | 400 | 795 | 0 | 4.00E-145 |
| hsa_circ_0002865 | 85 | 1 | 542 | 400 | 935 | 0.02 | 6.00E-154 |
| hsa_circ_0064330 | 85 | 1 | 102 | 6312 | 6212 | 0.05 | 4.00E-18 |
| hsa_circ_0064331 | 91 | 1 | 397 | 1 | 397 | 0 | 6.00E-149 |
| hsa_circ_0064331 | 91 | 1 | 603 | 1 | 603 | 0 | 0 |
| hsa_circ_0064331 | 91 | 1 | 931 | 1 | 931 | 0 | 0 |
| hsa_circ_0064331 | 90 | 1 | 545 | 59 | 603 | 0 | 0 |
| hsa_circ_0064353 | 80 | 1 | 320 | 83 | 393 | 0.03 | 2.00E-58 |
| hsa_circ_0064353 | 84 | 1 | 939 | 83 | 1024 | 0.02 | 0 |
| hsa_circ_0064368 | 95 | 1 | 261 | 360 | 100 | 0 | 3.00E-115 |
| hsa_circ_0064368 | 95 | 1 | 374 | 473 | 100 | 0 | 1.00E-164 |
| hsa_circ_0064368 | 95 | 364 | 738 | 474 | 100 | 0 | 8.00E-165 |
| hsa_circ_0064368 | 95 | 367 | 741 | 474 | 100 | 0 | 8.00E-165 |
| hsa_circ_0064368 | 94 | 13 | 236 | 697 | 474 | 0 | 5.00E-93 |
| hsa_circ_0067292 | 90 | 1 | 1625 | 193 | 1834 | 0.01 | 0 |
| hsa_circ_0067292 | 91 | 1 | 997 | 838 | 1834 | 0 | 0 |
| hsa_circ_0003665 | 93 | 1 | 935 | 935 | 1 | 0 | 0 |
| hsa_circ_0067324 | 94 | 132 | 797 | 708 | 41 | 0.01 | 0 |
| hsa_circ_0067324 | 94 | 206 | 871 | 708 | 41 | 0.01 | 0 |
| hsa_circ_0067324 | 94 | 456 | 1121 | 708 | 41 | 0.01 | 0 |
| hsa_circ_0067324 | 94 | 459 | 1124 | 708 | 41 | 0.01 | 0 |
| hsa_circ_0001340 | 89 | 4 | 250 | 248 | 1 | 0.02 | 3.00E-82 |
| hsa_circ_0001340 | 89 | 4 | 250 | 248 | 1 | 0.02 | 2.00E-82 |
| hsa_circ_0001340 | 89 | 4 | 250 | 248 | 1 | 0.02 | 2.00E-82 |
| hsa_circ_0064448 | 93 | 110 | 1655 | 167 | 1712 | 0 | 0 |
| hsa_circ_0064448 | 93 | 194 | 1739 | 167 | 1712 | 0 | 0 |
| hsa_circ_0064448 | 92 | 110 | 415 | 167 | 472 | 0 | 5.00E-120 |
| hsa_circ_0064426 | 91 | 1 | 610 | 1 | 610 | 0 | 0 |
| hsa_circ_0064426 | 92 | 1 | 373 | 1 | 373 | 0 | 3.00E-147 |
| hsa_circ_0064423 | 81 | 1 | 1052 | 1043 | 1 | 0.01 | 0 |
| hsa_circ_0001946 | 84 | 1 | 221 | 1283 | 1485 | 0.08 | 4.00E-47 |
| hsa_circ_0001946 | 78 | 1 | 1304 | 1 | 1344 | 0.05 | 0 |
| hsa_circ_0001946 | 78 | 1 | 1304 | 1 | 1344 | 0.05 | 0 |
| hsa_circ_0001946 | 84 | 1 | 221 | 1283 | 1485 | 0.08 | 3.00E-47 |
| hsa_circ_0001946 | 78 | 1 | 1304 | 1 | 1344 | 0.05 | 0 |
| hsa_circ_0001946 | 84 | 1 | 221 | 1283 | 1485 | 0.08 | 4.00E-48 |
| hsa_circ_0001946 | 78 | 1 | 1304 | 1 | 1344 | 0.05 | 0 |

novel_circ_024785 novel_circ_024786 novel_circ_024787 novel_circ_024788 novel_circ_024789 novel_circ_024790 novel_circ_024792 novel_circ_024793 novel_circ_024794 novel_circ_024795 novel_circ_024796 novel_circ_024797 novel_circ_024803 novel_circ_024804 novel_circ_024805 novel_circ_024806 novel_circ_024807 novel_circ_024808 novel_circ_024809 novel_circ_024810 novel_circ_024811 novel_circ_024812 novel_circ_024813 novel_circ_024814 novel_circ_024820 novel_circ_024821 novel_circ_024822 novel_circ_024823 novel_circ_024824 novel_circ_024825 novel_circ_024826 novel_circ_024827 novel_circ_024828 novel_circ_024829 novel_circ_024830 novel_circ_024831 novel_circ_024832 novel_circ_024833 novel_circ_024834 novel_circ_024835 novel_circ_024836 novel_circ_024837 novel_circ_024838 novel_circ_024839 novel_circ_024840 novel_circ_024841 novel_circ_024843 novel_circ_024844 novel_circ_024845 novel_circ_024846 novel_circ_024847 novel_circ_024848 novel_circ_024849 novel_circ_024850

| hsa_circ_0001946 | 86 | 3 | 174 | 449 | 622 | 0.01 | 8.00E-45 |
| --- | --- | --- | --- | --- | --- | --- | --- |
| hsa_circ_0001946 | 83 | 1 | 218 | 592 | 371 | 0.04 | 2.00E-47 |
| hsa_circ_0001946 | 88 | 3 | 126 | 449 | 572 | 0 | 2.00E-34 |
| hsa_circ_0001946 | 82 | 1 | 218 | 353 | 572 | 0.03 | 3.00E-45 |
| hsa_circ_0001946 | 82 | 2 | 200 | 572 | 371 | 0.03 | 2.00E-41 |
| hsa_circ_0001946 | 83 | 4 | 182 | 552 | 371 | 0.04 | 5.00E-37 |
| hsa_circ_0001946 | 75 | 5 | 214 | 749 | 536 | 0.06 | 2.00E-16 |
| hsa_circ_0001946 | 85 | 21 | 82 | 599 | 658 | 0.03 | 3.00E-09 |
| hsa_circ_0001946 | 77 | 1 | 209 | 447 | 658 | 0.04 | 4.00E-25 |
| hsa_circ_0001946 | 79 | 1 | 116 | 542 | 658 | 0.04 | 3.00E-13 |
| hsa_circ_0001946 | 77 | 1 | 209 | 447 | 658 | 0.04 | 3.00E-25 |
| hsa_circ_0001946 | 77 | 1 | 209 | 447 | 658 | 0.04 | 3.00E-25 |
| hsa_circ_0091664 | 95 | 1 | 528 | 632 | 105 | 0 | 0 |
| hsa_circ_0091664 | 95 | 1 | 632 | 632 | 1 | 0 | 0 |
| hsa_circ_0091656 | 95 | 1 | 1179 | 1179 | 1 | 0 | 0 |
| hsa_circ_0091654 | 92 | 1 | 460 | 1494 | 1035 | 0 | 0 |
| hsa_circ_0091654 | 93 | 1 | 1188 | 1494 | 310 | 0 | 0 |
| hsa_circ_0091654 | 93 | 1 | 751 | 748 | 1 | 0 | 0 |
| hsa_circ_0091649 | 95 | 1 | 1185 | 1336 | 155 | 0 | 0 |
| hsa_circ_0091649 | 94 | 1 | 1339 | 1336 | 1 | 0 | 0 |
| hsa_circ_0091649 | 96 | 1 | 639 | 897 | 259 | 0 | 0 |
| hsa_circ_0091649 | 95 | 1 | 897 | 897 | 1 | 0 | 0 |
| hsa_circ_0091649 | 96 | 1 | 536 | 794 | 259 | 0 | 0 |
| hsa_circ_0091649 | 97 | 1 | 434 | 588 | 155 | 0 | 0 |
| hsa_circ_0091631 | 95 | 1501 | 1686 | 315 | 500 | 0 | 5.00E-76 |
| hsa_circ_0091631 | 96 | 2 | 297 | 205 | 500 | 0 | 7.00E-133 |
| hsa_circ_0008579 | 95 | 1 | 300 | 1 | 300 | 0 | 7.00E-132 |
| hsa_circ_0091597 | 96 | 1 | 1003 | 553 | 1555 | 0 | 0 |
| hsa_circ_0091597 | 96 | 1 | 869 | 553 | 1421 | 0 | 0 |
| hsa_circ_0091597 | 96 | 1 | 717 | 553 | 1269 | 0 | 0 |
| hsa_circ_0091597 | 97 | 1 | 553 | 553 | 1105 | 0 | 0 |
| hsa_circ_0084859 | 94 | 1 | 374 | 374 | 1 | 0 | 3.00E-161 |
| hsa_circ_0084858 | 94 | 1 | 513 | 780 | 268 | 0 | 0 |
| hsa_circ_0084858 | 94 | 1 | 664 | 780 | 117 | 0 | 0 |
| hsa_circ_0084845 | 93 | 1 | 1179 | 475 | 1653 | 0 | 0 |
| hsa_circ_0084845 | 93 | 1 | 1359 | 295 | 1653 | 0 | 0 |
| hsa_circ_0084845 | 93 | 5 | 1499 | 159 | 1653 | 0 | 0 |
| hsa_circ_0084845 | 93 | 5 | 1128 | 159 | 1282 | 0 | 0 |
| hsa_circ_0084851 | 96 | 2 | 281 | 2 | 281 | 0 | 9.00E-126 |
| hsa_circ_0084849 | 95 | 1 | 380 | 1 | 380 | 0 | 7.00E-168 |
| hsa_circ_0084846 | 91 | 5 | 551 | 45 | 591 | 0 | 0 |
| hsa_circ_0003175 | 92 | 8 | 456 | 449 | 1 | 0 | 2.00E-179 |
| hsa_circ_0003175 | 94 | 1 | 338 | 338 | 1 | 0 | 6.00E-143 |
| hsa_circ_0003175 | 94 | 1 | 338 | 338 | 1 | 0 | 9.00E-143 |
| hsa_circ_0003175 | 94 | 1 | 338 | 338 | 1 | 0 | 9.00E-142 |
| hsa_circ_0003175 | 93 | 1 | 90 | 90 | 1 | 0 | 3.00E-30 |
| hsa_circ_0084842 | 97 | 532 | 1192 | 1 | 661 | 0 | 0 |
| hsa_circ_0084842 | 96 | 532 | 1087 | 1 | 556 | 0 | 0 |
| hsa_circ_0084842 | 96 | 4654 | 5209 | 1 | 556 | 0 | 0 |
| hsa_circ_0084832 | 94 | 1 | 540 | 1 | 540 | 0 | 0 |
| hsa_circ_0084824 | 89 | 1 | 991 | 1 | 992 | 0.01 | 0 |
| hsa_circ_0084819 | 90 | 1 | 1082 | 1 | 1083 | 0 | 0 |
| hsa_circ_0084824 | 91 | 1 | 654 | 1 | 654 | 0 | 0 |
| hsa_circ_0084824 | 91 | 1 | 469 | 1 | 469 | 0 | 0 |

novel_circ_024851 novel_circ_024852 novel_circ_024853 novel_circ_024854 novel_circ_024856 novel_circ_024857 novel_circ_024858 novel_circ_024860 novel_circ_024861 novel_circ_024862 novel_circ_024863 novel_circ_024864 novel_circ_024865 novel_circ_024866 novel_circ_024867 novel_circ_024868 novel_circ_024869 novel_circ_024870 novel_circ_024871 novel_circ_024872 novel_circ_024873 novel_circ_024874 novel_circ_024875 novel_circ_024876 novel_circ_024877 novel_circ_024878 novel_circ_024879 novel_circ_024880 novel_circ_024881 novel_circ_024882 novel_circ_024883 novel_circ_024884 novel_circ_024885 novel_circ_024886 novel_circ_024888 novel_circ_024889 novel_circ_024890 novel_circ_024891 novel_circ_024892 novel_circ_024893 novel_circ_024896 novel_circ_024897 novel_circ_024898 novel_circ_024900 novel_circ_024902 novel_circ_024903 novel_circ_024904 novel_circ_024905 novel_circ_024906 novel_circ_024907 novel_circ_024908 novel_circ_024909 novel_circ_024910 novel_circ_024911

| hsa_circ_0068257 | 91 | 4 | 565 | 845 | 284 | 0 | 0 |
| --- | --- | --- | --- | --- | --- | --- | --- |
| hsa_circ_0068258 | 91 | 1 | 813 | 1891 | 1079 | 0 | 0 |
| hsa_circ_0068263 | 91 | 491 | 1351 | 2 | 862 | 0 | 0 |
| hsa_circ_0068279 | 97 | 987 | 1105 | 113 | 231 | 0 | 5.00E-49 |
| hsa_circ_0068356 | 93 | 1 | 627 | 1 | 623 | 0.01 | 0 |
| hsa_circ_0068356 | 91 | 1 | 758 | 1 | 754 | 0.01 | 0 |
| hsa_circ_0068356 | 91 | 1 | 444 | 311 | 754 | 0 | 2.00E-173 |
| hsa_circ_0068363 | 92 | 1 | 1463 | 1 | 1463 | 0 | 0 |
| hsa_circ_0068363 | 93 | 1 | 1068 | 126 | 1193 | 0 | 0 |
| hsa_circ_0068365 | 91 | 1 | 360 | 215 | 574 | 0 | 4.00E-135 |
| hsa_circ_0068365 | 93 | 1 | 246 | 404 | 649 | 0 | 6.00E-97 |
| hsa_circ_0068363 | 93 | 1 | 510 | 684 | 1193 | 0 | 0 |
| hsa_circ_0068363 | 94 | 1 | 609 | 855 | 1463 | 0 | 0 |
| hsa_circ_0068374 | 92 | 1 | 401 | 178 | 578 | 0 | 3.00E-161 |
| hsa_circ_0068374 | 90 | 1 | 549 | 178 | 729 | 0.01 | 0 |
| hsa_circ_0068379 | 87 | 1 | 379 | 1 | 385 | 0.02 | 3.00E-117 |
| hsa_circ_0068374 | 90 | 3 | 517 | 868 | 1382 | 0 | 0 |
| hsa_circ_0068374 | 90 | 3 | 683 | 868 | 1548 | 0 | 0 |
| hsa_circ_0068374 | 90 | 3 | 737 | 868 | 1602 | 0 | 0 |
| hsa_circ_0068374 | 90 | 1 | 582 | 967 | 1548 | 0 | 0 |
| hsa_circ_0068374 | 90 | 1 | 636 | 967 | 1602 | 0 | 0 |
| hsa_circ_0068374 | 91 | 1 | 500 | 1049 | 1548 | 0 | 0 |
| hsa_circ_0068374 | 91 | 1 | 417 | 1132 | 1548 | 0 | 5.00E-160 |
| hsa_circ_0068382 | 87 | 60 | 729 | 670 | 1 | 0 | 0 |
| hsa_circ_0068382 | 87 | 164 | 833 | 670 | 1 | 0 | 0 |
| hsa_circ_0068382 | 88 | 60 | 387 | 670 | 343 | 0 | 3.00E-107 |
| hsa_circ_0068382 | 88 | 164 | 491 | 670 | 343 | 0 | 4.00E-107 |
| hsa_circ_0068389 | 92 | 450 | 825 | 1 | 376 | 0 | 6.00E-147 |
| hsa_circ_0068389 | 93 | 450 | 1252 | 1 | 803 | 0 | 0 |
| hsa_circ_0068389 | 92 | 1 | 376 | 1 | 376 | 0 | 3.00E-147 |
| hsa_circ_0068389 | 92 | 1 | 535 | 1 | 535 | 0 | 0 |
| hsa_circ_0068389 | 92 | 1 | 537 | 1 | 537 | 0 | 0 |
| hsa_circ_0068389 | 93 | 1 | 803 | 1 | 803 | 0 | 0 |
| hsa_circ_0068390 | 91 | 111 | 365 | 1 | 255 | 0 | 2.00E-93 |
| hsa_circ_0068396 | 84 | 4 | 286 | 283 | 1 | 0 | 2.00E-72 |
| hsa_circ_0068404 | 97 | 1 | 292 | 1 | 292 | 0 | 4.00E-139 |
| hsa_circ_0068404 | 90 | 1 | 364 | 202 | 565 | 0 | 2.00E-132 |
| hsa_circ_0068404 | 89 | 1 | 752 | 202 | 953 | 0 | 0 |
| hsa_circ_0068404 | 88 | 1 | 273 | 293 | 565 | 0 | 7.00E-87 |
| hsa_circ_0068404 | 88 | 1 | 792 | 293 | 1084 | 0 | 0 |
| hsa_circ_0068409 | 93 | 1 | 246 | 246 | 1 | 0 | 6.00E-97 |
| hsa_circ_0068412 | 95 | 1 | 397 | 408 | 1 | 0.03 | 4.00E-180 |
| hsa_circ_0068412 | 95 | 1 | 461 | 472 | 1 | 0.02 | 0 |
| hsa_circ_0068421 | 97 | 1 | 533 | 5804 | 5276 | 0.01 | 0 |
| hsa_circ_0022173 | 93 | 62 | 175 | 139 | 252 | 0 | 2.00E-40 |
| hsa_circ_0022174 | 93 | 1 | 380 | 1 | 380 | 0 | 1.00E-159 |
| hsa_circ_0022174 | 93 | 1 | 303 | 78 | 380 | 0 | 2.00E-123 |
| hsa_circ_0022181 | 94 | 1 | 1223 | 290 | 1512 | 0 | 0 |
| hsa_circ_0022181 | 94 | 1 | 1409 | 290 | 1698 | 0 | 0 |
| hsa_circ_0022181 | 94 | 1 | 2443 | 290 | 2732 | 0 | 0 |
| hsa_circ_0022182 | 94 | 1 | 1151 | 1 | 1151 | 0 | 0 |
| hsa_circ_0022182 | 94 | 1 | 1337 | 1 | 1337 | 0 | 0 |
| hsa_circ_0022181 | 94 | 1 | 1034 | 1699 | 2732 | 0 | 0 |
| hsa_circ_0022181 | 94 | 1 | 916 | 1817 | 2732 | 0 | 0 |

novel_circ_024912 novel_circ_024913 novel_circ_024914 novel_circ_024916 novel_circ_024917 novel_circ_024918 novel_circ_024919 novel_circ_024920 novel_circ_024921 novel_circ_024922 novel_circ_024923 novel_circ_024924 novel_circ_024925 novel_circ_024926 novel_circ_024927 novel_circ_024928 novel_circ_024929 novel_circ_024930 novel_circ_024931 novel_circ_024932 novel_circ_024933 novel_circ_024934 novel_circ_024935 novel_circ_024936 novel_circ_024937 novel_circ_024938 novel_circ_024939 novel_circ_024940 novel_circ_024941 novel_circ_024942 novel_circ_024943 novel_circ_024944 novel_circ_024945 novel_circ_024959 novel_circ_024960 novel_circ_024961 novel_circ_024962 novel_circ_024963 novel_circ_024964 novel_circ_024965 novel_circ_024966 novel_circ_024967 novel_circ_024968 novel_circ_024969 novel_circ_024970 novel_circ_024971 novel_circ_024972 novel_circ_024973 novel_circ_024974 novel_circ_024975 novel_circ_024976 novel_circ_024977 novel_circ_024978 novel_circ_024979

| hsa_circ_0022181 | 95 | 1 | 396 | 2337 | 2732 | 0 | 4.00E-175 |
| --- | --- | --- | --- | --- | --- | --- | --- |
| hsa_circ_0022189 | 90 | 1 | 250 | 1 | 247 | 0.01 | 5.00E-88 |
| hsa_circ_0009016 | 73 | 5318 | 5754 | 7245 | 6813 | 0.04 | 3.00E-28 |
| hsa_circ_0034396 | 79 | 14566 | 14769 | 440 | 237 | 0.04 | 2.00E-29 |
| hsa_circ_0015356 | 96 | 141 | 374 | 234 | 1 | 0 | 2.00E-103 |
| hsa_circ_0015356 | 96 | 28455 | 28688 | 234 | 1 | 0 | 2.00E-101 |
| hsa_circ_0015356 | 96 | 29329 | 29562 | 234 | 1 | 0 | 2.00E-101 |
| hsa_circ_0015356 | 96 | 1 | 445 | 712 | 268 | 0 | 0 |
| hsa_circ_0015361 | 96 | 5812 | 6122 | 311 | 1 | 0 | 6.00E-140 |
| hsa_circ_0015361 | 96 | 1 | 452 | 452 | 1 | 0 | 0 |
| hsa_circ_0015369 | 94 | 1 | 251 | 251 | 1 | 0 | 1.00E-104 |
| hsa_circ_0015369 | 95 | 1 | 309 | 309 | 1 | 0 | 7.00E-137 |
| hsa_circ_0015369 | 96 | 1 | 386 | 386 | 1 | 0 | 3.00E-176 |
| hsa_circ_0015369 | 96 | 1 | 446 | 446 | 1 | 0 | 0 |
| hsa_circ_0015369 | 96 | 6851 | 7298 | 448 | 1 | 0 | 0 |
| hsa_circ_0015369 | 96 | 1 | 515 | 515 | 1 | 0 | 0 |
| hsa_circ_0015369 | 96 | 1 | 712 | 712 | 1 | 0 | 0 |
| hsa_circ_0015369 | 96 | 1 | 810 | 810 | 1 | 0 | 0 |
| hsa_circ_0015369 | 96 | 1 | 870 | 870 | 1 | 0 | 0 |
| hsa_circ_0015369 | 96 | 12692 | 13561 | 870 | 1 | 0 | 0 |
| hsa_circ_0015369 | 96 | 15056 | 15925 | 870 | 1 | 0 | 0 |
| hsa_circ_0015369 | 96 | 23698 | 24567 | 870 | 1 | 0 | 0 |
| hsa_circ_0015369 | 97 | 1 | 734 | 870 | 137 | 0 | 0 |
| hsa_circ_0004436 | 97 | 1 | 424 | 424 | 1 | 0 | 0 |
| hsa_circ_0004436 | 97 | 1 | 424 | 424 | 1 | 0 | 0 |
| hsa_circ_0004436 | 97 | 1 | 364 | 364 | 1 | 0 | 1.00E-175 |
| hsa_circ_0004436 | 97 | 1 | 424 | 424 | 1 | 0 | 0 |
| hsa_circ_0009064 | 98 | 1 | 295 | 295 | 1 | 0 | 2.00E-142 |
| hsa_circ_0009064 | 97 | 1 | 355 | 355 | 1 | 0 | 2.00E-172 |
| hsa_circ_0009064 | 97 | 23698 | 24052 | 355 | 1 | 0 | 2.00E-170 |
| hsa_circ_0015379 | 99 | 1 | 158 | 158 | 1 | 0 | 6.00E-75 |
| hsa_circ_0015379 | 99 | 23698 | 23855 | 158 | 1 | 0 | 1.00E-72 |
| hsa_circ_0015380 | 97 | 1 | 60 | 60 | 1 | 0 | 1.00E-19 |
| hsa_circ_0003086 | 90 | 756 | 796 | 128237 | 128197 | 0 | 1.00E-05 |
| hsa_circ_0049577 | 91 | 1 | 380 | 380 | 1 | 0 | 3.00E-141 |
| hsa_circ_0049585 | 92 | 1 | 565 | 565 | 1 | 0 | 0 |
| hsa_circ_0049595 | 91 | 1 | 606 | 1 | 606 | 0 | 0 |
| hsa_circ_0049645 | 91 | 1 | 563 | 1 | 563 | 0 | 0 |
| hsa_circ_0005660 | 98 | 1 | 532 | 1 | 532 | 0 | 0 |
| hsa_circ_0049674 | 91 | 1 | 279 | 691 | 413 | 0 | 3.00E-105 |
| hsa_circ_0049674 | 92 | 1 | 578 | 990 | 413 | 0 | 0 |
| hsa_circ_0049674 | 92 | 12222 | 12800 | 991 | 413 | 0 | 0 |
| hsa_circ_0049674 | 91 | 2001 | 2941 | 1348 | 413 | 0.01 | 0 |
| hsa_circ_0049674 | 92 | 1 | 1146 | 1553 | 413 | 0 | 0 |
| hsa_circ_0049675 | 90 | 1 | 333 | 333 | 1 | 0 | 2.00E-118 |
| hsa_circ_0049675 | 91 | 2001 | 2813 | 808 | 1 | 0.01 | 0 |
| hsa_circ_0049675 | 91 | 1 | 1018 | 1013 | 1 | 0 | 0 |
| hsa_circ_0049675 | 92 | 12222 | 12521 | 451 | 152 | 0 | 3.00E-115 |
| hsa_circ_0049676 | 92 | 2001 | 2273 | 273 | 1 | 0 | 1.00E-102 |
| hsa_circ_0049676 | 93 | 1 | 478 | 478 | 1 | 0 | 0 |
| hsa_circ_0049676 | 92 | 2 | 616 | 615 | 1 | 0 | 0 |
| hsa_circ_0049676 | 92 | 140 | 754 | 615 | 1 | 0 | 0 |
| hsa_circ_0049676 | 92 | 301 | 915 | 615 | 1 | 0 | 0 |
| hsa_circ_0049676 | 92 | 401 | 1015 | 615 | 1 | 0 | 0 |

novel_circ_024980 novel_circ_024981 novel_circ_024982 novel_circ_024983 novel_circ_024984 novel_circ_024985 novel_circ_024986 novel_circ_024987 novel_circ_024988 novel_circ_024989 novel_circ_024990 novel_circ_024991 novel_circ_024993 novel_circ_024994 novel_circ_024995 novel_circ_024996 novel_circ_024997 novel_circ_024998 novel_circ_024999 novel_circ_025000 novel_circ_025001 novel_circ_025002 novel_circ_025003 novel_circ_025004 novel_circ_025005 novel_circ_025006 novel_circ_025007 novel_circ_025008 novel_circ_025009 novel_circ_025010 novel_circ_025011 novel_circ_025012 novel_circ_025013 novel_circ_025014 novel_circ_025015 novel_circ_025016 novel_circ_025017 novel_circ_025018 novel_circ_025019 novel_circ_025020 novel_circ_025021 novel_circ_025022 novel_circ_025023 novel_circ_025024 novel_circ_025025 novel_circ_025026 novel_circ_025027 novel_circ_025029 novel_circ_025030 novel_circ_025031 novel_circ_025032 novel_circ_025034 novel_circ_025035 novel_circ_025036

| hsa_circ_0049675 | 91 | 927 | 2093 | 2178 | 1012 | 0 | 0 |
| --- | --- | --- | --- | --- | --- | --- | --- |
| hsa_circ_0049676 | 93 | 301 | 804 | 615 | 112 | 0 | 0 |
| hsa_circ_0049676 | 93 | 1 | 261 | 877 | 617 | 0 | 3.00E-105 |
| hsa_circ_0049675 | 93 | 1 | 697 | 1848 | 1152 | 0 | 0 |
| hsa_circ_0049675 | 92 | 927 | 1953 | 2178 | 1152 | 0 | 0 |
| hsa_circ_0049675 | 94 | 1 | 186 | 3424 | 3239 | 0 | 2.00E-75 |
| hsa_circ_0049674 | 94 | 1 | 580 | 4975 | 4396 | 0 | 0 |
| hsa_circ_0049674 | 94 | 1 | 370 | 4975 | 4606 | 0 | 2.00E-157 |
| hsa_circ_0049674 | 95 | 1 | 277 | 4975 | 4699 | 0 | 4.00E-119 |
| hsa_circ_0002933 | 96 | 1 | 347 | 347 | 1 | 0 | 1.00E-159 |
| hsa_circ_0002933 | 96 | 1 | 439 | 439 | 1 | 0 | 0 |
| hsa_circ_0002933 | 96 | 40368 | 40807 | 440 | 1 | 0 | 0 |
| hsa_circ_0049736 | 91 | 1 | 379 | 379 | 1 | 0.01 | 1.00E-140 |
| hsa_circ_0090133 | 87 | 127 | 696 | 1 | 588 | 0.03 | 0 |
| hsa_circ_0090129 | 91 | 127 | 2109 | 436 | 2433 | 0.02 | 0 |
| hsa_circ_0090133 | 87 | 1 | 570 | 1 | 588 | 0.03 | 0 |
| hsa_circ_0090133 | 87 | 1 | 570 | 1 | 588 | 0.03 | 0 |
| hsa_circ_0090133 | 88 | 1 | 861 | 1 | 882 | 0.03 | 0 |
| hsa_circ_0090129 | 91 | 1 | 1983 | 436 | 2433 | 0.02 | 0 |
| hsa_circ_0092225 | 90 | 1 | 591 | 574 | 1161 | 0.02 | 0 |
| hsa_circ_0090129 | 93 | 1 | 1413 | 1024 | 2433 | 0.01 | 0 |
| hsa_circ_0064842 | 92 | 1 | 292 | 1052 | 1343 | 0 | 1.00E-115 |
| hsa_circ_0064842 | 92 | 1 | 359 | 1 | 359 | 0 | 1.00E-144 |
| hsa_circ_0064836 | 90 | 1 | 280 | 5668 | 5947 | 0 | 7.00E-99 |
| hsa_circ_0064836 | 91 | 1 | 529 | 5419 | 5947 | 0 | 0 |
| hsa_circ_0064836 | 91 | 1 | 533 | 5415 | 5947 | 0 | 0 |
| hsa_circ_0064836 | 90 | 1 | 280 | 5668 | 5947 | 0 | 6.00E-99 |
| hsa_circ_0064836 | 90 | 1 | 280 | 5668 | 5947 | 0 | 4.00E-99 |
| hsa_circ_0064836 | 92 | 1 | 1017 | 1 | 1020 | 0 | 0 |
| hsa_circ_0064837 | 94 | 1 | 327 | 273 | 599 | 0 | 8.00E-137 |
| hsa_circ_0064836 | 91 | 619 | 1503 | 1 | 888 | 0 | 0 |
| hsa_circ_0064836 | 92 | 1 | 649 | 58 | 709 | 0 | 0 |
| hsa_circ_0064836 | 92 | 1 | 706 | 1 | 709 | 0 | 0 |
| hsa_circ_0064836 | 92 | 49 | 754 | 1 | 709 | 0 | 0 |
| hsa_circ_0064836 | 92 | 430 | 1135 | 1 | 709 | 0 | 0 |
| hsa_circ_0064836 | 92 | 619 | 1324 | 1 | 709 | 0 | 0 |
| hsa_circ_0064836 | 90 | 1 | 501 | 58 | 561 | 0.01 | 0 |
| hsa_circ_0064836 | 90 | 1 | 558 | 1 | 561 | 0.01 | 0 |
| hsa_circ_0064836 | 90 | 430 | 987 | 1 | 561 | 0.01 | 0 |
| hsa_circ_0064836 | 90 | 619 | 1176 | 1 | 561 | 0.01 | 0 |
| hsa_circ_0064836 | 89 | 1 | 474 | 1 | 477 | 0.01 | 3.00E-167 |
| hsa_circ_0064836 | 89 | 430 | 903 | 1 | 477 | 0.01 | 6.00E-167 |
| hsa_circ_0064836 | 89 | 619 | 1092 | 1 | 477 | 0.01 | 7.00E-167 |
| hsa_circ_0064836 | 91 | 1 | 229 | 58 | 289 | 0.01 | 1.00E-84 |
| hsa_circ_0064836 | 91 | 1 | 229 | 58 | 289 | 0.01 | 1.00E-84 |
| hsa_circ_0064836 | 91 | 1 | 286 | 1 | 289 | 0.01 | 5.00E-108 |
| hsa_circ_0008654 | 86 | 191 | 570 | 2 | 381 | 0 | 1.00E-112 |
| hsa_circ_0008386 | 92 | 1 | 102 | 102 | 1 | 0 | 5.00E-34 |
| hsa_circ_0064825 | 93 | 123 | 710 | 764 | 177 | 0 | 0 |
| hsa_circ_0064825 | 89 | 1 | 406 | 406 | 1 | 0 | 7.00E-144 |
| hsa_circ_0064822 | 84 | 584 | 1359 | 1 | 767 | 0.03 | 0 |
| hsa_circ_0007714 | 100 | 24369 | 24439 | 320 | 390 | 0 | 2.00E-27 |
| hsa_circ_0067574 | 93 | 377 | 643 | 292 | 558 | 0 | 4.00E-108 |
| hsa_circ_0067574 | 96 | 245 | 371 | 292 | 418 | 0 | 3.00E-52 |

novel_circ_025037 novel_circ_025038 novel_circ_025039 novel_circ_025040 novel_circ_025048 novel_circ_025049 novel_circ_025050 novel_circ_025051 novel_circ_025052 novel_circ_025056 novel_circ_025058 novel_circ_025062 novel_circ_025063 novel_circ_025064 novel_circ_025065 novel_circ_025066 novel_circ_025067 novel_circ_025070 novel_circ_025071 novel_circ_025072 novel_circ_025073 novel_circ_025074 novel_circ_025075 novel_circ_025076 novel_circ_025077 novel_circ_025078 novel_circ_025079 novel_circ_025080 novel_circ_025081 novel_circ_025082 novel_circ_025083 novel_circ_025084 novel_circ_025085 novel_circ_025086 novel_circ_025087 novel_circ_025088 novel_circ_025089 novel_circ_025090 novel_circ_025091 novel_circ_025092 novel_circ_025094 novel_circ_025095 novel_circ_025097 novel_circ_025098 novel_circ_025099 novel_circ_025100 novel_circ_025101 novel_circ_025104 novel_circ_025105 novel_circ_025106 novel_circ_025107 novel_circ_025108 novel_circ_025109 novel_circ_025110

| hsa_circ_0067574 | 87 | 2 | 294 | 2 | 291 | 0.01 | 1.00E-86 |
| --- | --- | --- | --- | --- | --- | --- | --- |
| hsa_circ_0047477 | 84 | 30071 | 30168 | 12734 | 12638 | 0.03 | 8.00E-15 |
| hsa_circ_0024151 | 84 | 14207 | 14283 | 123617 | 123693 | 0 | 6.00E-11 |
| hsa_circ_0055147 | 84 | 40402 | 40515 | 43759 | 43868 | 0.05 | 4.00E-20 |
| hsa_circ_0038440 | 96 | 1 | 395 | 1 | 395 | 0 | 0 |
| hsa_circ_0038438 | 96 | 3067 | 3462 | 191 | 586 | 0 | 0 |
| hsa_circ_0038438 | 96 | 1 | 586 | 1 | 586 | 0 | 0 |
| hsa_circ_0005343 | 93 | 2971 | 3277 | 144520 | 144826 | 0.01 | 5.00E-123 |
| hsa_circ_0038438 | 97 | 1 | 401 | 1 | 401 | 0 | 0 |
| hsa_circ_0062467 | 74 | 15985 | 16407 | 12172 | 11746 | 0.06 | 8.00E-31 |
| hsa_circ_0062467 | 74 | 15985 | 16407 | 12172 | 11746 | 0.06 | 8.00E-31 |
| hsa_circ_0055804 | 87 | 40885 | 40954 | 34951 | 35018 | 0.03 | 4.00E-11 |
| hsa_circ_0073563 | 90 | 1 | 432 | 1527 | 1958 | 0 | 5.00E-155 |
| hsa_circ_0073563 | 90 | 510 | 1052 | 1416 | 1958 | 0 | 0 |
| hsa_circ_0073566 | 92 | 1 | 204 | 597 | 800 | 0 | 5.00E-75 |
| hsa_circ_0073563 | 93 | 1 | 769 | 220 | 988 | 0 | 0 |
| hsa_circ_0073559 | 95 | 1 | 237 | 44 | 280 | 0 | 3.00E-100 |
| hsa_circ_0069996 | 73 | 20504 | 20809 | 43639 | 43957 | 0.08 | 2.00E-16 |
| hsa_circ_0073555 | 85 | 1 | 605 | 116 | 720 | 0 | 1.00E-177 |
| hsa_circ_0073554 | 84 | 1 | 1228 | 1 | 1228 | 0 | 0 |
| hsa_circ_0073554 | 84 | 1 | 1029 | 1 | 1029 | 0 | 0 |
| hsa_circ_0073554 | 82 | 1 | 618 | 1 | 618 | 0.01 | 3.00E-148 |
| hsa_circ_0073551 | 82 | 1 | 1196 | 179 | 1365 | 0.02 | 0 |
| hsa_circ_0073544 | 85 | 1 | 1863 | 573 | 2426 | 0.01 | 0 |
| hsa_circ_0004768 | 82 | 1 | 578 | 1 | 569 | 0.02 | 3.00E-133 |
| hsa_circ_0073548 | 87 | 1 | 1245 | 1 | 1236 | 0.01 | 0 |
| hsa_circ_0073545 | 88 | 1 | 1417 | 146 | 1553 | 0.01 | 0 |
| hsa_circ_0073544 | 88 | 1 | 1817 | 1 | 1808 | 0.01 | 0 |
| hsa_circ_0073544 | 91 | 1 | 572 | 1 | 572 | 0.01 | 0 |
| hsa_circ_0073544 | 88 | 1 | 400 | 1 | 400 | 0.01 | 6.00E-129 |
| hsa_circ_0073537 | 84 | 1 | 1366 | 1370 | 1 | 0.01 | 0 |
| hsa_circ_0073536 | 85 | 1 | 1552 | 1739 | 184 | 0.01 | 0 |
| hsa_circ_0073536 | 86 | 1 | 1738 | 1739 | 1 | 0.01 | 0 |
| hsa_circ_0073535 | 86 | 1 | 1850 | 2088 | 238 | 0.01 | 0 |
| hsa_circ_0073537 | 85 | 1 | 1249 | 1252 | 1 | 0.02 | 0 |
| hsa_circ_0073536 | 86 | 1 | 1435 | 1621 | 184 | 0.01 | 0 |
| hsa_circ_0073536 | 91 | 1 | 372 | 369 | 1 | 0.01 | 1.00E-140 |
| hsa_circ_0073535 | 91 | 1 | 484 | 718 | 238 | 0.01 | 0 |
| hsa_circ_0073535 | 90 | 1 | 721 | 718 | 1 | 0 | 0 |
| hsa_circ_0073535 | 89 | 1 | 535 | 532 | 1 | 0.01 | 0 |
| hsa_circ_0073529 | 91 | 1 | 268 | 1 | 268 | 0 | 4.00E-99 |
| hsa_circ_0090873 | 92 | 95 | 1243 | 7 | 1157 | 0.01 | 0 |
| hsa_circ_0090930 | 94 | 1 | 410 | 410 | 1 | 0 | 7.00E-178 |
| hsa_circ_0081957 | 89 | 1 | 1165 | 1162 | 1 | 0 | 0 |
| hsa_circ_0081957 | 89 | 1 | 1165 | 1162 | 1 | 0 | 0 |
| hsa_circ_0081955 | 94 | 153 | 672 | 716 | 197 | 0 | 0 |
| hsa_circ_0081955 | 94 | 153 | 868 | 716 | 1 | 0 | 0 |
| hsa_circ_0009016 | 74 | 24138 | 24484 | 1E+06 | 1480227 | 0.05 | 3.00E-24 |
| hsa_circ_0081951 | 93 | 202 | 522 | 1929 | 1609 | 0 | 3.00E-133 |
| hsa_circ_0081951 | 93 | 202 | 645 | 1929 | 1486 | 0 | 0 |
| hsa_circ_0081951 | 92 | 202 | 1302 | 1929 | 829 | 0 | 0 |
| hsa_circ_0081951 | 91 | 1 | 780 | 1608 | 829 | 0 | 0 |
| hsa_circ_0081951 | 91 | 1 | 1125 | 1257 | 133 | 0 | 0 |
| hsa_circ_0081945 | 91 | 1 | 1526 | 2820 | 1295 | 0 | 0 |

novel_circ_025111 novel_circ_025112 novel_circ_025113 novel_circ_025114 novel_circ_025115 novel_circ_025117 novel_circ_025118 novel_circ_025122 novel_circ_025125 novel_circ_025126 novel_circ_025127 novel_circ_025128 novel_circ_025129 novel_circ_025130 novel_circ_025131 novel_circ_025132 novel_circ_025133 novel_circ_025134 novel_circ_025135 novel_circ_025136 novel_circ_025137 novel_circ_025138 novel_circ_025139 novel_circ_025140 novel_circ_025141 novel_circ_025142 novel_circ_025143 novel_circ_025144 novel_circ_025145 novel_circ_025146 novel_circ_025147 novel_circ_025148 novel_circ_025149 novel_circ_025150 novel_circ_025151 novel_circ_025152 novel_circ_025167 novel_circ_025169 novel_circ_025170 novel_circ_025171 novel_circ_025172 novel_circ_025173 novel_circ_025174 novel_circ_025175 novel_circ_025176 novel_circ_025177 novel_circ_025178 novel_circ_025179 novel_circ_025180 novel_circ_025181 novel_circ_025182 novel_circ_025183 novel_circ_025184 novel_circ_025185

| hsa_circ_0081951 | 94 | 1 | 571 | 828 | 258 | 0 | 0 |
| --- | --- | --- | --- | --- | --- | --- | --- |
| hsa_circ_0081951 | 92 | 1 | 572 | 572 | 1 | 0 | 0 |
| hsa_circ_0081945 | 91 | 1 | 1373 | 1820 | 448 | 0 | 0 |
| hsa_circ_0081945 | 93 | 1 | 1674 | 1665 | 1 | 0.01 | 0 |
| hsa_circ_0081945 | 95 | 3 | 132 | 130 | 1 | 0 | 3.00E-52 |
| hsa_circ_0081905 | 92 | 1 | 315 | 229 | 543 | 0 | 4.00E-125 |
| hsa_circ_0081904 | 96 | 169 | 815 | 1 | 647 | 0 | 0 |
| hsa_circ_0081900 | 89 | 253 | 598 | 1 | 346 | 0 | 9.00E-119 |
| hsa_circ_0081894 | 95 | 1 | 252 | 1158 | 907 | 0 | 3.00E-110 |
| hsa_circ_0001738 | 95 | 1 | 488 | 488 | 1 | 0 | 0 |
| hsa_circ_0001738 | 95 | 1 | 357 | 357 | 1 | 0 | 4.00E-160 |
| hsa_circ_0081894 | 93 | 1 | 549 | 549 | 1 | 0 | 0 |
| hsa_circ_0081894 | 93 | 1 | 467 | 467 | 1 | 0 | 0 |
| hsa_circ_0081888 | 92 | 1 | 745 | 745 | 1 | 0 | 0 |
| hsa_circ_0081886 | 93 | 1 | 983 | 1060 | 78 | 0 | 0 |
| hsa_circ_0081886 | 93 | 1 | 1060 | 1060 | 1 | 0 | 0 |
| hsa_circ_0003932 | 95 | 1 | 1400 | 1 | 1400 | 0 | 0 |
| hsa_circ_0003932 | 95 | 1 | 1216 | 185 | 1400 | 0 | 0 |
| hsa_circ_0003932 | 97 | 1 | 415 | 668 | 1082 | 0 | 0 |
| hsa_circ_0003932 | 96 | 1 | 527 | 556 | 1082 | 0 | 0 |
| hsa_circ_0003932 | 94 | 1 | 898 | 185 | 1082 | 0 | 0 |
| hsa_circ_0003932 | 94 | 1 | 1082 | 1 | 1082 | 0 | 0 |
| hsa_circ_0003932 | 92 | 1 | 596 | 185 | 780 | 0 | 0 |
| hsa_circ_0003932 | 93 | 1 | 780 | 1 | 780 | 0 | 0 |
| hsa_circ_0003932 | 93 | 149 | 815 | 1 | 667 | 0 | 0 |
| hsa_circ_0003932 | 92 | 1 | 407 | 1 | 407 | 0 | 8.00E-163 |
| hsa_circ_0005343 | 76 | 338 | 630 | 421088 | 421409 | 0.11 | 3.00E-30 |
| hsa_circ_0009378 | 91 | 786 | 1157 | 1 | 372 | 0 | 6.00E-138 |
| hsa_circ_0009378 | 91 | 1 | 372 | 1 | 372 | 0 | 2.00E-138 |
| hsa_circ_0009424 | 89 | 4 | 336 | 1540 | 1208 | 0 | 2.00E-113 |
| hsa_circ_0009426 | 91 | 4 | 492 | 702 | 214 | 0 | 0 |
| hsa_circ_0009427 | 88 | 2 | 1170 | 1553 | 385 | 0 | 0 |
| hsa_circ_0009427 | 89 | 1 | 1372 | 1756 | 385 | 0 | 0 |
| hsa_circ_0009427 | 90 | 128 | 1673 | 1930 | 385 | 0 | 0 |
| hsa_circ_0009427 | 90 | 128 | 1498 | 1930 | 560 | 0 | 0 |
| hsa_circ_0009427 | 91 | 128 | 1133 | 1930 | 925 | 0 | 0 |
| hsa_circ_0083832 | 89 | 4 | 1668 | 1665 | 1 | 0 | 0 |
| hsa_circ_0083832 | 90 | 1 | 2116 | 2116 | 1 | 0 | 0 |
| hsa_circ_0083830 | 90 | 1 | 594 | 768 | 178 | 0.01 | 0 |
| hsa_circ_0083830 | 90 | 2 | 1057 | 1227 | 178 | 0.01 | 0 |
| hsa_circ_0083832 | 89 | 2 | 592 | 588 | 1 | 0.01 | 0 |
| hsa_circ_0083832 | 89 | 4 | 1668 | 1665 | 1 | 0 | 0 |
| hsa_circ_0083832 | 90 | 1 | 2116 | 2116 | 1 | 0 | 0 |
| hsa_circ_0083832 | 90 | 2 | 463 | 588 | 130 | 0.01 | 3.00E-167 |
| hsa_circ_0083832 | 91 | 3 | 2168 | 2295 | 130 | 0 | 0 |
| hsa_circ_0083832 | 92 | 4 | 677 | 1665 | 992 | 0 | 0 |
| hsa_circ_0083832 | 93 | 3 | 1306 | 2295 | 992 | 0 | 0 |
| hsa_circ_0083832 | 93 | 3 | 1399 | 2388 | 992 | 0 | 0 |
| hsa_circ_0083832 | 92 | 4 | 590 | 1665 | 1079 | 0 | 0 |
| hsa_circ_0083832 | 92 | 1 | 1038 | 2116 | 1079 | 0 | 0 |
| hsa_circ_0083832 | 93 | 3 | 1219 | 2295 | 1079 | 0 | 0 |
| hsa_circ_0083832 | 92 | 4 | 459 | 1665 | 1210 | 0 | 5.00E-180 |
| hsa_circ_0083832 | 92 | 4 | 819 | 2025 | 1210 | 0 | 0 |
| hsa_circ_0083832 | 93 | 3 | 1088 | 2295 | 1210 | 0 | 0 |

novel_circ_025186 novel_circ_025187 novel_circ_025188 novel_circ_025189 novel_circ_025190 novel_circ_025191 novel_circ_025192 novel_circ_025193 novel_circ_025194 novel_circ_025195 novel_circ_025196 novel_circ_025197 novel_circ_025198 novel_circ_025199 novel_circ_025200 novel_circ_025208 novel_circ_025209 novel_circ_025210 novel_circ_025211 novel_circ_025212 novel_circ_025213 novel_circ_025214 novel_circ_025218 novel_circ_025219 novel_circ_025220 novel_circ_025221 novel_circ_025222 novel_circ_025223 novel_circ_025224 novel_circ_025225 novel_circ_025226 novel_circ_025227 novel_circ_025228 novel_circ_025229 novel_circ_025230 novel_circ_025231 novel_circ_025237 novel_circ_025238 novel_circ_025239 novel_circ_025240 novel_circ_025241 novel_circ_025242 novel_circ_025243 novel_circ_025245 novel_circ_025246 novel_circ_025247 novel_circ_025248 novel_circ_025249 novel_circ_025250 novel_circ_025251 novel_circ_025252 novel_circ_025253 novel_circ_025254 novel_circ_025255

| hsa_circ_0083832 | 93 | 3 | 1181 | 2388 | 1210 | 0 | 0 |
| --- | --- | --- | --- | --- | --- | --- | --- |
| hsa_circ_0083832 | 93 | 1 | 448 | 2116 | 1669 | 0 | 0 |
| hsa_circ_0083832 | 93 | 3 | 629 | 2295 | 1669 | 0 | 0 |
| hsa_circ_0083832 | 94 | 3 | 722 | 2388 | 1669 | 0 | 0 |
| hsa_circ_0083832 | 93 | 3 | 404 | 2295 | 1894 | 0 | 2.00E-168 |
| hsa_circ_0083832 | 94 | 3 | 497 | 2388 | 1894 | 0 | 0 |
| hsa_circ_0083832 | 95 | 3 | 269 | 2295 | 2029 | 0 | 3.00E-115 |
| hsa_circ_0002546 | 94 | 35806 | 35994 | 1 | 189 | 0.01 | 5.00E-73 |
| hsa_circ_0002546 | 94 | 1 | 189 | 1 | 189 | 0.01 | 2.00E-75 |
| hsa_circ_0002546 | 90 | 2561 | 2896 | 66 | 401 | 0.01 | 4.00E-119 |
| hsa_circ_0002546 | 90 | 1 | 332 | 70 | 401 | 0.01 | 1.00E-119 |
| hsa_circ_0002546 | 90 | 2 | 212 | 191 | 401 | 0 | 1.00E-72 |
| hsa_circ_0002546 | 90 | 2 | 212 | 191 | 401 | 0 | 4.00E-70 |
| hsa_circ_0002546 | 90 | 2 | 212 | 191 | 401 | 0 | 5.00E-70 |
| hsa_circ_0064026 | 82 | 16807 | 17836 | 248718 | 247672 | 0.03 | 0 |
| hsa_circ_0083336 | 91 | 1 | 392 | 1 | 392 | 0 | 2.00E-149 |
| hsa_circ_0083336 | 92 | 1 | 554 | 1 | 554 | 0 | 0 |
| hsa_circ_0083336 | 93 | 2 | 525 | 394 | 917 | 0 | 0 |
| hsa_circ_0083336 | 92 | 1 | 363 | 555 | 917 | 0 | 2.00E-143 |
| hsa_circ_0083348 | 89 | 235 | 624 | 214 | 603 | 0 | 3.00E-138 |
| hsa_circ_0083348 | 90 | 235 | 954 | 214 | 933 | 0 | 0 |
| hsa_circ_0083348 | 90 | 1 | 651 | 283 | 933 | 0 | 0 |
| hsa_circ_0006364 | 84 | 236 | 484 | 192 | 440 | 0 | 1.00E-61 |
| hsa_circ_0090458 | 85 | 1 | 223 | 1 | 223 | 0 | 3.00E-59 |
| hsa_circ_0079395 | 74 | 381 | 854 | 3148 | 2675 | 0.04 | 3.00E-46 |
| hsa_circ_0006742 | 80 | 3235 | 3338 | 14922 | 15026 | 0.03 | 2.00E-11 |
| hsa_circ_0078030 | 79 | 1978 | 2348 | 454 | 84 | 0.03 | 2.00E-65 |
| hsa_circ_0090368 | 89 | 1 | 1159 | 1 | 1159 | 0 | 0 |
| hsa_circ_0090368 | 91 | 1 | 688 | 1 | 688 | 0 | 0 |
| hsa_circ_0090367 | 92 | 1 | 846 | 1 | 846 | 0 | 0 |
| hsa_circ_0090368 | 91 | 1 | 571 | 1 | 571 | 0 | 0 |
| hsa_circ_0090367 | 92 | 1 | 729 | 1 | 729 | 0 | 0 |
| hsa_circ_0090365 | 91 | 1 | 866 | 1 | 866 | 0 | 0 |
| hsa_circ_0090368 | 91 | 1 | 403 | 1 | 403 | 0 | 6.00E-154 |
| hsa_circ_0090365 | 93 | 1 | 295 | 1 | 295 | 0 | 4.00E-119 |
| hsa_circ_0005316 | 92 | 1 | 796 | 1 | 781 | 0.02 | 0 |
| hsa_circ_0084887 | 92 | 1 | 417 | 139 | 555 | 0 | 5.00E-165 |
| hsa_circ_0084887 | 91 | 1 | 804 | 139 | 942 | 0 | 0 |
| hsa_circ_0084885 | 93 | 1 | 2193 | 403 | 2595 | 0 | 0 |
| hsa_circ_0084891 | 93 | 1 | 747 | 288 | 1034 | 0 | 0 |
| hsa_circ_0084891 | 94 | 1 | 1332 | 288 | 1619 | 0 | 0 |
| hsa_circ_0084891 | 93 | 1 | 462 | 573 | 1034 | 0 | 0 |
| hsa_circ_0084891 | 94 | 1 | 585 | 1035 | 1619 | 0 | 0 |
| hsa_circ_0084893 | 91 | 1 | 1298 | 1571 | 268 | 0 | 0 |
| hsa_circ_0084893 | 91 | 1 | 1938 | 2211 | 268 | 0 | 0 |
| hsa_circ_0084893 | 91 | 1677 | 3614 | 2211 | 268 | 0 | 0 |
| hsa_circ_0084893 | 91 | 1 | 1938 | 2211 | 268 | 0 | 0 |
| hsa_circ_0084893 | 91 | 1677 | 3614 | 2211 | 268 | 0 | 0 |
| hsa_circ_0084893 | 92 | 1 | 1019 | 2016 | 998 | 0 | 0 |
| hsa_circ_0084893 | 92 | 1 | 1214 | 2211 | 998 | 0 | 0 |
| hsa_circ_0084893 | 92 | 1677 | 2890 | 2211 | 998 | 0 | 0 |
| hsa_circ_0084897 | 94 | 12 | 597 | 586 | 1 | 0 | 0 |
| hsa_circ_0084897 | 92 | 1 | 879 | 879 | 1 | 0 | 0 |
| hsa_circ_0084897 | 92 | 1 | 1074 | 1074 | 1 | 0 | 0 |

novel_circ_025256 novel_circ_025257 novel_circ_025258 novel_circ_025259 novel_circ_025260 novel_circ_025261 novel_circ_025262 novel_circ_025263 novel_circ_025264 novel_circ_025265 novel_circ_025266 novel_circ_025267 novel_circ_025268 novel_circ_025269 novel_circ_025270 novel_circ_025271 novel_circ_025272 novel_circ_025273 novel_circ_025274 novel_circ_025275 novel_circ_025276 novel_circ_025277 novel_circ_025278 novel_circ_025279 novel_circ_025280 novel_circ_025281 novel_circ_025282 novel_circ_025283 novel_circ_025284 novel_circ_025285 novel_circ_025286 novel_circ_025287 novel_circ_025288 novel_circ_025289 novel_circ_025290 novel_circ_025291 novel_circ_025292 novel_circ_025293 novel_circ_025294 novel_circ_025295 novel_circ_025296 novel_circ_025297 novel_circ_025298 novel_circ_025299 novel_circ_025300 novel_circ_025301 novel_circ_025302 novel_circ_025303 novel_circ_025304 novel_circ_025305 novel_circ_025306 novel_circ_025307 novel_circ_025308 novel_circ_025309

| hsa_circ_0084897 | 92 | 1675 | 2750 | 1076 | 1 | 0 | 0 |
| --- | --- | --- | --- | --- | --- | --- | --- |
| hsa_circ_0084897 | 90 | 1 | 640 | 1074 | 435 | 0 | 0 |
| hsa_circ_0084897 | 90 | 1675 | 2316 | 1076 | 435 | 0 | 0 |
| hsa_circ_0084899 | 94 | 1 | 643 | 643 | 1 | 0 | 0 |
| hsa_circ_0084899 | 95 | 1 | 910 | 1054 | 145 | 0 | 0 |
| hsa_circ_0084899 | 95 | 4 | 1261 | 1402 | 145 | 0 | 0 |
| hsa_circ_0084901 | 94 | 4 | 762 | 926 | 168 | 0 | 0 |
| hsa_circ_0084901 | 96 | 1 | 1973 | 2140 | 168 | 0 | 0 |
| hsa_circ_0084901 | 96 | 1 | 2128 | 2295 | 168 | 0 | 0 |
| hsa_circ_0084901 | 94 | 4 | 665 | 926 | 265 | 0 | 0 |
| hsa_circ_0084903 | 93 | 4 | 350 | 348 | 2 | 0 | 1.00E-139 |
| hsa_circ_0084903 | 96 | 1 | 1478 | 1479 | 2 | 0 | 0 |
| hsa_circ_0084903 | 96 | 1 | 1561 | 1562 | 2 | 0 | 0 |
| hsa_circ_0084903 | 96 | 1 | 1716 | 1717 | 2 | 0 | 0 |
| hsa_circ_0084914 | 93 | 2 | 2486 | 2488 | 1 | 0 | 0 |
| hsa_circ_0084932 | 91 | 1 | 236 | 1 | 236 | 0 | 2.00E-86 |
| hsa_circ_0084932 | 93 | 1 | 595 | 1 | 595 | 0 | 0 |
| hsa_circ_0084932 | 93 | 1 | 719 | 1 | 719 | 0 | 0 |
| hsa_circ_0084932 | 94 | 1 | 854 | 1 | 854 | 0 | 0 |
| hsa_circ_0084932 | 93 | 1 | 1085 | 1 | 1085 | 0 | 0 |
| hsa_circ_0084932 | 94 | 1 | 1144 | 1 | 1144 | 0 | 0 |
| hsa_circ_0084932 | 92 | 1 | 1318 | 1 | 1318 | 0 | 0 |
| hsa_circ_0084934 | 94 | 1 | 484 | 1 | 484 | 0 | 0 |
| hsa_circ_0084939 | 97 | 1 | 259 | 1 | 259 | 0 | 4.00E-119 |
| hsa_circ_0084934 | 92 | 1 | 964 | 485 | 1448 | 0 | 0 |
| hsa_circ_0084932 | 92 | 1 | 965 | 596 | 1560 | 0 | 0 |
| hsa_circ_0084934 | 93 | 1 | 241 | 1208 | 1448 | 0 | 8.00E-96 |
| hsa_circ_0084932 | 95 | 1 | 514 | 1319 | 1832 | 0 | 0 |
| hsa_circ_0084932 | 95 | 1 | 514 | 1319 | 1832 | 0 | 0 |
| hsa_circ_0084940 | 94 | 1 | 388 | 1 | 388 | 0 | 1.00E-165 |
| hsa_circ_0084940 | 94 | 1 | 623 | 1 | 623 | 0 | 0 |
| hsa_circ_0084943 | 92 | 1 | 448 | 1 | 448 | 0 | 0 |
| hsa_circ_0084940 | 93 | 1 | 1771 | 176 | 1946 | 0 | 0 |
| hsa_circ_0084940 | 93 | 1 | 1956 | 176 | 2131 | 0 | 0 |
| hsa_circ_0084940 | 93 | 1 | 1743 | 389 | 2131 | 0 | 0 |
| hsa_circ_0084946 | 93 | 1 | 291 | 1 | 291 | 0 | 7.00E-117 |
| hsa_circ_0084946 | 93 | 1 | 548 | 1 | 548 | 0 | 0 |
| hsa_circ_0084943 | 94 | 1 | 1182 | 266 | 1447 | 0 | 0 |
| hsa_circ_0084943 | 93 | 1 | 1382 | 266 | 1647 | 0 | 0 |
| hsa_circ_0084940 | 93 | 1 | 1678 | 441 | 2118 | 0 | 0 |
| hsa_circ_0084940 | 93 | 1 | 1691 | 441 | 2131 | 0 | 0 |
| hsa_circ_0084947 | 93 | 1 | 365 | 1 | 365 | 0 | 7.00E-153 |
| hsa_circ_0084947 | 93 | 1 | 507 | 1 | 507 | 0 | 0 |
| hsa_circ_0084947 | 94 | 1 | 999 | 1 | 999 | 0 | 0 |
| hsa_circ_0084947 | 94 | 1 | 1199 | 1 | 1199 | 0 | 0 |
| hsa_circ_0084940 | 93 | 1 | 1508 | 624 | 2131 | 0 | 0 |
| hsa_circ_0084940 | 93 | 1 | 1400 | 732 | 2131 | 0 | 0 |
| hsa_circ_0084940 | 93 | 1 | 1244 | 888 | 2131 | 0 | 0 |
| hsa_circ_0084940 | 93 | 1 | 1143 | 989 | 2131 | 0 | 0 |
| hsa_circ_0084949 | 94 | 1 | 816 | 400 | 1215 | 0 | 0 |
| hsa_circ_0084940 | 93 | 1 | 1001 | 1131 | 2131 | 0 | 0 |
| hsa_circ_0084951 | 93 | 3 | 245 | 3 | 245 | 0 | 1.00E-98 |
| hsa_circ_0084951 | 92 | 3 | 569 | 3 | 569 | 0 | 0 |
| hsa_circ_0084951 | 92 | 3 | 754 | 3 | 754 | 0 | 0 |

novel_circ_025310 novel_circ_025311 novel_circ_025312 novel_circ_025313 novel_circ_025314 novel_circ_025315 novel_circ_025319 novel_circ_025320 novel_circ_025322 novel_circ_025323 novel_circ_025324 novel_circ_025326 novel_circ_025327 novel_circ_025328 novel_circ_025329 novel_circ_025331 novel_circ_025332 novel_circ_025333 novel_circ_025334 novel_circ_025335 novel_circ_025336 novel_circ_025337 novel_circ_025338 novel_circ_025339 novel_circ_025340 novel_circ_025341 novel_circ_025342 novel_circ_025343 novel_circ_025344 novel_circ_025345 novel_circ_025346 novel_circ_025347 novel_circ_025348 novel_circ_025349 novel_circ_025350 novel_circ_025351 novel_circ_025352 novel_circ_025353 novel_circ_025354 novel_circ_025355 novel_circ_025356 novel_circ_025359 novel_circ_025360 novel_circ_025361 novel_circ_025362 novel_circ_025363 novel_circ_025364 novel_circ_025365 novel_circ_025366 novel_circ_025367 novel_circ_025371 novel_circ_025372 novel_circ_025373 novel_circ_025374

| hsa_circ_0084951 | 92 | 3 | 907 | 3 | 907 | 0 | 0 |
| --- | --- | --- | --- | --- | --- | --- | --- |
| hsa_circ_0084951 | 92 | 1 | 460 | 448 | 907 | 0 | 0 |
| hsa_circ_0084951 | 92 | 1 | 460 | 448 | 907 | 0 | 0 |
| hsa_circ_0084951 | 92 | 1 | 460 | 448 | 907 | 0 | 0 |
| hsa_circ_0084953 | 92 | 1 | 338 | 1 | 338 | 0 | 1.00E-134 |
| hsa_circ_0084953 | 92 | 1 | 338 | 1 | 338 | 0 | 2.00E-134 |
| hsa_circ_0084955 | 95 | 1 | 666 | 666 | 1 | 0 | 0 |
| hsa_circ_0084955 | 95 | 1 | 514 | 514 | 1 | 0 | 0 |
| hsa_circ_0084957 | 92 | 260 | 617 | 5156 | 4796 | 0.01 | 1.00E-142 |
| hsa_circ_0084961 | 93 | 1 | 417 | 1 | 417 | 0 | 2.00E-173 |
| hsa_circ_0084962 | 92 | 1 | 293 | 161 | 453 | 0 | 3.00E-116 |
| hsa_circ_0084965 | 91 | 1 | 725 | 725 | 1 | 0 | 0 |
| hsa_circ_0084965 | 90 | 1 | 1068 | 1069 | 1 | 0 | 0 |
| hsa_circ_0084974 | 91 | 1 | 566 | 1 | 566 | 0 | 0 |
| hsa_circ_0084974 | 92 | 1 | 732 | 1 | 732 | 0 | 0 |
| hsa_circ_0004762 | 90 | 1 | 416 | 1 | 416 | 0 | 8.00E-153 |
| hsa_circ_0002961 | 97 | 1 | 565 | 1 | 565 | 0 | 0 |
| hsa_circ_0084996 | 97 | 1 | 664 | 103 | 766 | 0 | 0 |
| hsa_circ_0084997 | 96 | 86 | 789 | 1 | 704 | 0 | 0 |
| hsa_circ_0091175 | 96 | 4 | 250 | 247 | 1 | 0 | 4.00E-109 |
| hsa_circ_0091174 | 95 | 4 | 633 | 903 | 274 | 0 | 0 |
| hsa_circ_0091174 | 94 | 1 | 383 | 656 | 274 | 0 | 1.00E-164 |
| hsa_circ_0091174 | 95 | 1 | 555 | 656 | 102 | 0 | 0 |
| hsa_circ_0091172 | 91 | 1 | 971 | 1614 | 659 | 0.02 | 0 |
| hsa_circ_0091172 | 92 | 1 | 1461 | 1614 | 169 | 0.01 | 0 |
| hsa_circ_0091172 | 94 | 4 | 564 | 955 | 395 | 0 | 0 |
| hsa_circ_0091172 | 95 | 1 | 259 | 917 | 659 | 0 | 4.00E-114 |
| hsa_circ_0091172 | 94 | 1 | 749 | 917 | 169 | 0 | 0 |
| hsa_circ_0091171 | 93 | 1 | 1098 | 1104 | 1 | 0.01 | 0 |
| hsa_circ_0091172 | 93 | 4 | 490 | 655 | 169 | 0 | 0 |
| hsa_circ_0091170 | 91 | 1 | 822 | 902 | 78 | 0.01 | 0 |
| hsa_circ_0091170 | 89 | 1 | 596 | 676 | 78 | 0.01 | 0 |
| hsa_circ_0091168 | 96 | 1 | 233 | 972 | 740 | 0 | 9.00E-105 |
| hsa_circ_0091168 | 97 | 1 | 534 | 739 | 206 | 0 | 0 |
| hsa_circ_0091168 | 96 | 1 | 205 | 205 | 1 | 0 | 3.00E-89 |
| hsa_circ_0091167 | 93 | 1 | 403 | 795 | 393 | 0 | 6.00E-169 |
| hsa_circ_0091167 | 91 | 1 | 478 | 795 | 318 | 0.01 | 0 |
| hsa_circ_0091167 | 92 | 1 | 631 | 795 | 165 | 0.01 | 0 |
| hsa_circ_0091165 | 92 | 1 | 1052 | 1052 | 1 | 0.01 | 0 |
| hsa_circ_0091167 | 90 | 4 | 369 | 530 | 165 | 0.02 | 9.00E-132 |
| hsa_circ_0091165 | 93 | 4 | 421 | 418 | 1 | 0 | 1.00E-175 |
| hsa_circ_0036113 | 93 | 1 | 148 | 63 | 210 | 0 | 7.00E-54 |
| hsa_circ_0036113 | 92 | 1 | 210 | 1 | 210 | 0 | 6.00E-80 |
| hsa_circ_0036112 | 91 | 302 | 778 | 1 | 477 | 0 | 0 |
| hsa_circ_0036112 | 91 | 390 | 866 | 1 | 477 | 0 | 0 |
| hsa_circ_0036112 | 91 | 1 | 415 | 1 | 415 | 0 | 1.00E-155 |
| hsa_circ_0036112 | 91 | 302 | 716 | 1 | 415 | 0 | 2.00E-155 |
| hsa_circ_0036112 | 91 | 390 | 804 | 1 | 415 | 0 | 3.00E-155 |
| hsa_circ_0036112 | 91 | 390 | 660 | 1 | 271 | 0 | 1.00E-98 |
| hsa_circ_0036112 | 91 | 390 | 593 | 1 | 204 | 0 | 3.00E-73 |
| hsa_circ_0064893 | 93 | 1 | 560 | 809 | 1368 | 0 | 0 |
| hsa_circ_0064893 | 94 | 1 | 301 | 604 | 904 | 0 | 4.00E-129 |
| hsa_circ_0001283 | 93 | 434 | 1633 | 7 | 1206 | 0 | 0 |
| hsa_circ_0001283 | 92 | 434 | 1332 | 7 | 905 | 0 | 0 |

novel_circ_025375 novel_circ_025376 novel_circ_025377 novel_circ_025378 novel_circ_025379 novel_circ_025381 novel_circ_025382 novel_circ_025383 novel_circ_025384 novel_circ_025385 novel_circ_025386 novel_circ_025387 novel_circ_025388 novel_circ_025389 novel_circ_025393 novel_circ_025394 novel_circ_025395 novel_circ_025396 novel_circ_025397 novel_circ_025398 novel_circ_025399 novel_circ_025400 novel_circ_025401 novel_circ_025402 novel_circ_025403 novel_circ_025404 novel_circ_025405 novel_circ_025406 novel_circ_025407 novel_circ_025408 novel_circ_025409 novel_circ_025410 novel_circ_025411 novel_circ_025412 novel_circ_025413 novel_circ_025414 novel_circ_025415 novel_circ_025416 novel_circ_025417 novel_circ_025418 novel_circ_025419 novel_circ_025420 novel_circ_025421 novel_circ_025422 novel_circ_025423 novel_circ_025424 novel_circ_025425 novel_circ_025426 novel_circ_025427 novel_circ_025428 novel_circ_025429 novel_circ_025430 novel_circ_025431 novel_circ_025432

| hsa_circ_0001283 | 91 | 434 | 1234 | 7 | 807 | 0 | 0 |
| --- | --- | --- | --- | --- | --- | --- | --- |
| hsa_circ_0001283 | 92 | 293 | 686 | 7 | 400 | 0 | 2.00E-155 |
| hsa_circ_0001283 | 92 | 434 | 827 | 7 | 400 | 0 | 3.00E-155 |
| hsa_circ_0009368 | 91 | 1 | 431 | 1 | 431 | 0 | 2.00E-164 |
| hsa_circ_0007120 | 88 | 1 | 798 | 1 | 798 | 0.01 | 0 |
| hsa_circ_0037025 | 93 | 1 | 546 | 1 | 546 | 0 | 0 |
| hsa_circ_0037028 | 90 | 1 | 607 | 1 | 607 | 0 | 0 |
| hsa_circ_0037028 | 91 | 1 | 949 | 1 | 949 | 0 | 0 |
| hsa_circ_0037032 | 91 | 1 | 581 | 1 | 581 | 0 | 0 |
| hsa_circ_0037033 | 88 | 400 | 1376 | 518 | 1494 | 0 | 0 |
| hsa_circ_0037033 | 88 | 1 | 489 | 535 | 1023 | 0 | 5.00E-165 |
| hsa_circ_0037033 | 89 | 1 | 619 | 535 | 1153 | 0 | 0 |
| hsa_circ_0037033 | 88 | 1 | 960 | 535 | 1494 | 0 | 0 |
| hsa_circ_0037036 | 89 | 1 | 414 | 1 | 414 | 0 | 2.00E-143 |
| hsa_circ_0087731 | 92 | 3 | 720 | 3 | 708 | 0.02 | 0 |
| hsa_circ_0087731 | 93 | 3 | 888 | 3 | 876 | 0.01 | 0 |
| hsa_circ_0087731 | 93 | 3 | 888 | 3 | 876 | 0.01 | 0 |
| hsa_circ_0013662 | 91 | 9 | 741 | 9 | 741 | 0 | 0 |
| hsa_circ_0013663 | 91 | 1 | 608 | 1 | 608 | 0 | 0 |
| hsa_circ_0013668 | 96 | 1 | 428 | 214 | 641 | 0 | 0 |
| hsa_circ_0013668 | 96 | 1 | 428 | 214 | 641 | 0 | 0 |
| hsa_circ_0013672 | 94 | 1 | 421 | 217 | 637 | 0 | 0 |
| hsa_circ_0013685 | 92 | 1 | 906 | 1 | 906 | 0 | 0 |
| hsa_circ_0013686 | 92 | 1 | 444 | 1 | 444 | 0 | 1.00E-176 |
| hsa_circ_0013685 | 92 | 1 | 637 | 270 | 906 | 0 | 0 |
| hsa_circ_0013704 | 92 | 1 | 846 | 382 | 1227 | 0 | 0 |
| hsa_circ_0013726 | 89 | 1 | 768 | 1 | 768 | 0 | 0 |
| hsa_circ_0013735 | 95 | 1 | 297 | 1 | 297 | 0 | 7.00E-132 |
| hsa_circ_0013746 | 95 | 96 | 938 | 1 | 843 | 0 | 0 |
| hsa_circ_0013746 | 96 | 1 | 334 | 1 | 334 | 0 | 5.00E-154 |
| hsa_circ_0013746 | 95 | 1 | 554 | 1 | 554 | 0 | 0 |
| hsa_circ_0013746 | 95 | 1 | 554 | 1 | 554 | 0 | 0 |
| hsa_circ_0013746 | 95 | 1 | 843 | 1 | 843 | 0 | 0 |
| hsa_circ_0013746 | 95 | 1 | 956 | 1 | 956 | 0 | 0 |
| hsa_circ_0013746 | 94 | 1 | 835 | 9 | 843 | 0 | 0 |
| hsa_circ_0013746 | 94 | 1 | 719 | 125 | 843 | 0 | 0 |
| hsa_circ_0013746 | 94 | 5977 | 6604 | 215 | 843 | 0 | 0 |
| hsa_circ_0013746 | 94 | 1 | 336 | 219 | 554 | 0 | 4.00E-145 |
| hsa_circ_0013746 | 94 | 1 | 625 | 219 | 843 | 0 | 0 |
| hsa_circ_0031204 | 94 | 1 | 444 | 553 | 110 | 0 | 0 |
| hsa_circ_0031202 | 95 | 2 | 288 | 287 | 1 | 0 | 3.00E-126 |
| hsa_circ_0031200 | 96 | 1 | 735 | 1 | 735 | 0 | 0 |
| hsa_circ_0031193 | 95 | 4 | 426 | 423 | 1 | 0 | 0 |
| hsa_circ_0031186 | 94 | 1 | 588 | 2909 | 2322 | 0 | 0 |
| hsa_circ_0031186 | 94 | 7 | 504 | 2819 | 2322 | 0 | 0 |
| hsa_circ_0031186 | 93 | 7 | 681 | 2819 | 2145 | 0 | 0 |
| hsa_circ_0031186 | 93 | 1 | 543 | 2687 | 2145 | 0 | 0 |
| hsa_circ_0031186 | 94 | 1 | 1350 | 2687 | 1338 | 0 | 0 |
| hsa_circ_0031186 | 94 | 1 | 400 | 2144 | 1745 | 0 | 3.00E-172 |
| hsa_circ_0031186 | 94 | 1 | 975 | 2144 | 1170 | 0 | 0 |
| hsa_circ_0031190 | 94 | 1 | 459 | 885 | 427 | 0 | 0 |
| hsa_circ_0031190 | 93 | 1 | 488 | 717 | 230 | 0 | 0 |
| hsa_circ_0031184 | 95 | 1 | 417 | 1697 | 1281 | 0 | 0 |
| hsa_circ_0031162 | 97 | 1 | 625 | 2084 | 1460 | 0 | 0 |

novel_circ_025433 novel_circ_025434 novel_circ_025435 novel_circ_025436 novel_circ_025437 novel_circ_025438 novel_circ_025439 novel_circ_025440 novel_circ_025441 novel_circ_025442 novel_circ_025443 novel_circ_025444 novel_circ_025445 novel_circ_025446 novel_circ_025447 novel_circ_025448 novel_circ_025449 novel_circ_025450 novel_circ_025451 novel_circ_025453 novel_circ_025454 novel_circ_025455 novel_circ_025456 novel_circ_025457 novel_circ_025458 novel_circ_025459 novel_circ_025460 novel_circ_025461 novel_circ_025462 novel_circ_025463 novel_circ_025464 novel_circ_025465 novel_circ_025466 novel_circ_025467 novel_circ_025468 novel_circ_025469 novel_circ_025470 novel_circ_025471 novel_circ_025472 novel_circ_025473 novel_circ_025474 novel_circ_025475 novel_circ_025476 novel_circ_025477 novel_circ_025478 novel_circ_025479 novel_circ_025480 novel_circ_025481 novel_circ_025482 novel_circ_025483 novel_circ_025484 novel_circ_025485 novel_circ_025486 novel_circ_025488

| hsa_circ_0031162 | 95 | 1 | 903 | 2084 | 1182 | 0 | 0 |
| --- | --- | --- | --- | --- | --- | --- | --- |
| hsa_circ_0031162 | 93 | 1 | 603 | 1784 | 1182 | 0 | 0 |
| hsa_circ_0031162 | 93 | 2 | 619 | 1367 | 750 | 0 | 0 |
| hsa_circ_0031162 | 93 | 1 | 270 | 1019 | 750 | 0 | 3.00E-110 |
| hsa_circ_0031161 | 94 | 1 | 315 | 485 | 171 | 0 | 2.00E-133 |
| hsa_circ_0031158 | 95 | 1 | 489 | 619 | 131 | 0 | 0 |
| hsa_circ_0031158 | 95 | 410 | 539 | 260 | 131 | 0 | 2.00E-50 |
| hsa_circ_0031152 | 96 | 1 | 516 | 519 | 1 | 0.01 | 0 |
| hsa_circ_0031152 | 96 | 1 | 516 | 519 | 1 | 0.01 | 0 |
| hsa_circ_0003196 | 93 | 35 | 396 | 362 | 1 | 0 | 7.00E-148 |
| hsa_circ_0071425 | 90 | 35 | 1315 | 1919 | 636 | 0 | 0 |
| hsa_circ_0071427 | 89 | 1 | 919 | 922 | 1 | 0.01 | 0 |
| hsa_circ_0071425 | 88 | 1 | 1209 | 1557 | 340 | 0.01 | 0 |
| hsa_circ_0071425 | 88 | 1 | 1213 | 1557 | 336 | 0.01 | 0 |
| hsa_circ_0071425 | 88 | 1 | 1300 | 1557 | 249 | 0.01 | 0 |
| hsa_circ_0071430 | 88 | 1 | 424 | 427 | 1 | 0.01 | 2.00E-139 |
| hsa_circ_0071427 | 88 | 1 | 577 | 580 | 1 | 0.01 | 0 |
| hsa_circ_0071425 | 87 | 1 | 871 | 1215 | 336 | 0.01 | 0 |
| hsa_circ_0071425 | 86 | 1 | 1206 | 1215 | 1 | 0.01 | 0 |
| hsa_circ_0071425 | 85 | 7 | 337 | 331 | 1 | 0 | 2.00E-92 |
| hsa_circ_0071424 | 88 | 7 | 489 | 823 | 341 | 0 | 5.00E-165 |
| hsa_circ_0071424 | 89 | 7 | 829 | 823 | 1 | 0 | 0 |
| hsa_circ_0071424 | 91 | 13 | 740 | 728 | 1 | 0 | 0 |
| hsa_circ_0071424 | 91 | 1 | 340 | 340 | 1 | 0 | 2.00E-125 |
| hsa_circ_0071422 | 96 | 4 | 372 | 369 | 1 | 0 | 2.00E-173 |
| hsa_circ_0071422 | 96 | 4 | 372 | 369 | 1 | 0 | 3.00E-173 |
| hsa_circ_0006366 | 95 | 4 | 303 | 300 | 1 | 0 | 5.00E-130 |
| hsa_circ_0071419 | 86 | 172 | 793 | 622 | 1 | 0 | 0 |
| hsa_circ_0071411 | 93 | 234 | 607 | 149 | 522 | 0 | 5.00E-151 |
| hsa_circ_0071411 | 92 | 98 | 321 | 149 | 372 | 0 | 1.00E-84 |
| hsa_circ_0071407 | 90 | 1 | 166 | 428 | 593 | 0 | 2.00E-54 |
| hsa_circ_0071406 | 84 | 5 | 990 | 140 | 1119 | 0.01 | 0 |
| hsa_circ_0071406 | 84 | 14198 | 15314 | 1 | 1119 | 0.02 | 0 |
| hsa_circ_0087471 | 87 | 7 | 1088 | 1084 | 3 | 0 | 0 |
| hsa_circ_0087471 | 88 | 1 | 1475 | 1477 | 3 | 0.01 | 0 |
| hsa_circ_0087475 | 89 | 3 | 2510 | 2513 | 6 | 0 | 0 |
| hsa_circ_0087475 | 88 | 7 | 788 | 787 | 6 | 0.01 | 0 |
| hsa_circ_0087475 | 88 | 1 | 1175 | 1180 | 6 | 0.01 | 0 |
| hsa_circ_0087477 | 88 | 7 | 667 | 661 | 1 | 0.01 | 0 |
| hsa_circ_0087485 | 90 | 1 | 387 | 387 | 1 | 0.01 | 9.00E-142 |
| hsa_circ_0087484 | 90 | 1 | 600 | 775 | 176 | 0 | 0 |
| hsa_circ_0087477 | 89 | 1 | 1503 | 2170 | 668 | 0 | 0 |
| hsa_circ_0087495 | 90 | 1 | 392 | 903 | 512 | 0 | 7.00E-143 |
| hsa_circ_0087495 | 90 | 1 | 318 | 903 | 586 | 0 | 8.00E-112 |
| hsa_circ_0087495 | 88 | 3 | 537 | 1120 | 586 | 0 | 0 |
| hsa_circ_0087502 | 91 | 1 | 994 | 1086 | 93 | 0 | 0 |
| hsa_circ_0087503 | 92 | 1 | 775 | 871 | 97 | 0 | 0 |
| hsa_circ_0087503 | 93 | 1 | 714 | 871 | 158 | 0 | 0 |
| hsa_circ_0087503 | 93 | 1 | 478 | 871 | 394 | 0 | 0 |
| hsa_circ_0087503 | 93 | 1 | 360 | 871 | 512 | 0 | 4.00E-150 |
| hsa_circ_0087507 | 94 | 1 | 277 | 277 | 1 | 0 | 9.00E-116 |
| hsa_circ_0087502 | 92 | 45 | 304 | 1190 | 930 | 0 | 6.00E-98 |
| hsa_circ_0087511 | 96 | 191 | 271 | 9 | 89 | 0 | 6.00E-30 |
| hsa_circ_0087538 | 88 | 1 | 556 | 1 | 556 | 0 | 0 |

novel_circ_025489 novel_circ_025490 novel_circ_025491 novel_circ_025492 novel_circ_025493 novel_circ_025494 novel_circ_025497 novel_circ_025498 novel_circ_025499 novel_circ_025500 novel_circ_025501 novel_circ_025502 novel_circ_025503 novel_circ_025504 novel_circ_025507 novel_circ_025509 novel_circ_025511 novel_circ_025513 novel_circ_025514 novel_circ_025515 novel_circ_025516 novel_circ_025517 novel_circ_025519 novel_circ_025520 novel_circ_025522 novel_circ_025523 novel_circ_025524 novel_circ_025525 novel_circ_025526 novel_circ_025527 novel_circ_025528 novel_circ_025529 novel_circ_025530 novel_circ_025531 novel_circ_025532 novel_circ_025533 novel_circ_025534 novel_circ_025539 novel_circ_025541 novel_circ_025542 novel_circ_025543 novel_circ_025544 novel_circ_025545 novel_circ_025546 novel_circ_025547 novel_circ_025548 novel_circ_025549 novel_circ_025550 novel_circ_025551 novel_circ_025552 novel_circ_025553 novel_circ_025554 novel_circ_025555 novel_circ_025556

| hsa_circ_0087544 | 90 | 3 | 1010 | 3 | 1013 | 0.01 | 0 |
| --- | --- | --- | --- | --- | --- | --- | --- |
| hsa_circ_0087548 | 92 | 1 | 226 | 1 | 226 | 0 | 2.00E-87 |
| hsa_circ_0087555 | 95 | 6 | 491 | 6 | 491 | 0 | 0 |
| hsa_circ_0087555 | 94 | 6 | 741 | 6 | 741 | 0 | 0 |
| hsa_circ_0087557 | 92 | 3 | 250 | 3 | 250 | 0 | 1.00E-94 |
| hsa_circ_0087548 | 90 | 3 | 1133 | 1108 | 2241 | 0 | 0 |
| hsa_circ_0087559 | 92 | 1 | 329 | 162 | 490 | 0 | 3.00E-131 |
| hsa_circ_0049373 | 95 | 330 | 628 | 1749 | 2047 | 0 | 6.00E-131 |
| hsa_circ_0049373 | 91 | 2 | 331 | 1094 | 1423 | 0 | 2.00E-123 |
| hsa_circ_0049367 | 93 | 1 | 128 | 1 | 128 | 0 | 4.00E-46 |
| hsa_circ_0049357 | 96 | 1 | 228 | 87 | 314 | 0 | 3.00E-100 |
| hsa_circ_0049340 | 95 | 1 | 386 | 250 | 635 | 0 | 3.00E-171 |
| hsa_circ_0049340 | 95 | 2 | 564 | 73 | 635 | 0 | 0 |
| hsa_circ_0049332 | 92 | 1 | 440 | 100 | 539 | 0 | 8.00E-178 |
| hsa_circ_0049271 | 91 | 3 | 685 | 684 | 2 | 0 | 0 |
| hsa_circ_0049247 | 84 | 1 | 796 | 1541 | 734 | 0.02 | 0 |
| hsa_circ_0049226 | 87 | 1 | 361 | 489 | 126 | 0.01 | 3.00E-112 |
| hsa_circ_0049208 | 89 | 3 | 524 | 525 | 1 | 0.01 | 0 |
| hsa_circ_0049204 | 89 | 3 | 877 | 1092 | 215 | 0 | 0 |
| hsa_circ_0049203 | 89 | 3 | 1225 | 1400 | 175 | 0 | 0 |
| hsa_circ_0049208 | 90 | 1 | 372 | 375 | 1 | 0.01 | 1.00E-135 |
| hsa_circ_0049204 | 90 | 1 | 725 | 942 | 215 | 0 | 0 |
| hsa_circ_0049165 | 93 | 1 | 122 | 1 | 122 | 0 | 5.00E-45 |
| hsa_circ_0049149 | 83 | 9 | 216 | 375 | 165 | 0.02 | 4.00E-48 |
| hsa_circ_0003357 | 91 | 1 | 589 | 1 | 589 | 0 | 0 |
| hsa_circ_0020007 | 90 | 1 | 891 | 384 | 1274 | 0 | 0 |
| hsa_circ_0020007 | 90 | 1 | 1041 | 234 | 1274 | 0 | 0 |
| hsa_circ_0020007 | 91 | 1 | 1274 | 1 | 1274 | 0 | 0 |
| hsa_circ_0020007 | 91 | 364 | 1637 | 1 | 1274 | 0 | 0 |
| hsa_circ_0020006 | 93 | 1 | 989 | 1 | 989 | 0 | 0 |
| hsa_circ_0020007 | 93 | 1 | 382 | 1 | 382 | 0 | 5.00E-159 |
| hsa_circ_0020006 | 94 | 1 | 745 | 1 | 745 | 0 | 0 |
| hsa_circ_0020006 | 94 | 1 | 515 | 1 | 515 | 0 | 0 |
| hsa_circ_0020001 | 92 | 5 | 214 | 210 | 1 | 0 | 1.00E-78 |
| hsa_circ_0077762 | 92 | 1 | 345 | 1545 | 1891 | 0.01 | 5.00E-134 |
| hsa_circ_0077762 | 93 | 1 | 456 | 1434 | 1891 | 0 | 0 |
| hsa_circ_0077762 | 93 | 1 | 631 | 1259 | 1891 | 0 | 0 |
| hsa_circ_0042238 | 89 | 1 | 879 | 1 | 882 | 0 | 0 |
| hsa_circ_0042254 | 91 | 283 | 700 | 418 | 1 | 0 | 1.00E-158 |
| hsa_circ_0042261 | 91 | 1 | 499 | 501 | 3 | 0 | 0 |
| hsa_circ_0042264 | 88 | 4 | 411 | 408 | 1 | 0 | 1.00E-131 |
| hsa_circ_0042264 | 87 | 1 | 561 | 561 | 1 | 0 | 0 |
| hsa_circ_0042264 | 89 | 1 | 640 | 640 | 1 | 0 | 0 |
| hsa_circ_0042264 | 89 | 1 | 725 | 725 | 1 | 0 | 0 |
| hsa_circ_0042268 | 89 | 2 | 229 | 228 | 1 | 0.01 | 1.00E-73 |
| hsa_circ_0042334 | 90 | 1 | 525 | 525 | 1 | 0 | 0 |
| hsa_circ_0042366 | 87 | 94 | 878 | 266 | 1043 | 0.01 | 0 |
| hsa_circ_0056648 | 88 | 1 | 249 | 1627 | 1379 | 0 | 3.00E-80 |
| hsa_circ_0056648 | 90 | 1 | 507 | 1627 | 1121 | 0 | 0 |
| hsa_circ_0056648 | 91 | 1 | 670 | 1627 | 958 | 0 | 0 |
| hsa_circ_0056648 | 95 | 1 | 776 | 957 | 182 | 0 | 0 |
| hsa_circ_0056646 | 94 | 1 | 1367 | 1631 | 265 | 0 | 0 |
| hsa_circ_0056646 | 94 | 1 | 1493 | 1631 | 142 | 0 | 0 |
| hsa_circ_0056645 | 93 | 1 | 1877 | 1874 | 1 | 0 | 0 |

novel_circ_025557 novel_circ_025558 novel_circ_025559 novel_circ_025560 novel_circ_025561 novel_circ_025562 novel_circ_025563 novel_circ_025564 novel_circ_025565 novel_circ_025566 novel_circ_025567 novel_circ_025568 novel_circ_025569 novel_circ_025570 novel_circ_025571 novel_circ_025572 novel_circ_025573 novel_circ_025574 novel_circ_025575 novel_circ_025576 novel_circ_025577 novel_circ_025578 novel_circ_025579 novel_circ_025580 novel_circ_025581 novel_circ_025582 novel_circ_025583 novel_circ_025584 novel_circ_025585 novel_circ_025586 novel_circ_025587 novel_circ_025588 novel_circ_025589 novel_circ_025590 novel_circ_025591 novel_circ_025592 novel_circ_025593 novel_circ_025594 novel_circ_025595 novel_circ_025596 novel_circ_025597 novel_circ_025598 novel_circ_025599 novel_circ_025600 novel_circ_025601 novel_circ_025602 novel_circ_025603 novel_circ_025604 novel_circ_025605 novel_circ_025606 novel_circ_025607 novel_circ_025608 novel_circ_025609 novel_circ_025610

| hsa_circ_0056645 | 92 | 1 | 1101 | 1098 | 1 | 0 | 0 |
| --- | --- | --- | --- | --- | --- | --- | --- |
| hsa_circ_0056647 | 93 | 1 | 536 | 533 | 1 | 0.01 | 0 |
| hsa_circ_0056645 | 92 | 1 | 920 | 917 | 1 | 0 | 0 |
| hsa_circ_0056643 | 91 | 1 | 756 | 1789 | 1034 | 0 | 0 |
| hsa_circ_0056644 | 93 | 1 | 249 | 249 | 1 | 0 | 3.00E-100 |
| hsa_circ_0056643 | 93 | 1 | 649 | 649 | 1 | 0 | 0 |
| hsa_circ_0056642 | 93 | 1 | 1057 | 1059 | 3 | 0 | 0 |
| hsa_circ_0056637 | 93 | 1 | 1594 | 1839 | 246 | 0 | 0 |
| hsa_circ_0056643 | 94 | 1 | 403 | 403 | 1 | 0 | 3.00E-172 |
| hsa_circ_0056642 | 94 | 1 | 811 | 813 | 3 | 0 | 0 |
| hsa_circ_0056640 | 94 | 1 | 1173 | 1173 | 1 | 0 | 0 |
| hsa_circ_0056637 | 94 | 1 | 1443 | 1593 | 151 | 0 | 0 |
| hsa_circ_0056637 | 94 | 1 | 1593 | 1593 | 1 | 0 | 0 |
| hsa_circ_0056642 | 94 | 1 | 408 | 410 | 3 | 0 | 2.00E-173 |
| hsa_circ_0056637 | 94 | 1 | 1040 | 1190 | 151 | 0 | 0 |
| hsa_circ_0056637 | 94 | 1 | 780 | 780 | 1 | 0 | 0 |
| hsa_circ_0056636 | 94 | 1 | 549 | 2433 | 1885 | 0 | 0 |
| hsa_circ_0056636 | 94 | 1 | 549 | 2433 | 1885 | 0 | 0 |
| hsa_circ_0056636 | 93 | 1 | 399 | 2283 | 1885 | 0 | 2.00E-168 |
| hsa_circ_0056636 | 93 | 1 | 399 | 2283 | 1885 | 0 | 8.00E-168 |
| hsa_circ_0056636 | 95 | 1 | 664 | 2283 | 1620 | 0 | 0 |
| hsa_circ_0056636 | 97 | 1 | 265 | 1884 | 1620 | 0 | 2.00E-122 |
| hsa_circ_0056636 | 95 | 1 | 1041 | 1884 | 844 | 0 | 0 |
| hsa_circ_0056636 | 95 | 3 | 404 | 1617 | 1216 | 0 | 9.00E-177 |
| hsa_circ_0056636 | 95 | 3 | 776 | 1617 | 844 | 0 | 0 |
| hsa_circ_0056636 | 93 | 1 | 392 | 843 | 452 | 0 | 7.00E-163 |
| hsa_circ_0056635 | 92 | 1 | 963 | 963 | 1 | 0 | 0 |
| hsa_circ_0056634 | 93 | 1 | 1470 | 1470 | 1 | 0 | 0 |
| hsa_circ_0056629 | 92 | 1 | 1599 | 2051 | 453 | 0 | 0 |
| hsa_circ_0056636 | 92 | 10 | 448 | 439 | 1 | 0 | 6.00E-174 |
| hsa_circ_0056635 | 92 | 10 | 568 | 559 | 1 | 0 | 0 |
| hsa_circ_0056634 | 93 | 10 | 955 | 1066 | 121 | 0 | 0 |
| hsa_circ_0056634 | 93 | 10 | 1075 | 1066 | 1 | 0 | 0 |
| hsa_circ_0056629 | 93 | 10 | 1204 | 1647 | 453 | 0 | 0 |
| hsa_circ_0056627 | 93 | 10 | 2431 | 3211 | 790 | 0 | 0 |
| hsa_circ_0056634 | 94 | 3 | 636 | 754 | 121 | 0 | 0 |
| hsa_circ_0056634 | 95 | 1 | 507 | 627 | 121 | 0 | 0 |
| hsa_circ_0056634 | 94 | 1 | 627 | 627 | 1 | 0 | 0 |
| hsa_circ_0056627 | 94 | 1 | 1227 | 2016 | 790 | 0 | 0 |
| hsa_circ_0056627 | 94 | 1 | 775 | 1564 | 790 | 0 | 0 |
| hsa_circ_0056627 | 96 | 1 | 430 | 789 | 360 | 0 | 0 |
| hsa_circ_0056627 | 92 | 3 | 355 | 355 | 3 | 0 | 3.00E-136 |
| hsa_circ_0056626 | 93 | 3 | 711 | 709 | 1 | 0 | 0 |
| hsa_circ_0056625 | 94 | 3 | 1074 | 1471 | 400 | 0 | 0 |
| hsa_circ_0056624 | 95 | 717 | 1601 | 1109 | 225 | 0 | 0 |
| hsa_circ_0056624 | 95 | 1 | 761 | 985 | 225 | 0 | 0 |
| hsa_circ_0056623 | 93 | 1 | 1836 | 2193 | 358 | 0 | 0 |
| hsa_circ_0056623 | 91 | 1 | 748 | 1432 | 685 | 0 | 0 |
| hsa_circ_0056623 | 91 | 1 | 911 | 1432 | 522 | 0 | 0 |
| hsa_circ_0056623 | 91 | 1 | 1075 | 1432 | 358 | 0 | 0 |
| hsa_circ_0056623 | 93 | 3 | 733 | 808 | 78 | 0 | 0 |
| hsa_circ_0075491 | 82 | 1 | 512 | 1 | 512 | 0.01 | 2.00E-124 |
| hsa_circ_0075492 | 82 | 1 | 347 | 1 | 347 | 0.01 | 2.00E-79 |
| hsa_circ_0003042 | 90 | 3 | 295 | 227 | 519 | 0 | 6.00E-103 |

novel_circ_025611 novel_circ_025613 novel_circ_025614 novel_circ_025615 novel_circ_025616 novel_circ_025617 novel_circ_025618 novel_circ_025620 novel_circ_025621 novel_circ_025622 novel_circ_025623 novel_circ_025624 novel_circ_025626 novel_circ_025627 novel_circ_025629 novel_circ_025630 novel_circ_025631 novel_circ_025632 novel_circ_025633 novel_circ_025634 novel_circ_025635 novel_circ_025636 novel_circ_025637 novel_circ_025639 novel_circ_025640 novel_circ_025641 novel_circ_025642 novel_circ_025643 novel_circ_025644 novel_circ_025651 novel_circ_025652 novel_circ_025653 novel_circ_025654 novel_circ_025655 novel_circ_025656 novel_circ_025658 novel_circ_025659 novel_circ_025664 novel_circ_025665 novel_circ_025666 novel_circ_025667 novel_circ_025668 novel_circ_025669 novel_circ_025670 novel_circ_025671 novel_circ_025672 novel_circ_025673 novel_circ_025674 novel_circ_025675 novel_circ_025676 novel_circ_025677 novel_circ_025679 novel_circ_025684 novel_circ_025685

| hsa_circ_0075497 | 80 | 248 | 629 | 136 | 517 | 0.03 | 2.00E-75 |
| --- | --- | --- | --- | --- | --- | --- | --- |
| hsa_circ_0075501 | 94 | 1 | 493 | 493 | 1 | 0 | 0 |
| hsa_circ_0075501 | 93 | 1 | 621 | 621 | 1 | 0 | 0 |
| hsa_circ_0075501 | 94 | 1 | 464 | 621 | 158 | 0 | 0 |
| hsa_circ_0075501 | 92 | 1 | 231 | 621 | 391 | 0 | 6.00E-87 |
| hsa_circ_0075503 | 96 | 1 | 260 | 428 | 169 | 0 | 2.00E-114 |
| hsa_circ_0075504 | 96 | 1 | 259 | 259 | 1 | 0 | 8.00E-116 |
| hsa_circ_0016756 | 73 | 870 | 1570 | 758 | 1460 | 0.04 | 5.00E-56 |
| hsa_circ_0016756 | 73 | 1093 | 1793 | 758 | 1460 | 0.04 | 5.00E-56 |
| hsa_circ_0050768 | 77 | 13 | 187 | 208 | 34 | 0.01 | 4.00E-19 |
| hsa_circ_0050769 | 75 | 1215 | 2197 | 4293 | 3320 | 0.04 | 6.00E-116 |
| hsa_circ_0050769 | 75 | 798 | 1780 | 4293 | 3320 | 0.04 | 5.00E-116 |
| hsa_circ_0050769 | 75 | 632 | 1614 | 4293 | 3320 | 0.04 | 5.00E-116 |
| hsa_circ_0050769 | 82 | 1 | 153 | 4066 | 4218 | 0 | 9.00E-29 |
| hsa_circ_0051056 | 82 | 99 | 284 | 211 | 29 | 0.02 | 1.00E-35 |
| hsa_circ_0050796 | 94 | 3 | 495 | 3 | 495 | 0 | 0 |
| hsa_circ_0050796 | 94 | 3 | 599 | 3 | 599 | 0 | 0 |
| hsa_circ_0050796 | 93 | 3 | 757 | 3 | 757 | 0 | 0 |
| hsa_circ_0050795 | 93 | 3 | 1334 | 1847 | 3178 | 0 | 0 |
| hsa_circ_0050795 | 93 | 3 | 1336 | 1847 | 3180 | 0 | 0 |
| hsa_circ_0050799 | 93 | 1 | 364 | 1 | 364 | 0 | 1.00E-150 |
| hsa_circ_0050808 | 92 | 1 | 793 | 1 | 793 | 0 | 0 |
| hsa_circ_0050892 | 94 | 1 | 141 | 1 | 141 | 0 | 8.00E-54 |
| hsa_circ_0032117 | 93 | 2 | 598 | 2 | 598 | 0 | 0 |
| hsa_circ_0032117 | 92 | 2 | 720 | 2 | 720 | 0 | 0 |
| hsa_circ_0032128 | 94 | 1 | 442 | 1 | 442 | 0 | 0 |
| hsa_circ_0032132 | 94 | 15788 | 16635 | 1 | 848 | 0 | 0 |
| hsa_circ_0032137 | 94 | 5 | 423 | 5 | 423 | 0 | 2.00E-179 |
| hsa_circ_0032137 | 94 | 5 | 568 | 5 | 568 | 0 | 0 |
| hsa_circ_0090356 | 92 | 36918 | 37073 | 1 | 156 | 0 | 6.00E-53 |
| hsa_circ_0004357 | 94 | 1 | 300 | 300 | 1 | 0 | 2.00E-128 |
| hsa_circ_0090351 | 94 | 1 | 773 | 773 | 1 | 0 | 0 |
| hsa_circ_0090351 | 93 | 1 | 473 | 473 | 1 | 0 | 0 |
| hsa_circ_0090356 | 92 | 1 | 154 | 154 | 1 | 0 | 2.00E-52 |
| hsa_circ_0090349 | 93 | 1 | 467 | 733 | 267 | 0 | 0 |
| hsa_circ_0089912 | 84 | 11 | 1551 | 1556 | 1 | 0.01 | 0 |
| hsa_circ_0089912 | 84 | 11 | 1551 | 1556 | 1 | 0.01 | 0 |
| hsa_circ_0066812 | 86 | 1 | 1260 | 1227 | 1 | 0.03 | 0 |
| hsa_circ_0066812 | 84 | 1 | 1069 | 1227 | 192 | 0.03 | 0 |
| hsa_circ_0066812 | 84 | 1 | 882 | 1227 | 376 | 0.03 | 0 |
| hsa_circ_0066812 | 83 | 1 | 400 | 1227 | 858 | 0.08 | 1.00E-90 |
| hsa_circ_0006163 | 85 | 1 | 326 | 320 | 1 | 0.05 | 1.00E-84 |
| hsa_circ_0006163 | 88 | 1 | 518 | 512 | 1 | 0.03 | 2.00E-169 |
| hsa_circ_0006163 | 88 | 1 | 617 | 611 | 1 | 0.03 | 0 |
| hsa_circ_0006163 | 88 | 2670 | 3286 | 611 | 1 | 0.03 | 0 |
| hsa_circ_0066815 | 89 | 1 | 501 | 492 | 1 | 0.02 | 2.00E-175 |
| hsa_circ_0066815 | 92 | 1 | 291 | 492 | 202 | 0 | 3.00E-115 |
| hsa_circ_0066818 | 89 | 1 | 292 | 1 | 292 | 0 | 2.00E-97 |
| hsa_circ_0066819 | 92 | 294 | 779 | 1 | 486 | 0 | 0 |
| hsa_circ_0066819 | 92 | 1 | 486 | 1 | 486 | 0 | 0 |
| hsa_circ_0029815 | 95 | 6967 | 7422 | 458 | 3 | 0 | 0 |
| hsa_circ_0066834 | 92 | 2 | 272 | 2 | 272 | 0 | 9.00E-106 |
| hsa_circ_0066845 | 94 | 1 | 1605 | 1605 | 1 | 0 | 0 |
| hsa_circ_0066846 | 100 | 313 | 500 | 188 | 1 | 0 | 2.00E-94 |

novel_circ_025686 novel_circ_025687 novel_circ_025688 novel_circ_025689 novel_circ_025690 novel_circ_025691 novel_circ_025692 novel_circ_025693 novel_circ_025694 novel_circ_025695 novel_circ_025696 novel_circ_025697 novel_circ_025698 novel_circ_025700 novel_circ_025701 novel_circ_025702 novel_circ_025703 novel_circ_025706 novel_circ_025707 novel_circ_025708 novel_circ_025709 novel_circ_025710 novel_circ_025714 novel_circ_025715 novel_circ_025716 novel_circ_025717 novel_circ_025718 novel_circ_025719 novel_circ_025720 novel_circ_025721 novel_circ_025722 novel_circ_025723 novel_circ_025724 novel_circ_025728 novel_circ_025729 novel_circ_025730 novel_circ_025731 novel_circ_025732 novel_circ_025733 novel_circ_025734 novel_circ_025735 novel_circ_025736 novel_circ_025737 novel_circ_025738 novel_circ_025739 novel_circ_025740 novel_circ_025741 novel_circ_025742 novel_circ_025743 novel_circ_025744 novel_circ_025745 novel_circ_025746 novel_circ_025747 novel_circ_025748

| hsa_circ_0091258 | 90 | 8 | 386 | 3011 | 2633 | 0.01 | 2.00E-135 |
| --- | --- | --- | --- | --- | --- | --- | --- |
| hsa_circ_0067436 | 93 | 1 | 403 | 403 | 1 | 0 | 6.00E-169 |
| hsa_circ_0067436 | 92 | 1 | 796 | 796 | 1 | 0 | 0 |
| hsa_circ_0067436 | 93 | 1 | 1130 | 1130 | 1 | 0 | 0 |
| hsa_circ_0067437 | 92 | 1 | 393 | 636 | 244 | 0 | 2.00E-153 |
| hsa_circ_0067436 | 90 | 1 | 538 | 941 | 404 | 0 | 0 |
| hsa_circ_0067436 | 94 | 1 | 757 | 1230 | 474 | 0 | 0 |
| hsa_circ_0067439 | 94 | 1 | 434 | 434 | 1 | 0 | 0 |
| hsa_circ_0067436 | 93 | 1 | 556 | 1352 | 797 | 0 | 0 |
| hsa_circ_0067439 | 98 | 1 | 189 | 334 | 146 | 0 | 1.00E-88 |
| hsa_circ_0067439 | 97 | 1 | 289 | 434 | 146 | 0 | 9.00E-136 |
| hsa_circ_0067436 | 95 | 1 | 411 | 1352 | 942 | 0 | 0 |
| hsa_circ_0067436 | 93 | 1 | 222 | 1352 | 1131 | 0 | 6.00E-87 |
| hsa_circ_0056620 | 99 | 140 | 413 | 1 | 274 | 0 | 3.00E-137 |
| hsa_circ_0056620 | 98 | 140 | 541 | 1 | 402 | 0 | 0 |
| hsa_circ_0056621 | 95 | 637 | 1192 | 127 | 682 | 0 | 0 |
| hsa_circ_0056621 | 94 | 224 | 582 | 127 | 485 | 0 | 1.00E-152 |
| hsa_circ_0064565 | 91 | 127 | 469 | 1 | 343 | 0 | 9.00E-129 |
| hsa_circ_0064565 | 91 | 847 | 1189 | 1 | 343 | 0 | 2.00E-128 |
| hsa_circ_0064565 | 94 | 127 | 263 | 1 | 137 | 0 | 5.00E-53 |
| hsa_circ_0005277 | 96 | 1 | 217 | 5206 | 5422 | 0 | 7.00E-96 |
| hsa_circ_0005277 | 96 | 4278 | 4655 | 5045 | 5422 | 0 | 1.00E-175 |
| hsa_circ_0064561 | 95 | 1 | 465 | 765 | 1229 | 0 | 0 |
| hsa_circ_0064561 | 93 | 1 | 665 | 565 | 1229 | 0 | 0 |
| hsa_circ_0064561 | 93 | 1 | 863 | 367 | 1229 | 0 | 0 |
| hsa_circ_0064561 | 92 | 1 | 1229 | 1 | 1229 | 0 | 0 |
| hsa_circ_0064561 | 91 | 1 | 562 | 1 | 562 | 0 | 0 |
| hsa_circ_0064561 | 90 | 1 | 364 | 1 | 364 | 0 | 2.00E-132 |
| hsa_circ_0064559 | 94 | 1 | 369 | 638 | 1006 | 0 | 4.00E-160 |
| hsa_circ_0064559 | 94 | 1 | 735 | 272 | 1006 | 0 | 0 |
| hsa_circ_0064559 | 93 | 1 | 366 | 272 | 637 | 0 | 2.00E-153 |
| hsa_circ_0015730 | 91 | 1 | 442 | 1 | 442 | 0 | 1.00E-170 |
| hsa_circ_0014873 | 86 | 26107 | 27133 | 6576 | 5550 | 0.01 | 0 |
| hsa_circ_0024399 | 93 | 1 | 285 | 662 | 378 | 0 | 7.00E-117 |
| hsa_circ_0024399 | 92 | 1 | 385 | 662 | 278 | 0 | 1.00E-155 |
| hsa_circ_0023691 | 85 | 1 | 534 | 917 | 372 | 0.02 | 6.00E-155 |
| hsa_circ_0023691 | 86 | 1 | 636 | 1019 | 372 | 0.02 | 0 |
| hsa_circ_0023691 | 87 | 1 | 773 | 1156 | 372 | 0.02 | 0 |
| hsa_circ_0023695 | 91 | 1 | 452 | 474 | 23 | 0 | 4.00E-171 |
| hsa_circ_0023695 | 90 | 1 | 589 | 611 | 23 | 0 | 0 |
| hsa_circ_0023696 | 91 | 1 | 521 | 521 | 1 | 0 | 0 |
| hsa_circ_0023697 | 90 | 1 | 345 | 347 | 3 | 0.01 | 2.00E-123 |
| hsa_circ_0023700 | 92 | 1 | 189 | 189 | 1 | 0 | 3.00E-68 |
| hsa_circ_0023700 | 92 | 1 | 796 | 796 | 1 | 0 | 0 |
| hsa_circ_0023701 | 93 | 1 | 607 | 607 | 1 | 0 | 0 |
| hsa_circ_0023701 | 93 | 2 | 662 | 661 | 1 | 0 | 0 |
| hsa_circ_0023712 | 89 | 1 | 2528 | 2528 | 1 | 0 | 0 |
| hsa_circ_0000345 | 88 | 1 | 1981 | 1982 | 2 | 0 | 0 |
| hsa_circ_0023712 | 89 | 1 | 2342 | 2528 | 187 | 0 | 0 |
| hsa_circ_0023712 | 94 | 1 | 454 | 2621 | 2168 | 0 | 0 |
| hsa_circ_0023712 | 94 | 1 | 546 | 2713 | 2168 | 0 | 0 |
| hsa_circ_0023716 | 88 | 3 | 246 | 1463 | 1706 | 0 | 2.00E-77 |
| hsa_circ_0023730 | 91 | 2 | 306 | 305 | 1 | 0 | 6.00E-113 |
| hsa_circ_0023733 | 90 | 77 | 460 | 384 | 1 | 0 | 1.00E-141 |

novel_circ_025749 novel_circ_025750 novel_circ_025751 novel_circ_025752 novel_circ_025753 novel_circ_025754 novel_circ_025755 novel_circ_025756 novel_circ_025757 novel_circ_025758 novel_circ_025759 novel_circ_025760 novel_circ_025761 novel_circ_025762 novel_circ_025763 novel_circ_025764 novel_circ_025765 novel_circ_025766 novel_circ_025767 novel_circ_025769 novel_circ_025770 novel_circ_025771 novel_circ_025772 novel_circ_025773 novel_circ_025774 novel_circ_025775 novel_circ_025776 novel_circ_025777 novel_circ_025778 novel_circ_025779 novel_circ_025780 novel_circ_025781 novel_circ_025782 novel_circ_025783 novel_circ_025784 novel_circ_025785 novel_circ_025787 novel_circ_025788 novel_circ_025789 novel_circ_025799 novel_circ_025800 novel_circ_025805 novel_circ_025807 novel_circ_025808 novel_circ_025809 novel_circ_025812 novel_circ_025813 novel_circ_025814 novel_circ_025815 novel_circ_025816 novel_circ_025817 novel_circ_025818 novel_circ_025819 novel_circ_025820

| hsa_circ_0023733 | 91 | 4 | 1026 | 1023 | 1 | 0 | 0 |
| --- | --- | --- | --- | --- | --- | --- | --- |
| hsa_circ_0023769 | 91 | 3 | 943 | 1327 | 387 | 0 | 0 |
| hsa_circ_0023794 | 93 | 1 | 391 | 1 | 391 | 0 | 1.00E-160 |
| hsa_circ_0023815 | 89 | 1 | 453 | 453 | 1 | 0 | 1.00E-161 |
| hsa_circ_0023819 | 92 | 1 | 878 | 1051 | 174 | 0 | 0 |
| hsa_circ_0023820 | 89 | 1 | 638 | 659 | 1 | 0.03 | 0 |
| hsa_circ_0023829 | 91 | 16 | 484 | 1393 | 925 | 0 | 1.00E-180 |
| hsa_circ_0023836 | 90 | 6 | 498 | 679 | 187 | 0 | 0 |
| hsa_circ_0023836 | 90 | 1 | 924 | 1110 | 187 | 0 | 0 |
| hsa_circ_0023836 | 90 | 1 | 424 | 1110 | 687 | 0 | 3.00E-157 |
| hsa_circ_0023836 | 92 | 1 | 595 | 1281 | 687 | 0 | 0 |
| hsa_circ_0023836 | 92 | 1321 | 2152 | 1518 | 687 | 0 | 0 |
| hsa_circ_0023836 | 91 | 1 | 256 | 1872 | 1617 | 0 | 4.00E-92 |
| hsa_circ_0023837 | 91 | 3 | 559 | 556 | 3 | 0.01 | 0 |
| hsa_circ_0023837 | 93 | 3 | 287 | 556 | 275 | 0.01 | 4.00E-114 |
| hsa_circ_0078055 | 98 | 1 | 169 | 83 | 251 | 0 | 2.00E-79 |
| hsa_circ_0078056 | 96 | 45 | 531 | 1 | 487 | 0 | 0 |
| hsa_circ_0078056 | 95 | 1 | 485 | 126 | 610 | 0 | 0 |
| hsa_circ_0078056 | 95 | 1 | 582 | 126 | 707 | 0 | 0 |
| hsa_circ_0078059 | 86 | 1 | 532 | 1 | 538 | 0.02 | 4.00E-162 |
| hsa_circ_0078059 | 86 | 1 | 532 | 1 | 538 | 0.02 | 2.00E-161 |
| hsa_circ_0078059 | 87 | 1 | 889 | 1 | 895 | 0.01 | 0 |
| hsa_circ_0078059 | 87 | 1 | 890 | 1 | 896 | 0.01 | 0 |
| hsa_circ_0078059 | 88 | 1 | 945 | 1 | 951 | 0.01 | 0 |
| hsa_circ_0078059 | 89 | 1 | 1222 | 1 | 1228 | 0.01 | 0 |
| hsa_circ_0078070 | 92 | 2 | 934 | 2 | 934 | 0 | 0 |
| hsa_circ_0078082 | 94 | 1 | 582 | 103 | 684 | 0 | 0 |
| hsa_circ_0078083 | 94 | 1 | 294 | 181 | 474 | 0 | 7.00E-122 |
| hsa_circ_0078085 | 86 | 1 | 1432 | 1 | 1435 | 0 | 0 |
| hsa_circ_0078090 | 84 | 1 | 324 | 1 | 327 | 0.01 | 5.00E-84 |
| hsa_circ_0078094 | 90 | 1 | 351 | 157 | 507 | 0 | 4.00E-125 |
| hsa_circ_0078094 | 91 | 1 | 1608 | 157 | 1764 | 0 | 0 |
| hsa_circ_0078103 | 91 | 1 | 810 | 163 | 972 | 0 | 0 |
| hsa_circ_0078106 | 89 | 1 | 333 | 130 | 459 | 0.01 | 5.00E-114 |
| hsa_circ_0078106 | 89 | 1 | 665 | 301 | 962 | 0 | 0 |
| hsa_circ_0078107 | 88 | 1 | 1054 | 1 | 1054 | 0 | 0 |
| hsa_circ_0078129 | 93 | 1 | 604 | 1 | 604 | 0 | 0 |
| hsa_circ_0078129 | 93 | 1 | 725 | 1 | 725 | 0 | 0 |
| hsa_circ_0078129 | 94 | 1 | 308 | 726 | 1033 | 0 | 3.00E-131 |
| hsa_circ_0077745 | 83 | 1318 | 1410 | 2210 | 2302 | 0.02 | 3.00E-14 |
| hsa_circ_0020988 | 92 | 10 | 2469 | 2439 | 1 | 0.01 | 0 |
| hsa_circ_0021017 | 85 | 3 | 1785 | 196 | 1981 | 0.01 | 0 |
| hsa_circ_0021019 | 86 | 1 | 1278 | 1 | 1281 | 0.01 | 0 |
| hsa_circ_0021025 | 88 | 2 | 630 | 2 | 630 | 0 | 0 |
| hsa_circ_0021031 | 87 | 1 | 502 | 1 | 502 | 0 | 7.00E-164 |
| hsa_circ_0061315 | 94 | 4 | 423 | 614 | 1033 | 0 | 2.00E-178 |
| hsa_circ_0061315 | 92 | 1 | 459 | 152 | 610 | 0 | 0 |
| hsa_circ_0061315 | 93 | 1 | 610 | 1 | 610 | 0 | 0 |
| hsa_circ_0061312 | 91 | 1 | 861 | 1 | 861 | 0 | 0 |
| hsa_circ_0061315 | 93 | 1 | 339 | 1 | 339 | 0 | 4.00E-140 |
| hsa_circ_0061312 | 90 | 1 | 576 | 1 | 576 | 0 | 0 |
| hsa_circ_0061312 | 88 | 1 | 425 | 1 | 425 | 0 | 8.00E-138 |
| hsa_circ_0061310 | 88 | 1 | 563 | 1 | 563 | 0 | 0 |
| hsa_circ_0061307 | 94 | 1 | 556 | 1 | 556 | 0 | 0 |

novel_circ_025821 novel_circ_025822 novel_circ_025823 novel_circ_025824 novel_circ_025825 novel_circ_025826 novel_circ_025827 novel_circ_025828 novel_circ_025829 novel_circ_025830 novel_circ_025831 novel_circ_025832 novel_circ_025833 novel_circ_025834 novel_circ_025835 novel_circ_025836 novel_circ_025837 novel_circ_025838 novel_circ_025839 novel_circ_025840 novel_circ_025844 novel_circ_025845 novel_circ_025850 novel_circ_025854 novel_circ_025855 novel_circ_025856 novel_circ_025857 novel_circ_025860 novel_circ_025861 novel_circ_025863 novel_circ_025867 novel_circ_025868 novel_circ_025869 novel_circ_025871 novel_circ_025874 novel_circ_025875 novel_circ_025876 novel_circ_025877 novel_circ_025878 novel_circ_025879 novel_circ_025880 novel_circ_025881 novel_circ_025882 novel_circ_025883 novel_circ_025884 novel_circ_025885 novel_circ_025886 novel_circ_025887 novel_circ_025888 novel_circ_025889 novel_circ_025890 novel_circ_025891 novel_circ_025892 novel_circ_025893

| hsa_circ_0061312 | 88 | 1 | 279 | 1 | 279 | 0 | 2.00E-88 |
| --- | --- | --- | --- | --- | --- | --- | --- |
| hsa_circ_0061307 | 94 | 1 | 556 | 1 | 556 | 0 | 0 |
| hsa_circ_0061307 | 94 | 1 | 556 | 1 | 556 | 0 | 0 |
| hsa_circ_0061307 | 94 | 1 | 426 | 1 | 426 | 0 | 0 |
| hsa_circ_0079431 | 95 | 1 | 1276 | 161 | 1436 | 0 | 0 |
| hsa_circ_0079429 | 95 | 1 | 1449 | 306 | 1754 | 0 | 0 |
| hsa_circ_0079434 | 95 | 1 | 857 | 1 | 857 | 0 | 0 |
| hsa_circ_0079434 | 95 | 1 | 1026 | 1 | 1026 | 0 | 0 |
| hsa_circ_0079429 | 95 | 1 | 1199 | 556 | 1754 | 0 | 0 |
| hsa_circ_0079427 | 95 | 1 | 1317 | 667 | 1983 | 0 | 0 |
| hsa_circ_0079429 | 95 | 1 | 781 | 974 | 1754 | 0 | 0 |
| hsa_circ_0079438 | 96 | 1 | 332 | 108 | 439 | 0 | 1.00E-154 |
| hsa_circ_0079438 | 95 | 1 | 501 | 108 | 608 | 0 | 0 |
| hsa_circ_0079440 | 94 | 1 | 173 | 1 | 173 | 0 | 2.00E-70 |
| hsa_circ_0079440 | 95 | 1 | 291 | 1 | 291 | 0 | 2.00E-127 |
| hsa_circ_0079443 | 95 | 6 | 459 | 454 | 1 | 0 | 0 |
| hsa_circ_0079443 | 94 | 13 | 696 | 684 | 1 | 0 | 0 |
| hsa_circ_0079443 | 92 | 2 | 812 | 811 | 1 | 0 | 0 |
| hsa_circ_0091281 | 95 | 249 | 669 | 1 | 421 | 0 | 0 |
| hsa_circ_0091281 | 95 | 1 | 421 | 1 | 421 | 0 | 0 |
| hsa_circ_0091282 | 93 | 119 | 404 | 1 | 286 | 0 | 9.00E-114 |
| hsa_circ_0091285 | 96 | 1 | 552 | 690 | 139 | 0 | 0 |
| hsa_circ_0021710 | 79 | 3 | 400 | 3 | 400 | 0.01 | 2.00E-74 |
| hsa_circ_0021637 | 87 | 1 | 605 | 1 | 605 | 0 | 0 |
| hsa_circ_0021642 | 88 | 1 | 352 | 1 | 352 | 0.01 | 2.00E-112 |
| hsa_circ_0021632 | 83 | 1 | 865 | 1 | 865 | 0 | 0 |
| hsa_circ_0021630 | 84 | 1 | 1015 | 18746 | 19760 | 0 | 0 |
| hsa_circ_0021630 | 83 | 1 | 750 | 18746 | 19495 | 0 | 0 |
| hsa_circ_0021631 | 83 | 1 | 610 | 1 | 610 | 0 | 2.00E-155 |
| hsa_circ_0021631 | 82 | 1 | 449 | 1 | 449 | 0 | 4.00E-106 |
| hsa_circ_0021617 | 84 | 1 | 840 | 165 | 1007 | 0.01 | 0 |
| hsa_circ_0021617 | 85 | 1 | 1004 | 1 | 1007 | 0.01 | 0 |
| hsa_circ_0021608 | 85 | 3333 | 4755 | 2547 | 3984 | 0.01 | 0 |
| hsa_circ_0021612 | 85 | 1 | 532 | 1 | 547 | 0.03 | 1.00E-152 |
| hsa_circ_0021602 | 84 | 772 | 1918 | 1899 | 3045 | 0 | 0 |
| hsa_circ_0021602 | 84 | 896 | 2042 | 1899 | 3045 | 0 | 0 |
| hsa_circ_0021602 | 90 | 1 | 1897 | 1 | 1897 | 0 | 0 |
| hsa_circ_0021602 | 85 | 772 | 1677 | 1899 | 2804 | 0 | 0 |
| hsa_circ_0000285 | 88 | 1 | 282 | 1 | 282 | 0 | 1.00E-91 |
| hsa_circ_0021603 | 91 | 1 | 674 | 1 | 674 | 0 | 0 |
| hsa_circ_0000285 | 88 | 1 | 282 | 1 | 282 | 0 | 1.00E-91 |
| hsa_circ_0021603 | 91 | 1 | 674 | 1 | 674 | 0 | 0 |
| hsa_circ_0021603 | 92 | 1 | 389 | 1 | 389 | 0 | 3.00E-156 |
| hsa_circ_0021602 | 91 | 1 | 513 | 1100 | 1612 | 0 | 0 |
| hsa_circ_0021602 | 89 | 1 | 1223 | 1 | 1223 | 0 | 0 |
| hsa_circ_0021602 | 89 | 1 | 1099 | 1 | 1099 | 0 | 0 |
| hsa_circ_0053364 | 90 | 151 | 1735 | 3 | 1590 | 0 | 0 |
| hsa_circ_0053364 | 90 | 2828 | 4412 | 3 | 1590 | 0 | 0 |
| hsa_circ_0003475 | 89 | 1 | 835 | 519 | 1356 | 0 | 0 |
| hsa_circ_0053364 | 90 | 151 | 1735 | 3 | 1590 | 0 | 0 |
| hsa_circ_0053364 | 90 | 2828 | 4412 | 3 | 1590 | 0 | 0 |
| hsa_circ_0003475 | 89 | 1 | 367 | 519 | 888 | 0.01 | 2.00E-124 |
| hsa_circ_0053364 | 91 | 151 | 1267 | 3 | 1122 | 0 | 0 |
| hsa_circ_0053364 | 91 | 2828 | 3944 | 3 | 1122 | 0 | 0 |

novel_circ_025894 novel_circ_025895 novel_circ_025896 novel_circ_025897 novel_circ_025898 novel_circ_025899 novel_circ_025900 novel_circ_025901 novel_circ_025902 novel_circ_025903 novel_circ_025904 novel_circ_025905 novel_circ_025906 novel_circ_025907 novel_circ_025908 novel_circ_025909 novel_circ_025910 novel_circ_025911 novel_circ_025912 novel_circ_025913 novel_circ_025914 novel_circ_025915 novel_circ_025916 novel_circ_025917 novel_circ_025918 novel_circ_025920 novel_circ_025921 novel_circ_025923 novel_circ_025924 novel_circ_025925 novel_circ_025927 novel_circ_025928 novel_circ_025929 novel_circ_025930 novel_circ_025931 novel_circ_025932 novel_circ_025933 novel_circ_025934 novel_circ_025935 novel_circ_025936 novel_circ_025938 novel_circ_025939 novel_circ_025940 novel_circ_025941 novel_circ_025942 novel_circ_025943 novel_circ_025944 novel_circ_025945 novel_circ_025946 novel_circ_025947 novel_circ_025948 novel_circ_025949 novel_circ_025950 novel_circ_025951

| hsa_circ_0053364 | 92 | 3 | 888 | 3 | 888 | 0 | 0 |
| --- | --- | --- | --- | --- | --- | --- | --- |
| hsa_circ_0053363 | 92 | 3 | 1036 | 3 | 1036 | 0 | 0 |
| hsa_circ_0053363 | 92 | 2680 | 3713 | 3 | 1036 | 0 | 0 |
| hsa_circ_0053363 | 92 | 3 | 900 | 3 | 900 | 0 | 0 |
| hsa_circ_0053363 | 92 | 2680 | 3577 | 3 | 900 | 0 | 0 |
| hsa_circ_0053363 | 93 | 3 | 663 | 3 | 663 | 0 | 0 |
| hsa_circ_0053363 | 93 | 2680 | 3340 | 3 | 663 | 0 | 0 |
| hsa_circ_0053341 | 93 | 1 | 486 | 1 | 486 | 0 | 0 |
| hsa_circ_0053338 | 93 | 1 | 624 | 1 | 624 | 0 | 0 |
| hsa_circ_0053341 | 94 | 1 | 383 | 1 | 383 | 0 | 3.00E-166 |
| hsa_circ_0002583 | 92 | 1 | 312 | 397 | 86 | 0 | 2.00E-123 |
| hsa_circ_0053326 | 96 | 1 | 1062 | 1 | 1062 | 0 | 0 |
| hsa_circ_0053326 | 96 | 1 | 928 | 1 | 928 | 0 | 0 |
| hsa_circ_0053326 | 96 | 1 | 827 | 1 | 827 | 0 | 0 |
| hsa_circ_0053326 | 96 | 1 | 692 | 1 | 692 | 0 | 0 |
| hsa_circ_0053326 | 98 | 1 | 336 | 133 | 468 | 0 | 4.00E-165 |
| hsa_circ_0053326 | 97 | 1 | 468 | 1 | 468 | 0 | 0 |
| hsa_circ_0053326 | 98 | 1 | 363 | 1 | 363 | 0 | 8.00E-177 |
| hsa_circ_0053314 | 92 | 1 | 271 | 619 | 889 | 0 | 3.00E-104 |
| hsa_circ_0053294 | 94 | 1 | 658 | 186 | 843 | 0 | 0 |
| hsa_circ_0053294 | 94 | 1 | 643 | 186 | 828 | 0 | 0 |
| hsa_circ_0053286 | 91 | 4 | 841 | 1119 | 282 | 0 | 0 |
| hsa_circ_0053286 | 92 | 4 | 1122 | 1119 | 1 | 0 | 0 |
| hsa_circ_0053286 | 92 | 1 | 406 | 687 | 282 | 0 | 3.00E-162 |
| hsa_circ_0053285 | 92 | 2 | 591 | 996 | 407 | 0 | 0 |
| hsa_circ_0053104 | 93 | 2 | 801 | 2 | 801 | 0 | 0 |
| hsa_circ_0053095 | 92 | 1 | 351 | 421 | 771 | 0 | 2.00E-138 |
| hsa_circ_0053087 | 96 | 1 | 602 | 254 | 855 | 0 | 0 |
| hsa_circ_0053087 | 96 | 1 | 625 | 1 | 625 | 0 | 0 |
| hsa_circ_0053087 | 93 | 1 | 253 | 1 | 253 | 0 | 2.00E-102 |
| hsa_circ_0004476 | 94 | 87 | 195 | 260 | 152 | 0 | 7.00E-41 |
| hsa_circ_0004476 | 97 | 87 | 346 | 260 | 1 | 0 | 7.00E-123 |
| hsa_circ_0004976 | 90 | 370 | 731 | 364 | 3 | 0 | 1.00E-132 |
| hsa_circ_0004976 | 90 | 370 | 731 | 364 | 3 | 0 | 2.00E-132 |
| hsa_circ_0053043 | 86 | 895 | 1977 | 1083 | 1 | 0.01 | 0 |
| hsa_circ_0004476 | 97 | 1 | 260 | 260 | 1 | 0 | 5.00E-123 |
| hsa_circ_0004476 | 97 | 1 | 260 | 260 | 1 | 0 | 7.00E-123 |
| hsa_circ_0004976 | 90 | 284 | 645 | 364 | 3 | 0 | 1.00E-132 |
| hsa_circ_0004976 | 90 | 284 | 645 | 364 | 3 | 0 | 2.00E-132 |
| hsa_circ_0004976 | 90 | 24 | 385 | 364 | 3 | 0 | 2.00E-132 |
| hsa_circ_0053043 | 92 | 3 | 367 | 1083 | 719 | 0 | 1.00E-144 |
| hsa_circ_0053037 | 93 | 69 | 449 | 809 | 429 | 0 | 2.00E-158 |
| hsa_circ_0053037 | 95 | 69 | 604 | 809 | 274 | 0 | 0 |
| hsa_circ_0053037 | 95 | 69 | 877 | 809 | 1 | 0 | 0 |
| hsa_circ_0053037 | 93 | 1 | 381 | 809 | 429 | 0 | 2.00E-158 |
| hsa_circ_0053037 | 95 | 1 | 536 | 809 | 274 | 0 | 0 |
| hsa_circ_0053037 | 95 | 1 | 809 | 809 | 1 | 0 | 0 |
| hsa_circ_0053040 | 93 | 1 | 300 | 300 | 1 | 0 | 3.00E-120 |
| hsa_circ_0053037 | 95 | 1 | 728 | 728 | 1 | 0 | 0 |
| hsa_circ_0053037 | 96 | 2 | 514 | 513 | 1 | 0 | 0 |
| hsa_circ_0053037 | 96 | 1 | 428 | 428 | 1 | 0 | 0 |
| hsa_circ_0053034 | 96 | 1 | 553 | 809 | 257 | 0 | 0 |
| hsa_circ_0053037 | 96 | 3 | 273 | 271 | 1 | 0 | 9.00E-121 |
| hsa_circ_0053034 | 96 | 3 | 398 | 652 | 257 | 0 | 0 |

novel_circ_025952 novel_circ_025954 novel_circ_025955 novel_circ_025956 novel_circ_025959 novel_circ_025960 novel_circ_025961 novel_circ_025962 novel_circ_025966 novel_circ_025967 novel_circ_025968 novel_circ_025969 novel_circ_025970 novel_circ_025971 novel_circ_025972 novel_circ_025973 novel_circ_025974 novel_circ_025975 novel_circ_025976 novel_circ_025977 novel_circ_025982 novel_circ_025983 novel_circ_025984 novel_circ_025985 novel_circ_025986 novel_circ_025987 novel_circ_025988 novel_circ_025989 novel_circ_025991 novel_circ_026003 novel_circ_026004 novel_circ_026005 novel_circ_026006 novel_circ_026007 novel_circ_026009 novel_circ_026011 novel_circ_026014 novel_circ_026016 novel_circ_026017 novel_circ_026018 novel_circ_026019 novel_circ_026020 novel_circ_026022 novel_circ_026023 novel_circ_026024 novel_circ_026025 novel_circ_026026 novel_circ_026027 novel_circ_026028 novel_circ_026029 novel_circ_026030 novel_circ_026031 novel_circ_026032 novel_circ_026033

| hsa_circ_0053034 | 95 | 1 | 381 | 381 | 1 | 0 | 2.00E-168 |
| --- | --- | --- | --- | --- | --- | --- | --- |
| hsa_circ_0053029 | 92 | 4 | 813 | 813 | 1 | 0.01 | 0 |
| hsa_circ_0053029 | 95 | 1 | 462 | 462 | 1 | 0 | 0 |
| hsa_circ_0053024 | 97 | 1 | 472 | 472 | 1 | 0 | 0 |
| hsa_circ_0053019 | 91 | 1 | 880 | 53 | 932 | 0 | 0 |
| hsa_circ_0053019 | 91 | 1 | 831 | 53 | 883 | 0 | 0 |
| hsa_circ_0053013 | 95 | 1 | 355 | 1 | 355 | 0 | 2.00E-157 |
| hsa_circ_0053013 | 94 | 1 | 205 | 1 | 205 | 0 | 1.00E-82 |
| hsa_circ_0070683 | 79 | 3284 | 4278 | 1115 | 118 | 0.01 | 0 |
| hsa_circ_0070683 | 80 | 4 | 336 | 593 | 261 | 0.01 | 2.00E-63 |
| hsa_circ_0070683 | 79 | 435 | 1286 | 1115 | 261 | 0.01 | 9.00E-162 |
| hsa_circ_0070683 | 79 | 2869 | 3720 | 1115 | 261 | 0.01 | 3.00E-161 |
| hsa_circ_0070683 | 79 | 3193 | 4044 | 1115 | 261 | 0.01 | 3.00E-161 |
| hsa_circ_0070683 | 79 | 3284 | 4135 | 1115 | 261 | 0.01 | 3.00E-161 |
| hsa_circ_0070693 | 74 | 796 | 1252 | 1476 | 1020 | 0.04 | 9.00E-40 |
| hsa_circ_0070693 | 74 | 796 | 1252 | 1476 | 1020 | 0.04 | 8.00E-40 |
| hsa_circ_0070693 | 74 | 979 | 1435 | 1476 | 1020 | 0.04 | 9.00E-40 |
| hsa_circ_0070693 | 78 | 1 | 336 | 2565 | 2225 | 0.03 | 3.00E-50 |
| hsa_circ_0070691 | 81 | 29 | 508 | 2844 | 2360 | 0.02 | 6.00E-102 |
| hsa_circ_0070693 | 74 | 955 | 1411 | 1020 | 1476 | 0.04 | 1.00E-39 |
| hsa_circ_0070708 | 87 | 1 | 297 | 1090 | 1386 | 0 | 8.00E-92 |
| hsa_circ_0070708 | 88 | 1 | 667 | 1189 | 1855 | 0 | 0 |
| hsa_circ_0070708 | 89 | 1 | 395 | 1387 | 1781 | 0 | 3.00E-136 |
| hsa_circ_0070708 | 90 | 1 | 469 | 1387 | 1855 | 0 | 7.00E-169 |
| hsa_circ_0070708 | 90 | 1 | 370 | 1486 | 1855 | 0 | 4.00E-135 |
| hsa_circ_0070708 | 90 | 1 | 370 | 1486 | 1855 | 0 | 3.00E-134 |
| hsa_circ_0070708 | 87 | 325 | 677 | 1855 | 2207 | 0 | 6.00E-111 |
| hsa_circ_0070708 | 87 | 127 | 479 | 1855 | 2207 | 0 | 4.00E-111 |
| hsa_circ_0070708 | 87 | 1 | 352 | 1856 | 2207 | 0 | 1.00E-110 |
| hsa_circ_0049503 | 73 | 615 | 2275 | 445 | 2140 | 0.06 | 1.00E-134 |
| hsa_circ_0049507 | 83 | 1 | 217 | 1 | 217 | 0.03 | 4.00E-47 |
| hsa_circ_0049507 | 83 | 1 | 217 | 1 | 217 | 0.03 | 2.00E-47 |
| hsa_circ_0049511 | 76 | 1 | 951 | 24274 | 23324 | 0.03 | 2.00E-139 |
| hsa_circ_0049511 | 76 | 1 | 951 | 24274 | 23324 | 0.03 | 1.00E-139 |
| hsa_circ_0049848 | 91 | 1 | 1523 | 1523 | 1 | 0 | 0 |
| hsa_circ_0042350 | 96 | 65150 | 65201 | 15062 | 15011 | 0 | 3.00E-13 |
| hsa_circ_0004659 | 75 | 12146 | 12969 | 164530 | 165354 | 0.05 | 8.00E-94 |
| hsa_circ_0006731 | 96 | 1 | 632 | 1 | 632 | 0 | 0 |
| hsa_circ_0006731 | 96 | 1 | 632 | 1 | 632 | 0 | 0 |
| hsa_circ_0006731 | 95 | 1 | 304 | 1 | 304 | 0 | 2.00E-132 |
| hsa_circ_0067675 | 92 | 6 | 358 | 3238 | 2886 | 0 | 1.00E-139 |
| hsa_circ_0067675 | 90 | 5 | 474 | 1810 | 1340 | 0 | 2.00E-169 |
| hsa_circ_0067669 | 94 | 1 | 567 | 532 | 1098 | 0 | 0 |
| hsa_circ_0067669 | 97 | 1 | 374 | 89 | 462 | 0 | 3.00E-176 |
| hsa_circ_0067666 | 97 | 46 | 175 | 1 | 130 | 0 | 6.00E-56 |
| hsa_circ_0067662 | 96 | 258 | 1000 | 1 | 743 | 0 | 0 |
| hsa_circ_0067662 | 96 | 1 | 743 | 1 | 743 | 0 | 0 |
| hsa_circ_0067662 | 96 | 1009 | 1751 | 1 | 743 | 0 | 0 |
| hsa_circ_0067662 | 96 | 103 | 845 | 1 | 743 | 0 | 0 |
| hsa_circ_0067662 | 96 | 258 | 1000 | 1 | 743 | 0 | 0 |
| hsa_circ_0067662 | 96 | 1 | 743 | 1 | 743 | 0 | 0 |
| hsa_circ_0067662 | 96 | 1009 | 1751 | 1 | 743 | 0 | 0 |
| hsa_circ_0067662 | 96 | 103 | 845 | 1 | 743 | 0 | 0 |
| hsa_circ_0067662 | 96 | 258 | 1000 | 1 | 743 | 0 | 0 |

novel_circ_026034 novel_circ_026035 novel_circ_026036 novel_circ_026037 novel_circ_026038 novel_circ_026039 novel_circ_026040 novel_circ_026041 novel_circ_026042 novel_circ_026043 novel_circ_026044 novel_circ_026045 novel_circ_026046 novel_circ_026047 novel_circ_026048 novel_circ_026049 novel_circ_026050 novel_circ_026051 novel_circ_026052 novel_circ_026053 novel_circ_026054 novel_circ_026055 novel_circ_026056 novel_circ_026057 novel_circ_026058 novel_circ_026059 novel_circ_026060 novel_circ_026061 novel_circ_026062 novel_circ_026063 novel_circ_026064 novel_circ_026065 novel_circ_026066 novel_circ_026067 novel_circ_026068 novel_circ_026069 novel_circ_026070 novel_circ_026071 novel_circ_026072 novel_circ_026073 novel_circ_026074 novel_circ_026075 novel_circ_026076 novel_circ_026077 novel_circ_026078 novel_circ_026079 novel_circ_026080 novel_circ_026081 novel_circ_026082 novel_circ_026083 novel_circ_026084 novel_circ_026085 novel_circ_026086 novel_circ_026087

| hsa_circ_0067664 | 96 | 247 | 624 | 572 | 949 | 0 | 7.00E-175 |
| --- | --- | --- | --- | --- | --- | --- | --- |
| hsa_circ_0067664 | 95 | 1 | 408 | 133 | 540 | 0 | 0 |
| hsa_circ_0067664 | 95 | 1 | 540 | 1 | 540 | 0 | 0 |
| hsa_circ_0067662 | 96 | 1 | 743 | 1 | 743 | 0 | 0 |
| hsa_circ_0067662 | 96 | 1009 | 1751 | 1 | 743 | 0 | 0 |
| hsa_circ_0067662 | 96 | 103 | 845 | 1 | 743 | 0 | 0 |
| hsa_circ_0067662 | 96 | 258 | 1000 | 1 | 743 | 0 | 0 |
| hsa_circ_0067664 | 95 | 1 | 540 | 1 | 540 | 0 | 0 |
| hsa_circ_0067662 | 96 | 1009 | 1751 | 1 | 743 | 0 | 0 |
| hsa_circ_0067662 | 96 | 103 | 845 | 1 | 743 | 0 | 0 |
| hsa_circ_0067662 | 96 | 258 | 1000 | 1 | 743 | 0 | 0 |
| hsa_circ_0067662 | 96 | 1 | 528 | 1 | 529 | 0 | 0 |
| hsa_circ_0067662 | 96 | 1009 | 1536 | 1 | 529 | 0 | 0 |
| hsa_circ_0067662 | 96 | 103 | 630 | 1 | 529 | 0 | 0 |
| hsa_circ_0067658 | 98 | 1 | 592 | 1 | 592 | 0 | 0 |
| hsa_circ_0067658 | 98 | 1 | 592 | 1 | 592 | 0 | 0 |
| hsa_circ_0006496 | 98 | 1 | 305 | 1 | 305 | 0 | 1.00E-149 |
| hsa_circ_0067658 | 98 | 1 | 460 | 1 | 460 | 0 | 0 |
| hsa_circ_0067655 | 93 | 2 | 219 | 232 | 14 | 0 | 2.00E-86 |
| hsa_circ_0067648 | 89 | 93 | 1679 | 1581 | 1 | 0.01 | 0 |
| hsa_circ_0067647 | 89 | 243 | 2479 | 2513 | 274 | 0 | 0 |
| hsa_circ_0067648 | 89 | 1 | 1587 | 1581 | 1 | 0.01 | 0 |
| hsa_circ_0067647 | 88 | 1 | 1587 | 2513 | 924 | 0 | 0 |
| hsa_circ_0067645 | 93 | 1 | 830 | 1109 | 280 | 0 | 0 |
| hsa_circ_0067645 | 93 | 1 | 1109 | 1109 | 1 | 0 | 0 |
| hsa_circ_0067644 | 93 | 1 | 1240 | 1811 | 572 | 0 | 0 |
| hsa_circ_0067644 | 94 | 1 | 1478 | 1811 | 334 | 0 | 0 |
| hsa_circ_0067633 | 93 | 1 | 2690 | 3880 | 1191 | 0 | 0 |
| hsa_circ_0067645 | 91 | 1 | 444 | 918 | 475 | 0 | 2.00E-173 |
| hsa_circ_0067645 | 93 | 1 | 639 | 918 | 280 | 0 | 0 |
| hsa_circ_0067644 | 93 | 1 | 1049 | 1620 | 572 | 0 | 0 |
| hsa_circ_0067644 | 94 | 1 | 1287 | 1620 | 334 | 0 | 0 |
| hsa_circ_0067640 | 93 | 1 | 1971 | 2320 | 350 | 0 | 0 |
| hsa_circ_0067644 | 93 | 1 | 776 | 1347 | 572 | 0 | 0 |
| hsa_circ_0067644 | 94 | 1 | 554 | 981 | 428 | 0 | 0 |
| hsa_circ_0067642 | 93 | 1 | 1211 | 1211 | 1 | 0 | 0 |
| hsa_circ_0067640 | 93 | 1 | 1681 | 1681 | 1 | 0 | 0 |
| hsa_circ_0067644 | 95 | 4 | 368 | 792 | 428 | 0.01 | 1.00E-159 |
| hsa_circ_0067642 | 92 | 1 | 801 | 801 | 1 | 0 | 0 |
| hsa_circ_0067640 | 93 | 1 | 1060 | 1271 | 212 | 0 | 0 |
| hsa_circ_0067642 | 91 | 1 | 437 | 437 | 1 | 0 | 8.00E-168 |
| hsa_circ_0067633 | 91 | 1 | 320 | 323 | 1 | 0.01 | 2.00E-118 |
| hsa_circ_0067629 | 92 | 1 | 345 | 354 | 1 | 0.03 | 1.00E-135 |
| hsa_circ_0067625 | 93 | 1 | 807 | 968 | 153 | 0.01 | 0 |
| hsa_circ_0067625 | 93 | 1 | 463 | 614 | 152 | 0 | 0 |
| hsa_circ_0067625 | 93 | 1 | 312 | 463 | 152 | 0 | 1.00E-126 |
| hsa_circ_0067620 | 96 | 234 | 552 | 319 | 1 | 0 | 8.00E-147 |
| hsa_circ_0067614 | 93 | 893 | 1638 | 3434 | 2689 | 0 | 0 |
| hsa_circ_0067614 | 94 | 258 | 463 | 3434 | 3229 | 0 | 2.00E-84 |
| hsa_circ_0067614 | 93 | 258 | 1003 | 3434 | 2689 | 0 | 0 |
| hsa_circ_0067614 | 93 | 258 | 1629 | 3434 | 2063 | 0 | 0 |
| hsa_circ_0067614 | 93 | 1 | 678 | 3366 | 2689 | 0 | 0 |
| hsa_circ_0067614 | 93 | 1 | 353 | 3161 | 2809 | 0 | 1.00E-144 |
| hsa_circ_0067614 | 93 | 1 | 473 | 3161 | 2689 | 0 | 0 |

novel_circ_026088 novel_circ_026089 novel_circ_026090 novel_circ_026091 novel_circ_026092 novel_circ_026093 novel_circ_026094 novel_circ_026095 novel_circ_026096 novel_circ_026097 novel_circ_026098 novel_circ_026099 novel_circ_026100 novel_circ_026101 novel_circ_026102 novel_circ_026103 novel_circ_026104 novel_circ_026105 novel_circ_026106 novel_circ_026107 novel_circ_026108 novel_circ_026109 novel_circ_026110 novel_circ_026111 novel_circ_026112 novel_circ_026113 novel_circ_026114 novel_circ_026115 novel_circ_026116 novel_circ_026117 novel_circ_026118 novel_circ_026119 novel_circ_026120 novel_circ_026121 novel_circ_026122 novel_circ_026123 novel_circ_026124 novel_circ_026125 novel_circ_026126 novel_circ_026127 novel_circ_026128 novel_circ_026129 novel_circ_026130 novel_circ_026131 novel_circ_026132 novel_circ_026135 novel_circ_026136 novel_circ_026137 novel_circ_026138 novel_circ_026141 novel_circ_026142 novel_circ_026143 novel_circ_026145 novel_circ_026146

| hsa_circ_0067614 | 93 | 1 | 967 | 3161 | 2195 | 0 | 0 |
| --- | --- | --- | --- | --- | --- | --- | --- |
| hsa_circ_0067614 | 93 | 1 | 1099 | 3161 | 2063 | 0 | 0 |
| hsa_circ_0067614 | 93 | 1 | 1376 | 3161 | 1786 | 0 | 0 |
| hsa_circ_0067614 | 94 | 1 | 1738 | 3161 | 1424 | 0 | 0 |
| hsa_circ_0067614 | 94 | 1 | 1964 | 3161 | 1198 | 0 | 0 |
| hsa_circ_0067614 | 94 | 1 | 2152 | 3161 | 1010 | 0 | 0 |
| hsa_circ_0067614 | 94 | 1 | 2432 | 3161 | 730 | 0 | 0 |
| hsa_circ_0067614 | 94 | 1 | 2528 | 3161 | 634 | 0 | 0 |
| hsa_circ_0067614 | 93 | 1 | 367 | 3055 | 2689 | 0 | 5.00E-149 |
| hsa_circ_0067614 | 93 | 4 | 667 | 2962 | 2299 | 0 | 0 |
| hsa_circ_0067614 | 93 | 4 | 771 | 2962 | 2195 | 0 | 0 |
| hsa_circ_0067614 | 93 | 4 | 1066 | 2962 | 1900 | 0 | 0 |
| hsa_circ_0067614 | 93 | 4 | 1542 | 2962 | 1424 | 0 | 0 |
| hsa_circ_0067614 | 94 | 4 | 1768 | 2962 | 1198 | 0 | 0 |
| hsa_circ_0067614 | 94 | 4 | 2332 | 2962 | 634 | 0 | 0 |
| hsa_circ_0067614 | 93 | 4 | 2506 | 2962 | 460 | 0 | 0 |
| hsa_circ_0067614 | 94 | 4 | 510 | 2805 | 2299 | 0 | 0 |
| hsa_circ_0067619 | 94 | 1 | 390 | 626 | 237 | 0 | 2.00E-168 |
| hsa_circ_0067619 | 93 | 1 | 494 | 626 | 133 | 0 | 0 |
| hsa_circ_0067619 | 93 | 1 | 626 | 626 | 1 | 0 | 0 |
| hsa_circ_0067618 | 93 | 1 | 1118 | 1681 | 564 | 0 | 0 |
| hsa_circ_0067618 | 94 | 1 | 1265 | 1681 | 417 | 0 | 0 |
| hsa_circ_0067618 | 94 | 1 | 1491 | 1681 | 191 | 0 | 0 |
| hsa_circ_0067614 | 94 | 1 | 1959 | 2688 | 730 | 0 | 0 |
| hsa_circ_0067618 | 92 | 1 | 277 | 1055 | 779 | 0 | 9.00E-106 |
| hsa_circ_0067618 | 94 | 1 | 639 | 1055 | 417 | 0 | 0 |
| hsa_circ_0067616 | 95 | 199 | 1155 | 1053 | 97 | 0 | 0 |
| hsa_circ_0067616 | 95 | 1 | 957 | 1053 | 97 | 0 | 0 |
| hsa_circ_0067618 | 98 | 1 | 226 | 416 | 191 | 0 | 3.00E-109 |
| hsa_circ_0067616 | 95 | 1 | 694 | 790 | 97 | 0 | 0 |
| hsa_circ_0067616 | 94 | 1 | 790 | 790 | 1 | 0 | 0 |
| hsa_circ_0067614 | 94 | 1 | 898 | 1423 | 526 | 0 | 0 |
| hsa_circ_0067614 | 94 | 1 | 961 | 1423 | 463 | 0 | 0 |
| hsa_circ_0067616 | 93 | 1 | 564 | 564 | 1 | 0 | 0 |
| hsa_circ_0067616 | 93 | 1 | 507 | 507 | 1 | 0 | 0 |
| hsa_circ_0067616 | 92 | 1 | 278 | 374 | 97 | 0 | 1.00E-109 |
| hsa_circ_0067616 | 93 | 1 | 374 | 374 | 1 | 0 | 3.00E-151 |
| hsa_circ_0067614 | 93 | 1 | 482 | 1007 | 526 | 0 | 0 |
| hsa_circ_0067614 | 92 | 1 | 548 | 1007 | 460 | 0 | 0 |
| hsa_circ_0067614 | 93 | 1 | 204 | 729 | 526 | 0 | 2.00E-80 |
| hsa_circ_0067614 | 92 | 1 | 267 | 729 | 463 | 0 | 7.00E-102 |
| hsa_circ_0067614 | 91 | 1 | 270 | 729 | 460 | 0 | 1.00E-101 |
| hsa_circ_0067614 | 91 | 1 | 270 | 729 | 460 | 0 | 2.00E-101 |
| hsa_circ_0067614 | 90 | 377 | 833 | 464 | 11 | 0.01 | 3.00E-164 |
| hsa_circ_0067607 | 89 | 377 | 1260 | 6088 | 5208 | 0.01 | 0 |
| hsa_circ_0067607 | 89 | 1 | 882 | 6086 | 5208 | 0.01 | 0 |
| hsa_circ_0067611 | 88 | 3 | 559 | 557 | 1 | 0 | 0 |
| hsa_circ_0067607 | 89 | 3 | 759 | 5964 | 5208 | 0 | 0 |
| hsa_circ_0067603 | 85 | 1 | 499 | 1582 | 1084 | 0 | 2.00E-140 |
| hsa_circ_0008304 | 93 | 48 | 189 | 141 | 1 | 0.01 | 5.00E-52 |
| hsa_circ_0008304 | 93 | 48 | 189 | 141 | 1 | 0.01 | 1.00E-51 |
| hsa_circ_0008304 | 93 | 48 | 189 | 141 | 1 | 0.01 | 2.00E-51 |
| hsa_circ_0067595 | 92 | 555 | 1034 | 480 | 1 | 0 | 0 |
| hsa_circ_0067595 | 92 | 1 | 480 | 480 | 1 | 0 | 0 |

novel_circ_026147 novel_circ_026148 novel_circ_026149 novel_circ_026150 novel_circ_026151 novel_circ_026152 novel_circ_026153 novel_circ_026154 novel_circ_026155 novel_circ_026156 novel_circ_026158 novel_circ_026159 novel_circ_026160 novel_circ_026162 novel_circ_026163 novel_circ_026164 novel_circ_026165 novel_circ_026167 novel_circ_026168 novel_circ_026172 novel_circ_026173 novel_circ_026174 novel_circ_026175 novel_circ_026176 novel_circ_026177 novel_circ_026178 novel_circ_026182 novel_circ_026183 novel_circ_026184 novel_circ_026186 novel_circ_026187 novel_circ_026188 novel_circ_026189 novel_circ_026190 novel_circ_026191 novel_circ_026192 novel_circ_026193 novel_circ_026194 novel_circ_026195 novel_circ_026196 novel_circ_026197 novel_circ_026198 novel_circ_026199 novel_circ_026200 novel_circ_026201 novel_circ_026202 novel_circ_026203 novel_circ_026204 novel_circ_026205 novel_circ_026206 novel_circ_026207 novel_circ_026208 novel_circ_026209 novel_circ_026210

| hsa_circ_0067595 | 92 | 1 | 480 | 480 | 1 | 0 | 0 |
| --- | --- | --- | --- | --- | --- | --- | --- |
| hsa_circ_0067594 | 91 | 171 | 701 | 698 | 168 | 0 | 0 |
| hsa_circ_0067594 | 91 | 171 | 868 | 698 | 1 | 0 | 0 |
| hsa_circ_0067594 | 91 | 1 | 531 | 698 | 168 | 0 | 0 |
| hsa_circ_0067594 | 91 | 1 | 698 | 698 | 1 | 0 | 0 |
| hsa_circ_0067594 | 90 | 1 | 388 | 388 | 1 | 0 | 6.00E-139 |
| hsa_circ_0067594 | 89 | 1 | 319 | 319 | 1 | 0 | 2.00E-107 |
| hsa_circ_0067590 | 92 | 83 | 1323 | 164 | 1407 | 0 | 0 |
| hsa_circ_0067590 | 92 | 83 | 1322 | 164 | 1406 | 0 | 0 |
| hsa_circ_0067590 | 92 | 160 | 1399 | 164 | 1406 | 0 | 0 |
| hsa_circ_0067585 | 94 | 1 | 472 | 1 | 472 | 0 | 0 |
| hsa_circ_0067585 | 94 | 1 | 394 | 1 | 394 | 0 | 3.00E-172 |
| hsa_circ_0006347 | 80 | 39699 | 40429 | 2181 | 2912 | 0.06 | 4.00E-144 |
| hsa_circ_0004190 | 93 | 1 | 335 | 1 | 335 | 0.01 | 3.00E-136 |
| hsa_circ_0004921 | 94 | 104 | 351 | 1 | 248 | 0 | 1.00E-104 |
| hsa_circ_0004921 | 94 | 1 | 248 | 1 | 248 | 0 | 1.00E-104 |
| hsa_circ_0071129 | 89 | 339 | 602 | 1 | 264 | 0 | 3.00E-88 |
| hsa_circ_0071129 | 89 | 1 | 264 | 1 | 264 | 0 | 1.00E-88 |
| hsa_circ_0071135 | 86 | 1 | 775 | 775 | 1 | 0 | 0 |
| hsa_circ_0071141 | 91 | 4 | 823 | 820 | 1 | 0 | 0 |
| hsa_circ_0071158 | 92 | 1 | 261 | 261 | 1 | 0 | 6.00E-102 |
| hsa_circ_0071158 | 91 | 1 | 711 | 711 | 1 | 0 | 0 |
| hsa_circ_0071163 | 90 | 2 | 229 | 228 | 1 | 0 | 3.00E-80 |
| hsa_circ_0071163 | 90 | 1 | 450 | 450 | 1 | 0 | 2.00E-168 |
| hsa_circ_0071169 | 94 | 4 | 284 | 281 | 1 | 0 | 5.00E-118 |
| hsa_circ_0071142 | 95 | 1 | 409 | 2093 | 1685 | 0 | 0 |
| hsa_circ_0004509 | 93 | 2 | 379 | 378 | 1 | 0 | 6.00E-153 |
| hsa_circ_0004509 | 93 | 2 | 379 | 378 | 1 | 0 | 3.00E-153 |
| hsa_circ_0004509 | 93 | 2 | 379 | 378 | 1 | 0 | 2.00E-153 |
| hsa_circ_0073653 | 95 | 1 | 277 | 1 | 277 | 0 | 9.00E-121 |
| hsa_circ_0073653 | 93 | 1 | 410 | 1 | 410 | 0 | 7.00E-168 |
| hsa_circ_0073653 | 94 | 1 | 656 | 1 | 656 | 0 | 0 |
| hsa_circ_0073657 | 90 | 1 | 284 | 1 | 284 | 0 | 5.00E-103 |
| hsa_circ_0073657 | 92 | 1 | 717 | 1 | 720 | 0 | 0 |
| hsa_circ_0073657 | 91 | 1 | 886 | 1 | 889 | 0 | 0 |
| hsa_circ_0073657 | 90 | 1 | 2353 | 1 | 2356 | 0 | 0 |
| hsa_circ_0073661 | 91 | 1 | 566 | 1 | 569 | 0.01 | 0 |
| hsa_circ_0073665 | 88 | 1 | 1274 | 1 | 1274 | 0 | 0 |
| hsa_circ_0073671 | 92 | 1 | 535 | 1 | 535 | 0 | 0 |
| hsa_circ_0073671 | 90 | 3 | 2028 | 196 | 2218 | 0 | 0 |
| hsa_circ_0073672 | 90 | 1 | 1794 | 223 | 2013 | 0 | 0 |
| hsa_circ_0073672 | 91 | 1 | 2490 | 223 | 2709 | 0 | 0 |
| hsa_circ_0073671 | 91 | 1 | 4409 | 536 | 4938 | 0 | 0 |
| hsa_circ_0073671 | 91 | 1 | 4955 | 536 | 5487 | 0 | 0 |
| hsa_circ_0073672 | 94 | 1 | 376 | 1906 | 2281 | 0 | 5.00E-159 |
| hsa_circ_0073672 | 93 | 1 | 542 | 1906 | 2447 | 0 | 0 |
| hsa_circ_0073672 | 92 | 1 | 1898 | 1906 | 3800 | 0 | 0 |
| hsa_circ_0073672 | 92 | 1 | 2090 | 1906 | 3992 | 0 | 0 |
| hsa_circ_0073671 | 92 | 1 | 2544 | 2219 | 4759 | 0 | 0 |
| hsa_circ_0073671 | 92 | 1 | 2949 | 2219 | 5167 | 0 | 0 |
| hsa_circ_0073671 | 92 | 1 | 3151 | 2219 | 5369 | 0 | 0 |
| hsa_circ_0073671 | 92 | 1 | 3269 | 2219 | 5487 | 0 | 0 |
| hsa_circ_0073671 | 92 | 1 | 3269 | 2219 | 5487 | 0 | 0 |
| hsa_circ_0073672 | 92 | 1 | 1982 | 2014 | 3992 | 0 | 0 |

novel_circ_026211 novel_circ_026212 novel_circ_026213 novel_circ_026214 novel_circ_026215 novel_circ_026216 novel_circ_026217 novel_circ_026218 novel_circ_026219 novel_circ_026220 novel_circ_026221 novel_circ_026222 novel_circ_026223 novel_circ_026224 novel_circ_026225 novel_circ_026226 novel_circ_026227 novel_circ_026228 novel_circ_026229 novel_circ_026230 novel_circ_026231 novel_circ_026232 novel_circ_026233 novel_circ_026234 novel_circ_026235 novel_circ_026236 novel_circ_026237 novel_circ_026238 novel_circ_026239 novel_circ_026240 novel_circ_026241 novel_circ_026242 novel_circ_026246 novel_circ_026248 novel_circ_026249 novel_circ_026250 novel_circ_026251 novel_circ_026252 novel_circ_026253 novel_circ_026254 novel_circ_026255 novel_circ_026256 novel_circ_026257 novel_circ_026258 novel_circ_026259 novel_circ_026260 novel_circ_026261 novel_circ_026262 novel_circ_026263 novel_circ_026264 novel_circ_026265 novel_circ_026266 novel_circ_026267 novel_circ_026269

| hsa_circ_0073671 | 92 | 1 | 2436 | 2327 | 4759 | 0 | 0 |
| --- | --- | --- | --- | --- | --- | --- | --- |
| hsa_circ_0073671 | 92 | 1 | 2615 | 2327 | 4938 | 0 | 0 |
| hsa_circ_0073671 | 92 | 1 | 2755 | 2327 | 5081 | 0 | 0 |
| hsa_circ_0073671 | 92 | 1 | 3161 | 2327 | 5487 | 0 | 0 |
| hsa_circ_0073671 | 92 | 1 | 2017 | 2497 | 4510 | 0 | 0 |
| hsa_circ_0073672 | 91 | 15 | 1094 | 2721 | 3800 | 0 | 0 |
| hsa_circ_0073671 | 92 | 15 | 2465 | 3034 | 5487 | 0 | 0 |
| hsa_circ_0073671 | 92 | 1 | 633 | 4306 | 4938 | 0 | 0 |
| hsa_circ_0073671 | 91 | 1 | 773 | 4306 | 5081 | 0 | 0 |
| hsa_circ_0073674 | 92 | 1 | 377 | 78 | 454 | 0 | 2.00E-149 |
| hsa_circ_0073671 | 92 | 3 | 974 | 4513 | 5487 | 0 | 0 |
| hsa_circ_0073675 | 91 | 1 | 725 | 1 | 728 | 0 | 0 |
| hsa_circ_0073675 | 91 | 1 | 725 | 1 | 728 | 0 | 0 |
| hsa_circ_0073675 | 91 | 1 | 725 | 1 | 728 | 0 | 0 |
| hsa_circ_0073675 | 91 | 1 | 725 | 1 | 728 | 0 | 0 |
| hsa_circ_0073675 | 91 | 1 | 546 | 180 | 728 | 0.01 | 0 |
| hsa_circ_0073675 | 91 | 1 | 546 | 180 | 728 | 0.01 | 0 |
| hsa_circ_0073675 | 91 | 1 | 546 | 180 | 728 | 0.01 | 0 |
| hsa_circ_0073671 | 92 | 604 | 1149 | 5482 | 6027 | 0 | 0 |
| hsa_circ_0073675 | 92 | 3 | 320 | 411 | 728 | 0 | 3.00E-123 |
| hsa_circ_0073675 | 91 | 378 | 734 | 723 | 1079 | 0 | 9.00E-135 |
| hsa_circ_0073671 | 92 | 378 | 923 | 5482 | 6027 | 0 | 0 |
| hsa_circ_0073671 | 93 | 378 | 1108 | 5482 | 6212 | 0 | 0 |
| hsa_circ_0073675 | 91 | 176 | 532 | 723 | 1079 | 0 | 6.00E-135 |
| hsa_circ_0073675 | 91 | 1 | 351 | 729 | 1079 | 0 | 2.00E-133 |
| hsa_circ_0073671 | 92 | 1 | 540 | 5488 | 6027 | 0 | 0 |
| hsa_circ_0073671 | 91 | 1 | 996 | 5488 | 6483 | 0 | 0 |
| hsa_circ_0073671 | 92 | 1 | 536 | 5492 | 6027 | 0 | 0 |
| hsa_circ_0073675 | 92 | 1 | 264 | 816 | 1079 | 0 | 1.00E-103 |
| hsa_circ_0073671 | 93 | 1 | 453 | 5575 | 6027 | 0 | 0 |
| hsa_circ_0073680 | 86 | 1 | 568 | 1 | 568 | 0 | 9.00E-174 |
| hsa_circ_0073683 | 85 | 1 | 273 | 1 | 273 | 0 | 3.00E-75 |
| hsa_circ_0073685 | 87 | 365 | 601 | 4 | 240 | 0 | 2.00E-71 |
| hsa_circ_0054470 | 94 | 1 | 424 | 2000 | 2423 | 0.01 | 1.00E-180 |
| hsa_circ_0054470 | 95 | 1 | 483 | 1066 | 1548 | 0 | 0 |
| hsa_circ_0054470 | 93 | 1 | 1393 | 156 | 1548 | 0 | 0 |
| hsa_circ_0054470 | 93 | 6 | 1548 | 5 | 1548 | 0 | 0 |
| hsa_circ_0054470 | 95 | 1 | 585 | 866 | 1450 | 0 | 0 |
| hsa_circ_0054470 | 93 | 1 | 1295 | 156 | 1450 | 0 | 0 |
| hsa_circ_0054470 | 93 | 6 | 1450 | 5 | 1450 | 0 | 0 |
| hsa_circ_0054470 | 94 | 4 | 865 | 438 | 1299 | 0 | 0 |
| hsa_circ_0054470 | 94 | 1 | 310 | 866 | 1175 | 0 | 1.00E-130 |
| hsa_circ_0054470 | 93 | 1 | 594 | 582 | 1175 | 0 | 0 |
| hsa_circ_0054470 | 93 | 6 | 1175 | 5 | 1175 | 0 | 0 |
| hsa_circ_0054470 | 93 | 4 | 631 | 438 | 1065 | 0 | 0 |
| hsa_circ_0054470 | 93 | 4 | 431 | 438 | 865 | 0 | 2.00E-174 |
| hsa_circ_0054470 | 93 | 6 | 865 | 5 | 865 | 0 | 0 |
| hsa_circ_0054470 | 93 | 6 | 581 | 5 | 581 | 0 | 0 |
| hsa_circ_0066989 | 95 | 302 | 867 | 566 | 1 | 0 | 0 |
| hsa_circ_0066991 | 95 | 194 | 680 | 487 | 1 | 0 | 0 |
| hsa_circ_0066985 | 95 | 196 | 1013 | 818 | 1 | 0 | 0 |
| hsa_circ_0066989 | 95 | 1 | 564 | 564 | 1 | 0 | 0 |
| hsa_circ_0066985 | 95 | 1 | 818 | 818 | 1 | 0 | 0 |
| hsa_circ_0066971 | 89 | 1 | 632 | 1 | 626 | 0.02 | 0 |

novel_circ_026270 novel_circ_026271 novel_circ_026272 novel_circ_026273 novel_circ_026274 novel_circ_026275 novel_circ_026276 novel_circ_026277 novel_circ_026278 novel_circ_026279 novel_circ_026280 novel_circ_026281 novel_circ_026282 novel_circ_026283 novel_circ_026284 novel_circ_026285 novel_circ_026286 novel_circ_026287 novel_circ_026288 novel_circ_026289 novel_circ_026290 novel_circ_026291 novel_circ_026292 novel_circ_026293 novel_circ_026294 novel_circ_026295 novel_circ_026296 novel_circ_026297 novel_circ_026298 novel_circ_026299 novel_circ_026300 novel_circ_026301 novel_circ_026302 novel_circ_026303 novel_circ_026304 novel_circ_026305 novel_circ_026306 novel_circ_026308 novel_circ_026309 novel_circ_026310 novel_circ_026311 novel_circ_026312 novel_circ_026314 novel_circ_026315 novel_circ_026316 novel_circ_026317 novel_circ_026318 novel_circ_026319 novel_circ_026320 novel_circ_026321 novel_circ_026322 novel_circ_026323 novel_circ_026324 novel_circ_026325

| hsa_circ_0066971 | 89 | 1 | 378 | 1 | 378 | 0 | 2.00E-133 |
| --- | --- | --- | --- | --- | --- | --- | --- |
| hsa_circ_0082021 | 95 | 1 | 225 | 4258 | 4034 | 0 | 6.00E-97 |
| hsa_circ_0082021 | 92 | 1 | 2589 | 4258 | 1670 | 0 | 0 |
| hsa_circ_0082022 | 92 | 1 | 2364 | 2758 | 395 | 0 | 0 |
| hsa_circ_0082022 | 94 | 1 | 707 | 1104 | 395 | 0 | 0 |
| hsa_circ_0082021 | 92 | 1 | 1461 | 2379 | 916 | 0 | 0 |
| hsa_circ_0082021 | 92 | 1 | 1461 | 2379 | 916 | 0 | 0 |
| hsa_circ_0082022 | 90 | 1 | 520 | 800 | 278 | 0.01 | 0 |
| hsa_circ_0082021 | 90 | 1 | 1157 | 2075 | 916 | 0 | 0 |
| hsa_circ_0082021 | 90 | 1 | 1157 | 2075 | 916 | 0 | 0 |
| hsa_circ_0082021 | 90 | 1 | 570 | 1669 | 1100 | 0 | 0 |
| hsa_circ_0082021 | 91 | 1 | 754 | 1669 | 916 | 0 | 0 |
| hsa_circ_0082021 | 90 | 1 | 452 | 1551 | 1100 | 0 | 8.00E-168 |
| hsa_circ_0082021 | 91 | 1 | 636 | 1551 | 916 | 0 | 0 |
| hsa_circ_0082021 | 91 | 1 | 636 | 1551 | 916 | 0 | 0 |
| hsa_circ_0082021 | 90 | 1 | 980 | 1551 | 572 | 0.01 | 0 |
| hsa_circ_0082021 | 90 | 1 | 1159 | 1551 | 393 | 0.01 | 0 |
| hsa_circ_0082021 | 90 | 1 | 1542 | 1551 | 7 | 0.01 | 0 |
| hsa_circ_0082021 | 90 | 1 | 360 | 1275 | 916 | 0.01 | 4.00E-130 |
| hsa_circ_0082021 | 90 | 1 | 360 | 1275 | 916 | 0.01 | 5.00E-129 |
| hsa_circ_0082021 | 90 | 2 | 341 | 911 | 572 | 0.01 | 1.00E-120 |
| hsa_circ_0082021 | 89 | 2 | 520 | 911 | 393 | 0.01 | 0 |
| hsa_circ_0082021 | 90 | 1 | 383 | 392 | 7 | 0.01 | 4.00E-140 |
| hsa_circ_0082016 | 97 | 1 | 383 | 614 | 996 | 0 | 0 |
| hsa_circ_0082016 | 95 | 1 | 674 | 323 | 996 | 0 | 0 |
| hsa_circ_0082016 | 95 | 1 | 442 | 323 | 764 | 0 | 0 |
| hsa_circ_0082016 | 94 | 1 | 291 | 323 | 613 | 0 | 7.00E-122 |
| hsa_circ_0082012 | 95 | 1 | 812 | 1 | 812 | 0 | 0 |
| hsa_circ_0082013 | 95 | 1 | 569 | 1 | 569 | 0 | 0 |
| hsa_circ_0082012 | 95 | 1 | 729 | 84 | 812 | 0 | 0 |
| hsa_circ_0082012 | 95 | 1 | 812 | 1 | 812 | 0 | 0 |
| hsa_circ_0082014 | 96 | 1 | 300 | 1 | 300 | 0 | 3.00E-135 |
| hsa_circ_0082012 | 95 | 1 | 714 | 1 | 714 | 0 | 0 |
| hsa_circ_0082012 | 94 | 1 | 414 | 1 | 414 | 0 | 1.00E-176 |
| hsa_circ_0082012 | 93 | 1 | 243 | 1 | 243 | 0 | 1.00E-98 |
| hsa_circ_0082012 | 93 | 113 | 355 | 1 | 243 | 0 | 2.00E-98 |
| hsa_circ_0082003 | 89 | 1 | 1409 | 1 | 1406 | 0 | 0 |
| hsa_circ_0079659 | 93 | 1 | 327 | 327 | 1 | 0 | 2.00E-133 |
| hsa_circ_0079659 | 93 | 1 | 327 | 327 | 1 | 0 | 3.00E-133 |
| hsa_circ_0008578 | 85 | 6019 | 6103 | 7553 | 7468 | 0.01 | 8.00E-14 |
| hsa_circ_0079637 | 90 | 1 | 618 | 618 | 1 | 0.01 | 0 |
| hsa_circ_0002097 | 87 | 11 | 423 | 413 | 1 | 0 | 4.00E-131 |
| hsa_circ_0079630 | 96 | 1 | 662 | 133 | 794 | 0 | 0 |
| hsa_circ_0079630 | 96 | 1 | 794 | 1 | 794 | 0 | 0 |
| hsa_circ_0079626 | 96 | 571 | 1367 | 532 | 1328 | 0 | 0 |
| hsa_circ_0079626 | 96 | 690 | 1486 | 532 | 1328 | 0 | 0 |
| hsa_circ_0079630 | 97 | 1 | 331 | 133 | 463 | 0 | 5.00E-159 |
| hsa_circ_0079630 | 97 | 1 | 463 | 1 | 463 | 0 | 0 |
| hsa_circ_0079628 | 97 | 571 | 1036 | 379 | 844 | 0 | 0 |
| hsa_circ_0079623 | 94 | 1 | 650 | 1 | 653 | 0 | 0 |
| hsa_circ_0079617 | 94 | 3 | 653 | 169 | 822 | 0 | 0 |
| hsa_circ_0079617 | 94 | 83 | 733 | 169 | 822 | 0 | 0 |
| hsa_circ_0079623 | 94 | 1 | 650 | 1 | 653 | 0 | 0 |
| hsa_circ_0079626 | 93 | 1 | 531 | 1 | 534 | 0.01 | 0 |

novel_circ_026326 novel_circ_026327 novel_circ_026328 novel_circ_026330 novel_circ_026333 novel_circ_026335 novel_circ_026336 novel_circ_026337 novel_circ_026338 novel_circ_026339 novel_circ_026340 novel_circ_026341 novel_circ_026342 novel_circ_026343 novel_circ_026344 novel_circ_026345 novel_circ_026346 novel_circ_026347 novel_circ_026348 novel_circ_026349 novel_circ_026350 novel_circ_026351 novel_circ_026353 novel_circ_026354 novel_circ_026355 novel_circ_026356 novel_circ_026358 novel_circ_026359 novel_circ_026360 novel_circ_026361 novel_circ_026362 novel_circ_026363 novel_circ_026364 novel_circ_026365 novel_circ_026366 novel_circ_026367 novel_circ_026368 novel_circ_026369 novel_circ_026370 novel_circ_026371 novel_circ_026372 novel_circ_026373 novel_circ_026374 novel_circ_026375 novel_circ_026376 novel_circ_026377 novel_circ_026378 novel_circ_026379 novel_circ_026380 novel_circ_026381 novel_circ_026382 novel_circ_026383 novel_circ_026384 novel_circ_026385

| hsa_circ_0079623 | 94 | 1 | 650 | 1 | 653 | 0 | 0 |
| --- | --- | --- | --- | --- | --- | --- | --- |
| hsa_circ_0079617 | 94 | 3 | 653 | 169 | 822 | 0 | 0 |
| hsa_circ_0079623 | 95 | 1 | 272 | 1 | 272 | 0 | 1.00E-119 |
| hsa_circ_0013230 | 100 | 9972 | 10004 | 253 | 221 | 0 | 1.00E-06 |
| hsa_circ_0024326 | 76 | 10688 | 10949 | 31198 | 30936 | 0.04 | 3.00E-25 |
| hsa_circ_0047474 | 84 | 1 | 676 | 1 | 658 | 0.03 | 7.00E-177 |
| hsa_circ_0047474 | 84 | 1 | 676 | 1 | 658 | 0.03 | 6.00E-177 |
| hsa_circ_0047474 | 84 | 1 | 676 | 1 | 658 | 0.03 | 5.00E-177 |
| hsa_circ_0047474 | 84 | 121 | 796 | 1 | 658 | 0.03 | 6.00E-177 |
| hsa_circ_0047474 | 84 | 1 | 674 | 1 | 656 | 0.03 | 6.00E-176 |
| hsa_circ_0047474 | 84 | 121 | 794 | 1 | 656 | 0.03 | 7.00E-176 |
| hsa_circ_0090808 | 92 | 1 | 498 | 848 | 351 | 0 | 0 |
| hsa_circ_0090803 | 94 | 1 | 1497 | 4607 | 3111 | 0 | 0 |
| hsa_circ_0090803 | 94 | 1 | 497 | 4320 | 3824 | 0 | 0 |
| hsa_circ_0009069 | 91 | 1 | 265 | 265 | 1 | 0 | 9.00E-96 |
| hsa_circ_0090803 | 92 | 1 | 713 | 3823 | 3111 | 0 | 0 |
| hsa_circ_0090803 | 93 | 1 | 809 | 3823 | 3015 | 0 | 0 |
| hsa_circ_0090803 | 93 | 1 | 919 | 3823 | 2905 | 0 | 0 |
| hsa_circ_0090803 | 94 | 1 | 534 | 3644 | 3111 | 0 | 0 |
| hsa_circ_0090803 | 93 | 1 | 206 | 3110 | 2905 | 0 | 2.00E-81 |
| hsa_circ_0090803 | 93 | 351 | 772 | 2904 | 2483 | 0 | 7.00E-176 |
| hsa_circ_0085986 | 85 | 6 | 197 | 199 | 390 | 0 | 2.00E-50 |
| hsa_circ_0086082 | 92 | 1 | 380 | 557 | 178 | 0 | 2.00E-149 |
| hsa_circ_0086084 | 92 | 1 | 227 | 227 | 1 | 0 | 9.00E-85 |
| hsa_circ_0086152 | 88 | 484 | 2474 | 2638 | 630 | 0.01 | 0 |
| hsa_circ_0086152 | 88 | 5926 | 7916 | 2638 | 630 | 0.01 | 0 |
| hsa_circ_0073366 | 93 | 1 | 601 | 601 | 1 | 0 | 0 |
| hsa_circ_0073366 | 94 | 1 | 823 | 823 | 1 | 0 | 0 |
| hsa_circ_0073366 | 92 | 1 | 1048 | 1048 | 1 | 0 | 0 |
| hsa_circ_0073366 | 91 | 11 | 1228 | 1217 | 1 | 0 | 0 |
| hsa_circ_0073366 | 89 | 1 | 1384 | 2740 | 1354 | 0.01 | 0 |
| hsa_circ_0073368 | 88 | 1 | 839 | 1096 | 258 | 0 | 0 |
| hsa_circ_0073368 | 88 | 148 | 1118 | 1228 | 258 | 0 | 0 |
| hsa_circ_0073368 | 89 | 148 | 279 | 1228 | 1097 | 0 | 2.00E-38 |
| hsa_circ_0073372 | 93 | 1 | 594 | 1 | 594 | 0 | 0 |
| hsa_circ_0073372 | 93 | 1 | 609 | 1 | 609 | 0 | 0 |
| hsa_circ_0073372 | 93 | 1 | 609 | 1 | 609 | 0 | 0 |
| hsa_circ_0073375 | 90 | 593 | 1386 | 149 | 945 | 0.01 | 0 |
| hsa_circ_0073375 | 91 | 593 | 1534 | 149 | 1093 | 0.01 | 0 |
| hsa_circ_0073375 | 91 | 162 | 1103 | 149 | 1093 | 0.01 | 0 |
| hsa_circ_0073375 | 90 | 1 | 792 | 151 | 945 | 0.01 | 0 |
| hsa_circ_0073382 | 96 | 3 | 622 | 621 | 2 | 0 | 0 |
| hsa_circ_0073382 | 96 | 1 | 716 | 717 | 2 | 0 | 0 |
| hsa_circ_0073387 | 97 | 3 | 503 | 501 | 1 | 0 | 0 |
| hsa_circ_0073387 | 95 | 1 | 784 | 784 | 1 | 0 | 0 |
| hsa_circ_0073387 | 95 | 1 | 915 | 915 | 1 | 0 | 0 |
| hsa_circ_0073387 | 94 | 1 | 1086 | 1086 | 1 | 0 | 0 |
| hsa_circ_0073387 | 95 | 1 | 1263 | 1263 | 1 | 0 | 0 |
| hsa_circ_0008417 | 92 | 1 | 302 | 302 | 1 | 0 | 6.00E-118 |
| hsa_circ_0073394 | 93 | 1 | 479 | 666 | 188 | 0 | 0 |
| hsa_circ_0073394 | 93 | 4 | 591 | 775 | 188 | 0 | 0 |
| hsa_circ_0073400 | 94 | 3 | 489 | 487 | 1 | 0 | 0 |
| hsa_circ_0073400 | 94 | 1 | 335 | 374 | 40 | 0 | 3.00E-141 |
| hsa_circ_0073400 | 94 | 3 | 450 | 487 | 40 | 0 | 0 |

novel_circ_026386 novel_circ_026388 novel_circ_026389 novel_circ_026390 novel_circ_026391 novel_circ_026392 novel_circ_026393 novel_circ_026394 novel_circ_026395 novel_circ_026396 novel_circ_026397 novel_circ_026401 novel_circ_026402 novel_circ_026403 novel_circ_026404 novel_circ_026405 novel_circ_026406 novel_circ_026407 novel_circ_026408 novel_circ_026409 novel_circ_026410 novel_circ_026411 novel_circ_026412 novel_circ_026413 novel_circ_026414 novel_circ_026415 novel_circ_026416 novel_circ_026417 novel_circ_026418 novel_circ_026419 novel_circ_026420 novel_circ_026421 novel_circ_026422 novel_circ_026423 novel_circ_026424 novel_circ_026425 novel_circ_026426 novel_circ_026427 novel_circ_026428 novel_circ_026429 novel_circ_026430 novel_circ_026431 novel_circ_026432 novel_circ_026433 novel_circ_026434 novel_circ_026435 novel_circ_026436 novel_circ_026437 novel_circ_026438 novel_circ_026439 novel_circ_026440 novel_circ_026441 novel_circ_026442 novel_circ_026443

| hsa_circ_0073400 | 94 | 3 | 258 | 487 | 232 | 0 | 2.00E-107 |
| --- | --- | --- | --- | --- | --- | --- | --- |
| hsa_circ_0024007 | 89 | 13 | 554 | 13 | 554 | 0 | 0 |
| hsa_circ_0024007 | 89 | 13 | 646 | 13 | 646 | 0 | 0 |
| hsa_circ_0024007 | 89 | 13 | 1363 | 13 | 1360 | 0 | 0 |
| hsa_circ_0024007 | 89 | 13 | 1363 | 13 | 1360 | 0 | 0 |
| hsa_circ_0024011 | 91 | 1 | 197 | 713 | 909 | 0 | 2.00E-71 |
| hsa_circ_0024011 | 87 | 1 | 449 | 713 | 1145 | 0.05 | 2.00E-138 |
| hsa_circ_0024011 | 83 | 1 | 2093 | 713 | 2804 | 0.02 | 0 |
| hsa_circ_0024013 | 90 | 1 | 236 | 1 | 236 | 0.03 | 1.00E-79 |
| hsa_circ_0024014 | 80 | 1653 | 3430 | 1811 | 3603 | 0.03 | 0 |
| hsa_circ_0024014 | 80 | 1653 | 3900 | 1811 | 4073 | 0.03 | 0 |
| hsa_circ_0024021 | 90 | 4 | 393 | 4 | 387 | 0.02 | 2.00E-138 |
| hsa_circ_0024021 | 93 | 1 | 506 | 388 | 893 | 0 | 0 |
| hsa_circ_0024021 | 93 | 1 | 506 | 388 | 893 | 0 | 0 |
| hsa_circ_0024025 | 88 | 139 | 416 | 1 | 278 | 0 | 8.00E-88 |
| hsa_circ_0024025 | 81 | 1 | 576 | 279 | 840 | 0.08 | 2.00E-115 |
| hsa_circ_0024036 | 92 | 3 | 1115 | 2080 | 970 | 0 | 0 |
| hsa_circ_0024047 | 83 | 3979 | 4405 | 6 | 426 | 0.04 | 7.00E-103 |
| hsa_circ_0024047 | 83 | 6 | 432 | 6 | 426 | 0.04 | 7.00E-104 |
| hsa_circ_0024049 | 91 | 1 | 584 | 2 | 584 | 0 | 0 |
| hsa_circ_0024053 | 94 | 1 | 296 | 1 | 296 | 0 | 3.00E-126 |
| hsa_circ_0024053 | 94 | 1 | 296 | 1 | 296 | 0 | 1.00E-125 |
| hsa_circ_0024060 | 94 | 1 | 310 | 310 | 1 | 0 | 1.00E-130 |
| hsa_circ_0024060 | 92 | 1 | 1169 | 1169 | 1 | 0 | 0 |
| hsa_circ_0024060 | 92 | 1 | 793 | 1103 | 311 | 0 | 0 |
| hsa_circ_0024060 | 92 | 1 | 859 | 1169 | 311 | 0 | 0 |
| hsa_circ_0024060 | 92 | 1 | 381 | 1103 | 723 | 0 | 9.00E-152 |
| hsa_circ_0024060 | 92 | 1 | 447 | 1169 | 723 | 0 | 0 |
| hsa_circ_0024068 | 95 | 1 | 381 | 1 | 381 | 0 | 2.00E-168 |
| hsa_circ_0024068 | 91 | 2832 | 3072 | 623 | 380 | 0.01 | 2.00E-88 |
| hsa_circ_0024072 | 94 | 1 | 1217 | 4080 | 2864 | 0 | 0 |
| hsa_circ_0024079 | 94 | 1 | 1124 | 1124 | 1 | 0 | 0 |
| hsa_circ_0024080 | 94 | 1 | 609 | 609 | 1 | 0 | 0 |
| hsa_circ_0024079 | 94 | 1 | 917 | 1124 | 208 | 0 | 0 |
| hsa_circ_0024079 | 94 | 1 | 993 | 1200 | 208 | 0 | 0 |
| hsa_circ_0024079 | 94 | 202 | 1196 | 1202 | 208 | 0 | 0 |
| hsa_circ_0024080 | 94 | 1 | 339 | 525 | 187 | 0 | 8.00E-147 |
| hsa_circ_0024080 | 94 | 1 | 423 | 609 | 187 | 0 | 0 |
| hsa_circ_0024079 | 94 | 4 | 636 | 1026 | 394 | 0 | 0 |
| hsa_circ_0024079 | 94 | 1 | 731 | 1124 | 394 | 0 | 0 |
| hsa_circ_0024079 | 94 | 1 | 807 | 1200 | 394 | 0 | 0 |
| hsa_circ_0024079 | 94 | 202 | 1010 | 1202 | 394 | 0 | 0 |
| hsa_circ_0024079 | 93 | 1 | 392 | 1124 | 733 | 0 | 2.00E-164 |
| hsa_circ_0024084 | 91 | 1 | 112 | 112 | 1 | 0 | 4.00E-35 |
| hsa_circ_0024084 | 91 | 205 | 316 | 112 | 1 | 0 | 5.00E-35 |
| hsa_circ_0024086 | 88 | 1 | 1581 | 1626 | 1 | 0.03 | 0 |
| hsa_circ_0066561 | 92 | 1 | 407 | 407 | 1 | 0 | 3.00E-161 |
| hsa_circ_0066564 | 91 | 7 | 949 | 1532 | 590 | 0 | 0 |
| hsa_circ_0066564 | 92 | 1 | 624 | 2284 | 1661 | 0 | 0 |
| hsa_circ_0066563 | 91 | 1 | 1318 | 3218 | 1910 | 0.01 | 0 |
| hsa_circ_0066566 | 92 | 1 | 694 | 694 | 1 | 0 | 0 |
| hsa_circ_0066561 | 92 | 1 | 852 | 3783 | 2941 | 0.01 | 0 |
| hsa_circ_0066568 | 92 | 1 | 388 | 388 | 1 | 0 | 6.00E-154 |
| hsa_circ_0066561 | 93 | 1 | 546 | 3783 | 3238 | 0 | 0 |

novel_circ_026444 novel_circ_026445 novel_circ_026447 novel_circ_026448 novel_circ_026449 novel_circ_026450 novel_circ_026451 novel_circ_026452 novel_circ_026454 novel_circ_026455 novel_circ_026456 novel_circ_026457 novel_circ_026458 novel_circ_026459 novel_circ_026460 novel_circ_026462 novel_circ_026463 novel_circ_026466 novel_circ_026468 novel_circ_026469 novel_circ_026472 novel_circ_026473 novel_circ_026474 novel_circ_026475 novel_circ_026476 novel_circ_026477 novel_circ_026478 novel_circ_026479 novel_circ_026481 novel_circ_026482 novel_circ_026483 novel_circ_026484 novel_circ_026485 novel_circ_026486 novel_circ_026487 novel_circ_026488 novel_circ_026489 novel_circ_026490 novel_circ_026497 novel_circ_026498 novel_circ_026499 novel_circ_026500 novel_circ_026504 novel_circ_026507 novel_circ_026508 novel_circ_026509 novel_circ_026510 novel_circ_026511 novel_circ_026512 novel_circ_026513 novel_circ_026514 novel_circ_026515 novel_circ_026516 novel_circ_026517

| hsa_circ_0066570 | 94 | 1 | 325 | 327 | 3 | 0 | 1.00E-140 |
| --- | --- | --- | --- | --- | --- | --- | --- |
| hsa_circ_0091563 | 95 | 1 | 629 | 1 | 629 | 0 | 0 |
| hsa_circ_0074029 | 76 | 1582 | 1852 | 207889 | 207611 | 0.04 | 2.00E-27 |
| hsa_circ_0066961 | 90 | 1 | 303 | 2201 | 1899 | 0 | 8.00E-107 |
| hsa_circ_0066961 | 90 | 1 | 2394 | 4292 | 1899 | 0 | 0 |
| hsa_circ_0066963 | 89 | 3 | 634 | 7986 | 7355 | 0 | 0 |
| hsa_circ_0066965 | 90 | 3 | 403 | 517 | 117 | 0 | 3.00E-146 |
| hsa_circ_0066965 | 88 | 1 | 893 | 1391 | 520 | 0.03 | 0 |
| hsa_circ_0066967 | 90 | 4 | 287 | 286 | 3 | 0 | 3.00E-101 |
| hsa_circ_0066968 | 91 | 1 | 599 | 1422 | 824 | 0 | 0 |
| hsa_circ_0066968 | 94 | 1 | 405 | 1422 | 1018 | 0 | 9.00E-172 |
| hsa_circ_0075848 | 95 | 228 | 363 | 136 | 1 | 0 | 6.00E-54 |
| hsa_circ_0075848 | 91 | 1 | 299 | 432 | 133 | 0 | 1.00E-112 |
| hsa_circ_0075848 | 91 | 1 | 302 | 435 | 133 | 0 | 2.00E-114 |
| hsa_circ_0075851 | 84 | 10 | 533 | 4872 | 4349 | 0 | 1.00E-137 |
| hsa_circ_0075903 | 87 | 7 | 212 | 217 | 12 | 0.01 | 2.00E-61 |
| hsa_circ_0075903 | 85 | 1 | 280 | 291 | 12 | 0.01 | 1.00E-75 |
| hsa_circ_0086176 | 72 | 250 | 891 | 3552 | 2911 | 0.03 | 8.00E-38 |
| hsa_circ_0081383 | 80 | 1002 | 1081 | 588 | 667 | 0.05 | 5.00E-07 |
| hsa_circ_0081383 | 80 | 366 | 445 | 588 | 667 | 0.05 | 3.00E-07 |
| hsa_circ_0068074 | 87 | 890 | 1637 | 1 | 726 | 0.03 | 0 |
| hsa_circ_0068074 | 87 | 890 | 1641 | 1 | 731 | 0.03 | 0 |
| hsa_circ_0068074 | 89 | 11171 | 11917 | 628 | 1374 | 0 | 0 |
| hsa_circ_0068074 | 87 | 252 | 1003 | 1 | 731 | 0.03 | 0 |
| hsa_circ_0068074 | 89 | 1 | 438 | 1 | 438 | 0 | 5.00E-155 |
| hsa_circ_0068074 | 89 | 308 | 778 | 628 | 1098 | 0 | 2.00E-161 |
| hsa_circ_0068074 | 90 | 308 | 882 | 628 | 1202 | 0 | 0 |
| hsa_circ_0068074 | 89 | 1 | 648 | 727 | 1374 | 0 | 0 |
| hsa_circ_0068077 | 92 | 268 | 1217 | 1 | 950 | 0 | 0 |
| hsa_circ_0068077 | 92 | 1 | 839 | 112 | 950 | 0 | 0 |
| hsa_circ_0068081 | 90 | 3 | 424 | 122 | 543 | 0 | 2.00E-154 |
| hsa_circ_0068081 | 89 | 3 | 533 | 122 | 652 | 0 | 0 |
| hsa_circ_0068081 | 88 | 3 | 1550 | 122 | 1669 | 0 | 0 |
| hsa_circ_0068081 | 89 | 1 | 288 | 256 | 543 | 0 | 2.00E-98 |
| hsa_circ_0068081 | 86 | 1 | 909 | 761 | 1669 | 0 | 0 |
| hsa_circ_0068081 | 86 | 1 | 687 | 983 | 1669 | 0 | 0 |
| hsa_circ_0068081 | 84 | 1 | 840 | 983 | 1822 | 0 | 0 |
| hsa_circ_0002219 | 81 | 7 | 677 | 7 | 677 | 0 | 3.00E-154 |
| hsa_circ_0068120 | 89 | 1 | 462 | 151 | 615 | 0.01 | 7.00E-164 |
| hsa_circ_0068117 | 89 | 1 | 544 | 861 | 1407 | 0.01 | 0 |
| hsa_circ_0068122 | 85 | 110 | 726 | 620 | 1 | 0 | 8.00E-180 |
| hsa_circ_0068123 | 86 | 5 | 422 | 524 | 104 | 0.01 | 8.00E-123 |
| hsa_circ_0037109 | 90 | 391 | 780 | 390 | 1 | 0 | 2.00E-141 |
| hsa_circ_0037109 | 91 | 125 | 415 | 390 | 100 | 0 | 1.00E-106 |
| hsa_circ_0037109 | 90 | 125 | 514 | 390 | 1 | 0 | 1.00E-141 |
| hsa_circ_0037106 | 89 | 125 | 681 | 1142 | 586 | 0 | 0 |
| hsa_circ_0037106 | 89 | 125 | 982 | 1142 | 285 | 0 | 0 |
| hsa_circ_0037106 | 89 | 125 | 1161 | 1142 | 106 | 0 | 0 |
| hsa_circ_0037109 | 91 | 1 | 291 | 390 | 100 | 0 | 7.00E-107 |
| hsa_circ_0037109 | 90 | 1 | 390 | 390 | 1 | 0 | 9.00E-142 |
| hsa_circ_0037109 | 90 | 1 | 325 | 325 | 1 | 0 | 1.00E-115 |
| hsa_circ_0037106 | 89 | 1 | 492 | 1077 | 586 | 0 | 5.00E-175 |
| hsa_circ_0037106 | 88 | 1 | 793 | 1077 | 285 | 0 | 0 |
| hsa_circ_0037106 | 89 | 1 | 972 | 1077 | 106 | 0 | 0 |

novel_circ_026518 novel_circ_026519 novel_circ_026520 novel_circ_026521 novel_circ_026522 novel_circ_026523 novel_circ_026524 novel_circ_026525 novel_circ_026526 novel_circ_026527 novel_circ_026528 novel_circ_026529 novel_circ_026530 novel_circ_026531 novel_circ_026532 novel_circ_026533 novel_circ_026534 novel_circ_026535 novel_circ_026536 novel_circ_026537 novel_circ_026538 novel_circ_026539 novel_circ_026540 novel_circ_026543 novel_circ_026544 novel_circ_026545 novel_circ_026546 novel_circ_026547 novel_circ_026548 novel_circ_026549 novel_circ_026550 novel_circ_026551 novel_circ_026552 novel_circ_026555 novel_circ_026556 novel_circ_026557 novel_circ_026558 novel_circ_026559 novel_circ_026560 novel_circ_026565 novel_circ_026566 novel_circ_026567 novel_circ_026568 novel_circ_026569 novel_circ_026572 novel_circ_026574 novel_circ_026575 novel_circ_026581 novel_circ_026582 novel_circ_026583 novel_circ_026584 novel_circ_026585 novel_circ_026586 novel_circ_026587

| hsa_circ_0037106 | 89 | 1 | 1077 | 1077 | 1 | 0 | 0 |
| --- | --- | --- | --- | --- | --- | --- | --- |
| hsa_circ_0037106 | 90 | 10 | 413 | 989 | 586 | 0 | 2.00E-144 |
| hsa_circ_0037106 | 88 | 10 | 576 | 989 | 423 | 0 | 0 |
| hsa_circ_0037106 | 89 | 10 | 998 | 989 | 1 | 0 | 0 |
| hsa_circ_0037106 | 88 | 4 | 746 | 848 | 106 | 0 | 0 |
| hsa_circ_0037107 | 88 | 31 | 677 | 647 | 1 | 0 | 0 |
| hsa_circ_0014026 | 76 | 127 | 317 | 178 | 368 | 0 | 3.00E-21 |
| hsa_circ_0037107 | 87 | 1 | 468 | 647 | 180 | 0 | 1.00E-151 |
| hsa_circ_0037107 | 88 | 1 | 647 | 647 | 1 | 0 | 0 |
| hsa_circ_0037107 | 88 | 5 | 480 | 476 | 1 | 0 | 9.00E-163 |
| hsa_circ_0037106 | 89 | 5 | 585 | 581 | 1 | 0 | 0 |
| hsa_circ_0037107 | 90 | 4 | 317 | 314 | 1 | 0 | 6.00E-113 |
| hsa_circ_0037106 | 91 | 4 | 422 | 419 | 1 | 0 | 8.00E-158 |
| hsa_circ_0037106 | 91 | 4 | 422 | 419 | 1 | 0 | 1.00E-157 |
| hsa_circ_0037106 | 91 | 1 | 284 | 284 | 1 | 0 | 1.00E-104 |
| hsa_circ_0037101 | 95 | 1 | 107 | 1013 | 907 | 0 | 2.00E-39 |
| hsa_circ_0037101 | 87 | 15752 | 16283 | 775 | 244 | 0 | 1.00E-170 |
| hsa_circ_0037102 | 89 | 1 | 410 | 552 | 143 | 0 | 3.00E-139 |
| hsa_circ_0037102 | 92 | 1 | 244 | 386 | 143 | 0 | 3.00E-92 |
| hsa_circ_0037102 | 92 | 1 | 244 | 386 | 143 | 0 | 4.00E-92 |
| hsa_circ_0037102 | 92 | 1 | 244 | 386 | 143 | 0 | 4.00E-92 |
| hsa_circ_0037102 | 92 | 1 | 244 | 386 | 143 | 0 | 6.00E-92 |
| hsa_circ_0037097 | 89 | 244 | 927 | 684 | 1 | 0 | 0 |
| hsa_circ_0037097 | 89 | 71 | 754 | 684 | 1 | 0 | 0 |
| hsa_circ_0037094 | 89 | 71 | 1468 | 1525 | 131 | 0 | 0 |
| hsa_circ_0037097 | 90 | 1 | 326 | 326 | 1 | 0 | 3.00E-116 |
| hsa_circ_0037092 | 91 | 1 | 227 | 900 | 674 | 0 | 4.00E-83 |
| hsa_circ_0037092 | 88 | 1 | 377 | 900 | 524 | 0 | 3.00E-126 |
| hsa_circ_0005019 | 86 | 1 | 496 | 496 | 1 | 0 | 3.00E-152 |
| hsa_circ_0037084 | 88 | 1 | 554 | 2147 | 2700 | 0 | 0 |
| hsa_circ_0037084 | 89 | 1 | 304 | 2147 | 2450 | 0 | 2.00E-102 |
| hsa_circ_0037084 | 90 | 1 | 449 | 1698 | 2146 | 0 | 4.00E-161 |
| hsa_circ_0037084 | 89 | 1 | 903 | 1244 | 2146 | 0 | 0 |
| hsa_circ_0037075 | 87 | 5 | 325 | 180 | 500 | 0 | 2.00E-98 |
| hsa_circ_0037075 | 85 | 3 | 497 | 3 | 500 | 0.01 | 3.00E-137 |
| hsa_circ_0037073 | 82 | 1 | 661 | 1 | 664 | 0.01 | 6.00E-156 |
| hsa_circ_0066588 | 94 | 1 | 562 | 563 | 2 | 0 | 0 |
| hsa_circ_0020066 | 96 | 250 | 742 | 6047 | 5555 | 0 | 0 |
| hsa_circ_0020066 | 91 | 1 | 396 | 5660 | 5265 | 0 | 4.00E-150 |
| hsa_circ_0006317 | 89 | 1 | 785 | 101 | 885 | 0.01 | 0 |
| hsa_circ_0006317 | 89 | 1 | 885 | 1 | 885 | 0 | 0 |
| hsa_circ_0006317 | 89 | 131 | 1015 | 1 | 885 | 0 | 0 |
| hsa_circ_0006317 | 92 | 1 | 553 | 101 | 654 | 0 | 0 |
| hsa_circ_0006317 | 91 | 131 | 783 | 1 | 654 | 0 | 0 |
| hsa_circ_0074544 | 77 | 41 | 542 | 41 | 542 | 0.01 | 9.00E-79 |
| hsa_circ_0070719 | 90 | 184 | 579 | 567 | 172 | 0.01 | 7.00E-145 |
| hsa_circ_0070719 | 89 | 1 | 299 | 470 | 172 | 0.01 | 6.00E-103 |
| hsa_circ_0070711 | 95 | 1 | 570 | 612 | 43 | 0 | 0 |
| hsa_circ_0004613 | 95 | 1 | 383 | 383 | 1 | 0 | 1.00E-167 |
| hsa_circ_0083469 | 90 | 1 | 447 | 447 | 1 | 0 | 1.00E-166 |
| hsa_circ_0083469 | 91 | 1 | 518 | 518 | 1 | 0 | 0 |
| hsa_circ_0083469 | 92 | 1 | 756 | 756 | 1 | 0 | 0 |
| hsa_circ_0083471 | 92 | 1 | 303 | 303 | 1 | 0 | 7.00E-117 |
| hsa_circ_0083472 | 94 | 1 | 309 | 309 | 1 | 0 | 3.00E-130 |

novel_circ_026588 novel_circ_026589 novel_circ_026590 novel_circ_026591 novel_circ_026592 novel_circ_026593 novel_circ_026594 novel_circ_026595 novel_circ_026596 novel_circ_026597 novel_circ_026598 novel_circ_026599 novel_circ_026601 novel_circ_026602 novel_circ_026603 novel_circ_026604 novel_circ_026605 novel_circ_026606 novel_circ_026607 novel_circ_026608 novel_circ_026609 novel_circ_026610 novel_circ_026611 novel_circ_026612 novel_circ_026613 novel_circ_026616 novel_circ_026617 novel_circ_026618 novel_circ_026619 novel_circ_026620 novel_circ_026621 novel_circ_026622 novel_circ_026623 novel_circ_026624 novel_circ_026625 novel_circ_026626 novel_circ_026627 novel_circ_026628 novel_circ_026629 novel_circ_026630 novel_circ_026631 novel_circ_026632 novel_circ_026633 novel_circ_026634 novel_circ_026635 novel_circ_026636 novel_circ_026637 novel_circ_026638 novel_circ_026639 novel_circ_026640 novel_circ_026641 novel_circ_026642 novel_circ_026643 novel_circ_026644

| hsa_circ_0083472 | 93 | 1 | 238 | 309 | 72 | 0 | 8.00E-96 |
| --- | --- | --- | --- | --- | --- | --- | --- |
| hsa_circ_0003737 | 86 | 1 | 755 | 68287 | 67532 | 0.01 | 0 |
| hsa_circ_0003737 | 86 | 1 | 755 | 68287 | 67532 | 0.01 | 0 |
| hsa_circ_0083478 | 91 | 1 | 538 | 538 | 1 | 0 | 0 |
| hsa_circ_0002189 | 81 | 10480 | 11576 | 1116 | 1 | 0.04 | 0 |
| hsa_circ_0083480 | 82 | 1 | 1501 | 1893 | 390 | 0.01 | 0 |
| hsa_circ_0083479 | 91 | 1 | 447 | 448 | 2 | 0 | 1.00E-171 |
| hsa_circ_0085131 | 95 | 4029 | 4528 | 1 | 511 | 0.02 | 0 |
| hsa_circ_0083480 | 82 | 1 | 1501 | 1893 | 390 | 0.01 | 0 |
| hsa_circ_0083480 | 82 | 1 | 1501 | 1893 | 390 | 0.01 | 0 |
| hsa_circ_0083480 | 82 | 286 | 1786 | 1893 | 390 | 0.01 | 0 |
| hsa_circ_0059411 | 93 | 1 | 161 | 1 | 161 | 0 | 1.00E-61 |
| hsa_circ_0059398 | 90 | 3 | 1238 | 3 | 1259 | 0.02 | 0 |
| hsa_circ_0059398 | 92 | 1 | 737 | 492 | 1228 | 0 | 0 |
| hsa_circ_0059398 | 91 | 1 | 970 | 259 | 1228 | 0 | 0 |
| hsa_circ_0059398 | 89 | 3 | 1011 | 3 | 1032 | 0.02 | 0 |
| hsa_circ_0059388 | 90 | 94 | 273 | 180 | 1 | 0 | 1.00E-59 |
| hsa_circ_0059388 | 92 | 1 | 118 | 180 | 63 | 0 | 4.00E-41 |
| hsa_circ_0059386 | 95 | 1 | 310 | 84 | 393 | 0 | 1.00E-135 |
| hsa_circ_0059380 | 92 | 1 | 702 | 1 | 702 | 0 | 0 |
| hsa_circ_0059380 | 93 | 1 | 282 | 333 | 614 | 0 | 3.00E-115 |
| hsa_circ_0059380 | 92 | 1 | 469 | 1 | 469 | 0 | 0 |
| hsa_circ_0059380 | 91 | 1 | 332 | 1 | 332 | 0 | 1.00E-124 |
| hsa_circ_0013361 | 95 | 1 | 545 | 1559 | 1015 | 0 | 0 |
| hsa_circ_0013361 | 92 | 115 | 363 | 888 | 640 | 0 | 9.00E-97 |
| hsa_circ_0000540 | 85 | 6276 | 6383 | 8491 | 8384 | 0.02 | 1.00E-21 |
| hsa_circ_0066555 | 87 | 3 | 128 | 218 | 93 | 0 | 2.00E-33 |
| hsa_circ_0018216 | 75 | 835 | 1869 | 3310 | 2274 | 0.02 | 5.00E-123 |
| hsa_circ_0034965 | 95 | 1 | 291 | 1 | 291 | 0 | 7.00E-127 |
| hsa_circ_0034971 | 90 | 1 | 452 | 1 | 452 | 0 | 8.00E-168 |
| hsa_circ_0034982 | 91 | 1 | 397 | 408 | 15 | 0.01 | 8.00E-153 |
| hsa_circ_0034981 | 90 | 31 | 1869 | 2081 | 231 | 0.01 | 0 |
| hsa_circ_0034983 | 90 | 31 | 1664 | 1649 | 1 | 0.01 | 0 |
| hsa_circ_0034984 | 93 | 1 | 1006 | 1006 | 1 | 0 | 0 |
| hsa_circ_0034984 | 90 | 31 | 1472 | 1457 | 1 | 0.01 | 0 |
| hsa_circ_0034985 | 86 | 31 | 619 | 604 | 1 | 0.03 | 0 |
| hsa_circ_0034985 | 84 | 35 | 715 | 705 | 1 | 0.04 | 0 |
| hsa_circ_0034986 | 83 | 31 | 466 | 451 | 1 | 0.04 | 9.00E-108 |
| hsa_circ_0034986 | 84 | 1 | 824 | 839 | 1 | 0.04 | 0 |
| hsa_circ_0034986 | 87 | 1 | 2572 | 2590 | 1 | 0.02 | 0 |
| hsa_circ_0034988 | 89 | 7 | 1915 | 2008 | 94 | 0 | 0 |
| hsa_circ_0034986 | 89 | 1 | 2010 | 2590 | 578 | 0 | 0 |
| hsa_circ_0034986 | 89 | 4 | 2140 | 2717 | 578 | 0 | 0 |
| hsa_circ_0034990 | 89 | 1 | 1645 | 1651 | 1 | 0 | 0 |
| hsa_circ_0034990 | 89 | 7 | 1857 | 1857 | 1 | 0 | 0 |
| hsa_circ_0034990 | 89 | 1 | 1952 | 1955 | 1 | 0 | 0 |
| hsa_circ_0034990 | 89 | 4 | 2082 | 2082 | 1 | 0 | 0 |
| hsa_circ_0034990 | 88 | 1 | 2379 | 2382 | 1 | 0.01 | 0 |
| hsa_circ_0034990 | 88 | 9 | 2520 | 2533 | 1 | 0.01 | 0 |
| hsa_circ_0034990 | 88 | 1 | 2648 | 2669 | 1 | 0.01 | 0 |
| hsa_circ_0006327 | 87 | 1 | 1321 | 1327 | 1 | 0 | 0 |
| hsa_circ_0034991 | 88 | 7 | 1533 | 1653 | 121 | 0 | 0 |
| hsa_circ_0034991 | 88 | 1 | 1628 | 1751 | 121 | 0.01 | 0 |
| hsa_circ_0034991 | 88 | 1 | 1755 | 1878 | 121 | 0.01 | 0 |

novel_circ_026645 novel_circ_026646 novel_circ_026647 novel_circ_026648 novel_circ_026649 novel_circ_026650 novel_circ_026656 novel_circ_026657 novel_circ_026661 novel_circ_026662 novel_circ_026663 novel_circ_026664 novel_circ_026665 novel_circ_026666 novel_circ_026667 novel_circ_026668 novel_circ_026669 novel_circ_026670 novel_circ_026672 novel_circ_026673 novel_circ_026674 novel_circ_026680 novel_circ_026681 novel_circ_026682 novel_circ_026684 novel_circ_026685 novel_circ_026686 novel_circ_026687 novel_circ_026688 novel_circ_026689 novel_circ_026690 novel_circ_026691 novel_circ_026692 novel_circ_026693 novel_circ_026694 novel_circ_026695 novel_circ_026696 novel_circ_026697 novel_circ_026698 novel_circ_026699 novel_circ_026700 novel_circ_026701 novel_circ_026702 novel_circ_026703 novel_circ_026704 novel_circ_026705 novel_circ_026706 novel_circ_026707 novel_circ_026708 novel_circ_026709 novel_circ_026710 novel_circ_026711 novel_circ_026712 novel_circ_026713

| hsa_circ_0034991 | 88 | 4 | 1758 | 1878 | 121 | 0.01 | 0 |
| --- | --- | --- | --- | --- | --- | --- | --- |
| hsa_circ_0034990 | 87 | 1 | 2055 | 2382 | 325 | 0.01 | 0 |
| hsa_circ_0034990 | 87 | 1 | 2324 | 2669 | 325 | 0.02 | 0 |
| hsa_circ_0034993 | 86 | 1 | 787 | 1018 | 214 | 0.03 | 0 |
| hsa_circ_0034993 | 84 | 1 | 566 | 1018 | 435 | 0.05 | 2.00E-154 |
| hsa_circ_0034993 | 84 | 180 | 745 | 1018 | 435 | 0.05 | 3.00E-154 |
| hsa_circ_0035006 | 97 | 431 | 712 | 282 | 1 | 0 | 3.00E-131 |
| hsa_circ_0035012 | 94 | 1 | 189 | 895 | 707 | 0 | 5.00E-77 |
| hsa_circ_0035034 | 93 | 1 | 426 | 1 | 426 | 0 | 1.00E-176 |
| hsa_circ_0035035 | 94 | 1 | 318 | 1 | 318 | 0 | 8.00E-137 |
| hsa_circ_0035043 | 90 | 1 | 459 | 1 | 459 | 0 | 5.00E-165 |
| hsa_circ_0035043 | 89 | 1 | 677 | 1 | 677 | 0 | 0 |
| hsa_circ_0035043 | 89 | 1 | 830 | 1 | 830 | 0 | 0 |
| hsa_circ_0035043 | 89 | 1 | 830 | 1 | 830 | 0 | 0 |
| hsa_circ_0035046 | 89 | 1 | 530 | 1 | 530 | 0 | 0 |
| hsa_circ_0035046 | 88 | 1 | 584 | 1 | 584 | 0 | 0 |
| hsa_circ_0035048 | 97 | 1 | 245 | 1683 | 1439 | 0 | 1.00E-114 |
| hsa_circ_0035049 | 93 | 1 | 325 | 325 | 1 | 0 | 2.00E-130 |
| hsa_circ_0000595 | 93 | 2 | 249 | 2 | 249 | 0 | 1.00E-99 |
| hsa_circ_0035052 | 93 | 1 | 194 | 1 | 194 | 0 | 8.00E-75 |
| hsa_circ_0035052 | 93 | 1 | 415 | 1 | 415 | 0 | 3.00E-172 |
| hsa_circ_0021077 | 87 | 1 | 544 | 1109 | 1655 | 0.02 | 6.00E-171 |
| hsa_circ_0021086 | 92 | 1 | 546 | 546 | 1 | 0 | 0 |
| hsa_circ_0021089 | 93 | 497 | 715 | 807 | 589 | 0 | 9.00E-85 |
| hsa_circ_0021096 | 95 | 226 | 1321 | 2683 | 1588 | 0 | 0 |
| hsa_circ_0021115 | 92 | 110 | 418 | 1318 | 1010 | 0 | 1.00E-121 |
| hsa_circ_0021115 | 92 | 202 | 510 | 1318 | 1010 | 0 | 1.00E-121 |
| hsa_circ_0021115 | 93 | 110 | 316 | 1318 | 1112 | 0 | 8.00E-82 |
| hsa_circ_0021115 | 93 | 202 | 408 | 1318 | 1112 | 0 | 1.00E-81 |
| hsa_circ_0021122 | 92 | 1 | 261 | 261 | 1 | 0 | 3.00E-100 |
| hsa_circ_0021123 | 94 | 1296 | 2696 | 1400 | 1 | 0 | 0 |
| hsa_circ_0021142 | 94 | 1 | 1299 | 1334 | 36 | 0 | 0 |
| hsa_circ_0021142 | 94 | 1 | 1299 | 1334 | 36 | 0 | 0 |
| hsa_circ_0021142 | 94 | 1 | 342 | 377 | 36 | 0 | 6.00E-145 |
| hsa_circ_0021142 | 94 | 1 | 1299 | 1334 | 36 | 0 | 0 |
| hsa_circ_0021148 | 94 | 1 | 769 | 769 | 1 | 0 | 0 |
| hsa_circ_0021148 | 94 | 1 | 957 | 957 | 1 | 0 | 0 |
| hsa_circ_0021148 | 94 | 1 | 1725 | 1725 | 1 | 0 | 0 |
| hsa_circ_0021148 | 93 | 1 | 534 | 769 | 236 | 0 | 0 |
| hsa_circ_0003546 | 95 | 1 | 956 | 956 | 1 | 0 | 0 |
| hsa_circ_0003546 | 95 | 1 | 1028 | 1028 | 1 | 0 | 0 |
| hsa_circ_0004099 | 94 | 1 | 658 | 658 | 1 | 0 | 0 |
| hsa_circ_0004099 | 94 | 1 | 768 | 768 | 1 | 0 | 0 |
| hsa_circ_0004099 | 94 | 1 | 840 | 840 | 1 | 0 | 0 |
| hsa_circ_0004099 | 94 | 142 | 981 | 840 | 1 | 0 | 0 |
| hsa_circ_0021156 | 96 | 1 | 330 | 467 | 138 | 0 | 6.00E-150 |
| hsa_circ_0021160 | 94 | 1 | 395 | 227 | 621 | 0 | 7.00E-173 |
| hsa_circ_0021160 | 95 | 1 | 1057 | 227 | 1283 | 0 | 0 |
| hsa_circ_0021165 | 94 | 1 | 470 | 1 | 470 | 0 | 0 |
| hsa_circ_0021165 | 94 | 1 | 740 | 1 | 740 | 0 | 0 |
| hsa_circ_0021165 | 95 | 1 | 975 | 1 | 975 | 0 | 0 |
| hsa_circ_0021165 | 95 | 1 | 1715 | 1 | 1715 | 0 | 0 |
| hsa_circ_0021166 | 95 | 1 | 821 | 1 | 821 | 0 | 0 |
| hsa_circ_0021169 | 95 | 1 | 662 | 1 | 662 | 0 | 0 |

novel_circ_026714 novel_circ_026715 novel_circ_026716 novel_circ_026717 novel_circ_026718 novel_circ_026719 novel_circ_026720 novel_circ_026721 novel_circ_026722 novel_circ_026723 novel_circ_026724 novel_circ_026725 novel_circ_026726 novel_circ_026727 novel_circ_026728 novel_circ_026729 novel_circ_026730 novel_circ_026731 novel_circ_026732 novel_circ_026733 novel_circ_026734 novel_circ_026735 novel_circ_026736 novel_circ_026737 novel_circ_026738 novel_circ_026739 novel_circ_026740 novel_circ_026741 novel_circ_026742 novel_circ_026743 novel_circ_026744 novel_circ_026745 novel_circ_026746 novel_circ_026747 novel_circ_026748 novel_circ_026749 novel_circ_026750 novel_circ_026758 novel_circ_026759 novel_circ_026760 novel_circ_026762 novel_circ_026764 novel_circ_026765 novel_circ_026766 novel_circ_026767 novel_circ_026768 novel_circ_026769 novel_circ_026770 novel_circ_026771 novel_circ_026774 novel_circ_026775 novel_circ_026779 novel_circ_026780 novel_circ_026781

| hsa_circ_0021169 | 96 | 1 | 1402 | 1 | 1402 | 0 | 0 |
| --- | --- | --- | --- | --- | --- | --- | --- |
| hsa_circ_0021169 | 95 | 1 | 505 | 158 | 662 | 0 | 0 |
| hsa_circ_0021176 | 95 | 1 | 415 | 1 | 415 | 0 | 0 |
| hsa_circ_0021180 | 95 | 1 | 235 | 1 | 235 | 0 | 7.00E-101 |
| hsa_circ_0021180 | 96 | 1 | 975 | 1 | 975 | 0 | 0 |
| hsa_circ_0021179 | 95 | 1 | 1787 | 86 | 1872 | 0 | 0 |
| hsa_circ_0021182 | 96 | 3 | 194 | 3 | 194 | 0 | 2.00E-85 |
| hsa_circ_0021180 | 96 | 3 | 740 | 238 | 975 | 0 | 0 |
| hsa_circ_0021179 | 95 | 3 | 1552 | 323 | 1872 | 0 | 0 |
| hsa_circ_0021185 | 96 | 1 | 546 | 1 | 546 | 0 | 0 |
| hsa_circ_0021187 | 95 | 1 | 1311 | 167 | 1477 | 0 | 0 |
| hsa_circ_0021187 | 94 | 1 | 812 | 457 | 1268 | 0 | 0 |
| hsa_circ_0021187 | 94 | 1 | 1021 | 457 | 1477 | 0 | 0 |
| hsa_circ_0021194 | 95 | 1 | 521 | 1 | 521 | 0 | 0 |
| hsa_circ_0021198 | 96 | 120 | 212 | 1 | 93 | 0 | 3.00E-35 |
| hsa_circ_0021198 | 92 | 120 | 377 | 1 | 261 | 0.01 | 1.00E-100 |
| hsa_circ_0021210 | 93 | 1 | 482 | 1 | 482 | 0 | 0 |
| hsa_circ_0021219 | 93 | 1 | 443 | 571 | 129 | 0 | 0 |
| hsa_circ_0021224 | 92 | 1 | 534 | 534 | 1 | 0 | 0 |
| hsa_circ_0021223 | 93 | 1 | 876 | 876 | 1 | 0 | 0 |
| hsa_circ_0021223 | 93 | 1 | 954 | 954 | 1 | 0 | 0 |
| hsa_circ_0021229 | 92 | 1 | 465 | 465 | 1 | 0 | 0 |
| hsa_circ_0021227 | 93 | 1 | 564 | 564 | 1 | 0 | 0 |
| hsa_circ_0021227 | 93 | 1 | 807 | 807 | 1 | 0 | 0 |
| hsa_circ_0021227 | 93 | 1 | 885 | 885 | 1 | 0 | 0 |
| hsa_circ_0021227 | 94 | 1 | 999 | 999 | 1 | 0 | 0 |
| hsa_circ_0021227 | 94 | 1 | 1241 | 1241 | 1 | 0 | 0 |
| hsa_circ_0021227 | 93 | 1 | 1719 | 1719 | 1 | 0 | 0 |
| hsa_circ_0021227 | 92 | 1 | 657 | 807 | 151 | 0 | 0 |
| hsa_circ_0021230 | 93 | 1 | 545 | 547 | 3 | 0 | 0 |
| hsa_circ_0021227 | 95 | 1 | 534 | 999 | 466 | 0 | 0 |
| hsa_circ_0021227 | 95 | 1 | 776 | 1241 | 466 | 0 | 0 |
| hsa_circ_0007055 | 95 | 1 | 356 | 356 | 1 | 0 | 1.00E-159 |
| hsa_circ_0021227 | 94 | 1 | 573 | 1458 | 886 | 0 | 0 |
| hsa_circ_0021236 | 95 | 1 | 242 | 242 | 1 | 0 | 1.00E-104 |
| hsa_circ_0021239 | 91 | 1 | 340 | 340 | 1 | 0 | 1.00E-130 |
| hsa_circ_0021239 | 92 | 1 | 478 | 478 | 1 | 0 | 0 |
| hsa_circ_0003518 | 91 | 1763 | 2009 | 393 | 147 | 0 | 3.00E-88 |
| hsa_circ_0087463 | 89 | 1 | 576 | 576 | 1 | 0 | 0 |
| hsa_circ_0087463 | 89 | 1 | 709 | 709 | 1 | 0 | 0 |
| hsa_circ_0068521 | 95 | 1 | 215 | 215 | 1 | 0 | 2.00E-91 |
| hsa_circ_0068523 | 90 | 2 | 306 | 305 | 1 | 0 | 2.00E-105 |
| hsa_circ_0068523 | 91 | 6930 | 7134 | 511 | 307 | 0 | 1.00E-72 |
| hsa_circ_0068523 | 91 | 370 | 574 | 511 | 307 | 0 | 9.00E-74 |
| hsa_circ_0001375 | 91 | 1 | 646 | 1 | 646 | 0 | 0 |
| hsa_circ_0001375 | 91 | 1 | 646 | 1 | 646 | 0 | 0 |
| hsa_circ_0068525 | 92 | 320 | 1273 | 1 | 954 | 0 | 0 |
| hsa_circ_0068525 | 93 | 1 | 122 | 206 | 327 | 0 | 2.00E-43 |
| hsa_circ_0068525 | 90 | 1 | 270 | 685 | 954 | 0 | 7.00E-97 |
| hsa_circ_0079395 | 76 | 671 | 794 | 3568 | 3445 | 0.02 | 2.00E-08 |
| hsa_circ_0003163 | 83 | 2660 | 2740 | 1E+06 | 1058644 | 0.01 | 1.00E-10 |
| hsa_circ_0067832 | 93 | 1 | 719 | 719 | 1 | 0 | 0 |
| hsa_circ_0067832 | 93 | 5 | 1023 | 1019 | 1 | 0 | 0 |
| hsa_circ_0067832 | 93 | 1 | 1322 | 1322 | 1 | 0 | 0 |

novel_circ_026782 novel_circ_026783 novel_circ_026784 novel_circ_026785 novel_circ_026786 novel_circ_026787 novel_circ_026788 novel_circ_026789 novel_circ_026790 novel_circ_026791 novel_circ_026792 novel_circ_026793 novel_circ_026794 novel_circ_026795 novel_circ_026796 novel_circ_026797 novel_circ_026798 novel_circ_026799 novel_circ_026800 novel_circ_026801 novel_circ_026802 novel_circ_026803 novel_circ_026805 novel_circ_026806 novel_circ_026807 novel_circ_026808 novel_circ_026809 novel_circ_026810 novel_circ_026811 novel_circ_026814 novel_circ_026815 novel_circ_026816 novel_circ_026817 novel_circ_026818 novel_circ_026819 novel_circ_026820 novel_circ_026821 novel_circ_026822 novel_circ_026823 novel_circ_026824 novel_circ_026826 novel_circ_026827 novel_circ_026834 novel_circ_026835 novel_circ_026836 novel_circ_026837 novel_circ_026838 novel_circ_026839 novel_circ_026840 novel_circ_026841 novel_circ_026842 novel_circ_026843 novel_circ_026845 novel_circ_026846

| hsa_circ_0067832 | 93 | 1 | 719 | 719 | 1 | 0 | 0 |
| --- | --- | --- | --- | --- | --- | --- | --- |
| hsa_circ_0067832 | 93 | 1 | 948 | 948 | 1 | 0 | 0 |
| hsa_circ_0067832 | 93 | 5 | 1023 | 1019 | 1 | 0 | 0 |
| hsa_circ_0067832 | 93 | 384 | 1840 | 1457 | 1 | 0 | 0 |
| hsa_circ_0067832 | 93 | 1 | 719 | 719 | 1 | 0 | 0 |
| hsa_circ_0067832 | 93 | 5 | 1023 | 1019 | 1 | 0 | 0 |
| hsa_circ_0067832 | 93 | 1 | 1322 | 1322 | 1 | 0 | 0 |
| hsa_circ_0067833 | 92 | 5 | 440 | 846 | 411 | 0 | 6.00E-174 |
| hsa_circ_0067832 | 93 | 1 | 537 | 1322 | 786 | 0 | 0 |
| hsa_circ_0067834 | 92 | 1 | 527 | 527 | 1 | 0 | 0 |
| hsa_circ_0067836 | 94 | 1 | 518 | 518 | 1 | 0 | 0 |
| hsa_circ_0067836 | 93 | 1 | 734 | 734 | 1 | 0 | 0 |
| hsa_circ_0067836 | 94 | 1 | 518 | 518 | 1 | 0 | 0 |
| hsa_circ_0067836 | 93 | 88 | 822 | 735 | 1 | 0 | 0 |
| hsa_circ_0067838 | 95 | 1 | 290 | 1 | 290 | 0 | 3.00E-126 |
| hsa_circ_0067841 | 92 | 1 | 582 | 458 | 1039 | 0 | 0 |
| hsa_circ_0067846 | 96 | 1 | 438 | 438 | 1 | 0 | 0 |
| hsa_circ_0067848 | 98 | 1 | 234 | 234 | 1 | 0 | 6.00E-112 |
| hsa_circ_0067848 | 96 | 1 | 366 | 366 | 1 | 0 | 4.00E-170 |
| hsa_circ_0067848 | 96 | 1 | 668 | 668 | 1 | 0 | 0 |
| hsa_circ_0067849 | 96 | 1 | 434 | 563 | 130 | 0 | 0 |
| hsa_circ_0067849 | 97 | 136 | 487 | 828 | 477 | 0 | 7.00E-169 |
| hsa_circ_0031112 | 84 | 1 | 1034 | 1680 | 656 | 0.01 | 0 |
| hsa_circ_0014970 | 95 | 1197 | 1416 | 105 | 325 | 0 | 1.00E-91 |
| hsa_circ_0014970 | 95 | 20 | 497 | 478 | 1 | 0 | 0 |
| hsa_circ_0014970 | 95 | 1004 | 1223 | 105 | 325 | 0 | 9.00E-92 |
| hsa_circ_0014970 | 95 | 20 | 497 | 478 | 1 | 0 | 0 |
| hsa_circ_0014970 | 95 | 220 | 439 | 105 | 325 | 0 | 3.00E-92 |
| hsa_circ_0014988 | 91 | 1 | 234 | 1 | 234 | 0 | 1.00E-83 |
| hsa_circ_0062070 | 87 | 1 | 1497 | 1497 | 1 | 0 | 0 |
| hsa_circ_0062070 | 88 | 1 | 877 | 1084 | 208 | 0 | 0 |
| hsa_circ_0062070 | 88 | 1 | 1290 | 1497 | 208 | 0 | 0 |
| hsa_circ_0062070 | 83 | 1 | 1642 | 3005 | 1344 | 0.02 | 0 |
| hsa_circ_0062075 | 89 | 1 | 1122 | 1567 | 446 | 0 | 0 |
| hsa_circ_0062075 | 91 | 4 | 623 | 1233 | 614 | 0 | 0 |
| hsa_circ_0062076 | 88 | 1 | 529 | 798 | 270 | 0 | 2.00E-175 |
| hsa_circ_0062075 | 87 | 1 | 750 | 1791 | 1039 | 0.01 | 0 |
| hsa_circ_0007126 | 87 | 1 | 331 | 331 | 1 | 0.01 | 5.00E-104 |
| hsa_circ_0007126 | 87 | 1 | 552 | 555 | 1 | 0.01 | 2.00E-175 |
| hsa_circ_0007126 | 86 | 225 | 445 | 555 | 332 | 0.01 | 1.00E-61 |
| hsa_circ_0062093 | 84 | 93 | 829 | 450 | 1186 | 0 | 0 |
| hsa_circ_0062093 | 84 | 93 | 834 | 450 | 1191 | 0 | 0 |
| hsa_circ_0062112 | 86 | 1 | 481 | 1 | 499 | 0.04 | 7.00E-149 |
| hsa_circ_0062112 | 87 | 1 | 801 | 1 | 819 | 0.03 | 0 |
| hsa_circ_0062110 | 87 | 1 | 893 | 193 | 1103 | 0.03 | 0 |
| hsa_circ_0062110 | 88 | 1 | 1004 | 193 | 1214 | 0.03 | 0 |
| hsa_circ_0062114 | 90 | 3 | 437 | 321 | 755 | 0 | 1.00E-161 |
| hsa_circ_0062114 | 90 | 1 | 345 | 411 | 755 | 0 | 8.00E-127 |
| hsa_circ_0062114 | 91 | 1 | 448 | 411 | 858 | 0 | 6.00E-169 |
| hsa_circ_0062114 | 91 | 1 | 982 | 411 | 1392 | 0 | 0 |
| hsa_circ_0062114 | 89 | 1 | 337 | 522 | 858 | 0 | 5.00E-114 |
| hsa_circ_0062114 | 90 | 1 | 871 | 522 | 1392 | 0 | 0 |
| hsa_circ_0062130 | 90 | 1 | 633 | 1 | 633 | 0 | 0 |
| hsa_circ_0062126 | 89 | 1 | 938 | 493 | 1430 | 0 | 0 |

novel_circ_026847 novel_circ_026850 novel_circ_026851 novel_circ_026852 novel_circ_026853 novel_circ_026854 novel_circ_026855 novel_circ_026856 novel_circ_026857 novel_circ_026862 novel_circ_026865 novel_circ_026866 novel_circ_026867 novel_circ_026868 novel_circ_026869 novel_circ_026870 novel_circ_026871 novel_circ_026872 novel_circ_026873 novel_circ_026874 novel_circ_026875 novel_circ_026876 novel_circ_026877 novel_circ_026878 novel_circ_026879 novel_circ_026880 novel_circ_026881 novel_circ_026882 novel_circ_026883 novel_circ_026884 novel_circ_026885 novel_circ_026886 novel_circ_026887 novel_circ_026888 novel_circ_026889 novel_circ_026890 novel_circ_026891 novel_circ_026895 novel_circ_026896 novel_circ_026897 novel_circ_026898 novel_circ_026899 novel_circ_026900 novel_circ_026901 novel_circ_026902 novel_circ_026911 novel_circ_026912 novel_circ_026913 novel_circ_026914 novel_circ_026915 novel_circ_026916 novel_circ_026917 novel_circ_026918 novel_circ_026919

| hsa_circ_0062126 | 89 | 1 | 938 | 493 | 1430 | 0 | 0 |
| --- | --- | --- | --- | --- | --- | --- | --- |
| hsa_circ_0007999 | 82 | 21050 | 21197 | 119700 | 119847 | 0 | 8.00E-27 |
| hsa_circ_0007999 | 82 | 21050 | 21197 | 119700 | 119847 | 0 | 9.00E-27 |
| hsa_circ_0026345 | 93 | 1 | 720 | 1 | 720 | 0 | 0 |
| hsa_circ_0026347 | 93 | 232 | 681 | 124 | 573 | 0 | 0 |
| hsa_circ_0026347 | 93 | 1 | 450 | 124 | 573 | 0 | 0 |
| hsa_circ_0026357 | 85 | 298 | 1920 | 46 | 1689 | 0.03 | 0 |
| hsa_circ_0069031 | 88 | 16 | 301 | 286 | 1 | 0.01 | 1.00E-90 |
| hsa_circ_0069036 | 87 | 10 | 1264 | 17 | 1274 | 0 | 0 |
| hsa_circ_0026804 | 86 | 22134 | 22546 | 666 | 257 | 0.01 | 1.00E-119 |
| hsa_circ_0069051 | 91 | 4 | 328 | 325 | 1 | 0 | 2.00E-122 |
| hsa_circ_0069051 | 88 | 1 | 943 | 943 | 1 | 0 | 0 |
| hsa_circ_0069075 | 94 | 3 | 143 | 210 | 70 | 0 | 7.00E-54 |
| hsa_circ_0069077 | 90 | 1 | 350 | 350 | 1 | 0 | 1.00E-127 |
| hsa_circ_0083278 | 89 | 1 | 1036 | 4881 | 3846 | 0 | 0 |
| hsa_circ_0083278 | 91 | 1 | 847 | 4692 | 3846 | 0 | 0 |
| hsa_circ_0083278 | 89 | 1 | 520 | 3843 | 3324 | 0 | 2.00E-180 |
| hsa_circ_0083278 | 88 | 1 | 520 | 3129 | 2610 | 0 | 3.00E-177 |
| hsa_circ_0083278 | 89 | 1 | 1380 | 3129 | 1750 | 0 | 0 |
| hsa_circ_0083278 | 88 | 1 | 909 | 2990 | 2082 | 0 | 0 |
| hsa_circ_0083278 | 88 | 1 | 1241 | 2990 | 1750 | 0 | 0 |
| hsa_circ_0083278 | 88 | 3 | 426 | 2800 | 2377 | 0 | 3.00E-137 |
| hsa_circ_0083278 | 88 | 3 | 522 | 2800 | 2281 | 0 | 7.00E-174 |
| hsa_circ_0083278 | 88 | 3 | 721 | 2800 | 2082 | 0 | 0 |
| hsa_circ_0083278 | 88 | 3 | 1053 | 2800 | 1750 | 0 | 0 |
| hsa_circ_0083278 | 88 | 3 | 1569 | 2800 | 1234 | 0 | 0 |
| hsa_circ_0083278 | 88 | 1 | 1047 | 2280 | 1234 | 0 | 0 |
| hsa_circ_0083278 | 88 | 3 | 843 | 2074 | 1234 | 0 | 0 |
| hsa_circ_0083278 | 89 | 3 | 1030 | 2074 | 1047 | 0 | 0 |
| hsa_circ_0083278 | 88 | 3 | 1210 | 2074 | 867 | 0 | 0 |
| hsa_circ_0083278 | 89 | 3 | 1551 | 2074 | 526 | 0 | 0 |
| hsa_circ_0083278 | 88 | 3 | 621 | 1852 | 1234 | 0 | 0 |
| hsa_circ_0083278 | 89 | 3 | 808 | 1852 | 1047 | 0 | 0 |
| hsa_circ_0083278 | 88 | 1 | 516 | 1749 | 1234 | 0 | 3.00E-173 |
| hsa_circ_0083278 | 89 | 1 | 703 | 1749 | 1047 | 0 | 0 |
| hsa_circ_0083278 | 89 | 1 | 1233 | 1233 | 1 | 0 | 0 |
| hsa_circ_0083278 | 89 | 1 | 639 | 639 | 1 | 0 | 0 |
| hsa_circ_0083273 | 88 | 1 | 301 | 1 | 301 | 0 | 2.00E-97 |
| hsa_circ_0083272 | 87 | 1 | 525 | 555 | 1079 | 0 | 3.00E-163 |
| hsa_circ_0083272 | 88 | 1 | 734 | 346 | 1079 | 0 | 0 |
| hsa_circ_0083265 | 88 | 1 | 1288 | 1 | 1288 | 0 | 0 |
| hsa_circ_0083269 | 87 | 1 | 317 | 94 | 410 | 0 | 1.00E-97 |
| hsa_circ_0083266 | 89 | 1 | 785 | 340 | 1124 | 0 | 0 |
| hsa_circ_0083265 | 88 | 1 | 1288 | 1 | 1288 | 0 | 0 |
| hsa_circ_0083264 | 84 | 1 | 992 | 157 | 1145 | 0.01 | 0 |
| hsa_circ_0024935 | 92 | 1411 | 2481 | 1077 | 1 | 0.01 | 0 |
| hsa_circ_0024935 | 94 | 1 | 696 | 1077 | 382 | 0 | 0 |
| hsa_circ_0024936 | 92 | 1 | 458 | 458 | 1 | 0 | 0 |
| hsa_circ_0024936 | 93 | 4 | 151 | 148 | 1 | 0 | 9.00E-56 |
| hsa_circ_0024936 | 92 | 1 | 458 | 458 | 1 | 0 | 0 |
| hsa_circ_0024936 | 93 | 4 | 653 | 650 | 1 | 0 | 0 |
| hsa_circ_0024941 | 92 | 1 | 1296 | 1296 | 1 | 0 | 0 |
| hsa_circ_0024941 | 92 | 136 | 1431 | 1296 | 1 | 0 | 0 |
| hsa_circ_0024936 | 92 | 1 | 458 | 458 | 1 | 0 | 0 |

novel_circ_026920 novel_circ_026921 novel_circ_026922 novel_circ_026923 novel_circ_026924 novel_circ_026925 novel_circ_026926 novel_circ_026927 novel_circ_026928 novel_circ_026929 novel_circ_026930 novel_circ_026931 novel_circ_026932 novel_circ_026933 novel_circ_026934 novel_circ_026935 novel_circ_026936 novel_circ_026937 novel_circ_026938 novel_circ_026939 novel_circ_026940 novel_circ_026941 novel_circ_026942 novel_circ_026943 novel_circ_026944 novel_circ_026945 novel_circ_026946 novel_circ_026947 novel_circ_026948 novel_circ_026949 novel_circ_026950 novel_circ_026952 novel_circ_026953 novel_circ_026954 novel_circ_026955 novel_circ_026956 novel_circ_026957 novel_circ_026958 novel_circ_026959 novel_circ_026960 novel_circ_026961 novel_circ_026962 novel_circ_026963 novel_circ_026964 novel_circ_026965 novel_circ_026966 novel_circ_026967 novel_circ_026968 novel_circ_026969 novel_circ_026972 novel_circ_026973 novel_circ_026974 novel_circ_026975 novel_circ_026976

| hsa_circ_0024937 | 92 | 1 | 307 | 307 | 1 | 0 | 1.00E-120 |
| --- | --- | --- | --- | --- | --- | --- | --- |
| hsa_circ_0024941 | 94 | 1 | 315 | 315 | 1 | 0 | 2.00E-133 |
| hsa_circ_0024941 | 92 | 1 | 478 | 478 | 1 | 0 | 0 |
| hsa_circ_0024941 | 91 | 7 | 660 | 654 | 1 | 0 | 0 |
| hsa_circ_0024941 | 91 | 4 | 1098 | 1095 | 1 | 0 | 0 |
| hsa_circ_0024941 | 92 | 1 | 1296 | 1296 | 1 | 0 | 0 |
| hsa_circ_0024941 | 92 | 136 | 1431 | 1296 | 1 | 0 | 0 |
| hsa_circ_0024943 | 92 | 1 | 1407 | 1407 | 1 | 0 | 0 |
| hsa_circ_0024943 | 92 | 4 | 1608 | 1605 | 1 | 0 | 0 |
| hsa_circ_0024945 | 91 | 1 | 818 | 981 | 164 | 0 | 0 |
| hsa_circ_0024943 | 92 | 4 | 1325 | 1605 | 284 | 0 | 0 |
| hsa_circ_0024943 | 92 | 1 | 783 | 1407 | 625 | 0 | 0 |
| hsa_circ_0024943 | 93 | 4 | 984 | 1605 | 625 | 0 | 0 |
| hsa_circ_0024948 | 92 | 1 | 306 | 306 | 1 | 0 | 2.00E-118 |
| hsa_circ_0024943 | 93 | 4 | 507 | 1605 | 1102 | 0 | 0 |
| hsa_circ_0024943 | 95 | 4 | 201 | 1605 | 1408 | 0 | 2.00E-85 |
| hsa_circ_0024954 | 85 | 1 | 534 | 1 | 531 | 0.01 | 4.00E-152 |
| hsa_circ_0024958 | 95 | 1 | 394 | 1 | 394 | 0 | 5.00E-174 |
| hsa_circ_0024958 | 94 | 1 | 548 | 1 | 548 | 0 | 0 |
| hsa_circ_0024958 | 94 | 1 | 548 | 1 | 548 | 0 | 0 |
| hsa_circ_0024960 | 95 | 1 | 221 | 1 | 221 | 0 | 9.00E-95 |
| hsa_circ_0024960 | 94 | 1 | 375 | 1 | 375 | 0 | 2.00E-163 |
| hsa_circ_0024960 | 94 | 1 | 375 | 1 | 375 | 0 | 3.00E-162 |
| hsa_circ_0024978 | 93 | 1 | 468 | 262 | 729 | 0 | 0 |
| hsa_circ_0024990 | 92 | 1 | 264 | 459 | 196 | 0 | 3.00E-100 |
| hsa_circ_0005534 | 87 | 6743 | 7559 | 29423 | 30249 | 0.02 | 0 |
| hsa_circ_0024994 | 87 | 1 | 815 | 1 | 825 | 0.02 | 0 |
| hsa_circ_0024994 | 87 | 1 | 1232 | 1 | 1242 | 0.01 | 0 |
| hsa_circ_0024994 | 88 | 1 | 1307 | 1 | 1317 | 0.01 | 0 |
| hsa_circ_0024994 | 88 | 1 | 1463 | 1 | 1473 | 0.01 | 0 |
| hsa_circ_0024994 | 88 | 1 | 1715 | 1 | 1725 | 0.01 | 0 |
| hsa_circ_0024994 | 90 | 1 | 900 | 826 | 1725 | 0 | 0 |
| hsa_circ_0024995 | 91 | 1 | 408 | 76 | 483 | 0 | 1.00E-156 |
| hsa_circ_0024995 | 92 | 1 | 576 | 76 | 651 | 0 | 0 |
| hsa_circ_0024993 | 91 | 239 | 996 | 1722 | 2479 | 0 | 0 |
| hsa_circ_0024993 | 90 | 239 | 1187 | 1722 | 2670 | 0 | 0 |
| hsa_circ_0024993 | 91 | 1 | 756 | 1724 | 2479 | 0 | 0 |
| hsa_circ_0024993 | 91 | 1 | 588 | 1892 | 2479 | 0 | 0 |
| hsa_circ_0024993 | 90 | 1 | 779 | 1892 | 2670 | 0 | 0 |
| hsa_circ_0024993 | 90 | 1 | 420 | 2060 | 2479 | 0 | 5.00E-150 |
| hsa_circ_0024993 | 89 | 1 | 611 | 2060 | 2670 | 0 | 0 |
| hsa_circ_0024993 | 89 | 1 | 627 | 2060 | 2686 | 0 | 0 |
| hsa_circ_0024993 | 89 | 1 | 762 | 2060 | 2821 | 0 | 0 |
| hsa_circ_0024993 | 89 | 1 | 767 | 2060 | 2826 | 0 | 0 |
| hsa_circ_0024993 | 90 | 1 | 894 | 2060 | 2953 | 0 | 0 |
| hsa_circ_0024993 | 90 | 1 | 896 | 2060 | 2955 | 0 | 0 |
| hsa_circ_0024993 | 89 | 1 | 332 | 2339 | 2670 | 0 | 3.00E-111 |
| hsa_circ_0024993 | 92 | 1 | 429 | 2686 | 3114 | 0 | 5.00E-170 |
| hsa_circ_0024993 | 93 | 1 | 293 | 2822 | 3114 | 0 | 1.00E-119 |
| hsa_circ_0025004 | 91 | 3 | 252 | 5 | 257 | 0.02 | 1.00E-89 |
| hsa_circ_0025004 | 91 | 3 | 252 | 5 | 257 | 0.02 | 3.00E-88 |
| hsa_circ_0025004 | 90 | 3 | 372 | 5 | 377 | 0.01 | 7.00E-133 |
| hsa_circ_0025004 | 90 | 3 | 544 | 5 | 549 | 0.01 | 0 |
| hsa_circ_0025004 | 90 | 3 | 551 | 5 | 557 | 0.01 | 0 |

novel_circ_026977 novel_circ_026978 novel_circ_026980 novel_circ_026981 novel_circ_026982 novel_circ_026986 novel_circ_026987 novel_circ_026988 novel_circ_026989 novel_circ_026990 novel_circ_026991 novel_circ_026992 novel_circ_026993 novel_circ_026994 novel_circ_026995 novel_circ_026996 novel_circ_026997 novel_circ_026998 novel_circ_026999 novel_circ_027000 novel_circ_027001 novel_circ_027002 novel_circ_027003 novel_circ_027004 novel_circ_027005 novel_circ_027006 novel_circ_027007 novel_circ_027008 novel_circ_027014 novel_circ_027015 novel_circ_027016 novel_circ_027017 novel_circ_027018 novel_circ_027019 novel_circ_027022 novel_circ_027023 novel_circ_027024 novel_circ_027025 novel_circ_027026 novel_circ_027027 novel_circ_027028 novel_circ_027029 novel_circ_027030 novel_circ_027031 novel_circ_027032 novel_circ_027033 novel_circ_027034 novel_circ_027036 novel_circ_027038 novel_circ_027039 novel_circ_027041 novel_circ_027042 novel_circ_027043 novel_circ_027044

| hsa_circ_0025004 | 92 | 3 | 919 | 5 | 924 | 0.01 | 0 |
| --- | --- | --- | --- | --- | --- | --- | --- |
| hsa_circ_0025004 | 92 | 3 | 1113 | 5 | 1118 | 0 | 0 |
| hsa_circ_0025016 | 92 | 1 | 428 | 1 | 428 | 0 | 2.00E-169 |
| hsa_circ_0085176 | 96 | 10676 | 11072 | 397 | 1 | 0 | 0 |
| hsa_circ_0061389 | 93 | 110 | 287 | 1 | 178 | 0 | 2.00E-67 |
| hsa_circ_0061361 | 94 | 1 | 534 | 1133 | 600 | 0 | 0 |
| hsa_circ_0061362 | 93 | 1 | 330 | 330 | 1 | 0 | 4.00E-135 |
| hsa_circ_0061361 | 93 | 1 | 564 | 929 | 366 | 0 | 0 |
| hsa_circ_0061361 | 92 | 1 | 738 | 929 | 192 | 0 | 0 |
| hsa_circ_0061361 | 92 | 1 | 929 | 929 | 1 | 0 | 0 |
| hsa_circ_0061357 | 92 | 1 | 930 | 3098 | 2169 | 0 | 0 |
| hsa_circ_0061357 | 92 | 1 | 930 | 3098 | 2169 | 0 | 0 |
| hsa_circ_0061357 | 90 | 1 | 1896 | 3098 | 1182 | 0.01 | 0 |
| hsa_circ_0061361 | 91 | 1 | 599 | 599 | 1 | 0 | 0 |
| hsa_circ_0061357 | 92 | 1 | 600 | 2768 | 2169 | 0 | 0 |
| hsa_circ_0061359 | 87 | 1 | 967 | 1572 | 585 | 0.02 | 0 |
| hsa_circ_0061357 | 90 | 8 | 1467 | 1457 | 1 | 0 | 0 |
| hsa_circ_0061357 | 90 | 1 | 958 | 1223 | 266 | 0 | 0 |
| hsa_circ_0061357 | 90 | 1 | 916 | 1181 | 266 | 0 | 0 |
| hsa_circ_0061357 | 90 | 1 | 1184 | 1181 | 1 | 0 | 0 |
| hsa_circ_0061357 | 91 | 1 | 332 | 597 | 266 | 0 | 6.00E-123 |
| hsa_circ_0061357 | 91 | 1 | 600 | 597 | 1 | 0.01 | 0 |
| hsa_circ_0061354 | 92 | 1 | 1531 | 1531 | 1 | 0 | 0 |
| hsa_circ_0061354 | 92 | 1 | 1531 | 1531 | 1 | 0 | 0 |
| hsa_circ_0061355 | 93 | 1 | 527 | 886 | 360 | 0 | 0 |
| hsa_circ_0061354 | 93 | 1 | 1107 | 1107 | 1 | 0 | 0 |
| hsa_circ_0061354 | 92 | 1 | 580 | 580 | 1 | 0 | 0 |
| hsa_circ_0061354 | 93 | 4 | 393 | 390 | 1 | 0 | 9.00E-160 |
| hsa_circ_0006020 | 83 | 6 | 393 | 6 | 396 | 0.02 | 4.00E-96 |
| hsa_circ_0092270 | 81 | 16 | 759 | 16 | 765 | 0.03 | 2.00E-161 |
| hsa_circ_0092270 | 82 | 16 | 954 | 16 | 960 | 0.03 | 0 |
| hsa_circ_0092270 | 82 | 16 | 1047 | 16 | 1053 | 0.02 | 0 |
| hsa_circ_0006931 | 86 | 1 | 366 | 1 | 366 | 0.01 | 7.00E-105 |
| hsa_circ_0092270 | 83 | 1 | 654 | 391 | 1053 | 0.01 | 7.00E-172 |
| hsa_circ_0029803 | 96 | 1 | 328 | 1 | 328 | 0 | 5.00E-149 |
| hsa_circ_0029805 | 96 | 77 | 518 | 1 | 442 | 0 | 0 |
| hsa_circ_0029805 | 96 | 1 | 252 | 1 | 252 | 0 | 6.00E-112 |
| hsa_circ_0029805 | 96 | 1 | 442 | 1 | 442 | 0 | 0 |
| hsa_circ_0029805 | 96 | 1 | 331 | 112 | 442 | 0 | 5.00E-154 |
| hsa_circ_0029805 | 98 | 1 | 618 | 112 | 729 | 0 | 0 |
| hsa_circ_0029805 | 98 | 1 | 716 | 112 | 827 | 0 | 0 |
| hsa_circ_0029805 | 98 | 1 | 716 | 112 | 827 | 0 | 0 |
| hsa_circ_0029806 | 96 | 1 | 190 | 1 | 190 | 0 | 3.00E-84 |
| hsa_circ_0029805 | 98 | 1 | 575 | 253 | 827 | 0 | 0 |
| hsa_circ_0029805 | 99 | 1 | 385 | 443 | 827 | 0 | 0 |
| hsa_circ_0003489 | 95 | 1 | 333 | 1 | 336 | 0.01 | 2.00E-147 |
| hsa_circ_0029808 | 90 | 989 | 1255 | 1 | 267 | 0 | 5.00E-91 |
| hsa_circ_0029808 | 90 | 1 | 267 | 1 | 267 | 0 | 2.00E-91 |
| hsa_circ_0029810 | 95 | 295 | 683 | 389 | 1 | 0 | 3.00E-174 |
| hsa_circ_0035404 | 97 | 1 | 206 | 206 | 1 | 0 | 4.00E-93 |
| hsa_circ_0004923 | 83 | 447 | 898 | 595015 | 594557 | 0.02 | 1.00E-109 |
| hsa_circ_0035388 | 94 | 1 | 491 | 1253 | 763 | 0 | 0 |
| hsa_circ_0035387 | 93 | 1 | 573 | 1068 | 496 | 0 | 0 |
| hsa_circ_0035385 | 89 | 1 | 879 | 1345 | 464 | 0.01 | 0 |

novel_circ_027045 novel_circ_027046 novel_circ_027047 novel_circ_027048 novel_circ_027049 novel_circ_027050 novel_circ_027051 novel_circ_027052 novel_circ_027054 novel_circ_027055 novel_circ_027056 novel_circ_027057 novel_circ_027058 novel_circ_027059 novel_circ_027060 novel_circ_027061 novel_circ_027063 novel_circ_027064 novel_circ_027065 novel_circ_027066 novel_circ_027067 novel_circ_027068 novel_circ_027069 novel_circ_027070 novel_circ_027071 novel_circ_027072 novel_circ_027073 novel_circ_027074 novel_circ_027075 novel_circ_027080 novel_circ_027081 novel_circ_027082 novel_circ_027083 novel_circ_027084 novel_circ_027087 novel_circ_027088 novel_circ_027090 novel_circ_027091 novel_circ_027092 novel_circ_027093 novel_circ_027094 novel_circ_027096 novel_circ_027097 novel_circ_027098 novel_circ_027099 novel_circ_027100 novel_circ_027101 novel_circ_027102 novel_circ_027103 novel_circ_027104 novel_circ_027105 novel_circ_027106 novel_circ_027107 novel_circ_027108

| hsa_circ_0035385 | 89 | 1 | 370 | 833 | 464 | 0 | 7.00E-129 |
| --- | --- | --- | --- | --- | --- | --- | --- |
| hsa_circ_0035385 | 90 | 1 | 260 | 723 | 464 | 0 | 4.00E-91 |
| hsa_circ_0035385 | 87 | 1 | 460 | 463 | 1 | 0.01 | 2.00E-147 |
| hsa_circ_0035385 | 86 | 7 | 391 | 388 | 1 | 0.01 | 8.00E-113 |
| hsa_circ_0035385 | 86 | 7 | 391 | 388 | 1 | 0.01 | 1.00E-112 |
| hsa_circ_0035385 | 86 | 7 | 391 | 388 | 1 | 0.01 | 2.00E-112 |
| hsa_circ_0035385 | 87 | 1 | 276 | 279 | 1 | 0.01 | 5.00E-82 |
| hsa_circ_0035385 | 90 | 3 | 199 | 200 | 1 | 0.02 | 7.00E-65 |
| hsa_circ_0035381 | 91 | 1 | 533 | 1 | 533 | 0 | 0 |
| hsa_circ_0035375 | 91 | 37333 | 37688 | 365 | 1 | 0.02 | 2.00E-131 |
| hsa_circ_0003863 | 91 | 37333 | 37812 | 489 | 1 | 0.02 | 0 |
| hsa_circ_0035375 | 92 | 1 | 190 | 190 | 1 | 0 | 3.00E-69 |
| hsa_circ_0049674 | 78 | 38 | 2138 | 2339 | 214 | 0.03 | 0 |
| hsa_circ_0089760 | 93 | 1 | 183 | 1 | 183 | 0 | 7.00E-70 |
| hsa_circ_0089760 | 93 | 1 | 183 | 1 | 183 | 0 | 5.00E-70 |
| hsa_circ_0049675 | 79 | 1114 | 2439 | 1799 | 471 | 0.02 | 0 |
| hsa_circ_0089759 | 92 | 3 | 718 | 213 | 928 | 0 | 0 |
| hsa_circ_0089759 | 91 | 1043 | 1801 | 1 | 759 | 0 | 0 |
| hsa_circ_0049674 | 82 | 16 | 245 | 4960 | 4731 | 0 | 2.00E-49 |
| hsa_circ_0009134 | 89 | 1 | 330 | 1 | 330 | 0 | 8.00E-112 |
| hsa_circ_0089726 | 84 | 1 | 470 | 1 | 473 | 0.01 | 6.00E-125 |
| hsa_circ_0089721 | 85 | 9 | 793 | 33 | 820 | 0 | 0 |
| hsa_circ_0089721 | 85 | 1 | 796 | 22 | 820 | 0 | 0 |
| hsa_circ_0089721 | 85 | 1 | 817 | 1 | 820 | 0 | 0 |
| hsa_circ_0089717 | 84 | 1 | 998 | 1 | 1001 | 0 | 0 |
| hsa_circ_0089721 | 86 | 1 | 647 | 1 | 650 | 0 | 0 |
| hsa_circ_0089721 | 89 | 1 | 299 | 22 | 320 | 0 | 1.00E-99 |
| hsa_circ_0089721 | 89 | 1 | 320 | 1 | 320 | 0 | 3.00E-111 |
| hsa_circ_0089717 | 85 | 1 | 501 | 1 | 501 | 0 | 6.00E-145 |
| hsa_circ_0089648 | 90 | 1 | 373 | 548 | 176 | 0 | 3.00E-132 |
| hsa_circ_0089635 | 86 | 3 | 338 | 3 | 338 | 0 | 4.00E-100 |
| hsa_circ_0089620 | 91 | 149 | 270 | 257 | 136 | 0 | 1.00E-39 |
| hsa_circ_0089576 | 93 | 1 | 328 | 1 | 328 | 0 | 5.00E-134 |
| hsa_circ_0089479 | 79 | 1555 | 2981 | 1731 | 287 | 0.03 | 0 |
| hsa_circ_0089497 | 89 | 6 | 425 | 420 | 1 | 0 | 5.00E-145 |
| hsa_circ_0089512 | 89 | 166 | 663 | 5692 | 5189 | 0.01 | 1.00E-174 |
| hsa_circ_0021070 | 96 | 350 | 896 | 1 | 547 | 0 | 0 |
| hsa_circ_0021070 | 96 | 1 | 547 | 1 | 547 | 0 | 0 |
| hsa_circ_0021070 | 96 | 1 | 547 | 1 | 547 | 0 | 0 |
| hsa_circ_0021071 | 96 | 1 | 423 | 1 | 423 | 0 | 0 |
| hsa_circ_0021071 | 96 | 1 | 423 | 1 | 423 | 0 | 0 |
| hsa_circ_0006891 | 94 | 1 | 210 | 1 | 210 | 0 | 2.00E-86 |
| hsa_circ_0030191 | 96 | 1 | 457 | 1921 | 1465 | 0 | 0 |
| hsa_circ_0030191 | 95 | 1 | 758 | 1921 | 1167 | 0 | 0 |
| hsa_circ_0030191 | 96 | 1 | 454 | 1918 | 1465 | 0 | 0 |
| hsa_circ_0030191 | 95 | 1 | 221 | 1685 | 1465 | 0 | 2.00E-96 |
| hsa_circ_0030191 | 94 | 1 | 770 | 1573 | 807 | 0 | 0 |
| hsa_circ_0030191 | 95 | 2 | 360 | 1165 | 807 | 0 | 2.00E-159 |
| hsa_circ_0030188 | 92 | 305 | 888 | 4946 | 4360 | 0.01 | 0 |
| hsa_circ_0026031 | 94 | 1 | 269 | 267 | 1 | 0.01 | 4.00E-112 |
| hsa_circ_0026029 | 91 | 1 | 264 | 264 | 1 | 0 | 6.00E-95 |
| hsa_circ_0006222 | 92 | 307 | 638 | 332 | 1 | 0 | 3.00E-129 |
| hsa_circ_0026023 | 92 | 307 | 1493 | 1187 | 1 | 0 | 0 |
| hsa_circ_0026028 | 90 | 259 | 418 | 160 | 1 | 0 | 3.00E-52 |

novel_circ_027109 novel_circ_027110 novel_circ_027111 novel_circ_027112 novel_circ_027113 novel_circ_027114 novel_circ_027116 novel_circ_027117 novel_circ_027122 novel_circ_027123 novel_circ_027124 novel_circ_027125 novel_circ_027126 novel_circ_027127 novel_circ_027128 novel_circ_027129 novel_circ_027130 novel_circ_027131 novel_circ_027132 novel_circ_027133 novel_circ_027134 novel_circ_027135 novel_circ_027136 novel_circ_027137 novel_circ_027138 novel_circ_027139 novel_circ_027140 novel_circ_027142 novel_circ_027143 novel_circ_027144 novel_circ_027145 novel_circ_027146 novel_circ_027147 novel_circ_027148 novel_circ_027149 novel_circ_027150 novel_circ_027151 novel_circ_027152 novel_circ_027153 novel_circ_027154 novel_circ_027156 novel_circ_027157 novel_circ_027158 novel_circ_027159 novel_circ_027163 novel_circ_027164 novel_circ_027165 novel_circ_027166 novel_circ_027167 novel_circ_027171 novel_circ_027172 novel_circ_027173 novel_circ_027174 novel_circ_027175

| hsa_circ_0026024 | 91 | 259 | 1033 | 1055 | 281 | 0 | 0 |
| --- | --- | --- | --- | --- | --- | --- | --- |
| hsa_circ_0026025 | 94 | 1 | 768 | 769 | 1 | 0 | 0 |
| hsa_circ_0026023 | 92 | 331 | 1519 | 1189 | 1 | 0 | 0 |
| hsa_circ_0007838 | 91 | 4 | 443 | 440 | 1 | 0 | 8.00E-168 |
| hsa_circ_0026023 | 92 | 4 | 855 | 852 | 1 | 0 | 0 |
| hsa_circ_0026023 | 93 | 3 | 412 | 410 | 1 | 0 | 7.00E-173 |
| hsa_circ_0003949 | 87 | 9 | 464 | 3 | 452 | 0.02 | 1.00E-139 |
| hsa_circ_0072070 | 84 | 356 | 1404 | 1565 | 2609 | 0.02 | 0 |
| hsa_circ_0081042 | 94 | 1 | 557 | 1 | 557 | 0 | 0 |
| hsa_circ_0081042 | 94 | 1 | 836 | 1 | 836 | 0 | 0 |
| hsa_circ_0081042 | 94 | 1 | 973 | 1 | 973 | 0 | 0 |
| hsa_circ_0081042 | 95 | 1 | 1259 | 1 | 1259 | 0 | 0 |
| hsa_circ_0081042 | 95 | 1 | 1527 | 1 | 1527 | 0 | 0 |
| hsa_circ_0081042 | 94 | 1 | 1646 | 1 | 1646 | 0 | 0 |
| hsa_circ_0081042 | 94 | 1 | 1753 | 1 | 1753 | 0 | 0 |
| hsa_circ_0081042 | 94 | 1 | 2078 | 1 | 2078 | 0 | 0 |
| hsa_circ_0081044 | 95 | 1 | 1207 | 198 | 1404 | 0 | 0 |
| hsa_circ_0081044 | 94 | 1 | 1196 | 435 | 1630 | 0 | 0 |
| hsa_circ_0081044 | 95 | 1 | 373 | 478 | 850 | 0 | 5.00E-164 |
| hsa_circ_0081044 | 95 | 1 | 465 | 478 | 942 | 0 | 0 |
| hsa_circ_0081044 | 95 | 1 | 659 | 478 | 1136 | 0 | 0 |
| hsa_circ_0081044 | 95 | 1 | 750 | 478 | 1227 | 0 | 0 |
| hsa_circ_0081044 | 95 | 1 | 927 | 478 | 1404 | 0 | 0 |
| hsa_circ_0081044 | 95 | 1 | 1046 | 478 | 1523 | 0 | 0 |
| hsa_circ_0081044 | 94 | 1 | 1275 | 478 | 1752 | 0 | 0 |
| hsa_circ_0081047 | 94 | 1 | 688 | 1 | 688 | 0 | 0 |
| hsa_circ_0005208 | 96 | 2 | 268 | 2 | 268 | 0 | 3.00E-120 |
| hsa_circ_0090543 | 92 | 1 | 371 | 386 | 1 | 0.04 | 2.00E-148 |
| hsa_circ_0076922 | 99 | 1 | 269 | 1 | 269 | 0 | 5.00E-138 |
| hsa_circ_0076922 | 99 | 1 | 431 | 1 | 431 | 0 | 0 |
| hsa_circ_0076925 | 92 | 1 | 494 | 1 | 491 | 0.01 | 0 |
| hsa_circ_0076925 | 92 | 1 | 799 | 1 | 793 | 0.01 | 0 |
| hsa_circ_0076925 | 92 | 1 | 916 | 1 | 910 | 0.01 | 0 |
| hsa_circ_0076925 | 92 | 1 | 1049 | 1 | 1043 | 0.01 | 0 |
| hsa_circ_0076925 | 92 | 1 | 1184 | 1 | 1178 | 0.01 | 0 |
| hsa_circ_0076925 | 92 | 1 | 1380 | 1 | 1374 | 0 | 0 |
| hsa_circ_0076925 | 92 | 1 | 1528 | 1 | 1522 | 0 | 0 |
| hsa_circ_0076925 | 93 | 1 | 1618 | 1 | 1612 | 0 | 0 |
| hsa_circ_0076925 | 91 | 1 | 305 | 492 | 793 | 0.01 | 3.00E-115 |
| hsa_circ_0059680 | 84 | 240 | 752 | 2904 | 2392 | 0.02 | 4.00E-133 |
| hsa_circ_0004812 | 87 | 1 | 277 | 278 | 2 | 0.01 | 4.00E-84 |
| hsa_circ_0059681 | 88 | 1 | 643 | 645 | 3 | 0.01 | 0 |
| hsa_circ_0059681 | 88 | 1 | 365 | 367 | 3 | 0 | 3.00E-121 |
| hsa_circ_0032555 | 84 | 589 | 692 | 1909 | 1806 | 0 | 3.00E-19 |
| hsa_circ_0006756 | 92 | 6 | 436 | 90 | 520 | 0 | 2.00E-169 |
| hsa_circ_0059605 | 91 | 6 | 178 | 6 | 178 | 0 | 7.00E-60 |
| hsa_circ_0059588 | 95 | 1 | 581 | 1 | 581 | 0 | 0 |
| hsa_circ_0059582 | 96 | 1 | 781 | 1 | 781 | 0 | 0 |
| hsa_circ_0059584 | 97 | 1 | 282 | 1 | 282 | 0 | 7.00E-132 |
| hsa_circ_0059575 | 91 | 16 | 637 | 16 | 637 | 0 | 0 |
| hsa_circ_0059575 | 91 | 16 | 637 | 16 | 637 | 0 | 0 |
| hsa_circ_0059575 | 91 | 109 | 730 | 16 | 637 | 0 | 0 |
| hsa_circ_0059575 | 91 | 272 | 893 | 16 | 637 | 0 | 0 |
| hsa_circ_0059571 | 93 | 2 | 683 | 2350 | 1669 | 0 | 0 |

novel_circ_027176 novel_circ_027177 novel_circ_027178 novel_circ_027179 novel_circ_027180 novel_circ_027181 novel_circ_027182 novel_circ_027183 novel_circ_027184 novel_circ_027185 novel_circ_027186 novel_circ_027187 novel_circ_027188 novel_circ_027189 novel_circ_027190 novel_circ_027191 novel_circ_027199 novel_circ_027201 novel_circ_027202 novel_circ_027210 novel_circ_027211 novel_circ_027212 novel_circ_027214 novel_circ_027215 novel_circ_027216 novel_circ_027217 novel_circ_027218 novel_circ_027219 novel_circ_027220 novel_circ_027221 novel_circ_027222 novel_circ_027223 novel_circ_027224 novel_circ_027225 novel_circ_027226 novel_circ_027227 novel_circ_027228 novel_circ_027229 novel_circ_027230 novel_circ_027231 novel_circ_027236 novel_circ_027237 novel_circ_027238 novel_circ_027239 novel_circ_027240 novel_circ_027241 novel_circ_027242 novel_circ_027243 novel_circ_027244 novel_circ_027245 novel_circ_027246 novel_circ_027247 novel_circ_027248 novel_circ_027249

| hsa_circ_0059571 | 91 | 1 | 1022 | 2235 | 1214 | 0 | 0 |
| --- | --- | --- | --- | --- | --- | --- | --- |
| hsa_circ_0059571 | 91 | 1 | 428 | 2096 | 1669 | 0 | 2.00E-164 |
| hsa_circ_0059571 | 91 | 1 | 596 | 2096 | 1501 | 0 | 0 |
| hsa_circ_0059571 | 90 | 1 | 734 | 2096 | 1363 | 0 | 0 |
| hsa_circ_0059571 | 90 | 1 | 737 | 2096 | 1360 | 0 | 0 |
| hsa_circ_0059571 | 90 | 1 | 883 | 2096 | 1214 | 0 | 0 |
| hsa_circ_0059571 | 91 | 1 | 995 | 2096 | 1102 | 0 | 0 |
| hsa_circ_0059571 | 90 | 1 | 1396 | 2096 | 701 | 0 | 0 |
| hsa_circ_0059571 | 90 | 1 | 1396 | 2096 | 701 | 0 | 0 |
| hsa_circ_0059571 | 90 | 1 | 1533 | 2096 | 561 | 0 | 0 |
| hsa_circ_0059571 | 89 | 1 | 1630 | 2096 | 464 | 0 | 0 |
| hsa_circ_0059563 | 89 | 201 | 2348 | 4652 | 2502 | 0 | 0 |
| hsa_circ_0059573 | 89 | 4 | 455 | 1203 | 752 | 0 | 8.00E-158 |
| hsa_circ_0059573 | 90 | 4 | 567 | 1203 | 640 | 0 | 0 |
| hsa_circ_0059563 | 87 | 1 | 950 | 3454 | 2502 | 0.01 | 0 |
| hsa_circ_0059565 | 89 | 1 | 615 | 615 | 1 | 0 | 0 |
| hsa_circ_0059562 | 94 | 1 | 240 | 240 | 1 | 0 | 6.00E-98 |
| hsa_circ_0007894 | 93 | 1 | 438 | 1 | 438 | 0 | 5.00E-180 |
| hsa_circ_0059553 | 92 | 1 | 194 | 1 | 193 | 0.01 | 6.00E-69 |
| hsa_circ_0059546 | 90 | 1 | 249 | 233 | 481 | 0 | 6.00E-87 |
| hsa_circ_0059536 | 94 | 1 | 304 | 87 | 390 | 0 | 2.00E-127 |
| hsa_circ_0059534 | 91 | 1 | 630 | 1 | 627 | 0 | 0 |
| hsa_circ_0059528 | 90 | 326 | 411 | 318 | 233 | 0 | 9.00E-23 |
| hsa_circ_0059518 | 90 | 6 | 1302 | 310 | 1606 | 0 | 0 |
| hsa_circ_0059518 | 91 | 6 | 1126 | 310 | 1430 | 0 | 0 |
| hsa_circ_0059518 | 90 | 871 | 2300 | 1 | 1430 | 0 | 0 |
| hsa_circ_0059517 | 82 | 1 | 753 | 198 | 911 | 0.07 | 7.00E-167 |
| hsa_circ_0047298 | 92 | 2 | 607 | 65 | 676 | 0.01 | 0 |
| hsa_circ_0047296 | 93 | 210 | 284 | 75 | 1 | 0 | 2.00E-23 |
| hsa_circ_0047293 | 93 | 113 | 999 | 1207 | 321 | 0 | 0 |
| hsa_circ_0047293 | 92 | 1 | 467 | 976 | 510 | 0 | 0 |
| hsa_circ_0047293 | 93 | 1 | 656 | 976 | 321 | 0 | 0 |
| hsa_circ_0047293 | 93 | 1 | 411 | 731 | 321 | 0 | 4.00E-170 |
| hsa_circ_0047293 | 93 | 1 | 312 | 632 | 321 | 0 | 4.00E-125 |
| hsa_circ_0047290 | 90 | 108 | 581 | 789 | 310 | 0.02 | 1.00E-171 |
| hsa_circ_0047293 | 93 | 1 | 320 | 320 | 1 | 0 | 6.00E-128 |
| hsa_circ_0047291 | 92 | 1 | 396 | 629 | 234 | 0 | 2.00E-158 |
| hsa_circ_0047287 | 93 | 1 | 416 | 614 | 199 | 0 | 2.00E-169 |
| hsa_circ_0047287 | 92 | 1 | 614 | 614 | 1 | 0 | 0 |
| hsa_circ_0047287 | 92 | 1 | 381 | 381 | 1 | 0 | 2.00E-153 |
| hsa_circ_0089874 | 94 | 258 | 989 | 732 | 1 | 0 | 0 |
| hsa_circ_0089874 | 94 | 258 | 989 | 732 | 1 | 0 | 0 |
| hsa_circ_0089874 | 96 | 258 | 453 | 732 | 537 | 0 | 2.00E-85 |
| hsa_circ_0016344 | 90 | 3 | 197 | 195 | 1 | 0 | 5.00E-67 |
| hsa_circ_0016344 | 92 | 6925 | 7376 | 452 | 1 | 0 | 1.00E-176 |
| hsa_circ_0016344 | 92 | 1 | 650 | 650 | 1 | 0 | 0 |
| hsa_circ_0016346 | 93 | 6925 | 7179 | 255 | 1 | 0 | 4.00E-102 |
| hsa_circ_0016346 | 93 | 1 | 453 | 453 | 1 | 0 | 0 |
| hsa_circ_0016347 | 95 | 1 | 430 | 430 | 1 | 0 | 0 |
| hsa_circ_0016347 | 95 | 1 | 904 | 904 | 1 | 0 | 0 |
| hsa_circ_0016349 | 95 | 1 | 474 | 474 | 1 | 0 | 0 |
| hsa_circ_0016349 | 95 | 1 | 593 | 593 | 1 | 0 | 0 |
| hsa_circ_0016349 | 96 | 1 | 722 | 722 | 1 | 0 | 0 |
| hsa_circ_0016350 | 94 | 1 | 393 | 393 | 1 | 0 | 9.00E-172 |

novel_circ_027250 novel_circ_027251 novel_circ_027252 novel_circ_027253 novel_circ_027254 novel_circ_027255 novel_circ_027256 novel_circ_027257 novel_circ_027258 novel_circ_027259 novel_circ_027263 novel_circ_027264 novel_circ_027265 novel_circ_027266 novel_circ_027267 novel_circ_027268 novel_circ_027269 novel_circ_027270 novel_circ_027271 novel_circ_027272 novel_circ_027273 novel_circ_027274 novel_circ_027275 novel_circ_027276 novel_circ_027277 novel_circ_027278 novel_circ_027279 novel_circ_027280 novel_circ_027281 novel_circ_027283 novel_circ_027284 novel_circ_027285 novel_circ_027286 novel_circ_027287 novel_circ_027288 novel_circ_027289 novel_circ_027290 novel_circ_027291 novel_circ_027292 novel_circ_027293 novel_circ_027294 novel_circ_027295 novel_circ_027296 novel_circ_027297 novel_circ_027298 novel_circ_027299 novel_circ_027301 novel_circ_027302 novel_circ_027307 novel_circ_027308 novel_circ_027309 novel_circ_027310 novel_circ_027311 novel_circ_027312

| hsa_circ_0016350 | 95 | 1 | 512 | 512 | 1 | 0 | 0 |
| --- | --- | --- | --- | --- | --- | --- | --- |
| hsa_circ_0016350 | 95 | 1 | 559 | 559 | 1 | 0 | 0 |
| hsa_circ_0016350 | 96 | 1 | 641 | 641 | 1 | 0 | 0 |
| hsa_circ_0016350 | 95 | 1 | 748 | 748 | 1 | 0 | 0 |
| hsa_circ_0016350 | 95 | 1 | 872 | 872 | 1 | 0 | 0 |
| hsa_circ_0016351 | 98 | 5201 | 5448 | 248 | 1 | 0 | 1.00E-116 |
| hsa_circ_0016354 | 96 | 1 | 293 | 1 | 293 | 0 | 1.00E-134 |
| hsa_circ_0016354 | 97 | 1 | 497 | 1 | 497 | 0 | 0 |
| hsa_circ_0016354 | 97 | 1 | 716 | 1 | 716 | 0 | 0 |
| hsa_circ_0016354 | 97 | 1 | 297 | 498 | 794 | 0 | 3.00E-140 |
| hsa_circ_0016365 | 96 | 1 | 401 | 401 | 1 | 0 | 0 |
| hsa_circ_0016365 | 96 | 1 | 616 | 616 | 1 | 0 | 0 |
| hsa_circ_0008745 | 95 | 1 | 370 | 370 | 1 | 0 | 1.00E-165 |
| hsa_circ_0016366 | 92 | 1 | 560 | 2131 | 1572 | 0 | 0 |
| hsa_circ_0016368 | 92 | 1 | 418 | 418 | 1 | 0 | 1.00E-165 |
| hsa_circ_0016368 | 94 | 7 | 501 | 780 | 286 | 0 | 0 |
| hsa_circ_0016369 | 92 | 3 | 1627 | 1625 | 1 | 0 | 0 |
| hsa_circ_0016369 | 92 | 1 | 1812 | 1812 | 1 | 0 | 0 |
| hsa_circ_0016373 | 91 | 3 | 674 | 912 | 241 | 0 | 0 |
| hsa_circ_0016373 | 92 | 1 | 859 | 1099 | 241 | 0 | 0 |
| hsa_circ_0016373 | 92 | 1 | 626 | 1099 | 474 | 0 | 0 |
| hsa_circ_0016374 | 92 | 1 | 508 | 508 | 1 | 0 | 0 |
| hsa_circ_0006862 | 91 | 3 | 408 | 3 | 408 | 0 | 3.00E-157 |
| hsa_circ_0016381 | 94 | 1 | 758 | 1 | 758 | 0 | 0 |
| hsa_circ_0002321 | 98 | 1 | 298 | 1 | 298 | 0 | 2.00E-147 |
| hsa_circ_0002321 | 98 | 1 | 298 | 1 | 298 | 0 | 3.00E-147 |
| hsa_circ_0016394 | 97 | 1 | 447 | 1 | 447 | 0 | 0 |
| hsa_circ_0016396 | 97 | 1 | 405 | 1 | 405 | 0 | 0 |
| hsa_circ_0016396 | 97 | 1 | 274 | 132 | 405 | 0 | 4.00E-129 |
| hsa_circ_0016400 | 98 | 1 | 186 | 186 | 1 | 0 | 4.00E-87 |
| hsa_circ_0016405 | 90 | 1 | 279 | 1 | 279 | 0 | 7.00E-97 |
| hsa_circ_0016415 | 88 | 3 | 418 | 3 | 424 | 0.02 | 4.00E-136 |
| hsa_circ_0016419 | 98 | 280 | 411 | 1 | 132 | 0 | 2.00E-58 |
| hsa_circ_0005841 | 94 | 6746 | 7439 | 1 | 694 | 0 | 0 |
| hsa_circ_0005841 | 95 | 1 | 331 | 1 | 331 | 0 | 2.00E-147 |
| hsa_circ_0005841 | 94 | 1 | 694 | 1 | 694 | 0 | 0 |
| hsa_circ_0005841 | 94 | 1 | 694 | 1 | 694 | 0 | 0 |
| hsa_circ_0005841 | 93 | 1 | 573 | 122 | 694 | 0 | 0 |
| hsa_circ_0005841 | 93 | 1 | 573 | 122 | 694 | 0 | 0 |
| hsa_circ_0002721 | 93 | 117 | 782 | 1 | 666 | 0 | 0 |
| hsa_circ_0016427 | 93 | 1 | 572 | 1 | 572 | 0 | 0 |
| hsa_circ_0005314 | 93 | 1 | 209 | 1 | 209 | 0 | 4.00E-83 |
| hsa_circ_0016427 | 94 | 1 | 390 | 364 | 753 | 0 | 4.00E-165 |
| hsa_circ_0016427 | 91 | 1 | 2067 | 364 | 2439 | 0.01 | 0 |
| hsa_circ_0016429 | 96 | 1 | 274 | 1 | 274 | 0 | 2.00E-122 |
| hsa_circ_0016432 | 90 | 1 | 1677 | 134 | 1819 | 0.01 | 0 |
| hsa_circ_0012380 | 91 | 1 | 657 | 741 | 85 | 0 | 0 |
| hsa_circ_0012380 | 91 | 1 | 741 | 741 | 1 | 0 | 0 |
| hsa_circ_0078342 | 75 | 7164 | 7369 | 6421 | 6626 | 0.05 | 3.00E-16 |
| hsa_circ_0070472 | 85 | 1846 | 2000 | 4439 | 4590 | 0.02 | 8.00E-33 |
| hsa_circ_0084772 | 91 | 2035 | 2825 | 794 | 3 | 0 | 0 |
| hsa_circ_0084772 | 91 | 2035 | 2825 | 794 | 3 | 0 | 0 |
| hsa_circ_0084772 | 91 | 2035 | 2825 | 794 | 3 | 0 | 0 |
| hsa_circ_0084772 | 91 | 2035 | 2825 | 794 | 3 | 0 | 0 |

novel_circ_027316 novel_circ_027319 novel_circ_027320 novel_circ_027321 novel_circ_027322 novel_circ_027323 novel_circ_027324 novel_circ_027329 novel_circ_027330 novel_circ_027331 novel_circ_027332 novel_circ_027333 novel_circ_027334 novel_circ_027335 novel_circ_027336 novel_circ_027337 novel_circ_027338 novel_circ_027339 novel_circ_027340 novel_circ_027341 novel_circ_027342 novel_circ_027343 novel_circ_027344 novel_circ_027345 novel_circ_027346 novel_circ_027347 novel_circ_027348 novel_circ_027349 novel_circ_027353 novel_circ_027354 novel_circ_027355 novel_circ_027356 novel_circ_027358 novel_circ_027359 novel_circ_027360 novel_circ_027361 novel_circ_027362 novel_circ_027363 novel_circ_027364 novel_circ_027365 novel_circ_027366 novel_circ_027369 novel_circ_027370 novel_circ_027371 novel_circ_027372 novel_circ_027373 novel_circ_027374 novel_circ_027375 novel_circ_027376 novel_circ_027377 novel_circ_027378 novel_circ_027379 novel_circ_027380 novel_circ_027382

| hsa_circ_0055921 | 86 | 1 | 350 | 775 | 426 | 0 | 3.00E-101 |
| --- | --- | --- | --- | --- | --- | --- | --- |
| hsa_circ_0090869 | 95 | 1 | 495 | 495 | 1 | 0 | 0 |
| hsa_circ_0090866 | 95 | 1 | 739 | 4050 | 3312 | 0 | 0 |
| hsa_circ_0090866 | 94 | 1 | 808 | 4050 | 3243 | 0 | 0 |
| hsa_circ_0090866 | 95 | 1 | 968 | 4050 | 3083 | 0 | 0 |
| hsa_circ_0090866 | 94 | 1 | 506 | 3817 | 3312 | 0 | 0 |
| hsa_circ_0090866 | 95 | 4 | 244 | 3552 | 3312 | 0 | 2.00E-102 |
| hsa_circ_0035409 | 93 | 77 | 663 | 1248 | 664 | 0 | 0 |
| hsa_circ_0035410 | 93 | 1 | 586 | 865 | 282 | 0 | 0 |
| hsa_circ_0035410 | 93 | 1 | 867 | 865 | 1 | 0 | 0 |
| hsa_circ_0035407 | 93 | 1 | 1497 | 2261 | 767 | 0 | 0 |
| hsa_circ_0035410 | 93 | 2 | 471 | 751 | 282 | 0 | 0 |
| hsa_circ_0035410 | 93 | 2 | 752 | 751 | 1 | 0 | 0 |
| hsa_circ_0035409 | 94 | 2 | 1134 | 1133 | 1 | 0 | 0 |
| hsa_circ_0035407 | 94 | 2 | 1382 | 2147 | 767 | 0 | 0 |
| hsa_circ_0035407 | 94 | 2 | 1966 | 2147 | 183 | 0 | 0 |
| hsa_circ_0035410 | 92 | 1 | 399 | 399 | 1 | 0 | 4.00E-160 |
| hsa_circ_0035409 | 93 | 1 | 781 | 781 | 1 | 0 | 0 |
| hsa_circ_0035407 | 93 | 1 | 1029 | 1795 | 767 | 0 | 0 |
| hsa_circ_0035407 | 94 | 1 | 911 | 1677 | 767 | 0 | 0 |
| hsa_circ_0035409 | 95 | 1 | 382 | 382 | 1 | 0 | 2.00E-167 |
| hsa_circ_0035408 | 94 | 1 | 630 | 1073 | 444 | 0 | 0 |
| hsa_circ_0035407 | 94 | 1 | 1214 | 1396 | 183 | 0 | 0 |
| hsa_circ_0035407 | 93 | 1 | 1396 | 1396 | 1 | 0 | 0 |
| hsa_circ_0035407 | 93 | 1 | 1396 | 1396 | 1 | 0 | 0 |
| hsa_circ_0035407 | 93 | 3 | 893 | 1012 | 122 | 0 | 0 |
| hsa_circ_0035407 | 93 | 1 | 645 | 766 | 122 | 0 | 0 |
| hsa_circ_0035407 | 92 | 1 | 766 | 766 | 1 | 0 | 0 |
| hsa_circ_0089867 | 95 | 1 | 314 | 1 | 314 | 0 | 3.00E-136 |
| hsa_circ_0089867 | 95 | 1 | 471 | 1 | 471 | 0 | 0 |
| hsa_circ_0089867 | 90 | 1 | 748 | 1 | 751 | 0 | 0 |
| hsa_circ_0089867 | 91 | 1 | 822 | 1 | 825 | 0 | 0 |
| hsa_circ_0086396 | 91 | 2 | 413 | 412 | 1 | 0.01 | 1.00E-155 |
| hsa_circ_0086396 | 91 | 2 | 413 | 412 | 1 | 0.01 | 2.00E-155 |
| hsa_circ_0086396 | 91 | 2 | 413 | 412 | 1 | 0.01 | 3.00E-155 |
| hsa_circ_0086393 | 91 | 703 | 1498 | 1153 | 358 | 0 | 0 |
| hsa_circ_0086393 | 91 | 1 | 728 | 1085 | 358 | 0 | 0 |
| hsa_circ_0086393 | 90 | 1 | 663 | 1020 | 358 | 0 | 0 |
| hsa_circ_0086393 | 90 | 55 | 310 | 613 | 358 | 0 | 1.00E-90 |
| hsa_circ_0086393 | 90 | 1 | 254 | 611 | 358 | 0 | 2.00E-89 |
| hsa_circ_0086393 | 90 | 1 | 254 | 611 | 358 | 0 | 2.00E-89 |
| hsa_circ_0086388 | 87 | 1 | 496 | 4113 | 3615 | 0.01 | 2.00E-154 |
| hsa_circ_0008952 | 92 | 1 | 684 | 796 | 118 | 0.01 | 0 |
| hsa_circ_0008952 | 92 | 1 | 801 | 796 | 1 | 0.01 | 0 |
| hsa_circ_0008952 | 94 | 3 | 549 | 664 | 118 | 0 | 0 |
| hsa_circ_0008952 | 93 | 1 | 467 | 467 | 1 | 0 | 0 |
| hsa_circ_0008952 | 93 | 1 | 368 | 368 | 1 | 0 | 7.00E-153 |
| hsa_circ_0025766 | 82 | 4694 | 4800 | 35422 | 35318 | 0.04 | 2.00E-15 |
| hsa_circ_0086372 | 99 | 1 | 532 | 1437 | 906 | 0 | 0 |
| hsa_circ_0086376 | 95 | 1 | 363 | 363 | 1 | 0 | 2.00E-163 |
| hsa_circ_0086372 | 96 | 1 | 683 | 905 | 223 | 0 | 0 |
| hsa_circ_0086376 | 95 | 3 | 309 | 307 | 1 | 0 | 4.00E-134 |
| hsa_circ_0086372 | 96 | 3 | 560 | 780 | 223 | 0 | 0 |
| hsa_circ_0086366 | 88 | 1 | 564 | 1107 | 544 | 0 | 0 |

novel_circ_027383 novel_circ_027384 novel_circ_027385 novel_circ_027386 novel_circ_027387 novel_circ_027388 novel_circ_027389 novel_circ_027390 novel_circ_027391 novel_circ_027392 novel_circ_027393 novel_circ_027394 novel_circ_027395 novel_circ_027396 novel_circ_027397 novel_circ_027400 novel_circ_027402 novel_circ_027403 novel_circ_027404 novel_circ_027405 novel_circ_027406 novel_circ_027407 novel_circ_027408 novel_circ_027409 novel_circ_027410 novel_circ_027411 novel_circ_027412 novel_circ_027413 novel_circ_027414 novel_circ_027415 novel_circ_027416 novel_circ_027417 novel_circ_027418 novel_circ_027419 novel_circ_027420 novel_circ_027421 novel_circ_027422 novel_circ_027423 novel_circ_027424 novel_circ_027425 novel_circ_027426 novel_circ_027429 novel_circ_027430 novel_circ_027431 novel_circ_027432 novel_circ_027433 novel_circ_027434 novel_circ_027437 novel_circ_027438 novel_circ_027439 novel_circ_027440 novel_circ_027441 novel_circ_027442 novel_circ_027443

| hsa_circ_0086366 | 89 | 1 | 903 | 1107 | 205 | 0 | 0 |
| --- | --- | --- | --- | --- | --- | --- | --- |
| hsa_circ_0086366 | 90 | 1 | 1107 | 1107 | 1 | 0 | 0 |
| hsa_circ_0086366 | 90 | 1 | 1107 | 1107 | 1 | 0 | 0 |
| hsa_circ_0086366 | 90 | 1 | 1107 | 1107 | 1 | 0 | 0 |
| hsa_circ_0086366 | 90 | 1 | 1107 | 1107 | 1 | 0 | 0 |
| hsa_circ_0086366 | 92 | 1 | 553 | 757 | 205 | 0 | 0 |
| hsa_circ_0086366 | 92 | 1 | 339 | 543 | 205 | 0 | 8.00E-132 |
| hsa_circ_0086366 | 92 | 1 | 543 | 543 | 1 | 0 | 0 |
| hsa_circ_0086366 | 92 | 1 | 543 | 543 | 1 | 0 | 0 |
| hsa_circ_0086364 | 87 | 403 | 1094 | 2589 | 1901 | 0 | 0 |
| hsa_circ_0086364 | 88 | 403 | 1295 | 2589 | 1700 | 0 | 0 |
| hsa_circ_0086364 | 89 | 403 | 1671 | 2589 | 1324 | 0 | 0 |
| hsa_circ_0086364 | 90 | 403 | 2502 | 2589 | 490 | 0 | 0 |
| hsa_circ_0086364 | 90 | 403 | 2670 | 2589 | 322 | 0 | 0 |
| hsa_circ_0086364 | 88 | 217 | 1214 | 2589 | 1595 | 0 | 0 |
| hsa_circ_0086364 | 91 | 1 | 1009 | 1501 | 490 | 0 | 0 |
| hsa_circ_0067891 | 92 | 1 | 1045 | 1045 | 1 | 0 | 0 |
| hsa_circ_0067902 | 94 | 1 | 1120 | 1270 | 154 | 0 | 0 |
| hsa_circ_0067902 | 95 | 1 | 1378 | 1528 | 154 | 0 | 0 |
| hsa_circ_0067897 | 90 | 1 | 565 | 565 | 1 | 0 | 0 |
| hsa_circ_0067902 | 94 | 1 | 1120 | 1270 | 154 | 0 | 0 |
| hsa_circ_0067902 | 95 | 1 | 1378 | 1528 | 154 | 0 | 0 |
| hsa_circ_0067891 | 94 | 1 | 1772 | 2819 | 1045 | 0.01 | 0 |
| hsa_circ_0067898 | 89 | 1 | 404 | 405 | 2 | 0 | 8.00E-143 |
| hsa_circ_0067902 | 94 | 1 | 1120 | 1270 | 154 | 0 | 0 |
| hsa_circ_0067902 | 94 | 1 | 873 | 1023 | 154 | 0.01 | 0 |
| hsa_circ_0067902 | 94 | 1 | 1120 | 1270 | 154 | 0 | 0 |
| hsa_circ_0067902 | 95 | 1 | 1378 | 1528 | 154 | 0 | 0 |
| hsa_circ_0067891 | 94 | 1 | 1772 | 2819 | 1045 | 0.01 | 0 |
| hsa_circ_0067910 | 97 | 1 | 505 | 505 | 1 | 0 | 0 |
| hsa_circ_0067915 | 99 | 1 | 258 | 258 | 1 | 0 | 1.00E-128 |
| hsa_circ_0067915 | 93 | 1 | 652 | 658 | 1 | 0.01 | 0 |
| hsa_circ_0067918 | 93 | 1 | 393 | 393 | 1 | 0 | 2.00E-163 |
| hsa_circ_0067918 | 92 | 1 | 553 | 559 | 1 | 0.01 | 0 |
| hsa_circ_0067921 | 95 | 1 | 263 | 1 | 263 | 0 | 1.00E-114 |
| hsa_circ_0067921 | 94 | 1 | 488 | 1 | 488 | 0 | 0 |
| hsa_circ_0067930 | 95 | 2 | 311 | 2 | 311 | 0 | 4.00E-139 |
| hsa_circ_0067930 | 94 | 1 | 520 | 88 | 607 | 0 | 0 |
| hsa_circ_0067932 | 93 | 1 | 296 | 1 | 296 | 0 | 1.00E-119 |
| hsa_circ_0067932 | 94 | 1 | 409 | 1 | 409 | 0 | 1.00E-175 |
| hsa_circ_0008056 | 89 | 1 | 233 | 1 | 233 | 0 | 7.00E-76 |
| hsa_circ_0066258 | 81 | 44573 | 45506 | 1 | 939 | 0.06 | 0 |
| hsa_circ_0008107 | 92 | 1235 | 1623 | 389 | 1 | 0 | 1.00E-155 |
| hsa_circ_0008107 | 91 | 1 | 388 | 389 | 2 | 0 | 3.00E-147 |
| hsa_circ_0066258 | 91 | 1 | 532 | 1325 | 794 | 0 | 0 |
| hsa_circ_0066259 | 94 | 1 | 111 | 182 | 72 | 0 | 2.00E-39 |
| hsa_circ_0008107 | 91 | 1 | 307 | 308 | 2 | 0 | 2.00E-112 |
| hsa_circ_0079504 | 94 | 1 | 215 | 1779 | 1565 | 0 | 9.00E-90 |
| hsa_circ_0079507 | 95 | 1 | 234 | 380 | 147 | 0 | 3.00E-100 |
| hsa_circ_0079507 | 96 | 278 | 1542 | 2006 | 742 | 0 | 0 |
| hsa_circ_0079510 | 96 | 1 | 1263 | 1405 | 143 | 0 | 0 |
| hsa_circ_0079513 | 96 | 1 | 532 | 532 | 1 | 0 | 0 |
| hsa_circ_0079513 | 96 | 679 | 1300 | 622 | 1 | 0 | 0 |
| hsa_circ_0079513 | 96 | 1 | 760 | 760 | 1 | 0 | 0 |

novel_circ_027444 novel_circ_027445 novel_circ_027446 novel_circ_027447 novel_circ_027448 novel_circ_027449 novel_circ_027450 novel_circ_027451 novel_circ_027452 novel_circ_027453 novel_circ_027454 novel_circ_027455 novel_circ_027456 novel_circ_027457 novel_circ_027458 novel_circ_027459 novel_circ_027460 novel_circ_027461 novel_circ_027462 novel_circ_027463 novel_circ_027464 novel_circ_027465 novel_circ_027466 novel_circ_027467 novel_circ_027468 novel_circ_027469 novel_circ_027470 novel_circ_027471 novel_circ_027472 novel_circ_027473 novel_circ_027474 novel_circ_027480 novel_circ_027481 novel_circ_027482 novel_circ_027483 novel_circ_027484 novel_circ_027485 novel_circ_027486 novel_circ_027487 novel_circ_027488 novel_circ_027489 novel_circ_027490 novel_circ_027492 novel_circ_027493 novel_circ_027495 novel_circ_027496 novel_circ_027497 novel_circ_027498 novel_circ_027499 novel_circ_027500 novel_circ_027501 novel_circ_027502 novel_circ_027503 novel_circ_027504

| hsa_circ_0079513 | 96 | 7719 | 8563 | 845 | 1 | 0 | 0 |
| --- | --- | --- | --- | --- | --- | --- | --- |
| hsa_circ_0079513 | 96 | 1 | 933 | 933 | 1 | 0 | 0 |
| hsa_circ_0079513 | 96 | 1 | 1157 | 1157 | 1 | 0 | 0 |
| hsa_circ_0079512 | 96 | 494 | 1652 | 1197 | 39 | 0 | 0 |
| hsa_circ_0079514 | 96 | 7719 | 8325 | 712 | 106 | 0 | 0 |
| hsa_circ_0079514 | 96 | 1 | 695 | 800 | 106 | 0 | 0 |
| hsa_circ_0079514 | 95 | 1 | 919 | 1024 | 106 | 0 | 0 |
| hsa_circ_0079514 | 95 | 278 | 1198 | 1026 | 106 | 0 | 0 |
| hsa_circ_0079515 | 97 | 679 | 868 | 190 | 1 | 0 | 3.00E-85 |
| hsa_circ_0079515 | 96 | 1 | 328 | 328 | 1 | 0 | 2.00E-152 |
| hsa_circ_0079515 | 96 | 1 | 412 | 412 | 1 | 0 | 0 |
| hsa_circ_0079515 | 96 | 7719 | 8131 | 413 | 1 | 0 | 0 |
| hsa_circ_0079515 | 96 | 1 | 501 | 501 | 1 | 0 | 0 |
| hsa_circ_0079515 | 96 | 1 | 725 | 725 | 1 | 0 | 0 |
| hsa_circ_0079515 | 96 | 494 | 1220 | 727 | 1 | 0 | 0 |
| hsa_circ_0079515 | 96 | 615 | 1341 | 727 | 1 | 0 | 0 |
| hsa_circ_0079515 | 95 | 1 | 625 | 725 | 101 | 0 | 0 |
| hsa_circ_0079515 | 95 | 278 | 904 | 727 | 101 | 0 | 0 |
| hsa_circ_0079515 | 95 | 381 | 1007 | 727 | 101 | 0 | 0 |
| hsa_circ_0004671 | 95 | 1 | 397 | 397 | 1 | 0 | 5.00E-179 |
| hsa_circ_0004671 | 95 | 278 | 676 | 399 | 1 | 0 | 7.00E-180 |
| hsa_circ_0004671 | 95 | 381 | 779 | 399 | 1 | 0 | 9.00E-180 |
| hsa_circ_0079523 | 95 | 1 | 433 | 11361 | 10929 | 0 | 0 |
| hsa_circ_0079523 | 95 | 122 | 554 | 11361 | 10929 | 0 | 0 |
| hsa_circ_0079523 | 96 | 4 | 217 | 11142 | 10929 | 0 | 2.00E-95 |
| hsa_circ_0079524 | 96 | 1 | 315 | 315 | 1 | 0 | 8.00E-142 |
| hsa_circ_0079524 | 96 | 1 | 428 | 428 | 1 | 0 | 0 |
| hsa_circ_0079524 | 96 | 122 | 549 | 428 | 1 | 0 | 0 |
| hsa_circ_0079524 | 96 | 749 | 1176 | 428 | 1 | 0 | 0 |
| hsa_circ_0079524 | 95 | 122 | 427 | 428 | 123 | 0 | 5.00E-135 |
| hsa_circ_0009016 | 80 | 234 | 330 | 1E+06 | 1120822 | 0.02 | 3.00E-10 |
| hsa_circ_0066631 | 93 | 1 | 366 | 366 | 1 | 0 | 2.00E-148 |
| hsa_circ_0066609 | 92 | 319 | 760 | 1 | 442 | 0 | 1.00E-173 |
| hsa_circ_0066609 | 91 | 18520 | 18865 | 1 | 346 | 0 | 6.00E-129 |
| hsa_circ_0066609 | 91 | 319 | 664 | 1 | 346 | 0 | 2.00E-130 |
| hsa_circ_0066606 | 92 | 1 | 829 | 2046 | 1218 | 0 | 0 |
| hsa_circ_0044757 | 92 | 1 | 1372 | 4123 | 2733 | 0.03 | 0 |
| hsa_circ_0091442 | 95 | 1 | 1217 | 1346 | 130 | 0 | 0 |
| hsa_circ_0091443 | 94 | 1 | 820 | 1096 | 277 | 0 | 0 |
| hsa_circ_0091443 | 97 | 1 | 535 | 1371 | 837 | 0 | 0 |
| hsa_circ_0091451 | 94 | 1 | 785 | 785 | 1 | 0 | 0 |
| hsa_circ_0005219 | 96 | 1 | 749 | 1 | 750 | 0 | 0 |
| hsa_circ_0059219 | 95 | 18 | 437 | 1 | 420 | 0 | 0 |
| hsa_circ_0059237 | 93 | 1 | 432 | 91 | 522 | 0 | 1.00E-176 |
| hsa_circ_0059246 | 90 | 4 | 741 | 4 | 744 | 0 | 0 |
| hsa_circ_0059246 | 95 | 1 | 323 | 422 | 744 | 0 | 3.00E-141 |
| hsa_circ_0059248 | 94 | 1 | 296 | 1 | 296 | 0 | 3.00E-126 |
| hsa_circ_0059243 | 93 | 1 | 306 | 2099 | 2404 | 0 | 3.00E-125 |
| hsa_circ_0059260 | 90 | 62 | 348 | 1 | 287 | 0 | 3.00E-101 |
| hsa_circ_0059281 | 90 | 1 | 824 | 830 | 1 | 0.01 | 0 |
| hsa_circ_0059291 | 95 | 1 | 655 | 948 | 294 | 0 | 0 |
| hsa_circ_0059287 | 95 | 1 | 780 | 1499 | 720 | 0 | 0 |
| hsa_circ_0059292 | 94 | 1 | 369 | 488 | 120 | 0 | 9.00E-157 |
| hsa_circ_0059287 | 95 | 1 | 506 | 1499 | 994 | 0 | 0 |

novel_circ_027505 novel_circ_027506 novel_circ_027507 novel_circ_027508 novel_circ_027509 novel_circ_027510 novel_circ_027511 novel_circ_027512 novel_circ_027513 novel_circ_027514 novel_circ_027515 novel_circ_027516 novel_circ_027517 novel_circ_027518 novel_circ_027519 novel_circ_027520 novel_circ_027521 novel_circ_027522 novel_circ_027523 novel_circ_027524 novel_circ_027525 novel_circ_027526 novel_circ_027527 novel_circ_027531 novel_circ_027532 novel_circ_027533 novel_circ_027535 novel_circ_027536 novel_circ_027537 novel_circ_027538 novel_circ_027540 novel_circ_027542 novel_circ_027543 novel_circ_027546 novel_circ_027547 novel_circ_027548 novel_circ_027549 novel_circ_027550 novel_circ_027551 novel_circ_027552 novel_circ_027553 novel_circ_027554 novel_circ_027555 novel_circ_027556 novel_circ_027557 novel_circ_027558 novel_circ_027559 novel_circ_027560 novel_circ_027561 novel_circ_027562 novel_circ_027563 novel_circ_027564 novel_circ_027565 novel_circ_027566

| hsa_circ_0059291 | 96 | 1 | 281 | 948 | 668 | 0 | 1.00E-129 |
| --- | --- | --- | --- | --- | --- | --- | --- |
| hsa_circ_0059287 | 96 | 1 | 406 | 1499 | 1094 | 0 | 0 |
| hsa_circ_0059296 | 93 | 1 | 1290 | 1 | 1290 | 0 | 0 |
| hsa_circ_0059296 | 93 | 1 | 1377 | 1 | 1377 | 0 | 0 |
| hsa_circ_0059297 | 92 | 1 | 335 | 1 | 335 | 0 | 6.00E-133 |
| hsa_circ_0059297 | 92 | 1 | 504 | 1 | 504 | 0 | 0 |
| hsa_circ_0059296 | 92 | 1 | 1263 | 115 | 1377 | 0 | 0 |
| hsa_circ_0059303 | 92 | 1 | 2072 | 170 | 2241 | 0 | 0 |
| hsa_circ_0059310 | 88 | 1 | 343 | 1 | 343 | 0 | 5.00E-114 |
| hsa_circ_0059310 | 94 | 1 | 970 | 344 | 1313 | 0 | 0 |
| hsa_circ_0059310 | 94 | 1 | 1108 | 344 | 1451 | 0 | 0 |
| hsa_circ_0059300 | 94 | 1 | 479 | 2586 | 3064 | 0 | 0 |
| hsa_circ_0059300 | 94 | 1 | 385 | 2680 | 3064 | 0 | 3.00E-167 |
| hsa_circ_0059300 | 95 | 1 | 447 | 2680 | 3126 | 0 | 0 |
| hsa_circ_0059300 | 95 | 1 | 634 | 2680 | 3313 | 0 | 0 |
| hsa_circ_0059300 | 94 | 1 | 332 | 2733 | 3064 | 0 | 6.00E-143 |
| hsa_circ_0059300 | 95 | 1 | 394 | 2733 | 3126 | 0 | 1.00E-175 |
| hsa_circ_0059300 | 95 | 1 | 581 | 2733 | 3313 | 0 | 0 |
| hsa_circ_0005650 | 95 | 1 | 781 | 1 | 781 | 0 | 0 |
| hsa_circ_0005650 | 96 | 1 | 1034 | 1 | 1034 | 0 | 0 |
| hsa_circ_0005650 | 96 | 1 | 1034 | 1 | 1034 | 0 | 0 |
| hsa_circ_0005650 | 95 | 1 | 428 | 354 | 781 | 0 | 0 |
| hsa_circ_0005650 | 95 | 1 | 681 | 354 | 1034 | 0 | 0 |
| hsa_circ_0059363 | 86 | 1 | 641 | 1256 | 621 | 0.01 | 0 |
| hsa_circ_0059368 | 83 | 1 | 366 | 478 | 118 | 0.02 | 4.00E-85 |
| hsa_circ_0059368 | 80 | 1 | 267 | 478 | 217 | 0.03 | 3.00E-45 |
| hsa_circ_0046756 | 94 | 6 | 348 | 343 | 1 | 0 | 2.00E-147 |
| hsa_circ_0046757 | 93 | 1 | 762 | 980 | 219 | 0 | 0 |
| hsa_circ_0046760 | 94 | 1 | 565 | 565 | 1 | 0 | 0 |
| hsa_circ_0046760 | 91 | 97 | 886 | 790 | 1 | 0 | 0 |
| hsa_circ_0046764 | 83 | 7 | 2935 | 2957 | 23 | 0.01 | 0 |
| hsa_circ_0046769 | 83 | 1 | 2473 | 2671 | 202 | 0.01 | 0 |
| hsa_circ_0046769 | 83 | 1 | 2654 | 2852 | 202 | 0.01 | 0 |
| hsa_circ_0046772 | 86 | 1 | 875 | 1256 | 382 | 0.01 | 0 |
| hsa_circ_0046772 | 85 | 1 | 1193 | 1574 | 382 | 0.01 | 0 |
| hsa_circ_0046767 | 87 | 6 | 3940 | 5730 | 1796 | 0 | 0 |
| hsa_circ_0046772 | 85 | 1 | 747 | 1256 | 510 | 0.01 | 0 |
| hsa_circ_0046785 | 87 | 2 | 297 | 298 | 3 | 0 | 3.00E-91 |
| hsa_circ_0046785 | 88 | 3 | 425 | 425 | 3 | 0 | 5.00E-140 |
| hsa_circ_0046782 | 87 | 1 | 2354 | 2781 | 428 | 0 | 0 |
| hsa_circ_0046791 | 87 | 1 | 1929 | 1929 | 1 | 0 | 0 |
| hsa_circ_0046793 | 90 | 1 | 403 | 981 | 579 | 0 | 1.00E-145 |
| hsa_circ_0046794 | 89 | 1 | 513 | 513 | 1 | 0 | 0 |
| hsa_circ_0046796 | 90 | 6 | 284 | 279 | 1 | 0.01 | 3.00E-100 |
| hsa_circ_0046804 | 93 | 1 | 467 | 1 | 467 | 0 | 0 |
| hsa_circ_0046804 | 93 | 3 | 292 | 178 | 467 | 0 | 6.00E-118 |
| hsa_circ_0046805 | 94 | 1 | 724 | 1 | 724 | 0 | 0 |
| hsa_circ_0046805 | 94 | 1 | 1168 | 1 | 1168 | 0 | 0 |
| hsa_circ_0046807 | 96 | 1 | 415 | 1 | 415 | 0 | 0 |
| hsa_circ_0046807 | 94 | 1 | 859 | 1 | 859 | 0 | 0 |
| hsa_circ_0046819 | 94 | 1 | 200 | 1 | 200 | 0 | 2.00E-81 |
| hsa_circ_0046819 | 94 | 1 | 395 | 1 | 395 | 0 | 3.00E-171 |
| hsa_circ_0046819 | 93 | 1 | 676 | 1 | 663 | 0.02 | 0 |
| hsa_circ_0046819 | 91 | 1 | 1639 | 1 | 1629 | 0.02 | 0 |

novel_circ_027568 novel_circ_027569 novel_circ_027570 novel_circ_027571 novel_circ_027572 novel_circ_027573 novel_circ_027574 novel_circ_027575 novel_circ_027576 novel_circ_027577 novel_circ_027578 novel_circ_027579 novel_circ_027580 novel_circ_027581 novel_circ_027582 novel_circ_027583 novel_circ_027584 novel_circ_027585 novel_circ_027586 novel_circ_027587 novel_circ_027588 novel_circ_027589 novel_circ_027590 novel_circ_027591 novel_circ_027592 novel_circ_027593 novel_circ_027594 novel_circ_027595 novel_circ_027596 novel_circ_027597 novel_circ_027598 novel_circ_027599 novel_circ_027600 novel_circ_027601 novel_circ_027602 novel_circ_027603 novel_circ_027604 novel_circ_027605 novel_circ_027606 novel_circ_027607 novel_circ_027608 novel_circ_027609 novel_circ_027610 novel_circ_027611 novel_circ_027612 novel_circ_027613 novel_circ_027614 novel_circ_027615 novel_circ_027616 novel_circ_027617 novel_circ_027618 novel_circ_027619 novel_circ_027621 novel_circ_027622

| hsa_circ_0046827 | 94 | 1 | 384 | 1 | 384 | 0 | 2.00E-163 |
| --- | --- | --- | --- | --- | --- | --- | --- |
| hsa_circ_0046827 | 88 | 1 | 1903 | 1 | 1906 | 0 | 0 |
| hsa_circ_0046829 | 87 | 1 | 1519 | 1 | 1522 | 0.01 | 0 |
| hsa_circ_0046827 | 88 | 1 | 2241 | 385 | 2631 | 0.01 | 0 |
| hsa_circ_0046830 | 86 | 1 | 1311 | 1 | 1314 | 0.01 | 0 |
| hsa_circ_0046830 | 86 | 1 | 1459 | 1 | 1462 | 0.01 | 0 |
| hsa_circ_0046830 | 87 | 1 | 2005 | 1 | 2008 | 0.01 | 0 |
| hsa_circ_0046827 | 88 | 1 | 2181 | 445 | 2631 | 0.01 | 0 |
| hsa_circ_0046827 | 88 | 1 | 2436 | 445 | 2886 | 0.01 | 0 |
| hsa_circ_0046827 | 91 | 1 | 969 | 1915 | 2886 | 0.01 | 0 |
| hsa_circ_0046827 | 89 | 1 | 591 | 2038 | 2631 | 0.02 | 0 |
| hsa_circ_0046827 | 90 | 1 | 846 | 2038 | 2886 | 0.01 | 0 |
| hsa_circ_0046831 | 87 | 1 | 1878 | 424 | 2286 | 0.02 | 0 |
| hsa_circ_0046834 | 93 | 1 | 180 | 67 | 246 | 0 | 2.00E-70 |
| hsa_circ_0000826 | 95 | 1 | 706 | 1 | 703 | 0 | 0 |
| hsa_circ_0000826 | 95 | 1 | 997 | 1 | 994 | 0 | 0 |
| hsa_circ_0000826 | 95 | 1 | 997 | 1 | 994 | 0 | 0 |
| hsa_circ_0046844 | 95 | 1 | 565 | 1 | 565 | 0 | 0 |
| hsa_circ_0046844 | 95 | 1 | 856 | 1 | 856 | 0 | 0 |
| hsa_circ_0046845 | 96 | 1 | 417 | 1 | 417 | 0 | 0 |
| hsa_circ_0046845 | 96 | 1 | 560 | 1 | 560 | 0 | 0 |
| hsa_circ_0046845 | 96 | 1 | 708 | 1 | 708 | 0 | 0 |
| hsa_circ_0008972 | 88 | 1 | 1058 | 1 | 1050 | 0.03 | 0 |
| hsa_circ_0046847 | 95 | 1 | 348 | 1 | 348 | 0 | 4.00E-155 |
| hsa_circ_0046847 | 95 | 1 | 639 | 1 | 639 | 0 | 0 |
| hsa_circ_0005715 | 87 | 1 | 989 | 1 | 981 | 0.03 | 0 |
| hsa_circ_0046850 | 96 | 1 | 344 | 1 | 344 | 0 | 1.00E-159 |
| hsa_circ_0046850 | 95 | 1 | 492 | 1 | 492 | 0 | 0 |
| hsa_circ_0005715 | 85 | 1 | 842 | 148 | 981 | 0.04 | 0 |
| hsa_circ_0046850 | 95 | 1 | 291 | 202 | 492 | 0 | 2.00E-128 |
| hsa_circ_0046854 | 95 | 1 | 243 | 1 | 243 | 0 | 6.00E-107 |
| hsa_circ_0046857 | 95 | 1 | 402 | 1792 | 2193 | 0 | 0 |
| hsa_circ_0046865 | 92 | 1 | 1116 | 1116 | 1 | 0 | 0 |
| hsa_circ_0046869 | 91 | 1 | 348 | 348 | 1 | 0 | 4.00E-130 |
| hsa_circ_0046869 | 91 | 1 | 444 | 444 | 1 | 0 | 1.00E-166 |
| hsa_circ_0046869 | 92 | 1 | 617 | 617 | 1 | 0 | 0 |
| hsa_circ_0046869 | 90 | 7 | 1144 | 1138 | 1 | 0 | 0 |
| hsa_circ_0046869 | 90 | 1 | 1269 | 1272 | 1 | 0 | 0 |
| hsa_circ_0046869 | 92 | 1 | 2084 | 2087 | 1 | 0 | 0 |
| hsa_circ_0046869 | 92 | 1 | 2135 | 2138 | 1 | 0 | 0 |
| hsa_circ_0046873 | 95 | 1 | 937 | 1643 | 706 | 0 | 0 |
| hsa_circ_0046873 | 95 | 1 | 988 | 1694 | 706 | 0 | 0 |
| hsa_circ_0003080 | 96 | 1 | 333 | 333 | 1 | 0 | 8.00E-152 |
| hsa_circ_0003080 | 95 | 1 | 815 | 815 | 1 | 0 | 0 |
| hsa_circ_0003080 | 95 | 1 | 866 | 866 | 1 | 0 | 0 |
| hsa_circ_0046884 | 95 | 1 | 482 | 482 | 1 | 0 | 0 |
| hsa_circ_0046884 | 95 | 1 | 533 | 533 | 1 | 0 | 0 |
| hsa_circ_0046909 | 98 | 1 | 338 | 1 | 338 | 0 | 6.00E-168 |
| hsa_circ_0046909 | 96 | 1 | 473 | 1 | 473 | 0 | 0 |
| hsa_circ_0046909 | 96 | 1 | 647 | 1 | 647 | 0 | 0 |
| hsa_circ_0046909 | 95 | 1 | 494 | 154 | 647 | 0 | 0 |
| hsa_circ_0081329 | 82 | 47572 | 48982 | 1427 | 1 | 0.04 | 0 |
| hsa_circ_0081329 | 82 | 43418 | 44828 | 1427 | 1 | 0.04 | 0 |
| hsa_circ_0081329 | 82 | 43418 | 44828 | 1427 | 1 | 0.04 | 0 |

novel_circ_027623 novel_circ_027624 novel_circ_027625 novel_circ_027629 novel_circ_027630 novel_circ_027631 novel_circ_027632 novel_circ_027634 novel_circ_027635 novel_circ_027636 novel_circ_027637 novel_circ_027638 novel_circ_027642 novel_circ_027643 novel_circ_027644 novel_circ_027645 novel_circ_027646 novel_circ_027647 novel_circ_027648 novel_circ_027649 novel_circ_027650 novel_circ_027651 novel_circ_027652 novel_circ_027653 novel_circ_027654 novel_circ_027655 novel_circ_027656 novel_circ_027657 novel_circ_027658 novel_circ_027659 novel_circ_027662 novel_circ_027663 novel_circ_027664 novel_circ_027665 novel_circ_027666 novel_circ_027667 novel_circ_027668 novel_circ_027669 novel_circ_027670 novel_circ_027671 novel_circ_027672 novel_circ_027673 novel_circ_027674 novel_circ_027675 novel_circ_027676 novel_circ_027677 novel_circ_027678 novel_circ_027679 novel_circ_027680 novel_circ_027681 novel_circ_027682 novel_circ_027683 novel_circ_027684 novel_circ_027685

| hsa_circ_0081329 | 82 | 1846 | 3256 | 1427 | 1 | 0.04 | 0 |
| --- | --- | --- | --- | --- | --- | --- | --- |
| hsa_circ_0081329 | 82 | 1846 | 3256 | 1427 | 1 | 0.04 | 0 |
| hsa_circ_0081329 | 82 | 1846 | 3256 | 1427 | 1 | 0.04 | 0 |
| hsa_circ_0002595 | 74 | 14413 | 14819 | 314710 | 314308 | 0.03 | 3.00E-38 |
| hsa_circ_0002595 | 74 | 14413 | 14819 | 314710 | 314308 | 0.03 | 3.00E-38 |
| hsa_circ_0002595 | 74 | 14413 | 14819 | 314710 | 314308 | 0.03 | 3.00E-38 |
| hsa_circ_0046910 | 91 | 1 | 716 | 1 | 716 | 0 | 0 |
| hsa_circ_0046922 | 91 | 3 | 2245 | 2246 | 1 | 0 | 0 |
| hsa_circ_0046928 | 90 | 3 | 1085 | 1354 | 269 | 0 | 0 |
| hsa_circ_0046932 | 91 | 1 | 1584 | 1954 | 380 | 0.01 | 0 |
| hsa_circ_0046934 | 90 | 1 | 461 | 610 | 150 | 0 | 8.00E-173 |
| hsa_circ_0046939 | 92 | 1 | 425 | 425 | 1 | 0 | 4.00E-166 |
| hsa_circ_0046956 | 90 | 1 | 662 | 662 | 1 | 0 | 0 |
| hsa_circ_0046966 | 93 | 111 | 504 | 397 | 4 | 0 | 3.00E-162 |
| hsa_circ_0046966 | 89 | 1 | 561 | 1050 | 502 | 0.02 | 0 |
| hsa_circ_0046966 | 89 | 1 | 562 | 1050 | 501 | 0.02 | 0 |
| hsa_circ_0006400 | 88 | 1 | 350 | 338 | 1 | 0.03 | 3.00E-111 |
| hsa_circ_0006400 | 88 | 1 | 450 | 438 | 1 | 0.03 | 5.00E-150 |
| hsa_circ_0046971 | 87 | 1 | 407 | 407 | 1 | 0.01 | 4.00E-126 |
| hsa_circ_0046971 | 91 | 1 | 1035 | 1035 | 1 | 0 | 0 |
| hsa_circ_0046971 | 91 | 235 | 1269 | 1035 | 1 | 0 | 0 |
| hsa_circ_0046971 | 84 | 1 | 262 | 407 | 146 | 0.02 | 9.00E-66 |
| hsa_circ_0046971 | 88 | 1 | 434 | 579 | 146 | 0.01 | 8.00E-143 |
| hsa_circ_0046971 | 91 | 235 | 1035 | 1035 | 235 | 0 | 0 |
| hsa_circ_0046971 | 93 | 1 | 584 | 1035 | 452 | 0 | 0 |
| hsa_circ_0046971 | 93 | 235 | 818 | 1035 | 452 | 0 | 0 |
| hsa_circ_0046972 | 93 | 235 | 690 | 456 | 1 | 0 | 0 |
| hsa_circ_0000829 | 93 | 235 | 603 | 369 | 1 | 0 | 3.00E-153 |
| hsa_circ_0000829 | 94 | 235 | 360 | 369 | 242 | 0.02 | 3.00E-47 |
| hsa_circ_0000829 | 94 | 270 | 395 | 369 | 242 | 0.02 | 3.00E-47 |
| hsa_circ_0046974 | 94 | 168 | 501 | 334 | 1 | 0 | 2.00E-140 |
| hsa_circ_0046976 | 95 | 195 | 909 | 715 | 1 | 0 | 0 |
| hsa_circ_0046975 | 95 | 1 | 167 | 167 | 1 | 0 | 3.00E-68 |
| hsa_circ_0046976 | 94 | 1 | 413 | 413 | 1 | 0 | 0 |
| hsa_circ_0046976 | 94 | 1 | 284 | 413 | 130 | 0 | 2.00E-121 |
| hsa_circ_0008710 | 95 | 336 | 698 | 363 | 1 | 0 | 2.00E-161 |
| hsa_circ_0046981 | 93 | 1 | 335 | 335 | 1 | 0 | 1.00E-139 |
| hsa_circ_0046981 | 95 | 1 | 426 | 426 | 1 | 0 | 0 |
| hsa_circ_0046981 | 95 | 1 | 291 | 426 | 136 | 0 | 3.00E-125 |
| hsa_circ_0046985 | 94 | 1630 | 2588 | 1 | 959 | 0 | 0 |
| hsa_circ_0046985 | 94 | 201 | 1008 | 1 | 808 | 0 | 0 |
| hsa_circ_0046985 | 94 | 1 | 650 | 1 | 650 | 0 | 0 |
| hsa_circ_0046985 | 94 | 1 | 808 | 1 | 808 | 0 | 0 |
| hsa_circ_0046985 | 94 | 1 | 959 | 1 | 959 | 0 | 0 |
| hsa_circ_0046991 | 94 | 1 | 311 | 1 | 311 | 0 | 3.00E-131 |
| hsa_circ_0046991 | 94 | 1 | 610 | 1 | 610 | 0 | 0 |
| hsa_circ_0047007 | 86 | 3 | 872 | 3 | 872 | 0 | 0 |
| hsa_circ_0047007 | 82 | 3 | 1568 | 3 | 1538 | 0.03 | 0 |
| hsa_circ_0047011 | 83 | 1 | 726 | 1 | 720 | 0.02 | 0 |
| hsa_circ_0047011 | 80 | 1 | 1262 | 13 | 1244 | 0.03 | 0 |
| hsa_circ_0047011 | 82 | 3 | 2611 | 179 | 2727 | 0.03 | 0 |
| hsa_circ_0047019 | 91 | 1 | 534 | 1 | 534 | 0 | 0 |
| hsa_circ_0047019 | 89 | 1 | 259 | 276 | 534 | 0 | 2.00E-87 |
| hsa_circ_0047022 | 81 | 1 | 953 | 1 | 950 | 0.02 | 0 |

novel_circ_027687 novel_circ_027688 novel_circ_027689 novel_circ_027690 novel_circ_027691 novel_circ_027692 novel_circ_027693 novel_circ_027694 novel_circ_027695 novel_circ_027698 novel_circ_027699 novel_circ_027700 novel_circ_027701 novel_circ_027703 novel_circ_027704 novel_circ_027706 novel_circ_027707 novel_circ_027708 novel_circ_027709 novel_circ_027710 novel_circ_027711 novel_circ_027712 novel_circ_027713 novel_circ_027714 novel_circ_027716 novel_circ_027717 novel_circ_027718 novel_circ_027743 novel_circ_027744 novel_circ_027749 novel_circ_027750 novel_circ_027751 novel_circ_027753 novel_circ_027754 novel_circ_027755 novel_circ_027756 novel_circ_027757 novel_circ_027758 novel_circ_027760 novel_circ_027761 novel_circ_027762 novel_circ_027763 novel_circ_027764 novel_circ_027765 novel_circ_027766 novel_circ_027767 novel_circ_027768 novel_circ_027769 novel_circ_027770 novel_circ_027771 novel_circ_027774 novel_circ_027775 novel_circ_027776 novel_circ_027778

| hsa_circ_0047029 | 87 | 1 | 797 | 335 | 1128 | 0.01 | 0 |
| --- | --- | --- | --- | --- | --- | --- | --- |
| hsa_circ_0047030 | 92 | 1 | 642 | 387 | 1028 | 0 | 0 |
| hsa_circ_0047030 | 91 | 1 | 906 | 387 | 1292 | 0 | 0 |
| hsa_circ_0047034 | 89 | 2 | 377 | 263 | 638 | 0 | 3.00E-127 |
| hsa_circ_0047035 | 87 | 1 | 943 | 111 | 1053 | 0 | 0 |
| hsa_circ_0047035 | 88 | 1 | 1064 | 111 | 1174 | 0 | 0 |
| hsa_circ_0047035 | 87 | 1 | 731 | 565 | 1295 | 0 | 0 |
| hsa_circ_0047035 | 88 | 1 | 383 | 671 | 1053 | 0.01 | 2.00E-124 |
| hsa_circ_0047035 | 87 | 1 | 625 | 671 | 1295 | 0 | 0 |
| hsa_circ_0000833 | 75 | 4455 | 6018 | 6012 | 7522 | 0.06 | 0 |
| hsa_circ_0047040 | 85 | 268 | 465 | 229 | 422 | 0.03 | 8.00E-49 |
| hsa_circ_0047040 | 85 | 268 | 468 | 229 | 426 | 0.03 | 7.00E-48 |
| hsa_circ_0047040 | 88 | 268 | 606 | 229 | 563 | 0.02 | 9.00E-109 |
| hsa_circ_0003979 | 81 | 1 | 475 | 501 | 6 | 0.05 | 1.00E-102 |
| hsa_circ_0009205 | 83 | 1 | 307 | 519 | 213 | 0.01 | 2.00E-72 |
| hsa_circ_0009307 | 86 | 167 | 1148 | 991 | 1 | 0.01 | 0 |
| hsa_circ_0009307 | 86 | 23627 | 24608 | 991 | 1 | 0.01 | 0 |
| hsa_circ_0000006 | 86 | 167 | 875 | 845 | 128 | 0.01 | 0 |
| hsa_circ_0009319 | 85 | 167 | 626 | 469 | 1 | 0.02 | 1.00E-127 |
| hsa_circ_0009319 | 85 | 22674 | 23133 | 469 | 1 | 0.02 | 5.00E-126 |
| hsa_circ_0009351 | 93 | 19 | 538 | 520 | 1 | 0 | 0 |
| hsa_circ_0009354 | 93 | 4 | 373 | 370 | 1 | 0 | 5.00E-154 |
| hsa_circ_0009354 | 92 | 19 | 471 | 453 | 1 | 0 | 0 |
| hsa_circ_0008702 | 91 | 19 | 244 | 226 | 1 | 0 | 2.00E-82 |
| hsa_circ_0071349 | 82 | 1 | 662 | 1 | 662 | 0 | 1.00E-162 |
| hsa_circ_0071348 | 83 | 256 | 1007 | 703 | 1454 | 0 | 0 |
| hsa_circ_0071348 | 83 | 862 | 1613 | 703 | 1454 | 0 | 0 |
| hsa_circ_0047394 | 74 | 2031 | 2594 | 1550 | 987 | 0.01 | 2.00E-57 |
| hsa_circ_0047394 | 74 | 810 | 1373 | 987 | 1550 | 0.01 | 9.00E-58 |
| hsa_circ_0012274 | 93 | 1 | 432 | 1017 | 586 | 0 | 5.00E-180 |
| hsa_circ_0012274 | 91 | 9 | 771 | 1351 | 586 | 0 | 0 |
| hsa_circ_0012278 | 90 | 1 | 287 | 1 | 287 | 0 | 6.00E-103 |
| hsa_circ_0012285 | 91 | 1 | 709 | 42048 | 42735 | 0.03 | 0 |
| hsa_circ_0012300 | 95 | 1 | 835 | 835 | 1 | 0 | 0 |
| hsa_circ_0012300 | 95 | 1 | 835 | 835 | 1 | 0 | 0 |
| hsa_circ_0012301 | 95 | 1 | 515 | 658 | 144 | 0 | 0 |
| hsa_circ_0003161 | 94 | 1 | 208 | 208 | 1 | 0 | 7.00E-86 |
| hsa_circ_0012336 | 88 | 1 | 499 | 2966 | 3464 | 0 | 2.00E-170 |
| hsa_circ_0012349 | 95 | 354 | 814 | 584 | 124 | 0 | 0 |
| hsa_circ_0012358 | 95 | 1 | 99 | 99 | 1 | 0 | 2.00E-37 |
| hsa_circ_0012360 | 90 | 1 | 733 | 733 | 1 | 0 | 0 |
| hsa_circ_0089369 | 80 | 1 | 750 | 1932 | 1162 | 0.06 | 2.00E-150 |
| hsa_circ_0089307 | 85 | 2 | 506 | 1283 | 779 | 0.01 | 7.00E-144 |
| hsa_circ_0089307 | 85 | 2 | 506 | 1283 | 779 | 0.01 | 1.00E-143 |
| hsa_circ_0089307 | 85 | 46 | 491 | 1224 | 779 | 0.01 | 4.00E-126 |
| hsa_circ_0089307 | 85 | 3 | 443 | 1219 | 779 | 0.01 | 5.00E-125 |
| hsa_circ_0089307 | 85 | 3 | 443 | 1219 | 779 | 0.01 | 8.00E-125 |
| hsa_circ_0089307 | 91 | 503 | 875 | 777 | 405 | 0 | 6.00E-142 |
| hsa_circ_0089306 | 90 | 1 | 520 | 1260 | 735 | 0.01 | 0 |
| hsa_circ_0089306 | 87 | 1 | 664 | 664 | 1 | 0 | 0 |
| hsa_circ_0089302 | 89 | 1 | 2035 | 1 | 2047 | 0.01 | 0 |
| hsa_circ_0089302 | 88 | 1 | 1815 | 1 | 1827 | 0.01 | 0 |
| hsa_circ_0089291 | 87 | 1 | 888 | 891 | 1 | 0.02 | 0 |
| hsa_circ_0089283 | 83 | 1 | 851 | 1097 | 250 | 0.01 | 0 |

novel_circ_027779 novel_circ_027780 novel_circ_027781 novel_circ_027782 novel_circ_027783 novel_circ_027784 novel_circ_027785 novel_circ_027786 novel_circ_027787 novel_circ_027788 novel_circ_027789 novel_circ_027790 novel_circ_027791 novel_circ_027792 novel_circ_027793 novel_circ_027794 novel_circ_027795 novel_circ_027796 novel_circ_027797 novel_circ_027798 novel_circ_027799 novel_circ_027800 novel_circ_027801 novel_circ_027802 novel_circ_027803 novel_circ_027804 novel_circ_027805 novel_circ_027806 novel_circ_027807 novel_circ_027812 novel_circ_027813 novel_circ_027814 novel_circ_027818 novel_circ_027819 novel_circ_027820 novel_circ_027821 novel_circ_027822 novel_circ_027823 novel_circ_027824 novel_circ_027825 novel_circ_027826 novel_circ_027827 novel_circ_027828 novel_circ_027829 novel_circ_027830 novel_circ_027831 novel_circ_027832 novel_circ_027833 novel_circ_027834 novel_circ_027835 novel_circ_027836 novel_circ_027837 novel_circ_027839 novel_circ_027840

| hsa_circ_0089283 | 83 | 1 | 948 | 1097 | 153 | 0.01 | 0 |
| --- | --- | --- | --- | --- | --- | --- | --- |
| hsa_circ_0089283 | 84 | 1 | 1014 | 1097 | 87 | 0 | 0 |
| hsa_circ_0089283 | 84 | 1 | 1100 | 1097 | 1 | 0 | 0 |
| hsa_circ_0089283 | 84 | 1 | 1100 | 1097 | 1 | 0 | 0 |
| hsa_circ_0089283 | 88 | 8 | 687 | 680 | 1 | 0 | 0 |
| hsa_circ_0089283 | 86 | 1 | 325 | 477 | 153 | 0 | 2.00E-97 |
| hsa_circ_0001898 | 89 | 1 | 393 | 395 | 3 | 0.01 | 4.00E-135 |
| hsa_circ_0089269 | 90 | 1 | 725 | 10840 | 10116 | 0.01 | 0 |
| hsa_circ_0089269 | 91 | 1 | 541 | 10656 | 10116 | 0 | 0 |
| hsa_circ_0089275 | 92 | 6 | 710 | 6542 | 5838 | 0 | 0 |
| hsa_circ_0089280 | 91 | 1 | 830 | 1380 | 551 | 0 | 0 |
| hsa_circ_0089280 | 91 | 1 | 1380 | 1380 | 1 | 0 | 0 |
| hsa_circ_0089275 | 90 | 1 | 1568 | 1661 | 94 | 0 | 0 |
| hsa_circ_0089269 | 90 | 1 | 2013 | 5559 | 3547 | 0 | 0 |
| hsa_circ_0089280 | 91 | 1 | 1022 | 1280 | 259 | 0 | 0 |
| hsa_circ_0089269 | 91 | 1 | 1913 | 5459 | 3547 | 0 | 0 |
| hsa_circ_0089280 | 92 | 1 | 848 | 1106 | 259 | 0 | 0 |
| hsa_circ_0089280 | 92 | 1 | 1106 | 1106 | 1 | 0 | 0 |
| hsa_circ_0089275 | 91 | 1 | 1294 | 1387 | 94 | 0 | 0 |
| hsa_circ_0089274 | 91 | 1 | 1552 | 1552 | 1 | 0 | 0 |
| hsa_circ_0089269 | 91 | 1 | 1739 | 5285 | 3547 | 0 | 0 |
| hsa_circ_0089275 | 90 | 1 | 736 | 829 | 94 | 0 | 0 |
| hsa_circ_0089269 | 90 | 1 | 1181 | 4727 | 3547 | 0 | 0 |
| hsa_circ_0089275 | 89 | 1 | 446 | 539 | 94 | 0 | 2.00E-159 |
| hsa_circ_0089271 | 90 | 1 | 891 | 1103 | 213 | 0 | 0 |
| hsa_circ_0089273 | 88 | 1 | 545 | 545 | 1 | 0 | 0 |
| hsa_circ_0089271 | 89 | 1 | 633 | 845 | 213 | 0 | 0 |
| hsa_circ_0089273 | 90 | 4 | 357 | 354 | 1 | 0 | 4.00E-130 |
| hsa_circ_0089271 | 91 | 4 | 445 | 654 | 213 | 0 | 3.00E-172 |
| hsa_circ_0089255 | 94 | 1 | 254 | 254 | 1 | 0 | 5.00E-108 |
| hsa_circ_0089253 | 93 | 1 | 590 | 590 | 1 | 0 | 0 |
| hsa_circ_0089253 | 92 | 4 | 336 | 333 | 1 | 0 | 8.00E-132 |
| hsa_circ_0089218 | 84 | 1 | 252 | 1 | 252 | 0 | 2.00E-63 |
| hsa_circ_0089198 | 95 | 1 | 1266 | 1 | 1266 | 0 | 0 |
| hsa_circ_0089209 | 94 | 1 | 651 | 74 | 724 | 0 | 0 |
| hsa_circ_0089203 | 95 | 1 | 827 | 1 | 827 | 0 | 0 |
| hsa_circ_0089198 | 95 | 1 | 1175 | 1 | 1175 | 0 | 0 |
| hsa_circ_0089198 | 96 | 1 | 520 | 5 | 524 | 0 | 0 |
| hsa_circ_0089198 | 96 | 1 | 524 | 1 | 524 | 0 | 0 |
| hsa_circ_0089173 | 87 | 1 | 515 | 1 | 515 | 0 | 1.00E-162 |
| hsa_circ_0089168 | 90 | 6 | 353 | 6 | 353 | 0 | 2.00E-123 |
| hsa_circ_0089164 | 89 | 1 | 446 | 1 | 446 | 0.01 | 2.00E-154 |
| hsa_circ_0089163 | 89 | 1 | 237 | 1 | 237 | 0 | 3.00E-80 |
| hsa_circ_0089158 | 86 | 1 | 980 | 1 | 983 | 0 | 0 |
| hsa_circ_0089158 | 86 | 1 | 472 | 1 | 475 | 0.01 | 4.00E-141 |
| hsa_circ_0089146 | 91 | 2 | 901 | 2 | 901 | 0 | 0 |
| hsa_circ_0089146 | 91 | 2 | 612 | 2 | 612 | 0 | 0 |
| hsa_circ_0089146 | 92 | 2 | 438 | 2 | 438 | 0 | 8.00E-178 |
| hsa_circ_0089146 | 92 | 2 | 334 | 2 | 334 | 0 | 8.00E-132 |
| hsa_circ_0089146 | 91 | 2 | 270 | 2 | 270 | 0 | 2.00E-101 |
| hsa_circ_0089143 | 91 | 1 | 422 | 1 | 422 | 0 | 4.00E-161 |
| hsa_circ_0089142 | 90 | 1 | 547 | 1 | 547 | 0 | 0 |
| hsa_circ_0089134 | 92 | 1 | 601 | 274 | 874 | 0 | 0 |
| hsa_circ_0089134 | 93 | 1 | 721 | 1 | 721 | 0 | 0 |

novel_circ_027841 novel_circ_027842 novel_circ_027843 novel_circ_027845 novel_circ_027846 novel_circ_027847 novel_circ_027848 novel_circ_027849 novel_circ_027850 novel_circ_027851 novel_circ_027852 novel_circ_027853 novel_circ_027854 novel_circ_027855 novel_circ_027856 novel_circ_027857 novel_circ_027858 novel_circ_027860 novel_circ_027861 novel_circ_027862 novel_circ_027863 novel_circ_027864 novel_circ_027865 novel_circ_027867 novel_circ_027868 novel_circ_027869 novel_circ_027870 novel_circ_027871 novel_circ_027872 novel_circ_027873 novel_circ_027874 novel_circ_027881 novel_circ_027882 novel_circ_027883 novel_circ_027884 novel_circ_027885 novel_circ_027887 novel_circ_027888 novel_circ_027889 novel_circ_027890 novel_circ_027892 novel_circ_027893 novel_circ_027894 novel_circ_027895 novel_circ_027896 novel_circ_027899 novel_circ_027903 novel_circ_027904 novel_circ_027905 novel_circ_027906 novel_circ_027907 novel_circ_027908 novel_circ_027909 novel_circ_027910

| hsa_circ_0089130 | 93 | 698 | 996 | 172 | 470 | 0 | 1.00E-119 |
| --- | --- | --- | --- | --- | --- | --- | --- |
| hsa_circ_0089130 | 93 | 1 | 743 | 1 | 743 | 0 | 0 |
| hsa_circ_0069749 | 91 | 394 | 600 | 40285 | 40079 | 0 | 1.00E-74 |
| hsa_circ_0069749 | 92 | 1 | 603 | 1 | 603 | 0 | 0 |
| hsa_circ_0069741 | 93 | 1 | 711 | 588 | 1298 | 0 | 0 |
| hsa_circ_0069741 | 93 | 1 | 821 | 478 | 1298 | 0 | 0 |
| hsa_circ_0069747 | 94 | 1 | 607 | 1 | 607 | 0 | 0 |
| hsa_circ_0069749 | 94 | 1 | 246 | 1 | 246 | 0 | 6.00E-102 |
| hsa_circ_0001412 | 94 | 1 | 354 | 1 | 354 | 0 | 4.00E-150 |
| hsa_circ_0069747 | 94 | 1 | 464 | 1 | 464 | 0 | 0 |
| hsa_circ_0069742 | 94 | 1 | 685 | 1 | 685 | 0 | 0 |
| hsa_circ_0069747 | 94 | 1 | 218 | 1 | 218 | 0 | 4.00E-88 |
| hsa_circ_0069741 | 95 | 1 | 695 | 1 | 695 | 0 | 0 |
| hsa_circ_0069745 | 96 | 1 | 310 | 1 | 310 | 0 | 1.00E-140 |
| hsa_circ_0069743 | 95 | 3 | 418 | 3 | 418 | 0 | 0 |
| hsa_circ_0069742 | 95 | 1 | 483 | 1 | 483 | 0 | 0 |
| hsa_circ_0069741 | 95 | 1 | 587 | 1 | 587 | 0 | 0 |
| hsa_circ_0069733 | 85 | 7 | 248 | 244 | 3 | 0 | 5.00E-63 |
| hsa_circ_0069725 | 92 | 4173 | 4616 | 444 | 1 | 0 | 1.00E-175 |
| hsa_circ_0069725 | 92 | 1 | 444 | 444 | 1 | 0 | 1.00E-176 |
| hsa_circ_0069724 | 92 | 231 | 589 | 359 | 1 | 0 | 1.00E-142 |
| hsa_circ_0069724 | 92 | 1 | 359 | 359 | 1 | 0 | 1.00E-142 |
| hsa_circ_0069724 | 92 | 1 | 359 | 359 | 1 | 0 | 1.00E-142 |
| hsa_circ_0036666 | 93 | 1 | 1001 | 1001 | 1 | 0 | 0 |
| hsa_circ_0036666 | 93 | 4 | 932 | 929 | 1 | 0 | 0 |
| hsa_circ_0036666 | 93 | 1 | 774 | 774 | 1 | 0 | 0 |
| hsa_circ_0036666 | 93 | 4 | 631 | 628 | 1 | 0 | 0 |
| hsa_circ_0036661 | 95 | 2 | 189 | 187 | 1 | 0.01 | 1.00E-77 |
| hsa_circ_0036657 | 92 | 1 | 548 | 548 | 1 | 0 | 0 |
| hsa_circ_0036657 | 92 | 1 | 548 | 548 | 1 | 0 | 0 |
| hsa_circ_0036657 | 92 | 1 | 417 | 417 | 1 | 0 | 2.00E-163 |
| hsa_circ_0020746 | 91 | 1 | 931 | 456 | 1392 | 0.01 | 0 |
| hsa_circ_0020748 | 92 | 1 | 406 | 282 | 687 | 0 | 1.00E-160 |
| hsa_circ_0020748 | 91 | 1 | 687 | 1 | 687 | 0 | 0 |
| hsa_circ_0020748 | 90 | 1 | 281 | 1 | 281 | 0 | 3.00E-101 |
| hsa_circ_0020743 | 89 | 1 | 492 | 1 | 492 | 0 | 5.00E-175 |
| hsa_circ_0003395 | 86 | 3 | 319 | 72 | 388 | 0.01 | 5.00E-91 |
| hsa_circ_0020672 | 89 | 6 | 266 | 744 | 1004 | 0.01 | 2.00E-88 |
| hsa_circ_0020672 | 86 | 1 | 364 | 641 | 1004 | 0.01 | 6.00E-109 |
| hsa_circ_0020669 | 83 | 1 | 1008 | 111 | 1115 | 0.02 | 0 |
| hsa_circ_0020649 | 98 | 1 | 151 | 103 | 253 | 0 | 2.00E-69 |
| hsa_circ_0020649 | 94 | 1 | 253 | 1 | 253 | 0 | 2.00E-107 |
| hsa_circ_0028415 | 84 | 6228 | 6766 | 17 | 553 | 0.02 | 1.00E-142 |
| hsa_circ_0020611 | 86 | 10 | 425 | 517 | 102 | 0 | 4.00E-121 |
| hsa_circ_0020611 | 86 | 10 | 523 | 517 | 3 | 0 | 8.00E-159 |
| hsa_circ_0070475 | 89 | 2 | 418 | 2 | 418 | 0 | 2.00E-148 |
| hsa_circ_0070462 | 92 | 1 | 1075 | 373 | 1453 | 0.01 | 0 |
| hsa_circ_0070462 | 92 | 92 | 1538 | 1 | 1453 | 0 | 0 |
| hsa_circ_0070461 | 92 | 1 | 1628 | 577 | 2210 | 0 | 0 |
| hsa_circ_0070463 | 92 | 1 | 663 | 137 | 805 | 0.01 | 0 |
| hsa_circ_0070463 | 92 | 1 | 799 | 1 | 805 | 0.01 | 0 |
| hsa_circ_0070462 | 92 | 1 | 935 | 101 | 1041 | 0.01 | 0 |
| hsa_circ_0070461 | 92 | 3 | 1126 | 779 | 1908 | 0.01 | 0 |
| hsa_circ_0070461 | 92 | 1 | 1326 | 577 | 1908 | 0 | 0 |

novel_circ_027911 novel_circ_027912 novel_circ_027913 novel_circ_027914 novel_circ_027915 novel_circ_027916 novel_circ_027917 novel_circ_027918 novel_circ_027919 novel_circ_027920 novel_circ_027921 novel_circ_027923 novel_circ_027924 novel_circ_027925 novel_circ_027926 novel_circ_027927 novel_circ_027928 novel_circ_027929 novel_circ_027931 novel_circ_027932 novel_circ_027933 novel_circ_027934 novel_circ_027935 novel_circ_027936 novel_circ_027937 novel_circ_027938 novel_circ_027939 novel_circ_027940 novel_circ_027941 novel_circ_027942 novel_circ_027943 novel_circ_027944 novel_circ_027945 novel_circ_027946 novel_circ_027947 novel_circ_027948 novel_circ_027949 novel_circ_027951 novel_circ_027952 novel_circ_027953 novel_circ_027954 novel_circ_027955 novel_circ_027956 novel_circ_027957 novel_circ_027958 novel_circ_027959 novel_circ_027960 novel_circ_027961 novel_circ_027962 novel_circ_027963 novel_circ_027964 novel_circ_027965 novel_circ_027968 novel_circ_027969

| hsa_circ_0070461 | 93 | 1 | 291 | 577 | 867 | 0 | 3.00E-120 |
| --- | --- | --- | --- | --- | --- | --- | --- |
| hsa_circ_0070456 | 92 | 1 | 913 | 418 | 1330 | 0 | 0 |
| hsa_circ_0070456 | 92 | 170 | 1076 | 424 | 1330 | 0 | 0 |
| hsa_circ_0070451 | 93 | 3 | 335 | 3 | 335 | 0 | 2.00E-138 |
| hsa_circ_0070447 | 95 | 1 | 648 | 1302 | 1949 | 0 | 0 |
| hsa_circ_0070447 | 95 | 1 | 452 | 1302 | 1753 | 0 | 0 |
| hsa_circ_0070447 | 96 | 1 | 313 | 1302 | 1614 | 0 | 4.00E-144 |
| hsa_circ_0070447 | 95 | 1 | 613 | 1002 | 1614 | 0 | 0 |
| hsa_circ_0070447 | 94 | 1 | 198 | 1104 | 1301 | 0 | 5.00E-82 |
| hsa_circ_0070447 | 94 | 1 | 300 | 1002 | 1301 | 0 | 7.00E-127 |
| hsa_circ_0018216 | 88 | 343 | 856 | 3610 | 3097 | 0 | 3.00E-170 |
| hsa_circ_0018233 | 93 | 11 | 812 | 337 | 1137 | 0 | 0 |
| hsa_circ_0018233 | 93 | 1 | 603 | 535 | 1137 | 0 | 0 |
| hsa_circ_0018235 | 94 | 1 | 332 | 81 | 412 | 0 | 6.00E-143 |
| hsa_circ_0018235 | 93 | 1 | 643 | 81 | 723 | 0 | 0 |
| hsa_circ_0018240 | 83 | 1 | 1167 | 189 | 1346 | 0.01 | 0 |
| hsa_circ_0018240 | 84 | 1 | 1249 | 189 | 1428 | 0.01 | 0 |
| hsa_circ_0019254 | 88 | 1 | 435 | 437 | 3 | 0 | 2.00E-143 |
| hsa_circ_0019255 | 92 | 1 | 309 | 309 | 1 | 0 | 2.00E-121 |
| hsa_circ_0019255 | 92 | 560 | 869 | 310 | 1 | 0 | 1.00E-121 |
| hsa_circ_0019255 | 88 | 3 | 413 | 1012 | 602 | 0 | 5.00E-136 |
| hsa_circ_0019255 | 86 | 1 | 898 | 1496 | 602 | 0.01 | 0 |
| hsa_circ_0019255 | 93 | 1 | 259 | 259 | 1 | 0 | 7.00E-104 |
| hsa_circ_0019255 | 93 | 1340 | 1602 | 262 | 1 | 0 | 3.00E-103 |
| hsa_circ_0019255 | 88 | 3 | 413 | 1012 | 602 | 0 | 4.00E-136 |
| hsa_circ_0019255 | 93 | 1 | 259 | 259 | 1 | 0 | 4.00E-104 |
| hsa_circ_0019255 | 88 | 3 | 413 | 1012 | 602 | 0 | 4.00E-136 |
| hsa_circ_0019255 | 88 | 3 | 413 | 1012 | 602 | 0 | 3.00E-136 |
| hsa_circ_0019255 | 88 | 3 | 413 | 1012 | 602 | 0 | 3.00E-136 |
| hsa_circ_0019255 | 88 | 3 | 413 | 1012 | 602 | 0 | 3.00E-136 |
| hsa_circ_0019255 | 88 | 3 | 413 | 1012 | 602 | 0 | 2.00E-136 |
| hsa_circ_0019255 | 86 | 1 | 898 | 1496 | 602 | 0.01 | 0 |
| hsa_circ_0019255 | 88 | 3 | 412 | 1012 | 603 | 0 | 4.00E-136 |
| hsa_circ_0019255 | 86 | 1 | 897 | 1496 | 603 | 0.01 | 0 |
| hsa_circ_0006024 | 92 | 121 | 322 | 202 | 1 | 0 | 1.00E-75 |
| hsa_circ_0006024 | 92 | 1 | 202 | 202 | 1 | 0 | 6.00E-76 |
| hsa_circ_0014355 | 89 | 13274 | 13584 | 1 | 304 | 0.02 | 4.00E-100 |
| hsa_circ_0008576 | 96 | 2225 | 2274 | 931056 | 931105 | 0 | 2.00E-12 |
| hsa_circ_0008576 | 96 | 2225 | 2274 | 931056 | 931105 | 0 | 2.00E-12 |
| hsa_circ_0002575 | 93 | 1 | 924 | 1 | 927 | 0 | 0 |
| hsa_circ_0011088 | 81 | 1 | 230 | 311 | 555 | 0.06 | 1.00E-43 |
| hsa_circ_0011088 | 84 | 1 | 468 | 311 | 793 | 0.03 | 4.00E-127 |
| hsa_circ_0011090 | 82 | 3 | 614 | 3 | 614 | 0 | 9.00E-145 |
| hsa_circ_0011093 | 81 | 1 | 544 | 1 | 544 | 0 | 5.00E-122 |
| hsa_circ_0011108 | 92 | 4 | 384 | 405 | 25 | 0 | 2.00E-148 |
| hsa_circ_0011111 | 88 | 1 | 648 | 4851 | 4204 | 0 | 0 |
| hsa_circ_0011111 | 86 | 1 | 396 | 4702 | 4307 | 0 | 5.00E-120 |
| hsa_circ_0011111 | 87 | 1 | 545 | 4851 | 4307 | 0 | 2.00E-174 |
| hsa_circ_0011114 | 87 | 1 | 314 | 373 | 60 | 0 | 6.00E-98 |
| hsa_circ_0011111 | 85 | 1 | 722 | 5483 | 4762 | 0 | 0 |
| hsa_circ_0011114 | 84 | 6 | 408 | 551 | 150 | 0 | 2.00E-103 |
| hsa_circ_0011116 | 79 | 6 | 262 | 405 | 149 | 0 | 3.00E-41 |
| hsa_circ_0011149 | 88 | 82 | 450 | 175 | 543 | 0 | 5.00E-120 |
| hsa_circ_0011154 | 96 | 1 | 100 | 1 | 100 | 0 | 2.00E-39 |

novel_circ_027970 novel_circ_027973 novel_circ_027974 novel_circ_027979 novel_circ_027980 novel_circ_027981 novel_circ_027984 novel_circ_027985 novel_circ_027986 novel_circ_027987 novel_circ_027989 novel_circ_027990 novel_circ_027991 novel_circ_027992 novel_circ_027993 novel_circ_027994 novel_circ_027995 novel_circ_027996 novel_circ_027997 novel_circ_027998 novel_circ_027999 novel_circ_028000 novel_circ_028001 novel_circ_028002 novel_circ_028003 novel_circ_028005 novel_circ_028006 novel_circ_028007 novel_circ_028008 novel_circ_028009 novel_circ_028011 novel_circ_028012 novel_circ_028013 novel_circ_028018 novel_circ_028019 novel_circ_028020 novel_circ_028021 novel_circ_028022 novel_circ_028023 novel_circ_028024 novel_circ_028025 novel_circ_028026 novel_circ_028027 novel_circ_028028 novel_circ_028030 novel_circ_028031 novel_circ_028032 novel_circ_028033 novel_circ_028034 novel_circ_028035 novel_circ_028036 novel_circ_028037 novel_circ_028038 novel_circ_028039

| hsa_circ_0011154 | 90 | 1 | 252 | 302 | 553 | 0 | 1.00E-88 |
| --- | --- | --- | --- | --- | --- | --- | --- |
| hsa_circ_0000039 | 92 | 1 | 1584 | 1 | 1584 | 0 | 0 |
| hsa_circ_0011165 | 90 | 1 | 859 | 1888 | 2730 | 0.03 | 0 |
| hsa_circ_0011166 | 82 | 3 | 1461 | 196 | 1663 | 0.01 | 0 |
| hsa_circ_0011166 | 82 | 3 | 1634 | 196 | 1836 | 0.01 | 0 |
| hsa_circ_0011166 | 82 | 3 | 1759 | 196 | 1964 | 0.01 | 0 |
| hsa_circ_0011169 | 89 | 1 | 634 | 917 | 1550 | 0 | 0 |
| hsa_circ_0011169 | 89 | 1 | 558 | 993 | 1550 | 0 | 0 |
| hsa_circ_0011166 | 85 | 1 | 637 | 1325 | 1964 | 0 | 0 |
| hsa_circ_0011169 | 88 | 1 | 424 | 1300 | 1723 | 0 | 1.00E-140 |
| hsa_circ_0011175 | 88 | 362 | 645 | 264 | 547 | 0 | 3.00E-89 |
| hsa_circ_0011179 | 87 | 144 | 471 | 328 | 1 | 0 | 3.00E-102 |
| hsa_circ_0011179 | 87 | 544 | 871 | 328 | 1 | 0 | 6.00E-102 |
| hsa_circ_0011198 | 88 | 9 | 307 | 9 | 310 | 0.01 | 4.00E-95 |
| hsa_circ_0011198 | 87 | 9 | 865 | 9 | 866 | 0.02 | 0 |
| hsa_circ_0002272 | 94 | 1 | 278 | 1 | 278 | 0 | 5.00E-118 |
| hsa_circ_0002272 | 95 | 1 | 448 | 1 | 448 | 0 | 0 |
| hsa_circ_0075673 | 97 | 1 | 312 | 1 | 312 | 0 | 2.00E-148 |
| hsa_circ_0075673 | 96 | 1 | 489 | 1 | 489 | 0 | 0 |
| hsa_circ_0075676 | 95 | 1 | 347 | 1 | 347 | 0 | 3.00E-156 |
| hsa_circ_0075683 | 88 | 1 | 298 | 299 | 2 | 0 | 1.00E-95 |
| hsa_circ_0075689 | 87 | 790 | 2016 | 1242 | 1 | 0.02 | 0 |
| hsa_circ_0075689 | 87 | 793 | 2019 | 1242 | 1 | 0.02 | 0 |
| hsa_circ_0063861 | 91 | 6 | 314 | 6 | 314 | 0 | 2.00E-113 |
| hsa_circ_0063855 | 85 | 8 | 417 | 9 | 417 | 0 | 3.00E-117 |
| hsa_circ_0063855 | 87 | 8 | 296 | 9 | 296 | 0 | 4.00E-85 |
| hsa_circ_0063855 | 85 | 8 | 192 | 9 | 192 | 0.01 | 1.00E-47 |
| hsa_circ_0003144 | 86 | 1 | 263 | 1 | 263 | 0 | 1.00E-74 |
| hsa_circ_0063848 | 85 | 1 | 570 | 1 | 573 | 0.01 | 4.00E-167 |
| hsa_circ_0063829 | 89 | 5 | 332 | 5 | 332 | 0 | 2.00E-112 |
| hsa_circ_0063772 | 80 | 2 | 477 | 339 | 817 | 0.01 | 1.00E-91 |
| hsa_circ_0063772 | 81 | 1 | 558 | 257 | 817 | 0.01 | 9.00E-124 |
| hsa_circ_0063772 | 84 | 1 | 814 | 1 | 817 | 0.01 | 0 |
| hsa_circ_0063772 | 83 | 1 | 682 | 98 | 782 | 0.01 | 3.00E-169 |
| hsa_circ_0063772 | 84 | 1 | 779 | 1 | 782 | 0.01 | 0 |
| hsa_circ_0008199 | 85 | 1 | 1054 | 1 | 1057 | 0.01 | 0 |
| hsa_circ_0008199 | 85 | 174 | 1227 | 1 | 1057 | 0.01 | 0 |
| hsa_circ_0008199 | 85 | 269 | 1322 | 1 | 1057 | 0.01 | 0 |
| hsa_circ_0008199 | 88 | 1 | 751 | 1 | 751 | 0 | 0 |
| hsa_circ_0008199 | 90 | 1 | 612 | 1 | 612 | 0 | 0 |
| hsa_circ_0008199 | 90 | 1 | 531 | 1 | 531 | 0 | 0 |
| hsa_circ_0008199 | 91 | 1 | 372 | 1 | 372 | 0 | 4.00E-140 |
| hsa_circ_0008199 | 91 | 1 | 275 | 1 | 275 | 0 | 2.00E-101 |
| hsa_circ_0008199 | 91 | 269 | 543 | 1 | 275 | 0 | 5.00E-101 |
| hsa_circ_0002077 | 87 | 188 | 853 | 1 | 663 | 0.01 | 0 |
| hsa_circ_0005447 | 94 | 2855 | 2960 | 7364 | 7469 | 0 | 2.00E-37 |
| hsa_circ_0004386 | 92 | 421 | 628 | 1 | 208 | 0 | 5.00E-77 |
| hsa_circ_0050774 | 83 | 4 | 125 | 4 | 125 | 0 | 5.00E-23 |
| hsa_circ_0050774 | 83 | 4 | 125 | 4 | 125 | 0 | 1.00E-21 |
| hsa_circ_0003971 | 74 | 7621 | 7910 | 132726 | 132437 | 0.02 | 4.00E-21 |
| hsa_circ_0050766 | 82 | 656 | 917 | 6673 | 6412 | 0.04 | 4.00E-55 |
| hsa_circ_0050766 | 82 | 1733 | 1994 | 6673 | 6412 | 0.04 | 8.00E-55 |
| hsa_circ_0050766 | 78 | 1733 | 2543 | 6673 | 5863 | 0.04 | 2.00E-136 |
| hsa_circ_0050766 | 82 | 436 | 697 | 6673 | 6412 | 0.04 | 3.00E-55 |

novel_circ_028040 novel_circ_028041 novel_circ_028042 novel_circ_028043 novel_circ_028044 novel_circ_028045 novel_circ_028046 novel_circ_028047 novel_circ_028048 novel_circ_028049 novel_circ_028050 novel_circ_028051 novel_circ_028053 novel_circ_028056 novel_circ_028057 novel_circ_028058 novel_circ_028061 novel_circ_028062 novel_circ_028063 novel_circ_028064 novel_circ_028065 novel_circ_028066 novel_circ_028067 novel_circ_028068 novel_circ_028069 novel_circ_028070 novel_circ_028071 novel_circ_028073 novel_circ_028074 novel_circ_028075 novel_circ_028076 novel_circ_028077 novel_circ_028078 novel_circ_028079 novel_circ_028080 novel_circ_028081 novel_circ_028082 novel_circ_028083 novel_circ_028084 novel_circ_028085 novel_circ_028086 novel_circ_028087 novel_circ_028094 novel_circ_028097 novel_circ_028099 novel_circ_028100 novel_circ_028101 novel_circ_028102 novel_circ_028103 novel_circ_028104 novel_circ_028105 novel_circ_028106 novel_circ_028108 novel_circ_028109

| hsa_circ_0050769 | 87 | 49 | 1504 | 4940 | 3487 | 0.02 | 0 |
| --- | --- | --- | --- | --- | --- | --- | --- |
| hsa_circ_0006486 | 84 | 3 | 122 | 362 | 242 | 0.02 | 3.00E-25 |
| hsa_circ_0050766 | 78 | 558 | 1257 | 6723 | 6022 | 0.05 | 2.00E-109 |
| hsa_circ_0050766 | 79 | 332 | 769 | 6723 | 6286 | 0.03 | 1.00E-79 |
| hsa_circ_0050766 | 78 | 332 | 1031 | 6723 | 6022 | 0.05 | 2.00E-109 |
| hsa_circ_0050766 | 78 | 332 | 1031 | 6723 | 6022 | 0.05 | 2.00E-109 |
| hsa_circ_0055512 | 88 | 21852 | 22634 | 1172 | 1958 | 0.01 | 0 |
| hsa_circ_0058709 | 89 | 18976 | 19916 | 51 | 1009 | 0.03 | 0 |
| hsa_circ_0050766 | 88 | 1 | 2288 | 7364 | 5098 | 0.02 | 0 |
| hsa_circ_0050766 | 88 | 2 | 2161 | 7239 | 5098 | 0.02 | 0 |
| hsa_circ_0050766 | 88 | 1 | 2158 | 7237 | 5098 | 0.02 | 0 |
| hsa_circ_0050766 | 88 | 1 | 2059 | 7138 | 5098 | 0.02 | 0 |
| hsa_circ_0079395 | 73 | 13693 | 14841 | 3664 | 2514 | 0.04 | 1.00E-95 |
| hsa_circ_0050766 | 76 | 138 | 891 | 6701 | 5948 | 0.03 | 3.00E-105 |
| hsa_circ_0050786 | 92 | 3 | 219 | 3 | 219 | 0 | 1.00E-80 |
| hsa_circ_0050786 | 92 | 3 | 219 | 3 | 219 | 0 | 7.00E-81 |
| hsa_circ_0050782 | 91 | 154 | 426 | 280 | 6 | 0.01 | 1.00E-100 |
| hsa_circ_0050782 | 93 | 1 | 218 | 223 | 6 | 0 | 2.00E-86 |
| hsa_circ_0054475 | 92 | 4 | 2910 | 1 | 2907 | 0 | 0 |
| hsa_circ_0054473 | 92 | 4 | 3392 | 413 | 3798 | 0 | 0 |
| hsa_circ_0054473 | 92 | 4 | 3865 | 413 | 4275 | 0 | 0 |
| hsa_circ_0054478 | 92 | 1 | 477 | 2546 | 3019 | 0.01 | 0 |
| hsa_circ_0054478 | 92 | 1 | 950 | 2546 | 3496 | 0.01 | 0 |
| hsa_circ_0054485 | 98 | 1 | 219 | 328 | 110 | 0 | 1.00E-103 |
| hsa_circ_0054491 | 97 | 1 | 681 | 681 | 1 | 0 | 0 |
| hsa_circ_0054491 | 97 | 1 | 1166 | 1166 | 1 | 0 | 0 |
| hsa_circ_0054501 | 96 | 1 | 485 | 569 | 85 | 0 | 0 |
| hsa_circ_0032848 | 91 | 3264 | 3547 | 537 | 254 | 0 | 2.00E-103 |
| hsa_circ_0032848 | 91 | 4 | 287 | 537 | 254 | 0 | 1.00E-104 |
| hsa_circ_0032845 | 88 | 1 | 498 | 2438 | 1945 | 0.01 | 7.00E-164 |
| hsa_circ_0032845 | 89 | 1 | 577 | 2438 | 1866 | 0.01 | 0 |
| hsa_circ_0058208 | 88 | 498 | 565 | 26481 | 26549 | 0.01 | 1.00E-13 |
| hsa_circ_0058208 | 88 | 498 | 565 | 26481 | 26549 | 0.01 | 9.00E-13 |
| hsa_circ_0008048 | 82 | 61111 | 61261 | 22177 | 22025 | 0.04 | 2.00E-25 |
| hsa_circ_0072742 | 94 | 1 | 749 | 676 | 1424 | 0 | 0 |
| hsa_circ_0072742 | 94 | 1 | 647 | 676 | 1322 | 0 | 0 |
| hsa_circ_0072743 | 95 | 1 | 348 | 288 | 635 | 0 | 4.00E-155 |
| hsa_circ_0072742 | 95 | 1 | 737 | 287 | 1023 | 0 | 0 |
| hsa_circ_0072742 | 94 | 159 | 1181 | 1 | 1023 | 0 | 0 |
| hsa_circ_0072742 | 94 | 159 | 833 | 1 | 675 | 0 | 0 |
| hsa_circ_0072742 | 93 | 159 | 679 | 1 | 521 | 0 | 0 |
| hsa_circ_0072742 | 93 | 159 | 444 | 1 | 286 | 0 | 3.00E-117 |
| hsa_circ_0055800 | 84 | 12141 | 12253 | 4867 | 4976 | 0.04 | 1.00E-19 |
| hsa_circ_0084053 | 80 | 1 | 1594 | 216 | 1821 | 0.02 | 0 |
| hsa_circ_0084056 | 80 | 111 | 723 | 18 | 633 | 0.02 | 2.00E-123 |
| hsa_circ_0084053 | 80 | 1 | 1594 | 216 | 1821 | 0.02 | 0 |
| hsa_circ_0084053 | 81 | 1 | 1809 | 1 | 1821 | 0.02 | 0 |
| hsa_circ_0084053 | 80 | 1 | 1537 | 216 | 1764 | 0.02 | 0 |
| hsa_circ_0084053 | 81 | 1 | 1269 | 216 | 1496 | 0.02 | 0 |
| hsa_circ_0084053 | 80 | 1 | 1163 | 216 | 1387 | 0.02 | 0 |
| hsa_circ_0084053 | 82 | 2 | 726 | 389 | 1119 | 0.02 | 3.00E-169 |
| hsa_circ_0084053 | 82 | 1 | 895 | 216 | 1119 | 0.03 | 0 |
| hsa_circ_0084053 | 84 | 1 | 666 | 216 | 884 | 0.02 | 2.00E-180 |
| hsa_circ_0084053 | 84 | 1 | 881 | 1 | 884 | 0.01 | 0 |

novel_circ_028110 novel_circ_028111 novel_circ_028112 novel_circ_028113 novel_circ_028114 novel_circ_028115 novel_circ_028116 novel_circ_028118 novel_circ_028119 novel_circ_028120 novel_circ_028121 novel_circ_028122 novel_circ_028123 novel_circ_028124 novel_circ_028126 novel_circ_028127 novel_circ_028128 novel_circ_028129 novel_circ_028132 novel_circ_028133 novel_circ_028136 novel_circ_028137 novel_circ_028138 novel_circ_028139 novel_circ_028140 novel_circ_028141 novel_circ_028142 novel_circ_028143 novel_circ_028144 novel_circ_028145 novel_circ_028146 novel_circ_028147 novel_circ_028148 novel_circ_028149 novel_circ_028150 novel_circ_028151 novel_circ_028152 novel_circ_028153 novel_circ_028154 novel_circ_028155 novel_circ_028156 novel_circ_028157 novel_circ_028158 novel_circ_028159 novel_circ_028160 novel_circ_028161 novel_circ_028162 novel_circ_028174 novel_circ_028175 novel_circ_028176 novel_circ_028177 novel_circ_028178 novel_circ_028179 novel_circ_028180

| hsa_circ_0084053 | 84 | 81 | 961 | 1 | 884 | 0.01 | 0 |
| --- | --- | --- | --- | --- | --- | --- | --- |
| hsa_circ_0084053 | 84 | 1403 | 2283 | 1 | 884 | 0.01 | 0 |
| hsa_circ_0084053 | 85 | 1 | 559 | 216 | 777 | 0.02 | 1.00E-157 |
| hsa_circ_0084053 | 85 | 1 | 774 | 1 | 777 | 0.01 | 0 |
| hsa_circ_0084053 | 86 | 1 | 453 | 1 | 456 | 0.01 | 7.00E-134 |
| hsa_circ_0084053 | 86 | 1403 | 1855 | 1 | 456 | 0.01 | 3.00E-133 |
| hsa_circ_0084053 | 85 | 1 | 376 | 1 | 379 | 0.01 | 2.00E-104 |
| hsa_circ_0084046 | 90 | 2 | 751 | 2 | 751 | 0 | 0 |
| hsa_circ_0084049 | 88 | 1 | 395 | 1 | 396 | 0 | 4.00E-131 |
| hsa_circ_0084046 | 91 | 2 | 567 | 2 | 568 | 0 | 0 |
| hsa_circ_0084049 | 88 | 1 | 289 | 1 | 289 | 0 | 2.00E-92 |
| hsa_circ_0084043 | 92 | 1 | 677 | 1 | 677 | 0 | 0 |
| hsa_circ_0084039 | 91 | 1 | 847 | 491 | 1337 | 0 | 0 |
| hsa_circ_0084038 | 86 | 1 | 215 | 1 | 215 | 0 | 9.00E-60 |
| hsa_circ_0084017 | 91 | 1 | 366 | 1 | 367 | 0 | 4.00E-140 |
| hsa_circ_0084015 | 91 | 1 | 707 | 1 | 707 | 0 | 0 |
| hsa_circ_0084014 | 90 | 1 | 508 | 62 | 569 | 0.01 | 0 |
| hsa_circ_0084012 | 78 | 1 | 1269 | 117 | 1412 | 0.04 | 0 |
| hsa_circ_0086430 | 93 | 24 | 1158 | 609 | 1743 | 0 | 0 |
| hsa_circ_0086427 | 92 | 1 | 411 | 1 | 411 | 0 | 1.00E-166 |
| hsa_circ_0025509 | 84 | 1 | 492 | 1 | 495 | 0.02 | 2.00E-137 |
| hsa_circ_0025509 | 84 | 1 | 492 | 1 | 495 | 0.02 | 1.00E-137 |
| hsa_circ_0025502 | 93 | 190 | 716 | 1 | 527 | 0 | 0 |
| hsa_circ_0002873 | 92 | 1 | 198 | 1 | 198 | 0 | 4.00E-75 |
| hsa_circ_0002873 | 92 | 1 | 198 | 1 | 198 | 0 | 2.00E-75 |
| hsa_circ_0025500 | 95 | 14 | 729 | 716 | 1 | 0 | 0 |
| hsa_circ_0025497 | 95 | 14 | 1053 | 1164 | 125 | 0 | 0 |
| hsa_circ_0025497 | 95 | 14 | 1177 | 1164 | 1 | 0 | 0 |
| hsa_circ_0025497 | 95 | 1 | 324 | 448 | 125 | 0 | 2.00E-143 |
| hsa_circ_0025497 | 94 | 1 | 448 | 448 | 1 | 0 | 0 |
| hsa_circ_0025494 | 97 | 1 | 394 | 394 | 1 | 0 | 0 |
| hsa_circ_0025493 | 97 | 1 | 396 | 592 | 197 | 0 | 0 |
| hsa_circ_0025485 | 96 | 1 | 1490 | 2224 | 735 | 0 | 0 |
| hsa_circ_0025489 | 95 | 1 | 898 | 898 | 1 | 0 | 0 |
| hsa_circ_0025485 | 94 | 1 | 1405 | 1632 | 228 | 0 | 0 |
| hsa_circ_0025489 | 96 | 3 | 701 | 699 | 1 | 0 | 0 |
| hsa_circ_0025489 | 96 | 1 | 569 | 569 | 1 | 0 | 0 |
| hsa_circ_0025485 | 92 | 1 | 507 | 734 | 228 | 0 | 0 |
| hsa_circ_0025485 | 92 | 1 | 507 | 734 | 228 | 0 | 0 |
| hsa_circ_0025485 | 93 | 1 | 734 | 734 | 1 | 0 | 0 |
| hsa_circ_0025484 | 94 | 1 | 1246 | 1246 | 1 | 0 | 0 |
| hsa_circ_0025475 | 94 | 1 | 1154 | 2495 | 1342 | 0 | 0 |
| hsa_circ_0025475 | 95 | 1 | 1347 | 2495 | 1149 | 0 | 0 |
| hsa_circ_0025475 | 95 | 1 | 1555 | 2495 | 941 | 0 | 0 |
| hsa_circ_0025475 | 94 | 1 | 1118 | 2268 | 1151 | 0 | 0 |
| hsa_circ_0025475 | 94 | 1 | 1120 | 2268 | 1149 | 0 | 0 |
| hsa_circ_0025468 | 99 | 3 | 244 | 549 | 790 | 0 | 1.00E-119 |
| hsa_circ_0090660 | 95 | 1 | 394 | 394 | 1 | 0 | 3.00E-177 |
| hsa_circ_0090682 | 96 | 1 | 561 | 561 | 1 | 0 | 0 |
| hsa_circ_0090737 | 98 | 1 | 1029 | 1029 | 1 | 0 | 0 |
| hsa_circ_0090737 | 97 | 1 | 1424 | 1424 | 1 | 0 | 0 |
| hsa_circ_0090740 | 99 | 1 | 303 | 303 | 1 | 0 | 7.00E-157 |
| hsa_circ_0090740 | 99 | 1 | 402 | 402 | 1 | 0 | 0 |
| hsa_circ_0090750 | 93 | 1 | 395 | 395 | 1 | 0 | 2.00E-164 |

novel_circ_028181 novel_circ_028182 novel_circ_028183 novel_circ_028184 novel_circ_028185 novel_circ_028186 novel_circ_028187 novel_circ_028188 novel_circ_028189 novel_circ_028190 novel_circ_028191 novel_circ_028192 novel_circ_028193 novel_circ_028194 novel_circ_028195 novel_circ_028196 novel_circ_028197 novel_circ_028198 novel_circ_028199 novel_circ_028200 novel_circ_028201 novel_circ_028202 novel_circ_028204 novel_circ_028205 novel_circ_028208 novel_circ_028209 novel_circ_028210 novel_circ_028217 novel_circ_028218 novel_circ_028219 novel_circ_028220 novel_circ_028221 novel_circ_028222 novel_circ_028223 novel_circ_028224 novel_circ_028225 novel_circ_028226 novel_circ_028227 novel_circ_028228 novel_circ_028229 novel_circ_028230 novel_circ_028231 novel_circ_028232 novel_circ_028233 novel_circ_028234 novel_circ_028235 novel_circ_028237 novel_circ_028239 novel_circ_028240 novel_circ_028242 novel_circ_028243 novel_circ_028244 novel_circ_028245 novel_circ_028246

| hsa_circ_0090752 | 94 | 1 | 3149 | 3361 | 213 | 0 | 0 |
| --- | --- | --- | --- | --- | --- | --- | --- |
| hsa_circ_0090771 | 92 | 1 | 809 | 809 | 1 | 0 | 0 |
| hsa_circ_0090771 | 95 | 1 | 361 | 592 | 232 | 0 | 5.00E-159 |
| hsa_circ_0003732 | 95 | 1 | 421 | 421 | 1 | 0 | 0 |
| hsa_circ_0003732 | 94 | 10 | 667 | 658 | 1 | 0 | 0 |
| hsa_circ_0090778 | 95 | 1 | 1572 | 1789 | 218 | 0 | 0 |
| hsa_circ_0090778 | 95 | 761 | 2435 | 1892 | 218 | 0 | 0 |
| hsa_circ_0003732 | 94 | 10 | 475 | 658 | 193 | 0 | 0 |
| hsa_circ_0090784 | 94 | 1 | 481 | 615 | 135 | 0 | 0 |
| hsa_circ_0090784 | 94 | 1 | 693 | 827 | 135 | 0 | 0 |
| hsa_circ_0090785 | 95 | 1 | 447 | 447 | 1 | 0 | 0 |
| hsa_circ_0090790 | 94 | 1 | 589 | 589 | 1 | 0 | 0 |
| hsa_circ_0090790 | 95 | 1 | 1757 | 1757 | 1 | 0 | 0 |
| hsa_circ_0090793 | 95 | 1 | 810 | 1095 | 286 | 0 | 0 |
| hsa_circ_0090799 | 95 | 1 | 985 | 985 | 1 | 0 | 0 |
| hsa_circ_0002724 | 92 | 1 | 360 | 360 | 1 | 0 | 9.00E-142 |
| hsa_circ_0002724 | 93 | 1 | 459 | 459 | 1 | 0 | 0 |
| hsa_circ_0002724 | 92 | 1 | 520 | 528 | 1 | 0.02 | 0 |
| hsa_circ_0002724 | 92 | 50533 | 51059 | 534 | 1 | 0.02 | 0 |
| hsa_circ_0021476 | 93 | 1 | 577 | 124 | 700 | 0 | 0 |
| hsa_circ_0021477 | 93 | 1 | 289 | 1 | 289 | 0 | 2.00E-117 |
| hsa_circ_0021477 | 90 | 1 | 807 | 1 | 807 | 0 | 0 |
| hsa_circ_0091250 | 94 | 113 | 525 | 1 | 413 | 0 | 0 |
| hsa_circ_0025440 | 84 | 1 | 1018 | 1180 | 163 | 0 | 0 |
| hsa_circ_0055443 | 96 | 1 | 290 | 96 | 385 | 0 | 1.00E-129 |
| hsa_circ_0055438 | 80 | 156 | 482 | 3812 | 3481 | 0.08 | 8.00E-57 |
| hsa_circ_0006188 | 92 | 1 | 367 | 383 | 17 | 0.01 | 2.00E-145 |
| hsa_circ_0013758 | 87 | 1 | 398 | 403 | 3 | 0.01 | 2.00E-121 |
| hsa_circ_0079838 | 81 | 4 | 497 | 597 | 104 | 0 | 2.00E-104 |
| hsa_circ_0079823 | 80 | 3 | 402 | 3 | 402 | 0.02 | 6.00E-79 |
| hsa_circ_0004750 | 81 | 101 | 332 | 1 | 232 | 0 | 6.00E-44 |
| hsa_circ_0079818 | 84 | 1 | 326 | 1 | 326 | 0 | 1.00E-84 |
| hsa_circ_0079817 | 83 | 1 | 563 | 1 | 563 | 0 | 2.00E-139 |
| hsa_circ_0079812 | 81 | 1 | 786 | 1 | 786 | 0 | 0 |
| hsa_circ_0079808 | 84 | 1 | 1498 | 290 | 1787 | 0 | 0 |
| hsa_circ_0079817 | 83 | 1 | 410 | 1 | 410 | 0 | 2.00E-104 |
| hsa_circ_0079817 | 81 | 1 | 228 | 1 | 228 | 0 | 3.00E-45 |
| hsa_circ_0079812 | 80 | 1 | 451 | 1 | 451 | 0 | 7.00E-89 |
| hsa_circ_0079809 | 82 | 1 | 764 | 315 | 1078 | 0 | 3.00E-179 |
| hsa_circ_0079809 | 87 | 2 | 573 | 2 | 573 | 0 | 0 |
| hsa_circ_0079809 | 88 | 2 | 314 | 2 | 314 | 0 | 2.00E-102 |
| hsa_circ_0079808 | 90 | 1 | 399 | 290 | 688 | 0 | 2.00E-143 |
| hsa_circ_0079808 | 87 | 12 | 574 | 126 | 688 | 0 | 1.00E-177 |
| hsa_circ_0079806 | 86 | 2 | 1027 | 2 | 1027 | 0 | 0 |
| hsa_circ_0079806 | 84 | 1 | 590 | 124 | 713 | 0 | 1.00E-157 |
| hsa_circ_0079806 | 85 | 2 | 713 | 2 | 713 | 0 | 0 |
| hsa_circ_0079806 | 83 | 2 | 625 | 2 | 625 | 0 | 7.00E-160 |
| hsa_circ_0079806 | 86 | 2 | 429 | 2 | 429 | 0 | 9.00E-128 |
| hsa_circ_0079806 | 86 | 2 | 339 | 2 | 339 | 0 | 1.00E-99 |
| hsa_circ_0079801 | 85 | 1 | 302 | 302 | 1 | 0 | 1.00E-84 |
| hsa_circ_0079799 | 86 | 1 | 392 | 555 | 164 | 0 | 8.00E-118 |
| hsa_circ_0079799 | 85 | 1 | 553 | 555 | 3 | 0 | 2.00E-160 |
| hsa_circ_0079797 | 86 | 694 | 1594 | 1 | 902 | 0.01 | 0 |
| hsa_circ_0079799 | 86 | 7 | 337 | 333 | 3 | 0 | 8.00E-99 |

novel_circ_028247 novel_circ_028248 novel_circ_028249 novel_circ_028250 novel_circ_028251 novel_circ_028252 novel_circ_028253 novel_circ_028254 novel_circ_028255 novel_circ_028256 novel_circ_028257 novel_circ_028258 novel_circ_028259 novel_circ_028261 novel_circ_028262 novel_circ_028263 novel_circ_028264 novel_circ_028269 novel_circ_028270 novel_circ_028271 novel_circ_028272 novel_circ_028273 novel_circ_028274 novel_circ_028275 novel_circ_028276 novel_circ_028280 novel_circ_028281 novel_circ_028282 novel_circ_028283 novel_circ_028284 novel_circ_028285 novel_circ_028290 novel_circ_028291 novel_circ_028292 novel_circ_028293 novel_circ_028298 novel_circ_028299 novel_circ_028300 novel_circ_028301 novel_circ_028302 novel_circ_028303 novel_circ_028304 novel_circ_028305 novel_circ_028306 novel_circ_028307 novel_circ_028308 novel_circ_028309 novel_circ_028310 novel_circ_028311 novel_circ_028312 novel_circ_028313 novel_circ_028314 novel_circ_028315 novel_circ_028316

| hsa_circ_0079797 | 86 | 694 | 1594 | 1 | 902 | 0.01 | 0 |
| --- | --- | --- | --- | --- | --- | --- | --- |
| hsa_circ_0079763 | 95 | 254 | 454 | 433817 | 433617 | 0 | 3.00E-83 |
| hsa_circ_0079797 | 87 | 1 | 731 | 147 | 878 | 0.01 | 0 |
| hsa_circ_0079797 | 86 | 1 | 877 | 1 | 878 | 0.01 | 0 |
| hsa_circ_0079797 | 86 | 694 | 1570 | 1 | 878 | 0.01 | 0 |
| hsa_circ_0079794 | 87 | 344 | 1026 | 177 | 859 | 0 | 0 |
| hsa_circ_0079794 | 86 | 344 | 839 | 177 | 672 | 0 | 6.00E-147 |
| hsa_circ_0079794 | 90 | 344 | 501 | 177 | 334 | 0 | 5.00E-51 |
| hsa_circ_0057328 | 92 | 1 | 760 | 1 | 769 | 0.01 | 0 |
| hsa_circ_0057328 | 92 | 1 | 760 | 1 | 769 | 0.01 | 0 |
| hsa_circ_0057328 | 91 | 1 | 646 | 1 | 655 | 0.01 | 0 |
| hsa_circ_0057328 | 95 | 1 | 486 | 1 | 486 | 0 | 0 |
| hsa_circ_0057328 | 94 | 1 | 327 | 1 | 327 | 0 | 8.00E-142 |
| hsa_circ_0057326 | 95 | 2 | 240 | 1024 | 1262 | 0 | 1.00E-104 |
| hsa_circ_0057326 | 92 | 1 | 352 | 603 | 954 | 0 | 1.00E-140 |
| hsa_circ_0057326 | 93 | 1 | 954 | 1 | 954 | 0 | 0 |
| hsa_circ_0057313 | 94 | 5 | 223 | 5 | 223 | 0 | 3.00E-90 |
| hsa_circ_0011960 | 96 | 1 | 386 | 386 | 1 | 0 | 7.00E-178 |
| hsa_circ_0011960 | 96 | 1 | 386 | 386 | 1 | 0 | 9.00E-177 |
| hsa_circ_0004416 | 96 | 1 | 461 | 461 | 1 | 0 | 0 |
| hsa_circ_0011960 | 96 | 1 | 325 | 325 | 1 | 0 | 5.00E-149 |
| hsa_circ_0004416 | 96 | 1 | 400 | 400 | 1 | 0 | 0 |
| hsa_circ_0011959 | 96 | 1 | 401 | 484 | 84 | 0 | 0 |
| hsa_circ_0011956 | 97 | 1 | 186 | 186 | 1 | 0 | 1.00E-83 |
| hsa_circ_0011953 | 94 | 305 | 721 | 417 | 1 | 0 | 9.00E-180 |
| hsa_circ_0003751 | 95 | 3 | 509 | 1082 | 569 | 0.01 | 0 |
| hsa_circ_0003751 | 95 | 3 | 509 | 1082 | 569 | 0.01 | 0 |
| hsa_circ_0011948 | 96 | 915 | 1482 | 568 | 1 | 0 | 0 |
| hsa_circ_0011948 | 98 | 1 | 333 | 333 | 1 | 0 | 4.00E-165 |
| hsa_circ_0011943 | 89 | 1 | 360 | 360 | 1 | 0 | 9.00E-127 |
| hsa_circ_0011939 | 91 | 1 | 753 | 753 | 1 | 0 | 0 |
| hsa_circ_0065022 | 87 | 440 | 491 | 603 | 654 | 0 | 5.00E-06 |
| hsa_circ_0065022 | 87 | 217 | 268 | 603 | 654 | 0 | 7.00E-07 |
| hsa_circ_0065022 | 87 | 313 | 364 | 603 | 654 | 0 | 8.00E-07 |
| hsa_circ_0065022 | 87 | 440 | 491 | 603 | 654 | 0 | 9.00E-07 |
| hsa_circ_0007973 | 93 | 1 | 762 | 1 | 762 | 0 | 0 |
| hsa_circ_0011893 | 95 | 1 | 336 | 1 | 336 | 0 | 2.00E-148 |
| hsa_circ_0011892 | 95 | 1 | 418 | 1 | 418 | 0 | 0 |
| hsa_circ_0011890 | 95 | 1 | 573 | 1 | 573 | 0 | 0 |
| hsa_circ_0011890 | 95 | 1 | 370 | 1 | 370 | 0 | 2.00E-167 |
| hsa_circ_0011885 | 90 | 3 | 412 | 410 | 1 | 0 | 8.00E-148 |
| hsa_circ_0011885 | 90 | 3 | 412 | 410 | 1 | 0 | 1.00E-147 |
| hsa_circ_0011885 | 89 | 4 | 302 | 299 | 1 | 0 | 3.00E-101 |
| hsa_circ_0011875 | 90 | 1 | 514 | 1 | 514 | 0 | 0 |
| hsa_circ_0011874 | 90 | 1 | 592 | 1 | 592 | 0 | 0 |
| hsa_circ_0011865 | 91 | 1 | 529 | 529 | 1 | 0 | 0 |
| hsa_circ_0011862 | 91 | 1 | 1058 | 1060 | 3 | 0 | 0 |
| hsa_circ_0011862 | 91 | 1 | 818 | 820 | 3 | 0 | 0 |
| hsa_circ_0011862 | 91 | 1 | 672 | 674 | 3 | 0 | 0 |
| hsa_circ_0091321 | 95 | 7 | 1200 | 1194 | 1 | 0 | 0 |
| hsa_circ_0091321 | 94 | 1 | 1449 | 1449 | 1 | 0 | 0 |
| hsa_circ_0091321 | 95 | 1 | 1867 | 1867 | 1 | 0 | 0 |
| hsa_circ_0091321 | 95 | 3 | 2157 | 2156 | 1 | 0 | 0 |
| hsa_circ_0091321 | 95 | 7 | 1042 | 1194 | 159 | 0 | 0 |

novel_circ_028317 novel_circ_028318 novel_circ_028319 novel_circ_028320 novel_circ_028321 novel_circ_028322 novel_circ_028323 novel_circ_028328 novel_circ_028329 novel_circ_028330 novel_circ_028331 novel_circ_028334 novel_circ_028338 novel_circ_028339 novel_circ_028340 novel_circ_028341 novel_circ_028342 novel_circ_028343 novel_circ_028344 novel_circ_028345 novel_circ_028346 novel_circ_028347 novel_circ_028348 novel_circ_028349 novel_circ_028350 novel_circ_028351 novel_circ_028352 novel_circ_028353 novel_circ_028354 novel_circ_028355 novel_circ_028356 novel_circ_028357 novel_circ_028359 novel_circ_028360 novel_circ_028361 novel_circ_028362 novel_circ_028363 novel_circ_028365 novel_circ_028366 novel_circ_028367 novel_circ_028368 novel_circ_028369 novel_circ_028370 novel_circ_028371 novel_circ_028372 novel_circ_028373 novel_circ_028374 novel_circ_028375 novel_circ_028376 novel_circ_028380 novel_circ_028386 novel_circ_028387 novel_circ_028391 novel_circ_028392

| hsa_circ_0091321 | 95 | 7 | 927 | 1194 | 274 | 0 | 0 |
| --- | --- | --- | --- | --- | --- | --- | --- |
| hsa_circ_0091321 | 94 | 1 | 1176 | 1449 | 274 | 0 | 0 |
| hsa_circ_0091321 | 95 | 7 | 735 | 1194 | 466 | 0 | 0 |
| hsa_circ_0091321 | 94 | 1 | 909 | 1449 | 541 | 0 | 0 |
| hsa_circ_0091326 | 94 | 7 | 275 | 269 | 1 | 0 | 2.00E-111 |
| hsa_circ_0091326 | 93 | 1 | 524 | 524 | 1 | 0 | 0 |
| hsa_circ_0013355 | 92 | 1 | 162 | 2532 | 2371 | 0.01 | 3.00E-58 |
| hsa_circ_0013344 | 93 | 1 | 901 | 1 | 901 | 0 | 0 |
| hsa_circ_0013353 | 91 | 1 | 331 | 1 | 331 | 0 | 2.00E-122 |
| hsa_circ_0013344 | 93 | 1 | 660 | 1 | 660 | 0 | 0 |
| hsa_circ_0013344 | 93 | 1 | 473 | 1 | 473 | 0 | 0 |
| hsa_circ_0055813 | 79 | 8595 | 8998 | 70419 | 70823 | 0.03 | 1.00E-69 |
| hsa_circ_0090500 | 93 | 129 | 317 | 1 | 189 | 0 | 2.00E-73 |
| hsa_circ_0087777 | 96 | 1 | 749 | 111 | 862 | 0 | 0 |
| hsa_circ_0087777 | 96 | 17503 | 18258 | 105 | 862 | 0.01 | 0 |
| hsa_circ_0087777 | 96 | 1 | 859 | 1 | 862 | 0 | 0 |
| hsa_circ_0087777 | 96 | 12442 | 13300 | 1 | 862 | 0 | 0 |
| hsa_circ_0087777 | 96 | 12447 | 13305 | 1 | 862 | 0 | 0 |
| hsa_circ_0087777 | 96 | 1 | 590 | 111 | 703 | 0.01 | 0 |
| hsa_circ_0087777 | 96 | 17503 | 18099 | 105 | 703 | 0.01 | 0 |
| hsa_circ_0087777 | 96 | 1 | 700 | 1 | 703 | 0 | 0 |
| hsa_circ_0087777 | 96 | 3523 | 4222 | 1 | 703 | 0 | 0 |
| hsa_circ_0087777 | 96 | 12447 | 13146 | 1 | 703 | 0 | 0 |
| hsa_circ_0087777 | 96 | 1 | 466 | 111 | 579 | 0.01 | 0 |
| hsa_circ_0087777 | 96 | 17503 | 17975 | 105 | 579 | 0.01 | 0 |
| hsa_circ_0087777 | 96 | 1 | 576 | 1 | 579 | 0.01 | 0 |
| hsa_circ_0087777 | 95 | 1 | 254 | 111 | 364 | 0 | 1.00E-109 |
| hsa_circ_0087777 | 95 | 17503 | 17763 | 105 | 364 | 0 | 2.00E-109 |
| hsa_circ_0087777 | 95 | 1 | 364 | 1 | 364 | 0 | 2.00E-162 |
| hsa_circ_0087777 | 95 | 3523 | 3886 | 1 | 364 | 0 | 3.00E-161 |
| hsa_circ_0087777 | 95 | 12442 | 12805 | 1 | 364 | 0 | 9.00E-161 |
| hsa_circ_0006388 | 91 | 1 | 451 | 1 | 451 | 0 | 3.00E-172 |
| hsa_circ_0087764 | 94 | 1150 | 1259 | 1536 | 1427 | 0 | 6.00E-39 |
| hsa_circ_0087764 | 92 | 1150 | 1810 | 1536 | 876 | 0 | 0 |
| hsa_circ_0087774 | 92 | 1 | 551 | 729 | 179 | 0 | 0 |
| hsa_circ_0087758 | 94 | 1 | 988 | 182 | 1169 | 0 | 0 |
| hsa_circ_0087754 | 91 | 177 | 473 | 1 | 297 | 0 | 3.00E-108 |
| hsa_circ_0087752 | 97 | 1 | 229 | 530 | 302 | 0 | 2.00E-107 |
| hsa_circ_0087752 | 94 | 1 | 530 | 530 | 1 | 0 | 0 |
| hsa_circ_0087750 | 93 | 117 | 592 | 476 | 1 | 0 | 0 |
| hsa_circ_0087749 | 94 | 117 | 840 | 730 | 7 | 0 | 0 |
| hsa_circ_0087752 | 92 | 1 | 301 | 301 | 1 | 0 | 2.00E-117 |
| hsa_circ_0087750 | 92 | 1 | 359 | 476 | 118 | 0 | 7.00E-143 |
| hsa_circ_0087750 | 93 | 1 | 476 | 476 | 1 | 0 | 0 |
| hsa_circ_0087749 | 94 | 1 | 724 | 730 | 7 | 0 | 0 |
| hsa_circ_0087749 | 94 | 1 | 724 | 730 | 7 | 0 | 0 |
| hsa_circ_0087748 | 96 | 1 | 425 | 3890 | 3466 | 0 | 0 |
| hsa_circ_0004825 | 94 | 1 | 247 | 1 | 251 | 0.02 | 2.00E-101 |
| hsa_circ_0004825 | 94 | 1 | 247 | 1 | 251 | 0.02 | 1.00E-101 |
| hsa_circ_0064027 | 79 | 1 | 847 | 170 | 1053 | 0.07 | 1.00E-153 |
| hsa_circ_0007389 | 90 | 68210 | 68261 | 1E+06 | 1257985 | 0.02 | 1.00E-07 |
| hsa_circ_0016177 | 88 | 21 | 77 | 14181 | 14236 | 0.02 | 2.00E-07 |
| hsa_circ_0055813 | 79 | 62315 | 62590 | 70510 | 70788 | 0.01 | 6.00E-45 |
| hsa_circ_0055813 | 79 | 62315 | 62590 | 70510 | 70788 | 0.01 | 6.00E-45 |

novel_circ_028397 novel_circ_028398 novel_circ_028399 novel_circ_028400 novel_circ_028401 novel_circ_028402 novel_circ_028403 novel_circ_028404 novel_circ_028405 novel_circ_028406 novel_circ_028407 novel_circ_028408 novel_circ_028410 novel_circ_028411 novel_circ_028412 novel_circ_028413 novel_circ_028414 novel_circ_028415 novel_circ_028416 novel_circ_028417 novel_circ_028419 novel_circ_028420 novel_circ_028421 novel_circ_028424 novel_circ_028425 novel_circ_028426 novel_circ_028427 novel_circ_028428 novel_circ_028429 novel_circ_028430 novel_circ_028432 novel_circ_028433 novel_circ_028434 novel_circ_028435 novel_circ_028436 novel_circ_028437 novel_circ_028438 novel_circ_028439 novel_circ_028440 novel_circ_028441 novel_circ_028442 novel_circ_028443 novel_circ_028444 novel_circ_028445 novel_circ_028446 novel_circ_028447 novel_circ_028455 novel_circ_028456 novel_circ_028457 novel_circ_028459 novel_circ_028460 novel_circ_028461 novel_circ_028462 novel_circ_028463

| hsa_circ_0007636 | 83 | 12931 | 13053 | 580597 | 580718 | 0.01 | 8.00E-21 |
| --- | --- | --- | --- | --- | --- | --- | --- |
| hsa_circ_0074838 | 84 | 3643 | 3893 | 3214 | 2957 | 0.04 | 9.00E-62 |
| hsa_circ_0074837 | 94 | 19507 | 20471 | 965 | 1 | 0.01 | 0 |
| hsa_circ_0074837 | 94 | 1 | 965 | 965 | 1 | 0.01 | 0 |
| hsa_circ_0074828 | 94 | 25 | 324 | 301 | 1 | 0 | 2.00E-128 |
| hsa_circ_0074827 | 95 | 25 | 416 | 3261 | 2869 | 0 | 7.00E-173 |
| hsa_circ_0074827 | 95 | 25 | 828 | 3261 | 2457 | 0 | 0 |
| hsa_circ_0074827 | 94 | 25 | 1156 | 3261 | 2133 | 0 | 0 |
| hsa_circ_0074827 | 95 | 1 | 613 | 3069 | 2457 | 0 | 0 |
| hsa_circ_0074827 | 94 | 1 | 941 | 3069 | 2133 | 0 | 0 |
| hsa_circ_0074827 | 96 | 1 | 504 | 2960 | 2457 | 0 | 0 |
| hsa_circ_0074819 | 96 | 1 | 485 | 482 | 1 | 0.01 | 0 |
| hsa_circ_0074813 | 97 | 1 | 971 | 971 | 1 | 0 | 0 |
| hsa_circ_0074804 | 94 | 2 | 544 | 2 | 544 | 0 | 0 |
| hsa_circ_0074781 | 93 | 1 | 366 | 1 | 366 | 0 | 9.00E-152 |
| hsa_circ_0074779 | 92 | 1 | 654 | 230 | 883 | 0 | 0 |
| hsa_circ_0074779 | 92 | 2402 | 3056 | 229 | 883 | 0 | 0 |
| hsa_circ_0074779 | 92 | 1 | 883 | 1 | 883 | 0 | 0 |
| hsa_circ_0074779 | 91 | 1 | 288 | 230 | 517 | 0 | 7.00E-107 |
| hsa_circ_0074779 | 91 | 2402 | 2690 | 229 | 517 | 0 | 2.00E-106 |
| hsa_circ_0074778 | 95 | 1 | 160 | 1 | 160 | 0 | 2.00E-64 |
| hsa_circ_0074777 | 93 | 1 | 560 | 470 | 1029 | 0 | 0 |
| hsa_circ_0074775 | 92 | 1 | 408 | 1 | 408 | 0 | 2.00E-163 |
| hsa_circ_0074735 | 90 | 1 | 1005 | 1016 | 12 | 0 | 0 |
| hsa_circ_0074742 | 91 | 1 | 533 | 533 | 1 | 0 | 0 |
| hsa_circ_0074735 | 88 | 1 | 389 | 400 | 12 | 0 | 8.00E-128 |
| hsa_circ_0074717 | 89 | 12 | 430 | 590 | 172 | 0 | 2.00E-144 |
| hsa_circ_0074704 | 93 | 1 | 947 | 1 | 941 | 0.01 | 0 |
| hsa_circ_0074686 | 92 | 1 | 409 | 1 | 409 | 0 | 6.00E-164 |
| hsa_circ_0074678 | 88 | 4 | 1328 | 1340 | 1 | 0.01 | 0 |
| hsa_circ_0020094 | 94 | 1 | 601 | 199 | 799 | 0 | 0 |
| hsa_circ_0020094 | 94 | 1 | 601 | 199 | 799 | 0 | 0 |
| hsa_circ_0020094 | 95 | 1 | 799 | 1 | 799 | 0 | 0 |
| hsa_circ_0020094 | 94 | 1 | 601 | 199 | 799 | 0 | 0 |
| hsa_circ_0020094 | 95 | 1 | 799 | 1 | 799 | 0 | 0 |
| hsa_circ_0020094 | 95 | 1 | 705 | 1 | 705 | 0 | 0 |
| hsa_circ_0020094 | 95 | 1 | 536 | 1 | 536 | 0 | 0 |
| hsa_circ_0020089 | 92 | 1 | 155 | 1 | 155 | 0 | 2.00E-56 |
| hsa_circ_0020081 | 91 | 1 | 477 | 1 | 477 | 0 | 0 |
| hsa_circ_0020075 | 93 | 1 | 429 | 429 | 1 | 0 | 5.00E-180 |
| hsa_circ_0020066 | 93 | 1 | 650 | 7327 | 6678 | 0 | 0 |
| hsa_circ_0007319 | 97 | 214 | 334 | 1E+06 | 1120045 | 0 | 1.00E-50 |
| hsa_circ_0041743 | 89 | 163 | 1382 | 1736 | 517 | 0 | 0 |
| hsa_circ_0041742 | 87 | 1 | 1832 | 1826 | 1 | 0 | 0 |
| hsa_circ_0041741 | 84 | 1 | 911 | 1682 | 778 | 0.01 | 0 |
| hsa_circ_0041741 | 84 | 1 | 1054 | 1682 | 638 | 0.01 | 0 |
| hsa_circ_0041738 | 94 | 237 | 734 | 1037 | 540 | 0 | 0 |
| hsa_circ_0041730 | 90 | 301 | 422 | 1 | 122 | 0.02 | 9.00E-38 |
| hsa_circ_0041730 | 90 | 967 | 1088 | 1 | 122 | 0.02 | 2.00E-37 |
| hsa_circ_0059036 | 88 | 3 | 550 | 1472 | 923 | 0 | 0 |
| hsa_circ_0059083 | 80 | 1 | 526 | 149 | 674 | 0.02 | 2.00E-100 |
| hsa_circ_0007647 | 80 | 1 | 1146 | 1163 | 30 | 0.02 | 0 |
| hsa_circ_0024339 | 94 | 1 | 948 | 954 | 1 | 0.01 | 0 |
| hsa_circ_0024339 | 94 | 1 | 648 | 654 | 1 | 0.01 | 0 |

novel_circ_028464 novel_circ_028465 novel_circ_028466 novel_circ_028467 novel_circ_028468 novel_circ_028469 novel_circ_028470 novel_circ_028471 novel_circ_028472 novel_circ_028473 novel_circ_028474 novel_circ_028475 novel_circ_028476 novel_circ_028477 novel_circ_028478 novel_circ_028479 novel_circ_028480 novel_circ_028481 novel_circ_028482 novel_circ_028483 novel_circ_028484 novel_circ_028486 novel_circ_028487 novel_circ_028488 novel_circ_028489 novel_circ_028490 novel_circ_028491 novel_circ_028492 novel_circ_028494 novel_circ_028495 novel_circ_028496 novel_circ_028497 novel_circ_028498 novel_circ_028499 novel_circ_028500 novel_circ_028501 novel_circ_028502 novel_circ_028503 novel_circ_028504 novel_circ_028505 novel_circ_028506 novel_circ_028507 novel_circ_028508 novel_circ_028509 novel_circ_028510 novel_circ_028511 novel_circ_028512 novel_circ_028513 novel_circ_028514 novel_circ_028515 novel_circ_028516 novel_circ_028517 novel_circ_028518 novel_circ_028519

| hsa_circ_0024339 | 94 | 1 | 948 | 954 | 1 | 0.01 | 0 |
| --- | --- | --- | --- | --- | --- | --- | --- |
| hsa_circ_0024339 | 94 | 1 | 570 | 654 | 85 | 0 | 0 |
| hsa_circ_0024339 | 94 | 1 | 870 | 954 | 85 | 0 | 0 |
| hsa_circ_0024340 | 92 | 1 | 297 | 297 | 1 | 0 | 7.00E-117 |
| hsa_circ_0024340 | 92 | 1 | 450 | 450 | 1 | 0 | 0 |
| hsa_circ_0024340 | 93 | 1 | 597 | 597 | 1 | 0 | 0 |
| hsa_circ_0024340 | 93 | 1 | 438 | 597 | 160 | 0 | 0 |
| hsa_circ_0091435 | 85 | 334 | 680 | 344 | 1 | 0.03 | 5.00E-93 |
| hsa_circ_0091434 | 89 | 681 | 1032 | 3152 | 2801 | 0 | 2.00E-123 |
| hsa_circ_0039843 | 95 | 1 | 1212 | 1 | 1212 | 0 | 0 |
| hsa_circ_0039846 | 96 | 3 | 1095 | 3 | 1095 | 0 | 0 |
| hsa_circ_0039846 | 96 | 3 | 1214 | 3 | 1214 | 0 | 0 |
| hsa_circ_0039846 | 96 | 3 | 1366 | 3 | 1366 | 0 | 0 |
| hsa_circ_0039846 | 97 | 1 | 576 | 791 | 1366 | 0 | 0 |
| hsa_circ_0039849 | 96 | 1 | 405 | 1 | 405 | 0 | 0 |
| hsa_circ_0039849 | 95 | 1 | 749 | 1 | 749 | 0 | 0 |
| hsa_circ_0039850 | 95 | 1 | 615 | 1 | 615 | 0 | 0 |
| hsa_circ_0039851 | 94 | 1 | 494 | 1 | 494 | 0 | 0 |
| hsa_circ_0039850 | 94 | 1 | 630 | 122 | 751 | 0 | 0 |
| hsa_circ_0000709 | 93 | 1 | 480 | 1 | 480 | 0 | 0 |
| hsa_circ_0008272 | 92 | 1 | 136 | 184 | 319 | 0 | 9.00E-48 |
| hsa_circ_0068033 | 81 | 23 | 558 | 23 | 558 | 0 | 2.00E-114 |
| hsa_circ_0072017 | 95 | 1 | 463 | 463 | 1 | 0 | 0 |
| hsa_circ_0072016 | 90 | 1 | 240 | 353 | 120 | 0.03 | 3.00E-80 |
| hsa_circ_0071999 | 91 | 1 | 1109 | 1228 | 126 | 0.01 | 0 |
| hsa_circ_0072011 | 92 | 1 | 560 | 560 | 1 | 0 | 0 |
| hsa_circ_0071998 | 92 | 1 | 990 | 990 | 1 | 0 | 0 |
| hsa_circ_0071981 | 91 | 1 | 345 | 919 | 575 | 0 | 8.00E-132 |
| hsa_circ_0071975 | 92 | 1 | 203 | 602 | 400 | 0 | 8.00E-75 |
| hsa_circ_0003057 | 92 | 1 | 698 | 698 | 1 | 0 | 0 |
| hsa_circ_0071975 | 92 | 1 | 306 | 399 | 94 | 0 | 7.00E-122 |
| hsa_circ_0005446 | 92 | 7 | 243 | 84 | 320 | 0 | 1.00E-89 |
| hsa_circ_0005446 | 92 | 2 | 320 | 2 | 320 | 0 | 6.00E-127 |
| hsa_circ_0005446 | 92 | 2 | 320 | 2 | 320 | 0 | 5.00E-127 |
| hsa_circ_0005446 | 92 | 71 | 389 | 2 | 320 | 0 | 6.00E-127 |
| hsa_circ_0005446 | 93 | 2 | 316 | 2 | 316 | 0 | 8.00E-127 |
| hsa_circ_0005446 | 93 | 2 | 170 | 2 | 170 | 0 | 6.00E-66 |
| hsa_circ_0071966 | 86 | 1 | 284 | 1 | 284 | 0 | 9.00E-83 |
| hsa_circ_0071966 | 86 | 1 | 284 | 1 | 284 | 0 | 6.00E-83 |
| hsa_circ_0071955 | 90 | 1 | 592 | 161 | 752 | 0 | 0 |
| hsa_circ_0071955 | 91 | 1 | 682 | 71 | 752 | 0 | 0 |
| hsa_circ_0071955 | 91 | 2 | 752 | 2 | 752 | 0 | 0 |
| hsa_circ_0071943 | 92 | 1 | 648 | 1 | 648 | 0 | 0 |
| hsa_circ_0071943 | 95 | 1 | 303 | 1 | 303 | 0 | 2.00E-133 |
| hsa_circ_0071900 | 92 | 1 | 1103 | 514 | 1616 | 0 | 0 |
| hsa_circ_0071922 | 92 | 1 | 302 | 1 | 302 | 0 | 4.00E-116 |
| hsa_circ_0071921 | 92 | 1 | 315 | 1 | 315 | 0 | 8.00E-122 |
| hsa_circ_0071909 | 90 | 1 | 678 | 1 | 678 | 0 | 0 |
| hsa_circ_0071902 | 91 | 1 | 870 | 1 | 870 | 0 | 0 |
| hsa_circ_0007638 | 91 | 1 | 993 | 1 | 993 | 0 | 0 |
| hsa_circ_0071897 | 92 | 1 | 1699 | 1 | 1699 | 0 | 0 |
| hsa_circ_0007638 | 92 | 1 | 801 | 1 | 801 | 0 | 0 |
| hsa_circ_0071900 | 93 | 1 | 1314 | 1 | 1314 | 0 | 0 |
| hsa_circ_0071902 | 90 | 1 | 555 | 1 | 555 | 0 | 0 |

novel_circ_028520 novel_circ_028521 novel_circ_028522 novel_circ_028523 novel_circ_028524 novel_circ_028525 novel_circ_028526 novel_circ_028527 novel_circ_028530 novel_circ_028531 novel_circ_028537 novel_circ_028539 novel_circ_028540 novel_circ_028541 novel_circ_028542 novel_circ_028543 novel_circ_028544 novel_circ_028545 novel_circ_028546 novel_circ_028547 novel_circ_028549 novel_circ_028553 novel_circ_028556 novel_circ_028561 novel_circ_028562 novel_circ_028568 novel_circ_028569 novel_circ_028570 novel_circ_028571 novel_circ_028572 novel_circ_028573 novel_circ_028574 novel_circ_028576 novel_circ_028577 novel_circ_028578 novel_circ_028579 novel_circ_028580 novel_circ_028581 novel_circ_028582 novel_circ_028583 novel_circ_028584 novel_circ_028585 novel_circ_028586 novel_circ_028587 novel_circ_028588 novel_circ_028589 novel_circ_028590 novel_circ_028591 novel_circ_028592 novel_circ_028593 novel_circ_028594 novel_circ_028595 novel_circ_028596 novel_circ_028597

| hsa_circ_0071890 | 93 | 1 | 1499 | 1 | 1499 | 0 | 0 |
| --- | --- | --- | --- | --- | --- | --- | --- |
| hsa_circ_0007638 | 91 | 1 | 447 | 1 | 447 | 0 | 2.00E-173 |
| hsa_circ_0071900 | 93 | 1 | 960 | 1 | 960 | 0 | 0 |
| hsa_circ_0071897 | 93 | 1 | 1153 | 1 | 1153 | 0 | 0 |
| hsa_circ_0071890 | 93 | 1 | 1268 | 1 | 1268 | 0 | 0 |
| hsa_circ_0007638 | 94 | 1 | 315 | 1 | 315 | 0 | 2.00E-133 |
| hsa_circ_0071900 | 94 | 1 | 828 | 1 | 828 | 0 | 0 |
| hsa_circ_0071890 | 94 | 1 | 308 | 1 | 308 | 0 | 6.00E-128 |
| hsa_circ_0071876 | 90 | 10 | 925 | 1644 | 729 | 0 | 0 |
| hsa_circ_0071874 | 94 | 247 | 550 | 304 | 1 | 0 | 6.00E-127 |
| hsa_circ_0088276 | 99 | 1 | 268 | 268 | 1 | 0 | 1.00E-133 |
| hsa_circ_0088276 | 99 | 1 | 268 | 268 | 1 | 0 | 9.00E-134 |
| hsa_circ_0088276 | 99 | 1 | 268 | 268 | 1 | 0 | 4.00E-134 |
| hsa_circ_0027680 | 94 | 7 | 125 | 755 | 873 | 0 | 1.00E-44 |
| hsa_circ_0027680 | 88 | 1 | 748 | 1 | 748 | 0 | 0 |
| hsa_circ_0027680 | 88 | 273 | 1020 | 1 | 748 | 0 | 0 |
| hsa_circ_0051208 | 89 | 1 | 491 | 1 | 491 | 0.04 | 2.00E-174 |
| hsa_circ_0051208 | 90 | 1 | 591 | 1 | 591 | 0.03 | 0 |
| hsa_circ_0051208 | 91 | 1 | 704 | 1 | 704 | 0.03 | 0 |
| hsa_circ_0051208 | 92 | 1 | 1223 | 1 | 1223 | 0.01 | 0 |
| hsa_circ_0000937 | 92 | 12 | 373 | 15 | 376 | 0 | 1.00E-144 |
| hsa_circ_0015392 | 95 | 1 | 255 | 1 | 255 | 0 | 6.00E-112 |
| hsa_circ_0083278 | 89 | 1 | 849 | 5730 | 4882 | 0 | 0 |
| hsa_circ_0091354 | 96 | 1 | 373 | 515 | 887 | 0 | 2.00E-170 |
| hsa_circ_0091354 | 97 | 1 | 249 | 515 | 763 | 0 | 6.00E-117 |
| hsa_circ_0086398 | 83 | 1 | 1335 | 1 | 1317 | 0.05 | 0 |
| hsa_circ_0086399 | 91 | 1 | 611 | 1 | 611 | 0 | 0 |
| hsa_circ_0086399 | 83 | 1 | 1250 | 1 | 1232 | 0.05 | 0 |
| hsa_circ_0086401 | 78 | 1 | 690 | 1 | 680 | 0.08 | 5.00E-107 |
| hsa_circ_0086401 | 77 | 1 | 747 | 1 | 729 | 0.09 | 2.00E-109 |
| hsa_circ_0086402 | 76 | 2 | 639 | 2 | 621 | 0.1 | 2.00E-75 |
| hsa_circ_0086406 | 93 | 1 | 307 | 2897 | 2591 | 0 | 2.00E-127 |
| hsa_circ_0069901 | 93 | 7 | 288 | 282 | 1 | 0 | 7.00E-112 |
| hsa_circ_0069898 | 93 | 7 | 533 | 1425 | 899 | 0 | 0 |
| hsa_circ_0069898 | 92 | 7 | 540 | 1425 | 893 | 0 | 0 |
| hsa_circ_0069896 | 91 | 7 | 846 | 984 | 145 | 0 | 0 |
| hsa_circ_0069888 | 91 | 7 | 1583 | 1578 | 2 | 0 | 0 |
| hsa_circ_0069898 | 93 | 10 | 252 | 1134 | 893 | 0 | 4.00E-95 |
| hsa_circ_0069896 | 90 | 4 | 311 | 452 | 145 | 0 | 6.00E-108 |
| hsa_circ_0069888 | 91 | 4 | 1048 | 1046 | 2 | 0 | 0 |
| hsa_circ_0069888 | 92 | 1 | 593 | 594 | 2 | 0 | 0 |
| hsa_circ_0069890 | 92 | 1 | 341 | 341 | 1 | 0 | 3.00E-136 |
| hsa_circ_0069888 | 93 | 1 | 446 | 447 | 2 | 0 | 0 |
| hsa_circ_0069888 | 93 | 3 | 442 | 441 | 2 | 0 | 0 |
| hsa_circ_0069878 | 95 | 1 | 1181 | 1181 | 1 | 0 | 0 |
| hsa_circ_0069878 | 95 | 1 | 623 | 623 | 1 | 0 | 0 |
| hsa_circ_0069869 | 91 | 1 | 118 | 118 | 1 | 0 | 2.00E-37 |
| hsa_circ_0069869 | 91 | 1 | 118 | 118 | 1 | 0 | 6.00E-37 |
| hsa_circ_0069869 | 90 | 1 | 71 | 71 | 1 | 0 | 6.00E-18 |
| hsa_circ_0069867 | 84 | 13 | 1061 | 1040 | 1 | 0.02 | 0 |
| hsa_circ_0069867 | 84 | 1 | 628 | 619 | 1 | 0.02 | 1.00E-172 |
| hsa_circ_0069867 | 87 | 1 | 485 | 485 | 1 | 0 | 1.00E-157 |
| hsa_circ_0069867 | 87 | 1 | 485 | 485 | 1 | 0 | 1.00E-157 |
| hsa_circ_0066215 | 91 | 1 | 297 | 297 | 1 | 0 | 3.00E-110 |

novel_circ_028603 novel_circ_028604 novel_circ_028605 novel_circ_028606 novel_circ_028607 novel_circ_028608 novel_circ_028609 novel_circ_028610 novel_circ_028611 novel_circ_028612 novel_circ_028615 novel_circ_028616 novel_circ_028617 novel_circ_028618 novel_circ_028619 novel_circ_028620 novel_circ_028621 novel_circ_028622 novel_circ_028623 novel_circ_028624 novel_circ_028625 novel_circ_028626 novel_circ_028627 novel_circ_028628 novel_circ_028629 novel_circ_028630 novel_circ_028631 novel_circ_028632 novel_circ_028633 novel_circ_028634 novel_circ_028635 novel_circ_028636 novel_circ_028637 novel_circ_028638 novel_circ_028639 novel_circ_028640 novel_circ_028641 novel_circ_028642 novel_circ_028643 novel_circ_028648 novel_circ_028650 novel_circ_028651 novel_circ_028652 novel_circ_028653 novel_circ_028654 novel_circ_028655 novel_circ_028656 novel_circ_028657 novel_circ_028658 novel_circ_028659 novel_circ_028660 novel_circ_028661 novel_circ_028689 novel_circ_028690

| hsa_circ_0066216 | 84 | 449 | 614 | 1 | 166 | 0 | 5.00E-37 |
| --- | --- | --- | --- | --- | --- | --- | --- |
| hsa_circ_0066216 | 86 | 449 | 718 | 1 | 270 | 0 | 9.00E-75 |
| hsa_circ_0066216 | 86 | 449 | 718 | 1 | 270 | 0 | 2.00E-74 |
| hsa_circ_0066216 | 88 | 449 | 840 | 1 | 392 | 0 | 8.00E-131 |
| hsa_circ_0066216 | 89 | 449 | 1308 | 1 | 860 | 0 | 0 |
| hsa_circ_0066219 | 88 | 853 | 1756 | 13 | 907 | 0.01 | 0 |
| hsa_circ_0066216 | 86 | 1 | 270 | 1 | 270 | 0 | 3.00E-75 |
| hsa_circ_0066216 | 88 | 1 | 392 | 1 | 392 | 0 | 3.00E-131 |
| hsa_circ_0066216 | 90 | 1 | 694 | 167 | 860 | 0 | 0 |
| hsa_circ_0066219 | 91 | 1 | 245 | 384 | 627 | 0 | 4.00E-89 |
| hsa_circ_0066221 | 96 | 1 | 936 | 4448 | 3513 | 0 | 0 |
| hsa_circ_0066221 | 90 | 1 | 2471 | 5980 | 3513 | 0.01 | 0 |
| hsa_circ_0066221 | 91 | 993 | 4517 | 7034 | 3513 | 0.01 | 0 |
| hsa_circ_0066222 | 95 | 1 | 736 | 736 | 1 | 0 | 0 |
| hsa_circ_0066222 | 89 | 1 | 2271 | 2268 | 1 | 0.01 | 0 |
| hsa_circ_0066222 | 91 | 993 | 4317 | 3322 | 1 | 0.01 | 0 |
| hsa_circ_0066225 | 88 | 1 | 1658 | 1815 | 161 | 0.02 | 0 |
| hsa_circ_0066226 | 87 | 1 | 1535 | 1532 | 1 | 0.02 | 0 |
| hsa_circ_0066227 | 87 | 1 | 552 | 549 | 1 | 0.01 | 8.00E-174 |
| hsa_circ_0066227 | 90 | 1 | 1399 | 1396 | 1 | 0 | 0 |
| hsa_circ_0066225 | 91 | 417 | 2286 | 3133 | 1267 | 0 | 0 |
| hsa_circ_0066225 | 91 | 729 | 2598 | 3133 | 1267 | 0 | 0 |
| hsa_circ_0066227 | 85 | 1 | 407 | 549 | 146 | 0.01 | 5.00E-115 |
| hsa_circ_0066227 | 90 | 1 | 1109 | 1251 | 146 | 0 | 0 |
| hsa_circ_0066231 | 94 | 253 | 1032 | 780 | 1 | 0 | 0 |
| hsa_circ_0066231 | 94 | 565 | 1344 | 780 | 1 | 0 | 0 |
| hsa_circ_0001315 | 95 | 419 | 1053 | 635 | 1 | 0 | 0 |
| hsa_circ_0001315 | 95 | 565 | 1199 | 635 | 1 | 0 | 0 |
| hsa_circ_0066236 | 95 | 253 | 679 | 531 | 105 | 0 | 0 |
| hsa_circ_0066241 | 94 | 1 | 566 | 691 | 126 | 0 | 0 |
| hsa_circ_0066242 | 93 | 1 | 258 | 258 | 1 | 0 | 1.00E-103 |
| hsa_circ_0066242 | 94 | 2344 | 2491 | 406 | 259 | 0 | 2.00E-56 |
| hsa_circ_0066243 | 91 | 4 | 258 | 255 | 1 | 0 | 5.00E-95 |
| hsa_circ_0066245 | 94 | 3 | 364 | 978 | 617 | 0 | 1.00E-154 |
| hsa_circ_0066252 | 93 | 1 | 348 | 290 | 637 | 0 | 8.00E-142 |
| hsa_circ_0066252 | 94 | 1 | 539 | 290 | 828 | 0 | 0 |
| hsa_circ_0066252 | 93 | 1 | 723 | 290 | 1015 | 0 | 0 |
| hsa_circ_0066252 | 93 | 1 | 776 | 290 | 1068 | 0 | 0 |
| hsa_circ_0066252 | 95 | 1 | 500 | 1069 | 1568 | 0 | 0 |
| hsa_circ_0021758 | 87 | 12 | 628 | 617 | 1 | 0.01 | 0 |
| hsa_circ_0033089 | 92 | 1 | 478 | 2516 | 2039 | 0 | 0 |
| hsa_circ_0033081 | 97 | 1 | 779 | 10084 | 9306 | 0 | 0 |
| hsa_circ_0033081 | 97 | 1 | 948 | 10084 | 9137 | 0 | 0 |
| hsa_circ_0033081 | 96 | 1 | 1421 | 10084 | 8664 | 0 | 0 |
| hsa_circ_0033081 | 95 | 1 | 1952 | 10084 | 8133 | 0 | 0 |
| hsa_circ_0033081 | 95 | 1 | 1232 | 9895 | 8664 | 0 | 0 |
| hsa_circ_0033081 | 94 | 1 | 1763 | 9895 | 8133 | 0 | 0 |
| hsa_circ_0033088 | 94 | 2 | 642 | 1172 | 532 | 0 | 0 |
| hsa_circ_0033085 | 93 | 4 | 1013 | 1010 | 1 | 0 | 0 |
| hsa_circ_0033085 | 91 | 1 | 289 | 465 | 177 | 0 | 4.00E-109 |
| hsa_circ_0033085 | 91 | 1 | 292 | 465 | 174 | 0 | 5.00E-109 |
| hsa_circ_0033085 | 92 | 1 | 465 | 465 | 1 | 0 | 0 |
| hsa_circ_0033061 | 86 | 727 | 1897 | 1271 | 77 | 0.03 | 0 |
| hsa_circ_0033061 | 86 | 1 | 1171 | 1271 | 77 | 0.03 | 0 |

novel_circ_028691 novel_circ_028692 novel_circ_028693 novel_circ_028694 novel_circ_028695 novel_circ_028696 novel_circ_028697 novel_circ_028698 novel_circ_028699 novel_circ_028700 novel_circ_028701 novel_circ_028702 novel_circ_028703 novel_circ_028704 novel_circ_028705 novel_circ_028706 novel_circ_028707 novel_circ_028708 novel_circ_028709 novel_circ_028710 novel_circ_028712 novel_circ_028713 novel_circ_028714 novel_circ_028715 novel_circ_028716 novel_circ_028717 novel_circ_028718 novel_circ_028719 novel_circ_028720 novel_circ_028721 novel_circ_028722 novel_circ_028723 novel_circ_028724 novel_circ_028725 novel_circ_028726 novel_circ_028727 novel_circ_028728 novel_circ_028729 novel_circ_028730 novel_circ_028731 novel_circ_028732 novel_circ_028733 novel_circ_028734 novel_circ_028735 novel_circ_028736 novel_circ_028737 novel_circ_028738 novel_circ_028739 novel_circ_028740 novel_circ_028741 novel_circ_028742 novel_circ_028743 novel_circ_028744 novel_circ_028745

| hsa_circ_0033055 | 91 | 1 | 463 | 1286 | 1748 | 0 | 6.00E-179 |
| --- | --- | --- | --- | --- | --- | --- | --- |
| hsa_circ_0033042 | 90 | 1 | 2549 | 4207 | 6755 | 0 | 0 |
| hsa_circ_0033042 | 90 | 1 | 3182 | 3574 | 6755 | 0 | 0 |
| hsa_circ_0033055 | 91 | 1 | 453 | 1020 | 1472 | 0 | 1.00E-171 |
| hsa_circ_0033042 | 90 | 1 | 2999 | 3574 | 6572 | 0 | 0 |
| hsa_circ_0033055 | 93 | 1 | 193 | 284 | 476 | 0 | 3.00E-74 |
| hsa_circ_0033053 | 95 | 12 | 406 | 12 | 406 | 0 | 7.00E-178 |
| hsa_circ_0033048 | 85 | 1 | 1329 | 3266 | 4594 | 0.01 | 0 |
| hsa_circ_0033048 | 88 | 1 | 1962 | 2633 | 4594 | 0 | 0 |
| hsa_circ_0033048 | 85 | 1 | 1221 | 3266 | 4486 | 0.01 | 0 |
| hsa_circ_0033048 | 88 | 1 | 1854 | 2633 | 4486 | 0 | 0 |
| hsa_circ_0033048 | 92 | 1 | 1380 | 1540 | 2919 | 0 | 0 |
| hsa_circ_0033048 | 93 | 1 | 340 | 1911 | 2250 | 0 | 1.00E-140 |
| hsa_circ_0033048 | 92 | 1 | 711 | 1540 | 2250 | 0 | 0 |
| hsa_circ_0033048 | 93 | 1 | 1508 | 403 | 1910 | 0 | 0 |
| hsa_circ_0033048 | 92 | 1 | 1910 | 1 | 1910 | 0 | 0 |
| hsa_circ_0033048 | 93 | 1 | 1137 | 403 | 1539 | 0 | 0 |
| hsa_circ_0033048 | 92 | 1 | 1539 | 1 | 1539 | 0 | 0 |
| hsa_circ_0033048 | 92 | 1 | 546 | 403 | 948 | 0 | 0 |
| hsa_circ_0033048 | 92 | 1 | 948 | 1 | 948 | 0 | 0 |
| hsa_circ_0033048 | 92 | 1 | 403 | 231 | 633 | 0 | 3.00E-157 |
| hsa_circ_0033048 | 91 | 1 | 633 | 1 | 633 | 0 | 0 |
| hsa_circ_0033043 | 93 | 1 | 857 | 187 | 1043 | 0 | 0 |
| hsa_circ_0033042 | 93 | 10030 | 11338 | 267 | 1574 | 0.01 | 0 |
| hsa_circ_0033048 | 91 | 1 | 402 | 1 | 402 | 0 | 2.00E-153 |
| hsa_circ_0033043 | 93 | 1 | 626 | 187 | 812 | 0 | 0 |
| hsa_circ_0033042 | 94 | 10030 | 11107 | 267 | 1343 | 0.01 | 0 |
| hsa_circ_0033042 | 95 | 10030 | 10705 | 267 | 941 | 0.01 | 0 |
| hsa_circ_0033042 | 95 | 10992 | 11667 | 267 | 941 | 0.01 | 0 |
| hsa_circ_0033042 | 94 | 10030 | 10481 | 267 | 717 | 0.02 | 0 |
| hsa_circ_0033042 | 94 | 10992 | 11443 | 267 | 717 | 0.02 | 0 |
| hsa_circ_0033042 | 93 | 10030 | 10295 | 267 | 531 | 0.03 | 8.00E-106 |
| hsa_circ_0033042 | 93 | 10992 | 11257 | 267 | 531 | 0.03 | 9.00E-106 |
| hsa_circ_0033042 | 96 | 6283 | 6607 | 117 | 436 | 0.02 | 5.00E-146 |
| hsa_circ_0000563 | 97 | 1 | 1268 | 1268 | 1 | 0 | 0 |
| hsa_circ_0000562 | 93 | 1 | 1620 | 2499 | 872 | 0 | 0 |
| hsa_circ_0000562 | 93 | 1 | 1620 | 2499 | 872 | 0 | 0 |
| hsa_circ_0000562 | 93 | 1 | 1620 | 2499 | 872 | 0 | 0 |
| hsa_circ_0000562 | 93 | 1 | 1620 | 2499 | 872 | 0 | 0 |
| hsa_circ_0033029 | 86 | 1 | 628 | 55 | 682 | 0 | 0 |
| hsa_circ_0033029 | 93 | 1 | 160 | 1 | 160 | 0 | 5.00E-61 |
| hsa_circ_0004080 | 93 | 1 | 195 | 1 | 195 | 0 | 8.00E-77 |
| hsa_circ_0042523 | 97 | 240 | 452 | 842 | 630 | 0 | 1.00E-96 |
| hsa_circ_0042540 | 92 | 952 | 1480 | 1 | 529 | 0 | 0 |
| hsa_circ_0042540 | 92 | 1 | 293 | 237 | 529 | 0 | 1.00E-114 |
| hsa_circ_0042548 | 91 | 7 | 461 | 2026 | 1572 | 0 | 2.00E-174 |
| hsa_circ_0042583 | 92 | 1 | 1722 | 1722 | 1 | 0 | 0 |
| hsa_circ_0042596 | 92 | 1 | 683 | 683 | 1 | 0 | 0 |
| hsa_circ_0042604 | 93 | 1 | 943 | 943 | 1 | 0 | 0 |
| hsa_circ_0042610 | 94 | 1 | 365 | 365 | 1 | 0 | 7.00E-158 |
| hsa_circ_0042610 | 94 | 1 | 297 | 365 | 69 | 0 | 3.00E-125 |
| hsa_circ_0042653 | 93 | 7 | 1392 | 630 | 1994 | 0.02 | 0 |
| hsa_circ_0042653 | 93 | 4 | 1389 | 630 | 1994 | 0.02 | 0 |
| hsa_circ_0042655 | 94 | 3 | 788 | 3 | 788 | 0 | 0 |

novel_circ_028746 novel_circ_028747 novel_circ_028748 novel_circ_028750 novel_circ_028751 novel_circ_028752 novel_circ_028753 novel_circ_028756 novel_circ_028757 novel_circ_028758 novel_circ_028759 novel_circ_028760 novel_circ_028761 novel_circ_028762 novel_circ_028763 novel_circ_028764 novel_circ_028765 novel_circ_028766 novel_circ_028767 novel_circ_028768 novel_circ_028769 novel_circ_028770 novel_circ_028771 novel_circ_028772 novel_circ_028773 novel_circ_028774 novel_circ_028775 novel_circ_028776 novel_circ_028777 novel_circ_028778 novel_circ_028779 novel_circ_028780 novel_circ_028781 novel_circ_028782 novel_circ_028783 novel_circ_028784 novel_circ_028785 novel_circ_028786 novel_circ_028787 novel_circ_028788 novel_circ_028789 novel_circ_028790 novel_circ_028791 novel_circ_028792 novel_circ_028793 novel_circ_028794 novel_circ_028798 novel_circ_028799 novel_circ_028800 novel_circ_028801 novel_circ_028802 novel_circ_028803 novel_circ_028804 novel_circ_028805

| hsa_circ_0042662 | 91 | 1 | 473 | 1 | 452 | 0.04 | 2.00E-173 |
| --- | --- | --- | --- | --- | --- | --- | --- |
| hsa_circ_0042663 | 92 | 2 | 880 | 235 | 1113 | 0 | 0 |
| hsa_circ_0042663 | 92 | 2 | 1351 | 235 | 1584 | 0 | 0 |
| hsa_circ_0042676 | 95 | 1 | 306 | 137 | 442 | 0 | 2.00E-133 |
| hsa_circ_0007831 | 95 | 1 | 813 | 813 | 1 | 0 | 0 |
| hsa_circ_0042734 | 94 | 1 | 648 | 648 | 1 | 0 | 0 |
| hsa_circ_0007831 | 94 | 1 | 721 | 886 | 166 | 0 | 0 |
| hsa_circ_0042746 | 89 | 1 | 1059 | 1272 | 196 | 0.02 | 0 |
| hsa_circ_0042746 | 89 | 1 | 1062 | 1275 | 196 | 0.02 | 0 |
| hsa_circ_0042757 | 94 | 2 | 225 | 2 | 226 | 0 | 4.00E-93 |
| hsa_circ_0042757 | 95 | 2 | 297 | 2 | 298 | 0 | 1.00E-129 |
| hsa_circ_0042757 | 95 | 2 | 399 | 2 | 400 | 0 | 0 |
| hsa_circ_0042757 | 95 | 2 | 542 | 2 | 543 | 0 | 0 |
| hsa_circ_0042757 | 95 | 2 | 656 | 2 | 657 | 0 | 0 |
| hsa_circ_0042757 | 95 | 2 | 748 | 2 | 749 | 0 | 0 |
| hsa_circ_0042757 | 96 | 2 | 842 | 2 | 843 | 0 | 0 |
| hsa_circ_0042757 | 96 | 2 | 924 | 2 | 925 | 0 | 0 |
| hsa_circ_0042757 | 96 | 2 | 1092 | 2 | 1093 | 0 | 0 |
| hsa_circ_0042757 | 96 | 2 | 1295 | 2 | 1296 | 0 | 0 |
| hsa_circ_0042757 | 95 | 2 | 2637 | 2 | 2638 | 0 | 0 |
| hsa_circ_0042757 | 95 | 1 | 396 | 4 | 400 | 0 | 4.00E-180 |
| hsa_circ_0042762 | 96 | 1 | 451 | 1 | 451 | 0 | 0 |
| hsa_circ_0042762 | 95 | 1 | 349 | 103 | 451 | 0 | 2.00E-157 |
| hsa_circ_0042765 | 97 | 1 | 382 | 1 | 382 | 0 | 0 |
| hsa_circ_0000752 | 97 | 1 | 550 | 512 | 1061 | 0 | 0 |
| hsa_circ_0000752 | 96 | 1 | 753 | 512 | 1264 | 0 | 0 |
| hsa_circ_0003928 | 99 | 1 | 268 | 1 | 268 | 0 | 2.00E-132 |
| hsa_circ_0003928 | 96 | 1 | 639 | 1 | 639 | 0 | 0 |
| hsa_circ_0003928 | 96 | 1 | 775 | 1 | 775 | 0 | 0 |
| hsa_circ_0003928 | 95 | 1 | 1012 | 1 | 1012 | 0 | 0 |
| hsa_circ_0003928 | 95 | 1 | 453 | 187 | 639 | 0 | 0 |
| hsa_circ_0042767 | 94 | 1 | 371 | 1 | 371 | 0 | 1.00E-159 |
| hsa_circ_0042767 | 94 | 1 | 744 | 1 | 744 | 0 | 0 |
| hsa_circ_0042767 | 95 | 1 | 339 | 169 | 507 | 0 | 4.00E-150 |
| hsa_circ_0042769 | 94 | 1 | 573 | 373 | 945 | 0 | 0 |
| hsa_circ_0042757 | 94 | 1 | 969 | 1670 | 2638 | 0 | 0 |
| hsa_circ_0042770 | 94 | 1 | 396 | 1 | 396 | 0 | 2.00E-173 |
| hsa_circ_0042796 | 92 | 1 | 835 | 835 | 1 | 0 | 0 |
| hsa_circ_0042803 | 95 | 1 | 395 | 395 | 1 | 0 | 3.00E-176 |
| hsa_circ_0042803 | 95 | 1 | 524 | 524 | 1 | 0 | 0 |
| hsa_circ_0042803 | 94 | 1 | 813 | 813 | 1 | 0 | 0 |
| hsa_circ_0042798 | 94 | 1 | 1239 | 1689 | 451 | 0 | 0 |
| hsa_circ_0042806 | 94 | 1 | 747 | 747 | 1 | 0 | 0 |
| hsa_circ_0042806 | 93 | 1 | 844 | 1038 | 195 | 0 | 0 |
| hsa_circ_0042821 | 93 | 1 | 426 | 426 | 1 | 0 | 5.00E-180 |
| hsa_circ_0042824 | 94 | 1 | 291 | 291 | 1 | 0 | 2.00E-123 |
| hsa_circ_0091182 | 93 | 1 | 507 | 642 | 124 | 0.02 | 0 |
| hsa_circ_0091182 | 91 | 4 | 360 | 492 | 124 | 0.03 | 2.00E-138 |
| hsa_circ_0005447 | 94 | 46532 | 46640 | 7361 | 7469 | 0 | 1.00E-36 |
| hsa_circ_0019014 | 88 | 1 | 525 | 2052 | 1528 | 0 | 7.00E-174 |
| hsa_circ_0019014 | 91 | 1 | 1684 | 2052 | 387 | 0.01 | 0 |
| hsa_circ_0019014 | 92 | 1 | 1044 | 1531 | 506 | 0.02 | 0 |
| hsa_circ_0019014 | 92 | 1 | 1163 | 1531 | 387 | 0.02 | 0 |
| hsa_circ_0019014 | 94 | 4 | 386 | 383 | 1 | 0 | 7.00E-163 |

novel_circ_028806 novel_circ_028807 novel_circ_028808 novel_circ_028809 novel_circ_028810 novel_circ_028812 novel_circ_028813 novel_circ_028815 novel_circ_028816 novel_circ_028818 novel_circ_028819 novel_circ_028820 novel_circ_028821 novel_circ_028822 novel_circ_028823 novel_circ_028824 novel_circ_028825 novel_circ_028826 novel_circ_028827 novel_circ_028828 novel_circ_028829 novel_circ_028830 novel_circ_028831 novel_circ_028832 novel_circ_028833 novel_circ_028835 novel_circ_028836 novel_circ_028837 novel_circ_028838 novel_circ_028839 novel_circ_028840 novel_circ_028842 novel_circ_028844 novel_circ_028845 novel_circ_028846 novel_circ_028847 novel_circ_028848 novel_circ_028849 novel_circ_028850 novel_circ_028852 novel_circ_028853 novel_circ_028854 novel_circ_028855 novel_circ_028859 novel_circ_028860 novel_circ_028861 novel_circ_028862 novel_circ_028863 novel_circ_028864 novel_circ_028865 novel_circ_028866 novel_circ_028867 novel_circ_028868 novel_circ_028869

| hsa_circ_0019009 | 93 | 4 | 726 | 1302 | 580 | 0 | 0 |
| --- | --- | --- | --- | --- | --- | --- | --- |
| hsa_circ_0019009 | 93 | 1 | 917 | 1209 | 293 | 0 | 0 |
| hsa_circ_0019006 | 86 | 1 | 457 | 457 | 1 | 0 | 7.00E-139 |
| hsa_circ_0019004 | 85 | 4 | 373 | 370 | 1 | 0 | 1.00E-105 |
| hsa_circ_0018999 | 92 | 1 | 283 | 716 | 434 | 0 | 2.00E-107 |
| hsa_circ_0067830 | 95 | 2 | 534 | 121 | 653 | 0 | 0 |
| hsa_circ_0067830 | 95 | 2 | 316 | 121 | 435 | 0 | 8.00E-137 |
| hsa_circ_0075797 | 91 | 5 | 367 | 5 | 367 | 0 | 4.00E-135 |
| hsa_circ_0075797 | 90 | 5 | 306 | 5 | 306 | 0 | 1.00E-109 |
| hsa_circ_0075791 | 89 | 1 | 519 | 76 | 594 | 0 | 0 |
| hsa_circ_0075790 | 87 | 125 | 854 | 5909 | 6638 | 0 | 0 |
| hsa_circ_0075790 | 87 | 435 | 1164 | 5909 | 6638 | 0 | 0 |
| hsa_circ_0075790 | 87 | 510 | 1239 | 5909 | 6638 | 0 | 0 |
| hsa_circ_0075790 | 87 | 687 | 1416 | 5909 | 6638 | 0 | 0 |
| hsa_circ_0075788 | 91 | 1 | 390 | 1 | 390 | 0 | 2.00E-146 |
| hsa_circ_0075788 | 91 | 1 | 390 | 1 | 390 | 0 | 2.00E-146 |
| hsa_circ_0075788 | 91 | 1 | 390 | 1 | 390 | 0 | 1.00E-146 |
| hsa_circ_0075788 | 91 | 1 | 390 | 1 | 390 | 0 | 9.00E-147 |
| hsa_circ_0075780 | 91 | 1 | 312 | 129 | 440 | 0 | 4.00E-115 |
| hsa_circ_0075779 | 89 | 4 | 539 | 5 | 540 | 0 | 0 |
| hsa_circ_0075779 | 91 | 4 | 429 | 5 | 430 | 0 | 5.00E-160 |
| hsa_circ_0075774 | 86 | 1 | 392 | 2859 | 2468 | 0.01 | 8.00E-118 |
| hsa_circ_0075767 | 95 | 1 | 274 | 2570 | 2297 | 0 | 2.00E-117 |
| hsa_circ_0075767 | 94 | 1 | 362 | 2570 | 2209 | 0 | 1.00E-154 |
| hsa_circ_0075767 | 92 | 1 | 610 | 2570 | 1961 | 0 | 0 |
| hsa_circ_0075764 | 88 | 1 | 432 | 432 | 1 | 0 | 1.00E-146 |
| hsa_circ_0075755 | 88 | 1 | 1428 | 1428 | 1 | 0 | 0 |
| hsa_circ_0075741 | 90 | 4 | 744 | 738 | 1 | 0 | 0 |
| hsa_circ_0075735 | 89 | 4 | 1104 | 1101 | 1 | 0.01 | 0 |
| hsa_circ_0075731 | 89 | 4 | 1147 | 1154 | 11 | 0.01 | 0 |
| hsa_circ_0003576 | 86 | 4 | 537 | 540 | 1 | 0.01 | 1.00E-166 |
| hsa_circ_0075719 | 82 | 1 | 1066 | 1069 | 1 | 0.01 | 0 |
| hsa_circ_0075707 | 85 | 1 | 1912 | 2542 | 631 | 0.01 | 0 |
| hsa_circ_0075719 | 82 | 65 | 744 | 683 | 1 | 0 | 8.00E-165 |
| hsa_circ_0075708 | 88 | 1 | 1080 | 1437 | 358 | 0.01 | 0 |
| hsa_circ_0075708 | 90 | 1 | 885 | 1242 | 358 | 0.01 | 0 |
| hsa_circ_0007960 | 88 | 1 | 207 | 367 | 573 | 0 | 2.00E-65 |
| hsa_circ_0007960 | 87 | 1 | 380 | 191 | 573 | 0.01 | 1.00E-116 |
| hsa_circ_0007960 | 86 | 1 | 570 | 1 | 573 | 0.01 | 2.00E-175 |
| hsa_circ_0075697 | 90 | 1 | 637 | 1 | 637 | 0 | 0 |
| hsa_circ_0075697 | 89 | 1 | 301 | 1 | 301 | 0 | 2.00E-102 |
| hsa_circ_0075697 | 88 | 1 | 219 | 1 | 219 | 0 | 1.00E-68 |
| hsa_circ_0064026 | 81 | 50369 | 51539 | 248752 | 247557 | 0.04 | 0 |
| hsa_circ_0007133 | 76 | 49930 | 50178 | 223986 | 223740 | 0.03 | 1.00E-23 |
| hsa_circ_0007133 | 76 | 36465 | 36713 | 223986 | 223740 | 0.03 | 9.00E-24 |
| hsa_circ_0001675 | 91 | 1 | 905 | 1 | 905 | 0 | 0 |
| hsa_circ_0067389 | 94 | 3 | 406 | 404 | 1 | 0 | 2.00E-174 |
| hsa_circ_0067387 | 94 | 3 | 510 | 767 | 260 | 0 | 0 |
| hsa_circ_0067387 | 93 | 3 | 769 | 767 | 1 | 0 | 0 |
| hsa_circ_0067387 | 93 | 3 | 769 | 767 | 1 | 0 | 0 |
| hsa_circ_0067387 | 93 | 1 | 612 | 612 | 1 | 0 | 0 |
| hsa_circ_0067387 | 94 | 1 | 278 | 537 | 260 | 0 | 5.00E-118 |
| hsa_circ_0067387 | 93 | 1 | 537 | 537 | 1 | 0 | 0 |
| hsa_circ_0067387 | 92 | 1 | 259 | 259 | 1 | 0 | 4.00E-99 |

novel_circ_028870 novel_circ_028871 novel_circ_028872 novel_circ_028873 novel_circ_028874 novel_circ_028875 novel_circ_028876 novel_circ_028877 novel_circ_028878 novel_circ_028879 novel_circ_028880 novel_circ_028881 novel_circ_028882 novel_circ_028883 novel_circ_028884 novel_circ_028889 novel_circ_028890 novel_circ_028891 novel_circ_028892 novel_circ_028893 novel_circ_028894 novel_circ_028895 novel_circ_028896 novel_circ_028897 novel_circ_028898 novel_circ_028899 novel_circ_028900 novel_circ_028901 novel_circ_028902 novel_circ_028905 novel_circ_028906 novel_circ_028907 novel_circ_028908 novel_circ_028909 novel_circ_028910 novel_circ_028911 novel_circ_028912 novel_circ_028913 novel_circ_028914 novel_circ_028917 novel_circ_028918 novel_circ_028919 novel_circ_028920 novel_circ_028921 novel_circ_028922 novel_circ_028923 novel_circ_028924 novel_circ_028925 novel_circ_028926 novel_circ_028927 novel_circ_028928 novel_circ_028929 novel_circ_028930 novel_circ_028931

| hsa_circ_0067385 | 91 | 546 | 1701 | 1523 | 368 | 0 | 0 |
| --- | --- | --- | --- | --- | --- | --- | --- |
| hsa_circ_0067385 | 90 | 287 | 806 | 1523 | 1004 | 0 | 0 |
| hsa_circ_0067385 | 90 | 283 | 802 | 1523 | 1004 | 0 | 0 |
| hsa_circ_0067385 | 90 | 86 | 605 | 1523 | 1004 | 0 | 0 |
| hsa_circ_0067386 | 90 | 7 | 383 | 1254 | 878 | 0 | 2.00E-139 |
| hsa_circ_0067386 | 92 | 2 | 636 | 876 | 242 | 0 | 0 |
| hsa_circ_0067383 | 94 | 77 | 183 | 1 | 107 | 0 | 8.00E-40 |
| hsa_circ_0067382 | 97 | 1 | 318 | 88 | 405 | 0 | 1.00E-151 |
| hsa_circ_0067382 | 96 | 111 | 515 | 1 | 405 | 0 | 0 |
| hsa_circ_0067382 | 96 | 111 | 515 | 1 | 405 | 0 | 0 |
| hsa_circ_0067381 | 90 | 3 | 264 | 732 | 474 | 0.01 | 5.00E-93 |
| hsa_circ_0067381 | 90 | 3 | 369 | 732 | 369 | 0.01 | 2.00E-134 |
| hsa_circ_0067381 | 88 | 3 | 498 | 732 | 240 | 0.01 | 9.00E-168 |
| hsa_circ_0067380 | 89 | 3 | 922 | 984 | 68 | 0 | 0 |
| hsa_circ_0007818 | 90 | 4 | 424 | 421 | 1 | 0 | 3.00E-152 |
| hsa_circ_0020095 | 96 | 1 | 728 | 340 | 1067 | 0 | 0 |
| hsa_circ_0020095 | 95 | 1 | 942 | 340 | 1281 | 0 | 0 |
| hsa_circ_0020095 | 94 | 1 | 1347 | 340 | 1689 | 0 | 0 |
| hsa_circ_0020095 | 94 | 1 | 1347 | 340 | 1689 | 0 | 0 |
| hsa_circ_0020095 | 94 | 1 | 934 | 753 | 1689 | 0 | 0 |
| hsa_circ_0020095 | 94 | 1 | 934 | 753 | 1689 | 0 | 0 |
| hsa_circ_0020095 | 94 | 1 | 934 | 753 | 1689 | 0 | 0 |
| hsa_circ_0020095 | 94 | 1 | 801 | 886 | 1689 | 0 | 0 |
| hsa_circ_0020095 | 93 | 1 | 619 | 1068 | 1689 | 0 | 0 |
| hsa_circ_0020095 | 93 | 1 | 619 | 1068 | 1689 | 0 | 0 |
| hsa_circ_0020095 | 92 | 1 | 216 | 1471 | 1689 | 0.01 | 3.00E-82 |
| hsa_circ_0020095 | 92 | 1 | 216 | 1471 | 1689 | 0.01 | 3.00E-82 |
| hsa_circ_0020095 | 92 | 1 | 216 | 1471 | 1689 | 0.01 | 4.00E-82 |
| hsa_circ_0026789 | 91 | 1 | 216 | 267 | 52 | 0 | 6.00E-78 |
| hsa_circ_0026823 | 95 | 1 | 269 | 1 | 269 | 0 | 2.00E-116 |
| hsa_circ_0026849 | 94 | 1 | 384 | 1 | 384 | 0 | 9.00E-167 |
| hsa_circ_0026905 | 94 | 1 | 301 | 301 | 1 | 0 | 9.00E-126 |
| hsa_circ_0026905 | 95 | 1757 | 1882 | 427 | 302 | 0 | 3.00E-49 |
| hsa_circ_0026905 | 92 | 1 | 449 | 750 | 302 | 0 | 2.00E-179 |
| hsa_circ_0026905 | 93 | 1 | 682 | 983 | 302 | 0 | 0 |
| hsa_circ_0026905 | 94 | 1 | 233 | 983 | 751 | 0 | 2.00E-96 |
| hsa_circ_0026915 | 97 | 1124 | 1238 | 1 | 115 | 0 | 9.00E-48 |
| hsa_circ_0026984 | 93 | 1 | 546 | 546 | 1 | 0 | 0 |
| hsa_circ_0026990 | 93 | 769 | 2621 | 1853 | 1 | 0 | 0 |
| hsa_circ_0027024 | 91 | 303 | 918 | 124 | 741 | 0.01 | 0 |
| hsa_circ_0027024 | 91 | 303 | 1249 | 124 | 1072 | 0 | 0 |
| hsa_circ_0027024 | 91 | 303 | 1438 | 124 | 1261 | 0 | 0 |
| hsa_circ_0027032 | 91 | 1 | 318 | 1 | 318 | 0 | 4.00E-120 |
| hsa_circ_0027032 | 92 | 1 | 476 | 1 | 476 | 0 | 0 |
| hsa_circ_0027035 | 94 | 1 | 309 | 1 | 309 | 0 | 2.00E-128 |
| hsa_circ_0027065 | 87 | 6 | 693 | 749 | 62 | 0 | 0 |
| hsa_circ_0027065 | 92 | 1 | 1611 | 1672 | 62 | 0 | 0 |
| hsa_circ_0027066 | 96 | 1 | 918 | 1137 | 220 | 0 | 0 |
| hsa_circ_0027110 | 94 | 159 | 1140 | 982 | 1 | 0 | 0 |
| hsa_circ_0041553 | 98 | 41456 | 41659 | 3807 | 3604 | 0 | 5.00E-93 |
| hsa_circ_0004805 | 96 | 1 | 504 | 504 | 1 | 0 | 0 |
| hsa_circ_0041553 | 96 | 1 | 506 | 3804 | 3299 | 0 | 0 |
| hsa_circ_0004805 | 96 | 1 | 401 | 401 | 1 | 0 | 0 |
| hsa_circ_0041553 | 96 | 1 | 403 | 3701 | 3299 | 0 | 0 |

novel_circ_028935 novel_circ_028938 novel_circ_028939 novel_circ_028940 novel_circ_028941 novel_circ_028942 novel_circ_028943 novel_circ_028944 novel_circ_028945 novel_circ_028946 novel_circ_028947 novel_circ_028948 novel_circ_028949 novel_circ_028950 novel_circ_028951 novel_circ_028952 novel_circ_028953 novel_circ_028959 novel_circ_028960 novel_circ_028961 novel_circ_028962 novel_circ_028963 novel_circ_028965 novel_circ_028966 novel_circ_028967 novel_circ_028968 novel_circ_028969 novel_circ_028970 novel_circ_028971 novel_circ_028972 novel_circ_028973 novel_circ_028974 novel_circ_028975 novel_circ_028976 novel_circ_028977 novel_circ_028978 novel_circ_028980 novel_circ_028981 novel_circ_028982 novel_circ_028983 novel_circ_028984 novel_circ_028985 novel_circ_028986 novel_circ_028987 novel_circ_028988 novel_circ_028989 novel_circ_028990 novel_circ_028991 novel_circ_028992 novel_circ_028993 novel_circ_028994 novel_circ_028995 novel_circ_028996 novel_circ_029009

| hsa_circ_0090586 | 86 | 1 | 795 | 804 | 1 | 0.01 | 0 |
| --- | --- | --- | --- | --- | --- | --- | --- |
| hsa_circ_0089856 | 84 | 3 | 260 | 3 | 260 | 0 | 6.00E-63 |
| hsa_circ_0089856 | 84 | 3 | 260 | 3 | 260 | 0 | 5.00E-63 |
| hsa_circ_0090818 | 90 | 1 | 656 | 3770 | 3115 | 0.02 | 0 |
| hsa_circ_0090820 | 94 | 8 | 379 | 1320 | 949 | 0 | 9.00E-162 |
| hsa_circ_0090820 | 91 | 1 | 948 | 948 | 1 | 0 | 0 |
| hsa_circ_0090818 | 89 | 1 | 2479 | 2473 | 1 | 0 | 0 |
| hsa_circ_0090818 | 89 | 1 | 2479 | 2473 | 1 | 0 | 0 |
| hsa_circ_0033396 | 83 | 22271 | 22972 | 774 | 1460 | 0.05 | 6.00E-172 |
| hsa_circ_0090818 | 89 | 587 | 2208 | 1616 | 1 | 0 | 0 |
| hsa_circ_0090818 | 89 | 587 | 2208 | 1616 | 1 | 0 | 0 |
| hsa_circ_0090818 | 89 | 1 | 1620 | 1614 | 1 | 0 | 0 |
| hsa_circ_0090818 | 89 | 1 | 1620 | 1614 | 1 | 0 | 0 |
| hsa_circ_0090818 | 92 | 1 | 265 | 1549 | 1285 | 0 | 9.00E-101 |
| hsa_circ_0090818 | 88 | 1 | 1555 | 1549 | 1 | 0.01 | 0 |
| hsa_circ_0090818 | 88 | 4 | 1290 | 1281 | 1 | 0.01 | 0 |
| hsa_circ_0090818 | 88 | 4 | 1290 | 1281 | 1 | 0.01 | 0 |
| hsa_circ_0061760 | 81 | 29045 | 30098 | 9 | 1085 | 0.06 | 0 |
| hsa_circ_0090816 | 91 | 1 | 1154 | 1154 | 1 | 0 | 0 |
| hsa_circ_0090813 | 93 | 173 | 949 | 777 | 1 | 0 | 0 |
| hsa_circ_0090813 | 93 | 1 | 527 | 527 | 1 | 0 | 0 |
| hsa_circ_0090813 | 92 | 1 | 412 | 412 | 1 | 0 | 4.00E-167 |
| hsa_circ_0091475 | 96 | 281 | 598 | 318 | 1 | 0 | 3.00E-143 |
| hsa_circ_0091475 | 96 | 1 | 318 | 318 | 1 | 0 | 2.00E-143 |
| hsa_circ_0091474 | 94 | 1 | 1062 | 1062 | 1 | 0 | 0 |
| hsa_circ_0091474 | 94 | 1 | 409 | 909 | 501 | 0 | 6.00E-174 |
| hsa_circ_0091474 | 94 | 1 | 909 | 909 | 1 | 0 | 0 |
| hsa_circ_0091474 | 95 | 1 | 498 | 498 | 1 | 0 | 0 |
| hsa_circ_0091473 | 96 | 1 | 186 | 186 | 1 | 0 | 9.00E-82 |
| hsa_circ_0091470 | 93 | 211 | 1224 | 1817 | 804 | 0 | 0 |
| hsa_circ_0091470 | 94 | 1 | 1044 | 1453 | 410 | 0 | 0 |
| hsa_circ_0091470 | 94 | 1 | 1453 | 1453 | 1 | 0 | 0 |
| hsa_circ_0091470 | 92 | 1 | 530 | 1333 | 804 | 0 | 0 |
| hsa_circ_0091470 | 94 | 1 | 1333 | 1333 | 1 | 0 | 0 |
| hsa_circ_0091470 | 94 | 1 | 409 | 409 | 1 | 0 | 1.00E-175 |
| hsa_circ_0091469 | 94 | 1 | 409 | 409 | 1 | 0 | 6.00E-174 |
| hsa_circ_0091466 | 96 | 1 | 414 | 696 | 1109 | 0 | 0 |
| hsa_circ_0091466 | 96 | 1 | 543 | 567 | 1109 | 0 | 0 |
| hsa_circ_0091466 | 96 | 1 | 794 | 316 | 1109 | 0 | 0 |
| hsa_circ_0091466 | 97 | 1 | 190 | 920 | 1109 | 0 | 2.00E-87 |
| hsa_circ_0091466 | 95 | 1 | 604 | 316 | 919 | 0 | 0 |
| hsa_circ_0091466 | 95 | 94 | 788 | 1 | 695 | 0 | 0 |
| hsa_circ_0091464 | 95 | 1 | 1103 | 626 | 1728 | 0 | 0 |
| hsa_circ_0091464 | 95 | 1 | 899 | 830 | 1728 | 0 | 0 |
| hsa_circ_0091464 | 95 | 1 | 1229 | 109 | 1337 | 0 | 0 |
| hsa_circ_0091458 | 95 | 1 | 377 | 1224 | 1600 | 0 | 1.00E-169 |
| hsa_circ_0091462 | 97 | 1 | 434 | 263 | 696 | 0 | 0 |
| hsa_circ_0091462 | 97 | 1 | 165 | 1 | 165 | 0 | 9.00E-74 |
| hsa_circ_0091456 | 89 | 1 | 317 | 1 | 323 | 0.02 | 6.00E-108 |
| hsa_circ_0091456 | 89 | 101 | 417 | 1 | 323 | 0.02 | 8.00E-108 |
| hsa_circ_0091456 | 89 | 786 | 1102 | 1 | 323 | 0.02 | 2.00E-107 |
| hsa_circ_0091456 | 89 | 1005 | 1321 | 1 | 323 | 0.02 | 3.00E-107 |
| hsa_circ_0091456 | 89 | 1010 | 1326 | 1 | 323 | 0.02 | 3.00E-107 |
| hsa_circ_0076797 | 94 | 601 | 1245 | 2578 | 1934 | 0 | 0 |

novel_circ_029010 novel_circ_029011 novel_circ_029012 novel_circ_029013 novel_circ_029014 novel_circ_029015 novel_circ_029016 novel_circ_029017 novel_circ_029018 novel_circ_029019 novel_circ_029020 novel_circ_029021 novel_circ_029022 novel_circ_029023 novel_circ_029024 novel_circ_029025 novel_circ_029026 novel_circ_029027 novel_circ_029028 novel_circ_029029 novel_circ_029033 novel_circ_029034 novel_circ_029035 novel_circ_029036 novel_circ_029037 novel_circ_029038 novel_circ_029039 novel_circ_029040 novel_circ_029041 novel_circ_029042 novel_circ_029043 novel_circ_029044 novel_circ_029045 novel_circ_029046 novel_circ_029047 novel_circ_029048 novel_circ_029049 novel_circ_029057 novel_circ_029058 novel_circ_029059 novel_circ_029060 novel_circ_029062 novel_circ_029063 novel_circ_029064 novel_circ_029065 novel_circ_029072 novel_circ_029073 novel_circ_029074 novel_circ_029075 novel_circ_029076 novel_circ_029077 novel_circ_029078 novel_circ_029079 novel_circ_029081

| hsa_circ_0076797 | 94 | 601 | 1780 | 2578 | 1399 | 0 | 0 |
| --- | --- | --- | --- | --- | --- | --- | --- |
| hsa_circ_0076795 | 94 | 1 | 254 | 710 | 457 | 0 | 1.00E-104 |
| hsa_circ_0076790 | 89 | 254 | 2191 | 2384 | 471 | 0.04 | 0 |
| hsa_circ_0076795 | 93 | 1 | 375 | 375 | 1 | 0 | 9.00E-157 |
| hsa_circ_0076781 | 95 | 1 | 400 | 405 | 804 | 0 | 1.00E-178 |
| hsa_circ_0076781 | 93 | 1 | 646 | 159 | 804 | 0 | 0 |
| hsa_circ_0076781 | 94 | 160 | 963 | 1 | 804 | 0 | 0 |
| hsa_circ_0076781 | 95 | 3 | 281 | 526 | 804 | 0 | 7.00E-122 |
| hsa_circ_0076779 | 93 | 1 | 773 | 1 | 773 | 0 | 0 |
| hsa_circ_0076782 | 91 | 1 | 365 | 101 | 465 | 0 | 7.00E-138 |
| hsa_circ_0076779 | 93 | 1 | 682 | 1 | 682 | 0 | 0 |
| hsa_circ_0076781 | 93 | 1 | 404 | 1 | 404 | 0 | 2.00E-164 |
| hsa_circ_0076779 | 92 | 1 | 563 | 1 | 563 | 0 | 0 |
| hsa_circ_0076779 | 92 | 1 | 448 | 1 | 448 | 0 | 3.00E-177 |
| hsa_circ_0076776 | 97 | 56 | 177 | 996 | 875 | 0 | 2.00E-51 |
| hsa_circ_0076775 | 91 | 203 | 1339 | 1263 | 130 | 0.01 | 0 |
| hsa_circ_0076775 | 90 | 203 | 1468 | 1263 | 1 | 0.01 | 0 |
| hsa_circ_0076776 | 94 | 1 | 305 | 794 | 490 | 0 | 1.00E-129 |
| hsa_circ_0076775 | 88 | 15 | 346 | 461 | 130 | 0 | 3.00E-106 |
| hsa_circ_0076775 | 88 | 15 | 475 | 461 | 1 | 0 | 4.00E-151 |
| hsa_circ_0056730 | 95 | 20836 | 20874 | 173 | 211 | 0 | 2.00E-06 |
| hsa_circ_0005720 | 85 | 196 | 679 | 1 | 484 | 0 | 1.00E-133 |
| hsa_circ_0005720 | 84 | 1 | 410 | 1 | 410 | 0 | 2.00E-109 |
| hsa_circ_0005720 | 84 | 196 | 605 | 1 | 410 | 0 | 3.00E-109 |
| hsa_circ_0013229 | 90 | 1 | 682 | 1070 | 1725 | 0.04 | 0 |
| hsa_circ_0013229 | 90 | 1 | 682 | 1070 | 1725 | 0.04 | 0 |
| hsa_circ_0013229 | 92 | 1 | 1502 | 250 | 1725 | 0.02 | 0 |
| hsa_circ_0013229 | 90 | 1 | 822 | 930 | 1725 | 0.03 | 0 |
| hsa_circ_0013229 | 90 | 1 | 1022 | 730 | 1725 | 0.03 | 0 |
| hsa_circ_0013232 | 95 | 1 | 381 | 137 | 517 | 0 | 2.00E-168 |
| hsa_circ_0013232 | 95 | 1 | 480 | 38 | 517 | 0 | 0 |
| hsa_circ_0013232 | 95 | 1 | 517 | 1 | 517 | 0 | 0 |
| hsa_circ_0003310 | 94 | 1 | 372 | 372 | 1 | 0 | 2.00E-158 |
| hsa_circ_0013225 | 92 | 1 | 1313 | 1313 | 1 | 0 | 0 |
| hsa_circ_0013225 | 92 | 1 | 1313 | 1313 | 1 | 0 | 0 |
| hsa_circ_0013225 | 91 | 1 | 584 | 584 | 1 | 0 | 0 |
| hsa_circ_0013225 | 91 | 1 | 584 | 584 | 1 | 0 | 0 |
| hsa_circ_0001809 | 95 | 1 | 351 | 351 | 1 | 0 | 2.00E-158 |
| hsa_circ_0084727 | 97 | 1 | 516 | 516 | 1 | 0 | 0 |
| hsa_circ_0084726 | 95 | 1 | 364 | 831 | 468 | 0 | 5.00E-164 |
| hsa_circ_0052227 | 79 | 183 | 992 | 2 | 817 | 0.03 | 6.00E-153 |
| hsa_circ_0020768 | 89 | 1 | 380 | 1414 | 1035 | 0 | 3.00E-131 |
| hsa_circ_0006209 | 90 | 2 | 456 | 2 | 456 | 0 | 2.00E-165 |
| hsa_circ_0006209 | 90 | 2 | 456 | 2 | 456 | 0 | 4.00E-166 |
| hsa_circ_0006209 | 89 | 2 | 305 | 2 | 305 | 0.01 | 1.00E-100 |
| hsa_circ_0079499 | 93 | 1 | 318 | 1 | 318 | 0 | 8.00E-132 |
| hsa_circ_0007102 | 95 | 1 | 306 | 1 | 306 | 0 | 2.00E-133 |
| hsa_circ_0079488 | 93 | 1 | 944 | 944 | 1 | 0 | 0 |
| hsa_circ_0079488 | 93 | 1 | 750 | 879 | 130 | 0 | 0 |
| hsa_circ_0079488 | 93 | 1 | 879 | 879 | 1 | 0 | 0 |
| hsa_circ_0079488 | 93 | 1 | 641 | 641 | 1 | 0 | 0 |
| hsa_circ_0090443 | 91 | 3 | 519 | 102 | 618 | 0 | 0 |
| hsa_circ_0061305 | 89 | 966 | 1090 | 155 | 279 | 0 | 1.00E-35 |
| hsa_circ_0029821 | 89 | 4 | 1279 | 1279 | 1 | 0 | 0 |

novel_circ_029082 novel_circ_029083 novel_circ_029084 novel_circ_029085 novel_circ_029086 novel_circ_029087 novel_circ_029088 novel_circ_029089 novel_circ_029090 novel_circ_029091 novel_circ_029092 novel_circ_029093 novel_circ_029094 novel_circ_029095 novel_circ_029096 novel_circ_029097 novel_circ_029098 novel_circ_029138 novel_circ_029139 novel_circ_029140 novel_circ_029141 novel_circ_029142 novel_circ_029143 novel_circ_029144 novel_circ_029145 novel_circ_029146 novel_circ_029147 novel_circ_029148 novel_circ_029149 novel_circ_029150 novel_circ_029151 novel_circ_029152 novel_circ_029153 novel_circ_029154 novel_circ_029155 novel_circ_029156 novel_circ_029157 novel_circ_029158 novel_circ_029159 novel_circ_029160 novel_circ_029161 novel_circ_029162 novel_circ_029163 novel_circ_029164 novel_circ_029165 novel_circ_029166 novel_circ_029167 novel_circ_029168 novel_circ_029169 novel_circ_029170 novel_circ_029171 novel_circ_029172 novel_circ_029173 novel_circ_029174

| hsa_circ_0029821 | 90 | 110 | 2043 | 1937 | 1 | 0 | 0 |
| --- | --- | --- | --- | --- | --- | --- | --- |
| hsa_circ_0029823 | 93 | 1 | 182 | 410 | 229 | 0 | 1.00E-68 |
| hsa_circ_0029823 | 91 | 1 | 410 | 410 | 1 | 0 | 4.00E-156 |
| hsa_circ_0029823 | 90 | 4 | 1120 | 1120 | 1 | 0 | 0 |
| hsa_circ_0029826 | 91 | 4 | 569 | 566 | 1 | 0 | 0 |
| hsa_circ_0000468 | 87 | 1 | 448 | 448 | 1 | 0 | 7.00E-144 |
| hsa_circ_0000468 | 91 | 110 | 964 | 855 | 1 | 0 | 0 |
| hsa_circ_0000468 | 90 | 176 | 830 | 855 | 201 | 0 | 0 |
| hsa_circ_0029827 | 94 | 110 | 516 | 407 | 1 | 0 | 9.00E-178 |
| hsa_circ_0029827 | 94 | 176 | 582 | 407 | 1 | 0 | 1.00E-177 |
| hsa_circ_0029829 | 92 | 252 | 463 | 212 | 1 | 0 | 6.00E-81 |
| hsa_circ_0029829 | 92 | 489 | 700 | 212 | 1 | 0 | 8.00E-81 |
| hsa_circ_0029829 | 92 | 1124 | 1335 | 212 | 1 | 0 | 1.00E-80 |
| hsa_circ_0029829 | 92 | 1800 | 2011 | 212 | 1 | 0 | 2.00E-80 |
| hsa_circ_0029829 | 92 | 600 | 811 | 212 | 1 | 0 | 8.00E-81 |
| hsa_circ_0029829 | 92 | 1124 | 1335 | 212 | 1 | 0 | 1.00E-80 |
| hsa_circ_0029829 | 92 | 1928 | 2139 | 212 | 1 | 0 | 2.00E-80 |
| hsa_circ_0029837 | 95 | 1 | 189 | 1 | 189 | 0 | 2.00E-80 |
| hsa_circ_0029837 | 95 | 1 | 260 | 1 | 260 | 0 | 1.00E-114 |
| hsa_circ_0029837 | 97 | 1 | 422 | 1 | 422 | 0 | 0 |
| hsa_circ_0029837 | 97 | 1 | 570 | 1 | 570 | 0 | 0 |
| hsa_circ_0029834 | 85 | 1 | 1279 | 1 | 1301 | 0.05 | 0 |
| hsa_circ_0029844 | 98 | 1 | 300 | 1 | 300 | 0 | 3.00E-145 |
| hsa_circ_0029844 | 98 | 1 | 448 | 1 | 448 | 0 | 0 |
| hsa_circ_0029834 | 85 | 1 | 1157 | 123 | 1301 | 0.05 | 0 |
| hsa_circ_0029844 | 97 | 1 | 696 | 1 | 696 | 0 | 0 |
| hsa_circ_0029847 | 98 | 1 | 381 | 1 | 381 | 0 | 0 |
| hsa_circ_0029834 | 84 | 1 | 1090 | 190 | 1301 | 0.06 | 0 |
| hsa_circ_0029849 | 98 | 1 | 310 | 1 | 310 | 0 | 4.00E-154 |
| hsa_circ_0006416 | 76 | 6634 | 8714 | 6027 | 8123 | 0.07 | 0 |
| hsa_circ_0029849 | 97 | 1 | 558 | 1 | 558 | 0 | 0 |
| hsa_circ_0029849 | 97 | 1 | 721 | 1 | 721 | 0 | 0 |
| hsa_circ_0029847 | 95 | 1 | 1102 | 72 | 1173 | 0 | 0 |
| hsa_circ_0029847 | 95 | 1 | 1265 | 72 | 1336 | 0 | 0 |
| hsa_circ_0029847 | 95 | 1 | 1359 | 72 | 1430 | 0 | 0 |
| hsa_circ_0029849 | 96 | 1 | 396 | 163 | 558 | 0 | 0 |
| hsa_circ_0029849 | 96 | 1 | 559 | 163 | 721 | 0 | 0 |
| hsa_circ_0029849 | 95 | 17203 | 17453 | 308 | 558 | 0 | 8.00E-108 |
| hsa_circ_0029847 | 94 | 17203 | 18254 | 379 | 1430 | 0 | 0 |
| hsa_circ_0029853 | 95 | 1 | 411 | 1 | 411 | 0 | 0 |
| hsa_circ_0029853 | 95 | 1 | 573 | 1 | 573 | 0 | 0 |
| hsa_circ_0029853 | 94 | 1 | 792 | 1 | 792 | 0 | 0 |
| hsa_circ_0029853 | 94 | 1 | 955 | 1 | 955 | 0 | 0 |
| hsa_circ_0029853 | 94 | 1 | 1049 | 1 | 1049 | 0 | 0 |
| hsa_circ_0029853 | 95 | 1 | 1189 | 1 | 1189 | 0 | 0 |
| hsa_circ_0029853 | 95 | 1 | 1319 | 1 | 1319 | 0 | 0 |
| hsa_circ_0029853 | 95 | 1 | 1384 | 1 | 1384 | 0 | 0 |
| hsa_circ_0029853 | 95 | 1 | 1384 | 1 | 1384 | 0 | 0 |
| hsa_circ_0002552 | 94 | 1 | 544 | 1 | 544 | 0 | 0 |
| hsa_circ_0002552 | 94 | 1 | 801 | 1 | 801 | 0 | 0 |
| hsa_circ_0029865 | 93 | 1 | 381 | 1 | 381 | 0 | 9.00E-157 |
| hsa_circ_0029865 | 93 | 1 | 544 | 1 | 544 | 0 | 0 |
| hsa_circ_0029865 | 95 | 1 | 778 | 1 | 778 | 0 | 0 |
| hsa_circ_0029865 | 95 | 1 | 616 | 163 | 778 | 0 | 0 |

novel_circ_029175 novel_circ_029176 novel_circ_029177 novel_circ_029178 novel_circ_029179 novel_circ_029180 novel_circ_029183 novel_circ_029184 novel_circ_029185 novel_circ_029186 novel_circ_029187 novel_circ_029188 novel_circ_029192 novel_circ_029193 novel_circ_029194 novel_circ_029195 novel_circ_029196 novel_circ_029197 novel_circ_029198 novel_circ_029199 novel_circ_029200 novel_circ_029201 novel_circ_029202 novel_circ_029203 novel_circ_029204 novel_circ_029205 novel_circ_029206 novel_circ_029207 novel_circ_029208 novel_circ_029209 novel_circ_029210 novel_circ_029211 novel_circ_029212 novel_circ_029213 novel_circ_029214 novel_circ_029215 novel_circ_029220 novel_circ_029221 novel_circ_029222 novel_circ_029223 novel_circ_029224 novel_circ_029225 novel_circ_029226 novel_circ_029227 novel_circ_029228 novel_circ_029229 novel_circ_029230 novel_circ_029231 novel_circ_029232 novel_circ_029233 novel_circ_029234 novel_circ_029236 novel_circ_029239 novel_circ_029240

| hsa_circ_0000470 | 96 | 1 | 397 | 1 | 397 | 0 | 0 |
| --- | --- | --- | --- | --- | --- | --- | --- |
| hsa_circ_0000470 | 97 | 1 | 527 | 1 | 527 | 0 | 0 |
| hsa_circ_0029870 | 99 | 1 | 67 | 1 | 67 | 0 | 3.00E-25 |
| hsa_circ_0029872 | 90 | 1 | 325 | 330 | 6 | 0 | 8.00E-119 |
| hsa_circ_0029872 | 89 | 10 | 1043 | 1039 | 6 | 0 | 0 |
| hsa_circ_0029872 | 89 | 1 | 1370 | 1372 | 6 | 0 | 0 |
| hsa_circ_0084797 | 94 | 3 | 482 | 121 | 603 | 0.01 | 0 |
| hsa_circ_0084797 | 93 | 3 | 582 | 121 | 706 | 0.01 | 0 |
| hsa_circ_0084797 | 93 | 3 | 682 | 121 | 806 | 0.01 | 0 |
| hsa_circ_0084797 | 93 | 3 | 682 | 121 | 806 | 0.01 | 0 |
| hsa_circ_0084798 | 93 | 1 | 608 | 1 | 611 | 0 | 0 |
| hsa_circ_0084798 | 91 | 1 | 349 | 260 | 611 | 0.01 | 2.00E-134 |
| hsa_circ_0091476 | 92 | 1 | 286 | 286 | 1 | 0 | 6.00E-112 |
| hsa_circ_0091476 | 92 | 122 | 408 | 287 | 1 | 0 | 2.00E-112 |
| hsa_circ_0091476 | 94 | 1 | 461 | 781 | 321 | 0 | 0 |
| hsa_circ_0091476 | 95 | 1 | 818 | 1138 | 321 | 0 | 0 |
| hsa_circ_0091476 | 94 | 460 | 1454 | 1315 | 321 | 0 | 0 |
| hsa_circ_0091476 | 94 | 543 | 1537 | 1315 | 321 | 0 | 0 |
| hsa_circ_0091476 | 95 | 1 | 818 | 1138 | 321 | 0 | 0 |
| hsa_circ_0091477 | 95 | 1 | 694 | 816 | 123 | 0 | 0 |
| hsa_circ_0091477 | 95 | 4 | 874 | 993 | 123 | 0 | 0 |
| hsa_circ_0091477 | 95 | 460 | 1330 | 993 | 123 | 0 | 0 |
| hsa_circ_0091477 | 95 | 543 | 1413 | 993 | 123 | 0 | 0 |
| hsa_circ_0091478 | 96 | 1 | 357 | 357 | 1 | 0 | 4.00E-165 |
| hsa_circ_0091480 | 92 | 1 | 456 | 636 | 181 | 0 | 0 |
| hsa_circ_0091480 | 92 | 88 | 539 | 632 | 181 | 0 | 0 |
| hsa_circ_0091484 | 94 | 1 | 1996 | 81 | 2076 | 0 | 0 |
| hsa_circ_0091486 | 93 | 1 | 1364 | 1 | 1364 | 0 | 0 |
| hsa_circ_0091486 | 94 | 1 | 1877 | 1 | 1877 | 0 | 0 |
| hsa_circ_0091490 | 96 | 1 | 246 | 797 | 1042 | 0 | 3.00E-110 |
| hsa_circ_0091488 | 96 | 1 | 759 | 918 | 1676 | 0 | 0 |
| hsa_circ_0091488 | 96 | 1 | 649 | 1028 | 1676 | 0 | 0 |
| hsa_circ_0091488 | 96 | 1 | 513 | 1164 | 1676 | 0 | 0 |
| hsa_circ_0091491 | 90 | 129 | 526 | 1 | 394 | 0.02 | 1.00E-141 |
| hsa_circ_0091496 | 94 | 4 | 404 | 3397 | 2997 | 0 | 2.00E-174 |
| hsa_circ_0091498 | 95 | 4 | 200 | 198 | 2 | 0 | 8.00E-85 |
| hsa_circ_0035408 | 88 | 227 | 983 | 1071 | 309 | 0.01 | 0 |
| hsa_circ_0091522 | 93 | 3 | 547 | 97 | 641 | 0 | 0 |
| hsa_circ_0091522 | 93 | 1 | 438 | 424 | 861 | 0 | 0 |
| hsa_circ_0091526 | 92 | 235 | 445 | 213 | 3 | 0 | 1.00E-78 |
| hsa_circ_0030150 | 75 | 156 | 1126 | 1365 | 395 | 0.01 | 1.00E-115 |
| hsa_circ_0091526 | 92 | 235 | 445 | 213 | 3 | 0 | 9.00E-79 |
| hsa_circ_0091526 | 92 | 733 | 943 | 213 | 3 | 0 | 2.00E-78 |
| hsa_circ_0091526 | 92 | 235 | 445 | 213 | 3 | 0 | 7.00E-79 |
| hsa_circ_0091526 | 92 | 733 | 943 | 213 | 3 | 0 | 2.00E-78 |
| hsa_circ_0091527 | 92 | 292 | 496 | 207 | 3 | 0 | 4.00E-77 |
| hsa_circ_0091528 | 95 | 3 | 135 | 133 | 1 | 0 | 2.00E-52 |
| hsa_circ_0055223 | 88 | 28379 | 29511 | 4 | 1143 | 0.02 | 0 |
| hsa_circ_0065316 | 83 | 245 | 494 | 28 | 271 | 0.03 | 6.00E-65 |
| hsa_circ_0065316 | 83 | 95 | 344 | 28 | 271 | 0.03 | 4.00E-65 |
| hsa_circ_0065337 | 77 | 7 | 528 | 531 | 1 | 0.04 | 4.00E-77 |
| hsa_circ_0065345 | 87 | 1 | 548 | 548 | 1 | 0 | 7.00E-179 |
| hsa_circ_0065354 | 93 | 1 | 120 | 255 | 136 | 0 | 2.00E-43 |
| hsa_circ_0018343 | 87 | 1 | 1232 | 1520 | 286 | 0.01 | 0 |

novel_circ_029241 novel_circ_029242 novel_circ_029243 novel_circ_029244 novel_circ_029245 novel_circ_029249 novel_circ_029250 novel_circ_029251 novel_circ_029252 novel_circ_029253 novel_circ_029254 novel_circ_029255 novel_circ_029256 novel_circ_029257 novel_circ_029258 novel_circ_029259 novel_circ_029260 novel_circ_029261 novel_circ_029262 novel_circ_029263 novel_circ_029265 novel_circ_029266 novel_circ_029267 novel_circ_029268 novel_circ_029269 novel_circ_029270 novel_circ_029271 novel_circ_029272 novel_circ_029273 novel_circ_029274 novel_circ_029275 novel_circ_029276 novel_circ_029277 novel_circ_029278 novel_circ_029279 novel_circ_029280 novel_circ_029281 novel_circ_029282 novel_circ_029283 novel_circ_029284 novel_circ_029285 novel_circ_029286 novel_circ_029287 novel_circ_029288 novel_circ_029289 novel_circ_029290 novel_circ_029292 novel_circ_029293 novel_circ_029294 novel_circ_029295 novel_circ_029296 novel_circ_029297 novel_circ_029298 novel_circ_029299

| hsa_circ_0018341 | 93 | 2 | 365 | 364 | 1 | 0 | 2.00E-152 |
| --- | --- | --- | --- | --- | --- | --- | --- |
| hsa_circ_0018334 | 92 | 81 | 659 | 579 | 1 | 0 | 0 |
| hsa_circ_0018341 | 89 | 1 | 137 | 285 | 149 | 0.04 | 1.00E-41 |
| hsa_circ_0018341 | 94 | 1 | 285 | 285 | 1 | 0 | 3.00E-120 |
| hsa_circ_0018341 | 97 | 24 | 224 | 201 | 1 | 0 | 3.00E-90 |
| hsa_circ_0012274 | 95 | 1 | 585 | 585 | 1 | 0 | 0 |
| hsa_circ_0012274 | 94 | 1 | 429 | 429 | 1 | 0 | 0 |
| hsa_circ_0012274 | 95 | 1 | 585 | 585 | 1 | 0 | 0 |
| hsa_circ_0012275 | 95 | 1 | 324 | 324 | 1 | 0 | 4.00E-145 |
| hsa_circ_0090931 | 94 | 1 | 323 | 824 | 502 | 0 | 3.00E-136 |
| hsa_circ_0003479 | 84 | 337 | 1640 | 1308 | 1 | 0.01 | 0 |
| hsa_circ_0007725 | 83 | 337 | 1467 | 1135 | 1 | 0.01 | 0 |
| hsa_circ_0008699 | 87 | 594 | 1969 | 1358 | 1 | 0.02 | 0 |
| hsa_circ_0047981 | 93 | 1 | 308 | 1 | 308 | 0 | 1.00E-124 |
| hsa_circ_0047981 | 93 | 1 | 308 | 1 | 308 | 0 | 2.00E-124 |
| hsa_circ_0047981 | 90 | 1 | 487 | 1 | 487 | 0 | 3.00E-177 |
| hsa_circ_0047981 | 88 | 1 | 892 | 1 | 892 | 0 | 0 |
| hsa_circ_0047981 | 86 | 1 | 1362 | 1 | 1362 | 0 | 0 |
| hsa_circ_0047986 | 85 | 1 | 584 | 1 | 584 | 0 | 1.00E-172 |
| hsa_circ_0047986 | 83 | 1 | 1054 | 1 | 1054 | 0.01 | 0 |
| hsa_circ_0047989 | 86 | 1 | 403 | 1228 | 1630 | 0 | 3.00E-122 |
| hsa_circ_0047989 | 82 | 1 | 622 | 1228 | 1873 | 0.04 | 9.00E-149 |
| hsa_circ_0007157 | 96 | 1 | 725 | 725 | 1 | 0 | 0 |
| hsa_circ_0066889 | 96 | 279 | 1047 | 868 | 100 | 0 | 0 |
| hsa_circ_0007157 | 96 | 4 | 531 | 528 | 1 | 0 | 0 |
| hsa_circ_0007157 | 96 | 1 | 336 | 336 | 1 | 0 | 2.00E-153 |
| hsa_circ_0066899 | 96 | 1 | 432 | 432 | 1 | 0 | 0 |
| hsa_circ_0066892 | 96 | 1 | 658 | 658 | 1 | 0 | 0 |
| hsa_circ_0066889 | 96 | 1 | 757 | 757 | 1 | 0 | 0 |
| hsa_circ_0066892 | 96 | 1 | 527 | 527 | 1 | 0 | 0 |
| hsa_circ_0066892 | 95 | 3 | 420 | 418 | 1 | 0 | 0 |
| hsa_circ_0066889 | 96 | 3 | 519 | 517 | 1 | 0 | 0 |
| hsa_circ_0066892 | 96 | 1 | 322 | 322 | 1 | 0 | 5.00E-149 |
| hsa_circ_0066889 | 96 | 1 | 421 | 421 | 1 | 0 | 0 |
| hsa_circ_0070511 | 92 | 1 | 258 | 2920 | 2663 | 0 | 1.00E-98 |
| hsa_circ_0004998 | 91 | 1 | 201 | 201 | 1 | 0 | 2.00E-70 |
| hsa_circ_0004998 | 95 | 1 | 510 | 510 | 1 | 0 | 0 |
| hsa_circ_0004998 | 95 | 1 | 514 | 514 | 1 | 0 | 0 |
| hsa_circ_0004998 | 95 | 1 | 772 | 772 | 1 | 0 | 0 |
| hsa_circ_0003818 | 97 | 1 | 313 | 313 | 1 | 0 | 4.00E-149 |
| hsa_circ_0003818 | 97 | 1 | 571 | 571 | 1 | 0 | 0 |
| hsa_circ_0003818 | 97 | 1 | 696 | 696 | 1 | 0 | 0 |
| hsa_circ_0070515 | 97 | 1 | 523 | 523 | 1 | 0 | 0 |
| hsa_circ_0070515 | 97 | 202 | 724 | 523 | 1 | 0 | 0 |
| hsa_circ_0070515 | 97 | 1 | 383 | 523 | 141 | 0 | 0 |
| hsa_circ_0070515 | 97 | 202 | 584 | 523 | 141 | 0 | 0 |
| hsa_circ_0084737 | 97 | 357 | 646 | 3713 | 3423 | 0 | 3.00E-134 |
| hsa_circ_0084737 | 97 | 210 | 499 | 3713 | 3423 | 0 | 2.00E-134 |
| hsa_circ_0084737 | 97 | 1 | 278 | 3700 | 3423 | 0 | 5.00E-133 |
| hsa_circ_0084737 | 94 | 1 | 995 | 3700 | 2706 | 0 | 0 |
| hsa_circ_0084743 | 92 | 3 | 435 | 433 | 1 | 0 | 3.00E-172 |
| hsa_circ_0084737 | 93 | 3 | 1853 | 3138 | 1288 | 0 | 0 |
| hsa_circ_0084742 | 93 | 6 | 1418 | 1413 | 1 | 0 | 0 |
| hsa_circ_0084740 | 94 | 1 | 764 | 764 | 1 | 0 | 0 |

novel_circ_029302 novel_circ_029303 novel_circ_029305 novel_circ_029306 novel_circ_029307 novel_circ_029308 novel_circ_029309 novel_circ_029310 novel_circ_029311 novel_circ_029312 novel_circ_029313 novel_circ_029314 novel_circ_029315 novel_circ_029316 novel_circ_029317 novel_circ_029318 novel_circ_029322 novel_circ_029323 novel_circ_029324 novel_circ_029325 novel_circ_029326 novel_circ_029327 novel_circ_029328 novel_circ_029329 novel_circ_029330 novel_circ_029331 novel_circ_029332 novel_circ_029333 novel_circ_029334 novel_circ_029335 novel_circ_029337 novel_circ_029338 novel_circ_029339 novel_circ_029340 novel_circ_029341 novel_circ_029342 novel_circ_029343 novel_circ_029344 novel_circ_029345 novel_circ_029346 novel_circ_029347 novel_circ_029348 novel_circ_029350 novel_circ_029351 novel_circ_029352 novel_circ_029353 novel_circ_029354 novel_circ_029355 novel_circ_029356 novel_circ_029357 novel_circ_029358 novel_circ_029359 novel_circ_029360 novel_circ_029361

| hsa_circ_0080024 | 95 | 1 | 138 | 950 | 813 | 0 | 2.00E-55 |
| --- | --- | --- | --- | --- | --- | --- | --- |
| hsa_circ_0080027 | 91 | 1 | 333 | 333 | 1 | 0 | 8.00E-127 |
| hsa_circ_0026804 | 86 | 7250 | 7625 | 635 | 260 | 0 | 8.00E-109 |
| hsa_circ_0005369 | 79 | 7 | 329 | 3987 | 3677 | 0.09 | 3.00E-51 |
| hsa_circ_0080055 | 92 | 1557 | 1841 | 248 | 532 | 0 | 5.00E-111 |
| hsa_circ_0080055 | 92 | 1557 | 1841 | 248 | 532 | 0 | 6.00E-111 |
| hsa_circ_0080055 | 92 | 1557 | 1841 | 248 | 532 | 0 | 5.00E-111 |
| hsa_circ_0080055 | 92 | 1294 | 1578 | 248 | 532 | 0 | 5.00E-111 |
| hsa_circ_0080068 | 91 | 331 | 1146 | 3 | 818 | 0 | 0 |
| hsa_circ_0080068 | 91 | 3 | 818 | 3 | 818 | 0 | 0 |
| hsa_circ_0080120 | 93 | 270 | 381 | 1 | 112 | 0 | 6.00E-39 |
| hsa_circ_0080120 | 91 | 270 | 509 | 1 | 240 | 0 | 3.00E-88 |
| hsa_circ_0080121 | 90 | 933 | 1517 | 1 | 585 | 0 | 0 |
| hsa_circ_0080121 | 90 | 1 | 585 | 1 | 585 | 0 | 0 |
| hsa_circ_0080121 | 91 | 2 | 457 | 130 | 585 | 0 | 2.00E-173 |
| hsa_circ_0080121 | 91 | 1 | 298 | 288 | 585 | 0 | 2.00E-112 |
| hsa_circ_0089087 | 91 | 1 | 385 | 26 | 410 | 0 | 2.00E-145 |
| hsa_circ_0089090 | 93 | 1 | 167 | 1 | 167 | 0 | 3.00E-63 |
| hsa_circ_0089081 | 91 | 1 | 200 | 937 | 1136 | 0 | 2.00E-71 |
| hsa_circ_0089081 | 90 | 221 | 560 | 359 | 698 | 0 | 2.00E-120 |
| hsa_circ_0089078 | 90 | 376 | 948 | 39 | 606 | 0.01 | 0 |
| hsa_circ_0089078 | 90 | 376 | 948 | 39 | 606 | 0.01 | 0 |
| hsa_circ_0089078 | 88 | 376 | 705 | 39 | 366 | 0.01 | 7.00E-106 |
| hsa_circ_0089076 | 91 | 18236 | 18555 | 489 | 170 | 0 | 8.00E-118 |
| hsa_circ_0089076 | 95 | 18233 | 18348 | 489 | 374 | 0 | 9.00E-43 |
| hsa_circ_0089076 | 91 | 18233 | 18552 | 489 | 170 | 0 | 8.00E-118 |
| hsa_circ_0089076 | 89 | 18233 | 18721 | 489 | 1 | 0 | 1.00E-166 |
| hsa_circ_0089076 | 91 | 1 | 320 | 489 | 170 | 0 | 1.00E-119 |
| hsa_circ_0089076 | 89 | 1 | 489 | 489 | 1 | 0 | 2.00E-168 |
| hsa_circ_0089069 | 85 | 174 | 764 | 2219 | 1629 | 0 | 2.00E-171 |
| hsa_circ_0089074 | 87 | 1 | 642 | 950 | 309 | 0 | 0 |
| hsa_circ_0089074 | 87 | 4 | 579 | 884 | 309 | 0 | 0 |
| hsa_circ_0089073 | 93 | 13 | 243 | 231 | 1 | 0 | 6.00E-92 |
| hsa_circ_0089071 | 87 | 1 | 262 | 259 | 1 | 0.02 | 5.00E-78 |
| hsa_circ_0089057 | 89 | 4 | 304 | 1582 | 1282 | 0 | 1.00E-100 |
| hsa_circ_0043333 | 95 | 130 | 415 | 1 | 287 | 0 | 2.00E-123 |
| hsa_circ_0043333 | 95 | 1 | 286 | 1 | 287 | 0 | 4.00E-124 |
| hsa_circ_0043331 | 95 | 1 | 565 | 208 | 772 | 0 | 0 |
| hsa_circ_0043331 | 97 | 1 | 636 | 1 | 636 | 0 | 0 |
| hsa_circ_0005693 | 97 | 1 | 701 | 1 | 701 | 0 | 0 |
| hsa_circ_0043331 | 98 | 1 | 507 | 1 | 507 | 0 | 0 |
| hsa_circ_0005693 | 98 | 1 | 572 | 1 | 572 | 0 | 0 |
| hsa_circ_0043314 | 82 | 113 | 398 | 9691 | 9404 | 0.03 | 7.00E-60 |
| hsa_circ_0044255 | 97 | 1 | 393 | 1 | 393 | 0 | 0 |
| hsa_circ_0043309 | 80 | 1 | 742 | 2920 | 2181 | 0.06 | 6.00E-141 |
| hsa_circ_0044257 | 98 | 1 | 886 | 201 | 1086 | 0 | 0 |
| hsa_circ_0044257 | 98 | 1 | 888 | 309 | 1196 | 0 | 0 |
| hsa_circ_0044268 | 96 | 1 | 270 | 1 | 270 | 0 | 7.00E-122 |
| hsa_circ_0044268 | 97 | 1 | 331 | 1 | 331 | 0 | 1.00E-155 |
| hsa_circ_0044268 | 97 | 1 | 441 | 1 | 441 | 0 | 0 |
| hsa_circ_0044268 | 95 | 1 | 505 | 1 | 505 | 0 | 0 |
| hsa_circ_0044268 | 95 | 1 | 645 | 1 | 645 | 0 | 0 |
| hsa_circ_0044268 | 96 | 1 | 1000 | 1 | 1000 | 0 | 0 |
| hsa_circ_0044268 | 95 | 1 | 1199 | 1 | 1199 | 0 | 0 |

novel_circ_029362 novel_circ_029363 novel_circ_029364 novel_circ_029365 novel_circ_029366 novel_circ_029367 novel_circ_029368 novel_circ_029369 novel_circ_029370 novel_circ_029371 novel_circ_029372 novel_circ_029373 novel_circ_029374 novel_circ_029375 novel_circ_029377 novel_circ_029378 novel_circ_029379 novel_circ_029380 novel_circ_029381 novel_circ_029384 novel_circ_029385 novel_circ_029386 novel_circ_029387 novel_circ_029388 novel_circ_029389 novel_circ_029390 novel_circ_029391 novel_circ_029395 novel_circ_029396 novel_circ_029397 novel_circ_029398 novel_circ_029401 novel_circ_029402 novel_circ_029404 novel_circ_029405 novel_circ_029406 novel_circ_029410 novel_circ_029411 novel_circ_029412 novel_circ_029413 novel_circ_029414 novel_circ_029415 novel_circ_029416 novel_circ_029417 novel_circ_029418 novel_circ_029419 novel_circ_029420 novel_circ_029421 novel_circ_029422 novel_circ_029423 novel_circ_029424 novel_circ_029425 novel_circ_029426 novel_circ_029427

| hsa_circ_0044271 | 96 | 1 | 669 | 1 | 669 | 0 | 0 |
| --- | --- | --- | --- | --- | --- | --- | --- |
| hsa_circ_0044271 | 95 | 1 | 868 | 1 | 868 | 0 | 0 |
| hsa_circ_0002347 | 97 | 3 | 495 | 3 | 495 | 0 | 0 |
| hsa_circ_0002347 | 96 | 3 | 694 | 3 | 694 | 0 | 0 |
| hsa_circ_0044271 | 95 | 3 | 803 | 177 | 977 | 0 | 0 |
| hsa_circ_0044273 | 92 | 1 | 199 | 356 | 554 | 0 | 6.00E-76 |
| hsa_circ_0044291 | 93 | 1 | 1236 | 202 | 1413 | 0.02 | 0 |
| hsa_circ_0044308 | 93 | 1 | 878 | 439 | 1316 | 0 | 0 |
| hsa_circ_0044308 | 94 | 1 | 1244 | 439 | 1682 | 0 | 0 |
| hsa_circ_0044308 | 93 | 1 | 555 | 762 | 1316 | 0 | 0 |
| hsa_circ_0044308 | 94 | 1 | 921 | 762 | 1682 | 0 | 0 |
| hsa_circ_0044308 | 93 | 1 | 335 | 982 | 1316 | 0 | 6.00E-138 |
| hsa_circ_0044308 | 94 | 1 | 701 | 982 | 1682 | 0 | 0 |
| hsa_circ_0044308 | 96 | 1 | 365 | 1318 | 1682 | 0 | 7.00E-168 |
| hsa_circ_0044350 | 96 | 1 | 450 | 1978 | 1529 | 0 | 0 |
| hsa_circ_0044364 | 94 | 104 | 363 | 1 | 260 | 0 | 2.00E-109 |
| hsa_circ_0044365 | 94 | 90 | 159 | 1 | 70 | 0 | 1.00E-22 |
| hsa_circ_0044365 | 91 | 3 | 212 | 73 | 282 | 0 | 3.00E-76 |
| hsa_circ_0044413 | 94 | 1 | 1560 | 1567 | 1 | 0.01 | 0 |
| hsa_circ_0056620 | 80 | 148 | 546 | 4 | 402 | 0.01 | 7.00E-75 |
| hsa_circ_0044436 | 96 | 149 | 565 | 1 | 417 | 0 | 0 |
| hsa_circ_0044436 | 96 | 149 | 565 | 1 | 417 | 0 | 0 |
| hsa_circ_0044437 | 96 | 326 | 837 | 1 | 512 | 0 | 0 |
| hsa_circ_0044437 | 96 | 326 | 837 | 1 | 512 | 0 | 0 |
| hsa_circ_0044436 | 96 | 1 | 417 | 1 | 417 | 0 | 0 |
| hsa_circ_0000784 | 96 | 1 | 272 | 1 | 272 | 0 | 2.00E-121 |
| hsa_circ_0000784 | 96 | 1 | 272 | 1 | 272 | 0 | 5.00E-121 |
| hsa_circ_0044448 | 94 | 1 | 250 | 1 | 250 | 0 | 8.00E-106 |
| hsa_circ_0072562 | 95 | 1 | 631 | 631 | 1 | 0 | 0 |
| hsa_circ_0072562 | 96 | 1 | 267 | 631 | 365 | 0 | 3.00E-120 |
| hsa_circ_0072568 | 95 | 1 | 353 | 353 | 1 | 0 | 3.00E-156 |
| hsa_circ_0063267 | 92 | 1 | 669 | 1 | 669 | 0 | 0 |
| hsa_circ_0063267 | 92 | 1 | 669 | 1 | 669 | 0 | 0 |
| hsa_circ_0006355 | 75 | 9909 | 10208 | 5924 | 5611 | 0.06 | 4.00E-27 |
| hsa_circ_0004955 | 89 | 296 | 370 | 75 | 1 | 0 | 2.00E-18 |
| hsa_circ_0056761 | 94 | 296 | 1567 | 1272 | 1 | 0 | 0 |
| hsa_circ_0018656 | 80 | 1 | 1997 | 1 | 2062 | 0.06 | 0 |
| hsa_circ_0018656 | 79 | 1 | 2950 | 1 | 3051 | 0.07 | 0 |
| hsa_circ_0013951 | 94 | 1 | 200 | 1328 | 1527 | 0 | 2.00E-81 |
| hsa_circ_0013949 | 90 | 1 | 1654 | 1 | 1645 | 0.01 | 0 |
| hsa_circ_0013949 | 94 | 1 | 249 | 164 | 412 | 0 | 3.00E-105 |
| hsa_circ_0013949 | 93 | 1 | 412 | 1 | 412 | 0 | 1.00E-170 |
| hsa_circ_0013948 | 93 | 2 | 494 | 149 | 641 | 0 | 0 |
| hsa_circ_0013783 | 94 | 3 | 726 | 953 | 230 | 0 | 0 |
| hsa_circ_0007526 | 92 | 1 | 719 | 719 | 1 | 0 | 0 |
| hsa_circ_0004694 | 92 | 1 | 208 | 208 | 1 | 0 | 3.00E-79 |
| hsa_circ_0013829 | 88 | 209 | 617 | 167431 | 166999 | 0.06 | 9.00E-134 |
| hsa_circ_0013829 | 88 | 153 | 561 | 167431 | 166999 | 0.06 | 8.00E-134 |
| hsa_circ_0013857 | 90 | 1 | 610 | 606 | 1 | 0.01 | 0 |
| hsa_circ_0013857 | 88 | 1 | 499 | 495 | 1 | 0.01 | 2.00E-169 |
| hsa_circ_0013851 | 90 | 1 | 627 | 627 | 1 | 0 | 0 |
| hsa_circ_0013850 | 88 | 1 | 617 | 622 | 9 | 0.01 | 0 |
| hsa_circ_0013848 | 90 | 1 | 1085 | 1082 | 1 | 0 | 0 |
| hsa_circ_0007636 | 92 | 169 | 239 | 607891 | 607821 | 0 | 1.00E-19 |

novel_circ_029428 novel_circ_029429 novel_circ_029430 novel_circ_029431 novel_circ_029432 novel_circ_029433 novel_circ_029434 novel_circ_029435 novel_circ_029436 novel_circ_029437 novel_circ_029438 novel_circ_029439 novel_circ_029440 novel_circ_029441 novel_circ_029442 novel_circ_029444 novel_circ_029445 novel_circ_029446 novel_circ_029447 novel_circ_029448 novel_circ_029449 novel_circ_029450 novel_circ_029451 novel_circ_029452 novel_circ_029453 novel_circ_029454 novel_circ_029456 novel_circ_029457 novel_circ_029459 novel_circ_029460 novel_circ_029461 novel_circ_029462 novel_circ_029463 novel_circ_029464 novel_circ_029465 novel_circ_029466 novel_circ_029467 novel_circ_029468 novel_circ_029469 novel_circ_029470 novel_circ_029471 novel_circ_029472 novel_circ_029473 novel_circ_029474 novel_circ_029475 novel_circ_029476 novel_circ_029477 novel_circ_029478 novel_circ_029479 novel_circ_029480 novel_circ_029481 novel_circ_029482 novel_circ_029483 novel_circ_029484

| hsa_circ_0013849 | 88 | 1 | 340 | 594 | 258 | 0.01 | 7.00E-108 |
| --- | --- | --- | --- | --- | --- | --- | --- |
| hsa_circ_0013848 | 91 | 1 | 710 | 707 | 1 | 0 | 0 |
| hsa_circ_0013848 | 93 | 1 | 368 | 368 | 1 | 0 | 1.00E-154 |
| hsa_circ_0013848 | 94 | 1 | 251 | 251 | 1 | 0 | 5.00E-103 |
| hsa_circ_0013844 | 94 | 1 | 468 | 701 | 234 | 0 | 0 |
| hsa_circ_0013840 | 87 | 287 | 1258 | 2019 | 1042 | 0.01 | 0 |
| hsa_circ_0013843 | 87 | 5 | 663 | 858 | 194 | 0.01 | 0 |
| hsa_circ_0013840 | 87 | 1 | 749 | 1790 | 1042 | 0 | 0 |
| hsa_circ_0013841 | 87 | 1 | 763 | 1041 | 279 | 0.01 | 0 |
| hsa_circ_0042113 | 95 | 4302 | 4342 | 11004 | 11045 | 0.02 | 6.00E-08 |
| hsa_circ_0042113 | 95 | 18450 | 18490 | 11004 | 11045 | 0.02 | 1.00E-07 |
| hsa_circ_0013760 | 97 | 1 | 117 | 119 | 3 | 0 | 7.00E-50 |
| hsa_circ_0019532 | 88 | 1 | 833 | 1 | 833 | 0.02 | 0 |
| hsa_circ_0019532 | 89 | 1 | 2009 | 1 | 2024 | 0.02 | 0 |
| hsa_circ_0019532 | 89 | 1 | 2602 | 1 | 2617 | 0.02 | 0 |
| hsa_circ_0019539 | 91 | 1 | 291 | 1070 | 1360 | 0 | 2.00E-108 |
| hsa_circ_0019539 | 93 | 1 | 604 | 1070 | 1673 | 0 | 0 |
| hsa_circ_0019537 | 92 | 1 | 715 | 1128 | 1842 | 0.01 | 0 |
| hsa_circ_0019539 | 94 | 1 | 481 | 1193 | 1673 | 0 | 0 |
| hsa_circ_0019537 | 93 | 1 | 592 | 1251 | 1842 | 0.01 | 0 |
| hsa_circ_0019537 | 93 | 1 | 867 | 1251 | 2117 | 0 | 0 |
| hsa_circ_0002045 | 92 | 1 | 321 | 1 | 321 | 0.01 | 8.00E-127 |
| hsa_circ_0002045 | 93 | 1 | 596 | 1 | 596 | 0 | 0 |
| hsa_circ_0002045 | 93 | 1 | 520 | 77 | 596 | 0 | 0 |
| hsa_circ_0002045 | 93 | 1 | 608 | 77 | 684 | 0 | 0 |
| hsa_circ_0019541 | 95 | 1 | 660 | 104 | 763 | 0 | 0 |
| hsa_circ_0019569 | 94 | 1 | 416 | 1 | 416 | 0 | 2.00E-179 |
| hsa_circ_0006555 | 84 | 1 | 478 | 481 | 1 | 0.01 | 4.00E-131 |
| hsa_circ_0019586 | 93 | 2 | 576 | 575 | 1 | 0 | 0 |
| hsa_circ_0019590 | 93 | 2 | 510 | 509 | 1 | 0 | 0 |
| hsa_circ_0019590 | 93 | 2 | 510 | 509 | 1 | 0 | 0 |
| hsa_circ_0019592 | 92 | 2 | 415 | 414 | 1 | 0 | 1.00E-166 |
| hsa_circ_0019592 | 92 | 41 | 454 | 414 | 1 | 0 | 1.00E-166 |
| hsa_circ_0019593 | 93 | 2 | 282 | 281 | 1 | 0 | 2.00E-111 |
| hsa_circ_0019593 | 93 | 41 | 321 | 281 | 1 | 0 | 3.00E-111 |
| hsa_circ_0019602 | 95 | 1 | 439 | 439 | 1 | 0 | 0 |
| hsa_circ_0019603 | 94 | 1 | 279 | 279 | 1 | 0 | 1.00E-118 |
| hsa_circ_0019603 | 94 | 1 | 1259 | 1259 | 1 | 0 | 0 |
| hsa_circ_0019603 | 94 | 1 | 1418 | 1418 | 1 | 0 | 0 |
| hsa_circ_0019607 | 94 | 1 | 980 | 980 | 1 | 0 | 0 |
| hsa_circ_0019607 | 93 | 2 | 786 | 975 | 191 | 0 | 0 |
| hsa_circ_0019610 | 93 | 2 | 785 | 784 | 1 | 0 | 0 |
| hsa_circ_0019610 | 93 | 1 | 789 | 789 | 1 | 0 | 0 |
| hsa_circ_0019610 | 93 | 1180 | 1972 | 792 | 1 | 0 | 0 |
| hsa_circ_0019610 | 93 | 1 | 948 | 948 | 1 | 0 | 0 |
| hsa_circ_0019610 | 94 | 1 | 1233 | 1233 | 1 | 0 | 0 |
| hsa_circ_0019610 | 94 | 1 | 1332 | 1332 | 1 | 0 | 0 |
| hsa_circ_0019610 | 94 | 1 | 1504 | 1504 | 1 | 0 | 0 |
| hsa_circ_0019610 | 94 | 306 | 1941 | 1636 | 1 | 0 | 0 |
| hsa_circ_0019615 | 95 | 254 | 941 | 847 | 160 | 0 | 0 |
| hsa_circ_0002888 | 97 | 968 | 1098 | 132 | 1 | 0.01 | 3.00E-54 |
| hsa_circ_0019631 | 98 | 1 | 286 | 286 | 1 | 0 | 5.00E-138 |
| hsa_circ_0002888 | 97 | 968 | 1098 | 132 | 1 | 0.01 | 2.00E-54 |
| hsa_circ_0019631 | 98 | 1 | 286 | 286 | 1 | 0 | 4.00E-139 |

novel_circ_029485 novel_circ_029486 novel_circ_029487 novel_circ_029488 novel_circ_029489 novel_circ_029490 novel_circ_029491 novel_circ_029492 novel_circ_029493 novel_circ_029495 novel_circ_029496 novel_circ_029497 novel_circ_029498 novel_circ_029499 novel_circ_029500 novel_circ_029501 novel_circ_029502 novel_circ_029503 novel_circ_029504 novel_circ_029505 novel_circ_029506 novel_circ_029507 novel_circ_029508 novel_circ_029509 novel_circ_029510 novel_circ_029511 novel_circ_029512 novel_circ_029513 novel_circ_029514 novel_circ_029515 novel_circ_029517 novel_circ_029518 novel_circ_029524 novel_circ_029525 novel_circ_029526 novel_circ_029527 novel_circ_029528 novel_circ_029535 novel_circ_029536 novel_circ_029537 novel_circ_029538 novel_circ_029539 novel_circ_029540 novel_circ_029541 novel_circ_029542 novel_circ_029543 novel_circ_029544 novel_circ_029545 novel_circ_029546 novel_circ_029549 novel_circ_029553 novel_circ_029554 novel_circ_029559 novel_circ_029561

| hsa_circ_0019631 | 96 | 1 | 375 | 375 | 1 | 0 | 2.00E-173 |
| --- | --- | --- | --- | --- | --- | --- | --- |
| hsa_circ_0019631 | 95 | 1 | 631 | 631 | 1 | 0 | 0 |
| hsa_circ_0019631 | 95 | 1 | 733 | 733 | 1 | 0 | 0 |
| hsa_circ_0019631 | 95 | 1 | 1055 | 1055 | 1 | 0 | 0 |
| hsa_circ_0019638 | 93 | 1 | 345 | 345 | 1 | 0 | 8.00E-142 |
| hsa_circ_0019638 | 93 | 1 | 447 | 447 | 1 | 0 | 0 |
| hsa_circ_0019642 | 94 | 1 | 254 | 256 | 3 | 0 | 5.00E-108 |
| hsa_circ_0019642 | 94 | 1 | 356 | 358 | 3 | 0 | 7.00E-153 |
| hsa_circ_0019642 | 94 | 3115 | 3757 | 899 | 257 | 0 | 0 |
| hsa_circ_0019701 | 90 | 1 | 541 | 1 | 541 | 0 | 0 |
| hsa_circ_0019756 | 93 | 1 | 459 | 1 | 456 | 0.01 | 0 |
| hsa_circ_0019756 | 93 | 1 | 571 | 1 | 568 | 0.01 | 0 |
| hsa_circ_0019760 | 96 | 1 | 478 | 694 | 1171 | 0 | 0 |
| hsa_circ_0019764 | 92 | 1 | 237 | 237 | 1 | 0 | 6.00E-92 |
| hsa_circ_0019764 | 93 | 1 | 354 | 354 | 1 | 0 | 2.00E-143 |
| hsa_circ_0019764 | 94 | 145 | 260 | 354 | 239 | 0 | 5.00E-43 |
| hsa_circ_0019774 | 89 | 587 | 943 | 1 | 357 | 0 | 3.00E-121 |
| hsa_circ_0019774 | 89 | 587 | 943 | 1 | 357 | 0 | 4.00E-121 |
| hsa_circ_0019774 | 89 | 71 | 427 | 1 | 357 | 0 | 1.00E-121 |
| hsa_circ_0019774 | 89 | 1 | 357 | 1 | 357 | 0 | 1.00E-121 |
| hsa_circ_0019775 | 90 | 1 | 143 | 1 | 143 | 0 | 1.00E-44 |
| hsa_circ_0019776 | 93 | 3 | 452 | 3 | 452 | 0 | 0 |
| hsa_circ_0019776 | 94 | 1 | 308 | 145 | 452 | 0 | 1.00E-129 |
| hsa_circ_0019778 | 83 | 1 | 1838 | 2528 | 671 | 0.06 | 0 |
| hsa_circ_0019790 | 92 | 4 | 318 | 1349 | 1038 | 0.01 | 1.00E-120 |
| hsa_circ_0019790 | 94 | 1 | 571 | 1605 | 1038 | 0.01 | 0 |
| hsa_circ_0019794 | 96 | 1 | 352 | 439 | 88 | 0 | 5.00E-164 |
| hsa_circ_0019798 | 96 | 264 | 491 | 228 | 1 | 0 | 1.00E-101 |
| hsa_circ_0019806 | 97 | 1 | 1105 | 583 | 1687 | 0 | 0 |
| hsa_circ_0019814 | 88 | 1 | 881 | 1 | 881 | 0 | 0 |
| hsa_circ_0019883 | 94 | 1 | 223 | 1 | 223 | 0 | 1.00E-90 |
| hsa_circ_0019883 | 94 | 1 | 223 | 1 | 223 | 0 | 9.00E-90 |
| hsa_circ_0019981 | 78 | 1 | 411 | 1070 | 660 | 0.01 | 3.00E-68 |
| hsa_circ_0019981 | 77 | 1 | 585 | 1070 | 486 | 0.02 | 7.00E-85 |
| hsa_circ_0019981 | 76 | 1 | 707 | 1070 | 364 | 0.02 | 3.00E-94 |
| hsa_circ_0019981 | 76 | 1 | 850 | 1070 | 220 | 0.02 | 3.00E-120 |
| hsa_circ_0019980 | 75 | 1 | 1169 | 1242 | 77 | 0.02 | 9.00E-138 |
| hsa_circ_0019979 | 90 | 1 | 256 | 258 | 3 | 0 | 3.00E-90 |
| hsa_circ_0019979 | 90 | 1 | 256 | 258 | 3 | 0 | 2.00E-90 |
| hsa_circ_0019979 | 90 | 1 | 256 | 258 | 3 | 0 | 2.00E-90 |
| hsa_circ_0019979 | 90 | 1 | 256 | 258 | 3 | 0 | 2.00E-90 |
| hsa_circ_0019981 | 94 | 1 | 563 | 563 | 1 | 0 | 0 |
| hsa_circ_0019981 | 94 | 1 | 629 | 743 | 115 | 0 | 0 |
| hsa_circ_0019986 | 90 | 1 | 389 | 389 | 1 | 0 | 7.00E-143 |
| hsa_circ_0030150 | 93 | 1 | 350 | 350 | 1 | 0 | 7.00E-143 |
| hsa_circ_0030150 | 95 | 1 | 788 | 788 | 1 | 0 | 0 |
| hsa_circ_0030150 | 95 | 1 | 623 | 788 | 166 | 0 | 0 |
| hsa_circ_0030150 | 94 | 1 | 828 | 1403 | 576 | 0 | 0 |
| hsa_circ_0030151 | 89 | 1 | 282 | 282 | 1 | 0 | 2.00E-93 |
| hsa_circ_0030152 | 92 | 615 | 1178 | 5 | 568 | 0 | 0 |
| hsa_circ_0030154 | 89 | 1 | 328 | 1 | 319 | 0.05 | 1.00E-109 |
| hsa_circ_0030154 | 87 | 1 | 649 | 1 | 616 | 0.06 | 0 |
| hsa_circ_0030174 | 92 | 2 | 181 | 180 | 1 | 0 | 5.00E-65 |
| hsa_circ_0030177 | 96 | 1 | 603 | 1 | 603 | 0 | 0 |

novel_circ_029562 novel_circ_029563 novel_circ_029564 novel_circ_029565 novel_circ_029566 novel_circ_029567 novel_circ_029568 novel_circ_029569 novel_circ_029570 novel_circ_029571 novel_circ_029572 novel_circ_029573 novel_circ_029574 novel_circ_029575 novel_circ_029576 novel_circ_029577 novel_circ_029578 novel_circ_029579 novel_circ_029580 novel_circ_029581 novel_circ_029582 novel_circ_029583 novel_circ_029584 novel_circ_029585 novel_circ_029586 novel_circ_029587 novel_circ_029588 novel_circ_029589 novel_circ_029590 novel_circ_029591 novel_circ_029592 novel_circ_029593 novel_circ_029594 novel_circ_029595 novel_circ_029596 novel_circ_029597 novel_circ_029599 novel_circ_029601 novel_circ_029602 novel_circ_029603 novel_circ_029604 novel_circ_029605 novel_circ_029606 novel_circ_029608 novel_circ_029611 novel_circ_029612 novel_circ_029613 novel_circ_029614 novel_circ_029617 novel_circ_029618 novel_circ_029630 novel_circ_029631 novel_circ_029633 novel_circ_029634

| hsa_circ_0030177 | 94 | 1 | 866 | 1 | 866 | 0 | 0 |
| --- | --- | --- | --- | --- | --- | --- | --- |
| hsa_circ_0030178 | 95 | 1 | 300 | 76 | 375 | 0 | 3.00E-130 |
| hsa_circ_0030178 | 93 | 1 | 703 | 76 | 778 | 0 | 0 |
| hsa_circ_0030178 | 93 | 1 | 864 | 76 | 939 | 0 | 0 |
| hsa_circ_0030181 | 92 | 1 | 564 | 1 | 564 | 0 | 0 |
| hsa_circ_0030181 | 92 | 1 | 520 | 45 | 564 | 0 | 0 |
| hsa_circ_0030181 | 92 | 1 | 1186 | 45 | 1230 | 0 | 0 |
| hsa_circ_0030181 | 91 | 1 | 393 | 172 | 564 | 0 | 9.00E-152 |
| hsa_circ_0030181 | 91 | 1 | 624 | 172 | 795 | 0 | 0 |
| hsa_circ_0030181 | 91 | 1 | 714 | 172 | 885 | 0 | 0 |
| hsa_circ_0030181 | 92 | 1 | 835 | 172 | 1006 | 0 | 0 |
| hsa_circ_0030181 | 92 | 1 | 1059 | 172 | 1230 | 0 | 0 |
| hsa_circ_0030181 | 93 | 1 | 1135 | 172 | 1306 | 0 | 0 |
| hsa_circ_0030182 | 93 | 93 | 1263 | 1 | 1171 | 0 | 0 |
| hsa_circ_0030182 | 92 | 1 | 407 | 1 | 407 | 0 | 3.00E-161 |
| hsa_circ_0030182 | 92 | 1 | 532 | 1 | 532 | 0 | 0 |
| hsa_circ_0030182 | 91 | 1 | 622 | 1 | 622 | 0 | 0 |
| hsa_circ_0030182 | 93 | 1 | 743 | 1 | 743 | 0 | 0 |
| hsa_circ_0030182 | 93 | 1 | 1171 | 1 | 1171 | 0 | 0 |
| hsa_circ_0005168 | 91 | 1 | 321 | 1 | 321 | 0 | 4.00E-120 |
| hsa_circ_0006607 | 95 | 1 | 345 | 1 | 345 | 0 | 2.00E-153 |
| hsa_circ_0006607 | 95 | 1 | 648 | 1 | 648 | 0 | 0 |
| hsa_circ_0030187 | 95 | 1 | 303 | 1 | 303 | 0 | 2.00E-133 |
| hsa_circ_0073634 | 94 | 1 | 476 | 472 | 1 | 0.01 | 0 |
| hsa_circ_0073633 | 94 | 1 | 707 | 703 | 1 | 0.01 | 0 |
| hsa_circ_0073633 | 94 | 1 | 707 | 703 | 1 | 0.01 | 0 |
| hsa_circ_0073633 | 95 | 1 | 584 | 584 | 1 | 0 | 0 |
| hsa_circ_0073632 | 95 | 1 | 693 | 693 | 1 | 0 | 0 |
| hsa_circ_0073633 | 96 | 3 | 331 | 514 | 186 | 0 | 1.00E-149 |
| hsa_circ_0073633 | 95 | 3 | 516 | 514 | 1 | 0 | 0 |
| hsa_circ_0073631 | 91 | 565 | 1058 | 1205 | 712 | 0 | 0 |
| hsa_circ_0073631 | 91 | 565 | 1183 | 1205 | 587 | 0 | 0 |
| hsa_circ_0073631 | 91 | 565 | 1471 | 1205 | 299 | 0 | 0 |
| hsa_circ_0073631 | 91 | 565 | 1769 | 1205 | 1 | 0 | 0 |
| hsa_circ_0073631 | 92 | 1 | 475 | 1061 | 587 | 0 | 0 |
| hsa_circ_0073630 | 96 | 1 | 381 | 420 | 800 | 0 | 4.00E-175 |
| hsa_circ_0073630 | 96 | 1 | 419 | 1 | 419 | 0 | 0 |
| hsa_circ_0052815 | 87 | 2391 | 4259 | 1 | 1866 | 0.01 | 0 |
| hsa_circ_0055628 | 93 | 1 | 344 | 344 | 1 | 0 | 2.00E-139 |
| hsa_circ_0055628 | 93 | 1 | 344 | 344 | 1 | 0 | 1.00E-139 |
| hsa_circ_0055628 | 92 | 1 | 553 | 553 | 1 | 0 | 0 |
| hsa_circ_0055628 | 91 | 1 | 453 | 553 | 101 | 0 | 5.00E-175 |
| hsa_circ_0055666 | 93 | 1 | 318 | 711 | 394 | 0 | 4.00E-130 |
| hsa_circ_0055680 | 94 | 10 | 233 | 646 | 423 | 0 | 2.00E-91 |
| hsa_circ_0055763 | 92 | 1 | 582 | 2269 | 1691 | 0.01 | 0 |
| hsa_circ_0055768 | 96 | 4 | 663 | 955 | 296 | 0 | 0 |
| hsa_circ_0055771 | 89 | 1 | 240 | 327 | 88 | 0 | 1.00E-78 |
| hsa_circ_0055538 | 75 | 8997 | 10244 | 46913 | 48168 | 0.06 | 3.00E-140 |
| hsa_circ_0055793 | 90 | 2 | 367 | 2 | 367 | 0 | 1.00E-131 |
| hsa_circ_0055793 | 92 | 366 | 1315 | 404 | 1353 | 0 | 0 |
| hsa_circ_0055799 | 94 | 794 | 1461 | 1629 | 962 | 0 | 0 |
| hsa_circ_0055800 | 96 | 1 | 271 | 271 | 1 | 0 | 2.00E-123 |
| hsa_circ_0055800 | 96 | 1 | 271 | 271 | 1 | 0 | 1.00E-123 |
| hsa_circ_0055800 | 92 | 1 | 401 | 672 | 272 | 0 | 7.00E-158 |

novel_circ_029635 novel_circ_029636 novel_circ_029637 novel_circ_029638 novel_circ_029639 novel_circ_029640 novel_circ_029641 novel_circ_029642 novel_circ_029643 novel_circ_029644 novel_circ_029645 novel_circ_029646 novel_circ_029647 novel_circ_029648 novel_circ_029649 novel_circ_029651 novel_circ_029652 novel_circ_029653 novel_circ_029654 novel_circ_029655 novel_circ_029656 novel_circ_029657 novel_circ_029658 novel_circ_029659 novel_circ_029660 novel_circ_029661 novel_circ_029662 novel_circ_029663 novel_circ_029664 novel_circ_029665 novel_circ_029666 novel_circ_029667 novel_circ_029668 novel_circ_029669 novel_circ_029670 novel_circ_029671 novel_circ_029673 novel_circ_029674 novel_circ_029675 novel_circ_029676 novel_circ_029677 novel_circ_029678 novel_circ_029679 novel_circ_029680 novel_circ_029681 novel_circ_029682 novel_circ_029683 novel_circ_029684 novel_circ_029685 novel_circ_029686 novel_circ_029687 novel_circ_029691 novel_circ_029692 novel_circ_029693

| hsa_circ_0055800 | 92 | 111 | 794 | 955 | 272 | 0 | 0 |
| --- | --- | --- | --- | --- | --- | --- | --- |
| hsa_circ_0055800 | 92 | 327 | 1010 | 955 | 272 | 0 | 0 |
| hsa_circ_0055800 | 92 | 456 | 1139 | 955 | 272 | 0 | 0 |
| hsa_circ_0055800 | 91 | 456 | 986 | 955 | 425 | 0 | 0 |
| hsa_circ_0055800 | 91 | 1 | 283 | 955 | 673 | 0 | 9.00E-106 |
| hsa_circ_0055800 | 91 | 111 | 393 | 955 | 673 | 0 | 1.00E-105 |
| hsa_circ_0055800 | 95 | 130 | 412 | 1237 | 955 | 0 | 5.00E-122 |
| hsa_circ_0055800 | 96 | 130 | 345 | 1237 | 1022 | 0 | 9.00E-97 |
| hsa_circ_0055814 | 88 | 1 | 486 | 1 | 486 | 0 | 2.00E-163 |
| hsa_circ_0004147 | 88 | 1 | 405 | 1 | 405 | 0 | 2.00E-133 |
| hsa_circ_0055820 | 90 | 1 | 255 | 255 | 1 | 0 | 1.00E-88 |
| hsa_circ_0055821 | 96 | 1 | 744 | 366 | 1109 | 0 | 0 |
| hsa_circ_0055821 | 96 | 1 | 315 | 795 | 1109 | 0 | 7.00E-145 |
| hsa_circ_0055821 | 96 | 1 | 315 | 795 | 1109 | 0 | 1.00E-144 |
| hsa_circ_0055827 | 86 | 1 | 739 | 821 | 89 | 0.01 | 0 |
| hsa_circ_0055829 | 87 | 1 | 437 | 431 | 1 | 0.01 | 2.00E-134 |
| hsa_circ_0055833 | 93 | 4 | 1130 | 1232 | 103 | 0 | 0 |
| hsa_circ_0055835 | 93 | 1 | 1021 | 1024 | 1 | 0 | 0 |
| hsa_circ_0055835 | 93 | 4 | 1129 | 1129 | 1 | 0 | 0 |
| hsa_circ_0055837 | 93 | 1 | 496 | 496 | 1 | 0 | 0 |
| hsa_circ_0055838 | 92 | 1 | 275 | 275 | 1 | 0 | 2.00E-106 |
| hsa_circ_0055838 | 92 | 2 | 404 | 403 | 1 | 0 | 3.00E-162 |
| hsa_circ_0055838 | 92 | 636 | 1038 | 403 | 1 | 0 | 7.00E-162 |
| hsa_circ_0055841 | 91 | 2 | 282 | 283 | 3 | 0 | 1.00E-104 |
| hsa_circ_0055843 | 92 | 1 | 235 | 235 | 1 | 0 | 8.00E-91 |
| hsa_circ_0055843 | 93 | 1 | 352 | 355 | 1 | 0.01 | 3.00E-146 |
| hsa_circ_0055843 | 94 | 4 | 460 | 460 | 1 | 0.01 | 0 |
| hsa_circ_0055844 | 94 | 1 | 223 | 226 | 1 | 0.01 | 2.00E-91 |
| hsa_circ_0055844 | 95 | 4 | 331 | 331 | 1 | 0.01 | 1.00E-144 |
| hsa_circ_0055844 | 93 | 1 | 1490 | 1493 | 1 | 0 | 0 |
| hsa_circ_0055846 | 93 | 1 | 1384 | 1384 | 1 | 0 | 0 |
| hsa_circ_0055846 | 93 | 1 | 1257 | 1257 | 1 | 0 | 0 |
| hsa_circ_0055847 | 93 | 1 | 1159 | 1159 | 1 | 0 | 0 |
| hsa_circ_0001053 | 96 | 1 | 191 | 191 | 1 | 0 | 8.00E-85 |
| hsa_circ_0001053 | 96 | 9974 | 10164 | 191 | 1 | 0 | 5.00E-83 |
| hsa_circ_0001053 | 96 | 20804 | 20994 | 191 | 1 | 0 | 1.00E-82 |
| hsa_circ_0055852 | 90 | 1 | 125 | 125 | 1 | 0 | 7.00E-38 |
| hsa_circ_0055852 | 89 | 541 | 1165 | 741 | 126 | 0.01 | 0 |
| hsa_circ_0055855 | 90 | 2 | 820 | 819 | 1 | 0 | 0 |
| hsa_circ_0055855 | 90 | 2319 | 3137 | 819 | 1 | 0 | 0 |
| hsa_circ_0003909 | 95 | 1 | 295 | 1 | 295 | 0 | 2.00E-127 |
| hsa_circ_0003909 | 95 | 1 | 385 | 1 | 385 | 0 | 1.00E-170 |
| hsa_circ_0003909 | 95 | 1 | 620 | 1 | 620 | 0 | 0 |
| hsa_circ_0055860 | 95 | 1 | 566 | 1 | 566 | 0 | 0 |
| hsa_circ_0055862 | 95 | 3 | 417 | 3 | 417 | 0 | 0 |
| hsa_circ_0003160 | 92 | 1 | 587 | 589 | 3 | 0 | 0 |
| hsa_circ_0055904 | 85 | 1 | 379 | 379 | 1 | 0 | 6.00E-109 |
| hsa_circ_0055904 | 87 | 1 | 699 | 699 | 1 | 0 | 0 |
| hsa_circ_0055905 | 92 | 1 | 500 | 500 | 1 | 0 | 0 |
| hsa_circ_0055906 | 91 | 1 | 403 | 403 | 1 | 0 | 6.00E-154 |
| hsa_circ_0001058 | 90 | 1 | 320 | 320 | 1 | 0 | 6.00E-113 |
| hsa_circ_0007933 | 96 | 1 | 716 | 716 | 1 | 0 | 0 |
| hsa_circ_0015684 | 90 | 1 | 861 | 838 | 1698 | 0 | 0 |
| hsa_circ_0015693 | 92 | 1 | 460 | 1 | 460 | 0 | 0 |

novel_circ_029694 novel_circ_029695 novel_circ_029696 novel_circ_029697 novel_circ_029698 novel_circ_029699 novel_circ_029700 novel_circ_029701 novel_circ_029702 novel_circ_029703 novel_circ_029704 novel_circ_029705 novel_circ_029706 novel_circ_029707 novel_circ_029708 novel_circ_029709 novel_circ_029710 novel_circ_029711 novel_circ_029713 novel_circ_029714 novel_circ_029715 novel_circ_029717 novel_circ_029718 novel_circ_029719 novel_circ_029720 novel_circ_029721 novel_circ_029722 novel_circ_029724 novel_circ_029726 novel_circ_029727 novel_circ_029728 novel_circ_029730 novel_circ_029732 novel_circ_029734 novel_circ_029735 novel_circ_029736 novel_circ_029737 novel_circ_029738 novel_circ_029739 novel_circ_029740 novel_circ_029741 novel_circ_029751 novel_circ_029752 novel_circ_029753 novel_circ_029754 novel_circ_029755 novel_circ_029756 novel_circ_029757 novel_circ_029758 novel_circ_029759 novel_circ_029760 novel_circ_029761 novel_circ_029762 novel_circ_029763

| hsa_circ_0015692 | 89 | 1 | 1254 | 4621 | 5874 | 0 | 0 |
| --- | --- | --- | --- | --- | --- | --- | --- |
| hsa_circ_0015692 | 89 | 4 | 1063 | 4815 | 5874 | 0 | 0 |
| hsa_circ_0015692 | 89 | 3 | 849 | 5028 | 5874 | 0 | 0 |
| hsa_circ_0015692 | 89 | 1 | 513 | 5875 | 6387 | 0 | 0 |
| hsa_circ_0015692 | 88 | 1 | 1005 | 5875 | 6879 | 0 | 0 |
| hsa_circ_0015692 | 92 | 1 | 150 | 6730 | 6879 | 0 | 2.00E-53 |
| hsa_circ_0015696 | 93 | 130 | 600 | 1 | 471 | 0 | 0 |
| hsa_circ_0015709 | 92 | 1 | 456 | 456 | 1 | 0 | 0 |
| hsa_circ_0015709 | 93 | 1 | 880 | 880 | 1 | 0 | 0 |
| hsa_circ_0015712 | 91 | 1 | 408 | 408 | 1 | 0 | 2.00E-153 |
| hsa_circ_0015713 | 93 | 1 | 715 | 1048 | 334 | 0 | 0 |
| hsa_circ_0015716 | 94 | 1 | 974 | 974 | 1 | 0 | 0 |
| hsa_circ_0015709 | 93 | 1 | 442 | 4234 | 3793 | 0 | 0 |
| hsa_circ_0015709 | 95 | 1 | 276 | 4234 | 3959 | 0 | 3.00E-120 |
| hsa_circ_0015722 | 93 | 1 | 625 | 747 | 123 | 0 | 0 |
| hsa_circ_0015721 | 93 | 1 | 858 | 1491 | 634 | 0 | 0 |
| hsa_circ_0007636 | 74 | 13681 | 13933 | 1E+06 | 1161543 | 0.04 | 9.00E-15 |
| hsa_circ_0070255 | 87 | 35930 | 36569 | 652 | 1 | 0.02 | 0 |
| hsa_circ_0005959 | 83 | 84 | 1461 | 99 | 1484 | 0.02 | 0 |
| hsa_circ_0052333 | 82 | 1 | 1684 | 1 | 1707 | 0.02 | 0 |
| hsa_circ_0049503 | 77 | 2027 | 2249 | 977 | 1199 | 0.05 | 2.00E-26 |
| hsa_circ_0007073 | 90 | 4 | 223 | 220 | 1 | 0 | 7.00E-76 |
| hsa_circ_0087366 | 83 | 4967 | 6142 | 1247 | 1 | 0.06 | 0 |
| hsa_circ_0052317 | 79 | 778 | 1370 | 3294 | 2701 | 0.02 | 1.00E-110 |
| hsa_circ_0052319 | 76 | 7335 | 8613 | 1817 | 548 | 0.03 | 2.00E-171 |
| hsa_circ_0052319 | 76 | 1008 | 2286 | 1817 | 548 | 0.03 | 5.00E-172 |
| hsa_circ_0052319 | 76 | 1008 | 2286 | 1817 | 548 | 0.03 | 1.00E-171 |
| hsa_circ_0052303 | 83 | 101 | 220 | 2203 | 2084 | 0 | 1.00E-23 |
| hsa_circ_0064665 | 93 | 5422 | 5782 | 509 | 150 | 0 | 3.00E-148 |
| hsa_circ_0052300 | 90 | 12 | 112 | 265 | 365 | 0 | 5.00E-29 |
| hsa_circ_0000962 | 92 | 1 | 130 | 32570 | 32698 | 0.01 | 3.00E-45 |
| hsa_circ_0042202 | 72 | 348 | 782 | 3251 | 2821 | 0.04 | 2.00E-23 |
| hsa_circ_0052095 | 76 | 8 | 197 | 8 | 197 | 0.04 | 6.00E-17 |
| hsa_circ_0052095 | 76 | 8 | 197 | 8 | 197 | 0.04 | 5.00E-17 |
| hsa_circ_0052095 | 76 | 8 | 197 | 8 | 197 | 0.04 | 4.00E-17 |
| hsa_circ_0091184 | 93 | 1 | 1263 | 1251 | 1 | 0.01 | 0 |
| hsa_circ_0091182 | 92 | 1 | 1170 | 1800 | 643 | 0.01 | 0 |
| hsa_circ_0091184 | 93 | 1 | 1263 | 1251 | 1 | 0.01 | 0 |
| hsa_circ_0091184 | 92 | 1 | 1116 | 1104 | 1 | 0.01 | 0 |
| hsa_circ_0091184 | 93 | 1 | 1263 | 1251 | 1 | 0.01 | 0 |
| hsa_circ_0004854 | 94 | 1 | 305 | 305 | 1 | 0 | 1.00E-129 |
| hsa_circ_0074019 | 93 | 1 | 364 | 1 | 364 | 0 | 2.00E-152 |
| hsa_circ_0074014 | 94 | 1 | 743 | 1 | 743 | 0 | 0 |
| hsa_circ_0074016 | 95 | 1 | 328 | 1 | 328 | 0 | 2.00E-142 |
| hsa_circ_0074013 | 95 | 1 | 490 | 123 | 612 | 0 | 0 |
| hsa_circ_0074009 | 94 | 1 | 1189 | 153 | 1341 | 0 | 0 |
| hsa_circ_0074009 | 94 | 1 | 1341 | 1 | 1341 | 0 | 0 |
| hsa_circ_0074007 | 94 | 1 | 1507 | 1 | 1507 | 0 | 0 |
| hsa_circ_0074012 | 93 | 1 | 419 | 1 | 419 | 0 | 4.00E-171 |
| hsa_circ_0074009 | 93 | 1 | 851 | 1 | 851 | 0 | 0 |
| hsa_circ_0003154 | 93 | 1 | 527 | 1 | 527 | 0 | 0 |
| hsa_circ_0073969 | 94 | 706 | 2087 | 1047 | 2428 | 0 | 0 |
| hsa_circ_0074004 | 92 | 1 | 198 | 315 | 512 | 0 | 2.00E-75 |
| hsa_circ_0074001 | 93 | 3 | 623 | 177 | 797 | 0 | 0 |

novel_circ_029764 novel_circ_029765 novel_circ_029766 novel_circ_029767 novel_circ_029768 novel_circ_029769 novel_circ_029770 novel_circ_029771 novel_circ_029772 novel_circ_029773 novel_circ_029774 novel_circ_029775 novel_circ_029776 novel_circ_029777 novel_circ_029778 novel_circ_029779 novel_circ_029780 novel_circ_029781 novel_circ_029782 novel_circ_029783 novel_circ_029784 novel_circ_029785 novel_circ_029786 novel_circ_029787 novel_circ_029788 novel_circ_029789 novel_circ_029790 novel_circ_029791 novel_circ_029792 novel_circ_029793 novel_circ_029794 novel_circ_029795 novel_circ_029796 novel_circ_029798 novel_circ_029799 novel_circ_029800 novel_circ_029801 novel_circ_029802 novel_circ_029803 novel_circ_029806 novel_circ_029807 novel_circ_029808 novel_circ_029809 novel_circ_029810 novel_circ_029811 novel_circ_029812 novel_circ_029813 novel_circ_029814 novel_circ_029815 novel_circ_029816 novel_circ_029817 novel_circ_029818 novel_circ_029823 novel_circ_029828

| hsa_circ_0073991 | 94 | 706 | 1983 | 7 | 1284 | 0 | 0 |
| --- | --- | --- | --- | --- | --- | --- | --- |
| hsa_circ_0074001 | 94 | 3 | 425 | 177 | 599 | 0 | 5.00E-180 |
| hsa_circ_0073999 | 94 | 1 | 758 | 1 | 758 | 0 | 0 |
| hsa_circ_0073991 | 94 | 706 | 1785 | 7 | 1086 | 0 | 0 |
| hsa_circ_0074002 | 92 | 3 | 287 | 3 | 287 | 0 | 7.00E-112 |
| hsa_circ_0073999 | 94 | 1 | 620 | 1 | 620 | 0 | 0 |
| hsa_circ_0073991 | 94 | 587 | 1528 | 7 | 948 | 0 | 0 |
| hsa_circ_0073991 | 94 | 706 | 1647 | 7 | 948 | 0 | 0 |
| hsa_circ_0073991 | 94 | 929 | 1870 | 7 | 948 | 0 | 0 |
| hsa_circ_0073991 | 94 | 587 | 1352 | 7 | 772 | 0 | 0 |
| hsa_circ_0073991 | 94 | 706 | 1471 | 7 | 772 | 0 | 0 |
| hsa_circ_0073977 | 92 | 1 | 1049 | 1 | 1047 | 0.02 | 0 |
| hsa_circ_0073999 | 95 | 1 | 333 | 1 | 333 | 0 | 2.00E-148 |
| hsa_circ_0073977 | 92 | 1 | 922 | 128 | 1047 | 0.03 | 0 |
| hsa_circ_0073977 | 92 | 1 | 1049 | 1 | 1047 | 0.02 | 0 |
| hsa_circ_0073977 | 90 | 1 | 580 | 470 | 1047 | 0.04 | 0 |
| hsa_circ_0073977 | 91 | 2 | 699 | 352 | 1047 | 0.04 | 0 |
| hsa_circ_0073977 | 91 | 1 | 812 | 238 | 1047 | 0.03 | 0 |
| hsa_circ_0073977 | 92 | 1 | 922 | 128 | 1047 | 0.03 | 0 |
| hsa_circ_0073977 | 92 | 1 | 922 | 128 | 1047 | 0.03 | 0 |
| hsa_circ_0073977 | 92 | 1 | 1049 | 1 | 1047 | 0.02 | 0 |
| hsa_circ_0073979 | 95 | 1 | 342 | 1 | 342 | 0 | 2.00E-153 |
| hsa_circ_0073971 | 94 | 1 | 906 | 1 | 906 | 0 | 0 |
| hsa_circ_0073966 | 92 | 2 | 1155 | 5 | 1158 | 0 | 0 |
| hsa_circ_0073977 | 94 | 1 | 350 | 1 | 350 | 0 | 3.00E-151 |
| hsa_circ_0073977 | 94 | 1 | 237 | 1 | 237 | 0 | 1.00E-98 |
| hsa_circ_0073974 | 94 | 3 | 513 | 4 | 513 | 0 | 0 |
| hsa_circ_0073966 | 92 | 2 | 923 | 5 | 926 | 0 | 0 |
| hsa_circ_0073971 | 93 | 1 | 559 | 1 | 559 | 0 | 0 |
| hsa_circ_0073966 | 91 | 2 | 808 | 5 | 811 | 0 | 0 |
| hsa_circ_0073971 | 94 | 1 | 431 | 1 | 431 | 0 | 0 |
| hsa_circ_0073958 | 91 | 1 | 465 | 1 | 468 | 0.01 | 6.00E-179 |
| hsa_circ_0073943 | 94 | 6571 | 6876 | 604 | 915 | 0.02 | 4.00E-127 |
| hsa_circ_0002602 | 97 | 1 | 636 | 755 | 120 | 0 | 0 |
| hsa_circ_0002602 | 97 | 1 | 755 | 755 | 1 | 0 | 0 |
| hsa_circ_0002602 | 97 | 1 | 426 | 545 | 120 | 0 | 0 |
| hsa_circ_0002602 | 97 | 1 | 545 | 545 | 1 | 0 | 0 |
| hsa_circ_0073927 | 93 | 1 | 379 | 379 | 1 | 0 | 5.00E-154 |
| hsa_circ_0003163 | 78 | 105 | 324 | 202477 | 202686 | 0.07 | 3.00E-29 |
| hsa_circ_0073915 | 94 | 1 | 479 | 1 | 479 | 0 | 0 |
| hsa_circ_0073913 | 94 | 1 | 743 | 1 | 743 | 0 | 0 |
| hsa_circ_0073912 | 94 | 1 | 801 | 1 | 801 | 0 | 0 |
| hsa_circ_0073899 | 95 | 93 | 1146 | 2736 | 1683 | 0 | 0 |
| hsa_circ_0073899 | 95 | 1 | 1054 | 2736 | 1683 | 0 | 0 |
| hsa_circ_0073904 | 94 | 4 | 840 | 924 | 88 | 0 | 0 |
| hsa_circ_0073904 | 95 | 4 | 927 | 924 | 1 | 0 | 0 |
| hsa_circ_0073899 | 95 | 4 | 964 | 2606 | 1646 | 0 | 0 |
| hsa_circ_0073899 | 95 | 1 | 342 | 1682 | 1344 | 0.01 | 1.00E-150 |
| hsa_circ_0073899 | 95 | 1 | 305 | 1645 | 1344 | 0.01 | 7.00E-132 |
| hsa_circ_0073901 | 94 | 1 | 1248 | 1248 | 1 | 0 | 0 |
| hsa_circ_0073900 | 92 | 1 | 1345 | 1595 | 245 | 0.01 | 0 |
| hsa_circ_0073886 | 92 | 1 | 493 | 3313 | 2821 | 0 | 0 |
| hsa_circ_0073881 | 90 | 1 | 125 | 125 | 1 | 0 | 8.00E-39 |
| hsa_circ_0008530 | 90 | 8 | 352 | 344 | 1 | 0 | 1.00E-124 |

novel_circ_029830 novel_circ_029831 novel_circ_029832 novel_circ_029833 novel_circ_029834 novel_circ_029835 novel_circ_029836 novel_circ_029837 novel_circ_029838 novel_circ_029839 novel_circ_029840 novel_circ_029841 novel_circ_029842 novel_circ_029843 novel_circ_029844 novel_circ_029845 novel_circ_029846 novel_circ_029847 novel_circ_029848 novel_circ_029849 novel_circ_029850 novel_circ_029851 novel_circ_029852 novel_circ_029853 novel_circ_029854 novel_circ_029855 novel_circ_029856 novel_circ_029857 novel_circ_029858 novel_circ_029859 novel_circ_029860 novel_circ_029861 novel_circ_029862 novel_circ_029863 novel_circ_029864 novel_circ_029865 novel_circ_029866 novel_circ_029867 novel_circ_029868 novel_circ_029869 novel_circ_029870 novel_circ_029871 novel_circ_029872 novel_circ_029873 novel_circ_029874 novel_circ_029875 novel_circ_029876 novel_circ_029877 novel_circ_029878 novel_circ_029879 novel_circ_029880 novel_circ_029881 novel_circ_029882 novel_circ_029883

| hsa_circ_0073868 | 95 | 1 | 262 | 262 | 1 | 0 | 4.00E-114 |
| --- | --- | --- | --- | --- | --- | --- | --- |
| hsa_circ_0003721 | 94 | 1 | 530 | 530 | 1 | 0 | 0 |
| hsa_circ_0073864 | 95 | 1 | 1110 | 1427 | 318 | 0 | 0 |
| hsa_circ_0073864 | 96 | 1 | 983 | 1300 | 318 | 0 | 0 |
| hsa_circ_0073864 | 96 | 1 | 848 | 1165 | 318 | 0 | 0 |
| hsa_circ_0073865 | 96 | 5 | 248 | 380 | 137 | 0 | 8.00E-111 |
| hsa_circ_0073865 | 96 | 5 | 384 | 380 | 1 | 0 | 3.00E-176 |
| hsa_circ_0073864 | 96 | 5 | 672 | 985 | 318 | 0 | 0 |
| hsa_circ_0073864 | 96 | 5 | 819 | 985 | 171 | 0 | 0 |
| hsa_circ_0073864 | 97 | 1 | 580 | 897 | 318 | 0 | 0 |
| hsa_circ_0073864 | 96 | 1 | 813 | 813 | 1 | 0 | 0 |
| hsa_circ_0009030 | 96 | 1 | 424 | 424 | 1 | 0 | 0 |
| hsa_circ_0073862 | 93 | 1 | 2161 | 2330 | 170 | 0 | 0 |
| hsa_circ_0073852 | 93 | 1 | 2528 | 2644 | 117 | 0 | 0 |
| hsa_circ_0009030 | 97 | 1 | 288 | 288 | 1 | 0 | 1.00E-133 |
| hsa_circ_0073852 | 93 | 1 | 2392 | 2508 | 117 | 0 | 0 |
| hsa_circ_0073855 | 92 | 1 | 1957 | 1957 | 1 | 0 | 0 |
| hsa_circ_0073855 | 92 | 1 | 1787 | 1787 | 1 | 0 | 0 |
| hsa_circ_0073836 | 95 | 1 | 212 | 426 | 215 | 0 | 2.00E-91 |
| hsa_circ_0073836 | 96 | 1 | 282 | 426 | 145 | 0 | 3.00E-130 |
| hsa_circ_0073836 | 95 | 1 | 426 | 426 | 1 | 0 | 0 |
| hsa_circ_0073836 | 95 | 1 | 214 | 214 | 1 | 0 | 1.00E-92 |
| hsa_circ_0073832 | 96 | 299 | 1057 | 1586 | 828 | 0 | 0 |
| hsa_circ_0073832 | 96 | 1 | 606 | 1586 | 981 | 0 | 0 |
| hsa_circ_0073832 | 96 | 1 | 759 | 1586 | 828 | 0 | 0 |
| hsa_circ_0073832 | 95 | 1 | 1345 | 1586 | 242 | 0 | 0 |
| hsa_circ_0073832 | 94 | 1 | 1586 | 1586 | 1 | 0 | 0 |
| hsa_circ_0073831 | 94 | 1 | 1744 | 1744 | 1 | 0 | 0 |
| hsa_circ_0073830 | 93 | 311 | 2384 | 2410 | 337 | 0 | 0 |
| hsa_circ_0073830 | 93 | 311 | 2596 | 2410 | 125 | 0 | 0 |
| hsa_circ_0073832 | 96 | 1 | 449 | 1276 | 828 | 0 | 0 |
| hsa_circ_0073832 | 93 | 1 | 586 | 827 | 242 | 0 | 0 |
| hsa_circ_0073830 | 92 | 1 | 1010 | 1134 | 125 | 0 | 0 |
| hsa_circ_0073830 | 93 | 1 | 1134 | 1134 | 1 | 0 | 0 |
| hsa_circ_0073830 | 92 | 1 | 837 | 961 | 125 | 0 | 0 |
| hsa_circ_0073830 | 92 | 1 | 961 | 961 | 1 | 0 | 0 |
| hsa_circ_0073828 | 93 | 1 | 1205 | 1759 | 555 | 0 | 0 |
| hsa_circ_0073828 | 92 | 1 | 1530 | 1759 | 230 | 0 | 0 |
| hsa_circ_0073830 | 94 | 1 | 336 | 336 | 1 | 0 | 2.00E-143 |
| hsa_circ_0073828 | 94 | 1 | 580 | 1134 | 555 | 0 | 0 |
| hsa_circ_0073828 | 93 | 1 | 905 | 1134 | 230 | 0 | 0 |
| hsa_circ_0073828 | 93 | 1 | 1134 | 1134 | 1 | 0 | 0 |
| hsa_circ_0073828 | 93 | 1 | 693 | 922 | 230 | 0 | 0 |
| hsa_circ_0073828 | 93 | 1 | 220 | 774 | 555 | 0 | 7.00E-86 |
| hsa_circ_0073828 | 92 | 1 | 545 | 774 | 230 | 0 | 0 |
| hsa_circ_0073828 | 92 | 1 | 774 | 774 | 1 | 0 | 0 |
| hsa_circ_0073828 | 93 | 3 | 324 | 551 | 230 | 0 | 1.00E-130 |
| hsa_circ_0073828 | 92 | 3 | 553 | 551 | 1 | 0 | 0 |
| hsa_circ_0073827 | 89 | 1 | 732 | 720 | 1 | 0.02 | 0 |
| hsa_circ_0031412 | 93 | 1 | 432 | 432 | 1 | 0 | 5.00E-180 |
| hsa_circ_0005185 | 93 | 607 | 1526 | 920 | 1 | 0 | 0 |
| hsa_circ_0005185 | 93 | 290 | 1209 | 920 | 1 | 0 | 0 |
| hsa_circ_0005185 | 93 | 290 | 1209 | 920 | 1 | 0 | 0 |
| hsa_circ_0005185 | 93 | 1 | 920 | 920 | 1 | 0 | 0 |

novel_circ_029884 novel_circ_029887 novel_circ_029888 novel_circ_029889 novel_circ_029890 novel_circ_029891 novel_circ_029892 novel_circ_029893 novel_circ_029894 novel_circ_029895 novel_circ_029896 novel_circ_029897 novel_circ_029898 novel_circ_029899 novel_circ_029901 novel_circ_029903 novel_circ_029904 novel_circ_029907 novel_circ_029908 novel_circ_029909 novel_circ_029910 novel_circ_029911 novel_circ_029912 novel_circ_029913 novel_circ_029914 novel_circ_029915 novel_circ_029916 novel_circ_029922 novel_circ_029924 novel_circ_029925 novel_circ_029926 novel_circ_029927 novel_circ_029928 novel_circ_029929 novel_circ_029931 novel_circ_029932 novel_circ_029936 novel_circ_029937 novel_circ_029938 novel_circ_029939 novel_circ_029940 novel_circ_029941 novel_circ_029942 novel_circ_029943 novel_circ_029944 novel_circ_029945 novel_circ_029946 novel_circ_029947 novel_circ_029948 novel_circ_029950 novel_circ_029951 novel_circ_029952 novel_circ_029953 novel_circ_029954

| hsa_circ_0031411 | 95 | 1 | 529 | 529 | 1 | 0 | 0 |
| --- | --- | --- | --- | --- | --- | --- | --- |
| hsa_circ_0072415 | 92 | 1 | 1670 | 1 | 1670 | 0 | 0 |
| hsa_circ_0072403 | 92 | 1 | 2672 | 16 | 2687 | 0 | 0 |
| hsa_circ_0072405 | 94 | 1 | 845 | 219 | 1063 | 0 | 0 |
| hsa_circ_0072404 | 95 | 6 | 813 | 6 | 813 | 0 | 0 |
| hsa_circ_0072403 | 93 | 1 | 1002 | 16 | 1017 | 0 | 0 |
| hsa_circ_0072404 | 93 | 6 | 445 | 6 | 445 | 0 | 0 |
| hsa_circ_0072403 | 91 | 1 | 634 | 16 | 649 | 0 | 0 |
| hsa_circ_0072403 | 91 | 15603 | 16236 | 16 | 649 | 0 | 0 |
| hsa_circ_0072396 | 95 | 1 | 299 | 299 | 1 | 0 | 1.00E-132 |
| hsa_circ_0078802 | 87 | 198 | 781 | 3184 | 2596 | 0.02 | 0 |
| hsa_circ_0072396 | 96 | 1 | 223 | 223 | 1 | 0 | 3.00E-97 |
| hsa_circ_0078802 | 86 | 1 | 657 | 2838 | 2156 | 0.05 | 0 |
| hsa_circ_0072394 | 88 | 25686 | 26237 | 651 | 100 | 0 | 0 |
| hsa_circ_0072394 | 88 | 1 | 552 | 651 | 100 | 0 | 0 |
| hsa_circ_0072383 | 96 | 1 | 974 | 3372 | 2399 | 0 | 0 |
| hsa_circ_0072390 | 96 | 1 | 915 | 1086 | 172 | 0 | 0 |
| hsa_circ_0004608 | 92 | 1 | 457 | 1 | 457 | 0 | 0 |
| hsa_circ_0004608 | 92 | 1 | 457 | 1 | 457 | 0 | 0 |
| hsa_circ_0004608 | 92 | 1 | 457 | 1 | 457 | 0 | 0 |
| hsa_circ_0072369 | 79 | 1 | 1262 | 116 | 1329 | 0.06 | 0 |
| hsa_circ_0072363 | 92 | 1 | 654 | 654 | 1 | 0 | 0 |
| hsa_circ_0072361 | 91 | 337 | 1170 | 834 | 1 | 0 | 0 |
| hsa_circ_0002121 | 92 | 1 | 286 | 286 | 1 | 0 | 4.00E-109 |
| hsa_circ_0072362 | 90 | 1 | 486 | 535 | 50 | 0 | 2.00E-178 |
| hsa_circ_0072361 | 90 | 1 | 516 | 516 | 1 | 0 | 0 |
| hsa_circ_0007742 | 90 | 18656 | 18713 | 393997 | 394054 | 0 | 8.00E-10 |
| hsa_circ_0058253 | 92 | 1 | 670 | 670 | 1 | 0 | 0 |
| hsa_circ_0086440 | 85 | 22 | 2528 | 5611 | 3100 | 0.01 | 0 |
| hsa_circ_0086440 | 88 | 22 | 1329 | 5611 | 4304 | 0 | 0 |
| hsa_circ_0086443 | 88 | 1 | 1501 | 1501 | 1 | 0 | 0 |
| hsa_circ_0086446 | 89 | 22 | 940 | 919 | 1 | 0 | 0 |
| hsa_circ_0086443 | 89 | 1 | 1248 | 1501 | 254 | 0 | 0 |
| hsa_circ_0086447 | 89 | 22 | 858 | 837 | 1 | 0 | 0 |
| hsa_circ_0086449 | 88 | 1 | 742 | 742 | 1 | 0 | 0 |
| hsa_circ_0086449 | 89 | 1 | 456 | 742 | 287 | 0 | 1.00E-156 |
| hsa_circ_0091200 | 91 | 1 | 772 | 772 | 1 | 0 | 0 |
| hsa_circ_0091200 | 91 | 1 | 848 | 848 | 1 | 0 | 0 |
| hsa_circ_0091200 | 91 | 1 | 852 | 852 | 1 | 0 | 0 |
| hsa_circ_0091199 | 91 | 107 | 1050 | 1191 | 248 | 0 | 0 |
| hsa_circ_0091199 | 91 | 174 | 1117 | 1191 | 248 | 0 | 0 |
| hsa_circ_0091203 | 89 | 1 | 198 | 198 | 1 | 0 | 2.00E-65 |
| hsa_circ_0091203 | 91 | 1 | 265 | 265 | 1 | 0 | 2.00E-97 |
| hsa_circ_0003306 | 81 | 7673 | 8130 | 1455 | 1900 | 0.03 | 5.00E-98 |
| hsa_circ_0045586 | 86 | 11532 | 12076 | 562 | 14 | 0.02 | 4.00E-167 |
| hsa_circ_0045586 | 86 | 13140 | 13684 | 562 | 14 | 0.02 | 4.00E-167 |
| hsa_circ_0045586 | 86 | 13911 | 14455 | 562 | 14 | 0.02 | 4.00E-167 |
| hsa_circ_0045586 | 86 | 13140 | 13684 | 562 | 14 | 0.02 | 2.00E-167 |
| hsa_circ_0045586 | 86 | 13911 | 14455 | 562 | 14 | 0.02 | 3.00E-167 |
| hsa_circ_0086585 | 93 | 1 | 314 | 1 | 314 | 0 | 3.00E-126 |
| hsa_circ_0004926 | 82 | 1 | 723 | 733 | 14 | 0.02 | 2.00E-170 |
| hsa_circ_0061133 | 79 | 45 | 1504 | 1900 | 441 | 0.01 | 0 |
| hsa_circ_0061137 | 79 | 160 | 1336 | 1601 | 428 | 0.02 | 0 |
| hsa_circ_0061148 | 86 | 1 | 1512 | 1521 | 1 | 0.01 | 0 |

novel_circ_029956 novel_circ_029957 novel_circ_029959 novel_circ_029960 novel_circ_029961 novel_circ_029962 novel_circ_029963 novel_circ_029965 novel_circ_029966 novel_circ_029967 novel_circ_029968 novel_circ_029969 novel_circ_029970 novel_circ_029971 novel_circ_029972 novel_circ_029973 novel_circ_029974 novel_circ_029975 novel_circ_029977 novel_circ_029978 novel_circ_029981 novel_circ_029982 novel_circ_029983 novel_circ_029984 novel_circ_029985 novel_circ_029986 novel_circ_029987 novel_circ_029988 novel_circ_029989 novel_circ_029990 novel_circ_029991 novel_circ_029992 novel_circ_029993 novel_circ_029994 novel_circ_029995 novel_circ_029996 novel_circ_029997 novel_circ_029998 novel_circ_029999 novel_circ_030000 novel_circ_030001 novel_circ_030002 novel_circ_030003 novel_circ_030004 novel_circ_030005 novel_circ_030006 novel_circ_030007 novel_circ_030008 novel_circ_030009 novel_circ_030010 novel_circ_030011 novel_circ_030012 novel_circ_030013 novel_circ_030029

| hsa_circ_0061164 | 89 | 687 | 1078 | 3853 | 3462 | 0 | 2.00E-137 |
| --- | --- | --- | --- | --- | --- | --- | --- |
| hsa_circ_0061170 | 85 | 18 | 201 | 18 | 201 | 0 | 7.00E-46 |
| hsa_circ_0061206 | 90 | 1 | 415 | 1 | 415 | 0 | 1.00E-150 |
| hsa_circ_0061206 | 90 | 1 | 753 | 1 | 753 | 0 | 0 |
| hsa_circ_0061206 | 90 | 1 | 876 | 1 | 876 | 0 | 0 |
| hsa_circ_0061206 | 90 | 1 | 995 | 1 | 995 | 0 | 0 |
| hsa_circ_0061214 | 90 | 1 | 899 | 1 | 899 | 0 | 0 |
| hsa_circ_0061231 | 92 | 86 | 806 | 186 | 906 | 0 | 0 |
| hsa_circ_0061231 | 92 | 1 | 315 | 188 | 502 | 0 | 2.00E-123 |
| hsa_circ_0061231 | 91 | 1 | 595 | 188 | 782 | 0 | 0 |
| hsa_circ_0061231 | 92 | 1 | 719 | 188 | 906 | 0 | 0 |
| hsa_circ_0061232 | 91 | 1 | 275 | 1 | 275 | 0.01 | 1.00E-99 |
| hsa_circ_0061232 | 92 | 1 | 399 | 1 | 399 | 0.01 | 4.00E-155 |
| hsa_circ_0091211 | 89 | 1 | 952 | 428 | 1388 | 0.03 | 0 |
| hsa_circ_0091211 | 89 | 862 | 1813 | 428 | 1388 | 0.03 | 0 |
| hsa_circ_0091211 | 90 | 1 | 1063 | 317 | 1388 | 0.03 | 0 |
| hsa_circ_0091209 | 95 | 1 | 642 | 316 | 957 | 0 | 0 |
| hsa_circ_0091209 | 95 | 1 | 531 | 316 | 846 | 0 | 0 |
| hsa_circ_0084575 | 92 | 4 | 321 | 318 | 1 | 0.01 | 8.00E-122 |
| hsa_circ_0036139 | 88 | 1 | 549 | 343 | 891 | 0.01 | 0 |
| hsa_circ_0012945 | 93 | 1 | 707 | 1 | 707 | 0 | 0 |
| hsa_circ_0012942 | 96 | 148 | 623 | 476 | 1 | 0 | 0 |
| hsa_circ_0012942 | 96 | 106 | 581 | 476 | 1 | 0 | 0 |
| hsa_circ_0012942 | 96 | 1 | 476 | 476 | 1 | 0 | 0 |
| hsa_circ_0000085 | 96 | 1 | 638 | 638 | 1 | 0 | 0 |
| hsa_circ_0012938 | 96 | 1 | 918 | 918 | 1 | 0 | 0 |
| hsa_circ_0012942 | 96 | 1 | 390 | 390 | 1 | 0 | 4.00E-180 |
| hsa_circ_0012942 | 96 | 1 | 344 | 344 | 1 | 0 | 3.00E-156 |
| hsa_circ_0012938 | 95 | 1 | 786 | 786 | 1 | 0 | 0 |
| hsa_circ_0012939 | 95 | 1 | 617 | 618 | 2 | 0 | 0 |
| hsa_circ_0012938 | 95 | 1 | 719 | 719 | 1 | 0 | 0 |
| hsa_circ_0012938 | 95 | 5 | 280 | 276 | 1 | 0 | 7.00E-122 |
| hsa_circ_0012935 | 94 | 481 | 912 | 388 | 819 | 0 | 0 |
| hsa_circ_0012935 | 96 | 1 | 388 | 1 | 388 | 0 | 2.00E-180 |
| hsa_circ_0012935 | 96 | 1 | 388 | 1 | 388 | 0 | 2.00E-180 |
| hsa_circ_0012935 | 96 | 1 | 337 | 1 | 337 | 0 | 5.00E-154 |
| hsa_circ_0012931 | 95 | 1 | 356 | 1 | 359 | 0.01 | 2.00E-158 |
| hsa_circ_0006450 | 90 | 1 | 386 | 386 | 1 | 0 | 3.00E-141 |
| hsa_circ_0006450 | 90 | 1 | 386 | 386 | 1 | 0 | 6.00E-141 |
| hsa_circ_0006450 | 90 | 1 | 386 | 386 | 1 | 0 | 7.00E-141 |
| hsa_circ_0012926 | 90 | 511 | 1069 | 559 | 1 | 0 | 0 |
| hsa_circ_0012926 | 90 | 511 | 1069 | 559 | 1 | 0 | 0 |
| hsa_circ_0012926 | 90 | 511 | 1069 | 559 | 1 | 0 | 0 |
| hsa_circ_0012926 | 90 | 511 | 1069 | 559 | 1 | 0 | 0 |
| hsa_circ_0012925 | 90 | 836 | 1552 | 1821 | 1105 | 0 | 0 |
| hsa_circ_0012926 | 90 | 444 | 1002 | 559 | 1 | 0 | 0 |
| hsa_circ_0006781 | 91 | 1 | 328 | 328 | 1 | 0 | 2.00E-120 |
| hsa_circ_0012926 | 90 | 255 | 813 | 559 | 1 | 0 | 0 |
| hsa_circ_0012926 | 90 | 1 | 559 | 559 | 1 | 0 | 0 |
| hsa_circ_0012926 | 90 | 1 | 559 | 559 | 1 | 0 | 0 |
| hsa_circ_0012926 | 91 | 10 | 416 | 407 | 1 | 0 | 2.00E-154 |
| hsa_circ_0012925 | 91 | 183 | 713 | 1821 | 1291 | 0 | 0 |
| hsa_circ_0012925 | 91 | 10 | 540 | 1821 | 1291 | 0 | 0 |
| hsa_circ_0029734 | 73 | 2594 | 2925 | 84690 | 85029 | 0.06 | 5.00E-22 |

novel_circ_030030 novel_circ_030031 novel_circ_030032 novel_circ_030033 novel_circ_030036 novel_circ_030040 novel_circ_030041 novel_circ_030042 novel_circ_030043 novel_circ_030044 novel_circ_030045 novel_circ_030046 novel_circ_030047 novel_circ_030048 novel_circ_030054 novel_circ_030055 novel_circ_030056 novel_circ_030057 novel_circ_030058 novel_circ_030059 novel_circ_030060 novel_circ_030061 novel_circ_030062 novel_circ_030065 novel_circ_030066 novel_circ_030067 novel_circ_030068 novel_circ_030069 novel_circ_030070 novel_circ_030071 novel_circ_030072 novel_circ_030073 novel_circ_030074 novel_circ_030075 novel_circ_030076 novel_circ_030077 novel_circ_030078 novel_circ_030079 novel_circ_030080 novel_circ_030081 novel_circ_030082 novel_circ_030083 novel_circ_030084 novel_circ_030085 novel_circ_030099 novel_circ_030100 novel_circ_030101 novel_circ_030102 novel_circ_030103 novel_circ_030104 novel_circ_030105 novel_circ_030106 novel_circ_030107 novel_circ_030108

| hsa_circ_0044308 | 80 | 59569 | 60009 | 2624 | 3078 | 0.09 | 1.00E-81 |
| --- | --- | --- | --- | --- | --- | --- | --- |
| hsa_circ_0074029 | 82 | 9904 | 10067 | 207775 | 207609 | 0.03 | 1.00E-27 |
| hsa_circ_0032905 | 90 | 1496 | 2280 | 1 | 795 | 0.01 | 0 |
| hsa_circ_0032905 | 90 | 7937 | 8721 | 1 | 795 | 0.01 | 0 |
| hsa_circ_0025615 | 93 | 155 | 238 | 193 | 110 | 0 | 6.00E-27 |
| hsa_circ_0025617 | 94 | 1 | 260 | 1 | 260 | 0 | 1.00E-109 |
| hsa_circ_0025617 | 95 | 1 | 789 | 1 | 789 | 0 | 0 |
| hsa_circ_0025620 | 95 | 1 | 529 | 1 | 529 | 0 | 0 |
| hsa_circ_0025620 | 95 | 1 | 245 | 285 | 529 | 0 | 1.00E-104 |
| hsa_circ_0090129 | 93 | 110 | 772 | 346 | 1023 | 0.03 | 0 |
| hsa_circ_0007108 | 93 | 1 | 659 | 1 | 674 | 0.03 | 0 |
| hsa_circ_0090133 | 93 | 1 | 570 | 1 | 588 | 0.03 | 0 |
| hsa_circ_0092225 | 92 | 1 | 294 | 574 | 867 | 0 | 3.00E-115 |
| hsa_circ_0091072 | 97 | 1 | 211 | 1 | 211 | 0 | 1.00E-97 |
| hsa_circ_0083882 | 79 | 8 | 1073 | 198 | 1257 | 0.01 | 0 |
| hsa_circ_0083878 | 79 | 1061 | 2391 | 2 | 1326 | 0.01 | 0 |
| hsa_circ_0083882 | 76 | 7 | 646 | 430 | 1069 | 0 | 1.00E-93 |
| hsa_circ_0083882 | 78 | 6 | 694 | 381 | 1069 | 0 | 3.00E-114 |
| hsa_circ_0083882 | 78 | 7 | 504 | 430 | 927 | 0 | 3.00E-83 |
| hsa_circ_0083882 | 79 | 6 | 552 | 381 | 927 | 0 | 9.00E-104 |
| hsa_circ_0083878 | 81 | 394 | 1391 | 2 | 996 | 0 | 0 |
| hsa_circ_0083878 | 81 | 1061 | 2058 | 2 | 996 | 0 | 0 |
| hsa_circ_0083885 | 79 | 6 | 453 | 191 | 638 | 0 | 3.00E-82 |
| hsa_circ_0083872 | 83 | 504 | 1305 | 913 | 1720 | 0.01 | 0 |
| hsa_circ_0083872 | 83 | 619 | 1420 | 913 | 1720 | 0.01 | 0 |
| hsa_circ_0083872 | 83 | 504 | 1125 | 913 | 1540 | 0.01 | 2.00E-153 |
| hsa_circ_0083872 | 83 | 619 | 1240 | 913 | 1540 | 0.01 | 2.00E-153 |
| hsa_circ_0083871 | 81 | 226 | 949 | 5 | 728 | 0 | 1.00E-161 |
| hsa_circ_0083872 | 82 | 504 | 881 | 913 | 1296 | 0.02 | 1.00E-85 |
| hsa_circ_0083872 | 82 | 504 | 881 | 913 | 1296 | 0.02 | 2.00E-86 |
| hsa_circ_0083872 | 87 | 504 | 748 | 913 | 1157 | 0 | 8.00E-71 |
| hsa_circ_0083872 | 87 | 422 | 586 | 912 | 1076 | 0 | 2.00E-46 |
| hsa_circ_0083871 | 81 | 118 | 841 | 5 | 728 | 0 | 9.00E-162 |
| hsa_circ_0083871 | 82 | 226 | 851 | 5 | 630 | 0 | 8.00E-146 |
| hsa_circ_0083871 | 82 | 226 | 736 | 5 | 515 | 0 | 1.00E-118 |
| hsa_circ_0083871 | 84 | 226 | 662 | 5 | 441 | 0 | 6.00E-116 |
| hsa_circ_0083871 | 85 | 226 | 516 | 5 | 295 | 0 | 3.00E-78 |
| hsa_circ_0007118 | 80 | 4 | 368 | 752 | 388 | 0 | 4.00E-71 |
| hsa_circ_0007118 | 83 | 4 | 636 | 752 | 120 | 0 | 2.00E-166 |
| hsa_circ_0007118 | 90 | 1 | 252 | 371 | 120 | 0 | 6.00E-87 |
| hsa_circ_0007118 | 88 | 1 | 371 | 371 | 1 | 0 | 2.00E-119 |
| hsa_circ_0083855 | 86 | 308 | 732 | 441 | 17 | 0 | 1.00E-127 |
| hsa_circ_0083851 | 89 | 1 | 200 | 200 | 1 | 0 | 8.00E-63 |
| hsa_circ_0083851 | 93 | 4 | 108 | 105 | 1 | 0 | 8.00E-37 |
| hsa_circ_0025895 | 90 | 1 | 570 | 1 | 572 | 0.01 | 0 |
| hsa_circ_0025895 | 91 | 1 | 1059 | 1 | 1061 | 0 | 0 |
| hsa_circ_0025895 | 92 | 1 | 1302 | 1 | 1304 | 0 | 0 |
| hsa_circ_0025895 | 91 | 1 | 1757 | 1 | 1759 | 0 | 0 |
| hsa_circ_0025895 | 91 | 1 | 1878 | 1 | 1880 | 0 | 0 |
| hsa_circ_0025895 | 91 | 1 | 1879 | 1 | 1881 | 0 | 0 |
| hsa_circ_0025895 | 94 | 1 | 609 | 171 | 779 | 0 | 0 |
| hsa_circ_0025895 | 93 | 1 | 709 | 171 | 879 | 0 | 0 |
| hsa_circ_0025895 | 93 | 1 | 891 | 171 | 1061 | 0 | 0 |
| hsa_circ_0025895 | 93 | 1 | 1134 | 171 | 1304 | 0 | 0 |

novel_circ_030109 novel_circ_030110 novel_circ_030111 novel_circ_030112 novel_circ_030113 novel_circ_030114 novel_circ_030115 novel_circ_030116 novel_circ_030117 novel_circ_030118 novel_circ_030119 novel_circ_030120 novel_circ_030121 novel_circ_030122 novel_circ_030123 novel_circ_030124 novel_circ_030125 novel_circ_030126 novel_circ_030127 novel_circ_030128 novel_circ_030129 novel_circ_030130 novel_circ_030131 novel_circ_030132 novel_circ_030133 novel_circ_030134 novel_circ_030135 novel_circ_030136 novel_circ_030137 novel_circ_030138 novel_circ_030139 novel_circ_030141 novel_circ_030142 novel_circ_030143 novel_circ_030144 novel_circ_030145 novel_circ_030146 novel_circ_030147 novel_circ_030148 novel_circ_030150 novel_circ_030151 novel_circ_030152 novel_circ_030153 novel_circ_030154 novel_circ_030155 novel_circ_030156 novel_circ_030157 novel_circ_030158 novel_circ_030159 novel_circ_030160 novel_circ_030161 novel_circ_030162 novel_circ_030163 novel_circ_030164

| hsa_circ_0025895 | 92 | 1 | 1589 | 171 | 1759 | 0 | 0 |
| --- | --- | --- | --- | --- | --- | --- | --- |
| hsa_circ_0025895 | 92 | 1 | 1710 | 171 | 1880 | 0 | 0 |
| hsa_circ_0025895 | 92 | 1 | 1711 | 171 | 1881 | 0 | 0 |
| hsa_circ_0025895 | 93 | 1 | 476 | 304 | 779 | 0 | 0 |
| hsa_circ_0025895 | 92 | 1 | 1577 | 304 | 1880 | 0 | 0 |
| hsa_circ_0025895 | 93 | 1 | 489 | 573 | 1061 | 0 | 0 |
| hsa_circ_0025895 | 93 | 1 | 732 | 573 | 1304 | 0 | 0 |
| hsa_circ_0025895 | 92 | 1 | 1187 | 573 | 1759 | 0 | 0 |
| hsa_circ_0025895 | 91 | 1 | 1308 | 573 | 1880 | 0 | 0 |
| hsa_circ_0025895 | 91 | 1 | 1309 | 573 | 1881 | 0 | 0 |
| hsa_circ_0025895 | 91 | 1 | 1309 | 573 | 1881 | 0 | 0 |
| hsa_circ_0025895 | 91 | 1 | 980 | 780 | 1759 | 0 | 0 |
| hsa_circ_0025895 | 93 | 1 | 243 | 1062 | 1304 | 0 | 6.00E-97 |
| hsa_circ_0025895 | 90 | 1 | 698 | 1062 | 1759 | 0 | 0 |
| hsa_circ_0025895 | 90 | 1 | 819 | 1062 | 1880 | 0 | 0 |
| hsa_circ_0025895 | 89 | 4 | 455 | 1308 | 1759 | 0 | 4.00E-161 |
| hsa_circ_0025895 | 89 | 4 | 576 | 1308 | 1880 | 0 | 0 |
| hsa_circ_0025895 | 89 | 4 | 577 | 1308 | 1881 | 0 | 0 |
| hsa_circ_0025895 | 89 | 4 | 577 | 1308 | 1881 | 0 | 0 |
| hsa_circ_0025895 | 88 | 1 | 426 | 1456 | 1881 | 0 | 2.00E-141 |
| hsa_circ_0025895 | 91 | 1 | 613 | 1881 | 2493 | 0 | 0 |
| hsa_circ_0025895 | 91 | 1 | 906 | 1881 | 2786 | 0 | 0 |
| hsa_circ_0025895 | 91 | 1 | 1176 | 1881 | 3056 | 0 | 0 |
| hsa_circ_0025895 | 91 | 1 | 454 | 2040 | 2493 | 0 | 1.00E-170 |
| hsa_circ_0025895 | 90 | 1 | 560 | 2040 | 2599 | 0 | 0 |
| hsa_circ_0025895 | 90 | 1 | 1017 | 2040 | 3056 | 0 | 0 |
| hsa_circ_0025895 | 90 | 1 | 867 | 2190 | 3056 | 0 | 0 |
| hsa_circ_0025895 | 90 | 1 | 291 | 2496 | 2786 | 0 | 2.00E-103 |
| hsa_circ_0025895 | 90 | 1 | 561 | 2496 | 3056 | 0 | 0 |
| hsa_circ_0025895 | 91 | 1 | 457 | 2600 | 3056 | 0 | 3.00E-172 |
| hsa_circ_0025895 | 91 | 1 | 270 | 2787 | 3056 | 0 | 1.00E-98 |
| hsa_circ_0025897 | 93 | 2 | 847 | 846 | 1 | 0 | 0 |
| hsa_circ_0025897 | 93 | 1 | 675 | 675 | 1 | 0 | 0 |
| hsa_circ_0025897 | 93 | 2 | 847 | 846 | 1 | 0 | 0 |
| hsa_circ_0025897 | 93 | 95 | 940 | 846 | 1 | 0 | 0 |
| hsa_circ_0025897 | 93 | 1499 | 2344 | 846 | 1 | 0 | 0 |
| hsa_circ_0025897 | 92 | 2 | 550 | 846 | 298 | 0 | 0 |
| hsa_circ_0025897 | 92 | 95 | 643 | 846 | 298 | 0 | 0 |
| hsa_circ_0003402 | 99 | 1405 | 1497 | 1806 | 1714 | 0 | 2.00E-39 |
| hsa_circ_0007161 | 95 | 1 | 265 | 265 | 1 | 0 | 6.00E-114 |
| hsa_circ_0007161 | 95 | 1 | 265 | 265 | 1 | 0 | 8.00E-116 |
| hsa_circ_0007161 | 96 | 1 | 326 | 326 | 1 | 0 | 1.00E-149 |
| hsa_circ_0007161 | 96 | 27169 | 27538 | 370 | 1 | 0 | 2.00E-170 |
| hsa_circ_0045009 | 85 | 1 | 523 | 526 | 1 | 0.01 | 3.00E-147 |
| hsa_circ_0045009 | 85 | 1 | 523 | 526 | 1 | 0.01 | 1.00E-147 |
| hsa_circ_0045009 | 88 | 1 | 805 | 808 | 1 | 0.01 | 0 |
| hsa_circ_0045014 | 94 | 1 | 282 | 282 | 1 | 0 | 7.00E-117 |
| hsa_circ_0045014 | 93 | 1 | 1039 | 1039 | 1 | 0 | 0 |
| hsa_circ_0045014 | 93 | 4 | 1752 | 1749 | 1 | 0 | 0 |
| hsa_circ_0045019 | 94 | 1 | 469 | 469 | 1 | 0 | 0 |
| hsa_circ_0045019 | 93 | 1 | 757 | 757 | 1 | 0 | 0 |
| hsa_circ_0045019 | 94 | 1 | 957 | 957 | 1 | 0 | 0 |
| hsa_circ_0045019 | 93 | 4 | 1179 | 1176 | 1 | 0 | 0 |
| hsa_circ_0045019 | 93 | 4 | 1470 | 1467 | 1 | 0 | 0 |

novel_circ_030165 novel_circ_030166 novel_circ_030167 novel_circ_030168 novel_circ_030169 novel_circ_030170 novel_circ_030171 novel_circ_030172 novel_circ_030173 novel_circ_030174 novel_circ_030175 novel_circ_030176 novel_circ_030177 novel_circ_030178 novel_circ_030179 novel_circ_030180 novel_circ_030181 novel_circ_030182 novel_circ_030183 novel_circ_030184 novel_circ_030185 novel_circ_030186 novel_circ_030187 novel_circ_030188 novel_circ_030189 novel_circ_030190 novel_circ_030191 novel_circ_030192 novel_circ_030193 novel_circ_030194 novel_circ_030195 novel_circ_030196 novel_circ_030197 novel_circ_030198 novel_circ_030199 novel_circ_030200 novel_circ_030201 novel_circ_030202 novel_circ_030203 novel_circ_030204 novel_circ_030205 novel_circ_030206 novel_circ_030207 novel_circ_030208 novel_circ_030209 novel_circ_030210 novel_circ_030211 novel_circ_030212 novel_circ_030213 novel_circ_030214 novel_circ_030215 novel_circ_030216 novel_circ_030217 novel_circ_030218

| hsa_circ_0045021 | 92 | 4 | 1001 | 1305 | 308 | 0 | 0 |
| --- | --- | --- | --- | --- | --- | --- | --- |
| hsa_circ_0045024 | 93 | 4 | 555 | 552 | 1 | 0 | 0 |
| hsa_circ_0045024 | 92 | 4 | 846 | 843 | 1 | 0 | 0 |
| hsa_circ_0045024 | 93 | 4 | 422 | 552 | 134 | 0 | 2.00E-174 |
| hsa_circ_0045036 | 94 | 1 | 1852 | 1849 | 1 | 0 | 0 |
| hsa_circ_0045036 | 93 | 1 | 692 | 941 | 253 | 0 | 0 |
| hsa_circ_0045036 | 94 | 1 | 797 | 1046 | 253 | 0 | 0 |
| hsa_circ_0045036 | 94 | 4 | 1083 | 1329 | 253 | 0 | 0 |
| hsa_circ_0045036 | 95 | 1 | 1600 | 1849 | 253 | 0 | 0 |
| hsa_circ_0045037 | 94 | 1 | 598 | 598 | 1 | 0 | 0 |
| hsa_circ_0045037 | 94 | 1 | 707 | 707 | 1 | 0 | 0 |
| hsa_circ_0045037 | 94 | 4 | 884 | 881 | 1 | 0 | 0 |
| hsa_circ_0045037 | 95 | 1 | 1275 | 1275 | 1 | 0 | 0 |
| hsa_circ_0045037 | 95 | 1 | 1401 | 1401 | 1 | 0 | 0 |
| hsa_circ_0045039 | 94 | 1 | 472 | 472 | 1 | 0 | 0 |
| hsa_circ_0045039 | 94 | 4 | 758 | 755 | 1 | 0 | 0 |
| hsa_circ_0045039 | 95 | 1 | 1275 | 1275 | 1 | 0 | 0 |
| hsa_circ_0045039 | 95 | 1 | 1502 | 1502 | 1 | 0 | 0 |
| hsa_circ_0045044 | 95 | 4 | 391 | 388 | 1 | 0 | 3.00E-172 |
| hsa_circ_0045044 | 95 | 1 | 908 | 908 | 1 | 0 | 0 |
| hsa_circ_0045049 | 94 | 4 | 286 | 283 | 1 | 0 | 9.00E-121 |
| hsa_circ_0045049 | 96 | 1 | 490 | 490 | 1 | 0 | 0 |
| hsa_circ_0045049 | 96 | 1 | 677 | 677 | 1 | 0 | 0 |
| hsa_circ_0045049 | 95 | 1 | 803 | 803 | 1 | 0 | 0 |
| hsa_circ_0000796 | 96 | 1 | 568 | 568 | 1 | 0 | 0 |
| hsa_circ_0000796 | 95 | 1 | 694 | 694 | 1 | 0 | 0 |
| hsa_circ_0045049 | 96 | 1 | 921 | 1030 | 110 | 0 | 0 |
| hsa_circ_0000796 | 96 | 1 | 517 | 694 | 178 | 0 | 0 |
| hsa_circ_0045049 | 96 | 1 | 744 | 1030 | 287 | 0 | 0 |
| hsa_circ_0045049 | 96 | 1 | 1266 | 1552 | 287 | 0 | 0 |
| hsa_circ_0045057 | 96 | 1 | 875 | 875 | 1 | 0 | 0 |
| hsa_circ_0045071 | 96 | 1 | 712 | 712 | 1 | 0 | 0 |
| hsa_circ_0045071 | 96 | 1 | 345 | 345 | 1 | 0 | 1.00E-156 |
| hsa_circ_0045071 | 96 | 1 | 488 | 488 | 1 | 0 | 0 |
| hsa_circ_0045071 | 95 | 1 | 1587 | 1587 | 1 | 0 | 0 |
| hsa_circ_0045071 | 96 | 1 | 345 | 345 | 1 | 0 | 8.00E-157 |
| hsa_circ_0045071 | 96 | 1 | 488 | 488 | 1 | 0 | 0 |
| hsa_circ_0045071 | 96 | 1 | 712 | 712 | 1 | 0 | 0 |
| hsa_circ_0045071 | 96 | 1 | 904 | 904 | 1 | 0 | 0 |
| hsa_circ_0045071 | 95 | 1 | 1124 | 1124 | 1 | 0 | 0 |
| hsa_circ_0045071 | 95 | 1 | 1587 | 1587 | 1 | 0 | 0 |
| hsa_circ_0045073 | 95 | 1 | 779 | 779 | 1 | 0 | 0 |
| hsa_circ_0045073 | 94 | 1 | 1242 | 1242 | 1 | 0 | 0 |
| hsa_circ_0045074 | 96 | 1 | 416 | 416 | 1 | 0 | 0 |
| hsa_circ_0045074 | 95 | 1 | 636 | 636 | 1 | 0 | 0 |
| hsa_circ_0045074 | 94 | 1 | 1099 | 1099 | 1 | 0 | 0 |
| hsa_circ_0045074 | 95 | 1 | 1513 | 1513 | 1 | 0 | 0 |
| hsa_circ_0045074 | 96 | 1 | 3095 | 3095 | 1 | 0 | 0 |
| hsa_circ_0045079 | 93 | 1 | 463 | 463 | 1 | 0 | 0 |
| hsa_circ_0045079 | 95 | 1 | 877 | 877 | 1 | 0 | 0 |
| hsa_circ_0045079 | 95 | 1 | 1039 | 1039 | 1 | 0 | 0 |
| hsa_circ_0045084 | 96 | 1 | 1079 | 1079 | 1 | 0 | 0 |
| hsa_circ_0045085 | 96 | 1 | 1418 | 1420 | 3 | 0 | 0 |
| hsa_circ_0045085 | 96 | 1 | 1879 | 2796 | 918 | 0 | 0 |

novel_circ_030219 novel_circ_030220 novel_circ_030221 novel_circ_030222 novel_circ_030223 novel_circ_030224 novel_circ_030225 novel_circ_030226 novel_circ_030227 novel_circ_030228 novel_circ_030229 novel_circ_030230 novel_circ_030231 novel_circ_030232 novel_circ_030233 novel_circ_030234 novel_circ_030235 novel_circ_030236 novel_circ_030237 novel_circ_030238 novel_circ_030239 novel_circ_030240 novel_circ_030241 novel_circ_030242 novel_circ_030243 novel_circ_030244 novel_circ_030245 novel_circ_030246 novel_circ_030247 novel_circ_030248 novel_circ_030249 novel_circ_030250 novel_circ_030251 novel_circ_030252 novel_circ_030253 novel_circ_030254 novel_circ_030255 novel_circ_030256 novel_circ_030257 novel_circ_030258 novel_circ_030260 novel_circ_030264 novel_circ_030265 novel_circ_030267 novel_circ_030268 novel_circ_030269 novel_circ_030272 novel_circ_030273 novel_circ_030275 novel_circ_030276 novel_circ_030277 novel_circ_030278 novel_circ_030279 novel_circ_030280

| hsa_circ_0045071 | 96 | 236 | 2822 | 5667 | 3081 | 0 | 0 |
| --- | --- | --- | --- | --- | --- | --- | --- |
| hsa_circ_0045086 | 95 | 1 | 418 | 724 | 307 | 0 | 0 |
| hsa_circ_0045086 | 95 | 1 | 1376 | 1682 | 307 | 0 | 0 |
| hsa_circ_0045071 | 95 | 236 | 2319 | 5667 | 3584 | 0 | 0 |
| hsa_circ_0045090 | 95 | 1 | 980 | 980 | 1 | 0 | 0 |
| hsa_circ_0045086 | 95 | 1 | 1254 | 1682 | 429 | 0 | 0 |
| hsa_circ_0045071 | 95 | 236 | 2197 | 5667 | 3706 | 0 | 0 |
| hsa_circ_0045092 | 95 | 1 | 898 | 898 | 1 | 0 | 0 |
| hsa_circ_0045086 | 95 | 1 | 1172 | 1682 | 511 | 0 | 0 |
| hsa_circ_0045071 | 95 | 236 | 2115 | 5667 | 3788 | 0 | 0 |
| hsa_circ_0045092 | 95 | 1 | 684 | 898 | 215 | 0 | 0 |
| hsa_circ_0045090 | 95 | 1 | 795 | 1091 | 297 | 0 | 0 |
| hsa_circ_0045086 | 95 | 1 | 958 | 1682 | 725 | 0 | 0 |
| hsa_circ_0045084 | 95 | 1 | 1497 | 3497 | 2001 | 0 | 0 |
| hsa_circ_0045071 | 95 | 236 | 1901 | 5667 | 4002 | 0 | 0 |
| hsa_circ_0045071 | 95 | 236 | 1449 | 5667 | 4454 | 0 | 0 |
| hsa_circ_0045096 | 95 | 1 | 274 | 274 | 1 | 0 | 9.00E-121 |
| hsa_circ_0045096 | 95 | 23 | 299 | 277 | 1 | 0 | 2.00E-122 |
| hsa_circ_0045096 | 95 | 3 | 469 | 467 | 1 | 0 | 0 |
| hsa_circ_0045096 | 95 | 1 | 667 | 667 | 1 | 0 | 0 |
| hsa_circ_0045096 | 96 | 1 | 813 | 813 | 1 | 0 | 0 |
| hsa_circ_0045071 | 95 | 1 | 982 | 5667 | 4686 | 0 | 0 |
| hsa_circ_0045071 | 95 | 236 | 1217 | 5667 | 4686 | 0 | 0 |
| hsa_circ_0045096 | 96 | 3 | 358 | 467 | 112 | 0 | 3.00E-161 |
| hsa_circ_0045096 | 96 | 1 | 702 | 813 | 112 | 0 | 0 |
| hsa_circ_0045099 | 96 | 1 | 943 | 943 | 1 | 0 | 0 |
| hsa_circ_0045099 | 94 | 3 | 195 | 193 | 1 | 0 | 3.00E-79 |
| hsa_circ_0045099 | 95 | 1 | 393 | 393 | 1 | 0 | 9.00E-177 |
| hsa_circ_0045099 | 96 | 1 | 539 | 539 | 1 | 0 | 0 |
| hsa_circ_0045099 | 96 | 1 | 943 | 943 | 1 | 0 | 0 |
| hsa_circ_0045101 | 97 | 1 | 344 | 344 | 1 | 0 | 6.00E-163 |
| hsa_circ_0045099 | 96 | 1 | 748 | 943 | 196 | 0 | 0 |
| hsa_circ_0045099 | 96 | 1 | 550 | 943 | 394 | 0 | 0 |
| hsa_circ_0045099 | 95 | 1 | 404 | 943 | 540 | 0 | 0 |
| hsa_circ_0044908 | 93 | 102 | 470 | 481 | 113 | 0 | 5.00E-150 |
| hsa_circ_0034296 | 93 | 1 | 228 | 505 | 278 | 0 | 3.00E-90 |
| hsa_circ_0034294 | 86 | 1 | 923 | 926 | 1 | 0.01 | 0 |
| hsa_circ_0034290 | 86 | 1 | 1169 | 1172 | 1 | 0 | 0 |
| hsa_circ_0034286 | 87 | 1 | 1760 | 1933 | 171 | 0 | 0 |
| hsa_circ_0034286 | 87 | 1 | 1760 | 1933 | 171 | 0 | 0 |
| hsa_circ_0034283 | 83 | 1 | 648 | 866 | 219 | 0 | 2.00E-161 |
| hsa_circ_0034309 | 75 | 6 | 938 | 6 | 938 | 0 | 1.00E-124 |
| hsa_circ_0034320 | 84 | 10 | 353 | 805 | 462 | 0 | 7.00E-88 |
| hsa_circ_0034104 | 90 | 1 | 299 | 405 | 706 | 0.02 | 2.00E-103 |
| hsa_circ_0002144 | 74 | 2850 | 3110 | 50961 | 50712 | 0.05 | 9.00E-17 |
| hsa_circ_0002144 | 74 | 2850 | 3110 | 50961 | 50712 | 0.05 | 1.00E-16 |
| hsa_circ_0034131 | 83 | 22858 | 23069 | 2948 | 3165 | 0.06 | 4.00E-44 |
| hsa_circ_0034131 | 83 | 22803 | 23014 | 2948 | 3165 | 0.06 | 4.00E-44 |
| hsa_circ_0034131 | 83 | 19464 | 19675 | 2948 | 3165 | 0.06 | 4.00E-44 |
| hsa_circ_0034152 | 80 | 52227 | 54315 | 82 | 2180 | 0.05 | 0 |
| hsa_circ_0034131 | 83 | 14420 | 14631 | 2948 | 3165 | 0.06 | 5.00E-44 |
| hsa_circ_0034152 | 80 | 52128 | 54216 | 82 | 2180 | 0.05 | 0 |
| hsa_circ_0034131 | 83 | 12735 | 12946 | 2948 | 3165 | 0.06 | 1.00E-44 |
| hsa_circ_0034131 | 83 | 12735 | 12946 | 2948 | 3165 | 0.06 | 1.00E-44 |

novel_circ_030281 novel_circ_030282 novel_circ_030283 novel_circ_030284 novel_circ_030285 novel_circ_030286 novel_circ_030287 novel_circ_030288 novel_circ_030289 novel_circ_030290 novel_circ_030291 novel_circ_030292 novel_circ_030293 novel_circ_030294 novel_circ_030295 novel_circ_030296 novel_circ_030297 novel_circ_030299 novel_circ_030301 novel_circ_030302 novel_circ_030303 novel_circ_030304 novel_circ_030307 novel_circ_030309 novel_circ_030310 novel_circ_030311 novel_circ_030312 novel_circ_030313 novel_circ_030315 novel_circ_030316 novel_circ_030317 novel_circ_030318 novel_circ_030319 novel_circ_030320 novel_circ_030321 novel_circ_030322 novel_circ_030323 novel_circ_030324 novel_circ_030326 novel_circ_030327 novel_circ_030328 novel_circ_030329 novel_circ_030330 novel_circ_030331 novel_circ_030332 novel_circ_030333 novel_circ_030334 novel_circ_030335 novel_circ_030336 novel_circ_030337 novel_circ_030338 novel_circ_030339 novel_circ_030340 novel_circ_030341

| hsa_circ_0034131 | 83 | 12735 | 12946 | 2948 | 3165 | 0.06 | 2.00E-44 |
| --- | --- | --- | --- | --- | --- | --- | --- |
| hsa_circ_0034131 | 83 | 12735 | 12946 | 2948 | 3165 | 0.06 | 2.00E-44 |
| hsa_circ_0034131 | 83 | 12735 | 12946 | 2948 | 3165 | 0.06 | 3.00E-44 |
| hsa_circ_0034131 | 83 | 12735 | 12946 | 2948 | 3165 | 0.06 | 5.00E-44 |
| hsa_circ_0034117 | 86 | 345 | 486 | 367 | 509 | 0.02 | 2.00E-34 |
| hsa_circ_0034131 | 83 | 10053 | 10264 | 2948 | 3165 | 0.06 | 3.00E-44 |
| hsa_circ_0034131 | 83 | 5749 | 5960 | 2948 | 3165 | 0.06 | 9.00E-45 |
| hsa_circ_0034131 | 83 | 5749 | 5960 | 2948 | 3165 | 0.06 | 1.00E-44 |
| hsa_circ_0034131 | 79 | 272 | 446 | 2944 | 3119 | 0.04 | 1.00E-24 |
| hsa_circ_0034131 | 83 | 3208 | 3419 | 2948 | 3165 | 0.06 | 6.00E-45 |
| hsa_circ_0034131 | 83 | 3208 | 3419 | 2948 | 3165 | 0.06 | 9.00E-45 |
| hsa_circ_0034131 | 83 | 292 | 503 | 2948 | 3165 | 0.06 | 2.00E-45 |
| hsa_circ_0034131 | 83 | 292 | 503 | 2948 | 3165 | 0.06 | 3.00E-45 |
| hsa_circ_0034131 | 83 | 292 | 503 | 2948 | 3165 | 0.06 | 6.00E-45 |
| hsa_circ_0034131 | 83 | 292 | 503 | 2948 | 3165 | 0.06 | 9.00E-45 |
| hsa_circ_0034131 | 83 | 292 | 503 | 2948 | 3165 | 0.06 | 2.00E-44 |
| hsa_circ_0034151 | 87 | 303 | 385 | 2640 | 2719 | 0.04 | 2.00E-15 |
| hsa_circ_0034151 | 84 | 776 | 869 | 2655 | 2751 | 0.13 | 5.00E-15 |
| hsa_circ_0034157 | 94 | 3617 | 3709 | 184 | 277 | 0.01 | 1.00E-29 |
| hsa_circ_0034157 | 94 | 3559 | 3651 | 184 | 277 | 0.01 | 1.00E-29 |
| hsa_circ_0034157 | 94 | 152 | 244 | 184 | 277 | 0.01 | 4.00E-30 |
| hsa_circ_0034157 | 94 | 152 | 244 | 184 | 277 | 0.01 | 1.00E-29 |
| hsa_circ_0077662 | 93 | 8087 | 8153 | 16113 | 16048 | 0.01 | 2.00E-16 |
| hsa_circ_0034162 | 87 | 1 | 746 | 746 | 1 | 0 | 0 |
| hsa_circ_0034165 | 91 | 189 | 1786 | 1586 | 1 | 0.01 | 0 |
| hsa_circ_0034170 | 97 | 1 | 184 | 1802 | 1618 | 0.01 | 1.00E-81 |
| hsa_circ_0034170 | 97 | 1 | 184 | 1802 | 1618 | 0.01 | 2.00E-82 |
| hsa_circ_0034181 | 89 | 1 | 609 | 619 | 11 | 0 | 0 |
| hsa_circ_0034240 | 83 | 1 | 2789 | 2793 | 5 | 0 | 0 |
| hsa_circ_0034256 | 87 | 1 | 395 | 395 | 1 | 0 | 8.00E-123 |
| hsa_circ_0034256 | 87 | 1 | 647 | 647 | 1 | 0 | 0 |
| hsa_circ_0005387 | 88 | 1 | 446 | 446 | 1 | 0 | 8.00E-148 |
| hsa_circ_0034066 | 88 | 1 | 906 | 1 | 906 | 0 | 0 |
| hsa_circ_0034062 | 86 | 1 | 212 | 373 | 584 | 0 | 1.00E-58 |
| hsa_circ_0034062 | 89 | 1663 | 2060 | 187 | 584 | 0 | 4.00E-135 |
| hsa_circ_0034062 | 86 | 3 | 584 | 3 | 584 | 0 | 2.00E-177 |
| hsa_circ_0034062 | 89 | 1663 | 2060 | 187 | 584 | 0 | 2.00E-135 |
| hsa_circ_0034062 | 86 | 3 | 584 | 3 | 584 | 0 | 4.00E-178 |
| hsa_circ_0036148 | 90 | 1 | 858 | 858 | 1 | 0 | 0 |
| hsa_circ_0036148 | 91 | 1 | 746 | 746 | 1 | 0 | 0 |
| hsa_circ_0036148 | 88 | 1 | 1695 | 1695 | 1 | 0 | 0 |
| hsa_circ_0036151 | 91 | 1 | 235 | 235 | 1 | 0 | 8.00E-86 |
| hsa_circ_0036153 | 90 | 1 | 255 | 255 | 1 | 0 | 1.00E-88 |
| hsa_circ_0036153 | 89 | 1 | 604 | 607 | 1 | 0 | 0 |
| hsa_circ_0036153 | 89 | 15 | 948 | 937 | 1 | 0 | 0 |
| hsa_circ_0000624 | 89 | 1 | 347 | 352 | 3 | 0.01 | 2.00E-118 |
| hsa_circ_0000624 | 88 | 15 | 691 | 682 | 3 | 0 | 0 |
| hsa_circ_0000624 | 87 | 1 | 834 | 836 | 3 | 0.01 | 0 |
| hsa_circ_0000624 | 87 | 1 | 947 | 949 | 3 | 0.01 | 0 |
| hsa_circ_0036156 | 87 | 1 | 789 | 789 | 1 | 0.01 | 0 |
| hsa_circ_0036156 | 87 | 316 | 1105 | 790 | 1 | 0.01 | 0 |
| hsa_circ_0036161 | 92 | 13 | 315 | 453 | 151 | 0 | 4.00E-120 |
| hsa_circ_0036159 | 89 | 1 | 622 | 2877 | 2256 | 0 | 0 |
| hsa_circ_0036159 | 89 | 2 | 698 | 2952 | 2256 | 0 | 0 |

novel_circ_030342 novel_circ_030343 novel_circ_030344 novel_circ_030345 novel_circ_030346 novel_circ_030347 novel_circ_030348 novel_circ_030349 novel_circ_030350 novel_circ_030351 novel_circ_030352 novel_circ_030353 novel_circ_030354 novel_circ_030355 novel_circ_030356 novel_circ_030357 novel_circ_030358 novel_circ_030359 novel_circ_030360 novel_circ_030361 novel_circ_030362 novel_circ_030363 novel_circ_030364 novel_circ_030365 novel_circ_030366 novel_circ_030367 novel_circ_030368 novel_circ_030369 novel_circ_030370 novel_circ_030374 novel_circ_030375 novel_circ_030376 novel_circ_030377 novel_circ_030378 novel_circ_030379 novel_circ_030380 novel_circ_030381 novel_circ_030382 novel_circ_030383 novel_circ_030384 novel_circ_030385 novel_circ_030386 novel_circ_030387 novel_circ_030388 novel_circ_030389 novel_circ_030390 novel_circ_030391 novel_circ_030392 novel_circ_030393 novel_circ_030394 novel_circ_030395 novel_circ_030403 novel_circ_030404 novel_circ_030405

| hsa_circ_0036156 | 91 | 4 | 1620 | 4548 | 2932 | 0 | 0 |
| --- | --- | --- | --- | --- | --- | --- | --- |
| hsa_circ_0036156 | 91 | 1 | 1747 | 4678 | 2932 | 0 | 0 |
| hsa_circ_0036156 | 91 | 1 | 1845 | 4776 | 2932 | 0 | 0 |
| hsa_circ_0036156 | 90 | 1 | 1513 | 4678 | 3166 | 0 | 0 |
| hsa_circ_0036166 | 92 | 4 | 998 | 1179 | 185 | 0 | 0 |
| hsa_circ_0036166 | 92 | 67 | 1066 | 1184 | 185 | 0 | 0 |
| hsa_circ_0036166 | 92 | 1 | 1125 | 1309 | 185 | 0 | 0 |
| hsa_circ_0036166 | 92 | 3 | 1226 | 1408 | 185 | 0 | 0 |
| hsa_circ_0036167 | 92 | 1 | 1049 | 1049 | 1 | 0 | 0 |
| hsa_circ_0036167 | 93 | 4 | 590 | 712 | 126 | 0 | 0 |
| hsa_circ_0036167 | 92 | 4 | 797 | 919 | 126 | 0 | 0 |
| hsa_circ_0036167 | 91 | 67 | 865 | 924 | 126 | 0 | 0 |
| hsa_circ_0036167 | 92 | 1 | 924 | 1049 | 126 | 0 | 0 |
| hsa_circ_0036167 | 92 | 1 | 1022 | 1147 | 126 | 0 | 0 |
| hsa_circ_0036166 | 92 | 1 | 1242 | 1627 | 386 | 0 | 0 |
| hsa_circ_0036170 | 89 | 4 | 1815 | 1790 | 1 | 0.01 | 0 |
| hsa_circ_0036167 | 92 | 1 | 591 | 1049 | 459 | 0 | 0 |
| hsa_circ_0036167 | 92 | 1 | 689 | 1147 | 459 | 0 | 0 |
| hsa_circ_0036166 | 93 | 1 | 909 | 1627 | 719 | 0 | 0 |
| hsa_circ_0036170 | 89 | 4 | 1815 | 1790 | 1 | 0.01 | 0 |
| hsa_circ_0036171 | 91 | 1 | 432 | 432 | 1 | 0 | 1.00E-166 |
| hsa_circ_0036174 | 94 | 1 | 444 | 445 | 2 | 0 | 0 |
| hsa_circ_0036174 | 88 | 4 | 1472 | 1448 | 2 | 0.02 | 0 |
| hsa_circ_0036176 | 94 | 1 | 220 | 220 | 1 | 0 | 7.00E-91 |
| hsa_circ_0036176 | 87 | 4 | 1248 | 1223 | 1 | 0.02 | 0 |
| hsa_circ_0000626 | 86 | 4 | 1091 | 1066 | 1 | 0.02 | 0 |
| hsa_circ_0006509 | 85 | 4 | 933 | 908 | 1 | 0.02 | 0 |
| hsa_circ_0036209 | 91 | 1 | 511 | 864 | 354 | 0 | 0 |
| hsa_circ_0036209 | 90 | 285 | 1326 | 1394 | 354 | 0 | 0 |
| hsa_circ_0000627 | 84 | 8387 | 8856 | 21020 | 21489 | 0.05 | 4.00E-118 |
| hsa_circ_0000629 | 98 | 1 | 213 | 1 | 213 | 0 | 2.00E-100 |
| hsa_circ_0000629 | 97 | 1 | 362 | 1 | 362 | 0 | 6.00E-173 |
| hsa_circ_0008808 | 96 | 69 | 530 | 1 | 462 | 0 | 0 |
| hsa_circ_0008808 | 95 | 69 | 651 | 1 | 583 | 0 | 0 |
| hsa_circ_0008808 | 95 | 69 | 652 | 1 | 584 | 0 | 0 |
| hsa_circ_0008808 | 95 | 69 | 840 | 1 | 772 | 0 | 0 |
| hsa_circ_0036227 | 95 | 214 | 1101 | 1 | 888 | 0 | 0 |
| hsa_circ_0036227 | 94 | 214 | 1414 | 1 | 1197 | 0 | 0 |
| hsa_circ_0008808 | 97 | 1 | 294 | 1 | 294 | 0 | 2.00E-138 |
| hsa_circ_0008808 | 96 | 1 | 462 | 1 | 462 | 0 | 0 |
| hsa_circ_0008808 | 95 | 1 | 583 | 1 | 583 | 0 | 0 |
| hsa_circ_0008808 | 95 | 1 | 584 | 1 | 584 | 0 | 0 |
| hsa_circ_0036227 | 95 | 146 | 1033 | 1 | 888 | 0 | 0 |
| hsa_circ_0036227 | 96 | 1 | 149 | 1 | 149 | 0 | 3.00E-63 |
| hsa_circ_0036227 | 95 | 1 | 317 | 1 | 317 | 0 | 1.00E-139 |
| hsa_circ_0036227 | 95 | 1 | 439 | 1 | 439 | 0 | 0 |
| hsa_circ_0036243 | 80 | 1 | 255 | 177 | 436 | 0.02 | 2.00E-46 |
| hsa_circ_0036243 | 80 | 1 | 257 | 177 | 438 | 0.02 | 2.00E-47 |
| hsa_circ_0036244 | 84 | 1 | 182 | 1 | 182 | 0 | 4.00E-43 |
| hsa_circ_0000631 | 88 | 1 | 289 | 289 | 1 | 0 | 4.00E-94 |
| hsa_circ_0005073 | 88 | 235 | 532 | 419 | 122 | 0 | 4.00E-97 |
| hsa_circ_0025541 | 92 | 1 | 604 | 1 | 604 | 0 | 0 |
| hsa_circ_0025541 | 91 | 1 | 477 | 1 | 477 | 0 | 0 |
| hsa_circ_0025538 | 94 | 1 | 390 | 1 | 390 | 0 | 2.00E-166 |

novel_circ_030406 novel_circ_030407 novel_circ_030408 novel_circ_030409 novel_circ_030410 novel_circ_030411 novel_circ_030412 novel_circ_030413 novel_circ_030414 novel_circ_030415 novel_circ_030416 novel_circ_030417 novel_circ_030418 novel_circ_030419 novel_circ_030421 novel_circ_030422 novel_circ_030423 novel_circ_030424 novel_circ_030425 novel_circ_030426 novel_circ_030427 novel_circ_030428 novel_circ_030429 novel_circ_030430 novel_circ_030431 novel_circ_030432 novel_circ_030433 novel_circ_030434 novel_circ_030435 novel_circ_030436 novel_circ_030437 novel_circ_030438 novel_circ_030439 novel_circ_030441 novel_circ_030442 novel_circ_030443 novel_circ_030444 novel_circ_030445 novel_circ_030446 novel_circ_030447 novel_circ_030448 novel_circ_030452 novel_circ_030453 novel_circ_030455 novel_circ_030456 novel_circ_030457 novel_circ_030458 novel_circ_030459 novel_circ_030460 novel_circ_030461 novel_circ_030462 novel_circ_030463 novel_circ_030464 novel_circ_030465

| hsa_circ_0025538 | 93 | 1 | 235 | 156 | 390 | 0 | 8.00E-94 |
| --- | --- | --- | --- | --- | --- | --- | --- |
| hsa_circ_0025538 | 94 | 1 | 390 | 1 | 390 | 0 | 2.00E-166 |
| hsa_circ_0025535 | 90 | 12 | 157 | 146 | 1 | 0 | 3.00E-48 |
| hsa_circ_0025531 | 91 | 158 | 1839 | 1685 | 1 | 0.01 | 0 |
| hsa_circ_0025534 | 94 | 1 | 457 | 677 | 221 | 0 | 0 |
| hsa_circ_0025531 | 91 | 1 | 1682 | 1685 | 1 | 0.01 | 0 |
| hsa_circ_0025532 | 91 | 1 | 514 | 698 | 185 | 0 | 0 |
| hsa_circ_0025532 | 91 | 1 | 698 | 698 | 1 | 0 | 0 |
| hsa_circ_0025531 | 89 | 1 | 829 | 1085 | 254 | 0.01 | 0 |
| hsa_circ_0025531 | 90 | 1 | 1082 | 1085 | 1 | 0.01 | 0 |
| hsa_circ_0025530 | 90 | 1 | 1303 | 1308 | 3 | 0.01 | 0 |
| hsa_circ_0025527 | 90 | 1 | 1486 | 1619 | 131 | 0.01 | 0 |
| hsa_circ_0025531 | 88 | 1 | 628 | 884 | 254 | 0.01 | 0 |
| hsa_circ_0025531 | 89 | 1 | 384 | 387 | 1 | 0.02 | 6.00E-134 |
| hsa_circ_0079675 | 95 | 1 | 238 | 1 | 238 | 0 | 2.00E-102 |
| hsa_circ_0044079 | 98 | 1 | 372 | 372 | 1 | 0 | 0 |
| hsa_circ_0044079 | 97 | 2099 | 2398 | 300 | 1 | 0 | 1.00E-142 |
| hsa_circ_0044079 | 97 | 2099 | 2398 | 300 | 1 | 0 | 1.00E-142 |
| hsa_circ_0044079 | 97 | 1 | 299 | 299 | 1 | 0 | 5.00E-143 |
| hsa_circ_0044047 | 95 | 1 | 279 | 279 | 1 | 0 | 7.00E-122 |
| hsa_circ_0043983 | 93 | 1 | 3086 | 160 | 3209 | 0.01 | 0 |
| hsa_circ_0043989 | 93 | 1 | 1097 | 1 | 1097 | 0 | 0 |
| hsa_circ_0043989 | 94 | 1 | 956 | 1 | 956 | 0 | 0 |
| hsa_circ_0043991 | 93 | 1 | 397 | 1 | 397 | 0 | 3.00E-167 |
| hsa_circ_0043985 | 93 | 1 | 1020 | 798 | 1817 | 0 | 0 |
| hsa_circ_0043985 | 94 | 1 | 516 | 710 | 1225 | 0 | 0 |
| hsa_circ_0043984 | 91 | 1 | 1189 | 87 | 1239 | 0.03 | 0 |
| hsa_circ_0043983 | 91 | 1 | 1200 | 1 | 1164 | 0.03 | 0 |
| hsa_circ_0043985 | 93 | 1 | 349 | 361 | 709 | 0 | 2.00E-142 |
| hsa_circ_0043984 | 90 | 1 | 855 | 87 | 905 | 0.04 | 0 |
| hsa_circ_0043983 | 90 | 1 | 1014 | 1 | 978 | 0.04 | 0 |
| hsa_circ_0018328 | 84 | 7 | 1878 | 1887 | 1 | 0.01 | 0 |
| hsa_circ_0018328 | 84 | 1 | 2226 | 2241 | 1 | 0.01 | 0 |
| hsa_circ_0006243 | 95 | 1 | 295 | 295 | 1 | 0 | 4.00E-129 |
| hsa_circ_0006243 | 92 | 1 | 421 | 424 | 1 | 0.01 | 4.00E-171 |
| hsa_circ_0021307 | 92 | 333 | 502 | 87 | 256 | 0 | 5.00E-61 |
| hsa_circ_0021290 | 87 | 1415 | 1722 | 1713 | 2020 | 0 | 8.00E-94 |
| hsa_circ_0021287 | 89 | 227 | 712 | 62 | 550 | 0.01 | 6.00E-170 |
| hsa_circ_0021286 | 94 | 1 | 228 | 1 | 228 | 0 | 1.00E-93 |
| hsa_circ_0021284 | 93 | 1 | 306 | 1 | 306 | 0 | 4.00E-125 |
| hsa_circ_0021284 | 93 | 1 | 306 | 1 | 306 | 0 | 3.00E-125 |
| hsa_circ_0021278 | 97 | 1 | 492 | 2123 | 2614 | 0 | 0 |
| hsa_circ_0021278 | 95 | 1 | 1932 | 683 | 2614 | 0 | 0 |
| hsa_circ_0021278 | 94 | 1 | 1440 | 683 | 2122 | 0 | 0 |
| hsa_circ_0021278 | 94 | 1 | 927 | 418 | 1344 | 0 | 0 |
| hsa_circ_0021278 | 94 | 1 | 542 | 683 | 1224 | 0 | 0 |
| hsa_circ_0021278 | 94 | 1 | 691 | 418 | 1108 | 0 | 0 |
| hsa_circ_0021278 | 94 | 1 | 483 | 418 | 900 | 0 | 0 |
| hsa_circ_0021278 | 94 | 1 | 265 | 418 | 682 | 0 | 4.00E-109 |
| hsa_circ_0021278 | 94 | 1 | 549 | 1 | 549 | 0 | 0 |
| hsa_circ_0021276 | 94 | 11477 | 12026 | 230 | 779 | 0 | 0 |
| hsa_circ_0021274 | 95 | 458 | 1479 | 1 | 1022 | 0 | 0 |
| hsa_circ_0021278 | 94 | 1 | 417 | 1 | 417 | 0 | 0 |
| hsa_circ_0021276 | 94 | 11477 | 11894 | 230 | 647 | 0 | 0 |

novel_circ_030466 novel_circ_030467 novel_circ_030468 novel_circ_030469 novel_circ_030470 novel_circ_030471 novel_circ_030472 novel_circ_030473 novel_circ_030474 novel_circ_030475 novel_circ_030476 novel_circ_030477 novel_circ_030479 novel_circ_030480 novel_circ_030481 novel_circ_030483 novel_circ_030484 novel_circ_030485 novel_circ_030486 novel_circ_030487 novel_circ_030488 novel_circ_030496 novel_circ_030497 novel_circ_030498 novel_circ_030499 novel_circ_030500 novel_circ_030501 novel_circ_030502 novel_circ_030503 novel_circ_030504 novel_circ_030508 novel_circ_030510 novel_circ_030511 novel_circ_030512 novel_circ_030513 novel_circ_030514 novel_circ_030515 novel_circ_030516 novel_circ_030517 novel_circ_030518 novel_circ_030519 novel_circ_030520 novel_circ_030521 novel_circ_030524 novel_circ_030525 novel_circ_030526 novel_circ_030527 novel_circ_030528 novel_circ_030529 novel_circ_030530 novel_circ_030531 novel_circ_030532 novel_circ_030533 novel_circ_030534

| hsa_circ_0021276 | 95 | 1 | 567 | 81 | 647 | 0 | 0 |
| --- | --- | --- | --- | --- | --- | --- | --- |
| hsa_circ_0021275 | 95 | 1 | 793 | 1 | 793 | 0 | 0 |
| hsa_circ_0021274 | 96 | 1 | 890 | 1 | 890 | 0 | 0 |
| hsa_circ_0021274 | 96 | 254 | 1143 | 1 | 890 | 0 | 0 |
| hsa_circ_0021275 | 96 | 1 | 625 | 1 | 625 | 0 | 0 |
| hsa_circ_0021274 | 96 | 254 | 975 | 1 | 722 | 0 | 0 |
| hsa_circ_0021276 | 97 | 1 | 230 | 1 | 230 | 0 | 9.00E-105 |
| hsa_circ_0021275 | 97 | 1 | 376 | 1 | 376 | 0 | 5.00E-179 |
| hsa_circ_0021274 | 97 | 254 | 726 | 1 | 473 | 0 | 0 |
| hsa_circ_0021274 | 97 | 458 | 930 | 1 | 473 | 0 | 0 |
| hsa_circ_0021274 | 98 | 458 | 780 | 1 | 323 | 0 | 3.00E-159 |
| hsa_circ_0021274 | 99 | 458 | 554 | 1 | 97 | 0 | 4.00E-42 |
| hsa_circ_0021272 | 92 | 1 | 497 | 917 | 421 | 0 | 0 |
| hsa_circ_0021272 | 93 | 1 | 420 | 420 | 1 | 0 | 2.00E-173 |
| hsa_circ_0001693 | 75 | 30009 | 30357 | 2171 | 1812 | 0.07 | 1.00E-34 |
| hsa_circ_0021252 | 97 | 1 | 1845 | 2681 | 837 | 0 | 0 |
| hsa_circ_0021243 | 95 | 1 | 611 | 916 | 1526 | 0 | 0 |
| hsa_circ_0021243 | 93 | 3 | 512 | 406 | 915 | 0 | 0 |
| hsa_circ_0021242 | 93 | 1184 | 2551 | 1 | 1368 | 0 | 0 |
| hsa_circ_0021242 | 94 | 845 | 1700 | 1 | 856 | 0 | 0 |
| hsa_circ_0021242 | 93 | 845 | 1297 | 1 | 453 | 0 | 0 |
| hsa_circ_0066258 | 92 | 514 | 1050 | 1325 | 789 | 0 | 0 |
| hsa_circ_0066258 | 92 | 3231 | 3767 | 1325 | 789 | 0 | 0 |
| hsa_circ_0066258 | 91 | 1 | 274 | 1062 | 789 | 0 | 9.00E-101 |
| hsa_circ_0073711 | 96 | 1 | 747 | 220 | 963 | 0 | 0 |
| hsa_circ_0073711 | 95 | 1 | 555 | 220 | 771 | 0.01 | 0 |
| hsa_circ_0073709 | 92 | 2 | 920 | 2 | 920 | 0 | 0 |
| hsa_circ_0073709 | 91 | 2 | 536 | 2 | 536 | 0 | 0 |
| hsa_circ_0073709 | 92 | 2 | 459 | 2 | 459 | 0 | 0 |
| hsa_circ_0073709 | 92 | 2 | 425 | 2 | 425 | 0 | 1.00E-165 |
| hsa_circ_0073703 | 90 | 1 | 489 | 717 | 229 | 0 | 2.00E-178 |
| hsa_circ_0073700 | 87 | 1 | 710 | 804 | 98 | 0.01 | 0 |
| hsa_circ_0073700 | 87 | 1 | 653 | 747 | 98 | 0.01 | 0 |
| hsa_circ_0073700 | 89 | 9 | 508 | 597 | 98 | 0 | 2.00E-174 |
| hsa_circ_0073698 | 89 | 1 | 1051 | 1150 | 100 | 0 | 0 |
| hsa_circ_0073698 | 90 | 1 | 1150 | 1150 | 1 | 0 | 0 |
| hsa_circ_0073698 | 90 | 1 | 477 | 477 | 1 | 0 | 5.00E-175 |
| hsa_circ_0073693 | 89 | 1 | 309 | 823 | 1119 | 0.04 | 8.00E-102 |
| hsa_circ_0073693 | 95 | 1 | 144 | 823 | 966 | 0 | 8.00E-59 |
| hsa_circ_0073693 | 98 | 1 | 490 | 333 | 822 | 0 | 0 |
| hsa_circ_0073693 | 98 | 3 | 569 | 256 | 822 | 0 | 0 |
| hsa_circ_0073691 | 94 | 1 | 535 | 1 | 535 | 0 | 0 |
| hsa_circ_0073691 | 94 | 1 | 535 | 1 | 535 | 0 | 0 |
| hsa_circ_0092271 | 92 | 442 | 782 | 342 | 2 | 0 | 3.00E-134 |
| hsa_circ_0090609 | 84 | 1135 | 2846 | 1703 | 1 | 0.01 | 0 |
| hsa_circ_0090612 | 85 | 1 | 722 | 770 | 49 | 0 | 0 |
| hsa_circ_0090609 | 84 | 790 | 2501 | 1703 | 1 | 0.01 | 0 |
| hsa_circ_0004467 | 96 | 336 | 620 | 285 | 1 | 0 | 2.00E-131 |
| hsa_circ_0041186 | 93 | 7 | 288 | 282 | 1 | 0 | 3.00E-115 |
| hsa_circ_0041180 | 92 | 4 | 353 | 350 | 1 | 0 | 7.00E-138 |
| hsa_circ_0004931 | 92 | 4 | 460 | 457 | 1 | 0 | 0 |
| hsa_circ_0041174 | 84 | 814 | 1315 | 1327 | 832 | 0.01 | 3.00E-136 |
| hsa_circ_0041173 | 91 | 51 | 654 | 604 | 1 | 0 | 0 |
| hsa_circ_0007645 | 92 | 1 | 403 | 403 | 1 | 0 | 1.00E-160 |

novel_circ_030535 novel_circ_030536 novel_circ_030537 novel_circ_030538 novel_circ_030539 novel_circ_030540 novel_circ_030541 novel_circ_030542 novel_circ_030543 novel_circ_030544 novel_circ_030545 novel_circ_030546 novel_circ_030547 novel_circ_030548 novel_circ_030549 novel_circ_030550 novel_circ_030551 novel_circ_030552 novel_circ_030553 novel_circ_030554 novel_circ_030555 novel_circ_030556 novel_circ_030557 novel_circ_030558 novel_circ_030559 novel_circ_030560 novel_circ_030561 novel_circ_030562 novel_circ_030563 novel_circ_030564 novel_circ_030565 novel_circ_030566 novel_circ_030567 novel_circ_030568 novel_circ_030569 novel_circ_030570 novel_circ_030571 novel_circ_030572 novel_circ_030573 novel_circ_030574 novel_circ_030575 novel_circ_030576 novel_circ_030577 novel_circ_030578 novel_circ_030579 novel_circ_030583 novel_circ_030584 novel_circ_030585 novel_circ_030586 novel_circ_030587 novel_circ_030588 novel_circ_030589 novel_circ_030590 novel_circ_030591

| hsa_circ_0041173 | 91 | 1 | 604 | 604 | 1 | 0 | 0 |
| --- | --- | --- | --- | --- | --- | --- | --- |
| hsa_circ_0041173 | 92 | 1 | 338 | 338 | 1 | 0 | 8.00E-133 |
| hsa_circ_0041173 | 92 | 1 | 338 | 338 | 1 | 0 | 2.00E-132 |
| hsa_circ_0041168 | 91 | 1 | 948 | 948 | 1 | 0 | 0 |
| hsa_circ_0041168 | 90 | 1 | 390 | 898 | 509 | 0 | 9.00E-142 |
| hsa_circ_0041168 | 90 | 1 | 469 | 898 | 430 | 0 | 7.00E-174 |
| hsa_circ_0041168 | 90 | 1 | 613 | 898 | 286 | 0 | 0 |
| hsa_circ_0041168 | 91 | 1 | 898 | 898 | 1 | 0 | 0 |
| hsa_circ_0041168 | 91 | 7 | 831 | 825 | 1 | 0 | 0 |
| hsa_circ_0041168 | 91 | 1 | 628 | 628 | 1 | 0 | 0 |
| hsa_circ_0041168 | 91 | 1 | 508 | 508 | 1 | 0 | 0 |
| hsa_circ_0041168 | 92 | 4 | 429 | 426 | 1 | 0 | 1.00E-166 |
| hsa_circ_0041165 | 91 | 4 | 626 | 623 | 1 | 0 | 0 |
| hsa_circ_0041168 | 92 | 1 | 285 | 285 | 1 | 0 | 3.00E-110 |
| hsa_circ_0041165 | 91 | 1 | 482 | 482 | 1 | 0 | 0 |
| hsa_circ_0041161 | 92 | 1 | 725 | 873 | 149 | 0 | 0 |
| hsa_circ_0002723 | 97 | 1 | 451 | 451 | 1 | 0 | 0 |
| hsa_circ_0004445 | 97 | 1 | 536 | 536 | 1 | 0 | 0 |
| hsa_circ_0041242 | 91 | 1 | 515 | 515 | 1 | 0 | 0 |
| hsa_circ_0041252 | 95 | 1 | 570 | 571 | 2 | 0 | 0 |
| hsa_circ_0041267 | 91 | 1 | 341 | 341 | 1 | 0 | 3.00E-131 |
| hsa_circ_0041289 | 93 | 1 | 288 | 288 | 1 | 0 | 2.00E-118 |
| hsa_circ_0041300 | 94 | 1 | 1364 | 1364 | 1 | 0 | 0 |
| hsa_circ_0041305 | 92 | 1 | 2676 | 2676 | 1 | 0 | 0 |
| hsa_circ_0041319 | 92 | 1 | 756 | 756 | 1 | 0 | 0 |
| hsa_circ_0041324 | 91 | 1 | 232 | 232 | 1 | 0 | 2.00E-82 |
| hsa_circ_0041325 | 89 | 1 | 1022 | 1239 | 2260 | 0 | 0 |
| hsa_circ_0041344 | 82 | 4 | 935 | 2626 | 1692 | 0.01 | 0 |
| hsa_circ_0041362 | 88 | 1 | 239 | 1 | 239 | 0 | 2.00E-76 |
| hsa_circ_0041362 | 88 | 1 | 240 | 1 | 240 | 0 | 9.00E-76 |
| hsa_circ_0041362 | 89 | 1 | 328 | 1 | 328 | 0 | 2.00E-112 |
| hsa_circ_0041362 | 89 | 1 | 329 | 1 | 329 | 0 | 4.00E-112 |
| hsa_circ_0003627 | 89 | 12 | 920 | 12 | 920 | 0 | 0 |
| hsa_circ_0041367 | 88 | 1 | 860 | 528 | 1387 | 0 | 0 |
| hsa_circ_0041378 | 93 | 1 | 448 | 448 | 1 | 0 | 0 |
| hsa_circ_0041380 | 96 | 1 | 324 | 478 | 155 | 0 | 4.00E-150 |
| hsa_circ_0041384 | 97 | 3 | 202 | 200 | 1 | 0 | 8.00E-90 |
| hsa_circ_0041384 | 93 | 1 | 371 | 371 | 1 | 0 | 3.00E-151 |
| hsa_circ_0041387 | 94 | 1 | 532 | 532 | 1 | 0 | 0 |
| hsa_circ_0041387 | 94 | 110 | 638 | 529 | 1 | 0 | 0 |
| hsa_circ_0041387 | 94 | 407 | 935 | 529 | 1 | 0 | 0 |
| hsa_circ_0041390 | 93 | 1 | 324 | 324 | 1 | 0 | 8.00E-132 |
| hsa_circ_0041390 | 93 | 110 | 430 | 321 | 1 | 0 | 5.00E-130 |
| hsa_circ_0041390 | 95 | 1 | 301 | 510 | 210 | 0 | 4.00E-132 |
| hsa_circ_0041390 | 95 | 1 | 412 | 621 | 210 | 0 | 0 |
| hsa_circ_0041407 | 93 | 107 | 623 | 518 | 1 | 0 | 0 |
| hsa_circ_0041409 | 94 | 15 | 560 | 5263 | 4718 | 0 | 0 |
| hsa_circ_0041409 | 93 | 2 | 760 | 5476 | 4718 | 0 | 0 |
| hsa_circ_0041412 | 94 | 1 | 259 | 259 | 1 | 0 | 4.00E-109 |
| hsa_circ_0041412 | 95 | 15 | 400 | 386 | 1 | 0 | 7.00E-173 |
| hsa_circ_0041412 | 94 | 2 | 600 | 599 | 1 | 0 | 0 |
| hsa_circ_0041412 | 94 | 130 | 728 | 599 | 1 | 0 | 0 |
| hsa_circ_0041418 | 89 | 1 | 220 | 1 | 222 | 0.02 | 7.00E-71 |
| hsa_circ_0041418 | 89 | 1 | 220 | 1 | 222 | 0.02 | 1.00E-70 |

novel_circ_030592 novel_circ_030593 novel_circ_030594 novel_circ_030595 novel_circ_030596 novel_circ_030597 novel_circ_030598 novel_circ_030599 novel_circ_030600 novel_circ_030601 novel_circ_030602 novel_circ_030603 novel_circ_030604 novel_circ_030605 novel_circ_030606 novel_circ_030607 novel_circ_030609 novel_circ_030610 novel_circ_030611 novel_circ_030612 novel_circ_030613 novel_circ_030614 novel_circ_030615 novel_circ_030617 novel_circ_030618 novel_circ_030619 novel_circ_030620 novel_circ_030621 novel_circ_030622 novel_circ_030623 novel_circ_030624 novel_circ_030625 novel_circ_030626 novel_circ_030627 novel_circ_030628 novel_circ_030629 novel_circ_030630 novel_circ_030631 novel_circ_030632 novel_circ_030633 novel_circ_030634 novel_circ_030635 novel_circ_030636 novel_circ_030637 novel_circ_030639 novel_circ_030640 novel_circ_030642 novel_circ_030643 novel_circ_030644 novel_circ_030645 novel_circ_030646 novel_circ_030647 novel_circ_030648 novel_circ_030649

| hsa_circ_0041418 | 92 | 1 | 380 | 1 | 382 | 0.01 | 9.00E-152 |
| --- | --- | --- | --- | --- | --- | --- | --- |
| hsa_circ_0041418 | 94 | 1 | 587 | 1 | 589 | 0.01 | 0 |
| hsa_circ_0041418 | 95 | 1 | 756 | 1 | 758 | 0.01 | 0 |
| hsa_circ_0041415 | 95 | 1 | 859 | 379 | 1239 | 0 | 0 |
| hsa_circ_0041415 | 95 | 1 | 1088 | 379 | 1468 | 0 | 0 |
| hsa_circ_0041415 | 96 | 1 | 1189 | 379 | 1569 | 0 | 0 |
| hsa_circ_0041418 | 97 | 1 | 367 | 223 | 589 | 0 | 2.00E-177 |
| hsa_circ_0041418 | 97 | 1 | 536 | 223 | 758 | 0 | 0 |
| hsa_circ_0041415 | 97 | 1 | 868 | 601 | 1468 | 0 | 0 |
| hsa_circ_0041419 | 97 | 1 | 282 | 1 | 282 | 0 | 1.00E-133 |
| hsa_circ_0041419 | 97 | 1 | 451 | 1 | 451 | 0 | 0 |
| hsa_circ_0041420 | 97 | 1 | 376 | 1 | 376 | 0 | 1.00E-180 |
| hsa_circ_0041423 | 97 | 1 | 332 | 170 | 501 | 0 | 6.00E-158 |
| hsa_circ_0041439 | 92 | 1 | 504 | 1 | 504 | 0 | 0 |
| hsa_circ_0041439 | 93 | 1 | 217 | 517 | 733 | 0 | 3.00E-84 |
| hsa_circ_0041440 | 92 | 102 | 459 | 1 | 358 | 0.01 | 1.00E-140 |
| hsa_circ_0041461 | 85 | 1 | 562 | 1240 | 679 | 0.01 | 2.00E-160 |
| hsa_circ_0041462 | 90 | 1 | 315 | 317 | 3 | 0 | 8.00E-112 |
| hsa_circ_0041463 | 93 | 6 | 301 | 305 | 1 | 0.03 | 7.00E-120 |
| hsa_circ_0041463 | 88 | 1 | 432 | 414 | 1 | 0.08 | 3.00E-133 |
| hsa_circ_0041463 | 89 | 110 | 641 | 514 | 1 | 0.07 | 0 |
| hsa_circ_0041463 | 91 | 1 | 332 | 988 | 657 | 0 | 6.00E-123 |
| hsa_circ_0041466 | 93 | 1 | 647 | 649 | 3 | 0 | 0 |
| hsa_circ_0041488 | 93 | 1 | 320 | 320 | 1 | 0 | 6.00E-128 |
| hsa_circ_0041498 | 94 | 1 | 305 | 305 | 1 | 0 | 1.00E-129 |
| hsa_circ_0041498 | 93 | 1 | 882 | 882 | 1 | 0 | 0 |
| hsa_circ_0041498 | 90 | 1 | 1351 | 1351 | 1 | 0 | 0 |
| hsa_circ_0041498 | 90 | 4 | 1525 | 1522 | 1 | 0 | 0 |
| hsa_circ_0041499 | 87 | 4 | 928 | 1356 | 432 | 0 | 0 |
| hsa_circ_0041500 | 86 | 1 | 634 | 634 | 1 | 0 | 0 |
| hsa_circ_0041500 | 87 | 4 | 808 | 805 | 1 | 0 | 0 |
| hsa_circ_0041503 | 93 | 1 | 1015 | 1484 | 470 | 0 | 0 |
[truncated: 283,251 more chars]
